# DHARANI: A 3D Developing Human‐Brain Atlas Resource to Advance Neuroscience Internationally Integrated Multimodal Imaging and High‐Resolution Histology of the Second Trimester

**DOI:** 10.1002/cne.70006

**Published:** 2025-02-04

**Authors:** Richa Verma, Mihail Bota, Keerthi Ram, Jaikishan Jayakumar, Rebecca Folkerth, Karthika Pandurangan, Jivitha Jyothi Ramesh, Moitrayee Majumder, Rakshika Raveendran, Reetuparna Nanda, Sivamani K, Amal Dhivahar S, Srinivasa Karthik, Ramdayalan Kumarasami, Suresh S, S. Lata, E. Harish Kumar, Rajeswaran Rangasami, Chitra Srinivasan, Jayaraman Kumutha, Sudha Vasudevan, Koushik Bhat, Chrisline Sam C, Sivathanu Neelakantan, Stephen Savoia, Partha P. Mitra, Jayaraj Joseph, Paul R. Manger, Mohanasankar Sivaprakasam

**Affiliations:** ^1^ Sudha Gopalakrishnan Brain Centre Indian Institute of Technology, Madras Chennai India; ^2^ Healthcare Technology Innovation Centre, Research Park Indian Institute of Technology, Madras Chennai India; ^3^ Center for Computational Brain Research Indian Institute of Technology, Madras Chennai India; ^4^ Brain Injury Research Center, Department of Rehabilitation and Human Potential, Icahn School of Medicine at Mount Sinai New York USA; ^5^ Department of Electrical Engineering Indian Institute of Technology, Madras Chennai India; ^6^ Mediscan Systems Chennai India; ^7^ Department of Perinatal Pathology Mediscan Chennai India; ^8^ Department of Radiology Sri Ramachandra Institute of Higher Education and Research Chennai Tamil Nadu India; ^9^ Department of Pathology Saveetha Medical College Thandalam, Chennai Tamil Nadu India; ^10^ Department of Neonatology Saveetha Medical College Thandalam, Chennai Tamil Nadu India; ^11^ Cold Spring Harbor Laboratory New York USA; ^12^ School of Anatomical Sciences, Faculty of Health Sciences University of the Witwatersrand Johannesburg South Africa

**Keywords:** fetal brain, high‐resolution histology, RRID:SCR_002285, RRID:SCR_007117, second‐trimester atlas, three‐dimensional (3D) reconstruction

## Abstract

We introduce DHARANI, the first online platform with three‐dimensional (3D) histological reconstructions of the developing human brain from 14 to 24 gestational weeks (GW) across the five fetal brains. DHARANI features 5132 Nissl, hematoxylin and eosin stained, 20 µm coronal and sagittal sections, postmortem MRI, and a neuroanatomical atlas with 466 annotated sections covering ∼500 brain structures. It is accessible online at https://brainportal.humanbrain.in/publicview/index.html. The 3D reconstruction enables a volumetric view of the fetal brain, allowing visualization in all three planes akin to MRI, previously unachievable with histological datasets from the fetal brain. This allowed qualitative assessment of the growth of brain regions and layers throughout the second trimester. “DHARANI” documents the initiation of sulci, with the lateral fissure, calcarine, parieto‐occipital, and cingulate sulci, at 14 GW. The central and postcentral sulci appear by 24 GW; however, cytoarchitectonic boundaries become visible before sulcal patterns. Cortical plate (CP) lamination begins at 24 GW in the parietal and occipital cortices. The frontal cortex lacks lamination at 24 GW, although putative Betz cells are already visible and show early patterning in the intermediate zone. The cell‐sparse layer between the CP and subplate, containing late migratory neurons, begins in the orbital cortex at 14 GW and reaches the frontal cortex by 17 GW. The appearance of the honeycomb pattern in the occipital and parietal cortex occurs after 14 GW. Additionally, we describe the development of the thalamic pregeniculate with the rotation of the lateral geniculate nucleus. Cerebellar nuclei and an early Purkinje cell layer appear by 21 GW in the already foliated cerebellar cortex.

## Graphical abstract

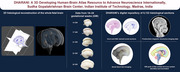

## 1 Introduction

The development of the human central nervous system is a complex biological process, with a greatly extended prenatal period and several key stages that result in various cell types, molecules, and intricate connections that cannot be directly compared with experimental animal models (Molnár 2011; Silbereis et al. 2016; Stiles and Jernigan 2010; Vasung et al. 2019). To date, openly accessible high‐resolution histology atlases for the entire human brain during development are scarce (Ding et al. 2022) compared to similar datasets in other species, such as laboratory rodents and nonhuman primates, where multimodal imaging and three‐dimensional (3D) reconstruction (Dong 2008; Woodward et al. 2018) have been possible. Advances in imaging and computational tools have facilitated the 3D reconstruction of the whole brain in animal models (Hawrylycz et al. 2011) through high‐resolution histology (Lin et al. 2019; Pinskiy et al. 2015) and the generation of multimodal and probabilistic atlases in adult humans (Amunts et al. 2013, Amunts et al. 2020; Amunts and Lippert 2021; Ding et al. 2016). Generating high‐quality histological datasets from the whole fetal human brain with minimal tissue loss and shrinkage has been challenging, even though histology remains the gold standard for the characterization of the transient features of the developing human brain (Altman and Bayer 2002; Vasung et al. 2019). The primary challenge contributing to this lies in the lack of methodological advancements (Crick and Jones 1993), hindering the processing of large‐volume brains with high throughput, and computational tools for analyzing terabytes and petabytes of data from high‐resolution imaging. Additionally, acquiring good‐quality postmortem tissue poses practical challenges (Molnár 2011; Vasung et al. 2019).

Over the last five decades, various tools and techniques have been employed to explore fundamental questions regarding the anatomical organization of the human brain, with seminal work on the organization of the fetal brain conducted using the Yakovlev and the Zagreb collections (Altman and Bayer 2002; Bayer and Altman 2005; Kostovic et al. 1991); however, most of these collections predate advanced imaging techniques like MRI and in utero ultrasonography (USG) and often lack detailed clinical information and prenatal history. Robust datasets integrating clinical information are essential for meaningful correlations between clinical findings, imaging, and histology. Studying the developing human brain offers insights into the organization of the adult human brain regarding cell types, neuronal density, connections, and laminar organization across brain regions (Altman and Bayer 1990; Bayer and Altman 2005; Bystron et al. 2005; Chi, Dooling, and Gilles 1977; Rakic 1968; Rakic 1988; Stiles and Jernigan 2010; Vasung et al. 2019; Verma et al. 2024). Advances in molecular techniques in the last two decades have provided a further understanding of the biological mechanisms and categorization of cell types with molecular signatures in the developing human brain (Bhaduri et al. 2021; Ding et al. 2022; Eze et al. 2021; Hansen et al. 2010; Kim et al. 2023); Additionally, generating robust spatiotemporal molecular maps relies on a high‐quality histological reference atlas, ideally with volume datasets. Whole‐brain atlases that provide volume datasets in fetal brains primarily rely on MRI (Gholipour et al. 2017; Vasung et al. 2016) and USG (Namburete et al. 2023), which lack cellular resolution and specificity, hampering the systematic evaluation of key transitional stages and the understanding of the neurogenic process.

The Sudha Gopalakrishnan Brain Centre (SGBC), at the Indian Institute of Technology, Madras (IITM), India, has taken the initiative to bridge the gap between whole‐volume datasets and cellular details in the developing human brain. The neuroanatomical investigation of serial fetal human brain sections, by freezing the whole brain as a single block, instead of using multiple slabs of tissue, was reported earlier for younger gestational ages (Verma et al. 2024) and was achieved using an engineering and technology‐driven approach, involving monitoring and optimizing parameters (Kumarasami et al. 2023) previously applied to rodent brains (Pinskiy et al. 2013).

DHARANI, digital histology datasets from the whole human brain in the second trimester, are accessible at https://brainportal.humanbrain.in/publicview/index.html along with an interactive interface that allows navigation through annotated representative sections with high sampling density. These histological datasets are complemented by postmortem MRI and block‐face imaging (BFI), facilitating multimodal comparison (Verma et al. 2024), thus enabling a comprehensive spatial and temporal visualization of the developing human brain. Compared to the earlier study by Verma et al. (2024) where histological characterization of early second‐trimester (13–16 gestational weeks [GW]) brains was reported, the current study expands to the entire second trimester, until 24 GW, hence allowing spatiotemporal characterization of the developing whole brain spanning the entire second trimester. Furthermore, the 3D reconstruction provided a unique perspective of the growth of brain regions from 14 to 24 GW, specifically the decrease in size of the lateral ventricles as the cerebral cortex develops, the development of sulci, and the corpus callosum.

**FIGURE 1:**
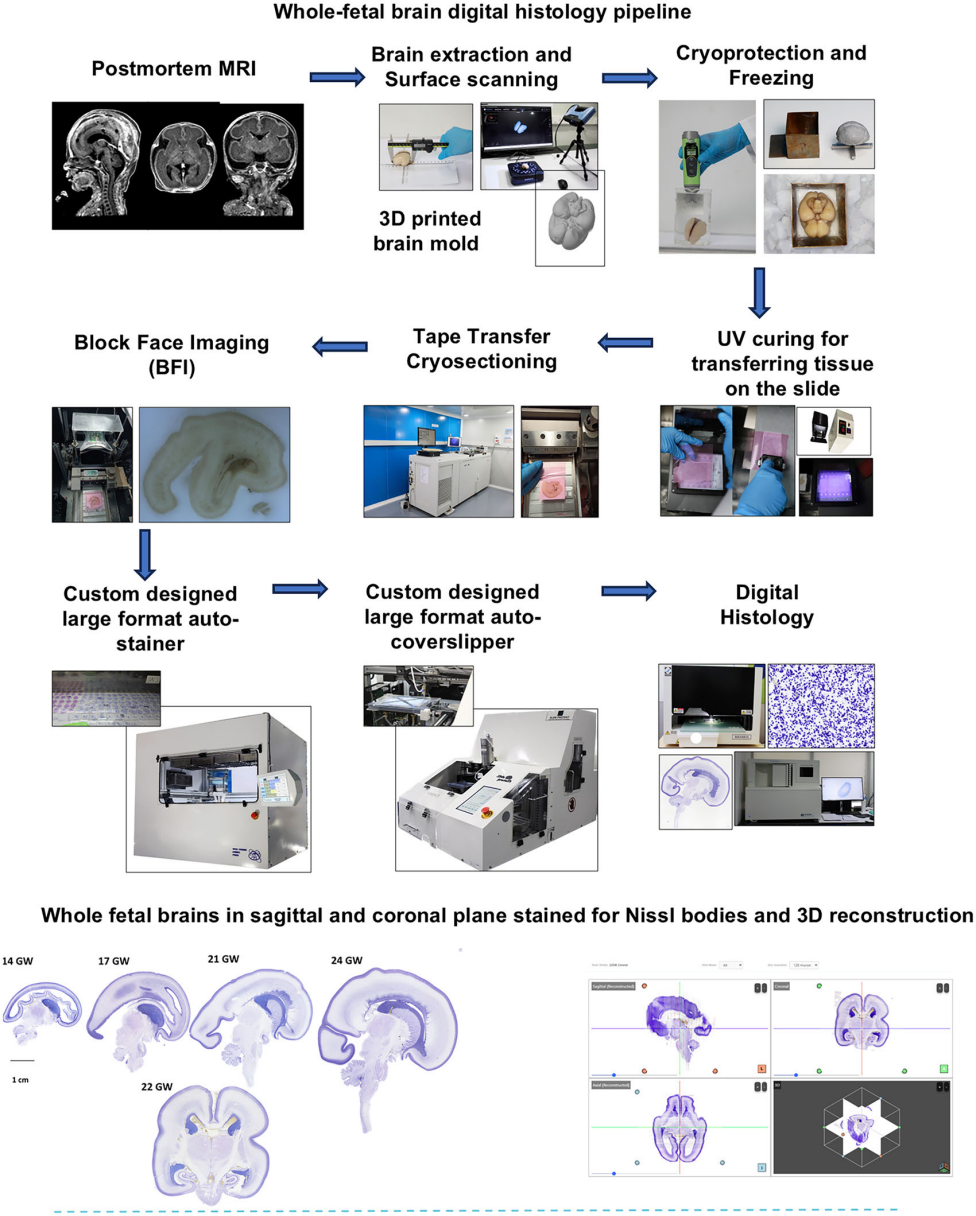
3D reconstruction of fetal brains at 14, 17, 22, and 24 gestational Weeks (GW).

A customized BFI technique aided in visualizing macro‐level features for the registration of high‐resolution histological tissue sections (Karthik et al. 2023). Histological processing of large whole brains was achieved through automated large‐format stainers (Sithambaram et al. 2023) and cover slippers for 6 × 8‐in. slides (Narayanan et al. 2023), ensuring consistent high‐quality results for thousands of sections per brain encompassing the early to late second trimester. The workflow for the whole‐fetal brain processing pipeline from postmortem MRI to digitization is depicted in Figure 1. The digitized histological sections were stack‐aligned and co‐registered to *in‐skull* MRIs to generate 3D volumes and visualization in all three planes. To summarize, Table 1 compares the DHARANI atlas from the second trimester to the currently available state‐of‐the‐art fetal brain histological atlases.

**TABLE 1:** Comparison of DHARANI to the currently available human developmental atlases.

Features of the currently available fetal atlases—second trimester	Bayer and Altman (2005)	Allen Brain Institute (2021)	DHARANI, IIT Madras (2024)
Details of dataset	2D Print Atlas (300 dpi) Nissl (88 sections from 9 brains)	2D Digital (1 µm/pixel) and Print Atlas (300 dpi) Nissl (168 sections from 3 brains)	**2D Digital (1 µm/pixel) and Print Atlas (300 dpi)** **3D reconstruction from histology and co‐registered to MRI** **Nissl and H&E (5132 sections from 5 brains)**
Multimodal imaging MRI and BFI	X	X	**MRI** **BFI**
Number of brains	9 Whole brains—13.5–24 GW	2 Half brains: 17 GW (15PCW), 23 GW (21PCW) and 1 brainstem 23 GW (21PCW)	**5 Whole brains—**14, 17, 21, 22 and 24 GW
Number of annotated sections (Nissl)	88 Annotated sections	168 Annotated sections	**466** Annotated sections
Maximum number of annotated sections—per brain	20	81	**124** Annotated sections
Thickness of each section	35 µm	20 µm	**20 µm**
Spacing between annotated sections	Uneven spacing	250–1200 µm spacing	**60–1000 **µm spacing
Embedding	9 Whole brain—embedded in celloidin	1 Hemisphere from 2 brains and 1 brainstem—embedded in OCT	**5 Whole brains** embedded in OCT as a single block
Staining series	2 Series—Nissl and Myelin	4 Series—Nissl, ISH, AChE, and Myelin	**3 Series—**Nissl, H&E, and Myelin/IHC^a^

Abbreviations: AChE, acetylcholinesterase; BFI, blockface imaging; GW, gestational weeks; ISH, in situ hybridization; OCT, optimal cutting temperature embedding medium; PCW, postconceptional week.

^a^Myelin and immunohistochemistry (IHC) datasets are not part of this study.

We use these high‐resolution histological datasets to construct 466 annotated sections (see Atlas Plates in Appendix A) from 5 brains with ∼60–120 sections per brain and to characterize the spatial‐temporal changes detectable across regions, fiber tracts, and transient structures from 14 to 24 GW. This is the largest publicly accessible digital dataset of the human fetal brain to date and to the best of our knowledge. This large cell‐level dataset from the second trimester developing human brain revealed several new findings in the cerebral cortex, thalamus, hypothalamus, and pretectum region. In the developing cerebral cortex, the cortical transient zones, sites for key neurogenic events, show early subregional boundaries. We describe the topographical relationship of the pregeniculate complex in the human fetal brain with the developing lateral geniculate nucleus (LGN), aligning with the rotation and maturation between 14 and 24 GW. Furthermore, we report the presence of several distinct white matter tracts in the thalamus and hypothalamus. In the pretectal area, we delineated key pretectal nuclei from 17 GW onward that have not been described previously in the developing human brain, to the best of our knowledge.

This large cell‐level dataset from the developing human brain revealed several new findings in the second trimester in the cerebral cortex, thalamus, hypothalamus, and pretectum of the developing human brain.

## 2 Methods

### 2.1 Specimen Procurement

De‐identified specimens (*n* = 5), with a postmortem interval (PMI) of 2–4 h, were collected after due consent from next of kin, in accordance with the declaration of Helsinki, along with prenatal ultrasound scans. Specimens from 14 to 24 GW (postconception week, PCW 12–22) were obtained from the Department of Pathology at Mediscan Systems Pvt. Ltd., Chennai, India (Mediscan). We use “GW” to facilitate comparison (Verma et al. 2024) with published atlases (Bayer and Altman 2003, 2005). On the basis of the prenatal USG, postmortem MRI, medical autopsy examination, and high‐resolution whole‐brain histology with hematoxylin and eosin (H&E) staining, the brain specimens were classified as non‐pathological. Post extraction, the mass and dimensions of each specimen were measured and recorded, as shown in Table 2.

**TABLE 2:** Specimen details.

		Dimensions (cm)		
Specimen	Mass (g)	A‐P	RL‐LL	D‐V
S1 (14 GW)	8.6	4.4	2.8	2
S2 (17 GW)	24.4	4.5	4	3.5
S3 (21 GW)	53.3	6	5	4.8
S4 (22 GW)	56.9	6.3	5.3	4.4
S5 (24 GW)	109.4	7.7	6.1	5.5

Abbreviations: A‐P, anterior to posterior; D‐V, dorsal to ventral; RL‐LL, right lateral to left lateral.

We describe below each of the processing steps and the several technologies developed at SGBC for processing the whole human brain as one single block, with large histological sections placed on 6 × 8‐in. slides.

### 2.2 Postmortem MRI

We performed a postmortem *in‐skull* MRI at the Department of Radiology, Sri Ramachandra Institute of Higher Education and Research. MRI was performed on the brains using 3 T (version DV28.0_R05_2.34.a, Signa Architect, GE medicals, USA) (Figure 1). The imaging utilized 16‐channel medium flex surface coils with an inner diameter of 30 cm. For all specimens, 14–24 GW (S1–S5), a 3D T1‐weighted gradient MP‐RAGE sequence was acquired with a matrix size of 512 × 512 with varying numbers of slices: 88, 84, 132, 108, and 152, respectively (parameters: repetition time 2500 ms; echo time 3 ms; flip angle 8°; field of view 140 mm; slice thickness 0.5 mm with no interleaving space; voxel spacing 0.23 × 0.23 × 0.5 mm^3^). The T1W MRI data was used as a reference during the 3D reconstructions of stacked histological sections.

### 2.3 Fixation, Cryoprotection, and Freezing

Brain specimens, following MR imaging and removal from the skull, were fixed in 4% paraformaldehyde (PFA) in 0.01 M phosphate buffer (PB) at room temperature, for a minimum of 2 weeks before histological processing. For cryosectioning large brains, cryoprotection was a critical step to minimize freezing artifacts and osmotic shock at the cellular level, in superficial as well as deeper structures (Kumarasami et al. 2023). Cryoprotection of the brains started with 10% sucrose in 4% PFA and 0.01 M PB, followed by 20% and 30% sucrose in 0.01 M PB at 4°C, until the specimen was equilibrated at each concentration. Given the wide range of brain size from 14 to 24 GW, the cryoprotection duration varied from 12 to 22 days. The volume of the immersion solution used was approximately five times that of the specimen.

A technology platform was designed to freeze large brains, where a custom‐designed brain master pattern (for alignment) and a copper base mold were generated (see Kumarasami et al. 2023). To design the custom brain master pattern for each specimen, 3D surface scans (Einscan Pro, SHINING 3D Technology GmbH, Germany) of the extracted brain were obtained and 3D‐printed (in metal) (Kumarasami et al. 2023). This allowed aligning the brain in anatomical coordinates during the preparation of the cryoblock, as closely as possible to that observed *in skull in the MRI* (Figure 1). Copper cubicles, designed to embed the specimen in optimal cutting temperature (OCT) embedding medium, were used to create cryoblocks. Each specimen was frozen using an isopentane dry ice bath, enabled with continuous temperature monitoring using a Re146 resistance temperature detector (RTD) sensor, with an accuracy of ±0.25°C. The freezing process was conducted in two stages: creating a cryomold and then freezing the brain tissue. First, the base mold, containing the brain master pattern and embedding medium, was placed in the isopentane container. Dry ice was added to the mixture as and when required to ensure minimal deviation from the desired −80°C isopentane temperature. The isopentane and dry ice mixture has been shown to freeze the brain tissue at a rate of 2–3°C/min uniformly for tissues with thicknesses up to 40 mm (Kumarasami et al. 2023). Once the embedding medium froze, the master pattern was removed with the aid of a heat gun, creating a negative impression of the brain surface in the frozen embedding medium with the desired alignment. The brain tissue was placed in the base mold and covered with the embedding medium. Once the embedding medium froze, heat was further applied to remove the frozen block from the base mold. The frozen block was then transferred to a −80°C refrigerator for storage until cryosectioning.

### 2.4 BFI and Cryosectioning Using the Tape Transfer Technique

Immediately prior to cryosectioning, the exposed surface of the cryoblock is imaged serially using a BFI camera (Figure 1) (Karthik et al. 2023). This series of images was used to reconstruct a 3D volume which can be used as a reference to quantify any distortions or shrinkage that may occur due to the histological processing and staining.

We custom‐designed the BFI setup for imaging large‐volume tissues (>600 cm^3^) and can image a very large field of view (upto 20 × 20 cm^2^) at a resolution of 70 µm/pixel under white light illumination. This system was also designed to work under extreme subzero temperatures of up to −30°C. Most importantly, it was designed to maintain its optical focus over a significant depth of tissue (∼7 cm) and over long periods of time without the need for readjustments. In addition, we have also used two adhesive fluorescent fiducial markers (1 × 1 cm^2^) which were used to align the images to compensate for any planar shifts that might occur during sectioning due to conveyor belt stoppage tolerances.

Using the Leica CM1520 and CM3600 (cryomacrotome) cryostats (Leica Biosystems, India), the frozen whole brains (S1: ∼1400, S2: ∼1700, S3: ∼2100, and S5: ∼2400, number of sections at 20 µm thickness) were serially sectioned in the sagittal plane, whereas S4 (∼2200) was serially sectioned in the coronal plane (20 µm thick sections). Prior to cryosectioning, the block was acclimatized for at least 30 min at −20°C (the chamber temperature of the cryostat). The tape transfer technique, which has been employed for small animal brains such as rodents and marmosets (Pinskiy et al. 2015), was optimized for use with the large 75 × 50 mm^2^ and 200 × 150 mm^2^ glass slides, specific to the human brains (Verma et al. 2024). The tape transfer technique employs ultraviolet (UV)‐sensitive adhesive tape (Custom Converting Inc., USA) that is wrapped onto the exposed surface of the frozen tissue block, and upon cryosectioning, the sliced section adheres to the tape. The tape with the section is placed on the polymer‐coated glass slide that is activated by UV light (Figure 1), resulting in a hands‐free transfer of the sections onto the glass slide, which minimizes tissue damage. For tape transfer sectioning, the slides were prepared before mounting the sections by coating them with a solution of (trimethoxysilyl) propyl methacrylate, 0.1 M acetic acid (ratio: 4:1) in acetone, for 24 h, followed by an application of a UV curable optical adhesive (Norland NOA 63, Edmund Optics) using Pasteur pipettes and a clean glass rod. The total volume required to coat one 75 × 50 mm^2^ and 200 × 150 mm^2^ slide was 40–50 and 200–250 µL, respectively.

### 2.5 Staining and Coverslipping

After cryosectioning, the sections were divided into three series and stained for Nissl substance, H&E, and the third series was kept for myelin and immunohistochemical staining (IHC) not reported herein. The sections were stained for Nissl substance using 0.2% thionine and for H&E using Harris’ regressive protocol. The Nissl and H&E staining were performed using an autostainer designed specifically to accommodate the large slides (Sithambaram et al. 2023). The developing human brain, specifically at a younger gestational stage, is high in water content, and hence, following staining, the sections were dried for 3 days in a temperature‐controlled chamber at 33°C. For high throughput of the thousands of sections per brain, an automated large‐format custom‐designed coverslipper was built that utilized DPX (Merck) mounting media to coverslip the sections immersed in xylene (Narayanan et al. 2023).

### 2.6 Scanning and Quality Check (QC)

Histological sections were digitized using a large format scanner (TissueScope LE120, Huron Digital Pathology, Canada) with an in‐plane resolution of 0.5 µm/pixel (Figure 1). The full‐resolution images underwent quality control to address issues related to imaging such as stitching, white balance, or focus in addition to any staining‐related artifacts. Only sections that passed these QCs were included in the analysis and ranged from 85% to 95% of the total sections across five brains.

### 2.7 Atlas Annotation

**FIGURE 2:**
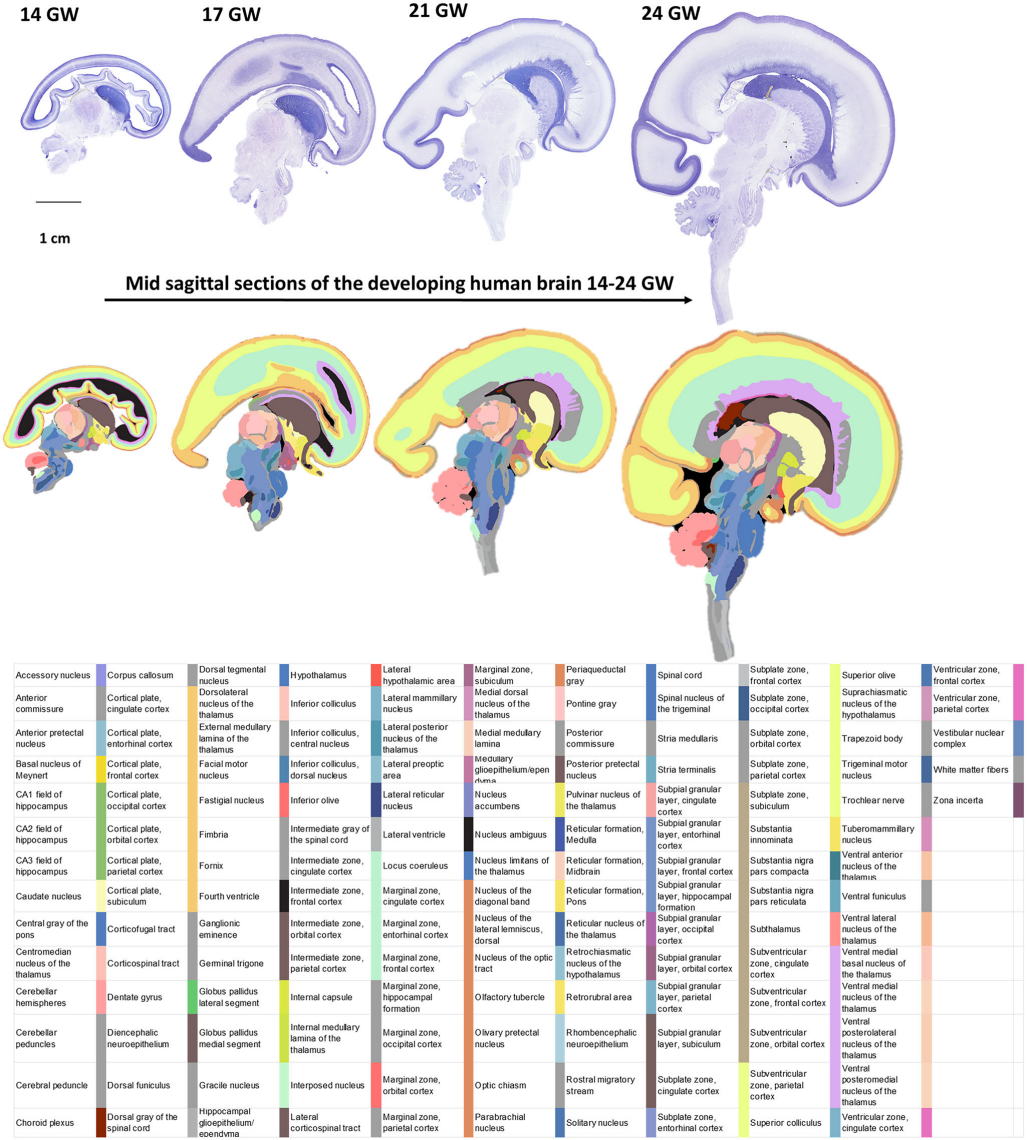
Whole fetal brain histology pipeline, starting from postmortem in‐skull MRI to digital histology datasets (20 µm section thickness) obtained using the tape transfer cryosectioning technique. Due to the large size of sections (2 × 3 to 6 × 8 in.) and high throughput, with 1400–2500 sections from each brain, a large format auto‐stainer and an automated cover‐slipper were custom‐built at the SGBC. Histological sections were digitized using a large format Huron, TissueScope LE120 Scanner (0.5 µm/pixel in‐plane resolution). The digitized images that passed quality checks were utilized for analysis.

Figure 2 shows Nissl‐stained representative sections from four different gestational ages sectioned in the sagittal plane from the right to the left hemisphere. Annotations were performed using an online custom‐designed atlas annotation tool (see Section 2.8.1) to mark identified structures, including brain regions, fiber tracts, ventricles, and other developmental structures such as the ganglionic eminence. The identification and naming of brain structures were performed using primarily the available human developmental atlases (Bayer and Altman 2005; Ding et al. 2022). Additionally, we have used the available adult human brain atlases (Ding et al. 2016; Paxinos et al. 2012) for regions that were ambiguous in the developmental atlases. In addition, we have also used the primate (marmoset) brain atlas of Paxinos et al. (2012) for detailed comparison. The identification and naming of brain structures is, therefore, a combination, as well as “common ground,” of several nomenclatures. Finally, the primary literature has been used extensively to identify and annotate structures for each basic division (see below). For example, the seven transient layers of the developing cerebral cortex were annotated using the classification and nomenclature provided by the Boulder committee (Bystron, Blakemore, and Rakic 2008) and include the ventricular zone (VZ), subventricular zone (SVZ), intermedial zone (IZ), subplate (SP), cortical plate (CP), marginal zone (MZ), and subgranular zone (SGZ). The abbreviations used for each identified structure follow the commonly employed approach: first letter upper case for gray matter regions and lower case for fiber tracts and transitory structures.

This composite nomenclature is hierarchically organized (mixed hierarchical approach), using the spatial partitioning relationship “part of.” Following previous approaches (Bota and Swanson 2008; Dong 2008; Swanson 2018), the most general categories of the hierarchically organized nomenclature are defined by the type of structure: gray matter, axonal pathway, migratory stream, neuro‐ or glioepithelium, or ventricles. Hence, these most general categories are the following: “brain,” “spinal cord,” “fiber tracts,” “developmental structures,” and “ventricles.” The first two categories include all identified and delineated gray matter parts and their parents in the brain and in the spinal cord of the human developing brain, respectively. The children of the “brain category” are the “telencephalon,” “diencephalon,” and “brainstem.” The “fiber tracts” category includes all axonal pathways identified in the literature used for annotations. The “developmental structures” include the migratory streams and the neuro‐ and glioepithelia, described in the literature and identified in our specimens. Finally, the category “ventricles” includes the lateral, third, the fourth ventricle, their parts, and the respective parts of the choroid plexus.

We aimed to identify and delineate the smallest brain regions (the leaves of the nomenclature tree) as defined and described in the used references. However, sometimes these regions could not be identified, because of objective reasons. For example, the individual raphe nuclei of the brainstem could not be identified in sagittal sections only based on cytoarchitecture and topographical positions. In such situations, we used the name of the parent structure to identify those regions. Therefore, the raphe nuclei are delineated as a set of nuclei in sagittal sections. Similarly, few sections had minor tissue damage. We annotated the boundaries in the damaged portion, with the parent structure, or with the name used in the adjacent section where the tissue was not damaged. We also had sections that showed thin portions like the SGZ, detached from the main section, and in such cases, we chose not to annotate as it was difficult to specify the small pieces of tissue with certainty. The annotation sampling across 5 brains ranged from 0.06 to 1 mm resulting in annotations of at least 1 section for every 50 sections. These annotated 466 Nissl sections across the five brains are also available online and at the end of this manuscript. The online repository has 5132 Nissl and H&E sections from five specimens, where 466 annotated sections are at 1 and 4666 4 µm/pixel in‐plane resolution.

These annotations along with the supporting histological sections result in the developing human brain atlas of the second trimester with 414 identified brain regions, fiber tracts, ventricles, and developmental structures (the smallest leaves). Overall, the developed hierarchically organized nomenclature includes about 500 terms to date.

### 2.8 Computational Processing of Brain Section Images

Each section image of DHARANI dataset was captured in 8‐bit 3‐channel (RGB) at 0.5 µm/pixel in‐plane imaging resolution. The image was subjected to a sequence of computational processing steps to extract geometric and photometric parameters, followed by the preparation of image tiles that are suitable for display on a web‐based high‐resolution image rendering software application. In the current data release, the pixel resolution of the histological sections with annotations is rendered as 1 µm/pixel.

#### 2.8.1 Digital Annotation

Following digitization of the images, we performed detailed anatomical delineation using cytoarchitectural boundaries ascertained from the staining for Nissl substance. These delineations were performed within 1 mm intervals across the whole brain (left to right in four samples and the full anterior–posterior extent for one sample). The data were stored in. json format for visualization and further analysis.

#### 2.8.2 Computation of 3D Geometric Parameters

The physical process of sectioning entails the geometrical transformation of the tissue slices with respect to the tissue block. We estimated the transformation parameters by computing the global 3° of freedom (rotation, shift in *x*‐, and *y*‐direction). The high‐resolution images were down‐sampled to 16 µm per pixel in‐plane resolution. The series of images were then stack‐aligned using the steps described in the following:

1. Alignment seeding: For the sagittal sections, an initial section *I*_0_ was selected at the approximate location of the midline where the brain stem could also be visualized, and also tissue area was maximal. The *I*_0_ section was manually rotated based on the anatomical landmarks (e.g., the angle of the brainstem to vertical) and padded so that the tissue region was centered in a square canvas of 5000 × 5000 pixels (80 × 80 mm^2^). This large padding allowed for any change in the size of the pixels during object rotation.
2. Alignment propagation: Neighboring sections were aligned to this initial seed section using automated image keypoint detection and descriptor matching on the luminosity channel, using Fiji Image processing plugin (SIFT: Lowe 2004; RRID: SCR_002285). A relative rotation and translation were computed for each section to align it to the seed section *I*_0_. These computed parameters were applied to create a 60‐µm isotropic Nissl stack. For sagittal sections, the direction cosines were *x*: AP, *y*: SI, *z*: RL. For coronal sections, the direction cosines are *x*: RL, *y*: SI, *z*: AP, anterior (A), posterior (P), superior (S), inferior (I), right (R), and left (L).
3. Registration to MRI: The postmortem in‐skull MRI was skull‐stripped and resampled to 60‐µm isotropic resolution using bicubic interpolation. The resampled MRI was aligned to the Nissl stack by using moments‐based registration with 6° of freedom (rotation and translation in AP, LR, and SI axes).
4. Alignment of interleaved H&E sections: Each H&E section was paired with the nearest Nissl section and aligned rigidly to it. The pairing was used in the viewer to display as overlays (H&E layer beneath the Nissl layer).

#### 2.8.3 3D Visualization

The histological (Nissl) 3D reconstructions computed at 16‐µm in‐plane resolution and 60‐µm slice separation (as described in Section 2.8.2) are saved as a 3D RGB volume in the same coordinate space of the MRI, in Nifti format (RRID: SCR_007117). A 4‐µm in‐plane resolution (and 60‐µm slice separation) volume was created in grayscale, inverting the contrast, and saved as raw array data. This raw volume is visualized using Nvidia IndeX software, with direct volume rendering and shaded rendering of orthogonal planes and pseudo coloring with a colormap theme representing cell density from blue (low) to orange (high) density (see Videos 1–8 in the Supporting Information). Videos 1–5 display 3D‐histological reconstructions of five brain specimens at 14, 17, 21, 22, and 24 GW. Videos 6–8 show 3D reconstructions in the coronal (video 6), axial (video 7) and sagittal (video 8) view of four brains at 14, 17, 22, and 24 GW.

The low‐footprint web viewer also has a 3D visualization at 128 and 64 µm in‐plane resolution to provide a quick 3D view of the whole dataset in RGB, and each plane in the 3D view is linked to the high‐resolution two‐dimensional (2D) viewer application. The quick 3D view shows a four‐quadrant display, with the sectioned view (same as the sectioning plane), reconstructed orthogonal views (digitally sectioned from the 3D reconstruction), and a three‐plane 3D view in the fourth quadrant. This 3D view is interactive, with digital re‐slicing happening based on cursor positions, enabling the user to see the same position in three views and also locate it in a 3D MRI reference space.

#### 2.8.4 Histogram Matching and Color Adjustments

Contrast and brightness computation for each section was done using reference image‐based calibration: A set of reference images was selected across the sections that had good brightness and contrast, and the nearby sections were color‐adjusted using linear and histogram operations (matching image intensity range, histogram mode, and spread). These effective correction parameters for each image were computed and stored at a lower resolution (16 µm per pixel). The parameters were applied at render time to produce color‐corrected views of the section images at arbitrary display resolutions.

#### 2.8.5 Low Footprint Web‐Based Viewer

**FIGURE 3:**
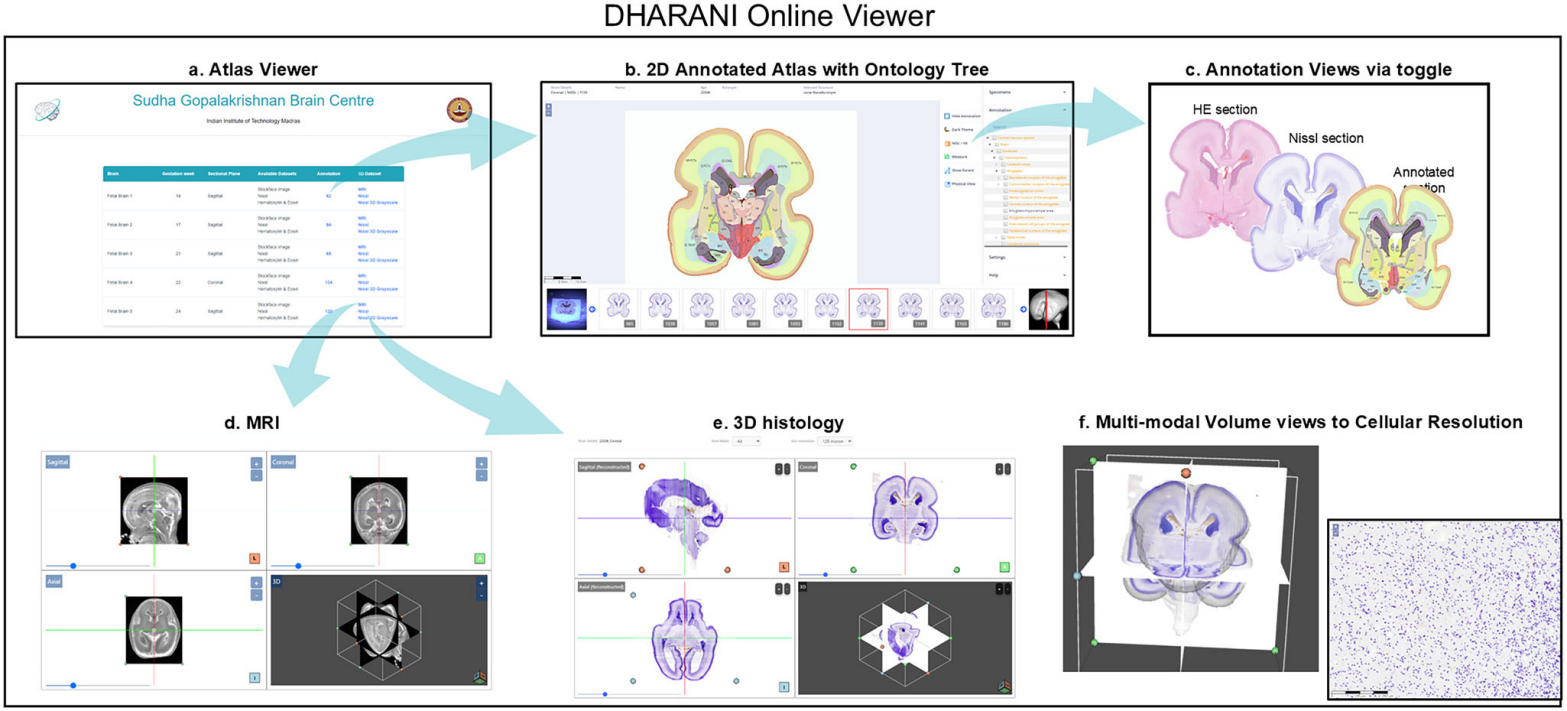
Representative sagittal sections, 20 µm thickness, stained with thionin to reveal Nissl bodies in 14–24 GW specimens. Using the Boulder Committee nomenclature (Bystron, Blakemore, and Rakic 2008), the cerebral cortex atlas annotation highlights the subpial granular zone (SGZ), the marginal zone (MZ), the cortical plate (CP), the subplate (SP), the intermediate zone (IZ), the subventricular zone (SVZ), and the ventricular zone (VZ) (see results for detailed laminar description and nomenclature used in different brain basic subdivision). The custom‐designed atlas annotation tool was employed for annotating over 466 histological sections from five brains. Any region that lacked distinct cytoarchitectural characteristics was categorized under the parent name.

It is important to note that individual section images were tens of GBs in data volume and required significant computation to render as a whole volume which consisted of hundreds of individual sections. We designed and developed an online viewer, where the acquired image from the scanner (tif format 8‐bit RGB) was loaded as a pixmap, transformed and color corrected, followed by conversion to deep zoom tiles of an image pyramid using the libvips (Martinez and Cupitt 2005) “dzsave” command. This step prepared an organized collection of image tiles at multiple resolutions that can be requested by the viewer application rendering the relatively low data usage to the user. DHARANI dataset display (Figure 3) is rendered using open‐source library, OpenLayers image tile layer with image source setup with the Zoomify protocol.

## 3 Results

We describe below the human fetal brain, from 14 to 24 GW, and based on high‐quality Nissl preparations. The online repository from the five specimens can be accessed at https://brainportal.humanbrain.in/publicview/index.html, and it includes the histological sections and their annotations but also includes 3D reconstructions of the five brains, providing visualizations and comparisons of each brain in all three standard planes. The online repository consists of 5132 Nissl‐ and H&E‐stained sections covering the whole brain of the 5 specimens, out of which 466 Nissl‐stained sections were annotated. The 466 Nissl and the corresponding H&E sections are available at 1 µm per pixel in‐plane resolution. The remaining sections are available at 4 µm per pixel in‐plane resolution. Our high‐quality Nissl preparations have allowed cytoarchitectural‐based annotations pioneered by Brodmann (detailed in Brodmann and Garey 2006). These annotations have been performed in 60–120 sections per brain, revealing region‐specific cytoarchitectural differences based on the density and morphology of cells, patterns of cell arrangement and orientation, the developing white matter, and developmental structures. The current reference atlases on the developing human brain are primarily detailed from a coronal perspective. The data presented here were sectioned in the sagittal plane (except one brain sectioned coronally). Hence, we provide brief descriptions in this manuscript to help navigate this large dataset. We present two specimens in the 21–22 GW, which have been sectioned in coronal and sagittal planes to facilitate comparisons between the two perspectives.

The regional description and analysis below follow the classic topographical arrangement of the mammalian brain. We make our observations starting with the oldest specimen, as the subregions and nuclei are more apparent, and we highlight differences from the younger specimens, including cytoarchitectural and shape changes that occur during development.

### 3.1 Gross Features

**FIGURE 4:**
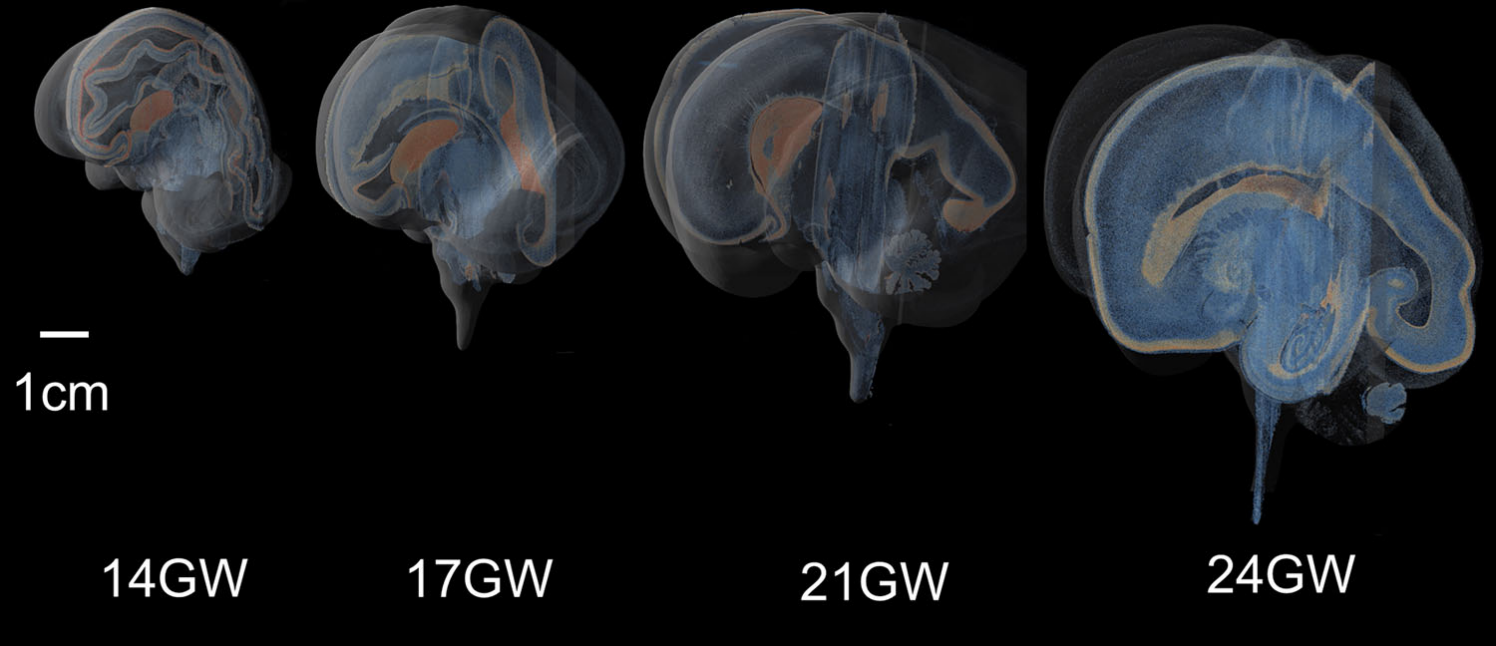
Summary of open‐access interactive online viewer https://brainportal.humanbrain.in/publicview/index.html.

We noted a 10× linear increase in brain size from 14 to 24 GW (Table 2, Figure 2). At early gestation, 14 GW, the lateral ventricle occupies most of the cerebral mantle, and the brain is mostly lissencephalic (Chi, Dooling, and Gilles 1977; Li et al. 2019). We observed the lateral fissure and early infolds of calcarine, parieto‐occipital, and cingulate sulci at 14 GW (Verma et al. 2024; Zhang et al. 2010, 2013). By 17 GW, the cingulate sulcus, including the marginal branch, and the calcarine sulcus show a more advanced stage of development than the parieto‐occipital sulcus (which becomes well‐defined by 21 GW). By 24 GW, we further note two developing sulci in the parietal cortex, the central sulcus and the postcentral sulcus. The corpus callosum is already partially developed by 14 GW, and by 21 GW, it has a well‐defined genu and splenium. Furthermore, at the midsagittal level, we note the relative rotation of key structures, such as the hypothalamus, amygdala, LGN, superior colliculus (SC), and inferior colliculus (IC), as the brain develops from the early to late second trimester. The 3D reconstructions from histology, in all five brains, allowed visualization and comparison of these key developmental features in all three planes, from 14 to 24 GW, as shown in Figure 4.

### 3.2 Telencephalon

#### 3.2.1 Developing Cerebral Cortex

##### 3.2.1.1 The Occipital Cortex

**FIGURE 5:**
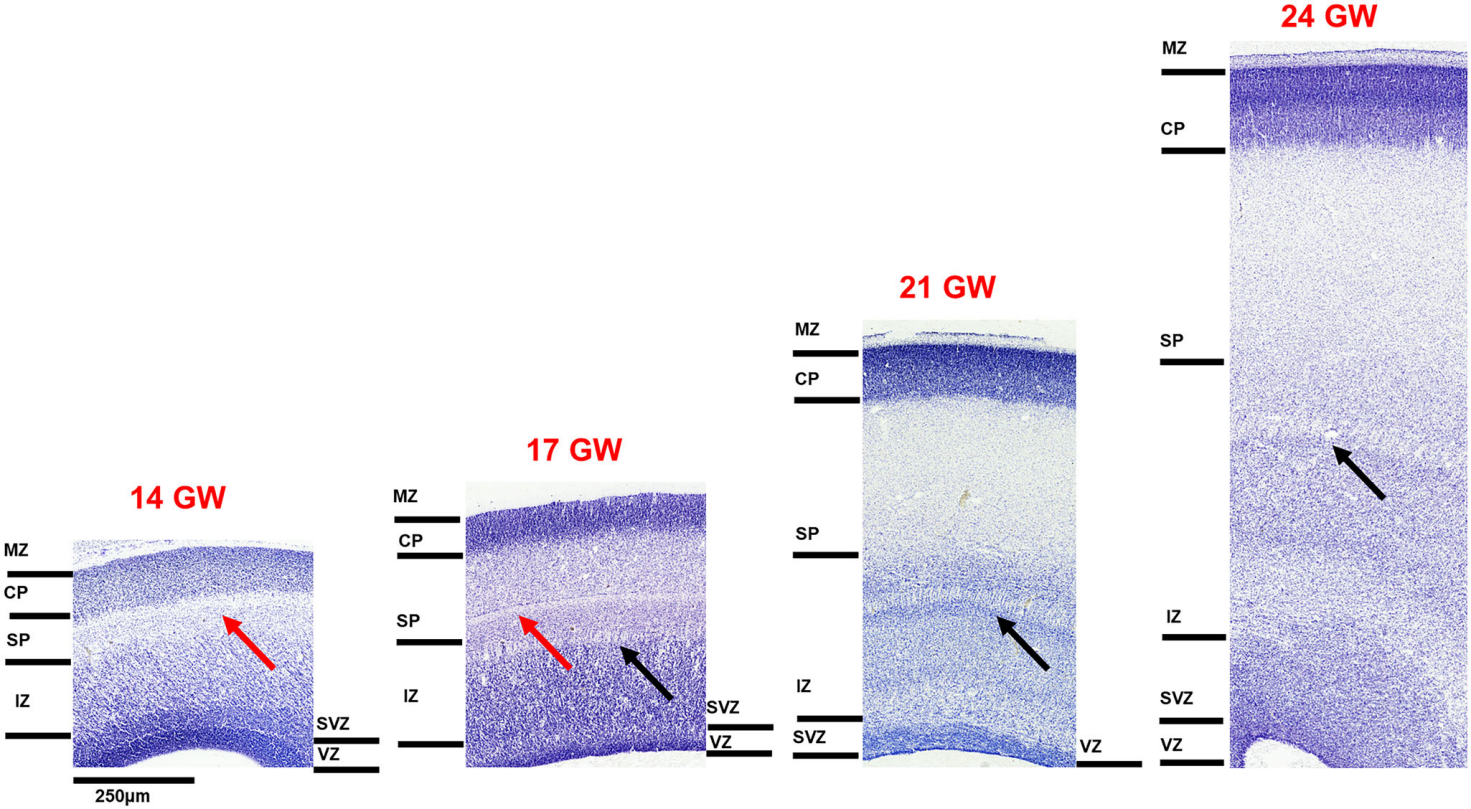
The laminar organization of the developing occipital cortex, revealed with Nissl staining, from 14 to 24 GW. The laminar organization from the ventricular zone (VZ) to the marginal zone (MZ) is readily demarcated. At 14 GW, the subplate (SP) shows the formation stage with an extra cell‐sparse layer (red arrow) that shifts ventral by 17 GW as the SP matures. The intermediate zone (IZ) lacks the honeycomb pattern at 14 GW, which appears at 17 GW and is more prominent by 24 GW (black arrow). The cortical plate (CP) shows lamination at 24 GW, along with an increase in thickness. The SP thickness increases significantly from 14 to 24 GW, attaining a thickness three times that of the CP.

During the early second trimester, the layers of the developing occipital cortex, from the SGZ to the VZ, are readily identified. To study the development of the occipital cortex, we used the developing calcarine sulcus at the midsagittal level as a central landmark. The CP appears to be the most cell‐dense layer with Nissl staining on the dorsal surface (Figure 5) just below the cell sparse, thin MZ. As the CP matured and thickness increased from 14 to 24 GW, we observed early signs of lamination in the CP in 24 GW. At this stage, CP comprises three sublayers, where the putative layer IV (based on cell density) and V can be well demarcated, and the future layer VI appears as a cell‐sparse layer, interposed between the upper thick CP and the SP (Figure 5). Despite the lamination observed at 24 GW, very few small scattered pyramidal‐like cells, based on the shape of the soma (Spruston 2008), were noted in this region. In comparison at 14 GW, the lower boundary of the CP is marked by a cell‐sparse layer within the SP, which is at the “formation stage” (Kostovic and Rakic 1990).

The SP formation stage in the occipital cortex is very specific and includes three sublayers in the occipital region; the outermost layer is cell‐dense, internal to which is a cell‐sparse layer, below which the cells are arranged horizontally (Figure 5, red arrows; Kostovic and Rakic 1990; Verma et al. 2024). This unique lamination of the SP was noted at 17 GW, but the cell‐sparse layer appears to move deeper as the CP and SP mature (see Figure 5, red arrows). The thickness measurements showed that the SP is twice the thickness of the CP at 14 GW, and as the brain develops, it expands to approximately three times the thickness of CP by 17 GW and tends to maintain that ratio until 24 GW, as has been previously reported (Kostovic and Rakic 1990).

The IZ in the occipital cortex shows the highest number of sub‐bands at all gestational ages, ranging from 4 at 14 GW to 6 sub‐bands by 24 GW. The peculiar feature of the occipital cortex IZ is the honeycomb pattern, with migrating cells arranged vertically as striations, distinctly from 17 to 24 GW compared with the 14 GW specimen (see Figure 5, black arrow). Previously, this honeycomb pattern has been reported at 13.5 GW (Bayer and Altman 2005). The honeycomb‐patterned STF3, part of the IZ, represents the developing thalamocortical connections specific to the granular cortex (Altman and Bayer 2002; Bayer and Altman 2005), although we observed this pattern from 17 GW. In other specimens (unpublished data), this pattern appears between 15 and 16 GW first in the caudal occipital cortex before expanding across the cortical mantle. Further investigation of this honeycomb pattern is needed, particularly regarding its correlation with the developmental timeline of thalamic nuclei such as the LGN, which matures between 21 and 24 GW, showing lamination (Hitchcock and Hickey 1980; Yamaguchi 2018) as observed in this study (see below). In order to address this discrepancy in the developmental timeline of this feature, investigation of more specimens between 13 and 17 GW is required.

In the most lateral part of the occipital cortex, the honeycomb pattern allows clear demarcation of its boundary with the adjacent temporal cortex and medially with the parietal cortex. The SVZ is readily identified as a thick darkly stained, cell‐dense layer and shows a distinct fiber layer between the IZ and SVZ. The thickness of this fiber layer increases from 14 to 24 GW. The VZ decreases in thickness with age and appears as a thin neuroepithelial lining by the end of the second trimester.

##### 3.2.1.2 The Parietal Cortex

**FIGURE 6:**
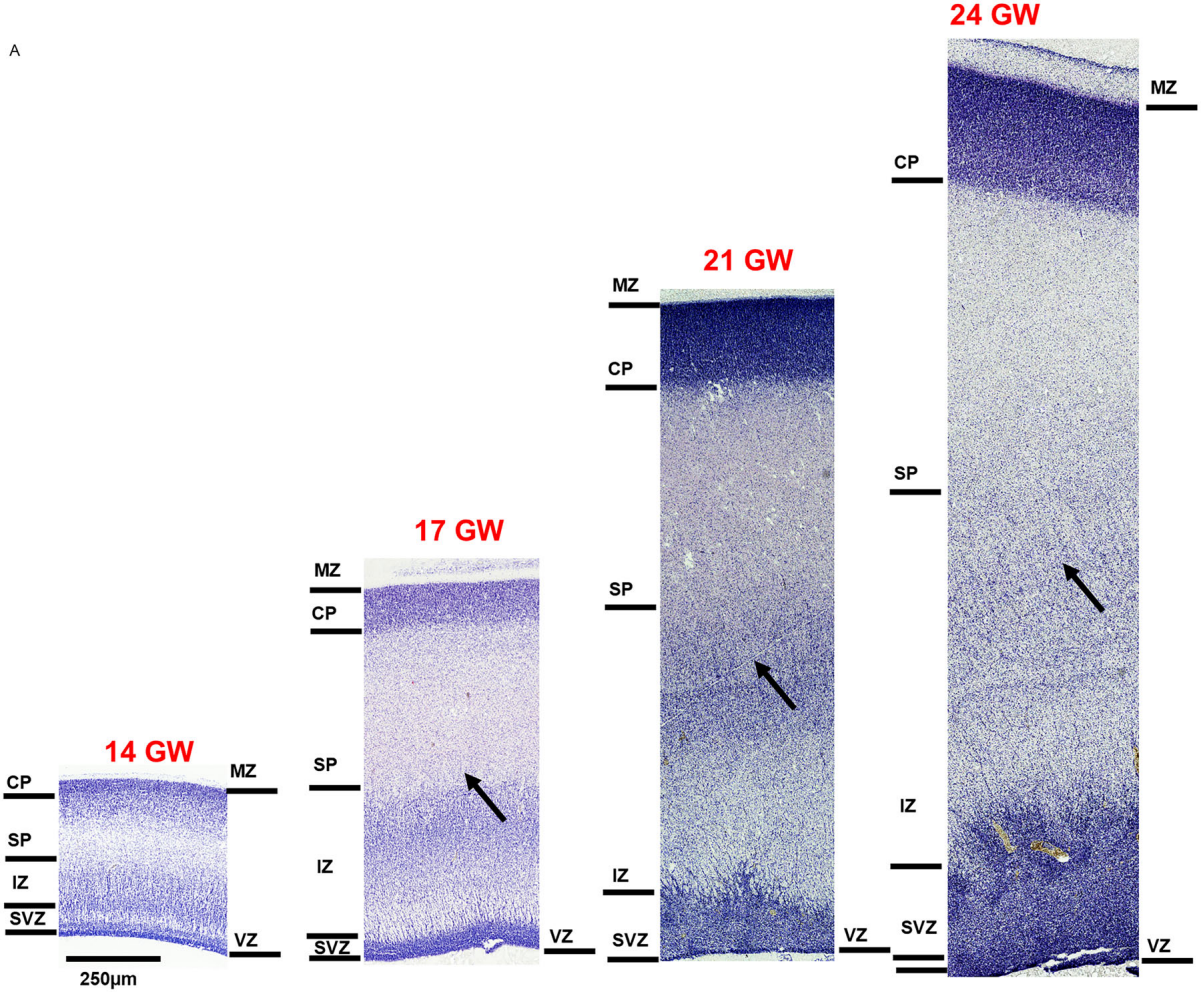
Laminar organization of the developing parietal cortex revealed with Nissl staining from 14 to 24 GW. The laminar organization from the ventricular zone (VZ) to the marginal zone (MZ) is readily demarcated where the CP is separated from the MG by densely labeled neurons. At 14 GW, similar to the occipital cortex, the subplate (SP) in the parietal cortex shows the formation stage. At 14 GW, the intermediate zone (IZ) lacks the honeycomb pattern that appears at 17 GW (black arrow) and is more prominent at 21–24 GW (black arrows).

Densely packed, strongly stained cell bodies define the CP in the parietal cortex. Between 14 and 21 GW, the CP shows that cells are round and evince an early developmental stage. As the CP matures, we observed several key changes specific to the parietal cortex by 24 GW. In 21–22 GW specimens, a few rare and scattered pyramidal cells are observed, and the majority of cells show well‐developed chromatin material. In 24 GW, the CP not only increases in thickness but also shows incipient lamination where adult‐like layers I, IV, and V are readily identified (see Figure 6) (Krsnik et al. 2017). At this stage, the developing somatosensory cortex (posterior to central sulcus) displays an additional band of densely packed cells, and well‐developed pyramidal cells are found below the developing layer IV. This pattern is consistent across all sections, with the number of pyramidal neurons being high compared to any other region at this stage. In addition, this dense cell band associated with the developing layer IV lies between the central sulcus and the posterior central sulcus, defining the anterior and posterior boundaries of the future somatosensory cortex. This relatively advanced morphogenesis of the CP in the parietal cortex, compared to adjacent regions, is possibly guided by synaptogenesis, with prior studies indicating that developing thalamocortical connections in the somatosensory cortex appear almost 2 weeks before those in the occipital and frontal cortices (Kostovic and Rakic 1990; Krsnik et al. 2017).

At early gestation ages, 14–17 GW, the parieto‐occipital sulcus is not completely formed; however, the boundary between the occipital cortex and the parietal cortex is marked by changes in the SP and the IZ. The IZ comprises three to four sublayers and exhibits a honeycomb pattern that is much broader than the occipital region (Figure 6, arrows). Furthermore, this honeycomb pattern is present only in the anterior parietal cortex, posterior to the incipient central sulcus, and is more specific to the developing somatosensory cortex in older gestational ages. Below the IZ, we observed a white fiber layer, which increases in thickness and appears more prominent as the brain develops. The SVZ and the VZ are densely packed layers that decrease in thickness with age. It is important to highlight that in the midsagittal level, on the ventral aspect, where we observed the appearance of the cingulate cortex, demarcation of the IZ was difficult.

##### 3.2.1.3 The Frontal Cortex

**FIGURE 7:**
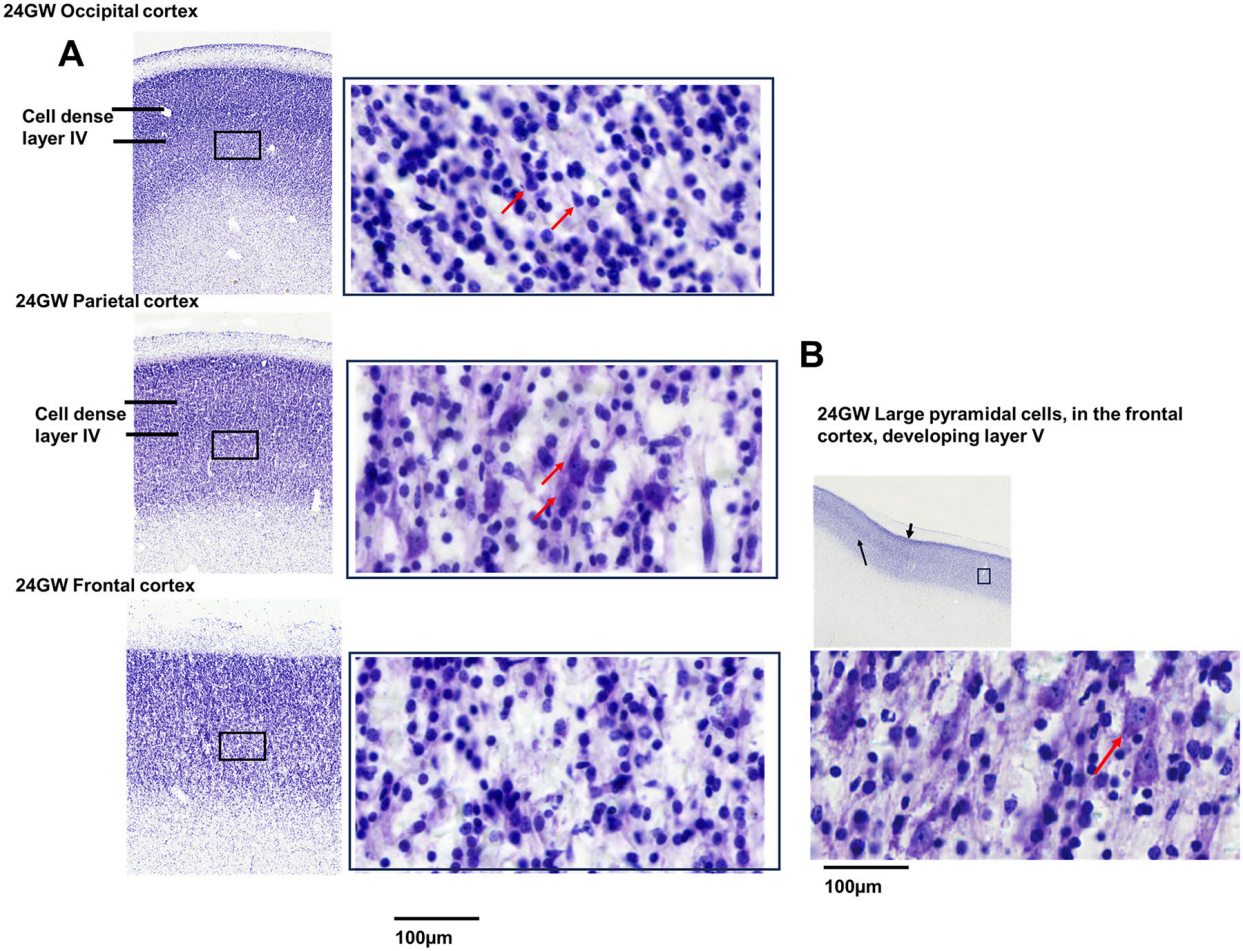
Development of cortical plate. Panel A shows the comparison of the development of lamination in the occipital, parietal, and frontal cortex at 24 GW. The zoomed‐in images show the cell type in the middle of the cortical plate, which has a cell‐dense layer, developing layer IV in the occipital and the parietal cortex, where red arrows point to the developing pyramidal‐like cells. The right Panel, B, shows the large pyramidal‐like cells, located anterior to the developing central sulcus, shown in the top panel black arrow where the developing layer IV ends.

**FIGURE 8:**
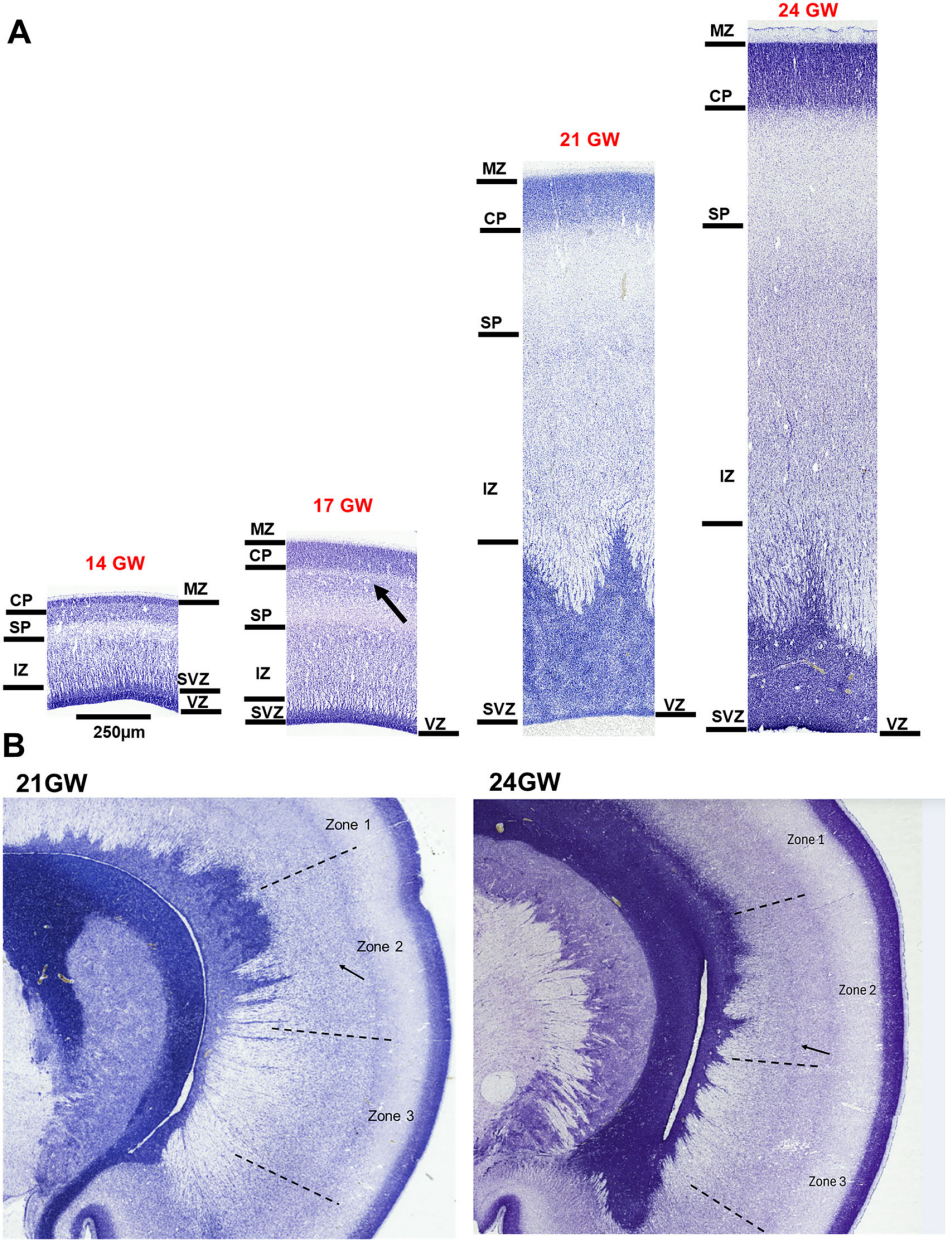
Development of the frontal cortex. Panel A shows the laminar organization of the developing frontal cortex revealed with Nissl staining from 14 to 24 GW. The laminar organization from the ventricular zone (VZ) to the marginal zone (MZ) is well demarcated and the CP shows dense packing of cells below the marginal zone (MZ). At 14 GW, the subplate (SP) shows the formation stage, with an extra cell‐sparse layer (black arrow) between the CP and the SP at 17 GW. The intermediate zone (IZ) lacks the honeycomb pattern. The cortical plate (CP) does not show lamination at 24 GW. Panel B shows the three zones of the developing frontal cortex in 21 and 24 GW that can be demarcated based on the changing pattern of the IZ. Zone 1 depicts the broad band of the IZ, Zone 2 shows the additional dense layer in the middle of the IZ that disappears in Zone 3. The subzones in the developing frontal cortex were observed as early as 21 GW.

In the developing frontal cortex, we observed maturation of the CP from 14 to 24 GW, indicated by an increase in thickness with dense CP. However, no clear lamination of the CP was observed (Figures 7 and 8). As the CP matures, in the dorsal region of the frontal cortex of the 24 GW specimen, we observed a few very large pyramidal‐like cells (see Figure 7B) located anterior to the developing central sulcus. On the basis of the size, morphology, and location, these are possibly the developing Betz cells. These cells have a width of 10–12 µm and are much larger than adjacent cells (Figure 7B, right panel). These cells are arranged in clusters most likely future layer V, similar to that observed in the giraffe primary motor cortex (Badlangana et al. 2007). Studies detailing the developing Betz cells in human fetuses are sparse. Previous studies (Nolan et al. 2024) have reported the presence of these cells at 18 GW, which we did not observe in the three specimens between the ages of 17–22 GW. These large pyramidal cells show a unique morphology as reported in several mammalian species (Jacobs et al. 2018), and this requires detailed characterizations to be made, like size and density in the developing human brain.

The boundary between the CP and the SP at 14 GW is difficult to delineate, but at 17 GW, the lower band of the CP shows cells with a cell‐sparse band, located internal to the CP (see Figure 8A, arrow, 17 GW) similar to the orbital cortex (described below). The cell‐sparse layer interposed between the CP and the SP was noted in the frontal cortex at 17 GW, extending from the orbital cortex and ending posteriorly at the boundary between the frontal and the parietal cortex. This cell‐sparse layer between CP and SP has been reported in the orbital cortex by Kopić et al. (2023) and Verma et al. (2024) at the early gestational stage, 13–15 GW. Previous work using deep projection neuron (DPN) markers (Kopić et al. 2023) showed that this cell‐sparse layer gives CP a “double plate” appearance, where the lower plate has migratory neurons that were densely labeled with Tbr1 and the cell‐sparse layer *labeled* with doublecortin, DCX. In this study, we observed the extension of this cell‐sparse layer from the orbital to the frontal cortex by 17 GW that disappeared by 21 GW when the SP is well developed. Here, by studying the older age group and the adjacent frontal cortex, we show that this additional layer extends to the frontal cortex by 17 GW, which might again represent the lower band of CP, as migrating cells which is more prominent at a later stage in this region. However, this needs to be confirmed using specific IHC or DPN markers. These temporal changes in the transient layers may be guided by new connections developing in the frontal and orbital cortices (Altman and Bayer 2002) by 20 GW. As reported previously, the SP at this stage expands (Kostovic and Rakic 1990). This cell‐sparse layer/double plate layer disappears, as observed in 21 GW, and most likely merges with the maturing SP.

As the central sulcus is absent at younger gestational ages (14 GW), the boundary of the frontal and parietal cortex is marked by changes in the appearance of the SP and the IZ, specifically, by the absence of the honeycomb pattern in the frontal cortex.

Specific to the developing frontal cortex and during 21–24 GW are the differences between the dorsal and the ventral aspects of the IZ. Close observation of the IZ reveals changes in the appearance of the sub‐bands of IZ. The frontal cortex region adjacent to the parietal cortex presents a broad upper band of the IZ that becomes sharper in the middle zone, with an additional thick middle sub‐band (Figure 7, black arrows, Panel B). The additional thick middle band is absent in the ventral portion of the frontal cortex, adjacent to the orbital cortex. Three subregions, which we refer to as Zones 1–3 within the developing frontal cortex, possibly representing the early patterning of developing motor, prefrontal, and orbitofrontal, respectively, can be identified at 21 GW and are more prominent at 24 GW. Previous studies have investigated the patterning in the frontal lobe with changes in the CP and the SP using the DPNs expression dynamics, from 9 to 15 PCW (11–17 GW) but not in IZ (Kopić et al. 2023). Other studies compared the overall IZ changing patterns of the adjacent parietal cortex and not the subzones in the frontal cortex (Altman and Bayer 2002; Bayer and Altman 2005). The boundary with the adjacent parietal and orbital cortex is marked by a disappearing honeycomb pattern in the IZ from the parietal cortex and the thick vertically oriented cells of the IZ in the orbital cortex. The boundary with the adjacent orbital cortex is demarcated by observing cell orientation in the IZ and the thinning of the SP.

The SVZ and the VZ are thick and densely labeled at early stages, with the white fiber layer superficial to the SVZ being prominent only in the dorsal portion of the developing frontal cortex at 17 GW when compared to the parietal and the occipital cortex. The SVZ and the VZ are still readily identified at 21–24 GW but appear significantly thinner than earlier in gestation.

##### 3.2.1.4 The Orbital Cortex

**FIGURE 9:**
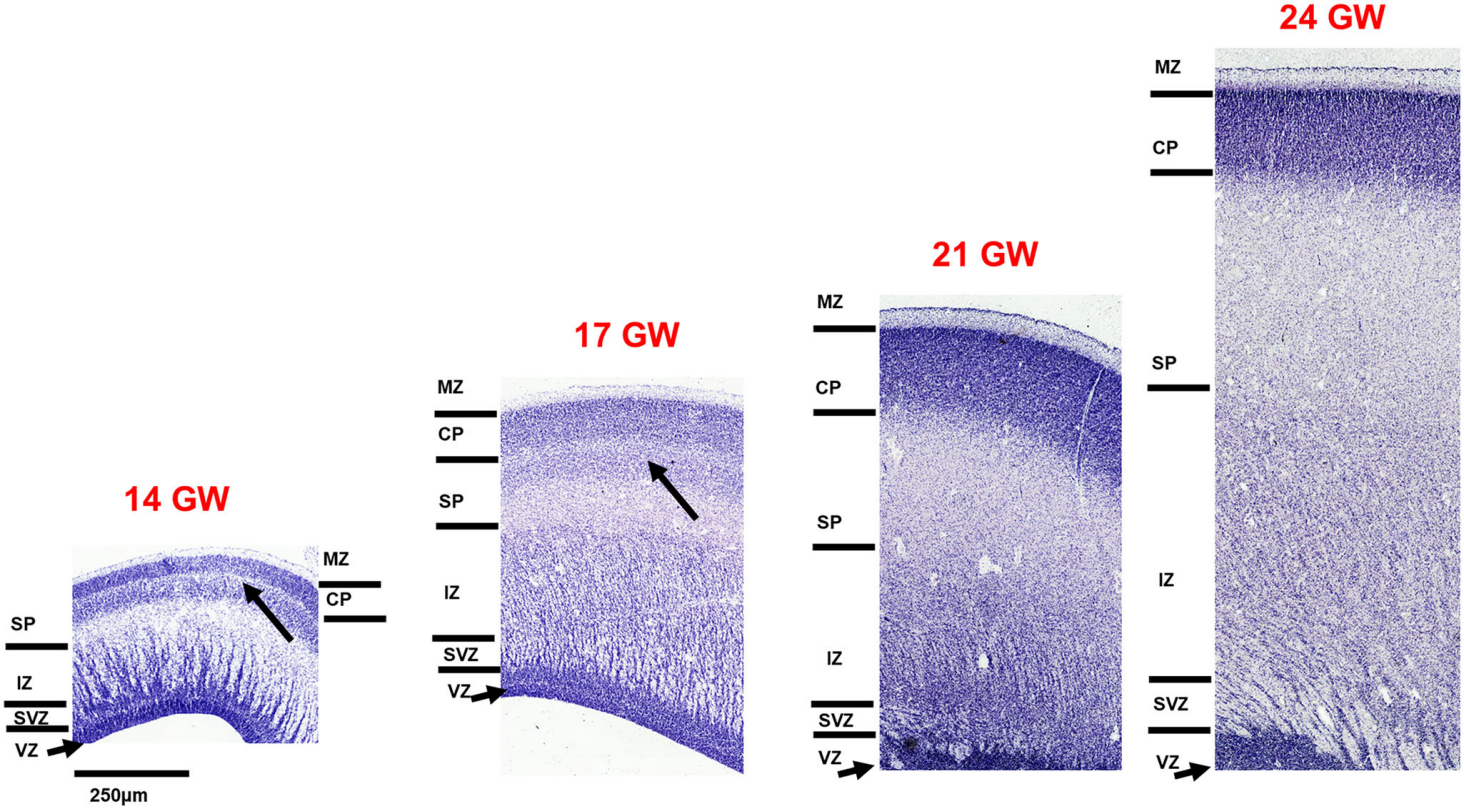
Representative sections demonstrating the development of the orbital cortex from 14 to 24 GW. The orbital cortex shows a cell‐dense, thick CP at all gestational ages, and uniquely depicts an extra cell‐sparse layer below the CP at 14 and 17 GW (black arrows), that disappears as the SP matures. The IZ shows dense vertically oriented cells that are prominent at all gestational ages.

From 14 to 24 GW, similar to other cortical regions, the MZ in the orbital cortex is a cell‐sparse layer internal to which is the CP (Figure 9). It forms a well‐defined layer with a dense uniform distribution of cells. Specific to the orbital region, at 14 GW, is a thin cell‐sparse band internally adjacent to the CP, which broadens by 17 GW (black arrow in Figure 9). As described above, this cell‐sparse layer extends toward the frontal cortex and ends at the boundary with the parietal cortex (Kopić et al. 2023; Verma et al. 2024). As described earlier, this cell‐sparse layer extends toward the frontal cortex and ends at the boundary with the parietal cortex. The CP and the SP show maturation by 21 GW, marked by the disappearance of the interposed cell‐sparse layer. However, the CP lacks lamination by the end of the second trimester as observed in 24 GW specimen.

From 14 GW, the IZ shows three sub‐bands and a distinct vertical arrangement of cells that are maintained throughout the second trimester. Changes in the pattern of IZ and the upper SP allowed the demarcation of the boundary between the orbital and the frontal cortex. At later gestational stages, the sub‐bands within the IZ are not as pronounced, but the SVZ and the VZ are thick cell‐dense layers between 14 and 17 GW and decrease in thickness with gestational age.

##### 3.2.1.5 The Temporal Cortex

**FIGURE 10:**
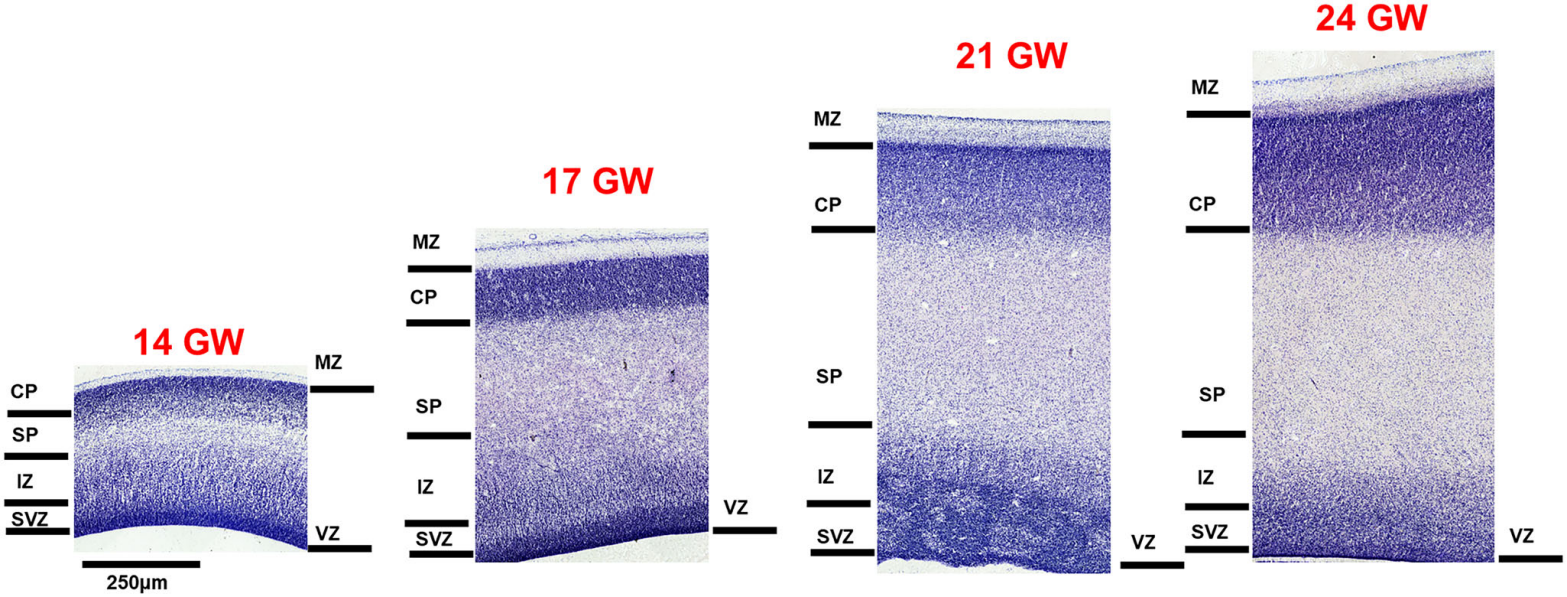
Representative sections showing the developing temporal cortex from 14 to 24 GW. The developing temporal cortex shows a dense thick upper cortical plate at all gestational ages, and with age shows an increase in thickness where the middle band is cell‐dense but does not show mature neurons at 24 GW. The SP increases in thickness with age and by 24 GW is twice the thickness of the CP. The IZ shows 2 sub‐bands at 14–17 GW, but these sub‐bands are difficult to demarcate at 24 GW.

In the developing temporal cortex, we observed an increase in the thickness of CP with age and presented with a very dense upper band of 21 GW (Figure 10). However, at 24 GW, the CP does not show a similar stage of lamination compared to the occipital and the parietal cortex. Moreover, the cells within the temporal CP are rounded and do not include pyramidal‐like cells. We noted in the more medial aspect of the temporal cortex at 24 GW, the upper band of CP is very distinct compared to the adjacent insula and entorhinal cortices. The SP has two bands in the formation stage, where the upper band does not have a well‐demarcated border with the CP, but the lower band is cell sparse and depicts a clear boundary with the IZ. The IZ shows two to three sub‐bands at 14 GW, but as early as 17 GW, their number decreases and shows a uniform pattern by 24 GW. Importantly, our observations in the temporal cortex highlight that the IZ lacks as many sub‐bands compared to other cortices, by 24 GW, and the CP does not show adult‐like lamination or mature neurons at 24 GW.

#### 3.2.2 The Claustrum (CLA) and the Insular Cortex

The CLA is well identified from 14 GW onward, adjacent to the insula and separated by the external capsule. CLA has a uniform distribution of cells, which are dense compared to the fibrous external capsule and the SP of the insula. Anteriorly, it shares a boundary with the insula SP and the external capsule. The posterior boundary of the CLA is marked by the thick fiber tract of the external capsule, and a large, oval‐shaped anterior commissure is readily identified on the ventral aspect. The insular cortex is bounded by the olfactory cortex on the ventral aspect and the frontal cortex on the dorsal aspect. In the 22 GW coronally cut specimen, the SP of the insular region is traversed by the CLA. The insula is readily identified from early gestation at 14 GW, adjacent to the lateral fissure. By 24 GW, the CP of the insula shows a cell‐dense band with lamination, relative to the adjacent orbital cortex both in the lateral and the medial aspect. The cell‐dense band predominantly shows small, rounded cells.

#### 3.2.3 The Olfactory Cortex

The olfactory cortex, also referred to as the piriform cortex, is located rostral to the entorhinal cortex that includes the endopiriform nucleus and the olfactory bulb. Rostrally, the olfactory cortex is bounded by the orbital cortex on the dorsal aspect and by the insula ventrally. The medial boundary is shared with the endopiriform nucleus. Delineation of the boundary with the adjacent orbital cortex is distinct as the seven layers—SG to VZ—of the orbital cortex are not discernible in the olfactory cortex noted in all the specimens. The three layers of the olfactory cortex are well defined by 21 GW where layer II shows thinning compared to the CP of the orbital cortex. Caudally, the olfactory cortex shares a boundary with the entorhinal cortex and the cortical amygdala, and on the medial aspect, it is bounded by the olfactory tubercle (OT). The boundaries, with the adjacent regions, are well demarcated from 14 GW onward. The endopiriform nucleus is a small telencephalic nucleus characterized by a distinct cytoarchitecture and cellular density with a columnar appearance. It shares the ventral boundary with the orbital cortex anteriorly and posteriorly with the cortical nucleus of the amygdala (COA). This small region was identified in 17–24 GW, but not in the 14 GW specimen.

#### 3.2.4 Amygdala

The cytoarchitectural description of the amygdala in this study employs the nomenclature from previous studies (Mulc et al. 2024; Sorvari et al. 1995) that parcel it into two main subdivisions: the basolateral complex of amygdala (BLA) and corticomedial complexes. We describe the organization of the developing amygdala from lateral to medial sections at 24 GW and highlight the still‐developing nuclei in younger age groups. The BLA comprises the lateral nucleus of the amygdala (LA), the basomedial nucleus of the amygdala (BM), and the basal nucleus of the amygdala (BA). In the most lateral aspect of the amygdala, the LA is readily identified in all ages and is positioned ventral from the anterior commissure, putamen (Put), and internal capsule. It exhibits a distinct vertical palisade arrangement of cells in the rostral region and a uniform clustered appearance caudally and ventrally. These cytoarchitectural variations may highlight possible subparts of the LA. At earlier gestational ages, 14 and 17 GW, the palisade pattern (Mulc et al. 2024; Ulfig, Setzer, and Bohl 1998) of the LA is less prominent and occupies a small amygdala region compared to older gestational ages. By mid‐gestation (21–22 GW), the LA of the amygdala shows a prominent palisade cytoarchitecture in both coronal and sagittal planes, and this pattern is more distinct in the coronal plane.

**FIGURE 11:**
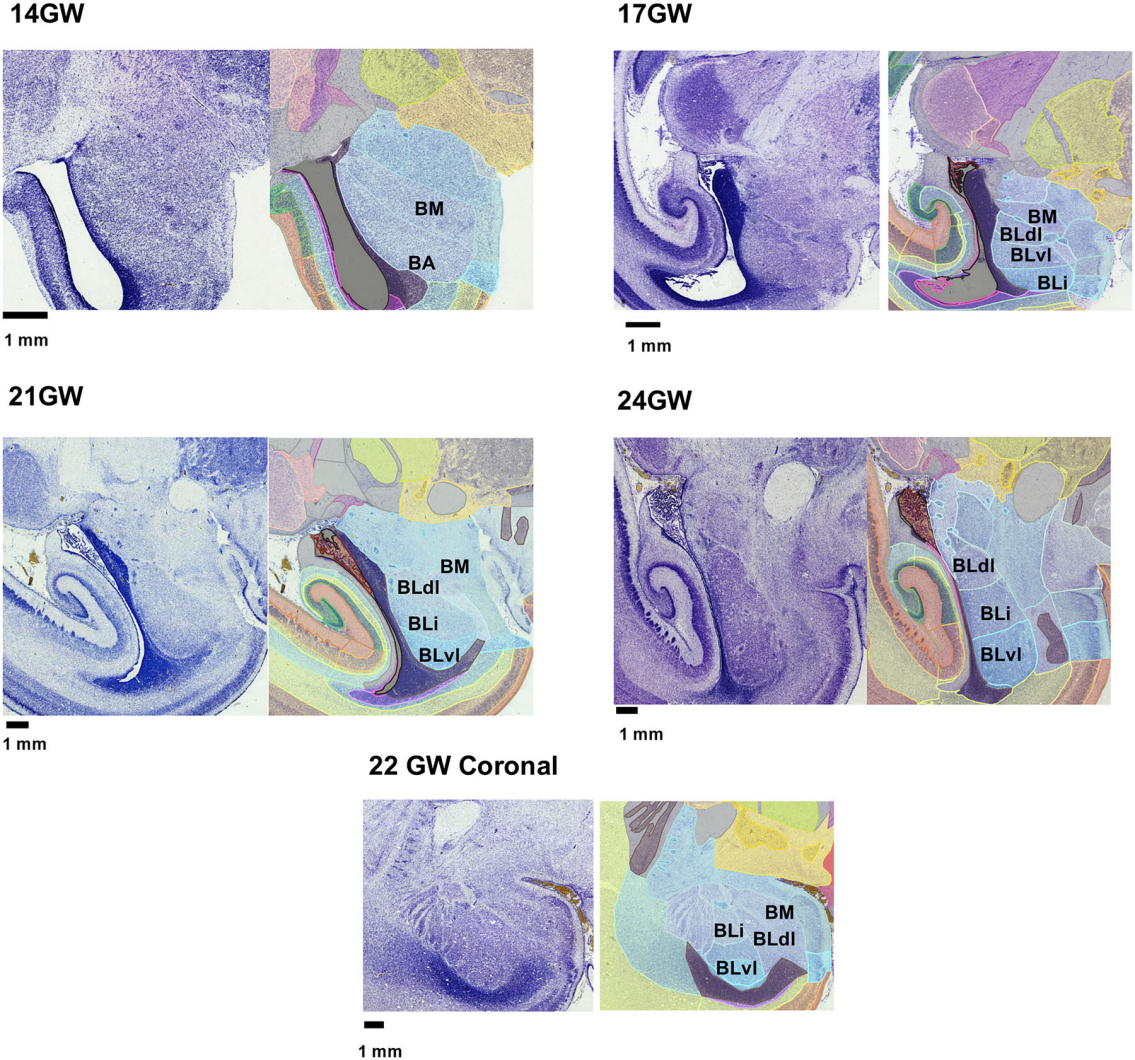
Development of the basal nucleus of the amygdala (BA), from 14 to 24 GW, sectioned in the sagittal plane, and 22 GW coronally. The subnuclei basal nucleus‐dorsolateral (BLdl), basal nucleus intermediate (BLi), and basal nucleus ventral (BLvl) were delineated in age group 17–24 GW. In 14 GW none of the subnuclei could be identified.

A prominent fiber tract in the BLA of the amygdala separates the BM and the BA (Ulfig, Setzer, and Bohl 1998). As the BLA expands, the ventral boundary above the ventricles shows a dense patch of migrating cells, and the developing paralaminar limitans nucleus (Mulc et al. 2024) may be identified above the ganglionic eminence from 21 GW onward, in both coronal and sagittal perspective. Within the BA and from 17 GW onward, we could identify the basal nucleus, dorsal part (BLdl), basal nucleus, intermediate part (BLi), and basal nucleus, ventral part (BLvl; Paxinos et al. 2012). The BA subdivisions are based on the variation in the overall cell density (see Figure 11). We also note that the BLdl is more cell‐dense by 24 GW as compared with the earlier stages. The BM appears more cell‐dense and includes several cell clusters. In the dorso‐caudal aspect of the amygdala, ventral to the anterior commissure, from 14 GW onward, the cell‐sparse region is identified as the amygdaloid striatal transitional area (ASTA). These nuclei are defined by the overall cell size and density and by the presence of fiber tracts, similar to those reported in adult humans (Ding et al. 2016) and in the marmoset (Paxinos et al. 2012) brains, but not in the developing fetal brain. Its boundary with the hippocampal formation is not well demarcated in the younger age groups 14–17 GW. In the dorsal aspect of the amygdala, ventral to the dorsal striatum, ASTA shows dense cell clusters from 17 to 24 GW.

In the dorsal aspect of the corticomedial complex, the central nucleus of the amygdala (CEA) was delineated from 14 GW onward as a distinct cell‐sparse, circular‐to‐oval region and surrounded by fibers. Furthermore, from 21 GW onward, several possible subdivisions may be identified in Nissl staining. Posteriorly, the corticomedial complex contains the periamygdaloid cortex (PAC), located adjacent to the entorhinal cortex that was readily delineated from 14 GW. The PAC has a cell‐dense thick layer II and the boundary with the entorhinal cortex and PAC is marked by the disappearance of the *lamina dissecans* of the entorhinal cortex. Dorsal to PAC is the COA, which shows thinning of the cell‐dense cortical layer II at 14–17 GW. COA forms an inverted “C” shaped discontinuous cortical layer that is thinner than the adjacent PAC and the medial cortex of the amygdala (MEA). In 21 GW and older specimens, the inverted “C” shape is maintained but is a continuous layer.

In the dorsal amygdala, the transition zone from the substantia innominata (SI) to the amygdala, specifically the anterior amygdaloid area (AAA), appears as a cell‐sparse region with uniformly distributed cells. At the mid‐sagittal level, ventral to the optic tract, the MEA of the amygdala is identified by loosely packed cells with a broad middle band, compared to the adjacent cortical amygdaloid nucleus. It is important to note that we observed the rotation of the amygdala from 14 to 24 GW, which results in changes in the orientation of the cortical regions of the amygdala with the adjacent olfactory cortex and the entorhinal cortex (Mulc et al. 2024; Nikolić and Kostović 1986). Our observations of subregions in the amygdala are made at an earlier stage compared to what has been previously reported (Mulc et al. 2024; Ulfig, Setzer, and Bohl 1998).

#### 3.2.5 The Hippocampal Formation and the Entorhinal Cortex

Ammon's horn (CA3, CA2, and CA1 fields) of the hippocampus is readily identified, exhibiting the developing transitional layers from the sub‐granular zone to the VZ from 14 GW onward. During early gestation, 14–17 GW, the boundaries between CA fields are delineated by variations in cell density, where the CA3 is a cell‐sparse region adjacent to the dentate gyrus (DG). The relative changes in the CP thickness allow demarcation of the boundary between CA3 and CA1, where CP is thicker in CA3, thinner in CA2, and becomes thicker again in CA1. The SP shows a similar thickness in all three CA fields but is not well‐defined in 14 and 17 GW, respectively. By 21 GW, the CP, SP, and IZ boundaries are readily identified. The DG is seen as a cell‐dense structure located within the curve of Ammon's horn, where at 14 GW, a dense layer of future granular cells is observed; however, the boundaries with the molecular layer (ML) and the SGZ cannot be delineated. By 21 GW, the boundaries between the three layers of DG are well defined. In CA1, CP shows several pyramidal cells adjacent to the SP by 21 GW and granular cells toward the broad MZ (Kostović et al. 1989). CA2–CA3 fields show similar patterns, but the identifiable pyramidal neurons are more numerous in CA1. We identified the subiculum, presubiculum, and parasubiculum at 14 GW (Hevner and Kinney 1996; Kier et al. 1997; Kostović et al. 1989; Šimić et al. 2022). The subiculum border with CA1 is marked by the diffuse appearance of the CP and a more well‐defined cell‐sparse SP, compared to CA1. The subiculum shows a clear SP only by 17 GW. Similarly, in 14 GW, the presubiculum is delineated by the broadening of the CP compared to its appearance in the CA1, and the upper layer of the CP in the presubiculum is cell sparse, revealing a split appearance of the CP. The typical *lamina dissecans* of the presubiculum can be readily identified by 17 GW. The parasubiculum has a cell‐dense and readily defined CP in the dorsal component. At 14 and 17 GW, the parasubiculum is adjacent to the entorhinal cortex, is a small region, and is difficult to delineate. Compared to the subicular complex, the lamination in the entorhinal cortex can be readily delineated at 14 GW, as described previously (Šimić et al. 2022). The entorhinal cortex reveals a distinct cytoarchitecture and shows the maturation of the CP with the formation of the *lamina dissecans* as early as 14 GW (Šimić et al. 2022). As early as 14 GW, we could delineate the CA fields and the subdivisions of the subicular complex. By comparison, most studies of the presubiculum and parasubiculum have been identified from 15 GW onward, and with specific IHC markers and Dil tracing (Hevner and Kinney 1996; Kier et al. 1997; Šimić et al. 2022).

#### 3.2.6 The Striatum

##### 3.2.6.1 Dorsal Striatum

The dorsal striatum consists of two large nuclei, the caudate (Cau) and the Put. The Cau is a cell‐dense, large, curved structure that courses along the ventral aspect of the lateral ventricle, from anterodorsal to posteroventral, and comprises a head, body, and tail. In the most medial aspect, Cau and Put are separated by the internal capsule (int). The Put occupies a large volume, is cell‐dense, with a patchy appearance (Kostović 1986; Mai and Ashwell 2004) within the sub‐cortical telencephalon lateral to the int, emerging from the head of the Cau nucleus. The patchy appearance of the Put is observed at an earlier age, 14 GW compared to the Cau (Mai and Ashwell 2004) which gets more prominent with a fan‐shaped expansion of the internal capsule over the Put. From 14 GW onward, both regions show round granular cells, and by 24 GW, there is an increase in mature cells with distinct cytoplasm and cell nucleus.

In the sagittal perspective, from 14 to 24 GW, specimens show prominent vertically oriented bands of three key structures—the anterior commissure (ac), Put (identified by its cell‐dense, but patchy appearance), and the vertical fiber tract of the int that can be readily demarcated throughout its antero‐posterior extent. The main difference noted with the increasing gestational age was that the size of these nuclei and the prominence of the white matter fiber tracts were more apparent, allowing more confident identification in the older specimens.

##### 3.2.6.2 Ventral Striatum

The nuclei included in the ventral striatum are the nucleus accumbens (NAc) and the OT. NAc is identified ventrally adjacent to the Put as a cell‐dense nucleus with several white matter tracts passing through, creating its patchy appearance. On the ventral aspect is the anterior commissure, which separates NAc from the SI, described in a later subsection. Anteriorly and coronally, NAc is observed on the dorsal aspect, and posteriorly, dorsal boundary is with the SI. The hypothalamus and amygdala share the boundary in the medial‐lateral aspect, respectively.

The OT in the sagittal perspective is bounded by the olfactory cortex anteriorly and by the SI posterio‐laterally and by the amygdala posterio‐medially. Cytoarchitecturally, the OT exhibits dense labeling, with layer II being much thicker than the olfactory cortex. We noted parts of the rostral migratory streams in OT across all ages.

##### 3.2.6.3 Pallidum

Bounded dorsally by the Put and ventrally by the anterior commissure are the two subdivisions of the globus pallidus (lateral and medial). The lateral division, which occupies the dorsal position, has a uniform density of cells and includes few large cells from 21 GW. The globus pallidus medial segment appears more cell sparse but the large cells are more prominent from 21 GW. At 17 GW, few scattered, large, dense labeled cells are observed in the medial segment of the globus pallidus. The medial medullary lamina distinctly separates the medial and lateral subdivisions from 14 GW onward.

#### 3.2.7 The Septal Region and the Bed Nucleus of the Stria Terminalis (BST)

Anteriorly and beneath the corpus callosum is the septum, which is seen as a cell‐sparse region occupying a position between the third ventricles medially and fornix laterally. Posteriorly, at the level of the third ventricle, the ventral boundary is with the hypothalamus. The ventral aspect shares a boundary with the arc or boomerang‐shaped (Kostović 1986) region, the nucleus of the diagonal band (NDB). NDB consists of large mature cells, identified distinctly from 21 GW onward, and is oriented in the direction of several fiber tracts, prominent in this region.

Lateral to the septal region is the cell‐dense BST, transected by the anterior commissure. The relative cell density, compared to the septum, is consistent from 14 to 24 GW, where mature cells are more prominent by 21 GW. However, it shows an indistinct boundary with the septum. In the sagittal perspective, at the most medial aspect, BST is located ventral to the stria terminalis (stt) and shares a boundary with SI ventrally. In the coronal perspective, the BST has a lateral border with the GP, and the internal capsule separates these two regions. Rostrally, the dorsal portion adjacent to the ganglionic eminence shows darker cell labeling, possibly indicating local migratory cell streams.

#### 3.2.8 The SI

The SI shows a heterogeneous distribution of cells with specific scattered islands of large, intensely stained cells identified as the basal nucleus of Meynert (BM) (Ding et al. 2016, 2022), from 17 GW onward. At 14 GW, the large, densely labeled cytoarchitecture of BM was not distinctly observed. Anteriorly, the SI is oriented in the dorsal‐ventral direction and ventrally borders the lateral hypothalamus. At the level where the lateral hypothalamus area (LHA) and SI are widest, both regions form an inverse arch, in the dorsal and ventral aspects, respectively. Posteriorly, the SI, at its lateral extent, borders the amygdala ventrally, where we identify a cell‐sparse region, the transition zone, or ASTA, delineating the SI from the amygdala.

The tenia tecta, considered a component of the cerebral cortex that extends into the septum (Dong 2008), was observed anteriorly below the corpus callosum and dorsal to the cingulate cortex. Posteriorly, it is positioned ventral to the septum. In the sagittal perspective, between the OT and the orbital cortex, it appears as a cell‐dense region between 14 and 24 GW.

#### 3.2.9 Migratory Streams

From 14 to 24 GW, we identified the lateral migratory stream in the CLA, endopiriform nucleus, and the LA (Bayer and Altman 2005), and the lateral migratory stream is observed with the receding SVZ, between the subcortical nuclei and the lateral cortex. The rostral migratory stream (Bayer and Altman 2005), also identified in our specimens, includes the mitotic and postmitotic cells of the forebrain, from the lateral ventricle and the olfactory bulb to the olfactory peduncles. Additionally, several other possible migratory streams, more localized, have been annotated under the general category “migratory stream, general.”

### 3.3 Diencephalon

The diencephalon is typically divided into three components: dorsal thalamus, ventral thalamus, and epithalamus, and it often includes the hypothalamus (Jones 2007). We acknowledge that gene expression studies of the developing brain reveal that the preoptic region and hypothalamus develop from a distinct segmental component of the prosencephalon, which also gives rise to the telencephalon and thus cannot be considered part of the traditionally defined diencephalon (Puelles et al. 2013). However, in the current description, we include the preoptic region and hypothalamus as separate divisions of the diencephalon, as done traditionally, for ease of comparison to other studies of human brain development.

#### 3.3.1 Preoptic Region

The preoptic region and the hypothalamic nuclei were identified and defined within four regions from anterior to posterior: the preoptic, anterior (referred to as supraoptic by Le Gros Clark 1938), tuberal, and mammillary body regions. Each of these regions was then analyzed from the lateral to medial zone, based on the three distinct longitudinal zones described by Crosby and Woodburne (1940): the lateral, medial, and periventricular zone (Simerly 2015). In the 22 GW, coronally sectioned specimen allowed the delineation of several nuclei based on their position and cytoarchitecture (Ding et al. 2022; Simerly 2015) as per the existing literature (Koutcherov et al. 2002).

In the oldest specimen, 24 GW, the most lateral sections have the optic tract ventrally, with an elongated supraoptic nucleus extending from the preoptic to the anterior region of the hypothalamus. At the level of the optic chiasm, in the rostral aspect, dorsal to the suprachiasmatic nucleus, we identified the medial preoptic nucleus as an elongated and dense patch of cells. Dorsal to the supraoptic nucleus, we identified the lateral hypothalamus (LHA) which extended throughout the anteroposterior extent of the hypothalamus. The lateral preoptic area (LPO) showed dense patches of cells that could be easily demarcated, from the relatively uniform and cell‐sparse LHA.

#### 3.3.2 Hypothalamus

We describe the hypothalamus in three regions: anterior, tuberal, and mammillary. The anterior region, from lateral to medial, shows the LHA, the anterior hypothalamic nucleus, and the paraventricular nucleus. In the most lateral aspect, the boundary between the LHA and the SI was marked by large cells that appeared in patches, the cells of Meynert, and a fiber tract that separated the two regions. Medially, in the periventricular zone, a dense nucleus adjacent to the ventricle is identified as the paraventricular nucleus of the hypothalamus, both in the anterior and posterior aspects. We observed the retrochiasmatic nucleus dorsal to the optic chiasm and ventral to the pituitary gland.

A dense patch of cells demarcated the boundary between the anterior hypothalamic nucleus and the tuberal region, further delineated by a fiber tract. In the most lateral aspect, the tuberal region has the LHA extending from the anterior region. Medially, the tuberal region contained the large ventromedial hypothalamic and dorsomedial hypothalamic nuclei. These nuclei are distinctly oriented at almost 135° to each other and separated from the mammillary region by a fiber tract.

The most caudo‐lateral aspect of the lateral hypothalamus, a cell‐dense vertically oriented patch in the mamillary region, was demarcated as the tuberomammillary nucleus. The mammillary body was readily identified in the mammillary region due to its oval shape and relatively high cell density. Dorsally, the posterior hypothalamic area is a distinct large region with a uniform distribution of cells located ventral to the field of Forel (FF) and is bordered laterally by the mammillothalamic tract. The supramammillary area was in the dorsal aspect, and the mammillothalamic tract was seen to exit the mammillary body. We also observed the dorsal, ventral, premammillary, and lateral mammillary nuclei in the mammillary region of the hypothalamus. We also identified the supramammillary decussation and the mammillary peduncles. All the nuclei reported here were identified across the five specimens (14–24 GW) examined.

#### 3.3.3 Dorsal Thalamus

We identified several nuclei in the dorsal thalamus, ventral thalamus (also referred to as prethalamus, e.g., Nagalski et al. 2016; Puelles et al. 2013), and the epithalamus, as well as the subthalamus and the surrounding white matter (Bayer and Altman 2005; Mai and Majtanik 2019) from 14 to 24 GW. Within the dorsal thalamus, nuclei were grouped into six complexes: dorsal, ventral, anterior, posterior, central, and periventricular. Our observations from sagittal sections are described from the lateral to medial aspect, and we report the changes observed with gestational age.

##### 3.3.3.1 Posterior Complex

**FIGURE 12:**
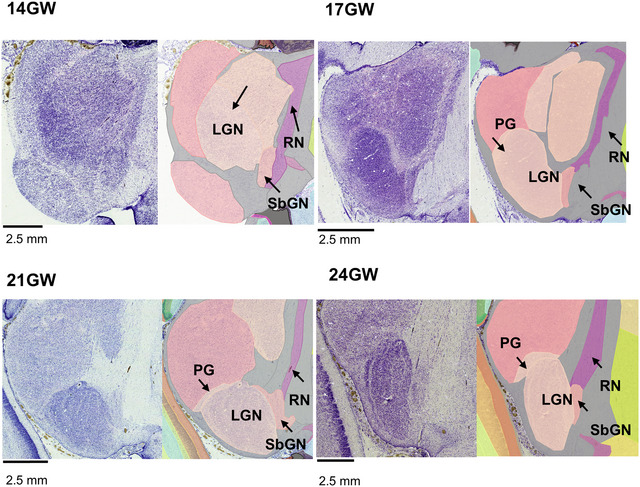
Topographical development of the pregeniculate complex with the changing position of dorsal lateral geniculate nucleus (dLGN) in the ventral thalamus from 14 to 24 GW. PG, pregeniculate; RN, reticular nucleus; SbGN, subgeniculate.

Within the posterior complex, the LGN, pulvinar, medial geniculate nucleus (MGN), lateral posterior nucleus, suprageniculate nucleus, and nucleus limitans of the thalamus were well delineated in all gestational ages examined (14–24 GW). In the oldest specimen, the LGN was identified about 1.5 cm from the midline, in the most lateral aspect of the gray matter mass forming the dorsal thalamus. In 14 GW LGN, it occupied a larger volumetric proportion of the dorsal thalamus in the early second trimester compared to other nuclei in this complex, as the pulvinar nucleus is not yet well developed. The LGN has an elongated shape and appears unlaminated but cell‐dense. Between 14 and 17 GW, we noted that the ventral half of the LGN showed a cell‐sparse region, which was included as a part of LGN as it was most likely at a different stage of development. By 21 GW, the LGN shows nascent signs of lamination but still contains immature cells. At 24 GW, the LGN is laminated, with four well‐defined layers, and prominent fiber tracts are present in the medial portion. Between 14 and 17 GW, the developing pregeniculate nucleus (described later in the ventral thalamus section) was observed in a position ventral and medial to the reticular nucleus (RT) and the LGN, respectively (see Figure 12). Note that as the LGN is located in a progressively ventrally location, we observed the pregeniculate nucleus appear as a cap superior to the LGN by 17 GW (see Figure 9 and details in the ventral thalamus section). From 17 to 24 GW, a cell‐sparse patch, the pregeniculate nucleus, appears like a cap surrounding the LGN that has moved from a vertical position to a ventral one, when compared to the 14 GW. The fan‐shaped fiber tract that was observed in the developing brain between 14 and 24 GW has also been studied in adult humans using imaging techniques and identified as thalamic prefrontal peduncles (Sun et al. 2018); however, this requires further detailed investigations in the developing human brain.

Surrounding the LGN, we observed the external medullary lamina, which runs adjacent and parallel to the RT and extends between the pulvinar and the LGN laterally. Medial and dorsal to the LGN, the pulvinar is identified as a cell‐sparse region, but the boundary with the LGN is well‐demarcated due to the characteristic intense labeling of the LGN. The pulvinar is a cell‐sparse region at gestational ages before 21 GW. However, it becomes more cell‐dense with increasing age and occupies a progressively larger proportional volume of the posterior complex. In contrast to the LGN, the MGN shows three subdivisions and a “blob‐like” cytoarchitecture in the central portion as early as 17 GW, which appears to be more cell‐dense by 24 GW. Furthermore, the MGN appears to be positioned more medially with the developing gestational ages. White matter separates the LGN and MGN throughout their extent, and the cell‐dense suprageniculate nucleus (SbGN) is identified posteriorly between the MGN and the pulvinar. In all specimens, in the medial and caudal portion of the developing dorsal thalamus, we delineated the nucleus limitans of the thalamus located between the pulvinar and the pretectal nuclear complex of the midbrain. It has a non‐homogenous cytoarchitecture with cell density higher than the pulvinar and the pretectum. Other atlases have grouped this under the SbGN (Ding et al. 2022). Lateral to the pulvinar complex and dorsal to the ventral posterolateral nucleus (VPL), we identified the lateral posterior nucleus of the thalamus in the lateral aspect of the dorsal thalamus.

##### 3.3.3.2 Ventral Complex

In the ventral complex, several nuclei were delineated under the ventral posterior group and the lateral group in all specimens from 14 to 24 GW. All the nuclei within the ventral complex showed high density with dense labeling from early 14 GW and were separated by prominent fiber tracts. CD15 labeling in the developing human brain has been used to indicate earlier maturation of these nuclei compared to the nuclei in the dorsal complex (Forutan et al. 2001). From the sagittal perspective, the VPL appears first in the most lateral sections and borders with the pulvinar dorsally and the LGN ventrally at 14 and 17 GW. At the level where the MGN is well delineated, the ventral posterior inferior nucleus (VPI) is located at the most inferior aspect of the MGN and appears as a cell‐sparse region. In the rostral aspect, most aspect is the ventral anterior nucleus (VA) caudal to which is the ventral lateral nucleus (VL). The ventral posteromedial nucleus (VPM) was observed more medially, whereas in the sagittal perspective, the centromedian nucleus was delineated in the caudal aspect in all specimens (14–24 GW). The VL, VP, and VPM appear as elongated structures in the sagittal perspective from 14 GW onward. The cell densities and shapes of these nuclei in the ventral complex were maintained from 14 to 24 GW.

##### 3.3.3.3 Central Complex

In the central complex of the thalamus, we identified the centromedian and central lateral nuclei. The centromedian nucleus appears as a distinct circular structure surrounded by a white matter tract, the internal medullary lamina, and it was observed in the youngest specimen (14 GW). In the coronally sectioned specimen, the central lateral nucleus delineated in 22 GW evinces a wing‐shaped appearance extending laterally from the central medial nucleus. The nucleus is not identified in the sagittal sectioned specimens.

##### 3.3.3.4 Dorsal Complex

In the dorsal complex, we identified the medial dorsal nucleus of the thalamus (MD) and the dorsolateral nucleus (DL), in examined ages (14–24 GW). Dorsal to the centromedian nucleus, MD was readily demarcated, bordering the subparafascicular tract caudally. At the mid‐sagittal plane, the ventral portion of the thalamus contains a region with clusters of cells identified as the nucleus limitans, whereas the most dorsal aspect reveals the crescent‐shaped DL nucleus, appearing as a uniform patch of cells separated from the anterior complex and centromedian nucleus by a thick fiber tract. The MD nucleus is a large nucleus, surrounded anteriorly by the ventral complex, posteriorly by the pulvinar, and ventrally by the centromedian nucleus and well demarcated even in 14 GW when the pulvinar appears as a cell‐sparse region.

##### 3.3.3.5 Anterior Complex

In the anterior complex of the dorsal thalamus, we identified the anterodorsal, anteroventral, and anteromedial nuclei in all ages examined (14–24 GW). The anteromedial nucleus is located adjacent to the third ventricle, and the anteroventral nucleus borders the lateral aspect of the anteromedial nucleus. Dorsally within this complex, we identified the anterodorsal nucleus, which occupies a more medial position in the rostral aspect of the dorsal thalamus. Rostrally, the mammillothalamic tract forms the boundary between the anterior and the ventral complexes.

##### 3.3.3.6 Periventricular Complex

The periventricular complex comprises several nuclei located adjacent or close to the lateral walls of the third ventricle. The nuclei identified in the older specimens (22–24 GW) are the paraventricular, paratenial, reuniens, central median, parafascicular nucleus, and rhomboid nuclei, which we could most readily identify in the coronally sectioned specimen. Between the paraventricular nucleus and the fiber bundle stria terminalis, there is a dense patch of cells identified as paratenial nucleus; however, in the 14–17 GW specimens with a thick neuroepithelium, it was difficult to delineate this nucleus with certainty near the ventricles. The boundaries with the surrounding gray matter are demarcated by a white matter tract that shows the distinct parafascicular nucleus on its lateral aspect. In the ventral aspect, the nucleus reuniens appears as a region with a dense patch of cells adjacent to the fornix. Posterior to the nucleus reuniens, a uniform cell‐sparse region is identified as the FF. In the sagittal plane, closer to the midline, the third ventricle appears to turn around the thalamus, this being more apparent in younger specimens when compared to the older specimens (24 GW), where the diencephalon and the brainstem have a more vertical, adult‐like orientation.

#### 3.3.4 Ventral Thalamus

The ventral thalamus, also referred to as the prethalamus (Paxinos et al. 2012; Puelles et al. 2013; Puelles and Rubenstein 1993), originates from prosomere 3, which includes the pregeniculate, subgeniculate, and reticular nuclei (Paxinos et al. 2012) and comprises the thalamic RT, the pregeniculate nucleus (LGv), zona incerta (ZI), and the FF. From 14 to 24 GW, RT was readily delineated, and it surrounds the rostral and the lateral boundaries of the dorsal thalamus, separated from the dorsal thalamus by the external medullary lamina. The external medullary lamina courses parallel to the RT and ventrally surrounds the LGN. The pregeniculate nucleus was observed in a position ventral to the RT in the 22 GW coronally sectioned specimen. The pregeniculate nucleus is referred to as the ventral LGN in non‐primates (Livingston and Mustari 2000). We observed the pregeniculate nucleus in ventral, medial, and dorsal aspects relative to the LGN, due to the developmental rotation of the LGN from the dorsomedial to the ventrolateral aspect of the dorsal thalamus (see Figure 12), as reported in monkeys (Livingston and Mustari 2000). Furthermore, at 14 GW, in the ventral aspect of the RT, a homogeneous cell‐dense region, compared to the RT, is delineated as the subgeniculate nucleus, similar to that described for the marmoset (Paxinos et al. 2012) but not described previously in the developing human brain. This was a consistent observation in all specimens (14–24 GW), where the subgeniculate nucleus was adjacent in its caudal aspect to the ZI.

In the sixth‐ to seventh‐month‐old fetal human brain, the pregeniculate nucleus was identified as a subregion of the RT (Ulfig, Nickel, and Bohl 1998). A much earlier study by Cooper (1945) described the pregeniculate nucleus as scattered cells around the LGN. Based on these reports and several descriptions of the pregeniculate and subgeniculate nuclei in primates (Paxinos et al. 2012; Ulfig, Nickel, and Bohl 1998), our observations show that the fetal pregeniculate nuclear complex can also be subdivided into dorsal, lateral, and medial subdivisions, similar to that described in monkeys and related to the rotation of the LGN. As the LGN matures and rotates, the pregeniculate region appears as a triangular cap at 22 GW in the sagittal and coronal planes and becomes thicker by 24 GW. This pregeniculate maturation is similar to observations made in macaques and marmosets (Livingston and Mustari 2000). However, the pregeniculate has not been studied in the developing fetal human brain at the start of the second trimester (Hitchcock and Hickey 1980).

The ZI, which appears as a palely stained cell‐sparse region, is often considered to be a rostral extension of the midbrain tegmentum. The ZI was located ventral to the dorsal thalamus, and the FF appeared as a medially located continuation of it.

#### 3.3.5 Epithalamus

**FIGURE 13:**
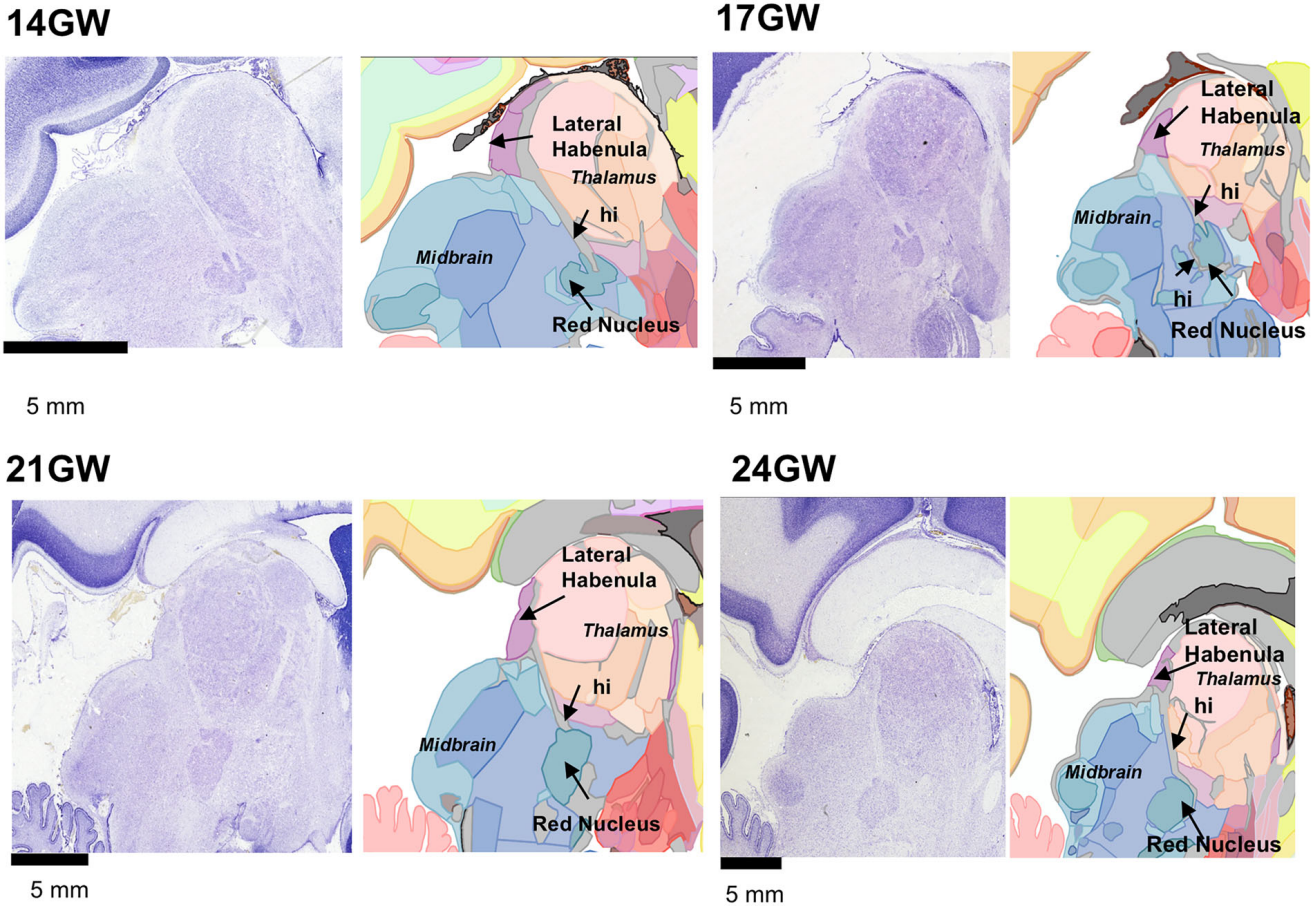
The development of the habenulo‐interpeduncular tract (hi) in the developing brain from 14 to 24 GW, in the sagittal plane. At 14 GW, hi extends rostrally from the lateral habenula, across its entire dorso‐ventral extent, and traverses the red nucleus. By 24 GW, the tract starts from the ventro‐rostral corner of the lateral habenula and curves around the red nucleus dorsally.

The epithalamus, depending on the used definition, comprises several nuclei, some of which are described elsewhere (see above, the paraventricular nuclei; Ding et al. 2022). The lateral habenula (LH) and medial habenula (MH) nuclei were observed adjacent to the third ventricle. MH is located posterior to the pineal gland. LH appears less cell‐dense than MH and is located more laterally and rostrally in sagittal sections. From 14 to 24 GW, we delineated the habenulo‐interpeduncular tract (hi) as a prominent tract coursing ventrally toward the red nucleus (Figure 13). This tract has been reported in the developing fetal human brain from 8 to 12 GW (Cho et al. 2014). As previously reported (Cho et al. 2014), we see IHT in older specimens (21–24 GW), adjacent, embedded, and coursing around the red nucleus.

### 3.4 Brainstem and Cerebellum

#### 3.4.1 Brainstem

The brainstem is a very complex central nervous system subdivision, which includes more than 100 nuclei. Therefore, for simplicity, we apply the classic topographical subdivisions of the midbrain, pons, and medulla to the developing brainstem, although we acknowledge that this terminology needs revision (Watson, Bartholomaeus, and Puelles 2019). The nuclei and fiber tracts identified in the brainstem are listed in Table 3. The nuclei are further grouped in three general categories: “cranial nerves nuclei,” “other nuclei” which include the brainstem intrinsic and neuromodulatory nuclei (Manger 2020), and the “undifferentiated gray matter,” which includes the reticular formation and the central gray of the pons. The annotations of the brainstem reticular formation are further subdivided by the brainstem classical divisions. The fiber tracts included in Table 3 are those pathways that are at least partially present in any of the topographical brainstem subdivisions. Each identified nucleus and fiber tract is also grouped by the age group (specimen) in which they were identified. Finally, those nuclei that extend both in the midbrain and pons, or pons and medulla, are in italic or bold italic, respectively.

**TABLE 3:** The list of brainstem nuclei and fiber tracts identified in our Nissl‐stained specimens and grouped by major topographical division (midbrain, pons, medulla), general category, and age groups (see text for details).

Brain part	14 GW	17 GW	21–22 GW	24 GW
**Cranial nerve nuclei**				
**Midbrain**				
Oculomotor nuclear complex (III)	+	+	+	+
Edinger–Westphal nucleus (III)			+	+
Trochlear nerve nucleus (IV)	+	+	+	+
Mesencephalic nucleus of trigeminal (V)	+	+	+	+
**Pons**				
Principal sensory nucleus of trigeminal (V)	+	+	+	+
Spinal nucleus of the trigeminal (V)^a^	+	+	+	+
Abducens nerve nucleus (VI)	+	+	+	+
Facial motor nucleus (VII)	+	+	+	+
**Cochlear nuclei (VIII)** ^a^	+	+	+	+
Dorsal cochlear nucleus^a^			+	+
Ventral cochlear nucleus^a^			+	+
**Medulla**				
Vestibular nuclear complex (VIII)^a^	+	+	+	+
Dorsal motor vagal nucleus (X)	+	+	+	+
Dorsal sensory vagal nucleus (X)	+	+	+	+
Solitary nuclear complex (X) (XI)	+	+	+	+
Nucleus ambiguous (X) (XI)	+	+	+	+
Hypoglossal nucleus (XI)	+	+	+	+
Accessory nucleus (XII)	+	+	+	+
**Other nuclei**				
**Midbrain**				
Anterior pretectal nucleus	+	+	+	+
Medial pretectal nucleus		+	+	+
Posterior pretectal nucleus	+	+	+	+
Nucleus of the optic tract		+	+	+
Olivary pretectal nucleus		+	+	+
Precommissural nucleus				+
Nucleus of the posterior commissure	+	+	+	+
Superior colliculus	+	+	+	+
Inferior colliculus	+	+	+	+
Inferior colliculus, central nucleus	+	+	+	+
Periaqueductal gray^b^	+	+	+	+
Parabigeminal nucleus	+	+	+	+
Interpeduncular nucleus	+	+	+	+
*Interpeduncular nucleus dorsomedial*			+	
*Interpeduncular nucleus, caudal*			+	
Substantia nigra pars compacta	+	+	+	+
Substantia nigra pars reticulata	+	+	+	+
Red nucleus	+	+	+	+
Ventral tegmental area	+	+	+	+
*Paranigral nucleus of the VTA*			+	
Retrorubral area (parabrachial pigmented nucleus)	+	+	+	+
Nucleus of the lateral lemniscus, dorsal^b^	+	+	+	+
Nucleus of the lateral lemniscus, ventral^b^	+	+	+	+
Parabrachial nucleus^b^	+	+	+	+
*Parabrachial nucleus, lateral part* ^b^			+	
*Parabrachial nucleus, medial part* ^b^			+	
Dorsal tegmental nucleus^b^	+	+	+	+
*Rhabdoid nucleus*			+	
Raphe nuclei^a^, ^b^	+	+	+	+
*Dorsal raphe nucleus*			+	
*Rostral linear raphe nucleus*			+	
*Caudal linear raphe nucleus*			+	
*Median raphe nucleus*			+	
Nucleus of Darkschewitsch			+	+
**Pons**				
Locus coeruleus	+	+	+	+
Superior olive	+	+	+	+
Reticular tegmental nucleus	+	+	+	+
Raphe Nuclei^a^, ^b^	+	+	+	+
*Raphe interpositus nucleus*			+	
*Raphe magnus nucleus* ^a^			+	
**Medulla**				
Area postrema	+	+	+	+
Cuneate nucleus	+	+	+	+
Gracile nucleus	+	+	+	+
Inferior olivary complex	+	+	+	+
Intercalated nucleus of medulla	+	+	+	+
Nucleus prepositus	+	+	+	+
Lateral reticular nucleus	+	+	+	+
Arcuate nucleus of the medulla	+	+	+	+
Nucleus of roller		+	+	+
Commissural nucleus		+	+	+
Raphe nuclei^a^, ^b^	+	+	+	+
*Raphe obscurus nucleus*			+	
*Raphe pallidus nucleus*			+	
**Undifferentiated gray matter**				
Reticular formation^a^, ^b^	+	+	+	+
Central gray of pons	+	+	+	+
**Major fiber tracts**				
Cerebral peduncles	+	+	+	+
Posterior commissure	+	+	+	+
Habenulo‐interpeduncular tract (fasciculus retroflexus)	+	+	+	+
Cerebellar peduncles	+	+	+	+
Trapezoid body	+	+	+	+
Corticofugal tract	+	+	+	+
Corticospinal tract	+	+	+	+
Pyramidal decussation	+	+	+	+
Dorsal funiculus	+	+	+	+
Brachium of superior colliculus			+	
Brachium of the inferior colliculus	+	+	+	+
Lateral lemniscus	+	+	+	+
Superior colliculus commissure			+	+
Lateral funiculus		+	+	+
Ventral funiculus		+	+	+
*Dorsal tegmental decussation*			+	
*Ventral tegmental decussation*			+	
*Superior cerebellar decussation*			+	

*Note: Italicized = observed in coronal plane*.

^a^ Extends into both Medulla and Pons.

^b^ Extends into both midbrain and Pons.

Overall, we have identified 74 brainstem nuclei and 29 fiber tracts in different age groups, as listed in Table 3. A comprehensive description of each identified brainstem nucleus and fiber tract is beyond the scope of this article; therefore, we briefly describe several key nuclei.

The pretectum (PRT) includes a collection of nuclei of the rostro‐dorsal brainstem, anterior to the SC, and in the transition zone between the midbrain and thalamus. Cytorchitectonically and topographically, the PRT is usually divided into five distinct nuclei: the nucleus of the optic tract (NOT), the olivary pretectal nucleus (OPT), the anterior pretectal nucleus (APT), medial pretectal nucleus (MPT), and posterior pretectal nucleus (PPT). At 14 GW, the APT and PPT are traversed by fiber tracts which may include migratory streams, and which are no longer visible at 24 GW. In the brainstem, we describe the pretectal nuclei NOT, APN, PPN, MPN, and OPN that have not been fully reported in the developing human brain, to the best of our knowledge, but have been described in adult humans (Borostyánkői‐Baldauf and Herczeg 2002).

The IC can be readily identified in Nissl stain from 14 GW onward, and it is located laterally from the periaqueductal gray and ventral from the SC. However, its subparts, the central nucleus of Inferior colliculus (ICc), the dorsal nucleus (ICd), and the external nucleus (ICe) (Geniec and Morest 1971; Tardif and Clarke 2001), can be reliably identified only in later stages of development, from 22 GW onward. The ICc has an ovoid shape, and it is Nissl‐stained more intensely. ICd appears like a cap of the ICc in sagittal cuts, and the ICe can be identified in the ventral IC, sharing a rather diffuse border with it.

The interpeduncular nucleus (IPN) is located rostrally to the interpeduncular fossa. IPN appears as a cluster of immature cells at 14 GW. At 22 GW, IPN can be subdivided into a dorsomedial and a caudal part, based on the cytoarchitecture. The dorsomedial part appears denser in Nissl stain than the caudal part. However, the two IPN parts could be identified only in the coronally cut specimen.

The pontine gray (PG) is composed of a dense aggregate of neurons in the ventral pons. This nucleus can be identified from 14 GW onward with corticofugal and transverse fibers serrating it. At 14 GW, the lateral area of this nucleus includes dense streams of neuro‐ and glioepithelia, which disappear by 17 GW. The PG cytoarchitecture becomes mostly homogeneous by 24 GW.

The superior olive (SO) is situated caudally near the facial nucleus, in the caudal pons. At 14 GW, this nuclear complex is small and cannot be subdivided into subparts. However, at 24 GW, the SO can be subdivided cytoarchitectonically into two parts.

The inferior olive (IO) nuclei are located in the superior medulla, just inferior to the pons. This complex is visible from 14 GW at the anterior tegmentum of the medulla. At 14 GW, the largest part of this complex appears only as a ring. IO complex appears corrugated at 24 GW.

The lateral reticular nuclei (LRN) are located as a complex of cellular aggregates in the medullary reticular formation, close to the inferior olivary complex. LRN appears as densely strained bands at 14 GW, which differentiate into aggregates of cells with different sizes by 22 GW. Subparts of this nucleus may be identified in Nissl stain by 24 GW.

The complex of the solitary nucleus (SOL) and tract can be identified from 14 GW onward, extending from the upper medulla to its lower part, medial to the vestibular nuclear complex at the top, and the cuneate and gracile nuclei at the bottom. Clusters of individual SOL nuclei are visible from 14 GW onward. A dense subnucleus, the commissural part of SOL, located medially, is also visible in Nissl preparation from 17 GW onward.

#### 3.4.2 Cerebellum

##### 3.4.2.1 Cerebellar Nuclei

In the developing cerebellar nuclei from 21 GW onward, we identified the dentate (DN), fastigial, and interposed nuclei. The cell‐dense DN is the largest of these three nuclei and is located in the middle of the cerebellar white matter, and it has its typical lobular appearance by 24 GW. The fastigial nucleus is located medial to the DN and appears as a cell‐dense patch but is smaller in size and does not have the lobular appearance of the DN. The interposed nucleus, found between the DN and fastigial nucleus, could be identified by its patchy appearance, at 24 GW with several fiber tracts passing through and around the cells forming the nucleus.

##### 3.4.2.2 Cerebellar Cortex

The lamination of the developing cerebellum is readily demarcated in all age groups; however, at younger gestational ages, the Purkinje cell layer was not identified. The foliation is seen as early as 17 GW. The Purkinje cell layer becomes well‐defined at 21 GW, containing a few large cells, but no well‐defined Purkinje cells were observed by the end of the second trimester. The cerebellar cortex showed an increased foliation with age.

The ventral aspect of the cerebellum housed the cell‐dense germinal trigone in all the specimens examined. The germinal trigone had a thick neuroepithelium in younger gestational ages (14–17 GW) and was found adjacent to the fourth ventricle.

## 4 Discussion

Compared to previous atlases (Bayer and Altman 2005; Ding et al. 2016), we have used in DHARANI high sampling density (60–1000 µm), annotated 466 plates (see Appendix A) from serial sections, and systematically reported our results from 60 to 120 serial sections per specimen. Although human fetal specimens offer crucial insights into developmental human neuroanatomy, the acquisition, digitization, and open online accessibility of this data posed several technological challenges. The work described herein highlights technological advances and interdisciplinary collaborative work, both nationally and internationally, between clinicians, neuroscientists, and engineers, to address the challenges of studying the whole human brain, and indeed human neuroanatomy, as highlighted by Crick and Jones (1993) three decades ago. In this study, the need to freeze and cryosection the whole brain as one single block was guided by computational requirements. Misalignment from 1000‐plus sections with nonuniform tissue shrinkage is often strenuous for 3D reconstruction, especially if the brain is slabbed in many pieces and each piece is processed separately (Amunts et al. 2020; Ding et al. 2016). This technological approach resulted in the first 3D reconstruction of the developing human brain in the second trimester.

These large, high‐quality histological datasets enabled us to present new insights in the telencephalon, diencephalon, and pretectum. The systematic investigation of the developing cerebral mantle from 14 to 24 GW reveals unique region‐specific morphogenesis throughout the cerebral cortex. We present our findings in region‐specific and layer wise and compare them with the previous reports (Altman and Bayer 2002; Bayer and Altman 2003; Kopić et al. 2023; Kostovic and Rakic 1990; Krsnik et al. 2017; Rakic 1988; Vasung et al. 2016; Verma et al. 2024). Recognizing these patterns is critical during the lissencephalic stage of the developing human brain as it allows a clear demarcation of the boundary with the adjacent cortices.

Key findings include the early patterning of the frontal cortex based on the intermediate zone (IZ) from 21 GW, the appearance of the honeycomb pattern in the occipital and parietal cortex after 14 GW, and the presence of putative Betz cells at 24 GW. The IZ in the second trimester is a crucial transitional layer where cells pause their migration and sort themselves among the developing fiber tracts, also referred to as a *sojourn* (a temporary stay) by Altman and Bayer (2002), before migrating toward their final destination in the cortex. Here, we specifically highlight the spatial‐temporal pattern of the IZ for the entire cerebral mantle. Compared to previous studies that delineated thalamic nuclei (histology) in the developing human brain in the second trimester (Bayer and Altman 2005; Ding et al. 2022; Forutan et al. 2001; Mai and Schönlau 1992), we outline in this study the developmental trajectory of the ventral thalamus, the fetal pregeniculate complex in relation to the LGN, and the RT.

The atlas includes delineations of the subregions of the subicular complex by 14 GW, identifies subnuclei within the BA from 17 to 24 GW, and describes the topographical development trajectory of the ventral thalamus. Furthermore, we report several fiber tracts, such as the fan‐shaped fiber tracts, referred to as the funnel of thalamocortical axons (Bayer and Altman 2007) in the dorsal thalamus in the first trimester, the development of the IHT from 14 to 24 GW, the commissure of the SC, and the supramammillary and mammillary peduncles in the hypothalamus. The fan‐shaped fiber tract that was observed in the developing brain between 14 and 24 GW has also been studied in adult humans using imaging techniques and identified as thalamic prefrontal peduncles (Sun et al. 2018); however, this requires further detailed investigations into the developing human brain.

In Nissl preparation, we identified and delineated about 74 cranial nerves, intrinsic, and neuromodulatory brainstem nuclei. Detailed IHC is required for the systematic mapping of the developing brainstem in humans. However, from 14 GW onward, we identify the pretectal nuclei: APN, PPN, and from 17 GW onward the NOT, MPN, and OPN. For brain parts classification and organization, we adopted a “common ground” approach across the already existent brain atlas nomenclatures (Bayer and Altman 2005; Ding et al. 2022). We acknowledge the recent advancement in understanding the spatial‐temporal molecular trajectories during development (Ding et al. 2022; Kim et al. 2023; Nagalski et al. 2016) and its importance in characterization of the fetal brain from 14 to 24 GW as presented in this study. However, given the uncertainty of applying the neuromeric model to the developing human brain at present, we have used the classical neuroanatomical subdivisions to avoid potential misassignment of specific nuclei to the incorrect neuromere (Puelles et al. 2013; Puelles and Rubenstein 1993). The alignment of the current demarcations applied to the developing human brain herein can be readily altered to reflect the neuromeric model, when greater precision of the neuromeric model in terms of which nuclei are precisely located in which neuromere is consolidated.

We do identify the current limitations in DHARANI, where in the future it will expand by adding other developmental ages and specific IHC markers to classify region‐ and layer‐specific cell types.

## 5 Conclusions

The current 3D reconstructed datasets provide a qualitative assessment of brain growth, but the potential lies in quantifying the changes in size and volume of brain structures across the entire prenatal stage. Our integrated histological neuroanatomical atlases with 3D reconstruction of the whole brain can be utilized for systematic evaluation and generation of morphometric data at different developmental stages, while simultaneously studying cellular organization. These data can be correlated with MRI and USG findings in both neurotypical and pathological conditions, contributing to a deeper understanding of human brain development and the deviations and insults that occur in cases of developmental disorders. Furthermore, correlating the histological datasets with spatial molecular maps will be crucial.

## Author Contributions

**Richa Verma**, **Mihail Bota**, **Keerthi Ram**, **Jaikishan Jayakumar**: conceptualization, data curation, formal analysis, methodology, project administration, visualization, writing–original draft. **Rebecca Folkerth**: writing–review and editing. **Karthika Pandurangan**, **Jivitha Jyothi Ramesh**, **Moitrayee Majumder**, **Rakshika Raveendran**, **Reetuparna Nanda**: investigation, formal analysis. **Sivamani K**., **Amal Dhivahar S**., **Srinivasa Karthik, Ramdayalan Kumarasami, Suresh S., S. Lata, E. Harish Kumar, Rajeswaran Rangasami, Chitra Srinivasan, Jayaraman Kumutha, Sudha Vasudevan, Koushik Bhat, Chrisline Sam C**., **Sivathanu Neelakantan, Stephen Savoia**: resources. **Partha P. Mitra**, **Jayaraj Joseph**: conceptualization, supervision, resources, methodology, funding. **Paul R. Manger**: conceptualization, data curation, investigation, writing–review and editing. **Mohanasankar Sivaprakasam**: conceptualization, supervision, resources, methodology, funding, writing–review and editing.

## Ethics Statement

The study was conducted according to the guidelines approved by the IITM Institutional Human Ethics Committee (EC/2021‐01/MS/06). All specimens were obtained based on the guidelines approved by the Review Board and Ethics Committee of Mediscan Systems Pvt. Ltd Chennai, India (Mediscan).

## Consent

All postmortem specimens were obtained after due consent was from the next of kin in accordance with the Declaration of Helsinki.

## Conflicts of Interest

The authors declare no conflicts of interest.

## Supporting information



Plates_14 to 24 gestational weeks (GW).

**Video 1**. 3D reconstruction from histological sections at 14 gestational weeks (GW).

**Video 2**. 3D reconstruction from histological sections at 17 gestational weeks (GW).

**Video 3**. 3D reconstruction from histological sections at 21 gestational weeks (GW).

**Video 4**. 3D reconstruction from histological sections at 22 gestational weeks (GW).

**Video 5**. 3D reconstruction from histological sections at 24 gestational weeks (GW).

**Video 6**. 3D reconstruction from histological sections from 14, 17, 22, and 24 gestational weeks (GW) in the coronal view.

**Video 7**. 3D reconstruction from histological sections from 14, 17, 22, and 24 gestational weeks (GW) in the axial view.

**Video 8**. 3D reconstruction from histological sections from 14, 17, 22, and 24 gestational weeks (GW) in the sagittal view.

## Data Availability

The data that support the findings of this study are available online https://brainportal.humanbrain.in/publicview/index.html and available from the corresponding author upon reasonable request.

## Appendix A

This article includes an online appendix which includes the Atlas Plates from 14 to 24 gestational weeks (GW). This plate section appears in the online PDF of the article (appended to the end of the article), and is also included as a standalone section as a PDF file in the supporting information.
