# Supplementary material for: DHARANI: A 3D Developing Human‐Brain Atlas Resource to Advance Neuroscience Internationally Integrated Multimodal Imaging and High‐Resolution Histology of the Second Trimester
Source: J Comp Neurol. 2025 Feb 4;533(2):e70006. doi: 10.1002/cne.70006 (PMC11794986; doi:10.1002/cne.70006)
Supplement: Supplementary file 1 — Plates_14 to 24 gestational weeks (GW). [file CNE-533-e70006-s003.pdf]

14 Gestational Week (GW)

Sagittal

This page is intentionally left blank

Age: 14 GW

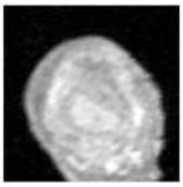

L-R Level: 12.78 mm

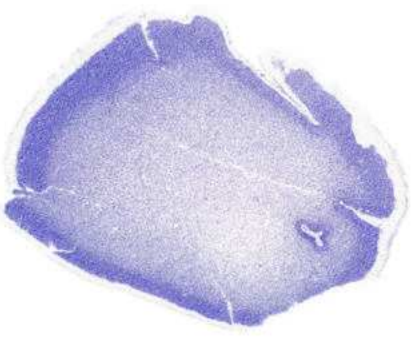

5 mm

Transient Layers

- SP
- CP
- MZ
- SGL

Cortical Areas

- TEMP

Age: 14 GW

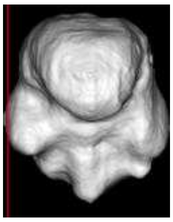

L-R Level: 12.78 mm

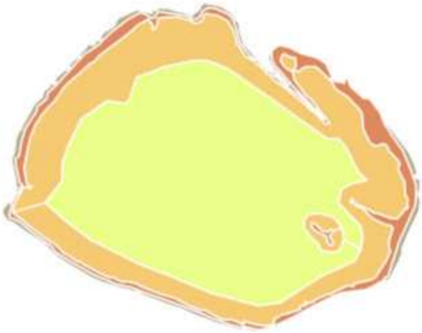

5 mm

Age: 14 GW

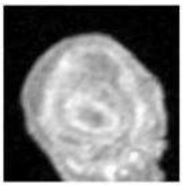

L-R Level: 12.3 mm

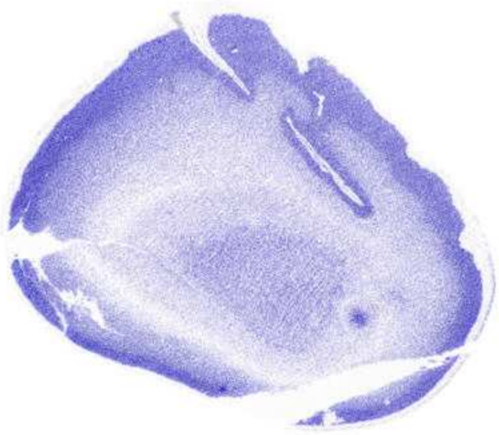

5 mm

Transient Layers

- IZ
- SP
- CP
- MZ
- SGL

Cortical Areas

- TEMP

Age: 14 GW

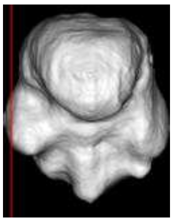

L-R Level: 12.3 mm

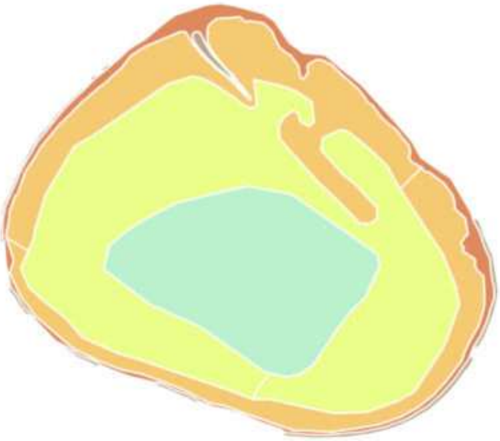

5 mm

Age: 14 GW

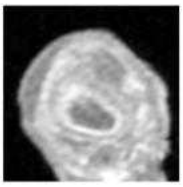

L-R Level: 11.7 mm

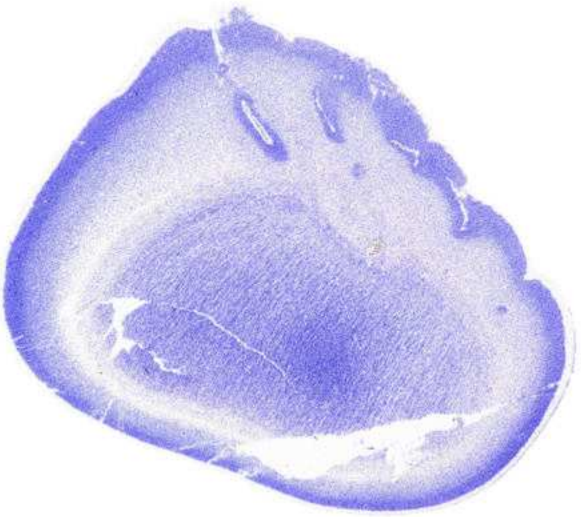

5 mm

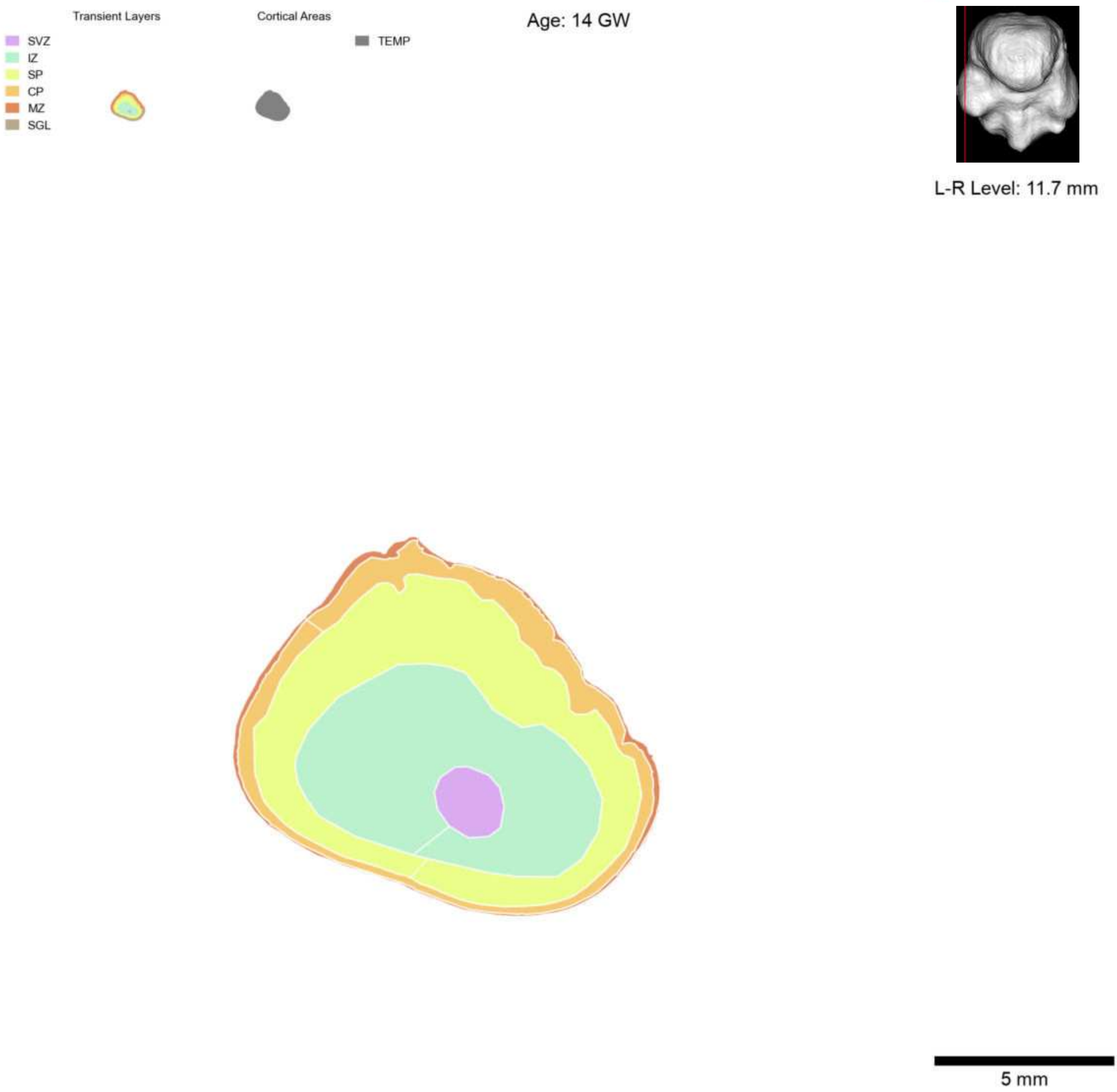

Transient Layers
SVZ
IZ
SP
CP
MZ
SGL
Cortical Areas
TEMP
Age: 14 GW
L-R Level: 11.7 mm
5 mm

Age: 14 GW

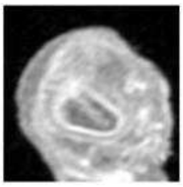

L-R Level: 11.28 mm

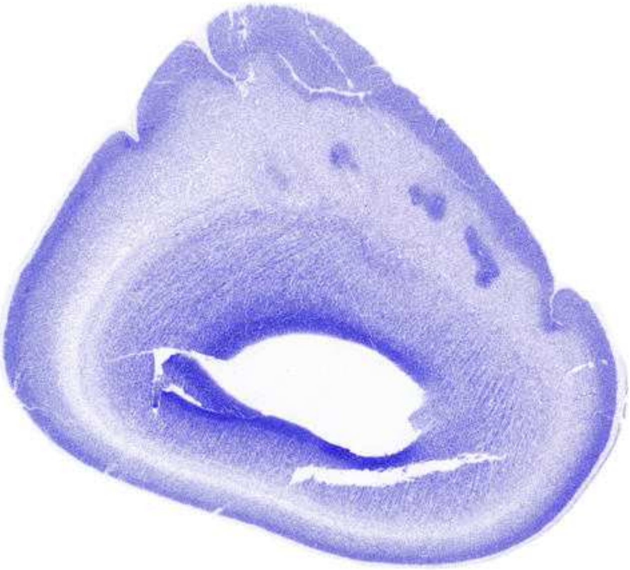

5 mm

Transient Layers

- VZ
- SVZ
- IZ
- SP
- CP
- MZ
- SGL

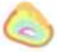

Cortical Areas

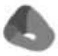

- PAR
- OCC
- TEMP

Age: 14 GW

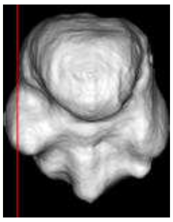

L-R Level: 11.28 mm

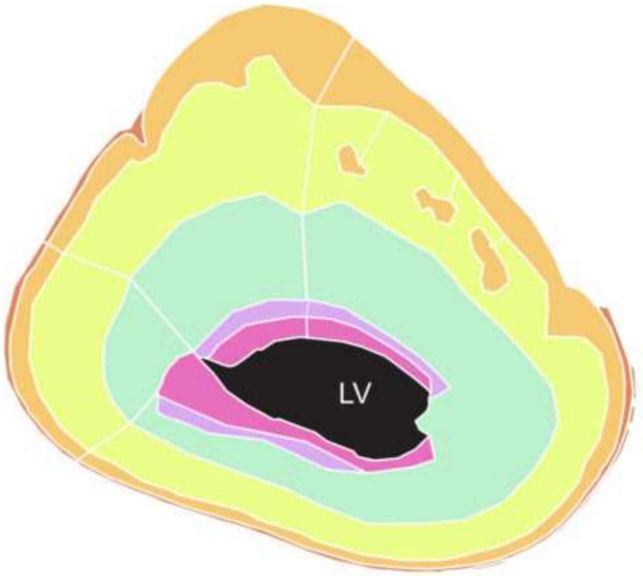

5 mm

LV: Lateral ventricle

Age: 14 GW

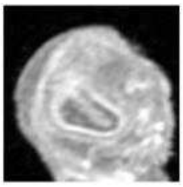

L-R Level: 11.04 mm

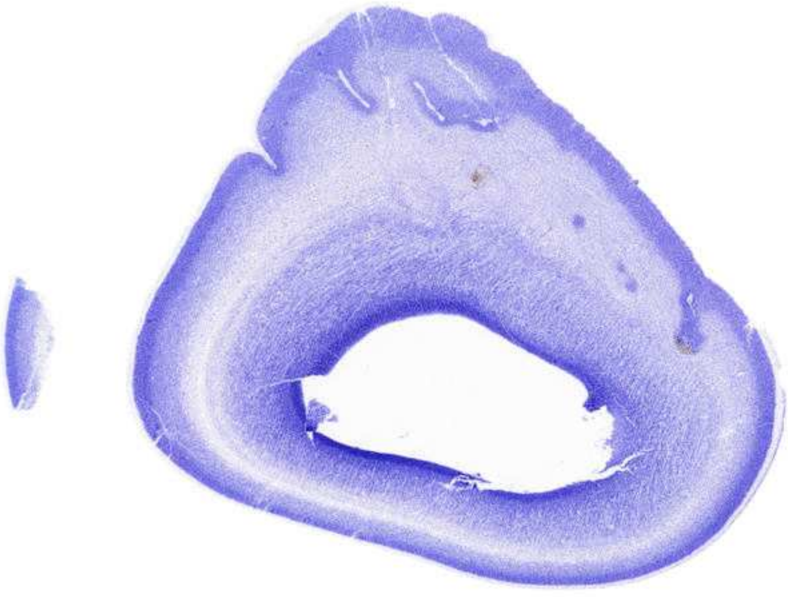

5 mm

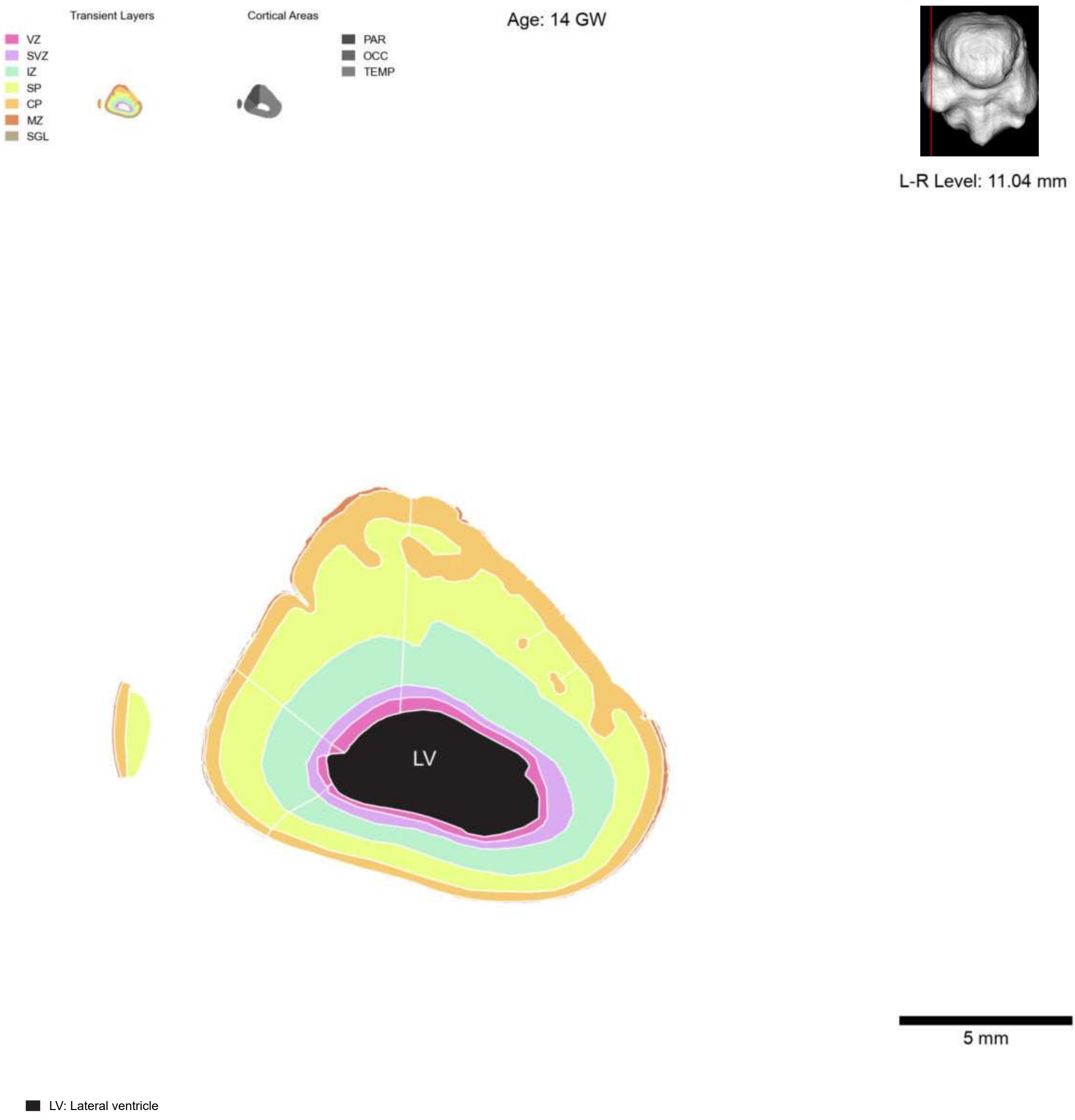

Transient Layers
VZ
SVZ
IZ
SP
CP
MZ
SGL
Cortical Areas
PAR
OCC
TEMP
Age: 14 GW
L-R Level: 11.04 mm
LV
5 mm
LV: Lateral ventricle

Age: 14 GW

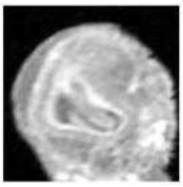

L-R Level: 10.5 mm

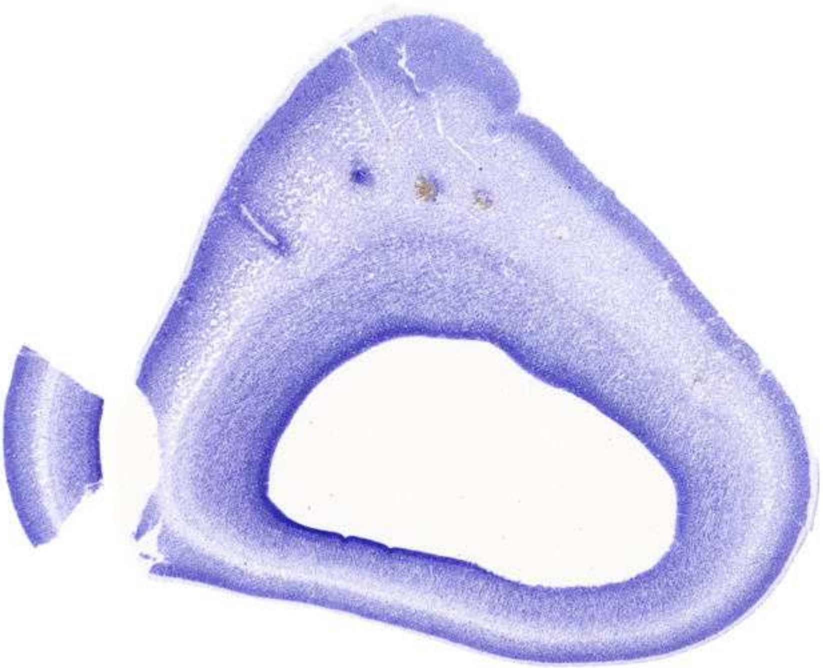

5 mm

Transient Layers

- VZ
- SVZ
- IZ
- SP
- CP
- MZ
- SGL

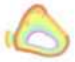

Cortical Areas

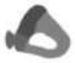

- PAR
- OCC
- TEMP

Age: 14 GW

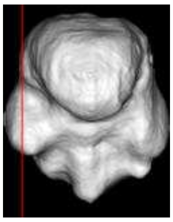

L-R Level: 10.5 mm

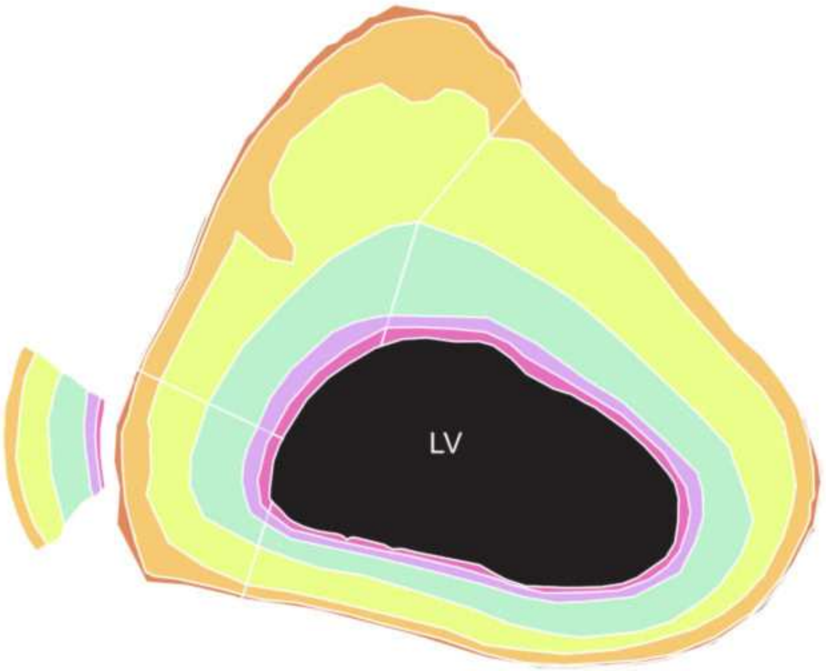

5 mm

LV: Lateral ventricle

Age: 14 GW

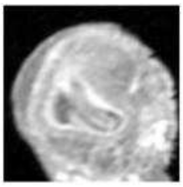

L-R Level: 10.38 mm

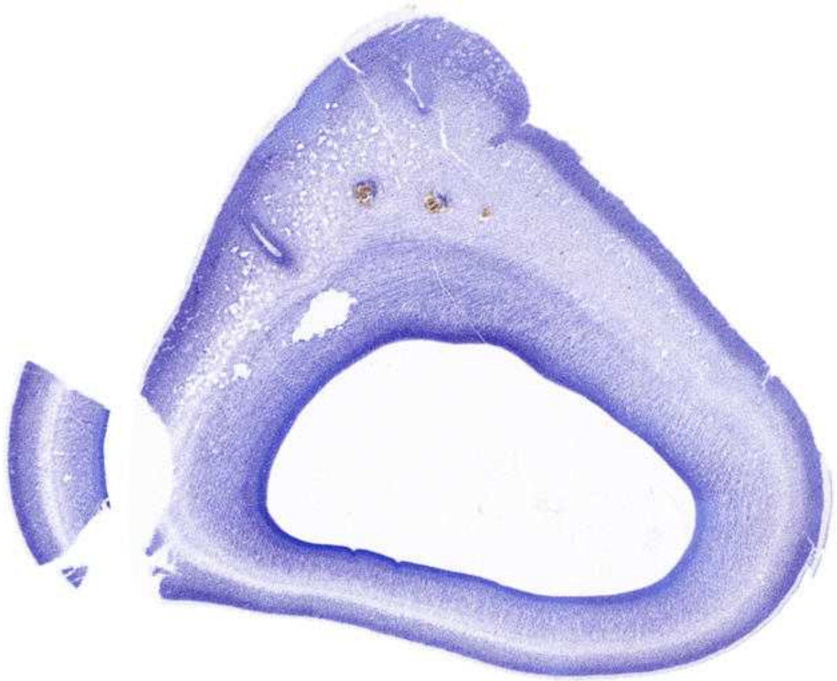

5 mm

Transient Layers

- VZ
- SVZ
- IZ
- SP
- CP
- MZ
- SGL

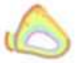

Cortical Areas

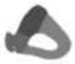

- PAR
- OCC
- TEMP

Age: 14 GW

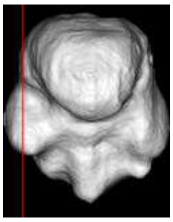

L-R Level: 10.38 mm

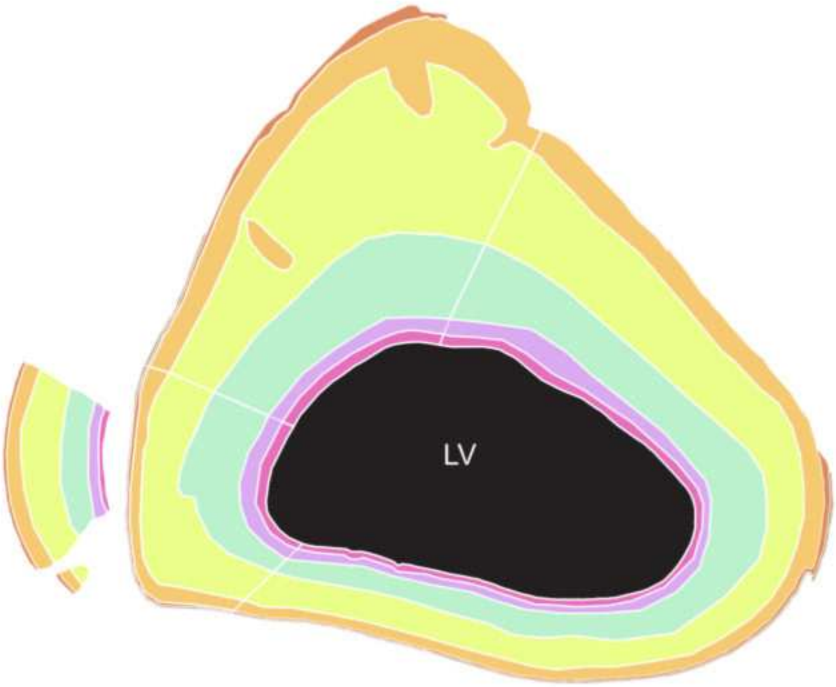

5 mm

LV: Lateral ventricle

Age: 14 GW

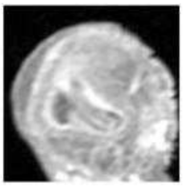

L-R Level: 10.26 mm

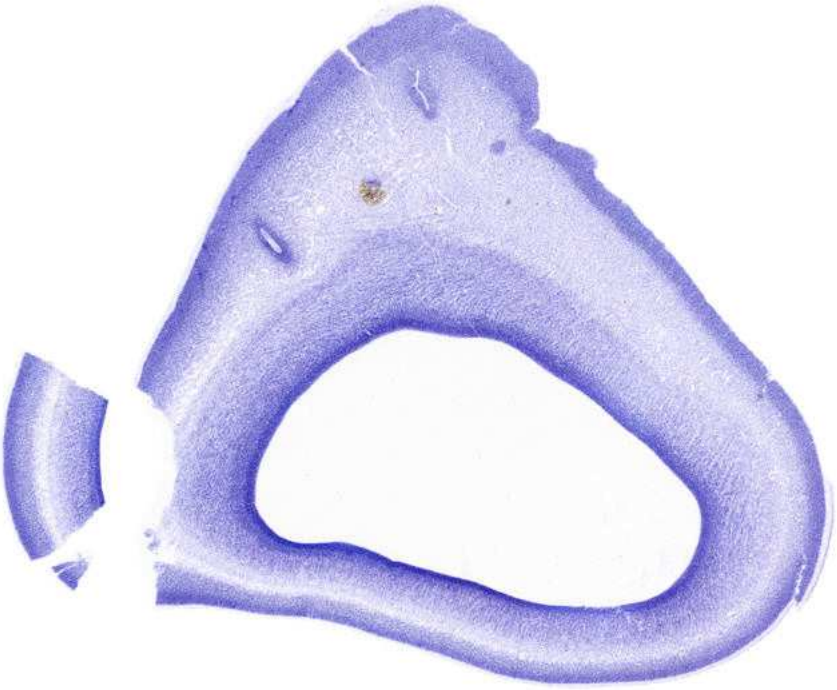

5 mm

Transient Layers

- VZ
- SVZ
- IZ
- SP
- CP
- MZ
- SGL

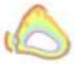

Cortical Areas

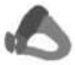

- PAR
- OCC
- TEMP

Age: 14 GW

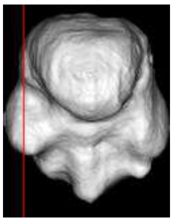

L-R Level: 10.26 mm

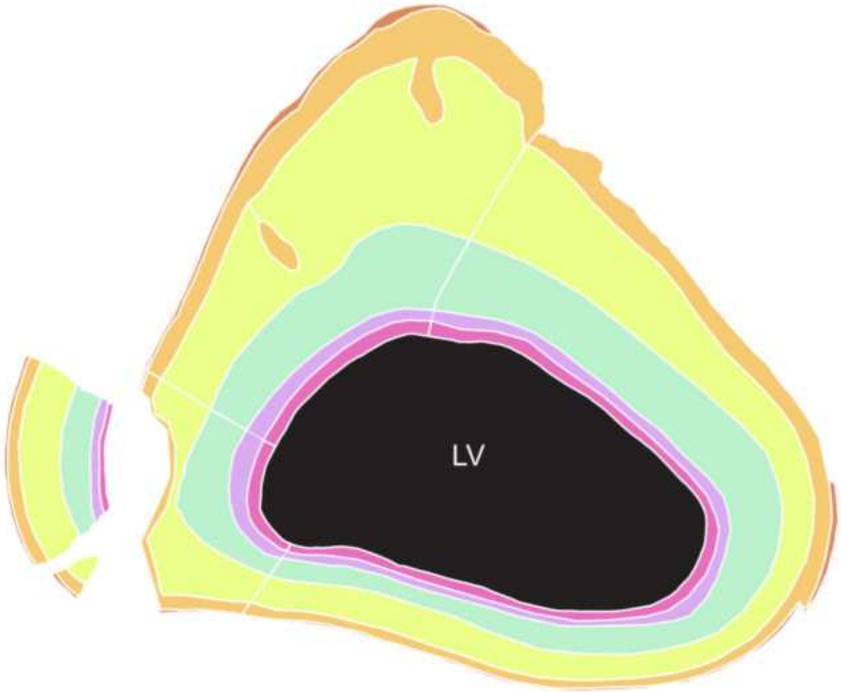

5 mm

LV: Lateral ventricle

Age: 14 GW

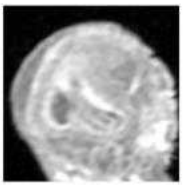

L-R Level: 10.14 mm

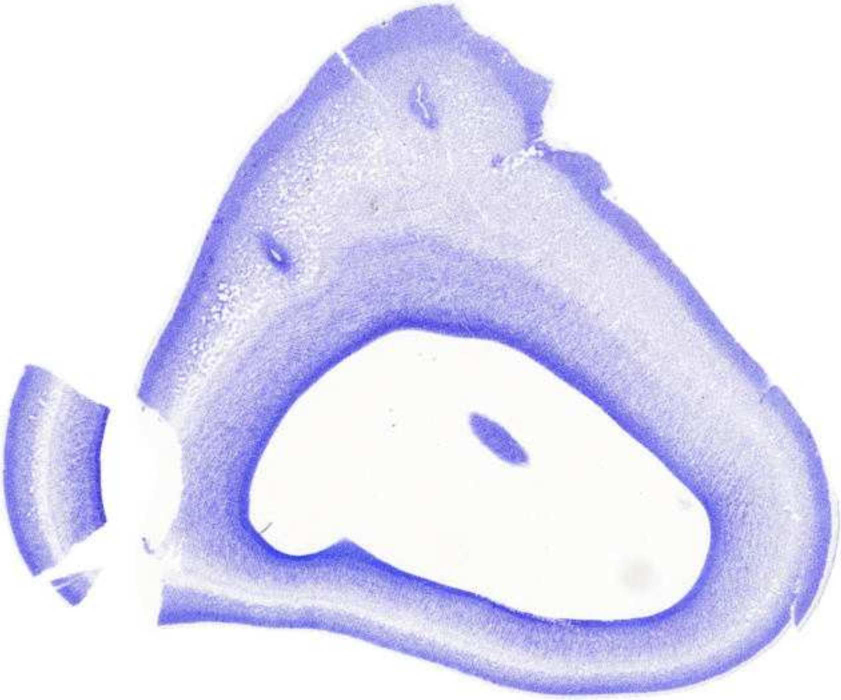

5 mm

Age: 14 GW

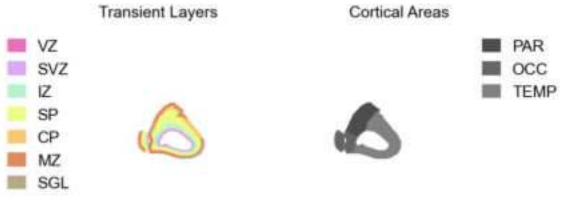

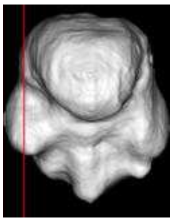

L-R Level: 10.14 mm

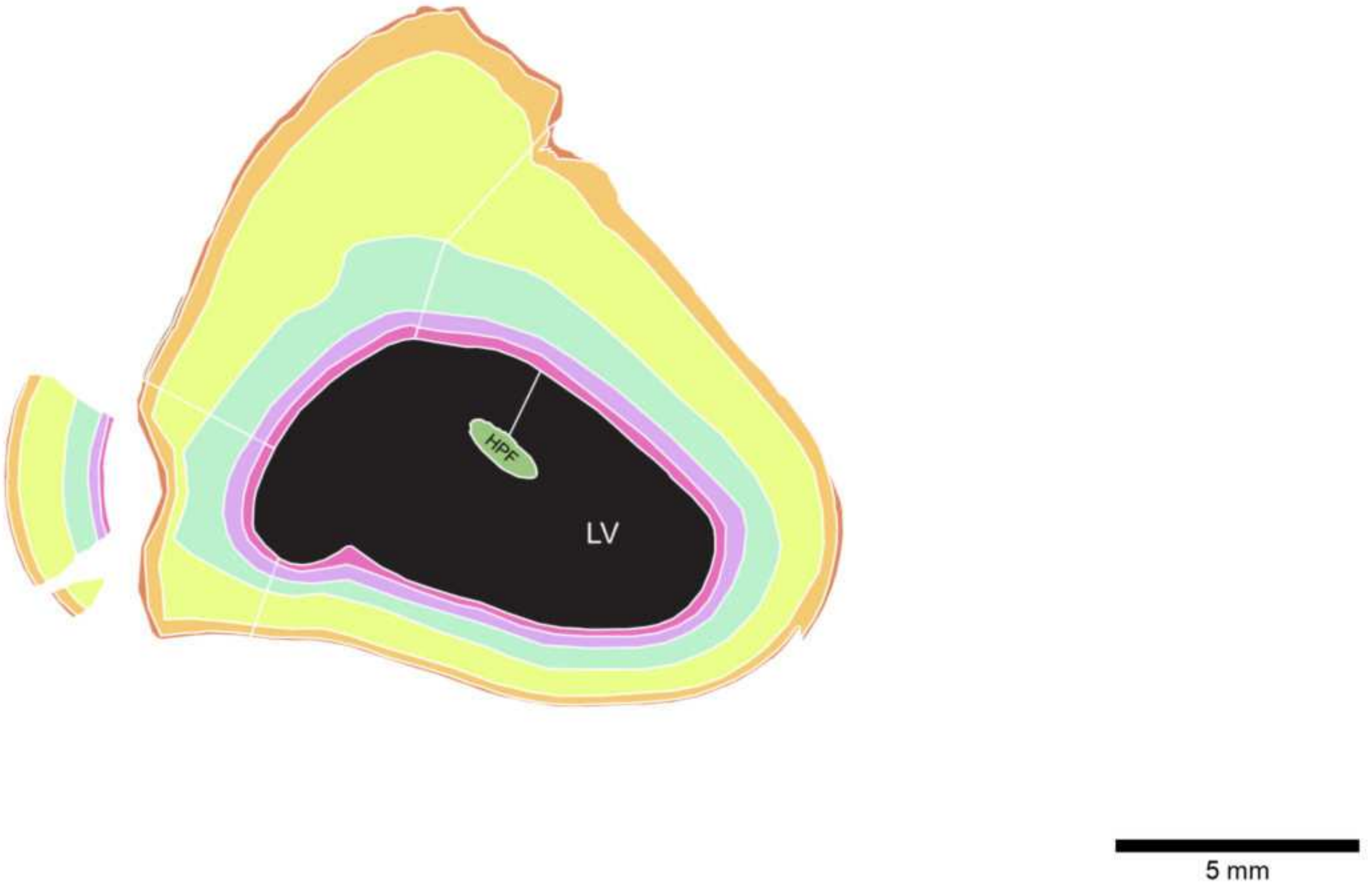

HPF: Hippocampal formation LV: Lateral ventricle

Age: 14 GW

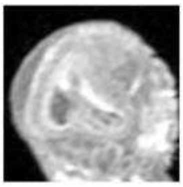

L-R Level: 9.96 mm

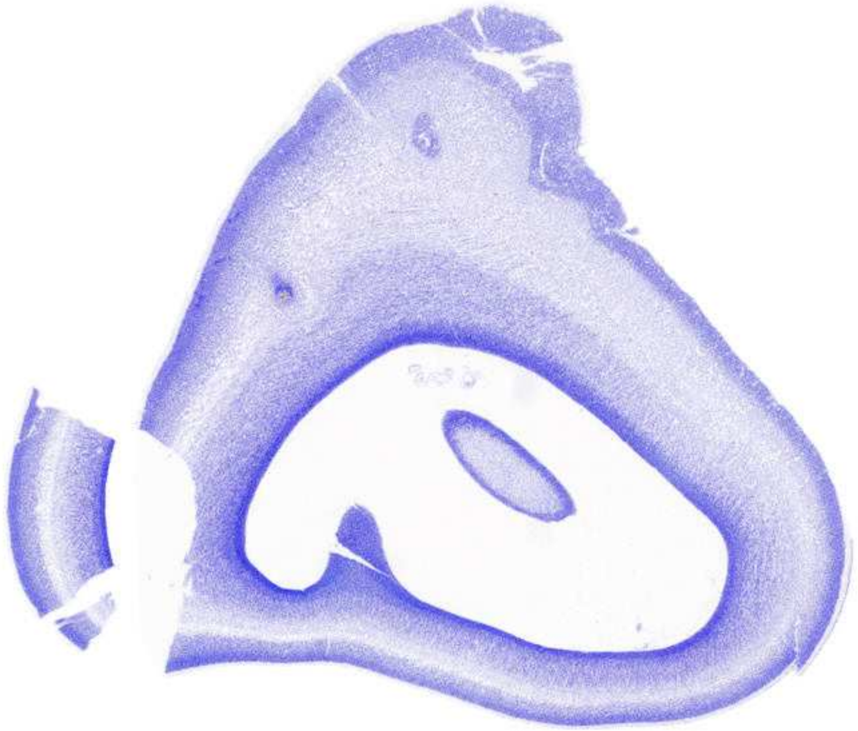

5 mm

Transient Layers

- VZ
- SVZ
- IZ
- SP
- CP
- MZ
- SGL

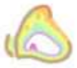

Cortical Areas

- PAR
- TEMP
- OCC
- INS

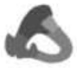

Age: 14 GW

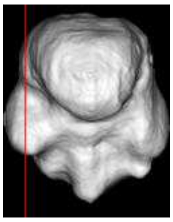

L-R Level: 9.96 mm

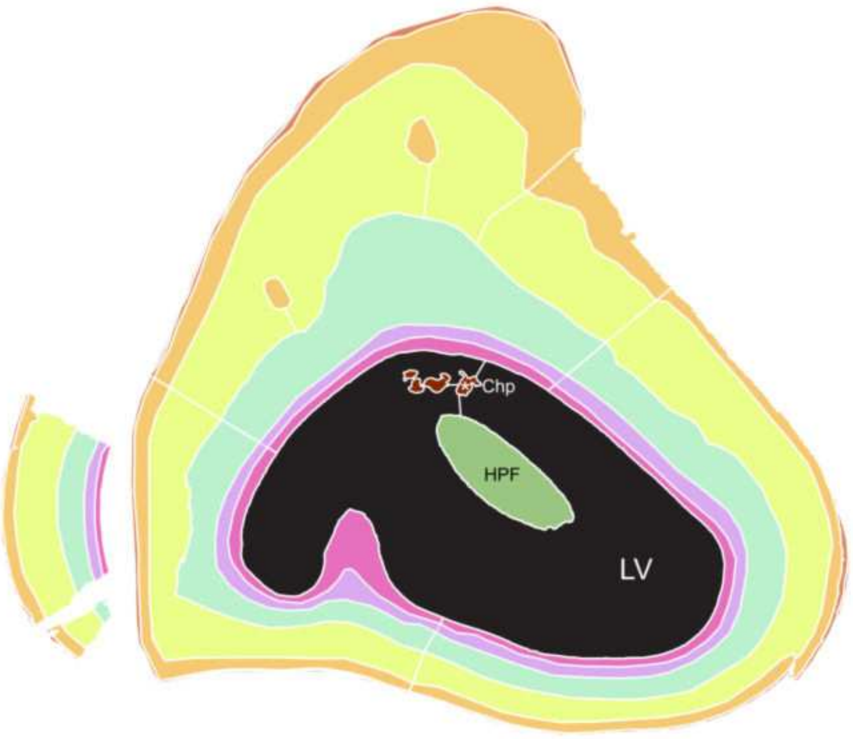

5 mm

Chp: Choroid plexus   HPF: Hippocampal formation   LV: Lateral ventricle

Age: 14 GW

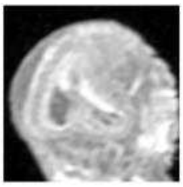

L-R Level: 9.84 mm

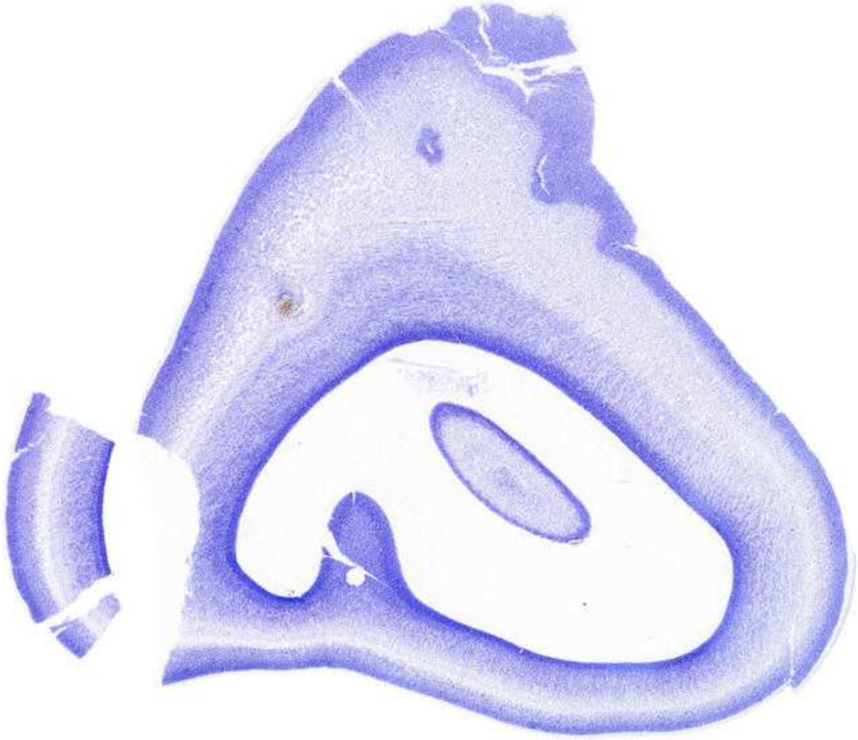

5 mm

Transient Layers

- VZ
- SVZ
- IZ
- SP
- CP
- MZ
- SGL

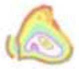

Cortical Areas

- PAR
- TEMP
- OCC
- INS

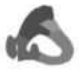

Age: 14 GW

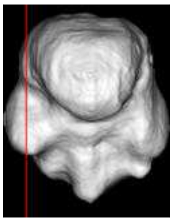

L-R Level: 9.84 mm

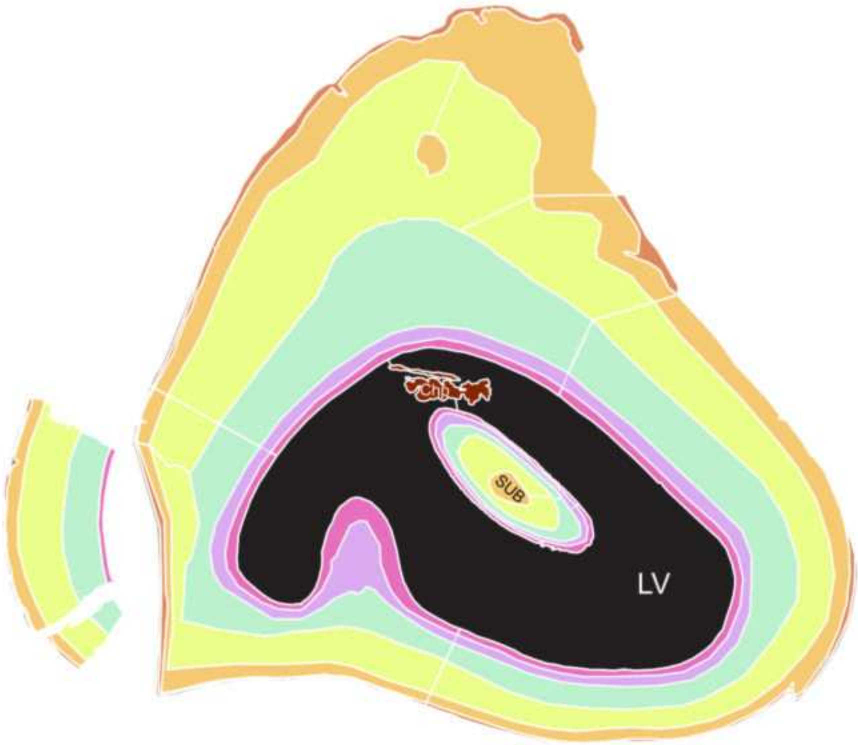

5 mm

Chp: Choroid plexus   LV: Lateral ventricle   SUB: Cortical plate, subiculum

Age: 14 GW

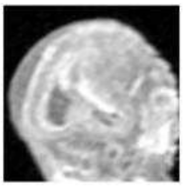

L-R Level: 9.6 mm

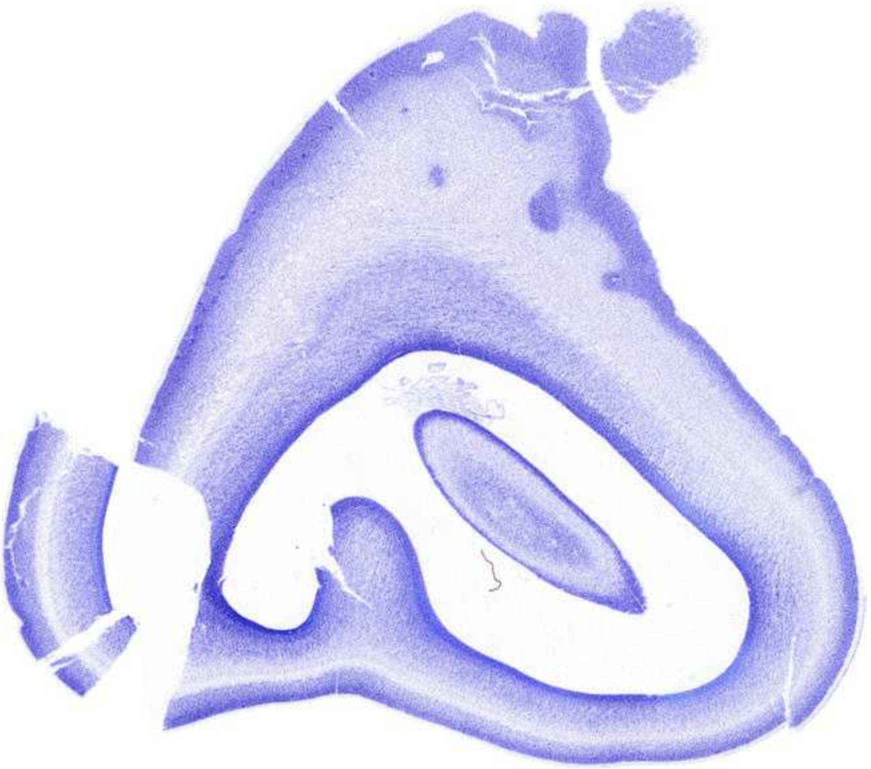

5 mm

Transient Layers

- VZ
- SVZ
- IZ
- SP
- CP
- MZ
- SGL

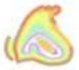

Cortical Areas

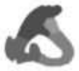

- PAR
- OCC
- TEMP
- INS

Age: 14 GW

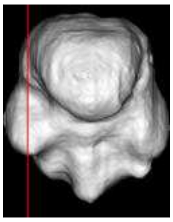

L-R Level: 9.6 mm

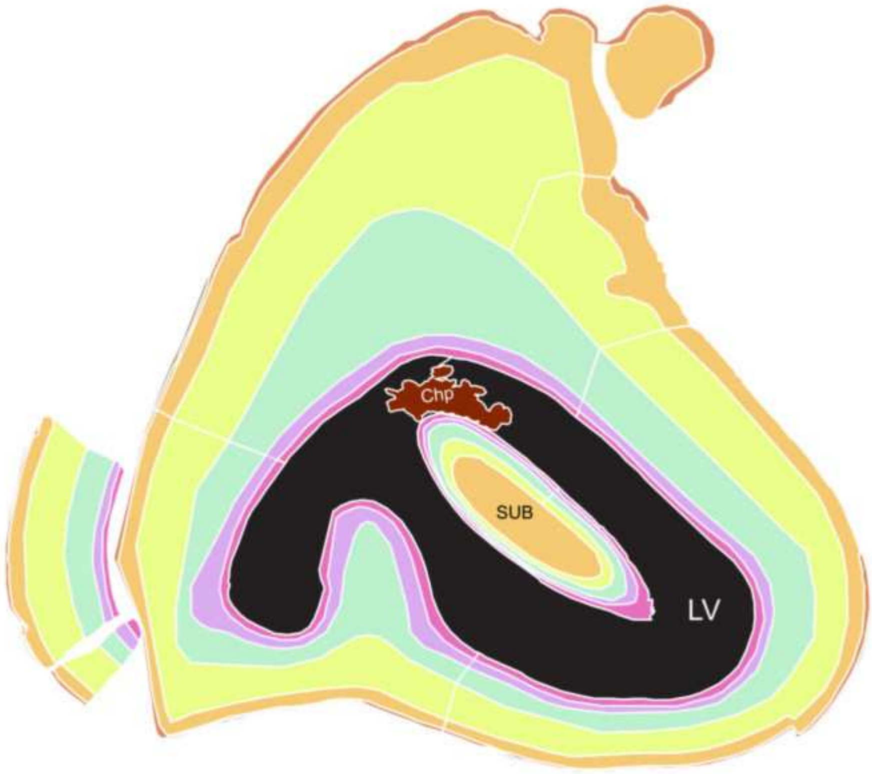

5 mm

Chp: Choroid plexus   LV: Lateral ventricle   SUB: Cortical plate, subiculum

Age: 14 GW

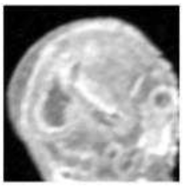

L-R Level: 9.24 mm

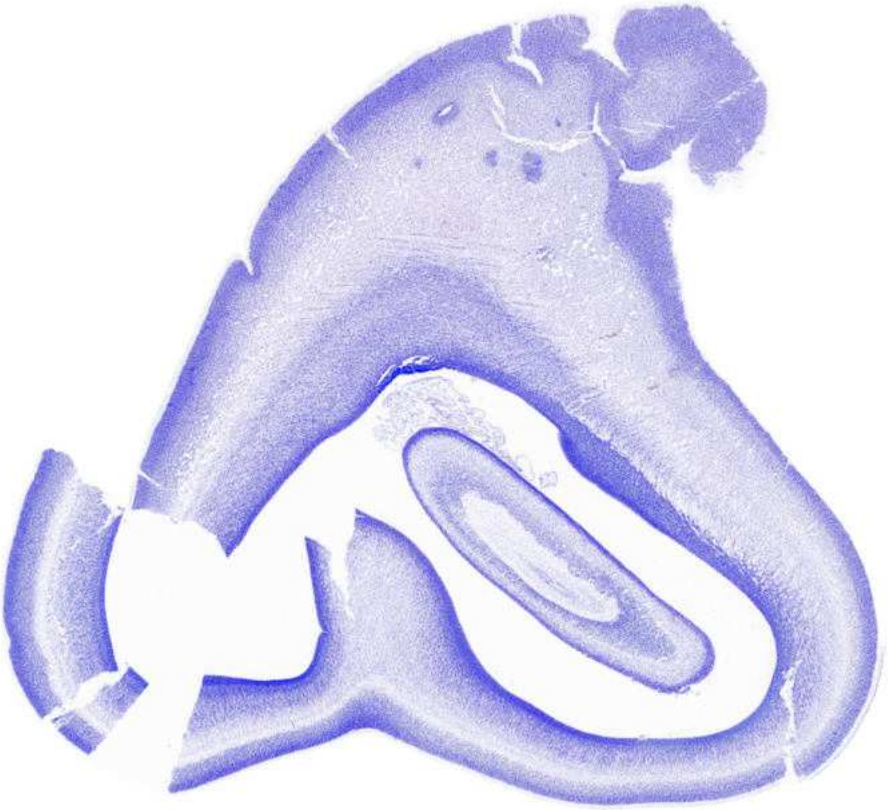

5 mm

Transient Layers

- VZ
- SVZ
- IZ
- SP
- CP
- MZ
- SGL

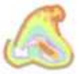

Cortical Areas

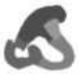

- PAR
- TEMP
- OCC
- INS

Age: 14 GW

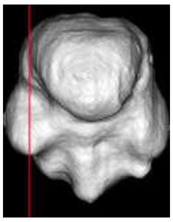

L-R Level: 9.24 mm

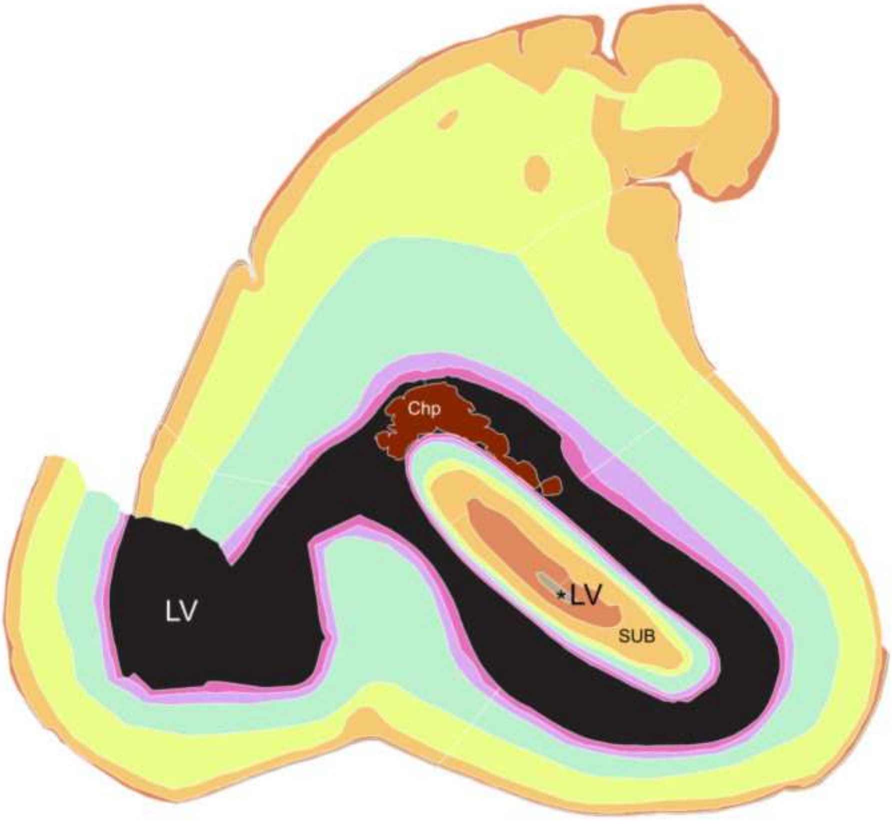

5 mm

Chp: Choroid plexus LV: Lateral ventricle SUB: Cortical plate, subiculum

Age: 14 GW

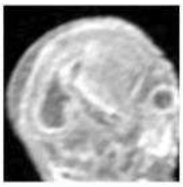

L-R Level: 9.0 mm

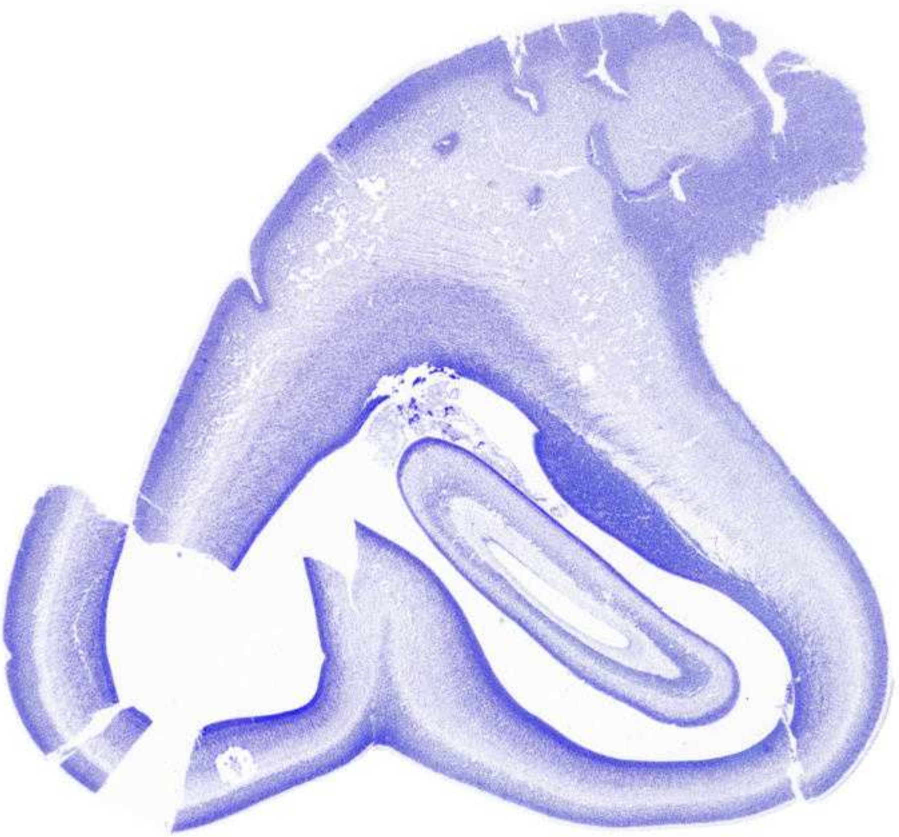

5 mm

Transient Layers

- VZ
- SVZ
- IZ
- SP
- CP
- MZ
- SGL

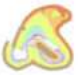

Cortical Areas

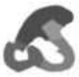

- PAR
- OCC
- TEMP
- INS

Age: 14 GW

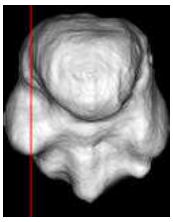

L-R Level: 9.0 mm

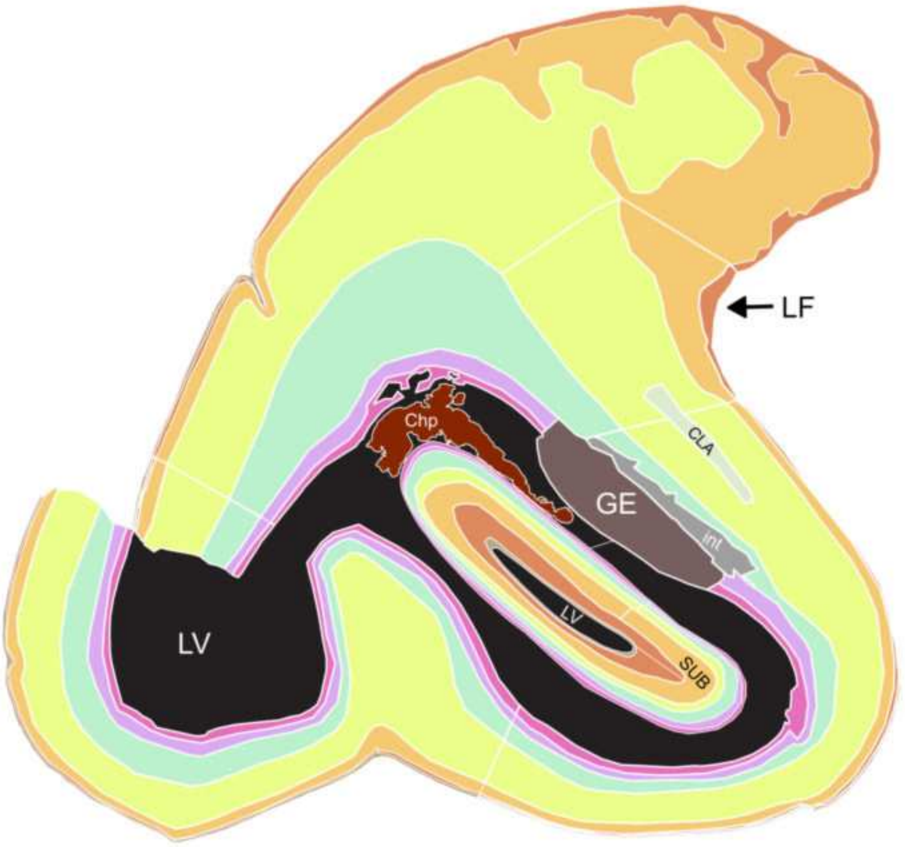

5 mm

CLA: Claustrum
Chp: Choroid plexus
GE: Ganglionic eminence
LV: Lateral ventricle
SUB: Cortical plate, subiculum
int: Internal capsule
LF: Lateral fissure

Age: 14 GW

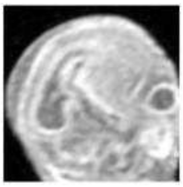

L-R Level: 8.58 mm

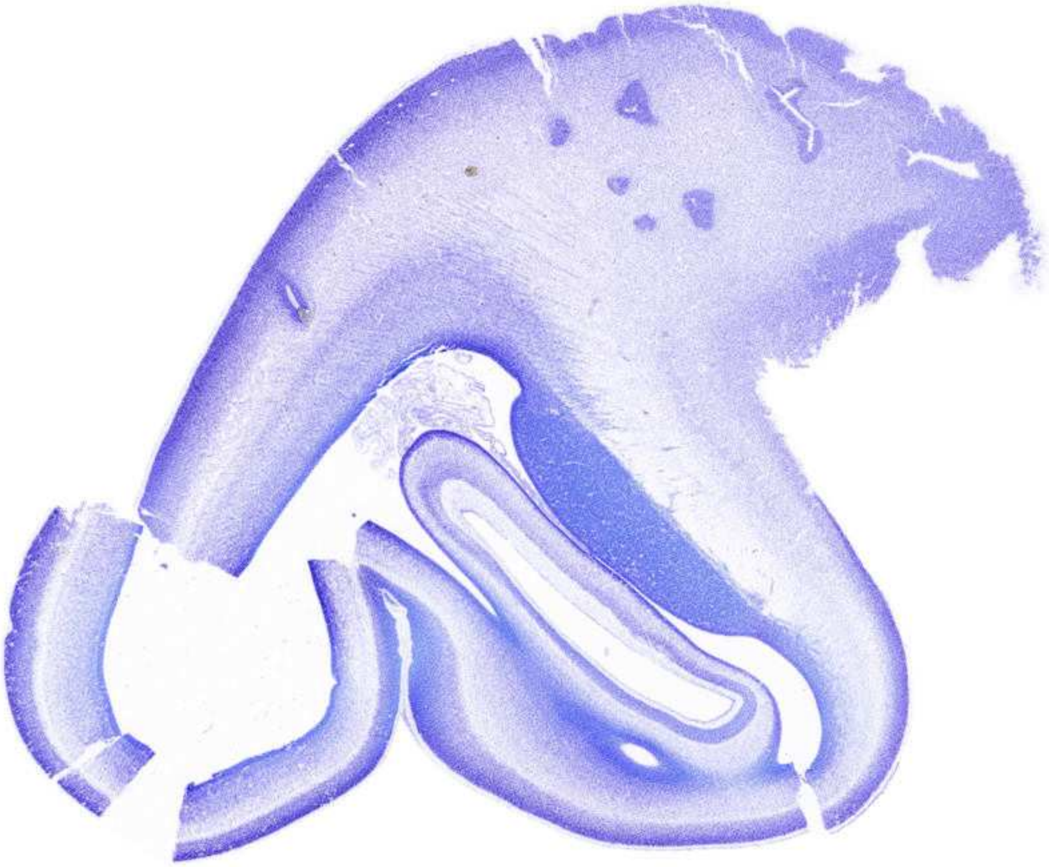

5 mm

Transient Layers

- VZ
- SVZ
- IZ
- SP
- CP
- MZ
- SGL

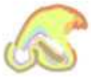

Cortical Areas

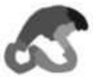

- FCTx
- PAR
- OCC
- TEMP
- INS

Age: 14 GW

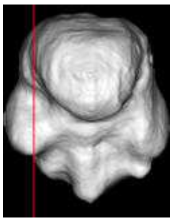

L-R Level: 8.58 mm

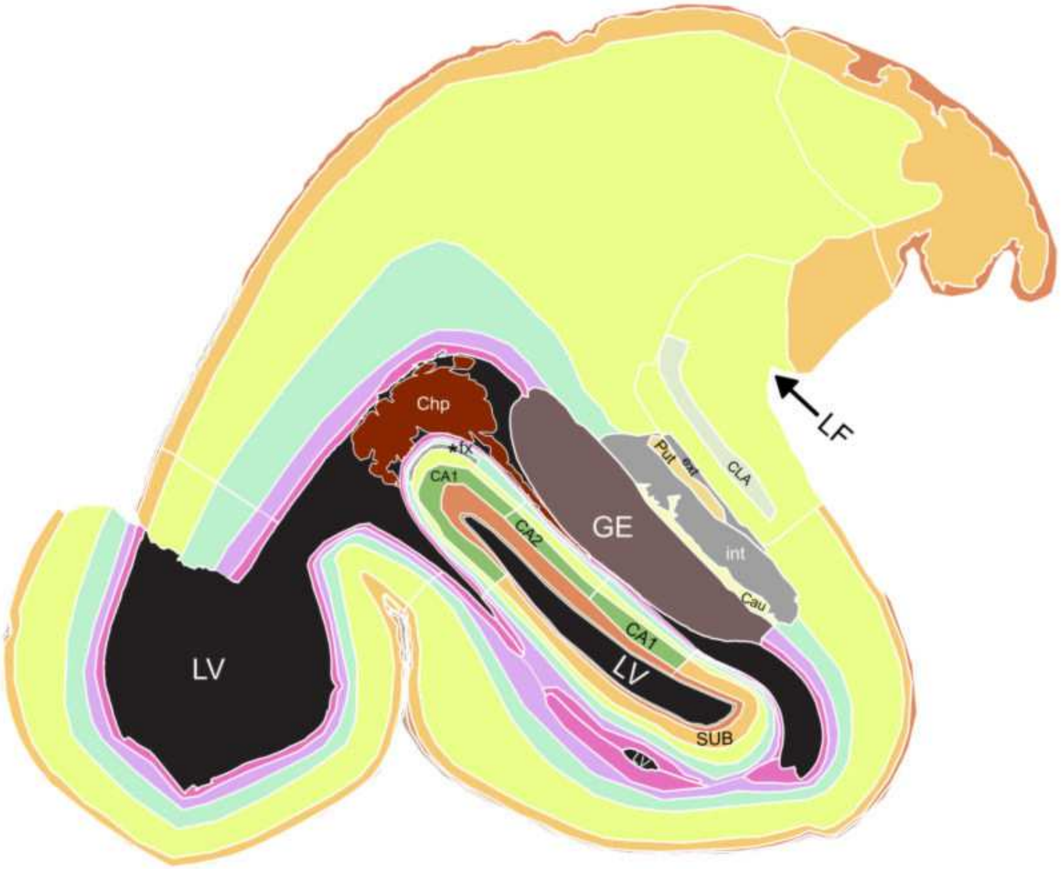

5 mm

- CA1: CA1 field [hippocampus]
- CA2: CA2 field [hippocampus]
- CLA: Claustrum
- Cau: Caudate nucleus
- Chp: Choroid plexus
- GE: Ganglionic eminence
- LV: Lateral ventricle
- Put: Putamen
- SUB: Cortical plate, subiculum
- ext: External capsule
- fx: Fornix
- int: Internal capsule
- → Lateral fissure

Age: 14 GW

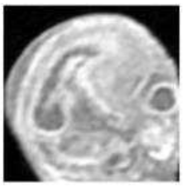

L-R Level: 8.16 mm

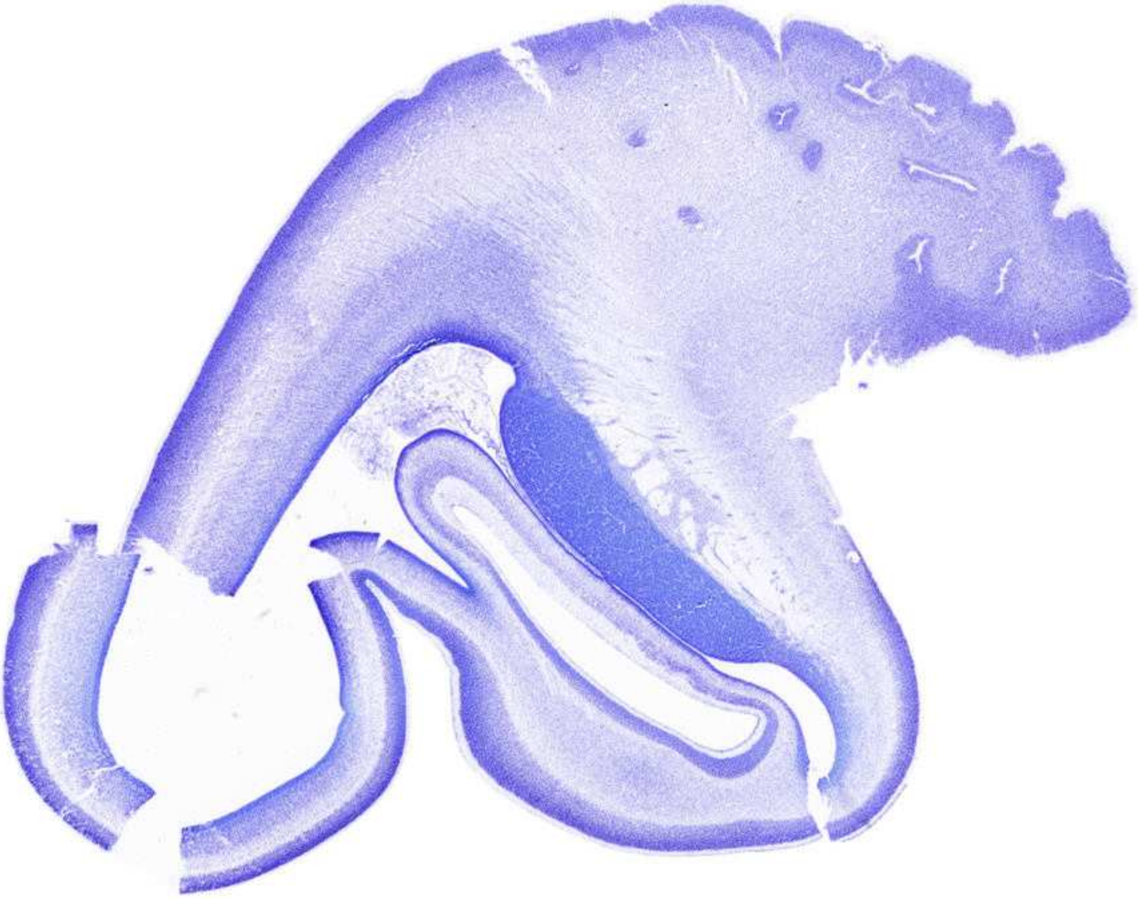

5 mm

Transient Layers

- VZ
- SVZ
- IZ
- SP
- CP
- MZ
- SGL

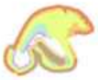

Cortical Areas

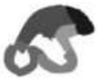

- FCTx
- PAR
- OCC
- TEMP
- INS

Age: 14 GW

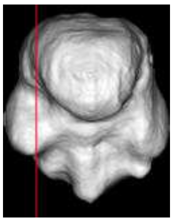

L-R Level: 8.16 mm

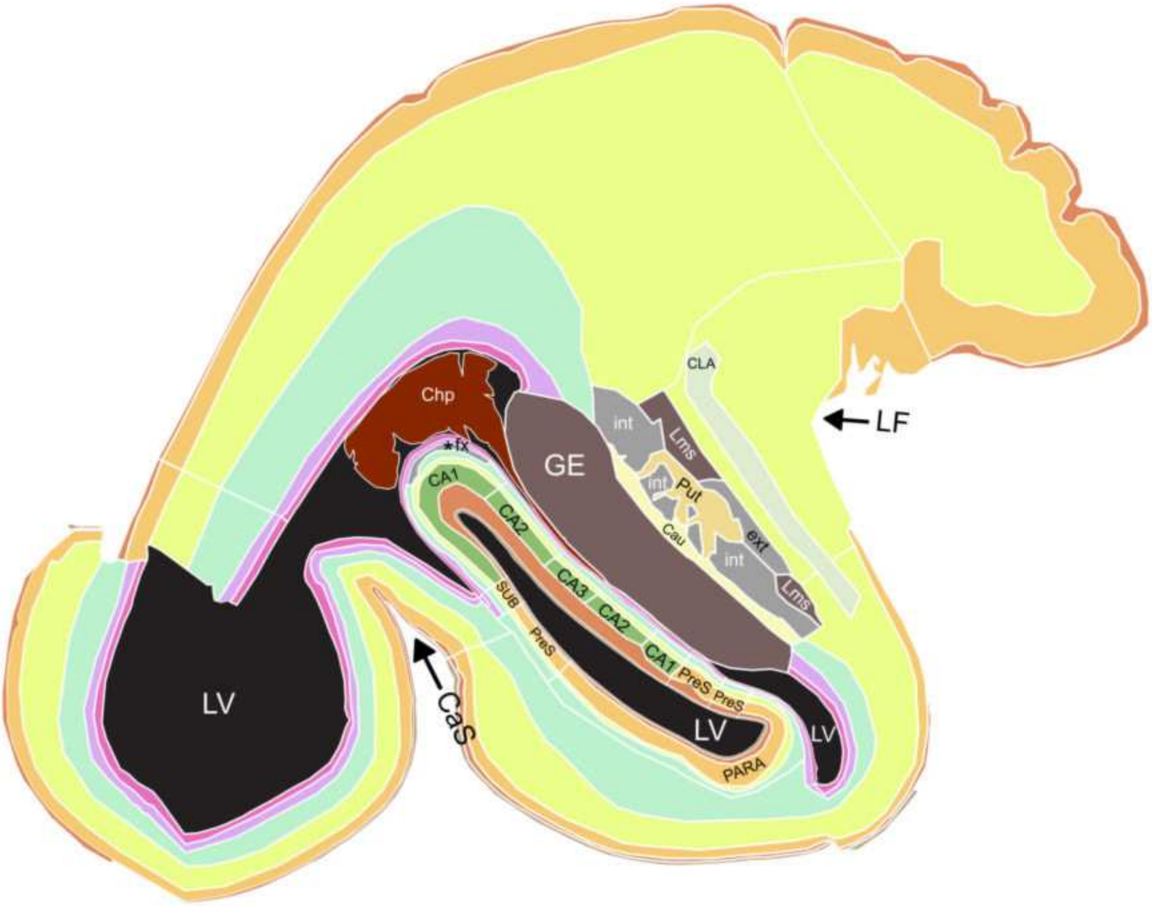

5 mm

- CA1: CA1 field [hippocampus]
- CA2: CA2 field [hippocampus]
- CA3: CA3 field [hippocampus]
- CLA: Claustrum
- Cau: Caudate nucleus
- Chp: Choroid plexus
- GE: Ganglionic eminence
- LV: Lateral ventricle
- Lms: Lateral migratory stream
- PARA: Cortical plate, parasubiculum
- PreS: Cortical plate, presubiculum
- Put: Putamen
- SUB: Cortical plate, subiculum
- ext: External capsule
- fx: Fornix
- int: Internal capsule
- → CaS: Calcarine Sulcus
- → LF: Lateral fissure

Age: 14 GW

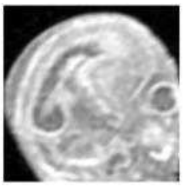

L-R Level: 7.86 mm

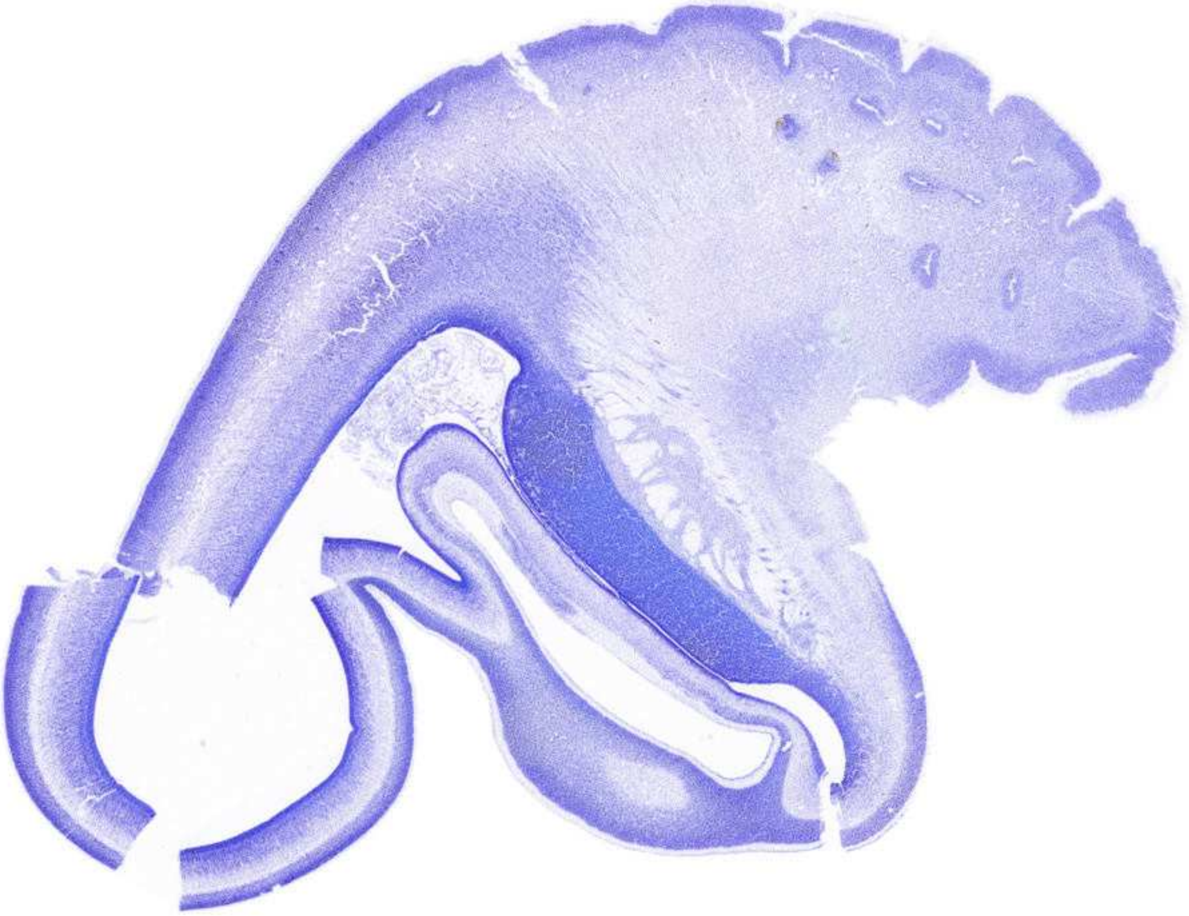

5 mm

Transient Layers

VZ
SVZ
IZ
SP
CP
MZ
SGL

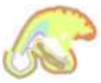

Cortical Areas

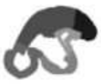

FCTx
PAR
OCC
TEMP
INS

Age: 14 GW

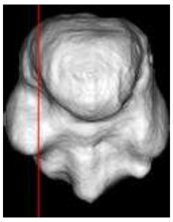

L-R Level: 7.86 mm

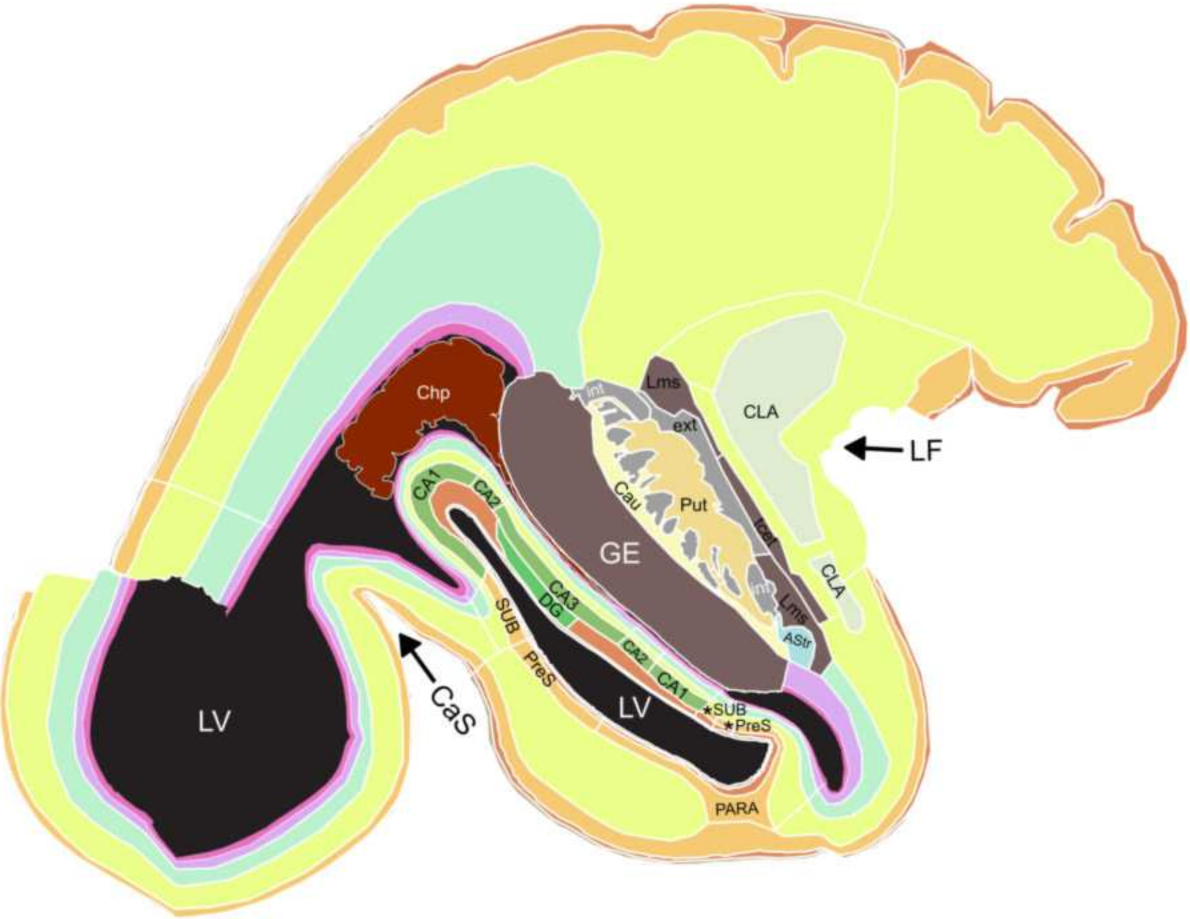

5 mm

AStr: Amygdalo-striatal area
CA1: CA1 field [hippocampus]
CA2: CA2 field [hippocampus]
CA3: CA3 field [hippocampus]
CLA: Claustrum

Cau: Caudate nucleus
Chp: Choroid plexus
DG: Dentate gyrus
GE: Ganglionic eminence
LV: Lateral ventricle

Lms: Lateral migratory stream
PARA: Cortical plate, parasubiculum
PreS: Cortical plate, presubiculum
Put: Putamen
SUB: Cortical plate, subiculum

ext: External capsule
int: Internal capsule
tcet: Transient cell zone in the external capsule
→ CaS: Calcarine Fissure
→ LF: Lateral Fissure

Age: 14 GW

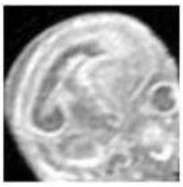

L-R Level: 7.74 mm

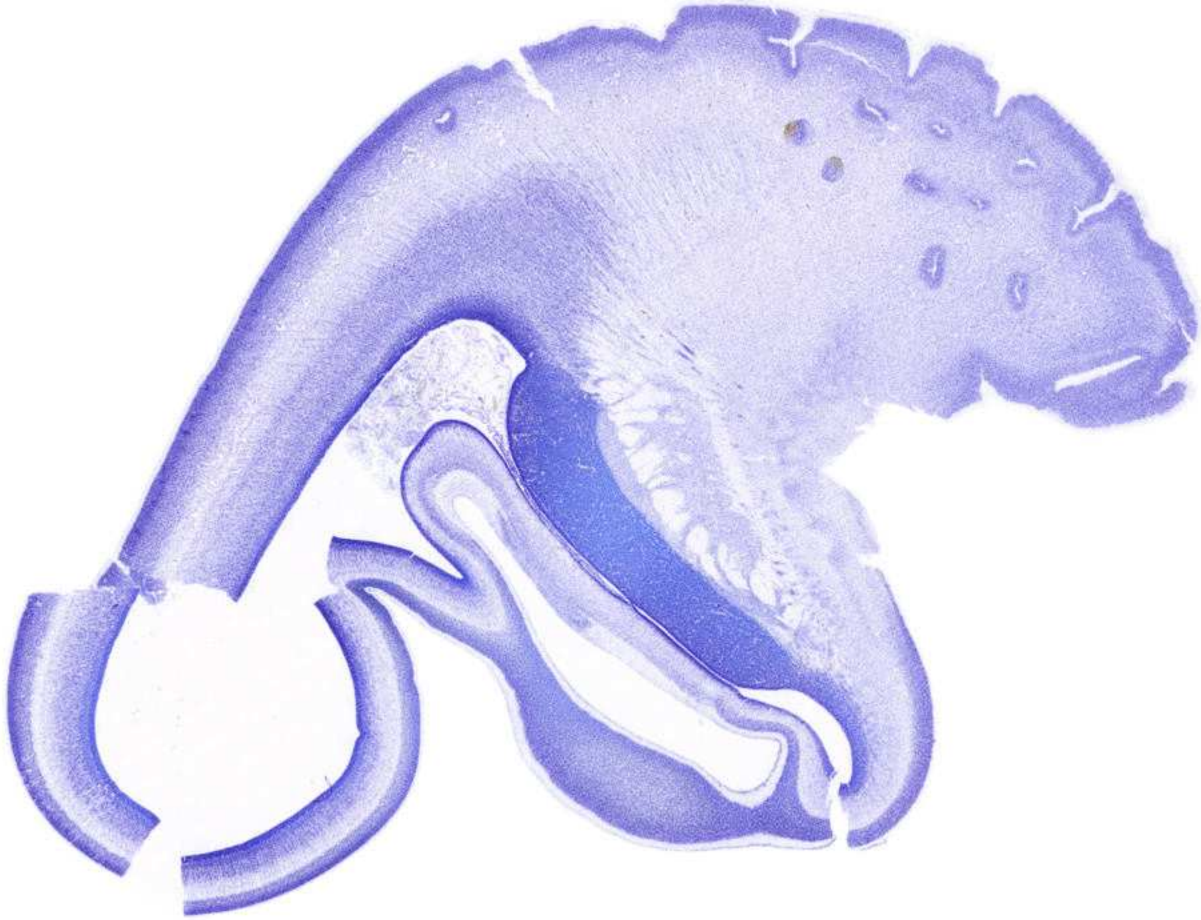

5 mm

Transient Layers

- VZ
- SVZ
- IZ
- SP
- CP
- MZ
- SGL

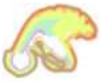

Cortical Areas

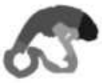

- FCTx
- PAR
- OCC
- TEMP
- INS

Age: 14 GW

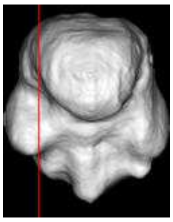

L-R Level: 7.74 mm

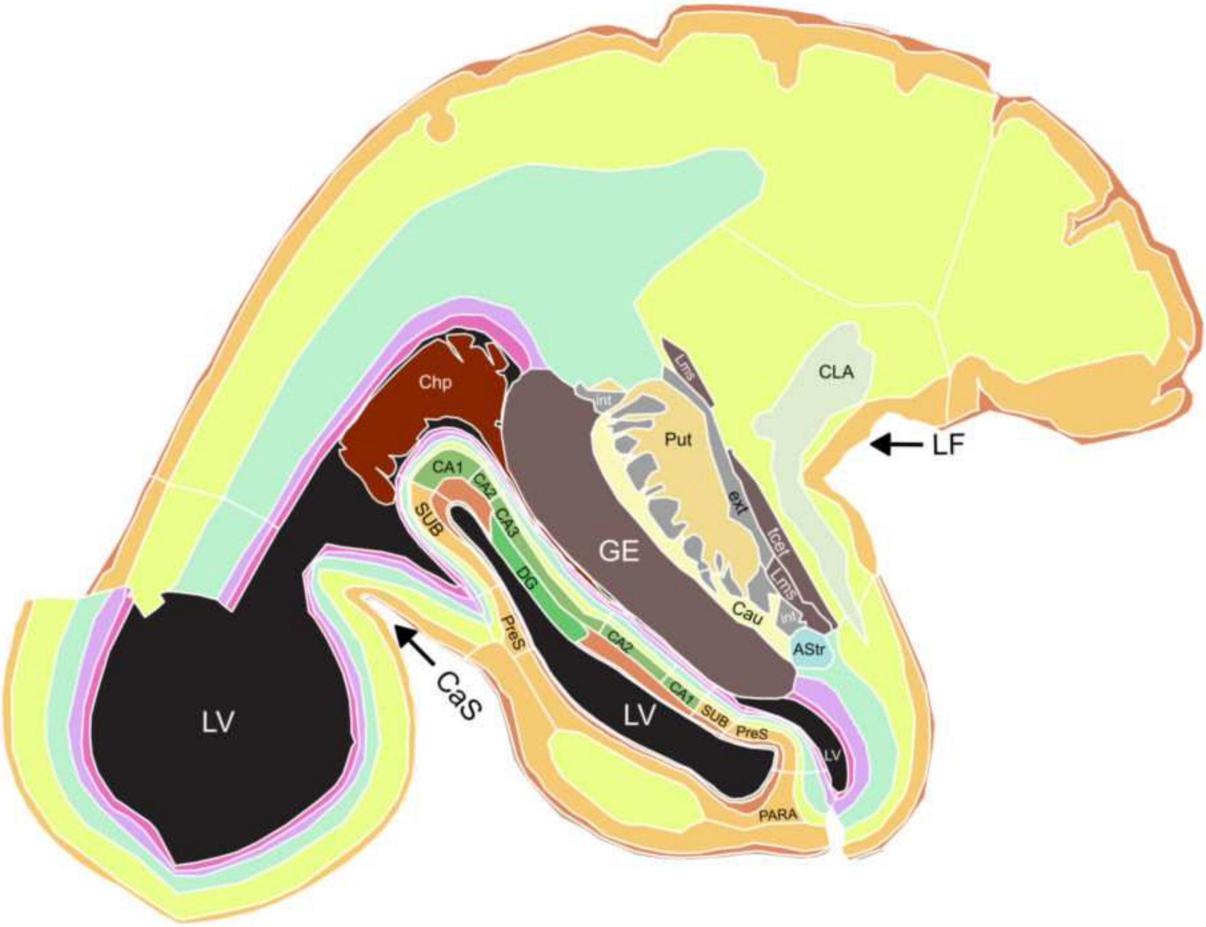

5 mm

- AStr: Amygdalo-striatal area
- CA1: CA1 field [hippocampus]
- CA2: CA2 field [hippocampus]
- CA3: CA3 field [hippocampus]
- CLA: Claustrum
- Cau: Caudate nucleus
- Chp: Choroid plexus
- DG: Dentate gyrus
- GE: Ganglionic eminence
- LV: Lateral ventricle
- Lms: Lateral migratory stream
- PARA: Cortical plate, parasubiculum
- PreS: Cortical plate, presubiculum
- Put: Putamen
- SUB: Cortical plate, subiculum
- ext: External capsule
- int: Internal capsule
- tcet: Transient cell zone in the external capsule
- → CaS: Calcarine sulcus
- → LF: Lateral fissure

Age: 14 GW

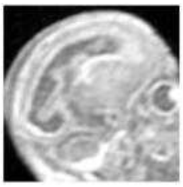

L-R Level: 7.08 mm

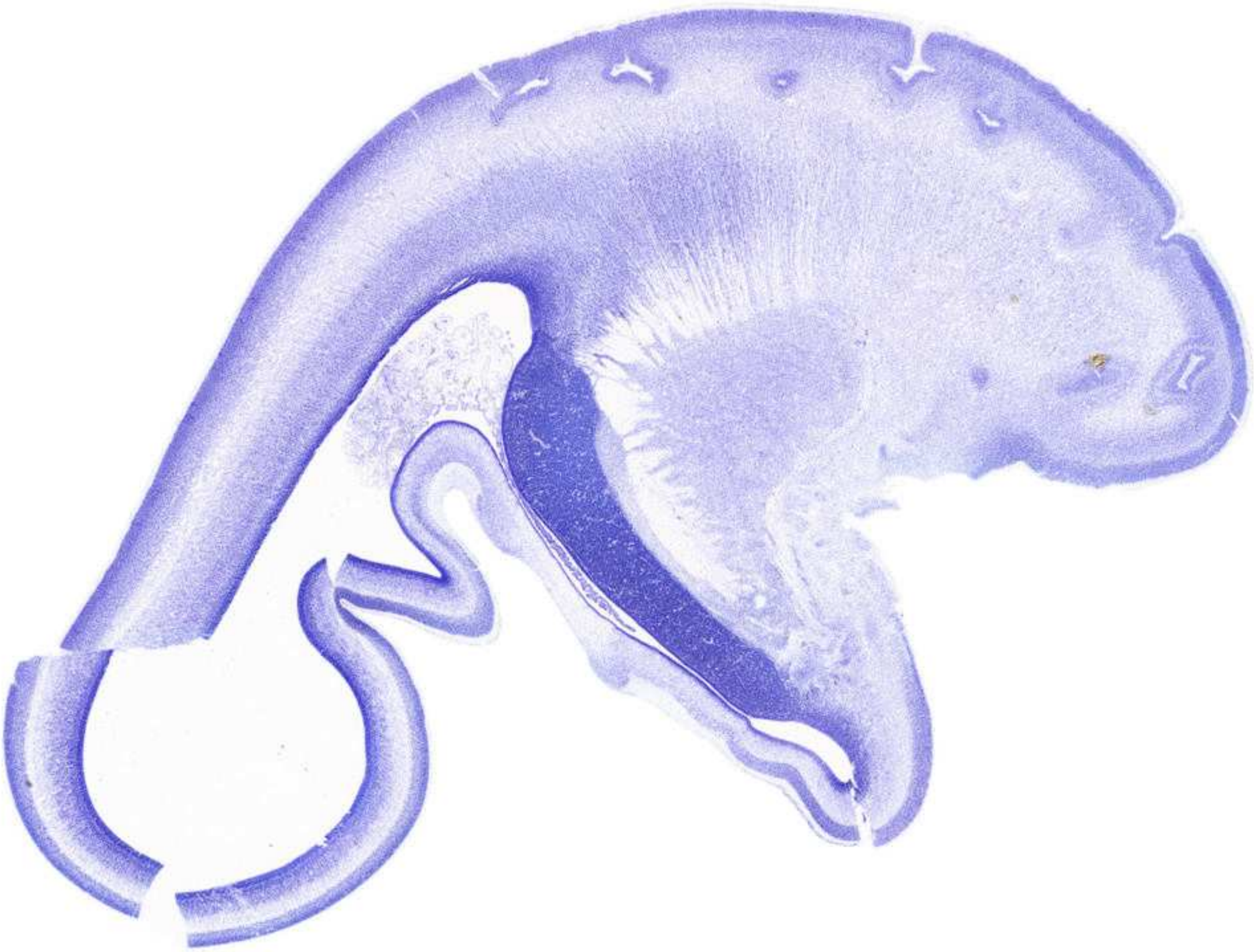

5 mm

Transient Layers

- VZ
- SVZ
- IZ
- SP
- CP
- MZ
- SGL

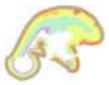

Cortical Areas

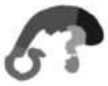

- FCTx
- PAR
- OCC
- TEMP
- INS

Age: 14 GW

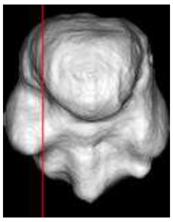

L-R Level: 7.08 mm

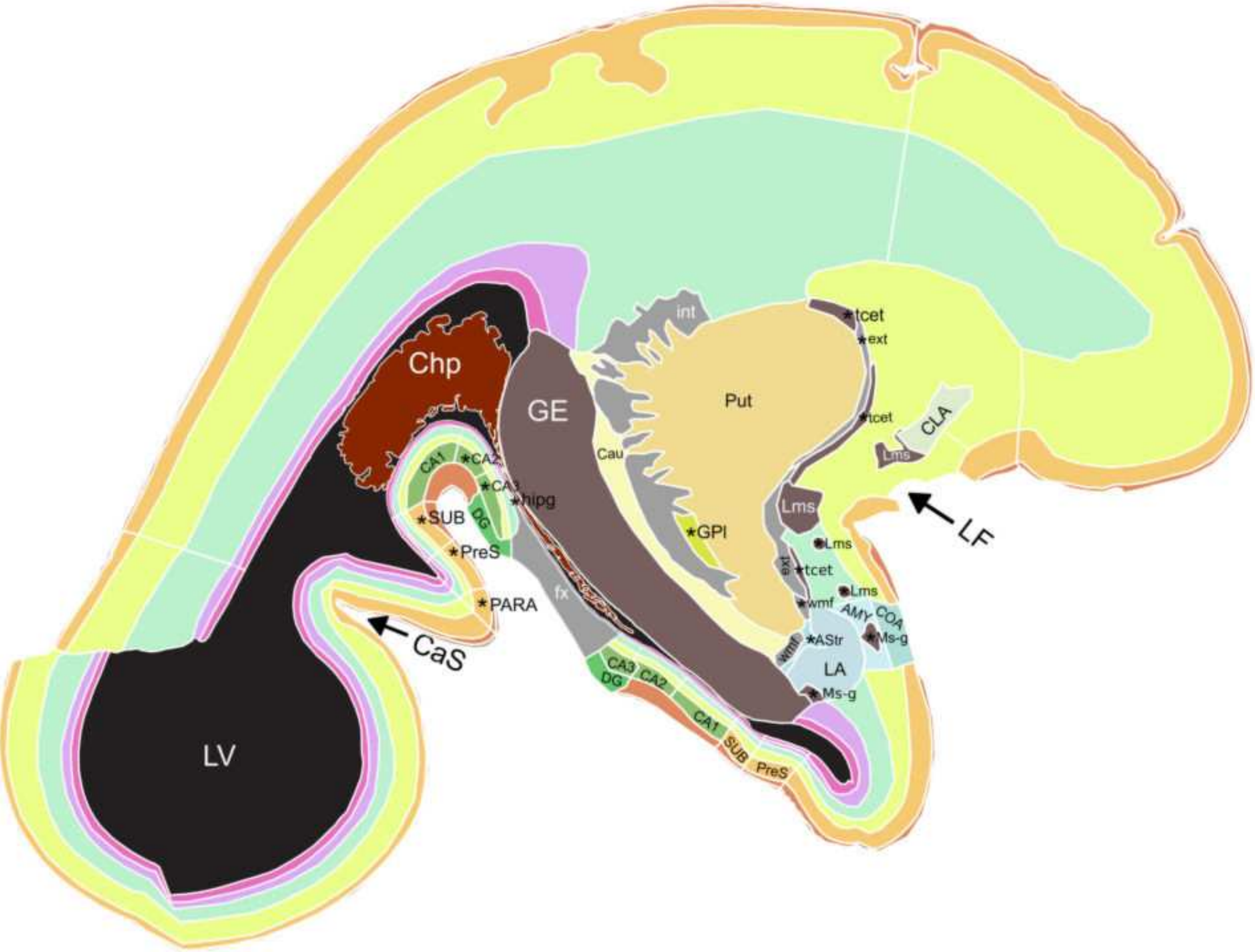

5 mm

- AMY: Amygdala
- AStr: Amygdalo-striatal area
- CA1: CA1 field [hippocampus]
- CA2: CA2 field [hippocampus]
- CA3: CA3 field [hippocampus]
- CLA: Claustrum
- COA: Cortical nucleus [amygdala]
- Cau: Caudate nucleus
- Chp: Choroid plexus
- DG: Dentate gyrus
- GE: Ganglionic eminence
- GPl: Globus pallidus lateral segment
- LA: Lateral nucleus [amygdala]
- LV: Lateral ventricle
- Lms: Lateral migratory stream
- Ms-g: Migratory stream, general
- PARA: Cortical plate, parasubiculum
- PreS: Cortical plate, presubiculum
- Put: Putamen
- SUB: Cortical plate, subiculum
- ext: External capsule
- fx: Fornix
- hipg: Hippocampal glioepithelium/ependyma
- int: Internal capsule
- tcet: Transient cell zone in the external capsule
- wmf: White matter fibers
- → CaS: Calcarine sulcus
- → LF: Lateral fissure

Age: 14 GW

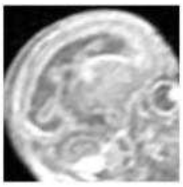

L-R Level: 6.66 mm

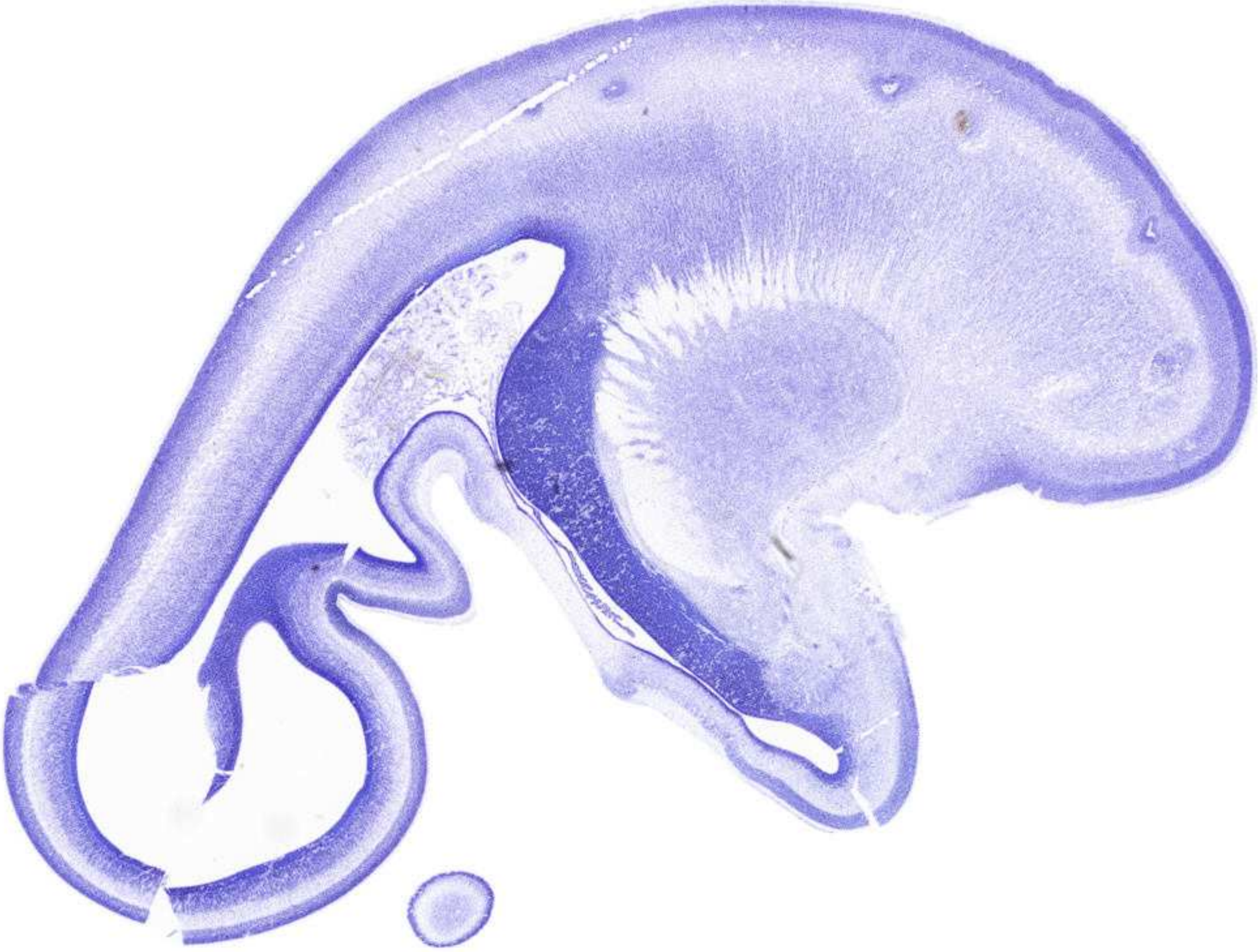

5 mm

Transient Layers

- VZ
- SVZ
- IZ
- SP
- CP
- MZ
- SGL

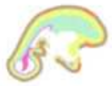

Cortical Areas

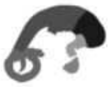

- FCTx
- PAR
- OCC
- TEMP
- INS

Age: 14 GW

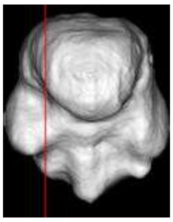

L-R Level: 6.66 mm

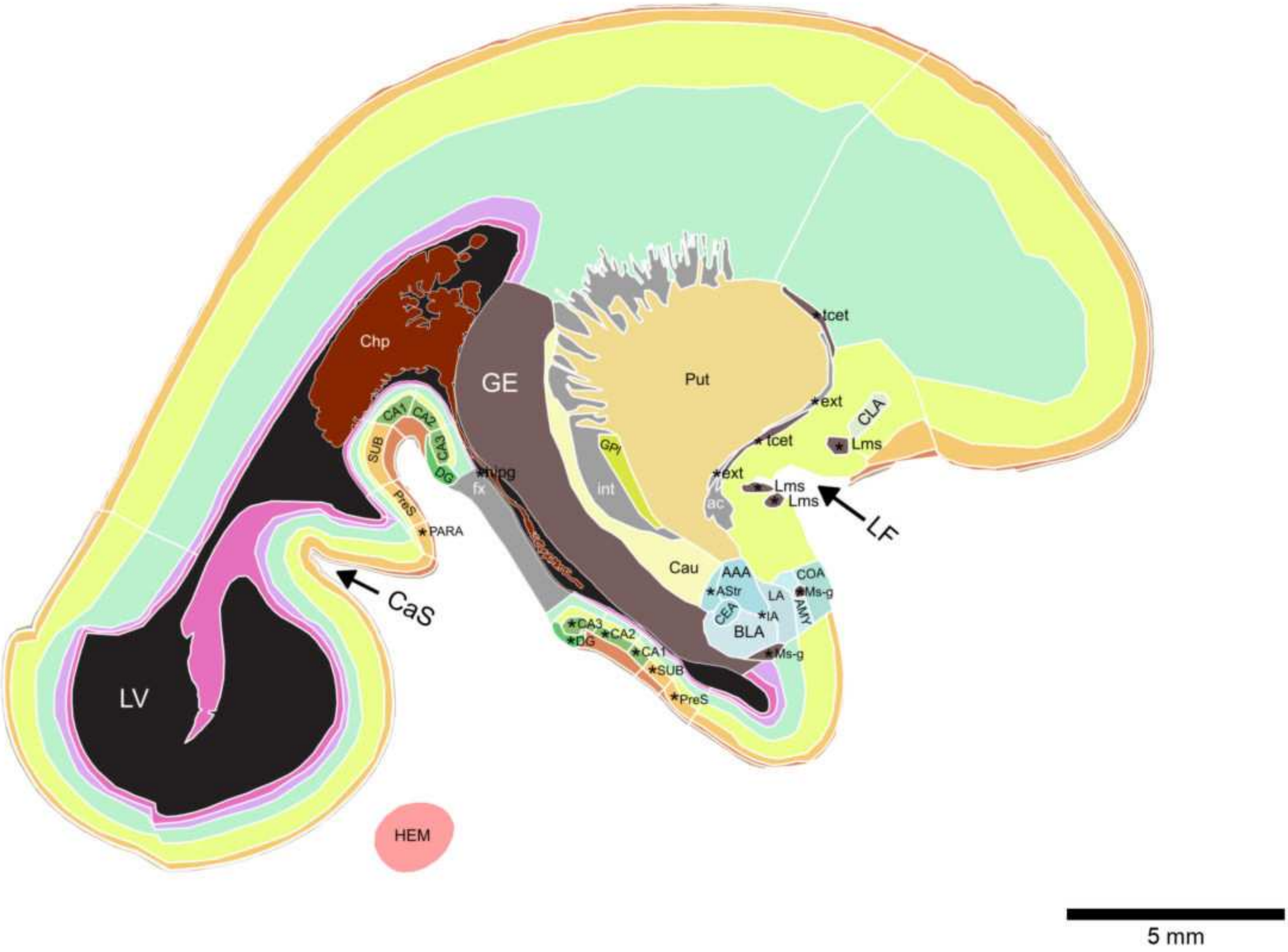

- AAA: Anterior amygdaloid area
- AMY: Amygdala
- AStr: Amygdalo-striatal area
- BL: Basal nucleus [amygdala]
- CA1: CA1 field [hippocampus]
- CA2: CA2 field [hippocampus]
- CA3: CA3 field [hippocampus]
- CEA: Central nucleus [amygdala]
- CLA: Claustrum
- COA: Cortical nucleus [amygdala]
- Cau: Caudate nucleus
- Chp: Choroid plexus
- DG: Dentate gyrus
- GE: Ganglionic eminence
- GPl: Globus pallidus lateral segment
- HEM: Cerebellar hemispheres
- IA: Intercalated cell groups [amygdala]
- LA: Lateral nucleus [amygdala]
- LV: Lateral ventricle
- Lms: Lateral migratory stream
- Ms-g: Migratory stream, general
- PARA: Cortical plate, parasubiculum
- PreS: Cortical plate, presubiculum
- Put: Putamen
- SUB: Cortical plate, subiculum
- ac: Anterior commissure
- ext: External capsule
- fx: Fornix
- hipg: Hippocampal glioepithelium/ependyma
- int: Internal capsule
- tcet: Transient cell zone in the external capsule
- → CaS: Calcarine sulcus
- → LF: Lateral fissure

Age: 14 GW

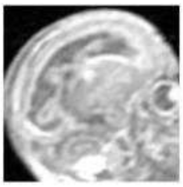

L-R Level: 6.48 mm

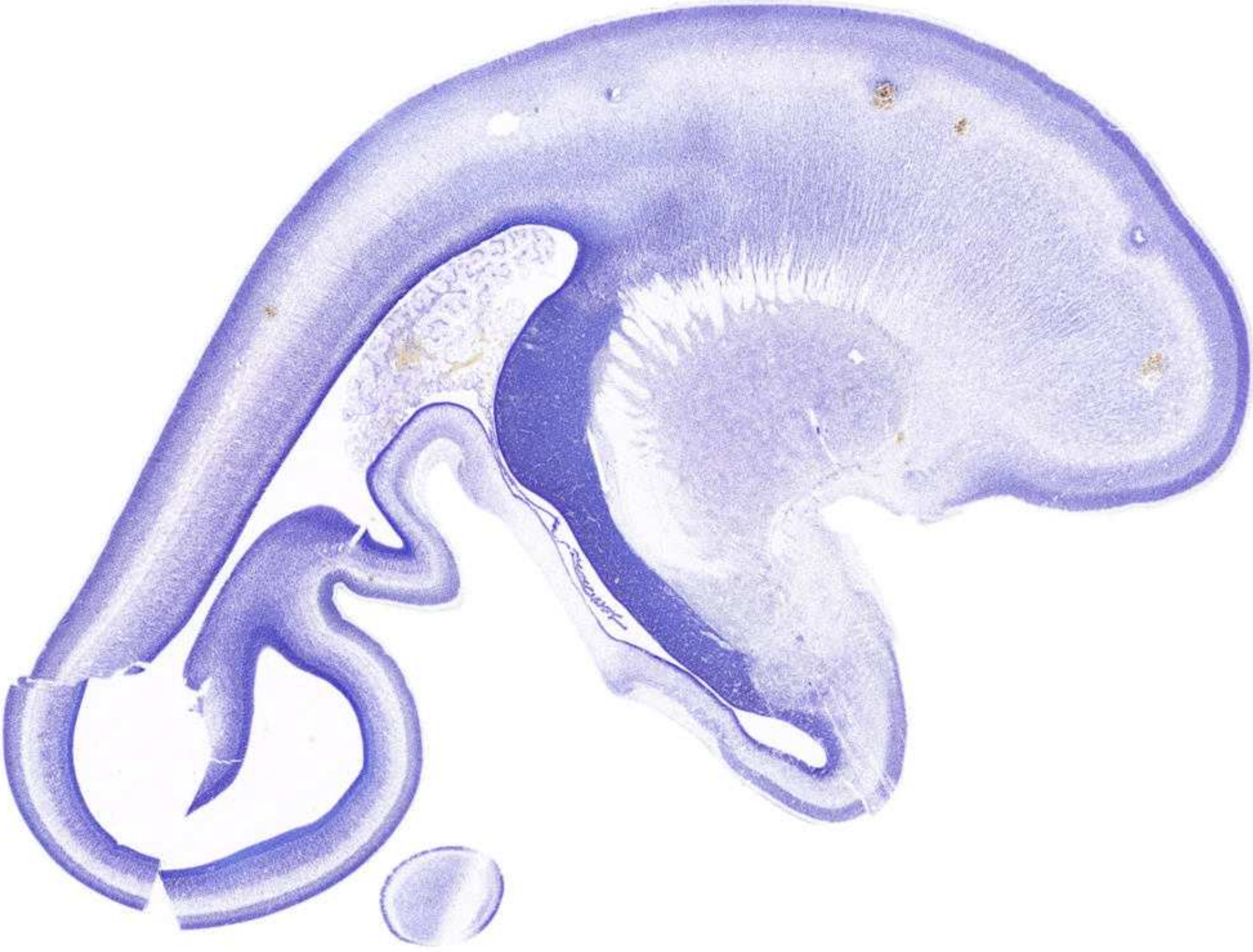

5 mm

Transient Layers

- VZ
- SVZ
- IZ
- SP
- CP
- MZ
- SGL

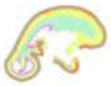

Cortical Areas

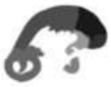

- FCTx
- ORB
- PAR
- OCC
- TEMP
- INS

Age: 14 GW

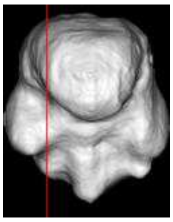

L-R Level: 6.48 mm

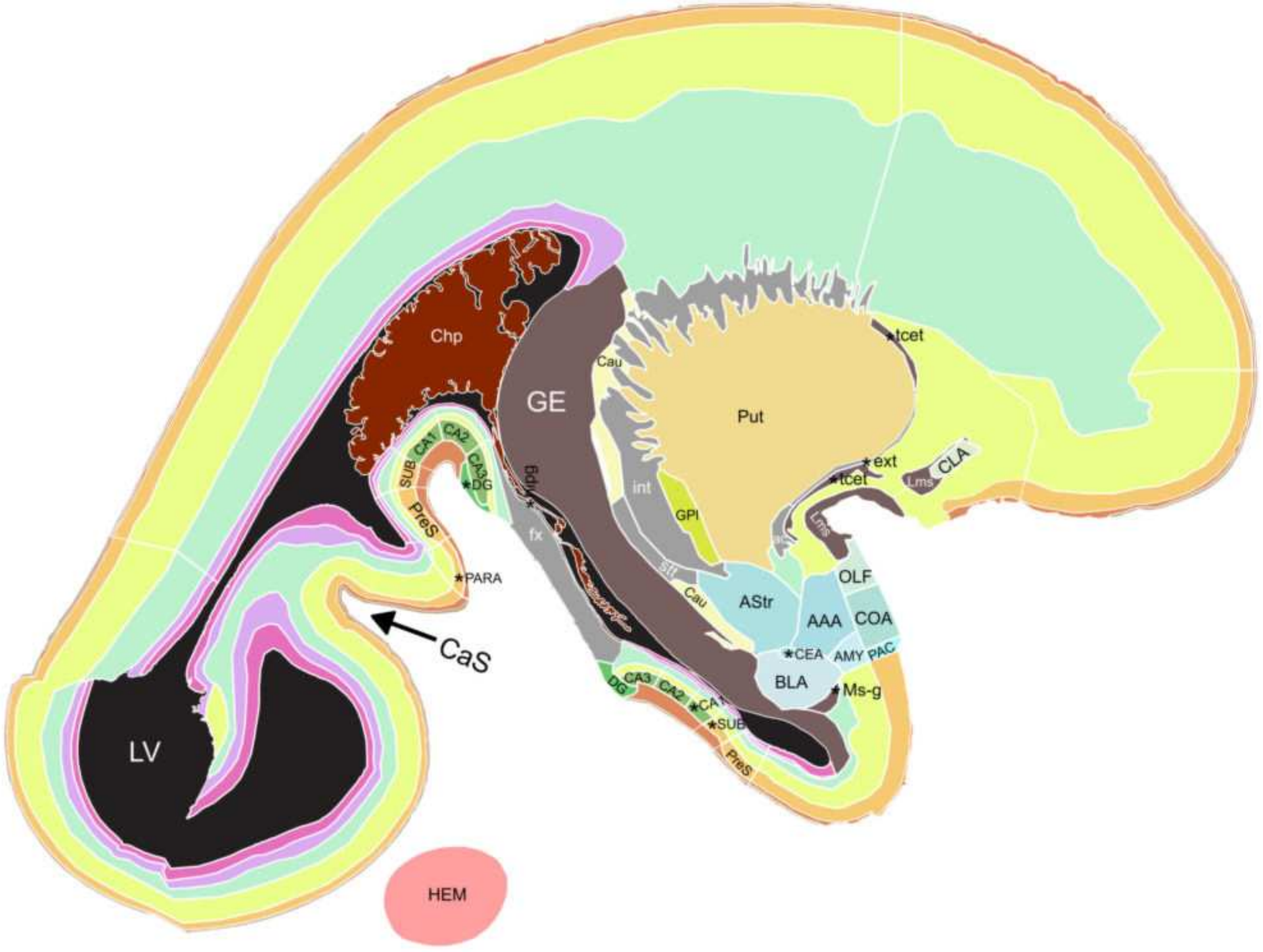

5 mm

- AAA: Anterior amygdaloid area
- AMY: Amygdala
- AStr: Amygdalo-striatal area
- BL: Basal nucleus [amygdala]
- CA1: CA1 field [hippocampus]
- CA2: CA2 field [hippocampus]
- CA3: CA3 field [hippocampus]
- CEA: Central nucleus [amygdala]
- CLA: Claustrum
- COA: Cortical nucleus [amygdala]
- Cau: Caudate nucleus
- Chp: Choroid plexus
- DG: Dentate gyrus
- GE: Ganglionic eminence
- GPl: Globus pallidus lateral segment
- HEM: Cerebellar hemispheres
- LV: Lateral ventricle
- Lms: Lateral migratory stream
- Ms-g: Migratory stream, general
- PARA: Cortical plate, parasubiculum
- PreS: Cortical plate, presubiculum
- Put: Putamen
- SUB: Cortical plate, subiculum
- ac: Anterior commissure
- ext: External capsule
- fx: Fornix
- hipg: Hippocampal glioepithelium/ependyma
- int: Internal capsule
- stt: Stria terminalis
- tcet: Transient cell zone in the external capsule
- → CaS: Calcarine sulcus

Age: 14 GW

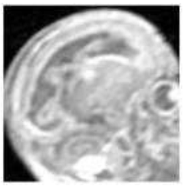

L-R Level: 6.42 mm

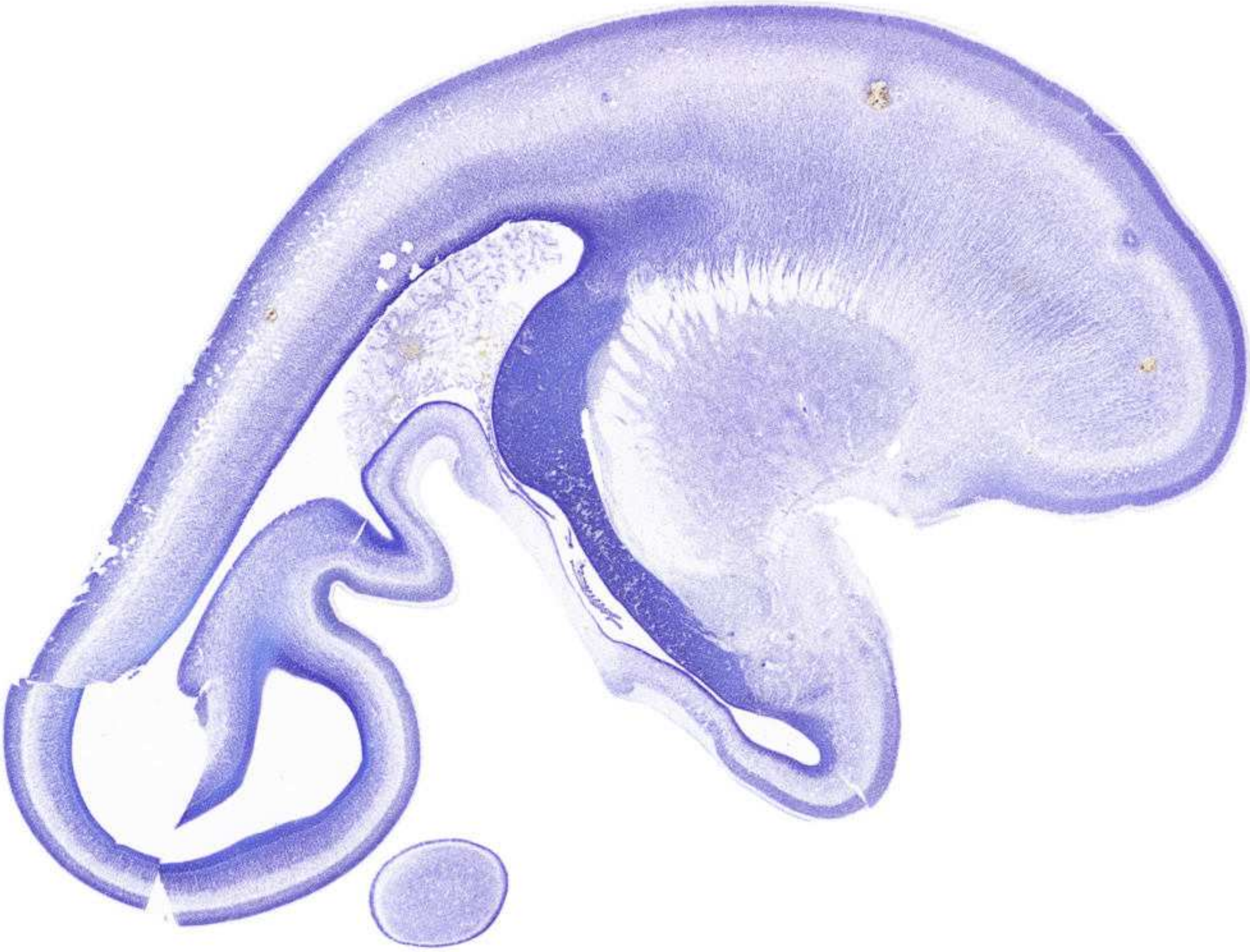

5 mm

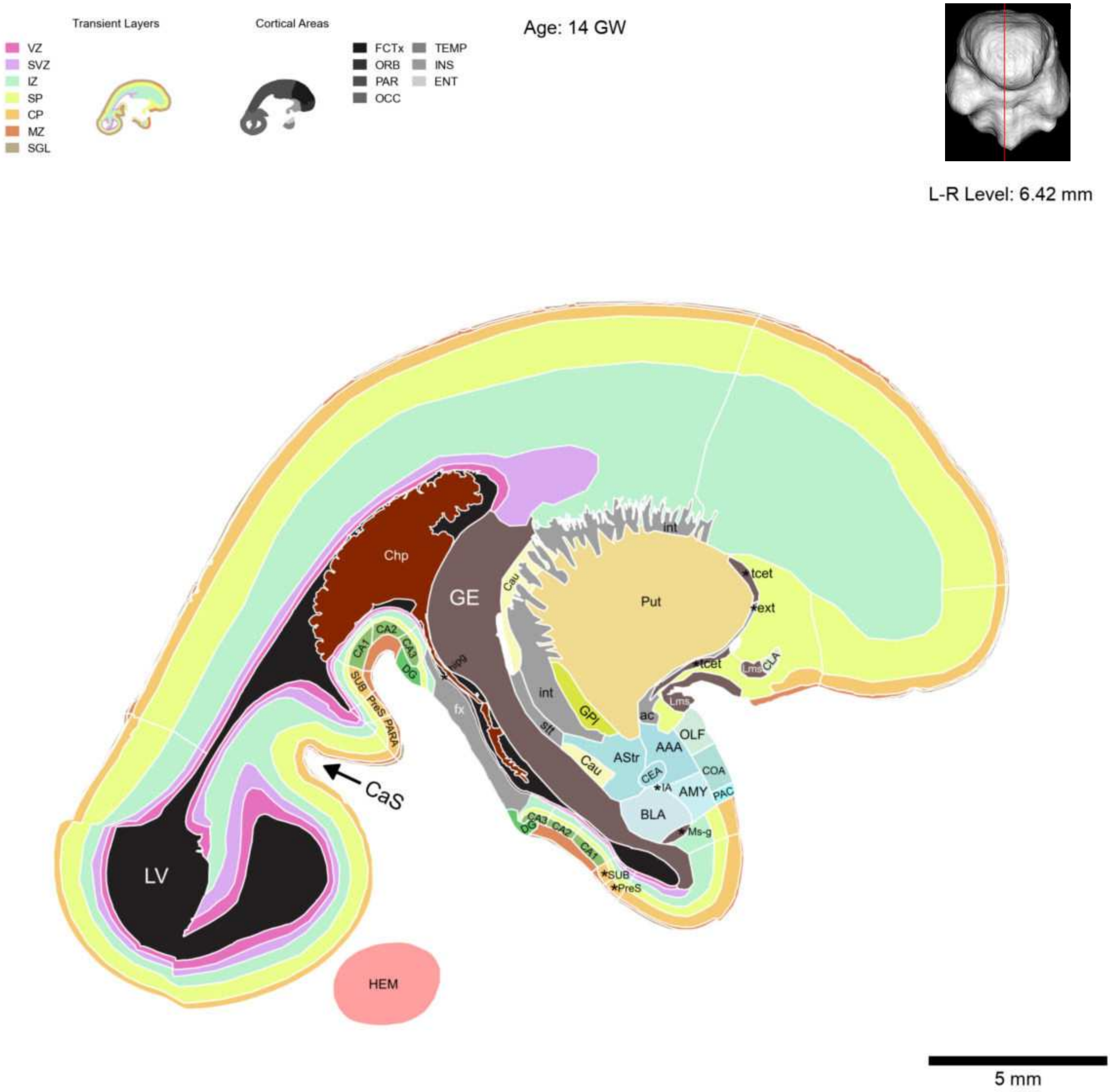

- AAA: Anterior amygdaloid area
- AMY: Amygdala
- AStr: Amygdalo-striatal area
- BL: Basal nucleus [amygdala]
- CA1: CA1 field [hippocampus]
- CA2: CA2 field [hippocampus]
- CA3: CA3 field [hippocampus]
- CEA: Central nucleus [amygdala]
- CLA: Claustrum
- COA: Cortical nucleus [amygdala]
- Cau: Caudate nucleus
- Chp: Choroid plexus
- DG: Dentate gyrus
- GE: Ganglionic eminence
- GPl: Globus pallidus lateral segment
- HEM: Cerebellar hemispheres
- IA: Intercalated cell groups [amygdala]
- LV: Lateral ventricle
- Lms: Lateral migratory stream
- Ms-g: Migratory stream, general
- PARA: Cortical plate, parasubiculum
- PreS: Cortical plate, presubiculum
- Put: Putamen
- SUB: Cortical plate, subiculum
- ac: Anterior commissure
- ext: External capsule
- fx: Fornix
- hipg: Hippocampal glioepithelium/ependyma
- int: Internal capsule
- stt: Stria terminalis
- tcet: Transient cell zone in the external capsule
- → CaS: Calcarine sulcus

Age: 14 GW

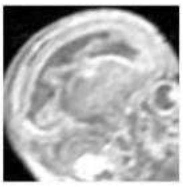

L-R Level: 6.18 mm

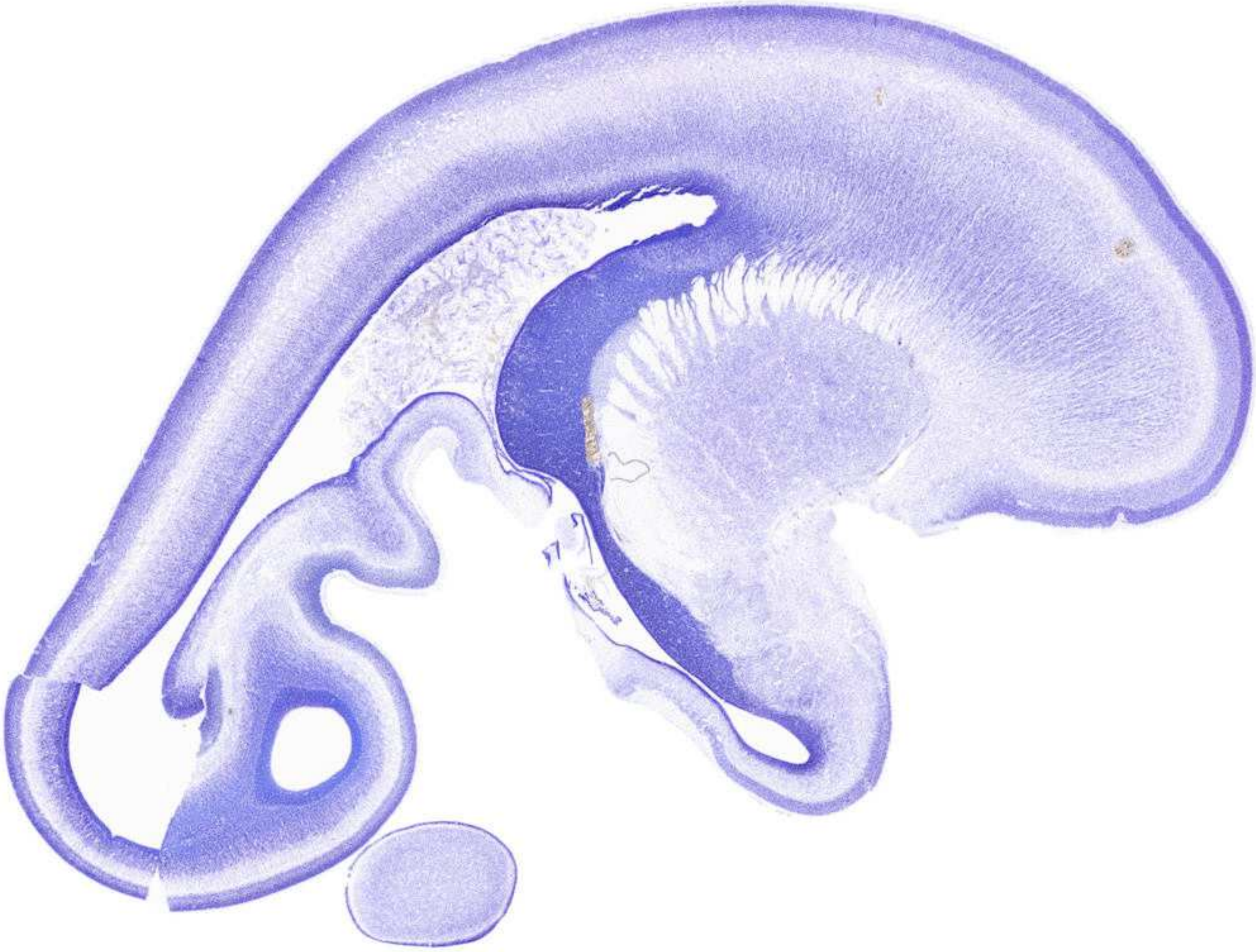

5 mm

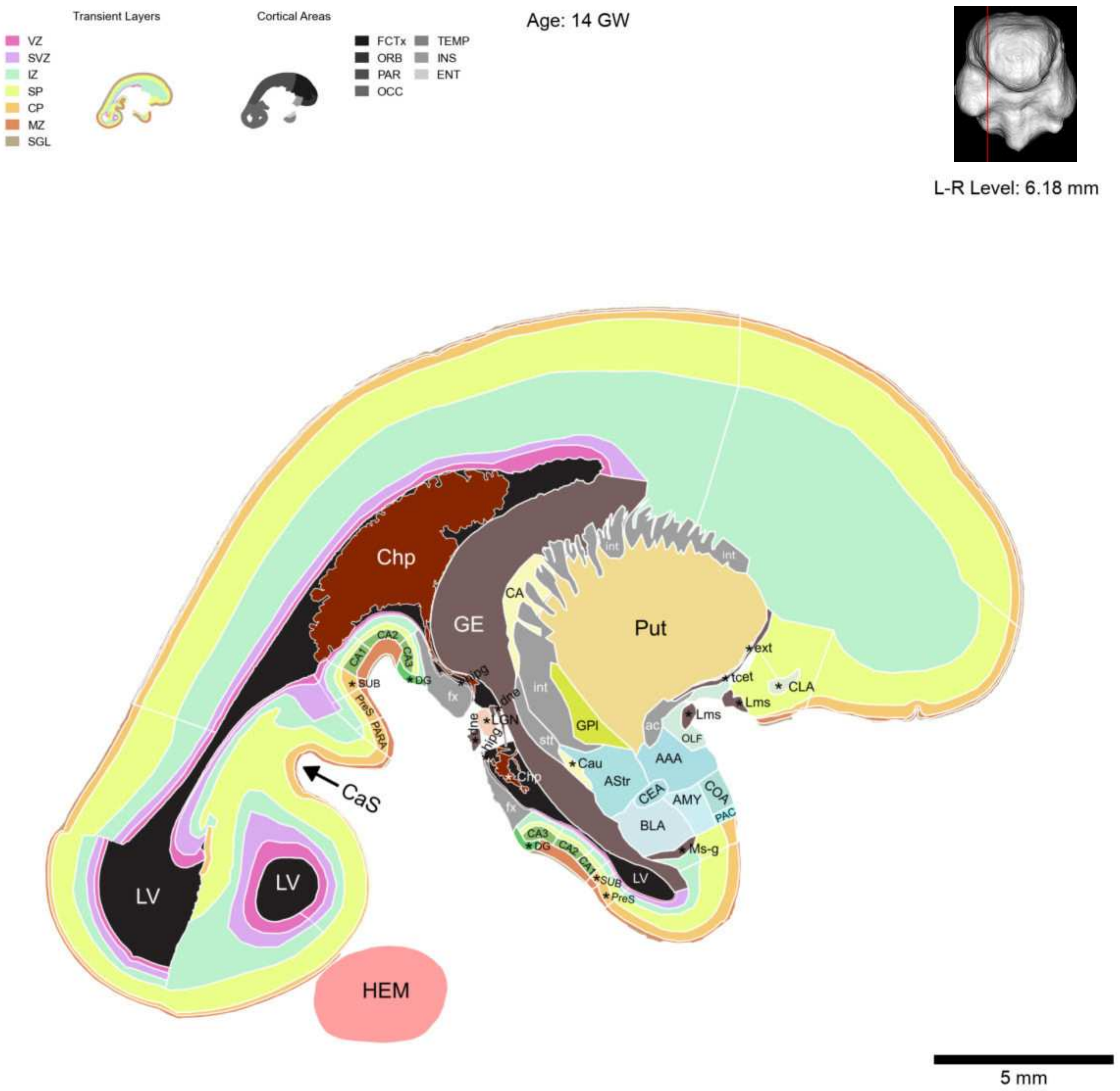

AAA: Anterior amygdaloid area
AMY: Amygdala
AStr: Amygdalo-striatal area
BL: Basal nucleus [amygdala]
CA1: CA1 field [hippocampus]
CA2: CA2 field [hippocampus]
CA3: CA3 field [hippocampus]
CEA: Central nucleus [amygdala]
CLA: Claustrum
COA: Cortical nucleus [amygdala]
Cau: Caudate nucleus
Chp: Choroid plexus
DG: Dentate gyrus
GE: Ganglionic eminence
GPl: Globus pallidus lateral segment
HEM: Cerebellar hemispheres
LGN: Lateral geniculate nucleus
LV: Lateral ventricle
Lms: Lateral migratory stream
Ms-g: Migratory stream, general
PARA: Cortical plate, parasubiculum
PreS: Cortical plate, presubiculum
Put: Putamen
SUB: Cortical plate, subiculum
ac: Anterior commissure
dne: Diencephalic neuroepithelium
ext: External capsule
fx: Fornix
hipg: Hippocampal glioepithelium/ependyma
int: Internal capsule
stt: Stria terminalis
tcet: Transient cell zone in the external capsule
→ CaS: Calcarine sulcus

Age: 14 GW

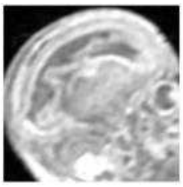

L-R Level: 6.06 mm

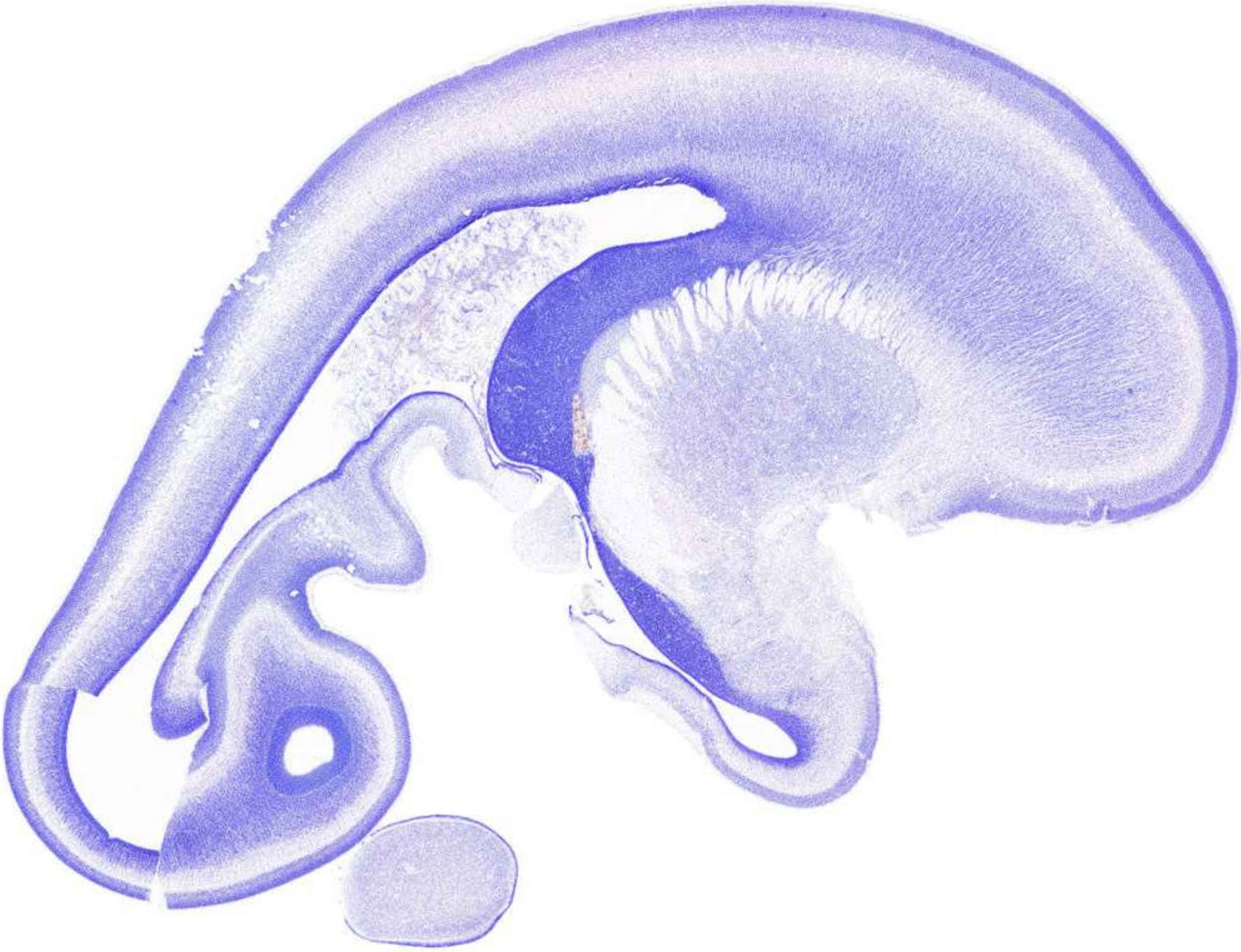

5 mm

Transient Layers

VZ
SVZ
IZ
SP
CP
MZ
SGL

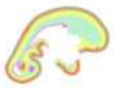

Cortical Areas

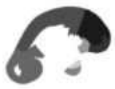

FCTx
ORB
PAR
OCC
TEMP
INS
ENT

Age: 14 GW

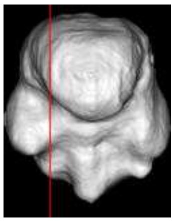

L-R Level: 6.06 mm

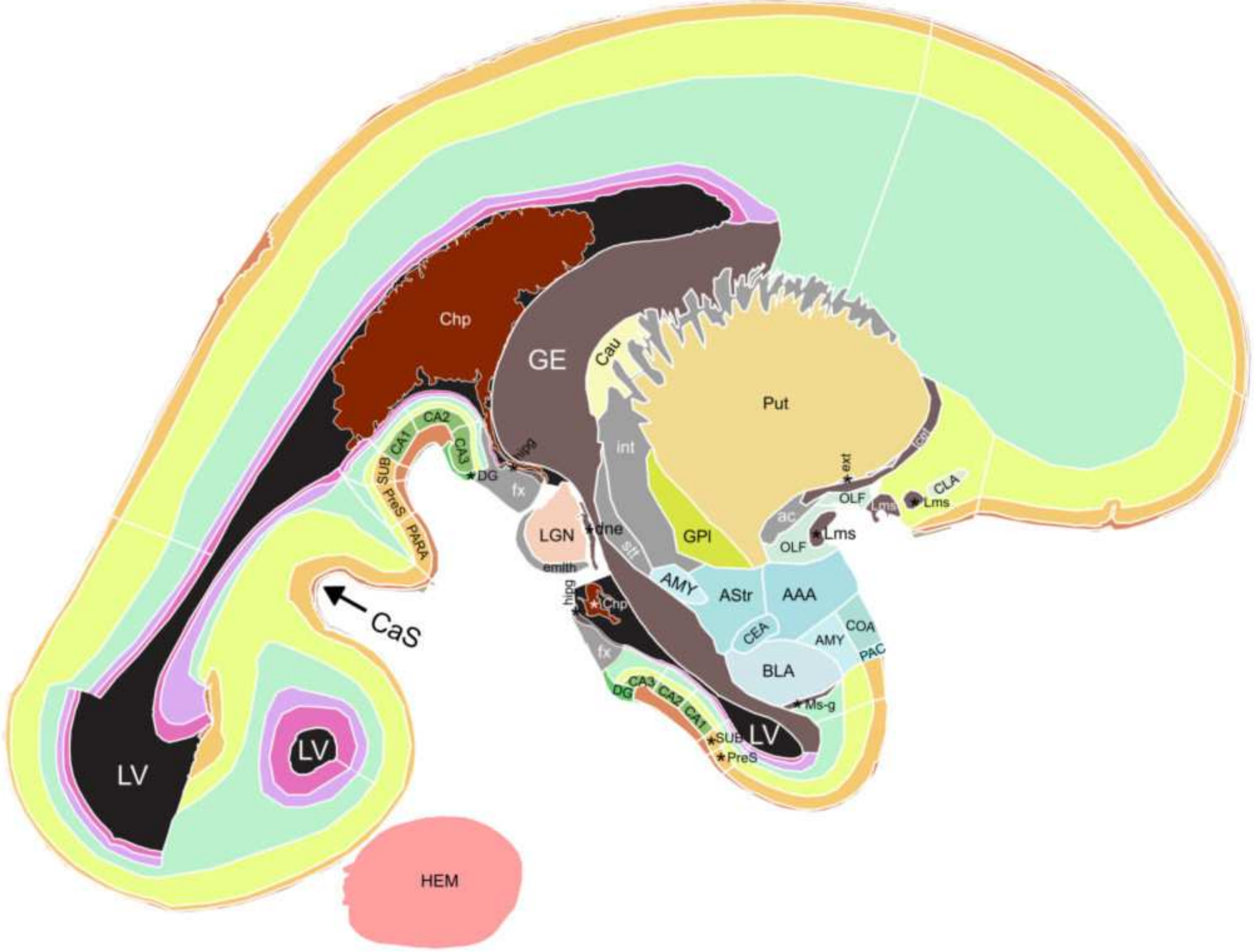

5 mm

AAA: Anterior amygdaloid area
AMY: Amygdala
AStr: Amygdalo-striatal area
BL: Basal nucleus [amygdala]
CA1: CA1 field [hippocampus]
CA2: CA2 field [hippocampus]
CA3: CA3 field [hippocampus]
CEA: Central nucleus [amygdala]
CLA: Claustrum
COA: Cortical nucleus [amygdala]
Cau: Caudate nucleus
Chp: Choroid plexus
DG: Dentate gyrus
GE: Ganglionic eminence
GPl: Globus pallidus lateral segment
HEM: Cerebellar hemispheres
LGN: Lateral geniculate nucleus
LV: Lateral ventricle
Lms: Lateral migratory stream
Ms-g: Migratory stream, general
PARA: Cortical plate, parasubiculum
PreS: Cortical plate, presubiculum
Put: Putamen
SUB: Cortical plate, subiculum
ac: Anterior commissure
dne: Diencephalic neuroepithelium
emlth: External medullary lamina [thalamus]
ext: External capsule
fx: Fornix
hipg: Hippocampal glioepithelium/ependyma
int: Internal capsule
stt: Stria terminalis
tcet: Transient cell zone in the external capsule
→ CaS: Calcarine sulcus

Age: 14 GW

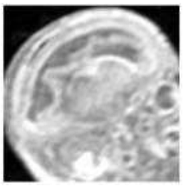

L-R Level: 5.7 mm

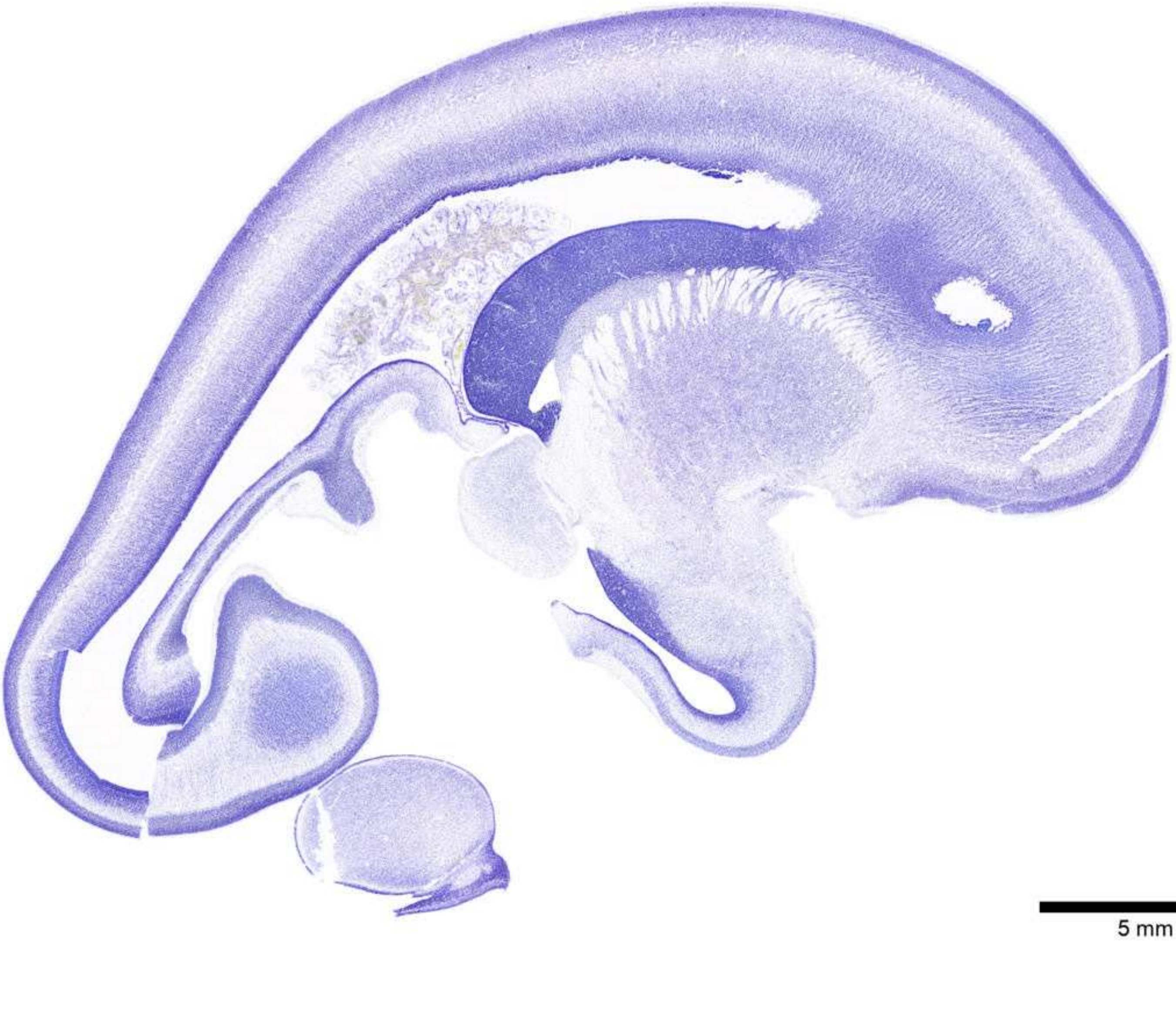

5 mm

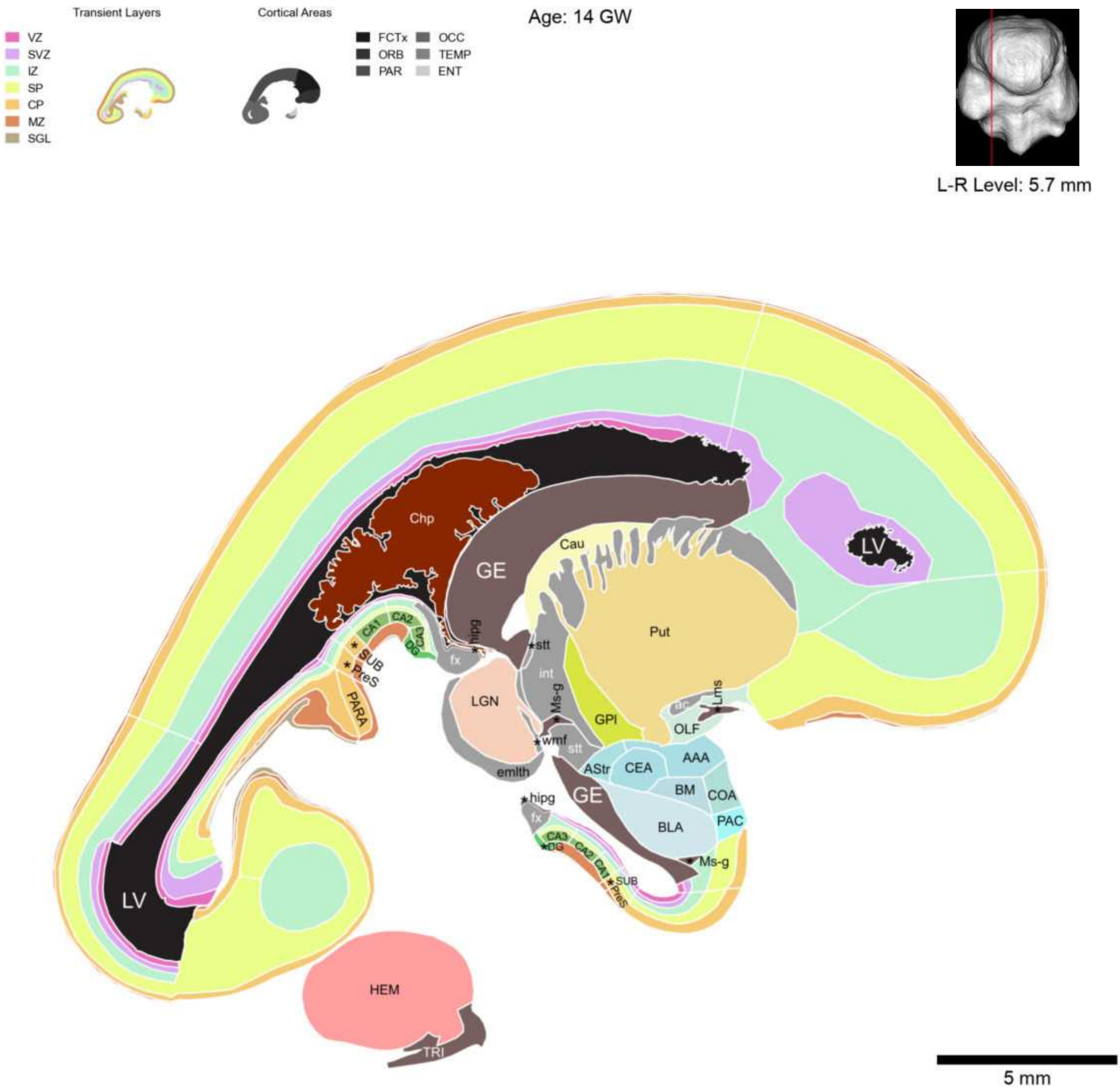

- AAA: Anterior amygdaloid area
- AStr: Amygdalo-striatal area
- BL: Basal nucleus [amygdala]
- BM: Basomedial nucleus [amygdala]
- CA1: CA1 field [hippocampus]
- CA2: CA2 field [hippocampus]
- CA3: CA3 field [hippocampus]
- CEA: Central nucleus [amygdala]
- COA: Cortical nucleus [amygdala]
- Cau: Caudate nucleus
- Chp: Choroid plexus
- DG: Dentate gyrus
- GE: Ganglionic eminence
- GPl: Globus pallidus lateral segment
- HEM: Cerebellar hemispheres
- LGN: Lateral geniculate nucleus
- LV: Lateral ventricle
- Lms: Lateral migratory stream
- Ms-g: Migratory stream, general
- PARA: Cortical plate, parasubiculum
- PreS: Cortical plate, presubiculum
- Put: Putamen
- SUB: Cortical plate, subiculum
- TRI: Germinal trigone
- ac: Anterior commissure
- emlth: External medullary lamina [thalamus]
- fx: Fornix
- hipg: Hippocampal glioepithelium/ependyma
- int: Internal capsule
- stt: Stria terminalis
- wmf: White matter fibers

Age: 14 GW

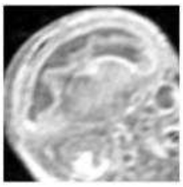

L-R Level: 5.58 mm

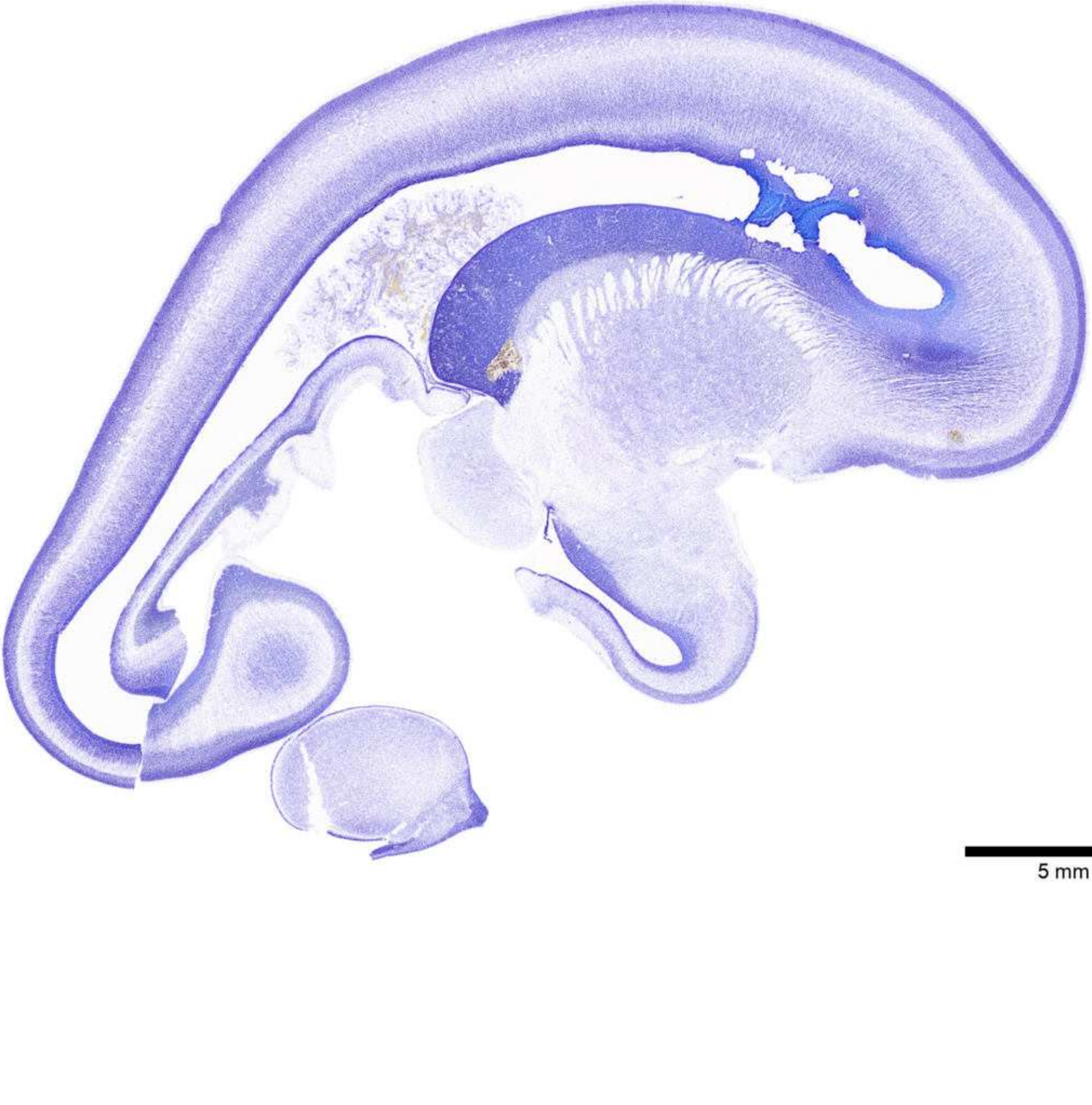

5 mm

Transient Layers

VZ
SVZ
IZ
SP
CP
MZ
SGL

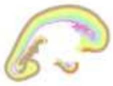

Cortical Areas

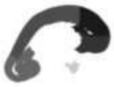

FCTx
ORB
PAR
OCC
ENT

Age: 14 GW

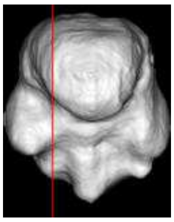

L-R Level: 5.58 mm

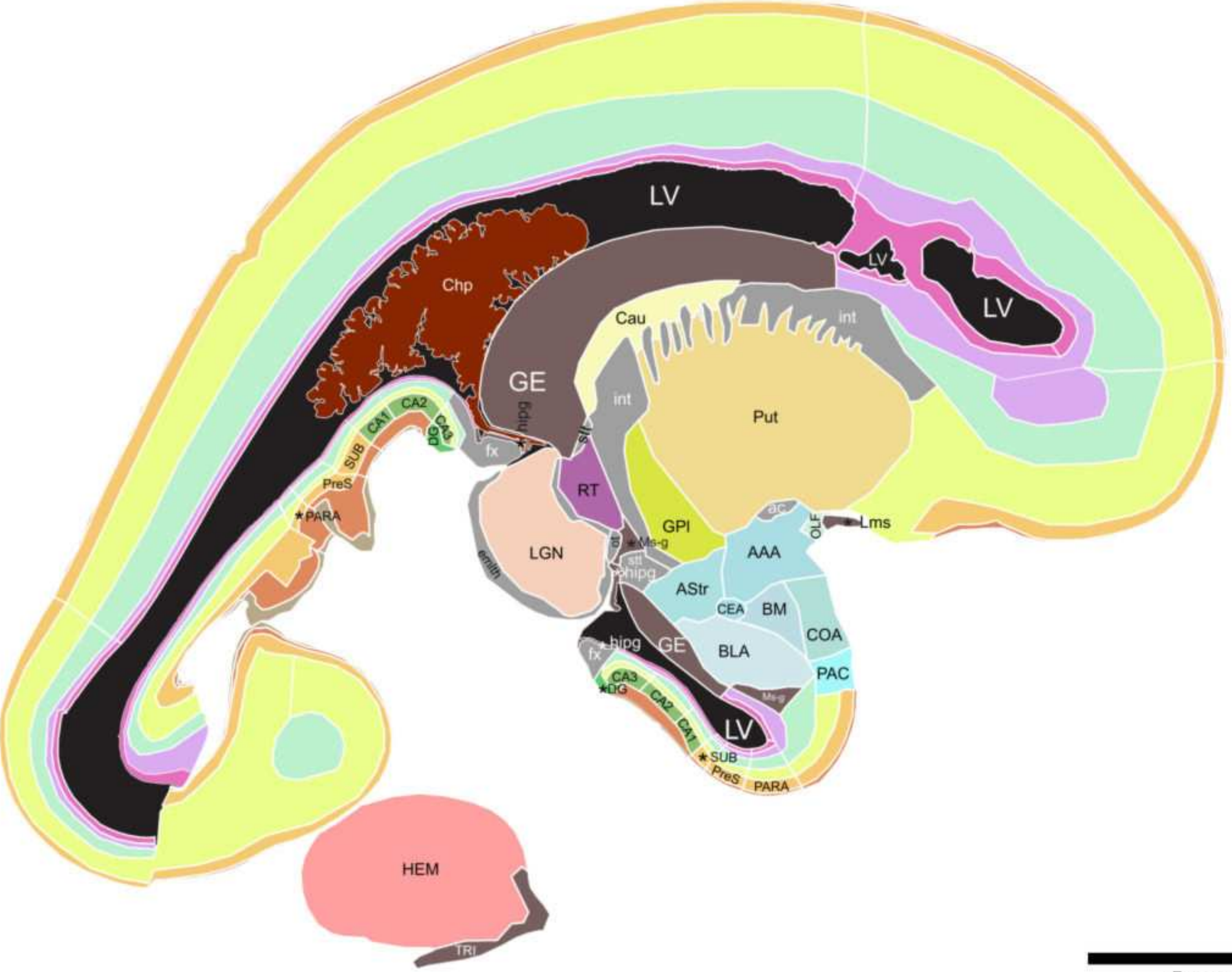

5 mm

AAA: Anterior amygdaloid area
AStr: Amygdalo-striatal area
BL: Basal nucleus [amygdala]
BM: Basomedial nucleus [amygdala]
CA1: CA1 field [hippocampus]
CA2: CA2 field [hippocampus]
CA3: CA3 field [hippocampus]
CEA: Central nucleus [amygdala]
COA: Cortical nucleus [amygdala]
Cau: Caudate nucleus
Chp: Choroid plexus
DG: Dentate gyrus
GE: Ganglionic eminence
GPl: Globus pallidus lateral segment
HEM: Cerebellar hemispheres
LGN: Lateral geniculate nucleus
LV: Lateral ventricle
Lms: Lateral migratory stream
Ms-g: Migratory stream, general
PARA: Cortical plate, parasubiculum
PreS: Cortical plate, presubiculum
Put: Putamen
RT: Reticular nucleus [thalamus]
SUB: Cortical plate, subiculum
TRI: Germinal trigone
ac: Anterior commissure
emlth: External medullary lamina [thalamus]
fx: Fornix
hipg: Hippocampal glioepithelium/ependyma
int: Internal capsule
ot: Optic tract
stt: Stria terminalis

Age: 14 GW

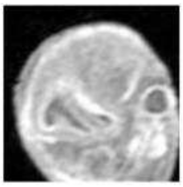

L-R Level: 4.8 mm

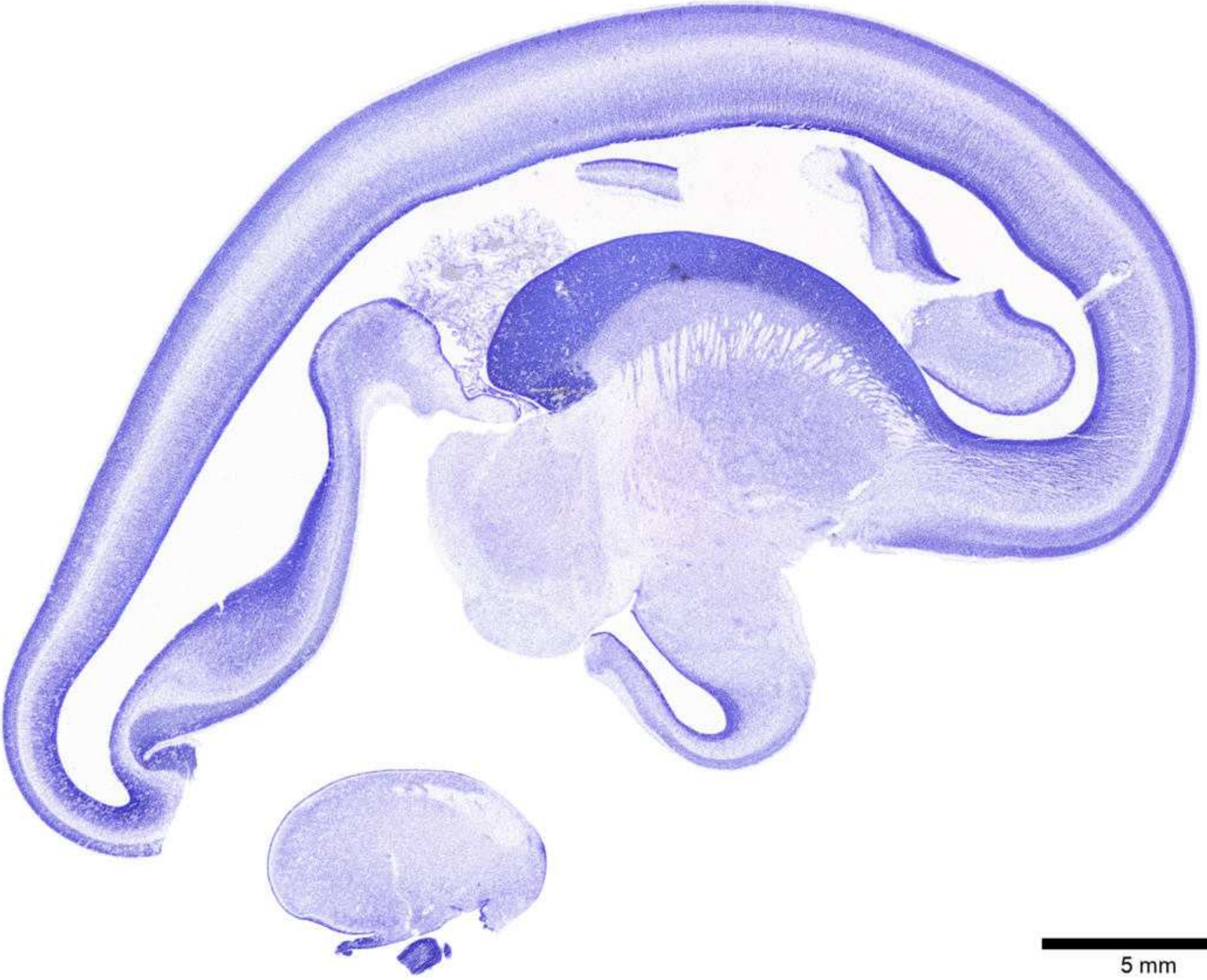

5 mm

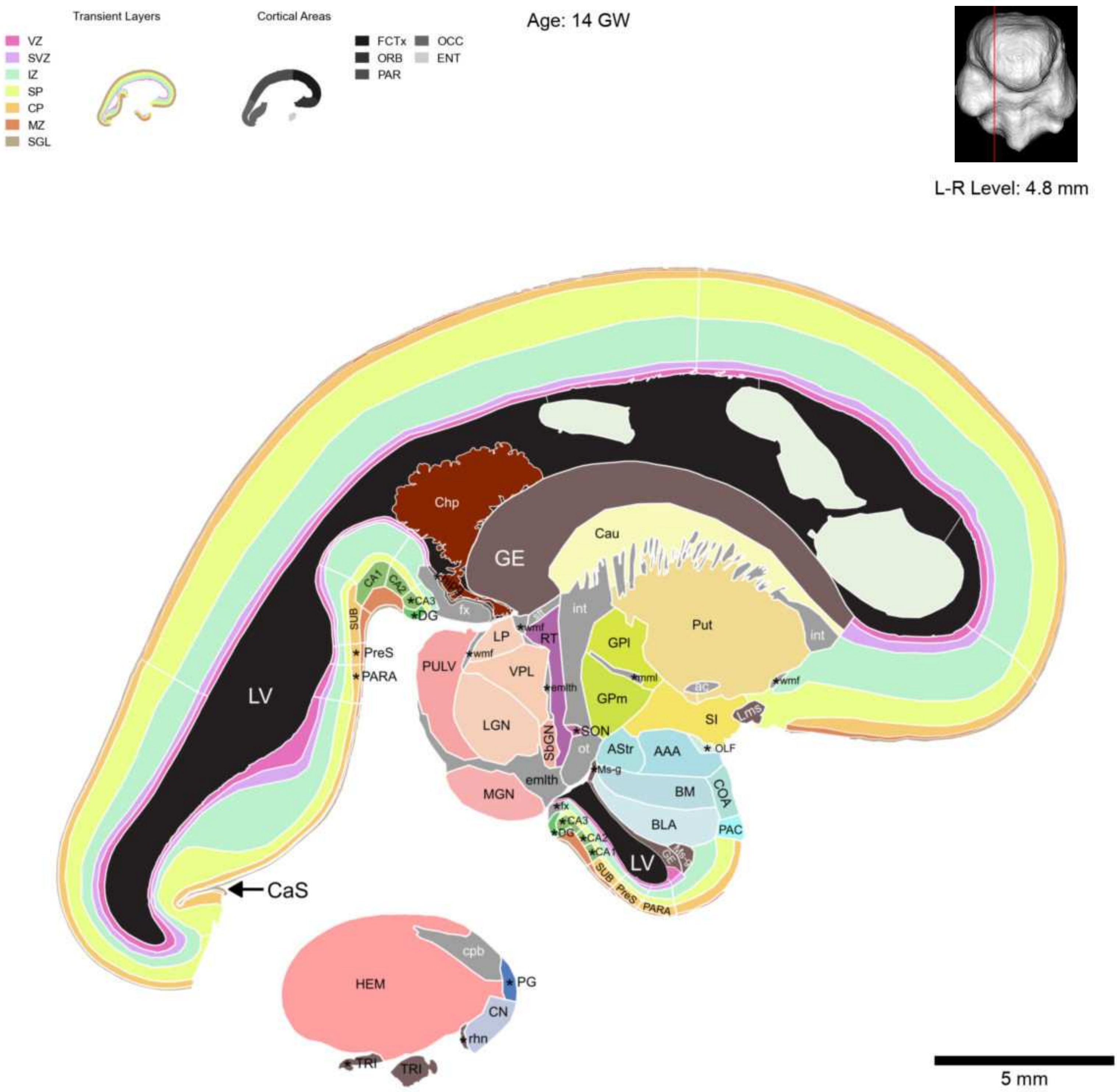

AAA: Anterior amygdaloid area
AStr: Amygdalo-striatal area
BL: Basal nucleus [amygdala]
BM: Basomedial nucleus [amygdala]
CA1: CA1 field [hippocampus]
CA2: CA2 field [hippocampus]
CA3: CA3 field [hippocampus]
CN: Cochlear nuclei
COA: Cortical nucleus [amygdala]
Cau: Caudate nucleus
Chp: Choroid plexus
DG: Dentate gyrus
GE: Ganglionic eminence
GPl: Globus pallidus lateral segment
GPm: Globus pallidus medial segment
HEM: Cerebellar hemispheres
LGN: Lateral geniculate nucleus
LP: Lateral posterior nucleus [thalamus]
LV: Lateral ventricle
Lms: Lateral migratory stream
MGN: Medial geniculate nucleus
Ms-g: Migratory stream, general
PARA: Cortical plate, parasubiculum
PG: Pontine gray
PULV: Pulvinar nucleus [thalamus]
PreS: Cortical plate, presubiculum
Put: Putamen
RT: Reticular nucleus [thalamus]
SI: Substantia innominata
SON: Supraoptic nucleus [hypothalamus]
SUB: Cortical plate, subiculum
SbGN: Subgeniculate nucleus
TRI: Germinal trigone
VPL: Ventral posterolateral nucleus [thalamus]
ac: Anterior commissure
cpb: Cerebellar peduncles
emlth: External medullary lamina [thalamus]
fx: Fornix
hipg: Hippocampal glioepithelium/ependyma
int: Internal capsule
mml: Medial medullary lamina
ot: Optic tract
rhn: Rhombencephalic neuroepithelium
stt: Stria terminalis
wmf: White matter fibers
CaS: Calcarine sulcus

Age: 14 GW

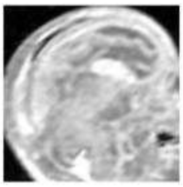

L-R Level: 3.96 mm

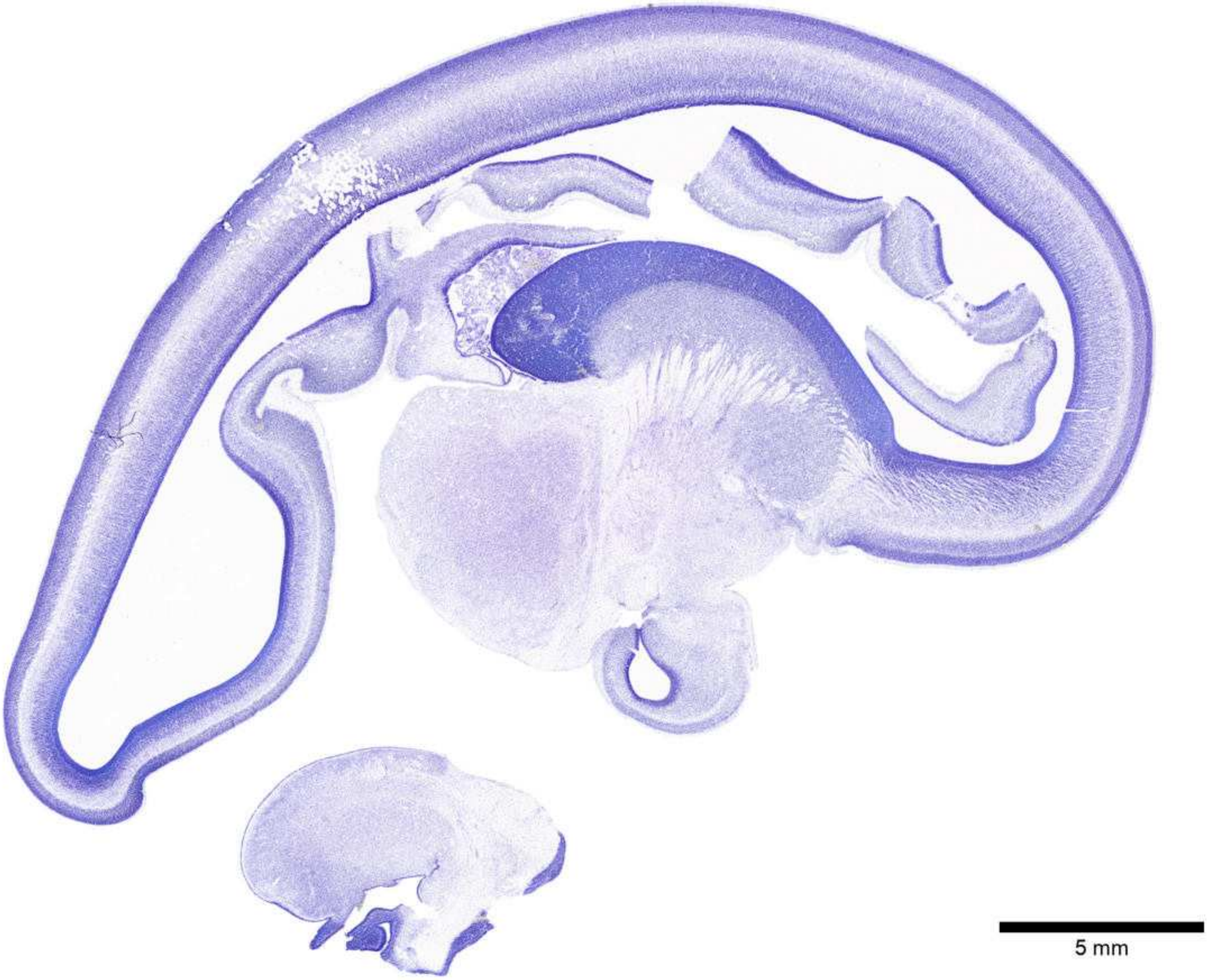

5 mm

Transient Layers

- VZ
- SVZ
- IZ
- SP
- CP
- MZ
- SGL

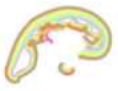

Cortical Areas

- FCTx
- ORB
- PAR
- OCC
- CING
- ENT

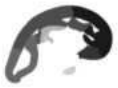

Age: 14 GW

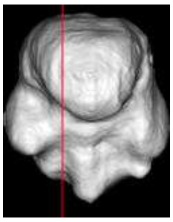

L-R Level: 3.96 mm

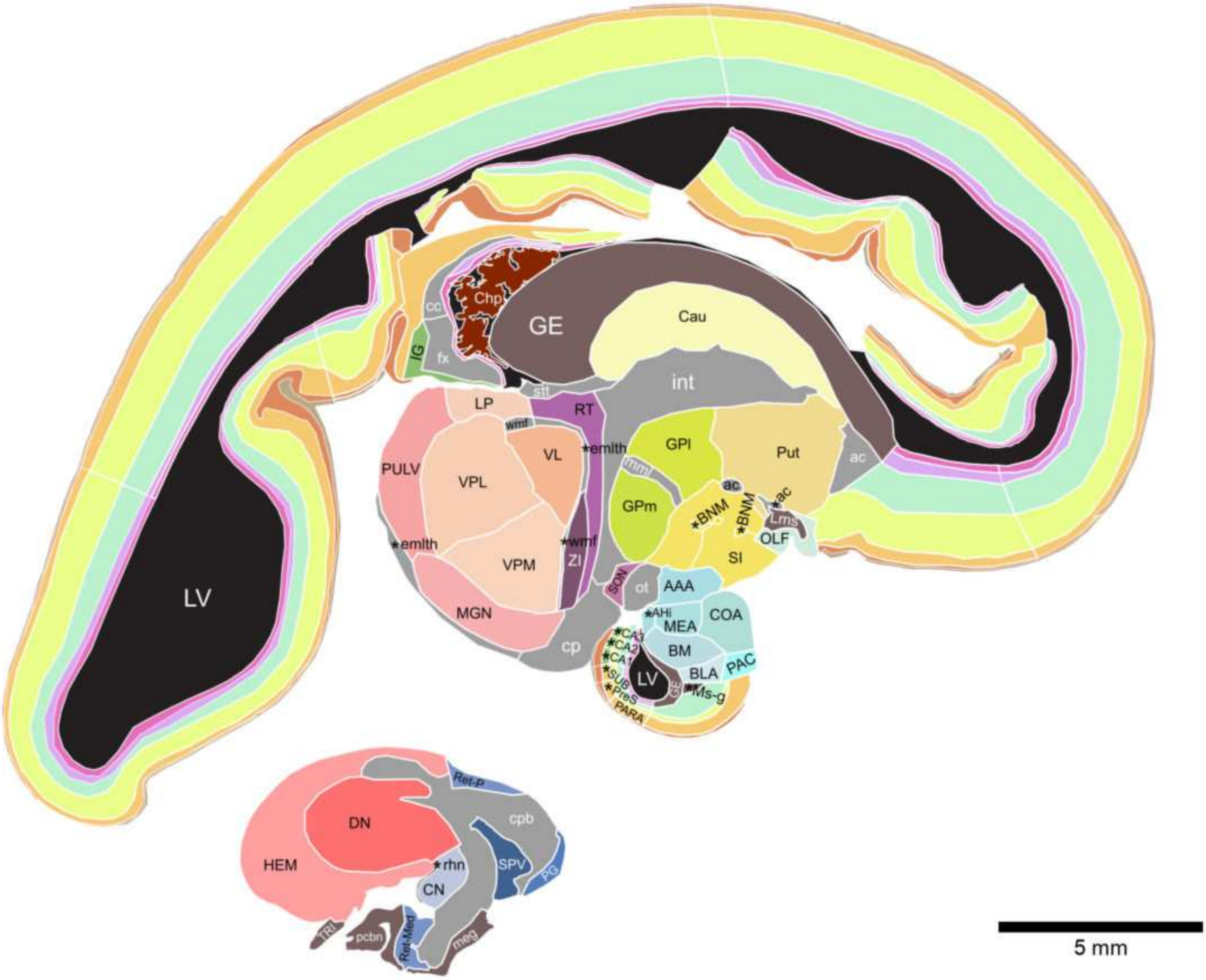

- AAA: Anterior amygdaloid area
- AHi: Amygdalo-hippocampal area
- BL: Basal nucleus [amygdala]
- BM: Basomedial nucleus [amygdala]
- BNM: Basal nucleus of Meynert
- CA1: CA1 field [hippocampus]
- CA2: CA2 field [hippocampus]
- CA3: CA3 field [hippocampus]
- CN: Cochlear nuclei
- COA: Cortical nucleus [amygdala]
- Cau: Caudate nucleus
- Chp: Choroid plexus
- DN: Dentate nucleus
- GE: Ganglionic eminence
- GPl: Globus pallidus lateral segment
- GPm: Globus pallidus medial segment
- HEM: Cerebellar hemispheres
- IG: Induseum griseum
- LP: Lateral posterior nucleus [thalamus]
- LV: Lateral ventricle
- Lms: Lateral migratory stream
- MEA: Medial nucleus [amygdala]
- MGN: Medial geniculate nucleus
- Ms-g: Migratory stream, general
- PARA: Cortical plate, parasubiculum
- PG: Pontine gray
- PULV: Pulvinar nucleus [thalamus]
- PreS: Cortical plate, presubiculum
- Put: Putamen
- RT: Reticular nucleus [thalamus]
- Ret-Med: Reticular formation, Medulla
- Ret-P: Reticular formation, Pons
- SI: Substantia innominata
- SON: Supraoptic nucleus [hypothalamus]
- SPV: Spinal nucleus of the trigeminal
- SUB: Cortical plate, subiculum
- TRI: Germinal trigone
- VL: Ventral lateral nucleus [thalamus]
- VPL: Ventral posterolateral nucleus [thalamus]
- VPM: Ventral posteromedial nucleus [thalamus]
- ZI: Zona incerta
- ac: Anterior commissure
- cc: Corpus callosum
- cp: Cerebral peduncle
- cpb: Cerebellar peduncles
- emlth: External medullary lamina [thalamus]
- fx: Fornix
- int: Internal capsule
- meg: Medullary glioepithelium/ependyma
- mml: Medial medullary lamina
- ot: Optic tract
- pcbn: Precerebellar neuroepithelium
- rhn: Rhombencephalic neuroepithelium
- stt: Stria terminalis
- wmf: White matter fibers

Age: 14 GW

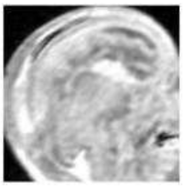

L-R Level: 3.6 mm

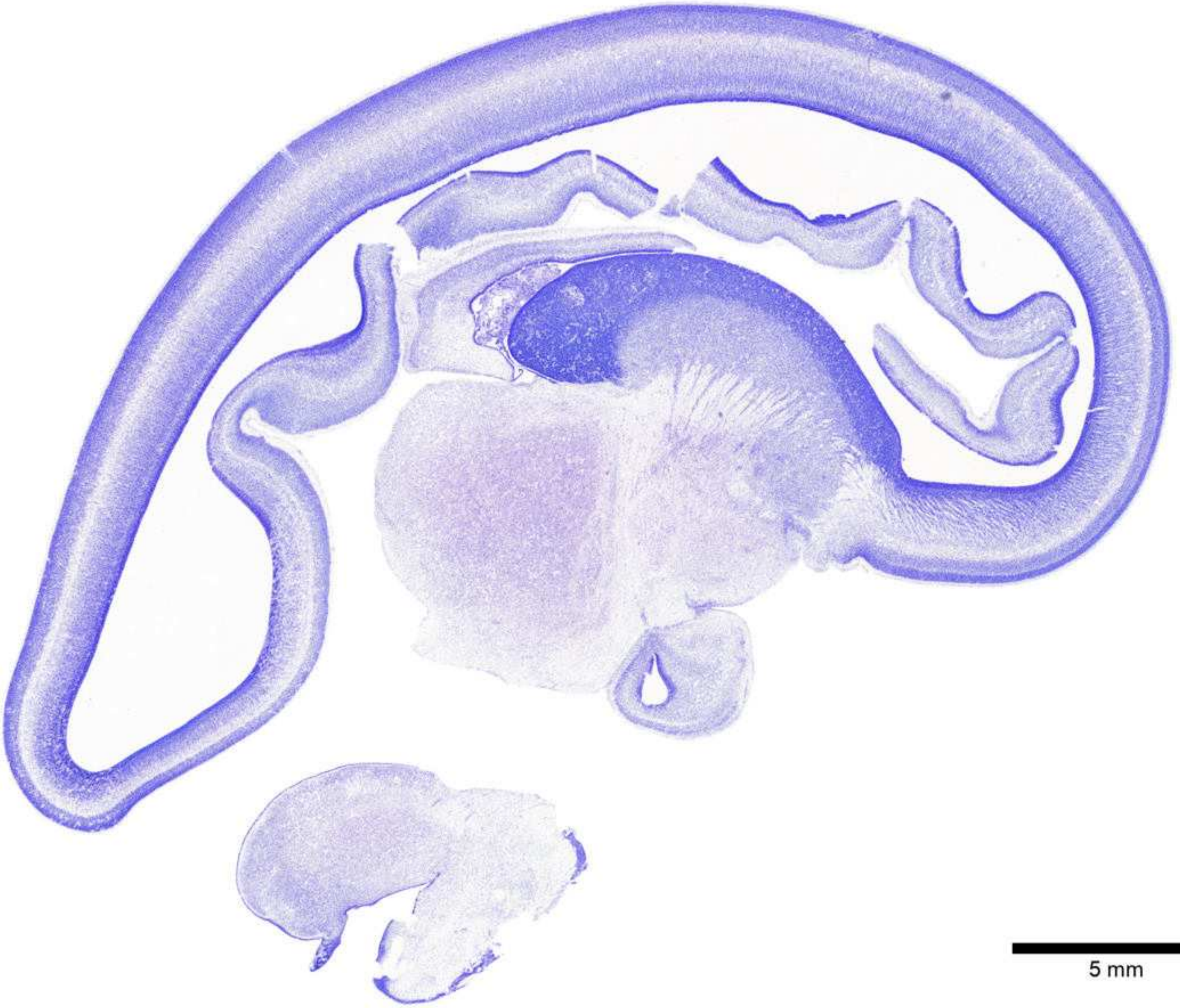

5 mm

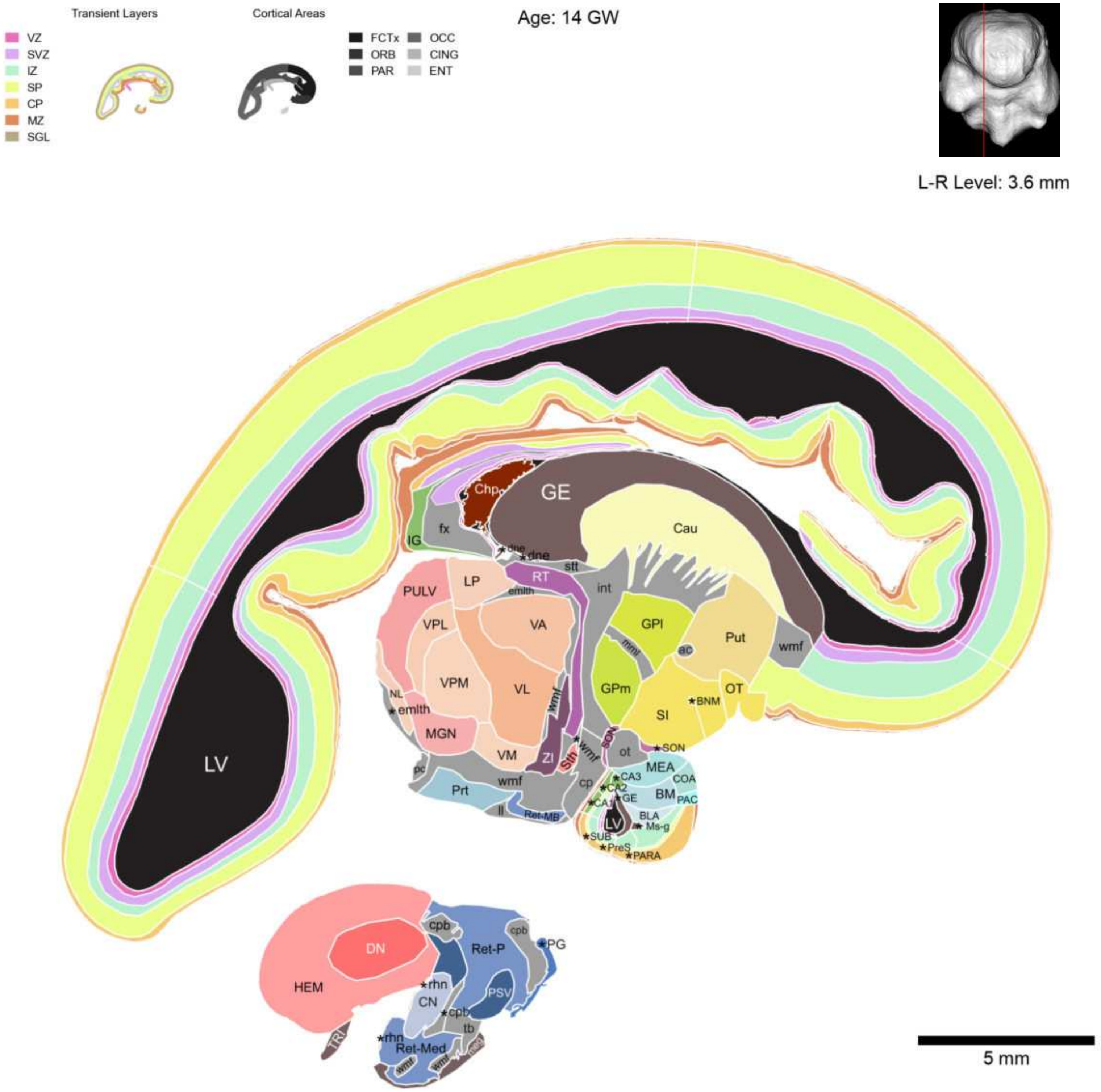

BL: Basal nucleus [amygdala]
BM: Basomedial nucleus [amygdala]
BNM: Basal nucleus of Meynert
CA1: CA1 field [hippocampus]
CA2: CA2 field [hippocampus]
CA3: CA3 field [hippocampus]
CN: Cochlear nuclei
COA: Cortical nucleus [amygdala]
Cau: Caudate nucleus
Chp: Choroid plexus
DN: Dentate nucleus
GE: Ganglionic eminence
GPl: Globus pallidus lateral segment
GPm: Globus pallidus medial segment
HEM: Cerebellar hemispheres
IG: Induseum griseum
LP: Lateral posterior nucleus [thalamus]
LV: Lateral ventricle
MEA: Medial nucleus [amygdala]
MGN: Medial geniculate nucleus
Ms-g: Migratory stream, general
NL: Nucleus limitans [thalamus]
OT: Olfactory tubercle
PARA: Cortical plate, parasubiculum
PG: Pontine gray
PSV: Principal sensory nucleus of the trigeminal
PULV: Pulvinar nucleus [thalamus]
PreS: Cortical plate, presubiculum
Prt: Pretectum
Put: Putamen
RT: Reticular nucleus [thalamus]
Ret-MB: Reticular formation, Midbrain
Ret-Med: Reticular formation, Medulla
Ret-P: Reticular formation, Pons
SI: Substantia innominata
SON: Supraoptic nucleus [hypothalamus]
SPV: Spinal nucleus of the trigeminal
SUB: Cortical plate, subiculum
Sth: Subthalamus
TRI: Germinal trigone
VA: Ventral anterior nucleus [thalamus]
VL: Ventral lateral nucleus [thalamus]
VM: Ventral medial nucleus [thalamus]
VPL: Ventral posterolateral nucleus [thalamus]
VPM: Ventral posteromedial nucleus [thalamus]
ZI: Zona incerta
ac: Anterior commissure
cp: Cerebral peduncle
cpb: Cerebellar peduncles
dne: Diencephalic neuroepithelium
emlth: External medullary lamina [thalamus]
fx: Fornix
int: Internal capsule
ll: Lateral lemniscus
meg: Medullary glioepithelium/ependyma
mml: Medial medullary lamina
ot: Optic tract
pc: Posterior commissure
rhn: Rhombencephalic neuroepithelium
stt: Stria terminalis
tb: Trapezoid body
wmf: White matter fibers

Age: 14 GW

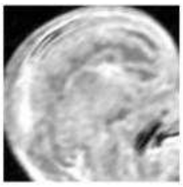

L-R Level: 3.06 mm

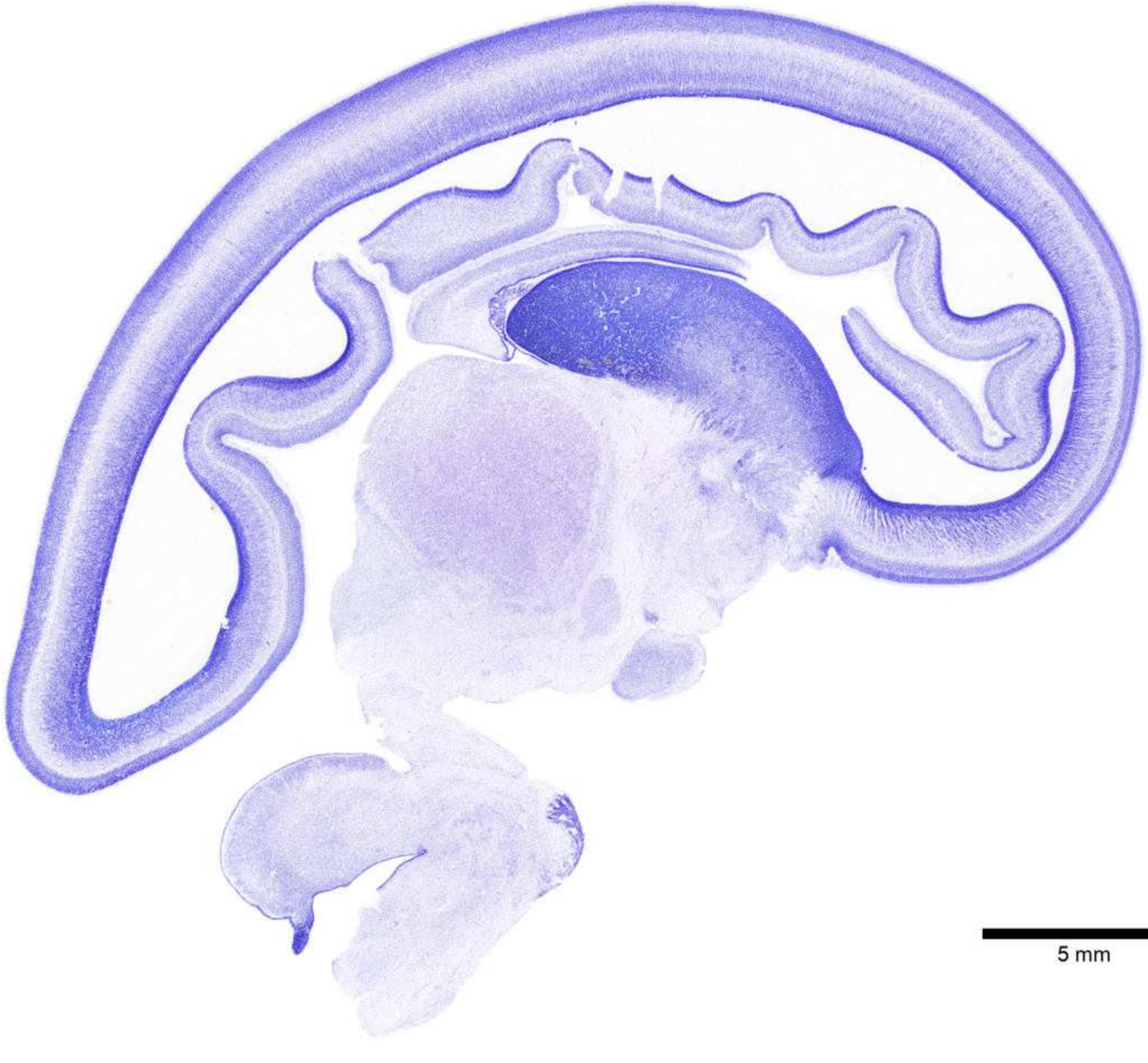

5 mm

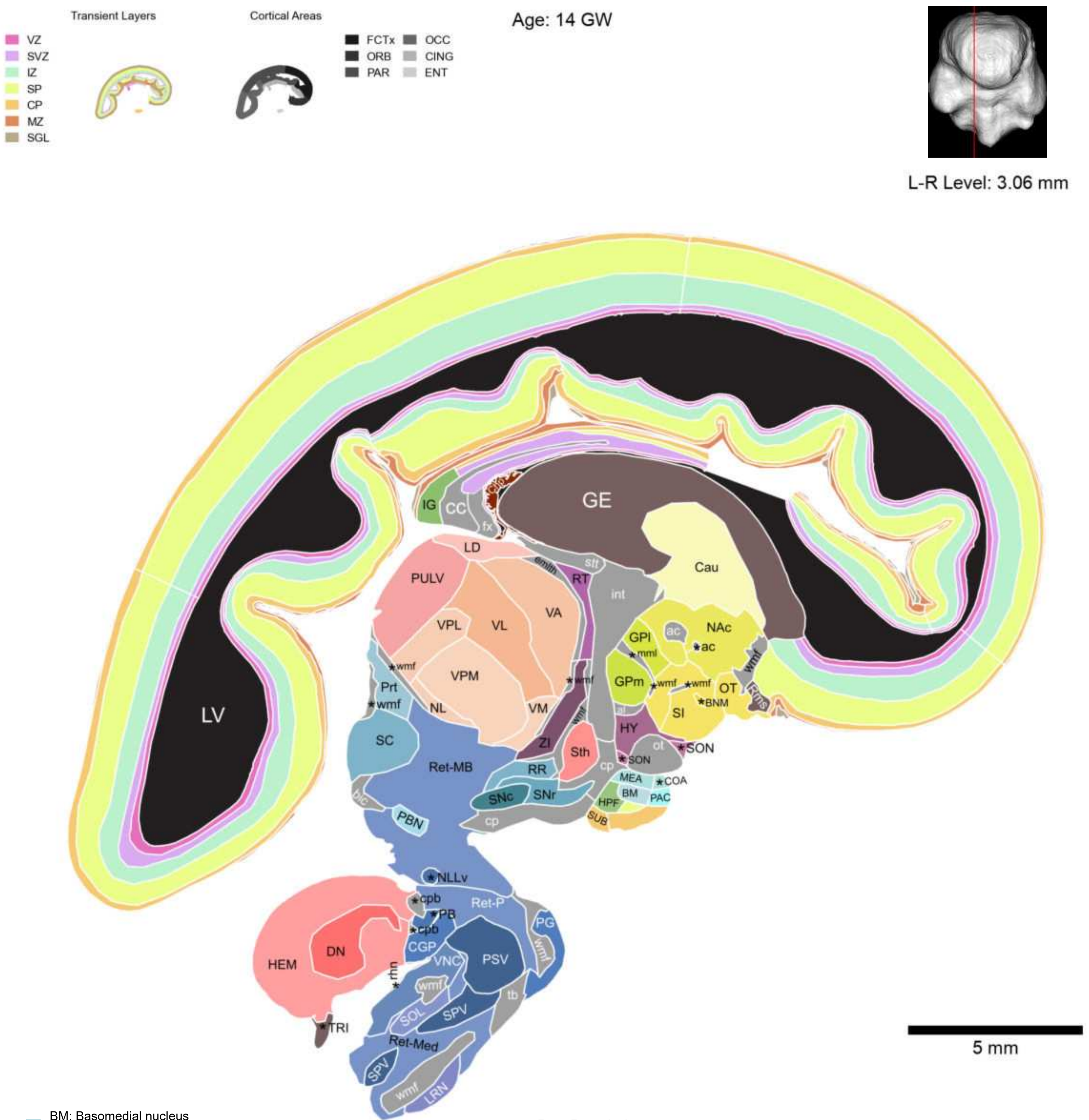

- BM: Basomedial nucleus [amygdala]
- BNM: Basal nucleus of Meynert
- CGP: Central gray of the pons
- COA: Cortical nucleus [amygdala]
- Cau: Caudate nucleus
- Chp: Choroid plexus
- DN: Dentate nucleus
- GE: Ganglionic eminence
- GPl: Globus pallidus lateral segment
- GPm: Globus pallidus medial segment
- HEM: Cerebellar hemispheres
- HPF: Hippocampal formation
- HY: Hypothalamus
- IG: Induseum griseum
- LD: Lateral dorsal nucleus [thalamus]
- LHA: Lateral hypothalamic area
- LRN: Lateral reticular nucleus
- LV: Lateral ventricle
- MEA: Medial nucleus [amygdala]
- NAc: Nucleus accumbens
- NL: Nucleus limitans [thalamus]
- NLLv: Nucleus of the lateral lemniscus, ventral
- OT: Olfactory tubercle
- PB: Parabrachial nucleus
- PBN: Parabigeminal nucleus
- PG: Pontine gray
- PSV: Principal sensory nucleus of the trigeminal
- PULV: Pulvinar nucleus [thalamus]
- Prt: Pretectum
- RR: Retrorubral area
- RT: Reticular nucleus [thalamus]
- Ret-MB: Reticular formation, Midbrain
- Ret-Med: Reticular formation, Medulla
- Ret-P: Reticular formation, Pons
- Rms: Rostral migratory stream
- SC: Superior colliculus
- SI: Substantia innominata
- SNc: Substantia nigra pars compacta
- SNr: Substantia nigra pars reticulata
- SOL: Solitary nucleus
- SON: Supraoptic nucleus [hypothalamus]
- SPV: Spinal nucleus of the trigeminal
- SUB: Cortical plate, subiculum
- Sth: Subthalamus
- TRI: Germinal trigone
- VA: Ventral anterior nucleus [thalamus]
- VL: Ventral lateral nucleus [thalamus]
- VM: Ventral medial nucleus [thalamus]
- VNC: Vestibular nuclear complex
- VPL: Ventral posterolateral nucleus [thalamus]
- VPM: Ventral posteromedial nucleus [thalamus]
- ZI: Zona incerta
- ac: Anterior commissure
- al: Ansa lenticularis
- bic: Brachium of the inferior colliculus
- cc: Corpus callosum
- cp: Cerebral peduncle
- cpb: Cerebellar peduncles
- emlth: External medullary lamina [thalamus]
- fx: Fornix
- int: Internal capsule
- mml: Medial medullary lamina
- ot: Optic tract
- rhn: Rhombencephalic neuroepithelium
- stt: Stria terminalis
- tb: Trapezoid body
- wmf: White matter fibers

Age: 14 GW

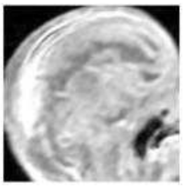

L-R Level: 2.64 mm

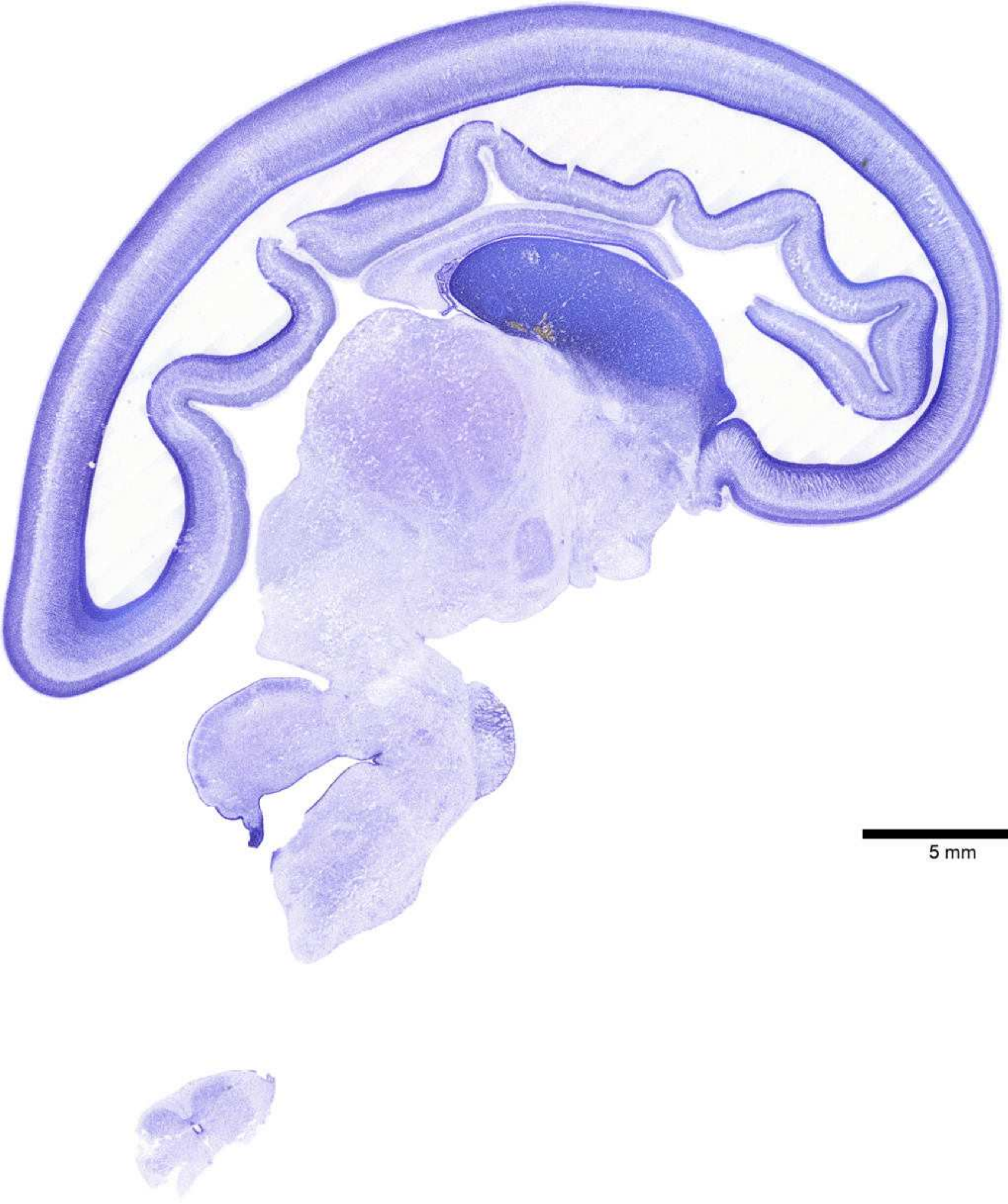

5 mm

Transient Layers

VZ
SVZ
IZ
SP
CP
MZ
SGL

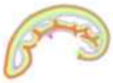

Cortical Areas

FCTx
ORB
PAR
OCC
CING

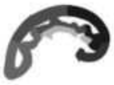

Age: 14 GW

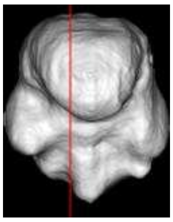

L-R Level: 2.64 mm

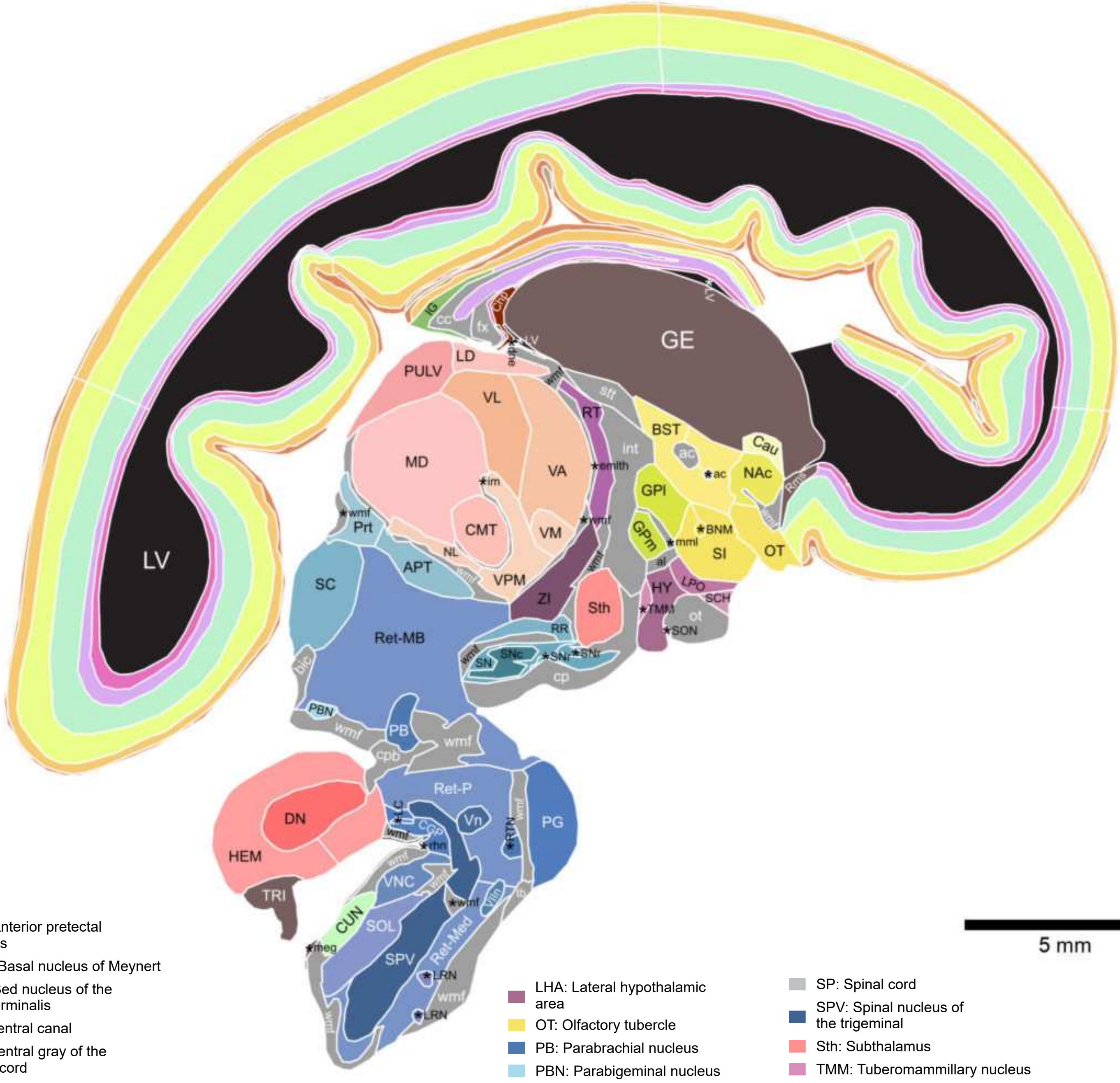

5 mm

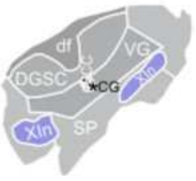

APT: Anterior pretectal nucleus
BNM: Basal nucleus of Meynert
BST: Bed nucleus of the stria terminalis
CC: Central canal
CG: Central gray of the spinal cord
CGP: Central gray of the pons
CMT: Centromedian nucleus [thalamus]
CUN: Cuneate nucleus
Cau: Caudate nucleus
Chp: Choroid plexus
DGSC: Dorsal gray of the spinal cord
DN: Dentate nucleus
GE: Ganglionic eminence
GPl: Globus pallidus lateral segment
GPm: Globus pallidus medial segment
HEM: Cerebellar hemispheres
HY: Hypothalamus
IG: Induseum griseum
LC: Locus coeruleus
LD: Lateral dorsal nucleus [thalamus]
LPO: Lateral preoptic area
LRN: Lateral reticular nucleus
LV: Lateral ventricle
MD: Medial dorsal nucleus [thalamus]
NAc: Nucleus accumbens
NL: Nucleus limitans [thalamus]
LHA: Lateral hypothalamic area
OT: Olfactory tubercle
PB: Parabrachial nucleus
PBN: Parabigeminal nucleus
PG: Pontine gray
PULV: Pulvinar nucleus [thalamus]
Prt: Pretectum
RR: Retrorubral area
RT: Reticular nucleus [thalamus]
RTN: Reticular tegmental nucleus
Ret-MB: Reticular formation, Midbrain
Ret-Med: Reticular formation, Medulla
Ret-P: Reticular formation, Pons
Rms: Rostral migratory stream
SC: Superior colliculus
SCH: Suprachiasmatic nucleus [hypothalamus]
SI: Substantia innominata
SN: Substantia nigra
SP: Spinal cord
SPV: Spinal nucleus of the trigeminal
Sth: Subthalamus
TMM: Tuberomammillary nucleus
TRI: Germinal trigone
VA: Ventral anterior nucleus [thalamus]
VG: Ventral gray of the spinal cord
VIIn: Facial motor nucleus
VL: Ventral lateral nucleus [thalamus]
VM: Ventral medial nucleus [thalamus]
VNC: Vestibular nuclear complex
VPM: Ventral posteromedial nucleus [thalamus]
Vn: Trigeminal motor nucleus
XIn: Accessory nucleus
ZI: Zona incerta
SNc: Substantia nigra pars compacta
SNr: Substantia nigra pars reticulata
SOL: Solitary nucleus
SON: Supraoptic nucleus [hypothalamus]

Age: 14 GW

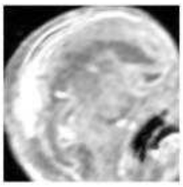

L-R Level: 2.28 mm

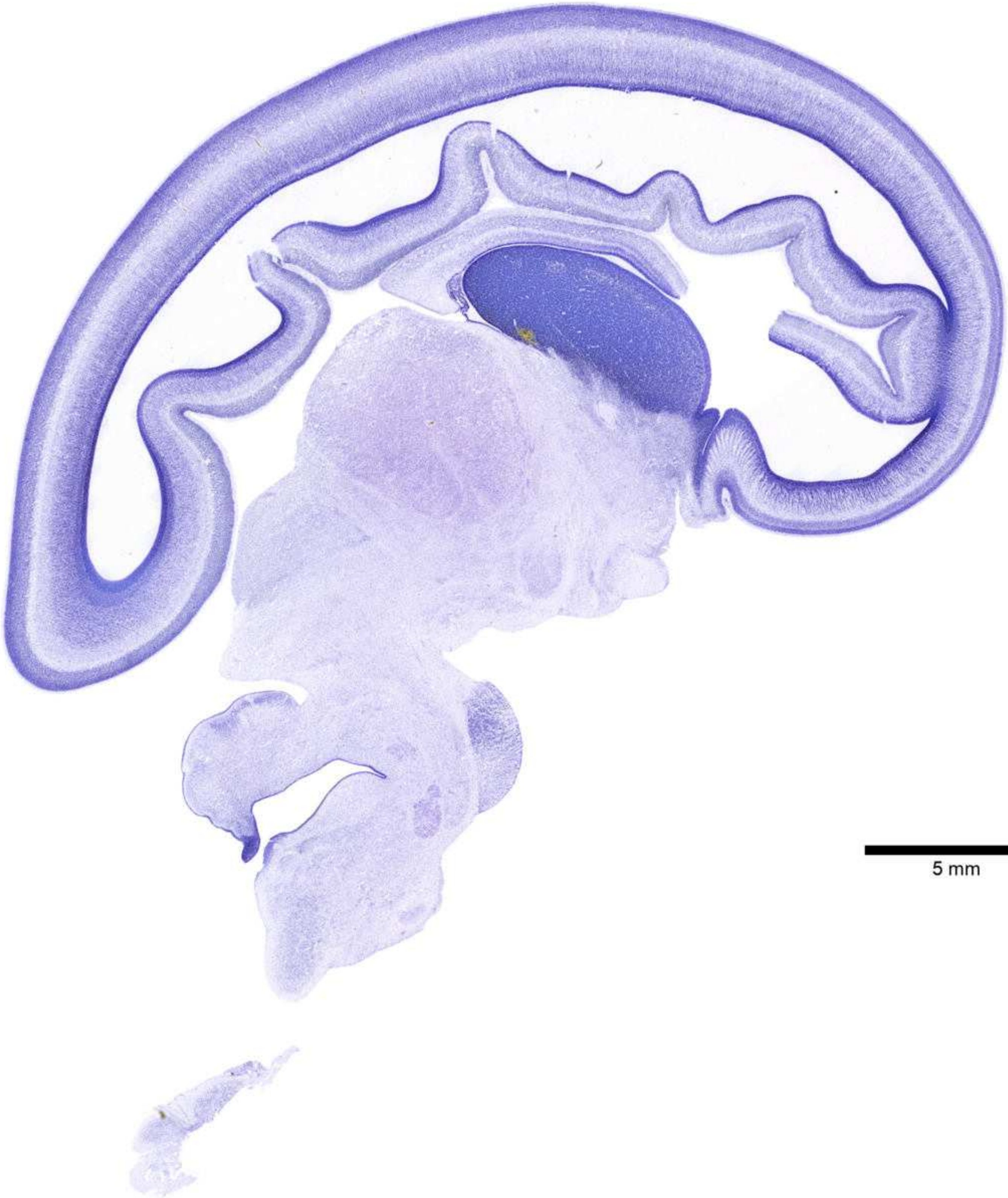

5 mm

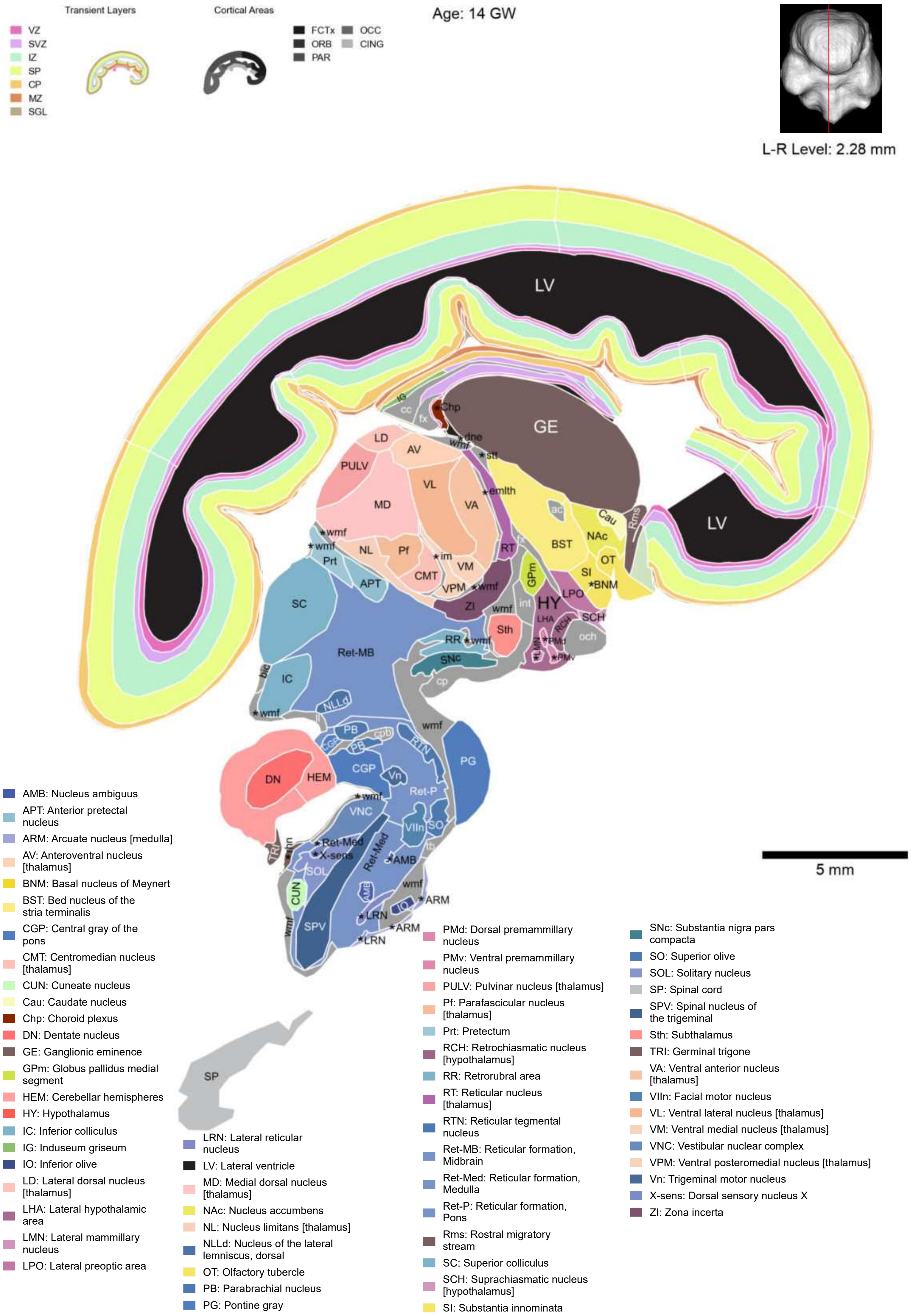

Transient Layers
VZ
SVZ
IZ
SP
CP
MZ
SGL
Cortical Areas
FCTx
ORB
PAR
OCC
CING
Age: 14 GW
L-R Level: 2.28 mm
5 mm
LV
GE
Chp
IG
cc
fx
dne
wmf
stt
LD
AV
PULV
VL
MD
VA
emlth
ac
Cau
Rms
NAc
BST
OT
SI
BNM
RT
GPm
LPO
HY
LHA
RCH
SCH
och
PMd
PMv
LMN
int
NL
Pf
im
VM
CMT
VPM
ZI
Prt
APT
SC
Ret-MB
RR
Sth
SNc
cp
bic
IC
NLLd
PB
cpb
CGP
RTN
PG
DN
HEM
Vn
Ret-P
VNC
VIIn
SO
Ret-Med
X-sens
SOL
AMB
CUN
IO
ARM
LRN
SPV
TRI
SP
AMB: Nucleus ambiguus
APT: Anterior pretectal nucleus
ARM: Arcuate nucleus [medulla]
AV: Anteroventral nucleus [thalamus]
BNM: Basal nucleus of Meynert
BST: Bed nucleus of the stria terminalis
CGP: Central gray of the pons
CMT: Centromedian nucleus [thalamus]
CUN: Cuneate nucleus
Cau: Caudate nucleus
Chp: Choroid plexus
DN: Dentate nucleus
GE: Ganglionic eminence
GPm: Globus pallidus medial segment
HEM: Cerebellar hemispheres
HY: Hypothalamus
IC: Inferior colliculus
IG: Induseum griseum
IO: Inferior olive
LD: Lateral dorsal nucleus [thalamus]
LHA: Lateral hypothalamic area
LMN: Lateral mammillary nucleus
LPO: Lateral preoptic area
LRN: Lateral reticular nucleus
LV: Lateral ventricle
MD: Medial dorsal nucleus [thalamus]
NAc: Nucleus accumbens
NL: Nucleus limitans [thalamus]
NLLd: Nucleus of the lateral lemniscus, dorsal
OT: Olfactory tubercle
PB: Parabrachial nucleus
PG: Pontine gray
PMd: Dorsal premammillary nucleus
PMv: Ventral premammillary nucleus
PULV: Pulvinar nucleus [thalamus]
Pf: Parafascicular nucleus [thalamus]
Prt: Pretectum
RCH: Retrochiasmatic nucleus [hypothalamus]
RR: Retrorubral area
RT: Reticular nucleus [thalamus]
RTN: Reticular tegmental nucleus
Ret-MB: Reticular formation, Midbrain
Ret-Med: Reticular formation, Medulla
Ret-P: Reticular formation, Pons
Rms: Rostral migratory stream
SC: Superior colliculus
SCH: Suprachiasmatic nucleus [hypothalamus]
SI: Substantia innominata
SNc: Substantia nigra pars compacta
SO: Superior olive
SOL: Solitary nucleus
SP: Spinal cord
SPV: Spinal nucleus of the trigeminal
Sth: Subthalamus
TRI: Germinal trigone
VA: Ventral anterior nucleus [thalamus]
VIIn: Facial motor nucleus
VL: Ventral lateral nucleus [thalamus]
VM: Ventral medial nucleus [thalamus]
VNC: Vestibular nuclear complex
VPM: Ventral posteromedial nucleus [thalamus]
Vn: Trigeminal motor nucleus
X-sens: Dorsal sensory nucleus X
ZI: Zona incerta

Age: 14 GW

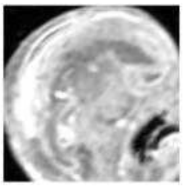

L-R Level: 2.1 mm

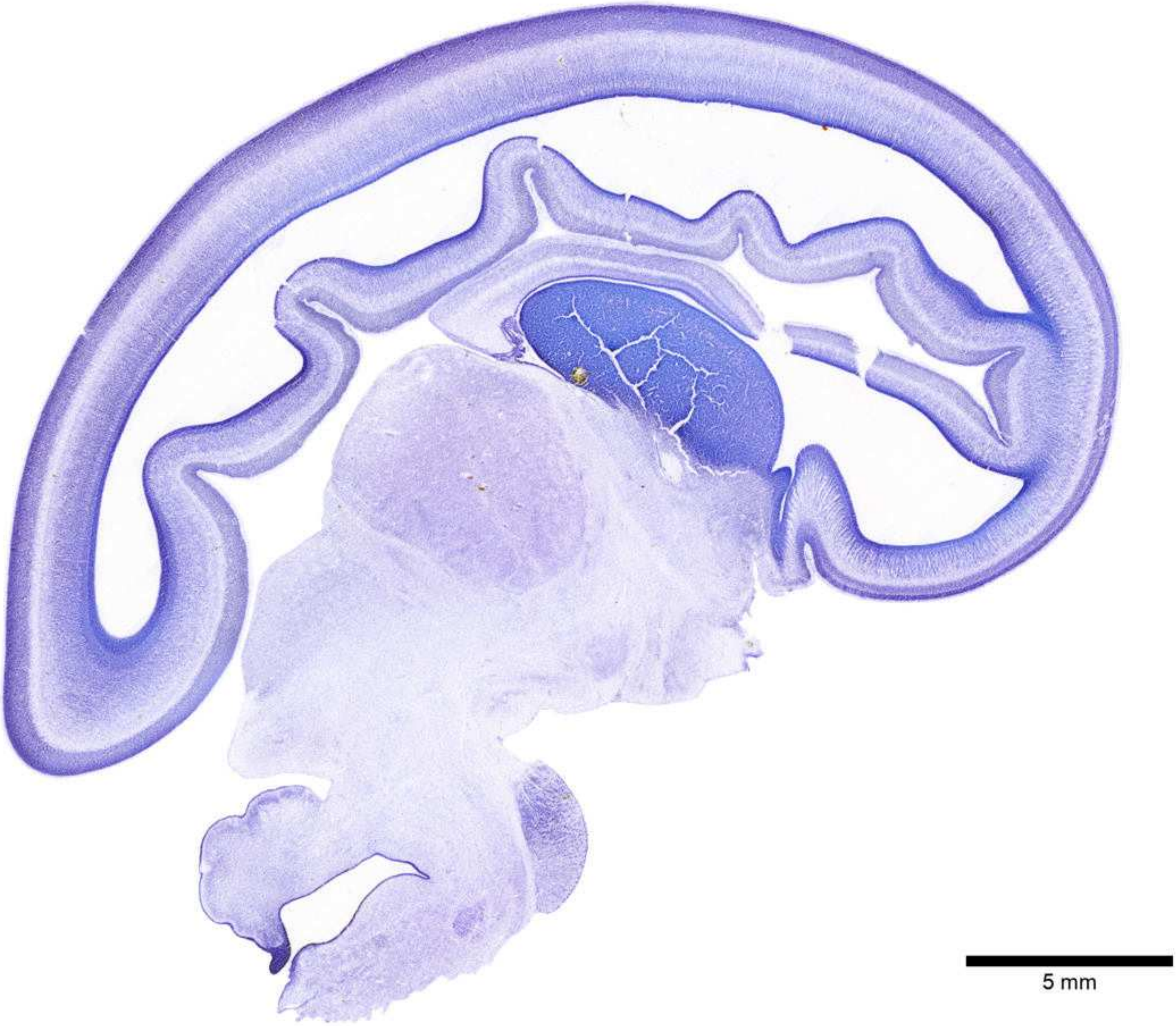

5 mm

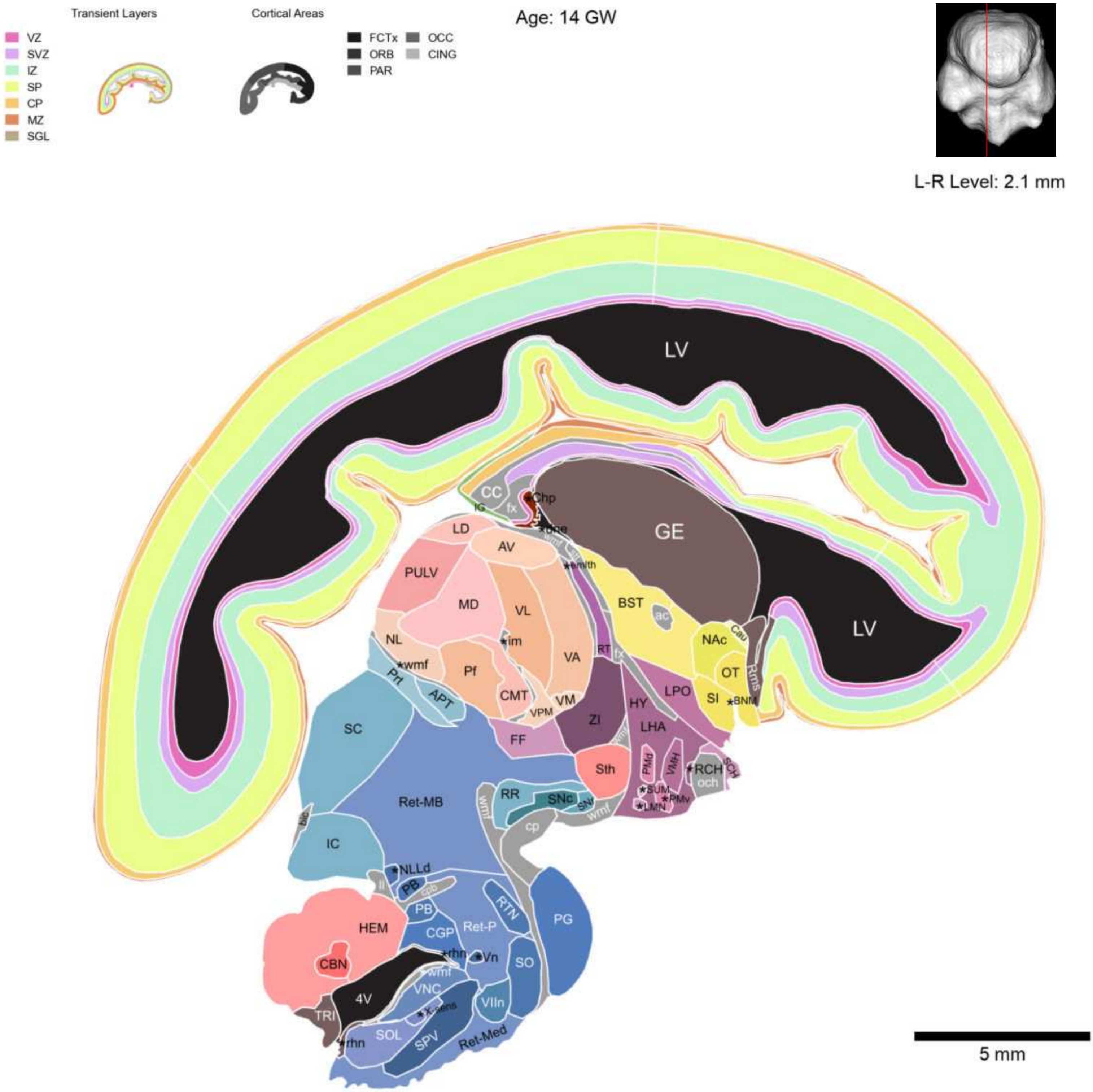

- 4V: Fourth ventricle
- APT: Anterior pretectal nucleus
- AV: Anteroventral nucleus [thalamus]
- BNM: Basal nucleus of Meynert
- BST: Bed nucleus of the stria terminalis
- CBN: Cerebellar nuclei
- CGP: Central gray of the pons
- CMT: Centromedian nucleus [thalamus]
- Cau: Caudate nucleus
- Chp: Choroid plexus
- FF: Field of Forel
- GE: Ganglionic eminence
- HEM: Cerebellar hemispheres
- HY: Hypothalamus
- IC: Inferior colliculus
- IG: Induseum griseum
- LD: Lateral dorsal nucleus [thalamus]
- LHA: Lateral hypothalamic area
- LMN: Lateral mammillary nucleus
- LPO: Lateral preoptic area
- LV: Lateral ventricle
- MD: Medial dorsal nucleus [thalamus]
- NAc: Nucleus accumbens
- NL: Nucleus limitans [thalamus]
- NLLd: Nucleus of the lateral lemniscus, dorsal
- OT: Olfactory tubercle
- PB: Parabrachial nucleus
- PG: Pontine gray
- PMd: Dorsal premammillary nucleus
- PMv: Ventral premammillary nucleus
- PULV: Pulvinar nucleus [thalamus]
- Pf: Parafascicular nucleus [thalamus]
- Prt: Pretectum
- RCH: Retrochiasmatic nucleus [hypothalamus]
- RR: Retrorubral area
- RT: Reticular nucleus [thalamus]
- RTN: Reticular tegmental nucleus
- Ret-MB: Reticular formation, Midbrain
- Ret-Med: Reticular formation, Medulla
- Ret-P: Reticular formation, Pons
- Rms: Rostral migratory stream
- SC: Superior colliculus
- SCH: Suprachiasmatic nucleus [hypothalamus]
- SI: Substantia innominata
- SNc: Substantia nigra pars compacta
- SNr: Substantia nigra pars reticulata
- SO: Superior olive
- SOL: Solitary nucleus
- SPV: Spinal nucleus of the trigeminal
- SUM: Supramammillary area
- Sth: Subthalamus
- TRI: Germinal trigone
- VA: Ventral anterior nucleus [thalamus]
- VIIn: Facial motor nucleus
- VL: Ventral lateral nucleus [thalamus]
- VM: Ventral medial nucleus [thalamus]
- VMH: Ventromedial nucleus [hypothalamus]
- VNC: Vestibular nuclear complex
- VPM: Ventral posteromedial nucleus [thalamus]
- Vn: Trigeminal motor nucleus
- X-sens: Dorsal sensory nucleus X
- ZI: Zona incerta

Age: 14 GW

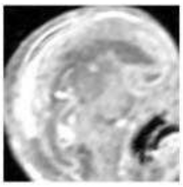

L-R Level: 2.04 mm

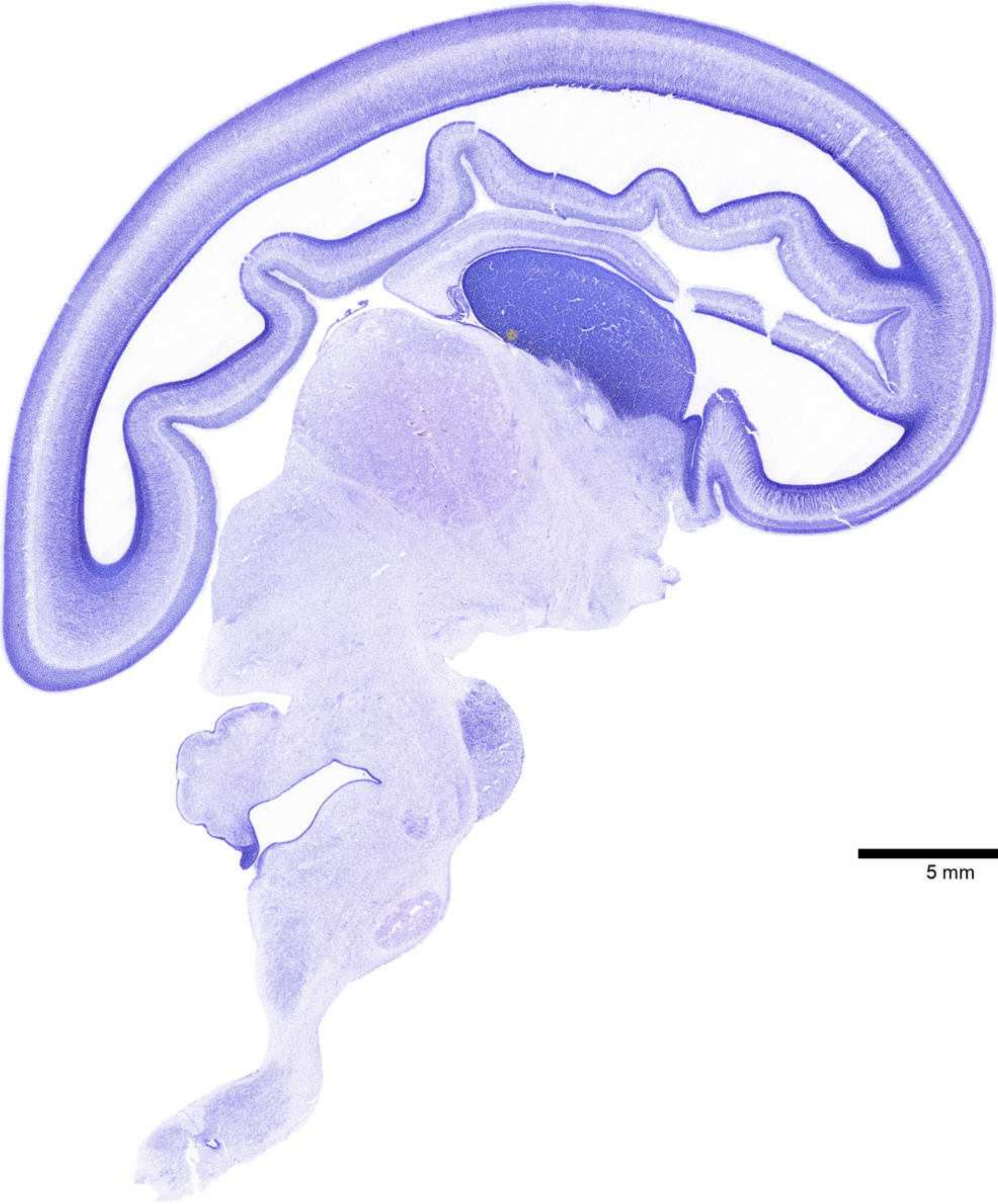

5 mm

Transient Layers

- VZ
- SVZ
- IZ
- SP
- CP
- MZ
- SGL

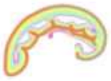

Cortical Areas

- FCTx
- ORB
- PAR
- OCC
- CING

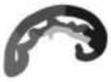

Age: 14 GW

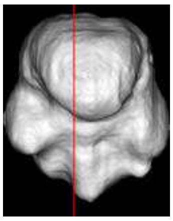

L-R Level: 2.04 mm

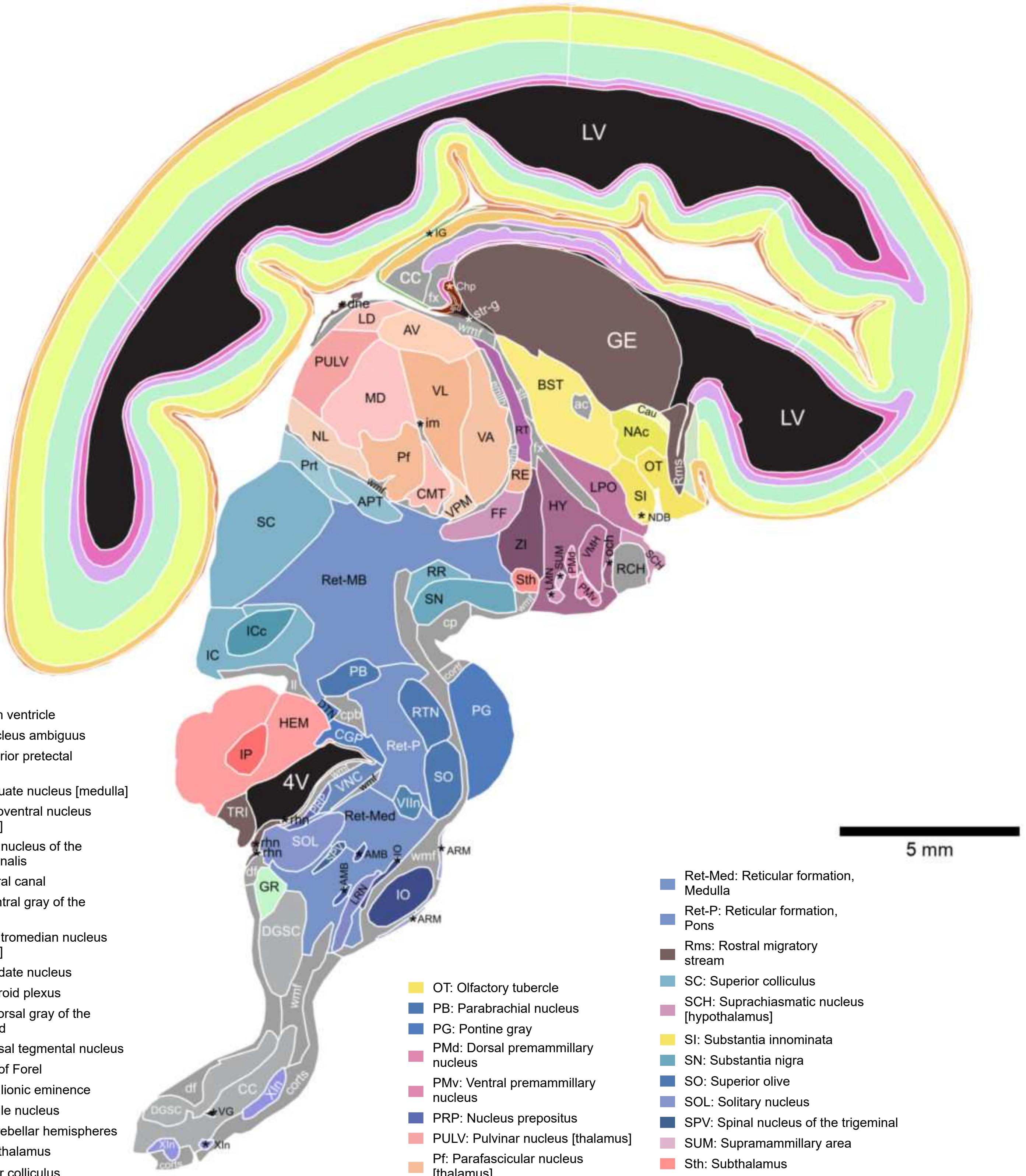

- 4V: Fourth ventricle
- AMB: Nucleus ambiguus
- APT: Anterior pretectal nucleus
- ARM: Arcuate nucleus [medulla]
- AV: Anteroventral nucleus [thalamus]
- BST: Bed nucleus of the stria terminalis
- CC: Central canal
- CGP: Central gray of the pons
- CMT: Centromedian nucleus [thalamus]
- Cau: Caudate nucleus
- Chp: Choroid plexus
- DGSC: Dorsal gray of the spinal cord
- DTN: Dorsal tegmental nucleus
- FF: Field of Forel
- GE: Ganglionic eminence
- GR: Gracile nucleus
- HEM: Cerebellar hemispheres
- HY: Hypothalamus
- IC: Inferior colliculus
- ICc: Inferior colliculus, central nucleus
- IG: Induseum griseum
- IO: Inferior olive
- IP: Interposed nucleus
- LD: Lateral dorsal nucleus [thalamus]
- LHA: Lateral hypothalamic area
- LMN: Lateral mammillary nucleus
- LPO: Lateral preoptic area
- LRN: Lateral reticular nucleus
- LV: Lateral ventricle
- MD: Medial dorsal nucleus [thalamus]
- NAc: Nucleus accumbens
- NDB: Nucleus of the diagonal band
- NL: Nucleus limitans [thalamus]
- OT: Olfactory tubercle
- PB: Parabrachial nucleus
- PG: Pontine gray
- PMd: Dorsal premammillary nucleus
- PMv: Ventral premammillary nucleus
- PRP: Nucleus prepositus
- PULV: Pulvinar nucleus [thalamus]
- Pf: Parafascicular nucleus [thalamus]
- Prt: Pretectum
- RCH: Retrochiasmatic nucleus [hypothalamus]
- RE: Nucleus reuniens
- RR: Retrorubral area
- RT: Reticular nucleus [thalamus]
- RTN: Reticular tegmental nucleus
- Ret-MB: Reticular formation, Midbrain
- Ret-Med: Reticular formation, Medulla
- Ret-P: Reticular formation, Pons
- Rms: Rostral migratory stream
- SC: Superior colliculus
- SCH: Suprachiasmatic nucleus [hypothalamus]
- SI: Substantia innominata
- SN: Substantia nigra
- SO: Superior olive
- SOL: Solitary nucleus
- SPV: Spinal nucleus of the trigeminal
- SUM: Supramammillary area
- Sth: Subthalamus
- TRI: Germinal trigone
- VA: Ventral anterior nucleus [thalamus]
- VG: Ventral gray of the spinal cord
- VIIn: Facial motor nucleus
- VL: Ventral lateral nucleus [thalamus]
- VMH: Ventromedial nucleus [hypothalamus]
- VNC: Vestibular nuclear complex
- VPM: Ventral posteromedial nucleus [thalamus]
- XIn: Accessory nucleus
- ZI: Zona incerta

Age: 14 GW

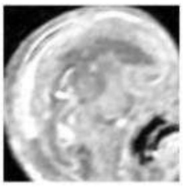

L-R Level: 1.86 mm

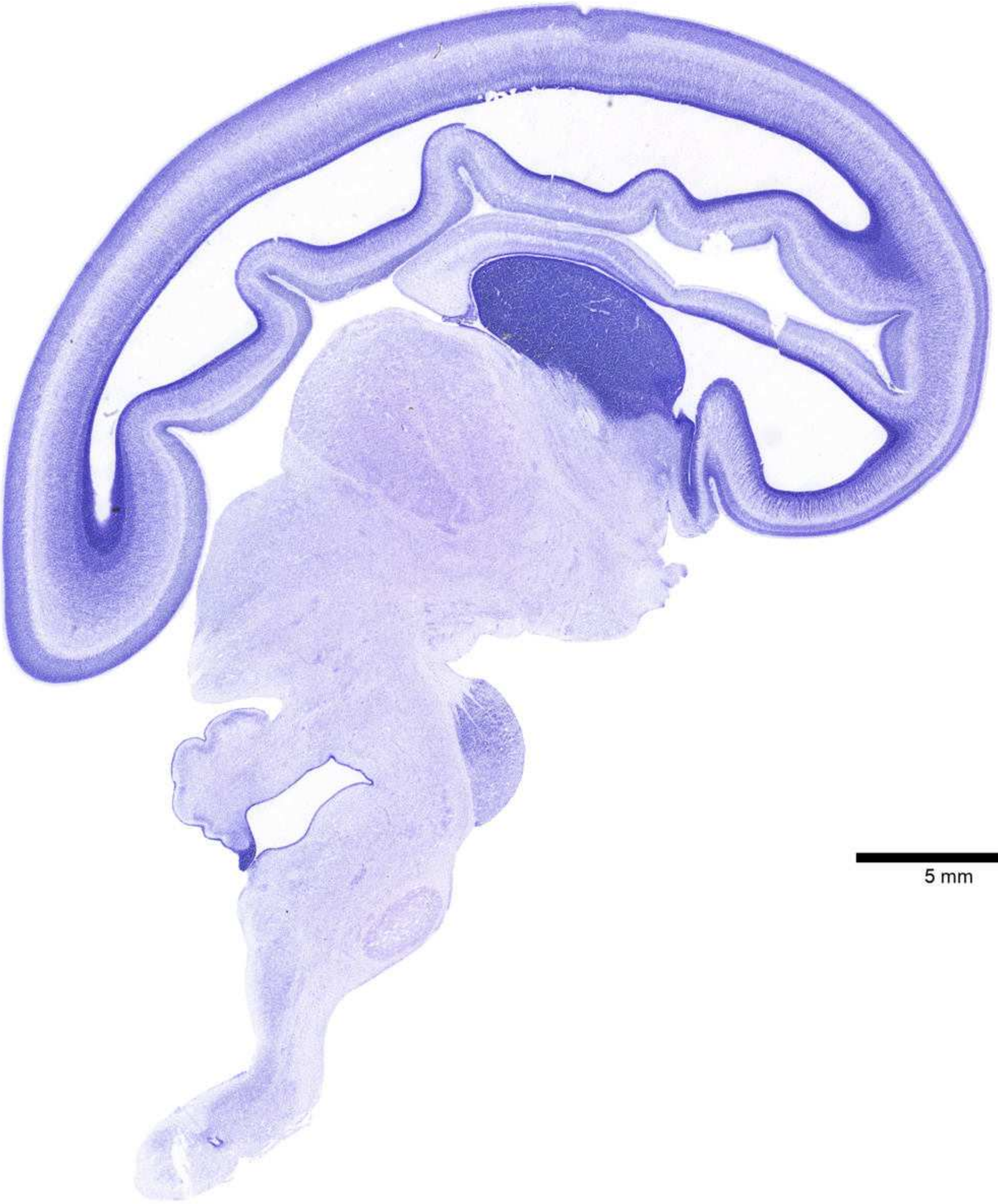

5 mm

Transient Layers

- VZ
- SVZ
- IZ
- SP
- CP
- MZ
- SGL

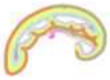

Cortical Areas

- FCTx
- ORB
- PAR
- OCC
- CING

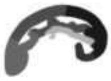

Age: 14 GW

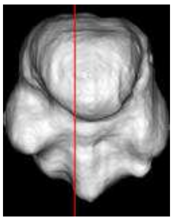

L-R Level: 1.86 mm

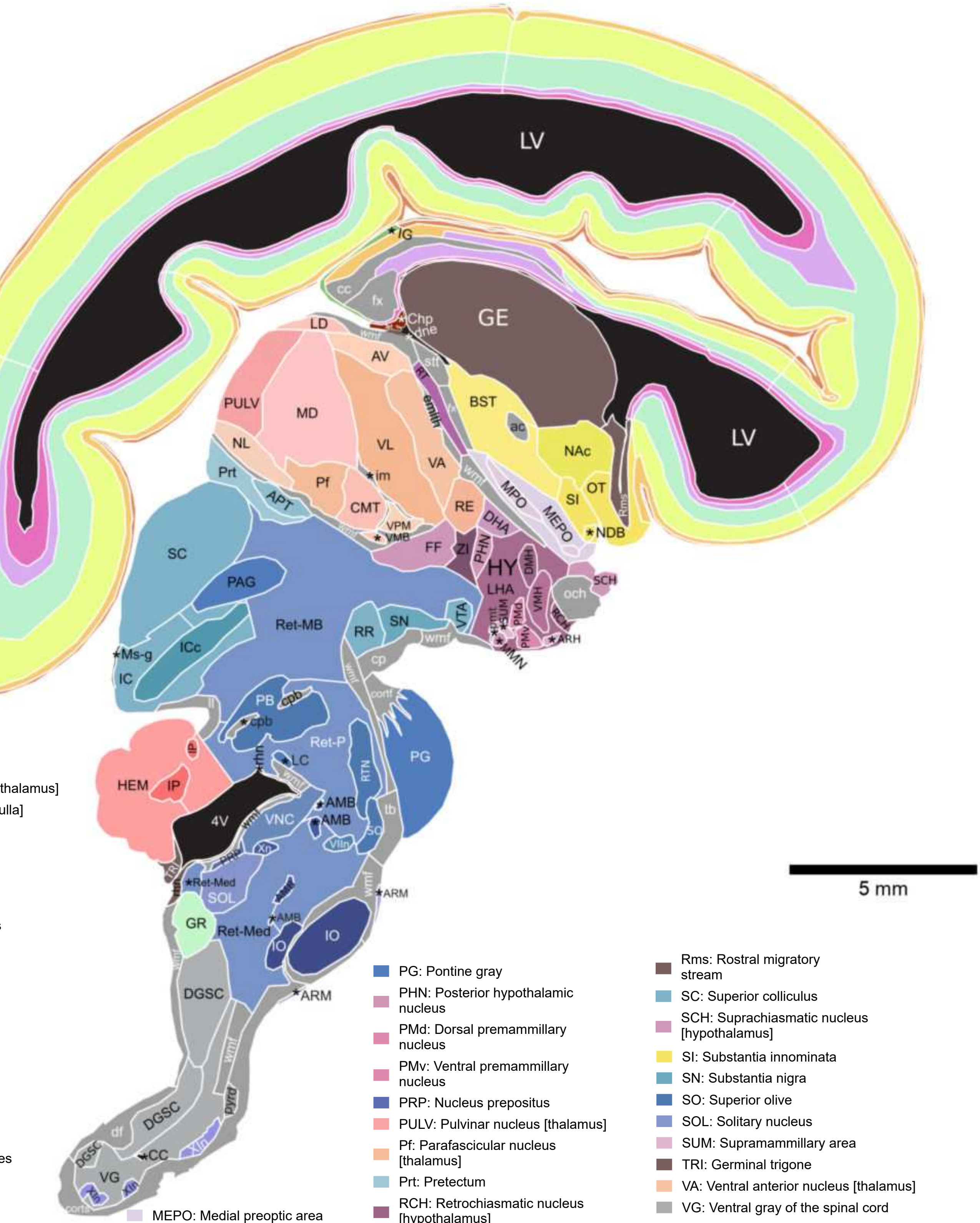

- 4V: Fourth ventricle
- AMB: Nucleus ambiguus
- APT: Anterior pretectal nucleus
- ARH: Arcuate nucleus [hypothalamus]
- ARM: Arcuate nucleus [medulla]
- AV: Anteroventral nucleus [thalamus]
- BST: Bed nucleus of the stria terminalis
- CC: Central canal
- CMT: Centromedian nucleus [thalamus]
- Chp: Choroid plexus
- DGSC: Dorsal gray of the spinal cord
- DHA: Dorsal hypothalamic area
- DMH: Dorsomedial nucleus [hypothalamus]
- FF: Field of Forel
- GE: Ganglionic eminence
- GR: Gracile nucleus
- HEM: Cerebellar hemispheres
- HY: Hypothalamus
- IC: Inferior colliculus
- ICc: Inferior colliculus, central nucleus
- IG: Induseum griseum
- IO: Inferior olive
- IP: Interposed nucleus
- LC: Locus coeruleus
- LD: Lateral dorsal nucleus [thalamus]
- LHA: Lateral hypothalamic area
- LV: Lateral ventricle
- MD: Medial dorsal nucleus [thalamus]
- MEPO: Medial preoptic area
- MMN: Medial mammillary nucleus
- MPO: Medial preoptic nucleus
- Ms-g: Migratory stream, general
- NAc: Nucleus accumbens
- NDB: Nucleus of the diagonal band
- NL: Nucleus limitans [thalamus]
- OT: Olfactory tubercle
- PAG: Periaqueductal gray
- PB: Parabrachial nucleus
- PG: Pontine gray
- PHN: Posterior hypothalamic nucleus
- PMd: Dorsal premammillary nucleus
- PMv: Ventral premammillary nucleus
- PRP: Nucleus prepositus
- PULV: Pulvinar nucleus [thalamus]
- Pf: Parafascicular nucleus [thalamus]
- Prt: Pretectum
- RCH: Retrochiasmatic nucleus [hypothalamus]
- RE: Nucleus reuniens
- RR: Retrorubral area
- RT: Reticular nucleus [thalamus]
- RTN: Reticular tegmental nucleus
- Ret-MB: Reticular formation, Midbrain
- Ret-Med: Reticular formation, Medulla
- Ret-P: Reticular formation, Pons
- Rms: Rostral migratory stream
- SC: Superior colliculus
- SCH: Suprachiasmatic nucleus [hypothalamus]
- SI: Substantia innominata
- SN: Substantia nigra
- SO: Superior olive
- SOL: Solitary nucleus
- SUM: Supramammillary area
- TRI: Germinal trigone
- VA: Ventral anterior nucleus [thalamus]
- VG: Ventral gray of the spinal cord
- VIIn: Facial motor nucleus
- VL: Ventral lateral nucleus [thalamus]
- VMB: Ventral medial basal nucleus [thalamus]
- VMH: Ventromedial nucleus [hypothalamus]
- VNC: Vestibular nuclear complex
- VPM: Ventral posteromedial nucleus [thalamus]
- VTA: Ventral tegmental area
- XIn: Accessory nucleus
- Xn: Dorsal motor nucleus
- ZI: Zona incerta

Age: 14 GW

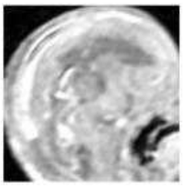

L-R Level: 1.68 mm

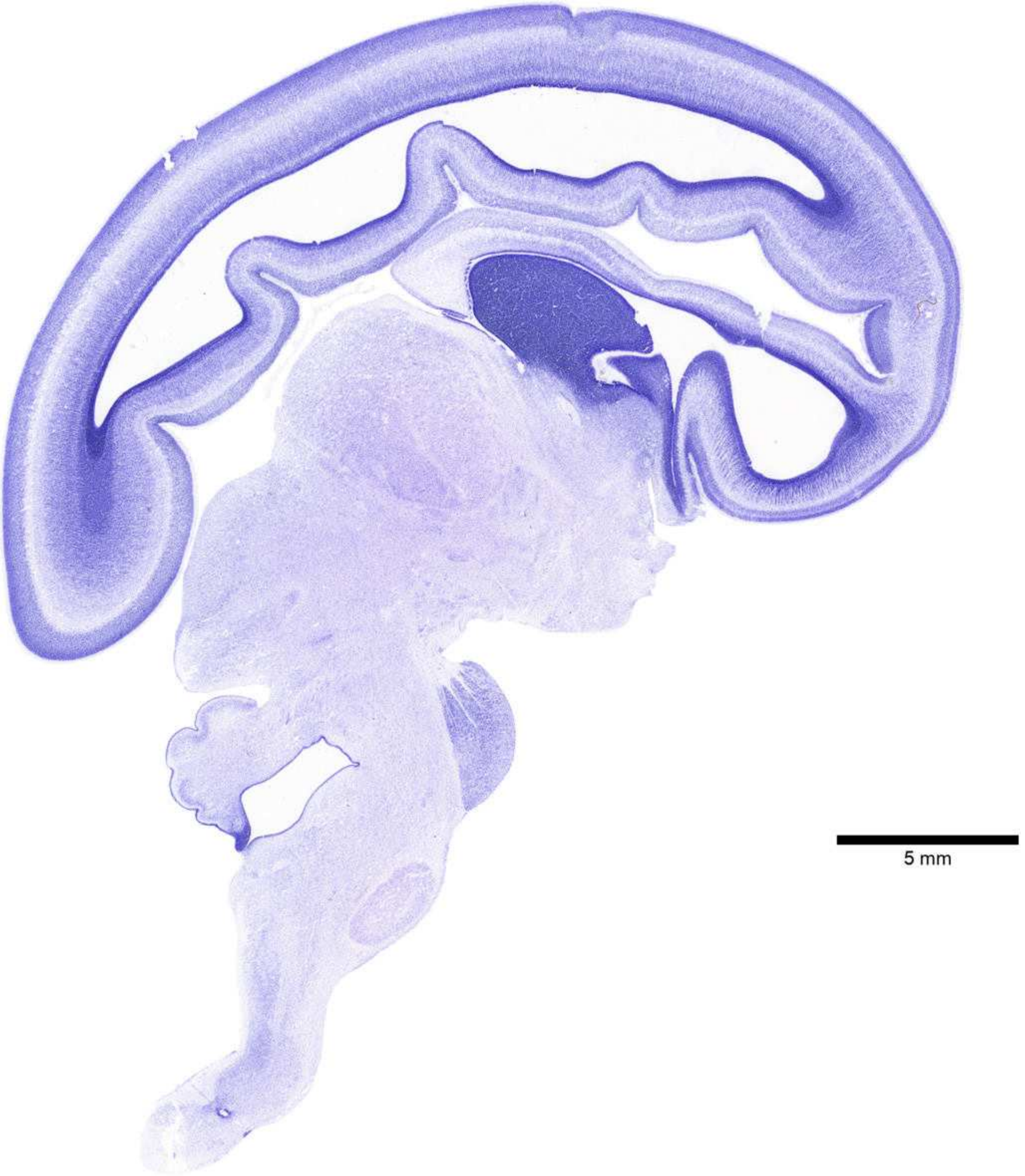

5 mm

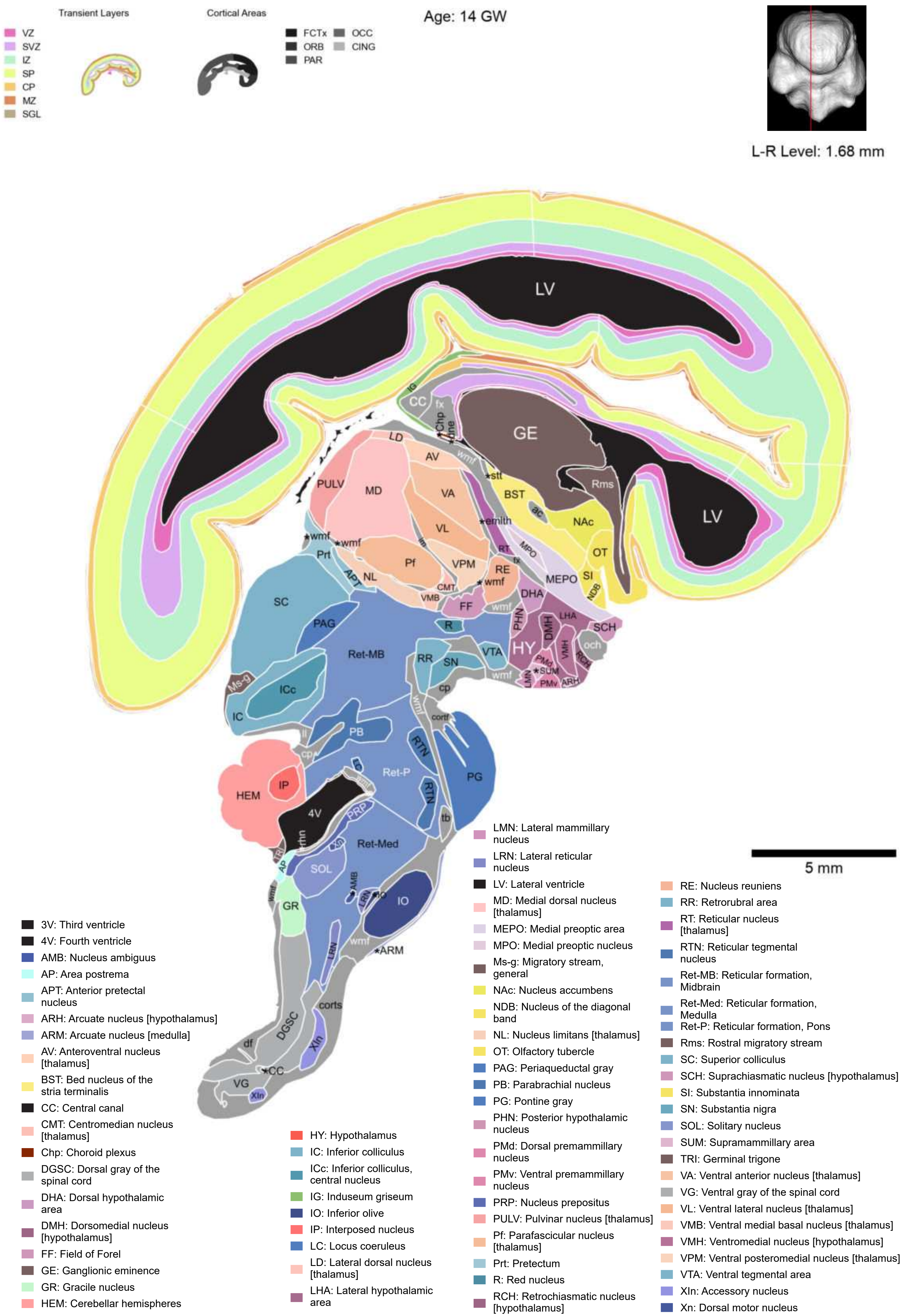
Age: 14 GW
L-R Level: 1.68 mm
Transient Layers
VZ
SVZ
IZ
SP
CP
MZ
SGL
Cortical Areas
FCTx
ORB
PAR
OCC
CING
5 mm
LV
GE
Rms
BST
NAc
OT
SI
NDB
MEPO
MPO
CC
IG
fx
Chp
LD
AV
VA
VL
MD
PULV
Pf
VPM
NL
APT
Prt
RE
RT
CMT
VMB
FF
DHA
PHN
DMH
LHA
SCH
och
HY
VMH
RCH
PMd
PMv
SUM
ARH
LMN
VTA
SN
RR
R
cp
SC
PAG
Ret-MB
Ms-g
ICc
IC
PB
RTN
Ret-P
PG
tb
HEM
IP
4V
PRP
Ret-Med
SOL
AP
TRI
GR
IO
AMB
LRN
ARM
corts
DGSC
Xln
df
CC
VG
XIn
3V: Third ventricle
4V: Fourth ventricle
AMB: Nucleus ambiguus
AP: Area postrema
APT: Anterior pretectal nucleus
ARH: Arcuate nucleus [hypothalamus]
ARM: Arcuate nucleus [medulla]
AV: Anteroventral nucleus [thalamus]
BST: Bed nucleus of the stria terminalis
CC: Central canal
CMT: Centromedian nucleus [thalamus]
Chp: Choroid plexus
DGSC: Dorsal gray of the spinal cord
DHA: Dorsal hypothalamic area
DMH: Dorsomedial nucleus [hypothalamus]
FF: Field of Forel
GE: Ganglionic eminence
GR: Gracile nucleus
HEM: Cerebellar hemispheres
HY: Hypothalamus
IC: Inferior colliculus
ICc: Inferior colliculus, central nucleus
IG: Induseum griseum
IO: Inferior olive
IP: Interposed nucleus
LC: Locus coeruleus
LD: Lateral dorsal nucleus [thalamus]
LHA: Lateral hypothalamic area
LMN: Lateral mammillary nucleus
LRN: Lateral reticular nucleus
LV: Lateral ventricle
MD: Medial dorsal nucleus [thalamus]
MEPO: Medial preoptic area
MPO: Medial preoptic nucleus
Ms-g: Migratory stream, general
NAc: Nucleus accumbens
NDB: Nucleus of the diagonal band
NL: Nucleus limitans [thalamus]
OT: Olfactory tubercle
PAG: Periaqueductal gray
PB: Parabrachial nucleus
PG: Pontine gray
PHN: Posterior hypothalamic nucleus
PMd: Dorsal premammillary nucleus
PMv: Ventral premammillary nucleus
PRP: Nucleus prepositus
PULV: Pulvinar nucleus [thalamus]
Pf: Parafascicular nucleus [thalamus]
Prt: Pretectum
R: Red nucleus
RCH: Retrochiasmatic nucleus [hypothalamus]
RE: Nucleus reuniens
RR: Retrorubral area
RT: Reticular nucleus [thalamus]
RTN: Reticular tegmental nucleus
Ret-MB: Reticular formation, Midbrain
Ret-Med: Reticular formation, Medulla
Ret-P: Reticular formation, Pons
Rms: Rostral migratory stream
SC: Superior colliculus
SCH: Suprachiasmatic nucleus [hypothalamus]
SI: Substantia innominata
SN: Substantia nigra
SOL: Solitary nucleus
SUM: Supramammillary area
TRI: Germinal trigone
VA: Ventral anterior nucleus [thalamus]
VG: Ventral gray of the spinal cord
VL: Ventral lateral nucleus [thalamus]
VMB: Ventral medial basal nucleus [thalamus]
VMH: Ventromedial nucleus [hypothalamus]
VPM: Ventral posteromedial nucleus [thalamus]
VTA: Ventral tegmental area
XIn: Accessory nucleus
Xn: Dorsal motor nucleus

Age: 14 GW

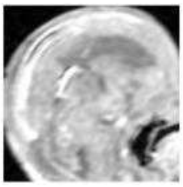

L-R Level: 1.26 mm

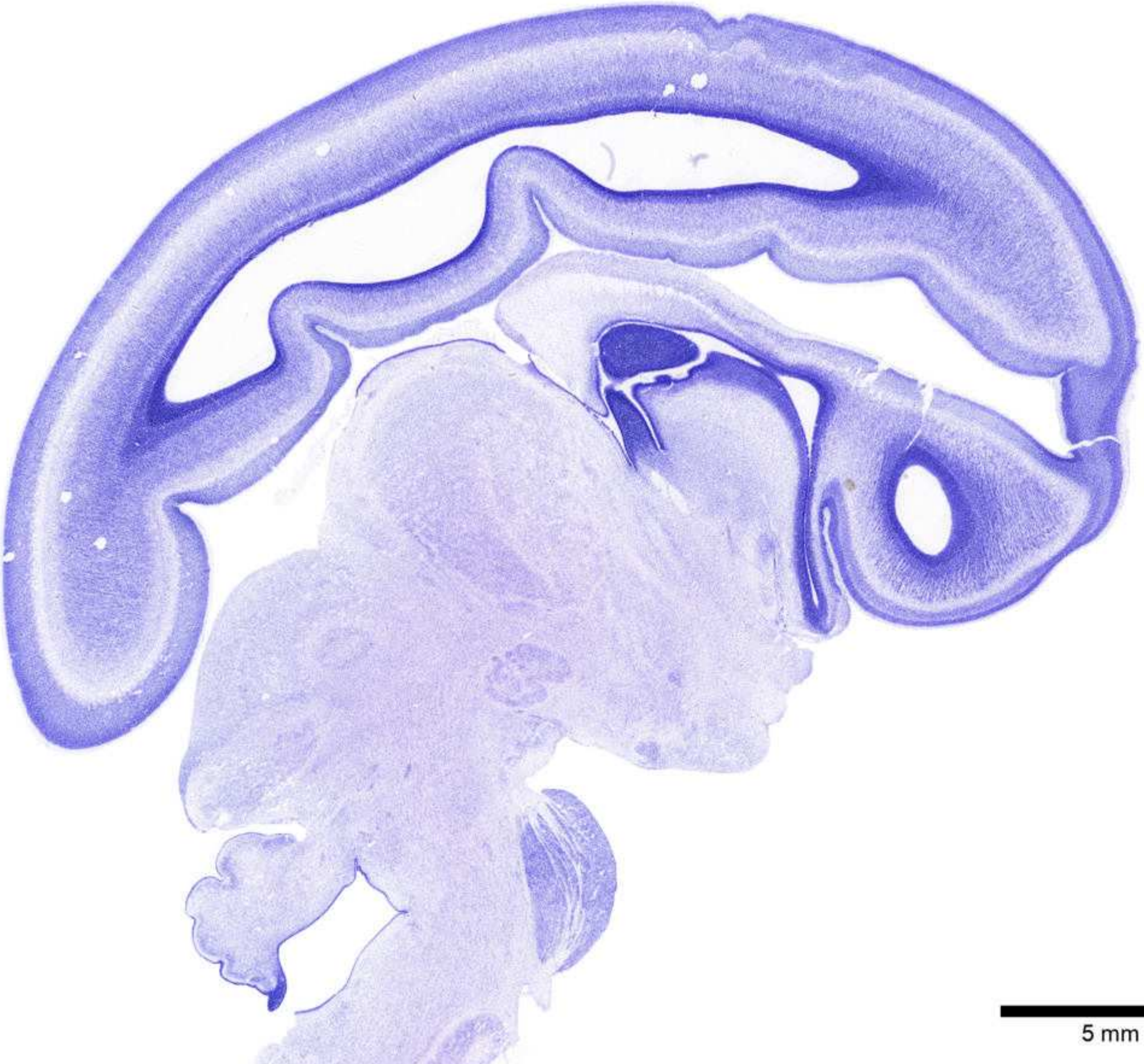

5 mm

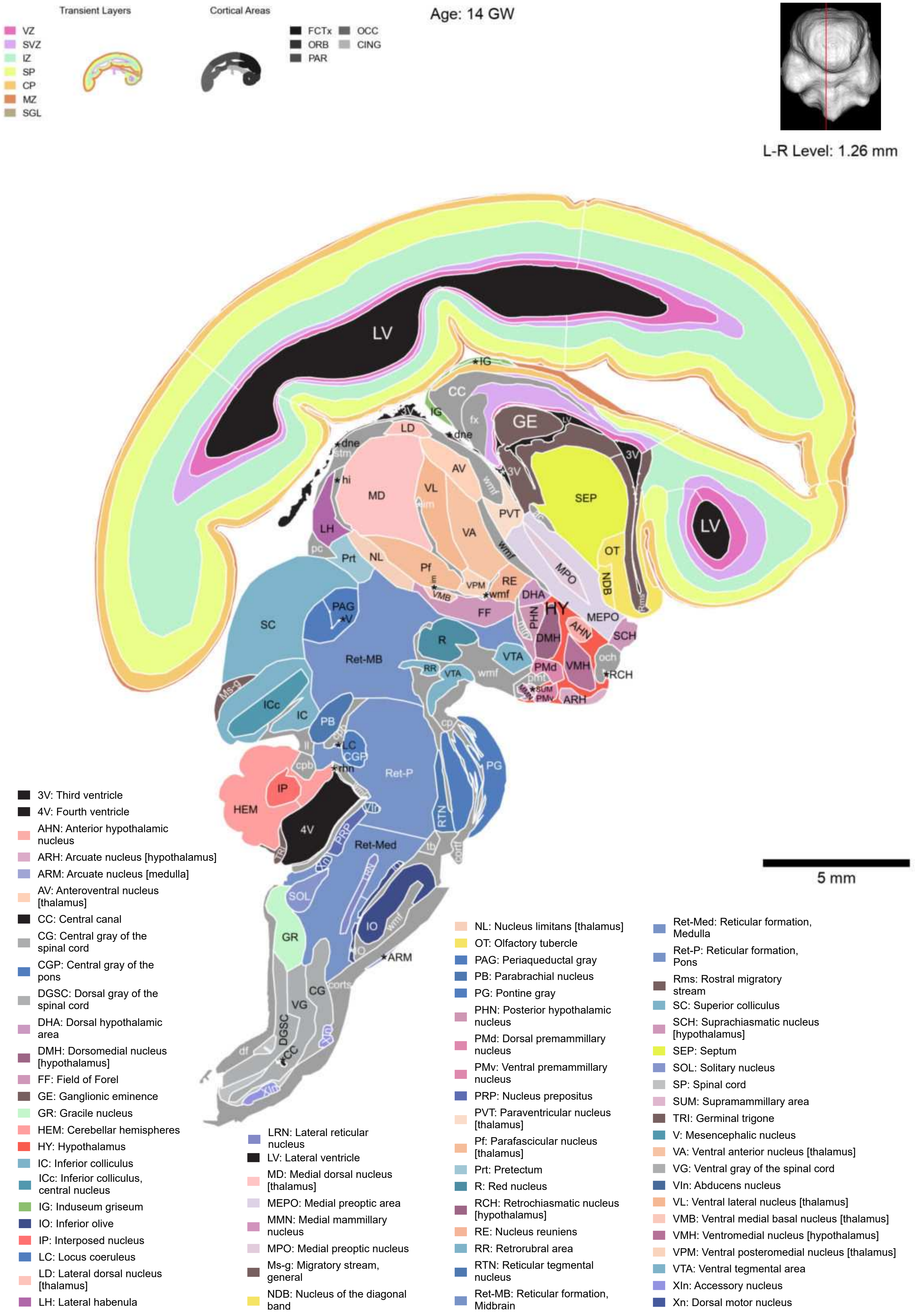

Transient Layers
VZ
SVZ
IZ
SP
CP
MZ
SGL
Cortical Areas
FCTx
ORB
PAR
OCC
CING
Age: 14 GW
L-R Level: 1.26 mm
5 mm
LV
IG
CC
fx
GE
3V
SEP
OT
NDB
Rms
LD
dne
stm
hi
MD
VL
AV
wmf
PVT
VA
LH
pc
Prt
NL
Pf
VPM
RE
VMB
MPO
DHA
HY
MEPO
PHN
AHN
SCH
DMH
VMH
och
RCH
PMd
SUM
PMv
ARH
FF
R
RR
VTA
PAG
SC
Ret-MB
Ms-g
ICc
IC
PB
LC
CGP
rhn
cpb
PG
Ret-P
RTN
cp
HEM
IP
4V
PRP
Ret-Med
tb
SOL
GR
IO
ARM
CG
VG
DGSC
CC
df
3V: Third ventricle
4V: Fourth ventricle
AHN: Anterior hypothalamic nucleus
ARH: Arcuate nucleus [hypothalamus]
ARM: Arcuate nucleus [medulla]
AV: Anteroventral nucleus [thalamus]
CC: Central canal
CG: Central gray of the spinal cord
CGP: Central gray of the pons
DGSC: Dorsal gray of the spinal cord
DHA: Dorsal hypothalamic area
DMH: Dorsomedial nucleus [hypothalamus]
FF: Field of Forel
GE: Ganglionic eminence
GR: Gracile nucleus
HEM: Cerebellar hemispheres
HY: Hypothalamus
IC: Inferior colliculus
ICc: Inferior colliculus, central nucleus
IG: Induseum griseum
IO: Inferior olive
IP: Interposed nucleus
LC: Locus coeruleus
LD: Lateral dorsal nucleus [thalamus]
LH: Lateral habenula
LRN: Lateral reticular nucleus
LV: Lateral ventricle
MD: Medial dorsal nucleus [thalamus]
MEPO: Medial preoptic area
MMN: Medial mammillary nucleus
MPO: Medial preoptic nucleus
Ms-g: Migratory stream, general
NDB: Nucleus of the diagonal band
NL: Nucleus limitans [thalamus]
OT: Olfactory tubercle
PAG: Periaqueductal gray
PB: Parabrachial nucleus
PG: Pontine gray
PHN: Posterior hypothalamic nucleus
PMd: Dorsal premammillary nucleus
PMv: Ventral premammillary nucleus
PRP: Nucleus prepositus
PVT: Paraventricular nucleus [thalamus]
Pf: Parafascicular nucleus [thalamus]
Prt: Pretectum
R: Red nucleus
RCH: Retrochiasmatic nucleus [hypothalamus]
RE: Nucleus reuniens
RR: Retrorubral area
RTN: Reticular tegmental nucleus
Ret-MB: Reticular formation, Midbrain
Ret-Med: Reticular formation, Medulla
Ret-P: Reticular formation, Pons
Rms: Rostral migratory stream
SC: Superior colliculus
SCH: Suprachiasmatic nucleus [hypothalamus]
SEP: Septum
SOL: Solitary nucleus
SP: Spinal cord
SUM: Supramammillary area
TRI: Germinal trigone
V: Mesencephalic nucleus
VA: Ventral anterior nucleus [thalamus]
VG: Ventral gray of the spinal cord
VIn: Abducens nucleus
VL: Ventral lateral nucleus [thalamus]
VMB: Ventral medial basal nucleus [thalamus]
VMH: Ventromedial nucleus [hypothalamus]
VPM: Ventral posteromedial nucleus [thalamus]
VTA: Ventral tegmental area
XIn: Accessory nucleus
Xn: Dorsal motor nucleus

Age: 14 GW

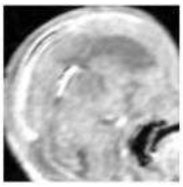

L-R Level: 1.08 mm

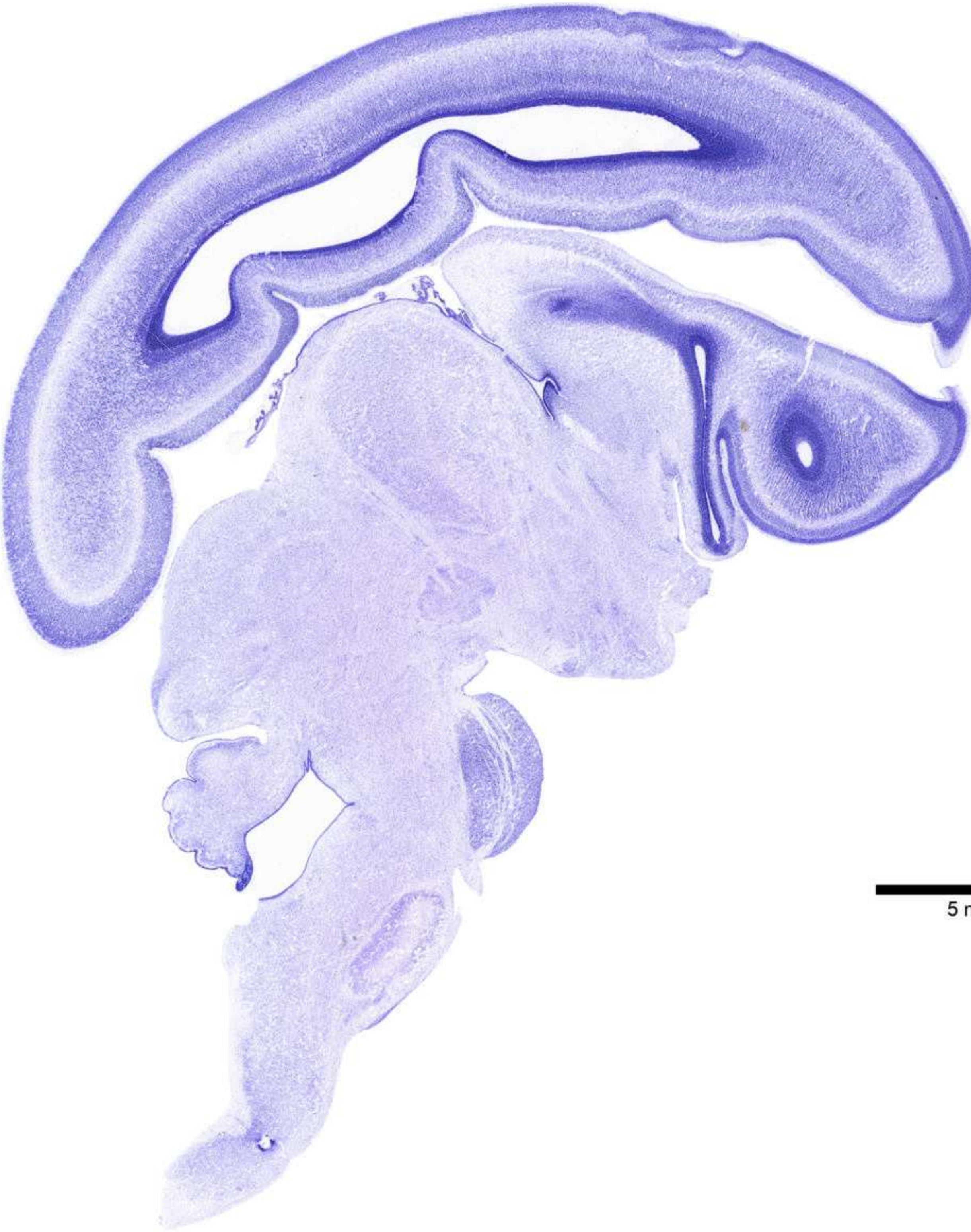

5 mm

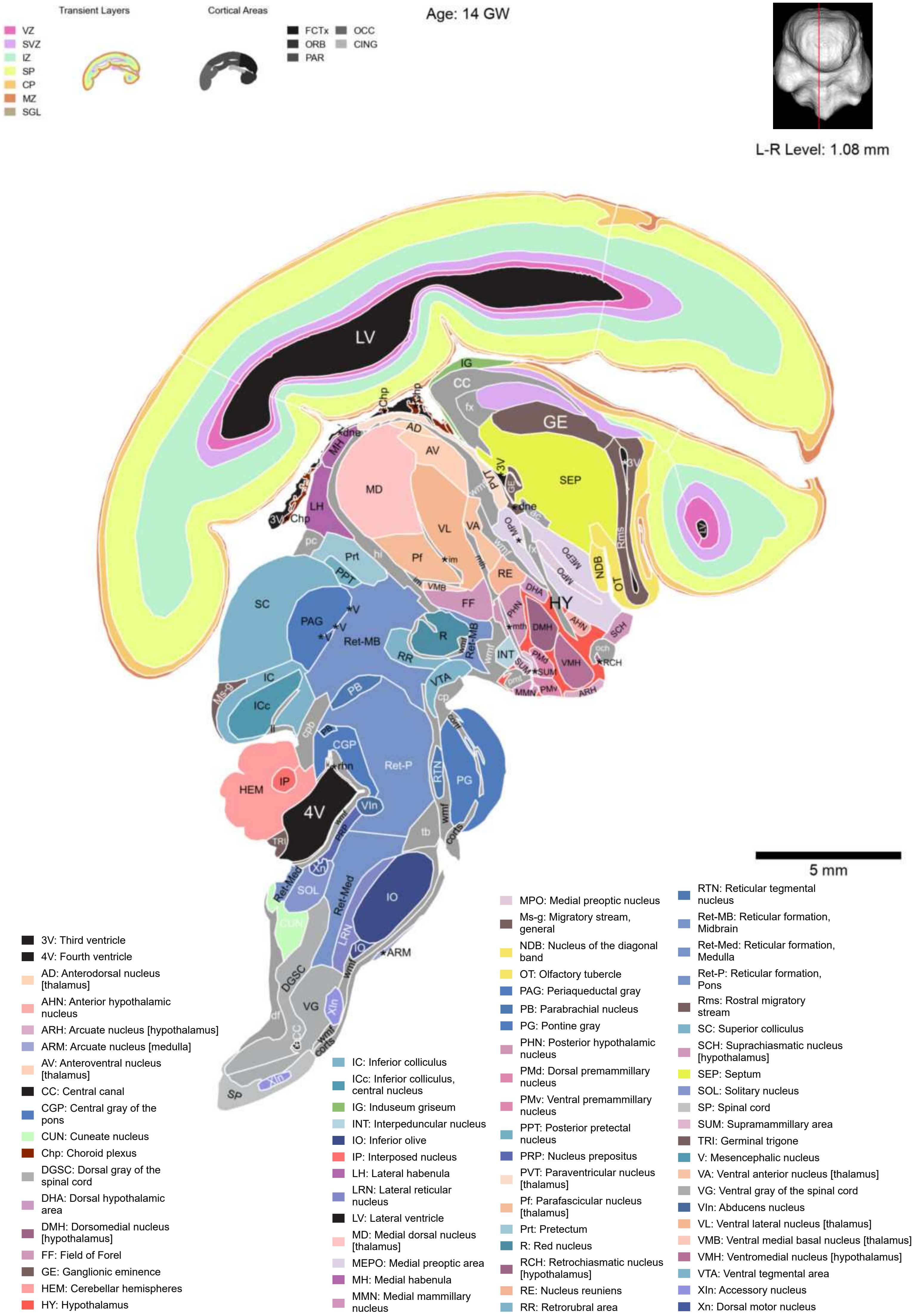

Transient Layers
VZ
SVZ
IZ
SP
CP
MZ
SGL
Cortical Areas
FCTx
ORB
PAR
OCC
CING
Age: 14 GW
L-R Level: 1.08 mm
5 mm
LV
IG
CC
fx
GE
Chp
AD
AV
MH
dne
MD
LH
3V
PVT
SEP
Rms
NDB
OT
VL
VA
MPO
MEPO
Pf
im
mth
RE
VMB
FF
DHA
HY
AHN
SCH
PHN
DMH
pc
Prt
PPT
SC
PAG
V
Ret-MB
R
RR
INT
SUM
PMd
VMH
och
RCH
MMN
PMv
ARH
IC
ICc
Ms-g
PB
VTA
cp
cpb
CGP
rhn
Ret-P
RTN
PG
HEM
IP
4V
VIn
PRP
wmf
tb
TRI
Xn
Ret-Med
SOL
IO
CUN
LRN
ARM
DGSC
VG
XIn
df
CC
corts
SP
3V: Third ventricle
4V: Fourth ventricle
AD: Anterodorsal nucleus [thalamus]
AHN: Anterior hypothalamic nucleus
ARH: Arcuate nucleus [hypothalamus]
ARM: Arcuate nucleus [medulla]
AV: Anteroventral nucleus [thalamus]
CC: Central canal
CGP: Central gray of the pons
CUN: Cuneate nucleus
Chp: Choroid plexus
DGSC: Dorsal gray of the spinal cord
DHA: Dorsal hypothalamic area
DMH: Dorsomedial nucleus [hypothalamus]
FF: Field of Forel
GE: Ganglionic eminence
HEM: Cerebellar hemispheres
HY: Hypothalamus
IC: Inferior colliculus
ICc: Inferior colliculus, central nucleus
IG: Induseum griseum
INT: Interpeduncular nucleus
IO: Inferior olive
IP: Interposed nucleus
LH: Lateral habenula
LRN: Lateral reticular nucleus
LV: Lateral ventricle
MD: Medial dorsal nucleus [thalamus]
MEPO: Medial preoptic area
MH: Medial habenula
MMN: Medial mammillary nucleus
MPO: Medial preoptic nucleus
Ms-g: Migratory stream, general
NDB: Nucleus of the diagonal band
OT: Olfactory tubercle
PAG: Periaqueductal gray
PB: Parabrachial nucleus
PG: Pontine gray
PHN: Posterior hypothalamic nucleus
PMd: Dorsal premammillary nucleus
PMv: Ventral premammillary nucleus
PPT: Posterior pretectal nucleus
PRP: Nucleus prepositus
PVT: Paraventricular nucleus [thalamus]
Pf: Parafascicular nucleus [thalamus]
Prt: Pretectum
R: Red nucleus
RCH: Retrochiasmatic nucleus [hypothalamus]
RE: Nucleus reuniens
RR: Retrorubral area
RTN: Reticular tegmental nucleus
Ret-MB: Reticular formation, Midbrain
Ret-Med: Reticular formation, Medulla
Ret-P: Reticular formation, Pons
Rms: Rostral migratory stream
SC: Superior colliculus
SCH: Suprachiasmatic nucleus [hypothalamus]
SEP: Septum
SOL: Solitary nucleus
SP: Spinal cord
SUM: Supramammillary area
TRI: Germinal trigone
V: Mesencephalic nucleus
VA: Ventral anterior nucleus [thalamus]
VG: Ventral gray of the spinal cord
VIn: Abducens nucleus
VL: Ventral lateral nucleus [thalamus]
VMB: Ventral medial basal nucleus [thalamus]
VMH: Ventromedial nucleus [hypothalamus]
VTA: Ventral tegmental area
XIn: Accessory nucleus
Xn: Dorsal motor nucleus

Age: 14 GW

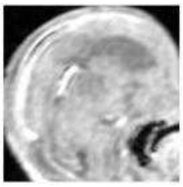

L-R Level: 0.96 mm

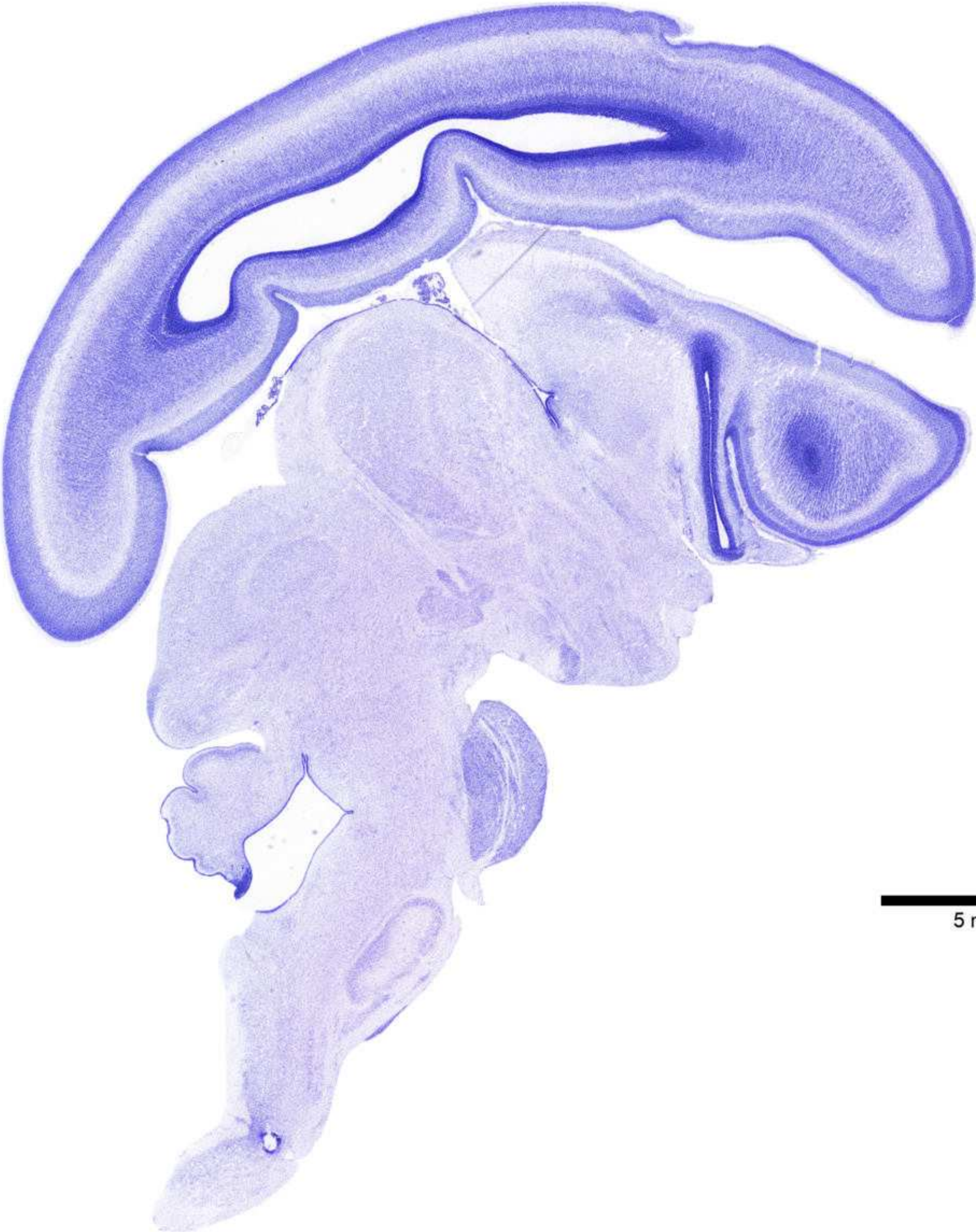

5 mm

Transient Layers

- VZ
- SVZ
- IZ
- SP
- CP
- MZ
- SGL

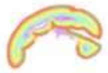

Cortical Areas

- FCTx
- ORB
- PAR
- OCC
- CING

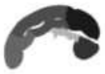

Age: 14 GW

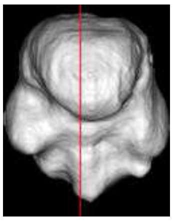

L-R Level: 0.96 mm

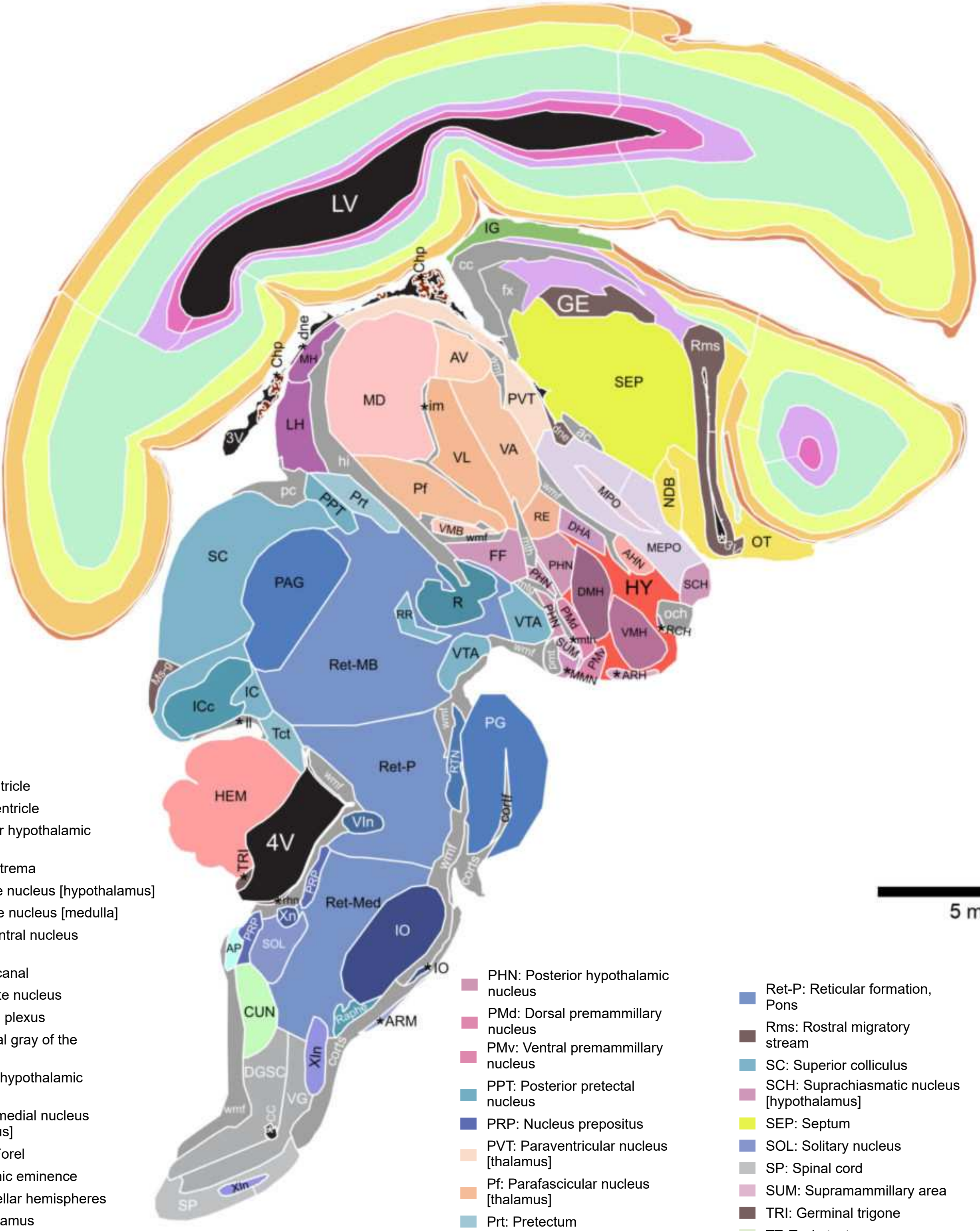

5 mm

- 3V: Third ventricle
- 4V: Fourth ventricle
- AHN: Anterior hypothalamic nucleus
- AP: Area postrema
- ARH: Arcuate nucleus [hypothalamus]
- ARM: Arcuate nucleus [medulla]
- AV: Anteroventral nucleus [thalamus]
- CC: Central canal
- CUN: Cuneate nucleus
- Chp: Choroid plexus
- DGSC: Dorsal gray of the spinal cord
- DHA: Dorsal hypothalamic area
- DMH: Dorsomedial nucleus [hypothalamus]
- FF: Field of Forel
- GE: Ganglionic eminence
- HEM: Cerebellar hemispheres
- HY: Hypothalamus
- IC: Inferior colliculus
- ICc: Inferior colliculus, central nucleus
- IG: Induseum griseum
- IO: Inferior olive
- LH: Lateral habenula
- LV: Lateral ventricle
- MD: Medial dorsal nucleus [thalamus]
- MEPO: Medial preoptic area
- MH: Medial habenula
- MMN: Medial mammillary nucleus
- MPO: Medial preoptic nucleus
- Ms-g: Migratory stream, general
- NDB: Nucleus of the diagonal band
- OT: Olfactory tubercle
- PAG: Periaqueductal gray
- PG: Pontine gray
- PHN: Posterior hypothalamic nucleus
- PMd: Dorsal premammillary nucleus
- PMv: Ventral premammillary nucleus
- PPT: Posterior pretectal nucleus
- PRP: Nucleus prepositus
- PVT: Paraventricular nucleus [thalamus]
- Pf: Parafascicular nucleus [thalamus]
- Prt: Pretectum
- R: Red nucleus
- RCH: Retrochiasmatic nucleus [hypothalamus]
- RE: Nucleus reuniens
- RR: Retrorubral area
- RTN: Reticular tegmental nucleus
- Raphe: Raphe nuclei
- Ret-MB: Reticular formation, Midbrain
- Ret-Med: Reticular formation, Medulla
- Ret-P: Reticular formation, Pons
- Rms: Rostral migratory stream
- SC: Superior colliculus
- SCH: Suprachiasmatic nucleus [hypothalamus]
- SEP: Septum
- SOL: Solitary nucleus
- SP: Spinal cord
- SUM: Supramammillary area
- TRI: Germinal trigone
- TT: Tenia tecta
- Tct: Tectum
- VA: Ventral anterior nucleus [thalamus]
- VG: Ventral gray of the spinal cord
- VIn: Abducens nucleus
- VL: Ventral lateral nucleus [thalamus]
- VMB: Ventral medial basal nucleus [thalamus]
- VMH: Ventromedial nucleus [hypothalamus]
- VTA: Ventral tegmental area
- XIn: Accessory nucleus
- Xn: Dorsal motor nucleus

Age: 14 GW

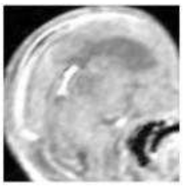

L-R Level: 0.72 mm

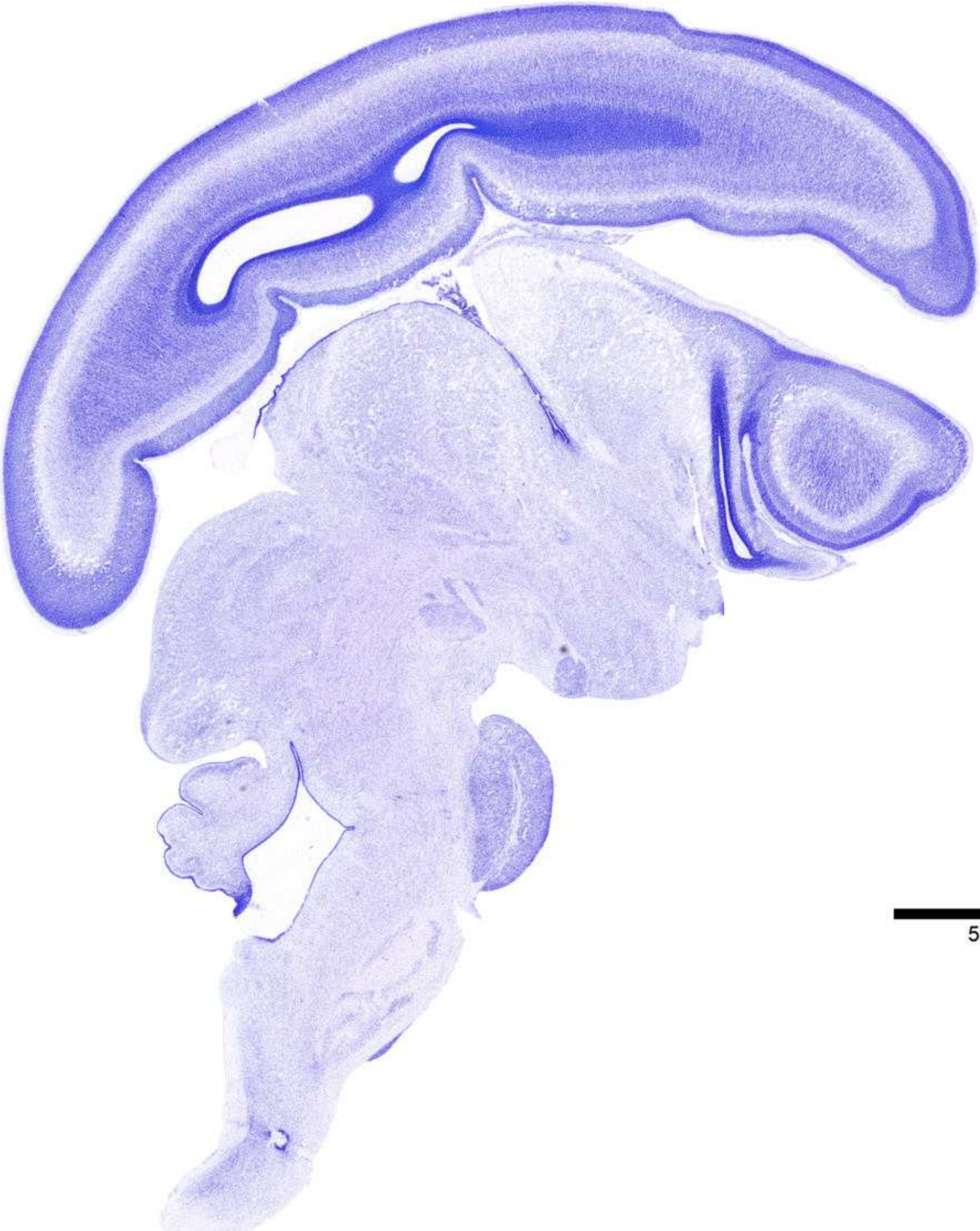

5 mm

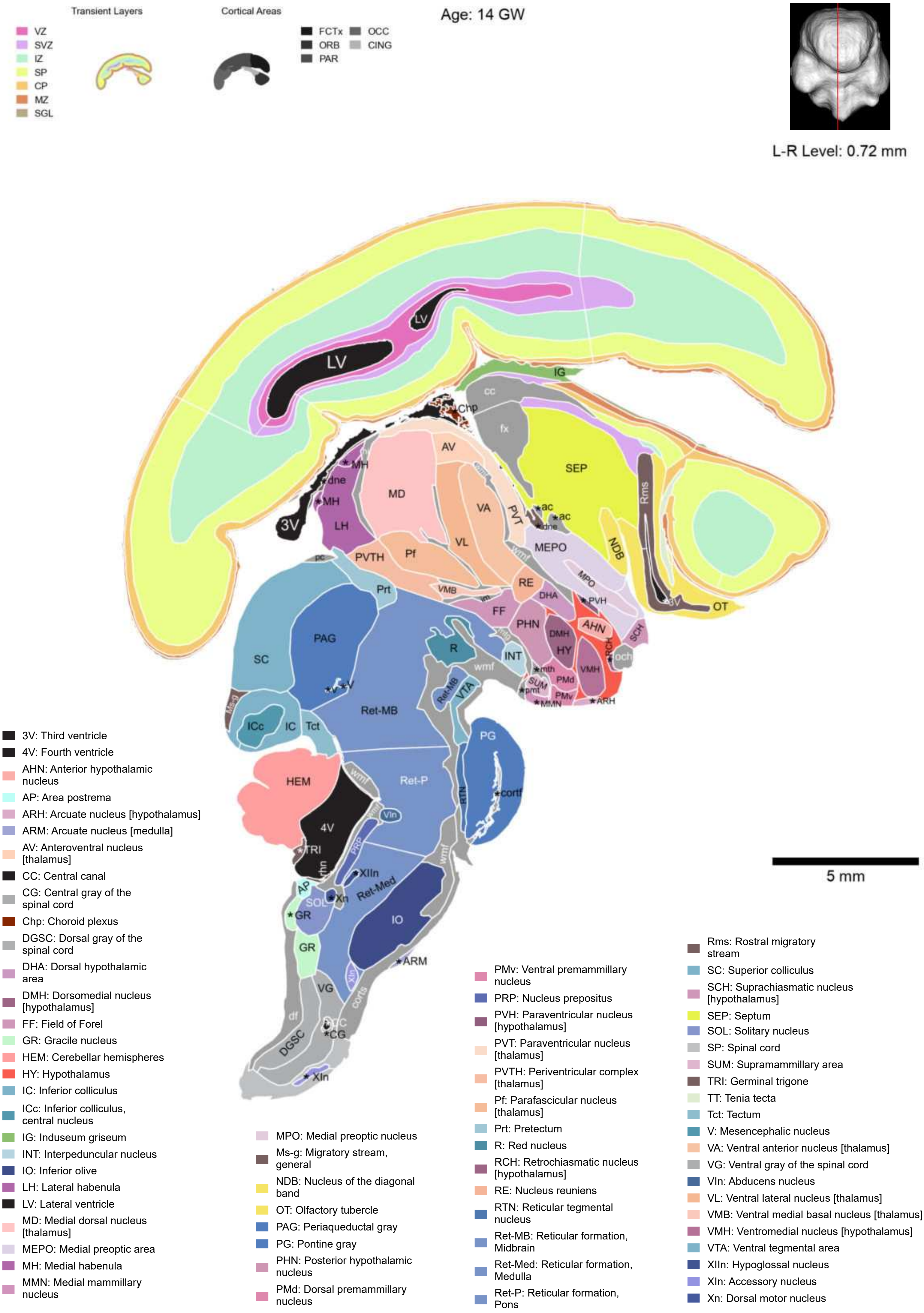
Transient Layers
VZ
SVZ
IZ
SP
CP
MZ
SGL
Cortical Areas
FCTx
ORB
PAR
OCC
CING
Age: 14 GW
L-R Level: 0.72 mm
5 mm
3V: Third ventricle
4V: Fourth ventricle
AHN: Anterior hypothalamic nucleus
AP: Area postrema
ARH: Arcuate nucleus [hypothalamus]
ARM: Arcuate nucleus [medulla]
AV: Anteroventral nucleus [thalamus]
CC: Central canal
CG: Central gray of the spinal cord
Chp: Choroid plexus
DGSC: Dorsal gray of the spinal cord
DHA: Dorsal hypothalamic area
DMH: Dorsomedial nucleus [hypothalamus]
FF: Field of Forel
GR: Gracile nucleus
HEM: Cerebellar hemispheres
HY: Hypothalamus
IC: Inferior colliculus
ICc: Inferior colliculus, central nucleus
IG: Induseum griseum
INT: Interpeduncular nucleus
IO: Inferior olive
LH: Lateral habenula
LV: Lateral ventricle
MD: Medial dorsal nucleus [thalamus]
MEPO: Medial preoptic area
MH: Medial habenula
MMN: Medial mammillary nucleus
MPO: Medial preoptic nucleus
Ms-g: Migratory stream, general
NDB: Nucleus of the diagonal band
OT: Olfactory tubercle
PAG: Periaqueductal gray
PG: Pontine gray
PHN: Posterior hypothalamic nucleus
PMd: Dorsal premammillary nucleus
PMv: Ventral premammillary nucleus
PRP: Nucleus prepositus
PVH: Paraventricular nucleus [hypothalamus]
PVT: Paraventricular nucleus [thalamus]
PVTH: Periventricular complex [thalamus]
Pf: Parafascicular nucleus [thalamus]
Prt: Pretectum
R: Red nucleus
RCH: Retrochiasmatic nucleus [hypothalamus]
RE: Nucleus reuniens
RTN: Reticular tegmental nucleus
Ret-MB: Reticular formation, Midbrain
Ret-Med: Reticular formation, Medulla
Ret-P: Reticular formation, Pons
Rms: Rostral migratory stream
SC: Superior colliculus
SCH: Suprachiasmatic nucleus [hypothalamus]
SEP: Septum
SOL: Solitary nucleus
SP: Spinal cord
SUM: Supramammillary area
TRI: Germinal trigone
TT: Tenia tecta
Tct: Tectum
V: Mesencephalic nucleus
VA: Ventral anterior nucleus [thalamus]
VG: Ventral gray of the spinal cord
VIn: Abducens nucleus
VL: Ventral lateral nucleus [thalamus]
VMB: Ventral medial basal nucleus [thalamus]
VMH: Ventromedial nucleus [hypothalamus]
VTA: Ventral tegmental area
XIIn: Hypoglossal nucleus
XIn: Accessory nucleus
Xn: Dorsal motor nucleus

Age: 14 GW

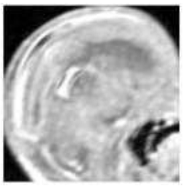

L-R Level: 0.3 mm

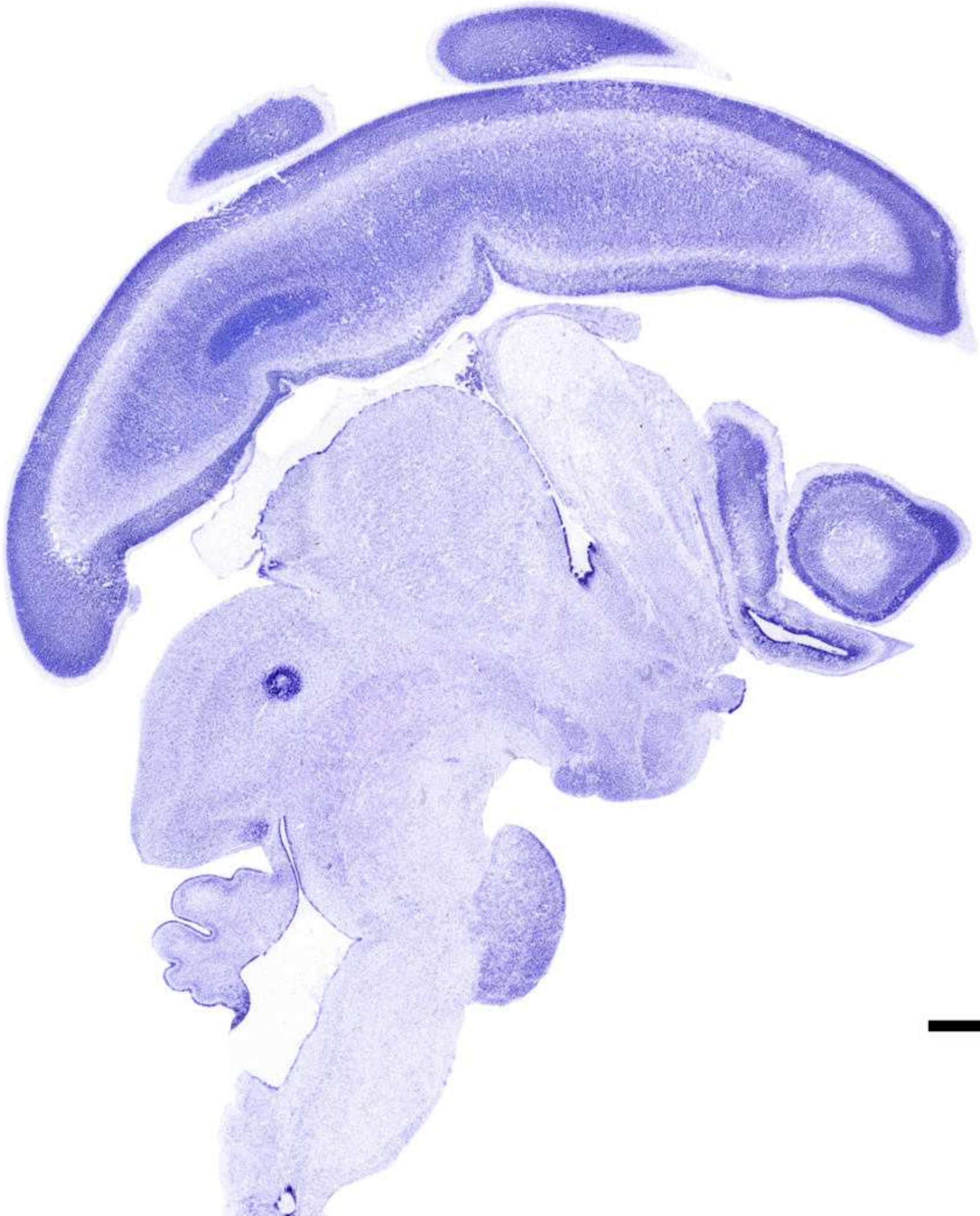

5 mm

Transient Layers

- SVZ
- IZ
- SP
- CP
- MZ
- SGL

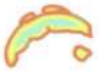

Cortical Areas

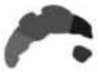

- FCTx
- ORB
- PAR
- OCC

Age: 14 GW

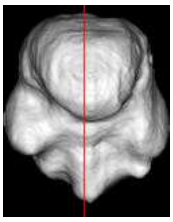

L-R Level: 0.3 mm

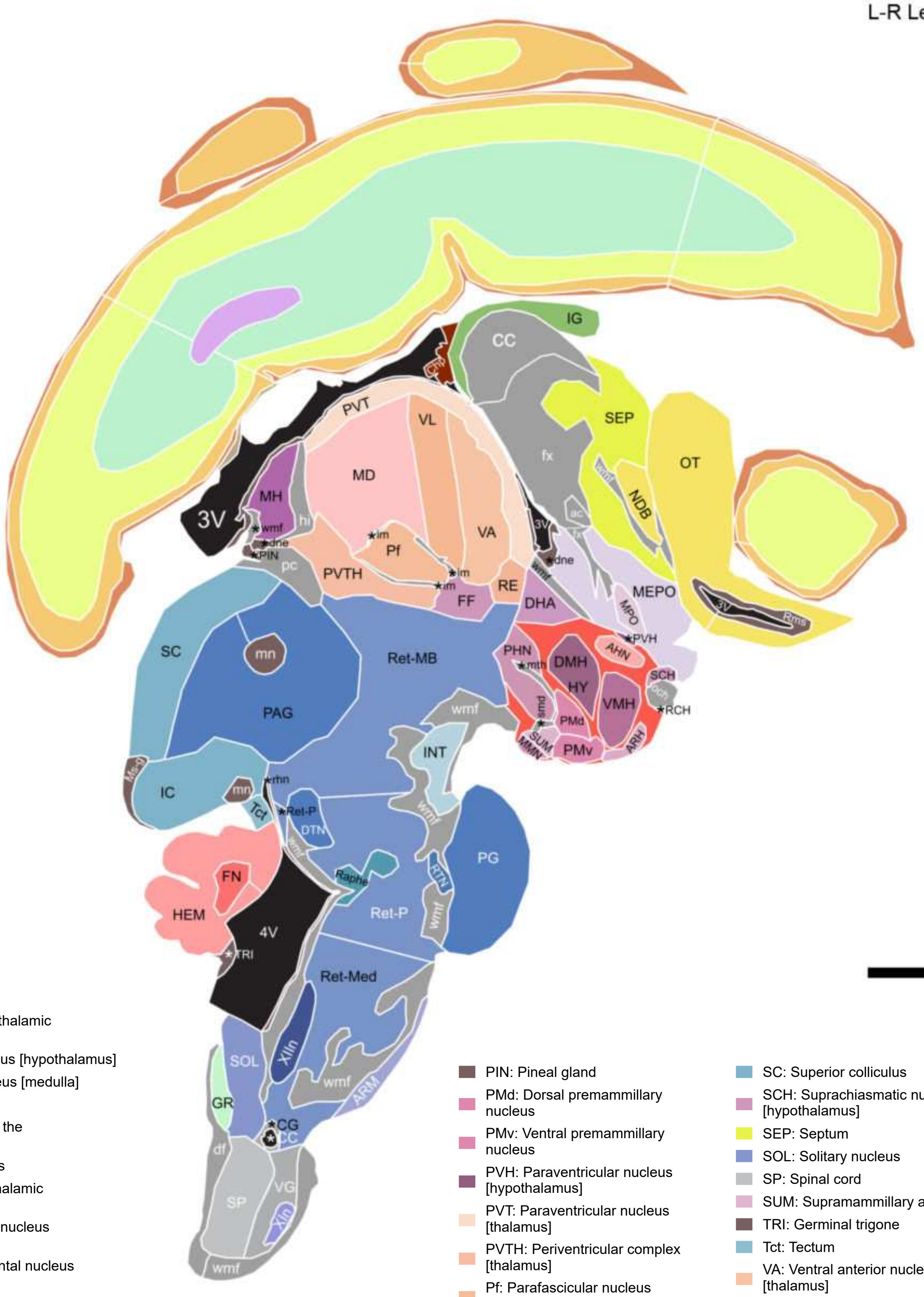

5 mm

- 3V: Third ventricle
- 4V: Fourth ventricle
- AHN: Anterior hypothalamic nucleus
- ARH: Arcuate nucleus [hypothalamus]
- ARM: Arcuate nucleus [medulla]
- CC: Central canal
- CG: Central gray of the spinal cord
- Chp: Choroid plexus
- DHA: Dorsal hypothalamic area
- DMH: Dorsomedial nucleus [hypothalamus]
- DTN: Dorsal tegmental nucleus
- FF: Field of Forel
- FN: Fastigial nucleus
- GR: Gracile nucleus
- HEM: Cerebellar hemispheres
- HY: Hypothalamus
- IC: Inferior colliculus
- IG: Induseum griseum
- INT: Interpeduncular nucleus
- MD: Medial dorsal nucleus [thalamus]
- MEPO: Medial preoptic area
- MH: Medial habenula
- MMN: Medial mammillary nucleus
- MPO: Medial preoptic nucleus
- Ms-g: Migratory stream, general
- NDB: Nucleus of the diagonal band
- OT: Olfactory tubercle
- PAG: Periaqueductal gray
- PG: Pontine gray
- PHN: Posterior hypothalamic nucleus
- PIN: Pineal gland
- PMd: Dorsal premammillary nucleus
- PMv: Ventral premammillary nucleus
- PVH: Paraventricular nucleus [hypothalamus]
- PVT: Paraventricular nucleus [thalamus]
- PVTH: Periventricular complex [thalamus]
- Pf: Parafascicular nucleus [thalamus]
- RCH: Retrochiasmatic nucleus [hypothalamus]
- RE: Nucleus reuniens
- RTN: Reticular tegmental nucleus
- Raphe: Raphe nuclei
- Ret-MB: Reticular formation, Midbrain
- Ret-Med: Reticular formation, Medulla
- Ret-P: Reticular formation, Pons
- Rms: Rostral migratory stream
- SC: Superior colliculus
- SCH: Suprachiasmatic nucleus [hypothalamus]
- SEP: Septum
- SOL: Solitary nucleus
- SP: Spinal cord
- SUM: Supramammillary area
- TRI: Germinal trigone
- Tct: Tectum
- VA: Ventral anterior nucleus [thalamus]
- VG: Ventral gray of the spinal cord
- VL: Ventral lateral nucleus [thalamus]
- VMH: Ventromedial nucleus [hypothalamus]
- XIIn: Hypoglossal nucleus
- XIn: Accessory nucleus

Age: 14 GW

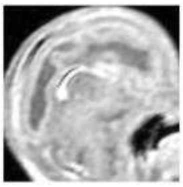

L-R Level: -0.6 mm

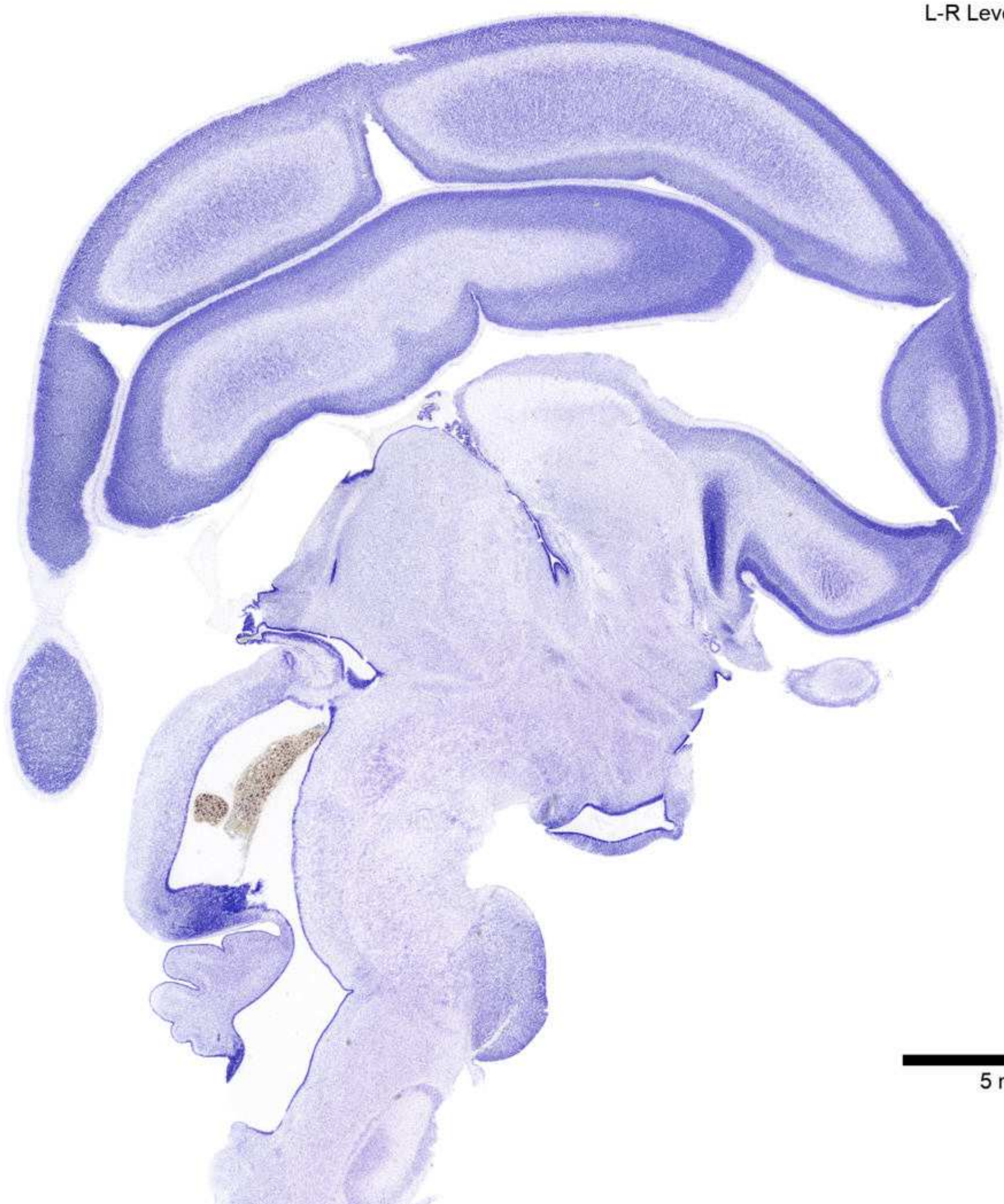

5 mm

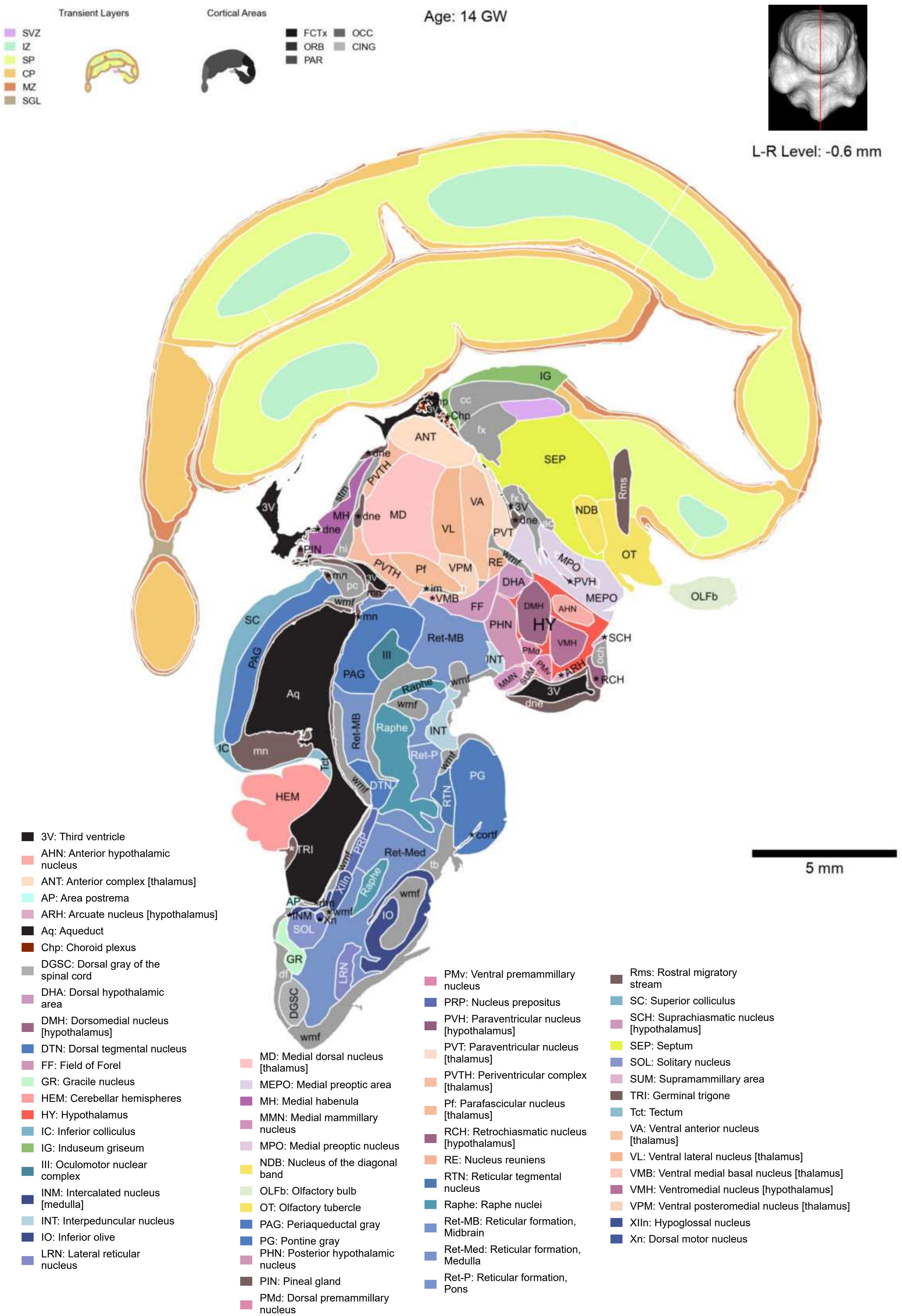

Transient Layers
SVZ
IZ
SP
CP
MZ
SGL
Cortical Areas
FCTx
ORB
PAR
OCC
CING
Age: 14 GW
L-R Level: -0.6 mm
5 mm
IG
cc
Chp
3V
fx
ANT
SEP
Rms
NDB
OT
OLFb
dne
PVTH
stm
MH
MD
VA
VL
PVT
3V
PIN
hi
Pf
VPM
RE
wmf
MPO
PVH
MEPO
DHA
DMH
AHN
HY
VMH
SCH
PHN
FF
mn
pc
im
VMB
Ret-MB
INT
PMd
PMv
ARH
MMN
SUM
och
RCH
SC
PAG
III
Aq
Raphe
Ret-MB
INT
Ret-P
IC
mn
Tct
HEM
DTN
RTN
PG
cortf
TRI
PRP
Ret-Med
XIIn
tb
AP
INM
Xn
SOL
IO
GR
df
LRN
DGSC
3V: Third ventricle
AHN: Anterior hypothalamic nucleus
ANT: Anterior complex [thalamus]
AP: Area postrema
ARH: Arcuate nucleus [hypothalamus]
Aq: Aqueduct
Chp: Choroid plexus
DGSC: Dorsal gray of the spinal cord
DHA: Dorsal hypothalamic area
DMH: Dorsomedial nucleus [hypothalamus]
DTN: Dorsal tegmental nucleus
FF: Field of Forel
GR: Gracile nucleus
HEM: Cerebellar hemispheres
HY: Hypothalamus
IC: Inferior colliculus
IG: Induseum griseum
III: Oculomotor nuclear complex
INM: Intercalated nucleus [medulla]
INT: Interpeduncular nucleus
IO: Inferior olive
LRN: Lateral reticular nucleus
MD: Medial dorsal nucleus [thalamus]
MEPO: Medial preoptic area
MH: Medial habenula
MMN: Medial mammillary nucleus
MPO: Medial preoptic nucleus
NDB: Nucleus of the diagonal band
OLFb: Olfactory bulb
OT: Olfactory tubercle
PAG: Periaqueductal gray
PG: Pontine gray
PHN: Posterior hypothalamic nucleus
PIN: Pineal gland
PMd: Dorsal premammillary nucleus
PMv: Ventral premammillary nucleus
PRP: Nucleus prepositus
PVH: Paraventricular nucleus [hypothalamus]
PVT: Paraventricular nucleus [thalamus]
PVTH: Periventricular complex [thalamus]
Pf: Parafascicular nucleus [thalamus]
RCH: Retrochiasmatic nucleus [hypothalamus]
RE: Nucleus reuniens
RTN: Reticular tegmental nucleus
Raphe: Raphe nuclei
Ret-MB: Reticular formation, Midbrain
Ret-Med: Reticular formation, Medulla
Ret-P: Reticular formation, Pons
Rms: Rostral migratory stream
SC: Superior colliculus
SCH: Suprachiasmatic nucleus [hypothalamus]
SEP: Septum
SOL: Solitary nucleus
SUM: Supramammillary area
TRI: Germinal trigone
Tct: Tectum
VA: Ventral anterior nucleus [thalamus]
VL: Ventral lateral nucleus [thalamus]
VMB: Ventral medial basal nucleus [thalamus]
VMH: Ventromedial nucleus [hypothalamus]
VPM: Ventral posteromedial nucleus [thalamus]
XIIn: Hypoglossal nucleus
Xn: Dorsal motor nucleus

Age: 14 GW

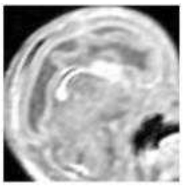

L-R Level: -0.96 mm

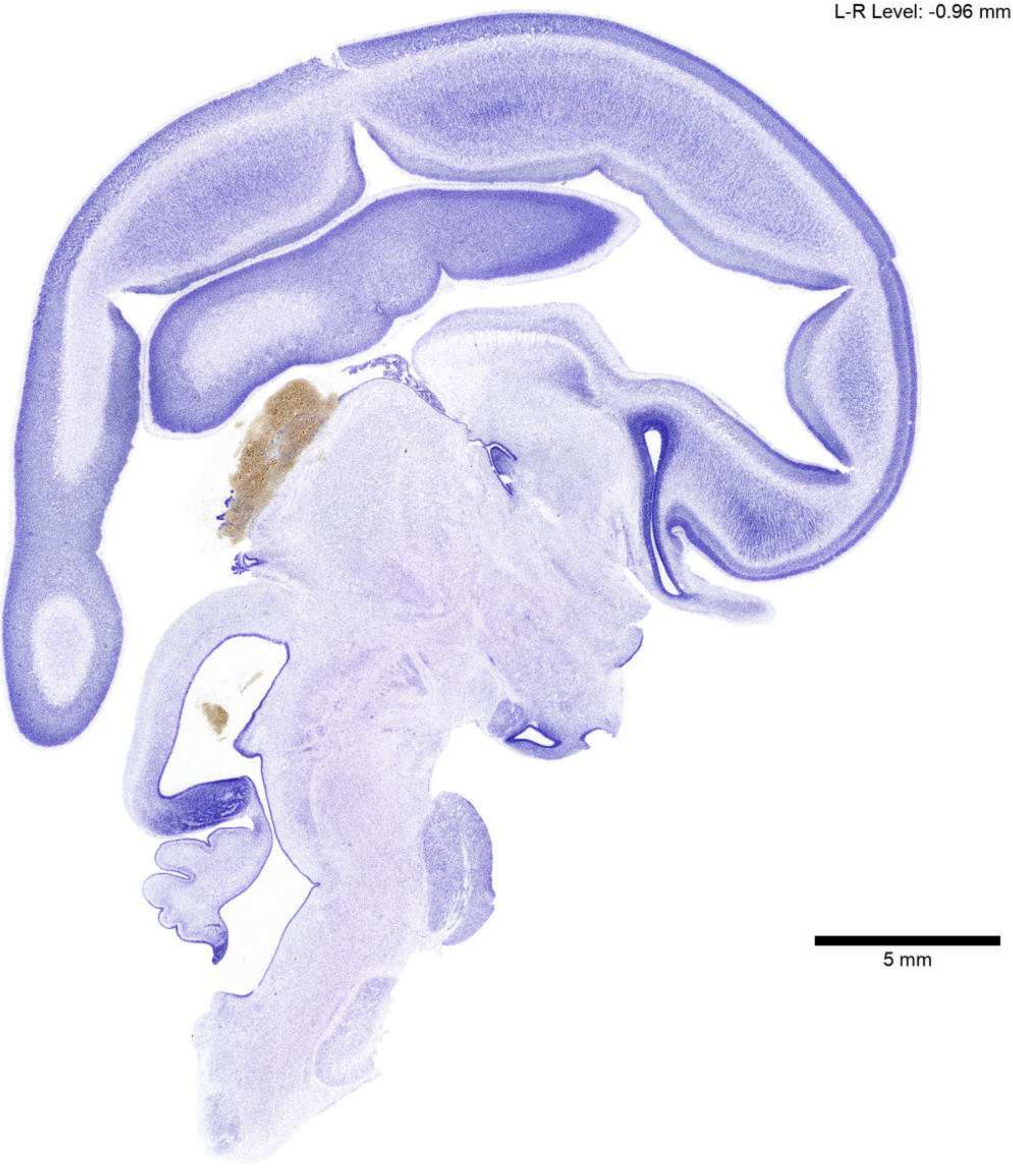

5 mm

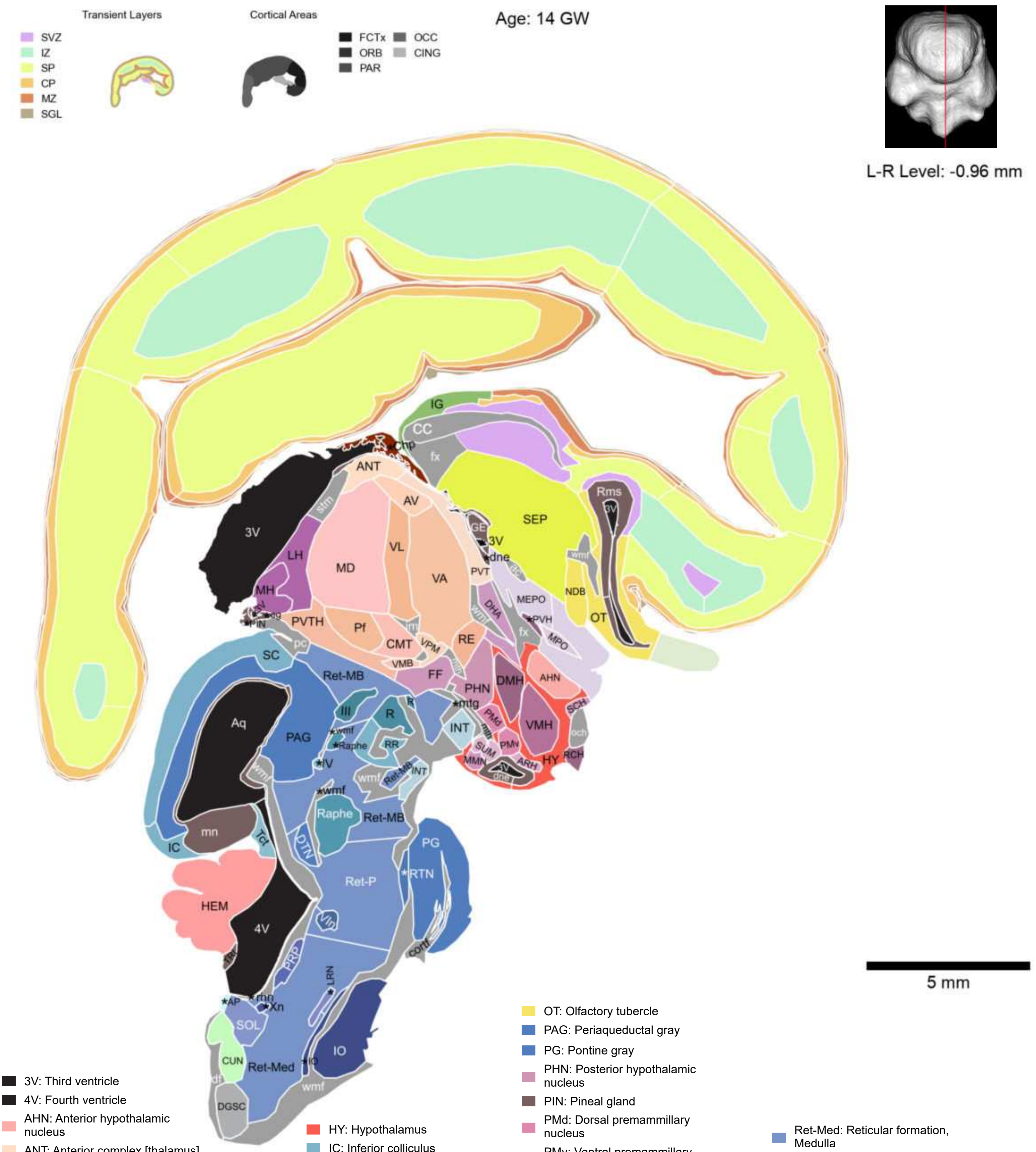

- 3V: Third ventricle
- 4V: Fourth ventricle
- AHN: Anterior hypothalamic nucleus
- ANT: Anterior complex [thalamus]
- AP: Area postrema
- ARH: Arcuate nucleus [hypothalamus]
- AV: Anteroventral nucleus [thalamus]
- Aq: Aqueduct
- CMT: Centromedian nucleus [thalamus]
- CUN: Cuneate nucleus
- Chp: Choroid plexus
- DGSC: Dorsal gray of the spinal cord
- DHA: Dorsal hypothalamic area
- DMH: Dorsomedial nucleus [hypothalamus]
- DTN: Dorsal tegmental nucleus
- FF: Field of Forel
- GE: Ganglionic eminence
- HEM: Cerebellar hemispheres
- HY: Hypothalamus
- IC: Inferior colliculus
- IG: Induseum griseum
- III: Oculomotor nuclear complex
- INT: Interpeduncular nucleus
- IO: Inferior olive
- IV: Trochlear nucleus
- LH: Lateral habenula
- LRN: Lateral reticular nucleus
- MD: Medial dorsal nucleus [thalamus]
- MEPO: Medial preoptic area
- MH: Medial habenula
- MMN: Medial mammillary nucleus
- MPO: Medial preoptic nucleus
- NDB: Nucleus of the diagonal band
- OLFb: Olfactory bulb
- OT: Olfactory tubercle
- PAG: Periaqueductal gray
- PG: Pontine gray
- PHN: Posterior hypothalamic nucleus
- PIN: Pineal gland
- PMd: Dorsal premammillary nucleus
- PMv: Ventral premammillary nucleus
- PRP: Nucleus prepositus
- PVH: Paraventricular nucleus [hypothalamus]
- PVT: Paraventricular nucleus [thalamus]
- PVTH: Periventricular complex [thalamus]
- Pf: Parafascicular nucleus [thalamus]
- R: Red nucleus
- RCH: Retrochiasmatic nucleus [hypothalamus]
- RE: Nucleus reuniens
- RR: Retrorubral area
- RTN: Reticular tegmental nucleus
- Raphe: Raphe nuclei
- Ret-MB: Reticular formation, Midbrain
- Ret-Med: Reticular formation, Medulla
- Ret-P: Reticular formation, Pons
- Rms: Rostral migratory stream
- SC: Superior colliculus
- SCH: Suprachiasmatic nucleus [hypothalamus]
- SEP: Septum
- SOL: Solitary nucleus
- SUM: Supramammillary area
- TRI: Germinal trigone
- Tct: Tectum
- VA: Ventral anterior nucleus [thalamus]
- VIn: Abducens nucleus
- VL: Ventral lateral nucleus [thalamus]
- VMB: Ventral medial basal nucleus [thalamus]
- VMH: Ventromedial nucleus [hypothalamus]
- VPM: Ventral posteromedial nucleus [thalamus]
- Xn: Dorsal motor nucleus

Age: 14 GW

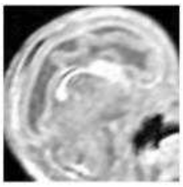

L-R Level: -1.08 mm

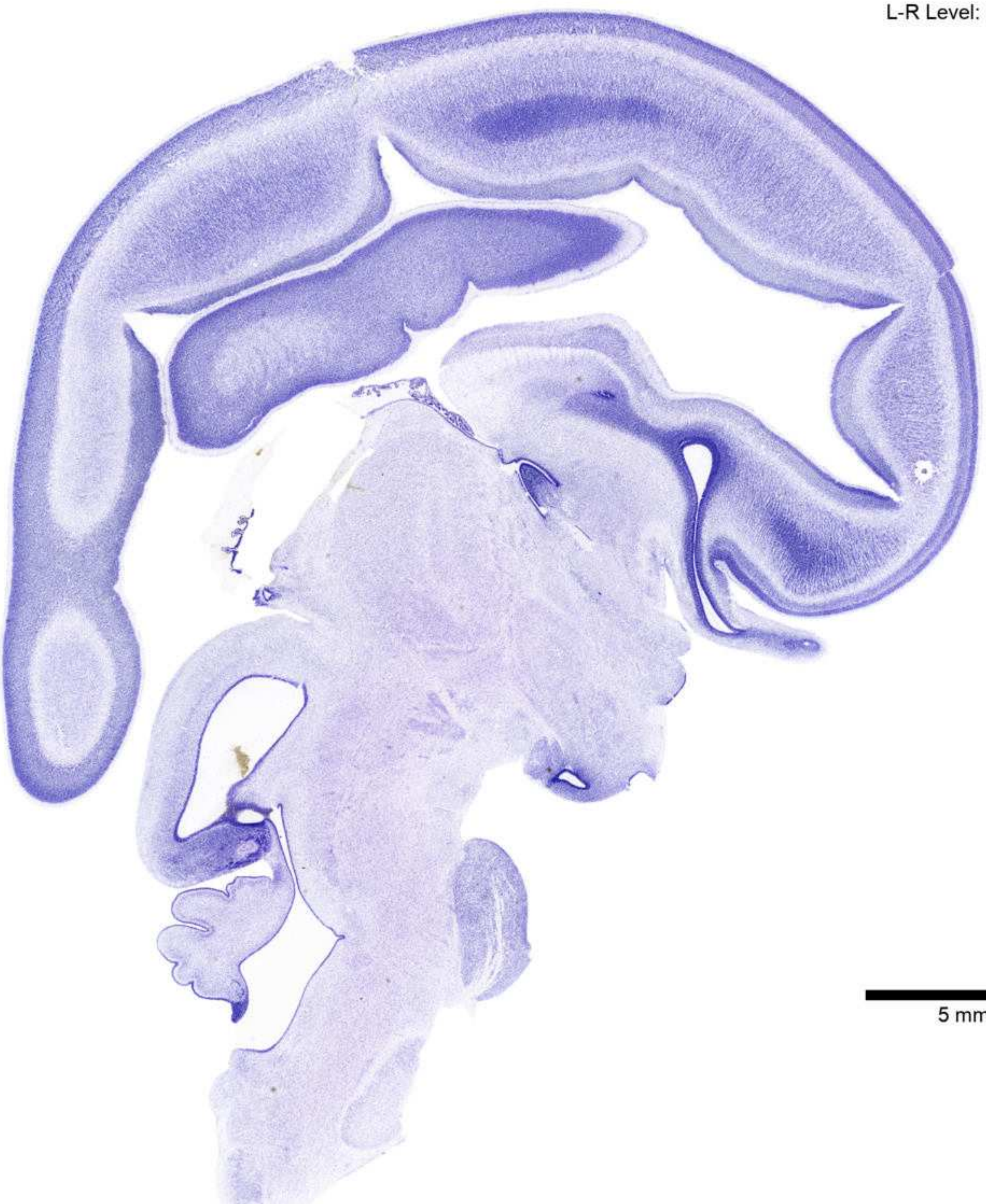

5 mm

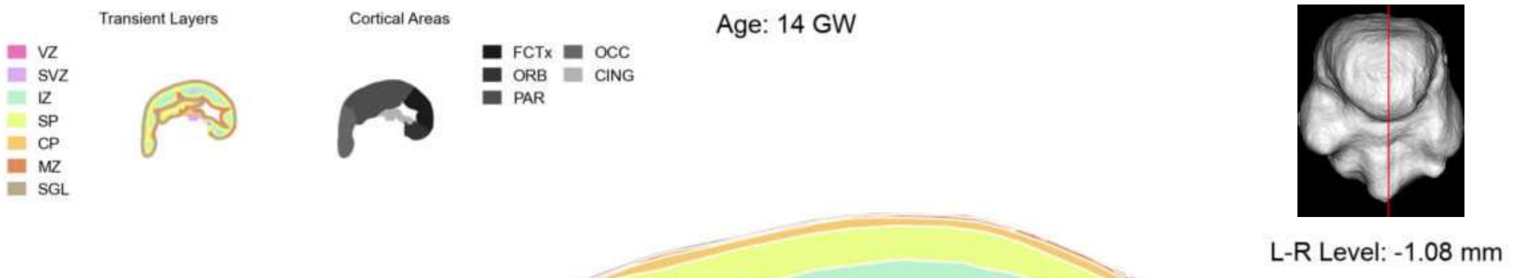

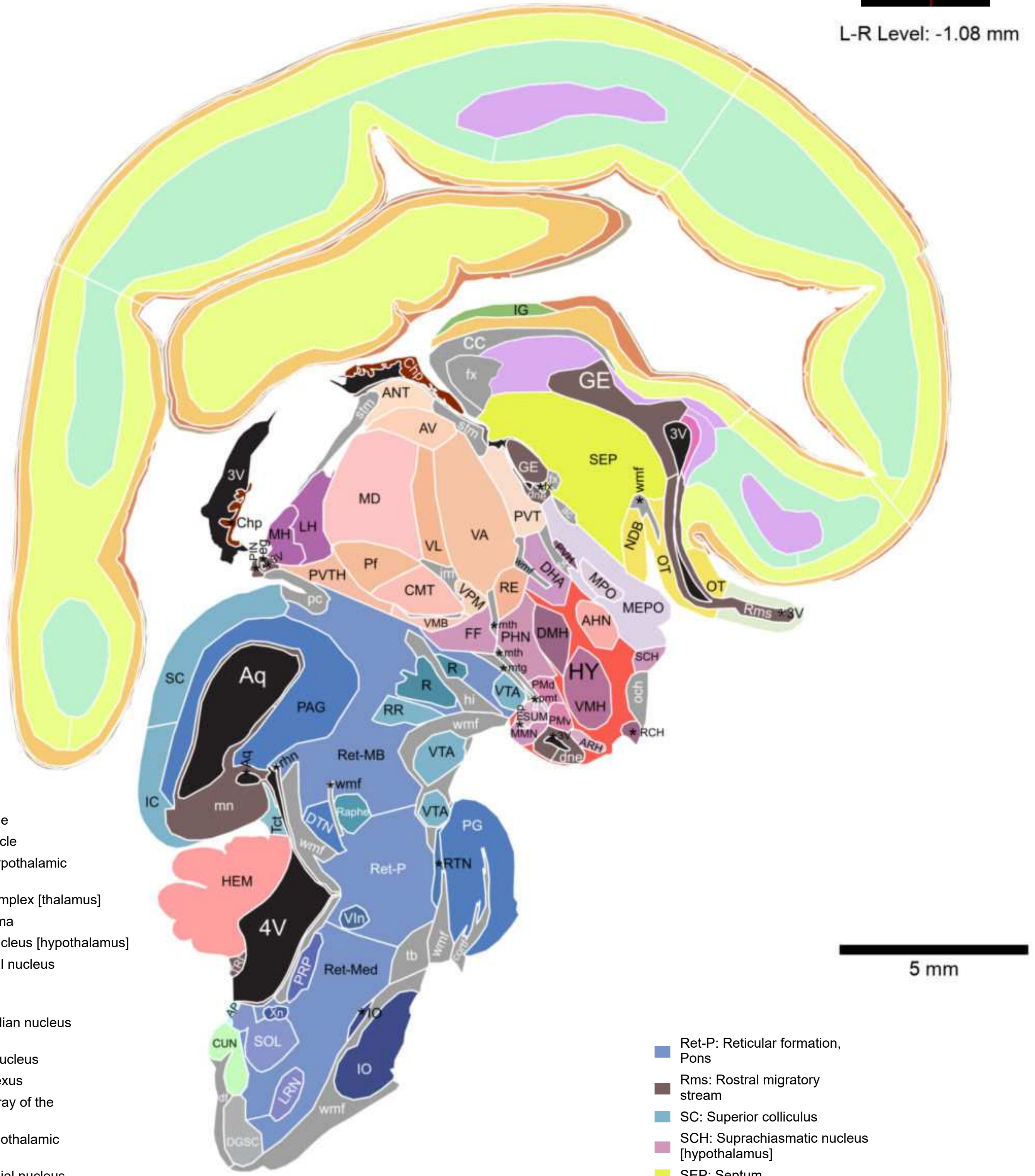

3V: Third ventricle
4V: Fourth ventricle
AHN: Anterior hypothalamic nucleus
ANT: Anterior complex [thalamus]
AP: Area postrema
ARH: Arcuate nucleus [hypothalamus]
AV: Anteroventral nucleus [thalamus]
Aq: Aqueduct
CMT: Centromedian nucleus [thalamus]
CUN: Cuneate nucleus
Chp: Choroid plexus
DGSC: Dorsal gray of the spinal cord
DHA: Dorsal hypothalamic area
DMH: Dorsomedial nucleus [hypothalamus]
DTN: Dorsal tegmental nucleus
FF: Field of Forel
GE: Ganglionic eminence
HEM: Cerebellar hemispheres
HY: Hypothalamus
IC: Inferior colliculus
IG: Induseum griseum
IO: Inferior olive
LH: Lateral habenula
LRN: Lateral reticular nucleus
MD: Medial dorsal nucleus [thalamus]
MEPO: Medial preoptic area
MH: Medial habenula
MMN: Medial mammillary nucleus
MPO: Medial preoptic nucleus
NDB: Nucleus of the diagonal band
OLFb: Olfactory bulb
OT: Olfactory tubercle
PAG: Periaqueductal gray
PG: Pontine gray
PHN: Posterior hypothalamic nucleus
PIN: Pineal gland
PMd: Dorsal premammillary nucleus
PMv: Ventral premammillary nucleus
PRP: Nucleus prepositus
PVH: Paraventricular nucleus [hypothalamus]
PVT: Paraventricular nucleus [thalamus]
PVTH: Periventricular complex [thalamus]
Pf: Parafascicular nucleus [thalamus]
R: Red nucleus
RCH: Retrochiasmatic nucleus [hypothalamus]
RE: Nucleus reuniens
RR: Retrorubral area
RTN: Reticular tegmental nucleus
Raphe: Raphe nuclei
Ret-MB: Reticular formation, Midbrain
Ret-Med: Reticular formation, Medulla
Ret-P: Reticular formation, Pons
Rms: Rostral migratory stream
SC: Superior colliculus
SCH: Suprachiasmatic nucleus [hypothalamus]
SEP: Septum
SOL: Solitary nucleus
SUM: Supramammillary area
TRI: Germinal trigone
Tct: Tectum
VA: Ventral anterior nucleus [thalamus]
VIn: Abducens nucleus
VL: Ventral lateral nucleus [thalamus]
VMB: Ventral medial basal nucleus [thalamus]
VMH: Ventromedial nucleus [hypothalamus]
VPM: Ventral posteromedial nucleus [thalamus]
VTA: Ventral tegmental area
Xn: Dorsal motor nucleus

Age: 14 GW

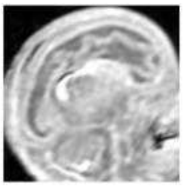

L-R Level: -1.98 mm

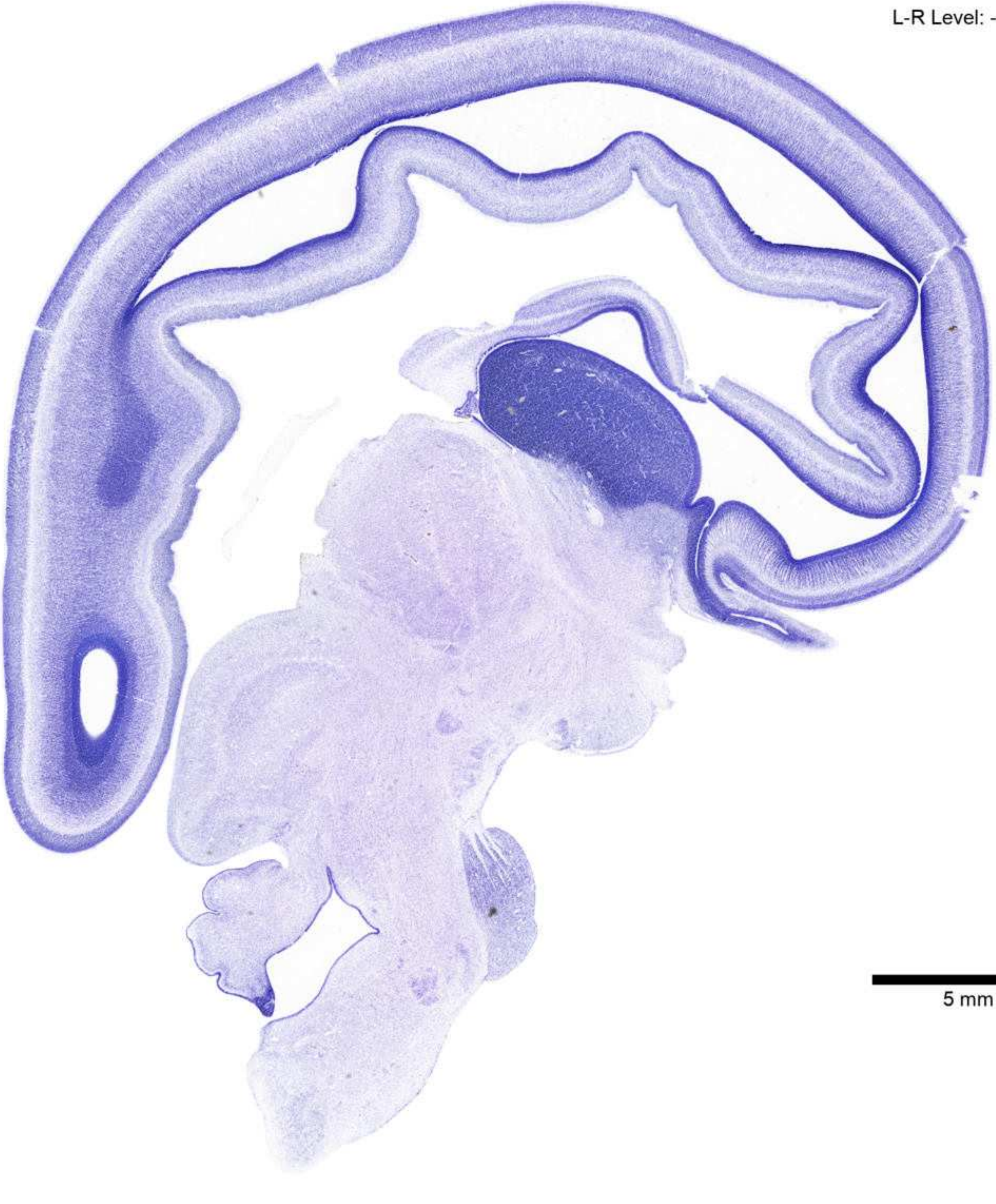

5 mm

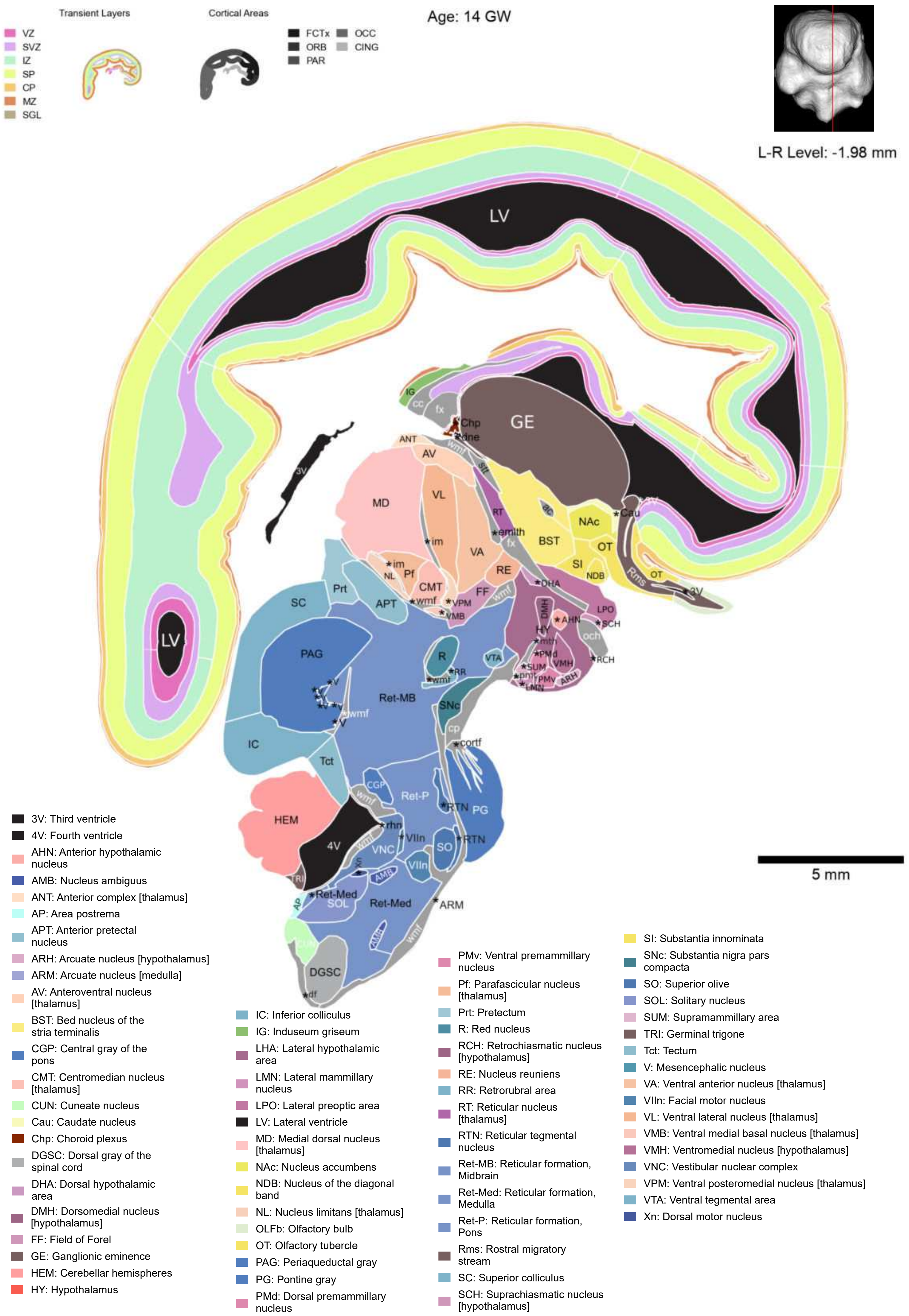

Transient Layers
VZ
SVZ
IZ
SP
CP
MZ
SGL
Cortical Areas
FCTx
ORB
PAR
OCC
CING
Age: 14 GW
L-R Level: -1.98 mm
5 mm
LV
GE
IG
cc
fx
Chp
dne
ANT
AV
wmf
stt
3V
MD
VL
RT
emlth
BST
NAc
Cau
OT
SI
NDB
Rms
im
VA
RE
NL
Pf
CMT
FF
DHA
Prt
SC
APT
VPM
VMB
DMH
AHN
LPO
SCH
HY
och
mth
PMd
VMH
RCH
SUM
pm
PMv
ARH
LMN
PAG
R
RR
VTA
V
Ret-MB
SNc
cp
cortf
IC
Tct
CGP
Ret-P
RTN
PG
HEM
rhn
VIIn
4V
VNC
SO
TRI
Xn
AMB
Ret-Med
SOL
ARM
AP
CUN
DGSC
df
3V: Third ventricle
4V: Fourth ventricle
AHN: Anterior hypothalamic nucleus
AMB: Nucleus ambiguus
ANT: Anterior complex [thalamus]
AP: Area postrema
APT: Anterior pretectal nucleus
ARH: Arcuate nucleus [hypothalamus]
ARM: Arcuate nucleus [medulla]
AV: Anteroventral nucleus [thalamus]
BST: Bed nucleus of the stria terminalis
CGP: Central gray of the pons
CMT: Centromedian nucleus [thalamus]
CUN: Cuneate nucleus
Cau: Caudate nucleus
Chp: Choroid plexus
DGSC: Dorsal gray of the spinal cord
DHA: Dorsal hypothalamic area
DMH: Dorsomedial nucleus [hypothalamus]
FF: Field of Forel
GE: Ganglionic eminence
HEM: Cerebellar hemispheres
HY: Hypothalamus
IC: Inferior colliculus
IG: Induseum griseum
LHA: Lateral hypothalamic area
LMN: Lateral mammillary nucleus
LPO: Lateral preoptic area
LV: Lateral ventricle
MD: Medial dorsal nucleus [thalamus]
NAc: Nucleus accumbens
NDB: Nucleus of the diagonal band
NL: Nucleus limitans [thalamus]
OLFb: Olfactory bulb
OT: Olfactory tubercle
PAG: Periaqueductal gray
PG: Pontine gray
PMd: Dorsal premammillary nucleus
PMv: Ventral premammillary nucleus
Pf: Parafascicular nucleus [thalamus]
Prt: Pretectum
R: Red nucleus
RCH: Retrochiasmatic nucleus [hypothalamus]
RE: Nucleus reuniens
RR: Retrorubral area
RT: Reticular nucleus [thalamus]
RTN: Reticular tegmental nucleus
Ret-MB: Reticular formation, Midbrain
Ret-Med: Reticular formation, Medulla
Ret-P: Reticular formation, Pons
Rms: Rostral migratory stream
SC: Superior colliculus
SCH: Suprachiasmatic nucleus [hypothalamus]
SI: Substantia innominata
SNc: Substantia nigra pars compacta
SO: Superior olive
SOL: Solitary nucleus
SUM: Supramammillary area
TRI: Germinal trigone
Tct: Tectum
V: Mesencephalic nucleus
VA: Ventral anterior nucleus [thalamus]
VIIn: Facial motor nucleus
VL: Ventral lateral nucleus [thalamus]
VMB: Ventral medial basal nucleus [thalamus]
VMH: Ventromedial nucleus [hypothalamus]
VNC: Vestibular nuclear complex
VPM: Ventral posteromedial nucleus [thalamus]
VTA: Ventral tegmental area
Xn: Dorsal motor nucleus

Age: 14 GW

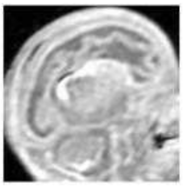

L-R Level: -2.22 mm

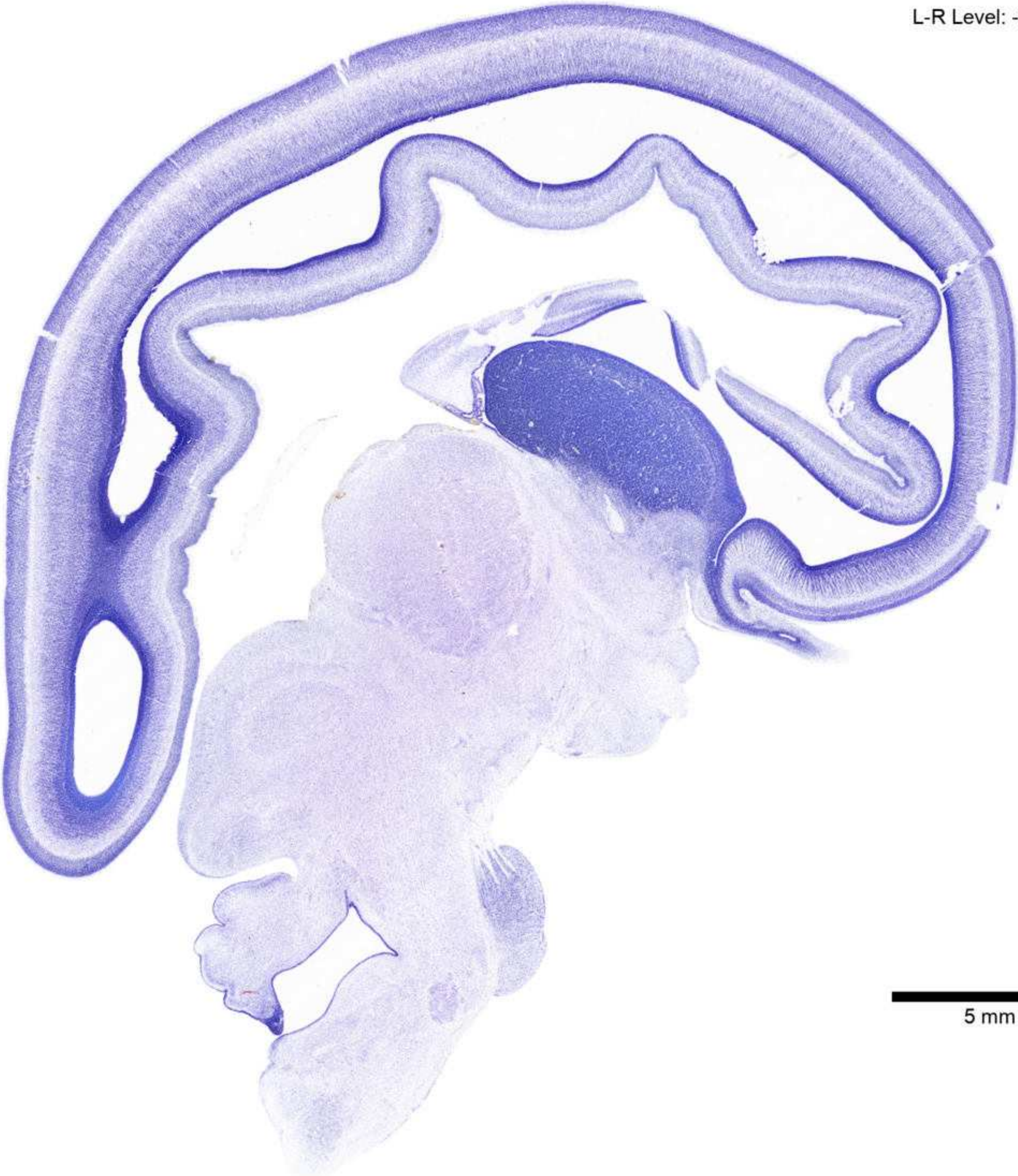

5 mm

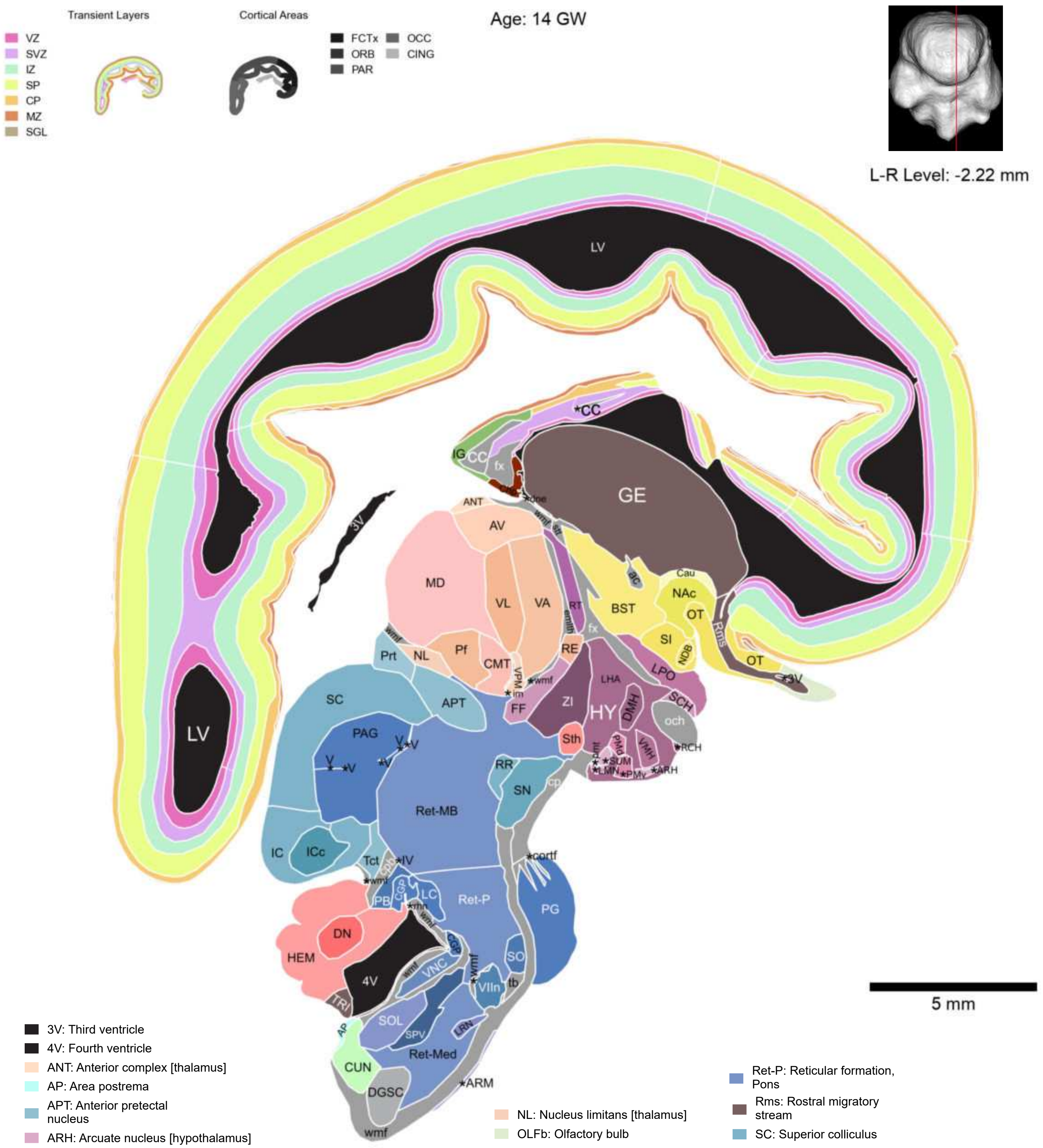

- 3V: Third ventricle
- 4V: Fourth ventricle
- ANT: Anterior complex [thalamus]
- AP: Area postrema
- APT: Anterior pretectal nucleus
- ARH: Arcuate nucleus [hypothalamus]
- ARM: Arcuate nucleus [medulla]
- AV: Anteroventral nucleus [thalamus]
- BST: Bed nucleus of the stria terminalis
- CGP: Central gray of the pons
- CMT: Centromedian nucleus [thalamus]
- CUN: Cuneate nucleus
- Cau: Caudate nucleus
- Chp: Choroid plexus
- DGSC: Dorsal gray of the spinal cord
- DMH: Dorsomedial nucleus [hypothalamus]
- DN: Dentate nucleus
- FF: Field of Forel
- GE: Ganglionic eminence
- HEM: Cerebellar hemispheres
- HY: Hypothalamus
- IC: Inferior colliculus
- ICc: Inferior colliculus, central nucleus
- IG: Induseum griseum
- IV: Trochlear nucleus
- LC: Locus coeruleus
- LHA: Lateral hypothalamic area
- LMN: Lateral mammillary nucleus
- LPO: Lateral preoptic area
- LRN: Lateral reticular nucleus
- LV: Lateral ventricle
- MD: Medial dorsal nucleus [thalamus]
- NAc: Nucleus accumbens
- NDB: Nucleus of the diagonal band
- NL: Nucleus limitans [thalamus]
- OLFb: Olfactory bulb
- OT: Olfactory tubercle
- PAG: Periaqueductal gray
- PB: Parabrachial nucleus
- PG: Pontine gray
- PMd: Dorsal premammillary nucleus
- PMv: Ventral premammillary nucleus
- Pf: Parafascicular nucleus [thalamus]
- Prt: Pretectum
- RCH: Retrochiasmatic nucleus [hypothalamus]
- RE: Nucleus reuniens
- RR: Retrorubral area
- RT: Reticular nucleus [thalamus]
- Ret-MB: Reticular formation, Midbrain
- Ret-Med: Reticular formation, Medulla
- Ret-P: Reticular formation, Pons
- Rms: Rostral migratory stream
- SC: Superior colliculus
- SCH: Suprachiasmatic nucleus [hypothalamus]
- SI: Substantia innominata
- SN: Substantia nigra
- SO: Superior olive
- SOL: Solitary nucleus
- SPV: Spinal nucleus of the trigeminal
- SUM: Supramammillary area
- Sth: Subthalamus
- TRI: Germinal trigone
- Tct: Tectum
- V: Mesencephalic nucleus
- VA: Ventral anterior nucleus [thalamus]
- VIIn: Facial motor nucleus
- VL: Ventral lateral nucleus [thalamus]
- VMH: Ventromedial nucleus [hypothalamus]
- VNC: Vestibular nuclear complex
- VPM: Ventral posteromedial nucleus [thalamus]
- ZI: Zona incerta

Age: 14 GW

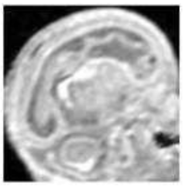

L-R Level: -2.88 mm

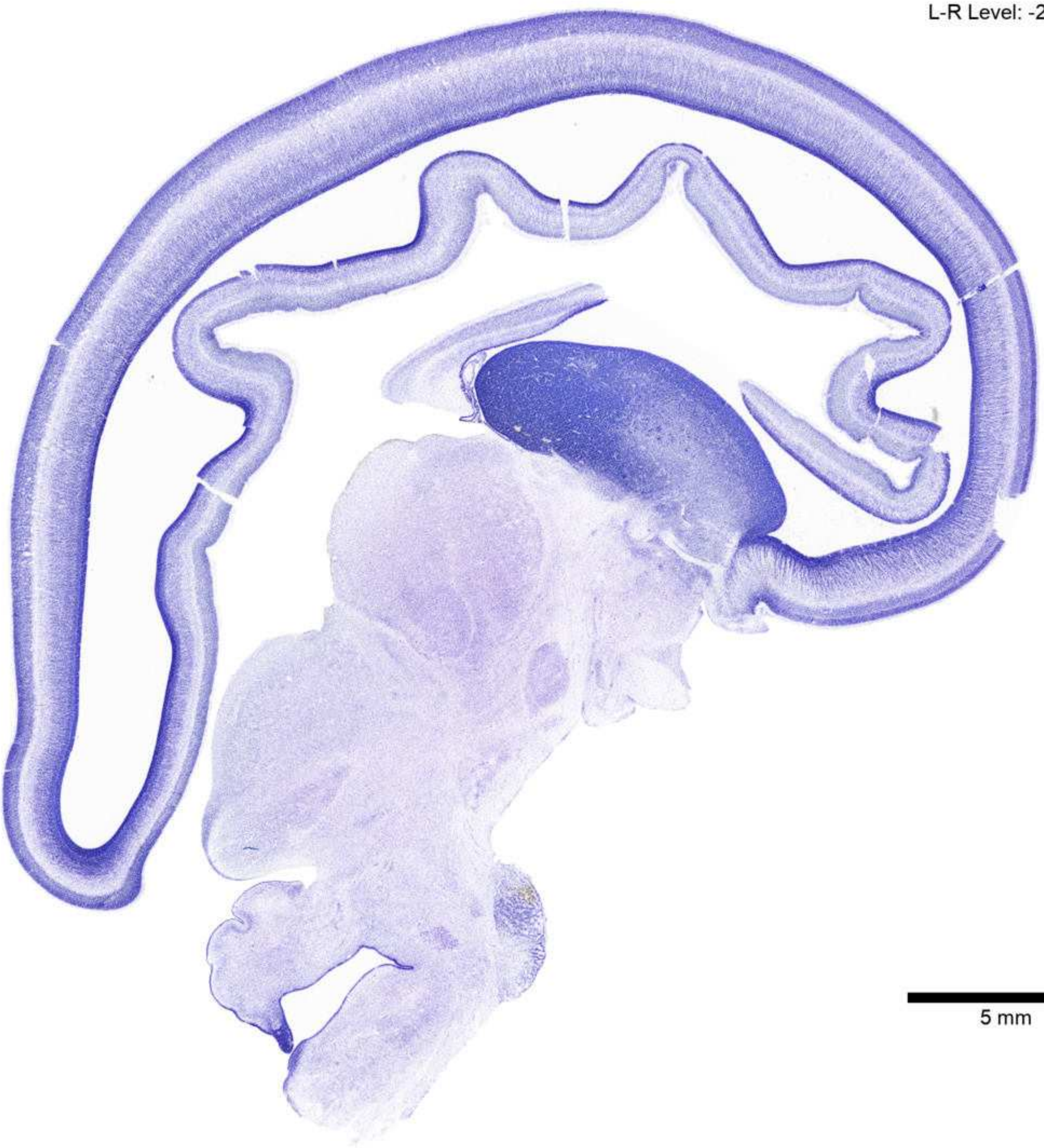

5 mm

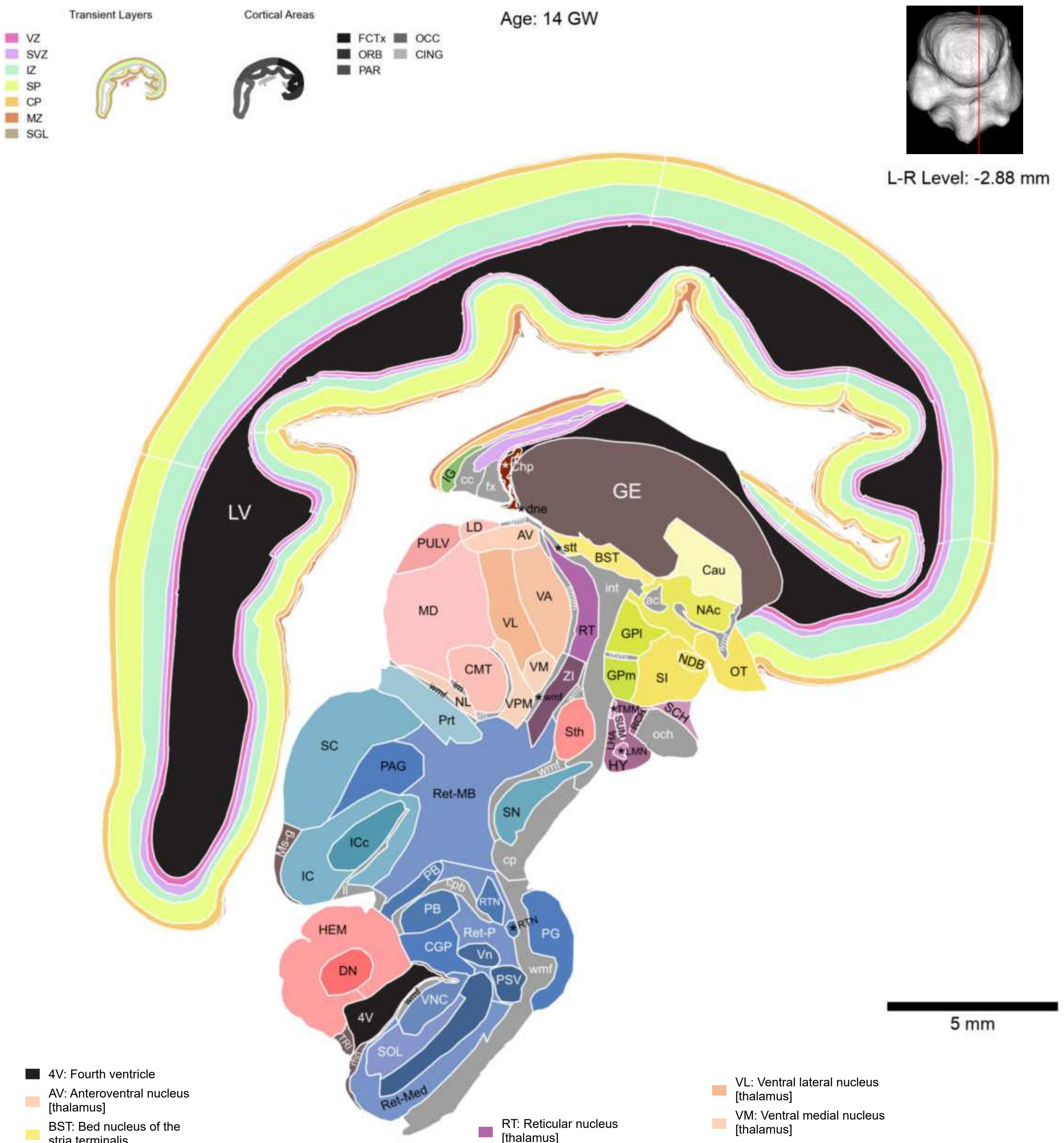

4V: Fourth ventricle
AV: Anteroventral nucleus [thalamus]
BST: Bed nucleus of the stria terminalis
CGP: Central gray of the pons
CMT: Centromedian nucleus [thalamus]
Cau: Caudate nucleus
Chp: Choroid plexus
DN: Dentate nucleus
GE: Ganglionic eminence
GPl: Globus pallidus lateral segment
GPm: Globus pallidus medial segment
HEM: Cerebellar hemispheres
HY: Hypothalamus
IC: Inferior colliculus
ICc: Inferior colliculus, central nucleus
IG: Induseum griseum
LD: Lateral dorsal nucleus [thalamus]
LHA: Lateral hypothalamic area
LMN: Lateral mammillary nucleus
LV: Lateral ventricle
MD: Medial dorsal nucleus [thalamus]
Ms-g: Migratory stream, general
NAc: Nucleus accumbens
NDB: Nucleus of the diagonal band
NL: Nucleus limitans [thalamus]
OT: Olfactory tubercle
PAG: Periaqueductal gray
PB: Parabrachial nucleus
PG: Pontine gray
PSV: Principal sensory nucleus of the trigeminal
PULV: Pulvinar nucleus [thalamus]
Prt: Pretectum
RCH: Retrochiasmatic nucleus [hypothalamus]
RT: Reticular nucleus [thalamus]
RTN: Reticular tegmental nucleus
Ret-MB: Reticular formation, Midbrain
Ret-Med: Reticular formation, Medulla
Ret-P: Reticular formation, Pons
SC: Superior colliculus
SCH: Suprachiasmatic nucleus [hypothalamus]
SI: Substantia innominata
SN: Substantia nigra
SOL: Solitary nucleus
SPV: Spinal nucleus of the trigeminal
SUM: Supramammillary area
Sth: Subthalamus
TMM: Tuberomammillary nucleus
TRI: Germinal trigone
VA: Ventral anterior nucleus [thalamus]
VL: Ventral lateral nucleus [thalamus]
VM: Ventral medial nucleus [thalamus]
VNC: Vestibular nuclear complex
VPM: Ventral posteromedial nucleus [thalamus]
Vn: Trigeminal motor nucleus
ZI: Zona incerta
ac: Anterior commissure
cc: Corpus callosum
cp: Cerebral peduncle
cpb: Cerebellar peduncles
dne: Diencephalic neuroepithelium
emlth: External medullary lamina [thalamus]
fx: Fornix
im: Internal medullary lamina [thalamus]
int: Internal capsule
ll: Lateral lemniscus
mml: Medial medullary lamina
och: Optic chiasm
rhn: Rhombencephalic neuroepithelium
stt: Stria terminalis
wmf: White matter fibers

Age: 14 GW

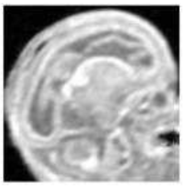

L-R Level: -3.72 mm

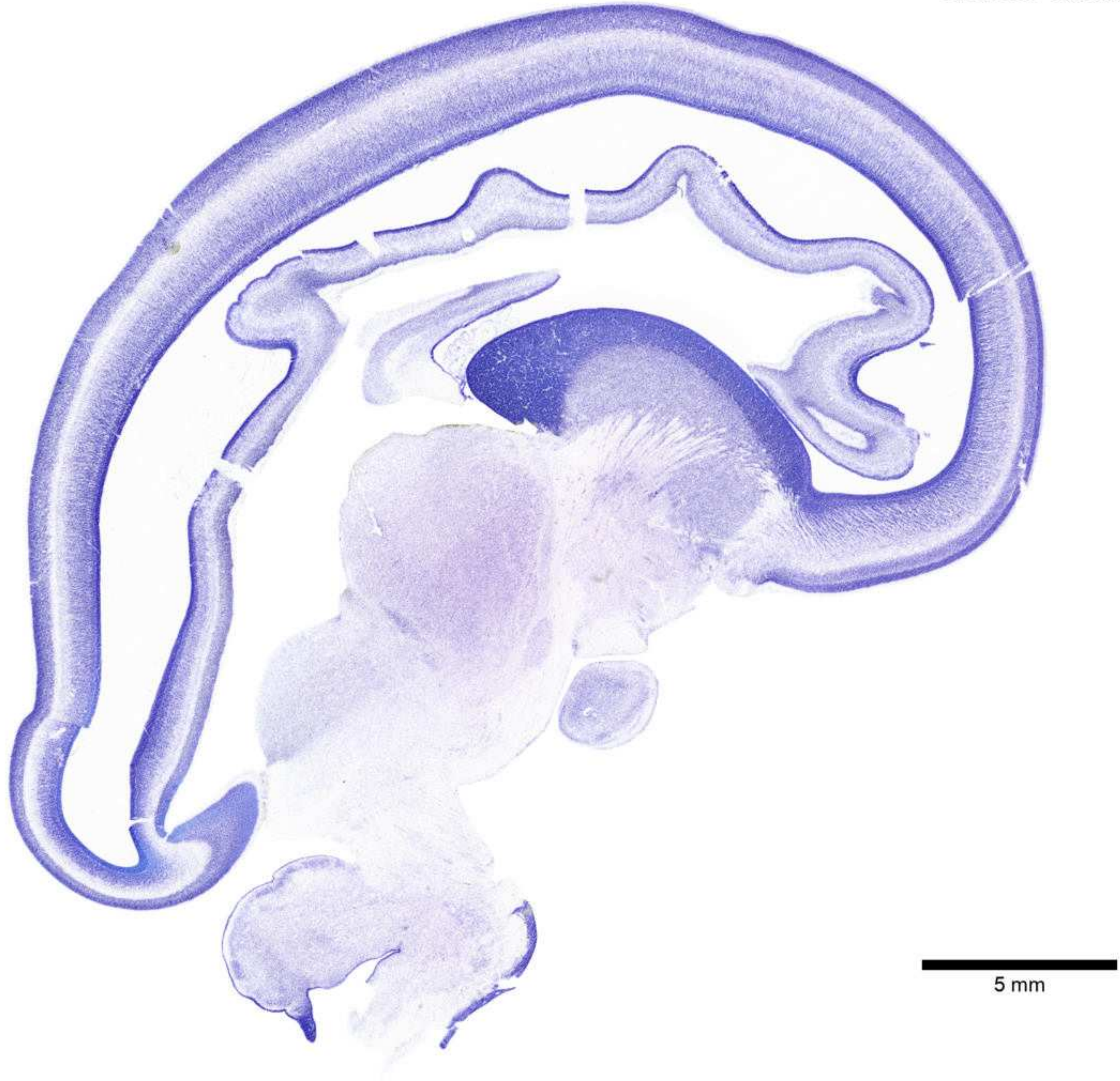

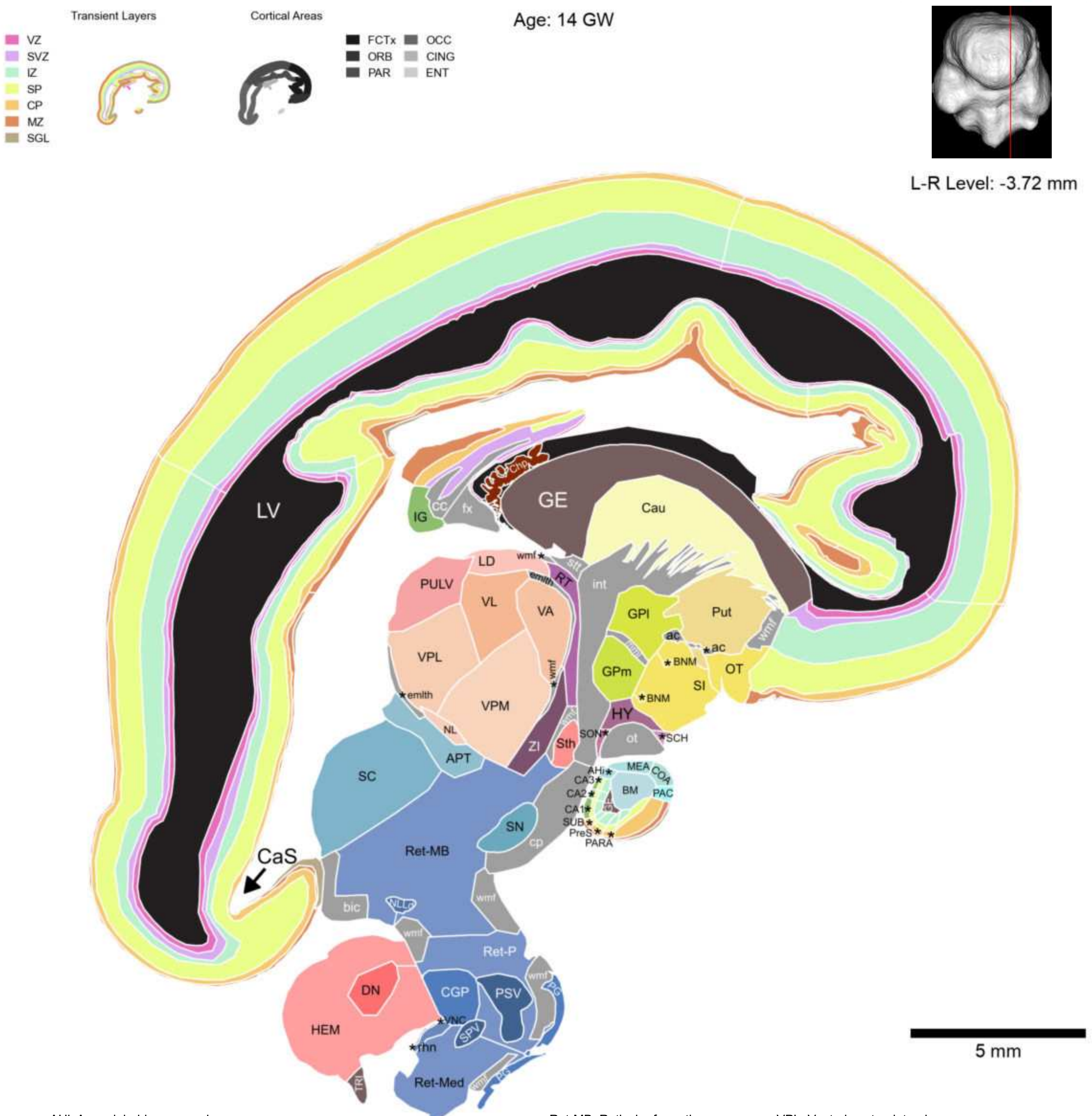

AHi: Amygdalo-hippocampal area
APT: Anterior pretectal nucleus
BM: Basomedial nucleus [amygdala]
BNM: Basal nucleus of Meynert
CA1: CA1 field [hippocampus]
CA2: CA2 field [hippocampus]
CA3: CA3 field [hippocampus]
CGP: Central gray of the pons
COA: Cortical nucleus [amygdala]
Cau: Caudate nucleus
Chp: Choroid plexus
DN: Dentate nucleus
GE: Ganglionic eminence
GPl: Globus pallidus lateral segment
GPm: Globus pallidus medial segment
HEM: Cerebellar hemispheres
HY: Hypothalamus
IG: Induseum griseum
LD: Lateral dorsal nucleus [thalamus]
LHA: Lateral hypothalamic area
LV: Lateral ventricle
MEA: Medial nucleus [amygdala]
NL: Nucleus limitans [thalamus]
NLLd: Nucleus of the lateral lemniscus, dorsal
OT: Olfactory tubercle
PARA: Cortical plate, parasubiculum
PG: Pontine gray
PSV: Principal sensory nucleus of the trigeminal
PULV: Pulvinar nucleus [thalamus]
PreS: Cortical plate, presubiculum
Put: Putamen
RT: Reticular nucleus [thalamus]
Ret-MB: Reticular formation, Midbrain
Ret-Med: Reticular formation, Medulla
Ret-P: Reticular formation, Pons
SC: Superior colliculus
SCH: Suprachiasmatic nucleus [hypothalamus]
SI: Substantia innominata
SN: Substantia nigra
SON: Supraoptic nucleus [hypothalamus]
SPV: Spinal nucleus of the trigeminal
SUB: Cortical plate, subiculum
Sth: Subthalamus
TRI: Germinal trigone
VA: Ventral anterior nucleus [thalamus]
VL: Ventral lateral nucleus [thalamus]
VNC: Vestibular nuclear complex
VPL: Ventral posterolateral nucleus [thalamus]
VPM: Ventral posteromedial nucleus [thalamus]
ZI: Zona incerta
ac: Anterior commissure
bic: Brachium of the inferior colliculus
cc: Corpus callosum
cp: Cerebral peduncle
emlth: External medullary lamina [thalamus]
fx: Fornix
int: Internal capsule
mml: Medial medullary lamina
ot: Optic tract
rhn: Rhombencephalic neuroepithelium
stt: Stria terminalis
wmf: White matter fibers

Age: 14 GW

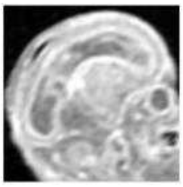

L-R Level: -4.32 mm

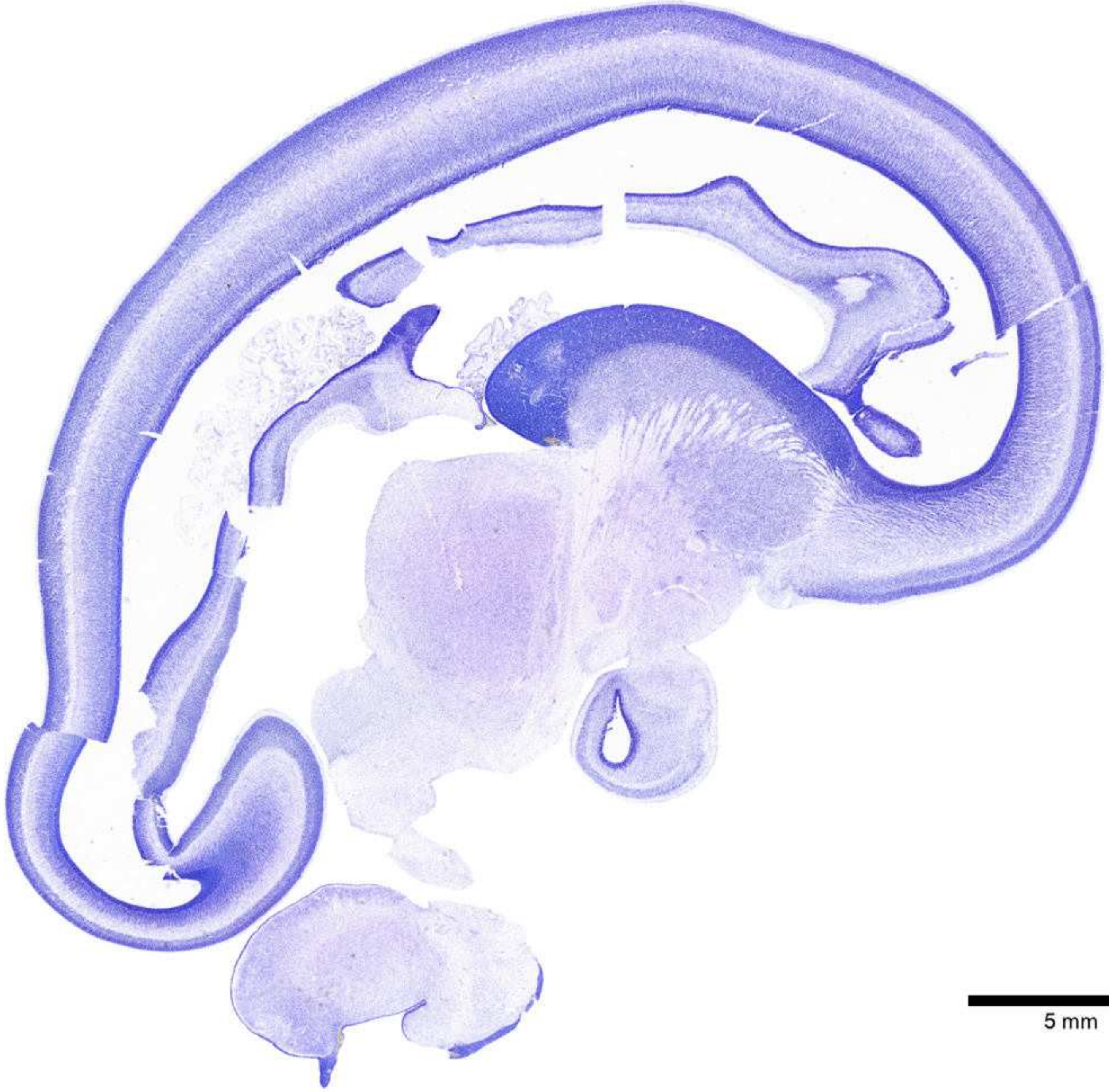

5 mm

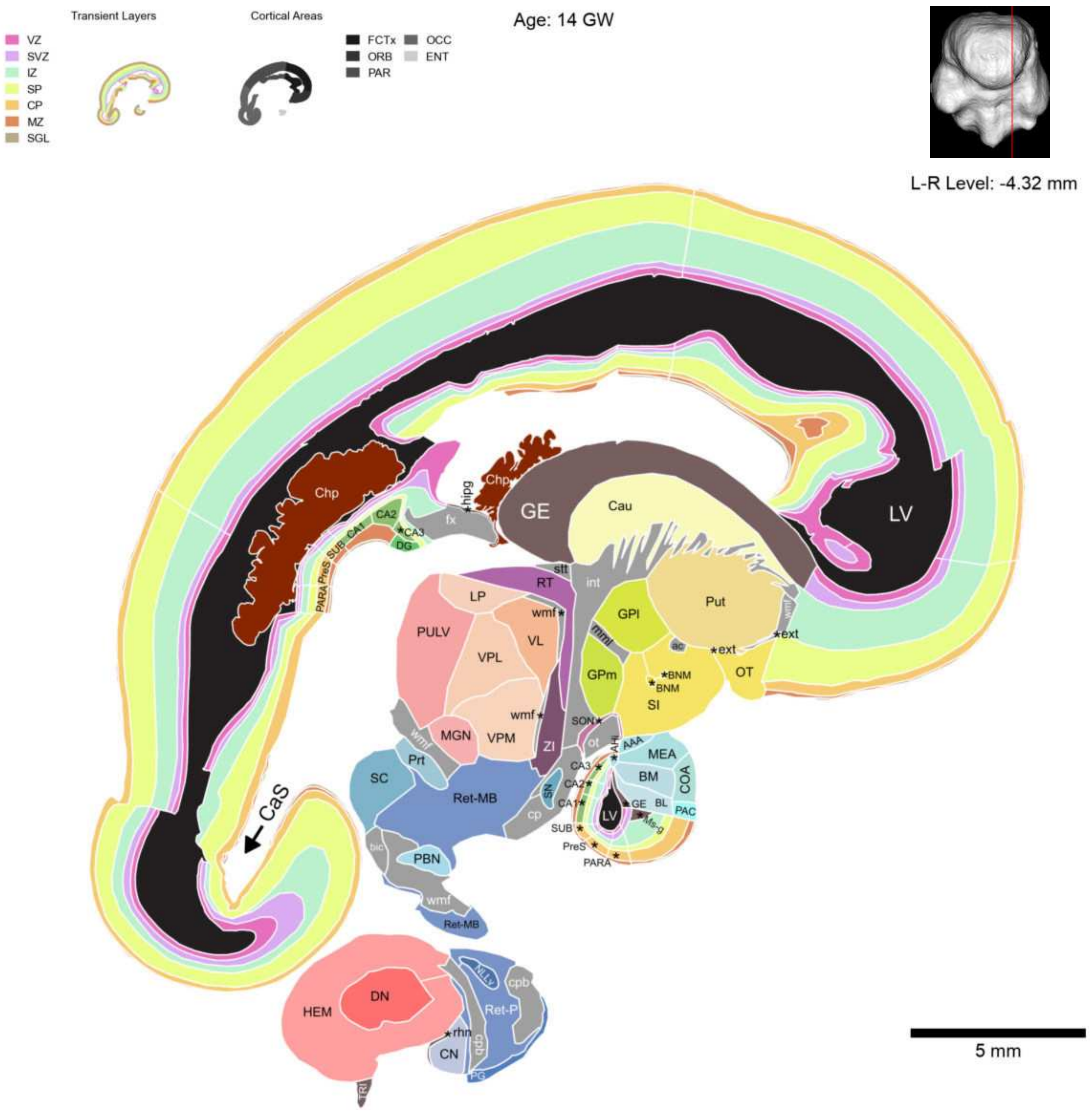

- AAA: Anterior amygdaloid area
- AHi: Amygdalo-hippocampal area
- BL: Basal nucleus [amygdala]
- BM: Basomedial nucleus [amygdala]
- BNM: Basal nucleus of Meynert
- CA1: CA1 field [hippocampus]
- CA2: CA2 field [hippocampus]
- CA3: CA3 field [hippocampus]
- CN: Cochlear nuclei
- COA: Cortical nucleus [amygdala]
- Cau: Caudate nucleus
- Chp: Choroid plexus
- DG: Dentate gyrus
- DN: Dentate nucleus
- GE: Ganglionic eminence
- GPl: Globus pallidus lateral segment
- GPm: Globus pallidus medial segment
- HEM: Cerebellar hemispheres
- LP: Lateral posterior nucleus [thalamus]
- LV: Lateral ventricle
- MEA: Medial nucleus [amygdala]
- MGN: Medial geniculate nucleus
- Ms-g: Migratory stream, general
- NLLv: Nucleus of the lateral lemniscus, ventral
- OT: Olfactory tubercle
- PARA: Cortical plate, parasubiculum
- PBN: Parabigeminal nucleus
- PG: Pontine gray
- PULV: Pulvinar nucleus [thalamus]
- PreS: Cortical plate, presubiculum
- Prt: Pretectum
- Put: Putamen
- RT: Reticular nucleus [thalamus]
- Ret-MB: Reticular formation, Midbrain
- Ret-P: Reticular formation, Pons
- SC: Superior colliculus
- SI: Substantia innominata
- SN: Substantia nigra
- SON: Supraoptic nucleus [hypothalamus]
- SUB: Cortical plate, subiculum
- TRI: Germinal trigone
- VL: Ventral lateral nucleus [thalamus]
- VPL: Ventral posterolateral nucleus [thalamus]
- VPM: Ventral posteromedial nucleus [thalamus]
- ZI: Zona incerta
- ac: Anterior commissure
- bic: Brachium of the inferior colliculus
- cp: Cerebral peduncle
- cpb: Cerebellar peduncles
- ext: External capsule
- fx: Fornix
- hipg: Hippocampal glioepithelium/ependyma
- int: Internal capsule
- mml: Medial medullary lamina
- ot: Optic tract
- rhn: Rhombencephalic neuroepithelium
- stt: Stria terminalis
- wmf: White matter fibers
- → CaS: Calcarine sulcus

Age: 14 GW

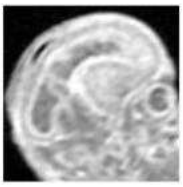

L-R Level: -4.8 mm

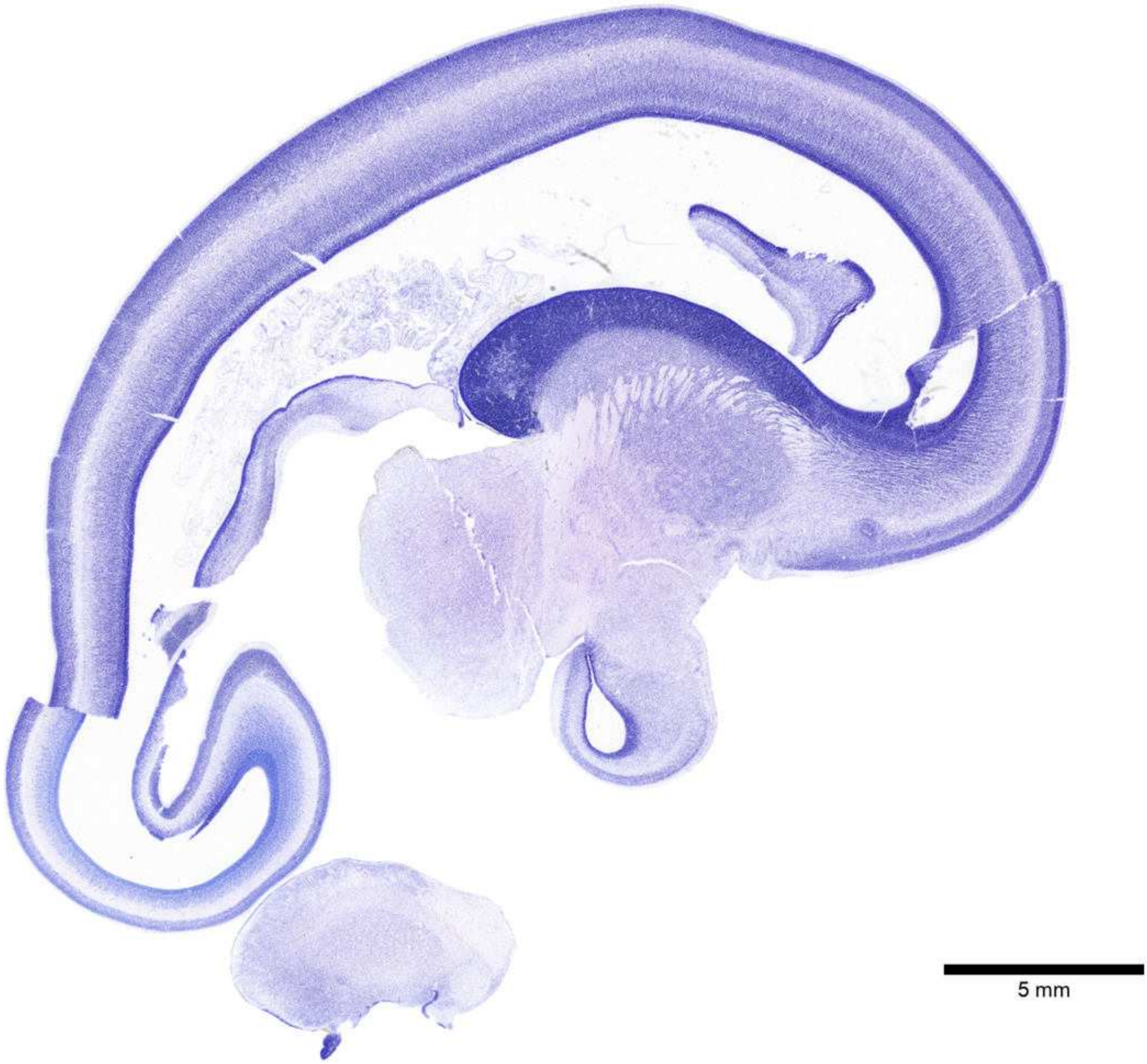

5 mm

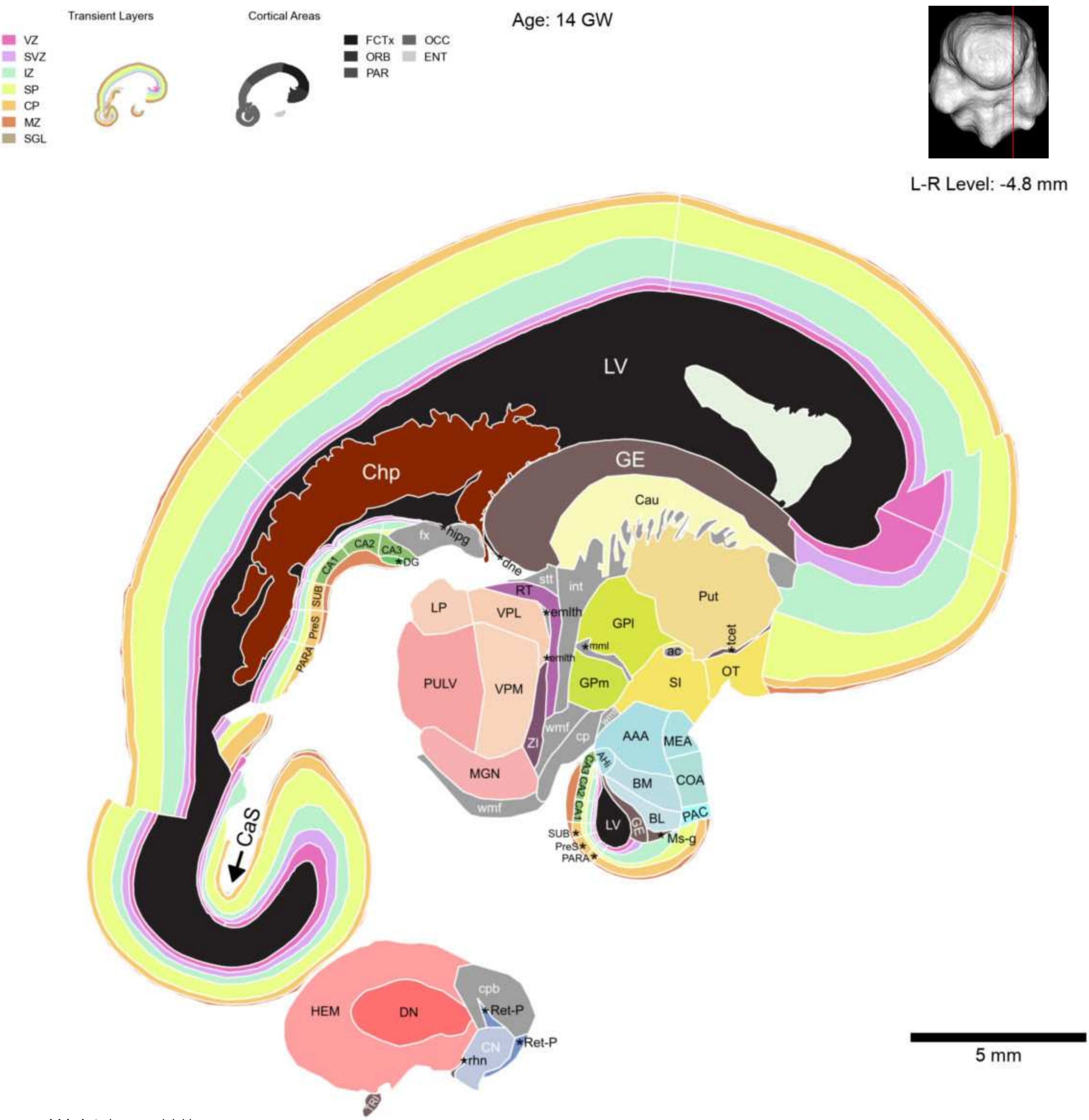

AAA: Anterior amygdaloid area
AHi: Amygdalo-hippocampal area
BL: Basal nucleus [amygdala]
BM: Basomedial nucleus [amygdala]
CA1: CA1 field [hippocampus]
CA2: CA2 field [hippocampus]
CA3: CA3 field [hippocampus]
CN: Cochlear nuclei
COA: Cortical nucleus [amygdala]
Cau: Caudate nucleus
Chp: Choroid plexus
DG: Dentate gyrus
DN: Dentate nucleus
GE: Ganglionic eminence
GPl: Globus pallidus lateral segment
GPm: Globus pallidus medial segment
HEM: Cerebellar hemispheres
LP: Lateral posterior nucleus [thalamus]
LV: Lateral ventricle
MEA: Medial nucleus [amygdala]
MGN: Medial geniculate nucleus
Ms-g: Migratory stream, general
OT: Olfactory tubercle
PARA: Cortical plate, parasubiculum
PULV: Pulvinar nucleus [thalamus]
PreS: Cortical plate, presubiculum
Put: Putamen
RT: Reticular nucleus [thalamus]
Ret-P: Reticular formation, Pons
SI: Substantia innominata
SUB: Cortical plate, subiculum
TRI: Germinal trigone
VPL: Ventral posterolateral nucleus [thalamus]
VPM: Ventral posteromedial nucleus [thalamus]
ZI: Zona incerta
ac: Anterior commissure
cp: Cerebral peduncle
cpb: Cerebellar peduncles
dne: Diencephalic neuroepithelium
emlth: External medullary lamina [thalamus]
fx: Fornix
hipg: Hippocampal glioepithelium/ependyma
int: Internal capsule
mml: Medial medullary lamina
rhn: Rhombencephalic neuroepithelium
stt: Stria terminalis
tcet: Transient cell zone in the external capsule
wmf: White matter fibers
→ CaS - Calcarine sulcus

Age: 14 GW

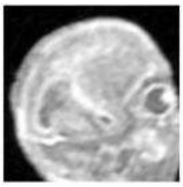

L-R Level: -6.18 mm

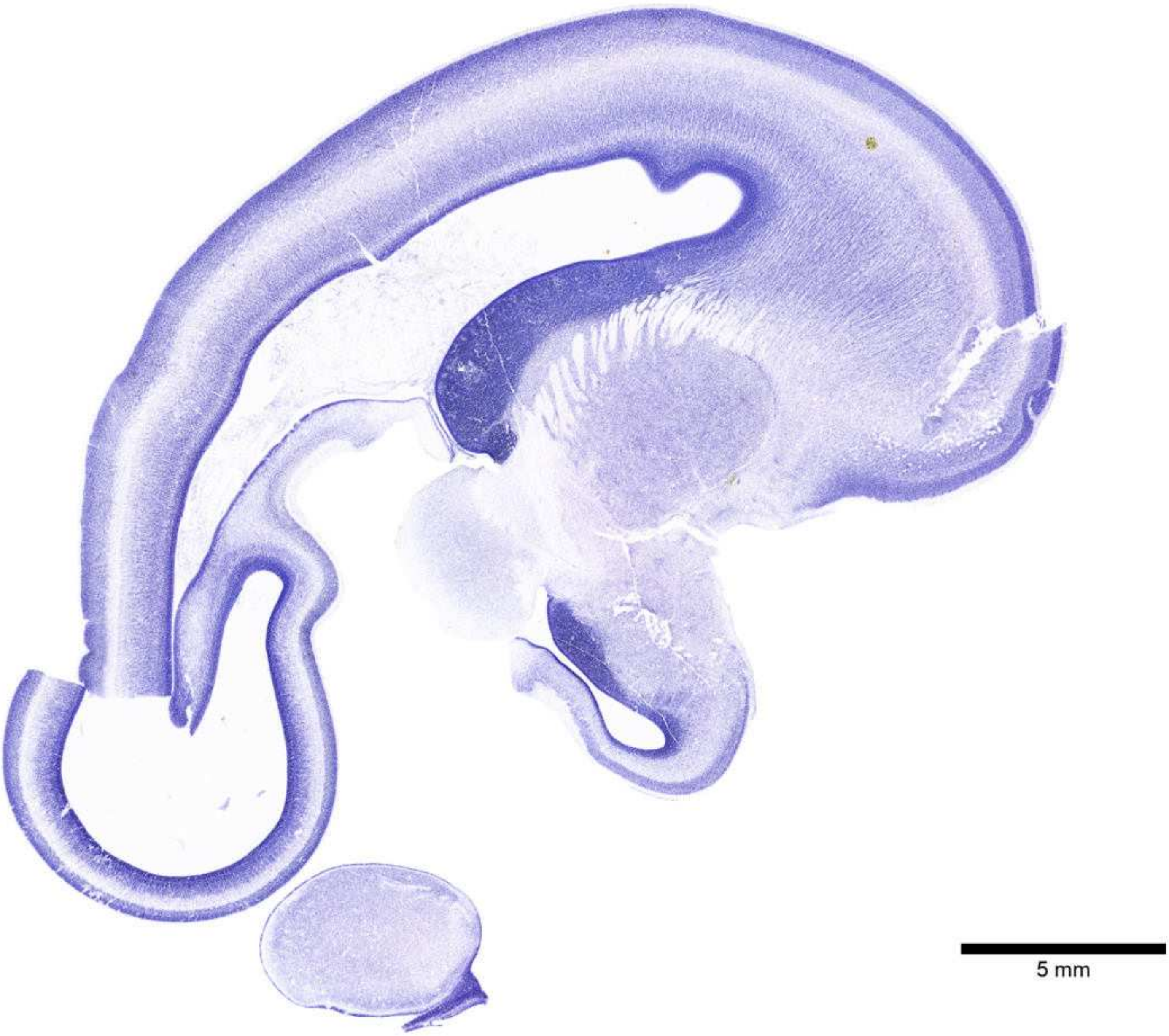

5 mm

Transient Layers

- VZ
- SVZ
- IZ
- SP
- CP
- MZ
- SGL

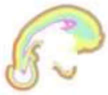

Cortical Areas

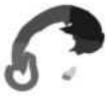

- FCTx
- ORB
- PAR
- OCC
- TEMP
- ENT

Age: 14 GW

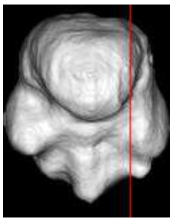

L-R Level: -6.18 mm

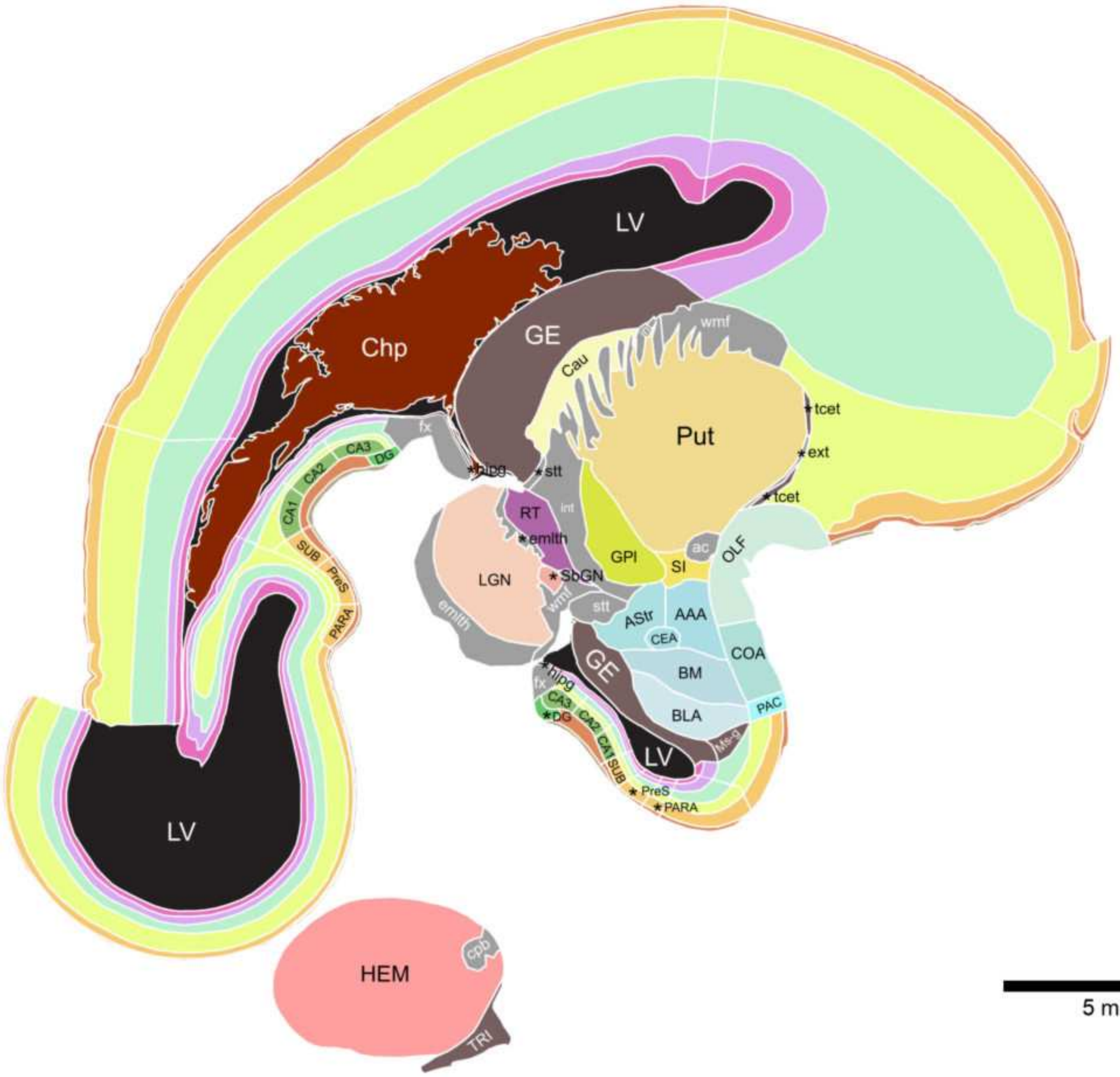

5 mm

- AAA: Anterior amygdaloid area
- AStr: Amygdalo-striatal area
- BL: Basal nucleus [amygdala]
- BM: Basomedial nucleus [amygdala]
- CA1: CA1 field [hippocampus]
- CA2: CA2 field [hippocampus]
- CA3: CA3 field [hippocampus]
- CEA: Central nucleus [amygdala]
- COA: Cortical nucleus [amygdala]
- Cau: Caudate nucleus
- Chp: Choroid plexus
- DG: Dentate gyrus
- GE: Ganglionic eminence
- GPl: Globus pallidus lateral segment
- HEM: Cerebellar hemispheres
- LGN: Lateral geniculate nucleus
- LV: Lateral ventricle
- Ms-g: Migratory stream, general
- PARA: Cortical plate, parasubiculum
- PreS: Cortical plate, presubiculum
- Put: Putamen
- RT: Reticular nucleus [thalamus]
- SI: Substantia innominata
- SUB: Cortical plate, subiculum
- SbGN: Subgeniculate nucleus
- TRI: Germinal trigone
- ac: Anterior commissure
- cpb: Cerebellar peduncles
- emlth: External medullary lamina [thalamus]
- ext: External capsule
- fx: Fornix
- hipg: Hippocampal glioepithelium/ependyma
- int: Internal capsule
- stt: Stria terminalis
- tcet: Transient cell zone in the external capsule
- wmf: White matter fibers

Age: 14 GW

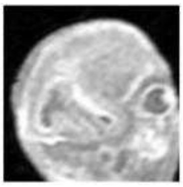

L-R Level: -6.54 mm

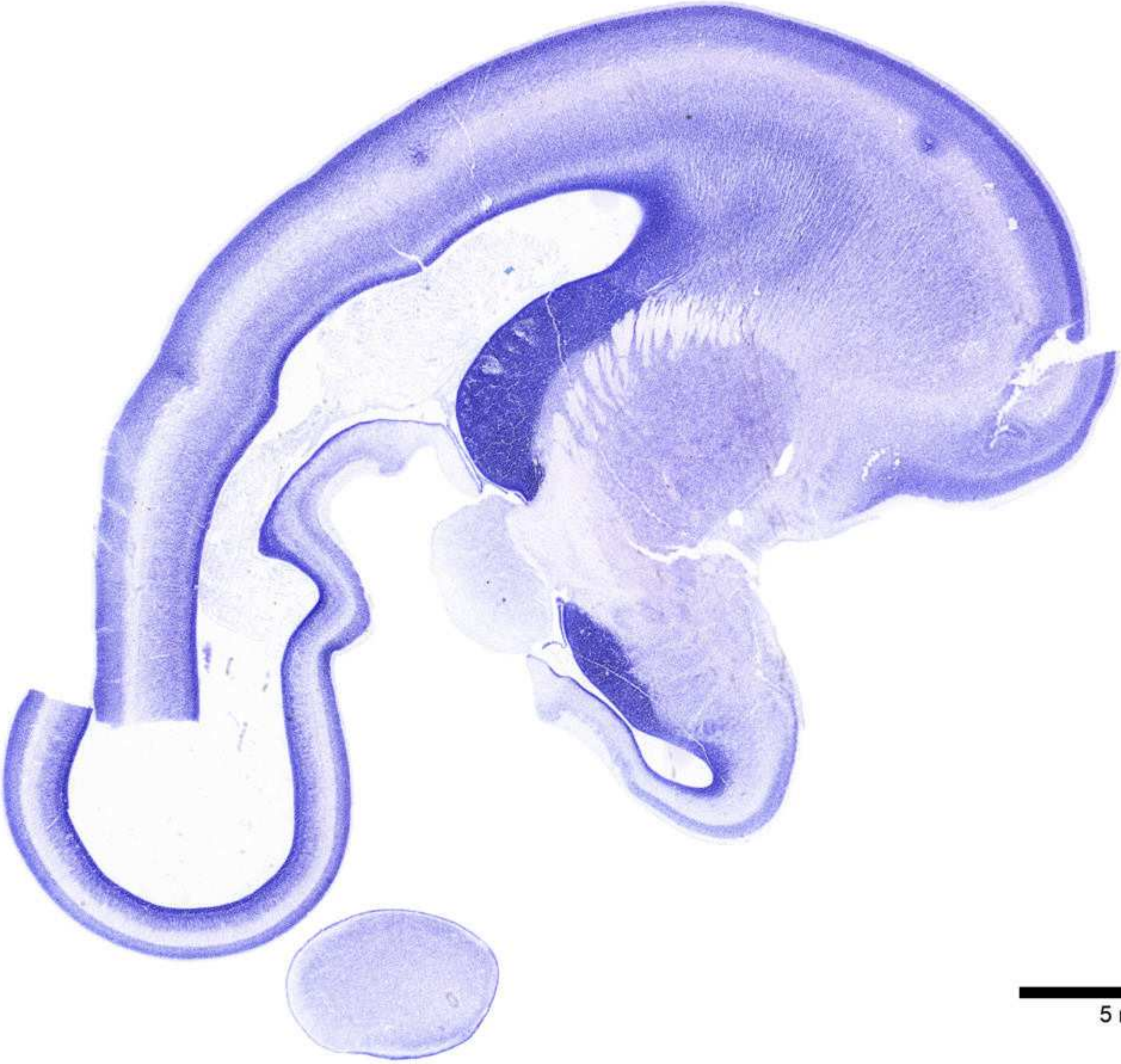

5 mm

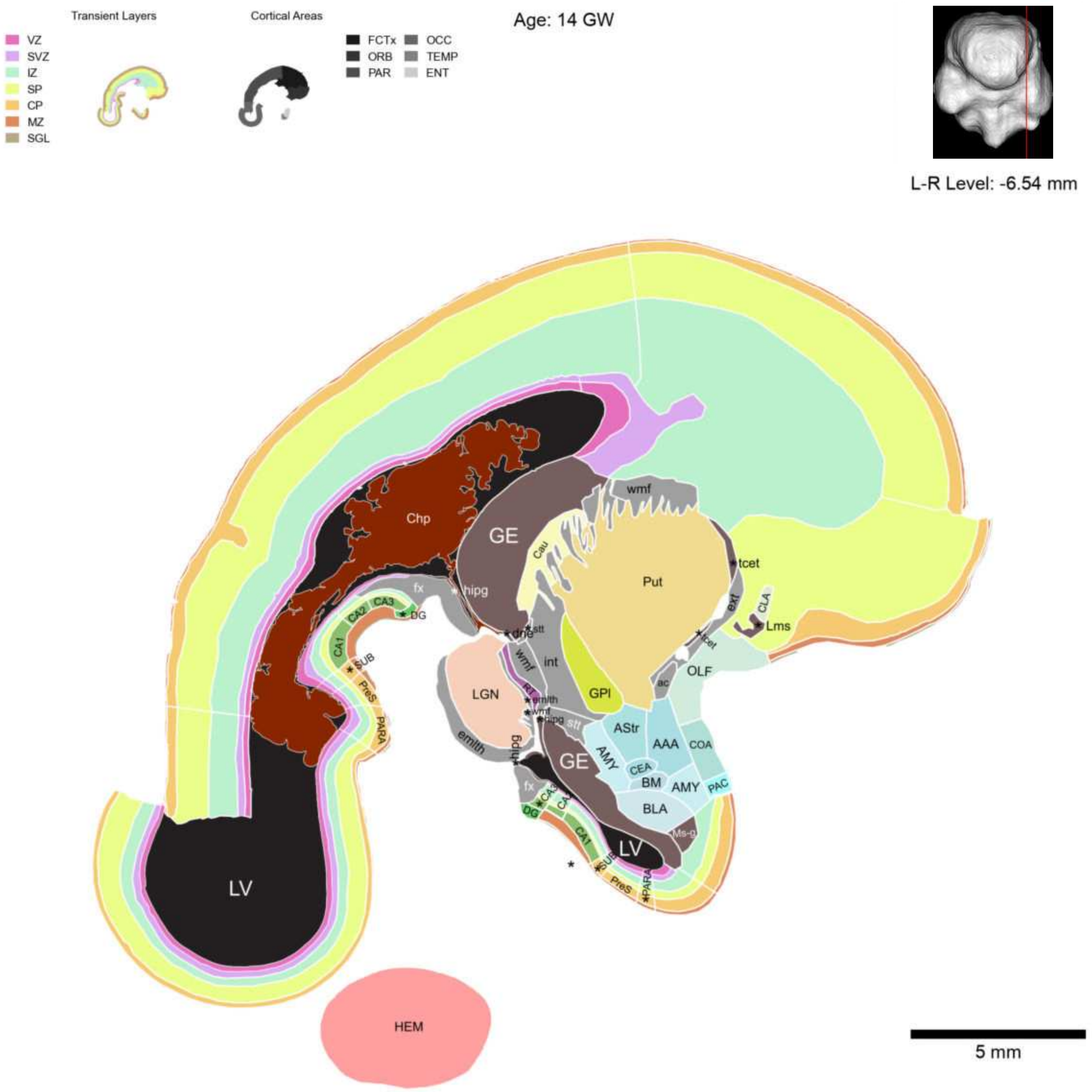

- AAA: Anterior amygdaloid area
- AMY: Amygdala
- AStr: Amygdalo-striatal area
- BL: Basal nucleus [amygdala]
- BM: Basomedial nucleus [amygdala]
- CA1: CA1 field [hippocampus]
- CA2: CA2 field [hippocampus]
- CA3: CA3 field [hippocampus]
- CEA: Central nucleus [amygdala]
- CLA: Claustrum
- COA: Cortical nucleus [amygdala]
- Cau: Caudate nucleus
- Chp: Choroid plexus
- DG: Dentate gyrus
- GE: Ganglionic eminence
- GPl: Globus pallidus lateral segment
- HEM: Cerebellar hemispheres
- LGN: Lateral geniculate nucleus
- LV: Lateral ventricle
- Lms: Lateral migratory stream
- Ms-g: Migratory stream, general
- PARA: Cortical plate, parasubiculum
- PreS: Cortical plate, presubiculum
- Put: Putamen
- RT: Reticular nucleus [thalamus]
- SUB: Cortical plate, subiculum
- ac: Anterior commissure
- dne: Diencephalic neuroepithelium
- emlth: External medullary lamina [thalamus]
- ext: External capsule
- fx: Fornix
- hipg: Hippocampal glioepithelium/ependyma
- int: Internal capsule
- stt: Stria terminalis
- tcet: Transient cell zone in the external capsule
- wmf: White matter fibers

Age: 14 GW

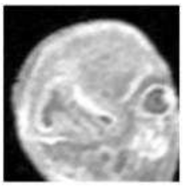

L-R Level: -6.66 mm

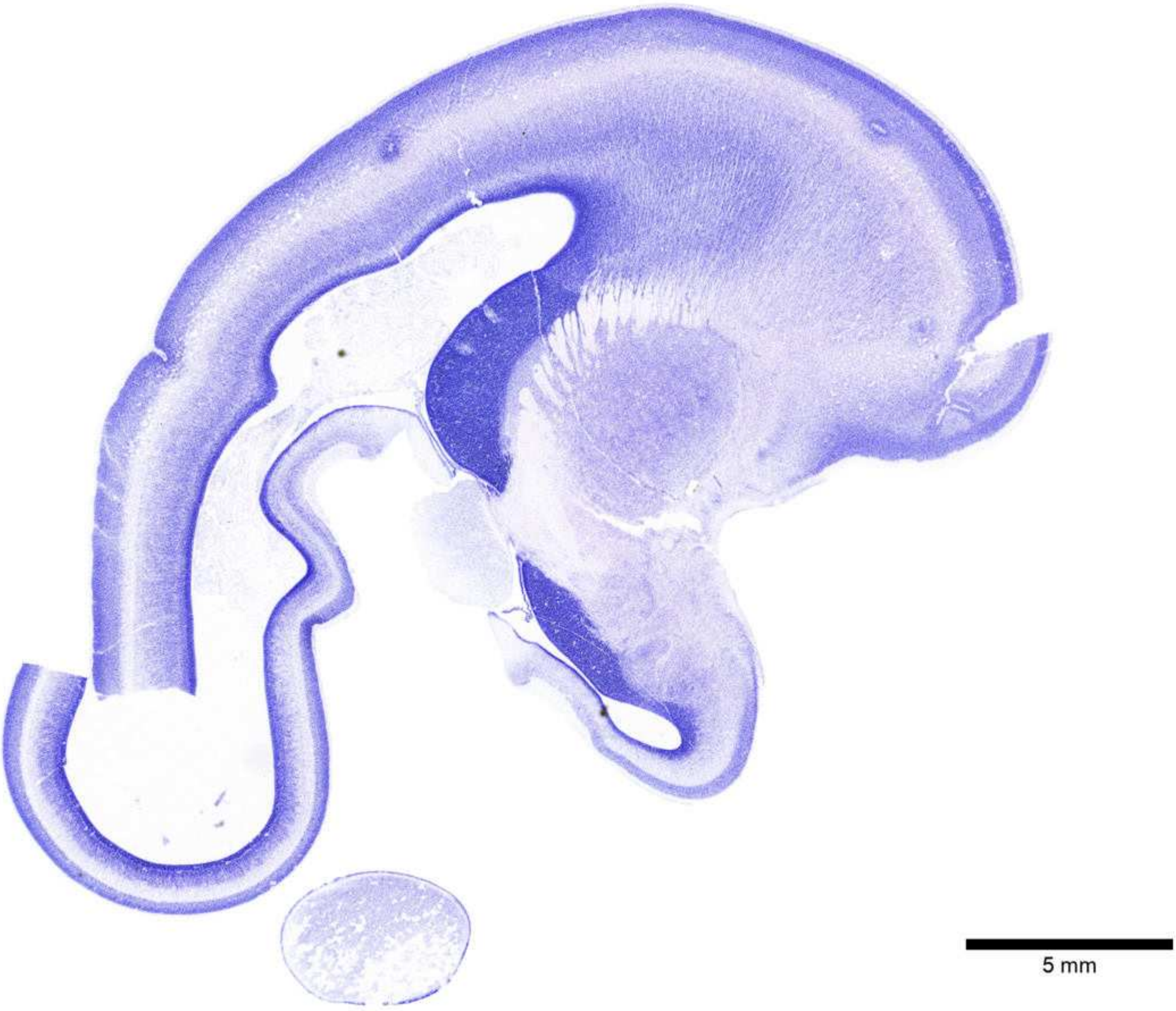

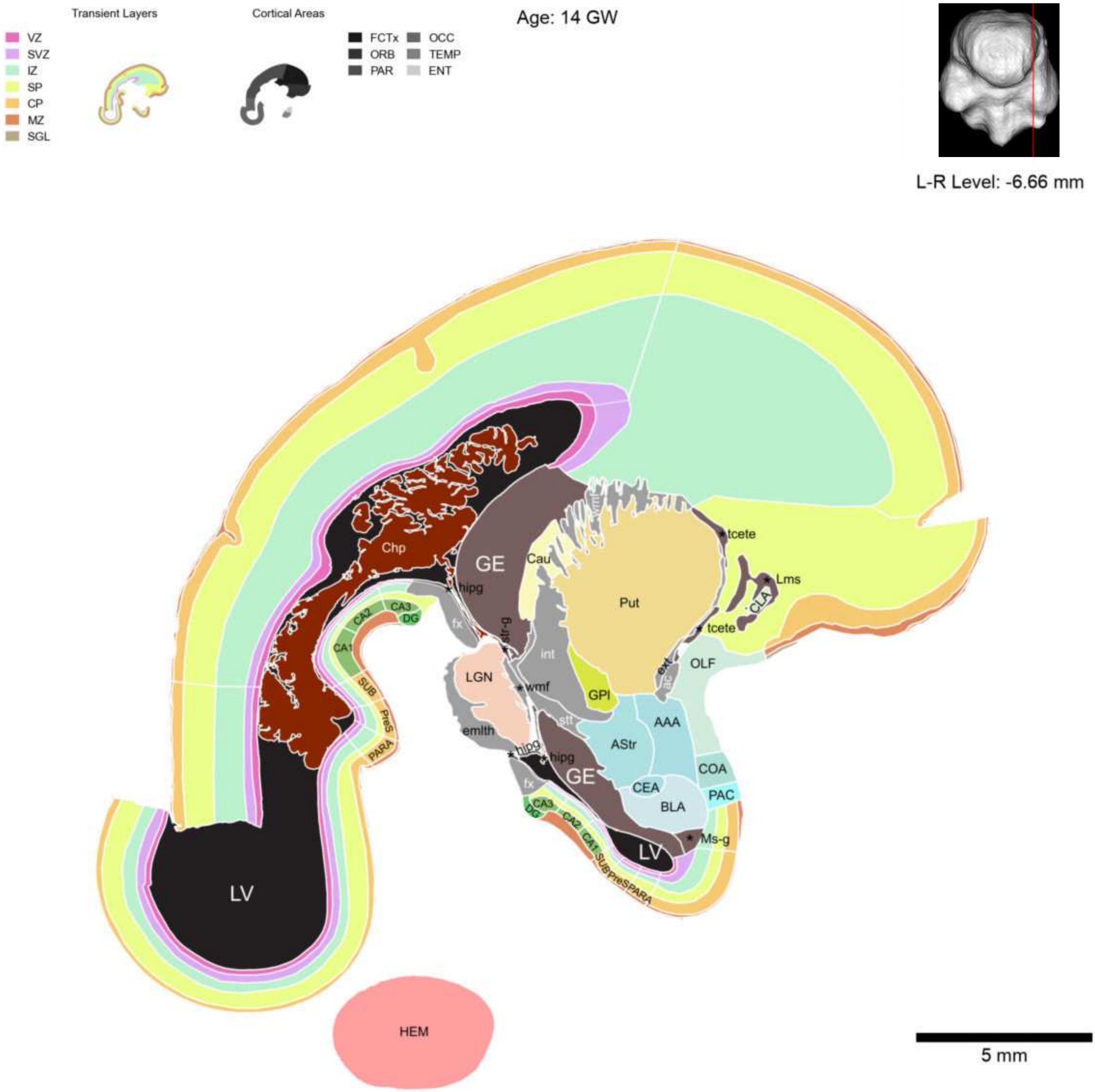

- AAA: Anterior amygdaloid area
- AStr: Amygdalo-striatal area
- BL: Basal nucleus [amygdala]
- CA1: CA1 field [hippocampus]
- CA2: CA2 field [hippocampus]
- CA3: CA3 field [hippocampus]
- CEA: Central nucleus [amygdala]
- CLA: Claustrum
- COA: Cortical nucleus [amygdala]
- Cau: Caudate nucleus
- Chp: Choroid plexus
- DG: Dentate gyrus
- GE: Ganglionic eminence
- GPl: Globus pallidus lateral segment
- HEM: Cerebellar hemispheres
- LGN: Lateral geniculate nucleus
- LV: Lateral ventricle
- Lms: Lateral migratory stream
- Ms-g: Migratory stream, general
- PARA: Cortical plate, parasubiculum
- PreS: Cortical plate, presubiculum
- Put: Putamen
- SUB: Cortical plate, subiculum
- ac: Anterior commissure
- emlth: External medullary lamina [thalamus]
- ext: External capsule
- fx: Fornix
- hipg: Hippocampal glioepithelium/ependyma
- int: Internal capsule
- str-g: Strionuclear glioepithelium
- stt: Stria terminalis
- tcete: Transient cell zone in the extreme capsule
- wmf: White matter fibers

Age: 14 GW

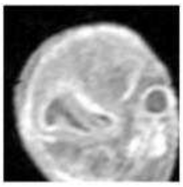

L-R Level: -7.26 mm

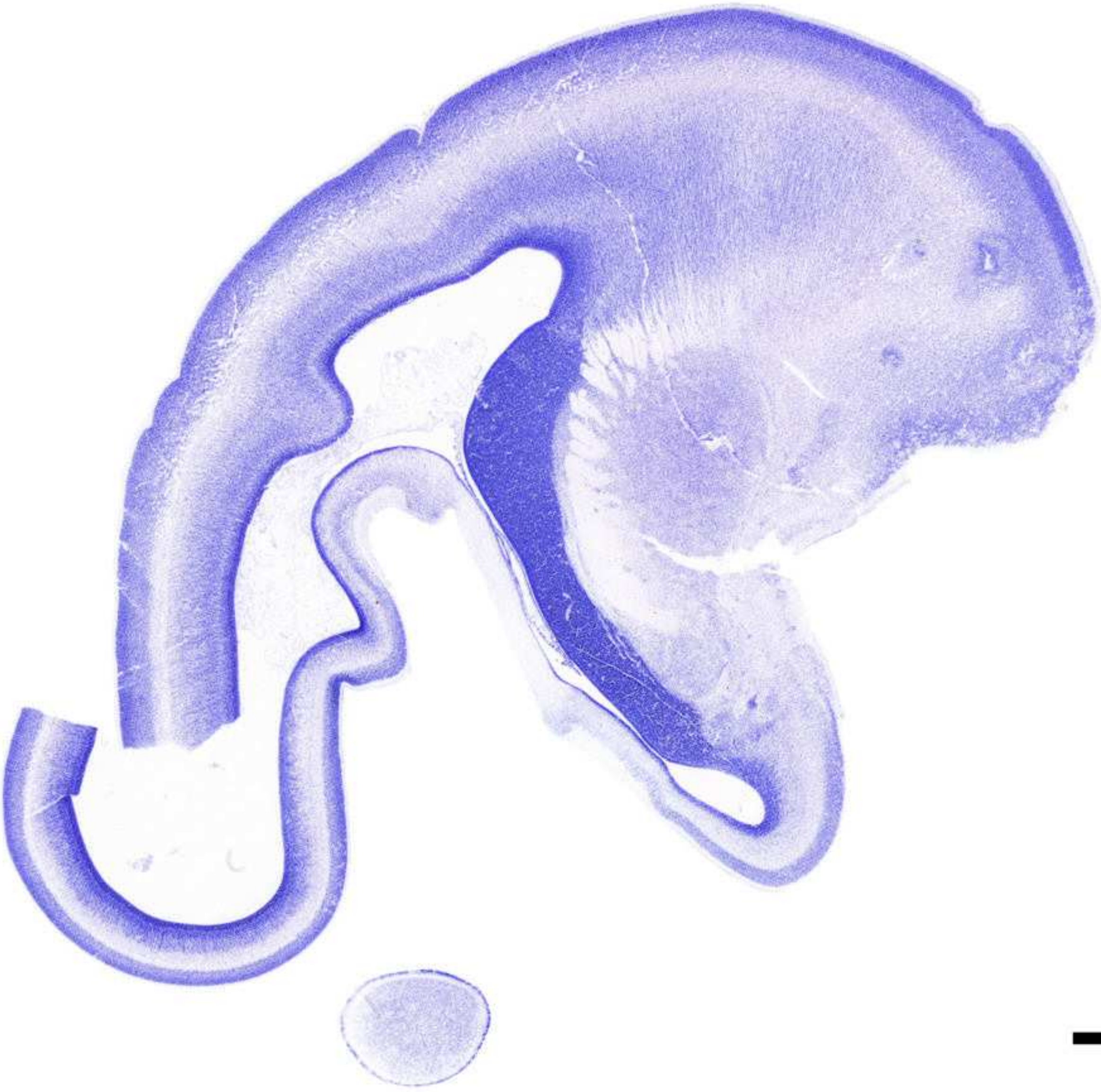

5 mm

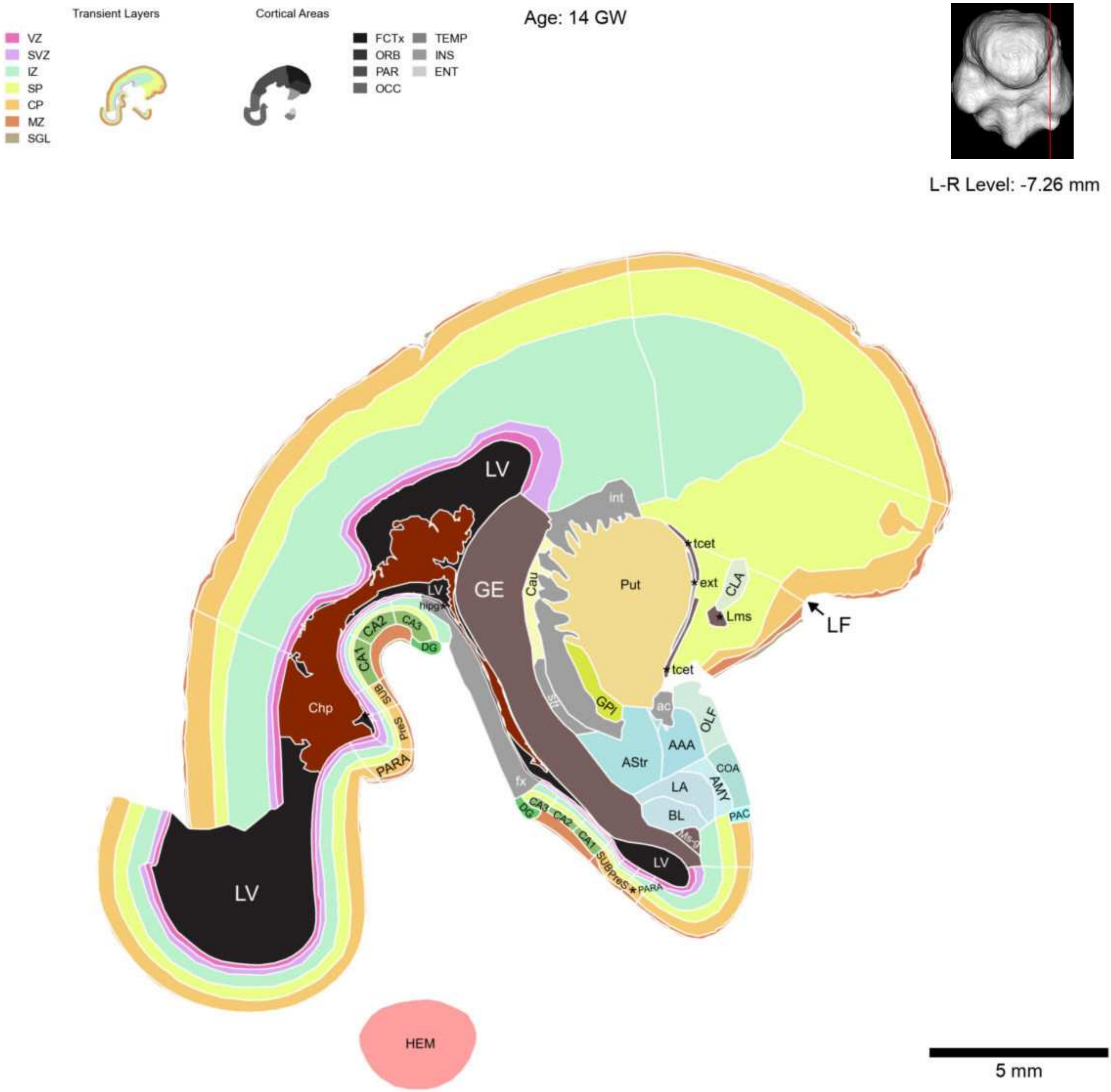

AAA: Anterior amygdaloid area
AMY: Amygdala
AStr: Amygdalo-striatal area
BL: Basal nucleus [amygdala]
CA1: CA1 field [hippocampus]
CA2: CA2 field [hippocampus]
CA3: CA3 field [hippocampus]
CLA: Claustrum
COA: Cortical nucleus [amygdala]
Cau: Caudate nucleus
Chp: Choroid plexus
DG: Dentate gyrus
GE: Ganglionic eminence
GPl: Globus pallidus lateral segment
HEM: Cerebellar hemispheres
LA: Lateral nucleus [amygdala]
LV: Lateral ventricle
Lms: Lateral migratory stream
Ms-g: Migratory stream, general
PARA: Cortical plate, parasubiculum
PreS: Cortical plate, presubiculum
Put: Putamen
SUB: Cortical plate, subiculum
ac: Anterior commissure
ext: External capsule
fx: Fornix
hipg: Hippocampal glioepithelium/ependyma
int: Internal capsule
stt: Stria terminalis
tcet: Transient cell zone in the external capsule
LF: Lateral Fissure

Age: 14 GW

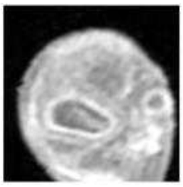

L-R Level: -8.28 mm

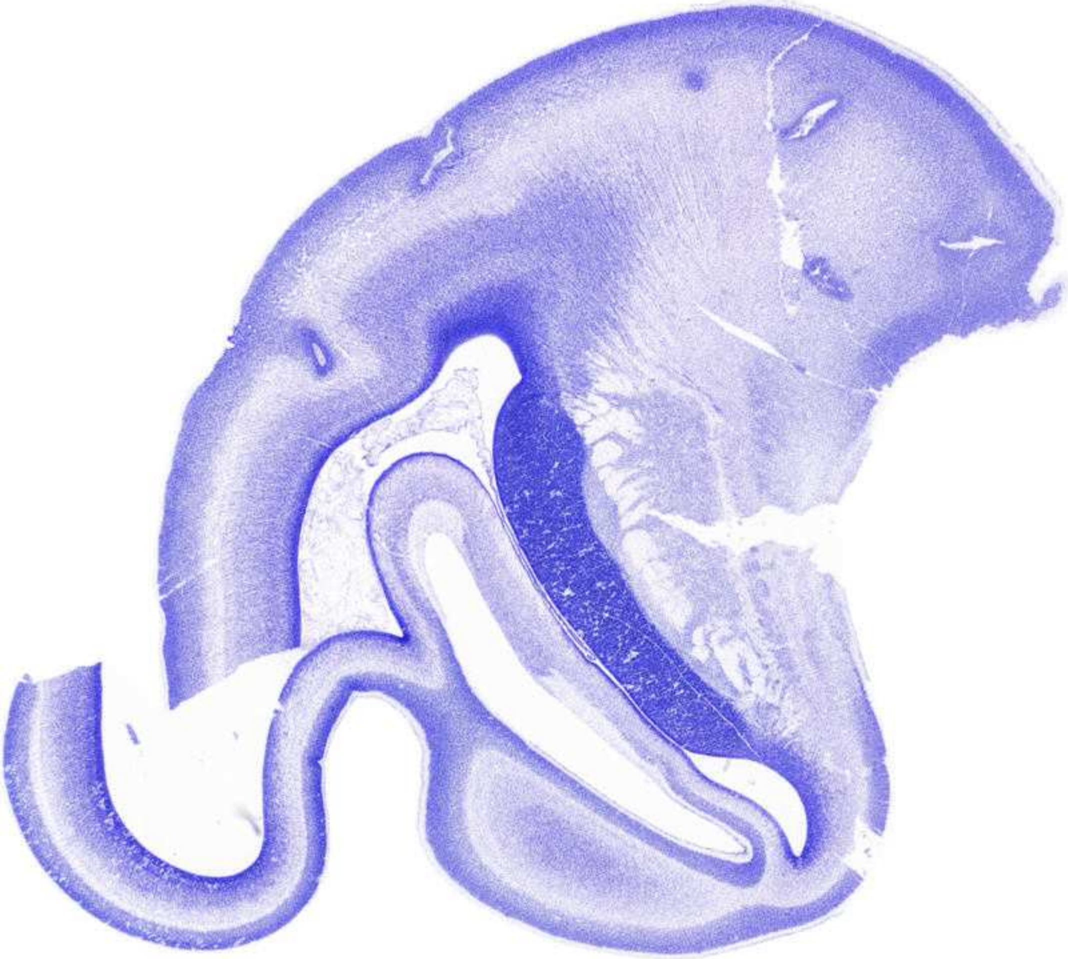

5 mm

Transient Layers

VZ
SVZ
IZ
SP
CP
MZ
SGL

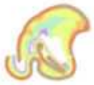

Cortical Areas

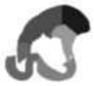

FCTx
PAR
OCC
TEMP
INS

Age: 14 GW

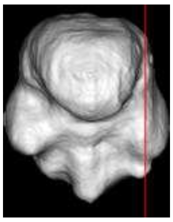

L-R Level: -8.28 mm

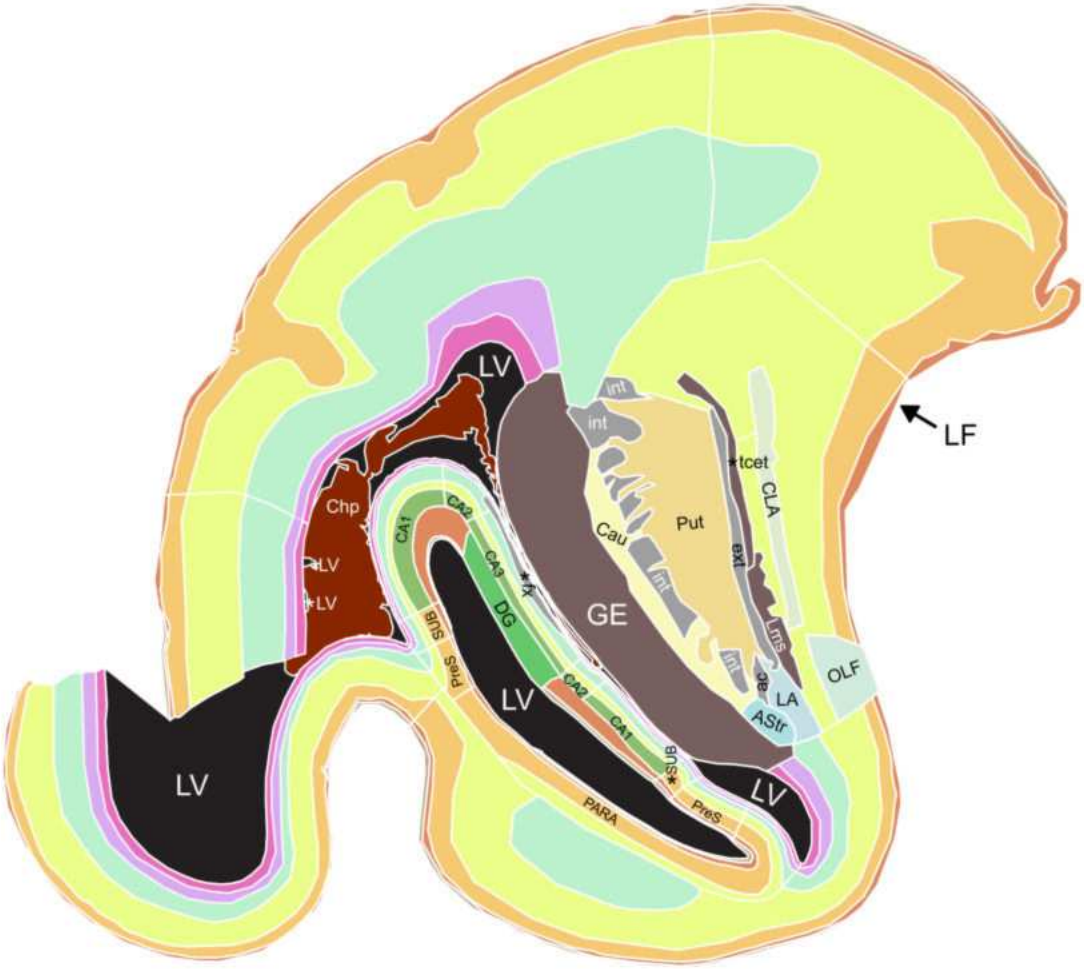

5 mm

AStr: Amygdalo-striatal area
CA1: CA1 field [hippocampus]
CA2: CA2 field [hippocampus]
CA3: CA3 field [hippocampus]
CLA: Claustrum
Cau: Caudate nucleus
Chp: Choroid plexus
DG: Dentate gyrus
GE: Ganglionic eminence
LA: Lateral nucleus [amygdala]
LV: Lateral ventricle
Lms: Lateral migratory stream
PARA: Cortical plate, parasubiculum
PreS: Cortical plate, presubiculum
Put: Putamen
SUB: Cortical plate, subiculum
ac: Anterior commissure
ext: External capsule
fx: Fornix
int: Internal capsule
tcet: Transient cell zone in the external capsule
LF: Lateral fissure

Age: 14 GW

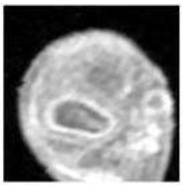

L-R Level: -8.4 mm

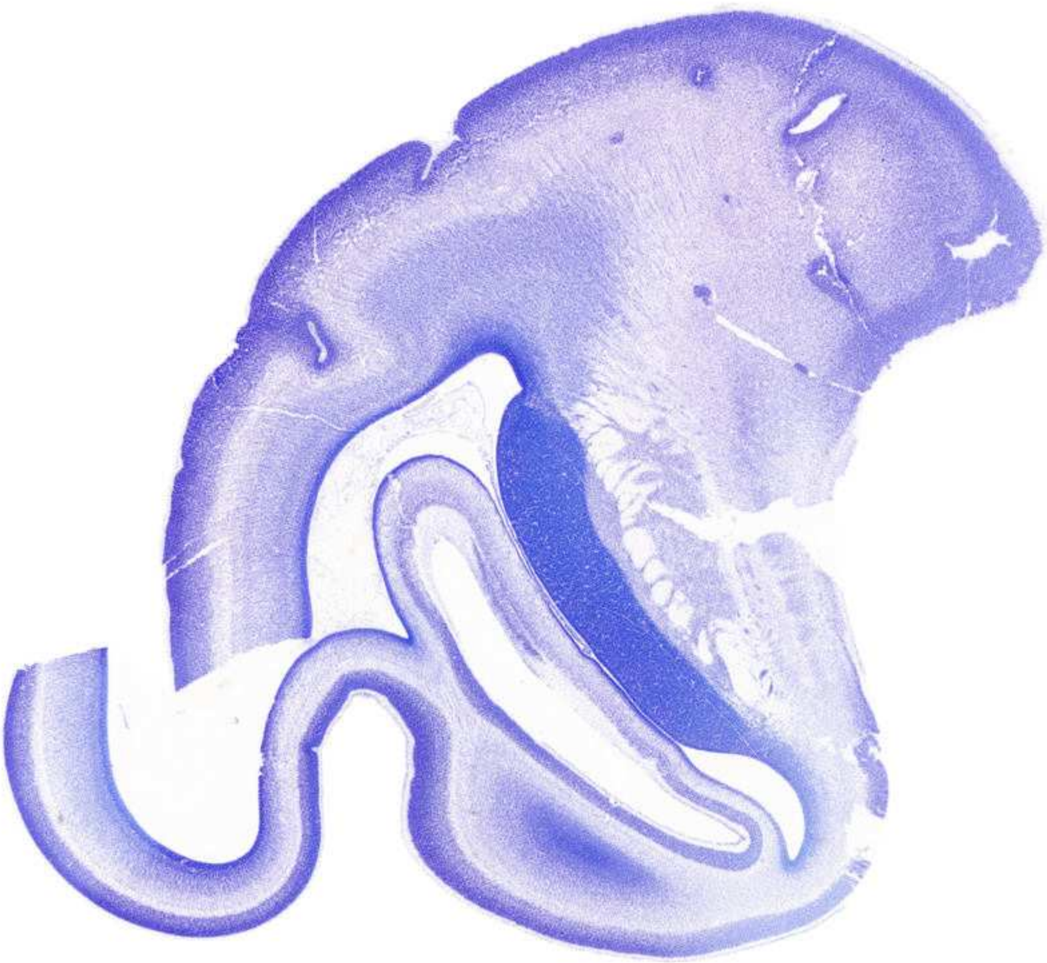

5 mm

Transient Layers

- VZ
- SVZ
- IZ
- SP
- CP
- MZ
- SGL

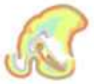

Cortical Areas

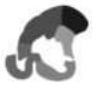

- FCTx
- PAR
- OCC
- TEMP
- INS

Age: 14 GW

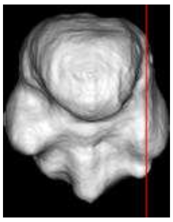

L-R Level: -8.4 mm

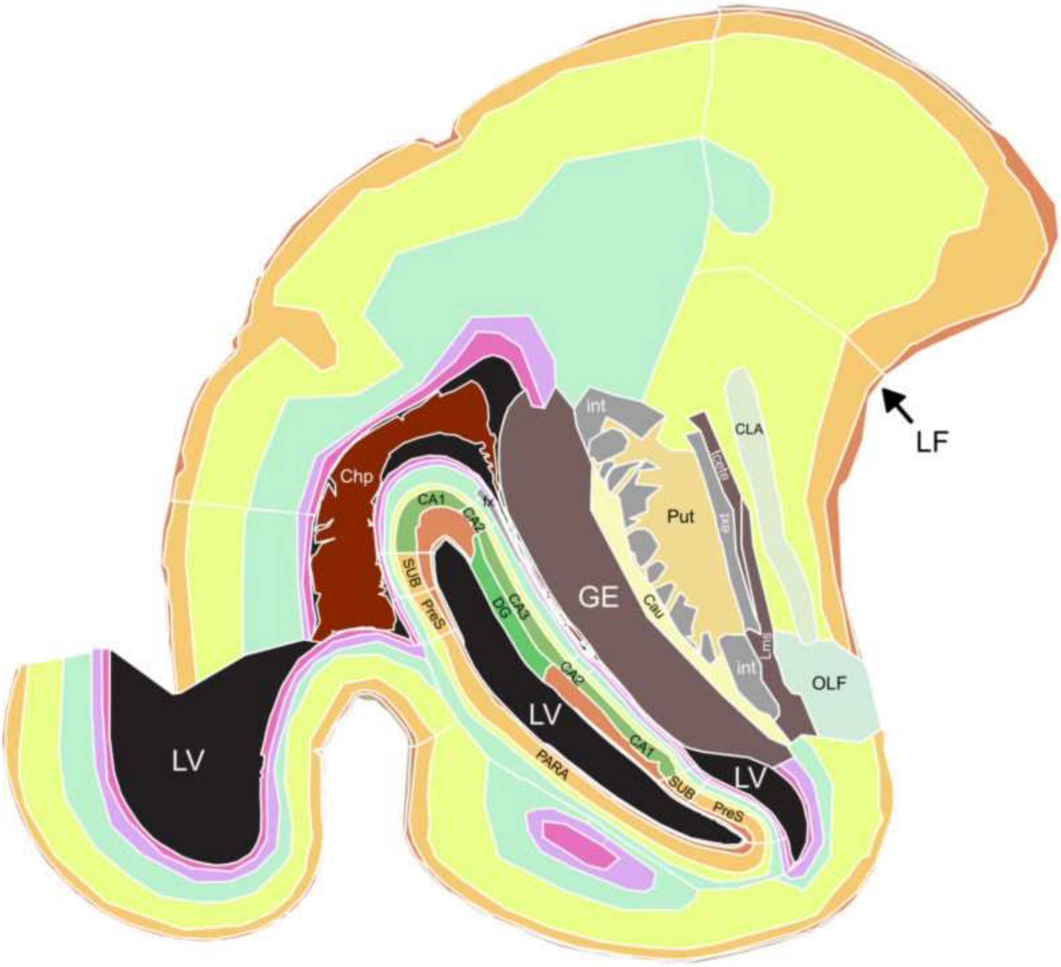

5 mm

- CA1: CA1 field [hippocampus]
- CA2: CA2 field [hippocampus]
- CA3: CA3 field [hippocampus]
- CLA: Claustrum
- Cau: Caudate nucleus
- Chp: Choroid plexus
- DG: Dentate gyrus
- GE: Ganglionic eminence
- LV: Lateral ventricle
- Lms: Lateral migratory stream
- PARA: Cortical plate, parasubiculum
- PreS: Cortical plate, presubiculum
- Put: Putamen
- SUB: Cortical plate, subiculum
- ext: External capsule
- fx: Fornix
- int: Internal capsule
- tcete: Transient cell zone in the extreme capsule
- LF: Lateral fissure

Age: 14 GW

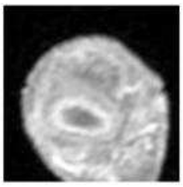

L-R Level: -9.18 mm

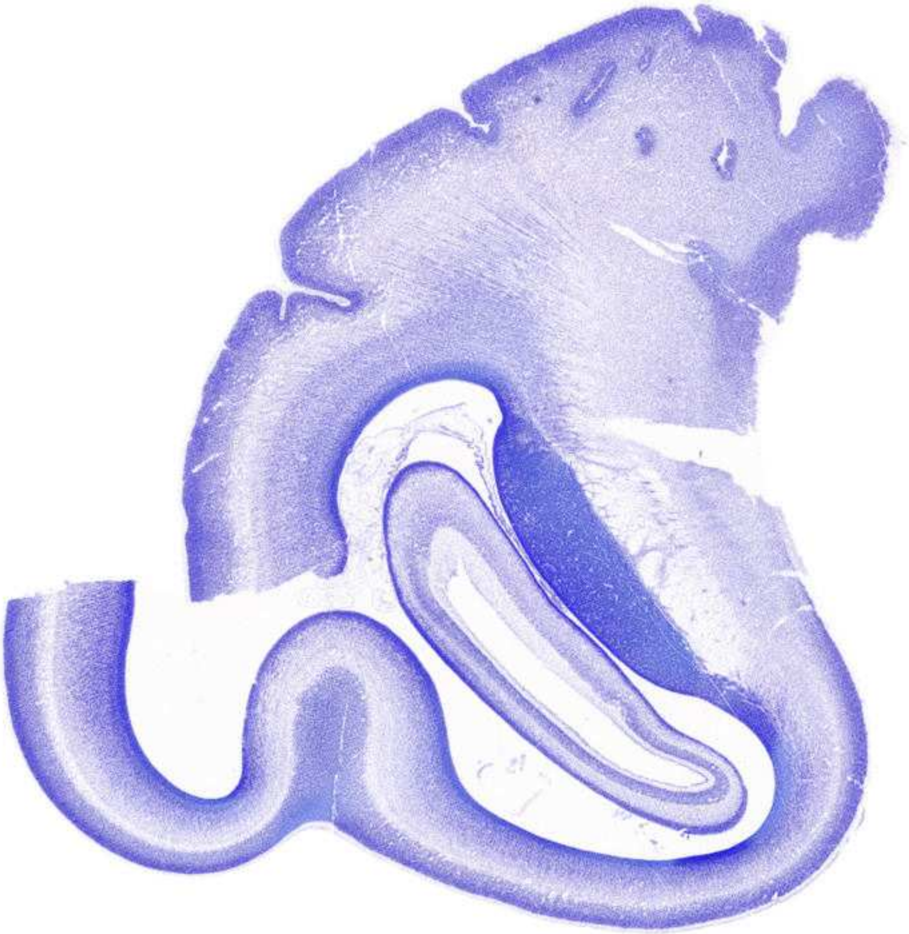

5 mm

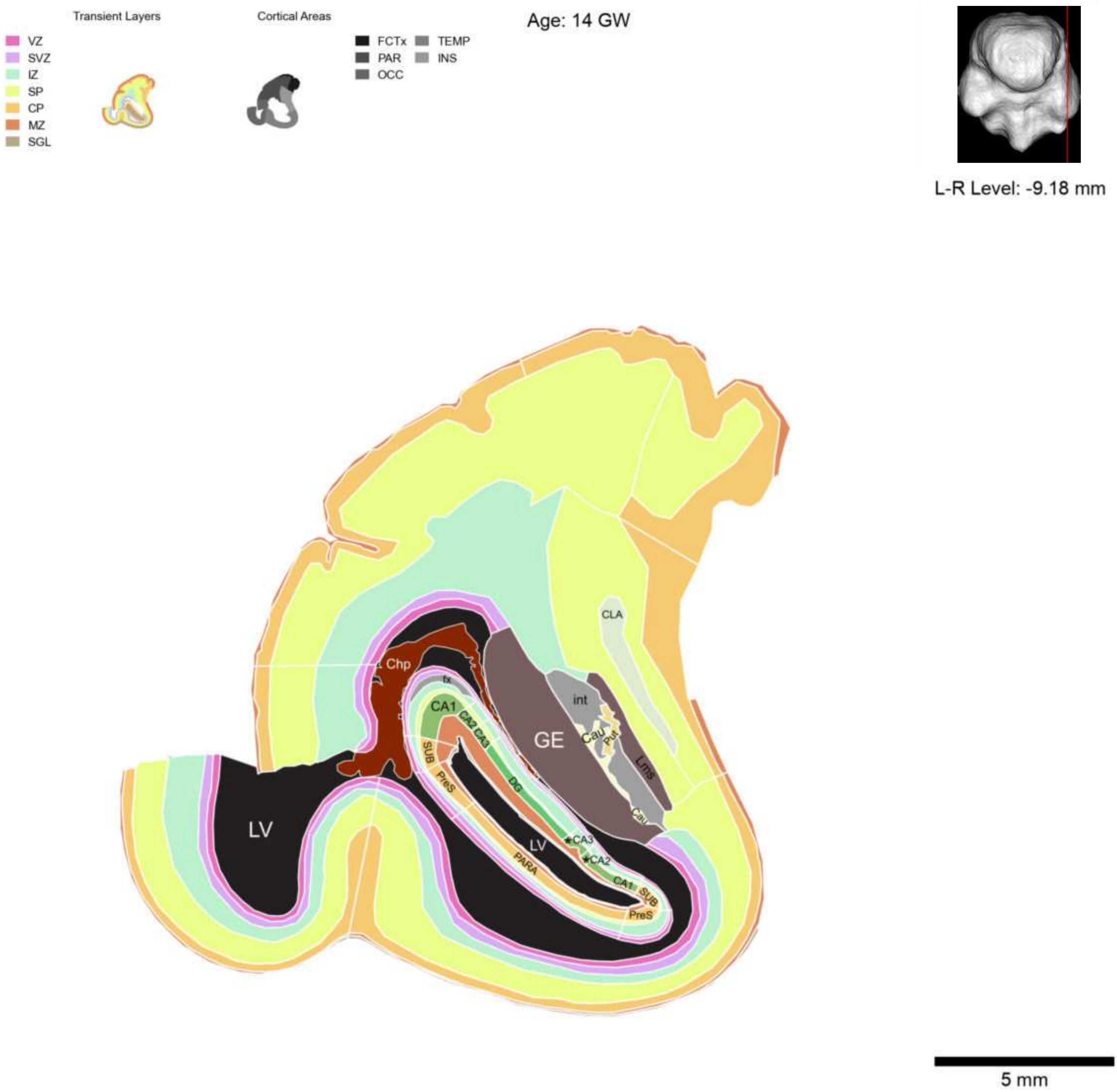

- CA1: CA1 field [hippocampus]
- CA2: CA2 field [hippocampus]
- CA3: CA3 field [hippocampus]
- CLA: Claustrum
- Cau: Caudate nucleus
- Chp: Choroid plexus
- DG: Dentate gyrus
- GE: Ganglionic eminence
- LV: Lateral ventricle
- Lms: Lateral migratory stream
- PARA: Cortical plate, parasubiculum
- PreS: Cortical plate, presubiculum
- Put: Putamen
- SUB: Cortical plate, subiculum
- fx: Fornix
- int: Internal capsule

Age: 14 GW

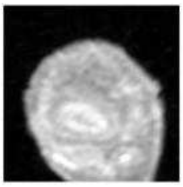

L-R Level: -9.78 mm

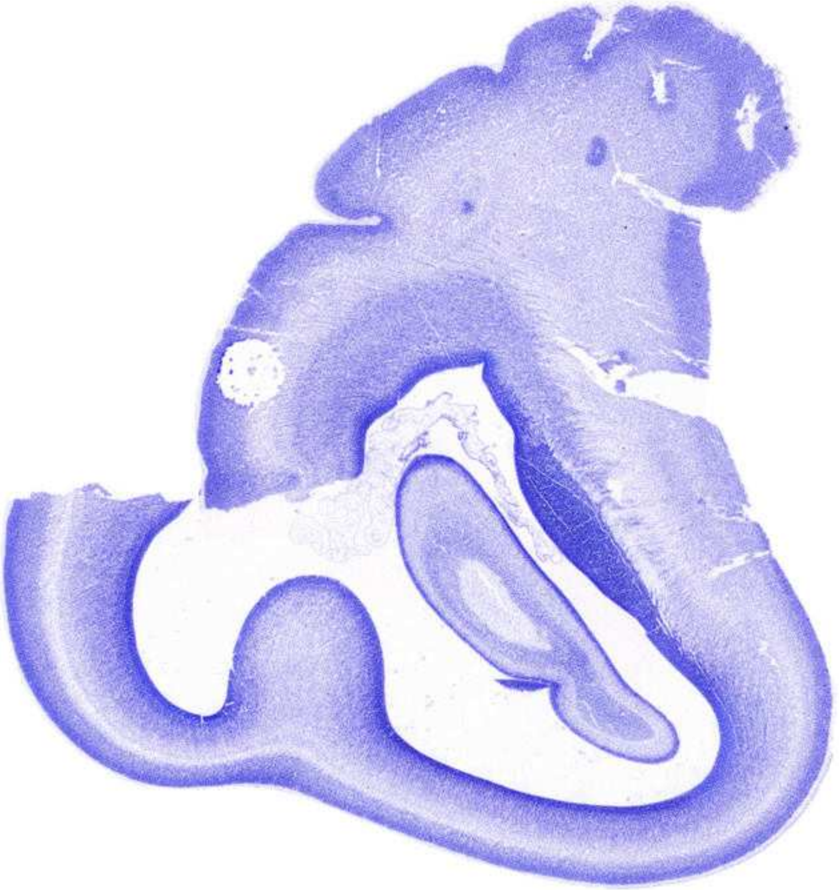

5 mm

Transient Layers

- VZ
- SVZ
- IZ
- SP
- CP
- MZ
- SGL

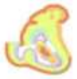

Cortical Areas

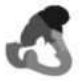

- FCTx
- PAR
- OCC
- TEMP
- INS

Age: 14 GW

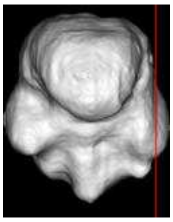

L-R Level: -9.78 mm

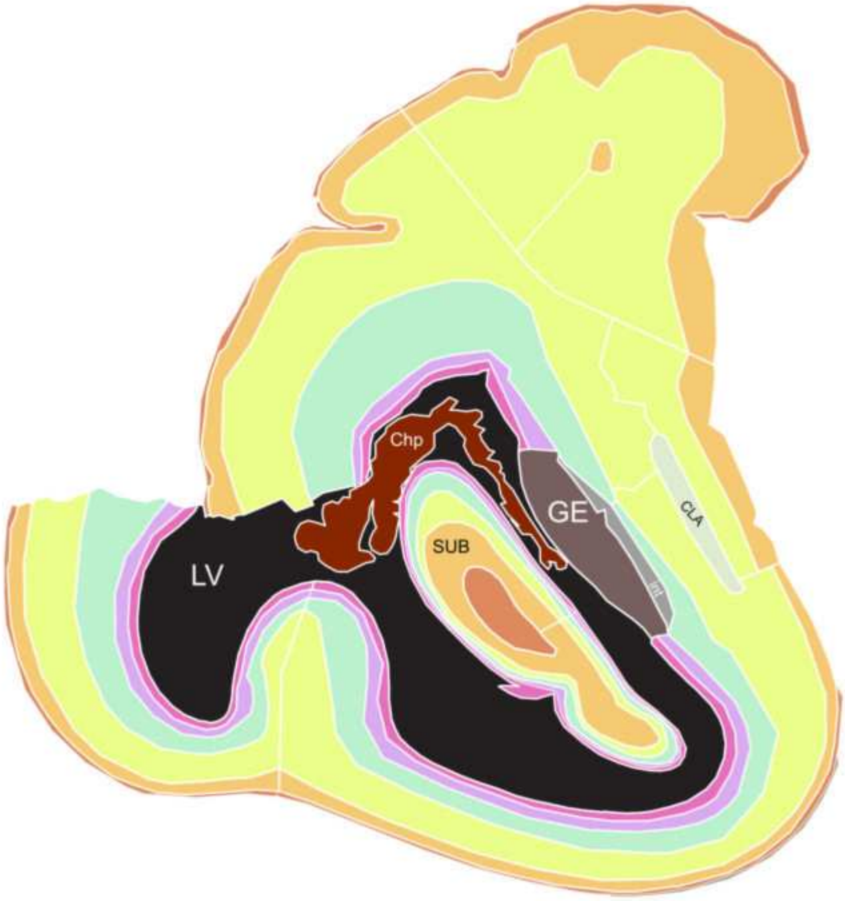

5 mm

- CLA: Claustrum
- GE: Ganglionic eminence
- SUB: Cortical plate, subiculum
- int: Internal capsule
- Chp: Choroid plexus
- LV: Lateral ventricle

Age: 14 GW

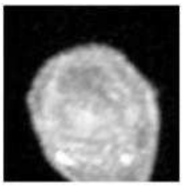

L-R Level: -10.38 mm

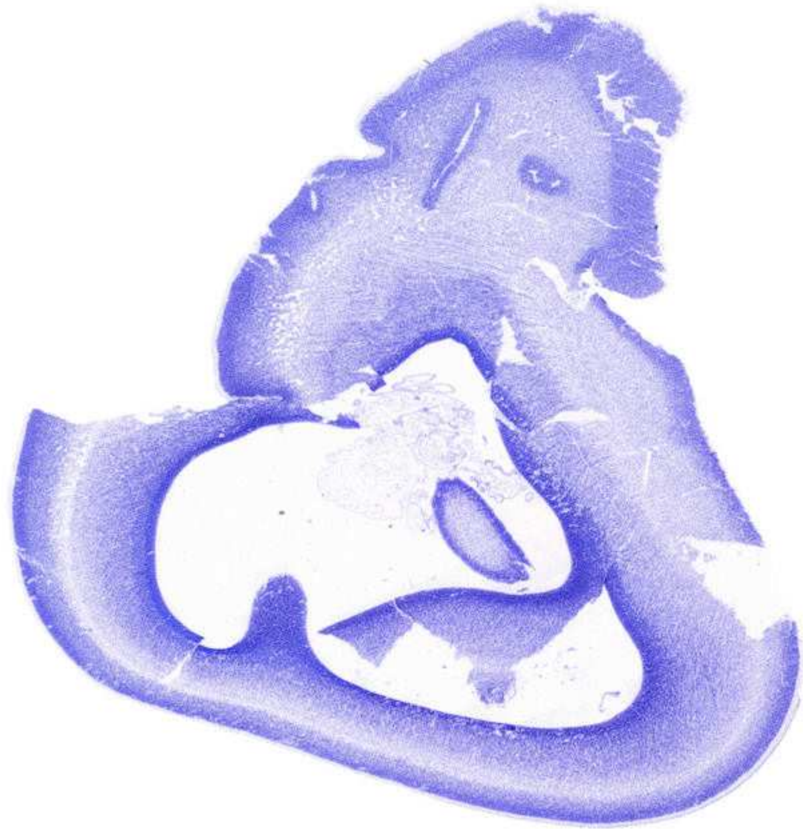

5 mm

Transient Layers

- VZ
- SVZ
- IZ
- SP
- CP
- MZ
- SGL

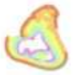

Cortical Areas

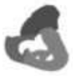

- PAR
- OCC
- TEMP

Age: 14 GW

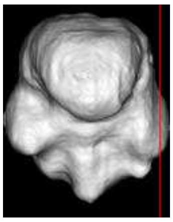

L-R Level: -10.38 mm

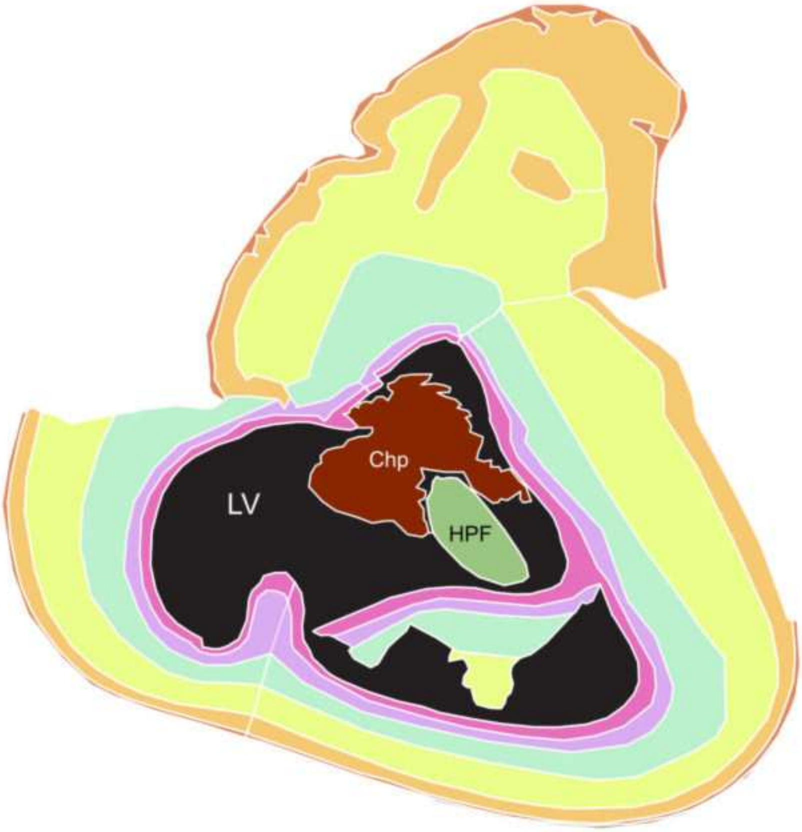

5 mm

Chp: Choroid plexus   HPF: Hippocampal formation   LV: Lateral ventricle

Age: 14 GW

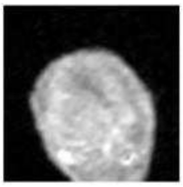

L-R Level: -10.86 mm

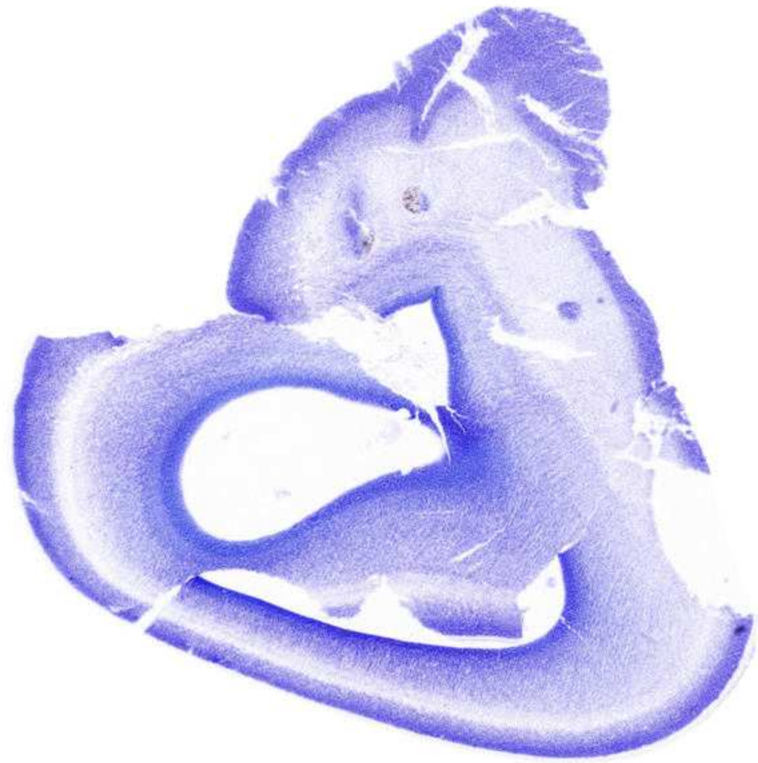

5 mm

Transient Layers

- VZ
- SVZ
- IZ
- SP
- CP
- MZ
- SGL

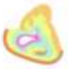

Cortical Areas

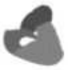

- PAR
- OCC
- TEMP

Age: 14 GW

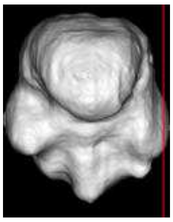

L-R Level: -10.86 mm

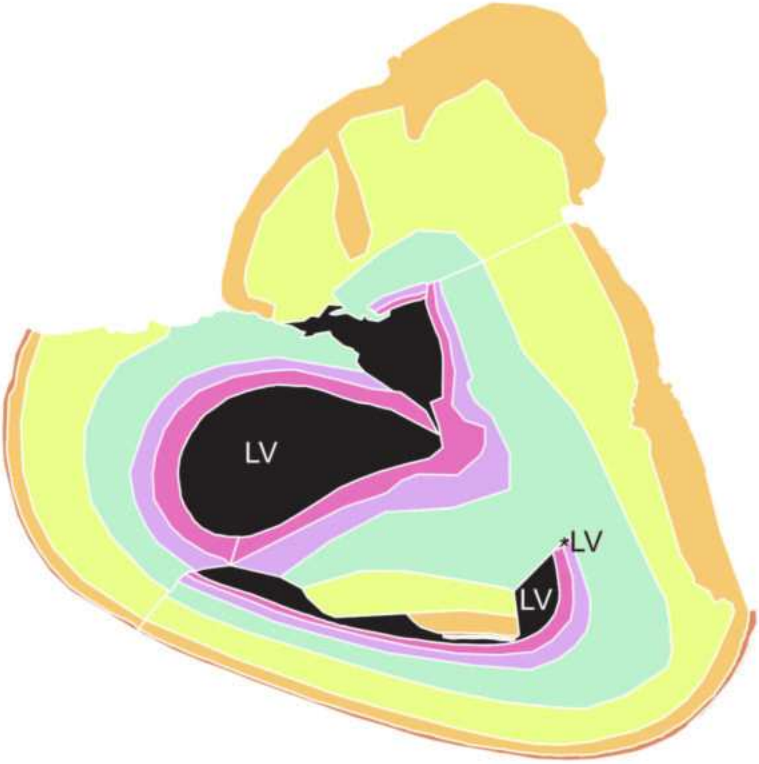

5 mm

LV: Lateral ventricle

Age: 14 GW

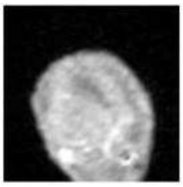

L-R Level: -11.04 mm

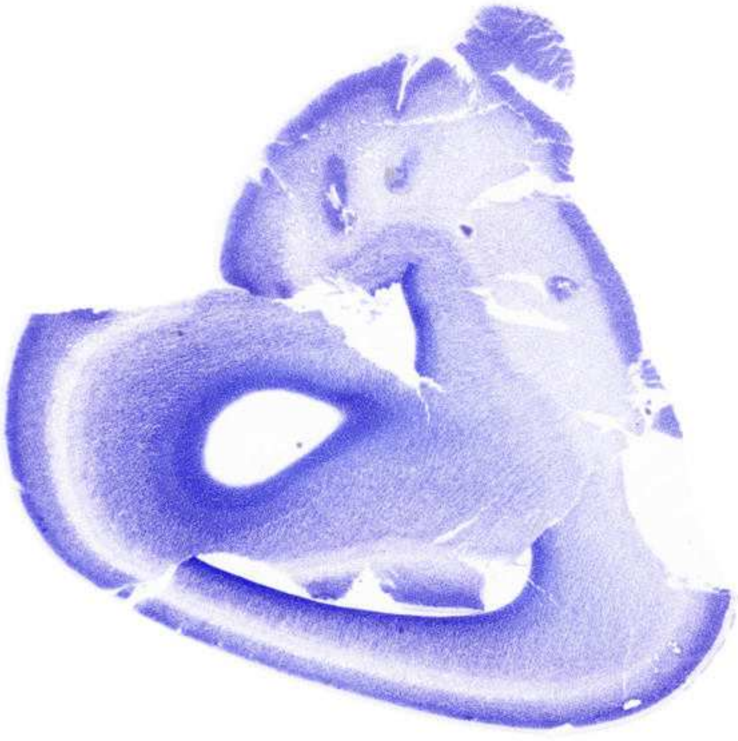

5 mm

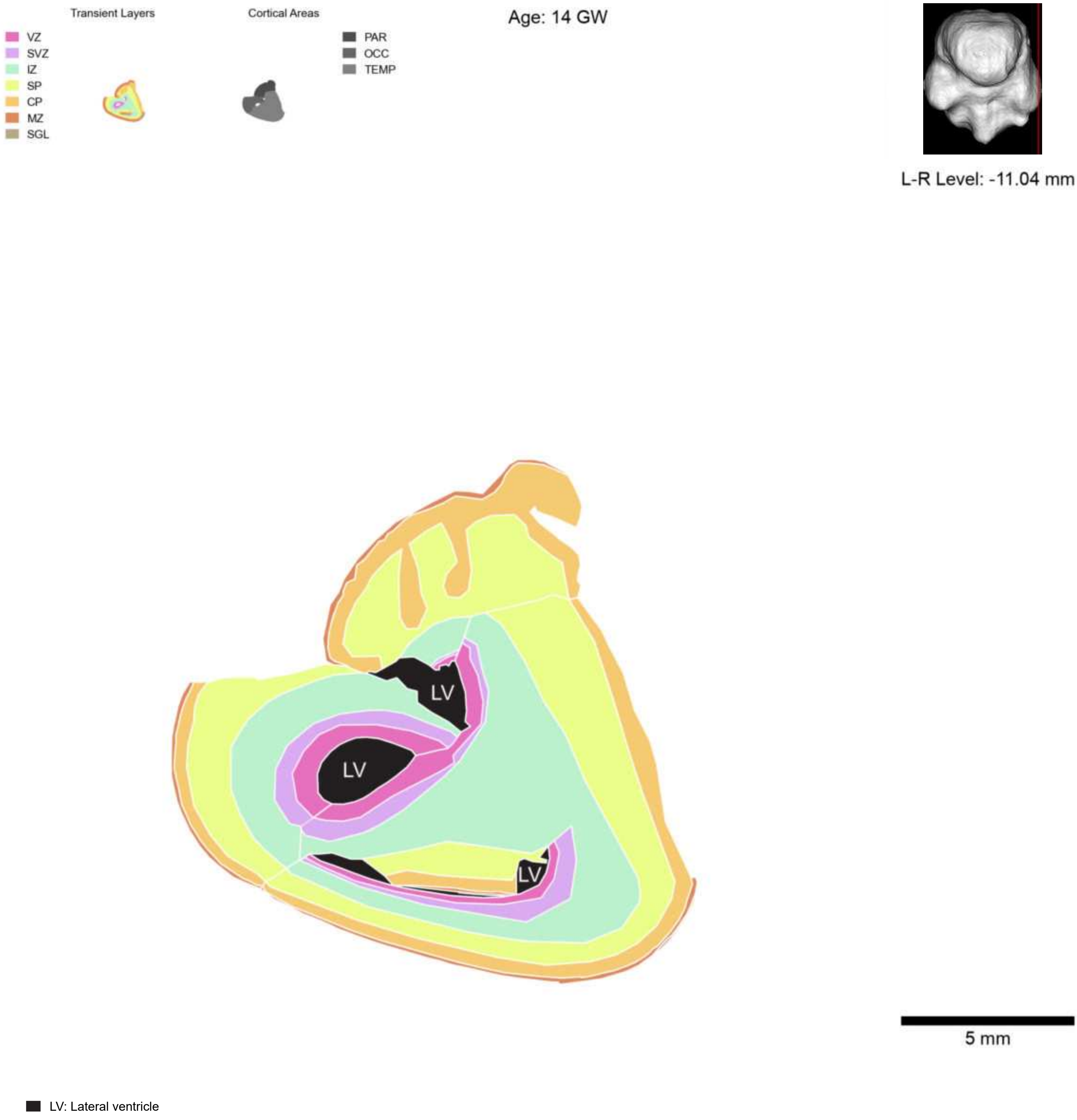
Transient Layers
VZ
SVZ
IZ
SP
CP
MZ
SGL
Cortical Areas
PAR
OCC
TEMP
Age: 14 GW
L-R Level: -11.04 mm
LV
LV
LV
5 mm
LV: Lateral ventricle

Age: 14 GW

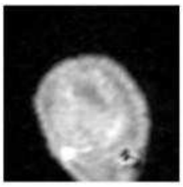

L-R Level: -11.46 mm

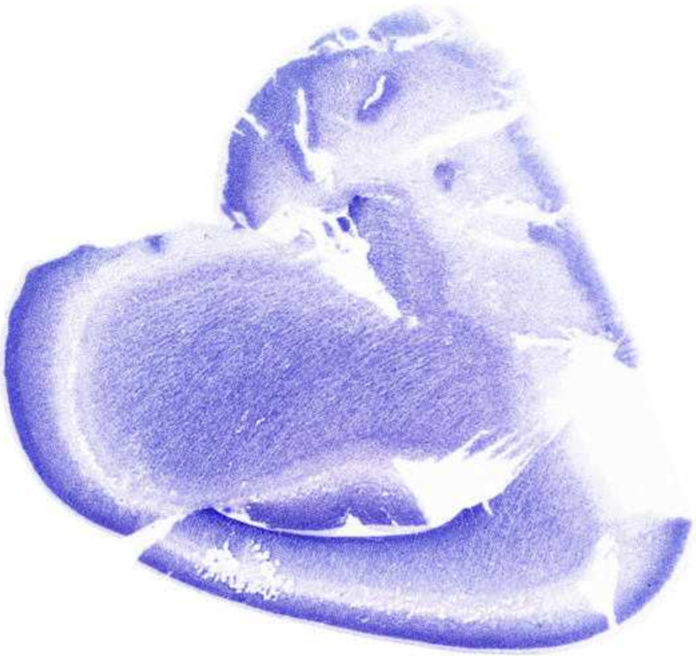

5 mm

Transient Layers

- VZ
- SVZ
- IZ
- SP
- CP
- MZ
- SGL

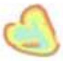

Cortical Areas

- PAR
- OCC
- TEMP

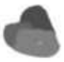

Age: 14 GW

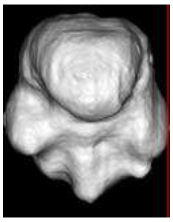

L-R Level: -11.46 mm

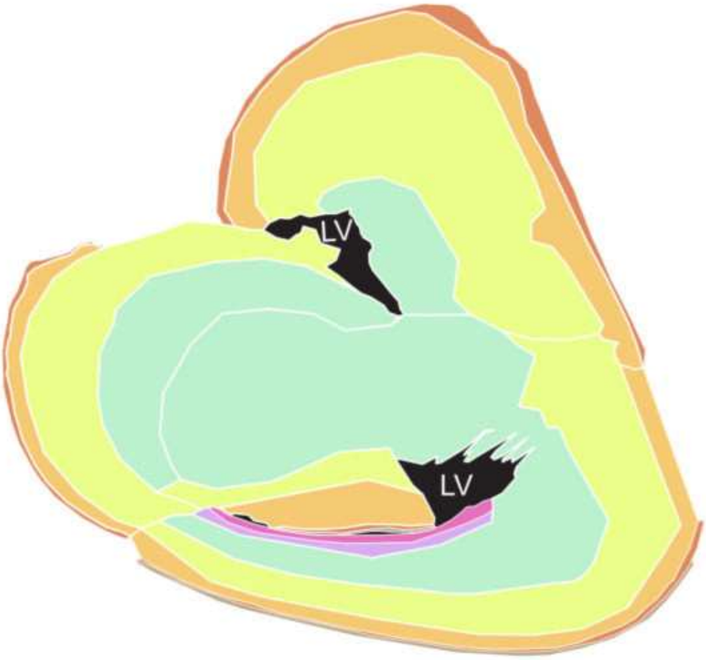

5 mm

LV: Lateral ventricle

Age: 14 GW

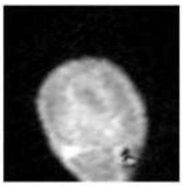

L-R Level: -11.76 mm

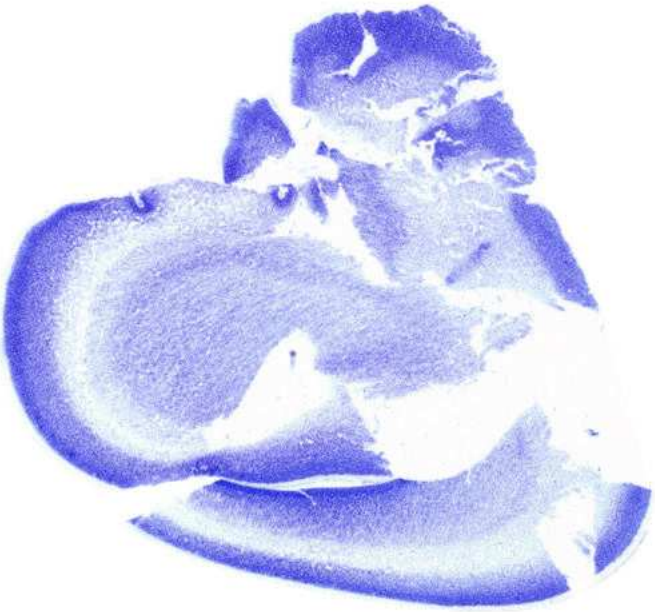

5 mm

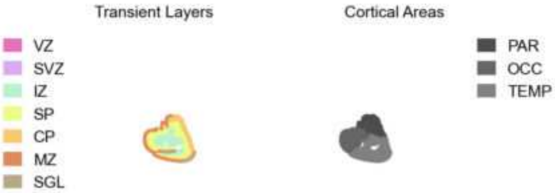

Age: 14 GW

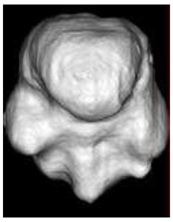

L-R Level: -11.76 mm

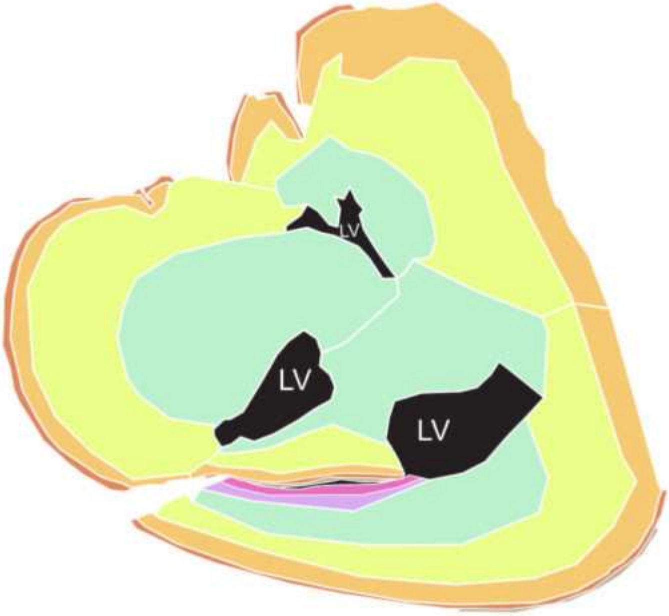

5 mm

LV: Lateral ventricle

17 Gestational Week (GW)

Sagittal

This page is intentionally left blank

Age: 17 GW

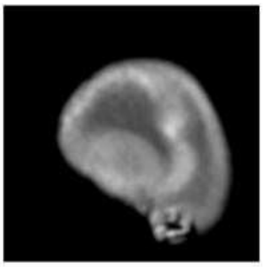

L-R Level: 16.02 mm

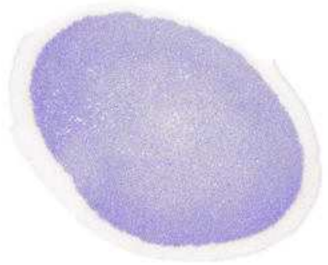

5 mm

Transient Layers

SP

CP

MZ

SGL

Cortical Areas

TEMP

Age: 17 GW

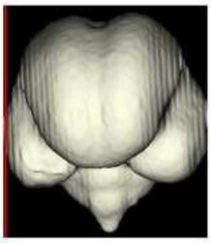

L-R Level: 16.02 mm

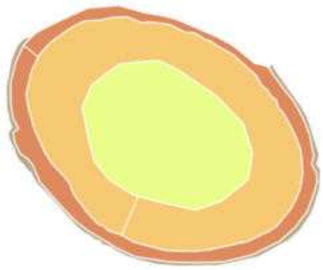

5 mm

Age: 17 GW

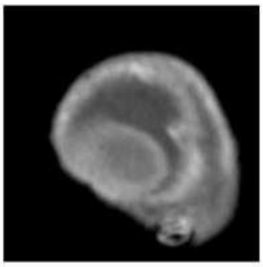

L-R Level: 15.12 mm

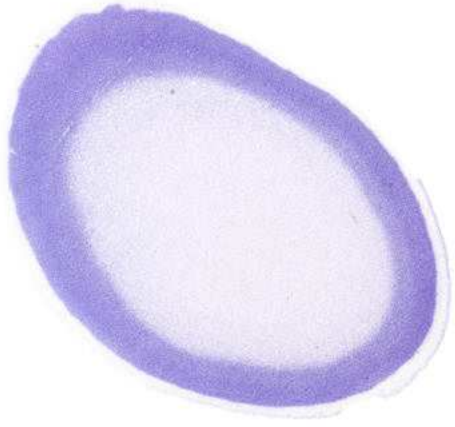

5 mm

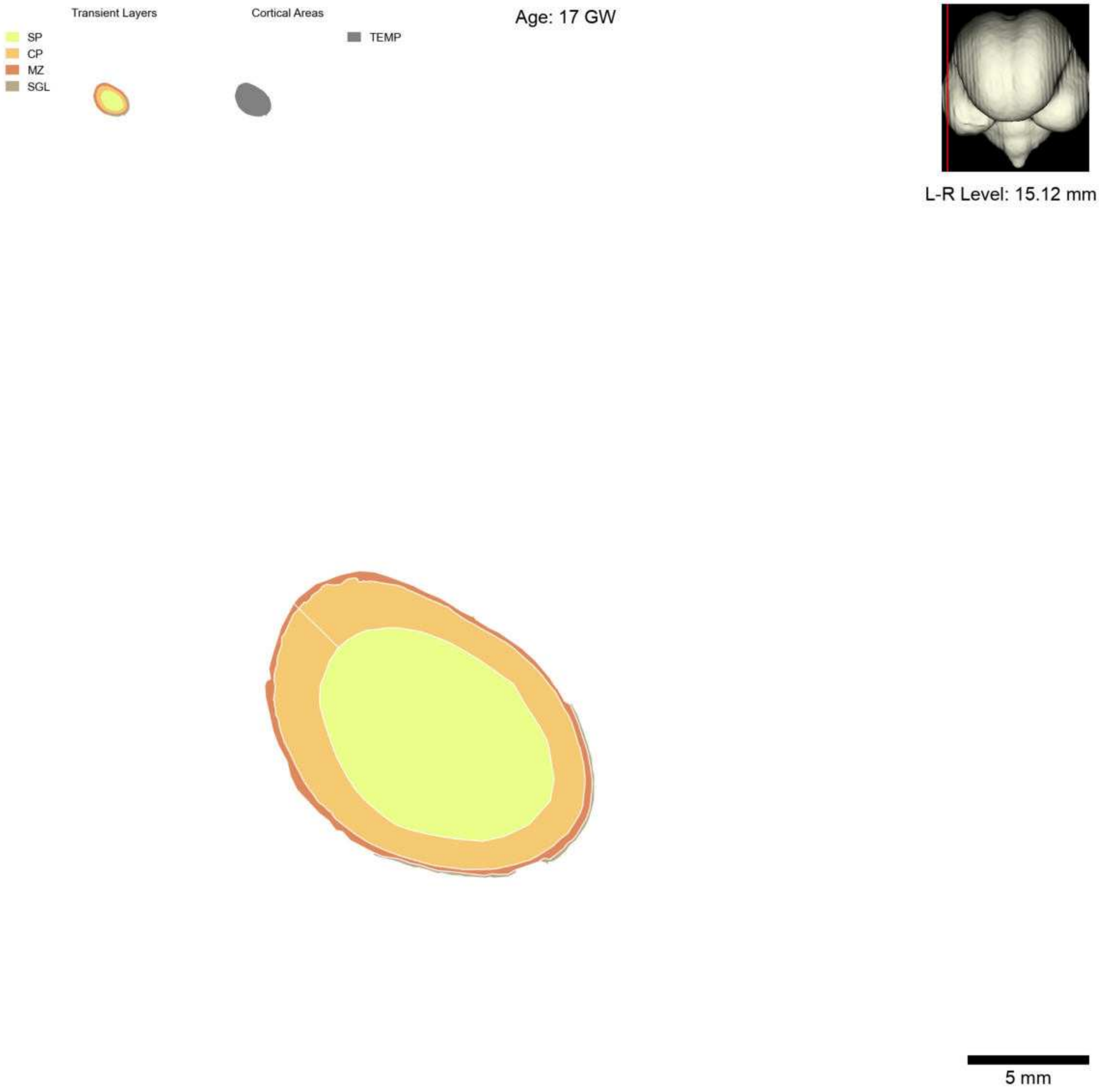
Transient Layers
SP
CP
MZ
SGL
Cortical Areas
TEMP
Age: 17 GW
L-R Level: 15.12 mm
5 mm

Age: 17 GW

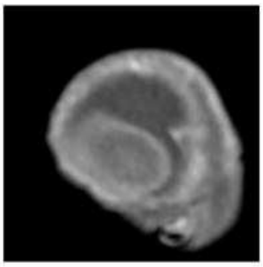

L-R Level: 14.64 mm

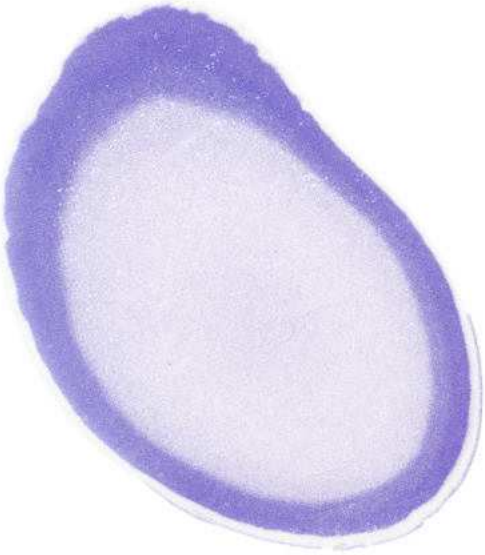

5 mm

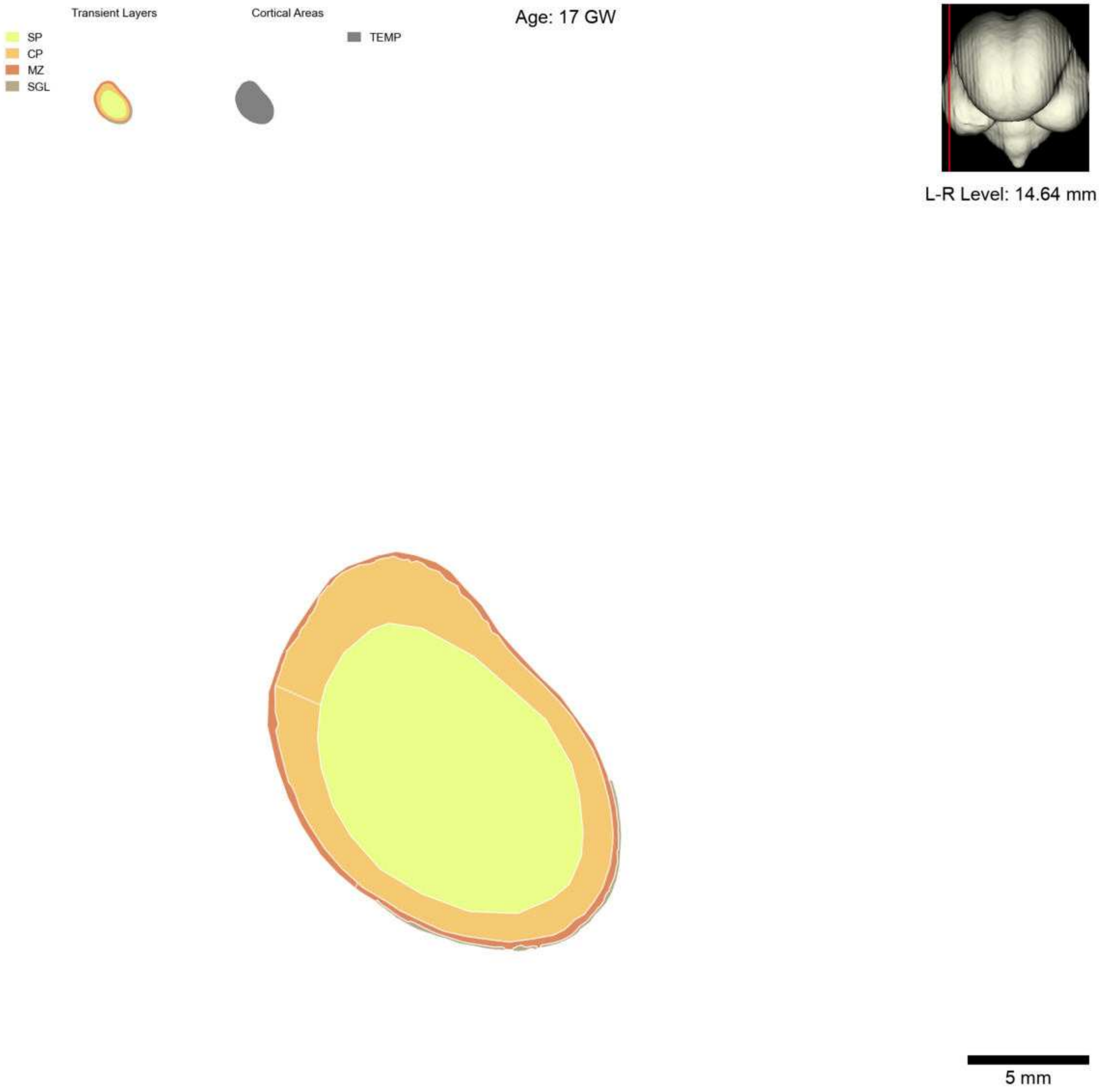
Transient Layers
Cortical Areas
Age: 17 GW
SP
CP
MZ
SGL
TEMP
L-R Level: 14.64 mm
5 mm

Age: 17 GW

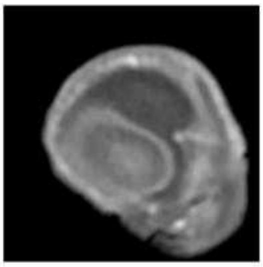

L-R Level: 13.92 mm

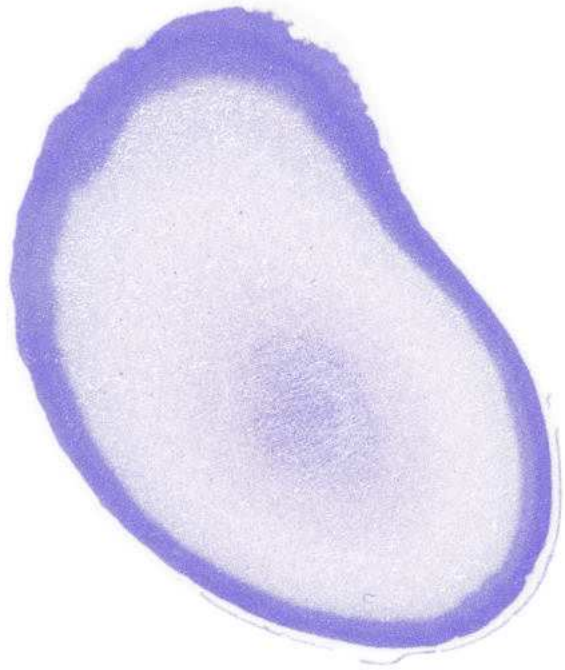

5 mm

Transient Layers

- IZ
- SP
- CP
- MZ
- SGL

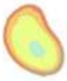

Cortical Areas

- TEMP

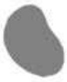

Age: 17 GW

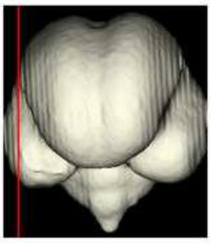

L-R Level: 13.92 mm

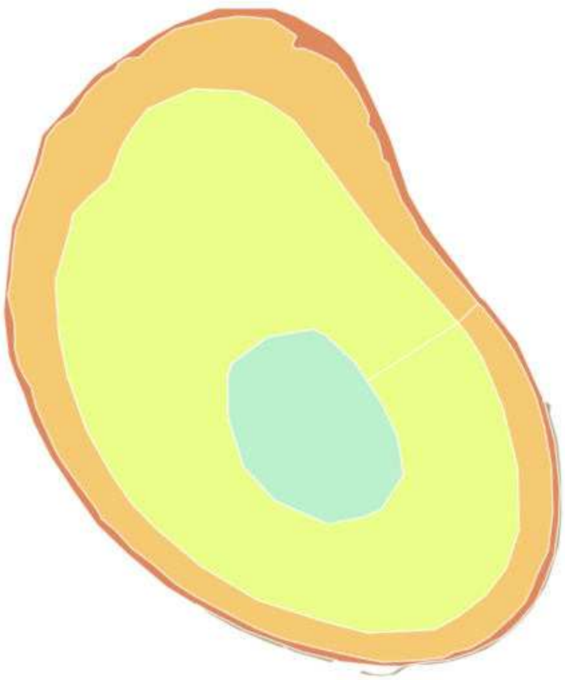

5 mm

Age: 17 GW

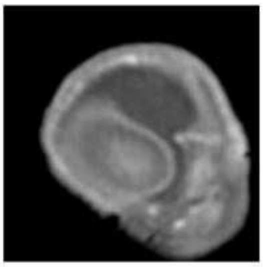

L-R Level: 13.62 mm

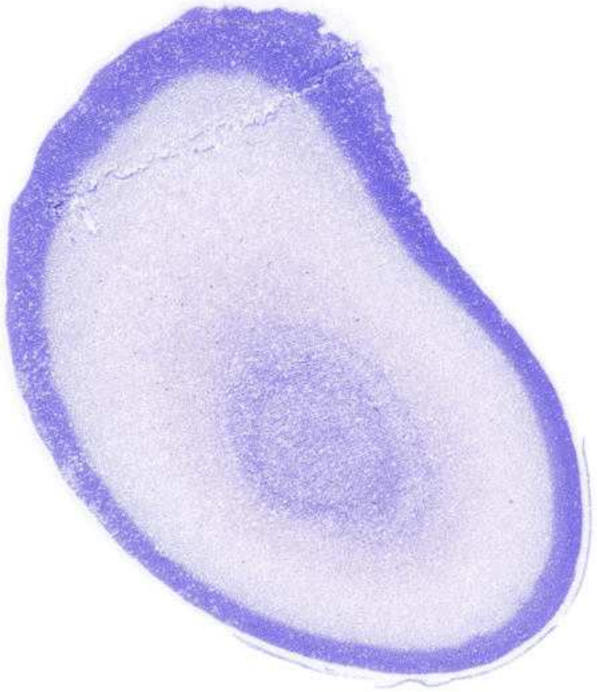

5 mm

Transient Layers

- IZ
- SP
- CP
- MZ
- SGL

Cortical Areas

- TEMP

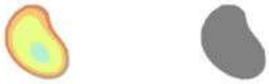

Age: 17 GW

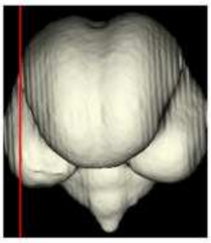

L-R Level: 13.62 mm

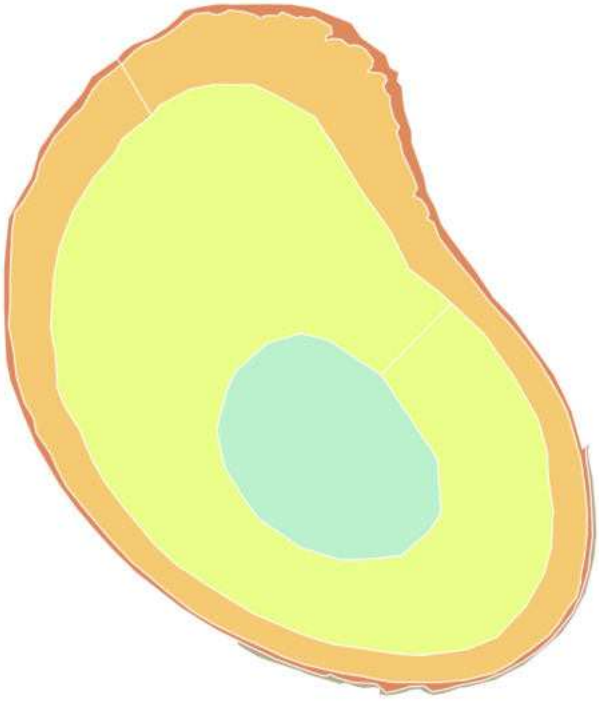

5 mm

Age: 17 GW

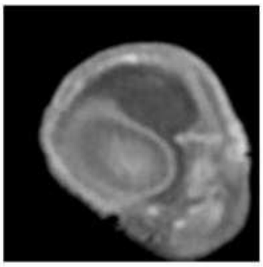

L-R Level: 13.44 mm

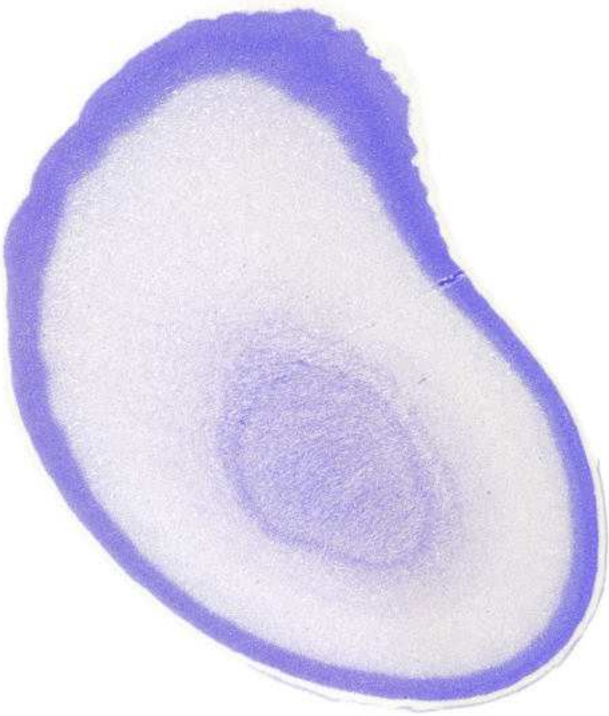

5 mm

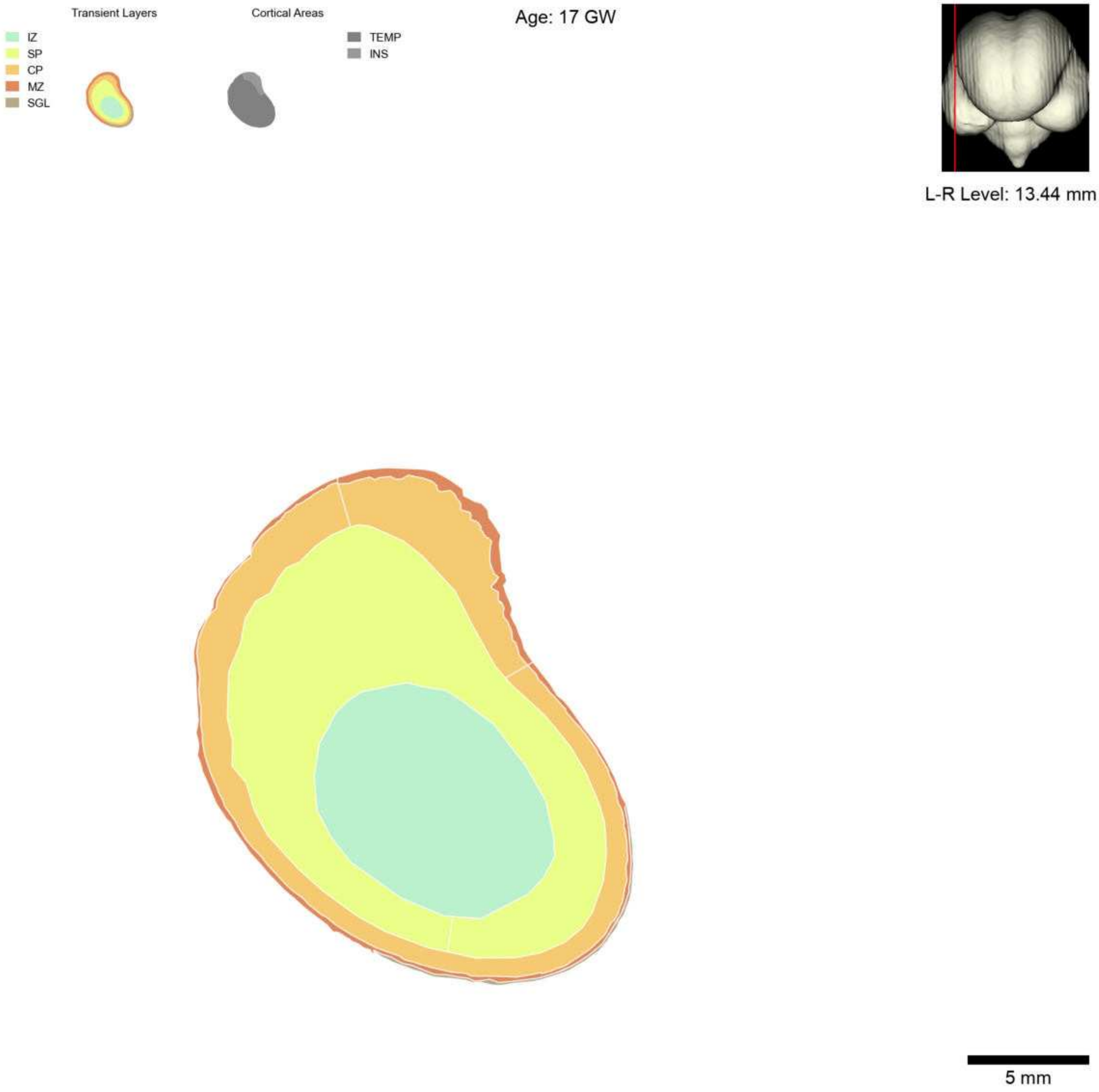
Transient Layers
IZ
SP
CP
MZ
SGL
Cortical Areas
TEMP
INS
Age: 17 GW
L-R Level: 13.44 mm
5 mm

Age: 17 GW

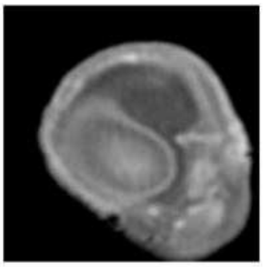

L-R Level: 13.32 mm

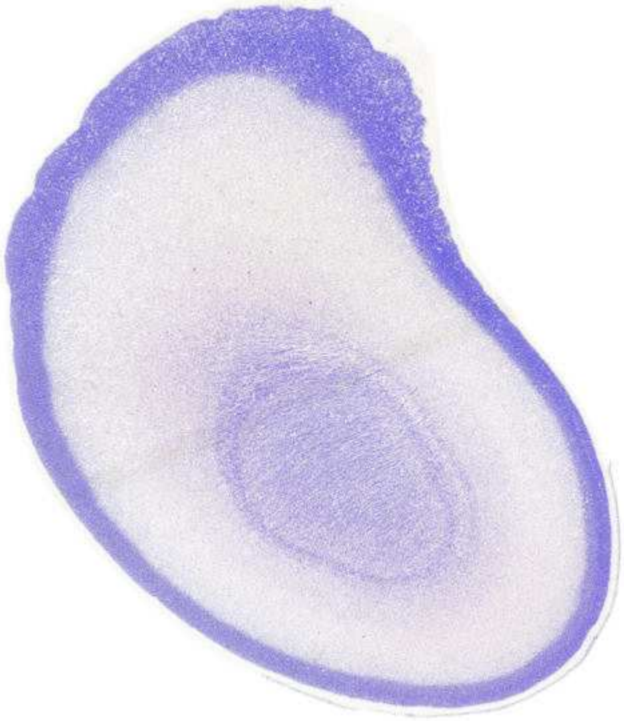

5 mm

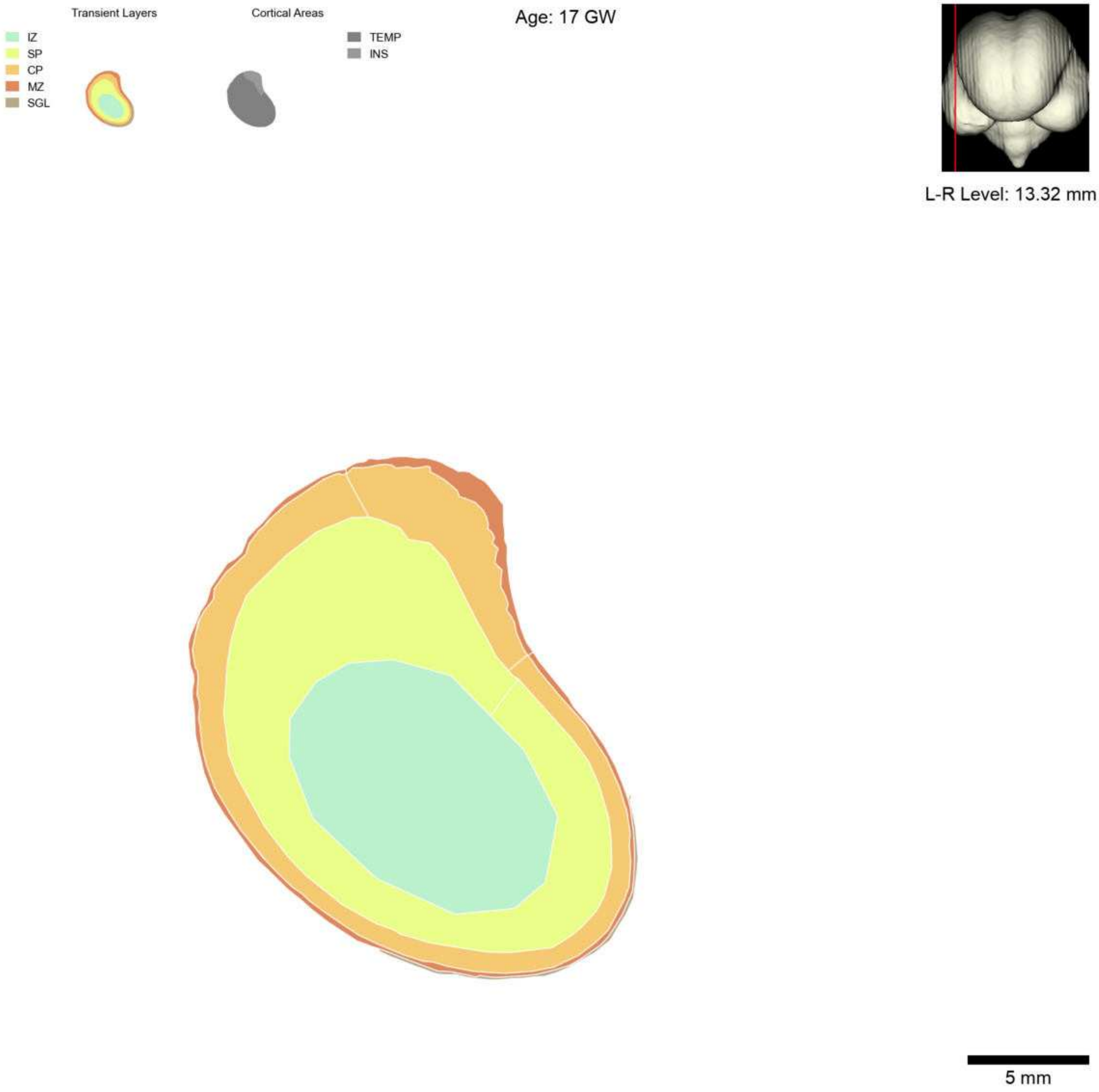
Transient Layers
IZ
SP
CP
MZ
SGL
Cortical Areas
TEMP
INS
Age: 17 GW
L-R Level: 13.32 mm
5 mm

Age: 17 GW

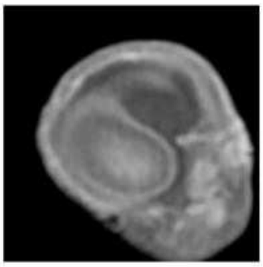

L-R Level: 13.02 mm

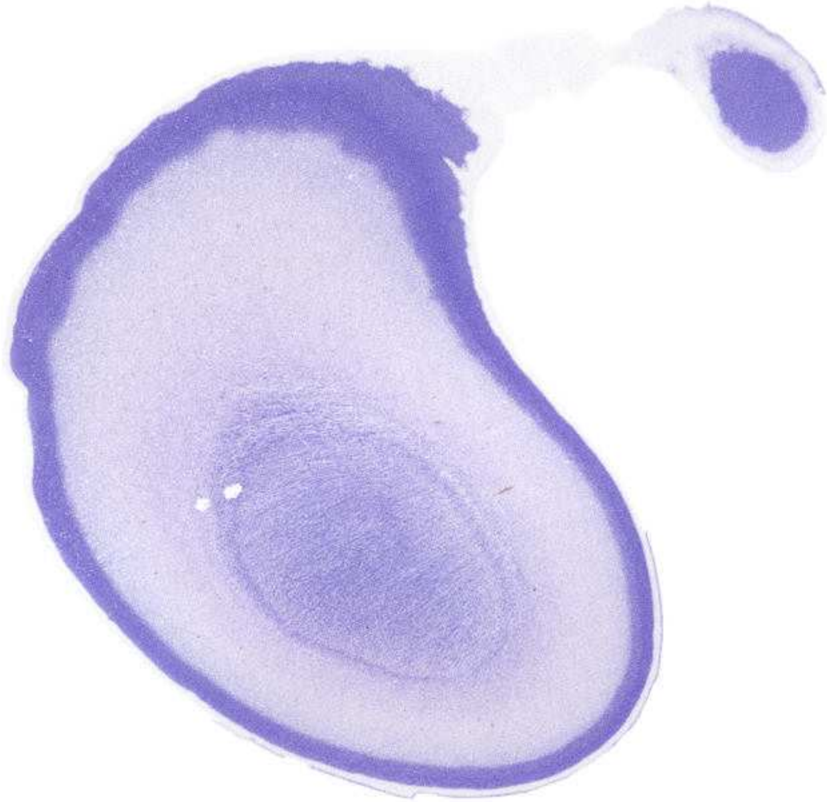

5 mm

Age: 17 GW

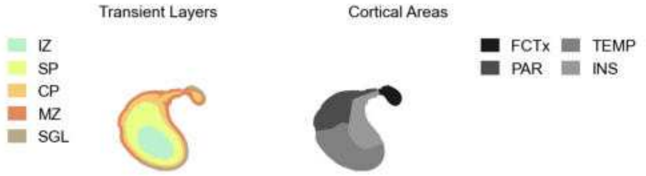

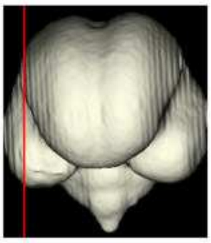

L-R Level: 13.02 mm

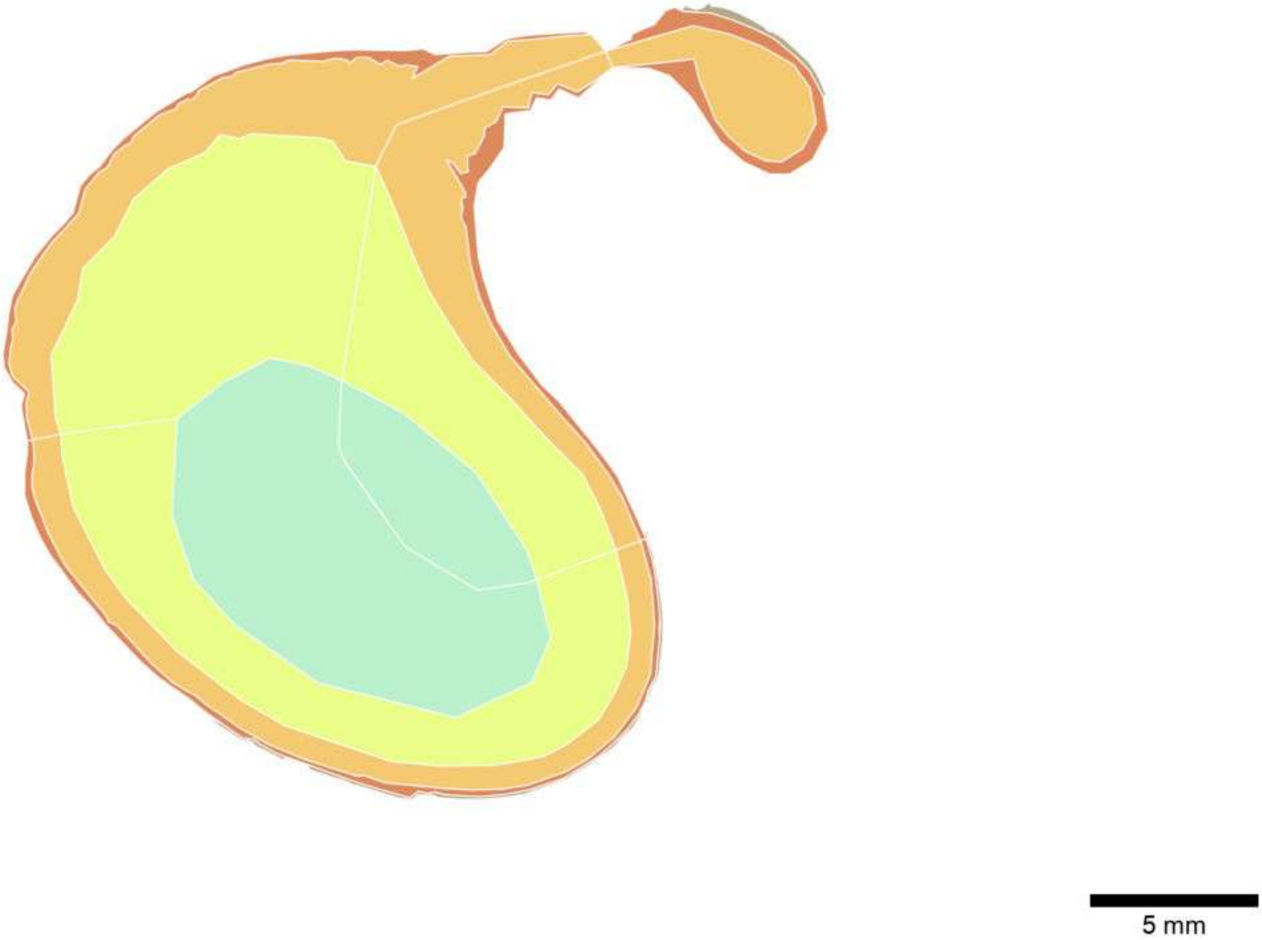

Age: 17 GW

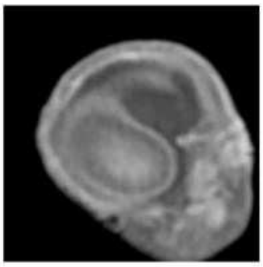

L-R Level: 12.96 mm

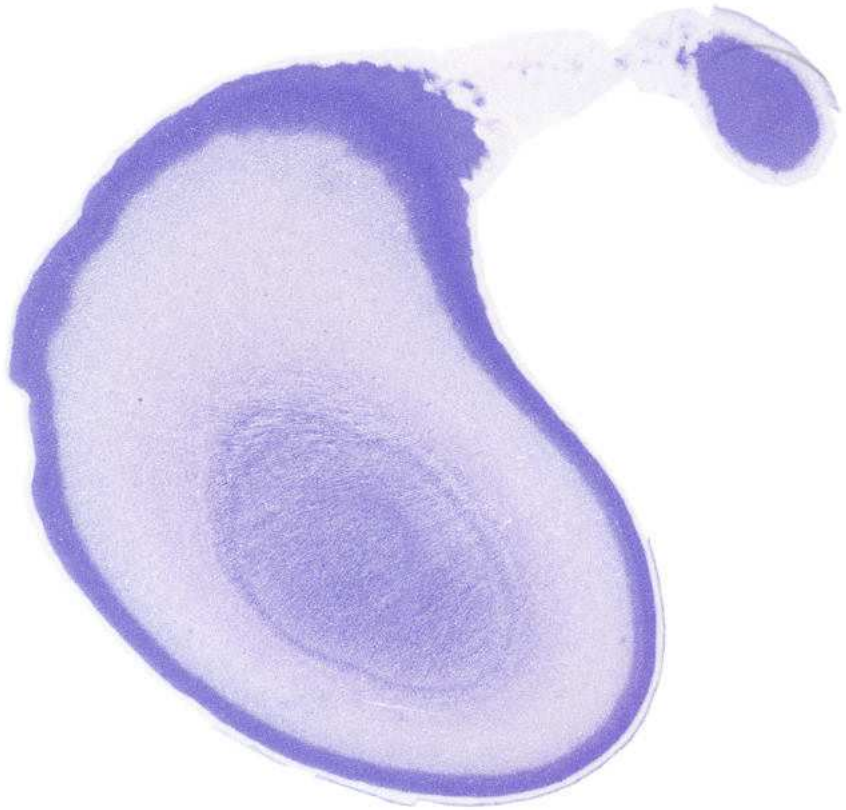

5 mm

Transient Layers

- IZ
- SP
- CP
- MZ
- SGL

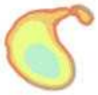

Cortical Areas

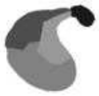

- FCTx
- PAR
- TEMP
- INS

Age: 17 GW

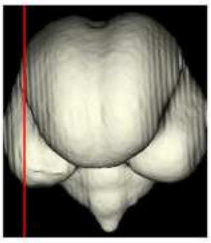

L-R Level: 12.96 mm

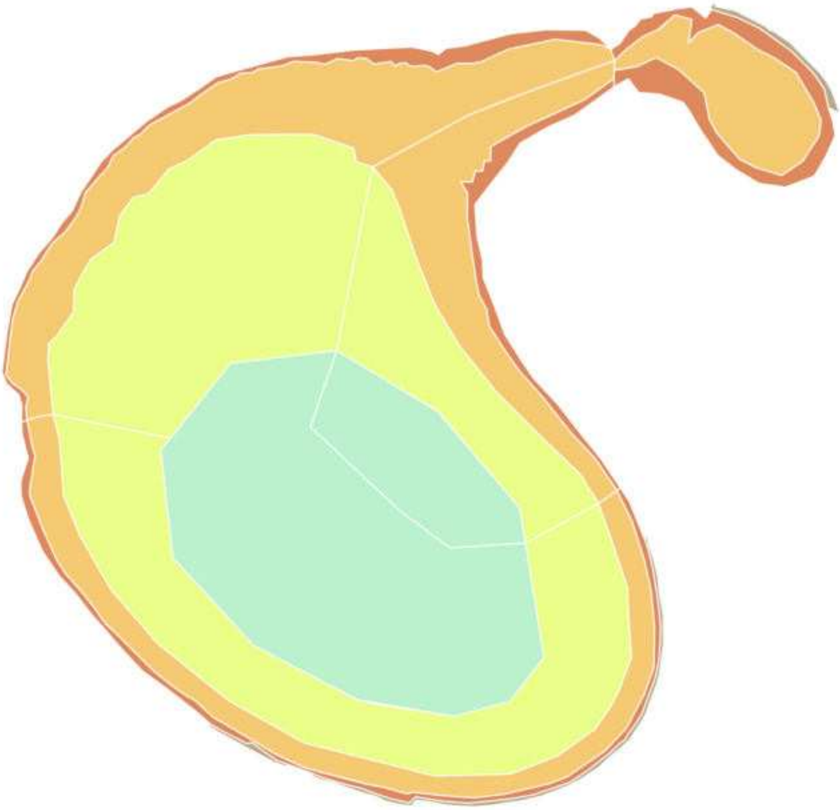

5 mm

Age: 17 GW

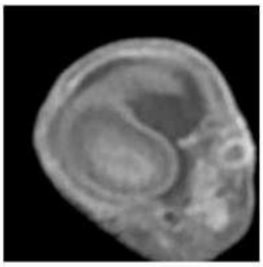

L-R Level: 12.48 mm

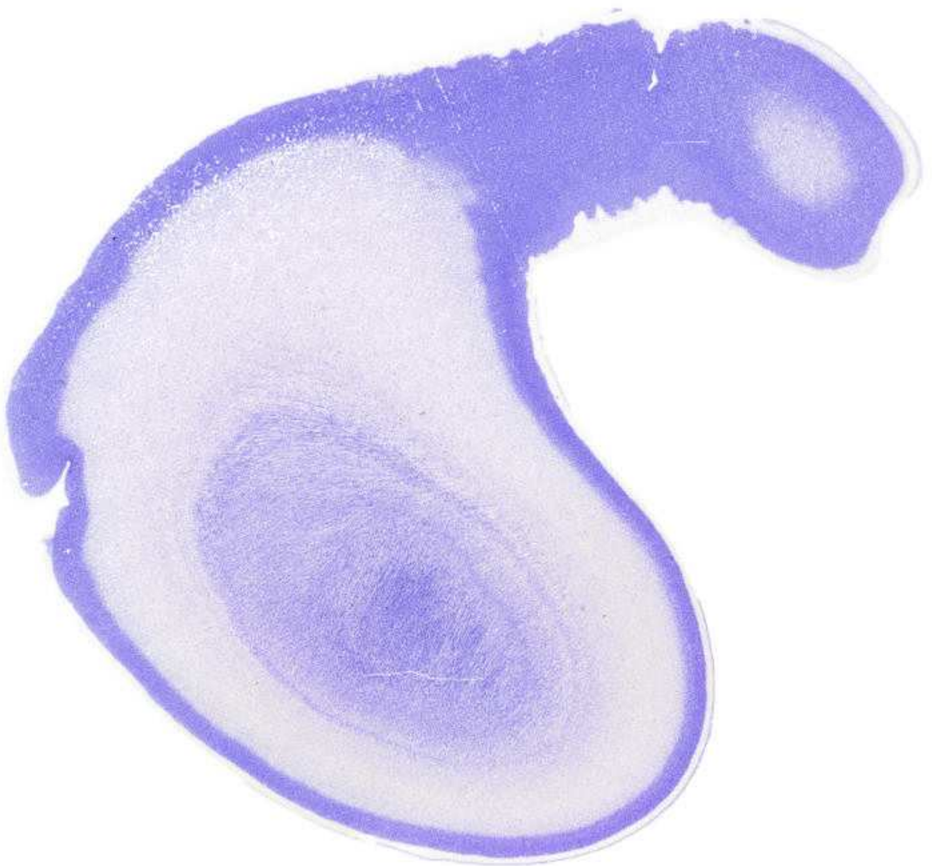

5 mm

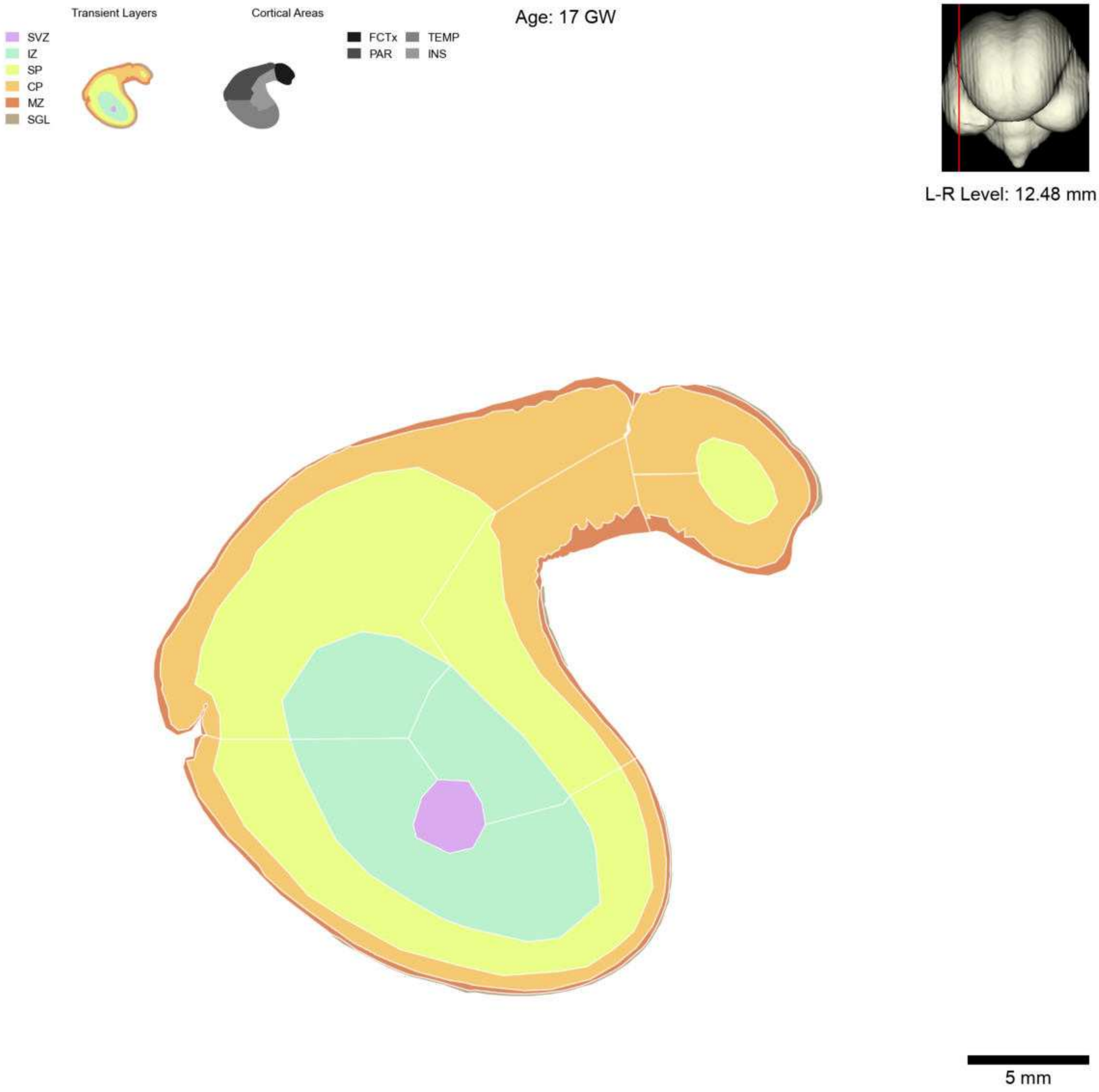
Transient Layers
SVZ
IZ
SP
CP
MZ
SGL
Cortical Areas
FCTx
PAR
TEMP
INS
Age: 17 GW
L-R Level: 12.48 mm
5 mm

Age: 17 GW

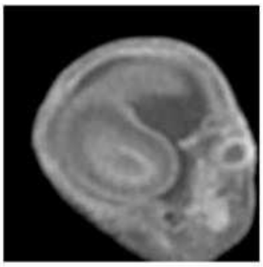

L-R Level: 12.24 mm

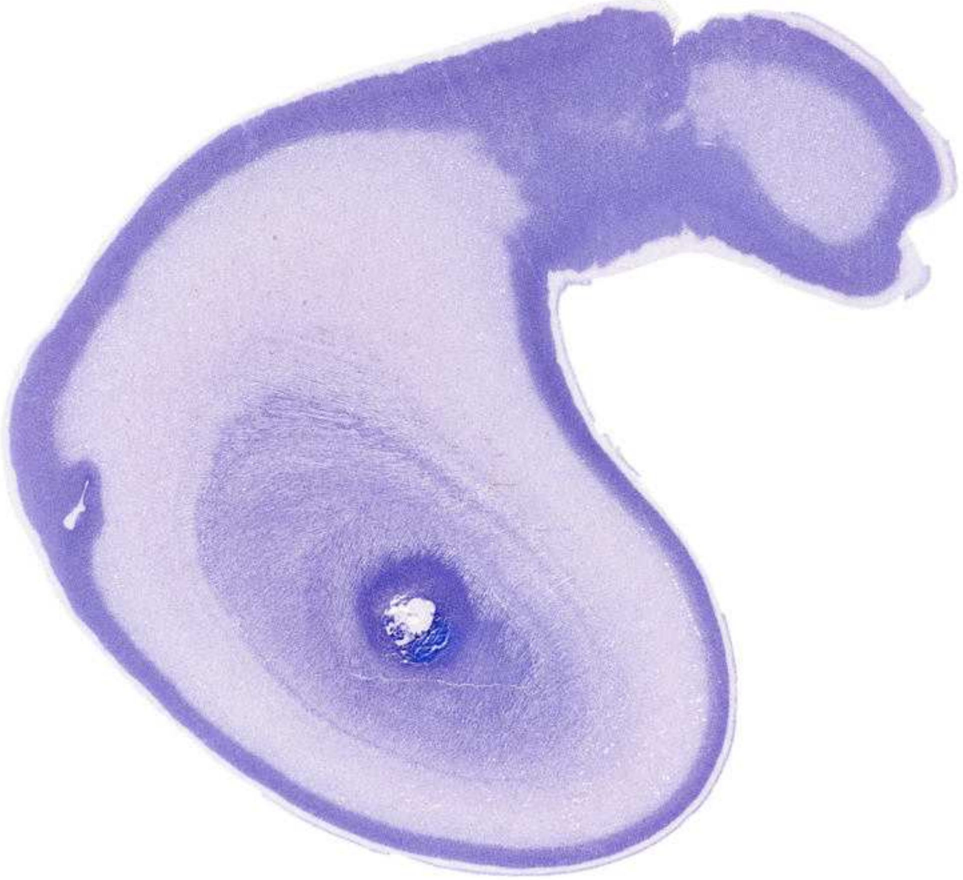

5 mm

Transient Layers

- VZ
- SVZ
- IZ
- SP
- CP
- MZ
- SGL

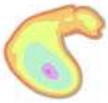

Cortical Areas

- FCTx
- PAR
- OCC
- TEMP
- INS

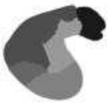

Age: 17 GW

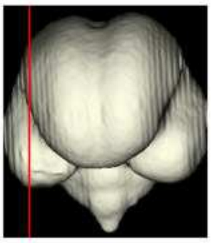

L-R Level: 12.24 mm

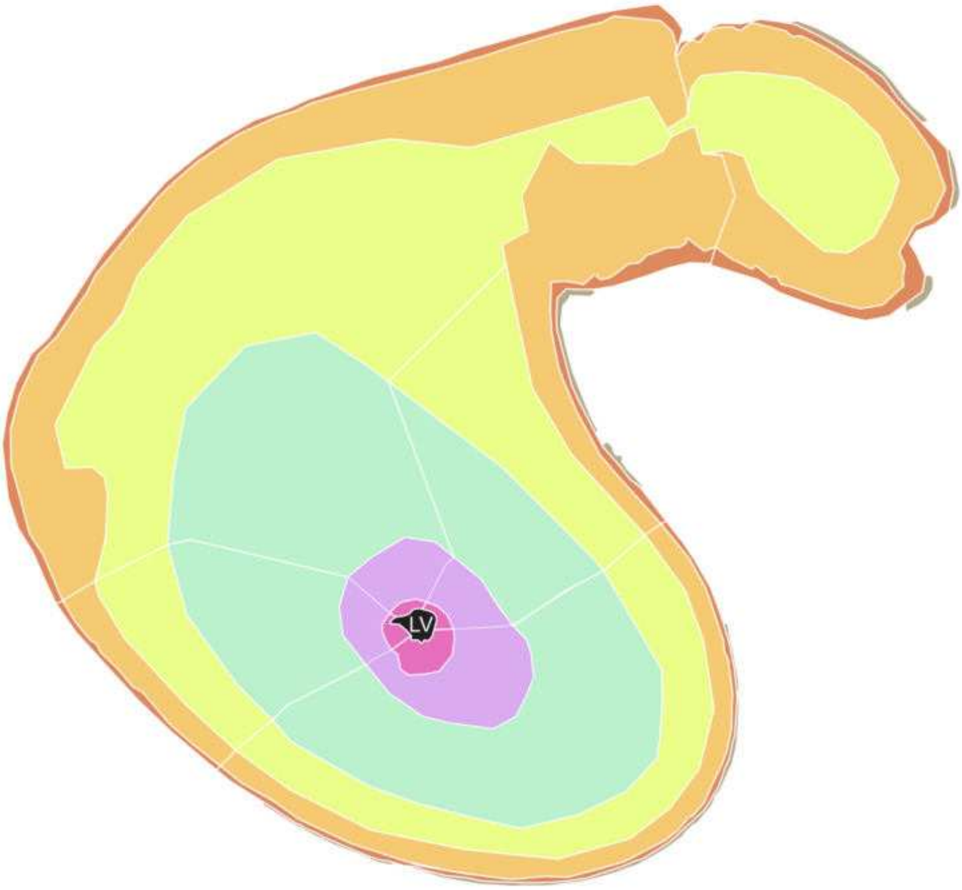

5 mm

LV: Lateral ventricle

Age: 17 GW

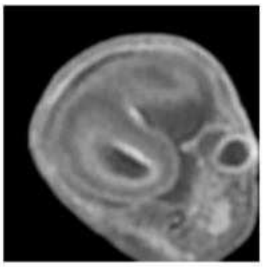

L-R Level: 11.4 mm

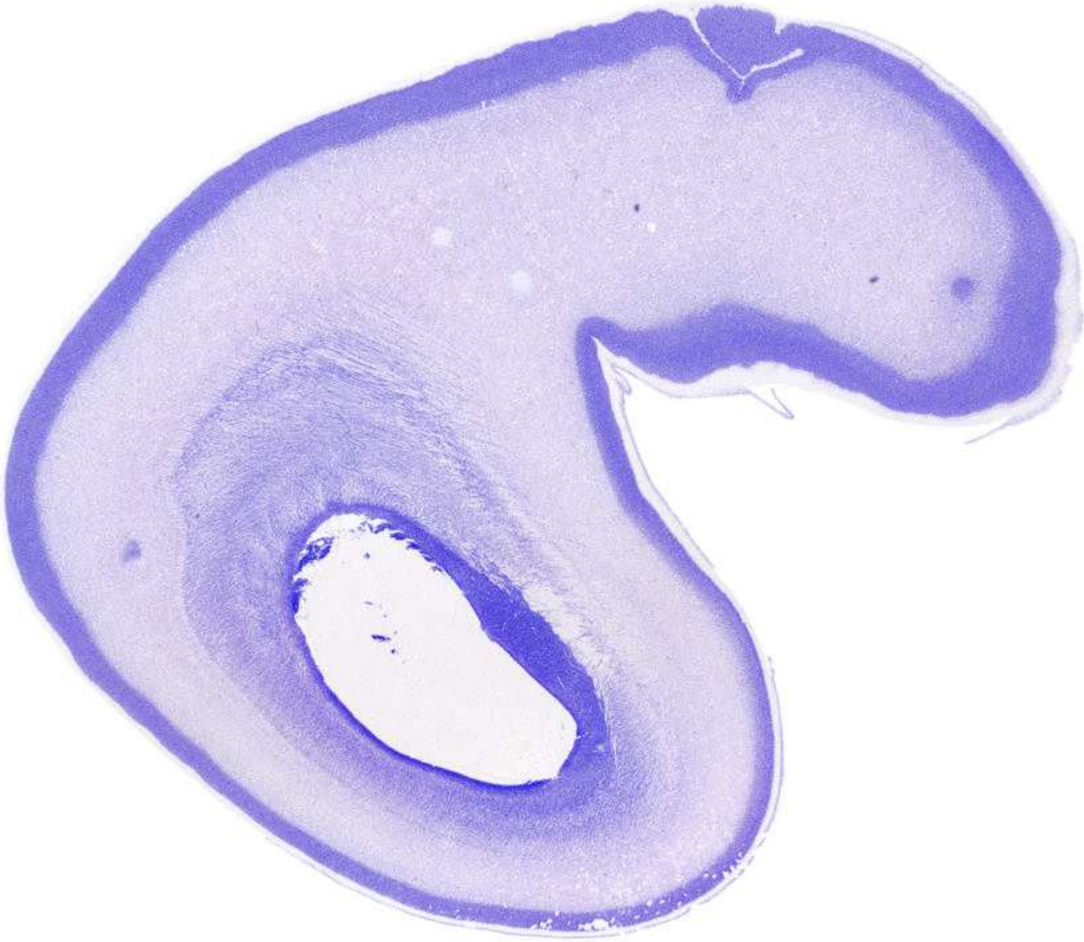

5 mm

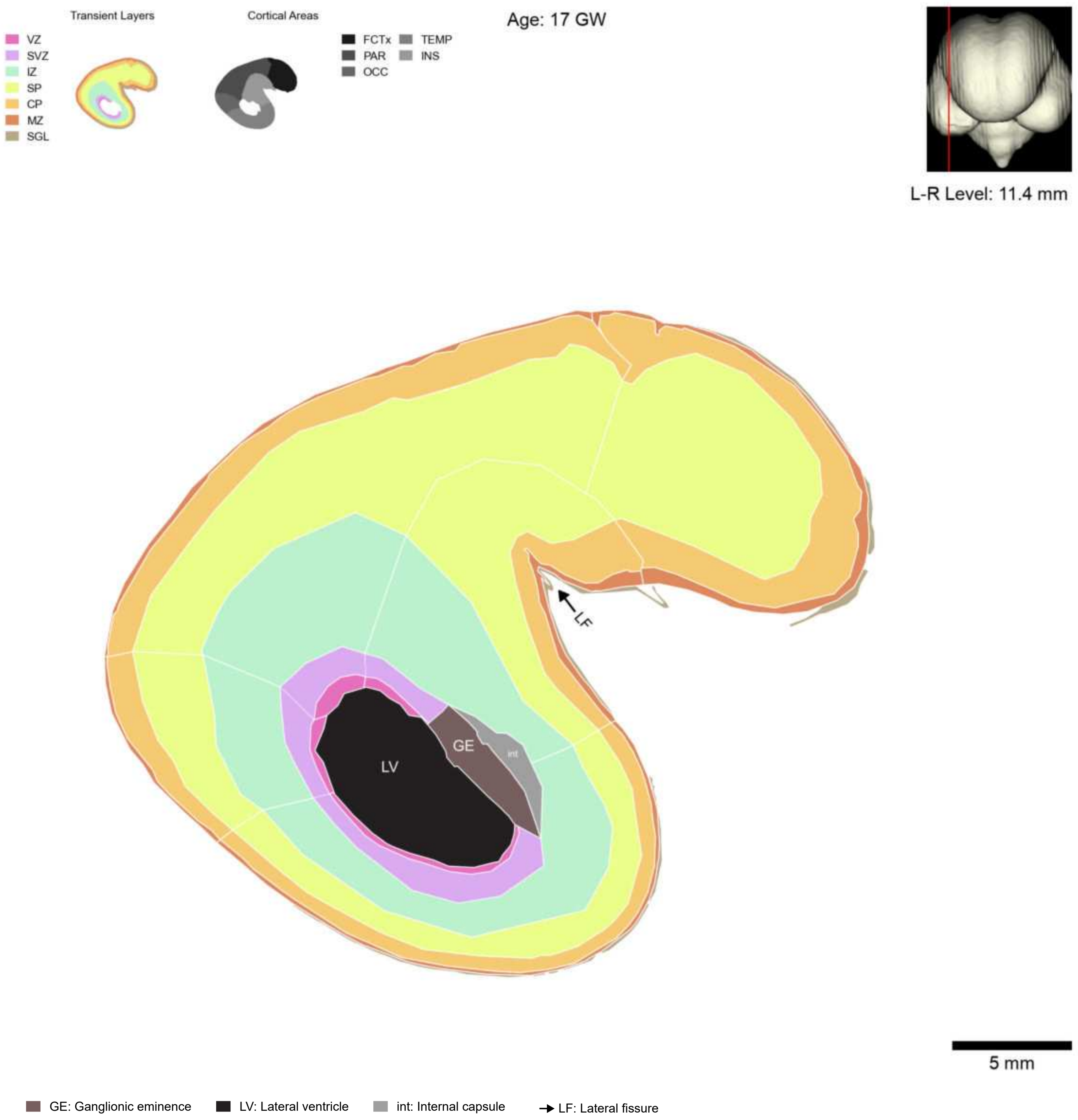
Transient Layers
VZ
SVZ
IZ
SP
CP
MZ
SGL
Cortical Areas
FCTx
PAR
OCC
TEMP
INS
Age: 17 GW
L-R Level: 11.4 mm
LV
GE
int
LF
5 mm
GE: Ganglionic eminence
LV: Lateral ventricle
int: Internal capsule
LF: Lateral fissure

Age: 17 GW

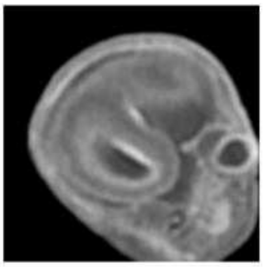

L-R Level: 11.28 mm

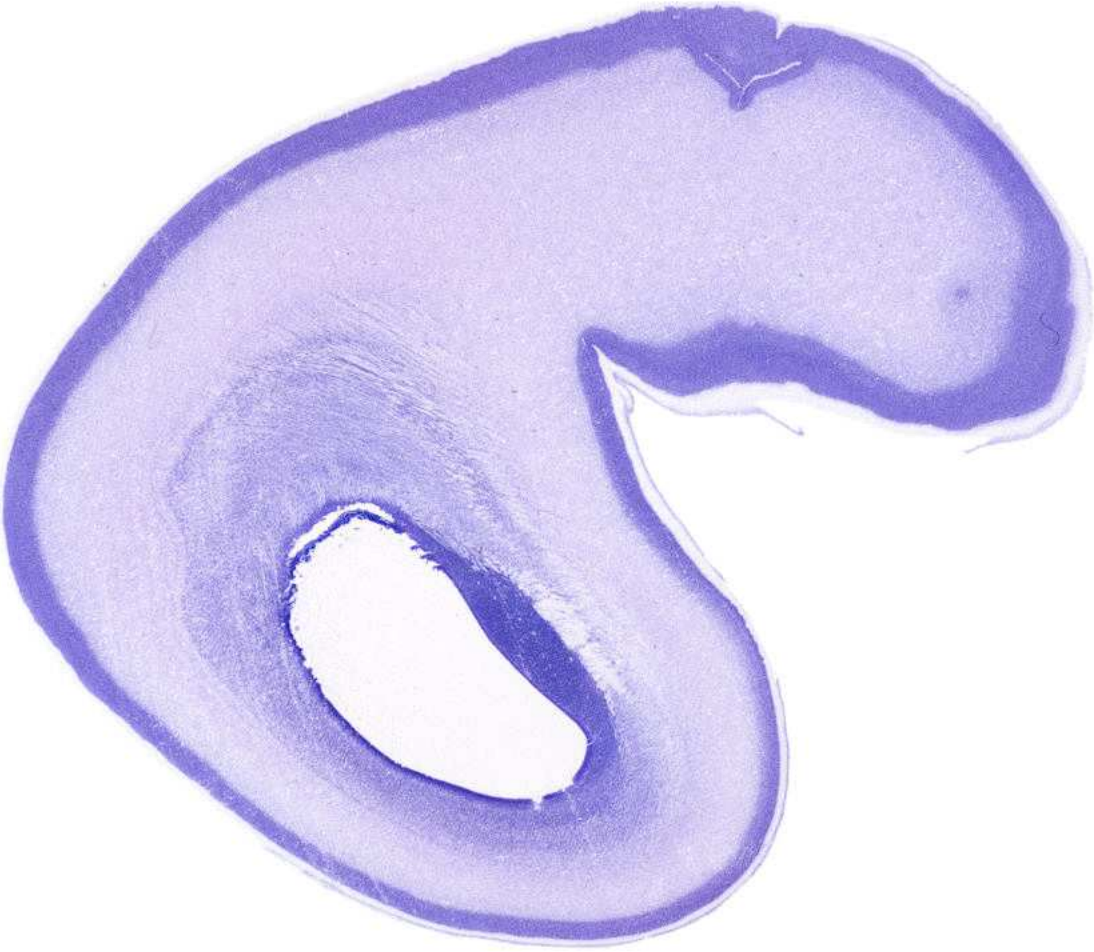

5 mm

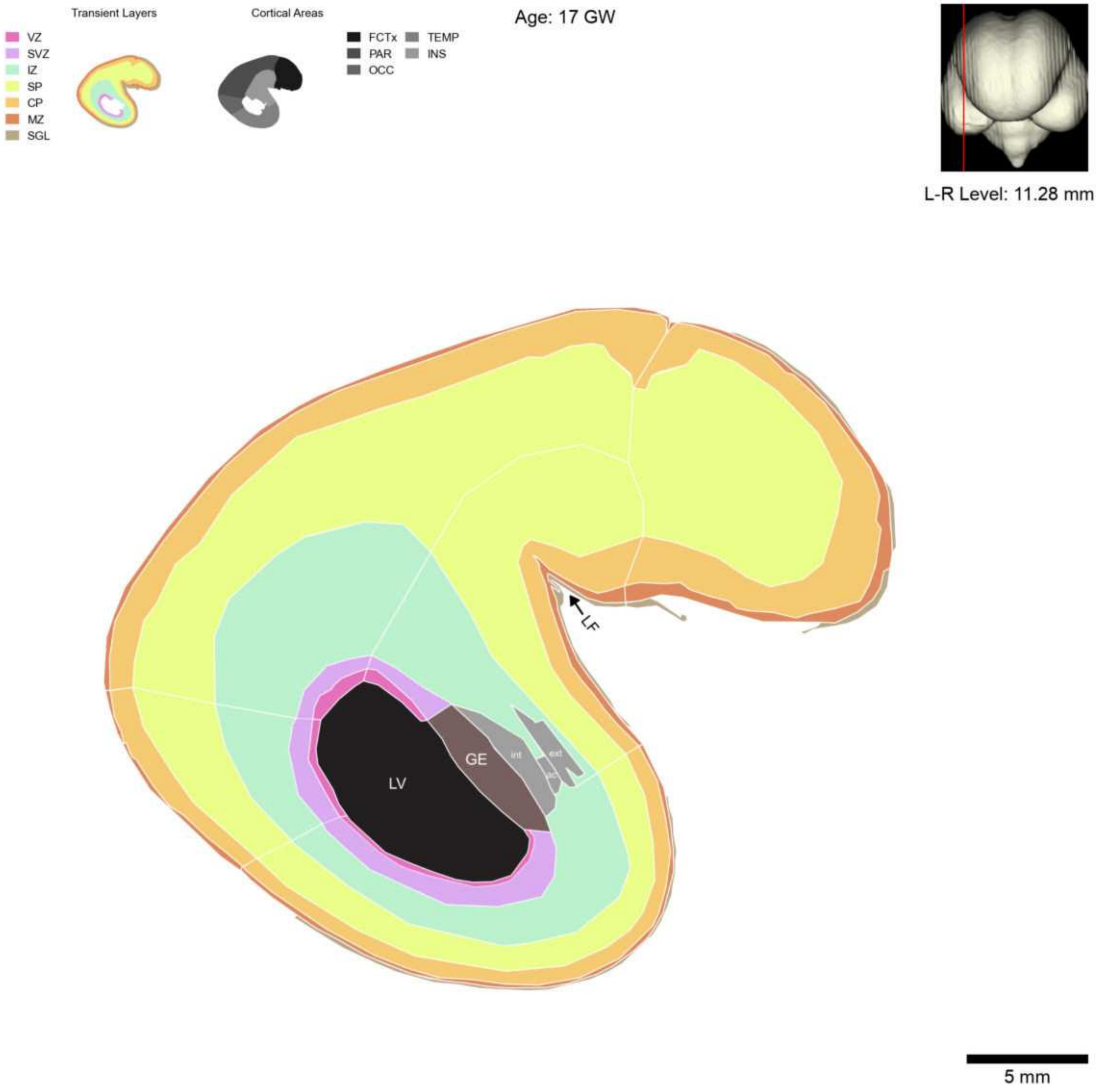

GE: Ganglionic eminence ac: Anterior commissure ext: External capsule int: Internal capsule

LV: Lateral ventricle → LF: Lateral fissure

Age: 17 GW

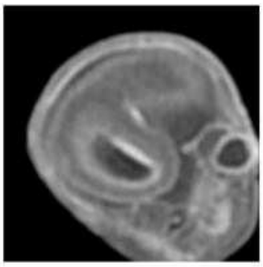

L-R Level: 11.04 mm

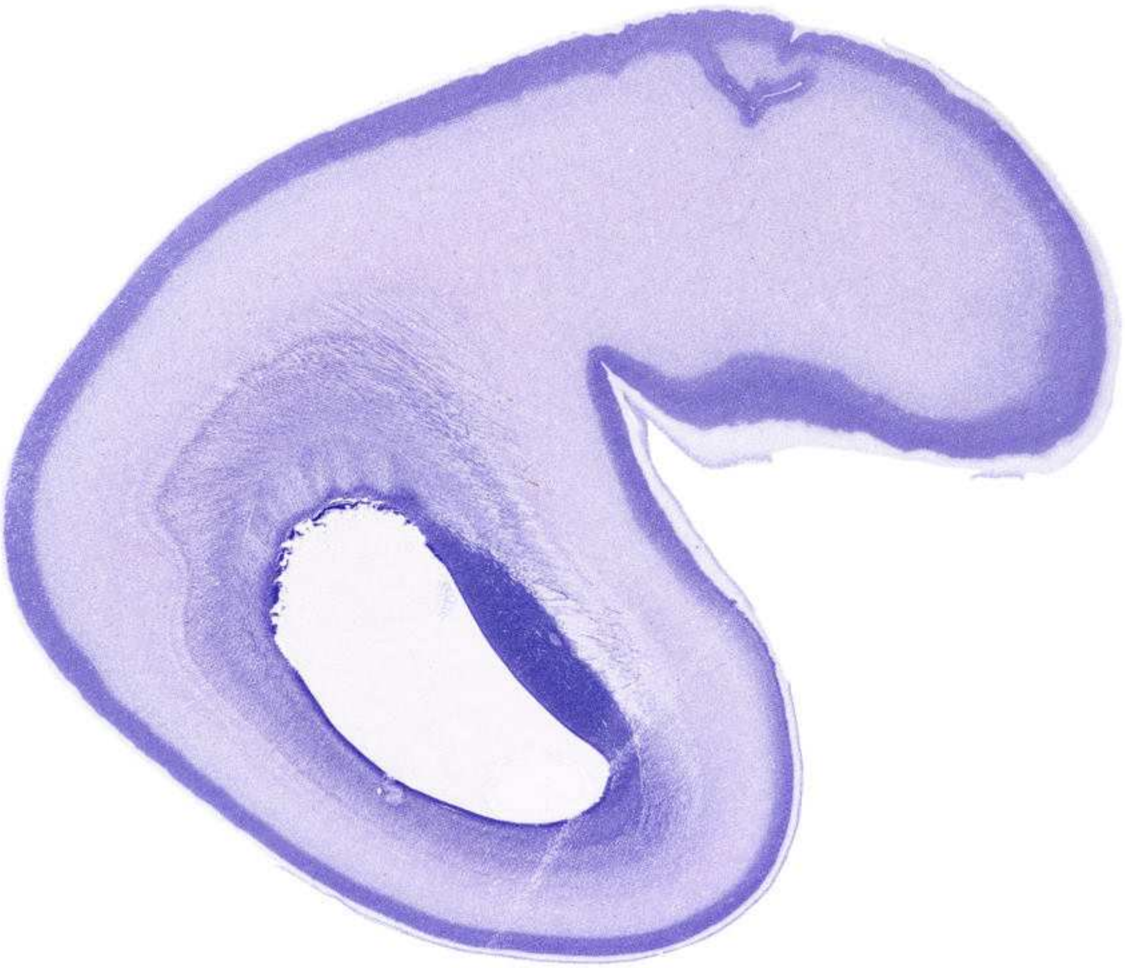

5 mm

Age: 17 GW

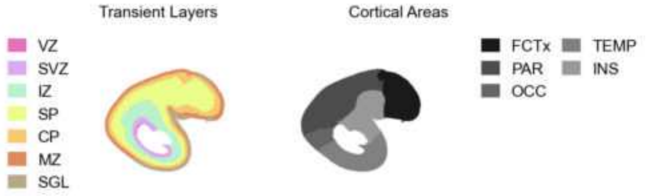

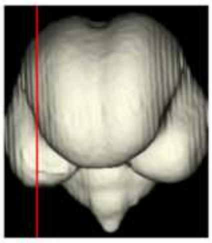

L-R Level: 11.04 mm

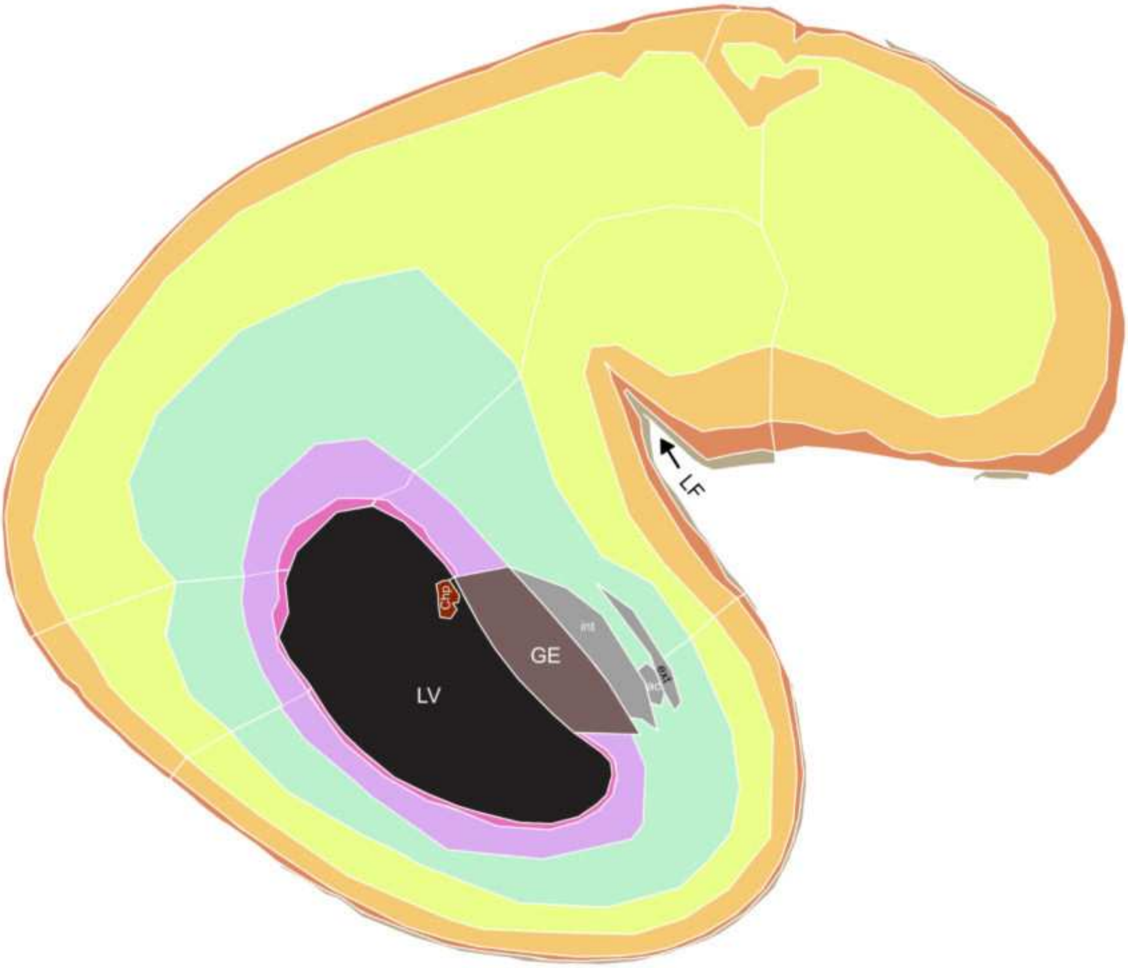

5 mm

Chp: Choroid plexus
GE: Ganglionic eminence
LV: Lateral ventricle
ac: Anterior commissure
ext: External capsule
→ LF: Lateral fissure
int: Internal capsule

Age: 17 GW

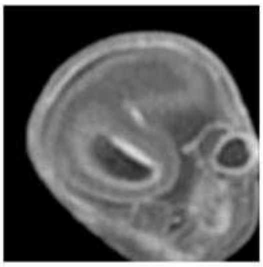

L-R Level: 10.86 mm

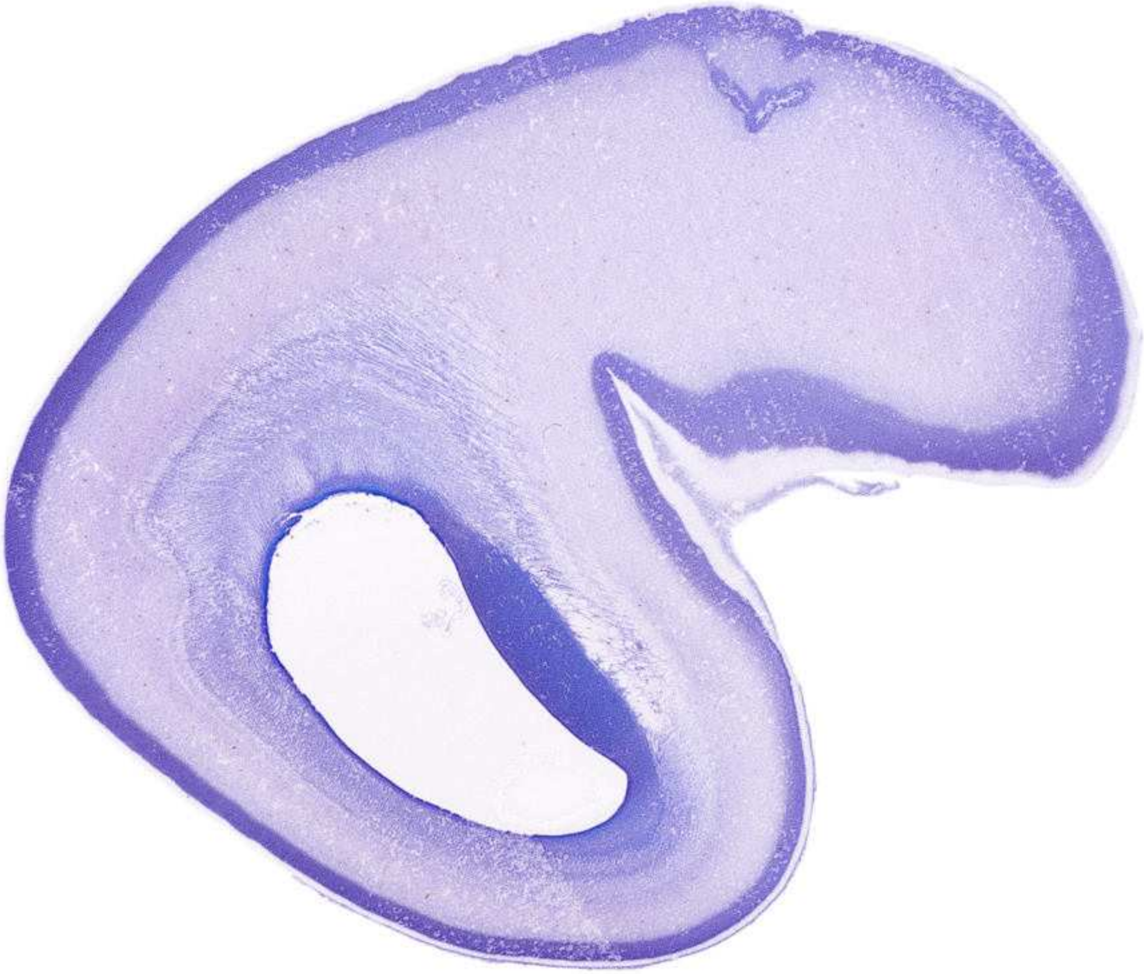

5 mm

Transient Layers

- VZ
- SVZ
- IZ
- SP
- CP
- MZ
- SGL

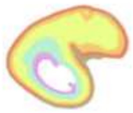

Cortical Areas

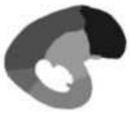

- FCTx
- PAR
- OCC
- TEMP
- INS

Age: 17 GW

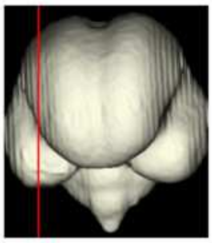

L-R Level: 10.86 mm

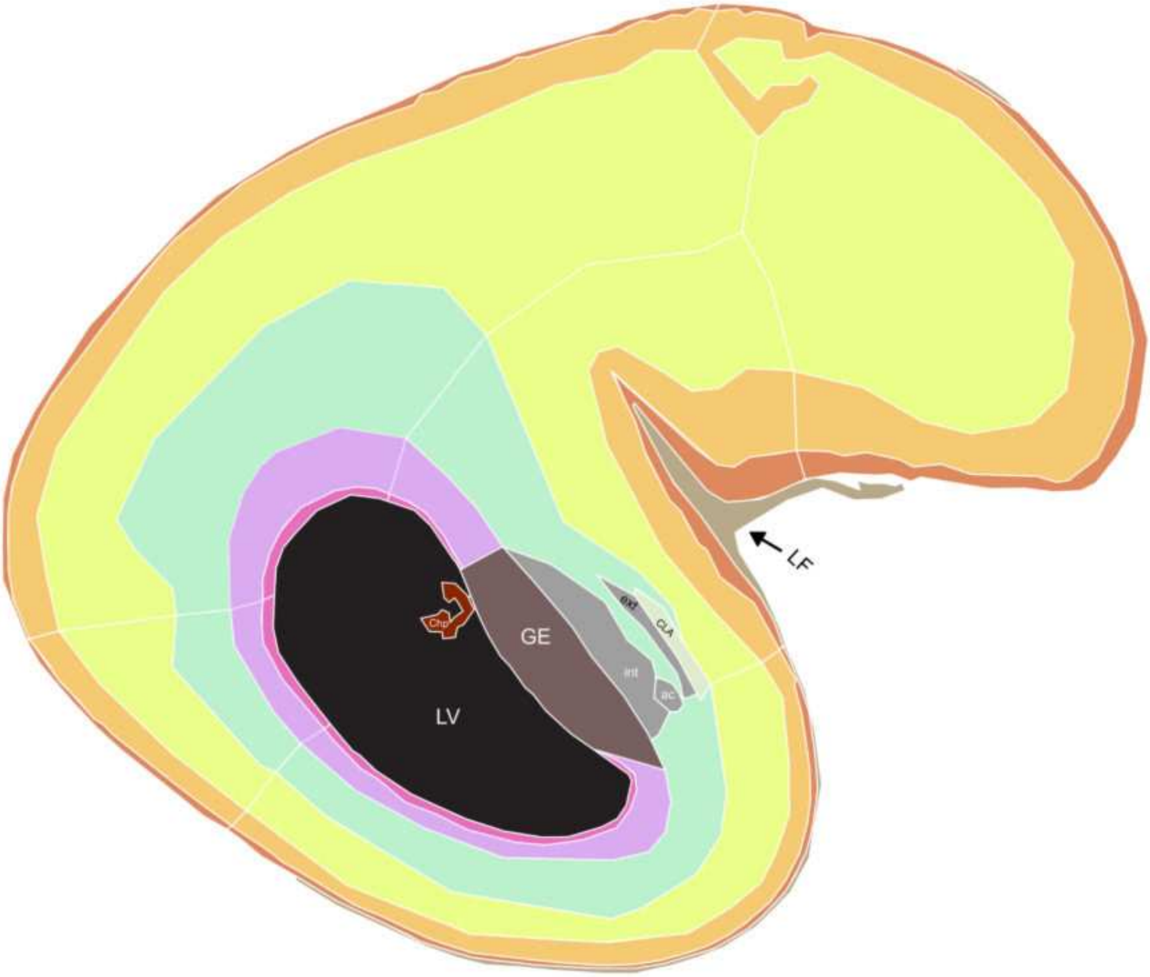

5 mm

- CLA: Claustrum
- Chp: Choroid plexus
- GE: Ganglionic eminence
- LV: Lateral ventricle
- ac: Anterior commissure
- ext: External capsule
- int: Internal capsule
- LF: Lateral fissure

Age: 17 GW

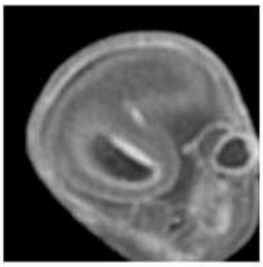

L-R Level: 10.8 mm

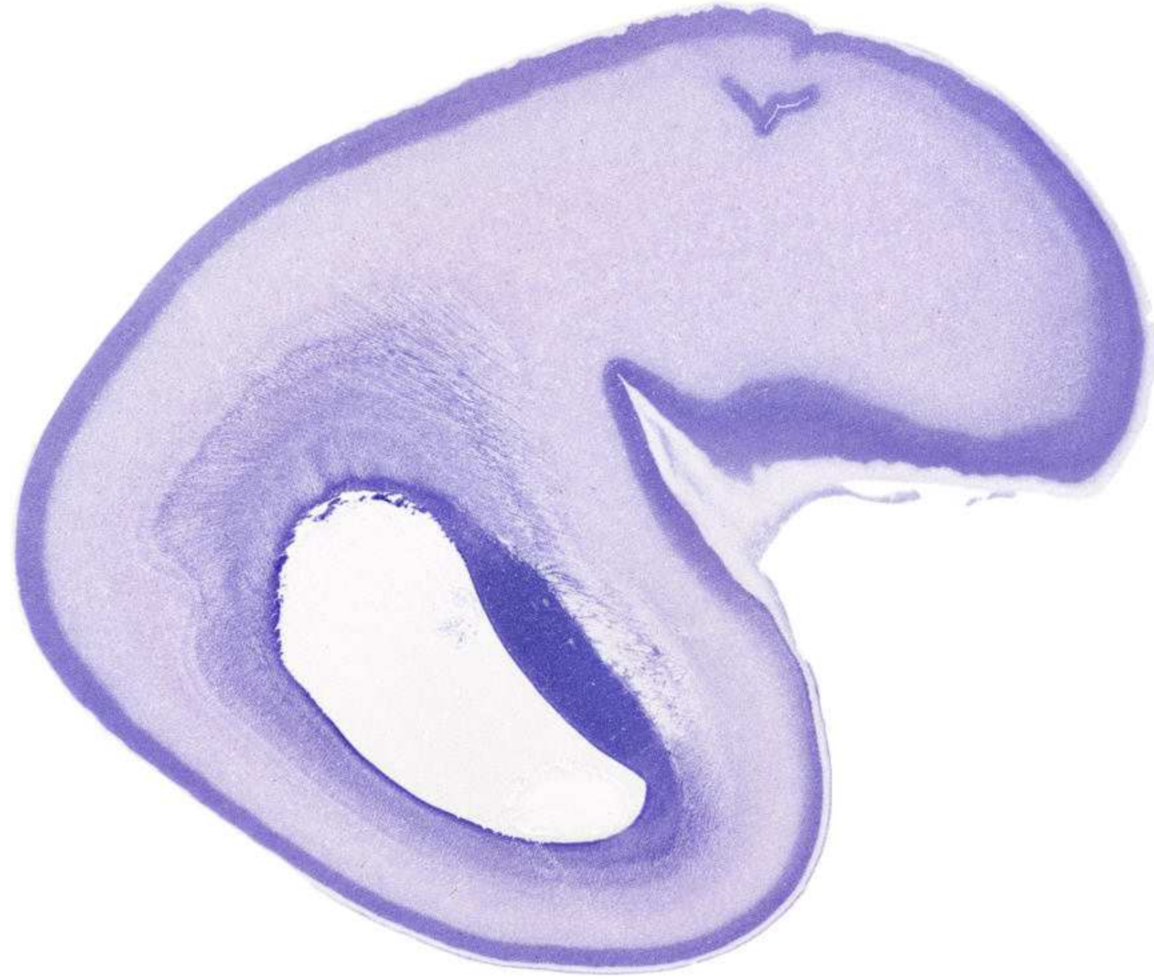

5 mm

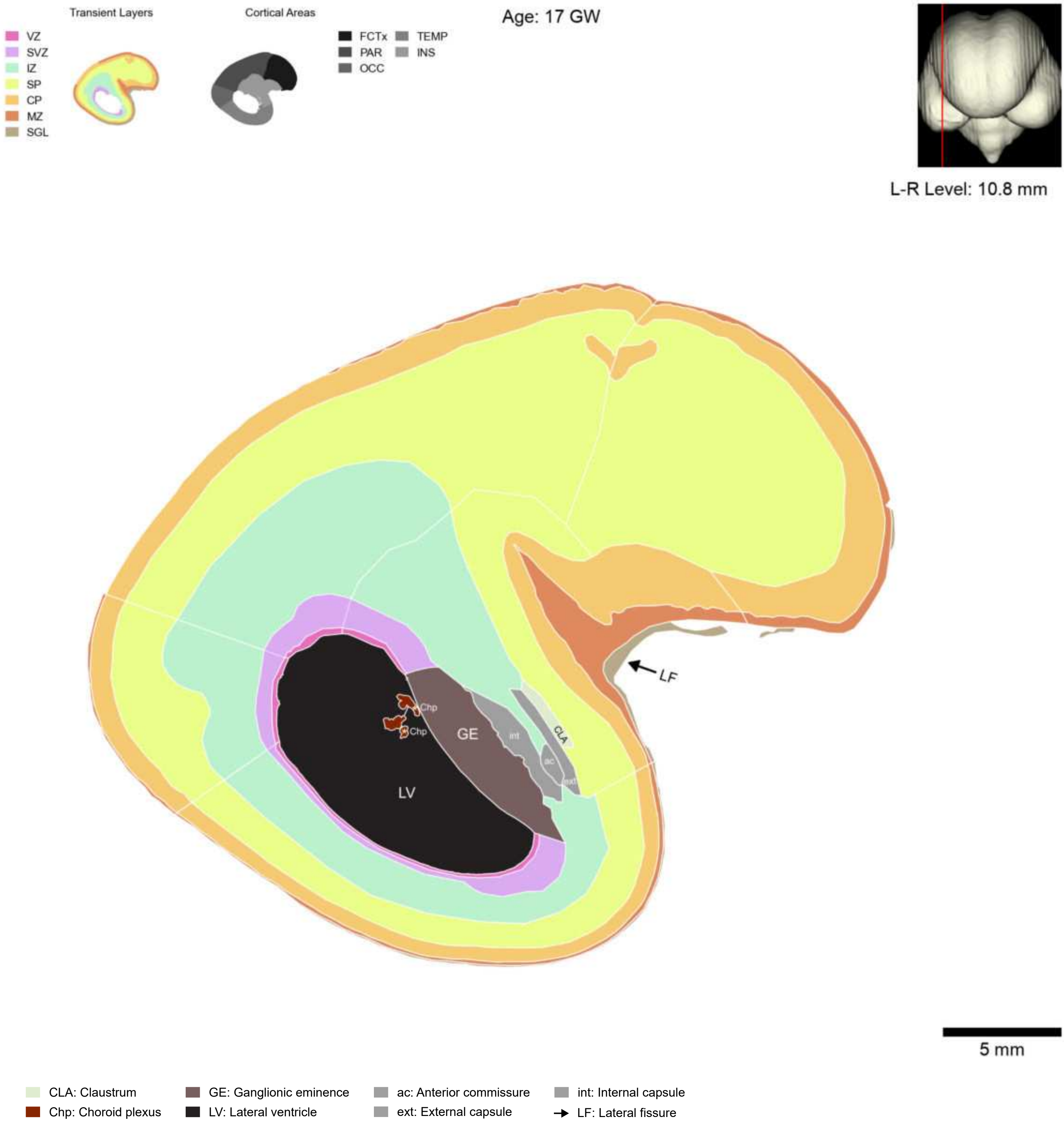
Transient Layers
VZ
SVZ
IZ
SP
CP
MZ
SGL
Cortical Areas
FCTx
PAR
OCC
TEMP
INS
Age: 17 GW
L-R Level: 10.8 mm
Chp
Chp
GE
int
CLA
ac
ext
LV
LF
5 mm
CLA: Claustrum
Chp: Choroid plexus
GE: Ganglionic eminence
LV: Lateral ventricle
ac: Anterior commissure
ext: External capsule
int: Internal capsule
LF: Lateral fissure

Age: 17 GW

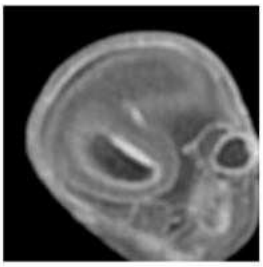

L-R Level: 10.74 mm

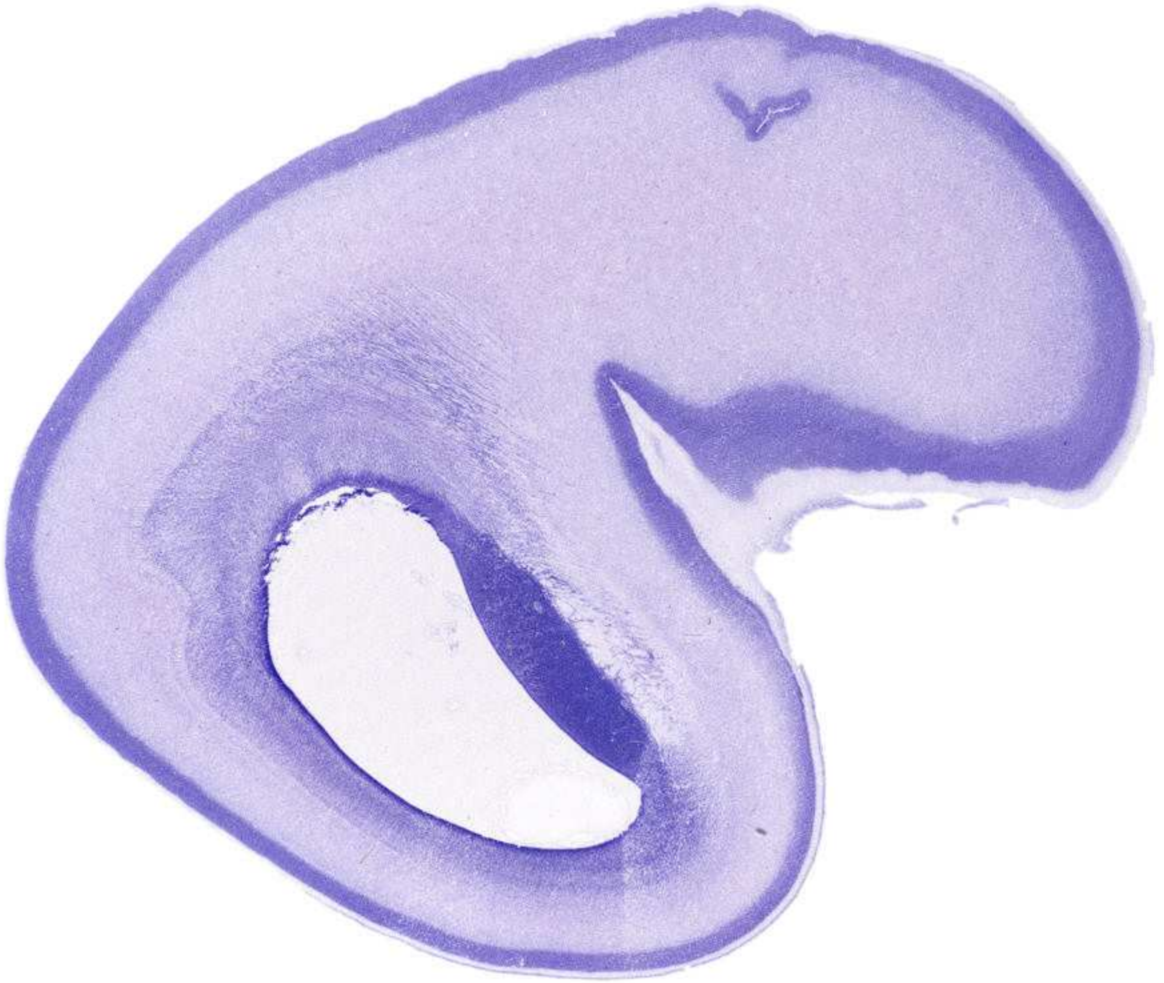

5 mm

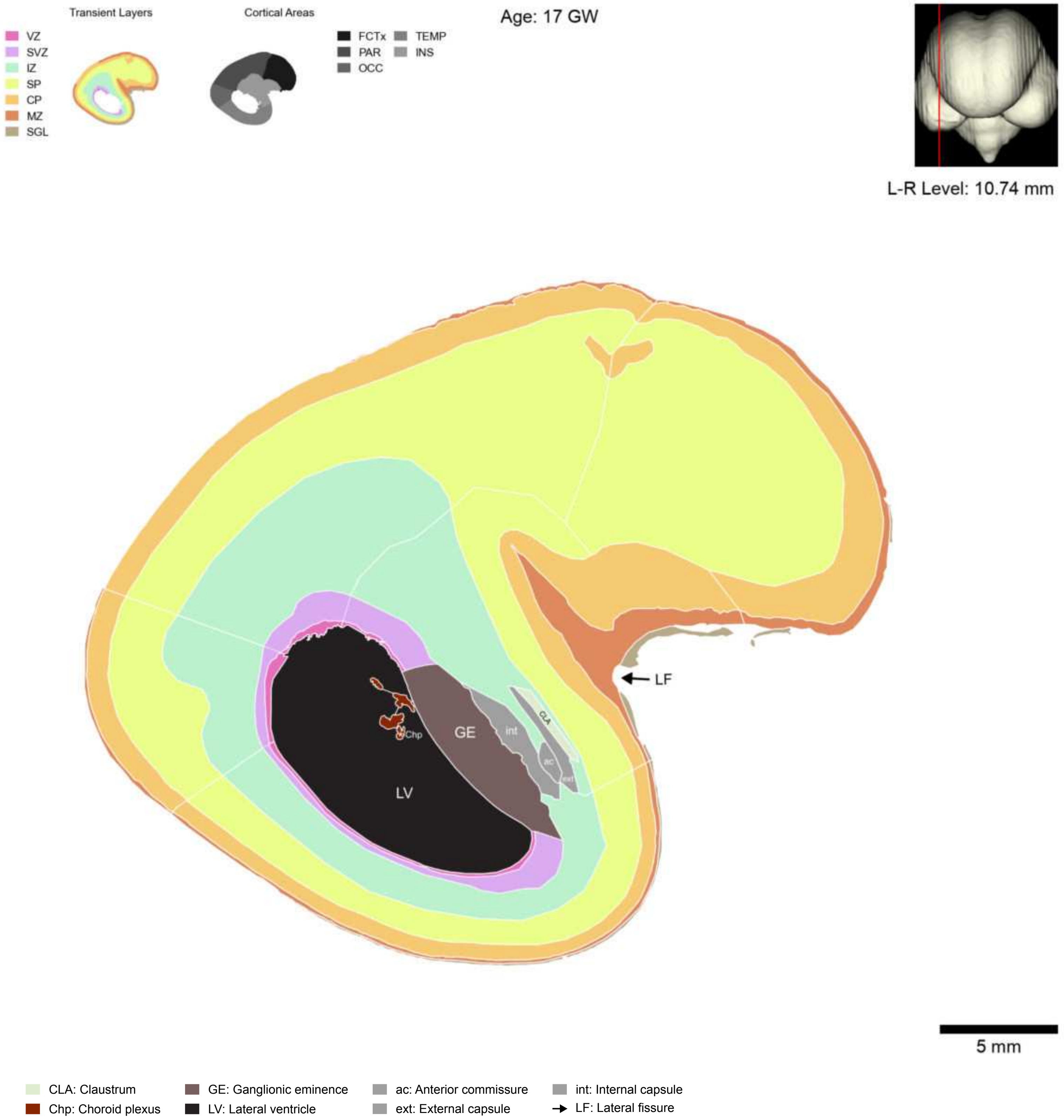
Transient Layers
VZ
SVZ
IZ
SP
CP
MZ
SGL
Cortical Areas
FCTx
PAR
OCC
TEMP
INS
Age: 17 GW
L-R Level: 10.74 mm
LF
GE
int
CLA
ac
ext
Chp
LV
5 mm
CLA: Claustrum
Chp: Choroid plexus
GE: Ganglionic eminence
LV: Lateral ventricle
ac: Anterior commissure
ext: External capsule
int: Internal capsule
LF: Lateral fissure

Age: 17 GW

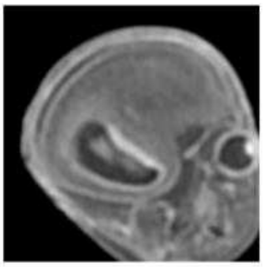

L-R Level: 9.42 mm

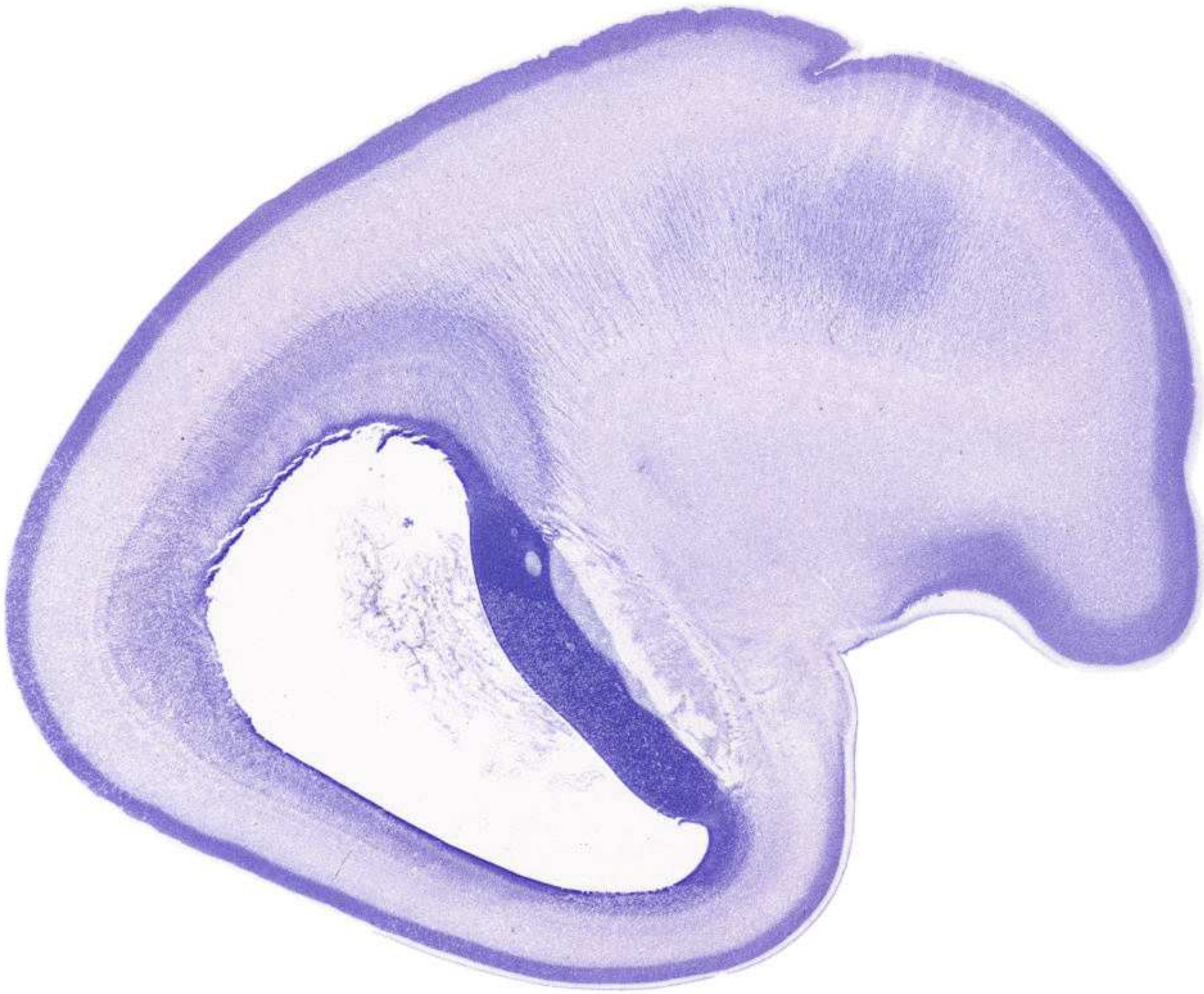

5 mm

Transient Layers

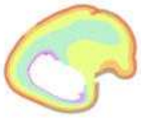

- VZ
- SVZ
- IZ
- SP
- CP
- MZ
- SGL

Cortical Areas

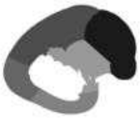

- FCTx
- PAR
- OCC
- TEMP
- INS

Age: 17 GW

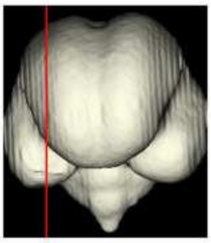

L-R Level: 9.42 mm

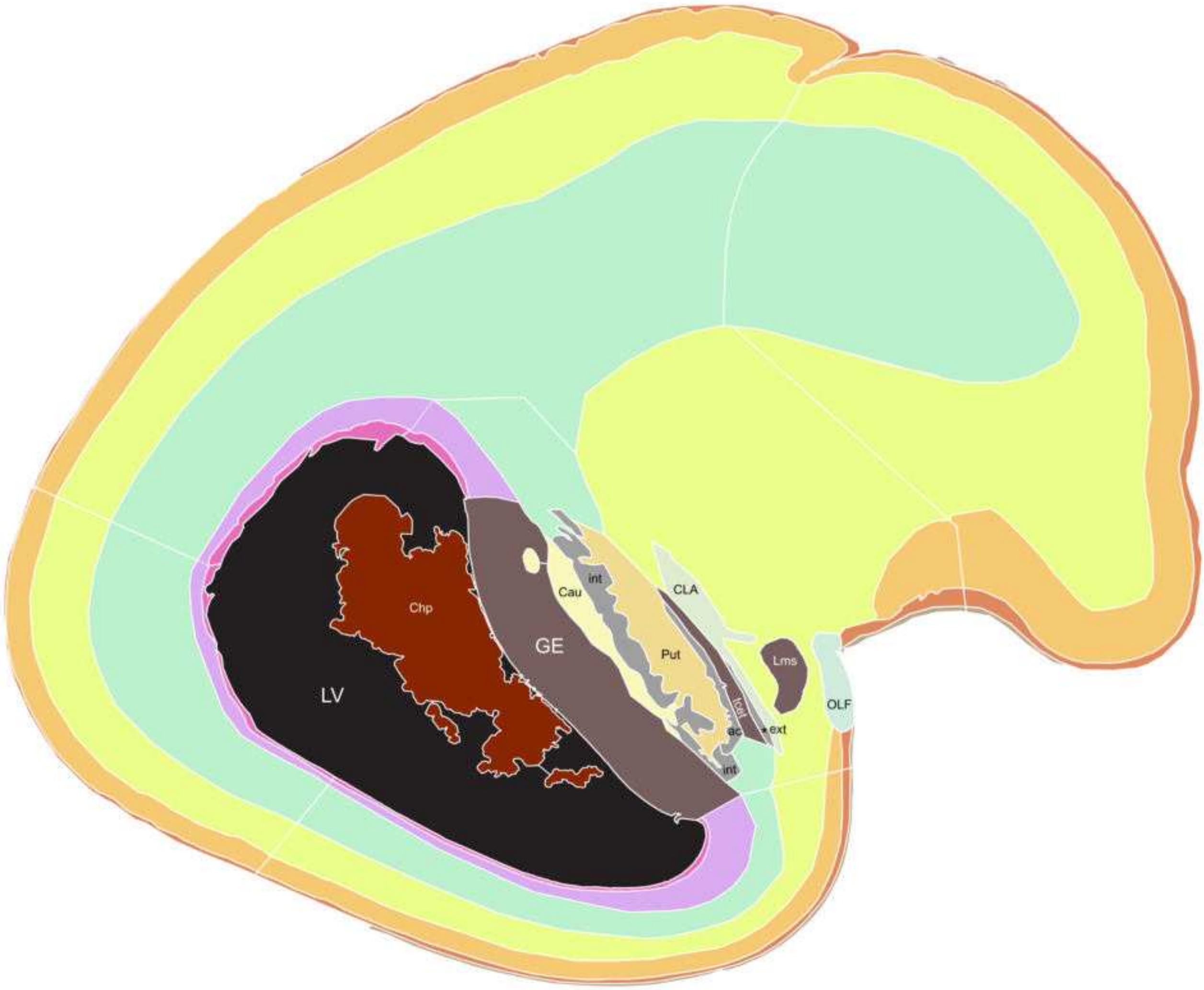

5 mm

- CLA: Claustrum
- Cau: Caudate nucleus
- Chp: Choroid plexus
- GE: Ganglionic eminence
- LV: Lateral ventricle
- Lms: Lateral migratory stream
- Put: Putamen
- ac: Anterior commissure
- ext: External capsule
- int: Internal capsule
- tcet: Transient cell zone in the external capsule

Age: 17 GW

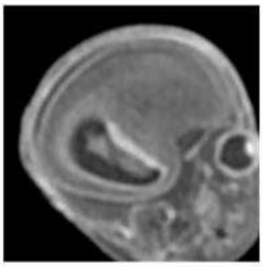

L-R Level: 8.94 mm

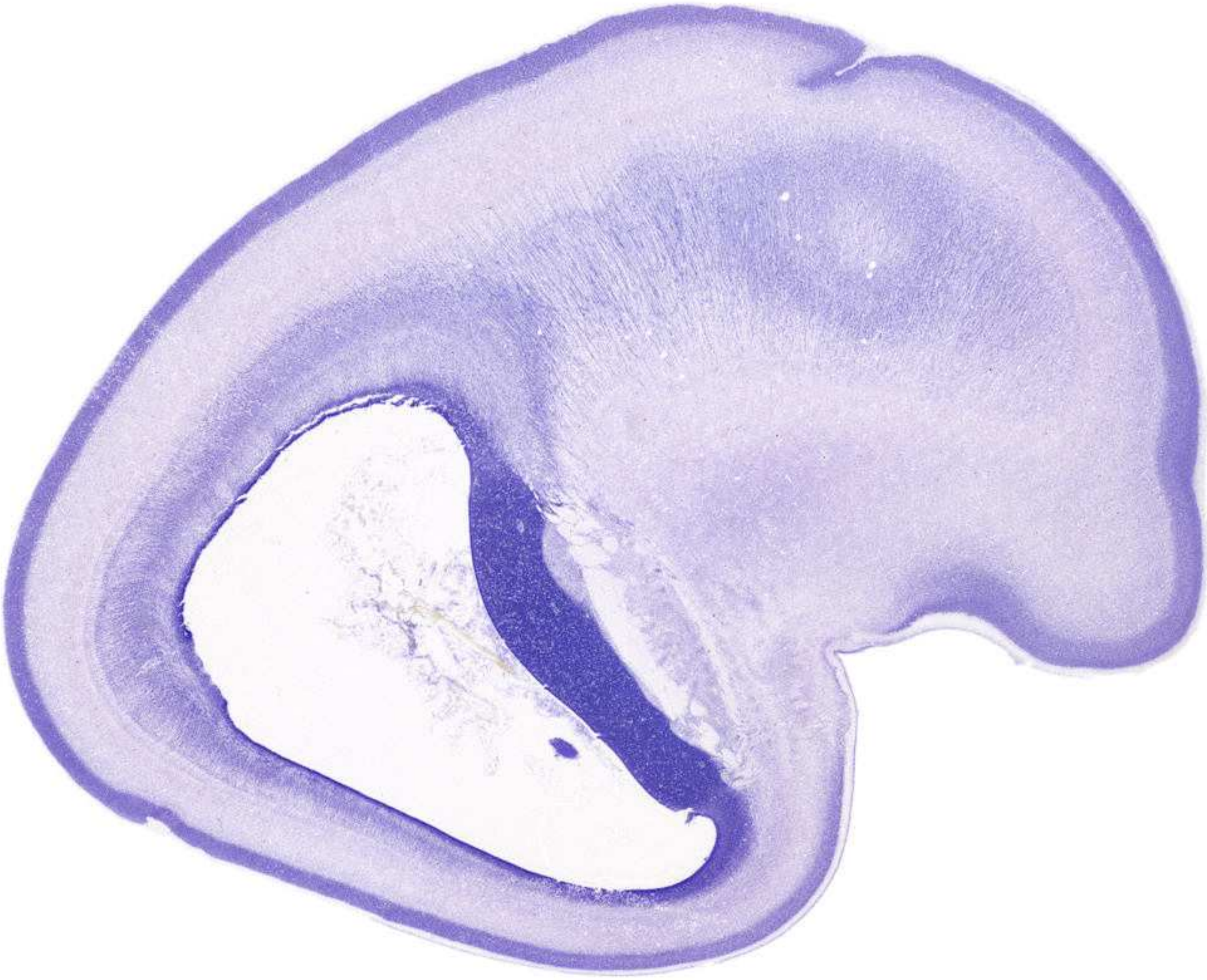

5 mm

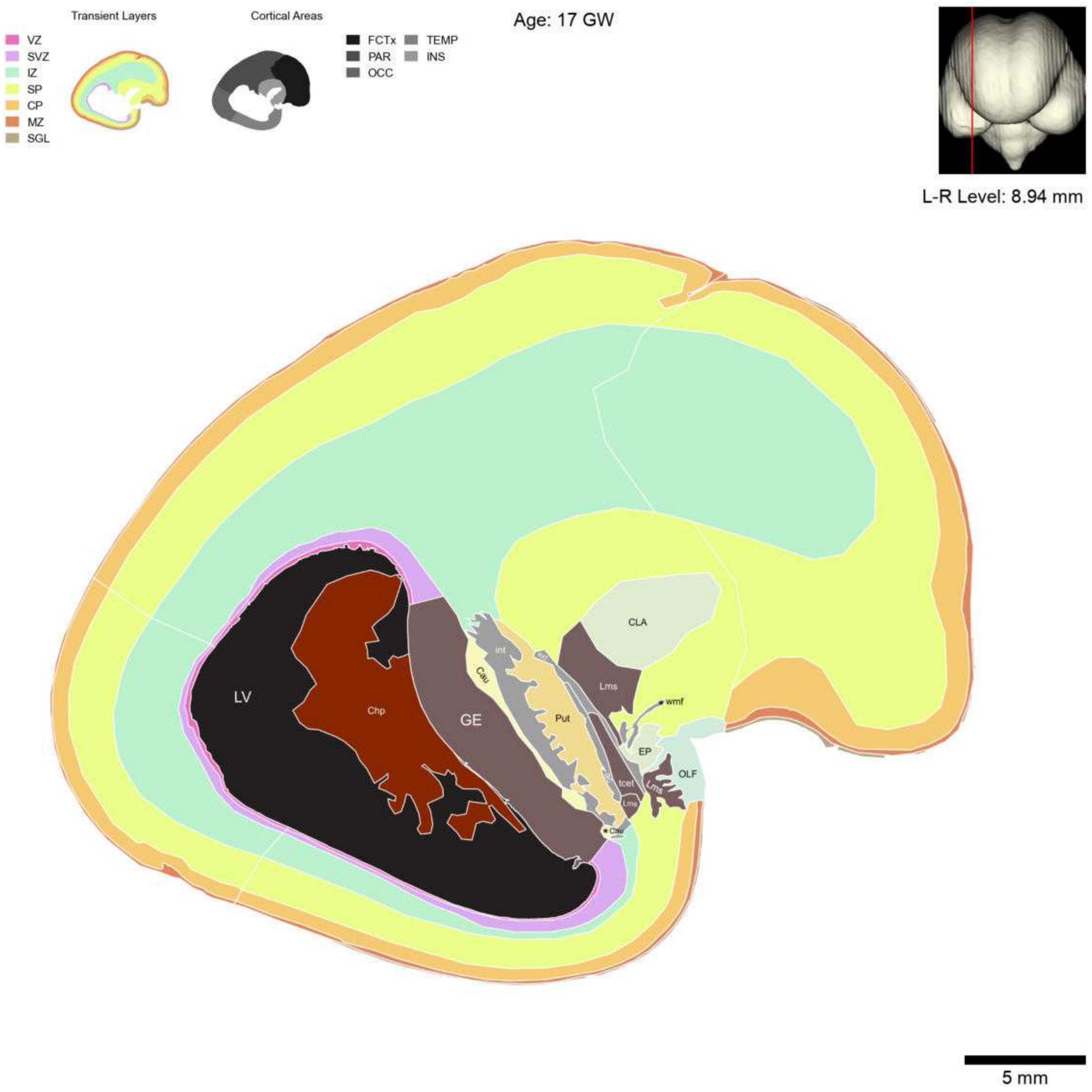

CLA: Claustrum
Cau: Caudate nucleus
Chp: Choroid plexus
EP: Endopiriform nucleus
GE: Ganglionic eminence
LV: Lateral ventricle
Lms: Lateral migratory stream
Put: Putamen
ac: Anterior commissure
ext: External capsule
int: Internal capsule
tcet: Transient cell zone in the external capsule
wmf: White matter fibers

Age: 17 GW

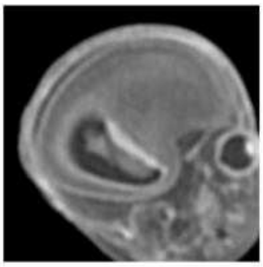

L-R Level: 8.76 mm

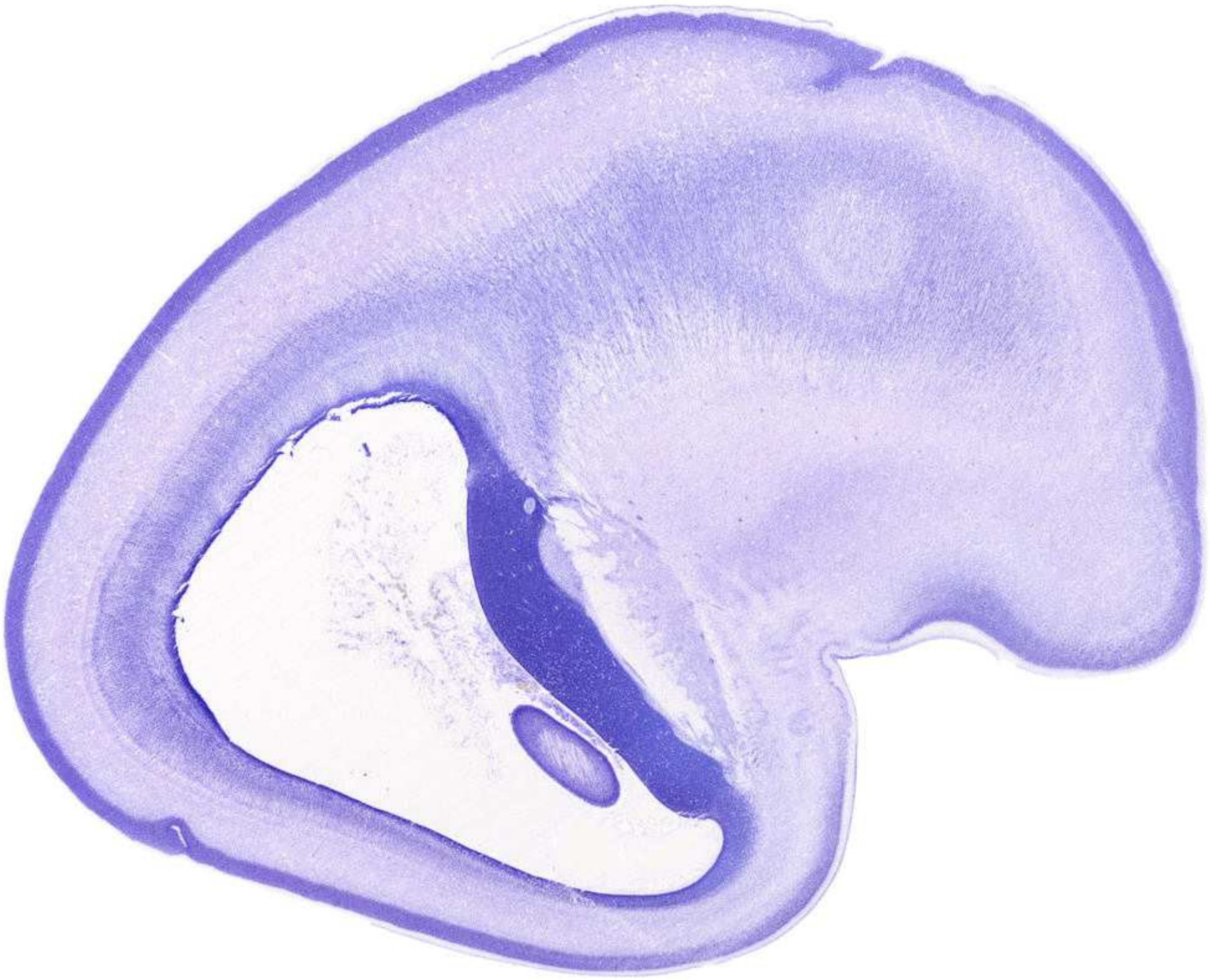

5 mm

Transient Layers

- VZ
- SVZ
- IZ
- SP
- CP
- MZ
- SGL

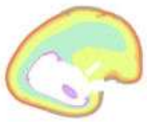

Cortical Areas

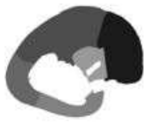

- FCTx
- PAR
- OCC
- TEMP
- INS

Age: 17 GW

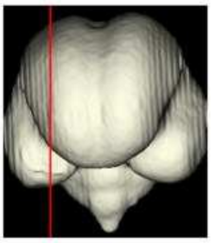

L-R Level: 8.76 mm

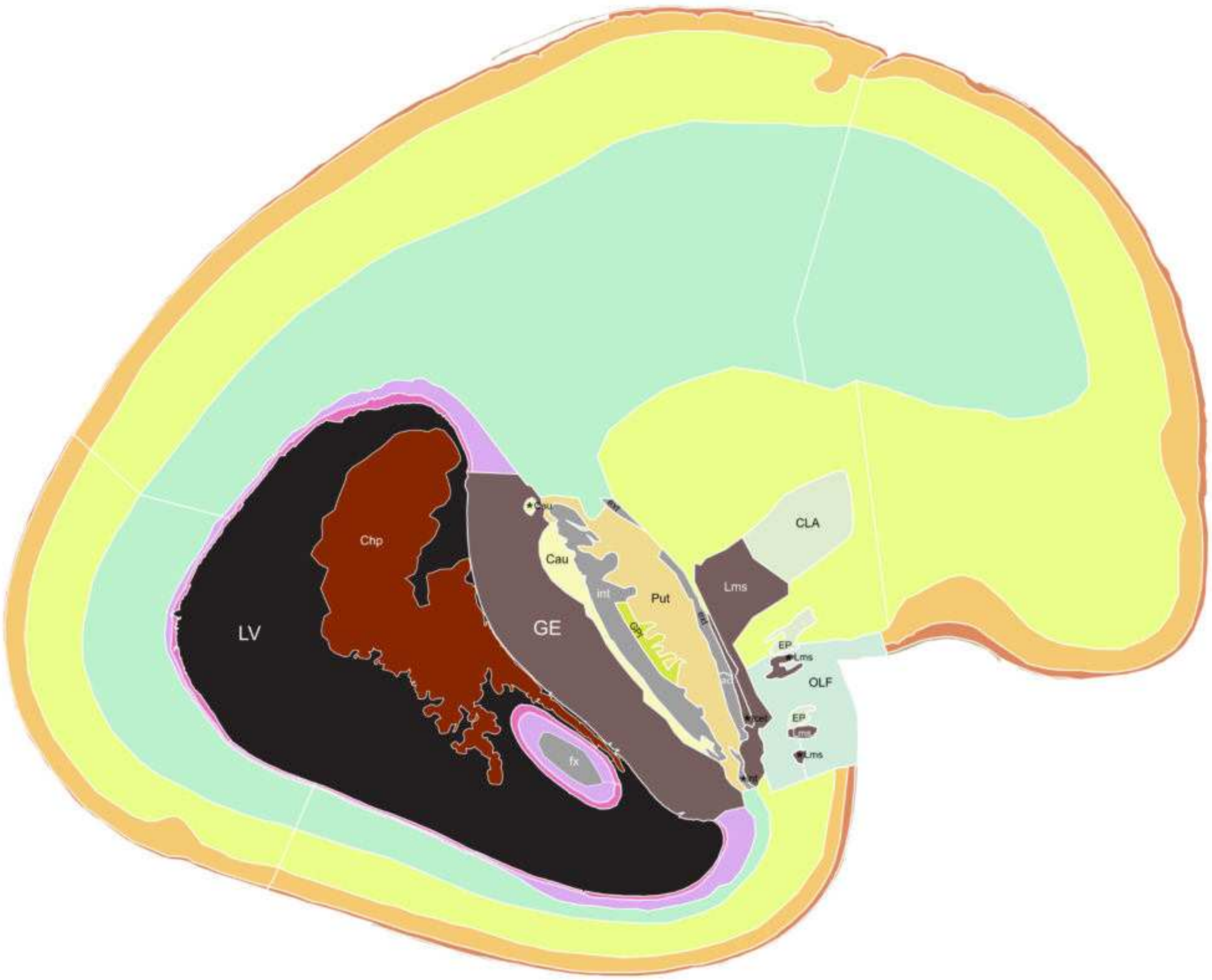

5 mm

- CLA: Claustrum
- Cau: Caudate nucleus
- Chp: Choroid plexus
- EP: Endopiriform nucleus
- GE: Ganglionic eminence
- GPl: Globus pallidus lateral segment
- LV: Lateral ventricle
- Lms: Lateral migratory stream
- Put: Putamen
- ac: Anterior commissure
- ext: External capsule
- fx: Fornix
- int: Internal capsule
- tcet: Transient cell zone in the external capsule

Age: 17 GW

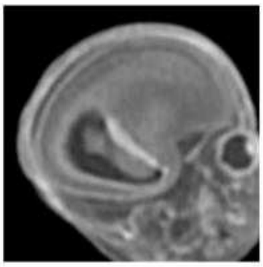

L-R Level: 8.34 mm

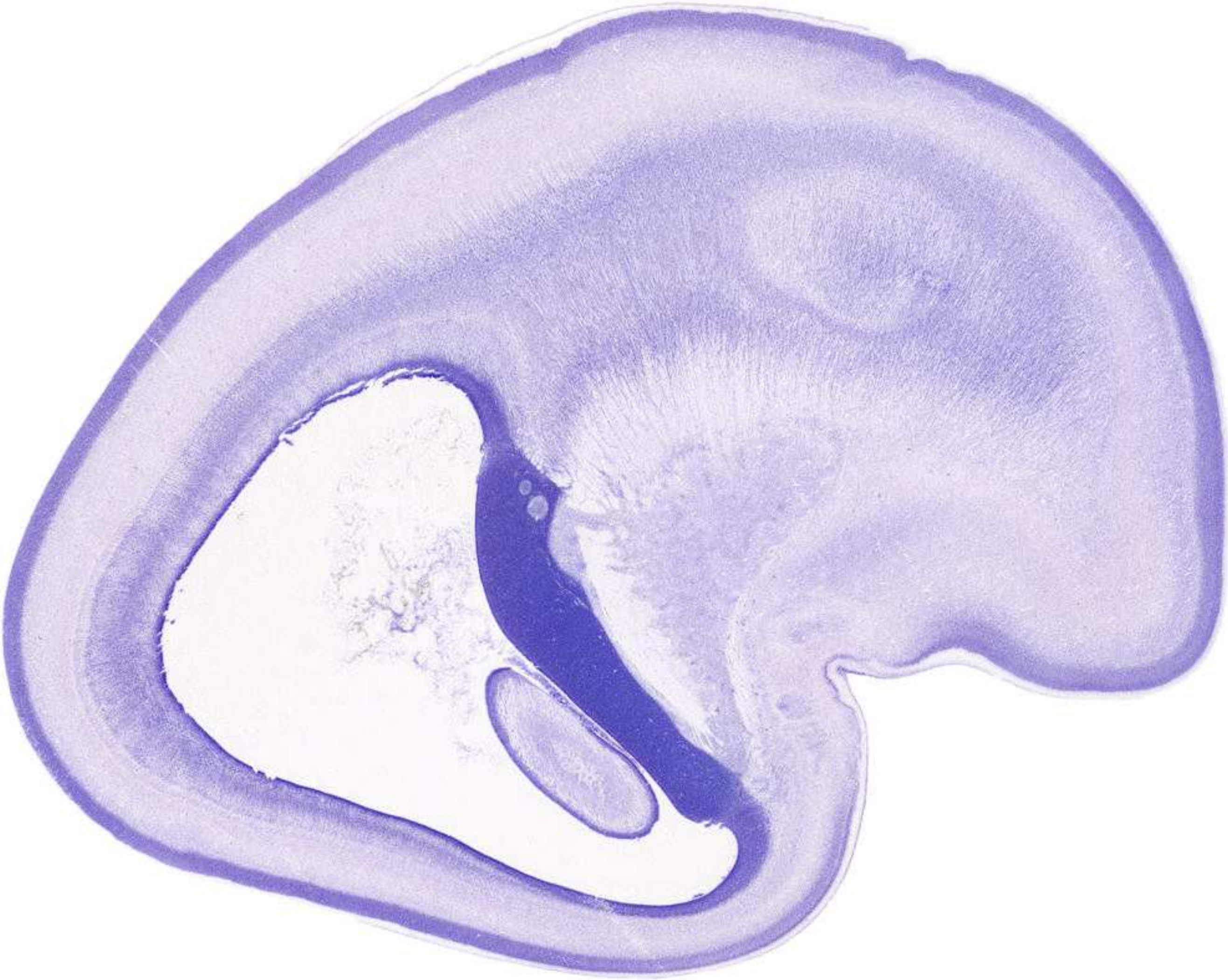

5 mm

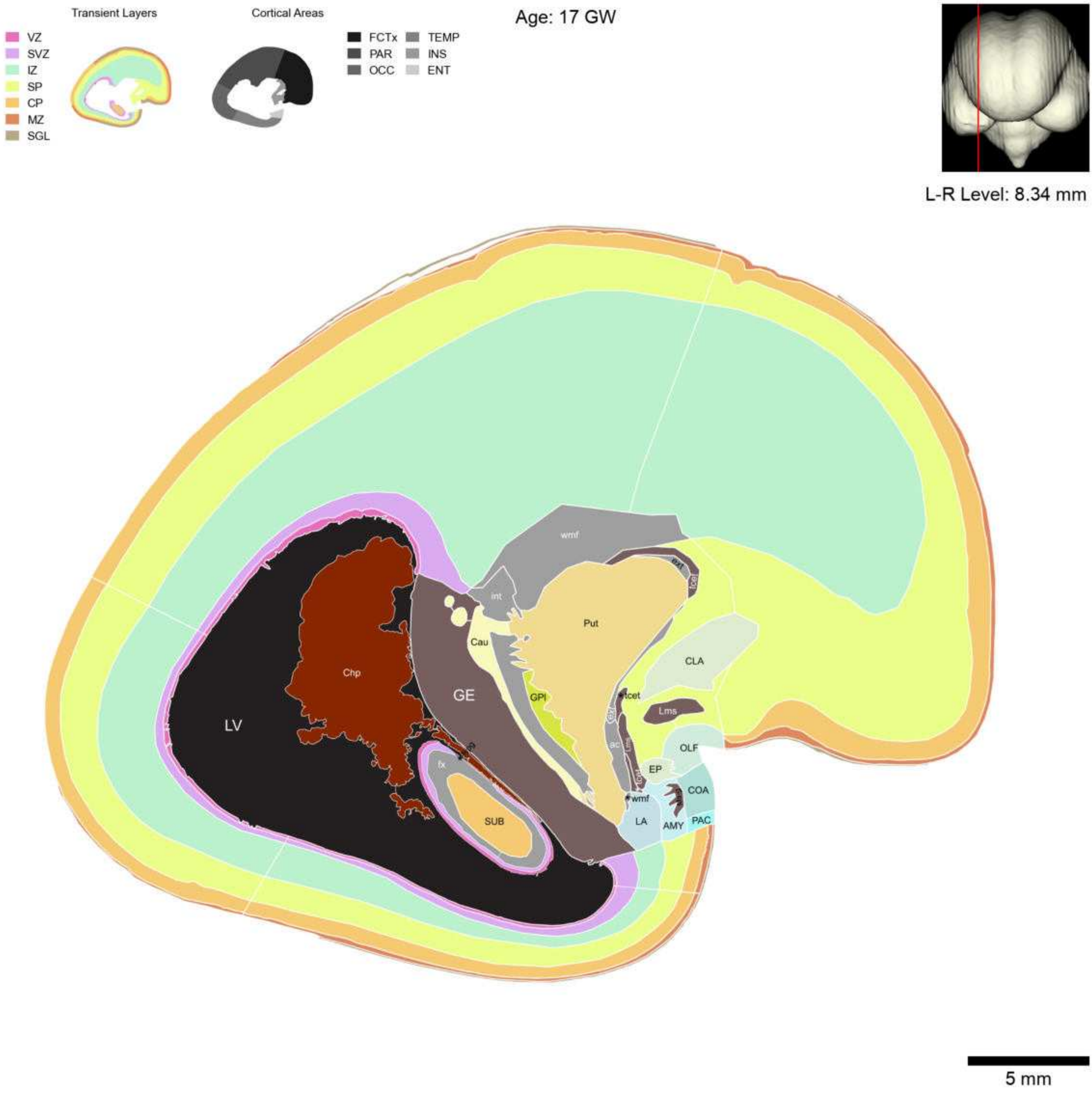

AMY: Amygdala
CLA: Claustrum
COA: Cortical nucleus [amygdala]
Cau: Caudate nucleus
Chp: Choroid plexus
EP: Endopiriform nucleus
GE: Ganglionic eminence
GPl: Globus pallidus lateral segment
LA: Lateral nucleus [amygdala]
LV: Lateral ventricle
Lms: Lateral migratory stream
Ms-g: Migratory stream, general
Put: Putamen
SUB: Cortical plate, subiculum
ac: Anterior commissure
ext: External capsule
fx: Fornix
hipg: Hippocampal glioepithelium/ependyma
int: Internal capsule
tcet: Transient cell zone in the external capsule
wmf: White matter fibers

Age: 17 GW

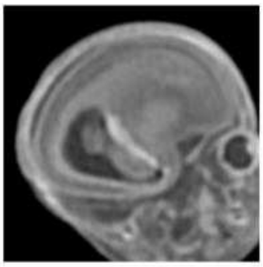

L-R Level: 7.98 mm

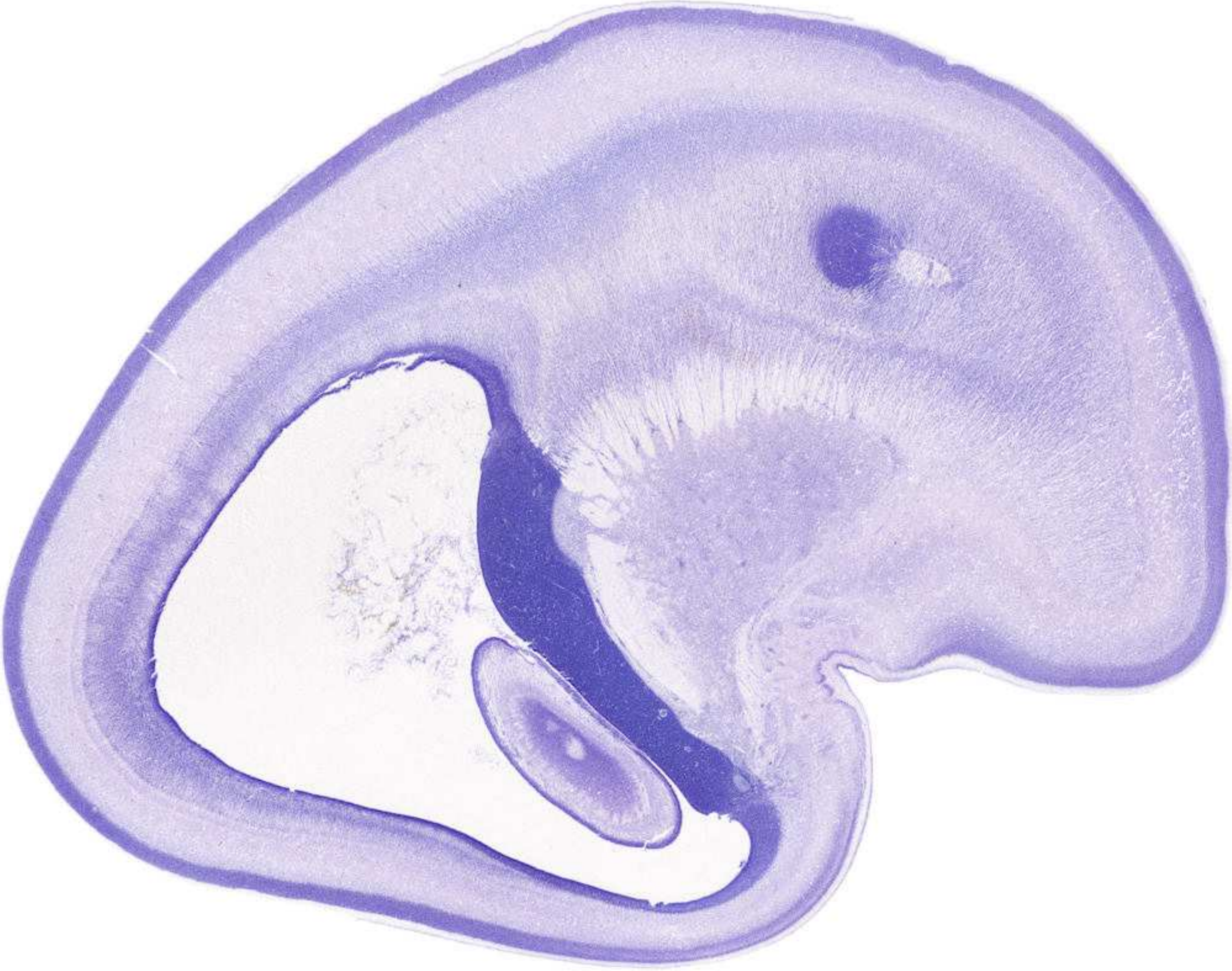

5 mm

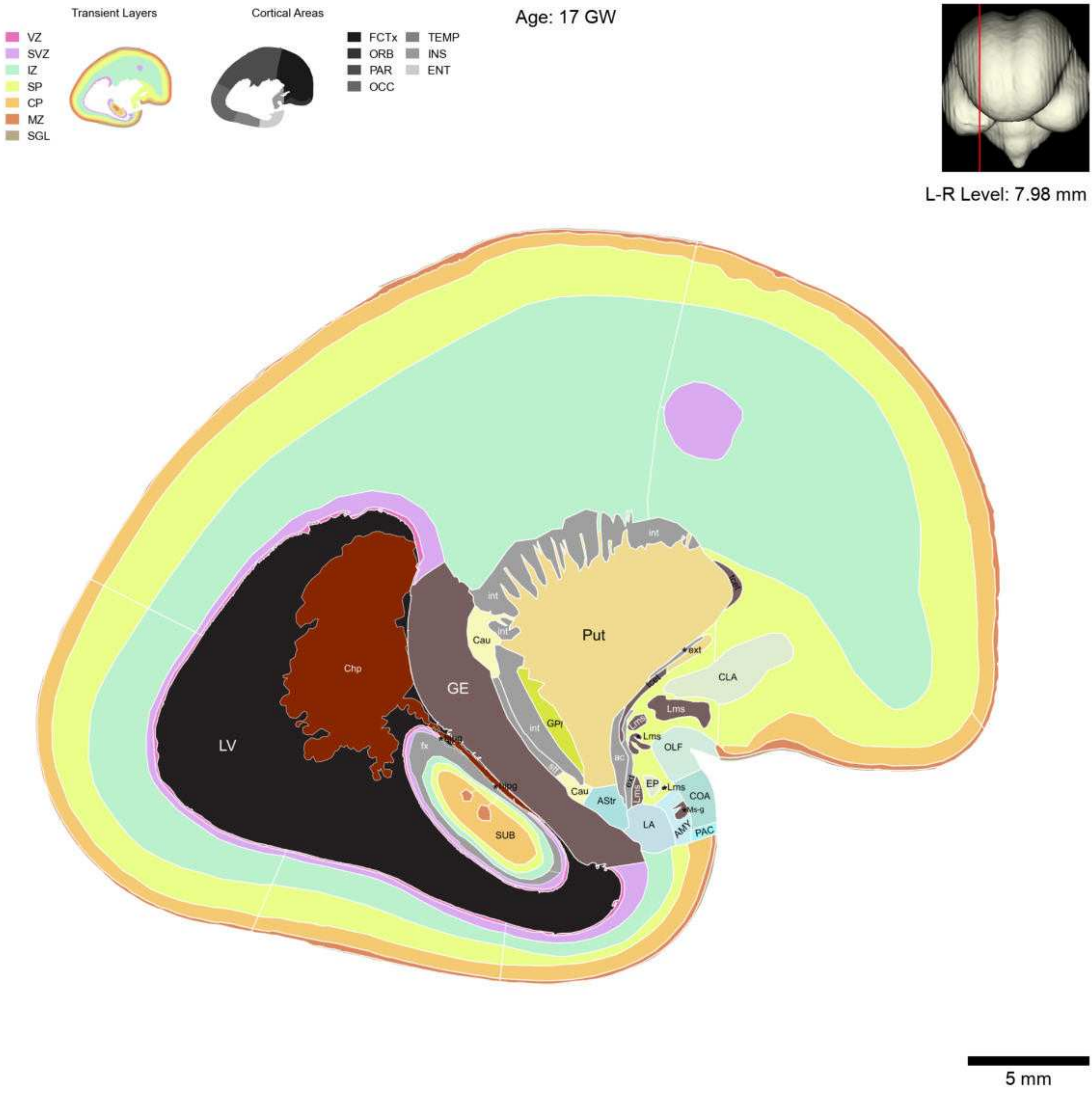

- AMY: Amygdala
- AStr: Amygdalo-striatal area
- CLA: Claustrum
- COA: Cortical nucleus [amygdala]
- Cau: Caudate nucleus
- Chp: Choroid plexus
- EP: Endopiriform nucleus
- GE: Ganglionic eminence
- GPl: Globus pallidus lateral segment
- LA: Lateral nucleus [amygdala]
- LV: Lateral ventricle
- Lms: Lateral migratory stream
- Ms-g: Migratory stream, general
- Put: Putamen
- SUB: Cortical plate, subiculum
- ac: Anterior commissure
- ext: External capsule
- fx: Fornix
- hipg: Hippocampal glioepithelium/ependyma
- int: Internal capsule
- stt: Stria terminalis
- tcet: Transient cell zone in the external capsule

Age: 17 GW

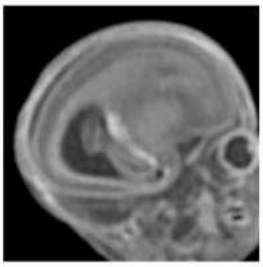

L-R Level: 7.62 mm

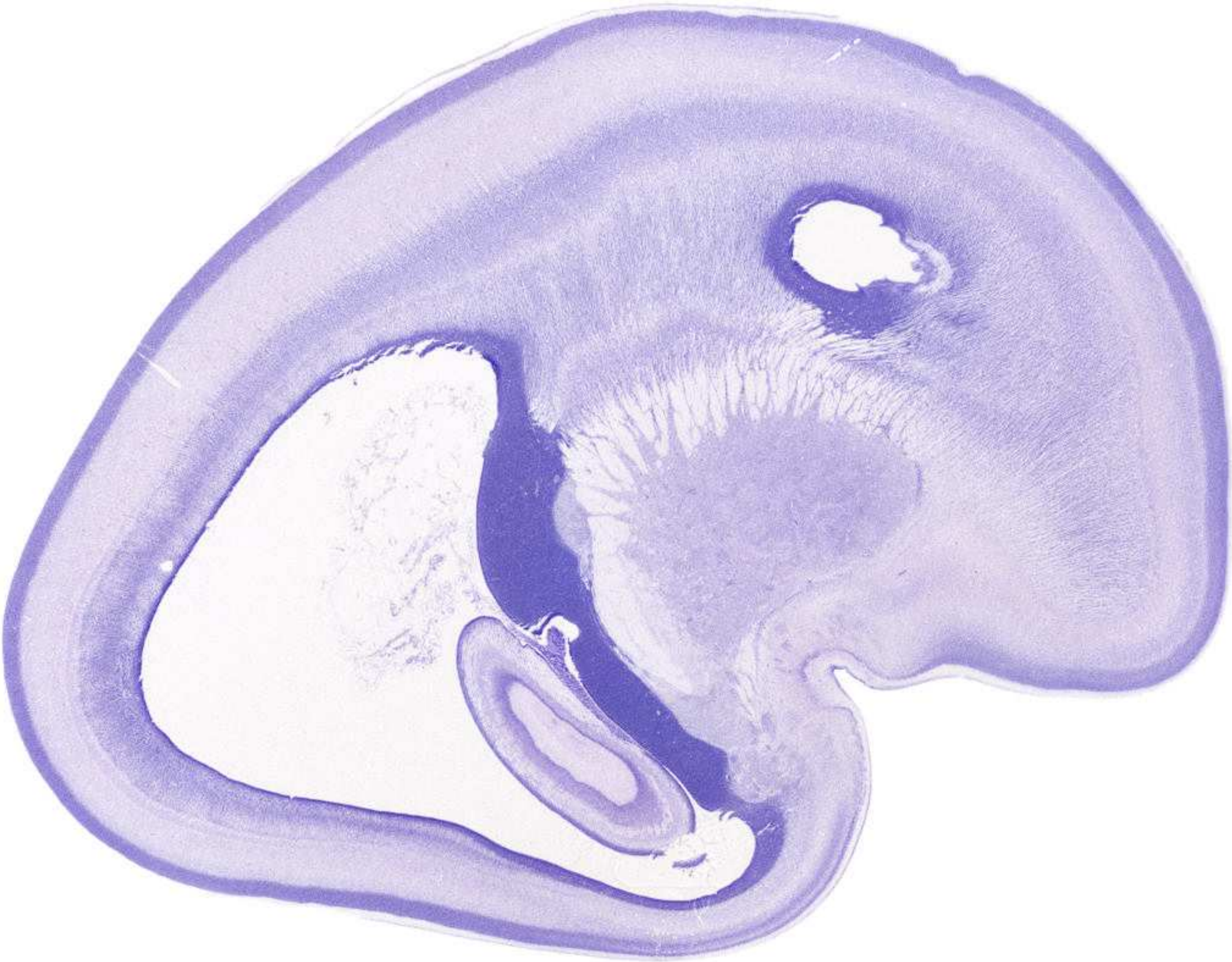

5 mm

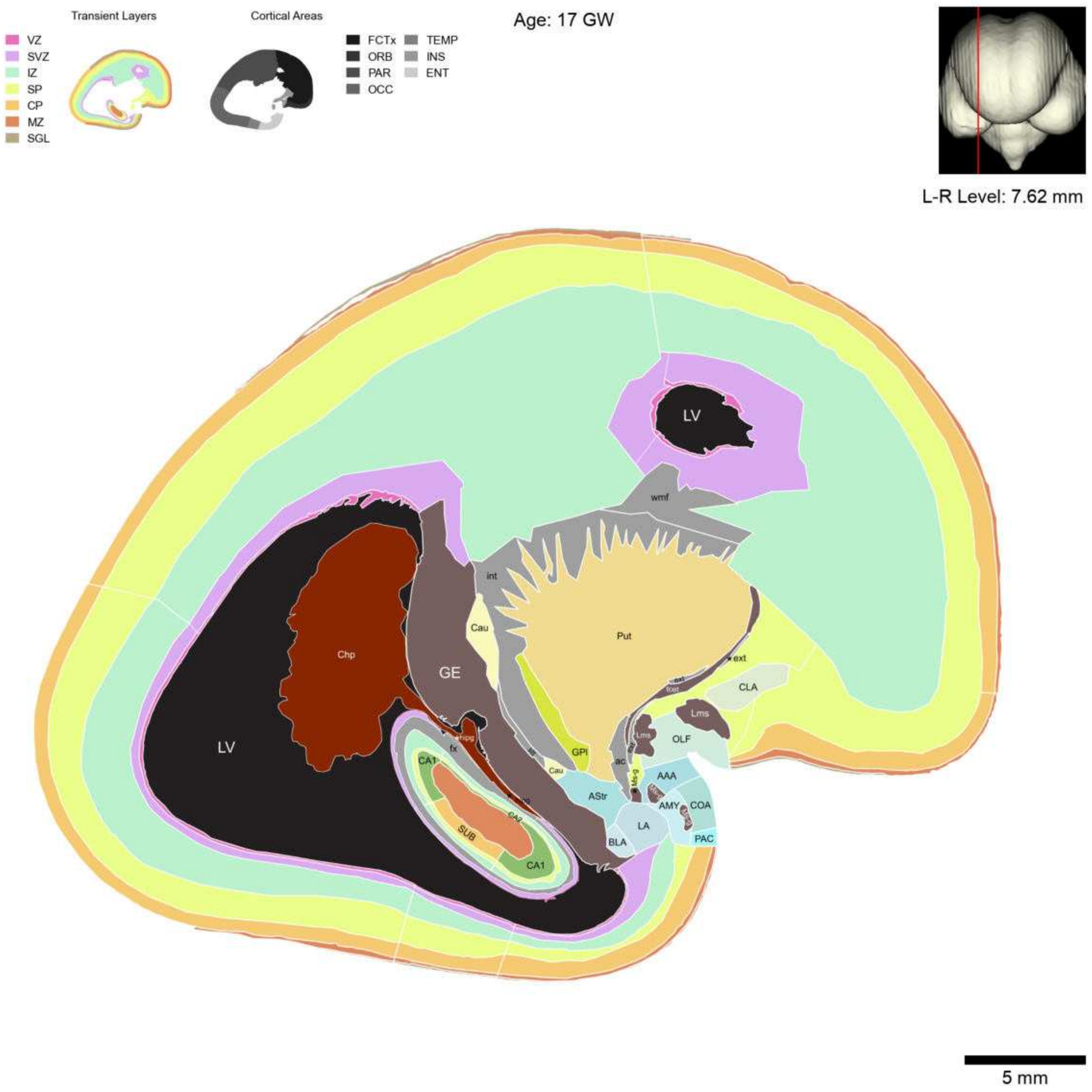

- AAA: Anterior amygdaloid area
- AMY: Amygdala
- AStr: Amygdalo-striatal area
- BL: Basal nucleus [amygdala]
- CA1: CA1 field [hippocampus]
- CA2: CA2 field [hippocampus]
- CLA: Claustrum
- COA: Cortical nucleus [amygdala]
- Cau: Caudate nucleus
- Chp: Choroid plexus
- GE: Ganglionic eminence
- GPl: Globus pallidus lateral segment
- LA: Lateral nucleus [amygdala]
- LV: Lateral ventricle
- Lms: Lateral migratory stream
- Ms-g: Migratory stream, general
- Put: Putamen
- SUB: Cortical plate, subiculum
- ac: Anterior commissure
- ext: External capsule
- fx: Fornix
- hipg: Hippocampal glioepithelium/ependyma
- int: Internal capsule
- stt: Stria terminalis
- tcet: Transient cell zone in the external capsule
- wmf: White matter fibers

Age: 17 GW

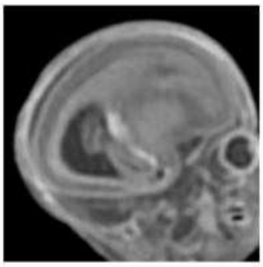

L-R Level: 7.5 mm

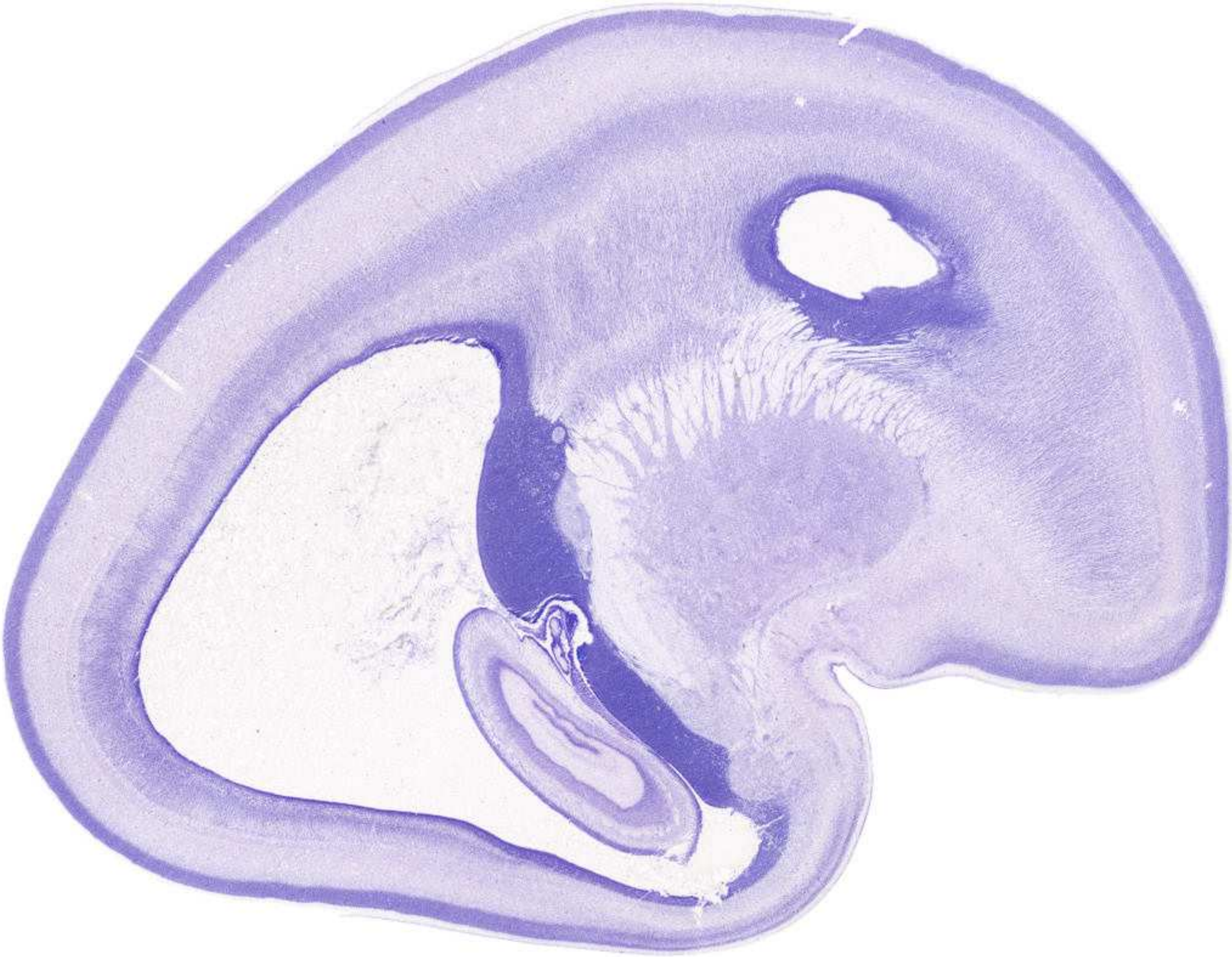

5 mm

Transient Layers

- VZ
- SVZ
- IZ
- SP
- CP
- MZ
- SGL

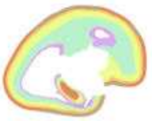

Cortical Areas

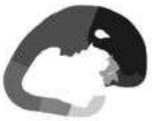

- FCTx
- ORB
- PAR
- OCC
- TEMP
- INS
- ENT

Age: 17 GW

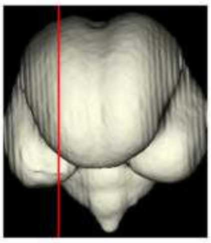

L-R Level: 7.5 mm

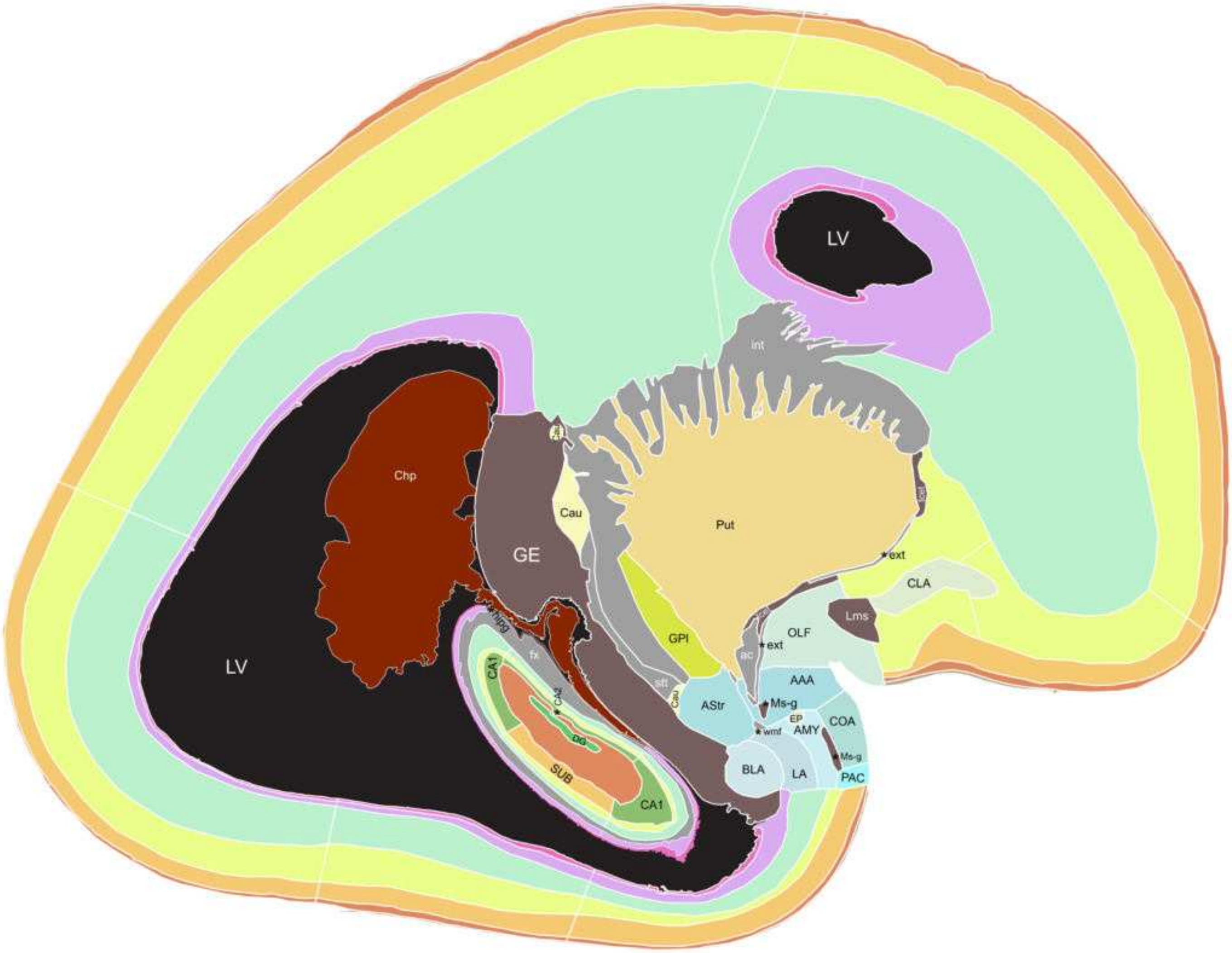

5 mm

- AAA: Anterior amygdaloid area
- AMY: Amygdala
- AStr: Amygdalo-striatal area
- BL: Basal nucleus [amygdala]
- CA1: CA1 field [hippocampus]
- CA2: CA2 field [hippocampus]
- CLA: Claustrum
- COA: Cortical nucleus [amygdala]
- Cau: Caudate nucleus
- Chp: Choroid plexus
- DG: Dentate gyrus
- EP: Endopiriform nucleus
- GE: Ganglionic eminence
- GPl: Globus pallidus lateral segment
- LA: Lateral nucleus [amygdala]
- LV: Lateral ventricle
- Lms: Lateral migratory stream
- Ms-g: Migratory stream, general
- Put: Putamen
- SUB: Cortical plate, subiculum
- ac: Anterior commissure
- ext: External capsule
- fx: Fornix
- hipg: Hippocampal glioepithelium/ependyma
- int: Internal capsule
- stt: Stria terminalis
- tcet: Transient cell zone in the external capsule
- wmf: White matter fibers

Age: 17 GW

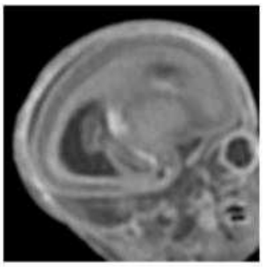

L-R Level: 7.2 mm

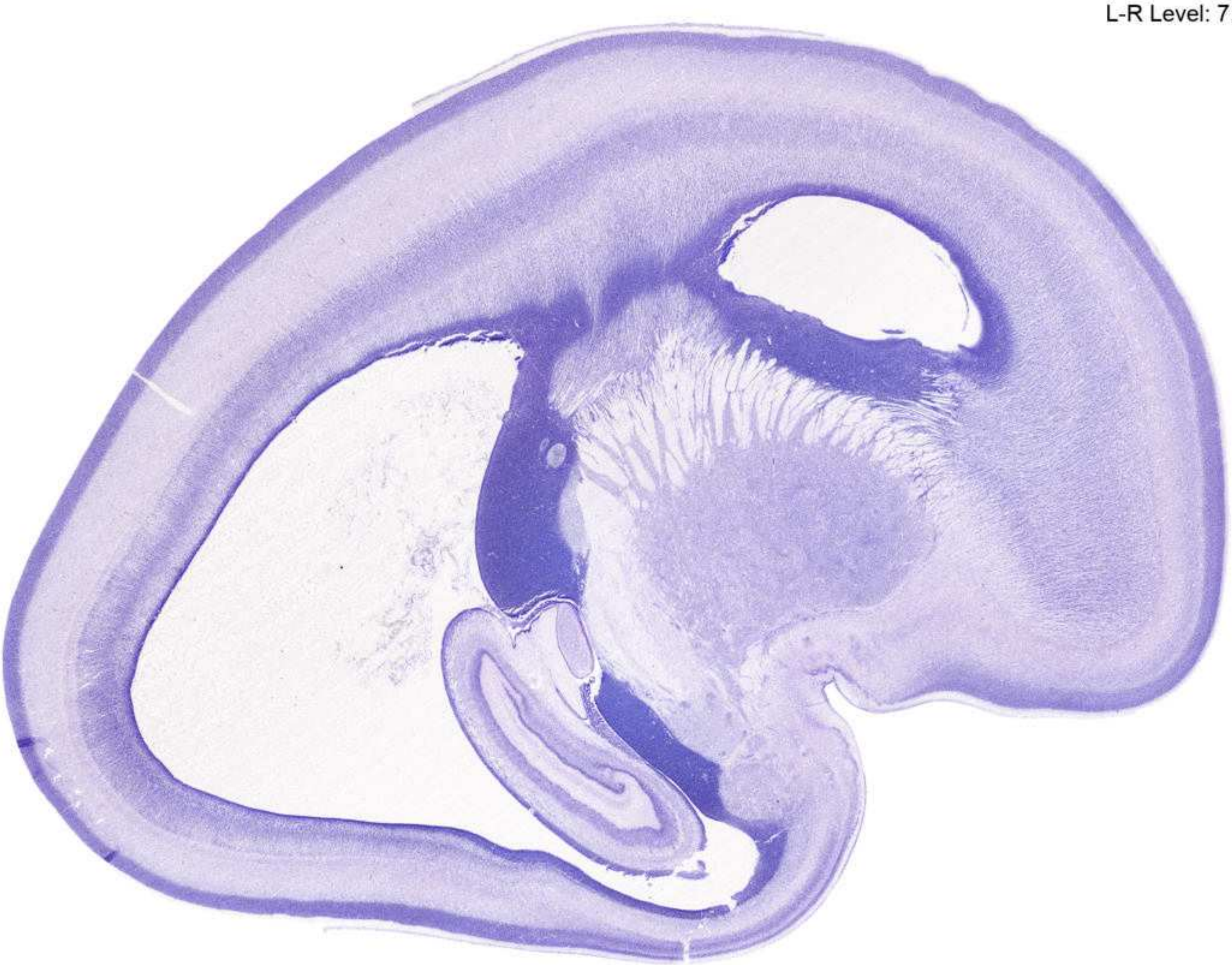

5 mm

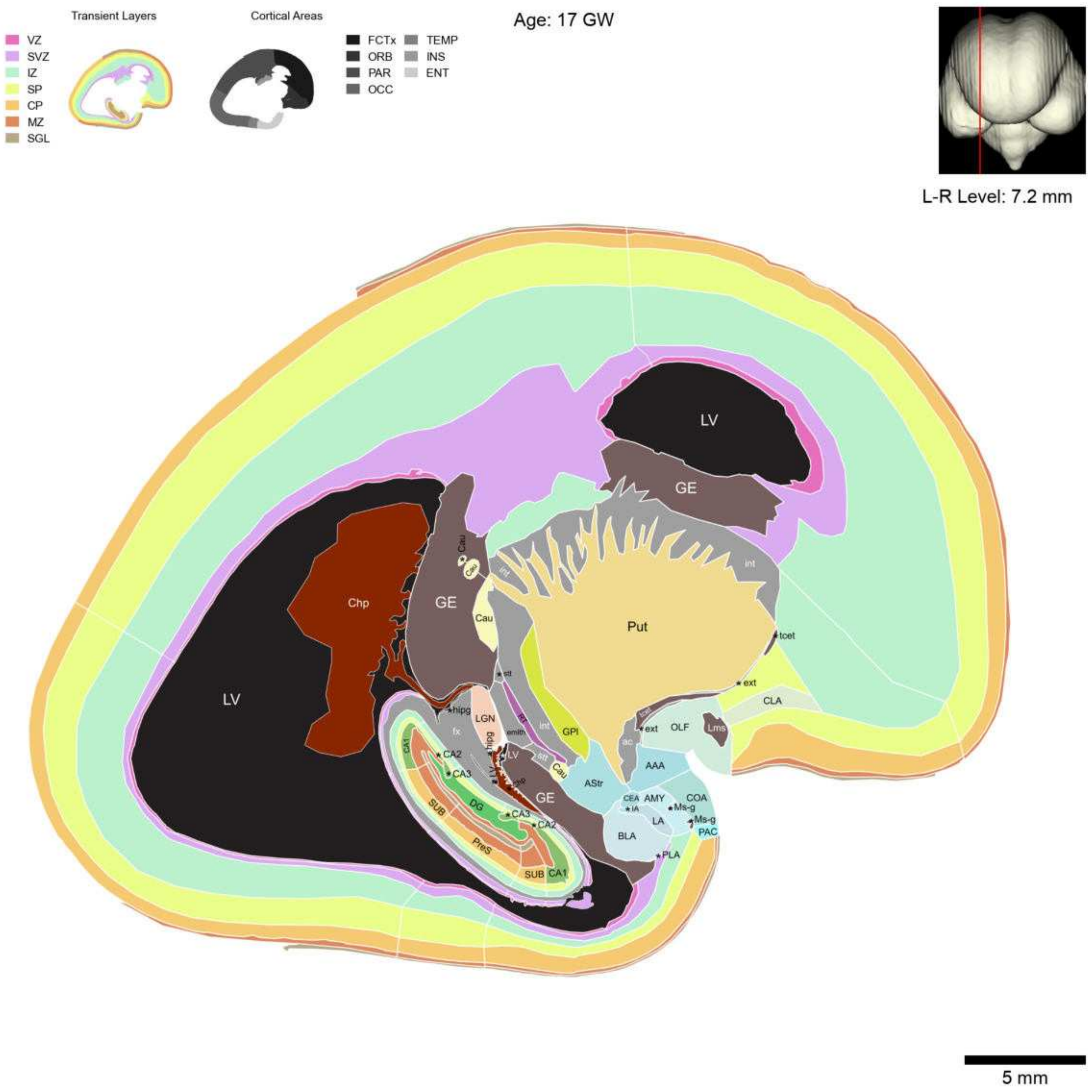

- AAA: Anterior amygdaloid area
- AMY: Amygdala
- AStr: Amygdalo-striatal area
- BL: Basal nucleus [amygdala]
- CA1: CA1 field [hippocampus]
- CA2: CA2 field [hippocampus]
- CA3: CA3 field [hippocampus]
- CEA: Central nucleus [amygdala]
- CLA: Claustrum
- COA: Cortical nucleus [amygdala]
- Cau: Caudate nucleus
- Chp: Choroid plexus
- DG: Dentate gyrus
- GE: Ganglionic eminence
- GPl: Globus pallidus lateral segment
- IA: Intercalated cell groups [amygdala]
- LA: Lateral nucleus [amygdala]
- LGN: Lateral geniculate nucleus
- LV: Lateral ventricle
- Lms: Lateral migratory stream
- Ms-g: Migratory stream, general
- PLA: Paralaminar nucleus [amygdala]
- PreS: Cortical plate, presubiculum
- Put: Putamen
- RT: Reticular nucleus [thalamus]
- SUB: Cortical plate, subiculum
- ac: Anterior commissure
- emlth: External medullary lamina [thalamus]
- ext: External capsule
- fx: Fornix
- hipg: Hippocampal glioepithelium/ependyma
- int: Internal capsule
- stt: Stria terminalis
- tcet: Transient cell zone in the external capsule

Age: 17 GW

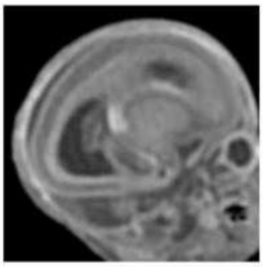

L-R Level: 6.84 mm

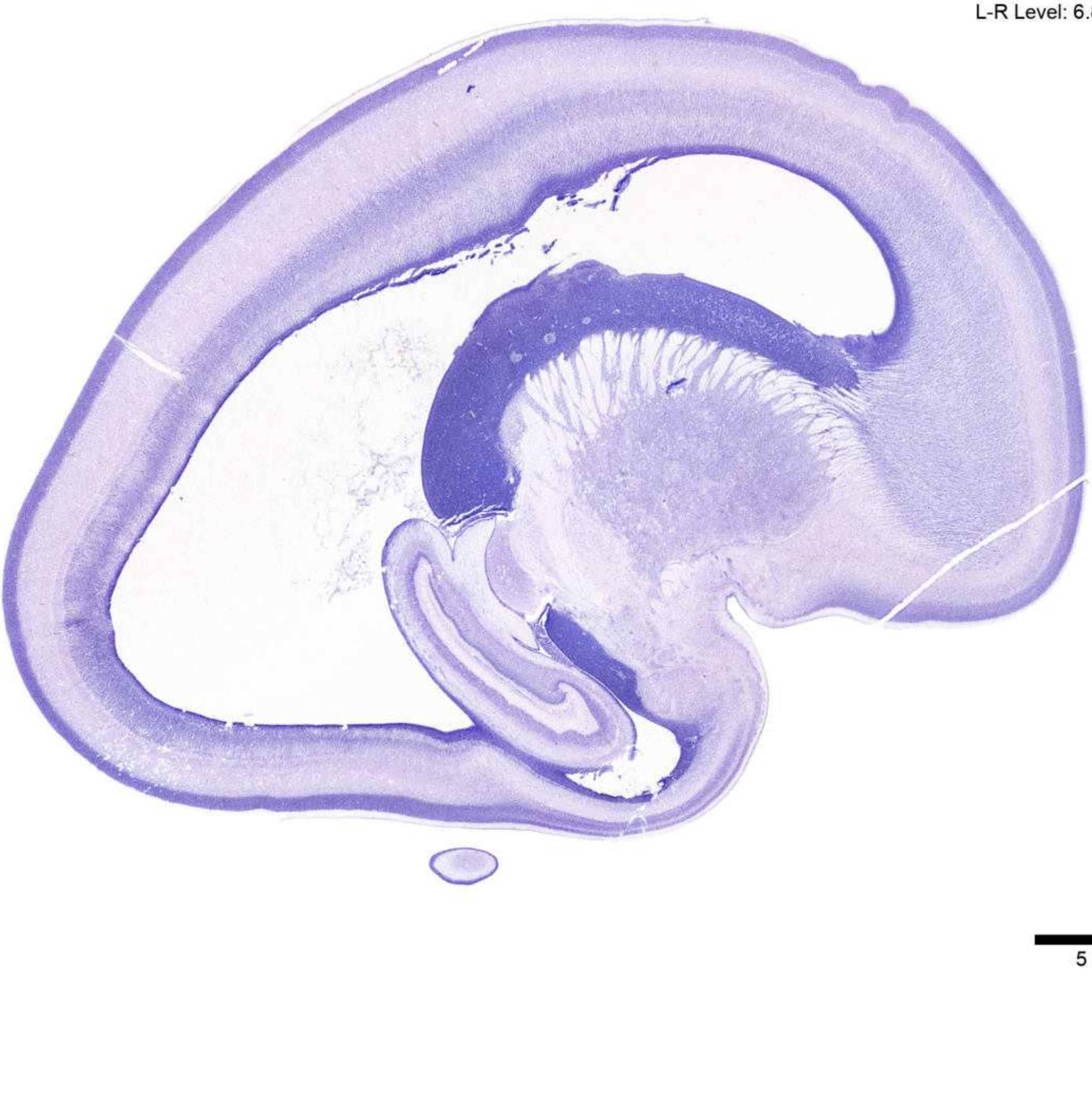

5 mm

Transient Layers

VZ
SVZ
IZ
SP
CP
MZ
SGL

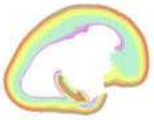

Cortical Areas

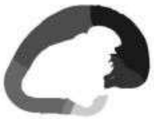

FCTx
ORB
PAR
OCC
TEMP
ENT

Age: 17 GW

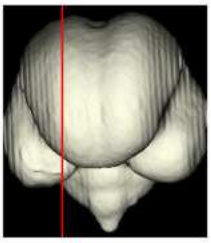

L-R Level: 6.84 mm

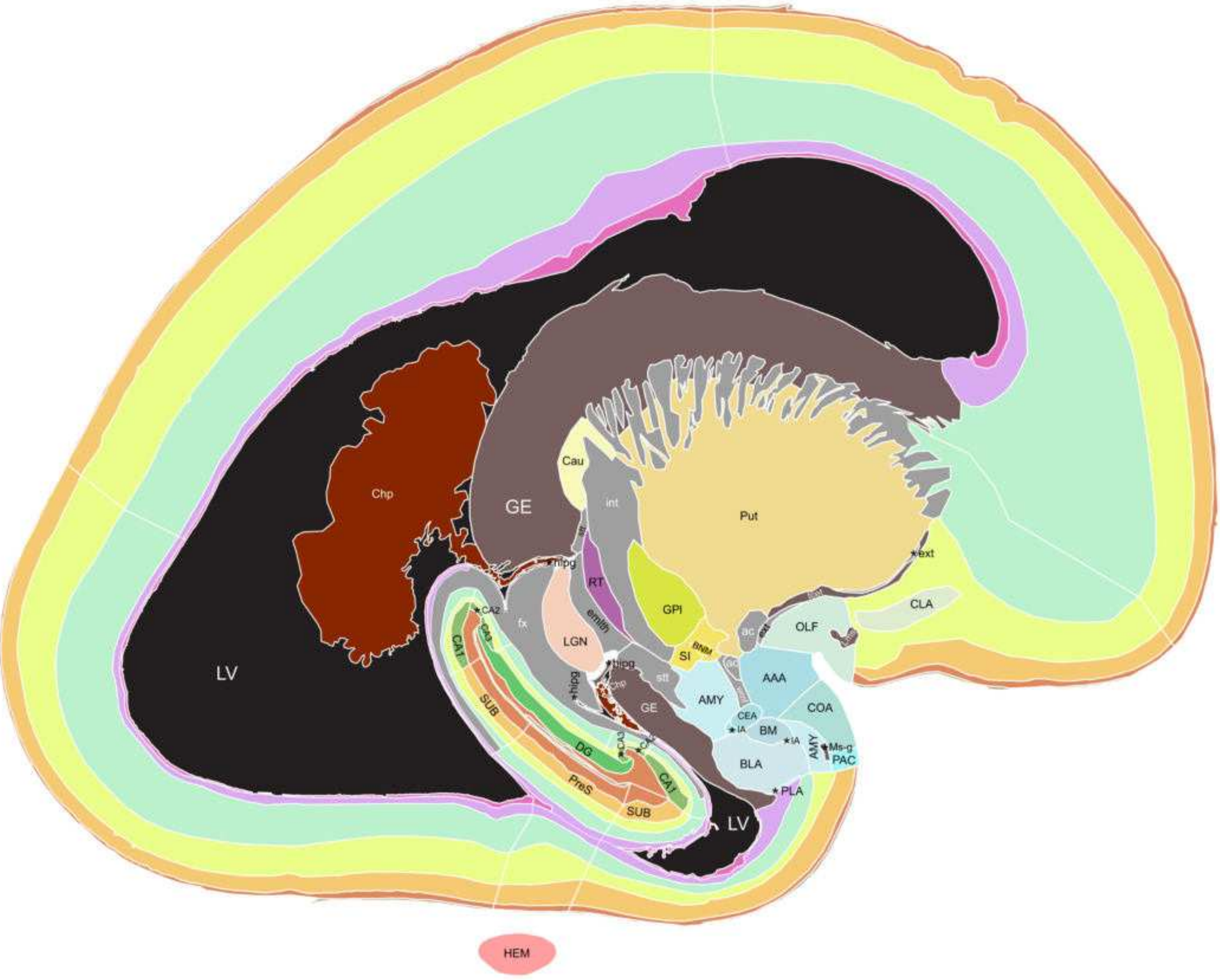

5 mm

AAA: Anterior amygdaloid area
AMY: Amygdala
BL: Basal nucleus [amygdala]
BM: Basomedial nucleus [amygdala]
BNM: Basal nucleus of Meynert
CA1: CA1 field [hippocampus]
CA2: CA2 field [hippocampus]
CA3: CA3 field [hippocampus]
CEA: Central nucleus [amygdala]
CLA: Claustrum
COA: Cortical nucleus [amygdala]
Cau: Caudate nucleus
Chp: Choroid plexus
DG: Dentate gyrus
GE: Ganglionic eminence
GPl: Globus pallidus lateral segment
HEM: Cerebellar hemispheres
IA: Intercalated cell groups [amygdala]
LGN: Lateral geniculate nucleus
LV: Lateral ventricle
Lms: Lateral migratory stream
Ms-g: Migratory stream, general
PLA: Paralaminar nucleus [amygdala]
PreS: Cortical plate, presubiculum
Put: Putamen
RT: Reticular nucleus [thalamus]
SI: Substantia innominata
SUB: Cortical plate, subiculum
ac: Anterior commissure
emlth: External medullary lamina [thalamus]
ext: External capsule
fx: Fornix
hipg: Hippocampal glioepithelium/ependyma
int: Internal capsule
stt: Stria terminalis
tcet: Transient cell zone in the external capsule
wmf: White matter fibers

Age: 17 GW

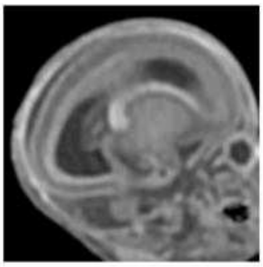

L-R Level: 6.42 mm

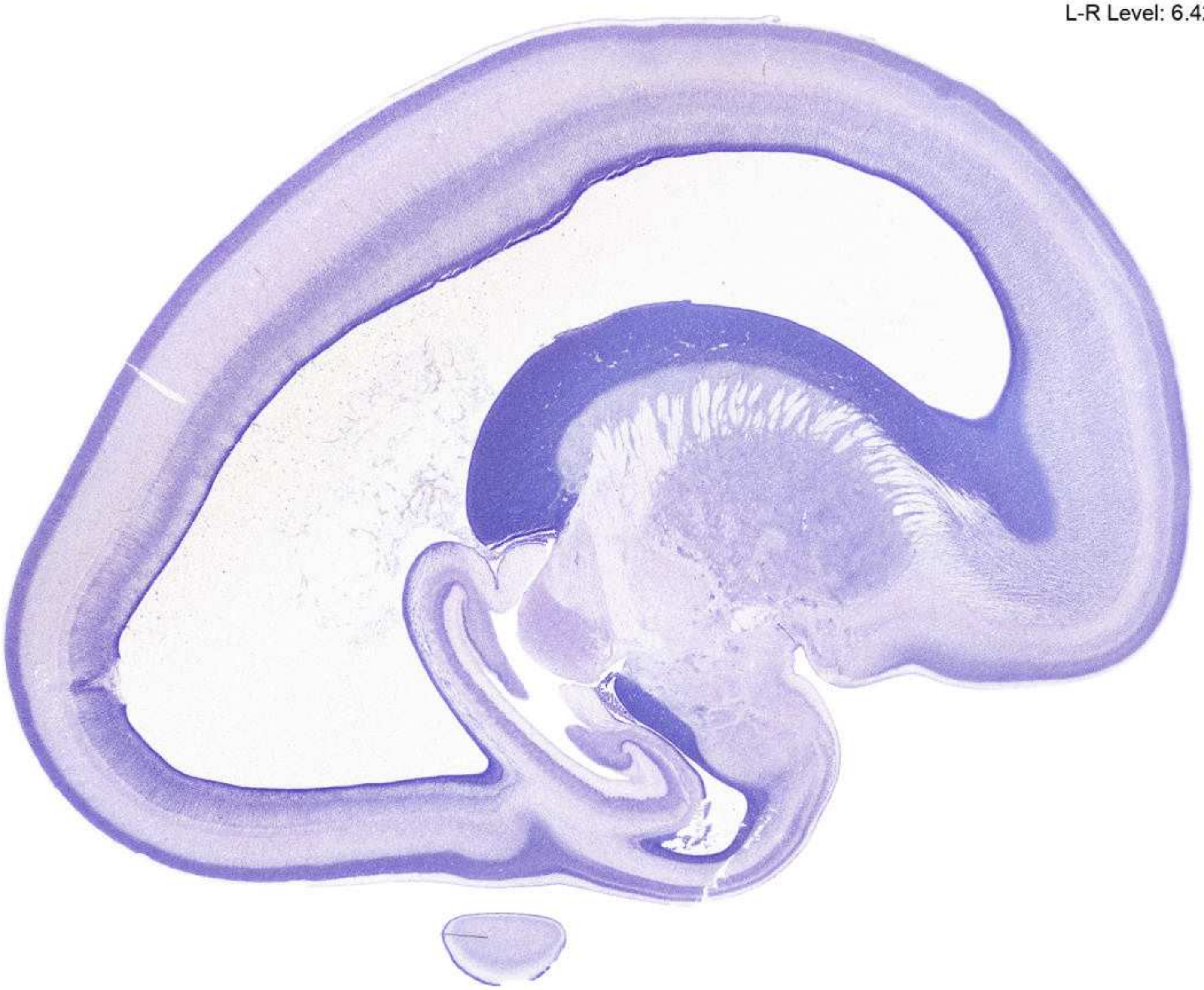

5 mm

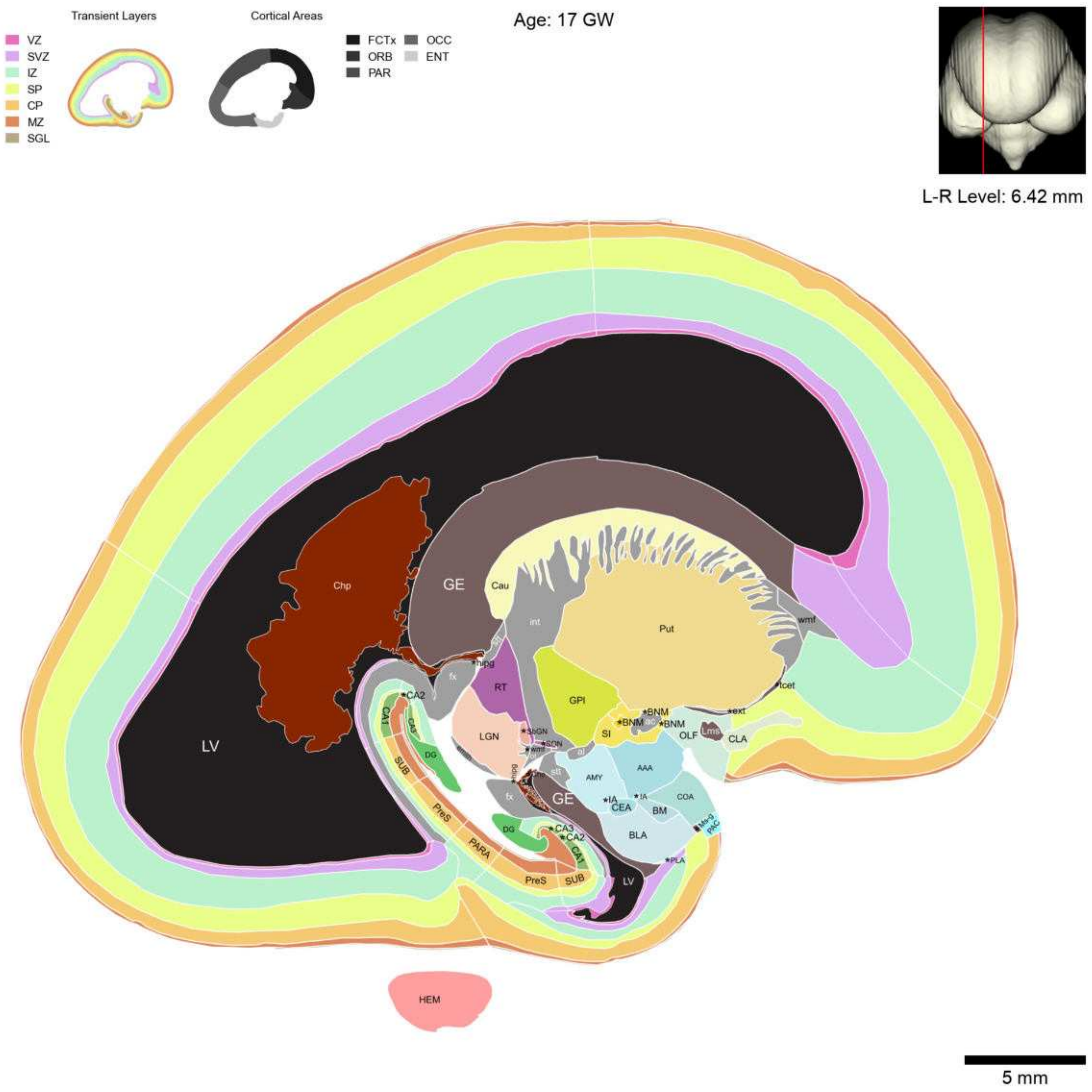

- AAA: Anterior amygdaloid area
- AMY: Amygdala
- BL: Basal nucleus [amygdala]
- BM: Basomedial nucleus [amygdala]
- BNM: Basal nucleus of Meynert
- CA1: CA1 field [hippocampus]
- CA2: CA2 field [hippocampus]
- CA3: CA3 field [hippocampus]
- CEA: Central nucleus [amygdala]
- CLA: Claustrum
- COA: Cortical nucleus [amygdala]
- Cau: Caudate nucleus
- Chp: Choroid plexus
- DG: Dentate gyrus
- GE: Ganglionic eminence
- GPl: Globus pallidus lateral segment
- HEM: Cerebellar hemispheres
- IA: Intercalated cell groups [amygdala]
- LGN: Lateral geniculate nucleus
- LV: Lateral ventricle
- Lms: Lateral migratory stream
- Ms-g: Migratory stream, general
- PARA: Cortical plate, parasubiculum
- PLA: Paralaminar nucleus [amygdala]
- PreS: Cortical plate, presubiculum
- Put: Putamen
- RT: Reticular nucleus [thalamus]
- SI: Substantia innominata
- SON: Supraoptic nucleus [hypothalamus]
- SUB: Cortical plate, subiculum
- SbGN: Subgeniculate nucleus
- ac: Anterior commissure
- al: Ansa lenticularis
- emlth: External medullary lamina [thalamus]
- ext: External capsule
- fx: Fornix
- hipg: Hippocampal glioepithelium/ependyma
- int: Internal capsule
- ot: Optic tract
- stt: Stria terminalis
- tcet: Transient cell zone in the external capsule
- wmf: White matter fibers

Age: 17 GW

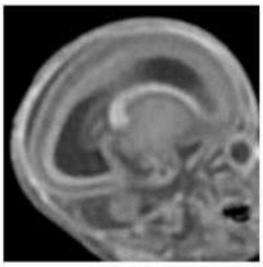

L-R Level: 6.18 mm

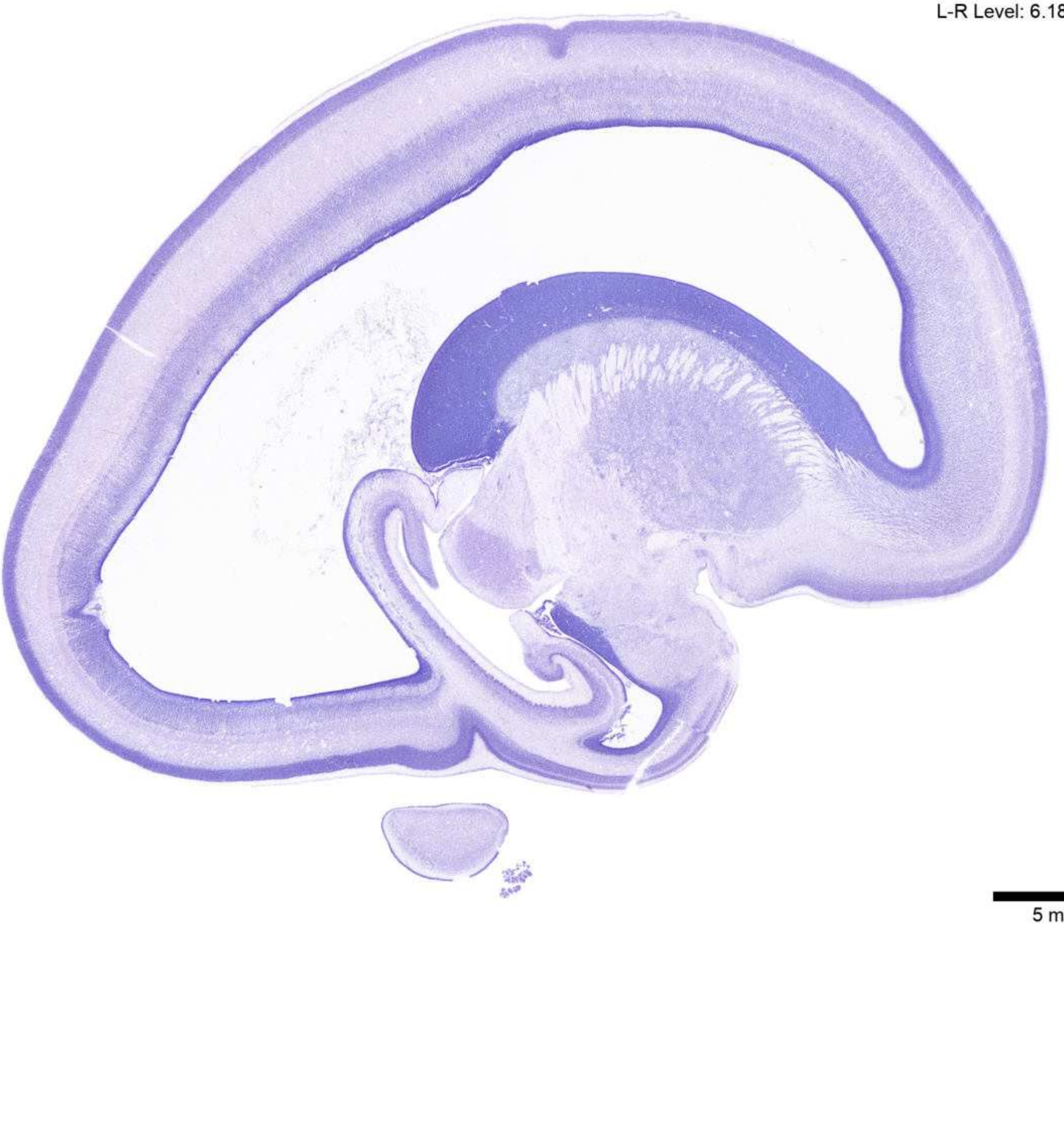

5 mm

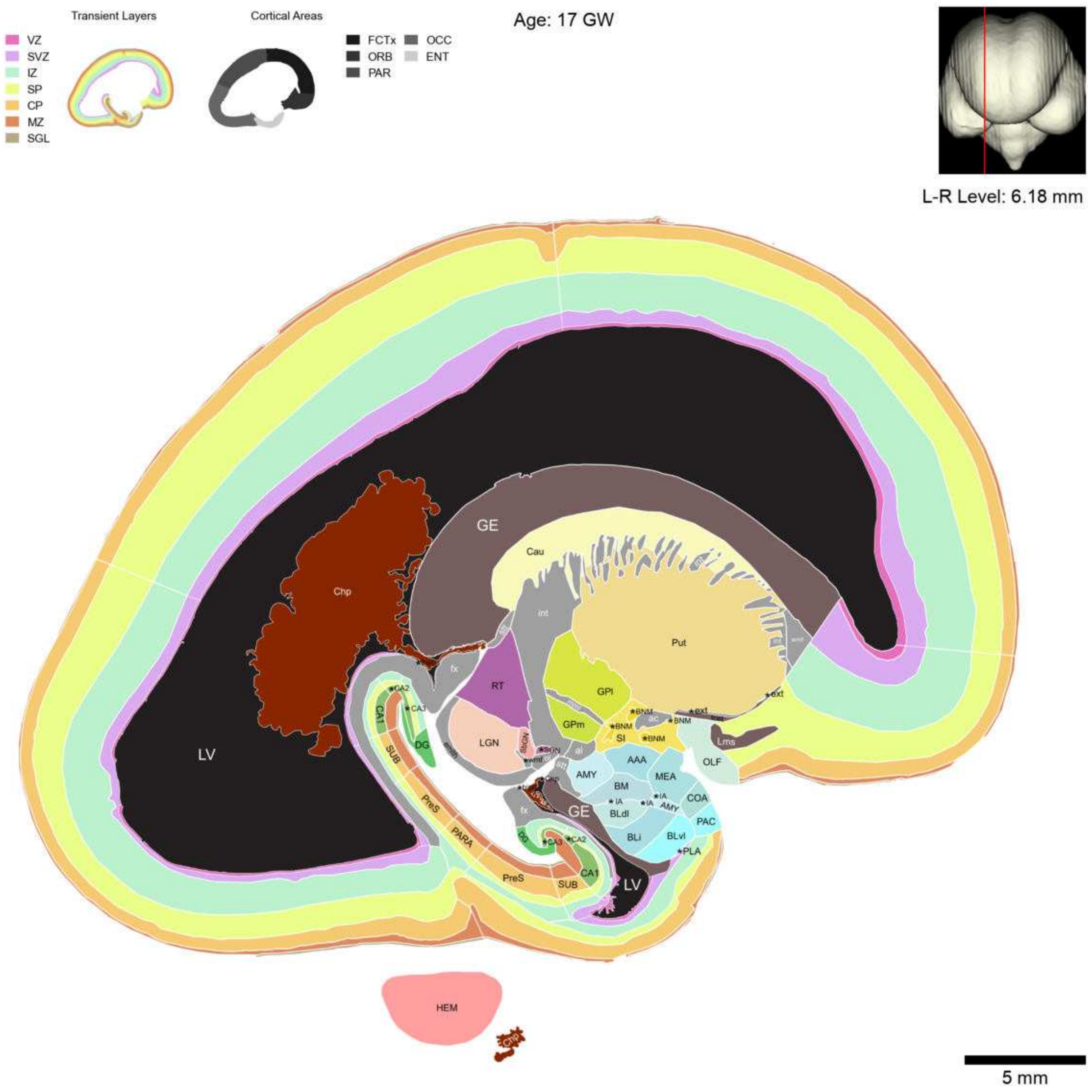

- AAA: Anterior amygdaloid area
- AMY: Amygdala
- BLdl: Basal nucleus [amygdala], dorsolateral part
- BLi: Basal nucleus [amygdala], intermediate part
- BLvl: Basal nucleus [amygdala], ventrolateral part
- BM: Basomedial nucleus [amygdala]
- BNM: Basal nucleus of Meynert
- CA1: CA1 field [hippocampus]
- CA2: CA2 field [hippocampus]
- CA3: CA3 field [hippocampus]
- COA: Cortical nucleus [amygdala]
- Cau: Caudate nucleus
- Chp: Choroid plexus
- DG: Dentate gyrus
- GE: Ganglionic eminence
- GPl: Globus pallidus lateral segment
- GPm: Globus pallidus medial segment
- HEM: Cerebellar hemispheres
- IA: Intercalated cell groups [amygdala]
- LGN: Lateral geniculate nucleus
- LV: Lateral ventricle
- Lms: Lateral migratory stream
- MEA: Medial nucleus [amygdala]
- PARA: Cortical plate, parasubiculum
- PLA: Paralaminar nucleus [amygdala]
- PreS: Cortical plate, presubiculum
- Put: Putamen
- RT: Reticular nucleus [thalamus]
- SI: Substantia innominata
- SON: Supraoptic nucleus [hypothalamus]
- SUB: Cortical plate, subiculum
- SbGN: Subgeniculate nucleus
- ac: Anterior commissure
- al: Ansa lenticularis
- emlth: External medullary lamina [thalamus]
- ext: External capsule
- fx: Fornix
- hipg: Hippocampal glioepithelium/ependyma
- int: Internal capsule
- mml: Medial medullary lamina
- ot: Optic tract
- stt: Stria terminalis
- tcet: Transient cell zone in the external capsule
- wmf: White matter fibers

Age: 17 GW

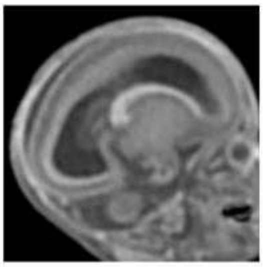

L-R Level: 5.82 mm

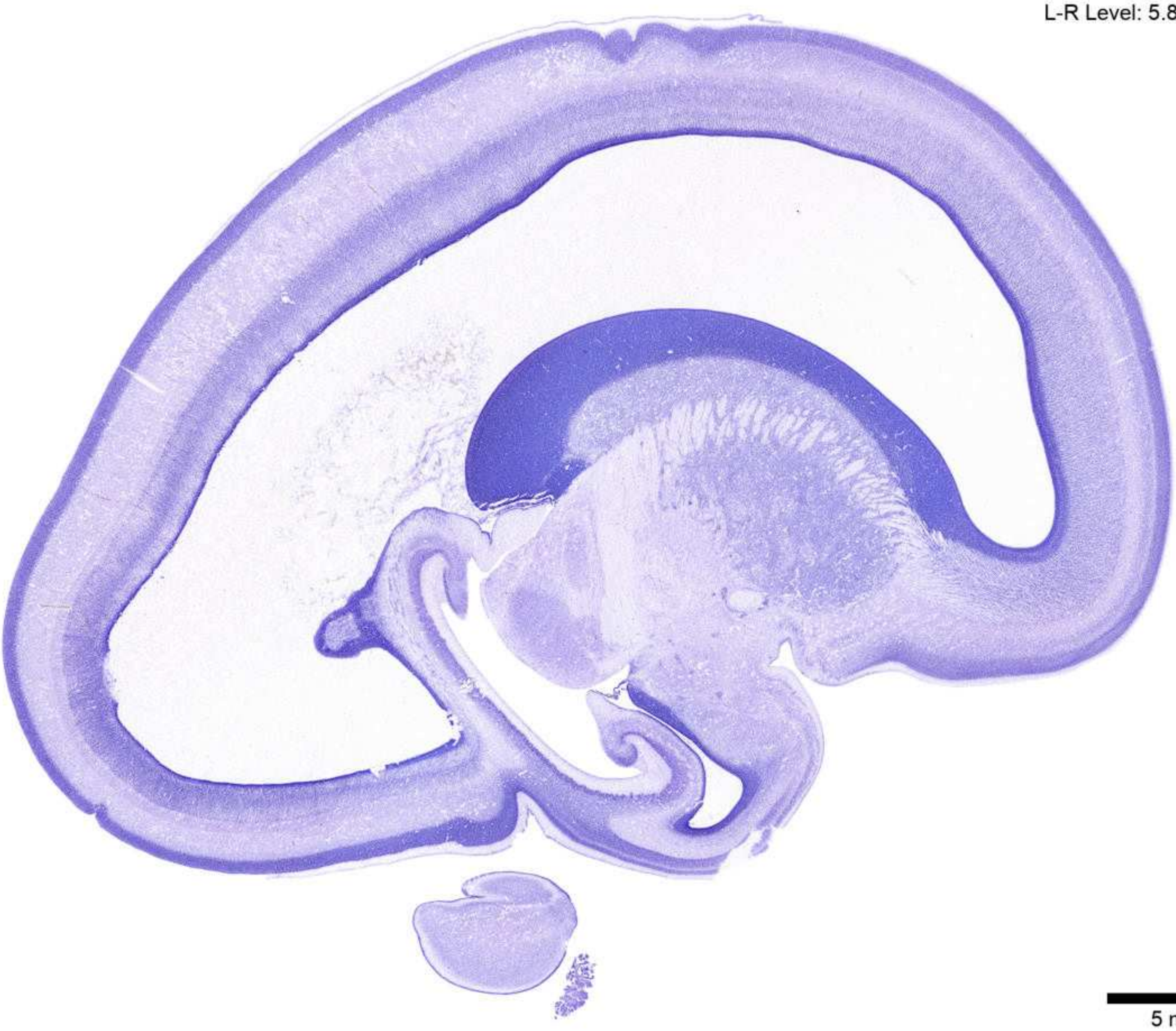

5 mm

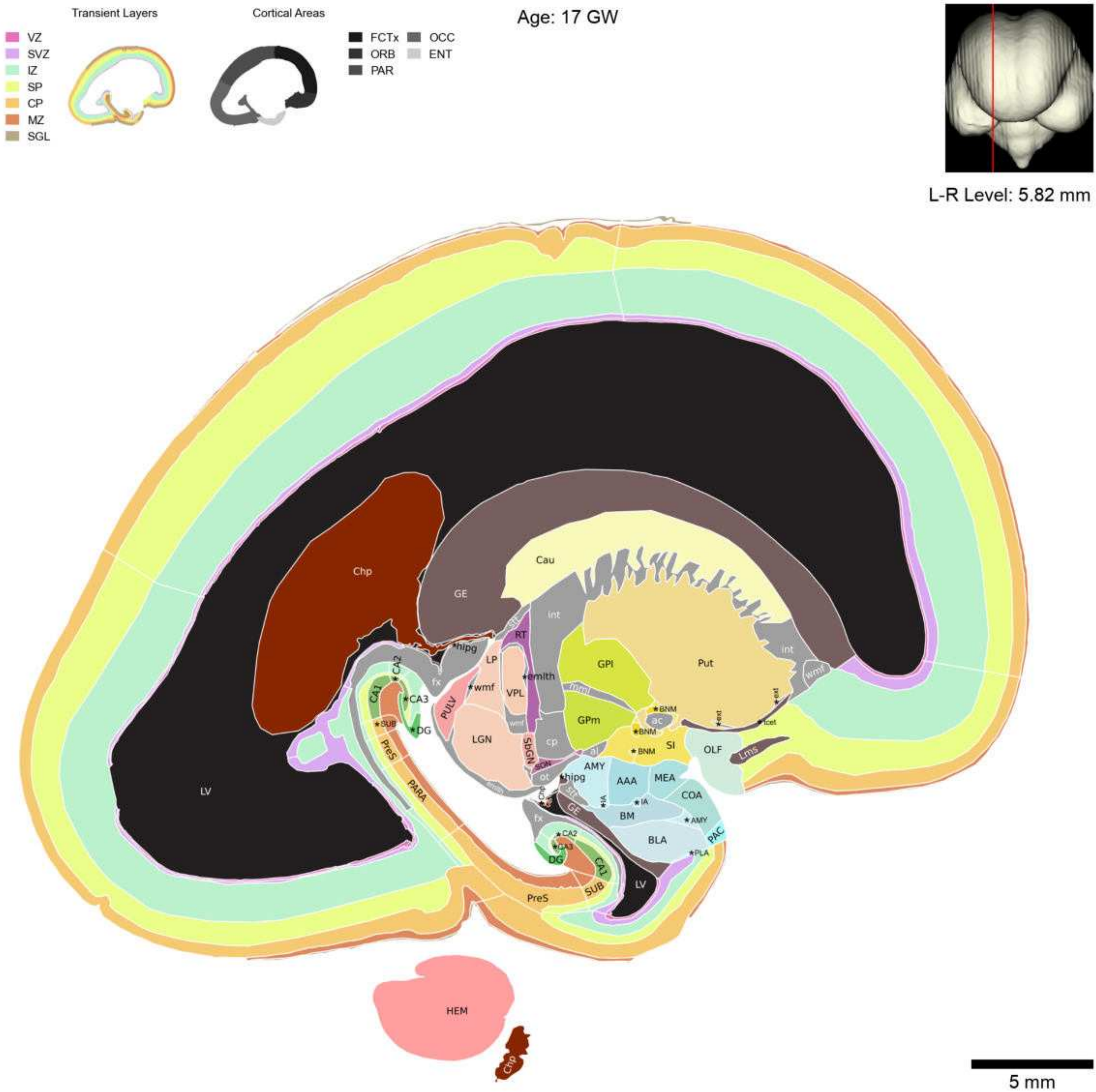

- AAA: Anterior amygdaloid area
- AMY: Amygdala
- BL: Basal nucleus [amygdala]
- BM: Basomedial nucleus [amygdala]
- BNM: Basal nucleus of Meynert
- CA1: CA1 field [hippocampus]
- CA2: CA2 field [hippocampus]
- CA3: CA3 field [hippocampus]
- COA: Cortical nucleus [amygdala]
- Cau: Caudate nucleus
- Chp: Choroid plexus
- DG: Dentate gyrus
- GE: Ganglionic eminence
- GPl: Globus pallidus lateral segment
- GPm: Globus pallidus medial segment
- HEM: Cerebellar hemispheres
- IA: Intercalated cell groups [amygdala]
- LGN: Lateral geniculate nucleus
- LP: Lateral posterior nucleus [thalamus]
- LV: Lateral ventricle
- Lms: Lateral migratory stream
- MEA: Medial nucleus [amygdala]
- PARA: Cortical plate, parasubiculum
- PLA: Paralaminar nucleus [amygdala]
- PULV: Pulvinar nucleus [thalamus]
- PreS: Cortical plate, presubiculum
- Put: Putamen
- RT: Reticular nucleus [thalamus]
- SI: Substantia innominata
- SON: Supraoptic nucleus [hypothalamus]
- SUB: Cortical plate, subiculum
- SbGN: Subgeniculate nucleus
- VPL: Ventral posterolateral nucleus [thalamus]
- ac: Anterior commissure
- al: Ansa lenticularis
- cp: Cerebral peduncle
- emlth: External medullary lamina [thalamus]
- ext: External capsule
- fx: Fornix
- hipg: Hippocampal glioepithelium/ependyma
- int: Internal capsule
- mml: Medial medullary lamina
- ot: Optic tract
- stt: Stria terminalis
- tcet: Transient cell zone in the external capsule
- wmf: White matter fibers

Age: 17 GW

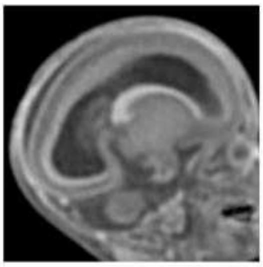

L-R Level: 5.58 mm

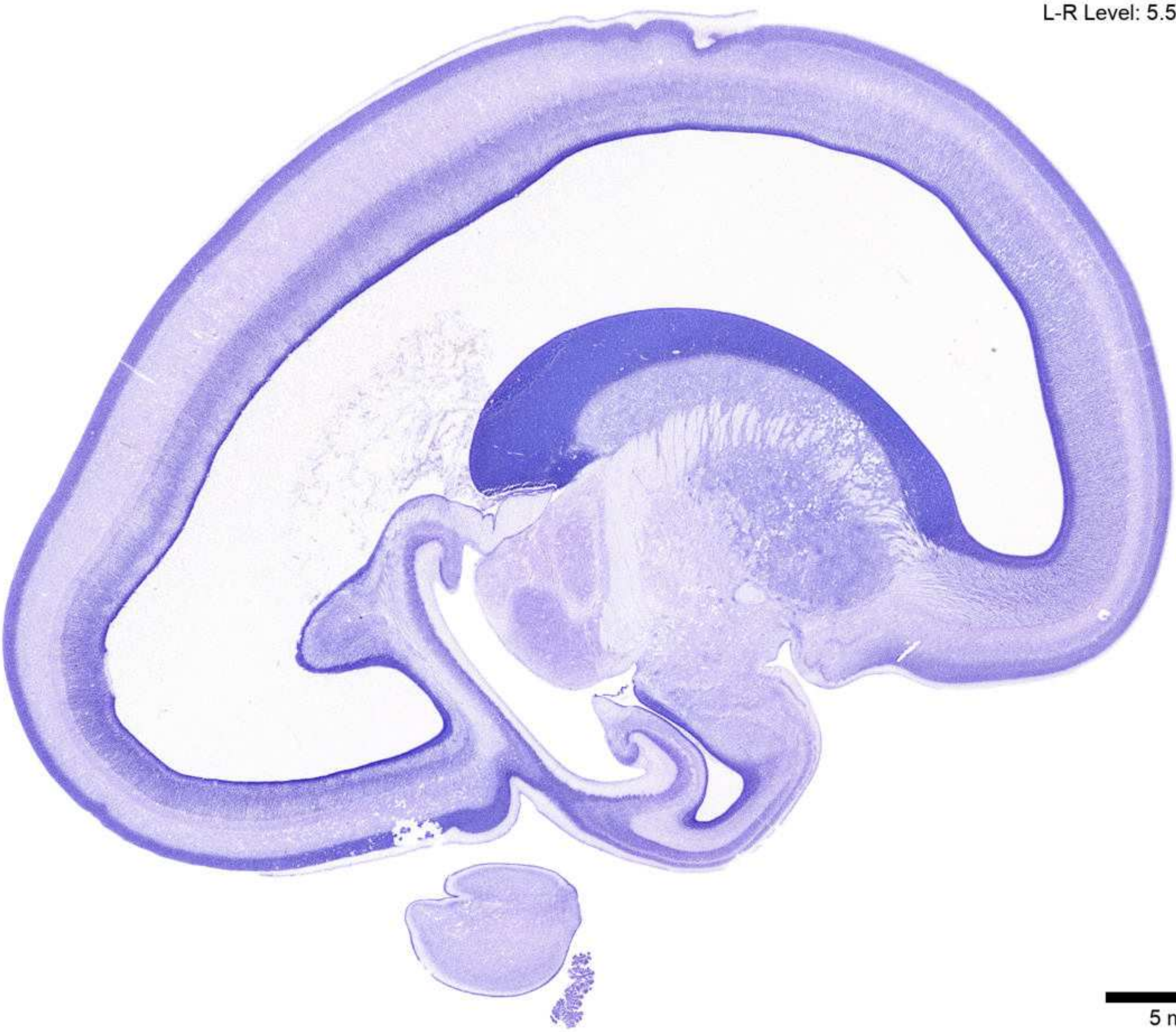

5 mm

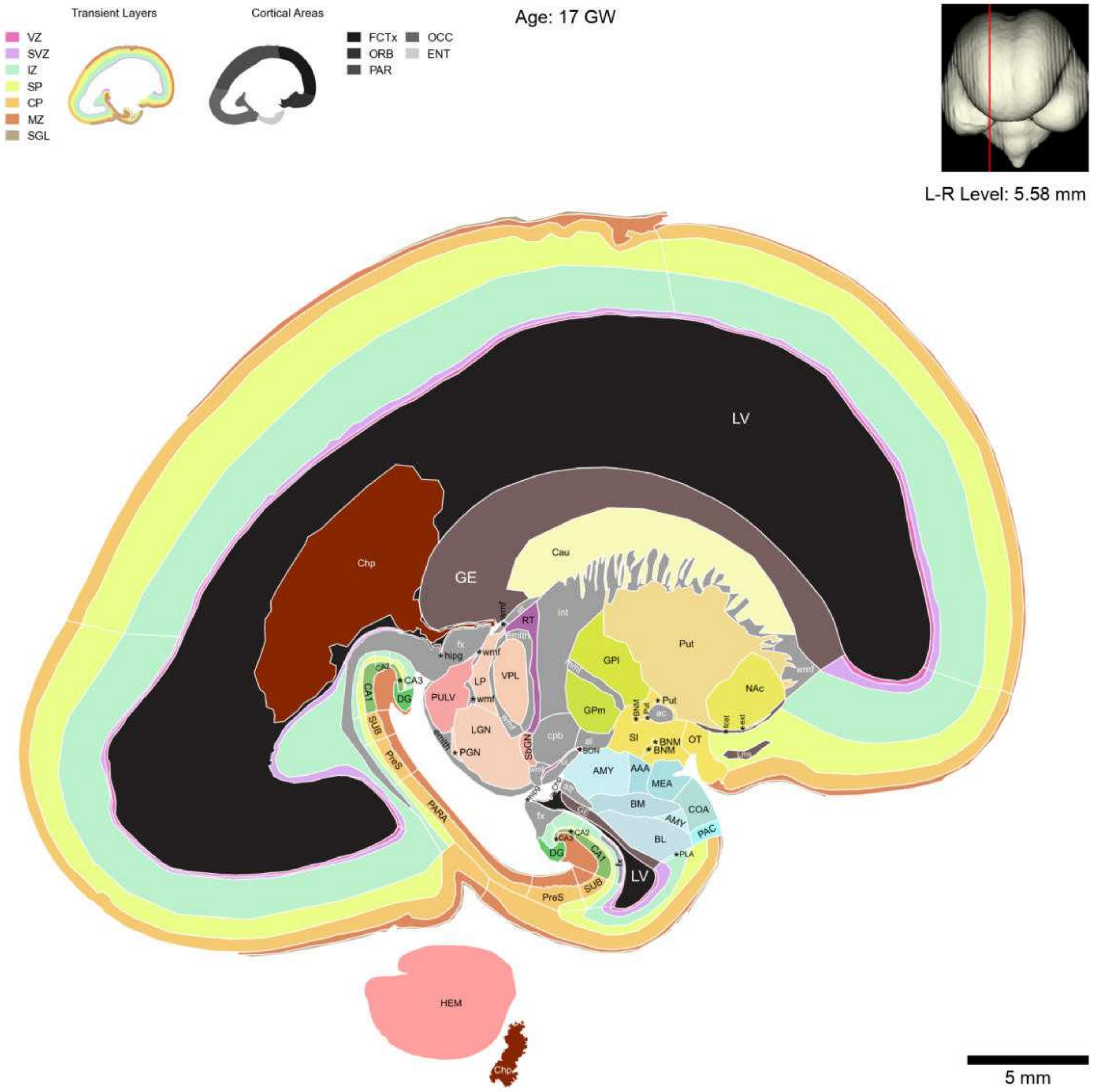

- AAA: Anterior amygdaloid area
- AMY: Amygdala
- BL: Basal nucleus [amygdala]
- BM: Basomedial nucleus [amygdala]
- BNM: Basal nucleus of Meynert
- CA1: CA1 field [hippocampus]
- CA2: CA2 field [hippocampus]
- CA3: CA3 field [hippocampus]
- COA: Cortical nucleus [amygdala]
- Cau: Caudate nucleus
- Chp: Choroid plexus
- DG: Dentate gyrus
- GE: Ganglionic eminence
- GPl: Globus pallidus lateral segment
- GPm: Globus pallidus medial segment
- HEM: Cerebellar hemispheres
- LGN: Lateral geniculate nucleus
- LP: Lateral posterior nucleus [thalamus]
- LV: Lateral ventricle
- Lms: Lateral migratory stream
- MEA: Medial nucleus [amygdala]
- NAc: Nucleus accumbens
- OT: Olfactory tubercle
- PARA: Cortical plate, parasubiculum
- PGN: Pregeniculate nucleus
- PLA: Paralaminar nucleus [amygdala]
- PULV: Pulvinar nucleus [thalamus]
- PreS: Cortical plate, presubiculum
- Put: Putamen
- RT: Reticular nucleus [thalamus]
- SI: Substantia innominata
- SON: Supraoptic nucleus [hypothalamus]
- SUB: Cortical plate, subiculum
- SbGN: Subgeniculate nucleus
- VPL: Ventral posterolateral nucleus [thalamus]
- ac: Anterior commissure
- al: Ansa lenticularis
- cpb: Cerebellar peduncles
- emlth: External medullary lamina [thalamus]
- ext: External capsule
- fx: Fornix
- hipg: Hippocampal glioepithelium/ependyma
- int: Internal capsule
- mml: Medial medullary lamina
- ot: Optic tract
- stt: Stria terminalis
- tcet: Transient cell zone in the external capsule
- wmf: White matter fibers

Age: 17 GW

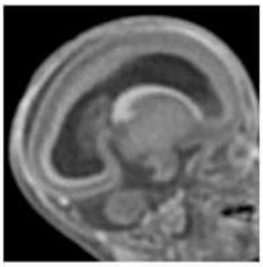

L-R Level: 5.34 mm

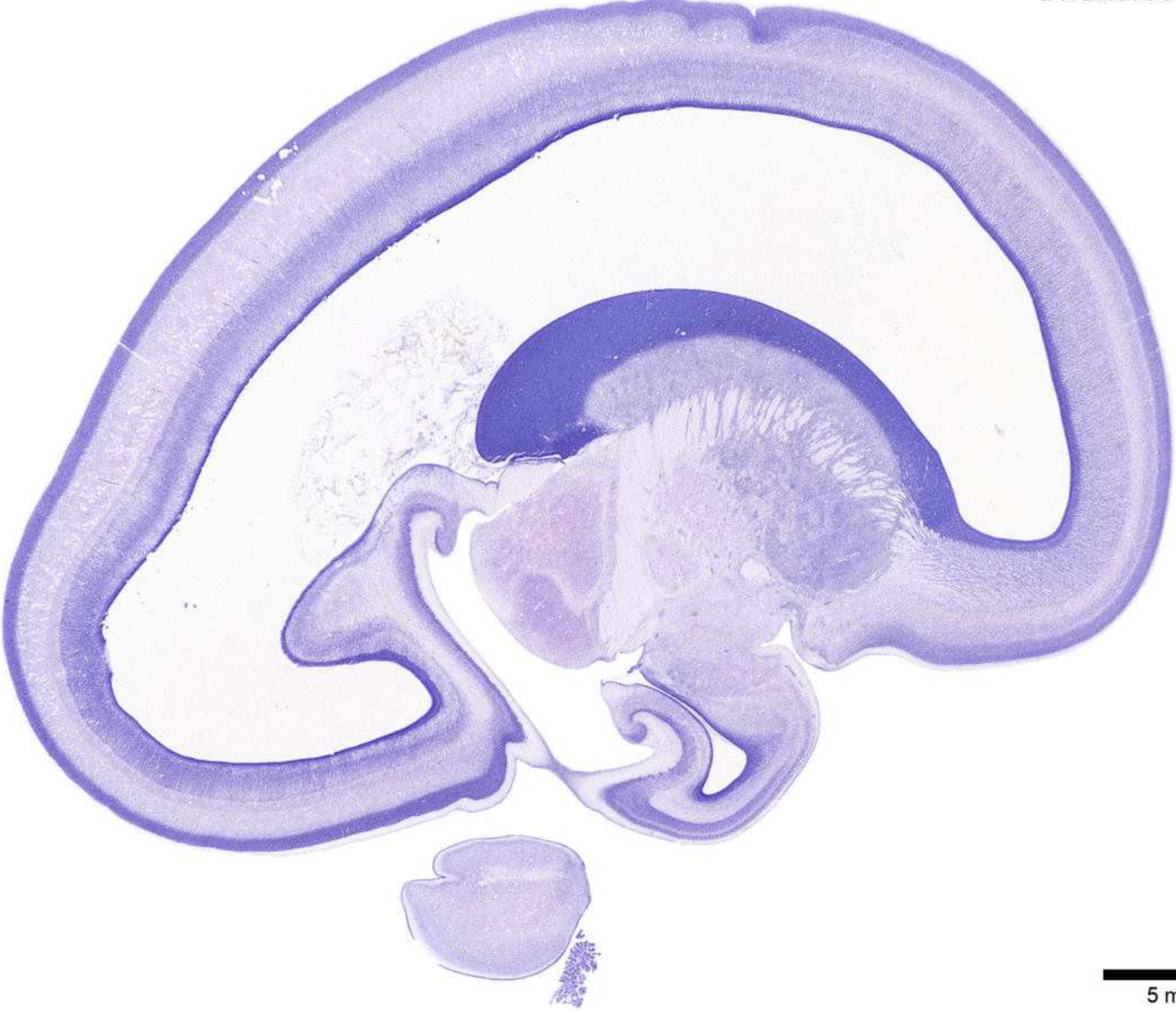

5 mm

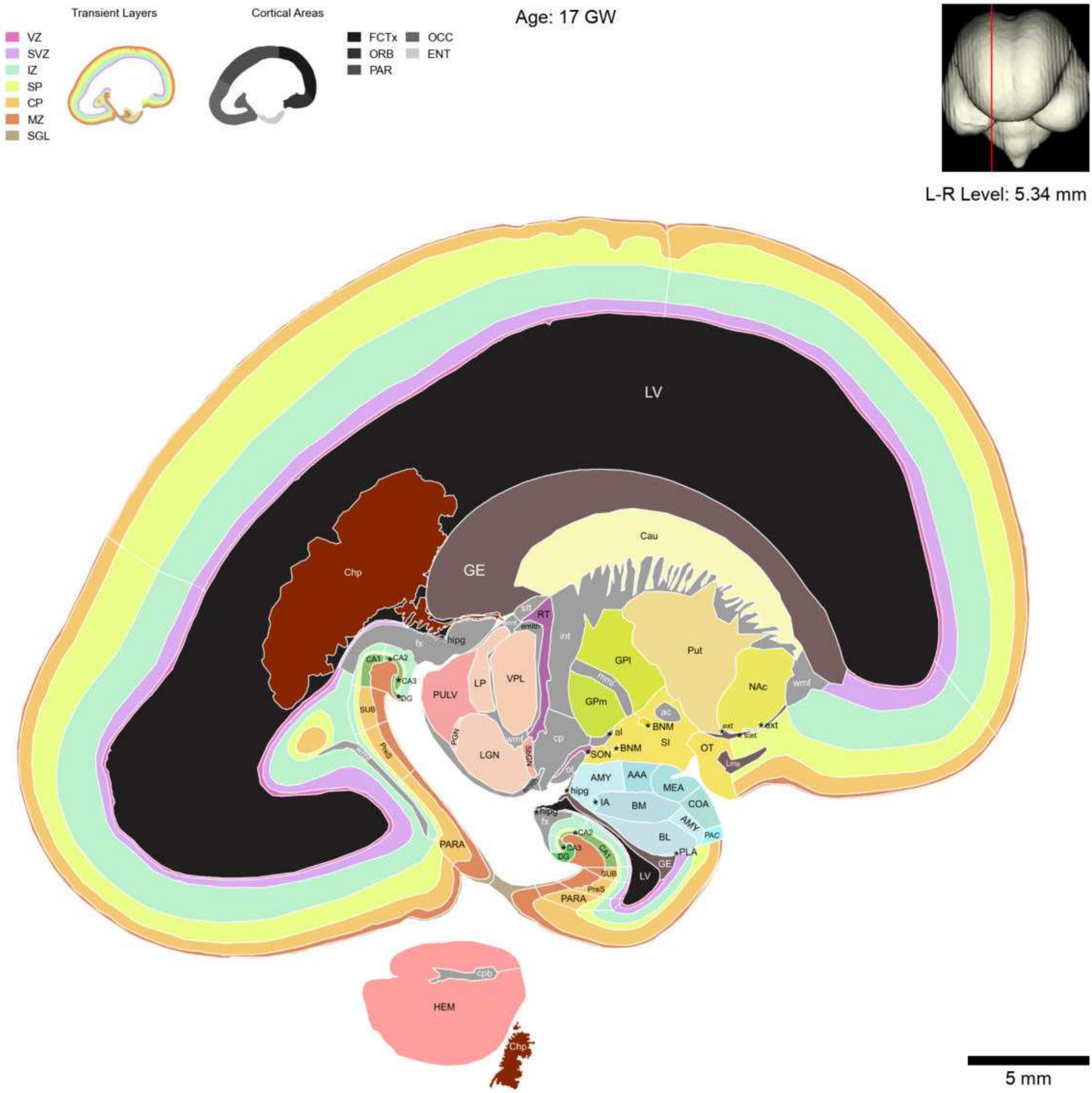

- AAA: Anterior amygdaloid area
- AMY: Amygdala
- BL: Basal nucleus [amygdala]
- BM: Basomedial nucleus [amygdala]
- BNM: Basal nucleus of Meynert
- CA1: CA1 field [hippocampus]
- CA2: CA2 field [hippocampus]
- CA3: CA3 field [hippocampus]
- COA: Cortical nucleus [amygdala]
- Cau: Caudate nucleus
- Chp: Choroid plexus
- DG: Dentate gyrus
- GE: Ganglionic eminence
- GPl: Globus pallidus lateral segment
- GPm: Globus pallidus medial segment
- HEM: Cerebellar hemispheres
- IA: Intercalated cell groups [amygdala]
- LGN: Lateral geniculate nucleus
- LP: Lateral posterior nucleus [thalamus]
- LV: Lateral ventricle
- Lms: Lateral migratory stream
- MEA: Medial nucleus [amygdala]
- NAc: Nucleus accumbens
- OT: Olfactory tubercle
- PARA: Cortical plate, parasubiculum
- PGN: Pregeniculate nucleus
- PLA: Paralaminar nucleus [amygdala]
- PULV: Pulvinar nucleus [thalamus]
- PreS: Cortical plate, presubiculum
- Put: Putamen
- RT: Reticular nucleus [thalamus]
- SI: Substantia innominata
- SON: Supraoptic nucleus [hypothalamus]
- SUB: Cortical plate, subiculum
- SbGN: Subgeniculate nucleus
- VPL: Ventral posterolateral nucleus [thalamus]
- ac: Anterior commissure
- al: Ansa lenticularis
- cp: Cerebral peduncle
- cpb: Cerebellar peduncles
- emlth: External medullary lamina [thalamus]
- ext: External capsule
- fx: Fornix
- hipg: Hippocampal glioepithelium/ependyma
- int: Internal capsule
- mml: Medial medullary lamina
- ot: Optic tract
- stt: Stria terminalis
- tcet: Transient cell zone in the external capsule
- wmf: White matter fibers

Age: 17 GW

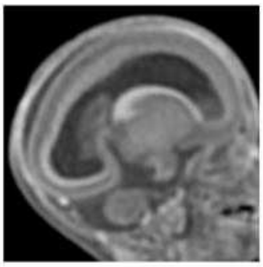

L-R Level: 5.16 mm

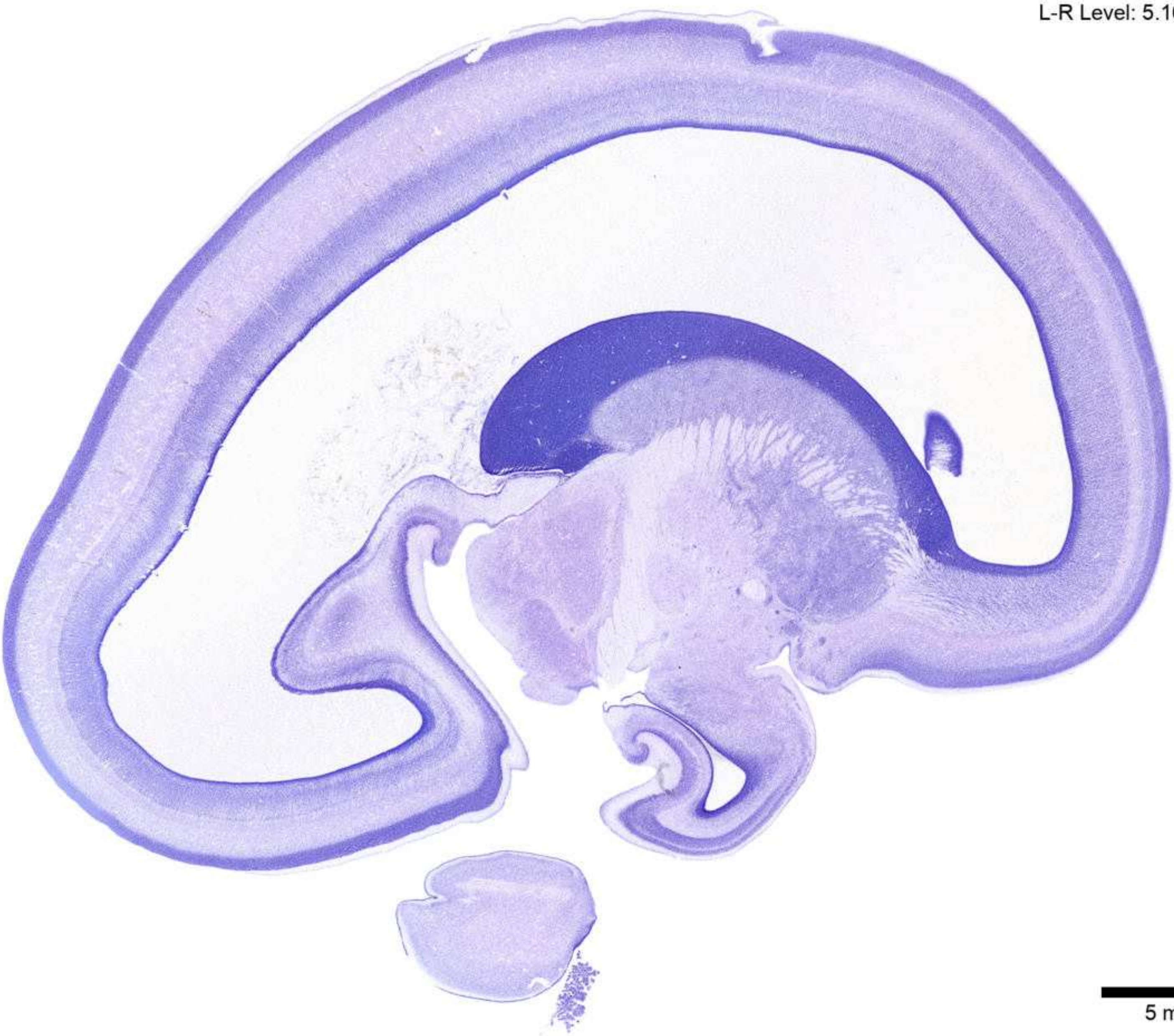

5 mm

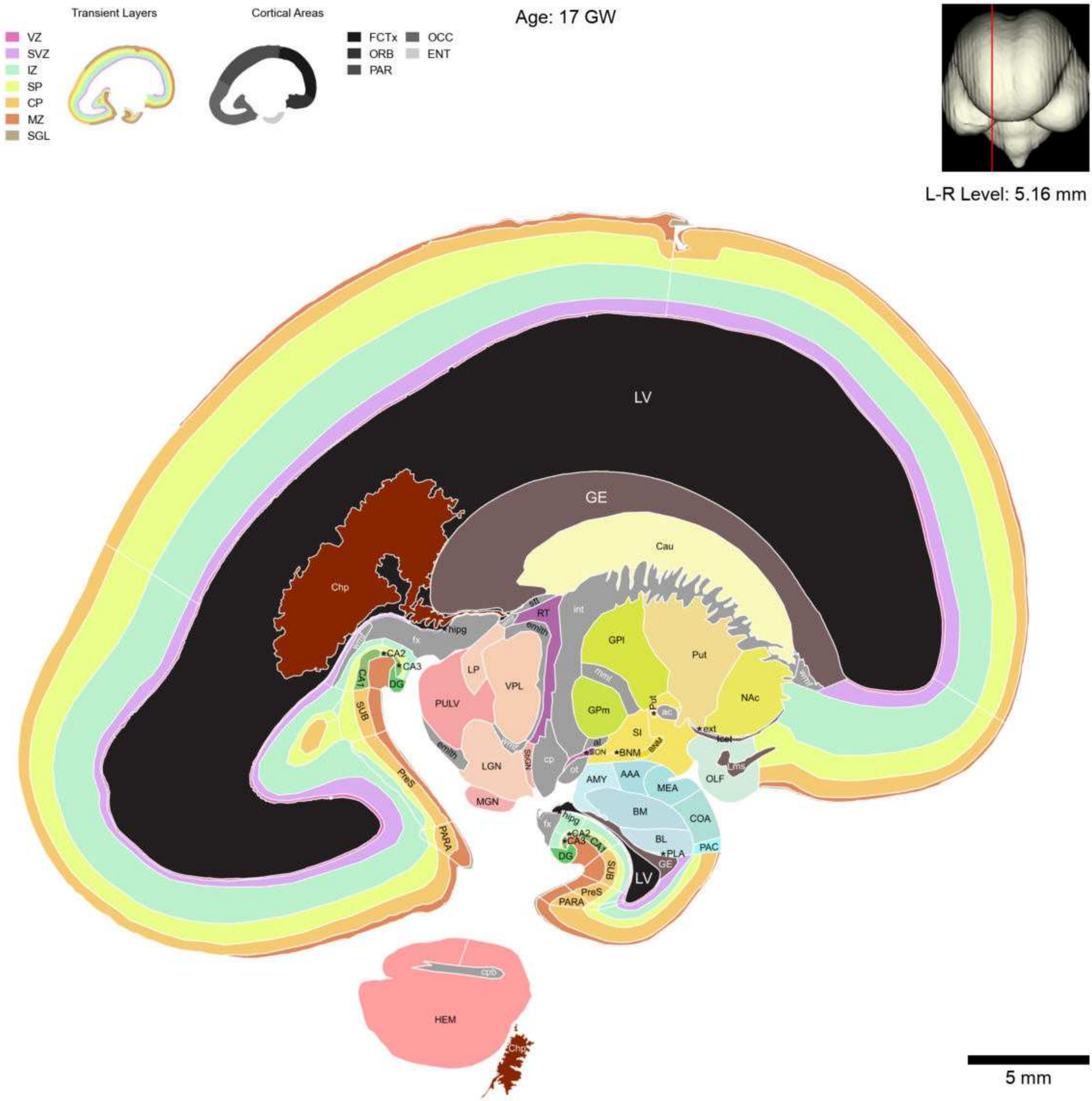

AAA: Anterior amygdaloid area
AMY: Amygdala
BL: Basal nucleus [amygdala]
BM: Basomedial nucleus [amygdala]
BNM: Basal nucleus of Meynert
CA1: CA1 field [hippocampus]
CA2: CA2 field [hippocampus]
CA3: CA3 field [hippocampus]
COA: Cortical nucleus [amygdala]
Cau: Caudate nucleus
Chp: Choroid plexus
DG: Dentate gyrus
GE: Ganglionic eminence
GPl: Globus pallidus lateral segment
GPm: Globus pallidus medial segment
HEM: Cerebellar hemispheres
LGN: Lateral geniculate nucleus
LP: Lateral posterior nucleus [thalamus]
LV: Lateral ventricle
Lms: Lateral migratory stream
MEA: Medial nucleus [amygdala]
MGN: Medial geniculate nucleus
NAc: Nucleus accumbens
PARA: Cortical plate, parasubiculum
PLA: Paralaminar nucleus [amygdala]
PULV: Pulvinar nucleus [thalamus]
PreS: Cortical plate, presubiculum
Put: Putamen
RT: Reticular nucleus [thalamus]
SI: Substantia innominata
SON: Supraoptic nucleus [hypothalamus]
SUB: Cortical plate, subiculum
SbGN: Subgeniculate nucleus
VPL: Ventral posterolateral nucleus [thalamus]
ac: Anterior commissure
al: Ansa lenticularis
cp: Cerebral peduncle
cpb: Cerebellar peduncles
emlth: External medullary lamina [thalamus]
ext: External capsule
fx: Fornix
hipg: Hippocampal glioepithelium/ependyma
int: Internal capsule
mml: Medial medullary lamina
ot: Optic tract
stt: Stria terminalis
tcet: Transient cell zone in the external capsule
wmf: White matter fibers

Age: 17 GW

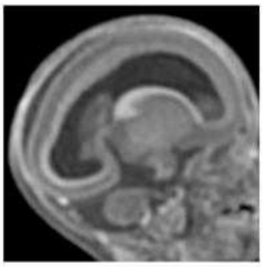

L-R Level: 4.92 mm

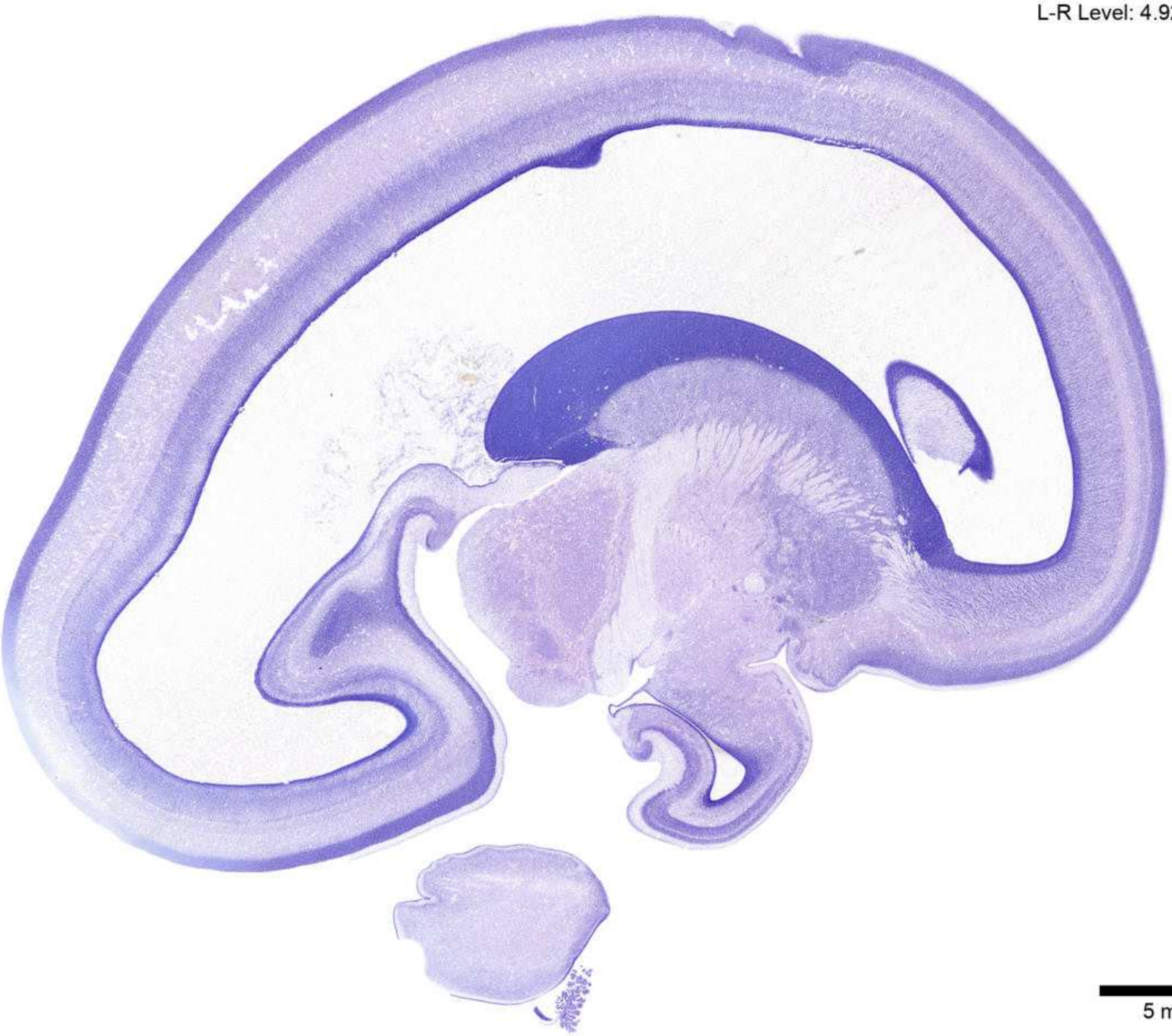

5 mm

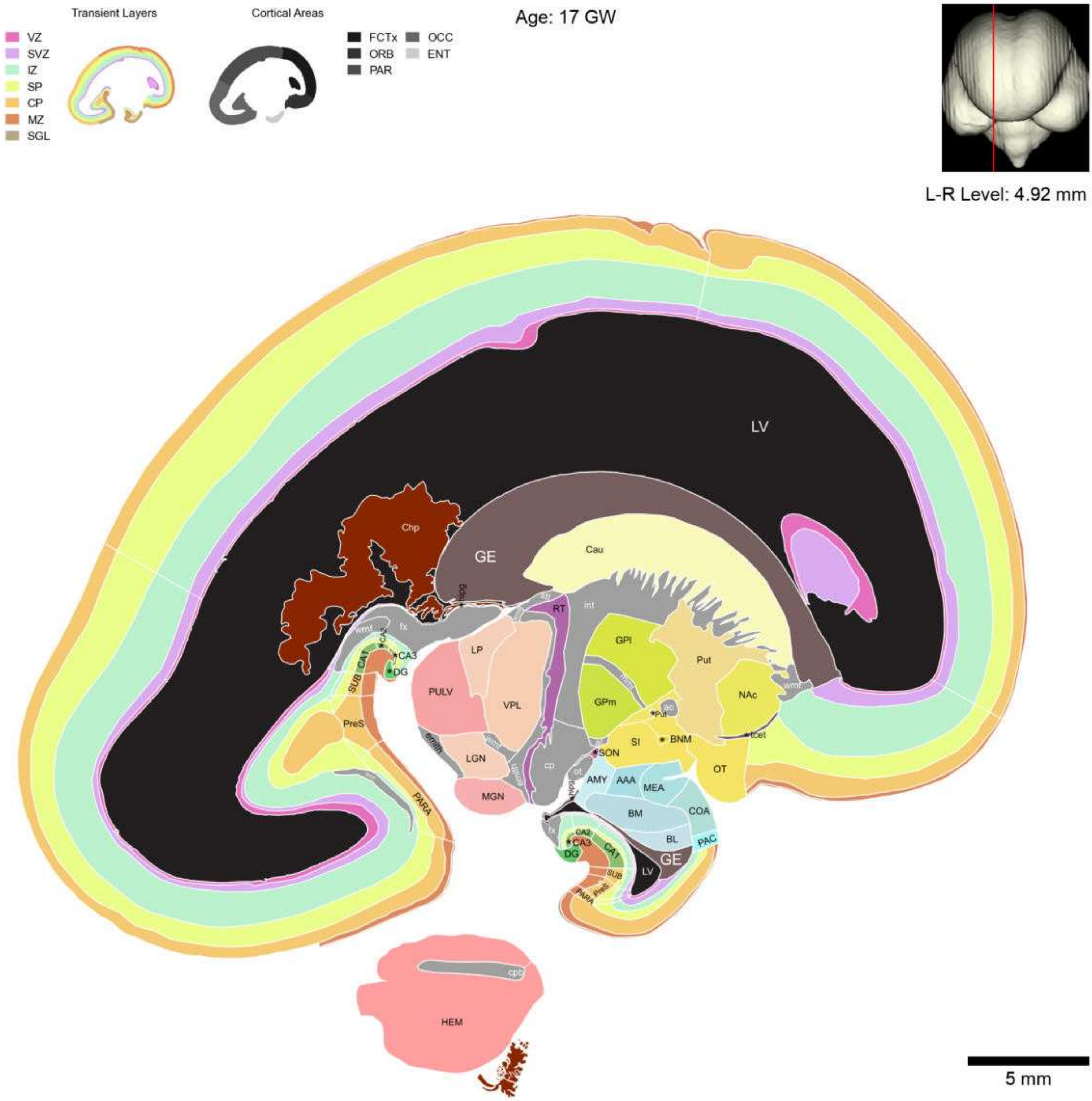

AAA: Anterior amygdaloid area
AMY: Amygdala
BL: Basal nucleus [amygdala]
BM: Basomedial nucleus [amygdala]
BNM: Basal nucleus of Meynert
CA1: CA1 field [hippocampus]
CA2: CA2 field [hippocampus]
CA3: CA3 field [hippocampus]
COA: Cortical nucleus [amygdala]
Cau: Caudate nucleus
Chp: Choroid plexus
DG: Dentate gyrus
GE: Ganglionic eminence
GPl: Globus pallidus lateral segment
GPm: Globus pallidus medial segment
HEM: Cerebellar hemispheres
LGN: Lateral geniculate nucleus
LP: Lateral posterior nucleus [thalamus]
LV: Lateral ventricle
MEA: Medial nucleus [amygdala]
MGN: Medial geniculate nucleus
NAc: Nucleus accumbens
OT: Olfactory tubercle
PARA: Cortical plate, parasubiculum
PULV: Pulvinar nucleus [thalamus]
PreS: Cortical plate, presubiculum
Put: Putamen
RT: Reticular nucleus [thalamus]
SI: Substantia innominata
SON: Supraoptic nucleus [hypothalamus]
SUB: Cortical plate, subiculum
VPL: Ventral posterolateral nucleus [thalamus]
ac: Anterior commissure
al: Ansa lenticularis
cp: Cerebral peduncle
cpb: Cerebellar peduncles
emlth: External medullary lamina [thalamus]
fx: Fornix
hipg: Hippocampal glioepithelium/ependyma
int: Internal capsule
mml: Medial medullary lamina
ot: Optic tract
stt: Stria terminalis
tcet: Transient cell zone in the external capsule
wmf: White matter fibers

Age: 17 GW

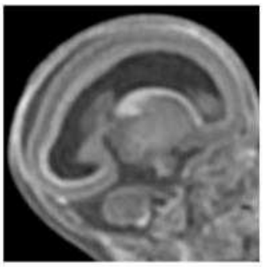

L-R Level: 4.44 mm

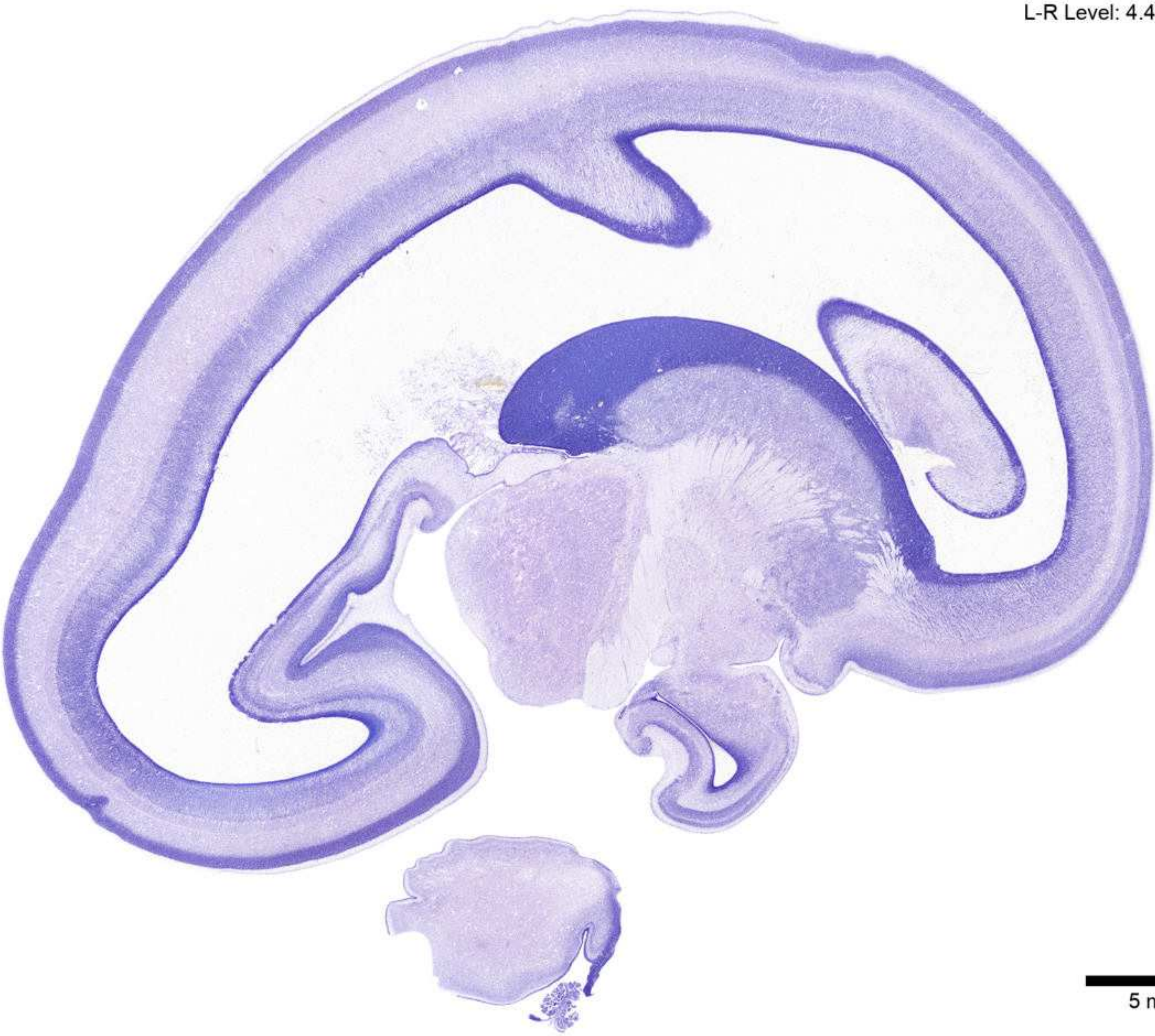

5 mm

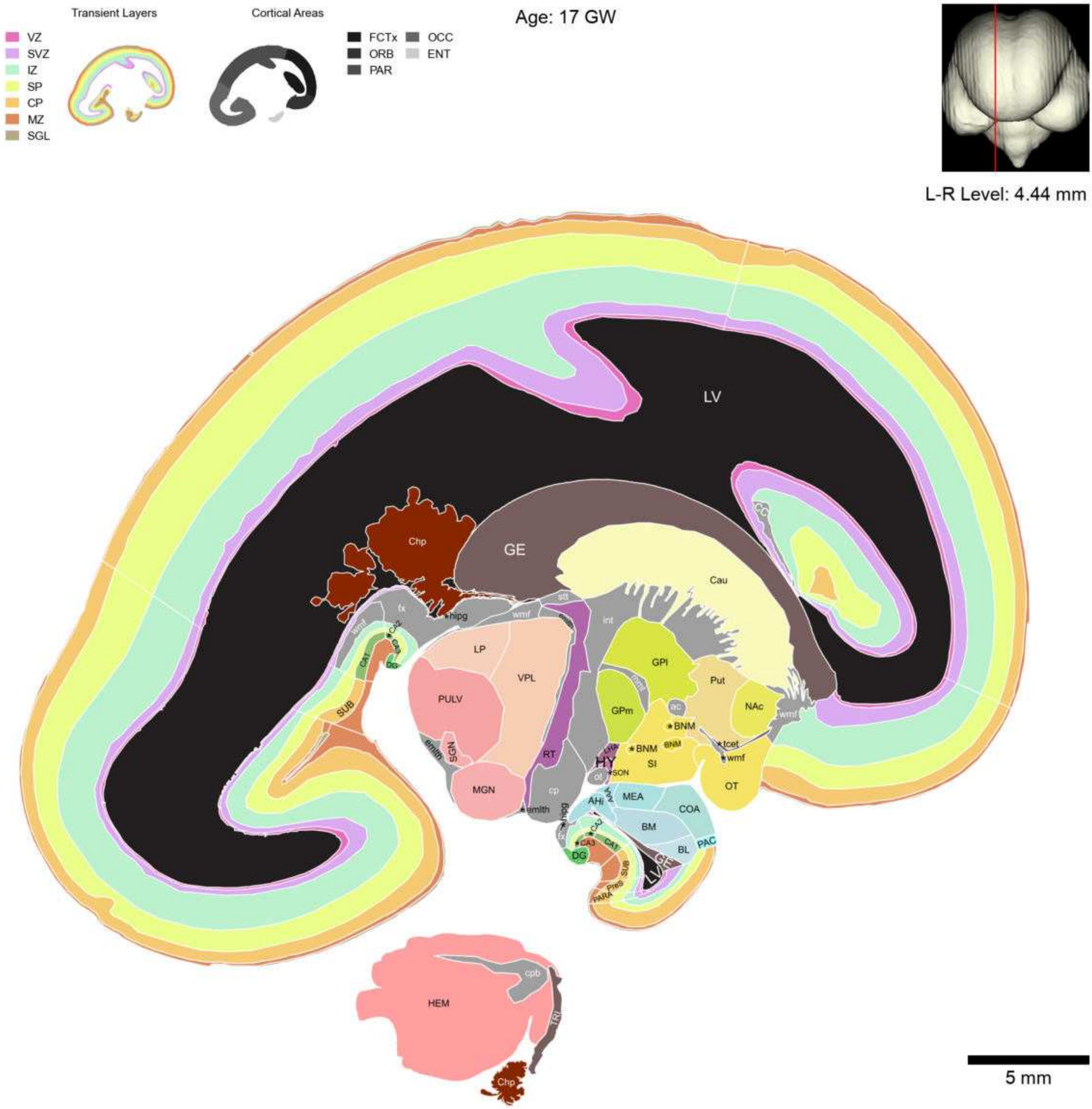

- AAA: Anterior amygdaloid area
- AHi: Amygdalo-hippocampal area
- BL: Basal nucleus [amygdala]
- BM: Basomedial nucleus [amygdala]
- BNM: Basal nucleus of Meynert
- CA1: CA1 field [hippocampus]
- CA2: CA2 field [hippocampus]
- CA3: CA3 field [hippocampus]
- COA: Cortical nucleus [amygdala]
- Cau: Caudate nucleus
- Chp: Choroid plexus
- DG: Dentate gyrus
- GE: Ganglionic eminence
- GPl: Globus pallidus lateral segment
- GPm: Globus pallidus medial segment
- HEM: Cerebellar hemispheres
- HY: Hypothalamus
- LHA: Lateral hypothalamic area
- LP: Lateral posterior nucleus [thalamus]
- LV: Lateral ventricle
- MEA: Medial nucleus [amygdala]
- MGN: Medial geniculate nucleus
- NAc: Nucleus accumbens
- OT: Olfactory tubercle
- PARA: Cortical plate, parasubiculum
- PULV: Pulvinar nucleus [thalamus]
- PreS: Cortical plate, presubiculum
- Put: Putamen
- RT: Reticular nucleus [thalamus]
- SGN: Suprageniculate nucleus
- SI: Substantia innominata
- SON: Supraoptic nucleus [hypothalamus]
- SUB: Cortical plate, subiculum
- TRI: Germinal trigone
- VPL: Ventral posterolateral nucleus [thalamus]
- ac: Anterior commissure
- cc: Corpus callosum
- cp: Cerebral peduncle
- cpb: Cerebellar peduncles
- emlth: External medullary lamina [thalamus]
- fx: Fornix
- hipg: Hippocampal glioepithelium/ependyma
- int: Internal capsule
- mml: Medial medullary lamina
- ot: Optic tract
- stt: Stria terminalis
- tcet: Transient cell zone in the external capsule
- wmf: White matter fibers

Age: 17 GW

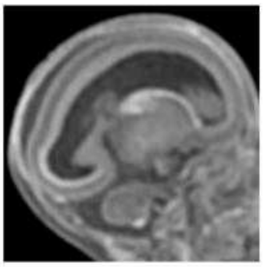

L-R Level: 4.02 mm

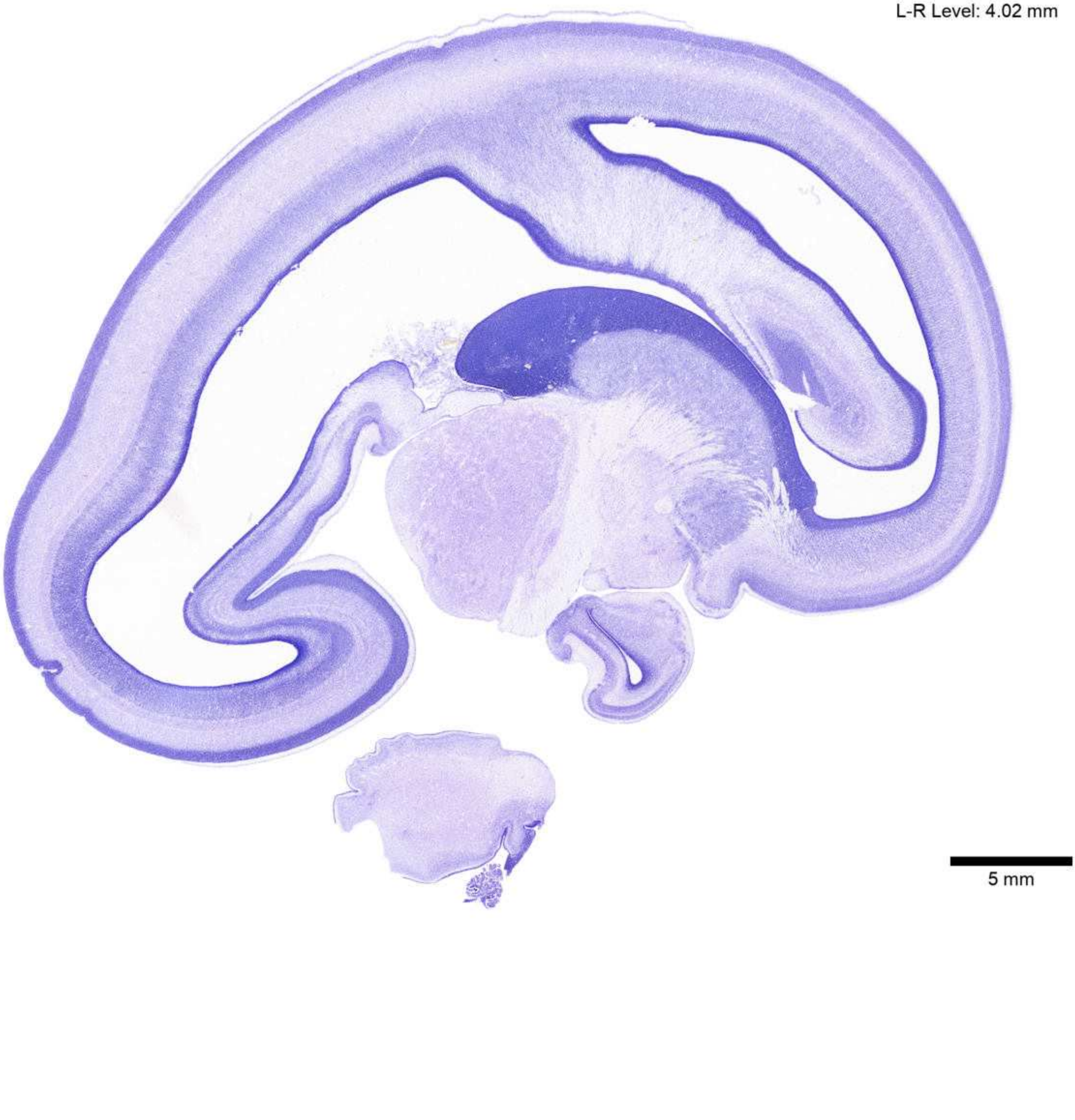

5 mm

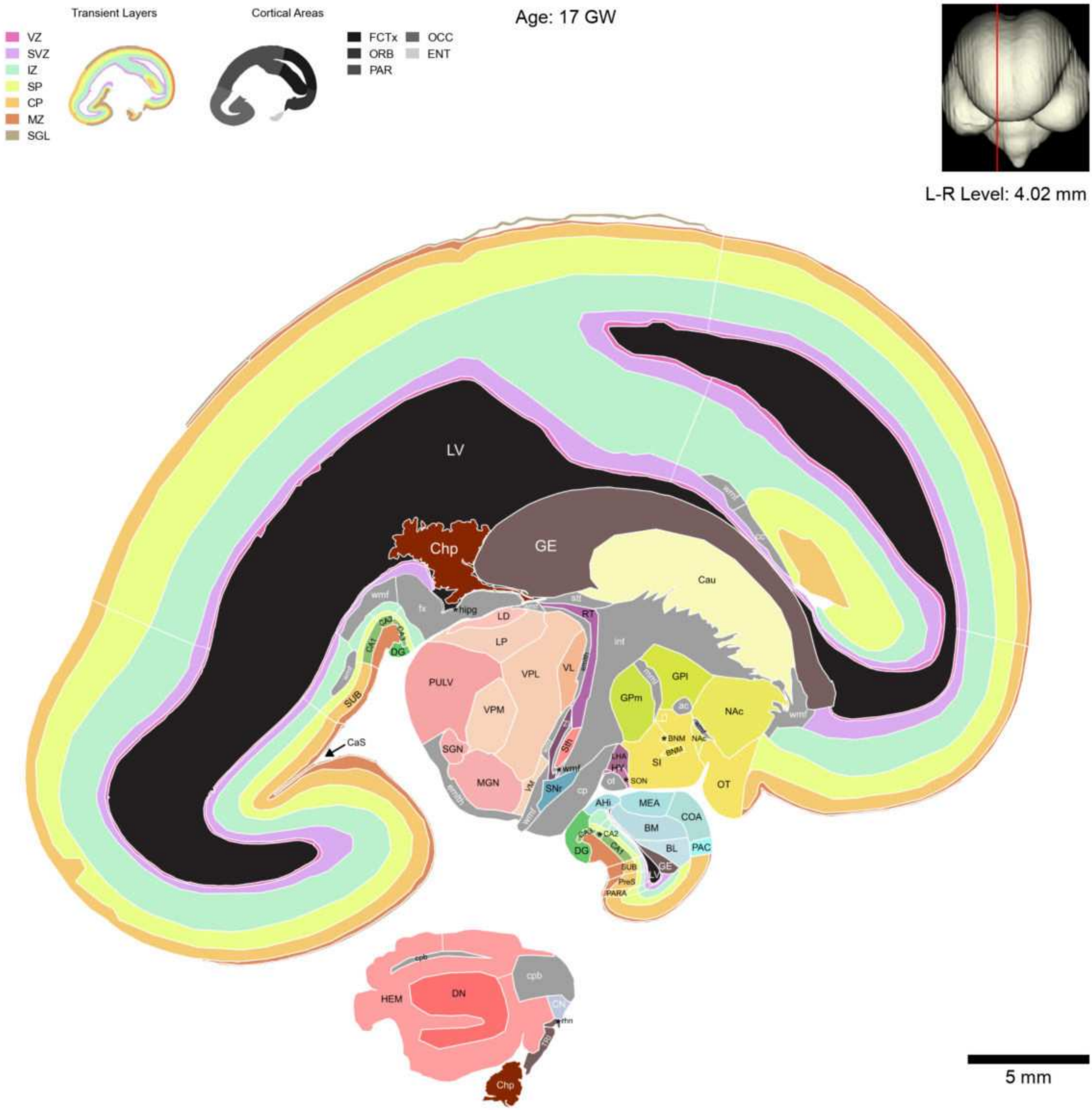

AHi: Amygdalo-hippocampal area
BL: Basal nucleus [amygdala]
BM: Basomedial nucleus [amygdala]
BNM: Basal nucleus of Meynert
CA1: CA1 field [hippocampus]
CA2: CA2 field [hippocampus]
CA3: CA3 field [hippocampus]
CN: Cochlear nuclei
COA: Cortical nucleus [amygdala]
Cau: Caudate nucleus
Chp: Choroid plexus
DG: Dentate gyrus
DN: Dentate nucleus
GE: Ganglionic eminence
GPl: Globus pallidus lateral segment
GPm: Globus pallidus medial segment
HEM: Cerebellar hemispheres
HY: Hypothalamus
LD: Lateral dorsal nucleus [thalamus]
LHA: Lateral hypothalamic area
LP: Lateral posterior nucleus [thalamus]
LV: Lateral ventricle
MEA: Medial nucleus [amygdala]
MGN: Medial geniculate nucleus
NAc: Nucleus accumbens
OT: Olfactory tubercle
PARA: Cortical plate, parasubiculum
PULV: Pulvinar nucleus [thalamus]
PreS: Cortical plate, presubiculum
RT: Reticular nucleus [thalamus]
SGN: Suprageniculate nucleus
SI: Substantia innominata
SNr: Substantia nigra pars reticulata
SON: Supraoptic nucleus [hypothalamus]
SUB: Cortical plate, subiculum
Sth: Subthalamus
TRI: Germinal trigone
VL: Ventral lateral nucleus [thalamus]
VM: Ventral medial nucleus [thalamus]
VPL: Ventral posterolateral nucleus [thalamus]
VPM: Ventral posteromedial nucleus [thalamus]
ZI: Zona incerta
ac: Anterior commissure
cc: Corpus callosum
cp: Cerebral peduncle
cpb: Cerebellar peduncles
emlth: External medullary lamina [thalamus]
fx: Fornix
hipg: Hippocampal glioepithelium/ependyma
int: Internal capsule
mml: Medial medullary lamina
ot: Optic tract
rhn: Rhombencephalic neuroepithelium
stt: Stria terminalis
wmf: White matter fibers
➔ CaS: Calcarine sulcus

Age: 17 GW

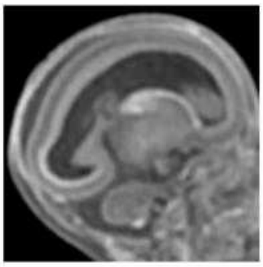

L-R Level: 3.9 mm

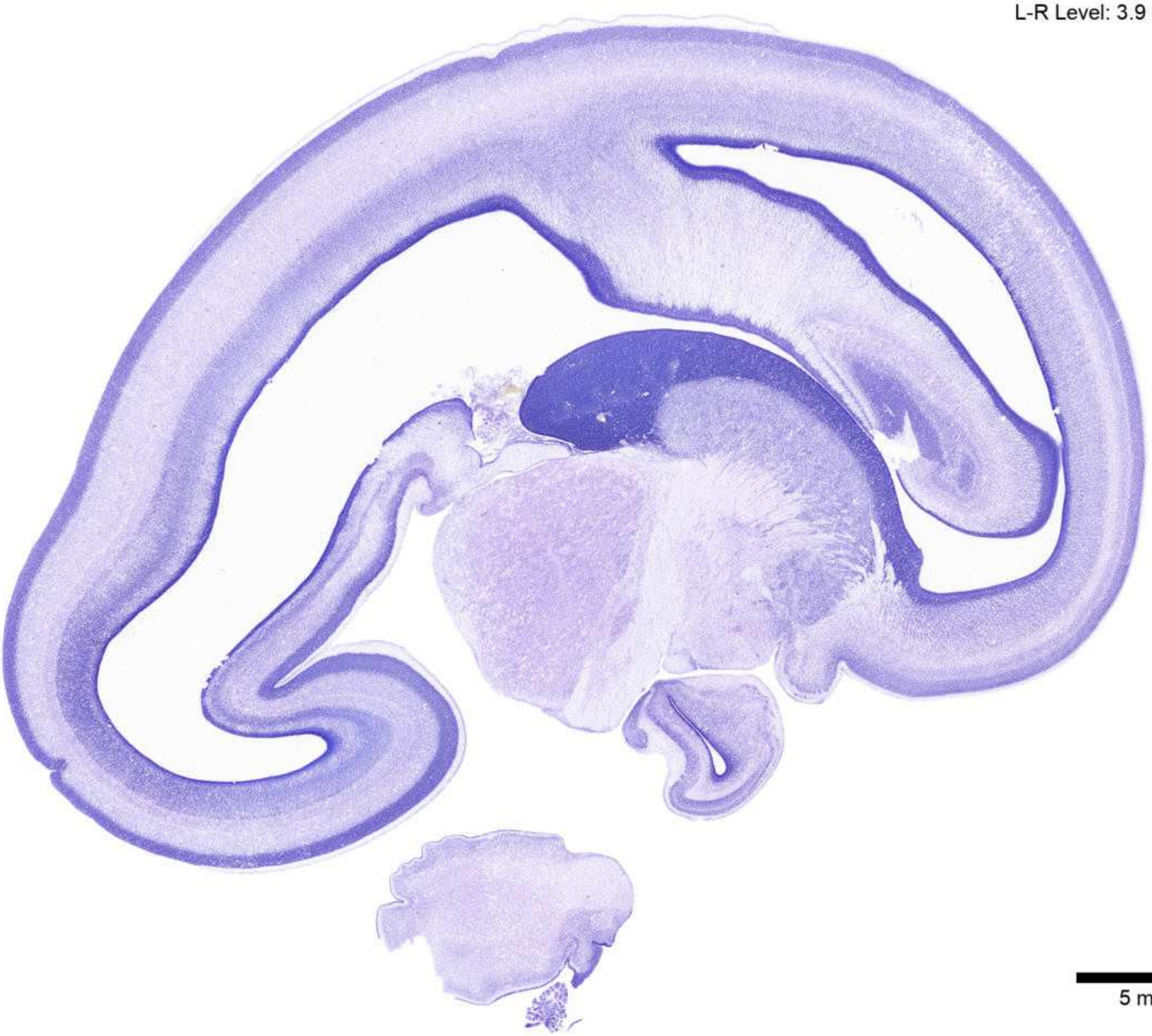

5 mm

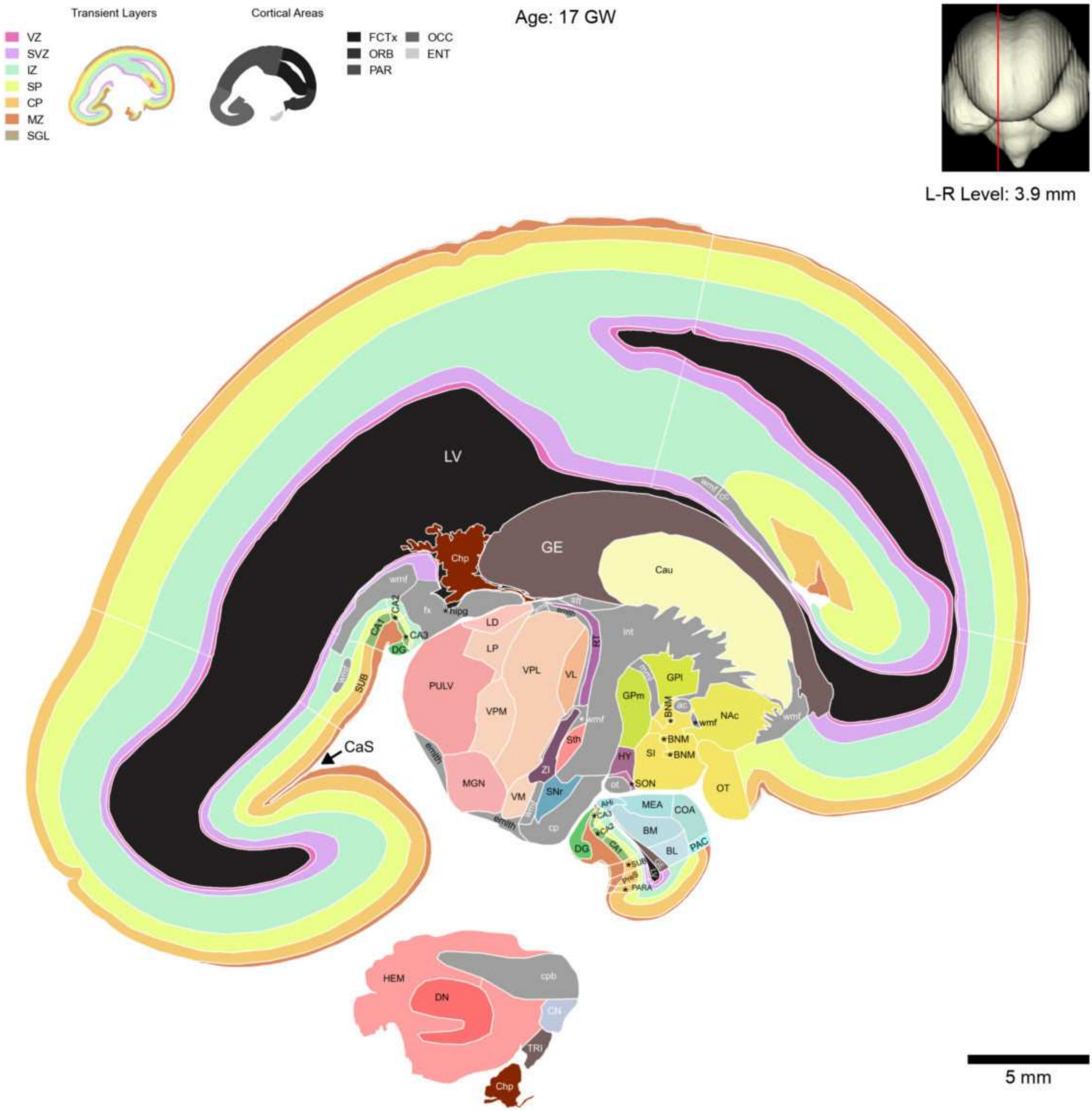

AHi: Amygdalo-hippocampal area
BL: Basal nucleus [amygdala]
BM: Basomedial nucleus [amygdala]
BNM: Basal nucleus of Meynert
CA1: CA1 field [hippocampus]
CA2: CA2 field [hippocampus]
CA3: CA3 field [hippocampus]
CN: Cochlear nuclei
COA: Cortical nucleus [amygdala]
Cau: Caudate nucleus
Chp: Choroid plexus
DG: Dentate gyrus
DN: Dentate nucleus
GE: Ganglionic eminence
GPl: Globus pallidus lateral segment
GPm: Globus pallidus medial segment
HEM: Cerebellar hemispheres
HY: Hypothalamus
LD: Lateral dorsal nucleus [thalamus]
LHA: Lateral hypothalamic area
LP: Lateral posterior nucleus [thalamus]
LV: Lateral ventricle
MEA: Medial nucleus [amygdala]
MGN: Medial geniculate nucleus
NAc: Nucleus accumbens
OT: Olfactory tubercle
PARA: Cortical plate, parasubiculum
PULV: Pulvinar nucleus [thalamus]
PreS: Cortical plate, presubiculum
RT: Reticular nucleus [thalamus]
SI: Substantia innominata
SNr: Substantia nigra pars reticulata
SON: Supraoptic nucleus [hypothalamus]
SUB: Cortical plate, subiculum
Sth: Subthalamus
TRI: Germinal trigone
VL: Ventral lateral nucleus [thalamus]
VM: Ventral medial nucleus [thalamus]
VPL: Ventral posterolateral nucleus [thalamus]
VPM: Ventral posteromedial nucleus [thalamus]
ZI: Zona incerta
ac: Anterior commissure
cc: Corpus callosum
cp: Cerebral peduncle
cpb: Cerebellar peduncles
emlth: External medullary lamina [thalamus]
fx: Fornix
hipg: Hippocampal glioepithelium/ependyma
int: Internal capsule
mml: Medial medullary lamina
ot: Optic tract
stt: Stria terminalis
wmf: White matter fibers
→ CaS: Calcarine sulcus

Age: 17 GW

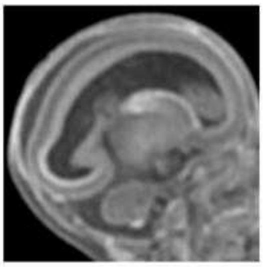

L-R Level: 3.72 mm

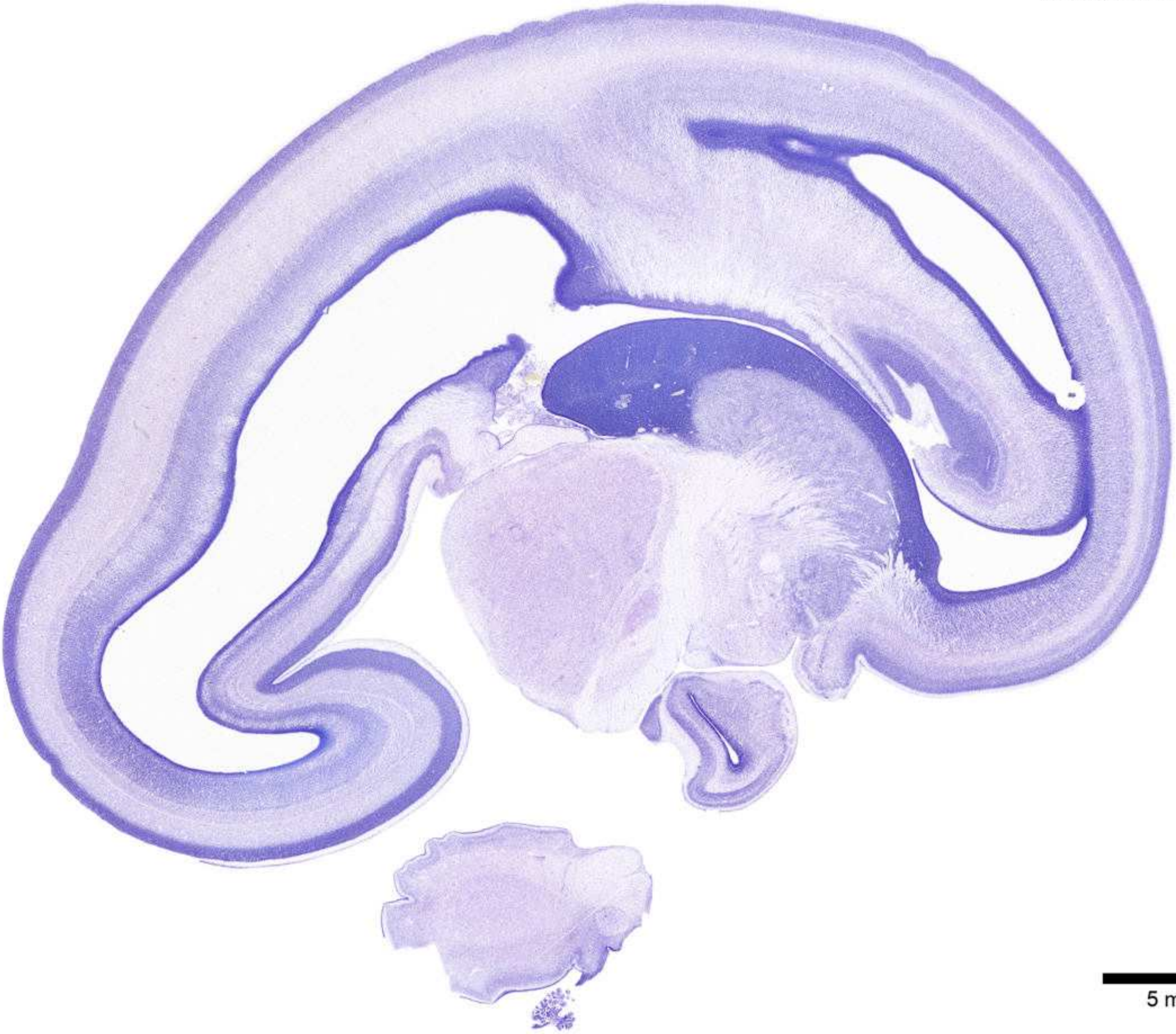

5 mm

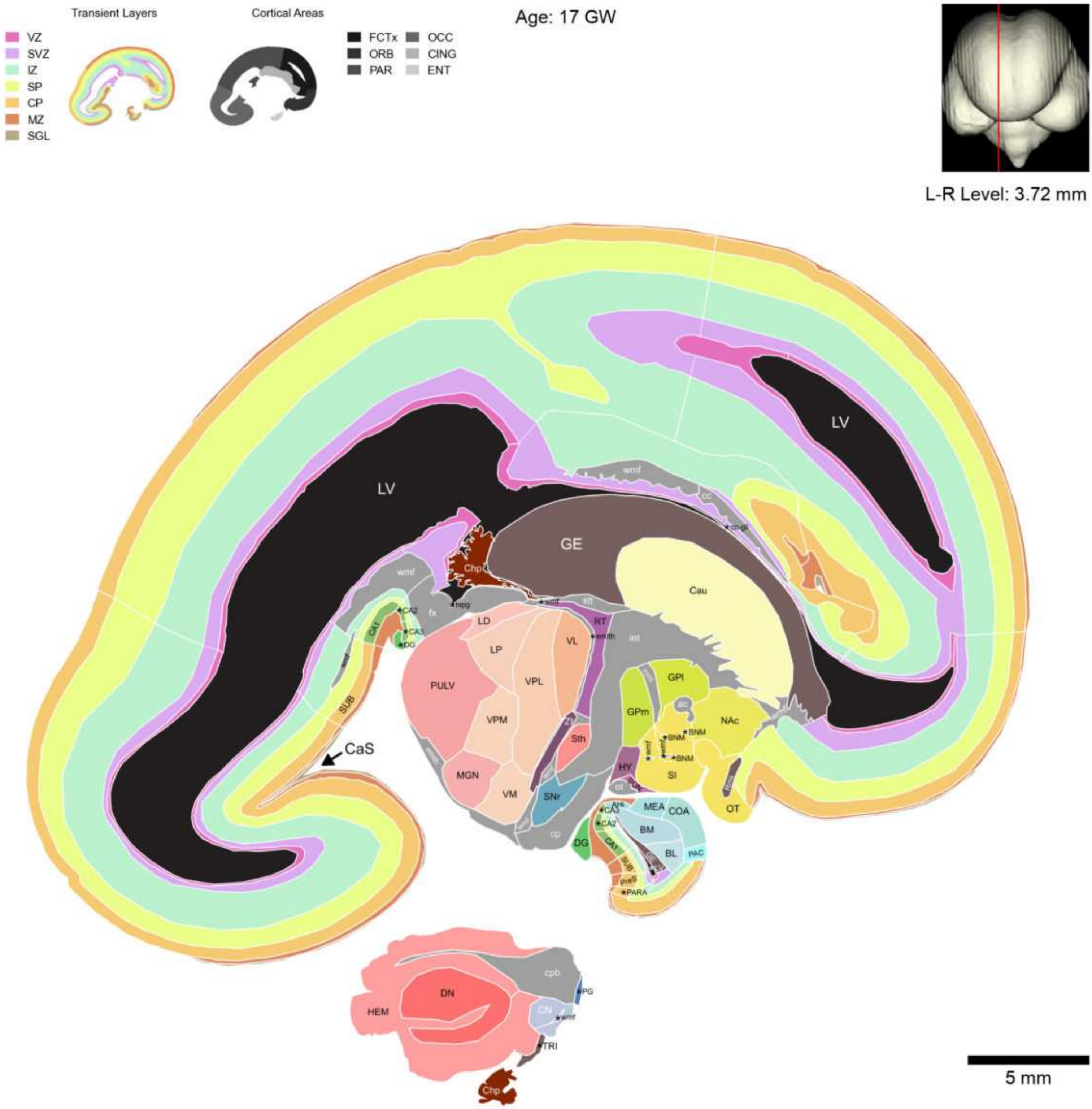

- AHi: Amygdalo-hippocampal area
- BL: Basal nucleus [amygdala]
- BM: Basomedial nucleus [amygdala]
- BNM: Basal nucleus of Meynert
- CA1: CA1 field [hippocampus]
- CA2: CA2 field [hippocampus]
- CA3: CA3 field [hippocampus]
- CN: Cochlear nuclei
- COA: Cortical nucleus [amygdala]
- Cau: Caudate nucleus
- Chp: Choroid plexus
- DG: Dentate gyrus
- DN: Dentate nucleus
- GE: Ganglionic eminence
- GPl: Globus pallidus lateral segment
- GPm: Globus pallidus medial segment
- HEM: Cerebellar hemispheres
- HY: Hypothalamus
- LD: Lateral dorsal nucleus [thalamus]
- LHA: Lateral hypothalamic area
- LP: Lateral posterior nucleus [thalamus]
- LV: Lateral ventricle
- MEA: Medial nucleus [amygdala]
- MGN: Medial geniculate nucleus
- NAc: Nucleus accumbens
- OT: Olfactory tubercle
- PARA: Cortical plate, parasubiculum
- PG: Pontine gray
- PULV: Pulvinar nucleus [thalamus]
- PreS: Cortical plate, presubiculum
- RT: Reticular nucleus [thalamus]
- Rms: Rostral migratory stream
- SI: Substantia innominata
- SNr: Substantia nigra pars reticulata
- SON: Supraoptic nucleus [hypothalamus]
- SUB: Cortical plate, subiculum
- Sth: Subthalamus
- TRI: Germinal trigone
- VL: Ventral lateral nucleus [thalamus]
- VM: Ventral medial nucleus [thalamus]
- VPL: Ventral posterolateral nucleus [thalamus]
- VPM: Ventral posteromedial nucleus [thalamus]
- ZI: Zona incerta
- ac: Anterior commissure
- cc: Corpus callosum
- cc-gli: Callosal glioepithelium
- cp: Cerebral peduncle
- cpb: Cerebellar peduncles
- emlth: External medullary lamina [thalamus]
- fx: Fornix
- hipg: Hippocampal glioepithelium/ependyma
- int: Internal capsule
- mml: Medial medullary lamina
- ot: Optic tract
- stt: Stria terminalis
- wmf: White matter fibers
- → CaS: Calcarine sulcus

Age: 17 GW

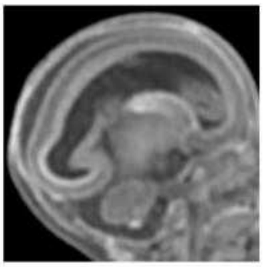

L-R Level: 3.36 mm

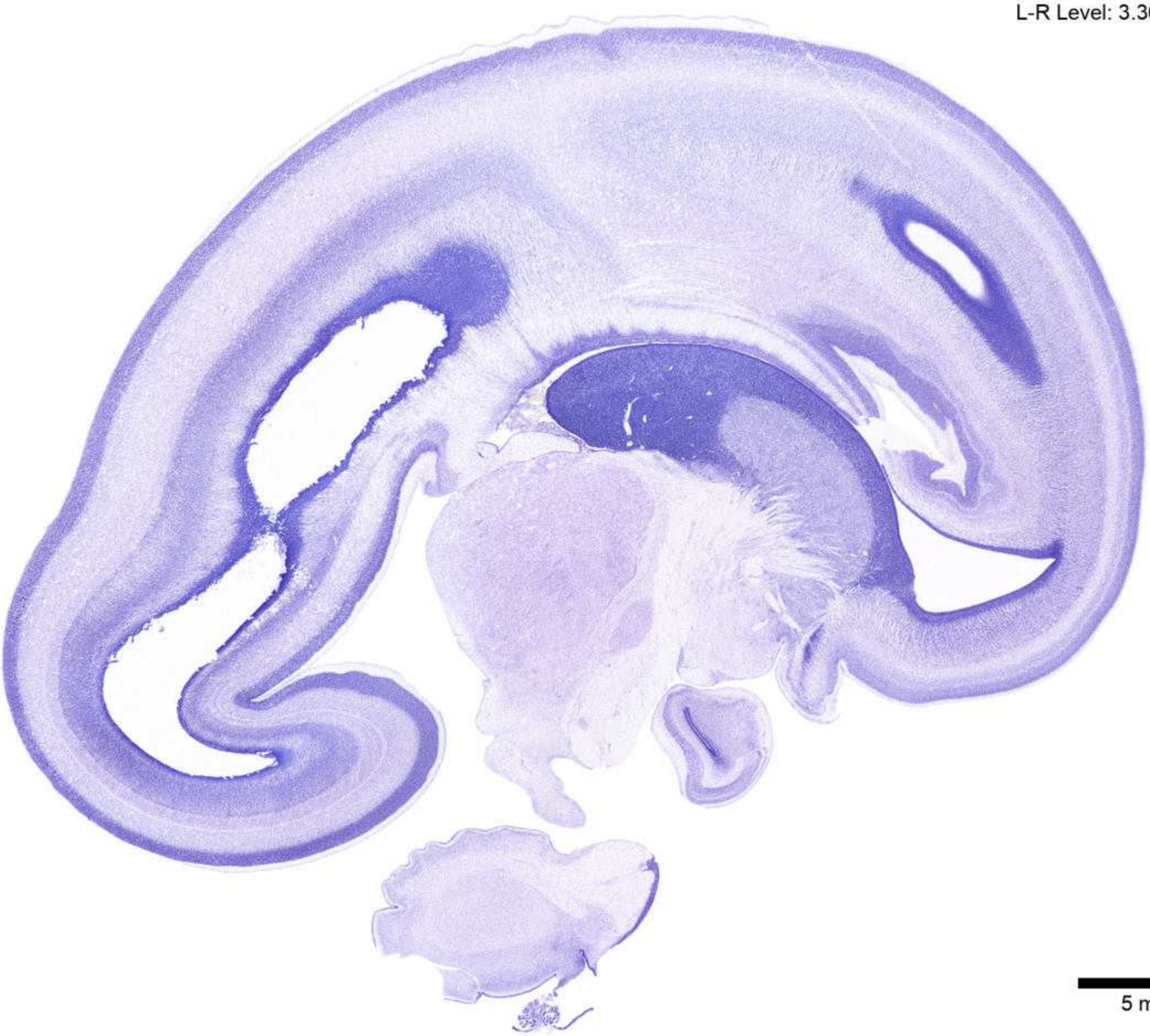

5 mm

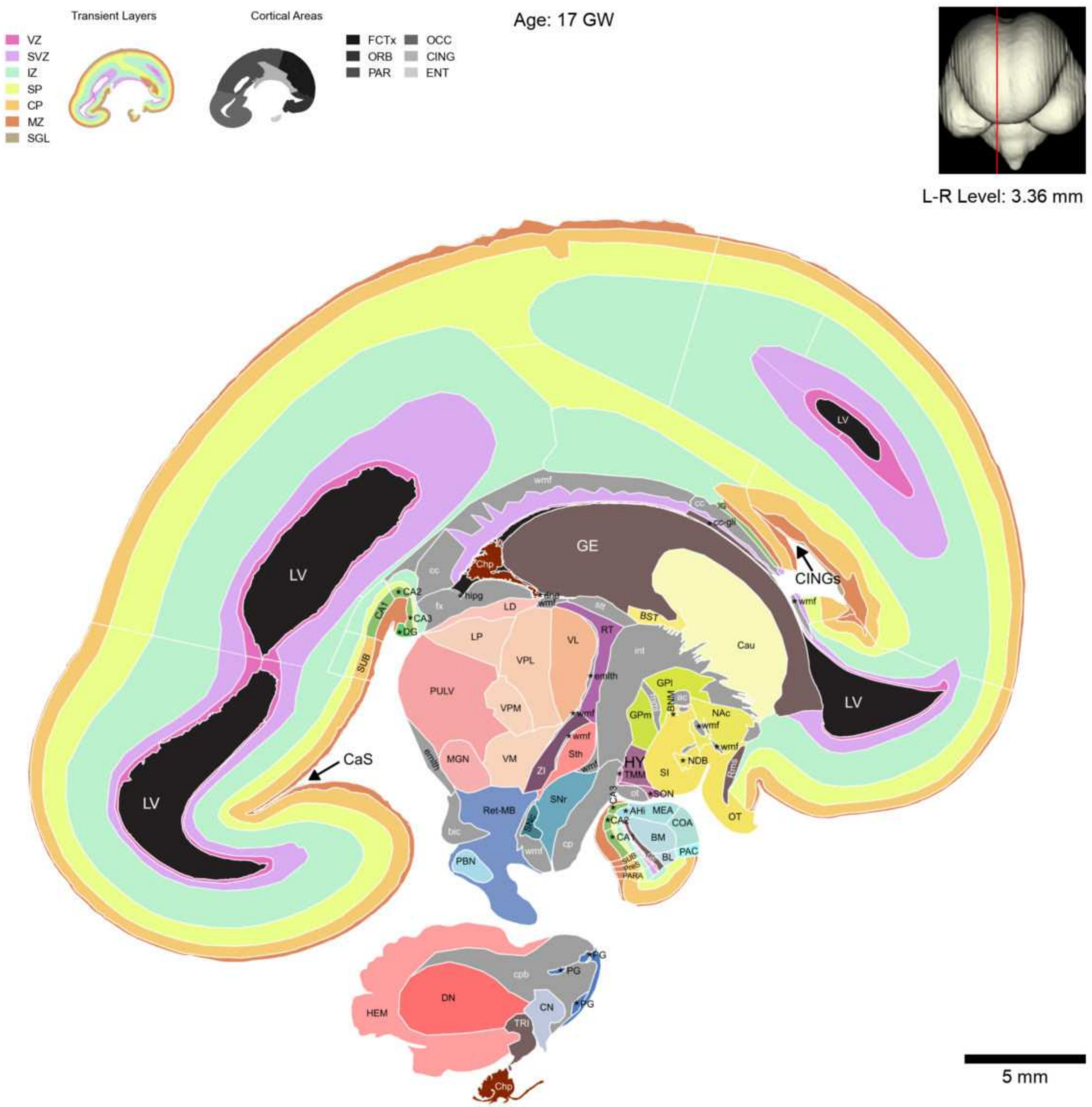

AHi: Amygdalo-hippocampal area
BL: Basal nucleus [amygdala]
BM: Basomedial nucleus [amygdala]
BNM: Basal nucleus of Meynert
BST: Bed nucleus of the stria terminalis
CA1: CA1 field [hippocampus]
CA2: CA2 field [hippocampus]
CA3: CA3 field [hippocampus]
CN: Cochlear nuclei
COA: Cortical nucleus [amygdala]
Cau: Caudate nucleus
Chp: Choroid plexus
DG: Dentate gyrus
DN: Dentate nucleus
GE: Ganglionic eminence
GPl: Globus pallidus lateral segment
GPm: Globus pallidus medial segment
HEM: Cerebellar hemispheres
HY: Hypothalamus
IG: Induseum griseum
LD: Lateral dorsal nucleus [thalamus]
LHA: Lateral hypothalamic area
LP: Lateral posterior nucleus [thalamus]
LV: Lateral ventricle
MEA: Medial nucleus [amygdala]
MGN: Medial geniculate nucleus
NAc: Nucleus accumbens
NDB: Nucleus of the diagonal band
OT: Olfactory tubercle
PARA: Cortical plate, parasubiculum
PBN: Parabigeminal nucleus
PG: Pontine gray
PULV: Pulvinar nucleus [thalamus]
PreS: Cortical plate, presubiculum
RT: Reticular nucleus [thalamus]
Ret-MB: Reticular formation, Midbrain
Rms: Rostral migratory stream
SI: Substantia innominata
SNc: Substantia nigra pars compacta
SNr: Substantia nigra pars reticulata
SON: Supraoptic nucleus [hypothalamus]
SUB: Cortical plate, subiculum
Sth: Subthalamus
TMM: Tuberomammillary nucleus
TRI: Germinal trigone
VL: Ventral lateral nucleus [thalamus]
VM: Ventral medial nucleus [thalamus]
VPL: Ventral posterolateral nucleus [thalamus]
VPM: Ventral posteromedial nucleus [thalamus]
ZI: Zona incerta
ac: Anterior commissure
bic: Brachium of the inferior colliculus
cc: Corpus callosum
cc-gli: Callosal glioepithelium
cp: Cerebral peduncle
cpb: Cerebellar peduncles
dne: Diencephalic neuroepithelium
emlth: External medullary lamina [thalamus]
fx: Fornix
hipg: Hippocampal glioepithelium/ependyma
int: Internal capsule
mml: Medial medullary lamina
ot: Optic tract
stt: Stria terminalis
wmf: White matter fibers
→ CaS: Calcarine sulcus
→ CINGs: Cingulate sulcus

Age: 17 GW

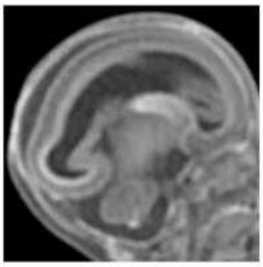

L-R Level: 3.06 mm

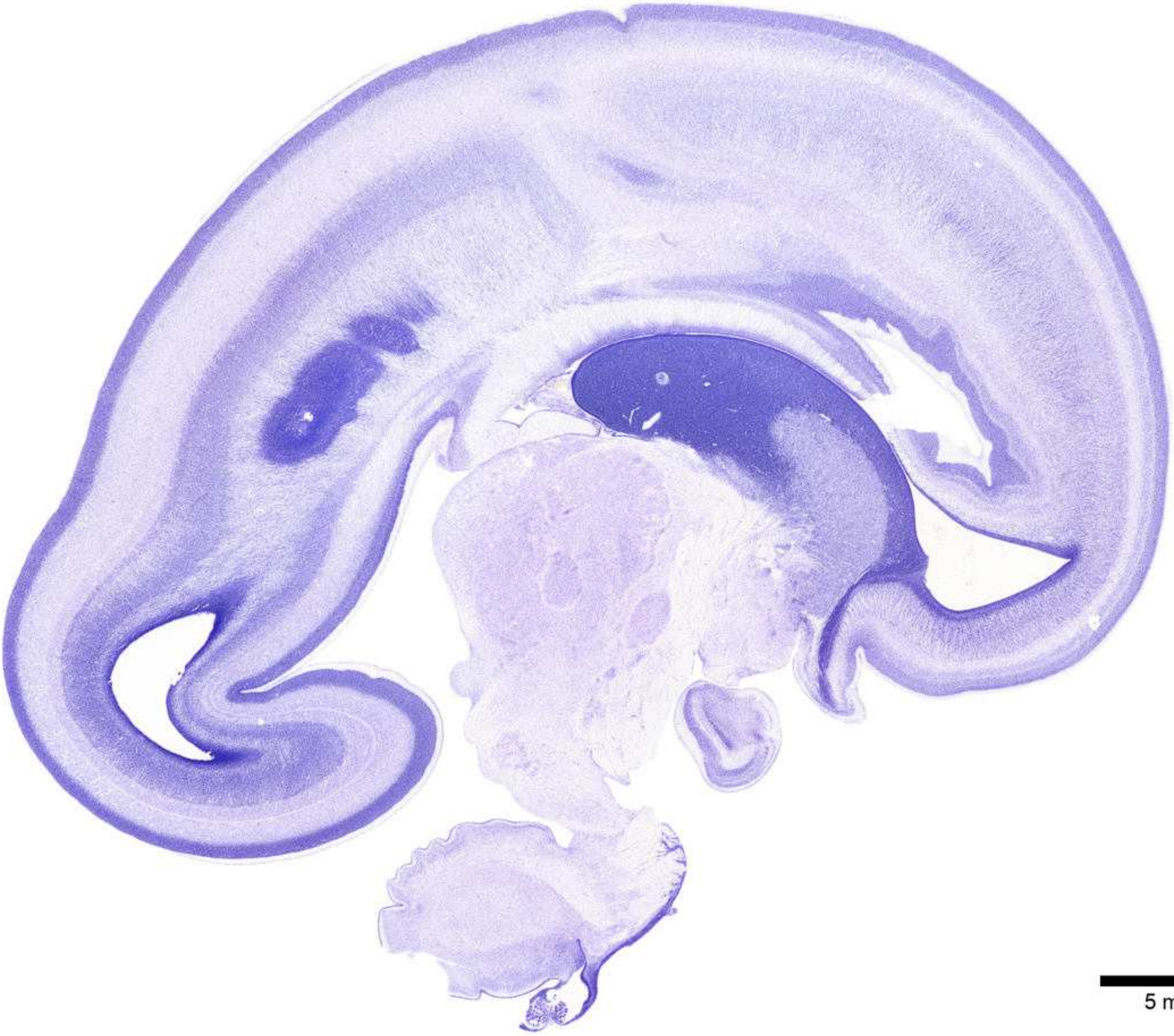

5 mm

Transient Layers

- VZ
- SVZ
- IZ
- SP
- CP
- MZ
- SGL

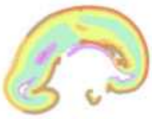

Cortical Areas

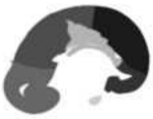

- FCTx
- ORB
- PAR
- OCC
- CING
- ENT

Age: 17 GW

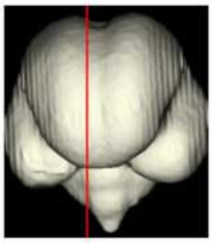

L-R Level: 3.06 mm

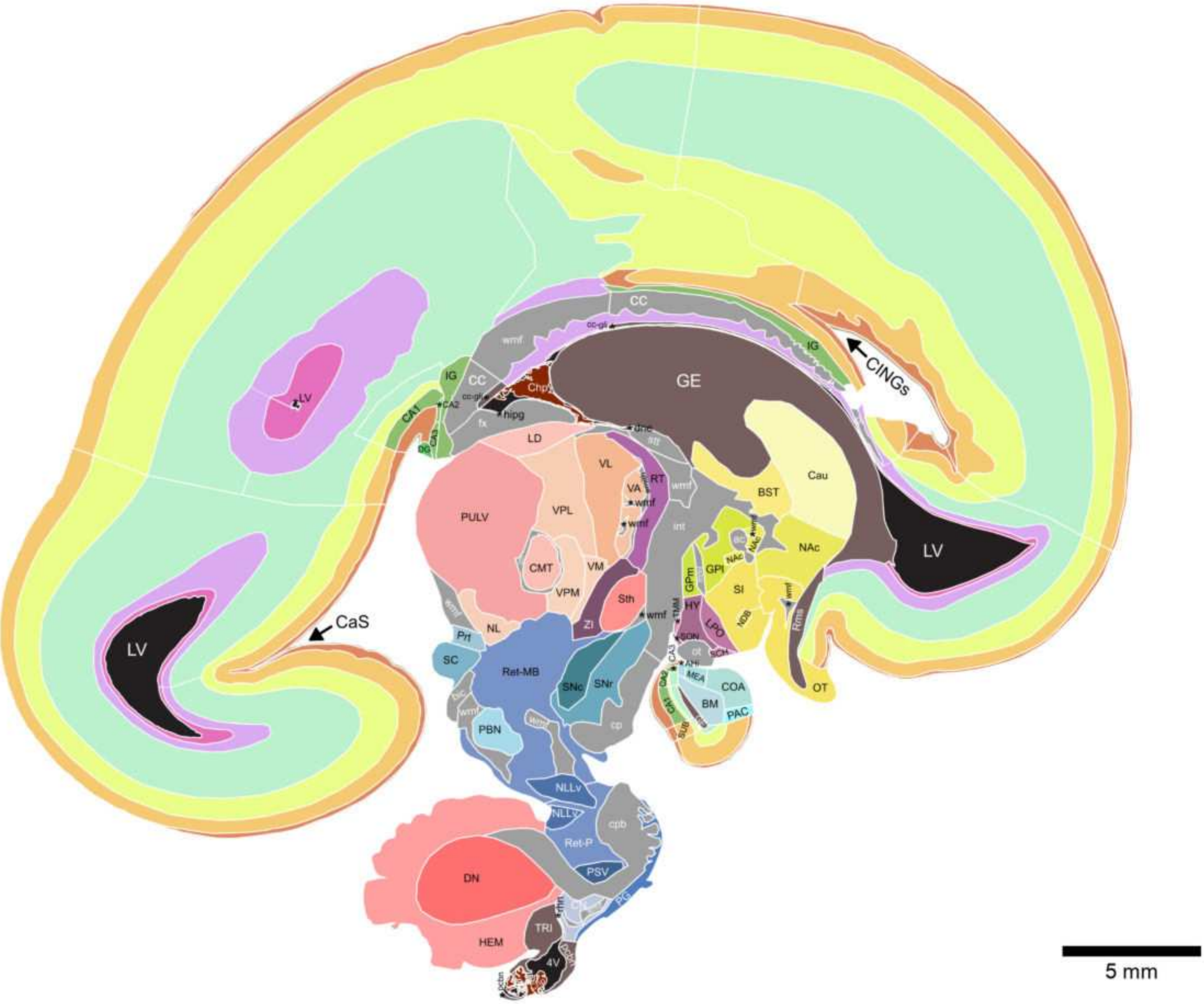

- 4V: Fourth ventricle
- AHi: Amygdalo-hippocampal area
- BM: Basomedial nucleus [amygdala]
- BST: Bed nucleus of the stria terminalis
- CA1: CA1 field [hippocampus]
- CA2: CA2 field [hippocampus]
- CA3: CA3 field [hippocampus]
- CMT: Centromedian nucleus [thalamus]
- CN: Cochlear nuclei
- COA: Cortical nucleus [amygdala]
- Cau: Caudate nucleus
- Chp: Choroid plexus
- DG: Dentate gyrus
- DN: Dentate nucleus
- GE: Ganglionic eminence
- GPl: Globus pallidus lateral segment
- GPm: Globus pallidus medial segment
- HEM: Cerebellar hemispheres
- HY: Hypothalamus
- IG: Induseum griseum
- LD: Lateral dorsal nucleus [thalamus]
- LHA: Lateral hypothalamic area
- LPO: Lateral preoptic area
- LV: Lateral ventricle
- MEA: Medial nucleus [amygdala]
- NAc: Nucleus accumbens
- NDB: Nucleus of the diagonal band
- NL: Nucleus limitans [thalamus]
- NLLv: Nucleus of the lateral lemniscus, ventral
- OT: Olfactory tubercle
- PBN: Parabigeminal nucleus
- PG: Pontine gray
- PSV: Principal sensory nucleus of the trigeminal
- PULV: Pulvinar nucleus [thalamus]
- Prt: Pretectum
- RT: Reticular nucleus [thalamus]
- Ret-MB: Reticular formation, Midbrain
- Ret-P: Reticular formation, Pons
- Rms: Rostral migratory stream
- SC: Superior colliculus
- SCH: Suprachiasmatic nucleus [hypothalamus]
- SI: Substantia innominata
- SNc: Substantia nigra pars compacta
- SNr: Substantia nigra pars reticulata
- SON: Supraoptic nucleus [hypothalamus]
- SUB: Cortical plate, subiculum
- Sth: Subthalamus
- TMM: Tuberomammillary nucleus
- TRI: Germinal trigone
- VA: Ventral anterior nucleus [thalamus]
- VL: Ventral lateral nucleus [thalamus]
- VM: Ventral medial nucleus [thalamus]
- VPL: Ventral posterolateral nucleus [thalamus]
- VPM: Ventral posteromedial nucleus [thalamus]
- ZI: Zona incerta
- → CaS: Calcarine sulcus
- → CINGs: Cingulate sulcus

Age: 17 GW

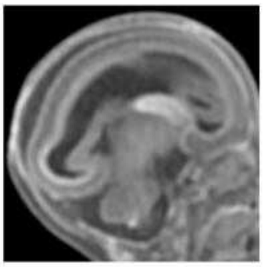

L-R Level: 2.76 mm

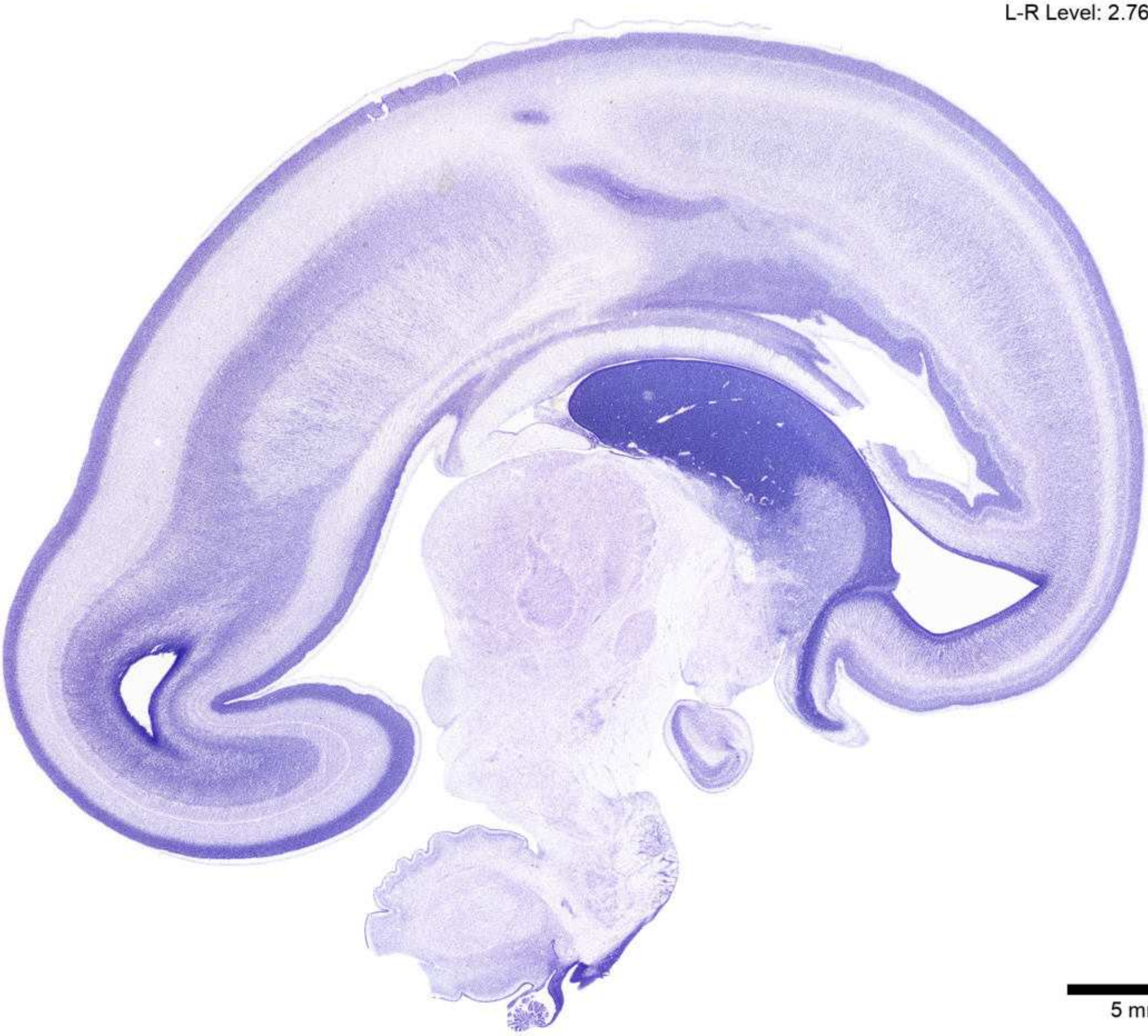

5 mm

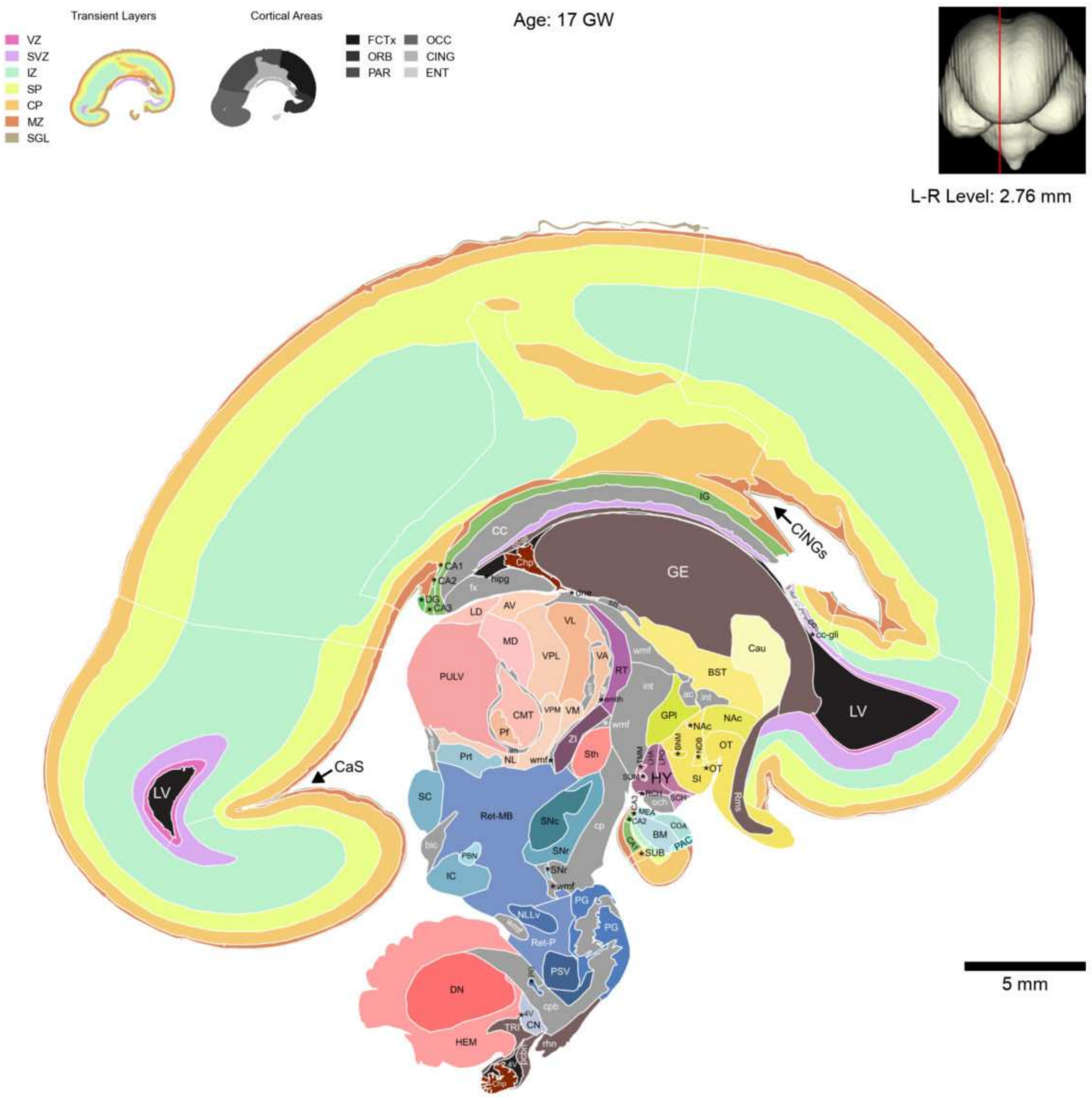

4V: Fourth ventricle
AV: Anteroventral nucleus [thalamus]
BM: Basomedial nucleus [amygdala]
BNM: Basal nucleus of Meynert
BST: Bed nucleus of the stria terminalis
CA1: CA1 field [hippocampus]
CA2: CA2 field [hippocampus]
CA3: CA3 field [hippocampus]
CMT: Centromedian nucleus [thalamus]
CN: Cochlear nuclei
COA: Cortical nucleus [amygdala]
Cau: Caudate nucleus
Chp: Choroid plexus
DG: Dentate gyrus
DN: Dentate nucleus
GE: Ganglionic eminence
GPl: Globus pallidus lateral segment
HEM: Cerebellar hemispheres
HY: Hypothalamus
IC: Inferior colliculus
IG: Induseum griseum
LD: Lateral dorsal nucleus [thalamus]
LHA: Lateral hypothalamic area
LPO: Lateral preoptic area
LV: Lateral ventricle
MD: Medial dorsal nucleus [thalamus]
MEA: Medial nucleus [amygdala]
NAc: Nucleus accumbens
NDB: Nucleus of the diagonal band
NL: Nucleus limitans [thalamus]
NLLv: Nucleus of the lateral lemniscus, ventral
OT: Olfactory tubercle
PBN: Parabigeminal nucleus
PG: Pontine gray
PSV: Principal sensory nucleus of the trigeminal
PULV: Pulvinar nucleus [thalamus]
Pf: Parafascicular nucleus [thalamus]
Prt: Pretectum
RCH: Retrochiasmatic nucleus [hypothalamus]
RT: Reticular nucleus [thalamus]
Ret-MB: Reticular formation, Midbrain
Ret-P: Reticular formation, Pons
Rms: Rostral migratory stream
SC: Superior colliculus
SCH: Suprachiasmatic nucleus [hypothalamus]
SI: Substantia innominata
SNc: Substantia nigra pars compacta
SNr: Substantia nigra pars reticulata
SPV: Spinal nucleus of the trigeminal
SUB: Cortical plate, subiculum
SUM: Supramammillary area
Sth: Subthalamus
TMM: Tuberomammillary nucleus
TRI: Germinal trigone
VA: Ventral anterior nucleus [thalamus]
VL: Ventral lateral nucleus [thalamus]
VM: Ventral medial nucleus [thalamus]
VPL: Ventral posterolateral nucleus [thalamus]
VPM: Ventral posteromedial nucleus [thalamus]
ZI: Zona incerta
→ CaS - Calcarine sulcus
→ CINGs- Cingulate sulcus

Age: 17 GW

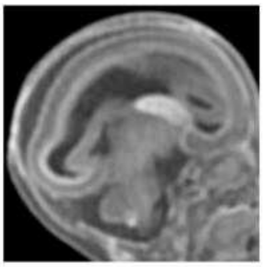

L-R Level: 2.52 mm

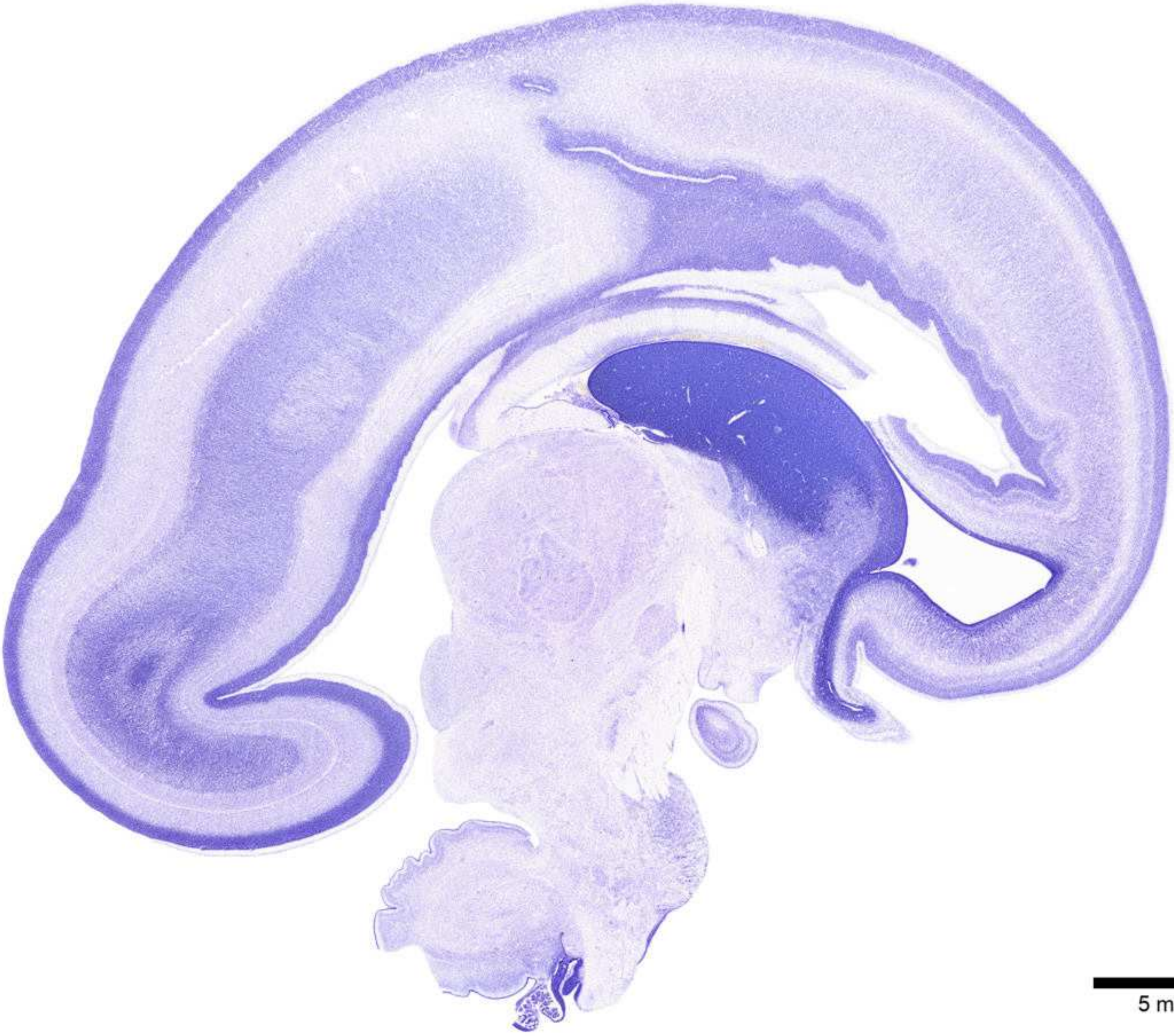

5 mm

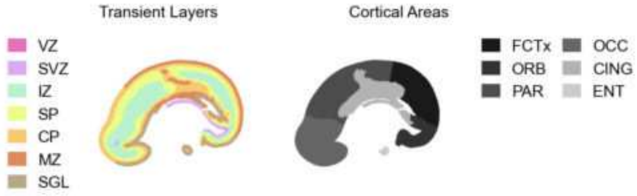

Age: 17 GW

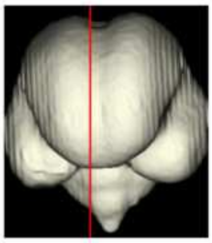

L-R Level: 2.52 mm

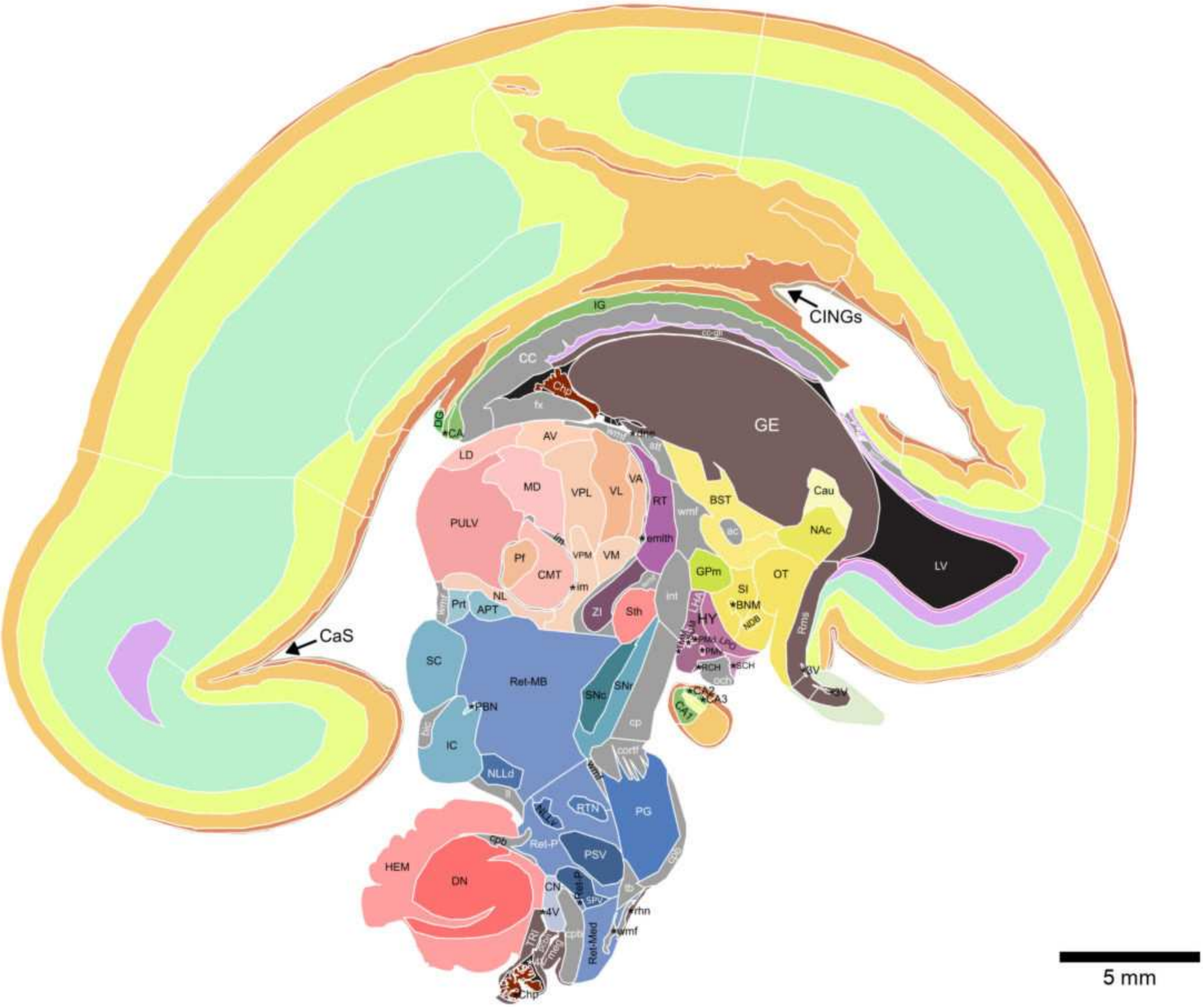

- 3V: Third ventricle
- 4V: Fourth ventricle
- APT: Anterior pretectal nucleus
- AV: Anteroventral nucleus [thalamus]
- BNM: Basal nucleus of Meynert
- BST: Bed nucleus of the stria terminalis
- CA: Ammon's horn
- CA1: CA1 field [hippocampus]
- CA2: CA2 field [hippocampus]
- CA3: CA3 field [hippocampus]
- CMT: Centromedian nucleus [thalamus]
- CN: Cochlear nuclei
- Cau: Caudate nucleus
- Chp: Choroid plexus
- DG: Dentate gyrus
- DN: Dentate nucleus
- GE: Ganglionic eminence
- GPm: Globus pallidus medial segment
- HEM: Cerebellar hemispheres
- HY: Hypothalamus
- IC: Inferior colliculus
- IG: Induseum griseum
- LD: Lateral dorsal nucleus [thalamus]
- LHA: Lateral hypothalamic area
- LPO: Lateral preoptic area
- LV: Lateral ventricle
- MD: Medial dorsal nucleus [thalamus]
- NAc: Nucleus accumbens
- NDB: Nucleus of the diagonal band
- NL: Nucleus limitans [thalamus]
- NLLd: Nucleus of the lateral lemniscus, dorsal
- NLLv: Nucleus of the lateral lemniscus, ventral
- OLFb: Olfactory bulb
- OT: Olfactory tubercle
- PBN: Parabigeminal nucleus
- PG: Pontine gray
- PMd: Dorsal premammillary nucleus
- PMv: Ventral premammillary nucleus
- PSV: Principal sensory nucleus of the trigeminal
- PULV: Pulvinar nucleus [thalamus]
- Pf: Parafascicular nucleus [thalamus]
- Prt: Pretectum
- RCH: Retrochiasmatic nucleus [hypothalamus]
- RT: Reticular nucleus [thalamus]
- RTN: Reticular tegmental nucleus
- Ret-MB: Reticular formation, Midbrain
- Ret-Med: Reticular formation, Medulla
- Ret-P: Reticular formation, Pons
- Rms: Rostral migratory stream
- SC: Superior colliculus
- SCH: Suprachiasmatic nucleus [hypothalamus]
- SI: Substantia innominata
- SNc: Substantia nigra pars compacta
- SNr: Substantia nigra pars reticulata
- SPV: Spinal nucleus of the trigeminal
- SUM: Supramammillary area
- Sth: Subthalamus
- TMM: Tuberomammillary nucleus
- TRI: Germinal trigone
- VA: Ventral anterior nucleus [thalamus]
- VL: Ventral lateral nucleus [thalamus]
- VM: Ventral medial nucleus [thalamus]
- VPL: Ventral posterolateral nucleus [thalamus]
- VPM: Ventral posteromedial nucleus [thalamus]
- ZI: Zona incerta
- → CaS: Calcarine sulcus
- → CINGs: Cingulate sulcus

Age: 17 GW

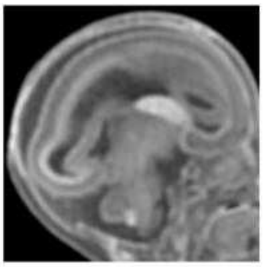

L-R Level: 2.22 mm

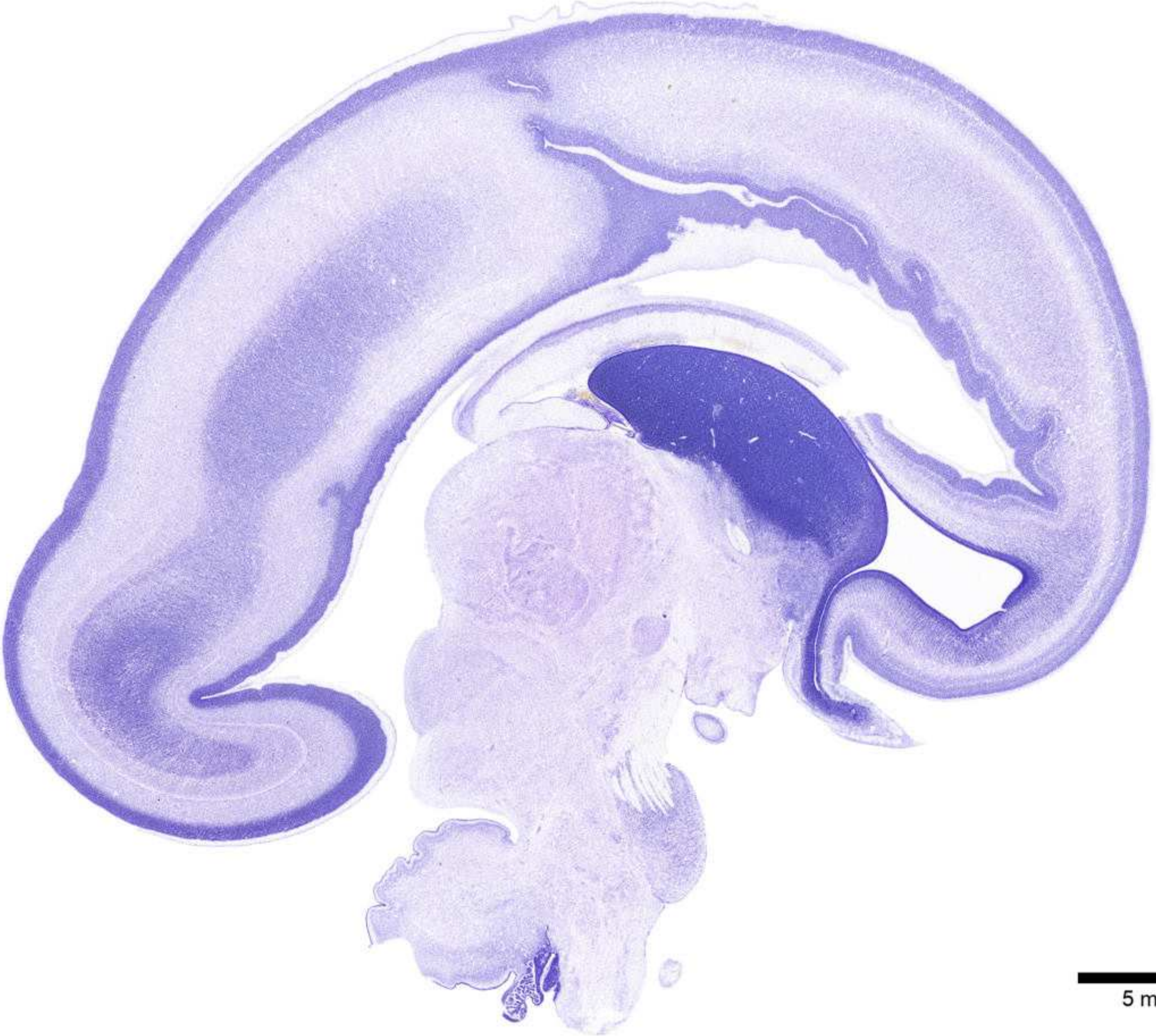

5 mm

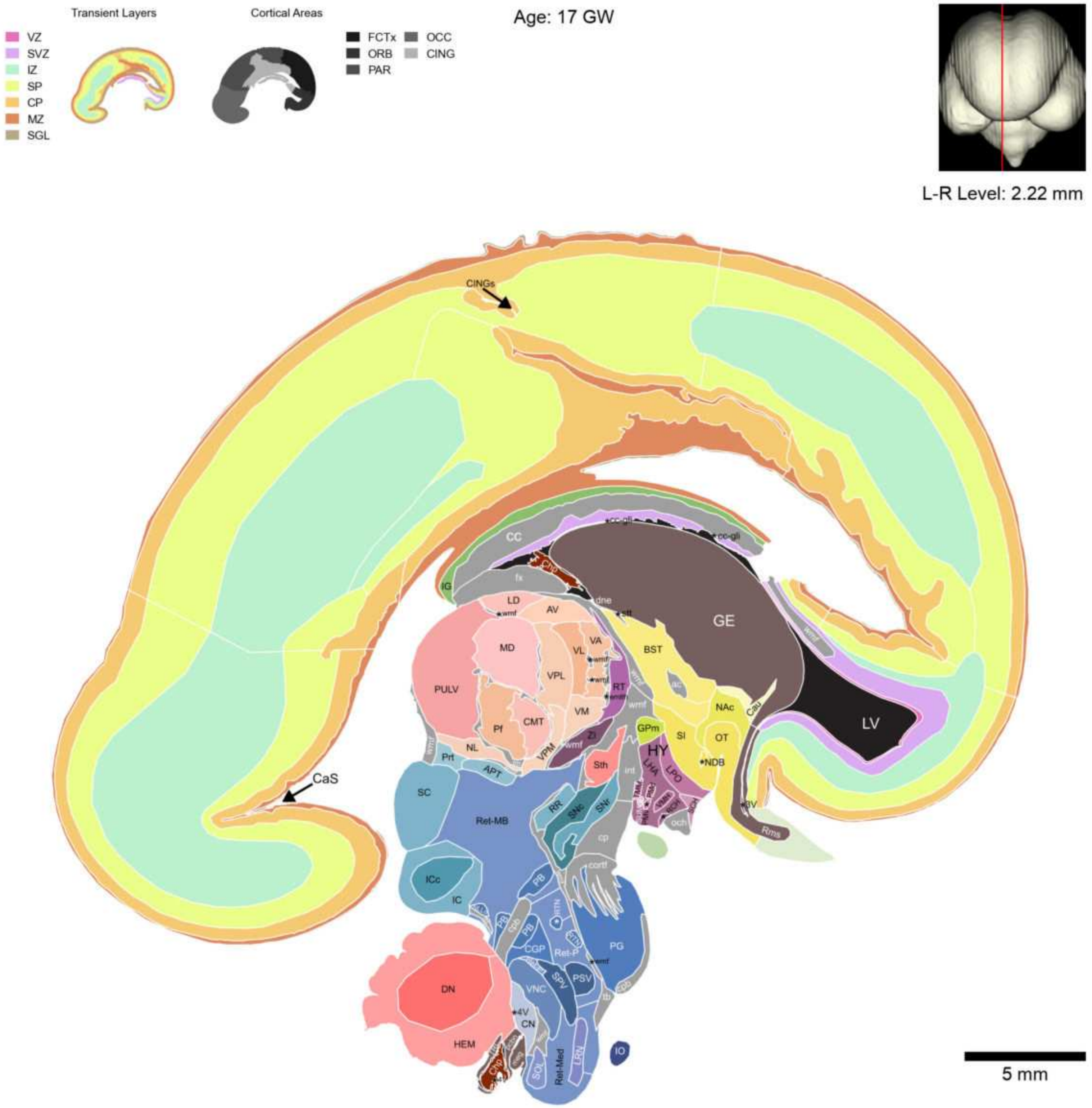

3V: Third ventricle
4V: Fourth ventricle
APT: Anterior pretectal nucleus
AV: Anteroventral nucleus [thalamus]
BST: Bed nucleus of the stria terminalis
CGP: Central gray of the pons
CMT: Centromedian nucleus [thalamus]
CN: Cochlear nuclei
Cau: Caudate nucleus
Chp: Choroid plexus
DN: Dentate nucleus
GE: Ganglionic eminence
GPm: Globus pallidus medial segment
HEM: Cerebellar hemispheres
HY: Hypothalamus
IC: Inferior colliculus
ICc: Inferior colliculus, central nucleus
IG: Induseum griseum
IO: Inferior olive
LD: Lateral dorsal nucleus [thalamus]
LHA: Lateral hypothalamic area
LPO: Lateral preoptic area
LRN: Lateral reticular nucleus
LV: Lateral ventricle
MD: Medial dorsal nucleus [thalamus]
NAc: Nucleus accumbens
NDB: Nucleus of the diagonal band
NL: Nucleus limitans [thalamus]
NLLd: Nucleus of the lateral lemniscus, dorsal
OLFb: Olfactory bulb
OT: Olfactory tubercle
PARG: Parahippocampal gyrus
PB: Parabrachial nucleus
PG: Pontine gray
PMd: Dorsal premammillary nucleus
PMv: Ventral premammillary nucleus
PSV: Principal sensory nucleus of the trigeminal
PULV: Pulvinar nucleus [thalamus]
Pf: Parafascicular nucleus [thalamus]
Prt: Pretectum
RCH: Retrochiasmatic nucleus [hypothalamus]
RR: Retrorubral area
RT: Reticular nucleus [thalamus]
RTN: Reticular tegmental nucleus
Ret-MB: Reticular formation, Midbrain
Ret-Med: Reticular formation, Medulla
Ret-P: Reticular formation, Pons
Rms: Rostral migratory stream
SC: Superior colliculus
SCH: Suprachiasmatic nucleus [hypothalamus]
SI: Substantia innominata
SNc: Substantia nigra pars compacta
SNr: Substantia nigra pars reticulata
SOL: Solitary nucleus
SPV: Spinal nucleus of the trigeminal
SUM: Supramammillary area
Sth: Subthalamus
TMM: Tuberomammillary nucleus
TRI: Germinal trigone
VA: Ventral anterior nucleus [thalamus]
VL: Ventral lateral nucleus [thalamus]
VM: Ventral medial nucleus [thalamus]
VMH: Ventromedial nucleus [hypothalamus]
VNC: Vestibular nuclear complex
VPL: Ventral posterolateral nucleus [thalamus]
VPM: Ventral posteromedial nucleus [thalamus]
ZI: Zona incerta
→ CaS: Calcarine sulcus
→ CINGs: Cingulate sulcus

Age: 17 GW

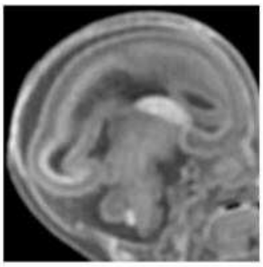

L-R Level: 2.04 mm

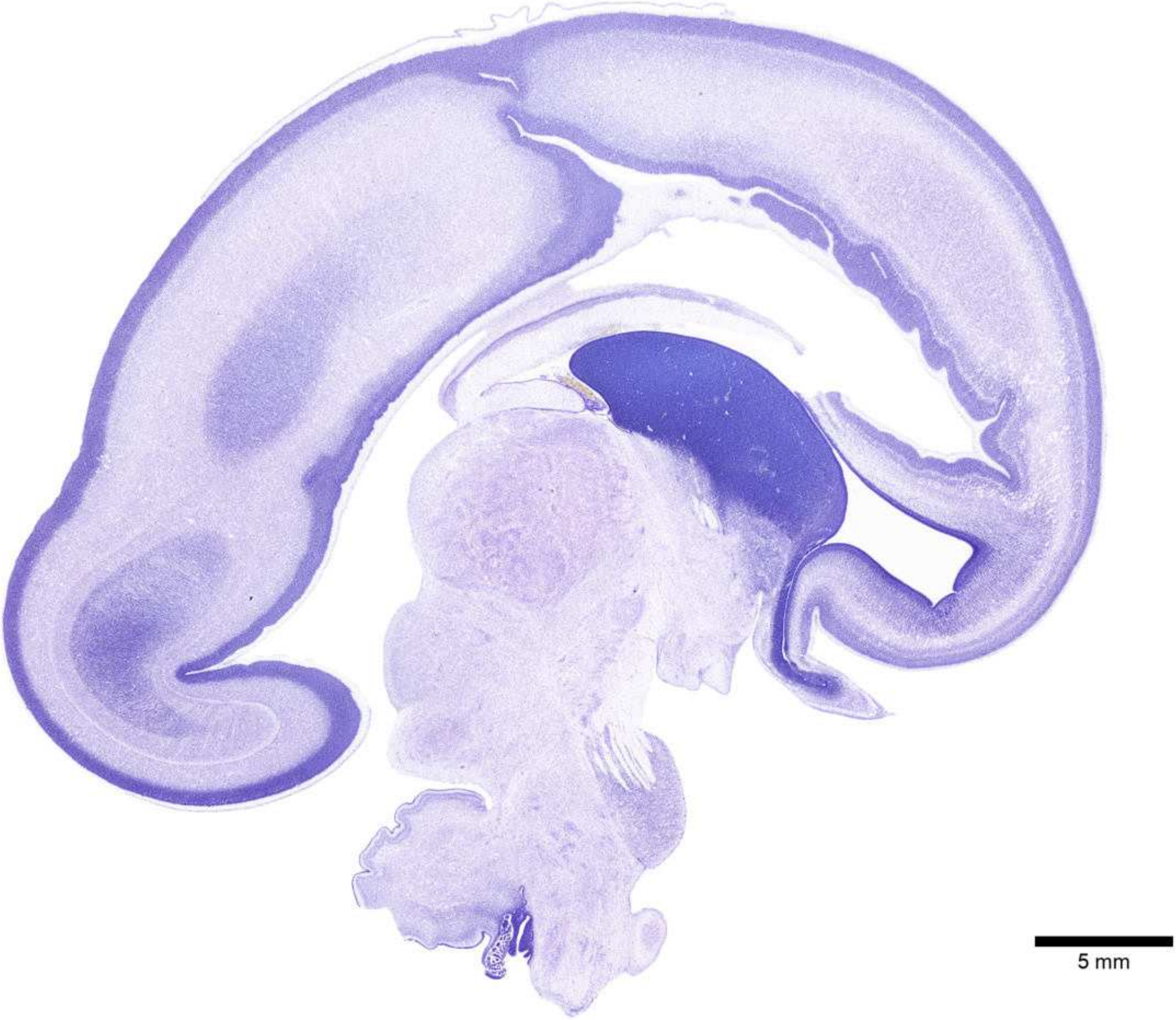

5 mm

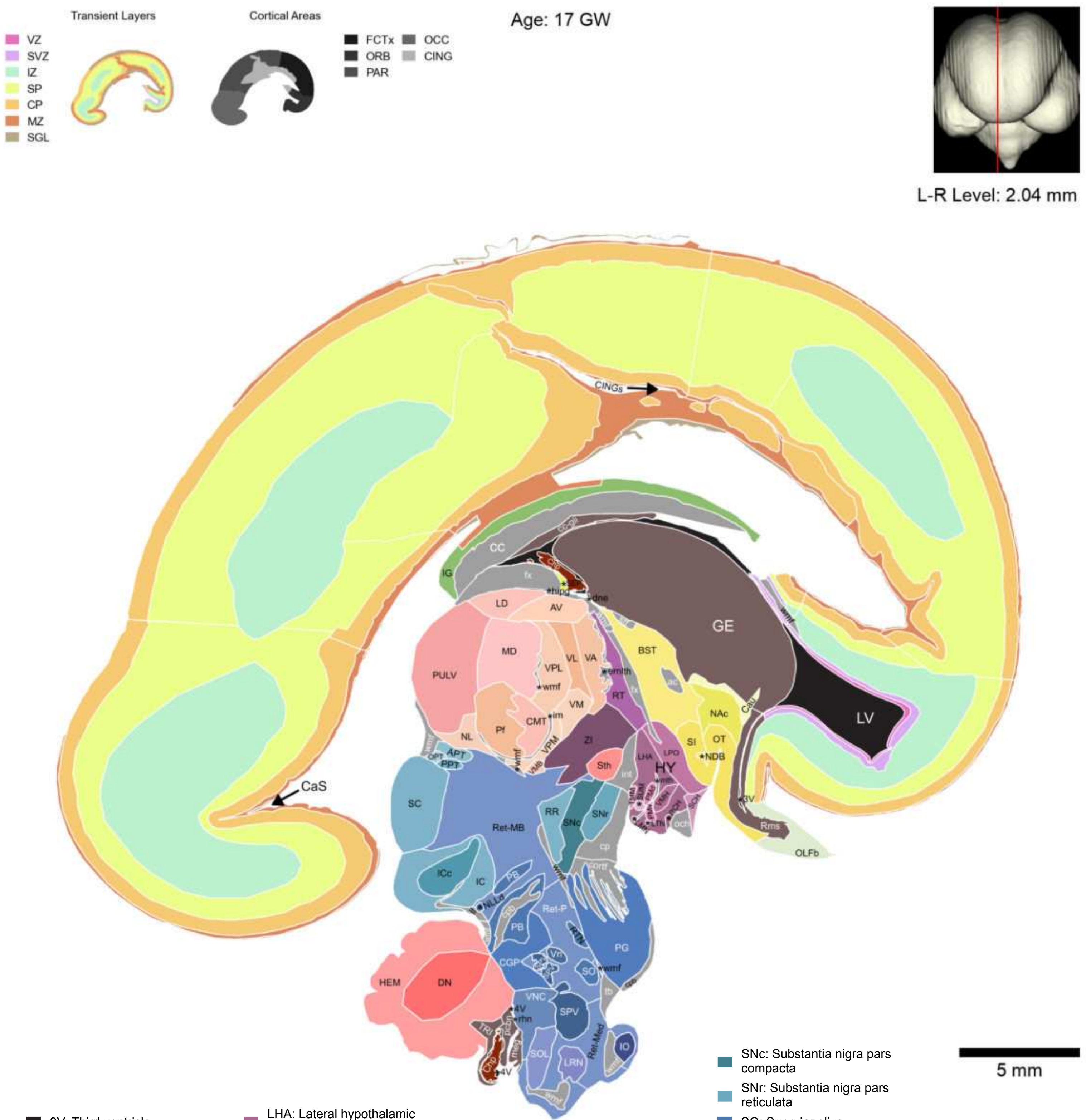

- 3V: Third ventricle
- 4V: Fourth ventricle
- APT: Anterior pretectal nucleus
- AV: Anteroventral nucleus [thalamus]
- BST: Bed nucleus of the stria terminalis
- CGP: Central gray of the pons
- CMT: Centromedian nucleus [thalamus]
- Cau: Caudate nucleus
- Chp: Choroid plexus
- DN: Dentate nucleus
- GE: Ganglionic eminence
- HEM: Cerebellar hemispheres
- HY: Hypothalamus
- IC: Inferior colliculus
- ICc: Inferior colliculus, central nucleus
- IG: Induseum griseum
- IO: Inferior olive
- LD: Lateral dorsal nucleus [thalamus]
- LHA: Lateral hypothalamic area
- LMN: Lateral mammillary nucleus
- LPO: Lateral preoptic area
- LRN: Lateral reticular nucleus
- LTN: Lateral tuberal nucleus
- LV: Lateral ventricle
- MD: Medial dorsal nucleus [thalamus]
- NAc: Nucleus accumbens
- NDB: Nucleus of the diagonal band
- NL: Nucleus limitans [thalamus]
- NLLd: Nucleus of the lateral lemniscus, dorsal
- OLFb: Olfactory bulb
- OPT: Olivary pretectal nucleus
- OT: Olfactory tubercle
- PB: Parabrachial nucleus
- PG: Pontine gray
- PMd: Dorsal premammillary nucleus
- PMv: Ventral premammillary nucleus
- PPT: Posterior pretectal nucleus
- PULV: Pulvinar nucleus [thalamus]
- Pf: Parafascicular nucleus [thalamus]
- RCH: Retrochiasmatic nucleus [hypothalamus]
- RR: Retrorubral area
- RT: Reticular nucleus [thalamus]
- RTN: Reticular tegmental nucleus
- Ret-MB: Reticular formation, Midbrain
- Ret-Med: Reticular formation, Medulla
- Ret-P: Reticular formation, Pons
- Rms: Rostral migratory stream
- SC: Superior colliculus
- SCH: Suprachiasmatic nucleus [hypothalamus]
- SEP: Septum
- SI: Substantia innominata
- SNc: Substantia nigra pars compacta
- SNr: Substantia nigra pars reticulata
- SO: Superior olive
- SOL: Solitary nucleus
- SPV: Spinal nucleus of the trigeminal
- SUM: Supramammillary area
- Sth: Subthalamus
- TMM: Tuberomammillary nucleus
- TRI: Germinal trigone
- VA: Ventral anterior nucleus [thalamus]
- VL: Ventral lateral nucleus [thalamus]
- VM: Ventral medial nucleus [thalamus]
- VMB: Ventral medial basal nucleus [thalamus]
- VMH: Ventromedial nucleus [hypothalamus]
- VNC: Vestibular nuclear complex
- VPL: Ventral posterolateral nucleus [thalamus]
- VPM: Ventral posteromedial nucleus [thalamus]
- Vn: Trigeminal motor nucleus
- ZI: Zona incerta
- → CaS: Calcarine sulcus
- → CINGs: Cingulate sulcus

Age: 17 GW

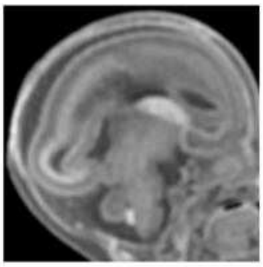

L-R Level: 1.8 mm

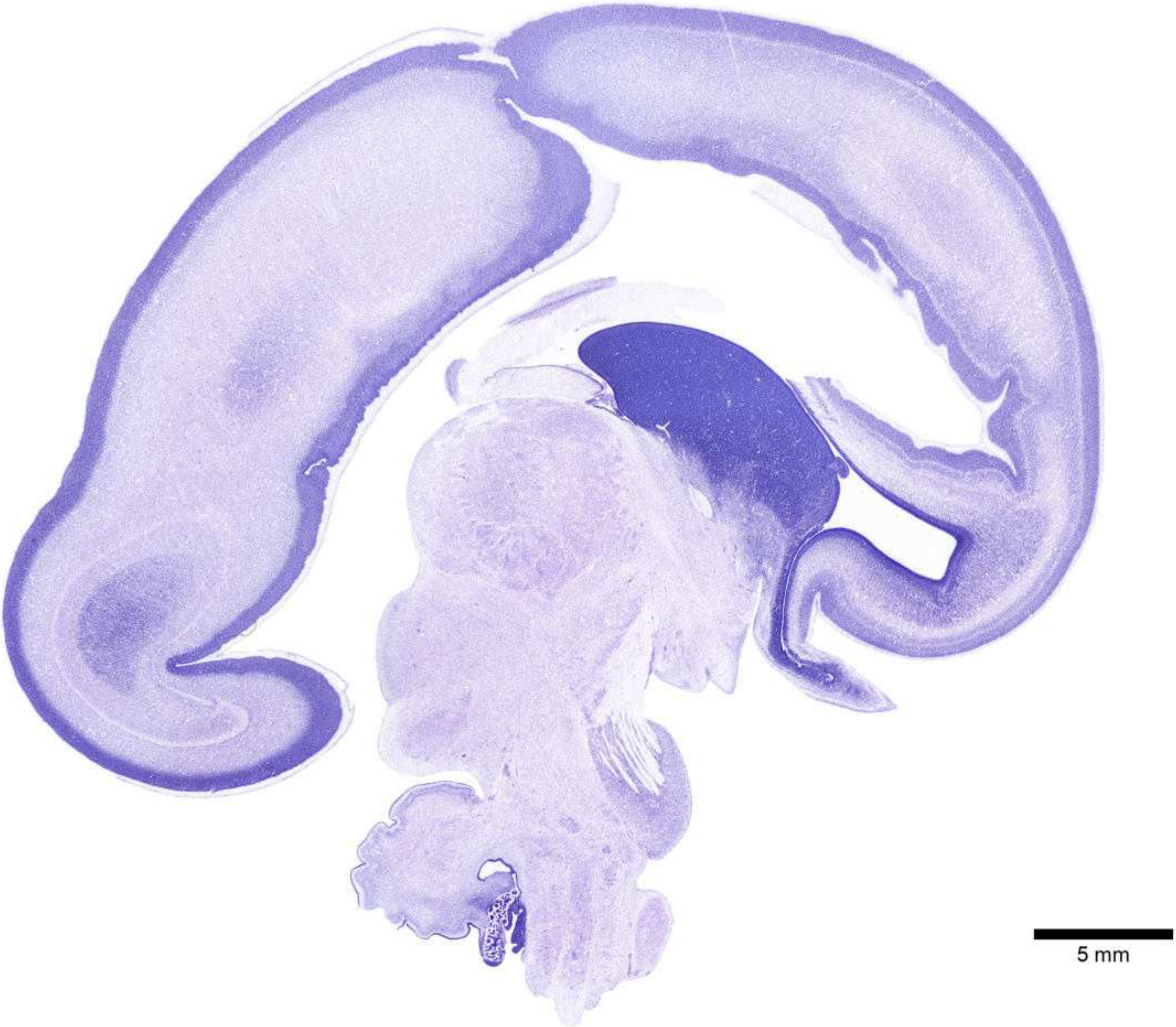

5 mm

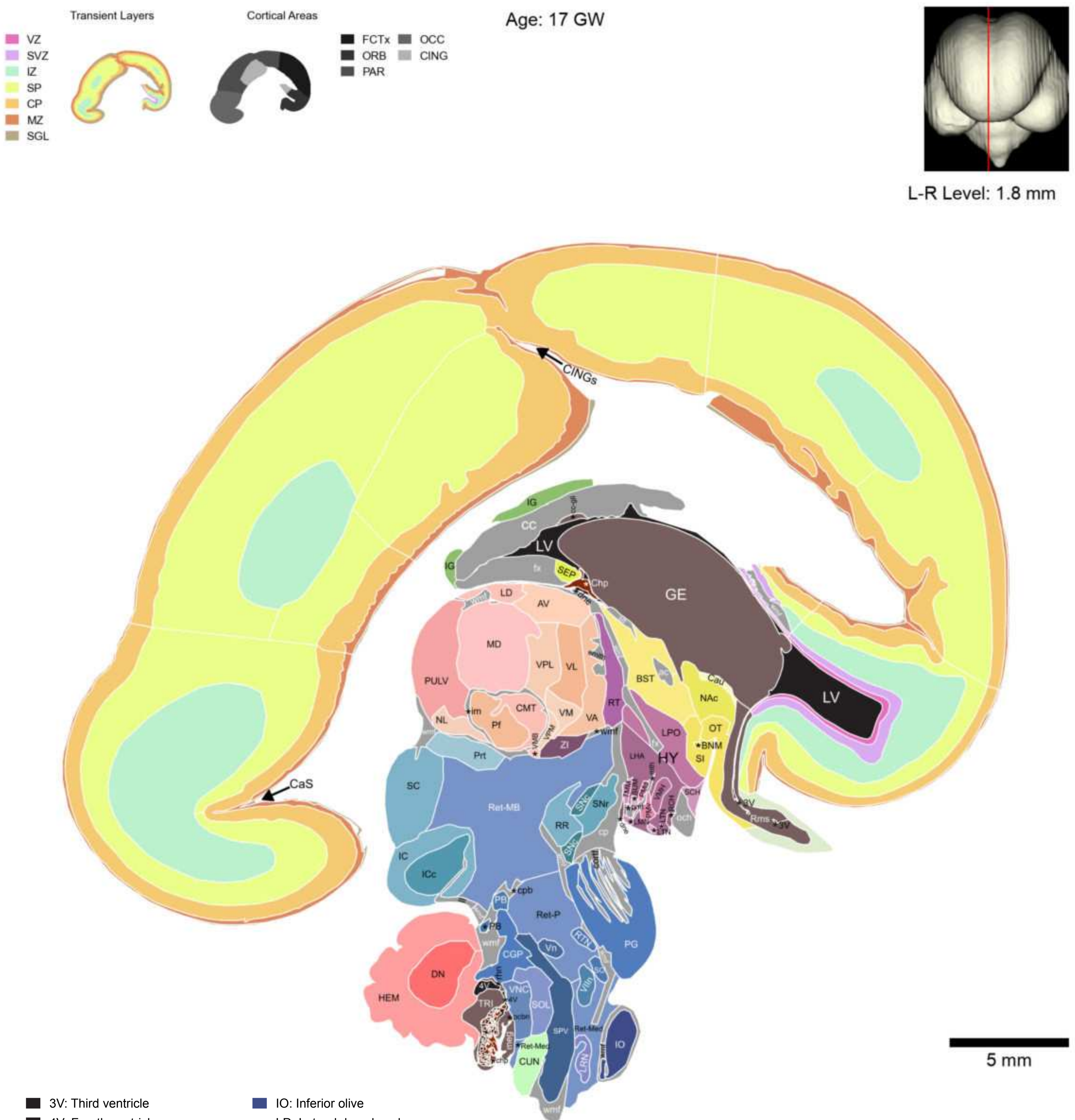

- 3V: Third ventricle
- 4V: Fourth ventricle
- AV: Anteroventral nucleus [thalamus]
- BNM: Basal nucleus of Meynert
- BST: Bed nucleus of the stria terminalis
- CGP: Central gray of the pons
- CMT: Centromedian nucleus [thalamus]
- CUN: Cuneate nucleus
- Cau: Caudate nucleus
- Chp: Choroid plexus
- DN: Dentate nucleus
- GE: Ganglionic eminence
- HEM: Cerebellar hemispheres
- HY: Hypothalamus
- IC: Inferior colliculus
- ICc: Inferior colliculus, central nucleus
- IG: Induseum griseum
- IO: Inferior olive
- LD: Lateral dorsal nucleus [thalamus]
- LHA: Lateral hypothalamic area
- LMN: Lateral mammillary nucleus
- LPO: Lateral preoptic area
- LRN: Lateral reticular nucleus
- LTN: Lateral tuberal nucleus
- LV: Lateral ventricle
- MD: Medial dorsal nucleus [thalamus]
- NAc: Nucleus accumbens
- NL: Nucleus limitans [thalamus]
- OLFb: Olfactory bulb
- OT: Olfactory tubercle
- PB: Parabrachial nucleus
- PG: Pontine gray
- PMd: Dorsal premammillary nucleus
- PMv: Ventral premammillary nucleus
- PULV: Pulvinar nucleus [thalamus]
- Pf: Parafascicular nucleus [thalamus]
- Prt: Pretectum
- RCH: Retrochiasmatic nucleus [hypothalamus]
- RR: Retrorubral area
- RT: Reticular nucleus [thalamus]
- RTN: Reticular tegmental nucleus
- Ret-MB: Reticular formation, Midbrain
- Ret-Med: Reticular formation, Medulla
- Ret-P: Reticular formation, Pons
- Rms: Rostral migratory stream
- SC: Superior colliculus
- SCH: Suprachiasmatic nucleus [hypothalamus]
- SEP: Septum
- SI: Substantia innominata
- SNc: Substantia nigra pars compacta
- SNr: Substantia nigra pars reticulata
- SO: Superior olive
- SOL: Solitary nucleus
- SPV: Spinal nucleus of the trigeminal
- SUM: Supramammillary area
- TMM: Tuberomammillary nucleus
- TRI: Germinal trigone
- VA: Ventral anterior nucleus [thalamus]
- VIIn: Facial motor nucleus
- VL: Ventral lateral nucleus [thalamus]
- VM: Ventral medial nucleus [thalamus]
- VMB: Ventral medial basal nucleus [thalamus]
- VMH: Ventromedial nucleus [hypothalamus]
- VNC: Vestibular nuclear complex
- VPL: Ventral posterolateral nucleus [thalamus]
- VPM: Ventral posteromedial nucleus [thalamus]
- Vn: Trigeminal motor nucleus
- ZI: Zona incerta
- → CaS: Calcarine sulcus
- → CINGs: Cingulate sulcus

Age: 17 GW

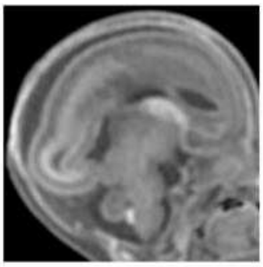

L-R Level: 1.56 mm

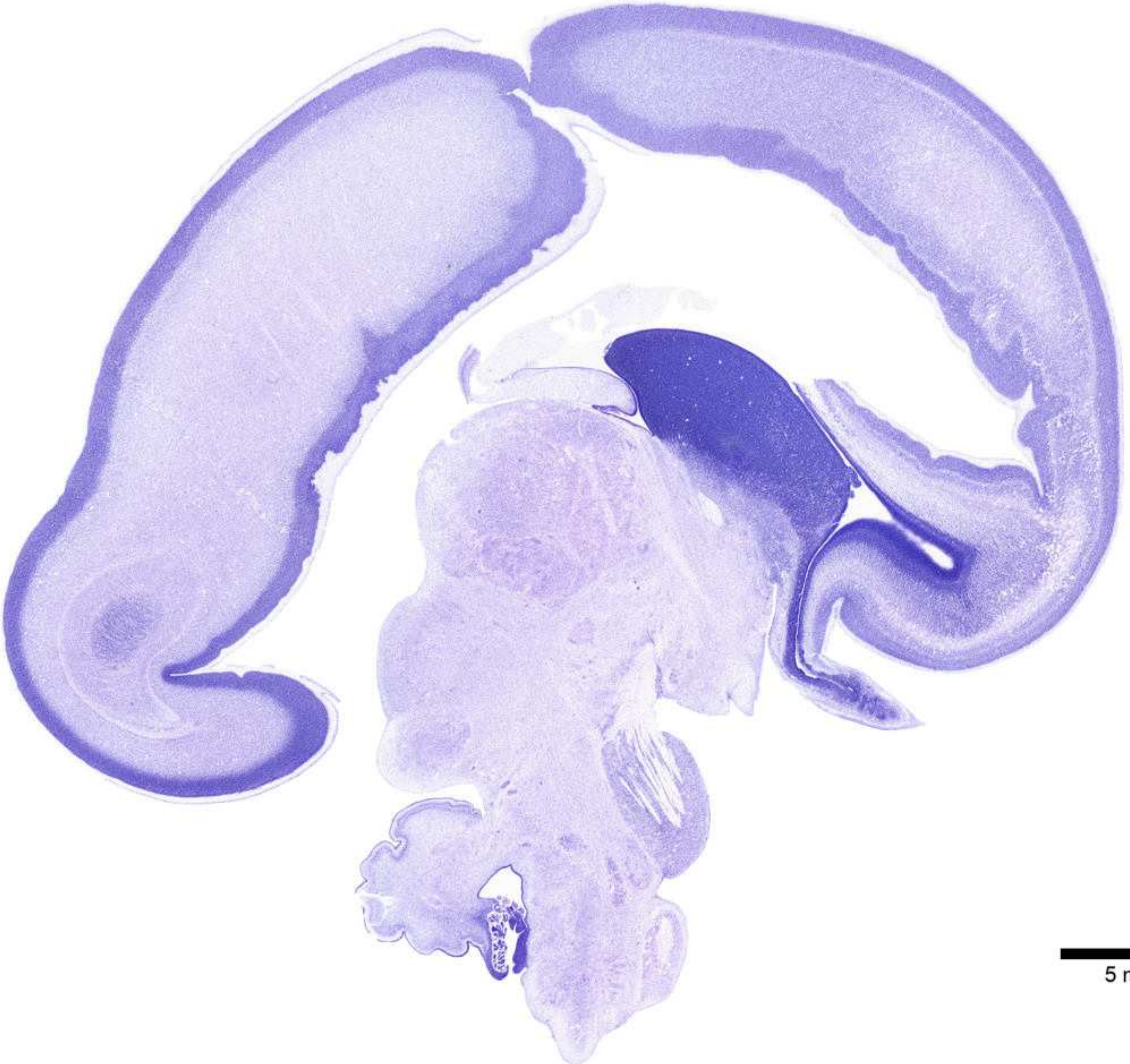

5 mm

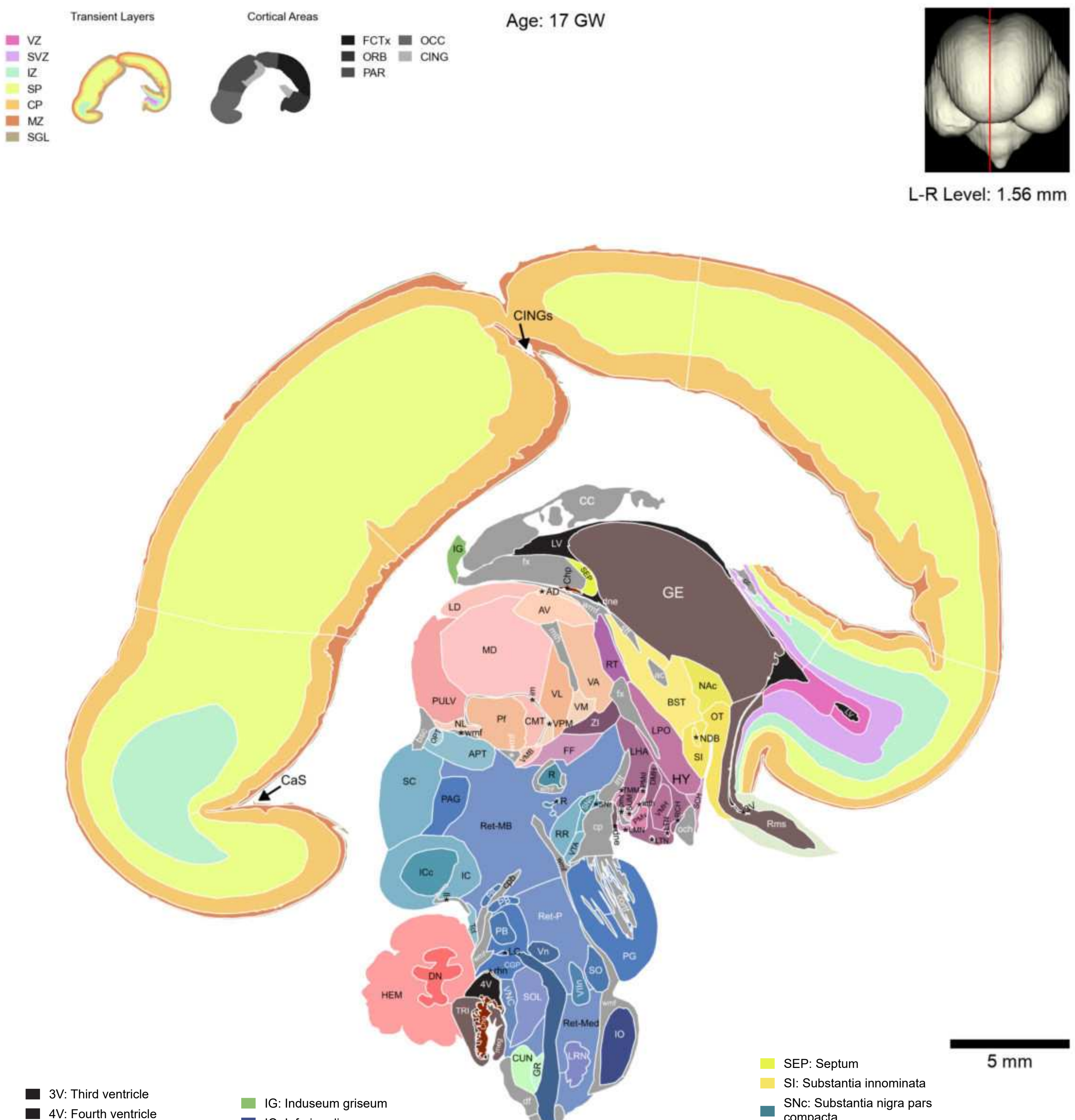

- 3V: Third ventricle
- 4V: Fourth ventricle
- AD: Anterodorsal nucleus [thalamus]
- APT: Anterior pretectal nucleus
- AV: Anteroventral nucleus [thalamus]
- BST: Bed nucleus of the stria terminalis
- CGP: Central gray of the pons
- CMT: Centromedian nucleus [thalamus]
- CUN: Cuneate nucleus
- Chp: Choroid plexus
- DMH: Dorsomedial nucleus [hypothalamus]
- DN: Dentate nucleus
- FF: Field of Forel
- GE: Ganglionic eminence
- GR: Gracile nucleus
- HEM: Cerebellar hemispheres
- HY: Hypothalamus
- IC: Inferior colliculus
- ICc: Inferior colliculus, central nucleus
- IG: Induseum griseum
- IO: Inferior olive
- LC: Locus coeruleus
- LD: Lateral dorsal nucleus [thalamus]
- LHA: Lateral hypothalamic area
- LMN: Lateral mammillary nucleus
- LPO: Lateral preoptic area
- LRN: Lateral reticular nucleus
- LTN: Lateral tuberal nucleus
- LV: Lateral ventricle
- MD: Medial dorsal nucleus [thalamus]
- NAc: Nucleus accumbens
- NDB: Nucleus of the diagonal band
- NL: Nucleus limitans [thalamus]
- OLFb: Olfactory bulb
- OPT: Olivary pretectal nucleus
- OT: Olfactory tubercle
- PAG: Periaqueductal gray
- PB: Parabrachial nucleus
- PG: Pontine gray
- PMd: Dorsal premammillary nucleus
- PMv: Ventral premammillary nucleus
- PULV: Pulvinar nucleus [thalamus]
- Pf: Parafascicular nucleus [thalamus]
- R: Red nucleus
- RCH: Retrochiasmatic nucleus [hypothalamus]
- RR: Retrorubral area
- RT: Reticular nucleus [thalamus]
- Ret-MB: Reticular formation, Midbrain
- Ret-Med: Reticular formation, Medulla
- Ret-P: Reticular formation, Pons
- Rms: Rostral migratory stream
- SC: Superior colliculus
- SCH: Suprachiasmatic nucleus [hypothalamus]
- SEP: Septum
- SI: Substantia innominata
- SNc: Substantia nigra pars compacta
- SNr: Substantia nigra pars reticulata
- SO: Superior olive
- SOL: Solitary nucleus
- SPV: Spinal nucleus of the trigeminal
- SUM: Supramammillary area
- TMM: Tuberomammillary nucleus
- TRI: Germinal trigone
- Tct: Tectum
- VA: Ventral anterior nucleus [thalamus]
- VIIn: Facial motor nucleus
- VL: Ventral lateral nucleus [thalamus]
- VM: Ventral medial nucleus [thalamus]
- VMB: Ventral medial basal nucleus [thalamus]
- VMH: Ventromedial nucleus [hypothalamus]
- VNC: Vestibular nuclear complex
- VPM: Ventral posteromedial nucleus [thalamus]
- VTA: Ventral tegmental area
- Vn: Trigeminal motor nucleus
- ZI: Zona incerta
- → CaS: Calcarine sulcus
- → CINGs: Cingulate sulcus

Age: 17 GW

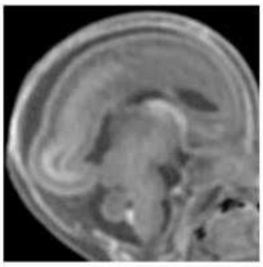

L-R Level: 1.26 mm

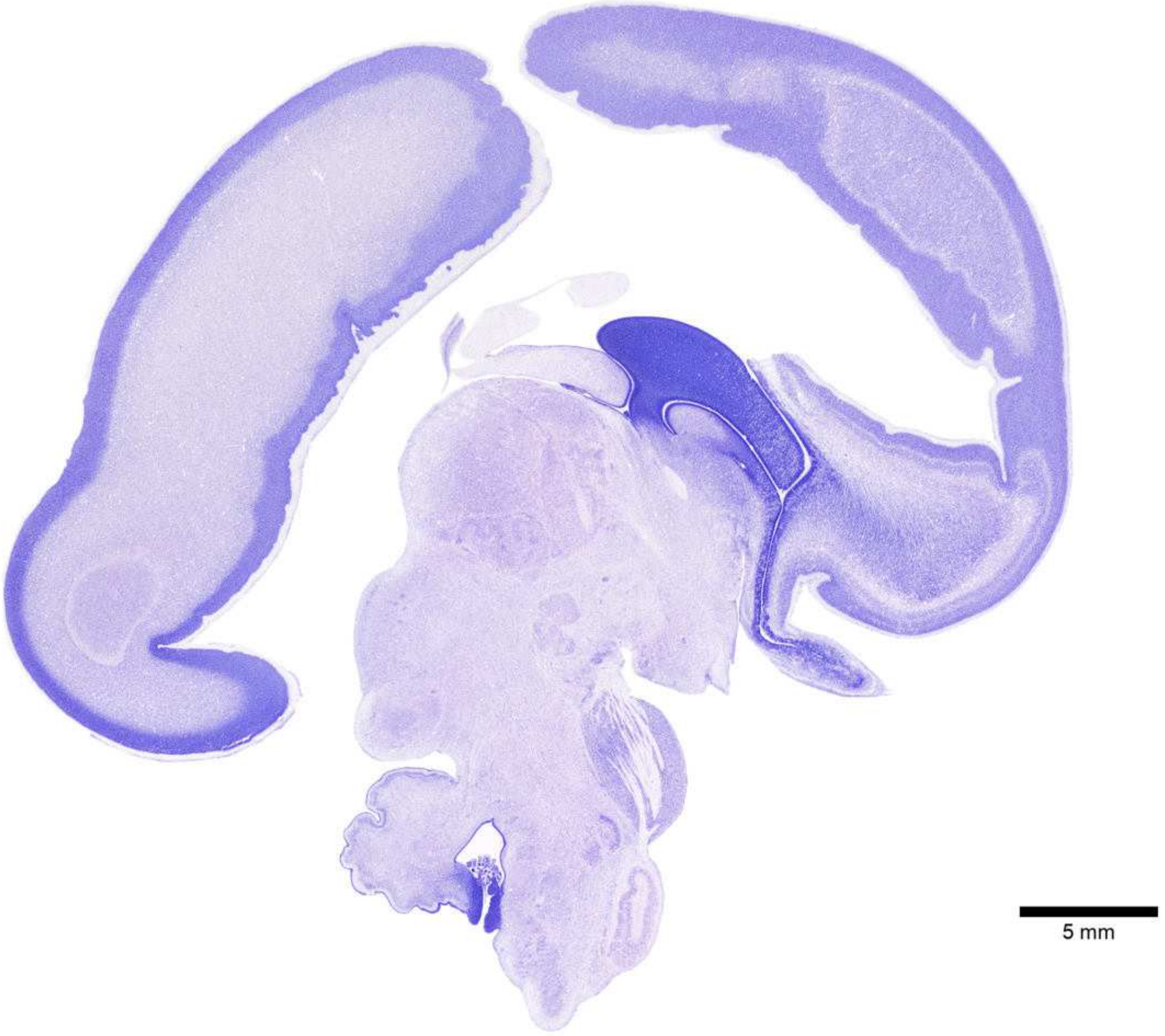

5 mm

Transient Layers

- VZ
- SVZ
- IZ
- SP
- CP
- MZ
- SGL

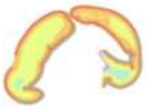

Cortical Areas

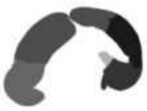

- FCTx
- ORB
- PAR
- OCC
- CING

Age: 17 GW

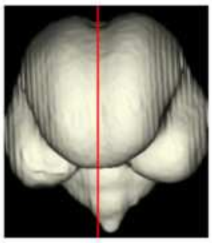

L-R Level: 1.26 mm

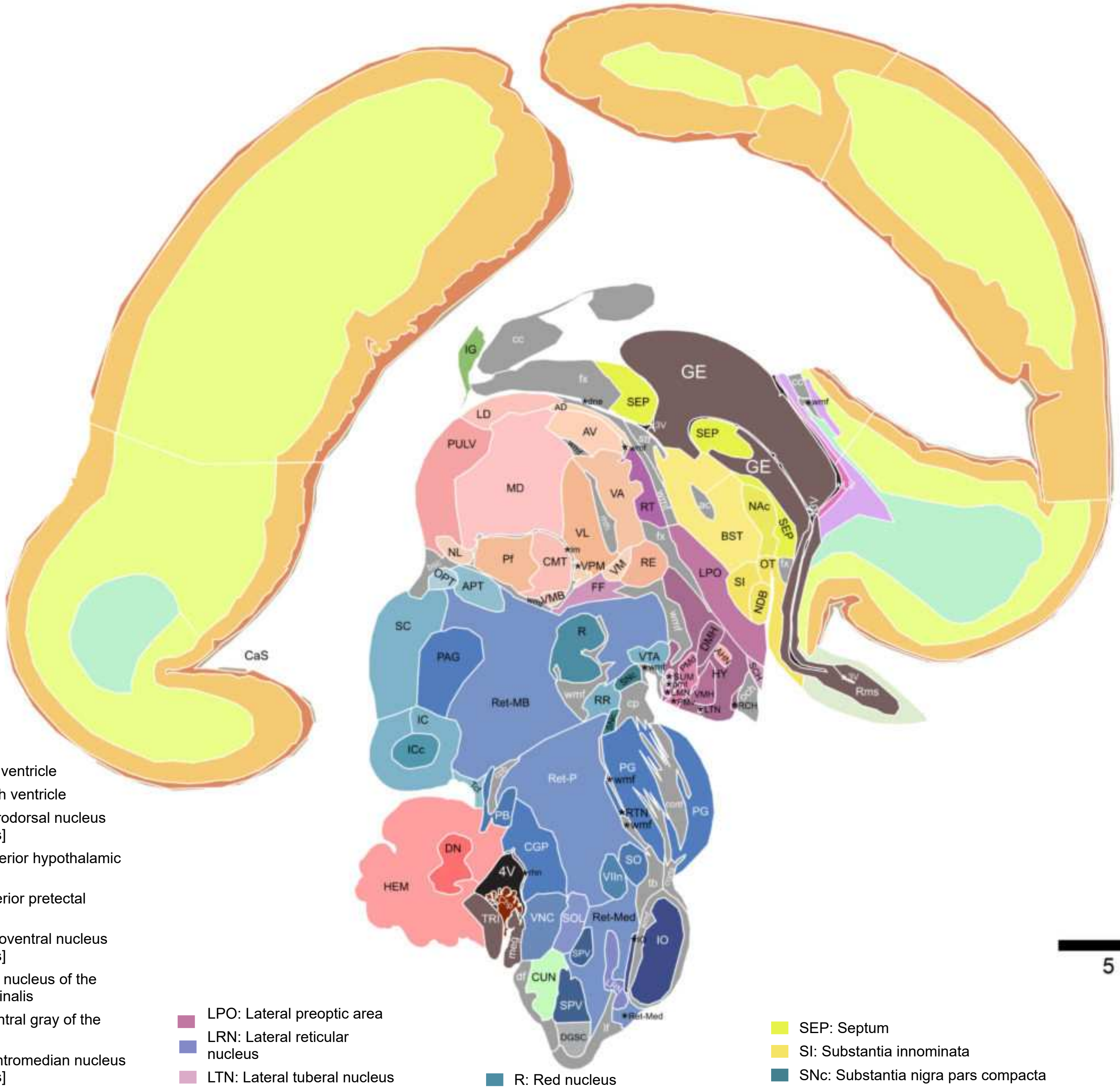

5 mm

- 3V: Third ventricle
- 4V: Fourth ventricle
- AD: Anterodorsal nucleus [thalamus]
- AHN: Anterior hypothalamic nucleus
- APT: Anterior pretectal nucleus
- AV: Anteroventral nucleus [thalamus]
- BST: Bed nucleus of the stria terminalis
- CGP: Central gray of the pons
- CMT: Centromedian nucleus [thalamus]
- CUN: Cuneate nucleus
- Chp: Choroid plexus
- DGSC: Dorsal gray of the spinal cord
- DMH: Dorsomedial nucleus [hypothalamus]
- DN: Dentate nucleus
- FF: Field of Forel
- GE: Ganglionic eminence
- HEM: Cerebellar hemispheres
- HY: Hypothalamus
- IC: Inferior colliculus
- ICc: Inferior colliculus, central nucleus
- IG: Induseum griseum
- IO: Inferior olive
- LD: Lateral dorsal nucleus [thalamus]
- LHA: Lateral hypothalamic area
- LMN: Lateral mammillary nucleus
- LPO: Lateral preoptic area
- LRN: Lateral reticular nucleus
- LTN: Lateral tuberal nucleus
- LV: Lateral ventricle
- MD: Medial dorsal nucleus [thalamus]
- NAc: Nucleus accumbens
- NDB: Nucleus of the diagonal band
- NL: Nucleus limitans [thalamus]
- OLFb: Olfactory bulb
- OPT: Olivary pretectal nucleus
- OT: Olfactory tubercle
- PAG: Periaqueductal gray
- PB: Parabrachial nucleus
- PG: Pontine gray
- PMd: Dorsal premammillary nucleus
- PMv: Ventral premammillary nucleus
- PULV: Pulvinar nucleus [thalamus]
- Pf: Parafascicular nucleus [thalamus]
- R: Red nucleus
- RCH: Retrochiasmatic nucleus [hypothalamus]
- RE: Nucleus reuniens
- RR: Retrorubral area
- RT: Reticular nucleus [thalamus]
- RTN: Reticular tegmental nucleus
- Ret-MB: Reticular formation, Midbrain
- Ret-Med: Reticular formation, Medulla
- Ret-P: Reticular formation, Pons
- Rms: Rostral migratory stream
- SC: Superior colliculus
- SCH: Suprachiasmatic nucleus [hypothalamus]
- SEP: Septum
- SI: Substantia innominata
- SNc: Substantia nigra pars compacta
- SO: Superior olive
- SOL: Solitary nucleus
- SPV: Spinal nucleus of the trigeminal
- SUM: Supramammillary area
- TRI: Germinal trigone
- Tct: Tectum
- VA: Ventral anterior nucleus [thalamus]
- VIIn: Facial motor nucleus
- VL: Ventral lateral nucleus [thalamus]
- VM: Ventral medial nucleus [thalamus]
- VMB: Ventral medial basal nucleus [thalamus]
- VMH: Ventromedial nucleus [hypothalamus]
- VNC: Vestibular nuclear complex
- VPM: Ventral posteromedial nucleus [thalamus]
- VTA: Ventral tegmental area
- → CaS: Calcarine sulcus

Age: 17 GW

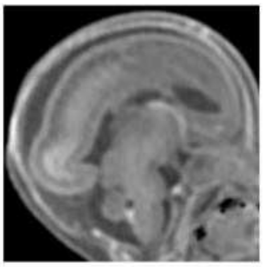

L-R Level: 0.96 mm

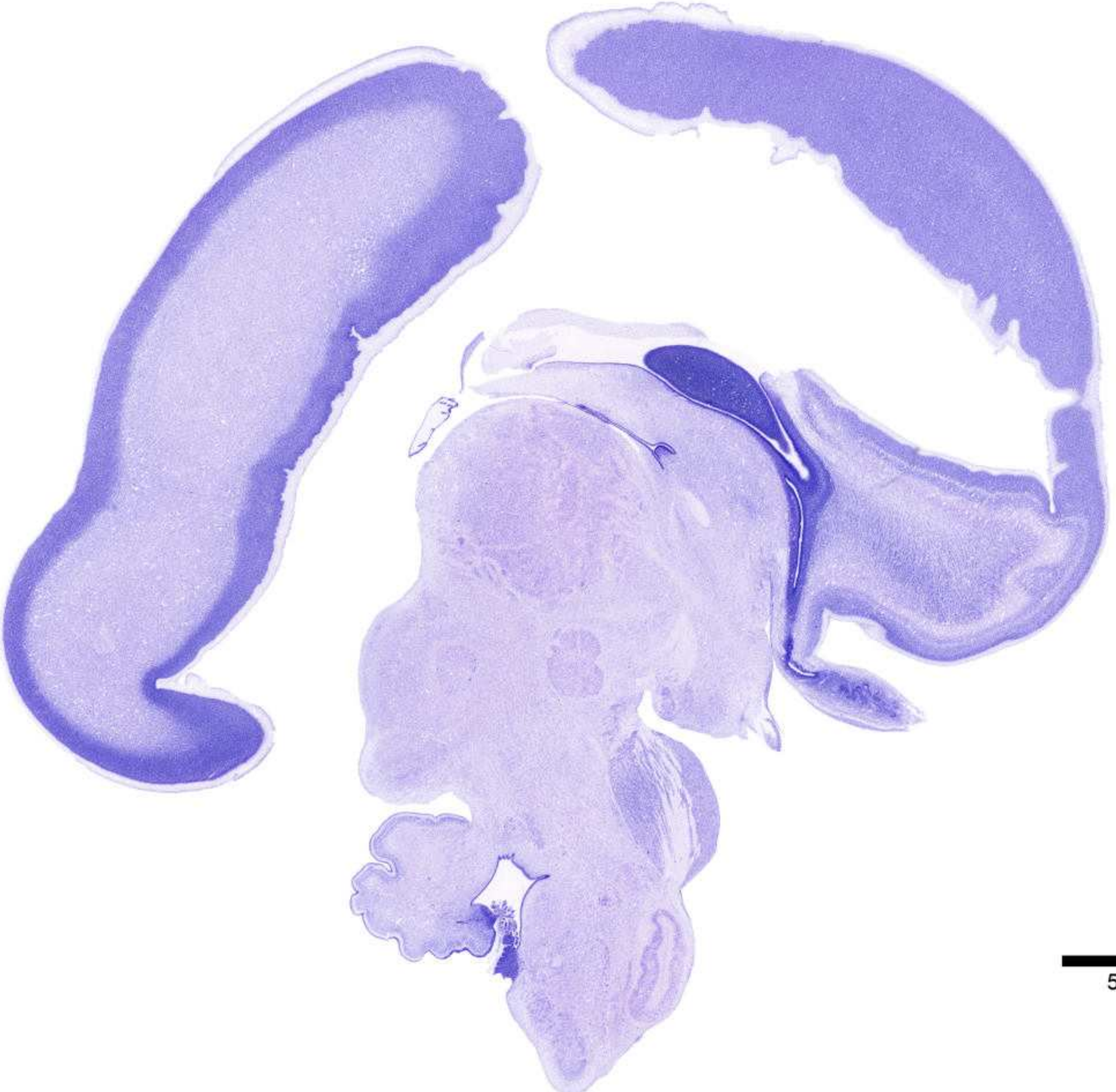

5 mm

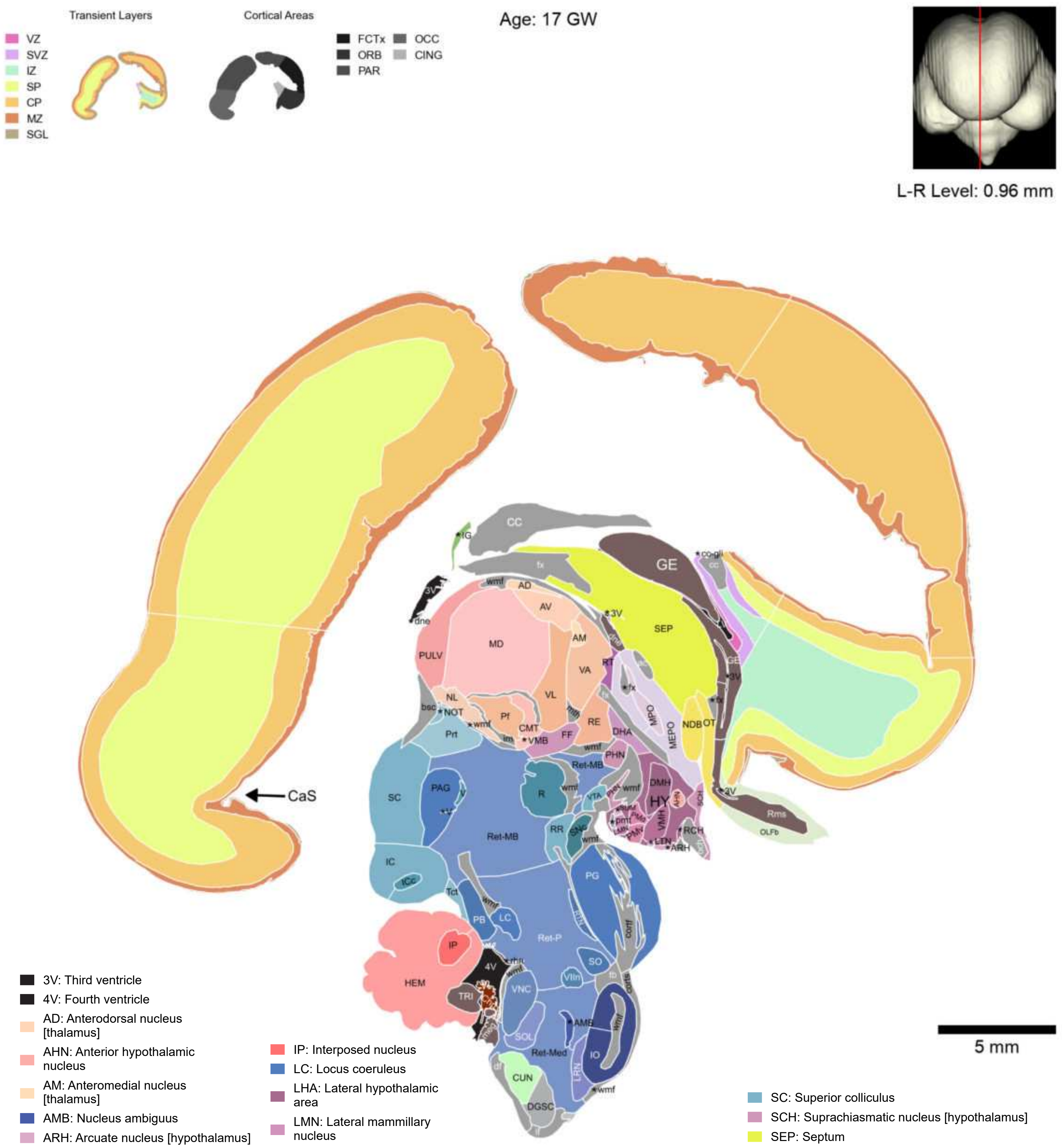

3V: Third ventricle
4V: Fourth ventricle
AD: Anterodorsal nucleus [thalamus]
AHN: Anterior hypothalamic nucleus
AM: Anteromedial nucleus [thalamus]
AMB: Nucleus ambiguus
ARH: Arcuate nucleus [hypothalamus]
AV: Anteroventral nucleus [thalamus]
CMT: Centromedian nucleus [thalamus]
CUN: Cuneate nucleus
Chp: Choroid plexus
DGSC: Dorsal gray of the spinal cord
DHA: Dorsal hypothalamic area
DMH: Dorsomedial nucleus [hypothalamus]
FF: Field of Forel
GE: Ganglionic eminence
HEM: Cerebellar hemispheres
HY: Hypothalamus
IC: Inferior colliculus
ICc: Inferior colliculus, central nucleus
IG: Induseum griseum
IO: Inferior olive
IP: Interposed nucleus
LC: Locus coeruleus
LHA: Lateral hypothalamic area
LMN: Lateral mammillary nucleus
LRN: Lateral reticular nucleus
LTN: Lateral tuberal nucleus
LV: Lateral ventricle
MD: Medial dorsal nucleus [thalamus]
MEPO: Medial preoptic area
MPO: Medial preoptic nucleus
NDB: Nucleus of the diagonal band
NL: Nucleus limitans [thalamus]
NOT: Nucleus of the optic tract
OLFb: Olfactory bulb
OT: Olfactory tubercle
PAG: Periaqueductal gray
PB: Parabrachial nucleus
PG: Pontine gray
PHN: Posterior hypothalamic nucleus
PMd: Dorsal premammillary nucleus
PMv: Ventral premammillary nucleus
PULV: Pulvinar nucleus [thalamus]
Pf: Parafascicular nucleus [thalamus]
Prt: Pretectum
R: Red nucleus
RCH: Retrochiasmatic nucleus [hypothalamus]
RE: Nucleus reuniens
RR: Retrorubral area
RT: Reticular nucleus [thalamus]
RTN: Reticular tegmental nucleus
Ret-MB: Reticular formation, Midbrain
Ret-Med: Reticular formation, Medulla
Ret-P: Reticular formation, Pons
Rms: Rostral migratory stream
SC: Superior colliculus
SCH: Suprachiasmatic nucleus [hypothalamus]
SEP: Septum
SNc: Substantia nigra pars compacta
SO: Superior olive
SOL: Solitary nucleus
SP: Spinal cord
SUM: Supramammillary area
TRI: Germinal trigone
Tct: Tectum
V: Mesencephalic nucleus
VA: Ventral anterior nucleus [thalamus]
VIIn: Facial motor nucleus
VL: Ventral lateral nucleus [thalamus]
VMB: Ventral medial basal nucleus [thalamus]
VMH: Ventromedial nucleus [hypothalamus]
VNC: Vestibular nuclear complex
VTA: Ventral tegmental area
→ CaS: Calcarine sulcus

Age: 17 GW

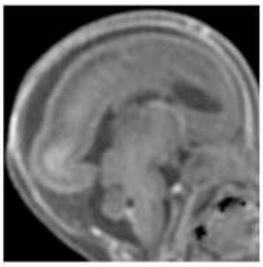

L-R Level: 0.72 mm

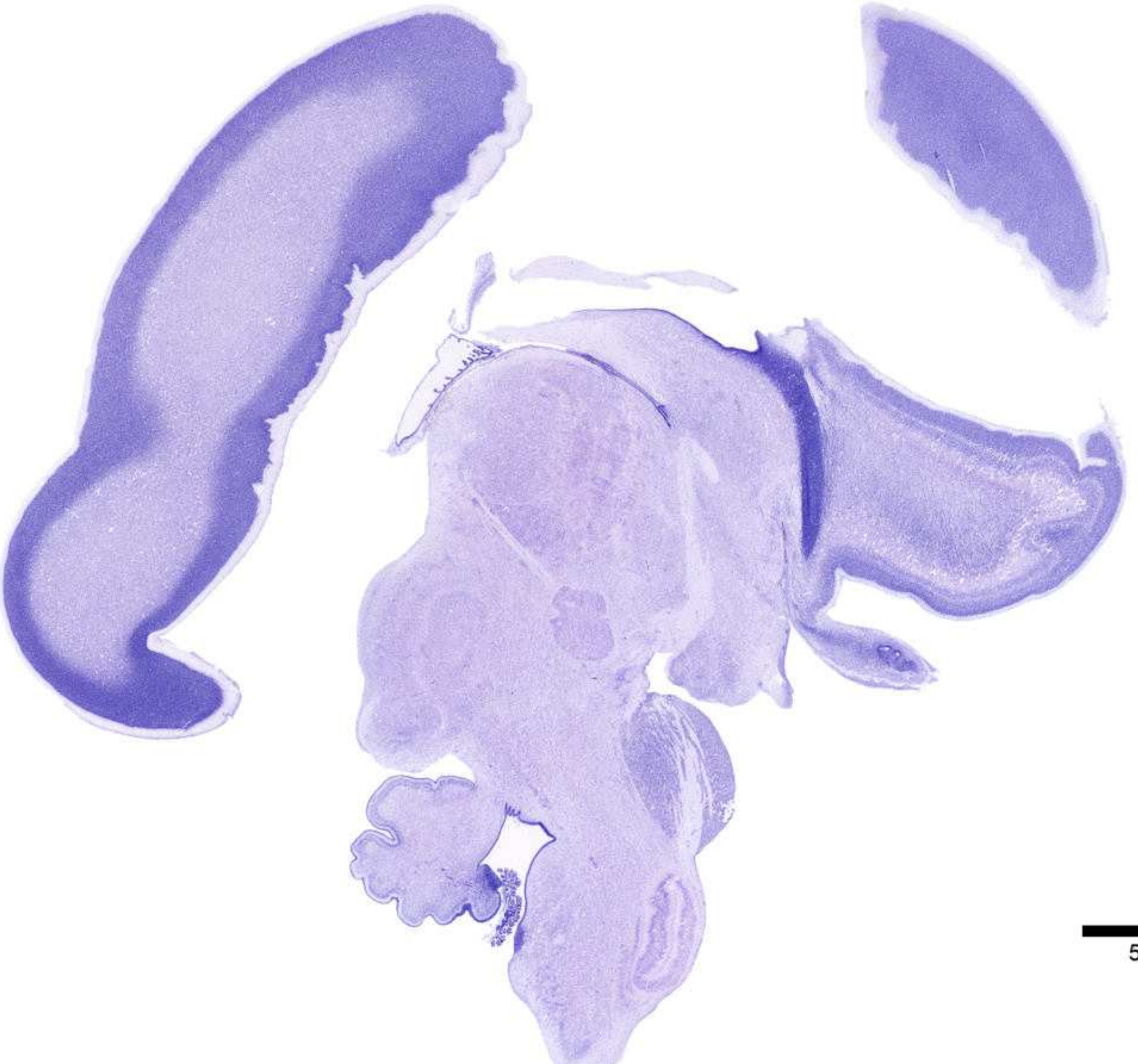

5 mm

Transient Layers

- SVZ
- IZ
- SP
- CP
- MZ
- SGL

Cortical Areas

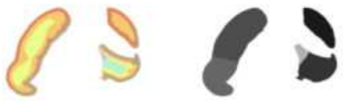

- FCTx
- ORB
- PAR
- OCC
- CING

Age: 17 GW

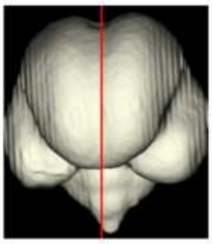

L-R Level: 0.72 mm

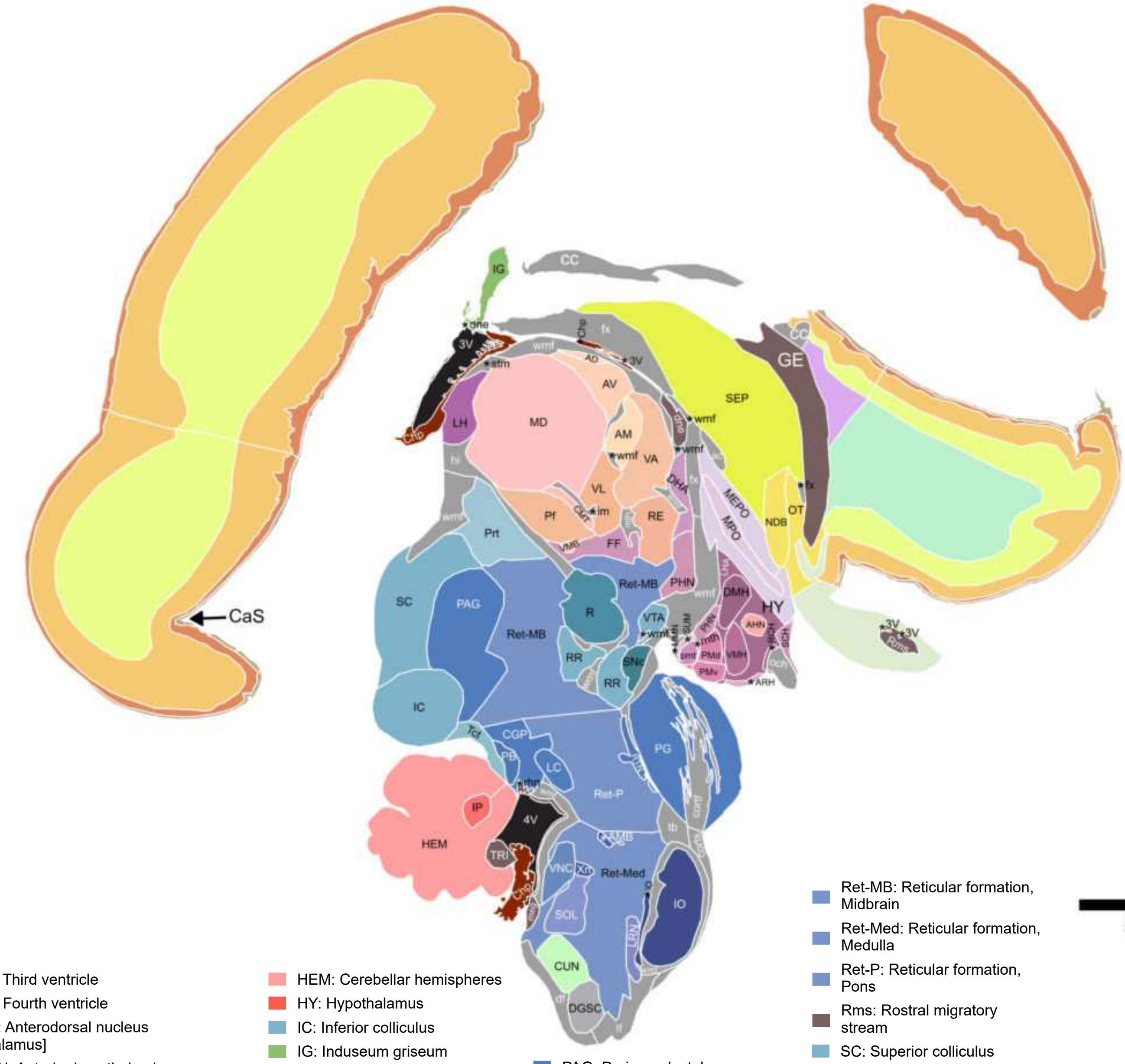

5 mm

- 3V: Third ventricle
- 4V: Fourth ventricle
- AD: Anterodorsal nucleus [thalamus]
- AHN: Anterior hypothalamic nucleus
- AM: Anteromedial nucleus [thalamus]
- AMB: Nucleus ambiguus
- ARH: Arcuate nucleus [hypothalamus]
- AV: Anteroventral nucleus [thalamus]
- CGP: Central gray of the pons
- CMT: Centromedian nucleus [thalamus]
- CUN: Cuneate nucleus
- Chp: Choroid plexus
- DGSC: Dorsal gray of the spinal cord
- DHA: Dorsal hypothalamic area
- DMH: Dorsomedial nucleus [hypothalamus]
- FF: Field of Forel
- GE: Ganglionic eminence
- HEM: Cerebellar hemispheres
- HY: Hypothalamus
- IC: Inferior colliculus
- IG: Induseum griseum
- IO: Inferior olive
- IP: Interposed nucleus
- LC: Locus coeruleus
- LH: Lateral habenula
- LHA: Lateral hypothalamic area
- LRN: Lateral reticular nucleus
- MD: Medial dorsal nucleus [thalamus]
- MEPO: Medial preoptic area
- MMN: Medial mammillary nucleus
- MPO: Medial preoptic nucleus
- NDB: Nucleus of the diagonal band
- OLFb: Olfactory bulb
- OT: Olfactory tubercle
- PAG: Periaqueductal gray
- PB: Parabrachial nucleus
- PG: Pontine gray
- PHN: Posterior hypothalamic nucleus
- PMd: Dorsal premammillary nucleus
- PMv: Ventral premammillary nucleus
- Pf: Parafascicular nucleus [thalamus]
- Prt: Pretectum
- R: Red nucleus
- RCH: Retrochiasmatic nucleus [hypothalamus]
- RE: Nucleus reuniens
- RR: Retrorubral area
- RTN: Reticular tegmental nucleus
- Ret-MB: Reticular formation, Midbrain
- Ret-Med: Reticular formation, Medulla
- Ret-P: Reticular formation, Pons
- Rms: Rostral migratory stream
- SC: Superior colliculus
- SCH: Suprachiasmatic nucleus [hypothalamus]
- SEP: Septum
- SNc: Substantia nigra pars compacta
- SOL: Solitary nucleus
- SUM: Supramammillary area
- TRI: Germinal trigone
- TT: Tenia tecta
- Tct: Tectum
- VA: Ventral anterior nucleus [thalamus]
- VL: Ventral lateral nucleus [thalamus]
- VMB: Ventral medial basal nucleus [thalamus]
- VMH: Ventromedial nucleus [hypothalamus]
- VNC: Vestibular nuclear complex
- VTA: Ventral tegmental area
- Xn: Dorsal motor nucleus
- → CaS: Calcarine sulcus

Age: 17 GW

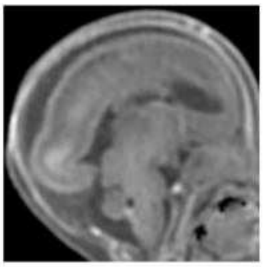

L-R Level: 0.6 mm

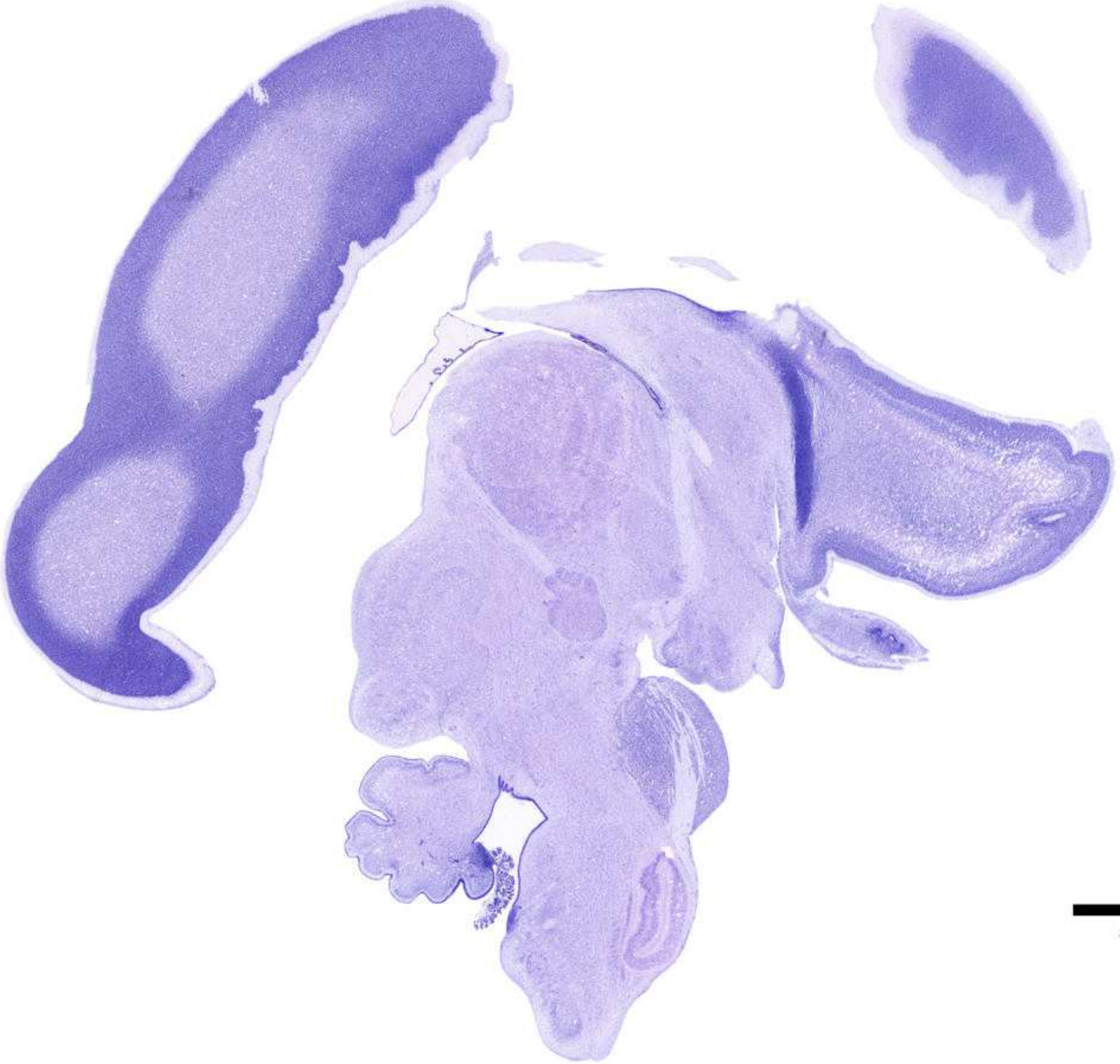

5 mm

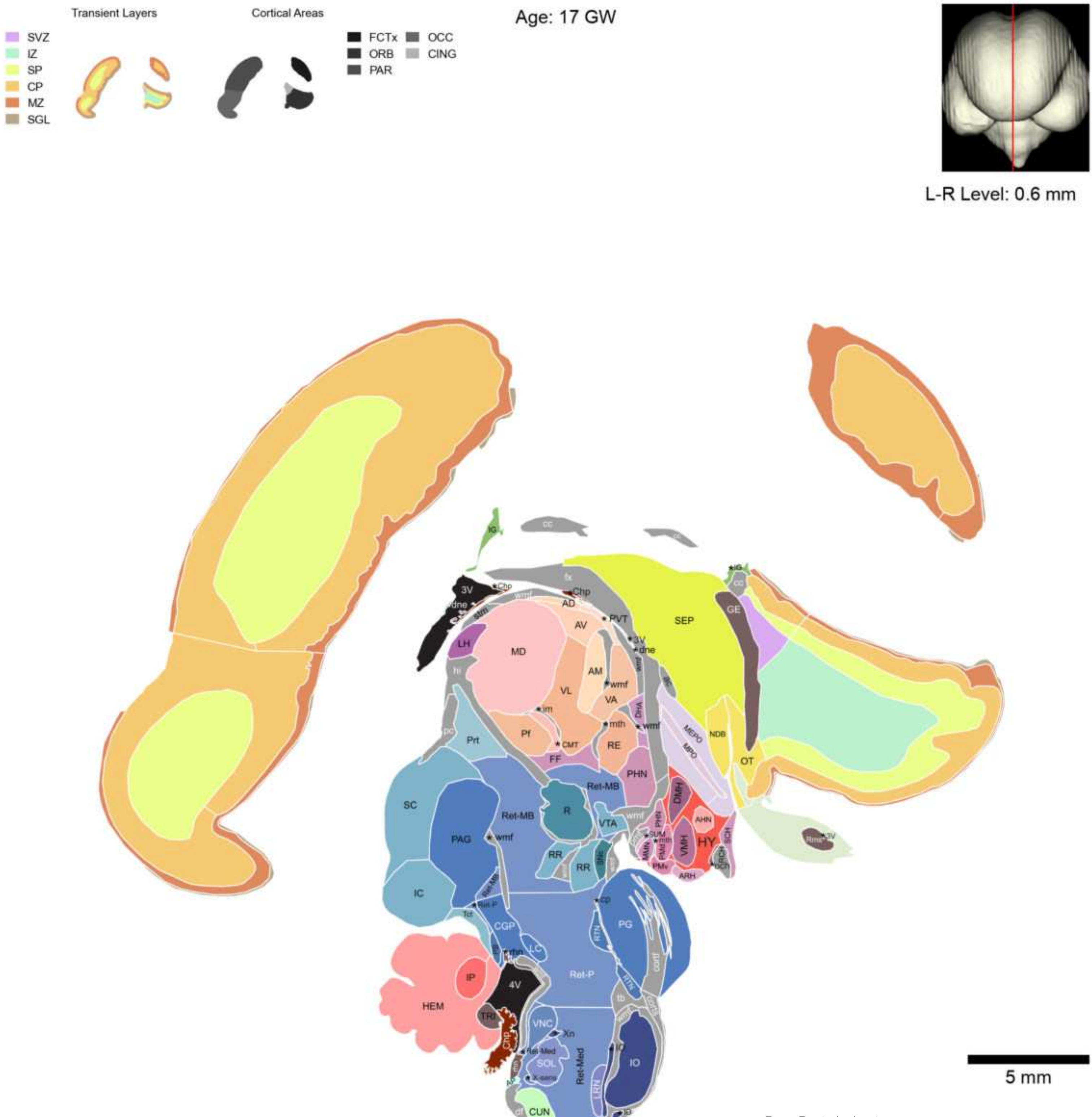

- 3V: Third ventricle
- 4V: Fourth ventricle
- AD: Anterodorsal nucleus [thalamus]
- AHN: Anterior hypothalamic nucleus
- AM: Anteromedial nucleus [thalamus]
- AP: Area postrema
- ARH: Arcuate nucleus [hypothalamus]
- ARM: Arcuate nucleus [medulla]
- AV: Anteroventral nucleus [thalamus]
- CGP: Central gray of the pons
- CMT: Centromedian nucleus [thalamus]
- CUN: Cuneate nucleus
- Chp: Choroid plexus
- DGSC: Dorsal gray of the spinal cord
- DHA: Dorsal hypothalamic area
- DMH: Dorsomedial nucleus [hypothalamus]
- FF: Field of Forel
- GE: Ganglionic eminence
- HEM: Cerebellar hemispheres
- HY: Hypothalamus
- IC: Inferior colliculus
- IG: Induseum griseum
- IO: Inferior olive
- IP: Interposed nucleus
- LC: Locus coeruleus
- LH: Lateral habenula
- LRN: Lateral reticular nucleus
- MD: Medial dorsal nucleus [thalamus]
- MEPO: Medial preoptic area
- MMN: Medial mammillary nucleus
- MPO: Medial preoptic nucleus
- NDB: Nucleus of the diagonal band
- OLFb: Olfactory bulb
- OT: Olfactory tubercle
- PAG: Periaqueductal gray
- PB: Parabrachial nucleus
- PG: Pontine gray
- PHN: Posterior hypothalamic nucleus
- PMd: Dorsal premammillary nucleus
- PMv: Ventral premammillary nucleus
- PVT: Paraventricular nucleus [thalamus]
- Pf: Parafascicular nucleus [thalamus]
- Prt: Pretectum
- R: Red nucleus
- RCH: Retrochiasmatic nucleus [hypothalamus]
- RE: Nucleus reuniens
- RR: Retrorubral area
- RTN: Reticular tegmental nucleus
- Ret-MB: Reticular formation, Midbrain
- Ret-Med: Reticular formation, Medulla
- Ret-P: Reticular formation, Pons
- Rms: Rostral migratory stream
- SC: Superior colliculus
- SCH: Suprachiasmatic nucleus [hypothalamus]
- SEP: Septum
- SNc: Substantia nigra pars compacta
- SOL: Solitary nucleus
- SUM: Supramammillary area
- TRI: Germinal trigone
- TT: Tenia tecta
- Tct: Tectum
- VA: Ventral anterior nucleus [thalamus]
- VL: Ventral lateral nucleus [thalamus]
- VMH: Ventromedial nucleus [hypothalamus]
- VNC: Vestibular nuclear complex
- VTA: Ventral tegmental area
- X-sens: Dorsal sensory nucleus X
- Xn: Dorsal motor nucleus

Age: 17 GW

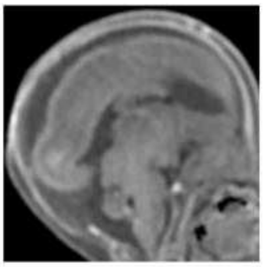

L-R Level: 0.18 mm

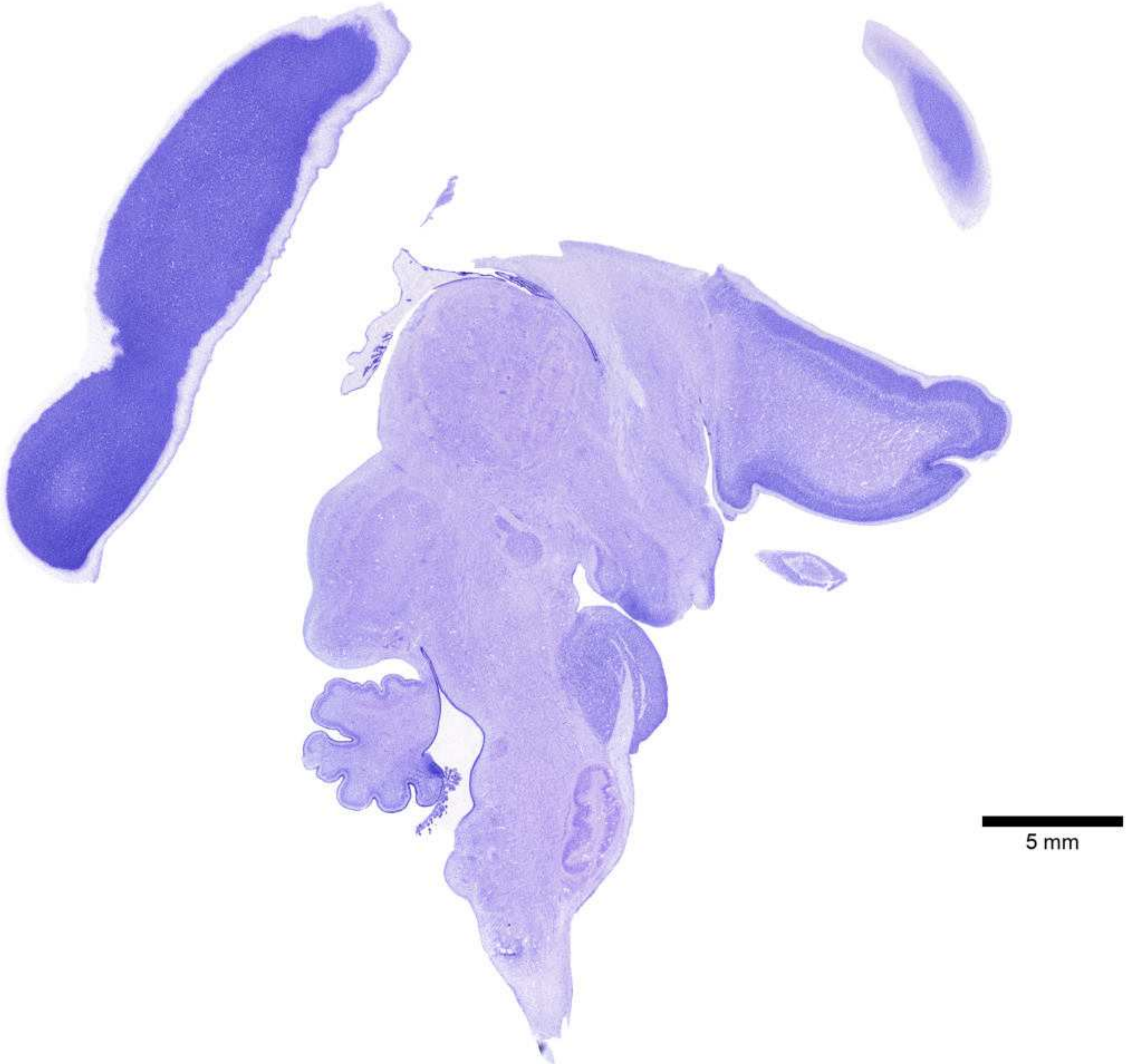

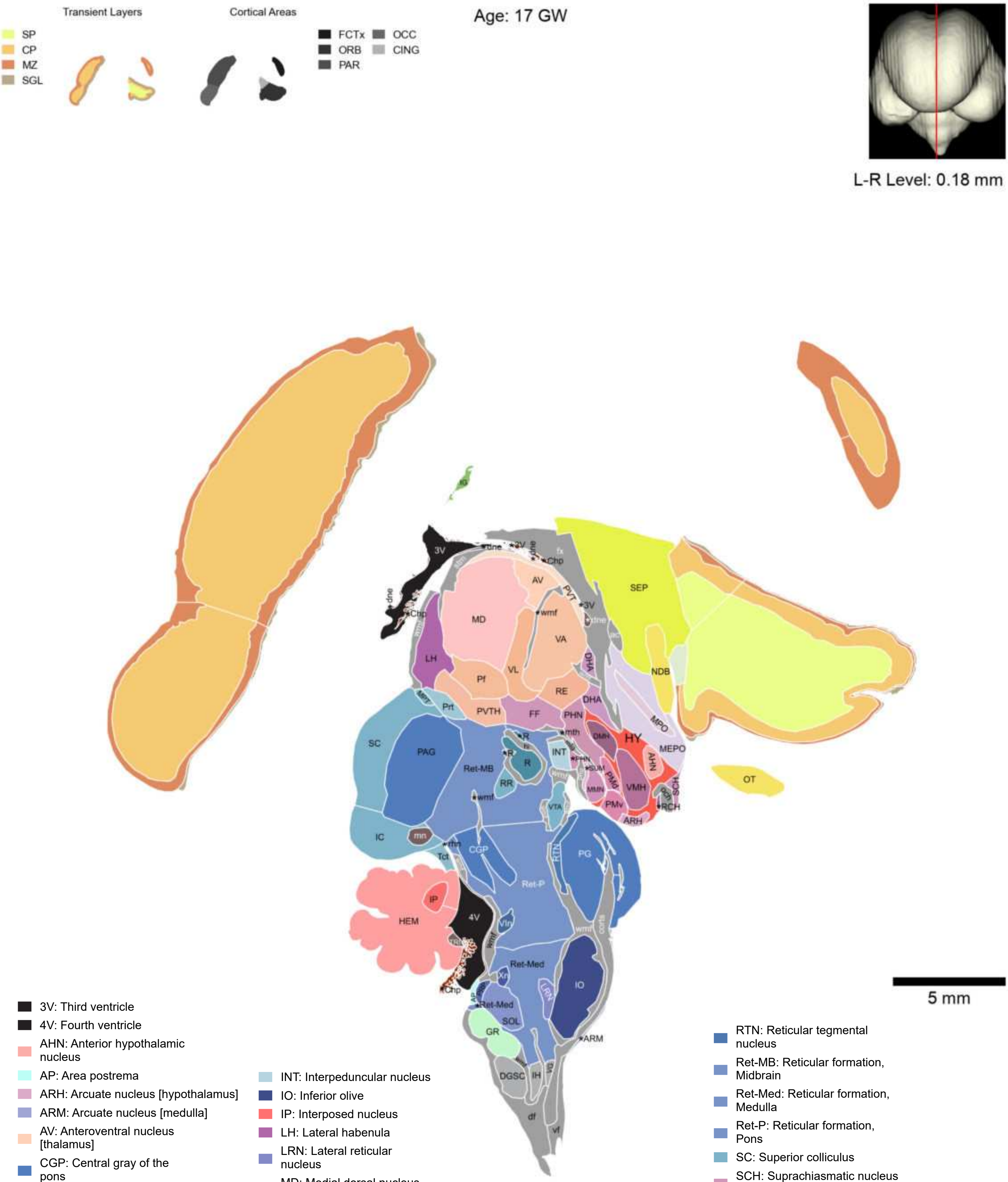

- 3V: Third ventricle
- 4V: Fourth ventricle
- AHN: Anterior hypothalamic nucleus
- AP: Area postrema
- ARH: Arcuate nucleus [hypothalamus]
- ARM: Arcuate nucleus [medulla]
- AV: Anteroventral nucleus [thalamus]
- CGP: Central gray of the pons
- Chp: Choroid plexus
- DGSC: Dorsal gray of the spinal cord
- DHA: Dorsal hypothalamic area
- DMH: Dorsomedial nucleus [hypothalamus]
- FF: Field of Forel
- GR: Gracile nucleus
- HEM: Cerebellar hemispheres
- HY: Hypothalamus
- IC: Inferior colliculus
- IG: Induseum griseum
- IH: Intermediate gray of the spinal cord
- INT: Interpeduncular nucleus
- IO: Inferior olive
- IP: Interposed nucleus
- LH: Lateral habenula
- LRN: Lateral reticular nucleus
- MD: Medial dorsal nucleus [thalamus]
- MEPO: Medial preoptic area
- MMN: Medial mammillary nucleus
- MPO: Medial preoptic nucleus
- MPT: Medial pretectal nucleus
- NDB: Nucleus of the diagonal band
- OT: Olfactory tubercle
- PAG: Periaqueductal gray
- PG: Pontine gray
- PHN: Posterior hypothalamic nucleus
- PMd: Dorsal premammillary nucleus
- PMv: Ventral premammillary nucleus
- PRP: Nucleus prepositus
- PVT: Paraventricular nucleus [thalamus]
- PVTH: Periventricular complex [thalamus]
- Pf: Parafascicular nucleus [thalamus]
- Prt: Pretectum
- R: Red nucleus
- RCH: Retrochiasmatic nucleus [hypothalamus]
- RE: Nucleus reuniens
- RR: Retrorubral area
- RTN: Reticular tegmental nucleus
- Ret-MB: Reticular formation, Midbrain
- Ret-Med: Reticular formation, Medulla
- Ret-P: Reticular formation, Pons
- SC: Superior colliculus
- SCH: Suprachiasmatic nucleus [hypothalamus]
- SEP: Septum
- SOL: Solitary nucleus
- SUM: Supramammillary area
- TRI: Germinal trigone
- TT: Tenia tecta
- Tct: Tectum
- VA: Ventral anterior nucleus [thalamus]
- VG: Ventral gray of the spinal cord
- VIn: Abducens nucleus
- VL: Ventral lateral nucleus [thalamus]
- VMH: Ventromedial nucleus [hypothalamus]
- VTA: Ventral tegmental area
- Xn: Dorsal motor nucleus

Age: 17 GW

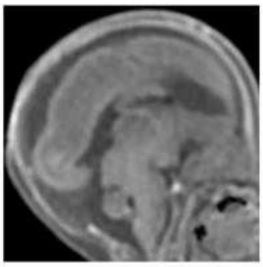

L-R Level: 0.0 mm

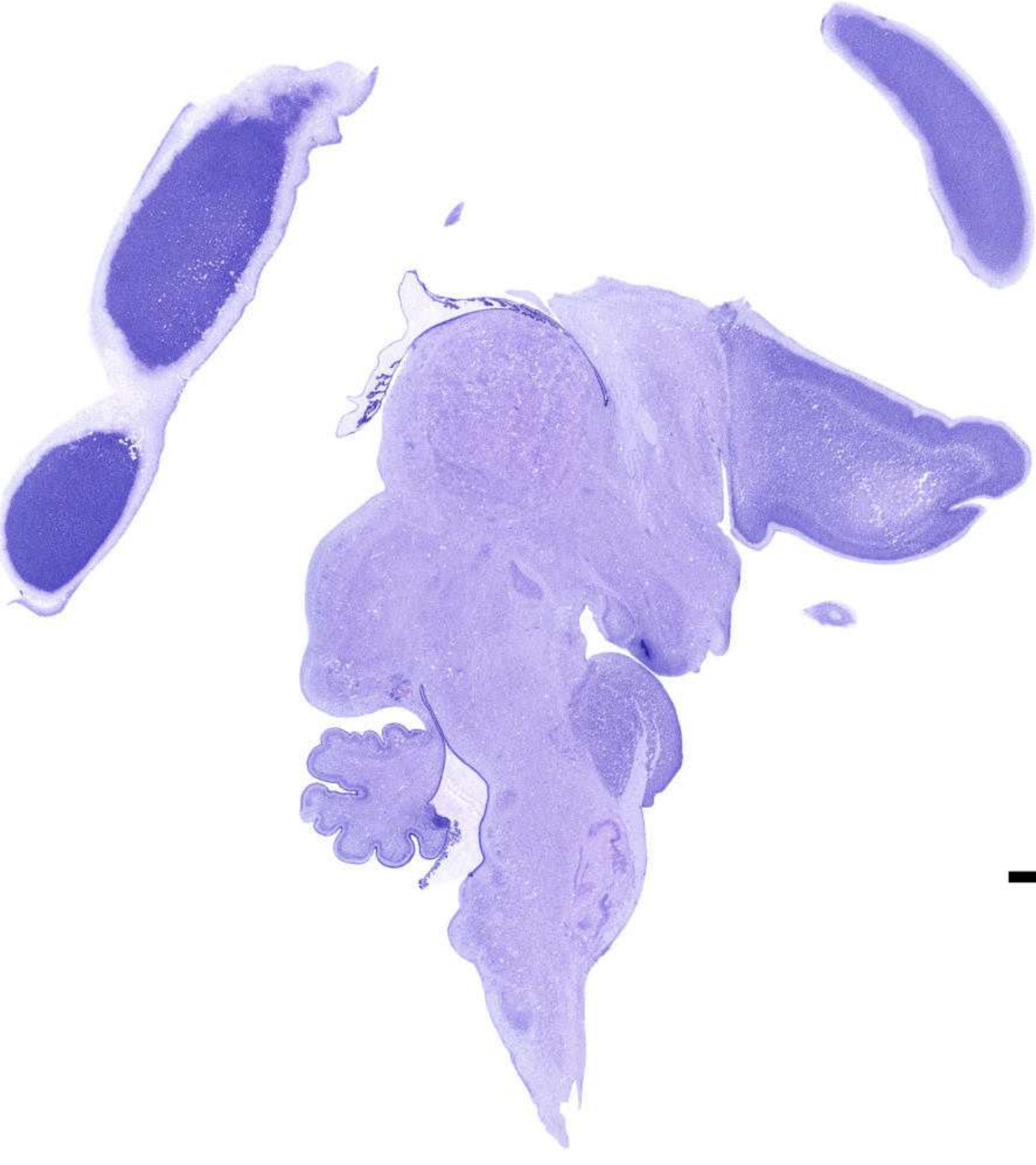

5 mm

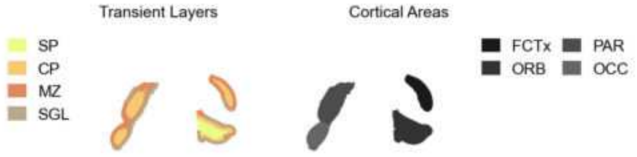

Age: 17 GW

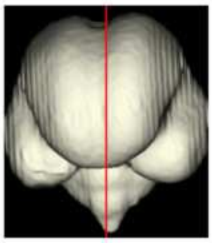

L-R Level: 0.0 mm

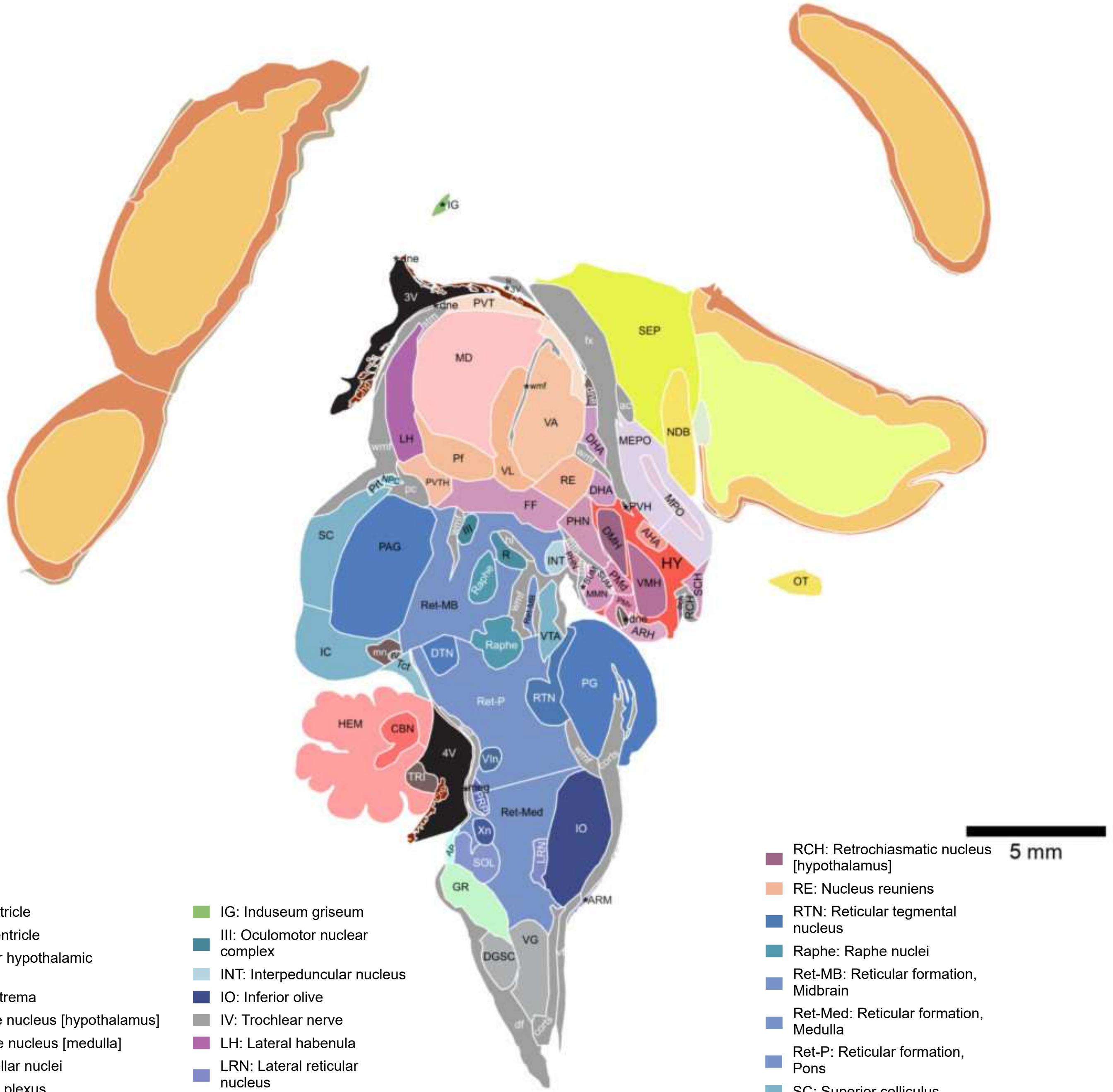

- 3V: Third ventricle
- 4V: Fourth ventricle
- AHA: Anterior hypothalamic area
- AP: Area postrema
- ARH: Arcuate nucleus [hypothalamus]
- ARM: Arcuate nucleus [medulla]
- CBN: Cerebellar nuclei
- Chp: Choroid plexus
- DGSC: Dorsal gray of the spinal cord
- DHA: Dorsal hypothalamic area
- DMH: Dorsomedial nucleus [hypothalamus]
- DTN: Dorsal tegmental nucleus
- FF: Field of Forel
- GR: Gracile nucleus
- HEM: Cerebellar hemispheres
- HY: Hypothalamus
- IC: Inferior colliculus
- IG: Induseum griseum
- III: Oculomotor nuclear complex
- INT: Interpeduncular nucleus
- IO: Inferior olive
- IV: Trochlear nerve
- LH: Lateral habenula
- LRN: Lateral reticular nucleus
- MD: Medial dorsal nucleus [thalamus]
- MEPO: Medial preoptic area
- MMN: Medial mammillary nucleus
- MPO: Medial preoptic nucleus
- NDB: Nucleus of the diagonal band
- NPC: Nucleus of the posterior commissure
- OT: Olfactory tubercle
- PAG: Periaqueductal gray
- PG: Pontine gray
- PHN: Posterior hypothalamic nucleus
- PMd: Dorsal premammillary nucleus
- PMv: Ventral premammillary nucleus
- PRP: Nucleus prepositus
- PVH: Paraventricular nucleus [hypothalamus]
- PVT: Paraventricular nucleus [thalamus]
- PVTH: Periventricular complex [thalamus]
- Pf: Parafascicular nucleus [thalamus]
- Prt: Pretectum
- R: Red nucleus
- RCH: Retrochiasmatic nucleus [hypothalamus]
- RE: Nucleus reuniens
- RTN: Reticular tegmental nucleus
- Raphe: Raphe nuclei
- Ret-MB: Reticular formation, Midbrain
- Ret-Med: Reticular formation, Medulla
- Ret-P: Reticular formation, Pons
- SC: Superior colliculus
- SCH: Suprachiasmatic nucleus [hypothalamus]
- SEP: Septum
- SOL: Solitary nucleus
- SUM: Supramammillary area
- TRI: Germinal trigone
- TT: Tenia tecta
- Tct: Tectum
- VA: Ventral anterior nucleus [thalamus]
- VG: Ventral gray of the spinal cord
- VIn: Abducens nucleus
- VL: Ventral lateral nucleus [thalamus]
- VMH: Ventromedial nucleus [hypothalamus]
- VTA: Ventral tegmental area
- Xn: Dorsal motor nucleus

Age: 17 GW

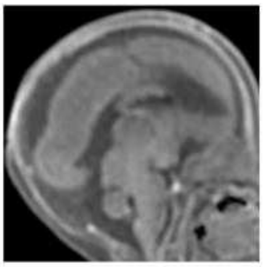

L-R Level: -0.24 mm

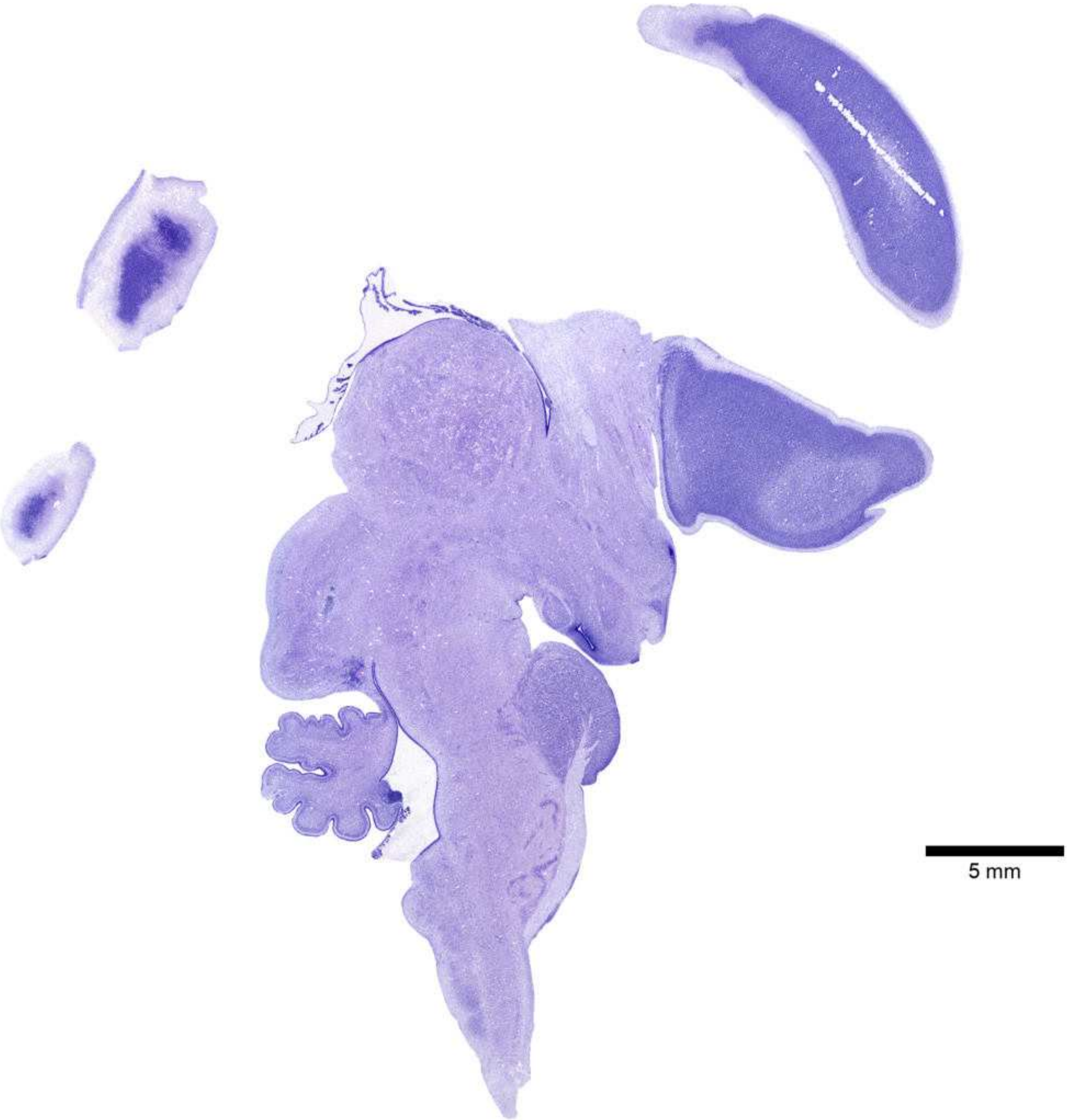

5 mm

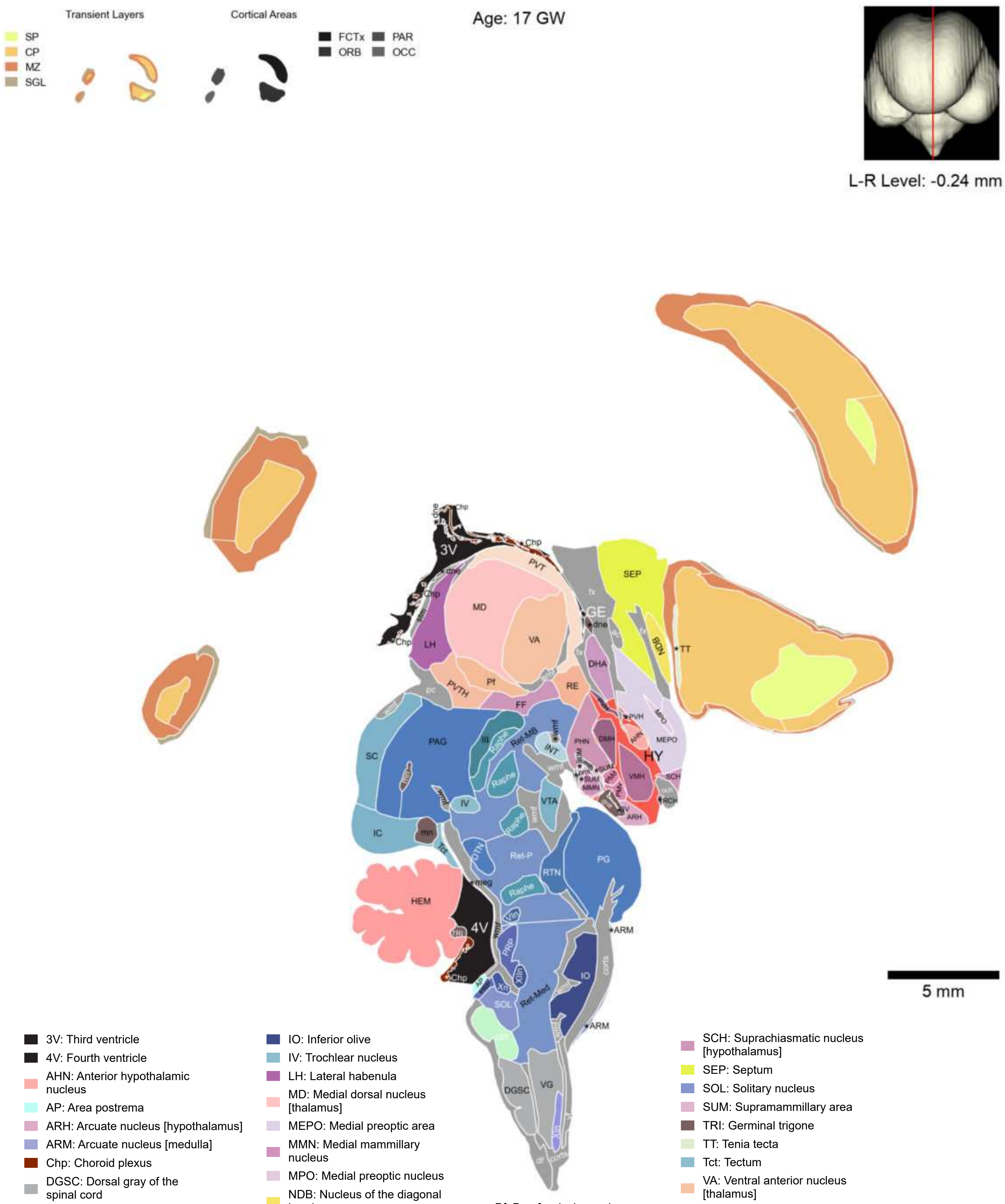

- 3V: Third ventricle
- 4V: Fourth ventricle
- AHN: Anterior hypothalamic nucleus
- AP: Area postrema
- ARH: Arcuate nucleus [hypothalamus]
- ARM: Arcuate nucleus [medulla]
- Chp: Choroid plexus
- DGSC: Dorsal gray of the spinal cord
- DHA: Dorsal hypothalamic area
- DMH: Dorsomedial nucleus [hypothalamus]
- DTN: Dorsal tegmental nucleus
- FF: Field of Forel
- GE: Ganglionic eminence
- GR: Gracile nucleus
- HEM: Cerebellar hemispheres
- HY: Hypothalamus
- IC: Inferior colliculus
- III: Oculomotor nuclear complex
- INT: Interpeduncular nucleus
- IO: Inferior olive
- IV: Trochlear nucleus
- LH: Lateral habenula
- MD: Medial dorsal nucleus [thalamus]
- MEPO: Medial preoptic area
- MMN: Medial mammillary nucleus
- MPO: Medial preoptic nucleus
- NDB: Nucleus of the diagonal band
- PAG: Periaqueductal gray
- PG: Pontine gray
- PHN: Posterior hypothalamic nucleus
- PMd: Dorsal premammillary nucleus
- PMv: Ventral premammillary nucleus
- PRP: Nucleus prepositus
- PVH: Paraventricular nucleus [hypothalamus]
- PVT: Paraventricular nucleus [thalamus]
- PVTH: Periventricular complex [thalamus]
- Pf: Parafascicular nucleus [thalamus]
- RCH: Retrochiasmatic nucleus [hypothalamus]
- RE: Nucleus reuniens
- RTN: Reticular tegmental nucleus
- Raphe: Raphe nuclei
- Ret-MB: Reticular formation, Midbrain
- Ret-Med: Reticular formation, Medulla
- Ret-P: Reticular formation, Pons
- SC: Superior colliculus
- SCH: Suprachiasmatic nucleus [hypothalamus]
- SEP: Septum
- SOL: Solitary nucleus
- SUM: Supramammillary area
- TRI: Germinal trigone
- TT: Tenia tecta
- Tct: Tectum
- VA: Ventral anterior nucleus [thalamus]
- VG: Ventral gray of the spinal cord
- VIn: Abducens nucleus
- VMH: Ventromedial nucleus [hypothalamus]
- VTA: Ventral tegmental area
- XIIn: Hypoglossal nucleus
- XIn: Accessory nucleus
- Xn: Dorsal motor nucleus

Age: 17 GW

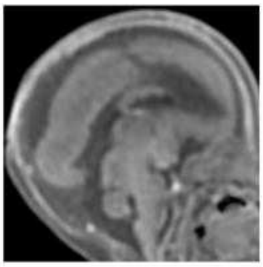

L-R Level: -0.48 mm

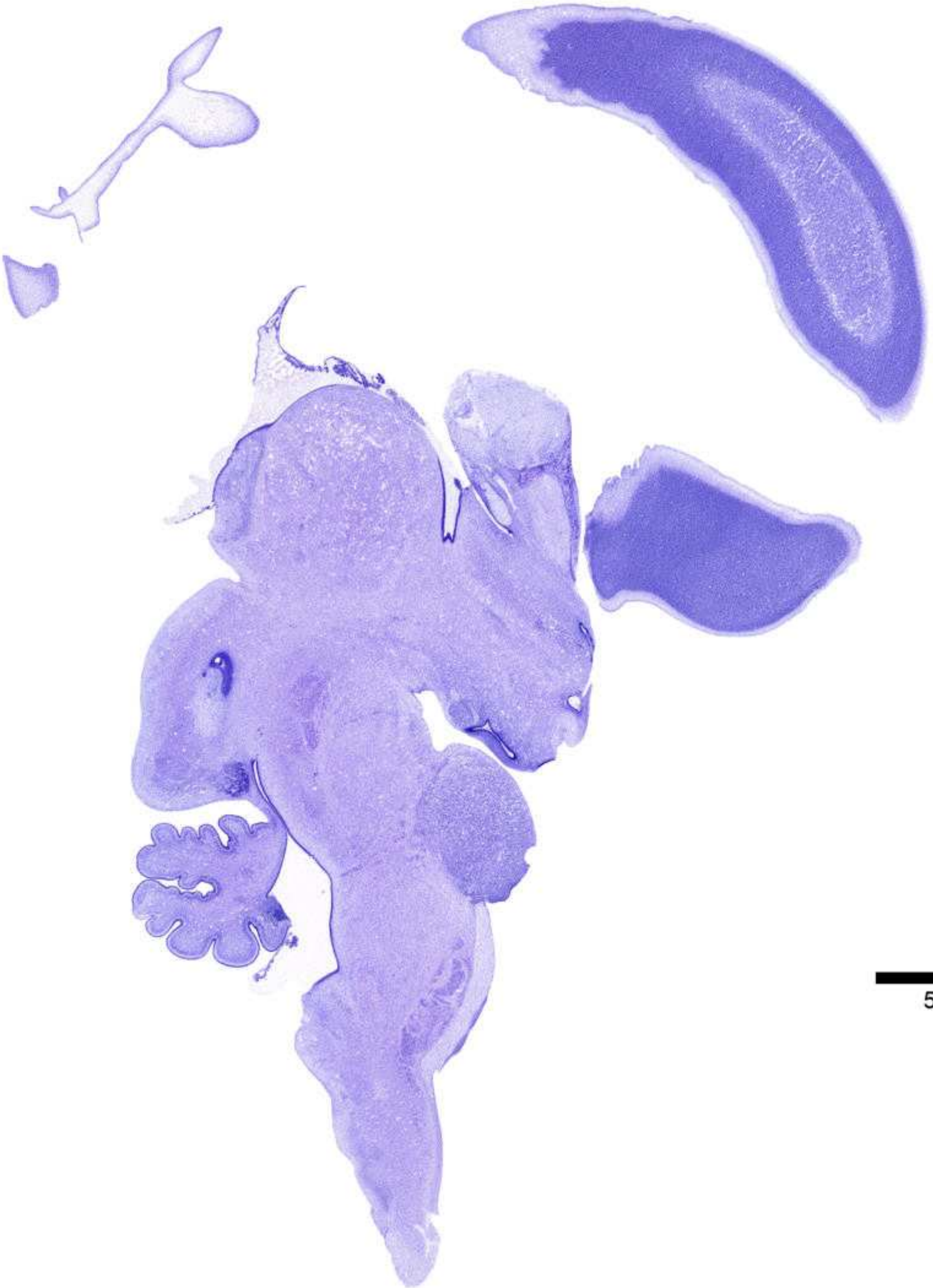

5 mm

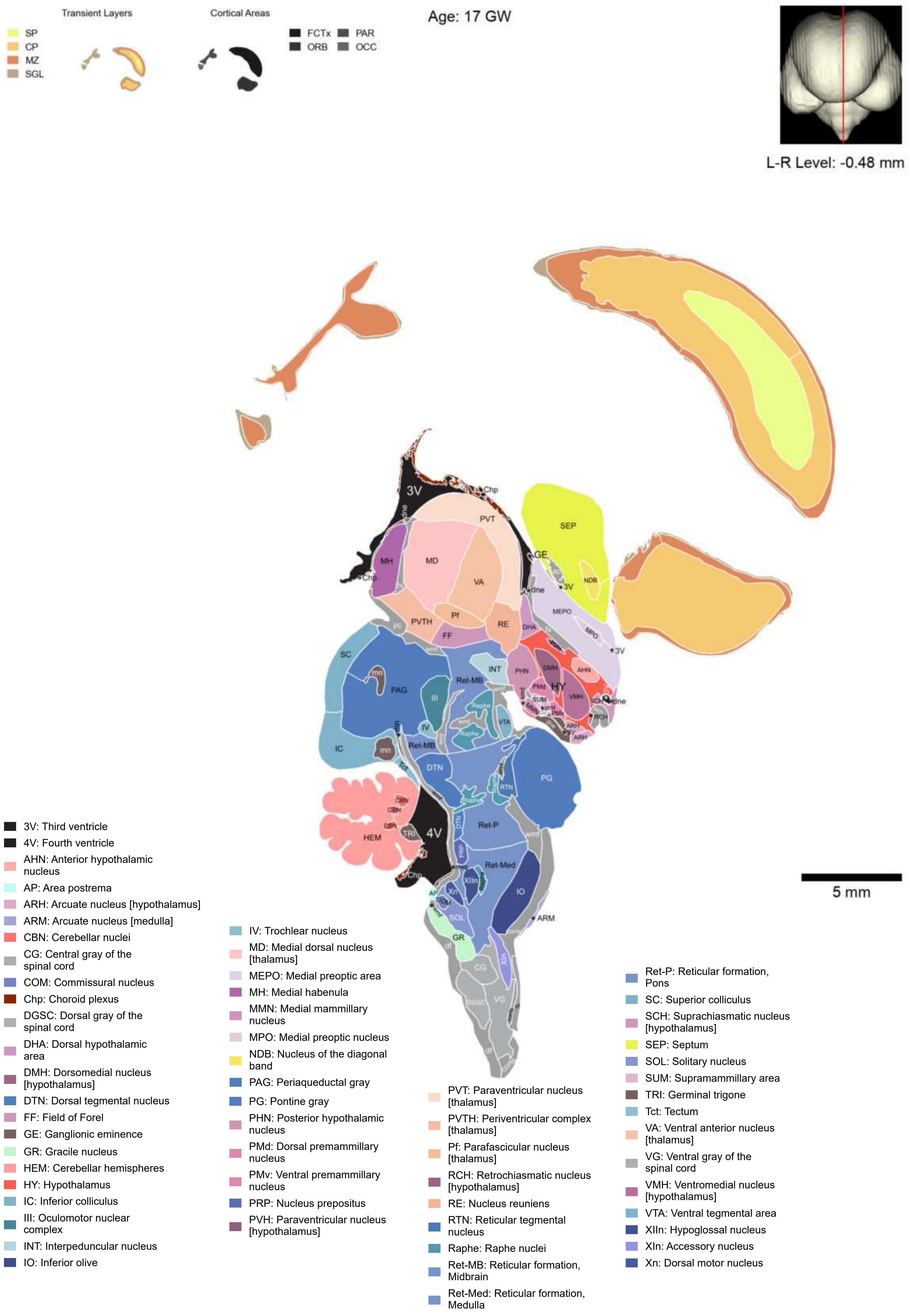
Transient Layers
SP
CP
MZ
SGL
Cortical Areas
FCTx
ORB
PAR
OCC
Age: 17 GW
L-R Level: -0.48 mm
5 mm
3V: Third ventricle
4V: Fourth ventricle
AHN: Anterior hypothalamic nucleus
AP: Area postrema
ARH: Arcuate nucleus [hypothalamus]
ARM: Arcuate nucleus [medulla]
CBN: Cerebellar nuclei
CG: Central gray of the spinal cord
COM: Commissural nucleus
Chp: Choroid plexus
DGSC: Dorsal gray of the spinal cord
DHA: Dorsal hypothalamic area
DMH: Dorsomedial nucleus [hypothalamus]
DTN: Dorsal tegmental nucleus
FF: Field of Forel
GE: Ganglionic eminence
GR: Gracile nucleus
HEM: Cerebellar hemispheres
HY: Hypothalamus
IC: Inferior colliculus
III: Oculomotor nuclear complex
INT: Interpeduncular nucleus
IO: Inferior olive
IV: Trochlear nucleus
MD: Medial dorsal nucleus [thalamus]
MEPO: Medial preoptic area
MH: Medial habenula
MMN: Medial mammillary nucleus
MPO: Medial preoptic nucleus
NDB: Nucleus of the diagonal band
PAG: Periaqueductal gray
PG: Pontine gray
PHN: Posterior hypothalamic nucleus
PMd: Dorsal premammillary nucleus
PMv: Ventral premammillary nucleus
PRP: Nucleus prepositus
PVH: Paraventricular nucleus [hypothalamus]
PVT: Paraventricular nucleus [thalamus]
PVTH: Periventricular complex [thalamus]
Pf: Parafascicular nucleus [thalamus]
RCH: Retrochiasmatic nucleus [hypothalamus]
RE: Nucleus reuniens
RTN: Reticular tegmental nucleus
Raphe: Raphe nuclei
Ret-MB: Reticular formation, Midbrain
Ret-Med: Reticular formation, Medulla
Ret-P: Reticular formation, Pons
SC: Superior colliculus
SCH: Suprachiasmatic nucleus [hypothalamus]
SEP: Septum
SOL: Solitary nucleus
SUM: Supramammillary area
TRI: Germinal trigone
Tct: Tectum
VA: Ventral anterior nucleus [thalamus]
VG: Ventral gray of the spinal cord
VMH: Ventromedial nucleus [hypothalamus]
VTA: Ventral tegmental area
XIIn: Hypoglossal nucleus
XIn: Accessory nucleus
Xn: Dorsal motor nucleus

Age: 17 GW

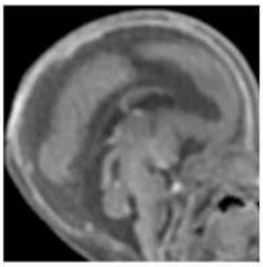

L-R Level: -1.14 mm

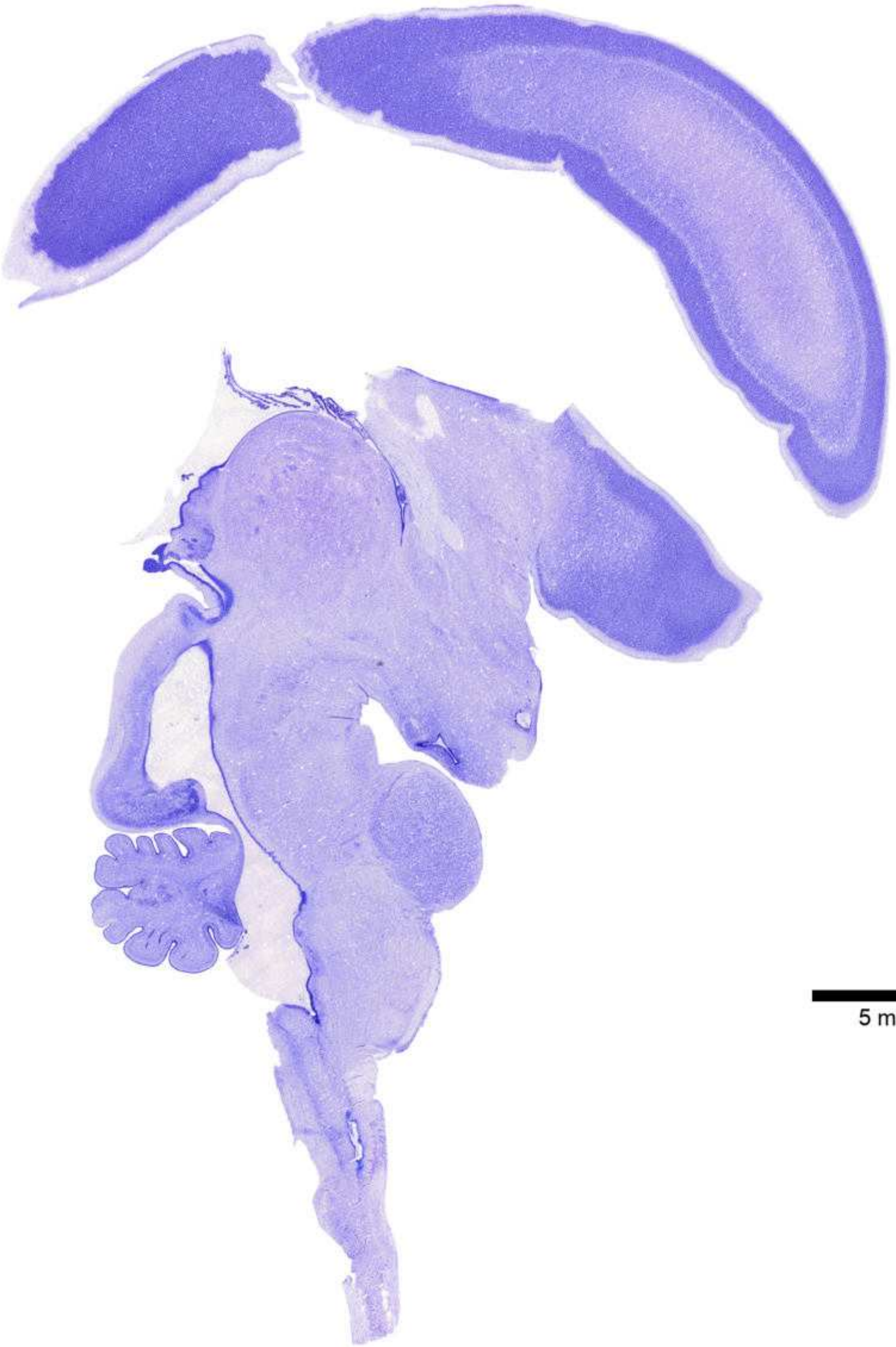

5 mm

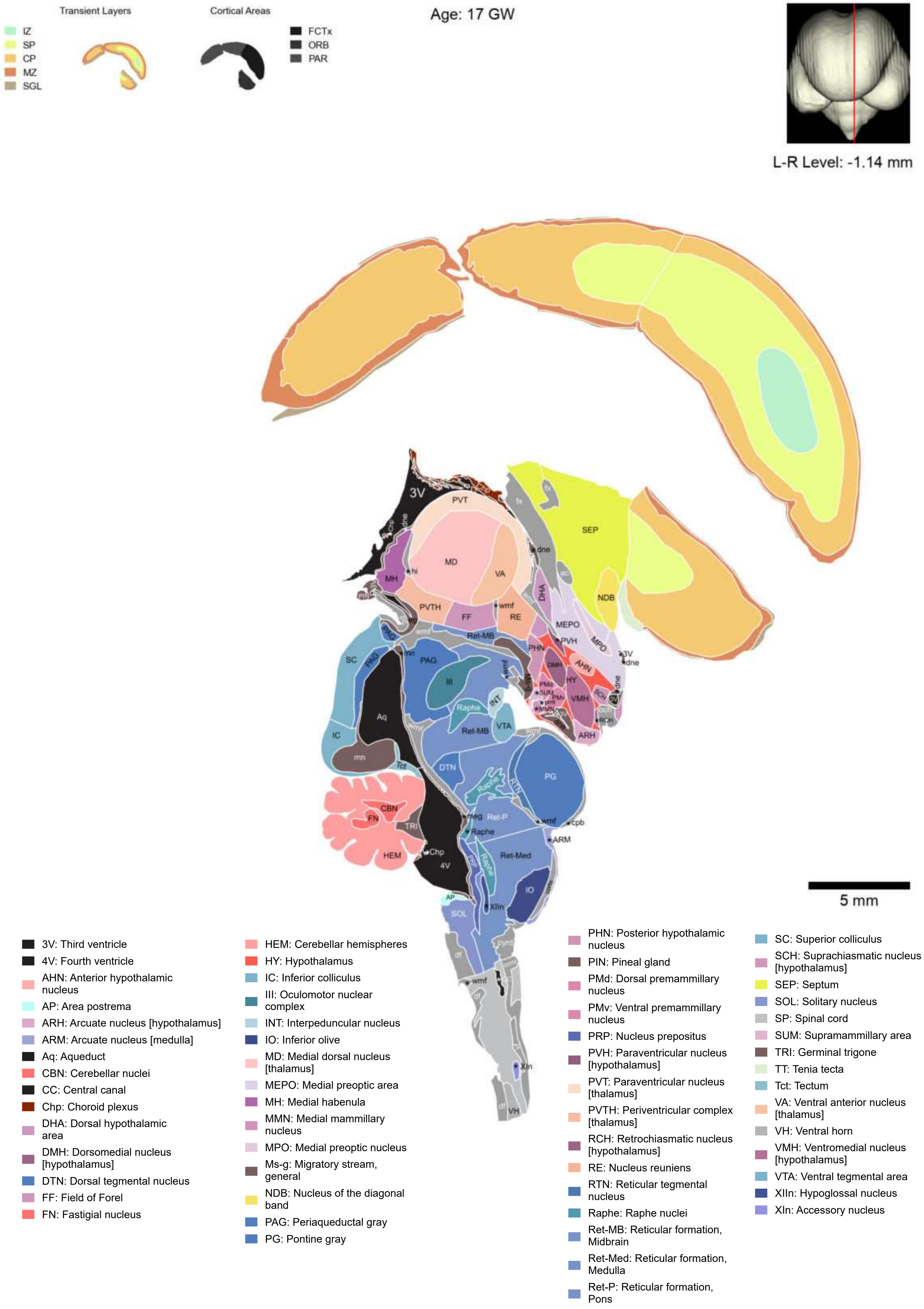
Transient Layers
IZ
SP
CP
MZ
SGL
Cortical Areas
FCTx
ORB
PAR
Age: 17 GW
L-R Level: -1.14 mm
5 mm
3V: Third ventricle
4V: Fourth ventricle
AHN: Anterior hypothalamic nucleus
AP: Area postrema
ARH: Arcuate nucleus [hypothalamus]
ARM: Arcuate nucleus [medulla]
Aq: Aqueduct
CBN: Cerebellar nuclei
CC: Central canal
Chp: Choroid plexus
DHA: Dorsal hypothalamic area
DMH: Dorsomedial nucleus [hypothalamus]
DTN: Dorsal tegmental nucleus
FF: Field of Forel
FN: Fastigial nucleus
HEM: Cerebellar hemispheres
HY: Hypothalamus
IC: Inferior colliculus
III: Oculomotor nuclear complex
INT: Interpeduncular nucleus
IO: Inferior olive
MD: Medial dorsal nucleus [thalamus]
MEPO: Medial preoptic area
MH: Medial habenula
MMN: Medial mammillary nucleus
MPO: Medial preoptic nucleus
Ms-g: Migratory stream, general
NDB: Nucleus of the diagonal band
PAG: Periaqueductal gray
PG: Pontine gray
PHN: Posterior hypothalamic nucleus
PIN: Pineal gland
PMd: Dorsal premammillary nucleus
PMv: Ventral premammillary nucleus
PRP: Nucleus prepositus
PVH: Paraventricular nucleus [hypothalamus]
PVT: Paraventricular nucleus [thalamus]
PVTH: Periventricular complex [thalamus]
RCH: Retrochiasmatic nucleus [hypothalamus]
RE: Nucleus reuniens
RTN: Reticular tegmental nucleus
Raphe: Raphe nuclei
Ret-MB: Reticular formation, Midbrain
Ret-Med: Reticular formation, Medulla
Ret-P: Reticular formation, Pons
SC: Superior colliculus
SCH: Suprachiasmatic nucleus [hypothalamus]
SEP: Septum
SOL: Solitary nucleus
SP: Spinal cord
SUM: Supramammillary area
TRI: Germinal trigone
TT: Tenia tecta
Tct: Tectum
VA: Ventral anterior nucleus [thalamus]
VH: Ventral horn
VMH: Ventromedial nucleus [hypothalamus]
VTA: Ventral tegmental area
XIIn: Hypoglossal nucleus
XIn: Accessory nucleus

Age: 17 GW

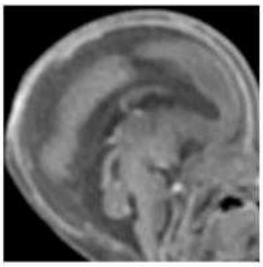

L-R Level: -1.44 mm

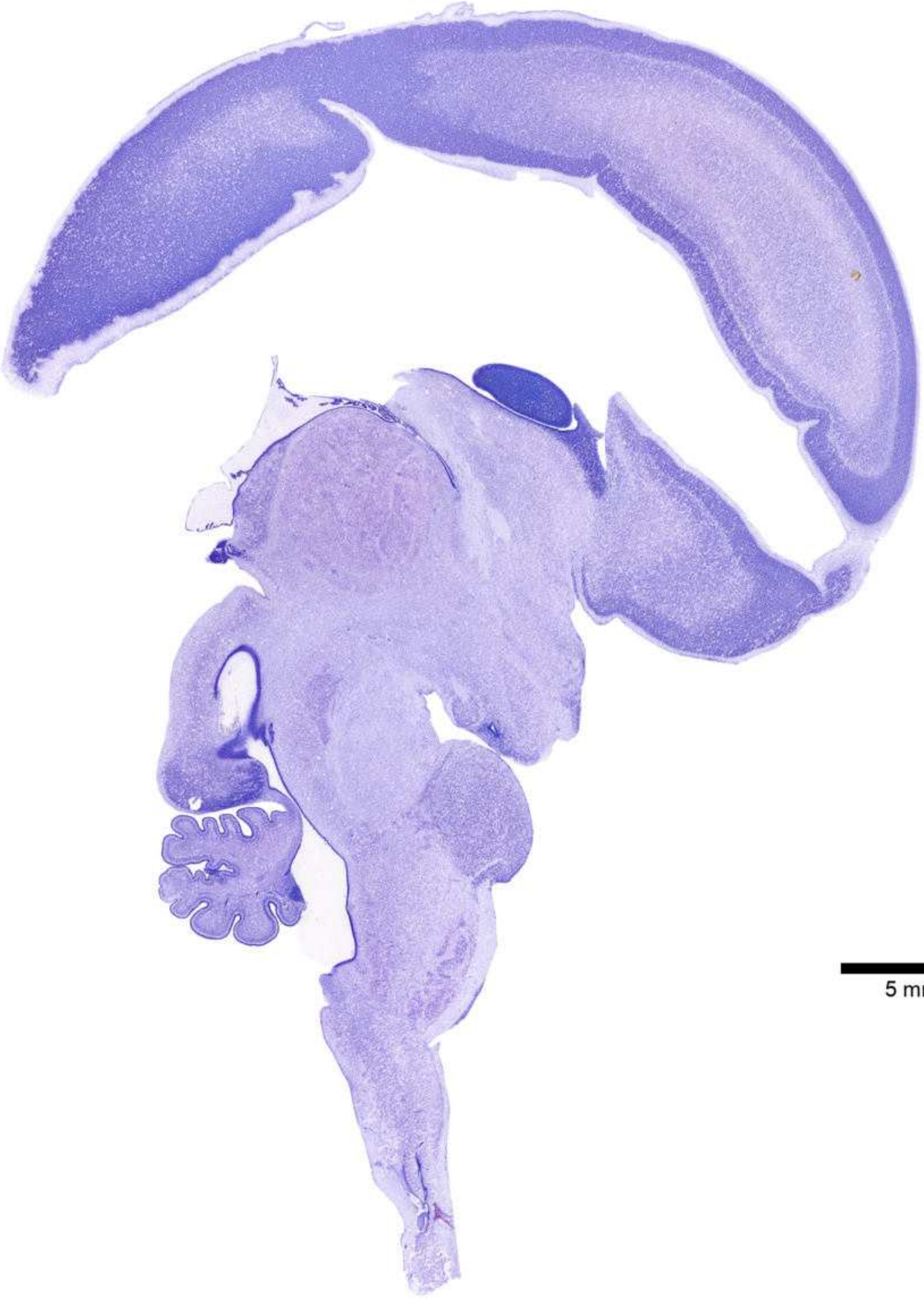

5 mm

Transient Layers

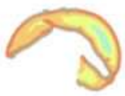

- IZ
- SP
- CP
- MZ
- SGL

Cortical Areas

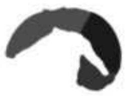

- FCTx
- ORB
- PAR

Age: 17 GW

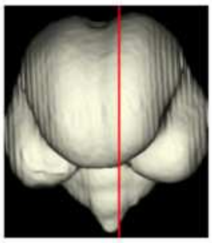

L-R Level: -1.44 mm

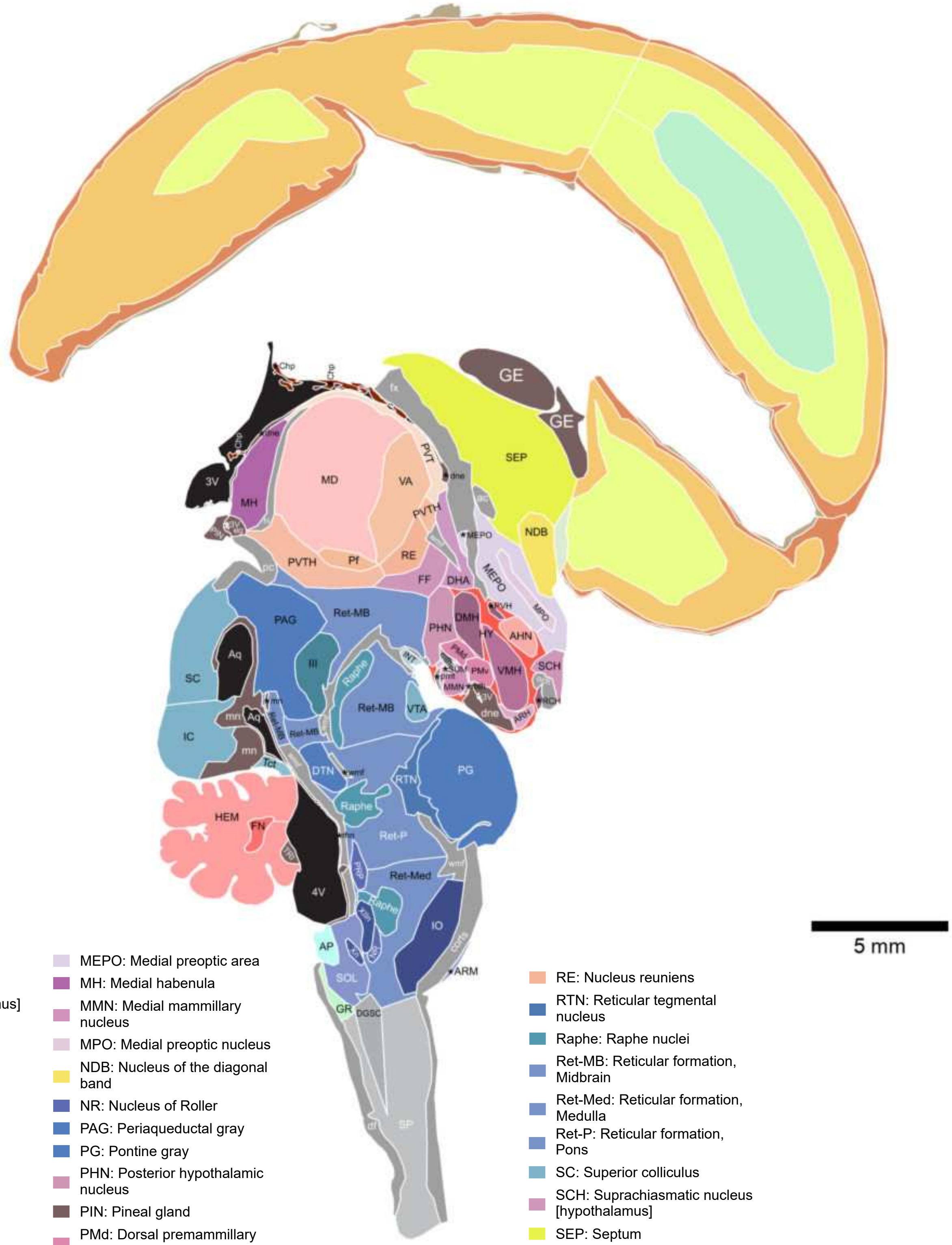

- 3V: Third ventricle
- 4V: Fourth ventricle
- AHN: Anterior hypothalamic nucleus
- AP: Area postrema
- ARH: Arcuate nucleus [hypothalamus]
- ARM: Arcuate nucleus [medulla]
- Aq: Aqueduct
- Chp: Choroid plexus
- DGSC: Dorsal gray of the spinal cord
- DHA: Dorsal hypothalamic area
- DMH: Dorsomedial nucleus [hypothalamus]
- DTN: Dorsal tegmental nucleus
- FF: Field of Forel
- FN: Fastigial nucleus
- GE: Ganglionic eminence
- GR: Gracile nucleus
- HEM: Cerebellar hemispheres
- HY: Hypothalamus
- IC: Inferior colliculus
- III: Oculomotor nuclear complex
- INT: Interpeduncular nucleus
- IO: Inferior olive
- MD: Medial dorsal nucleus [thalamus]
- MEPO: Medial preoptic area
- MH: Medial habenula
- MMN: Medial mammillary nucleus
- MPO: Medial preoptic nucleus
- NDB: Nucleus of the diagonal band
- NR: Nucleus of Roller
- PAG: Periaqueductal gray
- PG: Pontine gray
- PHN: Posterior hypothalamic nucleus
- PIN: Pineal gland
- PMd: Dorsal premammillary nucleus
- PMv: Ventral premammillary nucleus
- PRP: Nucleus prepositus
- PVH: Paraventricular nucleus [hypothalamus]
- PVT: Paraventricular nucleus [thalamus]
- PVTH: Periventricular complex [thalamus]
- Pf: Parafascicular nucleus [thalamus]
- RCH: Retrochiasmatic nucleus [hypothalamus]
- RE: Nucleus reuniens
- RTN: Reticular tegmental nucleus
- Raphe: Raphe nuclei
- Ret-MB: Reticular formation, Midbrain
- Ret-Med: Reticular formation, Medulla
- Ret-P: Reticular formation, Pons
- SC: Superior colliculus
- SCH: Suprachiasmatic nucleus [hypothalamus]
- SEP: Septum
- SOL: Solitary nucleus
- SP: Spinal cord
- SUM: Supramammillary area
- TRI: Germinal trigone
- TT: Tenia tecta
- Tct: Tectum
- VA: Ventral anterior nucleus [thalamus]
- VMH: Ventromedial nucleus [hypothalamus]
- VTA: Ventral tegmental area
- XIIn: Hypoglossal nucleus
- Xn: Dorsal motor nucleus

Age: 17 GW

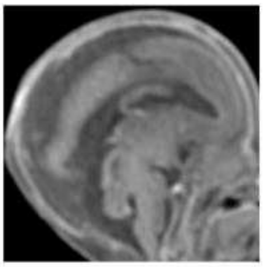

L-R Level: -1.92 mm

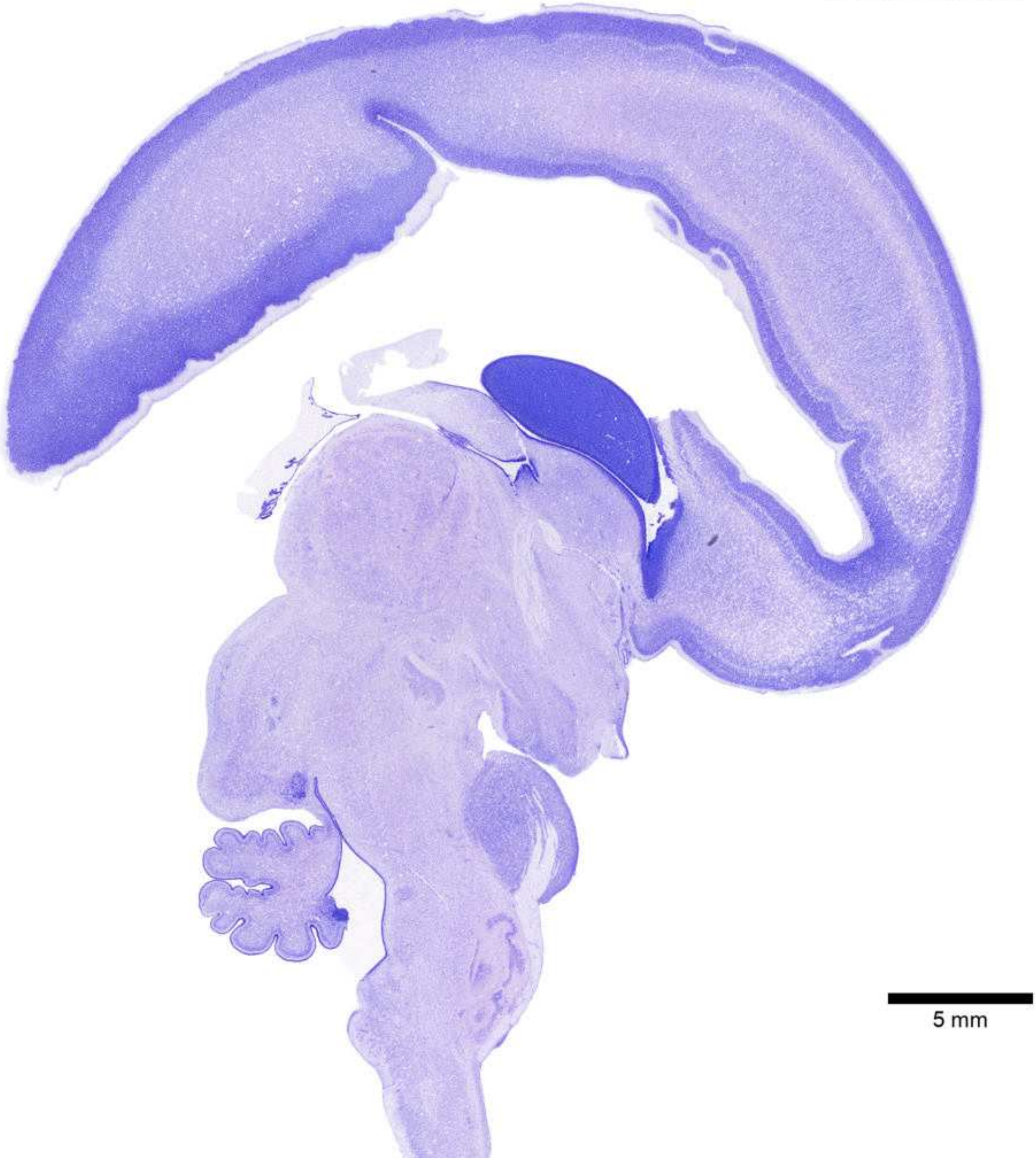

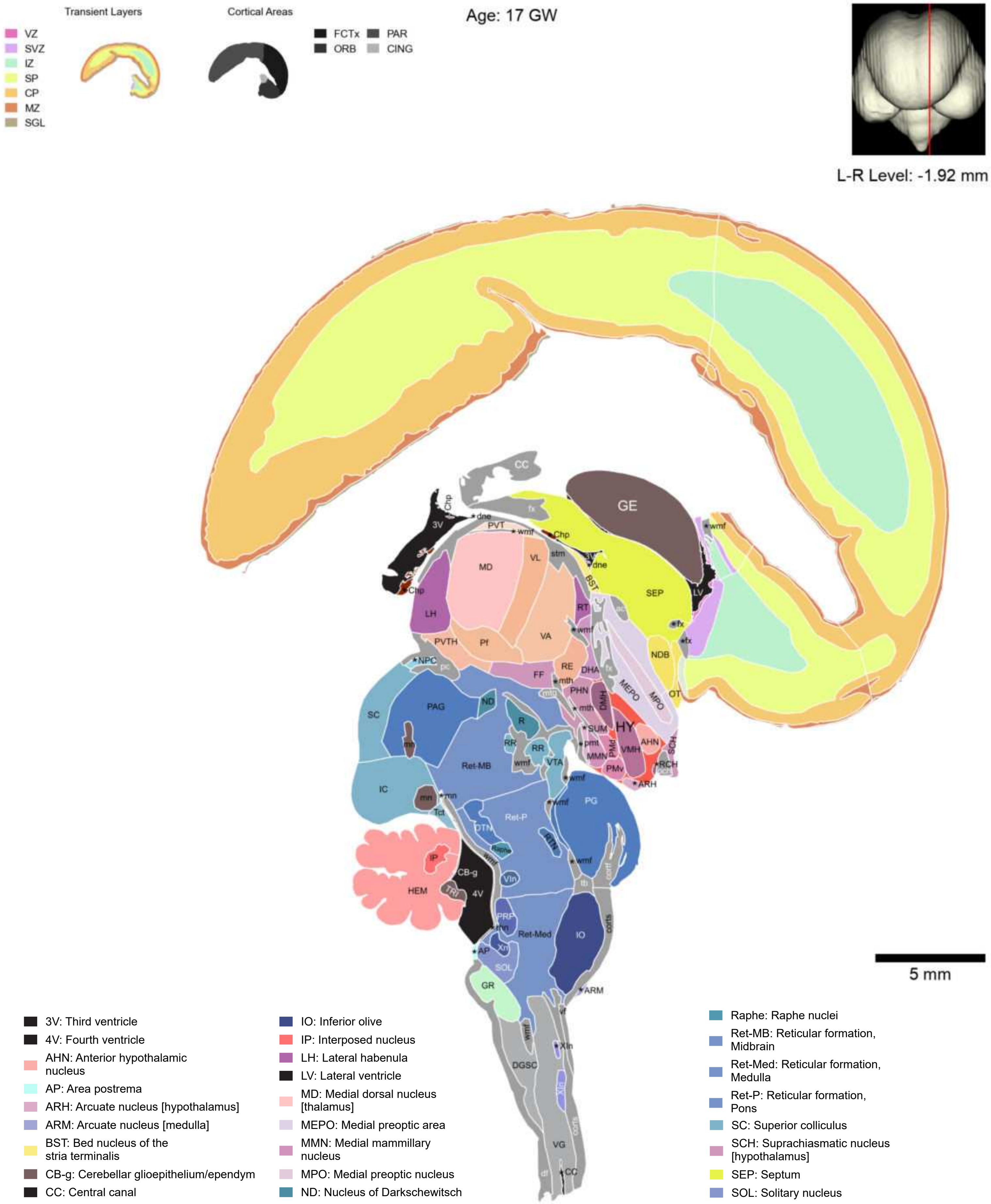

- 3V: Third ventricle
- 4V: Fourth ventricle
- AHN: Anterior hypothalamic nucleus
- AP: Area postrema
- ARH: Arcuate nucleus [hypothalamus]
- ARM: Arcuate nucleus [medulla]
- BST: Bed nucleus of the stria terminalis
- CB-g: Cerebellar glioepithelium/ependym
- CC: Central canal
- Chp: Choroid plexus
- DGSC: Dorsal gray of the spinal cord
- DHA: Dorsal hypothalamic area
- DMH: Dorsomedial nucleus [hypothalamus]
- DTN: Dorsal tegmental nucleus
- FF: Field of Forel
- GE: Ganglionic eminence
- GR: Gracile nucleus
- HEM: Cerebellar hemispheres
- HY: Hypothalamus
- IC: Inferior colliculus
- IO: Inferior olive
- IP: Interposed nucleus
- LH: Lateral habenula
- LV: Lateral ventricle
- MD: Medial dorsal nucleus [thalamus]
- MEPO: Medial preoptic area
- MMN: Medial mammillary nucleus
- MPO: Medial preoptic nucleus
- ND: Nucleus of Darkschewitsch
- NDB: Nucleus of the diagonal band
- NPC: Nucleus of the posterior commissure
- OT: Olfactory tubercle
- PAG: Periaqueductal gray
- PG: Pontine gray
- PHN: Posterior hypothalamic nucleus
- PMd: Dorsal premammillary nucleus
- PMv: Ventral premammillary nucleus
- PRP: Nucleus prepositus
- PVT: Paraventricular nucleus [thalamus]
- PVTH: Periventricular complex [thalamus]
- Pf: Parafascicular nucleus [thalamus]
- R: Red nucleus
- RCH: Retrochiasmatic nucleus [hypothalamus]
- RE: Nucleus reuniens
- RR: Retrorubral area
- RT: Reticular nucleus [thalamus]
- RTN: Reticular tegmental nucleus
- Raphe: Raphe nuclei
- Ret-MB: Reticular formation, Midbrain
- Ret-Med: Reticular formation, Medulla
- Ret-P: Reticular formation, Pons
- SC: Superior colliculus
- SCH: Suprachiasmatic nucleus [hypothalamus]
- SEP: Septum
- SOL: Solitary nucleus
- SUM: Supramammillary area
- TRI: Germinal trigone
- TT: Tenia tecta
- Tct: Tectum
- VA: Ventral anterior nucleus [thalamus]
- VG: Ventral gray of the spinal cord
- VIn: Abducens nucleus
- VL: Ventral lateral nucleus [thalamus]
- VMH: Ventromedial nucleus [hypothalamus]
- VTA: Ventral tegmental area
- XIn: Accessory nucleus
- Xn: Dorsal motor nucleus

Age: 17 GW

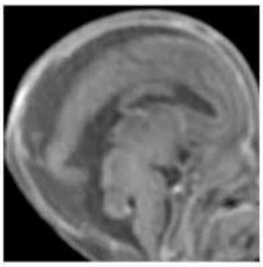

L-R Level: -2.28 mm

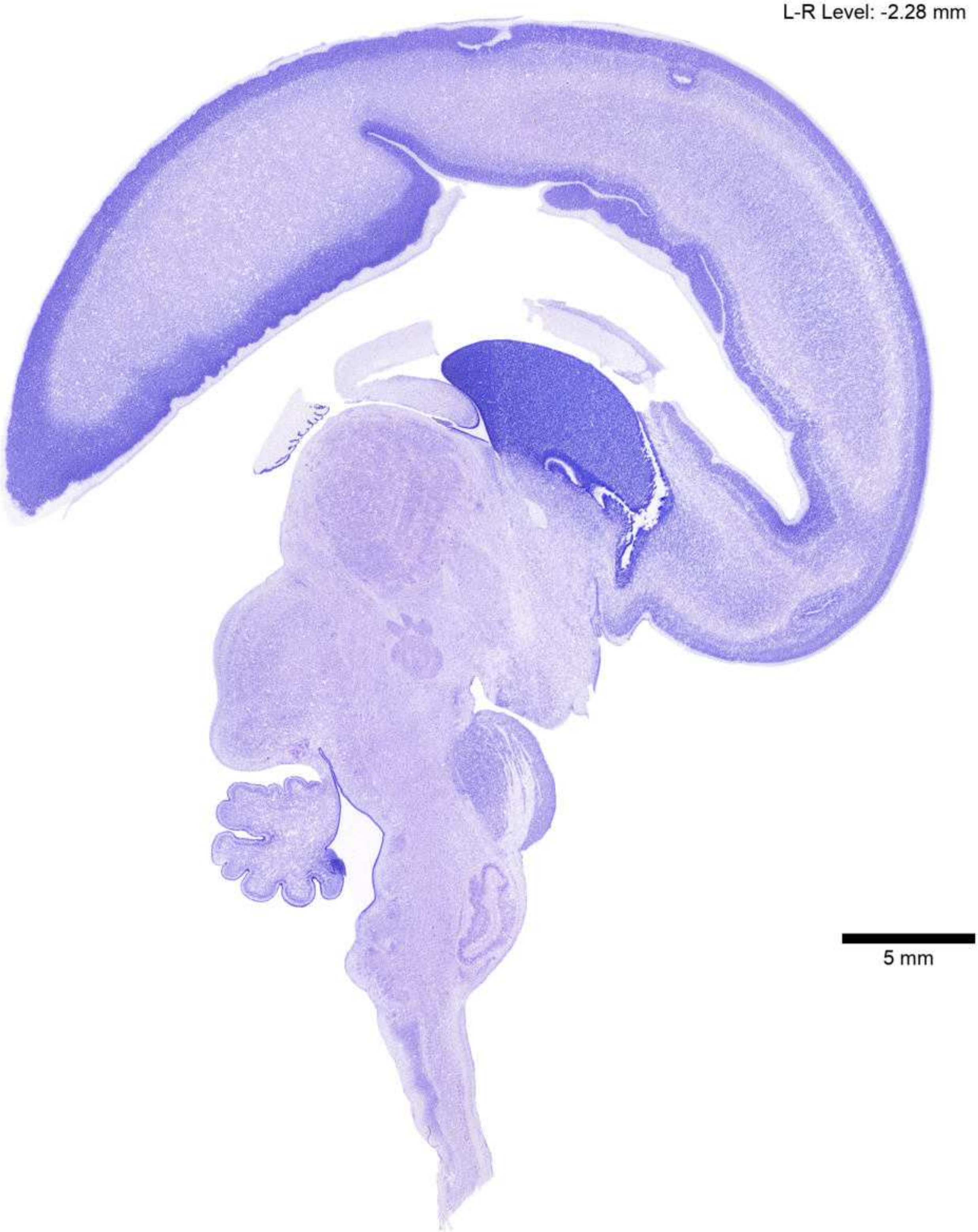

5 mm

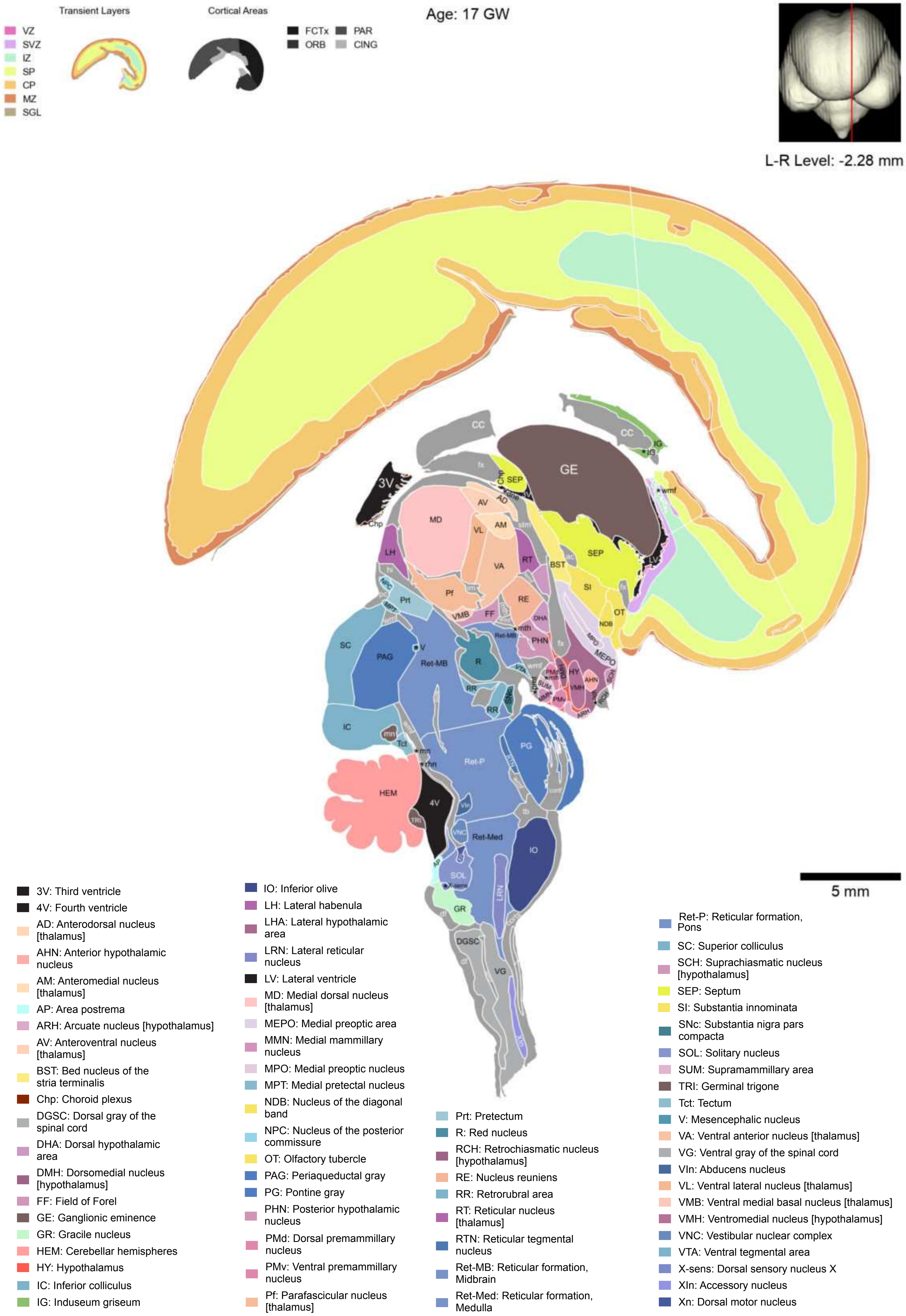
Transient Layers
VZ
SVZ
IZ
SP
CP
MZ
SGL
Cortical Areas
FCTx
ORB
PAR
CING
Age: 17 GW
L-R Level: -2.28 mm
5 mm
3V: Third ventricle
4V: Fourth ventricle
AD: Anterodorsal nucleus [thalamus]
AHN: Anterior hypothalamic nucleus
AM: Anteromedial nucleus [thalamus]
AP: Area postrema
ARH: Arcuate nucleus [hypothalamus]
AV: Anteroventral nucleus [thalamus]
BST: Bed nucleus of the stria terminalis
Chp: Choroid plexus
DGSC: Dorsal gray of the spinal cord
DHA: Dorsal hypothalamic area
DMH: Dorsomedial nucleus [hypothalamus]
FF: Field of Forel
GE: Ganglionic eminence
GR: Gracile nucleus
HEM: Cerebellar hemispheres
HY: Hypothalamus
IC: Inferior colliculus
IG: Induseum griseum
IO: Inferior olive
LH: Lateral habenula
LHA: Lateral hypothalamic area
LRN: Lateral reticular nucleus
LV: Lateral ventricle
MD: Medial dorsal nucleus [thalamus]
MEPO: Medial preoptic area
MMN: Medial mammillary nucleus
MPO: Medial preoptic nucleus
MPT: Medial pretectal nucleus
NDB: Nucleus of the diagonal band
NPC: Nucleus of the posterior commissure
OT: Olfactory tubercle
PAG: Periaqueductal gray
PG: Pontine gray
PHN: Posterior hypothalamic nucleus
PMd: Dorsal premammillary nucleus
PMv: Ventral premammillary nucleus
Pf: Parafascicular nucleus [thalamus]
Prt: Pretectum
R: Red nucleus
RCH: Retrochiasmatic nucleus [hypothalamus]
RE: Nucleus reuniens
RR: Retrorubral area
RT: Reticular nucleus [thalamus]
RTN: Reticular tegmental nucleus
Ret-MB: Reticular formation, Midbrain
Ret-Med: Reticular formation, Medulla
Ret-P: Reticular formation, Pons
SC: Superior colliculus
SCH: Suprachiasmatic nucleus [hypothalamus]
SEP: Septum
SI: Substantia innominata
SNc: Substantia nigra pars compacta
SOL: Solitary nucleus
SUM: Supramammillary area
TRI: Germinal trigone
Tct: Tectum
V: Mesencephalic nucleus
VA: Ventral anterior nucleus [thalamus]
VG: Ventral gray of the spinal cord
VIn: Abducens nucleus
VL: Ventral lateral nucleus [thalamus]
VMB: Ventral medial basal nucleus [thalamus]
VMH: Ventromedial nucleus [hypothalamus]
VNC: Vestibular nuclear complex
VTA: Ventral tegmental area
X-sens: Dorsal sensory nucleus X
XIn: Accessory nucleus
Xn: Dorsal motor nucleus

Age: 17 GW

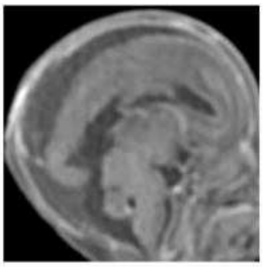

L-R Level: -2.52 mm

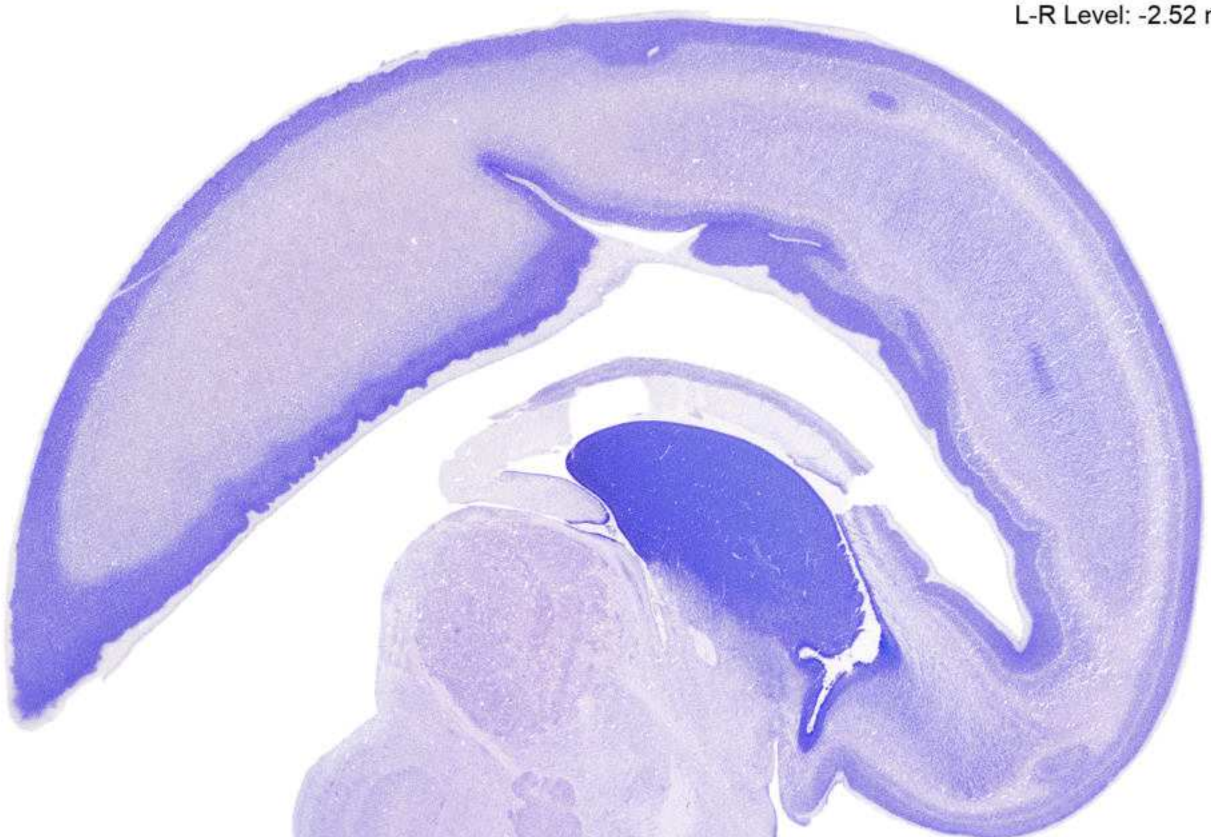

5 mm

Transient Layers

VZ
SVZ
IZ
SP
CP
MZ
SGL

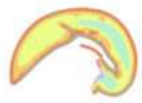

Cortical Areas

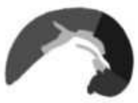

FCTx
ORB
PAR
CING

Age: 17 GW

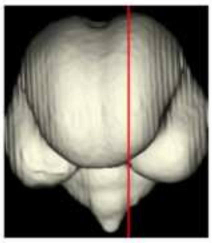

L-R Level: -2.52 mm

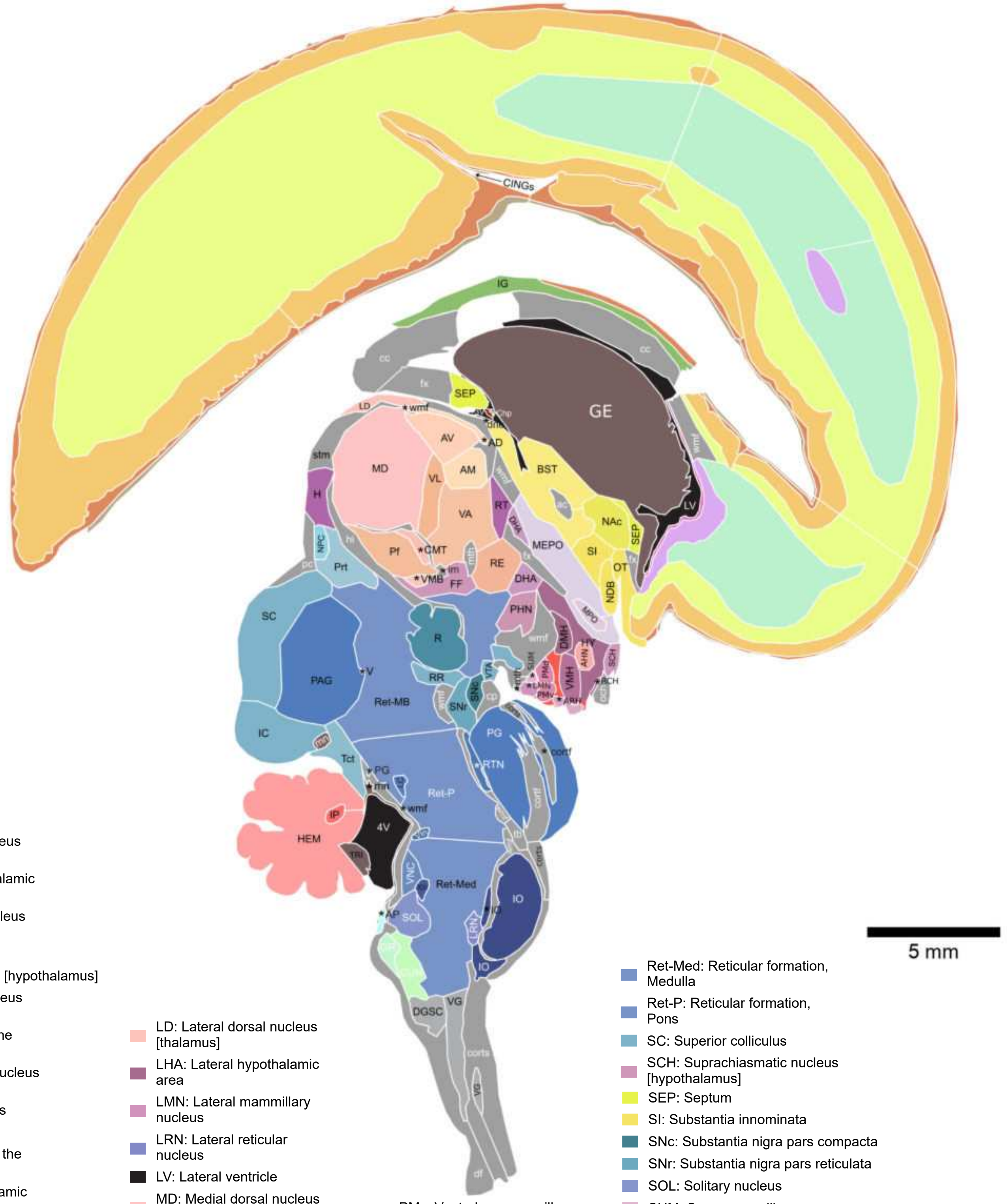

- 3V: Third ventricle
- 4V: Fourth ventricle
- AD: Anterodorsal nucleus [thalamus]
- AHN: Anterior hypothalamic nucleus
- AM: Anteromedial nucleus [thalamus]
- AP: Area postrema
- ARH: Arcuate nucleus [hypothalamus]
- AV: Anteroventral nucleus [thalamus]
- BST: Bed nucleus of the stria terminalis
- CMT: Centromedian nucleus [thalamus]
- CUN: Cuneate nucleus
- Chp: Choroid plexus
- DGSC: Dorsal gray of the spinal cord
- DHA: Dorsal hypothalamic area
- DMH: Dorsomedial nucleus [hypothalamus]
- FF: Field of Forel
- GE: Ganglionic eminence
- GR: Gracile nucleus
- H: Habenula
- HEM: Cerebellar hemispheres
- HY: Hypothalamus
- IC: Inferior colliculus
- IG: Induseum griseum
- IO: Inferior olive
- IP: Interposed nucleus
- LC: Locus coeruleus
- LD: Lateral dorsal nucleus [thalamus]
- LHA: Lateral hypothalamic area
- LMN: Lateral mammillary nucleus
- LRN: Lateral reticular nucleus
- LV: Lateral ventricle
- MD: Medial dorsal nucleus [thalamus]
- MEPO: Medial preoptic area
- MPO: Medial preoptic nucleus
- NAc: Nucleus accumbens
- NDB: Nucleus of the diagonal band
- NPC: Nucleus of the posterior commissure
- OT: Olfactory tubercle
- PAG: Periaqueductal gray
- PG: Pontine gray
- PHN: Posterior hypothalamic nucleus
- PMd: Dorsal premammillary nucleus
- PMv: Ventral premammillary nucleus
- Pf: Parafascicular nucleus [thalamus]
- Prt: Pretectum
- R: Red nucleus
- RCH: Retrochiasmatic nucleus [hypothalamus]
- RE: Nucleus reuniens
- RR: Retrorubral area
- RT: Reticular nucleus [thalamus]
- RTN: Reticular tegmental nucleus
- Ret-MB: Reticular formation, Midbrain
- Ret-Med: Reticular formation, Medulla
- Ret-P: Reticular formation, Pons
- SC: Superior colliculus
- SCH: Suprachiasmatic nucleus [hypothalamus]
- SEP: Septum
- SI: Substantia innominata
- SNc: Substantia nigra pars compacta
- SNr: Substantia nigra pars reticulata
- SOL: Solitary nucleus
- SUM: Supramammillary area
- TRI: Germinal trigone
- Tct: Tectum
- V: Mesencephalic nucleus
- VA: Ventral anterior nucleus [thalamus]
- VG: Ventral gray of the spinal cord
- VIn: Abducens nucleus
- VL: Ventral lateral nucleus [thalamus]
- VMB: Ventral medial basal nucleus [thalamus]
- VMH: Ventromedial nucleus [hypothalamus]
- VNC: Vestibular nuclear complex
- VTA: Ventral tegmental area
- Xn: Dorsal motor nucleus
- → CINGs: Cingulate sulcus

Age: 17 GW

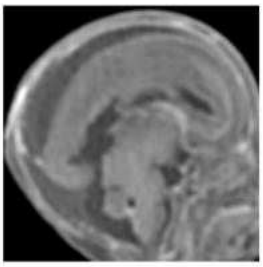

L-R Level: -2.88 mm

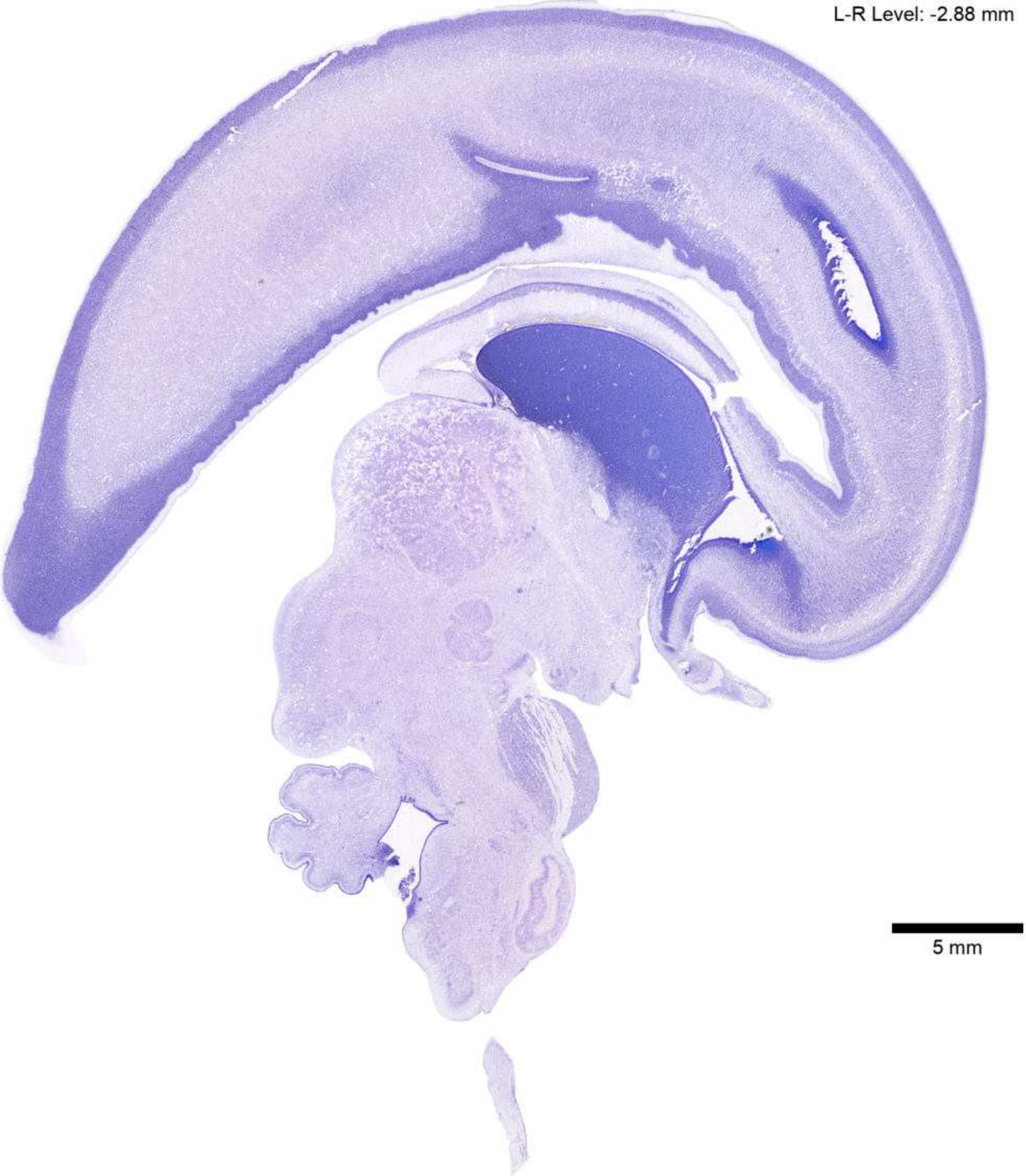

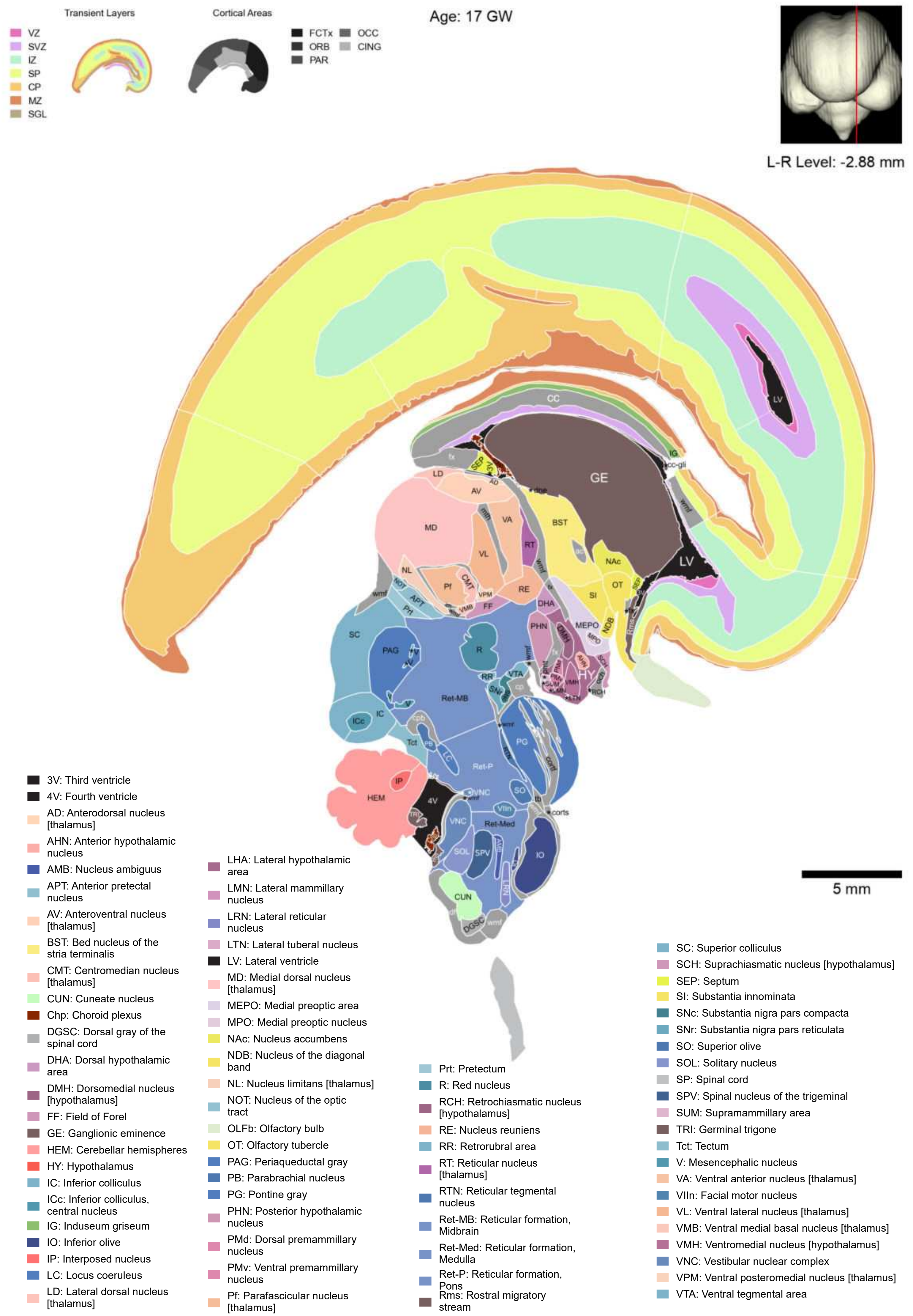

Transient Layers
VZ
SVZ
IZ
SP
CP
MZ
SGL
Cortical Areas
FCTx
ORB
PAR
OCC
CING
Age: 17 GW
L-R Level: -2.88 mm
5 mm
CC
LV
IG
cc-gli
GE
wmf
SEP
LD
AV
AD
MD
VA
VL
RT
BST
NAc
NL
Pf
CMT
VPM
RE
OT
SI
NDB
DHA
MEPO
MPO
PHN
DMH
HY
SC
PAG
R
RR
VTA
Ret-MB
IC
ICc
Tct
PB
LC
PG
Ret-P
HEM
IP
4V
VNC
SO
VIIn
Ret-Med
SOL
SPV
IO
LRN
CUN
DGSC
corts
3V: Third ventricle
4V: Fourth ventricle
AD: Anterodorsal nucleus [thalamus]
AHN: Anterior hypothalamic nucleus
AMB: Nucleus ambiguus
APT: Anterior pretectal nucleus
AV: Anteroventral nucleus [thalamus]
BST: Bed nucleus of the stria terminalis
CMT: Centromedian nucleus [thalamus]
CUN: Cuneate nucleus
Chp: Choroid plexus
DGSC: Dorsal gray of the spinal cord
DHA: Dorsal hypothalamic area
DMH: Dorsomedial nucleus [hypothalamus]
FF: Field of Forel
GE: Ganglionic eminence
HEM: Cerebellar hemispheres
HY: Hypothalamus
IC: Inferior colliculus
ICc: Inferior colliculus, central nucleus
IG: Induseum griseum
IO: Inferior olive
IP: Interposed nucleus
LC: Locus coeruleus
LD: Lateral dorsal nucleus [thalamus]
LHA: Lateral hypothalamic area
LMN: Lateral mammillary nucleus
LRN: Lateral reticular nucleus
LTN: Lateral tuberal nucleus
LV: Lateral ventricle
MD: Medial dorsal nucleus [thalamus]
MEPO: Medial preoptic area
MPO: Medial preoptic nucleus
NAc: Nucleus accumbens
NDB: Nucleus of the diagonal band
NL: Nucleus limitans [thalamus]
NOT: Nucleus of the optic tract
OLFb: Olfactory bulb
OT: Olfactory tubercle
PAG: Periaqueductal gray
PB: Parabrachial nucleus
PG: Pontine gray
PHN: Posterior hypothalamic nucleus
PMd: Dorsal premammillary nucleus
PMv: Ventral premammillary nucleus
Pf: Parafascicular nucleus [thalamus]
Prt: Pretectum
R: Red nucleus
RCH: Retrochiasmatic nucleus [hypothalamus]
RE: Nucleus reuniens
RR: Retrorubral area
RT: Reticular nucleus [thalamus]
RTN: Reticular tegmental nucleus
Ret-MB: Reticular formation, Midbrain
Ret-Med: Reticular formation, Medulla
Ret-P: Reticular formation, Pons
Rms: Rostral migratory stream
SC: Superior colliculus
SCH: Suprachiasmatic nucleus [hypothalamus]
SEP: Septum
SI: Substantia innominata
SNc: Substantia nigra pars compacta
SNr: Substantia nigra pars reticulata
SO: Superior olive
SOL: Solitary nucleus
SP: Spinal cord
SPV: Spinal nucleus of the trigeminal
SUM: Supramammillary area
TRI: Germinal trigone
Tct: Tectum
V: Mesencephalic nucleus
VA: Ventral anterior nucleus [thalamus]
VIIn: Facial motor nucleus
VL: Ventral lateral nucleus [thalamus]
VMB: Ventral medial basal nucleus [thalamus]
VMH: Ventromedial nucleus [hypothalamus]
VNC: Vestibular nuclear complex
VPM: Ventral posteromedial nucleus [thalamus]
VTA: Ventral tegmental area

Age: 17 GW

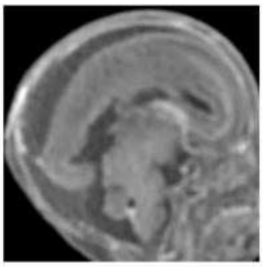

L-R Level: -3.12 mm

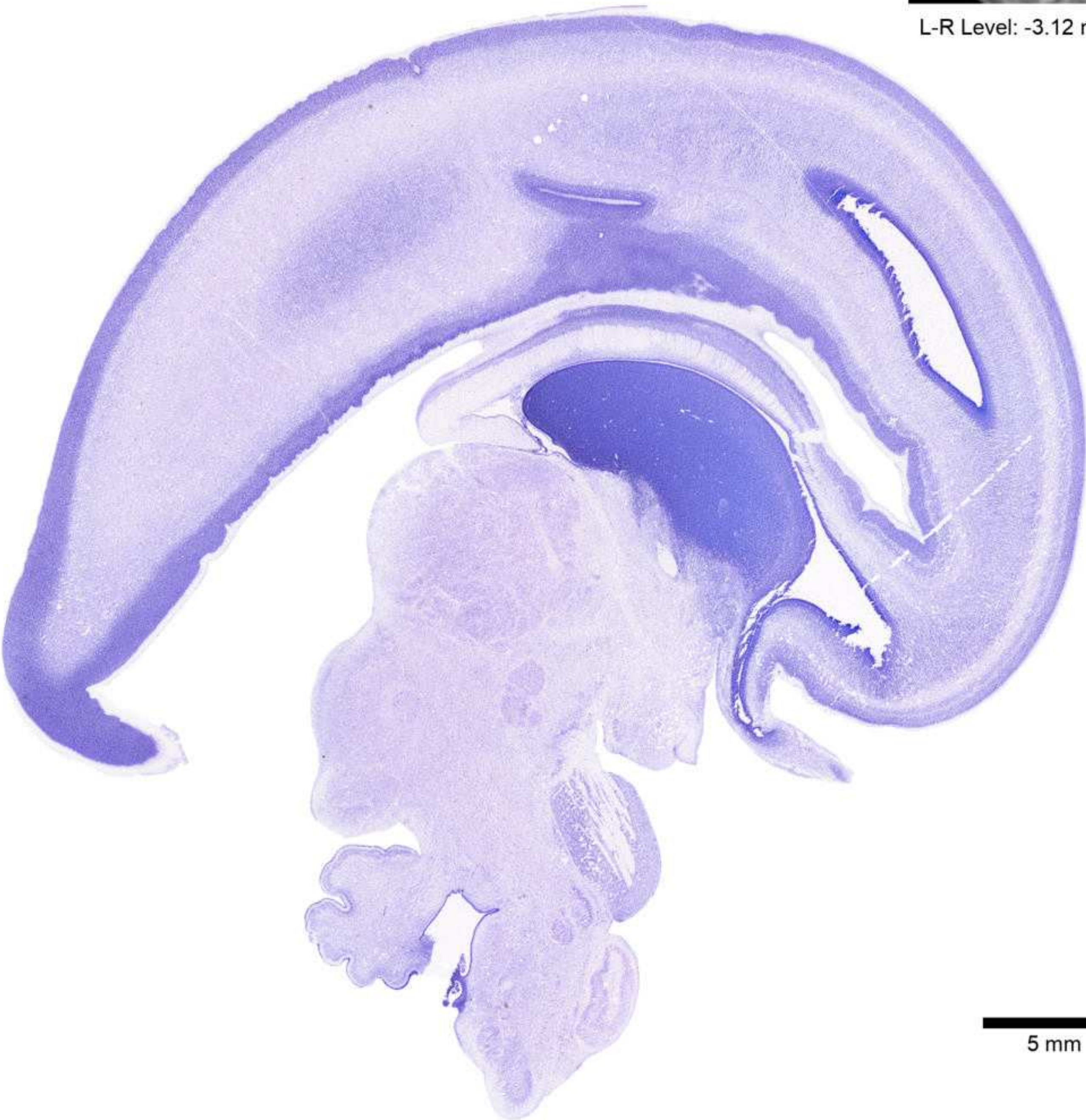

5 mm

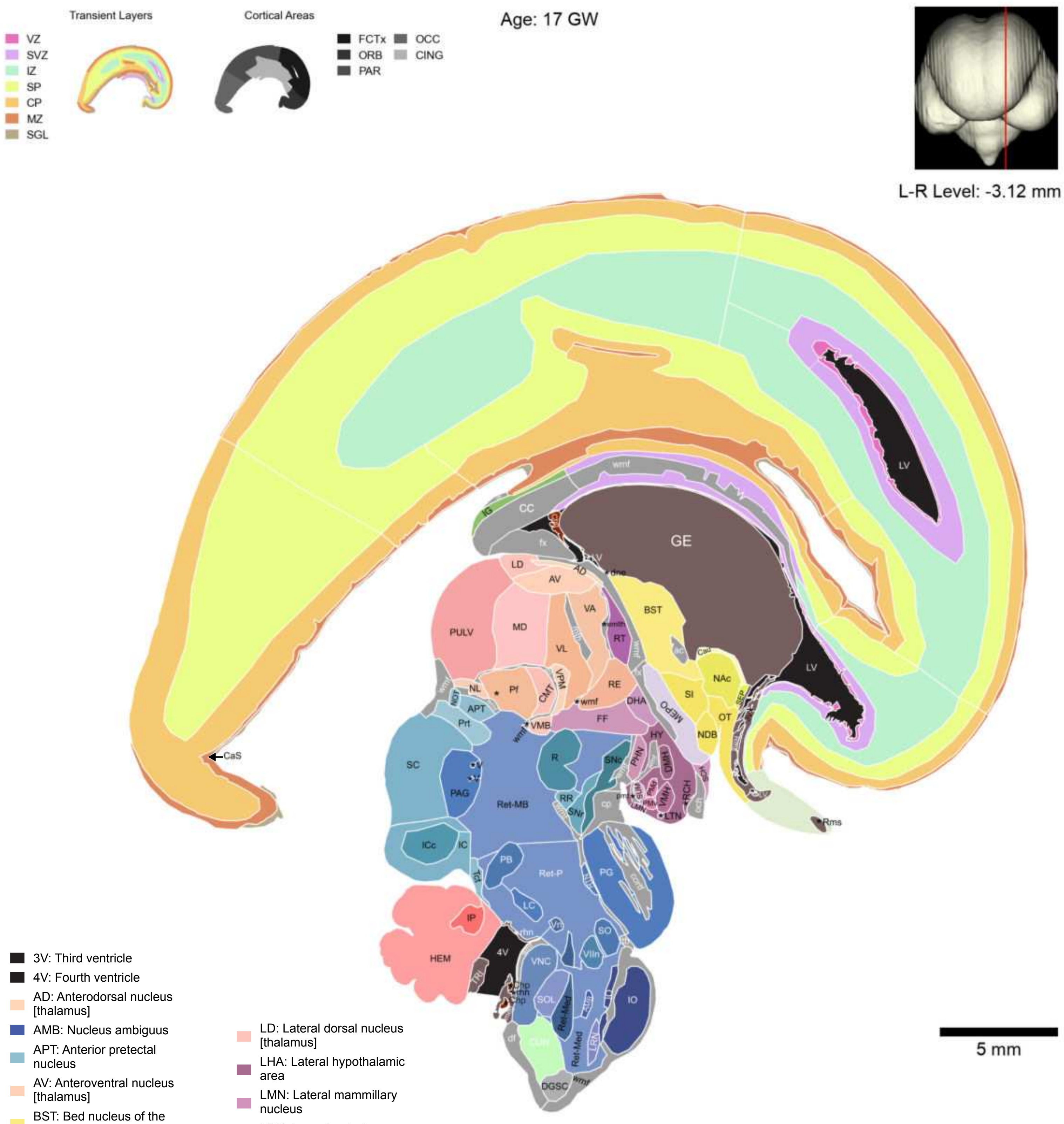

- 3V: Third ventricle
- 4V: Fourth ventricle
- AD: Anterodorsal nucleus [thalamus]
- AMB: Nucleus ambiguus
- APT: Anterior pretectal nucleus
- AV: Anteroventral nucleus [thalamus]
- BST: Bed nucleus of the stria terminalis
- CMT: Centromedian nucleus [thalamus]
- CUN: Cuneate nucleus
- Cau: Caudate nucleus
- Chp: Choroid plexus
- DGSC: Dorsal gray of the spinal cord
- DHA: Dorsal hypothalamic area
- DMH: Dorsomedial nucleus [hypothalamus]
- FF: Field of Forel
- GE: Ganglionic eminence
- HEM: Cerebellar hemispheres
- HY: Hypothalamus
- IC: Inferior colliculus
- ICc: Inferior colliculus, central nucleus
- IG: Induseum griseum
- IO: Inferior olive
- IP: Interposed nucleus
- LC: Locus coeruleus
- LD: Lateral dorsal nucleus [thalamus]
- LHA: Lateral hypothalamic area
- LMN: Lateral mammillary nucleus
- LRN: Lateral reticular nucleus
- LTN: Lateral tuberal nucleus
- LV: Lateral ventricle
- MD: Medial dorsal nucleus [thalamus]
- MEPO: Medial preoptic area
- NAc: Nucleus accumbens
- NDB: Nucleus of the diagonal band
- NL: Nucleus limitans [thalamus]
- NOT: Nucleus of the optic tract
- OLFb: Olfactory bulb
- OT: Olfactory tubercle
- PAG: Periaqueductal gray
- PB: Parabrachial nucleus
- PG: Pontine gray
- PHN: Posterior hypothalamic nucleus
- PMd: Dorsal premammillary nucleus
- PMv: Ventral premammillary nucleus
- PULV: Pulvinar nucleus [thalamus]
- Pf: Parafascicular nucleus [thalamus]
- Prt: Pretectum
- R: Red nucleus
- RCH: Retrochiasmatic nucleus [hypothalamus]
- RE: Nucleus reuniens
- RR: Retrorubral area
- RT: Reticular nucleus [thalamus]
- RTN: Reticular tegmental nucleus
- Ret-MB: Reticular formation, Midbrain
- Ret-Med: Reticular formation, Medulla
- Ret-P: Reticular formation, Pons
- Rms: Rostral migratory stream
- SC: Superior colliculus
- SCH: Suprachiasmatic nucleus [hypothalamus]
- SEP: Septum
- SI: Substantia innominata
- SNc: Substantia nigra pars compacta
- SNr: Substantia nigra pars reticulata
- SO: Superior olive
- SOL: Solitary nucleus
- SPV: Spinal nucleus of the trigeminal
- SUM: Supramammillary area
- TRI: Germinal trigone
- Tct: Tectum
- V: Mesencephalic nucleus
- VA: Ventral anterior nucleus [thalamus]
- VIIn: Facial motor nucleus
- VL: Ventral lateral nucleus [thalamus]
- VMB: Ventral medial basal nucleus [thalamus]
- VMH: Ventromedial nucleus [hypothalamus]
- VNC: Vestibular nuclear complex
- VPM: Ventral posteromedial nucleus [thalamus]
- Vn: Trigeminal motor nucleus
- CaS: Calcarine sulcus

Age: 17 GW

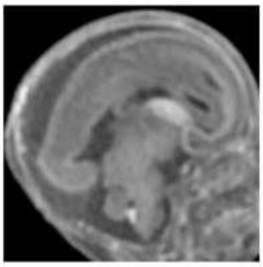

L-R Level: -3.6 mm

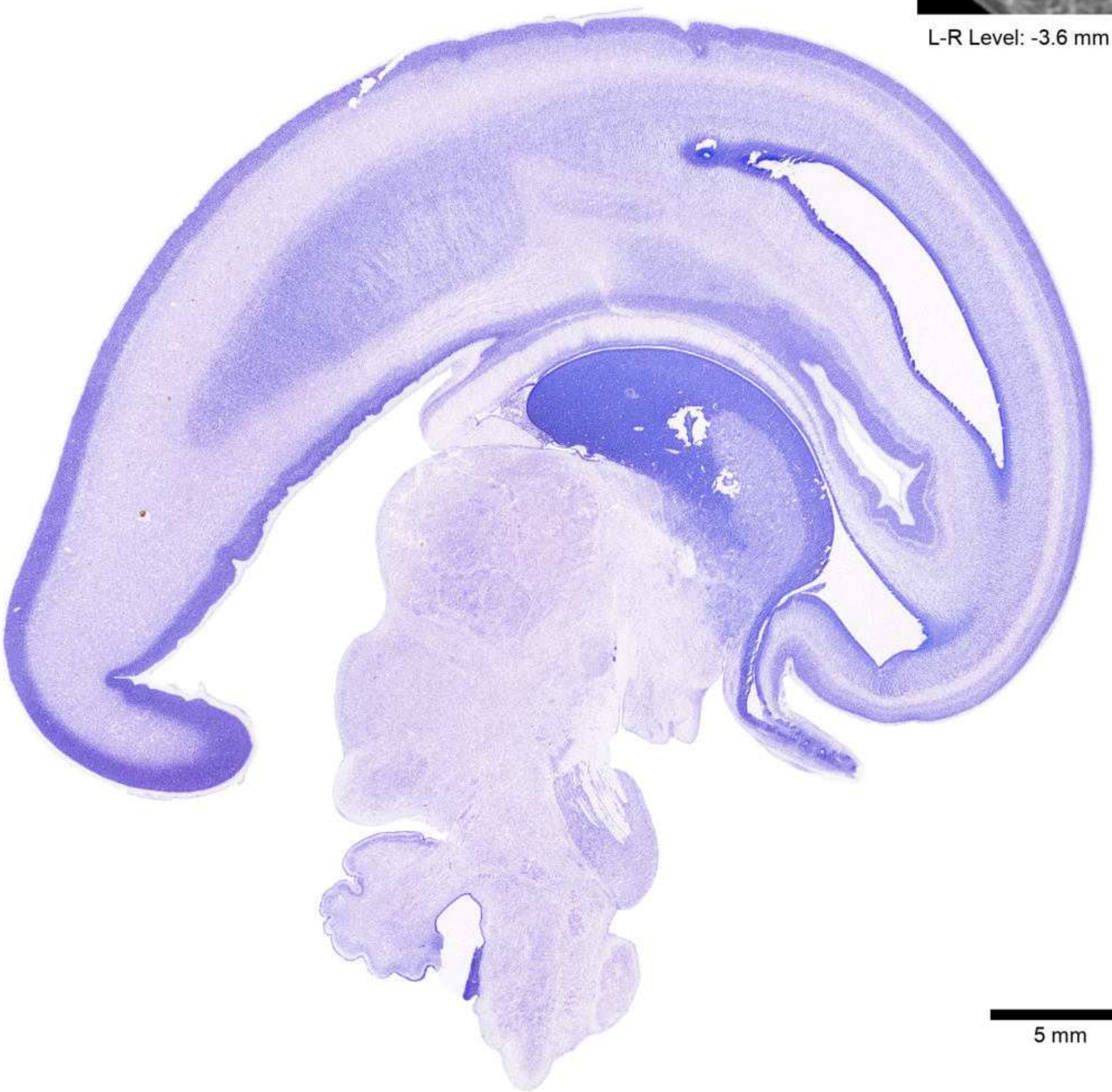

5 mm

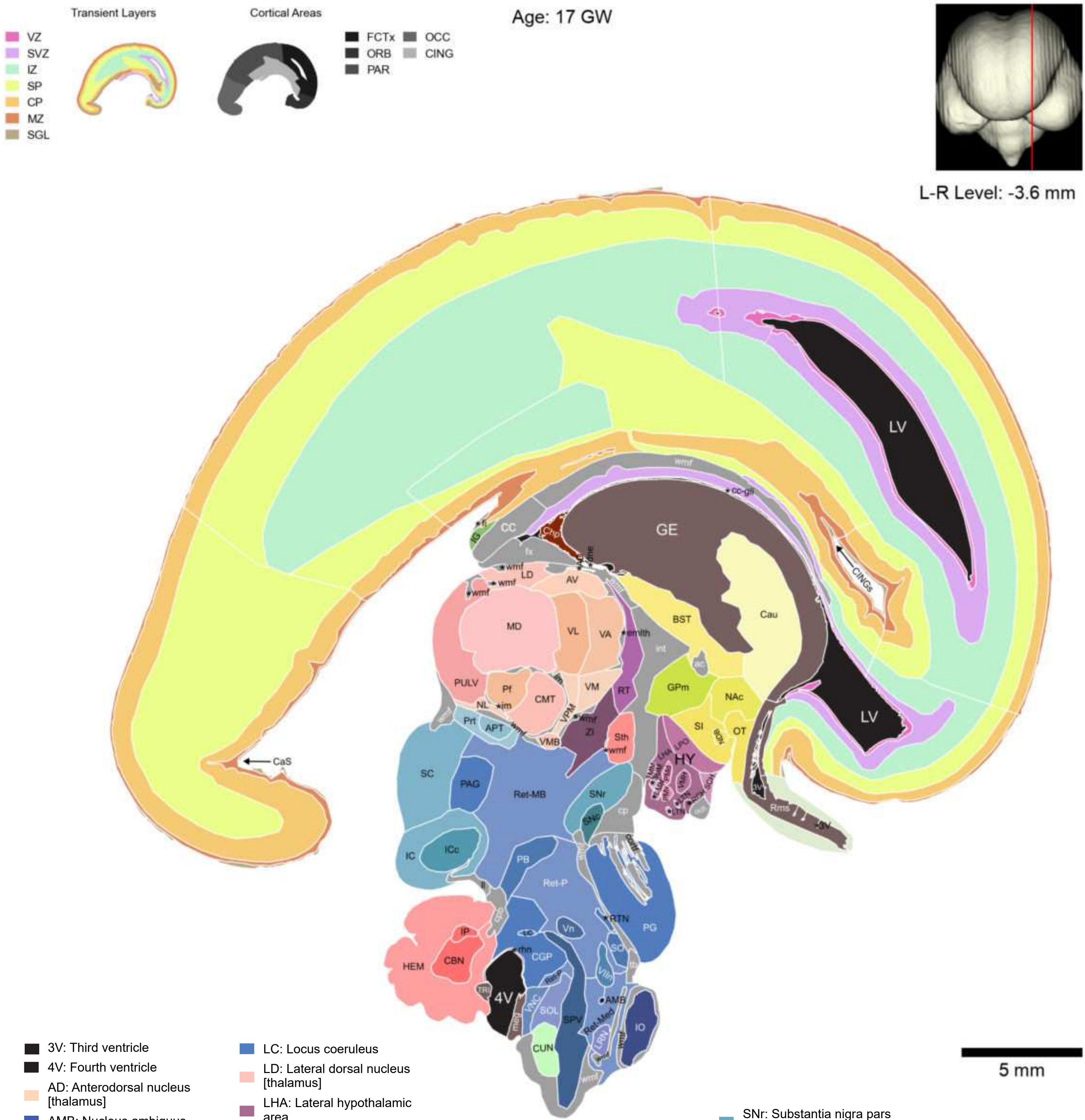

- 3V: Third ventricle
- 4V: Fourth ventricle
- AD: Anterodorsal nucleus [thalamus]
- AMB: Nucleus ambiguus
- APT: Anterior pretectal nucleus
- AV: Anteroventral nucleus [thalamus]
- BST: Bed nucleus of the stria terminalis
- CBN: Cerebellar nuclei
- CGP: Central gray of the pons
- CMT: Centromedian nucleus [thalamus]
- CUN: Cuneate nucleus
- Cau: Caudate nucleus
- Chp: Choroid plexus
- GE: Ganglionic eminence
- GPm: Globus pallidus medial segment
- HEM: Cerebellar hemispheres
- HY: Hypothalamus
- IC: Inferior colliculus
- ICc: Inferior colliculus, central nucleus
- IG: Induseum griseum
- IO: Inferior olive
- IP: Interposed nucleus
- LC: Locus coeruleus
- LD: Lateral dorsal nucleus [thalamus]
- LHA: Lateral hypothalamic area
- LMN: Lateral mammillary nucleus
- LPO: Lateral preoptic area
- LRN: Lateral reticular nucleus
- LTN: Lateral tuberal nucleus
- LV: Lateral ventricle
- MD: Medial dorsal nucleus [thalamus]
- NAc: Nucleus accumbens
- NDB: Nucleus of the diagonal band
- NL: Nucleus limitans [thalamus]
- OLFb: Olfactory bulb
- OT: Olfactory tubercle
- PAG: Periaqueductal gray
- PB: Parabrachial nucleus
- PG: Pontine gray
- PMd: Dorsal premammillary nucleus
- PMv: Ventral premammillary nucleus
- PULV: Pulvinar nucleus [thalamus]
- Pf: Parafascicular nucleus [thalamus]
- Prt: Pretectum
- RCH: Retrochiasmatic nucleus [hypothalamus]
- RT: Reticular nucleus [thalamus]
- RTN: Reticular tegmental nucleus
- Ret-MB: Reticular formation, Midbrain
- Ret-Med: Reticular formation, Medulla
- Ret-P: Reticular formation, Pons
- Rms: Rostral migratory stream
- SC: Superior colliculus
- SCH: Suprachiasmatic nucleus [hypothalamus]
- SI: Substantia innominata
- SNc: Substantia nigra pars compacta
- SNr: Substantia nigra pars reticulata
- SO: Superior olive
- SOL: Solitary nucleus
- SPV: Spinal nucleus of the trigeminal
- SUM: Supramammillary area
- Sth: Subthalamus
- TMM: Tuberomammillary nucleus
- TRI: Germinal trigone
- VA: Ventral anterior nucleus [thalamus]
- VIIn: Facial motor nucleus
- VL: Ventral lateral nucleus [thalamus]
- VM: Ventral medial nucleus [thalamus]
- VMB: Ventral medial basal nucleus [thalamus]
- VMH: Ventromedial nucleus [hypothalamus]
- VNC: Vestibular nuclear complex
- VPM: Ventral posteromedial nucleus [thalamus]
- Vn: Trigeminal motor nucleus
- ZI: Zona incerta
- → CaS: Calcarine sulcus
- → CINGs: Cingulate sulcus

Age: 17 GW

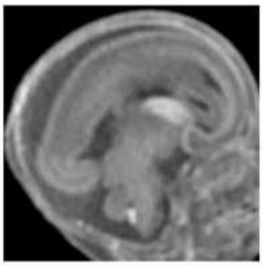

L-R Level: -3.9 mm

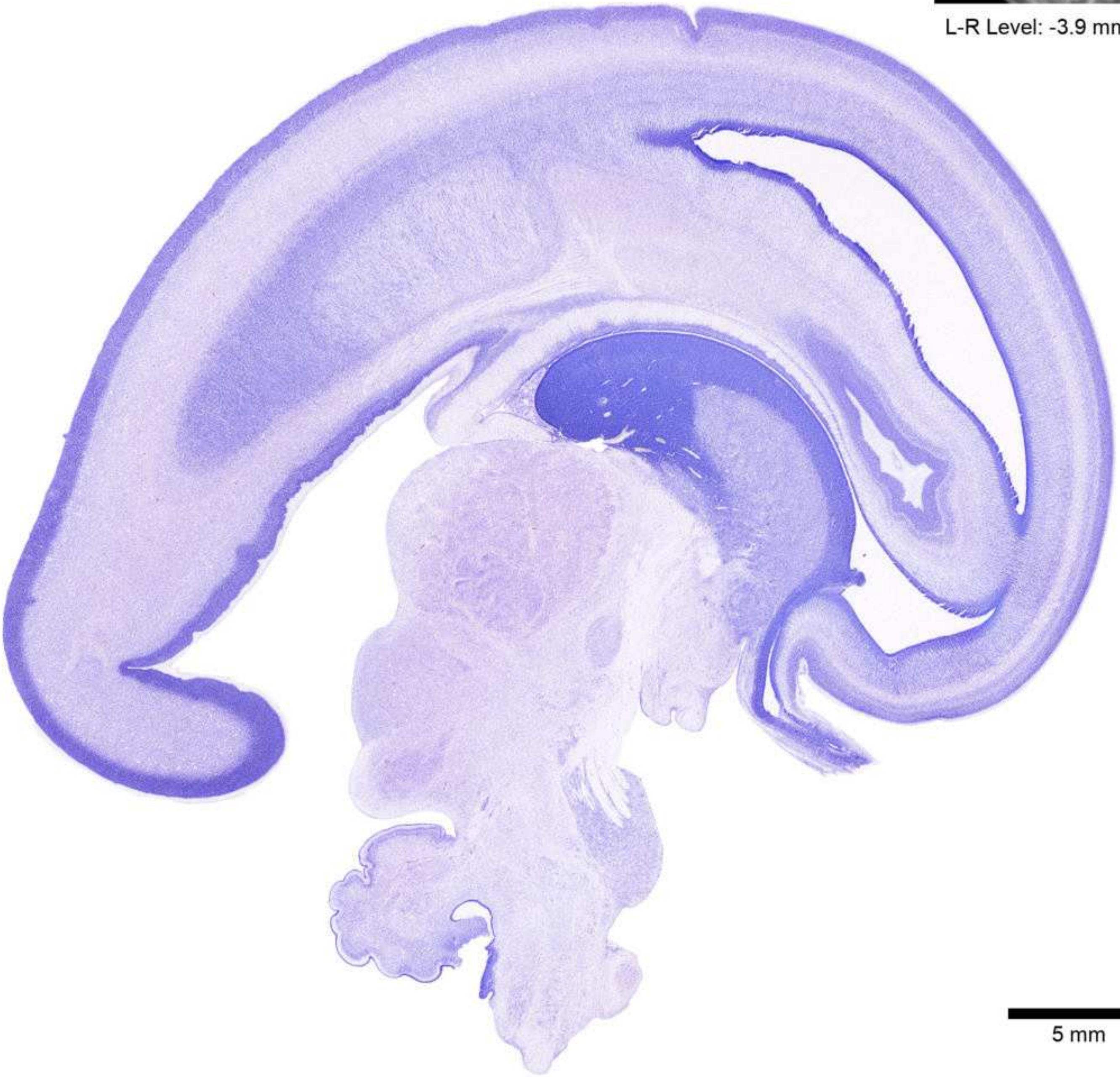

5 mm

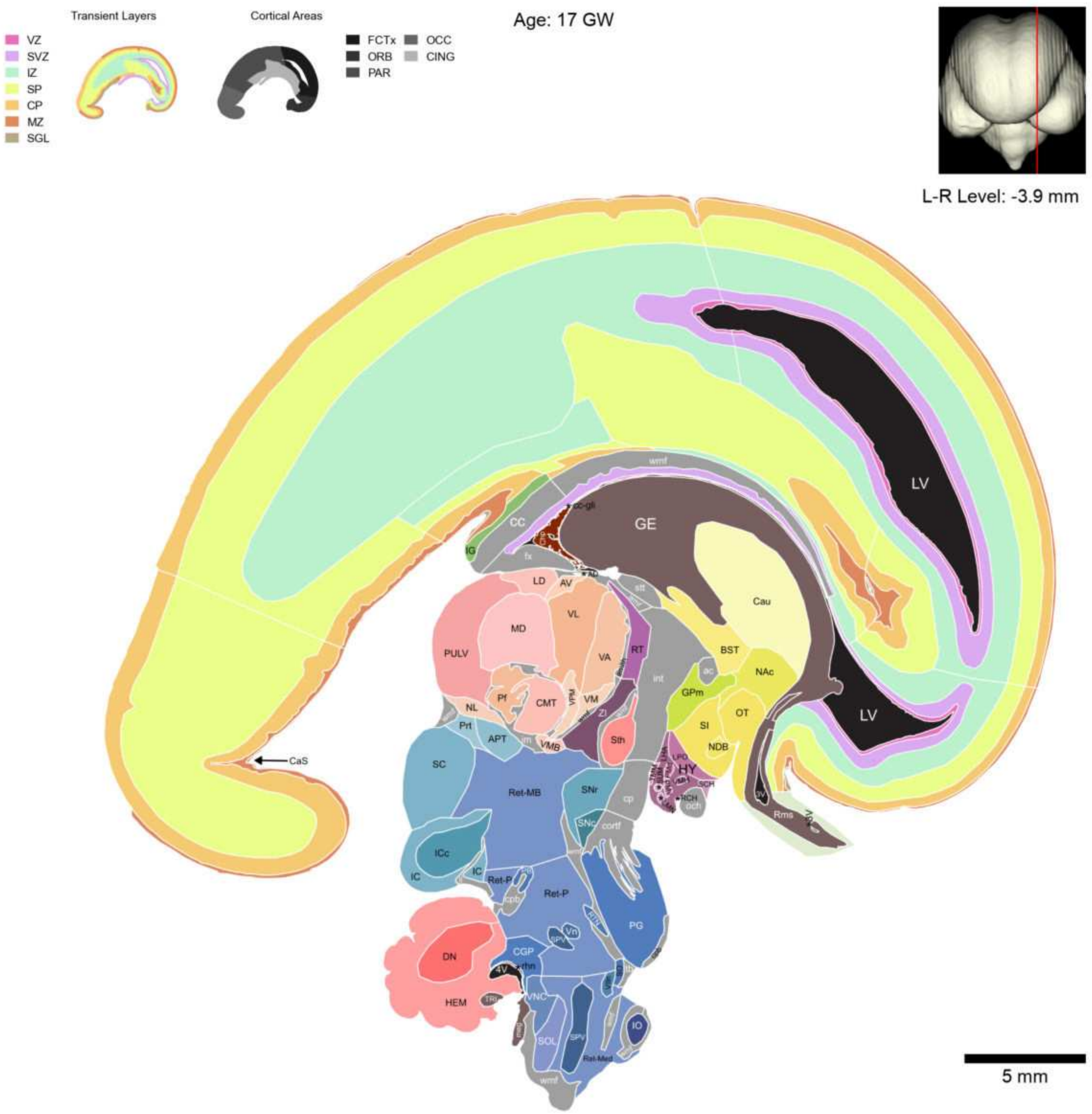

- 3V: Third ventricle
- 4V: Fourth ventricle
- AD: Anterodorsal nucleus [thalamus]
- APT: Anterior pretectal nucleus
- AV: Anteroventral nucleus [thalamus]
- BST: Bed nucleus of the stria terminalis
- CGP: Central gray of the pons
- CMT: Centromedian nucleus [thalamus]
- Cau: Caudate nucleus
- Chp: Choroid plexus
- DN: Dentate nucleus
- GE: Ganglionic eminence
- GPm: Globus pallidus medial segment
- HEM: Cerebellar hemispheres
- HY: Hypothalamus
- IC: Inferior colliculus
- ICc: Inferior colliculus, central nucleus
- IG: Induseum griseum
- IO: Inferior olive
- LD: Lateral dorsal nucleus [thalamus]
- LHA: Lateral hypothalamic area
- LMN: Lateral mammillary nucleus
- LPO: Lateral preoptic area
- LV: Lateral ventricle
- MD: Medial dorsal nucleus [thalamus]
- NAc: Nucleus accumbens
- NDB: Nucleus of the diagonal band
- NL: Nucleus limitans [thalamus]
- OLFb: Olfactory bulb
- OT: Olfactory tubercle
- PB: Parabrachial nucleus
- PG: Pontine gray
- PMd: Dorsal premammillary nucleus
- PMv: Ventral premammillary nucleus
- PULV: Pulvinar nucleus [thalamus]
- Pf: Parafascicular nucleus [thalamus]
- Prt: Pretectum
- RCH: Retrochiasmatic nucleus [hypothalamus]
- RT: Reticular nucleus [thalamus]
- RTN: Reticular tegmental nucleus
- Ret-MB: Reticular formation, Midbrain
- Ret-Med: Reticular formation, Medulla
- Ret-P: Reticular formation, Pons
- Rms: Rostral migratory stream
- SC: Superior colliculus
- SCH: Suprachiasmatic nucleus [hypothalamus]
- SI: Substantia innominata
- SNc: Substantia nigra pars compacta
- SNr: Substantia nigra pars reticulata
- SO: Superior olive
- SOL: Solitary nucleus
- SPV: Spinal nucleus of the trigeminal
- SUM: Supramammillary area
- Sth: Subthalamus
- TMM: Tuberomammillary nucleus
- TRI: Germinal trigone
- VA: Ventral anterior nucleus [thalamus]
- VIIn: Facial motor nucleus
- VL: Ventral lateral nucleus [thalamus]
- VM: Ventral medial nucleus [thalamus]
- VMB: Ventral medial basal nucleus [thalamus]
- VMH: Ventromedial nucleus [hypothalamus]
- VNC: Vestibular nuclear complex
- VPM: Ventral posteromedial nucleus [thalamus]
- Vn: Trigeminal motor nucleus
- ZI: Zona incerta
- → CaS: Calcarine sulcus

Age: 17 GW

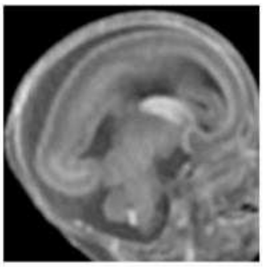

L-R Level: -4.26 mm

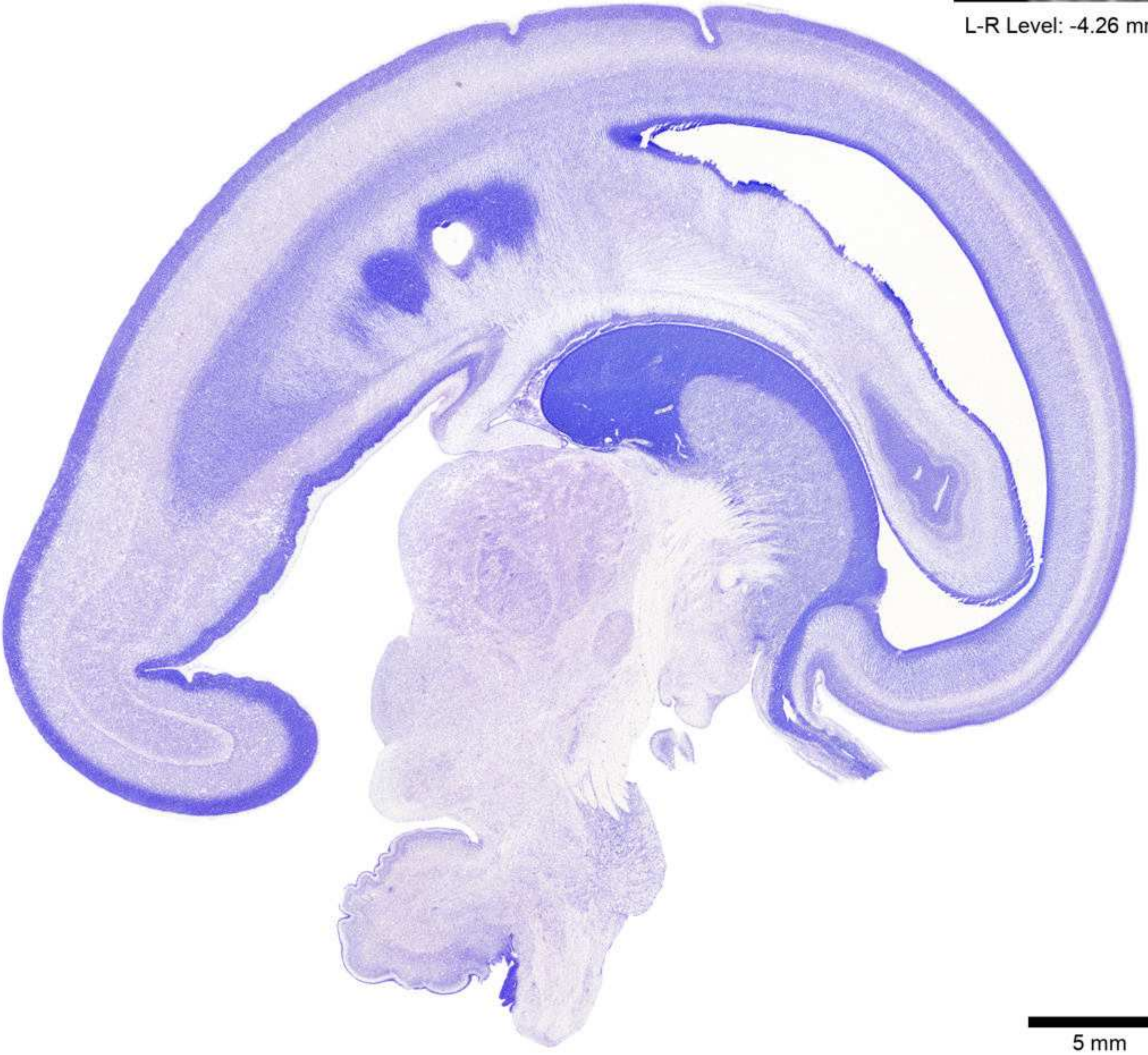

5 mm

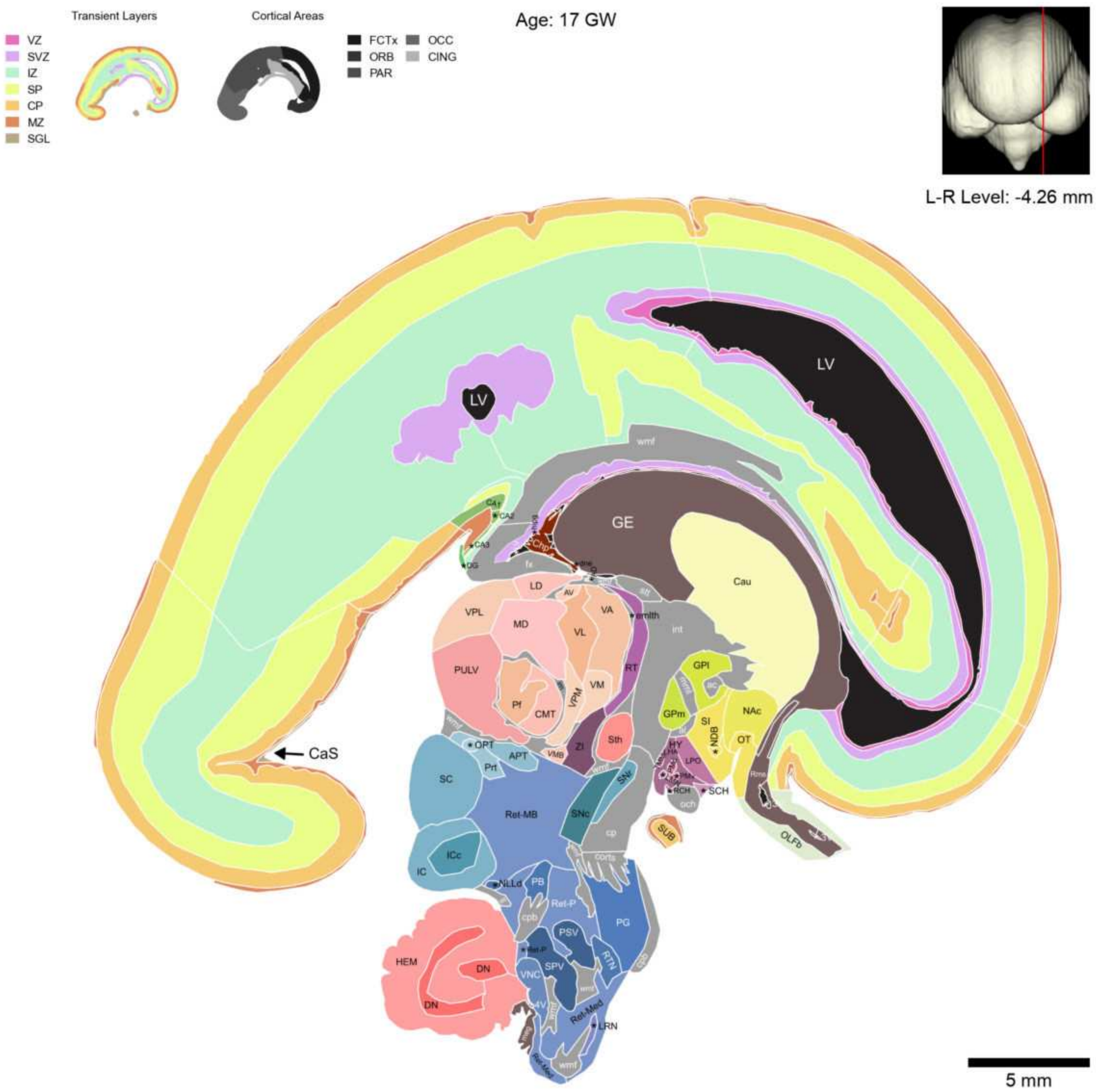

- 3V: Third ventricle
- 4V: Fourth ventricle
- AD: Anterodorsal nucleus [thalamus]
- APT: Anterior pretectal nucleus
- AV: Anteroventral nucleus [thalamus]
- CA1: CA1 field [hippocampus]
- CA2: CA2 field [hippocampus]
- CA3: CA3 field [hippocampus]
- CMT: Centromedian nucleus [thalamus]
- Cau: Caudate nucleus
- Chp: Choroid plexus
- DG: Dentate gyrus
- DN: Dentate nucleus
- GE: Ganglionic eminence
- GPl: Globus pallidus lateral segment
- GPm: Globus pallidus medial segment
- HEM: Cerebellar hemispheres
- HY: Hypothalamus
- IC: Inferior colliculus
- ICc: Inferior colliculus, central nucleus
- LD: Lateral dorsal nucleus [thalamus]
- LHA: Lateral hypothalamic area
- LMN: Lateral mammillary nucleus
- LPO: Lateral preoptic area
- LRN: Lateral reticular nucleus
- LV: Lateral ventricle
- MD: Medial dorsal nucleus [thalamus]
- NAc: Nucleus accumbens
- NDB: Nucleus of the diagonal band
- NLLd: Nucleus of the lateral lemniscus, dorsal
- OLFb: Olfactory bulb
- OPT: Olivary pretectal nucleus
- OT: Olfactory tubercle
- PB: Parabrachial nucleus
- PG: Pontine gray
- PMd: Dorsal premammillary nucleus
- PMv: Ventral premammillary nucleus
- PSV: Principal sensory nucleus of the trigeminal
- PULV: Pulvinar nucleus [thalamus]
- Pf: Parafascicular nucleus [thalamus]
- Prt: Pretectum
- RCH: Retrochiasmatic nucleus [hypothalamus]
- RT: Reticular nucleus [thalamus]
- RTN: Reticular tegmental nucleus
- Ret-MB: Reticular formation, Midbrain
- Ret-Med: Reticular formation, Medulla
- Ret-P: Reticular formation, Pons
- Rms: Rostral migratory stream
- SC: Superior colliculus
- SCH: Suprachiasmatic nucleus [hypothalamus]
- SI: Substantia innominata
- SNc: Substantia nigra pars compacta
- SNr: Substantia nigra pars reticulata
- SPV: Spinal nucleus of the trigeminal
- SUB: Cortical plate, subiculum
- Sth: Subthalamus
- TMM: Tuberomammillary nucleus
- VA: Ventral anterior nucleus [thalamus]
- VL: Ventral lateral nucleus [thalamus]
- VM: Ventral medial nucleus [thalamus]
- VMB: Ventral medial basal nucleus [thalamus]
- VNC: Vestibular nuclear complex
- VPL: Ventral posterolateral nucleus [thalamus]
- VPM: Ventral posteromedial nucleus [thalamus]
- ZI: Zona incerta
- → CaS: Calcarine sulcus

Age: 17 GW

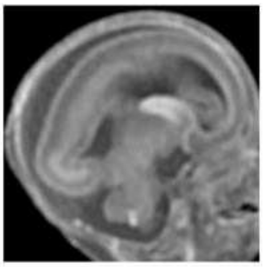

L-R Level: -4.44 mm

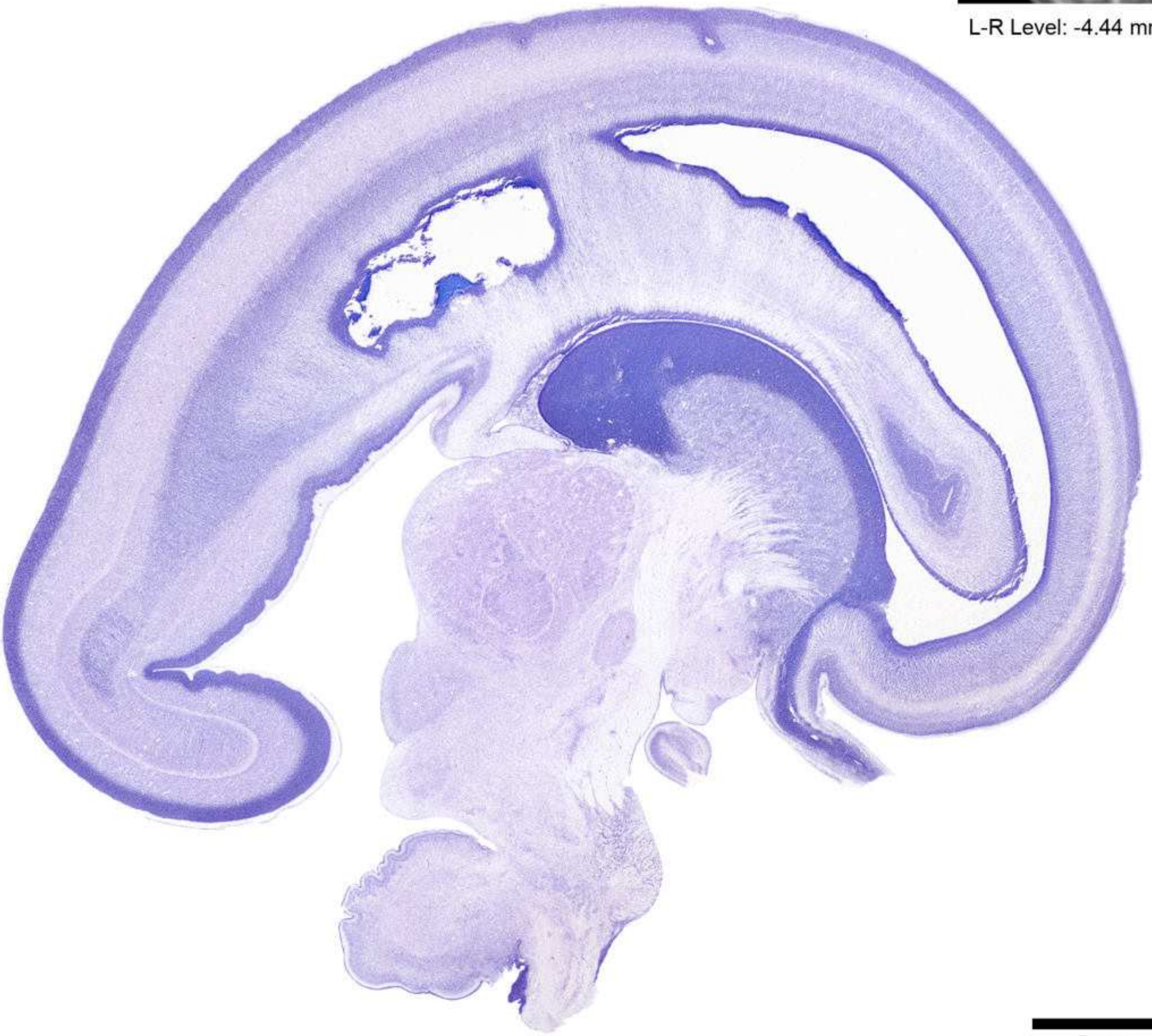

5 mm

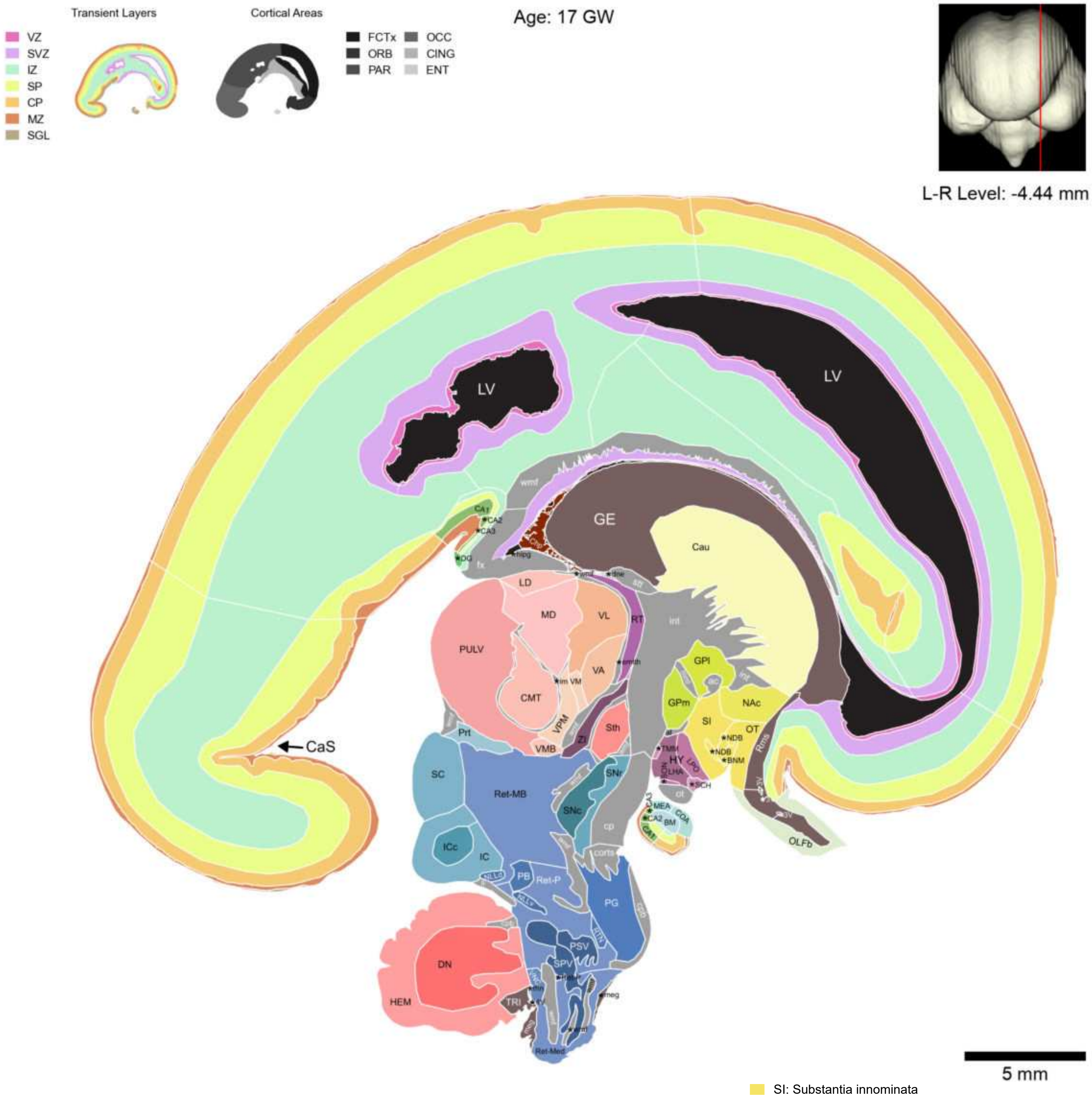

- 3V: Third ventricle
- 4V: Fourth ventricle
- BM: Basomedial nucleus [amygdala]
- BNM: Basal nucleus of Meynert
- CA1: CA1 field [hippocampus]
- CA2: CA2 field [hippocampus]
- CA3: CA3 field [hippocampus]
- CMT: Centromedian nucleus [thalamus]
- COA: Cortical nucleus [amygdala]
- Cau: Caudate nucleus
- Chp: Choroid plexus
- DG: Dentate gyrus
- DN: Dentate nucleus
- GE: Ganglionic eminence
- GPl: Globus pallidus lateral segment
- GPm: Globus pallidus medial segment
- HEM: Cerebellar hemispheres
- HY: Hypothalamus
- IC: Inferior colliculus
- ICc: Inferior colliculus, central nucleus
- LD: Lateral dorsal nucleus [thalamus]
- LHA: Lateral hypothalamic area
- LPO: Lateral preoptic area
- LV: Lateral ventricle
- MD: Medial dorsal nucleus [thalamus]
- MEA: Medial nucleus [amygdala]
- NAc: Nucleus accumbens
- NDB: Nucleus of the diagonal band
- NLLd: Nucleus of the lateral lemniscus, dorsal
- NLLv: Nucleus of the lateral lemniscus, ventral
- OLFb: Olfactory bulb
- OT: Olfactory tubercle
- PB: Parabrachial nucleus
- PG: Pontine gray
- PSV: Principal sensory nucleus of the trigeminal
- PULV: Pulvinar nucleus [thalamus]
- Prt: Pretectum
- RT: Reticular nucleus [thalamus]
- RTN: Reticular tegmental nucleus
- Ret-MB: Reticular formation, Midbrain
- Ret-Med: Reticular formation, Medulla
- Ret-P: Reticular formation, Pons
- Rms: Rostral migratory stream
- SC: Superior colliculus
- SCH: Suprachiasmatic nucleus [hypothalamus]
- SI: Substantia innominata
- SNc: Substantia nigra pars compacta
- SNr: Substantia nigra pars reticulata
- SON: Supraoptic nucleus [hypothalamus]
- SPV: Spinal nucleus of the trigeminal
- Sth: Subthalamus
- TMM: Tuberomammillary nucleus
- TRI: Germinal trigone
- VA: Ventral anterior nucleus [thalamus]
- VL: Ventral lateral nucleus [thalamus]
- VM: Ventral medial nucleus [thalamus]
- VMB: Ventral medial basal nucleus [thalamus]
- VNC: Vestibular nuclear complex
- VPM: Ventral posteromedial nucleus [thalamus]
- ZI: Zona incerta
- → CaS: Calcarine sulcus

Age: 17 GW

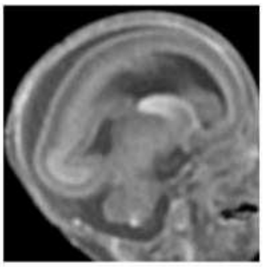

L-R Level: -4.86 mm

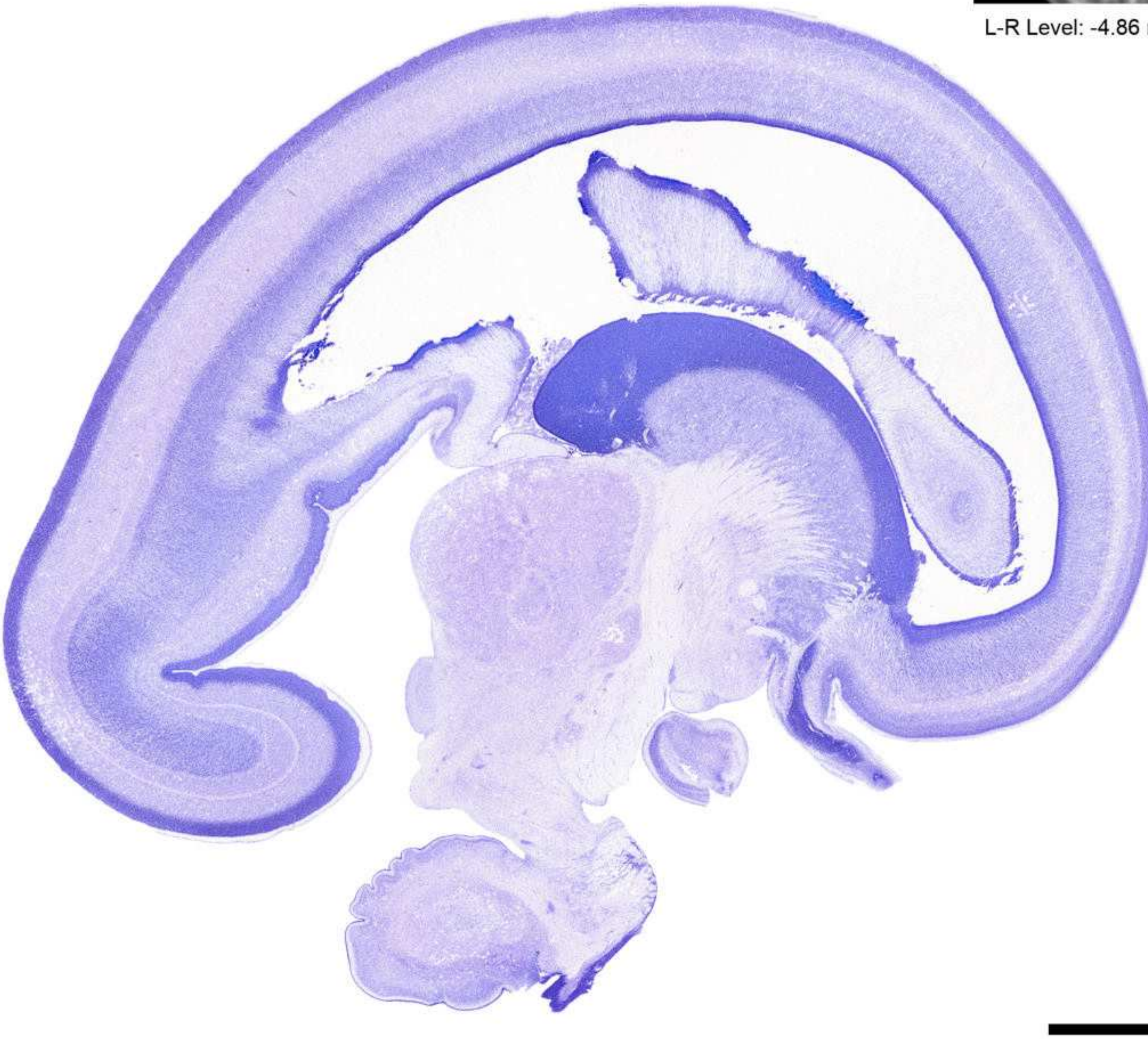

5 mm

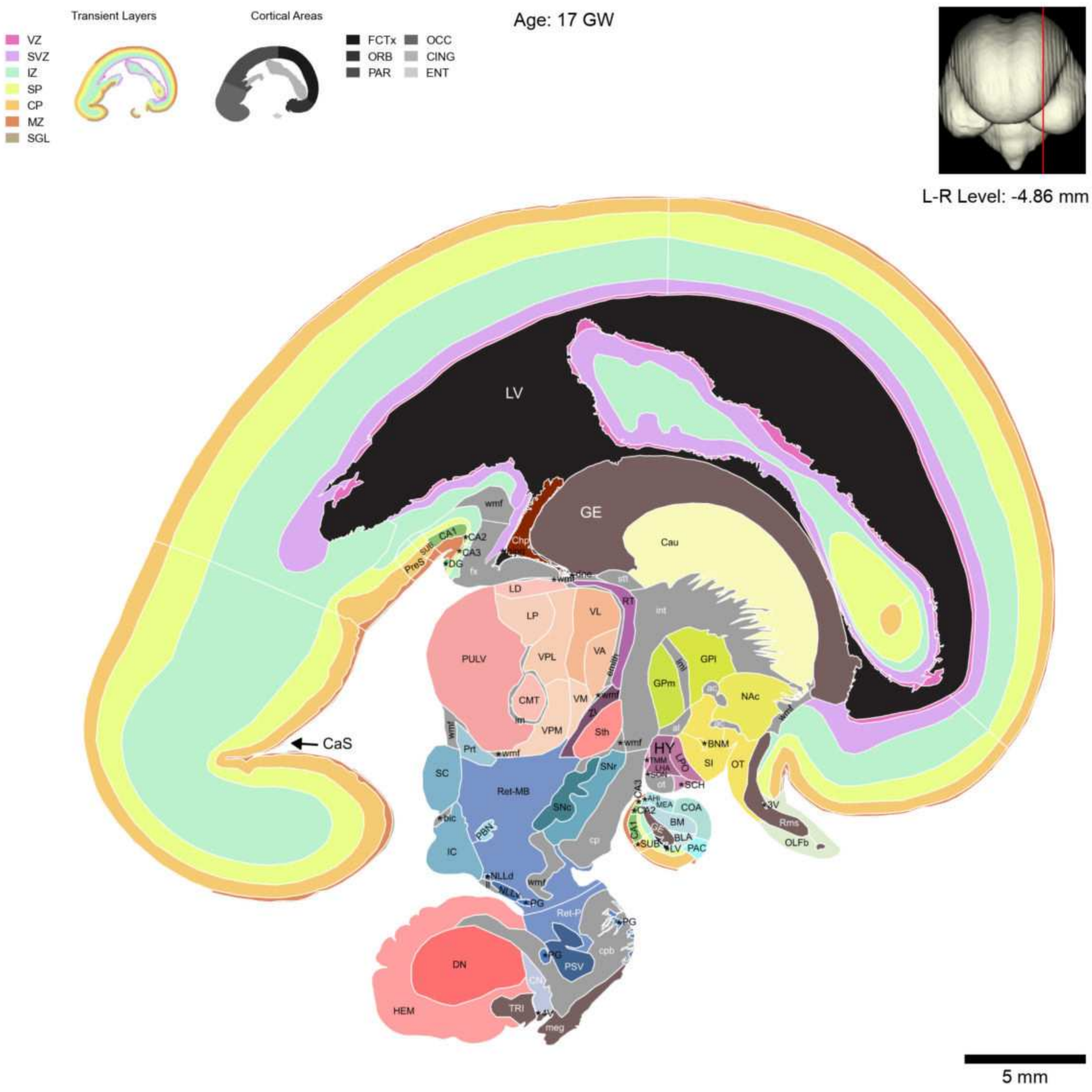

- 3V: Third ventricle
- 4V: Fourth ventricle
- AHi: Amygdalo-hippocampal area
- BLA: Basolateral complex [amygdala]
- BM: Basomedial nucleus [amygdala]
- BNM: Basal nucleus of Meynert
- CA1: CA1 field [hippocampus]
- CA2: CA2 field [hippocampus]
- CA3: CA3 field [hippocampus]
- CMT: Centromedian nucleus [thalamus]
- CN: Cochlear nuclei
- COA: Cortical nucleus [amygdala]
- Cau: Caudate nucleus
- Chp: Choroid plexus
- DG: Dentate gyrus
- DN: Dentate nucleus
- GE: Ganglionic eminence
- GPl: Globus pallidus lateral segment
- GPm: Globus pallidus medial segment
- HEM: Cerebellar hemispheres
- HY: Hypothalamus
- IC: Inferior colliculus
- LD: Lateral dorsal nucleus [thalamus]
- LHA: Lateral hypothalamic area
- LP: Lateral posterior nucleus [thalamus]
- LPO: Lateral preoptic area
- LV: Lateral ventricle
- MEA: Medial nucleus [amygdala]
- NAc: Nucleus accumbens
- NLLd: Nucleus of the lateral lemniscus, dorsal
- NLLv: Nucleus of the lateral lemniscus, ventral
- OLFb: Olfactory bulb
- OT: Olfactory tubercle
- PBN: Parabigeminal nucleus
- PG: Pontine gray
- PSV: Principal sensory nucleus of the trigeminal
- PULV: Pulvinar nucleus [thalamus]
- PreS: Cortical plate, presubiculum
- Prt: Pretectum
- RT: Reticular nucleus [thalamus]
- Ret-MB: Reticular formation, Midbrain
- Ret-P: Reticular formation, Pons
- Rms: Rostral migratory stream
- SC: Superior colliculus
- SCH: Suprachiasmatic nucleus [hypothalamus]
- SI: Substantia innominata
- SNc: Substantia nigra pars compacta
- SNr: Substantia nigra pars reticulata
- SON: Supraoptic nucleus [hypothalamus]
- SUB: Cortical plate, subiculum
- Sth: Subthalamus
- TMM: Tuberomammillary nucleus
- TRI: Germinal trigone
- VA: Ventral anterior nucleus [thalamus]
- VL: Ventral lateral nucleus [thalamus]
- VM: Ventral medial nucleus [thalamus]
- VPL: Ventral posterolateral nucleus [thalamus]
- VPM: Ventral posteromedial nucleus [thalamus]
- ZI: Zona incerta
- → CaS: Calcarine sulcus

Age: 17 GW

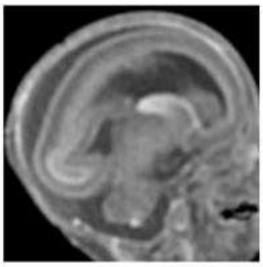

L-R Level: -5.04 mm

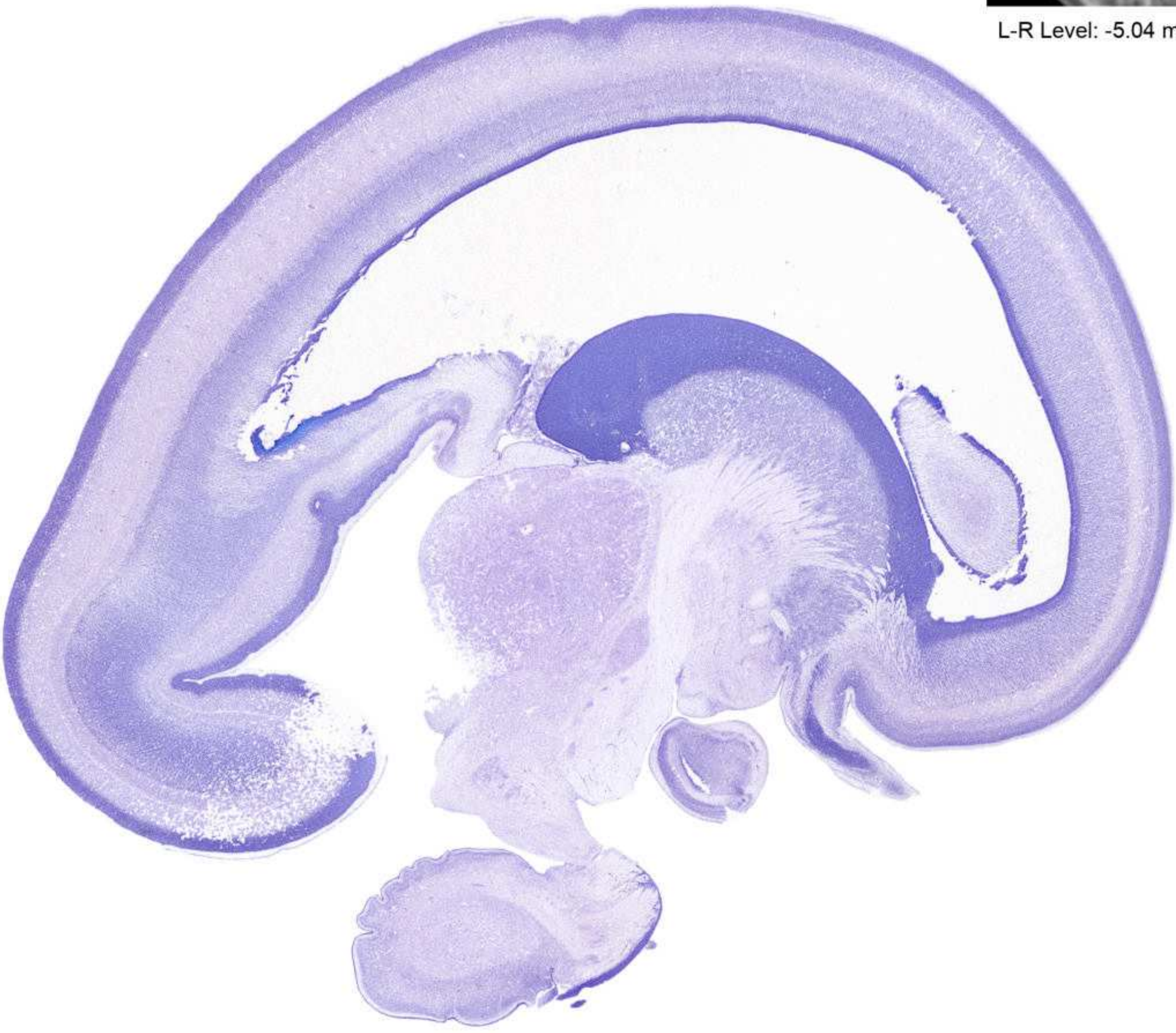

5 mm

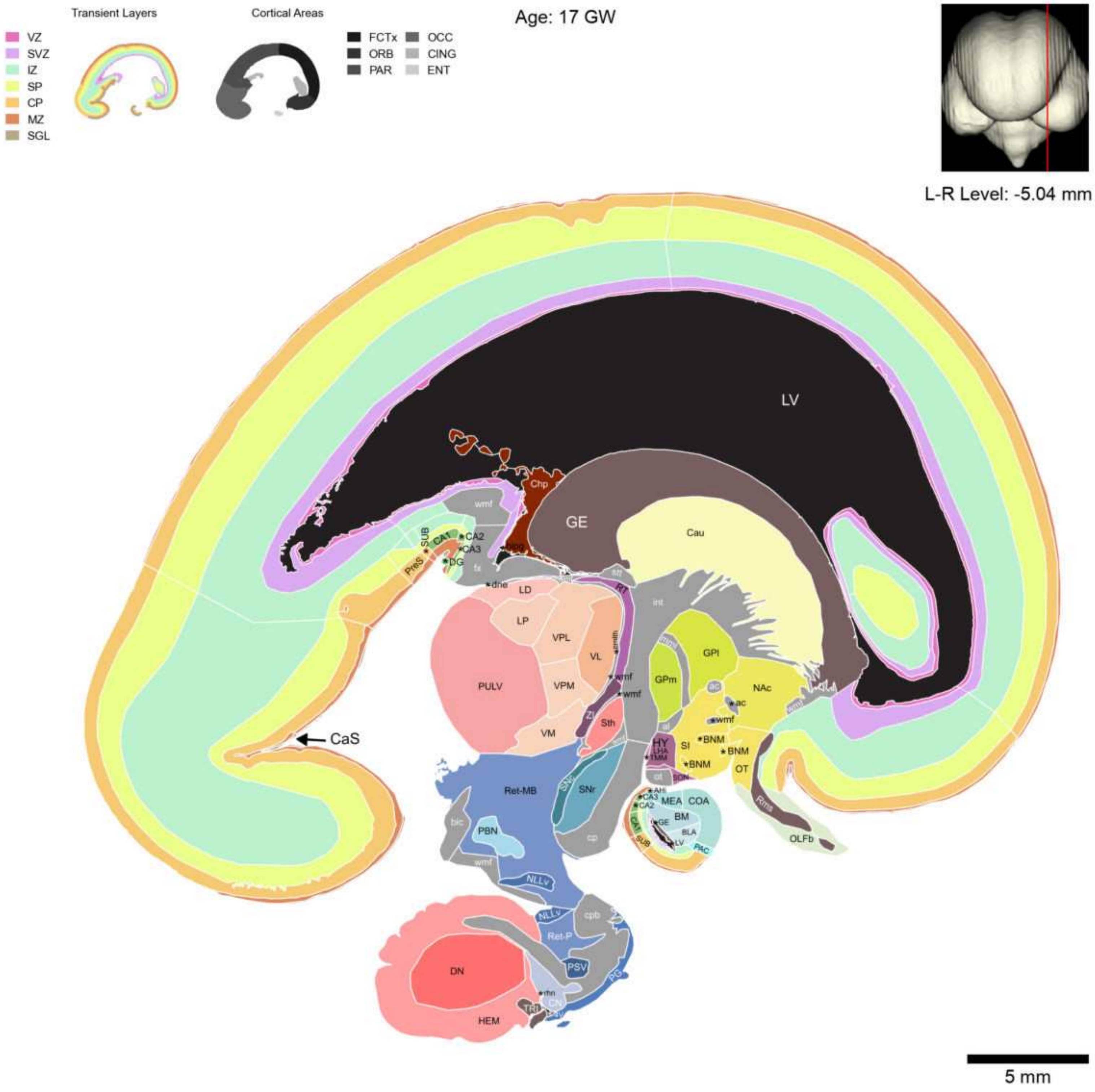

- 4V: Fourth ventricle
- AHi: Amygdalo-hippocampal area
- BLA: Basolateral complex [amygdala]
- BM: Basomedial nucleus [amygdala]
- BNM: Basal nucleus of Meynert
- CA1: CA1 field [hippocampus]
- CA2: CA2 field [hippocampus]
- CA3: CA3 field [hippocampus]
- CN: Cochlear nuclei
- COA: Cortical nucleus [amygdala]
- Cau: Caudate nucleus
- Chp: Choroid plexus
- DG: Dentate gyrus
- DN: Dentate nucleus
- GE: Ganglionic eminence
- GPl: Globus pallidus lateral segment
- GPm: Globus pallidus medial segment
- HEM: Cerebellar hemispheres
- HY: Hypothalamus
- LD: Lateral dorsal nucleus [thalamus]
- LHA: Lateral hypothalamic area
- LP: Lateral posterior nucleus [thalamus]
- LV: Lateral ventricle
- MEA: Medial nucleus [amygdala]
- NAc: Nucleus accumbens
- NLLv: Nucleus of the lateral lemniscus, ventral
- OLFb: Olfactory bulb
- OT: Olfactory tubercle
- PBN: Parabigeminal nucleus
- PG: Pontine gray
- PSV: Principal sensory nucleus of the trigeminal
- PULV: Pulvinar nucleus [thalamus]
- PreS: Cortical plate, presubiculum
- RT: Reticular nucleus [thalamus]
- Ret-MB: Reticular formation, Midbrain
- Ret-P: Reticular formation, Pons
- Rms: Rostral migratory stream
- SI: Substantia innominata
- SNc: Substantia nigra pars compacta
- SNr: Substantia nigra pars reticulata
- SON: Supraoptic nucleus [hypothalamus]
- SUB: Cortical plate, subiculum
- Sth: Subthalamus
- TMM: Tuberomammillary nucleus
- TRI: Germinal trigone
- VL: Ventral lateral nucleus [thalamus]
- VM: Ventral medial nucleus [thalamus]
- VPL: Ventral posterolateral nucleus [thalamus]
- VPM: Ventral posteromedial nucleus [thalamus]
- ZI: Zona incerta
- ac: Anterior commissure
- al: Ansa lenticularis
- bic: Brachium of the inferior colliculus
- cp: Cerebral peduncle
- cpb: Cerebellar peduncles
- dne: Diencephalic neuroepithelium
- emlth: External medullary lamina [thalamus]
- fx: Fornix
- hipg: Hippocampal glioepithelium/ependyma
- int: Internal capsule
- mml: Medial medullary lamina
- ot: Optic tract
- rhn: Rhombencephalic neuroepithelium
- stt: Stria terminalis
- wmf: White matter fibers
- → CaS: Calcarine sulcus

Age: 17 GW

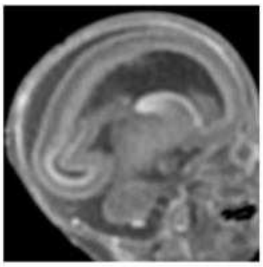

L-R Level: -5.52 mm

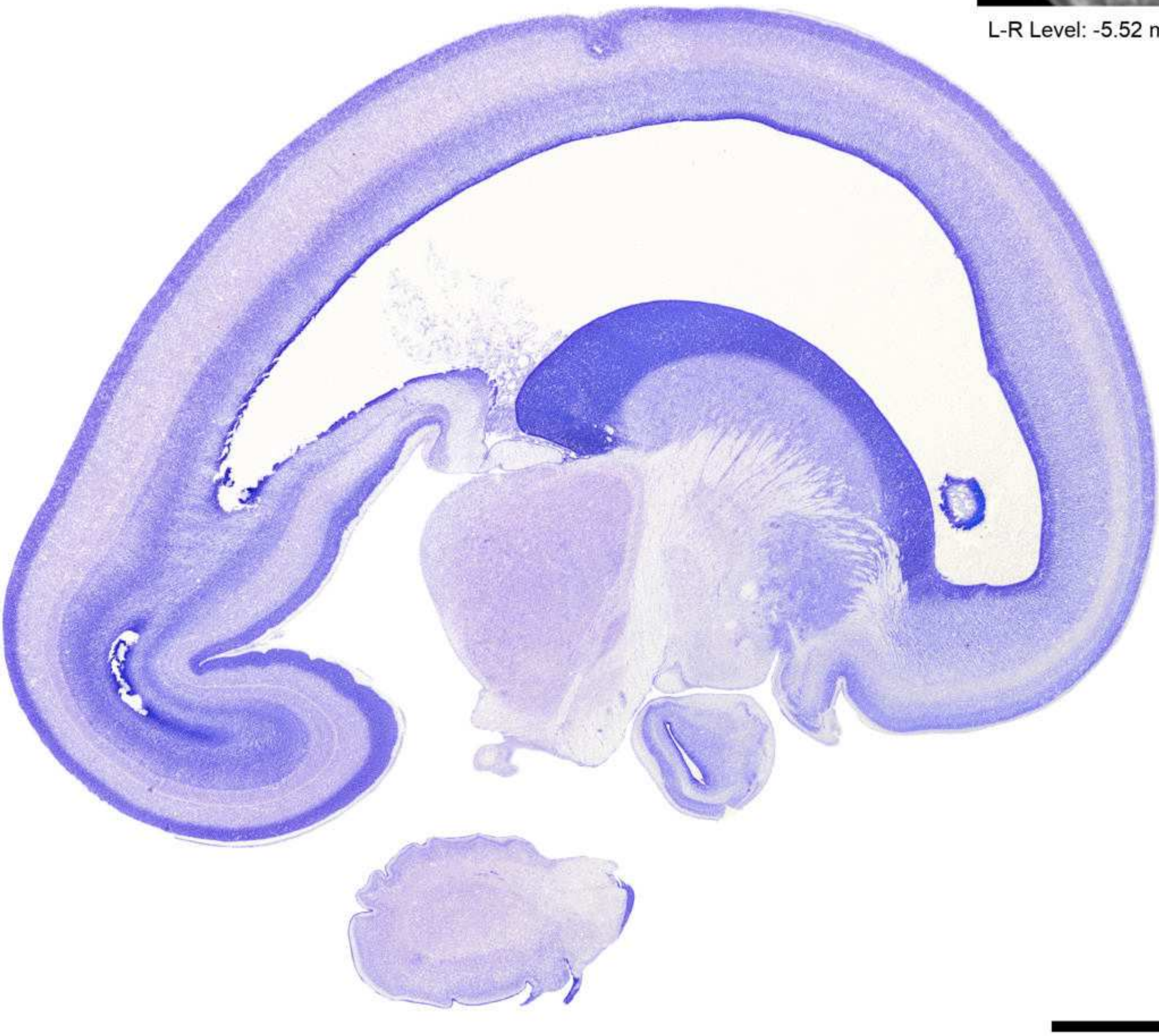

5 mm

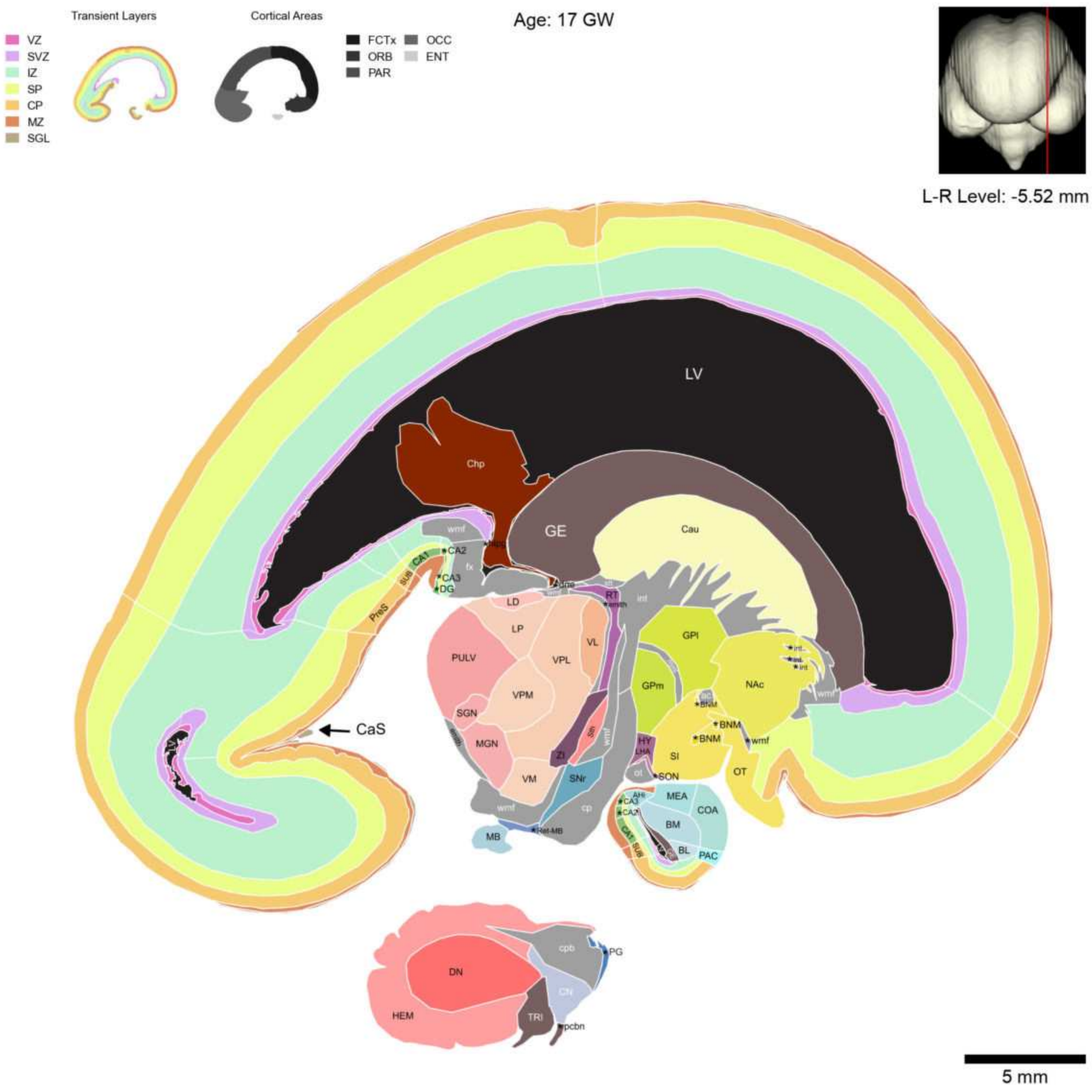

- AHi: Amygdalo-hippocampal area
- BL: Basal nucleus [amygdala]
- BM: Basomedial nucleus [amygdala]
- BNM: Basal nucleus of Meynert
- CA1: CA1 field [hippocampus]
- CA2: CA2 field [hippocampus]
- CA3: CA3 field [hippocampus]
- CN: Cochlear nuclei
- COA: Cortical nucleus [amygdala]
- Cau: Caudate nucleus
- Chp: Choroid plexus
- DG: Dentate gyrus
- DN: Dentate nucleus
- GE: Ganglionic eminence
- GPl: Globus pallidus lateral segment
- GPm: Globus pallidus medial segment
- HEM: Cerebellar hemispheres
- HY: Hypothalamus
- LD: Lateral dorsal nucleus [thalamus]
- LHA: Lateral hypothalamic area
- LP: Lateral posterior nucleus [thalamus]
- LV: Lateral ventricle
- MB: Midbrain
- MEA: Medial nucleus [amygdala]
- MGN: Medial geniculate nucleus
- NAc: Nucleus accumbens
- OT: Olfactory tubercle
- PG: Pontine gray
- PULV: Pulvinar nucleus [thalamus]
- PreS: Cortical plate, presubiculum
- RT: Reticular nucleus [thalamus]
- Ret-MB: Reticular formation, Midbrain
- SGN: Suprageniculate nucleus
- SI: Substantia innominata
- SNr: Substantia nigra pars reticulata
- SON: Supraoptic nucleus [hypothalamus]
- SUB: Cortical plate, subiculum
- Sth: Subthalamus
- TRI: Germinal trigone
- VL: Ventral lateral nucleus [thalamus]
- VM: Ventral medial nucleus [thalamus]
- VPL: Ventral posterolateral nucleus [thalamus]
- VPM: Ventral posteromedial nucleus [thalamus]
- ZI: Zona incerta
- ac: Anterior commissure
- cp: Cerebral peduncle
- cpb: Cerebellar peduncles
- dne: Diencephalic neuroepithelium
- emlth: External medullary lamina [thalamus]
- fx: Fornix
- hipg: Hippocampal glioepithelium/ependyma
- int: Internal capsule
- mml: Medial medullary lamina
- ot: Optic tract
- pcbn: Precerebellar neuroepithelium
- stt: Stria terminalis
- wmf: White matter fibers
- → CaS: Calcarine sulcus

Age: 17 GW

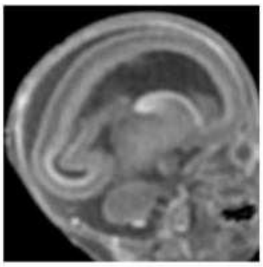

L-R Level: -5.7 mm

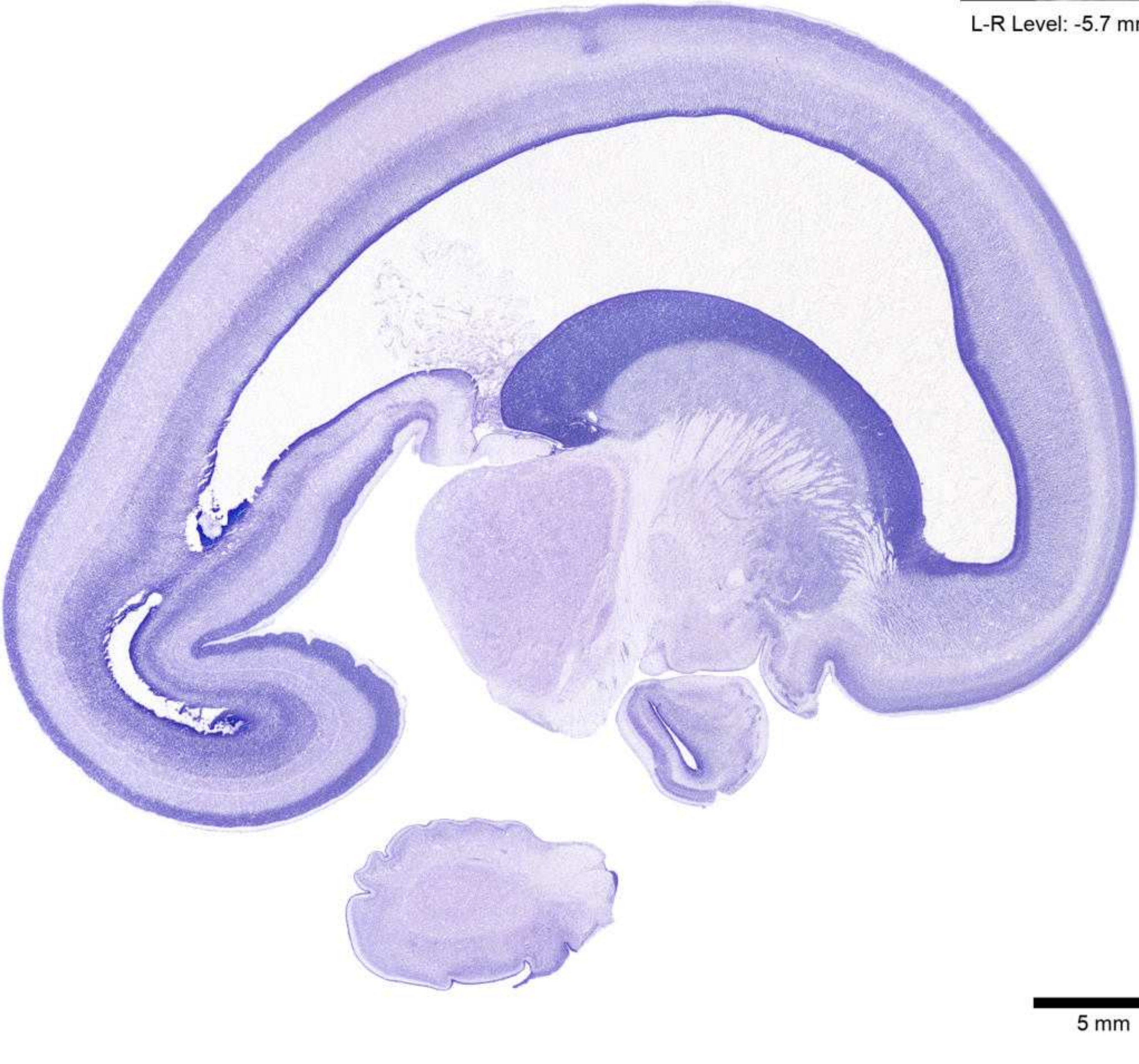

5 mm

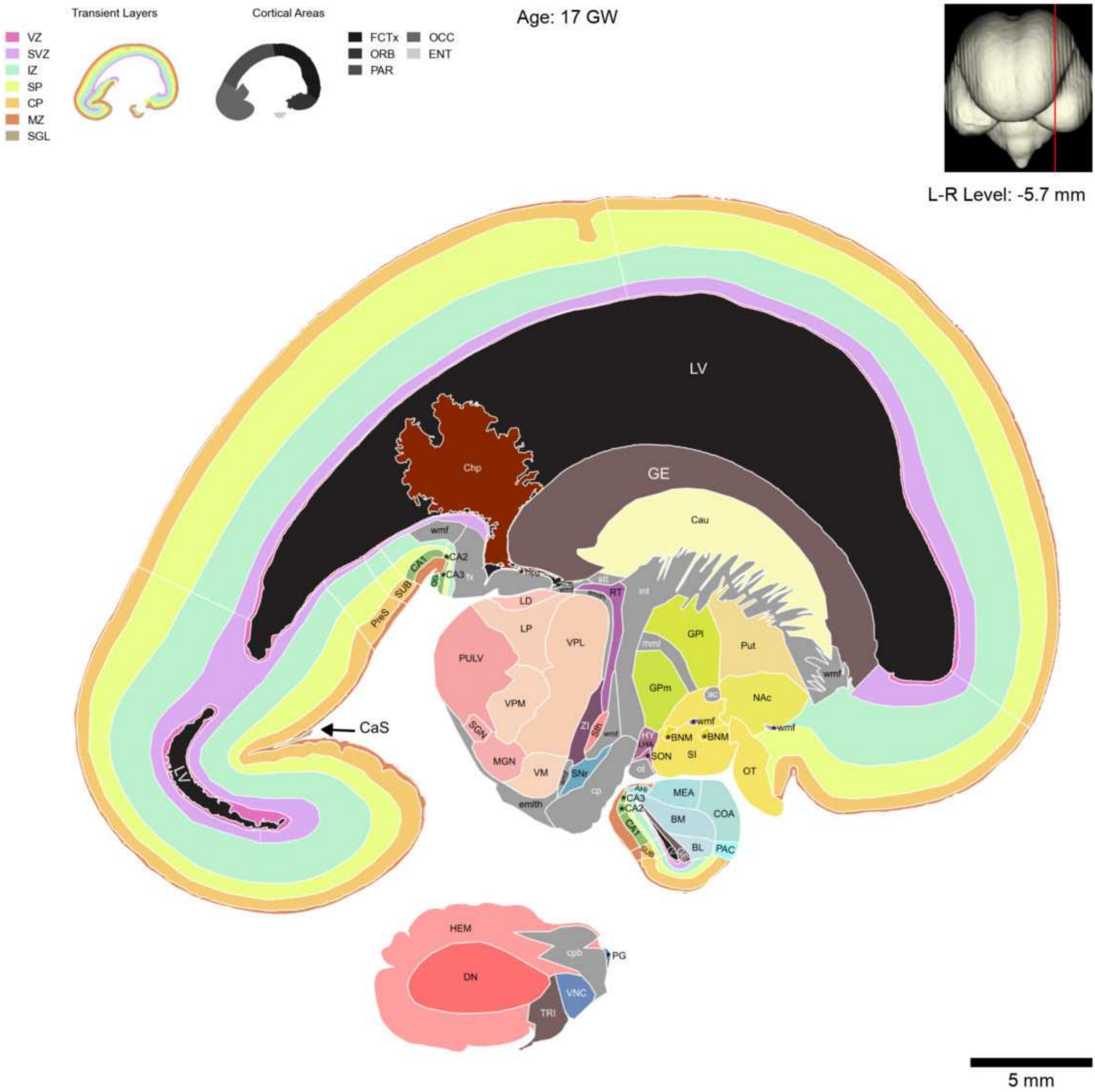

- AHi: Amygdalo-hippocampal area
- BL: Basal nucleus [amygdala]
- BM: Basomedial nucleus [amygdala]
- BNM: Basal nucleus of Meynert
- CA1: CA1 field [hippocampus]
- CA2: CA2 field [hippocampus]
- CA3: CA3 field [hippocampus]
- COA: Cortical nucleus [amygdala]
- Cau: Caudate nucleus
- Chp: Choroid plexus
- DG: Dentate gyrus
- DN: Dentate nucleus
- GE: Ganglionic eminence
- GPl: Globus pallidus lateral segment
- GPm: Globus pallidus medial segment
- HEM: Cerebellar hemispheres
- HY: Hypothalamus
- LD: Lateral dorsal nucleus [thalamus]
- LHA: Lateral hypothalamic area
- LP: Lateral posterior nucleus [thalamus]
- LV: Lateral ventricle
- MEA: Medial nucleus [amygdala]
- MGN: Medial geniculate nucleus
- NAc: Nucleus accumbens
- OT: Olfactory tubercle
- PG: Pontine gray
- PULV: Pulvinar nucleus [thalamus]
- PreS: Cortical plate, presubiculum
- Put: Putamen
- RT: Reticular nucleus [thalamus]
- SGN: Suprageniculate nucleus
- SI: Substantia innominata
- SNr: Substantia nigra pars reticulata
- SON: Supraoptic nucleus [hypothalamus]
- SUB: Cortical plate, subiculum
- Sth: Subthalamus
- TRI: Germinal trigone
- VM: Ventral medial nucleus [thalamus]
- VNC: Vestibular nuclear complex
- VPL: Ventral posterolateral nucleus [thalamus]
- VPM: Ventral posteromedial nucleus [thalamus]
- ZI: Zona incerta
- ac: Anterior commissure
- cp: Cerebral peduncle
- cpb: Cerebellar peduncles
- dne: Diencephalic neuroepithelium
- emlth: External medullary lamina [thalamus]
- fx: Fornix
- hipg: Hippocampal glioepithelium/ependyma
- int: Internal capsule
- mml: Medial medullary lamina
- ot: Optic tract
- stt: Stria terminalis
- wmf: White matter fibers
- → CaS: Calcarine sulcus

Age: 17 GW

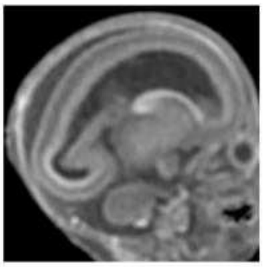

L-R Level: -6.12 mm

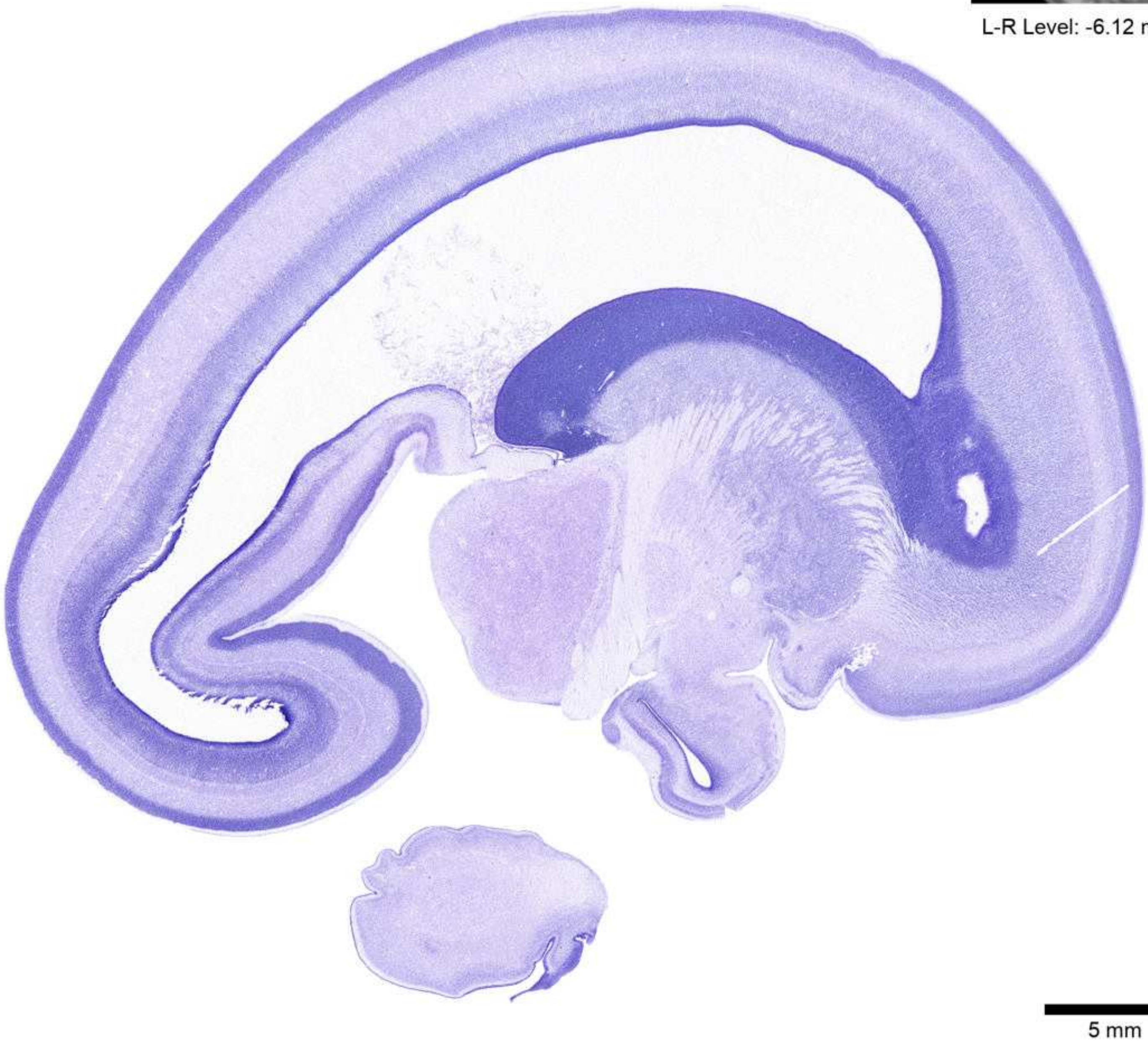

5 mm

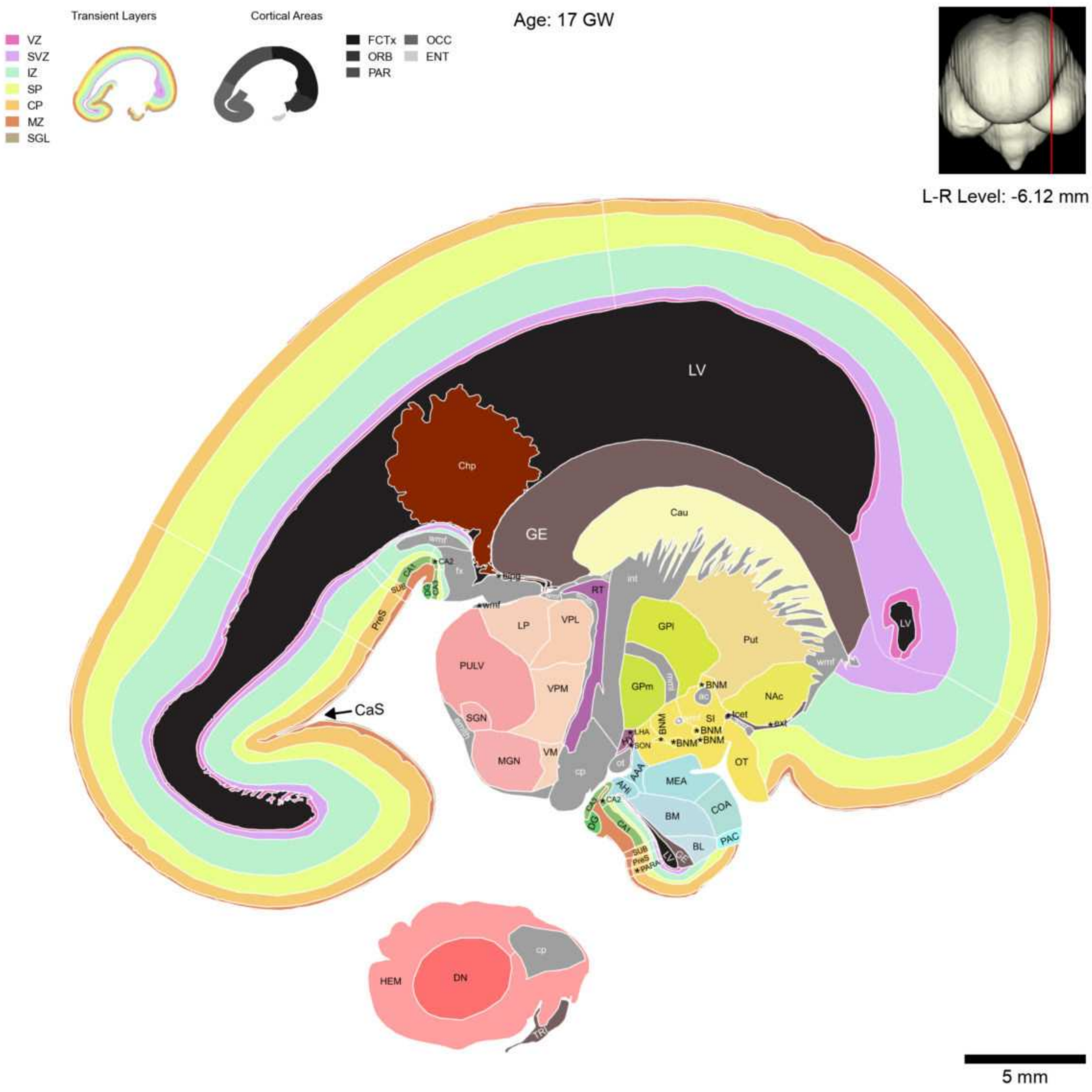

- AAA: Anterior amygdaloid area
- AHi: Amygdalo-hippocampal area
- BL: Basal nucleus [amygdala]
- BM: Basomedial nucleus [amygdala]
- BNM: Basal nucleus of Meynert
- CA1: CA1 field [hippocampus]
- CA2: CA2 field [hippocampus]
- CA3: CA3 field [hippocampus]
- COA: Cortical nucleus [amygdala]
- Cau: Caudate nucleus
- Chp: Choroid plexus
- DG: Dentate gyrus
- DN: Dentate nucleus
- GE: Ganglionic eminence
- GPl: Globus pallidus lateral segment
- GPm: Globus pallidus medial segment
- HEM: Cerebellar hemispheres
- HY: Hypothalamus
- LHA: Lateral hypothalamic area
- LP: Lateral posterior nucleus [thalamus]
- LV: Lateral ventricle
- MEA: Medial nucleus [amygdala]
- MGN: Medial geniculate nucleus
- NAc: Nucleus accumbens
- OT: Olfactory tubercle
- PARA: Cortical plate, parasubiculum
- PULV: Pulvinar nucleus [thalamus]
- PreS: Cortical plate, presubiculum
- Put: Putamen
- RT: Reticular nucleus [thalamus]
- SGN: Suprageniculate nucleus
- SI: Substantia innominata
- SON: Supraoptic nucleus [hypothalamus]
- SUB: Cortical plate, subiculum
- TRI: Germinal trigone
- VM: Ventral medial nucleus [thalamus]
- VPL: Ventral posterolateral nucleus [thalamus]
- VPM: Ventral posteromedial nucleus [thalamus]
- ac: Anterior commissure
- cp: Cerebral peduncle
- emlth: External medullary lamina [thalamus]
- ext: External capsule
- fx: Fornix
- hipg: Hippocampal glioepithelium/ependyma
- int: Internal capsule
- mml: Medial medullary lamina
- ot: Optic tract
- stt: Stria terminalis
- tcet: Transient cell zone in the external capsule
- wmf: White matter fibers
- → CaS: Calcarine sulcus

Age: 17 GW

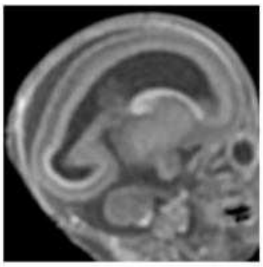

L-R Level: -6.48 mm

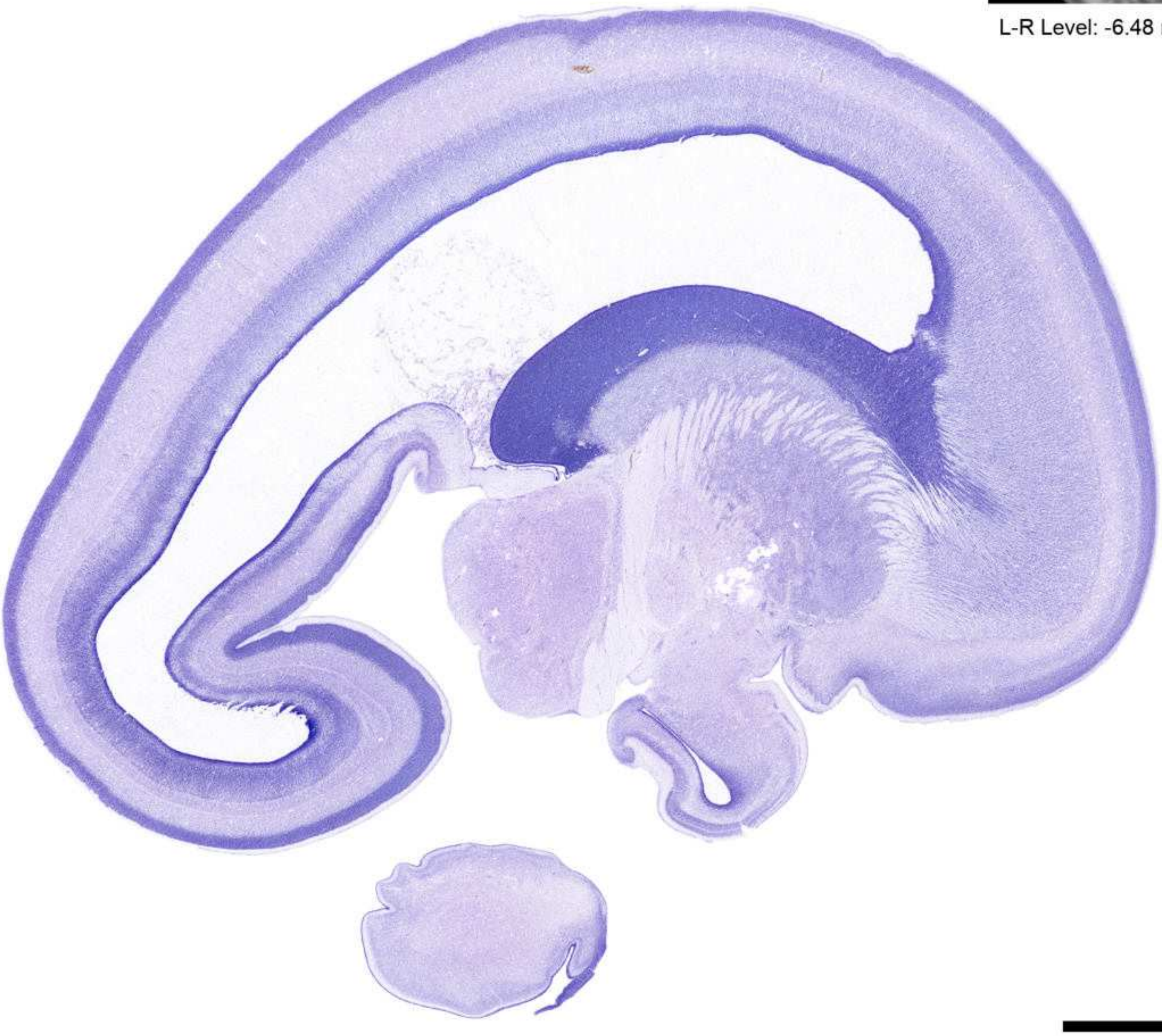

5 mm

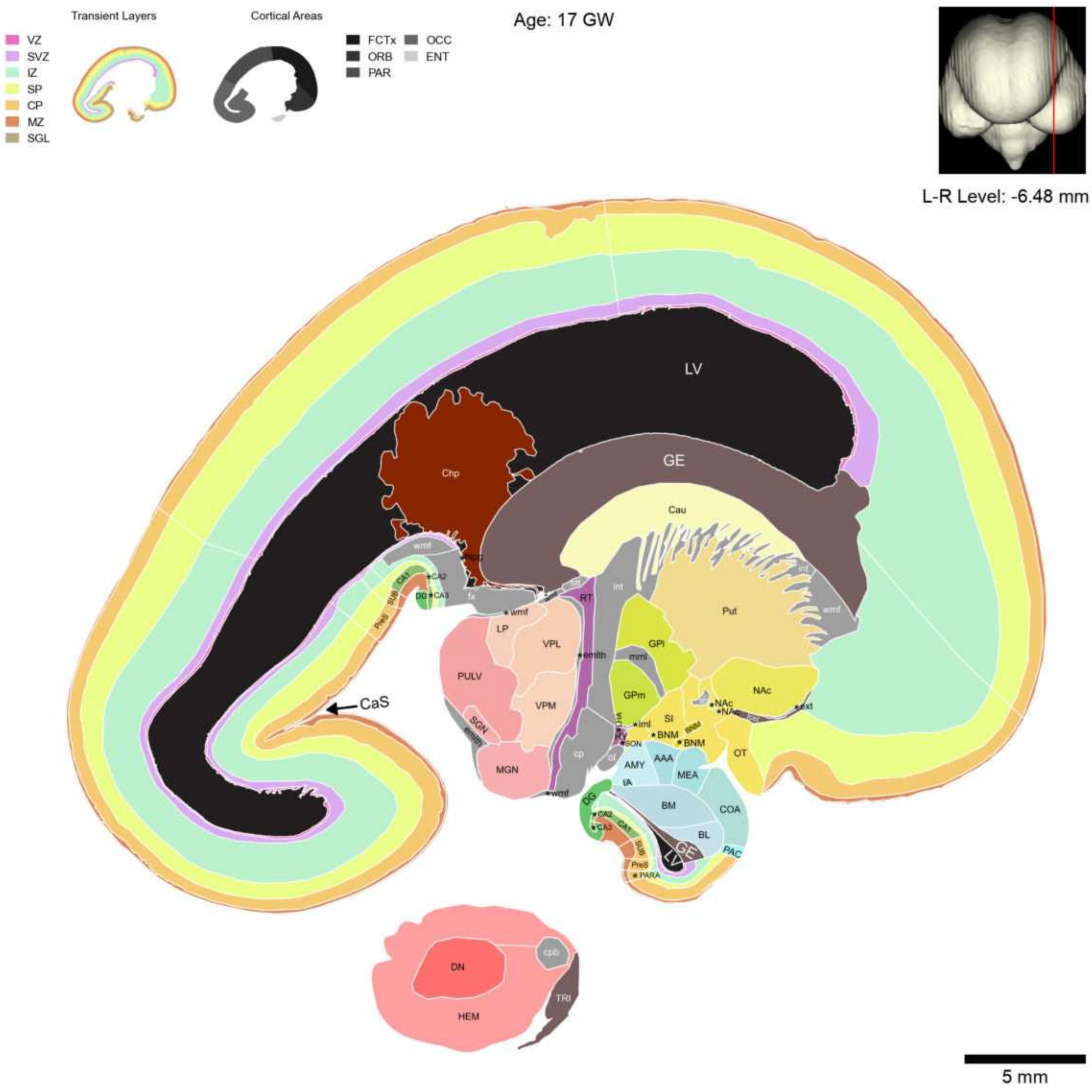

- AAA: Anterior amygdaloid area
- AMY: Amygdala
- BL: Basal nucleus [amygdala]
- BM: Basomedial nucleus [amygdala]
- BNM: Basal nucleus of Meynert
- CA1: CA1 field [hippocampus]
- CA2: CA2 field [hippocampus]
- CA3: CA3 field [hippocampus]
- COA: Cortical nucleus [amygdala]
- Cau: Caudate nucleus
- Chp: Choroid plexus
- DG: Dentate gyrus
- DN: Dentate nucleus
- GE: Ganglionic eminence
- GPl: Globus pallidus lateral segment
- GPm: Globus pallidus medial segment
- HEM: Cerebellar hemispheres
- HY: Hypothalamus
- IA: Intercalated cell groups [amygdala]
- LHA: Lateral hypothalamic area
- LP: Lateral posterior nucleus [thalamus]
- LV: Lateral ventricle
- MEA: Medial nucleus [amygdala]
- MGN: Medial geniculate nucleus
- NAc: Nucleus accumbens
- OT: Olfactory tubercle
- PARA: Cortical plate, parasubiculum
- PULV: Pulvinar nucleus [thalamus]
- PreS: Cortical plate, presubiculum
- Put: Putamen
- RT: Reticular nucleus [thalamus]
- SGN: Suprageniculate nucleus
- SI: Substantia innominata
- SON: Supraoptic nucleus [hypothalamus]
- SUB: Cortical plate, subiculum
- TRI: Germinal trigone
- VPL: Ventral posterolateral nucleus [thalamus]
- VPM: Ventral posteromedial nucleus [thalamus]
- ac: Anterior commissure
- cp: Cerebral peduncle
- cpb: Cerebellar peduncles
- emlth: External medullary lamina [thalamus]
- ext: External capsule
- fx: Fornix
- hipg: Hippocampal glioepithelium/ependyma
- int: Internal capsule
- lml: Lateral medullary lamina
- ot: Optic tract
- stt: Stria terminalis
- tcet: Transient cell zone in the external capsule
- wmf: White matter fibers
- → CaS: Calcarine sulcus

Age: 17 GW

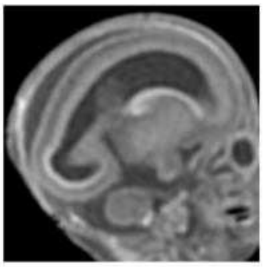

L-R Level: -6.78 mm

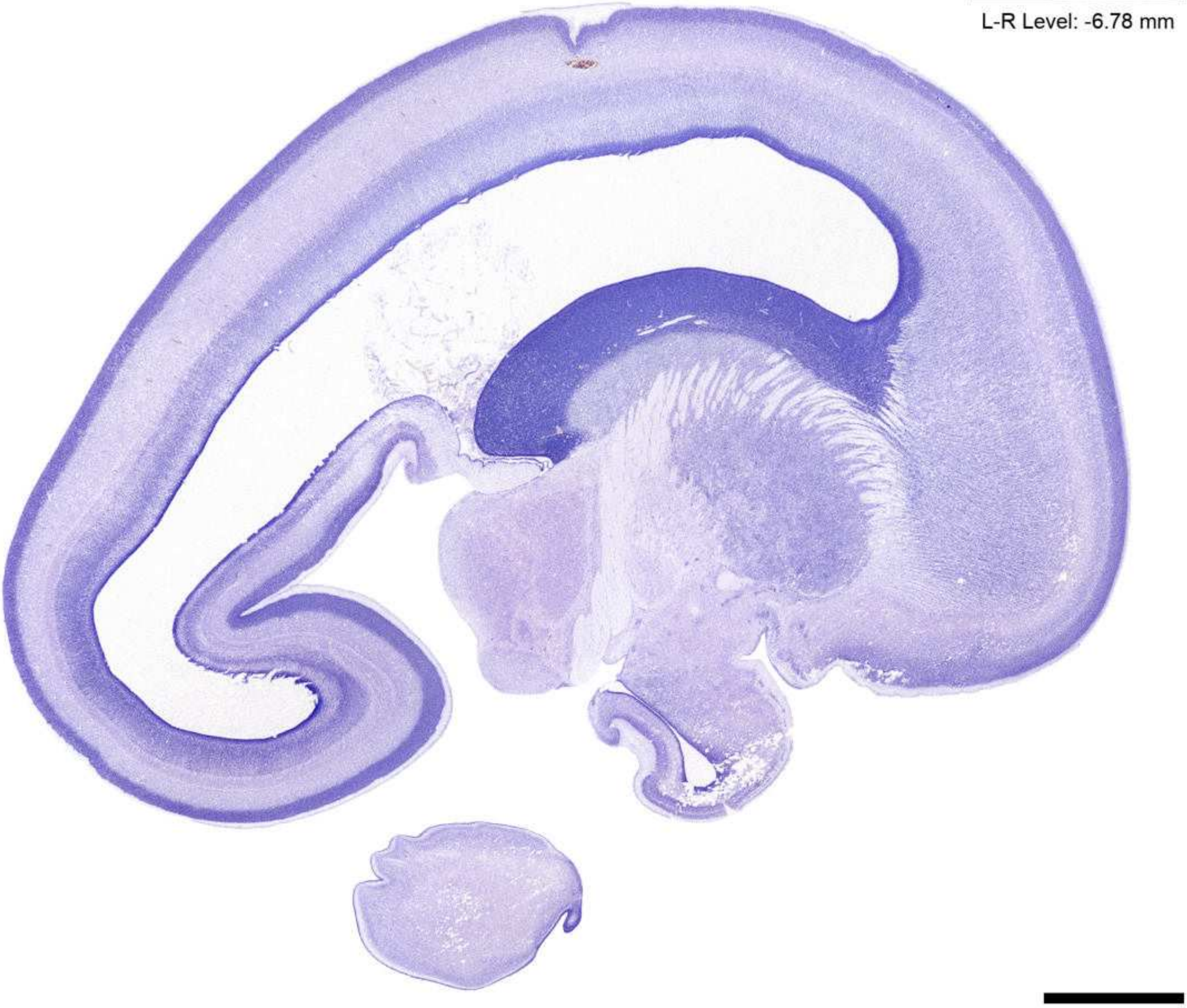

5 mm

Age: 17 GW

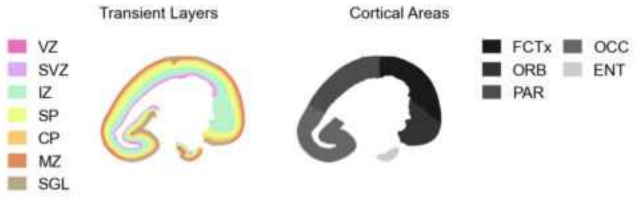

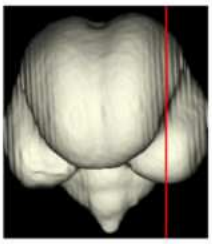

L-R Level: -6.78 mm

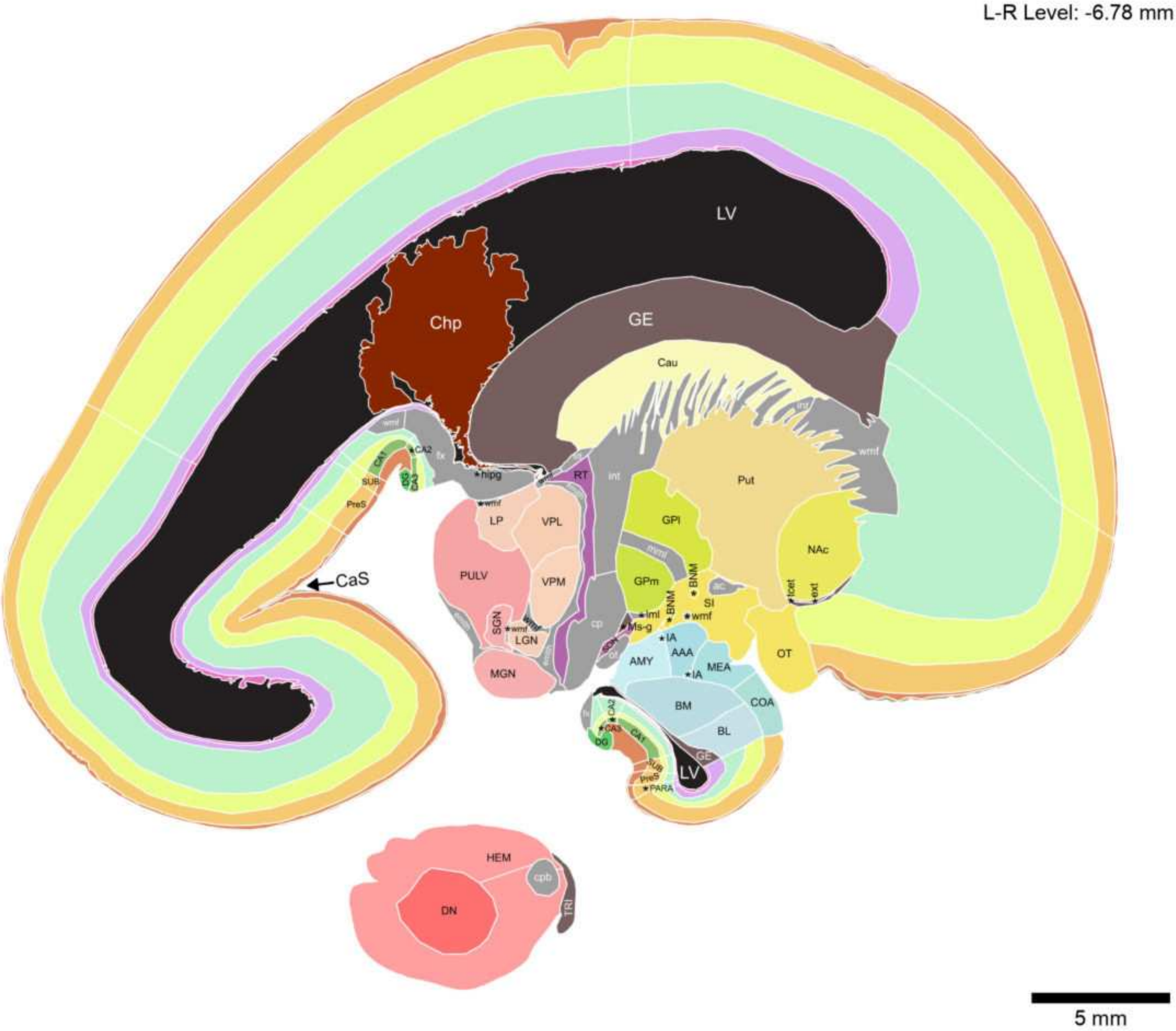

- AAA: Anterior amygdaloid area
- AMY: Amygdala
- BL: Basal nucleus [amygdala]
- BM: Basomedial nucleus [amygdala]
- BNM: Basal nucleus of Meynert
- CA1: CA1 field [hippocampus]
- CA2: CA2 field [hippocampus]
- CA3: CA3 field [hippocampus]
- COA: Cortical nucleus [amygdala]
- Cau: Caudate nucleus
- Chp: Choroid plexus
- DG: Dentate gyrus
- DN: Dentate nucleus
- GE: Ganglionic eminence
- GPl: Globus pallidus lateral segment
- GPm: Globus pallidus medial segment
- HEM: Cerebellar hemispheres
- IA: Intercalated cell groups [amygdala]
- LGN: Lateral geniculate nucleus
- LP: Lateral posterior nucleus [thalamus]
- LV: Lateral ventricle
- MEA: Medial nucleus [amygdala]
- MGN: Medial geniculate nucleus
- Ms-g: Migratory stream, general
- NAc: Nucleus accumbens
- OT: Olfactory tubercle
- PARA: Cortical plate, parasubiculum
- PULV: Pulvinar nucleus [thalamus]
- PreS: Cortical plate, presubiculum
- Put: Putamen
- RT: Reticular nucleus [thalamus]
- SGN: Suprageniculate nucleus
- SI: Substantia innominata
- SON: Supraoptic nucleus [hypothalamus]
- SUB: Cortical plate, subiculum
- TRI: Germinal trigone
- VPL: Ventral posterolateral nucleus [thalamus]
- VPM: Ventral posteromedial nucleus [thalamus]
- ac: Anterior commissure
- cp: Cerebral peduncle
- cpb: Cerebellar peduncles
- emlth: External medullary lamina [thalamus]
- ext: External capsule
- fx: Fornix
- hipg: Hippocampal glioepithelium/ependyma
- int: Internal capsule
- lml: Lateral medullary lamina
- mml: Medial medullary lamina
- ot: Optic tract
- stt: Stria terminalis
- tcet: Transient cell zone in the external capsule
- wmf: White matter fibers
- → CaS: Calcarine sulcus

Age: 17 GW

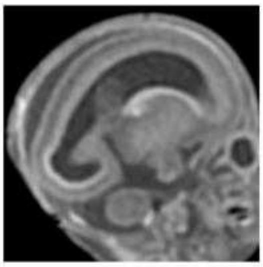

L-R Level: -7.02 mm

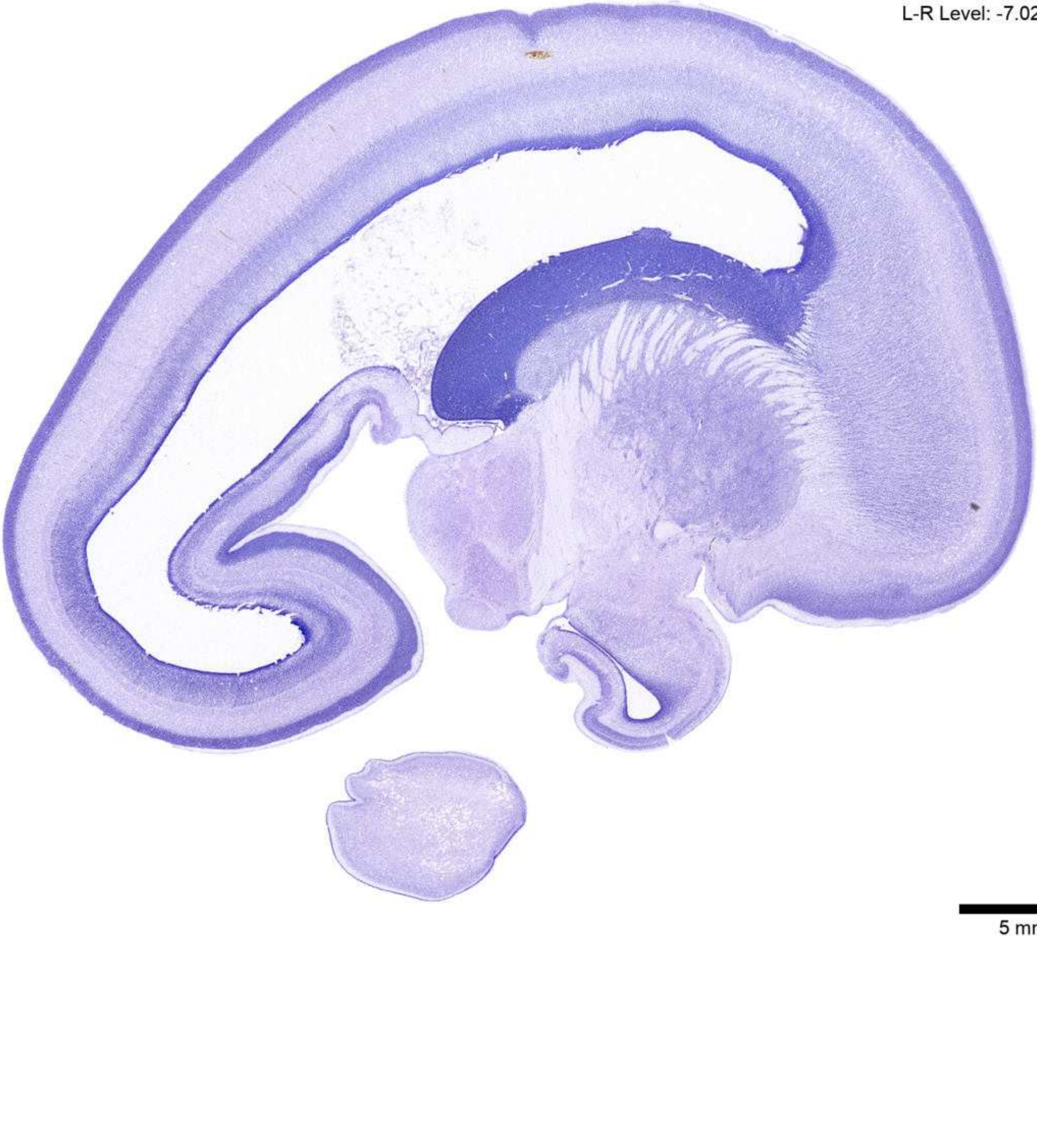

5 mm

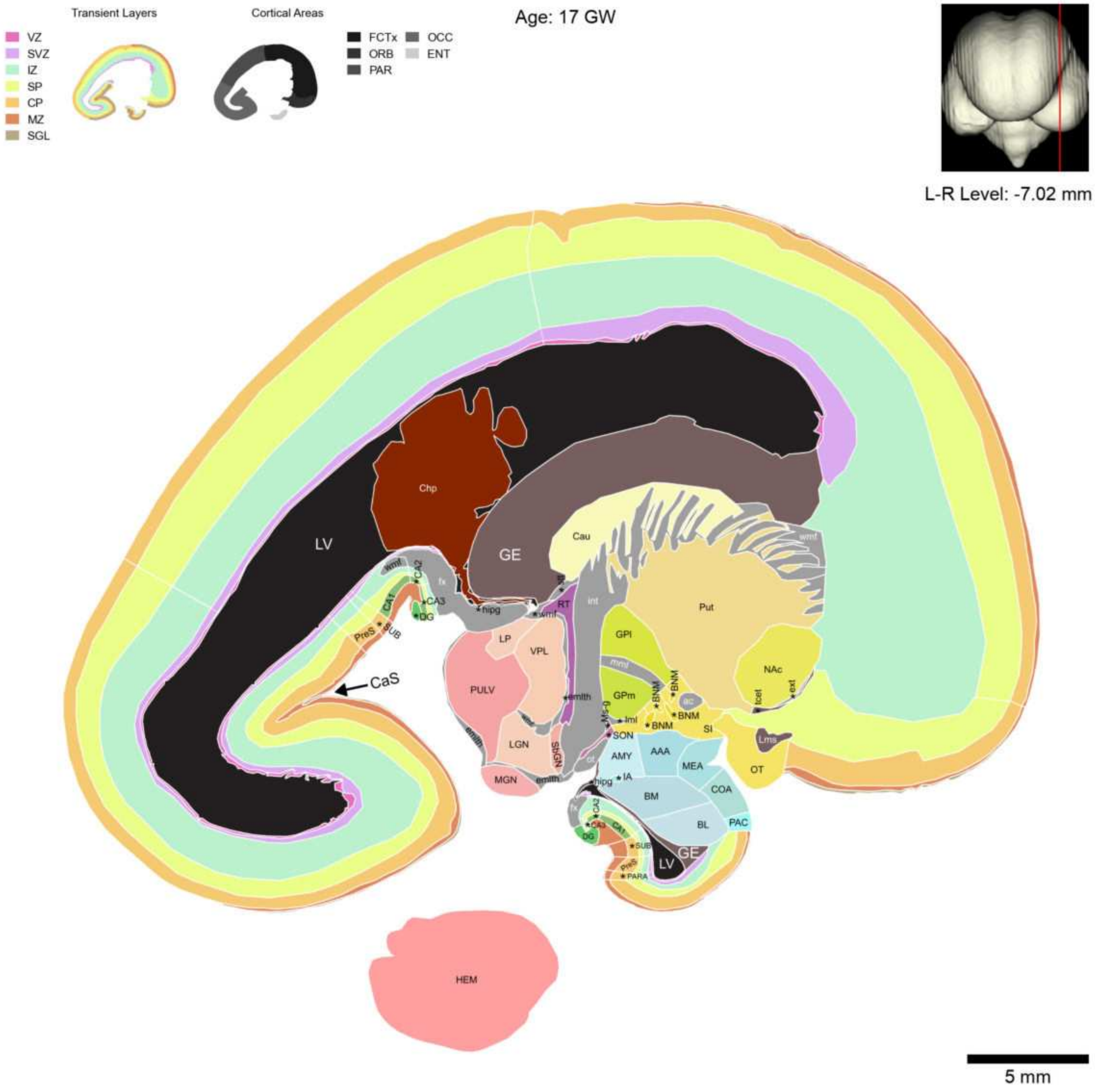

- AAA: Anterior amygdaloid area
- AMY: Amygdala
- BL: Basal nucleus [amygdala]
- BM: Basomedial nucleus [amygdala]
- BNM: Basal nucleus of Meynert
- CA1: CA1 field [hippocampus]
- CA2: CA2 field [hippocampus]
- CA3: CA3 field [hippocampus]
- COA: Cortical nucleus [amygdala]
- Cau: Caudate nucleus
- Chp: Choroid plexus
- DG: Dentate gyrus
- GE: Ganglionic eminence
- GPl: Globus pallidus lateral segment
- GPm: Globus pallidus medial segment
- HEM: Cerebellar hemispheres
- IA: Intercalated cell groups [amygdala]
- LGN: Lateral geniculate nucleus
- LP: Lateral posterior nucleus [thalamus]
- LV: Lateral ventricle
- Lms: Lateral migratory stream
- MEA: Medial nucleus [amygdala]
- MGN: Medial geniculate nucleus
- Ms-g: Migratory stream, general
- NAc: Nucleus accumbens
- OT: Olfactory tubercle
- PARA: Cortical plate, parasubiculum
- PULV: Pulvinar nucleus [thalamus]
- PreS: Cortical plate, presubiculum
- Put: Putamen
- RT: Reticular nucleus [thalamus]
- SI: Substantia innominata
- SON: Supraoptic nucleus [hypothalamus]
- SUB: Cortical plate, subiculum
- SbGN: Subgeniculate nucleus
- VPL: Ventral posterolateral nucleus [thalamus]
- ac: Anterior commissure
- emlth: External medullary lamina [thalamus]
- ext: External capsule
- fx: Fornix
- hipg: Hippocampal glioepithelium/ependyma
- int: Internal capsule
- lml: Lateral medullary lamina
- mml: Medial medullary lamina
- ot: Optic tract
- stt: Stria terminalis
- tcet: Transient cell zone in the external capsule
- wmf: White matter fibers
- → CaS: Calcarine sulcus

Age: 17 GW

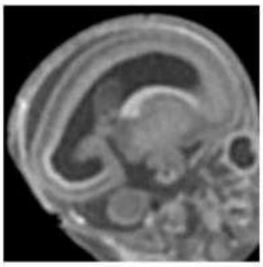

L-R Level: -7.5 mm

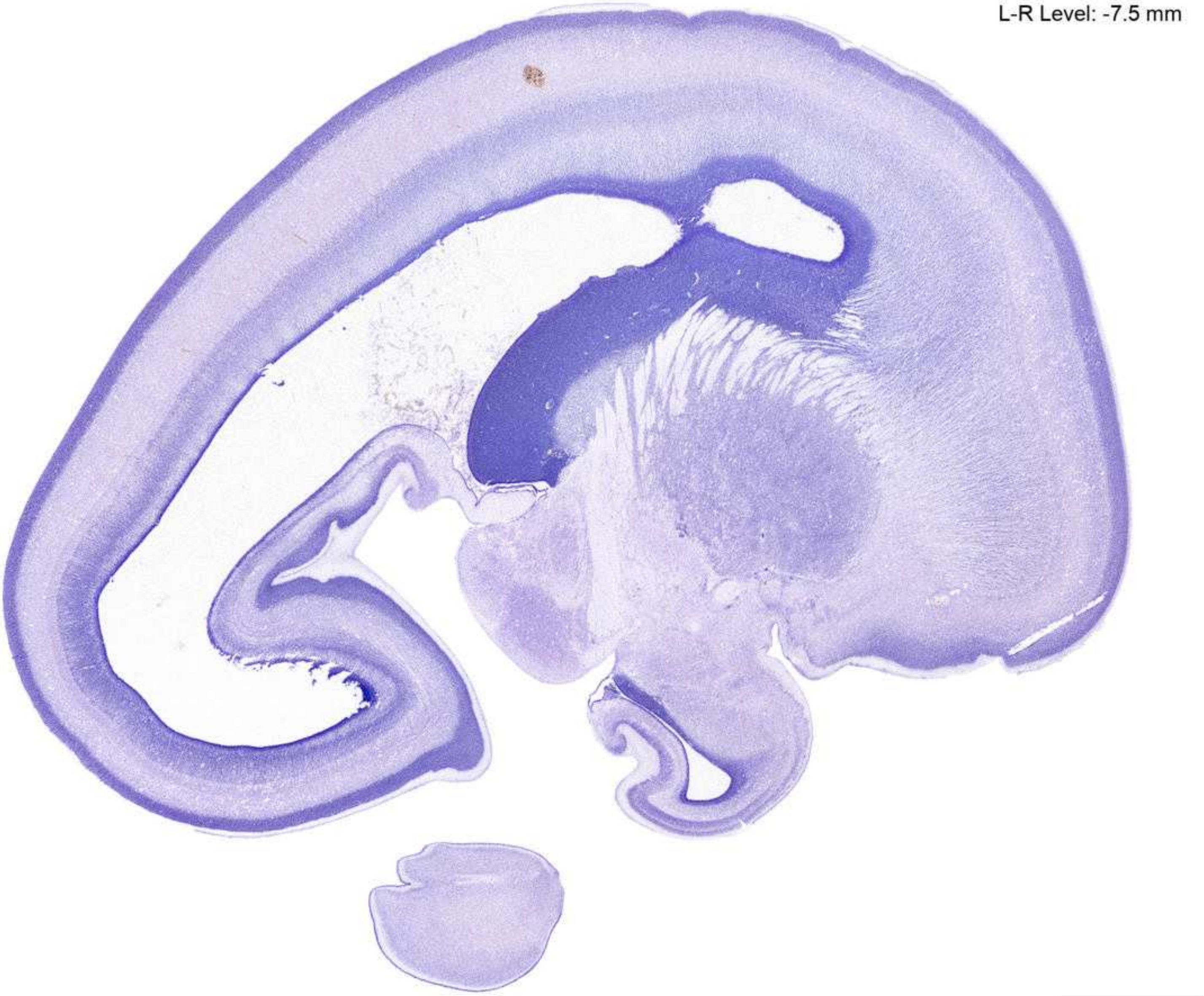

5 mm

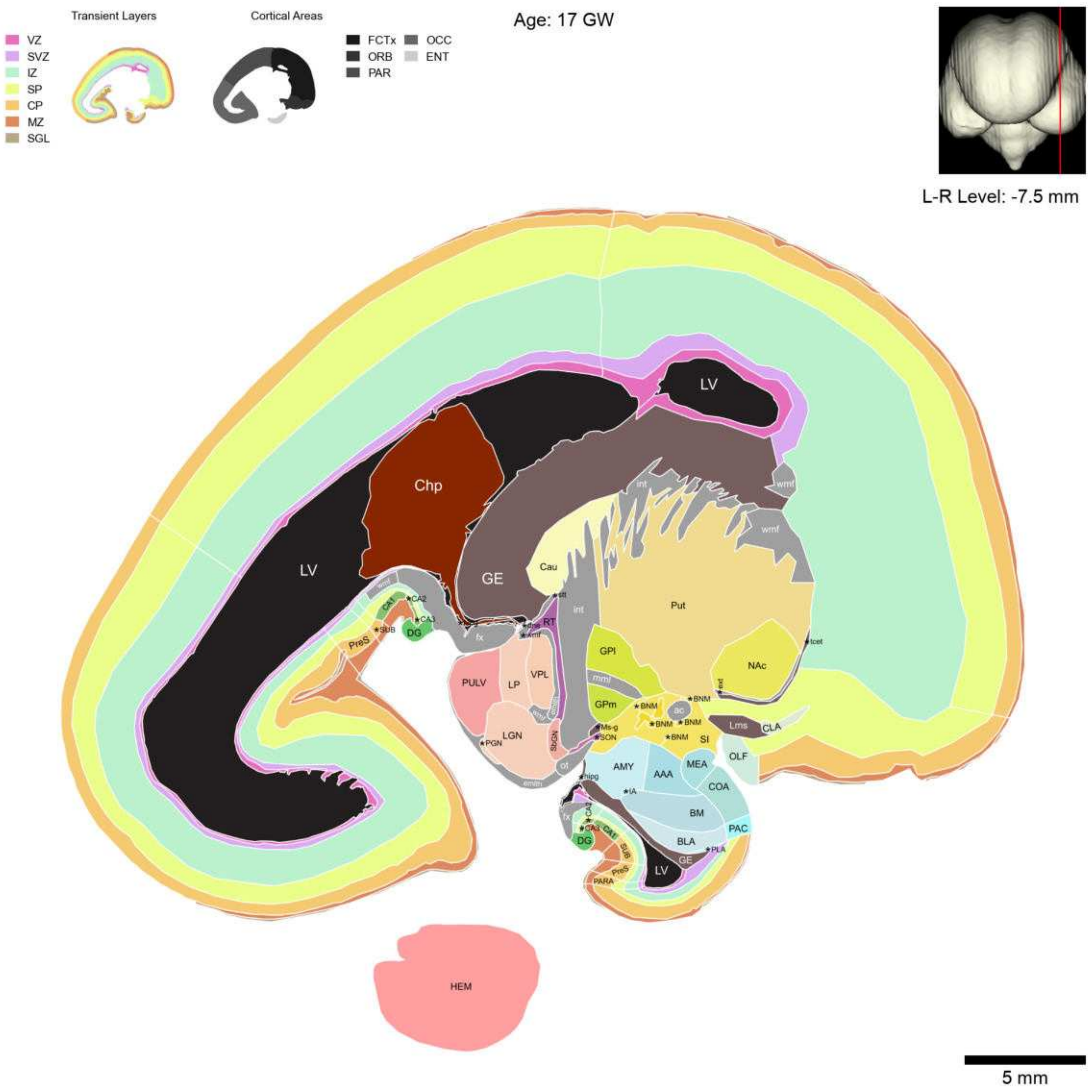

AAA: Anterior amygdaloid area
AMY: Amygdala
BLA: Basolateral complex [amygdala]
BM: Basomedial nucleus [amygdala]
BNM: Basal nucleus of Meynert
CA1: CA1 field [hippocampus]
CA2: CA2 field [hippocampus]
CA3: CA3 field [hippocampus]
CLA: Claustrum
COA: Cortical nucleus [amygdala]
Cau: Caudate nucleus
Chp: Choroid plexus
DG: Dentate gyrus
GE: Ganglionic eminence
GPl: Globus pallidus lateral segment
GPm: Globus pallidus medial segment
HEM: Cerebellar hemispheres
IA: Intercalated cell groups [amygdala]
LGN: Lateral geniculate nucleus
LP: Lateral posterior nucleus [thalamus]
LV: Lateral ventricle
Lms: Lateral migratory stream
MEA: Medial nucleus [amygdala]
Ms-g: Migratory stream, general
NAc: Nucleus accumbens
PARA: Cortical plate, parasubiculum
PGN: Pregeniculate nucleus
PLA: Paralaminar nucleus [amygdala]
PULV: Pulvinar nucleus [thalamus]
PreS: Cortical plate, presubiculum
Put: Putamen
RT: Reticular nucleus [thalamus]
SI: Substantia innominata
SON: Supraoptic nucleus [hypothalamus]
SUB: Cortical plate, subiculum
SbGN: Subgeniculate nucleus
VPL: Ventral posterolateral nucleus [thalamus]
ac: Anterior commissure
dne: Diencephalic neuroepithelium
emlth: External medullary lamina [thalamus]
ext: External capsule
fx: Fornix
hipg: Hippocampal glioepithelium/ependyma
int: Internal capsule
mml: Medial medullary lamina
ot: Optic tract
stt: Stria terminalis
tcet: Transient cell zone in the external capsule
wmf: White matter fibers

Age: 17 GW

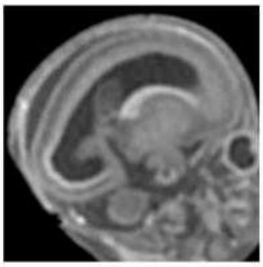

L-R Level: -7.68 mm

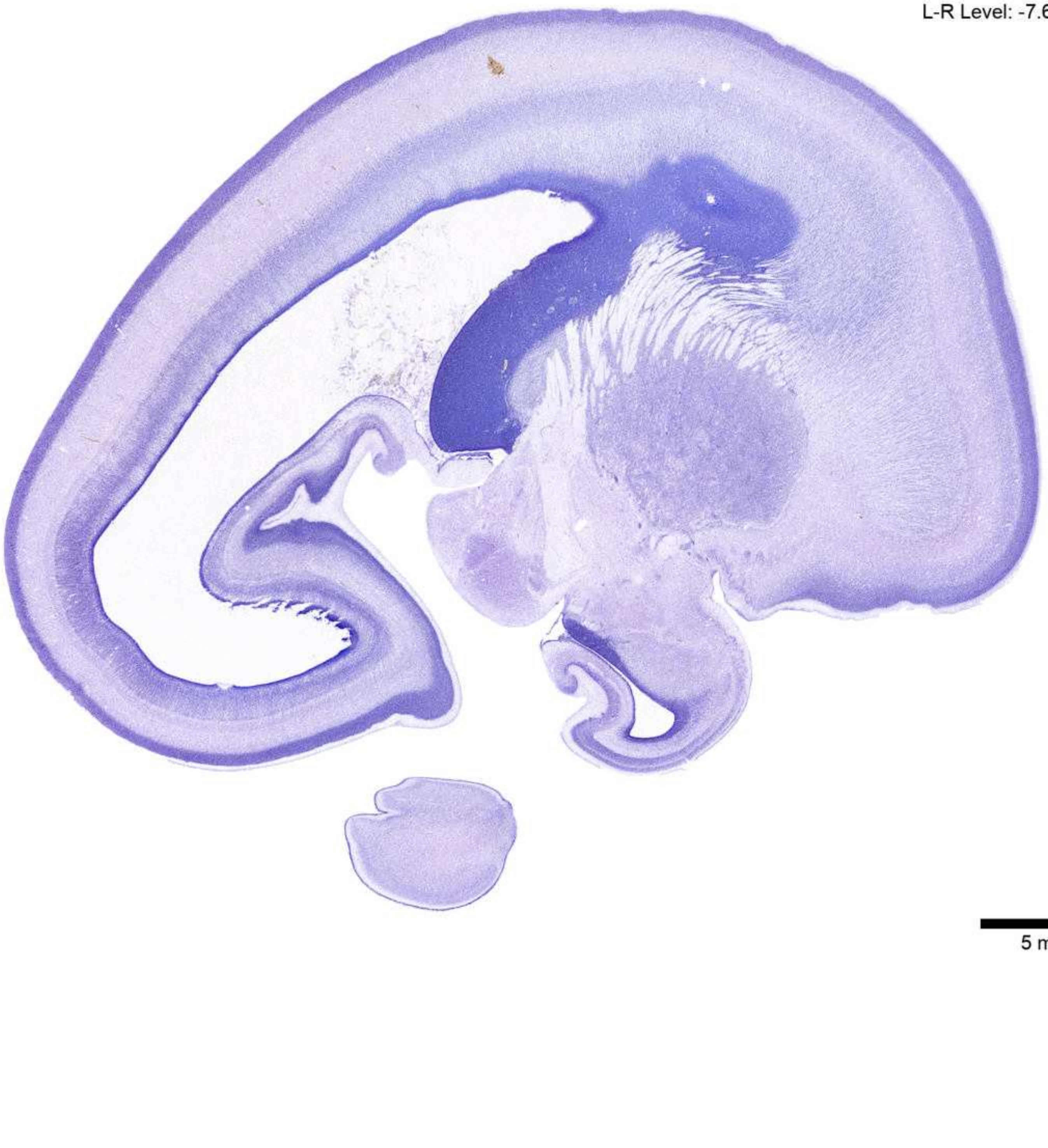

5 mm

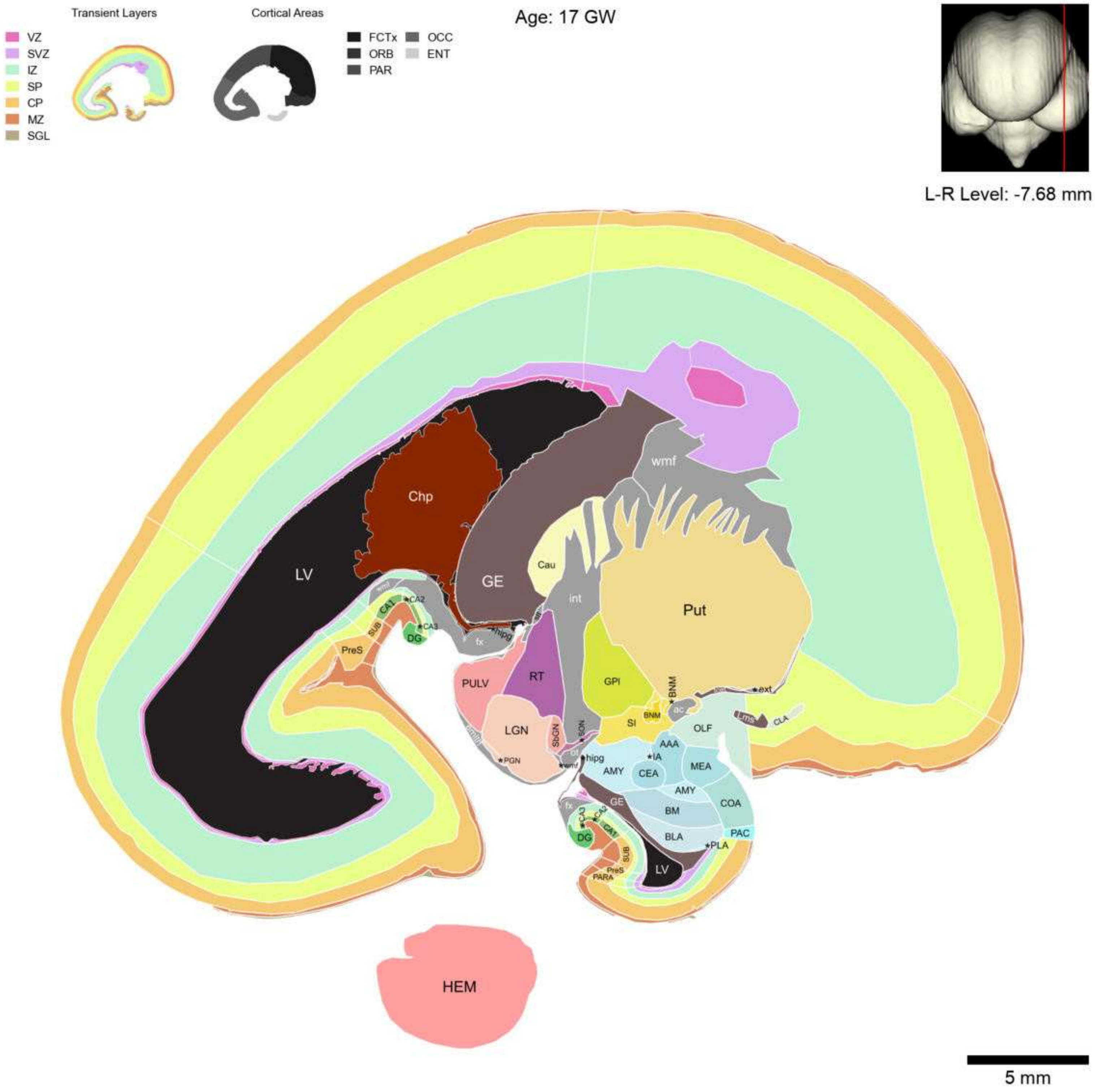

AAA: Anterior amygdaloid area
AMY: Amygdala
BLA: Basolateral complex [amygdala]
BM: Basomedial nucleus [amygdala]
BNM: Basal nucleus of Meynert
CA1: CA1 field [hippocampus]
CA2: CA2 field [hippocampus]
CA3: CA3 field [hippocampus]
CEA: Central nucleus [amygdala]
CLA: Claustrum
COA: Cortical nucleus [amygdala]
Cau: Caudate nucleus
Chp: Choroid plexus
DG: Dentate gyrus
GE: Ganglionic eminence
GPl: Globus pallidus lateral segment
HEM: Cerebellar hemispheres
IA: Intercalated cell groups [amygdala]
LGN: Lateral geniculate nucleus
LV: Lateral ventricle
Lms: Lateral migratory stream
MEA: Medial nucleus [amygdala]
PARA: Cortical plate, parasubiculum
PGN: Pregeniculate nucleus
PLA: Paralaminar nucleus [amygdala]
PULV: Pulvinar nucleus [thalamus]
PreS: Cortical plate, presubiculum
Put: Putamen
RT: Reticular nucleus [thalamus]
SI: Substantia innominata
SON: Supraoptic nucleus [hypothalamus]
SUB: Cortical plate, subiculum
SbGN: Subgeniculate nucleus
ac: Anterior commissure
emlth: External medullary lamina [thalamus]
ext: External capsule
fx: Fornix
hipg: Hippocampal glioepithelium/ependyma
int: Internal capsule
ot: Optic tract
stt: Stria terminalis
tcet: Transient cell zone in the external capsule
wmf: White matter fibers

Age: 17 GW

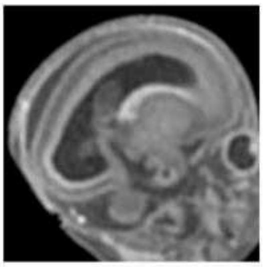

L-R Level: -8.04 mm

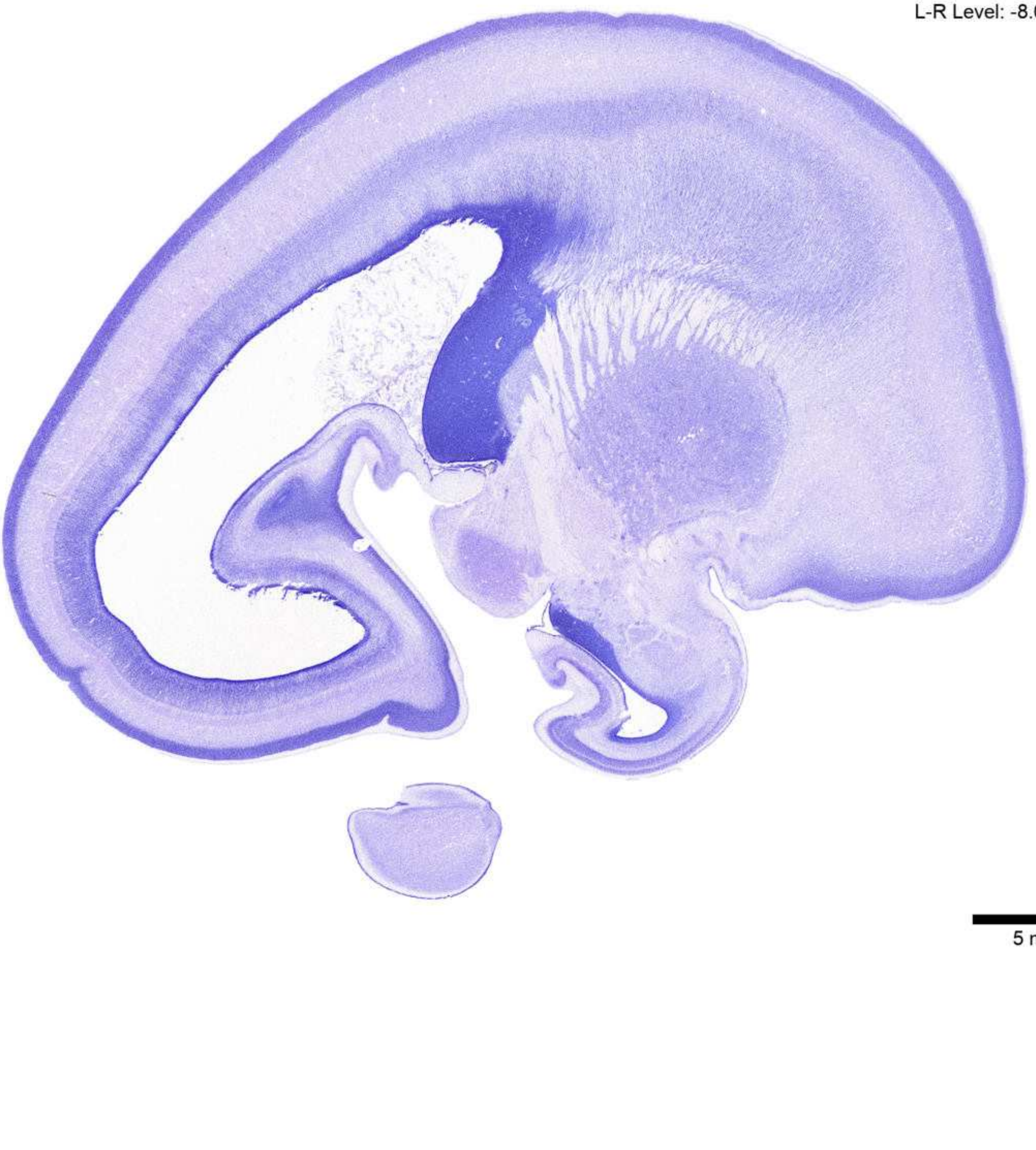

5 mm

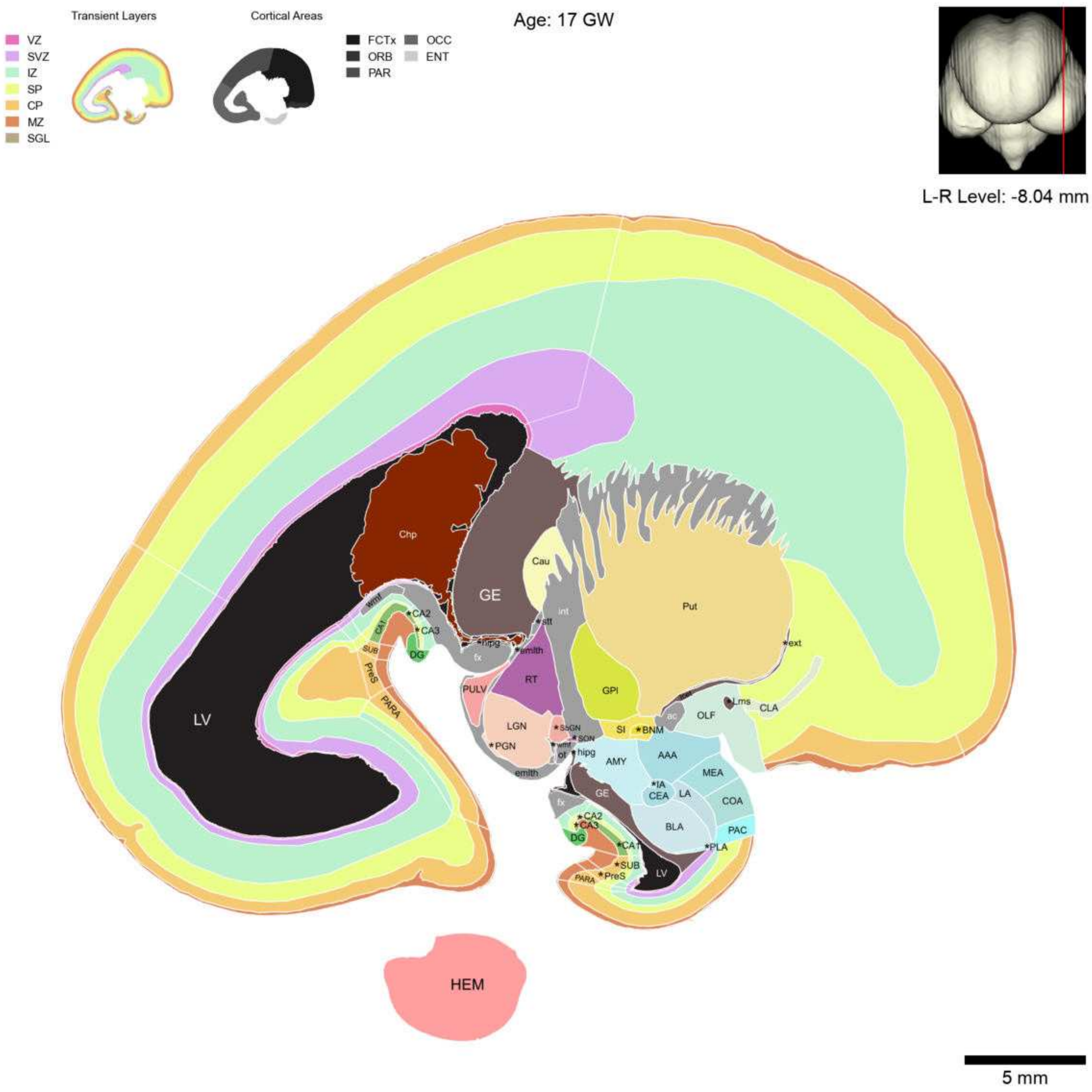

AAA: Anterior amygdaloid area
AMY: Amygdala
BL: Basal nucleus [amygdala]
BNM: Basal nucleus of Meynert
CA1: CA1 field [hippocampus]
CA2: CA2 field [hippocampus]
CA3: CA3 field [hippocampus]
CEA: Central nucleus [amygdala]
CLA: Claustrum
COA: Cortical nucleus [amygdala]
Cau: Caudate nucleus
Chp: Choroid plexus
DG: Dentate gyrus
GE: Ganglionic eminence
GPl: Globus pallidus lateral segment
HEM: Cerebellar hemispheres
IA: Intercalated cell groups [amygdala]
LA: Lateral nucleus [amygdala]
LGN: Lateral geniculate nucleus
LV: Lateral ventricle
Lms: Lateral migratory stream
MEA: Medial nucleus [amygdala]
PARA: Cortical plate, parasubiculum
PGN: Pregeniculate nucleus
PLA: Paralaminar nucleus [amygdala]
PULV: Pulvinar nucleus [thalamus]
PreS: Cortical plate, presubiculum
Put: Putamen
RT: Reticular nucleus [thalamus]
SI: Substantia innominata
SON: Supraoptic nucleus [hypothalamus]
SUB: Cortical plate, subiculum
SbGN: Subgeniculate nucleus
ac: Anterior commissure
emlth: External medullary lamina [thalamus]
ext: External capsule
fx: Fornix
hipg: Hippocampal glioepithelium/ependyma
int: Internal capsule
ot: Optic tract
stt: Stria terminalis
tcet: Transient cell zone in the external capsule
wmf: White matter fibers

Age: 17 GW

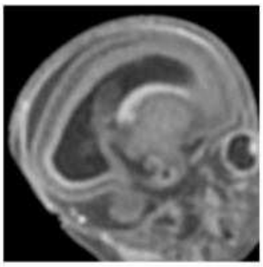

L-R Level: -8.28 mm

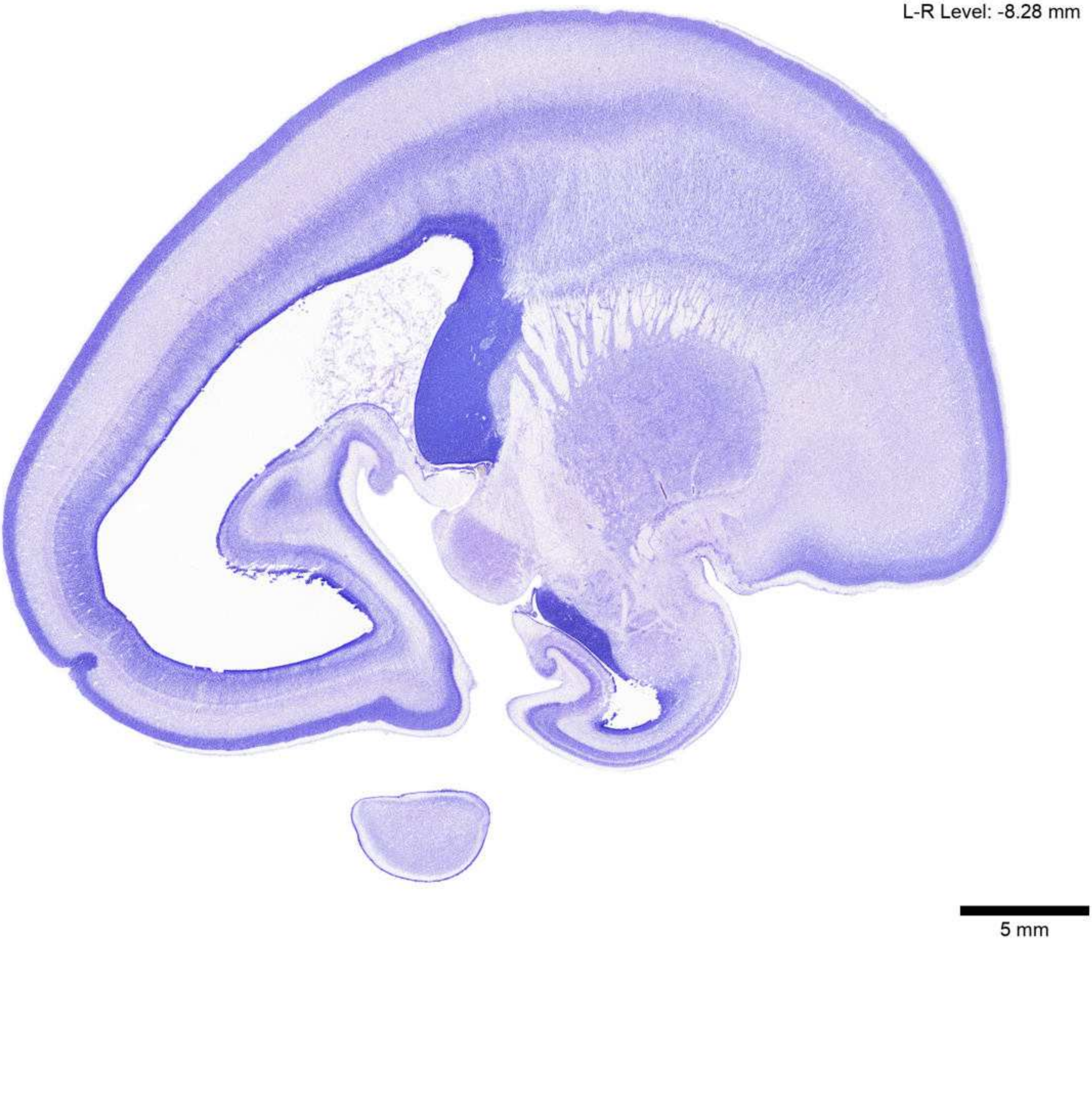

5 mm

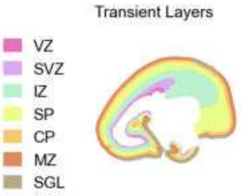

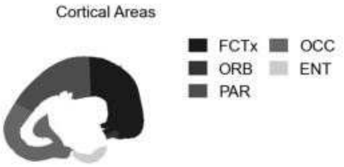

Age: 17 GW

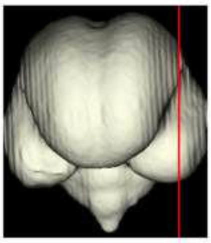

L-R Level: -8.28 mm

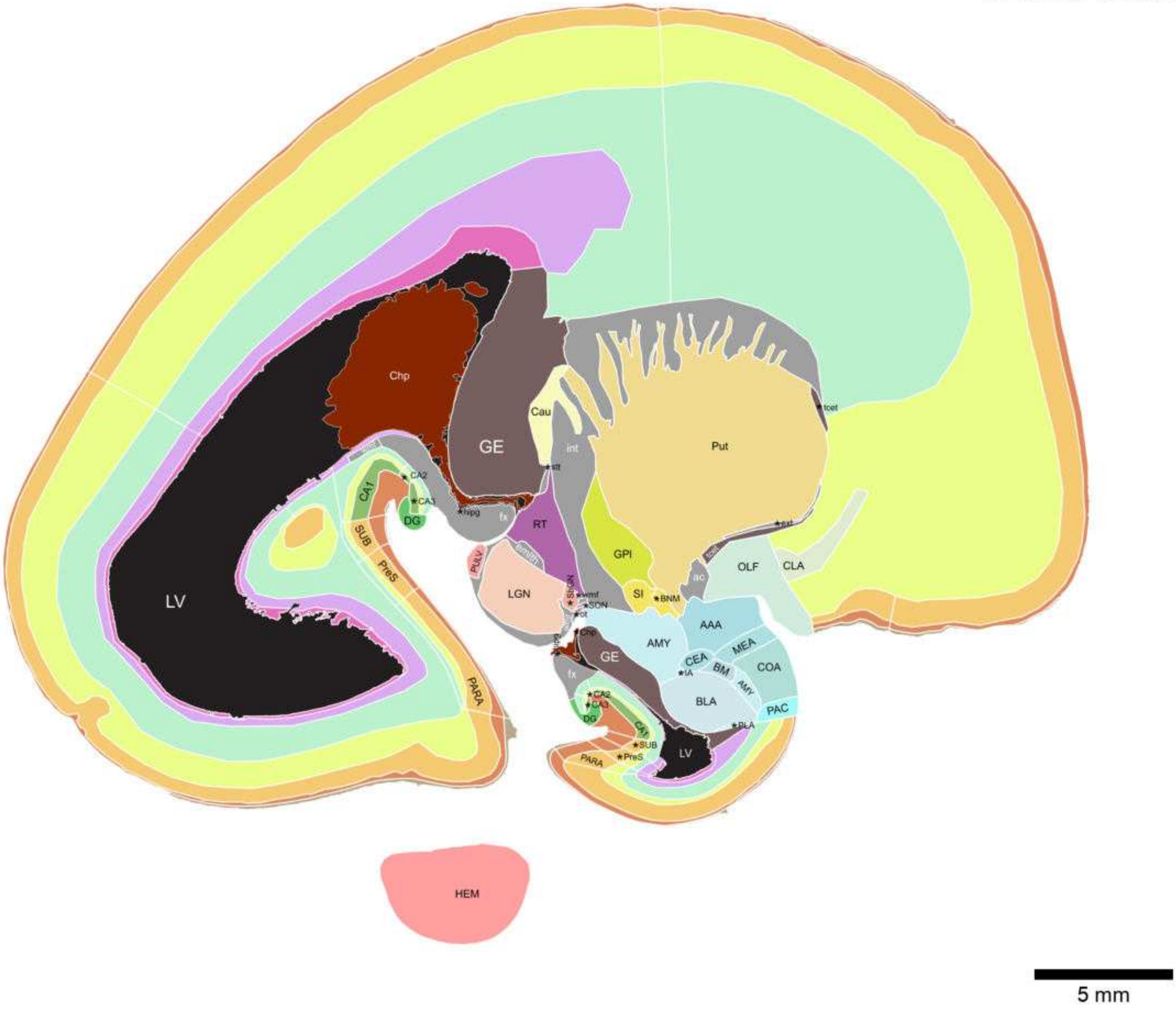

- AAA: Anterior amygdaloid area
- AMY: Amygdala
- BL: Basal nucleus [amygdala]
- BM: Basomedial nucleus [amygdala]
- BNM: Basal nucleus of Meynert
- CA1: CA1 field [hippocampus]
- CA2: CA2 field [hippocampus]
- CA3: CA3 field [hippocampus]
- CEA: Central nucleus [amygdala]
- CLA: Claustrum
- COA: Cortical nucleus [amygdala]
- Cau: Caudate nucleus
- Chp: Choroid plexus
- DG: Dentate gyrus
- GE: Ganglionic eminence
- GPl: Globus pallidus lateral segment
- HEM: Cerebellar hemispheres
- IA: Intercalated cell groups [amygdala]
- LGN: Lateral geniculate nucleus
- LV: Lateral ventricle
- MEA: Medial nucleus [amygdala]
- PARA: Cortical plate, parasubiculum
- PLA: Paralaminar nucleus [amygdala]
- PULV: Pulvinar nucleus [thalamus]
- PreS: Cortical plate, presubiculum
- Put: Putamen
- RT: Reticular nucleus [thalamus]
- SI: Substantia innominata
- SON: Supraoptic nucleus [hypothalamus]
- SUB: Cortical plate, subiculum
- SbGN: Subgeniculate nucleus
- ac: Anterior commissure
- emlth: External medullary lamina [thalamus]
- ext: External capsule
- fx: Fornix
- hipg: Hippocampal glioepithelium/ependyma
- int: Internal capsule
- ot: Optic tract
- stt: Stria terminalis
- tcet: Transient cell zone in the external capsule
- wmf: White matter fibers

Age: 17 GW

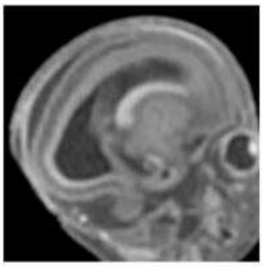

L-R Level: -8.76 mm

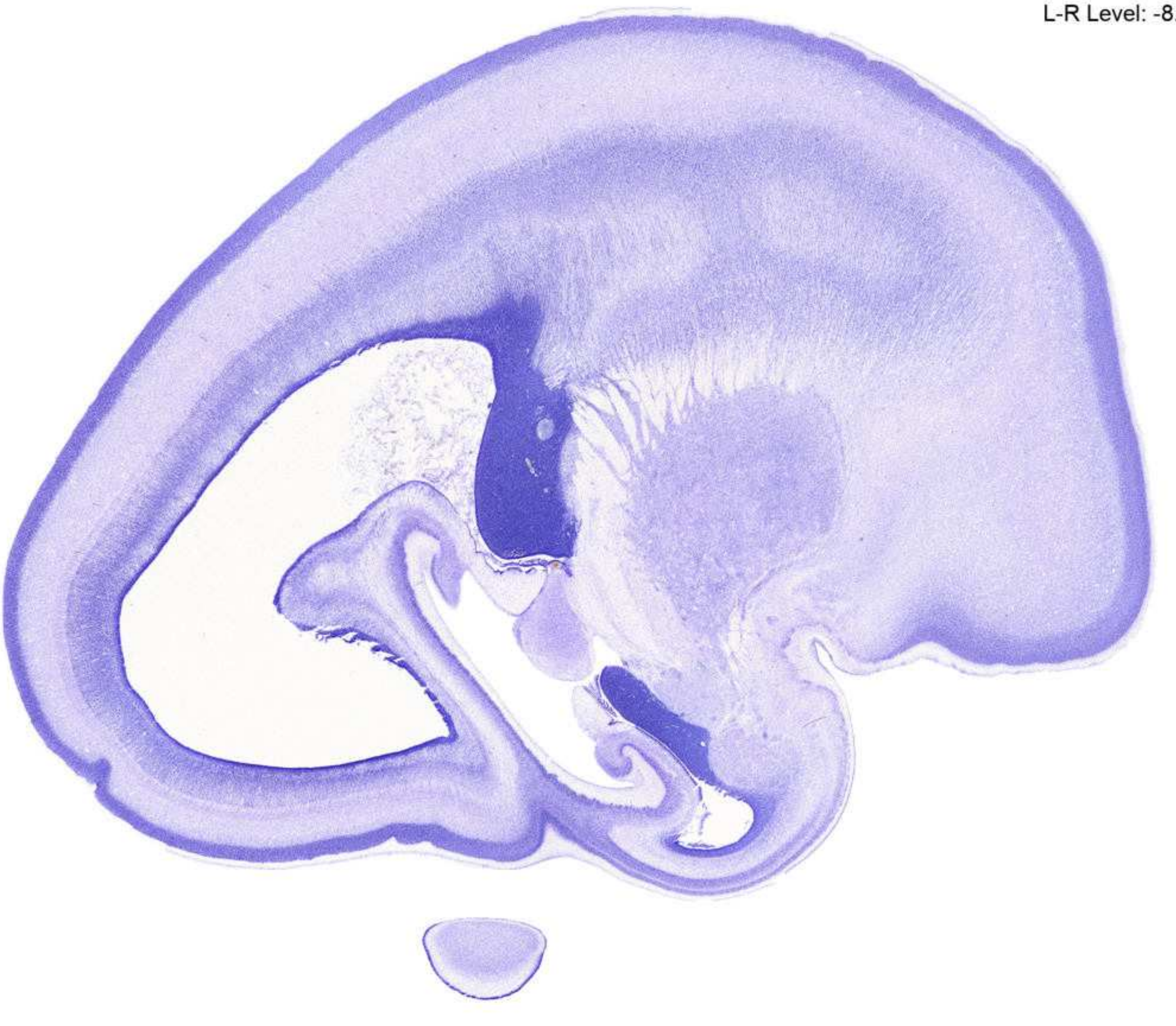

5 mm

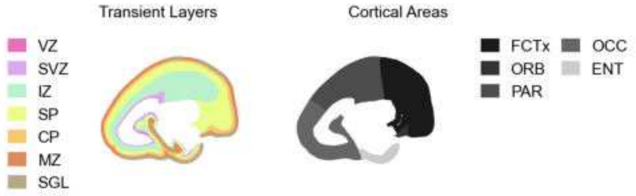

Age: 17 GW

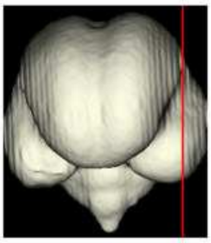

L-R Level: -8.76 mm

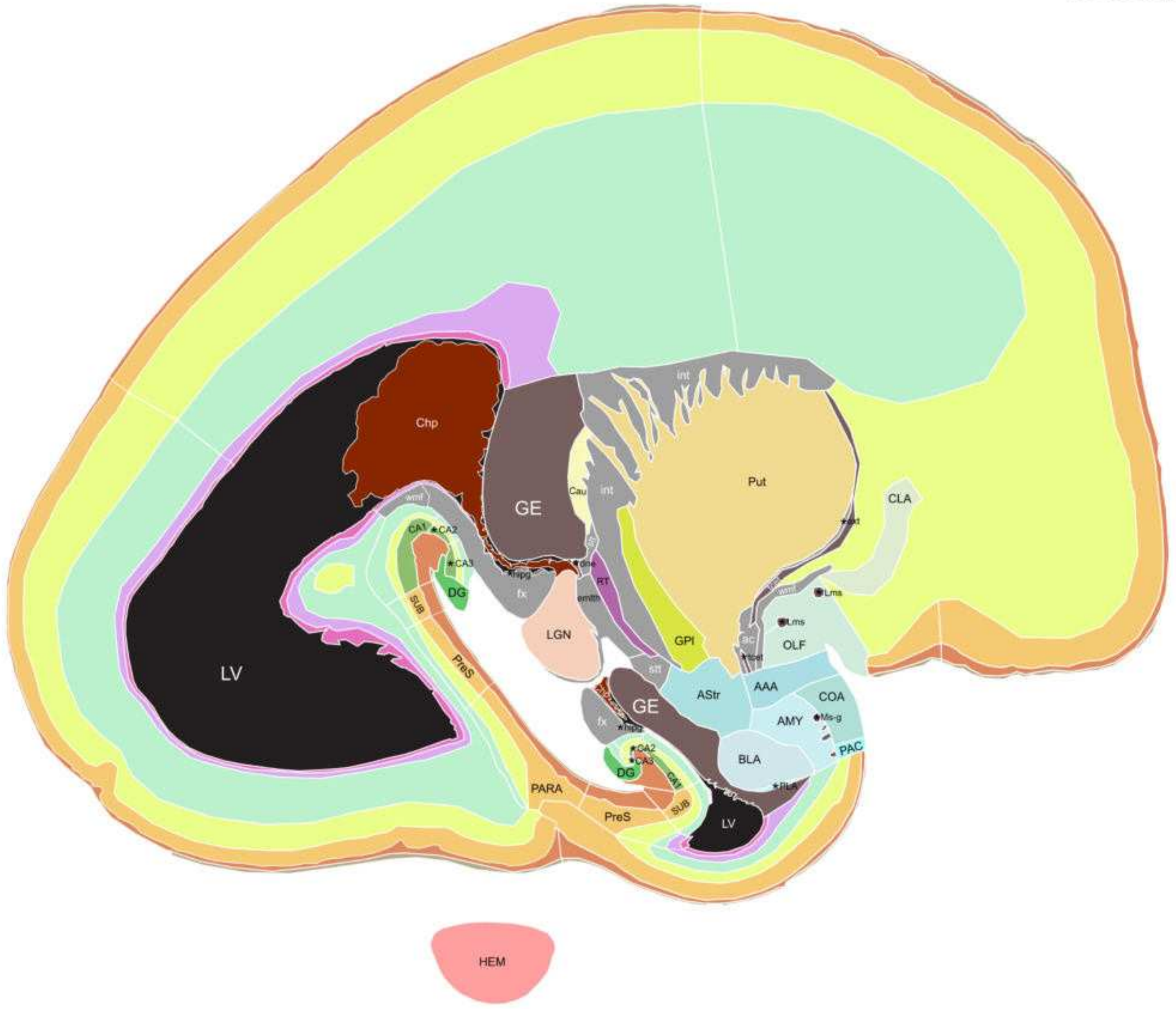

5 mm

- AAA: Anterior amygdaloid area
- AMY: Amygdala
- AStr: Amygdalo-striatal area
- BL: Basal nucleus [amygdala]
- CA1: CA1 field [hippocampus]
- CA2: CA2 field [hippocampus]
- CA3: CA3 field [hippocampus]
- CLA: Claustrum
- COA: Cortical nucleus [amygdala]
- Cau: Caudate nucleus
- Chp: Choroid plexus
- DG: Dentate gyrus
- GE: Ganglionic eminence
- GPl: Globus pallidus lateral segment
- HEM: Cerebellar hemispheres
- LGN: Lateral geniculate nucleus
- LV: Lateral ventricle
- Lms: Lateral migratory stream
- Ms-g: Migratory stream, general
- PARA: Cortical plate, parasubiculum
- PLA: Paralaminar nucleus [amygdala]
- PreS: Cortical plate, presubiculum
- Put: Putamen
- RT: Reticular nucleus [thalamus]
- SUB: Cortical plate, subiculum
- ac: Anterior commissure
- dne: Diencephalic neuroepithelium
- emlth: External medullary lamina [thalamus]
- ext: External capsule
- fx: Fornix
- hipg: Hippocampal glioepithelium/ependyma
- int: Internal capsule
- stt: Stria terminalis
- tcet: Transient cell zone in the external capsule
- wmf: White matter fibers

Age: 17 GW

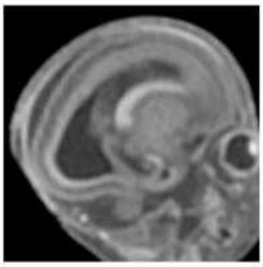

L-R Level: -8.94 mm

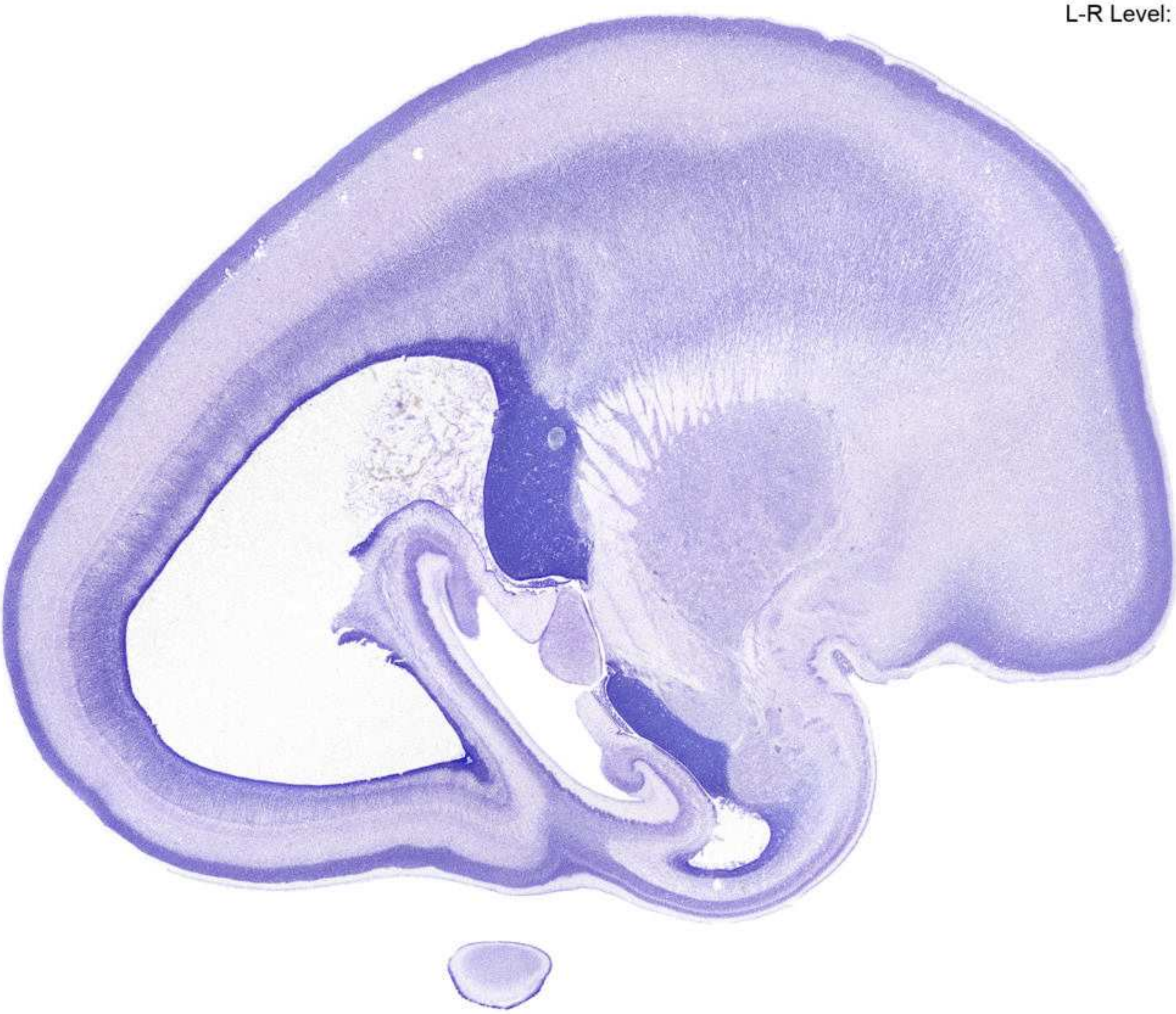

5 mm

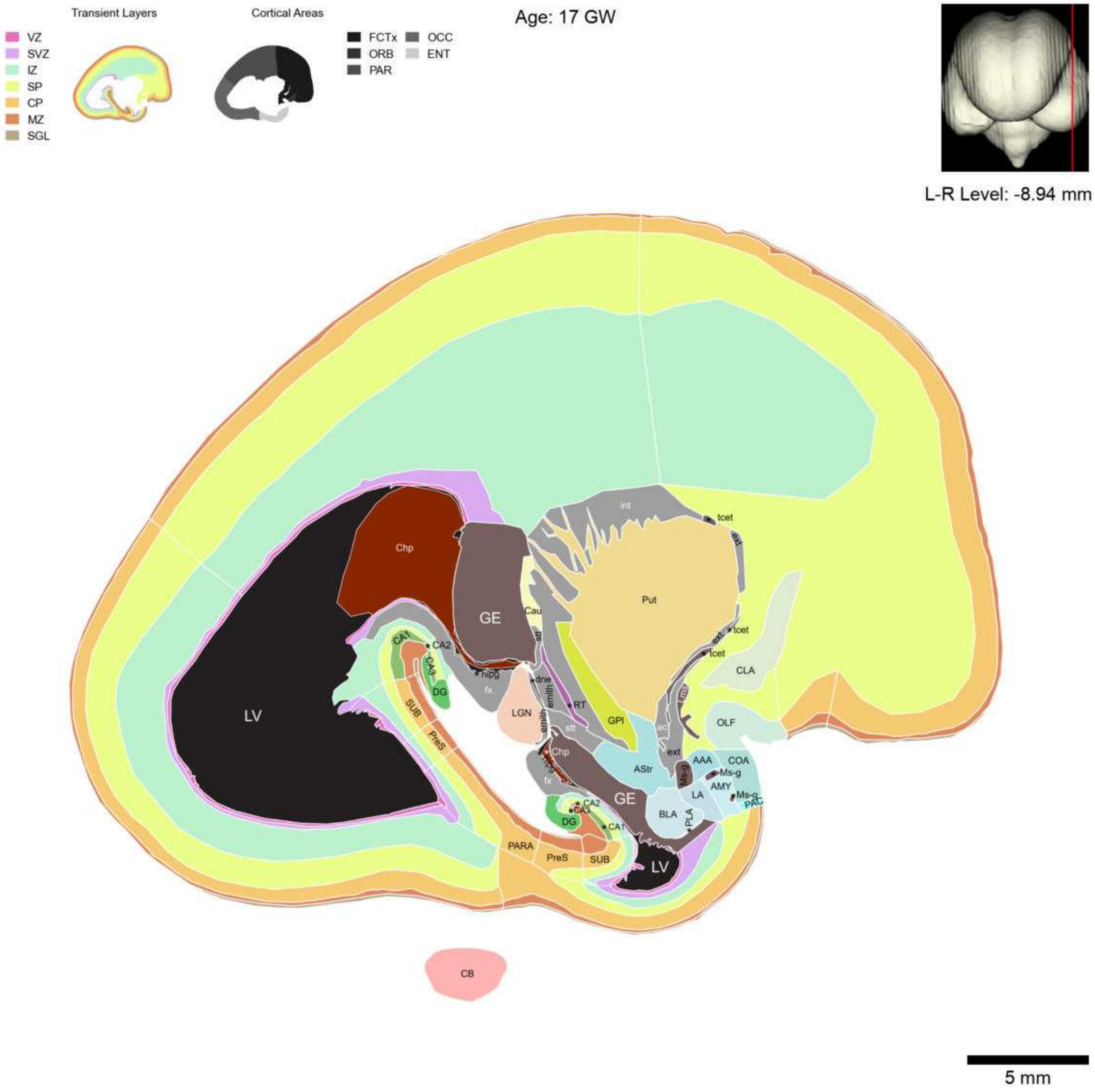

AAA: Anterior amygdaloid area
AMY: Amygdala
AStr: Amygdalo-striatal area
BL: Basal nucleus [amygdala]
CA1: CA1 field [hippocampus]
CA2: CA2 field [hippocampus]
CA3: CA3 field [hippocampus]
CB: Cerebellum
CLA: Claustrum
COA: Cortical nucleus [amygdala]
Cau: Caudate nucleus
Chp: Choroid plexus
DG: Dentate gyrus
GE: Ganglionic eminence
GPl: Globus pallidus lateral segment
LA: Lateral nucleus [amygdala]
LGN: Lateral geniculate nucleus
LV: Lateral ventricle
Lms: Lateral migratory stream
Ms-g: Migratory stream, general
PARA: Cortical plate, parasubiculum
PLA: Paralaminar nucleus [amygdala]
PreS: Cortical plate, presubiculum
Put: Putamen
RT: Reticular nucleus [thalamus]
SUB: Cortical plate, subiculum
ac: Anterior commissure
dne: Diencephalic neuroepithelium
emlth: External medullary lamina [thalamus]
ext: External capsule
fx: Fornix
hipg: Hippocampal glioepithelium/ependyma
int: Internal capsule
stt: Stria terminalis
tcet: Transient cell zone in the external capsule

Age: 17 GW

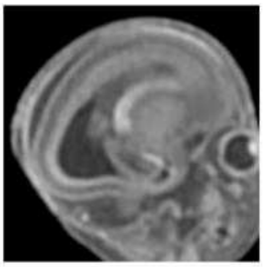

L-R Level: -9.42 mm

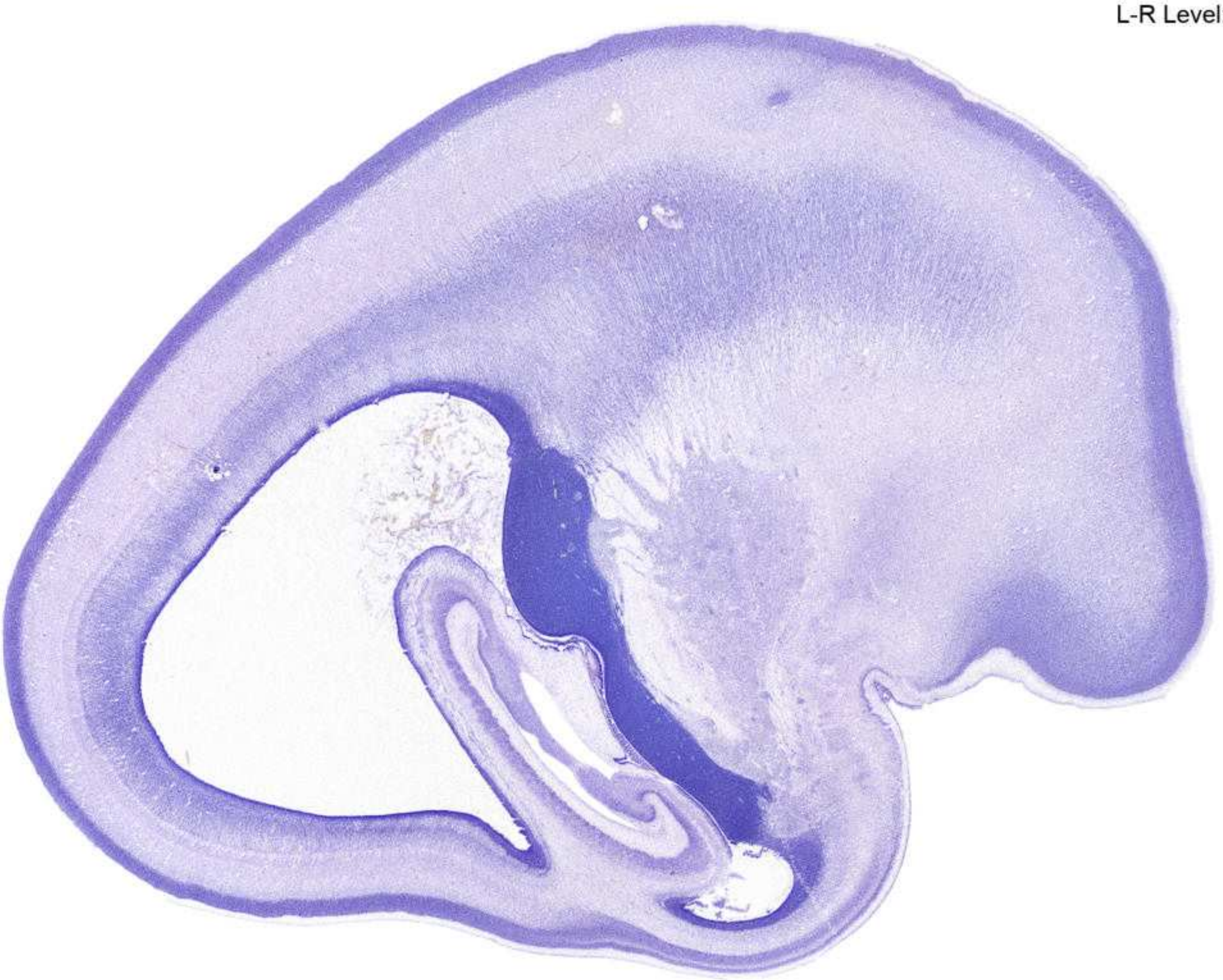

5 mm

Transient Layers

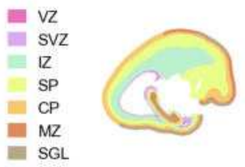

Cortical Areas

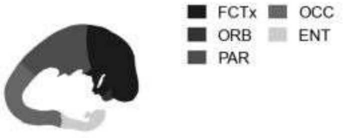

Age: 17 GW

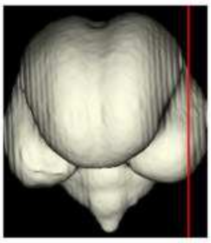

L-R Level: -9.42 mm

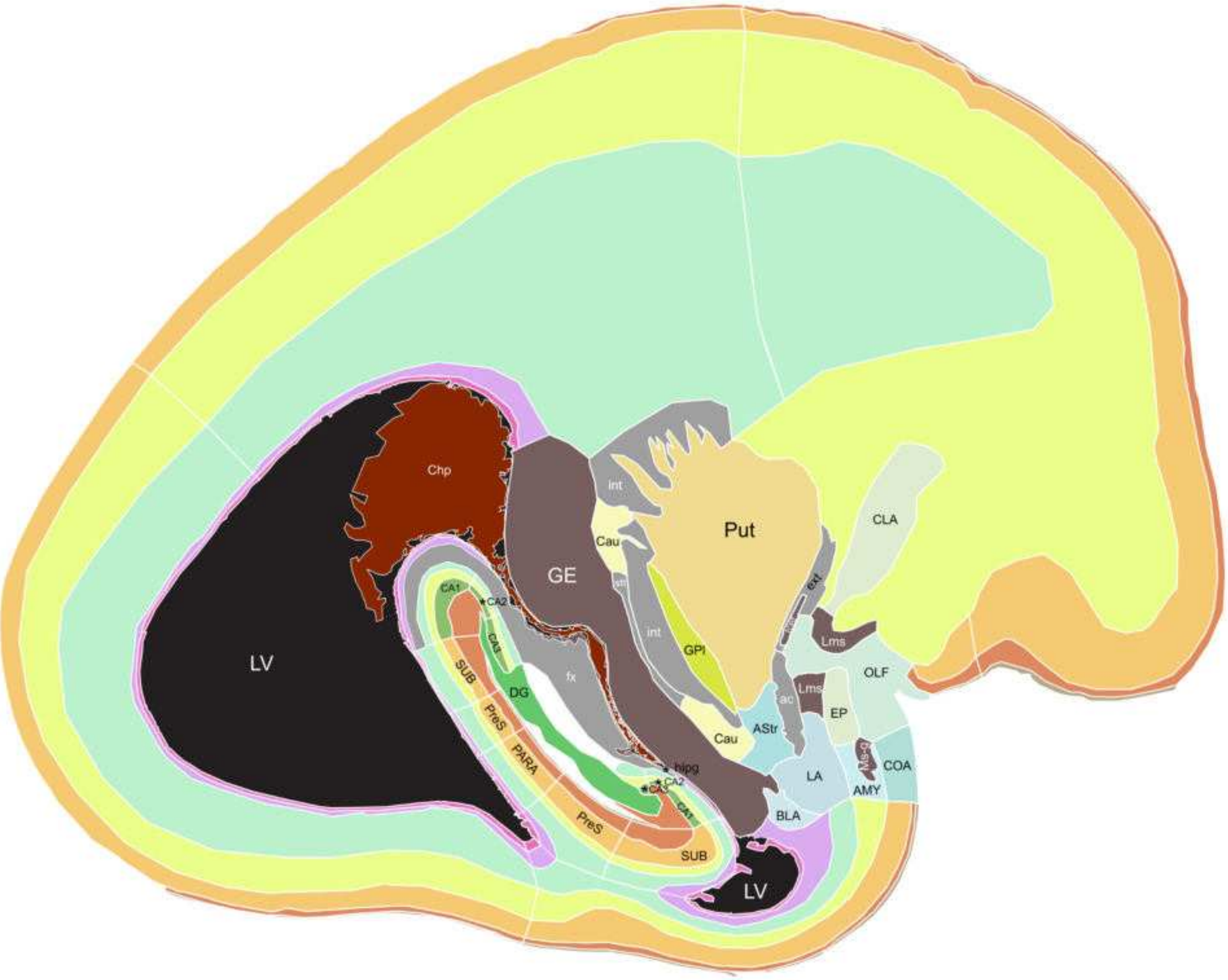

5 mm

- AMY: Amygdala
- AStr: Amygdalo-striatal area
- BL: Basal nucleus [amygdala]
- CA1: CA1 field [hippocampus]
- CA2: CA2 field [hippocampus]
- CA3: CA3 field [hippocampus]
- CLA: Claustrum
- COA: Cortical nucleus [amygdala]
- Cau: Caudate nucleus
- Chp: Choroid plexus
- DG: Dentate gyrus
- EP: Endopiriform nucleus
- GE: Ganglionic eminence
- GPl: Globus pallidus lateral segment
- LA: Lateral nucleus [amygdala]
- LV: Lateral ventricle
- Lms: Lateral migratory stream
- Ms-g: Migratory stream, general
- PARA: Cortical plate, parasubiculum
- PreS: Cortical plate, presubiculum
- Put: Putamen
- SUB: Cortical plate, subiculum
- ac: Anterior commissure
- ext: External capsule
- fx: Fornix
- hipg: Hippocampal glioepithelium/ependyma
- int: Internal capsule
- stt: Stria terminalis
- tcet: Transient cell zone in the external capsule

Age: 17 GW

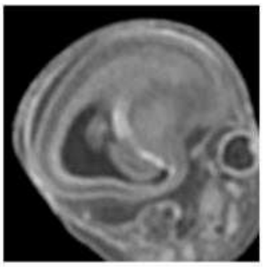

L-R Level: -10.14 mm

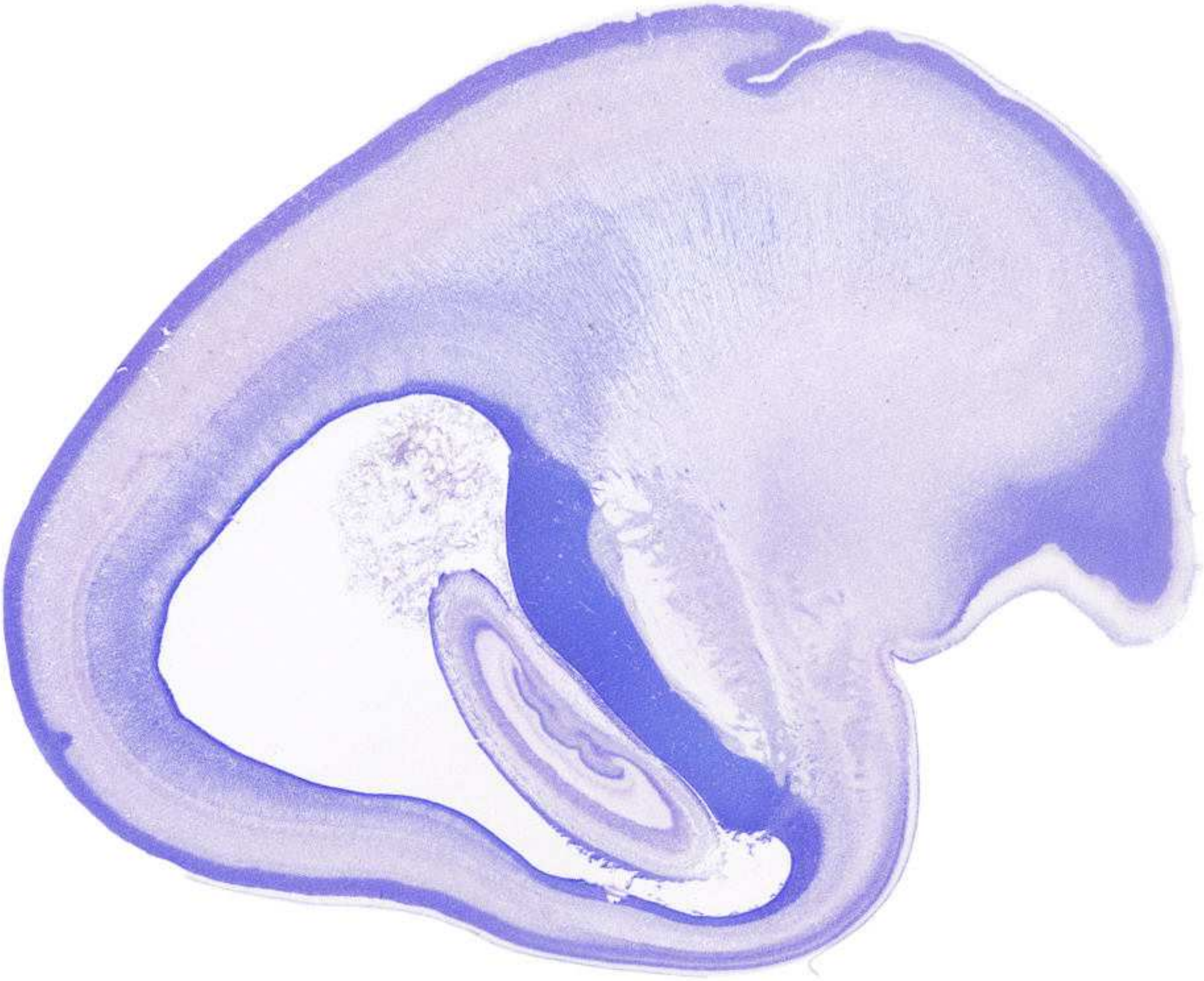

5 mm

Transient Layers

- VZ
- SVZ
- IZ
- SP
- CP
- MZ
- SGL

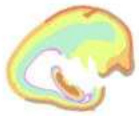

Cortical Areas

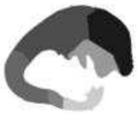

- FCTx
- PAR
- OCC
- TEMP
- INS
- ENT

Age: 17 GW

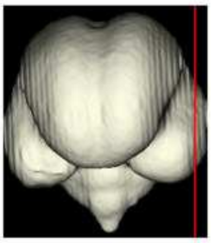

L-R Level: -10.14 mm

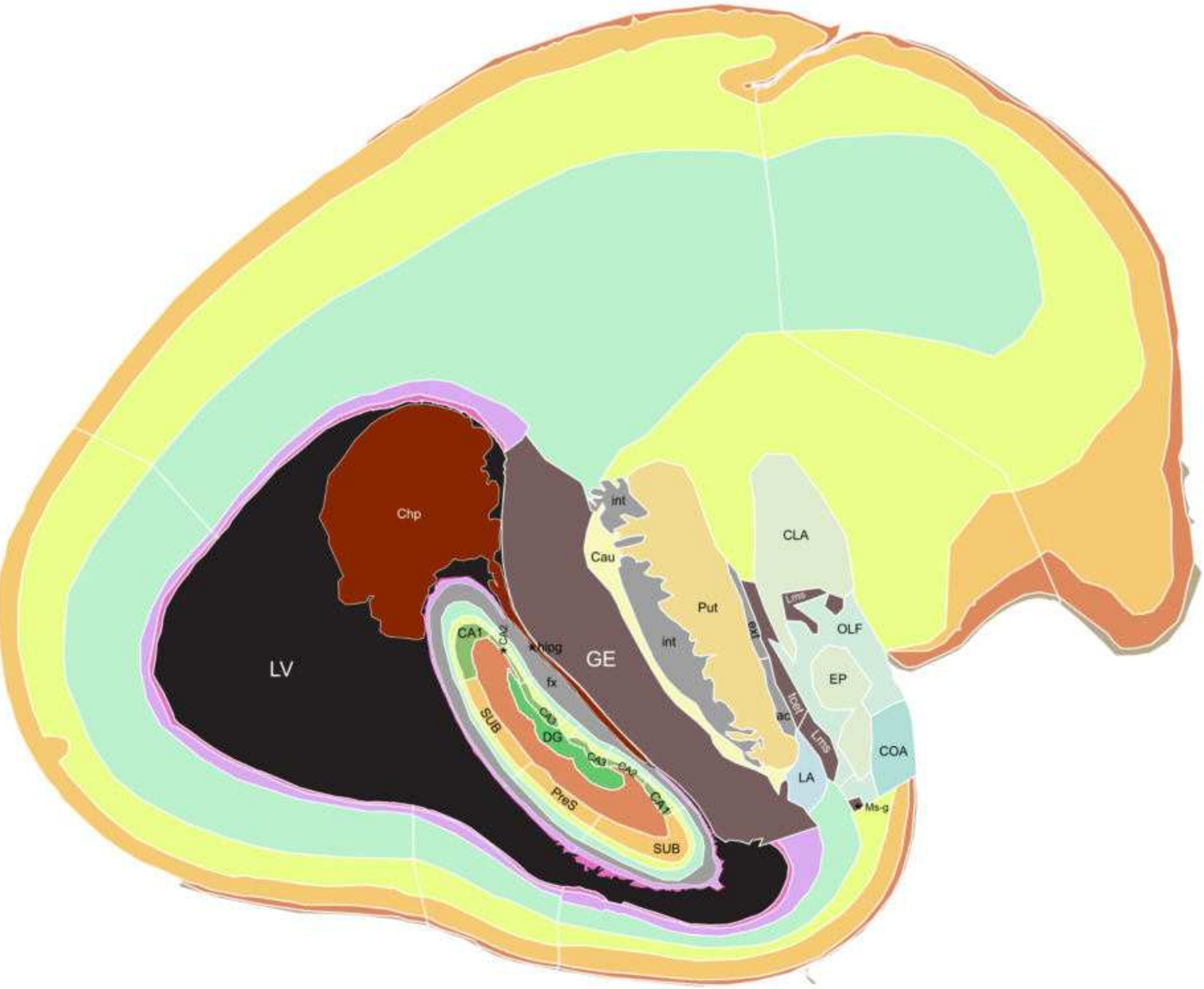

5 mm

- CA1: CA1 field [hippocampus]
- CA2: CA2 field [hippocampus]
- CA3: CA3 field [hippocampus]
- CLA: Claustrum
- COA: Cortical nucleus [amygdala]
- Cau: Caudate nucleus
- Chp: Choroid plexus
- DG: Dentate gyrus
- EP: Endopiriform nucleus
- GE: Ganglionic eminence
- LA: Lateral nucleus [amygdala]
- LV: Lateral ventricle
- Lms: Lateral migratory stream
- Ms-g: Migratory stream, general
- PreS: Cortical plate, presubiculum
- Put: Putamen
- SUB: Cortical plate, subiculum
- ac: Anterior commissure
- ext: External capsule
- fx: Fornix
- hipg: Hippocampal glioepithelium/ependyma
- int: Internal capsule
- tcet: Transient cell zone in the external capsule

Age: 17 GW

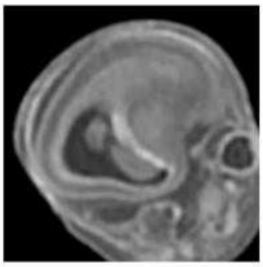

L-R Level: -10.5 mm

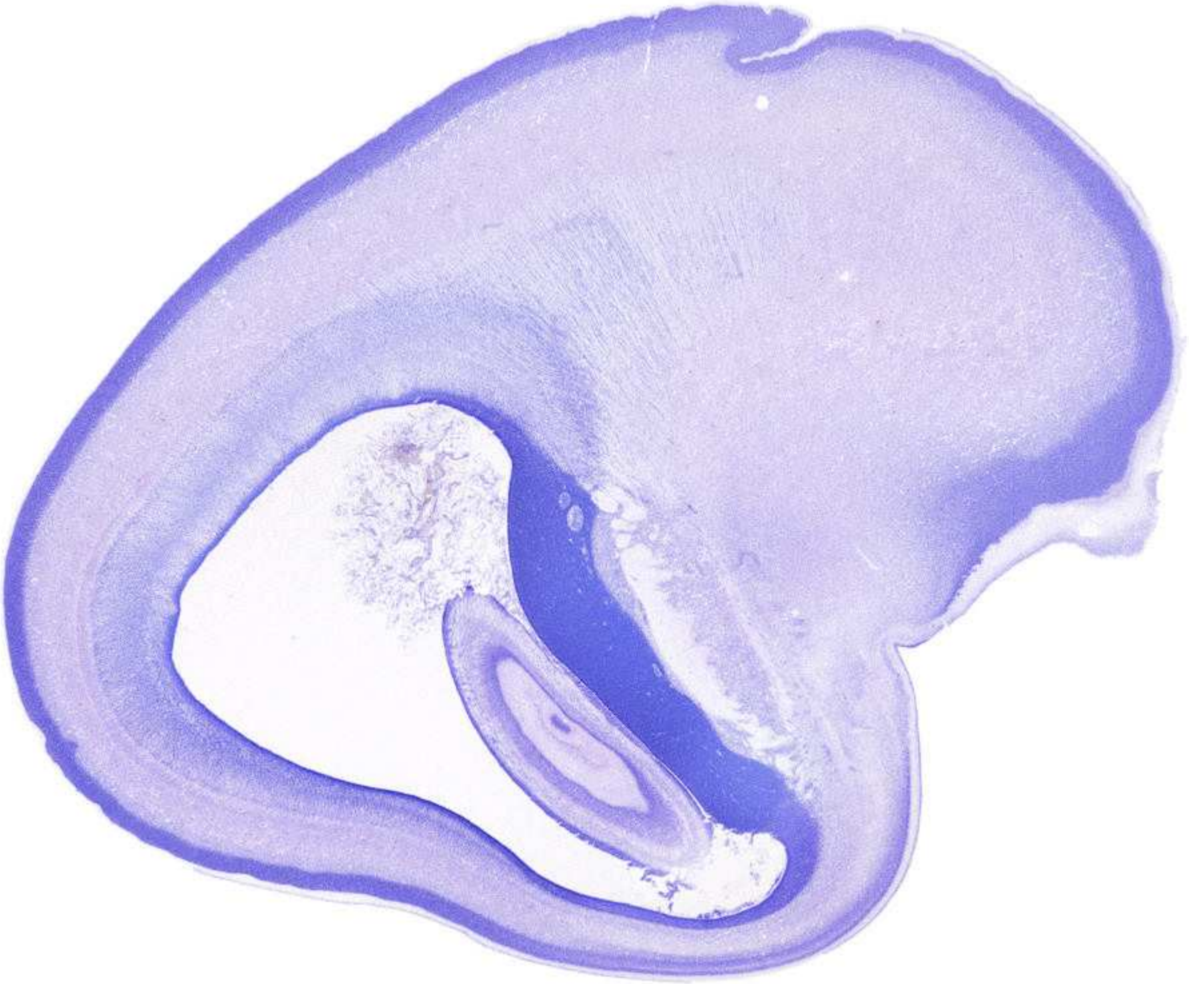

5 mm

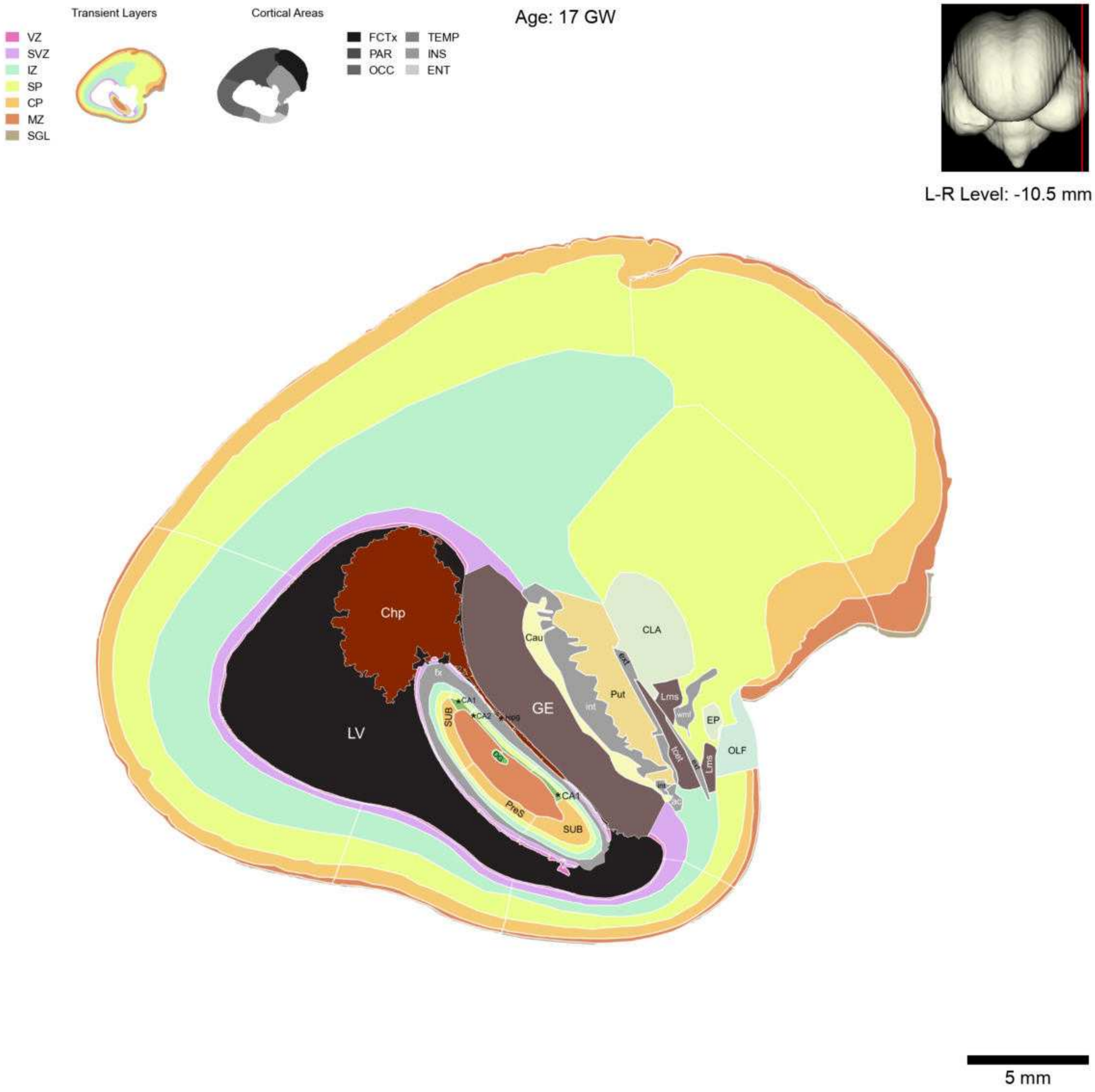

CA1: CA1 field [hippocampus]
CA2: CA2 field [hippocampus]
CLA: Claustrum
Cau: Caudate nucleus
Chp: Choroid plexus
DG: Dentate gyrus
EP: Endopiriform nucleus
GE: Ganglionic eminence
LV: Lateral ventricle
Lms: Lateral migratory stream
PreS: Cortical plate, presubiculum
Put: Putamen
SUB: Cortical plate, subiculum
ac: Anterior commissure
ext: External capsule
fx: Fornix
hipg: Hippocampal glioepithelium/ependyma
int: Internal capsule
tcet: Transient cell zone in the external capsule
wmf: White matter fibers

Age: 17 GW

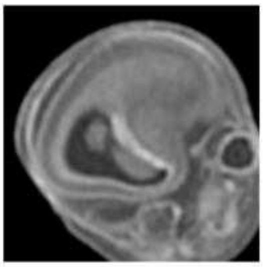

L-R Level: -10.8 mm

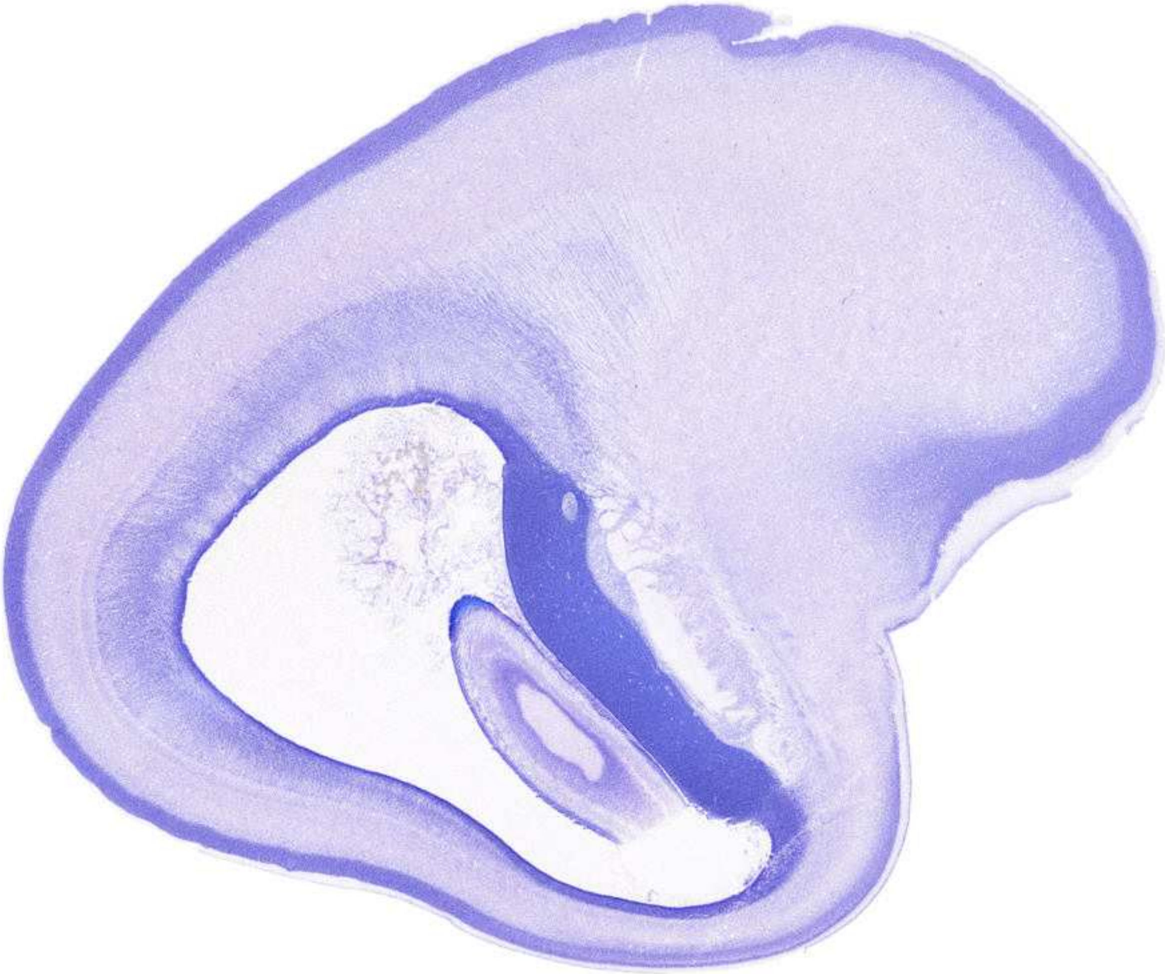

5 mm

Transient Layers

- VZ
- SVZ
- IZ
- SP
- CP
- MZ
- SGL

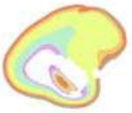

Cortical Areas

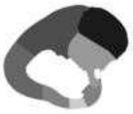

- FCTx
- PAR
- OCC
- TEMP
- INS
- ENT

Age: 17 GW

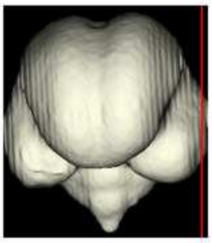

L-R Level: -10.8 mm

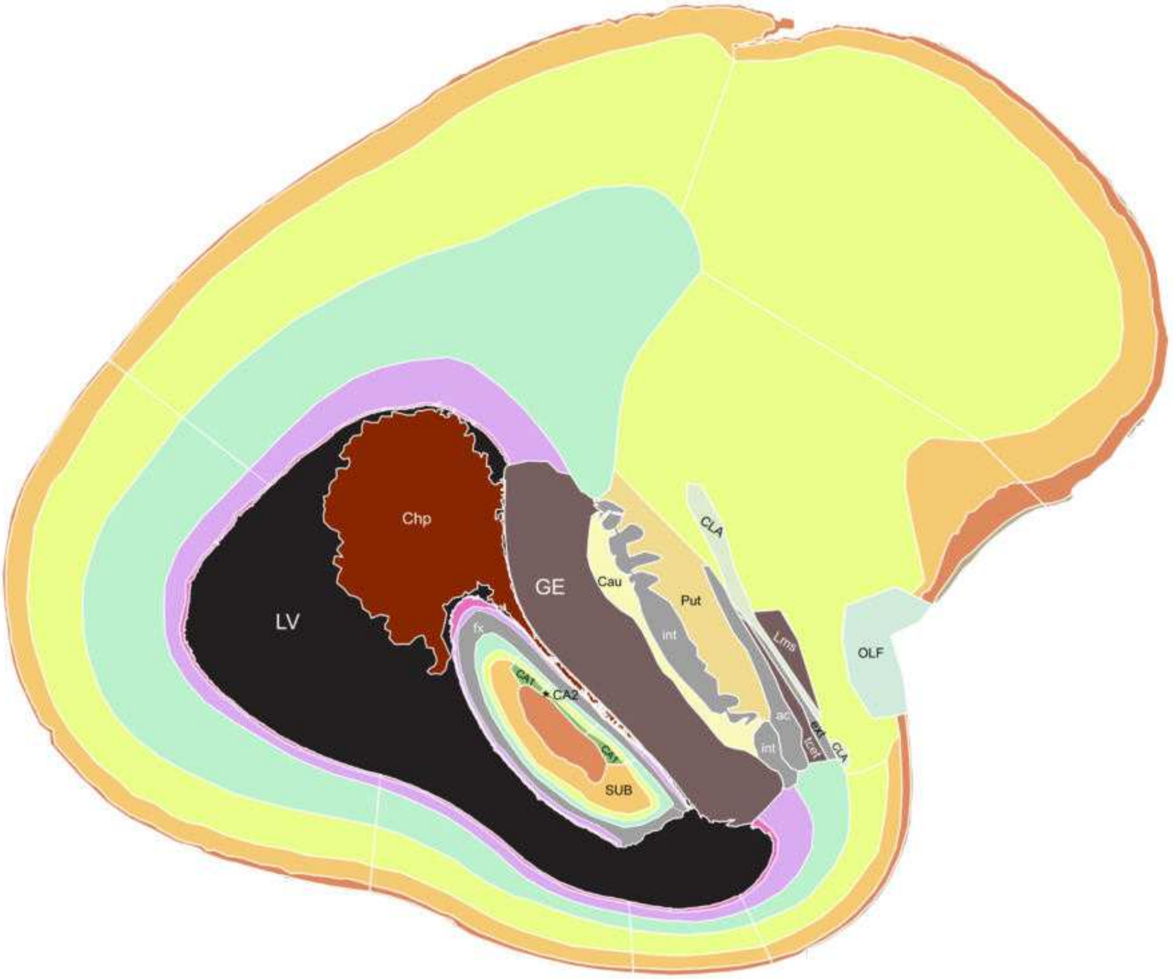

5 mm

- CA1: CA1 field [hippocampus]
- CA2: CA2 field [hippocampus]
- CLA: Claustrum
- Cau: Caudate nucleus
- Chp: Choroid plexus
- GE: Ganglionic eminence
- LV: Lateral ventricle
- Lms: Lateral migratory stream
- Put: Putamen
- SUB: Cortical plate, subiculum
- ac: Anterior commissure
- ext: External capsule
- fx: Fornix
- int: Internal capsule
- tcet: Transient cell zone in the external capsule

Age: 17 GW

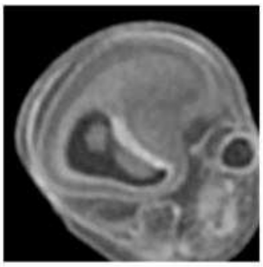

L-R Level: -11.04 mm

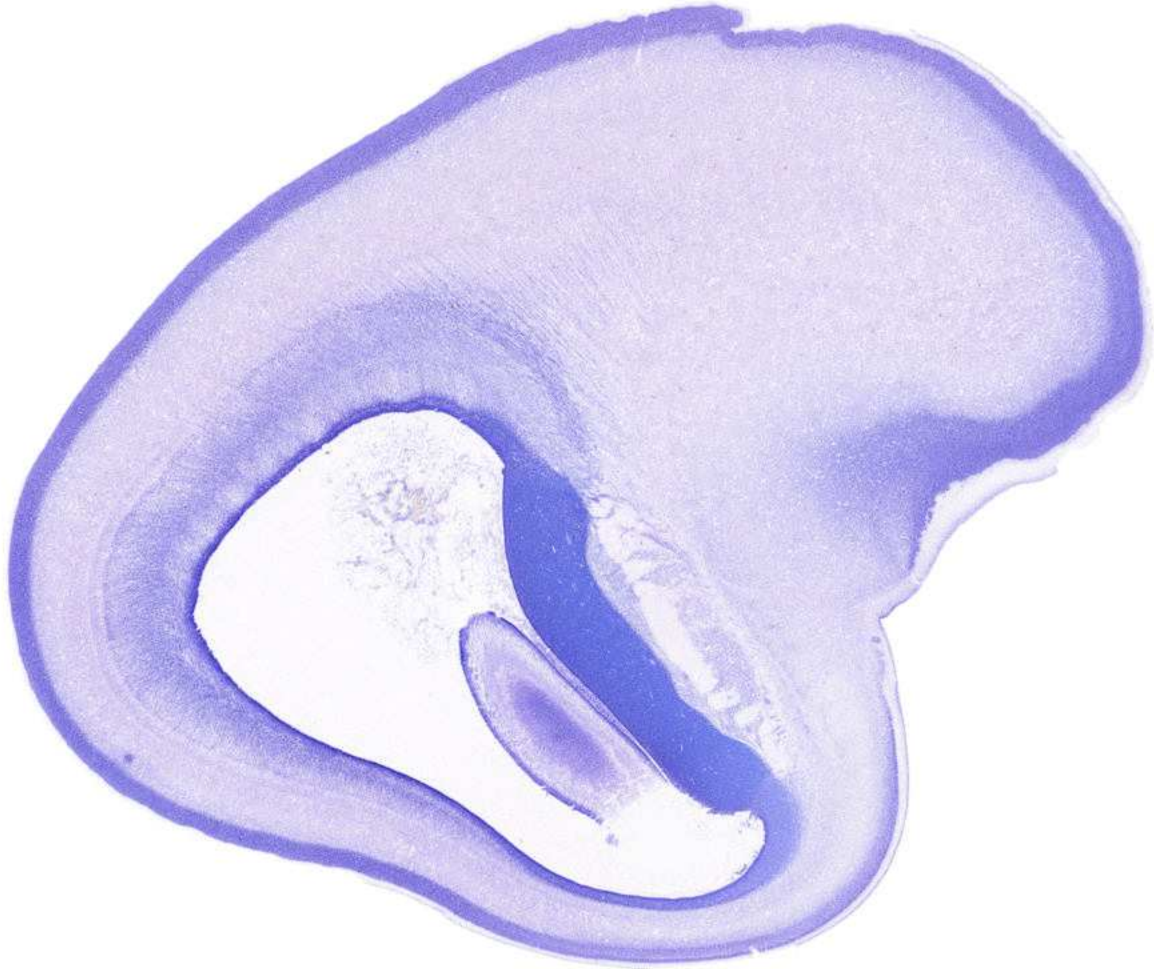

5 mm

Transient Layers

- VZ
- SVZ
- IZ
- SP
- CP
- MZ
- SGL

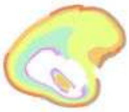

Cortical Areas

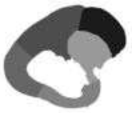

- FCTx
- PAR
- OCC
- TEMP
- INS

Age: 17 GW

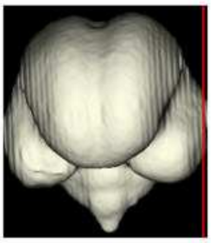

L-R Level: -11.04 mm

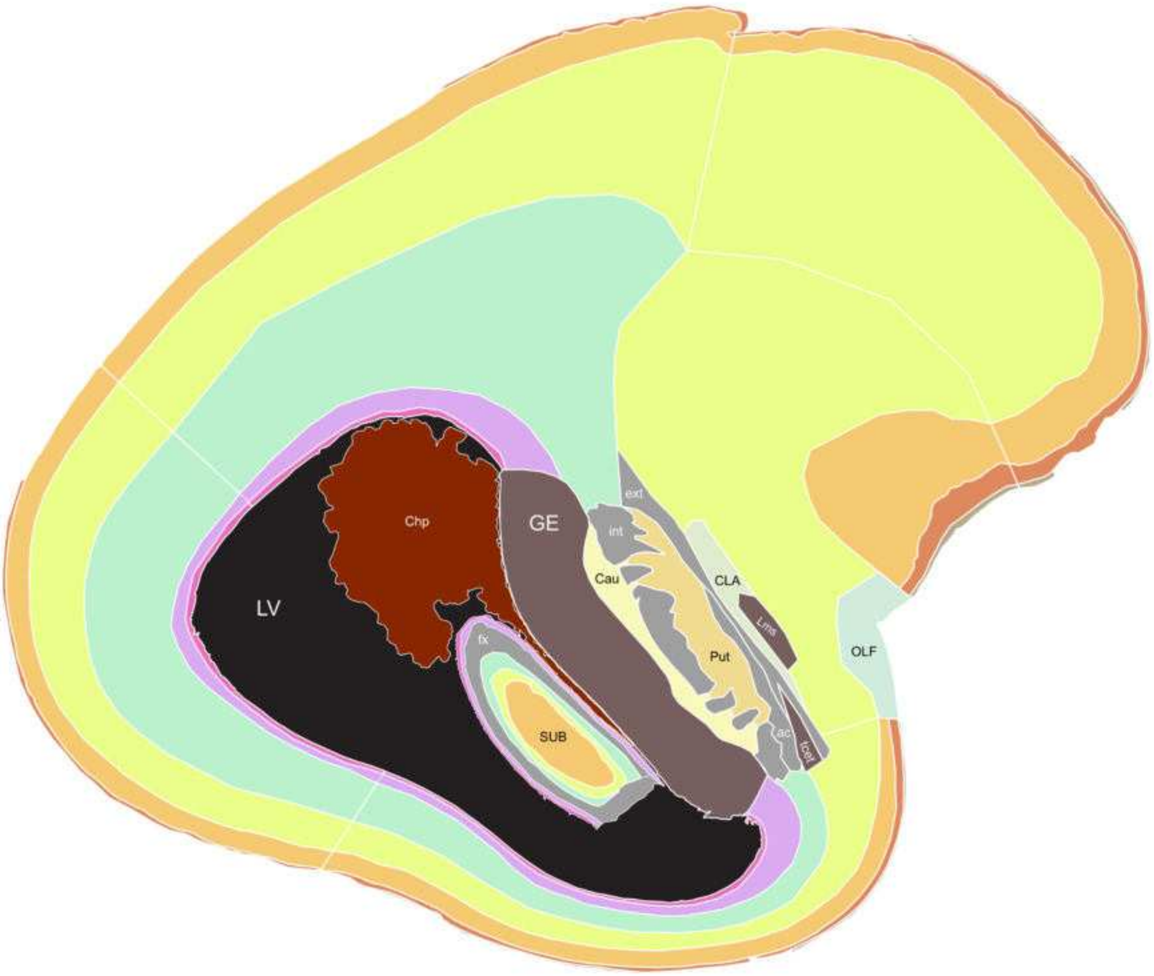

5 mm

- CLA: Claustrum
- Cau: Caudate nucleus
- Chp: Choroid plexus
- GE: Ganglionic eminence
- LV: Lateral ventricle
- Lms: Lateral migratory stream
- Put: Putamen
- SUB: Cortical plate, subiculum
- ac: Anterior commissure
- ext: External capsule
- fx: Fornix
- int: Internal capsule
- tcet: Transient cell zone in the external capsule

Age: 17 GW

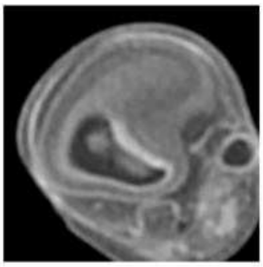

L-R Level: -11.52 mm

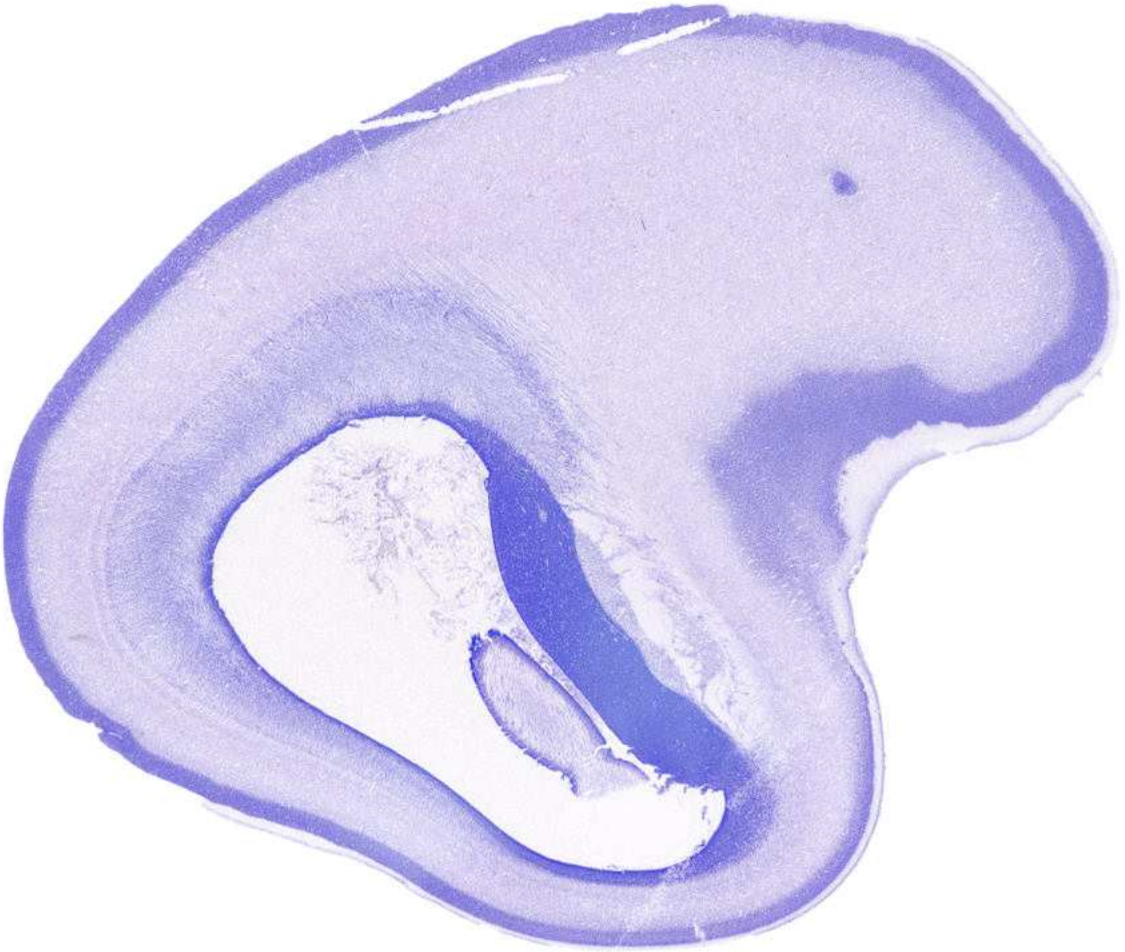

5 mm

Transient Layers

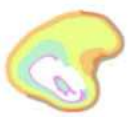

- VZ
- SVZ
- IZ
- SP
- CP
- MZ
- SGL

Cortical Areas

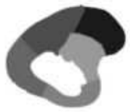

- FCTx
- PAR
- OCC
- TEMP
- INS

Age: 17 GW

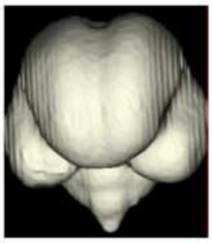

L-R Level: -11.52 mm

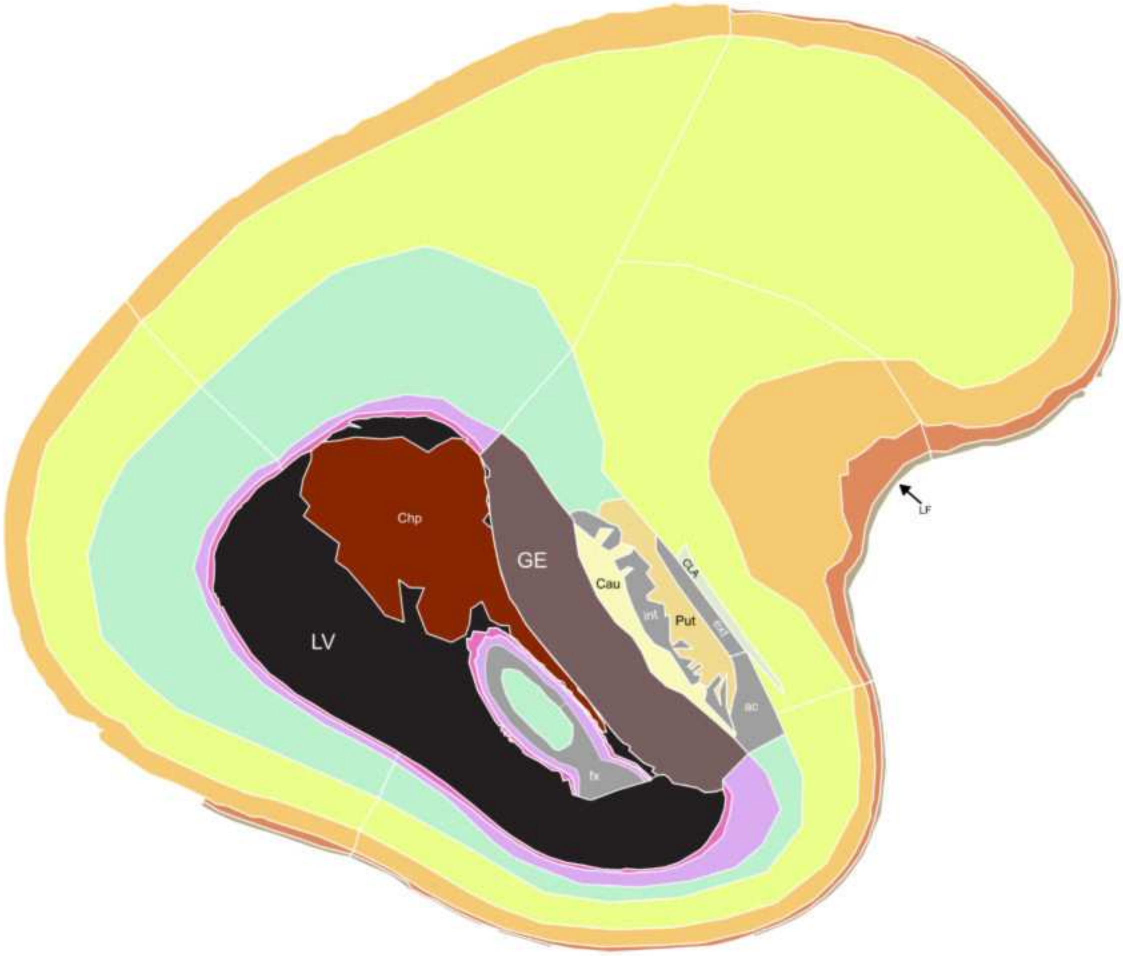

5 mm

- CLA: Claustrum
- Cau: Caudate nucleus
- Chp: Choroid plexus
- GE: Ganglionic eminence
- LV: Lateral ventricle
- Put: Putamen
- ac: Anterior commissure
- ext: External capsule
- → LF: Lateral fissure
- fx: Fornix
- int: Internal capsule

Age: 17 GW

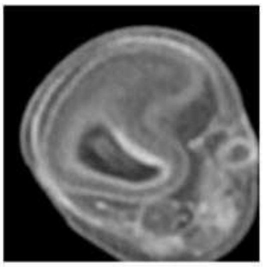

L-R Level: -12.6 mm

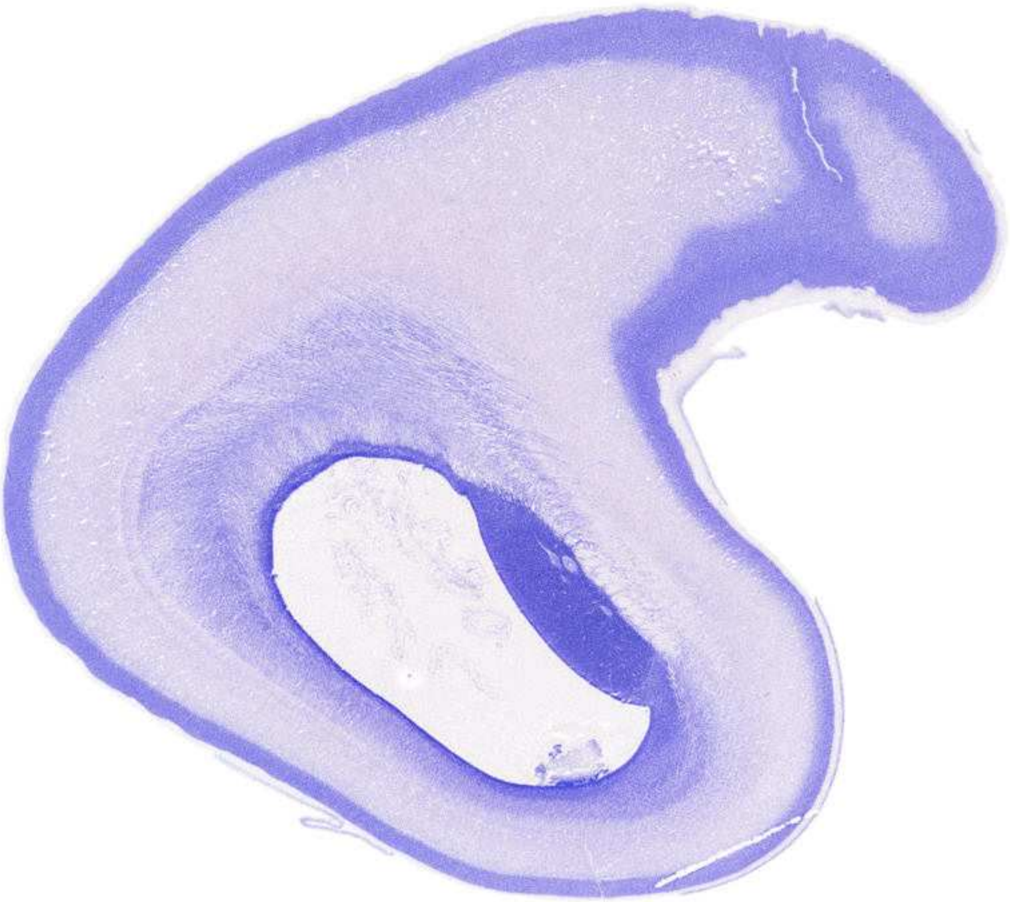

5 mm

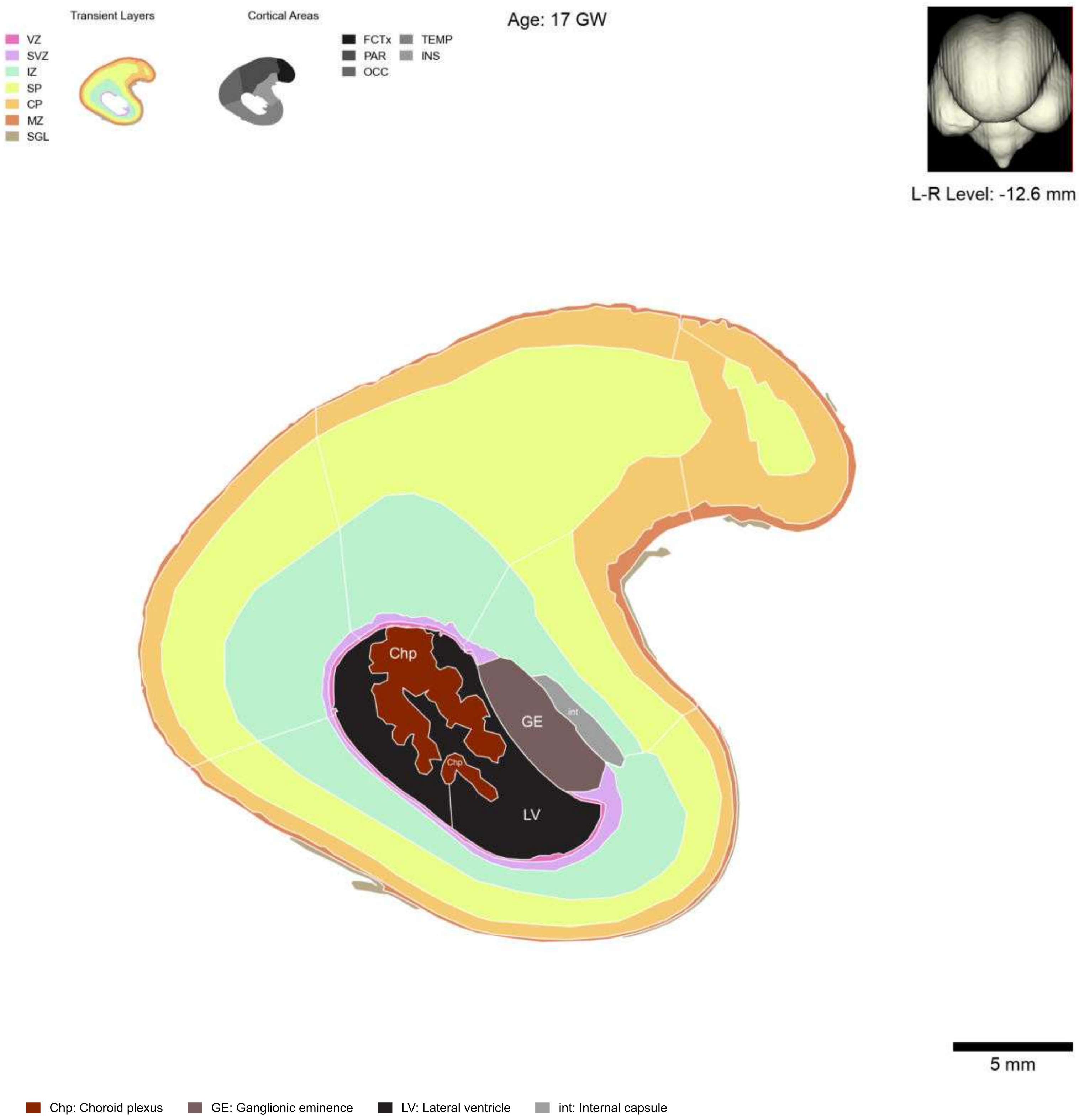
Transient Layers
VZ
SVZ
IZ
SP
CP
MZ
SGL
Cortical Areas
FCTx
PAR
OCC
TEMP
INS
Age: 17 GW
L-R Level: -12.6 mm
Chp
GE
int
Chp
LV
5 mm
Chp: Choroid plexus
GE: Ganglionic eminence
LV: Lateral ventricle
int: Internal capsule

Age: 17 GW

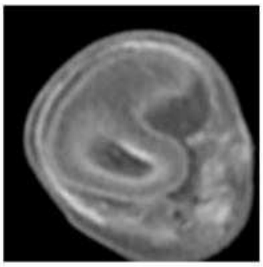

L-R Level: -13.74 mm

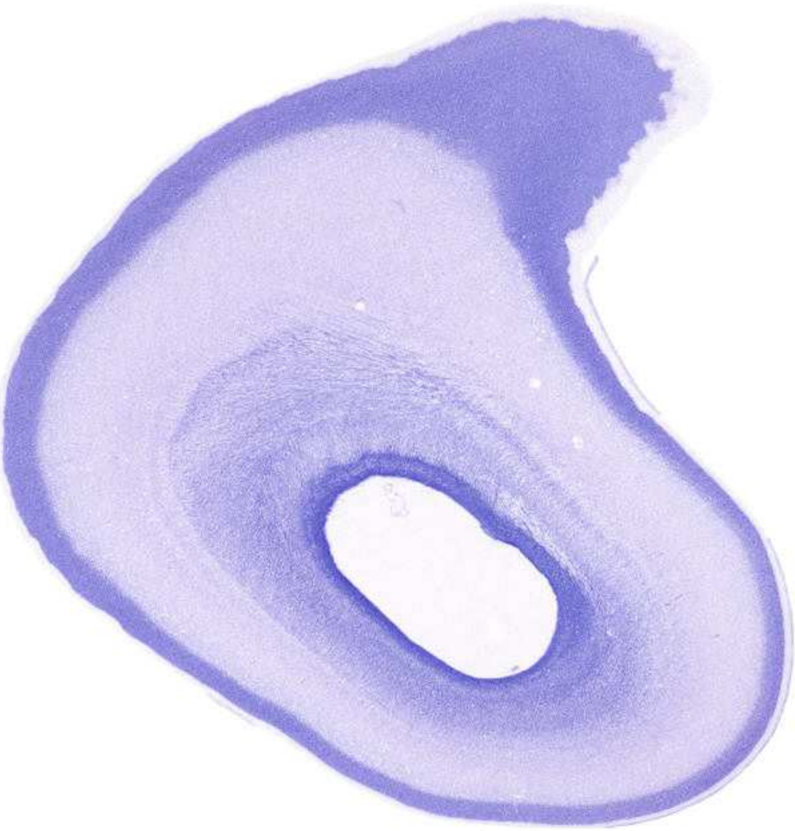

5 mm

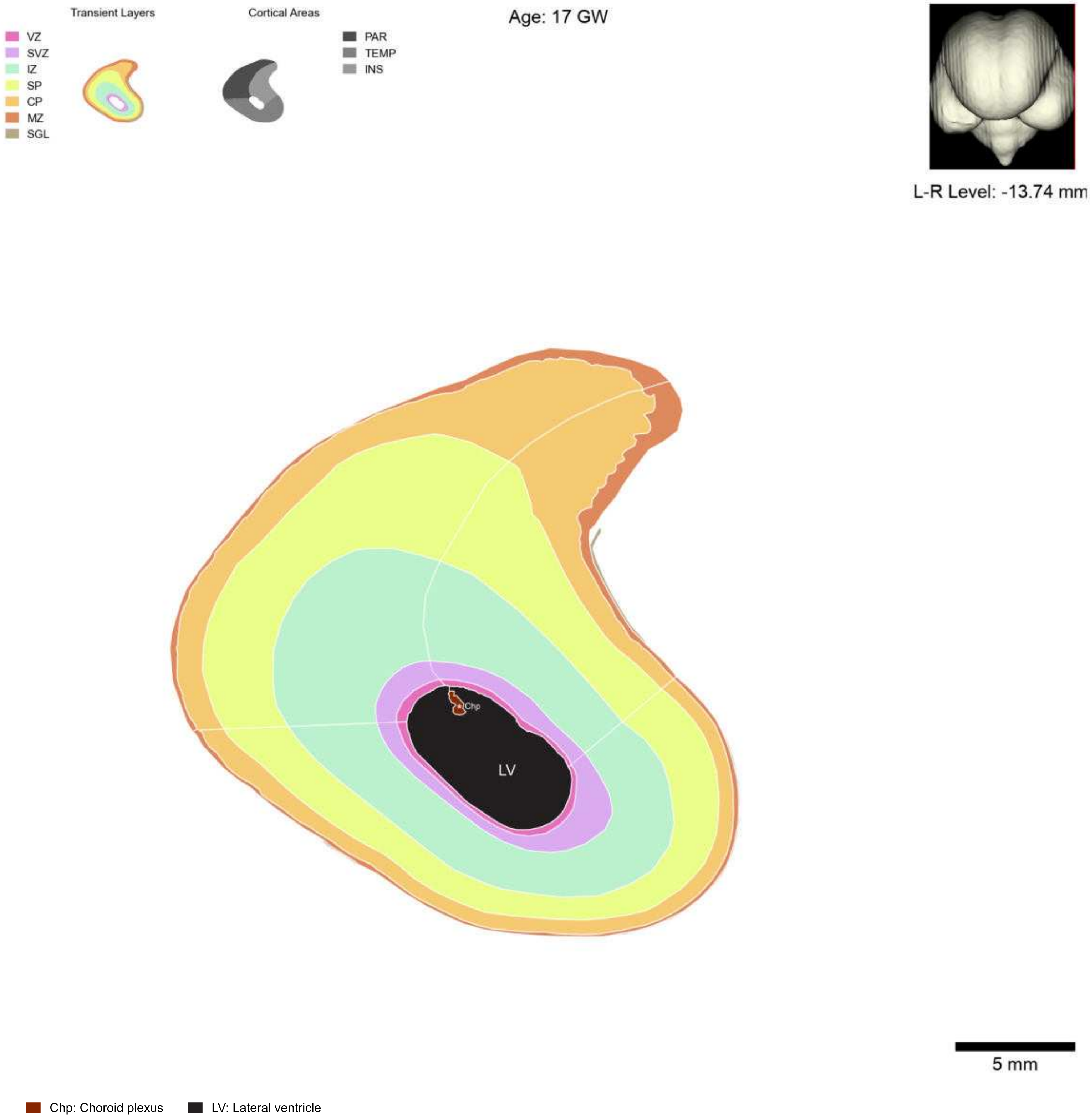
Transient Layers
VZ
SVZ
IZ
SP
CP
MZ
SGL
Cortical Areas
PAR
TEMP
INS
Age: 17 GW
L-R Level: -13.74 mm
Chp
LV
5 mm
Chp: Choroid plexus
LV: Lateral ventricle

Age: 17 GW

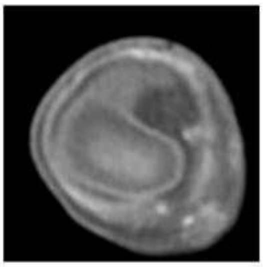

L-R Level: -15.3 mm

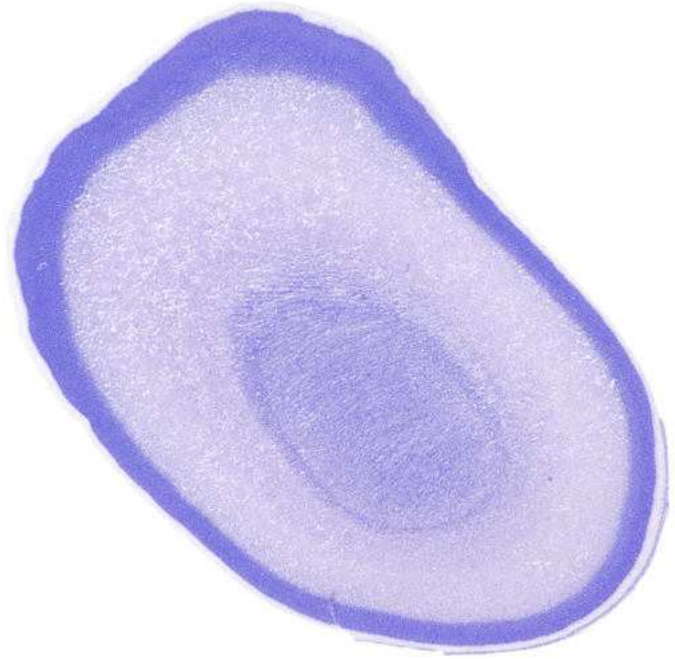

5 mm

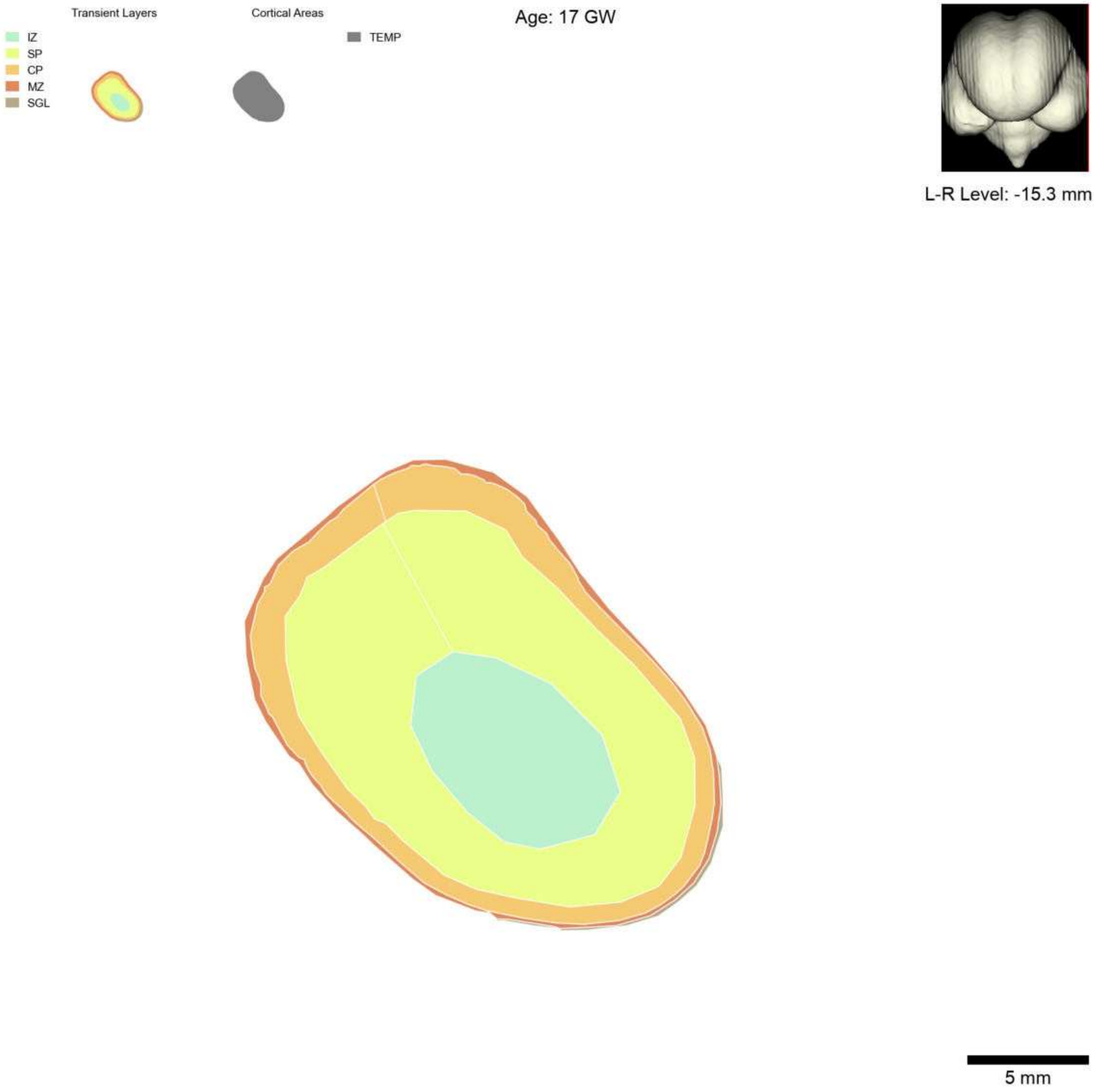

Transient Layers
IZ
SP
CP
MZ
SGL
Cortical Areas
TEMP
Age: 17 GW
L-R Level: -15.3 mm
5 mm

Age: 17 GW

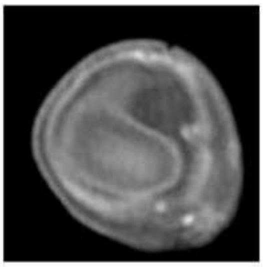

L-R Level: -15.78 mm

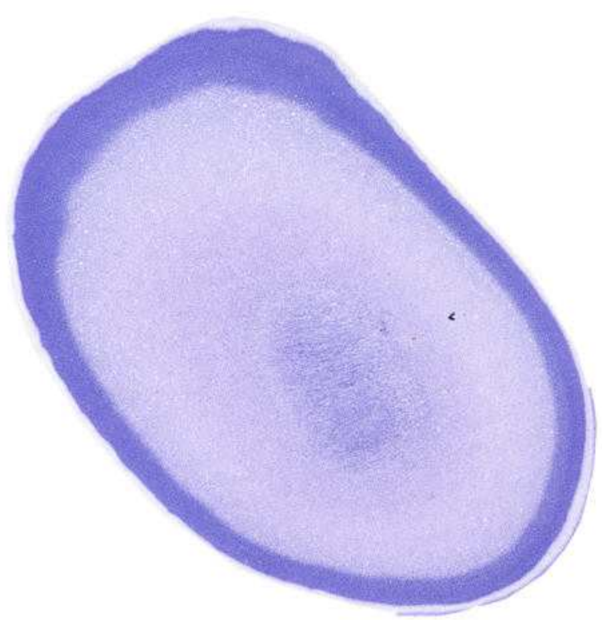

5 mm

Transient Layers

- IZ
- SP
- CP
- MZ
- SGL

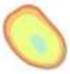

Cortical Areas

- TEMP

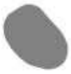

Age: 17 GW

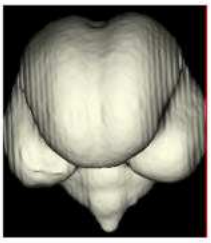

L-R Level: -15.78 mm

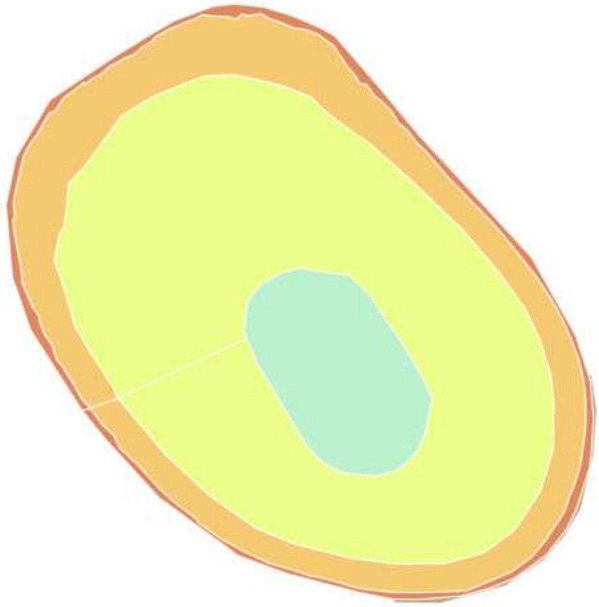

5 mm

Age: 17 GW

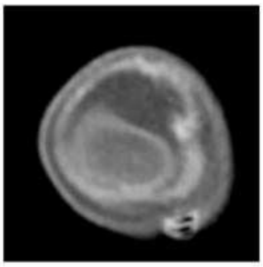

L-R Level: -17.1 mm

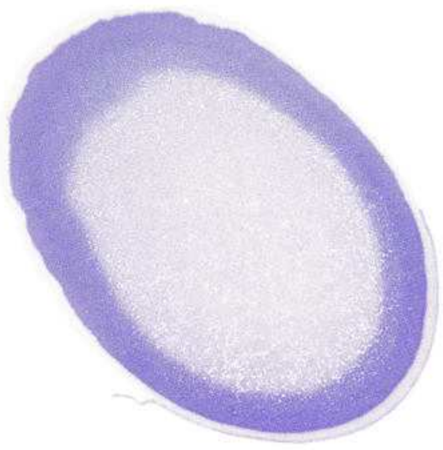

5 mm

Transient Layers

- SP
- CP
- MZ
- SGL

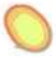

Cortical Areas

- TEMP

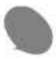

Age: 17 GW

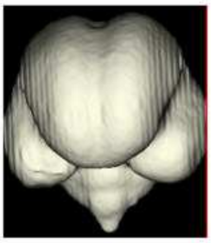

L-R Level: -17.1 mm

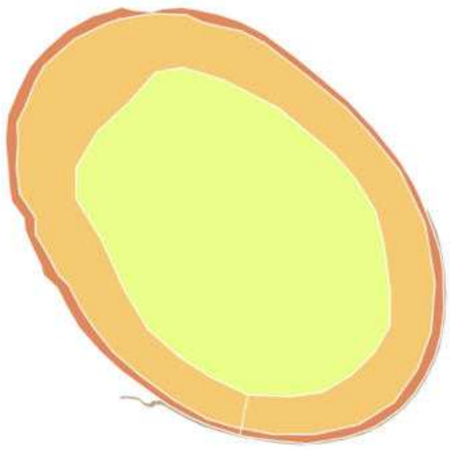

5 mm

Age: 17 GW

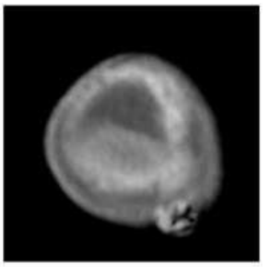

L-R Level: -18.24 mm

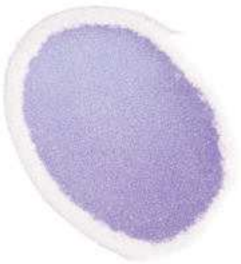

5 mm

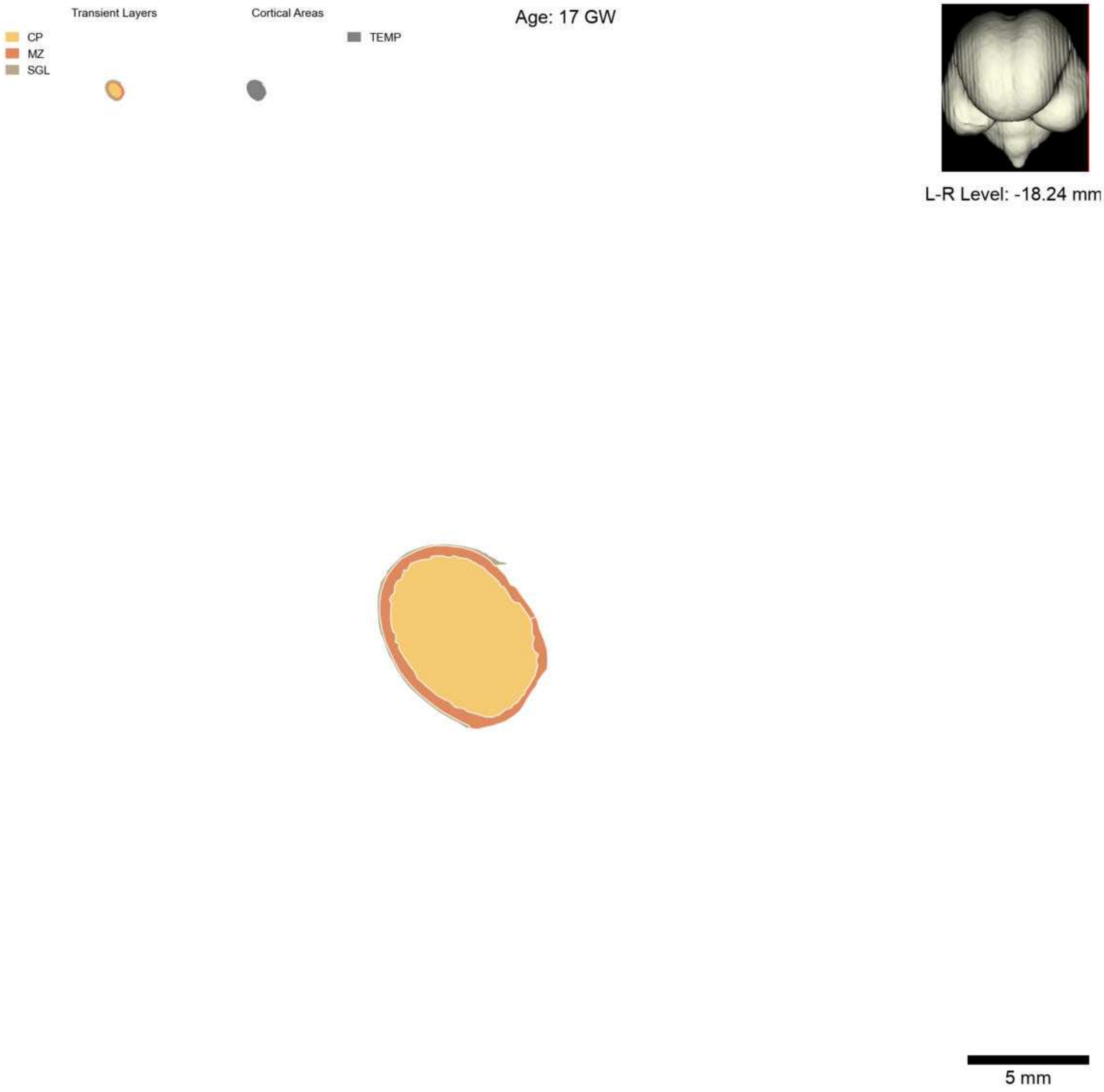
Transient Layers
CP
MZ
SGL
Cortical Areas
TEMP
Age: 17 GW
L-R Level: -18.24 mm
5 mm

21 Gestational Week (GW)

Sagittal

This page is intentionally left blank

Age: 21 GW

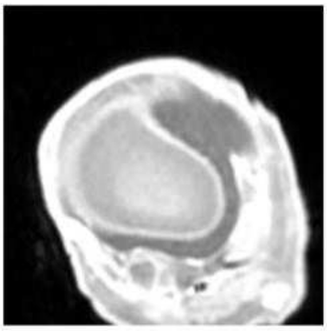

L-R Level: 19.8 mm

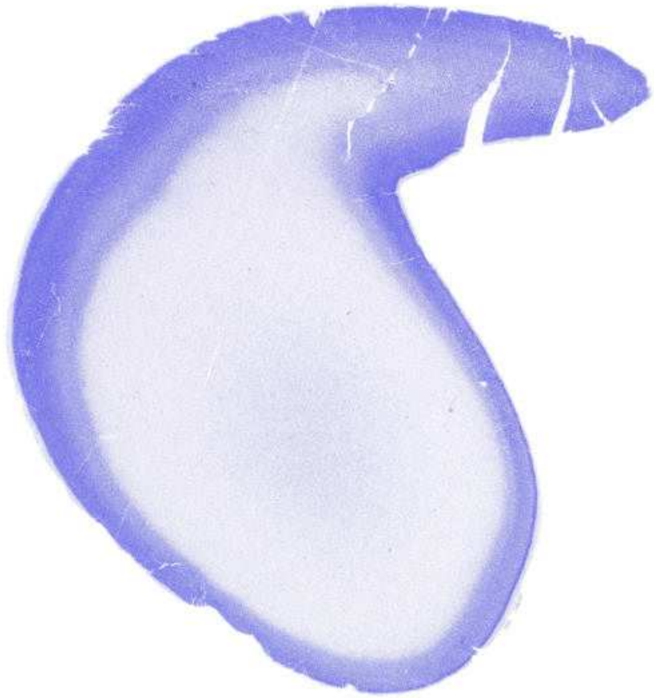

5 mm

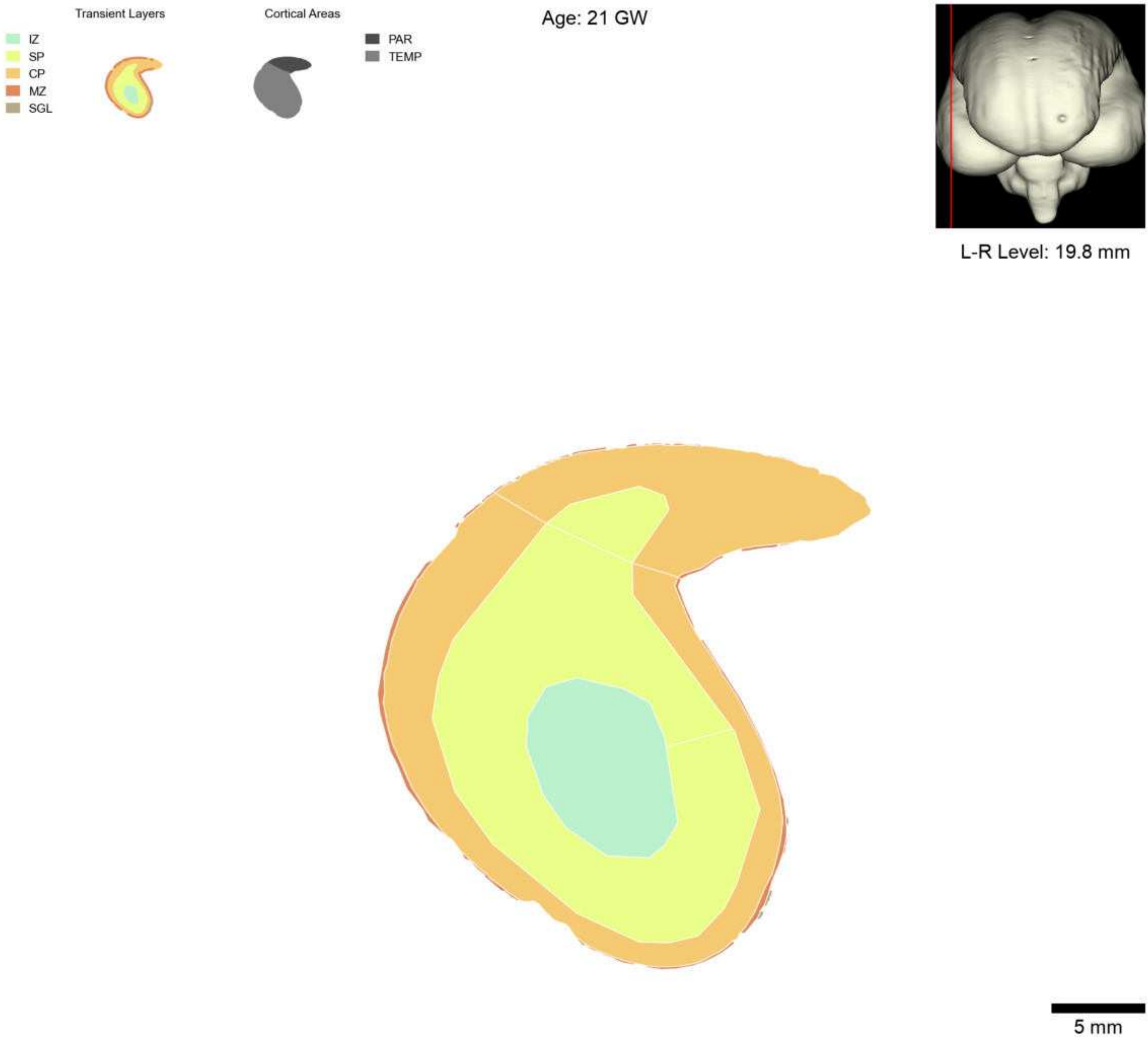
Transient Layers
IZ
SP
CP
MZ
SGL
Cortical Areas
PAR
TEMP
Age: 21 GW
L-R Level: 19.8 mm
5 mm

Age: 21 GW

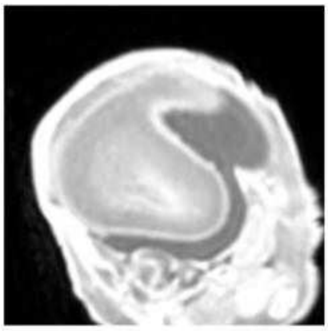

L-R Level: 18.18 mm

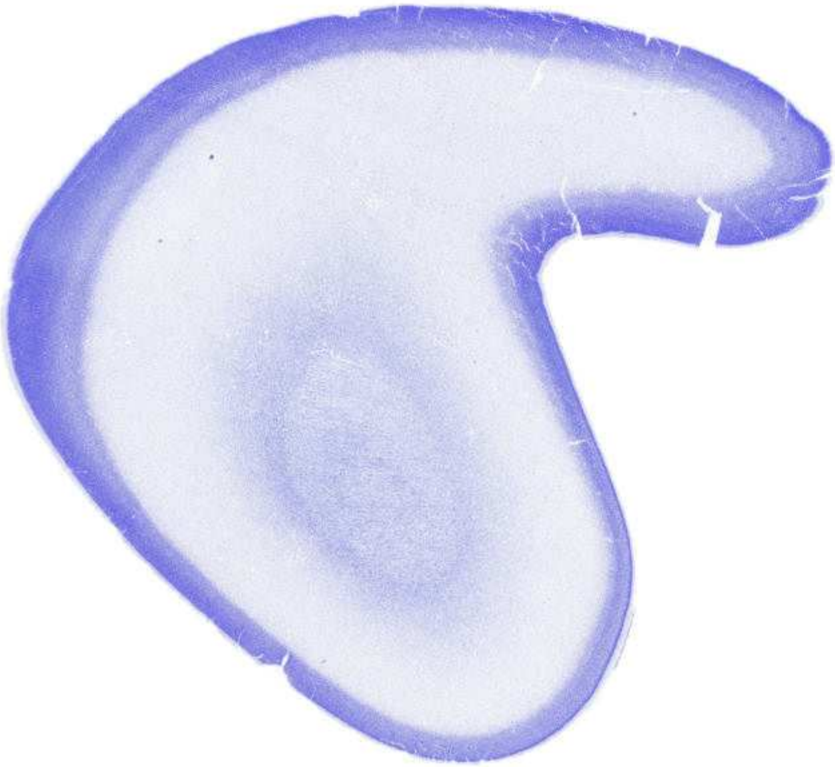

5 mm

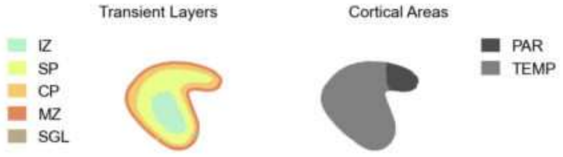

Age: 21 GW

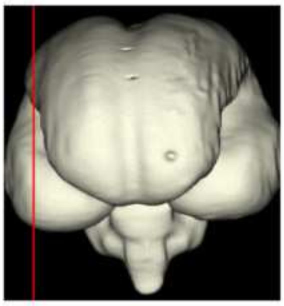

L-R Level: 18.18 mm

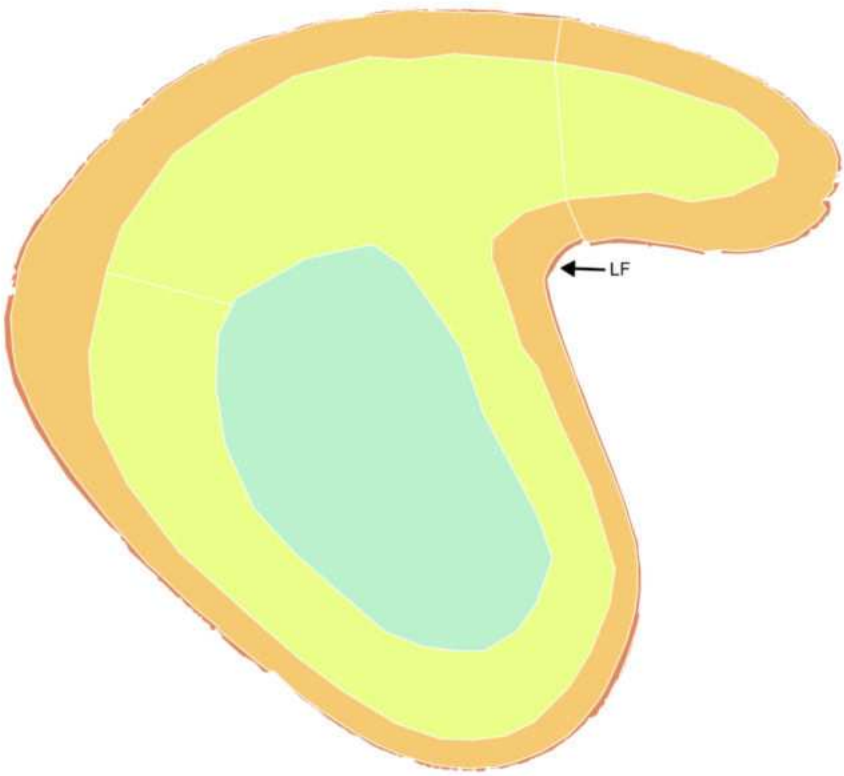

5 mm

→ LF: Lateral fissure

Age: 21 GW

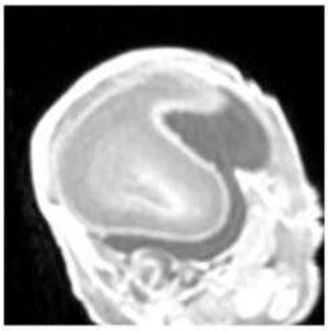

L-R Level: 16.74 mm

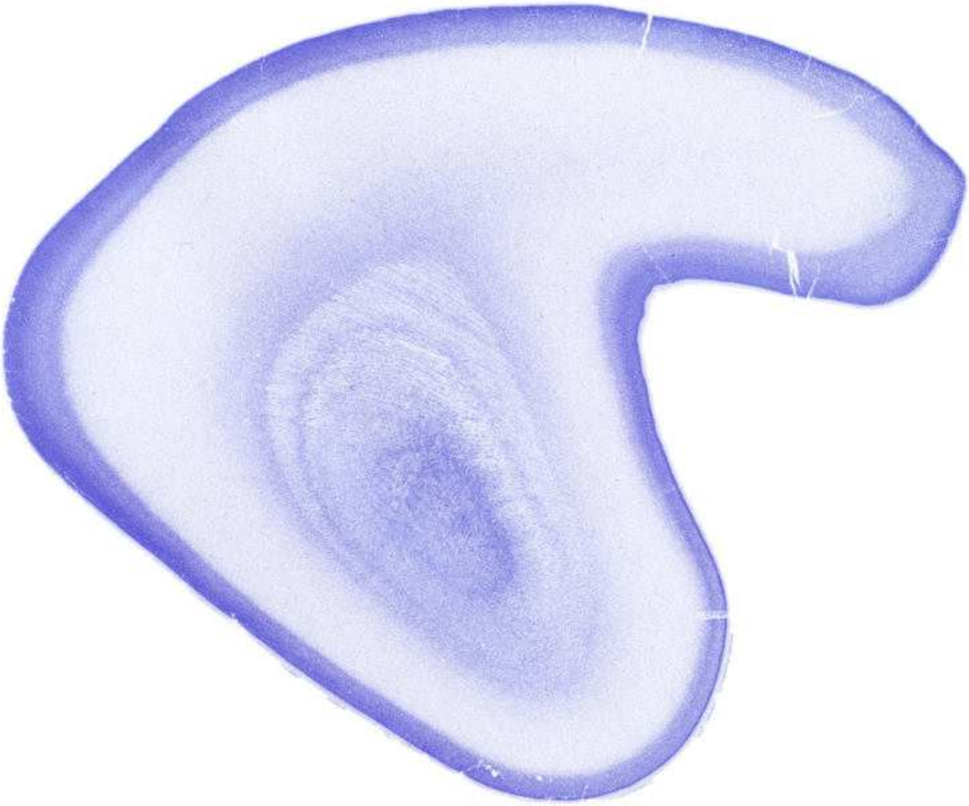

5 mm

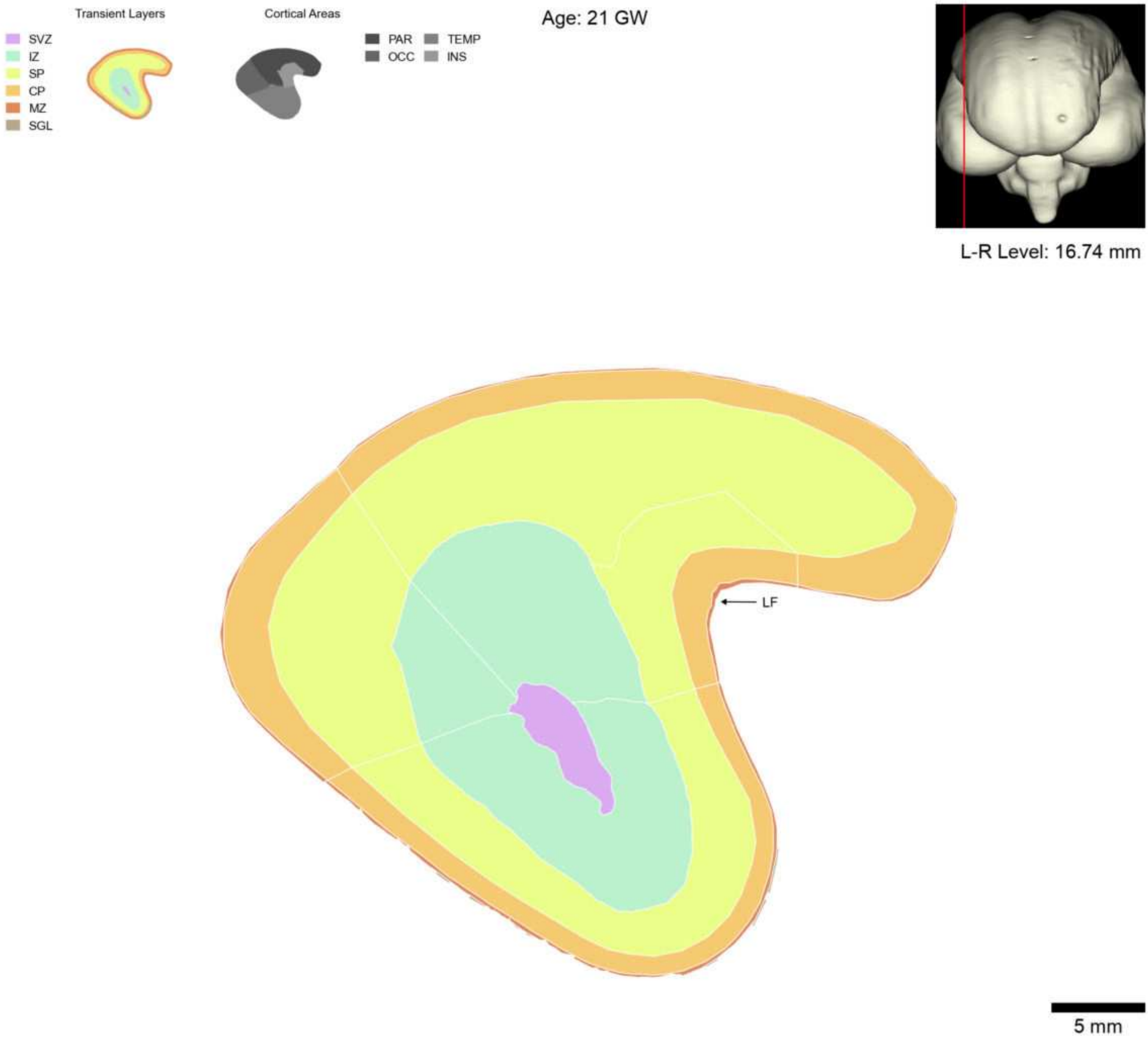

→ LF: Lateral fissure

Age: 21 GW

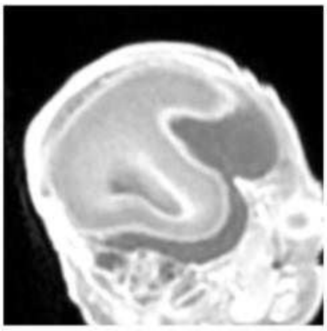

L-R Level: 14.46 mm

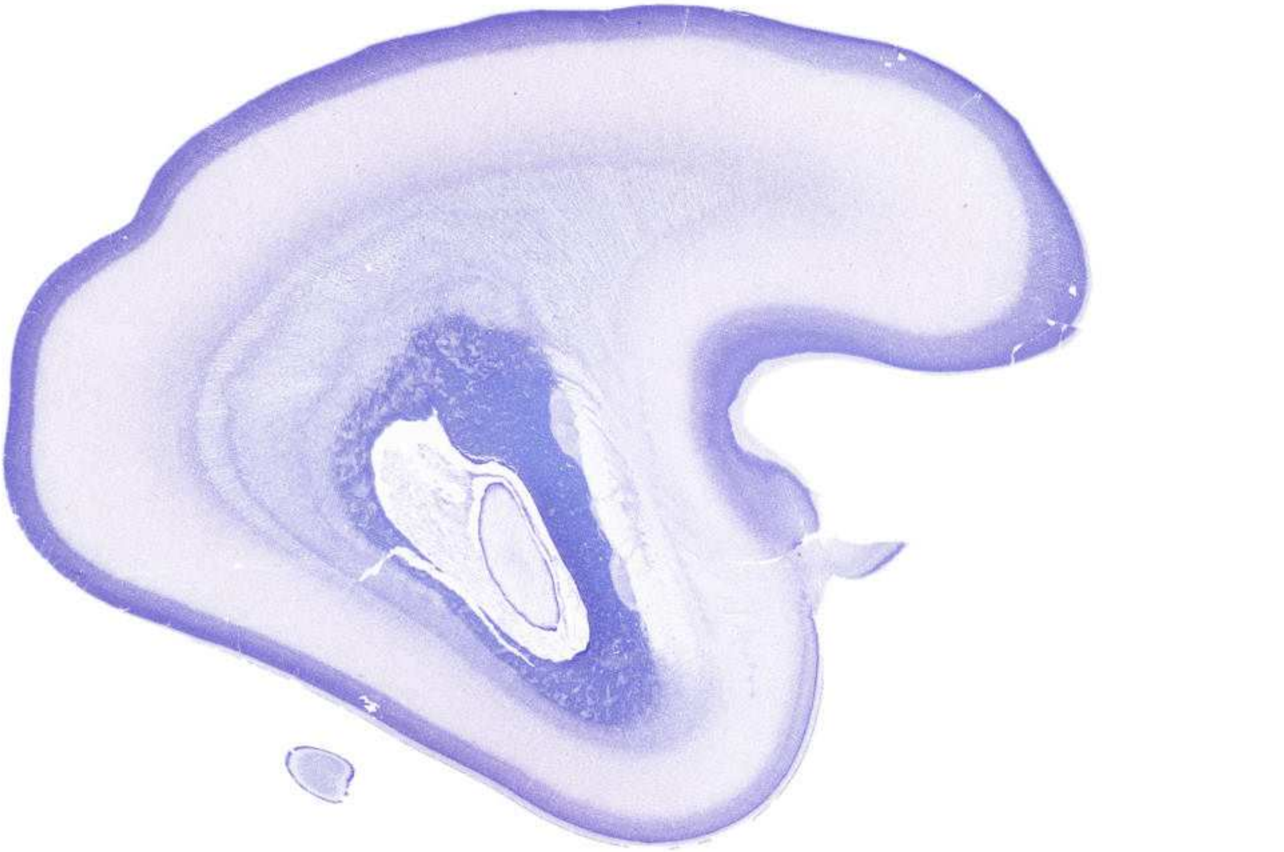

5 mm

Transient Layers

- VZ
- SVZ
- IZ
- SP
- CP
- MZ
- SGL

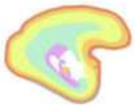

Cortical Areas

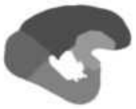

- PAR
- OCC
- TEMP
- INS

Age: 21 GW

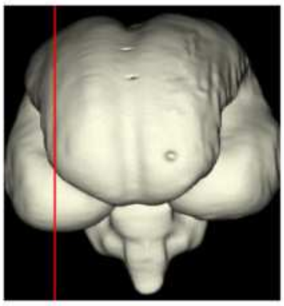

L-R Level: 14.46 mm

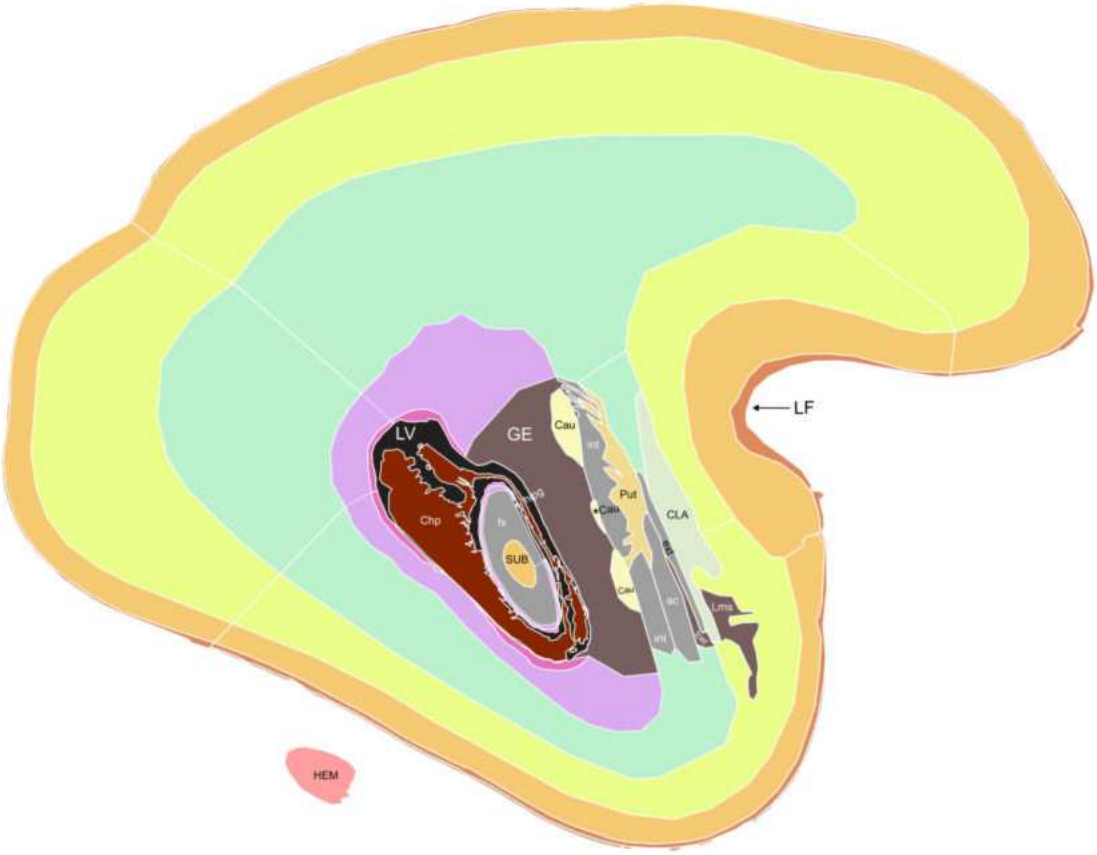

5 mm

- CLA: Claustrum
- Cau: Caudate nucleus
- Chp: Choroid plexus
- GE: Ganglionic eminence
- HEM: Cerebellar hemispheres
- LV: Lateral ventricle
- Lms: Lateral migratory stream
- Put: Putamen
- SUB: Cortical plate, subiculum
- ac: Anterior commissure
- ext: External capsule
- fx: Fornix
- hipg: Hippocampal glioepithelium/ependyma
- int: Internal capsule
- tcet: Transient cell zone in the external capsule
- → LF: Lateral fissure

Age: 21 GW

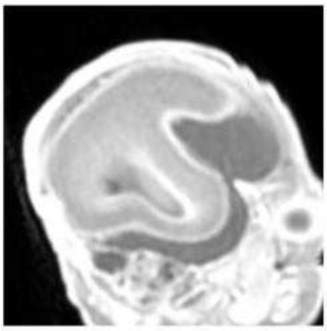

L-R Level: 14.28 mm

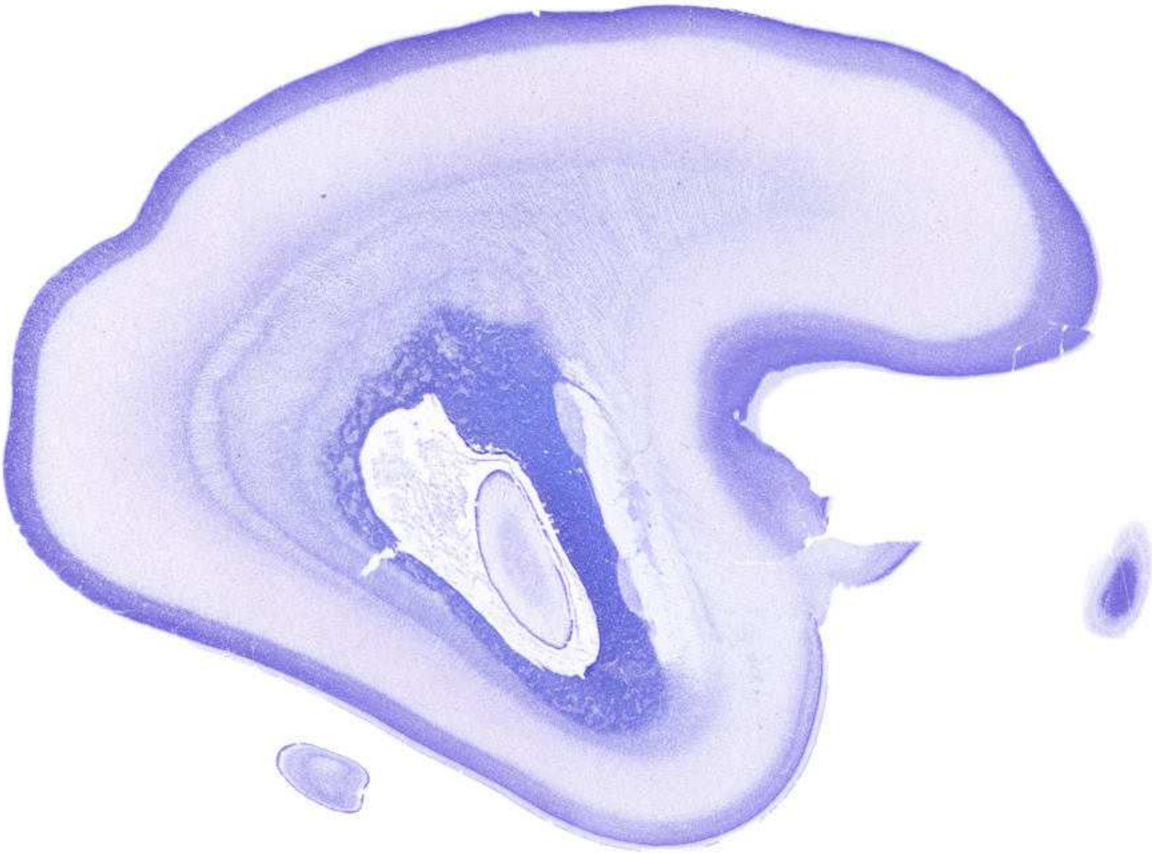

5 mm

Transient Layers

- VZ
- SVZ
- IZ
- SP
- CP
- MZ
- SGL

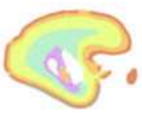

Cortical Areas

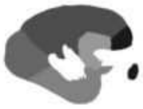

- FCTx
- PAR
- OCC
- TEMP
- INS

Age: 21 GW

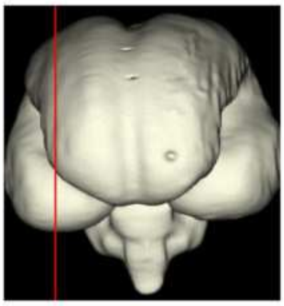

L-R Level: 14.28 mm

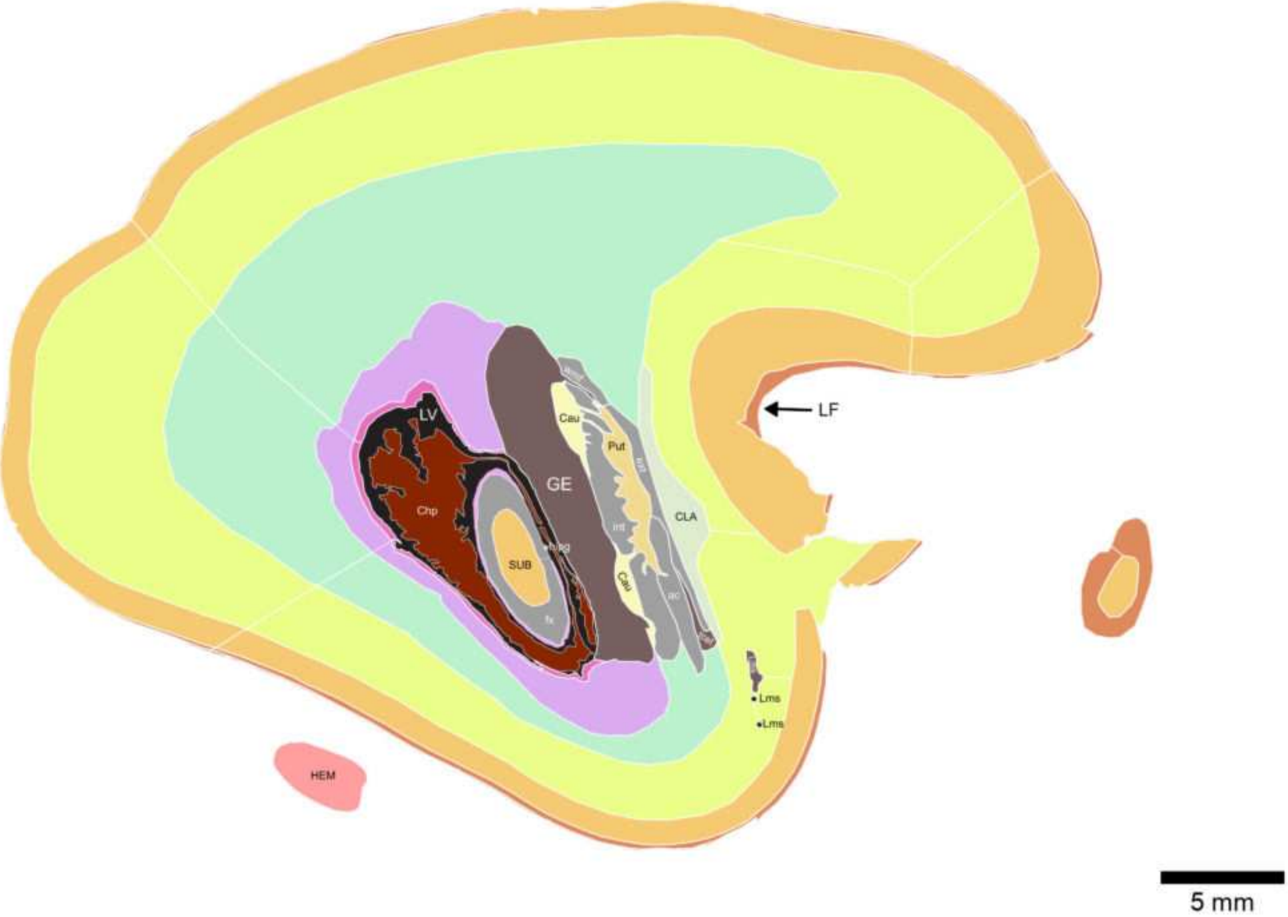

- CLA: Claustrum
- Cau: Caudate nucleus
- Chp: Choroid plexus
- GE: Ganglionic eminence
- HEM: Cerebellar hemispheres
- LV: Lateral ventricle
- Lms: Lateral migratory stream
- Put: Putamen
- SUB: Cortical plate, subiculum
- ac: Anterior commissure
- ext: External capsule
- fx: Fornix
- hipg: Hippocampal glioepithelium/ependyma
- int: Internal capsule
- tcet: Transient cell zone in the external capsule
- wmf: White matter fibers
- → LF: Lateral fissure

Age: 21 GW

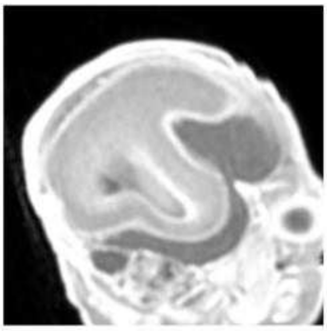

L-R Level: 13.98 mm

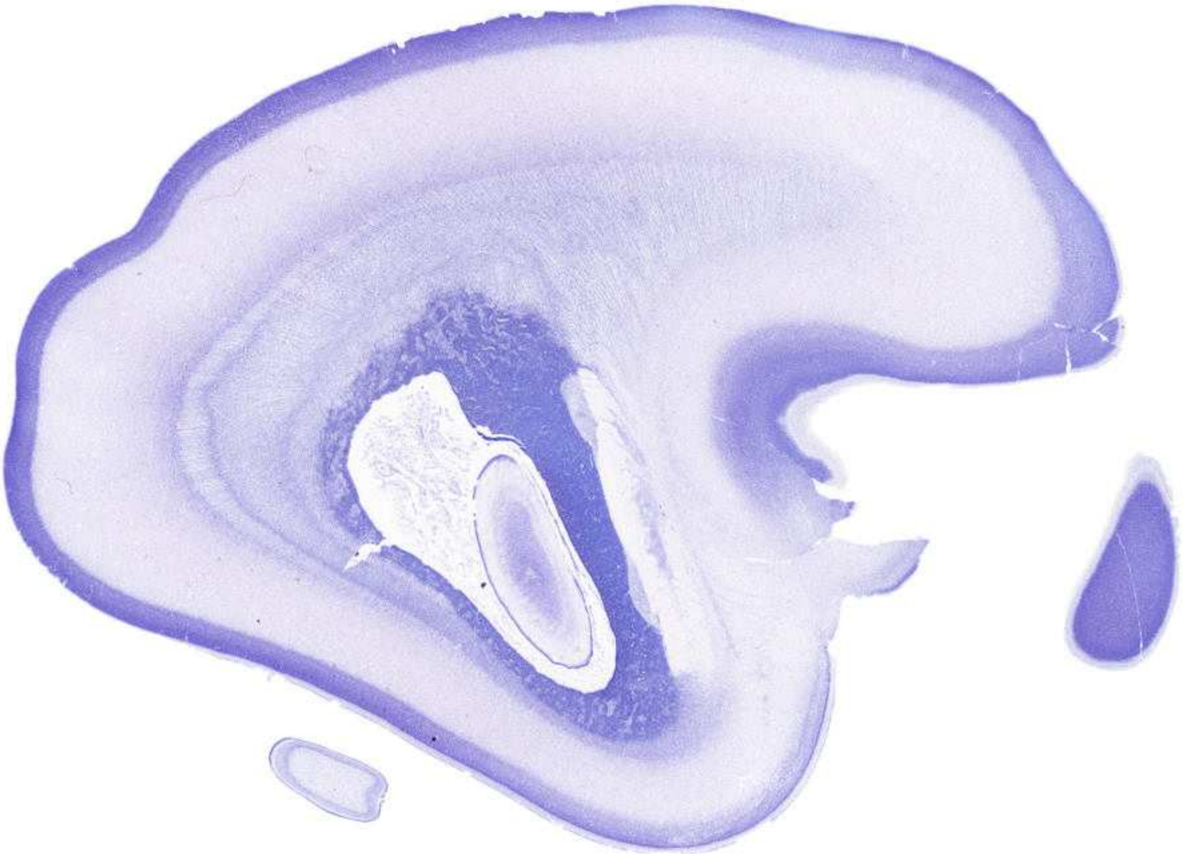

5 mm

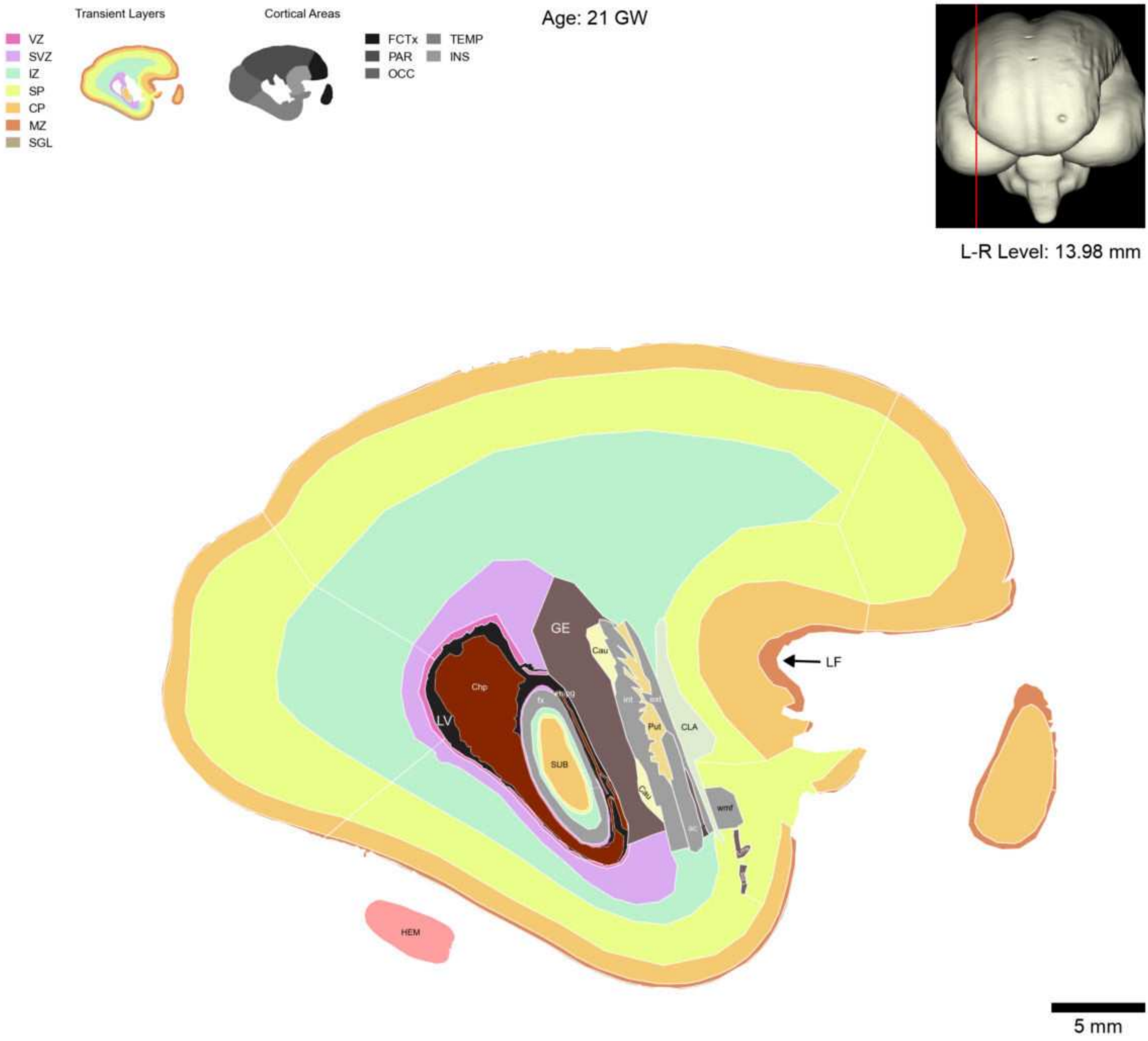

- CLA: Claustrum
- Cau: Caudate nucleus
- Chp: Choroid plexus
- GE: Ganglionic eminence
- HEM: Cerebellar hemispheres
- LV: Lateral ventricle
- Lms: Lateral migratory stream
- Put: Putamen
- SUB: Cortical plate, subiculum
- ac: Anterior commissure
- ext: External capsule
- fx: Fornix
- hipg: Hippocampal glioepithelium/ependyma
- int: Internal capsule
- tcet: Transient cell zone in the external capsule
- wmf: White matter fibers
- → LF: Lateral fissure

Age: 21 GW

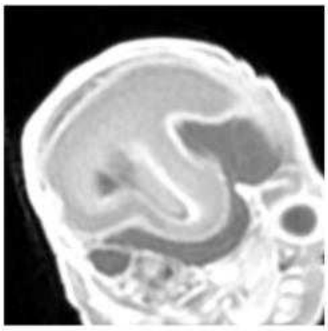

L-R Level: 13.68 mm

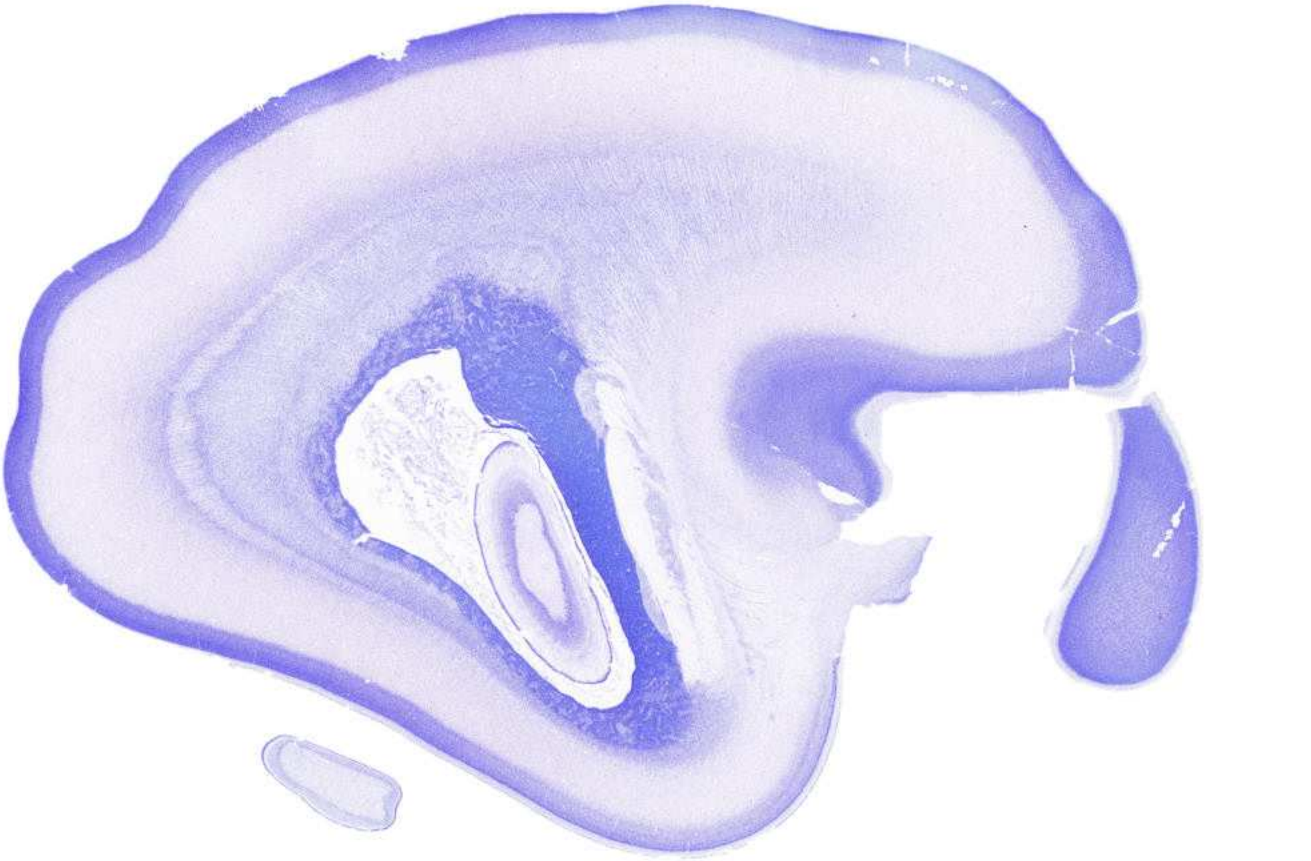

5 mm

Transient Layers

- VZ
- SVZ
- IZ
- SP
- CP
- MZ
- SGL

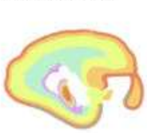

Cortical Areas

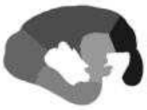

- FCTx
- PAR
- OCC
- TEMP
- INS

Age: 21 GW

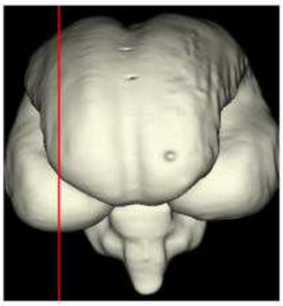

L-R Level: 13.68 mm

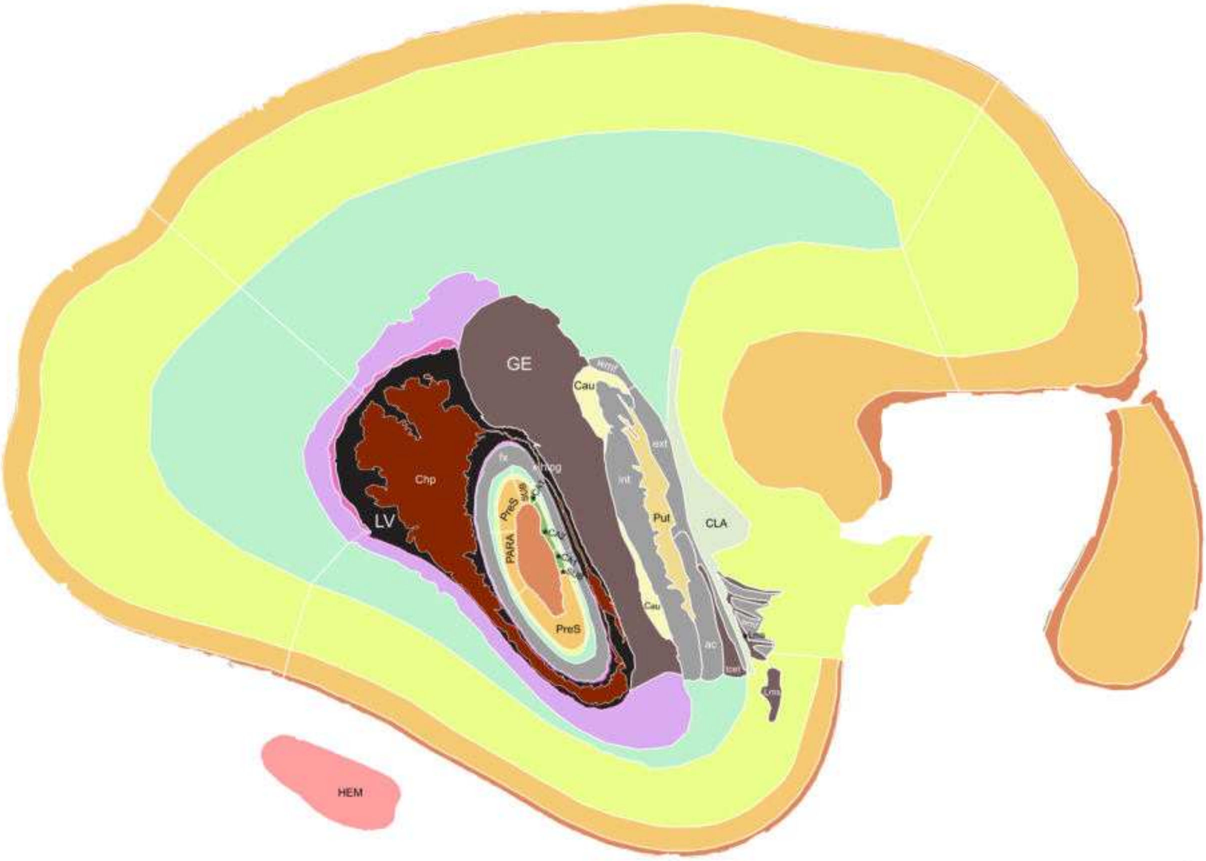

5 mm

- CA1: CA1 field [hippocampus]
- CA2: CA2 field [hippocampus]
- CLA: Claustrum
- Cau: Caudate nucleus
- Chp: Choroid plexus
- GE: Ganglionic eminence
- HEM: Cerebellar hemispheres
- LV: Lateral ventricle
- Lms: Lateral migratory stream
- PARA: Cortical plate, parasubiculum
- PreS: Cortical plate, presubiculum
- Put: Putamen
- SUB: Cortical plate, subiculum
- ac: Anterior commissure
- ext: External capsule
- fx: Fornix
- hipg: Hippocampal glioepithelium/ependyma
- int: Internal capsule
- tcet: Transient cell zone in the external capsule
- wmf: White matter fibers

Age: 21 GW

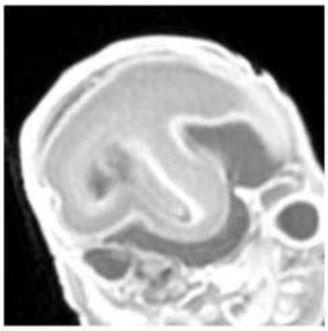

L-R Level: 13.32 mm

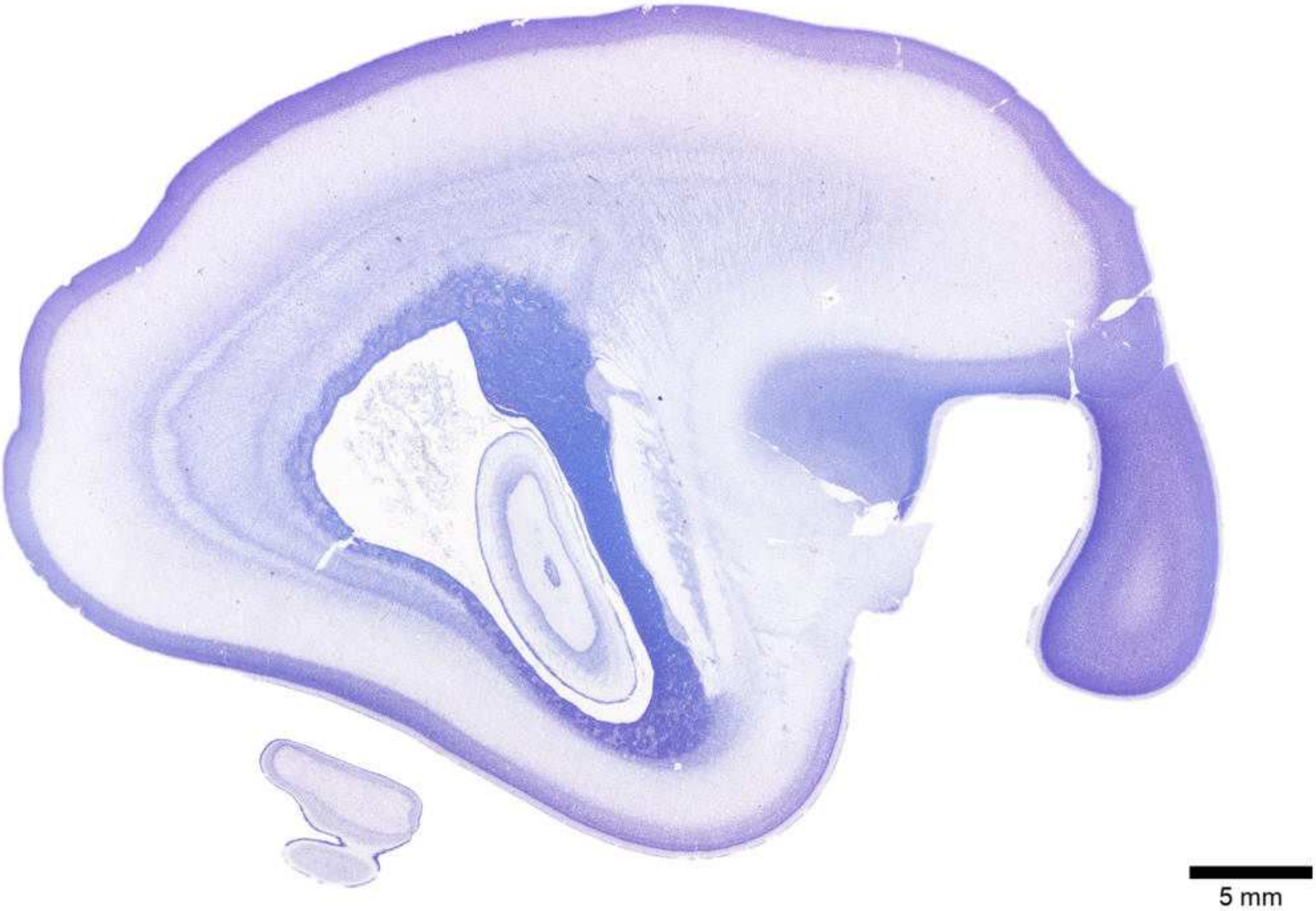

5 mm

Transient Layers

- VZ
- SVZ
- IZ
- SP
- CP
- MZ
- SGL

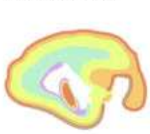

Cortical Areas

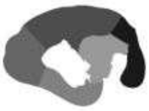

- FCTx
- PAR
- OCC
- TEMP
- INS

Age: 21 GW

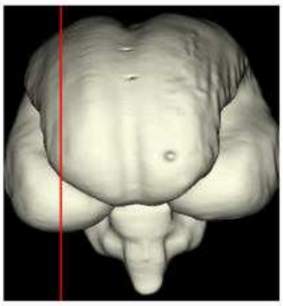

L-R Level: 13.32 mm

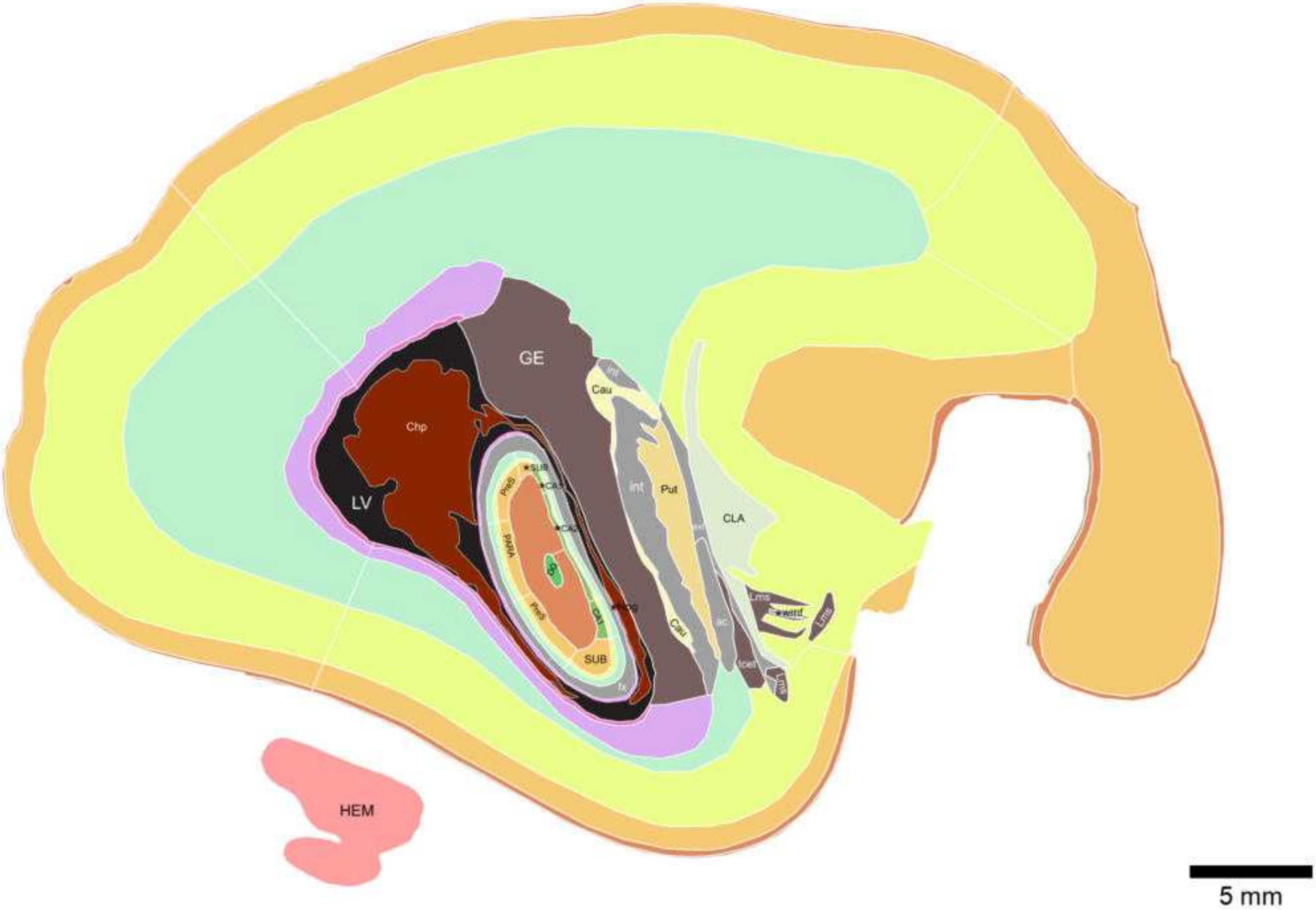

- CA1: CA1 field [hippocampus]
- CA2: CA2 field [hippocampus]
- CLA: Claustrum
- Cau: Caudate nucleus
- Chp: Choroid plexus
- DG: Dentate gyrus
- GE: Ganglionic eminence
- HEM: Cerebellar hemispheres
- LV: Lateral ventricle
- Lms: Lateral migratory stream
- PARA: Cortical plate, parasubiculum
- PreS: Cortical plate, presubiculum
- Put: Putamen
- SUB: Cortical plate, subiculum
- ac: Anterior commissure
- ext: External capsule
- fx: Fornix
- hipg: Hippocampal glioepithelium/ependyma
- int: Internal capsule
- tcet: Transient cell zone in the external capsule
- wmf: White matter fibers

Age: 21 GW

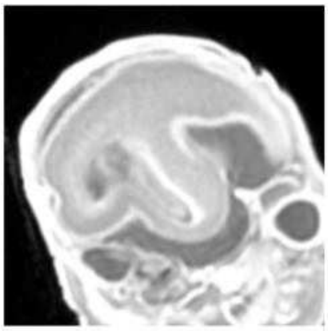

L-R Level: 12.96 mm

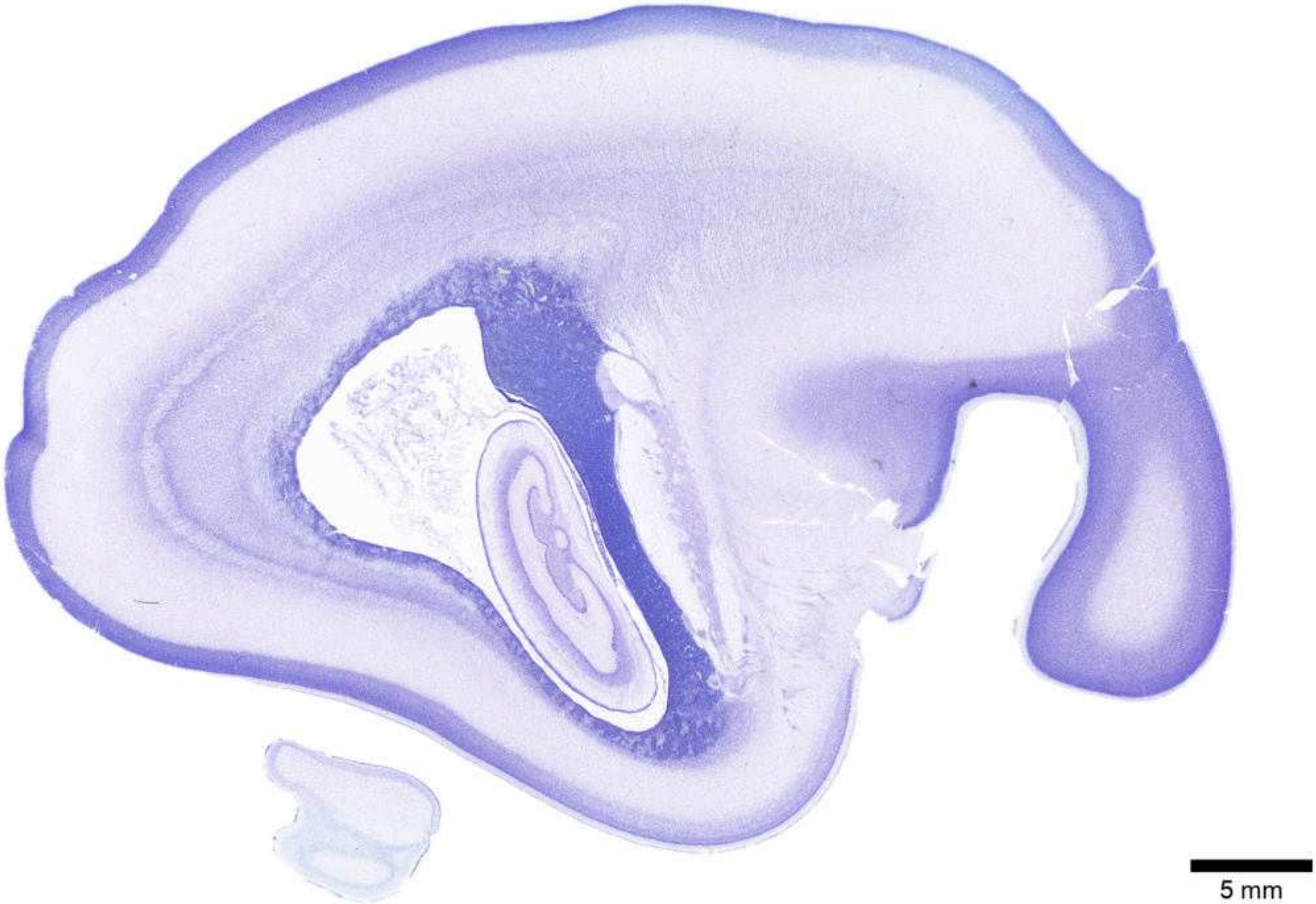

5 mm

Transient Layers

- VZ
- SVZ
- IZ
- SP
- CP
- MZ
- SGL

Cortical Areas

- FCTx
- PAR
- OCC
- TEMP
- INS

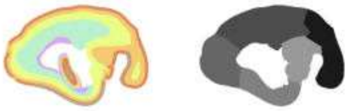

Age: 21 GW

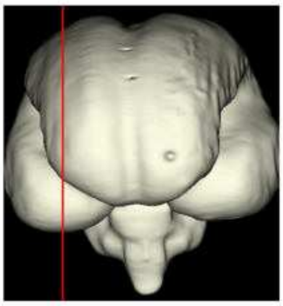

L-R Level: 12.96 mm

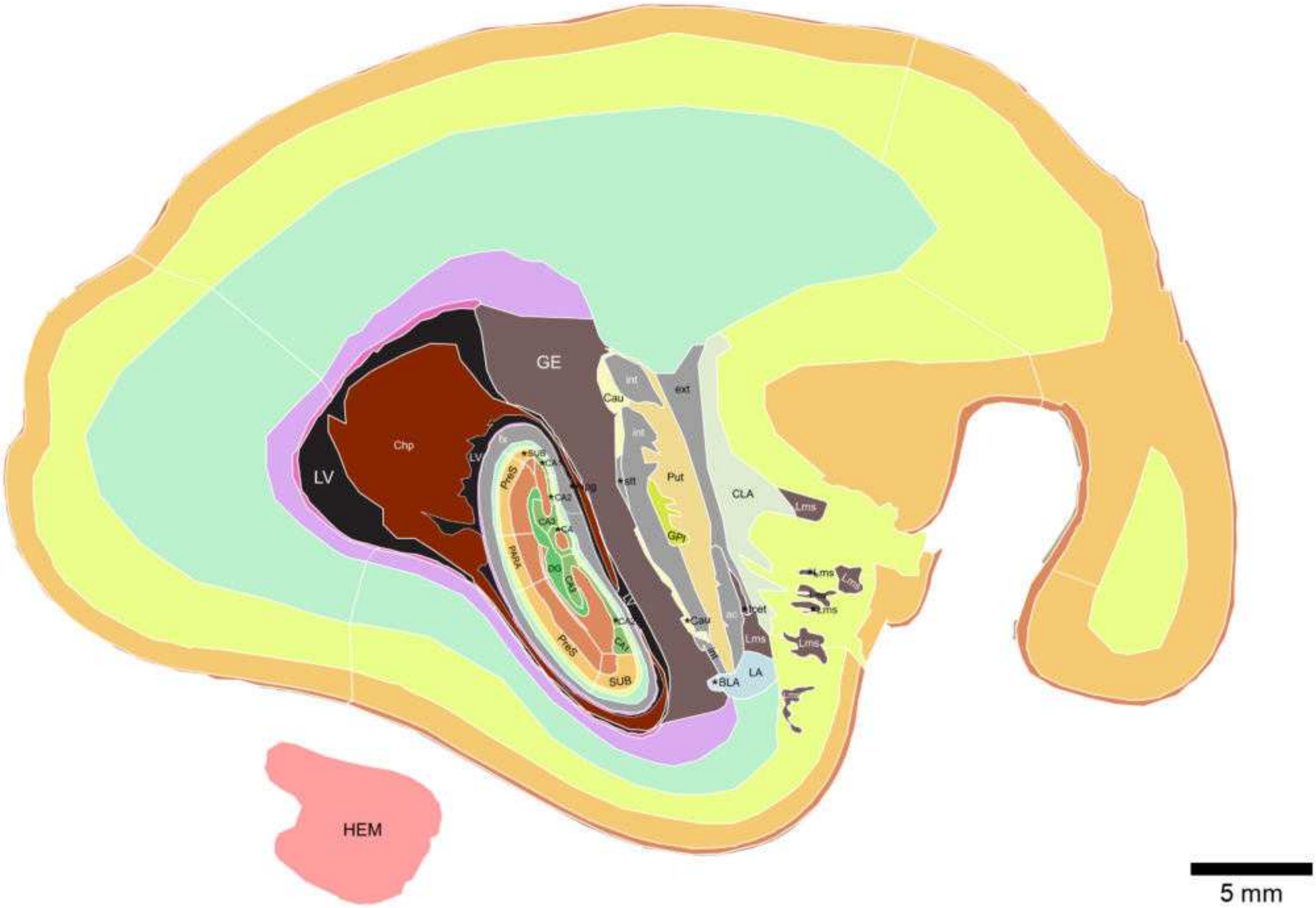

- BL: Basal nucleus [amygdala]
- CA: Ammon's horn
- CA1: CA1 field [hippocampus]
- CA2: CA2 field [hippocampus]
- CA3: CA3 field [hippocampus]
- CLA: Claustrum
- Cau: Caudate nucleus
- Chp: Choroid plexus
- DG: Dentate gyrus
- GE: Ganglionic eminence
- GPl: Globus pallidus lateral segment
- HEM: Cerebellar hemispheres
- LA: Lateral nucleus [amygdala]
- LV: Lateral ventricle
- Lms: Lateral migratory stream
- PARA: Cortical plate, parasubiculum
- PreS: Cortical plate, presubiculum
- Put: Putamen
- SUB: Cortical plate, subiculum
- ac: Anterior commissure
- ext: External capsule
- fx: Fornix
- hipg: Hippocampal glioepithelium/ependyma
- int: Internal capsule
- stt: Stria terminalis
- tcet: Transient cell zone in the external capsule

Age: 21 GW

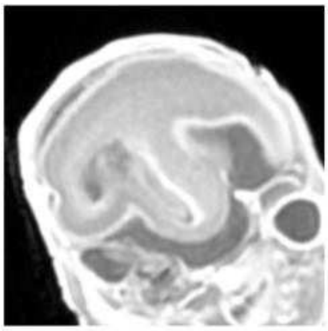

L-R Level: 12.72 mm

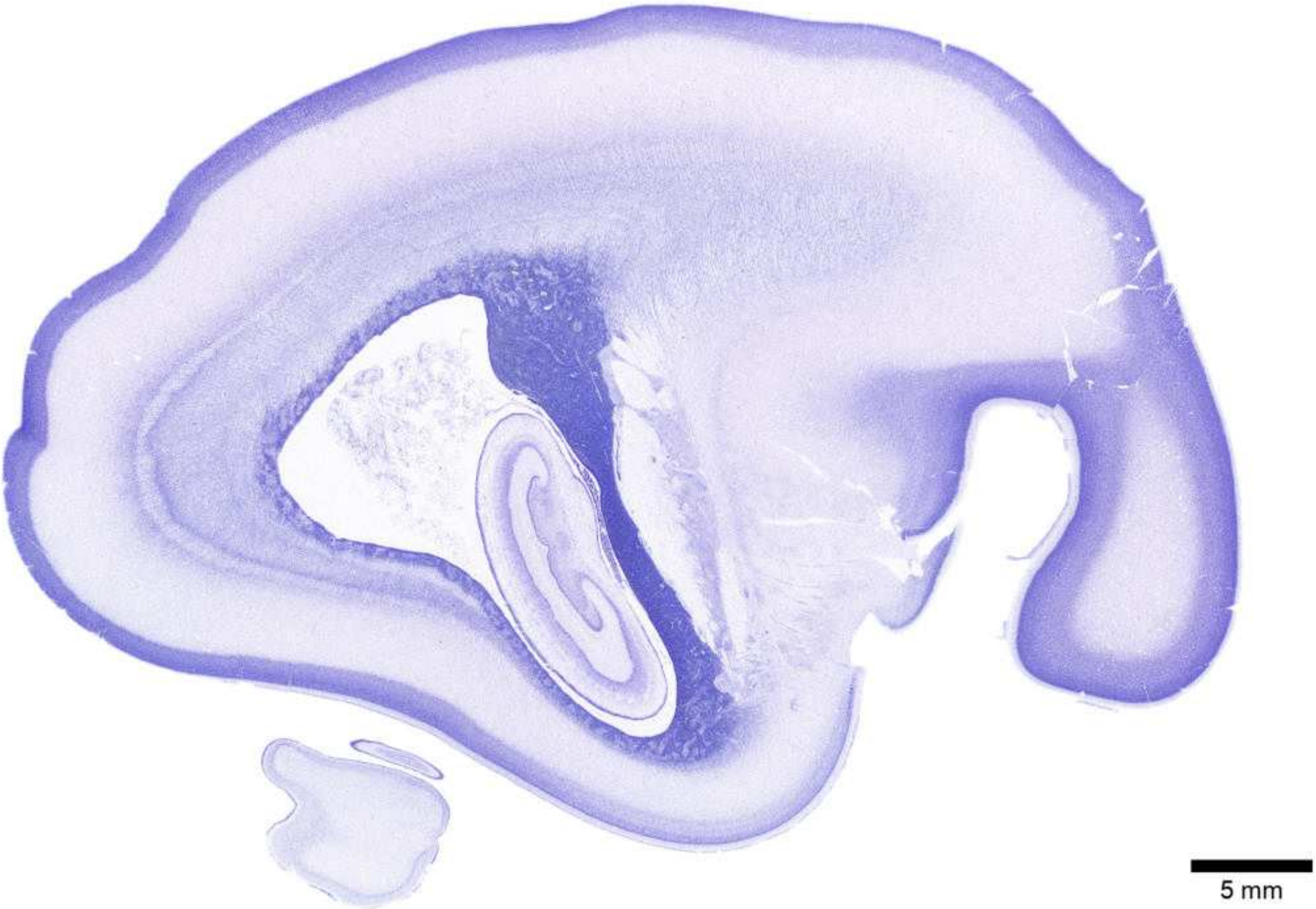

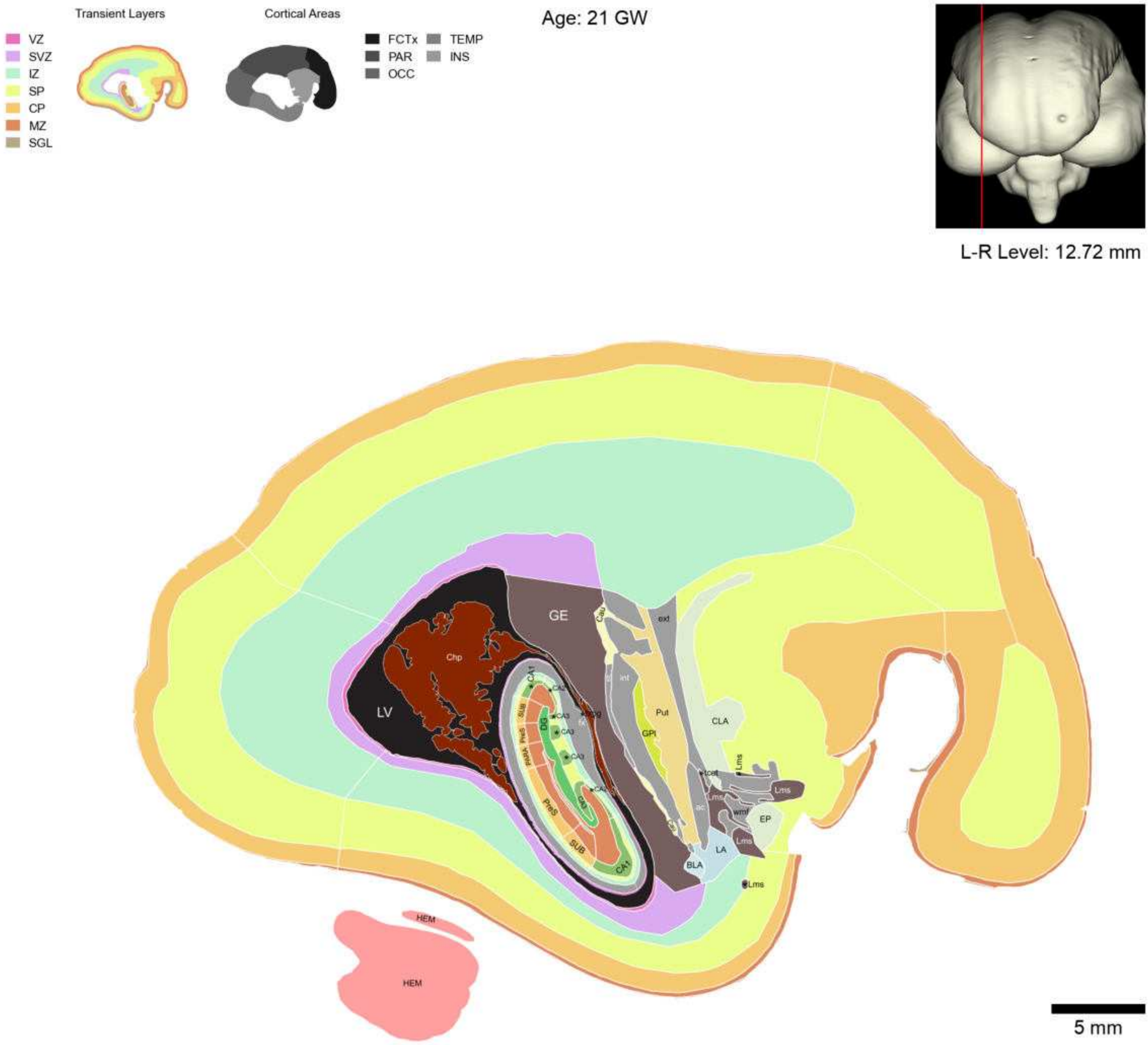

- BL: Basal nucleus [amygdala]
- CA1: CA1 field [hippocampus]
- CA2: CA2 field [hippocampus]
- CA3: CA3 field [hippocampus]
- CLA: Claustrum
- Cau: Caudate nucleus
- Chp: Choroid plexus
- DG: Dentate gyrus
- EP: Endopiriform nucleus
- GE: Ganglionic eminence
- GPl: Globus pallidus lateral segment
- HEM: Cerebellar hemispheres
- LA: Lateral nucleus [amygdala]
- LV: Lateral ventricle
- Lms: Lateral migratory stream
- PARA: Cortical plate, parasubiculum
- PreS: Cortical plate, presubiculum
- Put: Putamen
- SUB: Cortical plate, subiculum
- ac: Anterior commissure
- ext: External capsule
- fx: Fornix
- hipg: Hippocampal glioepithelium/ependyma
- int: Internal capsule
- stt: Stria terminalis
- tcet: Transient cell zone in the external capsule
- wmf: White matter fibers

Age: 21 GW

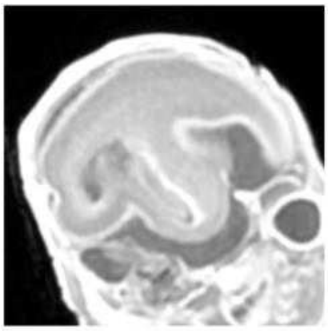

L-R Level: 12.54 mm

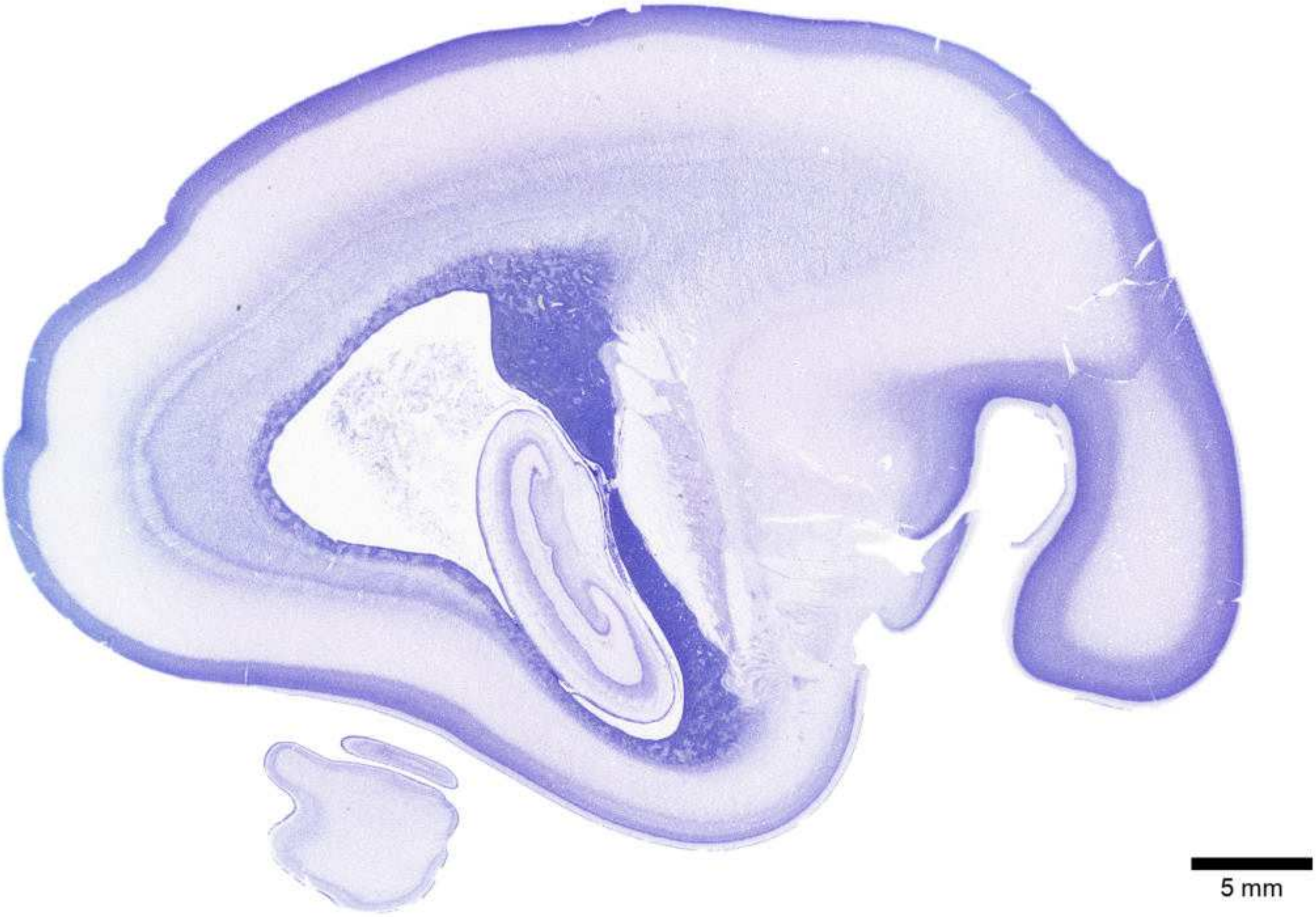

Transient Layers

- VZ
- SVZ
- IZ
- SP
- CP
- MZ
- SGL

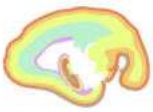

Cortical Areas

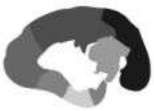

- FCTx
- PAR
- OCC
- TEMP
- INS
- ENT

Age: 21 GW

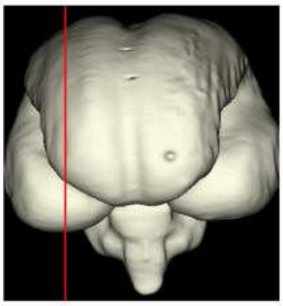

L-R Level: 12.54 mm

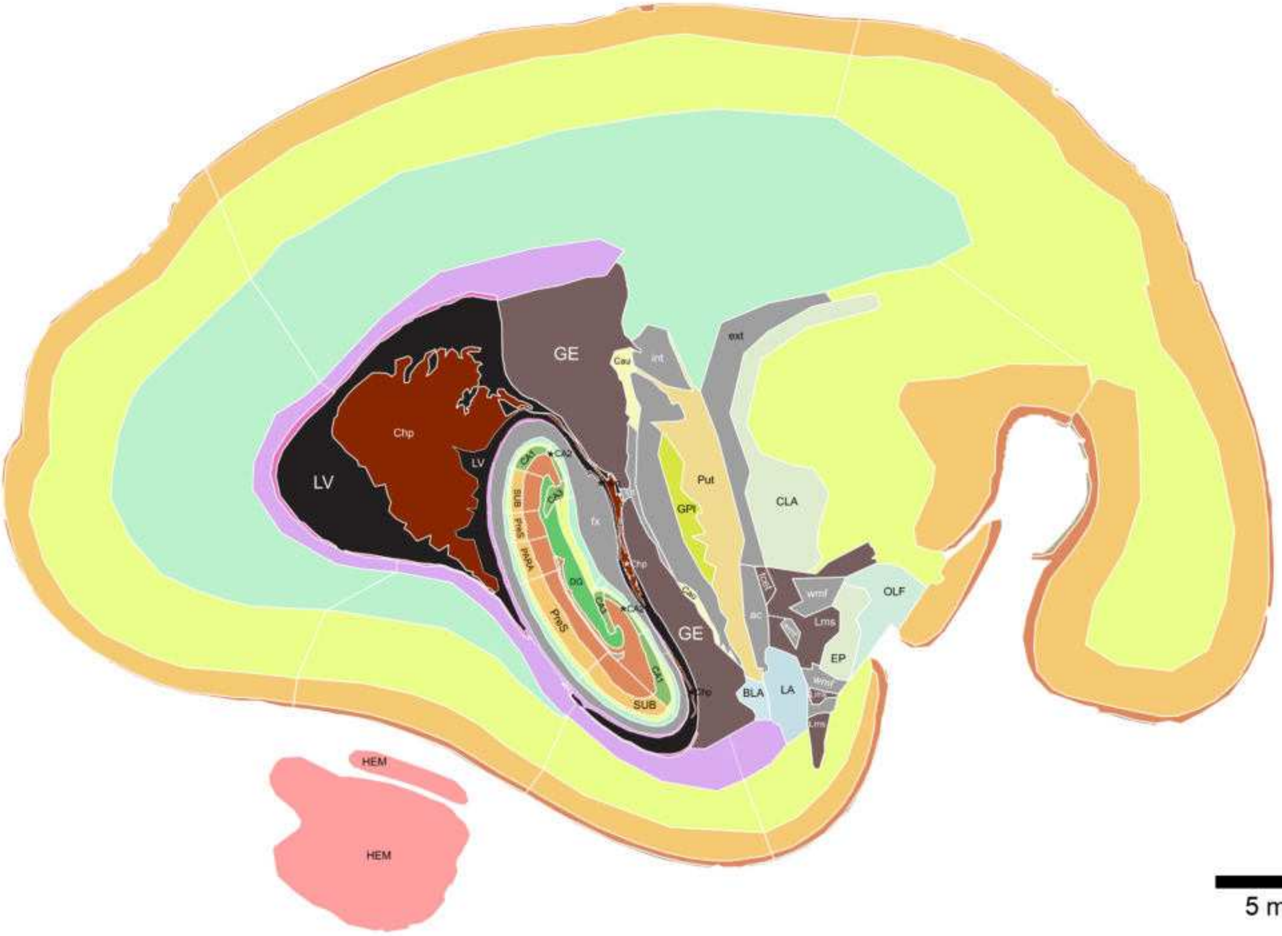

5 mm

- BL: Basal nucleus [amygdala]
- CA1: CA1 field [hippocampus]
- CA2: CA2 field [hippocampus]
- CA3: CA3 field [hippocampus]
- CLA: Claustrum
- Cau: Caudate nucleus
- Chp: Choroid plexus
- DG: Dentate gyrus
- EP: Endopiriform nucleus
- GE: Ganglionic eminence
- GPl: Globus pallidus lateral segment
- HEM: Cerebellar hemispheres
- LA: Lateral nucleus [amygdala]
- LV: Lateral ventricle
- Lms: Lateral migratory stream
- PARA: Cortical plate, parasubiculum
- PreS: Cortical plate, presubiculum
- Put: Putamen
- SUB: Cortical plate, subiculum
- ac: Anterior commissure
- ext: External capsule
- fx: Fornix
- hipg: Hippocampal glioepithelium/ependyma
- int: Internal capsule
- stt: Stria terminalis
- tcet: Transient cell zone in the external capsule
- wmf: White matter fibers

Age: 21 GW

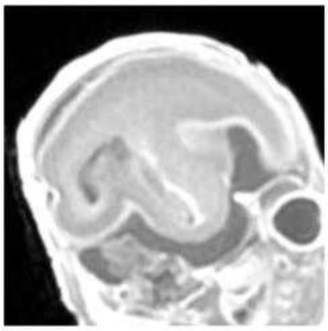

L-R Level: 12.36 mm

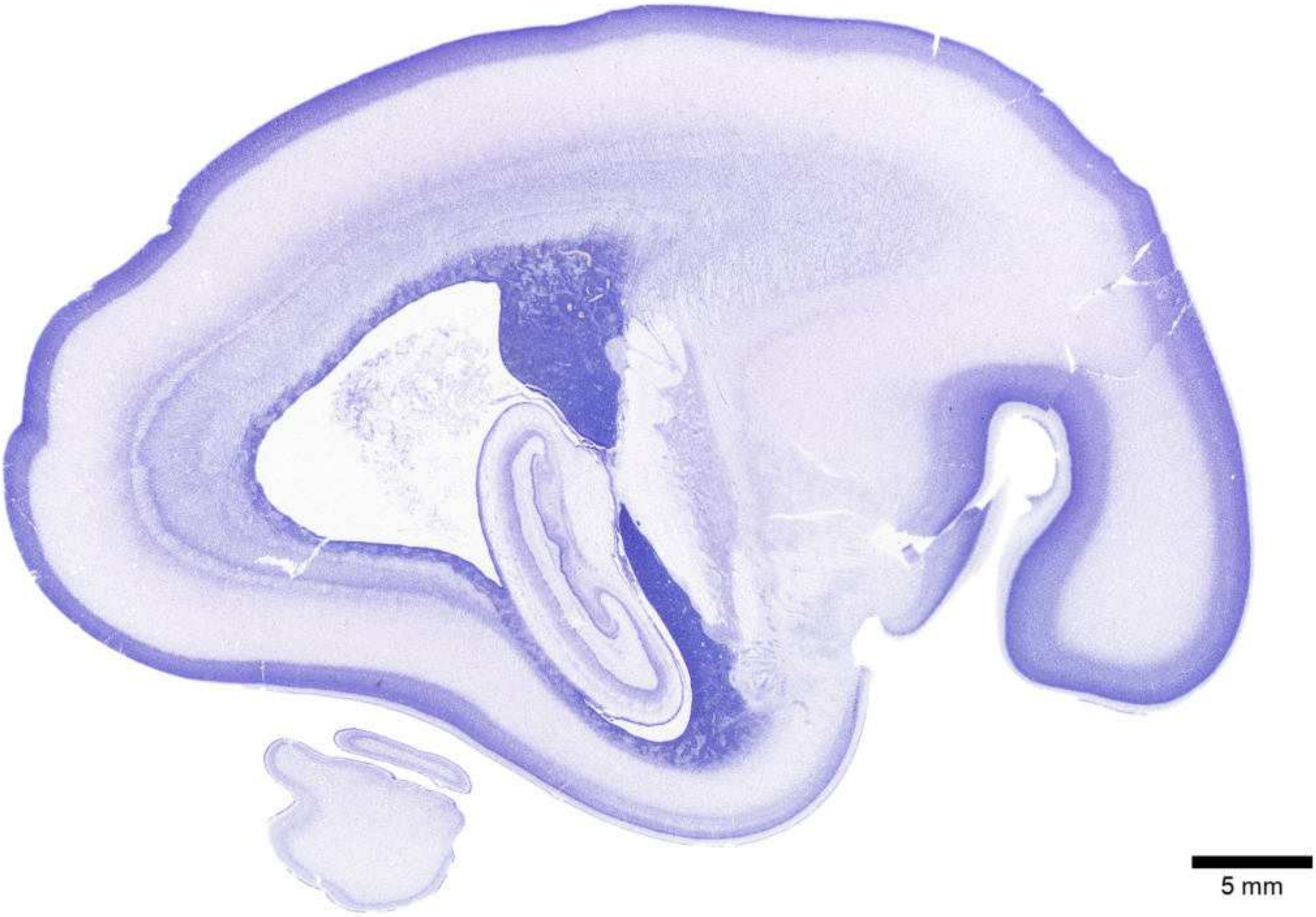

Transient Layers

VZ
SVZ
IZ
SP
CP
MZ
SGL

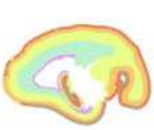

Cortical Areas

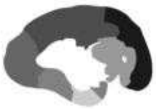

FCTx
PAR
OCC
TEMP
INS
ENT

Age: 21 GW

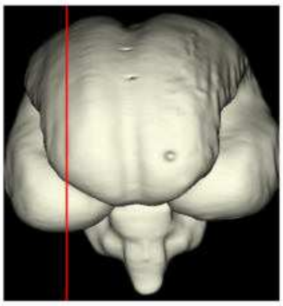

L-R Level: 12.36 mm

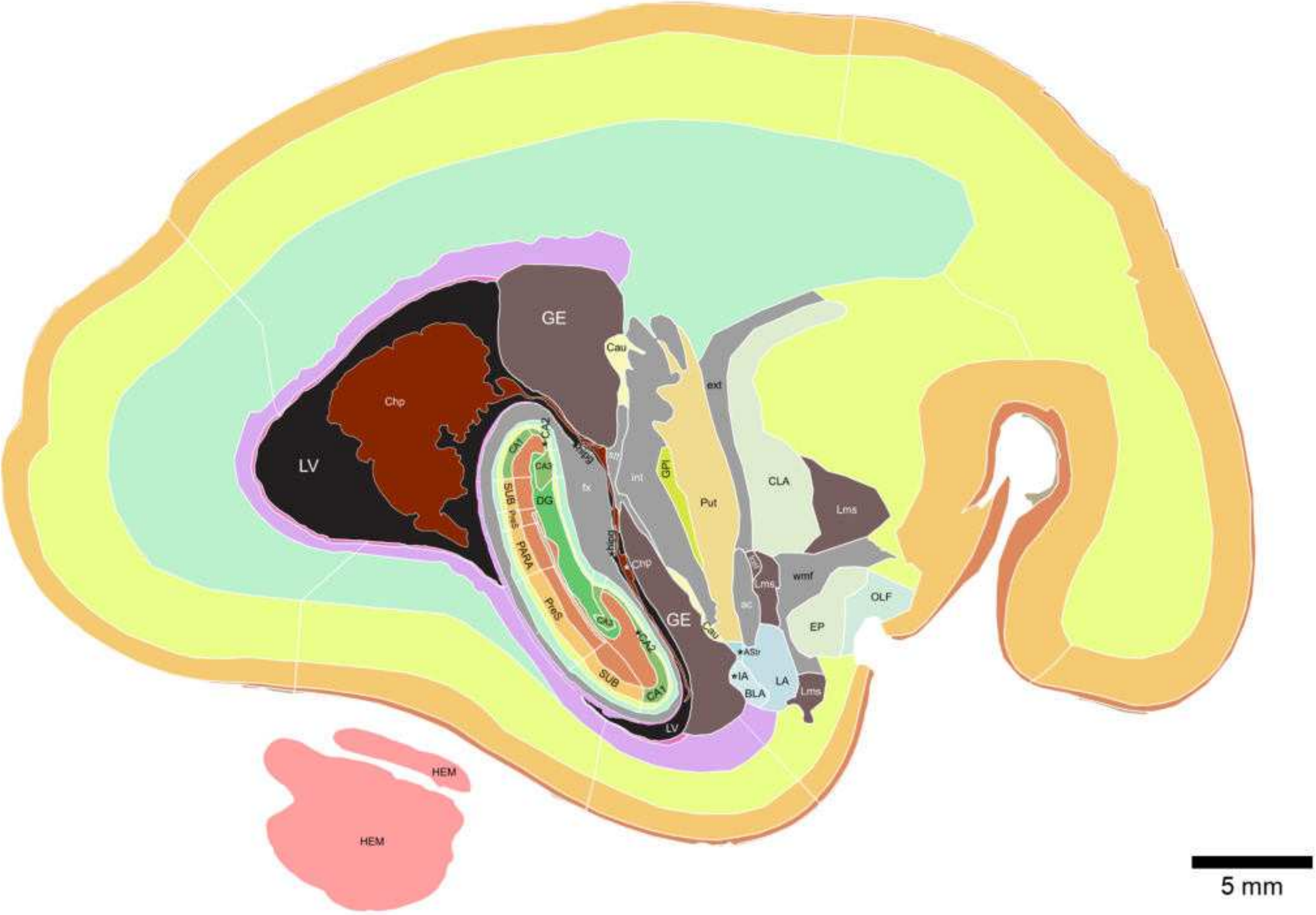

AStr: Amygdalo-striatal area
BL: Basal nucleus [amygdala]
CA1: CA1 field [hippocampus]
CA2: CA2 field [hippocampus]
CA3: CA3 field [hippocampus]
CLA: Claustrum
Cau: Caudate nucleus
Chp: Choroid plexus

DG: Dentate gyrus
EP: Endopiriform nucleus
GE: Ganglionic eminence
GPl: Globus pallidus lateral segment
HEM: Cerebellar hemispheres
IA: Intercalated cell groups [amygdala]
LA: Lateral nucleus [amygdala]

LV: Lateral ventricle
Lms: Lateral migratory stream
PARA: Cortical plate, parasubiculum
PreS: Cortical plate, presubiculum
Put: Putamen
SUB: Cortical plate, subiculum
ac: Anterior commissure

ext: External capsule
fx: Fornix
hipg: Hippocampal glioepithelium/ependyma
int: Internal capsule
stt: Stria terminalis
tcet: Transient cell zone in the external capsule
wmf: White matter fibers

Age: 21 GW

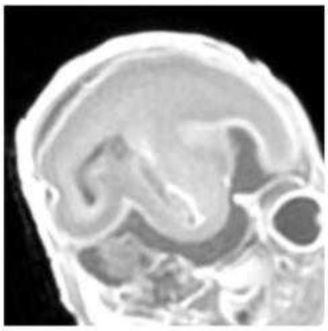

L-R Level: 11.76 mm

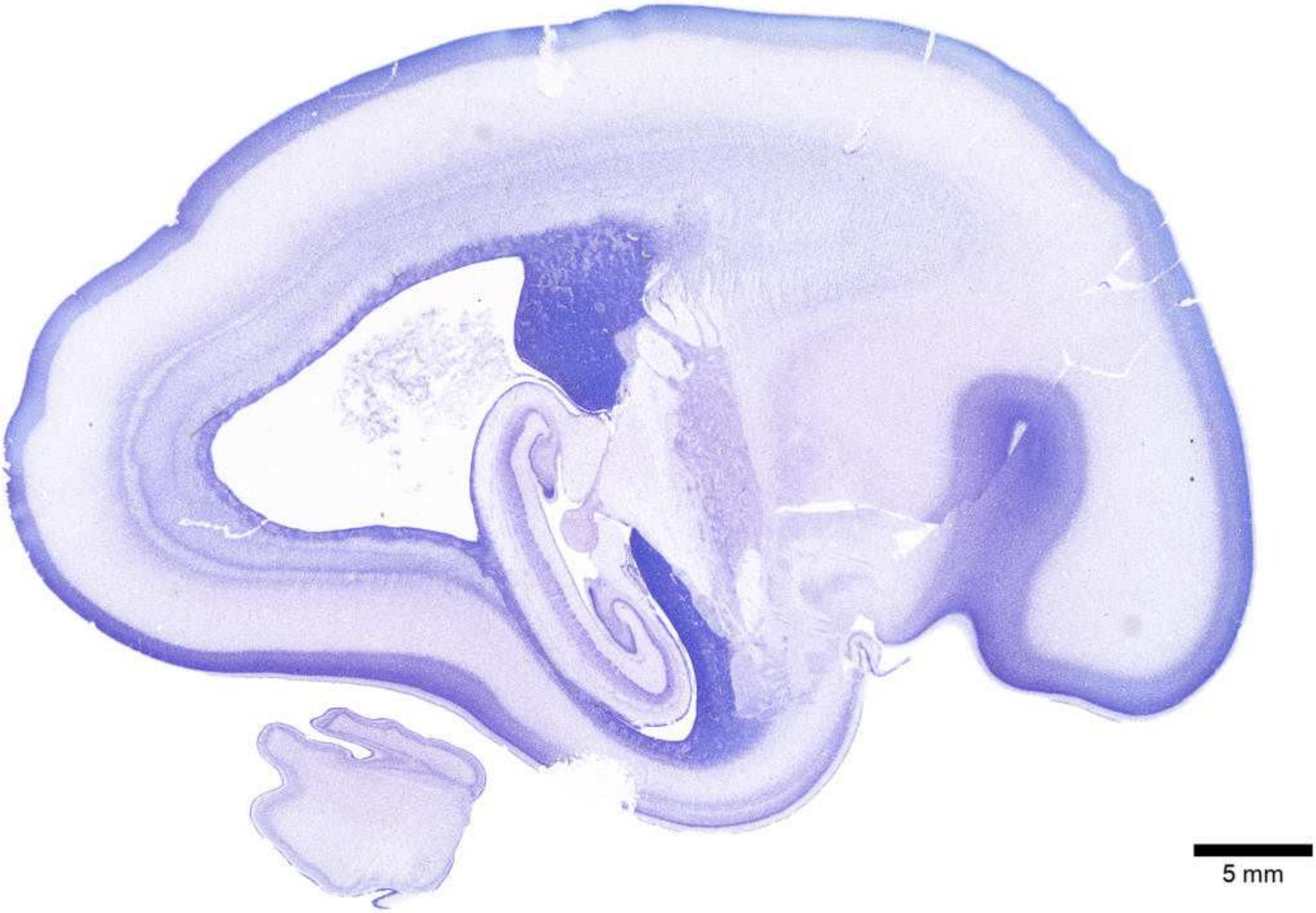

Transient Layers

- VZ
- SVZ
- IZ
- SP
- CP
- MZ
- SGL

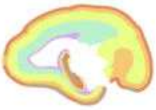

Cortical Areas

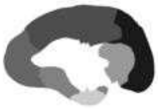

- FCTx
- PAR
- OCC
- TEMP
- INS
- ENT

Age: 21 GW

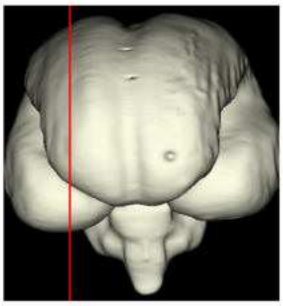

L-R Level: 11.76 mm

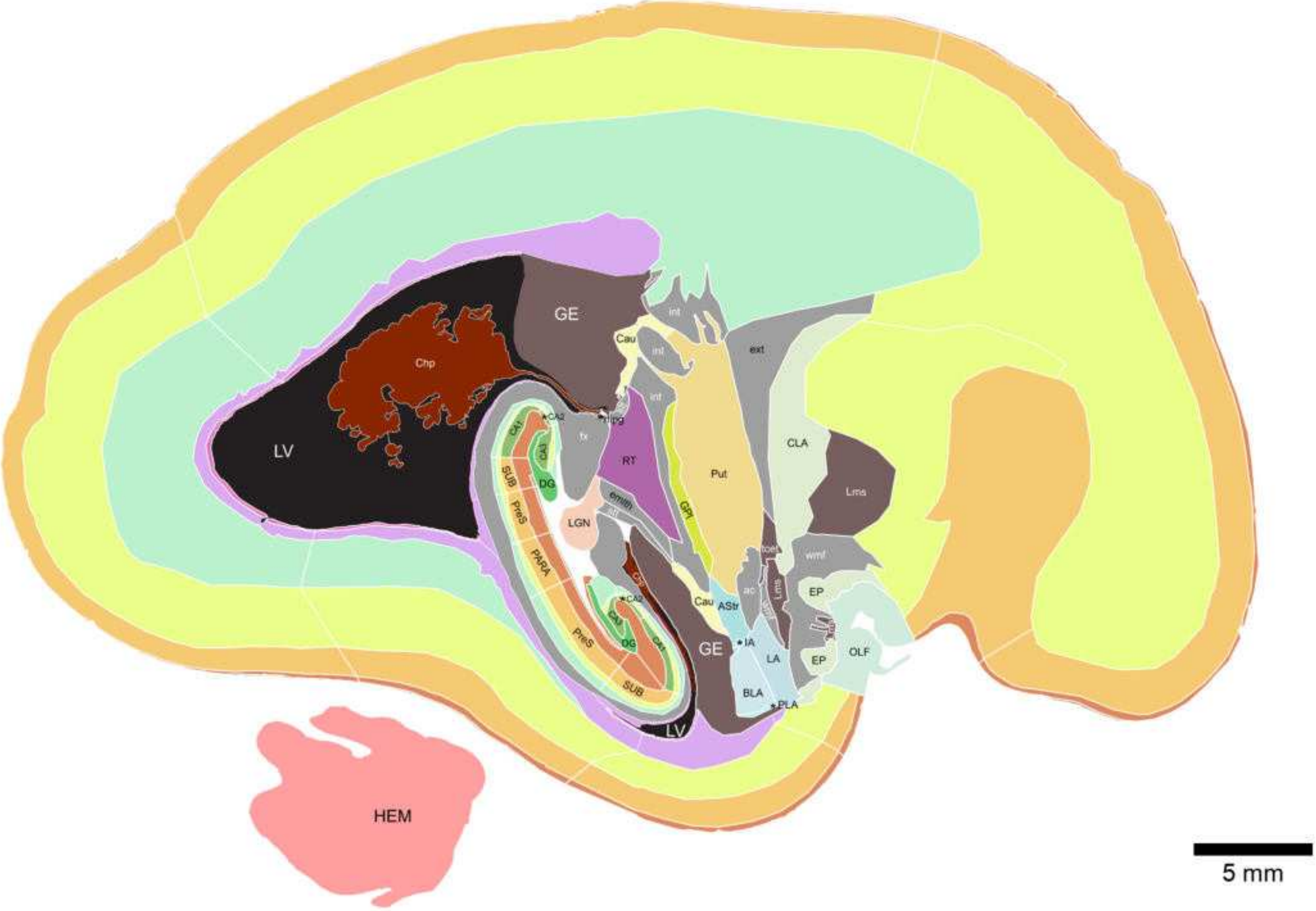

- AStr: Amygdalo-striatal area
- BL: Basal nucleus [amygdala]
- CA1: CA1 field [hippocampus]
- CA2: CA2 field [hippocampus]
- CA3: CA3 field [hippocampus]
- CLA: Claustrum
- Cau: Caudate nucleus
- Chp: Choroid plexus
- DG: Dentate gyrus
- EP: Endopiriform nucleus
- GE: Ganglionic eminence
- GPl: Globus pallidus lateral segment
- HEM: Cerebellar hemispheres
- IA: Intercalated cell groups [amygdala]
- LA: Lateral nucleus [amygdala]
- LGN: Lateral geniculate nucleus
- LV: Lateral ventricle
- Lms: Lateral migratory stream
- PARA: Cortical plate, parasubiculum
- PLA: Paralaminar nucleus [amygdala]
- PreS: Cortical plate, presubiculum
- Put: Putamen
- RT: Reticular nucleus [thalamus]
- SUB: Cortical plate, subiculum
- ac: Anterior commissure
- emlth: External medullary lamina [thalamus]
- ext: External capsule
- fx: Fornix
- hipg: Hippocampal glioepithelium/ependyma
- int: Internal capsule
- stt: Stria terminalis
- tcet: Transient cell zone in the external capsule
- wmf: White matter fibers

Age: 21 GW

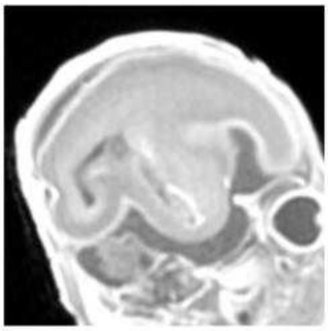

L-R Level: 11.28 mm

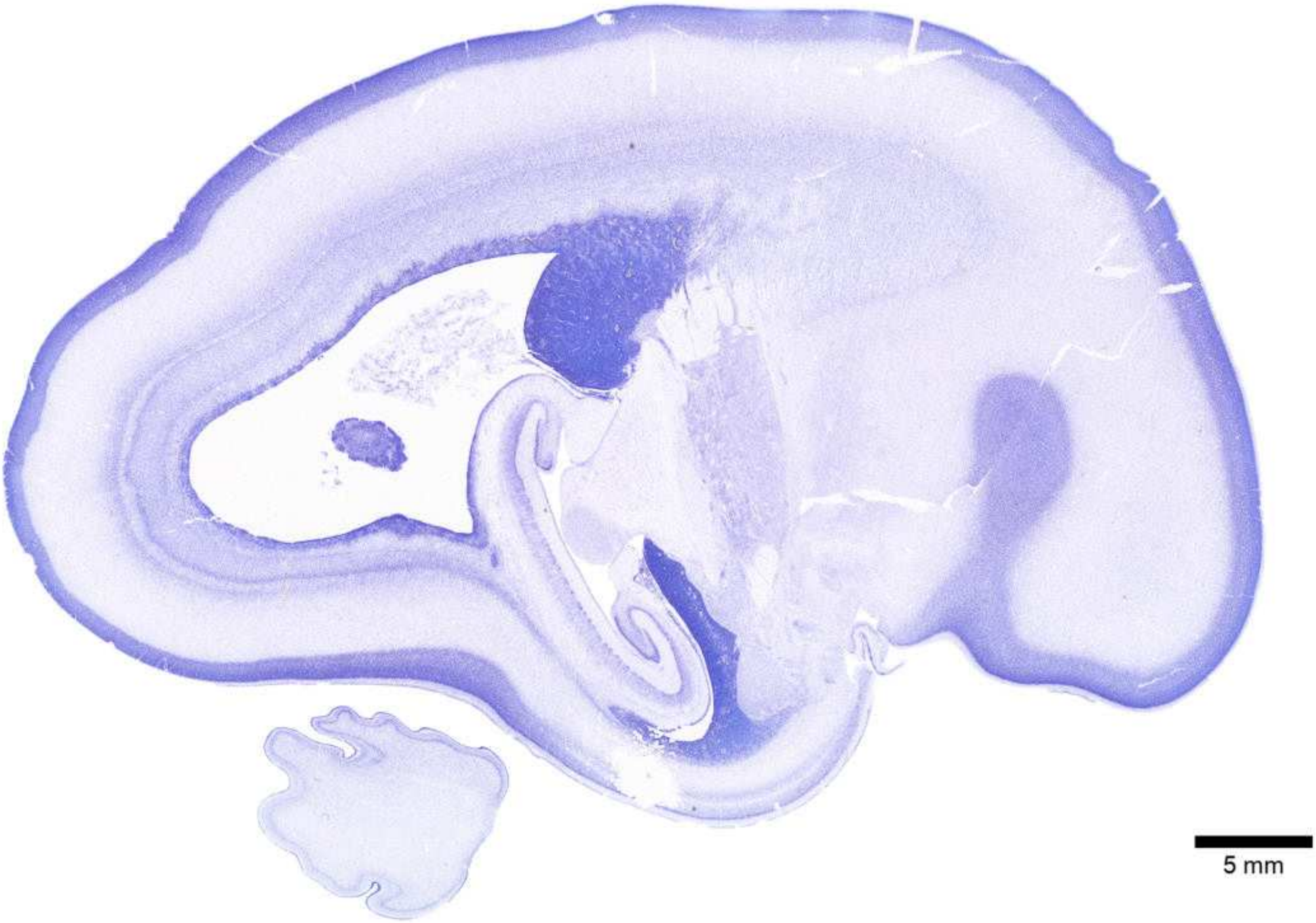

5 mm

Transient Layers

- VZ
- SVZ
- IZ
- SP
- CP
- MZ
- SGL

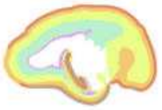

Cortical Areas

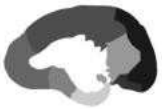

- FCTx
- ORB
- PAR
- OCC
- TEMP
- INS
- ENT

Age: 21 GW

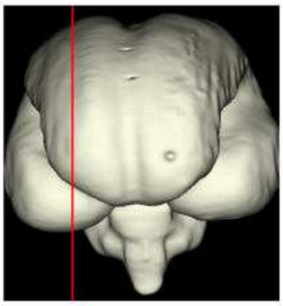

L-R Level: 11.28 mm

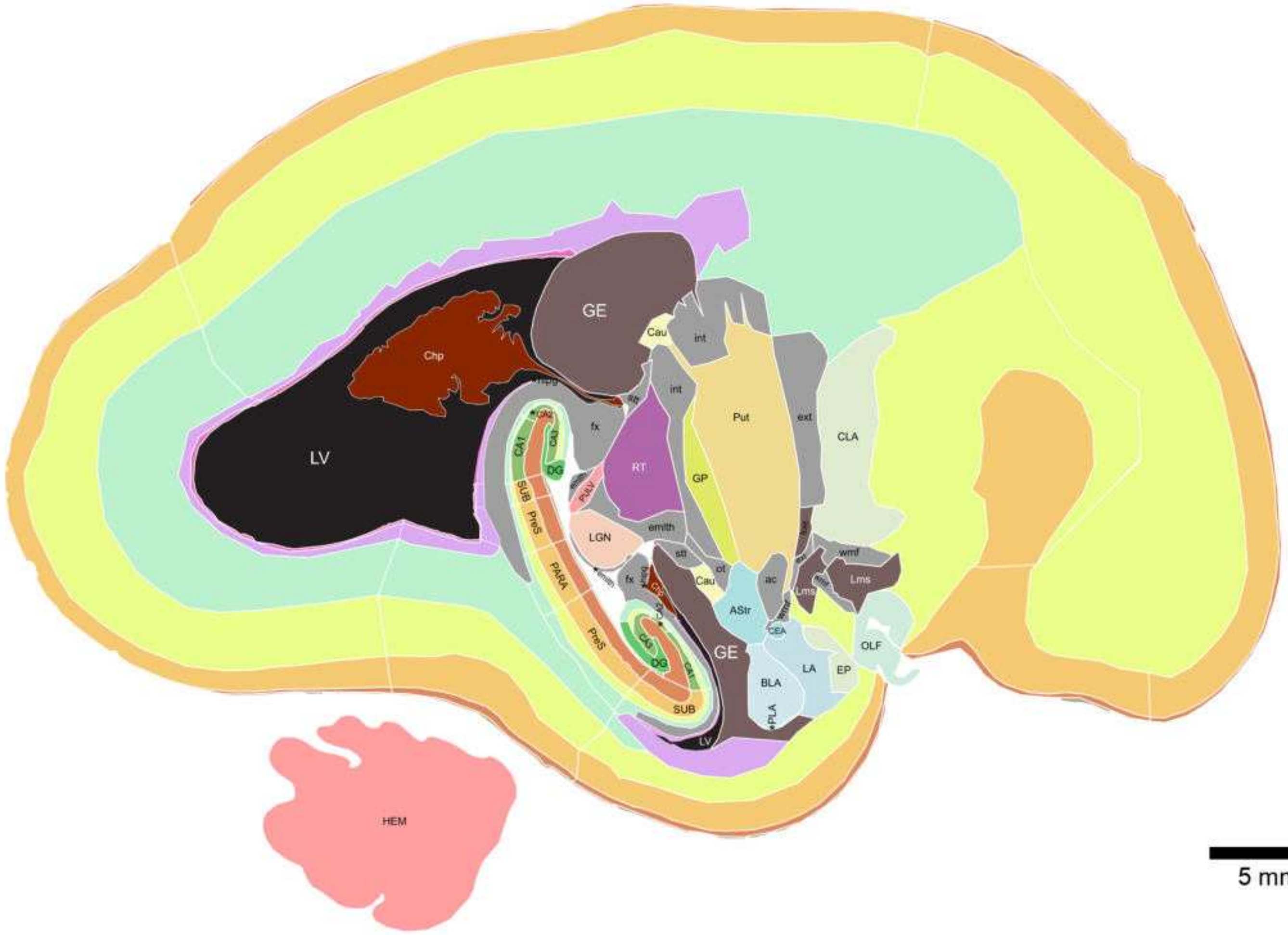

5 mm

- AStr: Amygdalo-striatal area
- BL: Basal nucleus [amygdala]
- CA1: CA1 field [hippocampus]
- CA2: CA2 field [hippocampus]
- CA3: CA3 field [hippocampus]
- CEA: Central nucleus [amygdala]
- CLA: Claustrum
- Cau: Caudate nucleus
- Chp: Choroid plexus
- DG: Dentate gyrus
- EP: Endopiriform nucleus
- GE: Ganglionic eminence
- GP: Globus pallidus
- HEM: Cerebellar hemispheres
- LA: Lateral nucleus [amygdala]
- LGN: Lateral geniculate nucleus
- LV: Lateral ventricle
- Lms: Lateral migratory stream
- PARA: Cortical plate, parasubiculum
- PLA: Paralaminar nucleus [amygdala]
- PULV: Pulvinar nucleus [thalamus]
- PreS: Cortical plate, presubiculum
- Put: Putamen
- RT: Reticular nucleus [thalamus]
- SUB: Cortical plate, subiculum
- ac: Anterior commissure
- emlth: External medullary lamina [thalamus]
- ext: External capsule
- fx: Fornix
- hipg: Hippocampal glioepithelium/ependyma
- int: Internal capsule
- ot: Optic tract
- stt: Stria terminalis
- tcet: Transient cell zone in the external capsule
- wmf: White matter fibers

Age: 21 GW

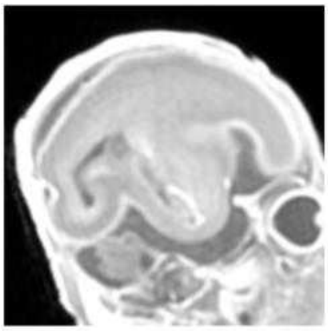

L-R Level: 11.1 mm

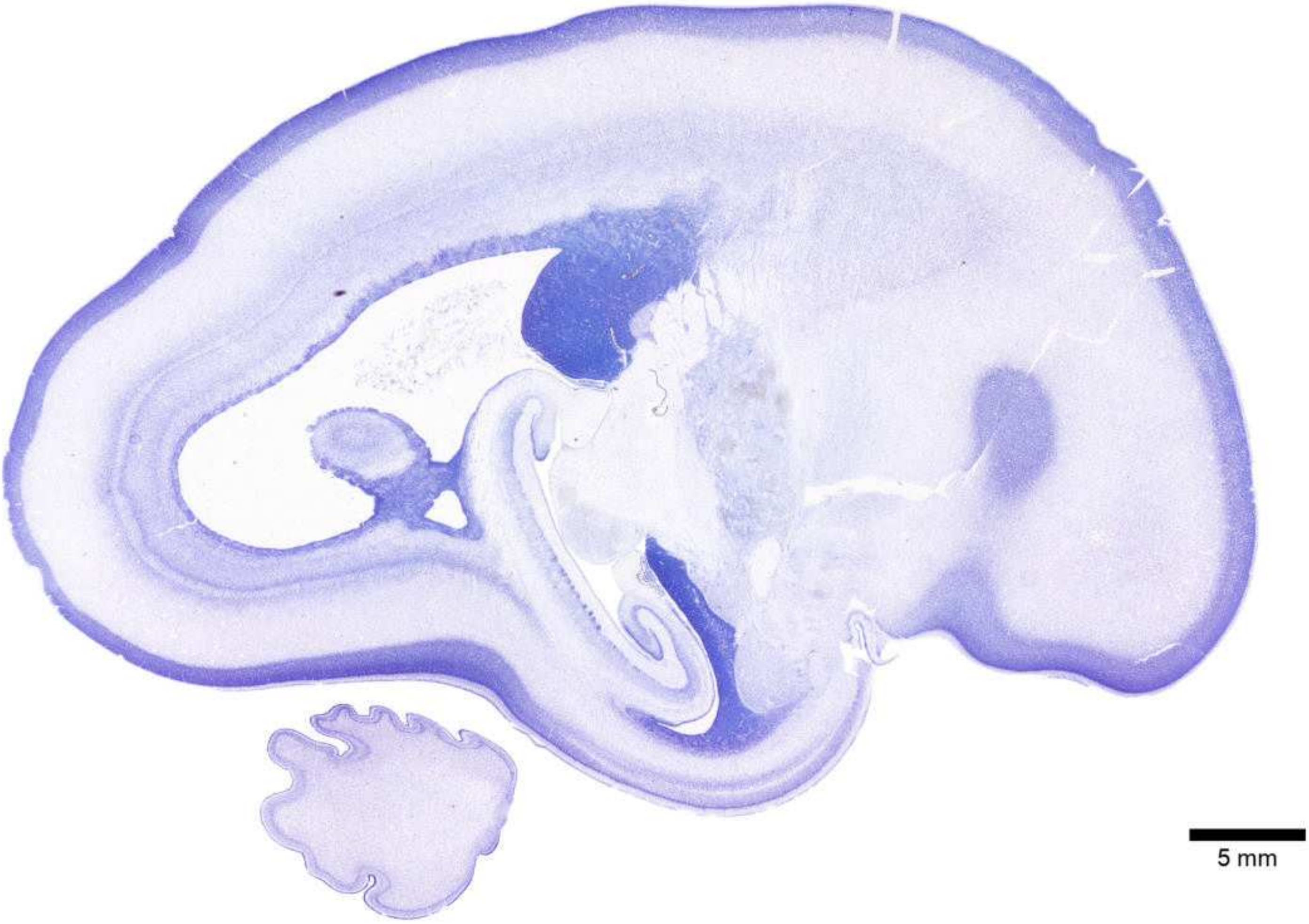

Transient Layers

- VZ
- SVZ
- IZ
- SP
- CP
- MZ
- SGL

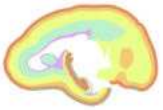

Cortical Areas

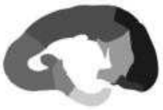

- FCTx
- ORB
- PAR
- OCC
- TEMP
- INS
- ENT

Age: 21 GW

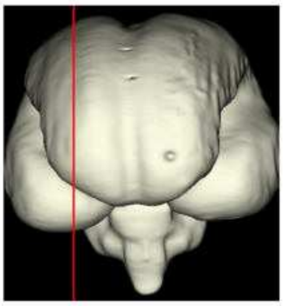

L-R Level: 11.1 mm

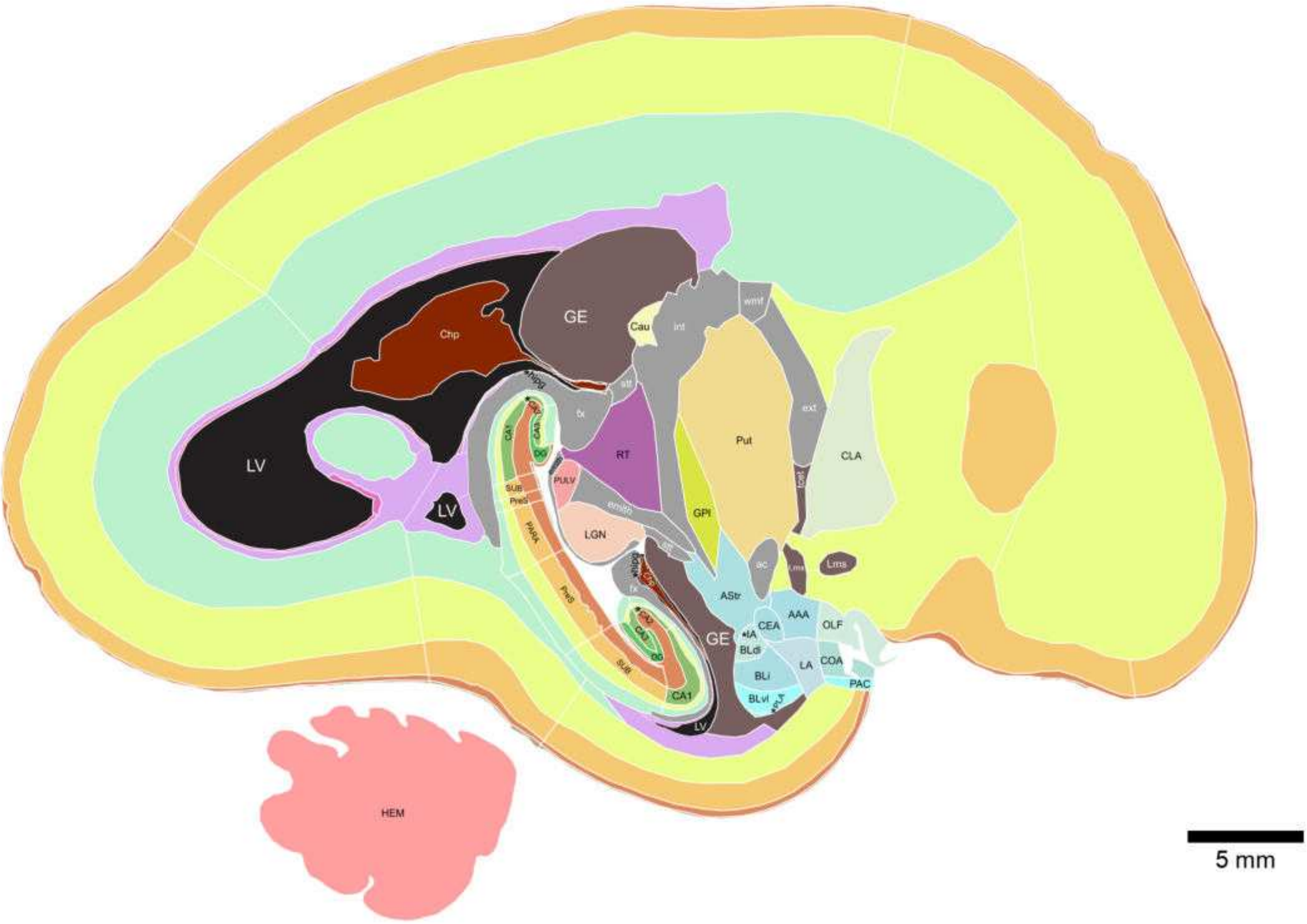

- AAA: Anterior amygdaloid area
- AStr: Amygdalo-striatal area
- BLdl: Basal nucleus [amygdala], dorsolateral part
- BLi: Basal nucleus [amygdala], intermediate part
- BLvl: Basal nucleus [amygdala], ventrolateral part
- CA1: CA1 field [hippocampus]
- CA2: CA2 field [hippocampus]
- CA3: CA3 field [hippocampus]
- CEA: Central nucleus [amygdala]
- CLA: Claustrum
- COA: Cortical nucleus [amygdala]
- Cau: Caudate nucleus
- Chp: Choroid plexus
- DG: Dentate gyrus
- GE: Ganglionic eminence
- GPl: Globus pallidus lateral segment
- HEM: Cerebellar hemispheres
- IA: Intercalated cell groups [amygdala]
- LA: Lateral nucleus [amygdala]
- LGN: Lateral geniculate nucleus
- LV: Lateral ventricle
- Lms: Lateral migratory stream
- PARA: Cortical plate, parasubiculum
- PLA: Paralaminar nucleus [amygdala]
- PULV: Pulvinar nucleus [thalamus]
- PreS: Cortical plate, presubiculum
- Put: Putamen
- RT: Reticular nucleus [thalamus]
- SUB: Cortical plate, subiculum
- ac: Anterior commissure
- emlth: External medullary lamina [thalamus]
- ext: External capsule
- fx: Fornix
- hipg: Hippocampal glioepithelium/ependyma
- int: Internal capsule
- stt: Stria terminalis
- tcet: Transient cell zone in the external capsule
- wmf: White matter fibers

Age: 21 GW

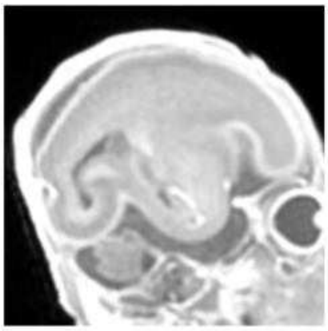

L-R Level: 10.86 mm

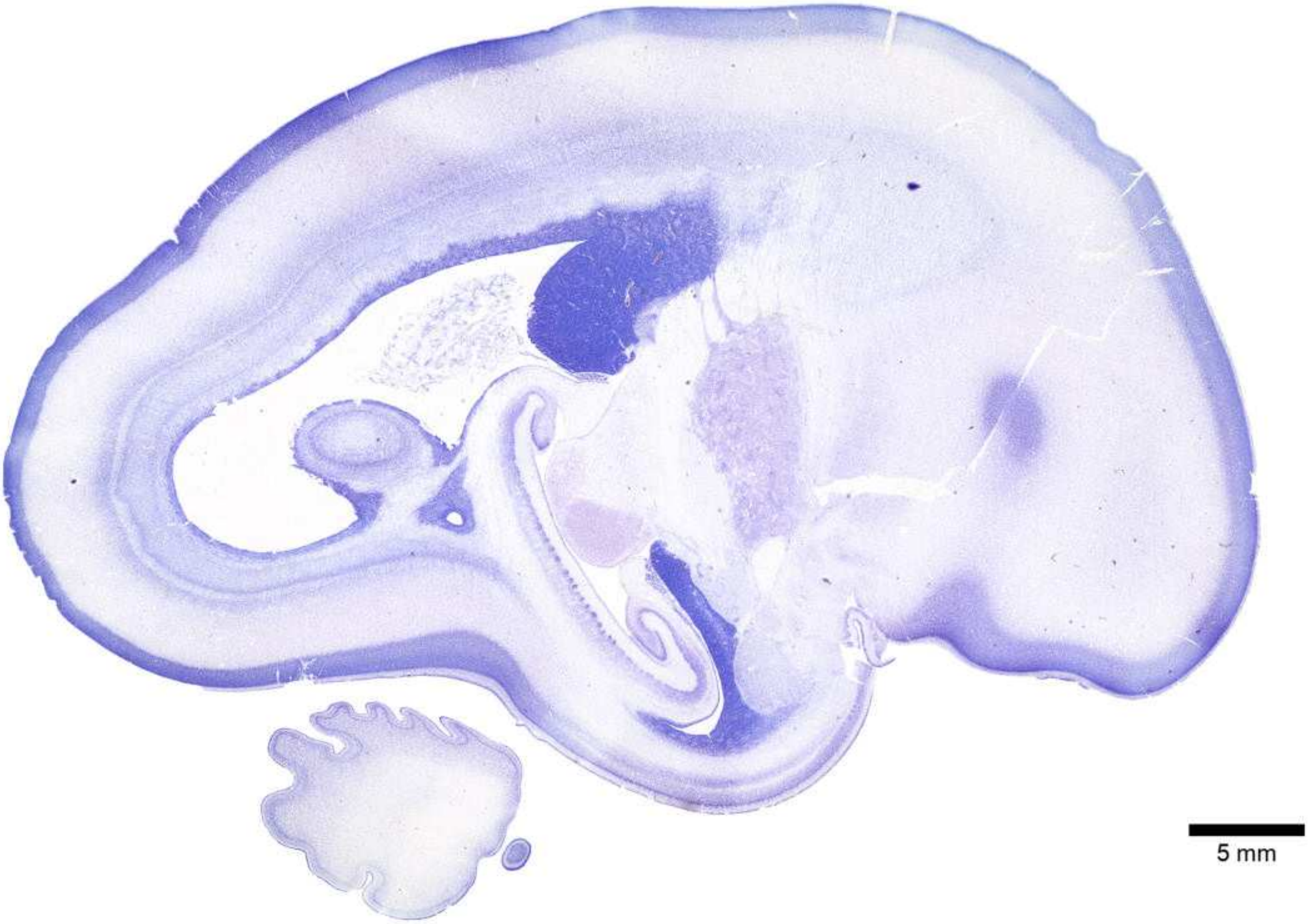

Transient Layers

- VZ
- SVZ
- IZ
- SP
- CP
- MZ
- SGL

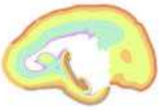

Cortical Areas

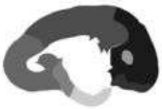

- FCTx
- ORB
- PAR
- OCC
- TEMP
- INS
- ENT

Age: 21 GW

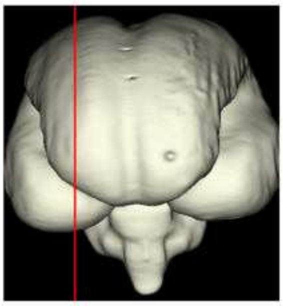

L-R Level: 10.86 mm

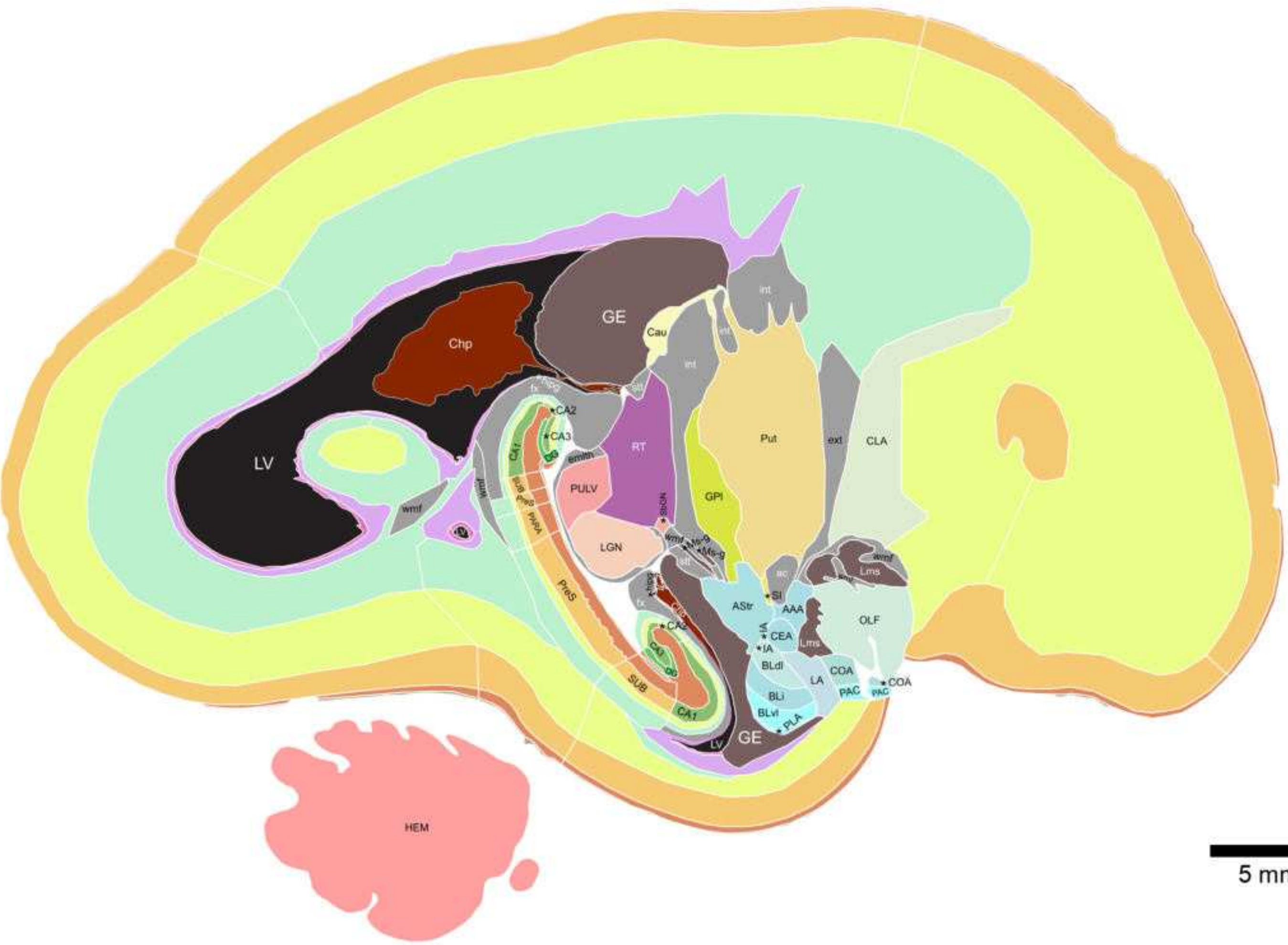

5 mm

- AAA: Anterior amygdaloid area
- AStr: Amygdalo-striatal area
- BLdl: Basal nucleus [amygdala], dorsolateral part
- BLi: Basal nucleus [amygdala], intermediate part
- BLvl: Basal nucleus [amygdala], ventrolateral part
- CA1: CA1 field [hippocampus]
- CA2: CA2 field [hippocampus]
- CA3: CA3 field [hippocampus]
- CEA: Central nucleus [amygdala]
- CLA: Claustrum
- COA: Cortical nucleus [amygdala]
- Cau: Caudate nucleus
- Chp: Choroid plexus
- DG: Dentate gyrus
- GE: Ganglionic eminence
- GPl: Globus pallidus lateral segment
- HEM: Cerebellar hemispheres
- IA: Intercalated cell groups [amygdala]
- LA: Lateral nucleus [amygdala]
- LGN: Lateral geniculate nucleus
- LV: Lateral ventricle
- Lms: Lateral migratory stream
- Ms-g: Migratory stream, general
- PARA: Cortical plate, parasubiculum
- PLA: Paralaminar nucleus [amygdala]
- PULV: Pulvinar nucleus [thalamus]
- PreS: Cortical plate, presubiculum
- Put: Putamen
- RT: Reticular nucleus [thalamus]
- SI: Substantia innominata
- SUB: Cortical plate, subiculum
- SbGN: Subgeniculate nucleus
- ac: Anterior commissure
- emlth: External medullary lamina [thalamus]
- ext: External capsule
- fx: Fornix
- hipg: Hippocampal glioepithelium/ependyma
- int: Internal capsule
- stt: Stria terminalis
- wmf: White matter fibers

Age: 21 GW

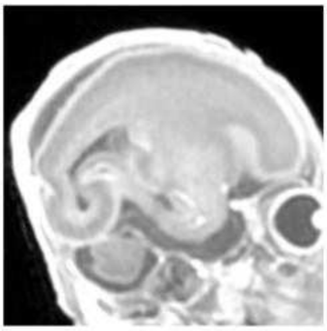

L-R Level: 10.08 mm

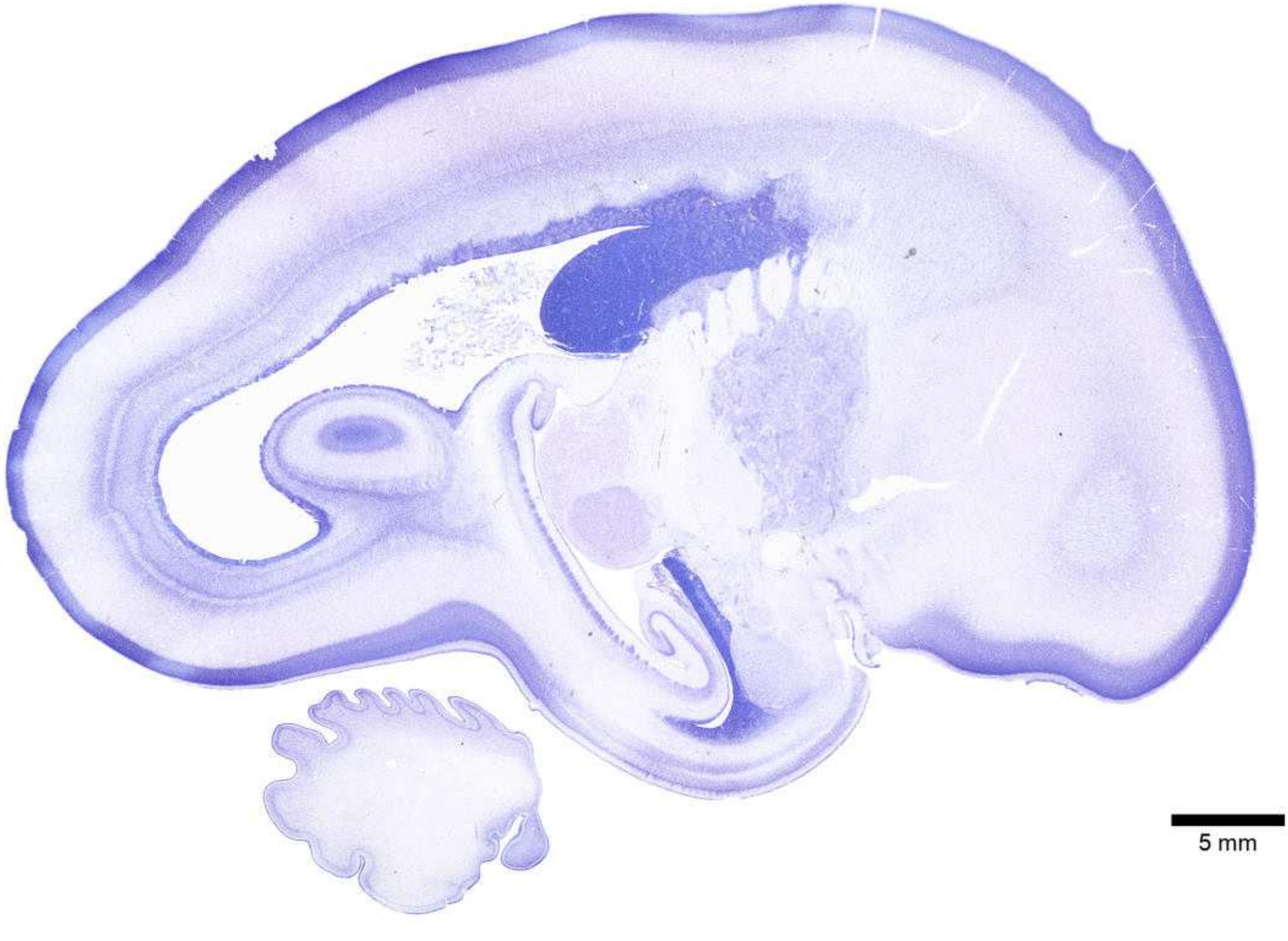

5 mm

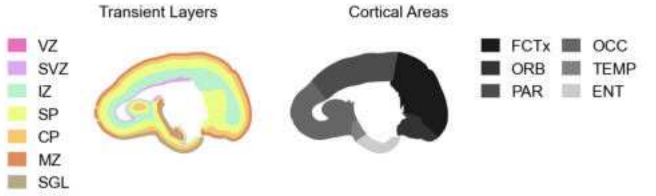

Age: 21 GW

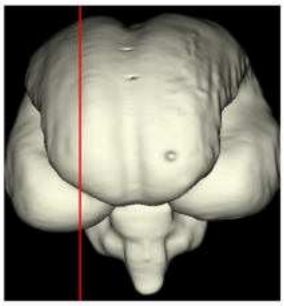

L-R Level: 10.08 mm

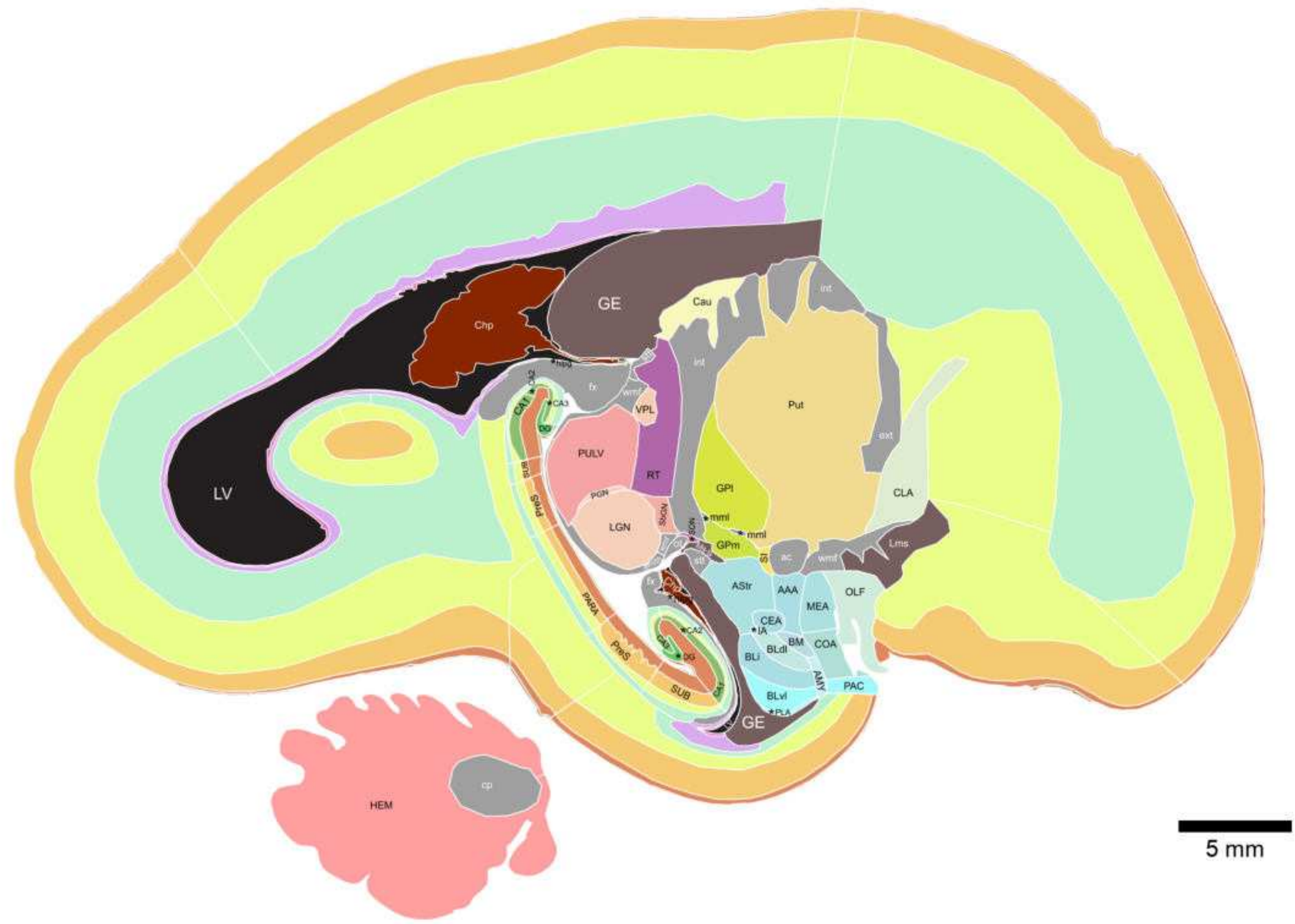

- AAA: Anterior amygdaloid area
- AMY: Amygdala
- AStr: Amygdalo-striatal area
- BLdl: Basal nucleus [amygdala], dorsolateral part
- BLi: Basal nucleus [amygdala], intermediate part
- BLvl: Basal nucleus [amygdala], ventrolateral part
- BM: Basomedial nucleus [amygdala]
- CA1: CA1 field [hippocampus]
- CA2: CA2 field [hippocampus]
- CA3: CA3 field [hippocampus]
- CEA: Central nucleus [amygdala]
- CLA: Claustrum
- COA: Cortical nucleus [amygdala]
- Cau: Caudate nucleus
- Chp: Choroid plexus
- DG: Dentate gyrus
- GE: Ganglionic eminence
- GPl: Globus pallidus lateral segment
- GPm: Globus pallidus medial segment
- HEM: Cerebellar hemispheres
- IA: Intercalated cell groups [amygdala]
- LGN: Lateral geniculate nucleus
- LV: Lateral ventricle
- Lms: Lateral migratory stream
- MEA: Medial nucleus [amygdala]
- Ms-g: Migratory stream, general
- PARA: Cortical plate, parasubiculum
- PGN: Pregeniculate nucleus
- PLA: Paralaminar nucleus [amygdala]
- PULV: Pulvinar nucleus [thalamus]
- PreS: Cortical plate, presubiculum
- Put: Putamen
- RT: Reticular nucleus [thalamus]
- SI: Substantia innominata
- SON: Supraoptic nucleus [hypothalamus]
- SUB: Cortical plate, subiculum
- SbGN: Subgeniculate nucleus
- VPL: Ventral posterolateral nucleus [thalamus]
- ac: Anterior commissure
- cp: Cerebral peduncle
- emlth: External medullary lamina [thalamus]
- ext: External capsule
- fx: Fornix
- hipg: Hippocampal glioepithelium/ependyma
- int: Internal capsule
- mml: Medial medullary lamina
- ot: Optic tract
- stt: Stria terminalis
- wmf: White matter fibers

Age: 21 GW

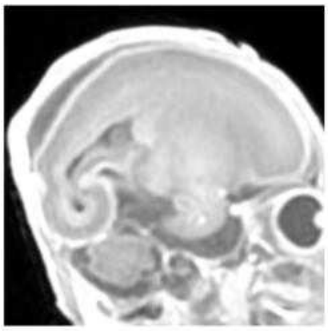

L-R Level: 9.42 mm

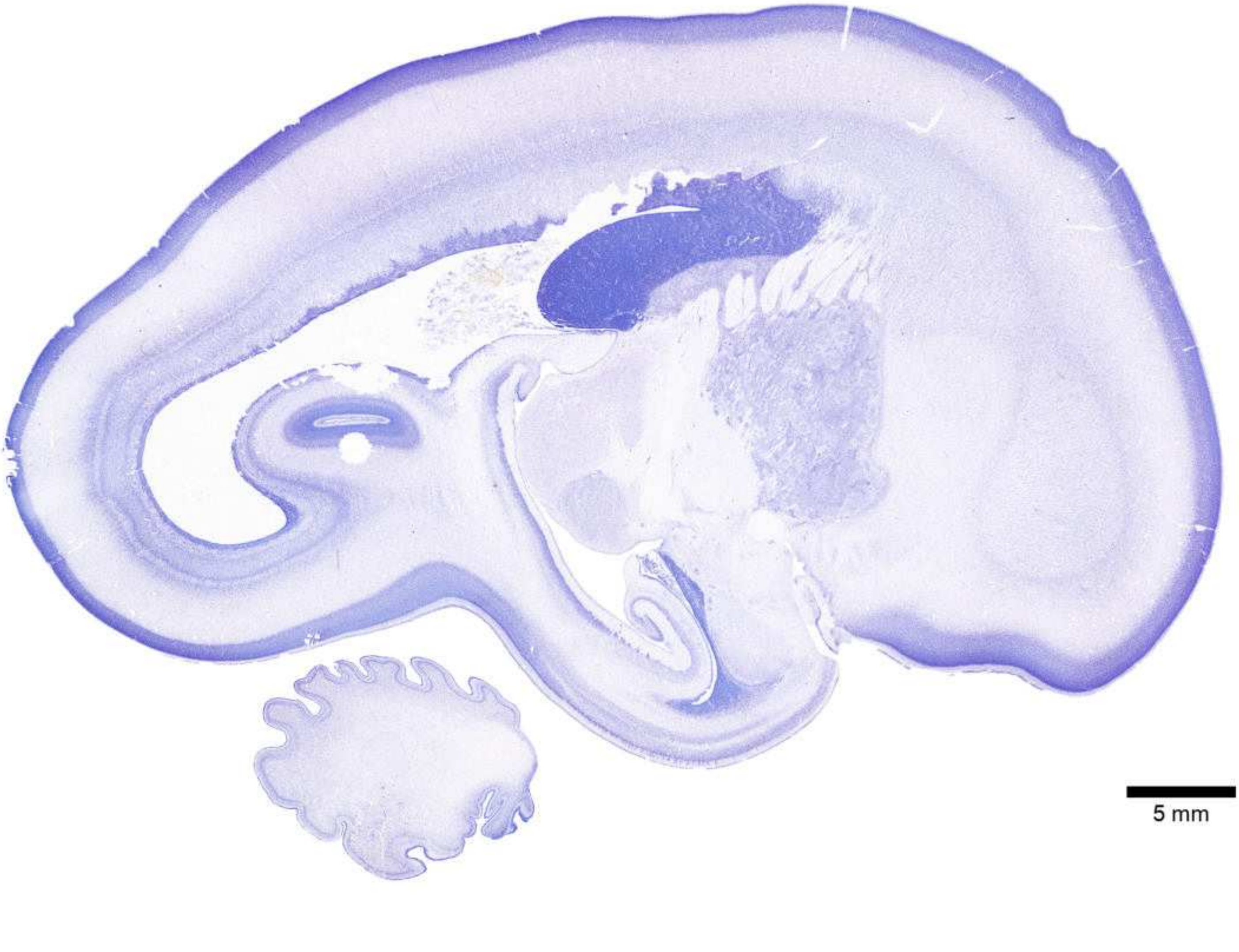

5 mm

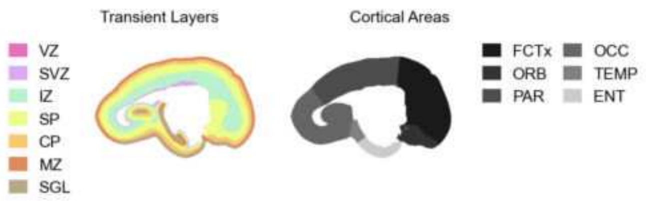

Age: 21 GW

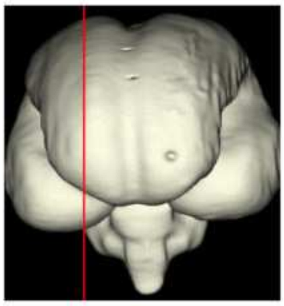

L-R Level: 9.42 mm

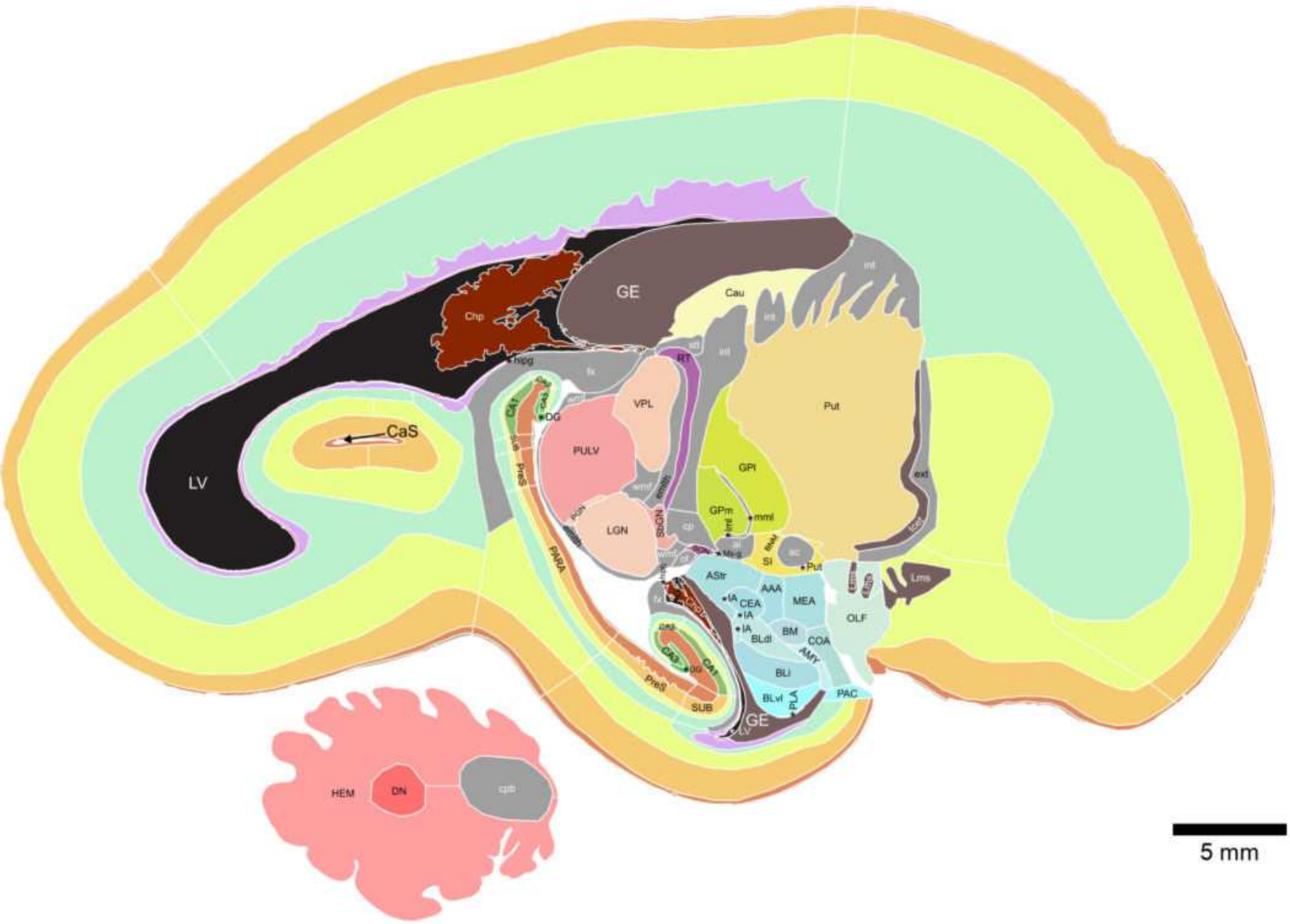

- AAA: Anterior amygdaloid area
- AMY: Amygdala
- AStr: Amygdalo-striatal area
- BLdl: Basal nucleus [amygdala], dorsolateral part
- BLi: Basal nucleus [amygdala], intermediate part
- BLvl: Basal nucleus [amygdala], ventrolateral part
- BM: Basomedial nucleus [amygdala]
- BNM: Basal nucleus of Meynert
- CA1: CA1 field [hippocampus]
- CA2: CA2 field [hippocampus]
- CA3: CA3 field [hippocampus]
- CEA: Central nucleus [amygdala]
- COA: Cortical nucleus [amygdala]
- Cau: Caudate nucleus
- Chp: Choroid plexus
- DG: Dentate gyrus
- DN: Dentate nucleus
- GE: Ganglionic eminence
- GPl: Globus pallidus lateral segment
- GPm: Globus pallidus medial segment
- HEM: Cerebellar hemispheres
- IA: Intercalated cell groups [amygdala]
- LGN: Lateral geniculate nucleus
- LV: Lateral ventricle
- Lms: Lateral migratory stream
- MEA: Medial nucleus [amygdala]
- Ms-g: Migratory stream, general
- PARA: Cortical plate, parasubiculum
- PGN: Pregeniculate nucleus
- PLA: Paralaminar nucleus [amygdala]
- PULV: Pulvinar nucleus [thalamus]
- PreS: Cortical plate, presubiculum
- Put: Putamen
- RT: Reticular nucleus [thalamus]
- SI: Substantia innominata
- SON: Supraoptic nucleus [hypothalamus]
- SUB: Cortical plate, subiculum
- SbGN: Subgeniculate nucleus
- VPL: Ventral posterolateral nucleus [thalamus]
- ac: Anterior commissure
- al: Ansa lenticularis
- cp: Cerebral peduncle
- cpb: Cerebellar peduncles
- emlth: External medullary lamina [thalamus]
- ext: External capsule
- fx: Fornix
- hipg: Hippocampal glioepithelium/ependyma
- int: Internal capsule
- lml: Lateral medullary lamina
- mml: Medial medullary lamina
- ot: Optic tract
- stt: Stria terminalis
- tcet: Transient cell zone in the external capsule
- wmf: White matter fibers
- → CaS - Calcarine sulcus

Age: 21 GW

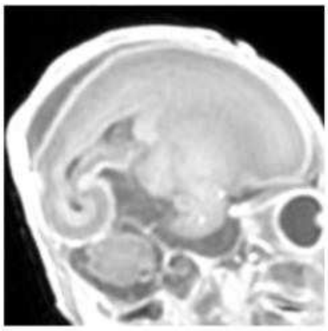

L-R Level: 8.94 mm

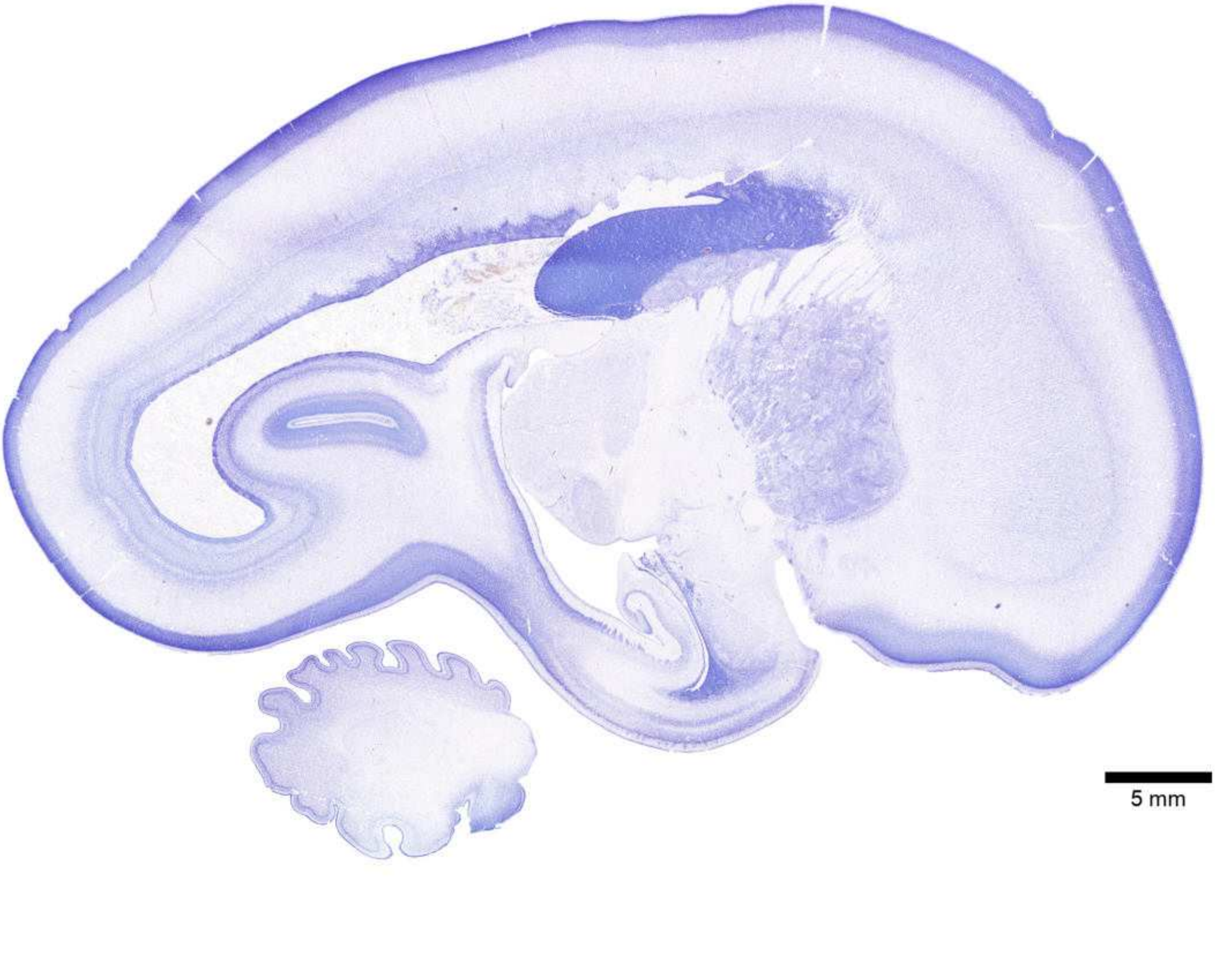

5 mm

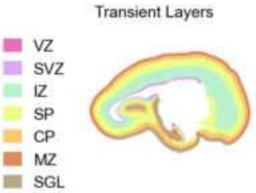

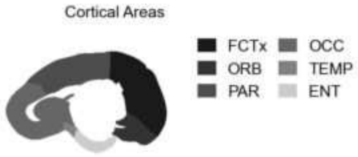

Age: 21 GW

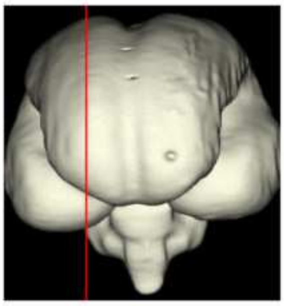

L-R Level: 8.94 mm

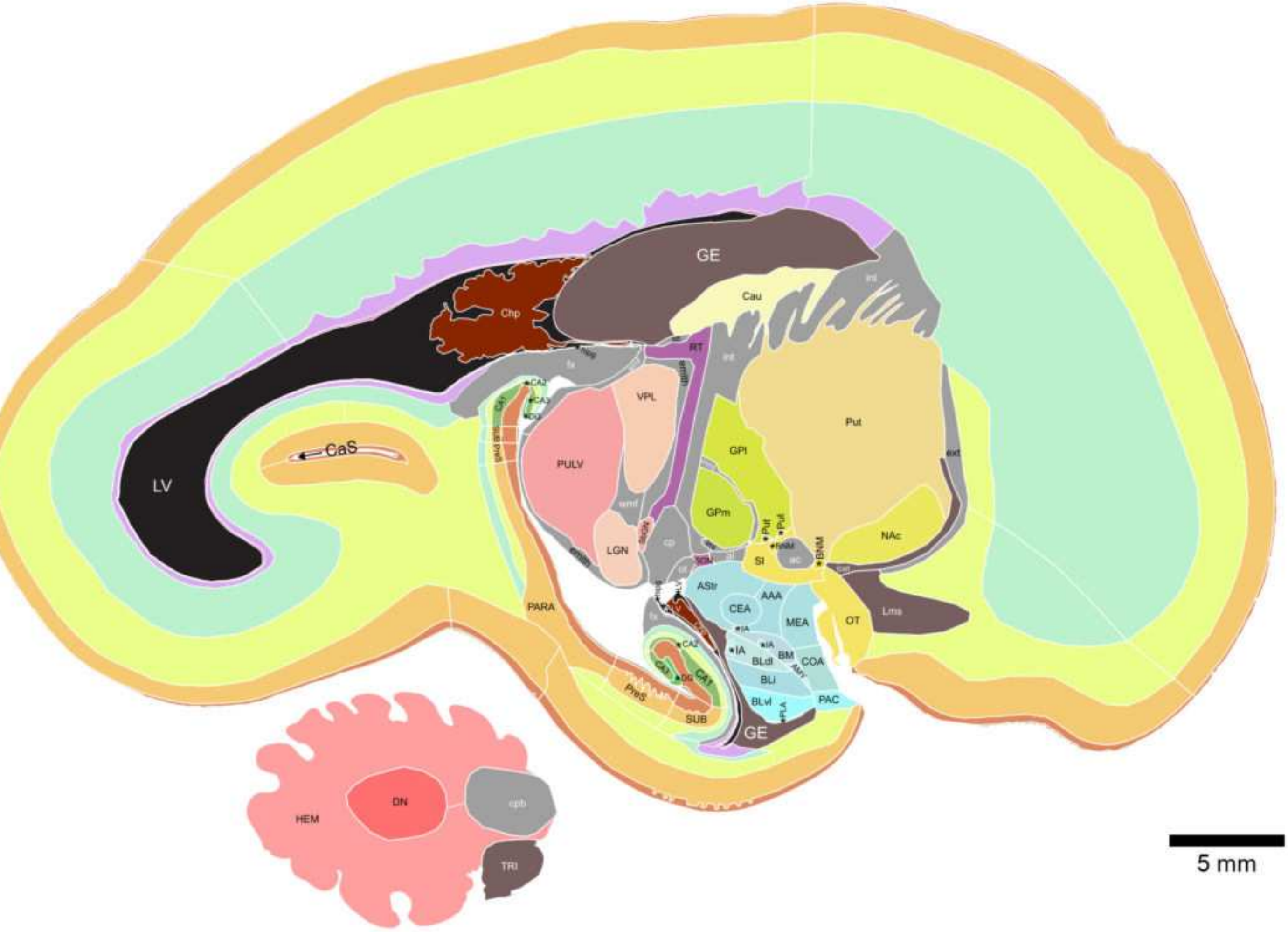

- AAA: Anterior amygdaloid area
- AMY: Amygdala
- AStr: Amygdalo-striatal area
- BLdl: Basal nucleus [amygdala], dorsolateral part
- BLi: Basal nucleus [amygdala], intermediate part
- BLvl: Basal nucleus [amygdala], ventrolateral part
- BM: Basomedial nucleus [amygdala]
- BNM: Basal nucleus of Meynert
- CA1: CA1 field [hippocampus]
- CA2: CA2 field [hippocampus]
- CA3: CA3 field [hippocampus]
- CEA: Central nucleus [amygdala]
- COA: Cortical nucleus [amygdala]
- Cau: Caudate nucleus
- Chp: Choroid plexus
- DG: Dentate gyrus
- DN: Dentate nucleus
- GE: Ganglionic eminence
- GPl: Globus pallidus lateral segment
- GPm: Globus pallidus medial segment
- HEM: Cerebellar hemispheres
- IA: Intercalated cell groups [amygdala]
- LGN: Lateral geniculate nucleus
- LV: Lateral ventricle
- Lms: Lateral migratory stream
- MEA: Medial nucleus [amygdala]
- NAc: Nucleus accumbens
- OT: Olfactory tubercle
- PARA: Cortical plate, parasubiculum
- PLA: Paralaminar nucleus [amygdala]
- PULV: Pulvinar nucleus [thalamus]
- PreS: Cortical plate, presubiculum
- Put: Putamen
- RT: Reticular nucleus [thalamus]
- SI: Substantia innominata
- SON: Supraoptic nucleus [hypothalamus]
- SUB: Cortical plate, subiculum
- SbGN: Subgeniculate nucleus
- TRI: Germinal trigone
- VPL: Ventral posterolateral nucleus [thalamus]
- ac: Anterior commissure
- al: Ansa lenticularis
- cp: Cerebral peduncle
- cpb: Cerebellar peduncles
- emlth: External medullary lamina [thalamus]
- ext: External capsule
- fx: Fornix
- hipg: Hippocampal glioepithelium/ependyma
- int: Internal capsule
- lml: Lateral medullary lamina
- mml: Medial medullary lamina
- ot: Optic tract
- stt: Stria terminalis
- tcet: Transient cell zone in the external capsule
- wmf: White matter fibers
- CaS: Calcarine sulcus

Age: 21 GW

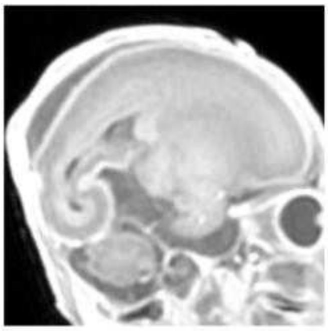

L-R Level: 8.7 mm

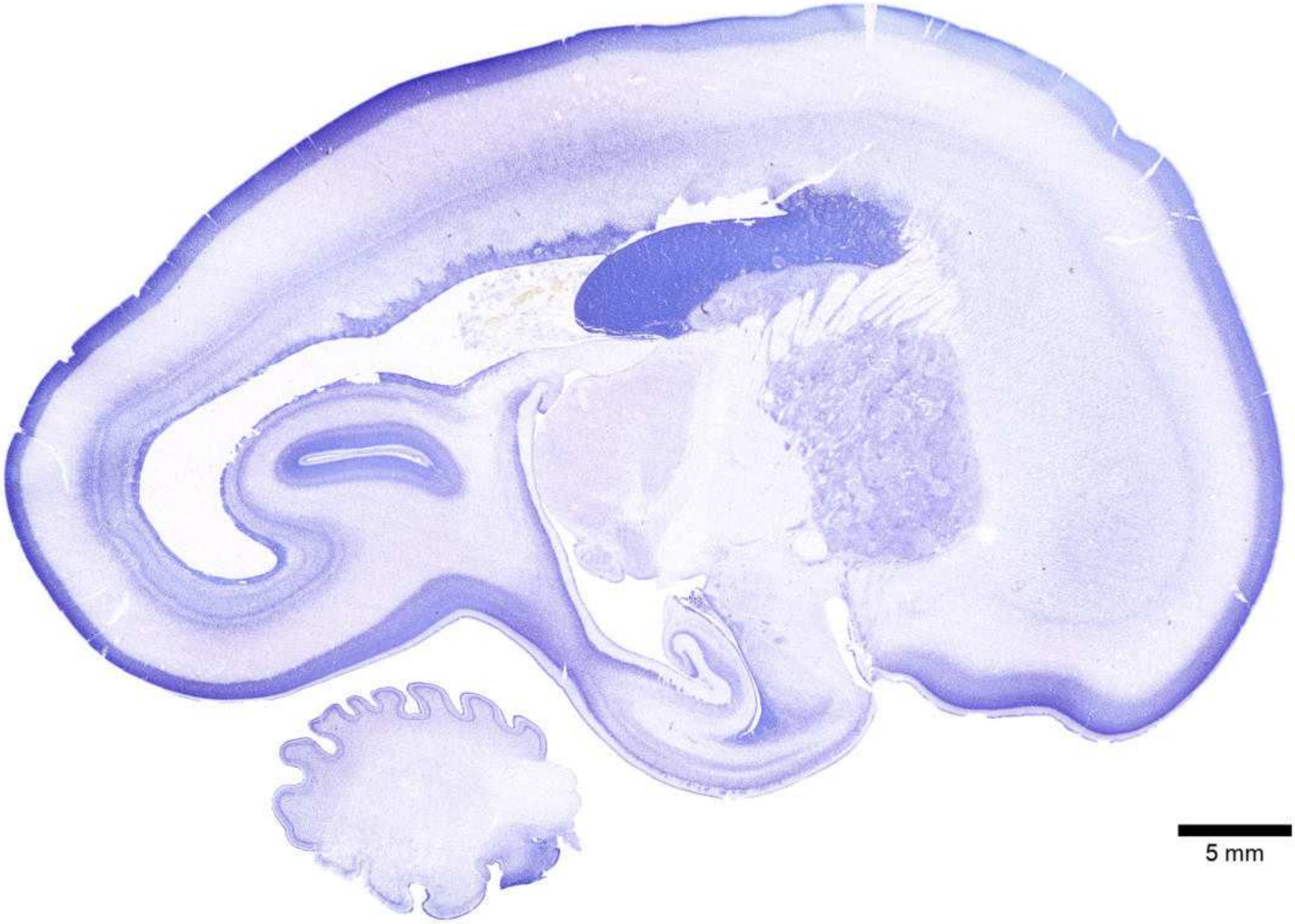

5 mm

Transient Layers

- VZ
- SVZ
- IZ
- SP
- CP
- MZ
- SGL

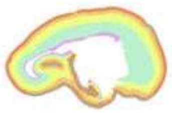

Cortical Areas

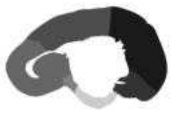

- FCTx
- ORB
- PAR
- OCC
- TEMP
- ENT

Age: 21 GW

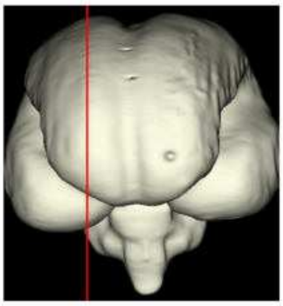

L-R Level: 8.7 mm

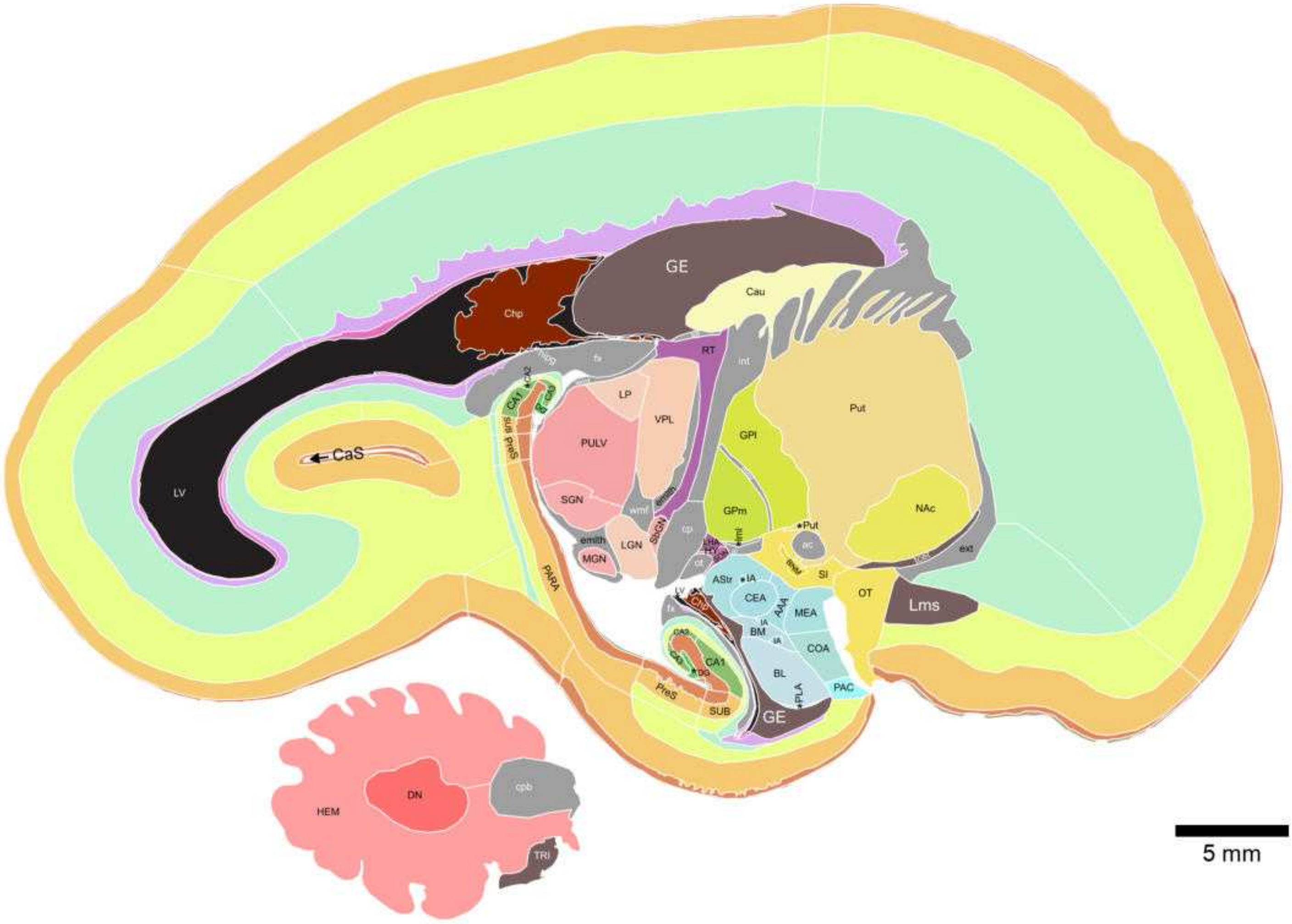

5 mm

- AAA: Anterior amygdaloid area
- AStr: Amygdalo-striatal area
- BL: Basal nucleus [amygdala]
- BM: Basomedial nucleus [amygdala]
- BNM: Basal nucleus of Meynert
- CA1: CA1 field [hippocampus]
- CA2: CA2 field [hippocampus]
- CA3: CA3 field [hippocampus]
- CEA: Central nucleus [amygdala]
- COA: Cortical nucleus [amygdala]
- Cau: Caudate nucleus
- Chp: Choroid plexus
- DG: Dentate gyrus
- DN: Dentate nucleus
- GE: Ganglionic eminence
- GPl: Globus pallidus lateral segment
- GPm: Globus pallidus medial segment
- HEM: Cerebellar hemispheres
- HY: Hypothalamus
- IA: Intercalated cell groups [amygdala]
- LGN: Lateral geniculate nucleus
- LHA: Lateral hypothalamic area
- LP: Lateral posterior nucleus [thalamus]
- LV: Lateral ventricle
- Lms: Lateral migratory stream
- MEA: Medial nucleus [amygdala]
- MGN: Medial geniculate nucleus
- NAc: Nucleus accumbens
- OT: Olfactory tubercle
- PARA: Cortical plate, parasubiculum
- PLA: Paralaminar nucleus [amygdala]
- PULV: Pulvinar nucleus [thalamus]
- PreS: Cortical plate, presubiculum
- Put: Putamen
- RT: Reticular nucleus [thalamus]
- SGN: Suprageniculate nucleus
- SI: Substantia innominata
- SON: Supraoptic nucleus [hypothalamus]
- SUB: Cortical plate, subiculum
- SbGN: Subgeniculate nucleus
- TRI: Germinal trigone
- VPL: Ventral posterolateral nucleus [thalamus]
- ac: Anterior commissure
- al: Ansa lenticularis
- cp: Cerebral peduncle
- cpb: Cerebellar peduncles
- emlth: External medullary lamina [thalamus]
- ext: External capsule
- fx: Fornix
- hipg: Hippocampal glioepithelium/ependyma
- int: Internal capsule
- lml: Lateral medullary lamina
- mml: Medial medullary lamina
- ot: Optic tract
- stt: Stria terminalis
- tcet: Transient cell zone in the external capsule
- wmf: White matter fibers

Age: 21 GW

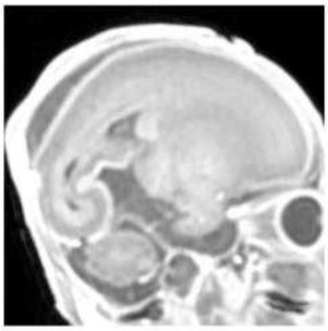

L-R Level: 8.34 mm

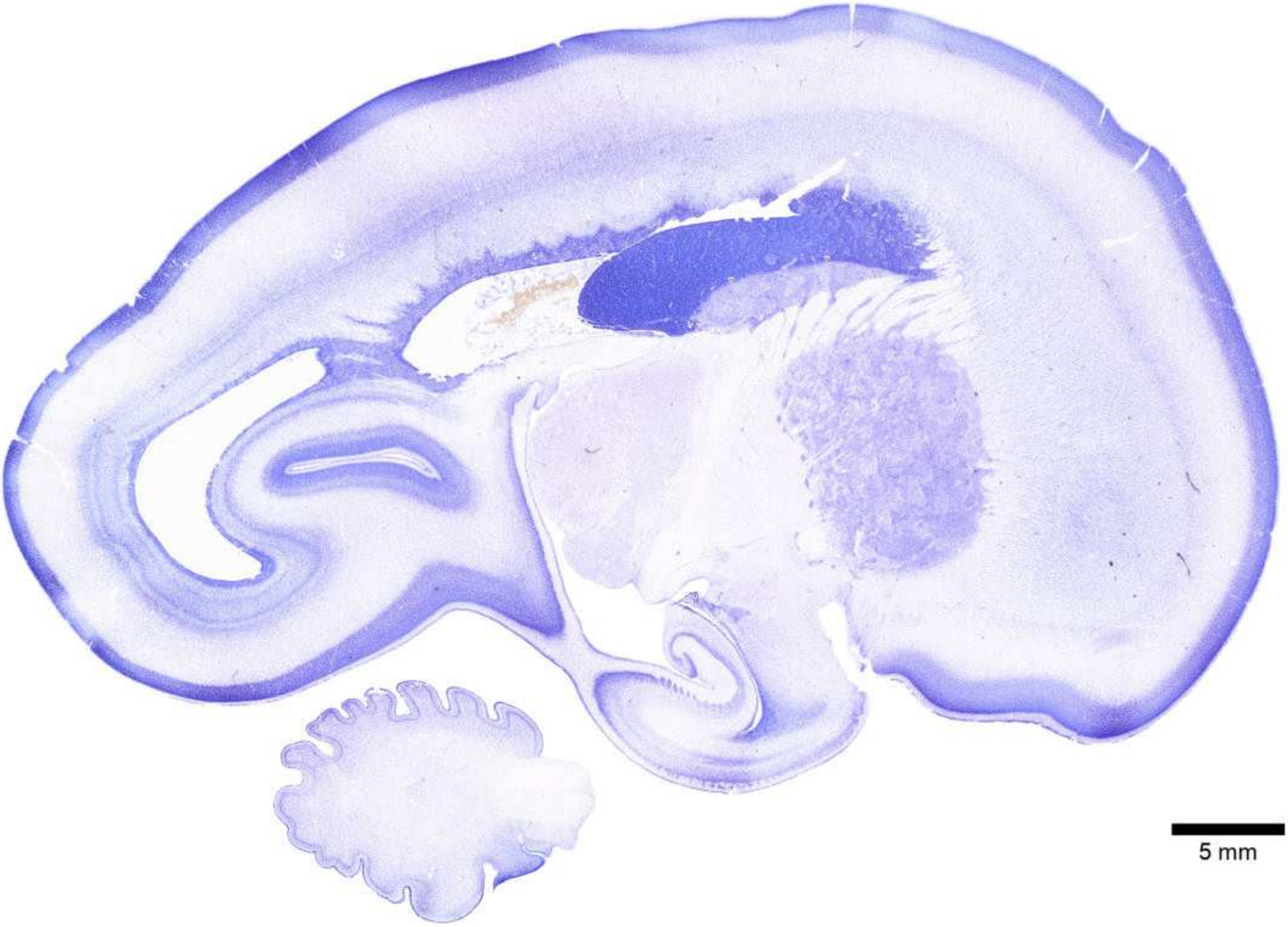

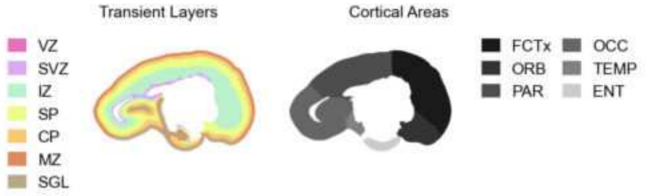

Age: 21 GW

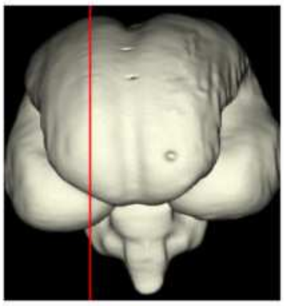

L-R Level: 8.34 mm

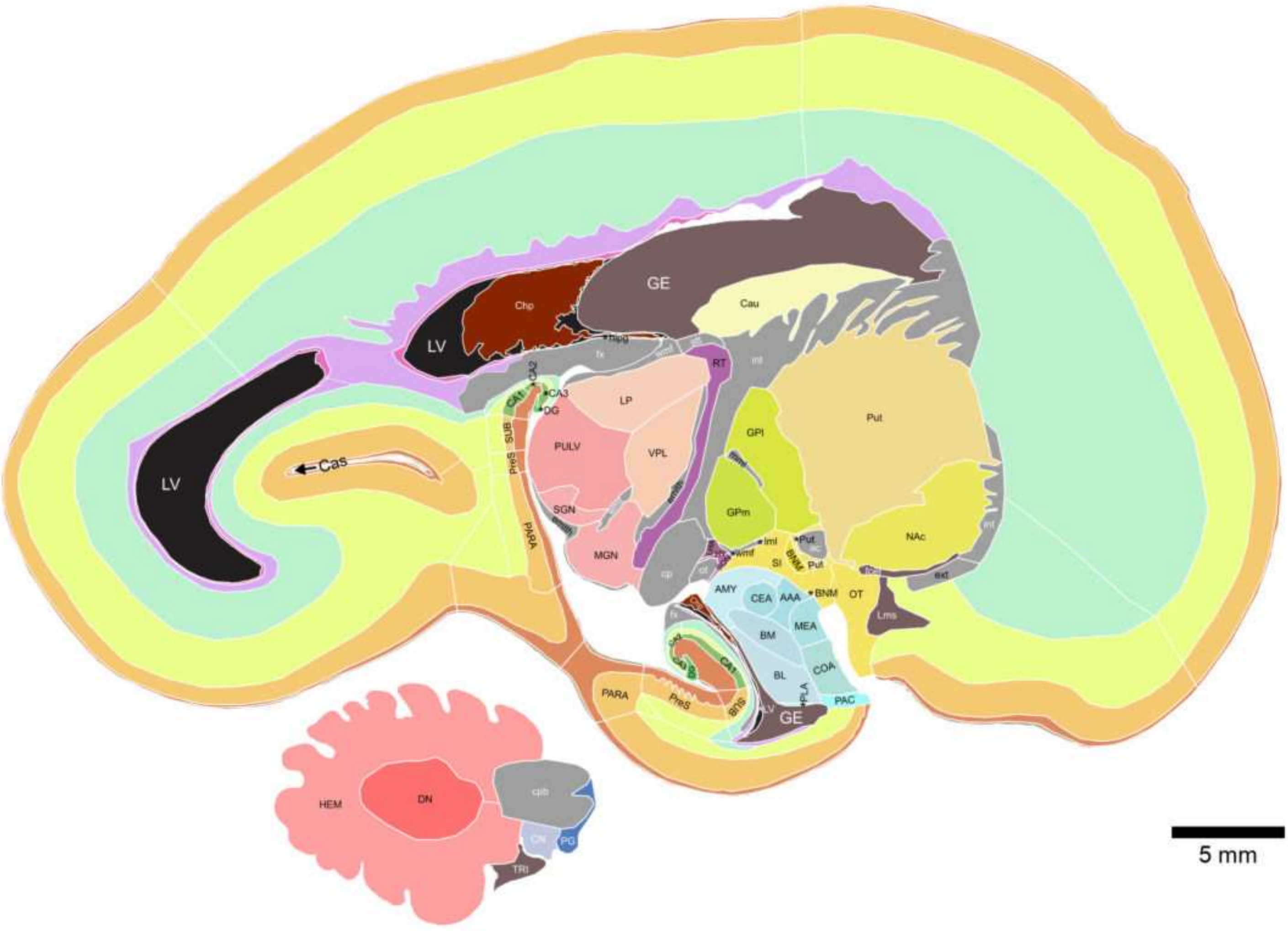

- AAA: Anterior amygdaloid area
- AMY: Amygdala
- BL: Basal nucleus [amygdala]
- BM: Basomedial nucleus [amygdala]
- BNM: Basal nucleus of Meynert
- CA1: CA1 field [hippocampus]
- CA2: CA2 field [hippocampus]
- CA3: CA3 field [hippocampus]
- CEA: Central nucleus [amygdala]
- CN: Cochlear nuclei
- COA: Cortical nucleus [amygdala]
- Cau: Caudate nucleus
- Chp: Choroid plexus
- DG: Dentate gyrus
- DN: Dentate nucleus
- GE: Ganglionic eminence
- GPl: Globus pallidus lateral segment
- GPm: Globus pallidus medial segment
- HEM: Cerebellar hemispheres
- HY: Hypothalamus
- LHA: Lateral hypothalamic area
- LP: Lateral posterior nucleus [thalamus]
- LV: Lateral ventricle
- Lms: Lateral migratory stream
- MEA: Medial nucleus [amygdala]
- MGN: Medial geniculate nucleus
- NAc: Nucleus accumbens
- OT: Olfactory tubercle
- PARA: Cortical plate, parasubiculum
- PG: Pontine gray
- PLA: Paralaminar nucleus [amygdala]
- PULV: Pulvinar nucleus [thalamus]
- PreS: Cortical plate, presubiculum
- Put: Putamen
- RT: Reticular nucleus [thalamus]
- SGN: Suprageniculate nucleus
- SI: Substantia innominata
- SON: Supraoptic nucleus [hypothalamus]
- SUB: Cortical plate, subiculum
- TRI: Germinal trigone
- VPL: Ventral posterolateral nucleus [thalamus]
- ac: Anterior commissure
- cp: Cerebral peduncle
- cpb: Cerebellar peduncles
- emlth: External medullary lamina [thalamus]
- ext: External capsule
- fx: Fornix
- hipg: Hippocampal glioepithelium/ependyma
- int: Internal capsule
- lml: Lateral medullary lamina
- mml: Medial medullary lamina
- ot: Optic tract
- stt: Stria terminalis
- tcet: Transient cell zone in the external capsule
- wmf: White matter fibers
- → CaS: Calcarine sulcus

Age: 21 GW

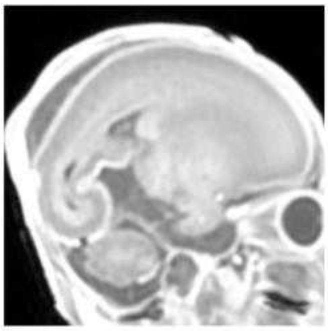

L-R Level: 7.8 mm

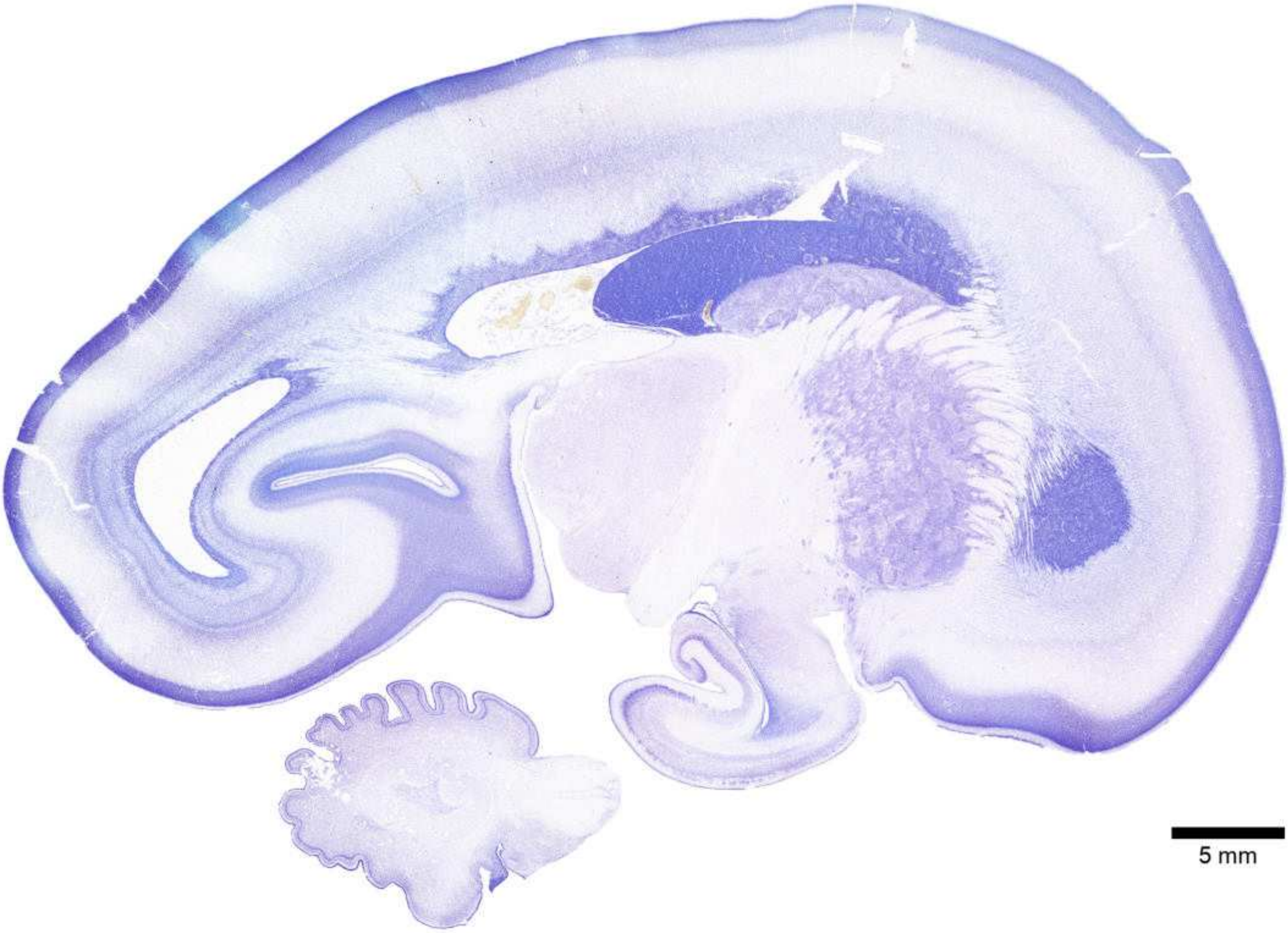

Transient Layers

- VZ
- SVZ
- IZ
- SP
- CP
- MZ
- SGL

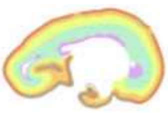

Cortical Areas

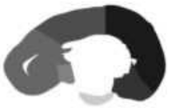

- FCTx
- ORB
- PAR
- OCC
- TEMP
- ENT

Age: 21 GW

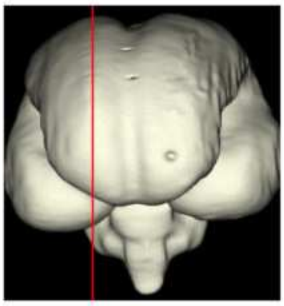

L-R Level: 7.8 mm

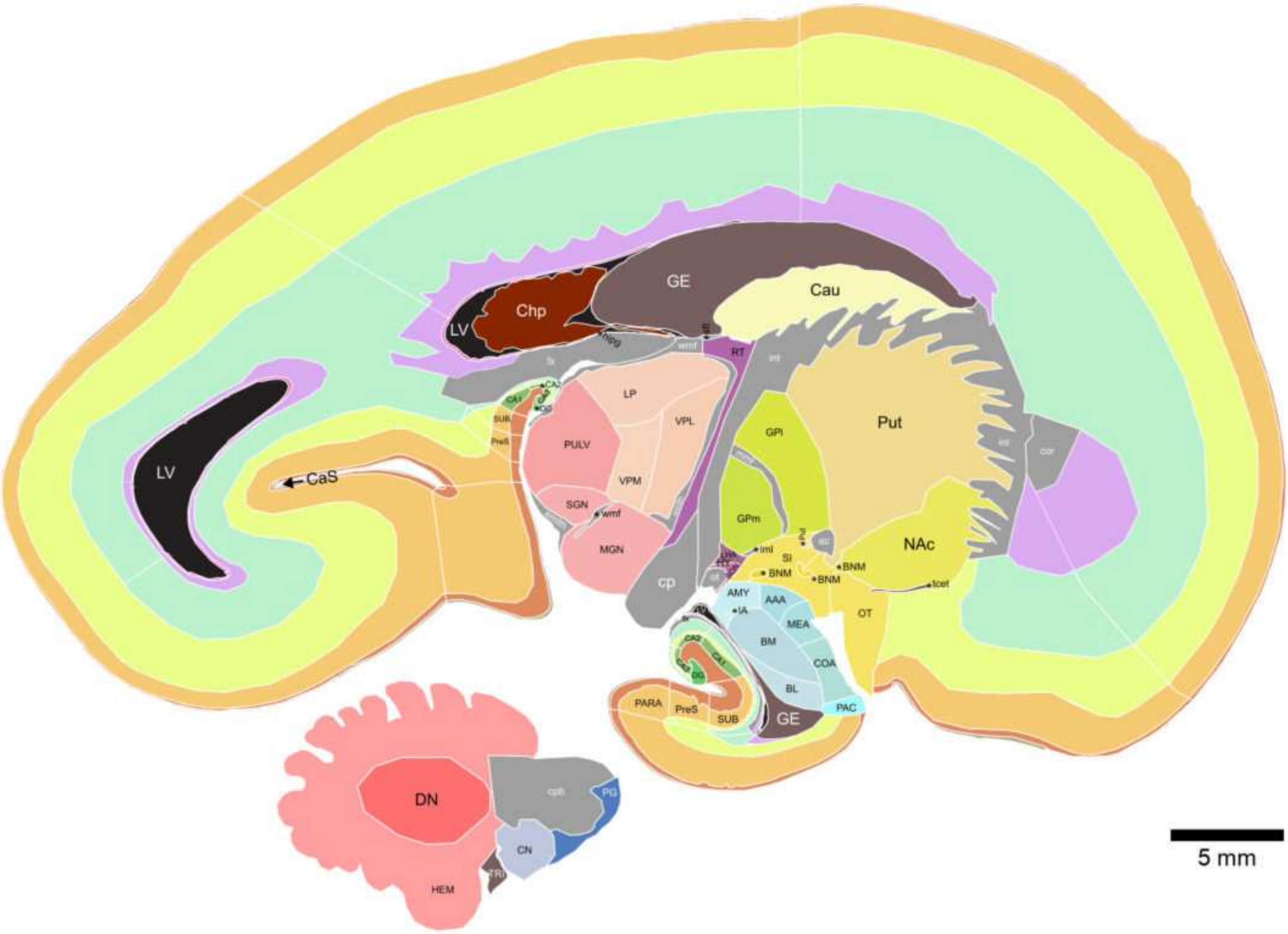

5 mm

- AAA: Anterior amygdaloid area
- AMY: Amygdala
- BL: Basal nucleus [amygdala]
- BM: Basomedial nucleus [amygdala]
- BNM: Basal nucleus of Meynert
- CA1: CA1 field [hippocampus]
- CA2: CA2 field [hippocampus]
- CA3: CA3 field [hippocampus]
- CN: Cochlear nuclei
- COA: Cortical nucleus [amygdala]
- Cau: Caudate nucleus
- Chp: Choroid plexus
- DG: Dentate gyrus
- DN: Dentate nucleus
- GE: Ganglionic eminence
- GPl: Globus pallidus lateral segment
- GPm: Globus pallidus medial segment
- HEM: Cerebellar hemispheres
- HY: Hypothalamus
- IA: Intercalated cell groups [amygdala]
- LHA: Lateral hypothalamic area
- LP: Lateral posterior nucleus [thalamus]
- LV: Lateral ventricle
- MEA: Medial nucleus [amygdala]
- MGN: Medial geniculate nucleus
- NAc: Nucleus accumbens
- OT: Olfactory tubercle
- PARA: Cortical plate, parasubiculum
- PG: Pontine gray
- PULV: Pulvinar nucleus [thalamus]
- PreS: Cortical plate, presubiculum
- Put: Putamen
- RT: Reticular nucleus [thalamus]
- SGN: Suprageniculate nucleus
- SI: Substantia innominata
- SON: Supraoptic nucleus [hypothalamus]
- SUB: Cortical plate, subiculum
- TRI: Germinal trigone
- VPL: Ventral posterolateral nucleus [thalamus]
- VPM: Ventral posteromedial nucleus [thalamus]
- ac: Anterior commissure
- cor: Corona radiata
- cp: Cerebral peduncle
- cpb: Cerebellar peduncles
- emlth: External medullary lamina [thalamus]
- fx: Fornix
- hipg: Hippocampal glioepithelium/ependyma
- int: Internal capsule
- lml: Lateral medullary lamina
- mml: Medial medullary lamina
- ot: Optic tract
- stt: Stria terminalis
- tcet: Transient cell zone in the external capsule
- wmf: White matter fibers
- → CaS: Calcarine sulcus

Age: 21 GW

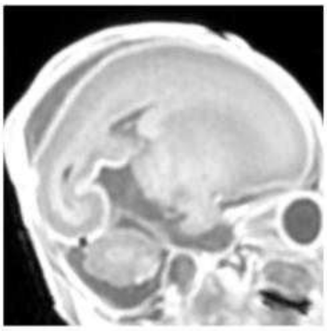

L-R Level: 7.56 mm

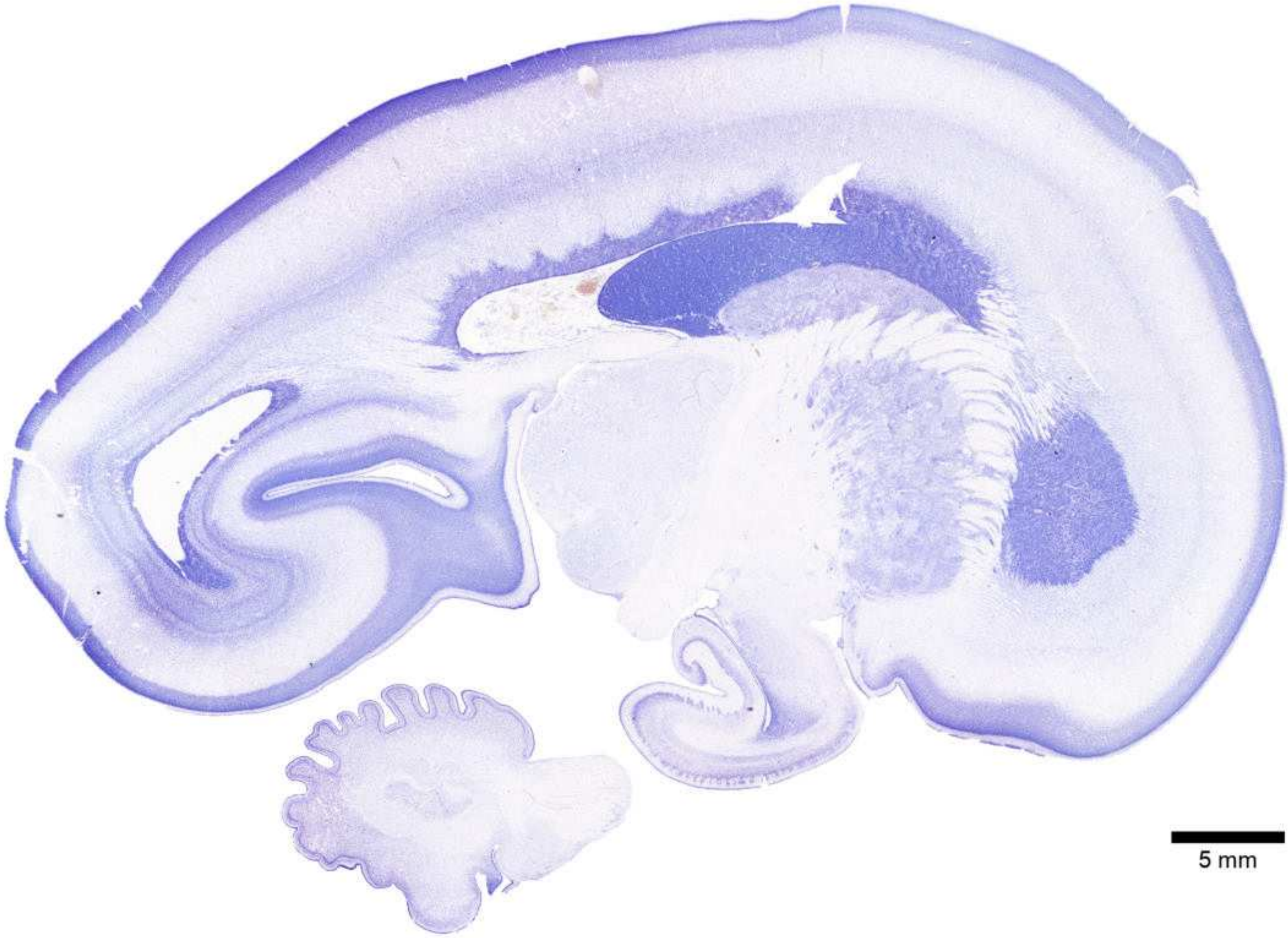

5 mm

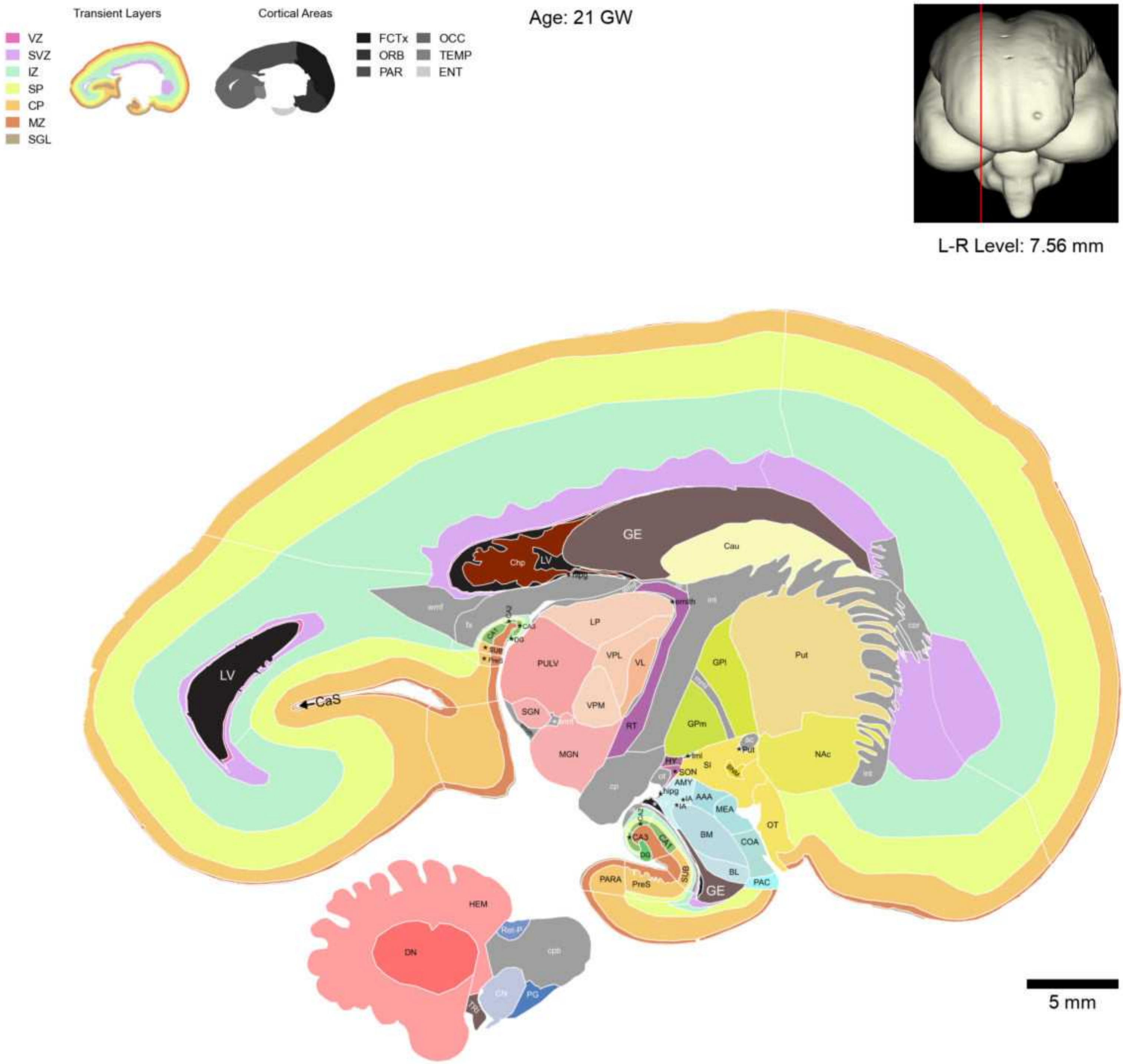

- AAA: Anterior amygdaloid area
- AMY: Amygdala
- BL: Basal nucleus [amygdala]
- BM: Basomedial nucleus [amygdala]
- BNM: Basal nucleus of Meynert
- CA1: CA1 field [hippocampus]
- CA2: CA2 field [hippocampus]
- CA3: CA3 field [hippocampus]
- CN: Cochlear nuclei
- COA: Cortical nucleus [amygdala]
- Cau: Caudate nucleus
- Chp: Choroid plexus
- DG: Dentate gyrus
- DN: Dentate nucleus
- GE: Ganglionic eminence
- GPl: Globus pallidus lateral segment
- GPm: Globus pallidus medial segment
- HEM: Cerebellar hemispheres
- HY: Hypothalamus
- IA: Intercalated cell groups [amygdala]
- LHA: Lateral hypothalamic area
- LP: Lateral posterior nucleus [thalamus]
- LV: Lateral ventricle
- MEA: Medial nucleus [amygdala]
- MGN: Medial geniculate nucleus
- NAc: Nucleus accumbens
- OT: Olfactory tubercle
- PARA: Cortical plate, parasubiculum
- PG: Pontine gray
- PULV: Pulvinar nucleus [thalamus]
- PreS: Cortical plate, presubiculum
- Put: Putamen
- RT: Reticular nucleus [thalamus]
- Ret-P: Reticular formation, Pons
- SGN: Suprageniculate nucleus
- SI: Substantia innominata
- SON: Supraoptic nucleus [hypothalamus]
- SUB: Cortical plate, subiculum
- TRI: Germinal trigone
- VL: Ventral lateral nucleus [thalamus]
- VPL: Ventral posterolateral nucleus [thalamus]
- VPM: Ventral posteromedial nucleus [thalamus]
- ac: Anterior commissure
- cor: Corona radiata
- cp: Cerebral peduncle
- cpb: Cerebellar peduncles
- emlth: External medullary lamina [thalamus]
- fx: Fornix
- hipg: Hippocampal glioepithelium/ependyma
- int: Internal capsule
- lml: Lateral medullary lamina
- mml: Medial medullary lamina
- ot: Optic tract
- stt: Stria terminalis
- wmf: White matter fibers
- → CaS: Calcarine sulcus

Age: 21 GW

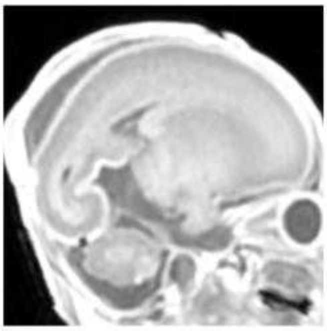

L-R Level: 7.32 mm

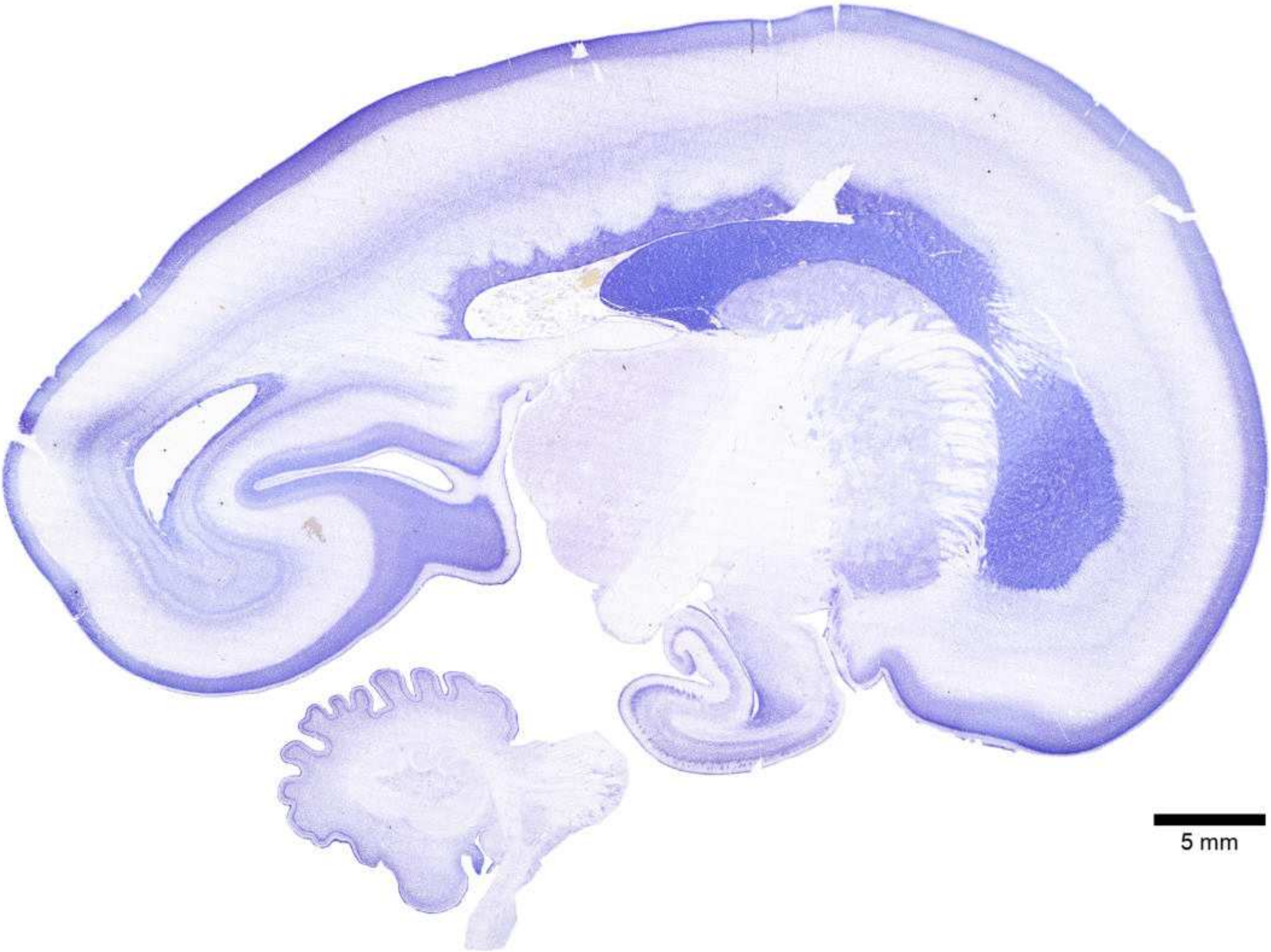

5 mm

Transient Layers

- VZ
- SVZ
- IZ
- SP
- CP
- MZ
- SGL

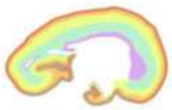

Cortical Areas

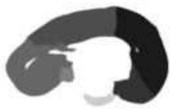

- FCTx
- ORB
- PAR
- OCC
- TEMP
- ENT

Age: 21 GW

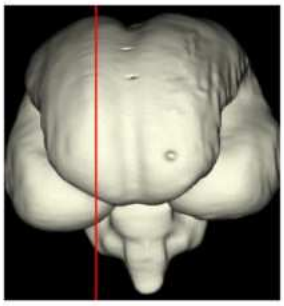

L-R Level: 7.32 mm

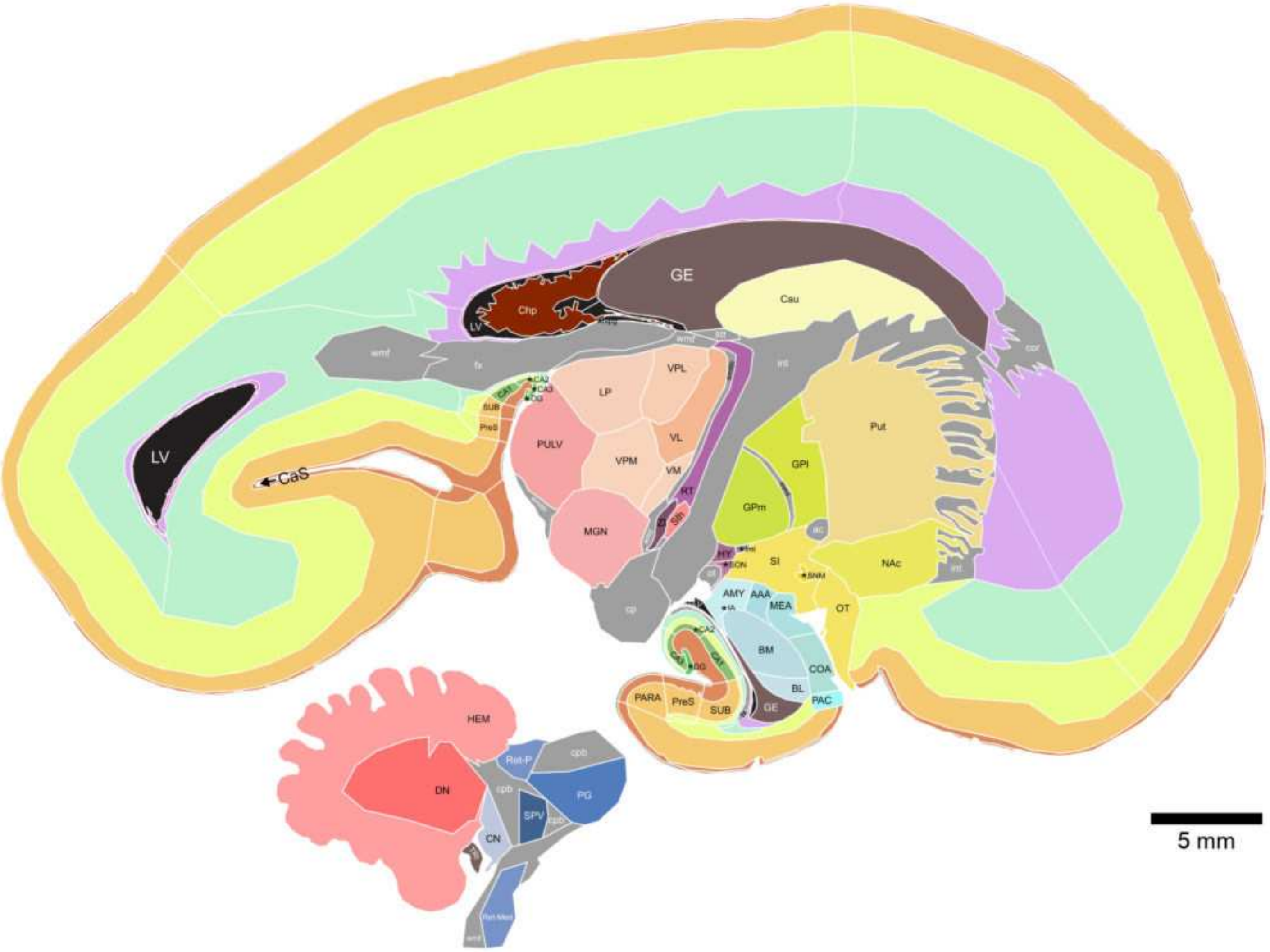

- AAA: Anterior amygdaloid area
- AMY: Amygdala
- BL: Basal nucleus [amygdala]
- BM: Basomedial nucleus [amygdala]
- BNM: Basal nucleus of Meynert
- CA1: CA1 field [hippocampus]
- CA2: CA2 field [hippocampus]
- CA3: CA3 field [hippocampus]
- CN: Cochlear nuclei
- COA: Cortical nucleus [amygdala]
- Cau: Caudate nucleus
- Chp: Choroid plexus
- DG: Dentate gyrus
- DN: Dentate nucleus
- GE: Ganglionic eminence
- GPl: Globus pallidus lateral segment
- GPm: Globus pallidus medial segment
- HEM: Cerebellar hemispheres
- HY: Hypothalamus
- IA: Intercalated cell groups [amygdala]
- LHA: Lateral hypothalamic area
- LP: Lateral posterior nucleus [thalamus]
- LV: Lateral ventricle
- MEA: Medial nucleus [amygdala]
- MGN: Medial geniculate nucleus
- NAc: Nucleus accumbens
- OT: Olfactory tubercle
- PARA: Cortical plate, parasubiculum
- PG: Pontine gray
- PULV: Pulvinar nucleus [thalamus]
- PreS: Cortical plate, presubiculum
- Put: Putamen
- RT: Reticular nucleus [thalamus]
- Ret-Med: Reticular formation, Medulla
- Ret-P: Reticular formation, Pons
- SI: Substantia innominata
- SON: Supraoptic nucleus [hypothalamus]
- SPV: Spinal nucleus of the trigeminal
- SUB: Cortical plate, subiculum
- Sth: Subthalamus
- TRI: Germinal trigone
- VL: Ventral lateral nucleus [thalamus]
- VM: Ventral medial nucleus [thalamus]
- VPL: Ventral posterolateral nucleus [thalamus]
- VPM: Ventral posteromedial nucleus [thalamus]
- ZI: Zona incerta
- ac: Anterior commissure
- cor: Corona radiata
- cp: Cerebral peduncle
- cpb: Cerebellar peduncles
- emlth: External medullary lamina [thalamus]
- fx: Fornix
- hipg: Hippocampal glioepithelium/ependyma
- int: Internal capsule
- lml: Lateral medullary lamina
- mml: Medial medullary lamina
- ot: Optic tract
- stt: Stria terminalis
- wmf: White matter fibers
- → CaS: Calcarine sulcus

Age: 21 GW

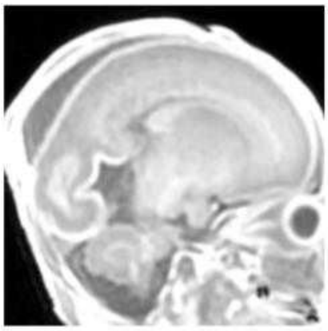

L-R Level: 6.66 mm

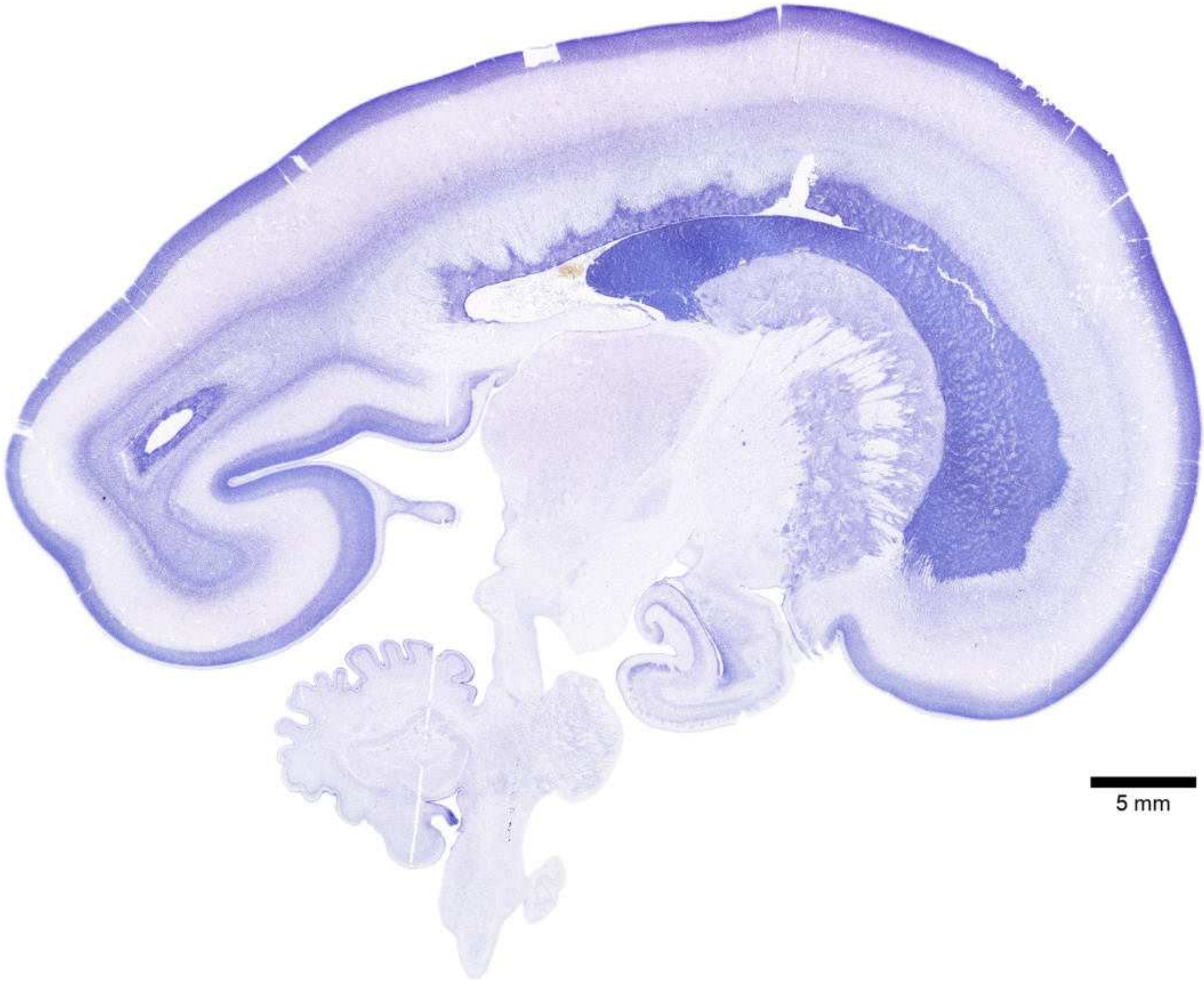

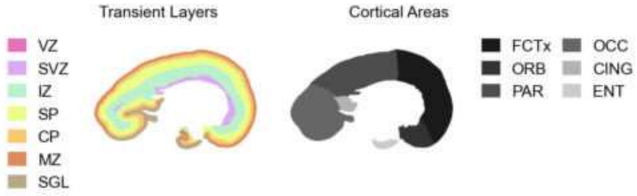

Age: 21 GW

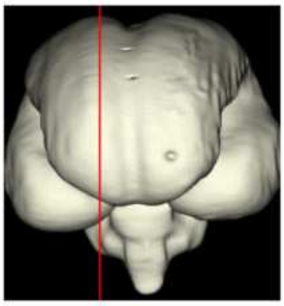

L-R Level: 6.66 mm

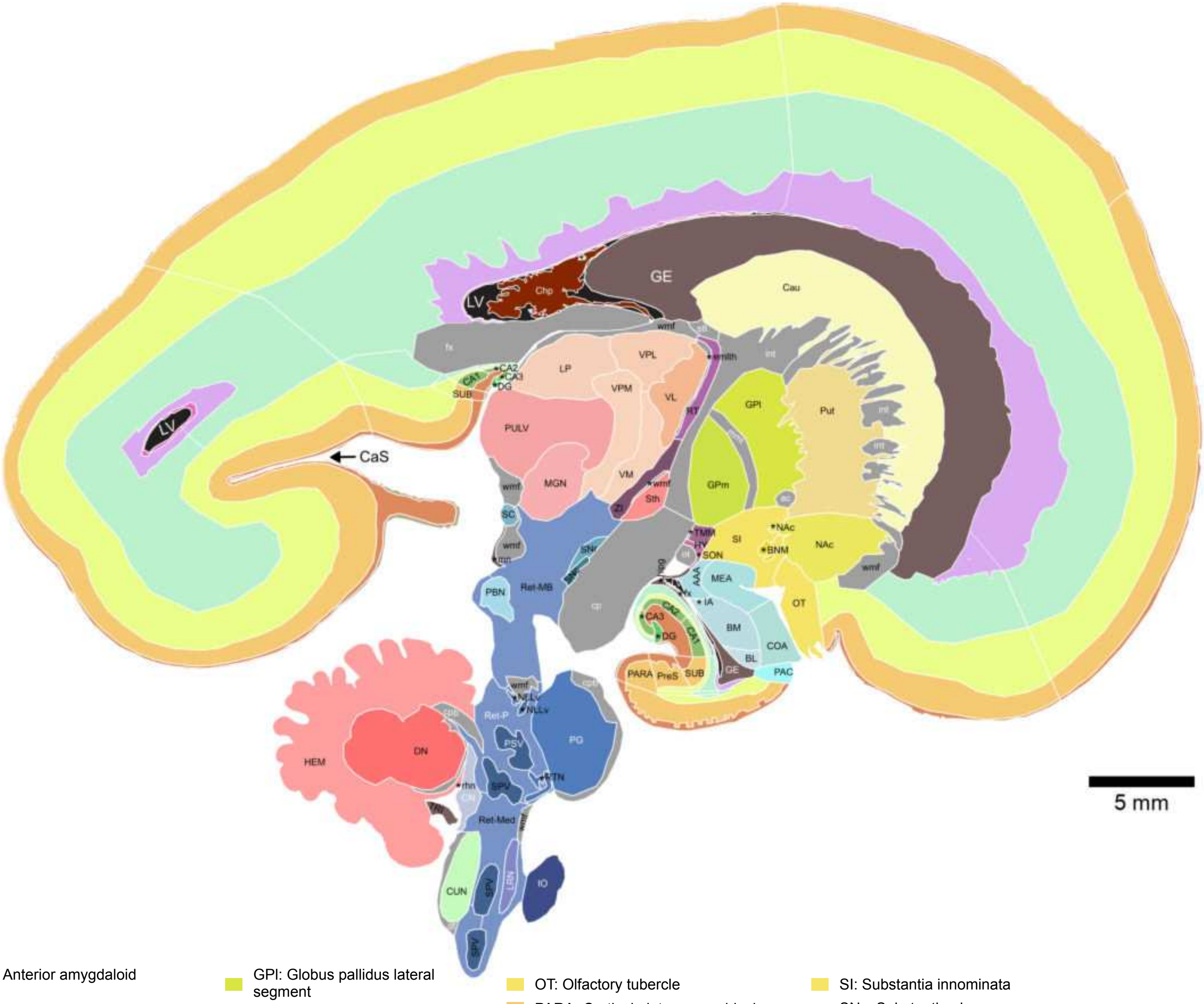

- AAA: Anterior amygdaloid area
- BL: Basal nucleus [amygdala]
- BM: Basomedial nucleus [amygdala]
- BNM: Basal nucleus of Meynert
- CA1: CA1 field [hippocampus]
- CA2: CA2 field [hippocampus]
- CA3: CA3 field [hippocampus]
- CN: Cochlear nuclei
- COA: Cortical nucleus [amygdala]
- CUN: Cuneate nucleus
- Cau: Caudate nucleus
- Chp: Choroid plexus
- DG: Dentate gyrus
- DN: Dentate nucleus
- GE: Ganglionic eminence
- GPl: Globus pallidus lateral segment
- GPm: Globus pallidus medial segment
- HEM: Cerebellar hemispheres
- HY: Hypothalamus
- IA: Intercalated cell groups [amygdala]
- IO: Inferior olive
- LHA: Lateral hypothalamic area
- LP: Lateral posterior nucleus [thalamus]
- LRN: Lateral reticular nucleus
- LV: Lateral ventricle
- MEA: Medial nucleus [amygdala]
- MGN: Medial geniculate nucleus
- NAc: Nucleus accumbens
- NLLv: Nucleus of the lateral lemniscus, ventral
- OT: Olfactory tubercle
- PARA: Cortical plate, parasubiculum
- PBN: Parabigeminal nucleus
- PG: Pontine gray
- PSV: Principal sensory nucleus of the trigeminal
- PULV: Pulvinar nucleus [thalamus]
- PreS: Cortical plate, presubiculum
- Put: Putamen
- RT: Reticular nucleus [thalamus]
- RTN: Reticular tegmental nucleus
- Ret-MB: Reticular formation, Midbrain
- Ret-Med: Reticular formation, Medulla
- Ret-P: Reticular formation, Pons
- SC: Superior colliculus
- SI: Substantia innominata
- SNc: Substantia nigra pars compacta
- SNr: Substantia nigra pars reticulata
- SON: Supraoptic nucleus [hypothalamus]
- SPV: Spinal nucleus of the trigeminal
- SUB: Cortical plate, subiculum
- Sth: Subthalamus
- TMM: Tuberomammillary nucleus
- TRI: Germinal trigone
- VL: Ventral lateral nucleus [thalamus]
- VM: Ventral medial nucleus [thalamus]
- VPL: Ventral posterolateral nucleus [thalamus]
- VPM: Ventral posteromedial nucleus [thalamus]
- ZI: Zona incerta
- → CaS: Calcarine sulcus

Age: 21 GW

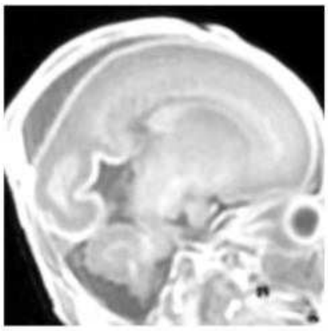

L-R Level: 6.48 mm

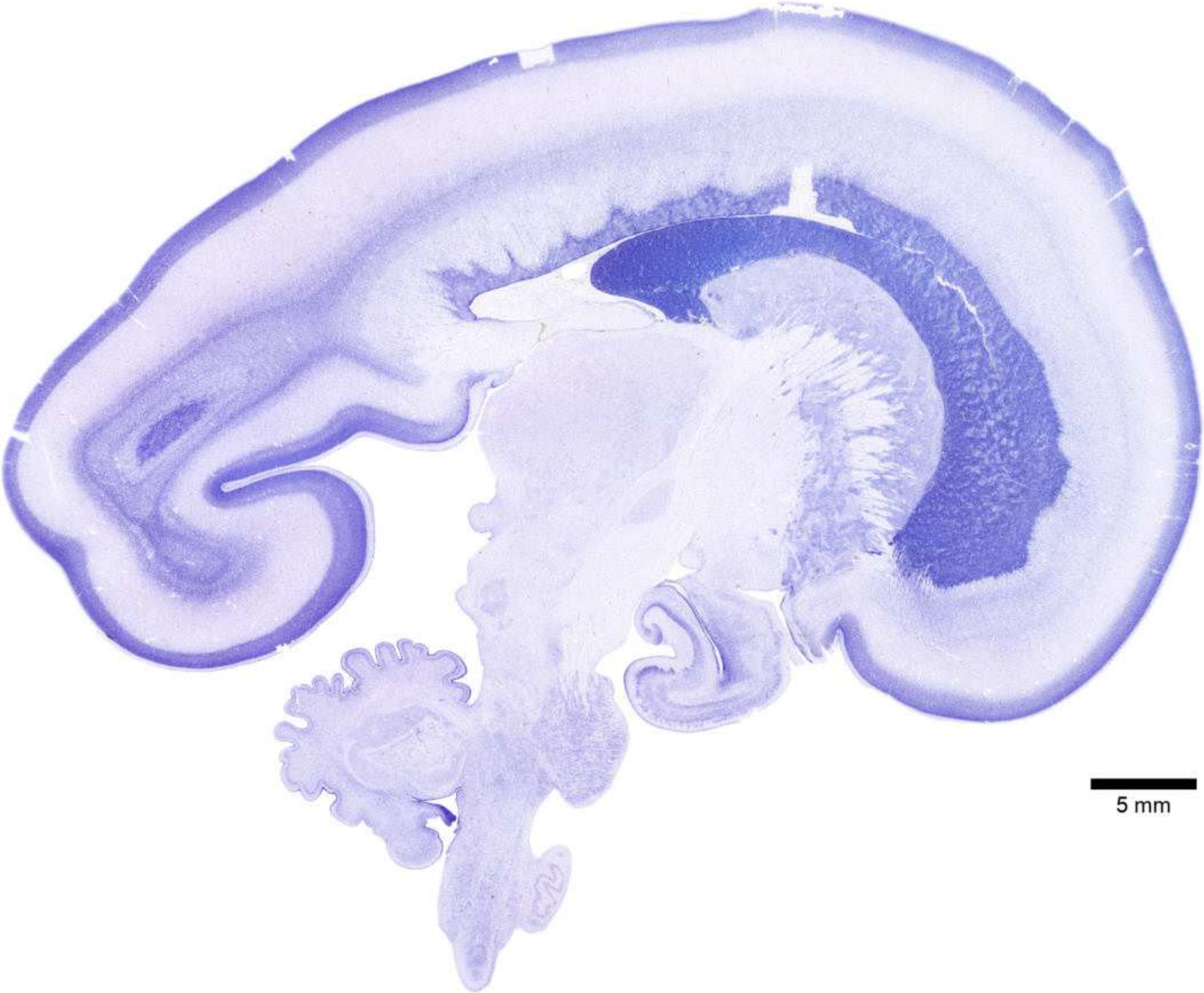

5 mm

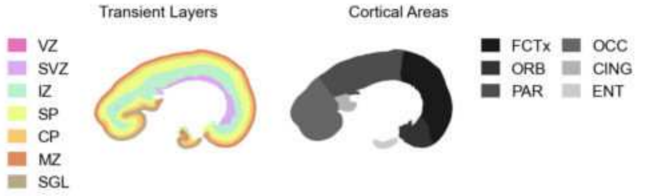

Age: 21 GW

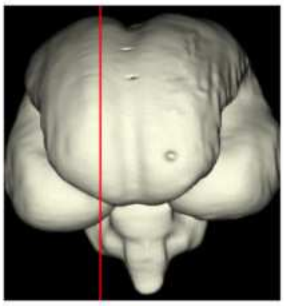

L-R Level: 6.48 mm

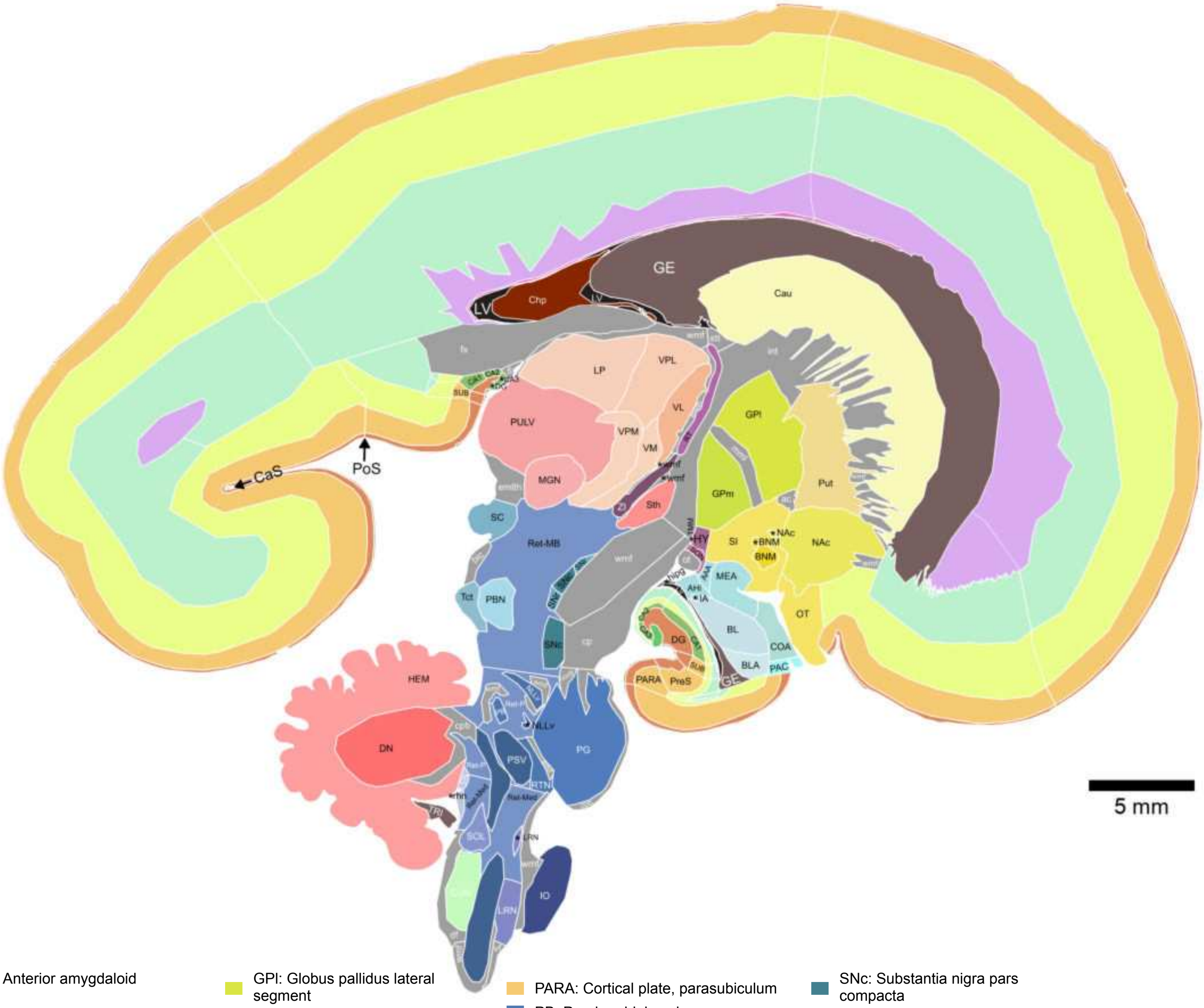

- AAA: Anterior amygdaloid area
- AHi: Amygdalo-hippocampal area
- BL: Basal nucleus [amygdala]
- BLA: Basolateral complex [amygdala]
- BNM: Basal nucleus of Meynert
- CA1: CA1 field [hippocampus]
- CA2: CA2 field [hippocampus]
- CA3: CA3 field [hippocampus]
- CN: Cochlear nuclei
- COA: Cortical nucleus [amygdala]
- CUN: Cuneate nucleus
- Cau: Caudate nucleus
- Chp: Choroid plexus
- DG: Dentate gyrus
- DN: Dentate nucleus
- GE: Ganglionic eminence
- GPl: Globus pallidus lateral segment
- GPm: Globus pallidus medial segment
- HEM: Cerebellar hemispheres
- HY: Hypothalamus
- IA: Intercalated cell groups [amygdala]
- IO: Inferior olive
- LHA: Lateral hypothalamic area
- LP: Lateral posterior nucleus [thalamus]
- LRN: Lateral reticular nucleus
- LV: Lateral ventricle
- MEA: Medial nucleus [amygdala]
- MGN: Medial geniculate nucleus
- NAc: Nucleus accumbens
- NLLv: Nucleus of the lateral lemniscus, ventral
- OT: Olfactory tubercle
- PARA: Cortical plate, parasubiculum
- PB: Parabrachial nucleus
- PBN: Parabigeminal nucleus
- PG: Pontine gray
- PSV: Principal sensory nucleus of the trigeminal
- PULV: Pulvinar nucleus [thalamus]
- PreS: Cortical plate, presubiculum
- Put: Putamen
- RT: Reticular nucleus [thalamus]
- RTN: Reticular tegmental nucleus
- Ret-MB: Reticular formation, Midbrain
- Ret-Med: Reticular formation, Medulla
- Ret-P: Reticular formation, Pons
- SC: Superior colliculus
- SI: Substantia innominata
- SNc: Substantia nigra pars compacta
- SNr: Substantia nigra pars reticulata
- SOL: Solitary nucleus
- SON: Supraoptic nucleus [hypothalamus]
- SPV: Spinal nucleus of the trigeminal
- SUB: Cortical plate, subiculum
- Sth: Subthalamus
- TMM: Tuberomammillary nucleus
- TRI: Germinal trigone
- Tct: Tectum
- VL: Ventral lateral nucleus [thalamus]
- VM: Ventral medial nucleus [thalamus]
- VPL: Ventral posterolateral nucleus [thalamus]
- VPM: Ventral posteromedial nucleus [thalamus]
- ZI: Zona incerta
- → CaS: Calcarine sulcus
- → POS: Parieto-occipital sulcus

Age: 21 GW

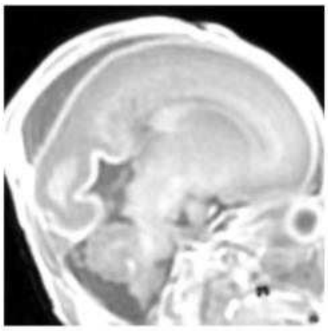

L-R Level: 6.3 mm

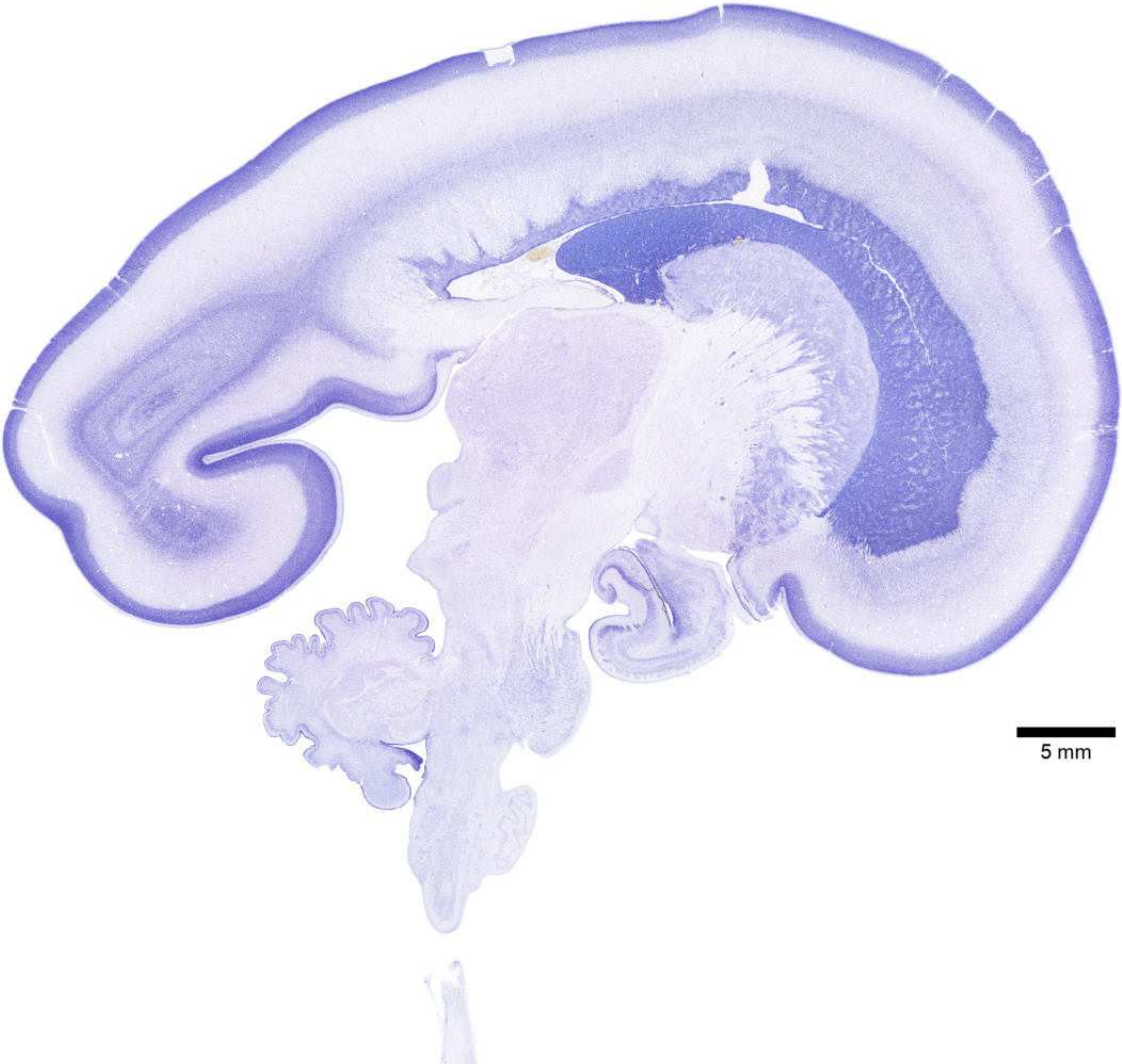

5 mm

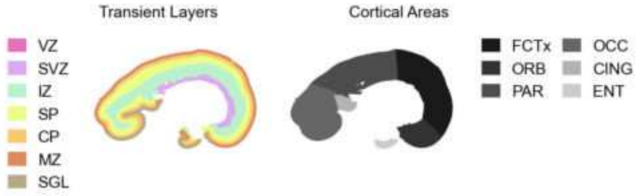

Age: 21 GW

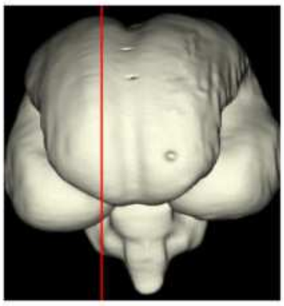

L-R Level: 6.3 mm

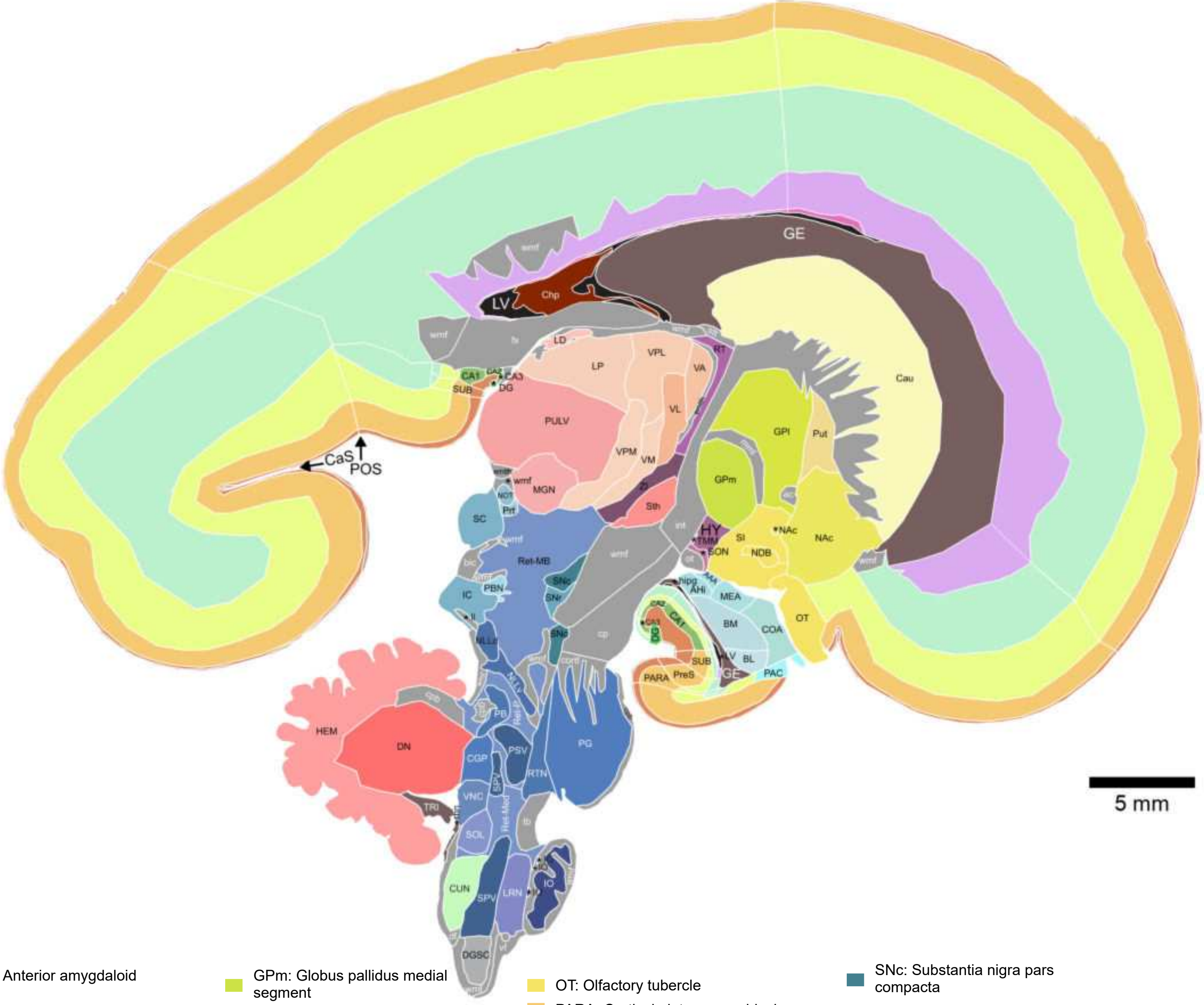

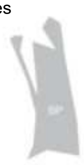

- AAA: Anterior amygdaloid area
- AHi: Amygdalo-hippocampal area
- BL: Basal nucleus [amygdala]
- BM: Basomedial nucleus [amygdala]
- CA1: CA1 field [hippocampus]
- CA2: CA2 field [hippocampus]
- CA3: CA3 field [hippocampus]
- CGP: Central gray of the pons
- COA: Cortical nucleus [amygdala]
- CUN: Cuneate nucleus
- Cau: Caudate nucleus
- Chp: Choroid plexus
- DG: Dentate gyrus
- DGSC: Dorsal gray of the spinal cord
- DN: Dentate nucleus
- GE: Ganglionic eminence
- GPl: Globus pallidus lateral segment
- GPm: Globus pallidus medial segment
- HEM: Cerebellar hemispheres
- HY: Hypothalamus
- IC: Inferior colliculus
- IO: Inferior olive
- LD: Lateral dorsal nucleus [thalamus]
- LHA: Lateral hypothalamic area
- LP: Lateral posterior nucleus [thalamus]
- LRN: Lateral reticular nucleus
- LV: Lateral ventricle
- MEA: Medial nucleus [amygdala]
- MGN: Medial geniculate nucleus
- NAc: Nucleus accumbens
- NDB: Nucleus of the diagonal band
- NLLd: Nucleus of the lateral lemniscus, dorsal
- NLLv: Nucleus of the lateral lemniscus, ventral
- NOT: Nucleus of the optic tract
- OT: Olfactory tubercle
- PARA: Cortical plate, parasubiculum
- PB: Parabrachial nucleus
- PBN: Parabigeminal nucleus
- PG: Pontine gray
- PSV: Principal sensory nucleus of the trigeminal
- PULV: Pulvinar nucleus [thalamus]
- PreS: Cortical plate, presubiculum
- Prt: Pretectum
- Put: Putamen
- RT: Reticular nucleus [thalamus]
- RTN: Reticular tegmental nucleus
- Ret-MB: Reticular formation, Midbrain
- Ret-Med: Reticular formation, Medulla
- Ret-P: Reticular formation, Pons
- SC: Superior colliculus
- SI: Substantia innominata
- SNc: Substantia nigra pars compacta
- SNr: Substantia nigra pars reticulata
- SOL: Solitary nucleus
- SON: Supraoptic nucleus [hypothalamus]
- SP: Spinal cord
- SPV: Spinal nucleus of the trigeminal
- SUB: Cortical plate, subiculum
- Sth: Subthalamus
- TMM: Tuberomammillary nucleus
- TRI: Germinal trigone
- VA: Ventral anterior nucleus [thalamus]
- VL: Ventral lateral nucleus [thalamus]
- VM: Ventral medial nucleus [thalamus]
- VNC: Vestibular nuclear complex
- VPL: Ventral posterolateral nucleus [thalamus]
- VPM: Ventral posteromedial nucleus [thalamus]
- ZI: Zona incerta
- → CaS: Calcarine sulcus
- → POS: Parieto-occipital sulcus

Age: 21 GW

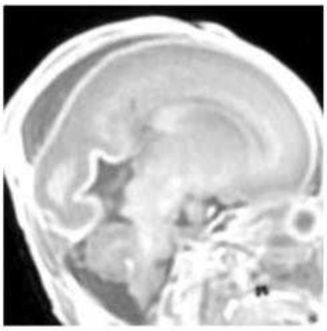

L-R Level: 5.88 mm

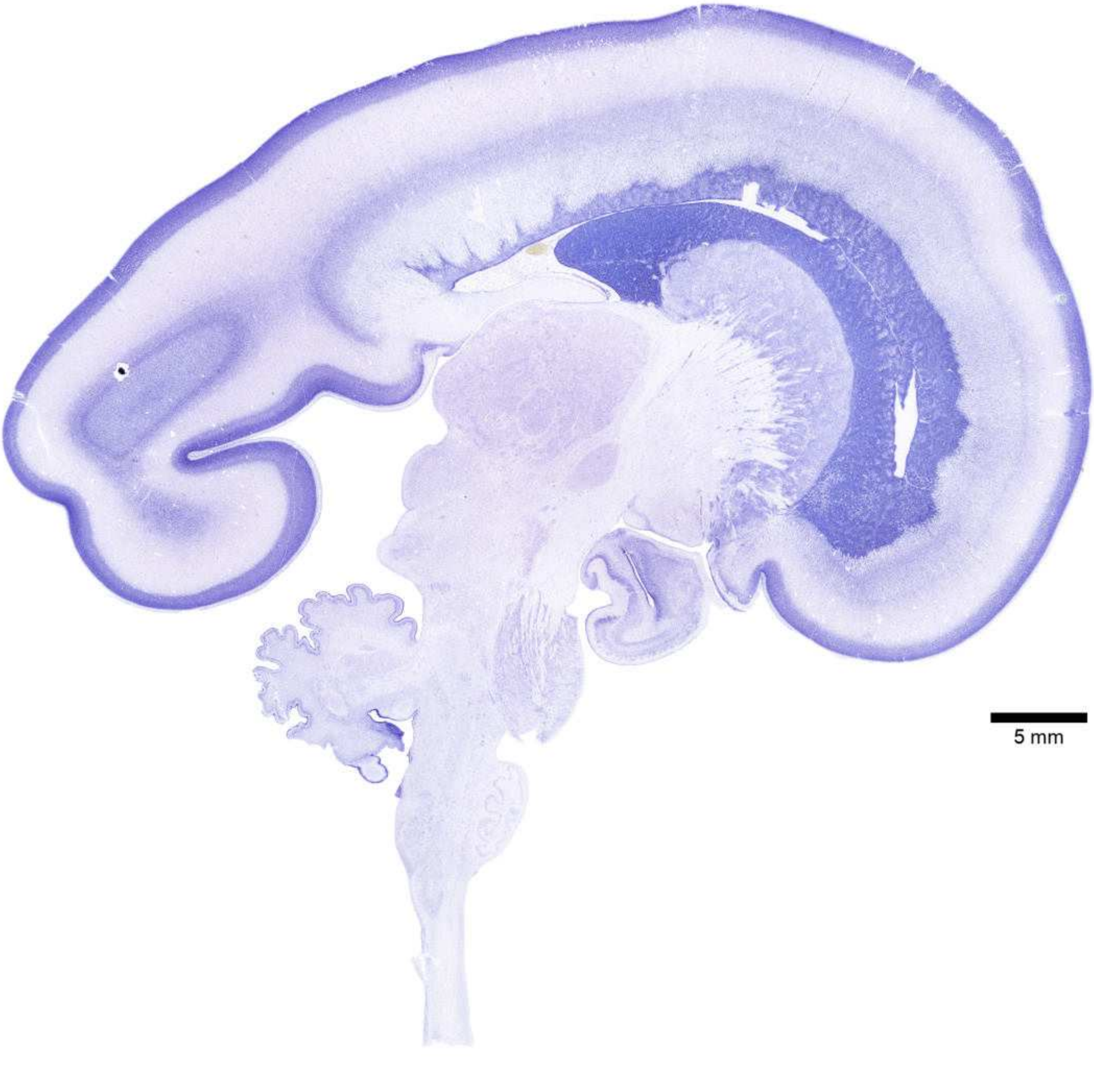

5 mm

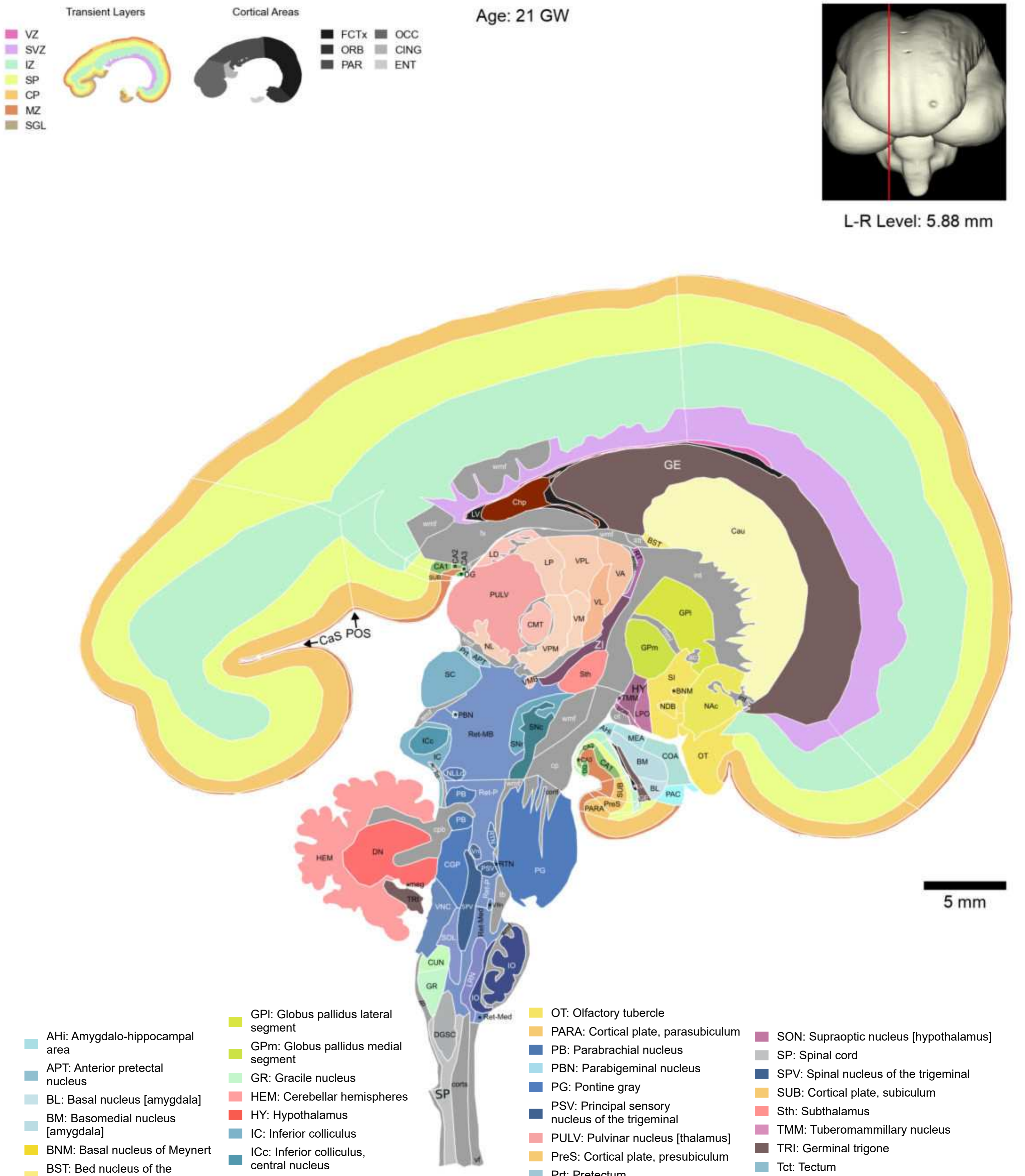

AHi: Amygdalo-hippocampal area
APT: Anterior pretectal nucleus
BL: Basal nucleus [amygdala]
BM: Basomedial nucleus [amygdala]
BNM: Basal nucleus of Meynert
BST: Bed nucleus of the stria terminalis
CA1: CA1 field [hippocampus]
CA2: CA2 field [hippocampus]
CA3: CA3 field [hippocampus]
CGP: Central gray of the pons
CMT: Centromedian nucleus [thalamus]
COA: Cortical nucleus [amygdala]
CUN: Cuneate nucleus
Cau: Caudate nucleus
Chp: Choroid plexus
DG: Dentate gyrus
DGSC: Dorsal gray of the spinal cord
DN: Dentate nucleus
GE: Ganglionic eminence
GPl: Globus pallidus lateral segment
GPm: Globus pallidus medial segment
GR: Gracile nucleus
HEM: Cerebellar hemispheres
HY: Hypothalamus
IC: Inferior colliculus
ICc: Inferior colliculus, central nucleus
IO: Inferior olive
LD: Lateral dorsal nucleus [thalamus]
LHA: Lateral hypothalamic area
LP: Lateral posterior nucleus [thalamus]
LPO: Lateral preoptic area
LRN: Lateral reticular nucleus
LV: Lateral ventricle
MEA: Medial nucleus [amygdala]
NAc: Nucleus accumbens
NDB: Nucleus of the diagonal band
NL: Nucleus limitans [thalamus]
NLLd: Nucleus of the lateral lemniscus, dorsal
OT: Olfactory tubercle
PARA: Cortical plate, parasubiculum
PB: Parabrachial nucleus
PBN: Parabigeminal nucleus
PG: Pontine gray
PSV: Principal sensory nucleus of the trigeminal
PULV: Pulvinar nucleus [thalamus]
PreS: Cortical plate, presubiculum
Prt: Pretectum
RT: Reticular nucleus [thalamus]
RTN: Reticular tegmental nucleus
Ret-MB: Reticular formation, Midbrain
Ret-Med: Reticular formation, Medulla
Ret-P: Reticular formation, Pons
SC: Superior colliculus
SI: Substantia innominata
SNc: Substantia nigra pars compacta
SNr: Substantia nigra pars reticulata
SOL: Solitary nucleus
SON: Supraoptic nucleus [hypothalamus]
SP: Spinal cord
SPV: Spinal nucleus of the trigeminal
SUB: Cortical plate, subiculum
Sth: Subthalamus
TMM: Tuberomammillary nucleus
TRI: Germinal trigone
Tct: Tectum
VA: Ventral anterior nucleus [thalamus]
VIIn: Facial motor nucleus
VL: Ventral lateral nucleus [thalamus]
VM: Ventral medial nucleus [thalamus]
VMB: Ventral medial basal nucleus [thalamus]
VNC: Vestibular nuclear complex
VPL: Ventral posterolateral nucleus [thalamus]
VPM: Ventral posteromedial nucleus [thalamus]
Vn: Trigeminal motor nucleus
ZI: Zona incerta
→ CaS: Calcarine sulcus
→ POS: Parieto-occipital sulcus

Age: 21 GW

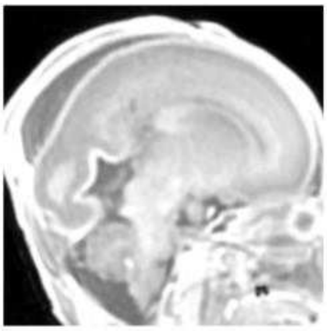

L-R Level: 5.58 mm

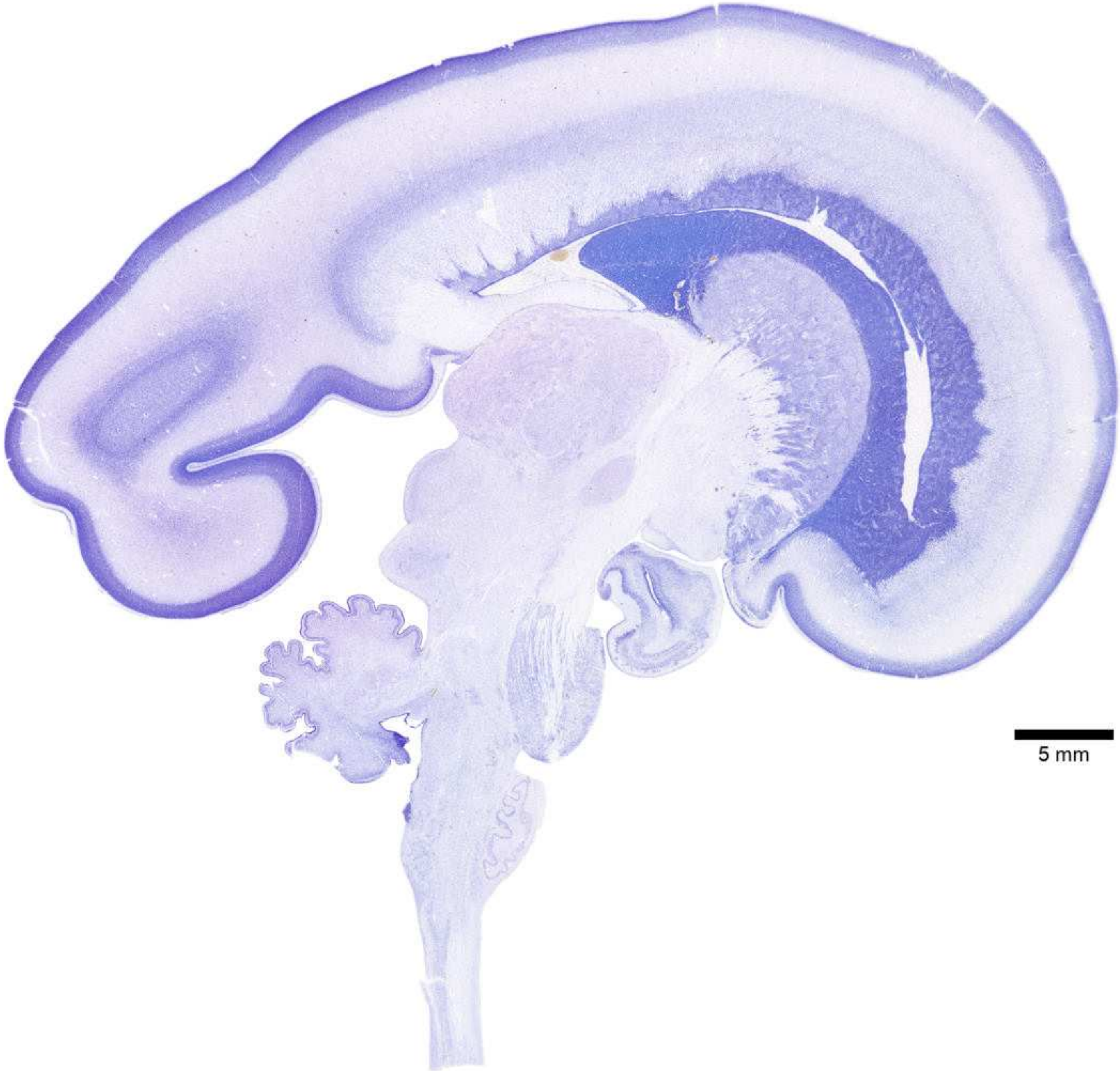

5 mm

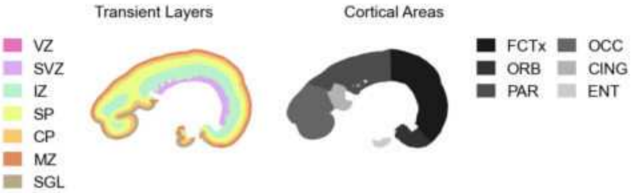

Age: 21 GW

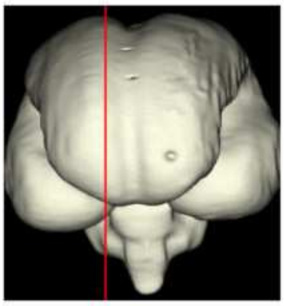

L-R Level: 5.58 mm

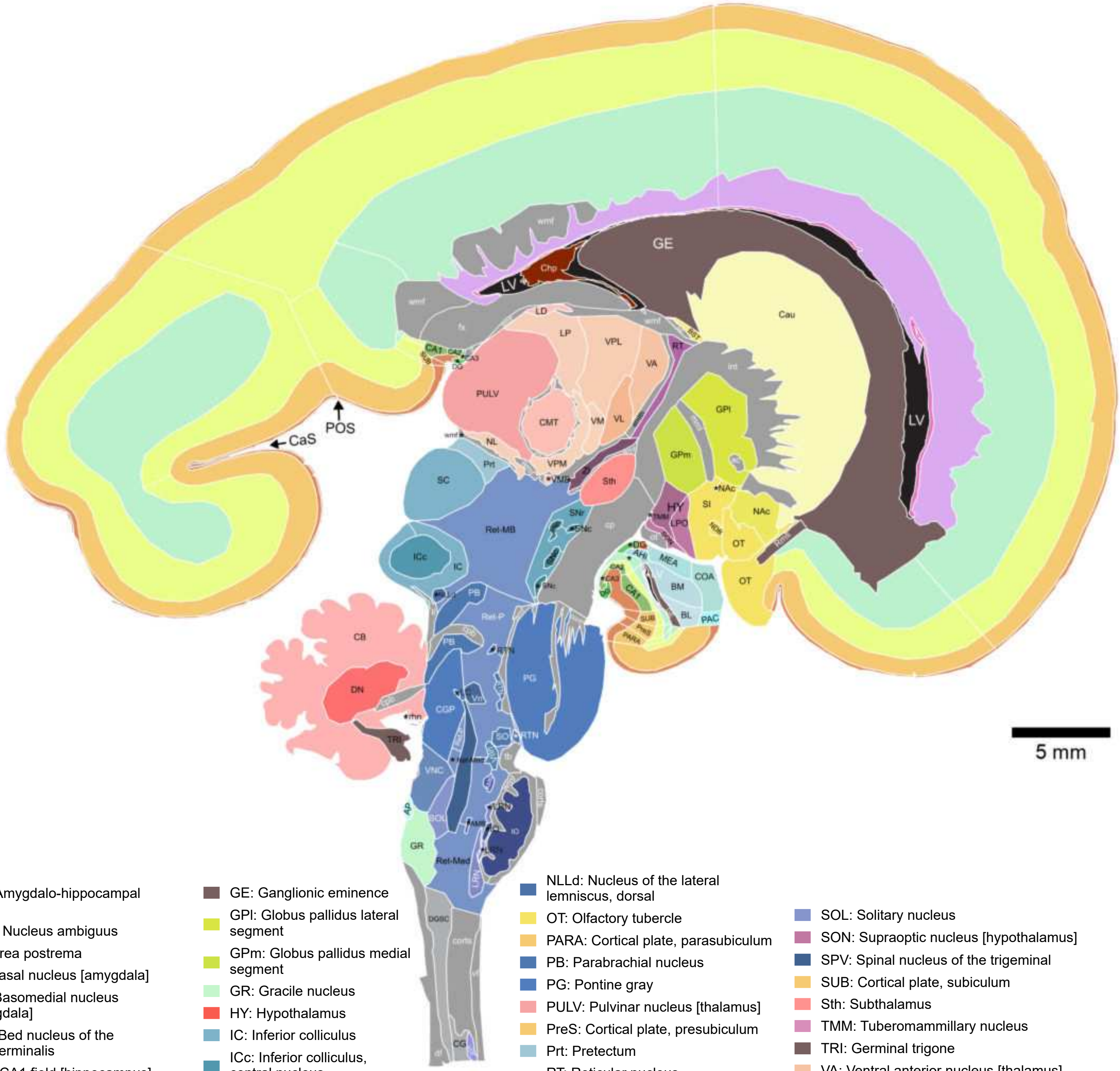

- AHi: Amygdalo-hippocampal area
- AMB: Nucleus ambiguus
- AP: Area postrema
- BL: Basal nucleus [amygdala]
- BM: Basomedial nucleus [amygdala]
- BST: Bed nucleus of the stria terminalis
- CA1: CA1 field [hippocampus]
- CA2: CA2 field [hippocampus]
- CA3: CA3 field [hippocampus]
- CB: Cerebellum
- CG: Central gray of the spinal cord
- CGP: Central gray of the pons
- CMT: Centromedian nucleus [thalamus]
- COA: Cortical nucleus [amygdala]
- Cau: Caudate nucleus
- Chp: Choroid plexus
- DG: Dentate gyrus
- DGSC: Dorsal gray of the spinal cord
- DN: Dentate nucleus
- GE: Ganglionic eminence
- GPl: Globus pallidus lateral segment
- GPm: Globus pallidus medial segment
- GR: Gracile nucleus
- HY: Hypothalamus
- IC: Inferior colliculus
- ICc: Inferior colliculus, central nucleus
- IO: Inferior olive
- LC: Locus coeruleus
- LD: Lateral dorsal nucleus [thalamus]
- LHA: Lateral hypothalamic area
- LP: Lateral posterior nucleus [thalamus]
- LPO: Lateral preoptic area
- LRN: Lateral reticular nucleus
- LV: Lateral ventricle
- MEA: Medial nucleus [amygdala]
- NAc: Nucleus accumbens
- NDB: Nucleus of the diagonal band
- NL: Nucleus limitans [thalamus]
- NLLd: Nucleus of the lateral lemniscus, dorsal
- OT: Olfactory tubercle
- PARA: Cortical plate, parasubiculum
- PB: Parabrachial nucleus
- PG: Pontine gray
- PULV: Pulvinar nucleus [thalamus]
- PreS: Cortical plate, presubiculum
- Prt: Pretectum
- RT: Reticular nucleus [thalamus]
- RTN: Reticular tegmental nucleus
- Ret-MB: Reticular formation, Midbrain
- Ret-Med: Reticular formation, Medulla
- Ret-P: Reticular formation, Pons
- Rms: Rostral migratory stream
- SC: Superior colliculus
- SI: Substantia innominata
- SNc: Substantia nigra pars compacta
- SNr: Substantia nigra pars reticulata
- SO: Superior olive
- SOL: Solitary nucleus
- SON: Supraoptic nucleus [hypothalamus]
- SPV: Spinal nucleus of the trigeminal
- SUB: Cortical plate, subiculum
- Sth: Subthalamus
- TMM: Tuberomammillary nucleus
- TRI: Germinal trigone
- VA: Ventral anterior nucleus [thalamus]
- VIIn: Facial motor nucleus
- VL: Ventral lateral nucleus [thalamus]
- VM: Ventral medial nucleus [thalamus]
- VMB: Ventral medial basal nucleus [thalamus]
- VNC: Vestibular nuclear complex
- VPL: Ventral posterolateral nucleus [thalamus]
- VPM: Ventral posteromedial nucleus [thalamus]
- Vn: Trigeminal motor nucleus
- XIn: Accessory nucleus
- ZI: Zona incerta
- → CaS: Calcarine sulcus
- → POS: Parieto-occipital sulcus

Age: 21 GW

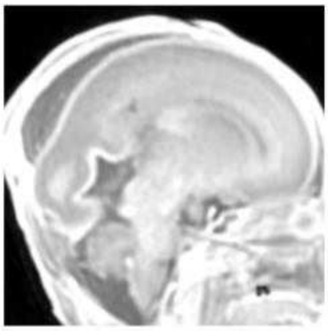

L-R Level: 4.62 mm

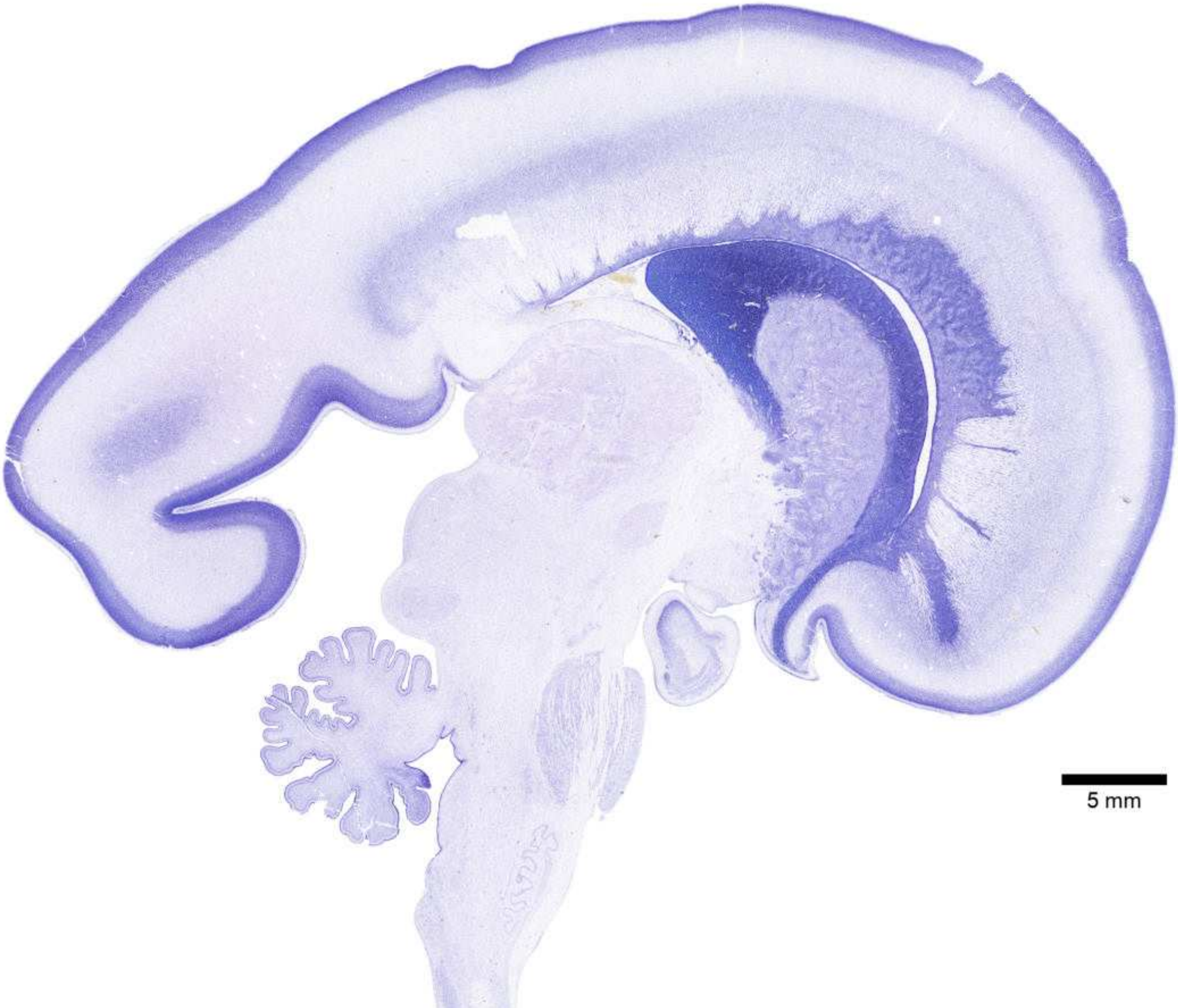

Transient Layers

- VZ
- SVZ
- IZ
- SP
- CP
- MZ
- SGL

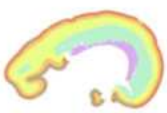

Cortical Areas

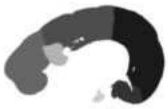

- FCTx
- ORB
- PAR
- OCC
- CING
- ENT

Age: 21 GW

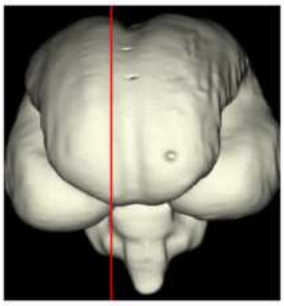

L-R Level: 4.62 mm

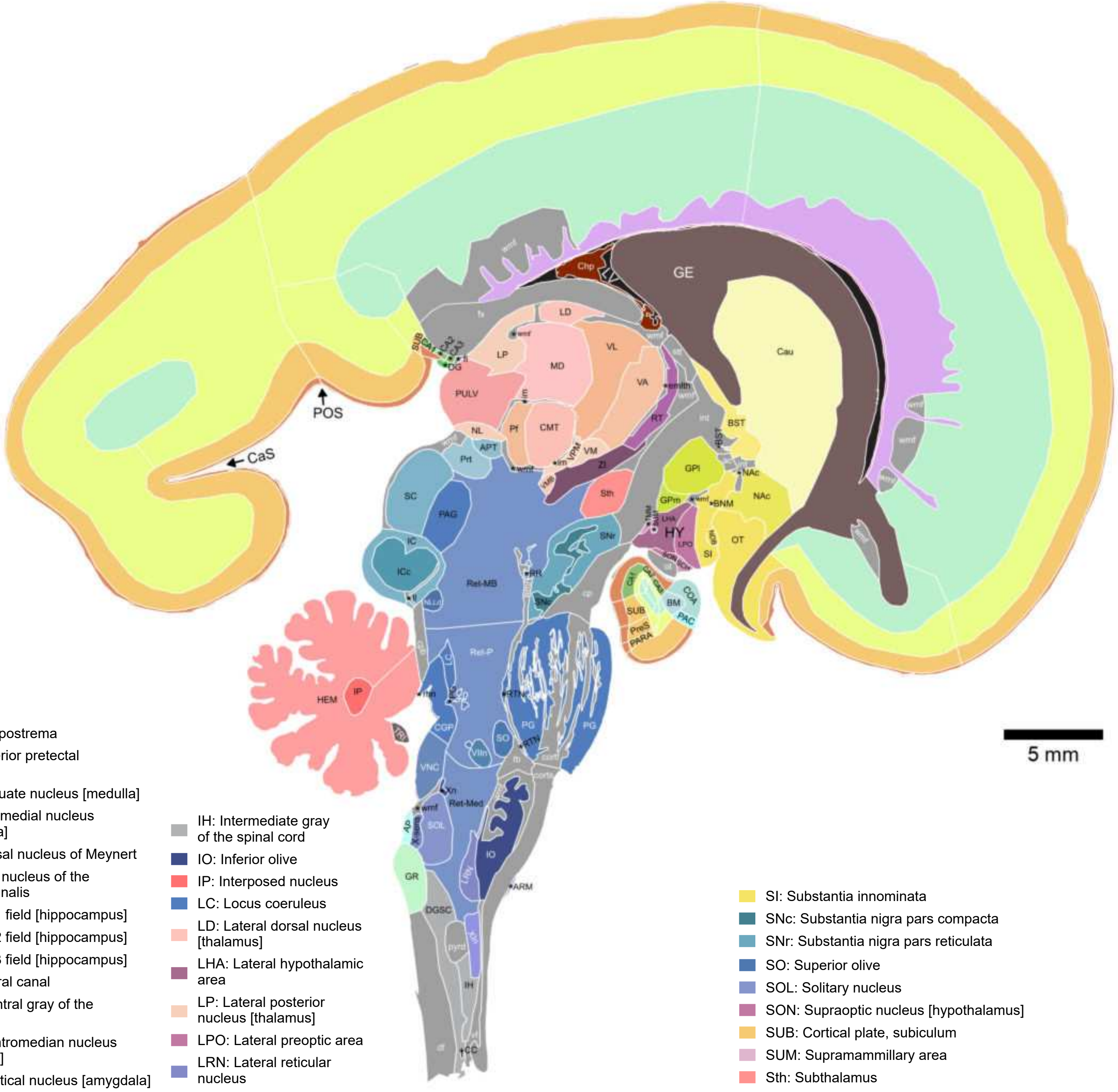

- AP: Area postrema
- APT: Anterior pretectal nucleus
- ARM: Arcuate nucleus [medulla]
- BM: Basomedial nucleus [amygdala]
- BNM: Basal nucleus of Meynert
- BST: Bed nucleus of the stria terminalis
- CA1: CA1 field [hippocampus]
- CA2: CA2 field [hippocampus]
- CA3: CA3 field [hippocampus]
- CC: Central canal
- CGP: Central gray of the pons
- CMT: Centromedian nucleus [thalamus]
- COA: Cortical nucleus [amygdala]
- Cau: Caudate nucleus
- Chp: Choroid plexus
- DG: Dentate gyrus
- DGSC: Dorsal gray of the spinal cord
- GE: Ganglionic eminence
- GPl: Globus pallidus lateral segment
- GPm: Globus pallidus medial segment
- GR: Gracile nucleus
- HEM: Cerebellar hemispheres
- HY: Hypothalamus
- IC: Inferior colliculus
- ICc: Inferior colliculus, central nucleus
- IH: Intermediate gray of the spinal cord
- IO: Inferior olive
- IP: Interposed nucleus
- LC: Locus coeruleus
- LD: Lateral dorsal nucleus [thalamus]
- LHA: Lateral hypothalamic area
- LP: Lateral posterior nucleus [thalamus]
- LPO: Lateral preoptic area
- LRN: Lateral reticular nucleus
- LV: Lateral ventricle
- MD: Medial dorsal nucleus [thalamus]
- NAc: Nucleus accumbens
- NDB: Nucleus of the diagonal band
- NL: Nucleus limitans [thalamus]
- NLLd: Nucleus of the lateral lemniscus, dorsal
- OT: Olfactory tubercle
- PAG: Periaqueductal gray
- PARA: Cortical plate, parasubiculum
- PG: Pontine gray
- PULV: Pulvinar nucleus [thalamus]
- Pf: Parafascicular nucleus [thalamus]
- PreS: Cortical plate, presubiculum
- Prt: Pretectum
- RR: Retrorubral area
- RT: Reticular nucleus [thalamus]
- RTN: Reticular tegmental nucleus
- Ret-MB: Reticular formation, Midbrain
- Ret-Med: Reticular formation, Medulla
- Ret-P: Reticular formation, Pons
- SC: Superior colliculus
- SCH: Suprachiasmatic nucleus [hypothalamus]
- SI: Substantia innominata
- SNc: Substantia nigra pars compacta
- SNr: Substantia nigra pars reticulata
- SO: Superior olive
- SOL: Solitary nucleus
- SON: Supraoptic nucleus [hypothalamus]
- SUB: Cortical plate, subiculum
- SUM: Supramammillary area
- Sth: Subthalamus
- TMM: Tuberomammillary nucleus
- TRI: Germinal trigone
- VA: Ventral anterior nucleus [thalamus]
- VIIn: Facial motor nucleus
- VL: Ventral lateral nucleus [thalamus]
- VM: Ventral medial nucleus [thalamus]
- VMB: Ventral medial basal nucleus [thalamus]
- VNC: Vestibular nuclear complex
- VPM: Ventral posteromedial nucleus [thalamus]
- X-sens: Dorsal sensory nucleus X
- XIn: Accessory nucleus
- Xn: Dorsal motor nucleus
- ZI: Zona incerta
- → CaS: Calcarine sulcus
- → POS: Parieto-occipital sulcus

Age: 21 GW

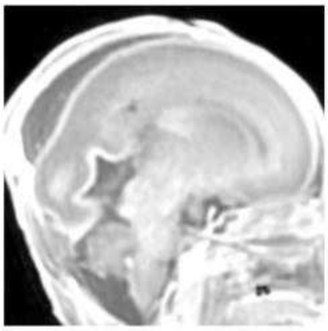

L-R Level: 4.5 mm

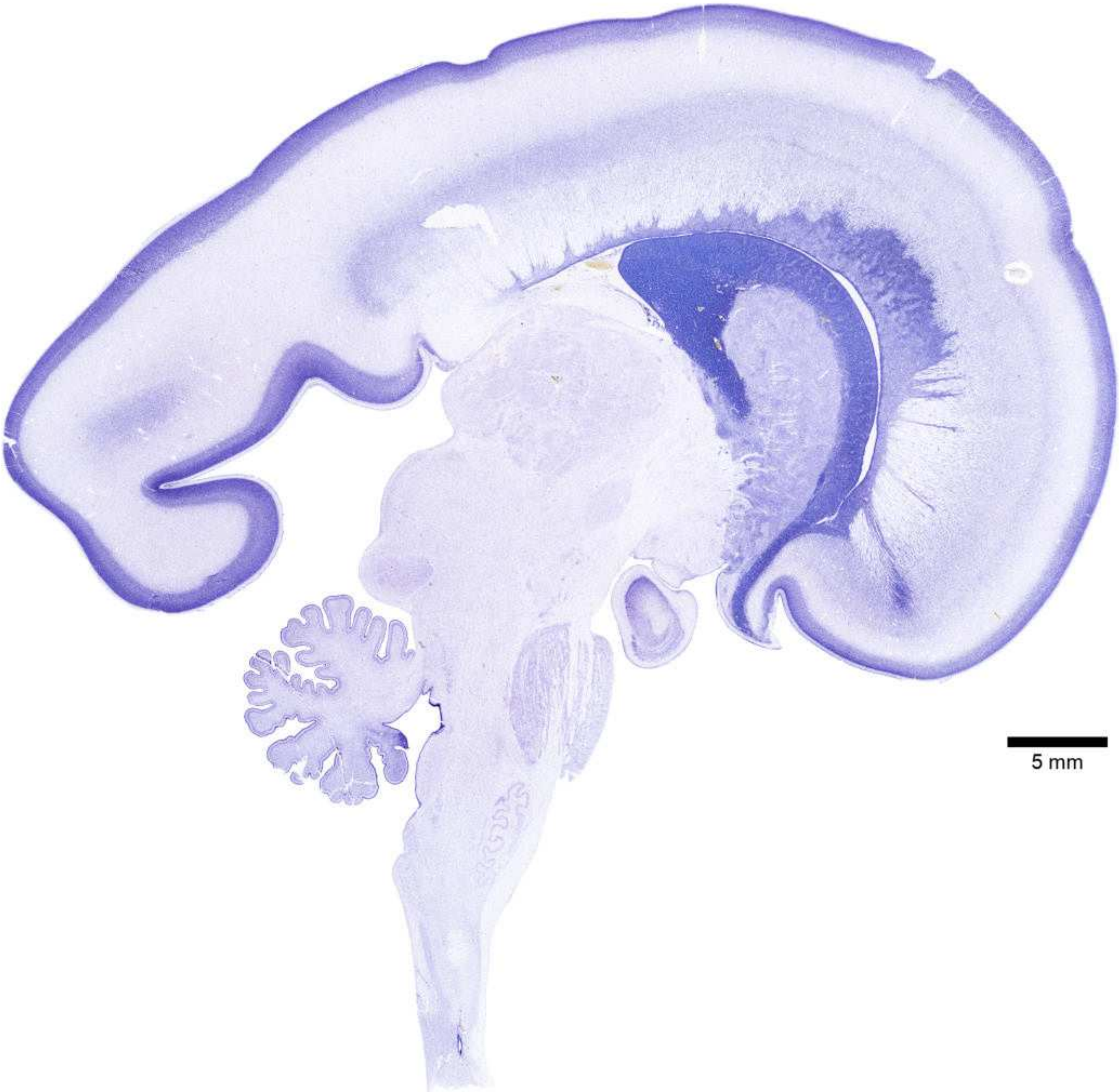

5 mm

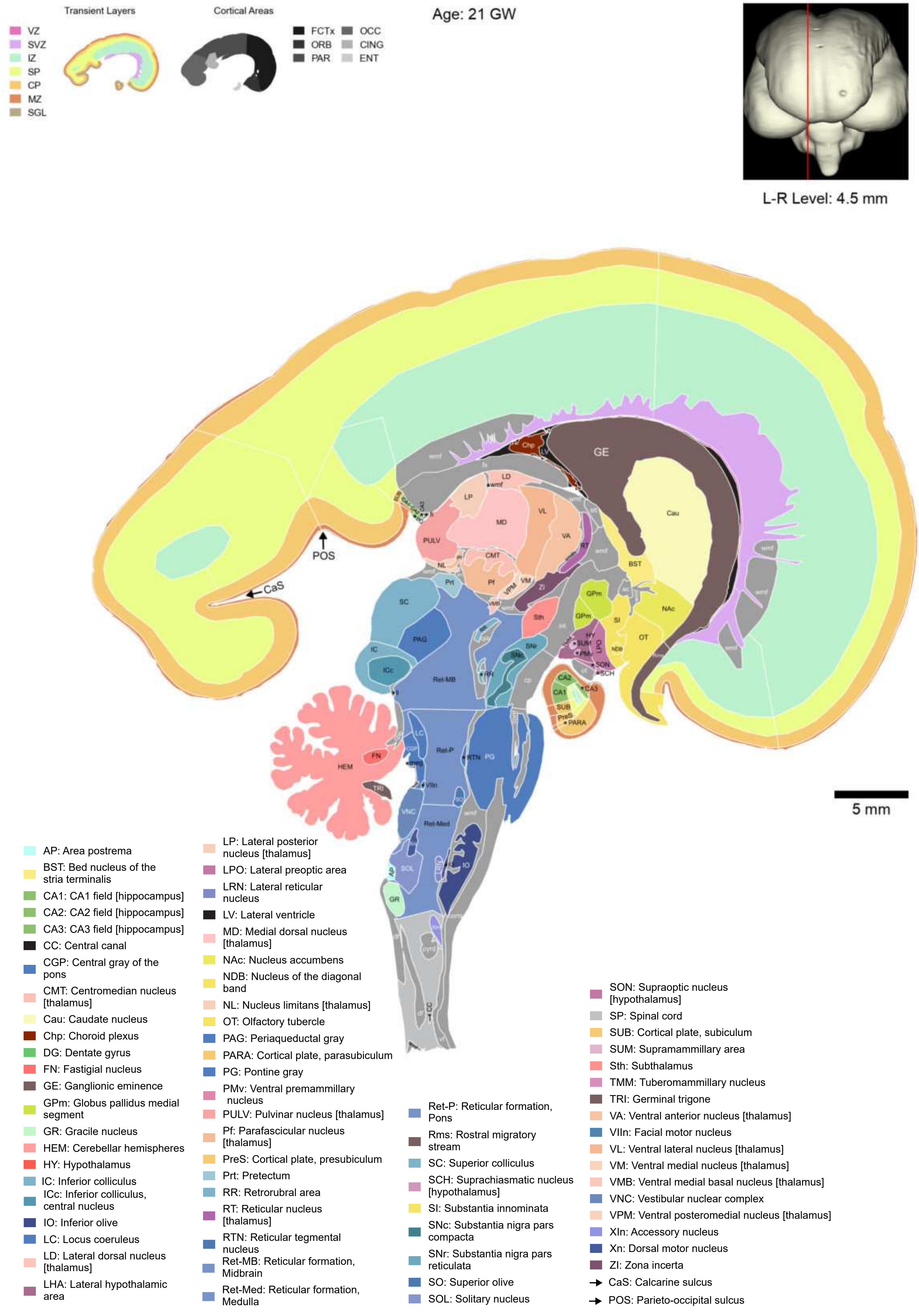
Transient Layers
VZ
SVZ
IZ
SP
CP
MZ
SGL
Cortical Areas
FCTx
ORB
PAR
OCC
CING
ENT
Age: 21 GW
L-R Level: 4.5 mm
GE
Cau
POS
CaS
5 mm
AP: Area postrema
BST: Bed nucleus of the stria terminalis
CA1: CA1 field [hippocampus]
CA2: CA2 field [hippocampus]
CA3: CA3 field [hippocampus]
CC: Central canal
CGP: Central gray of the pons
CMT: Centromedian nucleus [thalamus]
Cau: Caudate nucleus
Chp: Choroid plexus
DG: Dentate gyrus
FN: Fastigial nucleus
GE: Ganglionic eminence
GPm: Globus pallidus medial segment
GR: Gracile nucleus
HEM: Cerebellar hemispheres
HY: Hypothalamus
IC: Inferior colliculus
ICc: Inferior colliculus, central nucleus
IO: Inferior olive
LC: Locus coeruleus
LD: Lateral dorsal nucleus [thalamus]
LHA: Lateral hypothalamic area
LP: Lateral posterior nucleus [thalamus]
LPO: Lateral preoptic area
LRN: Lateral reticular nucleus
LV: Lateral ventricle
MD: Medial dorsal nucleus [thalamus]
NAc: Nucleus accumbens
NDB: Nucleus of the diagonal band
NL: Nucleus limitans [thalamus]
OT: Olfactory tubercle
PAG: Periaqueductal gray
PARA: Cortical plate, parasubiculum
PG: Pontine gray
PMv: Ventral premammillary nucleus
PULV: Pulvinar nucleus [thalamus]
Pf: Parafascicular nucleus [thalamus]
PreS: Cortical plate, presubiculum
Prt: Pretectum
RR: Retrorubral area
RT: Reticular nucleus [thalamus]
RTN: Reticular tegmental nucleus
Ret-MB: Reticular formation, Midbrain
Ret-Med: Reticular formation, Medulla
Ret-P: Reticular formation, Pons
Rms: Rostral migratory stream
SC: Superior colliculus
SCH: Suprachiasmatic nucleus [hypothalamus]
SI: Substantia innominata
SNc: Substantia nigra pars compacta
SNr: Substantia nigra pars reticulata
SO: Superior olive
SOL: Solitary nucleus
SON: Supraoptic nucleus [hypothalamus]
SP: Spinal cord
SUB: Cortical plate, subiculum
SUM: Supramammillary area
Sth: Subthalamus
TMM: Tuberomammillary nucleus
TRI: Germinal trigone
VA: Ventral anterior nucleus [thalamus]
VIIn: Facial motor nucleus
VL: Ventral lateral nucleus [thalamus]
VM: Ventral medial nucleus [thalamus]
VMB: Ventral medial basal nucleus [thalamus]
VNC: Vestibular nuclear complex
VPM: Ventral posteromedial nucleus [thalamus]
XIn: Accessory nucleus
Xn: Dorsal motor nucleus
ZI: Zona incerta
CaS: Calcarine sulcus
POS: Parieto-occipital sulcus

Age: 21 GW

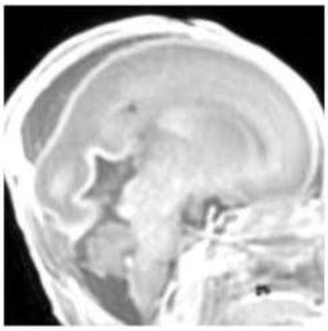

L-R Level: 4.26 mm

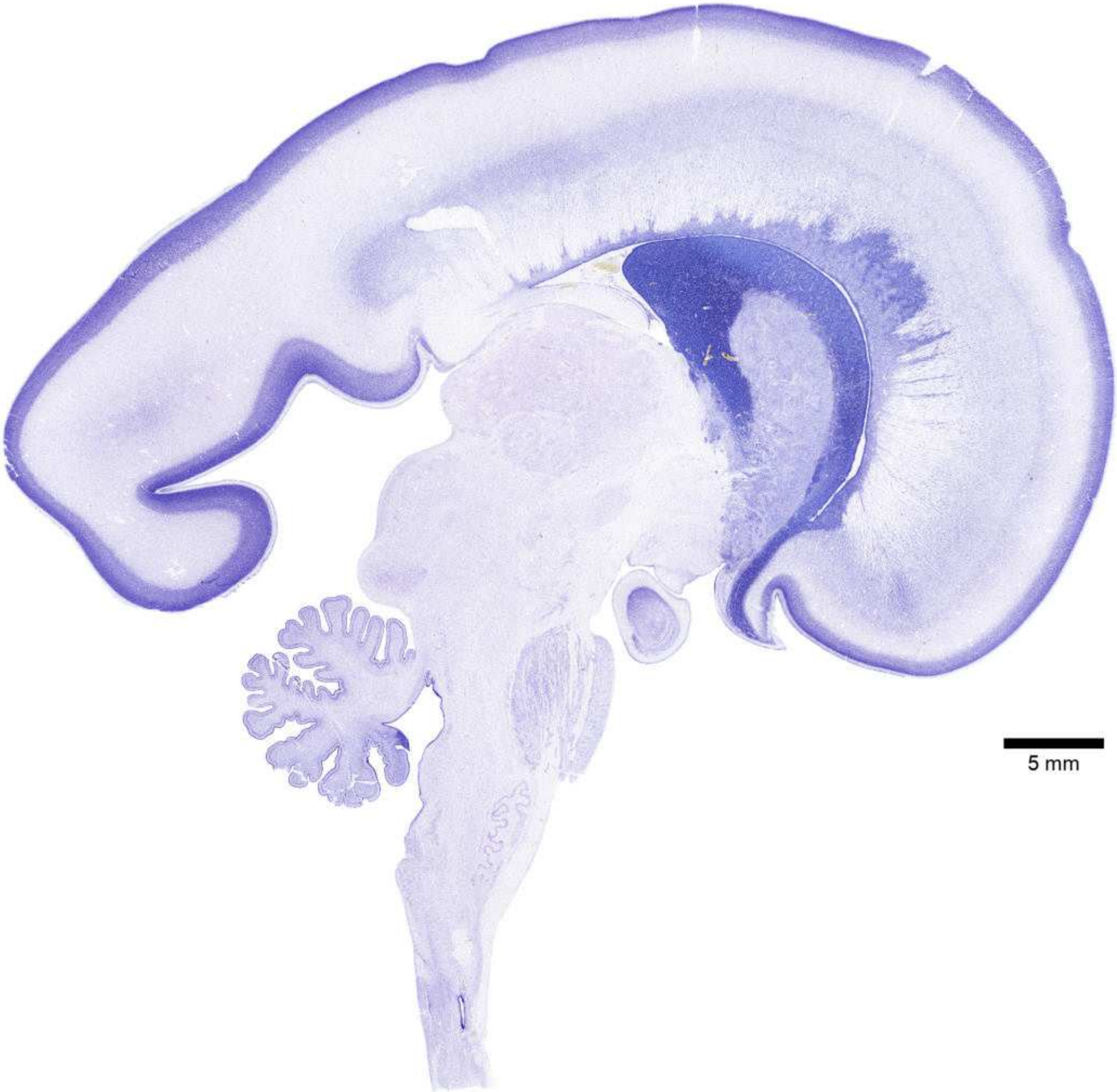

5 mm

Transient Layers

- VZ
- SVZ
- IZ
- SP
- CP
- MZ
- SGL

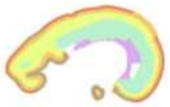

Cortical Areas

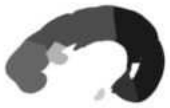

- FCTx
- ORB
- PAR
- OCC
- CING
- ENT

Age: 21 GW

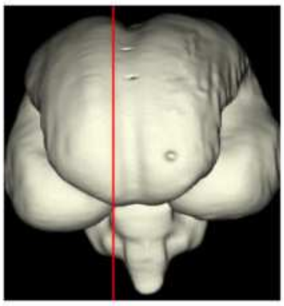

L-R Level: 4.26 mm

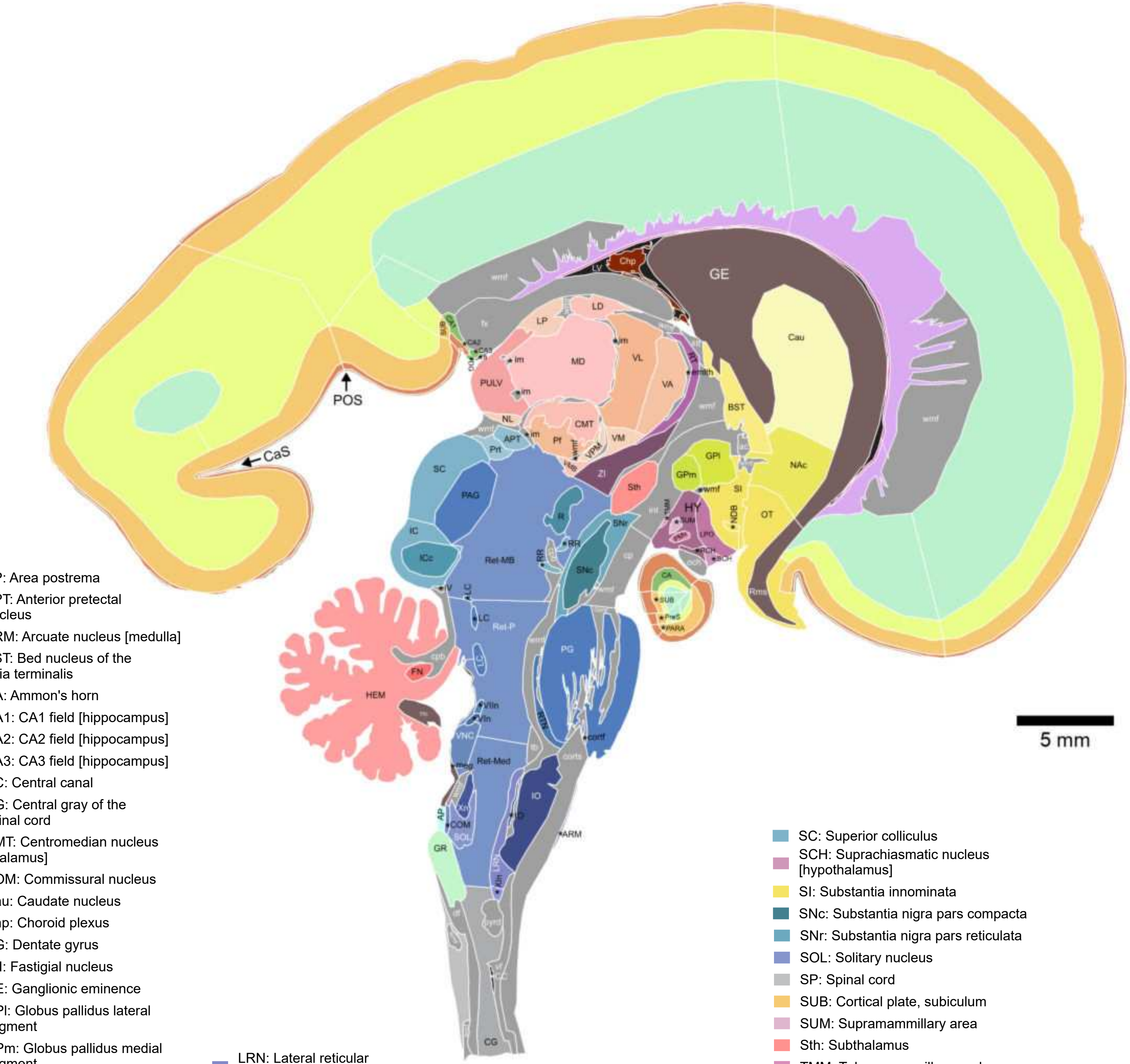

- AP: Area postrema
- APT: Anterior pretectal nucleus
- ARM: Arcuate nucleus [medulla]
- BST: Bed nucleus of the stria terminalis
- CA: Ammon's horn
- CA1: CA1 field [hippocampus]
- CA2: CA2 field [hippocampus]
- CA3: CA3 field [hippocampus]
- CC: Central canal
- CG: Central gray of the spinal cord
- CMT: Centromedian nucleus [thalamus]
- COM: Commissural nucleus
- Cau: Caudate nucleus
- Chp: Choroid plexus
- DG: Dentate gyrus
- FN: Fastigial nucleus
- GE: Ganglionic eminence
- GPl: Globus pallidus lateral segment
- GPm: Globus pallidus medial segment
- GR: Gracile nucleus
- HEM: Cerebellar hemispheres
- HY: Hypothalamus
- IC: Inferior colliculus
- ICc: Inferior colliculus, central nucleus
- IO: Inferior olive
- IV: Trochlear nerve
- LC: Locus coeruleus
- LD: Lateral dorsal nucleus [thalamus]
- LHA: Lateral hypothalamic area
- LP: Lateral posterior nucleus [thalamus]
- LPO: Lateral preoptic area
- LRN: Lateral reticular nucleus
- LV: Lateral ventricle
- MD: Medial dorsal nucleus [thalamus]
- NAc: Nucleus accumbens
- NDB: Nucleus of the diagonal band
- NL: Nucleus limitans [thalamus]
- OT: Olfactory tubercle
- PAG: Periaqueductal gray
- PARA: Cortical plate, parasubiculum
- PG: Pontine gray
- PMv: Ventral premammillary nucleus
- PULV: Pulvinar nucleus [thalamus]
- Pf: Parafascicular nucleus [thalamus]
- PreS: Cortical plate, presubiculum
- Prt: Pretectum
- R: Red nucleus
- RCH: Retrochiasmatic nucleus [hypothalamus]
- RR: Retrorubral area
- RT: Reticular nucleus [thalamus]
- RTN: Reticular tegmental nucleus
- Ret-MB: Reticular formation, Midbrain
- Ret-Med: Reticular formation, Medulla
- Ret-P: Reticular formation, Pons
- Rms: Rostral migratory stream
- SC: Superior colliculus
- SCH: Suprachiasmatic nucleus [hypothalamus]
- SI: Substantia innominata
- SNc: Substantia nigra pars compacta
- SNr: Substantia nigra pars reticulata
- SOL: Solitary nucleus
- SP: Spinal cord
- SUB: Cortical plate, subiculum
- SUM: Supramammillary area
- Sth: Subthalamus
- TMM: Tuberomammillary nucleus
- TRI: Germinal trigone
- VA: Ventral anterior nucleus [thalamus]
- VIIn: Facial motor nucleus
- VIn: Abducens nucleus
- VL: Ventral lateral nucleus [thalamus]
- VM: Ventral medial nucleus [thalamus]
- VMB: Ventral medial basal nucleus [thalamus]
- VNC: Vestibular nuclear complex
- VPM: Ventral posteromedial nucleus [thalamus]
- XIn: Accessory nucleus
- Xn: Dorsal motor nucleus
- ZI: Zona incerta
- → CaS: Calcarine sulcus
- → POS: Parieto-occipital sulcus

Age: 21 GW

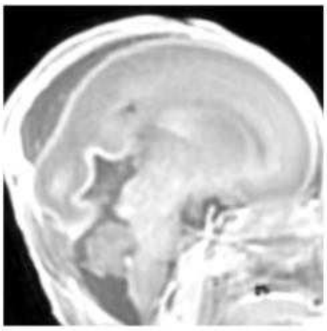

L-R Level: 4.14 mm

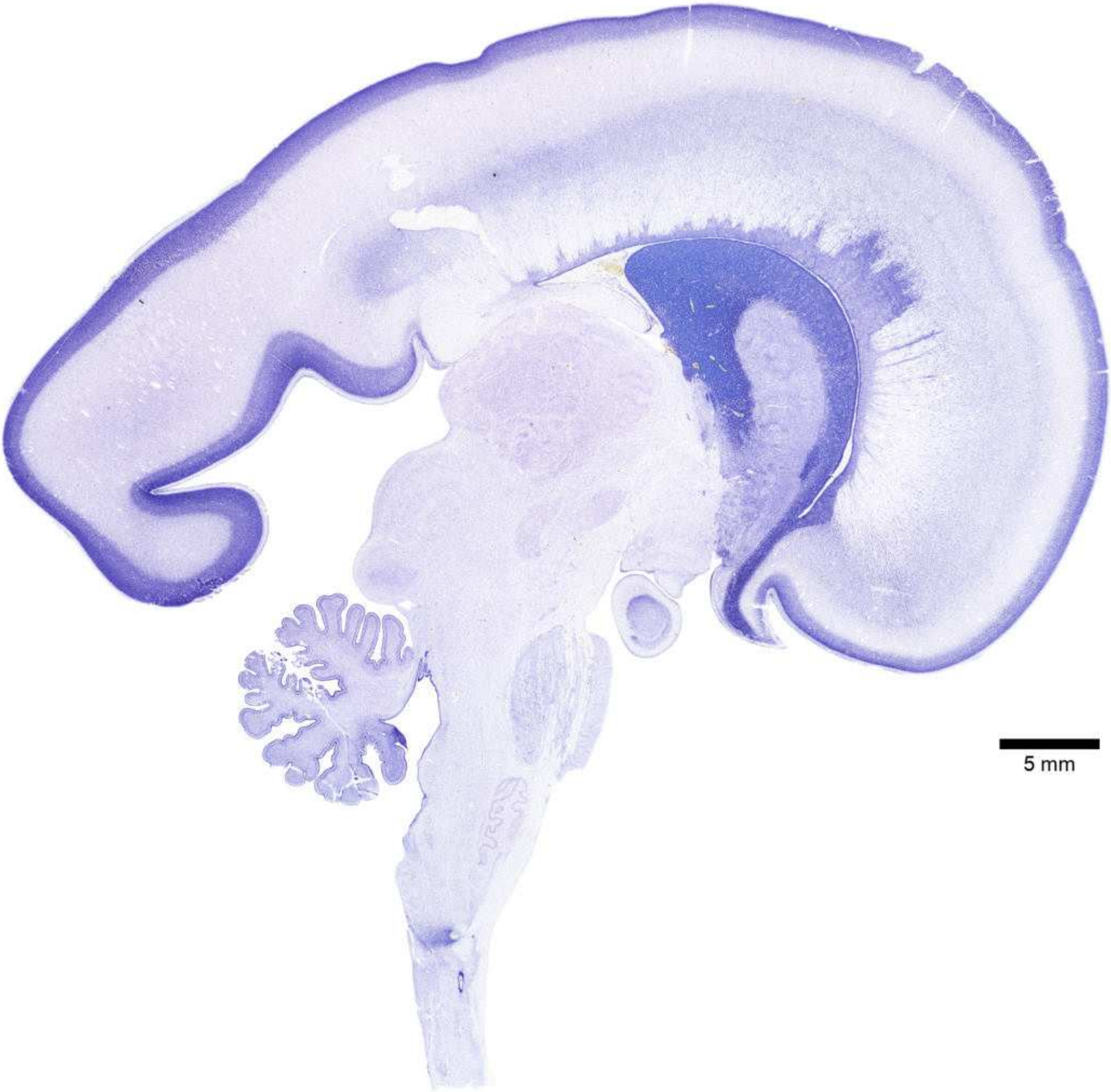

5 mm

Transient Layers

- VZ
- SVZ
- IZ
- SP
- CP
- MZ
- SGL

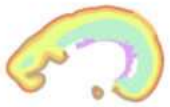

Cortical Areas

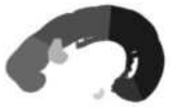

- FCTx
- ORB
- PAR
- OCC
- CING
- ENT

Age: 21 GW

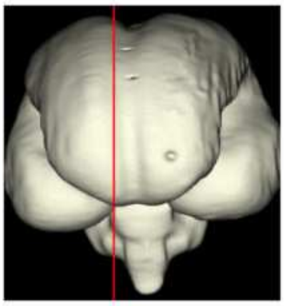

L-R Level: 4.14 mm

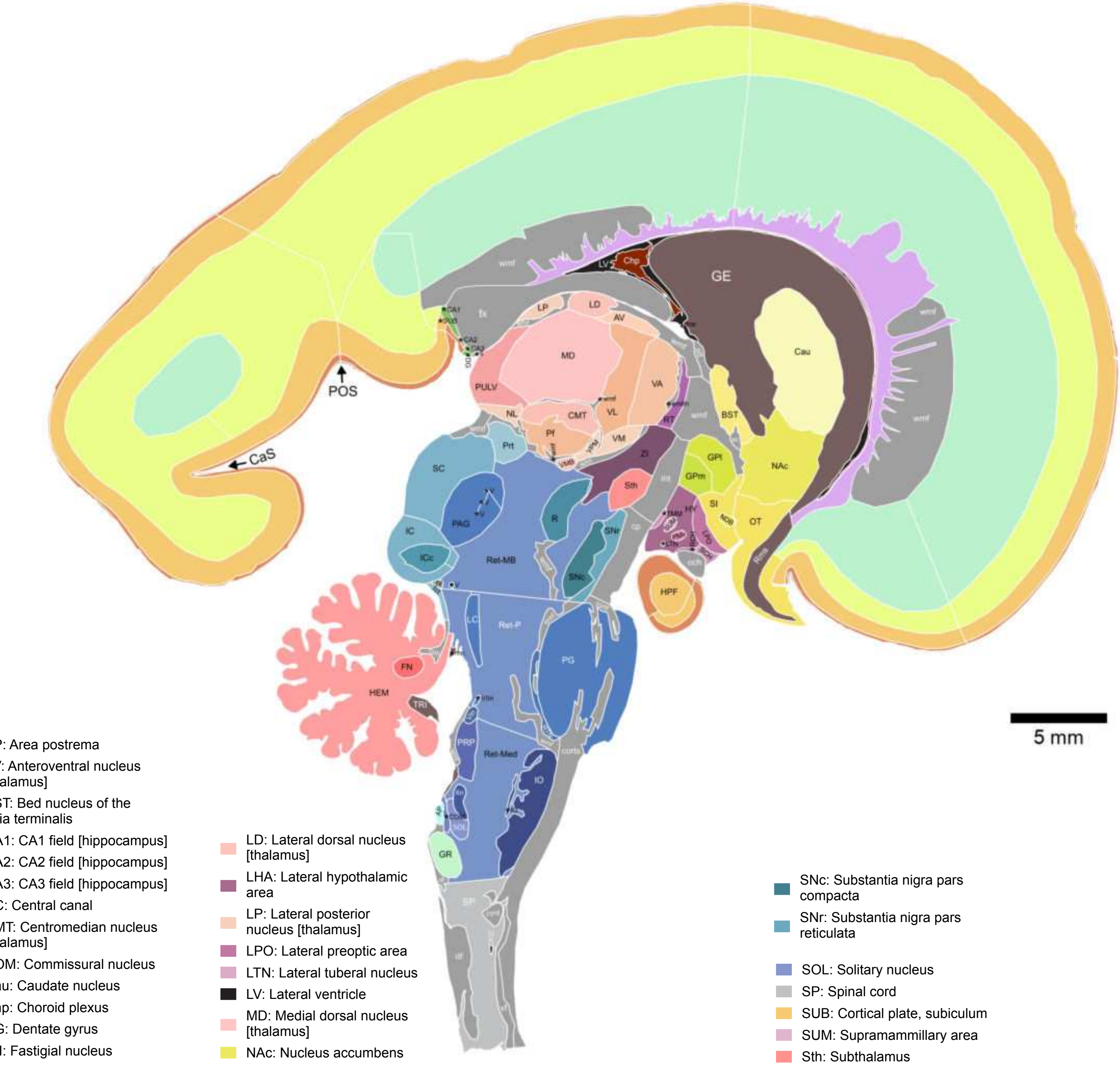

5 mm

- AP: Area postrema
- AV: Anteroventral nucleus [thalamus]
- BST: Bed nucleus of the stria terminalis
- CA1: CA1 field [hippocampus]
- CA2: CA2 field [hippocampus]
- CA3: CA3 field [hippocampus]
- CC: Central canal
- CMT: Centromedian nucleus [thalamus]
- COM: Commissural nucleus
- Cau: Caudate nucleus
- Chp: Choroid plexus
- DG: Dentate gyrus
- FN: Fastigial nucleus
- GE: Ganglionic eminence
- GPl: Globus pallidus lateral segment
- GPm: Globus pallidus medial segment
- GR: Gracile nucleus
- HEM: Cerebellar hemispheres
- HPF: Cortical plate, hippocampal formation
- HY: Hypothalamus
- IC: Inferior colliculus
- ICc: Inferior colliculus, central nucleus
- IO: Inferior olive
- IV: Trochlear nerve
- LC: Locus coeruleus
- LD: Lateral dorsal nucleus [thalamus]
- LHA: Lateral hypothalamic area
- LP: Lateral posterior nucleus [thalamus]
- LPO: Lateral preoptic area
- LTN: Lateral tuberal nucleus
- LV: Lateral ventricle
- MD: Medial dorsal nucleus [thalamus]
- NAc: Nucleus accumbens
- NDB: Nucleus of the diagonal band
- NL: Nucleus limitans [thalamus]
- OT: Olfactory tubercle
- PAG: Periaqueductal gray
- PG: Pontine gray
- PMv: Ventral premammillary nucleus
- PRP: Nucleus prepositus
- PULV: Pulvinar nucleus [thalamus]
- Pf: Parafascicular nucleus [thalamus]
- Prt: Pretectum
- R: Red nucleus
- RCH: Retrochiasmatic nucleus [hypothalamus]
- RT: Reticular nucleus [thalamus]
- RTN: Reticular tegmental nucleus
- Ret-MB: Reticular formation, Midbrain
- Ret-Med: Reticular formation, Medulla
- Ret-P: Reticular formation, Pons
- Rms: Rostral migratory stream
- SC: Superior colliculus
- SCH: Suprachiasmatic nucleus [hypothalamus]
- SI: Substantia innominata
- SNc: Substantia nigra pars compacta
- SNr: Substantia nigra pars reticulata
- SOL: Solitary nucleus
- SP: Spinal cord
- SUB: Cortical plate, subiculum
- SUM: Supramammillary area
- Sth: Subthalamus
- TMM: Tuberomammillary nucleus
- TRI: Germinal trigone
- Tct: Tectum
- V: Mesencephalic nucleus
- VA: Ventral anterior nucleus [thalamus]
- VIIn: Facial motor nucleus
- VIn: Abducens nucleus
- VL: Ventral lateral nucleus [thalamus]
- VM: Ventral medial nucleus [thalamus]
- VMB: Ventral medial basal nucleus [thalamus]
- VPM: Ventral posteromedial nucleus [thalamus]
- Xn: Dorsal motor nucleus
- ZI: Zona incerta
- → CaS: Calcarine sulcus
- → POS: Parieto-occipital sulcus

Age: 21 GW

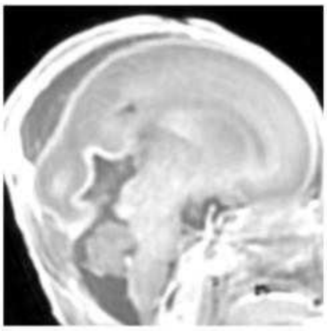

L-R Level: 3.66 mm

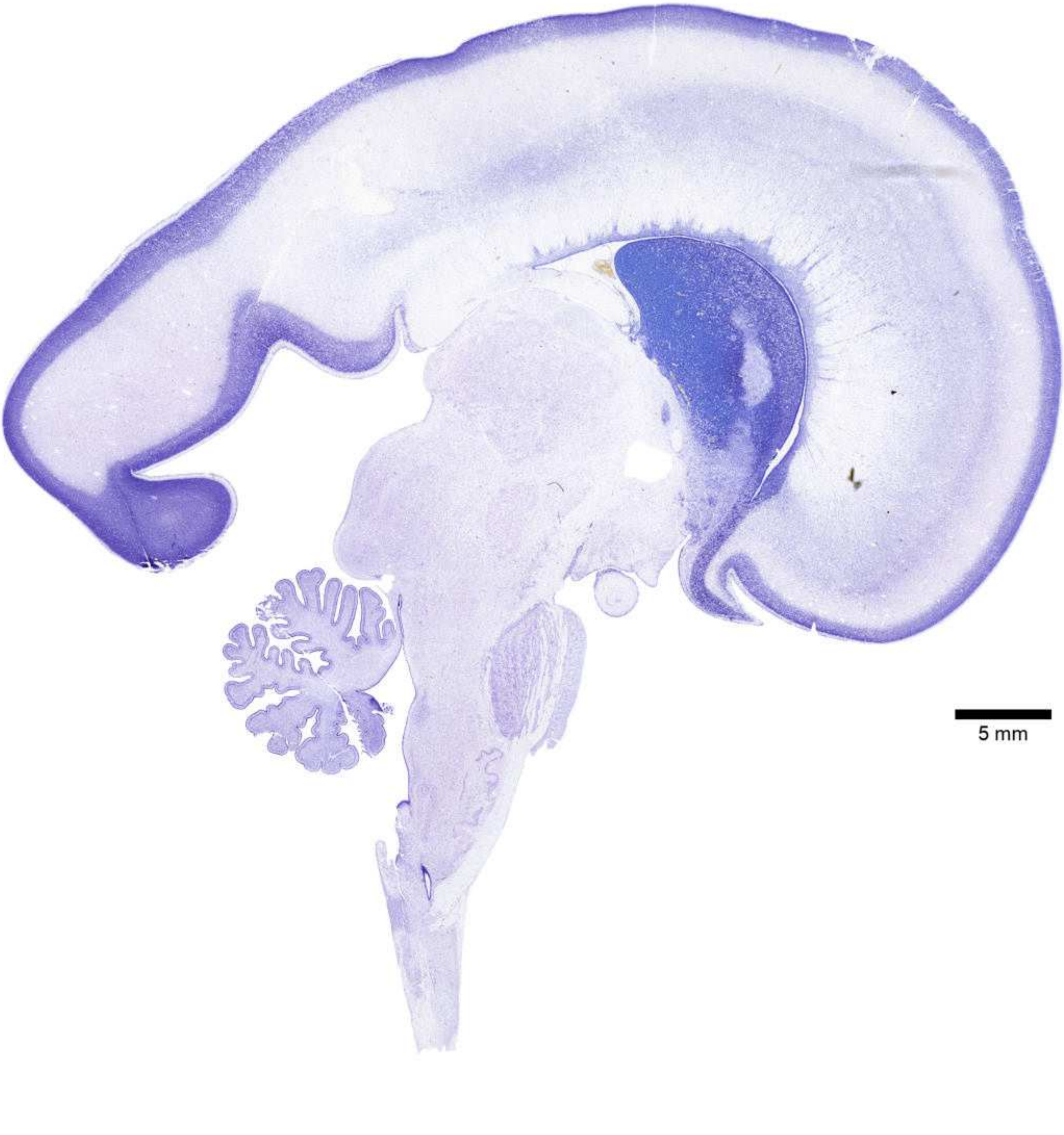

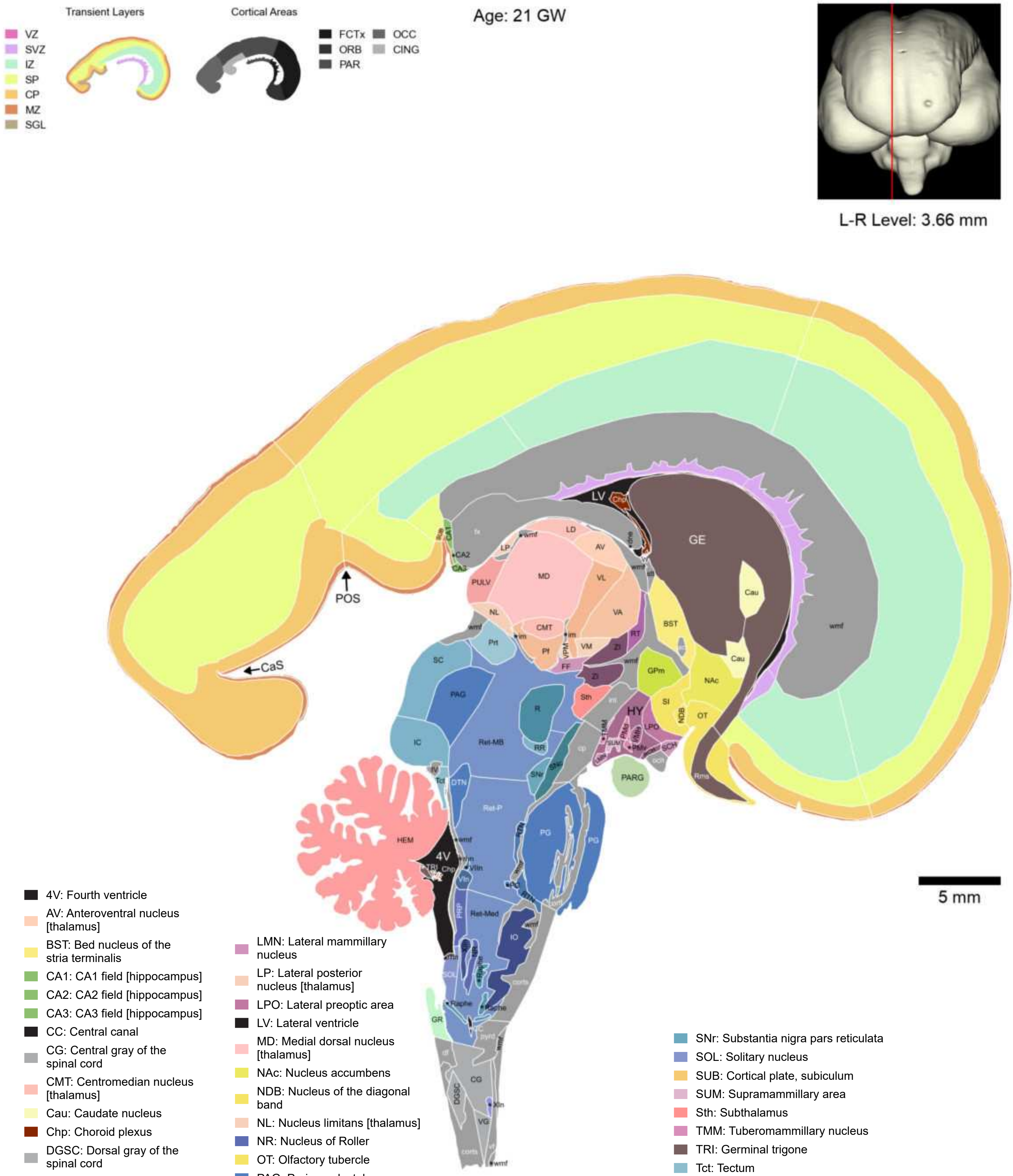

4V: Fourth ventricle
AV: Anteroventral nucleus [thalamus]
BST: Bed nucleus of the stria terminalis
CA1: CA1 field [hippocampus]
CA2: CA2 field [hippocampus]
CA3: CA3 field [hippocampus]
CC: Central canal
CG: Central gray of the spinal cord
CMT: Centromedian nucleus [thalamus]
Cau: Caudate nucleus
Chp: Choroid plexus
DGSC: Dorsal gray of the spinal cord
DTN: Dorsal tegmental nucleus
FF: Field of Forel
GE: Ganglionic eminence
GPm: Globus pallidus medial segment
GR: Gracile nucleus
HEM: Cerebellar hemispheres
HY: Hypothalamus
IC: Inferior colliculus
IO: Inferior olive
IV: Trochlear nerve
LD: Lateral dorsal nucleus [thalamus]
LHA: Lateral hypothalamic area
LMN: Lateral mammillary nucleus
LP: Lateral posterior nucleus [thalamus]
LPO: Lateral preoptic area
LV: Lateral ventricle
MD: Medial dorsal nucleus [thalamus]
NAc: Nucleus accumbens
NDB: Nucleus of the diagonal band
NL: Nucleus limitans [thalamus]
NR: Nucleus of Roller
OT: Olfactory tubercle
PAG: Periaqueductal gray
PARG: Parahippocampal gyrus
PG: Pontine gray
PMd: Dorsal premammillary nucleus
PMv: Ventral premammillary nucleus
PRP: Nucleus prepositus
PULV: Pulvinar nucleus [thalamus]
Pf: Parafascicular nucleus [thalamus]
Prt: Pretectum
R: Red nucleus
RCH: Retrochiasmatic nucleus [hypothalamus]
RR: Retrorubral area
RT: Reticular nucleus [thalamus]
RTN: Reticular tegmental nucleus
Raphe: Raphe nuclei
Ret-MB: Reticular formation, Midbrain
Ret-Med: Reticular formation, Medulla
Ret-P: Reticular formation, Pons
Rms: Rostral migratory stream
SC: Superior colliculus
SCH: Suprachiasmatic nucleus [hypothalamus]
SI: Substantia innominata
SNc: Substantia nigra pars compacta
SNr: Substantia nigra pars reticulata
SOL: Solitary nucleus
SUB: Cortical plate, subiculum
SUM: Supramammillary area
Sth: Subthalamus
TMM: Tuberomammillary nucleus
TRI: Germinal trigone
Tct: Tectum
VA: Ventral anterior nucleus [thalamus]
VG: Ventral gray of the spinal cord
VIIn: Facial motor nucleus
VIn: Abducens nucleus
VL: Ventral lateral nucleus [thalamus]
VM: Ventral medial nucleus [thalamus]
VMH: Ventromedial nucleus [hypothalamus]
VPM: Ventral posteromedial nucleus [thalamus]
XIIn: Hypoglossal nucleus
XIn: Accessory nucleus
ZI: Zona incerta
→ CaS: Calcarine sulcus
→ POS: Parieto-occipital sulcus

Age: 21 GW

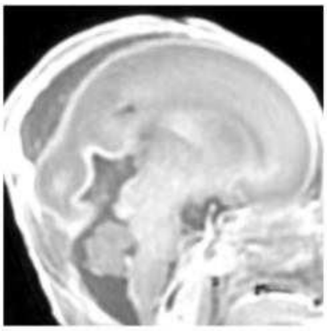

L-R Level: 3.36 mm

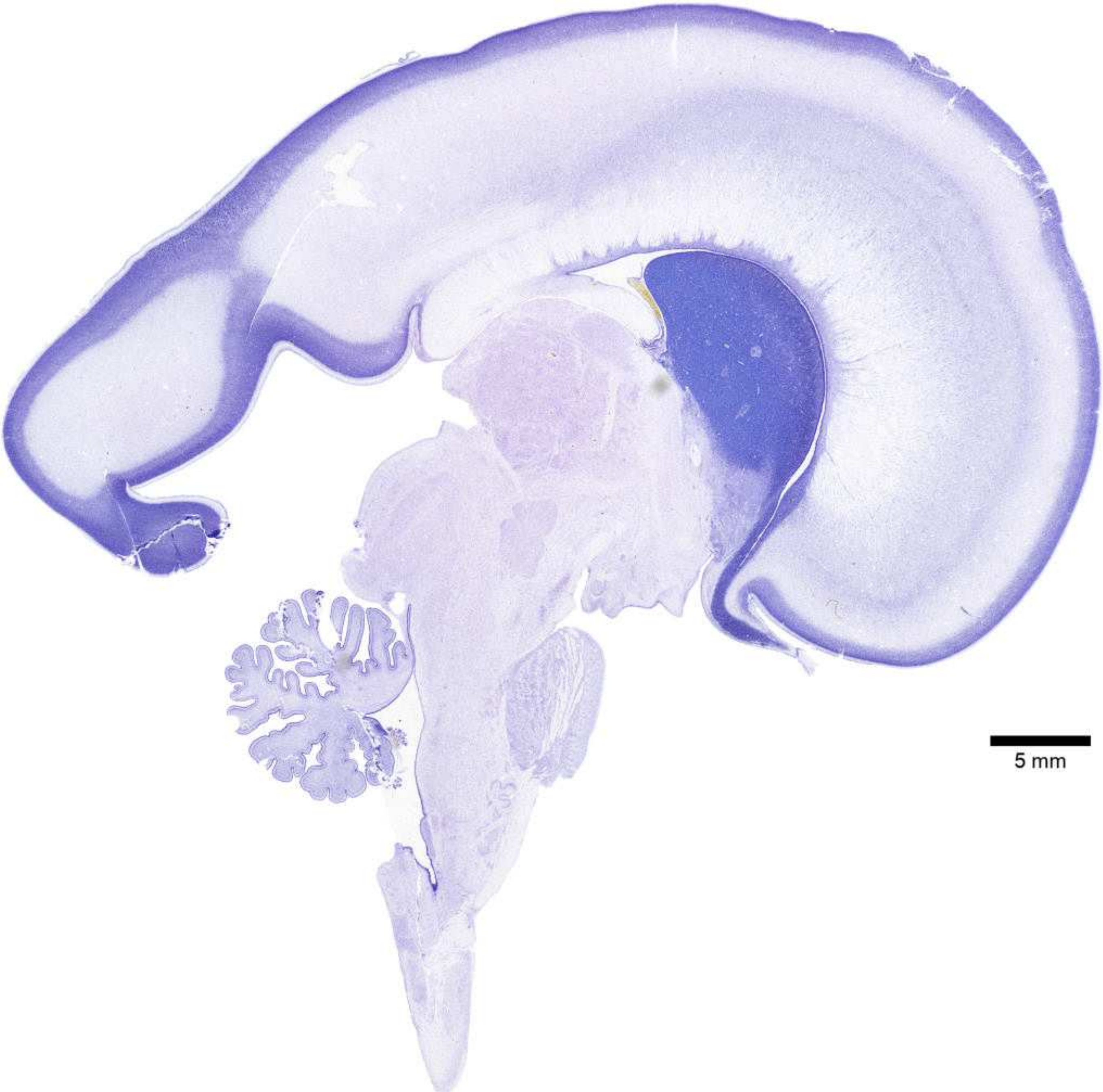

5 mm

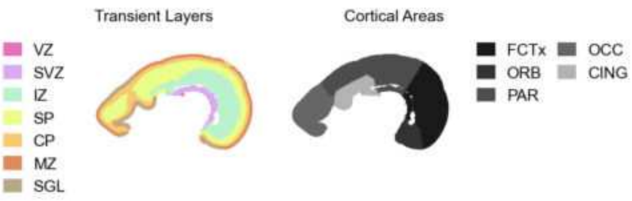

Age: 21 GW

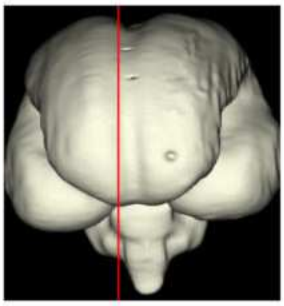

L-R Level: 3.36 mm

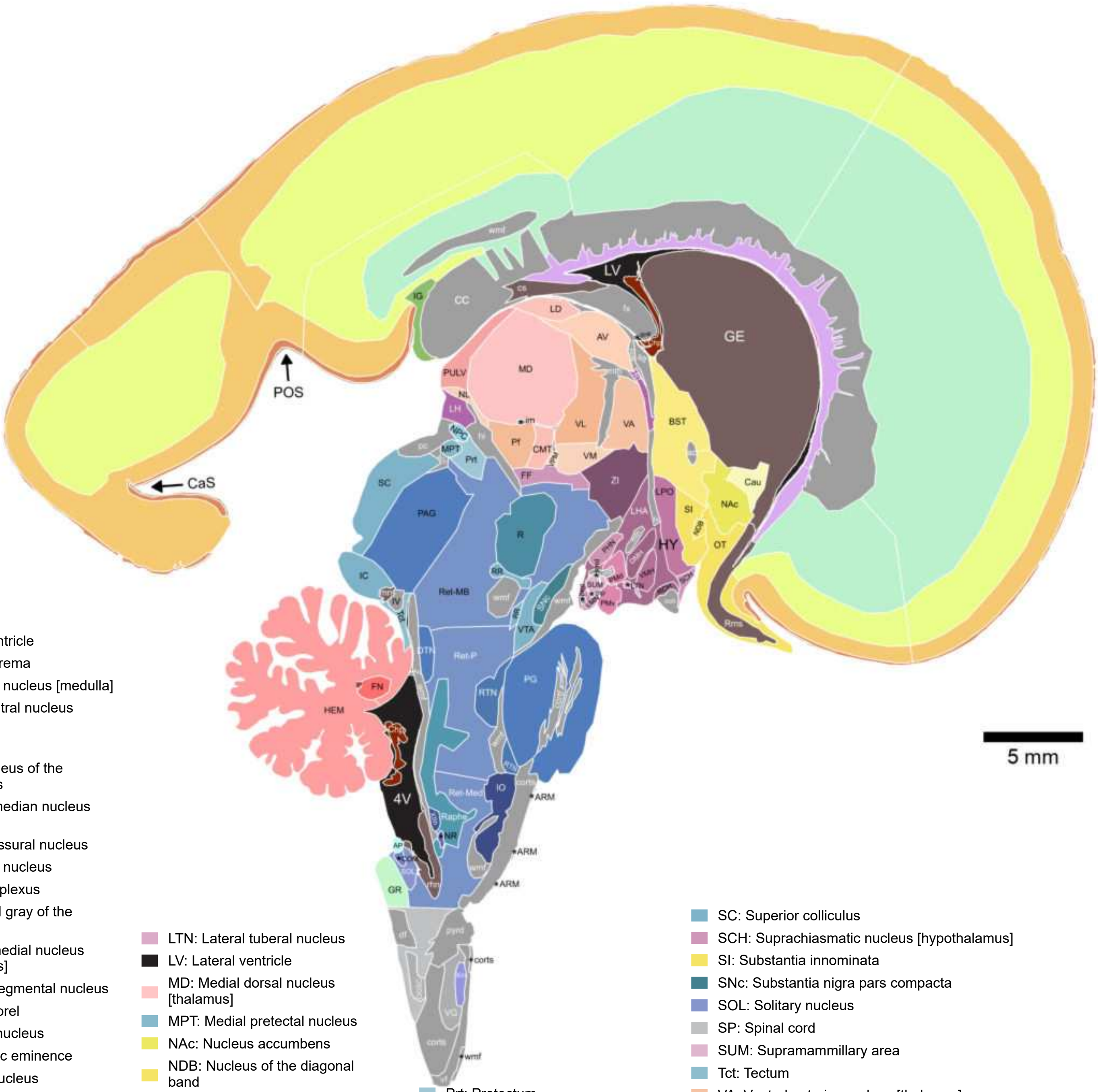

4V: Fourth ventricle
AP: Area postrema
ARM: Arcuate nucleus [medulla]
AV: Anteroventral nucleus [thalamus]
Aq: Aqueduct
BST: Bed nucleus of the stria terminalis
CMT: Centromedian nucleus [thalamus]
COM: Commissural nucleus
Cau: Caudate nucleus
Chp: Choroid plexus
DGSC: Dorsal gray of the spinal cord
DMH: Dorsomedial nucleus [hypothalamus]
DTN: Dorsal tegmental nucleus
FF: Field of Forel
FN: Fastigial nucleus
GE: Ganglionic eminence
GR: Gracile nucleus
HEM: Cerebellar hemispheres
HY: Hypothalamus
IC: Inferior colliculus
IG: Induseum griseum
IO: Inferior olive
IP: Interposed nucleus
IV: Trochlear nerve
LD: Lateral dorsal nucleus [thalamus]
LH: Lateral habenula
LHA: Lateral hypothalamic area
LMN: Lateral mammillary nucleus
LPO: Lateral preoptic area
LTN: Lateral tuberal nucleus
LV: Lateral ventricle
MD: Medial dorsal nucleus [thalamus]
MPT: Medial pretectal nucleus
NAc: Nucleus accumbens
NDB: Nucleus of the diagonal band
NL: Nucleus limitans [thalamus]
NPC: Nucleus of the posterior commissure
NR: Nucleus of Roller
OT: Olfactory tubercle
PAG: Periaqueductal gray
PG: Pontine gray
PHN: Posterior hypothalamic nucleus
PMd: Dorsal premammillary nucleus
PMv: Ventral premammillary nucleus
PULV: Pulvinar nucleus [thalamus]
Pf: Parafascicular nucleus [thalamus]
Prt: Pretectum
R: Red nucleus
RCH: Retrochiasmatic nucleus [hypothalamus]
RR: Retrorubral area
RT: Reticular nucleus [thalamus]
RTN: Reticular tegmental nucleus
Raphe: Raphe nuclei
Ret-MB: Reticular formation, Midbrain
Ret-Med: Reticular formation, Medulla
Ret-P: Reticular formation, Pons
Rms: Rostral migratory stream
SC: Superior colliculus
SCH: Suprachiasmatic nucleus [hypothalamus]
SI: Substantia innominata
SNc: Substantia nigra pars compacta
SOL: Solitary nucleus
SP: Spinal cord
SUM: Supramammillary area
Tct: Tectum
VA: Ventral anterior nucleus [thalamus]
VG: Ventral gray of the spinal cord
VL: Ventral lateral nucleus [thalamus]
VM: Ventral medial nucleus [thalamus]
VMH: Ventromedial nucleus [hypothalamus]
VPM: Ventral posteromedial nucleus [thalamus]
VTA: Ventral tegmental area
XIIn: Hypoglossal nucleus
XIn: Accessory nucleus
ZI: Zona incerta
→ CaS: Calcarine sulcus
→ POS: Parieto-occipital sulcus

Age: 21 GW

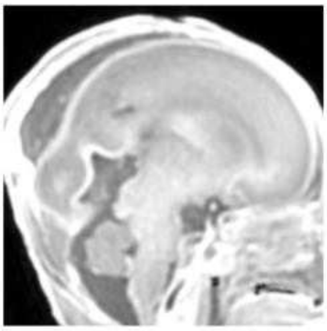

L-R Level: 3.0 mm

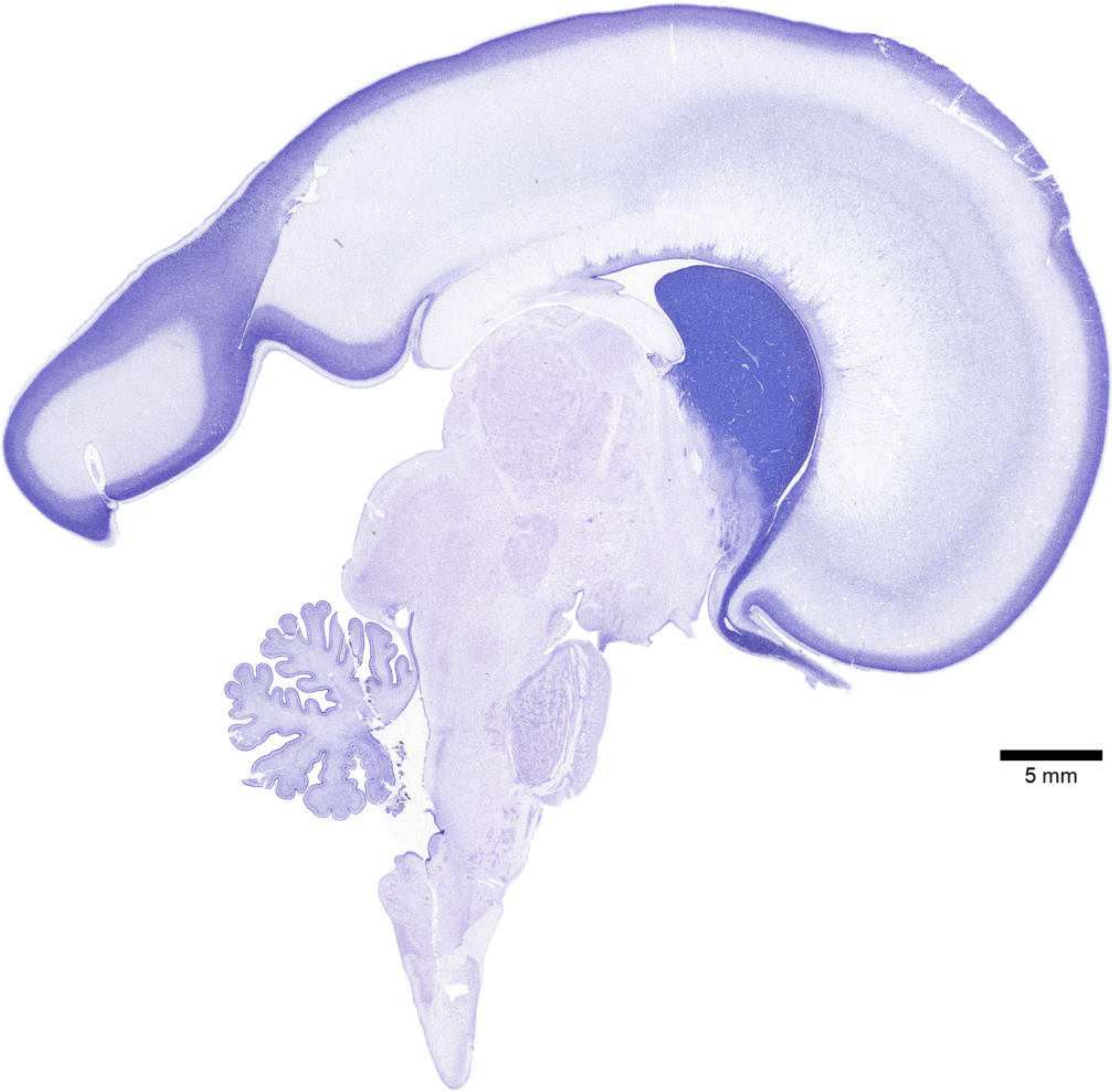

5 mm

Transient Layers

- VZ
- SVZ
- IZ
- SP
- CP
- MZ
- SGL

Cortical Areas

- FCTx
- ORB
- PAR
- OCC
- CING

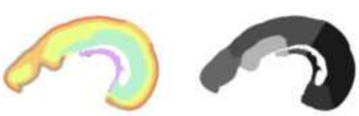

Age: 21 GW

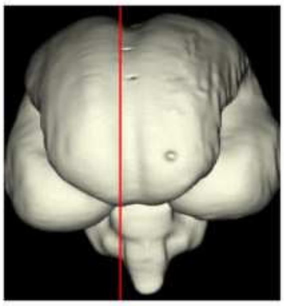

L-R Level: 3.0 mm

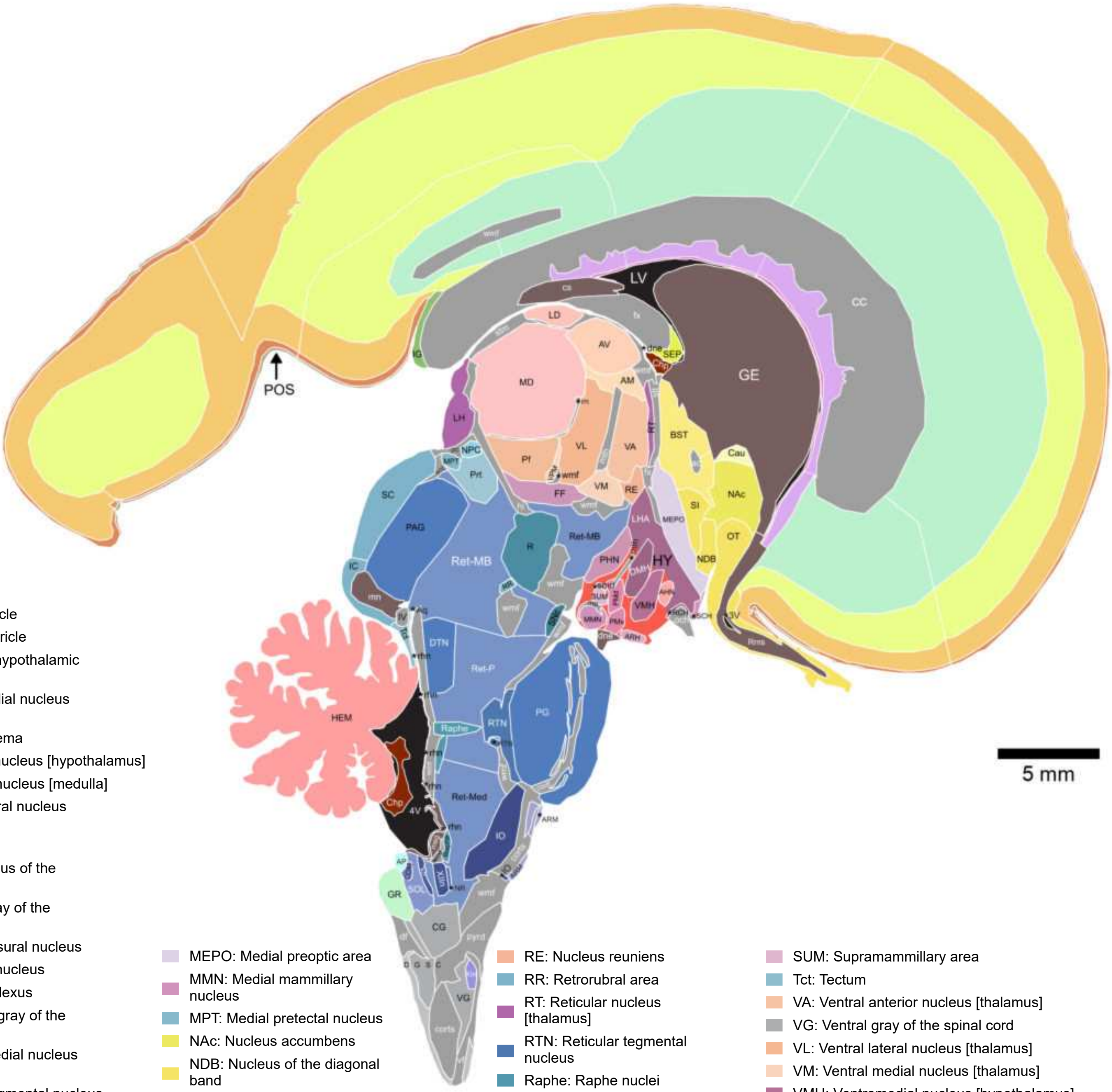

- 3V: Third ventricle
- 4V: Fourth ventricle
- AHN: Anterior hypothalamic nucleus
- AM: Anteromedial nucleus [thalamus]
- AP: Area postrema
- ARH: Arcuate nucleus [hypothalamus]
- ARM: Arcuate nucleus [medulla]
- AV: Anteroventral nucleus [thalamus]
- Aq: Aqueduct
- BST: Bed nucleus of the stria terminalis
- CG: Central gray of the spinal cord
- COM: Commissural nucleus
- Cau: Caudate nucleus
- Chp: Choroid plexus
- DGSC: Dorsal gray of the spinal cord
- DMH: Dorsomedial nucleus [hypothalamus]
- DTN: Dorsal tegmental nucleus
- FF: Field of Forel
- GE: Ganglionic eminence
- GR: Gracile nucleus
- HEM: Cerebellar hemispheres
- HY: Hypothalamus
- IC: Inferior colliculus
- IG: Induseum griseum
- IO: Inferior olive
- IV: Trochlear nerve
- LD: Lateral dorsal nucleus [thalamus]
- LH: Lateral habenula
- LHA: Lateral hypothalamic area
- LV: Lateral ventricle
- MD: Medial dorsal nucleus [thalamus]
- MEPO: Medial preoptic area
- MMN: Medial mammillary nucleus
- MPT: Medial pretectal nucleus
- NAc: Nucleus accumbens
- NDB: Nucleus of the diagonal band
- NPC: Nucleus of the posterior commissure
- NR: Nucleus of Roller
- OT: Olfactory tubercle
- PAG: Periaqueductal gray
- PG: Pontine gray
- PHN: Posterior hypothalamic nucleus
- PMd: Dorsal premammillary nucleus
- PMv: Ventral premammillary nucleus
- Pf: Parafascicular nucleus [thalamus]
- Prt: Pretectum
- R: Red nucleus
- RCH: Retrochiasmatic nucleus [hypothalamus]
- RE: Nucleus reuniens
- RR: Retrorubral area
- RT: Reticular nucleus [thalamus]
- RTN: Reticular tegmental nucleus
- Raphe: Raphe nuclei
- Ret-MB: Reticular formation, Midbrain
- Ret-Med: Reticular formation, Medulla
- Ret-P: Reticular formation, Pons
- Rms: Rostral migratory stream
- SC: Superior colliculus
- SCH: Suprachiasmatic nucleus [hypothalamus]
- SEP: Septum
- SI: Substantia innominata
- SNc: Substantia nigra pars compacta
- SOL: Solitary nucleus
- SP: Spinal cord
- SUM: Supramammillary area
- Tct: Tectum
- VA: Ventral anterior nucleus [thalamus]
- VG: Ventral gray of the spinal cord
- VL: Ventral lateral nucleus [thalamus]
- VM: Ventral medial nucleus [thalamus]
- VMH: Ventromedial nucleus [hypothalamus]
- VPM: Ventral posteromedial nucleus [thalamus]
- XIIn: Hypoglossal nucleus
- XIn: Accessory nucleus
- Xn: Dorsal motor nucleus
- ➔ POS: Parieto-occipital sulcus

Age: 21 GW

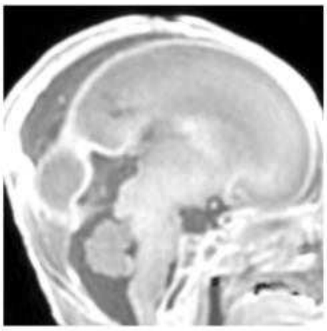

L-R Level: 2.58 mm

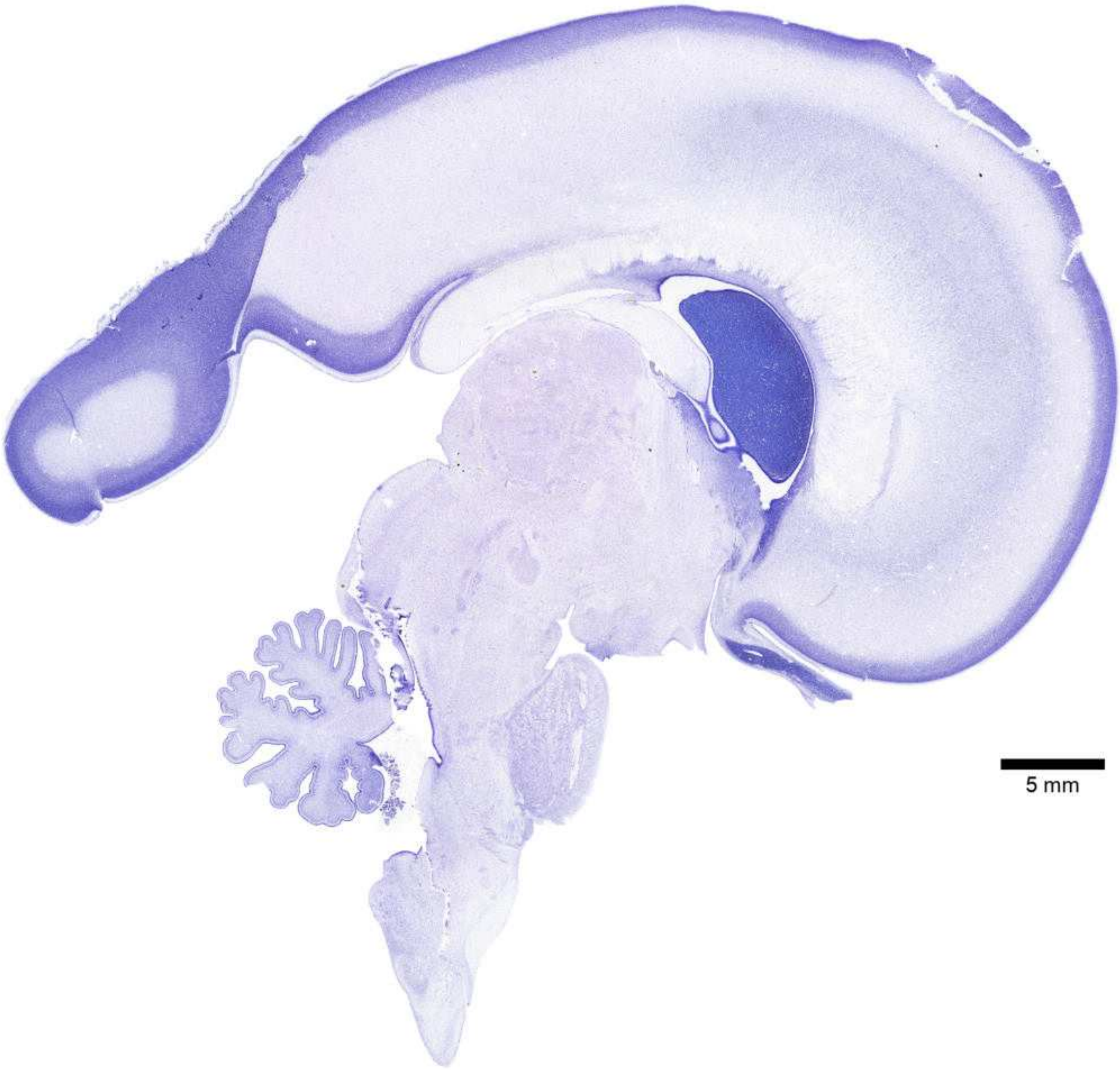

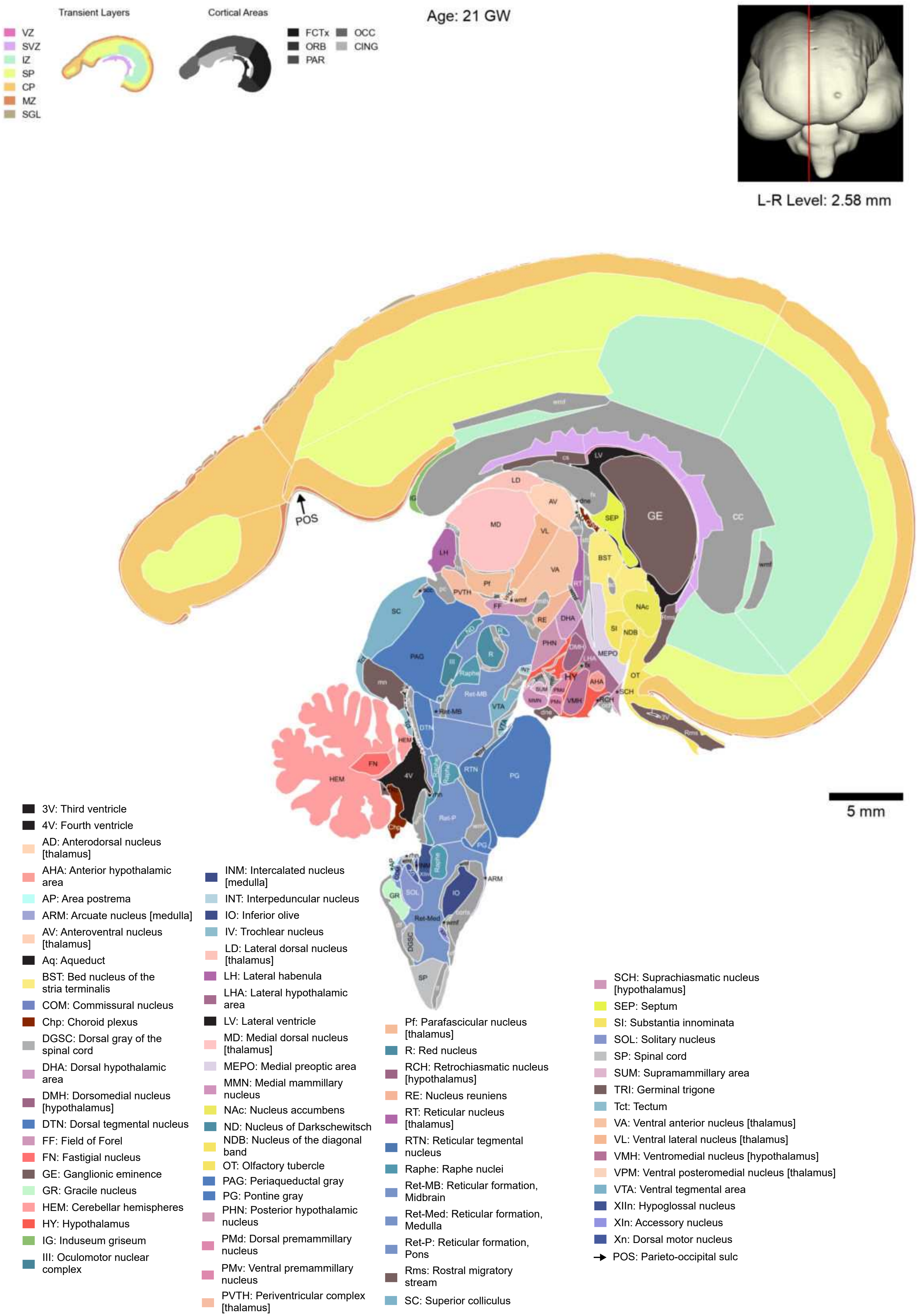
Transient Layers
VZ
SVZ
IZ
SP
CP
MZ
SGL
Cortical Areas
FCTx
ORB
PAR
OCC
CING
Age: 21 GW
L-R Level: 2.58 mm
5 mm
POS
GE
CC
LV
LD
AV
MD
VL
VA
LH
SEP
BST
NAc
SC
PAG
PVTH
Pf
FF
RE
DHA
PHN
MEPO
HY
AHA
OT
VMH
Ret-MB
VTA
DTN
FN
HEM
4V
RTN
PG
Ret-P
IO
SOL
GR
Ret-Med
DGSC
SP
3V: Third ventricle
4V: Fourth ventricle
AD: Anterodorsal nucleus [thalamus]
AHA: Anterior hypothalamic area
AP: Area postrema
ARM: Arcuate nucleus [medulla]
AV: Anteroventral nucleus [thalamus]
Aq: Aqueduct
BST: Bed nucleus of the stria terminalis
COM: Commissural nucleus
Chp: Choroid plexus
DGSC: Dorsal gray of the spinal cord
DHA: Dorsal hypothalamic area
DMH: Dorsomedial nucleus [hypothalamus]
DTN: Dorsal tegmental nucleus
FF: Field of Forel
FN: Fastigial nucleus
GE: Ganglionic eminence
GR: Gracile nucleus
HEM: Cerebellar hemispheres
HY: Hypothalamus
IG: Induseum griseum
III: Oculomotor nuclear complex
INM: Intercalated nucleus [medulla]
INT: Interpeduncular nucleus
IO: Inferior olive
IV: Trochlear nucleus
LD: Lateral dorsal nucleus [thalamus]
LH: Lateral habenula
LHA: Lateral hypothalamic area
LV: Lateral ventricle
MD: Medial dorsal nucleus [thalamus]
MEPO: Medial preoptic area
MMN: Medial mammillary nucleus
NAc: Nucleus accumbens
ND: Nucleus of Darkschewitsch
NDB: Nucleus of the diagonal band
OT: Olfactory tubercle
PAG: Periaqueductal gray
PG: Pontine gray
PHN: Posterior hypothalamic nucleus
PMd: Dorsal premammillary nucleus
PMv: Ventral premammillary nucleus
PVTH: Periventricular complex [thalamus]
Pf: Parafascicular nucleus [thalamus]
R: Red nucleus
RCH: Retrochiasmatic nucleus [hypothalamus]
RE: Nucleus reuniens
RT: Reticular nucleus [thalamus]
RTN: Reticular tegmental nucleus
Raphe: Raphe nuclei
Ret-MB: Reticular formation, Midbrain
Ret-Med: Reticular formation, Medulla
Ret-P: Reticular formation, Pons
Rms: Rostral migratory stream
SC: Superior colliculus
SCH: Suprachiasmatic nucleus [hypothalamus]
SEP: Septum
SI: Substantia innominata
SOL: Solitary nucleus
SP: Spinal cord
SUM: Supramammillary area
TRI: Germinal trigone
Tct: Tectum
VA: Ventral anterior nucleus [thalamus]
VL: Ventral lateral nucleus [thalamus]
VMH: Ventromedial nucleus [hypothalamus]
VPM: Ventral posteromedial nucleus [thalamus]
VTA: Ventral tegmental area
XIIn: Hypoglossal nucleus
XIn: Accessory nucleus
Xn: Dorsal motor nucleus
POS: Parieto-occipital sulc

Age: 21 GW

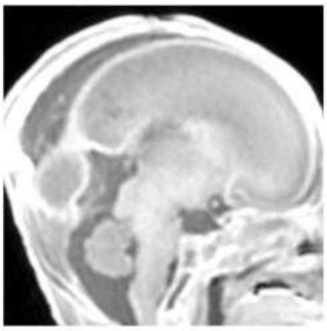

L-R Level: 2.28 mm

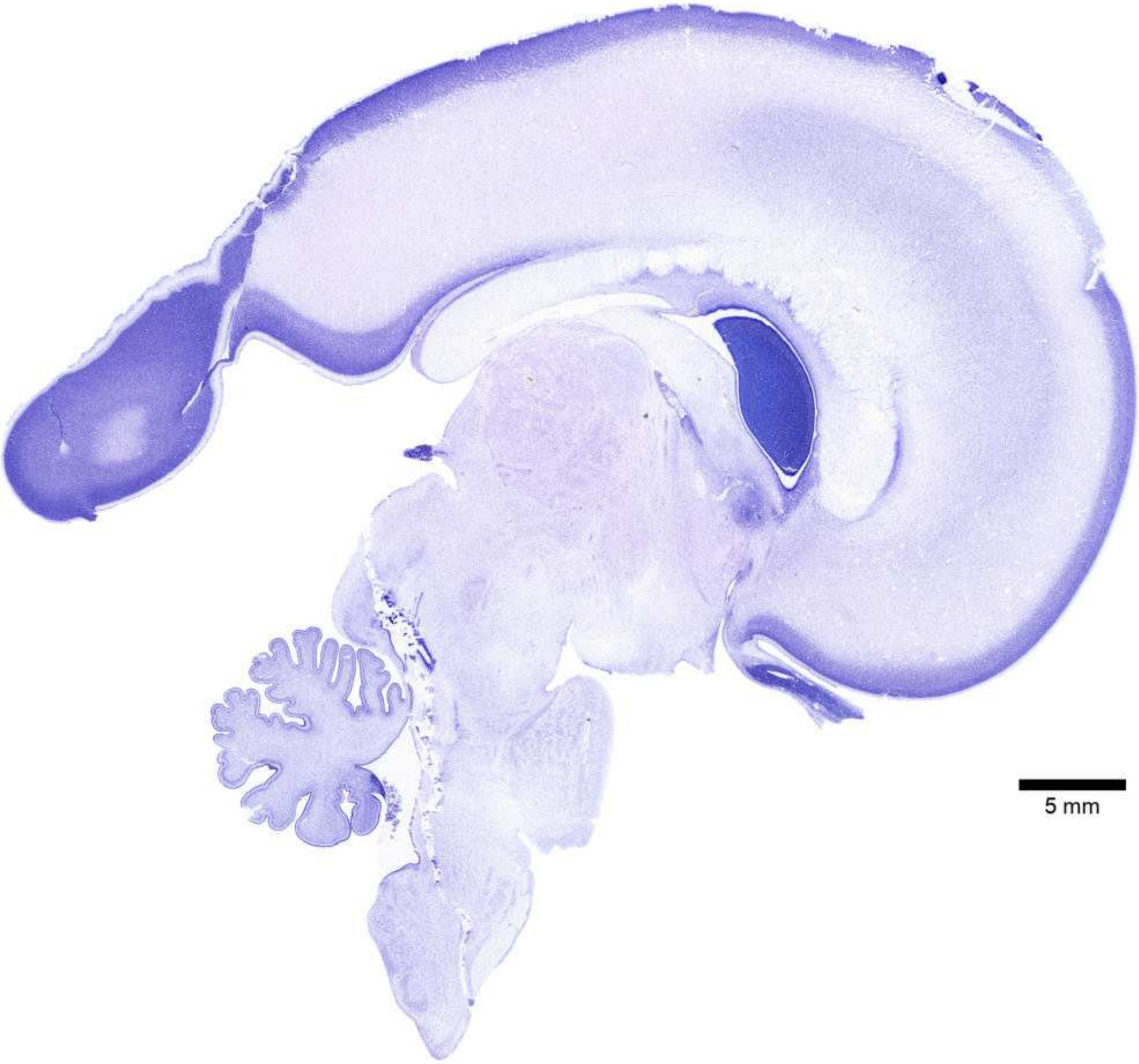

5 mm

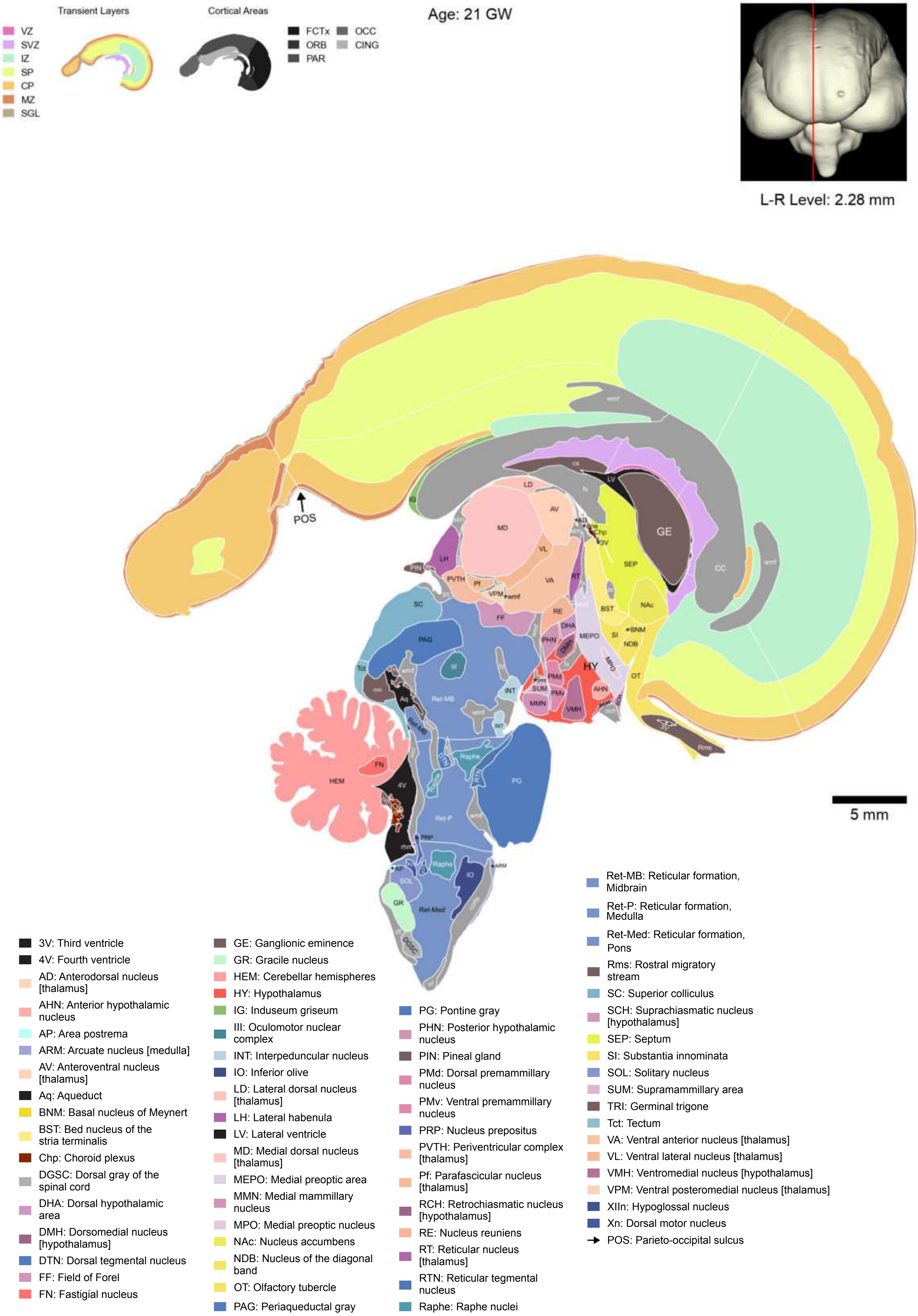

Transient Layers
VZ
SVZ
IZ
SP
CP
MZ
SGL
Cortical Areas
FCTx
ORB
PAR
OCC
CING
Age: 21 GW
L-R Level: 2.28 mm
POS
GE
SEP
CC
NAc
HY
MD
AV
LD
VL
VA
LH
PVTH
Pf
VPM
RE
DHA
PHN
MEPO
BST
SI
BNM
NDB
OT
SC
PAG
FF
III
Tct
Aq
Ret-MB
INT
SUM
PMd
PMv
MMN
VMH
AHN
HEM
FN
4V
Raphe
RTN
DTN
PG
Ret-P
PRP
AP
SOL
IO
GR
Ret-Med
DGSC
ARM
Rms
5 mm
3V: Third ventricle
4V: Fourth ventricle
AD: Anterodorsal nucleus [thalamus]
AHN: Anterior hypothalamic nucleus
AP: Area postrema
ARM: Arcuate nucleus [medulla]
AV: Anteroventral nucleus [thalamus]
Aq: Aqueduct
BNM: Basal nucleus of Meynert
BST: Bed nucleus of the stria terminalis
Chp: Choroid plexus
DGSC: Dorsal gray of the spinal cord
DHA: Dorsal hypothalamic area
DMH: Dorsomedial nucleus [hypothalamus]
DTN: Dorsal tegmental nucleus
FF: Field of Forel
FN: Fastigial nucleus
GE: Ganglionic eminence
GR: Gracile nucleus
HEM: Cerebellar hemispheres
HY: Hypothalamus
IG: Induseum griseum
III: Oculomotor nuclear complex
INT: Interpeduncular nucleus
IO: Inferior olive
LD: Lateral dorsal nucleus [thalamus]
LH: Lateral habenula
LV: Lateral ventricle
MD: Medial dorsal nucleus [thalamus]
MEPO: Medial preoptic area
MMN: Medial mammillary nucleus
MPO: Medial preoptic nucleus
NAc: Nucleus accumbens
NDB: Nucleus of the diagonal band
OT: Olfactory tubercle
PAG: Periaqueductal gray
PG: Pontine gray
PHN: Posterior hypothalamic nucleus
PIN: Pineal gland
PMd: Dorsal premammillary nucleus
PMv: Ventral premammillary nucleus
PRP: Nucleus prepositus
PVTH: Periventricular complex [thalamus]
Pf: Parafascicular nucleus [thalamus]
RCH: Retrochiasmatic nucleus [hypothalamus]
RE: Nucleus reuniens
RT: Reticular nucleus [thalamus]
RTN: Reticular tegmental nucleus
Raphe: Raphe nuclei
Ret-MB: Reticular formation, Midbrain
Ret-P: Reticular formation, Medulla
Ret-Med: Reticular formation, Pons
Rms: Rostral migratory stream
SC: Superior colliculus
SCH: Suprachiasmatic nucleus [hypothalamus]
SEP: Septum
SI: Substantia innominata
SOL: Solitary nucleus
SUM: Supramammillary area
TRI: Germinal trigone
Tct: Tectum
VA: Ventral anterior nucleus [thalamus]
VL: Ventral lateral nucleus [thalamus]
VMH: Ventromedial nucleus [hypothalamus]
VPM: Ventral posteromedial nucleus [thalamus]
XIIn: Hypoglossal nucleus
Xn: Dorsal motor nucleus
POS: Parieto-occipital sulcus

Age: 21 GW

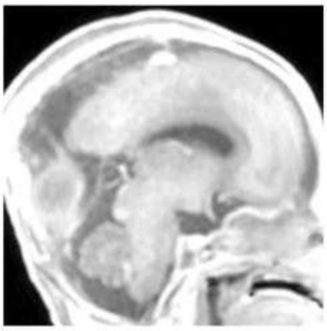

L-R Level: -0.48 mm

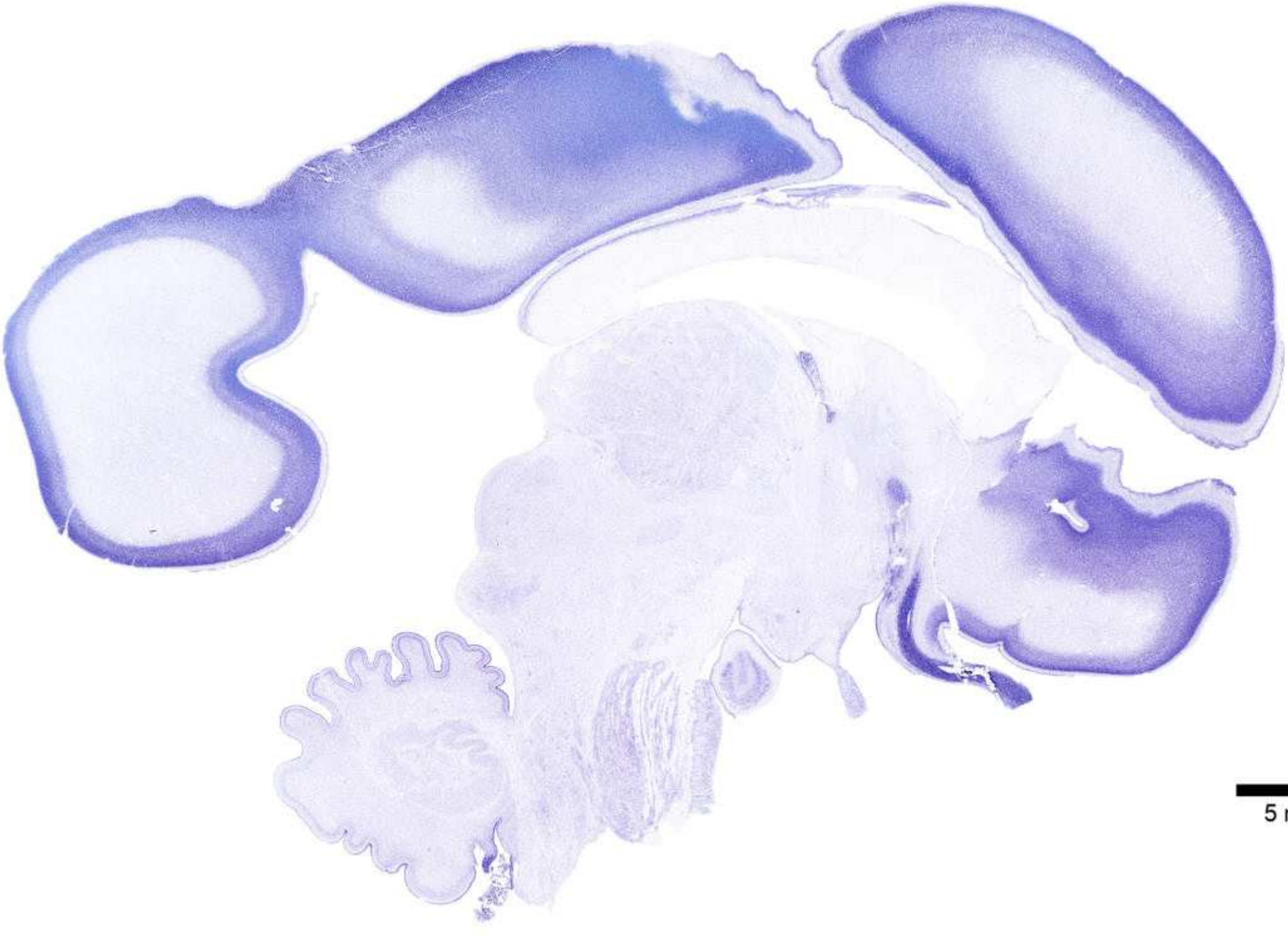

5 mm

Age: 21 GW

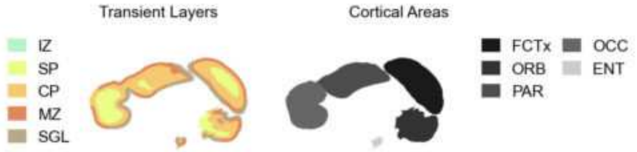

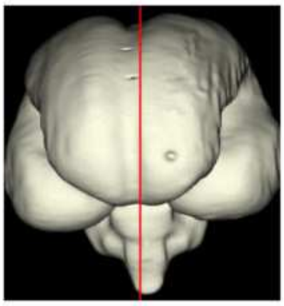

L-R Level: -0.48 mm

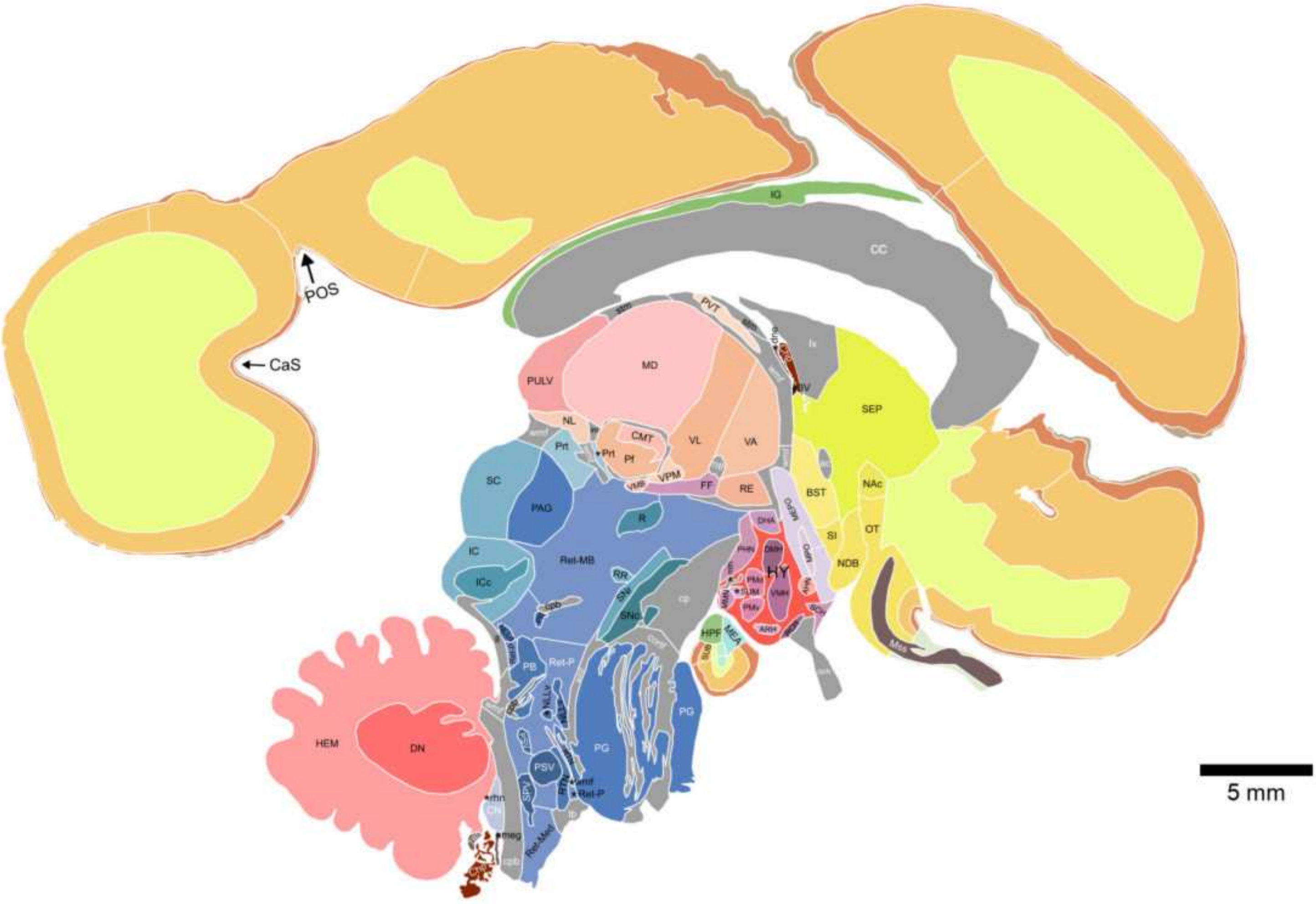

- 3V: Third ventricle
- AHN: Anterior hypothalamic nucleus
- ARH: Arcuate nucleus [hypothalamus]
- BST: Bed nucleus of the stria terminalis
- CMT: Centromedian nucleus [thalamus]
- CN: Cochlear nuclei
- Chp: Choroid plexus
- DHA: Dorsal hypothalamic area
- DMH: Dorsomedial nucleus [hypothalamus]
- DN: Dentate nucleus
- FF: Field of Forel
- HEM: Cerebellar hemispheres
- HPF: Hippocampal formation
- HY: Hypothalamus
- IC: Inferior colliculus
- ICc: Inferior colliculus, central nucleus
- IG: Induseum griseum
- MD: Medial dorsal nucleus [thalamus]
- MEA: Medial nucleus [amygdala]
- MEPO: Medial preoptic area
- MMN: Medial mammillary nucleus
- MPO: Medial preoptic nucleus
- Mss: Migratory streams
- NAc: Nucleus accumbens
- NDB: Nucleus of the diagonal band
- NL: Nucleus limitans [thalamus]
- NLLd: Nucleus of the lateral lemniscus, dorsal
- NLLv: Nucleus of the lateral lemniscus, ventral
- OLFb: Olfactory bulb
- OT: Olfactory tubercle
- PAG: Periaqueductal gray
- PB: Parabrachial nucleus
- PG: Pontine gray
- PHN: Posterior hypothalamic nucleus
- PMd: Dorsal premammillary nucleus
- PMv: Ventral premammillary nucleus
- PSV: Principal sensory nucleus of the trigeminal
- PULV: Pulvinar nucleus [thalamus]
- PVT: Paraventricular nucleus [thalamus]
- Pf: Parafascicular nucleus [thalamus]
- Prt: Pretectum
- R: Red nucleus
- RCH: Retrochiasmatic nucleus [hypothalamus]
- RE: Nucleus reuniens
- RR: Retrorubral area
- RTN: Reticular tegmental nucleus
- Ret-MB: Reticular formation, Midbrain
- Ret-Med: Reticular formation, Medulla
- Ret-P: Reticular formation, Pons
- SC: Superior colliculus
- SCH: Suprachiasmatic nucleus [hypothalamus]
- SEP: Septum
- SI: Substantia innominata
- SNc: Substantia nigra pars compacta
- SNr: Substantia nigra pars reticulata
- SPV: Spinal nucleus of the trigeminal
- SUB: Cortical plate, subiculum
- SUM: Supramammillary area
- TRI: Germinal trigone
- VA: Ventral anterior nucleus [thalamus]
- VL: Ventral lateral nucleus [thalamus]
- VMB: Ventral medial basal nucleus [thalamus]
- VMH: Ventromedial nucleus [hypothalamus]
- VPM: Ventral posteromedial nucleus [thalamus]
- → CaS: Calcarine sulcus
- → POS: Parieto-occipital sulcus

Age: 21 GW

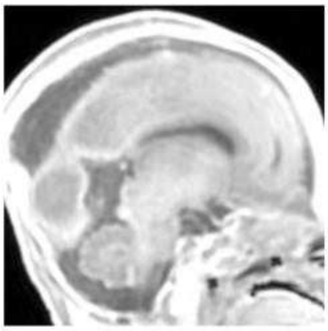

L-R Level: -1.56 mm

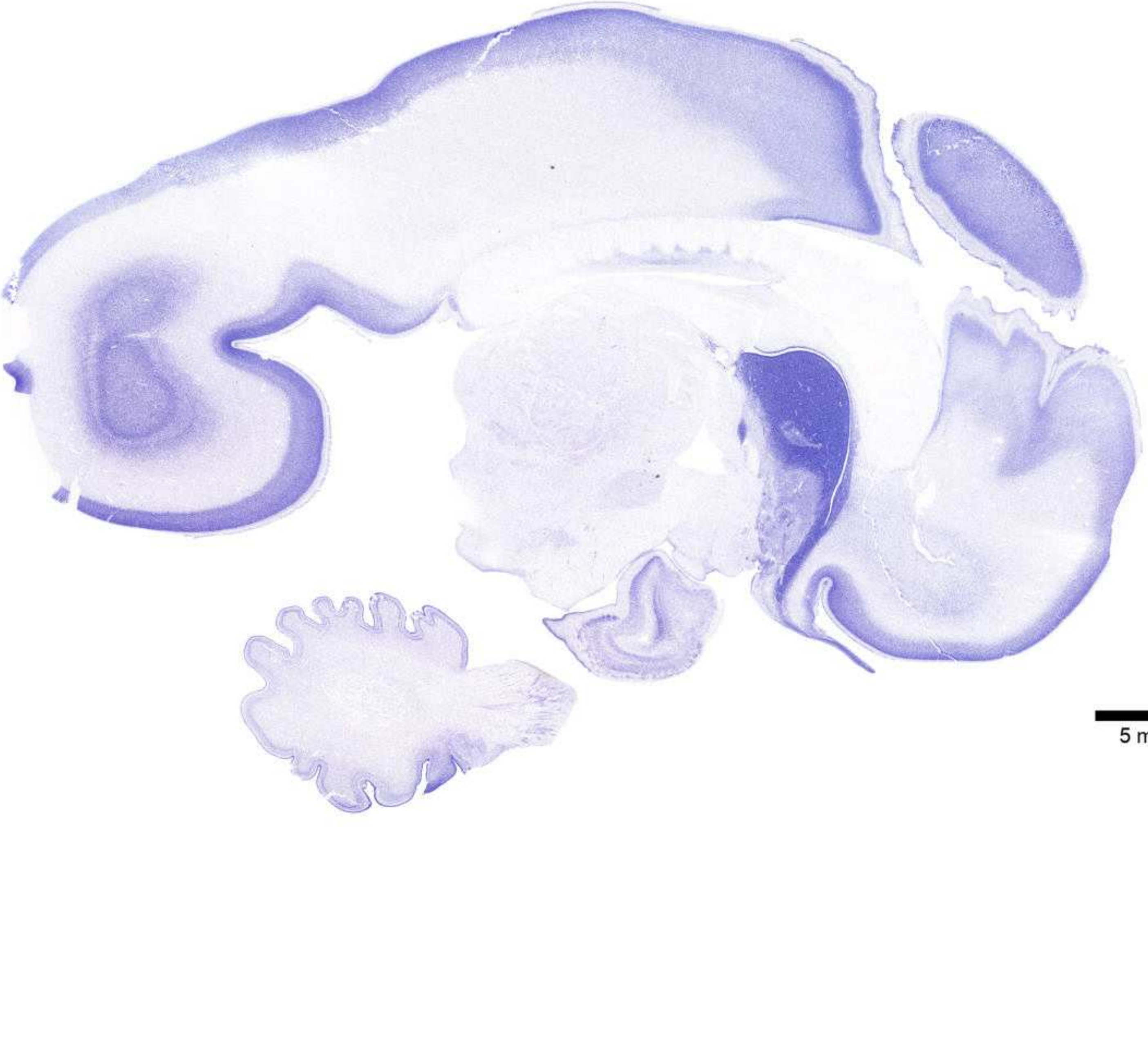

5 mm

Transient Layers

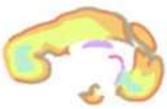

VZ
SVZ
IZ
SP
CP
MZ
SGL

Cortical Areas

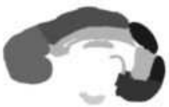

FCTx
ORB
PAR
OCC
CING
ENT

Age: 21 GW

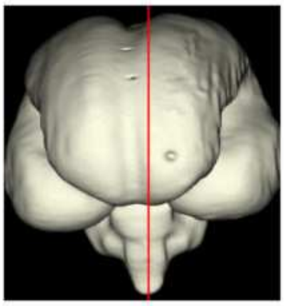

L-R Level: -1.56 mm

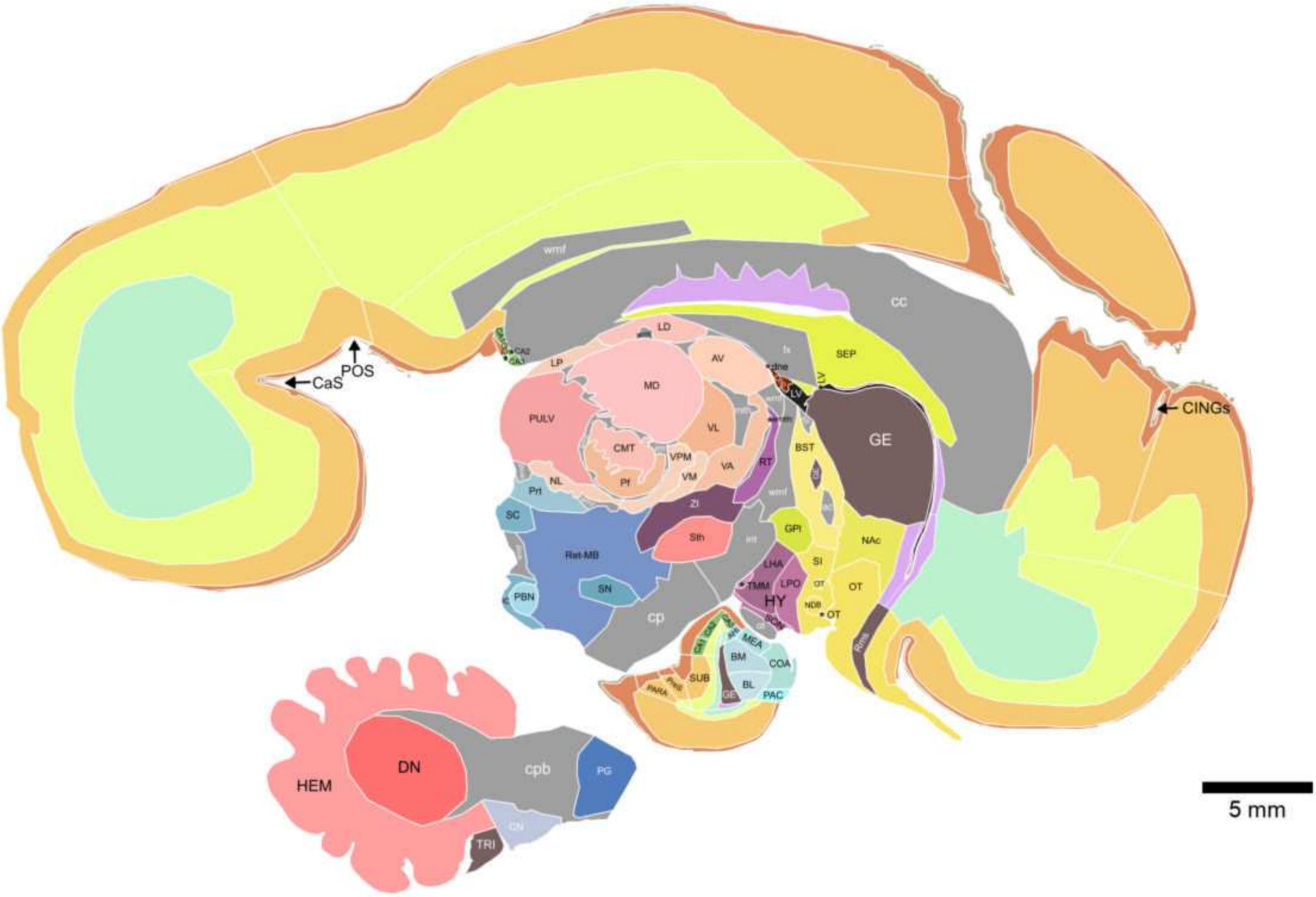

AHi: Amygdalo-hippocampal area
AV: Anteroventral nucleus [thalamus]
BL: Basal nucleus [amygdala]
BM: Basomedial nucleus [amygdala]
BST: Bed nucleus of the stria terminalis
CA1: CA1 field [hippocampus]
CA2: CA2 field [hippocampus]
CA3: CA3 field [hippocampus]
CMT: Centromedian nucleus [thalamus]
CN: Cochlear nuclei
COA: Cortical nucleus [amygdala]
Chp: Choroid plexus
DG: Dentate gyrus
DN: Dentate nucleus
GE: Ganglionic eminence
GPl: Globus pallidus lateral segment
HEM: Cerebellar hemispheres
HY: Hypothalamus
IC: Inferior colliculus
LD: Lateral dorsal nucleus [thalamus]
LHA: Lateral hypothalamic area
LP: Lateral posterior nucleus [thalamus]
LPO: Lateral preoptic area
LV: Lateral ventricle
MD: Medial dorsal nucleus [thalamus]
MEA: Medial nucleus [amygdala]
NAc: Nucleus accumbens
NDB: Nucleus of the diagonal band
NL: Nucleus limitans [thalamus]
OT: Olfactory tubercle
PARA: Cortical plate, parasubiculum
PBN: Parabigeminal nucleus
PG: Pontine gray
PULV: Pulvinar nucleus [thalamus]
Pf: Parafascicular nucleus [thalamus]
PreS: Cortical plate, presubiculum
Prt: Pretectum
RT: Reticular nucleus [thalamus]
Ret-MB: Reticular formation, Midbrain
Rms: Rostral migratory stream
SC: Superior colliculus
SEP: Septum
SI: Substantia innominata
SN: Substantia nigra
SON: Supraoptic nucleus [hypothalamus]
SUB: Cortical plate, subiculum
Sth: Subthalamus
TMM: Tuberomammillary nucleus
TRI: Germinal trigone
VA: Ventral anterior nucleus [thalamus]
VL: Ventral lateral nucleus [thalamus]
VM: Ventral medial nucleus [thalamus]
VPM: Ventral posteromedial nucleus [thalamus]
ZI: Zona incerta
ac: Anterior commissure
cc: Corpus callosum
cp: Cerebral peduncle
cpb: Cerebellar peduncles
dne: Diencephalic neuroepithelium
emlth: External medullary lamina [thalamus]
fx: Fornix
im: Internal medullary lamina [thalamus]
int: Internal capsule
mth: Mammillothalamic tract
ot: Optic tract
stt: Stria terminalis
wmf: White matter fibers
→ CaS: Calcarine sulcus
→ CINGs: Cingulate sulcus
→ POS: Parieto-occipital sulcus

Age: 21 GW

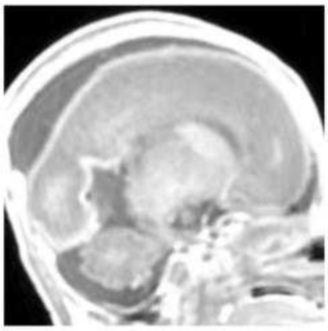

L-R Level: -2.04 mm

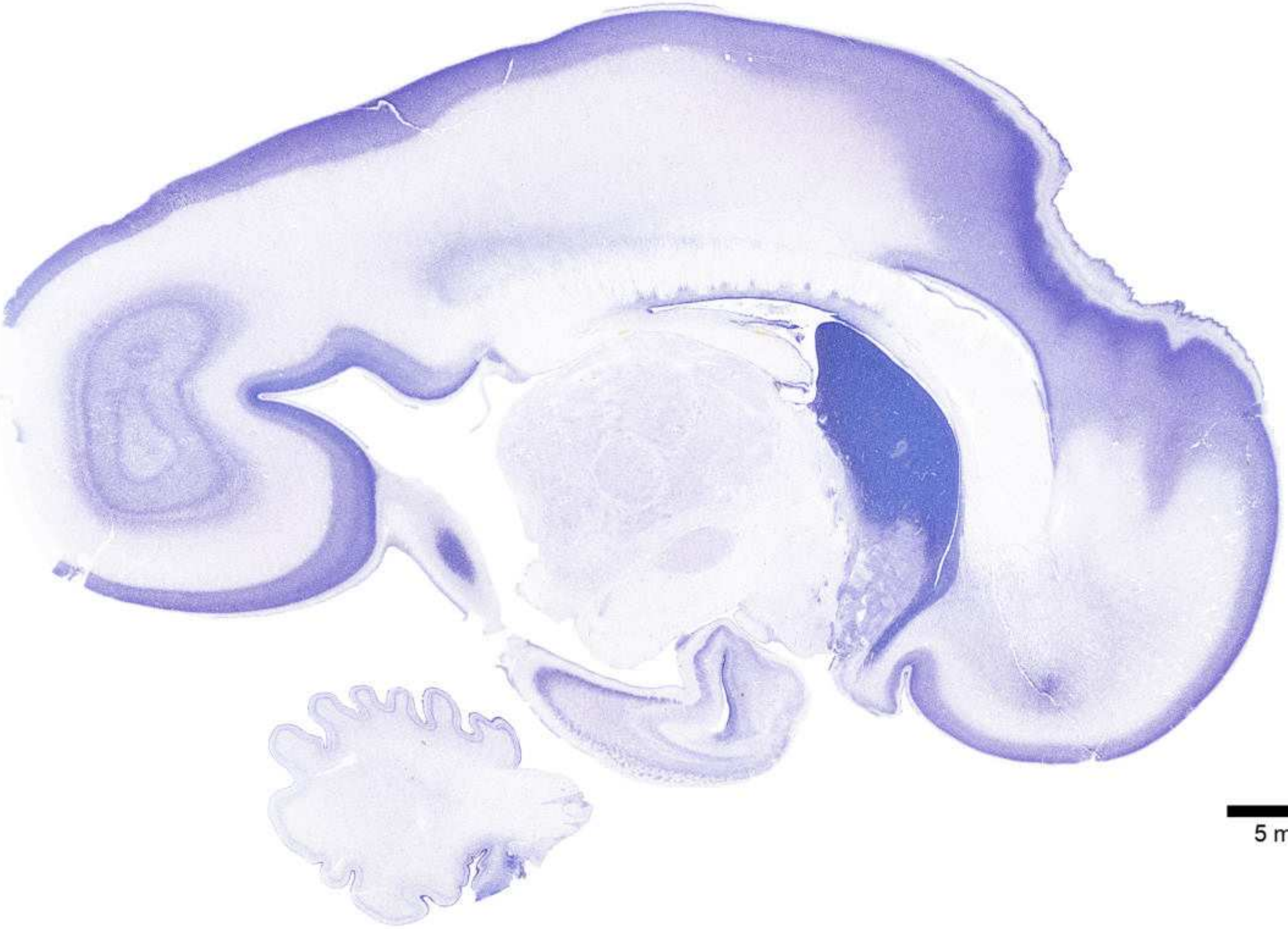

5 mm

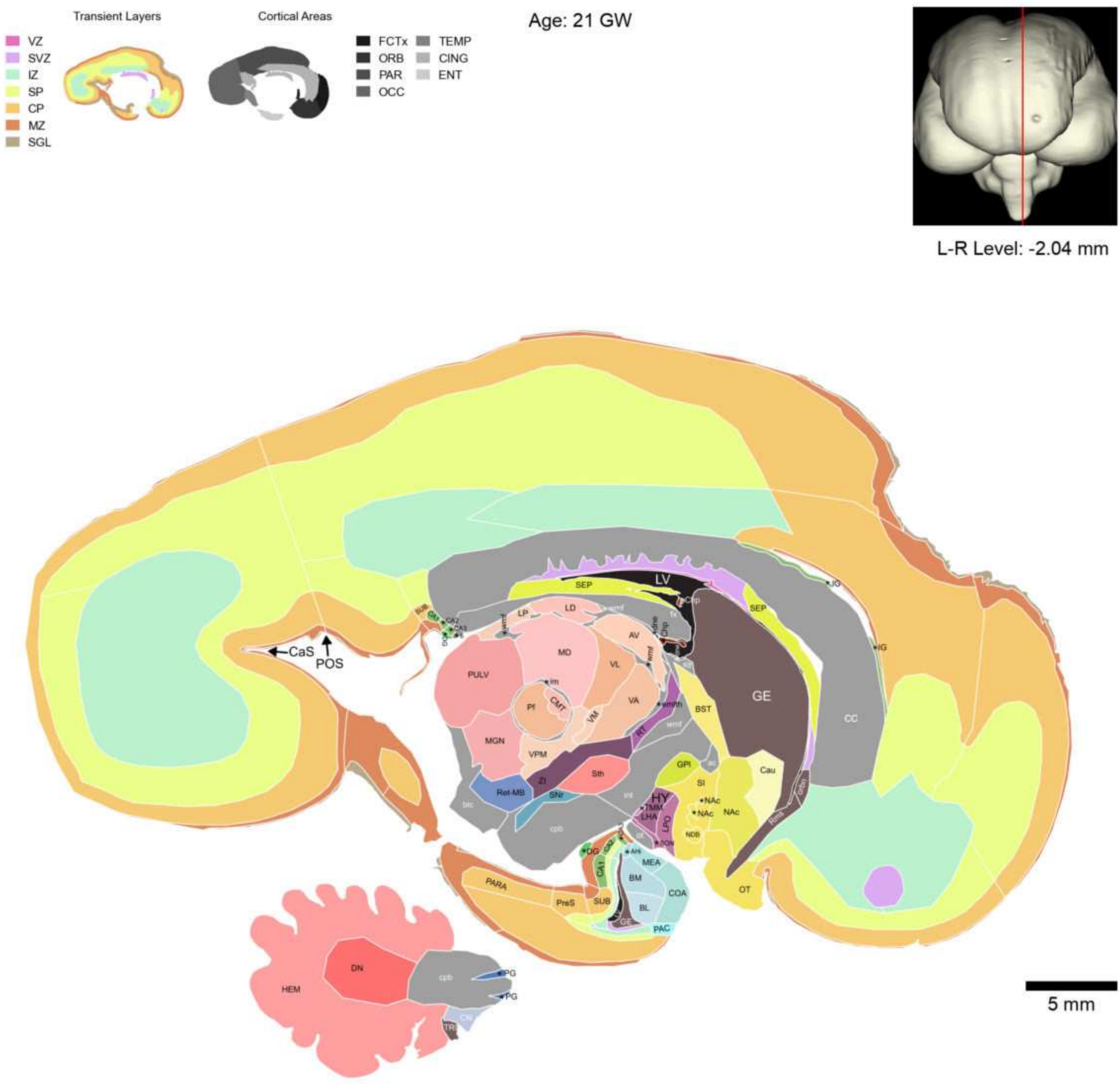

- AHi: Amygdalo-hippocampal area
- AV: Anteroventral nucleus [thalamus]
- BL: Basal nucleus [amygdala]
- BM: Basomedial nucleus [amygdala]
- BST: Bed nucleus of the stria terminalis
- CA1: CA1 field [hippocampus]
- CA2: CA2 field [hippocampus]
- CA3: CA3 field [hippocampus]
- CMT: Centromedian nucleus [thalamus]
- CN: Cochlear nuclei
- COA: Cortical nucleus [amygdala]
- Cau: Caudate nucleus
- Chp: Choroid plexus
- DG: Dentate gyrus
- DN: Dentate nucleus
- GE: Ganglionic eminence
- GPl: Globus pallidus lateral segment
- HEM: Cerebellar hemispheres
- HY: Hypothalamus
- IG: Induseum griseum
- LD: Lateral dorsal nucleus [thalamus]
- LHA: Lateral hypothalamic area
- LP: Lateral posterior nucleus [thalamus]
- LPO: Lateral preoptic area
- LV: Lateral ventricle
- MD: Medial dorsal nucleus [thalamus]
- MEA: Medial nucleus [amygdala]
- MGN: Medial geniculate nucleus
- NAc: Nucleus accumbens
- NDB: Nucleus of the diagonal band
- OT: Olfactory tubercle
- PARA: Cortical plate, parasubiculum
- PG: Pontine gray
- PULV: Pulvinar nucleus [thalamus]
- Pf: Parafascicular nucleus [thalamus]
- PreS: Cortical plate, presubiculum
- RT: Reticular nucleus [thalamus]
- Ret-MB: Reticular formation, Midbrain
- Rms: Rostral migratory stream
- SEP: Septum
- SI: Substantia innominata
- SNr: Substantia nigra pars reticulata
- SON: Supraoptic nucleus [hypothalamus]
- SUB: Cortical plate, subiculum
- Sth: Subthalamus
- TMM: Tuberomammillary nucleus
- TRI: Germinal trigone
- VA: Ventral anterior nucleus [thalamus]
- VL: Ventral lateral nucleus [thalamus]
- VM: Ventral medial nucleus [thalamus]
- VPM: Ventral posteromedial nucleus [thalamus]
- ZI: Zona incerta
- ac: Anterior commissure
- bic: Brachium of the inferior colliculus
- cc: Corpus callosum
- cpb: Cerebellar peduncles
- dne: Diencephalic neuroepithelium
- emlth: External medullary lamina [thalamus]
- fi: Fimbria
- fx: Fornix
- im: Internal medullary lamina [thalamus]
- int: Internal capsule
- orbn: Orbitofrontal neuroepithelium
- ot: Optic tract
- stt: Stria terminalis
- wmf: White matter fibers
- CaS: Calcarine sulcus
- POS: Parieto-occipital sulcus

Age: 21 GW

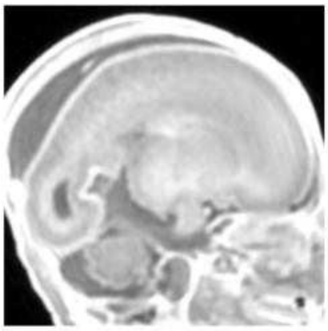

L-R Level: -3.0 mm

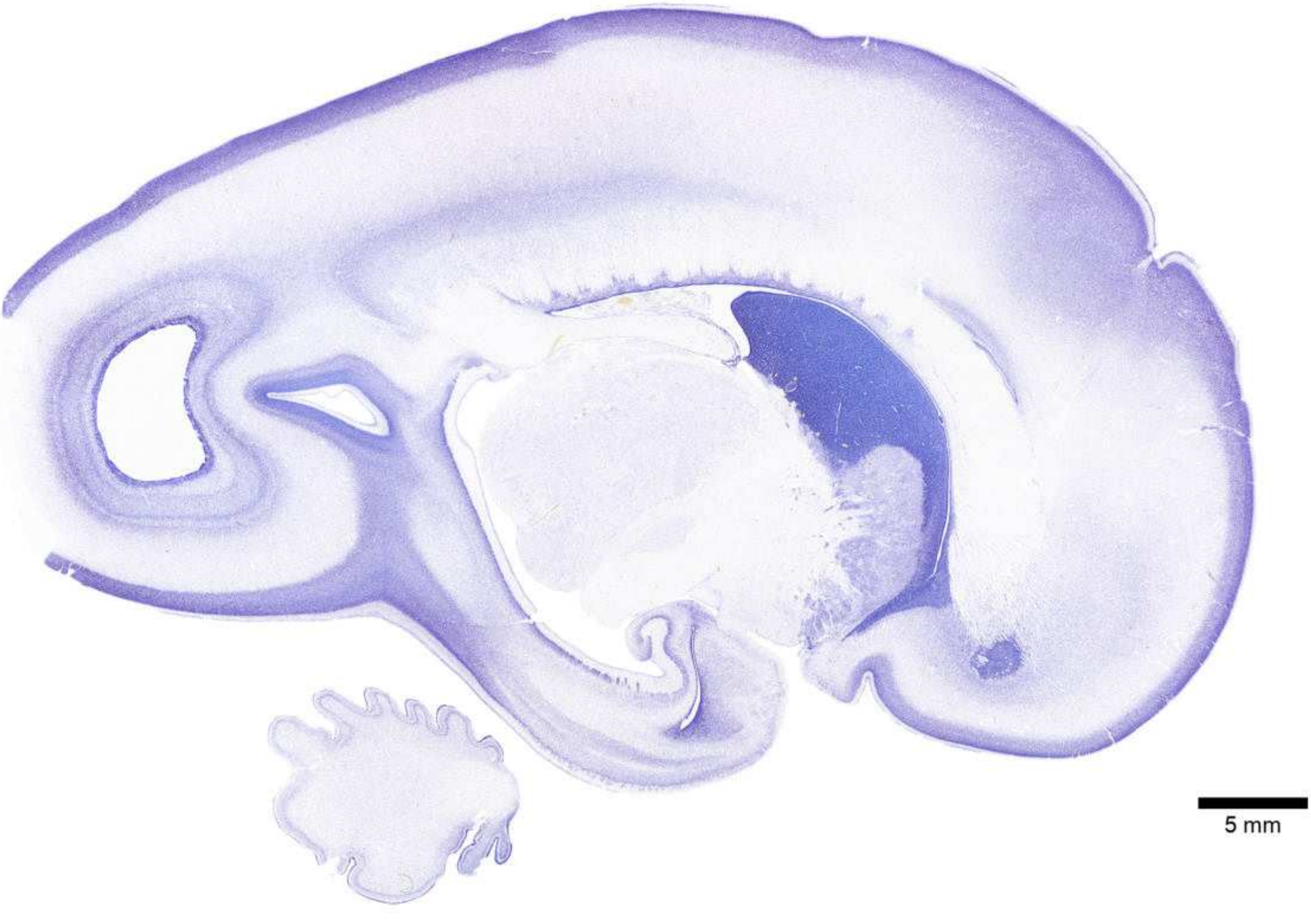

5 mm

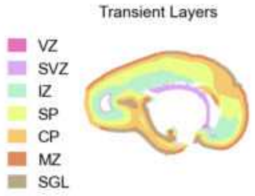

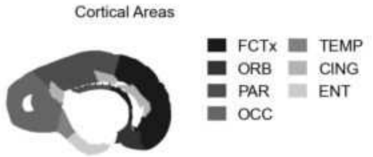

Age: 21 GW

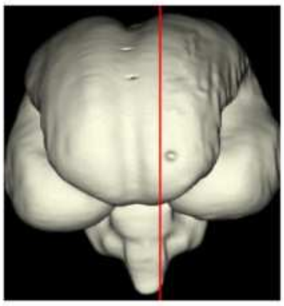

L-R Level: -3.0 mm

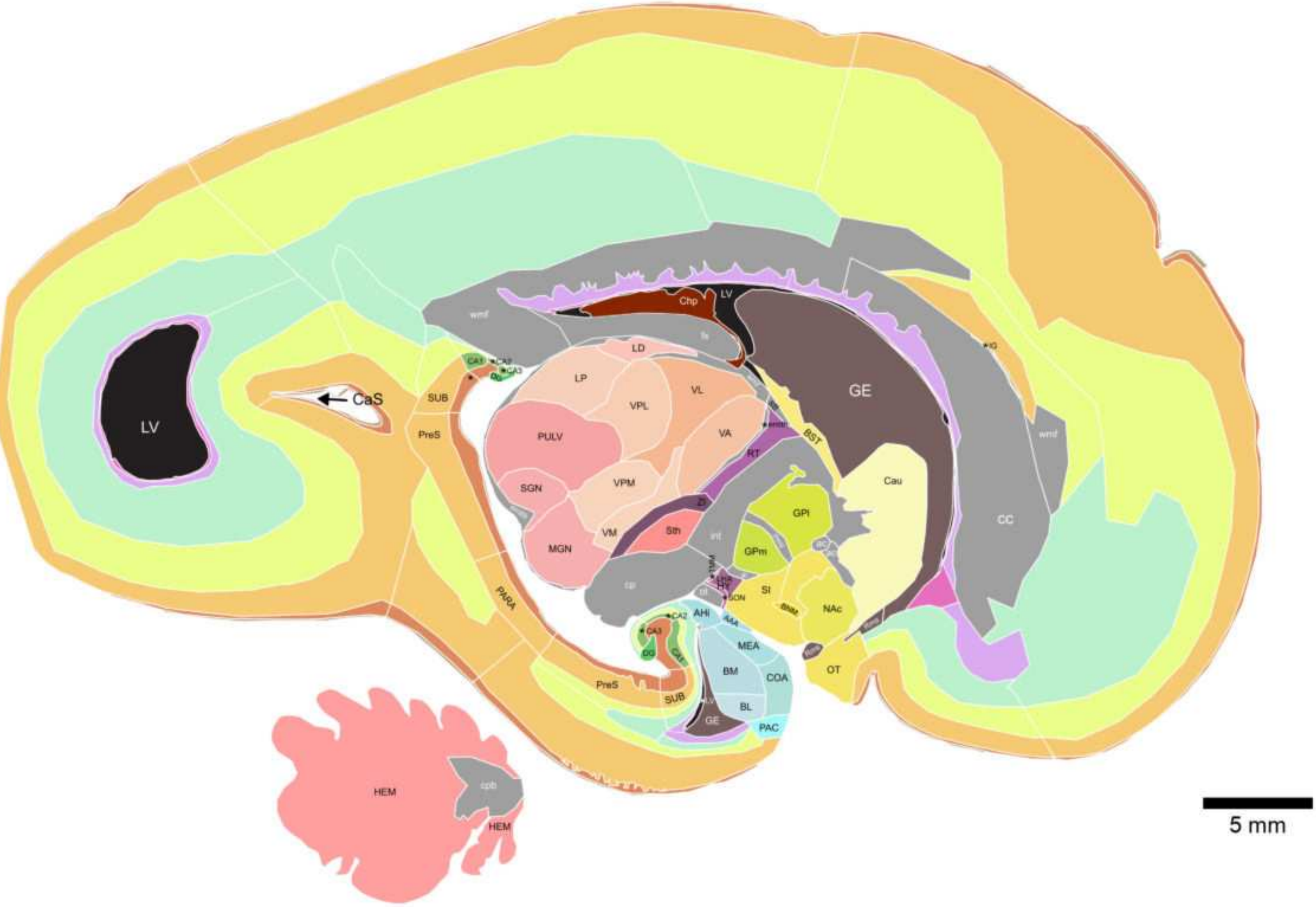

AAA: Anterior amygdaloid area
AHi: Amygdalo-hippocampal area
BL: Basal nucleus [amygdala]
BM: Basomedial nucleus [amygdala]
BNM: Basal nucleus of Meynert
BST: Bed nucleus of the stria terminalis
CA1: CA1 field [hippocampus]
CA2: CA2 field [hippocampus]
CA3: CA3 field [hippocampus]
COA: Cortical nucleus [amygdala]
Cau: Caudate nucleus
Chp: Choroid plexus
DG: Dentate gyrus
GE: Ganglionic eminence
GPl: Globus pallidus lateral segment
GPm: Globus pallidus medial segment
HEM: Cerebellar hemispheres
HY: Hypothalamus
IG: Induseum griseum
LD: Lateral dorsal nucleus [thalamus]
LHA: Lateral hypothalamic area
LP: Lateral posterior nucleus [thalamus]
LV: Lateral ventricle
MEA: Medial nucleus [amygdala]
MGN: Medial geniculate nucleus
NAc: Nucleus accumbens
OT: Olfactory tubercle
PARA: Cortical plate, parasubiculum
PULV: Pulvinar nucleus [thalamus]
PreS: Cortical plate, presubiculum
RT: Reticular nucleus [thalamus]
Rms: Rostral migratory stream
SGN: Suprageniculate nucleus
SI: Substantia innominata
SON: Supraoptic nucleus [hypothalamus]
SUB: Cortical plate, subiculum
Sth: Subthalamus
TMM: Tuberomammillary nucleus
VA: Ventral anterior nucleus [thalamus]
VL: Ventral lateral nucleus [thalamus]
VM: Ventral medial nucleus [thalamus]
VPL: Ventral posterolateral nucleus [thalamus]
VPM: Ventral posteromedial nucleus [thalamus]
ZI: Zona incerta
ac: Anterior commissure
al: Ansa lenticularis
cc: Corpus callosum
cp: Cerebral peduncle
cpb: Cerebellar peduncles
emlth: External medullary lamina [thalamus]
fx: Fornix
int: Internal capsule
mml: Medial medullary lamina
ot: Optic tract
stt: Stria terminalis
wmf: White matter fibers
➔ CaS: Calcarine sulcus

Age: 21 GW

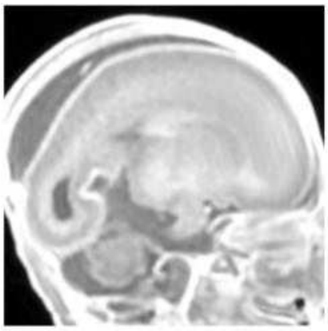

L-R Level: -3.54 mm

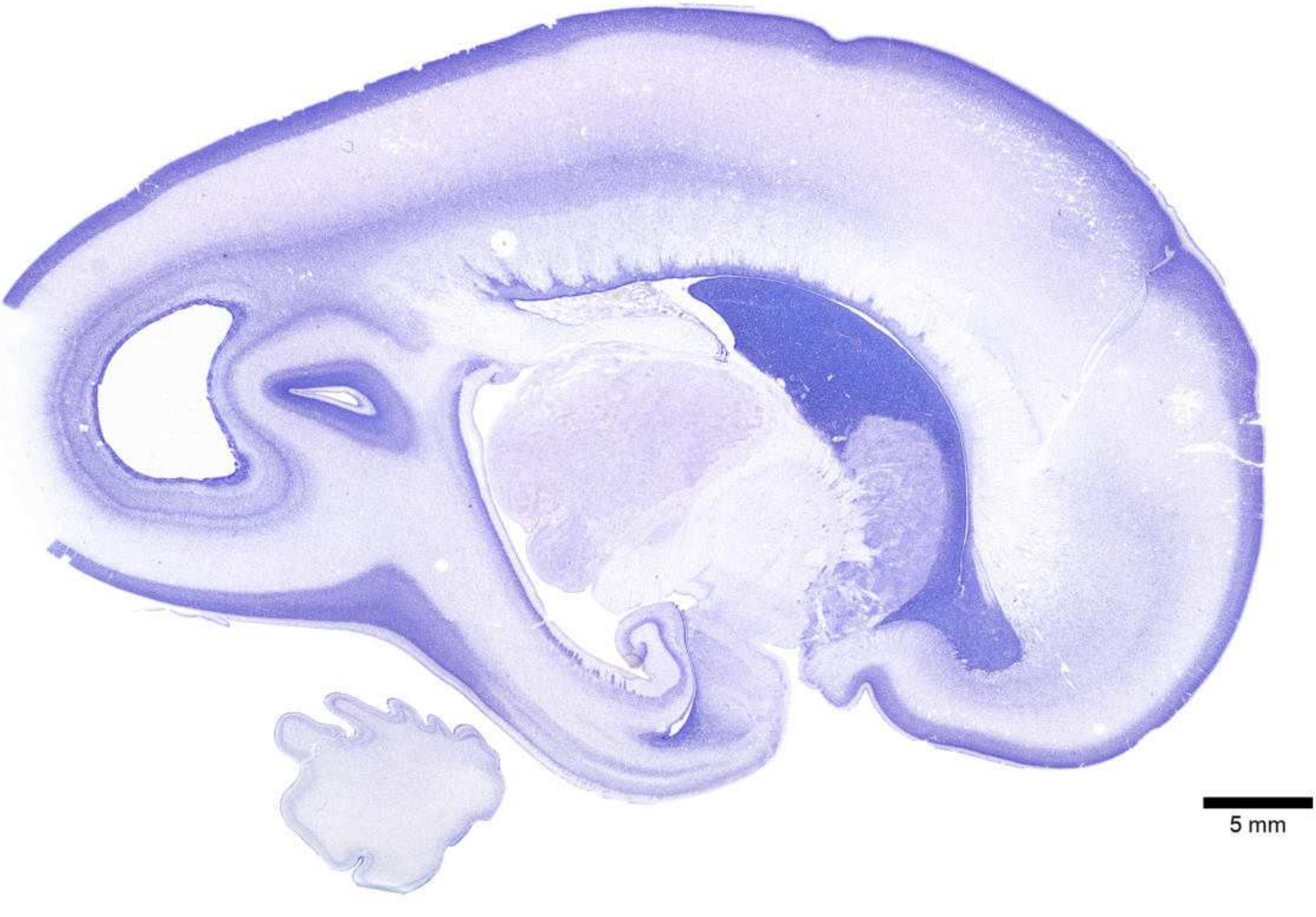

5 mm

Transient Layers

- VZ
- SVZ
- IZ
- SP
- CP
- MZ
- SGL

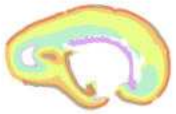

Cortical Areas

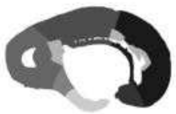

- FCTx
- ORB
- PAR
- OCC
- TEMP
- CING
- ENT

Age: 21 GW

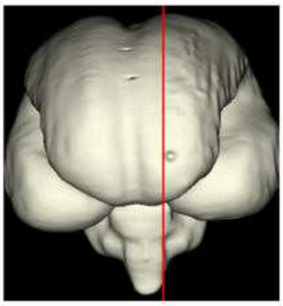

L-R Level: -3.54 mm

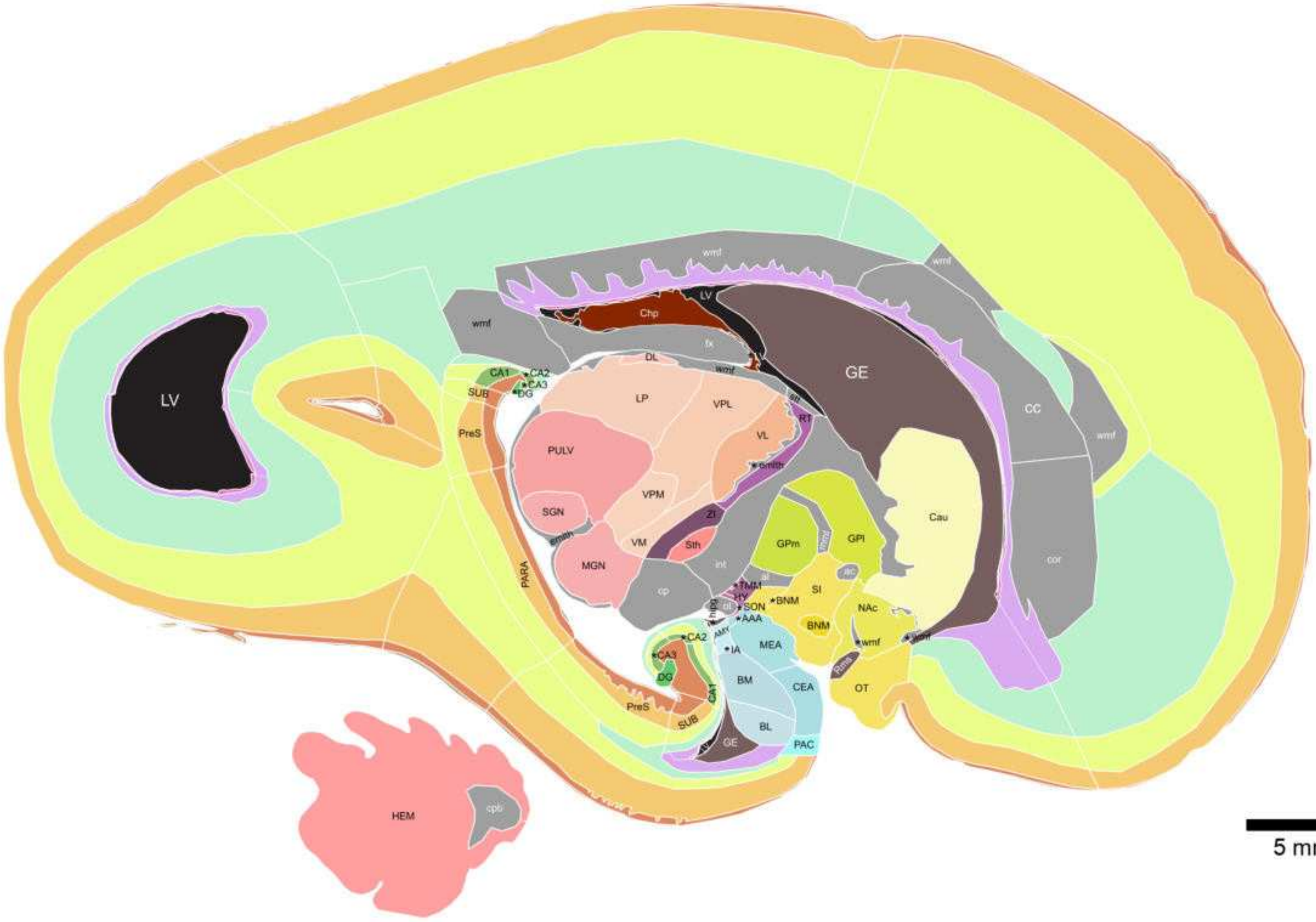

5 mm

AAA: Anterior amygdaloid area
AMY: Amygdala
BL: Basal nucleus [amygdala]
BM: Basomedial nucleus [amygdala]
BNM: Basal nucleus of Meynert
CA1: CA1 field [hippocampus]
CA2: CA2 field [hippocampus]
CA3: CA3 field [hippocampus]
CEA: Central nucleus [amygdala]
Cau: Caudate nucleus
Chp: Choroid plexus
DG: Dentate gyrus
DL: Dorsolateral nucleus [thalamus]
GE: Ganglionic eminence
GPl: Globus pallidus lateral segment
GPm: Globus pallidus medial segment
HEM: Cerebellar hemispheres
HY: Hypothalamus
IA: Intercalated cell groups [amygdala]
LHA: Lateral hypothalamic area
LP: Lateral posterior nucleus [thalamus]
LV: Lateral ventricle
MEA: Medial nucleus [amygdala]
MGN: Medial geniculate nucleus
NAc: Nucleus accumbens
OT: Olfactory tubercle
PARA: Cortical plate, parasubiculum
PULV: Pulvinar nucleus [thalamus]
PreS: Cortical plate, presubiculum
RT: Reticular nucleus [thalamus]
Rms: Rostral migratory stream
SGN: Suprageniculate nucleus
SI: Substantia innominata
SON: Supraoptic nucleus [hypothalamus]
SUB: Cortical plate, subiculum
Sth: Subthalamus
TMM: Tuberomammillary nucleus
VL: Ventral lateral nucleus [thalamus]
VM: Ventral medial nucleus [thalamus]
VPL: Ventral posterolateral nucleus [thalamus]
VPM: Ventral posteromedial nucleus [thalamus]
ZI: Zona incerta
ac: Anterior commissure
al: Ansa lenticularis
cc: Corpus callosum
cor: Corona radiata
cp: Cerebral peduncle
cpb: Cerebellar peduncles
emlth: External medullary lamina [thalamus]
fx: Fornix
hipg: Hippocampal glioepithelium/ependyma
int: Internal capsule
mml: Medial medullary lamina
ot: Optic tract
stt: Stria terminalis
wmf: White matter fibers

Age: 21 GW

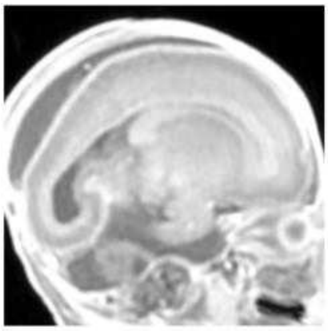

L-R Level: -4.32 mm

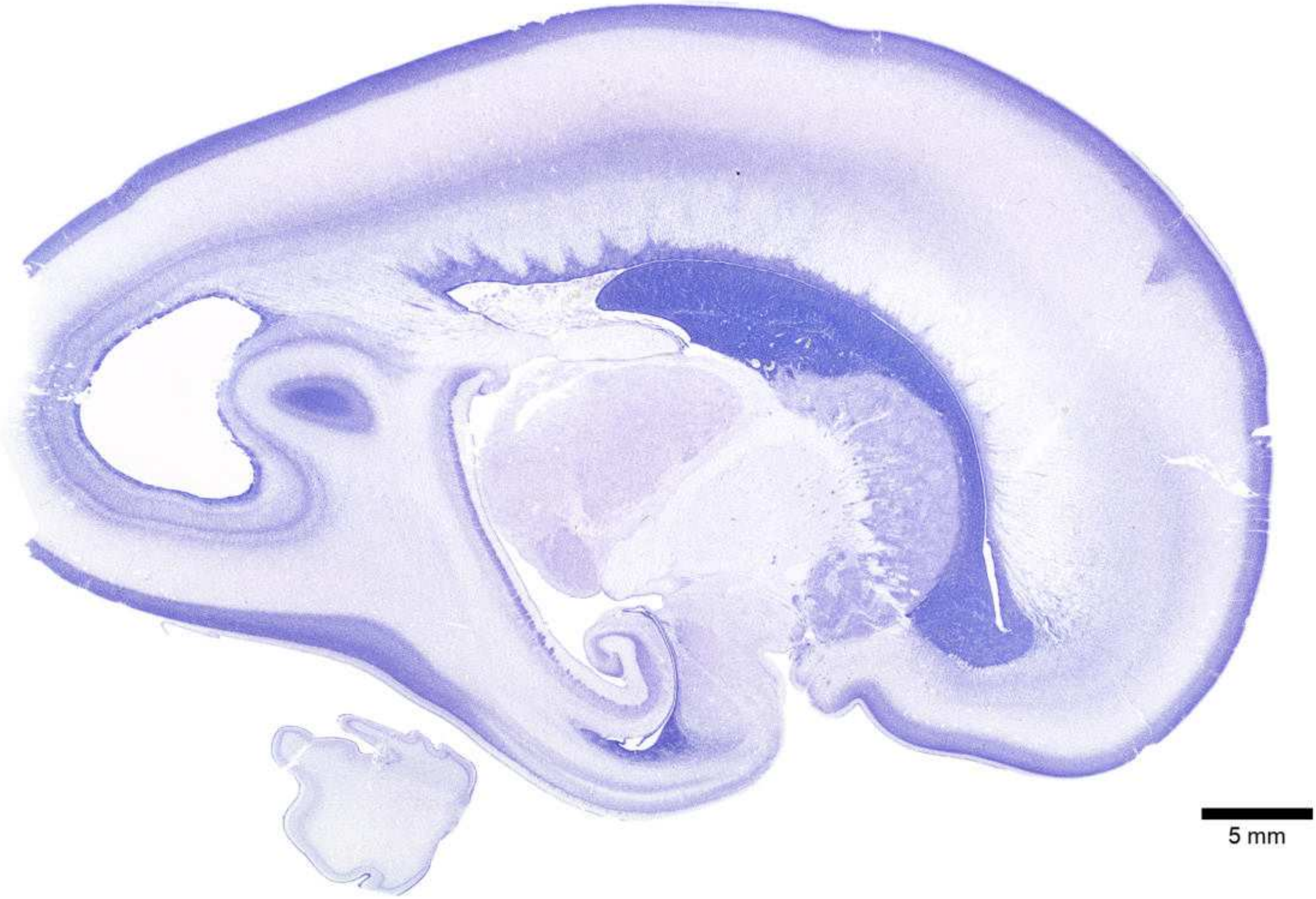

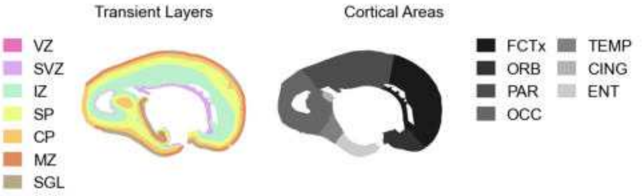

Age: 21 GW

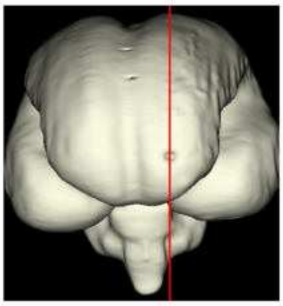

L-R Level: -4.32 mm

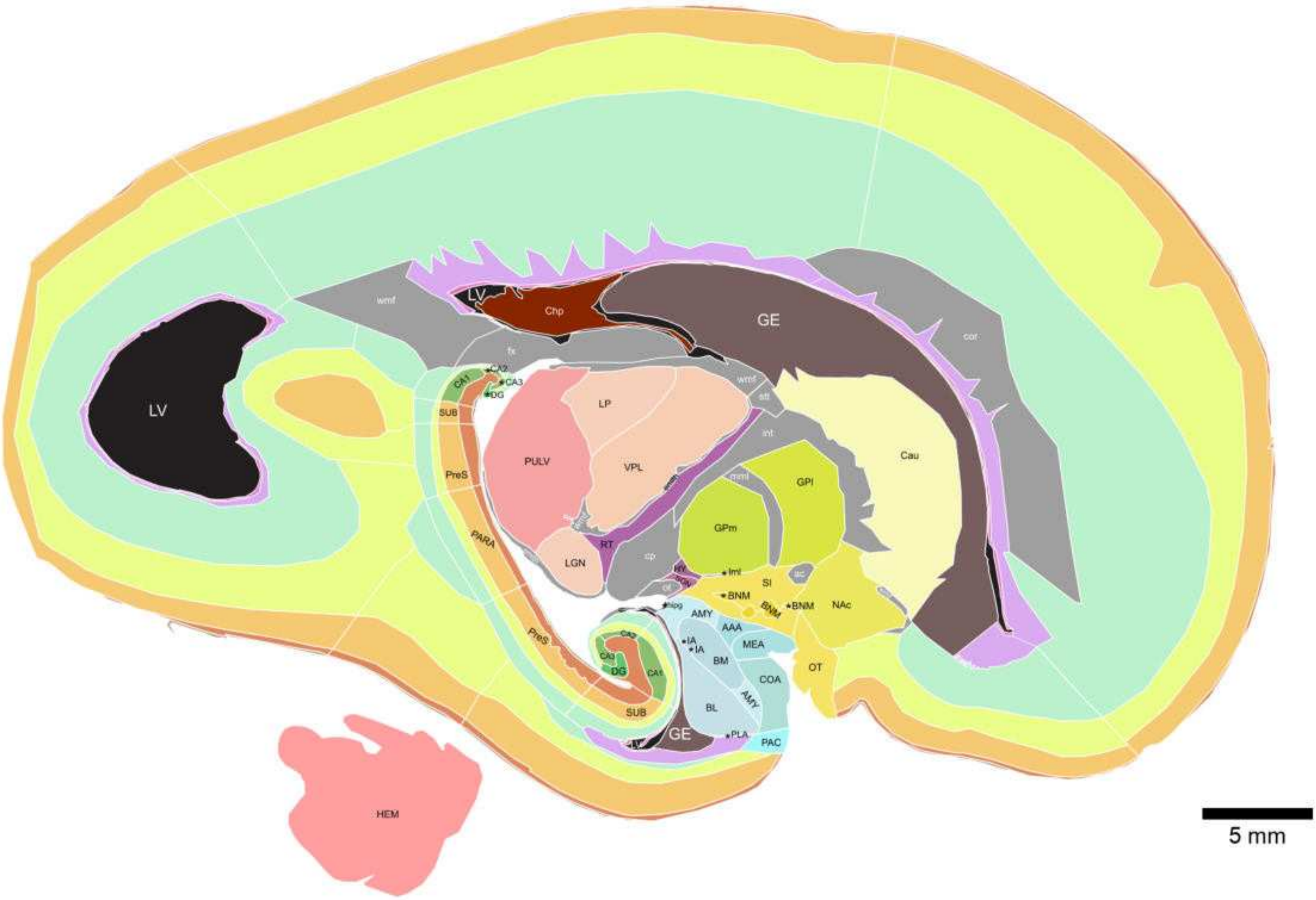

AAA: Anterior amygdaloid area
AMY: Amygdala
BL: Basal nucleus [amygdala]
BM: Basomedial nucleus [amygdala]
BNM: Basal nucleus of Meynert
CA1: CA1 field [hippocampus]
CA2: CA2 field [hippocampus]
CA3: CA3 field [hippocampus]
COA: Cortical nucleus [amygdala]
Cau: Caudate nucleus
Chp: Choroid plexus
DG: Dentate gyrus
GE: Ganglionic eminence
GPl: Globus pallidus lateral segment
GPm: Globus pallidus medial segment
HEM: Cerebellar hemispheres
HY: Hypothalamus
IA: Intercalated cell groups [amygdala]
LGN: Lateral geniculate nucleus
LHA: Lateral hypothalamic area
LP: Lateral posterior nucleus [thalamus]
LV: Lateral ventricle
MEA: Medial nucleus [amygdala]
NAc: Nucleus accumbens
OT: Olfactory tubercle
PARA: Cortical plate, parasubiculum
PLA: Paralaminar nucleus [amygdala]
PULV: Pulvinar nucleus [thalamus]
PreS: Cortical plate, presubiculum
RT: Reticular nucleus [thalamus]
SI: Substantia innominata
SON: Supraoptic nucleus [hypothalamus]
SUB: Cortical plate, subiculum
VPL: Ventral posterolateral nucleus [thalamus]
ac: Anterior commissure
cor: Corona radiata
cp: Cerebral peduncle
emlth: External medullary lamina [thalamus]
fi: Fimbria
fx: Fornix
hipg: Hippocampal glioepithelium/ependyma
int: Internal capsule
lml: Lateral medullary lamina
mml: Medial medullary lamina
ot: Optic tract
stt: Stria terminalis
wmf: White matter fibers

Age: 21 GW

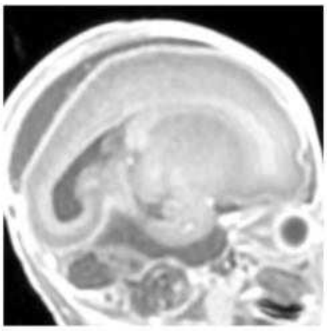

L-R Level: -4.8 mm

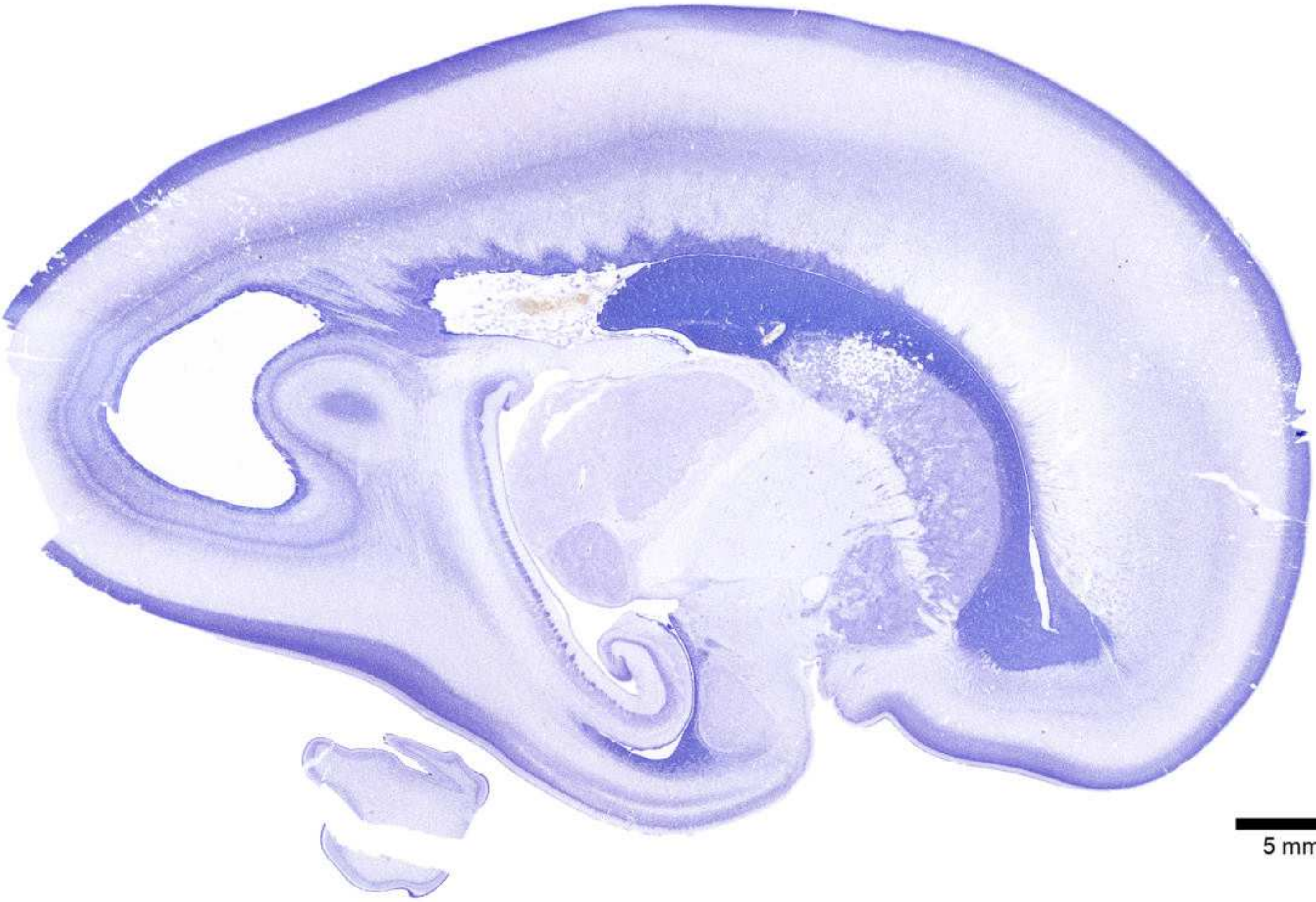

5 mm

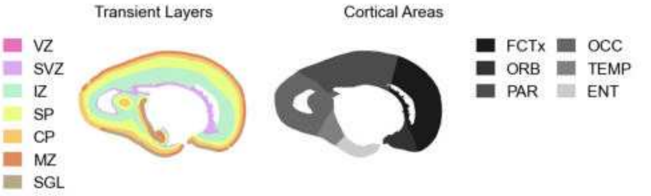

Age: 21 GW

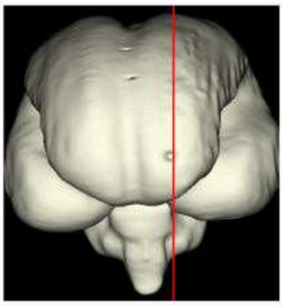

L-R Level: -4.8 mm

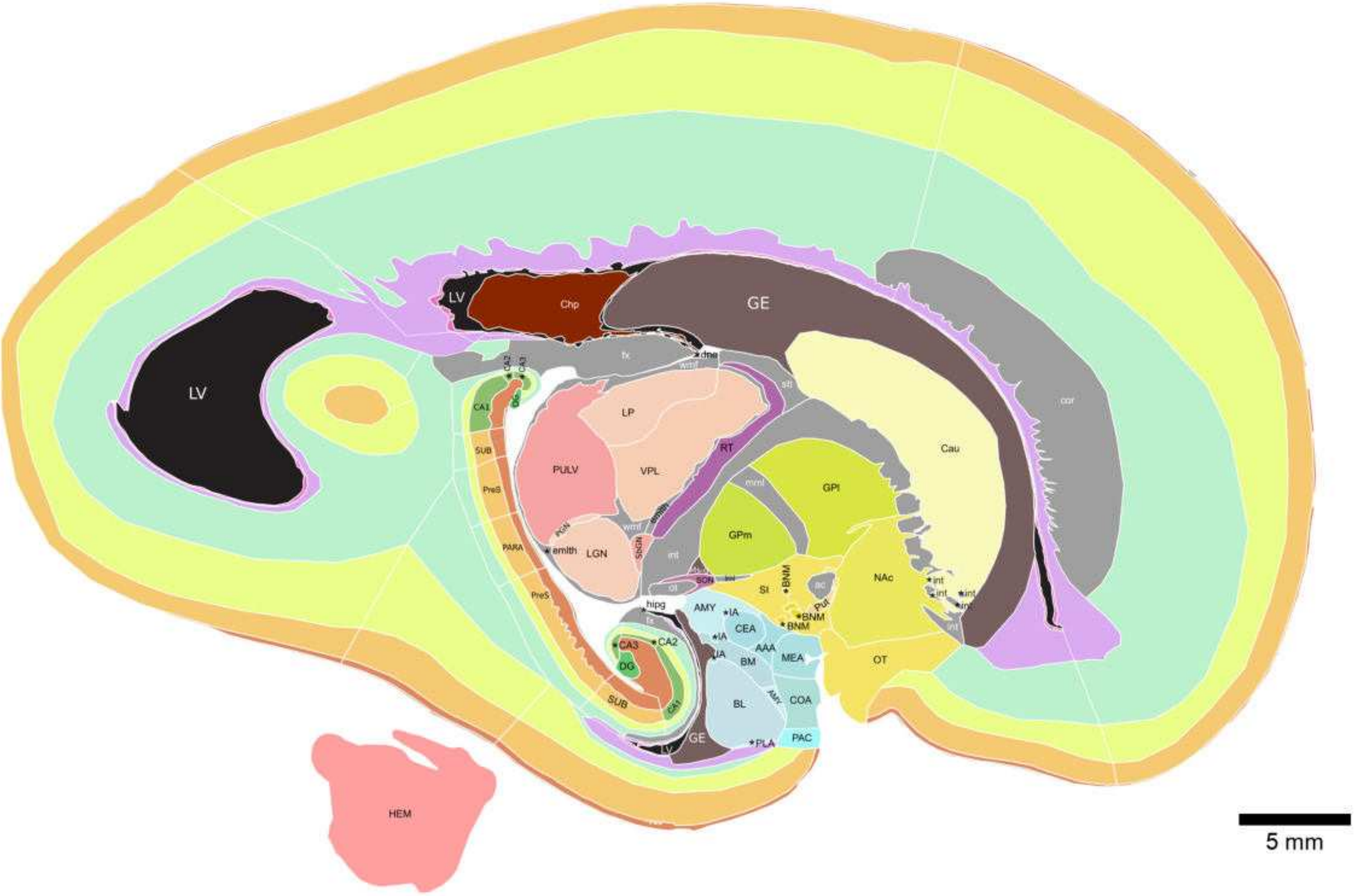

- AAA: Anterior amygdaloid area
- AMY: Amygdala
- BL: Basal nucleus [amygdala]
- BM: Basomedial nucleus [amygdala]
- BNM: Basal nucleus of Meynert
- CA1: CA1 field [hippocampus]
- CA2: CA2 field [hippocampus]
- CA3: CA3 field [hippocampus]
- CEA: Central nucleus [amygdala]
- COA: Cortical nucleus [amygdala]
- Cau: Caudate nucleus
- Chp: Choroid plexus
- DG: Dentate gyrus
- GE: Ganglionic eminence
- GPl: Globus pallidus lateral segment
- GPm: Globus pallidus medial segment
- HEM: Cerebellar hemispheres
- IA: Intercalated cell groups [amygdala]
- LGN: Lateral geniculate nucleus
- LP: Lateral posterior nucleus [thalamus]
- LV: Lateral ventricle
- MEA: Medial nucleus [amygdala]
- Ms-g: Migratory stream, general
- NAc: Nucleus accumbens
- OT: Olfactory tubercle
- PARA: Cortical plate, parasubiculum
- PGN: Pregeniculate nucleus
- PLA: Paralaminar nucleus [amygdala]
- PULV: Pulvinar nucleus [thalamus]
- PreS: Cortical plate, presubiculum
- Put: Putamen
- RT: Reticular nucleus [thalamus]
- SI: Substantia innominata
- SON: Supraoptic nucleus [hypothalamus]
- SUB: Cortical plate, subiculum
- SbGN: Subgeniculate nucleus
- VPL: Ventral posterolateral nucleus [thalamus]
- ac: Anterior commissure
- cor: Corona radiata
- dne: Diencephalic neuroepithelium
- emlth: External medullary lamina [thalamus]
- fx: Fornix
- hipg: Hippocampal glioepithelium/ependyma
- int: Internal capsule
- lml: Lateral medullary lamina
- mml: Medial medullary lamina
- ot: Optic tract
- stt: Stria terminalis
- wmf: White matter fibers

Age: 21 GW

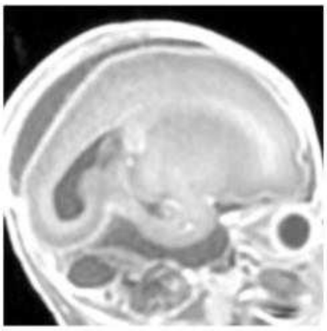

L-R Level: -5.28 mm

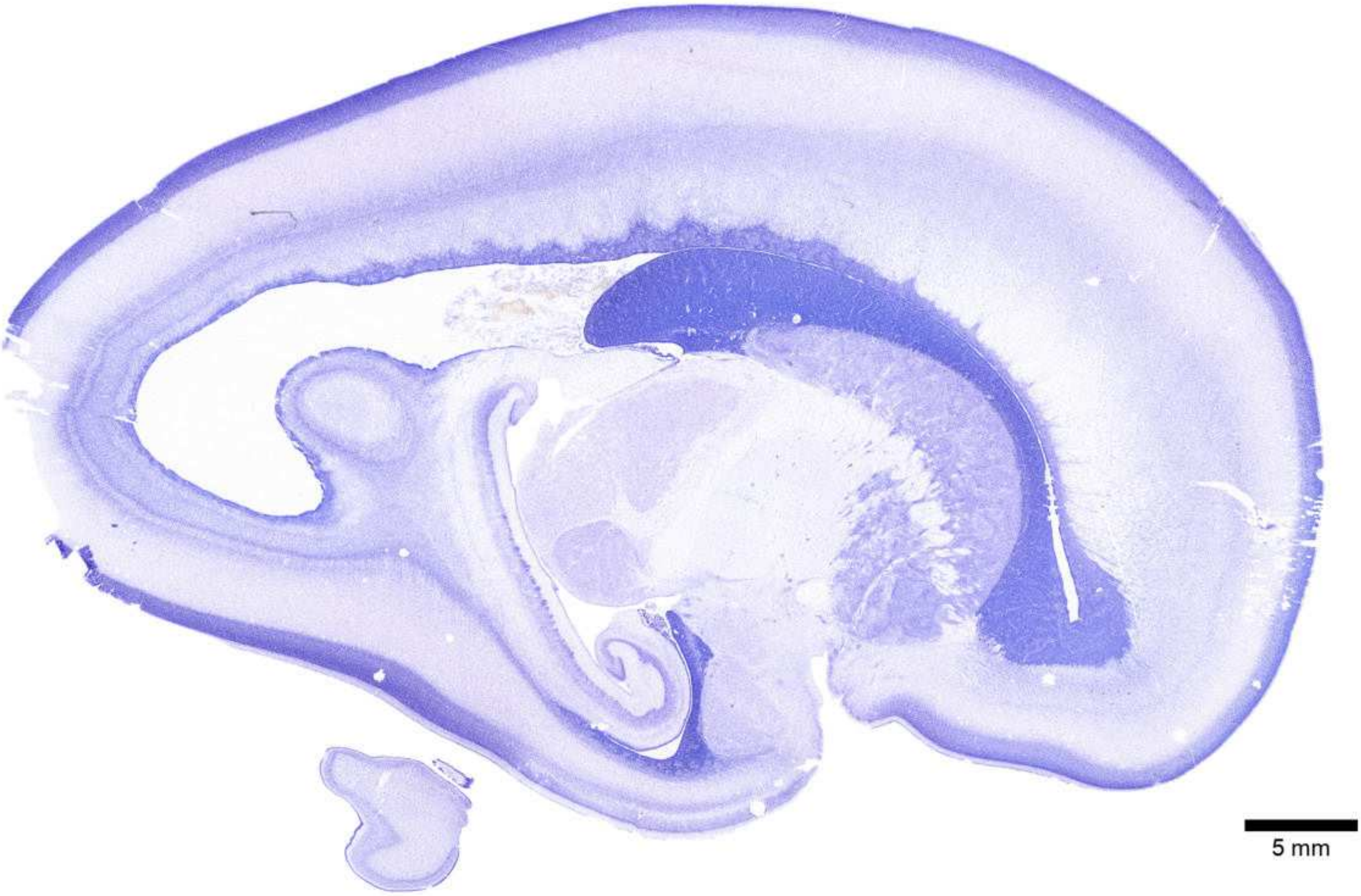

5 mm

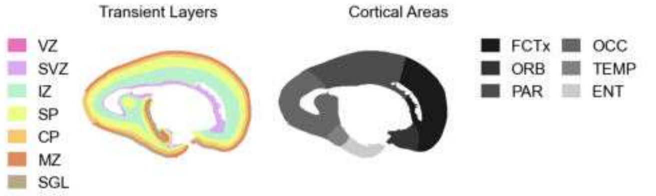

Age: 21 GW

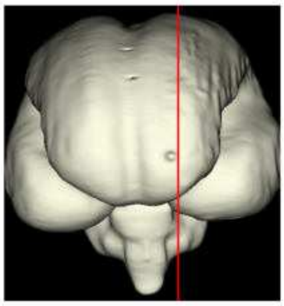

L-R Level: -5.28 mm

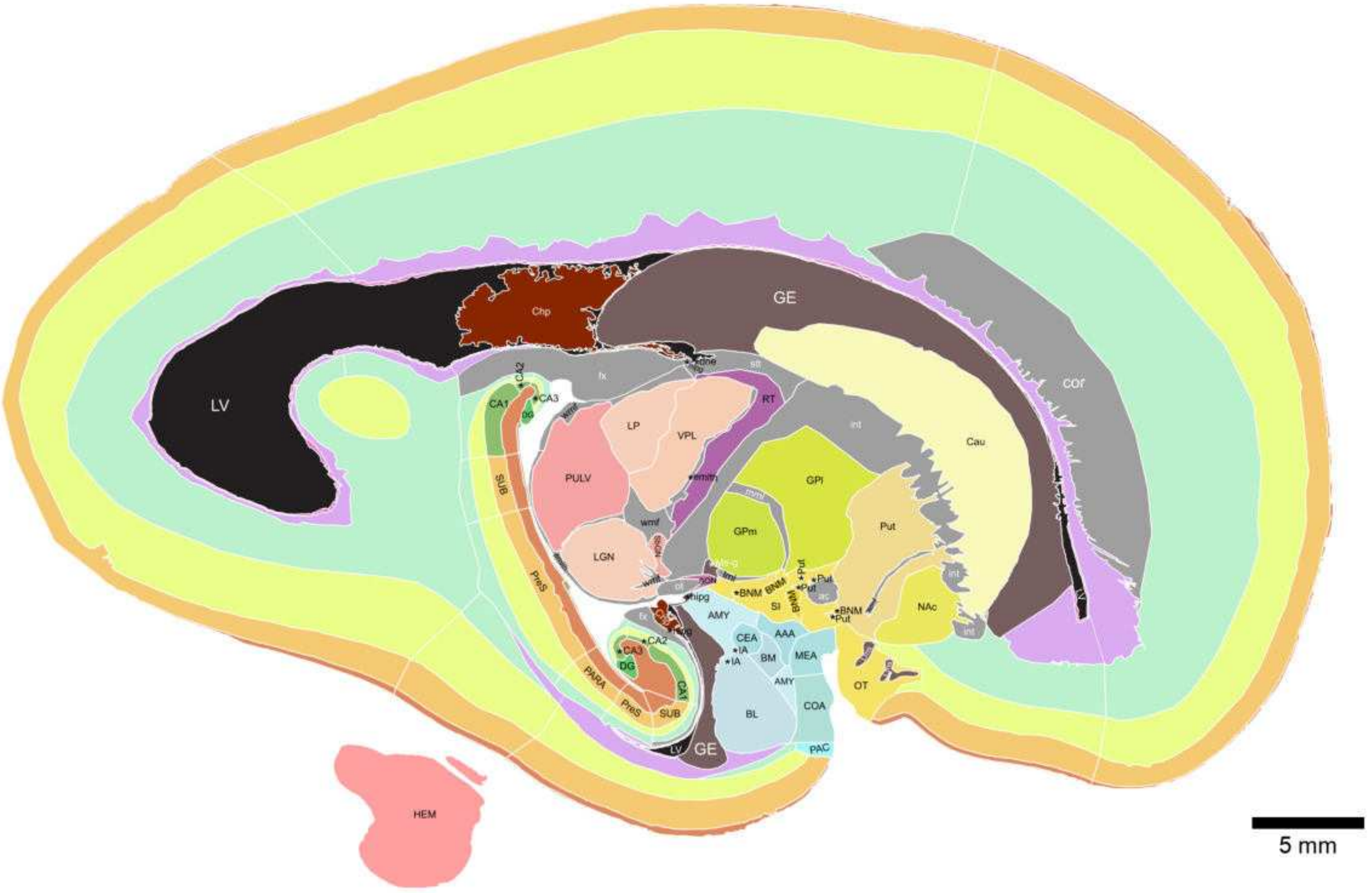

AAA: Anterior amygdaloid area
AMY: Amygdala
BL: Basal nucleus [amygdala]
BM: Basomedial nucleus [amygdala]
BNM: Basal nucleus of Meynert
CA1: CA1 field [hippocampus]
CA2: CA2 field [hippocampus]
CA3: CA3 field [hippocampus]
CEA: Central nucleus [amygdala]
COA: Cortical nucleus [amygdala]
Cau: Caudate nucleus
Chp: Choroid plexus

DG: Dentate gyrus
GE: Ganglionic eminence
GPl: Globus pallidus lateral segment
GPm: Globus pallidus medial segment
HEM: Cerebellar hemispheres
IA: Intercalated cell groups [amygdala]
LGN: Lateral geniculate nucleus
LP: Lateral posterior nucleus [thalamus]
LV: Lateral ventricle
Lms: Lateral migratory stream
MEA: Medial nucleus [amygdala]
Ms-g: Migratory stream, general

NAc: Nucleus accumbens
OT: Olfactory tubercle
PARA: Cortical plate, parasubiculum
PULV: Pulvinar nucleus [thalamus]
PreS: Cortical plate, presubiculum
Put: Putamen
RT: Reticular nucleus [thalamus]
SI: Substantia innominata
SON: Supraoptic nucleus [hypothalamus]
SUB: Cortical plate, subiculum
SbGN: Subgeniculate nucleus
VPL: Ventral posterolateral nucleus [thalamus]

ac: Anterior commissure
cor: Corona radiata
dne: Diencephalic neuroepithelium
emlth: External medullary lamina [thalamus]
fx: Fornix
hipg: Hippocampal glioepithelium/ependyma
int: Internal capsule
lml: Lateral medullary lamina
mml: Medial medullary lamina
ot: Optic tract
stt: Stria terminalis
wmf: White matter fibers

Age: 21 GW

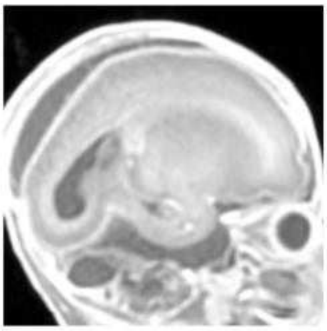

L-R Level: -5.52 mm

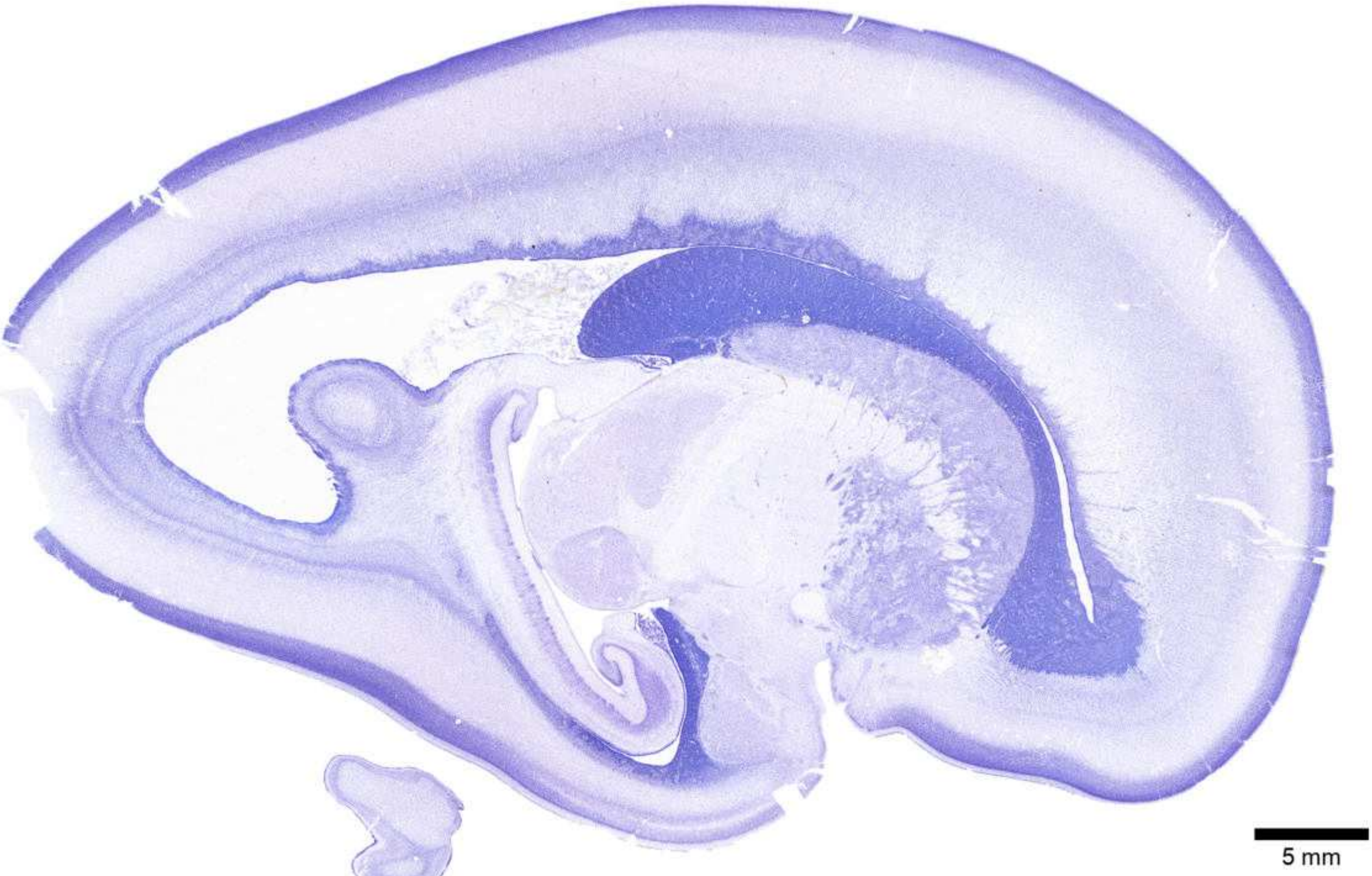

5 mm

Transient Layers

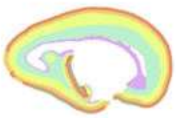

- VZ
- SVZ
- IZ
- SP
- CP
- MZ
- SGL

Cortical Areas

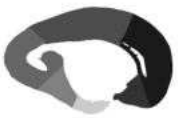

- FCTx
- ORB
- PAR
- OCC
- TEMP
- ENT

Age: 21 GW

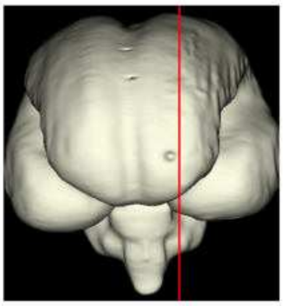

L-R Level: -5.52 mm

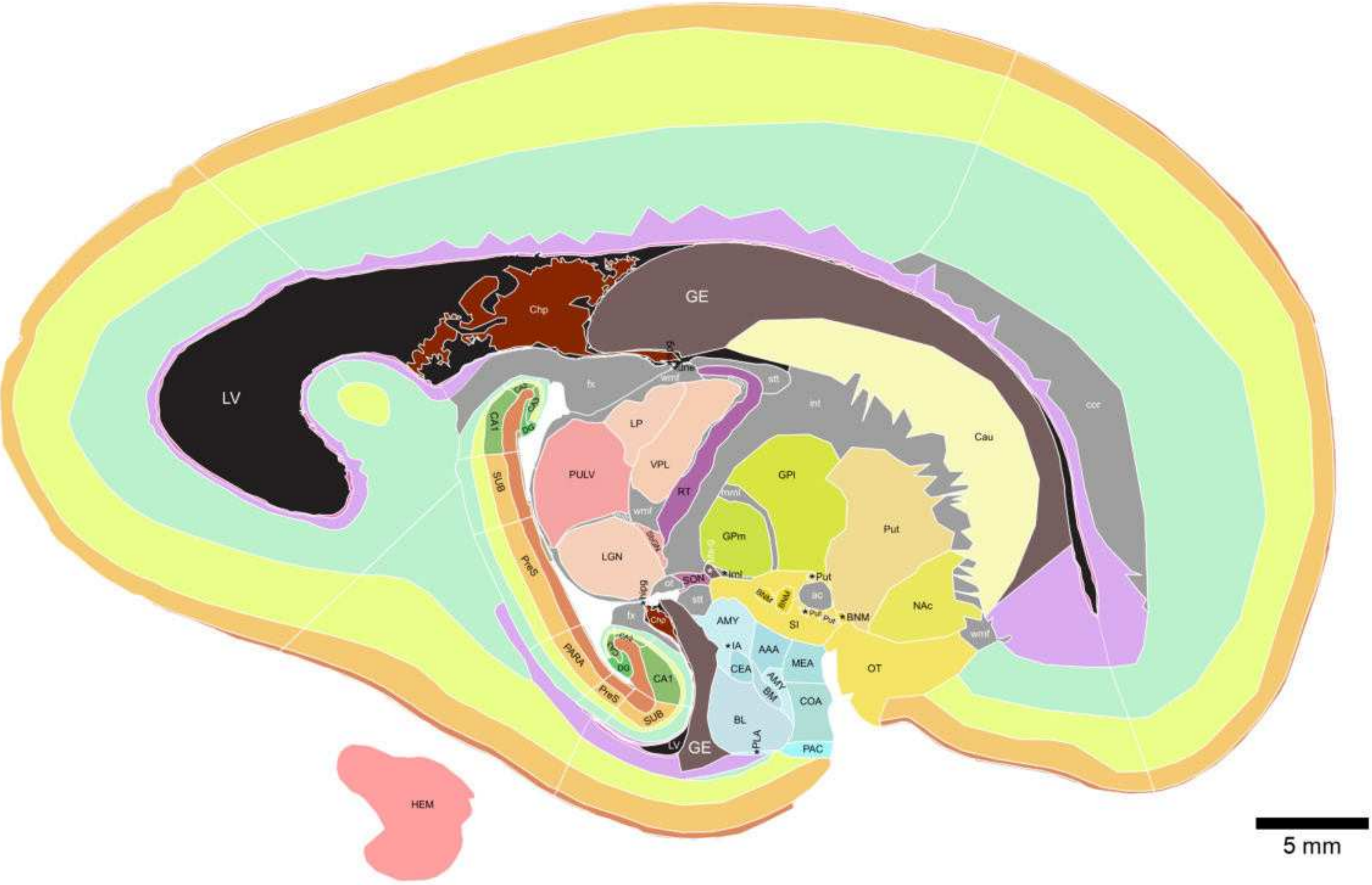

- AAA: Anterior amygdaloid area
- AMY: Amygdala
- BL: Basal nucleus [amygdala]
- BM: Basomedial nucleus [amygdala]
- BNM: Basal nucleus of Meynert
- CA1: CA1 field [hippocampus]
- CA2: CA2 field [hippocampus]
- CA3: CA3 field [hippocampus]
- CEA: Central nucleus [amygdala]
- COA: Cortical nucleus [amygdala]
- Cau: Caudate nucleus
- Chp: Choroid plexus
- DG: Dentate gyrus
- GE: Ganglionic eminence
- GPl: Globus pallidus lateral segment
- GPm: Globus pallidus medial segment
- HEM: Cerebellar hemispheres
- IA: Intercalated cell groups [amygdala]
- LGN: Lateral geniculate nucleus
- LP: Lateral posterior nucleus [thalamus]
- LV: Lateral ventricle
- MEA: Medial nucleus [amygdala]
- Ms-g: Migratory stream, general
- NAc: Nucleus accumbens
- OT: Olfactory tubercle
- PARA: Cortical plate, parasubiculum
- PLA: Paralaminar nucleus [amygdala]
- PULV: Pulvinar nucleus [thalamus]
- PreS: Cortical plate, presubiculum
- Put: Putamen
- RT: Reticular nucleus [thalamus]
- SI: Substantia innominata
- SON: Supraoptic nucleus [hypothalamus]
- SUB: Cortical plate, subiculum
- SbGN: Subgeniculate nucleus
- VPL: Ventral posterolateral nucleus [thalamus]
- ac: Anterior commissure
- cor: Corona radiata
- dne: Diencephalic neuroepithelium
- emlth: External medullary lamina [thalamus]
- fx: Fornix
- hipg: Hippocampal glioepithelium/ependyma
- int: Internal capsule
- lml: Lateral medullary lamina
- mml: Medial medullary lamina
- ot: Optic tract
- stt: Stria terminalis
- wmf: White matter fibers

Age: 21 GW

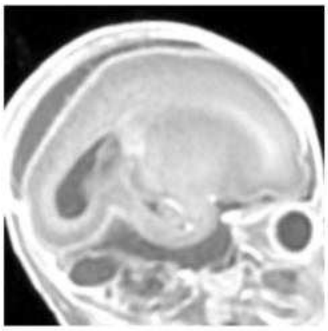

L-R Level: -5.82 mm

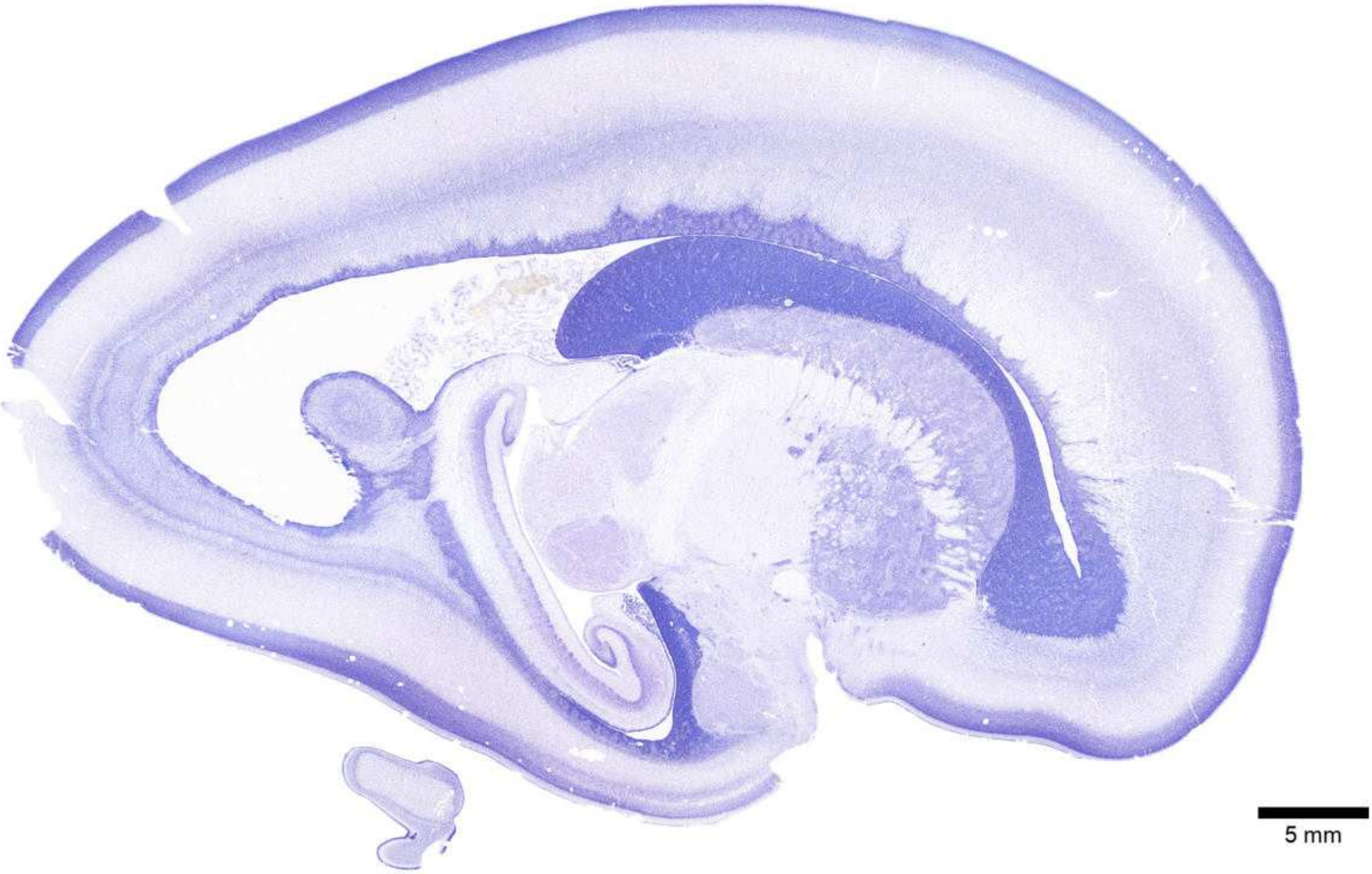

5 mm

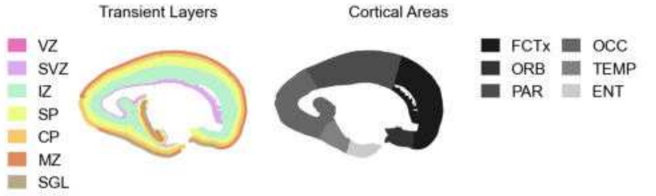

Age: 21 GW

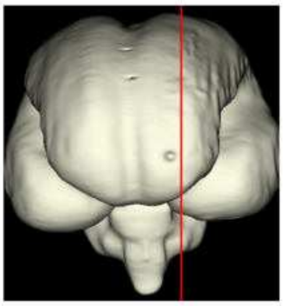

L-R Level: -5.82 mm

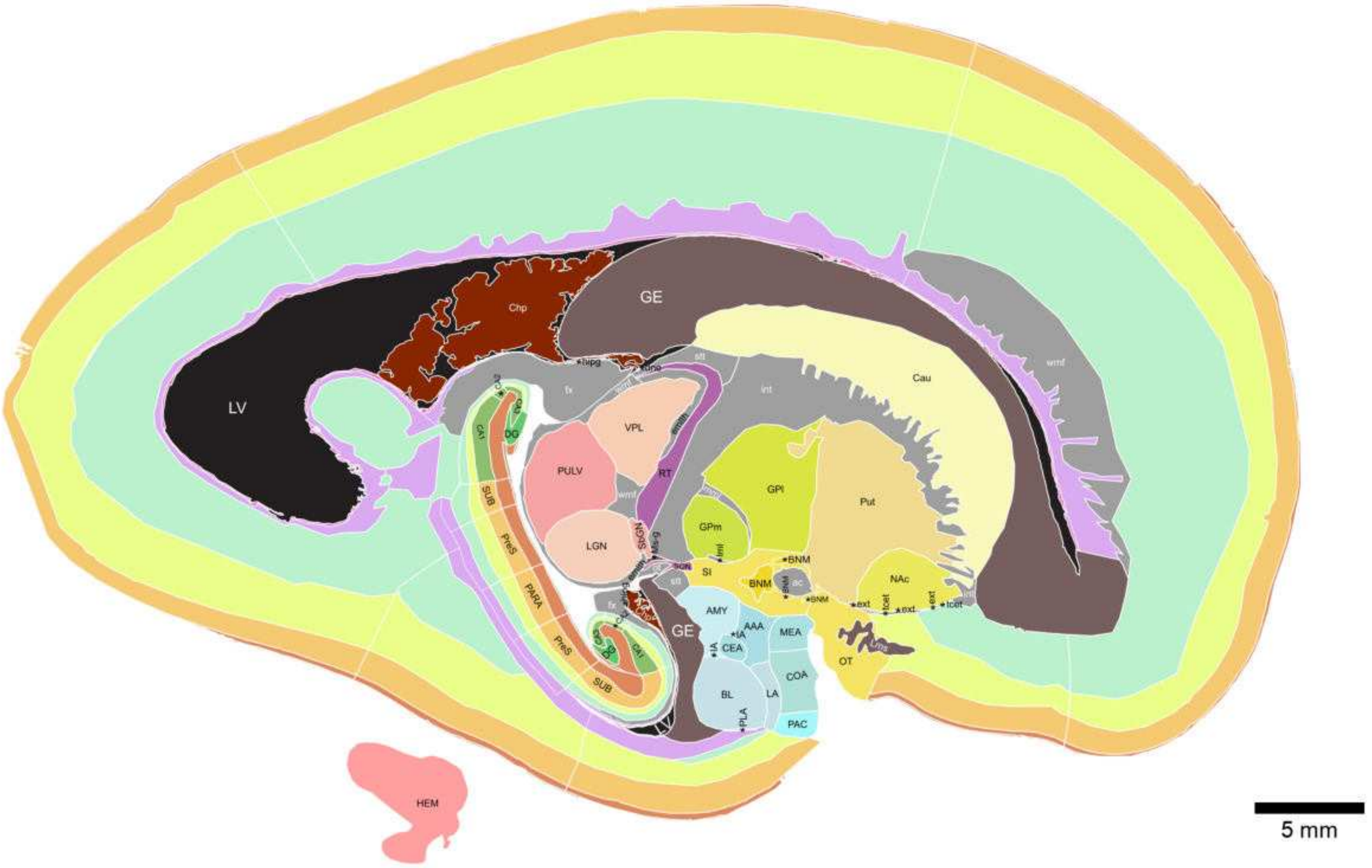

AAA: Anterior amygdaloid area
AMY: Amygdala
BL: Basal nucleus [amygdala]
BNM: Basal nucleus of Meynert
CA1: CA1 field [hippocampus]
CA2: CA2 field [hippocampus]
CA3: CA3 field [hippocampus]
CEA: Central nucleus [amygdala]
COA: Cortical nucleus [amygdala]
Cau: Caudate nucleus
Chp: Choroid plexus
DG: Dentate gyrus
GE: Ganglionic eminence
GPl: Globus pallidus lateral segment
GPm: Globus pallidus medial segment
HEM: Cerebellar hemispheres
IA: Intercalated cell groups [amygdala]
LA: Lateral nucleus [amygdala]
LGN: Lateral geniculate nucleus
LV: Lateral ventricle
Lms: Lateral migratory stream
MEA: Medial nucleus [amygdala]
Ms-g: Migratory stream, general
NAc: Nucleus accumbens
OT: Olfactory tubercle
PARA: Cortical plate, parasubiculum
PLA: Paralaminar nucleus [amygdala]
PULV: Pulvinar nucleus [thalamus]
PreS: Cortical plate, presubiculum
Put: Putamen
RT: Reticular nucleus [thalamus]
SI: Substantia innominata
SON: Supraoptic nucleus [hypothalamus]
SUB: Cortical plate, subiculum
SbGN: Subgeniculate nucleus
VPL: Ventral posterolateral nucleus [thalamus]
ac: Anterior commissure
dne: Diencephalic neuroepithelium
emlth: External medullary lamina [thalamus]
ext: External capsule
fx: Fornix
hipg: Hippocampal glioepithelium/ependyma
int: Internal capsule
lml: Lateral medullary lamina
mml: Medial medullary lamina
ot: Optic tract
stt: Stria terminalis
tcet: Transient cell zone in the external capsule
wmf: White matter fibers

Age: 21 GW

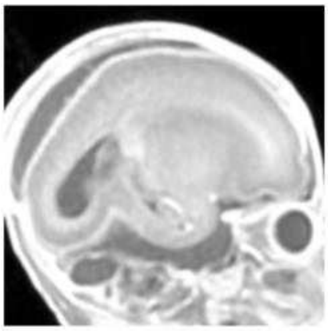

L-R Level: -6.84 mm

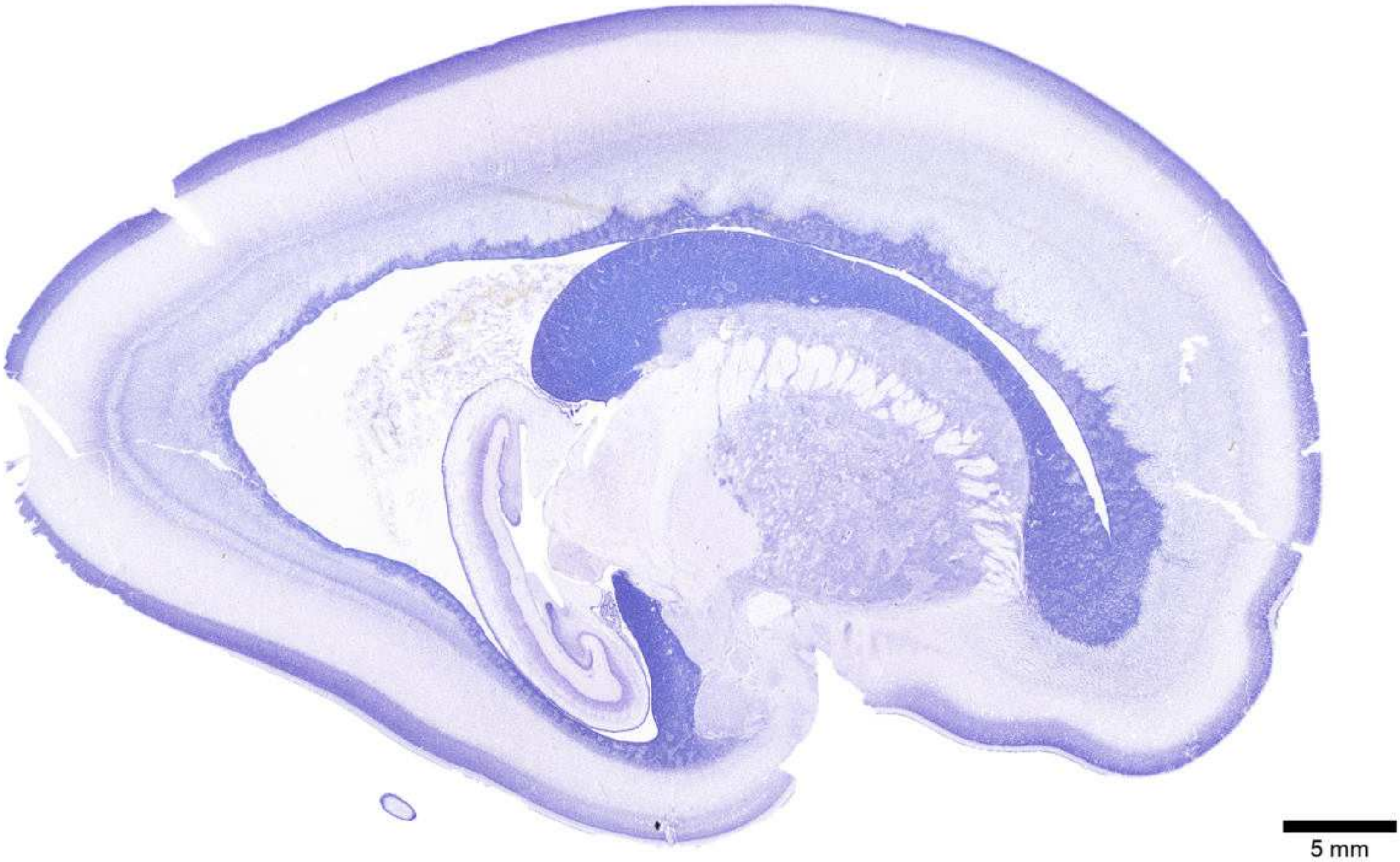

Transient Layers

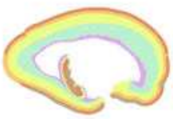

VZ
SVZ
IZ
SP
CP
MZ
SGL

Cortical Areas

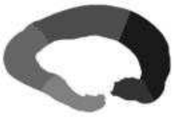

FCTx
ORB
PAR
OCC
TEMP

Age: 21 GW

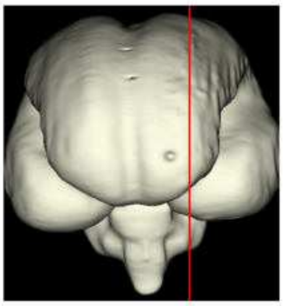

L-R Level: -6.84 mm

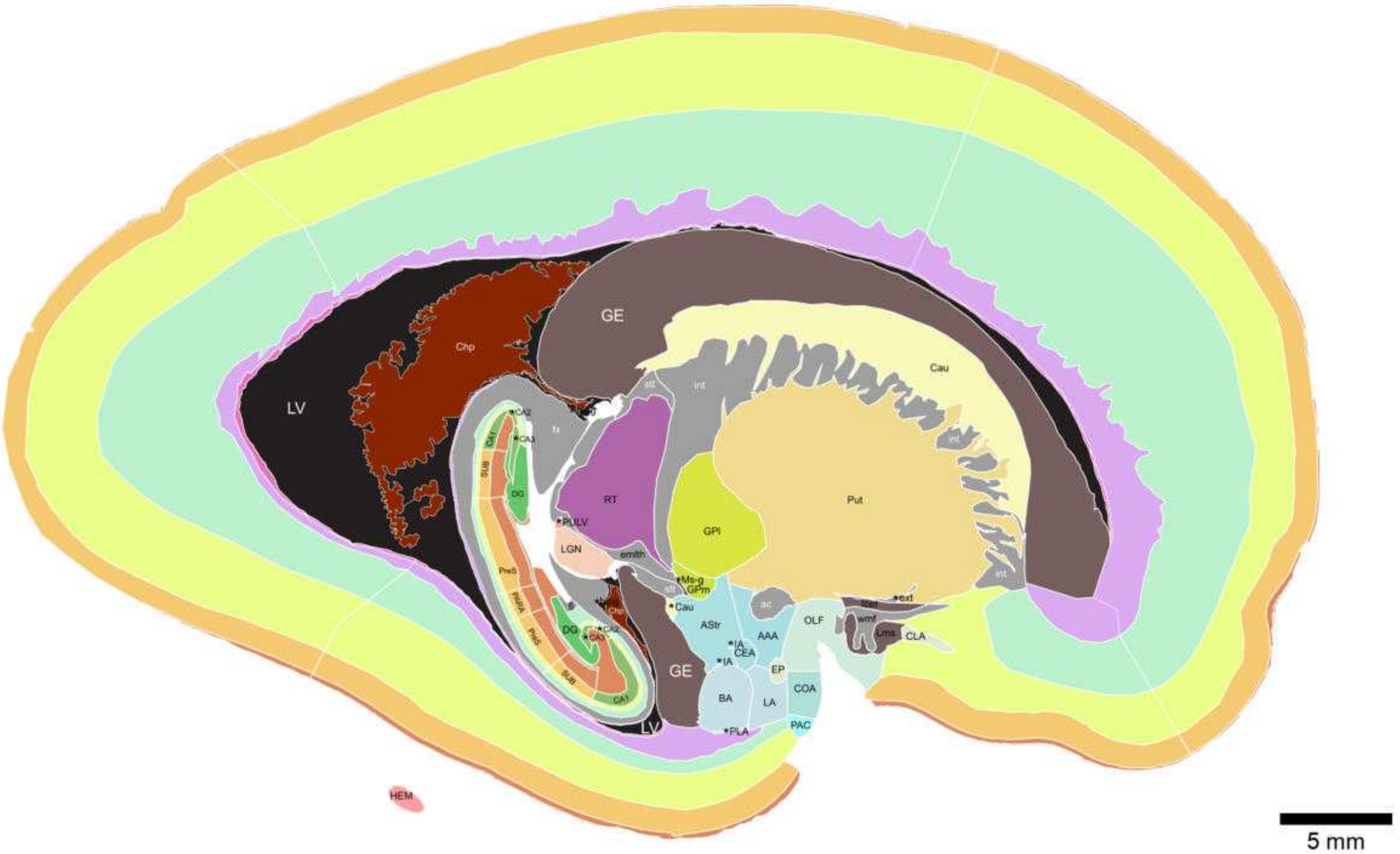

- AAA: Anterior amygdaloid area
- AStr: Amygdalo-striatal area
- BA: Basal nucleus [amygdala]
- CA1: CA1 field [hippocampus]
- CA2: CA2 field [hippocampus]
- CA3: CA3 field [hippocampus]
- CEA: Central nucleus [amygdala]
- CLA: Claustrum
- COA: Cortical nucleus [amygdala]
- Cau: Caudate nucleus
- Chp: Choroid plexus
- DG: Dentate gyrus
- EP: Endopiriform nucleus
- GE: Ganglionic eminence
- GPl: Globus pallidus lateral segment
- GPm: Globus pallidus medial segment
- HEM: Cerebellar hemispheres
- IA: Intercalated cell groups [amygdala]
- LA: Lateral nucleus [amygdala]
- LGN: Lateral geniculate nucleus
- LV: Lateral ventricle
- Lms: Lateral migratory stream
- Ms-g: Migratory stream, general
- PARA: Cortical plate, parasubiculum
- PLA: Paralaminar nucleus [amygdala]
- PULV: Pulvinar nucleus [thalamus]
- PreS: Cortical plate, presubiculum
- Put: Putamen
- RT: Reticular nucleus [thalamus]
- SUB: Cortical plate, subiculum
- ac: Anterior commissure
- emlth: External medullary lamina [thalamus]
- ext: External capsule
- fi: Fimbria
- fx: Fornix
- hipg: Hippocampal glioepithelium/ependyma
- int: Internal capsule
- stt: Stria terminalis
- tcet: Transient cell zone in the external capsule
- wmf: White matter fibers

Age: 21 GW

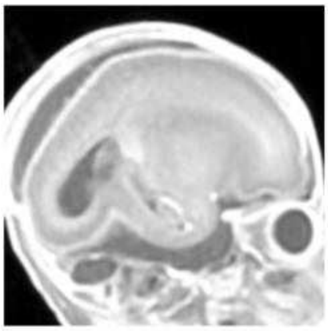

L-R Level: -7.2 mm

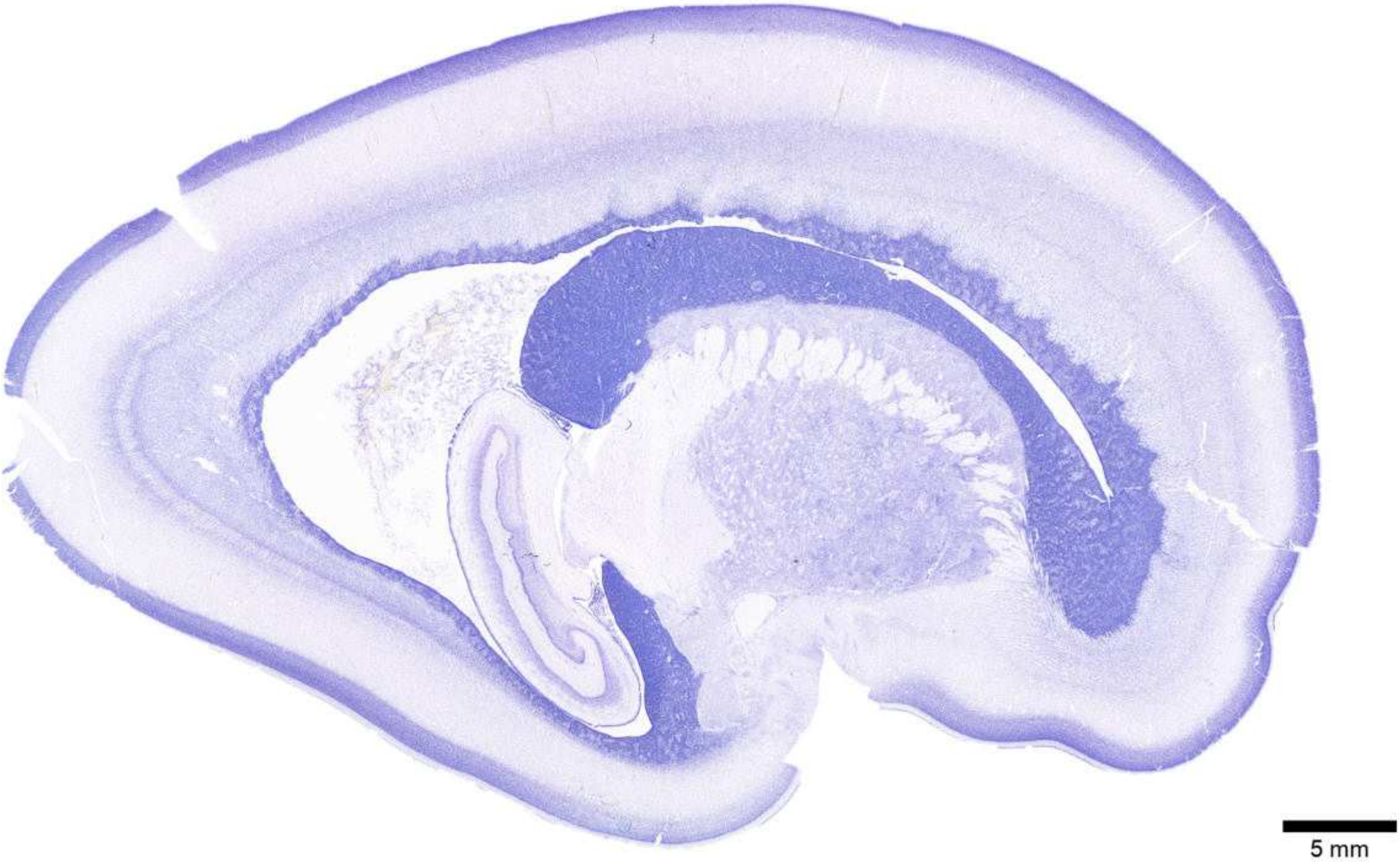

5 mm

Transient Layers

- VZ
- SVZ
- IZ
- SP
- CP
- MZ
- SGL

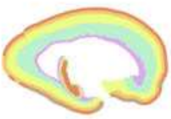

Cortical Areas

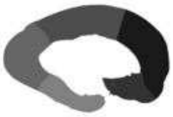

- FCTx
- ORB
- PAR
- OCC
- TEMP

Age: 21 GW

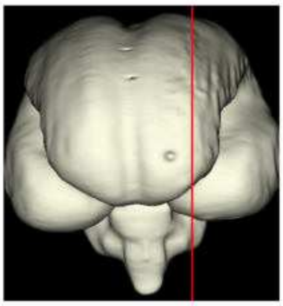

L-R Level: -7.2 mm

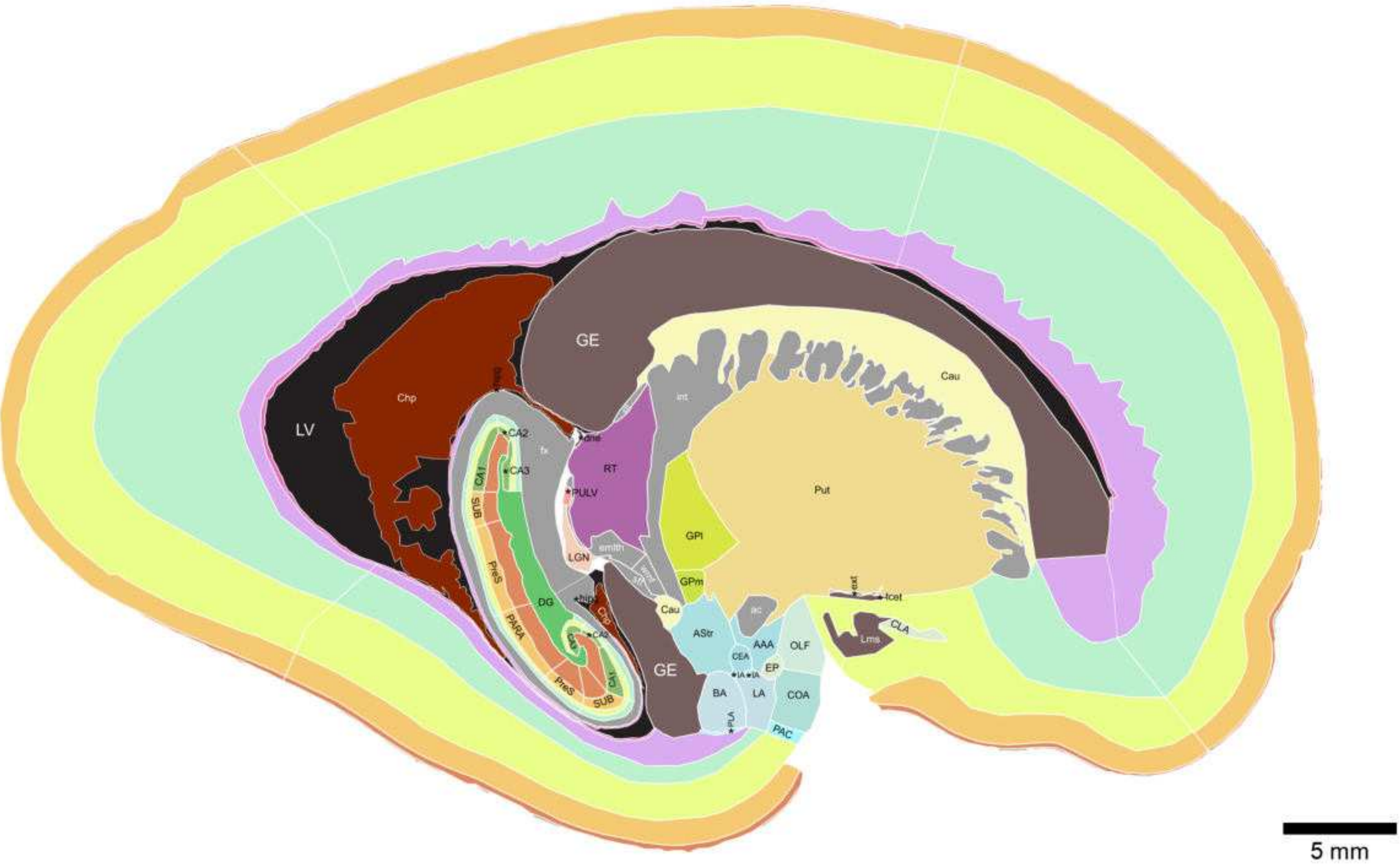

5 mm

- AAA: Anterior amygdaloid area
- AStr: Amygdalo-striatal area
- BA: Basal nucleus [amygdala]
- CA1: CA1 field [hippocampus]
- CA2: CA2 field [hippocampus]
- CA3: CA3 field [hippocampus]
- CEA: Central nucleus [amygdala]
- CLA: Claustrum
- COA: Cortical nucleus [amygdala]
- Cau: Caudate nucleus
- Chp: Choroid plexus
- DG: Dentate gyrus
- EP: Endopiriform nucleus
- GE: Ganglionic eminence
- GPl: Globus pallidus lateral segment
- GPm: Globus pallidus medial segment
- IA: Intercalated cell groups [amygdala]
- LA: Lateral nucleus [amygdala]
- LGN: Lateral geniculate nucleus
- LV: Lateral ventricle
- Lms: Lateral migratory stream
- PARA: Cortical plate, parasubiculum
- PLA: Paralaminar nucleus [amygdala]
- PULV: Pulvinar nucleus [thalamus]
- PreS: Cortical plate, presubiculum
- Put: Putamen
- RT: Reticular nucleus [thalamus]
- SUB: Cortical plate, subiculum
- ac: Anterior commissure
- dne: Diencephalic neuroepithelium
- emlth: External medullary lamina [thalamus]
- ext: External capsule
- fx: Fornix
- hipg: Hippocampal glioepithelium/ependyma
- int: Internal capsule
- stt: Stria terminalis
- tcet: Transient cell zone in the external capsule
- wmf: White matter fibers

Age: 21 GW

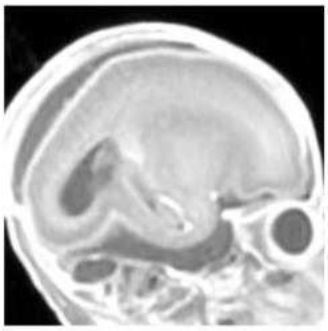

L-R Level: -7.38 mm

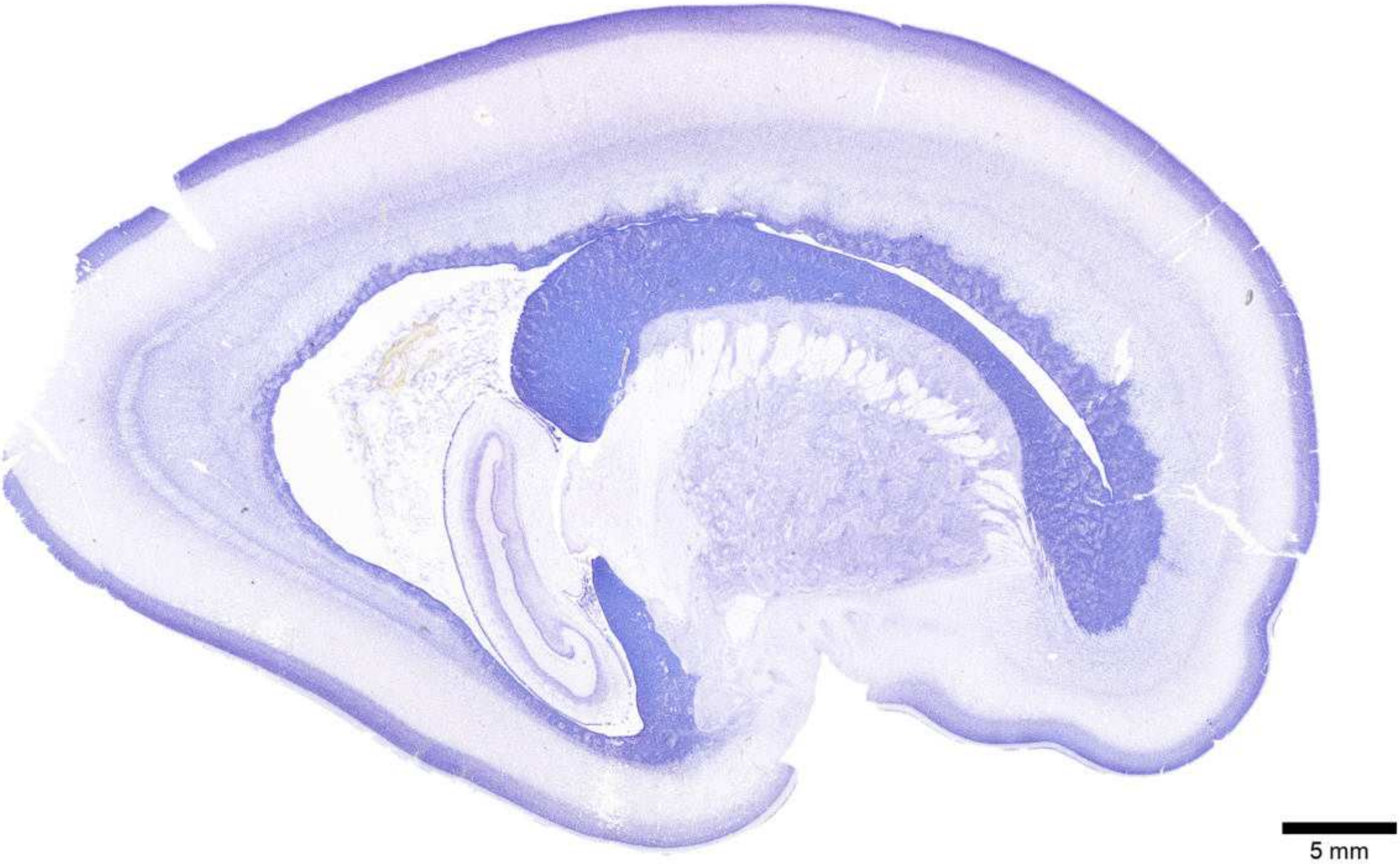

5 mm

Transient Layers

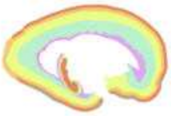

VZ
SVZ
IZ
SP
CP
MZ
SGL

Cortical Areas

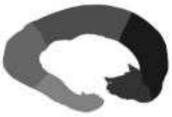

FCTx
ORB
PAR
OCC
TEMP

Age: 21 GW

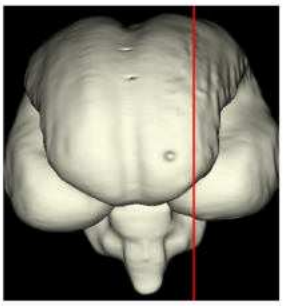

L-R Level: -7.38 mm

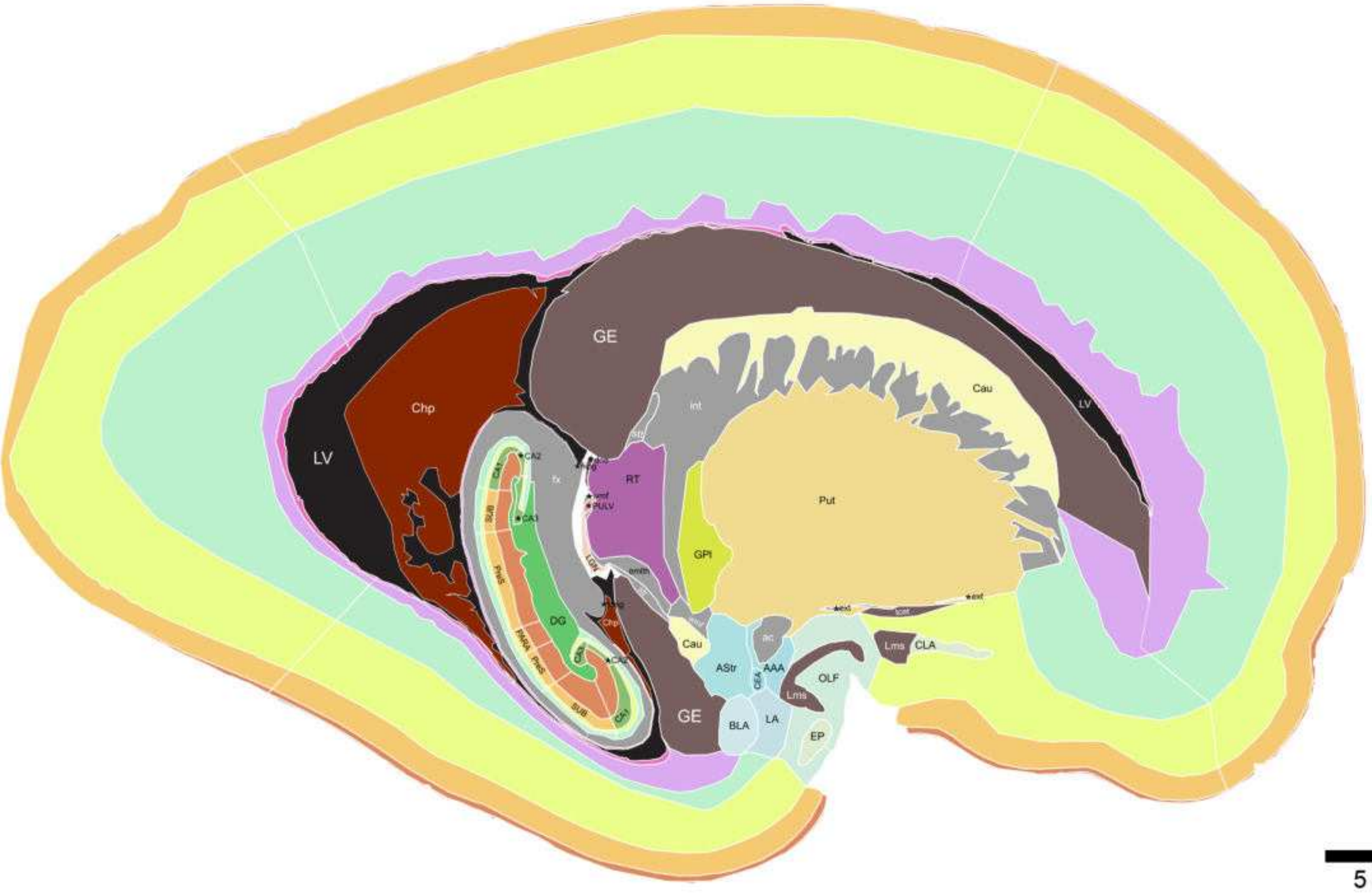

5 mm

- AAA: Anterior amygdaloid area
- AStr: Amygdalo-striatal area
- BL: Basal nucleus [amygdala]
- CA1: CA1 field [hippocampus]
- CA2: CA2 field [hippocampus]
- CA3: CA3 field [hippocampus]
- CEA: Central nucleus [amygdala]
- CLA: Claustrum
- Cau: Caudate nucleus
- Chp: Choroid plexus
- DG: Dentate gyrus
- EP: Endopiriform nucleus
- GE: Ganglionic eminence
- GPl: Globus pallidus lateral segment
- LA: Lateral nucleus [amygdala]
- LGN: Lateral geniculate nucleus
- LV: Lateral ventricle
- Lms: Lateral migratory stream
- PARA: Cortical plate, parasubiculum
- PULV: Pulvinar nucleus [thalamus]
- PreS: Cortical plate, presubiculum
- Put: Putamen
- RT: Reticular nucleus [thalamus]
- SUB: Cortical plate, subiculum
- ac: Anterior commissure
- dne: Diencephalic neuroepithelium
- emlth: External medullary lamina [thalamus]
- ext: External capsule
- fx: Fornix
- hipg: Hippocampal glioepithelium/ependyma
- int: Internal capsule
- stt: Stria terminalis
- tcet: Transient cell zone in the external capsule
- wmf: White matter fibers

Age: 21 GW

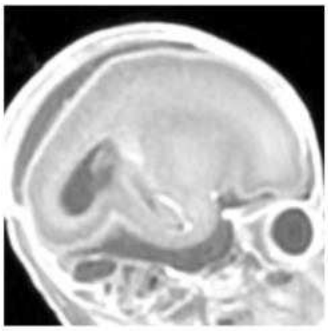

L-R Level: -7.56 mm

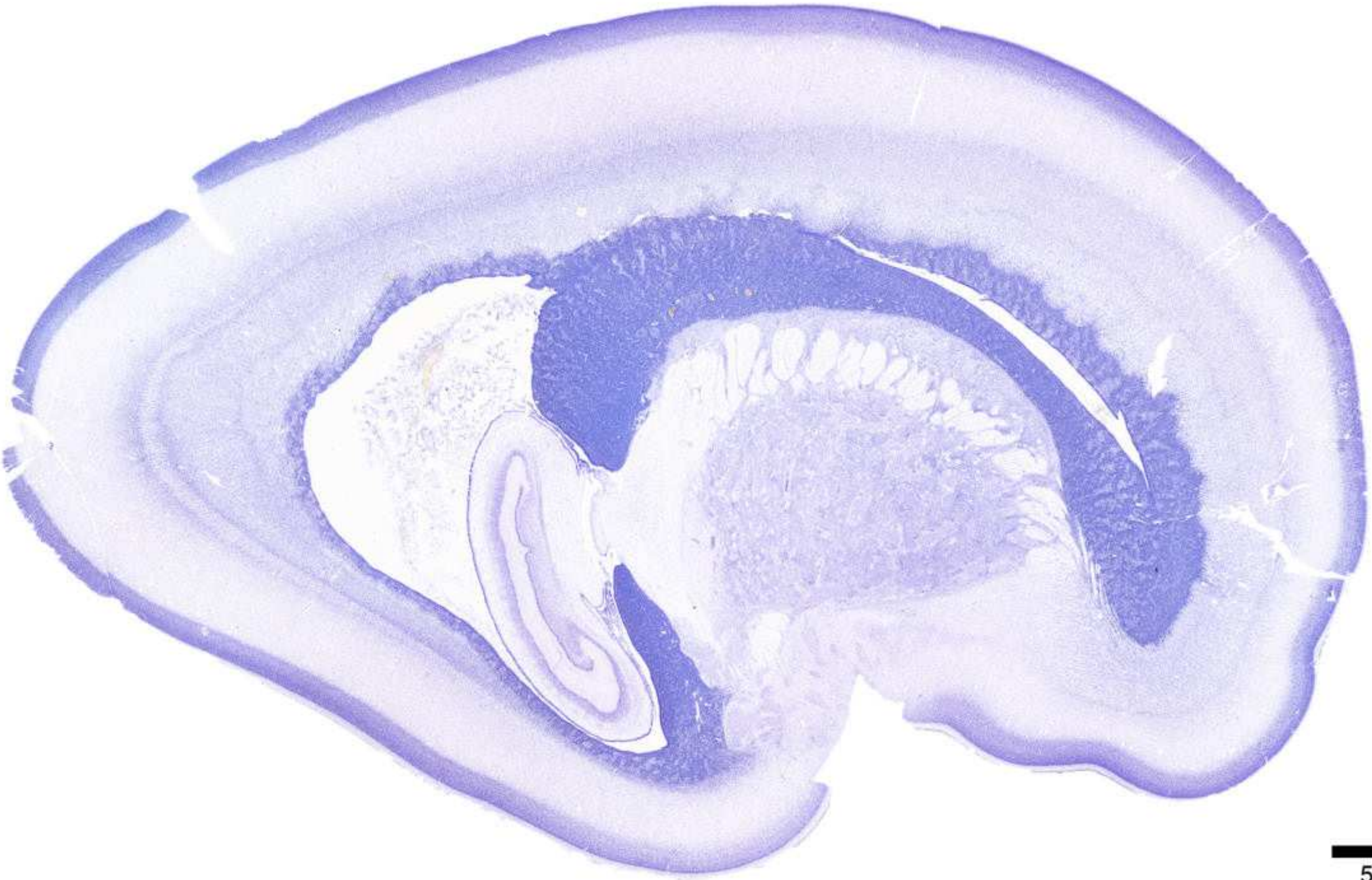

5 mm

Transient Layers

- VZ
- SVZ
- IZ
- SP
- CP
- MZ
- SGL

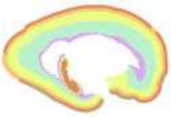

Cortical Areas

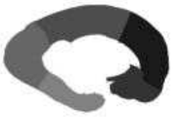

- FCTx
- ORB
- PAR
- OCC
- TEMP

Age: 21 GW

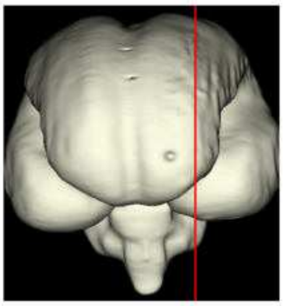

L-R Level: -7.56 mm

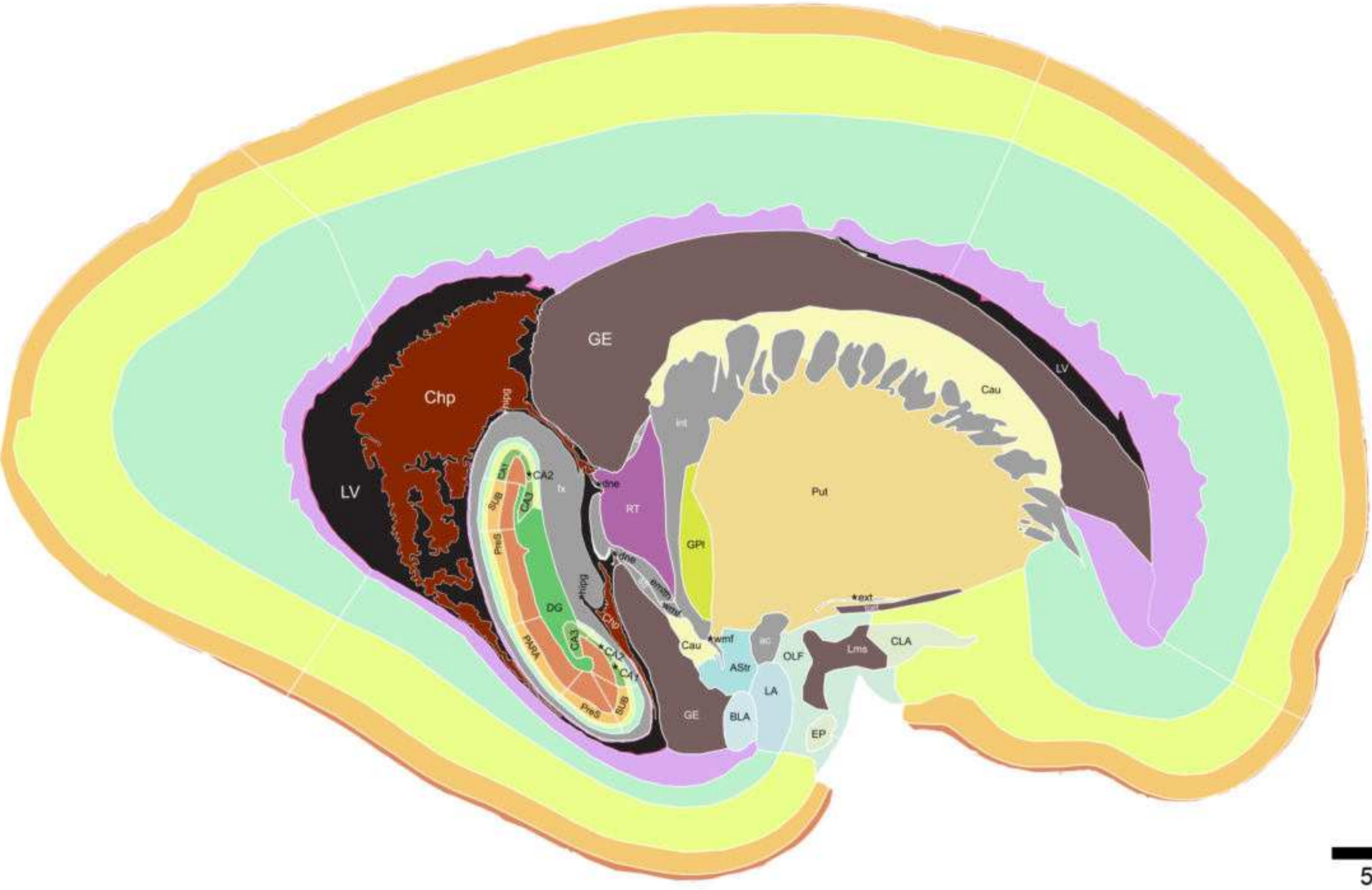

5 mm

- AStr: Amygdalo-striatal area
- BL: Basal nucleus [amygdala]
- CA1: CA1 field [hippocampus]
- CA2: CA2 field [hippocampus]
- CA3: CA3 field [hippocampus]
- CLA: Claustrum
- Cau: Caudate nucleus
- Chp: Choroid plexus
- DG: Dentate gyrus
- EP: Endopiriform nucleus
- GE: Ganglionic eminence
- GPl: Globus pallidus lateral segment
- LA: Lateral nucleus [amygdala]
- LV: Lateral ventricle
- Lms: Lateral migratory stream
- PARA: Cortical plate, parasubiculum
- PreS: Cortical plate, presubiculum
- Put: Putamen
- RT: Reticular nucleus [thalamus]
- SUB: Cortical plate, subiculum
- ac: Anterior commissure
- dne: Diencephalic neuroepithelium
- emlth: External medullary lamina [thalamus]
- ext: External capsule
- fx: Fornix
- hipg: Hippocampal glioepithelium/ependyma
- int: Internal capsule
- stt: Stria terminalis
- tcet: Transient cell zone in the external capsule
- wmf: White matter fibers

Age: 21 GW

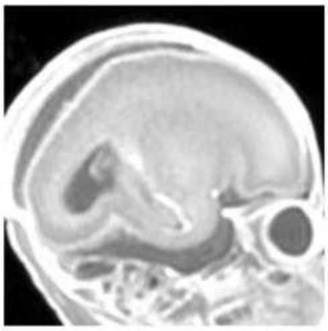

L-R Level: -7.92 mm

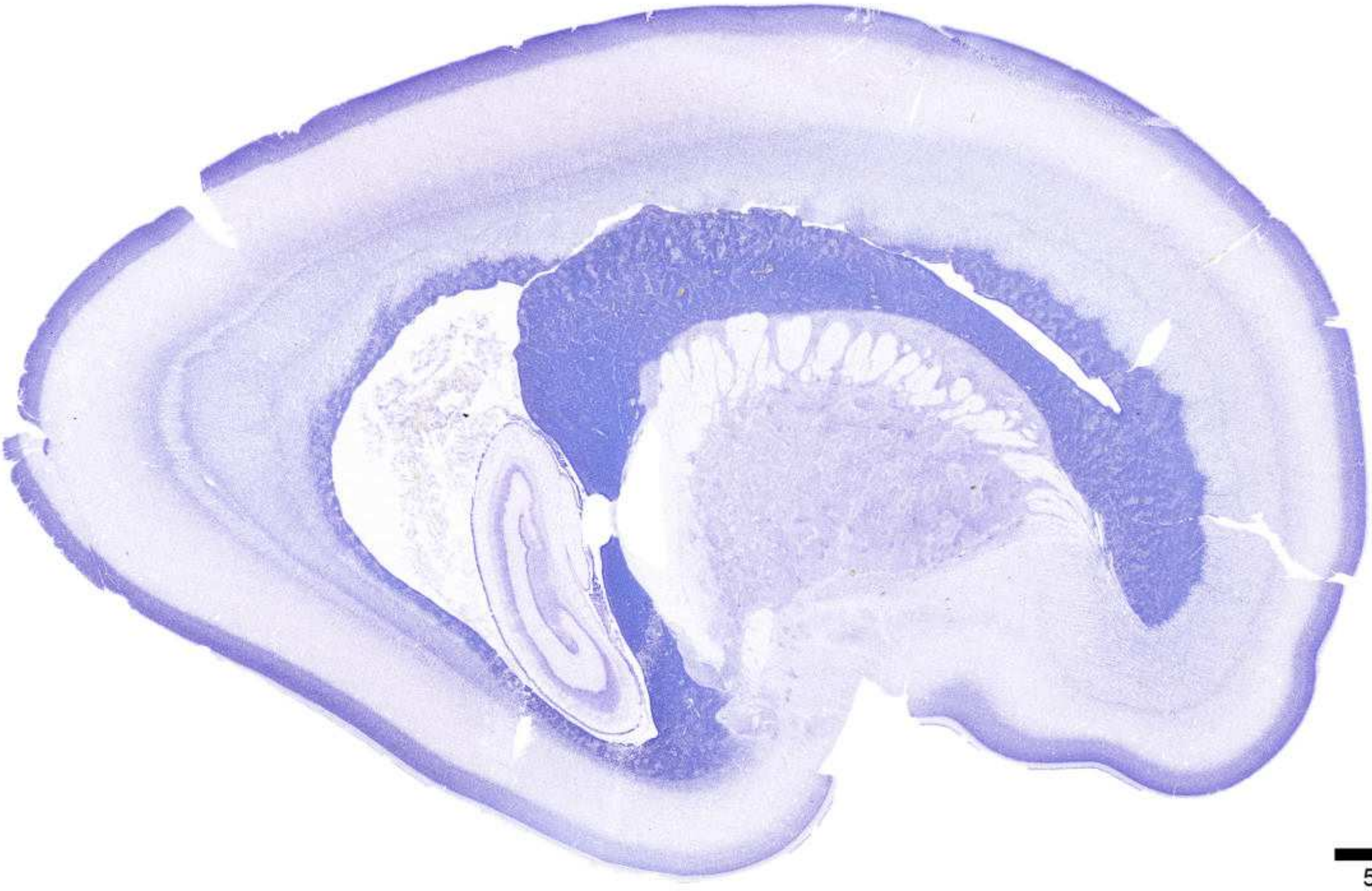

5 mm

Transient Layers

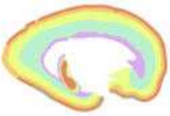

- VZ
- SVZ
- IZ
- SP
- CP
- MZ
- SGL

Cortical Areas

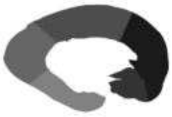

- FCTx
- ORB
- PAR
- OCC
- TEMP

Age: 21 GW

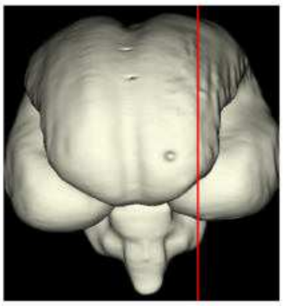

L-R Level: -7.92 mm

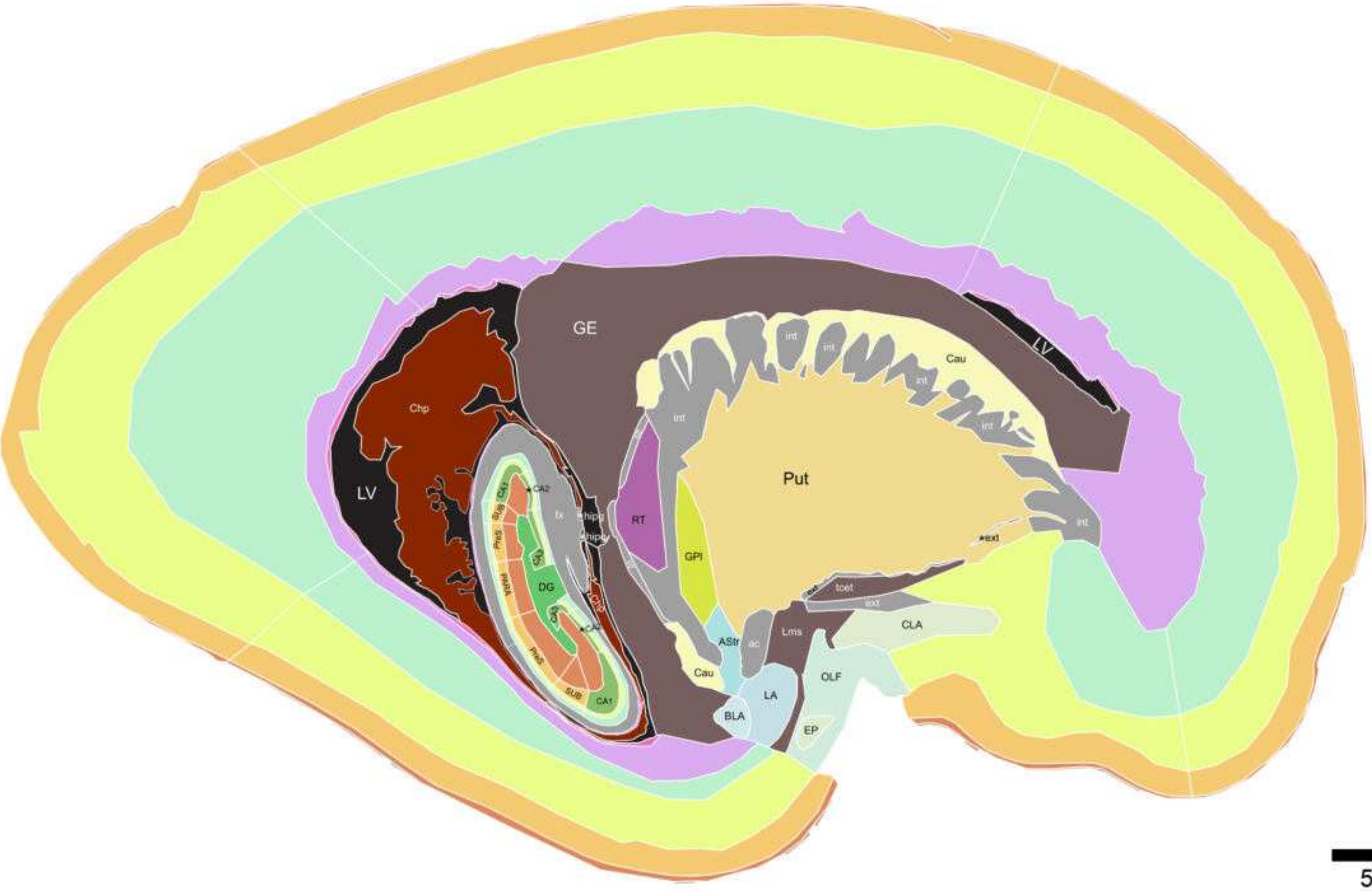

5 mm

- AStr: Amygdalo-striatal area
- BL: Basal nucleus [amygdala]
- CA1: CA1 field [hippocampus]
- CA2: CA2 field [hippocampus]
- CA3: CA3 field [hippocampus]
- CLA: Claustrum
- Cau: Caudate nucleus
- Chp: Choroid plexus
- DG: Dentate gyrus
- EP: Endopiriform nucleus
- GE: Ganglionic eminence
- GPl: Globus pallidus lateral segment
- LA: Lateral nucleus [amygdala]
- LV: Lateral ventricle
- Lms: Lateral migratory stream
- PARA: Cortical plate, parasubiculum
- PreS: Cortical plate, presubiculum
- Put: Putamen
- RT: Reticular nucleus [thalamus]
- SUB: Cortical plate, subiculum
- ac: Anterior commissure
- ext: External capsule
- fx: Fornix
- hipg: Hippocampal glioepithelium/ependyma
- int: Internal capsule
- stt: Stria terminalis
- tcet: Transient cell zone in the external capsule

Age: 21 GW

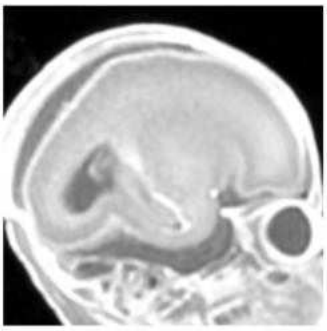

L-R Level: -8.22 mm

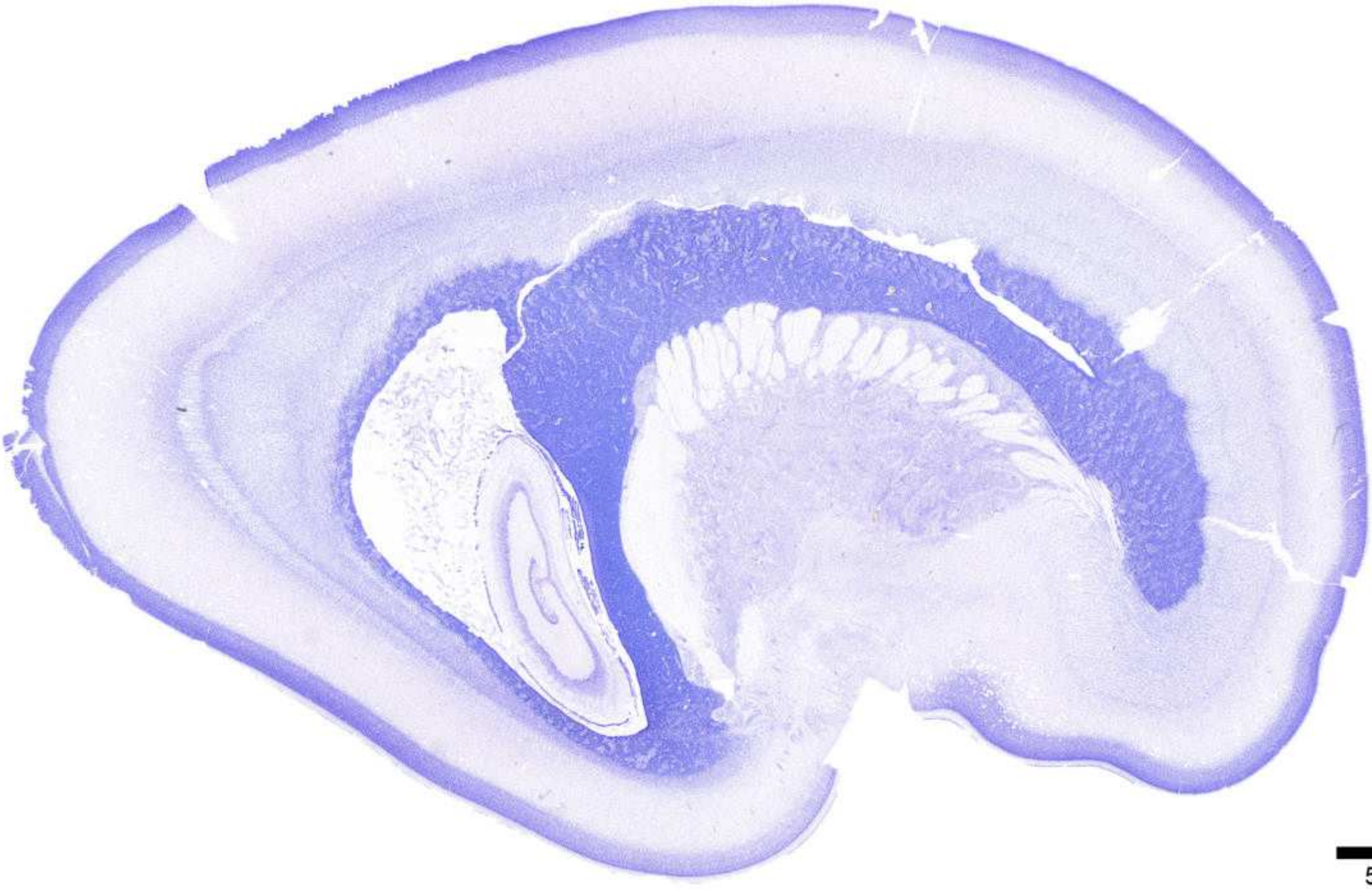

5 mm

Transient Layers

VZ
SVZ
IZ
SP
CP
MZ
SGL

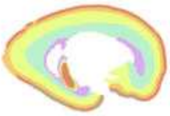

Cortical Areas

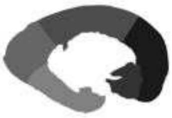

FCTx
ORB
PAR
OCC
TEMP

Age: 21 GW

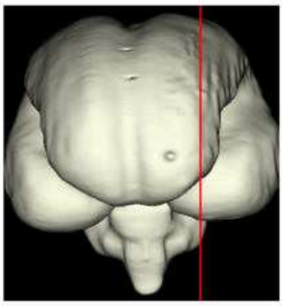

L-R Level: -8.22 mm

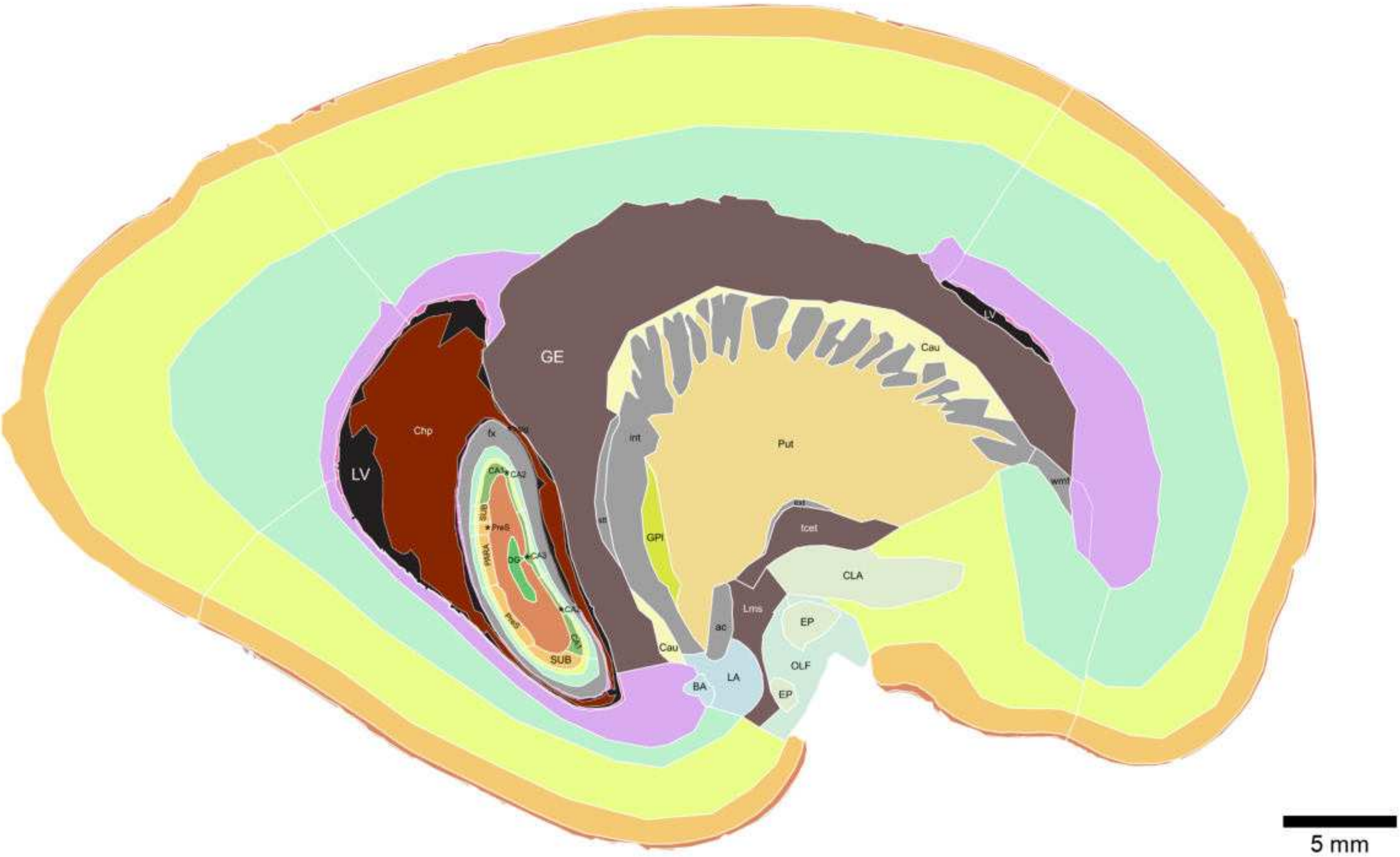

- BA: Basal nucleus [amygdala]
- CA1: CA1 field [hippocampus]
- CA2: CA2 field [hippocampus]
- CA3: CA3 field [hippocampus]
- CLA: Claustrum
- Cau: Caudate nucleus
- Chp: Choroid plexus
- DG: Dentate gyrus
- EP: Endopiriform nucleus
- GE: Ganglionic eminence
- GPl: Globus pallidus lateral segment
- LA: Lateral nucleus [amygdala]
- LV: Lateral ventricle
- Lms: Lateral migratory stream
- PARA: Cortical plate, parasubiculum
- PreS: Cortical plate, presubiculum
- Put: Putamen
- SUB: Cortical plate, subiculum
- ac: Anterior commissure
- ext: External capsule
- fx: Fornix
- hipg: Hippocampal glioepithelium/ependyma
- int: Internal capsule
- stt: Stria terminalis
- tcet: Transient cell zone in the external capsule
- wmf: White matter fibers

Age: 21 GW

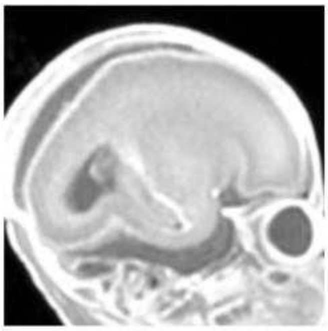

L-R Level: -8.34 mm

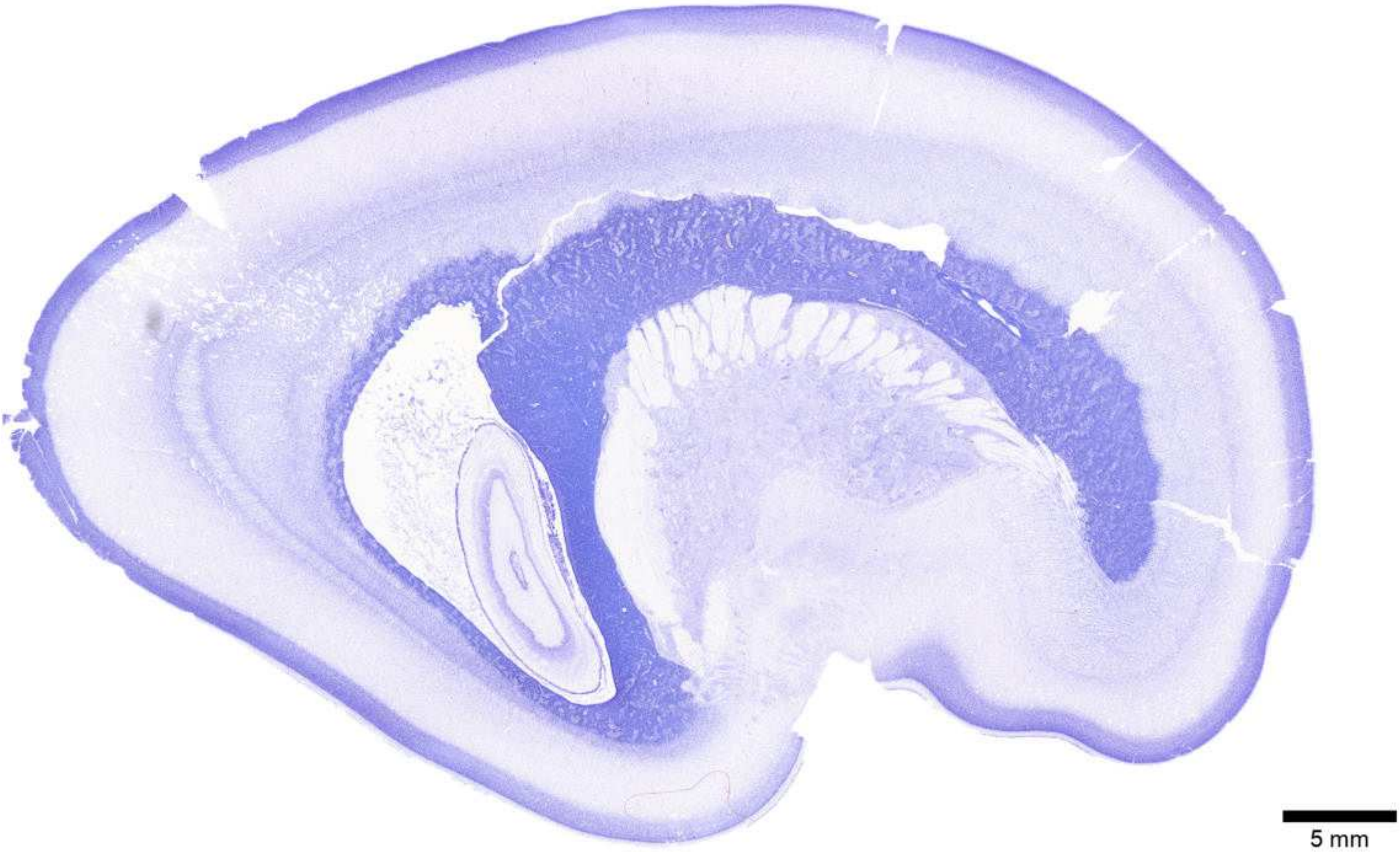

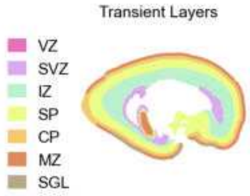

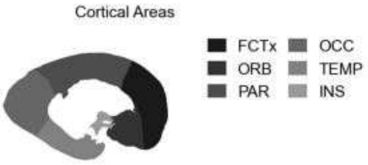

Age: 21 GW

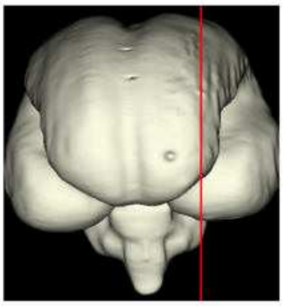

L-R Level: -8.34 mm

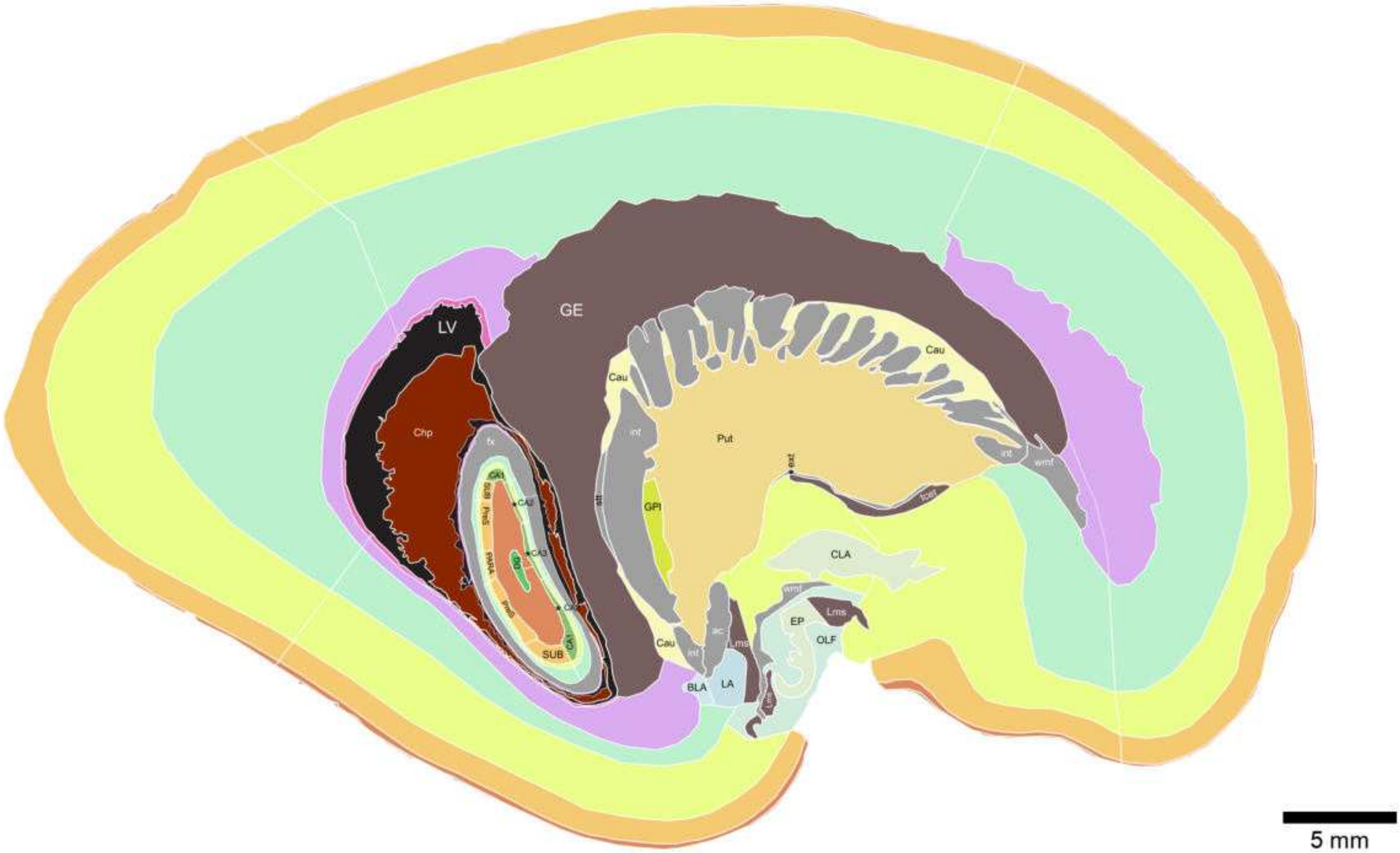

- BL: Basal nucleus [amygdala]
- CA1: CA1 field [hippocampus]
- CA2: CA2 field [hippocampus]
- CA3: CA3 field [hippocampus]
- CLA: Claustrum
- Cau: Caudate nucleus
- Chp: Choroid plexus
- DG: Dentate gyrus
- EP: Endopiriform nucleus
- GE: Ganglionic eminence
- GPl: Globus pallidus lateral segment
- LA: Lateral nucleus [amygdala]
- LV: Lateral ventricle
- Lms: Lateral migratory stream
- PARA: Cortical plate, parasubiculum
- PreS: Cortical plate, presubiculum
- Put: Putamen
- SUB: Cortical plate, subiculum
- ac: Anterior commissure
- ext: External capsule
- fx: Fornix
- int: Internal capsule
- stt: Stria terminalis
- tcet: Transient cell zone in the external capsule
- wmf: White matter fibers

Age: 21 GW

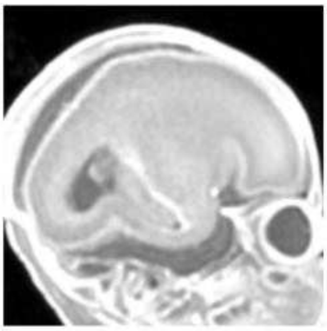

L-R Level: -9.12 mm

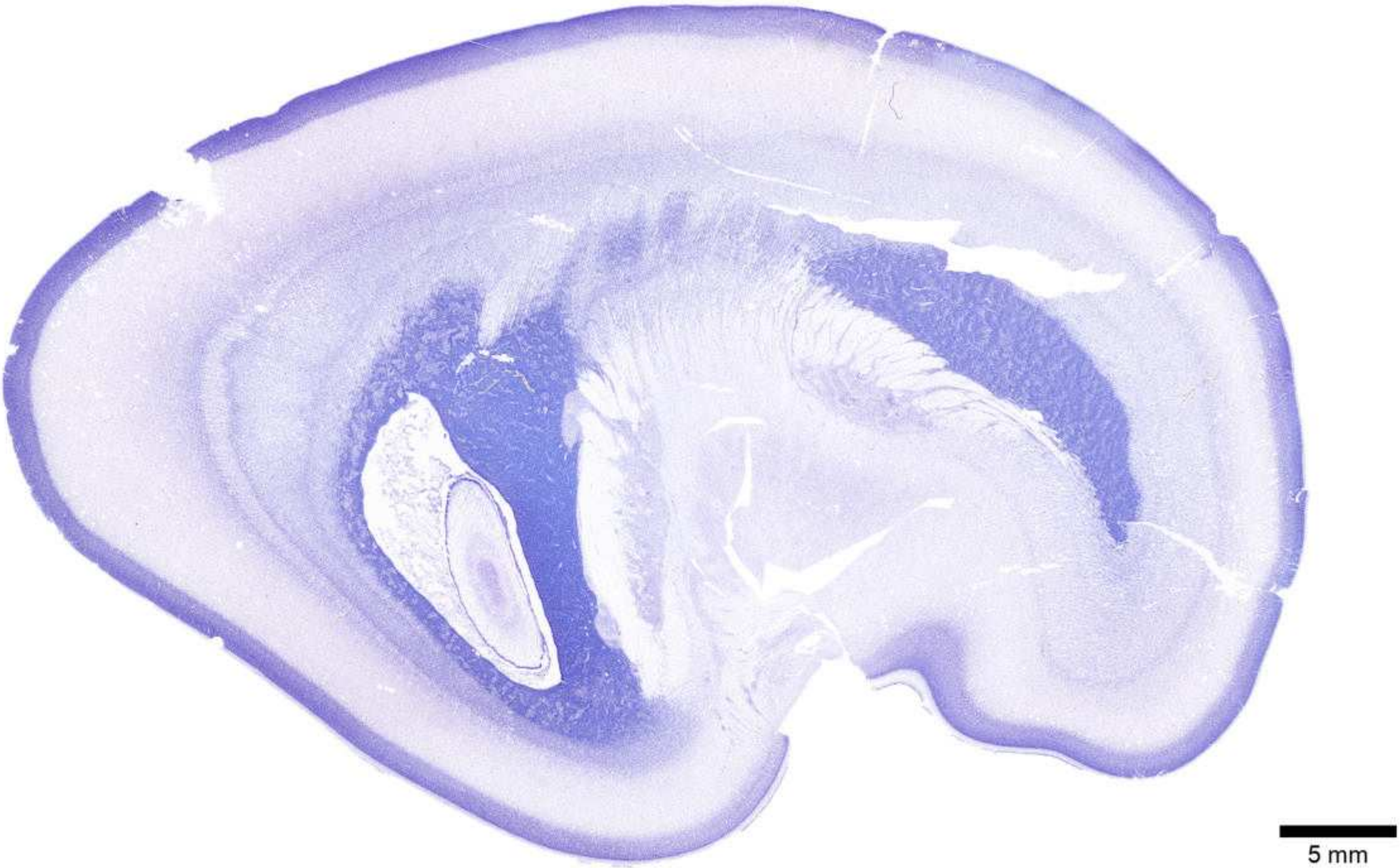

5 mm

Transient Layers

VZ
SVZ
IZ
SP
CP
MZ
SGL

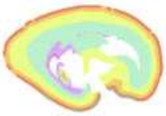

Cortical Areas

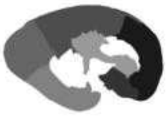

FCTx
ORB
PAR
OCC
TEMP
INS

Age: 21 GW

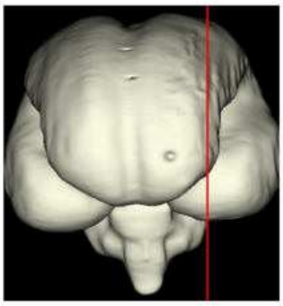

L-R Level: -9.12 mm

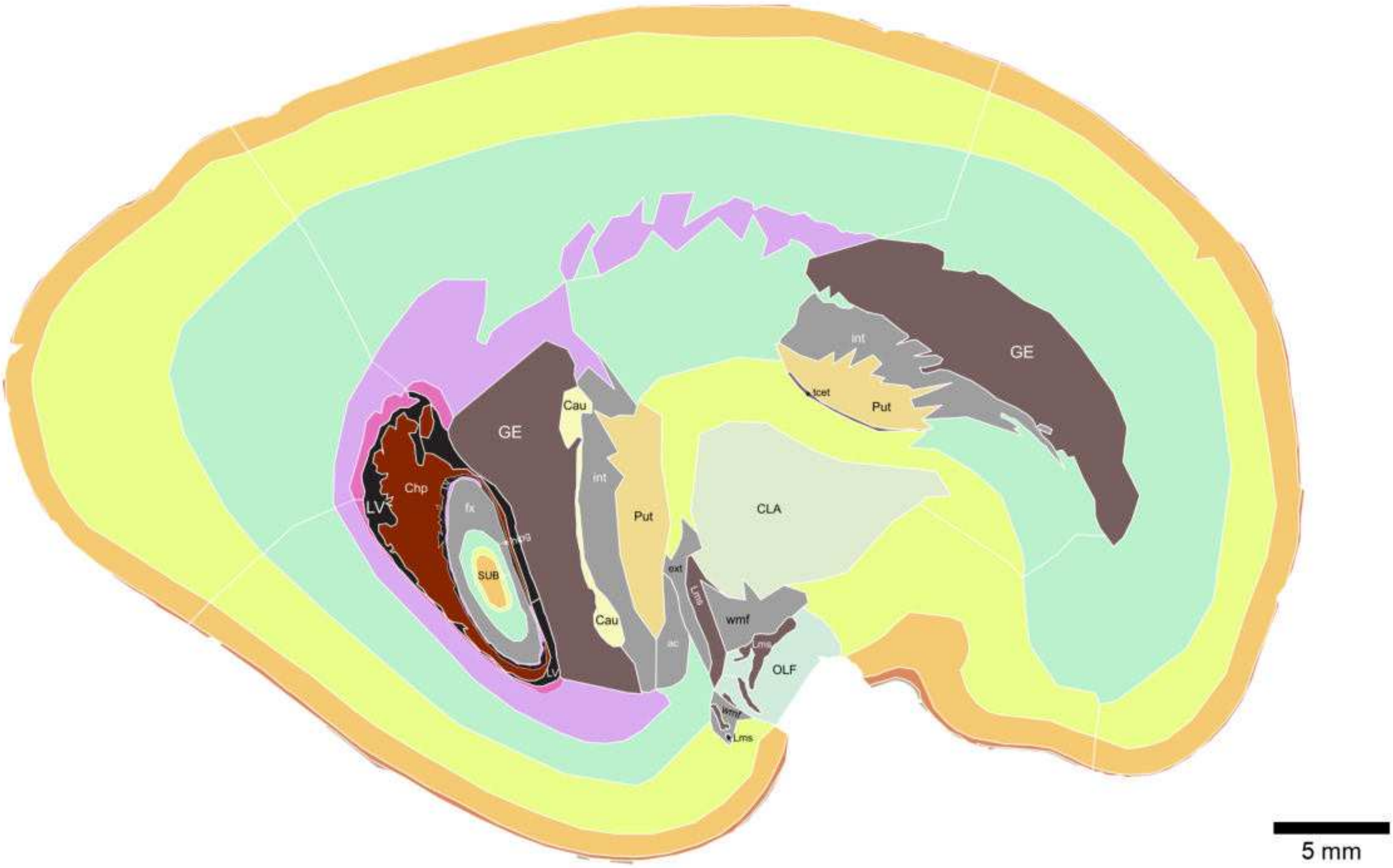

CLA: Claustrum
Cau: Caudate nucleus
Chp: Choroid plexus
GE: Ganglionic eminence
LV: Lateral ventricle
Lms: Lateral migratory stream
Put: Putamen
SUB: Cortical plate, subiculum
ac: Anterior commissure
ext: External capsule
fx: Fornix
hipg: Hippocampal glioepithelium/ependyma
int: Internal capsule
tcet: Transient cell zone in the external capsule
wmf: White matter fibers

Age: 21 GW

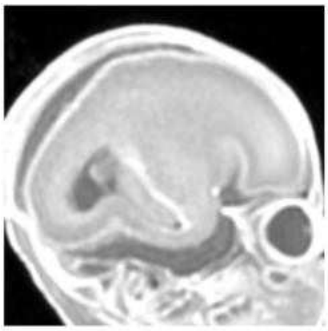

L-R Level: -9.66 mm

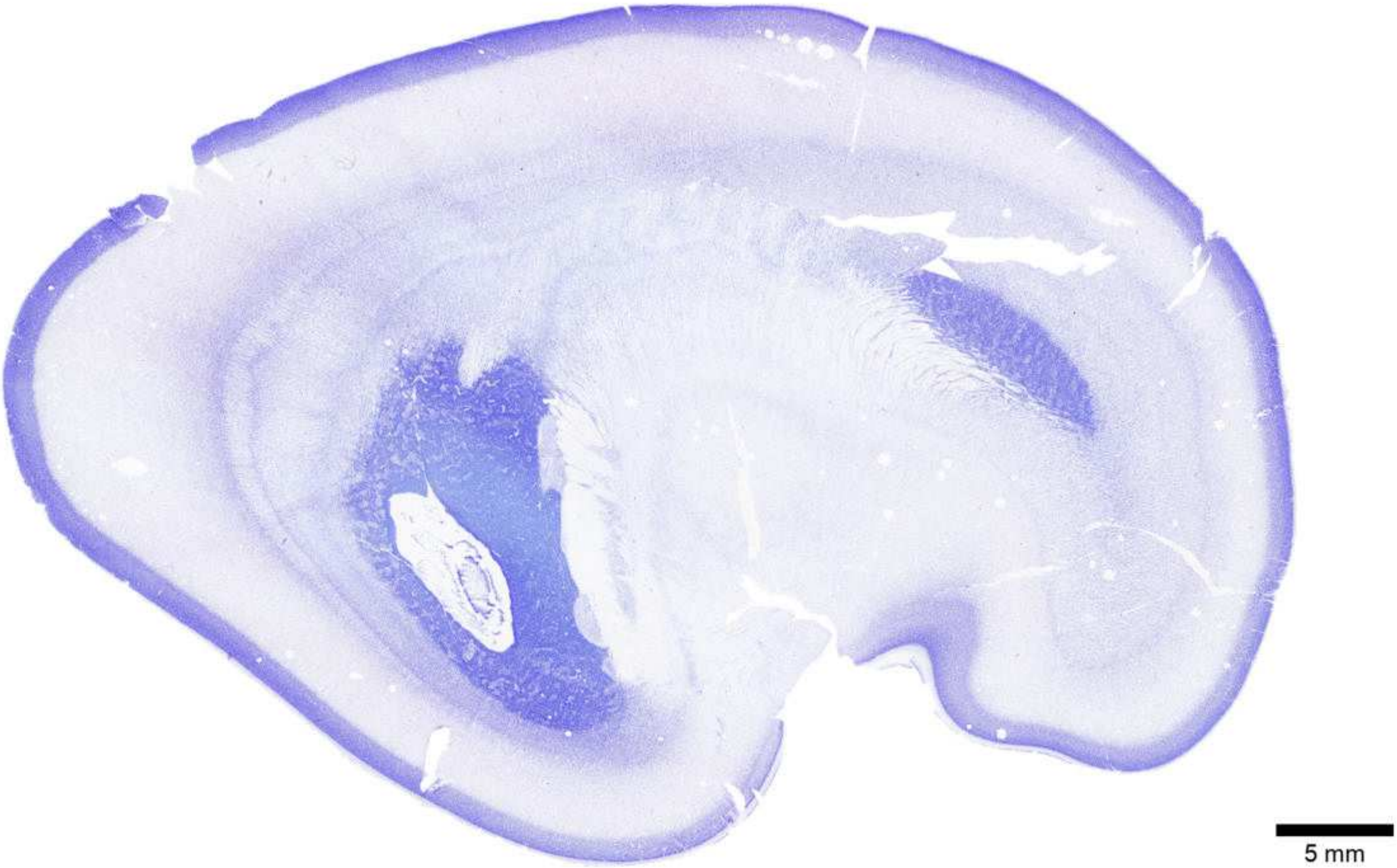

5 mm

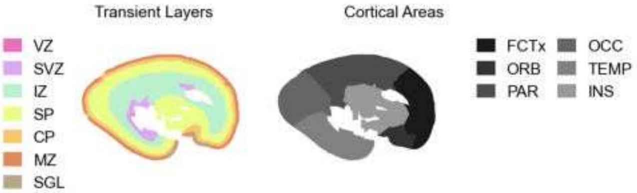

Age: 21 GW

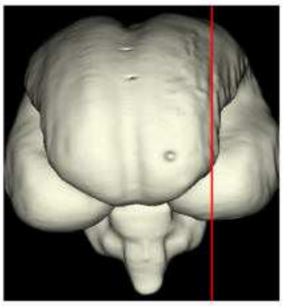

L-R Level: -9.66 mm

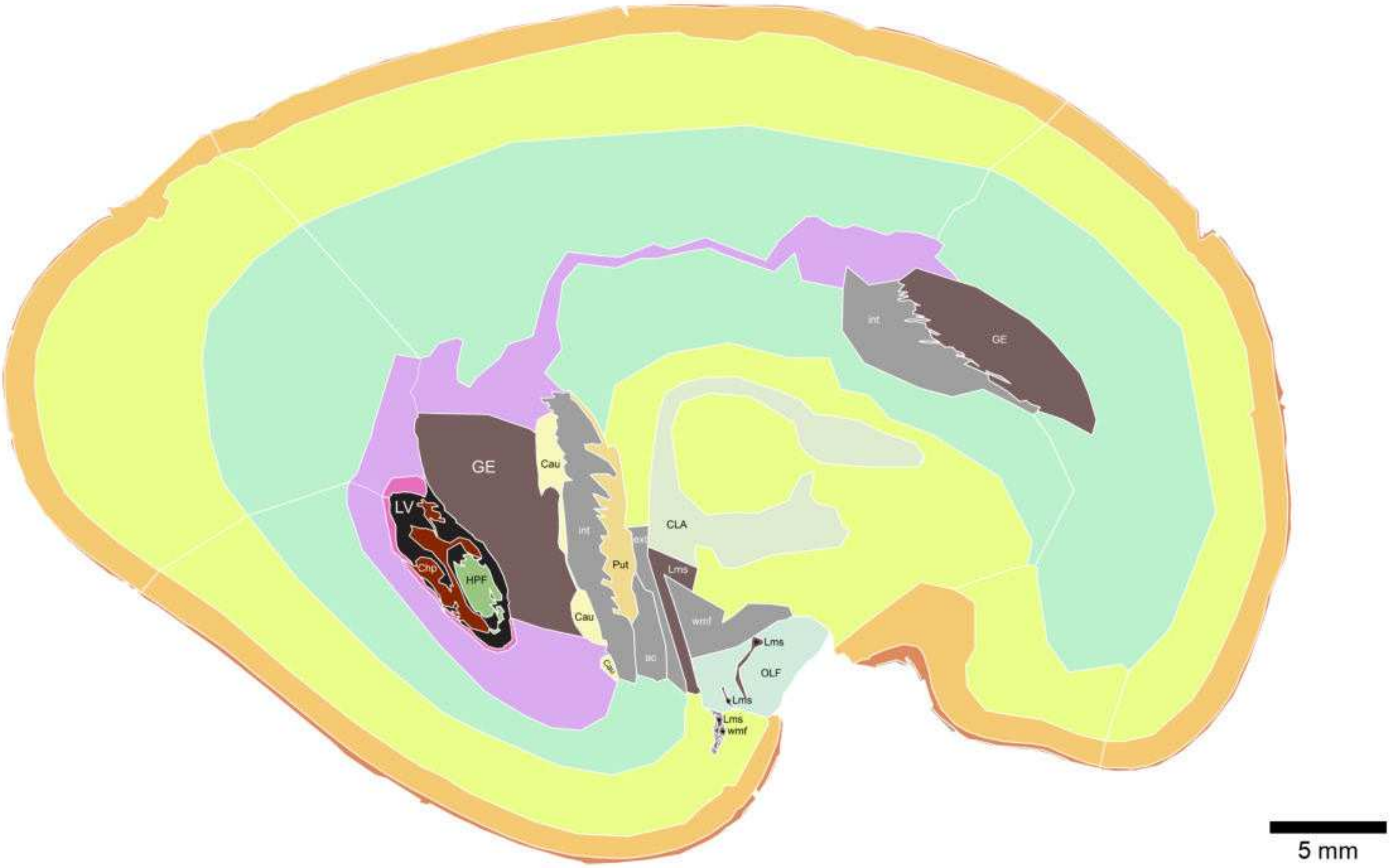

CLA: Claustrum
Cau: Caudate nucleus
Chp: Choroid plexus
GE: Ganglionic eminence
HPF: Hippocampal formation
LV: Lateral ventricle
Lms: Lateral migratory stream
Put: Putamen
ac: Anterior commissure
ext: External capsule
int: Internal capsule
wmf: White matter fibers

Age: 21 GW

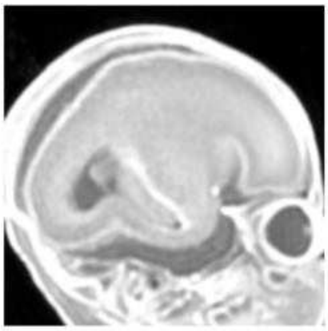

L-R Level: -9.84 mm

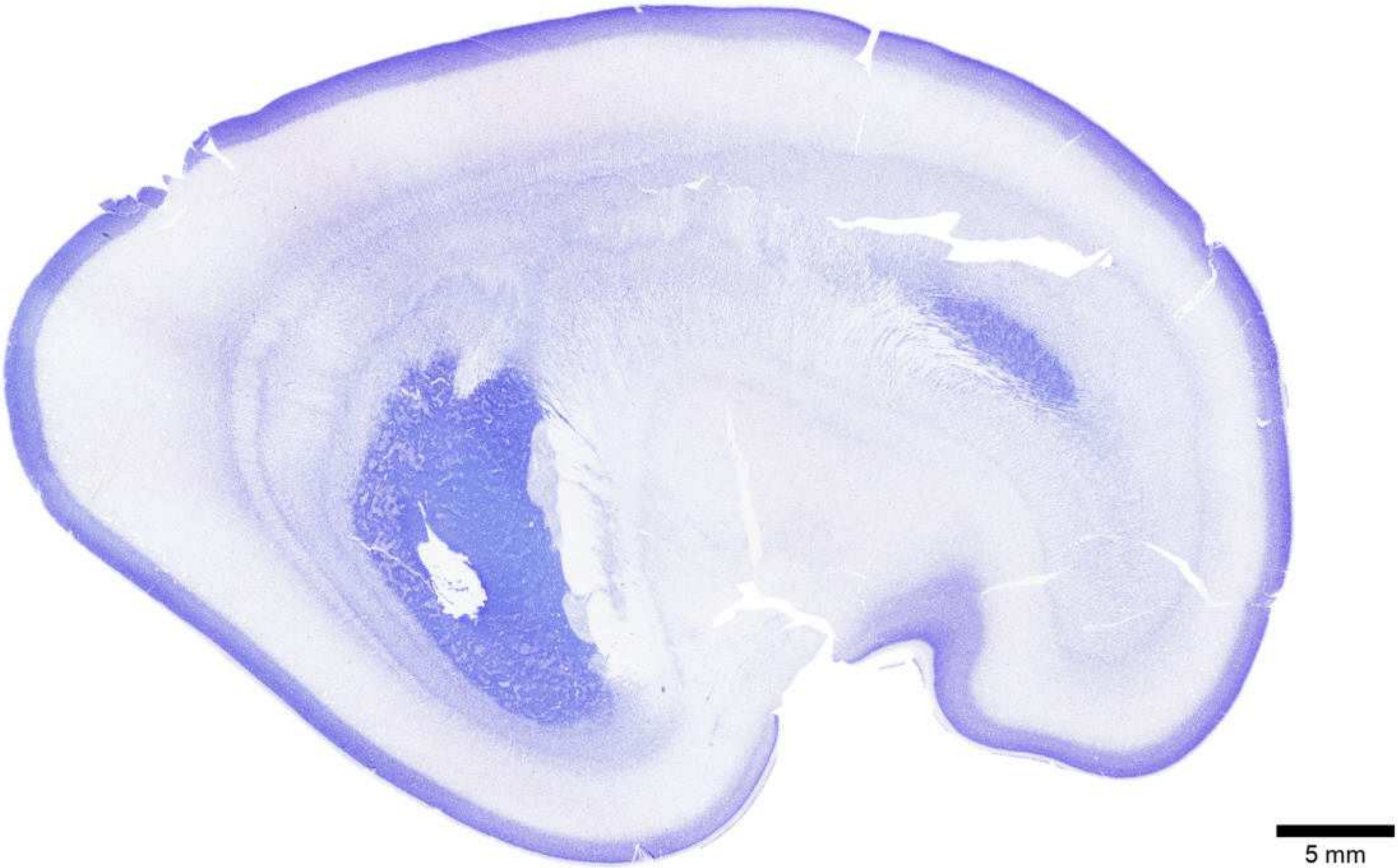

Transient Layers

- SVZ
- IZ
- SP
- CP
- MZ
- SGL

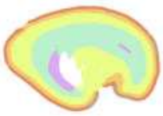

Cortical Areas

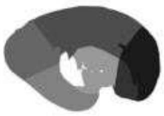

- FCTx
- ORB
- PAR
- OCC
- TEMP
- INS

Age: 21 GW

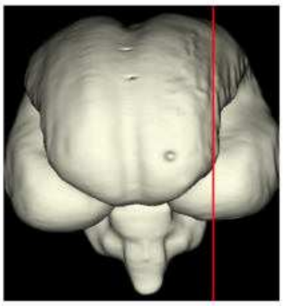

L-R Level: -9.84 mm

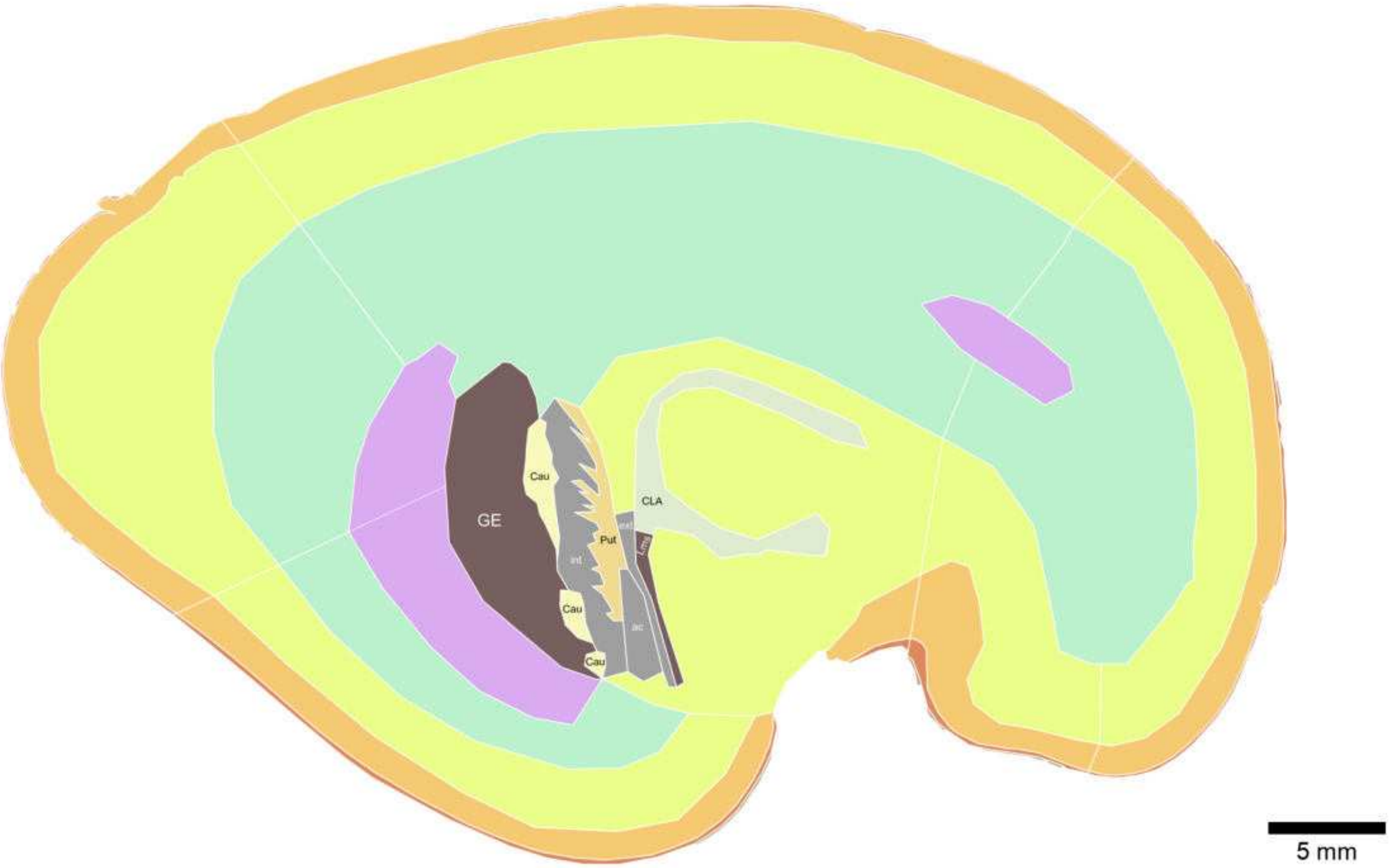

- CLA: Claustrum
- Cau: Caudate nucleus
- GE: Ganglionic eminence
- Lms: Lateral migratory stream
- Put: Putamen
- ac: Anterior commissure
- ext: External capsule
- int: Internal capsule

Age: 21 GW

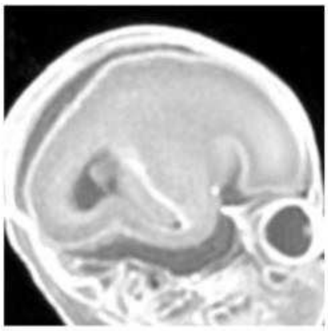

L-R Level: -10.08 mm

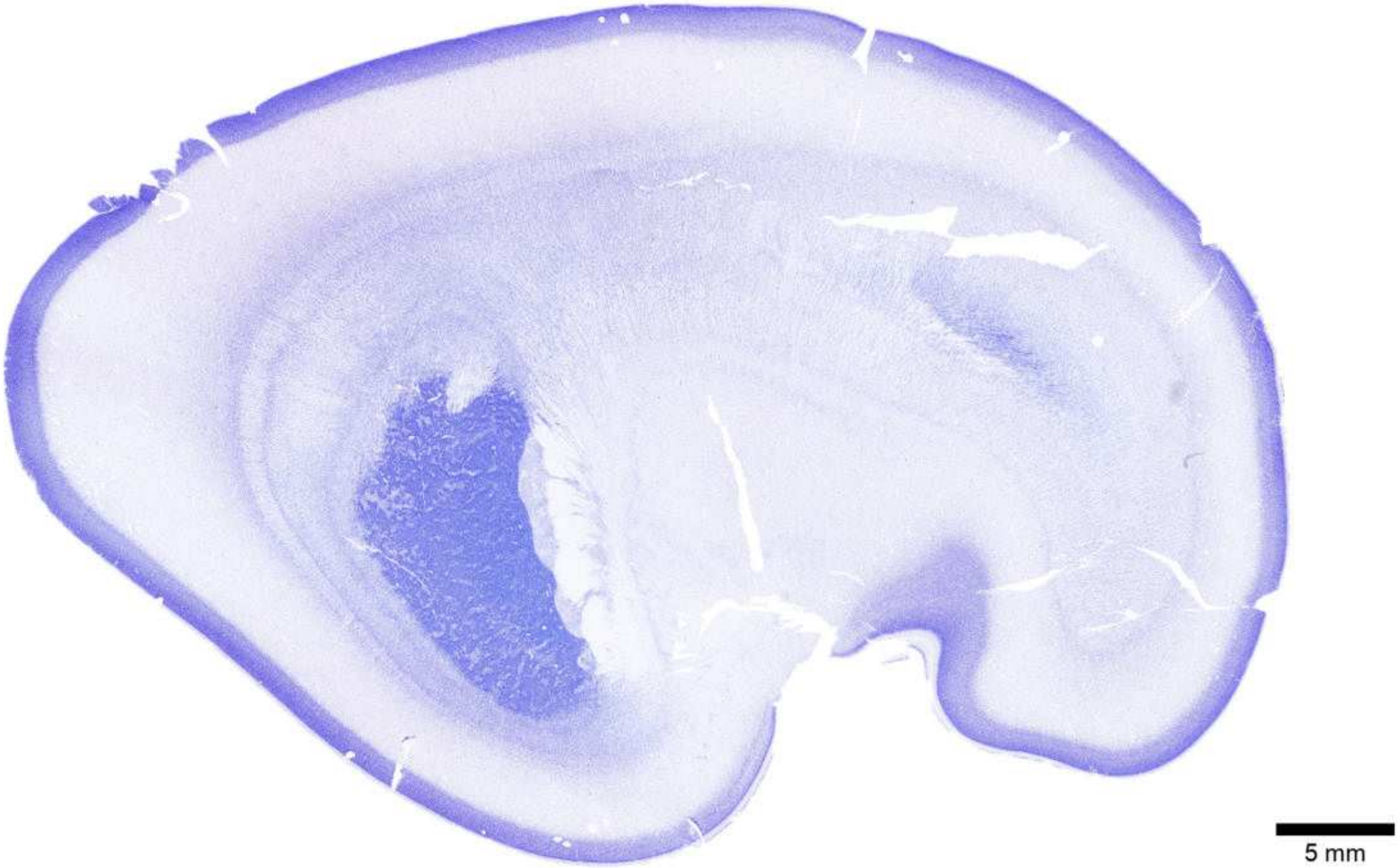

Transient Layers

- SVZ
- IZ
- SP
- CP
- MZ
- SGL

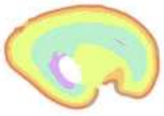

Cortical Areas

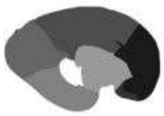

- FCTx
- ORB
- PAR
- OCC
- TEMP
- INS

Age: 21 GW

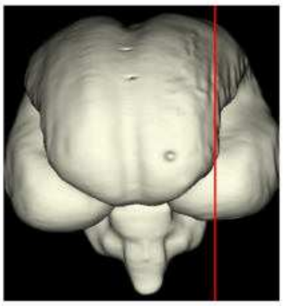

L-R Level: -10.08 mm

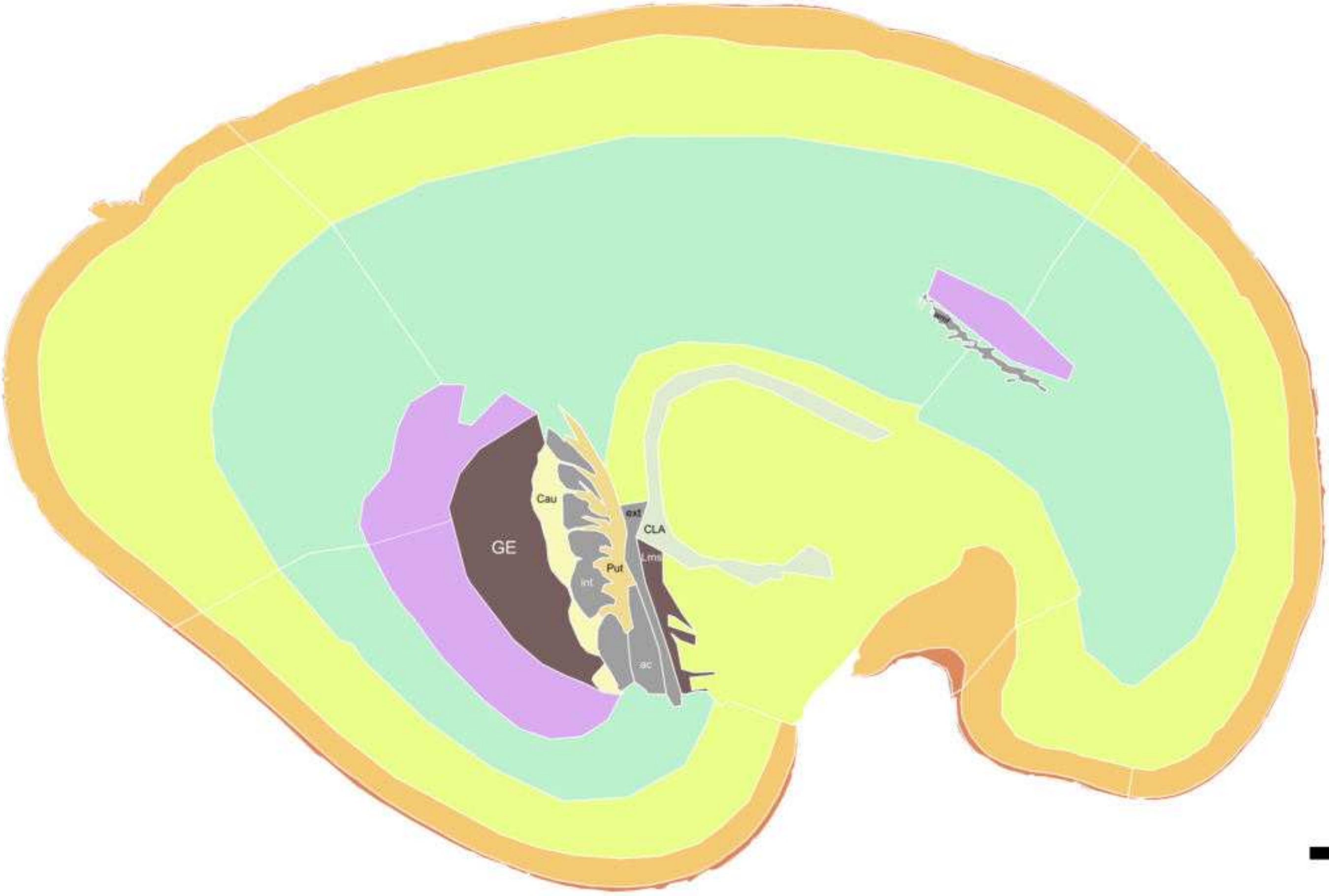

5 mm

- CLA: Claustrum
- Cau: Caudate nucleus
- GE: Ganglionic eminence
- Lms: Lateral migratory stream
- Put: Putamen
- ac: Anterior commissure
- ext: External capsule
- int: Internal capsule
- wmf: White matter fibers

Age: 21 GW

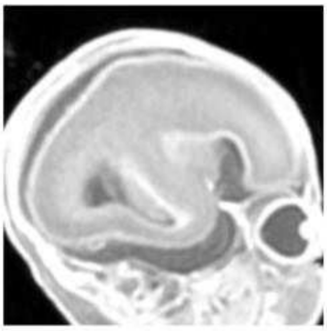

L-R Level: -10.8 mm

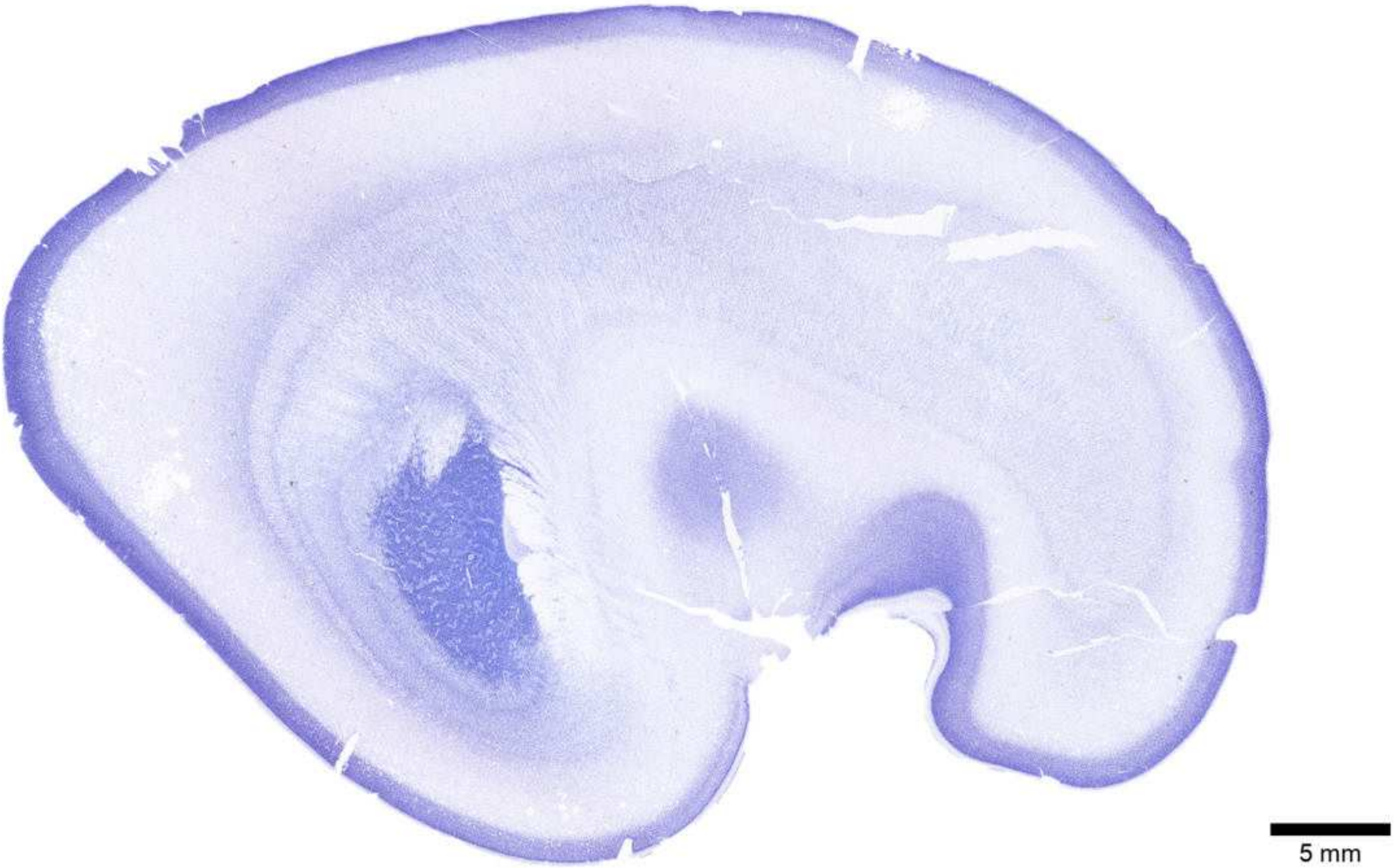

Transient Layers

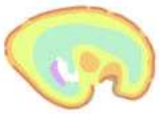

SVZ
IZ
SP
CP
MZ
SGL

Cortical Areas

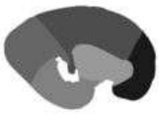

FCTx
PAR
OCC
TEMP
INS

Age: 21 GW

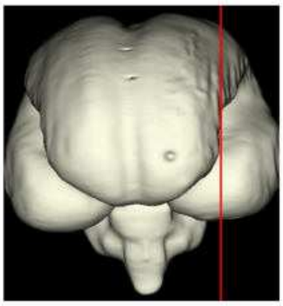

L-R Level: -10.8 mm

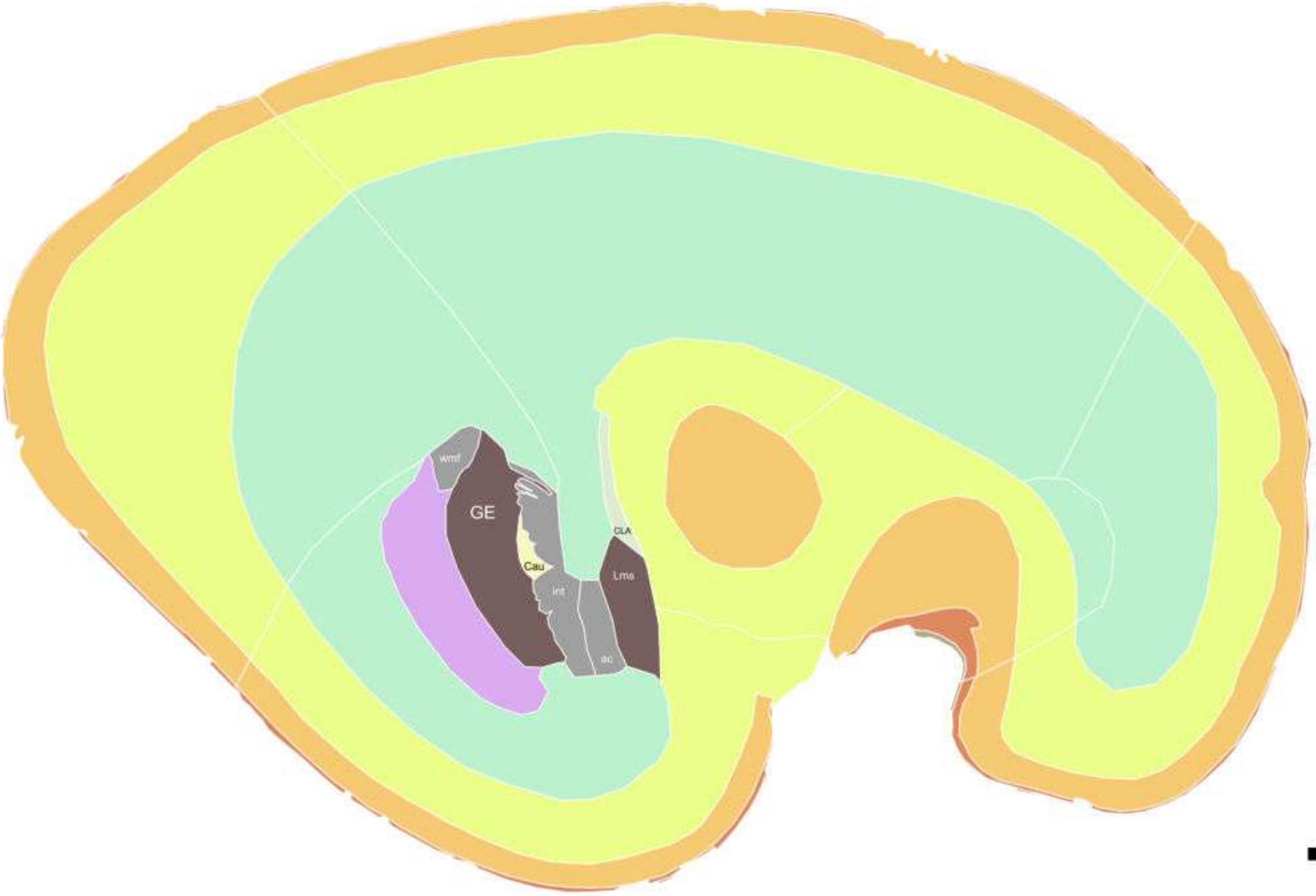

5 mm

CLA: Claustrum
Cau: Caudate nucleus
GE: Ganglionic eminence
Lms: Lateral migratory stream
ac: Anterior commissure
int: Internal capsule
wmf: White matter fibers

Age: 21 GW

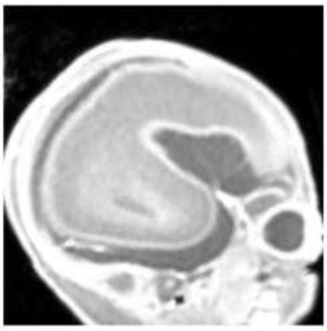

L-R Level: -12.42 mm

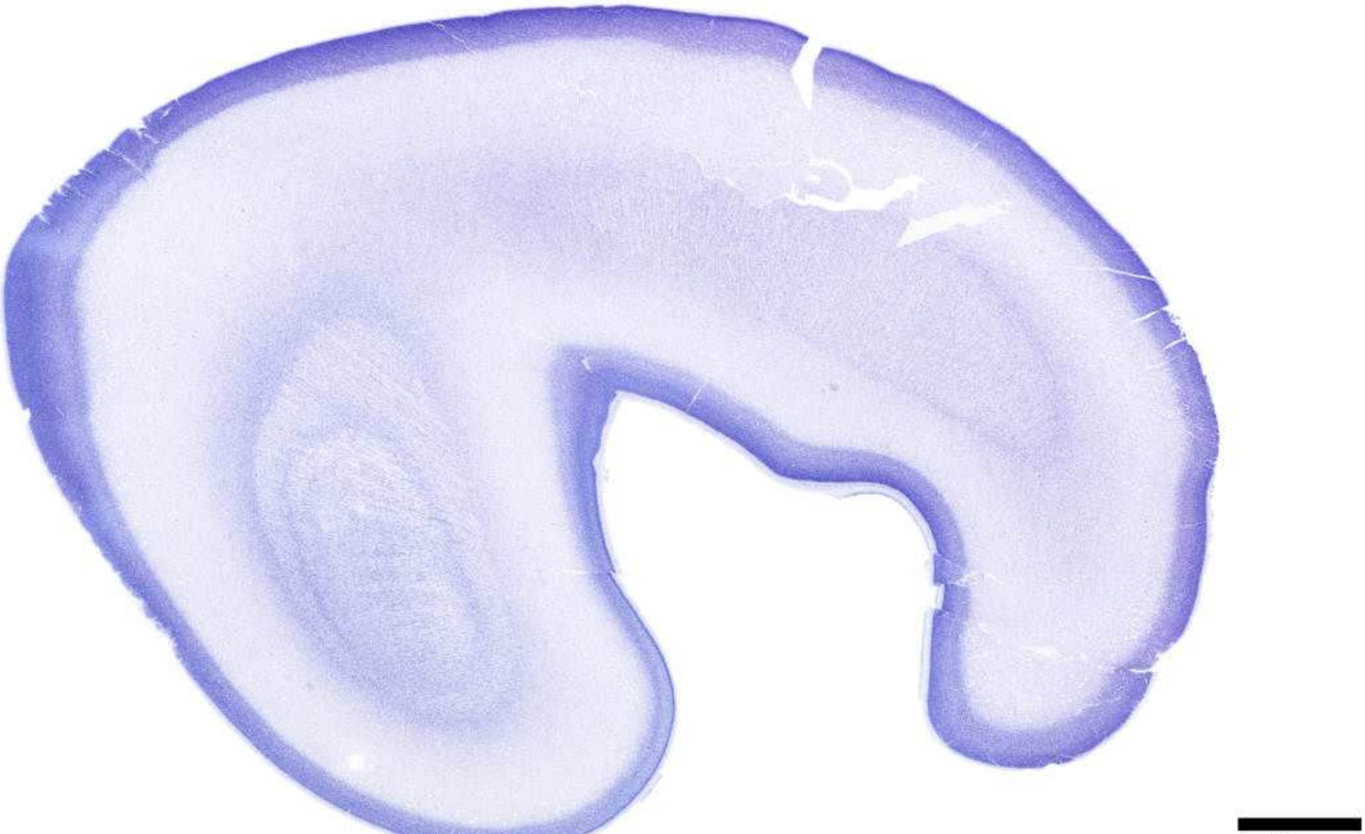

5 mm

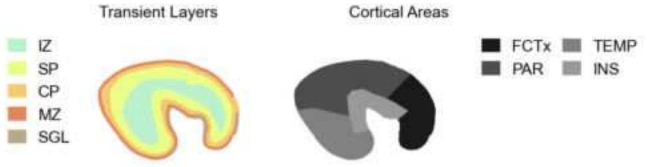

Age: 21 GW

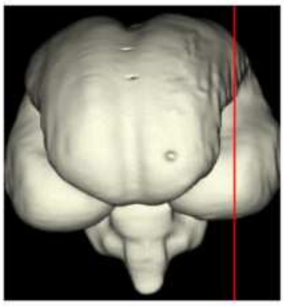

L-R Level: -12.42 mm

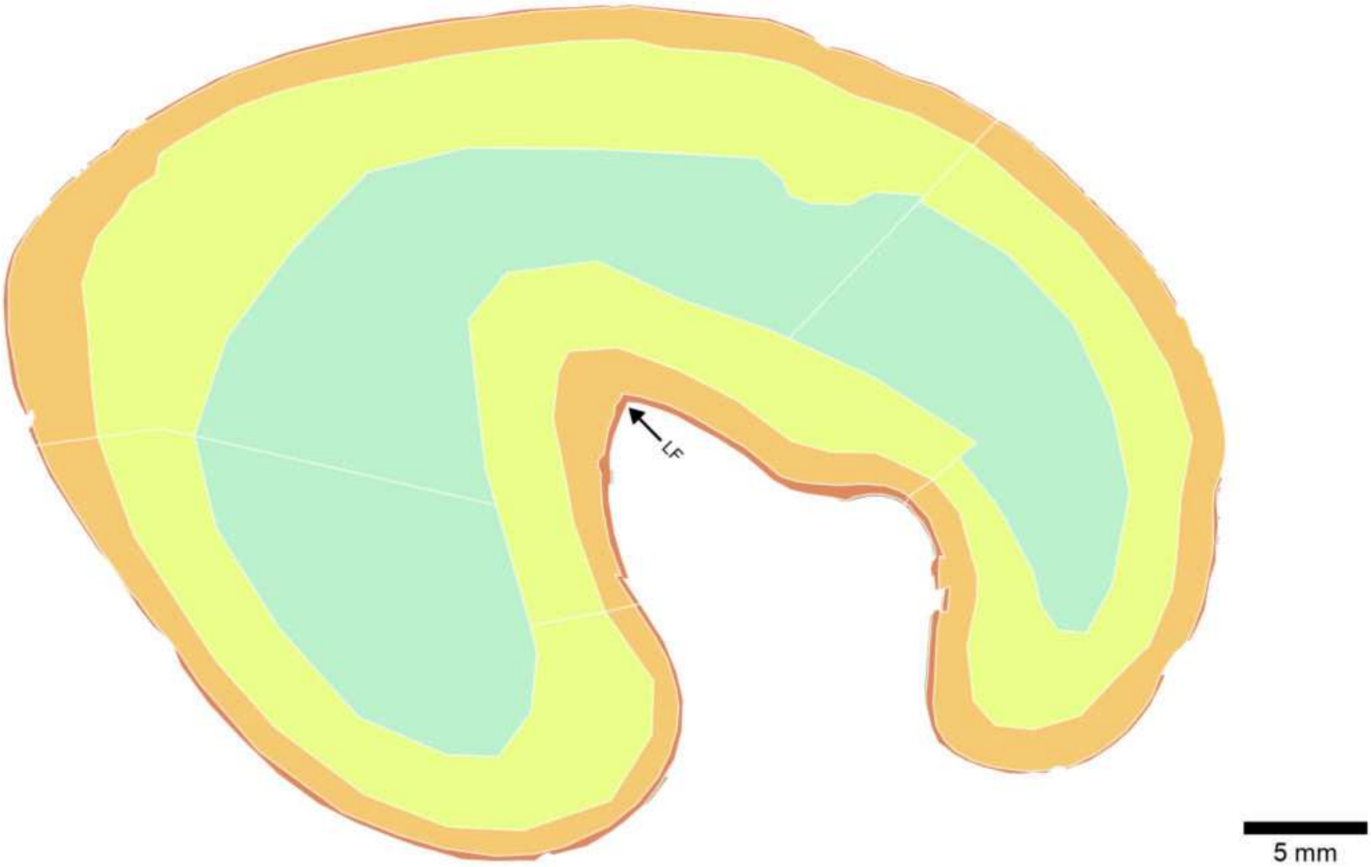

→ LF: Lateral fissure

Age: 21 GW

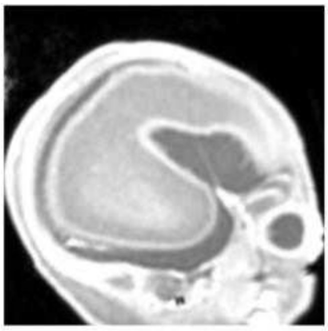

L-R Level: -14.34 mm

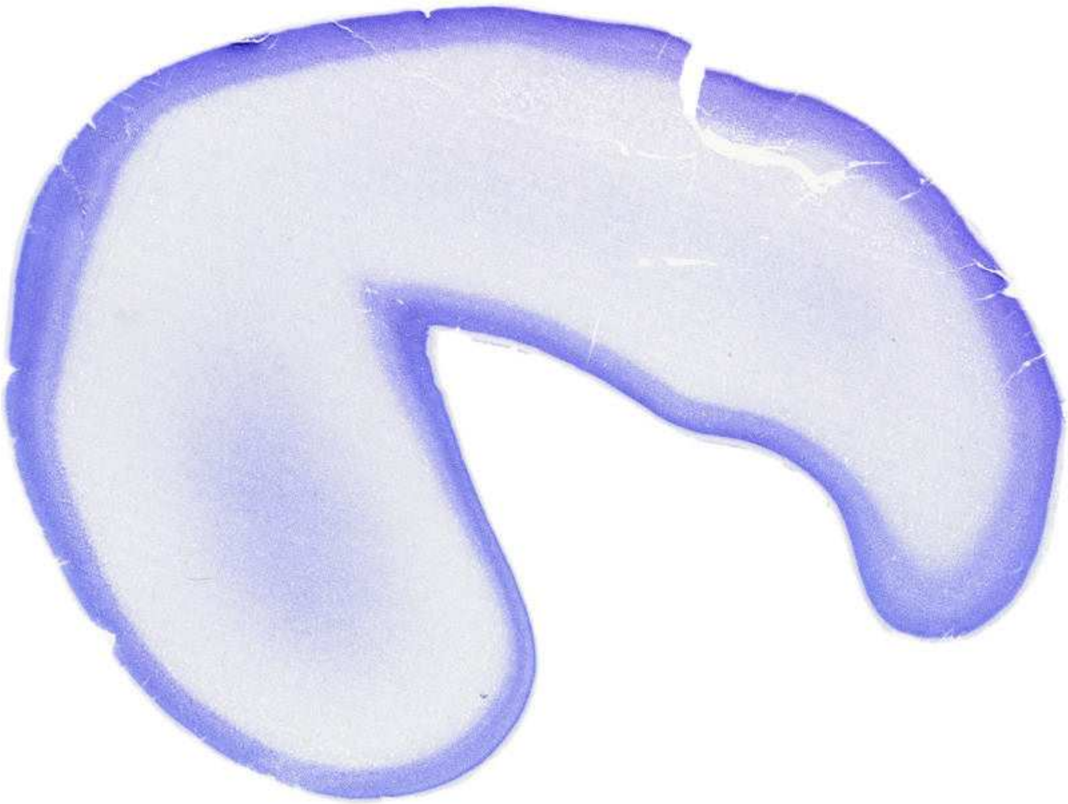

5 mm

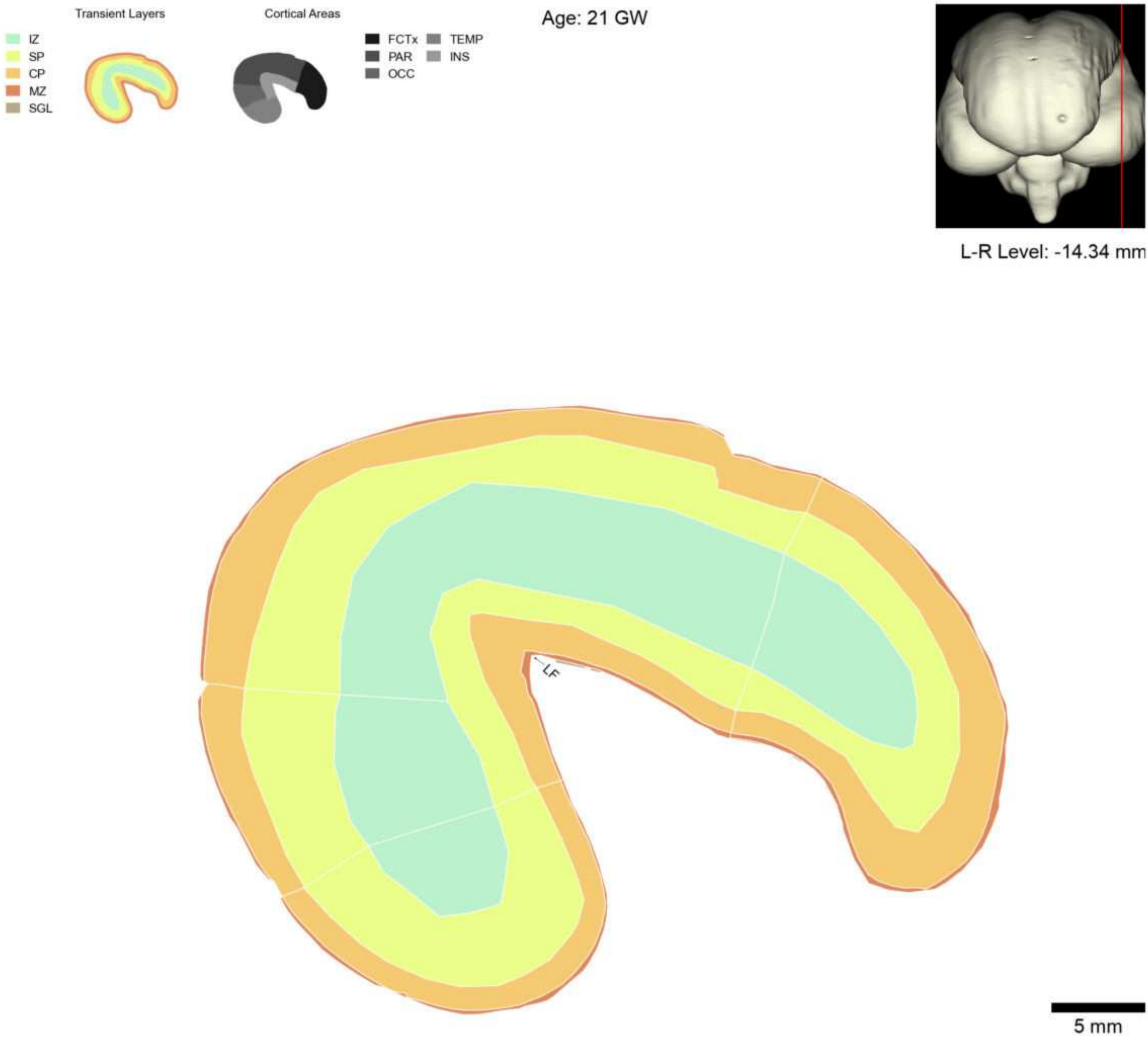

→ LF - Lateral fissure

Age: 21 GW

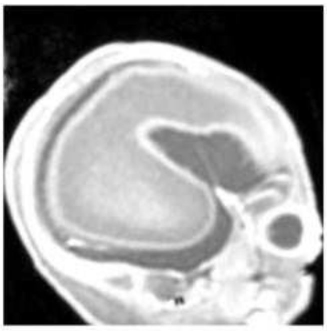

L-R Level: -14.88 mm

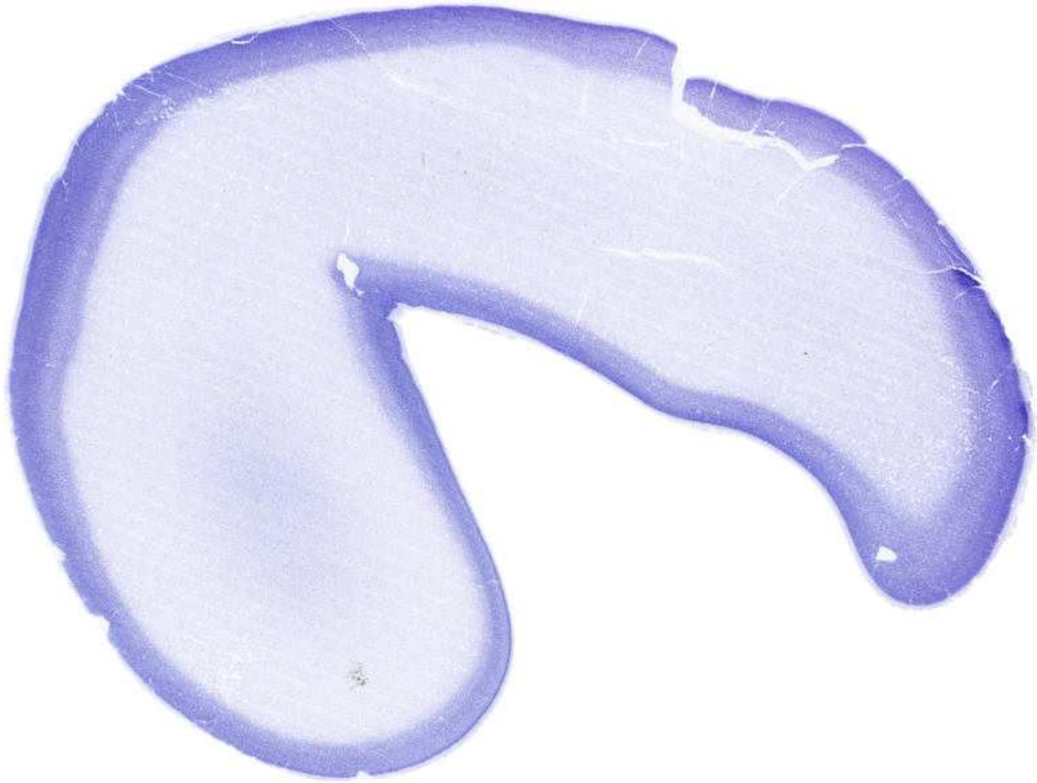

5 mm

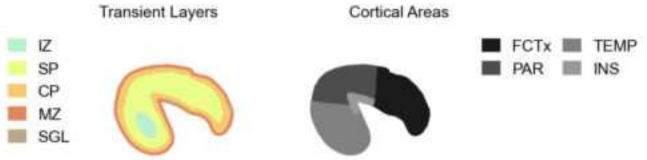

Age: 21 GW

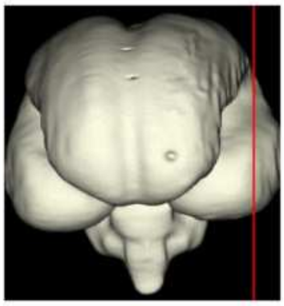

L-R Level: -14.88 mm

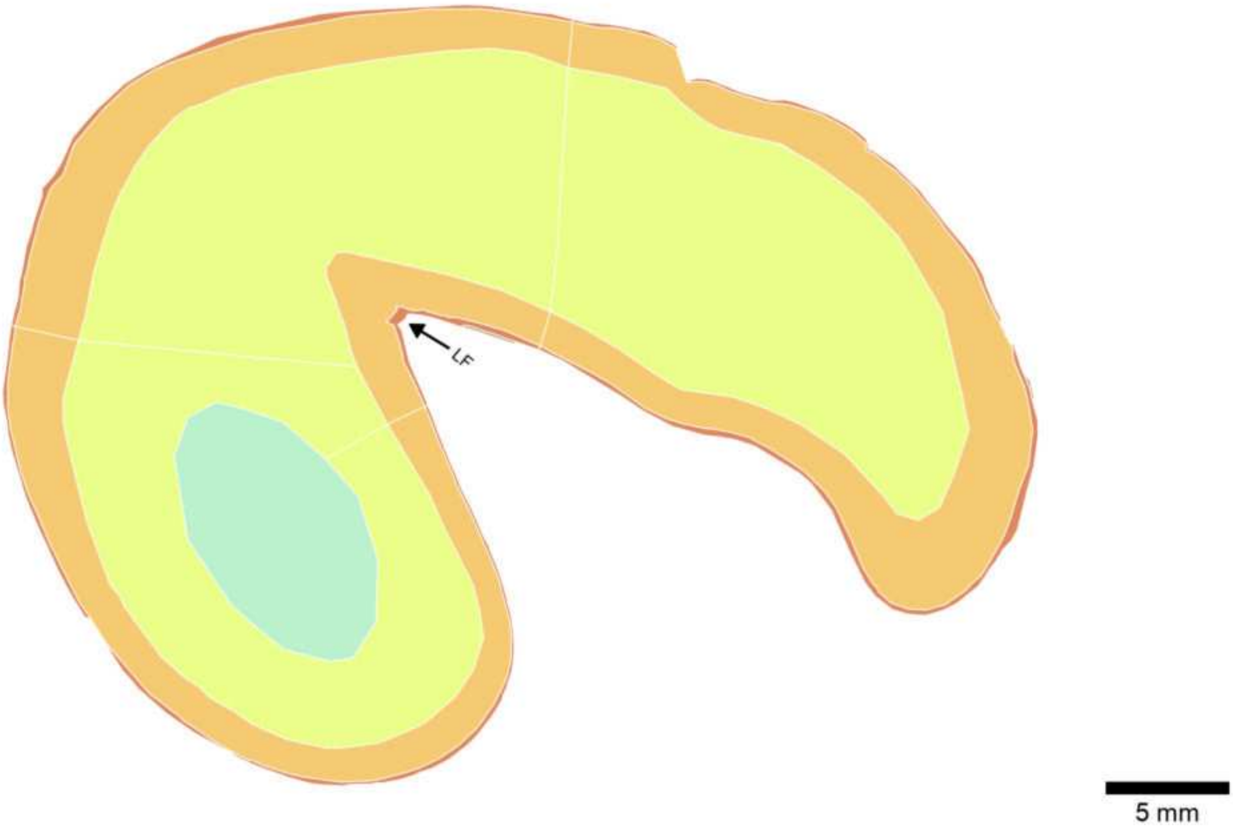

→ LF: Lateral fissure

Age: 21 GW

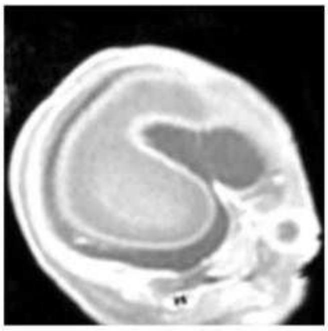

L-R Level: -15.78 mm

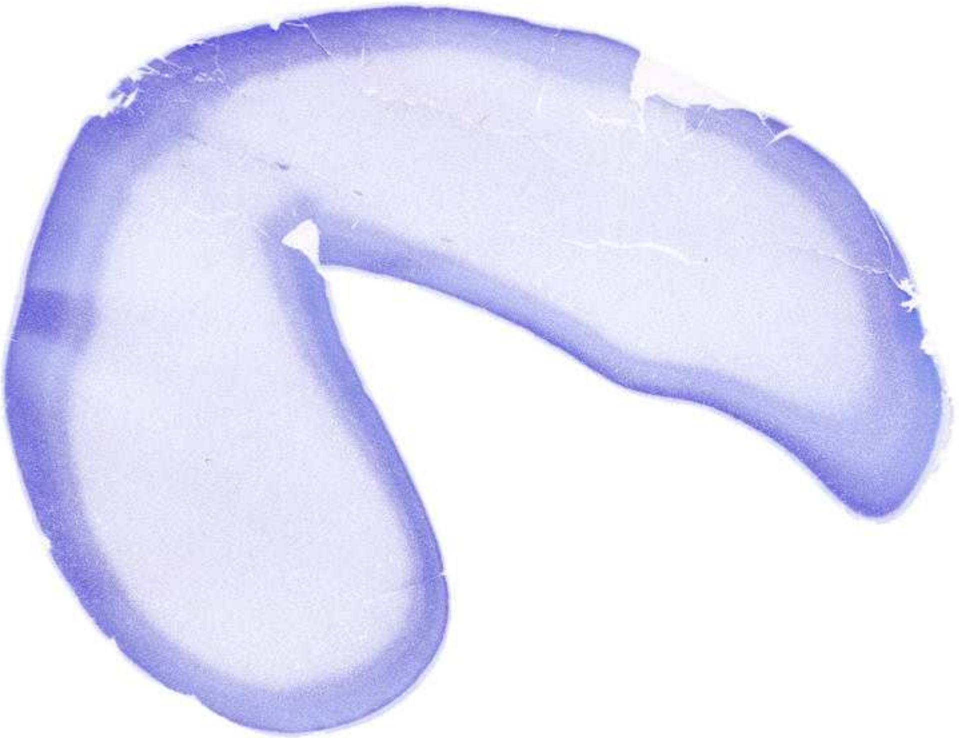

5 mm

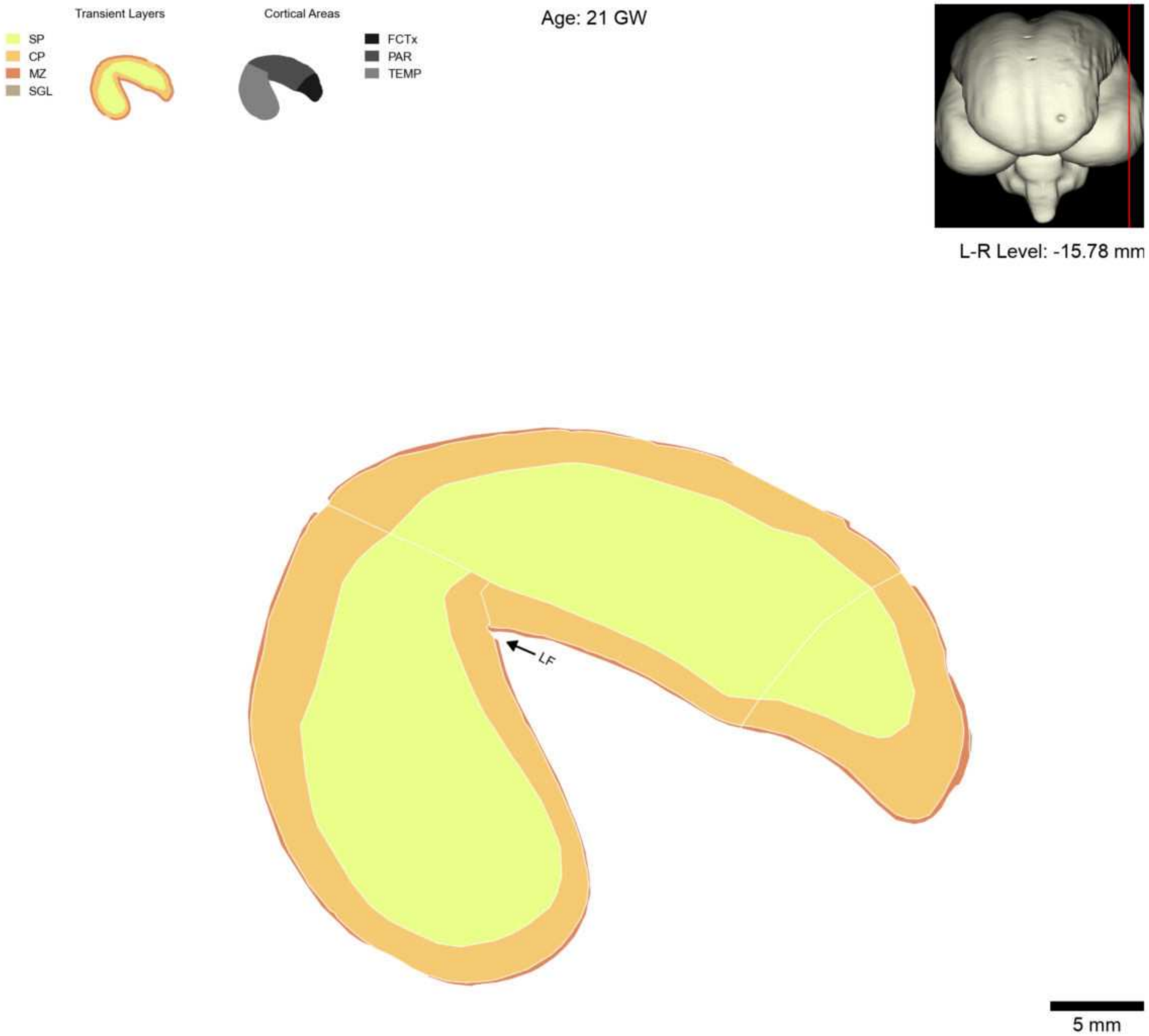

→ LF: Lateral fissure

Age: 21 GW

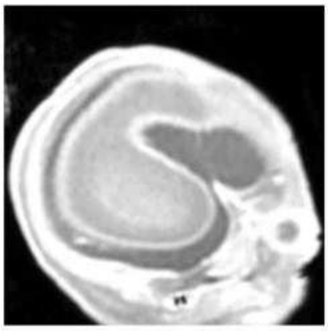

L-R Level: -16.14 mm

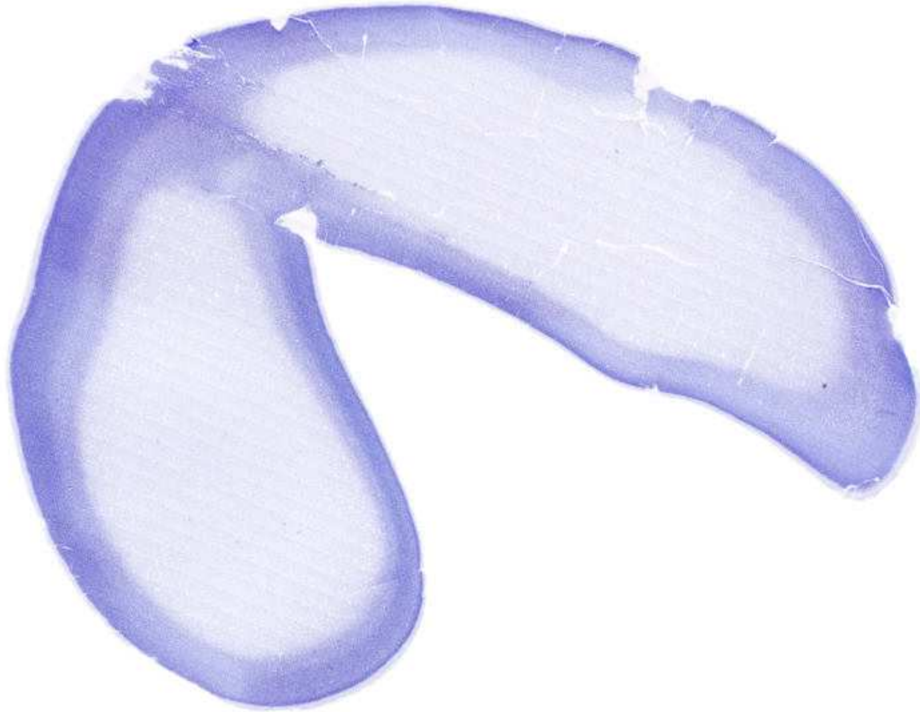

5 mm

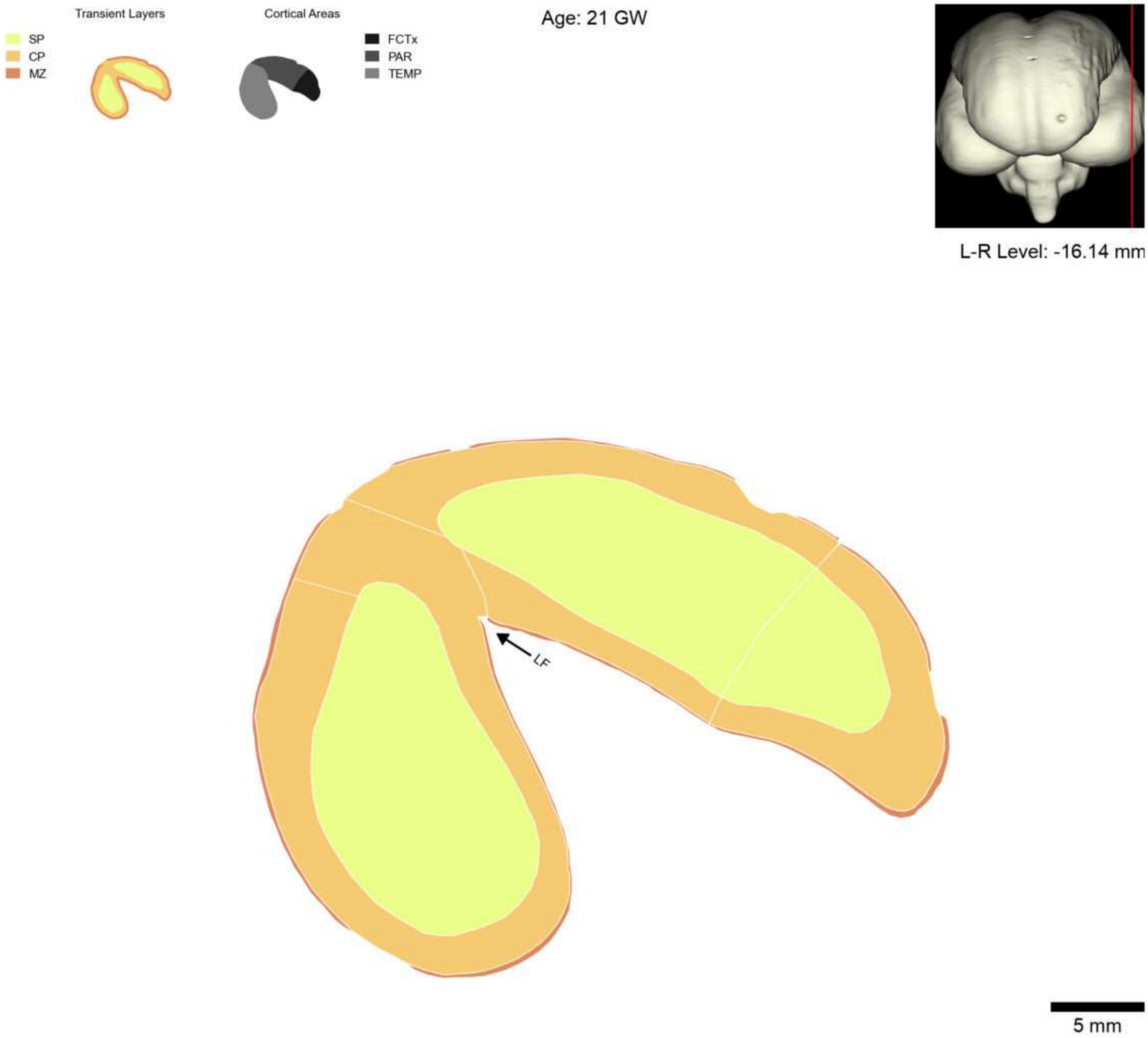

→ LF: Lateral fissure

Age: 21 GW

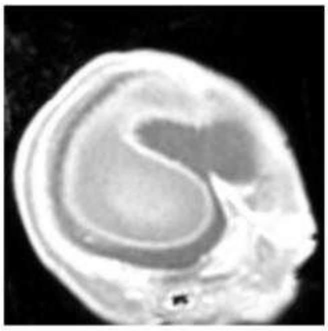

L-R Level: -16.68 mm

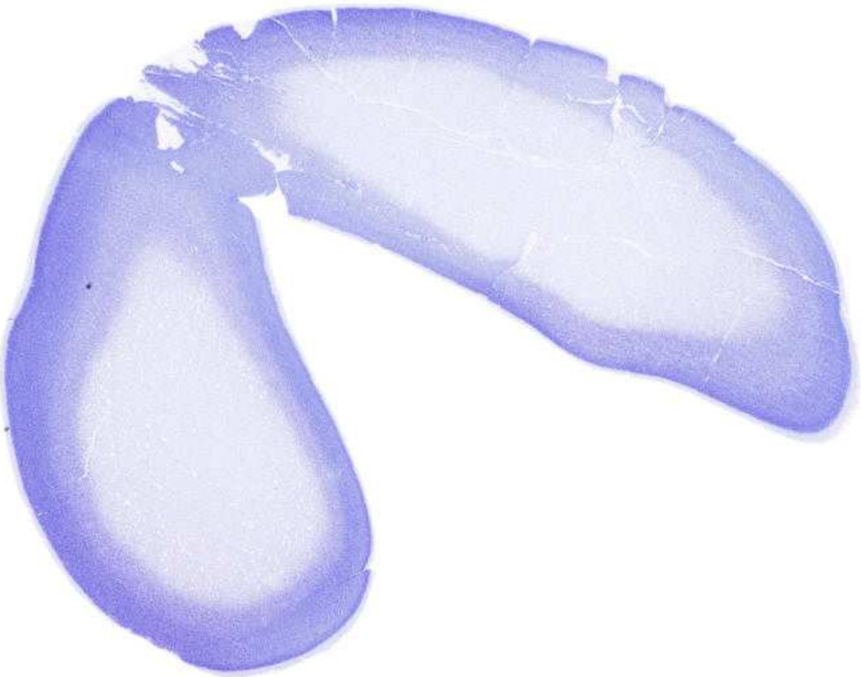

5 mm

Age: 21 GW

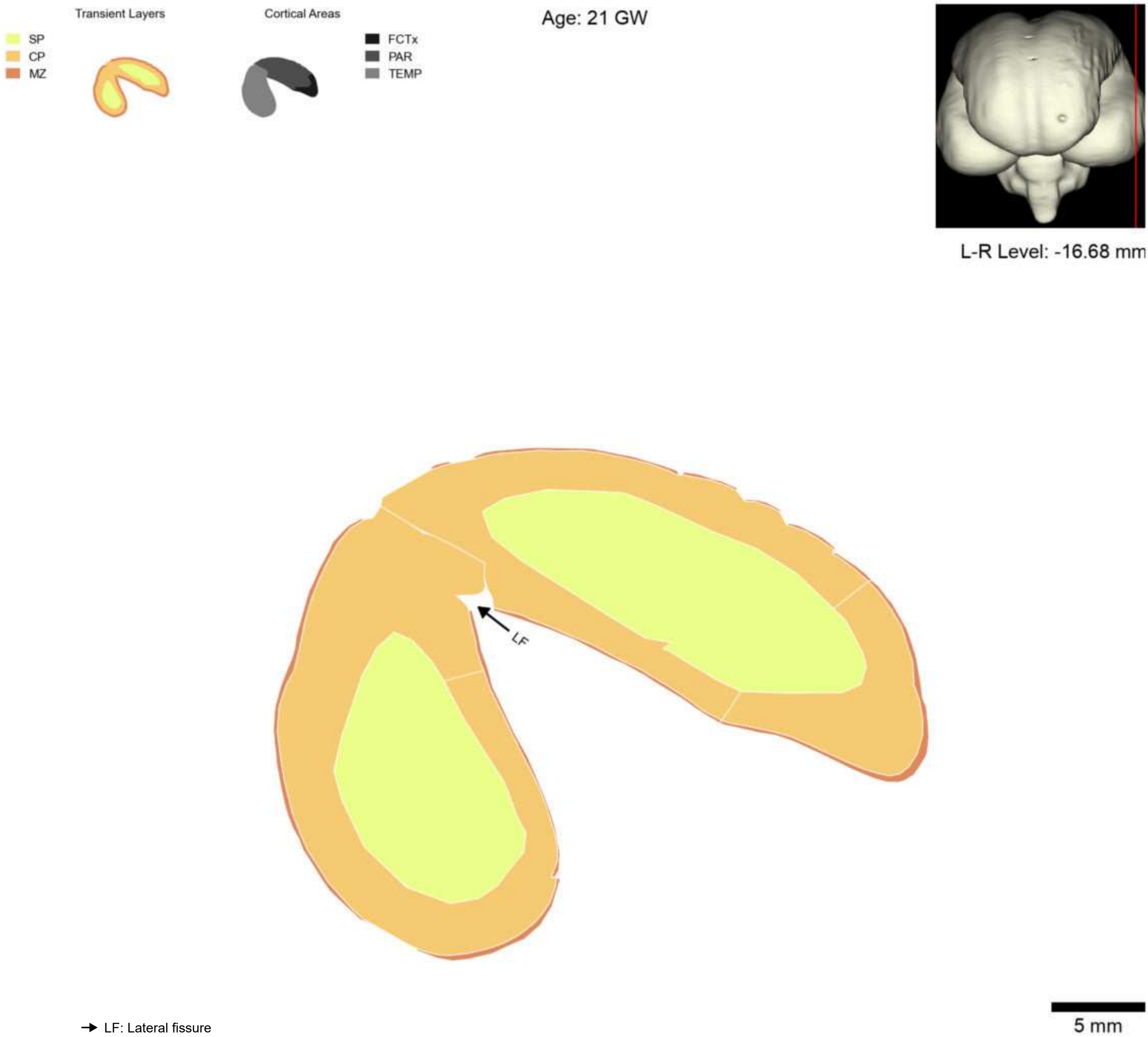

→ LF: Lateral fissure

Age: 21 GW

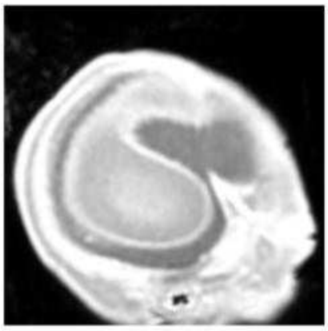

L-R Level: -16.86 mm

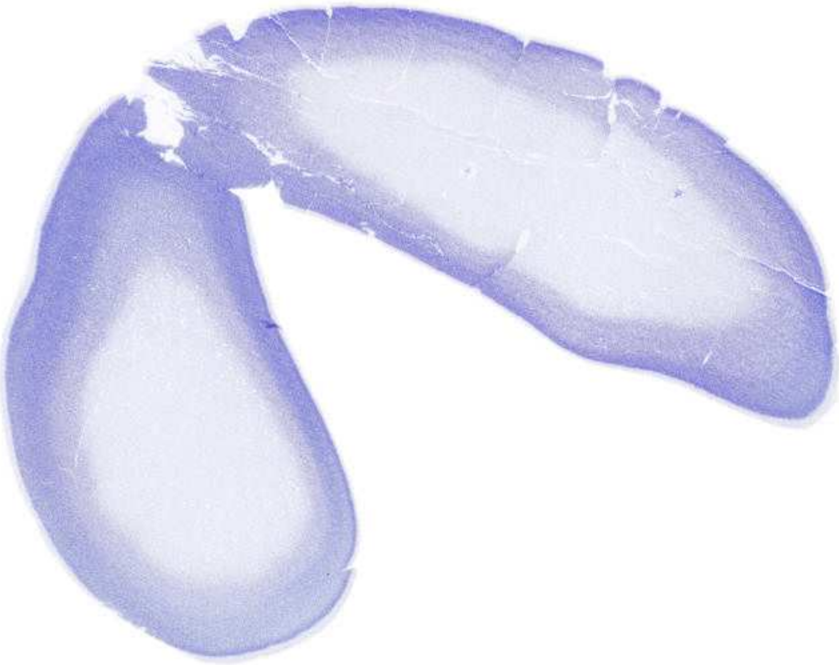

5 mm

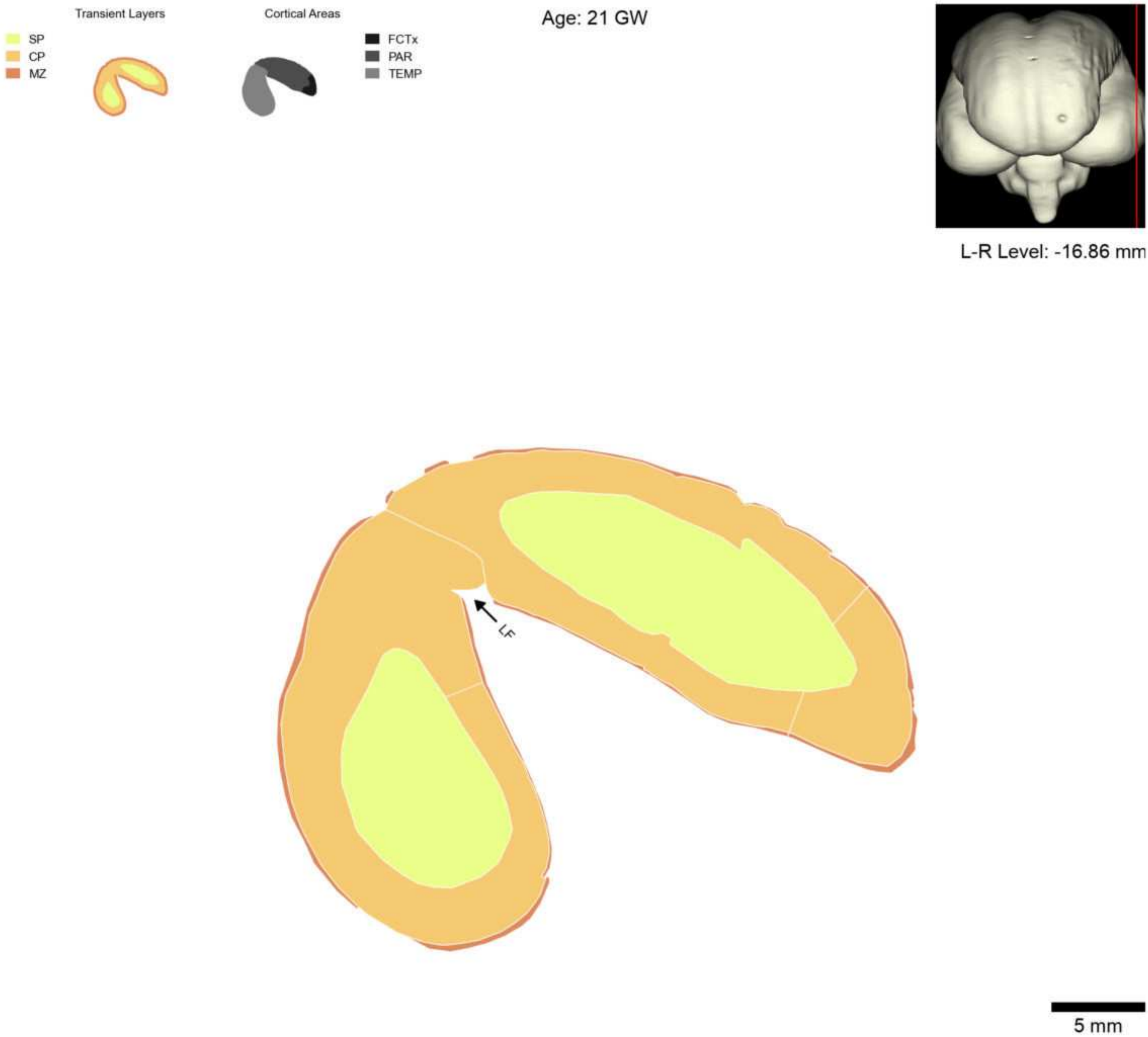

→ LF: Lateral fissure

Age: 21 GW

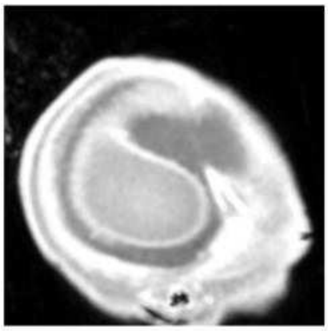

L-R Level: -17.52 mm

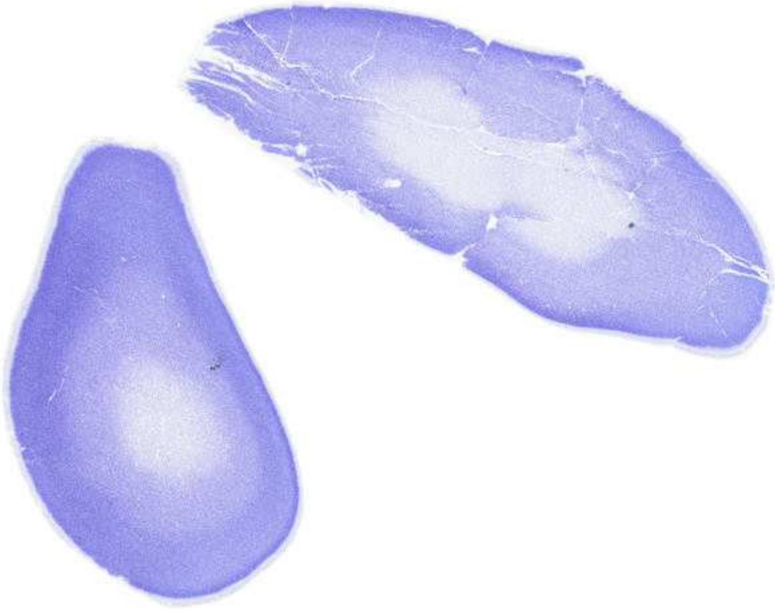

5 mm

Transient Layers

- SP
- CP
- MZ

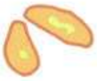

Cortical Areas

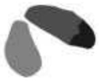

- FCTx
- PAR
- TEMP

Age: 21 GW

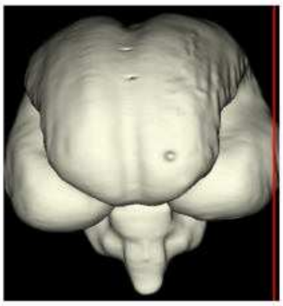

L-R Level: -17.52 mm

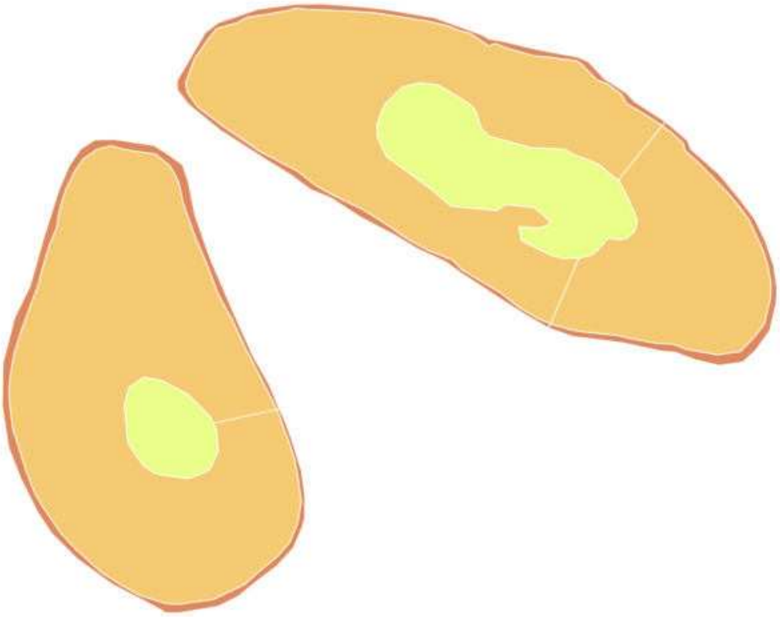

5 mm

Age: 21 GW

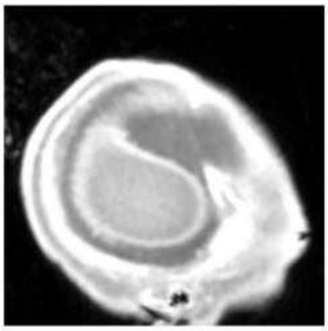

L-R Level: -18.24 mm

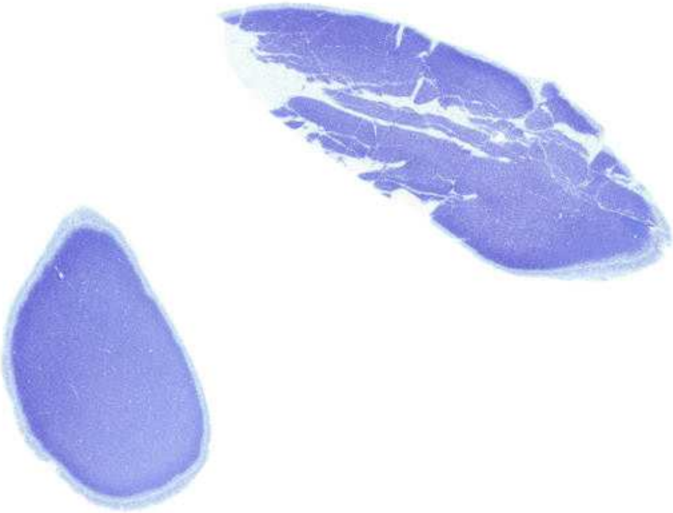

5 mm

Age: 21 GW

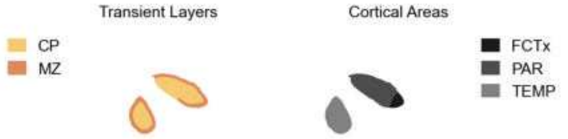

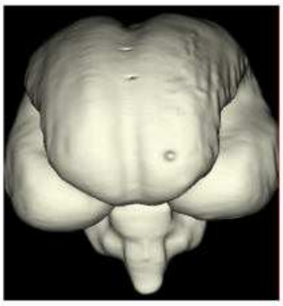

L-R Level: -18.24 mm

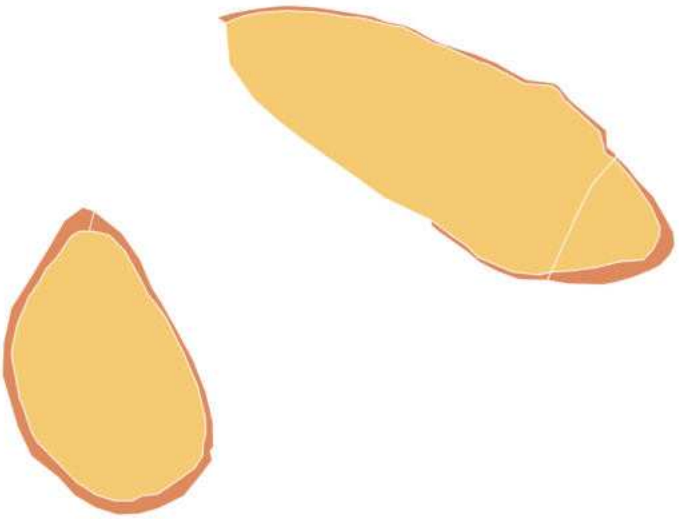

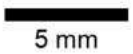

22 Gestational Week (GW)

Coronal

This page is intentionally left blank

Age: 22 GW

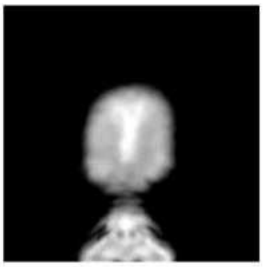

A-P Level: 22.5 mm

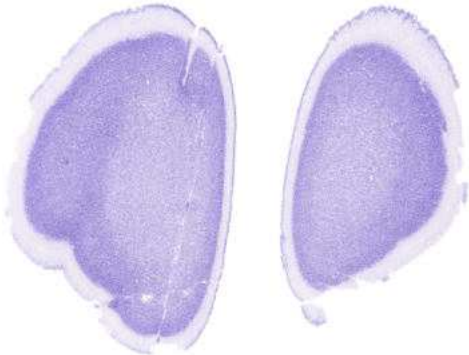

5 mm

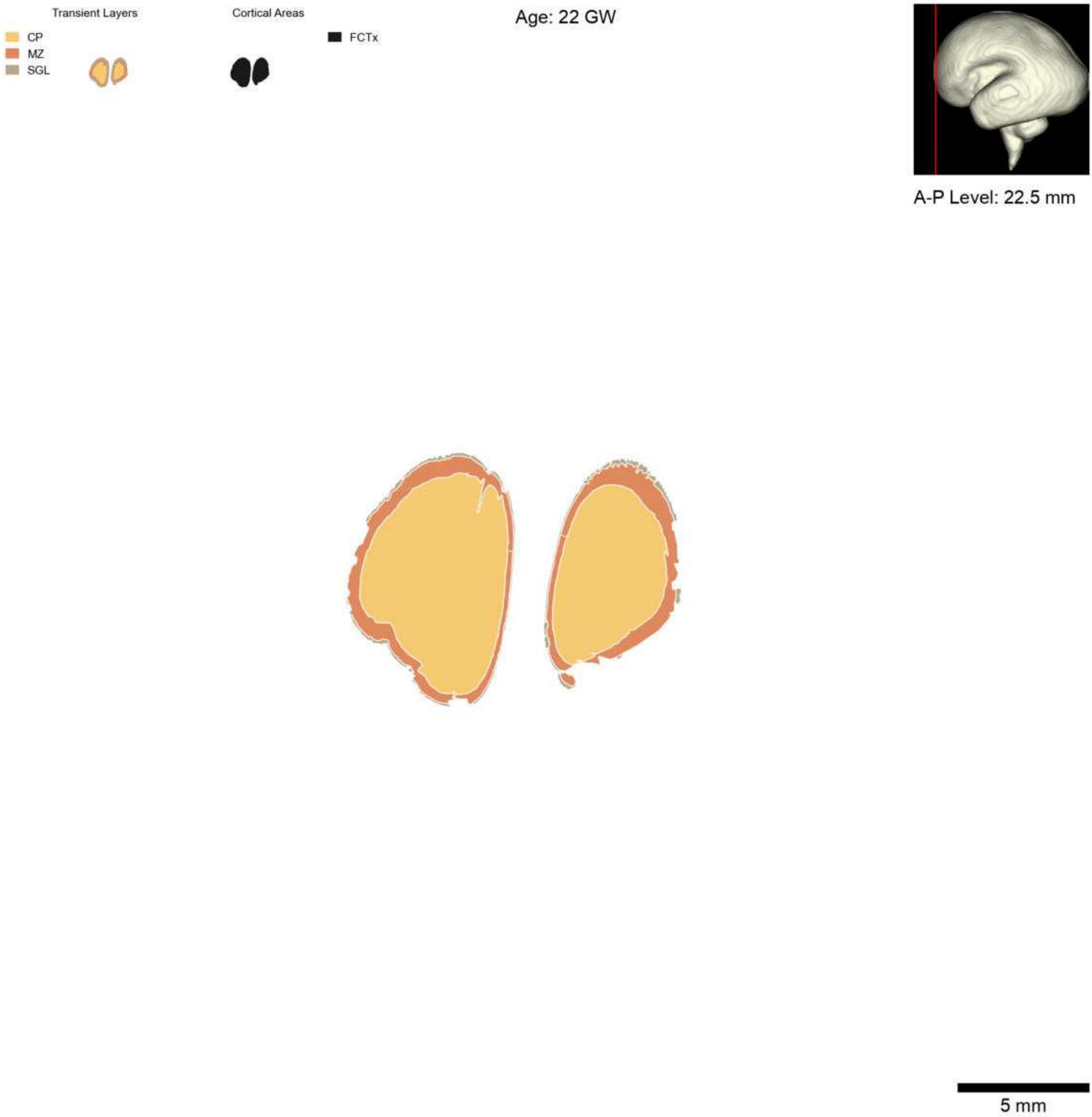
Transient Layers
CP
MZ
SGL
Cortical Areas
FCTx
Age: 22 GW
A-P Level: 22.5 mm
5 mm

Age: 22 GW

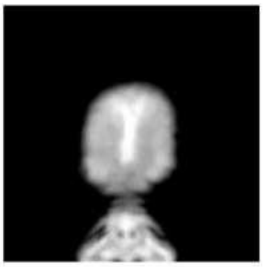

A-P Level: 22.32 mm

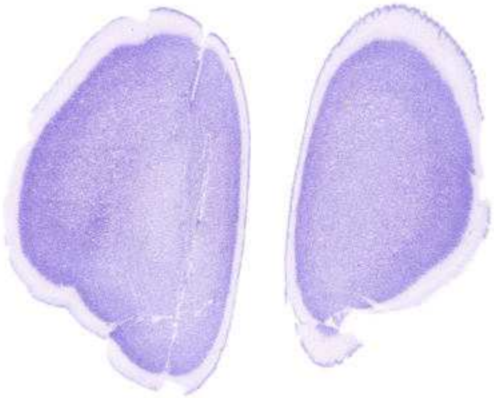

5 mm

Transient Layers

- CP
- MZ
- SGL

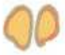

Cortical Areas

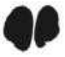

- FCTx

Age: 22 GW

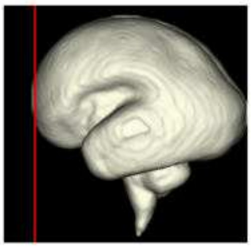

A-P Level: 22.32 mm

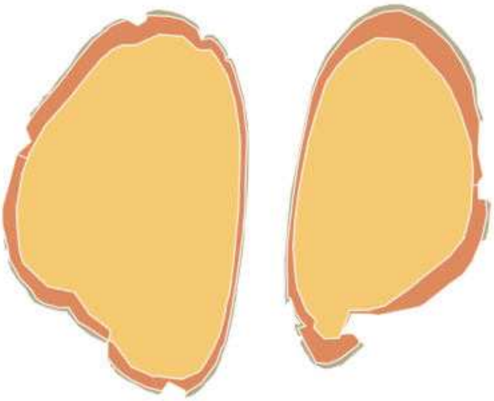

5 mm

Age: 22 GW

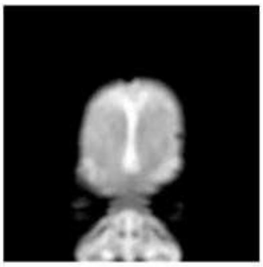

A-P Level: 21.78 mm

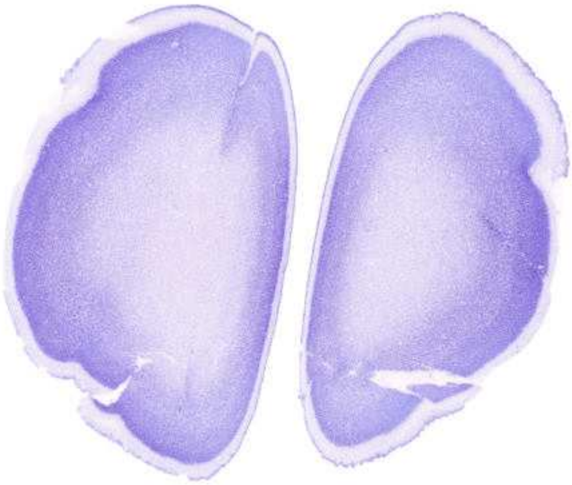

5 mm

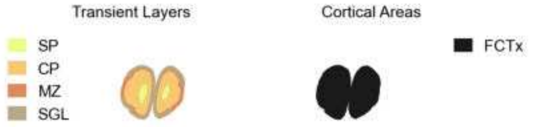

Age: 22 GW

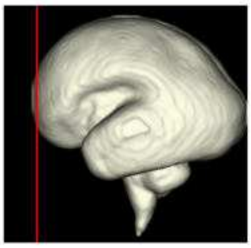

A-P Level: 21.78 mm

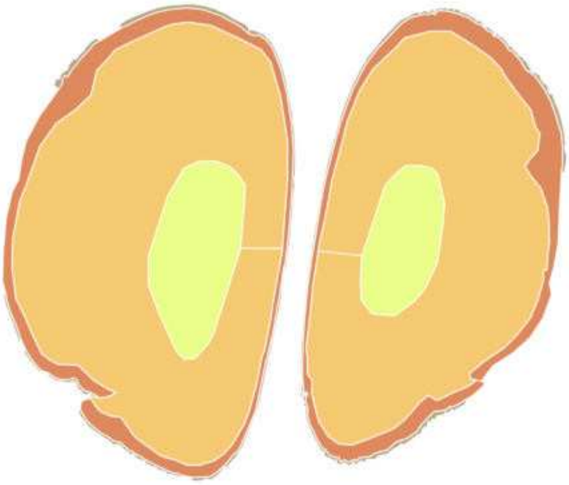

5 mm

Age: 22 GW

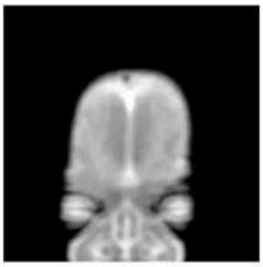

A-P Level: 20.94 mm

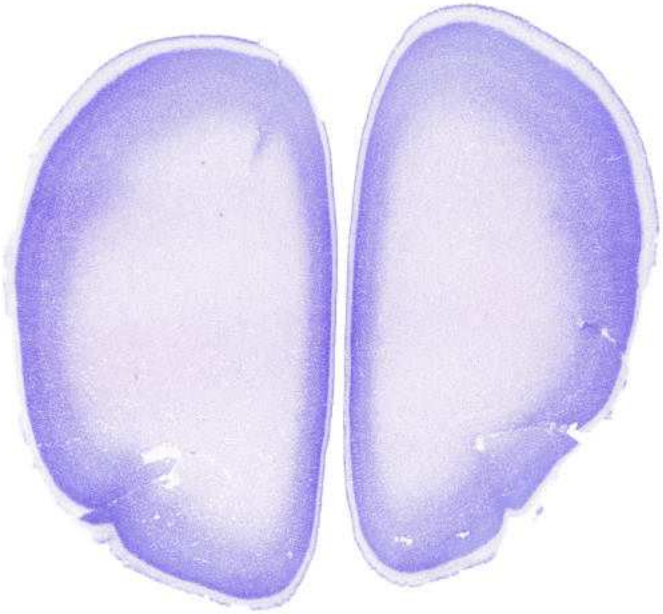

5 mm

Transient Layers

- SP
- CP
- MZ
- SGL

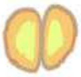

Cortical Areas

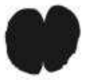

- FCTx

Age: 22 GW

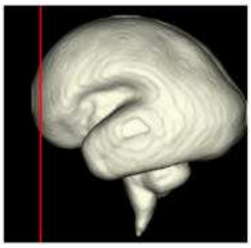

A-P Level: 20.94 mm

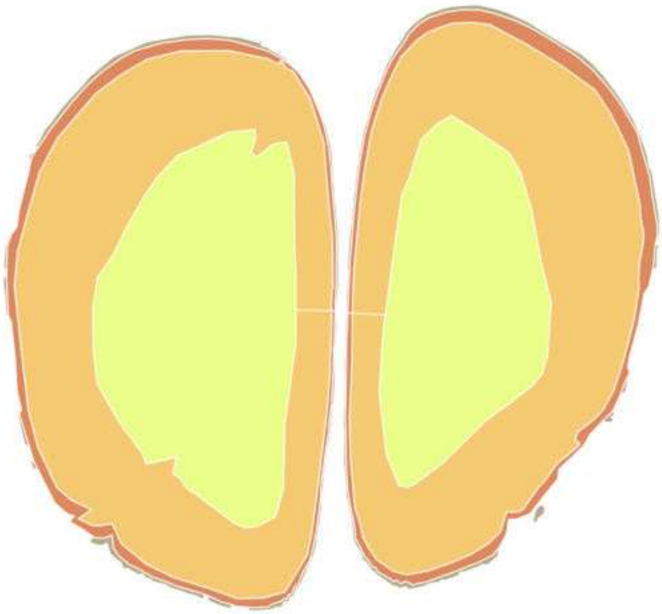

5 mm

Age: 22 GW

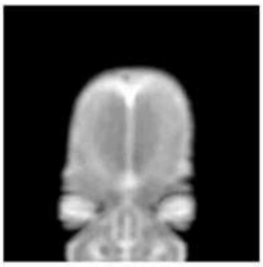

A-P Level: 20.22 mm

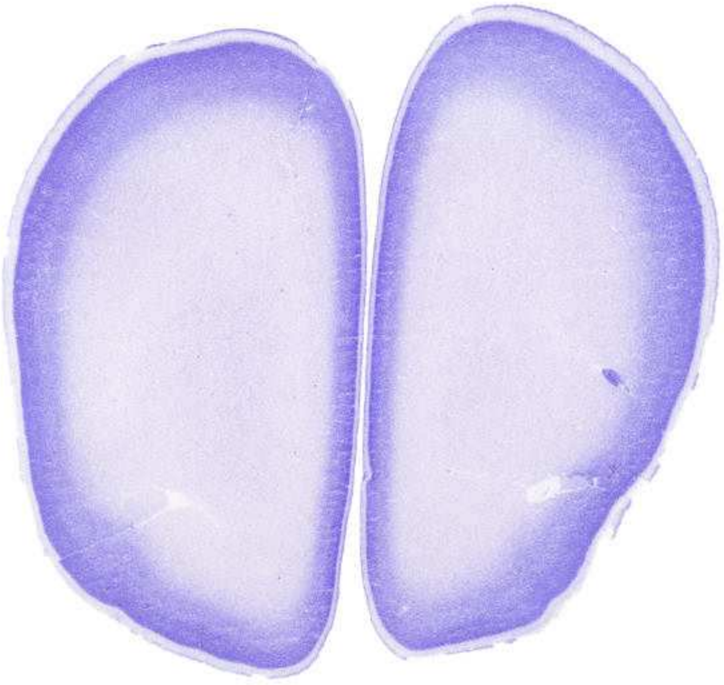

5 mm

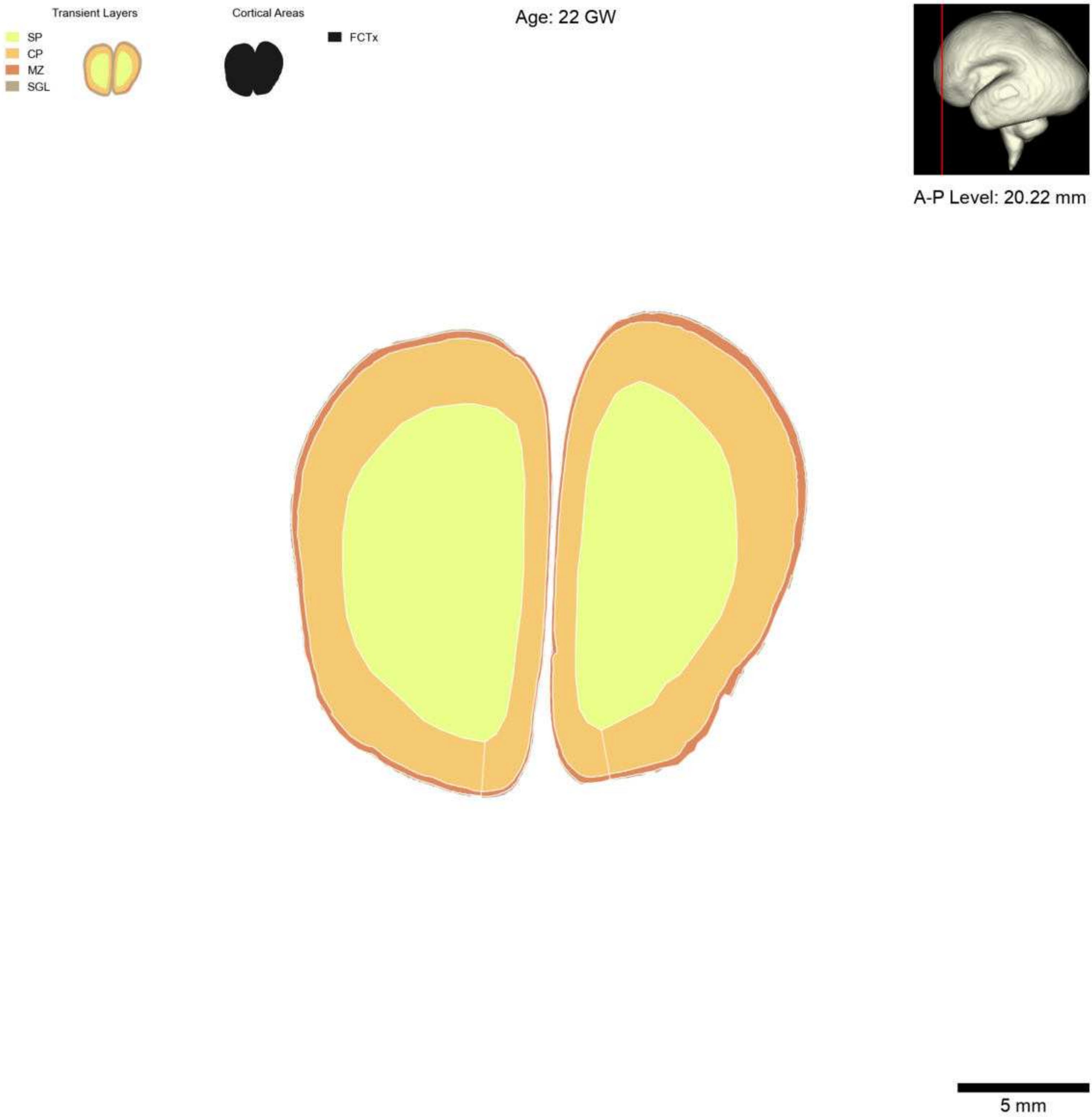
Transient Layers
SP
CP
MZ
SGL
Cortical Areas
FCTx
Age: 22 GW
A-P Level: 20.22 mm
5 mm

Age: 22 GW

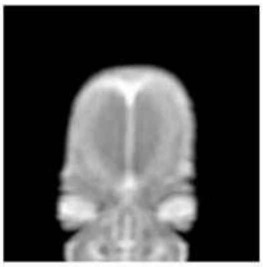

A-P Level: 19.98 mm

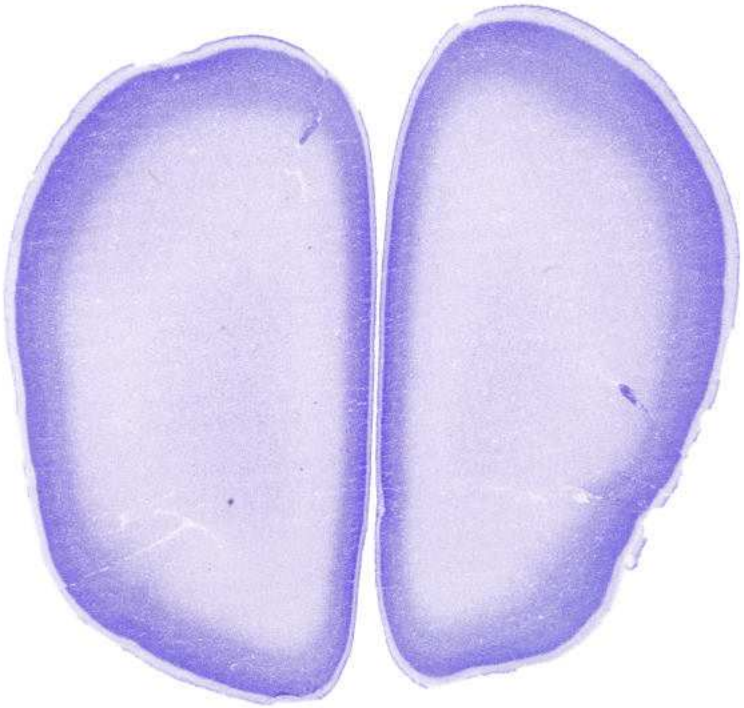

5 mm

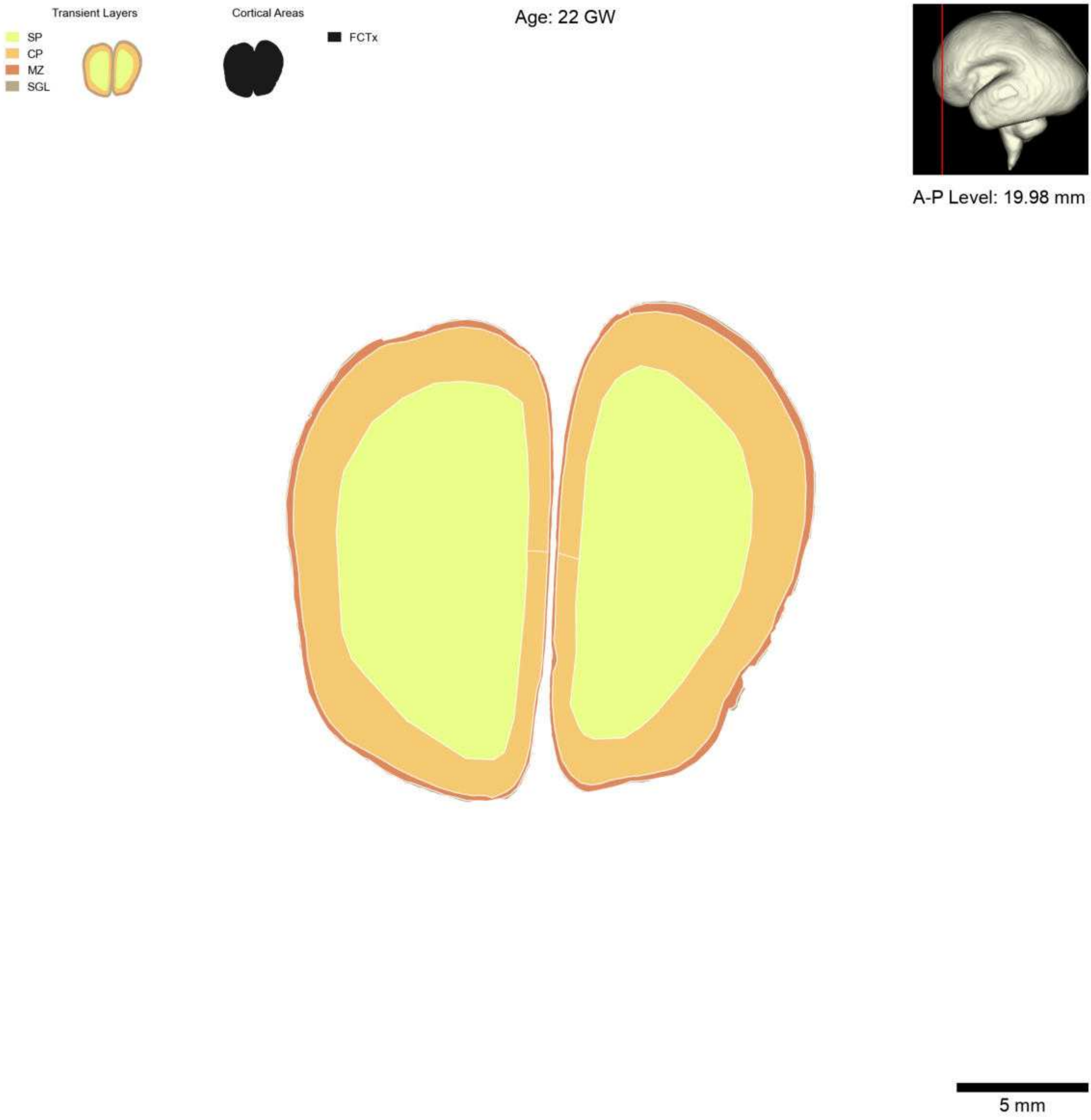
Transient Layers
SP
CP
MZ
SGL
Cortical Areas
FCTx
Age: 22 GW
A-P Level: 19.98 mm
5 mm

Age: 22 GW

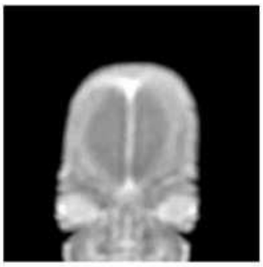

A-P Level: 19.5 mm

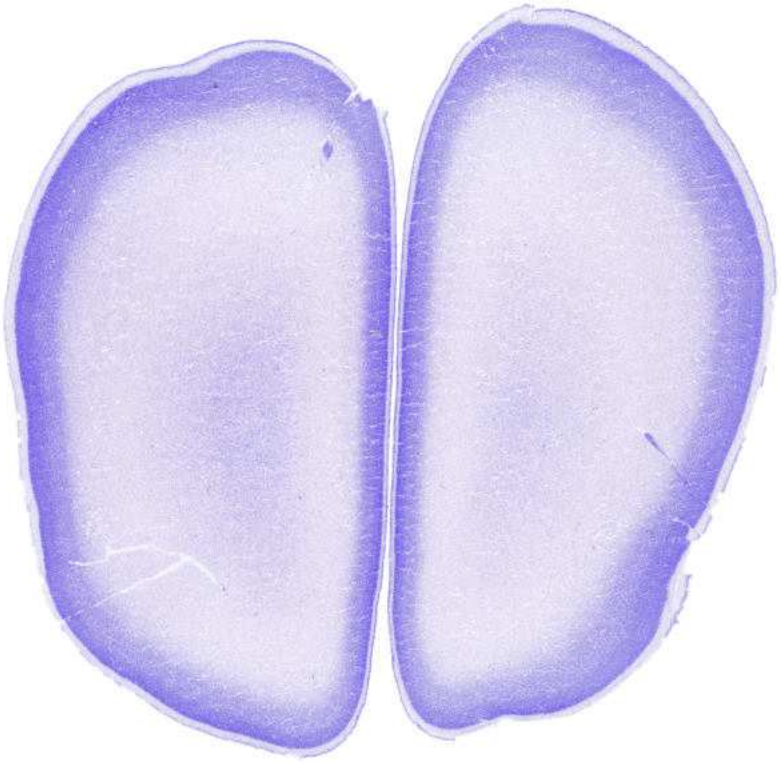

5 mm

Age: 22 GW

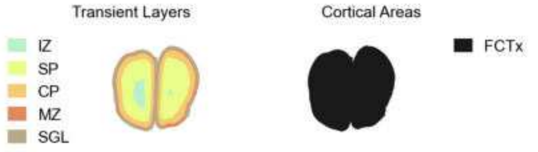

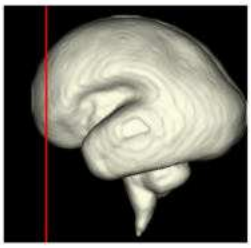

A-P Level: 19.5 mm

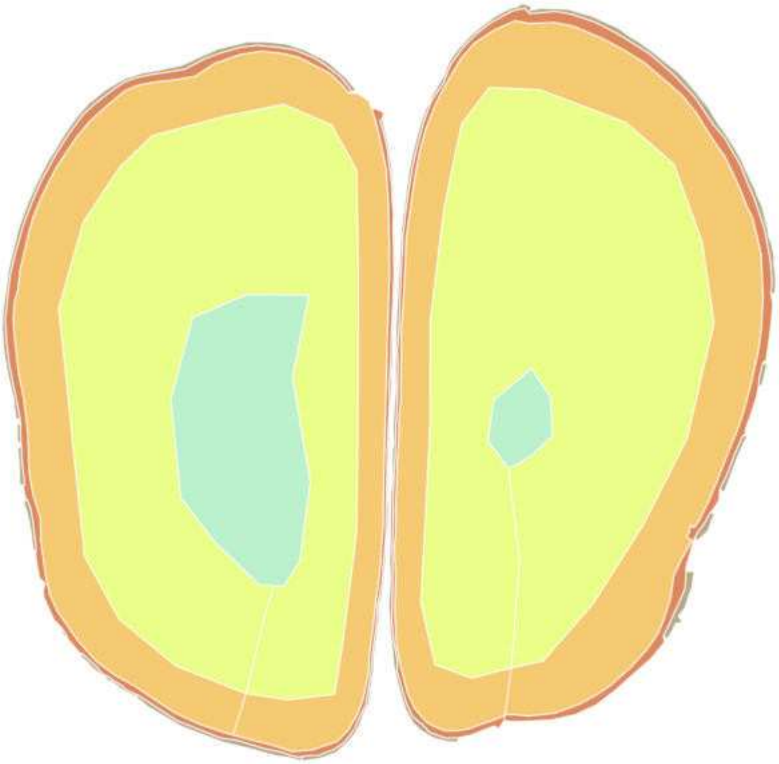

Age: 22 GW

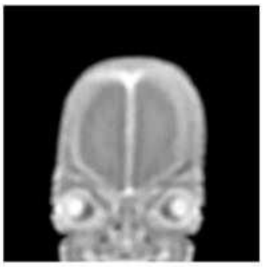

A-P Level: 18.72 mm

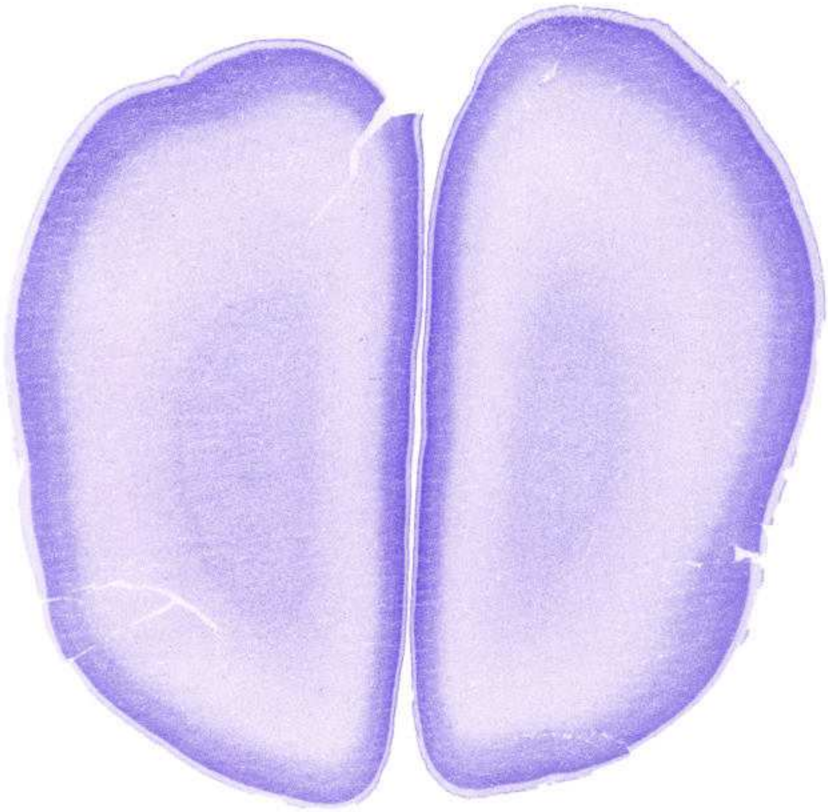

5 mm

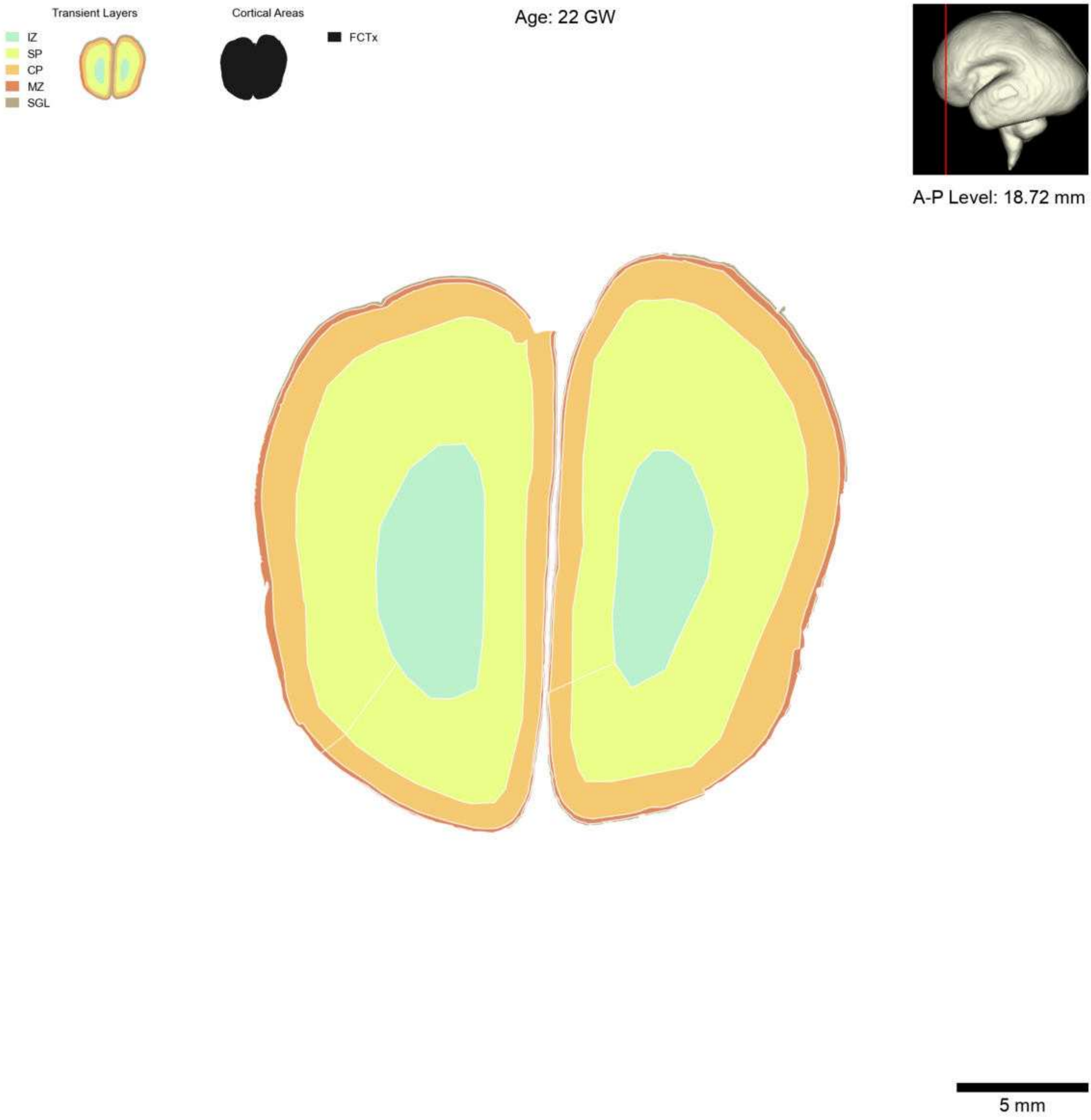
Transient Layers
IZ
SP
CP
MZ
SGL
Cortical Areas
FCTx
Age: 22 GW
A-P Level: 18.72 mm
5 mm

Age: 22 GW

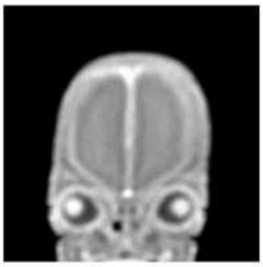

A-P Level: 18.24 mm

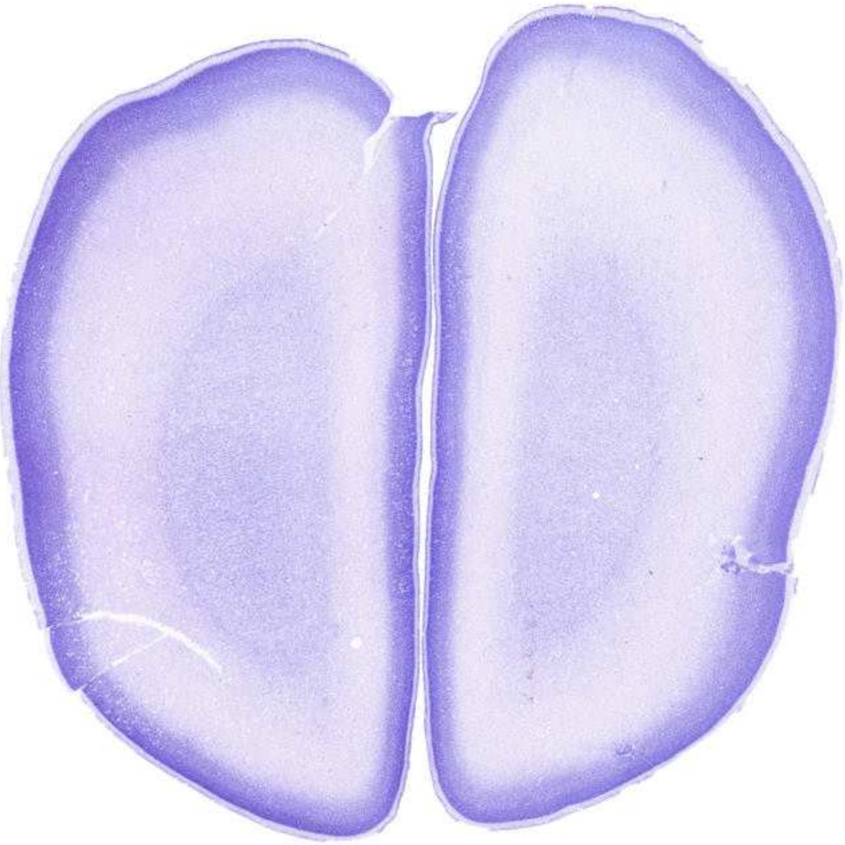

5 mm

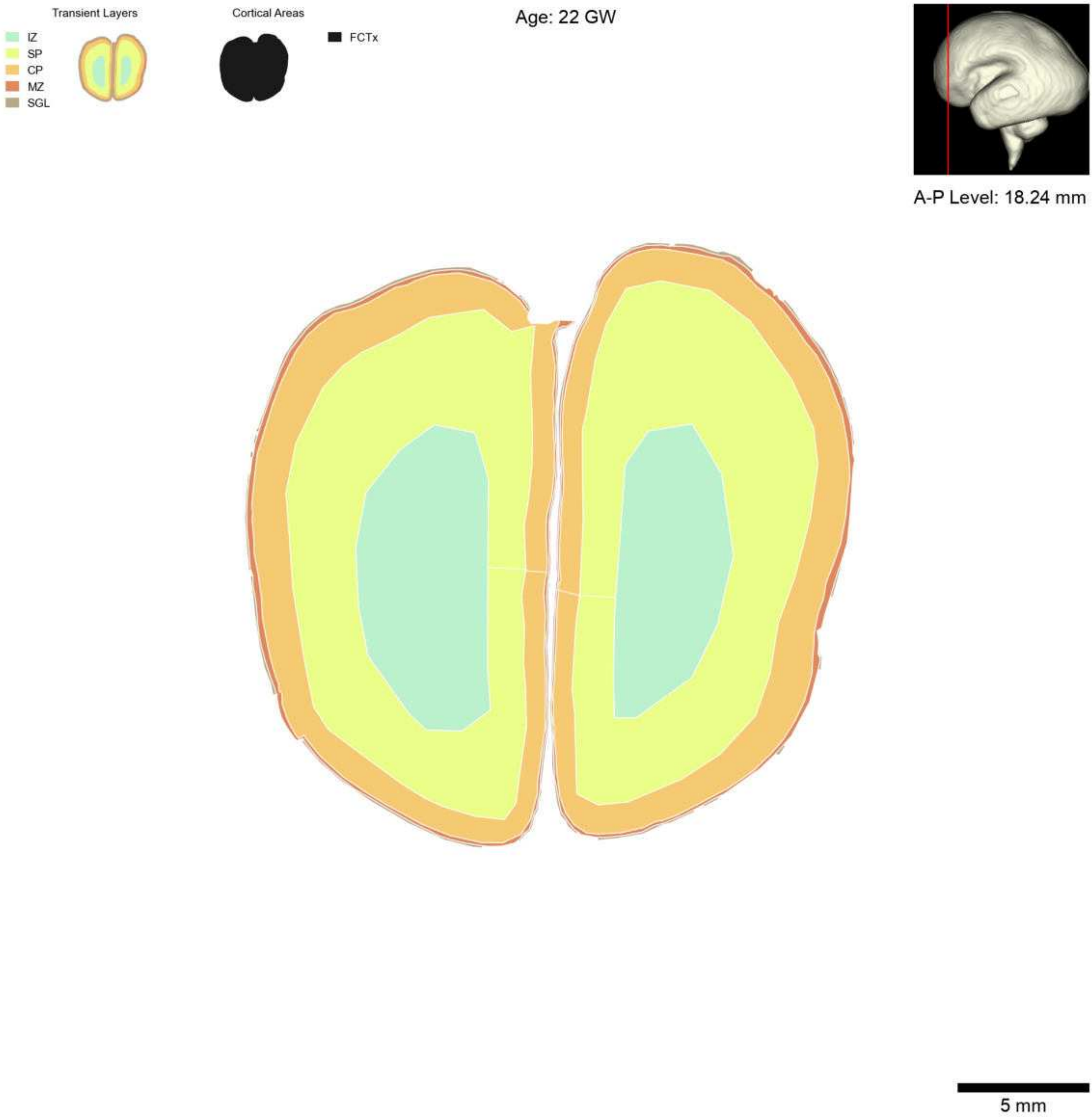

Transient Layers
IZ
SP
CP
MZ
SGL
Cortical Areas
FCTx
Age: 22 GW
A-P Level: 18.24 mm
5 mm

Age: 22 GW

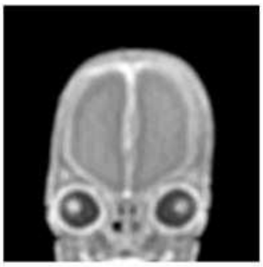

A-P Level: 17.82 mm

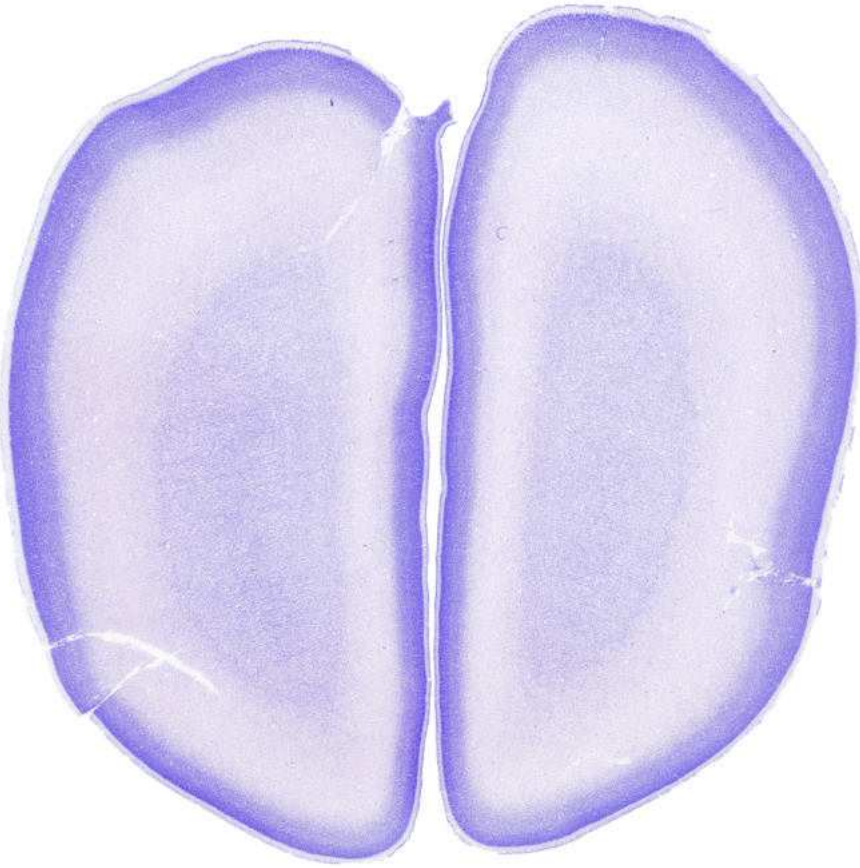

5 mm

Transient Layers

- IZ
- SP
- CP
- MZ
- SGL

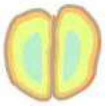

Cortical Areas

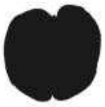

- FCTx

Age: 22 GW

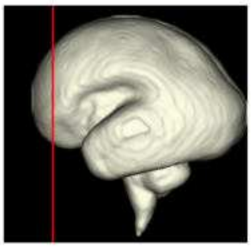

A-P Level: 17.82 mm

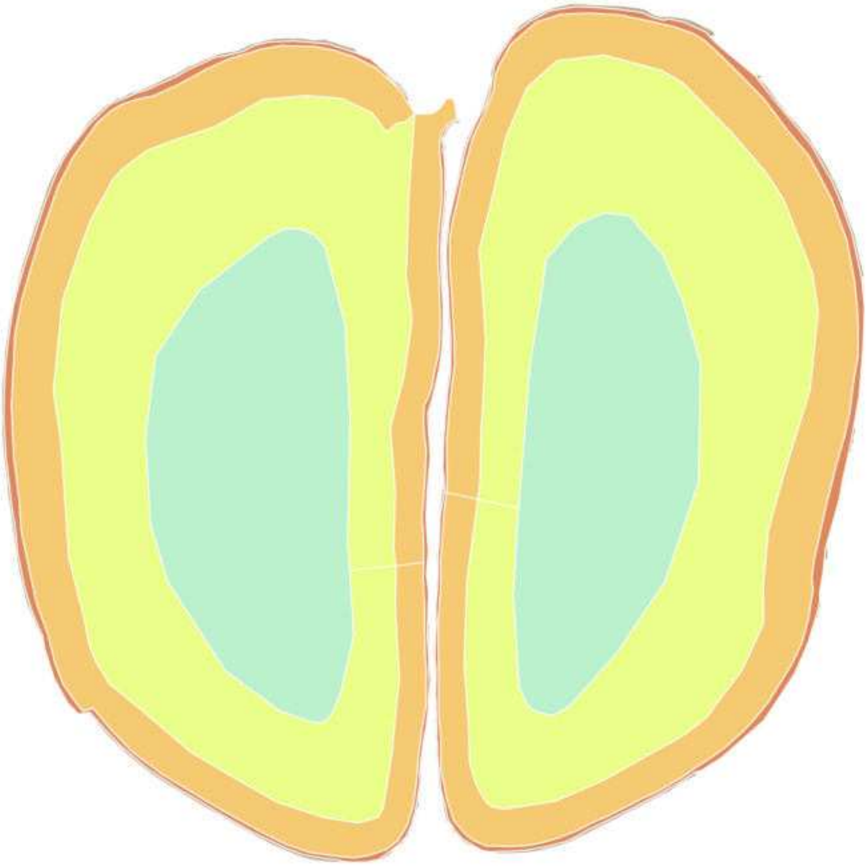

5 mm

Age: 22 GW

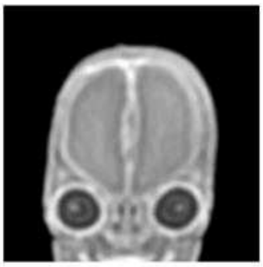
A-P Level: 17.4 mm

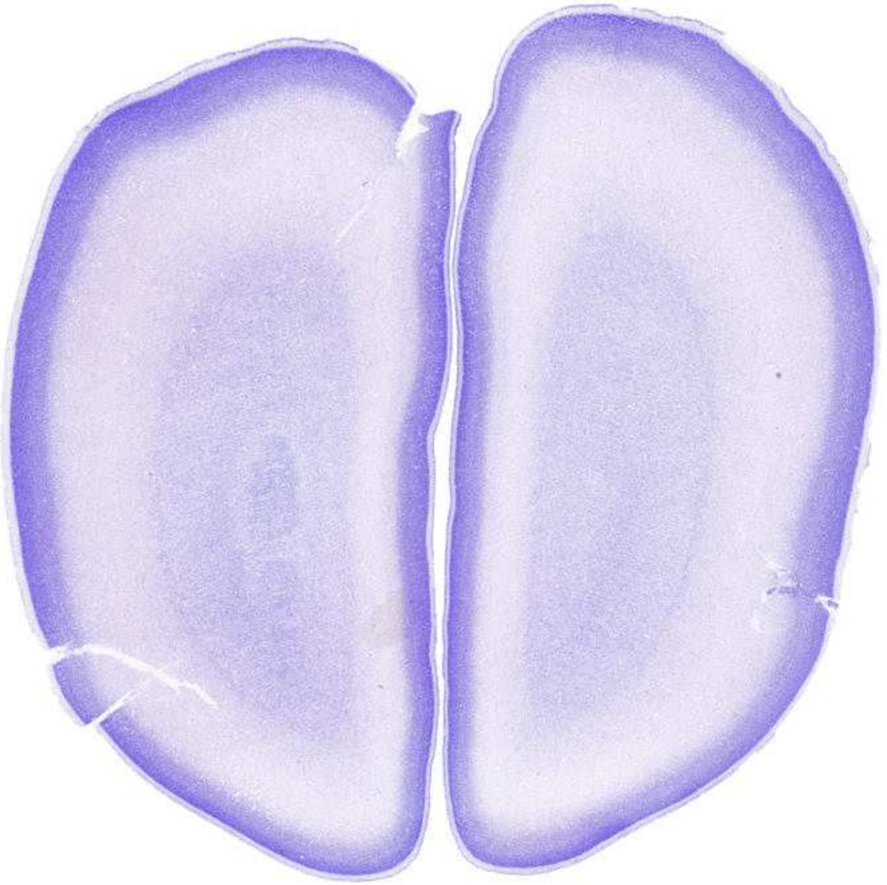

5 mm

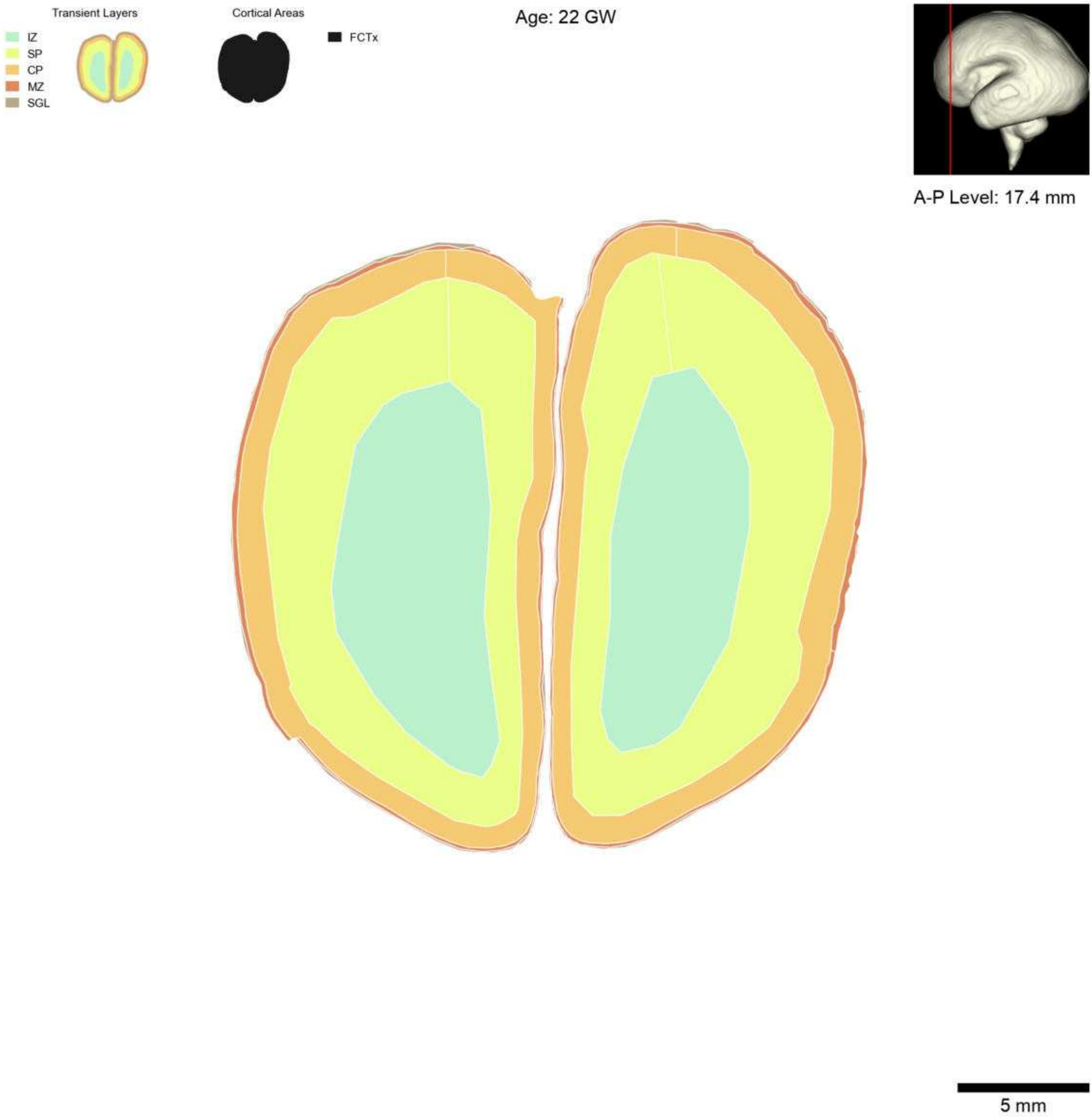

Transient Layers
IZ
SP
CP
MZ
SGL
Cortical Areas
FCTx
Age: 22 GW
A-P Level: 17.4 mm
5 mm

Age: 22 GW

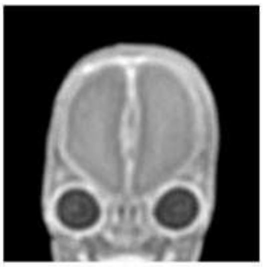

A-P Level: 16.98 mm

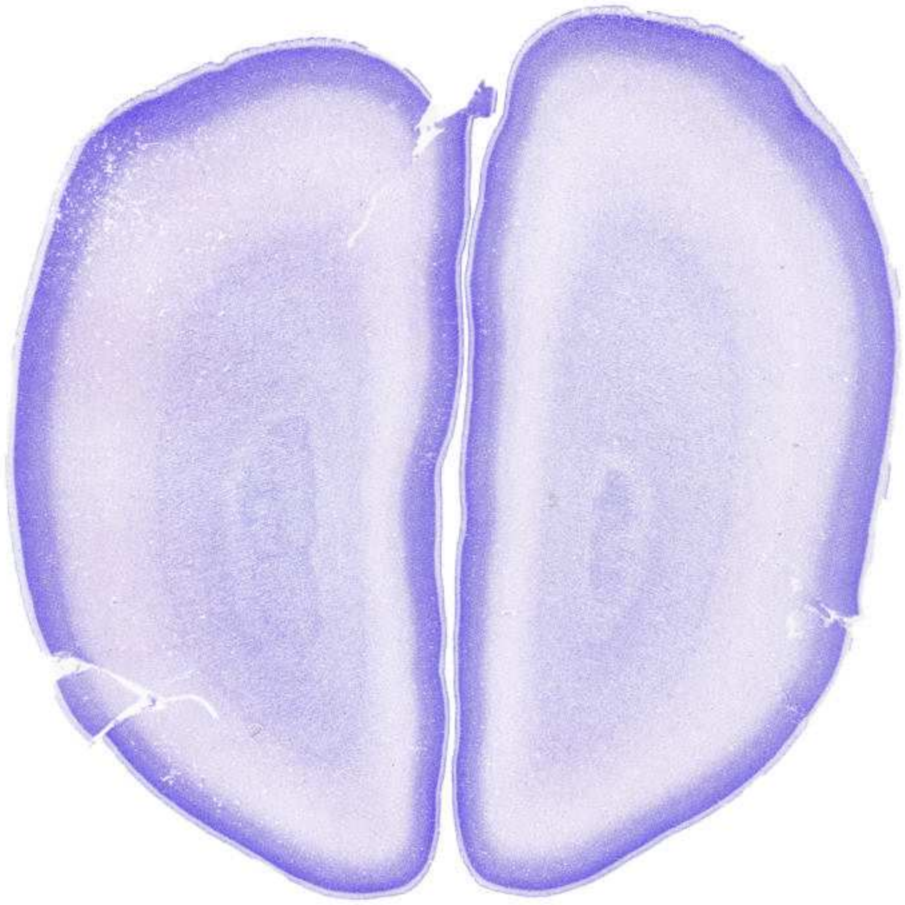

5 mm

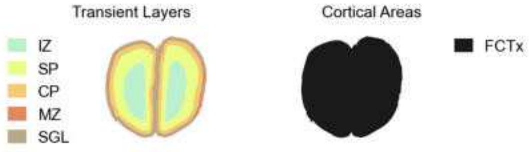

Age: 22 GW

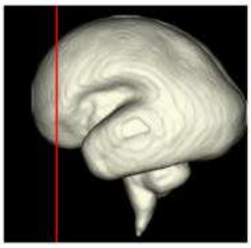

A-P Level: 16.98 mm

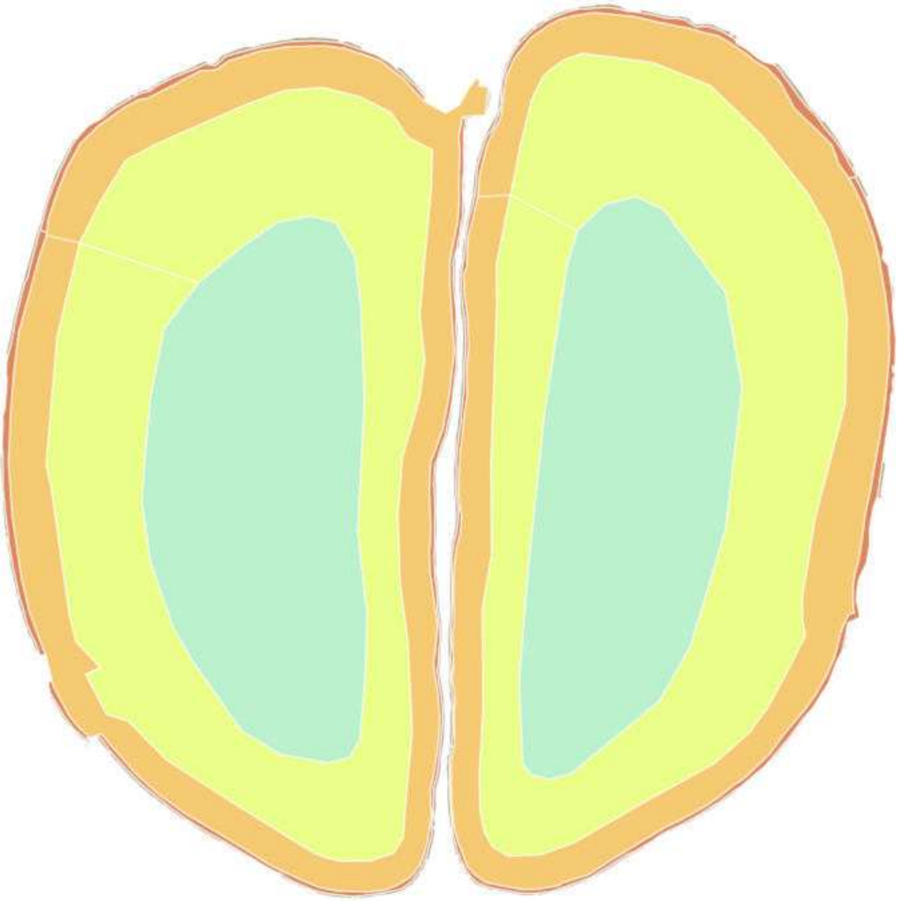

5 mm

Age: 22 GW

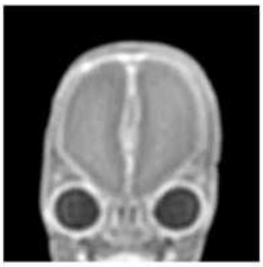

A-P Level: 16.56 mm

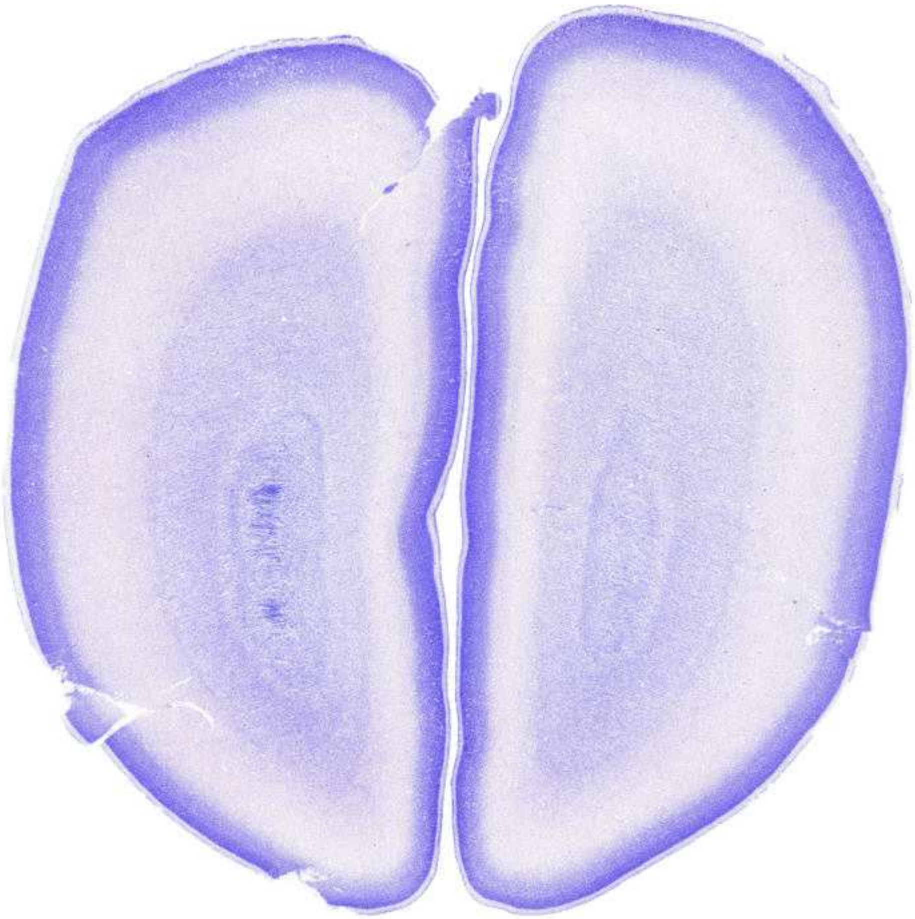

5 mm

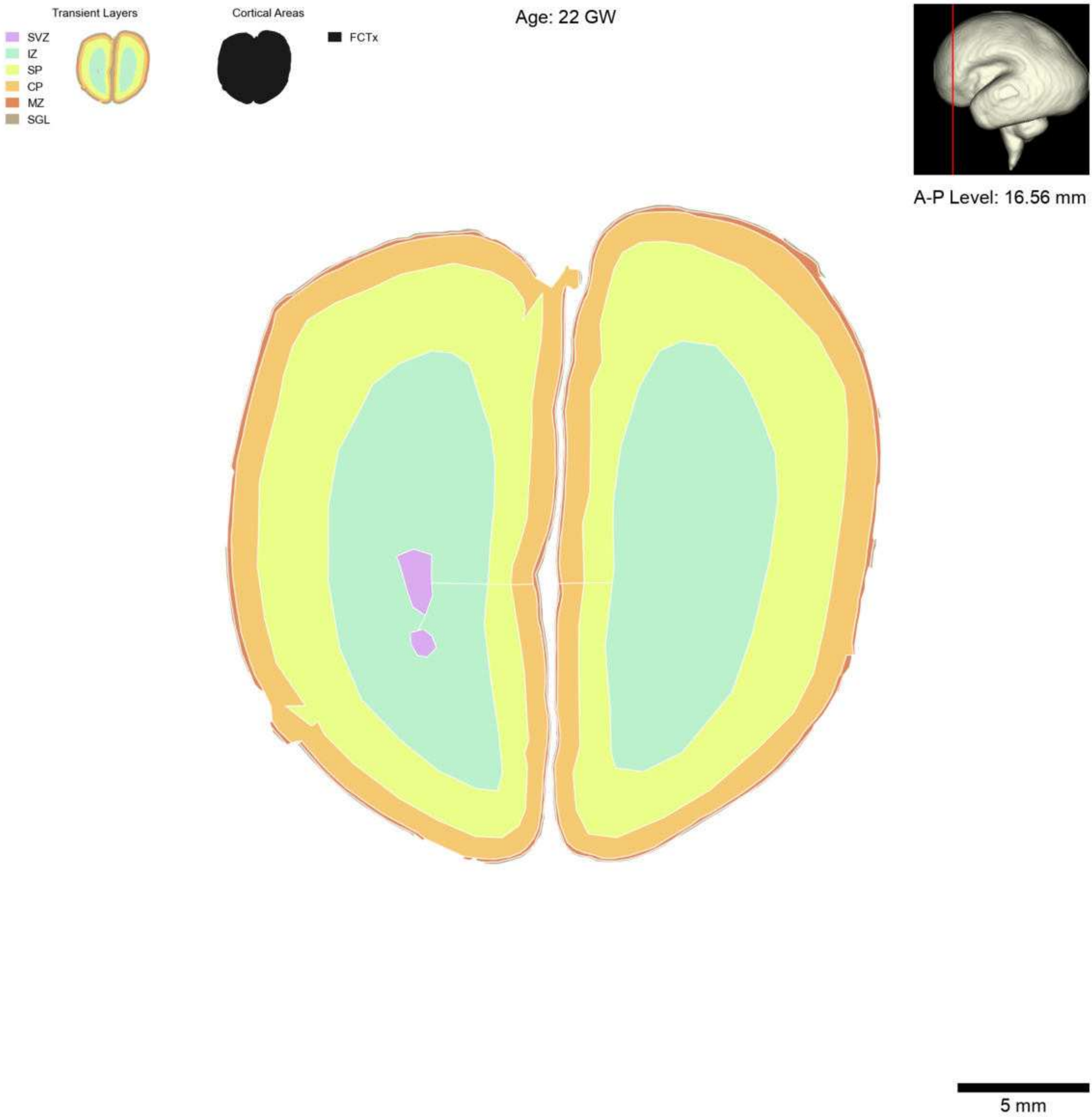

Transient Layers
SVZ
IZ
SP
CP
MZ
SGL
Cortical Areas
FCTx
Age: 22 GW
A-P Level: 16.56 mm
5 mm

Age: 22 GW

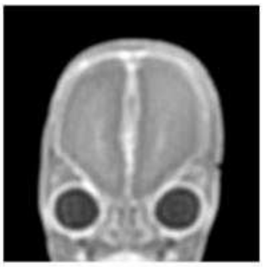

A-P Level: 16.02 mm

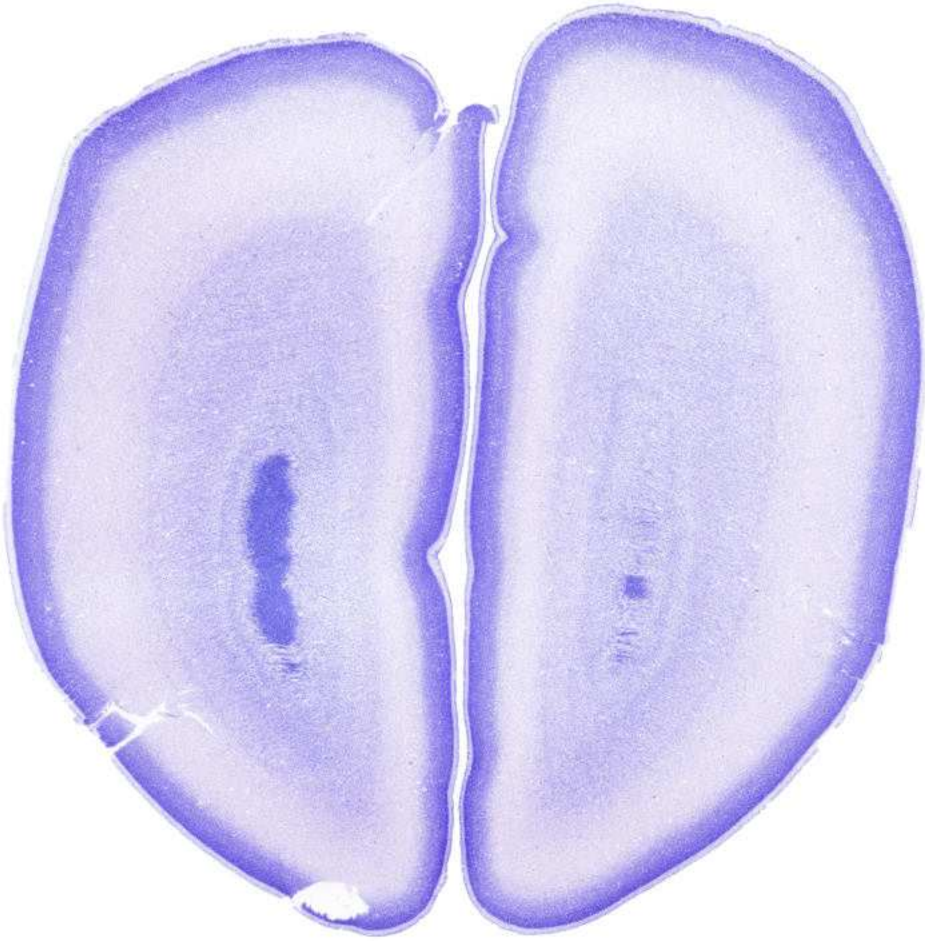

5 mm

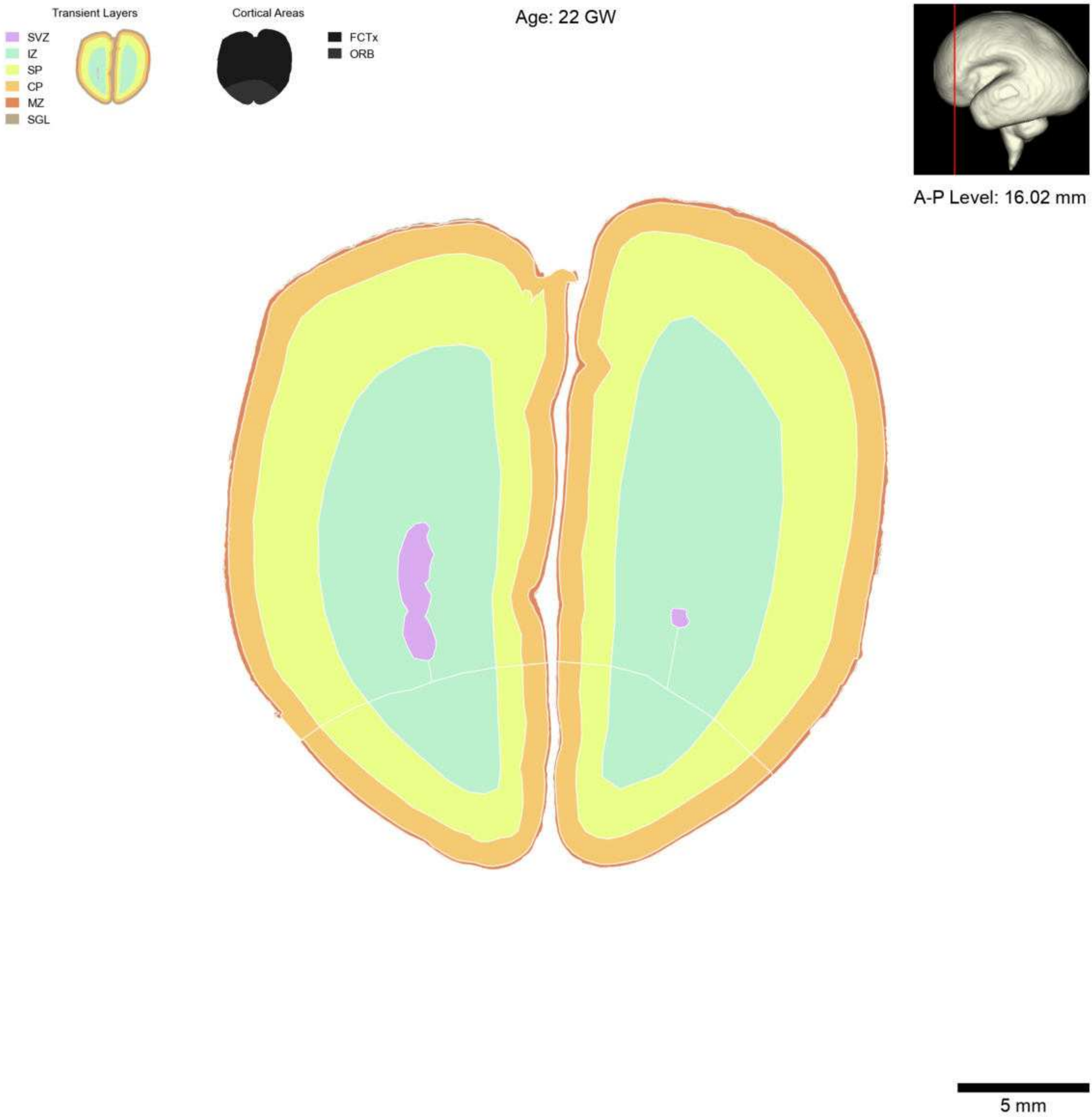

Transient Layers
SVZ
IZ
SP
CP
MZ
SGL
Cortical Areas
FCTx
ORB
Age: 22 GW
A-P Level: 16.02 mm
5 mm

Age: 22 GW

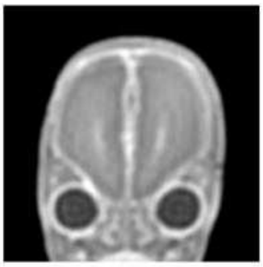

A-P Level: 15.66 mm

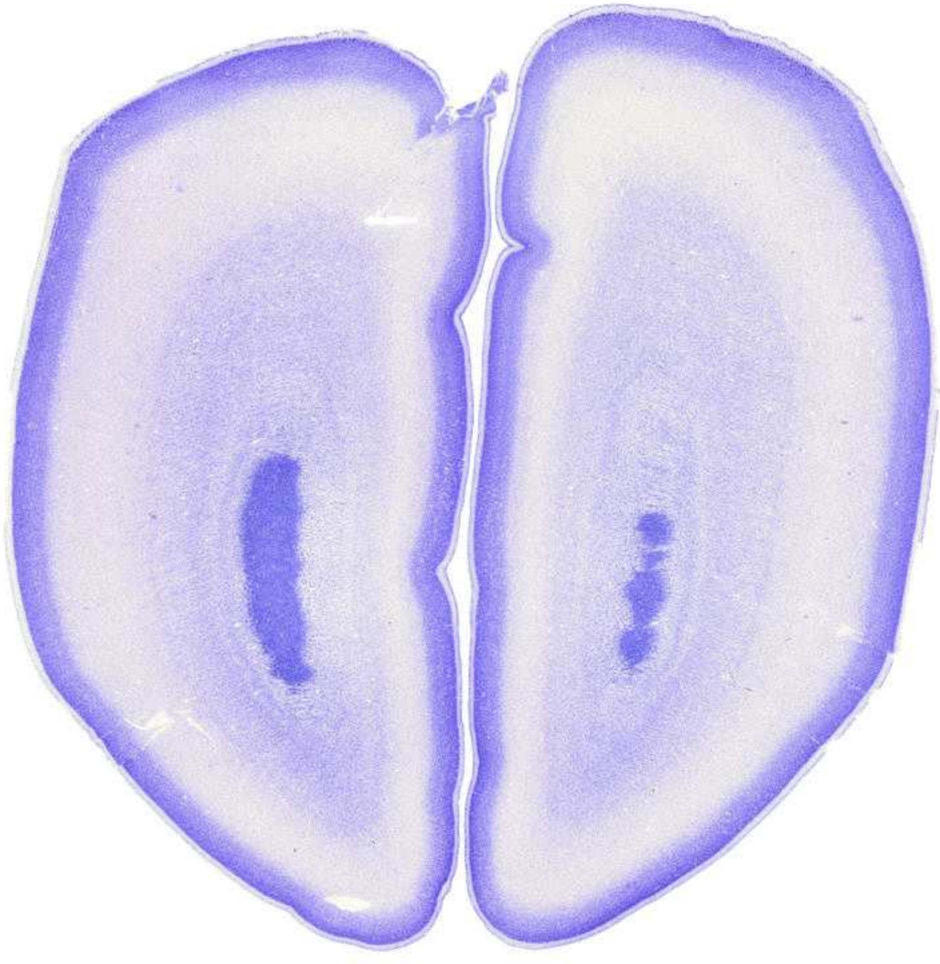

5 mm

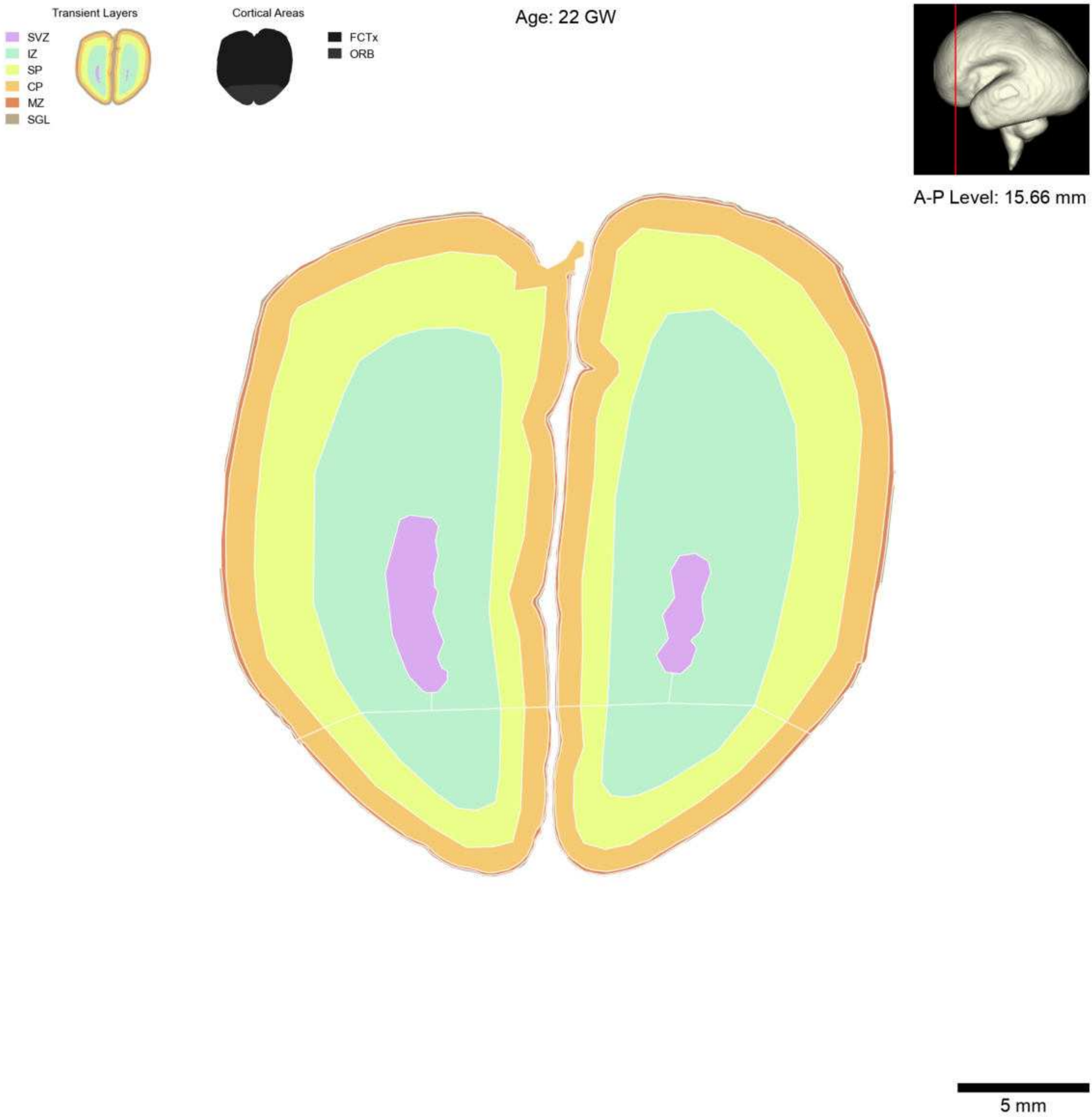

Transient Layers
SVZ
IZ
SP
CP
MZ
SGL
Cortical Areas
FCTx
ORB
Age: 22 GW
A-P Level: 15.66 mm
5 mm

Age: 22 GW

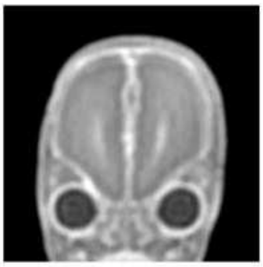

A-P Level: 15.48 mm

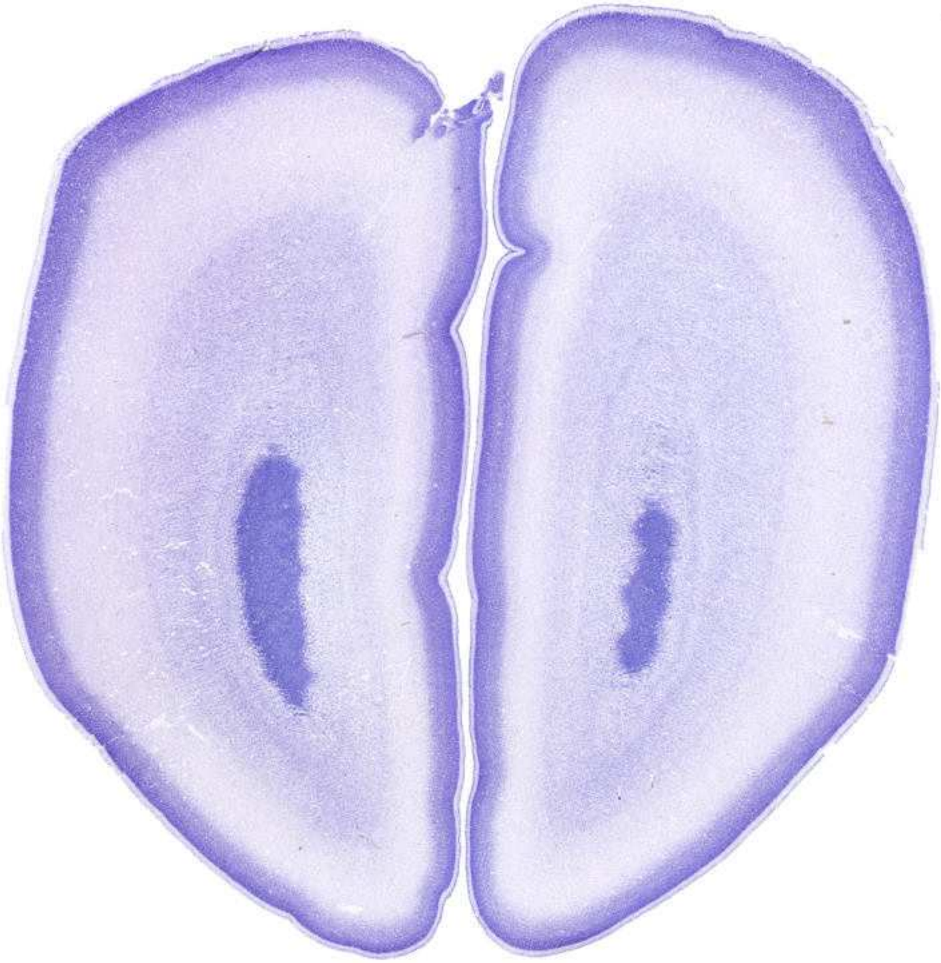

5 mm

Transient Layers

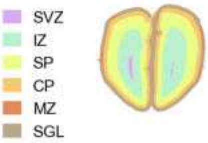

Cortical Areas

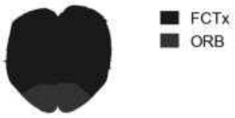

Age: 22 GW

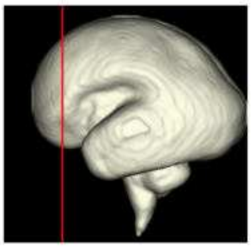

A-P Level: 15.48 mm

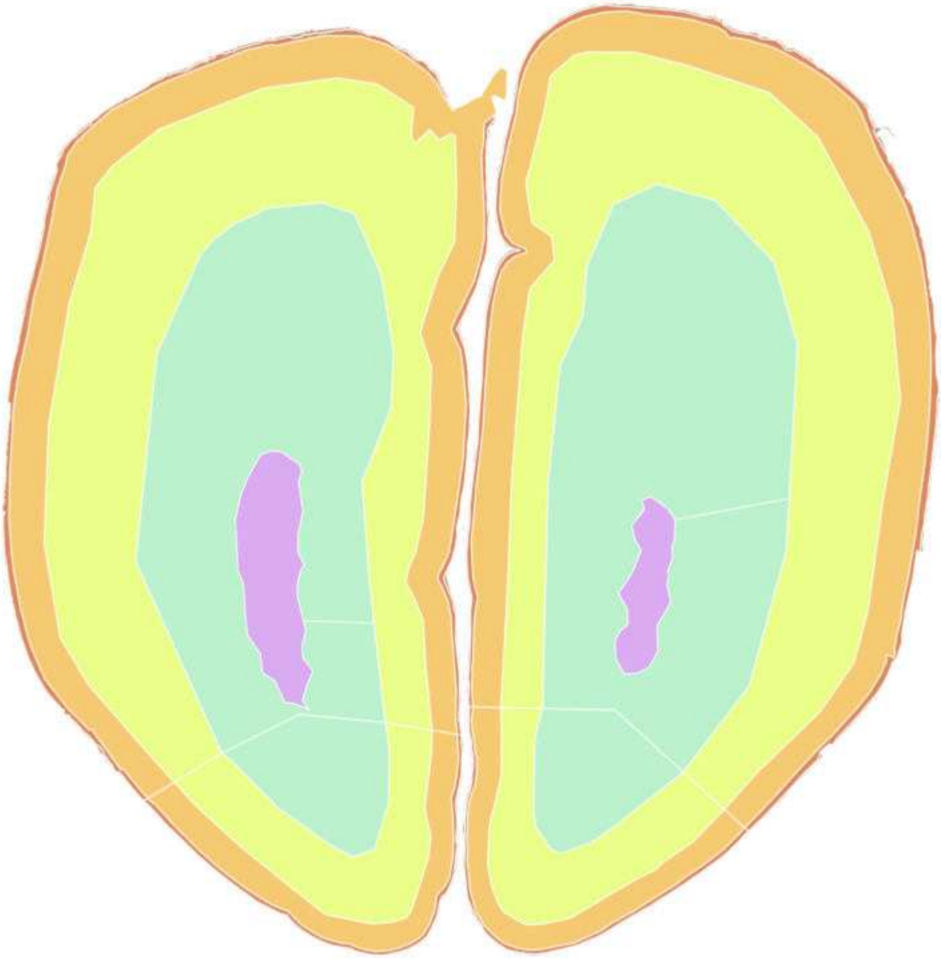

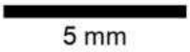

Age: 22 GW

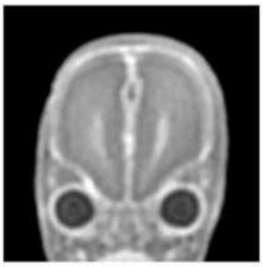

A-P Level: 15.12 mm

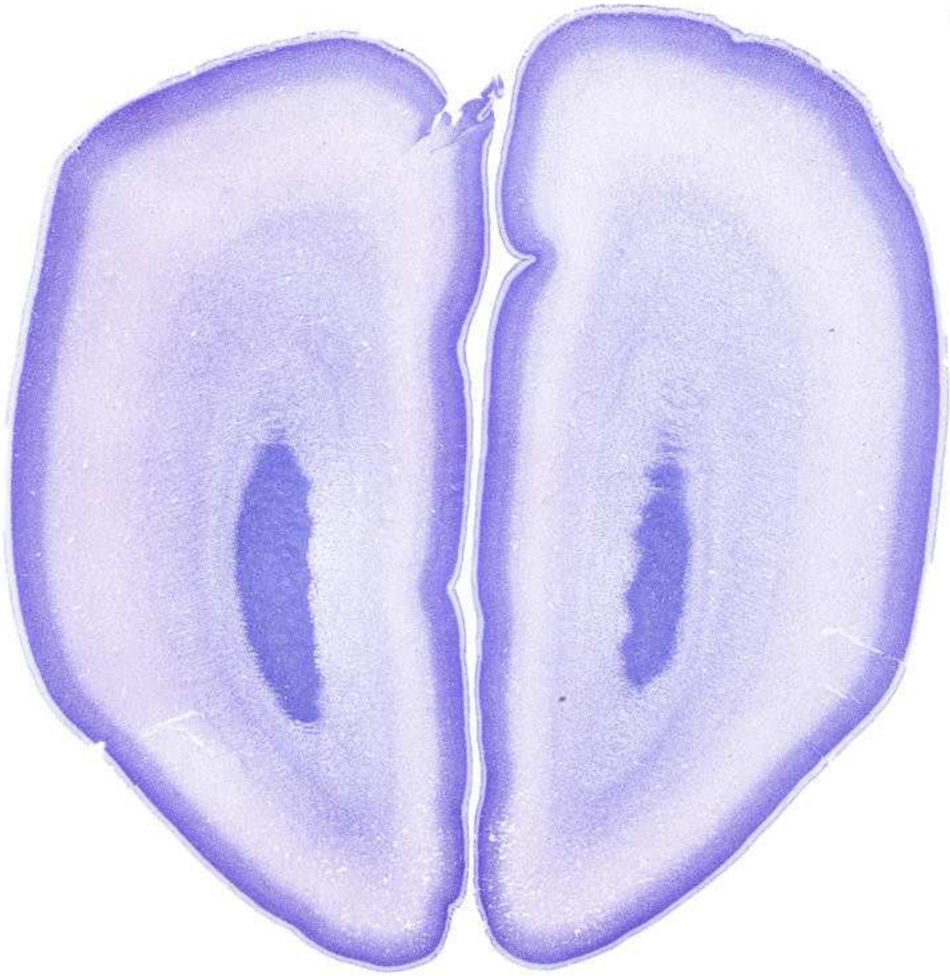

5 mm

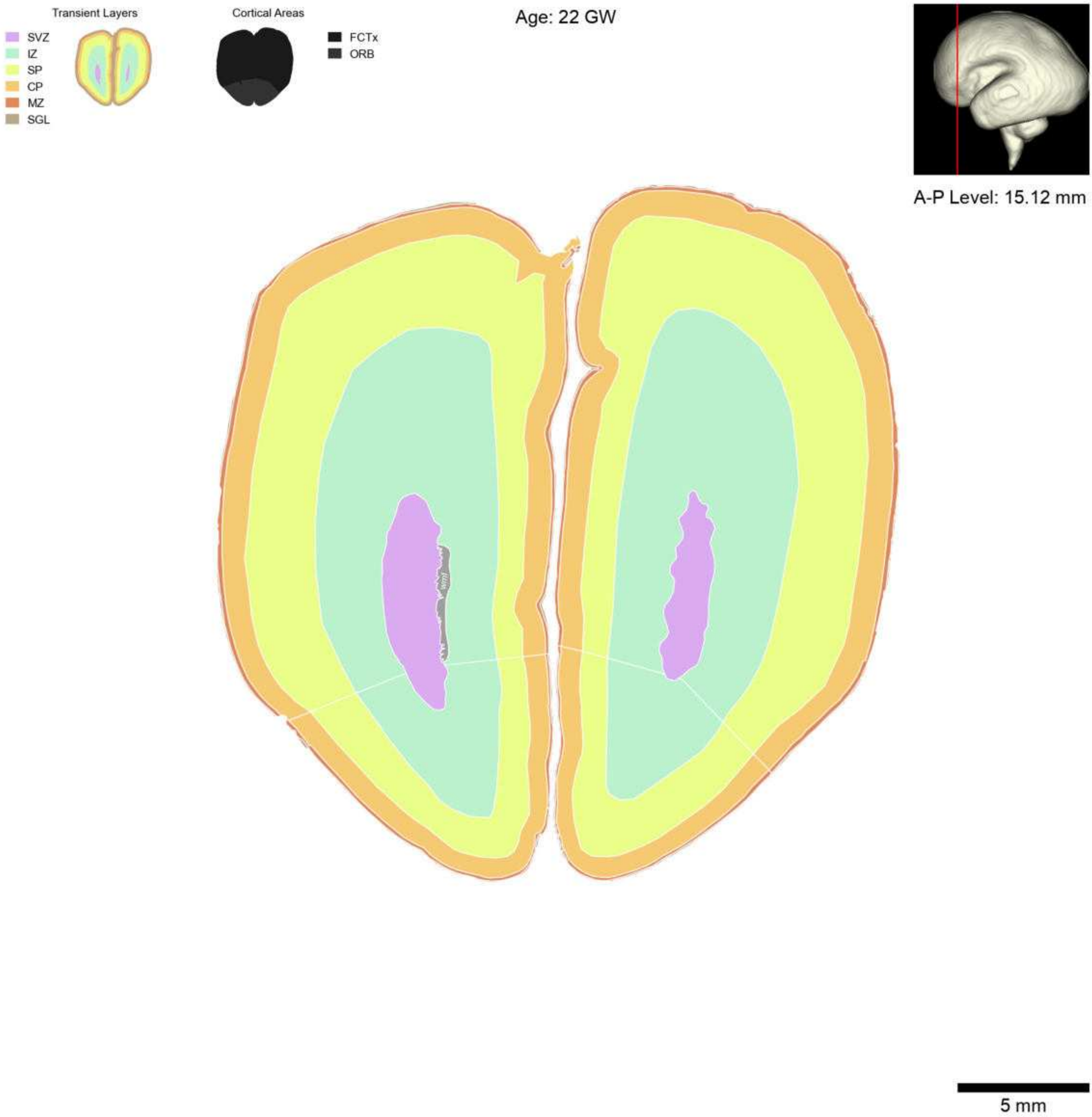

wmf: White matter fibers

Age: 22 GW

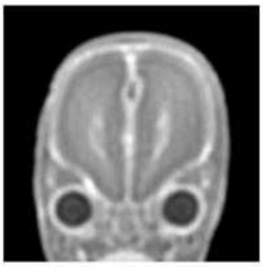

A-P Level: 14.82 mm

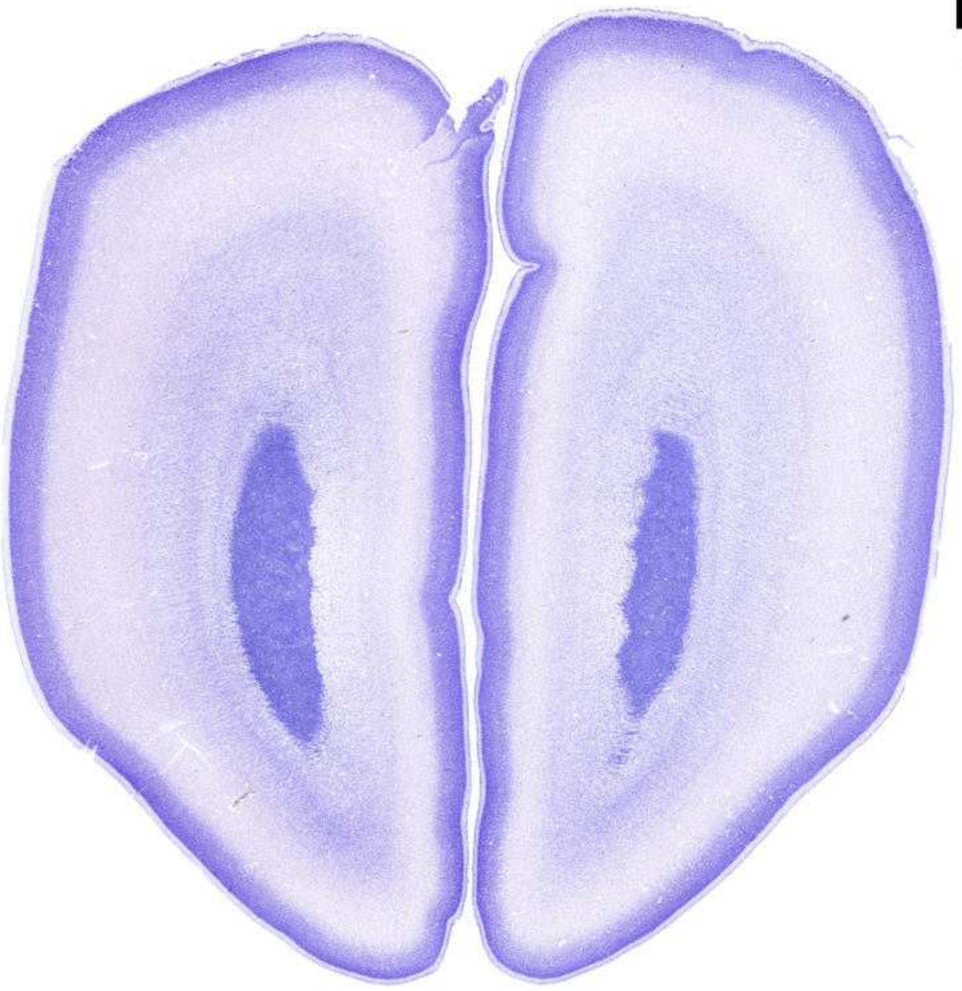

5 mm

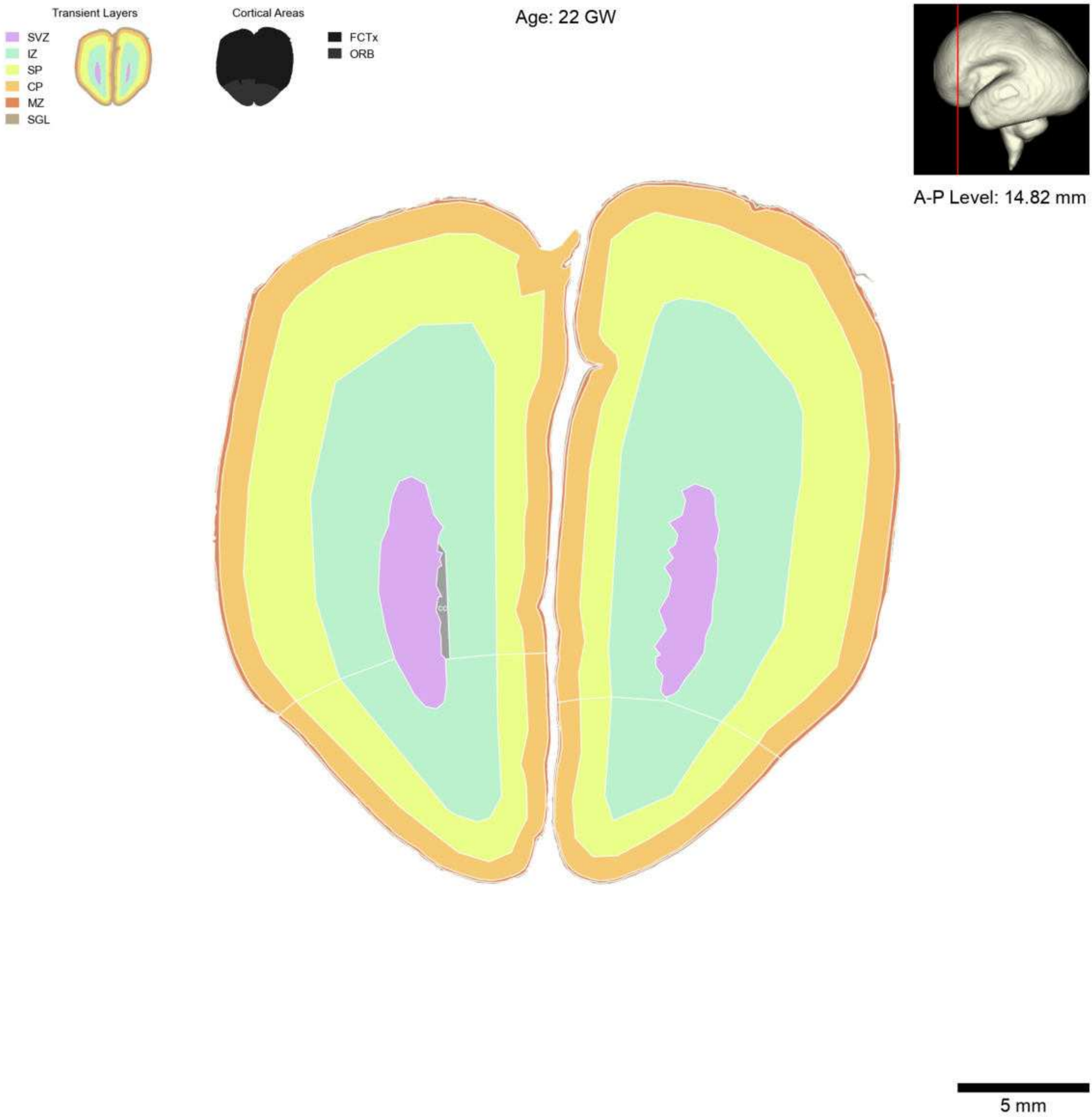

cc: Corpus callosum

Age: 22 GW

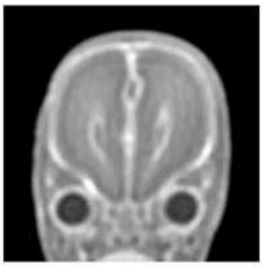

A-P Level: 14.46 mm

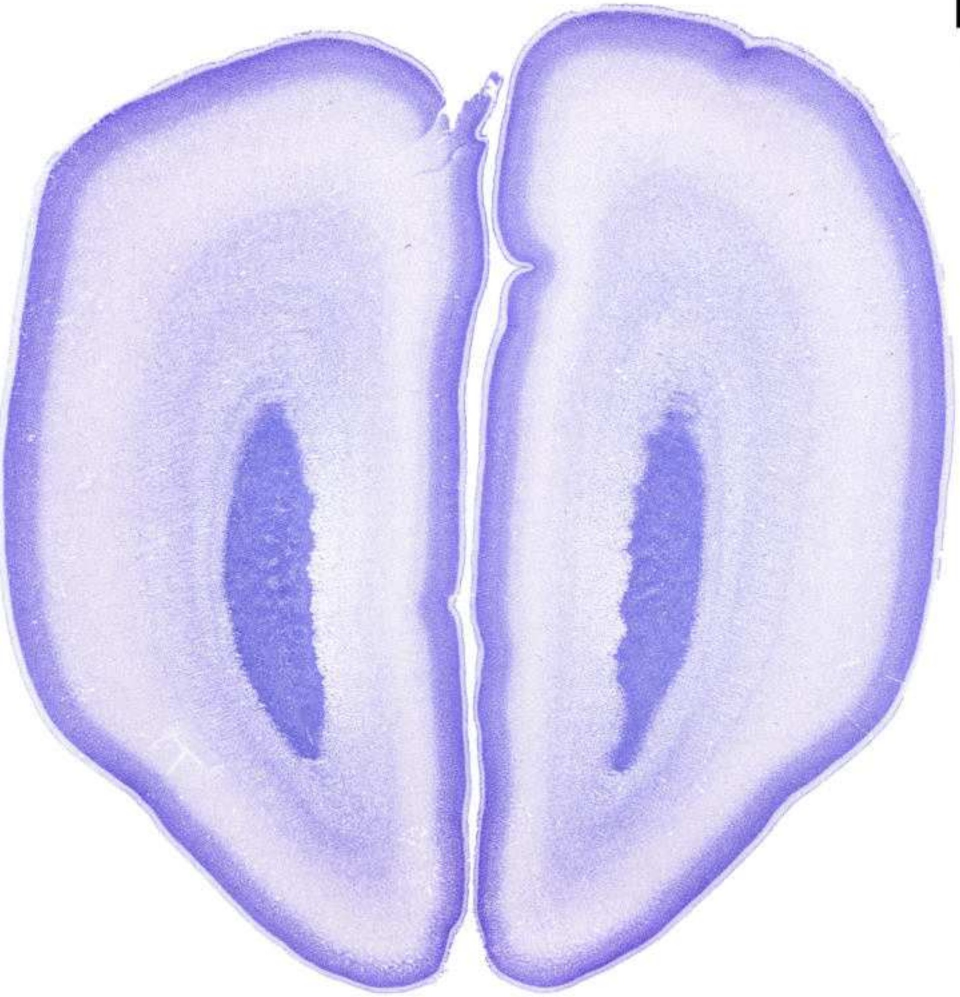

5 mm

Transient Layers

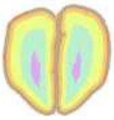

- SVZ
- IZ
- SP
- CP
- MZ
- SGL

Cortical Areas

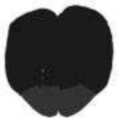

- FCTx
- ORB

Age: 22 GW

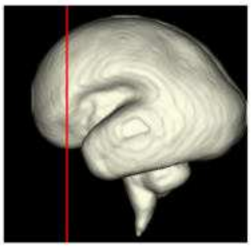

A-P Level: 14.46 mm

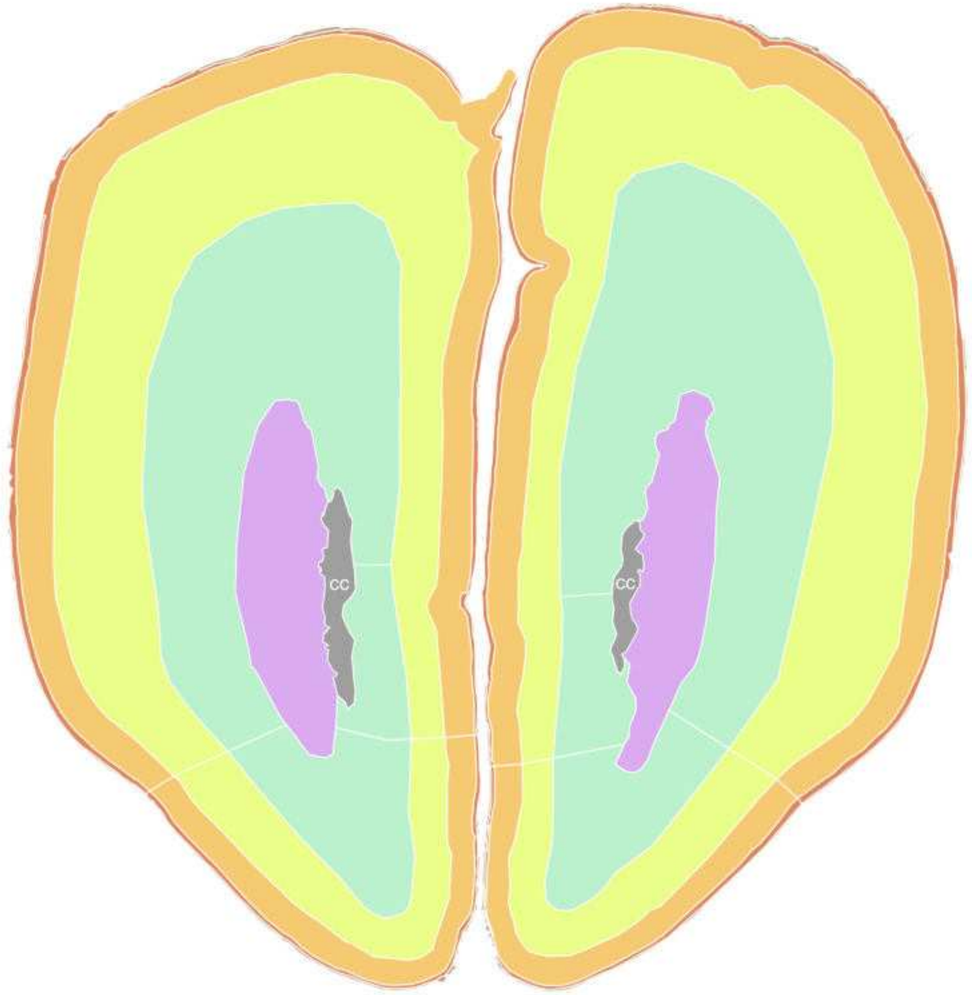

5 mm

cc: Corpus callosum

Age: 22 GW

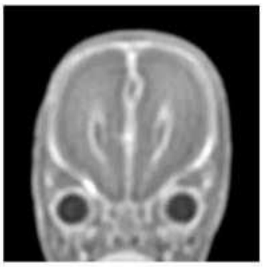

A-P Level: 14.28 mm

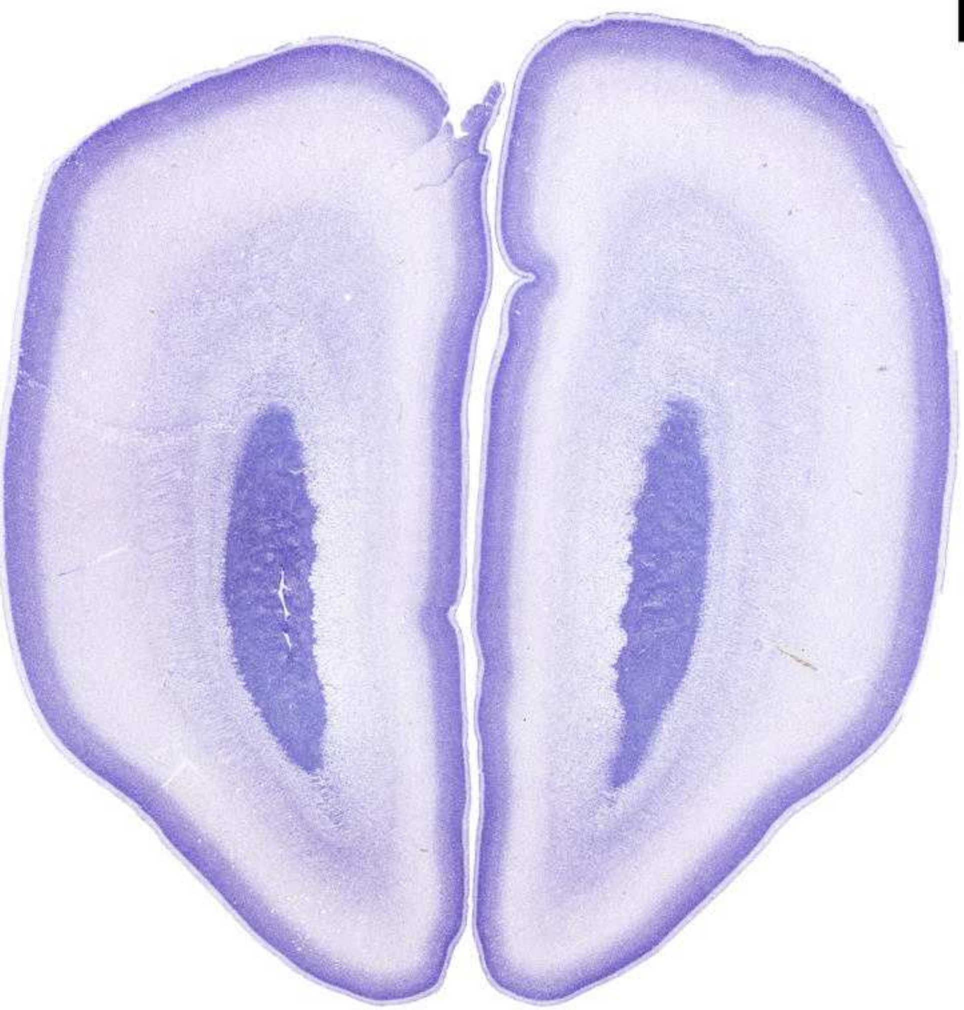

5 mm

Transient Layers

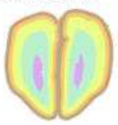

- VZ
- SVZ
- IZ
- SP
- CP
- MZ
- SGL

Cortical Areas

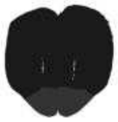

- FCTx
- ORB

Age: 22 GW

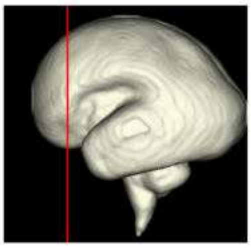

A-P Level: 14.28 mm

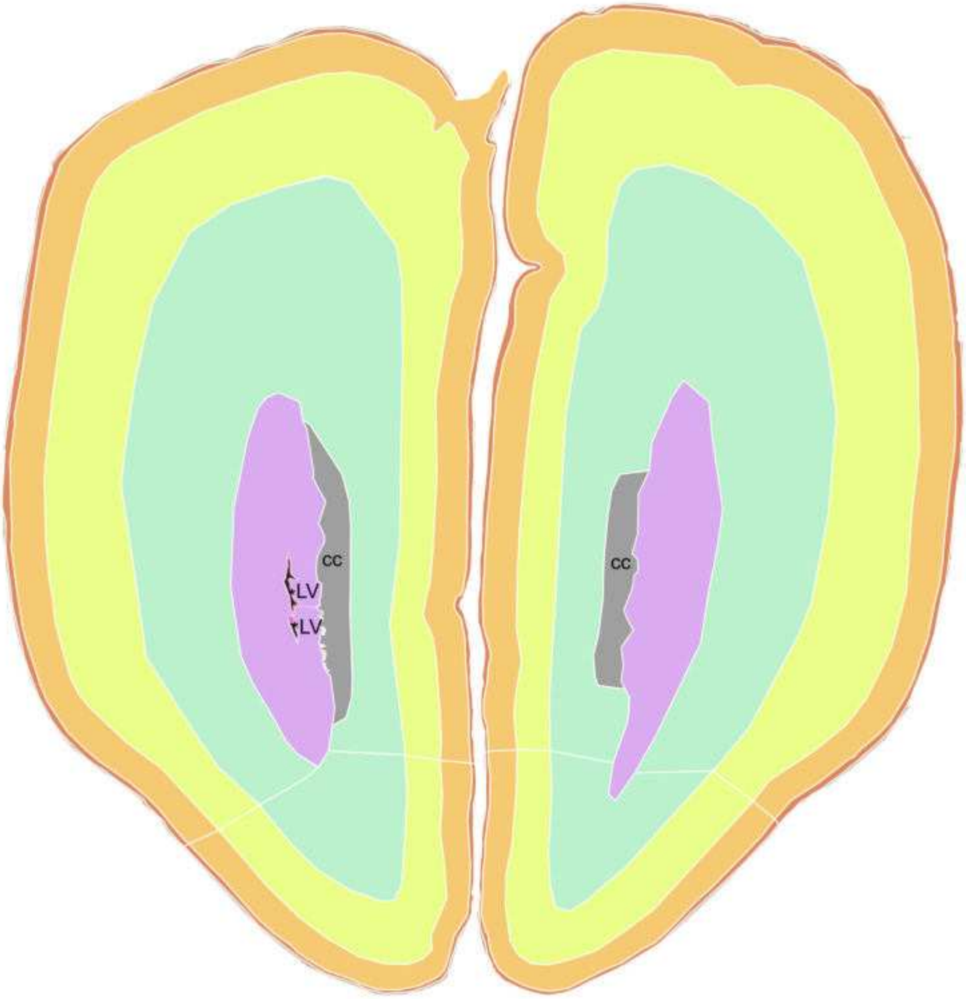

5 mm

LV: Lateral ventricle   cc: Corpus callosum

Age: 22 GW

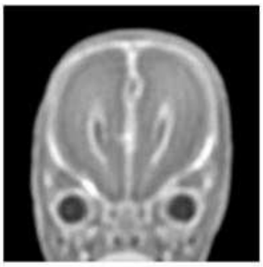

A-P Level: 13.92 mm

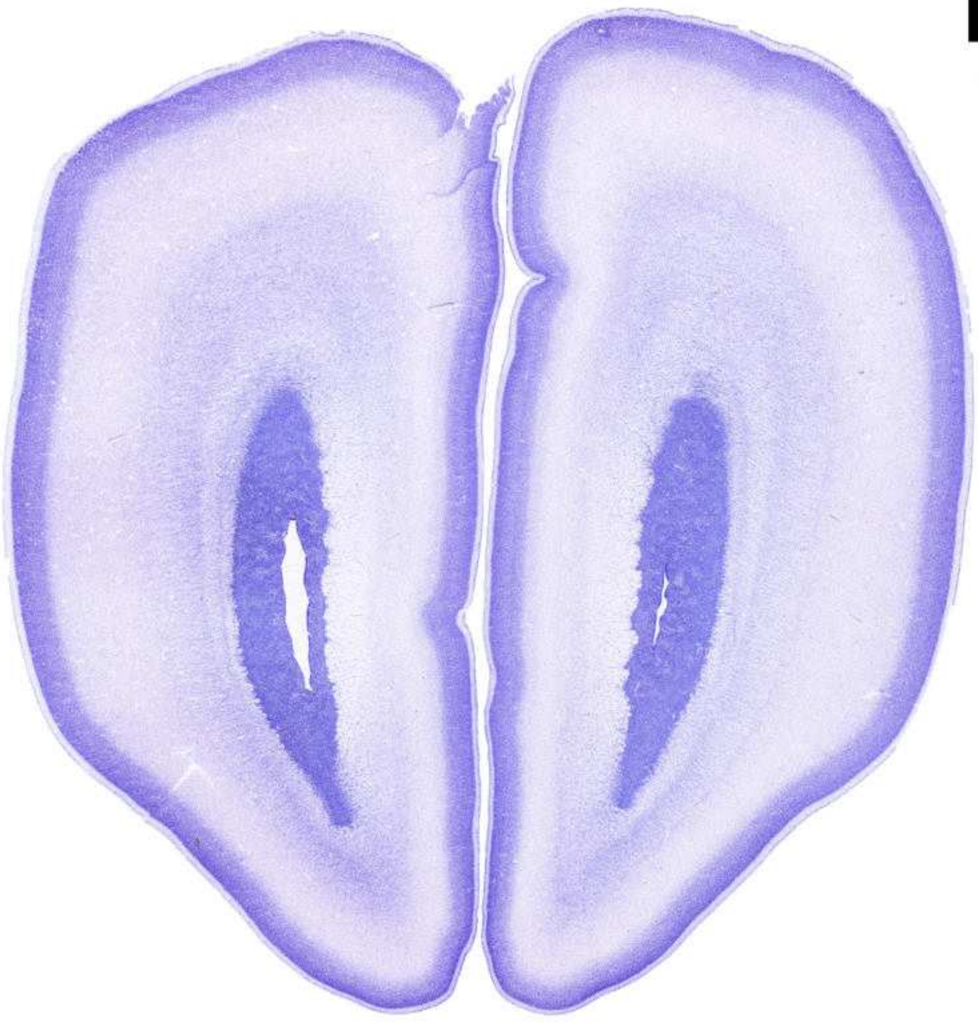

5 mm

Transient Layers

- SVZ
- IZ
- SP
- CP
- MZ
- SGL

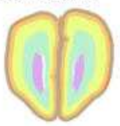

Cortical Areas

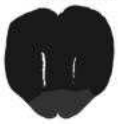

- FCTx
- ORB

Age: 22 GW

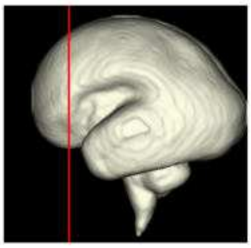

A-P Level: 13.92 mm

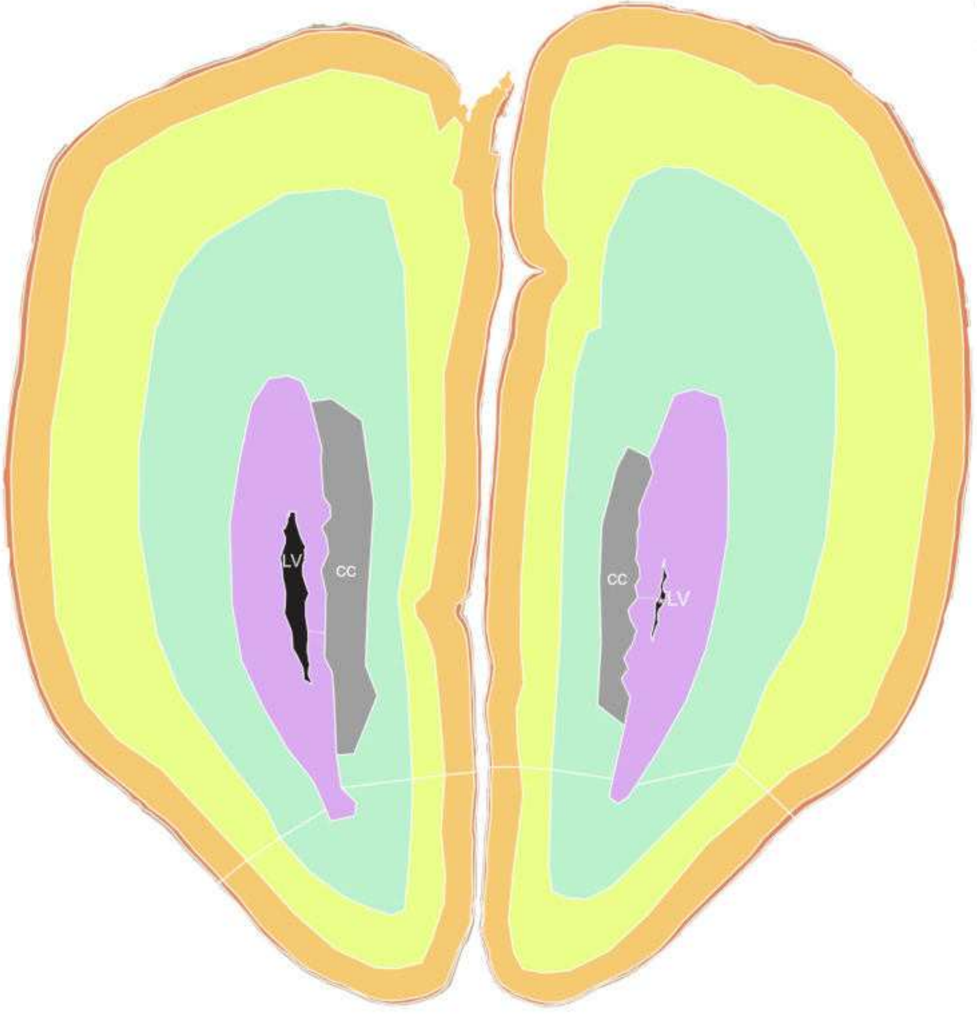

5 mm

LV: Lateral ventricle   cc: Corpus callosum

Age: 22 GW

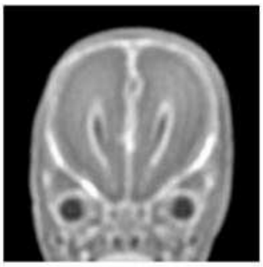

A-P Level: 13.56 mm

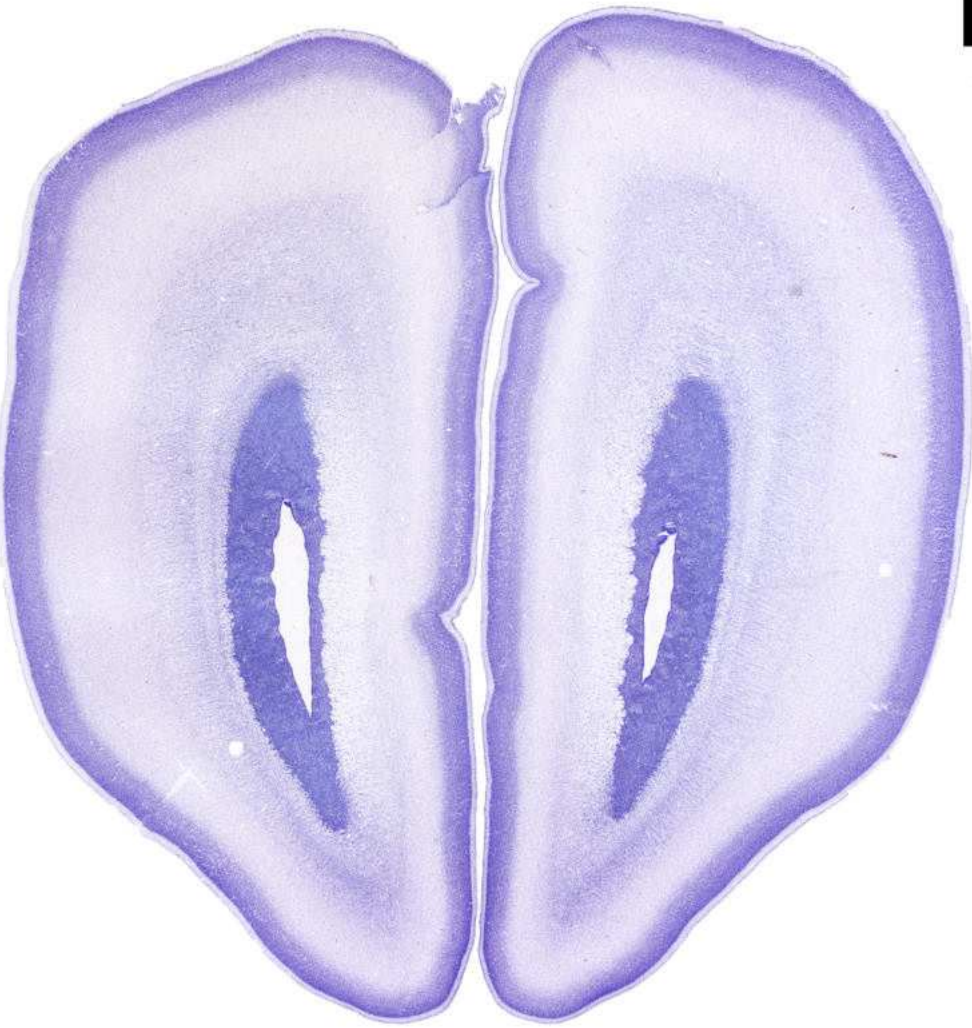

5 mm

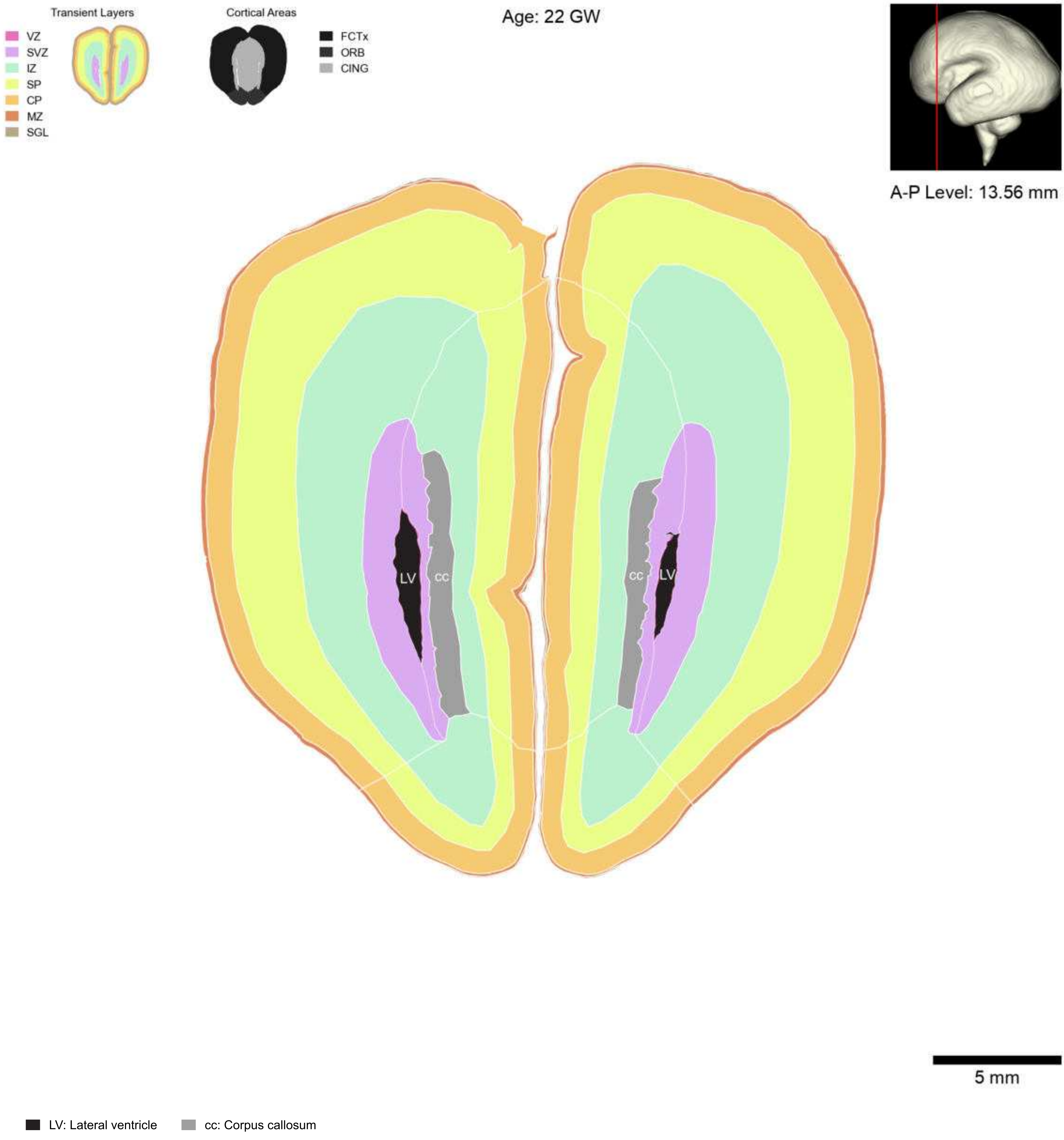
Transient Layers
VZ
SVZ
IZ
SP
CP
MZ
SGL
Cortical Areas
FCTx
ORB
CING
Age: 22 GW
A-P Level: 13.56 mm
LV
cc
cc
LV
5 mm
LV: Lateral ventricle
cc: Corpus callosum

Age: 22 GW

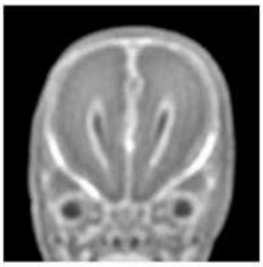

A-P Level: 13.14 mm

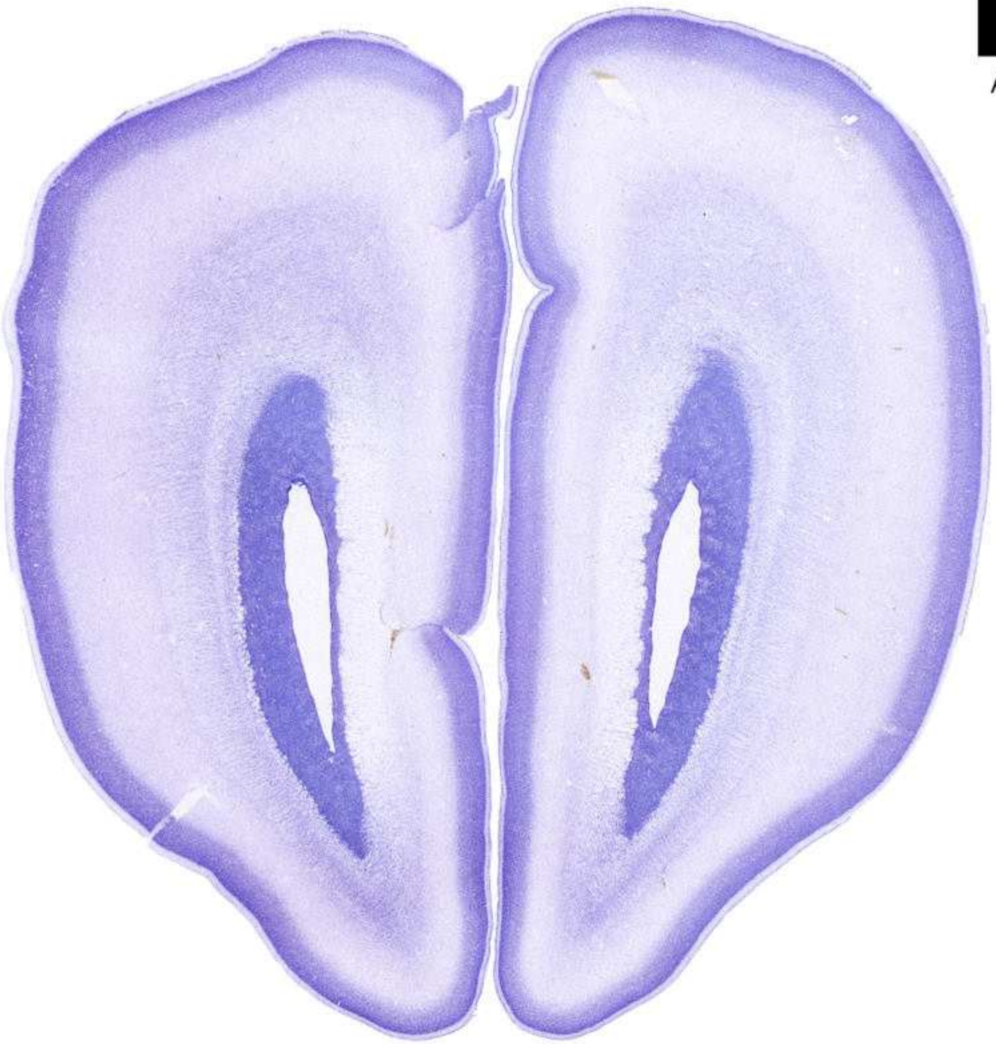

5 mm

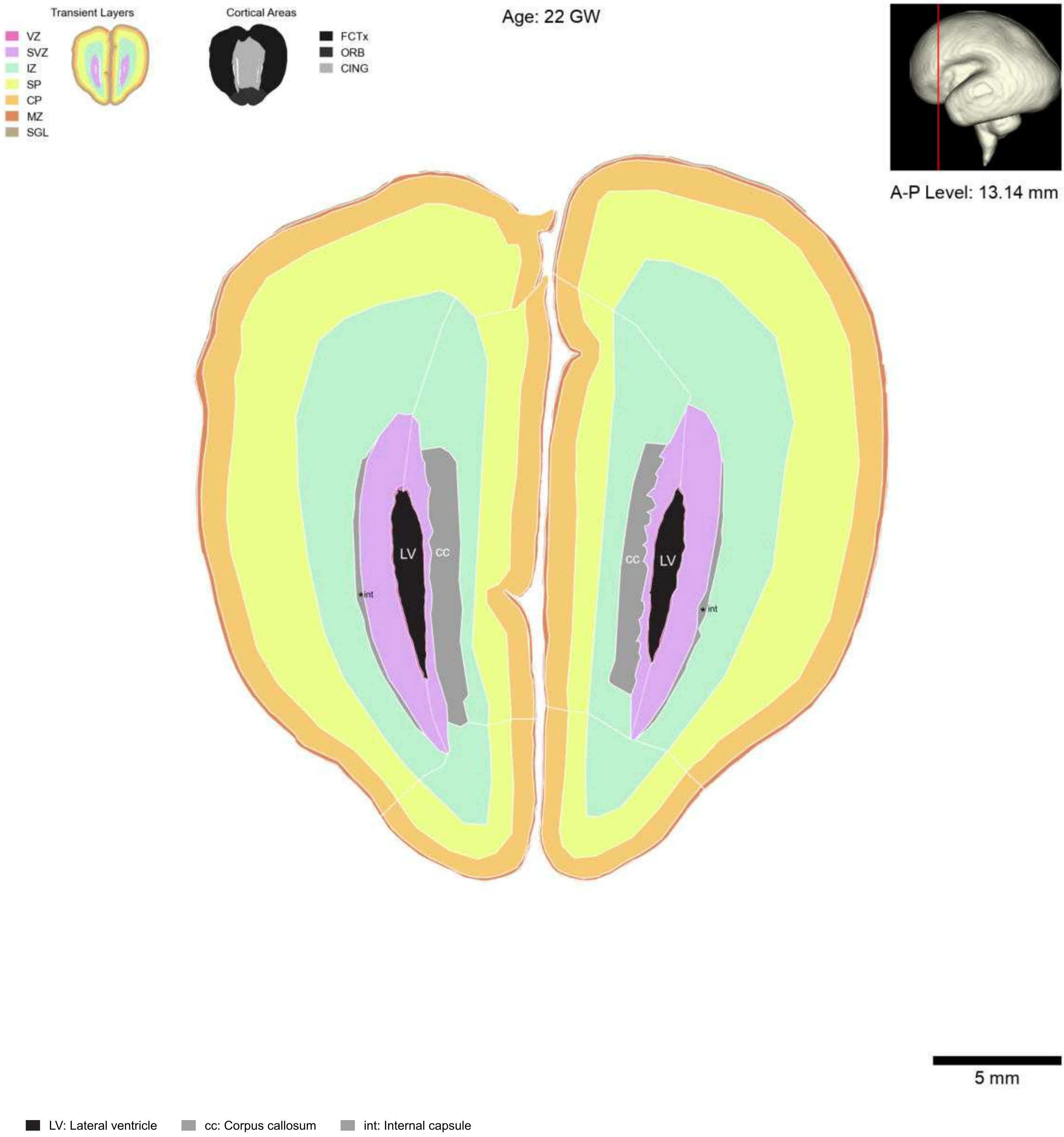
Transient Layers
VZ
SVZ
IZ
SP
CP
MZ
SGL
Cortical Areas
FCTx
ORB
CING
Age: 22 GW
A-P Level: 13.14 mm
LV
cc
int
cc
LV
int
5 mm
LV: Lateral ventricle
cc: Corpus callosum
int: Internal capsule

Age: 22 GW

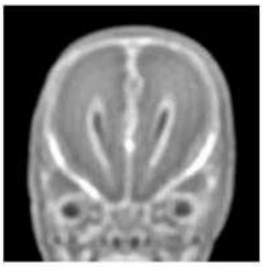

A-P Level: 12.96 mm

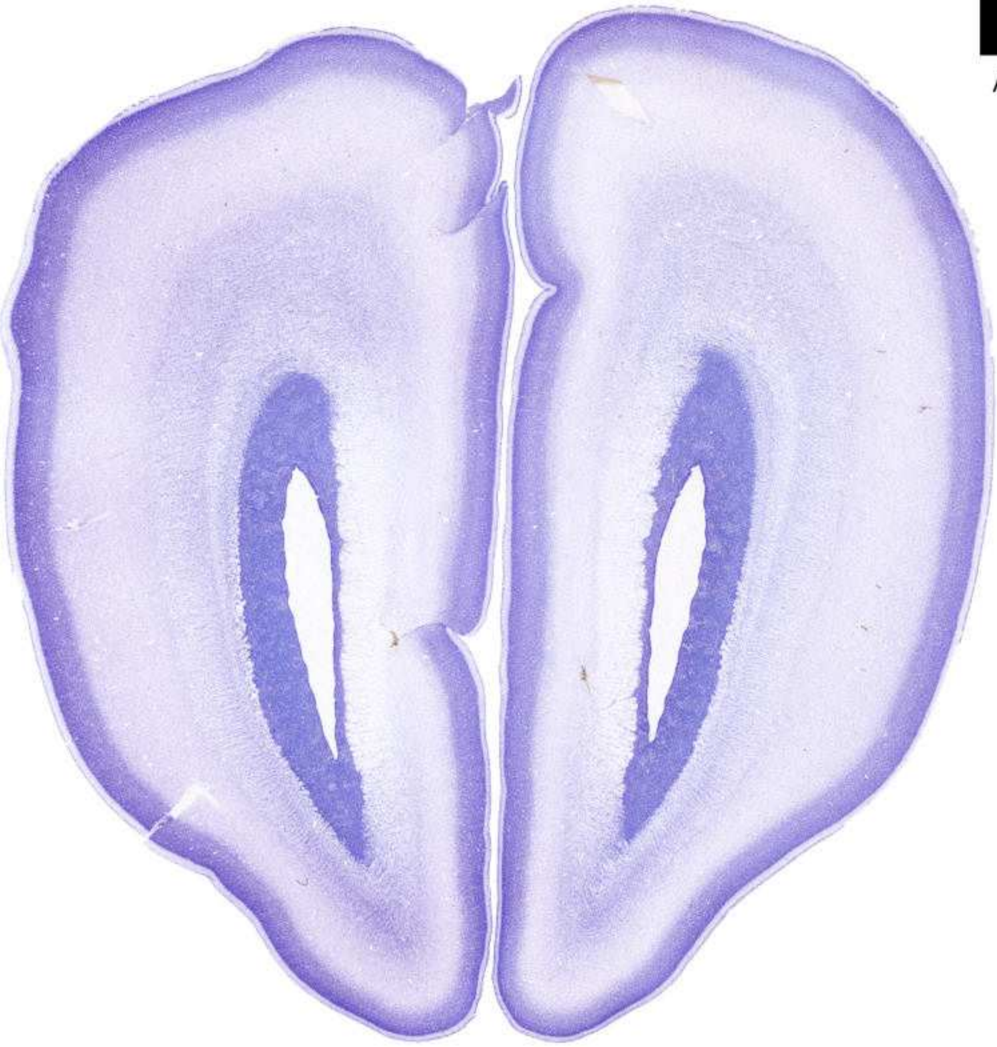

5 mm

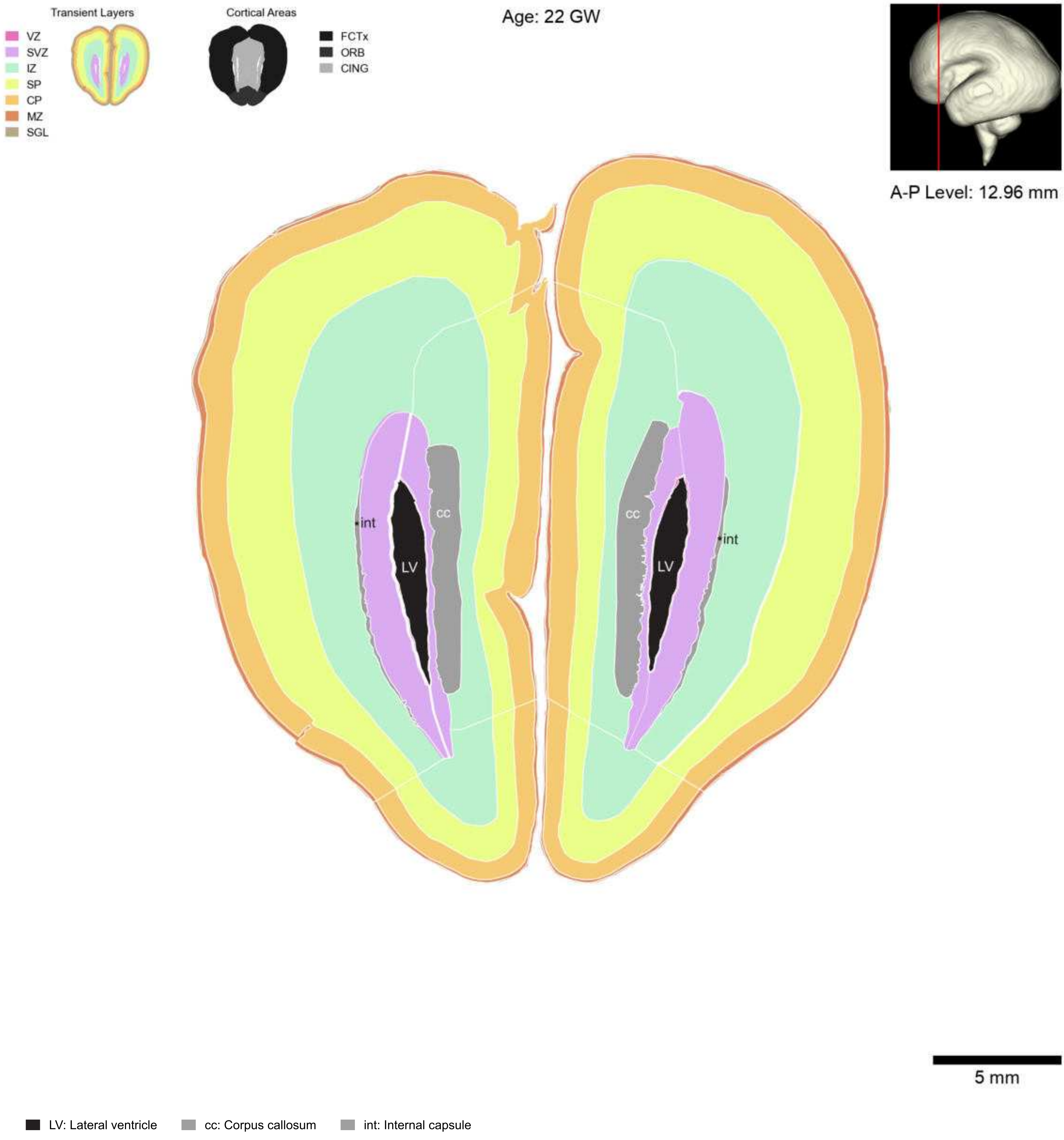
Transient Layers
VZ
SVZ
IZ
SP
CP
MZ
SGL
Cortical Areas
FCTx
ORB
CING
Age: 22 GW
A-P Level: 12.96 mm
int
cc
LV
cc
LV
int
5 mm
LV: Lateral ventricle
cc: Corpus callosum
int: Internal capsule

Age: 22 GW

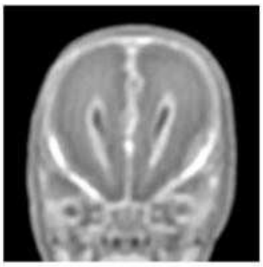

A-P Level: 12.36 mm

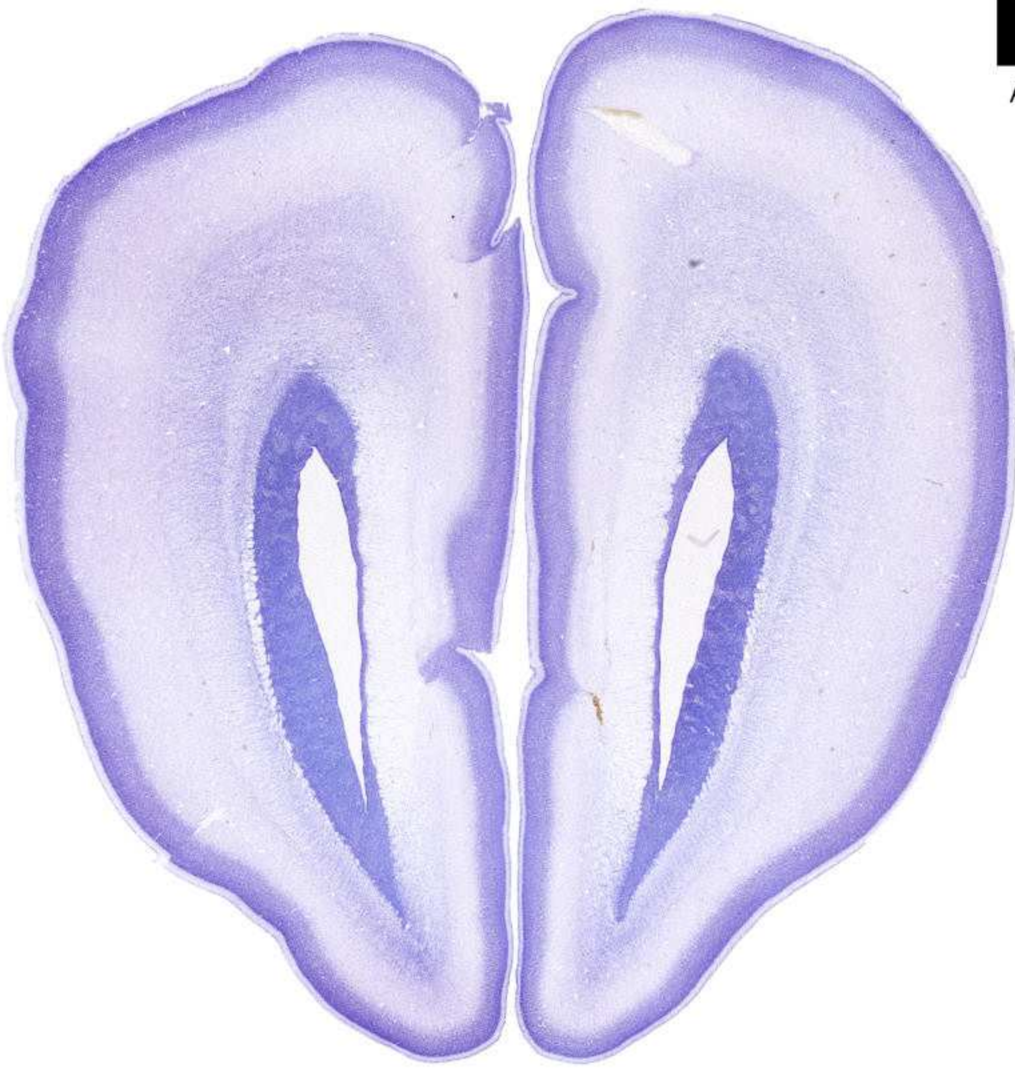

5 mm

Transient Layers

- VZ
- SVZ
- IZ
- SP
- CP
- MZ
- SGL

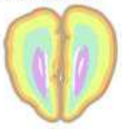

Cortical Areas

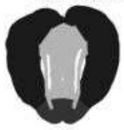

- FCTx
- ORB
- CING

Age: 22 GW

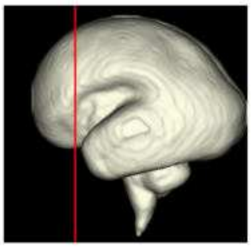

A-P Level: 12.36 mm

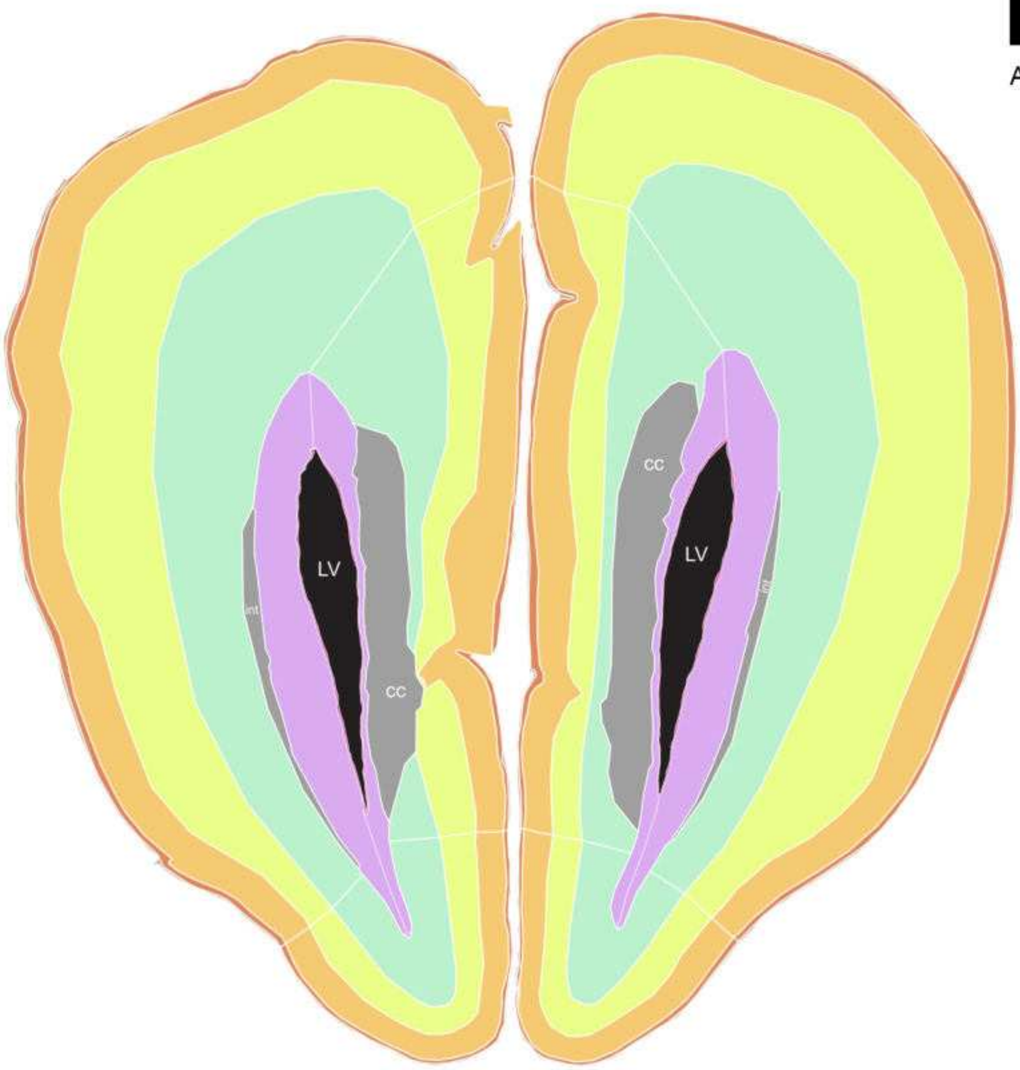

5 mm

LV: Lateral ventricle   cc: Corpus callosum   int: Internal capsule

Age: 22 GW

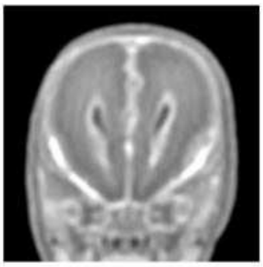

A-P Level: 12.06 mm

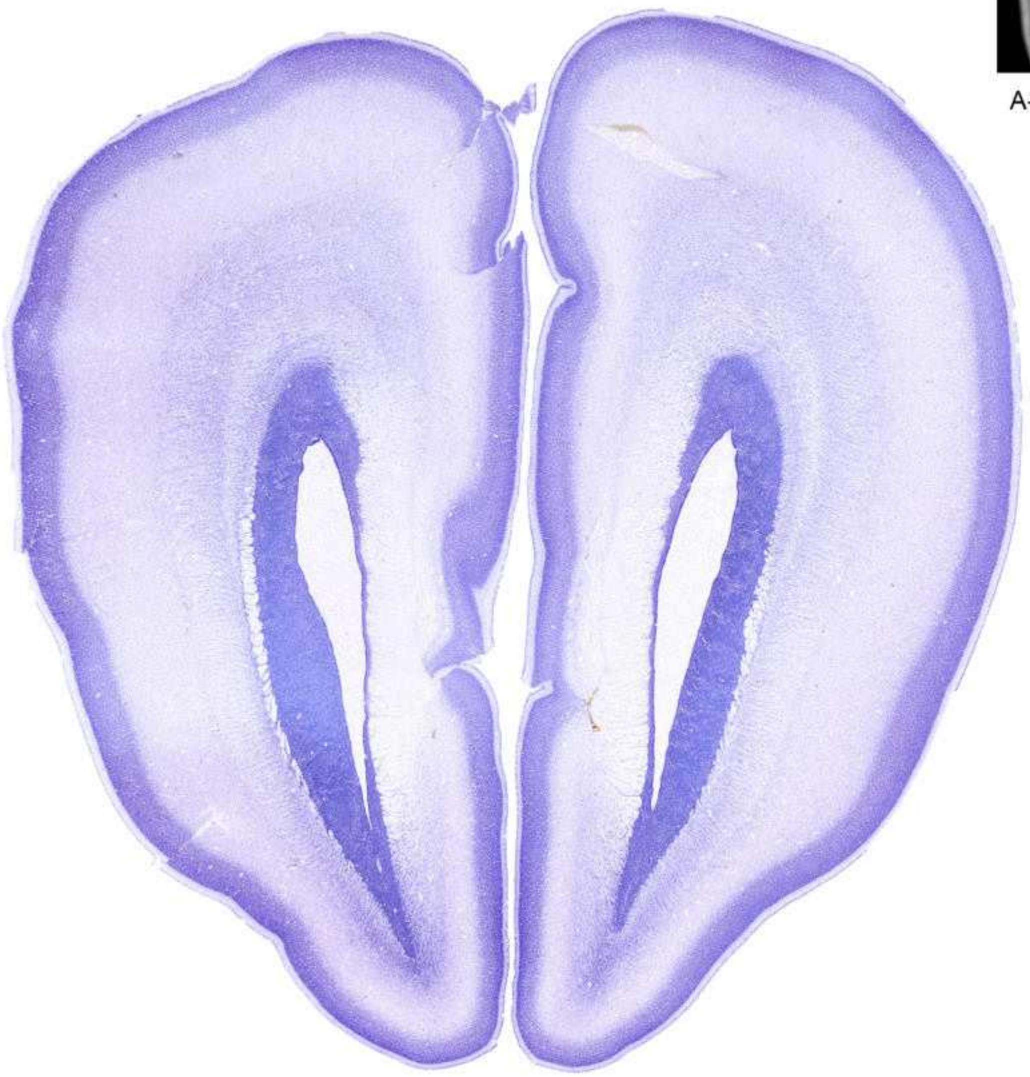

5 mm

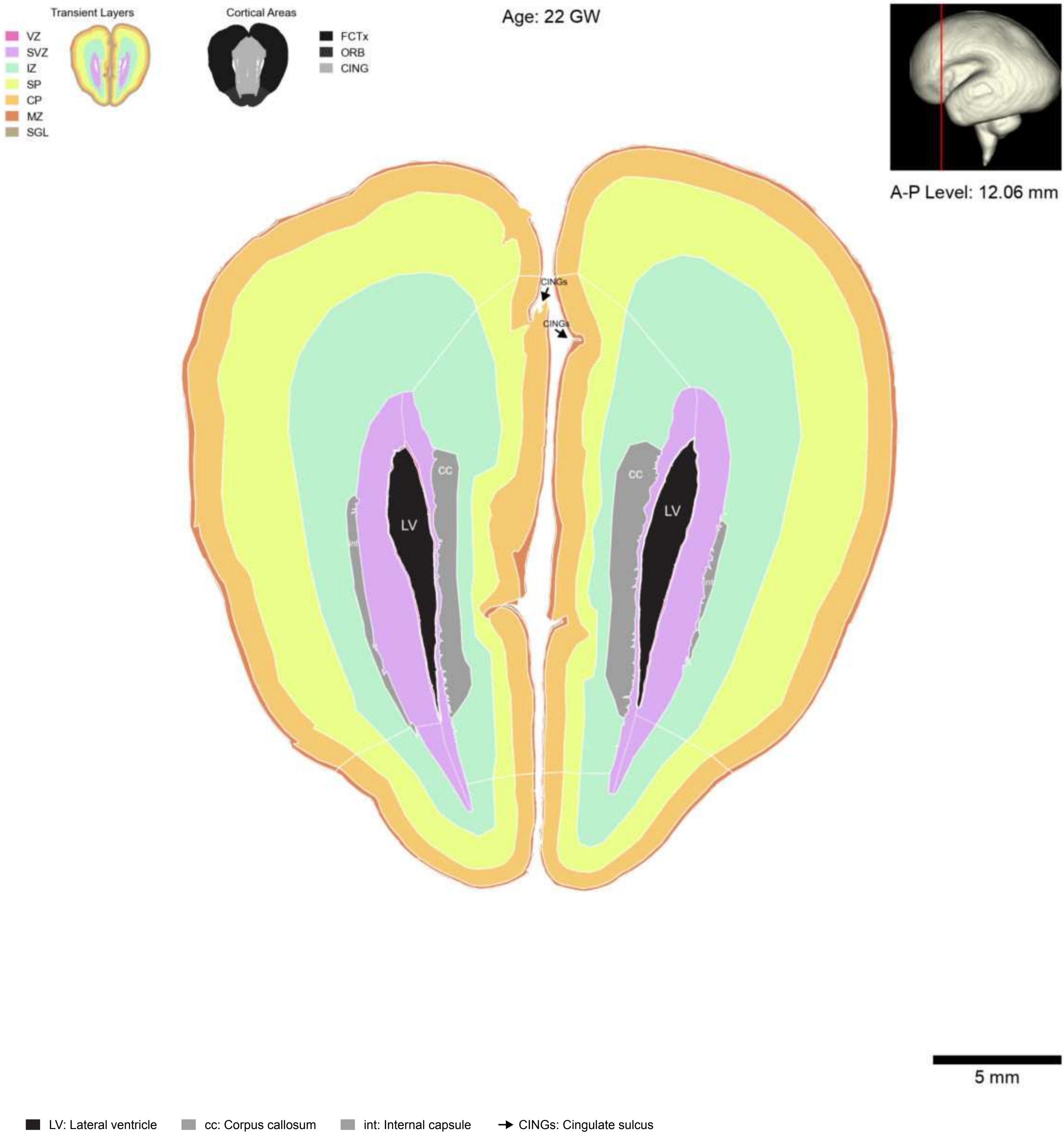
Transient Layers
VZ
SVZ
IZ
SP
CP
MZ
SGL
Cortical Areas
FCTx
ORB
CING
Age: 22 GW
A-P Level: 12.06 mm
CINGs
CINGs
cc
cc
LV
LV
int
int
5 mm
LV: Lateral ventricle
cc: Corpus callosum
int: Internal capsule
CINGs: Cingulate sulcus

Age: 22 GW

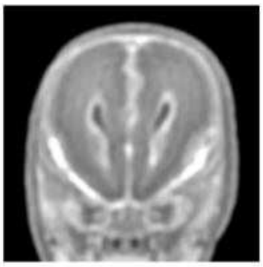

A-P Level: 11.64 mm

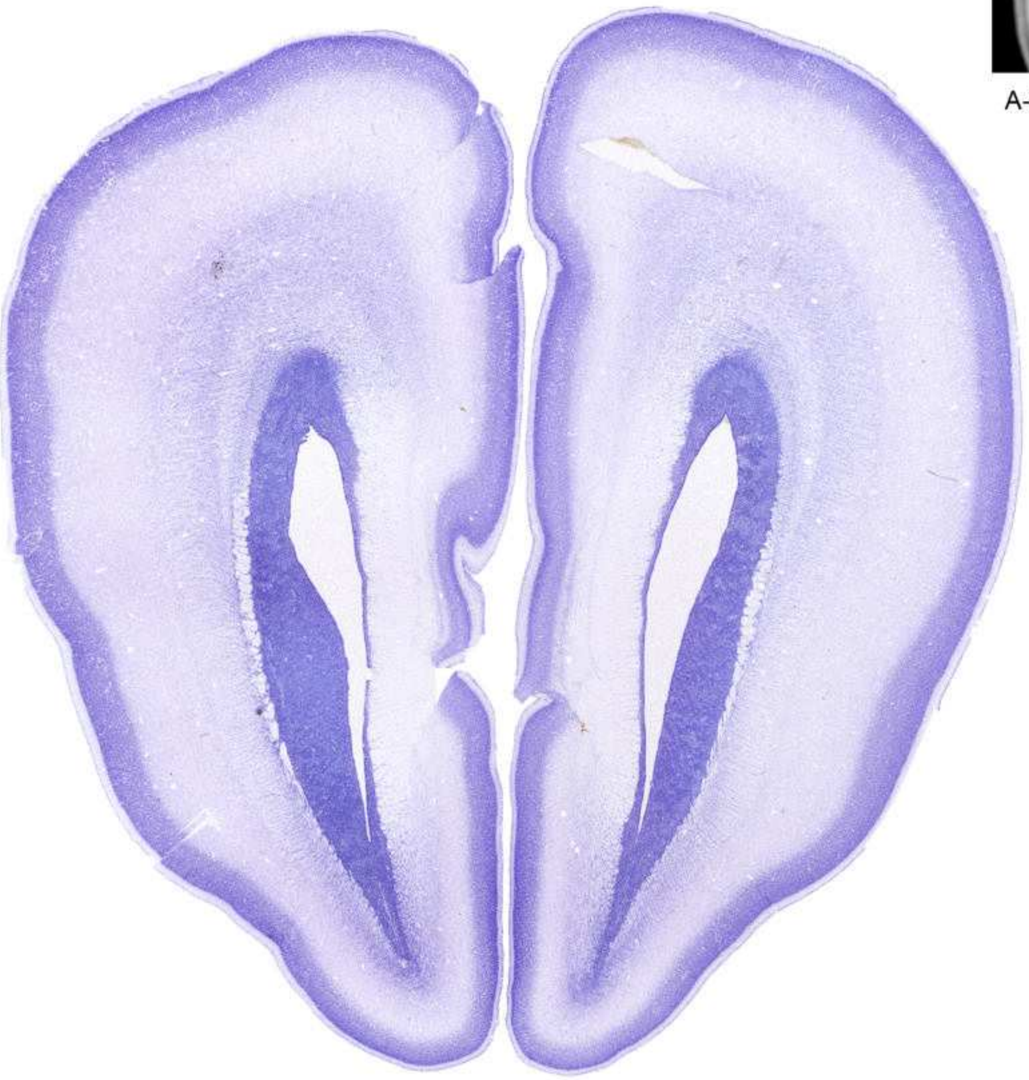

5 mm

Age: 22 GW

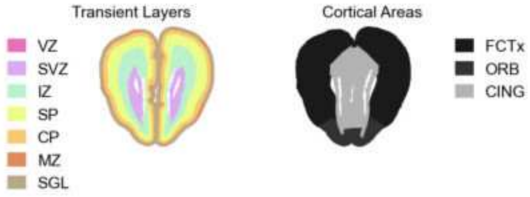

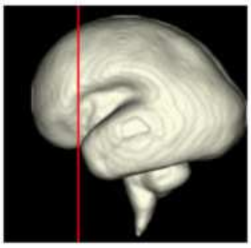

A-P Level: 11.64 mm

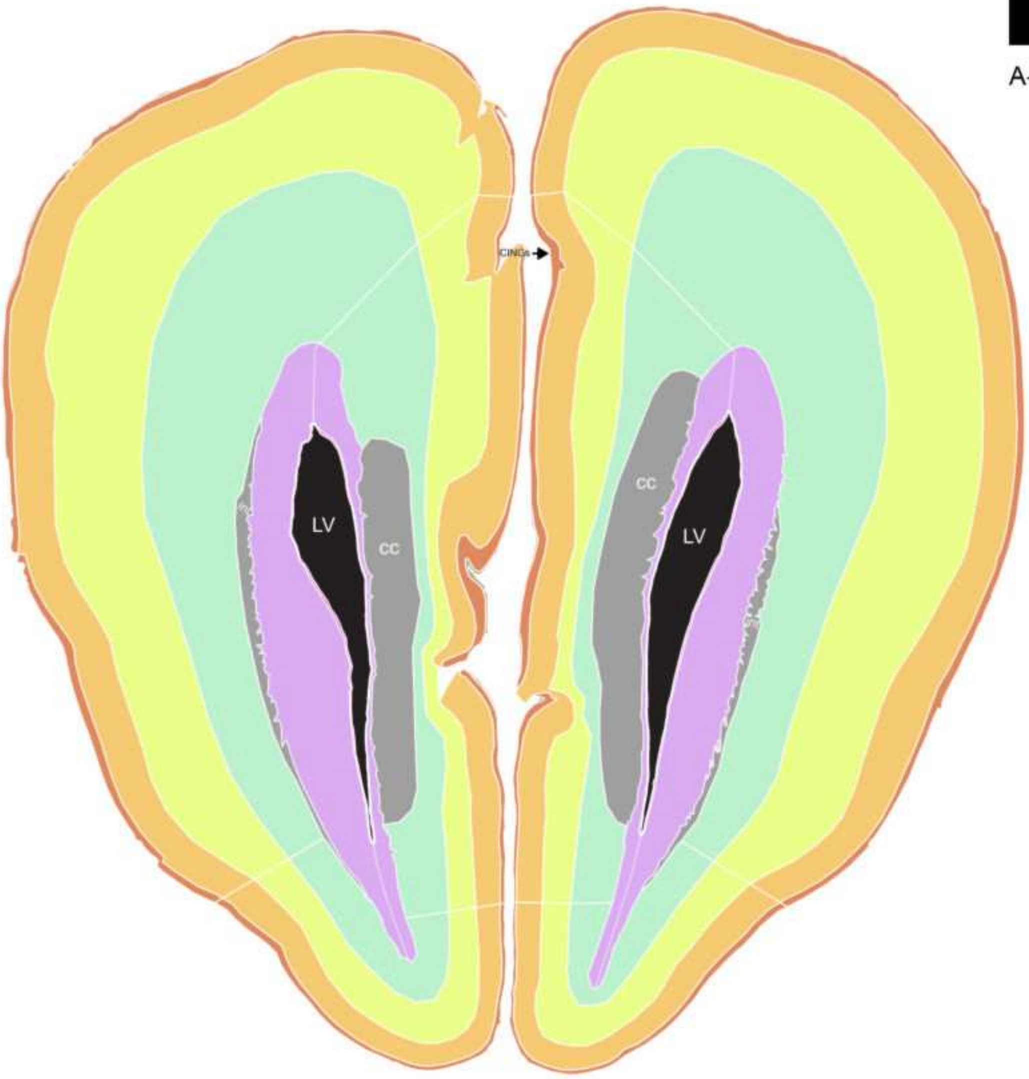

5 mm

LV: Lateral ventricle cc: Corpus callosum int: Internal capsule → CINGs: Cingulate sulcus

Age: 22 GW

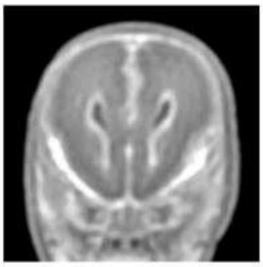

A-P Level: 11.4 mm

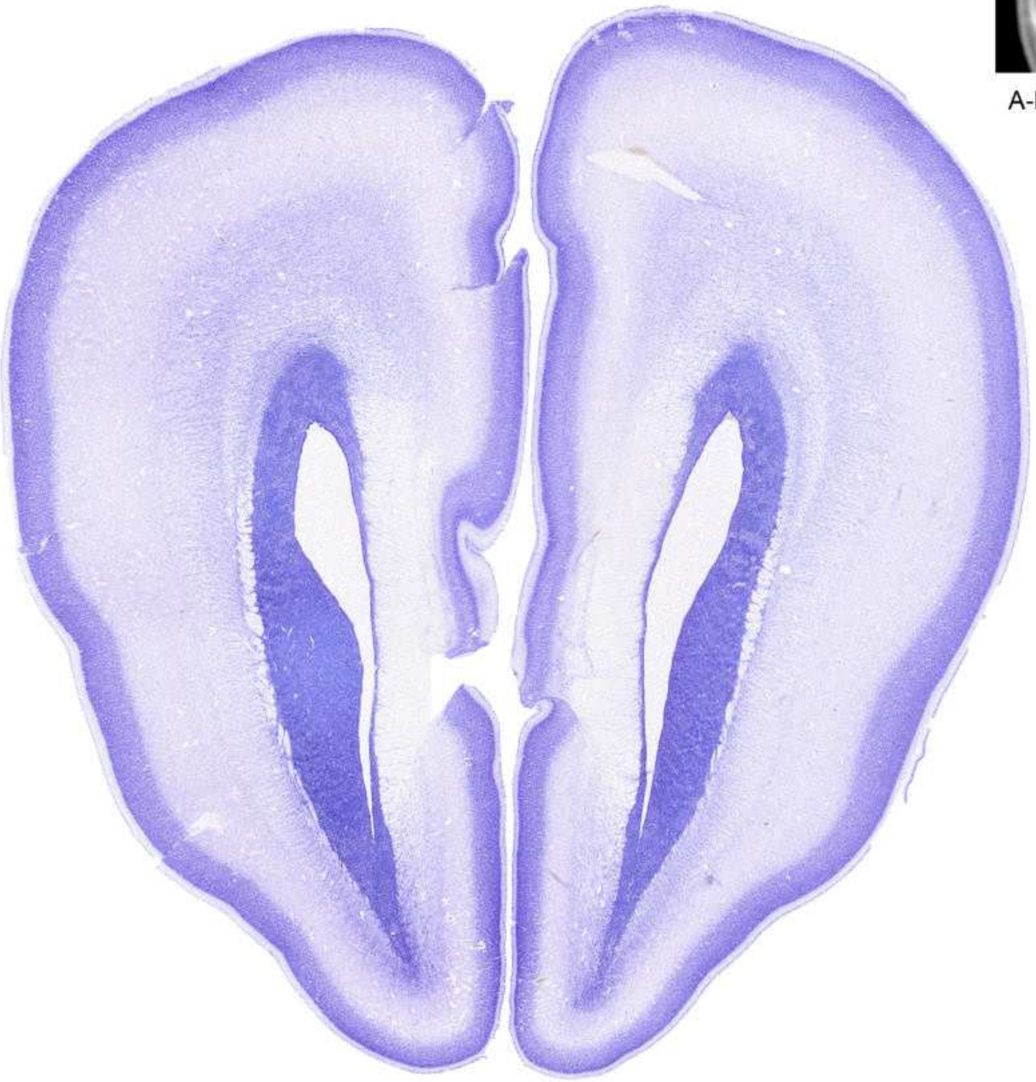

5 mm

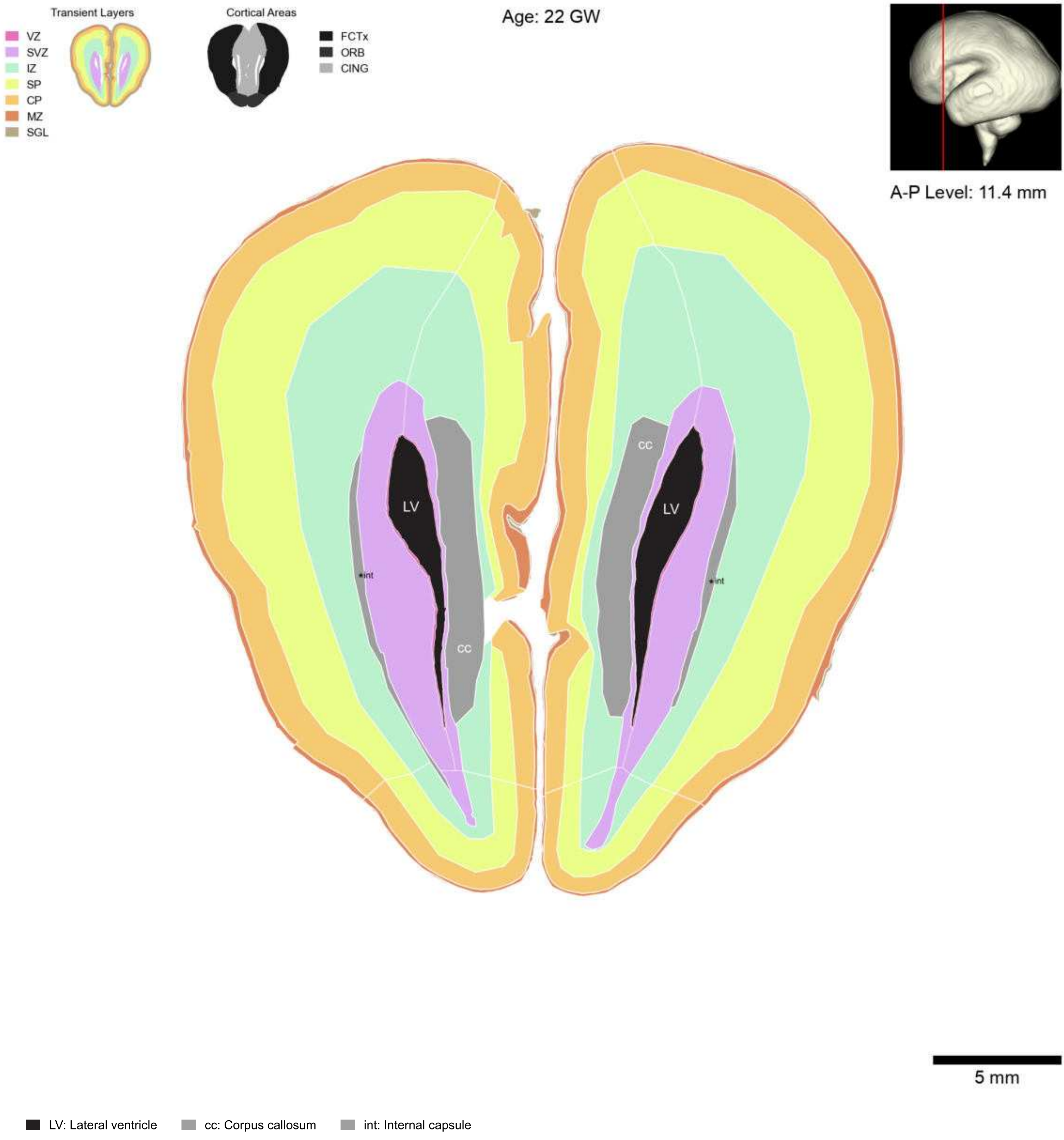

Transient Layers
VZ
SVZ
IZ
SP
CP
MZ
SGL
Cortical Areas
FCTx
ORB
CING
Age: 22 GW
A-P Level: 11.4 mm
LV
LV
cc
cc
int
int
5 mm
LV: Lateral ventricle
cc: Corpus callosum
int: Internal capsule

Age: 22 GW

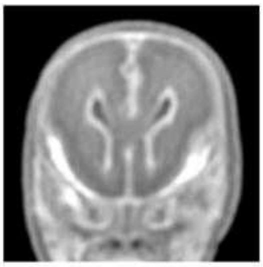

A-P Level: 10.86 mm

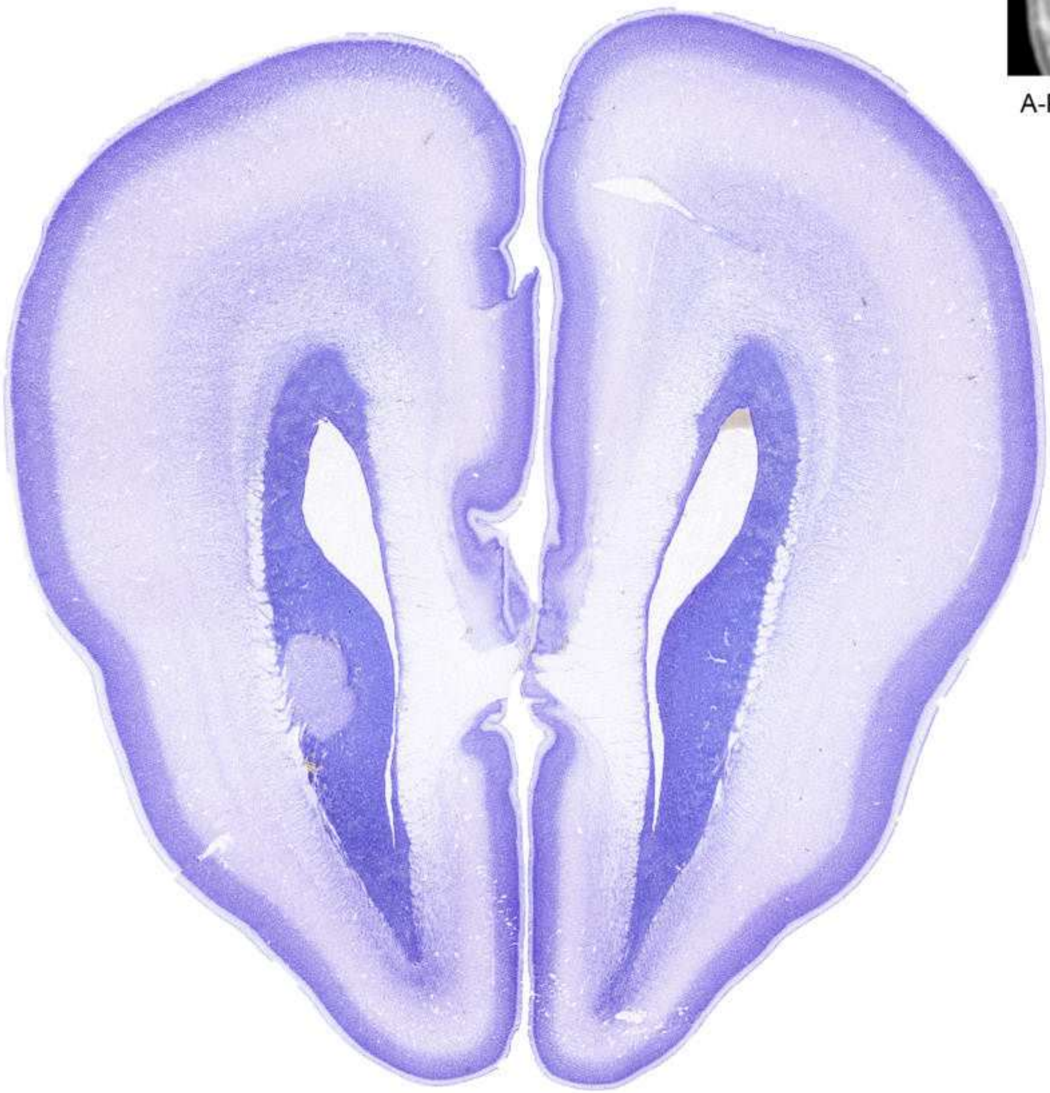

5 mm

Transient Layers

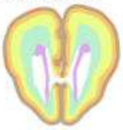

- VZ
- SVZ
- IZ
- SP
- CP
- MZ
- SGL

Cortical Areas

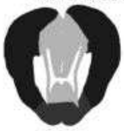

- FCTx
- ORB
- CING

Age: 22 GW

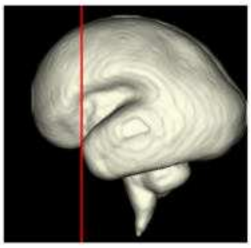

A-P Level: 10.86 mm

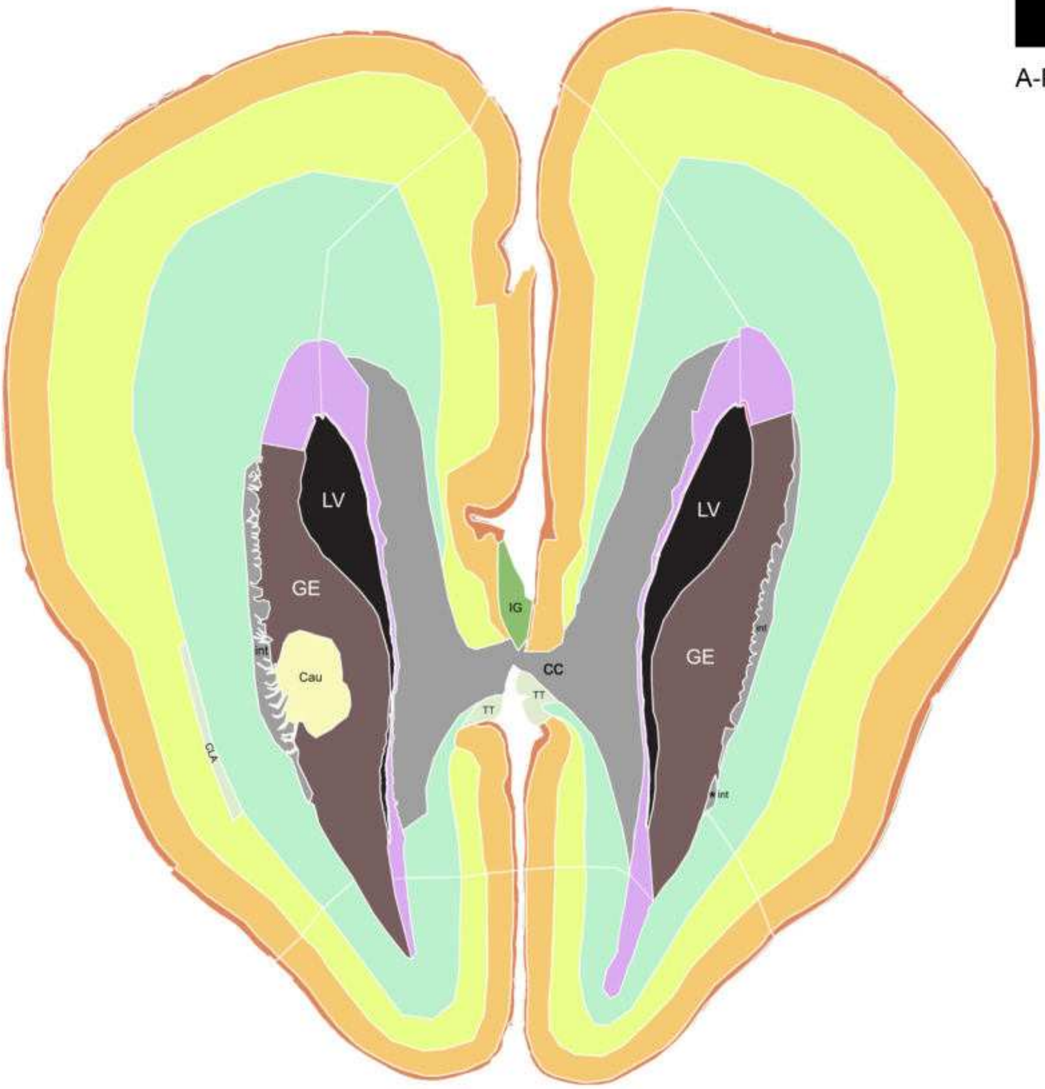

5 mm

CLA: Claustrum	GE: Ganglionic eminence	LV: Lateral ventricle	cc: Corpus callosum
Cau: Caudate nucleus	IG: Induseum griseum	TT: Tenia tecta	int: Internal capsule

Age: 22 GW

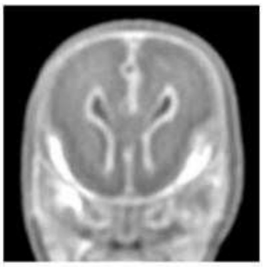

A-P Level: 10.5 mm

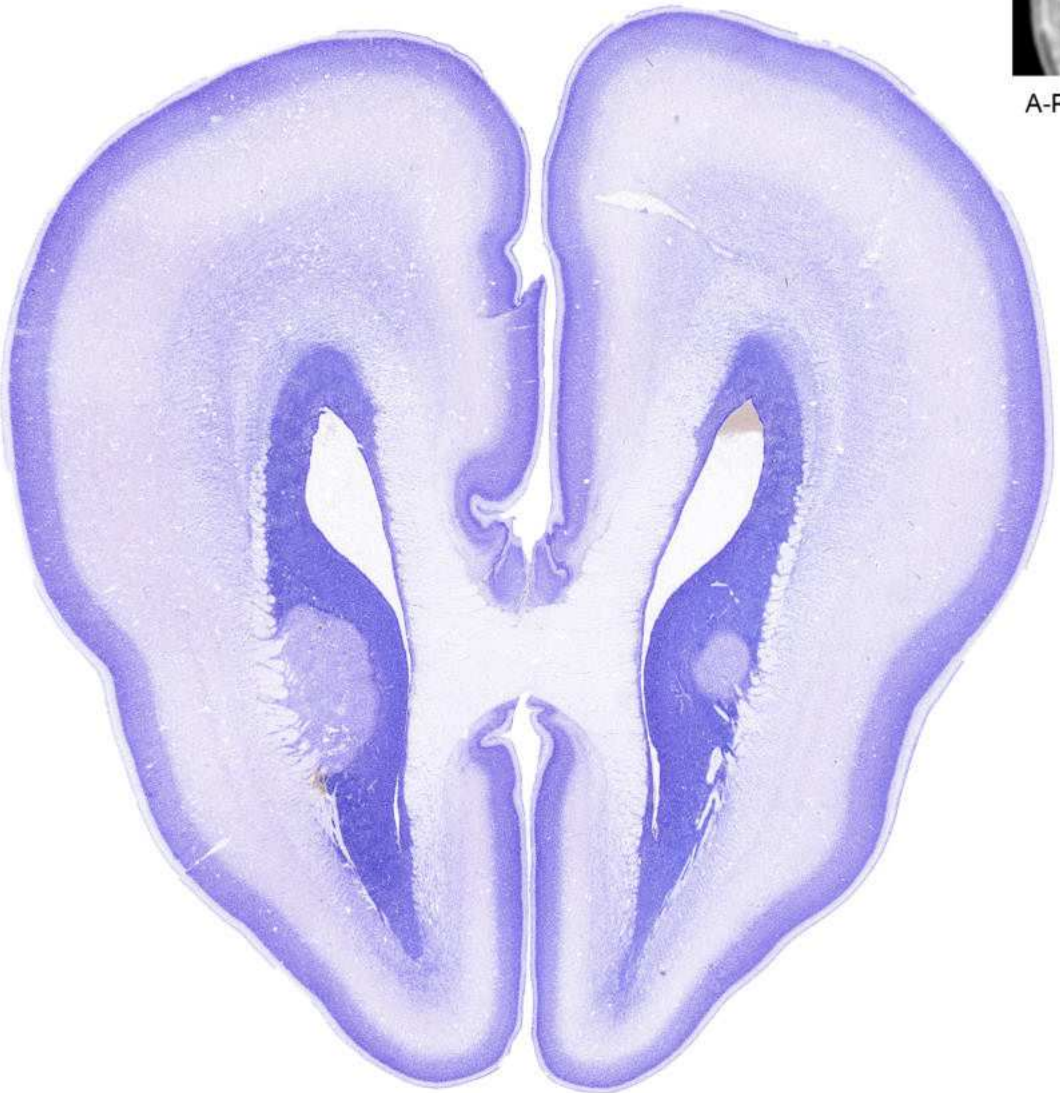

5 mm

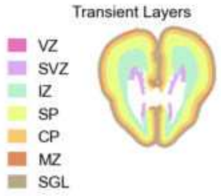

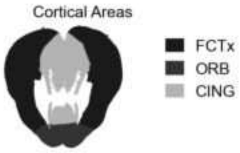

Age: 22 GW

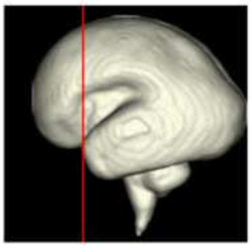

A-P Level: 10.5 mm

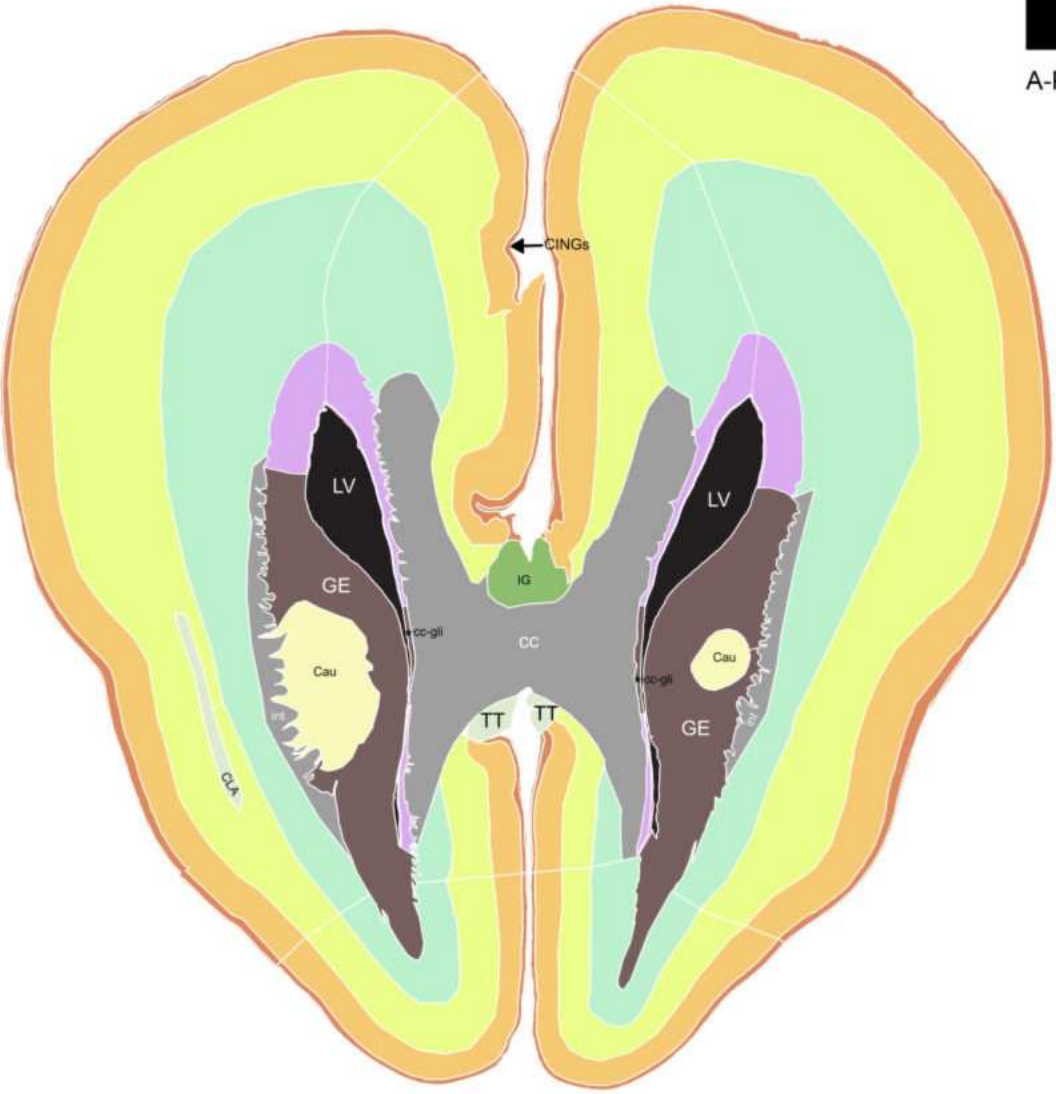

5 mm

CLA: Claustrum
Cau: Caudate nucleus
GE: Ganglionic eminence
IG: Induseum griseum
LV: Lateral ventricle
TT: Tenia tecta
cc: Corpus callosum
cc-gli: Callosal glioepithelium
int: Internal capsule
CINGs: Cingulate sulcus

Age: 22 GW

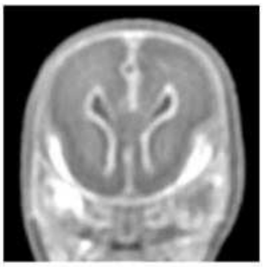

A-P Level: 10.26 mm

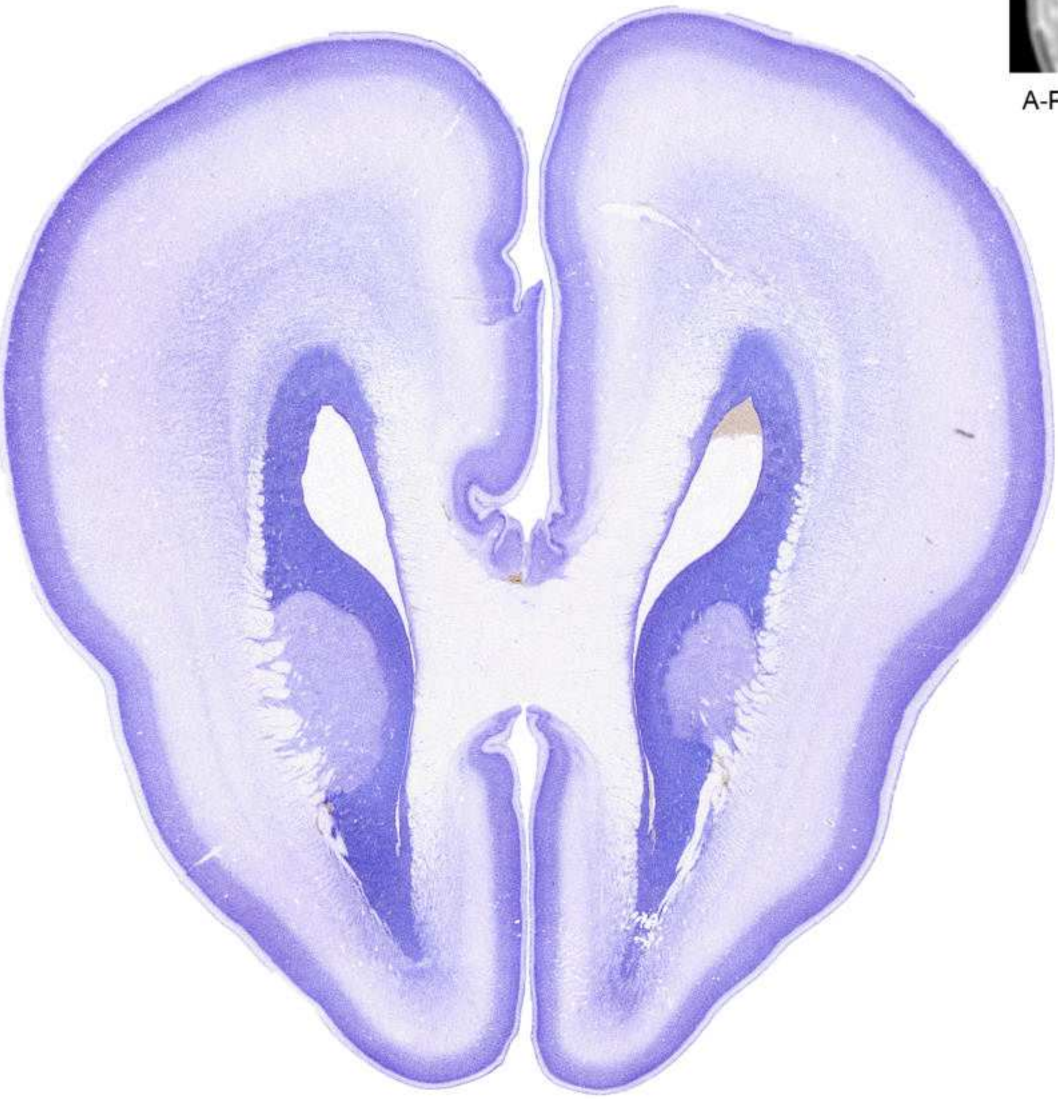

5 mm

Age: 22 GW

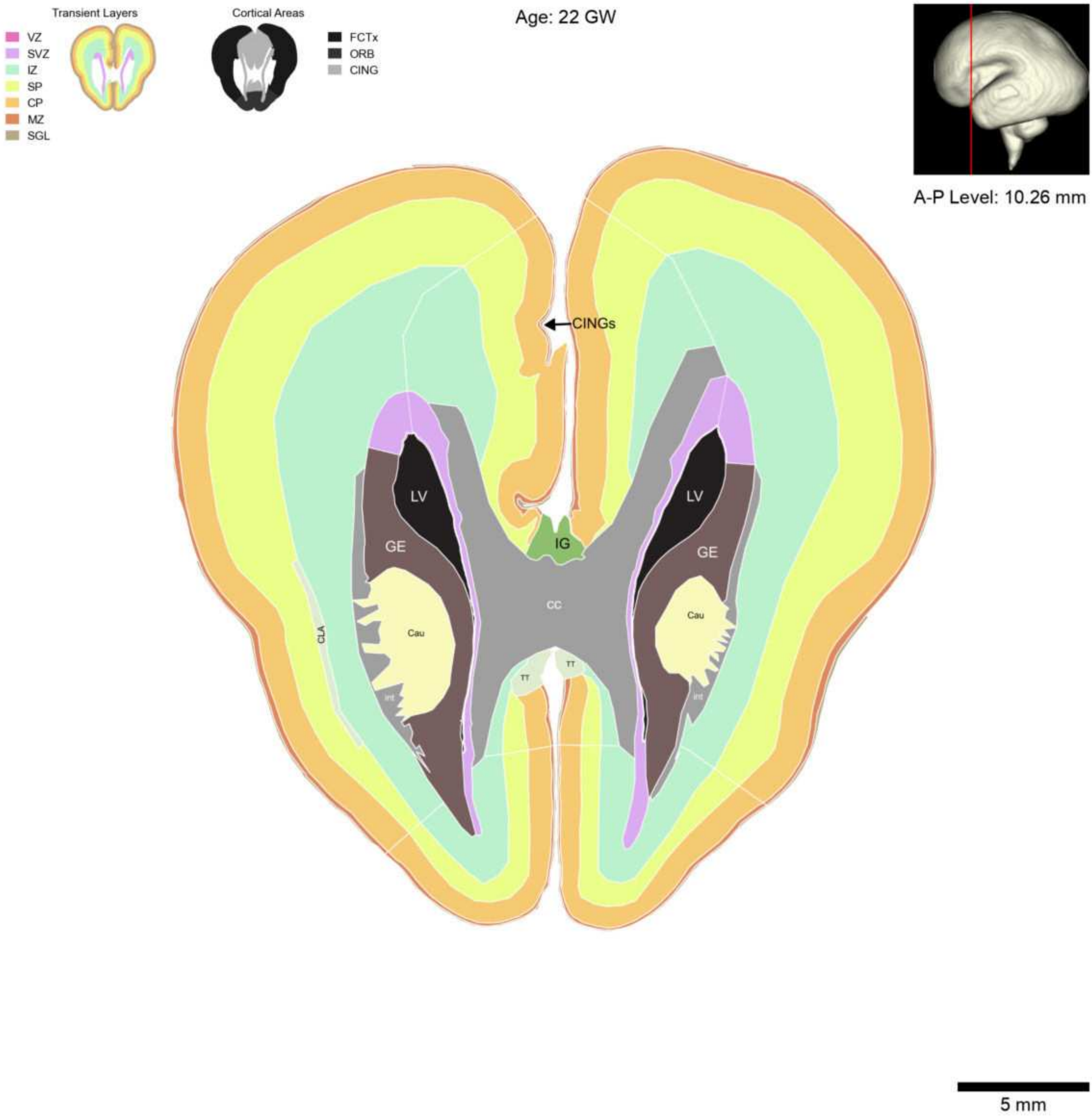

CLA: Claustrum
Cau: Caudate nucleus
GE: Ganglionic eminence
IG: Induseum griseum
LV: Lateral ventricle
TT: Tenia tecta
cc: Corpus callosum
int: Internal capsule
CINGs: Cingulate sulcus

Age: 22 GW

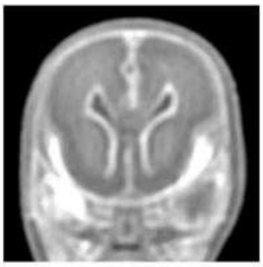

A-P Level: 9.9 mm

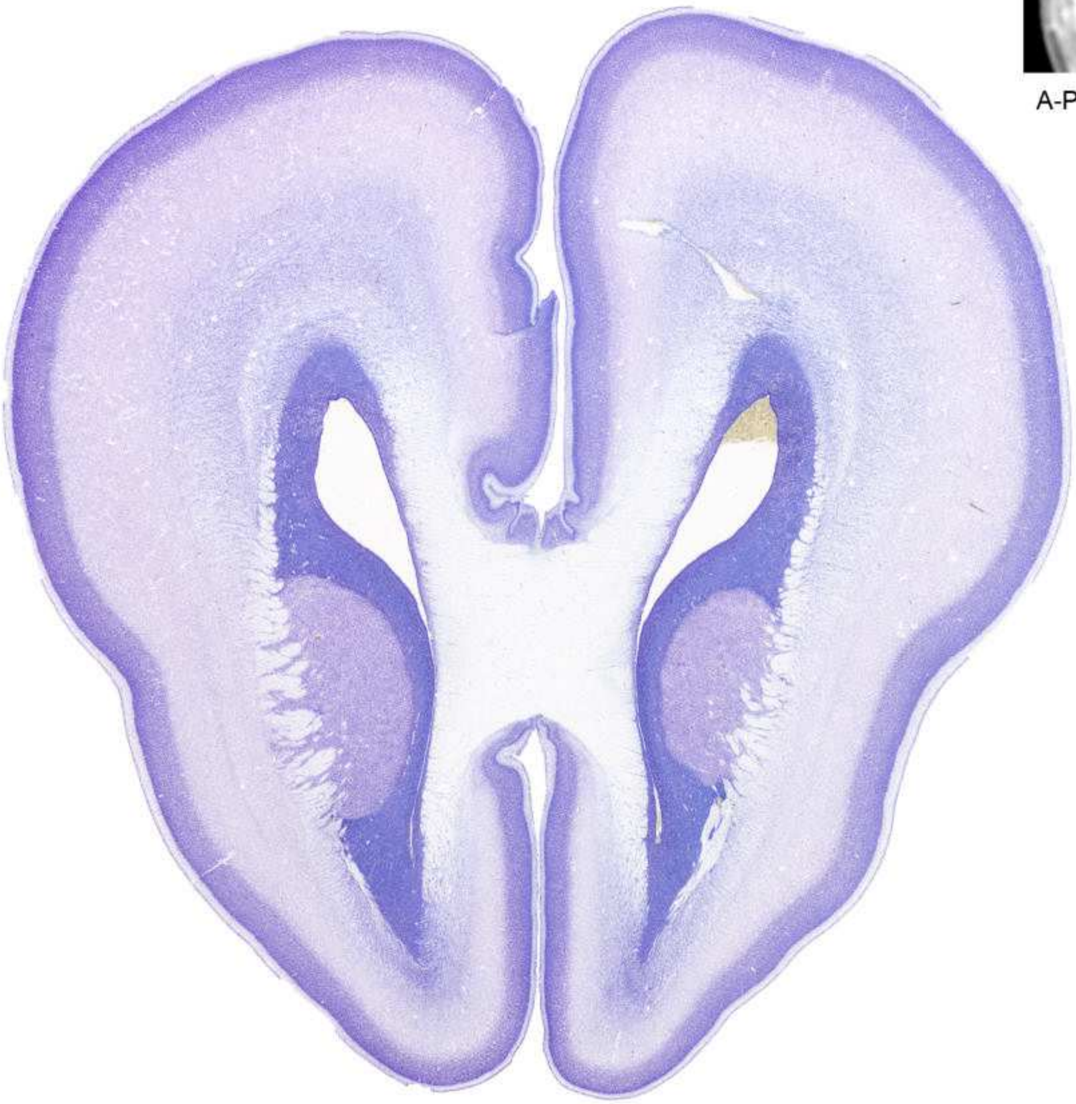

5 mm

Transient Layers

- VZ
- SVZ
- IZ
- SP
- CP
- MZ
- SGL

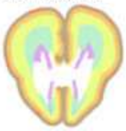

Cortical Areas

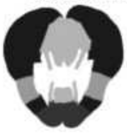

- FCTx
- ORB
- INS
- CING

Age: 22 GW

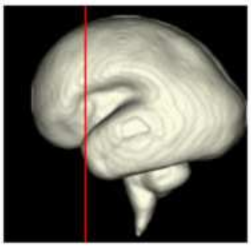

A-P Level: 9.9 mm

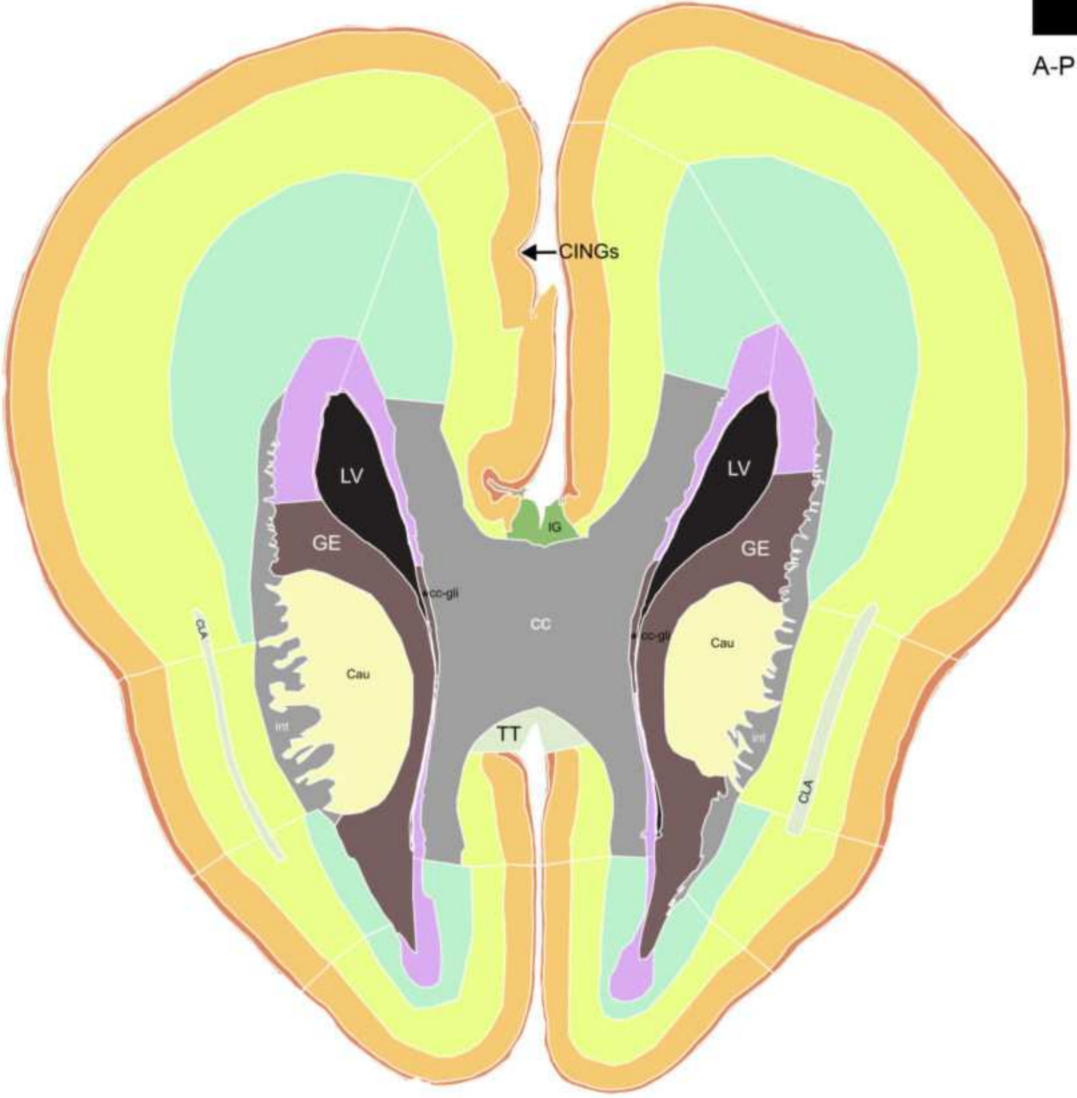

5 mm

CLA: Claustrum

Cau: Caudate nucleus

GE: Ganglionic eminence

IG: Induseum griseum

LV: Lateral ventricle

→ CINGs: Cingulate sulcus

TT: Tenia tecta

cc: Corpus callosum

cc-gli: Callosal glioepithelium

int: Internal capsule

Age: 22 GW

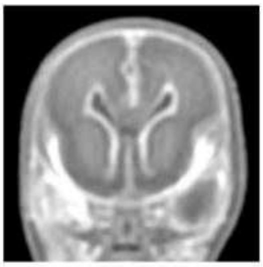

A-P Level: 9.48 mm

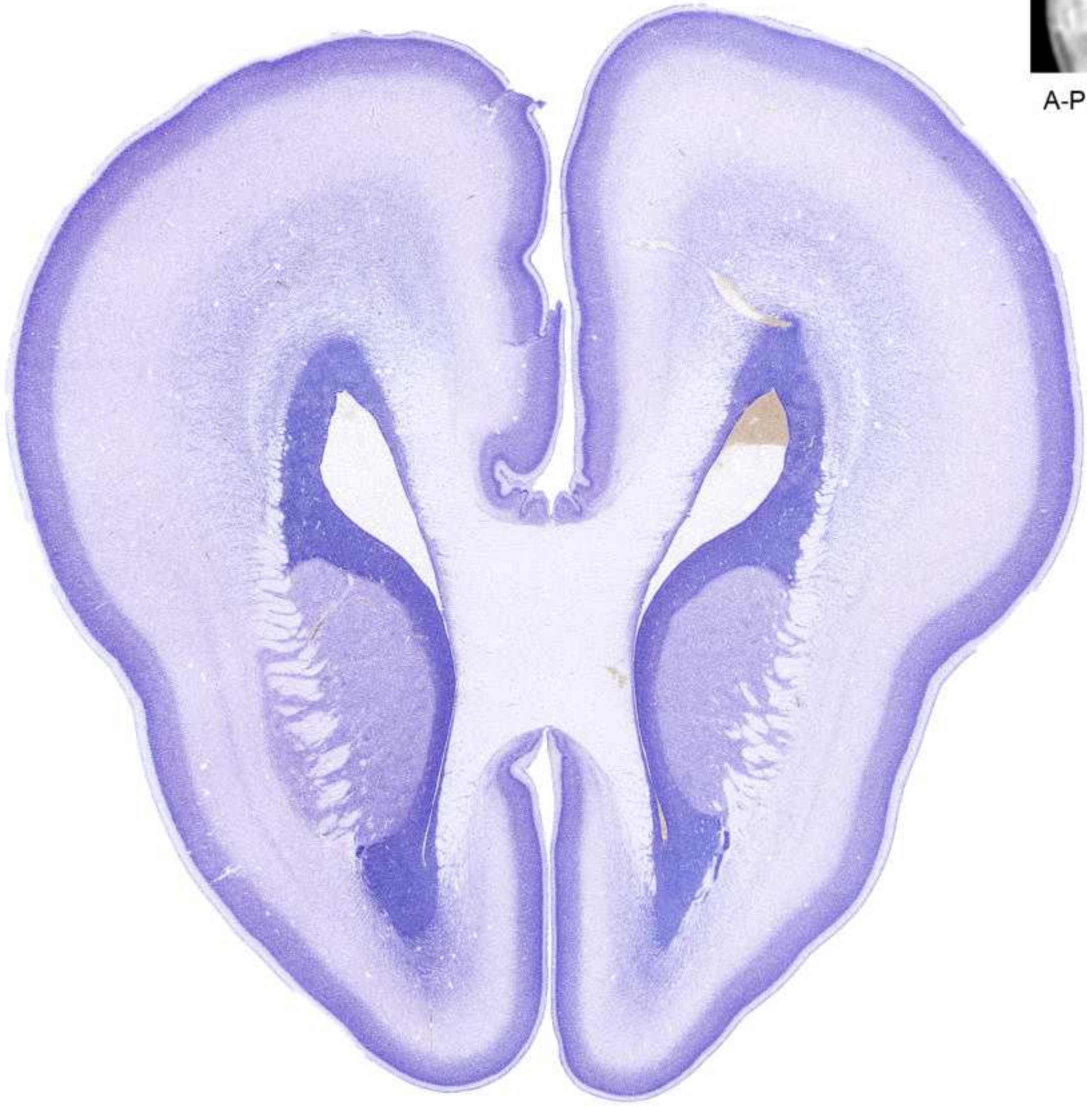

5 mm

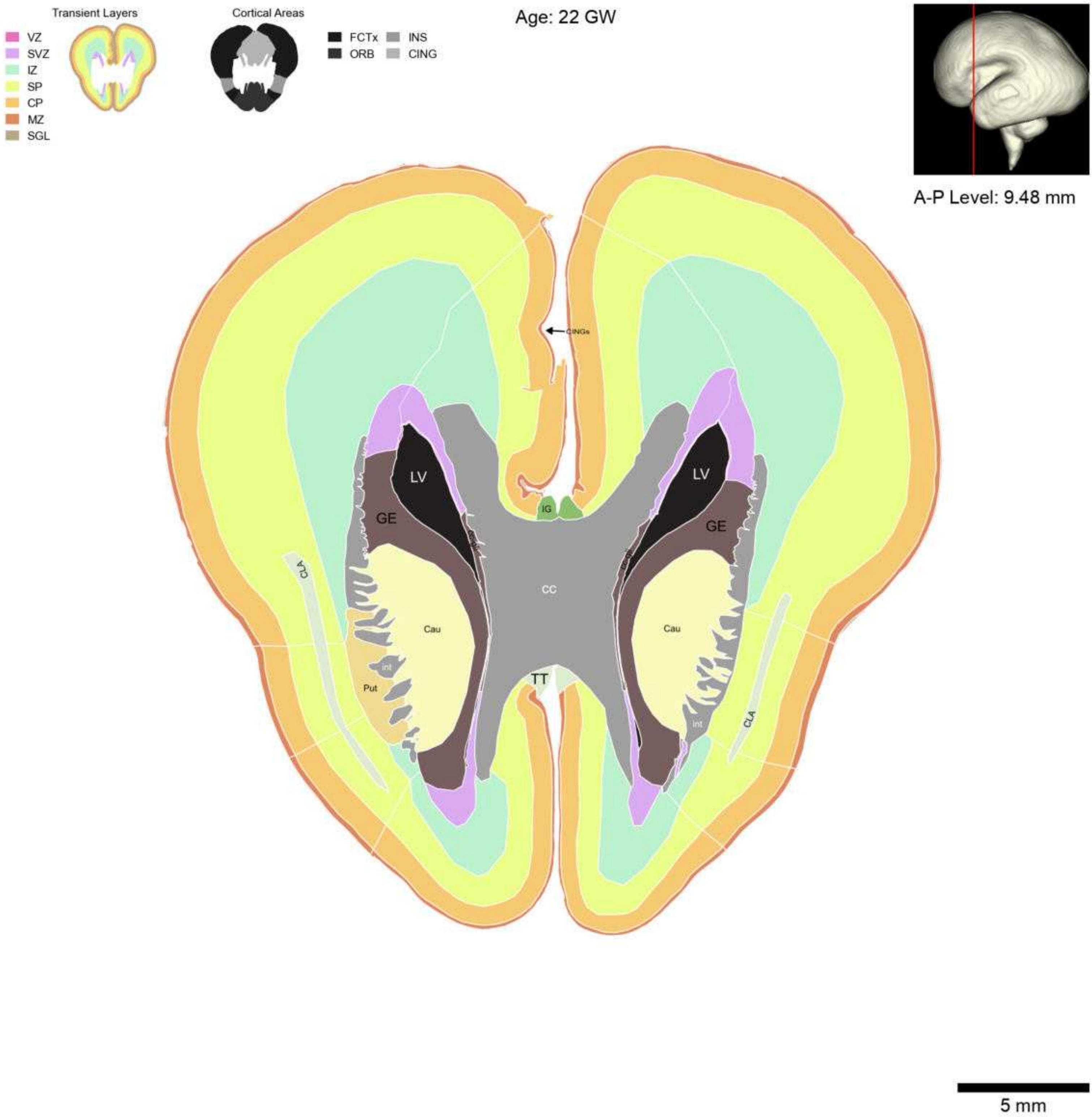

CLA: Claustrum
Cau: Caudate nucleus
GE: Ganglionic eminence
IG: Induseum griseum
LV: Lateral ventricle
Put: Putamen
TT: Tenia tecta
cc: Corpus callosum
cc-gli: Callosal glioepithelium
int: Internal capsule

Age: 22 GW

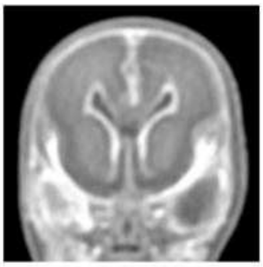

A-P Level: 9.12 mm

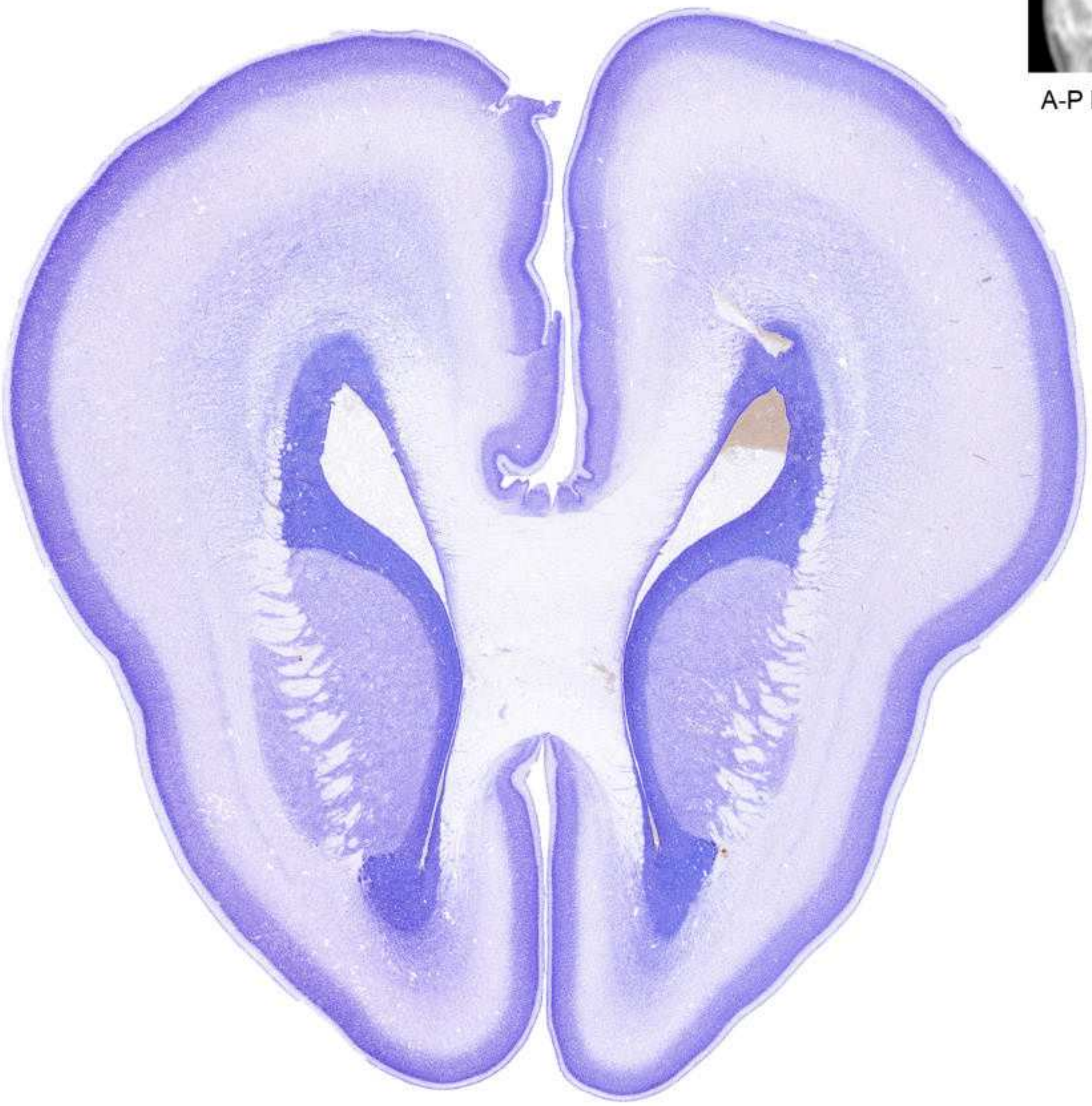

5 mm

Transient Layers

- VZ
- SVZ
- IZ
- SP
- CP
- MZ
- SGL

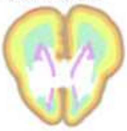

Cortical Areas

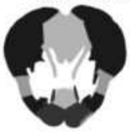

- FCTx
- ORB
- TEMP
- INS
- CING

Age: 22 GW

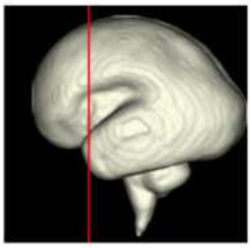

A-P Level: 9.12 mm

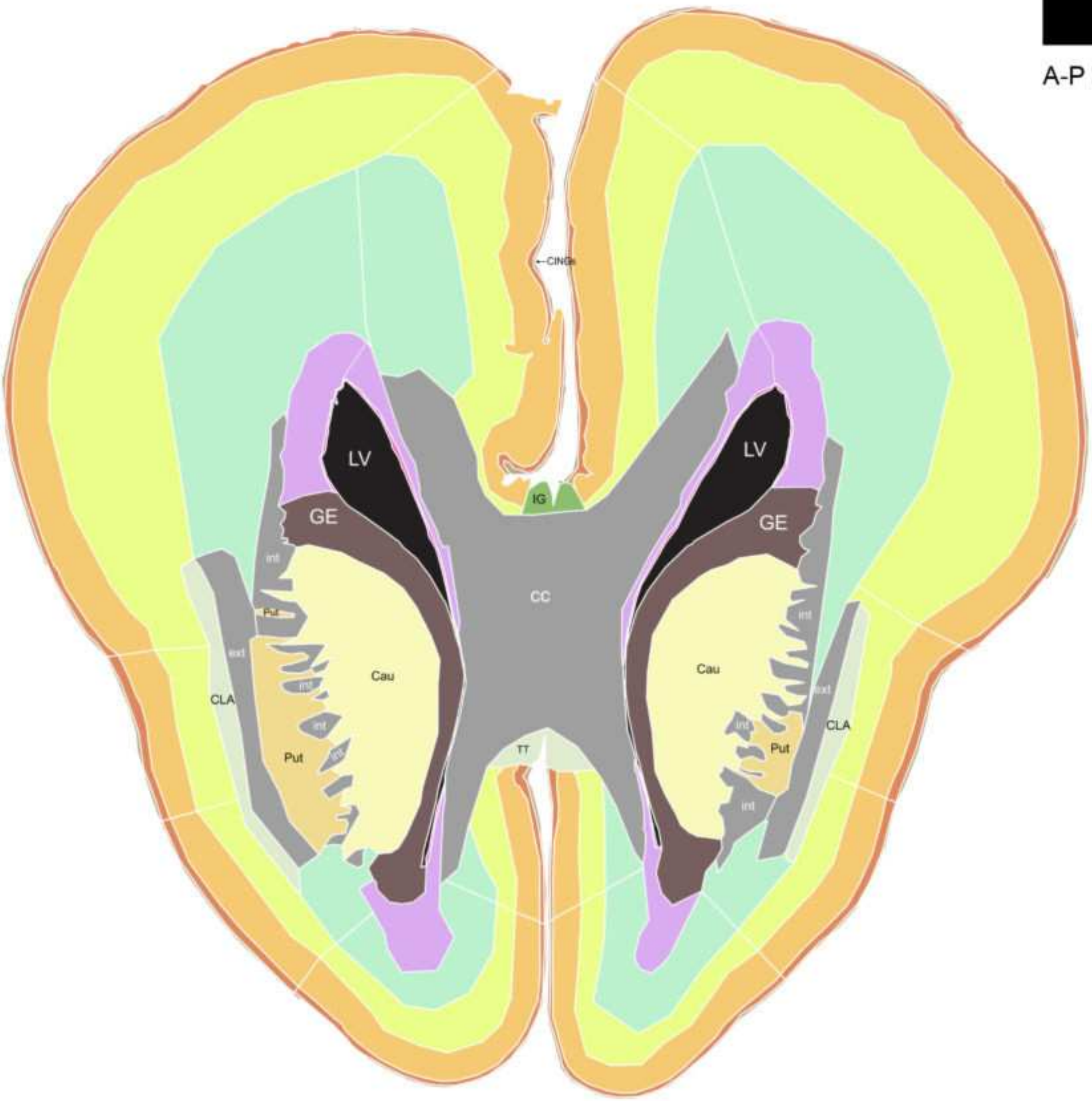

5 mm

- CLA: Claustrum
- Cau: Caudate nucleus
- GE: Ganglionic eminence
- IG: Induseum griseum
- LV: Lateral ventricle
- Put: Putamen
- TT: Tenia tecta
- cc: Corpus callosum
- → CINGs: Cingulate sulcus
- ext: External capsule
- int: Internal capsule

Age: 22 GW

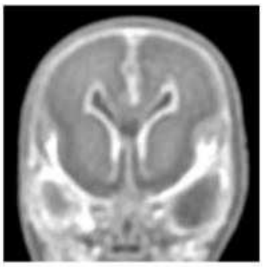

A-P Level: 8.76 mm

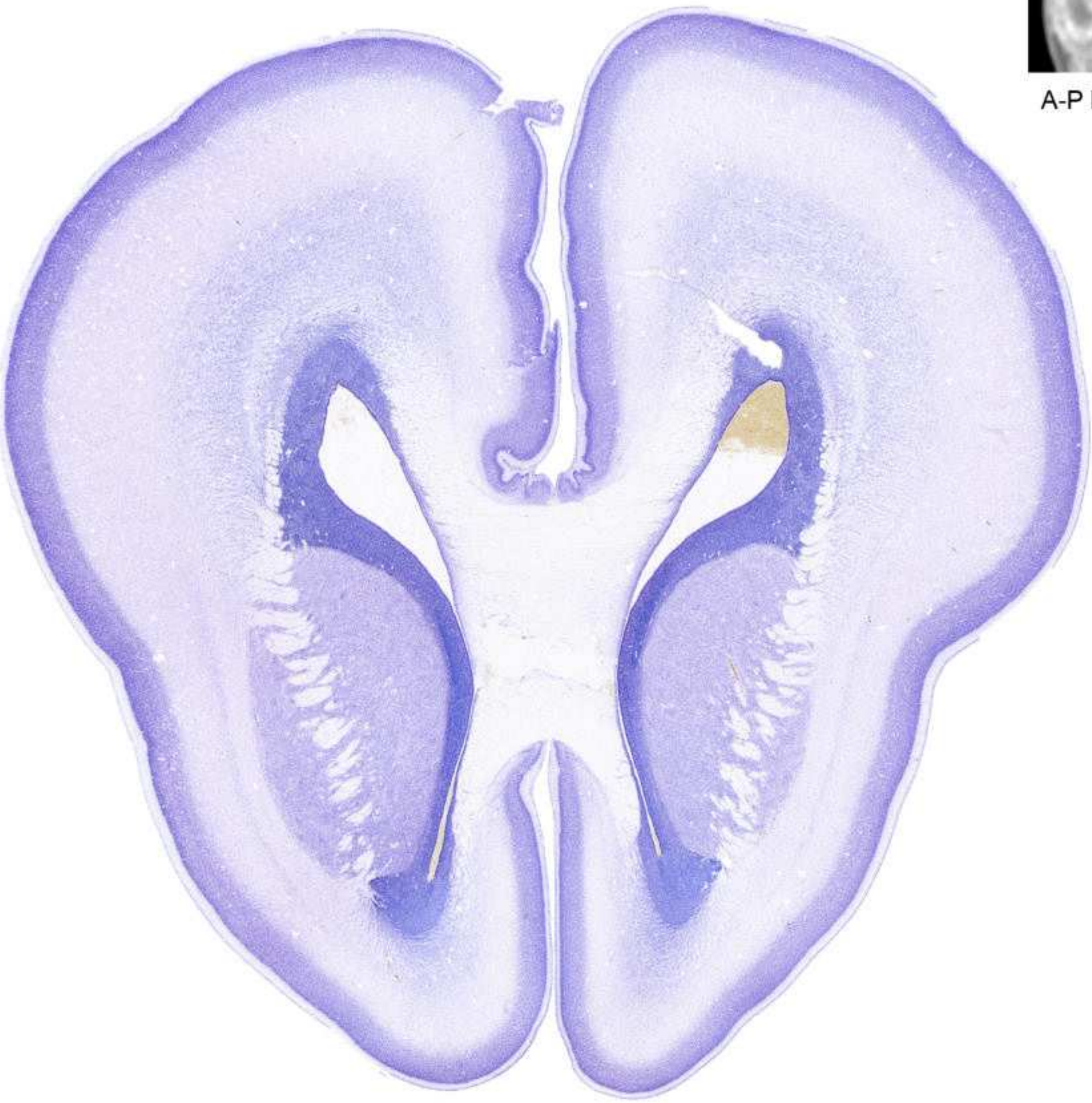

5 mm

Transient Layers

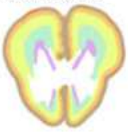

VZ
SVZ
IZ
SP
CP
MZ
SGL

Cortical Areas

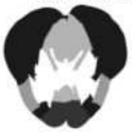

FCTx INS
ORB CING

Age: 22 GW

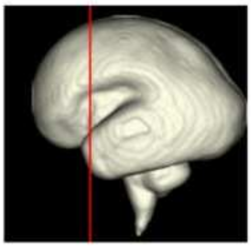

A-P Level: 8.76 mm

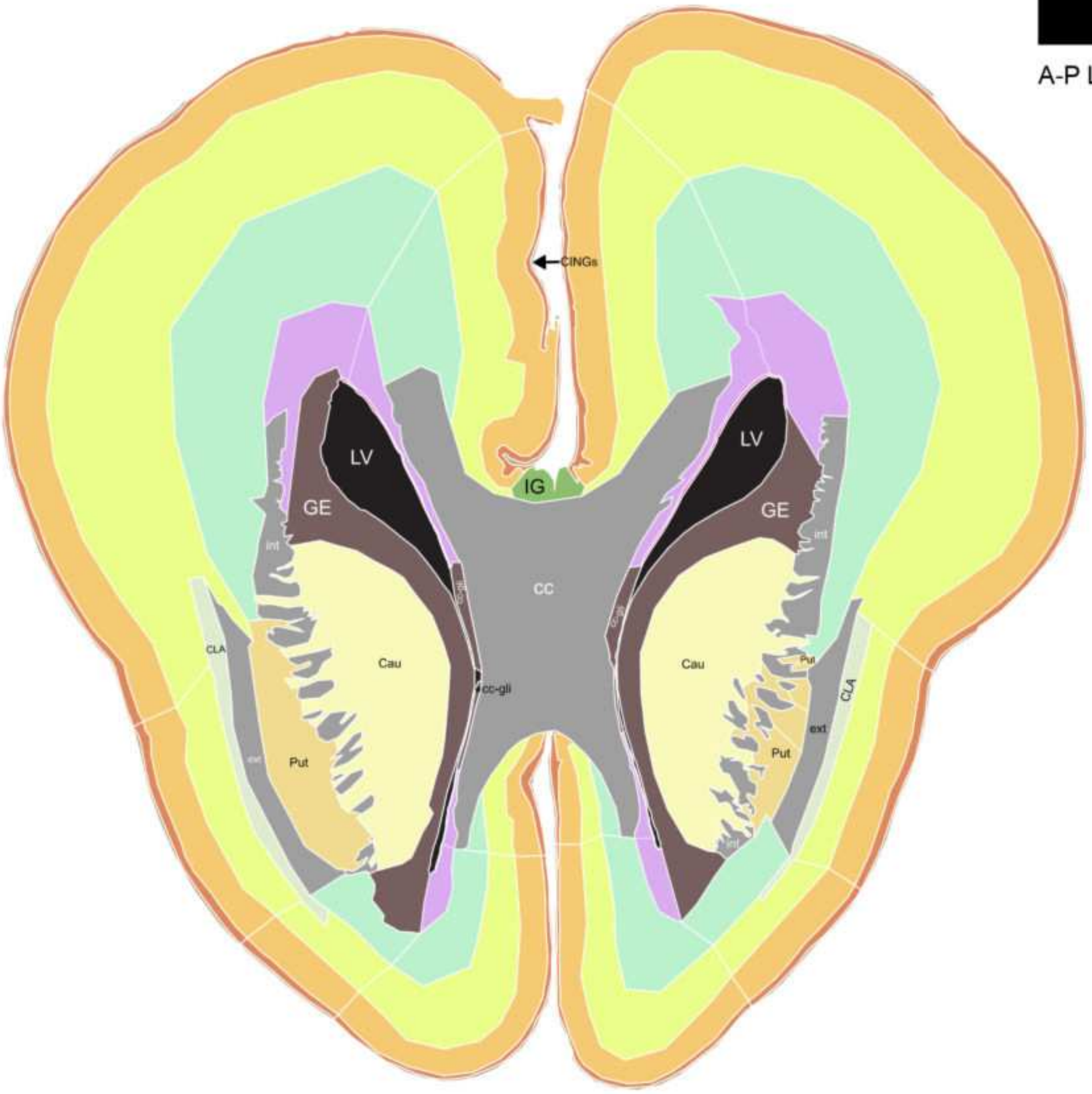

5 mm

CLA: Claustrum
Cau: Caudate nucleus
GE: Ganglionic eminence
IG: Induseum griseum
LV: Lateral ventricle
Put: Putamen
cc: Corpus callosum
cc-gli: Callosal glioepithelium
→ CINGs: Cingulate sulcus
ext: External capsule
int: Internal capsule

Age: 22 GW

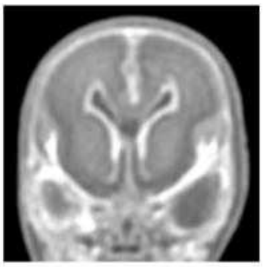

A-P Level: 8.4 mm

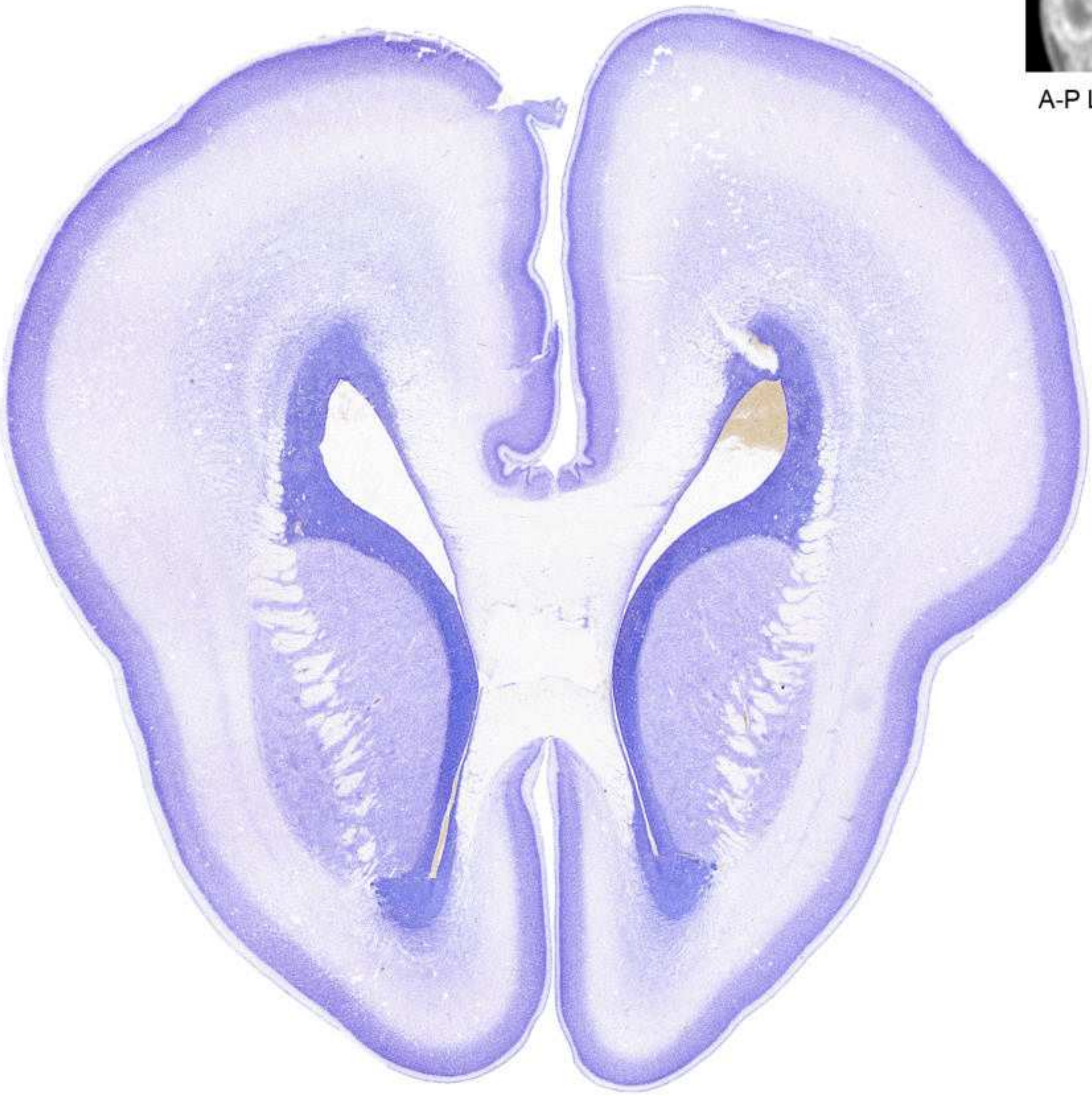

5 mm

Transient Layers

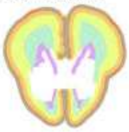

- VZ
- SVZ
- IZ
- SP
- CP
- MZ
- SGL

Cortical Areas

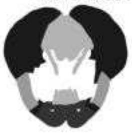

- FCTx
- ORB
- INS
- CING

Age: 22 GW

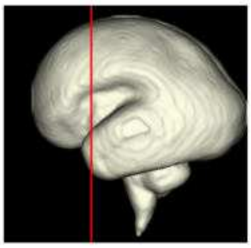

A-P Level: 8.4 mm

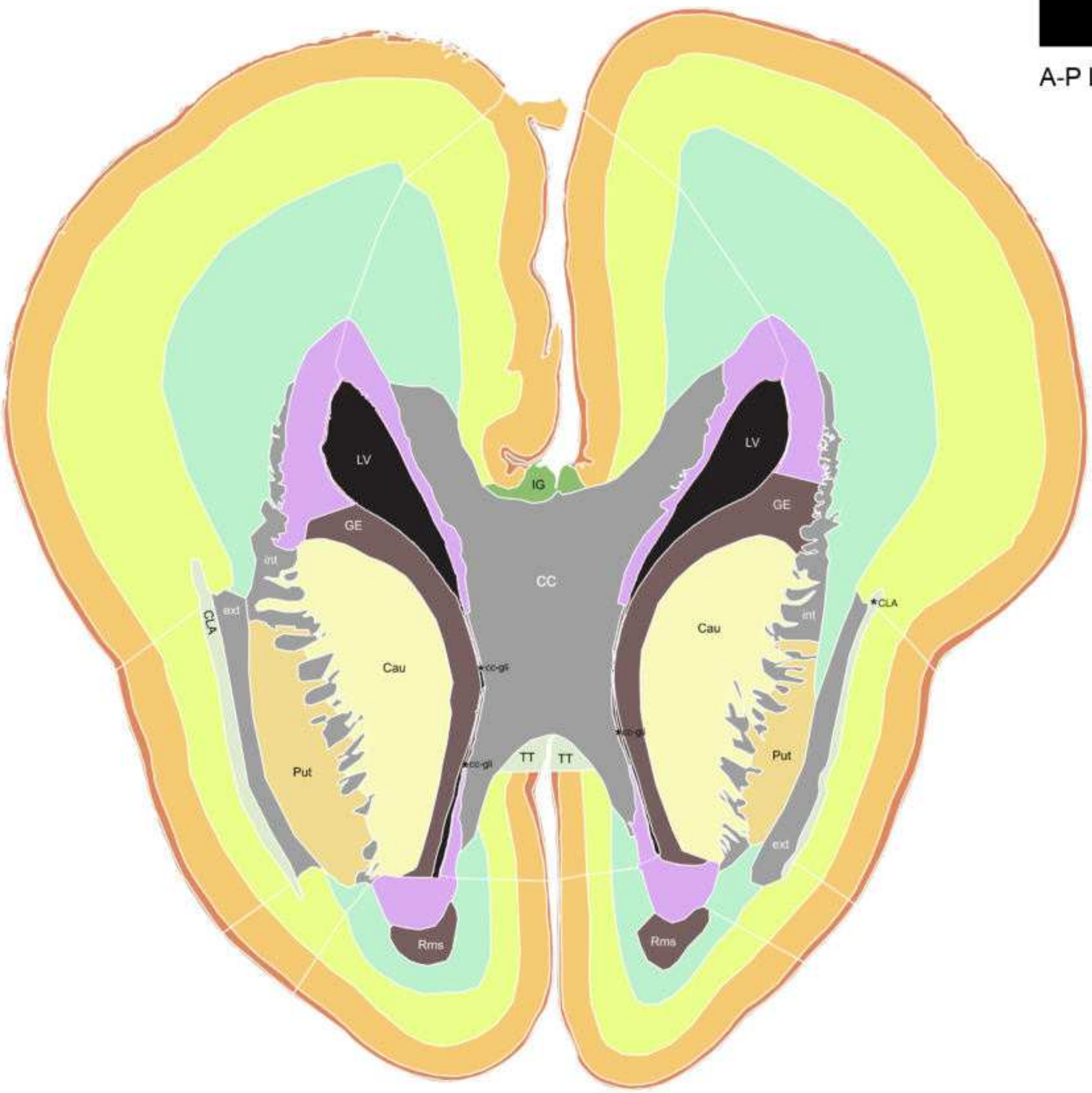

5 mm

- CLA: Claustrum
- Cau: Caudate nucleus
- GE: Ganglionic eminence
- IG: Induseum griseum
- LV: Lateral ventricle
- Put: Putamen
- Rms: Rostral migratory stream
- TT: Tenia tecta
- cc: Corpus callosum
- cc-gli: Callosal glioepithelium
- ext: External capsule
- int: Internal capsule

Age: 22 GW

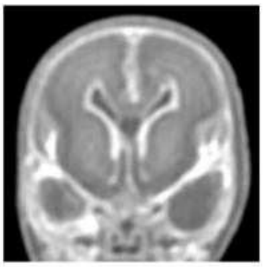

A-P Level: 7.86 mm

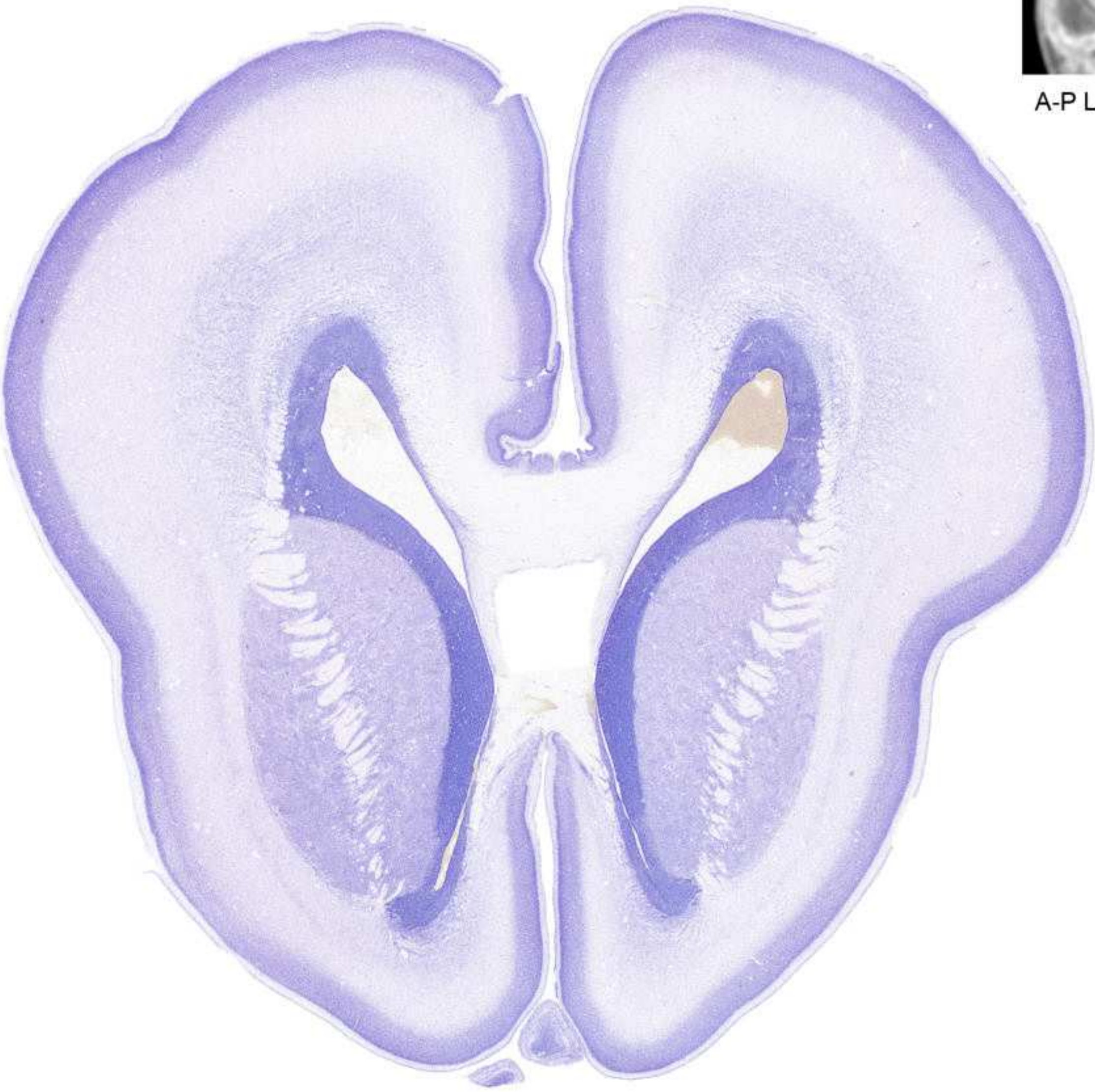

5 mm

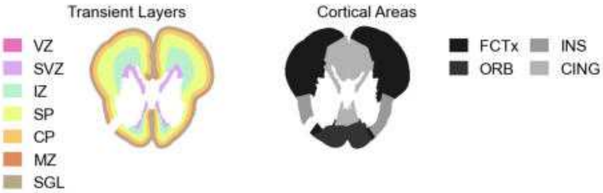

Age: 22 GW

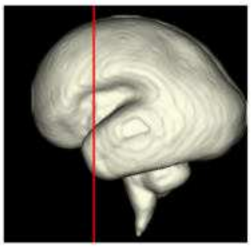

A-P Level: 7.86 mm

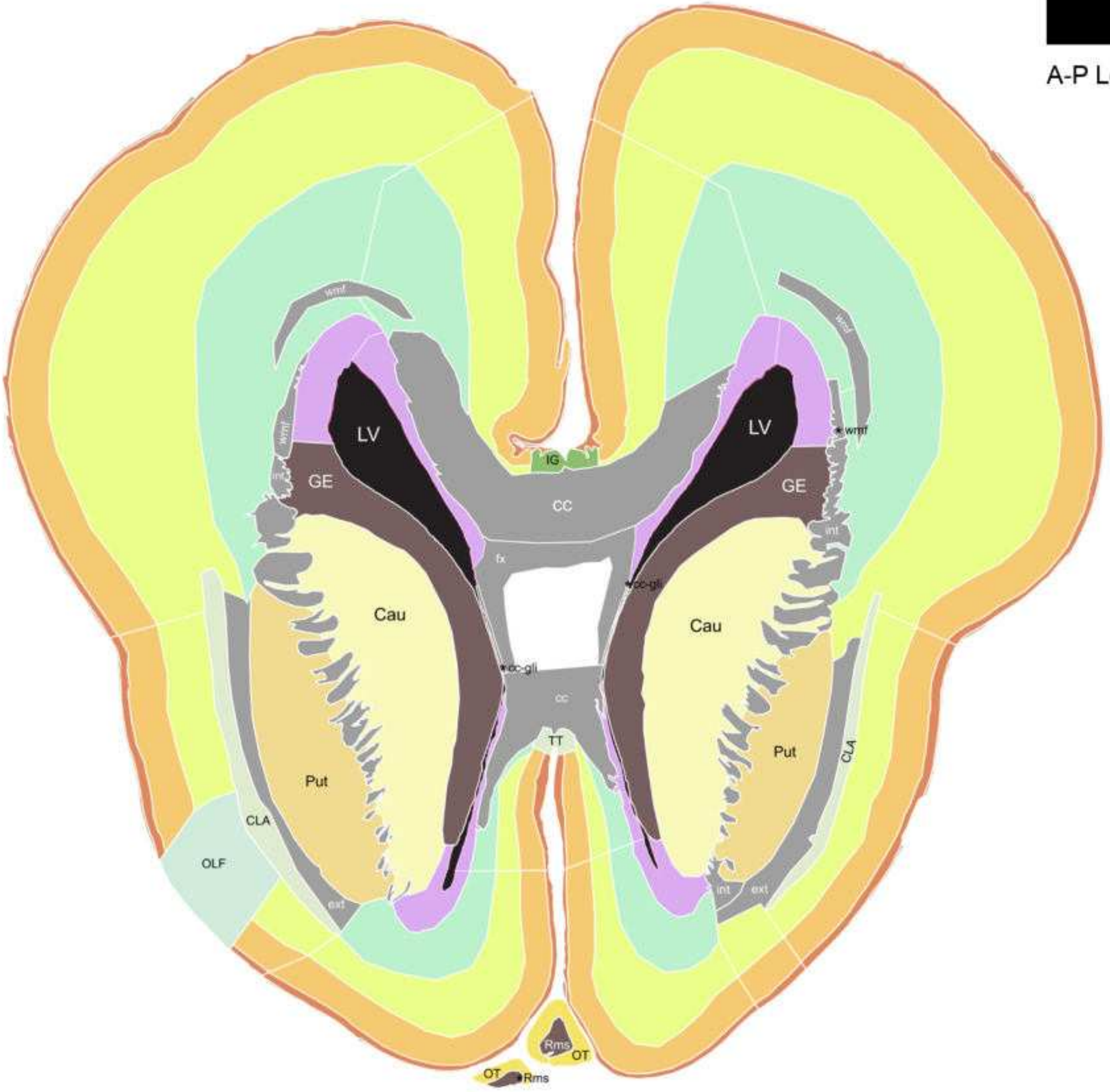

5 mm

CLA: Claustrum	LV: Lateral ventricle	TT: Tenia tecta	fx: Fornix
Cau: Caudate nucleus	OT: Olfactory tubercle	cc: Corpus callosum	int: Internal capsule
GE: Ganglionic eminence	Put: Putamen	cc-gli: Callosal glioepithelium	wmf: White matter fibers
IG: Induseum griseum	Rms: Rostral migratory stream	ext: External capsule	

Age: 22 GW

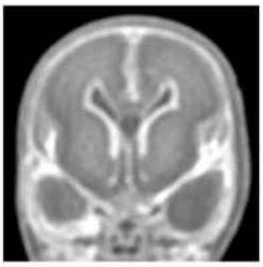

A-P Level: 7.44 mm

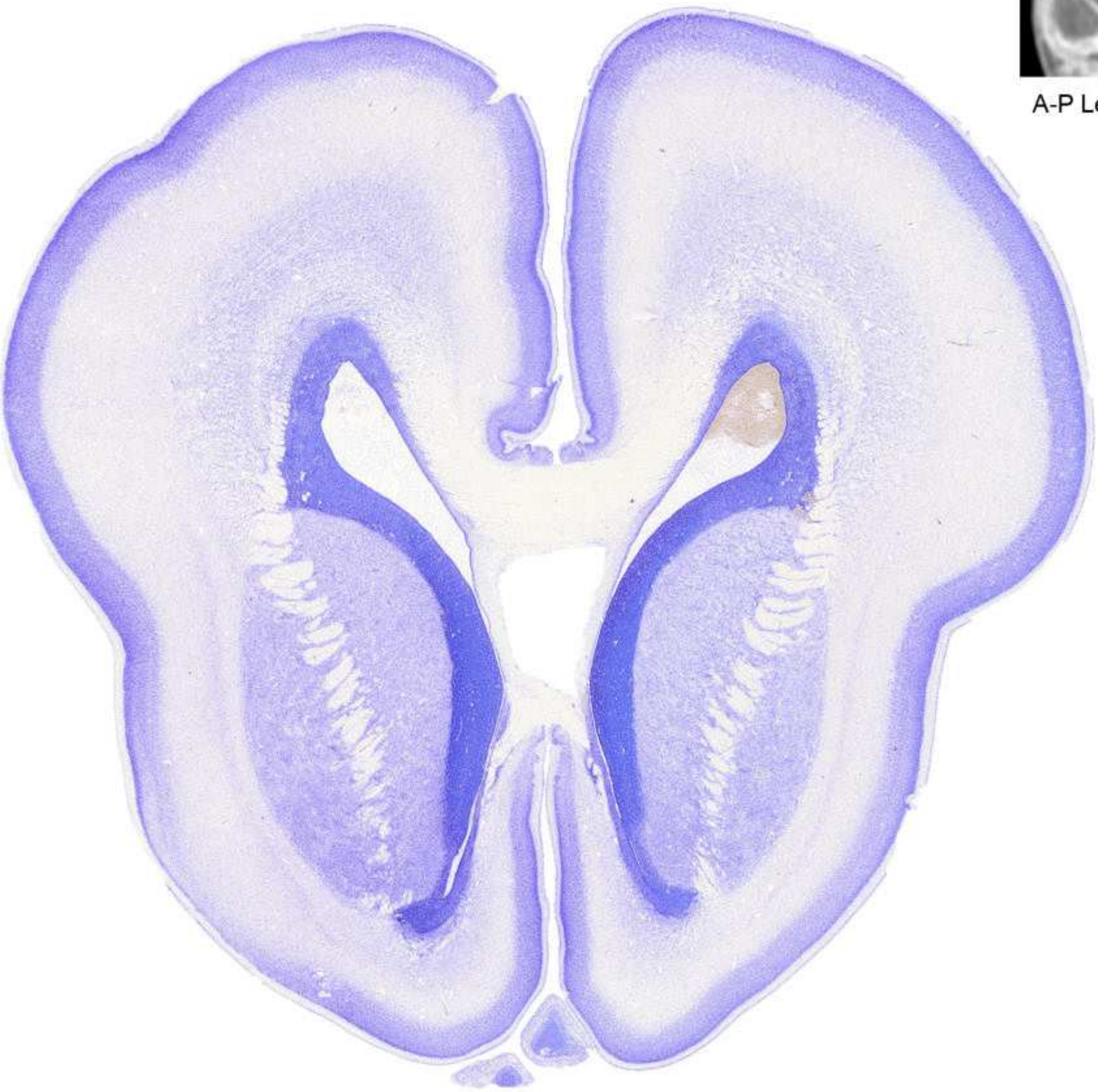

5 mm

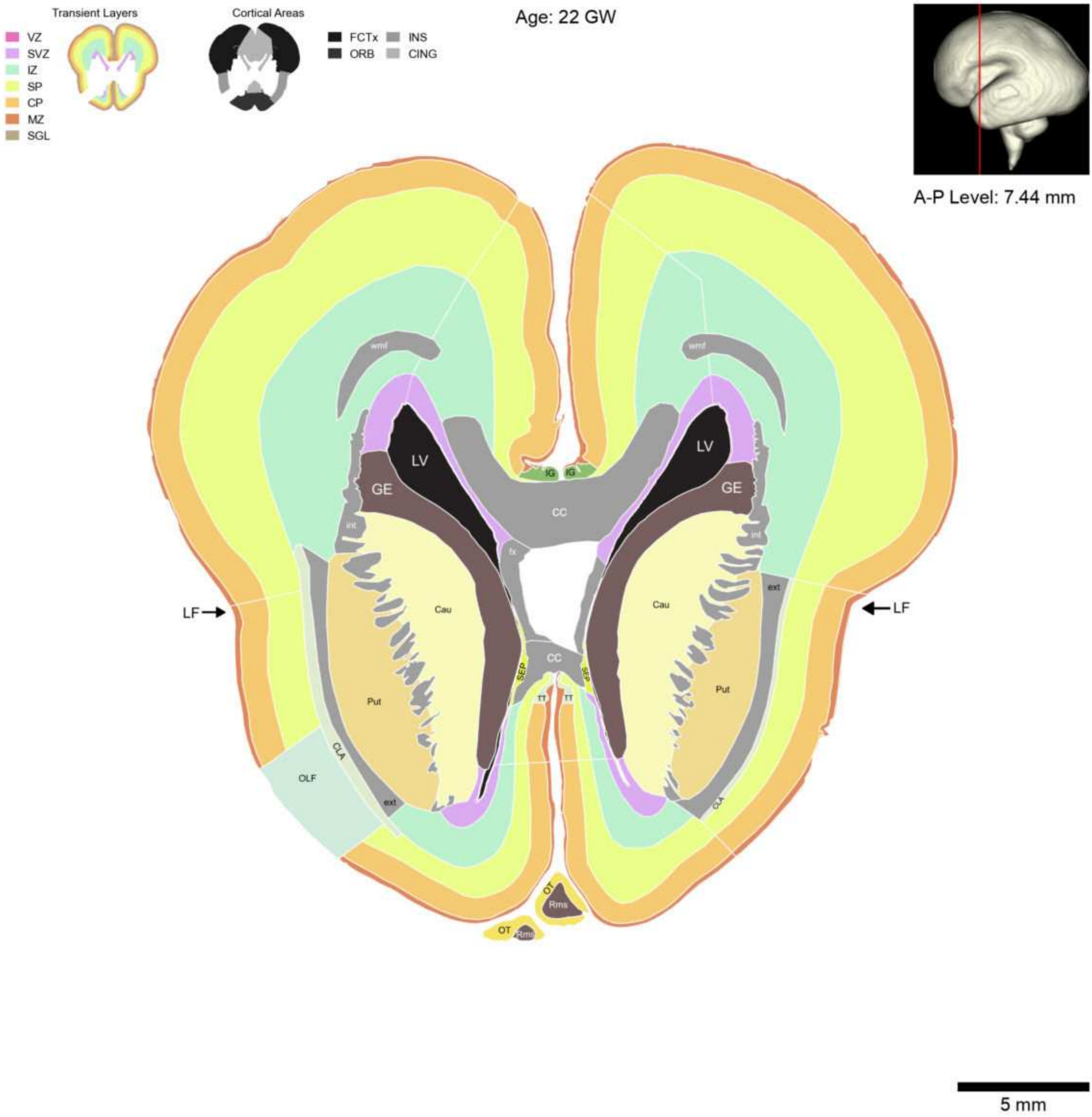

- CLA: Claustrum
- Cau: Caudate nucleus
- GE: Ganglionic eminence
- IG: Induseum griseum
- LV: Lateral ventricle
- OT: Olfactory tubercle
- Put: Putamen
- Rms: Rostral migratory stream
- SEP: Septum
- TT: Tenia tecta
- cc: Corpus callosum
- ext: External capsule
- fx: Fornix
- int: Internal capsule
- wmf: White matter fibers
- → LF: Lateral fissure

Age: 22 GW

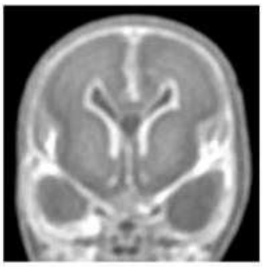

A-P Level: 6.96 mm

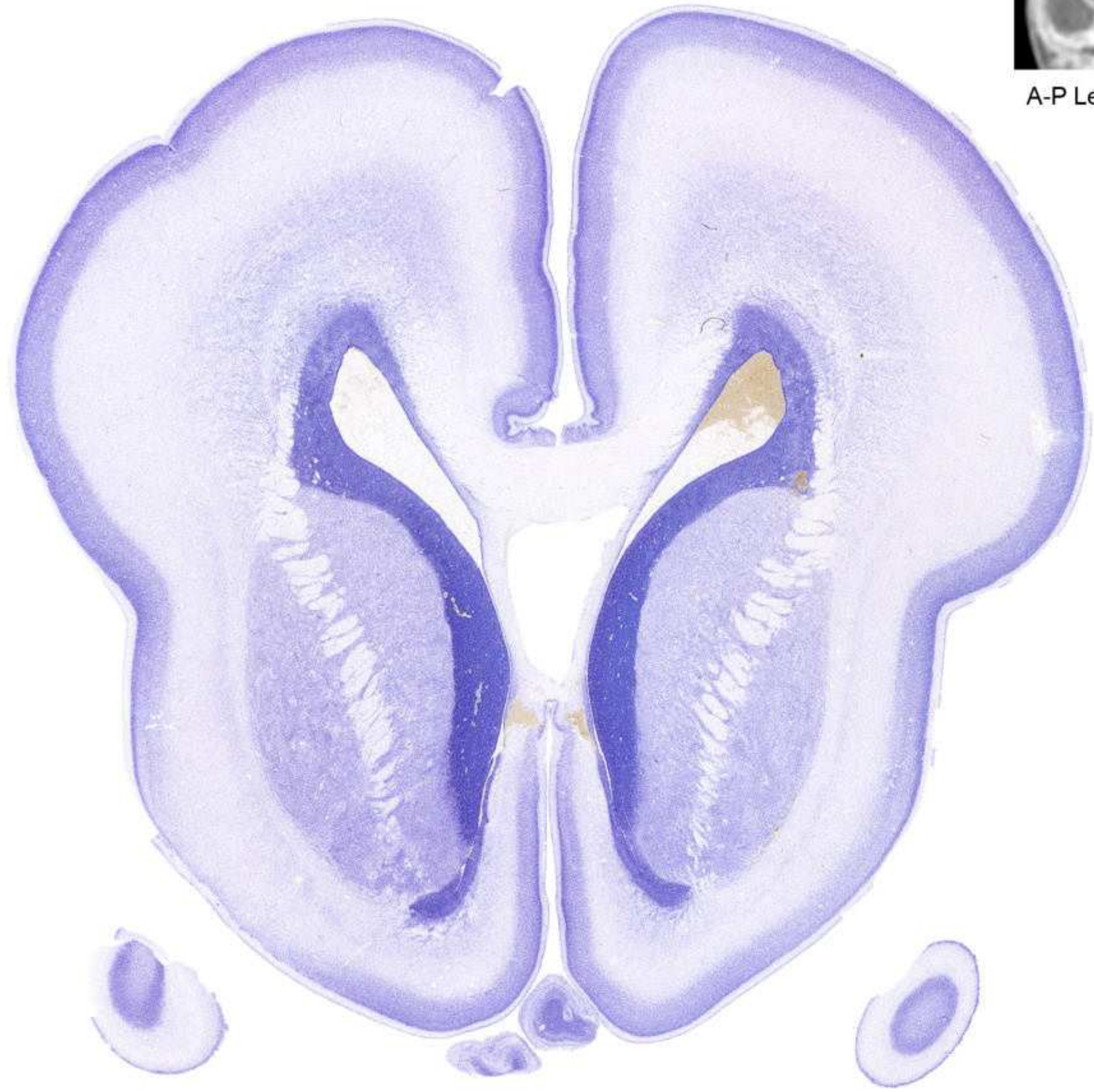

5 mm

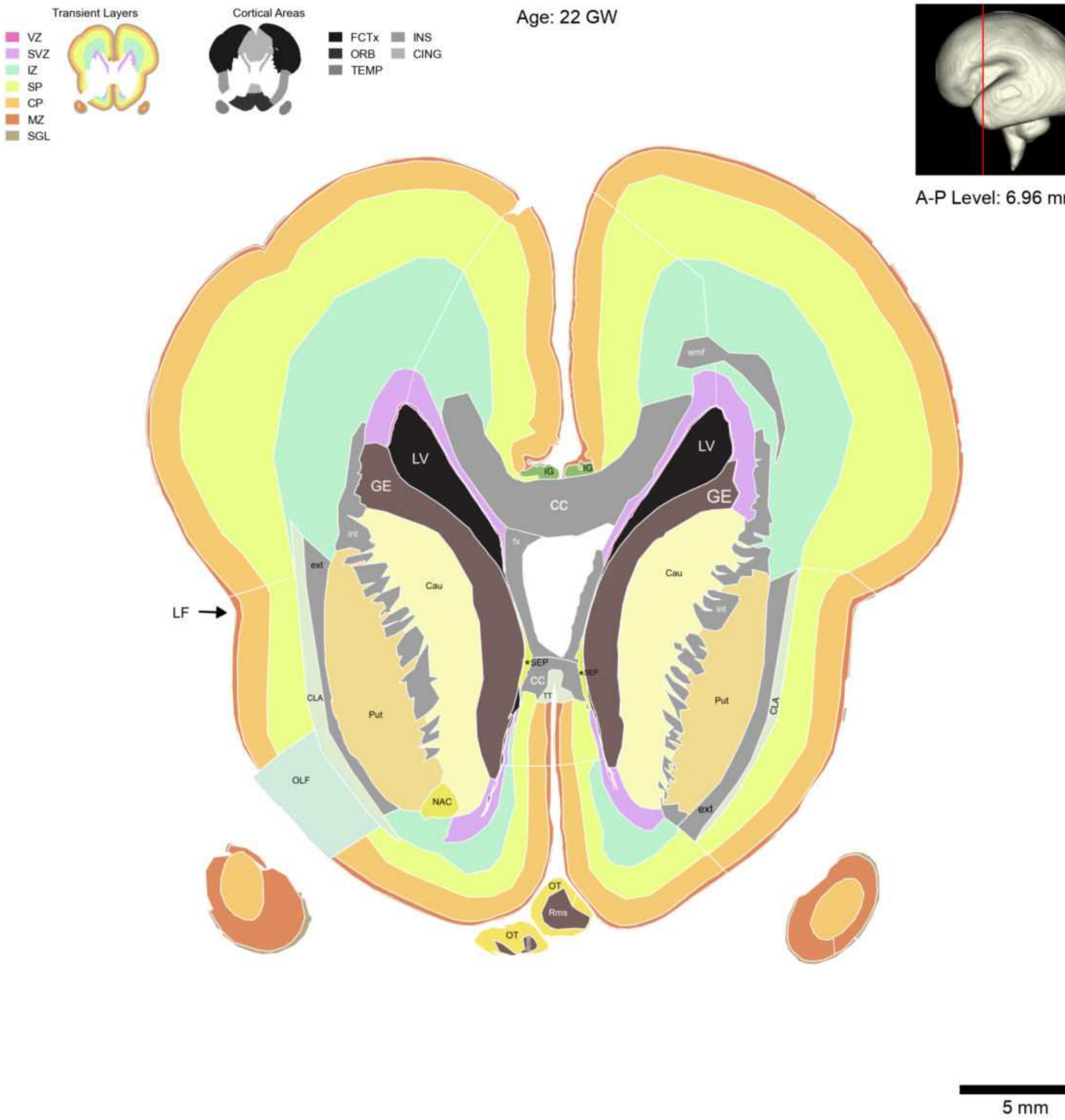

- CLA: Claustrum
- Cau: Caudate nucleus
- GE: Ganglionic eminence
- IG: Induseum griseum
- LV: Lateral ventricle
- NAc: Nucleus accumbens
- OT: Olfactory tubercle
- Put: Putamen
- Rms: Rostral migratory stream
- SEP: Septum
- TT: Tenia tecta
- cc: Corpus callosum
- ext: External capsule
- fx: Fornix
- int: Internal capsule
- wmf: White matter fibers
- → LF: Lateral fissure

Age: 22 GW

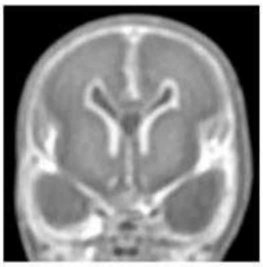

A-P Level: 6.6 mm

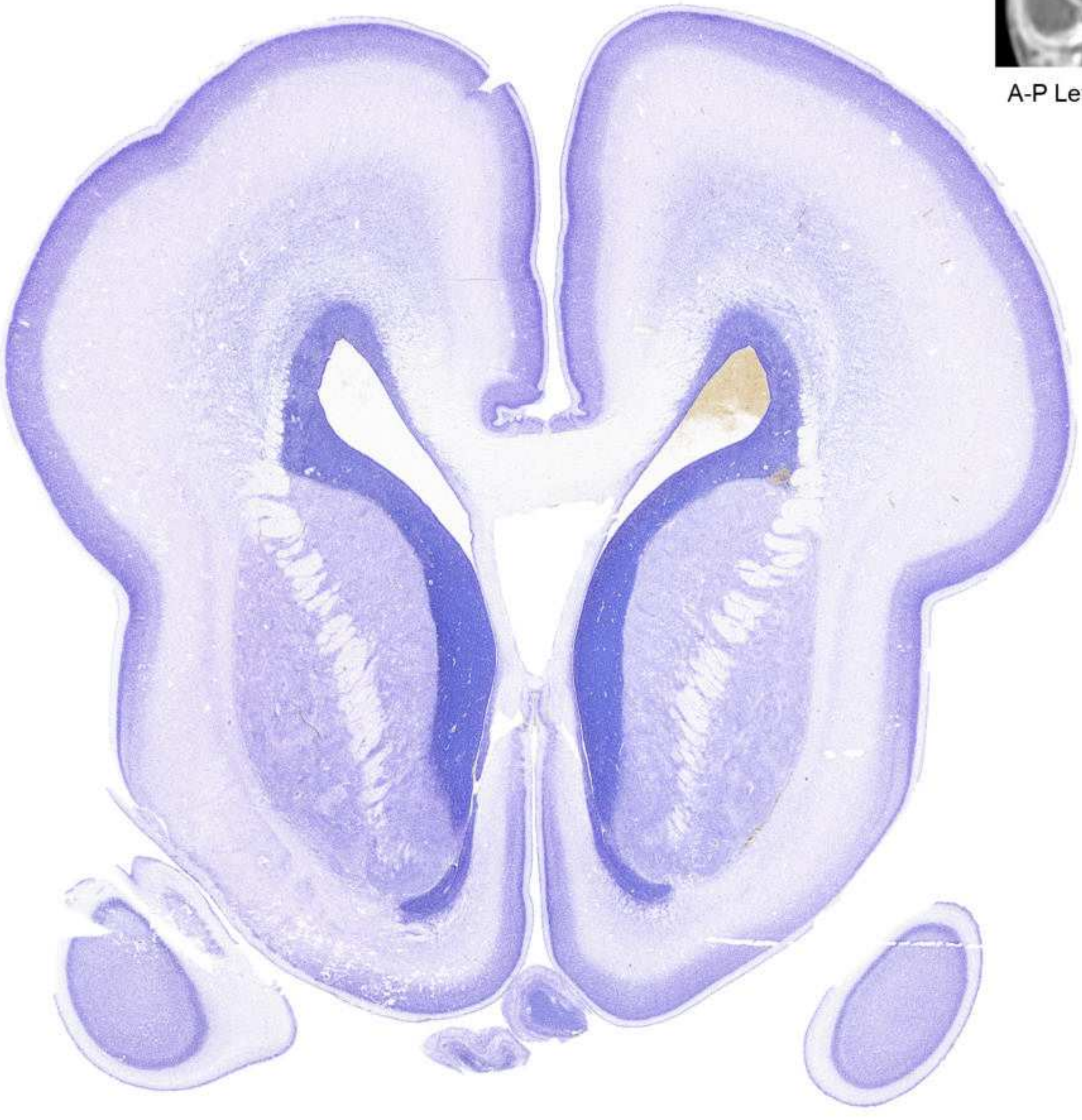

5 mm

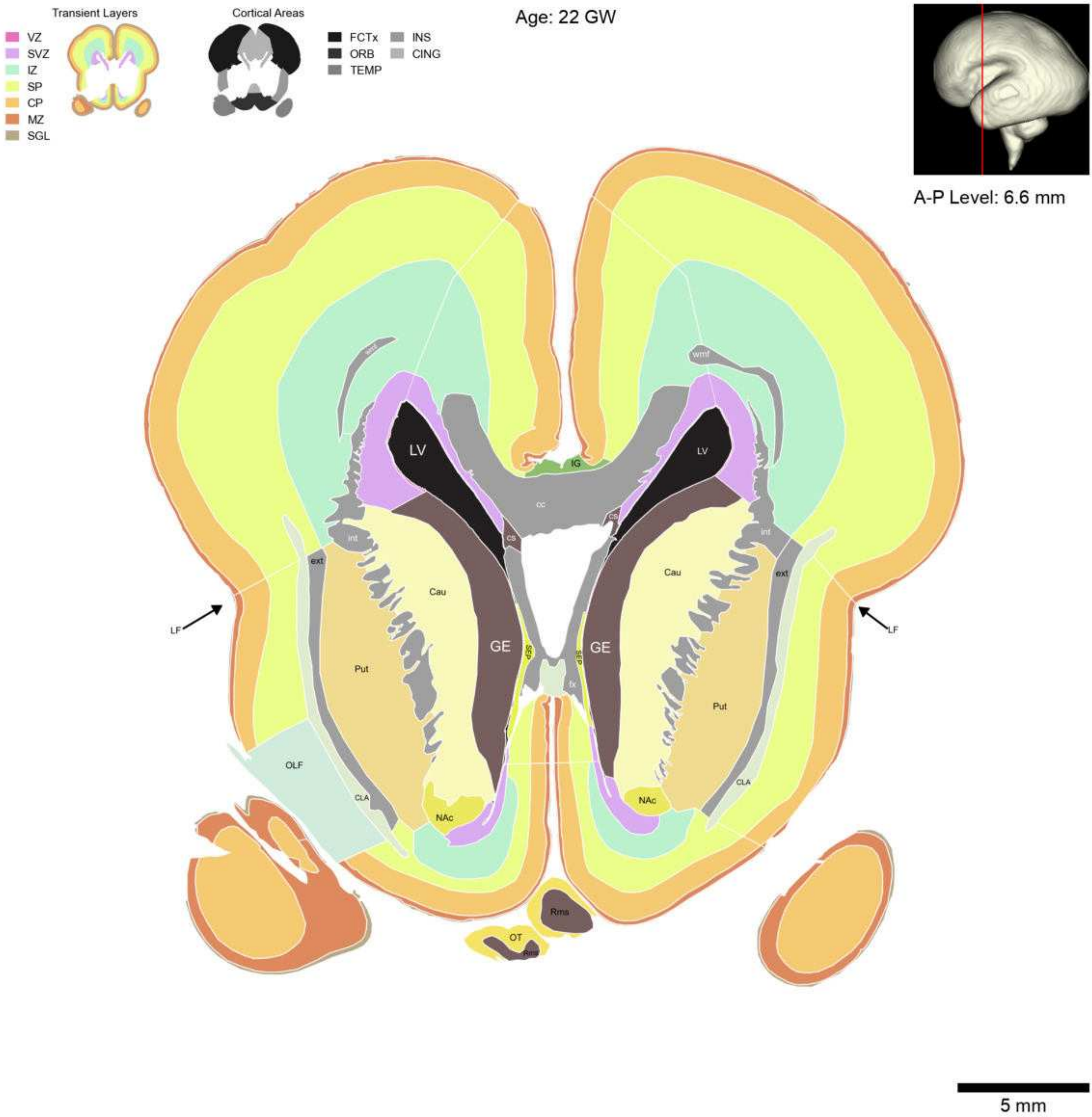

- CLA: Claustrum
- Cau: Caudate nucleus
- GE: Ganglionic eminence
- IG: Induseum griseum
- LV: Lateral ventricle
- NAc: Nucleus accumbens
- OT: Olfactory tubercle
- Put: Putamen
- Rms: Rostral migratory stream
- SEP: Septum
- TT: Tenia tecta
- cc: Corpus callosum
- cs: Calossal sling
- ext: External capsule
- fx: Fornix
- int: Internal capsule
- wmf: White matter fibers

Age: 22 GW

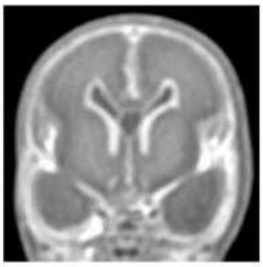

A-P Level: 6.18 mm

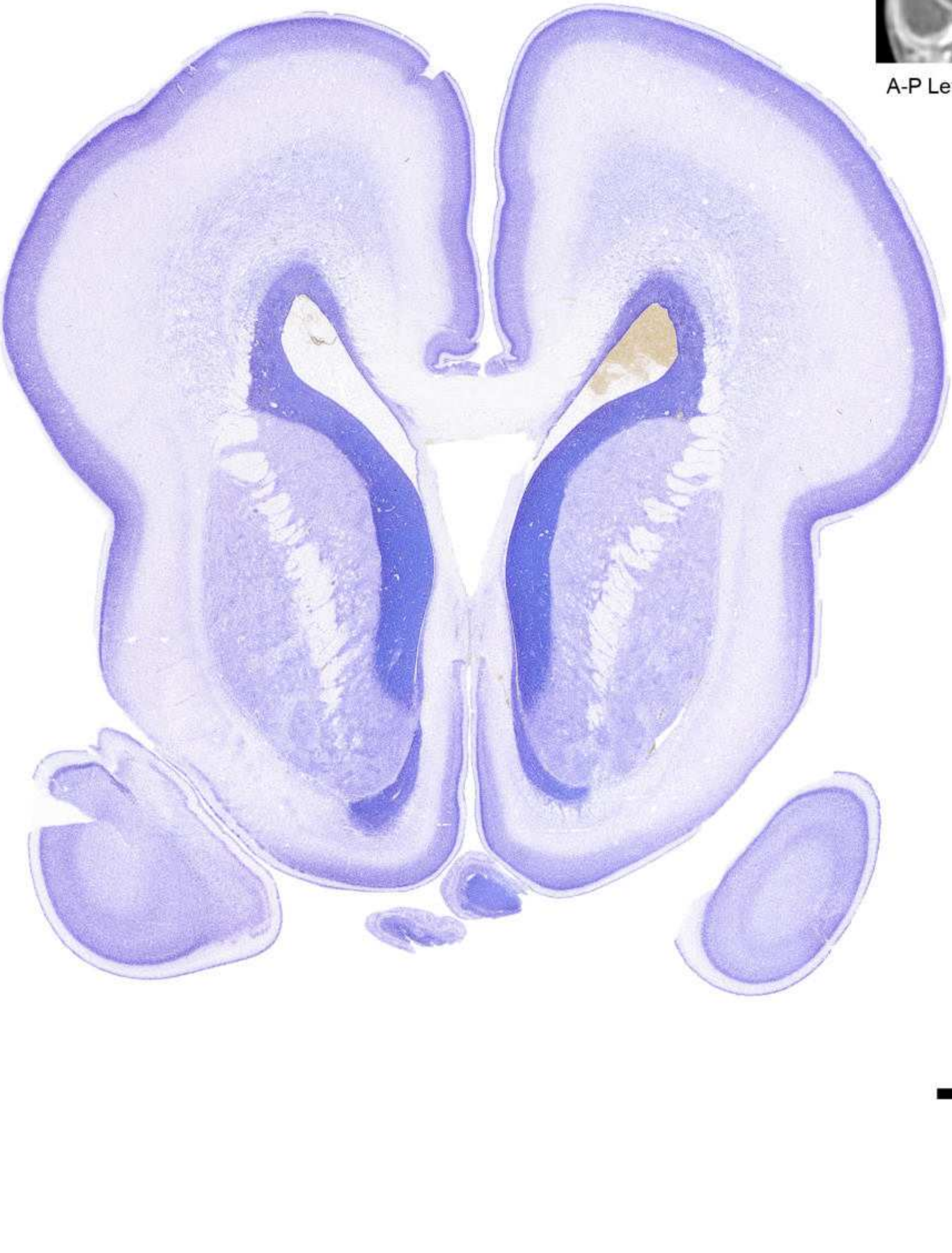

5 mm

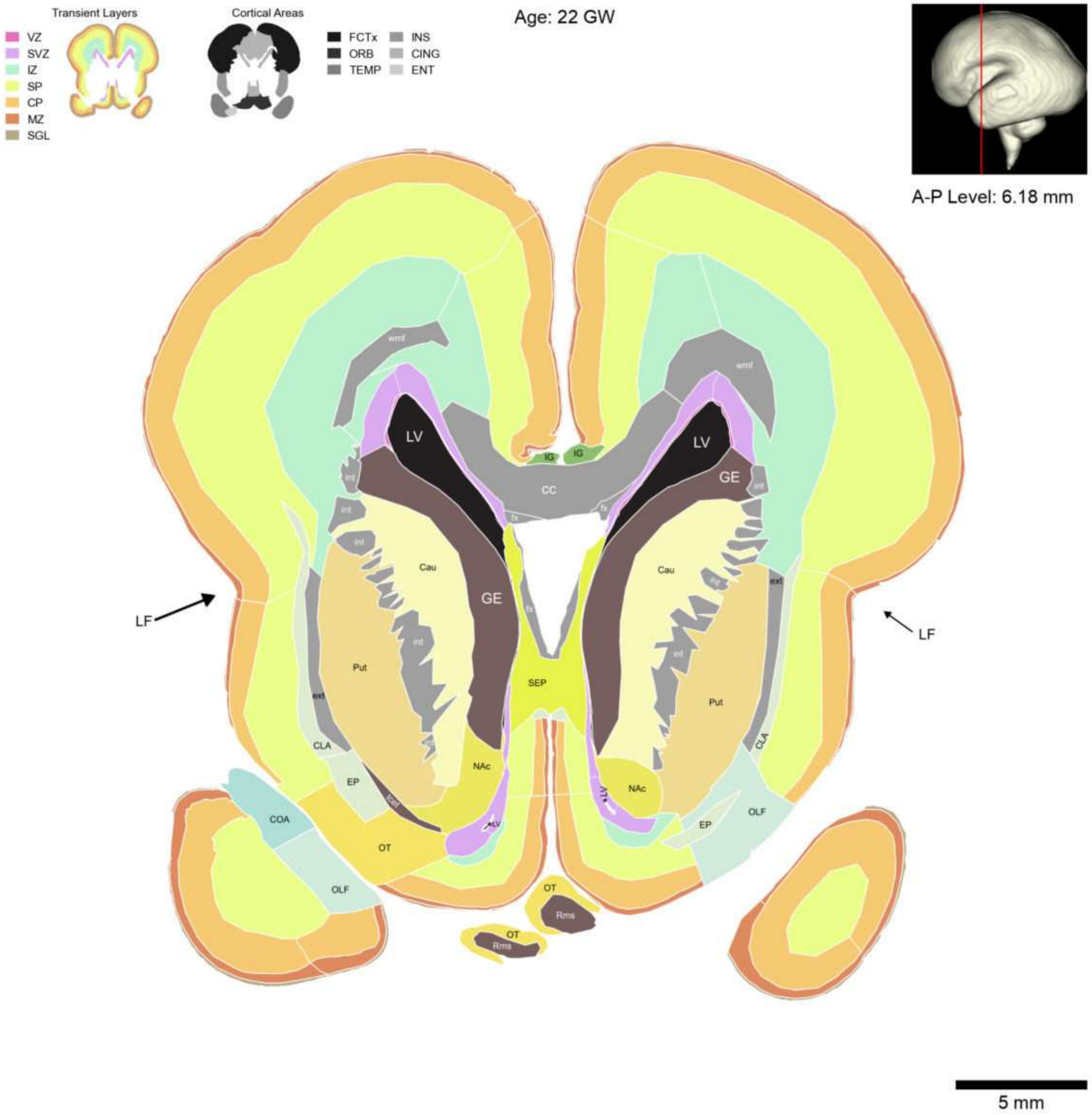

CLA: Claustrum
COA: Cortical nucleus [amygdala]
Cau: Caudate nucleus
EP: Endopiriform nucleus
GE: Ganglionic eminence
IG: Induseum griseum
LV: Lateral ventricle
NAc: Nucleus accumbens
OT: Olfactory tubercle
Put: Putamen
Rms: Rostral migratory stream
SEP: Septum
TT: Tenia tecta
cc: Corpus callosum
ext: External capsule
fx: Fornix
int: Internal capsule
tcet: Transient cell zone in the external capsule
wmf: White matter fibers
→ LF: Lateral fissure

Age: 22 GW

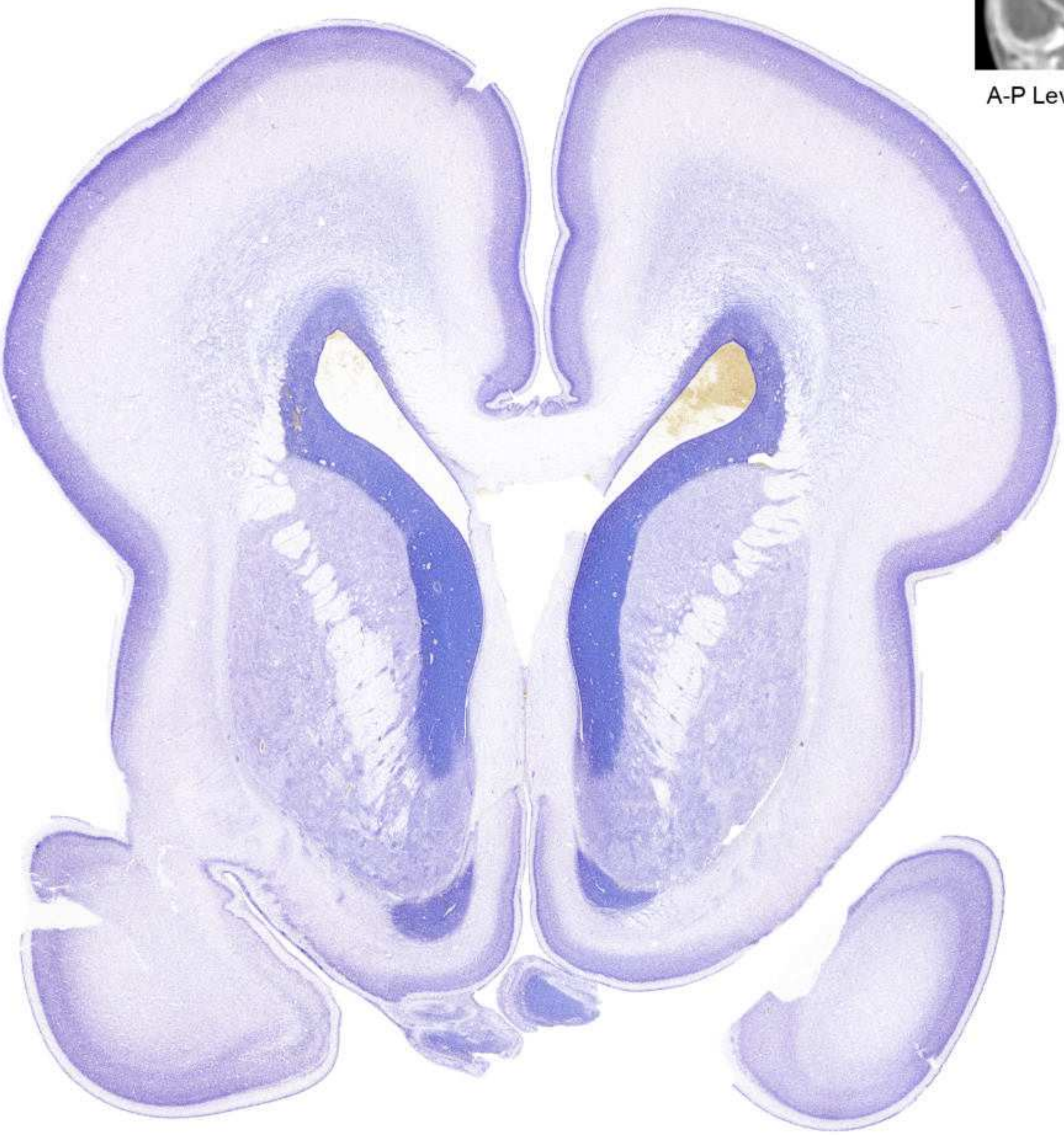

A-P Level: 5.94 mm

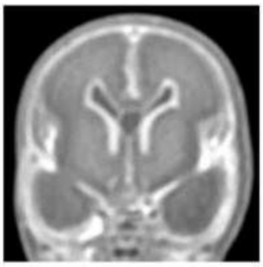

5 mm

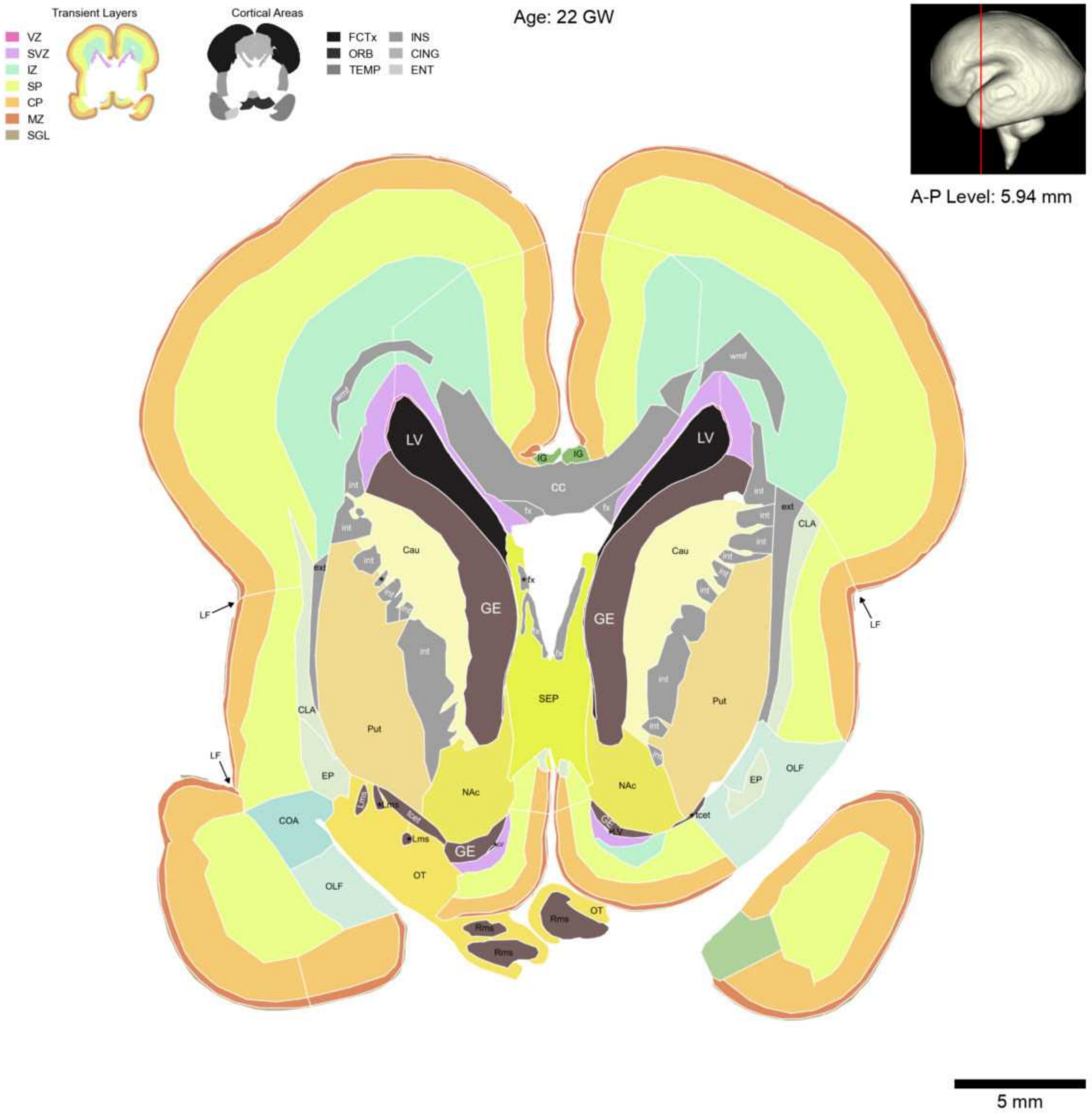

CLA: Claustrum
COA: Cortical nucleus [amygdala]
Cau: Caudate nucleus
EP: Endopiriform nucleus
GE: Ganglionic eminence
IG: Induseum griseum
LV: Lateral ventricle
Lms: Lateral migratory stream
NAc: Nucleus accumbens
OT: Olfactory tubercle
Put: Putamen
Rms: Rostral migratory stream
SEP: Septum
TT: Tenia tecta
cc: Corpus callosum
ext: External capsule
fx: Fornix
int: Internal capsule
tcet: Transient cell zone in the external capsule
wmf: White matter fibers
LF: Lateral fissure

Age: 22 GW

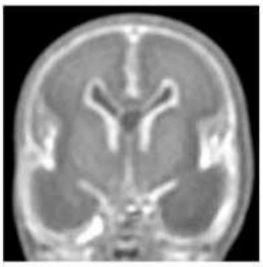

A-P Level: 5.34 mm

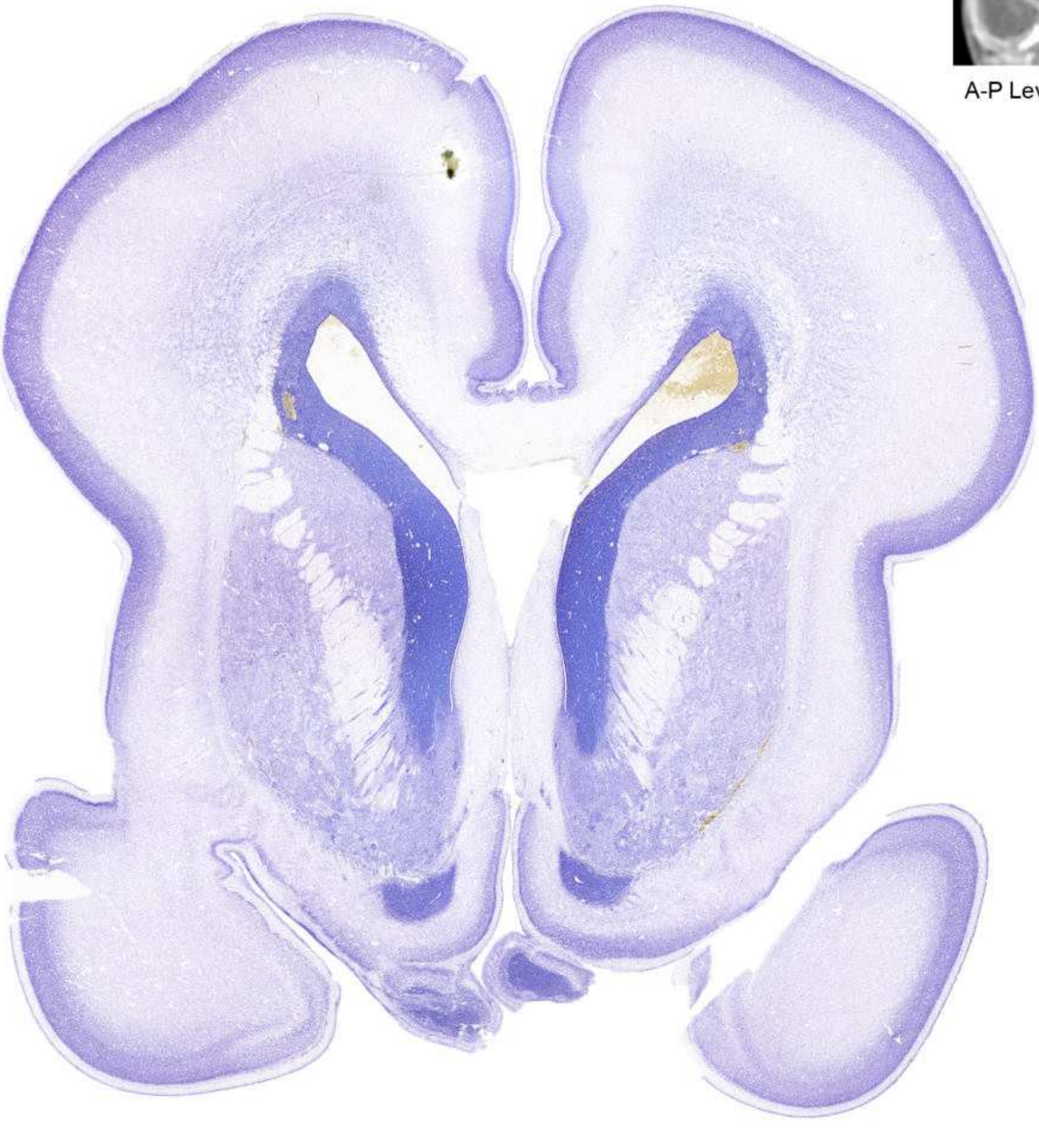

5 mm

Transient Layers

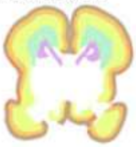

VZ
SVZ
IZ
SP
CP
MZ
SGL

Cortical Areas

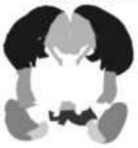

FCTx
ORB
TEMP
INS
CING
ENT

Age: 22 GW

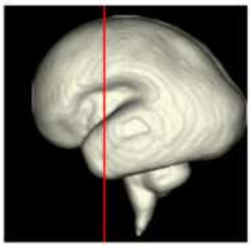

A-P Level: 5.34 mm

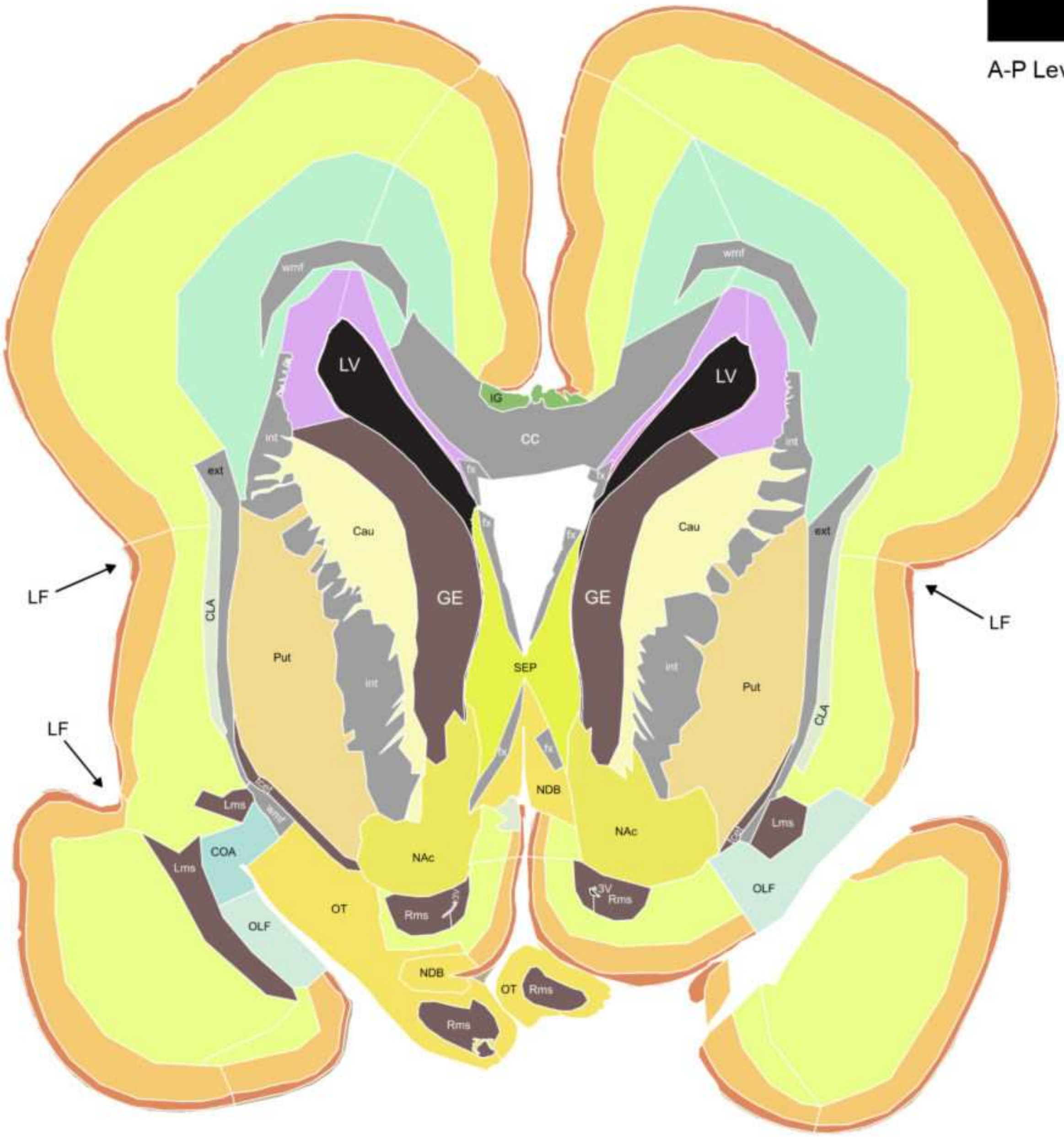

5 mm

3V: Third ventricle	LV: Lateral ventricle	Put: Putamen	ext: External capsule
CLA: Claustrum	Lms: Lateral migratory stream	Rms: Rostral migratory stream	fx: Fornix
COA: Cortical nucleus [amygdala]	NAc: Nucleus accumbens	SEP: Septum	int: Internal capsule
Cau: Caudate nucleus	NDB: Nucleus of the diagonal band	TT: Tenia tecta	tcet: Transient cell zone in the external capsule
GE: Ganglionic eminence	OT: Olfactory tubercle	cc: Corpus callosum	wmf: White matter fibers
IG: Induseum griseum			LF: Lateral fissure

Age: 22 GW

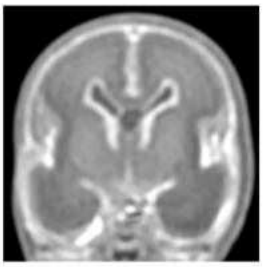

A-P Level: 4.98 mm

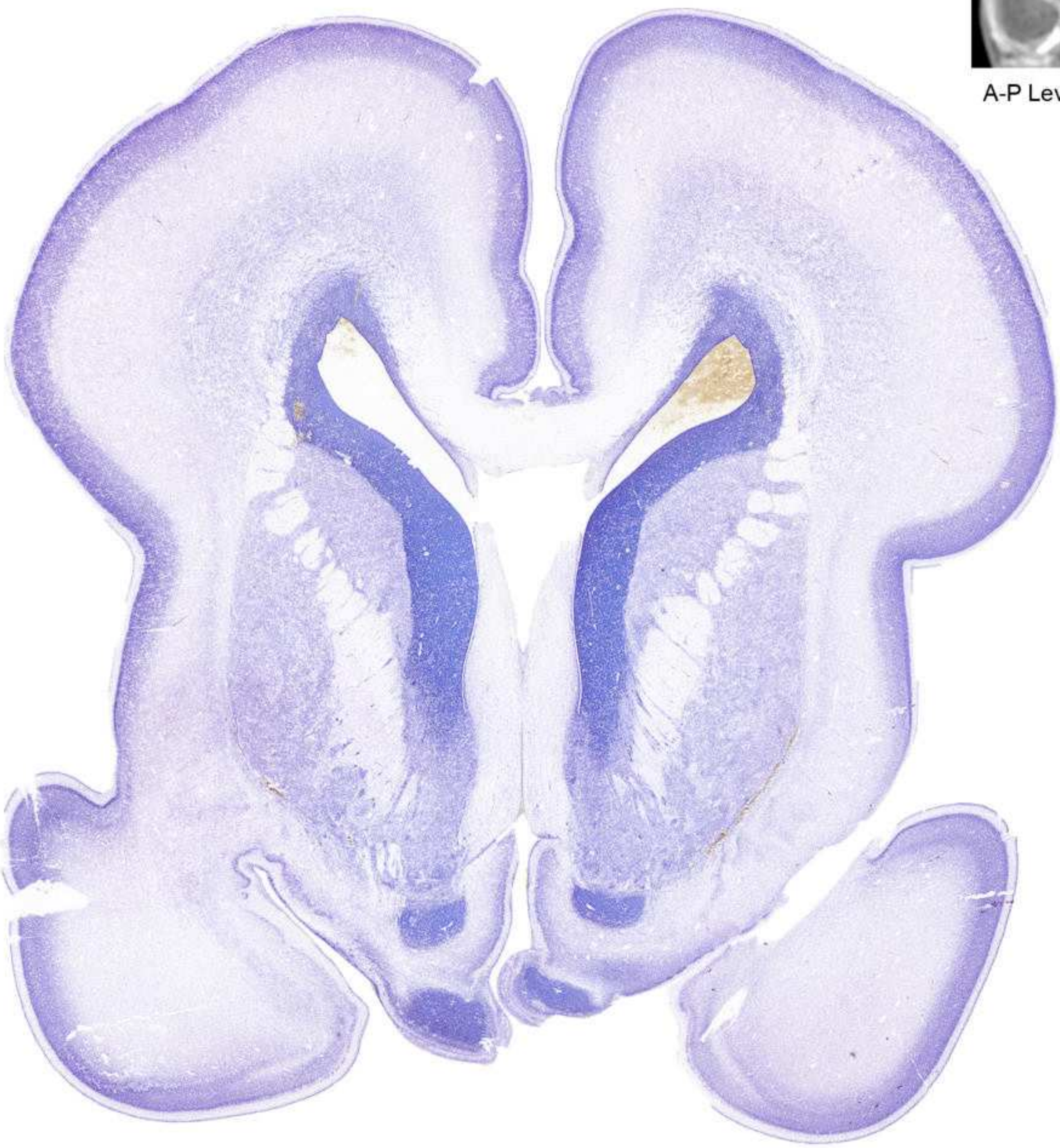

5 mm

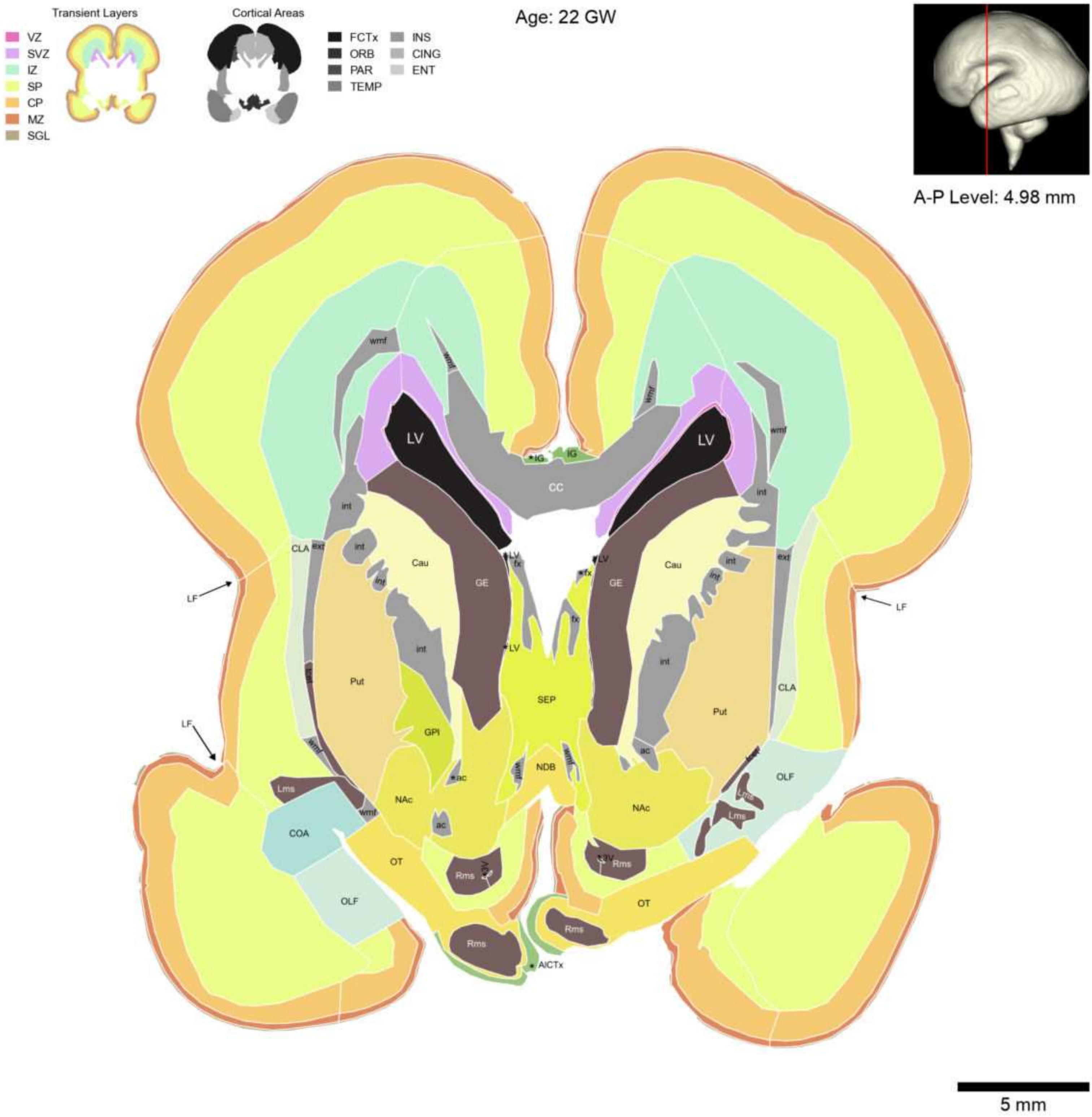

- 3V: Third ventricle
- AlCTx: Allocortex
- CLA: Claustrum
- COA: Cortical nucleus [amygdala]
- Cau: Caudate nucleus
- GE: Ganglionic eminence
- GPl: Globus pallidus lateral segment
- IG: Induseum griseum
- LV: Lateral ventricle
- Lms: Lateral migratory stream
- NAc: Nucleus accumbens
- NDB: Nucleus of the diagonal band
- OT: Olfactory tubercle
- Put: Putamen
- Rms: Rostral migratory stream
- SEP: Septum
- ac: Anterior commissure
- cc: Corpus callosum
- ext: External capsule
- fx: Fornix
- int: Internal capsule
- tcet: Transient cell zone in the external capsule
- wmf: White matter fibers
- LF: Lateral fissure

Age: 22 GW

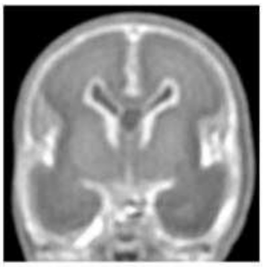

A-P Level: 4.68 mm

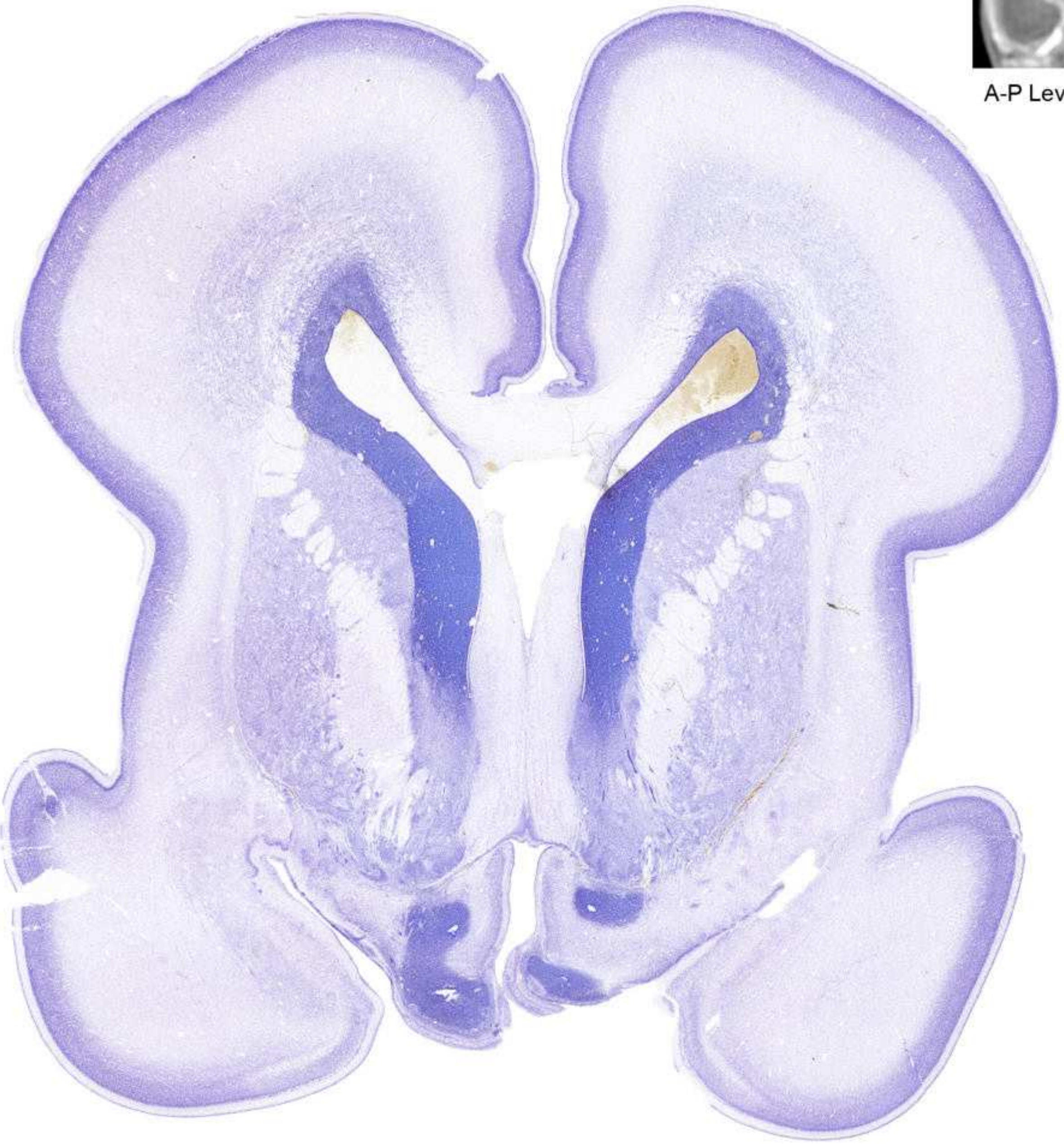

5 mm

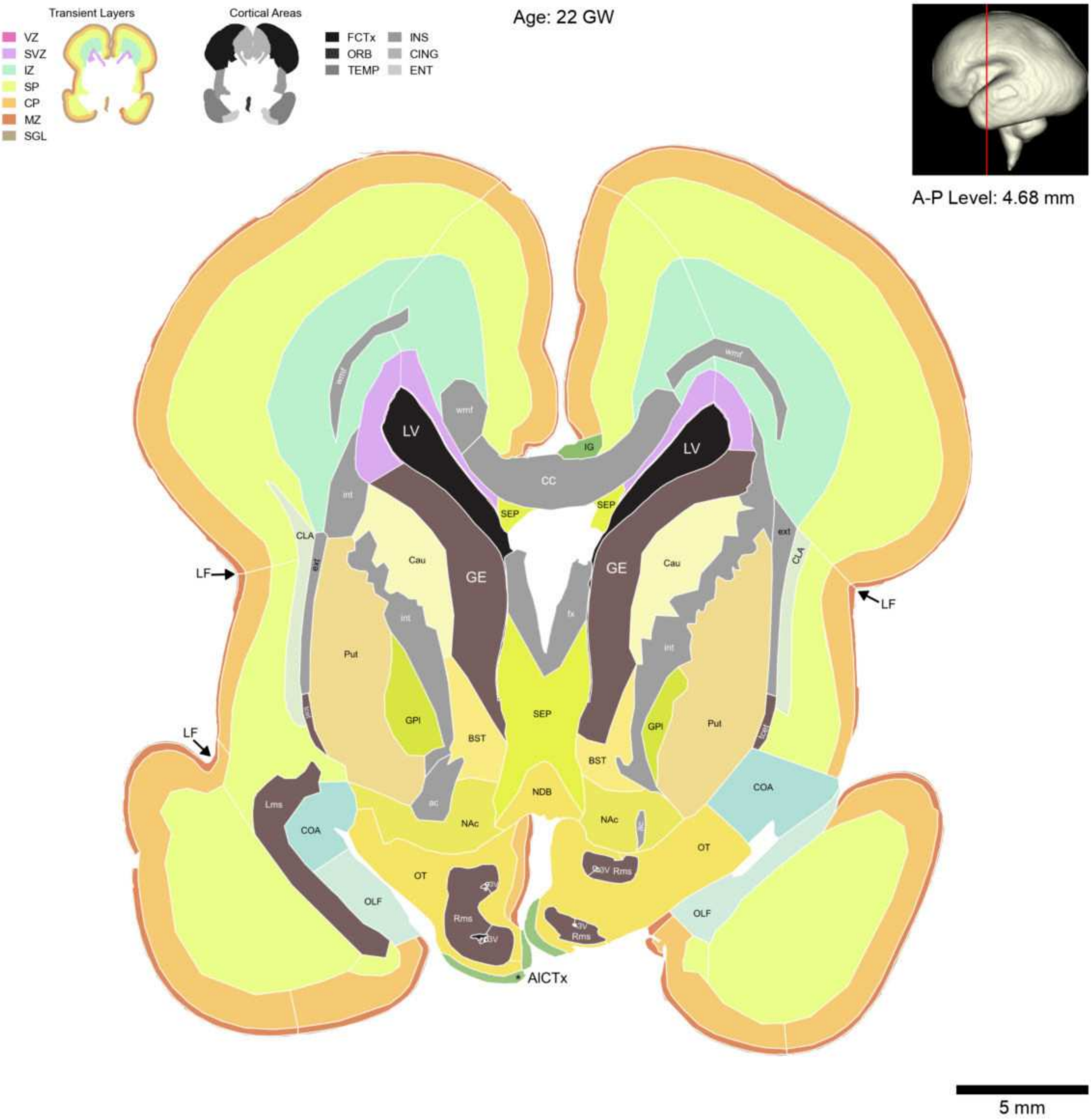

- 3V: Third ventricle
- AICTx: Allocortex
- BST: Bed nucleus of the stria terminalis
- CLA: Claustrum
- COA: Cortical nucleus [amygdala]
- Cau: Caudate nucleus
- GE: Ganglionic eminence
- GPl: Globus pallidus lateral segment
- IG: Induseum griseum
- LV: Lateral ventricle
- Lms: Lateral migratory stream
- NAc: Nucleus accumbens
- NDB: Nucleus of the diagonal band
- OT: Olfactory tubercle
- Put: Putamen
- Rms: Rostral migratory stream
- SEP: Septum
- ac: Anterior commissure
- cc: Corpus callosum
- ext: External capsule
- fx: Fornix
- int: Internal capsule
- tcet: Transient cell zone in the external capsule
- wmf: White matter fibers
- LF: Lateral fissure

Age: 22 GW

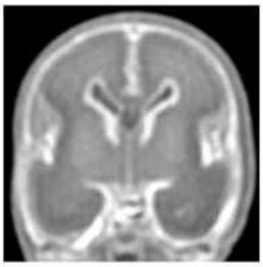

A-P Level: 4.26 mm

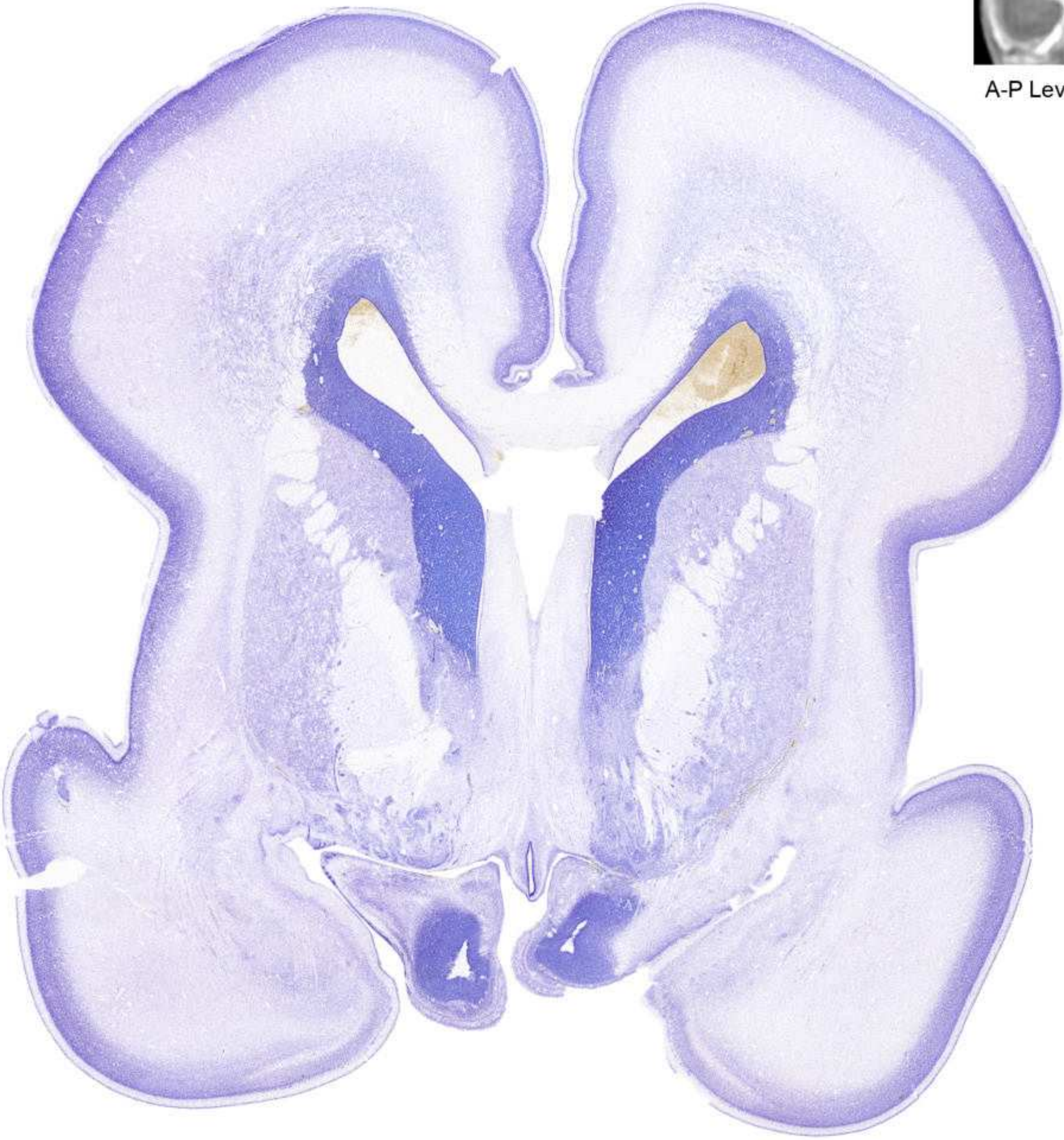

5 mm

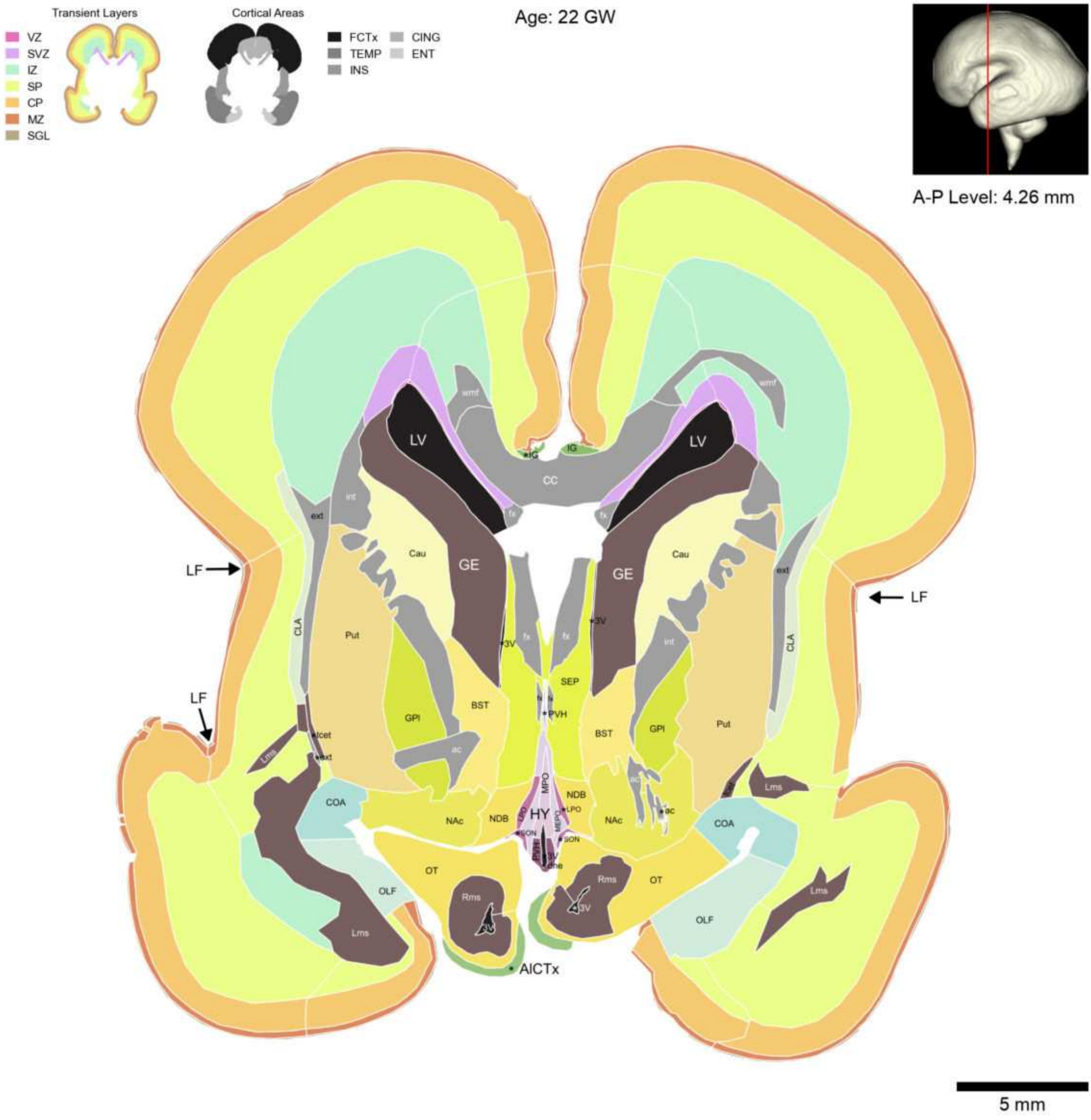

- 3V: Third ventricle
- AlCTx: Allocortex
- BST: Bed nucleus of the stria terminalis
- CLA: Claustrum
- COA: Cortical nucleus [amygdala]
- Cau: Caudate nucleus
- GE: Ganglionic eminence
- GPl: Globus pallidus lateral segment
- HY: Hypothalamus
- IG: Induseum griseum
- LPO: Lateral preoptic area
- LV: Lateral ventricle
- Lms: Lateral migratory stream
- MEPO: Medial preoptic area
- MPO: Medial preoptic nucleus
- NAc: Nucleus accumbens
- NDB: Nucleus of the diagonal band
- OT: Olfactory tubercle
- PVH: Paraventricular nucleus [hypothalamus]
- Put: Putamen
- Rms: Rostral migratory stream
- SEP: Septum
- SON: Supraoptic nucleus [hypothalamus]
- ac: Anterior commissure
- cc: Corpus callosum
- dne: Diencephalic neuroepithelium
- ext: External capsule
- fx: Fornix
- int: Internal capsule
- tcet: Transient cell zone in the external capsule
- wmf: White matter fibers
- → LF: Lateral fissure

Age: 22 GW

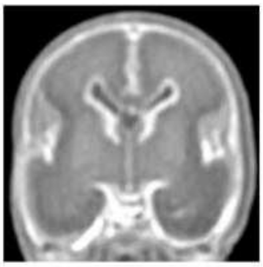

A-P Level: 3.78 mm

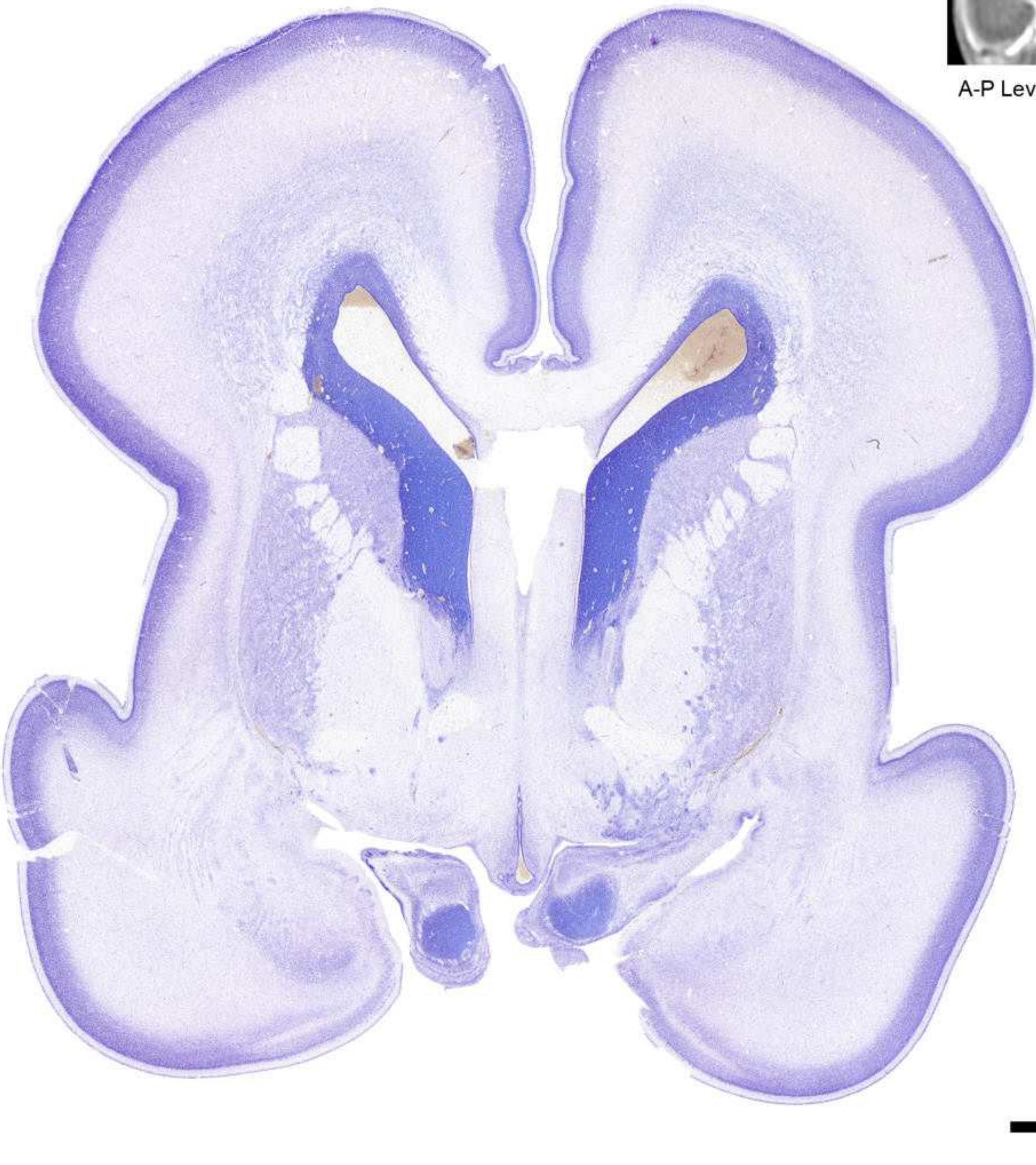

5 mm

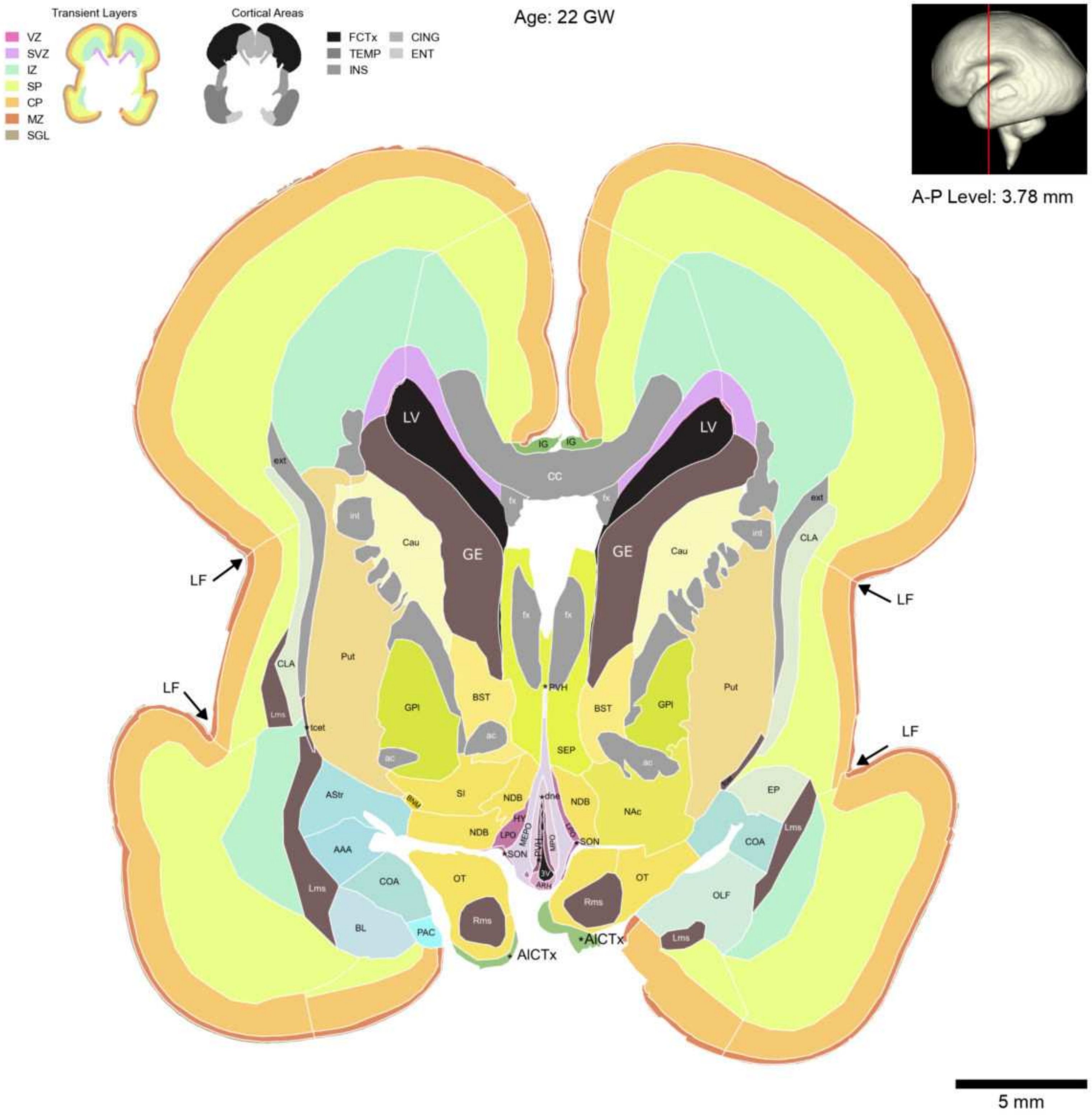

- 3V: Third ventricle
- AAA: Anterior amygdaloid area
- ARH: Arcuate nucleus [hypothalamus]
- AStr: Amygdalo-striatal area
- AICTx: Allocortex
- BL: Basal nucleus [amygdala]
- BNM: Basal nucleus of Meynert
- BST: Bed nucleus of the stria terminalis
- CLA: Claustrum
- COA: Cortical nucleus [amygdala]
- Cau: Caudate nucleus
- EP: Endopiriform nucleus
- GE: Ganglionic eminence
- GPl: Globus pallidus lateral segment
- HY: Hypothalamus
- IG: Induseum griseum
- LPO: Lateral preoptic area
- LV: Lateral ventricle
- Lms: Lateral migratory stream
- MEPO: Medial preoptic area
- MPO: Medial preoptic nucleus
- NAc: Nucleus accumbens
- NDB: Nucleus of the diagonal band
- OT: Olfactory tubercle
- PVH: Paraventricular nucleus [hypothalamus]
- Put: Putamen
- Rms: Rostral migratory stream
- SEP: Septum
- SI: Substantia innominata
- SON: Supraoptic nucleus [hypothalamus]
- ac: Anterior commissure
- cc: Corpus callosum
- dne: Diencephalic neuroepithelium
- ext: External capsule
- fx: Fornix
- int: Internal capsule
- tcet: Transient cell zone in the external capsule
- → LF: Lateral fissure

Age: 22 GW

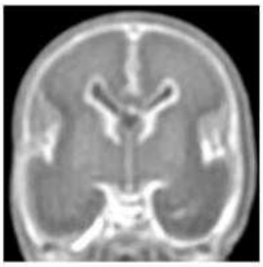

A-P Level: 3.48 mm

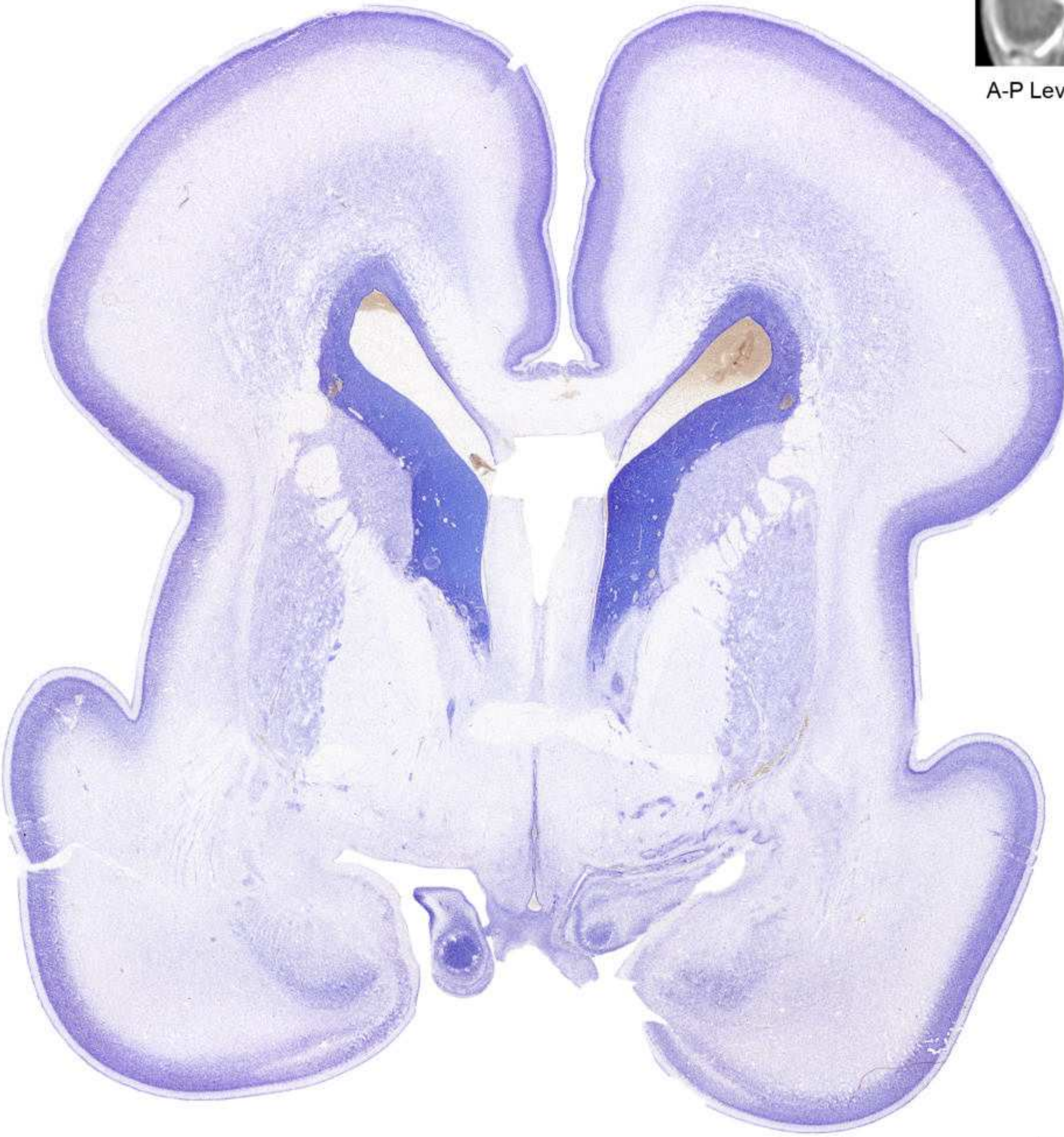

5 mm

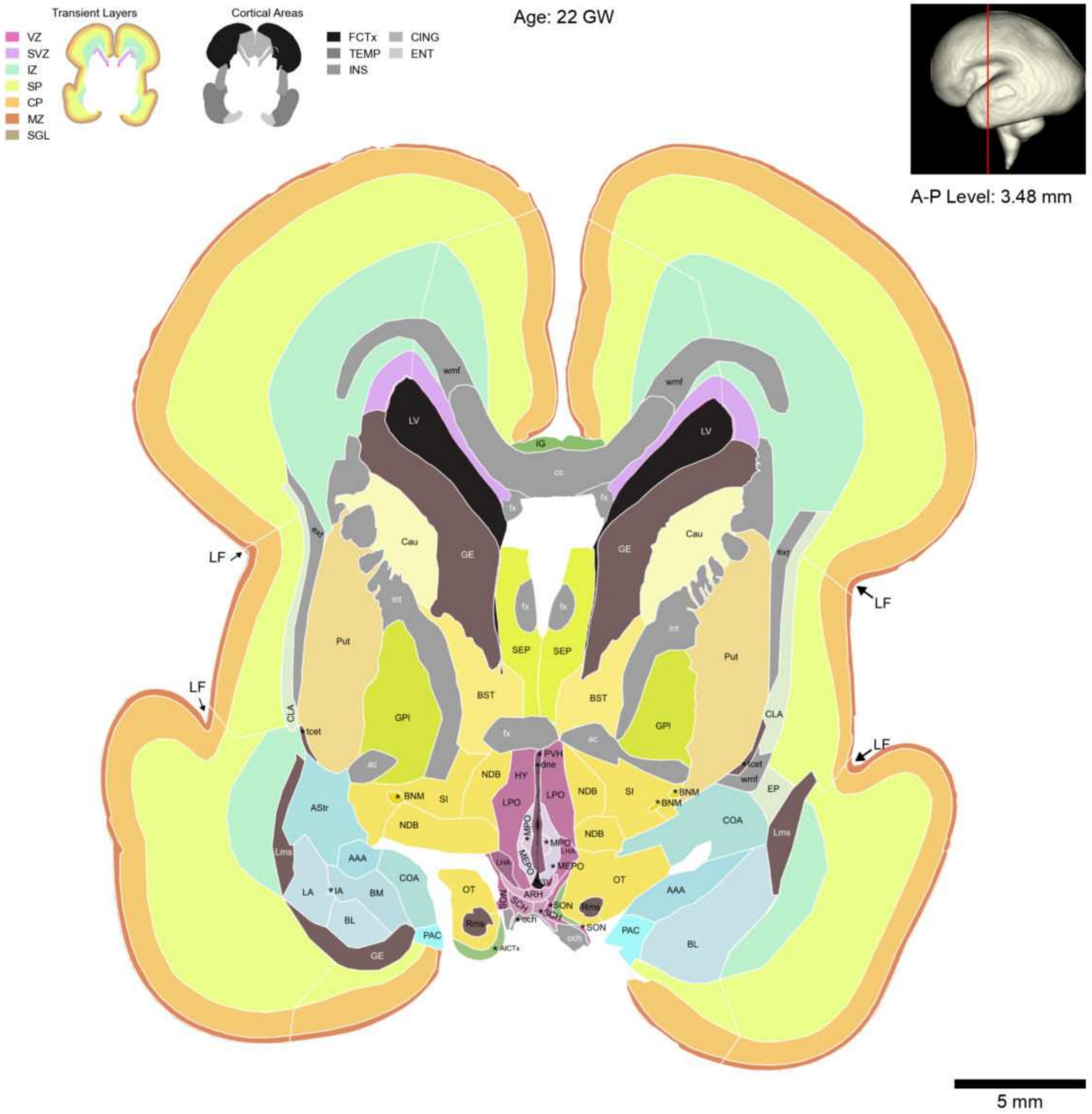

- 3V: Third ventricle
- AAA: Anterior amygdaloid area
- ARH: Arcuate nucleus [hypothalamus]
- AStr: Amygdalo-striatal area
- AlCTx: Allocortex
- BL: Basal nucleus [amygdala]
- BM: Basomedial nucleus [amygdala]
- BNM: Basal nucleus of Meynert
- BST: Bed nucleus of the stria terminalis
- CLA: Claustrum
- COA: Cortical nucleus [amygdala]
- Cau: Caudate nucleus
- EP: Endopiriform nucleus
- GE: Ganglionic eminence
- GPl: Globus pallidus lateral segment
- HY: Hypothalamus
- IA: Intercalated cell groups [amygdala]
- IG: Induseum griseum
- LA: Lateral nucleus [amygdala]
- LHA: Lateral hypothalamic area
- LPO: Lateral preoptic area
- LV: Lateral ventricle
- Lms: Lateral migratory stream
- MEPO: Medial preoptic area
- MPO: Medial preoptic nucleus
- NDB: Nucleus of the diagonal band
- OT: Olfactory tubercle
- PVH: Paraventricular nucleus [hypothalamus]
- Put: Putamen
- Rms: Rostral migratory stream
- SCH: Suprachiasmatic nucleus [hypothalamus]
- SEP: Septum
- SI: Substantia innominata
- SON: Supraoptic nucleus [hypothalamus]
- ac: Anterior commissure
- cc: Corpus callosum
- dne: Diencephalic neuroepithelium
- ext: External capsule
- fx: Fornix
- int: Internal capsule
- och: Optic chiasm
- tcet: Transient cell zone in the external capsule
- wmf: White matter fibers
- LF: Lateral fissure

Age: 22 GW

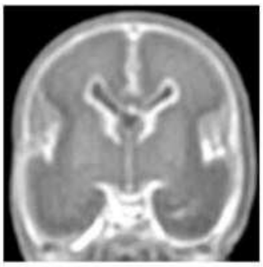

A-P Level: 2.4 mm

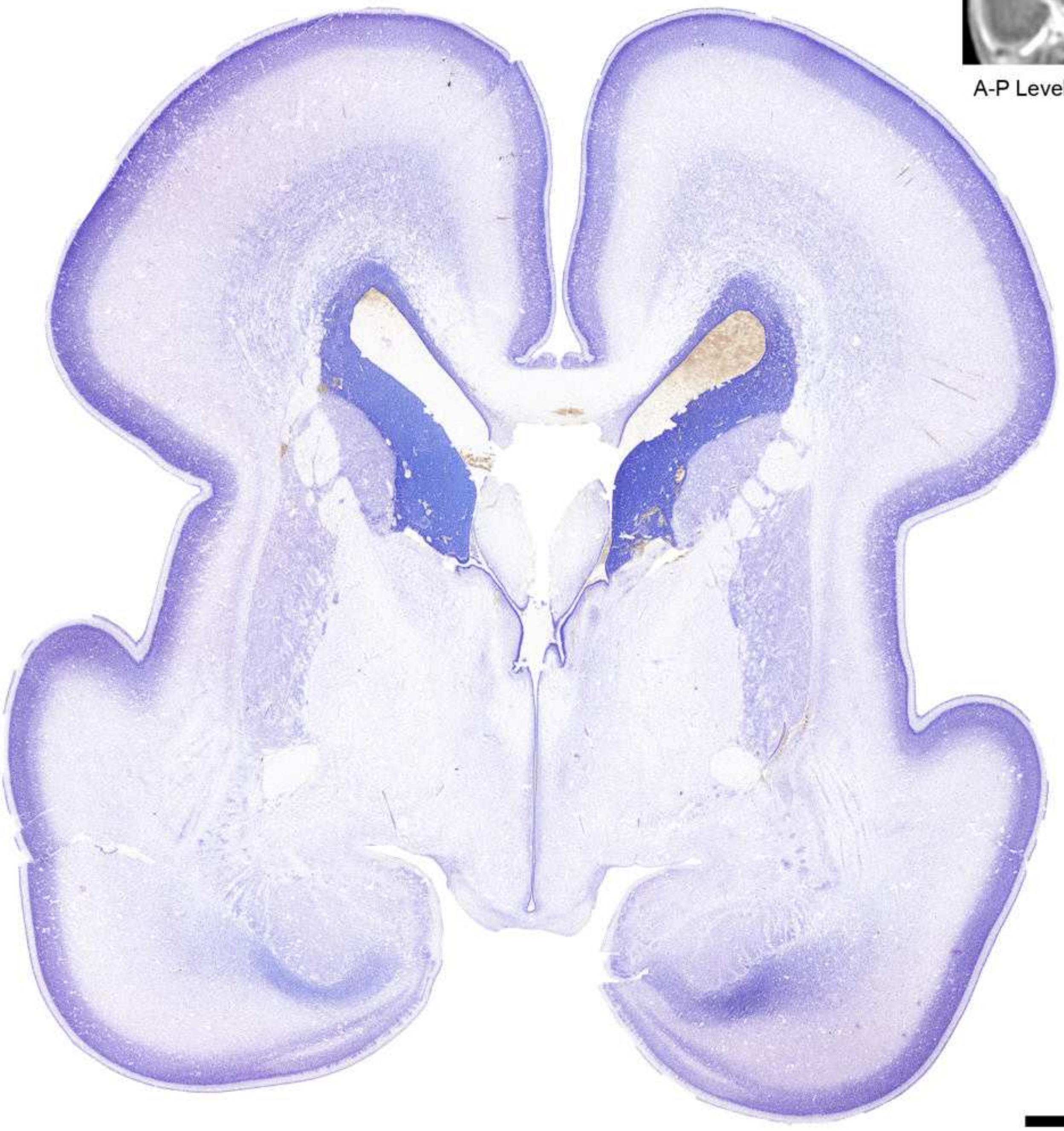

5 mm

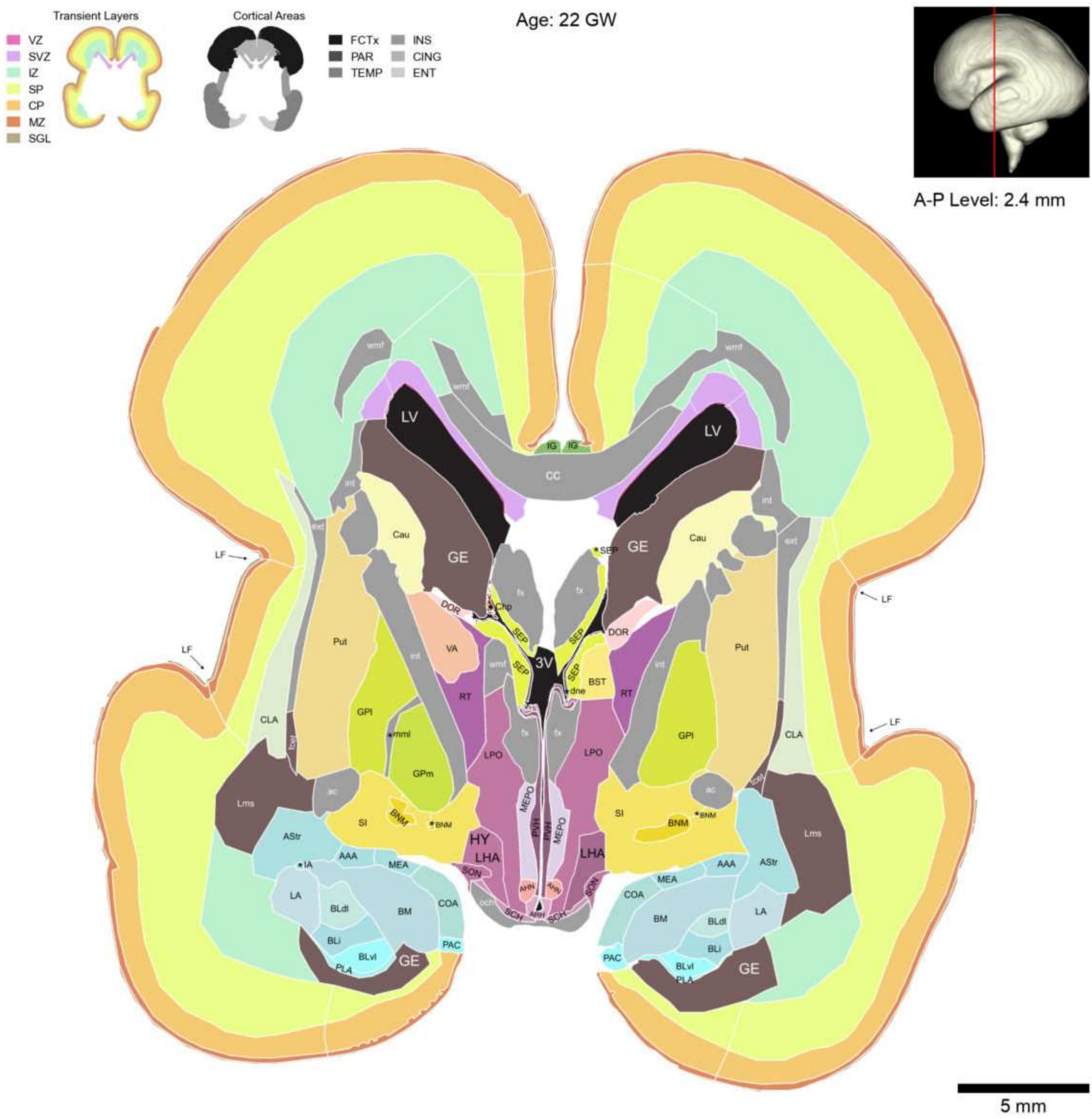

- 3V: Third ventricle
- AAA: Anterior amygdaloid area
- AHN: Anterior hypothalamic nucleus
- ARH: Arcuate nucleus [hypothalamus]
- AStr: Amygdalo-striatal area
- BLdl: Basal nucleus [amygdala], dorsolateral part
- BLi: Basal nucleus [amygdala], intermediate part
- BLvl: Basal nucleus [amygdala], ventrolateral part
- BM: Basomedial nucleus [amygdala]
- BNM: Basal nucleus of Meynert
- BST: Bed nucleus of the stria terminalis
- CLA: Claustrum
- COA: Cortical nucleus [amygdala]
- Cau: Caudate nucleus
- Chp: Choroid plexus
- DOR: Dorsal complex [thalamus]
- GE: Ganglionic eminence
- GPl: Globus pallidus lateral segment
- GPm: Globus pallidus medial segment
- HY: Hypothalamus
- IA: Intercalated cell groups [amygdala]
- IG: Induseum griseum
- LA: Lateral nucleus [amygdala]
- LHA: Lateral hypothalamic area
- LPO: Lateral preoptic area
- LV: Lateral ventricle
- Lms: Lateral migratory stream
- MEA: Medial nucleus [amygdala]
- MEPO: Medial preoptic area
- PLA: Paralaminar nucleus [amygdala]
- PVH: Paraventricular nucleus [hypothalamus]
- Put: Putamen
- RT: Reticular nucleus [thalamus]
- SCH: Suprachiasmatic nucleus [hypothalamus]
- SEP: Septum
- SI: Substantia innominata
- SON: Supraoptic nucleus [hypothalamus]
- VA: Ventral anterior nucleus [thalamus]
- ac: Anterior commissure
- cc: Corpus callosum
- dne: Diencephalic neuroepithelium
- ext: External capsule
- fx: Fornix
- int: Internal capsule
- mml: Medial medullary lamina
- och: Optic chiasm
- tcet: Transient cell zone in the external capsule
- wmf: White matter fibers
- → LF: Lateral fissure

Age: 22 GW

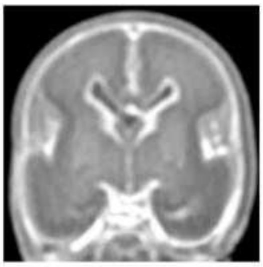

A-P Level: 2.04 mm

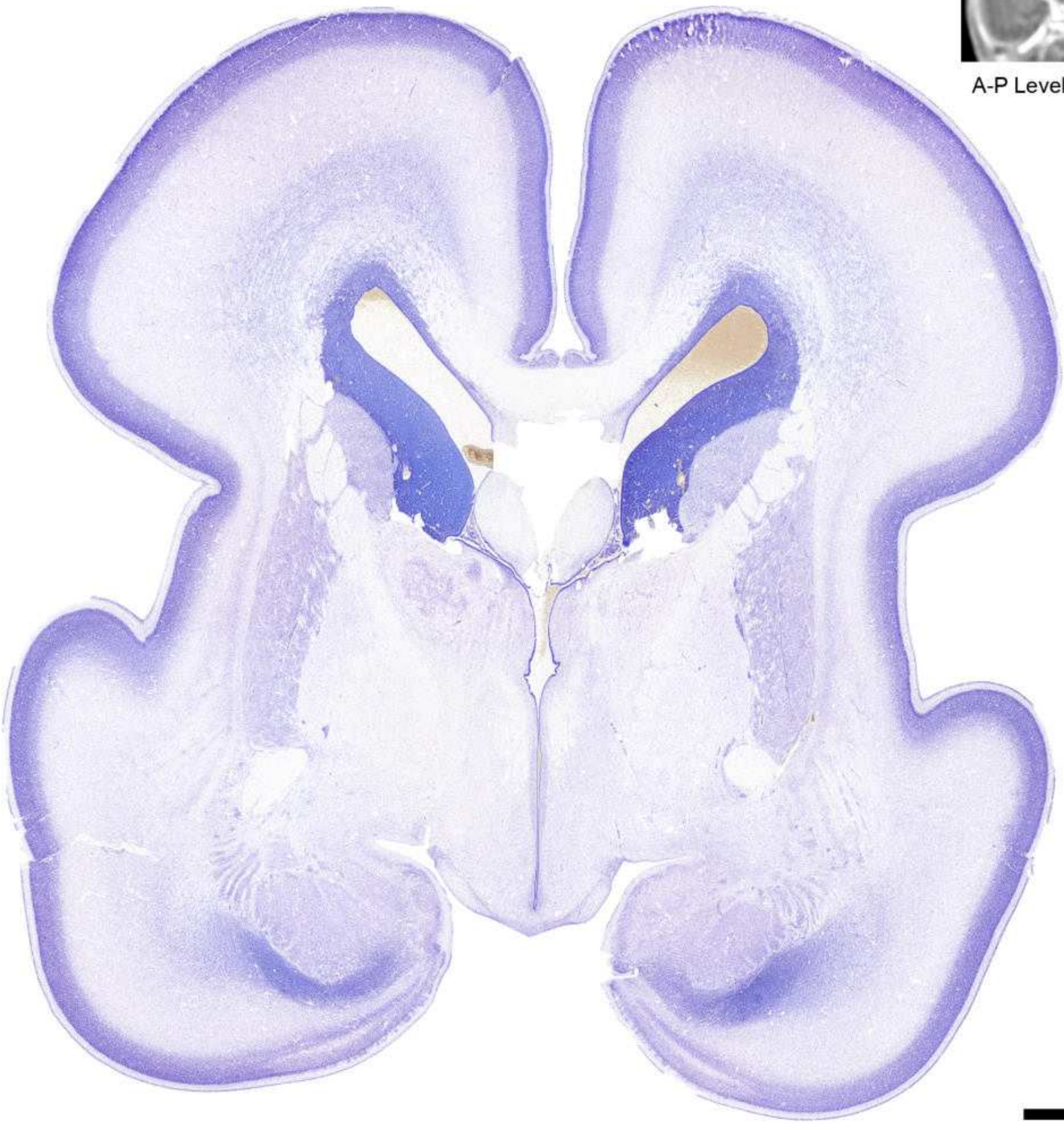

5 mm

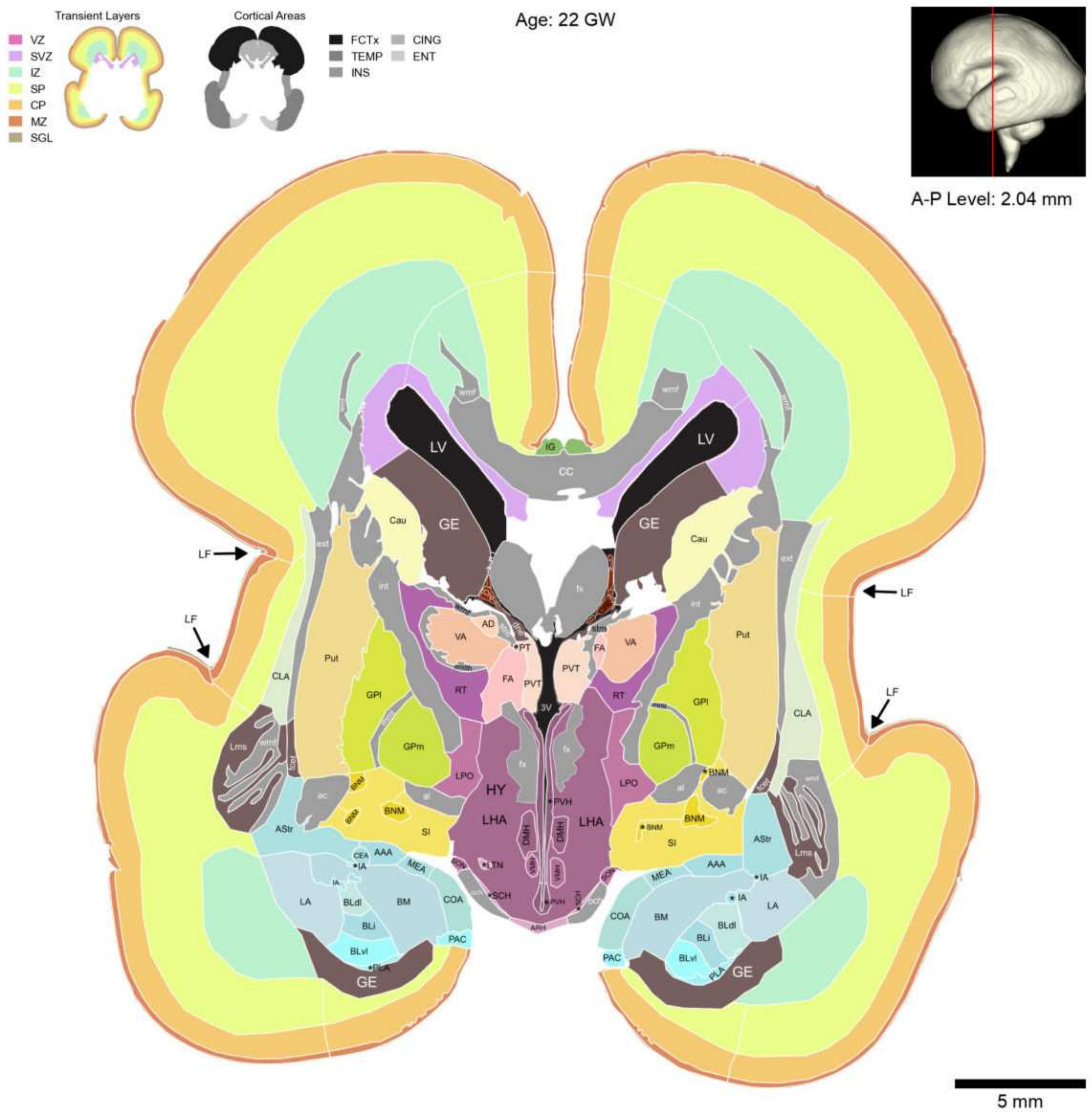

- 3V: Third ventricle
- AAA: Anterior amygdaloid area
- AD: Anterodorsal nucleus [thalamus]
- ARH: Arcuate nucleus [hypothalamus]
- AStr: Amygdalo-striatal area
- BLdl: Basal nucleus [amygdala], dorsolateral part
- BLi: Basal nucleus [amygdala], intermediate part
- BLvl: Basal nucleus [amygdala], ventrolateral part
- BM: Basomedial nucleus [amygdala]
- BNM: Basal nucleus of Meynert
- CEA: Central nucleus [amygdala]
- CLA: Claustrum
- COA: Cortical nucleus [amygdala]
- Cau: Caudate nucleus
- Chp: Choroid plexus
- DMH: Dorsomedial nucleus [hypothalamus]
- FA: Nucleus fasciculosus [thalamus]
- GE: Ganglionic eminence
- GPl: Globus pallidus lateral segment
- GPm: Globus pallidus medial segment
- HY: Hypothalamus
- IA: Intercalated cell groups [amygdala]
- IG: Induseum griseum
- LA: Lateral nucleus [amygdala]
- LHA: Lateral hypothalamic area
- LPO: Lateral preoptic area
- LTN: Lateral tuberal nucleus
- LV: Lateral ventricle
- Lms: Lateral migratory stream
- MEA: Medial nucleus [amygdala]
- PLA: Paralaminar nucleus [amygdala]
- PT: Paratenial nucleus [thalamus]
- PVH: Paraventricular nucleus [hypothalamus]
- PVT: Paraventricular nucleus [thalamus]
- Put: Putamen
- RT: Reticular nucleus [thalamus]
- SCH: Suprachiasmatic nucleus [hypothalamus]
- SI: Substantia innominata
- SON: Supraoptic nucleus [hypothalamus]
- VA: Ventral anterior nucleus [thalamus]
- VMH: Ventromedial nucleus [hypothalamus]
- ac: Anterior commissure
- al: Ansa lenticularis
- cc: Corpus callosum
- dne: Diencephalic neuroepithelium
- emlth: External medullary lamina [thalamus]
- ext: External capsule
- fx: Fornix
- int: Internal capsule
- mml: Medial medullary lamina
- och: Optic chiasm
- stm: Stria medullaris
- tcet: Transient cell zone in the external capsule
- wmf: White matter fibers
- LF: Lateral fissure

Age: 22 GW

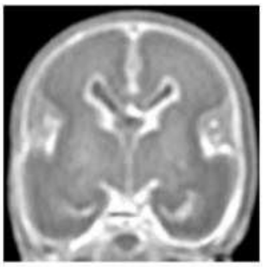

A-P Level: 1.56 mm

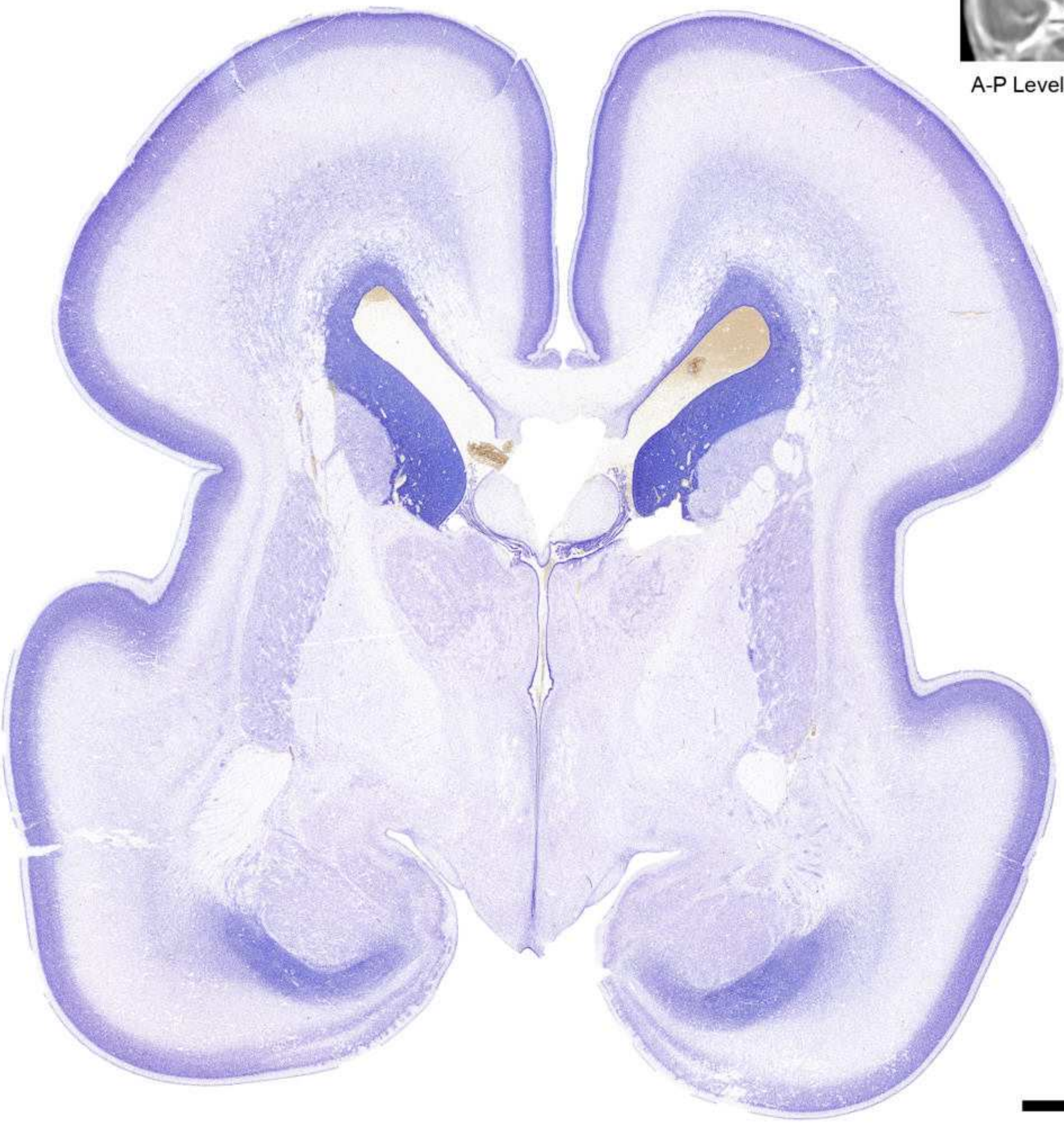

5 mm

Age: 22 GW

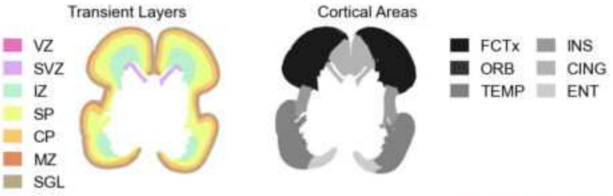

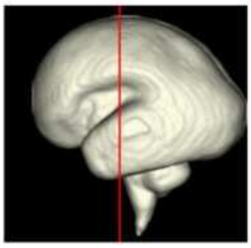

A-P Level: 1.56 mm

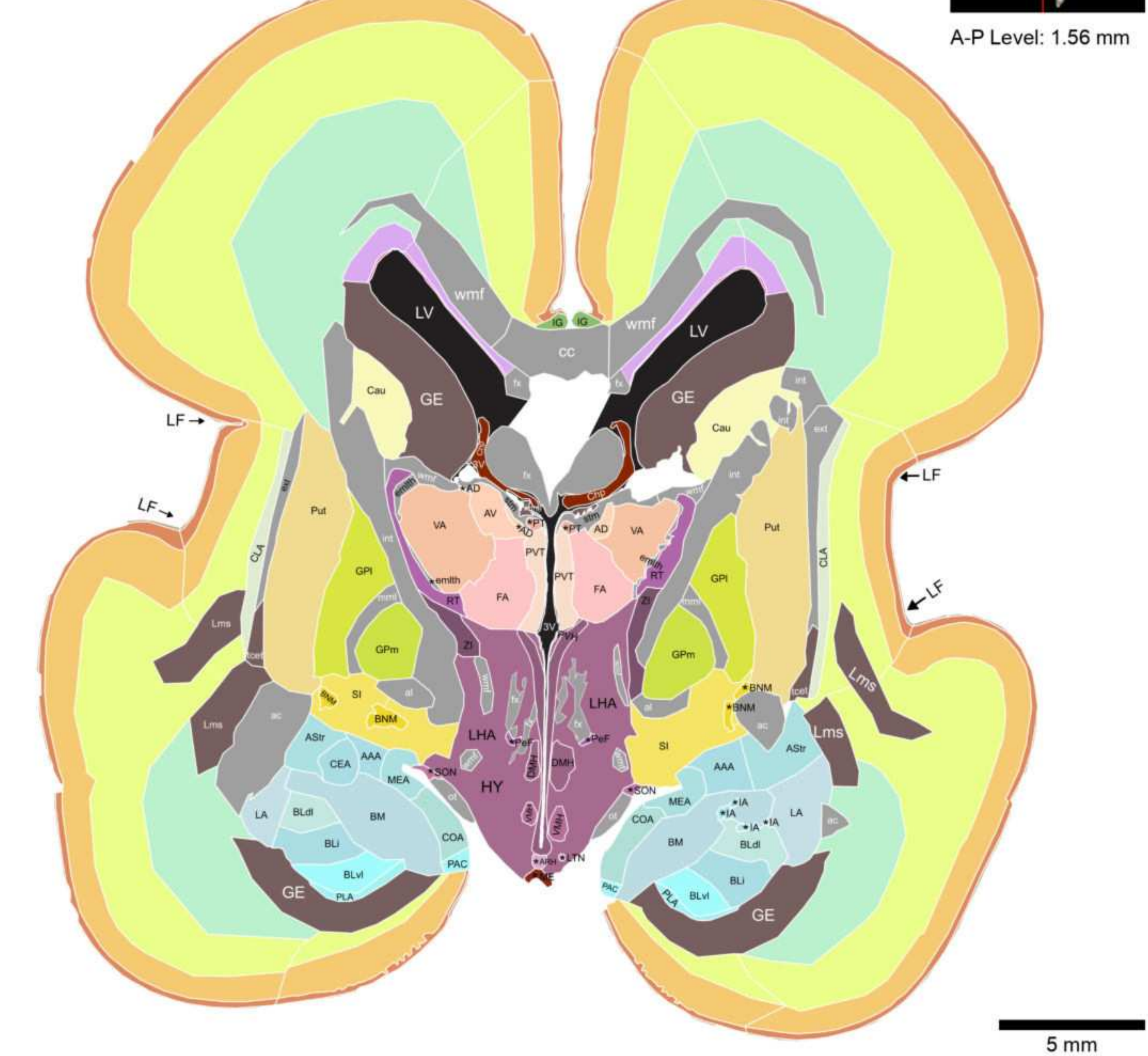

- 3V: Third ventricle
- AAA: Anterior amygdaloid area
- AD: Anterodorsal nucleus [thalamus]
- ARH: Arcuate nucleus [hypothalamus]
- AStr: Amygdalo-striatal area
- AV: Anteroventral nucleus [thalamus]
- BLdl: Basal nucleus [amygdala], dorsolateral part
- BLi: Basal nucleus [amygdala], intermediate part
- BLvl: Basal nucleus [amygdala], ventrolateral part
- BM: Basomedial nucleus [amygdala]
- BNM: Basal nucleus of Meynert
- CEA: Central nucleus [amygdala]
- CLA: Claustrum
- COA: Cortical nucleus [amygdala]
- Cau: Caudate nucleus
- Chp: Choroid plexus
- DMH: Dorsomedial nucleus [hypothalamus]
- FA: Nucleus fasciculosus [thalamus]
- GE: Ganglionic eminence
- GPl: Globus pallidus lateral segment
- GPm: Globus pallidus medial segment
- HY: Hypothalamus
- IA: Intercalated cell groups [amygdala]
- IG: Induseum griseum
- LA: Lateral nucleus [amygdala]
- LHA: Lateral hypothalamic area
- LTN: Lateral tuberal nucleus
- LV: Lateral ventricle
- Lms: Lateral migratory stream
- ME: Median eminence
- MEA: Medial nucleus [amygdala]
- PLA: Paralaminar nucleus [amygdala]
- PT: Paratenial nucleus [thalamus]
- PVH: Paraventricular nucleus [hypothalamus]
- PVT: Paraventricular nucleus [thalamus]
- PeF: Perifornical nucleus
- Put: Putamen
- RT: Reticular nucleus [thalamus]
- SI: Substantia innominata
- SON: Supraoptic nucleus [hypothalamus]
- VA: Ventral anterior nucleus [thalamus]
- VMH: Ventromedial nucleus [hypothalamus]
- ZI: Zona incerta
- ac: Anterior commissure
- al: Ansa lenticularis
- cc: Corpus callosum
- dne: Diencephalic neuroepithelium
- emlth: External medullary lamina [thalamus]
- ext: External capsule
- fx: Fornix
- int: Internal capsule
- mml: Medial medullary lamina
- ot: Optic tract
- stm: Stria medullaris
- tcet: Transient cell zone in the external capsule
- wmf: White matter fibers
- → LF: Lateral fissure

Age: 22 GW

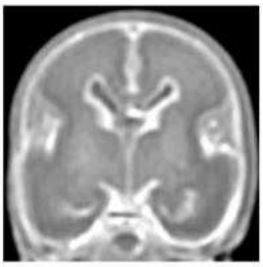

A-P Level: 1.32 mm

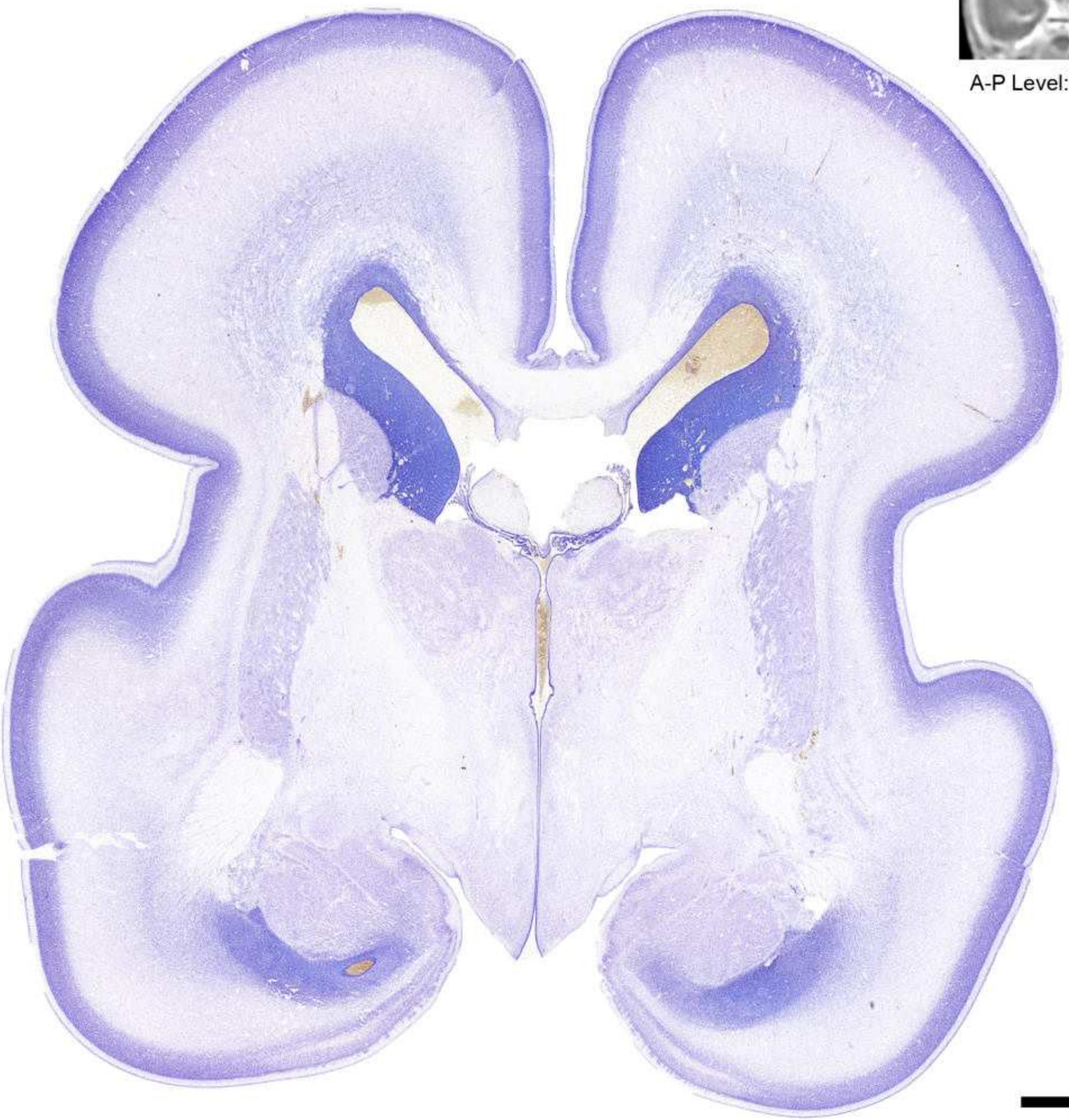

5 mm

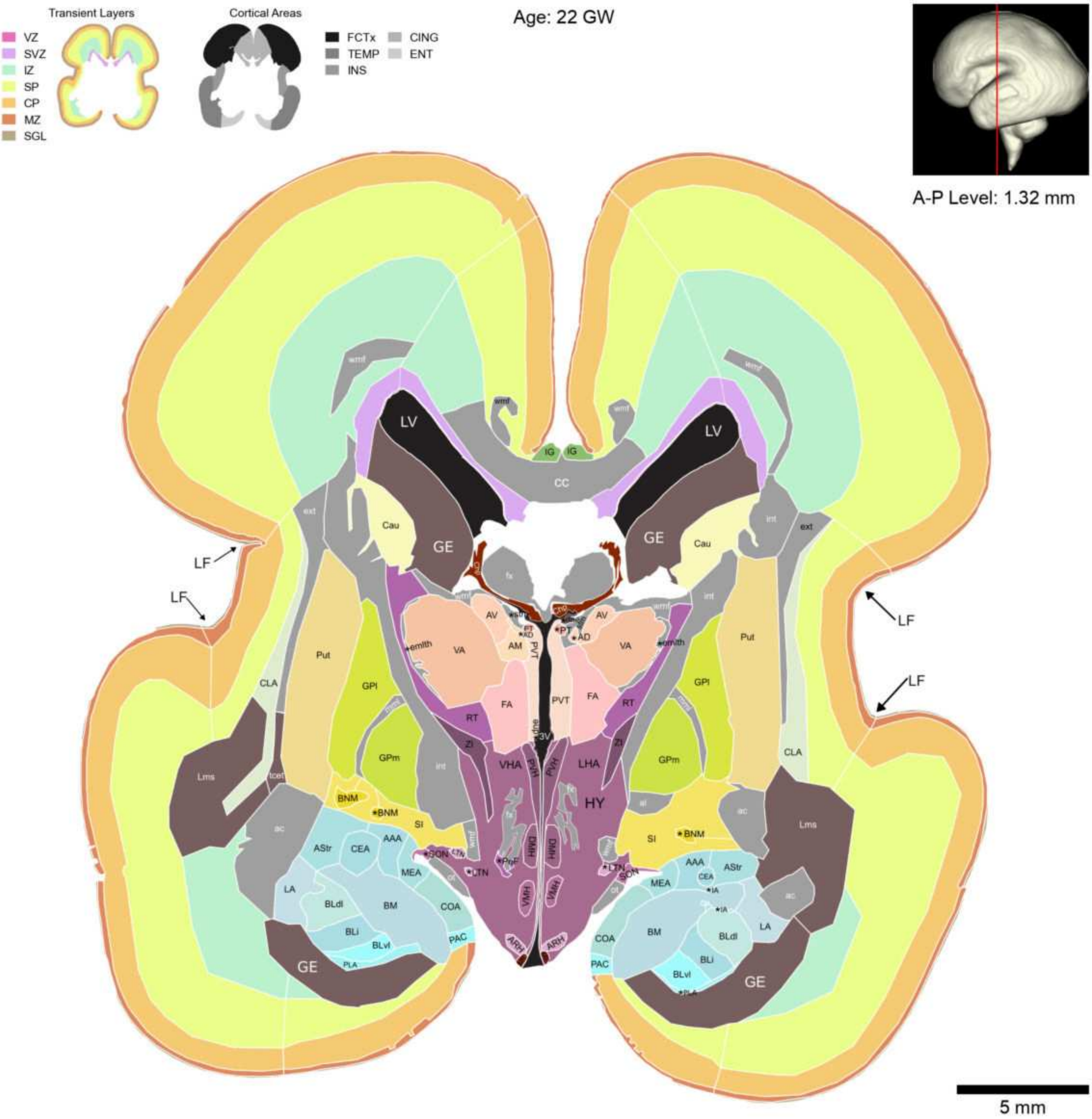

- 3V: Third ventricle
- AAA: Anterior amygdaloid area
- AD: Anterodorsal nucleus [thalamus]
- AM: Anteromedial nucleus [thalamus]
- ARH: Arcuate nucleus [hypothalamus]
- AStr: Amygdalo-striatal area
- AV: Anteroventral nucleus [thalamus]
- BLdl: Basal nucleus [amygdala], dorsolateral part
- BLi: Basal nucleus [amygdala], intermediate part
- BLvl: Basal nucleus [amygdala], ventrolateral part
- BM: Basomedial nucleus [amygdala]
- BNM: Basal nucleus of Meynert
- CEA: Central nucleus [amygdala]
- CLA: Claustrum
- COA: Cortical nucleus [amygdala]
- Cau: Caudate nucleus
- Chp: Choroid plexus
- DMH: Dorsomedial nucleus [hypothalamus]
- FA: Nucleus fasciculosus [thalamus]
- GE: Ganglionic eminence
- GPl: Globus pallidus lateral segment
- GPm: Globus pallidus medial segment
- HY: Hypothalamus
- IA: Intercalated cell groups [amygdala]
- IG: Induseum griseum
- LA: Lateral nucleus [amygdala]
- LHA: Lateral hypothalamic area
- LTN: Lateral tuberal nucleus
- LV: Lateral ventricle
- Lms: Lateral migratory stream
- ME: Median eminence
- MEA: Medial nucleus [amygdala]
- PLA: Paralaminar nucleus [amygdala]
- PT: Paratenial nucleus [thalamus]
- PVH: Paraventricular nucleus [hypothalamus]
- PVT: Paraventricular nucleus [thalamus]
- PeF: Perifornical nucleus
- Put: Putamen
- RT: Reticular nucleus [thalamus]
- SI: Substantia innominata
- SON: Supraoptic nucleus [hypothalamus]
- VA: Ventral anterior nucleus [thalamus]
- VMH: Ventromedial nucleus [hypothalamus]
- ZI: Zona incerta
- ac: Anterior commissure
- al: Ansa lenticularis
- cc: Corpus callosum
- dne: Diencephalic neuroepithelium
- emlth: External medullary lamina [thalamus]
- ext: External capsule
- fx: Fornix
- int: Internal capsule
- mml: Medial medullary lamina
- ot: Optic tract
- stm: Stria medullaris
- tcet: Transient cell zone in the external capsule
- wmf: White matter fibers
- → LF: Lateral fissure

Age: 22 GW

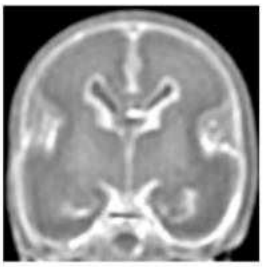

A-P Level: 1.14 mm

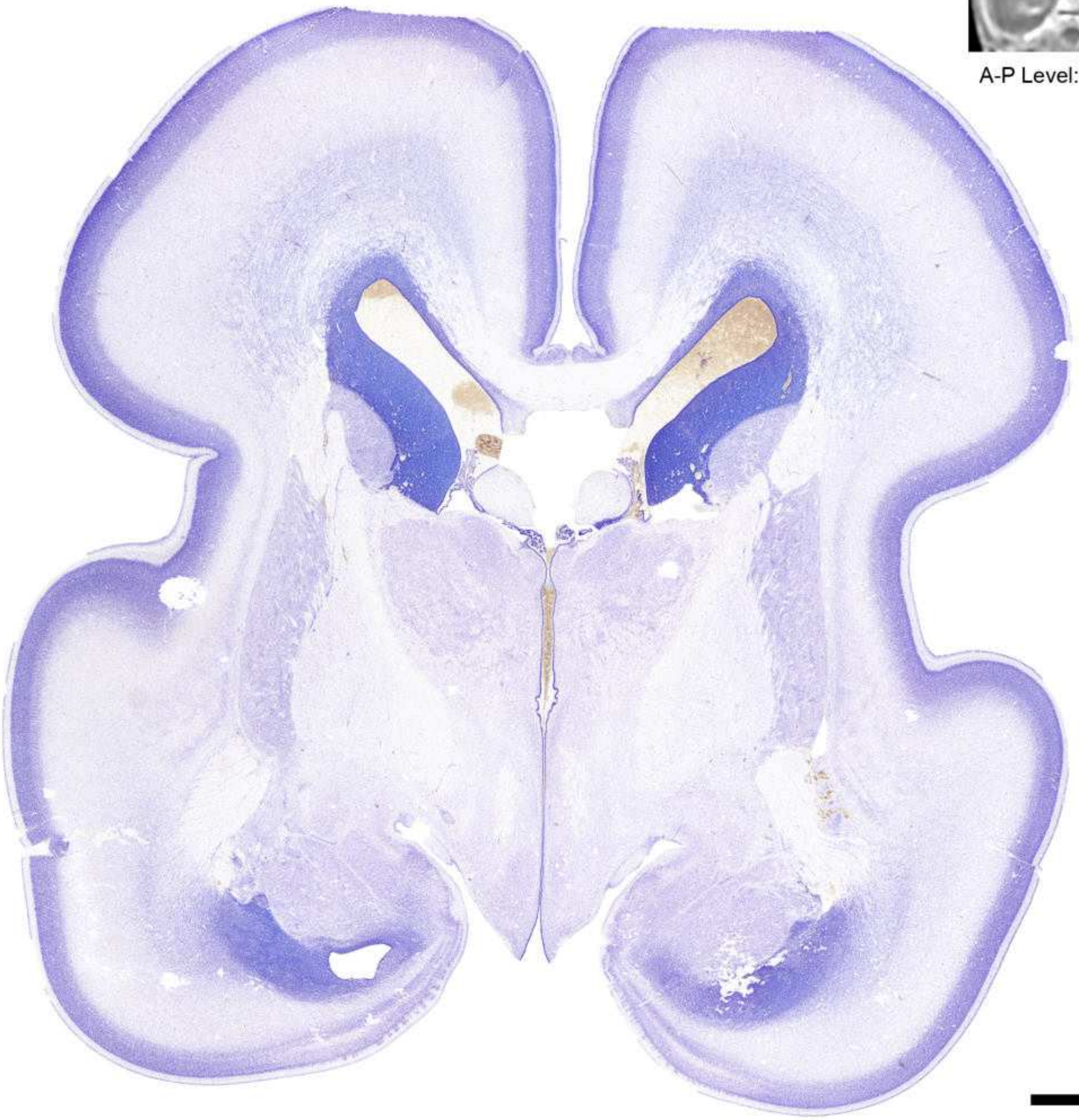

5 mm

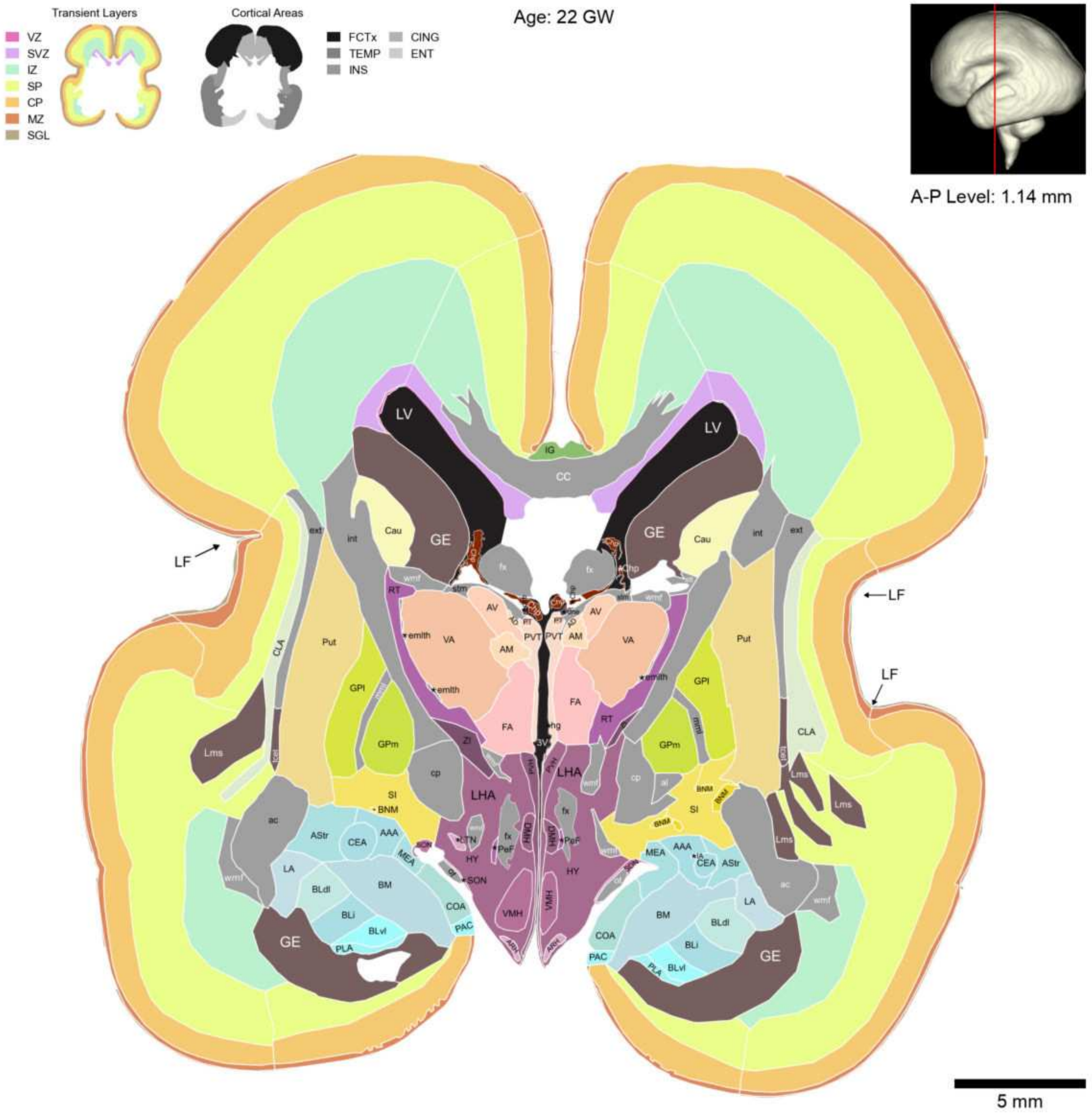

- 3V: Third ventricle
- AAA: Anterior amygdaloid area
- AD: Anterodorsal nucleus [thalamus]
- AM: Anteromedial nucleus [thalamus]
- ARH: Arcuate nucleus [hypothalamus]
- AStr: Amygdalo-striatal area
- AV: Anteroventral nucleus [thalamus]
- BLdl: Basal nucleus [amygdala], dorsolateral part
- BLi: Basal nucleus [amygdala], intermediate part
- BLvl: Basal nucleus [amygdala], ventrolateral part
- BM: Basomedial nucleus [amygdala]
- BNM: Basal nucleus of Meynert
- CEA: Central nucleus [amygdala]
- CLA: Claustrum
- COA: Cortical nucleus [amygdala]
- Cau: Caudate nucleus
- Chp: Choroid plexus
- DMH: Dorsomedial nucleus [hypothalamus]
- FA: Nucleus fasciculosus [thalamus]
- GE: Ganglionic eminence
- GPl: Globus pallidus lateral segment
- GPm: Globus pallidus medial segment
- HY: Hypothalamus
- IA: Intercalated cell groups [amygdala]
- IG: Induseum griseum
- LA: Lateral nucleus [amygdala]
- LHA: Lateral hypothalamic area
- LTN: Lateral tuberal nucleus
- LV: Lateral ventricle
- Lms: Lateral migratory stream
- MEA: Medial nucleus [amygdala]
- PLA: Paralaminar nucleus [amygdala]
- PT: Paratenial nucleus [thalamus]
- PVH: Paraventricular nucleus [hypothalamus]
- PVT: Paraventricular nucleus [thalamus]
- PeF: Perifornical nucleus
- Put: Putamen
- RT: Reticular nucleus [thalamus]
- SI: Substantia innominata
- SON: Supraoptic nucleus [hypothalamus]
- VA: Ventral anterior nucleus [thalamus]
- VMH: Ventromedial nucleus [hypothalamus]
- ZI: Zona incerta
- ac: Anterior commissure
- al: Ansa lenticularis
- cc: Corpus callosum
- cp: Cerebral peduncle
- dne: Diencephalic neuroepithelium
- emlth: External medullary lamina [thalamus]
- ext: External capsule
- fx: Fornix
- hg: Hypothalamic glioepithelium/ependyma
- int: Internal capsule
- mml: Medial medullary lamina
- ot: Optic tract
- stm: Stria medullaris
- stt: Stria terminalis
- tcet: Transient cell zone in the external capsule
- wmf: White matter fibers
- → LF: Lateral fissure

Age: 22 GW

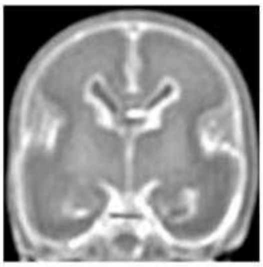

A-P Level: 0.48 mm

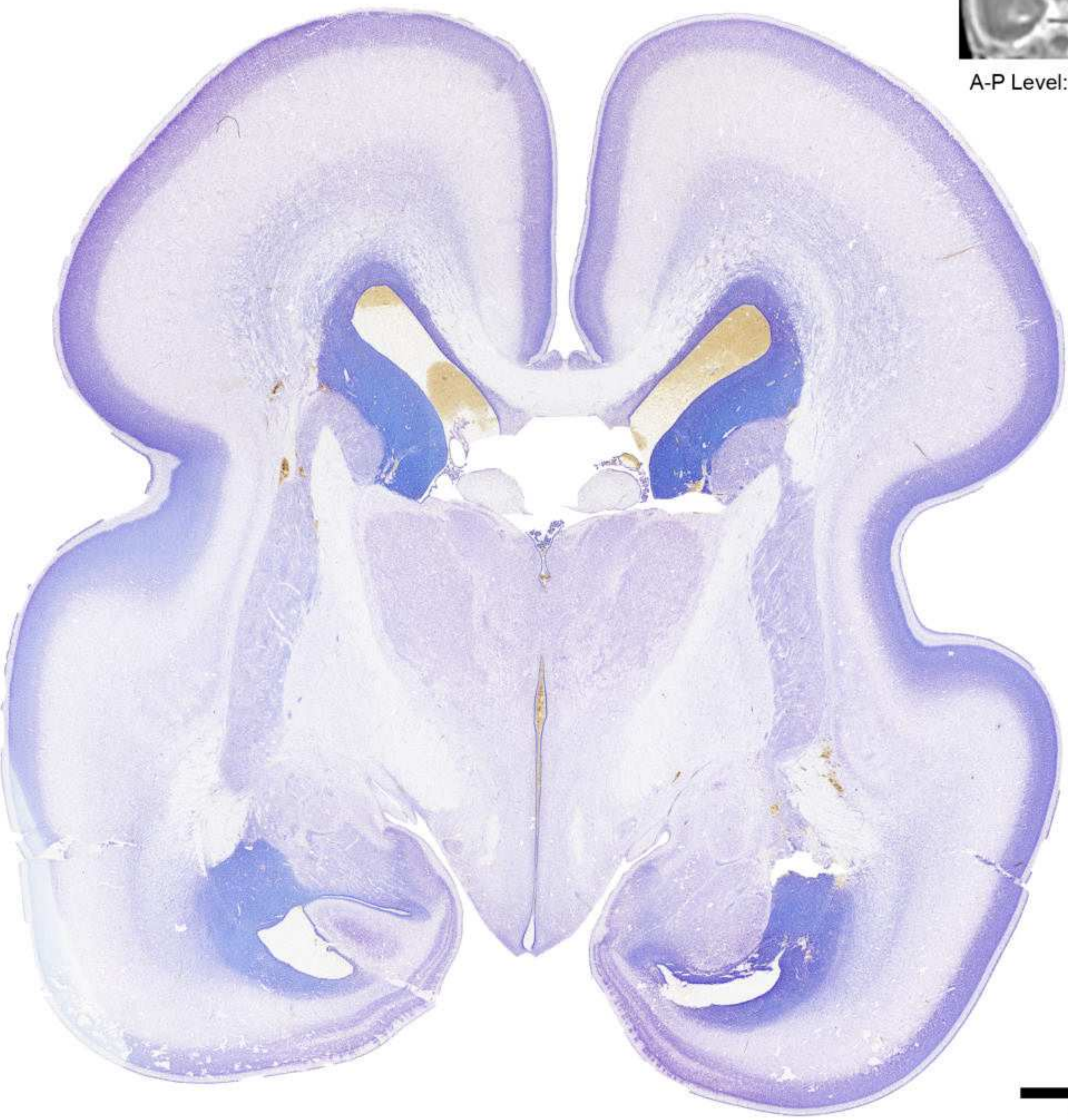

5 mm

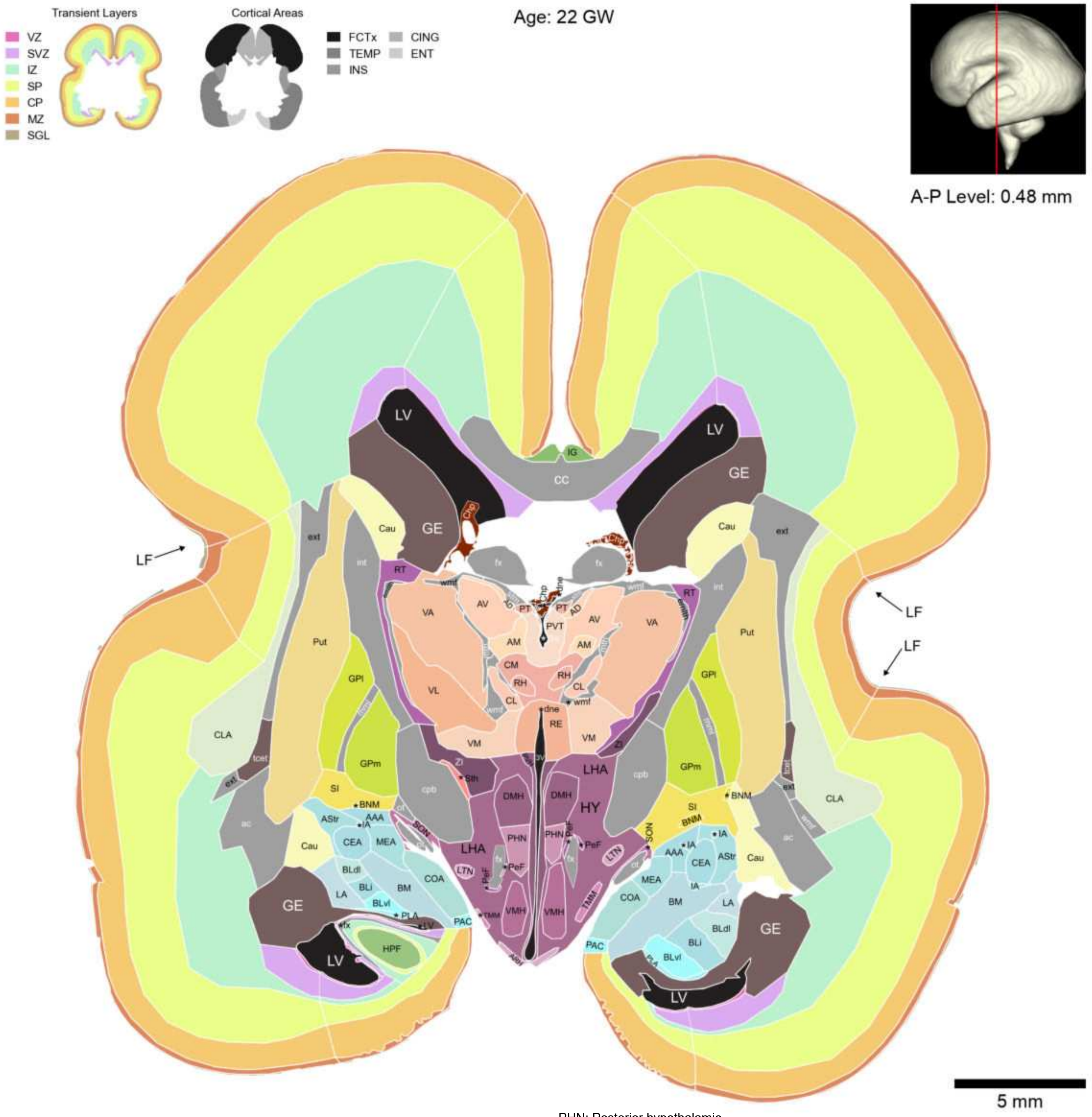

- 3V: Third ventricle
- AAA: Anterior amygdaloid area
- AD: Anterodorsal nucleus [thalamus]
- AM: Anteromedial nucleus [thalamus]
- ARH: Arcuate nucleus [hypothalamus]
- AStr: Amygdalo-striatal area
- AV: Anteroventral nucleus [thalamus]
- BLdl: Basal nucleus [amygdala], dorsolateral part
- BLi: Basal nucleus [amygdala], intermediate part
- BLvl: Basal nucleus [amygdala], ventrolateral part
- BM: Basomedial nucleus [amygdala]
- BNM: Basal nucleus of Meynert
- CEA: Central nucleus [amygdala]
- CL: Central lateral nucleus [thalamus]
- CLA: Claustrum
- CM: Central medial nucleus [thalamus]
- COA: Cortical nucleus [amygdala]
- Cau: Caudate nucleus
- Chp: Choroid plexus
- DMH: Dorsomedial nucleus [hypothalamus]
- GE: Ganglionic eminence
- GPl: Globus pallidus lateral segment
- GPm: Globus pallidus medial segment
- HPF: Hippocampal formation
- HY: Hypothalamus
- IA: Intercalated cell groups [amygdala]
- IG: Induseum griseum
- LA: Lateral nucleus [amygdala]
- LHA: Lateral hypothalamic area
- LTN: Lateral tuberal nucleus
- LV: Lateral ventricle
- MEA: Medial nucleus [amygdala]
- PHN: Posterior hypothalamic nucleus
- PLA: Paralaminar nucleus [amygdala]
- PT: Paratenial nucleus [thalamus]
- PVH: Paraventricular nucleus [hypothalamus]
- PVT: Paraventricular nucleus [thalamus]
- PeF: Perifornical nucleus
- Put: Putamen
- RE: Nucleus reuniens
- RH: Rhomboid nucleus [thalamus]
- RT: Reticular nucleus [thalamus]
- SI: Substantia innominata
- SON: Supraoptic nucleus [hypothalamus]
- Sth: Subthalamus
- TMM: Tuberomammillary nucleus
- VA: Ventral anterior nucleus [thalamus]
- VL: Ventral lateral nucleus [thalamus]
- VM: Ventral medial nucleus [thalamus]
- VMH: Ventromedial nucleus [hypothalamus]
- ZI: Zona incerta
- ac: Anterior commissure
- cc: Corpus callosum
- cpb: Cerebellar peduncles
- dne: Diencephalic neuroepithelium
- emlth: External medullary lamina [thalamus]
- ext: External capsule
- fx: Fornix
- int: Internal capsule
- mml: Medial medullary lamina
- mth: Mammillothalamic tract
- ot: Optic tract
- stm: Stria medullaris
- tcet: Transient cell zone in the external capsule
- wmf: White matter fibers
- → LF: Lateral fissure

Age: 22 GW

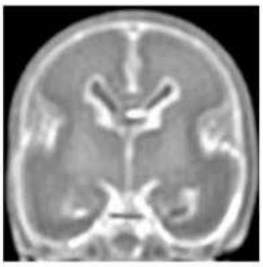

A-P Level: 0.36 mm

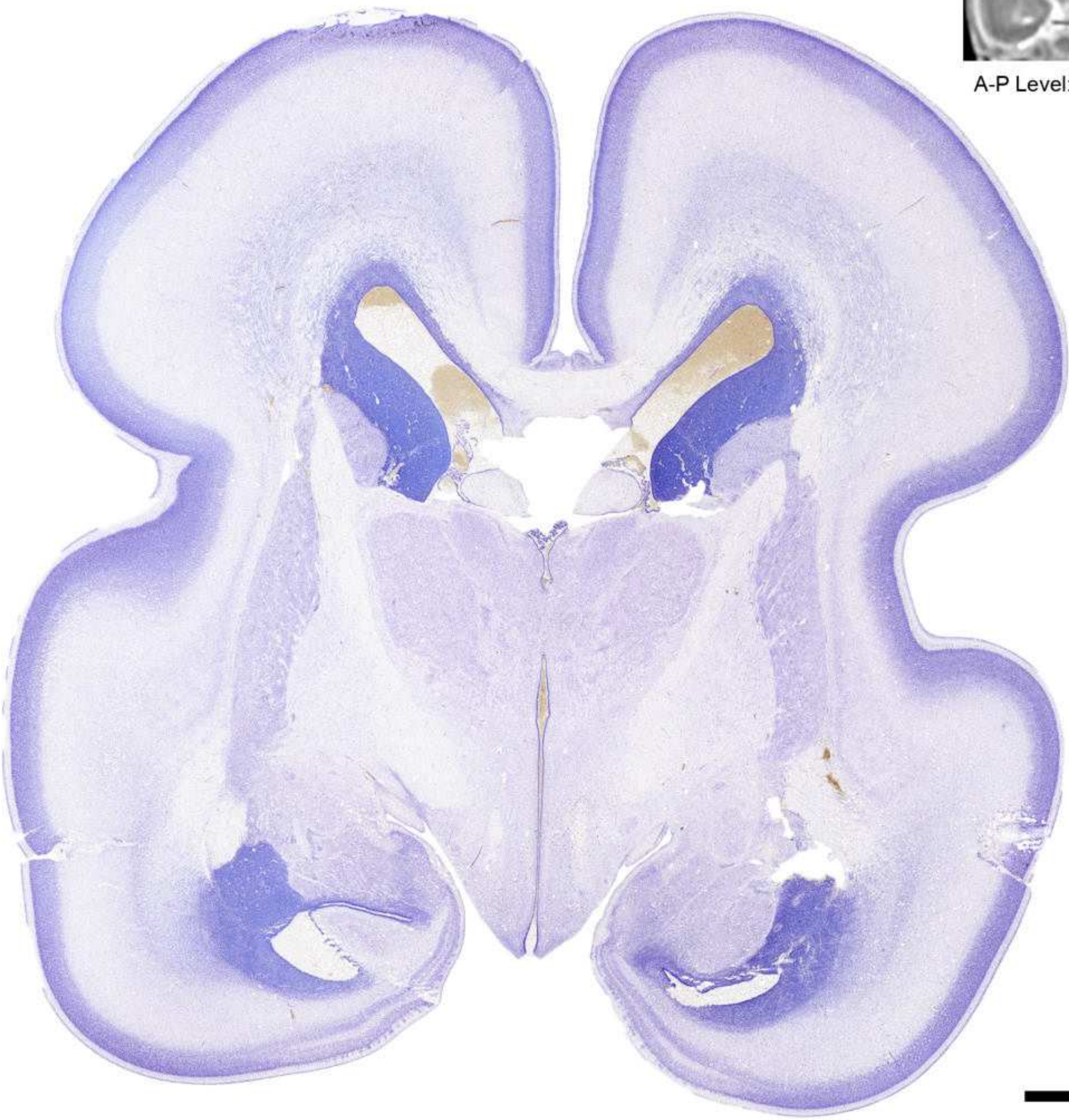

5 mm

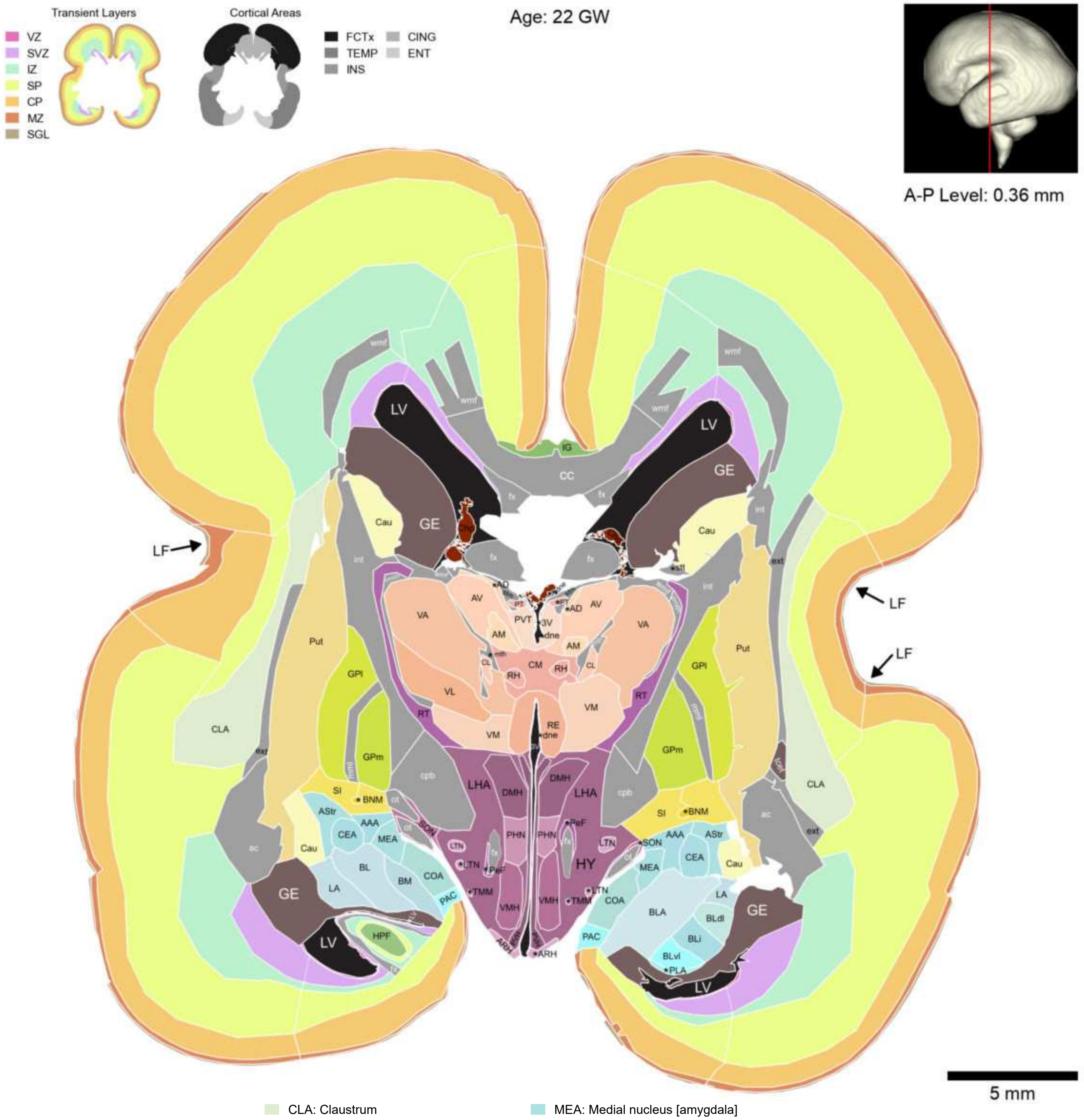

- 3V: Third ventricle
- AAA: Anterior amygdaloid area
- AD: Anterodorsal nucleus [thalamus]
- AM: Anteromedial nucleus [thalamus]
- ARH: Arcuate nucleus [hypothalamus]
- AStr: Amygdalo-striatal area
- AV: Anteroventral nucleus [thalamus]
- BL: Basal nucleus [amygdala]
- BLA: Basolateral complex [amygdala]
- BLdl: Basal nucleus [amygdala], dorsolateral part
- BLi: Basal nucleus [amygdala], intermediate part
- BLvl: Basal nucleus [amygdala], ventrolateral part
- BM: Basomedial nucleus [amygdala]
- BNM: Basal nucleus of Meynert
- CEA: Central nucleus [amygdala]
- CL: Central lateral nucleus [thalamus]
- CLA: Claustrum
- CM: Central medial nucleus [thalamus]
- COA: Cortical nucleus [amygdala]
- Cau: Caudate nucleus
- Chp: Choroid plexus
- DMH: Dorsomedial nucleus [hypothalamus]
- GE: Ganglionic eminence
- GPl: Globus pallidus lateral segment
- GPm: Globus pallidus medial segment
- HPF: Hippocampal formation
- HY: Hypothalamus
- IG: Induseum griseum
- LA: Lateral nucleus [amygdala]
- LHA: Lateral hypothalamic area
- LTN: Lateral tuberal nucleus
- LV: Lateral ventricle
- MEA: Medial nucleus [amygdala]
- PHN: Posterior hypothalamic nucleus
- PLA: Paralaminar nucleus [amygdala]
- PT: Paratenial nucleus [thalamus]
- PVH: Paraventricular nucleus [hypothalamus]
- PVT: Paraventricular nucleus [thalamus]
- PeF: Perifornical nucleus
- Put: Putamen
- RE: Nucleus reuniens
- RH: Rhomboid nucleus [thalamus]
- RT: Reticular nucleus [thalamus]
- SI: Substantia innominata
- SON: Supraoptic nucleus [hypothalamus]
- TMM: Tuberomammillary nucleus
- VA: Ventral anterior nucleus [thalamus]
- VL: Ventral lateral nucleus [thalamus]
- VM: Ventral medial nucleus [thalamus]
- VMH: Ventromedial nucleus [hypothalamus]
- ac: Anterior commissure
- cc: Corpus callosum
- cpb: Cerebellar peduncles
- dne: Diencephalic neuroepithelium
- emlth: External medullary lamina [thalamus]
- ext: External capsule
- fx: Fornix
- int: Internal capsule
- mml: Medial medullary lamina
- mth: Mammillothalamic tract
- ot: Optic tract
- stm: Stria medullaris
- stt: Stria terminalis
- tcet: Transient cell zone in the external capsule
- wmf: White matter fibers
- → LF: Lateral fissure

Age: 22 GW

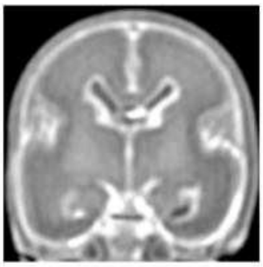

A-P Level: -0.12 mm

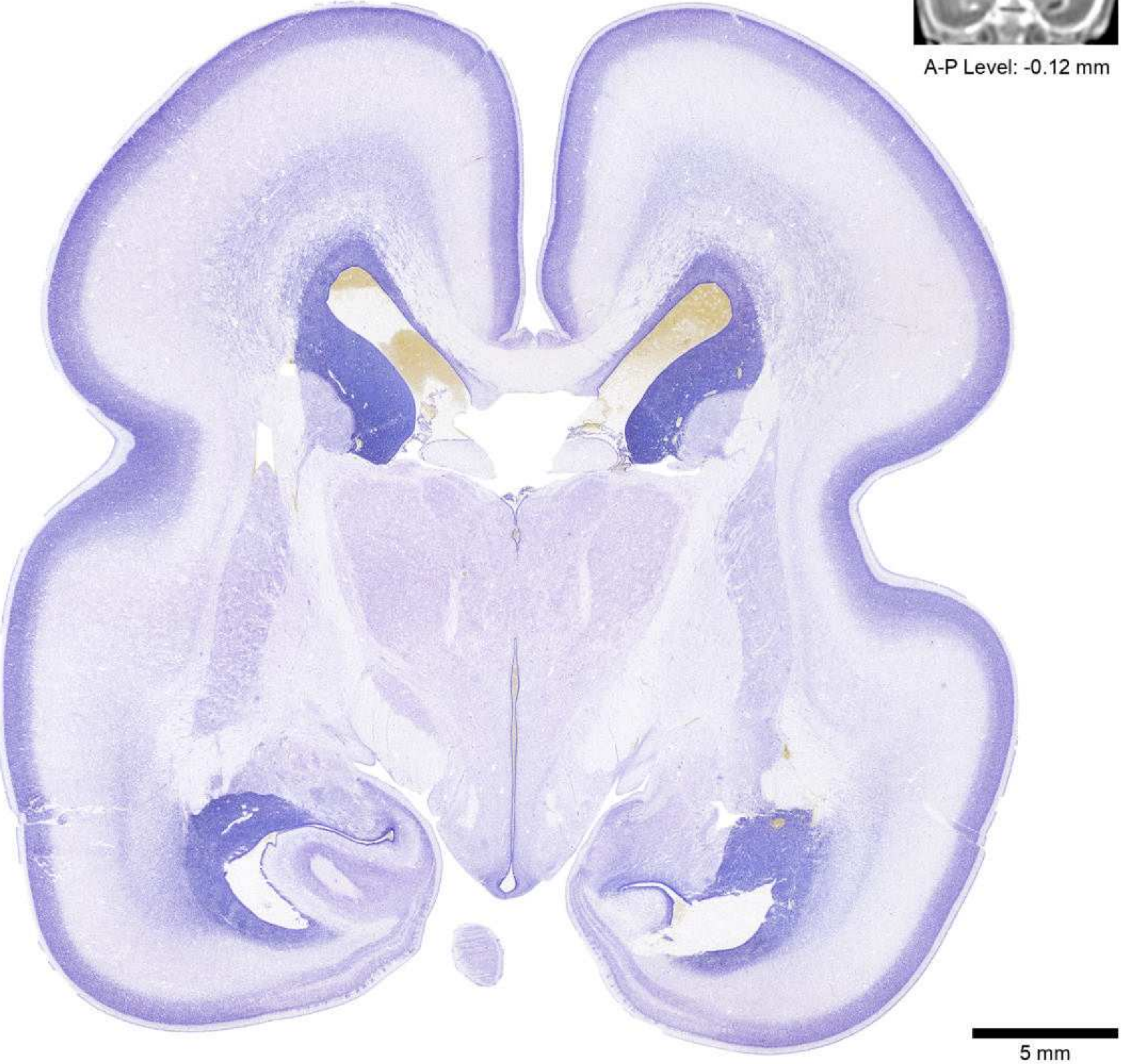

5 mm

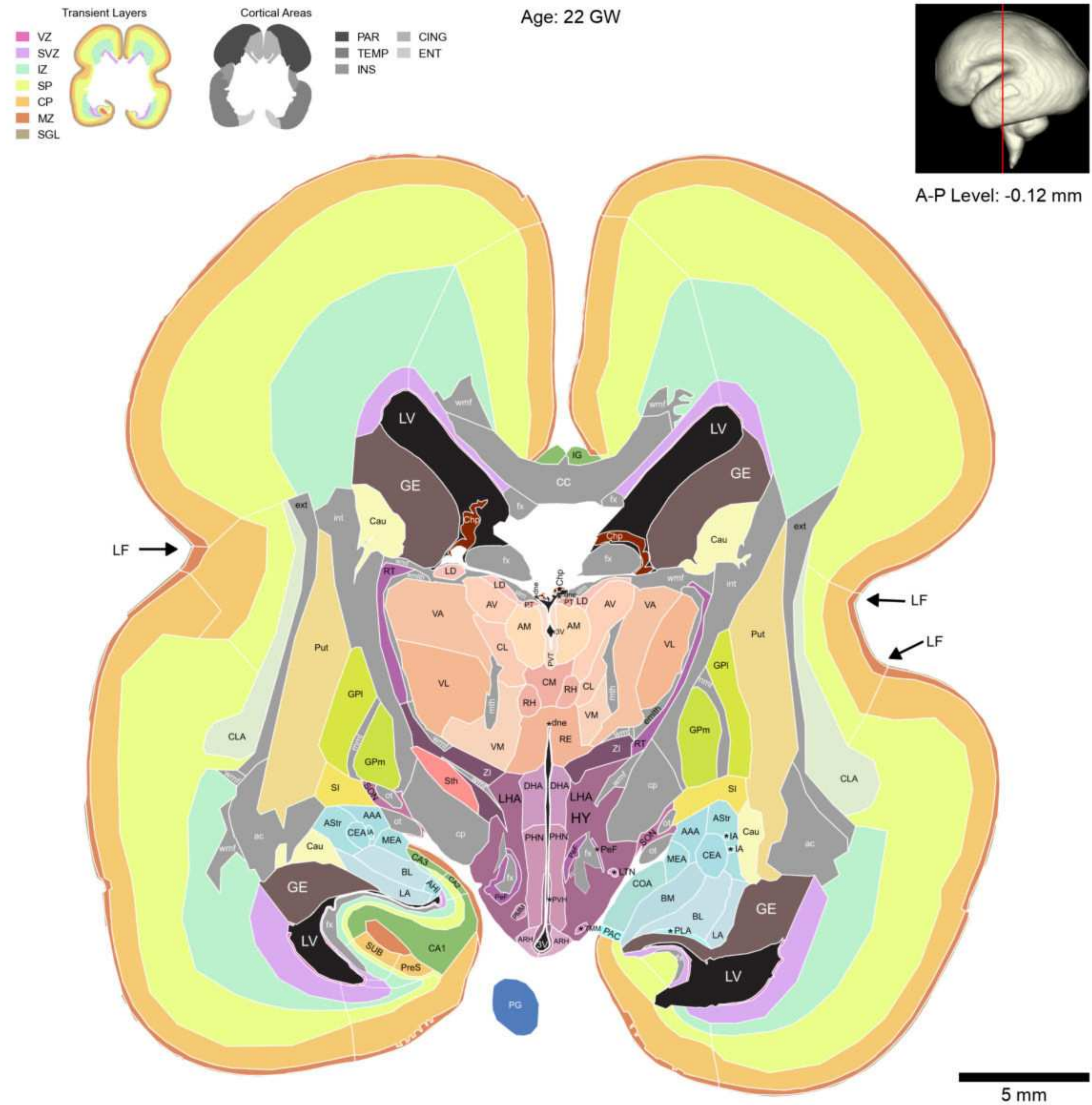

- 3V: Third ventricle
- AAA: Anterior amygdaloid area
- AHi: Amygdalo-hippocampal area
- AM: Anteromedial nucleus [thalamus]
- ARH: Arcuate nucleus [hypothalamus]
- AStr: Amygdalo-striatal area
- AV: Anteroventral nucleus [thalamus]
- BL: Basal nucleus [amygdala]
- BM: Basomedial nucleus [amygdala]
- CA1: CA1 field [hippocampus]
- CA2: CA2 field [hippocampus]
- CA3: CA3 field [hippocampus]
- CEA: Central nucleus [amygdala]
- CL: Central lateral nucleus [thalamus]
- CLA: Claustrum
- CM: Central medial nucleus [thalamus]
- COA: Cortical nucleus [amygdala]
- Cau: Caudate nucleus
- Chp: Choroid plexus
- DHA: Dorsal hypothalamic area
- GE: Ganglionic eminence
- GPl: Globus pallidus lateral segment
- GPm: Globus pallidus medial segment
- HY: Hypothalamus
- IA: Intercalated cell groups [amygdala]
- IG: Induseum griseum
- LA: Lateral nucleus [amygdala]
- LD: Lateral dorsal nucleus [thalamus]
- LHA: Lateral hypothalamic area
- LTN: Lateral tuberal nucleus
- LV: Lateral ventricle
- MEA: Medial nucleus [amygdala]
- PG: Pontine gray
- PHN: Posterior hypothalamic nucleus
- PLA: Paralaminar nucleus [amygdala]
- PMM: Premammillary area
- PT: Paratenial nucleus [thalamus]
- PVH: Paraventricular nucleus [hypothalamus]
- PVT: Paraventricular nucleus [thalamus]
- PeF: Perifornical nucleus
- PreS: Cortical plate, presubiculum
- Put: Putamen
- RE: Nucleus reuniens
- RH: Rhomboid nucleus [thalamus]
- RT: Reticular nucleus [thalamus]
- SI: Substantia innominata
- SON: Supraoptic nucleus [hypothalamus]
- SUB: Cortical plate, subiculum
- Sth: Subthalamus
- TMM: Tuberomammillary nucleus
- VA: Ventral anterior nucleus [thalamus]
- VL: Ventral lateral nucleus [thalamus]
- VM: Ventral medial nucleus [thalamus]
- ZI: Zona incerta
- ac: Anterior commissure
- cc: Corpus callosum
- cp: Cerebral peduncle
- dne: Diencephalic neuroepithelium
- emlth: External medullary lamina [thalamus]
- ext: External capsule
- fx: Fornix
- int: Internal capsule
- mml: Medial medullary lamina
- mth: Mammillothalamic tract
- ot: Optic tract
- stm: Stria medullaris
- wmf: White matter fibers
- → LF: Lateral fissure

Age: 22 GW

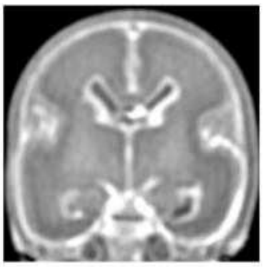

A-P Level: -0.54 mm

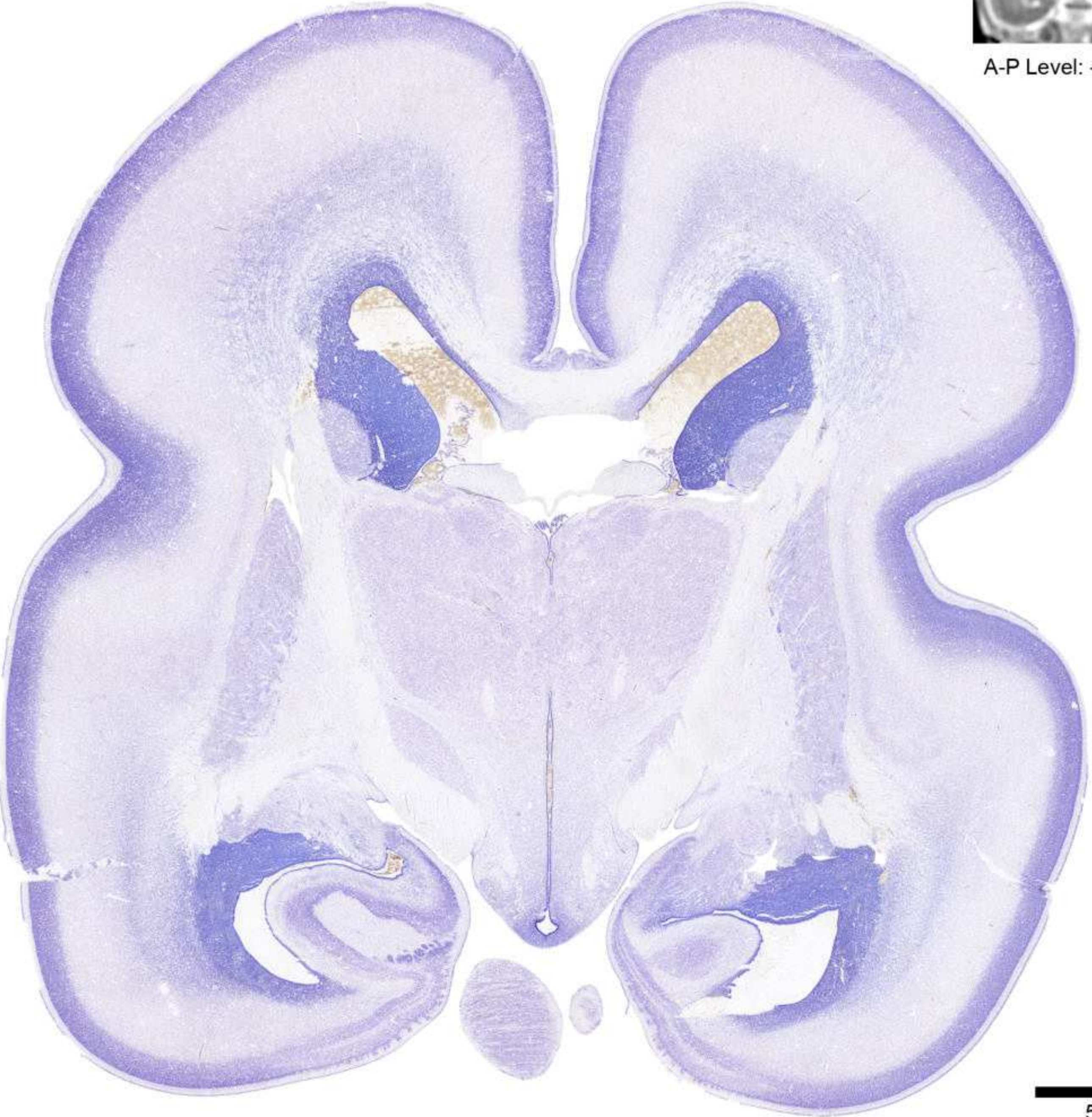

5 mm

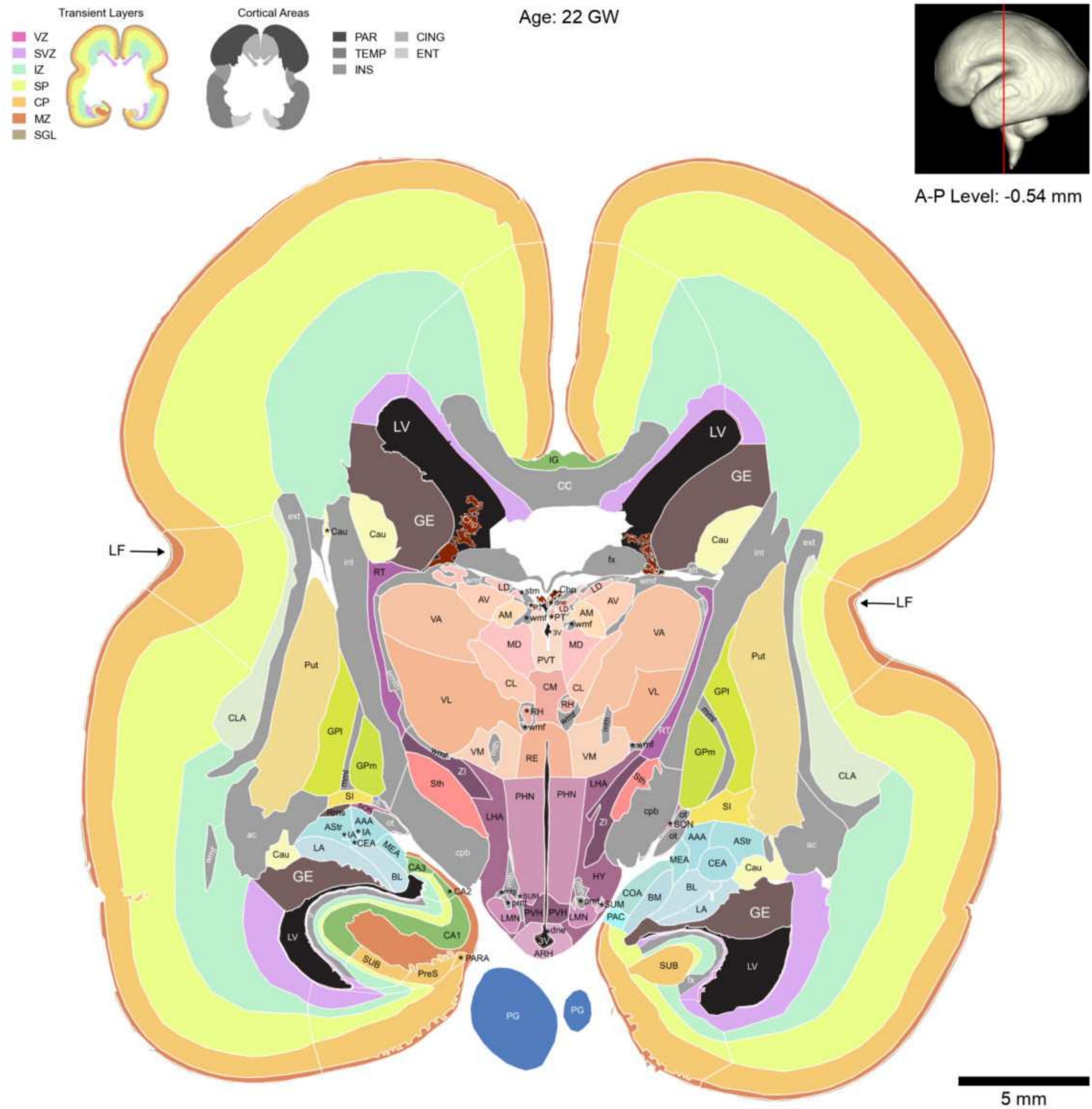

- 3V: Third ventricle
- AAA: Anterior amygdaloid area
- AM: Anteromedial nucleus [thalamus]
- ARH: Arcuate nucleus [hypothalamus]
- AStr: Amygdalo-striatal area
- AV: Anteroventral nucleus [thalamus]
- BL: Basal nucleus [amygdala]
- BM: Basomedial nucleus [amygdala]
- CA1: CA1 field [hippocampus]
- CA2: CA2 field [hippocampus]
- CA3: CA3 field [hippocampus]
- CEA: Central nucleus [amygdala]
- CL: Central lateral nucleus [thalamus]
- CLA: Claustrum
- CM: Central medial nucleus [thalamus]
- COA: Cortical nucleus [amygdala]
- Cau: Caudate nucleus
- Chp: Choroid plexus
- GE: Ganglionic eminence
- GPl: Globus pallidus lateral segment
- GPm: Globus pallidus medial segment
- HY: Hypothalamus
- IA: Intercalated cell groups [amygdala]
- IG: Induseum griseum
- LA: Lateral nucleus [amygdala]
- LD: Lateral dorsal nucleus [thalamus]
- LHA: Lateral hypothalamic area
- LMN: Lateral mammillary nucleus
- LV: Lateral ventricle
- MD: Medial dorsal nucleus [thalamus]
- MEA: Medial nucleus [amygdala]
- PARA: Cortical plate, parasubiculum
- PG: Pontine gray
- PHN: Posterior hypothalamic nucleus
- PT: Paratenial nucleus [thalamus]
- PVH: Paraventricular nucleus [hypothalamus]
- PVT: Paraventricular nucleus [thalamus]
- PreS: Cortical plate, presubiculum
- Put: Putamen
- RE: Nucleus reuniens
- RH: Rhomboid nucleus [thalamus]
- RT: Reticular nucleus [thalamus]
- Rms: Rostral migratory stream
- SI: Substantia innominata
- SON: Supraoptic nucleus [hypothalamus]
- SUB: Cortical plate, subiculum
- SUM: Supramammillary area
- Sth: Subthalamus
- VA: Ventral anterior nucleus [thalamus]
- VL: Ventral lateral nucleus [thalamus]
- VM: Ventral medial nucleus [thalamus]
- ZI: Zona incerta
- ac: Anterior commissure
- cc: Corpus callosum
- cpb: Cerebellar peduncles
- dne: Diencephalic neuroepithelium
- emlth: External medullary lamina [thalamus]
- ext: External capsule
- fx: Fornix
- int: Internal capsule
- mml: Medial medullary lamina
- mtg: Mammillotegmental tract
- mth: Mammillothalamic tract
- ot: Optic tract
- pmt: Principal mammillary tract
- stm: Stria medullaris
- stt: Stria terminalis
- wmf: White matter fibers
- → LF: Lateral fissure

Age: 22 GW

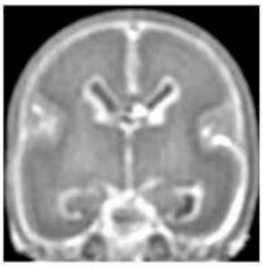

A-P Level: -0.9 mm

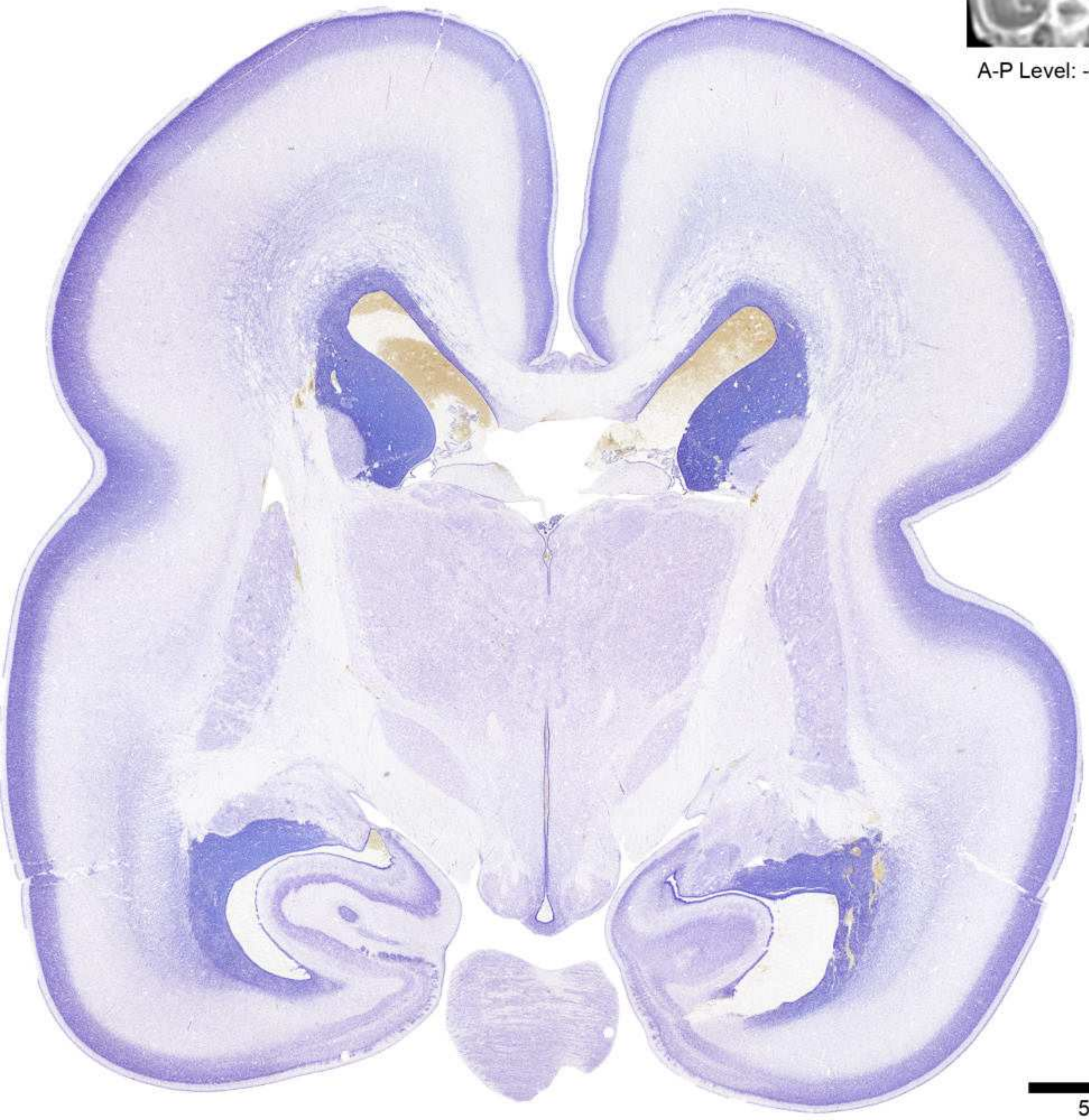

5 mm

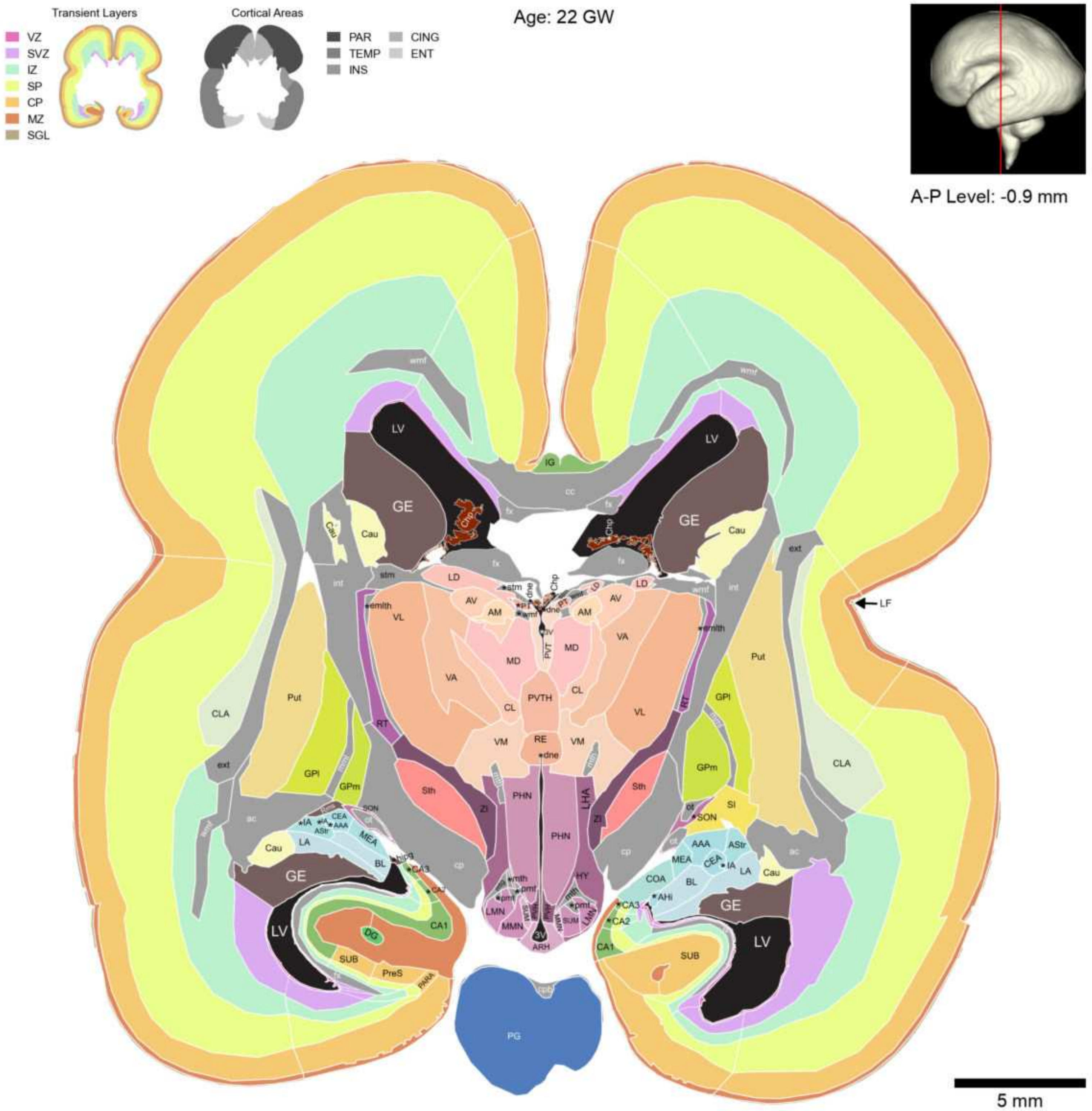

- 3V: Third ventricle
- AAA: Anterior amygdaloid area
- AHi: Amygdalo-hippocampal area
- AM: Anteromedial nucleus [thalamus]
- ARH: Arcuate nucleus [hypothalamus]
- AStr: Amygdalo-striatal area
- AV: Anteroventral nucleus [thalamus]
- BL: Basal nucleus [amygdala]
- CA1: CA1 field [hippocampus]
- CA2: CA2 field [hippocampus]
- CA3: CA3 field [hippocampus]
- CEA: Central nucleus [amygdala]
- CL: Central lateral nucleus [thalamus]
- CLA: Claustrum
- COA: Cortical nucleus [amygdala]
- Cau: Caudate nucleus
- Chp: Choroid plexus
- DG: Dentate gyrus
- GE: Ganglionic eminence
- GPl: Globus pallidus lateral segment
- GPm: Globus pallidus medial segment
- HY: Hypothalamus
- IA: Intercalated cell groups [amygdala]
- IG: Induseum griseum
- LA: Lateral nucleus [amygdala]
- LD: Lateral dorsal nucleus [thalamus]
- LHA: Lateral hypothalamic area
- LMN: Lateral mammillary nucleus
- LV: Lateral ventricle
- MD: Medial dorsal nucleus [thalamus]
- MEA: Medial nucleus [amygdala]
- MMN: Medial mammillary nucleus
- PARA: Cortical plate, parasubiculum
- PG: Pontine gray
- PHN: Posterior hypothalamic nucleus
- PT: Paratenial nucleus [thalamus]
- PVH: Paraventricular nucleus [hypothalamus]
- PVT: Paraventricular nucleus [thalamus]
- PVTH: Periventricular complex [thalamus]
- PreS: Cortical plate, presubiculum
- Put: Putamen
- RE: Nucleus reuniens
- RT: Reticular nucleus [thalamus]
- Rms: Rostral migratory stream
- SI: Substantia innominata
- SON: Supraoptic nucleus [hypothalamus]
- SUB: Cortical plate, subiculum
- SUM: Supramammillary area
- Sth: Subthalamus
- VA: Ventral anterior nucleus [thalamus]
- VL: Ventral lateral nucleus [thalamus]
- VM: Ventral medial nucleus [thalamus]
- ZI: Zona incerta
- ac: Anterior commissure
- cc: Corpus callosum
- cp: Cerebral peduncle
- cpb: Cerebellar peduncles
- dne: Diencephalic neuroepithelium
- emlth: External medullary lamina [thalamus]
- ext: External capsule
- fx: Fornix
- hipg: Hippocampal glioepithelium/ependyma
- int: Internal capsule
- mml: Medial medullary lamina
- mtg: Mammillotegmental tract
- mth: Mammillothalamic tract
- ot: Optic tract
- pmt: Principal mammillary tract
- stm: Stria medullaris
- wmf: White matter fibers
- → LF: Lateral fissure

Age: 22 GW

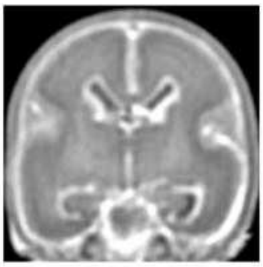

A-P Level: -1.2 mm

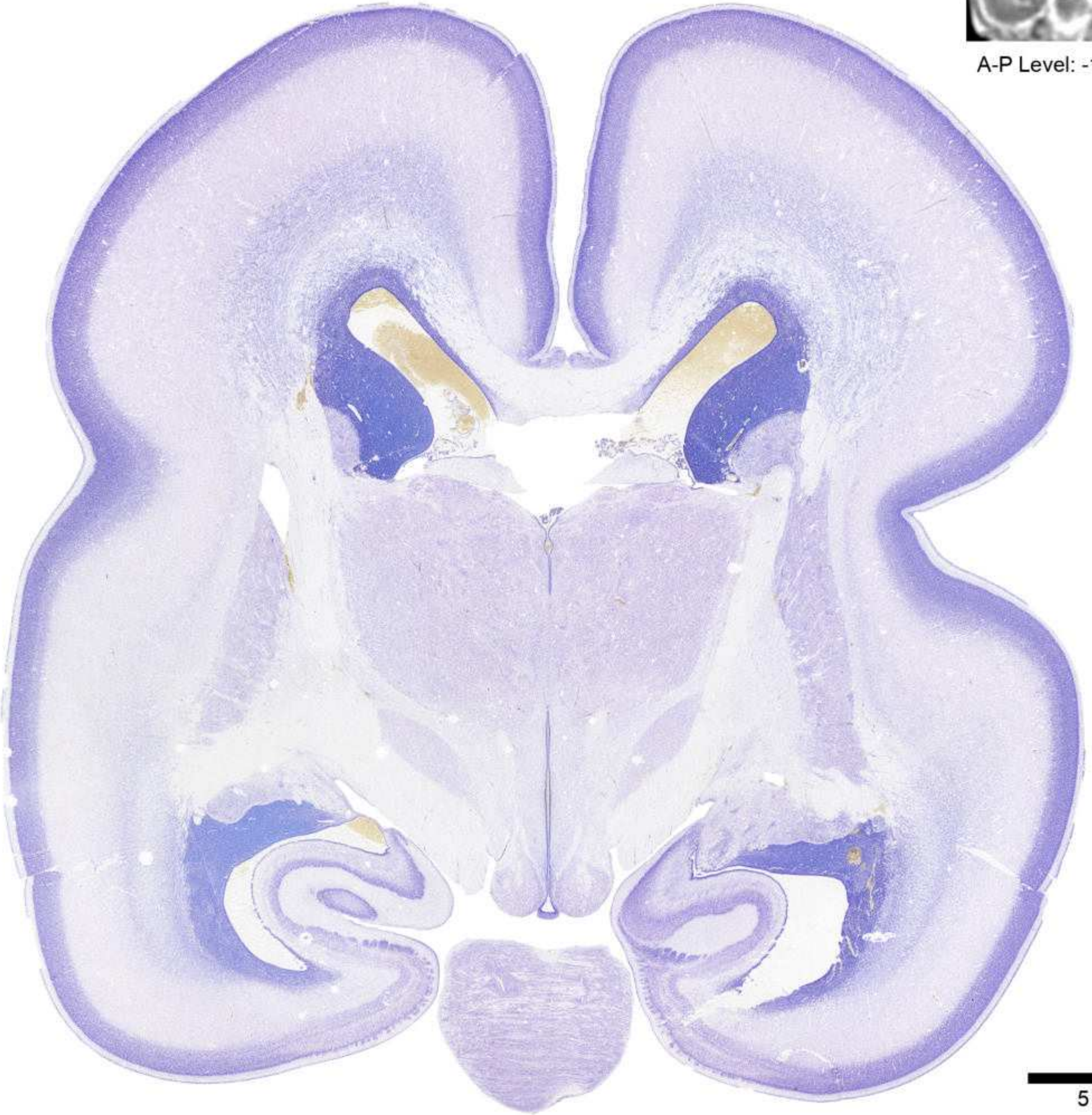

5 mm

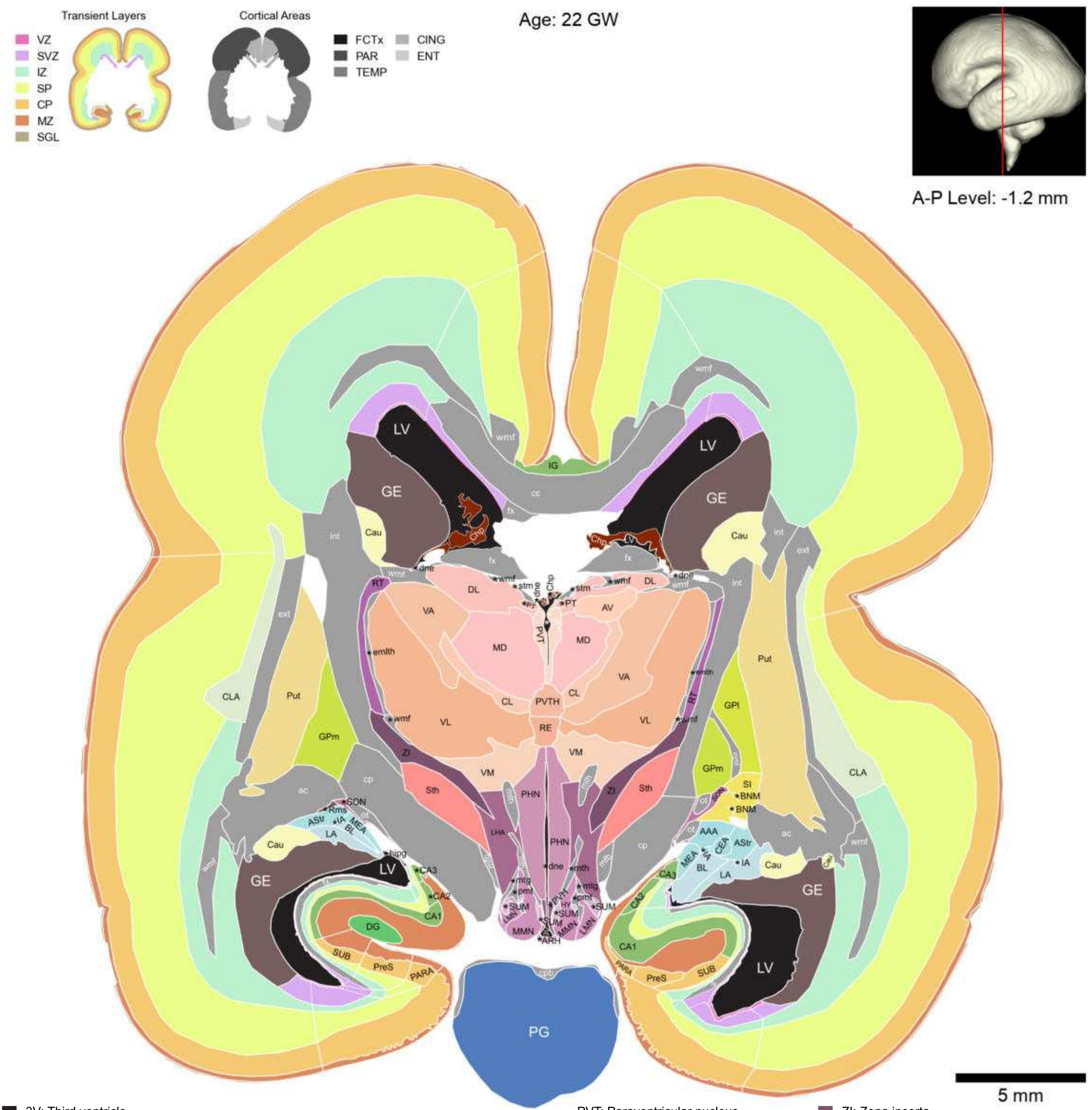

- 3V: Third ventricle
- AAA: Anterior amygdaloid area
- ARH: Arcuate nucleus [hypothalamus]
- AStr: Amygdalo-striatal area
- AV: Anteroventral nucleus [thalamus]
- BL: Basal nucleus [amygdala]
- BNM: Basal nucleus of Meynert
- CA1: CA1 field [hippocampus]
- CA2: CA2 field [hippocampus]
- CA3: CA3 field [hippocampus]
- CEA: Central nucleus [amygdala]
- CL: Central lateral nucleus [thalamus]
- CLA: Claustrum
- Cau: Caudate nucleus
- Chp: Choroid plexus
- DG: Dentate gyrus
- DL: Dorsolateral nucleus [thalamus]
- GE: Ganglionic eminence
- GPl: Globus pallidus lateral segment
- GPm: Globus pallidus medial segment
- HY: Hypothalamus
- IA: Intercalated cell groups [amygdala]
- IG: Induseum griseum
- LA: Lateral nucleus [amygdala]
- LHA: Lateral hypothalamic area
- LMN: Lateral mammillary nucleus
- LV: Lateral ventricle
- MD: Medial dorsal nucleus [thalamus]
- MEA: Medial nucleus [amygdala]
- MMN: Medial mammillary nucleus
- PARA: Cortical plate, parasubiculum
- PG: Pontine gray
- PHN: Posterior hypothalamic nucleus
- PT: Paratenial nucleus [thalamus]
- PVH: Paraventricular nucleus [hypothalamus]
- PVT: Paraventricular nucleus [thalamus]
- PVTH: Periventricular complex [thalamus]
- PreS: Cortical plate, presubiculum
- Put: Putamen
- RE: Nucleus reuniens
- RT: Reticular nucleus [thalamus]
- Rms: Rostral migratory stream
- SI: Substantia innominata
- SON: Supraoptic nucleus [hypothalamus]
- SUB: Cortical plate, subiculum
- SUM: Supramammillary area
- Sth: Subthalamus
- VA: Ventral anterior nucleus [thalamus]
- VL: Ventral lateral nucleus [thalamus]
- VM: Ventral medial nucleus [thalamus]
- ZI: Zona incerta
- ac: Anterior commissure
- cc: Corpus callosum
- cp: Cerebral peduncle
- cpb: Cerebellar peduncles
- dne: Diencephalic neuroepithelium
- emlth: External medullary lamina [thalamus]
- ext: External capsule
- fx: Fornix
- hipg: Hippocampal glioepithelium/ependyma
- int: Internal capsule
- mfb: Medial forebrain bundle
- mml: Medial medullary lamina
- mtg: Mammillotegmental tract
- mth: mammillothalamic tract
- ot: Optic tract
- pmt: Principal mammillary tract
- stm: Stria medullaris
- wmf: White matter fibers

Age: 22 GW

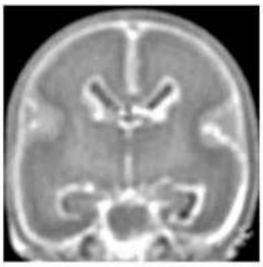

A-P Level: -1.62 mm

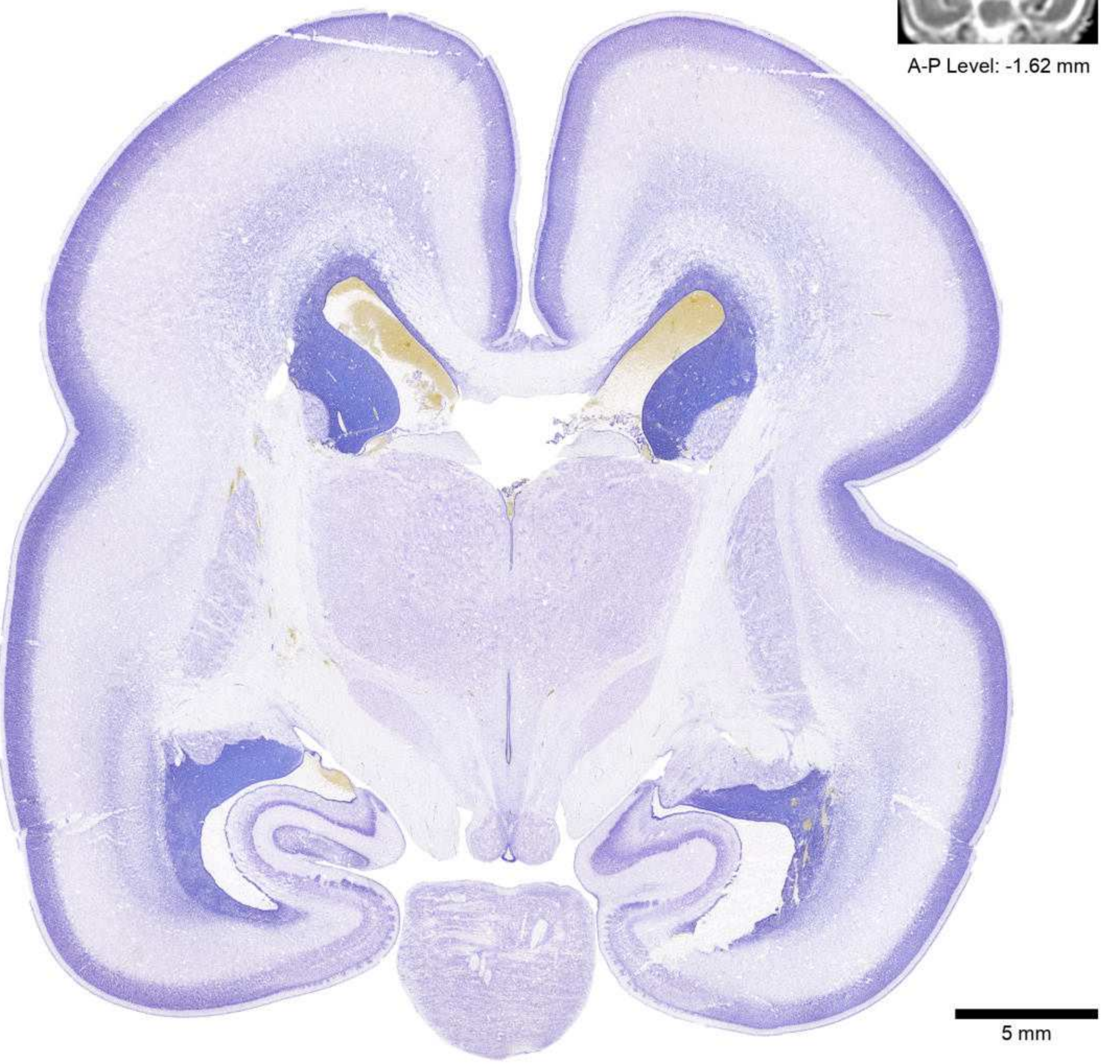

5 mm

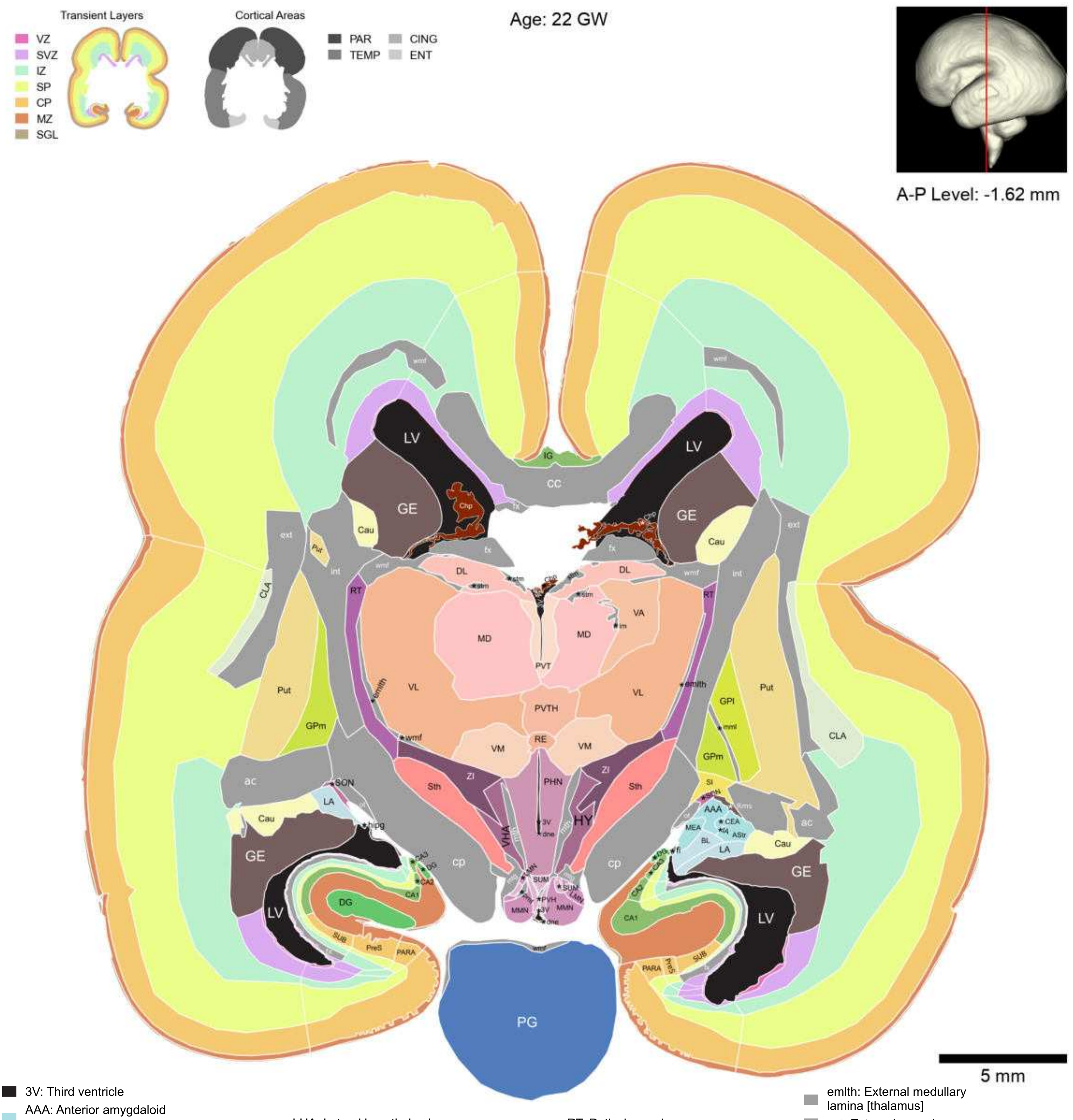

- 3V: Third ventricle
- AAA: Anterior amygdaloid area
- AStr: Amygdalo-striatal area
- BL: Basal nucleus [amygdala]
- CA1: CA1 field [hippocampus]
- CA2: CA2 field [hippocampus]
- CA3: CA3 field [hippocampus]
- CEA: Central nucleus [amygdala]
- CLA: Claustrum
- Cau: Caudate nucleus
- Chp: Choroid plexus
- DG: Dentate gyrus
- DL: Dorsolateral nucleus [thalamus]
- GE: Ganglionic eminence
- GPl: Globus pallidus lateral segment
- GPm: Globus pallidus medial segment
- HY: Hypothalamus
- IA: Intercalated cell groups [amygdala]
- IG: Induseum griseum
- LA: Lateral nucleus [amygdala]
- LHA: Lateral hypothalamic area
- LMN: Lateral mammillary nucleus
- LV: Lateral ventricle
- MD: Medial dorsal nucleus [thalamus]
- MEA: Medial nucleus [amygdala]
- MMN: Medial mammillary nucleus
- PARA: Cortical plate, parasubiculum
- PG: Pontine gray
- PHN: Posterior hypothalamic nucleus
- PVH: Paraventricular nucleus [hypothalamus]
- PVT: Paraventricular nucleus [thalamus]
- PVTH: Periventricular complex [thalamus]
- PreS: Cortical plate, presubiculum
- Put: Putamen
- RE: Nucleus reuniens
- RT: Reticular nucleus [thalamus]
- Rms: Rostral migratory stream
- SI: Substantia innominata
- SON: Supraoptic nucleus [hypothalamus]
- SUB: Cortical plate, subiculum
- SUM: Supramammillary area
- Sth: Subthalamus
- VA: Ventral anterior nucleus [thalamus]
- VL: Ventral lateral nucleus [thalamus]
- VM: Ventral medial nucleus [thalamus]
- ZI: Zona incerta
- ac: Anterior commissure
- cc: Corpus callosum
- cp: Cerebral peduncle
- dne: Diencephalic neuroepithelium
- emlth: External medullary lamina [thalamus]
- ext: External capsule
- fi: Fimbria
- fx: Fornix
- hipg: Hippocampal glioepithelium/ependyma
- im: Internal medullary lamina [thalamus]
- int: Internal capsule
- mml: Medial medullary lamina
- mtg: Mammillotegmental tract
- mth: mammillothalamic tract
- ot: Optic tract
- pmt: Principal mammillary tract
- stm: Stria medullaris
- wmf: White matter fibers

Age: 22 GW

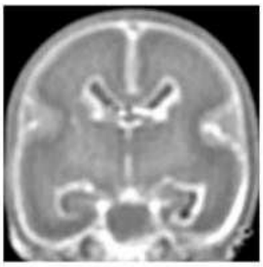

A-P Level: -2.04 mm

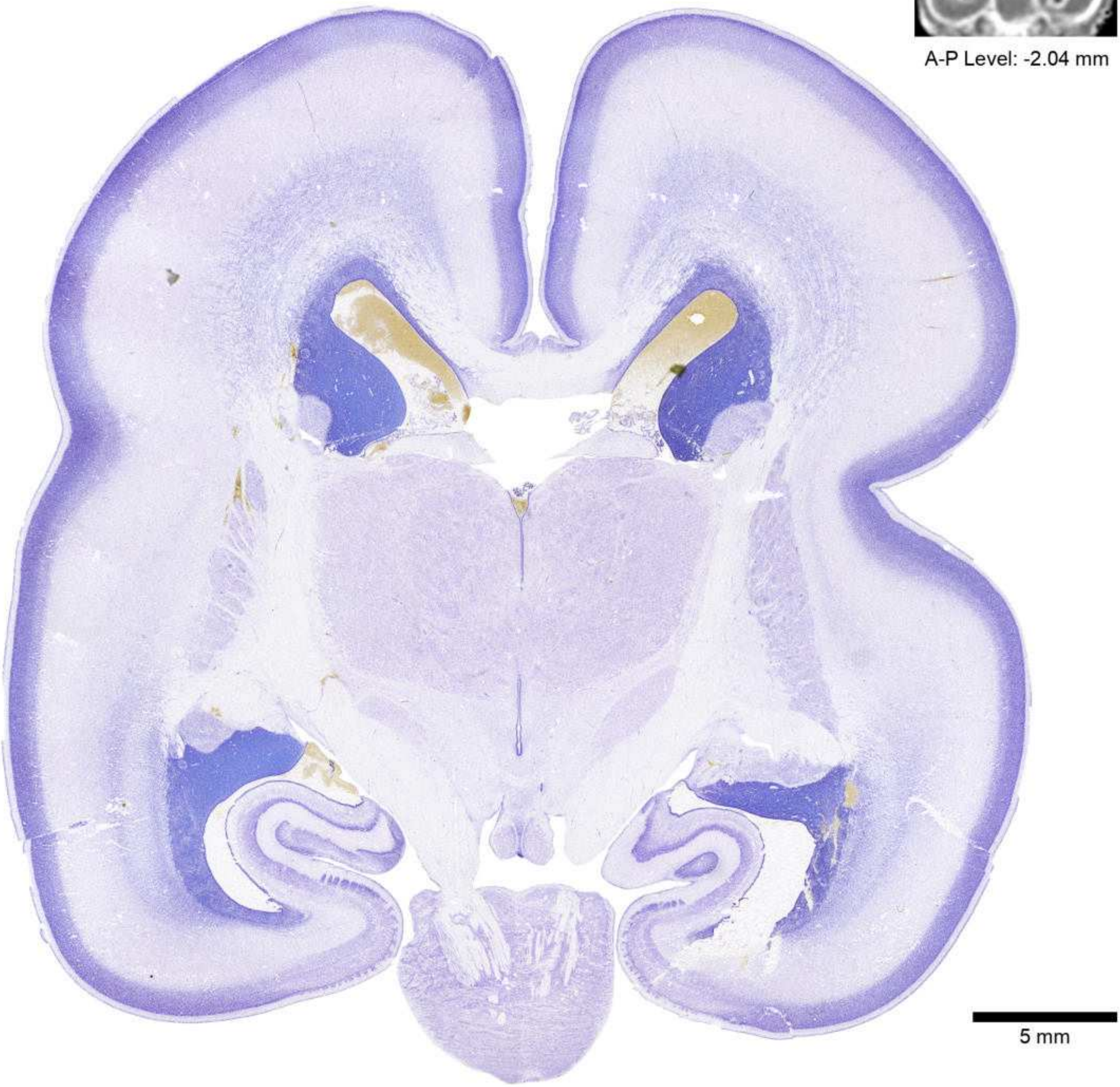

5 mm

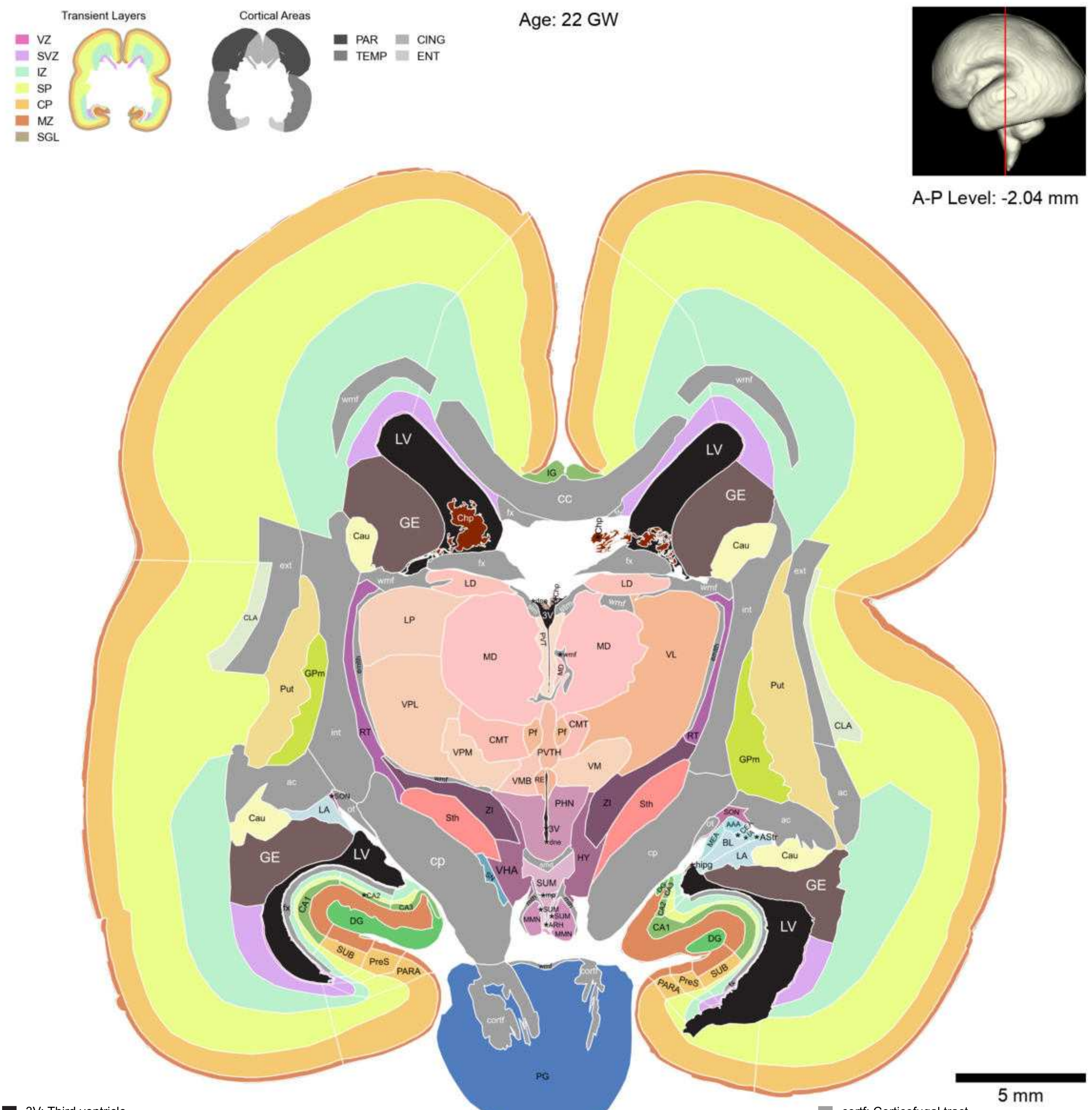

- 3V: Third ventricle
- AAA: Anterior amygdaloid area
- ARH: Arcuate nucleus [hypothalamus]
- AStr: Amygdalo-striatal area
- BL: Basal nucleus [amygdala]
- CA1: CA1 field [hippocampus]
- CA2: CA2 field [hippocampus]
- CA3: CA3 field [hippocampus]
- CEA: Central nucleus [amygdala]
- CLA: Claustrum
- CMT: Centromedian nucleus [thalamus]
- Cau: Caudate nucleus
- Chp: Choroid plexus
- DG: Dentate gyrus
- GE: Ganglionic eminence
- GPm: Globus pallidus medial segment
- HY: Hypothalamus
- IA: Intercalated cell groups [amygdala]
- IG: Induseum griseum
- LA: Lateral nucleus [amygdala]
- LD: Lateral dorsal nucleus [thalamus]
- LHA: Lateral hypothalamic area
- LP: Lateral posterior nucleus [thalamus]
- LV: Lateral ventricle
- MD: Medial dorsal nucleus [thalamus]
- MEA: Medial nucleus [amygdala]
- MMN: Medial mammillary nucleus
- PARA: Cortical plate, parasubiculum
- PG: Pontine gray
- PHN: Posterior hypothalamic nucleus
- PVT: Paraventricular nucleus [thalamus]
- PVTH: Periventricular complex [thalamus]
- Pf: Parafascicular nucleus [thalamus]
- PreS: Cortical plate, presubiculum
- Put: Putamen
- RE: Nucleus reuniens
- RT: Reticular nucleus [thalamus]
- SN: Substantia nigra
- SON: Supraoptic nucleus [hypothalamus]
- SUB: Cortical plate, subiculum
- SUM: Supramammillary area
- Sth: Subthalamus
- VL: Ventral lateral nucleus [thalamus]
- VM: Ventral medial nucleus [thalamus]
- VMB: Ventral medial basal nucleus [thalamus]
- VPL: Ventral posterolateral nucleus [thalamus]
- VPM: Ventral posteromedial nucleus [thalamus]
- ZI: Zona incerta
- ac: Anterior commissure
- cc: Corpus callosum
- cortf: Corticofugal tract
- cp: Cerebral peduncle
- dne: Diencephalic neuroepithelium
- emlth: External medullary lamina [thalamus]
- ext: External capsule
- fx: Fornix
- hipg: Hippocampal glioepithelium/ependyma
- int: Internal capsule
- mp: Mammillary peduncle
- mth: Mammillothalamic tract
- ot: Optic tract
- smd: Supramammillary decussation
- stm: Stria medullaris
- wmf: White matter fibers

Age: 22 GW

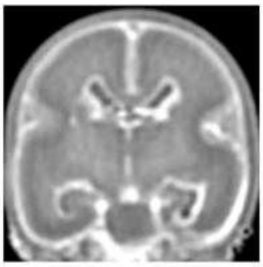

A-P Level: -2.64 mm

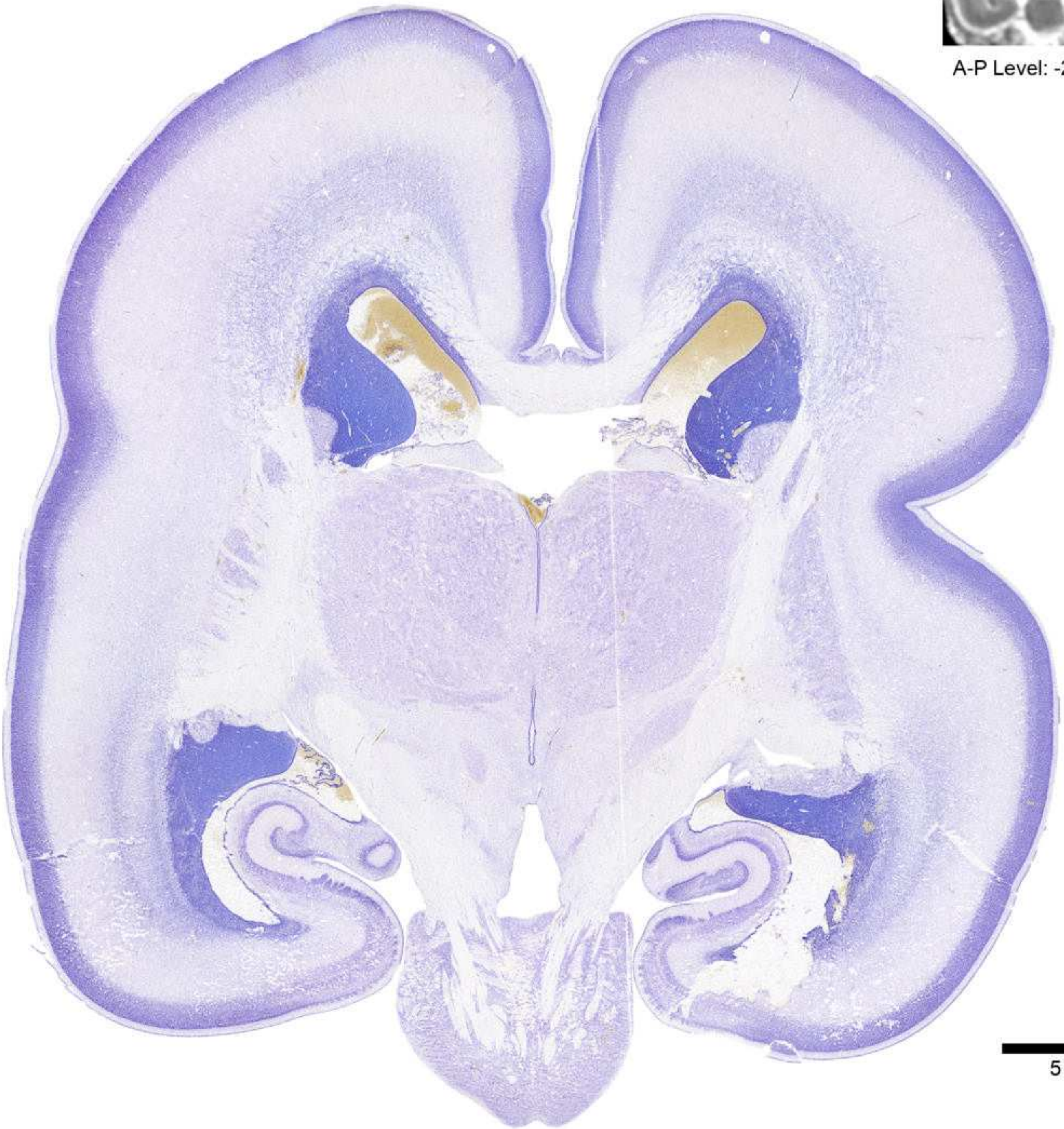

5 mm

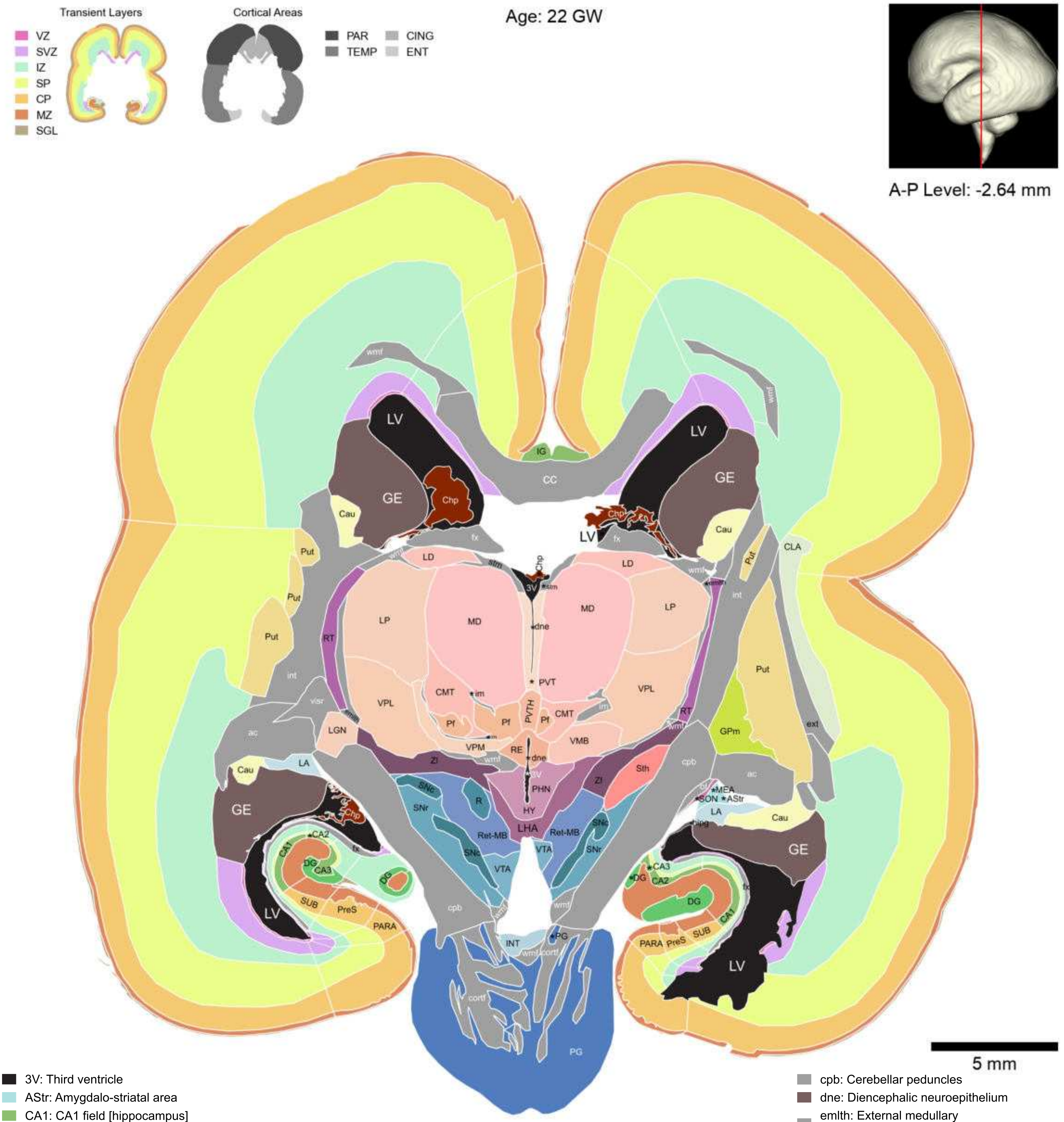

- 3V: Third ventricle
- AStr: Amygdalo-striatal area
- CA1: CA1 field [hippocampus]
- CA2: CA2 field [hippocampus]
- CA3: CA3 field [hippocampus]
- CLA: Claustrum
- CMT: Centromedian nucleus [thalamus]
- Cau: Caudate nucleus
- Chp: Choroid plexus
- DG: Dentate gyrus
- GE: Ganglionic eminence
- GPm: Globus pallidus medial segment
- HY: Hypothalamus
- IG: Induseum griseum
- INT: Interpeduncular nucleus
- LA: Lateral nucleus [amygdala]
- LD: Lateral dorsal nucleus [thalamus]
- LGN: Lateral geniculate nucleus
- LHA: Lateral hypothalamic area
- LP: Lateral posterior nucleus [thalamus]
- LV: Lateral ventricle
- MD: Medial dorsal nucleus [thalamus]
- MEA: Medial nucleus [amygdala]
- PARA: Cortical plate, parasubiculum
- PG: Pontine gray
- PHN: Posterior hypothalamic nucleus
- PVT: Paraventricular nucleus [thalamus]
- PVTH: Periventricular complex [thalamus]
- Pf: Parafascicular nucleus [thalamus]
- PreS: Cortical plate, presubiculum
- Put: Putamen
- R: Red nucleus
- RE: Nucleus reuniens
- RT: Reticular nucleus [thalamus]
- Ret-MB: Reticular formation, Midbrain
- SNc: Substantia nigra pars compacta
- SNr: Substantia nigra pars reticulata
- SON: Supraoptic nucleus [hypothalamus]
- SUB: Cortical plate, subiculum
- Sth: Subthalamus
- VMB: Ventral medial basal nucleus [thalamus]
- VPL: Ventral posterolateral nucleus [thalamus]
- VPM: Ventral posteromedial nucleus [thalamus]
- VTA: Ventral tegmental area
- ZI: Zona incerta
- ac: Anterior commissure
- cc: Corpus callosum
- cortf: Corticofugal tract
- cpb: Cerebellar peduncles
- dne: Diencephalic neuroepithelium
- emlth: External medullary lamina [thalamus]
- ext: External capsule
- fx: Fornix
- hipg: Hippocampal glioepithelium/ependyma
- im: Internal medullary lamina [thalamus]
- int: Internal capsule
- ot: Optic tract
- stm: Stria medullaris
- visr: Visual radiation
- wmf: White matter fibers

Age: 22 GW

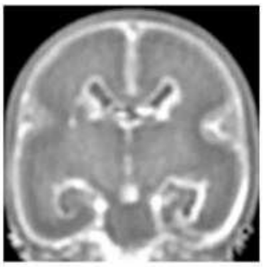

A-P Level: -3.06 mm

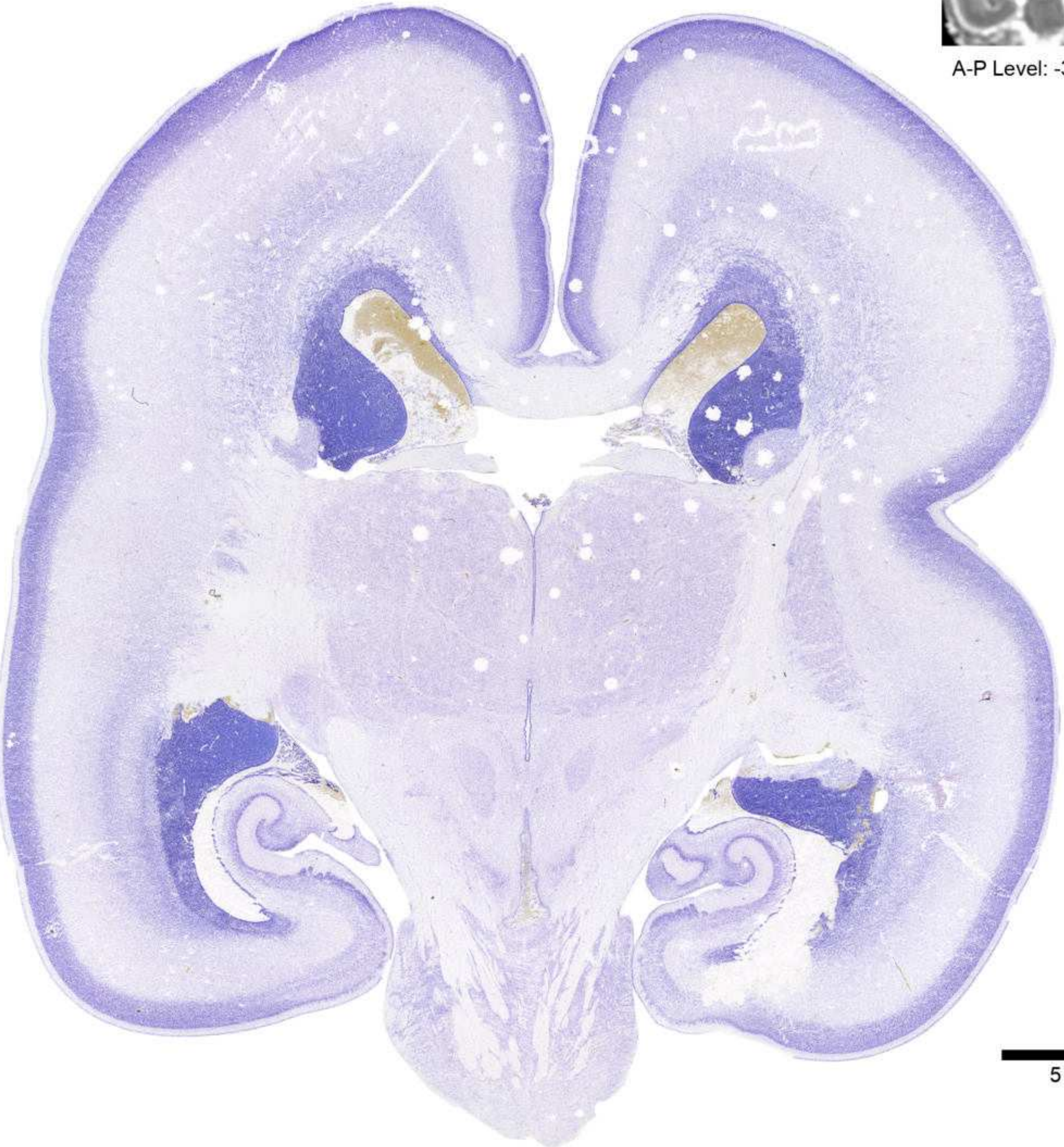

5 mm

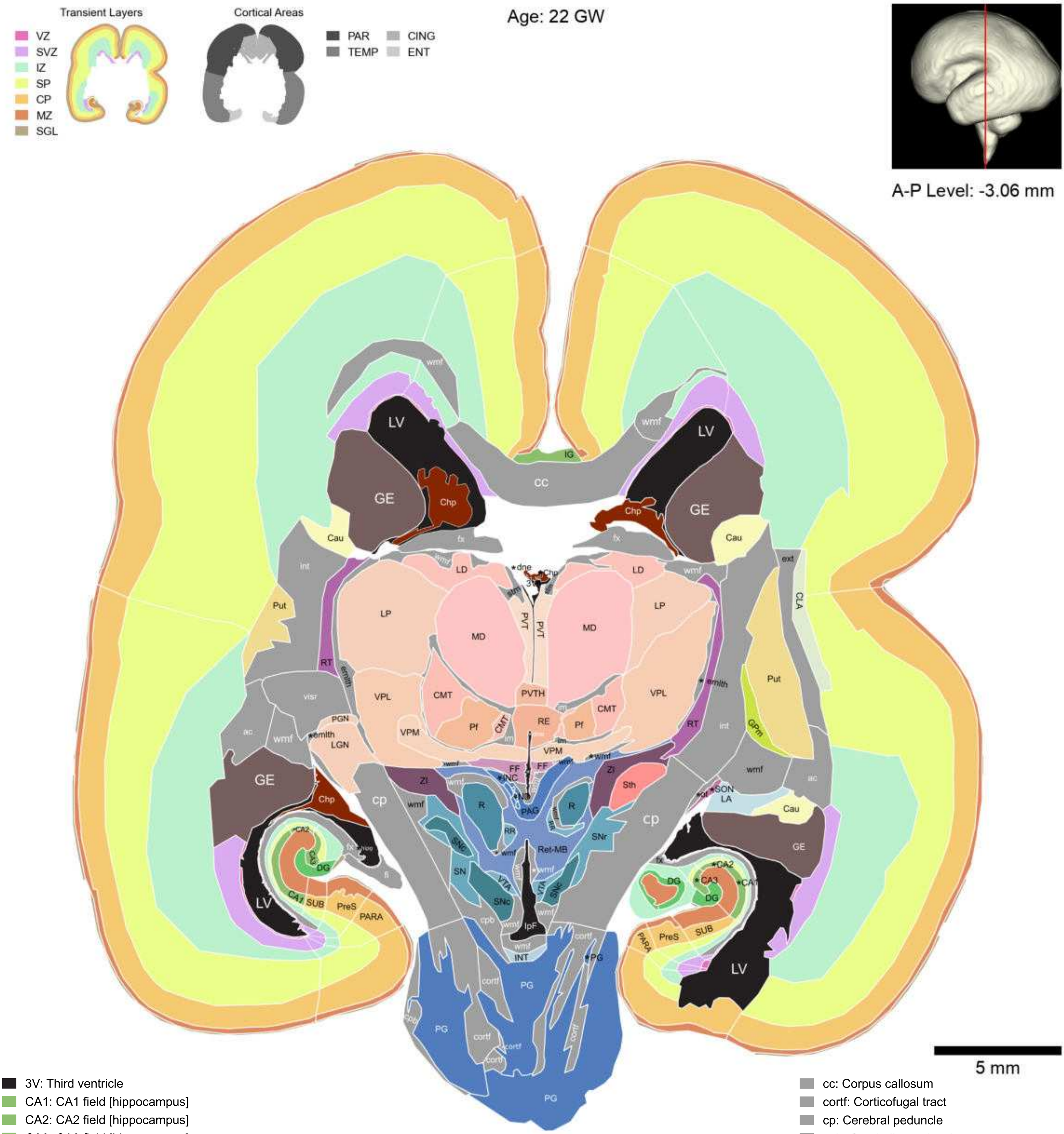

- 3V: Third ventricle
- CA1: CA1 field [hippocampus]
- CA2: CA2 field [hippocampus]
- CA3: CA3 field [hippocampus]
- CLA: Claustrum
- CMT: Centromedian nucleus [thalamus]
- Cau: Caudate nucleus
- Chp: Choroid plexus
- DG: Dentate gyrus
- FF: Field of Forel
- GE: Ganglionic eminence
- GPm: Globus pallidus medial segment
- IG: Induseum griseum
- INC: Interstitial nucleus of Cajal
- INT: Interpeduncular nucleus
- IpF: Interpeduncular fossa
- LA: Lateral nucleus [amygdala]
- LD: Lateral dorsal nucleus [thalamus]
- LGN: Lateral geniculate nucleus
- LP: Lateral posterior nucleus [thalamus]
- LV: Lateral ventricle
- MD: Medial dorsal nucleus [thalamus]
- ND: Nucleus of Darkschewitsch
- PAG: Periaqueductal gray
- PARA: Cortical plate, parasubiculum
- PG: Pontine gray
- PGN: Pregeniculate nucleus
- PVT: Paraventricular nucleus [thalamus]
- PVTH: Periventricular complex [thalamus]
- Pf: Parafascicular nucleus [thalamus]
- PreS: Cortical plate, presubiculum
- Put: Putamen
- R: Red nucleus
- RE: Nucleus reuniens
- RR: Retrorubral area
- RT: Reticular nucleus [thalamus]
- Ret-MB: Reticular formation, Midbrain
- SN: Substantia nigra
- SNc: Substantia nigra pars compacta
- SNr: Substantia nigra pars reticulata
- SON: Supraoptic nucleus [hypothalamus]
- SUB: Cortical plate, subiculum
- Sth: Subthalamus
- VPL: Ventral posterolateral nucleus [thalamus]
- VPM: Ventral posteromedial nucleus [thalamus]
- VTA: Ventral tegmental area
- ZI: Zona incerta
- ac: Anterior commissure
- cc: Corpus callosum
- cortf: Corticofugal tract
- cp: Cerebral peduncle
- cpb: Cerebellar peduncles
- dne: Diencephalic neuroepithelium
- emlth: External medullary lamina [thalamus]
- ext: External capsule
- fi: Fimbria
- fx: Fornix
- hipg: Hippocampal glioepithelium/ependyma
- im: Internal medullary lamina [thalamus]
- int: Internal capsule
- ot: Optic tract
- stm: Stria medullaris
- visr: Visual radiation
- wmf: White matter fibers

Age: 22 GW

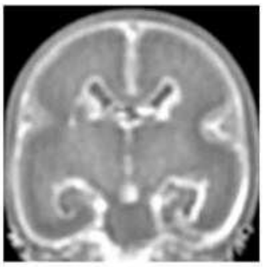

A-P Level: -3.48 mm

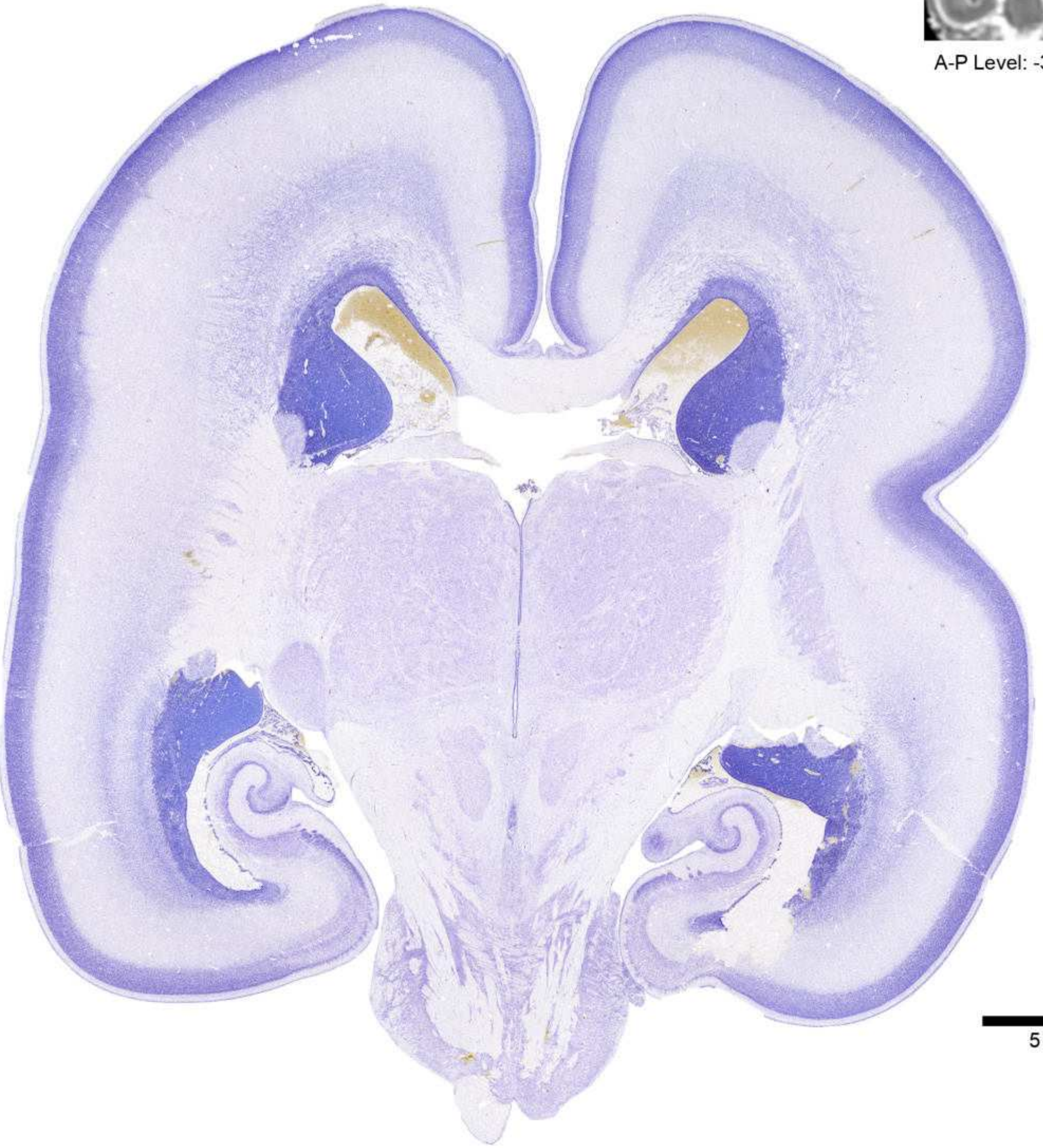

5 mm

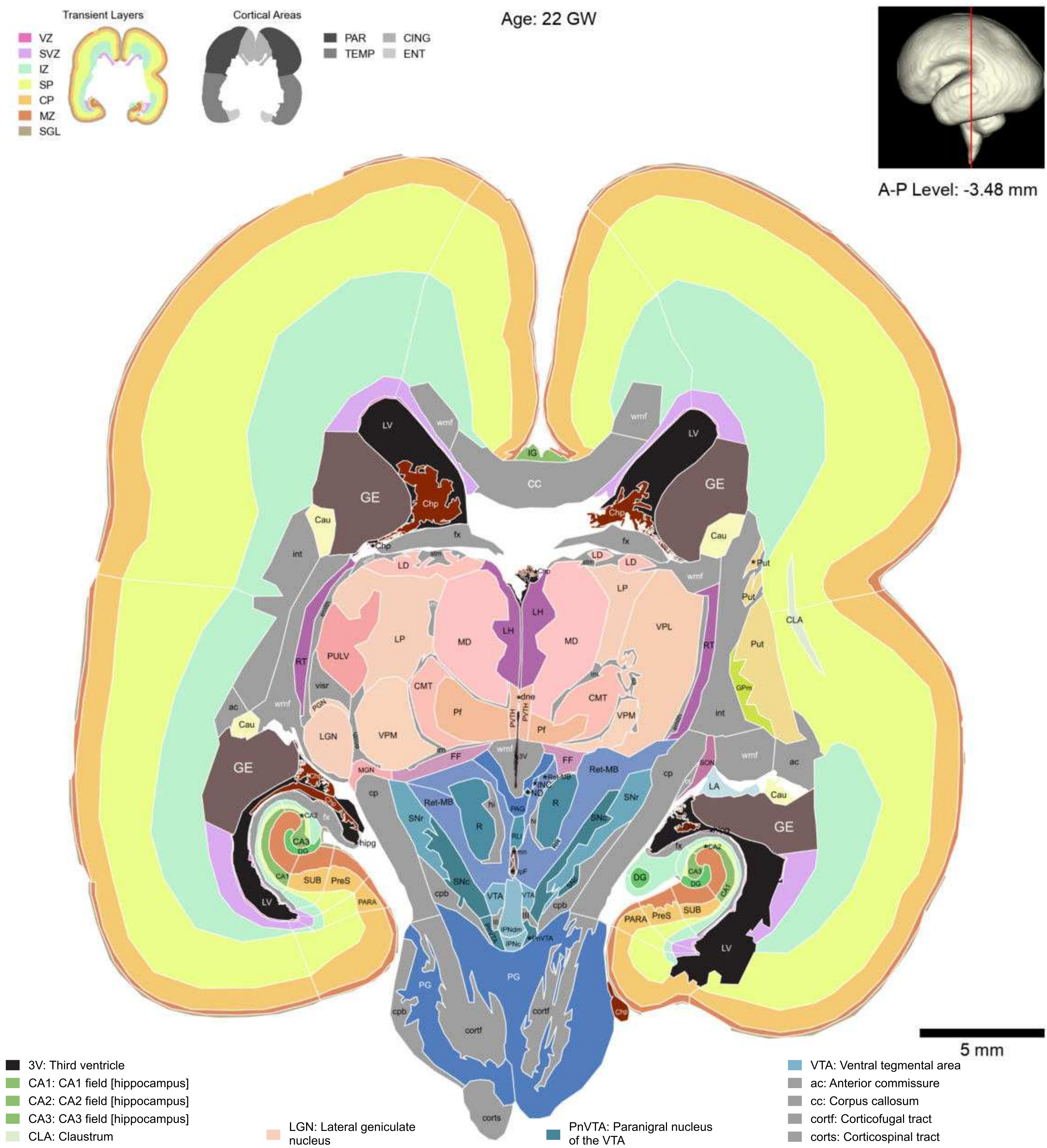

3V: Third ventricle
CA1: CA1 field [hippocampus]
CA2: CA2 field [hippocampus]
CA3: CA3 field [hippocampus]
CLA: Claustrum
CMT: Centromedian nucleus [thalamus]
Cau: Caudate nucleus
Chp: Choroid plexus
DG: Dentate gyrus
FF: Field of Forel
GE: Ganglionic eminence
GPm: Globus pallidus medial segment
IG: Induseum griseum
III: Oculomotor nerve
INC: Interstitial nucleus of Cajal
IPNc: Interpeduncular nucleu, caudal part
IPNdm: Interpeduncular nucleu, dorsomedial part
IpF: Interpeduncular fossa
LA: Lateral nucleus [amygdala]
LD: Lateral dorsal nucleus [thalamus]

LGN: Lateral geniculate nucleus
LH: Lateral habenula
LP: Lateral posterior nucleus [thalamus]
LV: Lateral ventricle
MD: Medial dorsal nucleus [thalamus]
MGN: Medial geniculate nucleus
ND: Nucleus of Darkschewitsch
PAG: Periaqueductal gray
PARA: Cortical plate, parasubiculum
PG: Pontine gray
PGN: Pregeniculate nucleus
PULV: Pulvinar nucleus [thalamus]
PVTH: Periventricular complex [thalamus]
Pf: Parafascicular nucleus [thalamus]

PnVTA: Paranigral nucleus of the VTA
PreS: Cortical plate, presubiculum
Put: Putamen
R: Red nucleus
RLI: Rostral linear raphe nucleus
RR: Retrorubral area
RT: Reticular nucleus [thalamus]
Ret-MB: Reticular formation, Midbrain
SNc: Substantia nigra pars compacta
SNr: Substantia nigra pars reticulata
SON: Supraoptic nucleus [hypothalamus]
SUB: Cortical plate, subiculum
VPL: Ventral posterolateral nucleus [thalamus]
VPM: Ventral posteromedial nucleus [thalamus]

VTA: Ventral tegmental area
ac: Anterior commissure
cc: Corpus callosum
cortf: Corticofugal tract
corts: Corticospinal tract
cp: Cerebral peduncle
cpb: Cerebellar peduncles
dne: Diencephalic neuroepithelium
emlth: External medullary lamina [thalamus]
fx: Fornix
hi: Habenulo-interpenduncular tract
hipg: Hippocampal glioepithelium/ependyma
im: Internal medullary lamina [thalamus]
int: Internal capsule
mn: Mesencephalic neuroepithelium
ot: Optic tract
stm: Stria medullaris
visr: Visual radiation
wmf: White matter fibers

Age: 22 GW

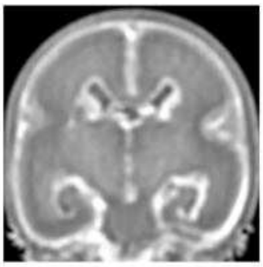

A-P Level: -3.84 mm

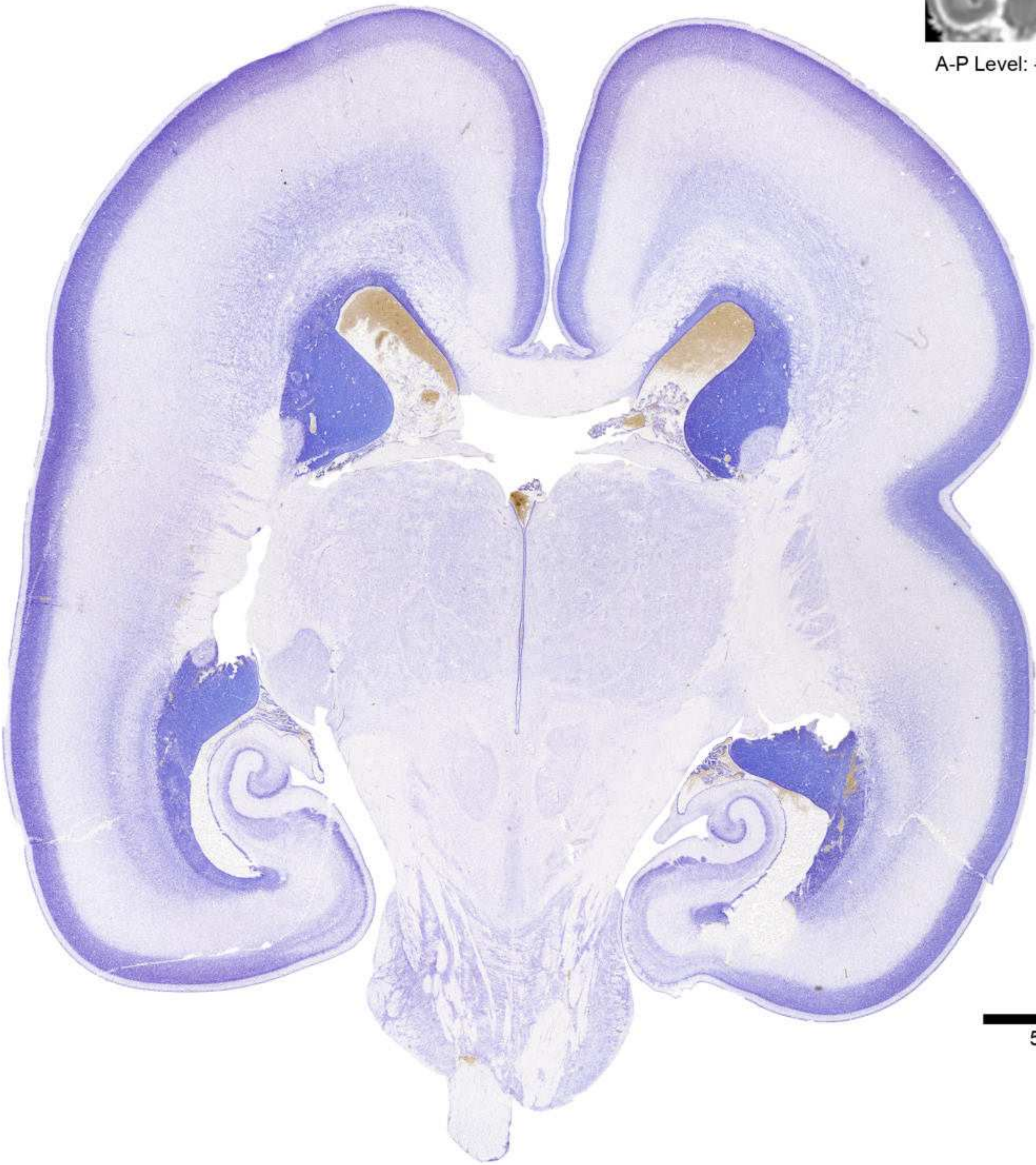

5 mm

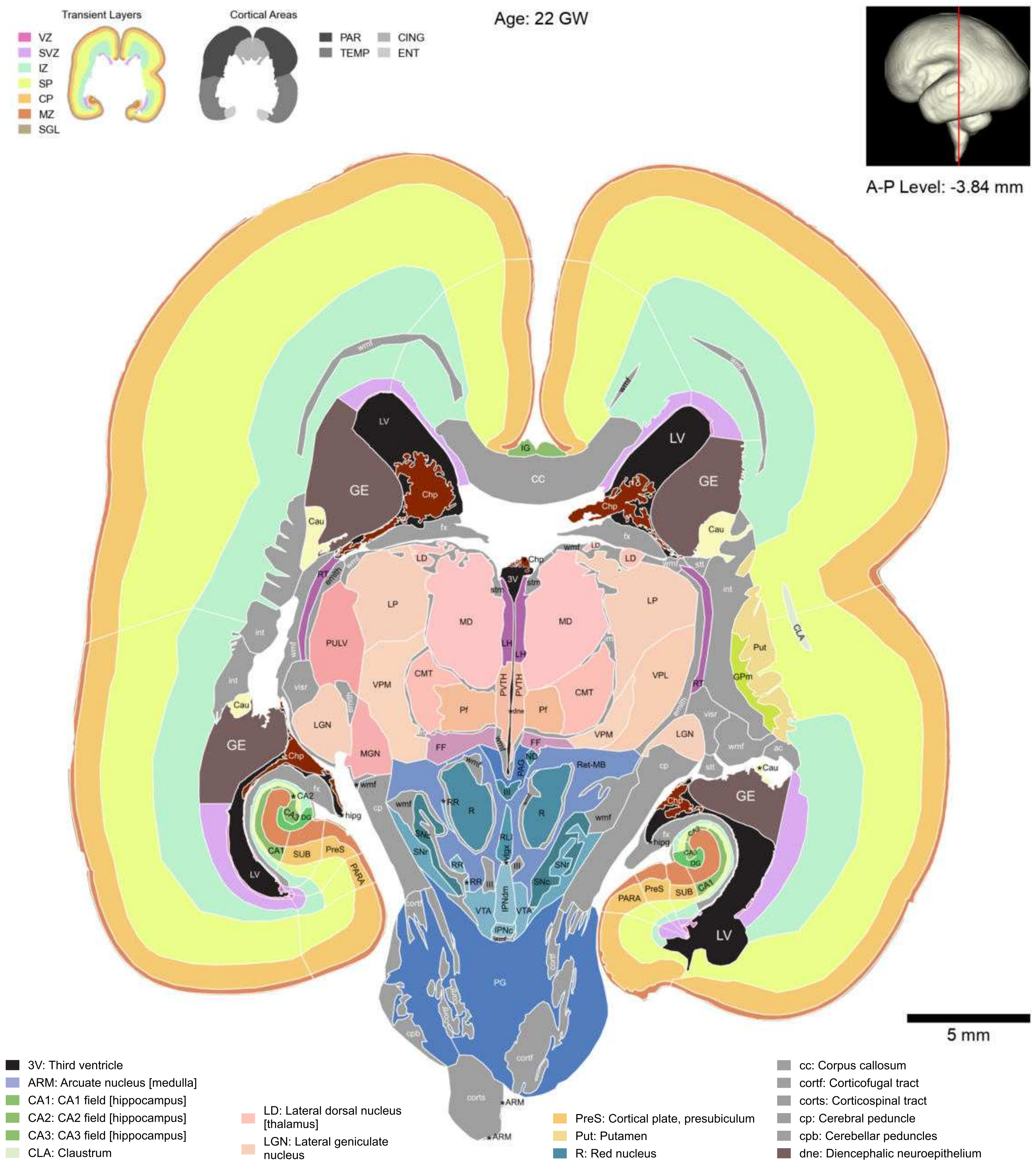

- 3V: Third ventricle
- ARM: Arcuate nucleus [medulla]
- CA1: CA1 field [hippocampus]
- CA2: CA2 field [hippocampus]
- CA3: CA3 field [hippocampus]
- CLA: Claustrum
- CMT: Centromedian nucleus [thalamus]
- Cau: Caudate nucleus
- Chp: Choroid plexus
- DG: Dentate gyrus
- FF: Field of Forel
- GE: Ganglionic eminence
- GPm: Globus pallidus medial segment
- IG: Induseum griseum
- III: Oculomotor nerve
- IPNc: Interpeduncular nucleu, caudal part
- IPNdm: Interpeduncular nucleu, dorsomedial part
- LD: Lateral dorsal nucleus [thalamus]
- LGN: Lateral geniculate nucleus
- LH: Lateral habenula
- LP: Lateral posterior nucleus [thalamus]
- LV: Lateral ventricle
- MD: Medial dorsal nucleus [thalamus]
- MGN: Medial geniculate nucleus
- ND: Nucleus of Darkschewitsch
- PAG: Periaqueductal gray
- PARA: Cortical plate, parasubiculum
- PG: Pontine gray
- PULV: Pulvinar nucleus [thalamus]
- PVTH: Periventricular complex [thalamus]
- Pf: Parafascicular nucleus [thalamus]
- PreS: Cortical plate, presubiculum
- Put: Putamen
- R: Red nucleus
- RLI: Rostral linear raphe nucleus
- RR: Retrorubral area
- RT: Reticular nucleus [thalamus]
- Ret-MB: Reticular formation, Midbrain
- SNc: Substantia nigra pars compacta
- SNr: Substantia nigra pars reticulata
- SUB: Cortical plate, subiculum
- VPL: Ventral posterolateral nucleus [thalamus]
- VPM: Ventral posteromedial nucleus [thalamus]
- VTA: Ventral tegmental area
- ac: Anterior commissure
- cc: Corpus callosum
- cortf: Corticofugal tract
- corts: Corticospinal tract
- cp: Cerebral peduncle
- cpb: Cerebellar peduncles
- dne: Diencephalic neuroepithelium
- emlth: External medullary lamina [thalamus]
- fx: Fornix
- hipg: Hippocampal glioepithelium/ependyma
- im: Internal medullary lamina [thalamus]
- int: Internal capsule
- stm: Stria medullaris
- stt: Stria terminalis
- visr: Visual radiation
- vtgx: Ventral tegmental decussation
- wmf: White matter fibers

Age: 22 GW

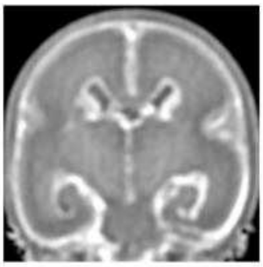

A-P Level: -4.2 mm

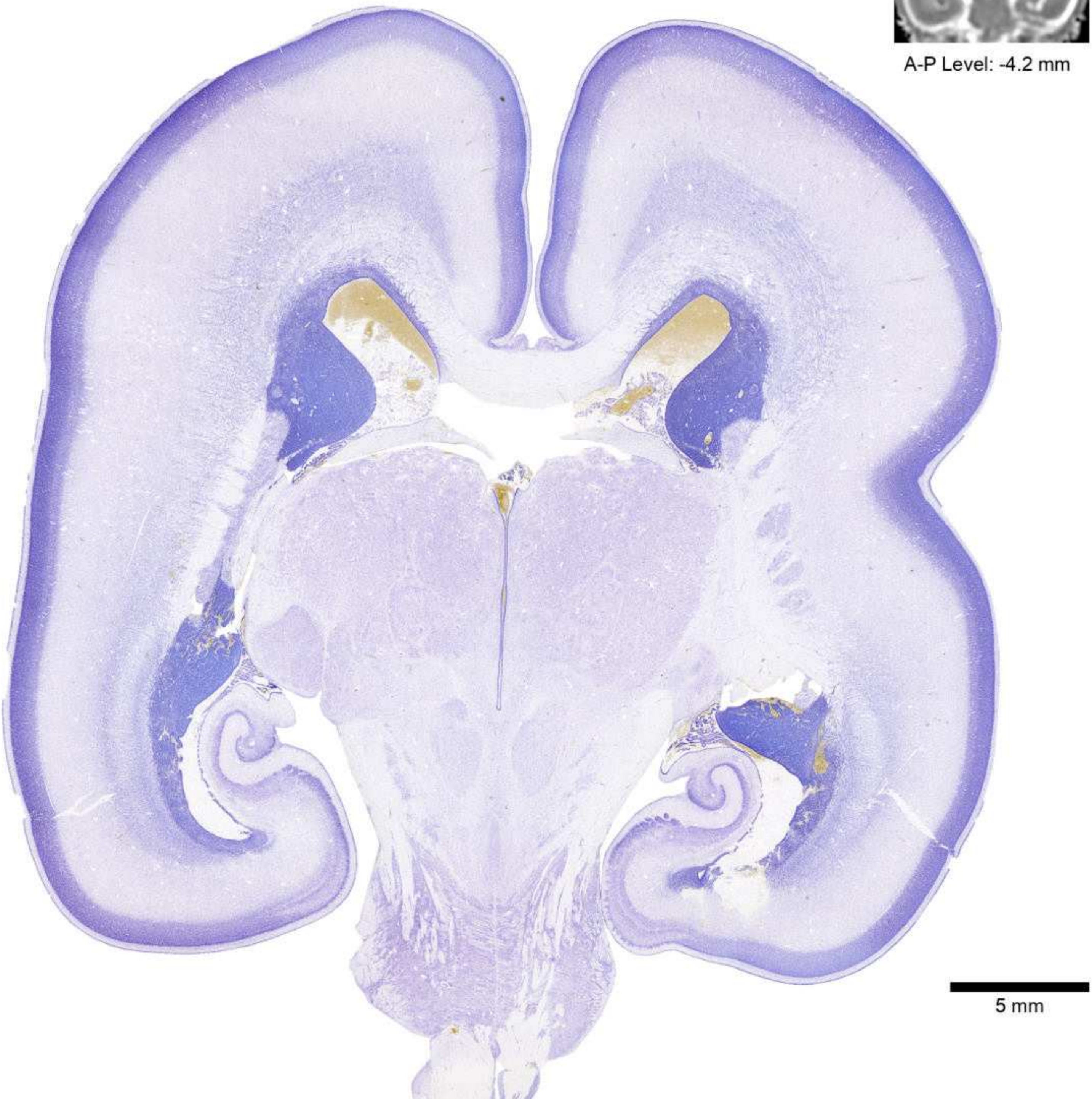

5 mm

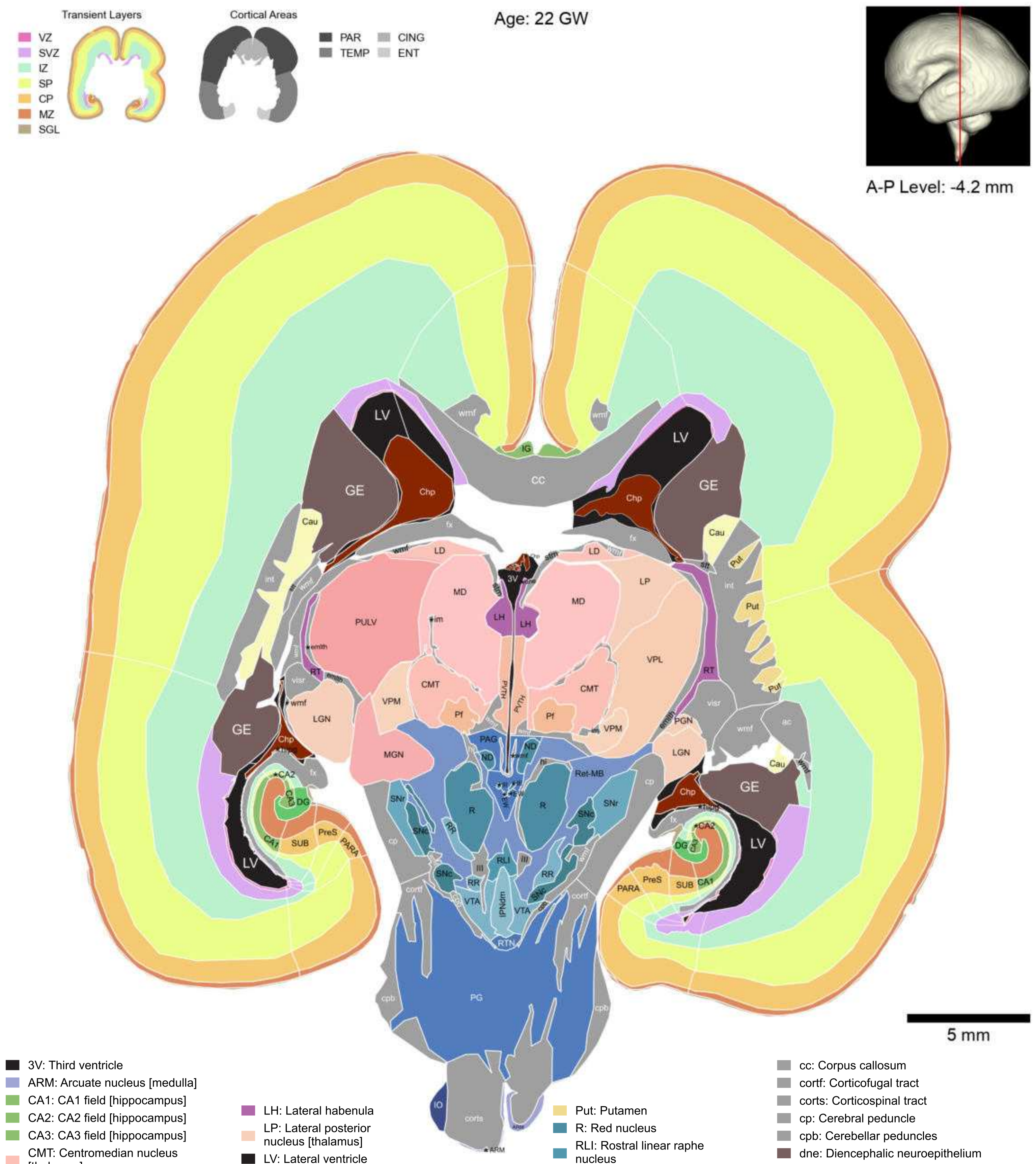

- 3V: Third ventricle
- ARM: Arcuate nucleus [medulla]
- CA1: CA1 field [hippocampus]
- CA2: CA2 field [hippocampus]
- CA3: CA3 field [hippocampus]
- CMT: Centromedian nucleus [thalamus]
- Cau: Caudate nucleus
- Chp: Choroid plexus
- DG: Dentate gyrus
- EW: Edinger-Westphal nucleus
- GE: Ganglionic eminence
- IG: Induseum griseum
- III: Oculomotor nerve
- IO: Inferior olive
- IPNdm: Interpeduncular nucleu, dorsomedial part
- LD: Lateral dorsal nucleus [thalamus]
- LGN: Lateral geniculate nucleus
- LH: Lateral habenula
- LP: Lateral posterior nucleus [thalamus]
- LV: Lateral ventricle
- MD: Medial dorsal nucleus [thalamus]
- MGN: Medial geniculate nucleus
- ND: Nucleus of Darkschewitsch
- PAG: Periaqueductal gray
- PARA: Cortical plate, parasubiculum
- PG: Pontine gray
- PGN: Pregeniculate nucleus
- PULV: Pulvinar nucleus [thalamus]
- PVTH: Periventricular complex [thalamus]
- Pf: Parafascicular nucleus [thalamus]
- PreS: Cortical plate, presubiculum
- Put: Putamen
- R: Red nucleus
- RLI: Rostral linear raphe nucleus
- RR: Retrorubral area
- RT: Reticular nucleus [thalamus]
- RTN: Reticular tegmental nucleus
- Ret-MB: Reticular formation, Midbrain
- SNc: Substantia nigra pars compacta
- SNr: Substantia nigra pars reticulata
- SUB: Cortical plate, subiculum
- VPL: Ventral posterolateral nucleus [thalamus]
- VPM: Ventral posteromedial nucleus [thalamus]
- VTA: Ventral tegmental area
- ac: Anterior commissure
- cc: Corpus callosum
- cortf: Corticofugal tract
- corts: Corticospinal tract
- cp: Cerebral peduncle
- cpb: Cerebellar peduncles
- dne: Diencephalic neuroepithelium
- emlth: External medullary lamina [thalamus]
- fx: Fornix
- hi: Habenulo-interpenduncular tract
- hipg: Hippocampal glioepithelium/ependyma
- im: Internal medullary lamina [thalamus]
- int: Internal capsule
- stm: Stria medullaris
- stt: Stria terminalis
- visr: Visual radiation
- wmf: White matter fibers

Age: 22 GW

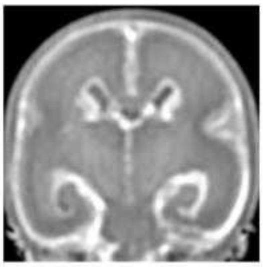

A-P Level: -4.56 mm

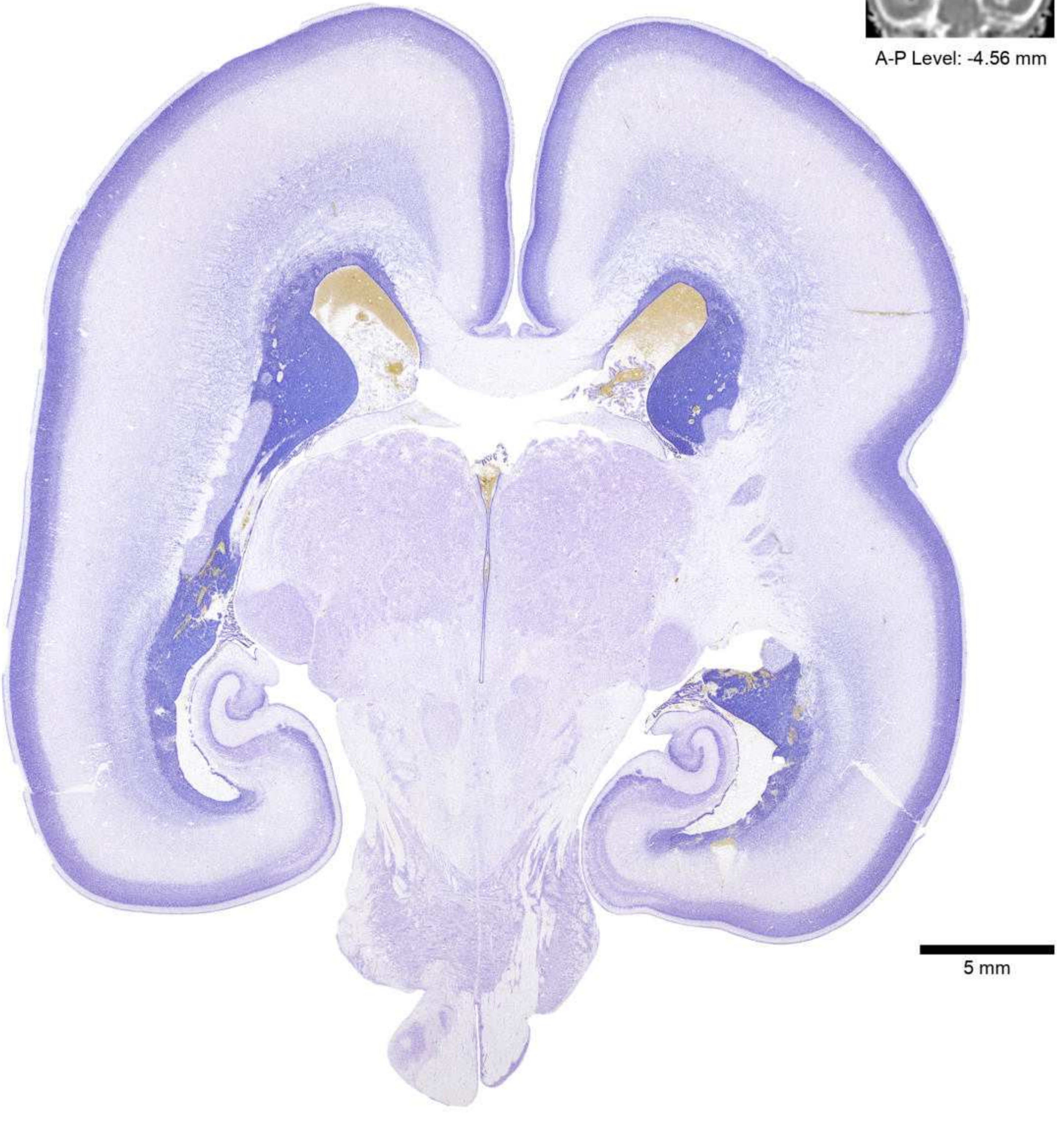

Age: 22 GW

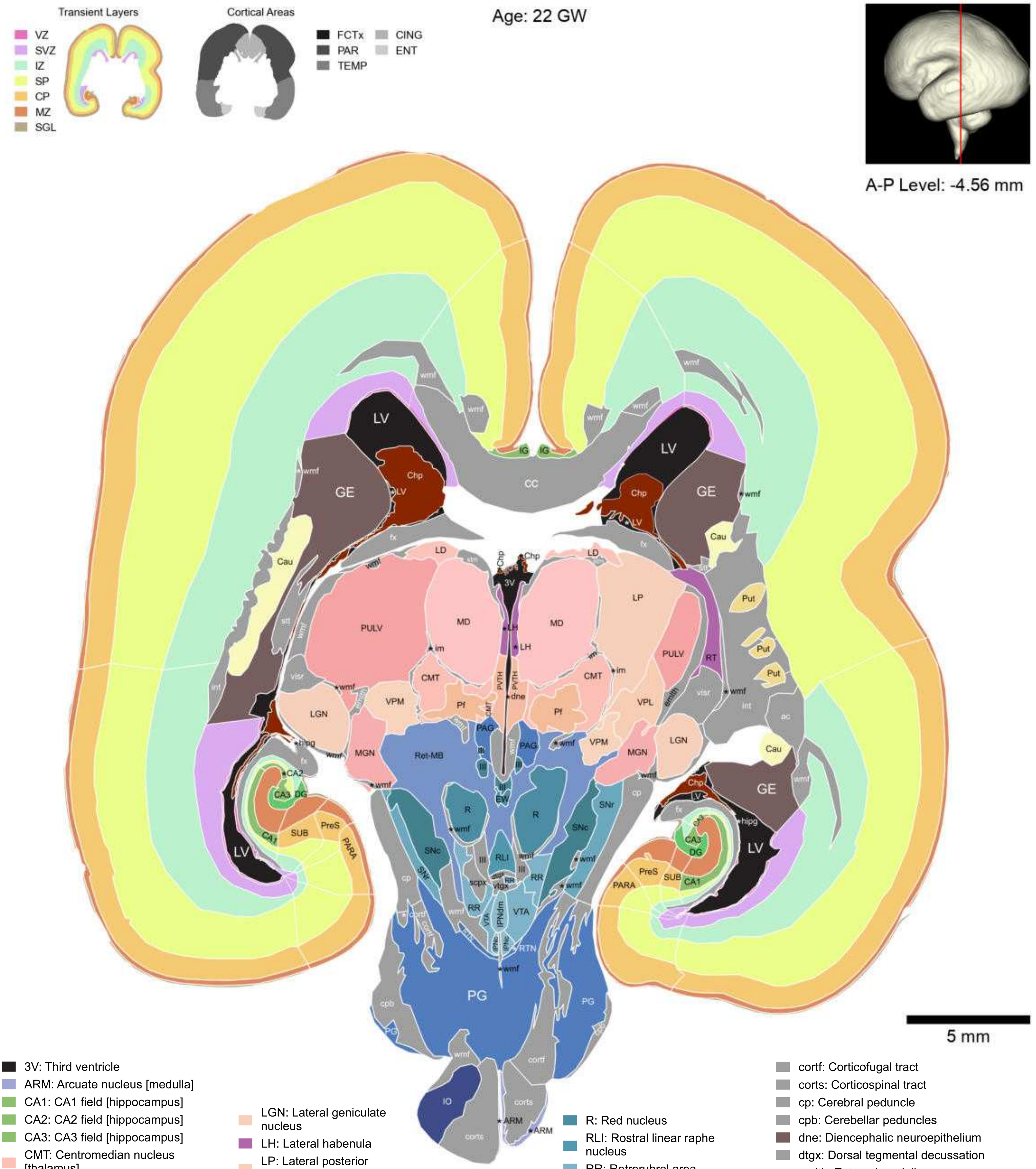

3V: Third ventricle
ARM: Arcuate nucleus [medulla]
CA1: CA1 field [hippocampus]
CA2: CA2 field [hippocampus]
CA3: CA3 field [hippocampus]
CMT: Centromedian nucleus [thalamus]
Cau: Caudate nucleus
Chp: Choroid plexus
DG: Dentate gyrus
EW: Edinger-Westphal nucleus
GE: Ganglionic eminence
IG: Induseum griseum
III: Oculomotor nerve
IO: Inferior olive
IPNc: Interpeduncular nucleu, caudal part
IPNdm: Interpeduncular nucleu, dorsomedial part
LD: Lateral dorsal nucleus [thalamus]
LGN: Lateral geniculate nucleus
LH: Lateral habenula
LP: Lateral posterior nucleus [thalamus]
LV: Lateral ventricle
MD: Medial dorsal nucleus [thalamus]
MGN: Medial geniculate nucleus
PAG: Periaqueductal gray
PARA: Cortical plate, parasubiculum
PG: Pontine gray
PULV: Pulvinar nucleus [thalamus]
PVTH: Periventricular complex [thalamus]
Pf: Parafascicular nucleus [thalamus]
PreS: Cortical plate, presubiculum
Put: Putamen
R: Red nucleus
RLI: Rostral linear raphe nucleus
RR: Retrorubral area
RT: Reticular nucleus [thalamus]
RTN: Reticular tegmental nucleus
Ret-MB: Reticular formation, Midbrain
SNc: Substantia nigra pars compacta
SNr: Substantia nigra pars reticulata
SUB: Cortical plate, subiculum
VPL: Ventral posterolateral nucleus [thalamus]
VPM: Ventral posteromedial nucleus [thalamus]
VTA: Ventral tegmental area
ac: Anterior commissure
cc: Corpus callosum
cortf: Corticofugal tract
corts: Corticospinal tract
cp: Cerebral peduncle
cpb: Cerebellar peduncles
dne: Diencephalic neuroepithelium
dtgx: Dorsal tegmental decussation
emlth: External medullary lamina [thalamus]
fx: Fornix
hipg: Hippocampal glioepithelium/ependyma
im: Internal medullary lamina [thalamus]
int: Internal capsule
scpx: Superior cerebellar peduncle decussation
stm: Stria medullaris
stt: Stria terminalis
visr: Visual radiation
vtgx: Ventral tegmental decussation
wmf: White matter fibers

Age: 22 GW

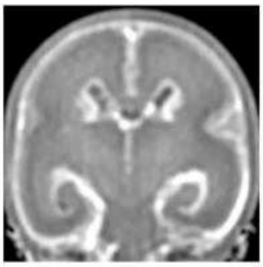

A-P Level: -5.22 mm

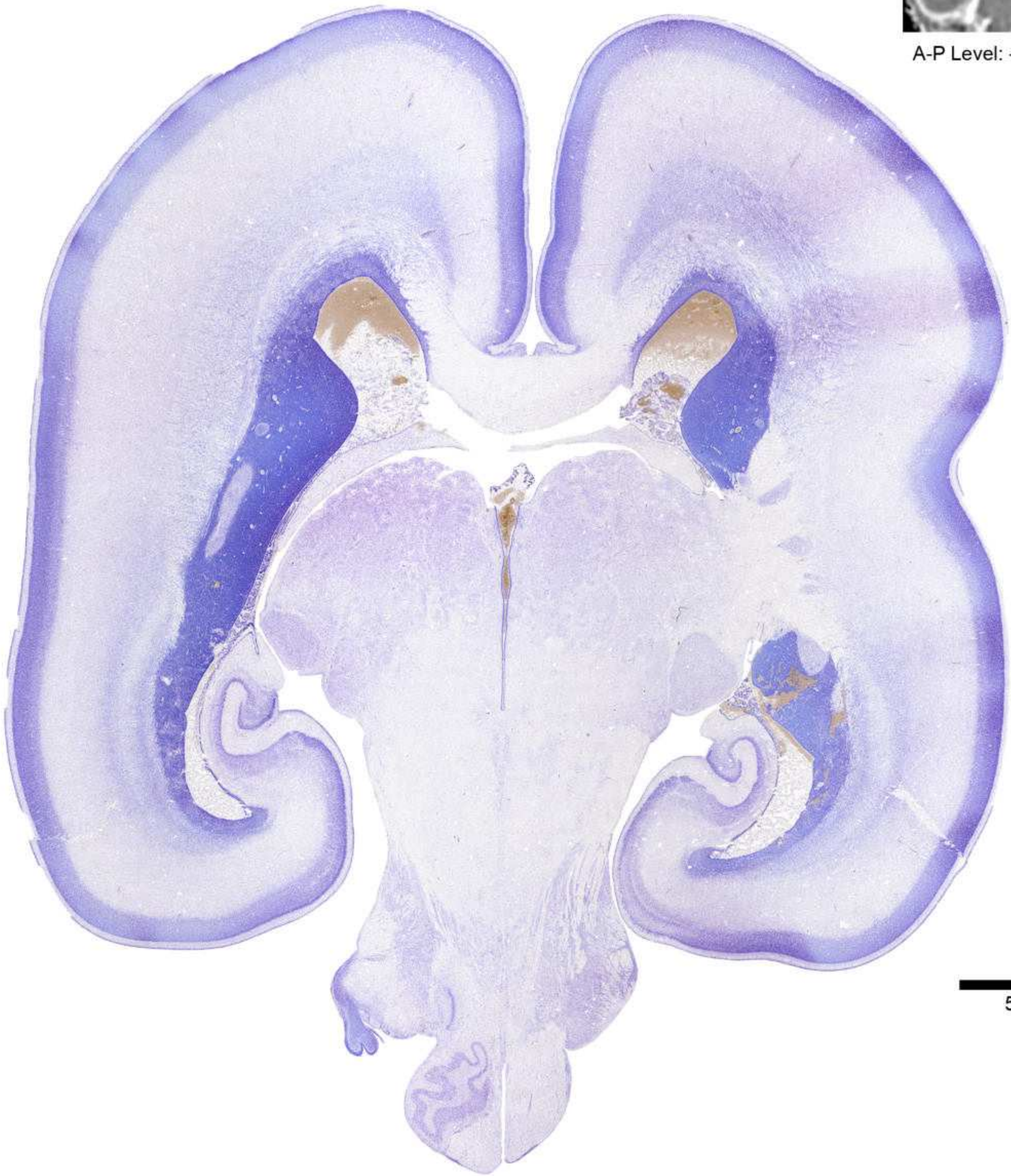

5 mm

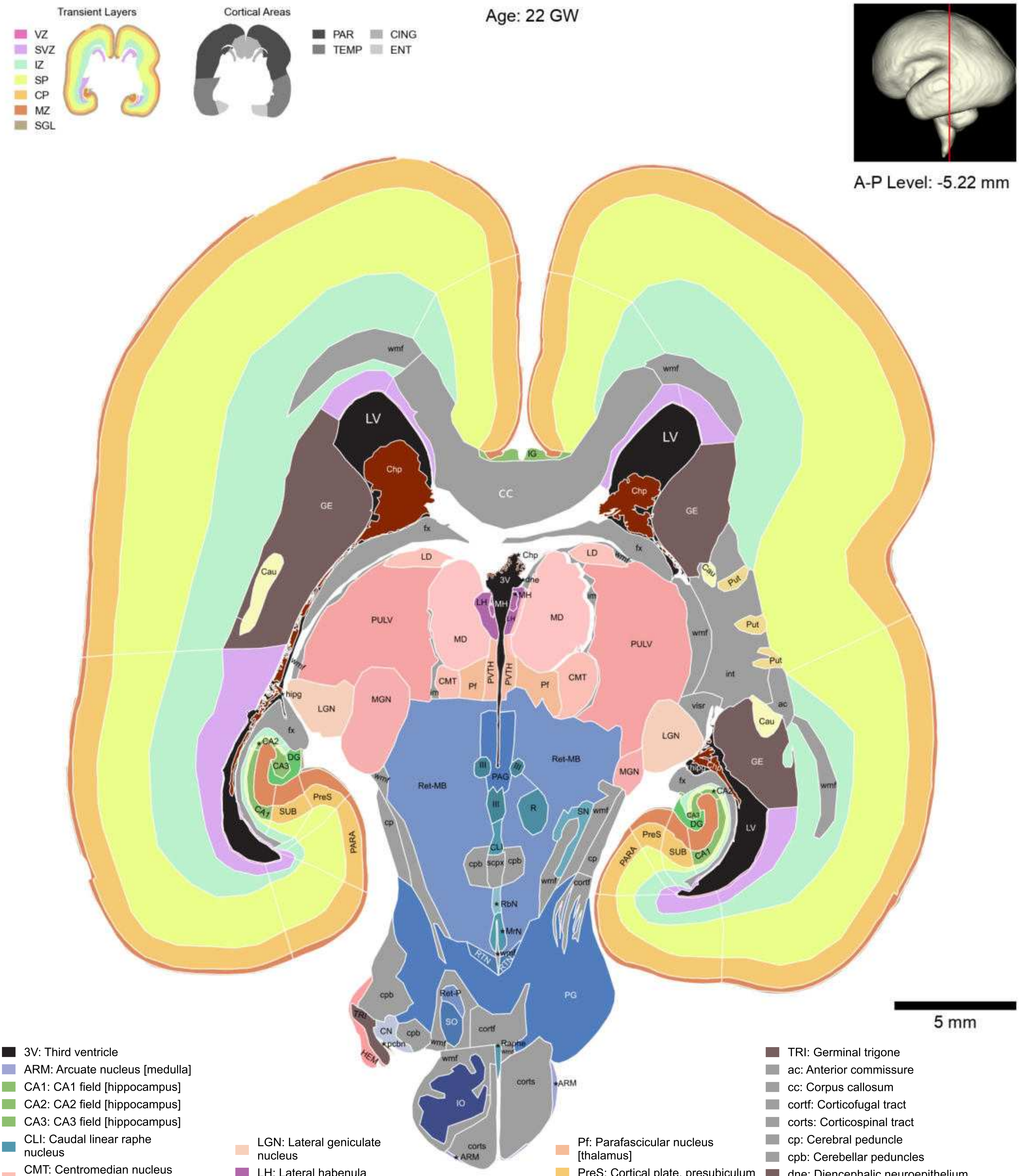

3V: Third ventricle
ARM: Arcuate nucleus [medulla]
CA1: CA1 field [hippocampus]
CA2: CA2 field [hippocampus]
CA3: CA3 field [hippocampus]
CLI: Caudal linear raphe nucleus
CMT: Centromedian nucleus [thalamus]
CN: Cochlear nuclei
Cau: Caudate nucleus
Chp: Choroid plexus
DG: Dentate gyrus
GE: Ganglionic eminence
HEM: Cerebellar hemispheres
IG: Induseum griseum
III: Oculomotor nuclear complex
IO: Inferior olive
LD: Lateral dorsal nucleus [thalamus]
LGN: Lateral geniculate nucleus
LH: Lateral habenula
LV: Lateral ventricle
MD: Medial dorsal nucleus [thalamus]
MGN: Medial geniculate nucleus
MH: Medial habenula
MrN: Median raphe nucleus
PAG: Periaqueductal gray
PARA: Cortical plate, parasubiculum
PG: Pontine gray
PULV: Pulvinar nucleus [thalamus]
PVTH: Periventricular complex [thalamus]
Pf: Parafascicular nucleus [thalamus]
PreS: Cortical plate, presubiculum
Put: Putamen
R: Red nucleus
RTN: Reticular tegmental nucleus
Raphe: Raphe nuclei
RbN: Rhabdoid nucleus
Ret-MB: Reticular formation, Midbrain
Ret-P: Reticular formation, Pons
SN: Substantia nigra
SO: Superior olive
SUB: Cortical plate, subiculum
TRI: Germinal trigone
ac: Anterior commissure
cc: Corpus callosum
cortf: Corticofugal tract
corts: Corticospinal tract
cp: Cerebral peduncle
cpb: Cerebellar peduncles
dne: Diencephalic neuroepithelium
fx: Fornix
hipg: Hippocampal glioepithelium/ependyma
im: Internal medullary lamina [thalamus]
int: Internal capsule
pcbn: Precerebellar neuroepithelium
scpx: Superior cerebellar peduncle decussation
visr: Visual radiation
wmf: White matter fibers

Age: 22 GW

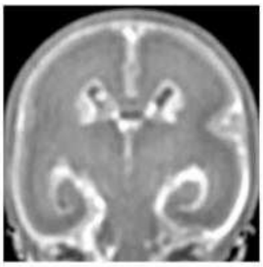

A-P Level: -5.88 mm

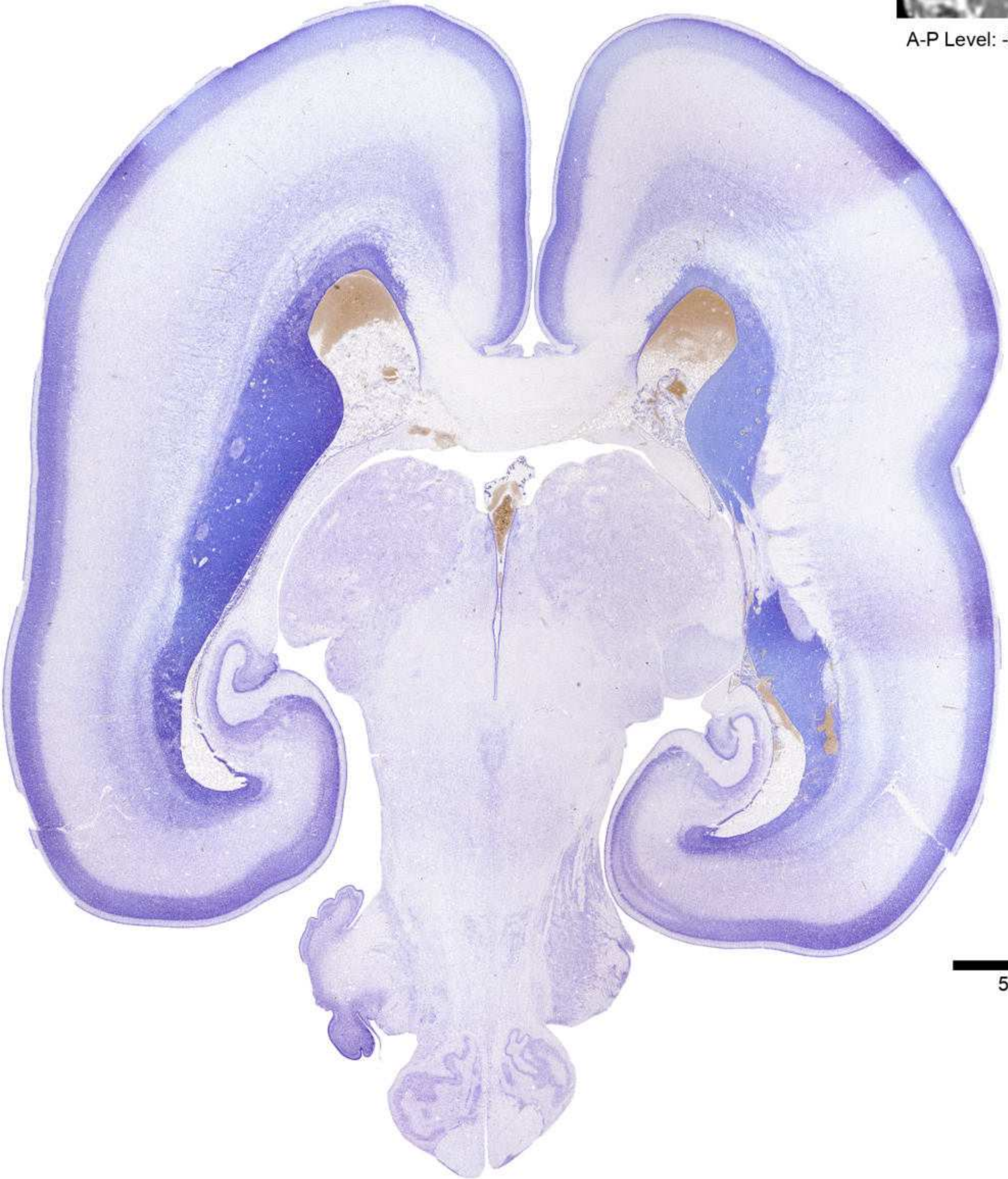

5 mm

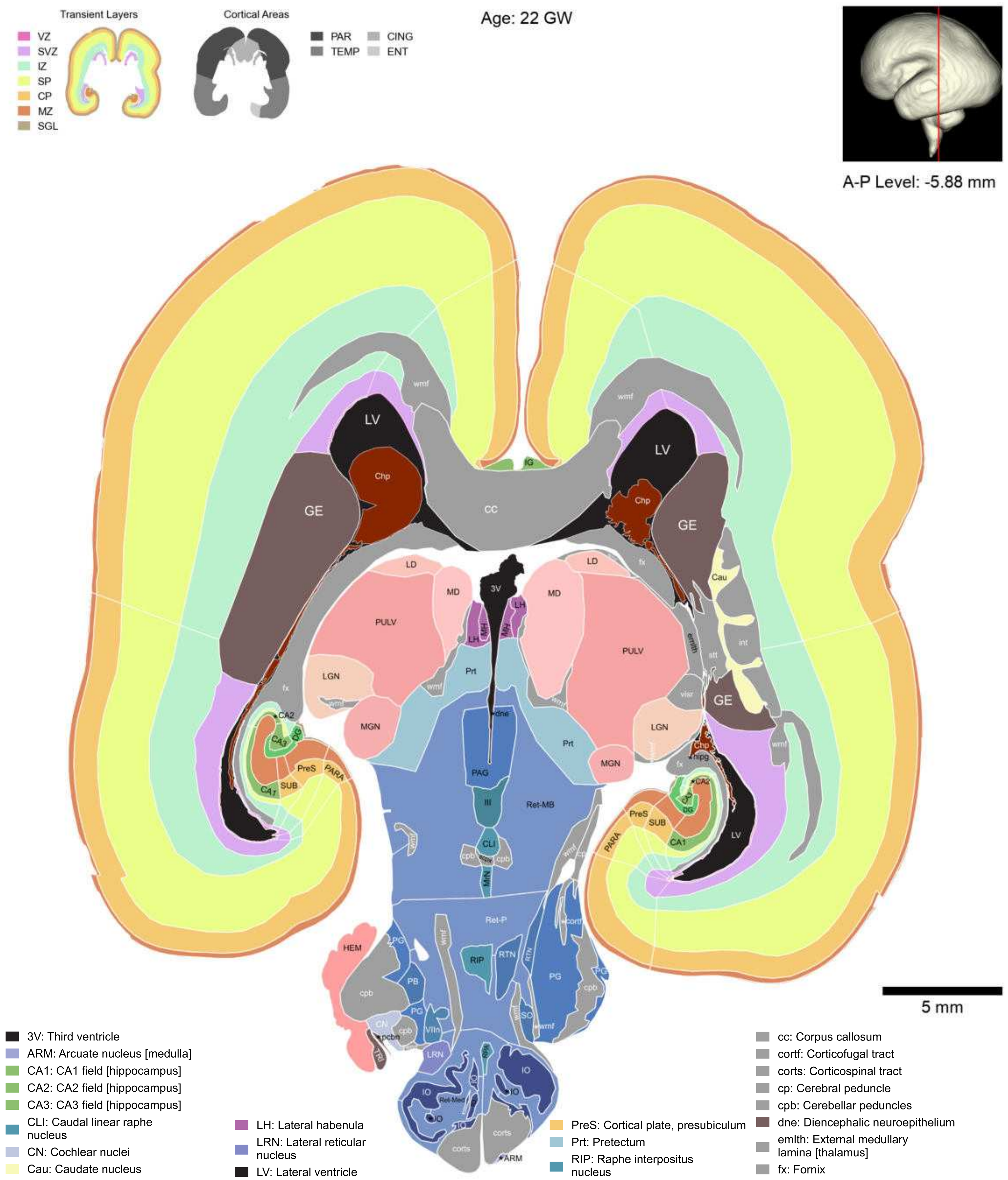

- 3V: Third ventricle
- ARM: Arcuate nucleus [medulla]
- CA1: CA1 field [hippocampus]
- CA2: CA2 field [hippocampus]
- CA3: CA3 field [hippocampus]
- CLI: Caudal linear raphe nucleus
- CN: Cochlear nuclei
- Cau: Caudate nucleus
- Chp: Choroid plexus
- DG: Dentate gyrus
- GE: Ganglionic eminence
- HEM: Cerebellar hemispheres
- IG: Induseum griseum
- III: Oculomotor nuclear complex
- IO: Inferior olive
- LD: Lateral dorsal nucleus [thalamus]
- LGN: Lateral geniculate nucleus
- LH: Lateral habenula
- LRN: Lateral reticular nucleus
- LV: Lateral ventricle
- MD: Medial dorsal nucleus [thalamus]
- MGN: Medial geniculate nucleus
- MH: Medial habenula
- MrN: Median raphe nucleus
- PAG: Periaqueductal gray
- PARA: Cortical plate, parasubiculum
- PB: Parabrachial nucleus
- PG: Pontine gray
- PULV: Pulvinar nucleus [thalamus]
- PreS: Cortical plate, presubiculum
- Prt: Pretectum
- RIP: Raphe interpositus nucleus
- RPA: Raphe pallidus nucleus
- RTN: Reticular tegmental nucleus
- Ret-MB: Reticular formation, Midbrain
- Ret-Med: Reticular formation, Medulla
- Ret-P: Reticular formation, Pons
- SO: Superior olive
- SUB: Cortical plate, subiculum
- TRI: Germinal trigone
- VIIn: Facial motor nucleus
- cc: Corpus callosum
- cortf: Corticofugal tract
- corts: Corticospinal tract
- cp: Cerebral peduncle
- cpb: Cerebellar peduncles
- dne: Diencephalic neuroepithelium
- emlth: External medullary lamina [thalamus]
- fx: Fornix
- hipg: Hippocampal glioepithelium/ependyma
- int: Internal capsule
- pcbn: Precerebellar neuroepithelium
- scpx: Superior cerebellar peduncle decussation
- stt: Stria terminalis
- visr: Visual radiation
- wmf: White matter fibers

Age: 22 GW

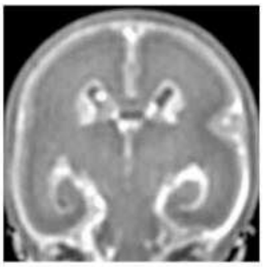

A-P Level: -6.12 mm

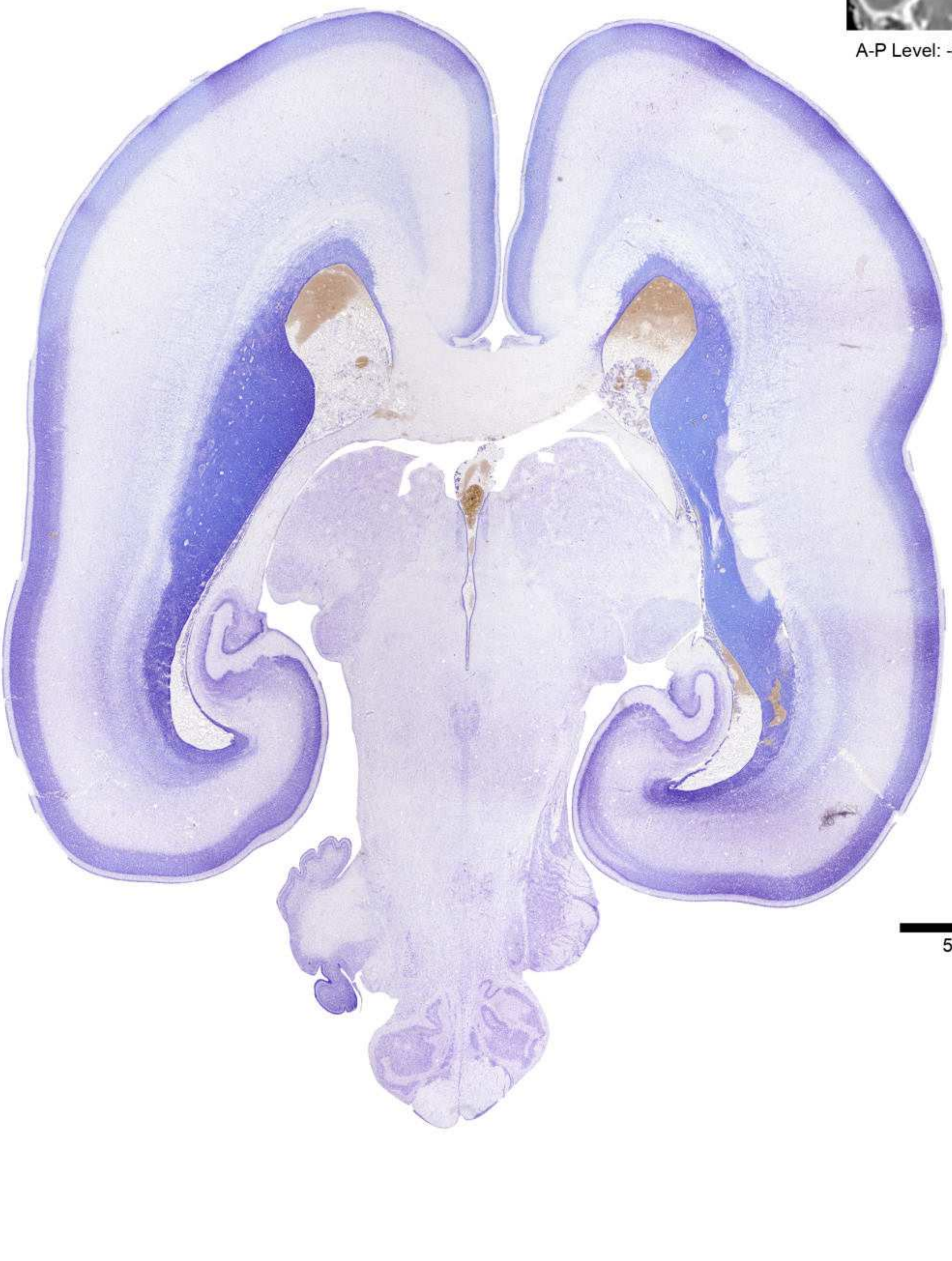

5 mm

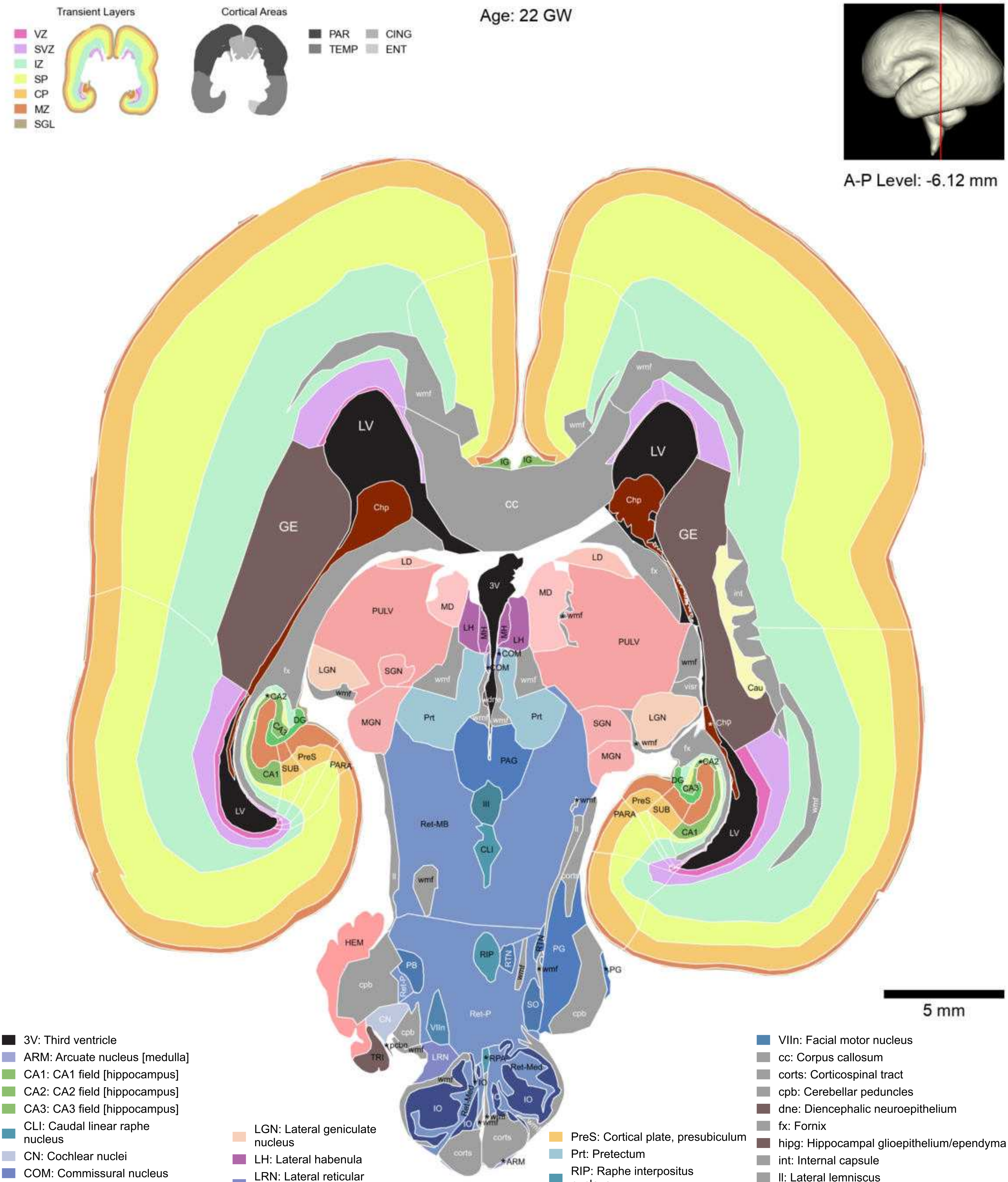

- 3V: Third ventricle
- ARM: Arcuate nucleus [medulla]
- CA1: CA1 field [hippocampus]
- CA2: CA2 field [hippocampus]
- CA3: CA3 field [hippocampus]
- CLI: Caudal linear raphe nucleus
- CN: Cochlear nuclei
- COM: Commissural nucleus
- Cau: Caudate nucleus
- Chp: Choroid plexus
- DG: Dentate gyrus
- GE: Ganglionic eminence
- HEM: Cerebellar hemispheres
- IG: Induseum griseum
- III: Oculomotor nuclear complex
- IO: Inferior olive
- LD: Lateral dorsal nucleus [thalamus]
- LGN: Lateral geniculate nucleus
- LH: Lateral habenula
- LRN: Lateral reticular nucleus
- LV: Lateral ventricle
- MD: Medial dorsal nucleus [thalamus]
- MGN: Medial geniculate nucleus
- MH: Medial habenula
- PAG: Periaqueductal gray
- PARA: Cortical plate, parasubiculum
- PB: Parabrachial nucleus
- PG: Pontine gray
- PULV: Pulvinar nucleus [thalamus]
- PreS: Cortical plate, presubiculum
- Prt: Pretectum
- RIP: Raphe interpositus nucleus
- RPA: Raphe pallidus nucleus
- RTN: Reticular tegmental nucleus
- Ret-MB: Reticular formation, Midbrain
- Ret-Med: Reticular formation, Medulla
- Ret-P: Reticular formation, Pons
- SGN: Suprageniculate nucleus
- SO: Superior olive
- SUB: Cortical plate, subiculum
- TRI: Germinal trigone
- VIIn: Facial motor nucleus
- cc: Corpus callosum
- corts: Corticospinal tract
- cpb: Cerebellar peduncles
- dne: Diencephalic neuroepithelium
- fx: Fornix
- hipg: Hippocampal glioepithelium/ependyma
- int: Internal capsule
- ll: Lateral lemniscus
- pcbn: Precerebellar neuroepithelium
- visr: Visual radiation
- wmf: White matter fibers

Age: 22 GW

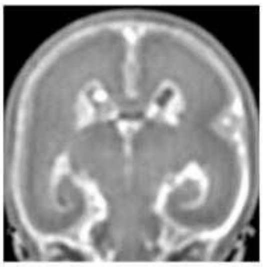

A-P Level: -6.66 mm

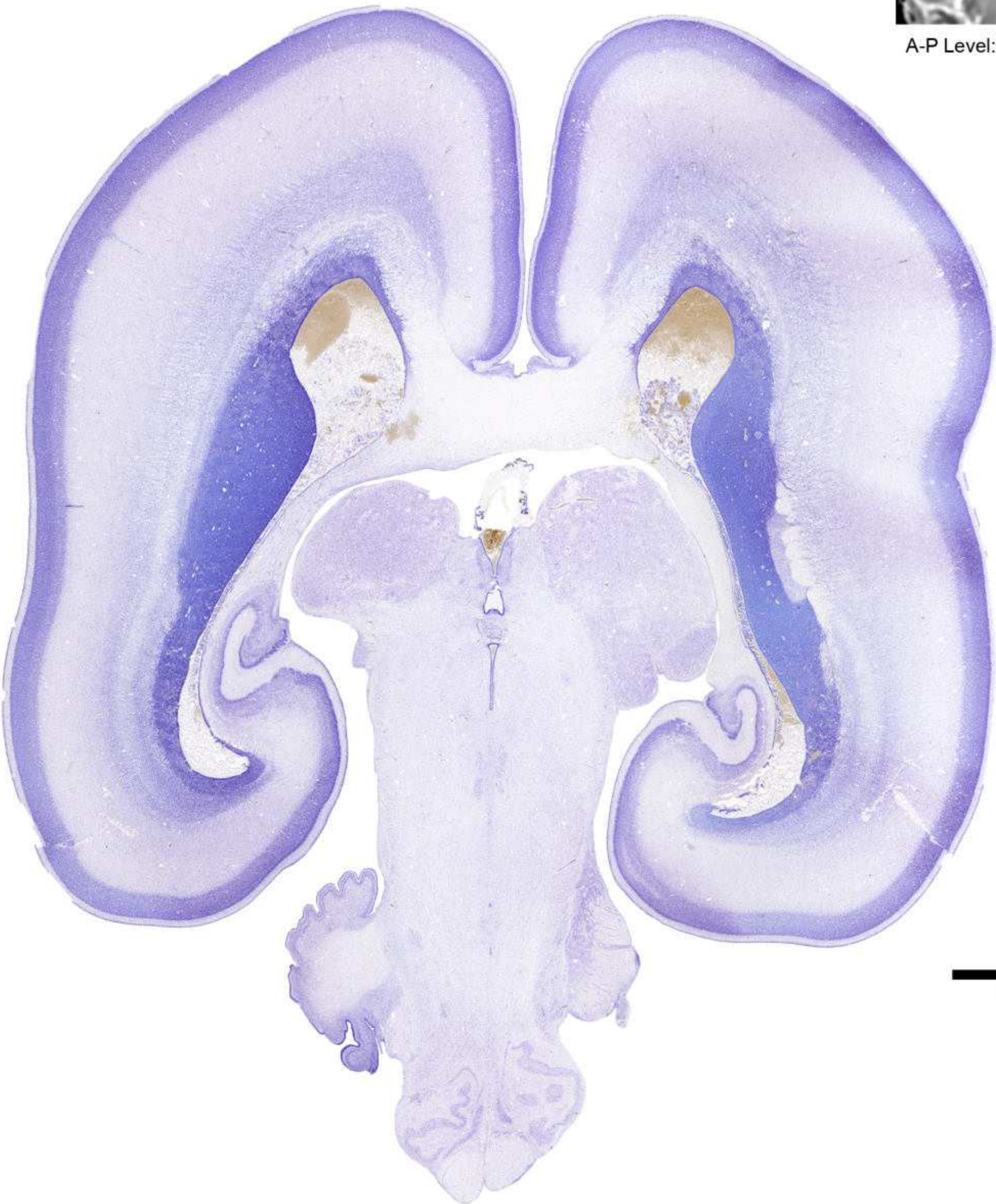

5 mm

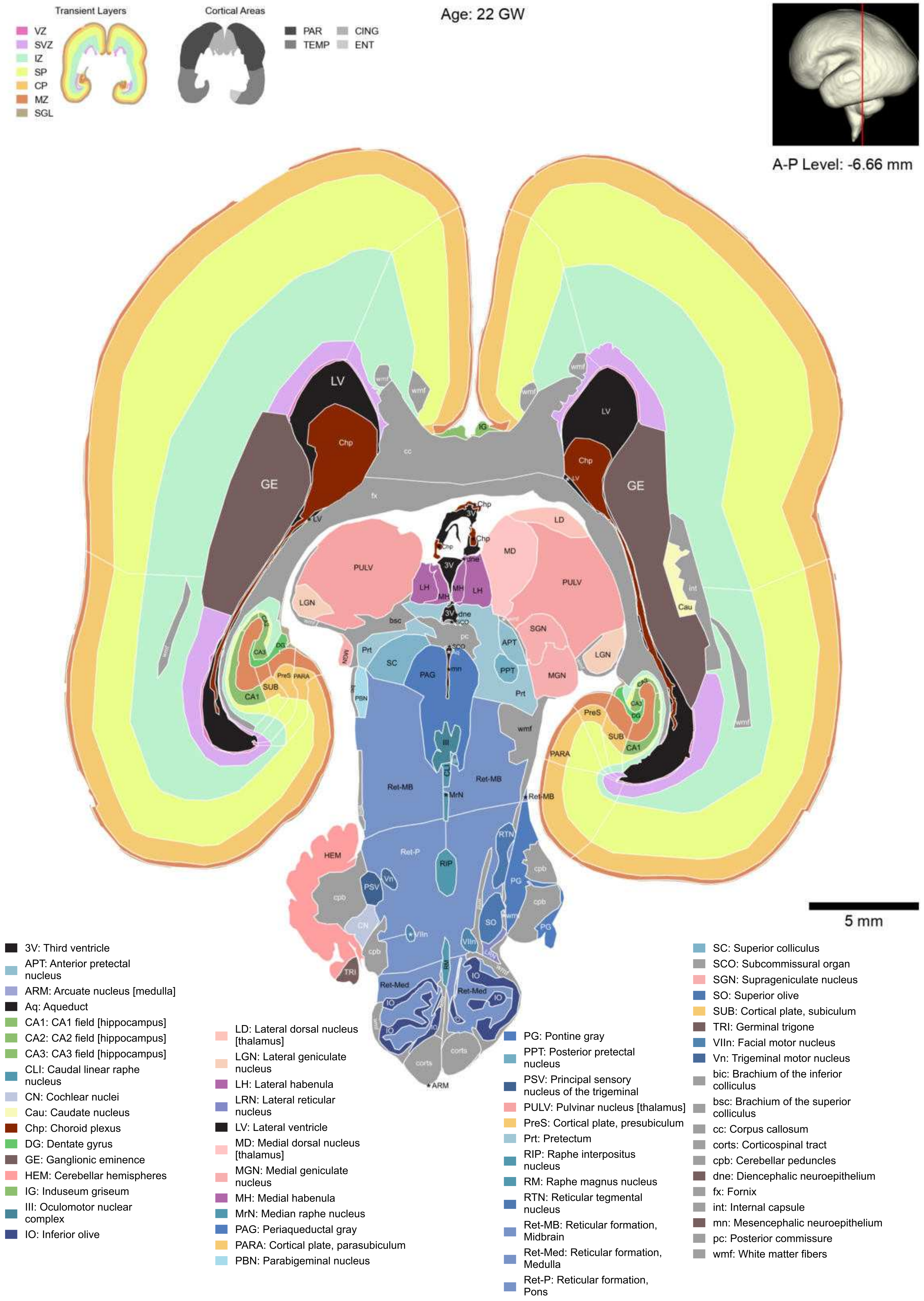

Transient Layers
VZ
SVZ
IZ
SP
CP
MZ
SGL
Cortical Areas
PAR
TEMP
CING
ENT
Age: 22 GW
A-P Level: -6.66 mm
5 mm
LV
Chp
GE
cc
fx
IG
wmf
PULV
LGN
LD
MD
LH
MH
3V
dne
SCO
pc
bsc
APT
SGN
MGN
Prt
SC
PAG
mn
PPT
PBN
CA1
CA2
CA3
DG
SUB
PreS
PARA
int
Cau
III
CLI
MrN
Ret-MB
Ret-P
RIP
RTN
PG
HEM
cpb
PSV
Vn
CN
VIIn
SO
LRN
RM
TRI
Ret-Med
IO
corts
ARM
3V: Third ventricle
APT: Anterior pretectal nucleus
ARM: Arcuate nucleus [medulla]
Aq: Aqueduct
CA1: CA1 field [hippocampus]
CA2: CA2 field [hippocampus]
CA3: CA3 field [hippocampus]
CLI: Caudal linear raphe nucleus
CN: Cochlear nuclei
Cau: Caudate nucleus
Chp: Choroid plexus
DG: Dentate gyrus
GE: Ganglionic eminence
HEM: Cerebellar hemispheres
IG: Induseum griseum
III: Oculomotor nuclear complex
IO: Inferior olive
LD: Lateral dorsal nucleus [thalamus]
LGN: Lateral geniculate nucleus
LH: Lateral habenula
LRN: Lateral reticular nucleus
LV: Lateral ventricle
MD: Medial dorsal nucleus [thalamus]
MGN: Medial geniculate nucleus
MH: Medial habenula
MrN: Median raphe nucleus
PAG: Periaqueductal gray
PARA: Cortical plate, parasubiculum
PBN: Parabigeminal nucleus
PG: Pontine gray
PPT: Posterior pretectal nucleus
PSV: Principal sensory nucleus of the trigeminal
PULV: Pulvinar nucleus [thalamus]
PreS: Cortical plate, presubiculum
Prt: Pretectum
RIP: Raphe interpositus nucleus
RM: Raphe magnus nucleus
RTN: Reticular tegmental nucleus
Ret-MB: Reticular formation, Midbrain
Ret-Med: Reticular formation, Medulla
Ret-P: Reticular formation, Pons
SC: Superior colliculus
SCO: Subcommissural organ
SGN: Suprageniculate nucleus
SO: Superior olive
SUB: Cortical plate, subiculum
TRI: Germinal trigone
VIIn: Facial motor nucleus
Vn: Trigeminal motor nucleus
bic: Brachium of the inferior colliculus
bsc: Brachium of the superior colliculus
cc: Corpus callosum
corts: Corticospinal tract
cpb: Cerebellar peduncles
dne: Diencephalic neuroepithelium
fx: Fornix
int: Internal capsule
mn: Mesencephalic neuroepithelium
pc: Posterior commissure
wmf: White matter fibers

Age: 22 GW

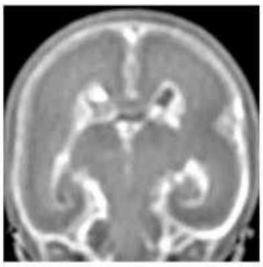

A-P Level: -7.2 mm

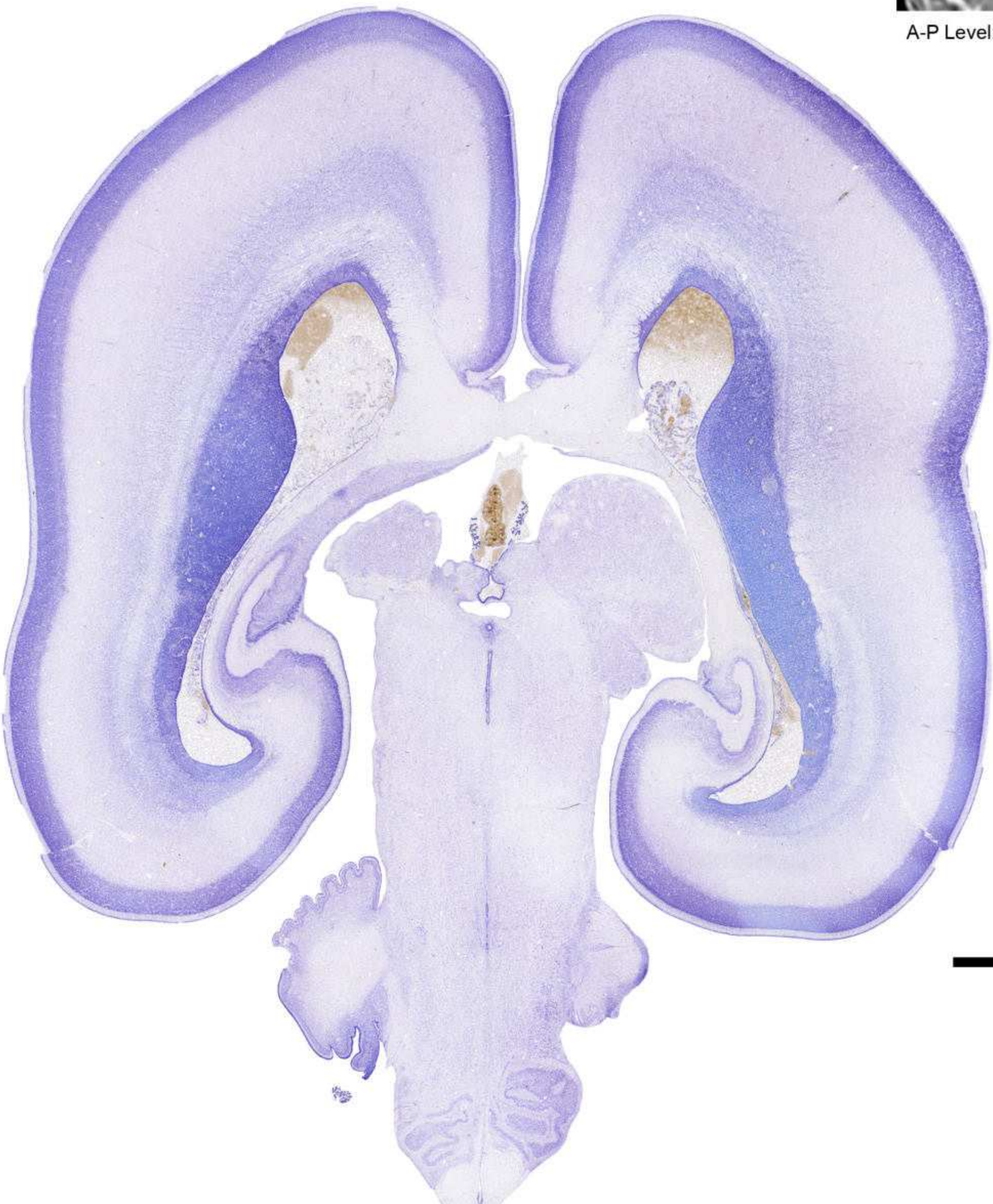

5 mm

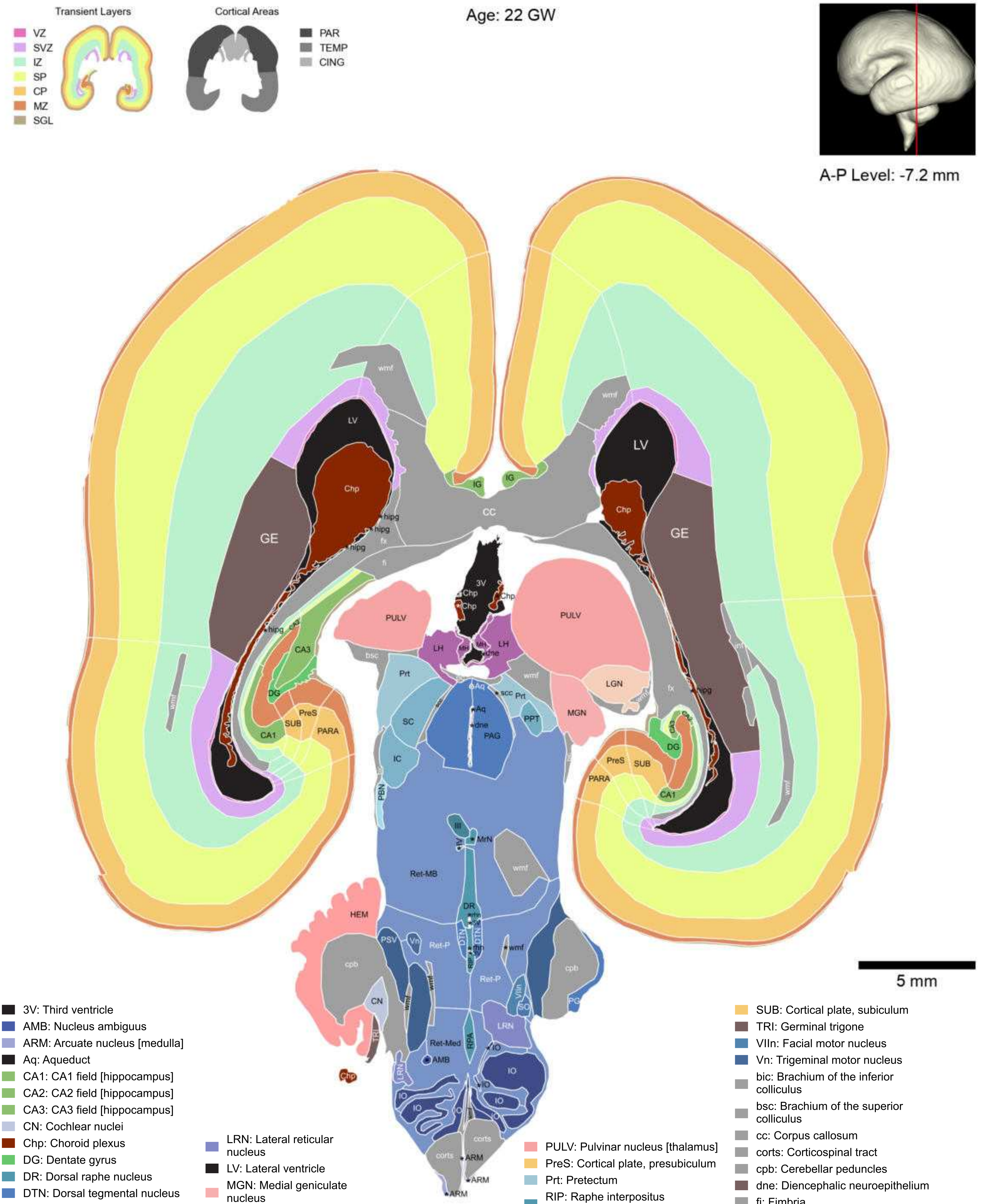

- 3V: Third ventricle
- AMB: Nucleus ambiguus
- ARM: Arcuate nucleus [medulla]
- Aq: Aqueduct
- CA1: CA1 field [hippocampus]
- CA2: CA2 field [hippocampus]
- CA3: CA3 field [hippocampus]
- CN: Cochlear nuclei
- Chp: Choroid plexus
- DG: Dentate gyrus
- DR: Dorsal raphe nucleus
- DTN: Dorsal tegmental nucleus
- GE: Ganglionic eminence
- HEM: Cerebellar hemispheres
- IC: Inferior colliculus
- IG: Induseum griseum
- III: Oculomotor nuclear complex
- IO: Inferior olive
- IV: Trochlear nucleus
- LGN: Lateral geniculate nucleus
- LH: Lateral habenula
- LRN: Lateral reticular nucleus
- LV: Lateral ventricle
- MGN: Medial geniculate nucleus
- MH: Medial habenula
- MrN: Median raphe nucleus
- PAG: Periaqueductal gray
- PARA: Cortical plate, parasubiculum
- PBN: Parabigeminal nucleus
- PG: Pontine gray
- PPT: Posterior pretectal nucleus
- PSV: Principal sensory nucleus of the trigeminal
- PULV: Pulvinar nucleus [thalamus]
- PreS: Cortical plate, presubiculum
- Prt: Pretectum
- RIP: Raphe interpositus nucleus
- RPA: Raphe pallidus nucleus
- Ret-MB: Reticular formation, Midbrain
- Ret-Med: Reticular formation, Medulla
- Ret-P: Reticular formation, Pons
- SC: Superior colliculus
- SO: Superior olive
- SPV: Spinal nucleus of the trigeminal
- SUB: Cortical plate, subiculum
- TRI: Germinal trigone
- VIIn: Facial motor nucleus
- Vn: Trigeminal motor nucleus
- bic: Brachium of the inferior colliculus
- bsc: Brachium of the superior colliculus
- cc: Corpus callosum
- corts: Corticospinal tract
- cpb: Cerebellar peduncles
- dne: Diencephalic neuroepithelium
- fi: Fimbria
- fx: Fornix
- hipg: Hippocampal glioepithelium/ependyma
- int: Internal capsule
- pc: Posterior commissure
- rhn: Rhombencephalic neuroepithelium
- scc: Superior colliculus commissure
- wmf: White matter fibers

Age: 22 GW

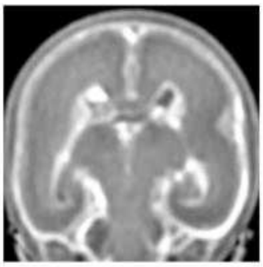

A-P Level: -7.5 mm

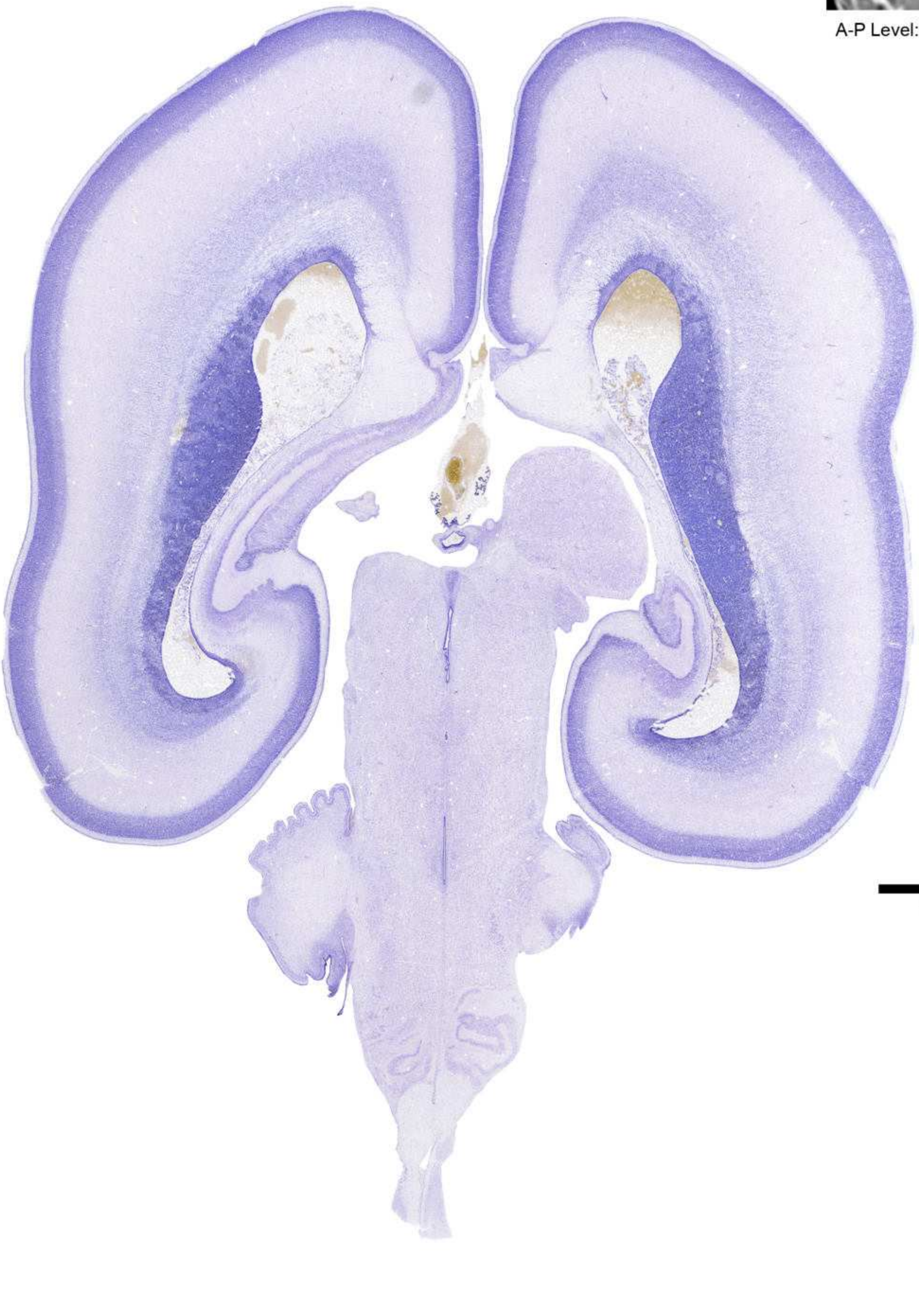

5 mm

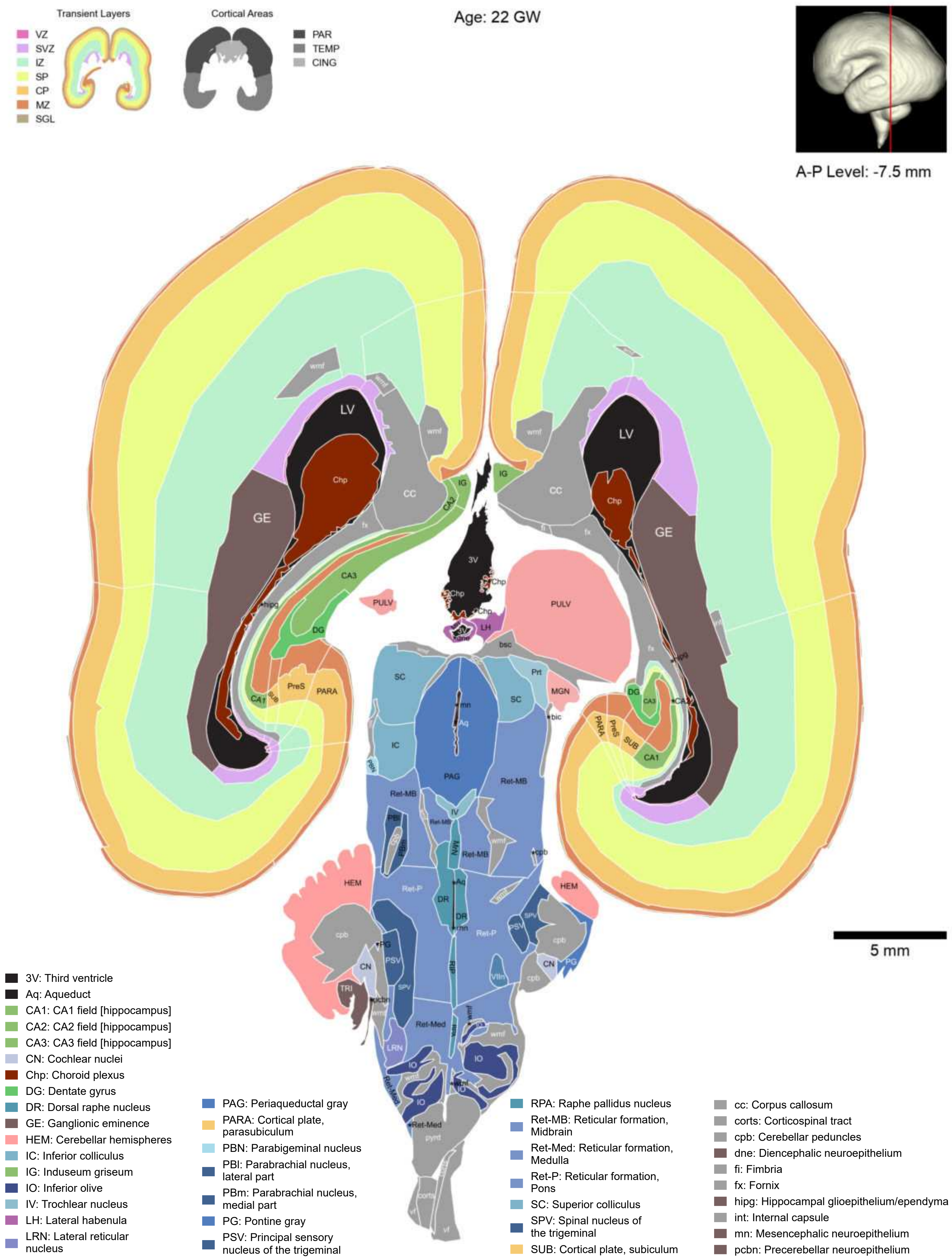

3V: Third ventricle
Aq: Aqueduct
CA1: CA1 field [hippocampus]
CA2: CA2 field [hippocampus]
CA3: CA3 field [hippocampus]
CN: Cochlear nuclei
Chp: Choroid plexus
DG: Dentate gyrus
DR: Dorsal raphe nucleus
GE: Ganglionic eminence
HEM: Cerebellar hemispheres
IC: Inferior colliculus
IG: Induseum griseum
IO: Inferior olive
IV: Trochlear nucleus
LH: Lateral habenula
LRN: Lateral reticular nucleus
LV: Lateral ventricle
MGN: Medial geniculate nucleus
MH: Medial habenula
MrN: Median raphe nucleus
PAG: Periaqueductal gray
PARA: Cortical plate, parasubiculum
PBN: Parabigeminal nucleus
PBl: Parabrachial nucleus, lateral part
PBm: Parabrachial nucleus, medial part
PG: Pontine gray
PSV: Principal sensory nucleus of the trigeminal
PULV: Pulvinar nucleus [thalamus]
PreS: Cortical plate, presubiculum
Prt: Pretectum
RIP: Raphe interpositus nucleus
RPA: Raphe pallidus nucleus
Ret-MB: Reticular formation, Midbrain
Ret-Med: Reticular formation, Medulla
Ret-P: Reticular formation, Pons
SC: Superior colliculus
SPV: Spinal nucleus of the trigeminal
SUB: Cortical plate, subiculum
TRI: Germinal trigone
VIIn: Facial motor nucleus
bic: Brachium of the inferior colliculus
bsc: Brachium of the superior colliculus
cc: Corpus callosum
corts: Corticospinal tract
cpb: Cerebellar peduncles
dne: Diencephalic neuroepithelium
fi: Fimbria
fx: Fornix
hipg: Hippocampal glioepithelium/ependyma
int: Internal capsule
mn: Mesencephalic neuroepithelium
pcbn: Precerebellar neuroepithelium
pyrd: Pyramidal decussation
rhn: Rhombencephalic neuroepithelium
scc: Superior colliculus commissure
vf: Ventral funiculus
wmf: White matter fibers

Age: 22 GW

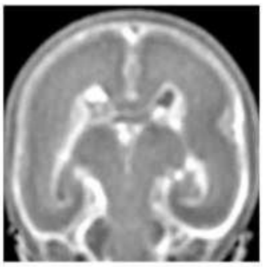

A-P Level: -7.68 mm

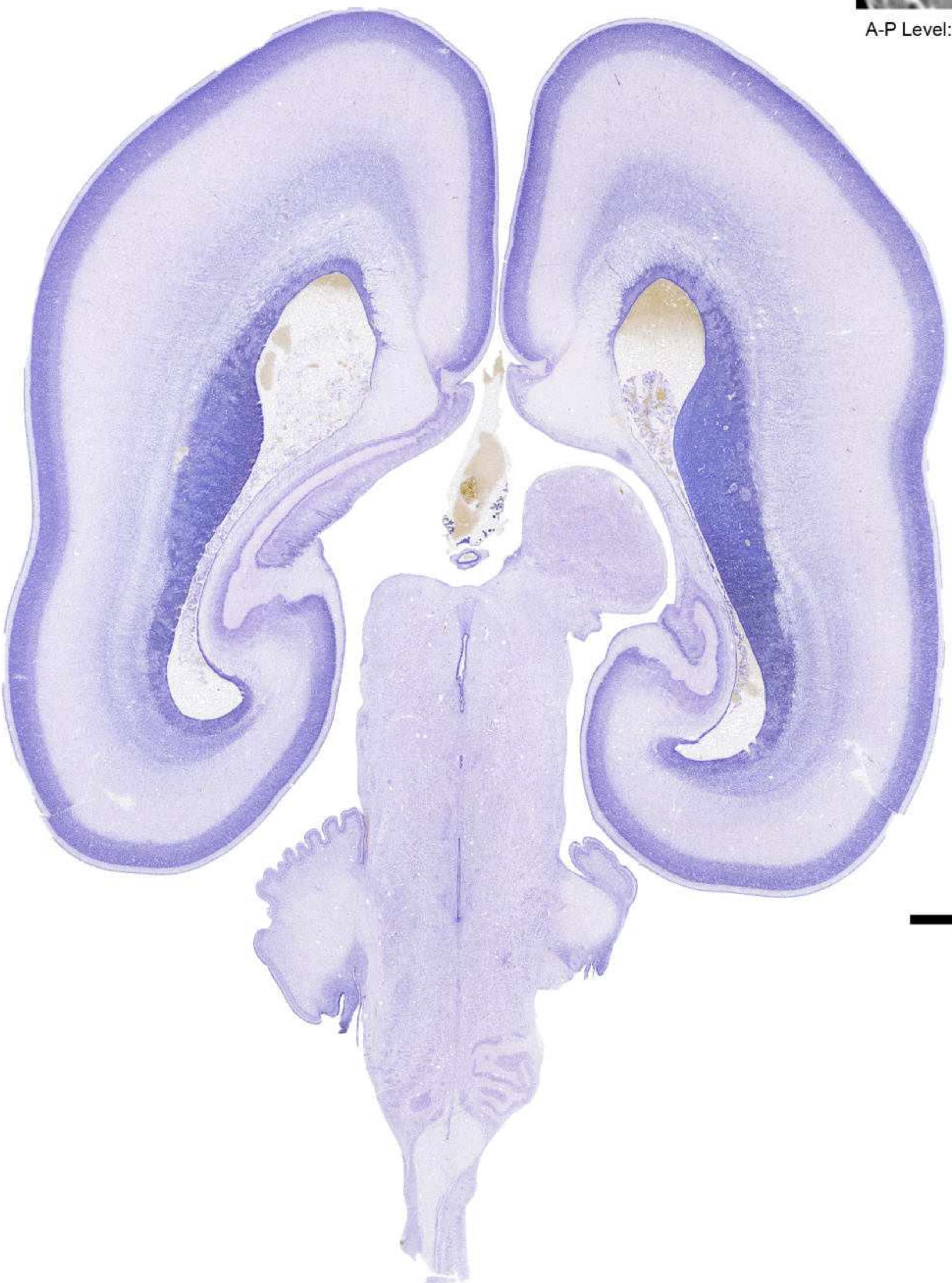

5 mm

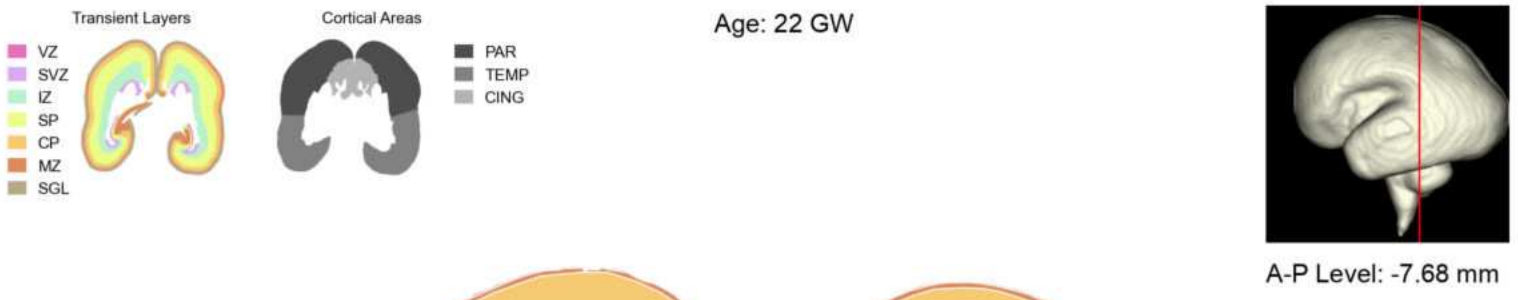

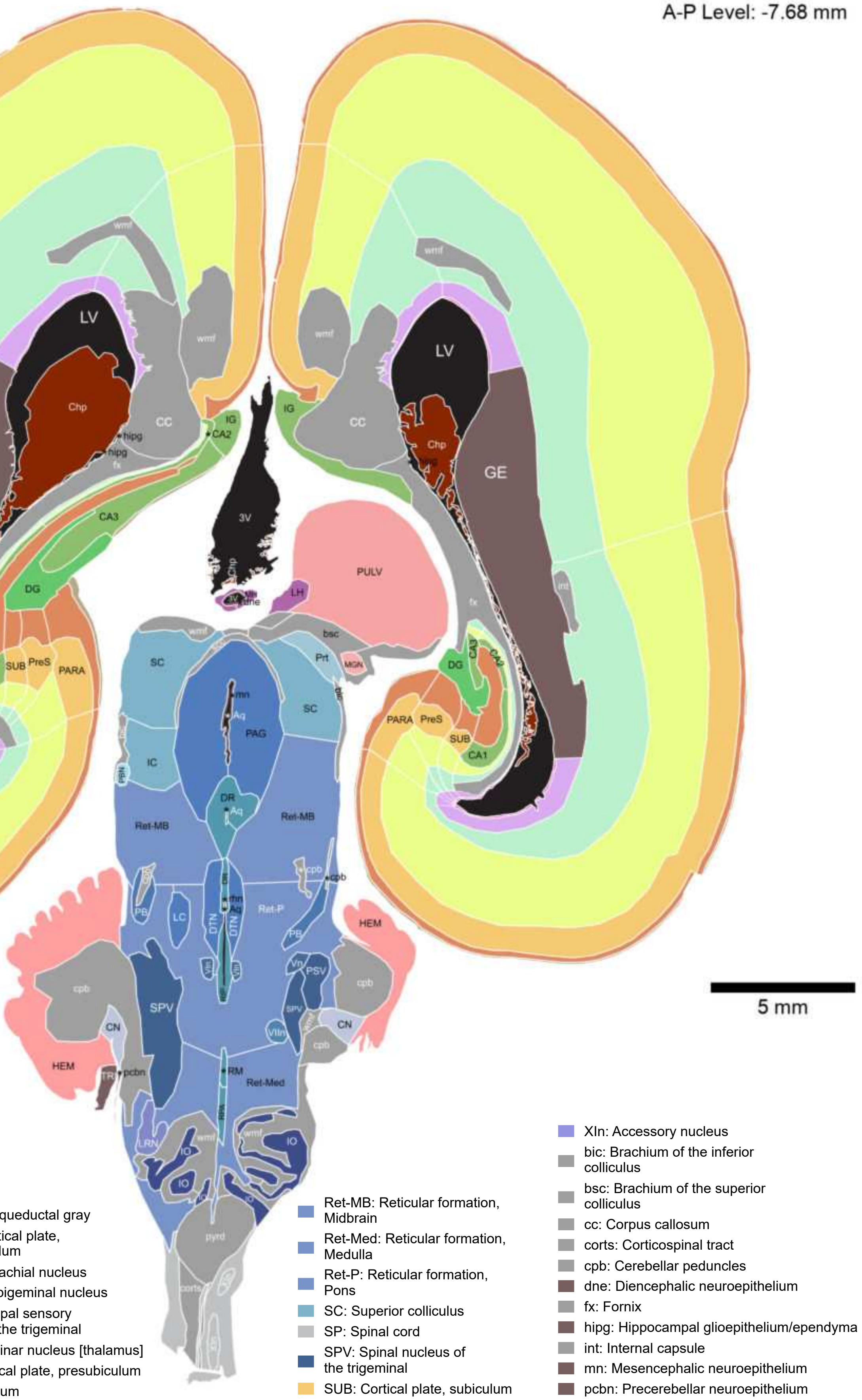

- 3V: Third ventricle
- Aq: Aqueduct
- CA1: CA1 field [hippocampus]
- CA2: CA2 field [hippocampus]
- CA3: CA3 field [hippocampus]
- CN: Cochlear nuclei
- Chp: Choroid plexus
- DG: Dentate gyrus
- DR: Dorsal raphe nucleus
- DTN: Dorsal tegmental nucleus
- GE: Ganglionic eminence
- HEM: Cerebellar hemispheres
- IC: Inferior colliculus
- IG: Induseum griseum
- IO: Inferior olive
- LC: Locus coeruleus
- LH: Lateral habenula
- LRN: Lateral reticular nucleus
- LV: Lateral ventricle
- MGN: Medial geniculate nucleus
- MH: Medial habenula
- PAG: Periaqueductal gray
- PARA: Cortical plate, parasubiculum
- PB: Parabrachial nucleus
- PBN: Parabigeminal nucleus
- PSV: Principal sensory nucleus of the trigeminal
- PULV: Pulvinar nucleus [thalamus]
- PreS: Cortical plate, presubiculum
- Prt: Pretectum
- RIP: Raphe interpositus nucleus
- RM: Raphe magnus nucleus
- RPA: Raphe pallidus nucleus
- Ret-MB: Reticular formation, Midbrain
- Ret-Med: Reticular formation, Medulla
- Ret-P: Reticular formation, Pons
- SC: Superior colliculus
- SP: Spinal cord
- SPV: Spinal nucleus of the trigeminal
- SUB: Cortical plate, subiculum
- TRI: Germinal trigone
- VIIn: Facial motor nucleus
- VIn: Abducens nucleus
- Vn: Trigeminal motor nucleus
- XIn: Accessory nucleus
- bic: Brachium of the inferior colliculus
- bsc: Brachium of the superior colliculus
- cc: Corpus callosum
- corts: Corticospinal tract
- cpb: Cerebellar peduncles
- dne: Diencephalic neuroepithelium
- fx: Fornix
- hipg: Hippocampal glioepithelium/ependyma
- int: Internal capsule
- mn: Mesencephalic neuroepithelium
- pcbn: Precerebellar neuroepithelium
- pyrd: Pyramidal decussation
- rhn: Rhombencephalic neuroepithelium
- scc: Superior colliculus commissure
- wmf: White matter fibers

Age: 22 GW

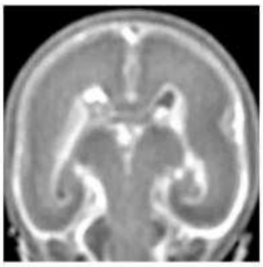

A-P Level: -8.28 mm

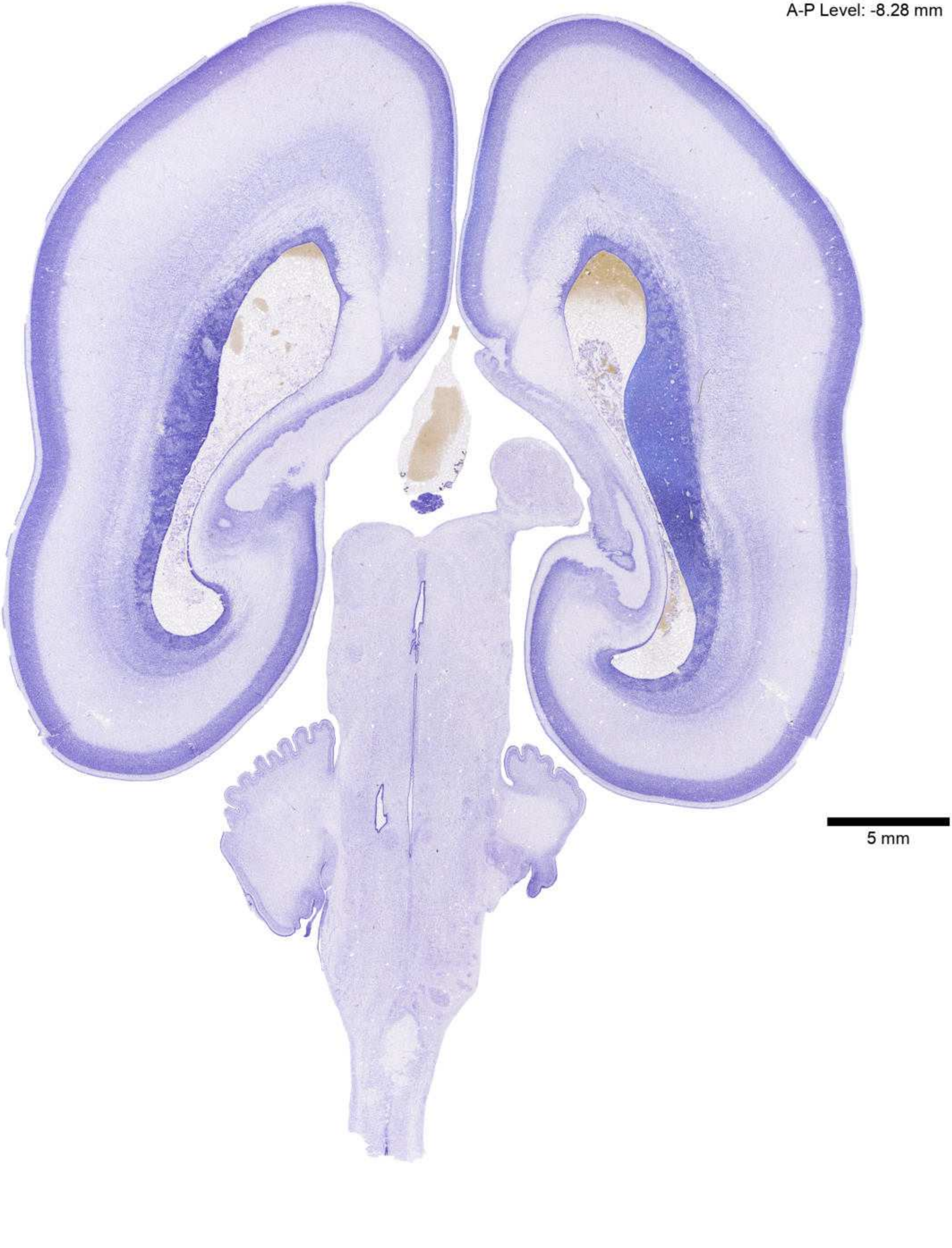

5 mm

Transient Layers

- VZ
- SVZ
- IZ
- SP
- CP
- MZ
- SGL

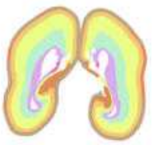

Cortical Areas

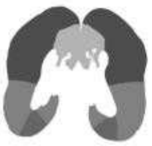

- PAR
- TEMP
- OCC
- CING

Age: 22 GW

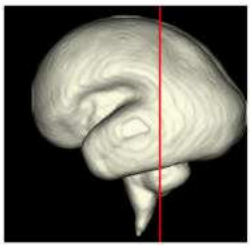

A-P Level: -8.28 mm

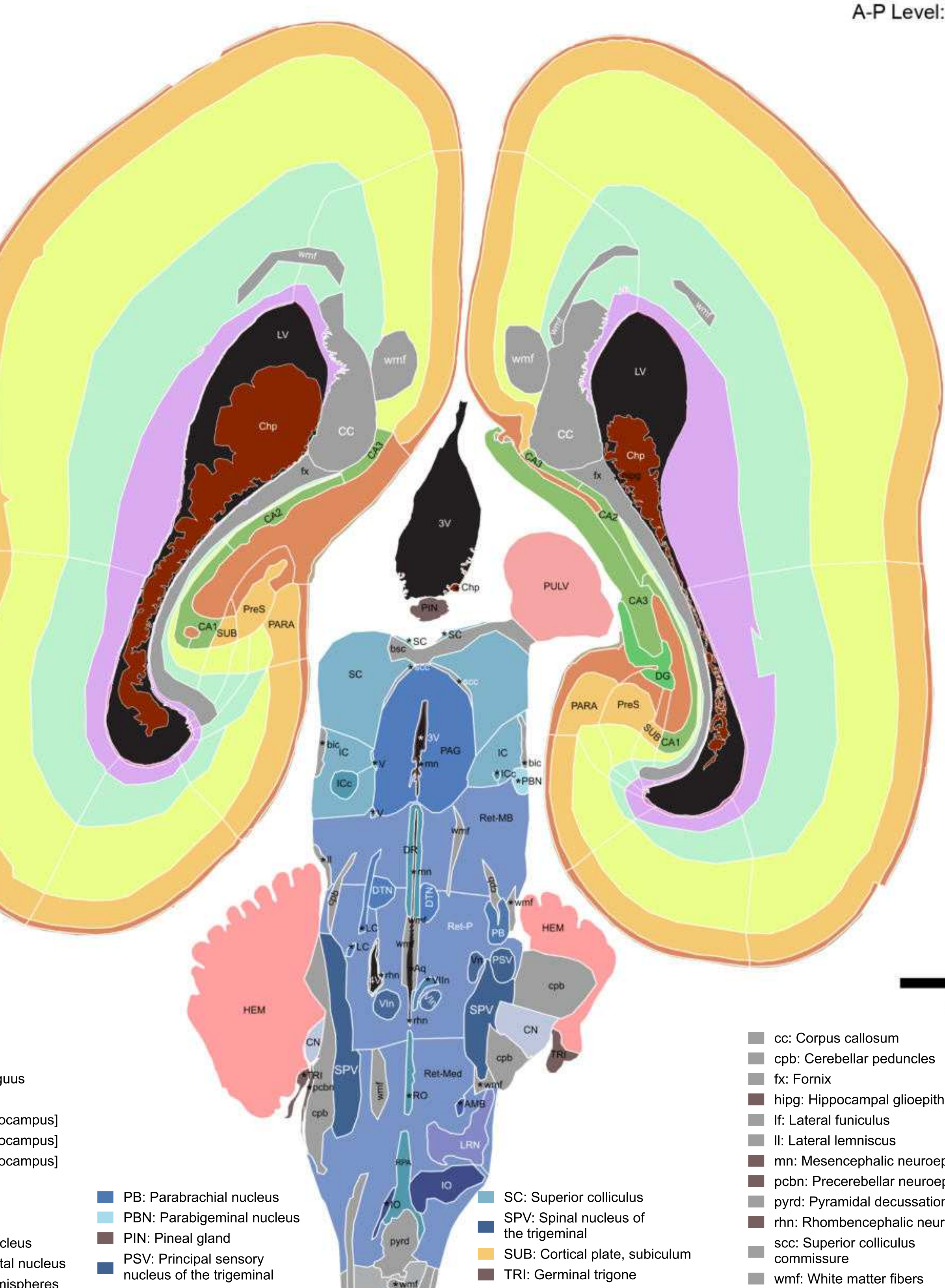

5 mm

- 3V: Third ventricle
- 4V: Fourth ventricle
- AMB: Nucleus ambiguus
- Aq: Aqueduct
- CA1: CA1 field [hippocampus]
- CA2: CA2 field [hippocampus]
- CA3: CA3 field [hippocampus]
- CN: Cochlear nuclei
- Chp: Choroid plexus
- DG: Dentate gyrus
- DR: Dorsal raphe nucleus
- DTN: Dorsal tegmental nucleus
- HEM: Cerebellar hemispheres
- IC: Inferior colliculus
- ICc: Inferior colliculus, central nucleus
- IO: Inferior olive
- LC: Locus coeruleus
- LRN: Lateral reticular nucleus
- LV: Lateral ventricle
- PAG: Periaqueductal gray
- PARA: Cortical plate, parasubiculum
- PB: Parabrachial nucleus
- PBN: Parabigeminal nucleus
- PIN: Pineal gland
- PSV: Principal sensory nucleus of the trigeminal
- PULV: Pulvinar nucleus [thalamus]
- PreS: Cortical plate, presubiculum
- RO: Raphe obscurus nucleus
- RPA: Raphe pallidus nucleus
- Ret-MB: Reticular formation, Midbrain
- Ret-Med: Reticular formation, Medulla
- Ret-P: Reticular formation, Pons
- SC: Superior colliculus
- SPV: Spinal nucleus of the trigeminal
- SUB: Cortical plate, subiculum
- TRI: Germinal trigone
- V: Mesencephalic nucleus
- VG: Ventral gray of the spinal cord
- VIIn: Facial motor nucleus
- VIn: Abducens nucleus
- Vn: Trigeminal motor nucleus
- bic: Brachium of the inferior colliculus
- bsc: Brachium of the superior colliculus
- cc: Corpus callosum
- cpb: Cerebellar peduncles
- fx: Fornix
- hipg: Hippocampal glioepithelium/ependyma
- lf: Lateral funiculus
- ll: Lateral lemniscus
- mn: Mesencephalic neuroepithelium
- pcbn: Precerebellar neuroepithelium
- pyrd: Pyramidal decussation
- rhn: Rhombencephalic neuroepithelium
- scc: Superior colliculus commissure
- wmf: White matter fibers

Age: 22 GW

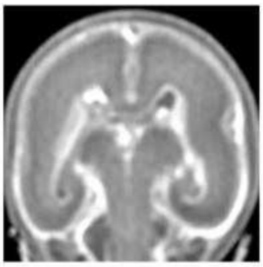

A-P Level: -8.58 mm

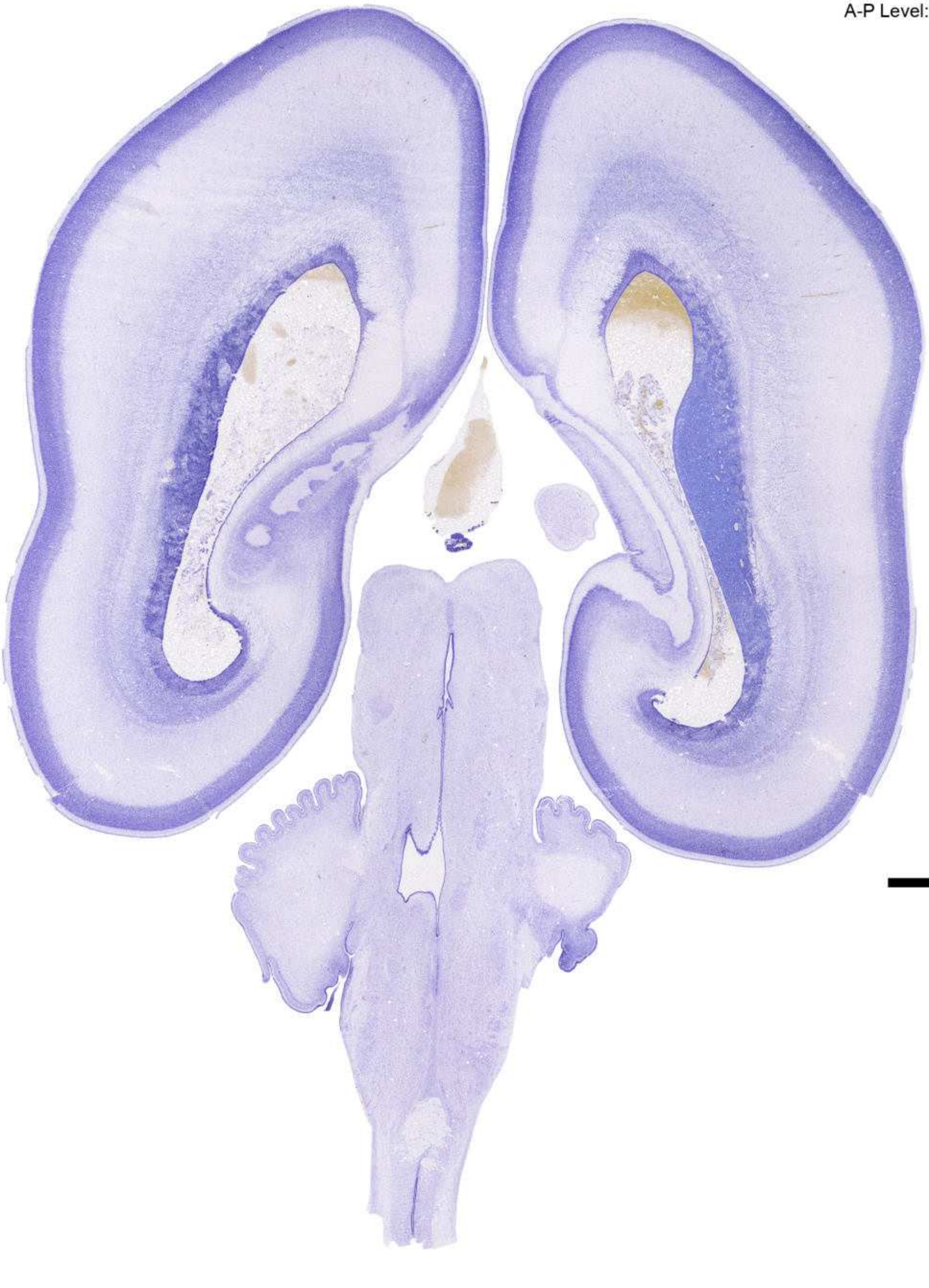

5 mm

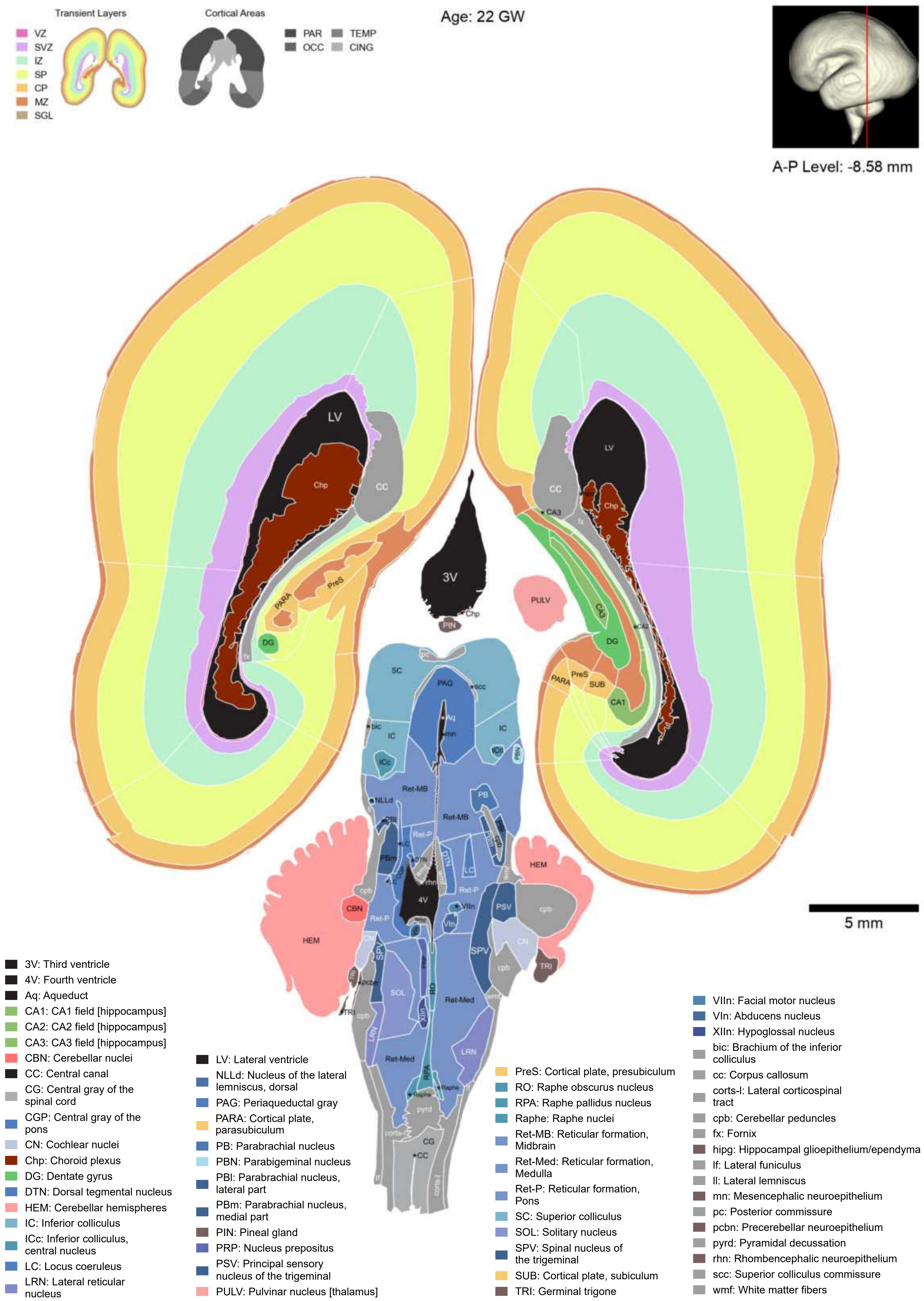
Transient Layers
VZ
SVZ
IZ
SP
CP
MZ
SGL
Cortical Areas
PAR
OCC
TEMP
CING
Age: 22 GW
A-P Level: -8.58 mm
5 mm
3V: Third ventricle
4V: Fourth ventricle
Aq: Aqueduct
CA1: CA1 field [hippocampus]
CA2: CA2 field [hippocampus]
CA3: CA3 field [hippocampus]
CBN: Cerebellar nuclei
CC: Central canal
CG: Central gray of the spinal cord
CGP: Central gray of the pons
CN: Cochlear nuclei
Chp: Choroid plexus
DG: Dentate gyrus
DTN: Dorsal tegmental nucleus
HEM: Cerebellar hemispheres
IC: Inferior colliculus
ICc: Inferior colliculus, central nucleus
LC: Locus coeruleus
LRN: Lateral reticular nucleus
LV: Lateral ventricle
NLLd: Nucleus of the lateral lemniscus, dorsal
PAG: Periaqueductal gray
PARA: Cortical plate, parasubiculum
PB: Parabrachial nucleus
PBN: Parabigeminal nucleus
PBl: Parabrachial nucleus, lateral part
PBm: Parabrachial nucleus, medial part
PIN: Pineal gland
PRP: Nucleus prepositus
PSV: Principal sensory nucleus of the trigeminal
PULV: Pulvinar nucleus [thalamus]
PreS: Cortical plate, presubiculum
RO: Raphe obscurus nucleus
RPA: Raphe pallidus nucleus
Raphe: Raphe nuclei
Ret-MB: Reticular formation, Midbrain
Ret-Med: Reticular formation, Medulla
Ret-P: Reticular formation, Pons
SC: Superior colliculus
SOL: Solitary nucleus
SPV: Spinal nucleus of the trigeminal
SUB: Cortical plate, subiculum
TRI: Germinal trigone
VIIn: Facial motor nucleus
VIn: Abducens nucleus
XIIn: Hypoglossal nucleus
bic: Brachium of the inferior colliculus
cc: Corpus callosum
corts-l: Lateral corticospinal tract
cpb: Cerebellar peduncles
fx: Fornix
hipg: Hippocampal glioepithelium/ependyma
lf: Lateral funiculus
ll: Lateral lemniscus
mn: Mesencephalic neuroepithelium
pc: Posterior commissure
pcbn: Precerebellar neuroepithelium
pyrd: Pyramidal decussation
rhn: Rhombencephalic neuroepithelium
scc: Superior colliculus commissure
wmf: White matter fibers

Age: 22 GW

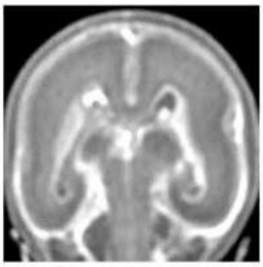

A-P Level: -8.94 mm

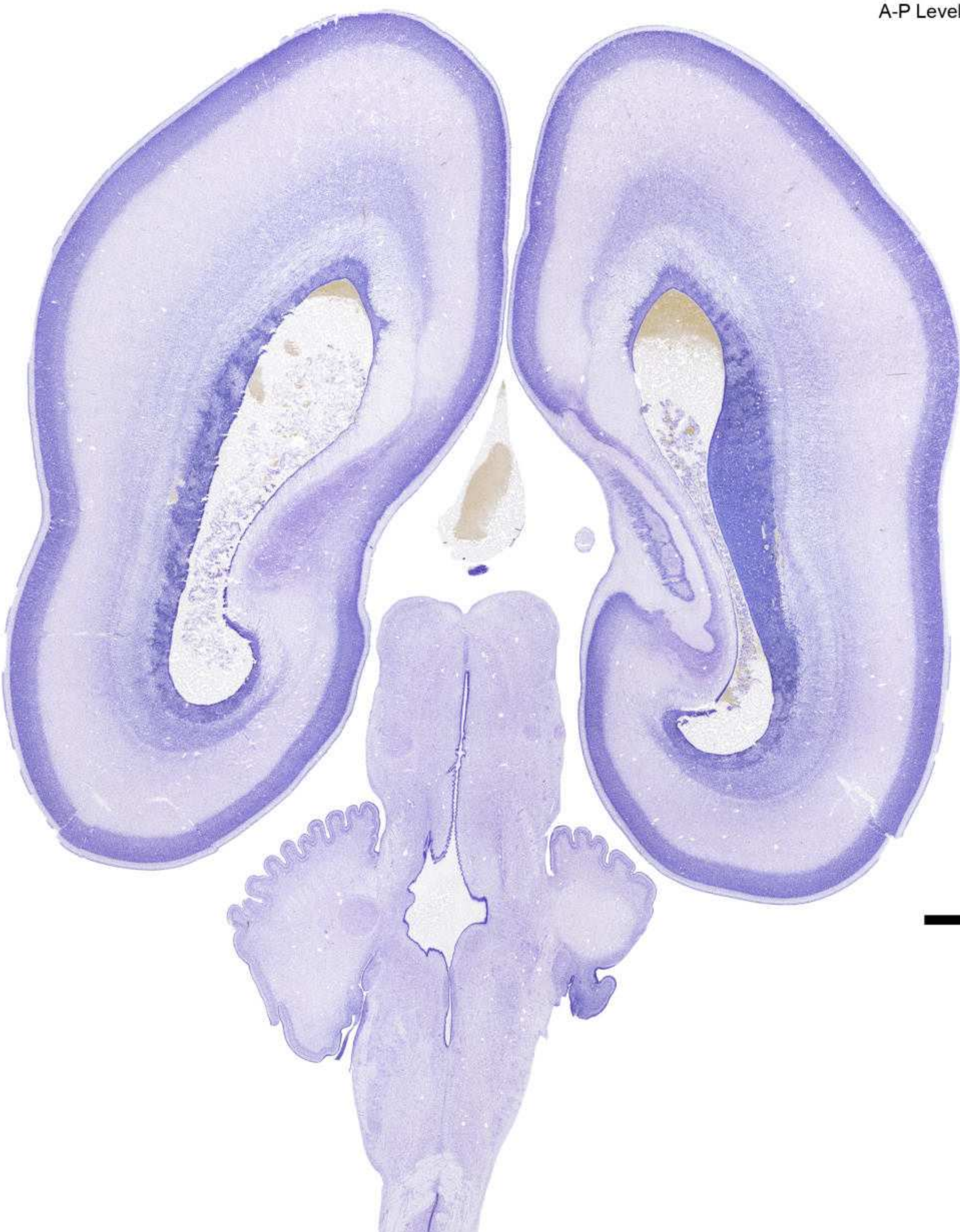

5 mm

Transient Layers

- VZ
- SVZ
- IZ
- SP
- CP
- MZ
- SGL

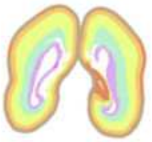

Cortical Areas

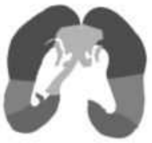

- PAR
- OCC
- TEMP
- CING

Age: 22 GW

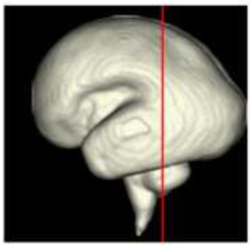

A-P Level: -8.94 mm

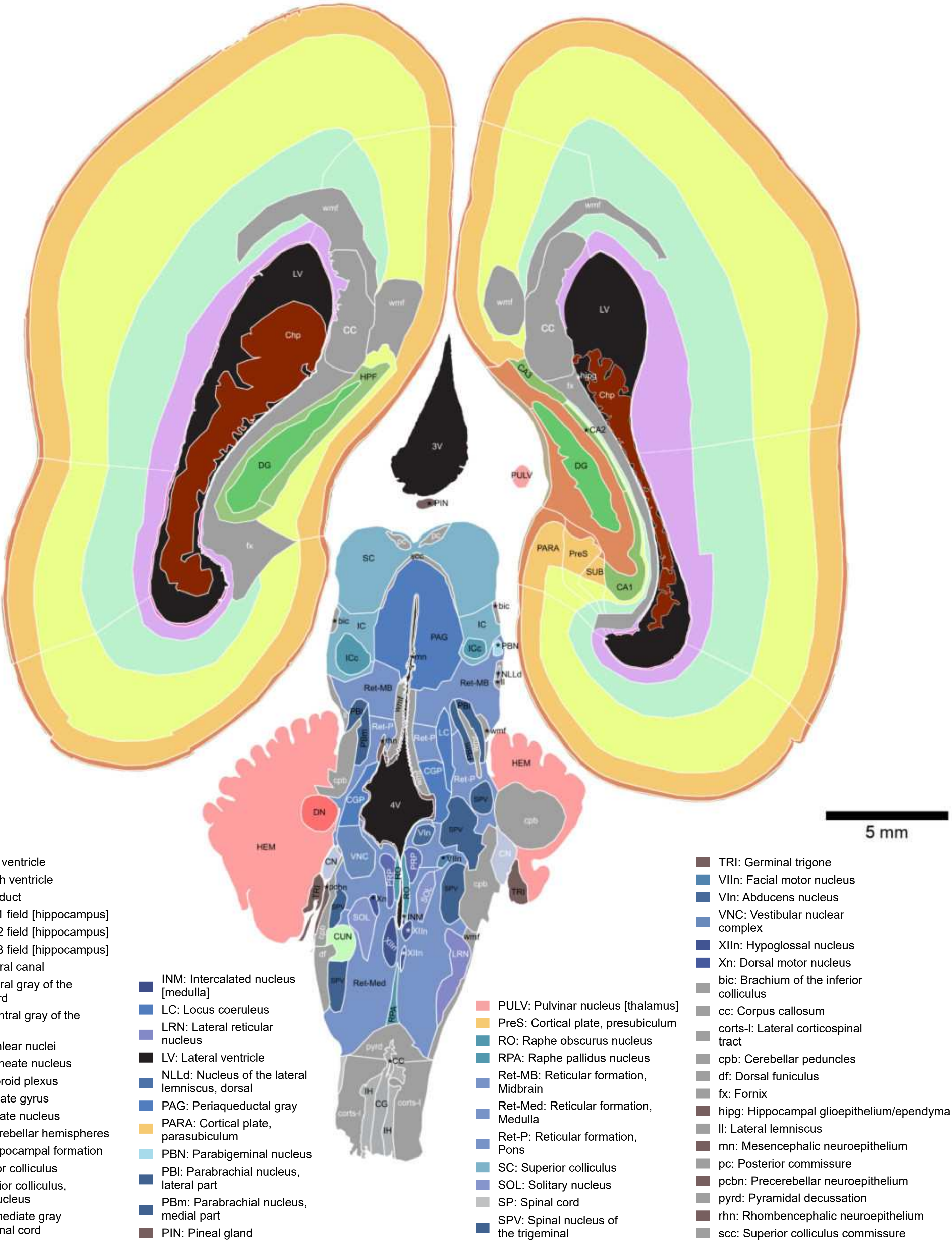

- 3V: Third ventricle
- 4V: Fourth ventricle
- Aq: Aqueduct
- CA1: CA1 field [hippocampus]
- CA2: CA2 field [hippocampus]
- CA3: CA3 field [hippocampus]
- CC: Central canal
- CG: Central gray of the spinal cord
- CGP: Central gray of the pons
- CN: Cochlear nuclei
- CUN: Cuneate nucleus
- Chp: Choroid plexus
- DG: Dentate gyrus
- DN: Dentate nucleus
- HEM: Cerebellar hemispheres
- HPF: Hippocampal formation
- IC: Inferior colliculus
- ICc: Inferior colliculus, central nucleus
- IH: Intermediate gray of the spinal cord
- INM: Intercalated nucleus [medulla]
- LC: Locus coeruleus
- LRN: Lateral reticular nucleus
- LV: Lateral ventricle
- NLLd: Nucleus of the lateral lemniscus, dorsal
- PAG: Periaqueductal gray
- PARA: Cortical plate, parasubiculum
- PBN: Parabigeminal nucleus
- PBl: Parabrachial nucleus, lateral part
- PBm: Parabrachial nucleus, medial part
- PIN: Pineal gland
- PRP: Nucleus prepositus
- PULV: Pulvinar nucleus [thalamus]
- PreS: Cortical plate, presubiculum
- RO: Raphe obscurus nucleus
- RPA: Raphe pallidus nucleus
- Ret-MB: Reticular formation, Midbrain
- Ret-Med: Reticular formation, Medulla
- Ret-P: Reticular formation, Pons
- SC: Superior colliculus
- SOL: Solitary nucleus
- SP: Spinal cord
- SPV: Spinal nucleus of the trigeminal
- SUB: Cortical plate, subiculum
- TRI: Germinal trigone
- VIIn: Facial motor nucleus
- VIn: Abducens nucleus
- VNC: Vestibular nuclear complex
- XIIn: Hypoglossal nucleus
- Xn: Dorsal motor nucleus
- bic: Brachium of the inferior colliculus
- cc: Corpus callosum
- corts-l: Lateral corticospinal tract
- cpb: Cerebellar peduncles
- df: Dorsal funiculus
- fx: Fornix
- hipg: Hippocampal glioepithelium/ependyma
- ll: Lateral lemniscus
- mn: Mesencephalic neuroepithelium
- pc: Posterior commissure
- pcbn: Precerebellar neuroepithelium
- pyrd: Pyramidal decussation
- rhn: Rhombencephalic neuroepithelium
- scc: Superior colliculus commissure
- wmf: White matter fibers

Age: 22 GW

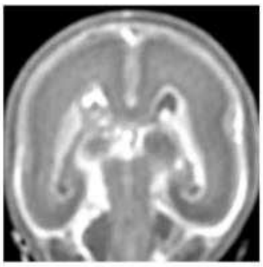

A-P Level: -9.3 mm

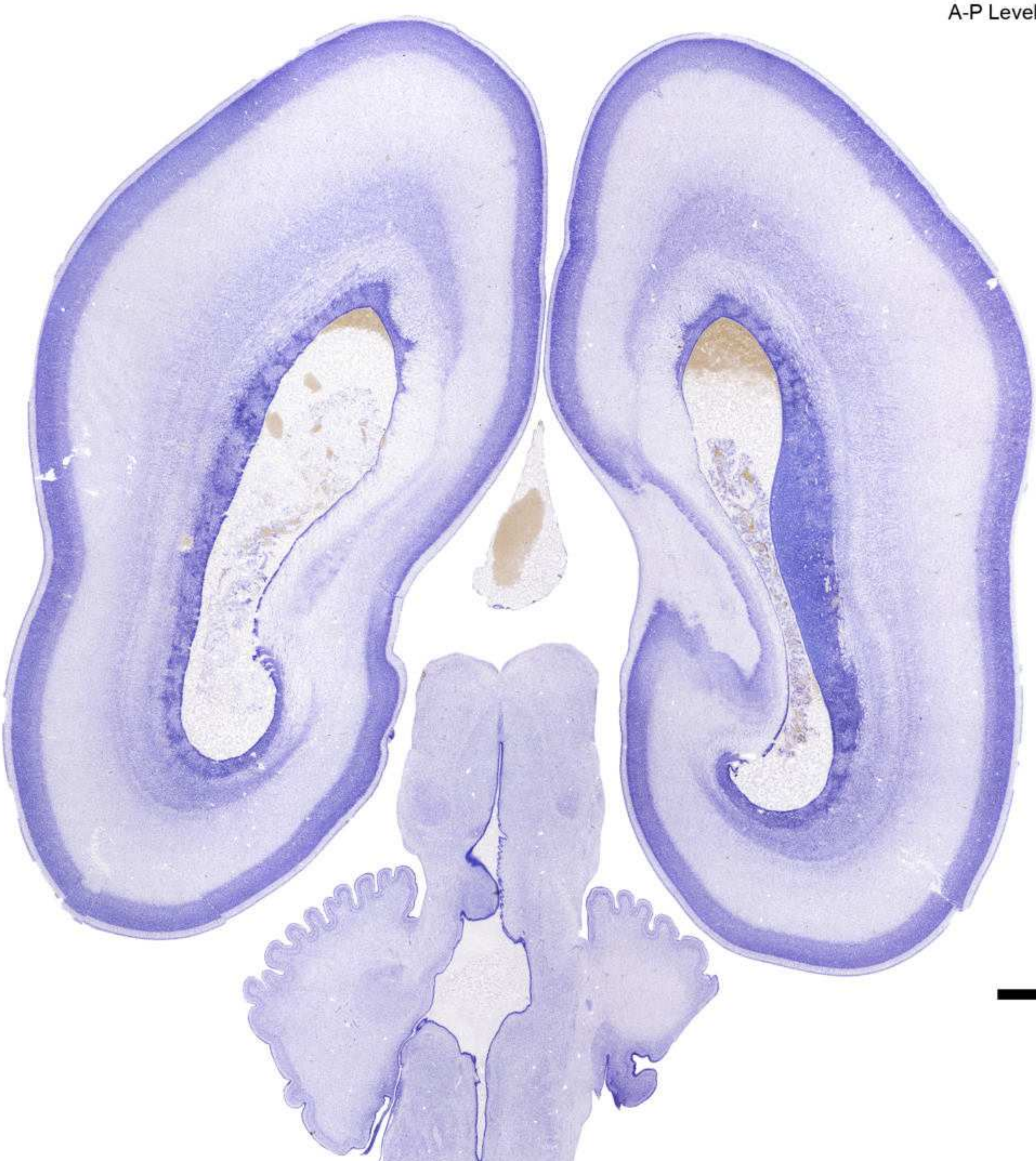

5 mm

Transient Layers

- VZ
- SVZ
- IZ
- SP
- CP
- MZ
- SGL

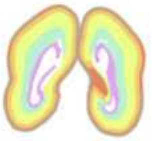

Cortical Areas

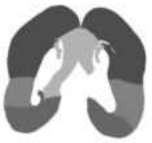

- PAR
- TEMP
- OCC
- CING

Age: 22 GW

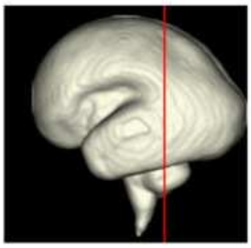

A-P Level: -9.3 mm

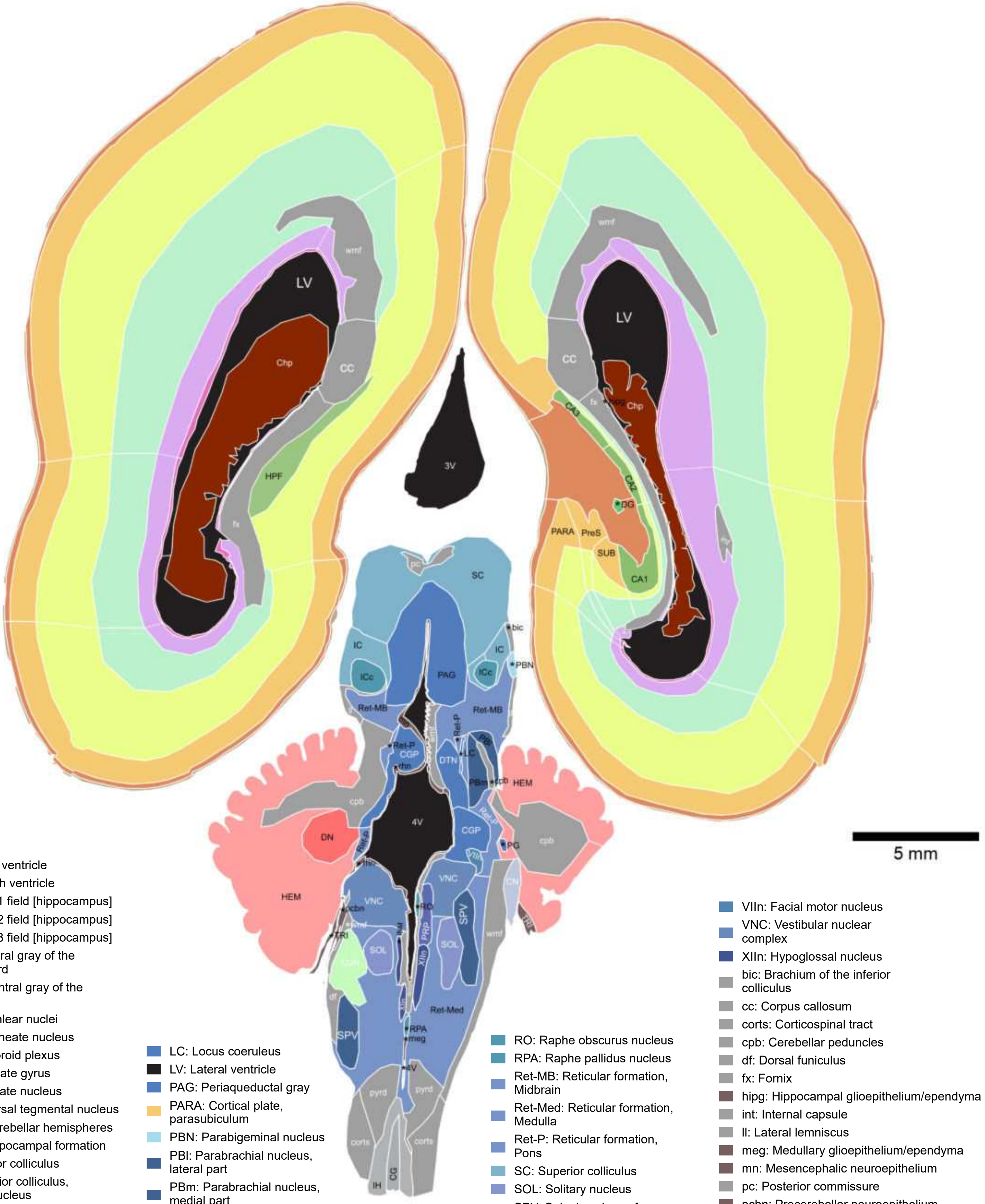

- 3V: Third ventricle
- 4V: Fourth ventricle
- CA1: CA1 field [hippocampus]
- CA2: CA2 field [hippocampus]
- CA3: CA3 field [hippocampus]
- CG: Central gray of the spinal cord
- CGP: Central gray of the pons
- CN: Cochlear nuclei
- CUN: Cuneate nucleus
- Chp: Choroid plexus
- DG: Dentate gyrus
- DN: Dentate nucleus
- DTN: Dorsal tegmental nucleus
- HEM: Cerebellar hemispheres
- HPF: Hippocampal formation
- IC: Inferior colliculus
- ICc: Inferior colliculus, central nucleus
- IH: Intermediate gray of the spinal cord
- INM: Intercalated nucleus [medulla]
- LC: Locus coeruleus
- LV: Lateral ventricle
- PAG: Periaqueductal gray
- PARA: Cortical plate, parasubiculum
- PBN: Parabigeminal nucleus
- PBl: Parabrachial nucleus, lateral part
- PBm: Parabrachial nucleus, medial part
- PG: Pontine gray
- PRP: Nucleus prepositus
- PreS: Cortical plate, presubiculum
- RO: Raphe obscurus nucleus
- RPA: Raphe pallidus nucleus
- Ret-MB: Reticular formation, Midbrain
- Ret-Med: Reticular formation, Medulla
- Ret-P: Reticular formation, Pons
- SC: Superior colliculus
- SOL: Solitary nucleus
- SPV: Spinal nucleus of the trigeminal
- SUB: Cortical plate, subiculum
- TRI: Germinal trigone
- VIIn: Facial motor nucleus
- VNC: Vestibular nuclear complex
- XIIn: Hypoglossal nucleus
- bic: Brachium of the inferior colliculus
- cc: Corpus callosum
- corts: Corticospinal tract
- cpb: Cerebellar peduncles
- df: Dorsal funiculus
- fx: Fornix
- hipg: Hippocampal glioepithelium/ependyma
- int: Internal capsule
- ll: Lateral lemniscus
- meg: Medullary glioepithelium/ependyma
- mn: Mesencephalic neuroepithelium
- pc: Posterior commissure
- pcbn: Precerebellar neuroepithelium
- pyrd: Pyramidal decussation
- rhn: Rhombencephalic neuroepithelium
- wmf: White matter fibers

Age: 22 GW

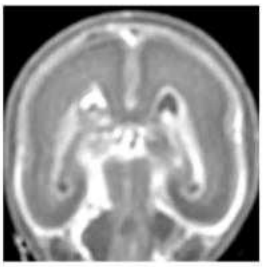

A-P Level: -9.84 mm

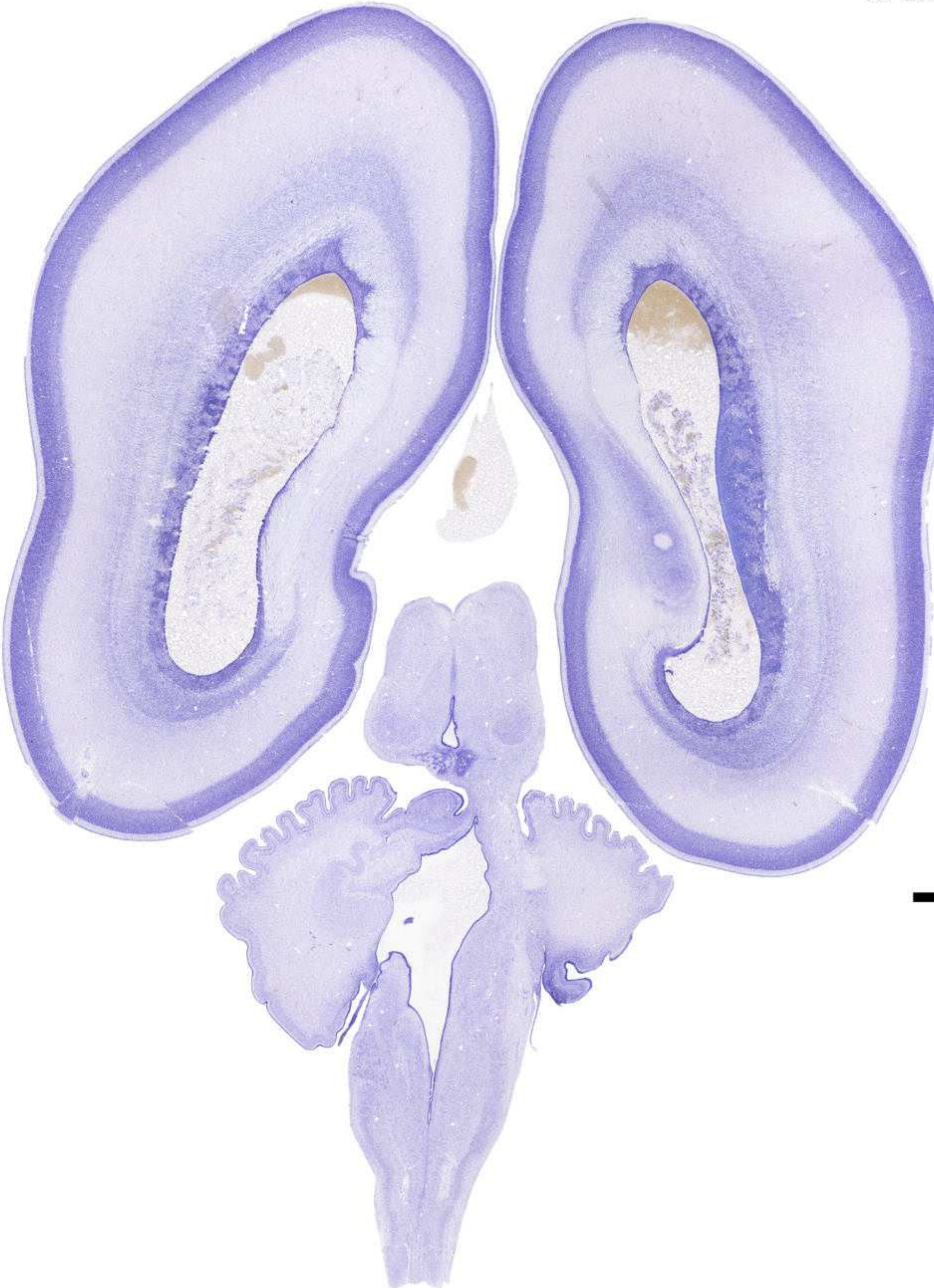

5 mm

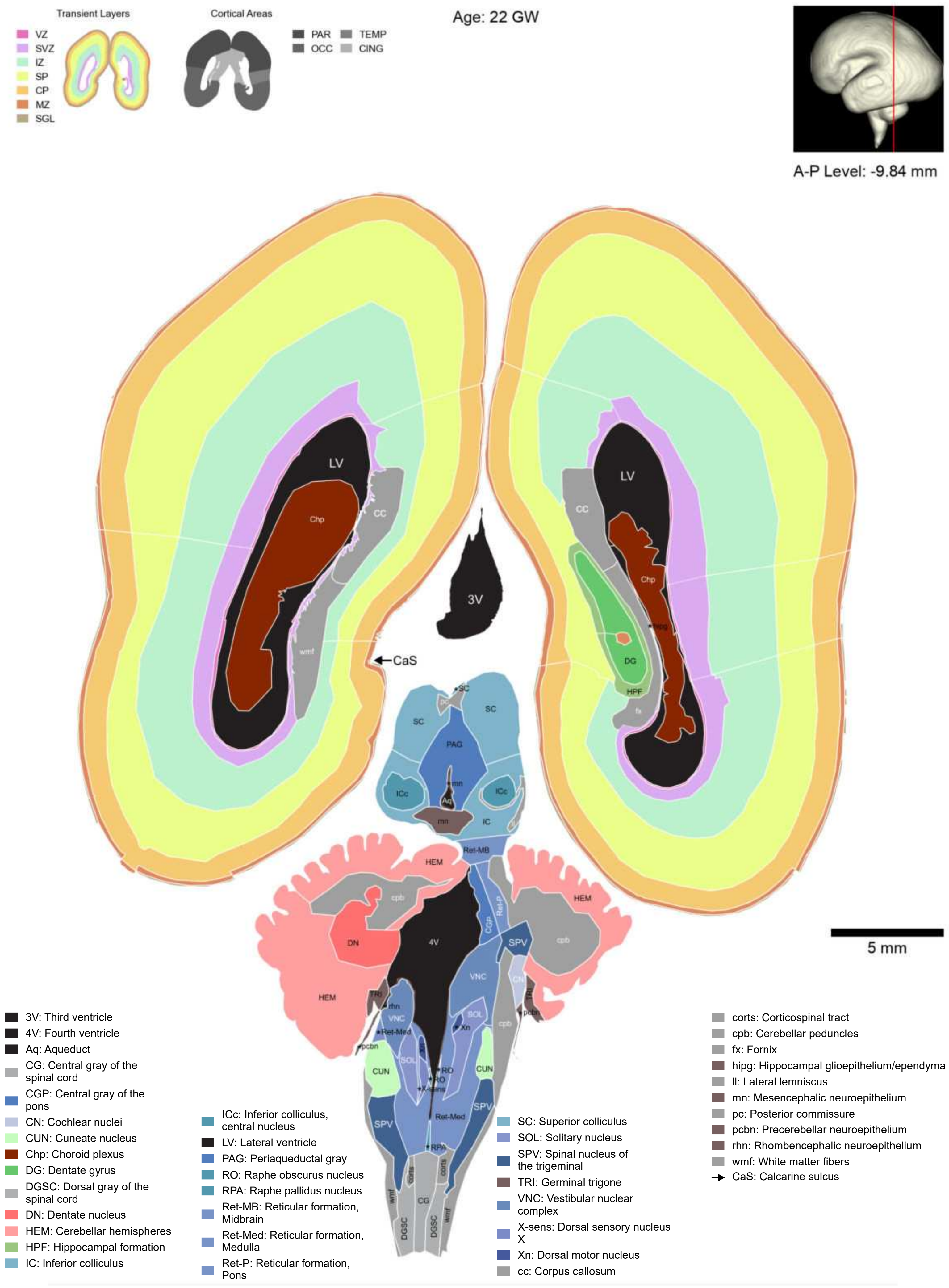

Transient Layers
VZ
SVZ
IZ
SP
CP
MZ
SGL
Cortical Areas
PAR
TEMP
OCC
CING
Age: 22 GW
A-P Level: -9.84 mm
LV
Chp
CC
3V
CaS
wmf
DG
HPF
hipg
fx
SC
pc
PAG
mn
Aq
ICc
IC
Ret-MB
HEM
cpb
DN
4V
CGP
Ret-P
SPV
VNC
CN
TRI
rhn
pcbn
SOL
Xn
Ret-Med
CUN
RO
X-sens
RPA
corts
CG
DGSC
5 mm
3V: Third ventricle
4V: Fourth ventricle
Aq: Aqueduct
CG: Central gray of the spinal cord
CGP: Central gray of the pons
CN: Cochlear nuclei
CUN: Cuneate nucleus
Chp: Choroid plexus
DG: Dentate gyrus
DGSC: Dorsal gray of the spinal cord
DN: Dentate nucleus
HEM: Cerebellar hemispheres
HPF: Hippocampal formation
IC: Inferior colliculus
ICc: Inferior colliculus, central nucleus
LV: Lateral ventricle
PAG: Periaqueductal gray
RO: Raphe obscurus nucleus
RPA: Raphe pallidus nucleus
Ret-MB: Reticular formation, Midbrain
Ret-Med: Reticular formation, Medulla
Ret-P: Reticular formation, Pons
SC: Superior colliculus
SOL: Solitary nucleus
SPV: Spinal nucleus of the trigeminal
TRI: Germinal trigone
VNC: Vestibular nuclear complex
X-sens: Dorsal sensory nucleus X
Xn: Dorsal motor nucleus
cc: Corpus callosum
corts: Corticospinal tract
cpb: Cerebellar peduncles
fx: Fornix
hipg: Hippocampal glioepithelium/ependyma
ll: Lateral lemniscus
mn: Mesencephalic neuroepithelium
pc: Posterior commissure
pcbn: Precerebellar neuroepithelium
rhn: Rhombencephalic neuroepithelium
wmf: White matter fibers
CaS: Calcarine sulcus

Age: 22 GW

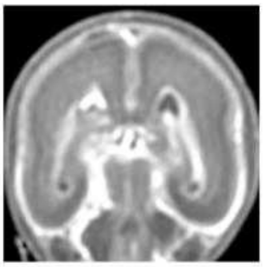

A-P Level: -10.32 mm

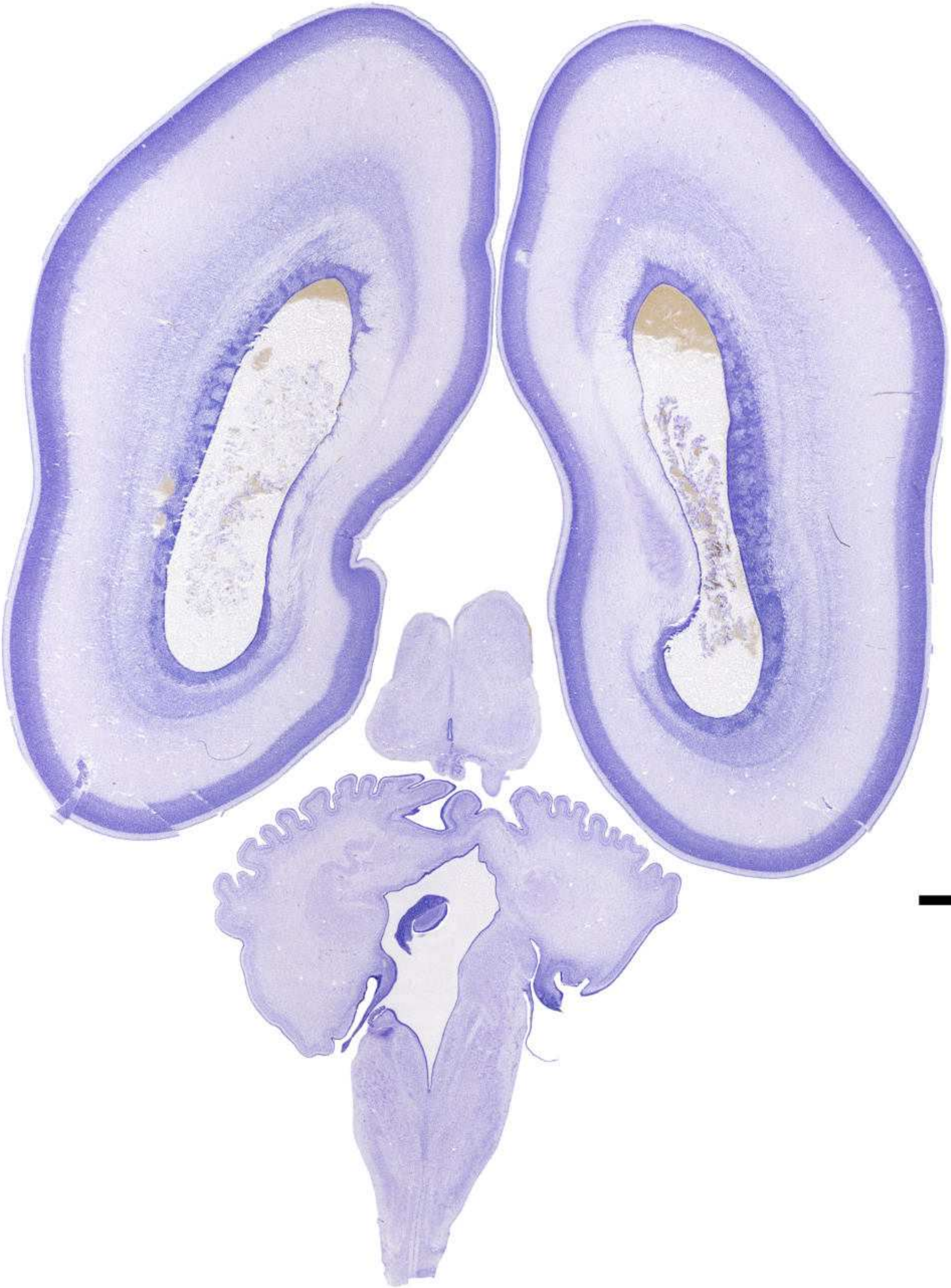

5 mm

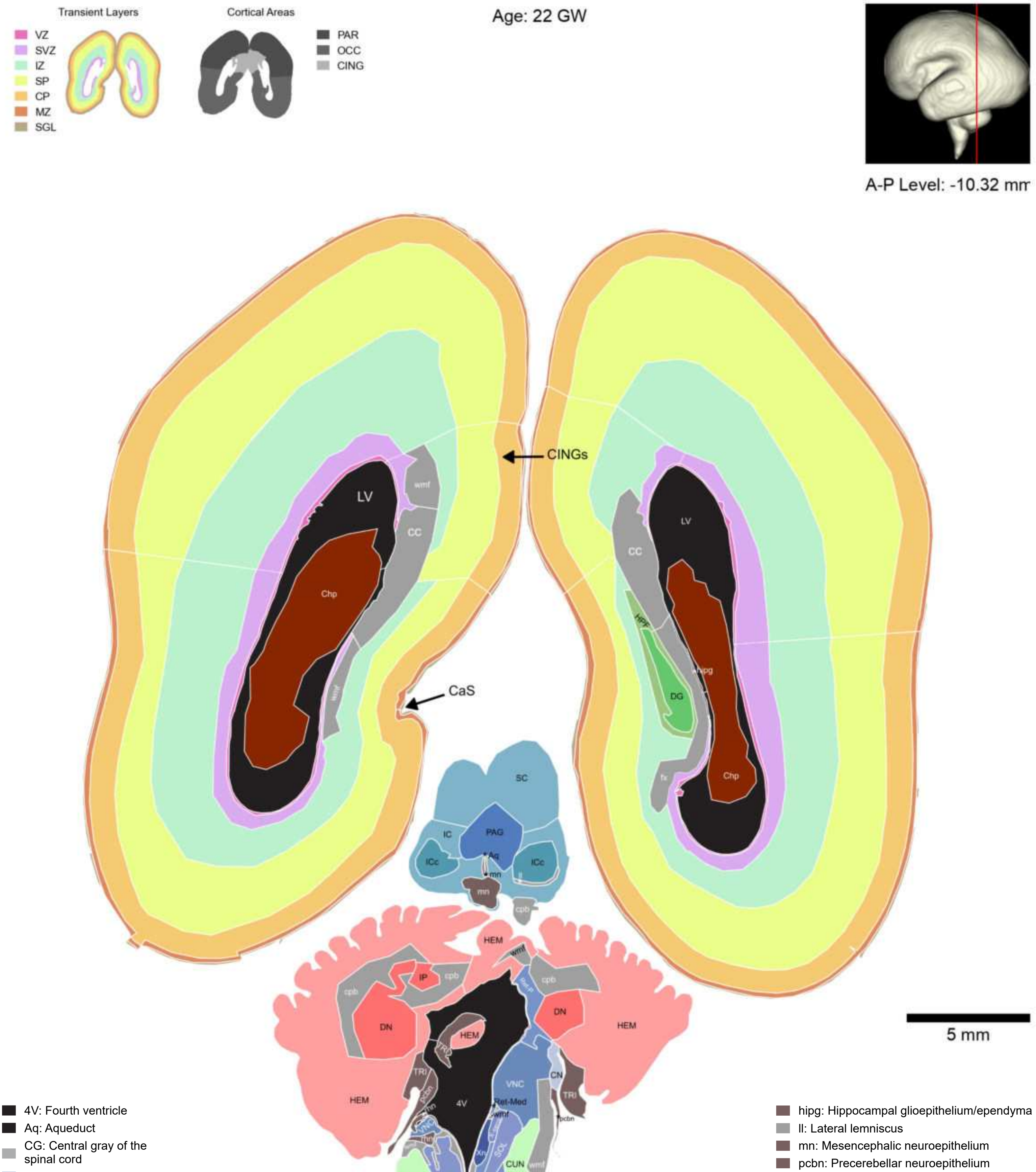

- 4V: Fourth ventricle
- Aq: Aqueduct
- CG: Central gray of the spinal cord
- CN: Cochlear nuclei
- CUN: Cuneate nucleus
- Chp: Choroid plexus
- DG: Dentate gyrus
- DGSC: Dorsal gray of the spinal cord
- DN: Dentate nucleus
- HEM: Cerebellar hemispheres
- HPF: Hippocampal formation
- IC: Inferior colliculus
- ICc: Inferior colliculus, central nucleus
- IP: Interposed nucleus
- LV: Lateral ventricle
- PAG: Periaqueductal gray
- RPA: Raphe pallidus nucleus
- Ret-Med: Reticular formation, Medulla
- Ret-P: Reticular formation, Pons
- SC: Superior colliculus
- SOL: Solitary nucleus
- SPV: Spinal nucleus of the trigeminal
- TRI: Germinal trigone
- VNC: Vestibular nuclear complex
- X-sens: Dorsal sensory nucleus X
- Xn: Dorsal motor nucleus
- cc: Corpus callosum
- cpb: Cerebellar peduncles
- df: Dorsal funiculus
- fx: Fornix
- hipg: Hippocampal glioepithelium/ependyma
- ll: Lateral lemniscus
- mn: Mesencephalic neuroepithelium
- pcbn: Precerebellar neuroepithelium
- rhn: Rhombencephalic neuroepithelium
- wmf: White matter fibers
- → CaS: Calcarine sulcus
- → CINGs: Cingulate sulcus

Age: 22 GW

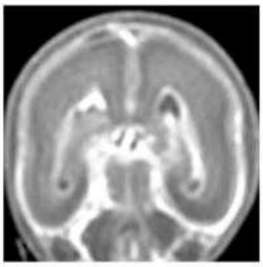

A-P Level: -10.68 mm

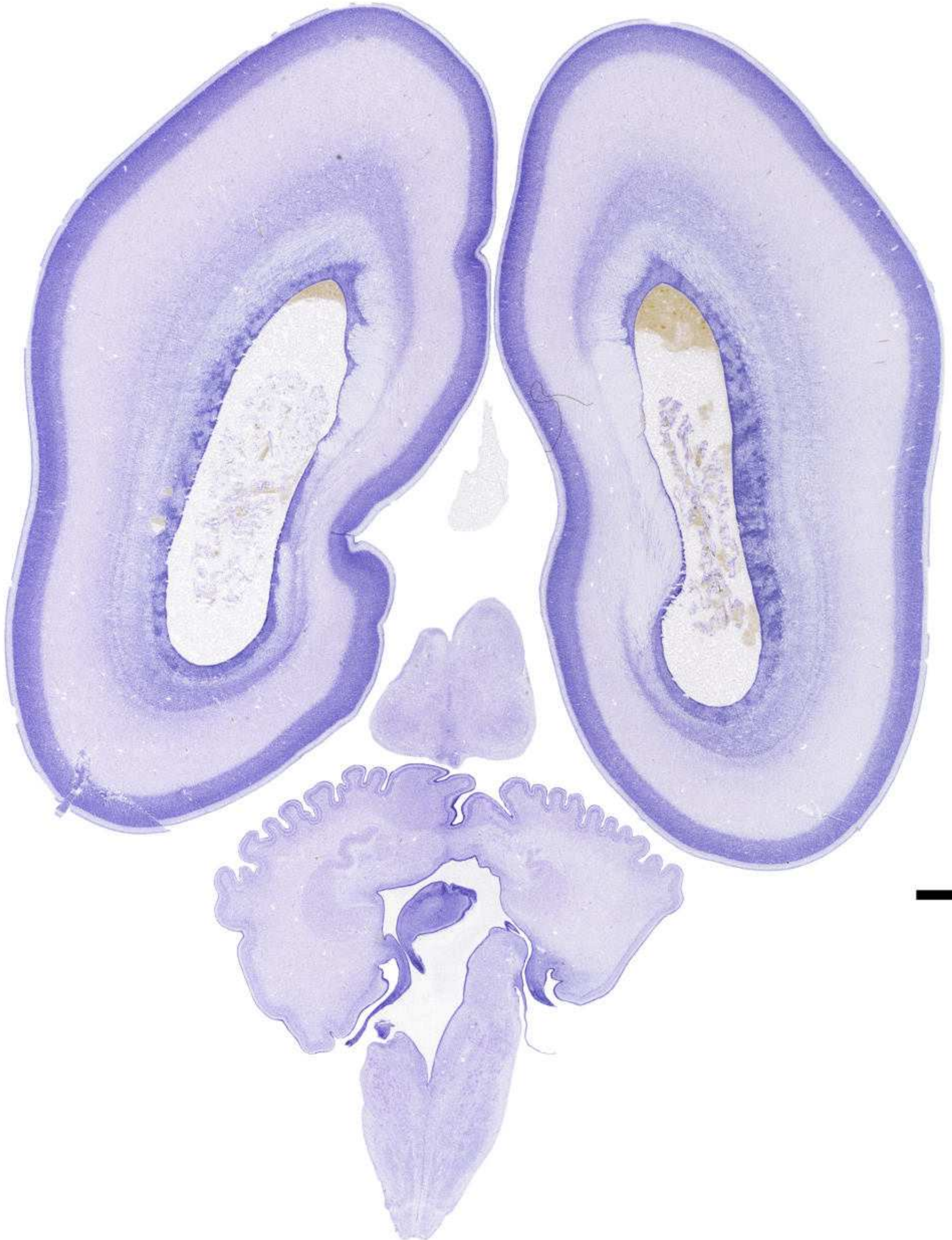

5 mm

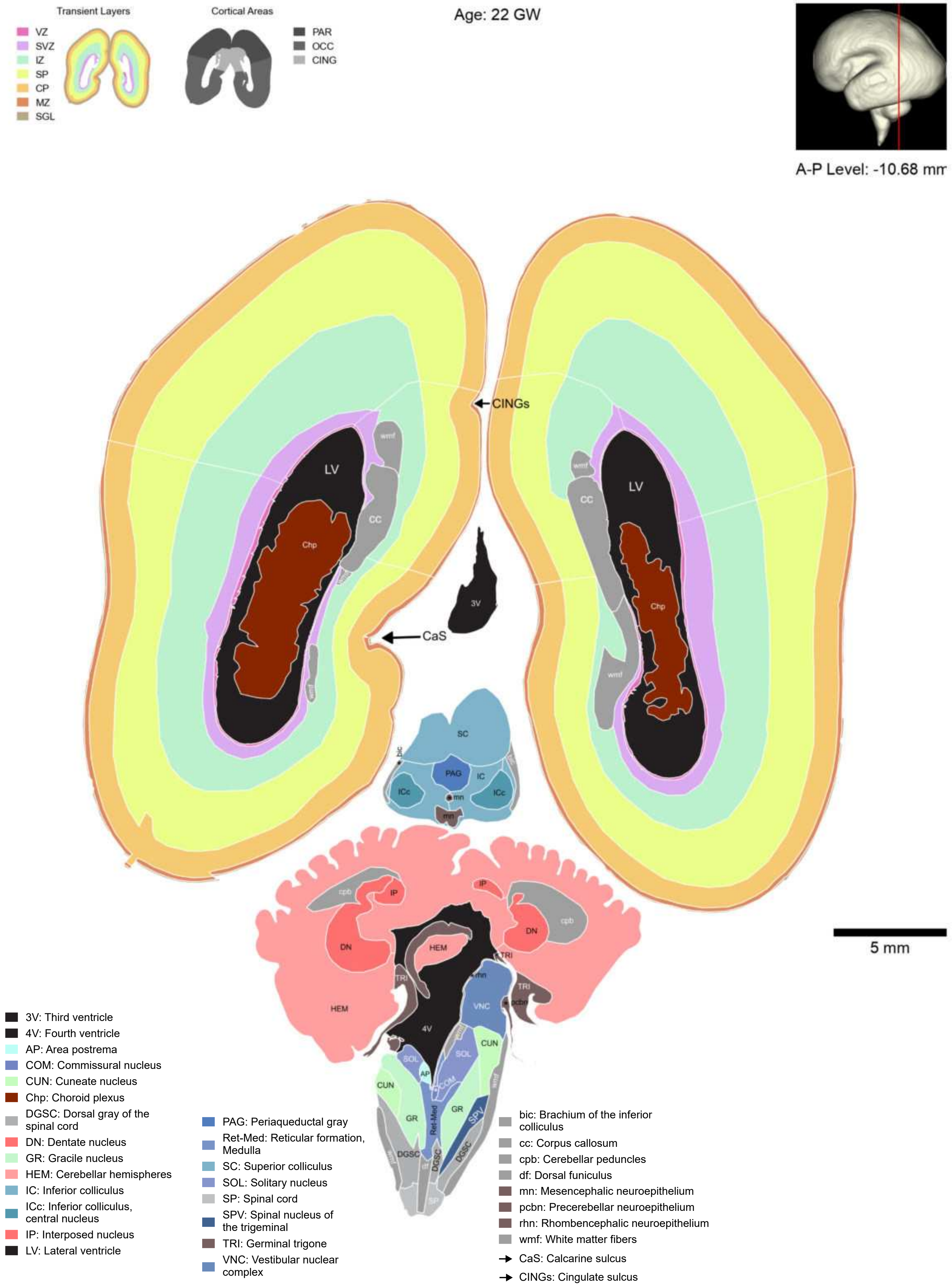

Age: 22 GW
A-P Level: -10.68 mm
Transient Layers
VZ
SVZ
IZ
SP
CP
MZ
SGL
Cortical Areas
PAR
OCC
CING
CINGs
CaS
LV
wmf
CC
Chp
3V
SC
bic
PAG
IC
ICc
mn
IP
cpb
DN
HEM
TRI
rhn
VNC
pcbn
4V
CUN
SOL
AP
COM
GR
Ret-Med
SPV
DGSC
df
SP
5 mm
3V: Third ventricle
4V: Fourth ventricle
AP: Area postrema
COM: Commissural nucleus
CUN: Cuneate nucleus
Chp: Choroid plexus
DGSC: Dorsal gray of the spinal cord
DN: Dentate nucleus
GR: Gracile nucleus
HEM: Cerebellar hemispheres
IC: Inferior colliculus
ICc: Inferior colliculus, central nucleus
IP: Interposed nucleus
LV: Lateral ventricle
PAG: Periaqueductal gray
Ret-Med: Reticular formation, Medulla
SC: Superior colliculus
SOL: Solitary nucleus
SP: Spinal cord
SPV: Spinal nucleus of the trigeminal
TRI: Germinal trigone
VNC: Vestibular nuclear complex
bic: Brachium of the inferior colliculus
cc: Corpus callosum
cpb: Cerebellar peduncles
df: Dorsal funiculus
mn: Mesencephalic neuroepithelium
pcbn: Precerebellar neuroepithelium
rhn: Rhombencephalic neuroepithelium
wmf: White matter fibers
CaS: Calcarine sulcus
CINGs: Cingulate sulcus

Age: 22 GW

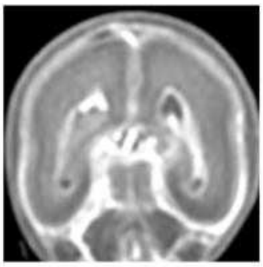

A-P Level: -10.98 mm

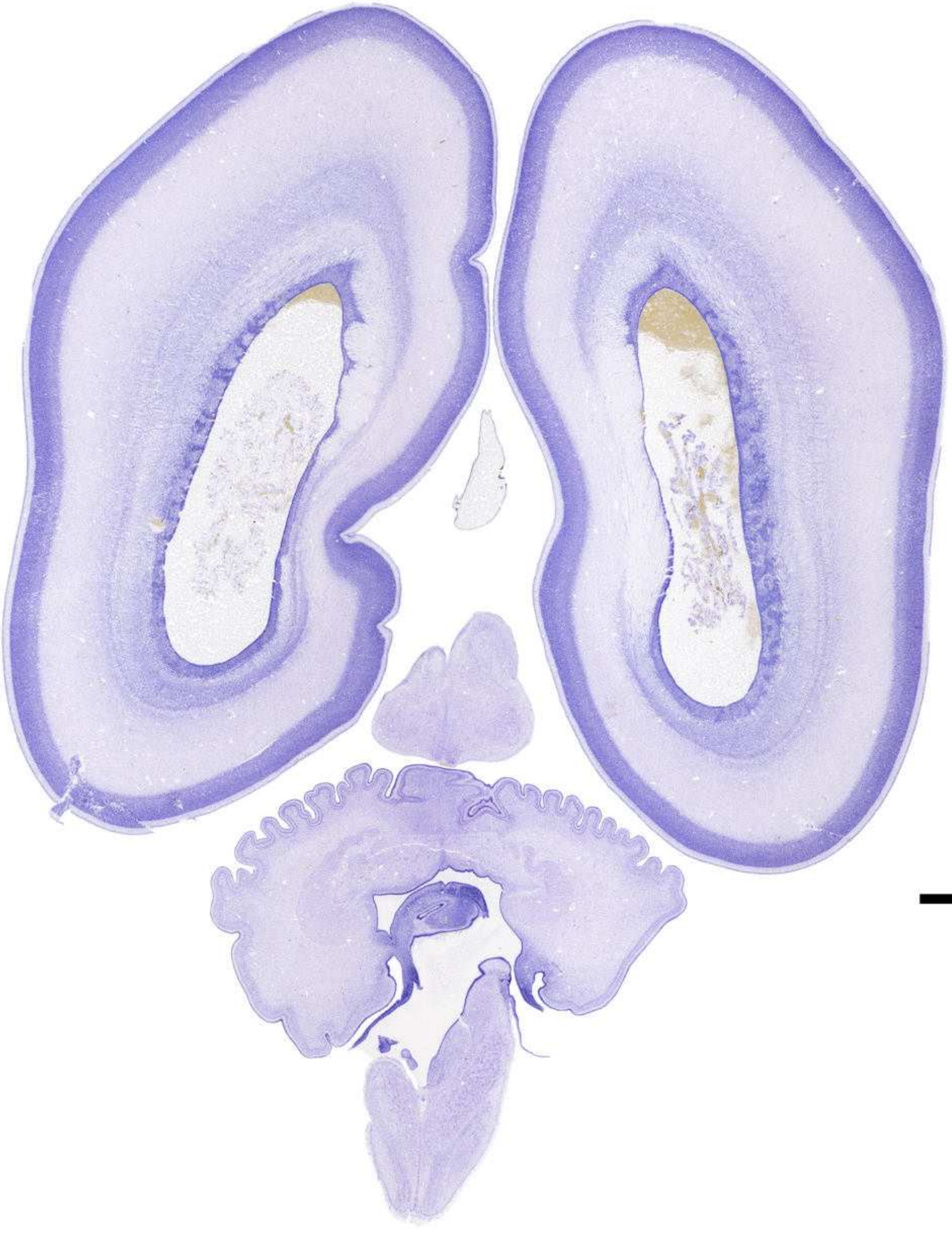

5 mm

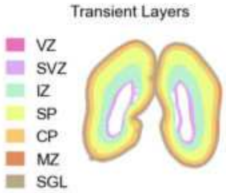

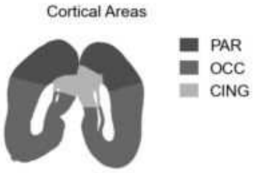

Age: 22 GW

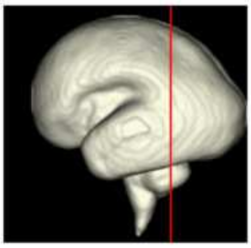

A-P Level: -10.98 mm

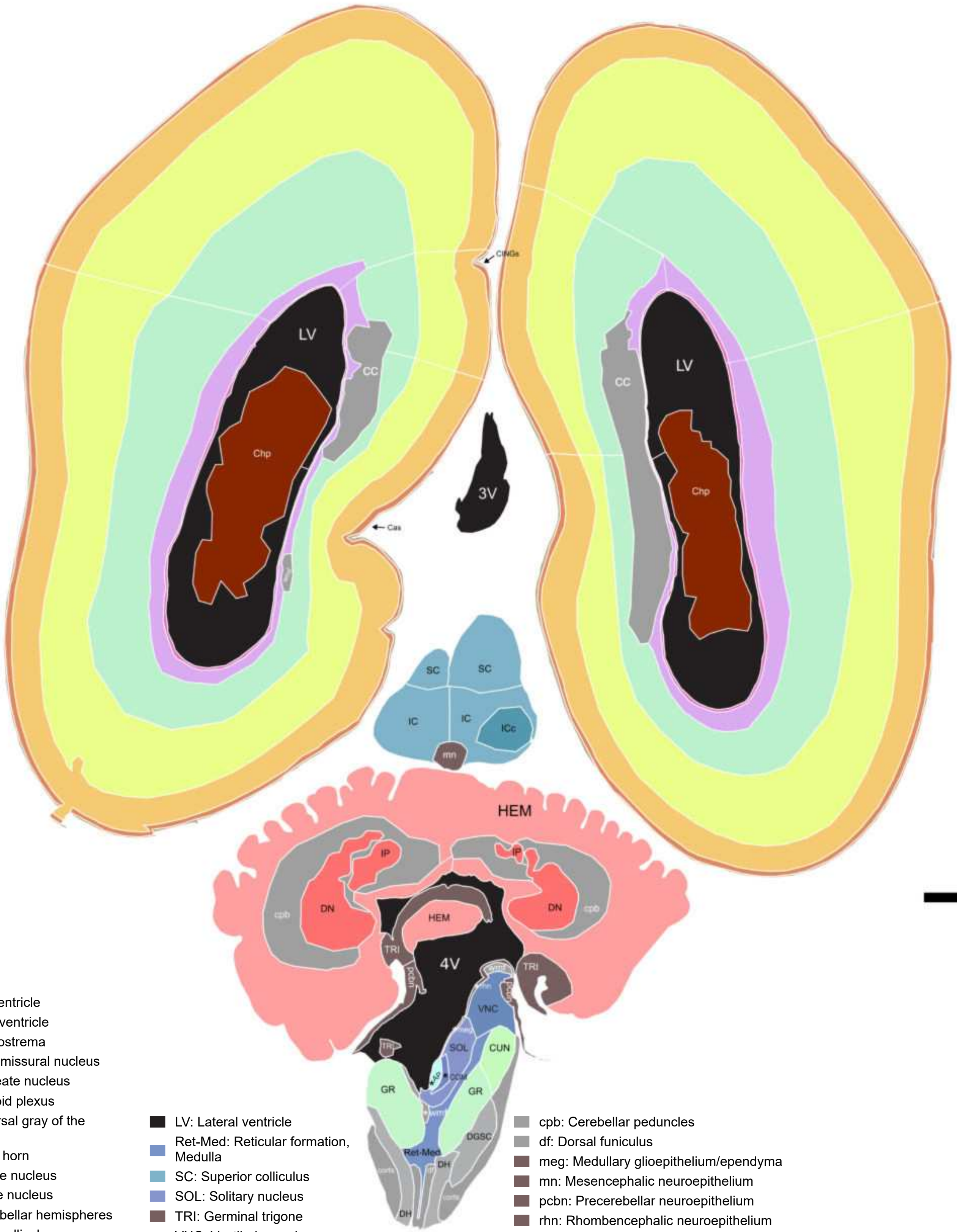

5 mm

- 3V: Third ventricle
- 4V: Fourth ventricle
- AP: Area postrema
- COM: Commissural nucleus
- CUN: Cuneate nucleus
- Chp: Choroid plexus
- DGSC: Dorsal gray of the spinal cord
- DH: Dorsal horn
- DN: Dentate nucleus
- GR: Gracile nucleus
- HEM: Cerebellar hemispheres
- IC: Inferior colliculus
- ICc: Inferior colliculus, central nucleus
- IP: Interposed nucleus
- LV: Lateral ventricle
- Ret-Med: Reticular formation, Medulla
- SC: Superior colliculus
- SOL: Solitary nucleus
- TRI: Germinal trigone
- VNC: Vestibular nuclear complex
- cc: Corpus callosum
- corts: Corticospinal tract
- cpb: Cerebellar peduncles
- df: Dorsal funiculus
- meg: Medullary glioepithelium/ependyma
- mn: Mesencephalic neuroepithelium
- pcbn: Precerebellar neuroepithelium
- rhn: Rhombencephalic neuroepithelium
- wmf: White matter fibers
- → CaS: Calcarine sulcus
- → CINGs: Cingulate sulcus

Age: 22 GW

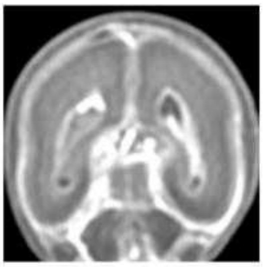

A-P Level: -11.4 mm

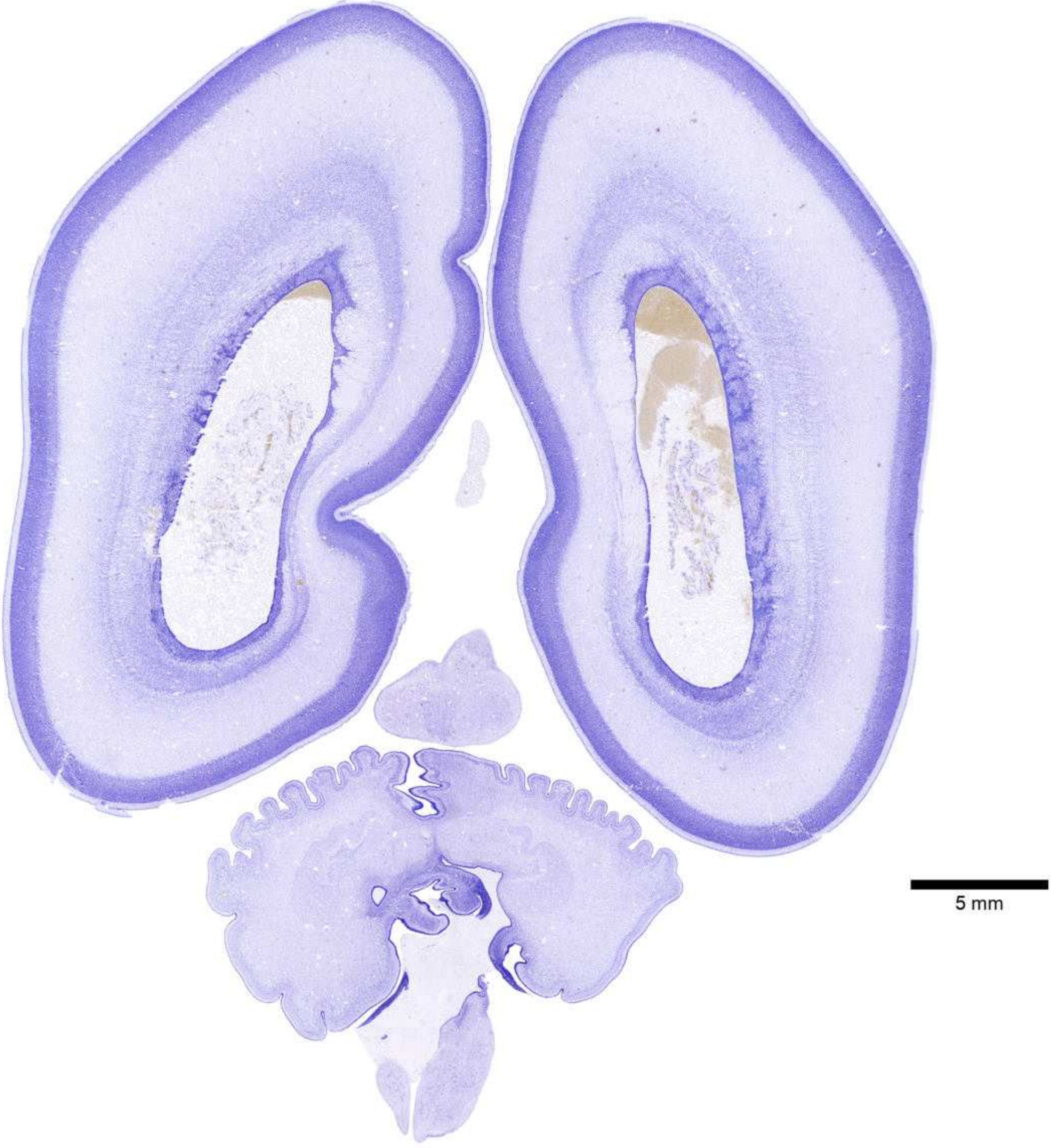

5 mm

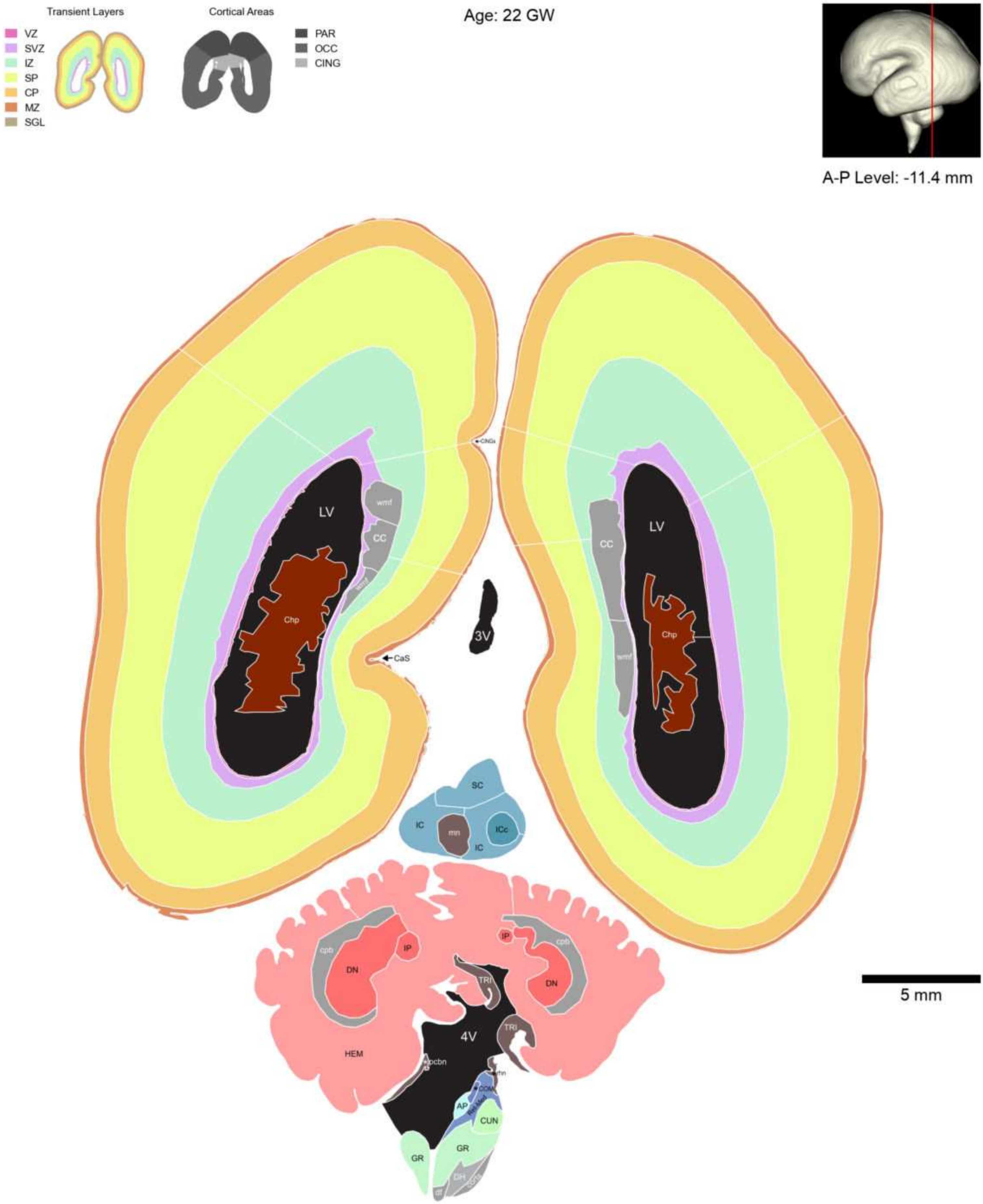

3V: Third ventricle
4V: Fourth ventricle
AP: Area postrema
COM: Commissural nucleus
CUN: Cuneate nucleus
Chp: Choroid plexus
DH: Dorsal horn
DN: Dentate nucleus
GR: Gracile nucleus
HEM: Cerebellar hemispheres
IC: Inferior colliculus
ICc: Inferior colliculus, central nucleus
IP: Interposed nucleus
LV: Lateral ventricle
Ret-Med: Reticular formation, Medulla
SC: Superior colliculus
TRI: Germinal trigone
cc: Corpus callosum
corts: Corticospinal tract
cpb: Cerebellar peduncles
df: Dorsal funiculus
mn: Mesencephalic neuroepithelium
pcbn: Precerebellar neuroepithelium
rhn: Rhombencephalic neuroepithelium
wmf: White matter fibers
CINGs: Cingulate sulcus
CaS: Calcarine sulcus

Age: 22 GW

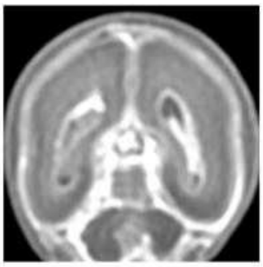

A-P Level: -11.82 mm

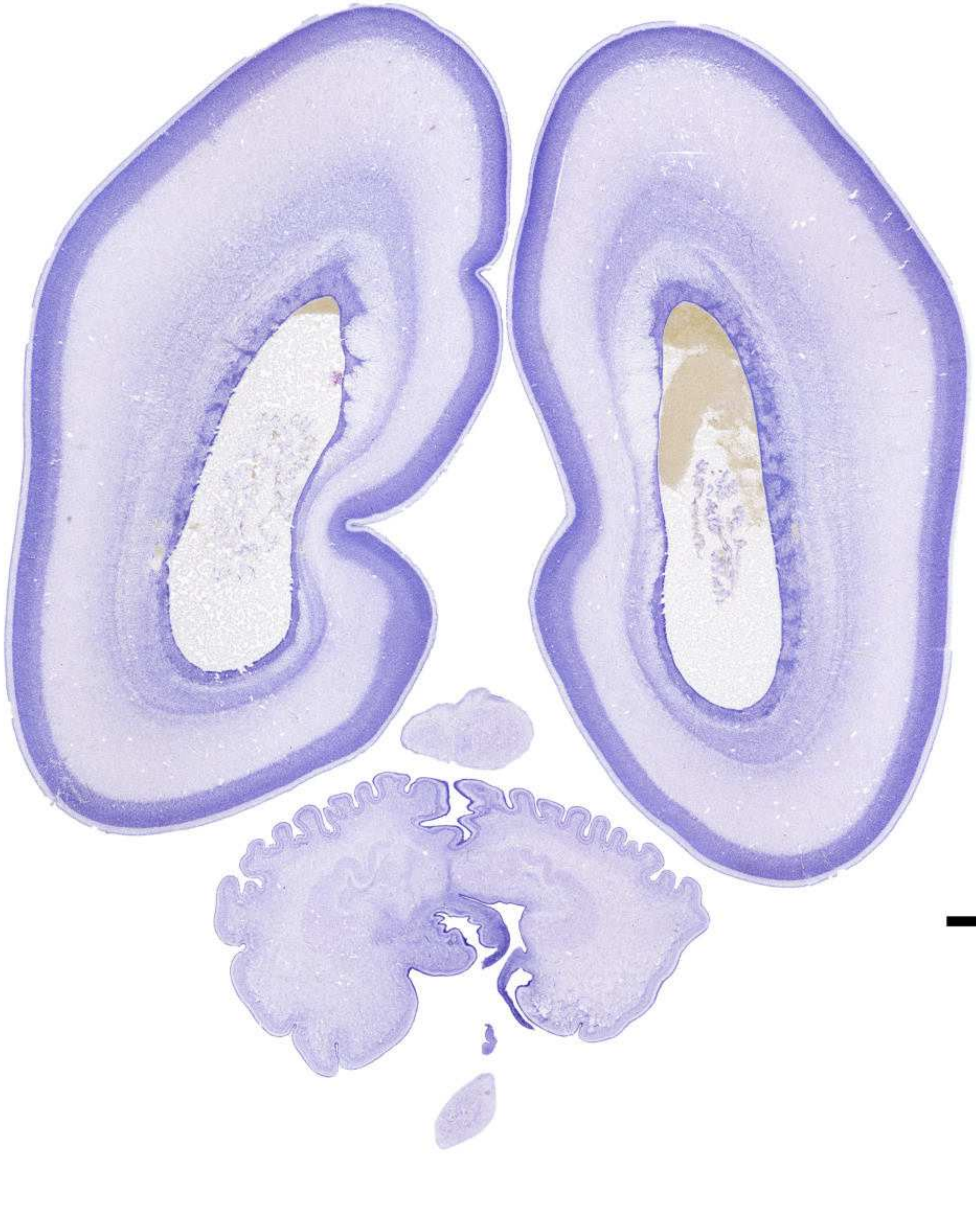

5 mm

Transient Layers

- VZ
- SVZ
- IZ
- SP
- CP
- MZ
- SGL

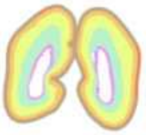

Cortical Areas

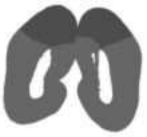

- PAR
- OCC

Age: 22 GW

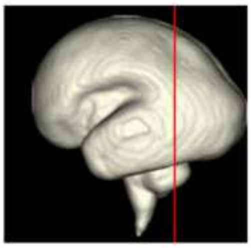

A-P Level: -11.82 mm

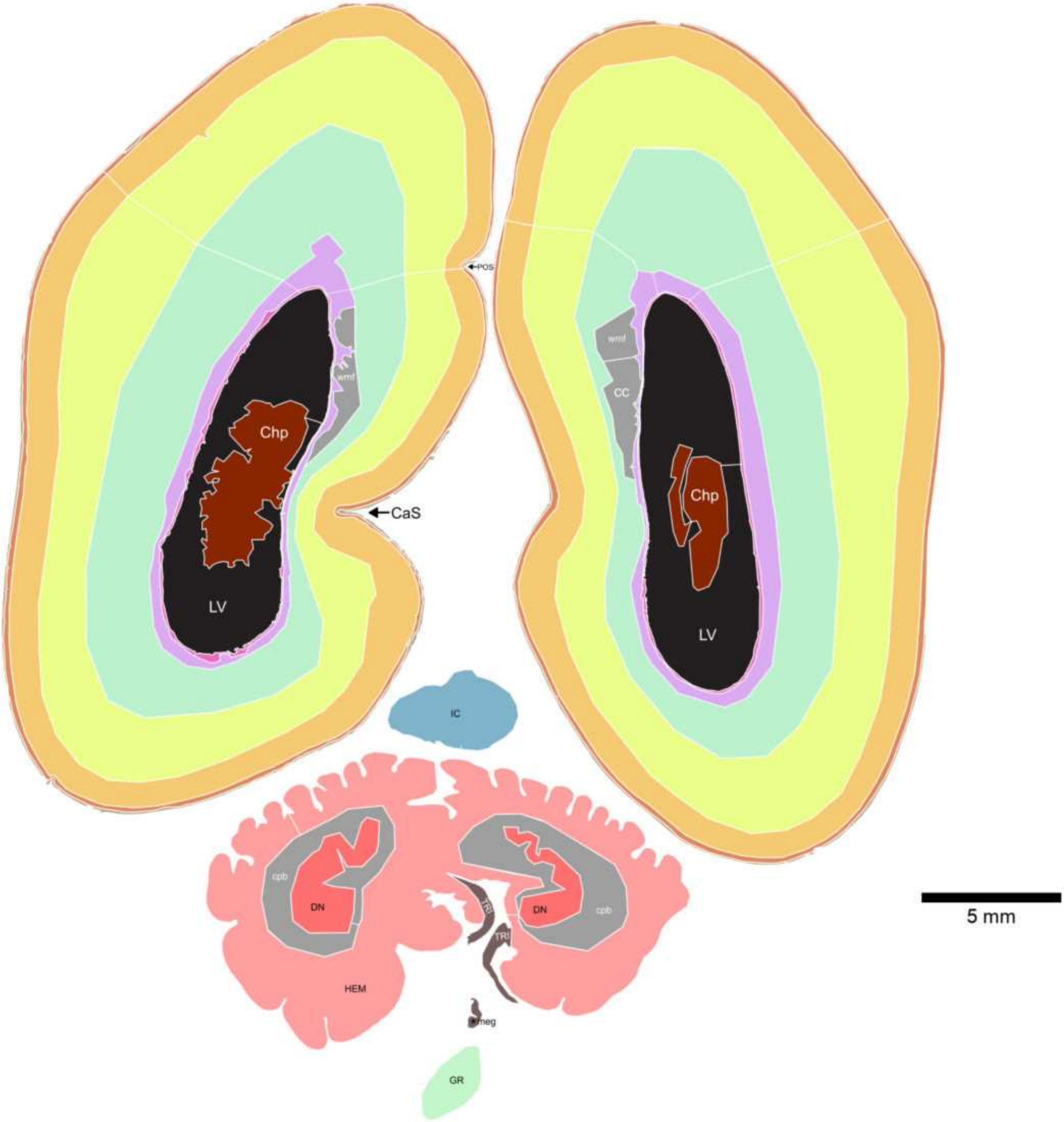

Chp: Choroid plexus	HEM: Cerebellar hemispheres	TRI: Germinal trigone	meg: Medullary glioepithelium/ependyma
DN: Dentate nucleus	IC: Inferior colliculus	cc: Corpus callosum	wmf: White matter fibers
GR: Gracile nucleus	LV: Lateral ventricle	cpb: Cerebellar peduncles	→ CaS: Calcarine sulcus
			→ POS: Parieto-occipital sulcus

Age: 22 GW

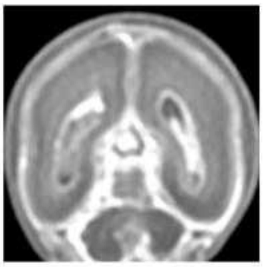

A-P Level: -12.18 mm

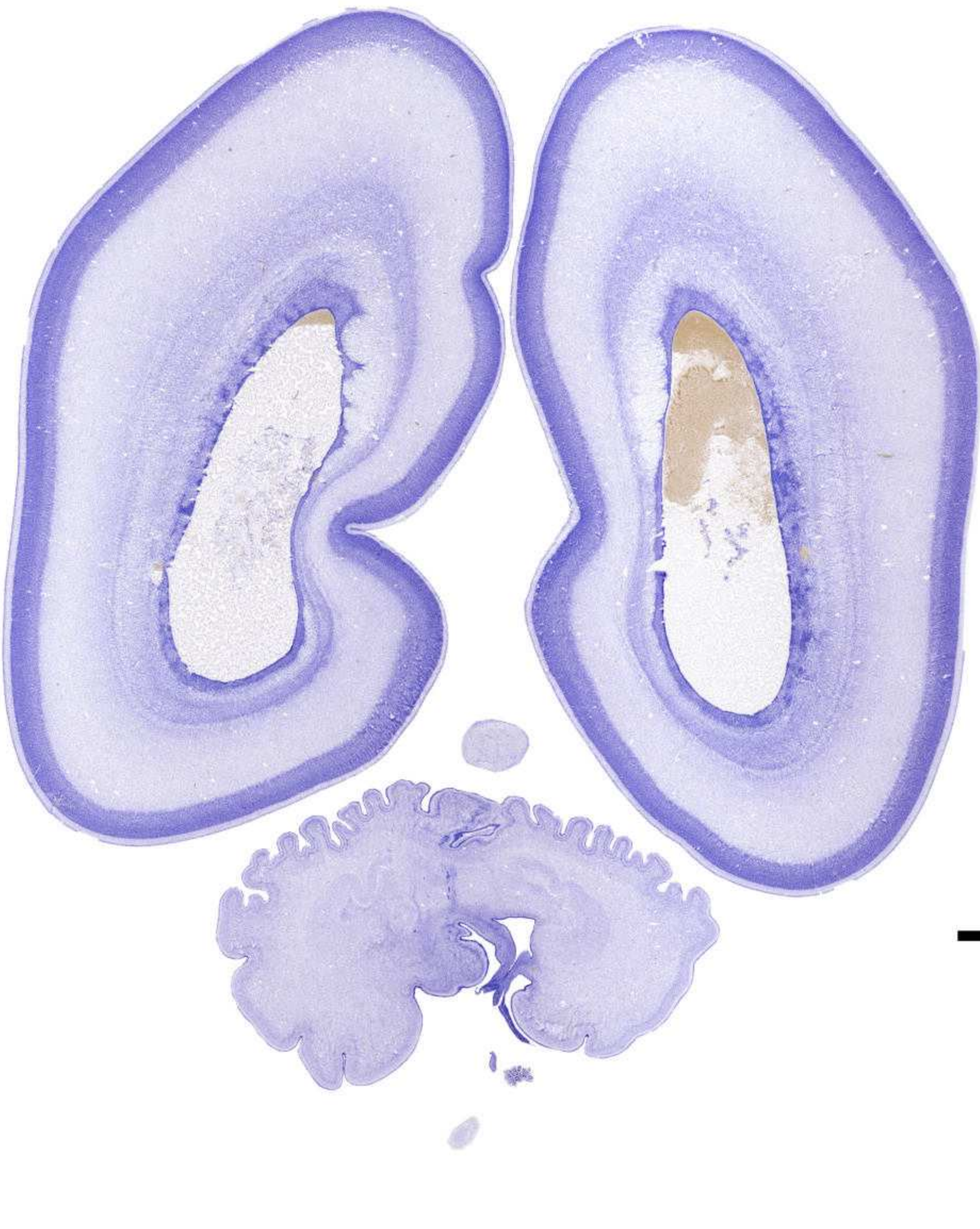

5 mm

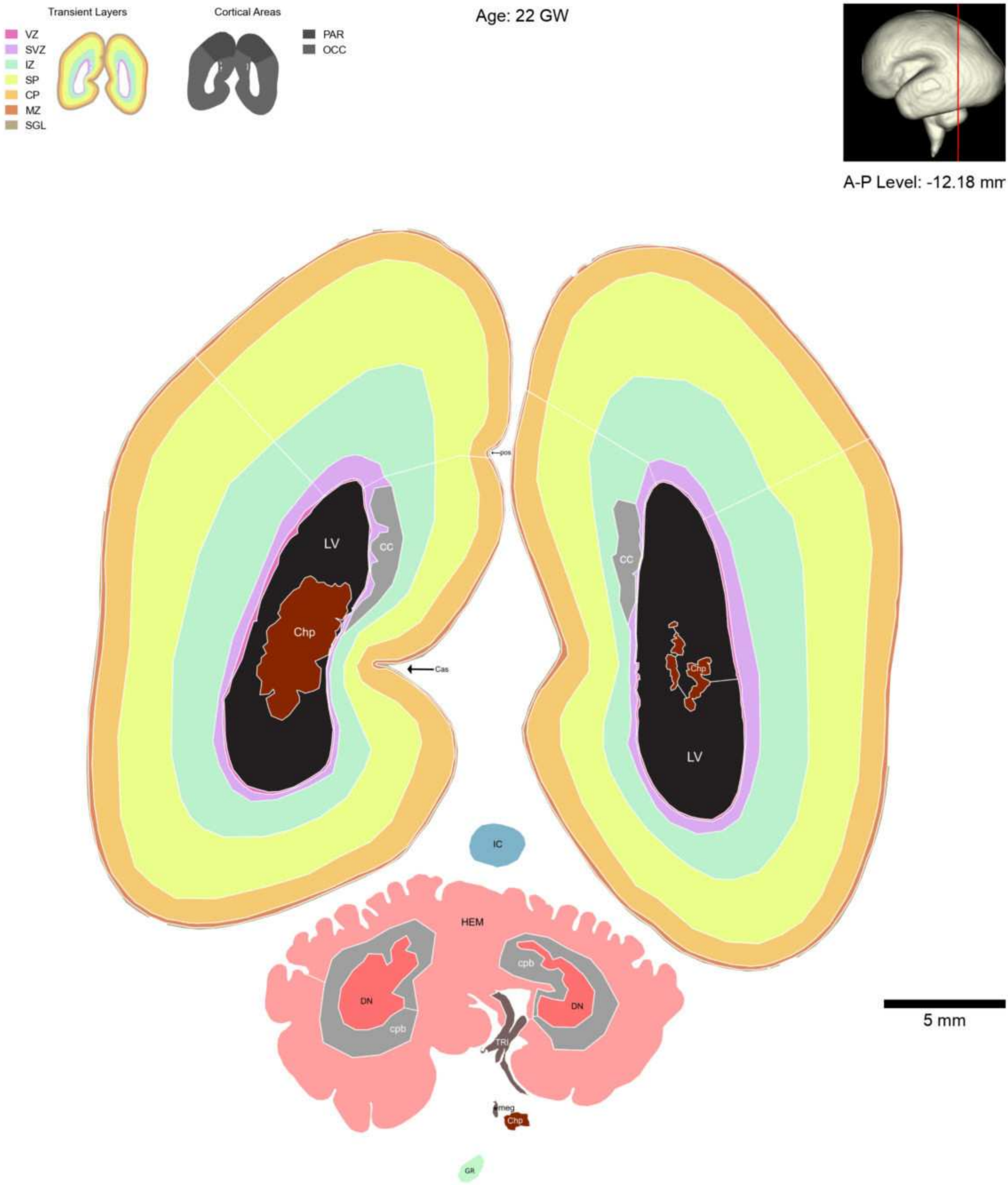

Chp: Choroid plexus
DN: Dentate nucleus
GR: Gracile nucleus
HEM: Cerebellar hemispheres
IC: Inferior colliculus
LV: Lateral ventricle
TRI: Germinal trigone
cc: Corpus callosum
cpb: Cerebellar peduncles
meg: Medullary glioepithelium/ependyma
→ CaS: Calcarine sulcus
→ POS : Parieto-occipital sulcus

Age: 22 GW

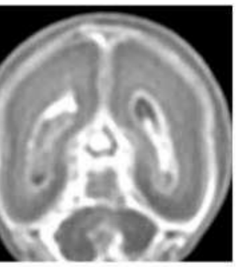

A-P Level: -12.54 mm

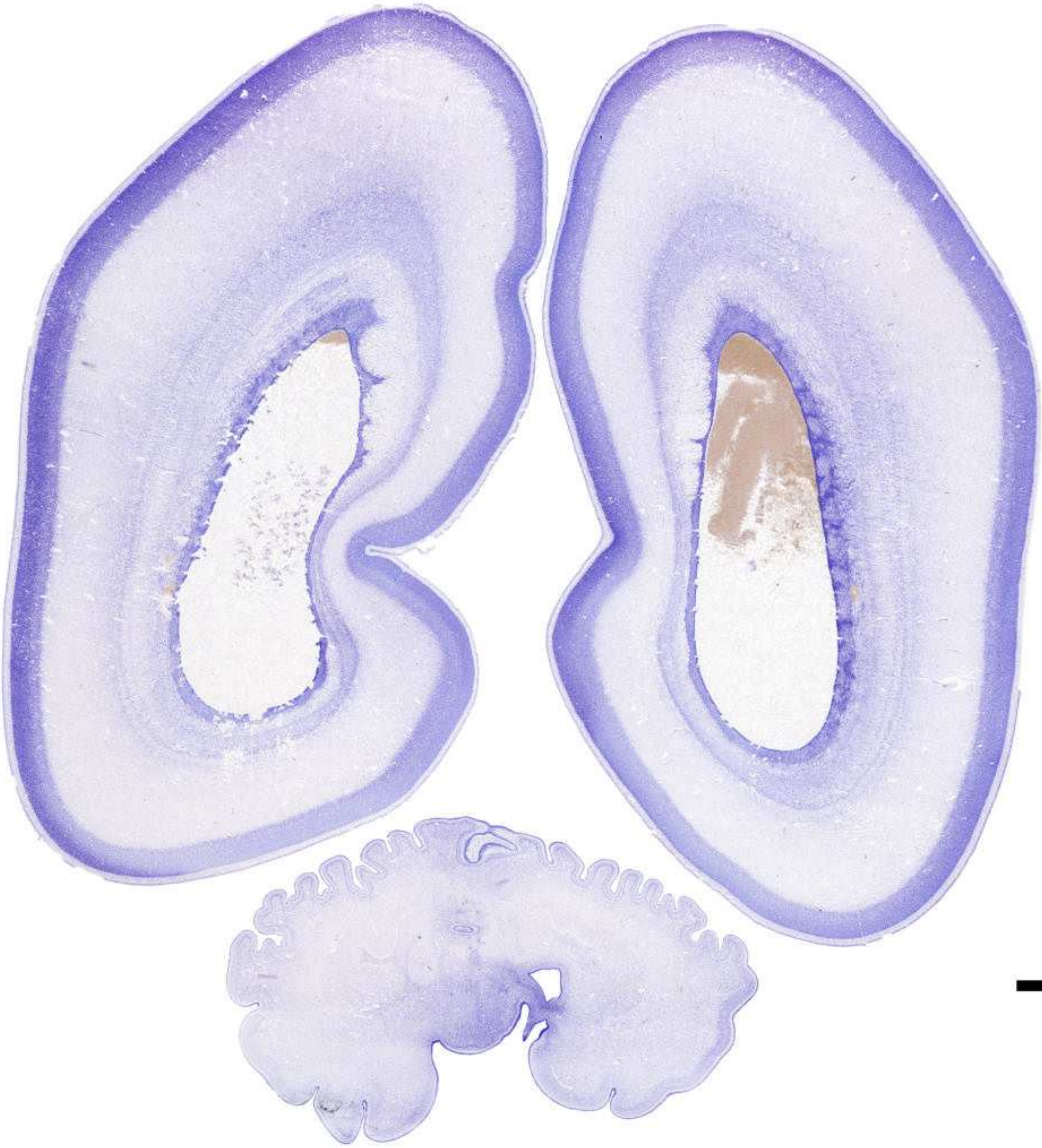

5 mm

Transient Layers

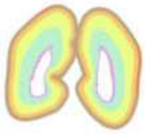

- VZ
- SVZ
- IZ
- SP
- CP
- MZ
- SGL

Cortical Areas

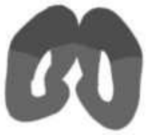

- PAR
- OCC

Age: 22 GW

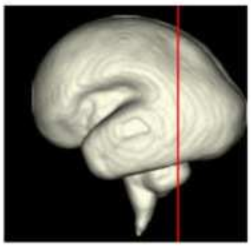

A-P Level: -12.54 mm

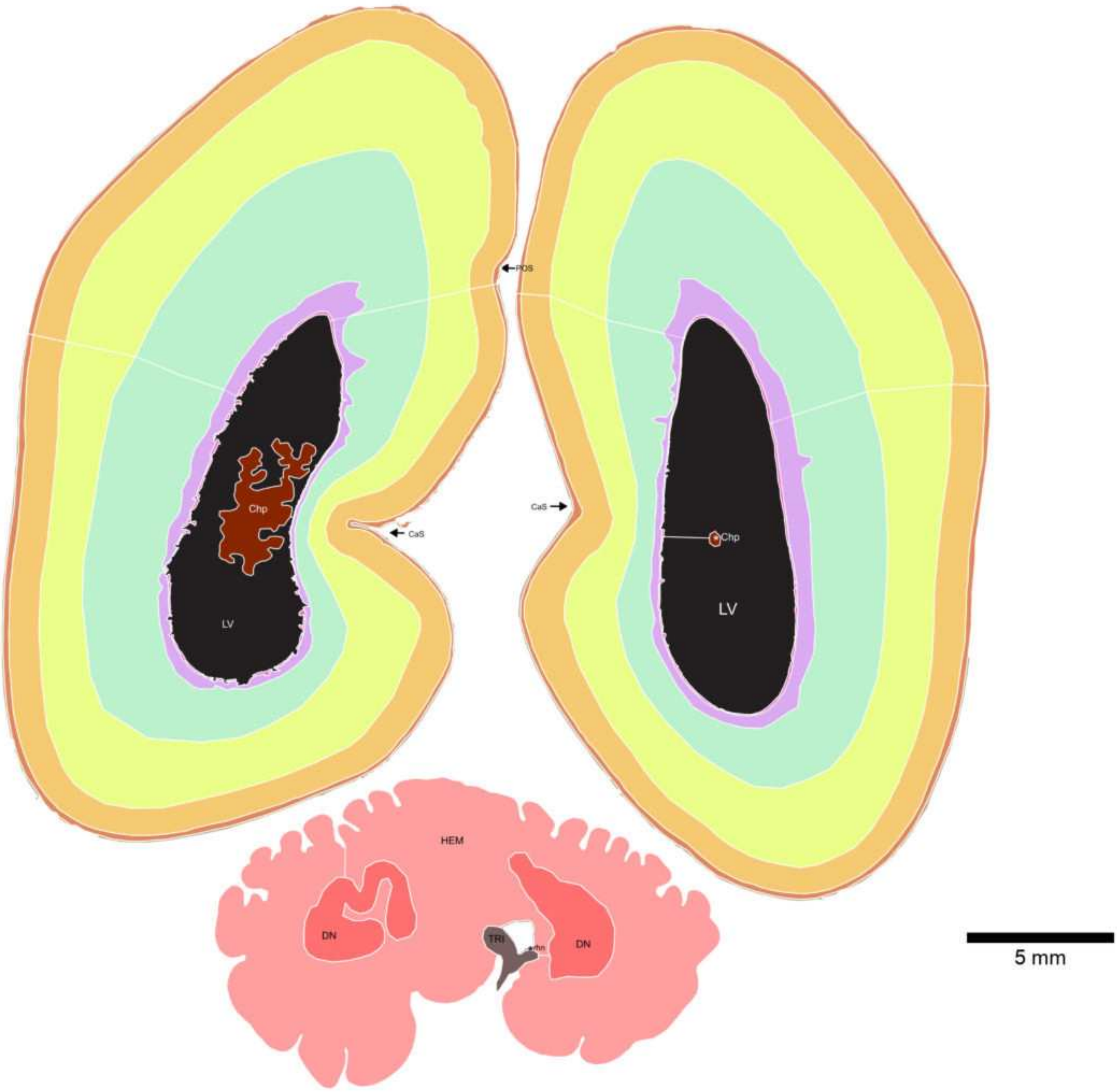

- Chp: Choroid plexus
- DN: Dentate nucleus
- HEM: Cerebellar hemispheres
- LV: Lateral ventricle
- TRI: Germinal trigone
- rhn: Rhombencephalic neuroepithelium
- CaS: Calcarine sulcus
- POS: Parieto-occipital sulcus

Age: 22 GW

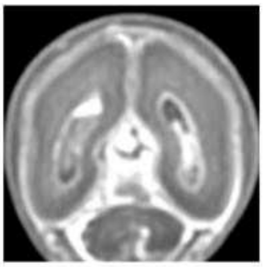

A-P Level: -13.2 mm

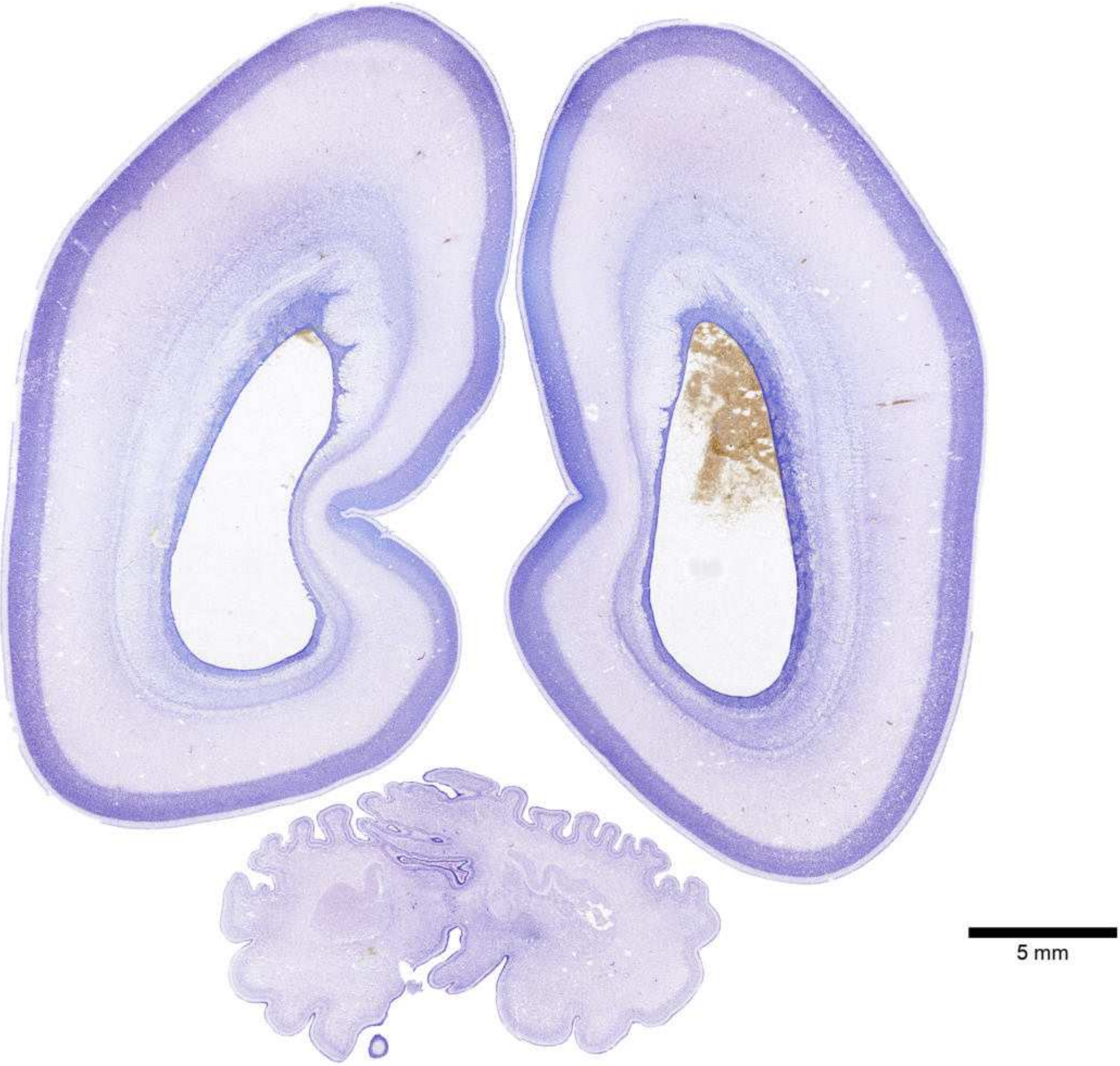

Age: 22 GW

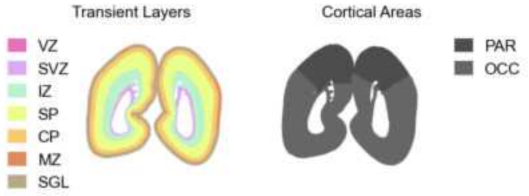

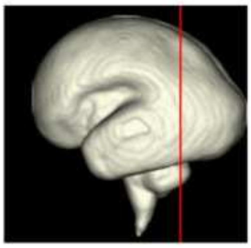

A-P Level: -13.2 mm

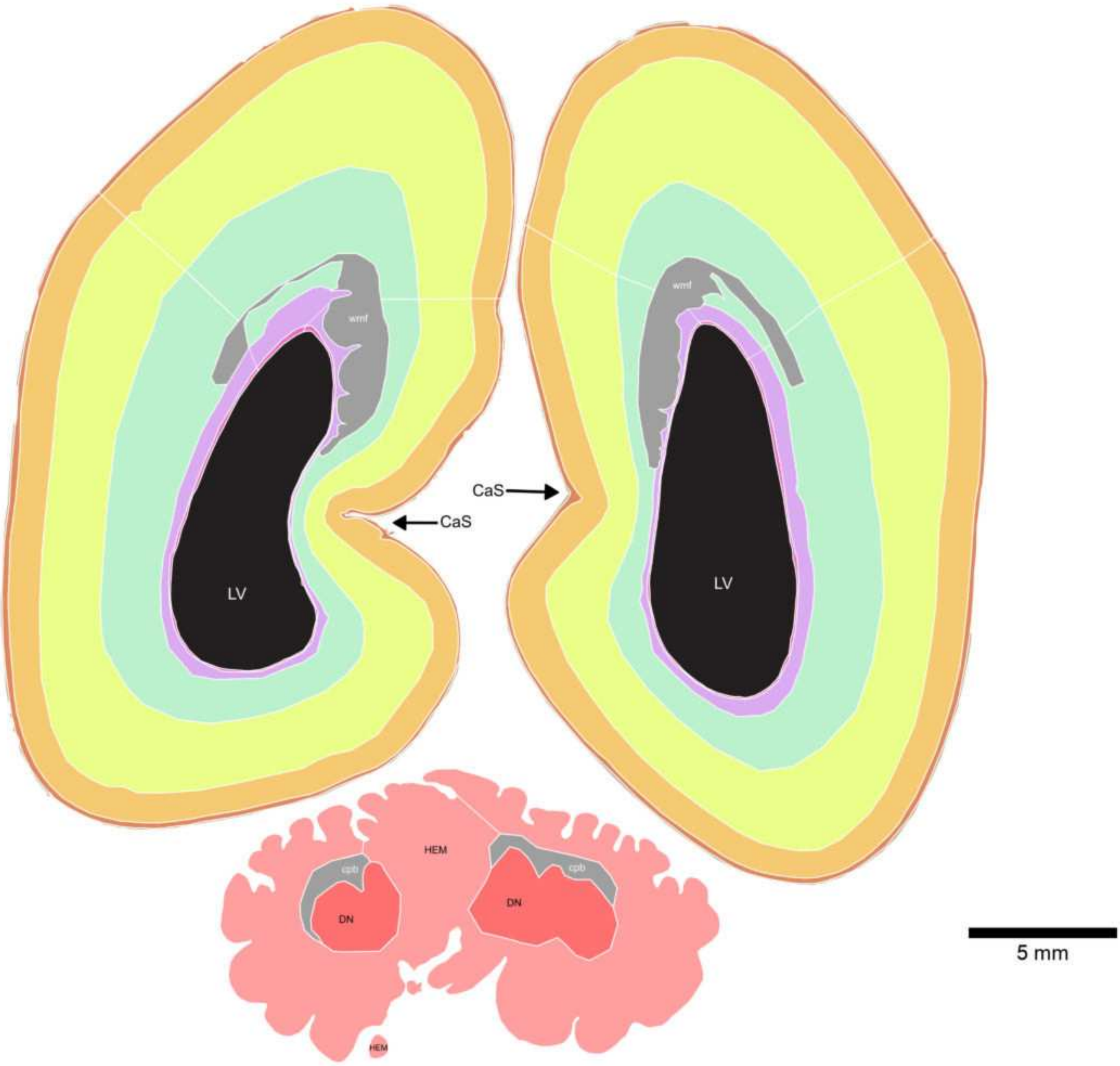

DN: Dentate nucleus
HEM: Cerebellar hemispheres
LV: Lateral ventricle
cpb: Cerebellar peduncles
wmf: White matter fibers
→ CaS: Calcarine sulcus

Age: 22 GW

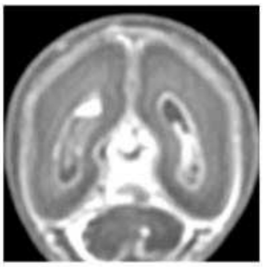

A-P Level: -13.56 mm

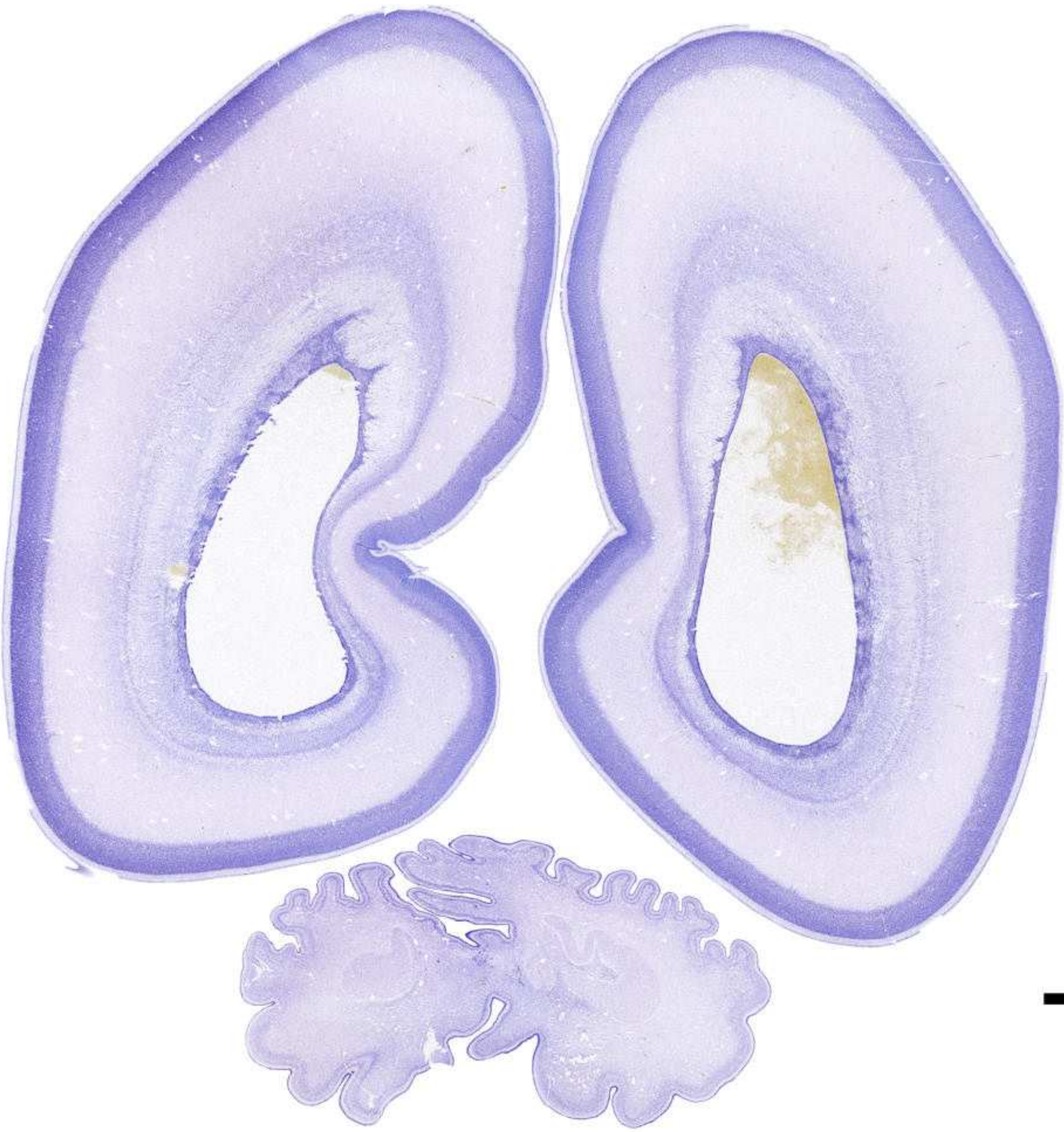

5 mm

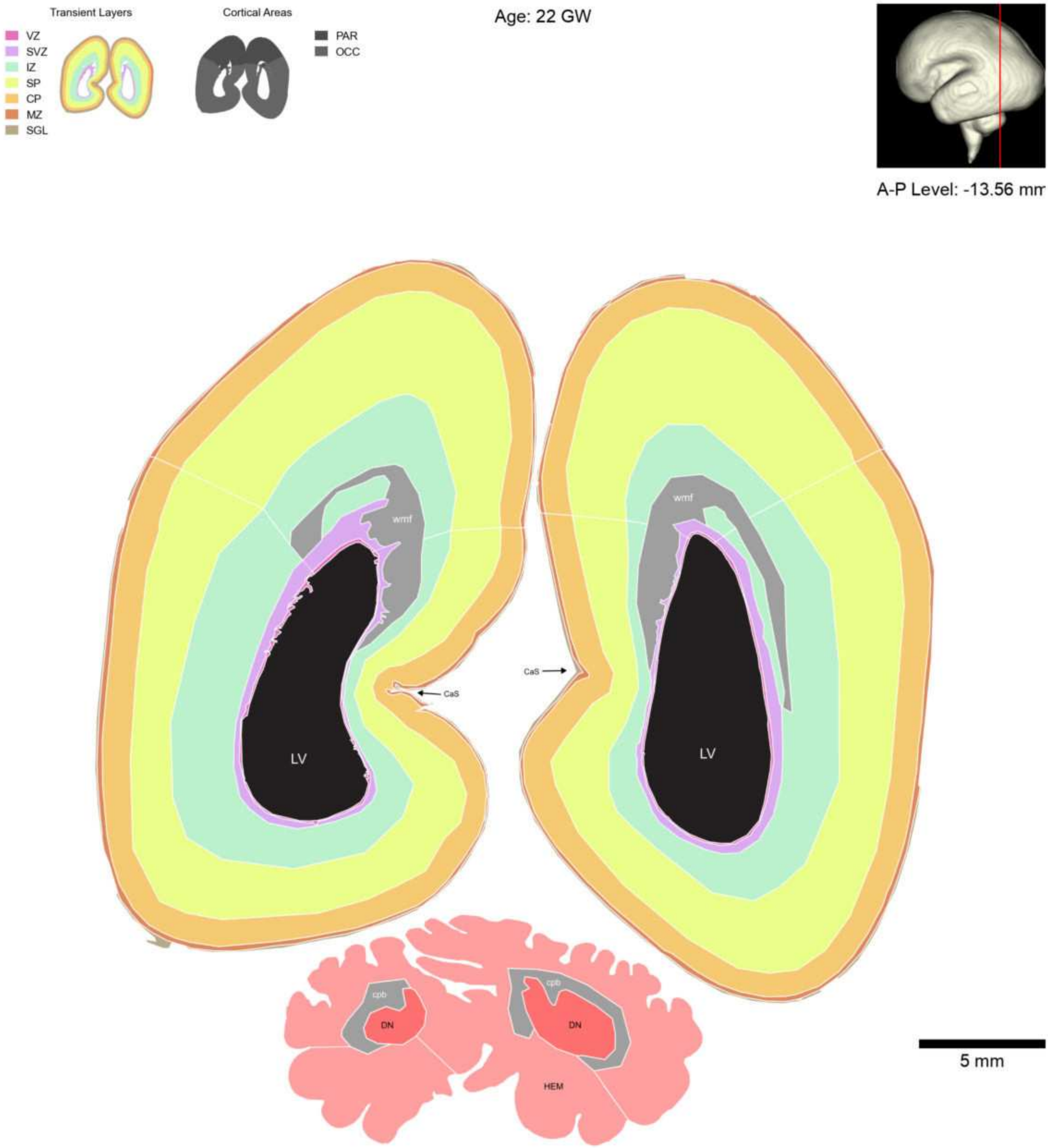

DN: Dentate nucleus
HEM: Cerebellar hemispheres
LV: Lateral ventricle
→ CaS: Calcarine sulcus
cpb: Cerebellar peduncles
wmf: White matter fibers

Age: 22 GW

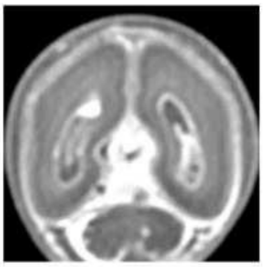

A-P Level: -13.86 mm

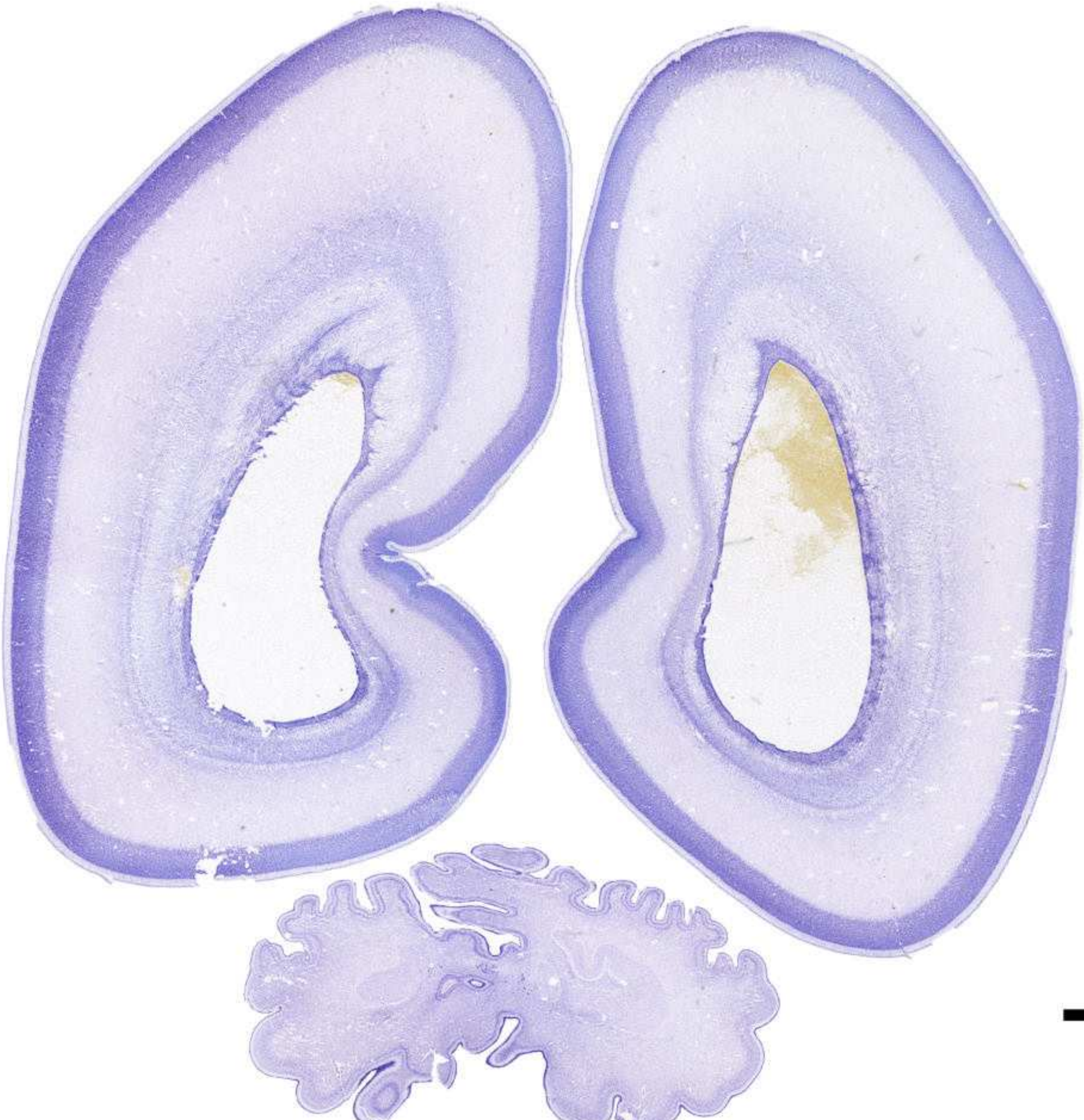

5 mm

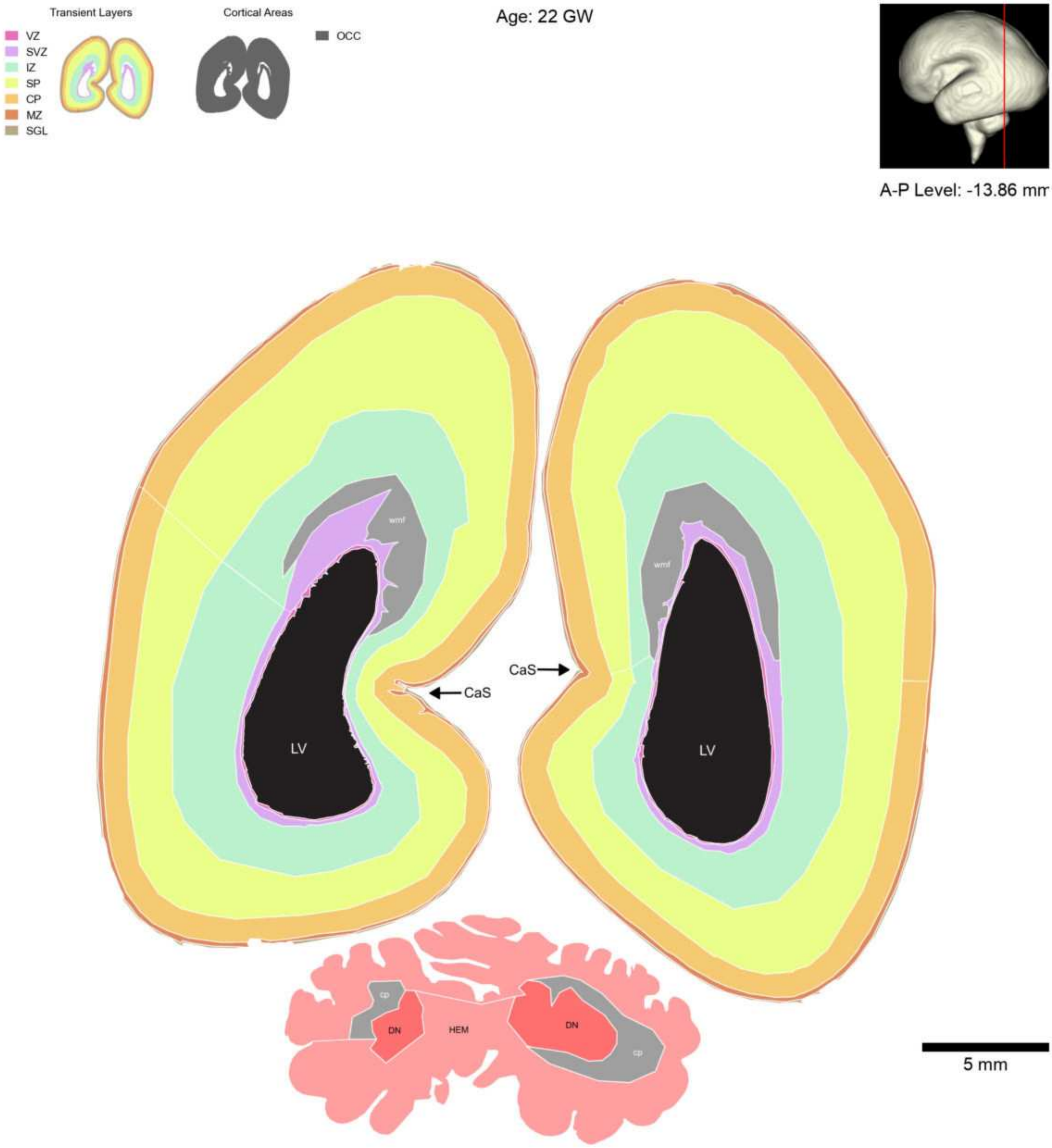

DN: Dentate nucleus
HEM: Cerebellar hemispheres
LV: Lateral ventricle
→ CaS: Calcarine sulcus
cp: Cerebral peduncle
wmf: White matter fibers

Age: 22 GW

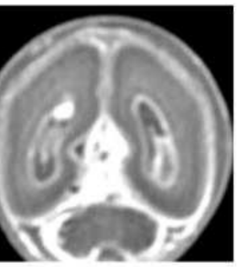

A-P Level: -14.28 mm

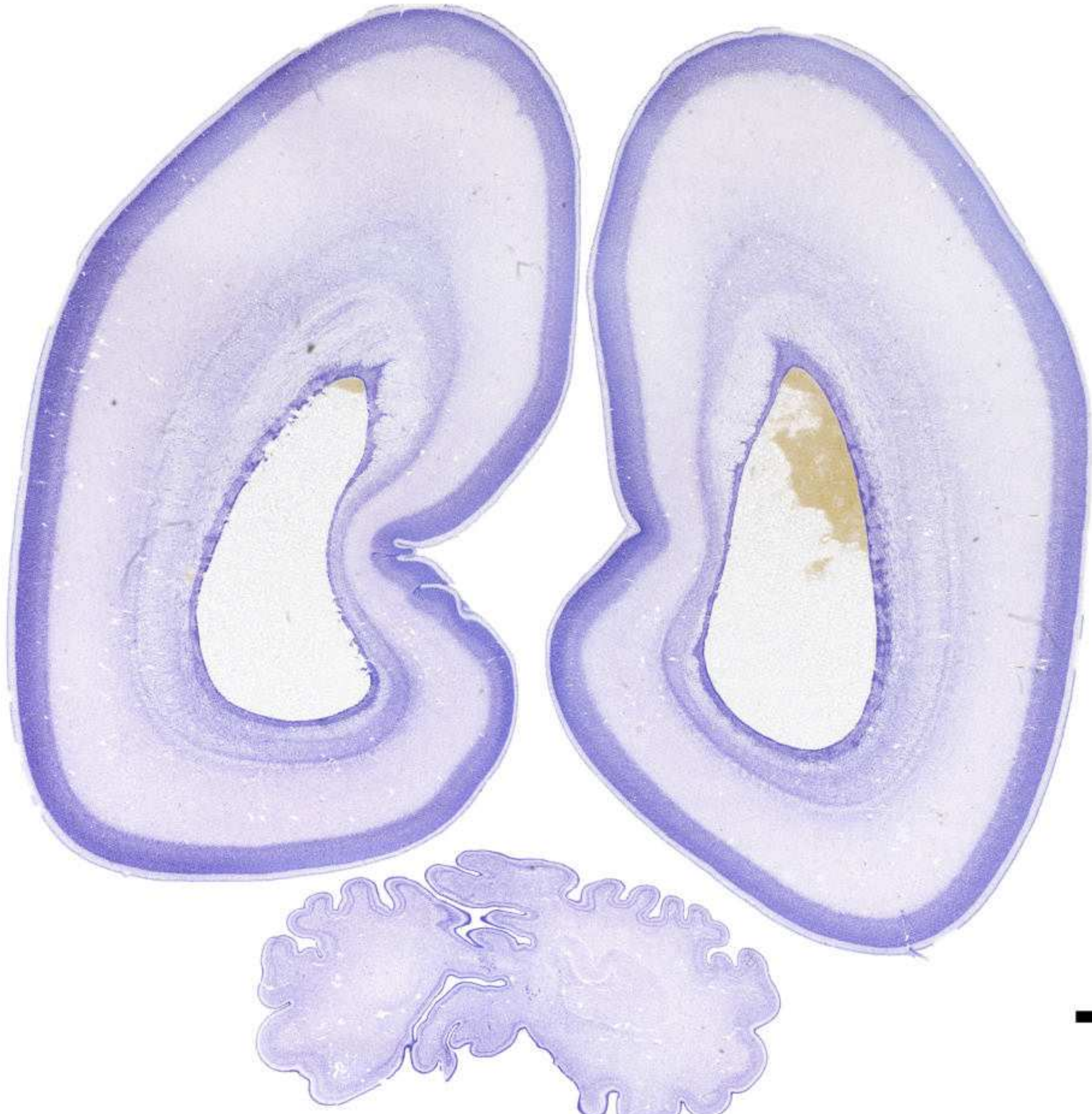

5 mm

Transient Layers

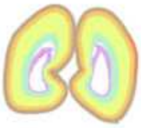

- VZ
- SVZ
- IZ
- SP
- CP
- MZ
- SGL

Cortical Areas

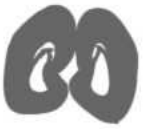

- OCC

Age: 22 GW

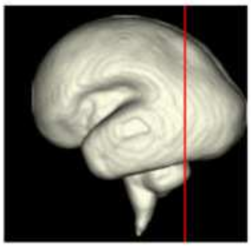

A-P Level: -14.28 mm

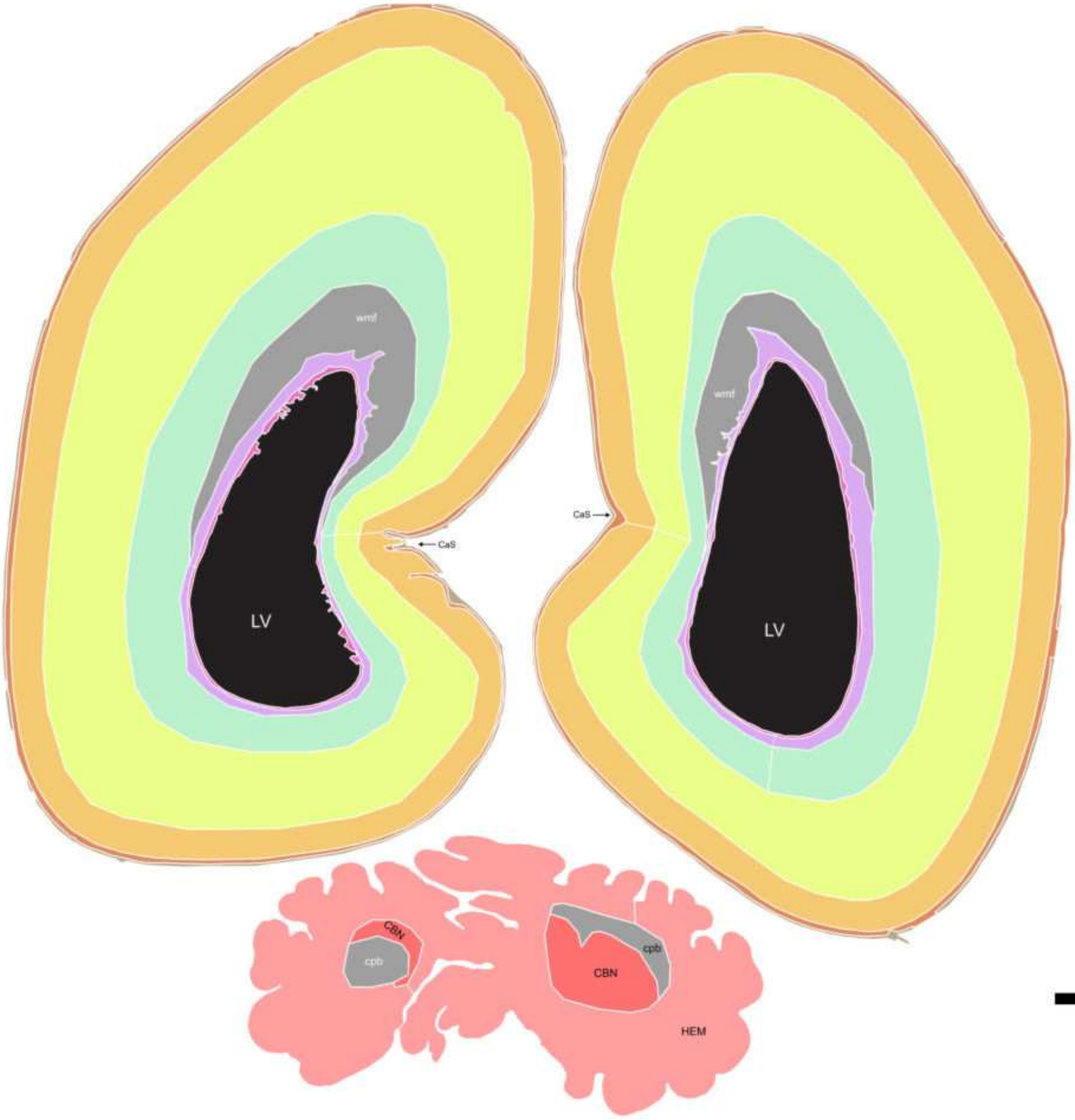

5 mm

- CBN: Cerebellar nuclei
- HEM: Cerebellar hemispheres
- LV: Lateral ventricle
- → CaS: Calcarine sulcus
- cpb: Cerebellar peduncles
- wmf: White matter fibers

Age: 22 GW

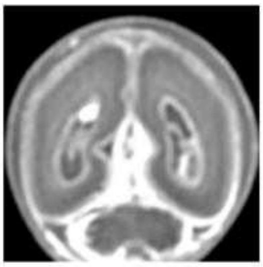

A-P Level: -14.88 mm

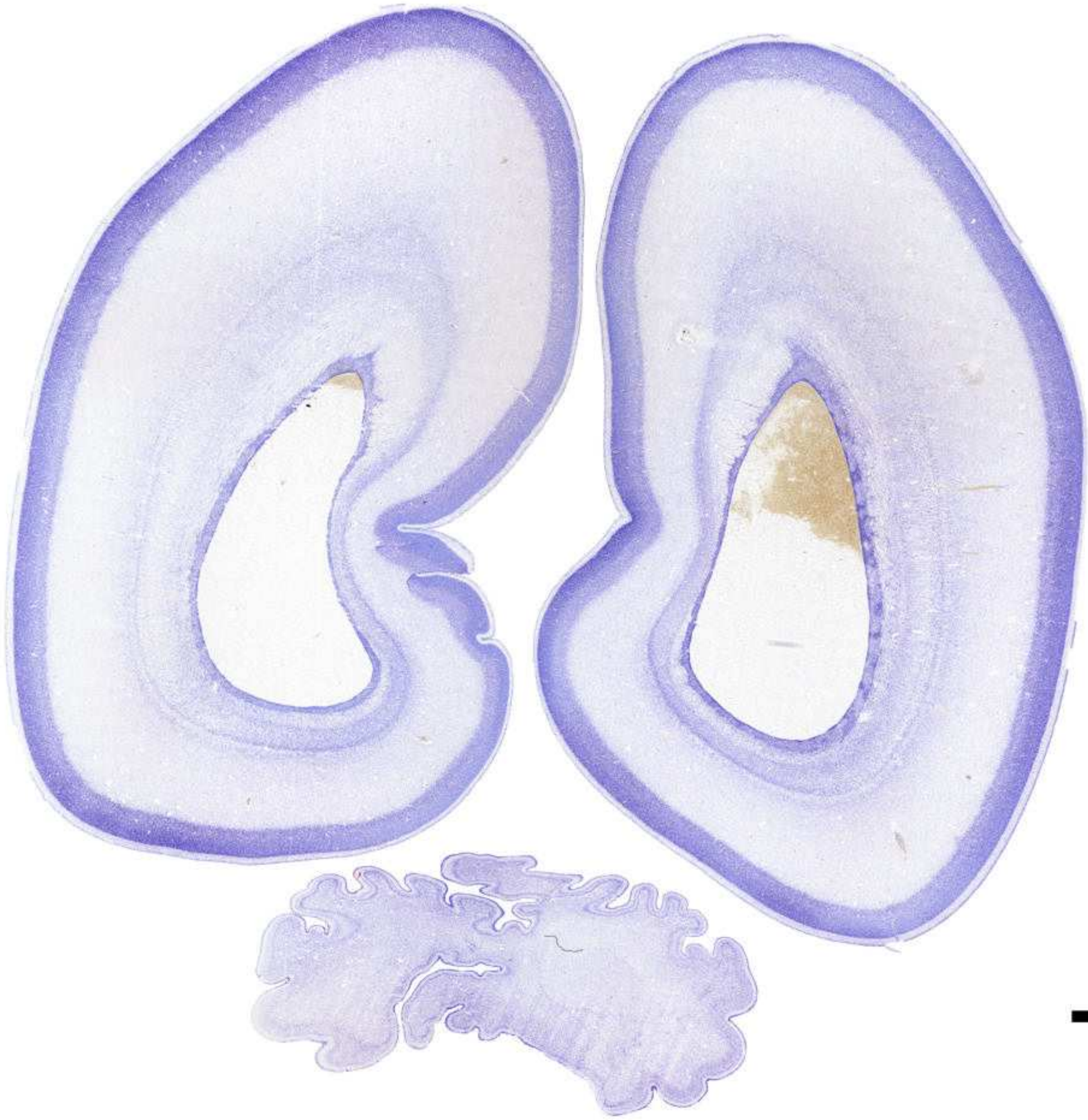

5 mm

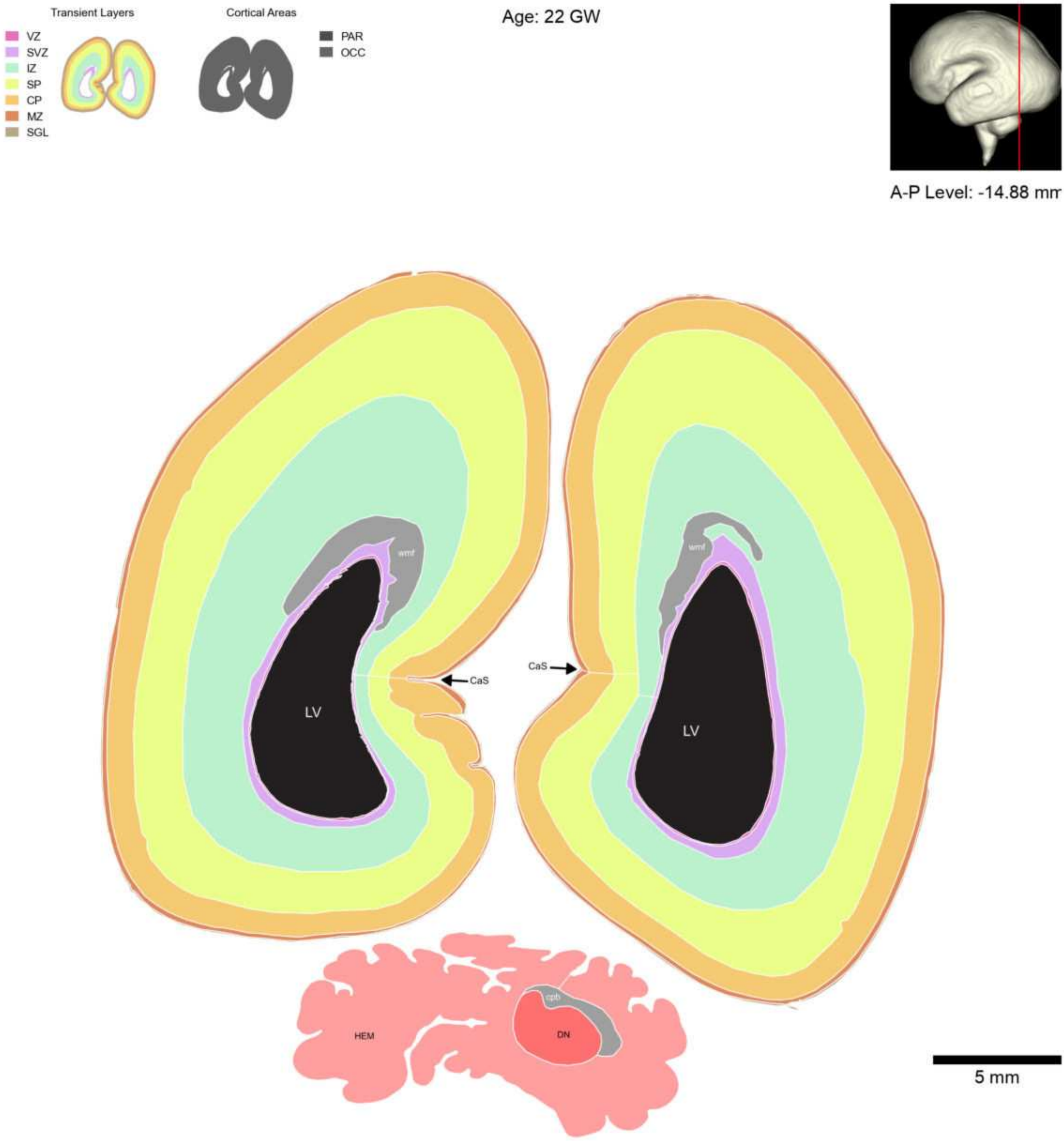

DN: Dentate nucleus
HEM: Cerebellar hemispheres
LV: Lateral ventricle
cpb: Cerebellar peduncles
wmf: White matter fibers
→ CaS: Calcarine sulcus

Age: 22 GW

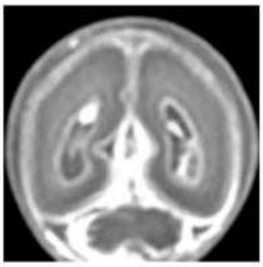

A-P Level: -15.12 mm

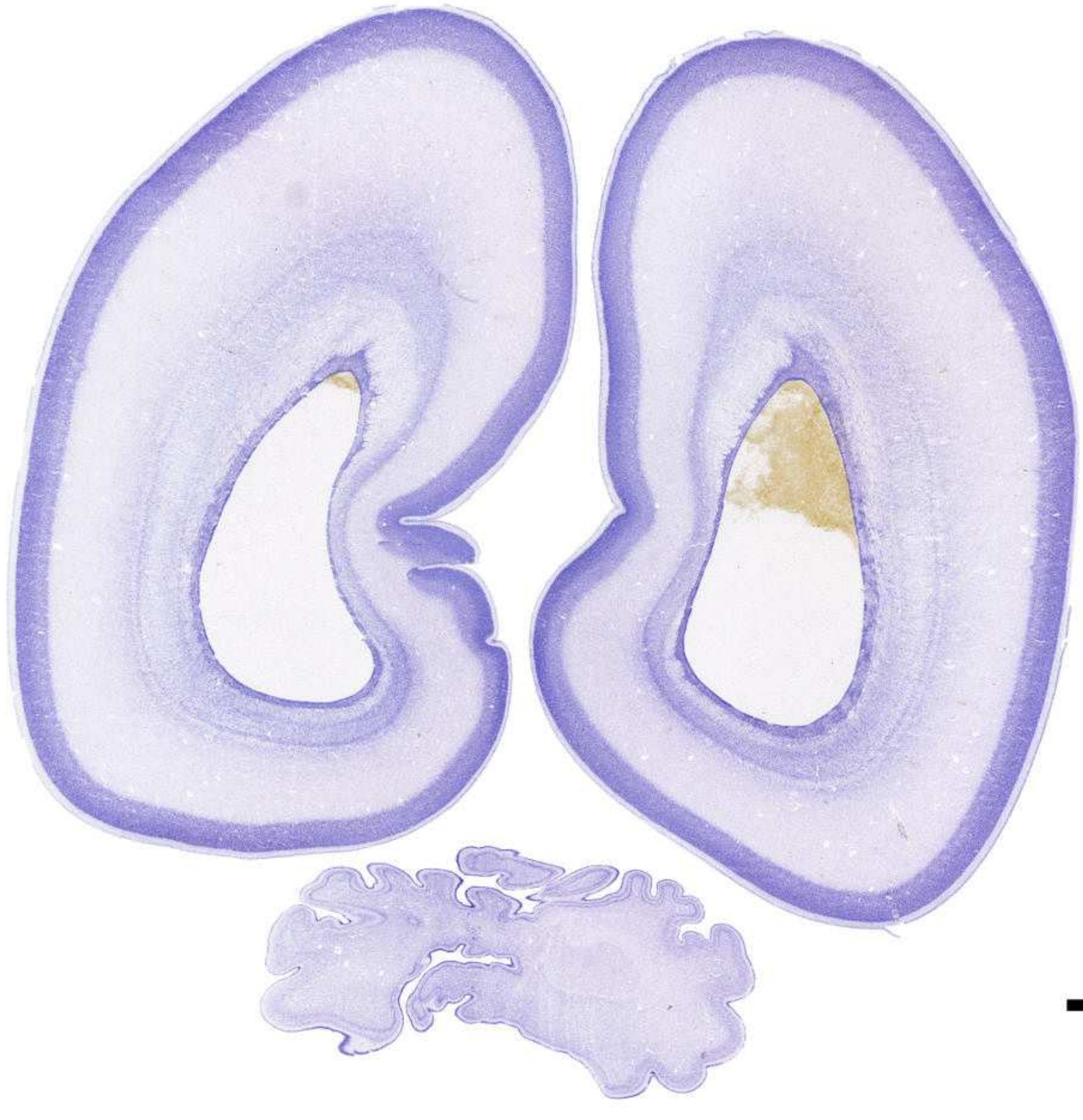

5 mm

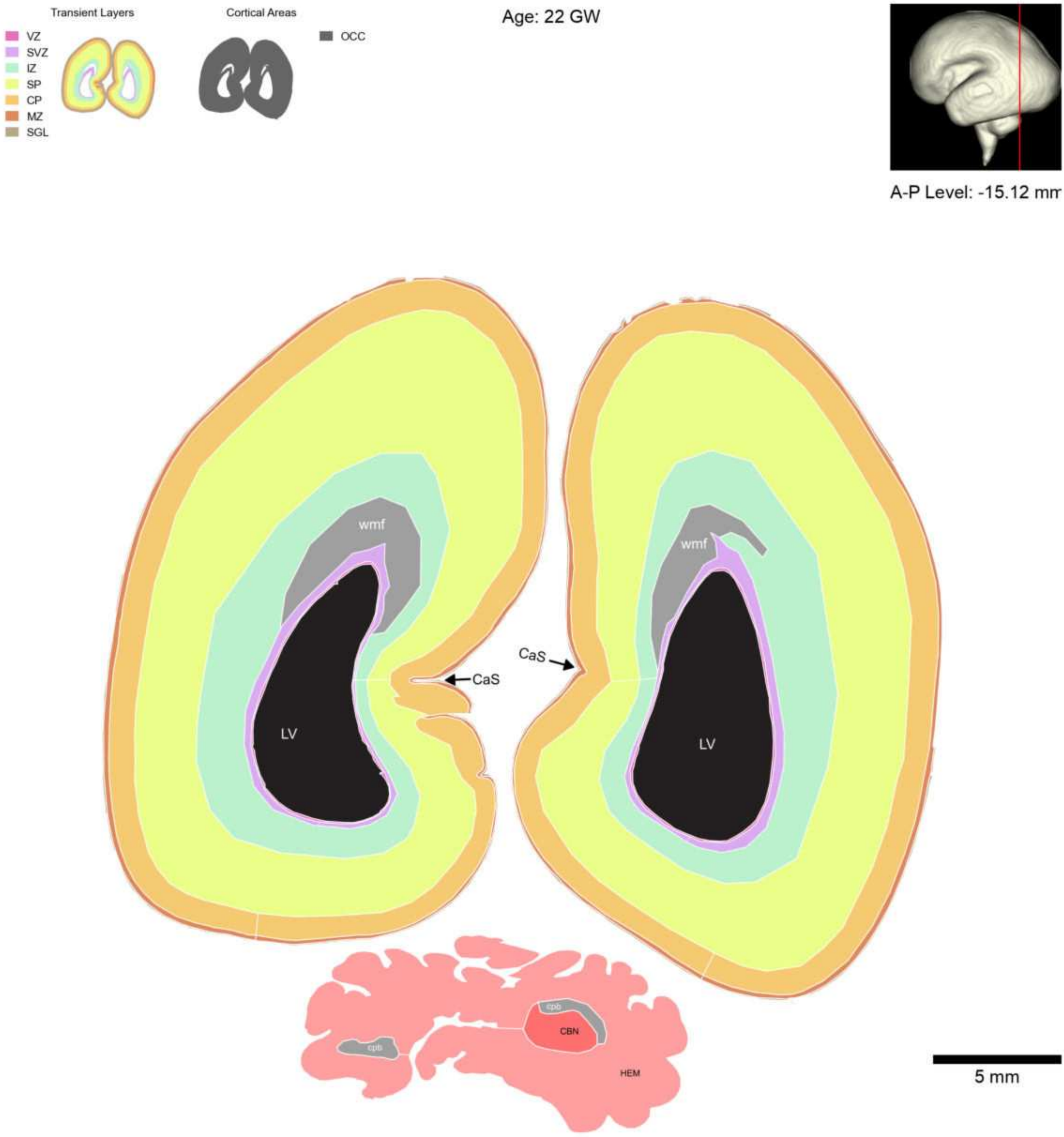

CBN: Cerebellar nuclei
HEM: Cerebellar hemispheres
LV: Lateral ventricle
cpb: Cerebellar peduncles
wmf: White matter fibers
CaS: Calcarine sulcus

Age: 22 GW

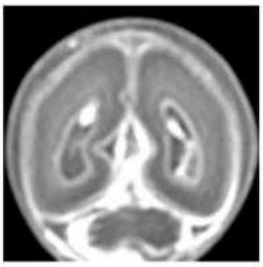

A-P Level: -15.42 mm

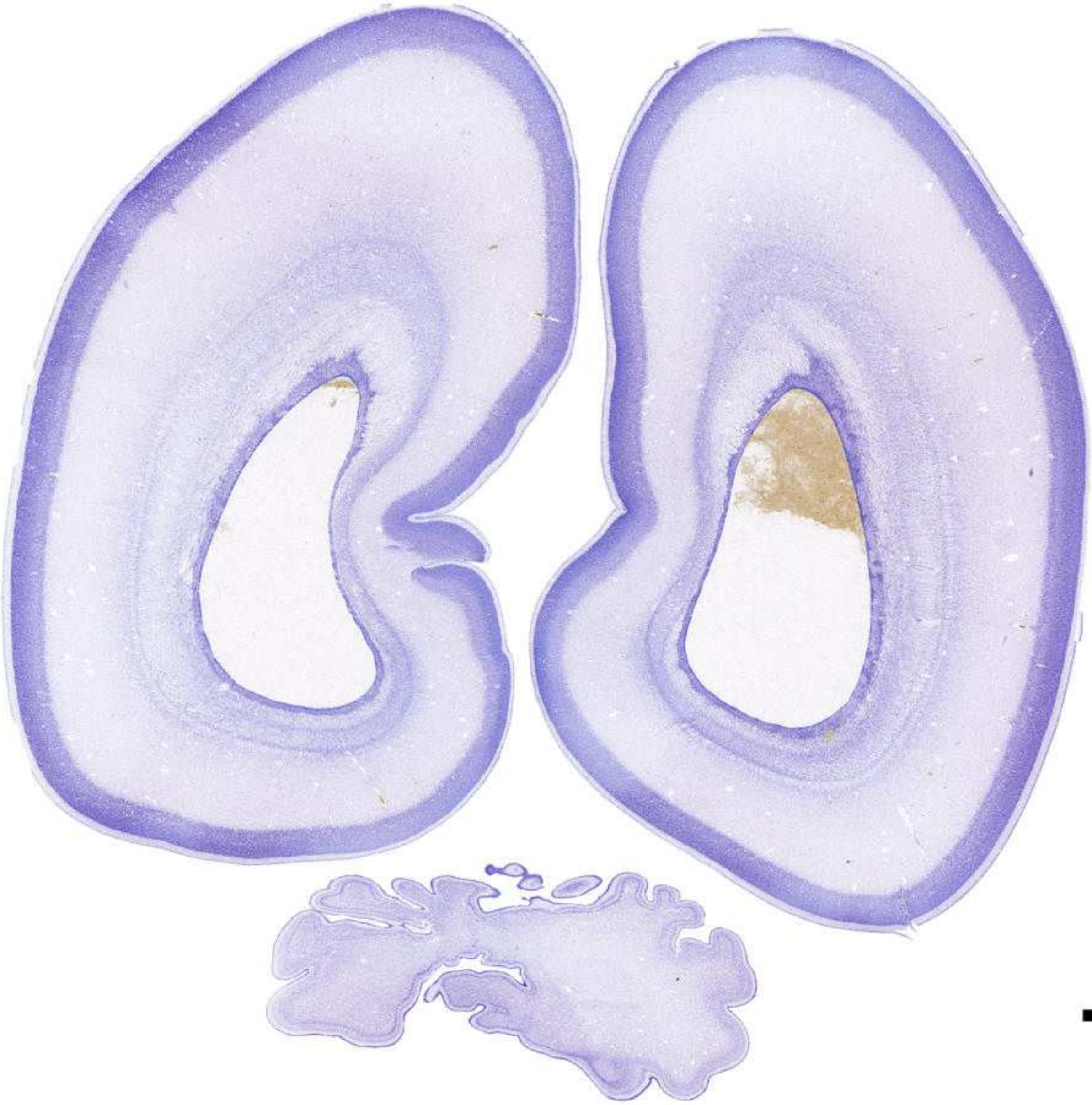

5 mm

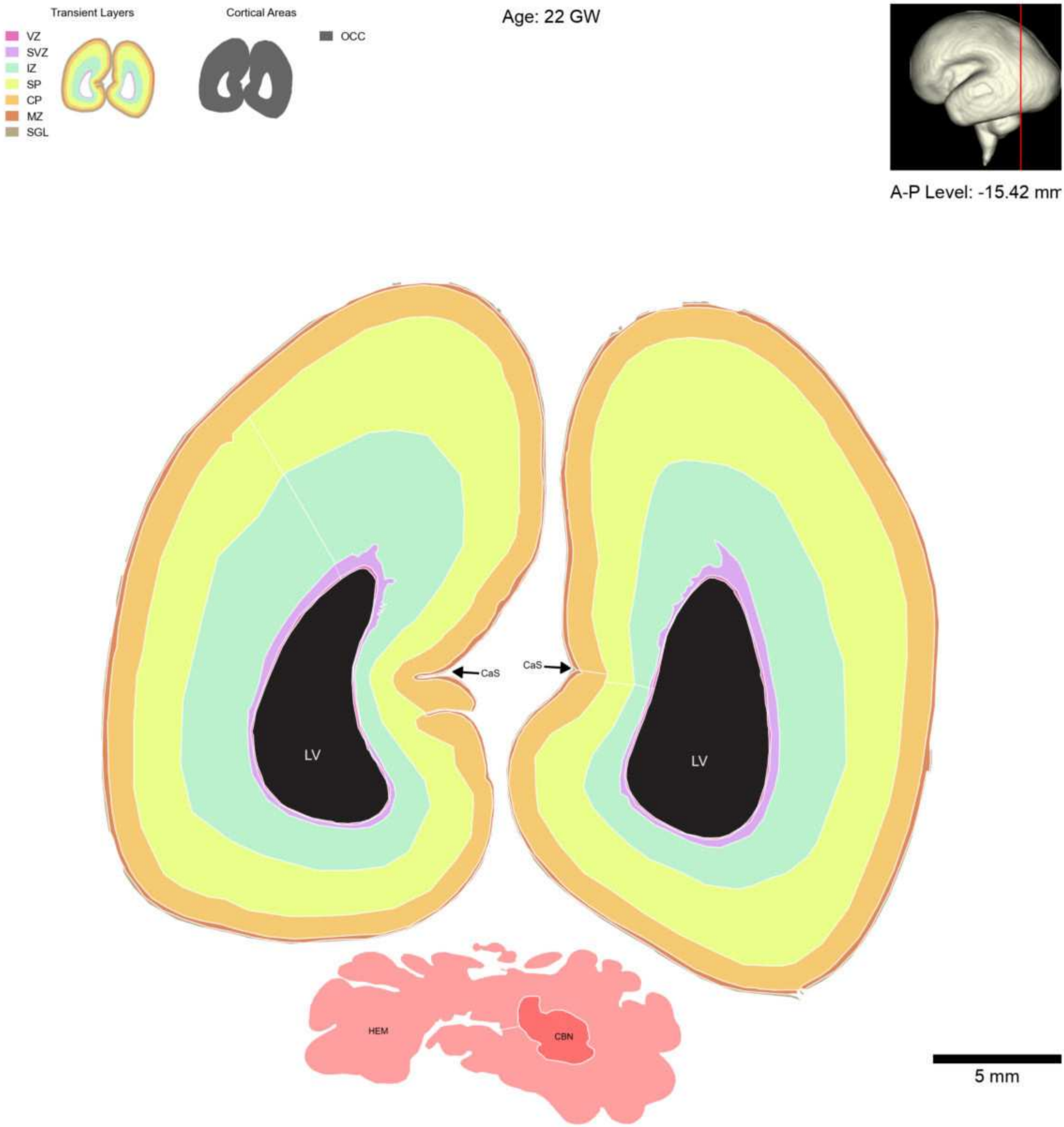

CBN: Cerebellar nuclei HEM: Cerebellar hemispheres LV: Lateral ventricle → CaS: Calcarine sulcus

Age: 22 GW

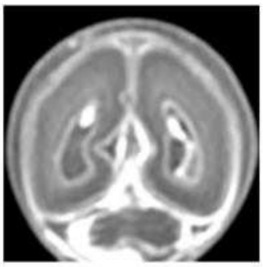

A-P Level: -15.84 mm

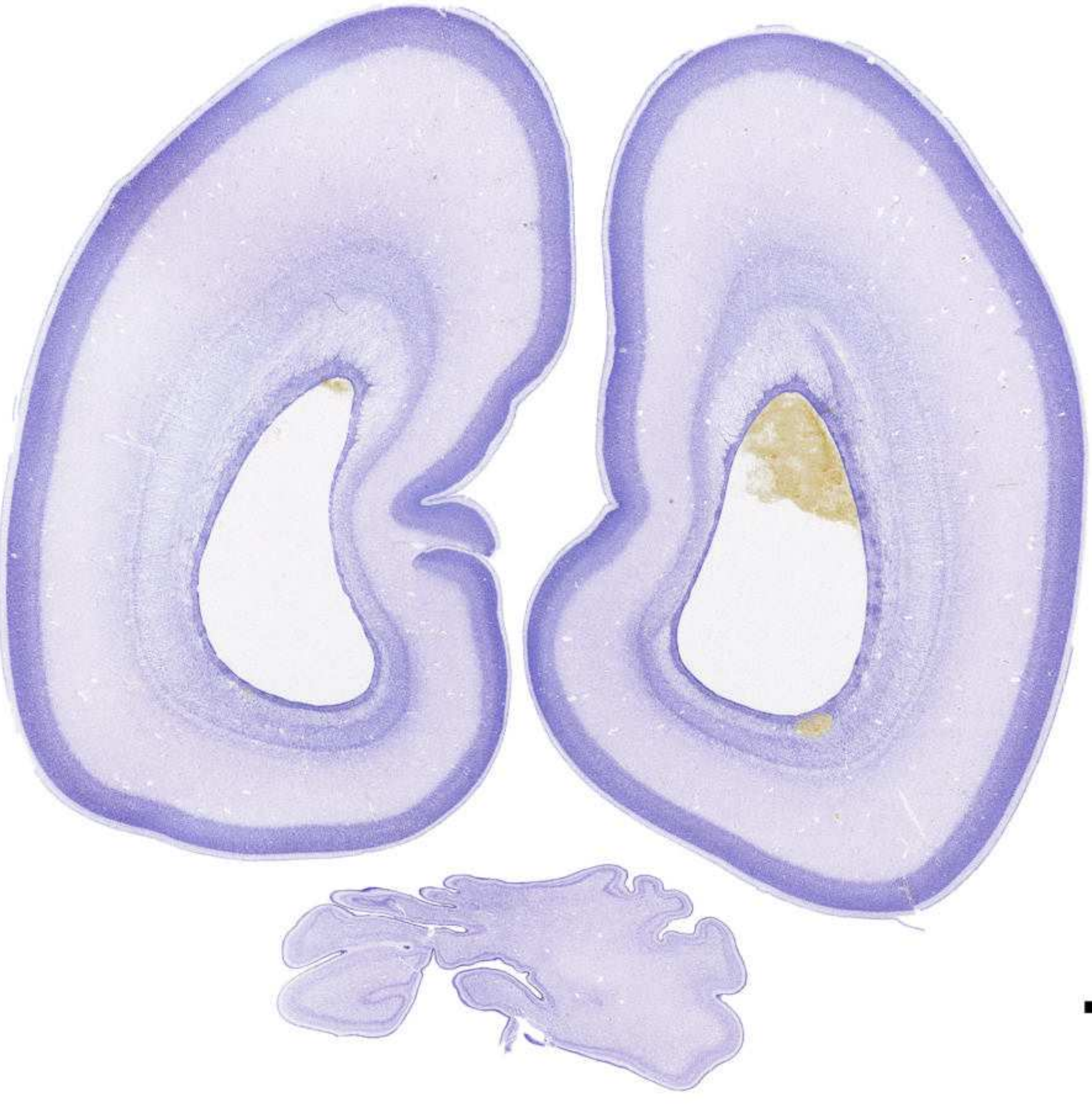

5 mm

Transient Layers

Cortical Areas

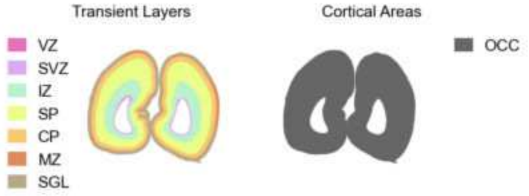

Age: 22 GW

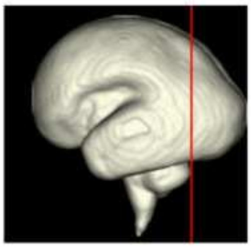

A-P Level: -15.84 mm

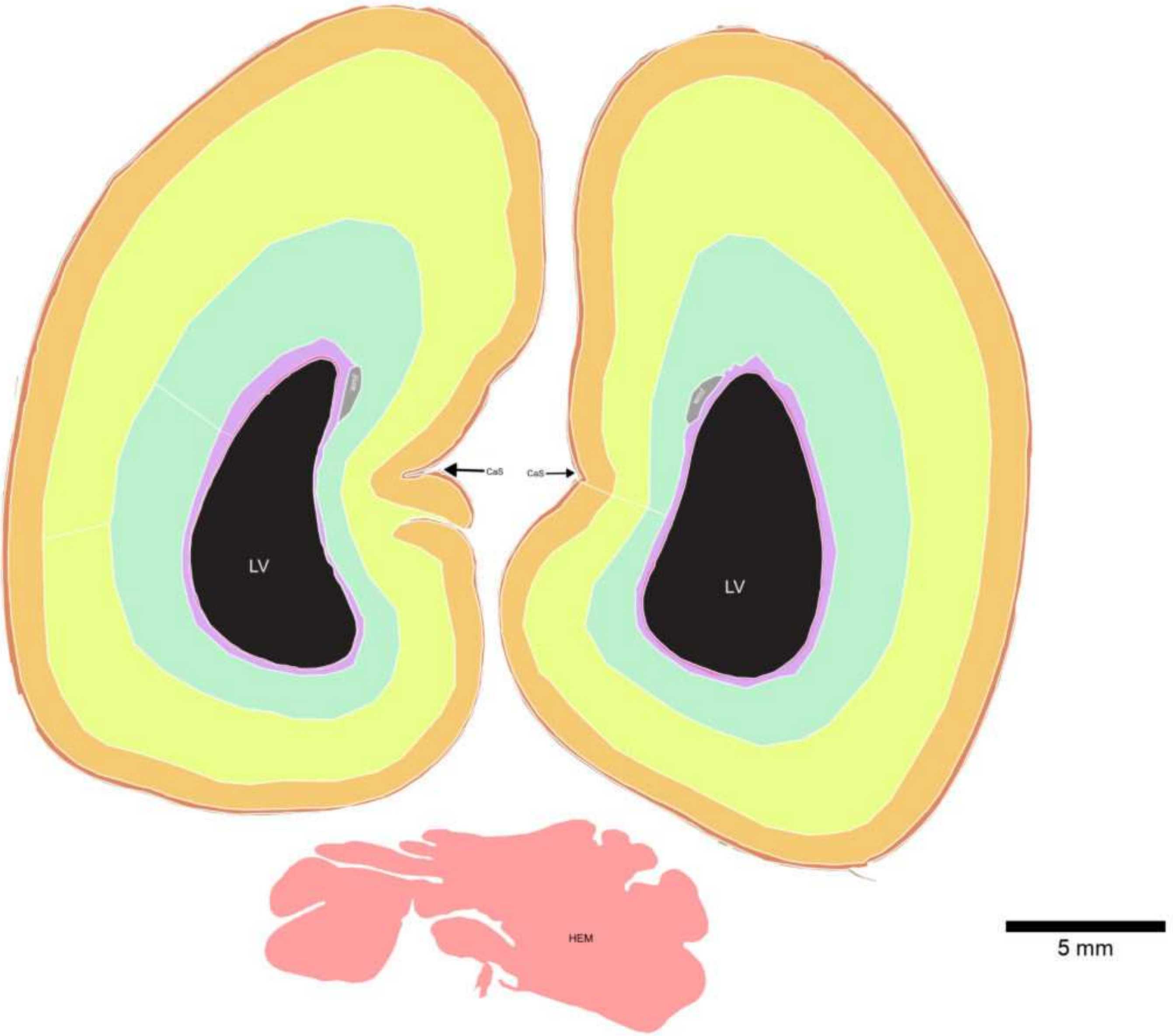

HEM: Cerebellar hemispheres LV: Lateral ventricle wmf: White matter fibers CaS: Calcarine sulcus

Age: 22 GW

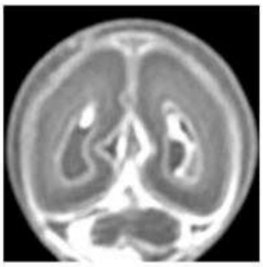

A-P Level: -16.02 mm

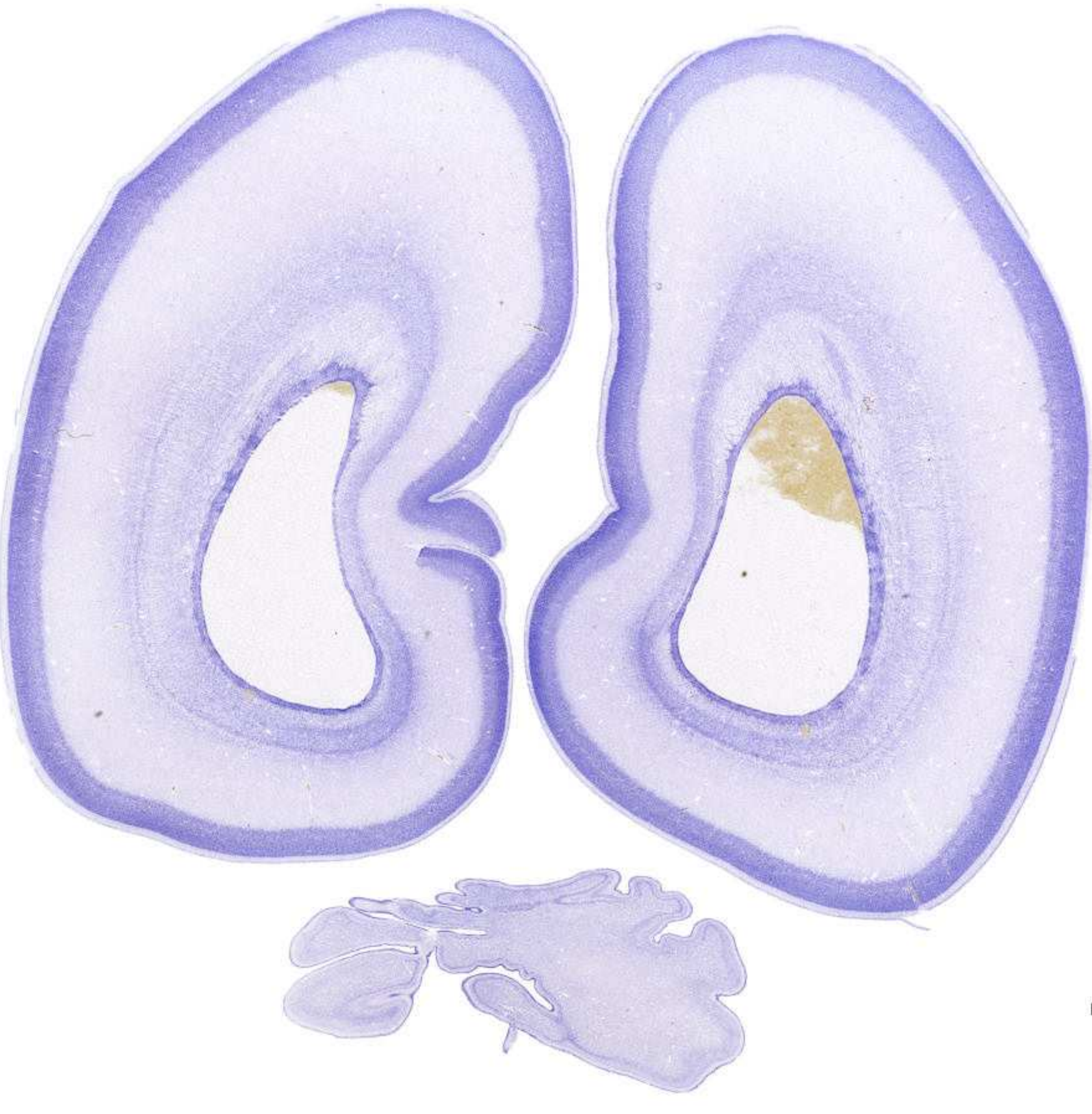

5 mm

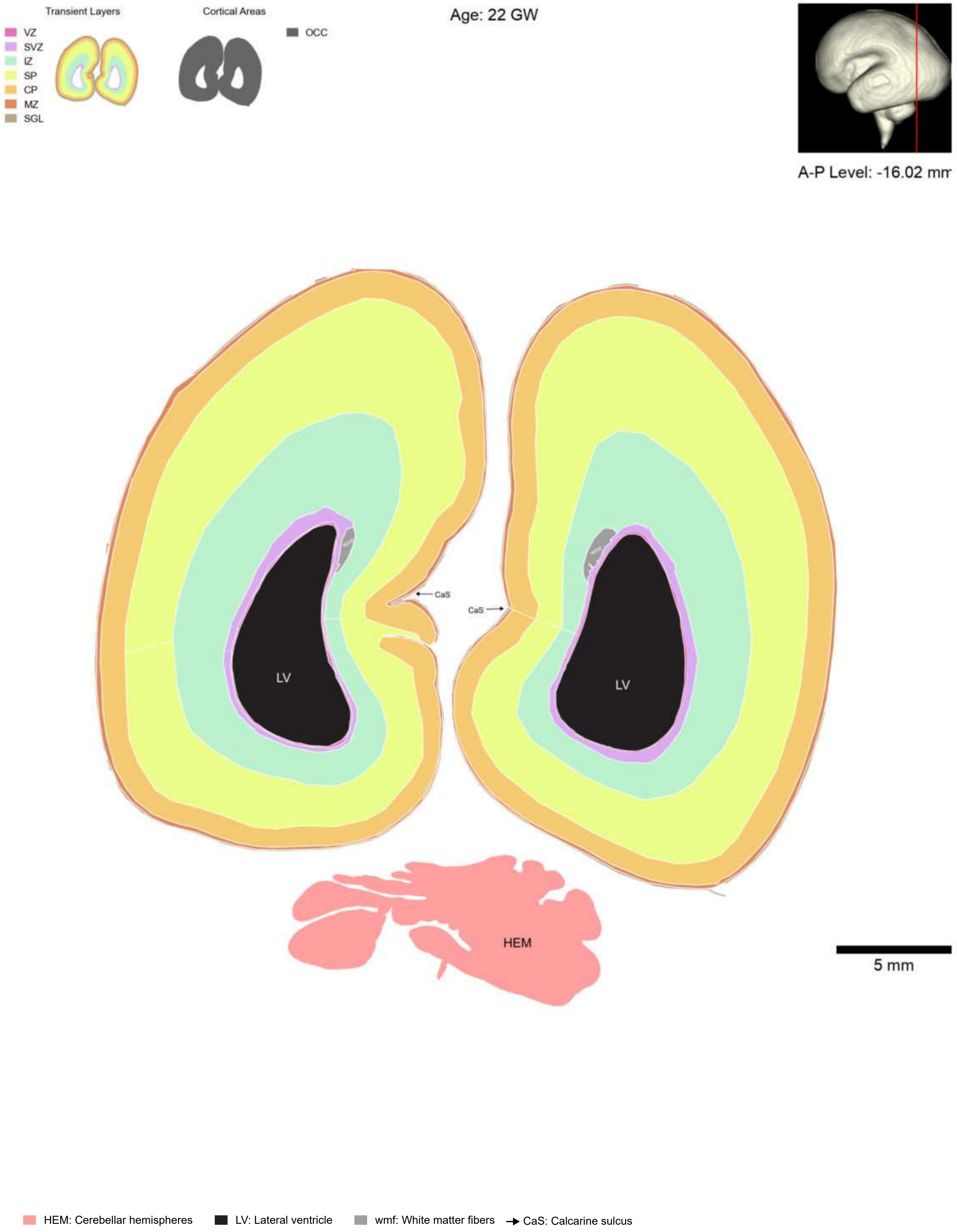
Transient Layers
VZ
SVZ
IZ
SP
CP
MZ
SGL
Cortical Areas
OCC
Age: 22 GW
A-P Level: -16.02 mm
CaS
CaS
LV
LV
HEM
5 mm
HEM: Cerebellar hemispheres
LV: Lateral ventricle
wmf: White matter fibers
CaS: Calcarine sulcus

Age: 22 GW

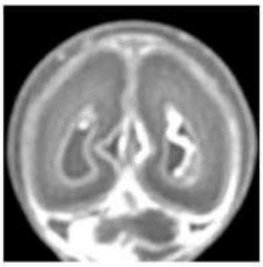

A-P Level: -16.8 mm

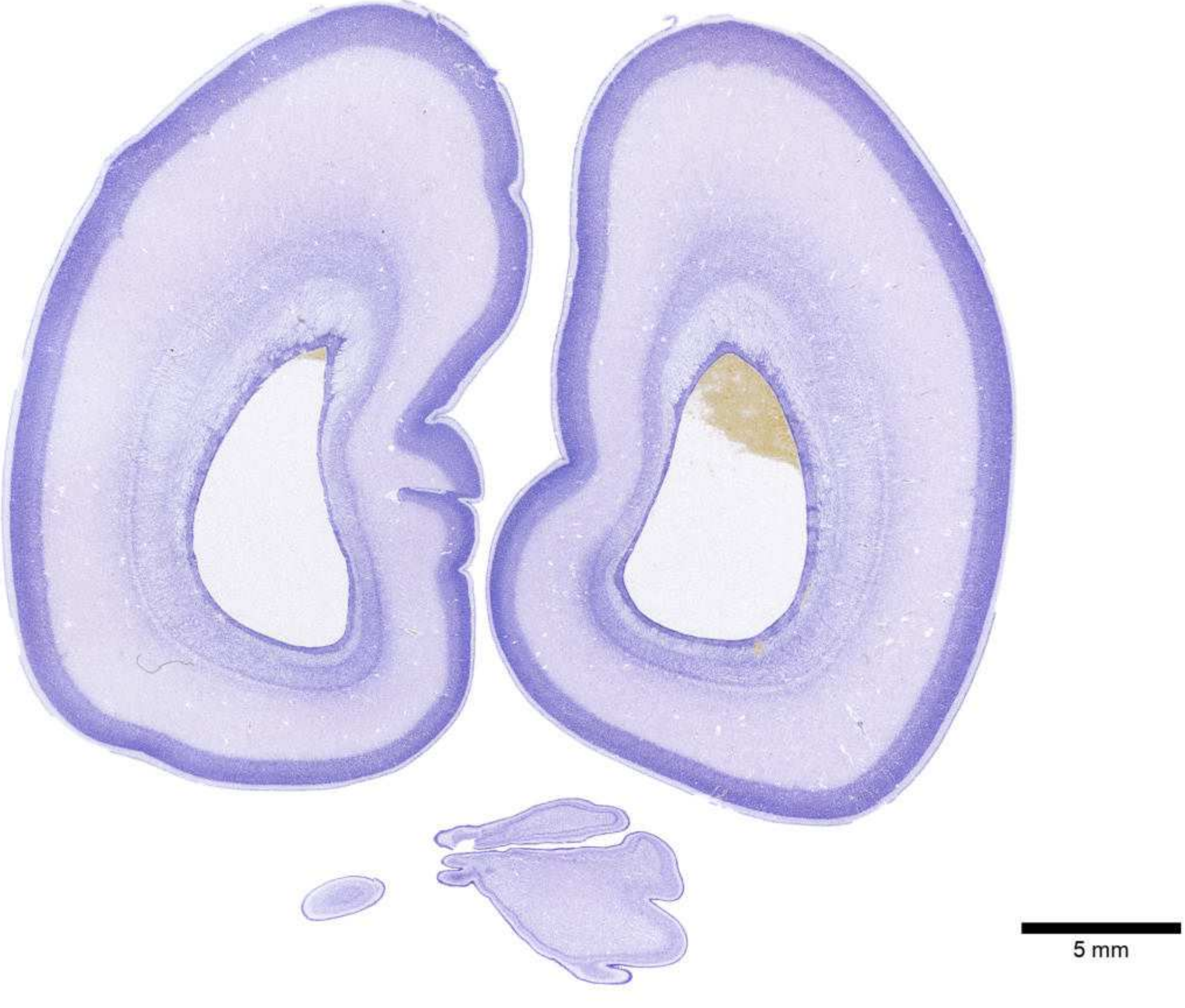

5 mm

Transient Layers

- VZ
- SVZ
- IZ
- SP
- CP
- MZ
- SGL

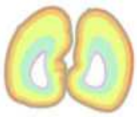

Cortical Areas

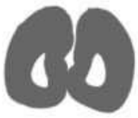

- OCC

Age: 22 GW

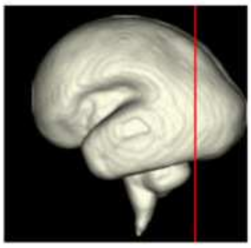

A-P Level: -16.8 mm

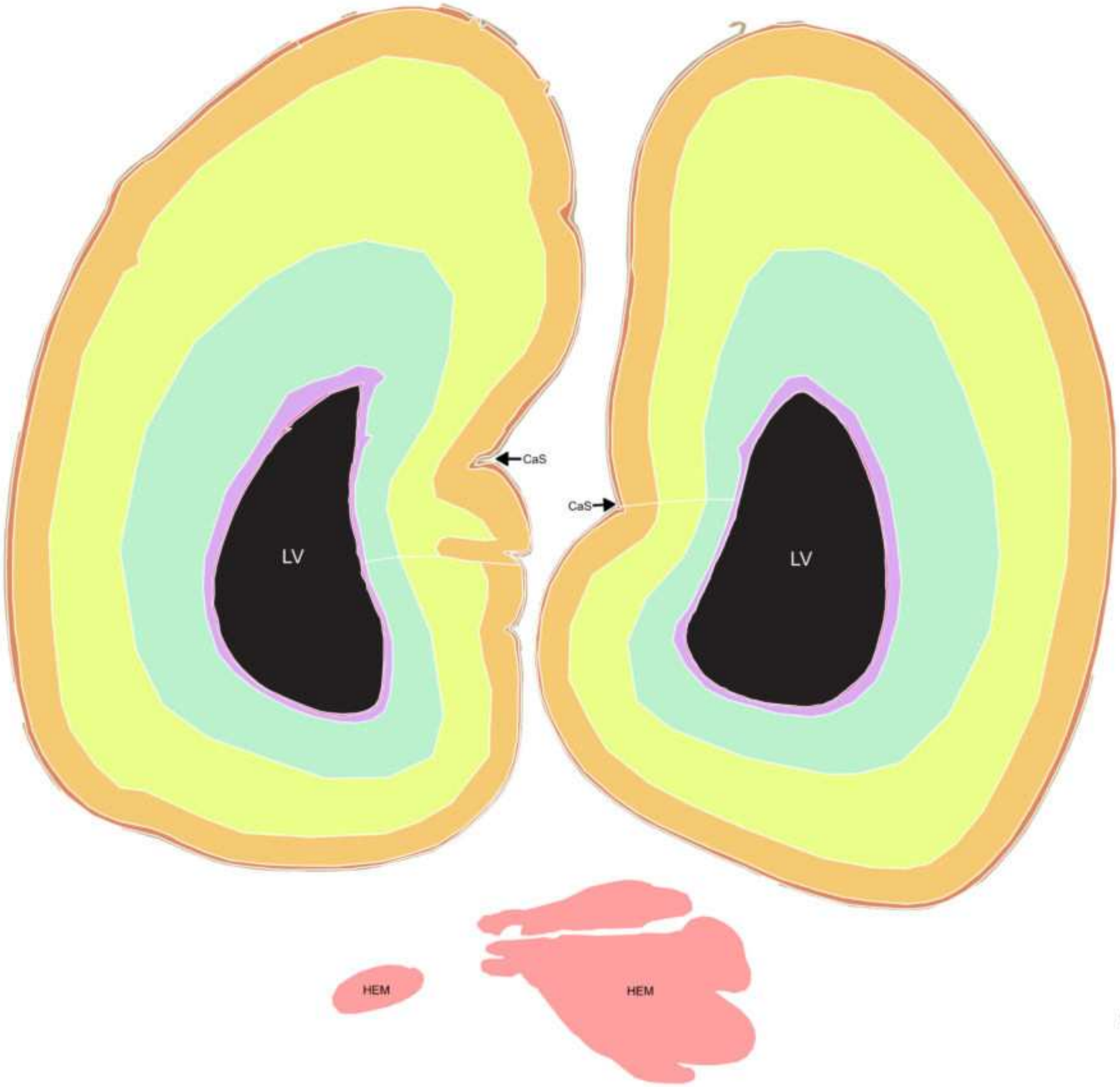

5 mm

HEM: Cerebellar hemispheres   LV: Lateral ventricle   → CaS: Calcarine sulcus

Age: 22 GW

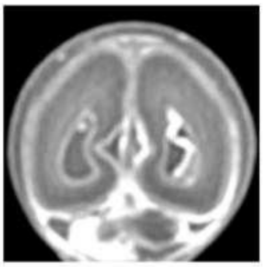

A-P Level: -17.16 mm

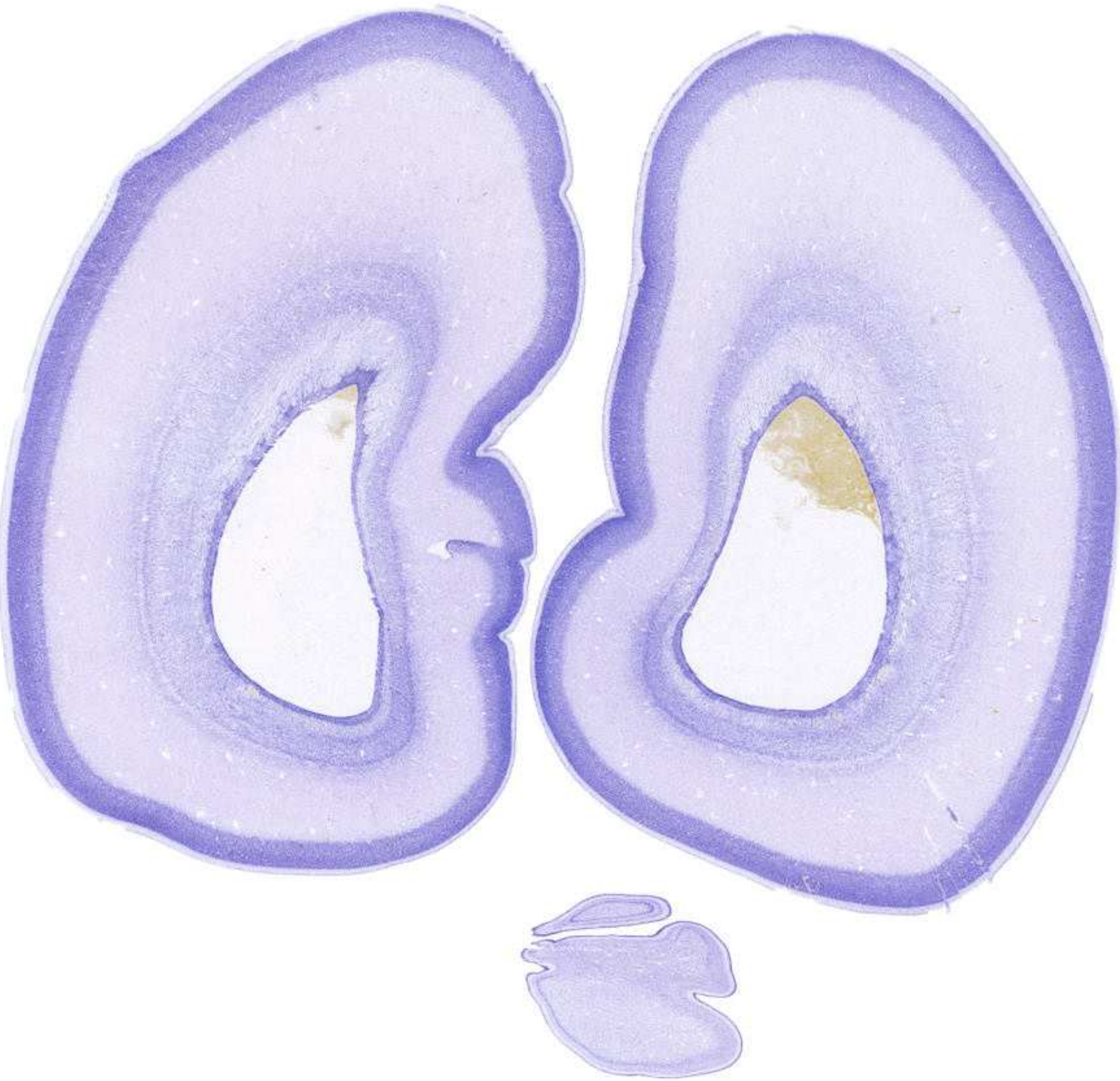

5 mm

Transient Layers

- VZ
- SVZ
- IZ
- SP
- CP
- MZ
- SGL

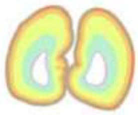

Cortical Areas

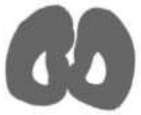

- OCC

Age: 22 GW

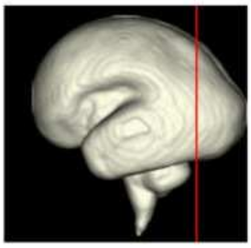

A-P Level: -17.16 mm

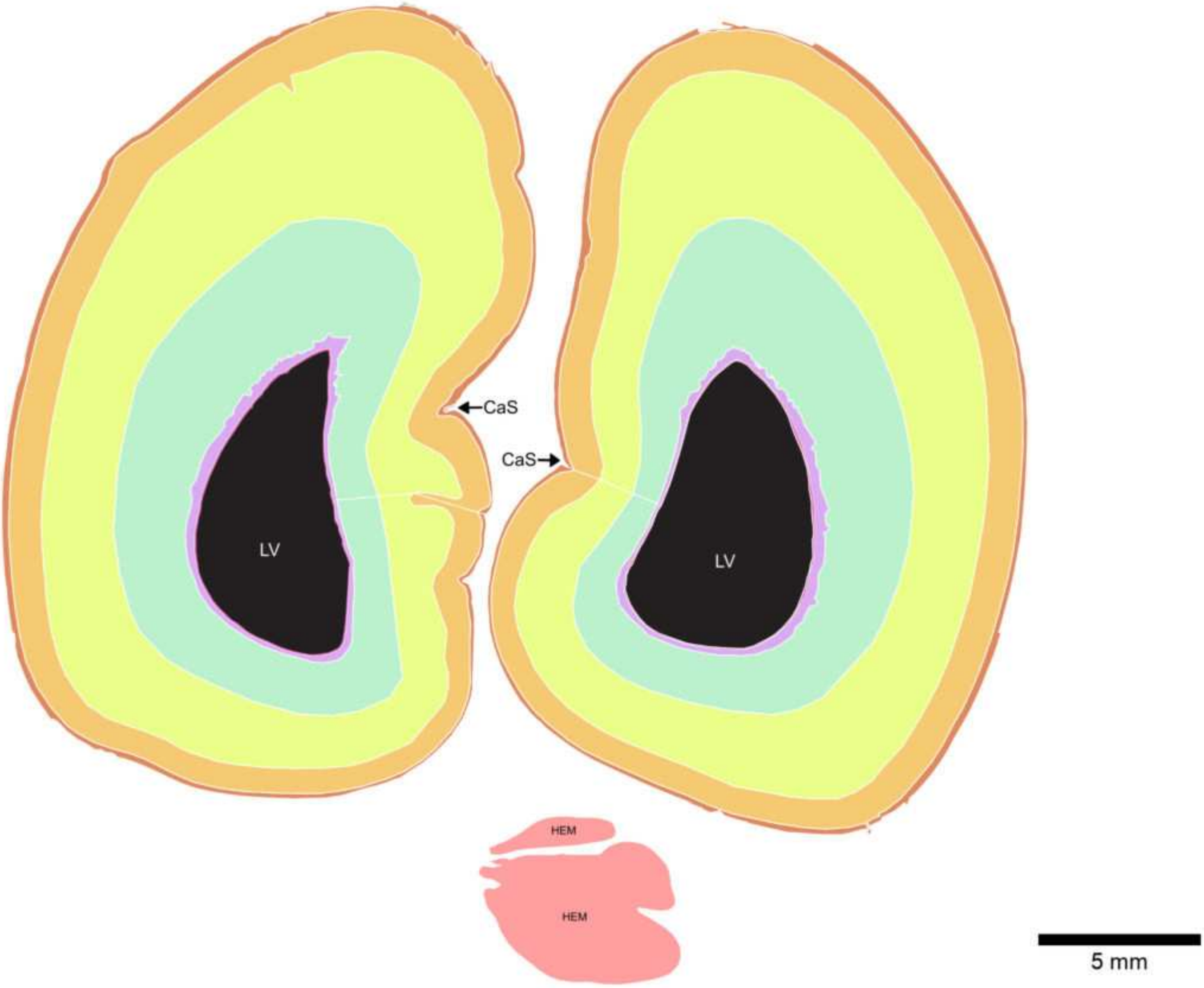

HEM: Cerebellar hemispheres   LV: Lateral ventricle   → CaS: Calcarine sulcus

Age: 22 GW

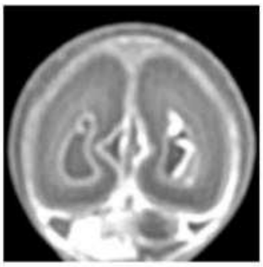

A-P Level: -17.64 mm

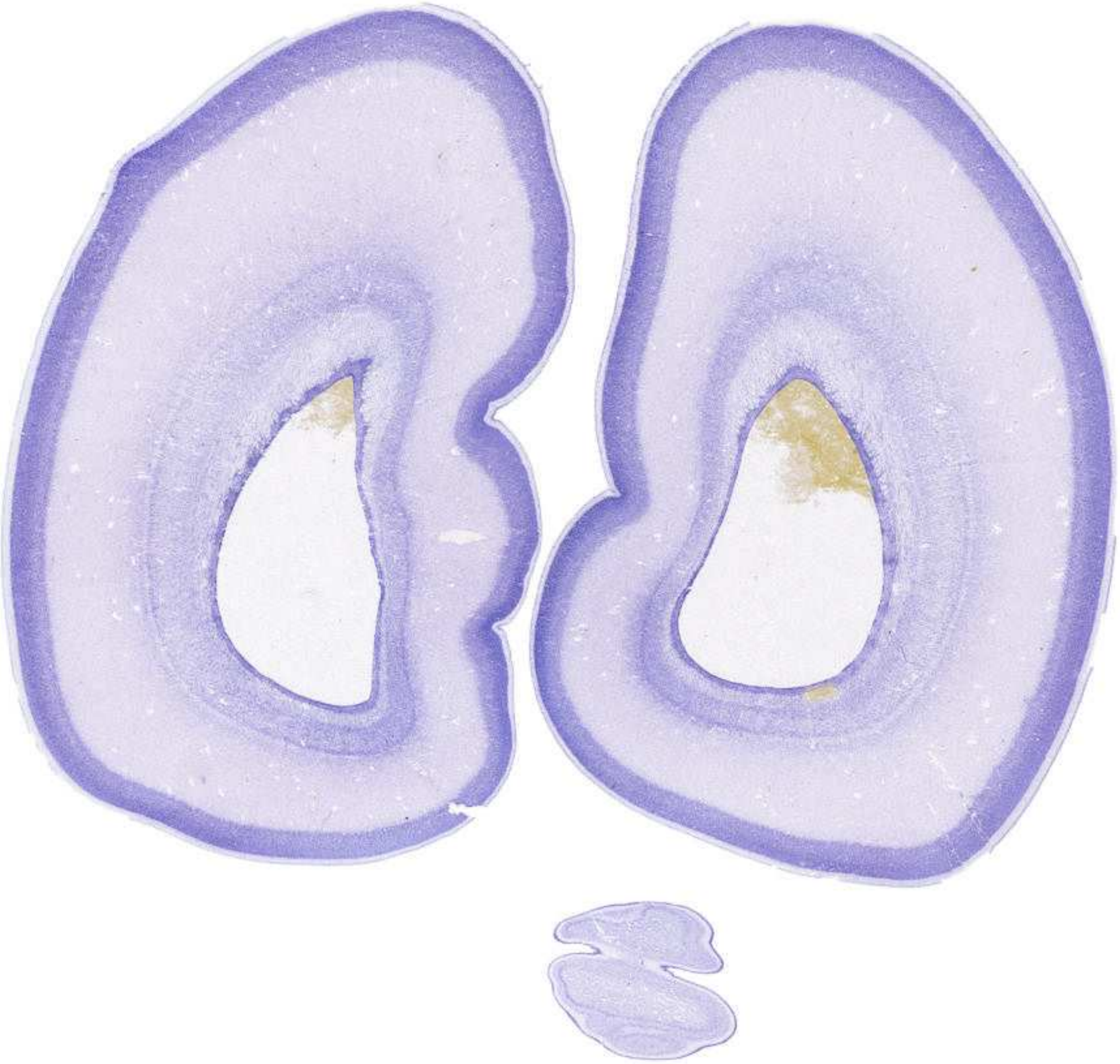

5 mm

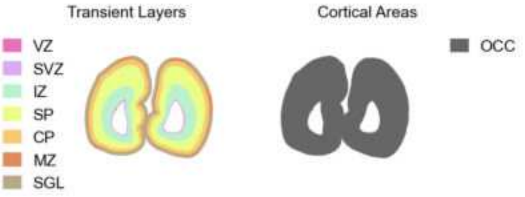

Age: 22 GW

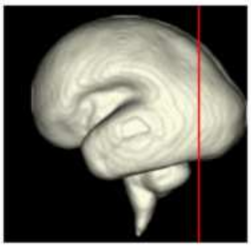

A-P Level: -17.64 mm

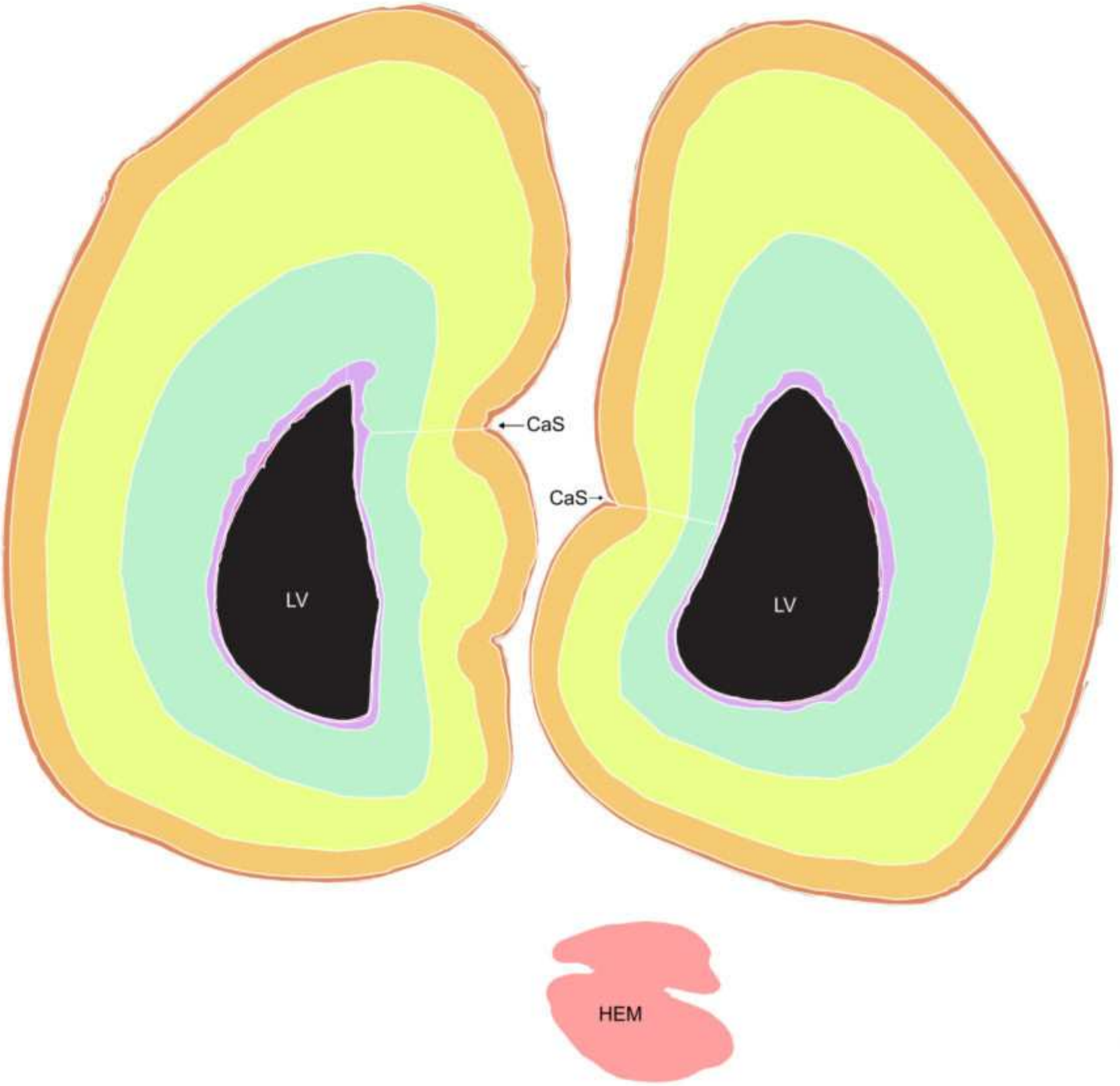

5 mm

HEM: Cerebellar hemispheres

LV: Lateral ventricle

CaS: Calcarine sulcus

Age: 22 GW

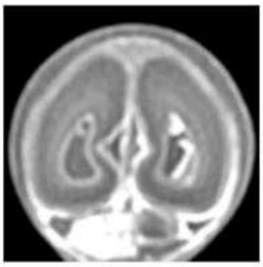

A-P Level: -18.12 mm

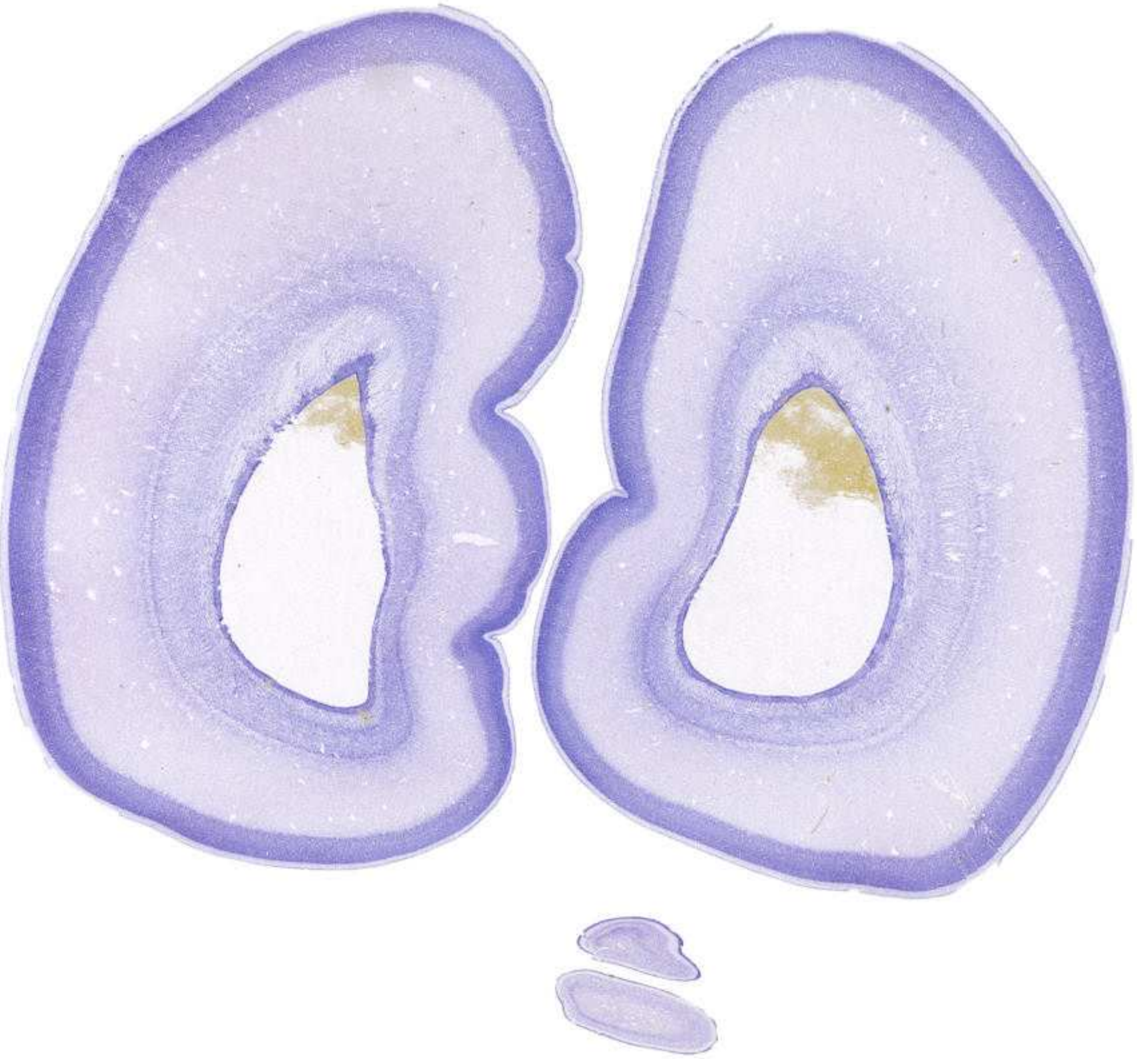

5 mm

Transient Layers

- VZ
- SVZ
- IZ
- SP
- CP
- MZ
- SGL

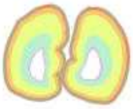

Cortical Areas

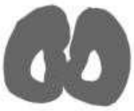

- OCC

Age: 22 GW

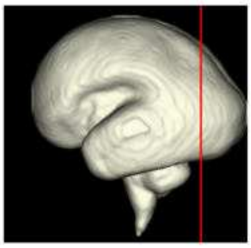

A-P Level: -18.12 mm

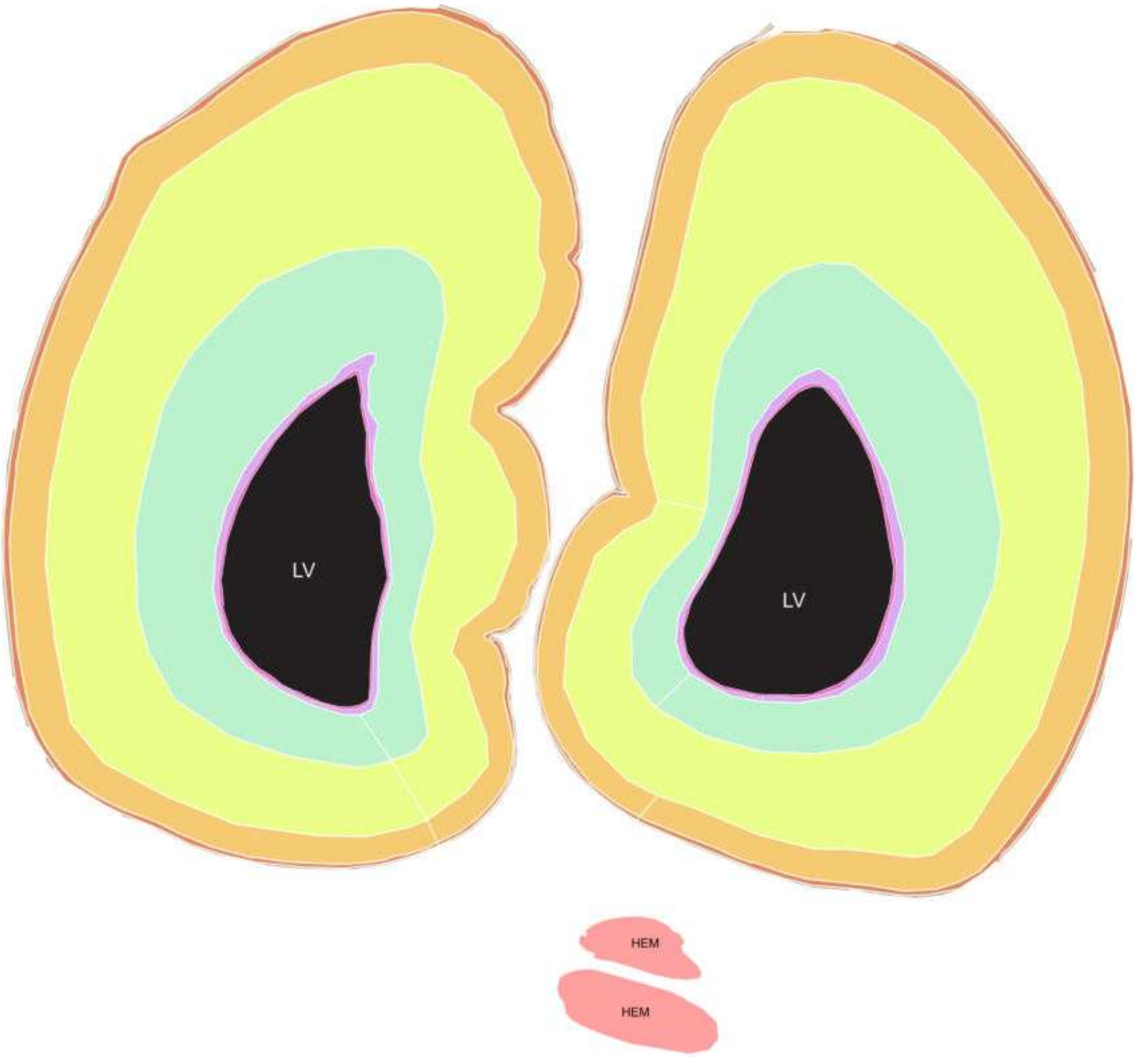

5 mm

HEM: Cerebellar hemispheres   LV: Lateral ventricle

Age: 22 GW

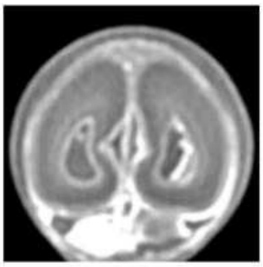

A-P Level: -18.72 mm

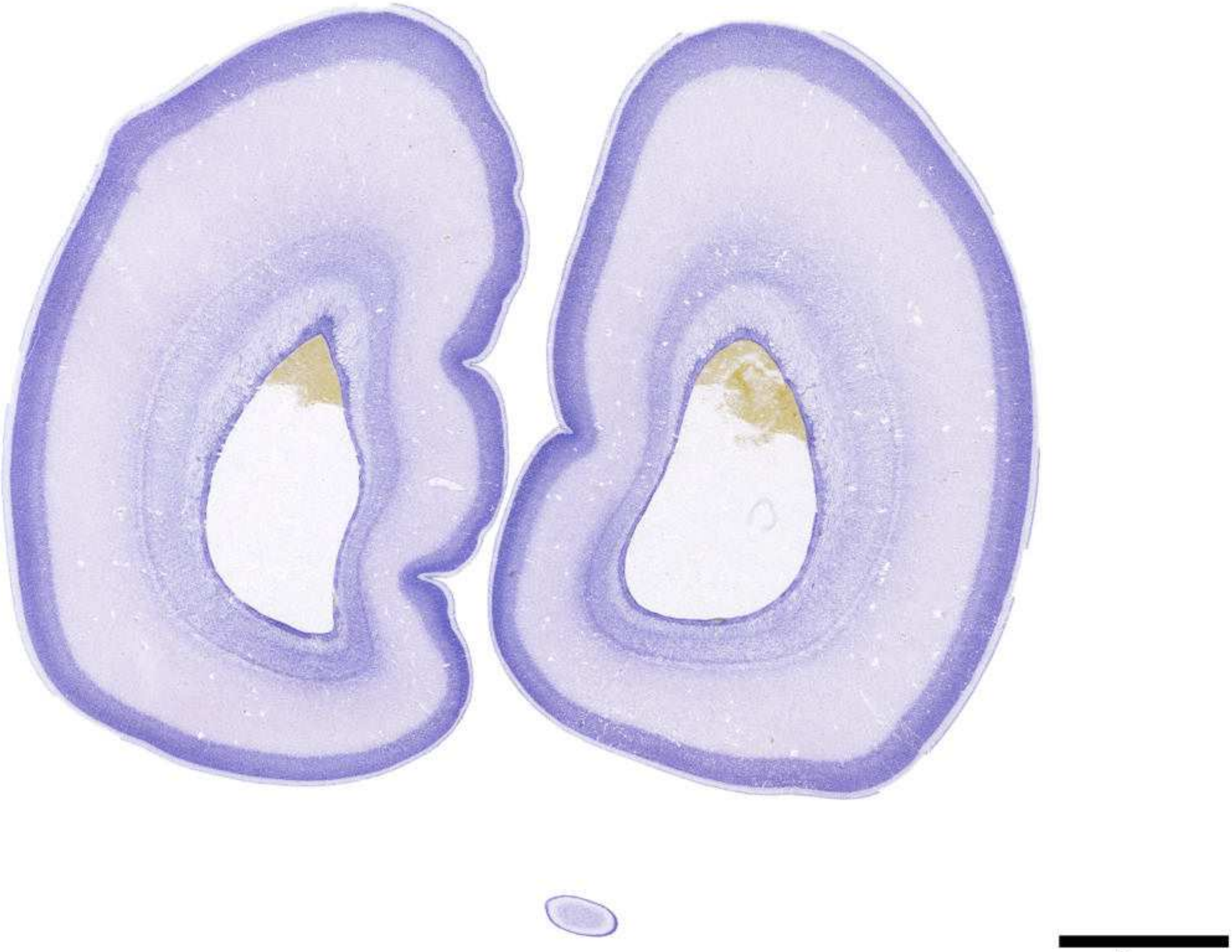

5 mm

Transient Layers

- VZ
- SVZ
- IZ
- SP
- CP
- MZ
- SGL

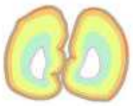

Cortical Areas

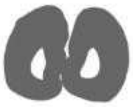

- OCC

Age: 22 GW

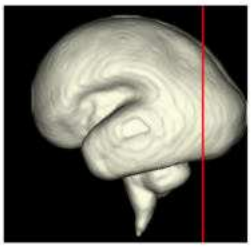

A-P Level: -18.72 mm

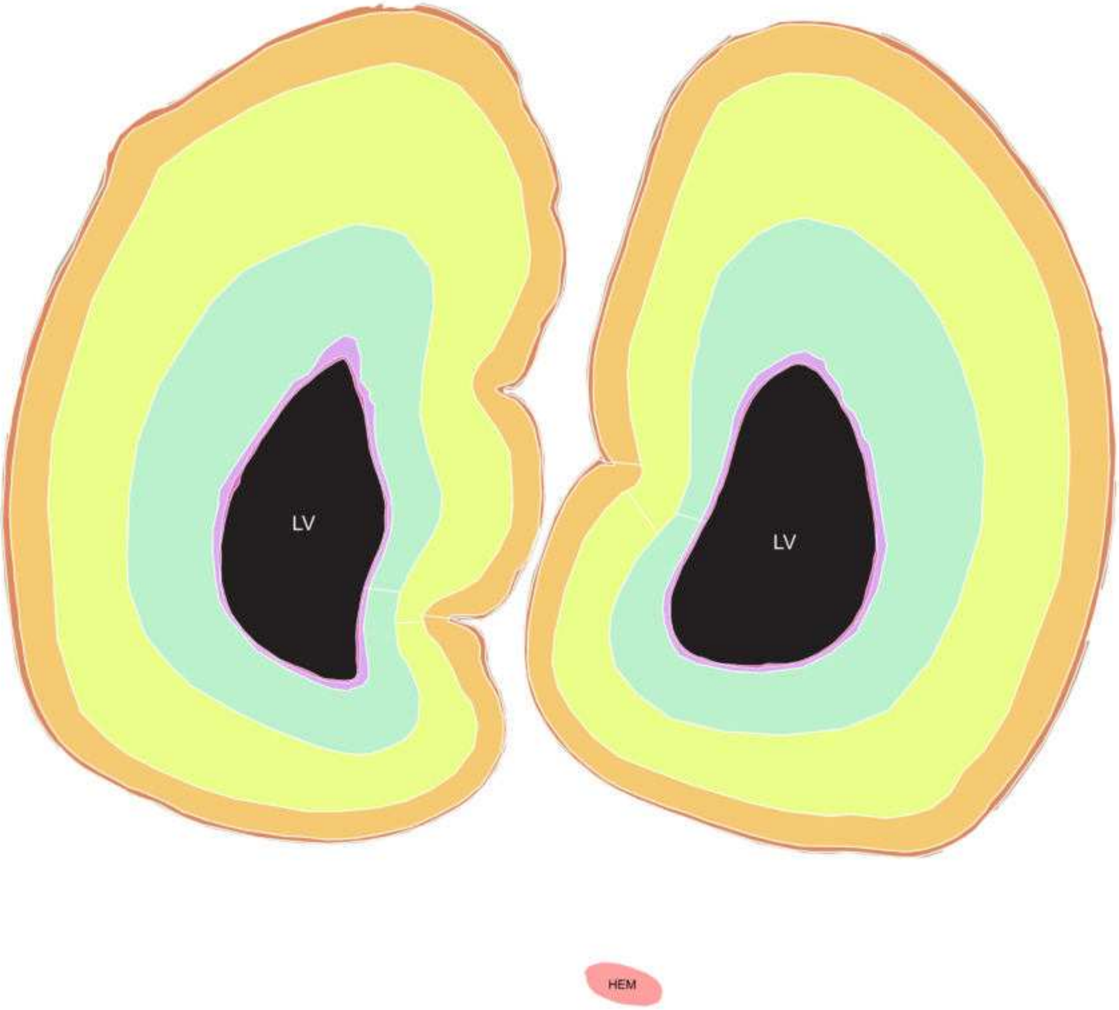

5 mm

HEM: Cerebellar hemispheres   LV: Lateral ventricle

Age: 22 GW

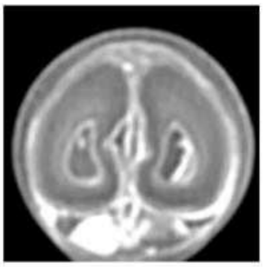

A-P Level: -19.26 mm

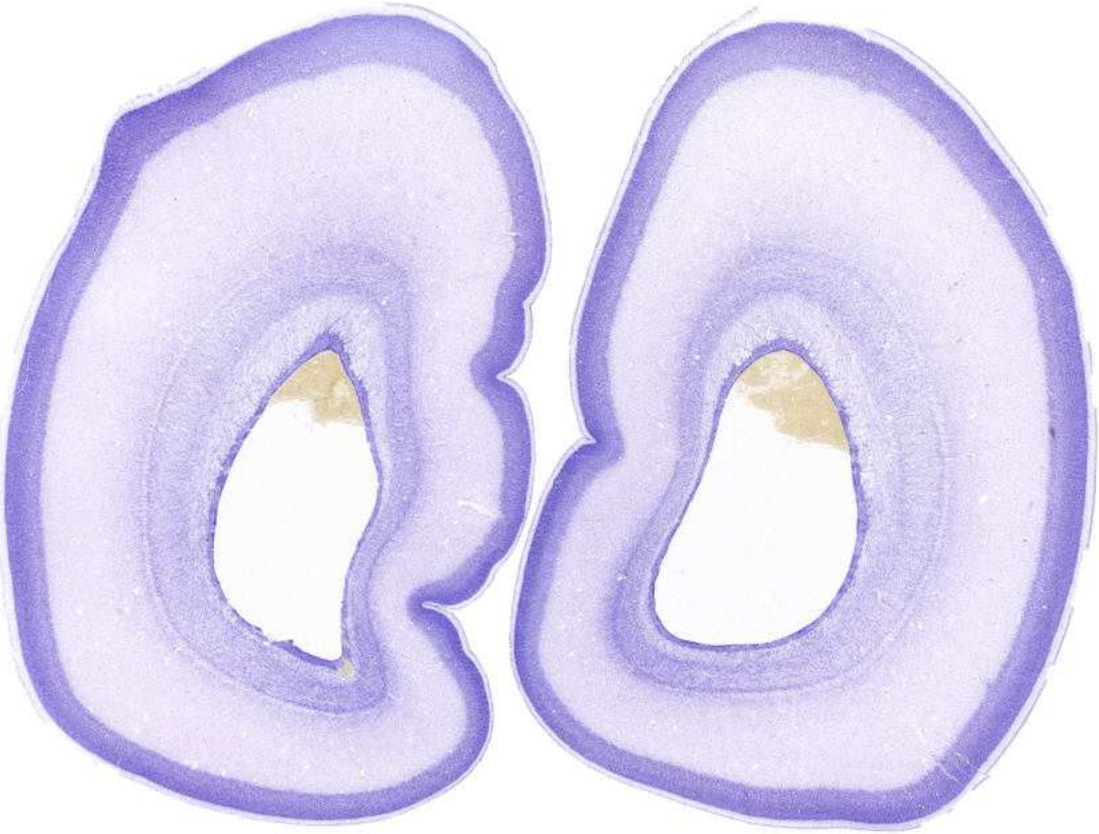

5 mm

Transient Layers

- VZ
- SVZ
- IZ
- SP
- CP
- MZ
- SGL

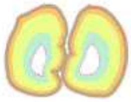

Cortical Areas

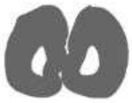

- OCC

Age: 22 GW

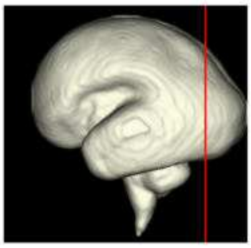

A-P Level: -19.26 mm

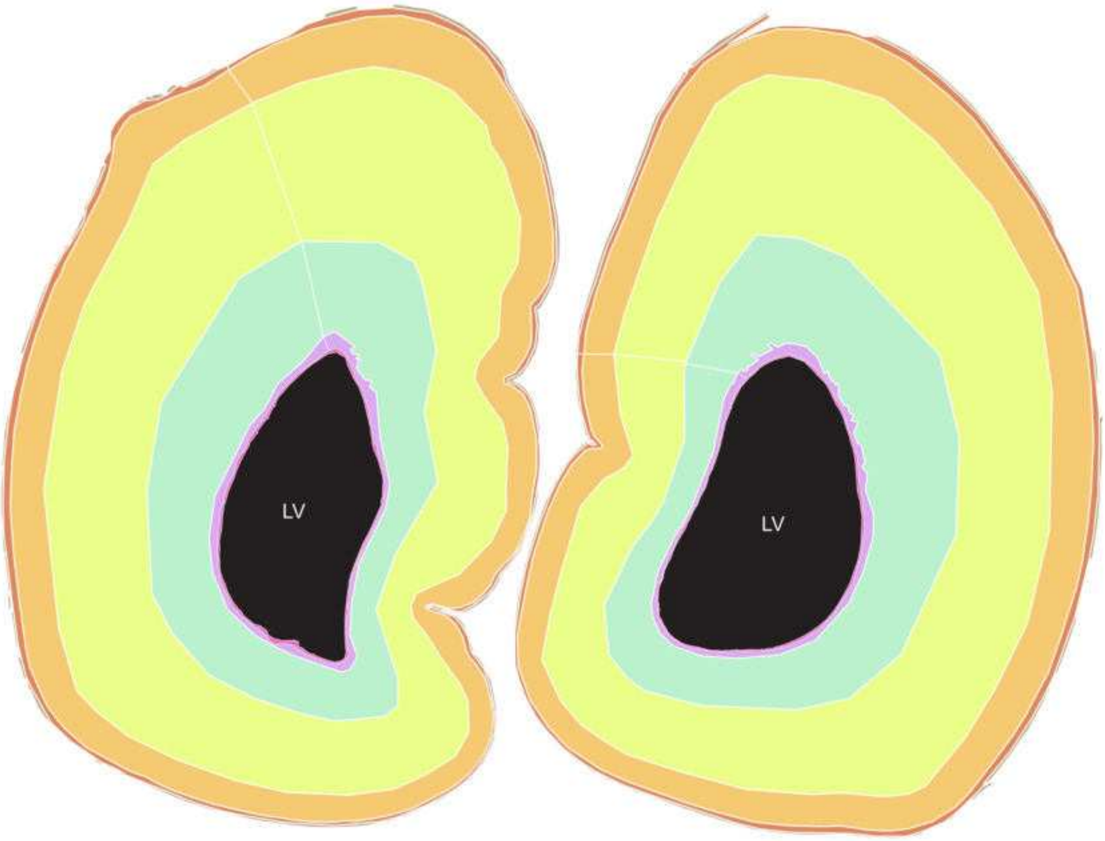

5 mm

LV: Lateral ventricle

Age: 22 GW

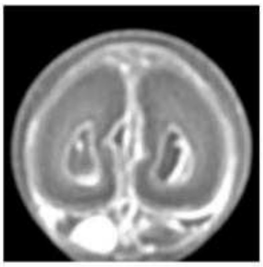

A-P Level: -19.62 mm

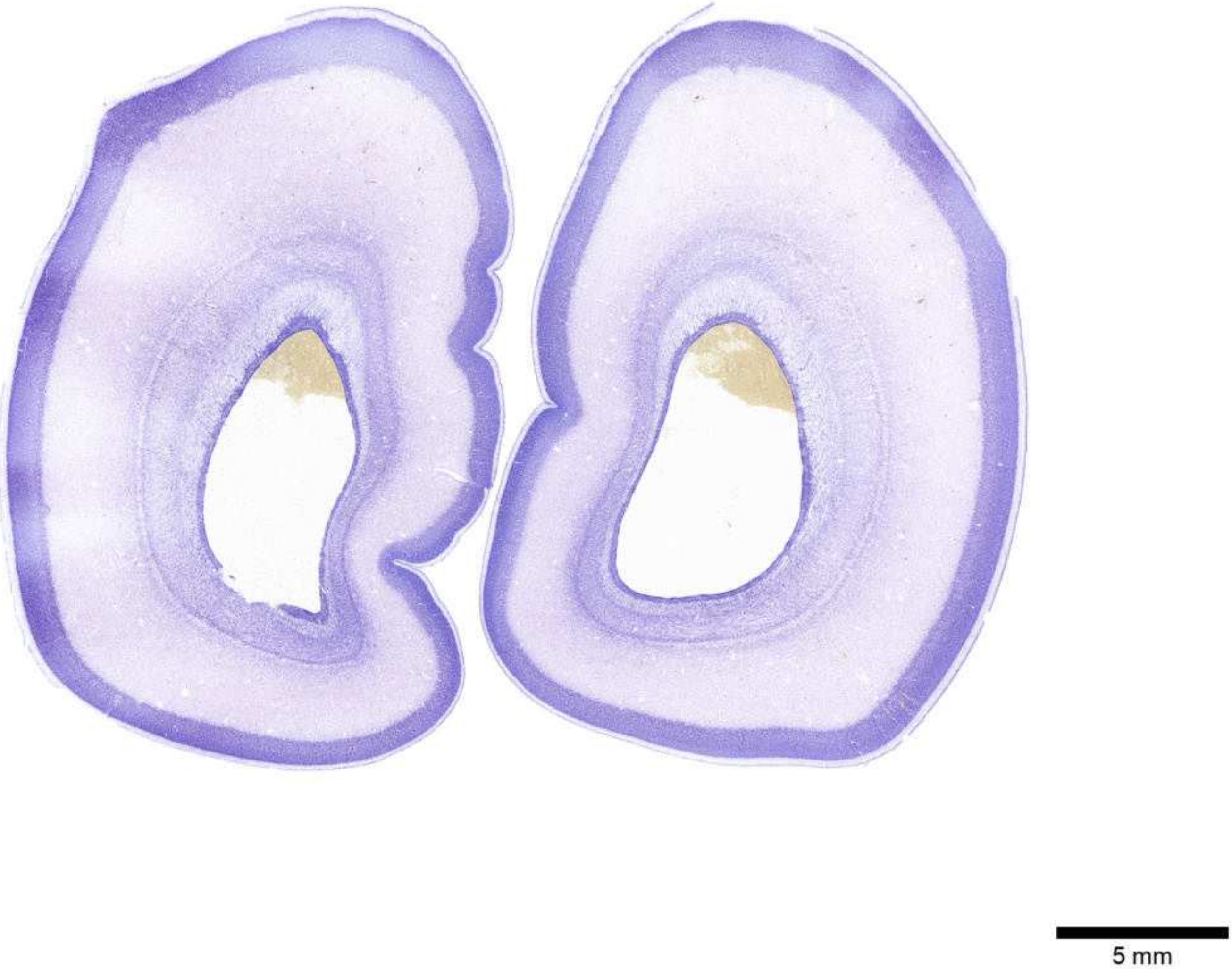

5 mm

Transient Layers

VZ
SVZ
IZ
SP
CP
MZ
SGL

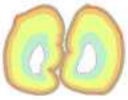

Cortical Areas

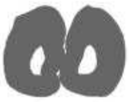

OCC

Age: 22 GW

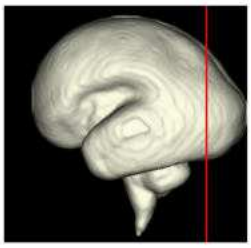

A-P Level: -19.62 mm

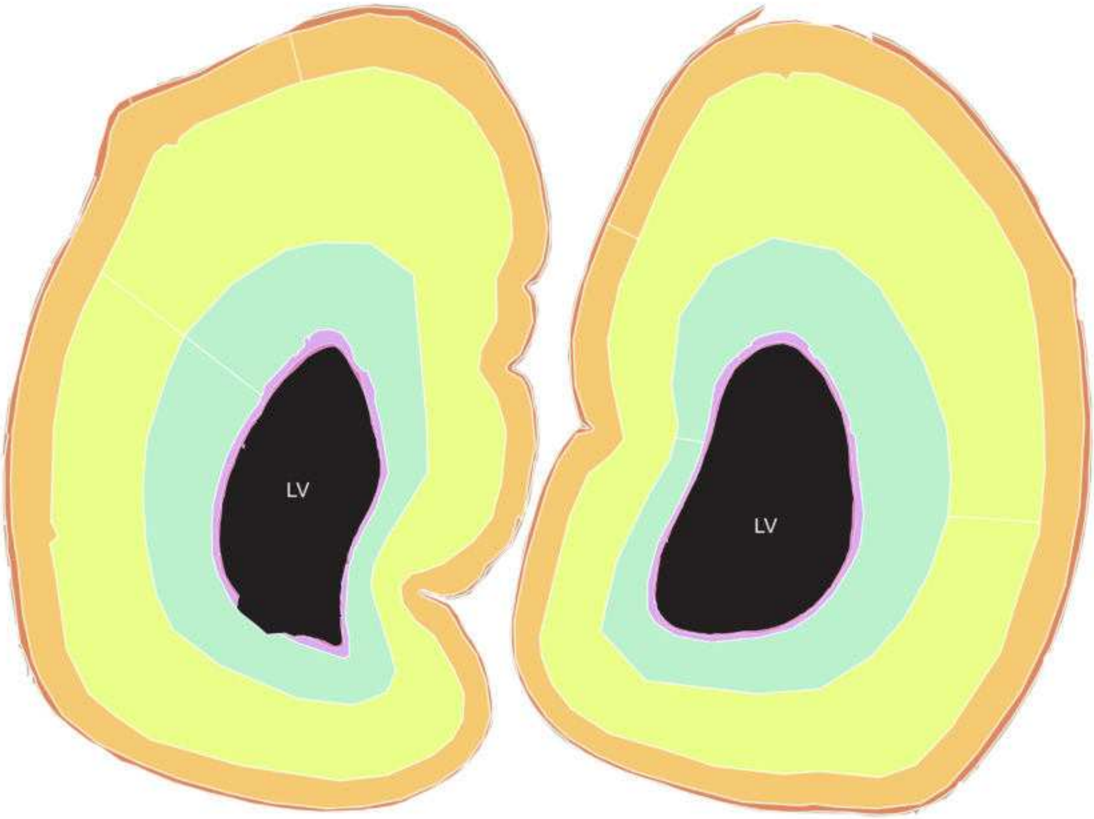

5 mm

LV: Lateral ventricle

Age: 22 GW

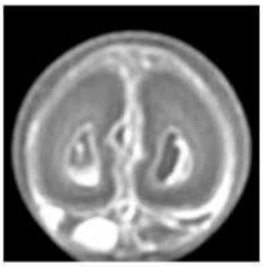

A-P Level: -19.98 mm

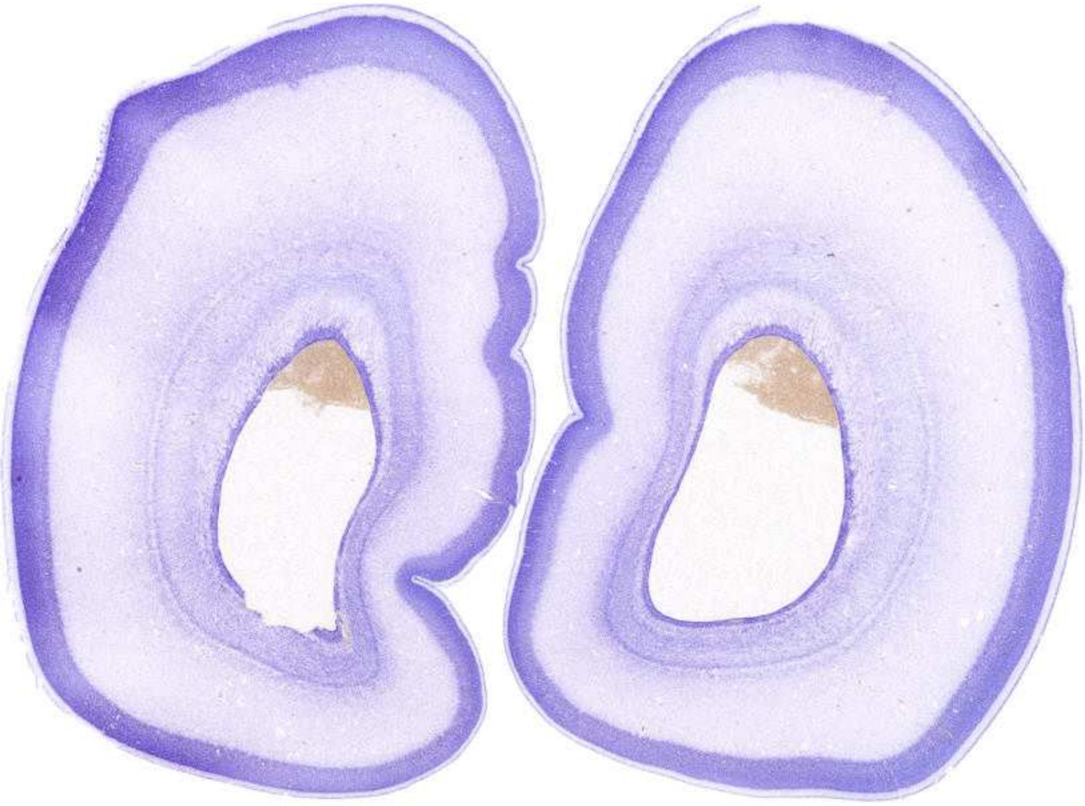

5 mm

Transient Layers

- VZ
- SVZ
- IZ
- SP
- CP
- MZ
- SGL

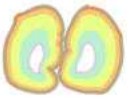

Cortical Areas

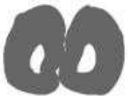

- OCC

Age: 22 GW

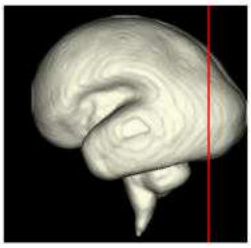

A-P Level: -19.98 mm

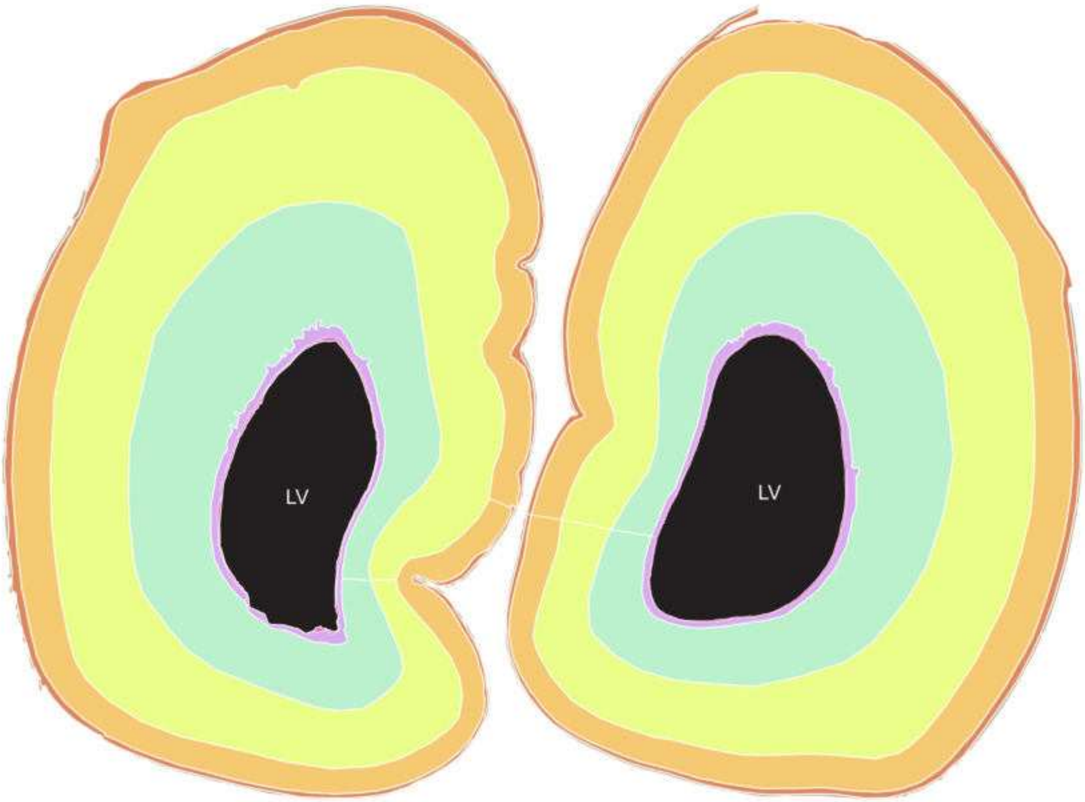

5 mm

LV: Lateral ventricle

Age: 22 GW

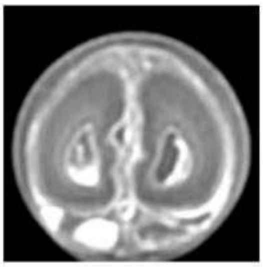

A-P Level: -20.16 mm

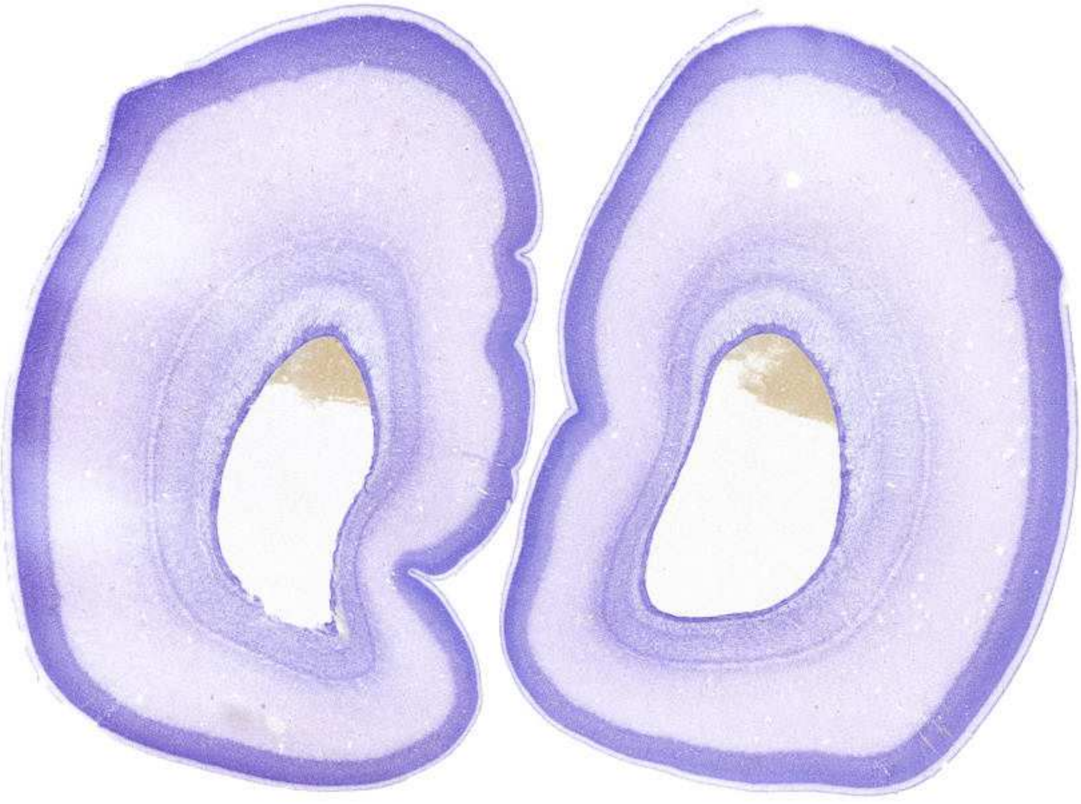

5 mm

Transient Layers

- VZ
- SVZ
- IZ
- SP
- CP
- MZ
- SGL

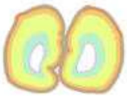

Cortical Areas

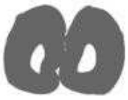

- OCC

Age: 22 GW

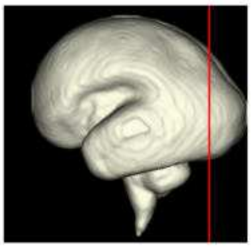

A-P Level: -20.16 mm

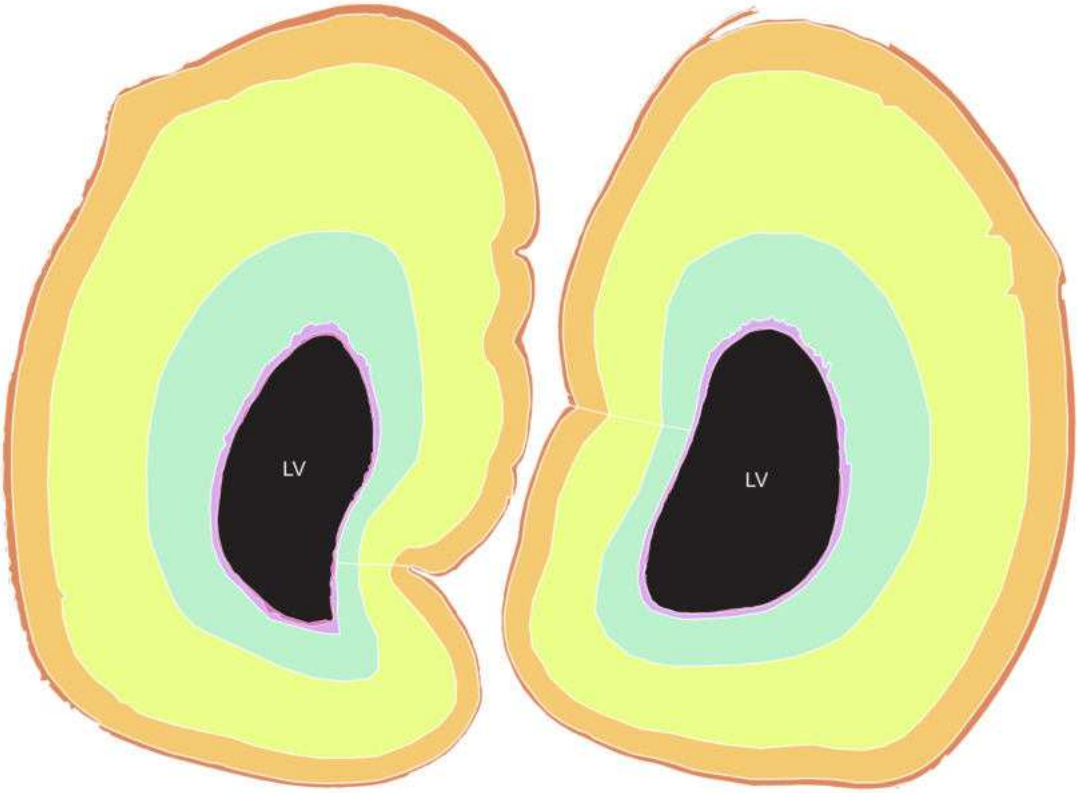

5 mm

LV: Lateral ventricle

Age: 22 GW

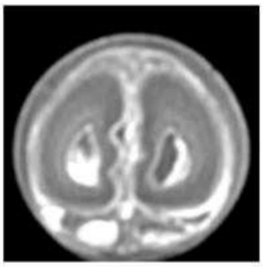

A-P Level: -20.58 mm

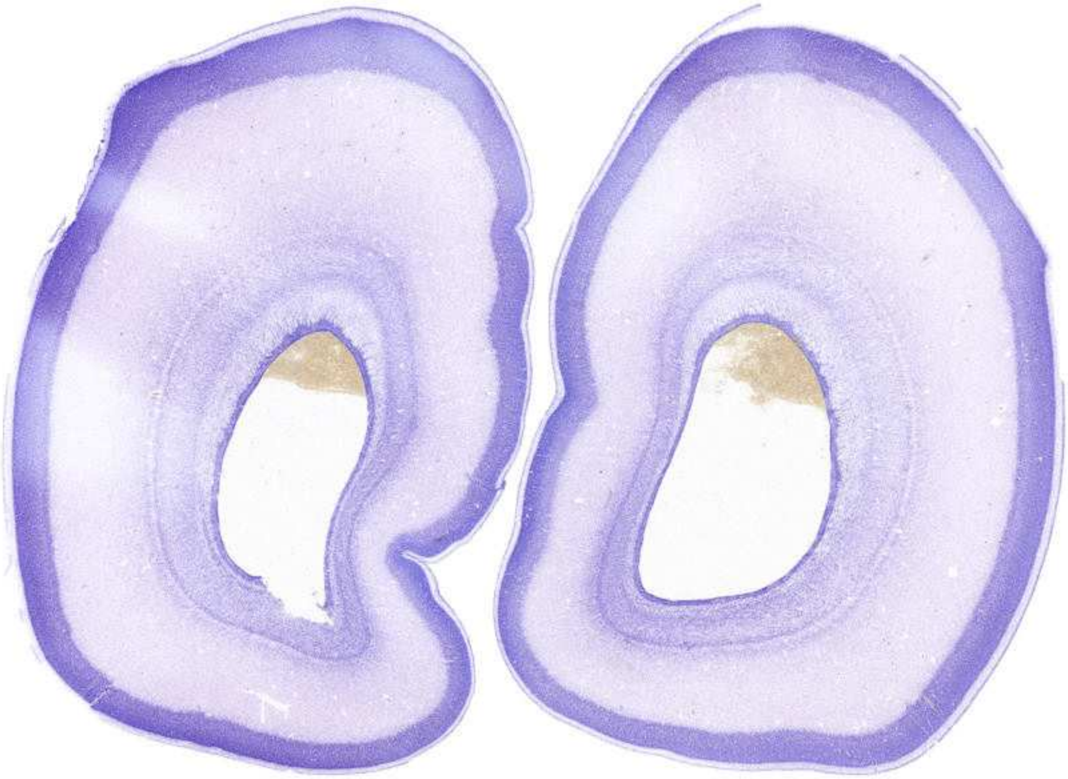

5 mm

Transient Layers

- VZ
- SVZ
- IZ
- SP
- CP
- MZ
- SGL

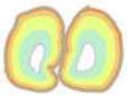

Cortical Areas

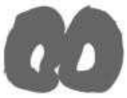

- OCC

Age: 22 GW

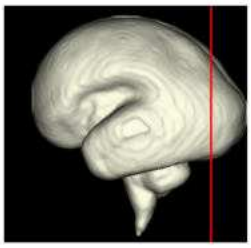

A-P Level: -20.58 mm

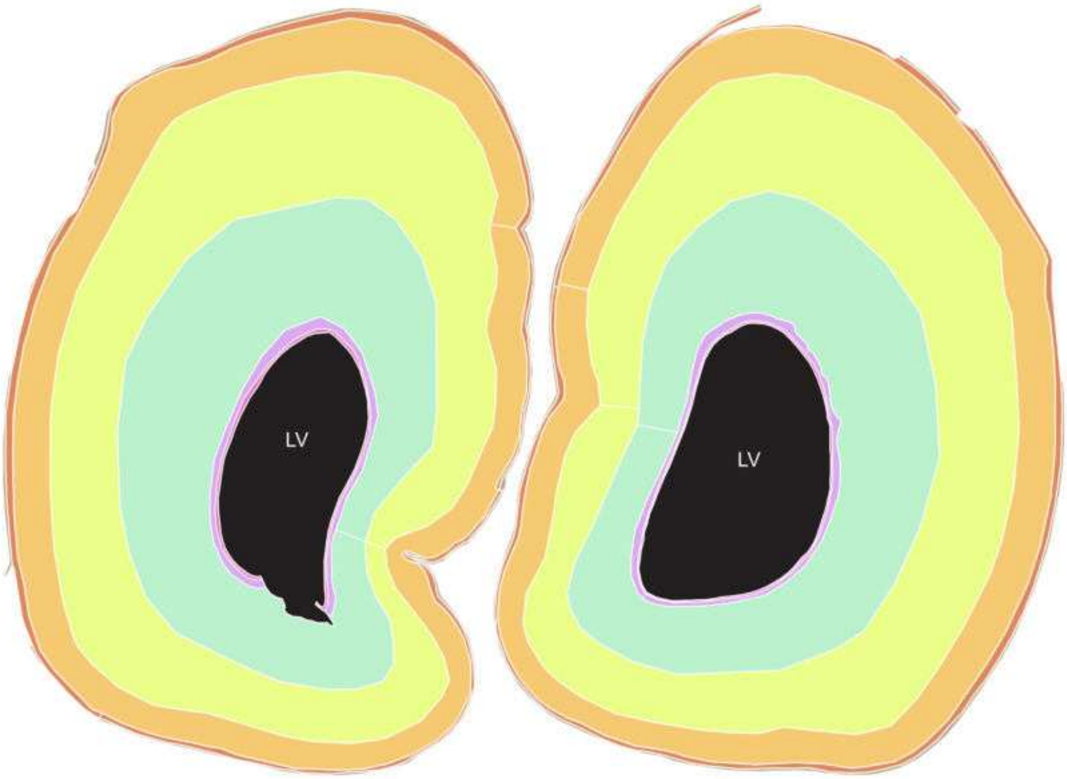

5 mm

LV: Lateral ventricle

Age: 22 GW

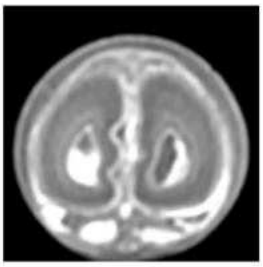

A-P Level: -20.82 mm

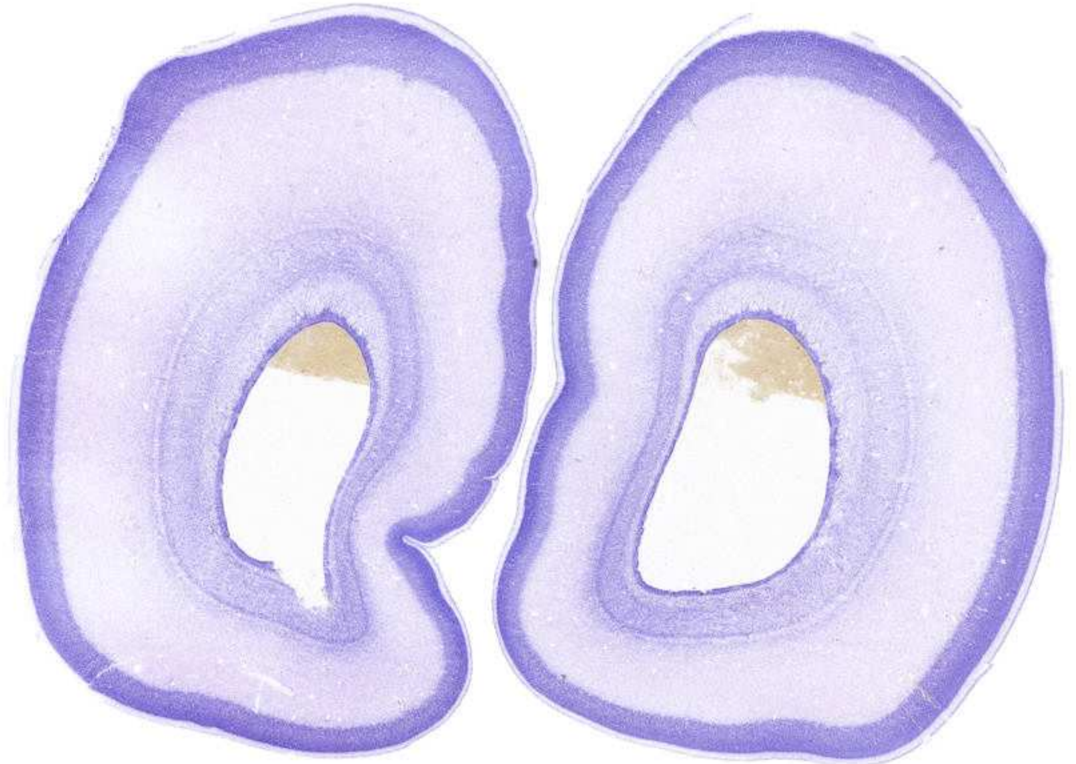

5 mm

Transient Layers

- VZ
- SVZ
- IZ
- SP
- CP
- MZ
- SGL

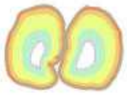

Cortical Areas

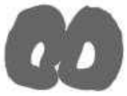

- OCC

Age: 22 GW

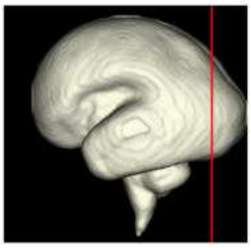

A-P Level: -20.82 mm

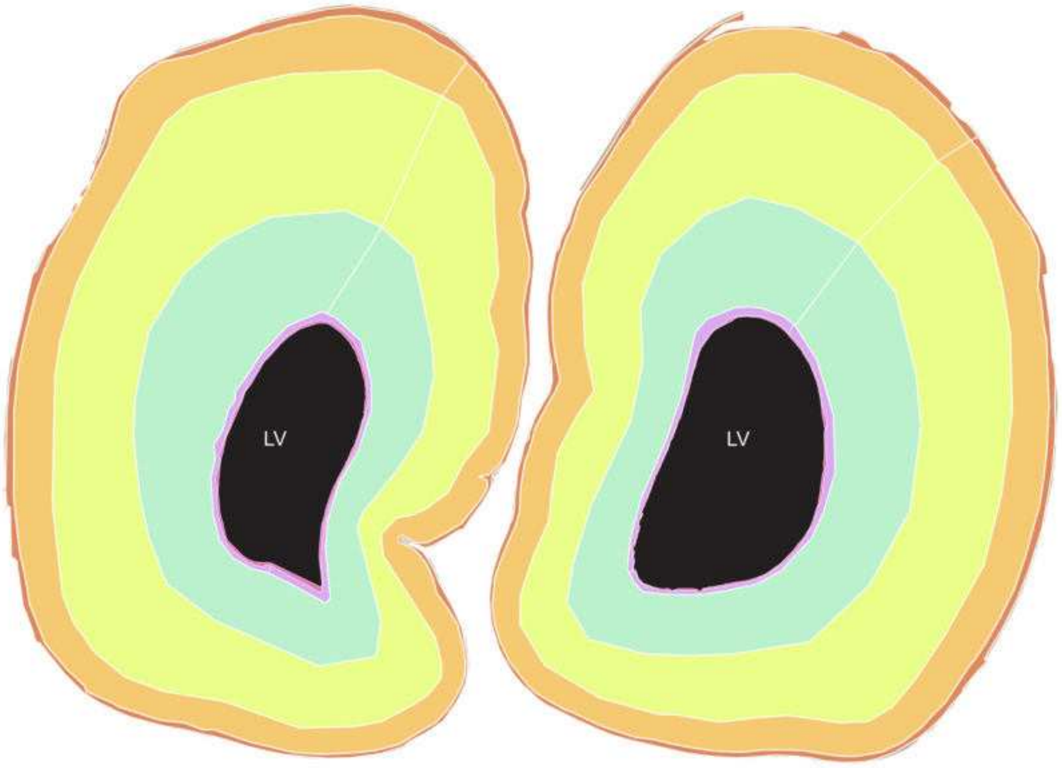

5 mm

LV: Lateral ventricle

Age: 22 GW

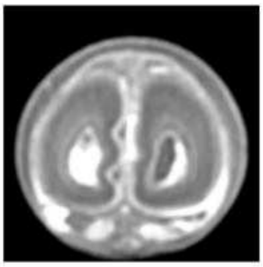

A-P Level: -21.42 mm

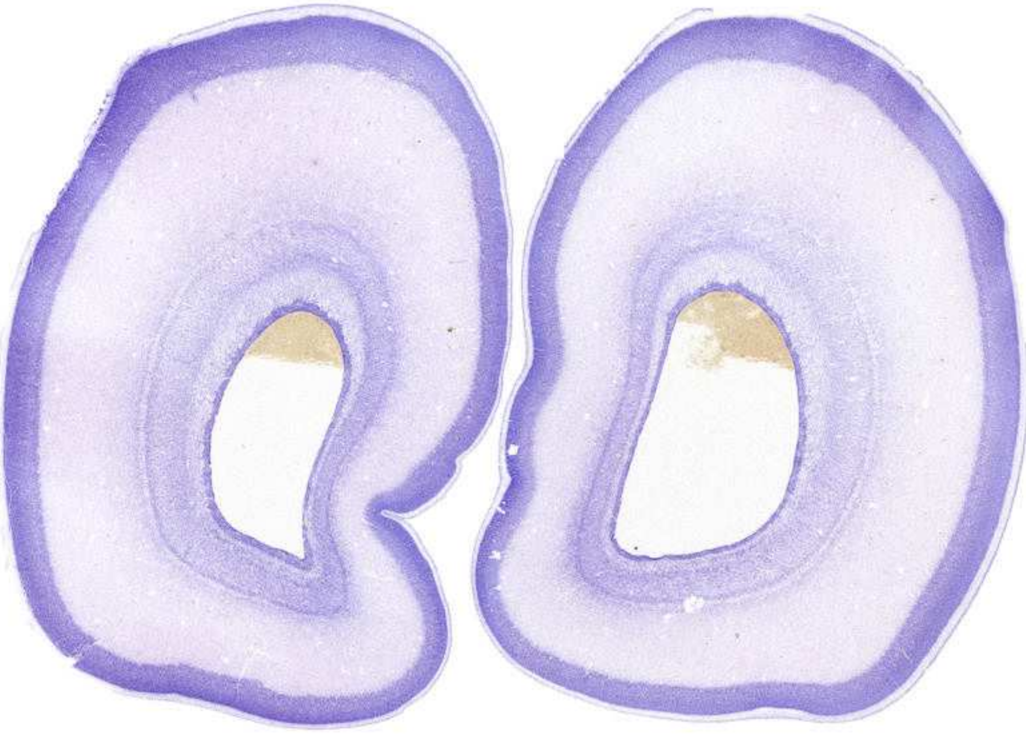

5 mm

Age: 22 GW

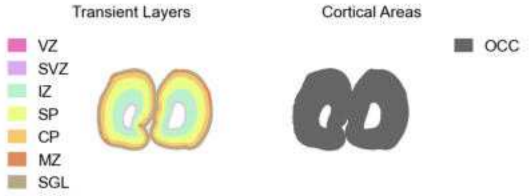

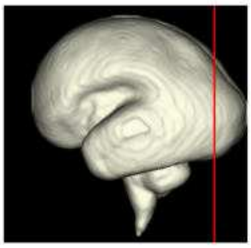

A-P Level: -21.42 mm

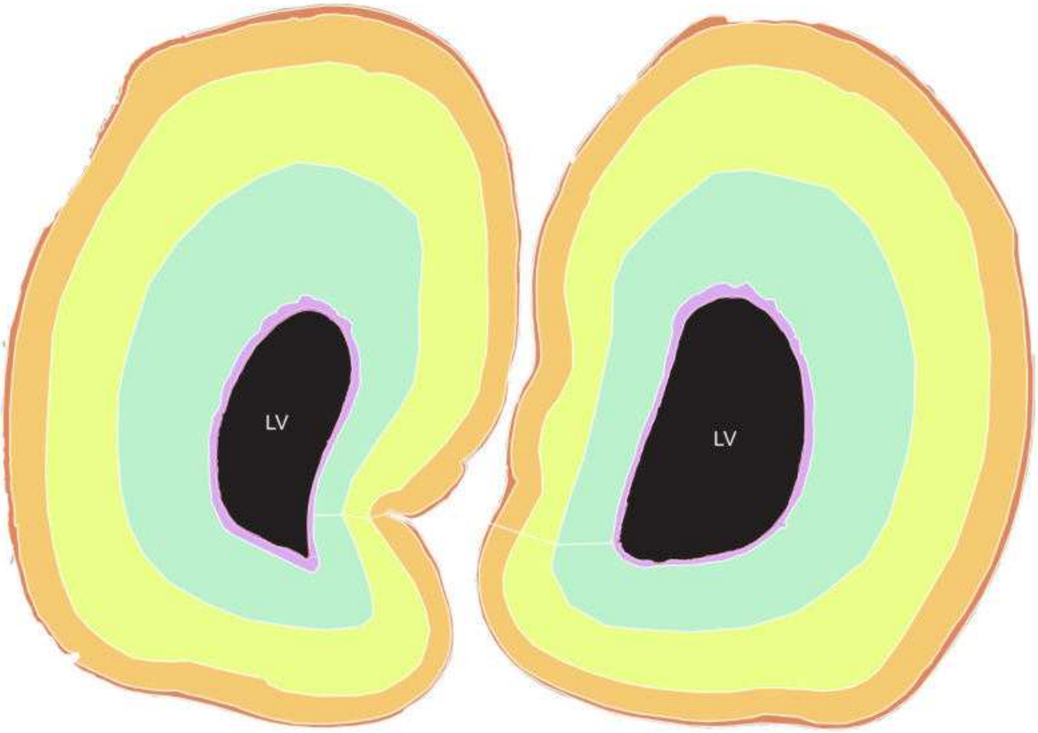

5 mm

LV: Lateral ventricle

Age: 22 GW

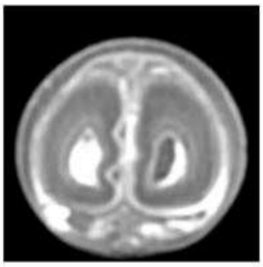

A-P Level: -21.6 mm

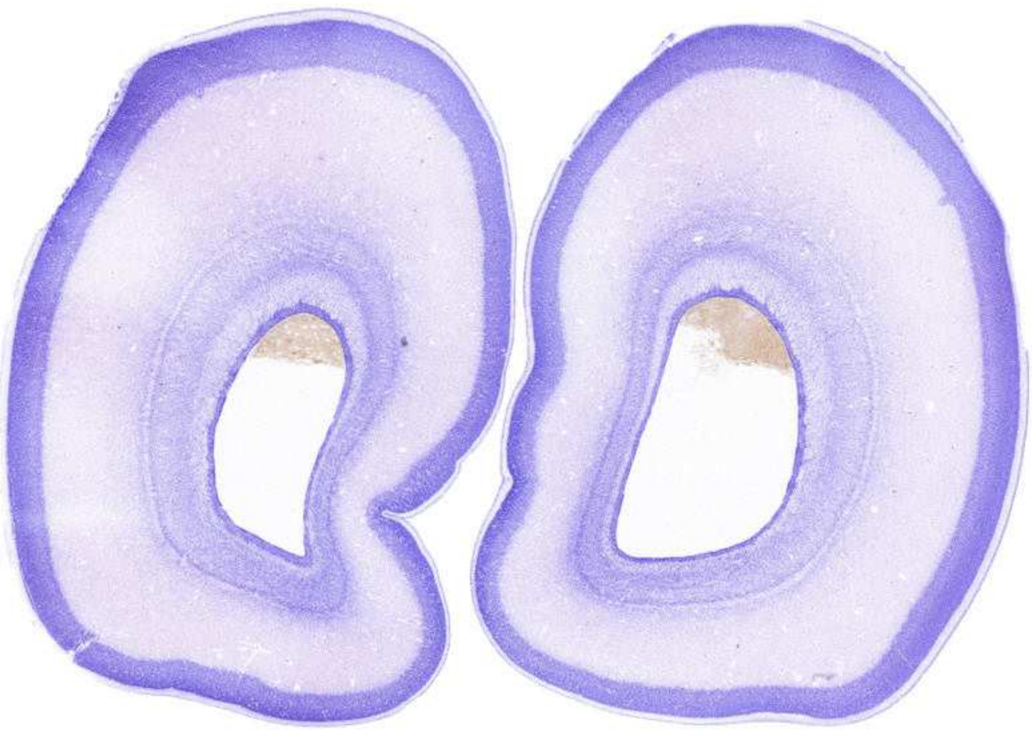

5 mm

Transient Layers

- VZ
- SVZ
- IZ
- SP
- CP
- MZ
- SGL

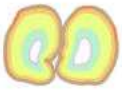

Cortical Areas

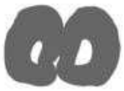

- OCC

Age: 22 GW

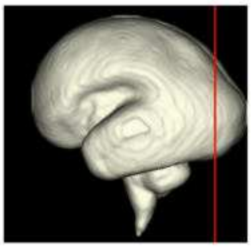

A-P Level: -21.6 mm

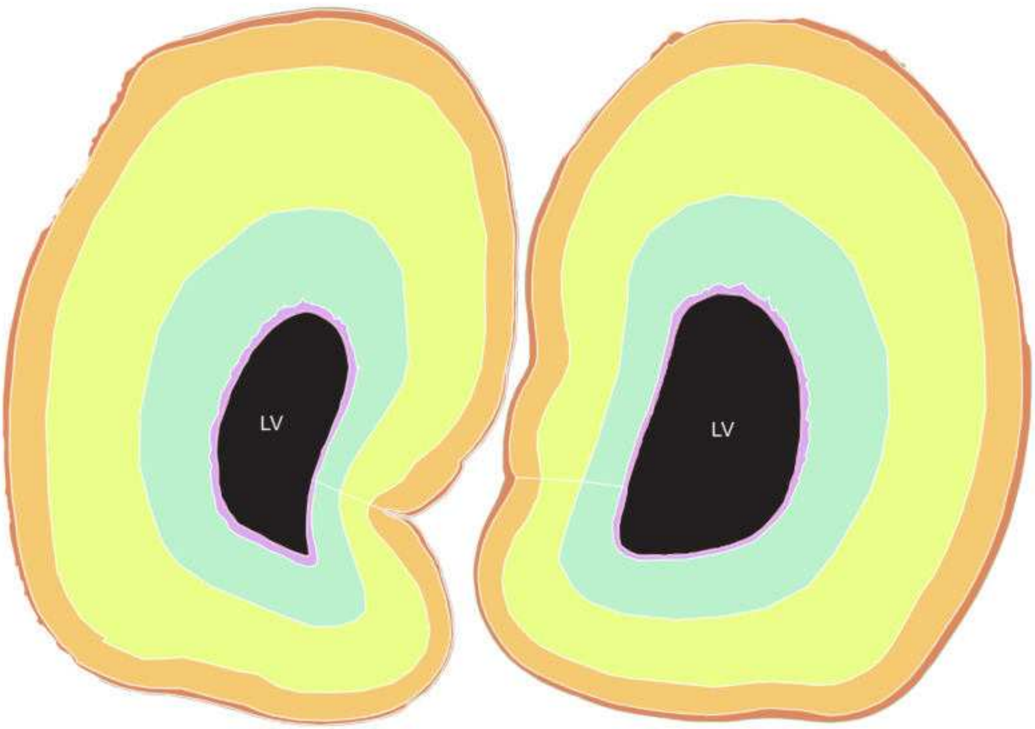

5 mm

LV: Lateral ventricle

Age: 22 GW

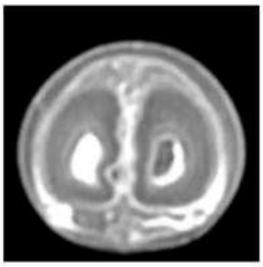

A-P Level: -22.26 mm

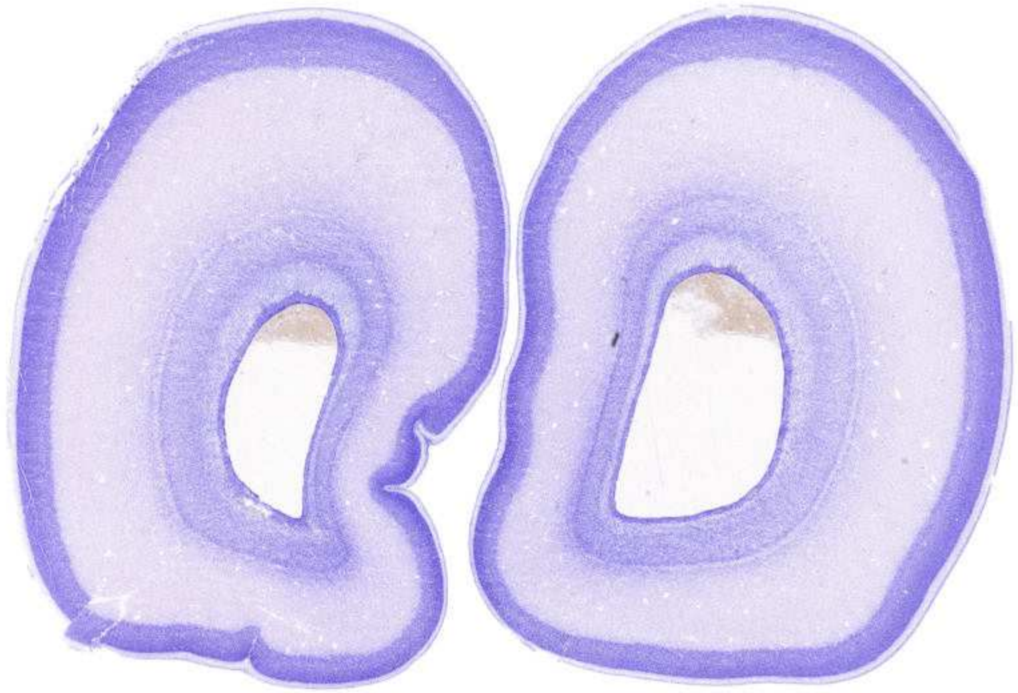

5 mm

Transient Layers

- VZ
- SVZ
- IZ
- SP
- CP
- MZ
- SGL

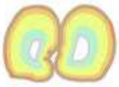

Cortical Areas

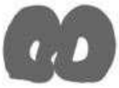

- OCC

Age: 22 GW

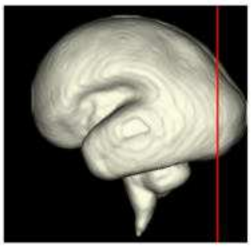

A-P Level: -22.26 mm

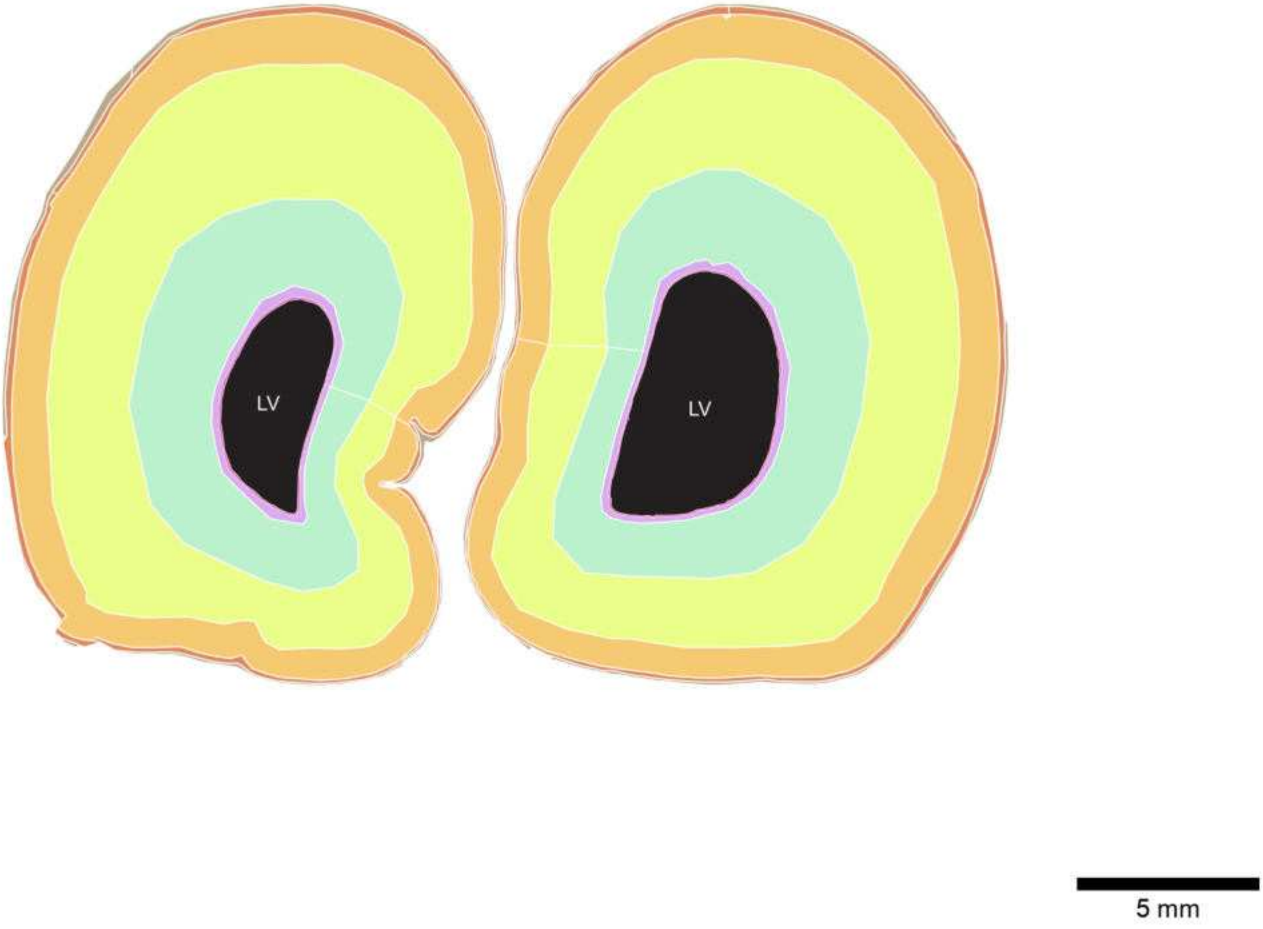

LV: Lateral ventricle

Age: 22 GW

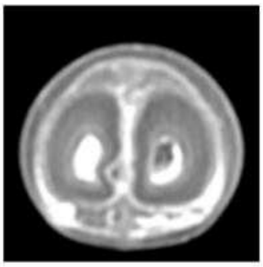

A-P Level: -22.74 mm

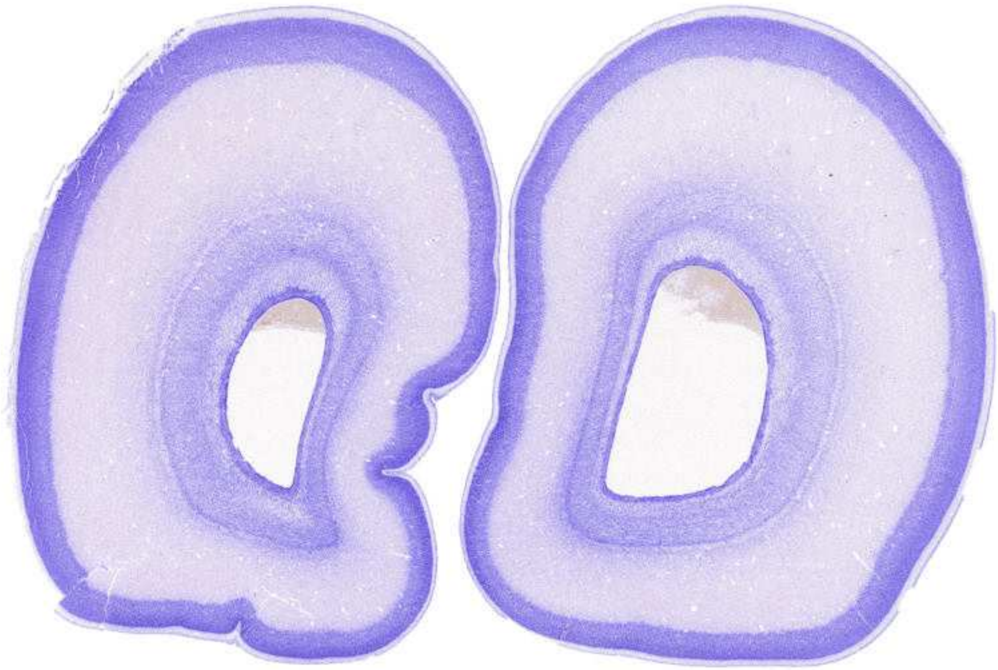

5 mm

Transient Layers

- VZ
- SVZ
- IZ
- SP
- CP
- MZ
- SGL

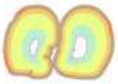

Cortical Areas

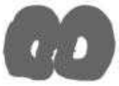

- OCC

Age: 22 GW

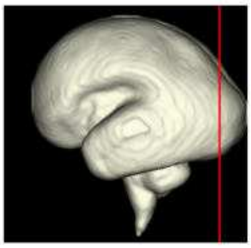

A-P Level: -22.74 mm

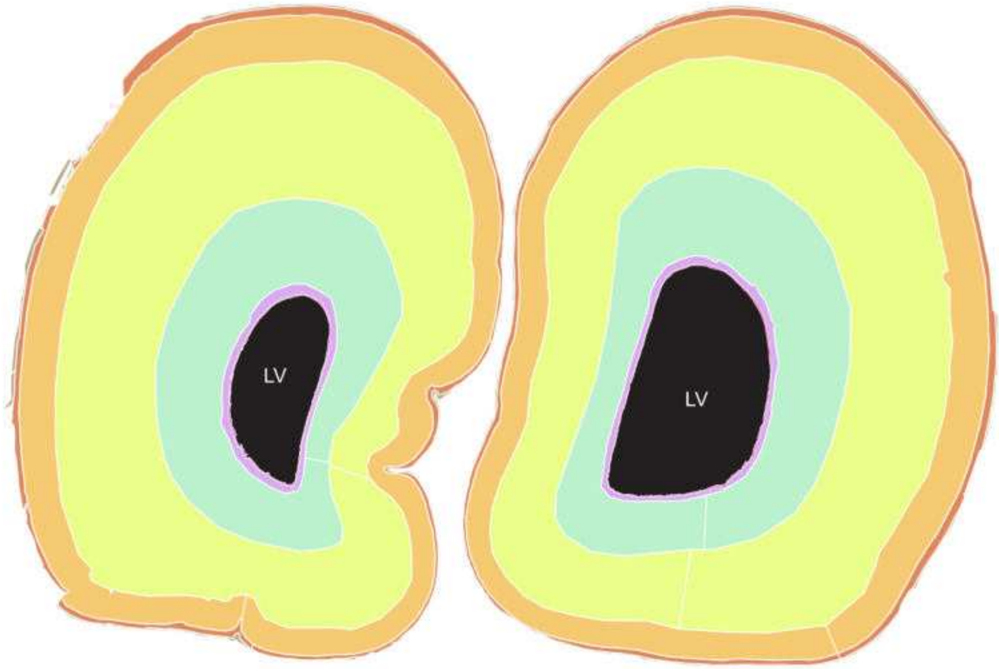

5 mm

LV: Lateral ventricle

Age: 22 GW

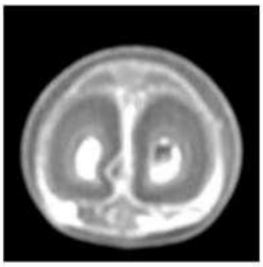

A-P Level: -23.1 mm

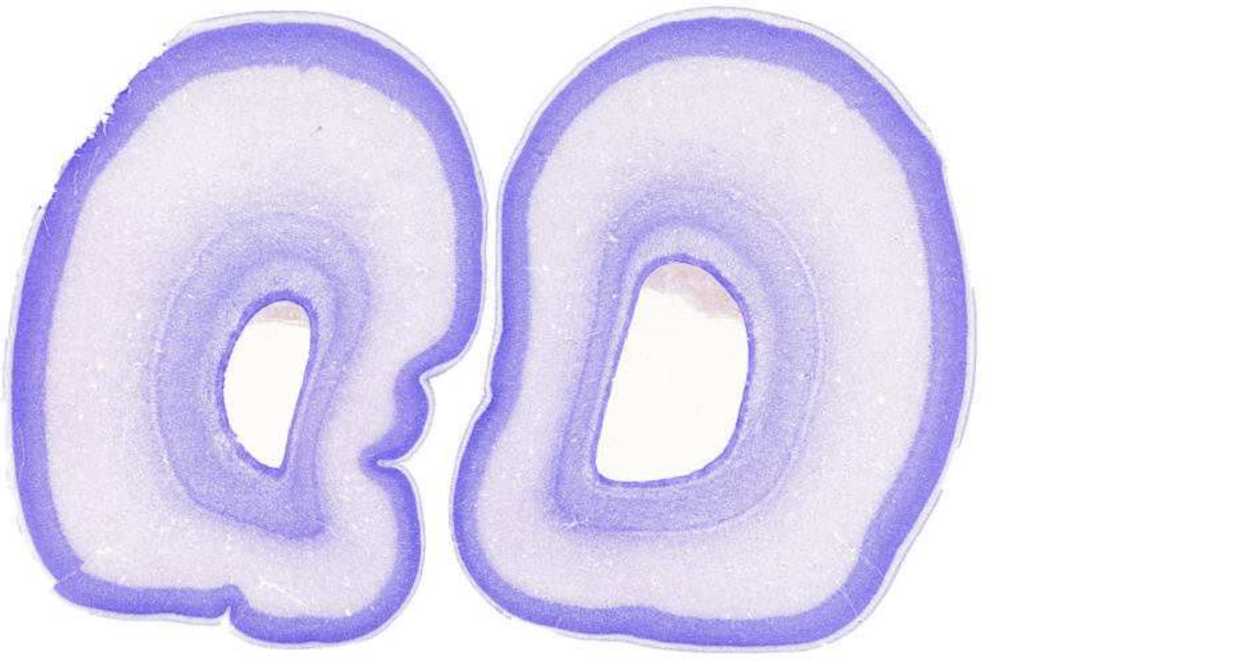

5 mm

Transient Layers

- VZ
- SVZ
- IZ
- SP
- CP
- MZ
- SGL

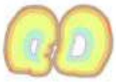

Cortical Areas

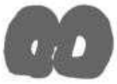

- OCC

Age: 22 GW

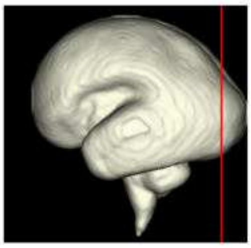

A-P Level: -23.1 mm

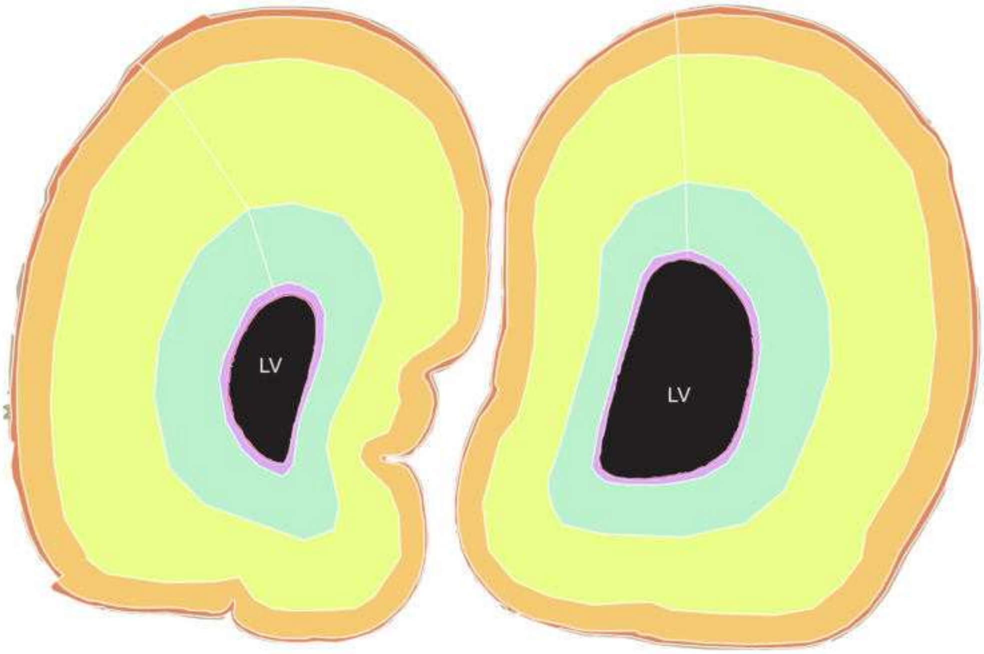

5 mm

LV: Lateral ventricle

Age: 22 GW

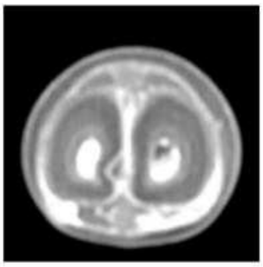

A-P Level: -23.34 mm

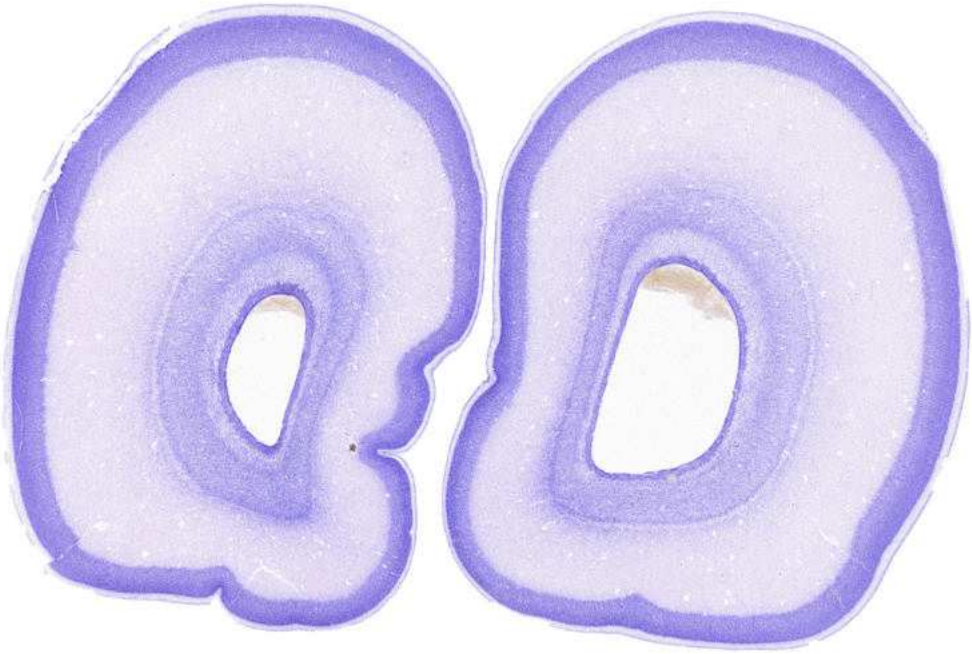

5 mm

Transient Layers

- VZ
- SVZ
- IZ
- SP
- CP
- MZ
- SGL

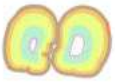

Cortical Areas

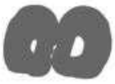

- OCC

Age: 22 GW

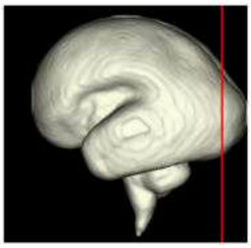

A-P Level: -23.34 mm

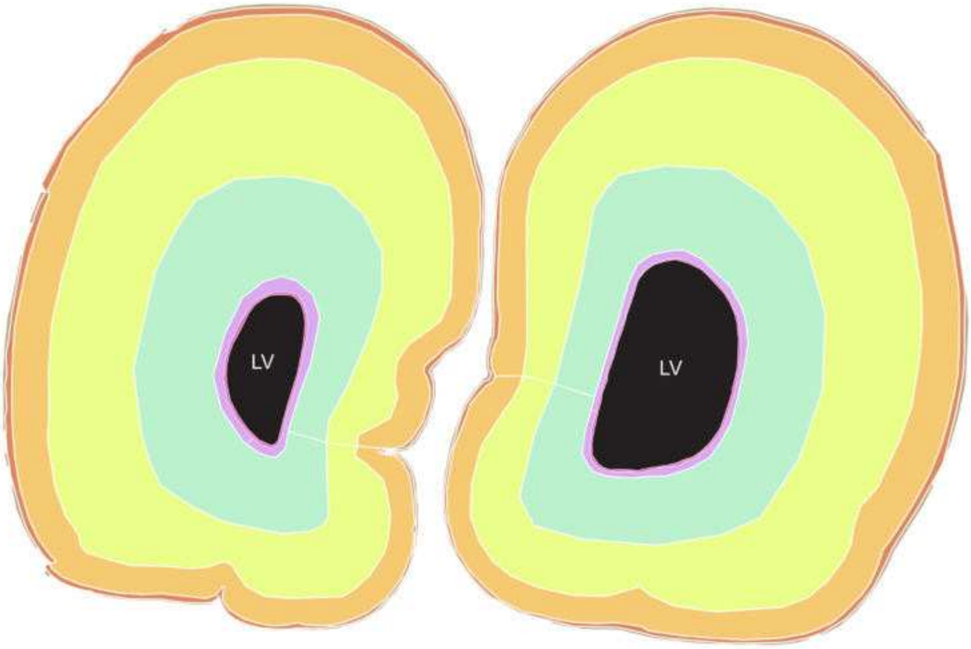

5 mm

LV: Lateral ventricle

Age: 22 GW

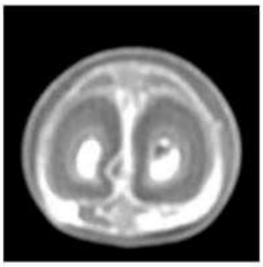

A-P Level: -23.46 mm

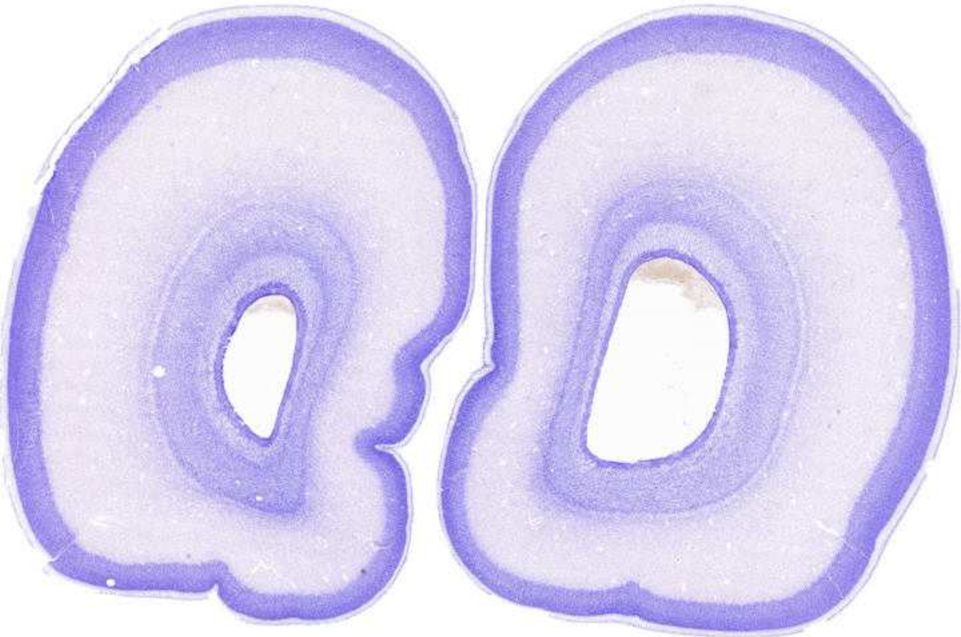

5 mm

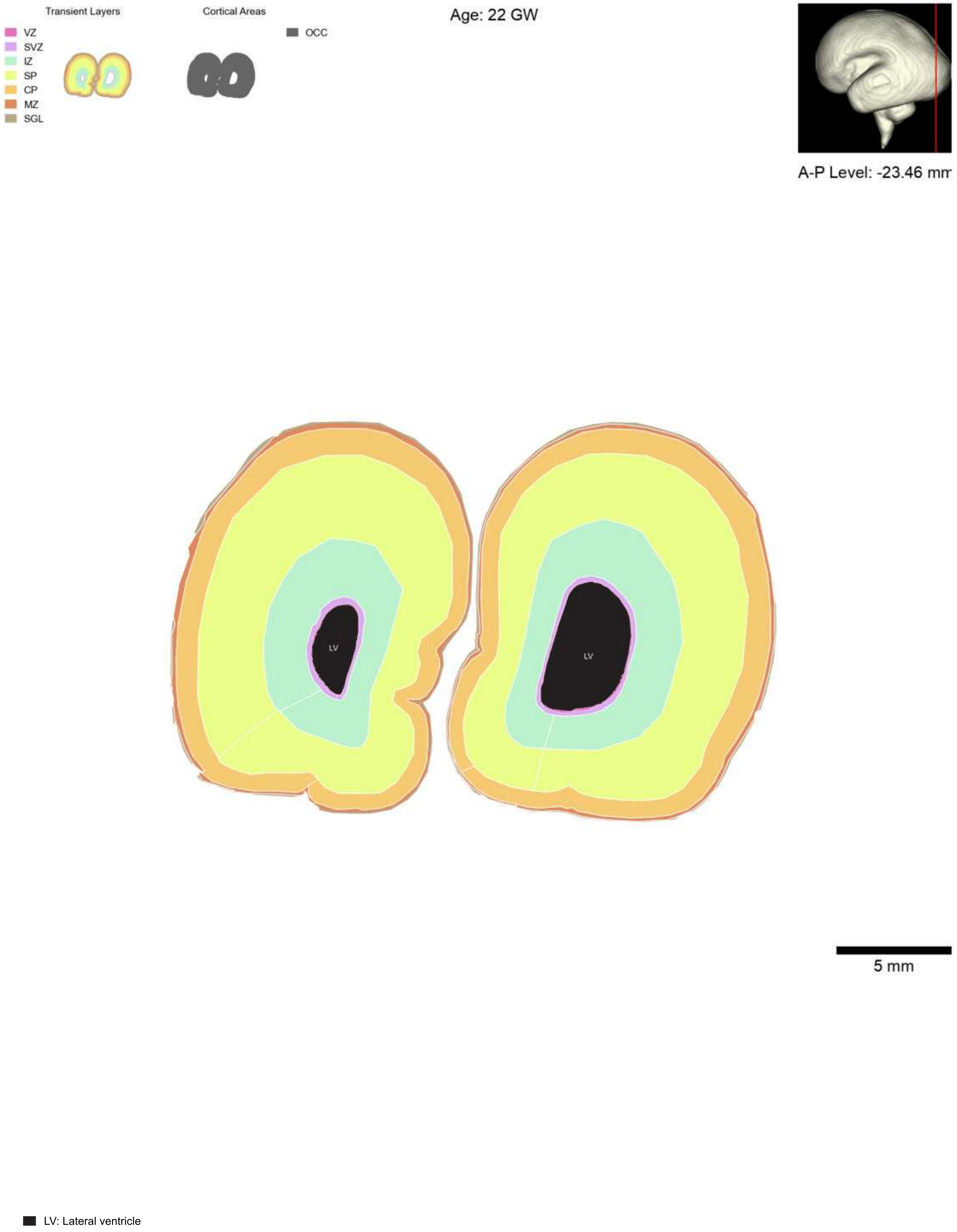
Transient Layers
VZ
SVZ
IZ
SP
CP
MZ
SGL
Cortical Areas
OCC
Age: 22 GW
A-P Level: -23.46 mm
LV
LV
5 mm
LV: Lateral ventricle

Age: 22 GW

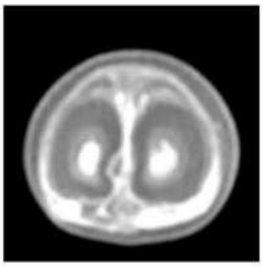

A-P Level: -23.88 mm

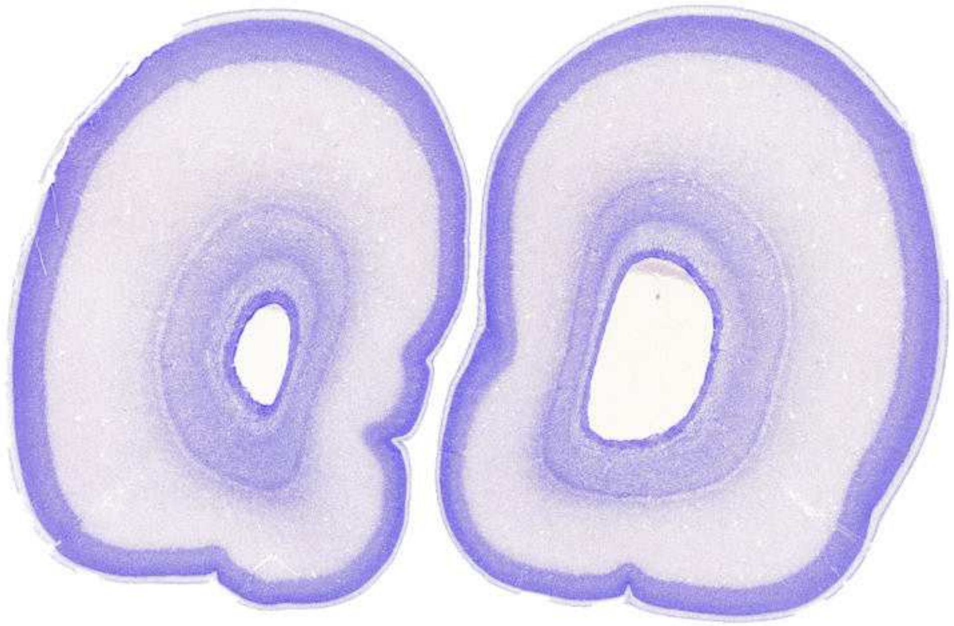

5 mm

Transient Layers

- VZ
- SVZ
- IZ
- SP
- CP
- MZ
- SGL

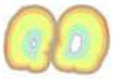

Cortical Areas

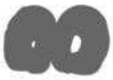

- OCC

Age: 22 GW

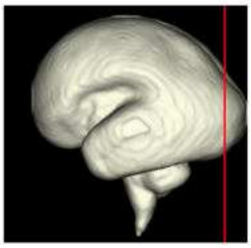

A-P Level: -23.88 mm

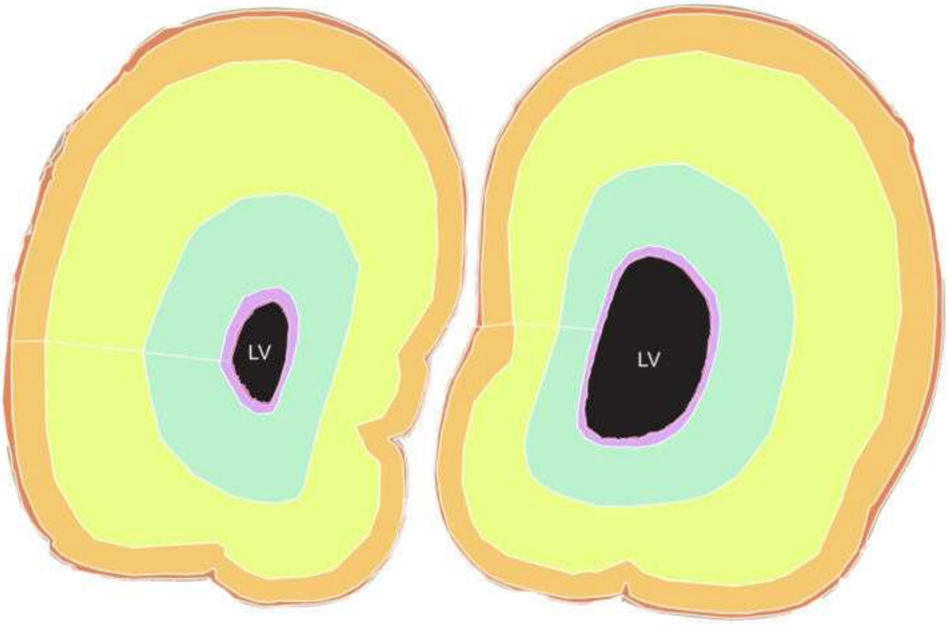

5 mm

LV: Lateral ventricle

Age: 22 GW

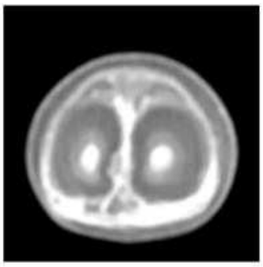

A-P Level: -24.3 mm

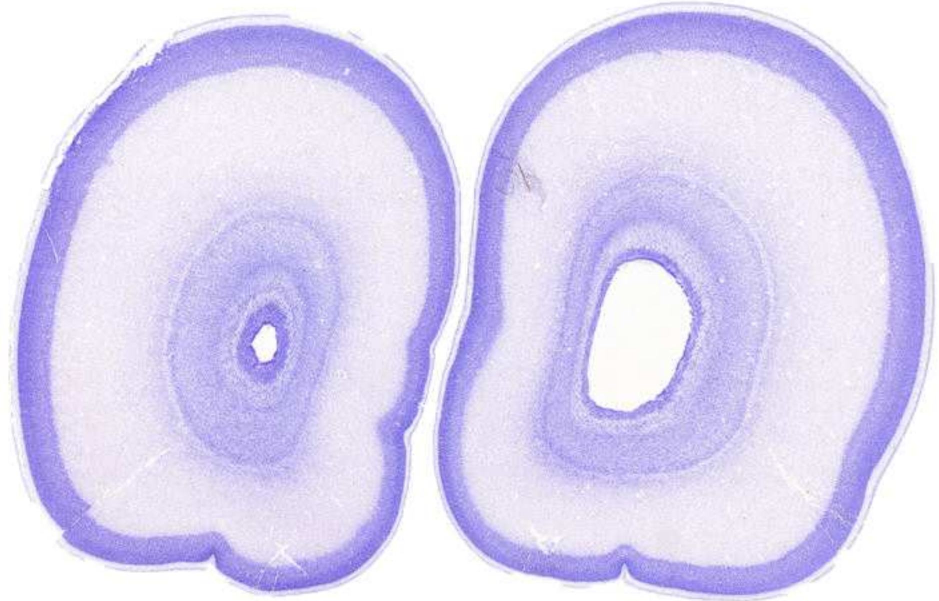

5 mm

Transient Layers

- VZ
- SVZ
- IZ
- SP
- CP
- MZ
- SGL

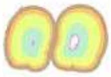

Cortical Areas

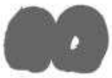

- OCC

Age: 22 GW

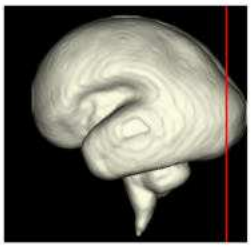

A-P Level: -24.3 mm

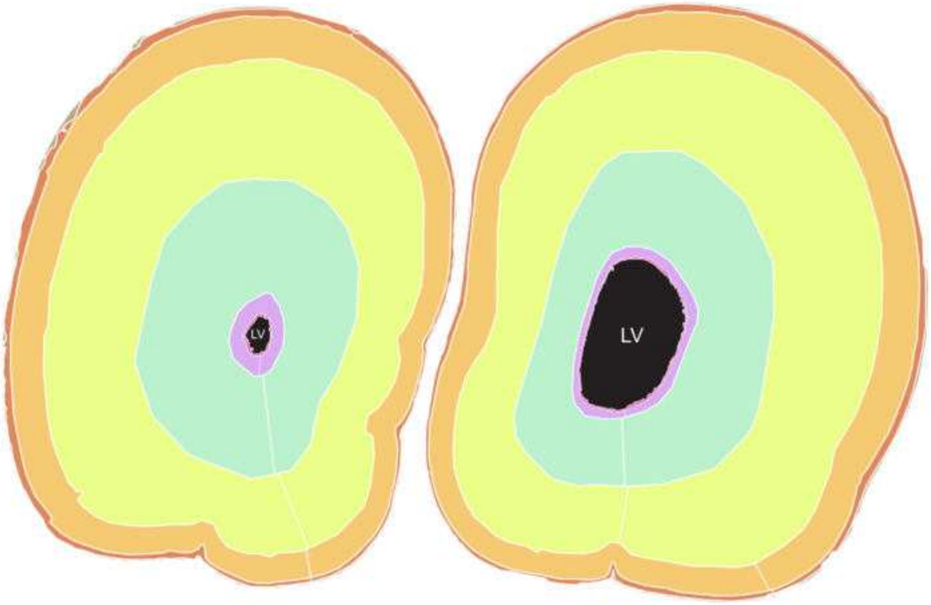

5 mm

LV: Lateral ventricle

Age: 22 GW

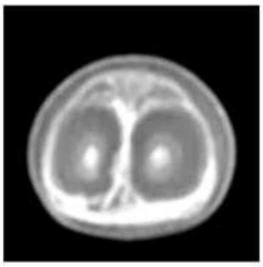

A-P Level: -24.66 mm

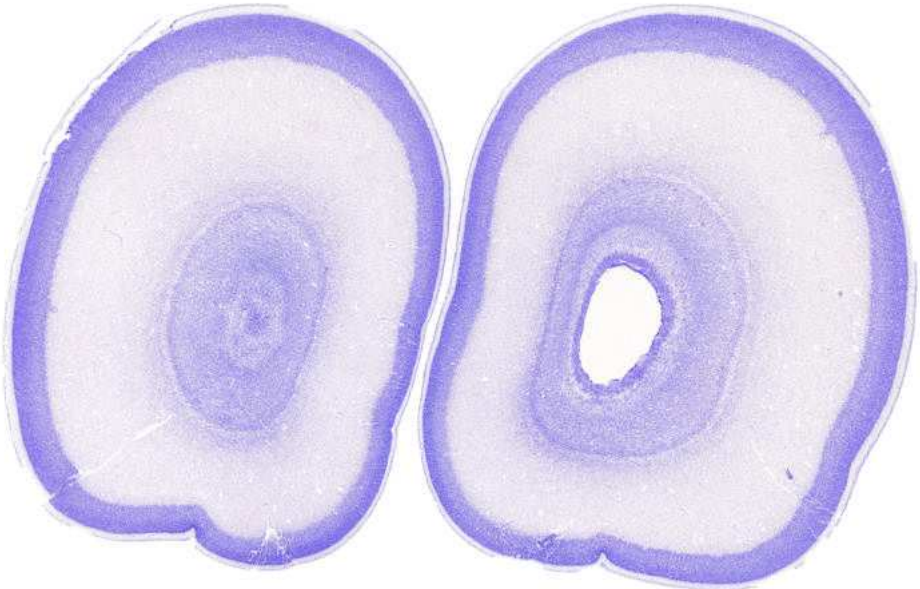

5 mm

Transient Layers

- VZ
- SVZ
- IZ
- SP
- CP
- MZ
- SGL

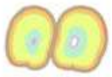

Cortical Areas

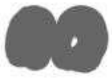

- OCC

Age: 22 GW

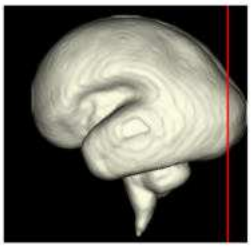

A-P Level: -24.66 mm

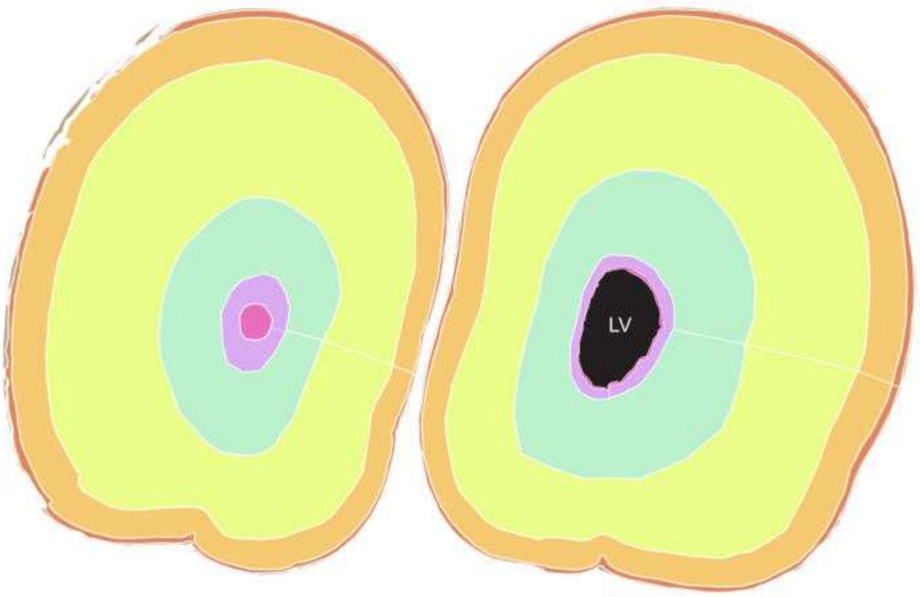

5 mm

LV: Lateral ventricle

Age: 22 GW

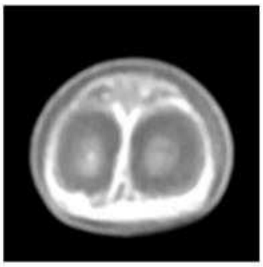

A-P Level: -25.2 mm

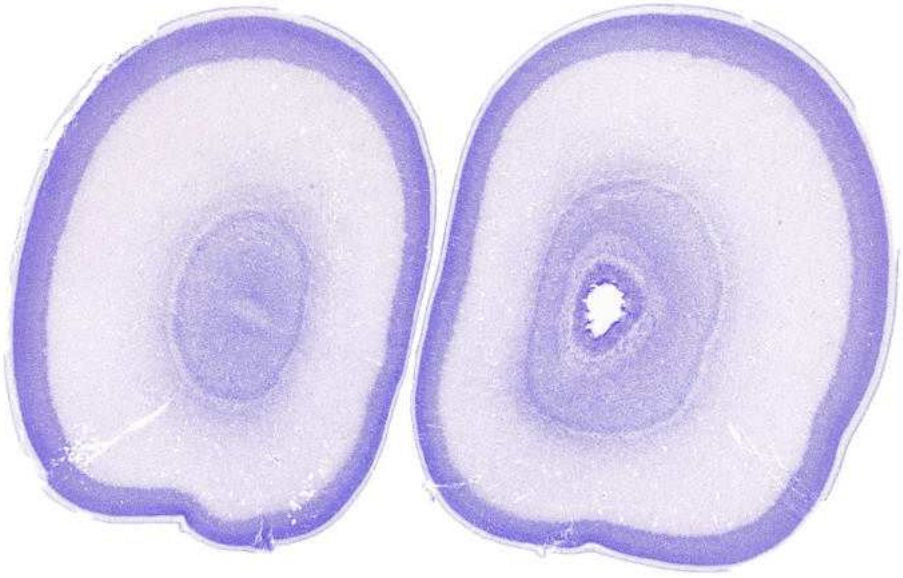

5 mm

Transient Layers

- VZ
- SVZ
- IZ
- SP
- CP
- MZ
- SGL

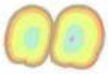

Cortical Areas

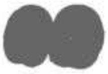

- OCC

Age: 22 GW

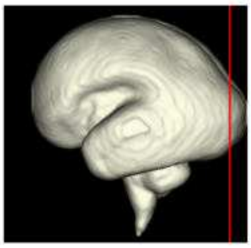

A-P Level: -25.2 mm

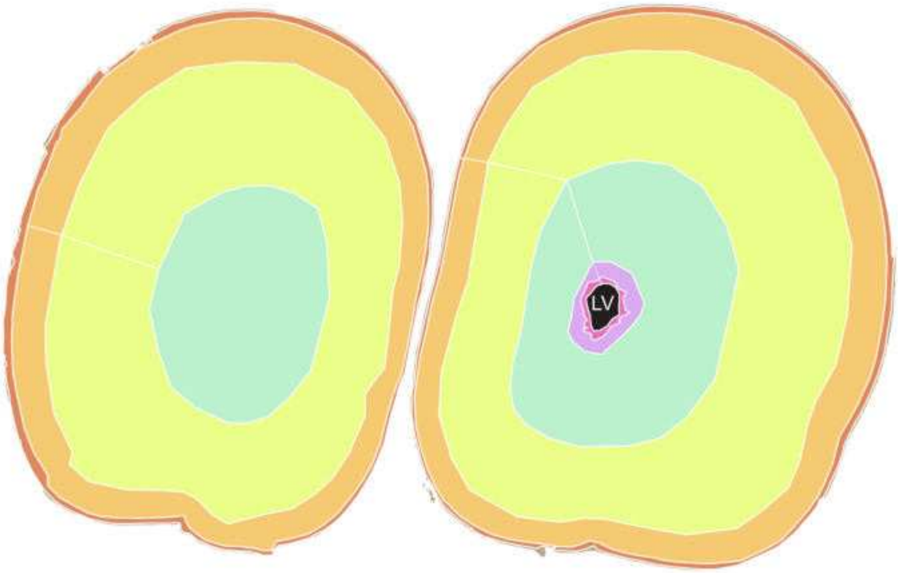

5 mm

- LV: Lateral ventricle

Age: 22 GW

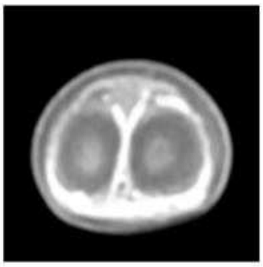

A-P Level: -25.44 mm

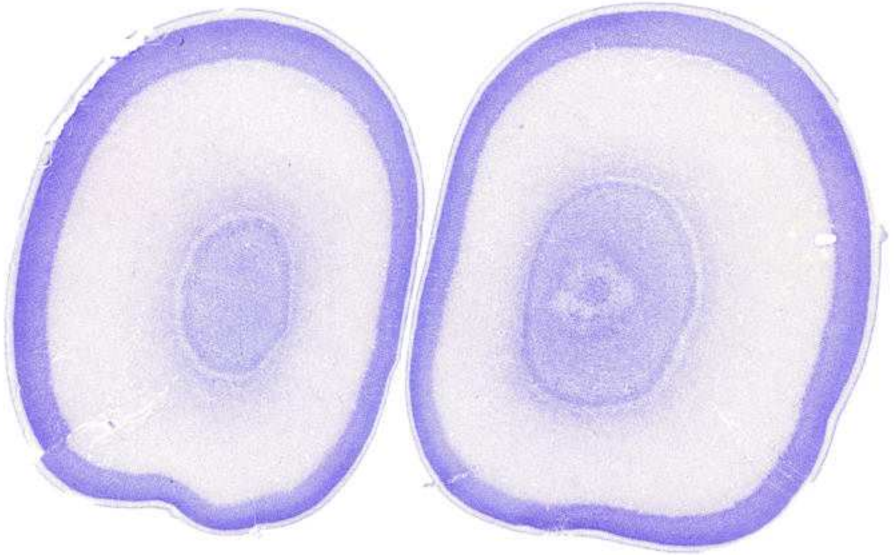

5 mm

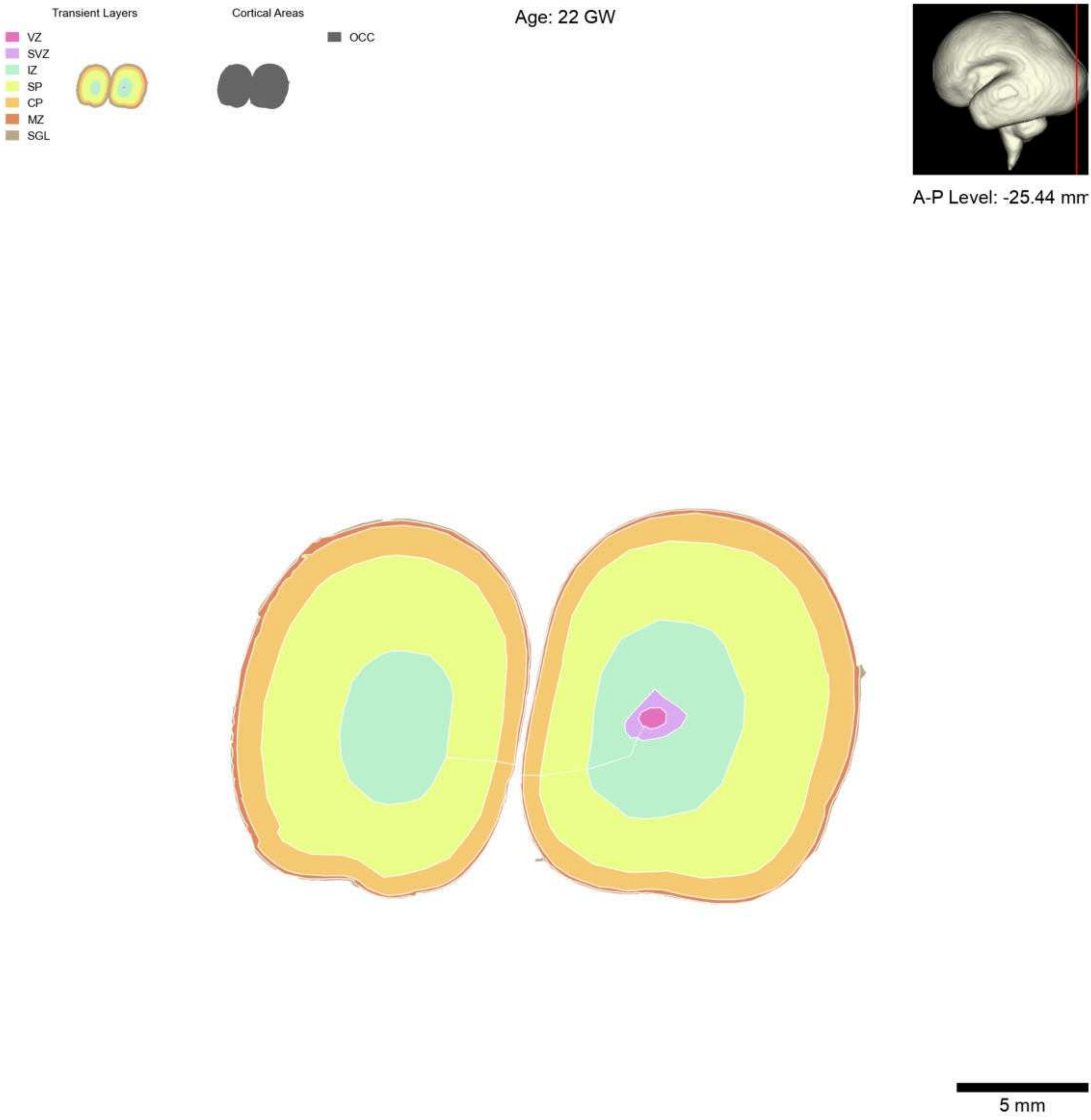

Transient Layers
VZ
SVZ
IZ
SP
CP
MZ
SGL
Cortical Areas
OCC
Age: 22 GW
A-P Level: -25.44 mm
5 mm

Age: 22 GW

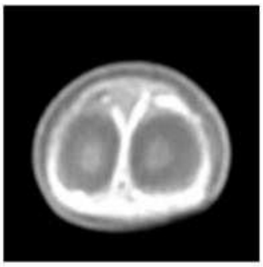

A-P Level: -25.68 mm

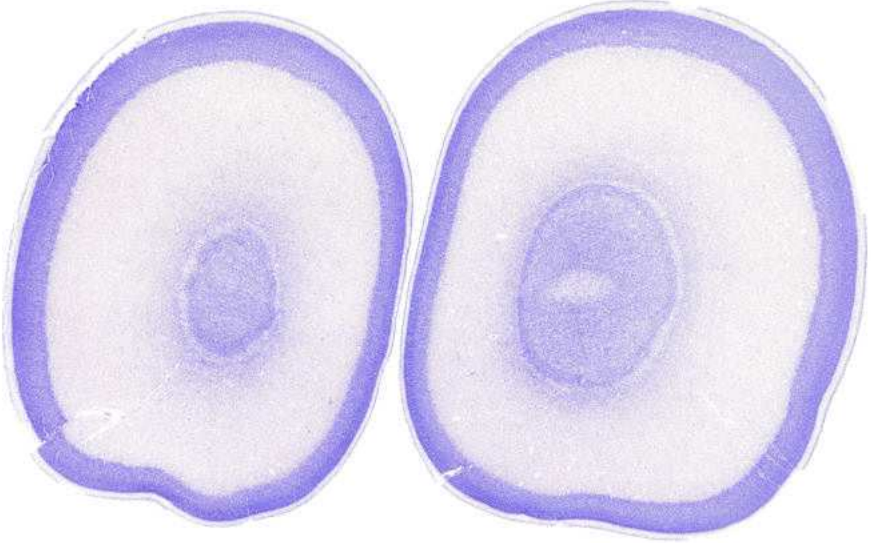

5 mm

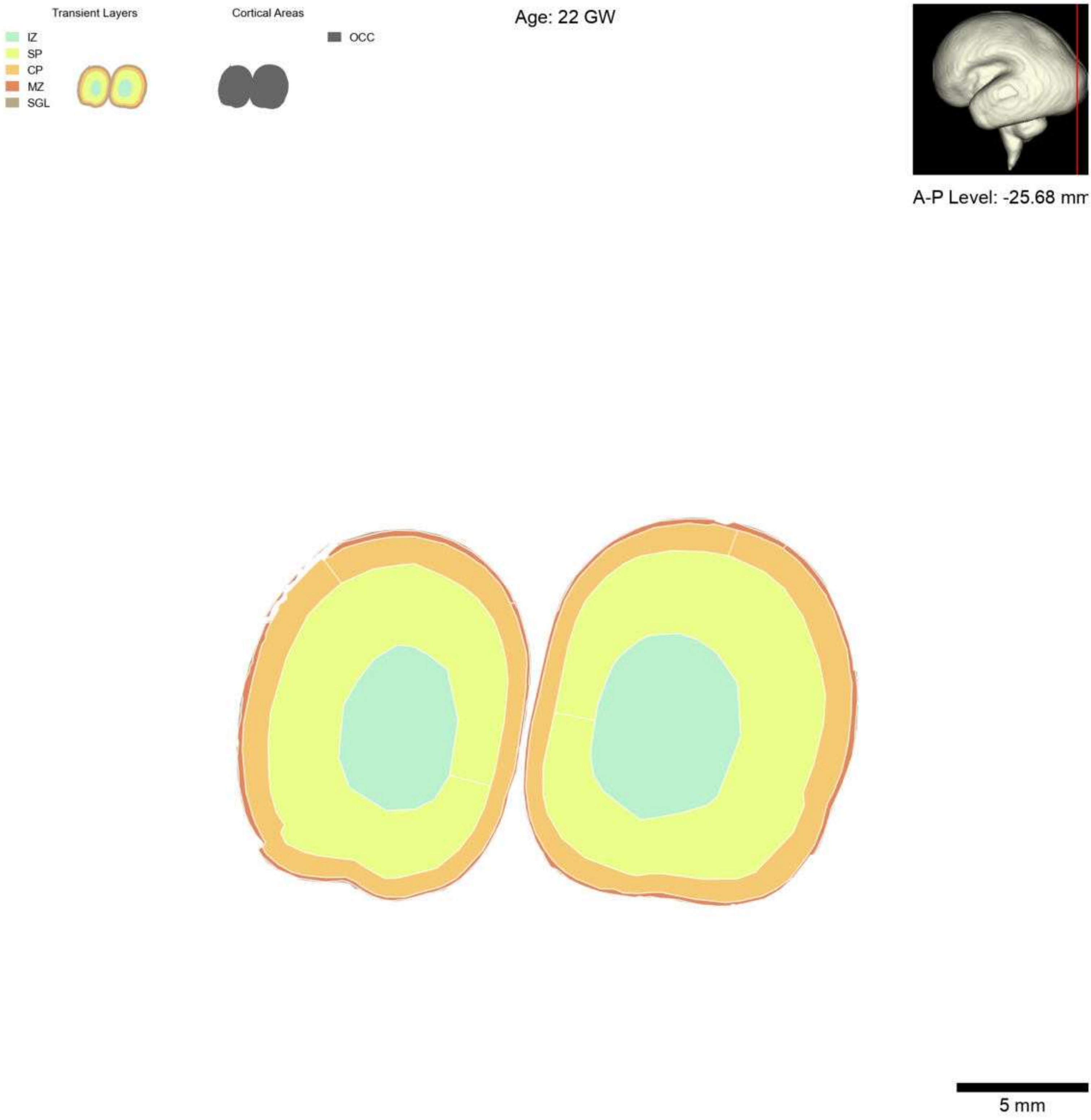
Transient Layers
IZ
SP
CP
MZ
SGL
Cortical Areas
OCC
Age: 22 GW
A-P Level: -25.68 mm
5 mm

Age: 22 GW

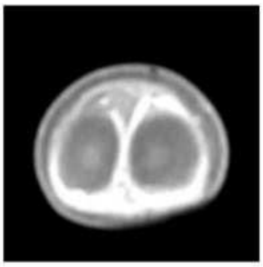

A-P Level: -25.98 mm

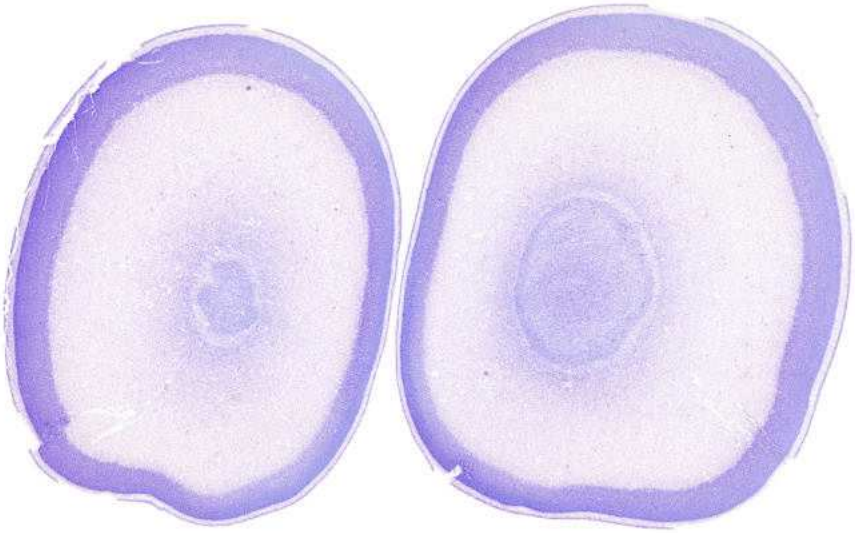

5 mm

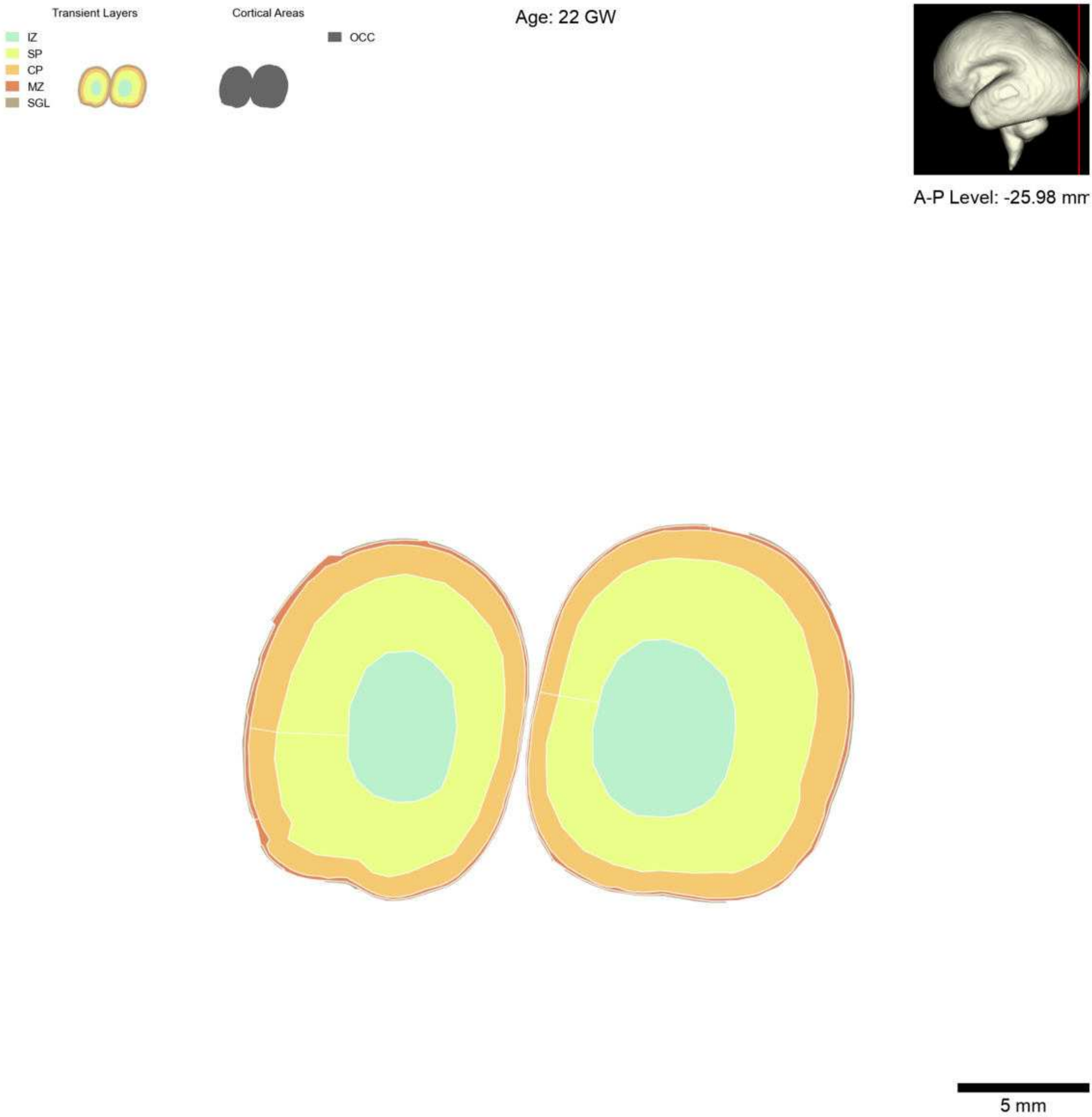
Transient Layers
IZ
SP
CP
MZ
SGL
Cortical Areas
OCC
Age: 22 GW
A-P Level: -25.98 mm
5 mm

Age: 22 GW

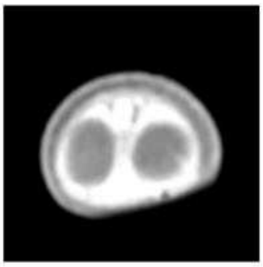

A-P Level: -27.42 mm

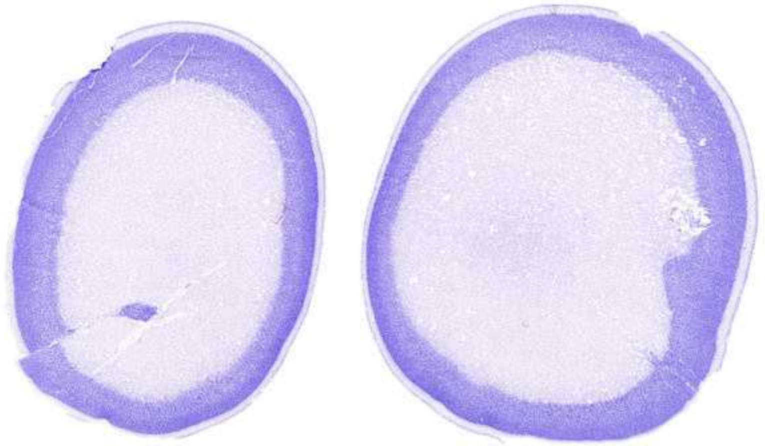

5 mm

Transient Layers

- SP
- CP
- MZ
- SGL

Cortical Areas

- OCC

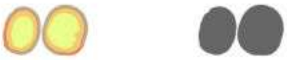

Age: 22 GW

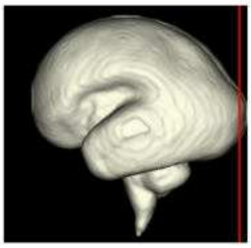

A-P Level: -27.42 mm

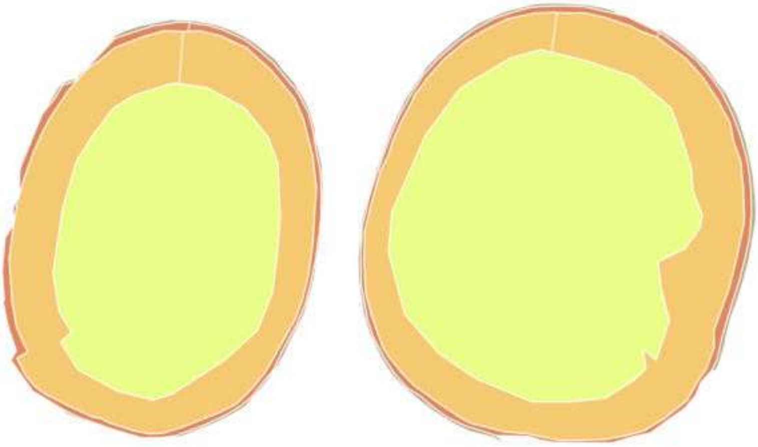

5 mm

Age: 22 GW

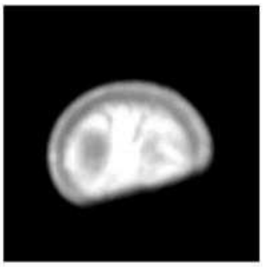

A-P Level: -28.62 mm

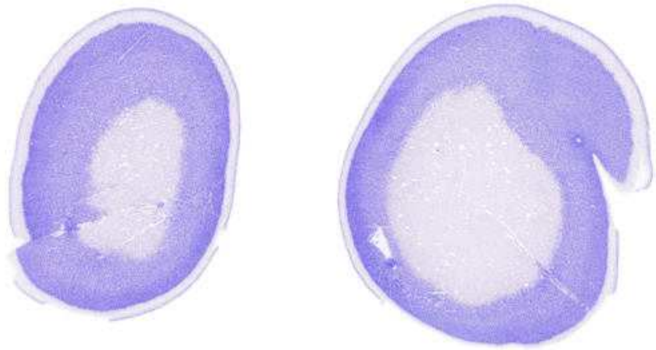

5 mm

Age: 22 GW

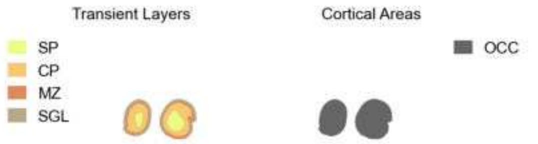

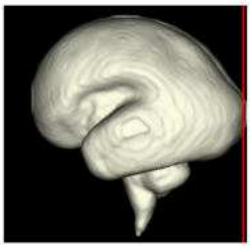

A-P Level: -28.62 mm

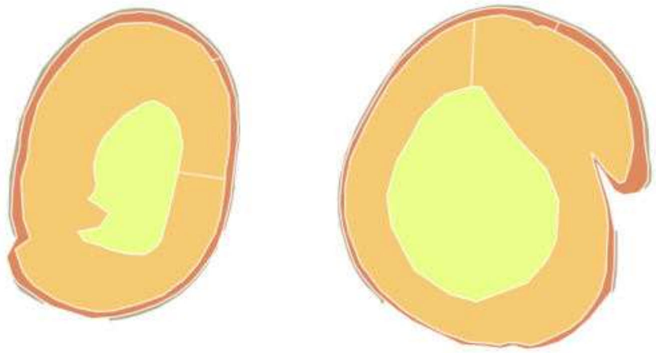

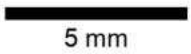

Age: 22 GW

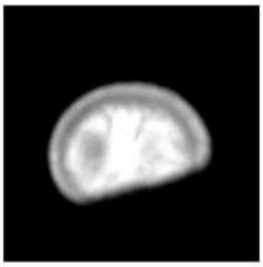

A-P Level: -29.16 mm

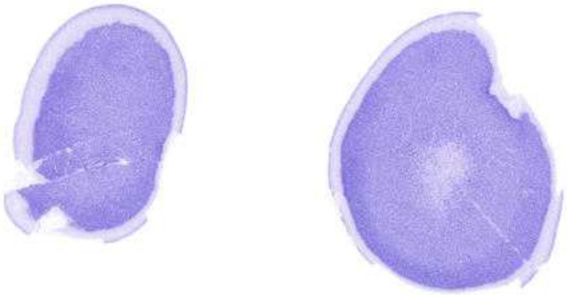

5 mm

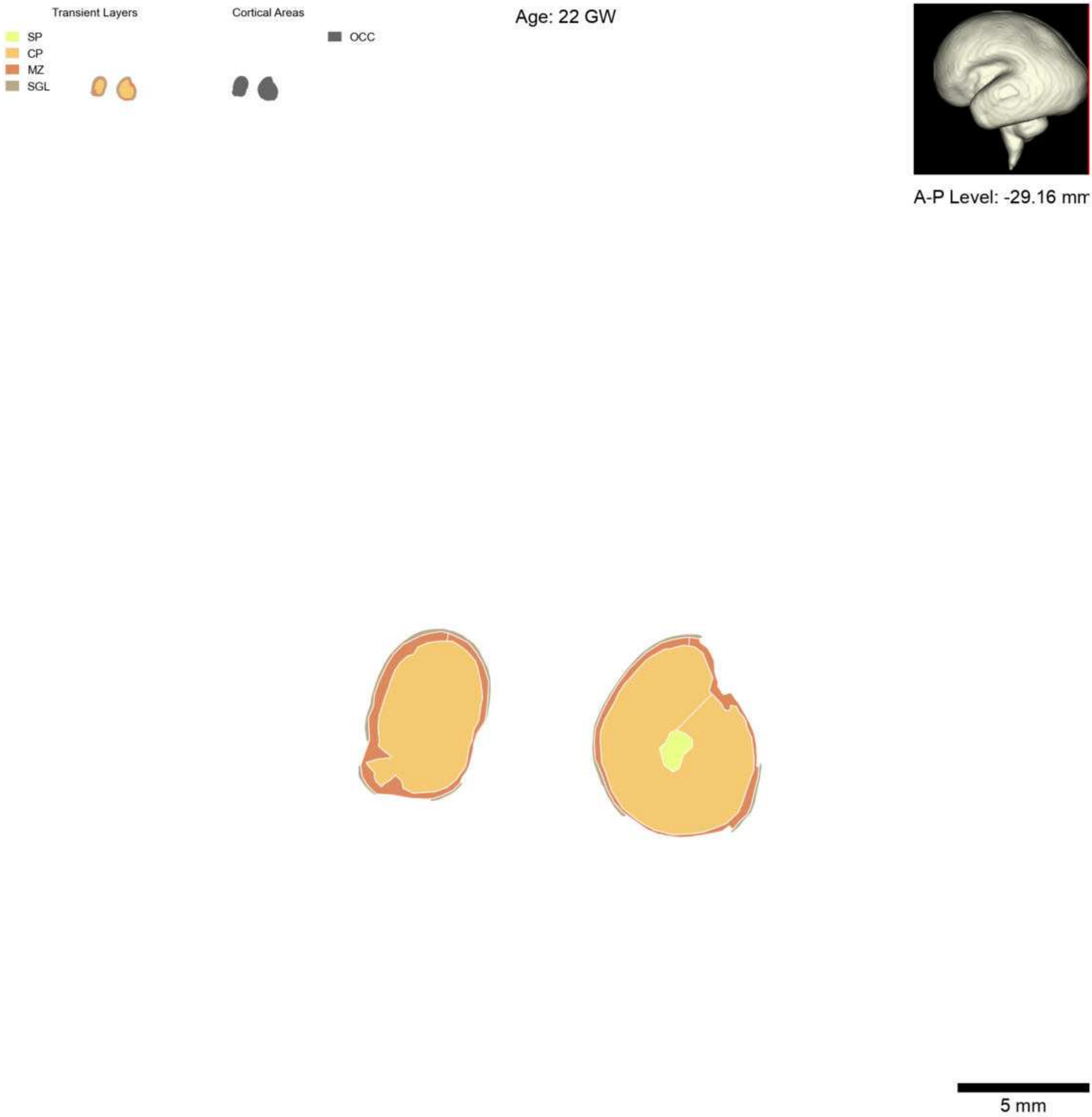
Transient Layers
SP
CP
MZ
SGL
Cortical Areas
OCC
Age: 22 GW
A-P Level: -29.16 mm
5 mm

Age: 22 GW

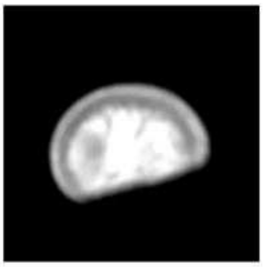

A-P Level: -29.4 mm

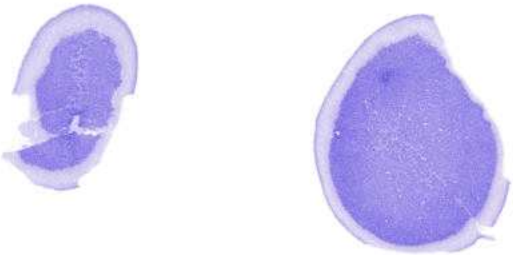

5 mm

Transient Layers

- CP
- MZ
- SGL

Cortical Areas

- OCC

Age: 22 GW

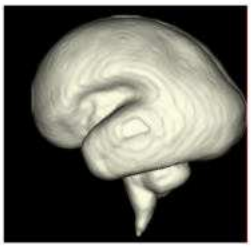

A-P Level: -29.4 mm

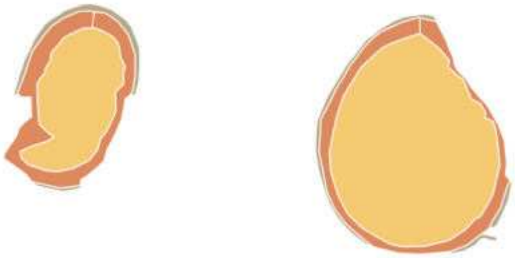

5 mm

24 Gestational Week (GW)

Sagittal

This page is intentionally left blank

Age: 24 GW

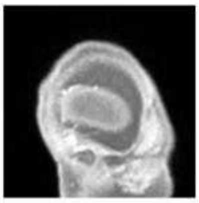

L-R Level: 28.86 mm

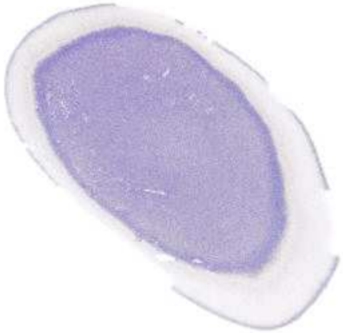

5 mm

Transient Layers

- CP
- MZ
- SGL

Cortical Areas

- TEMP

Age: 24 GW

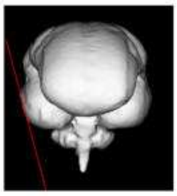

L-R Level: 28.86 mm

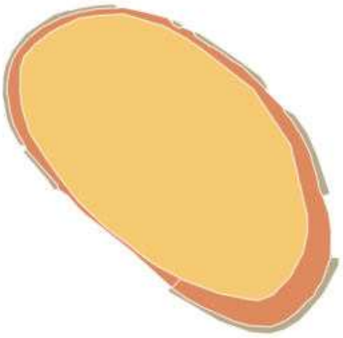

5 mm

Age: 24 GW

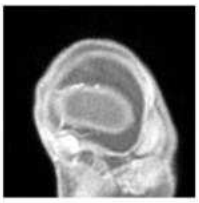

L-R Level: 28.02 mm

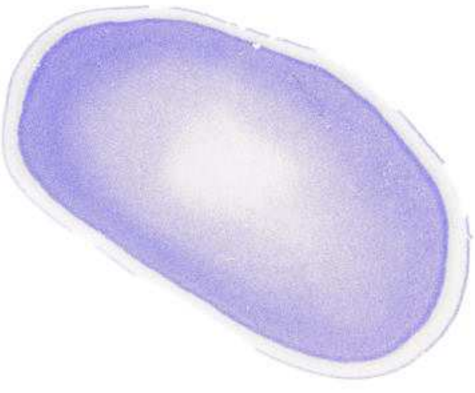

5 mm

Transient Layers

- SP
- CP
- MZ
- SGL

Cortical Areas

- TEMP

Age: 24 GW

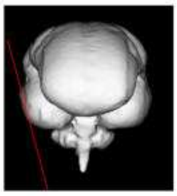

L-R Level: 28.02 mm

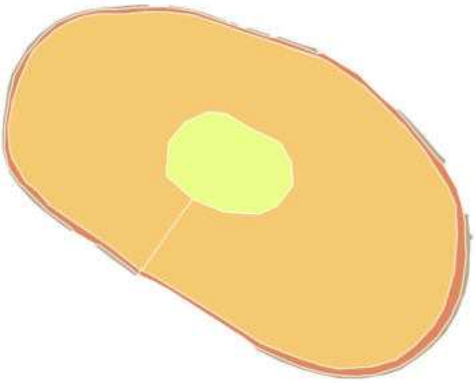

5 mm

Age: 24 GW

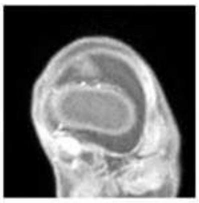

L-R Level: 27.6 mm

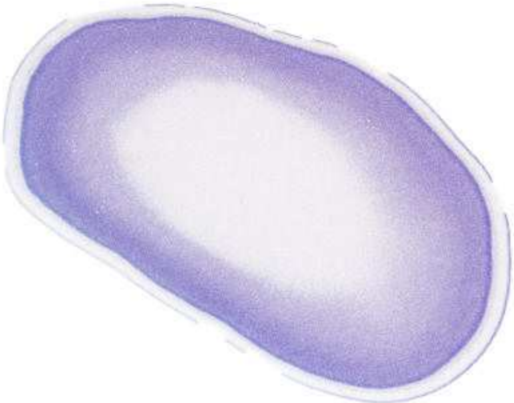

5 mm

Age: 24 GW

L-R Level: 27.6 mm

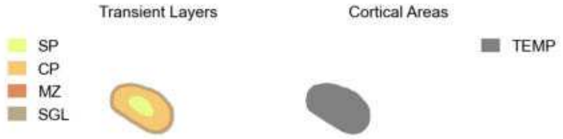

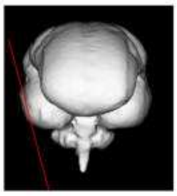

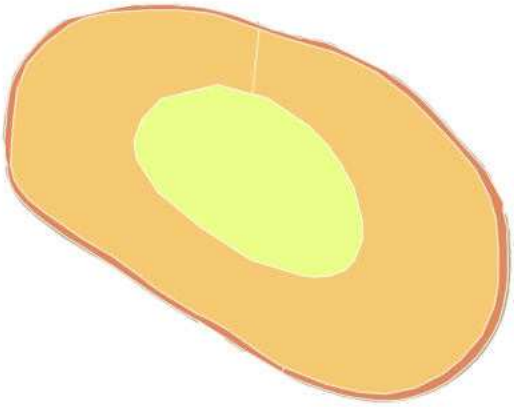

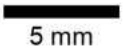

Age: 24 GW

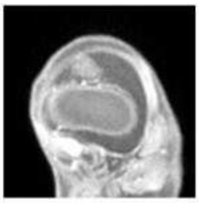

L-R Level: 27.12 mm

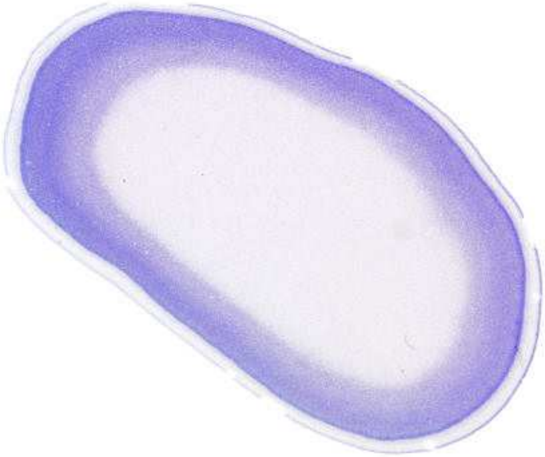

5 mm

Transient Layers

- SP
- CP
- MZ
- SGL

Cortical Areas

- TEMP

Age: 24 GW

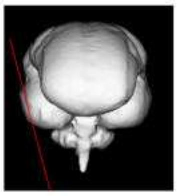

L-R Level: 27.12 mm

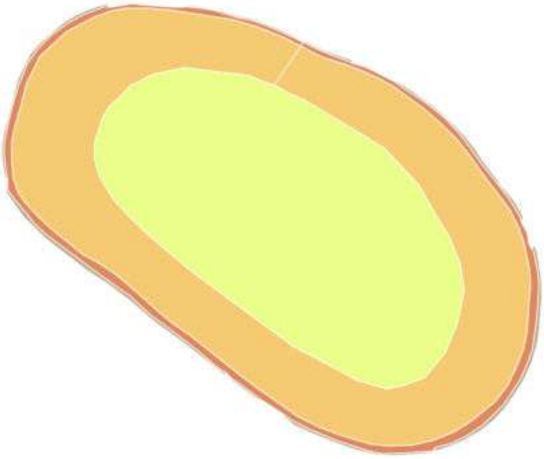

5 mm

Age: 24 GW

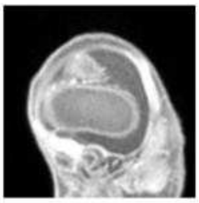

L-R Level: 26.46 mm

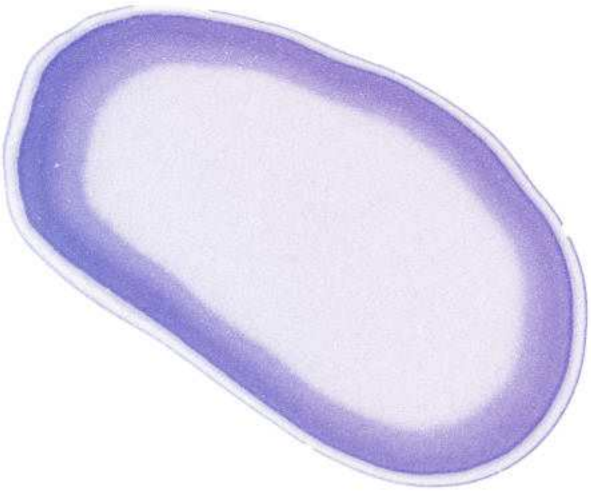

5 mm

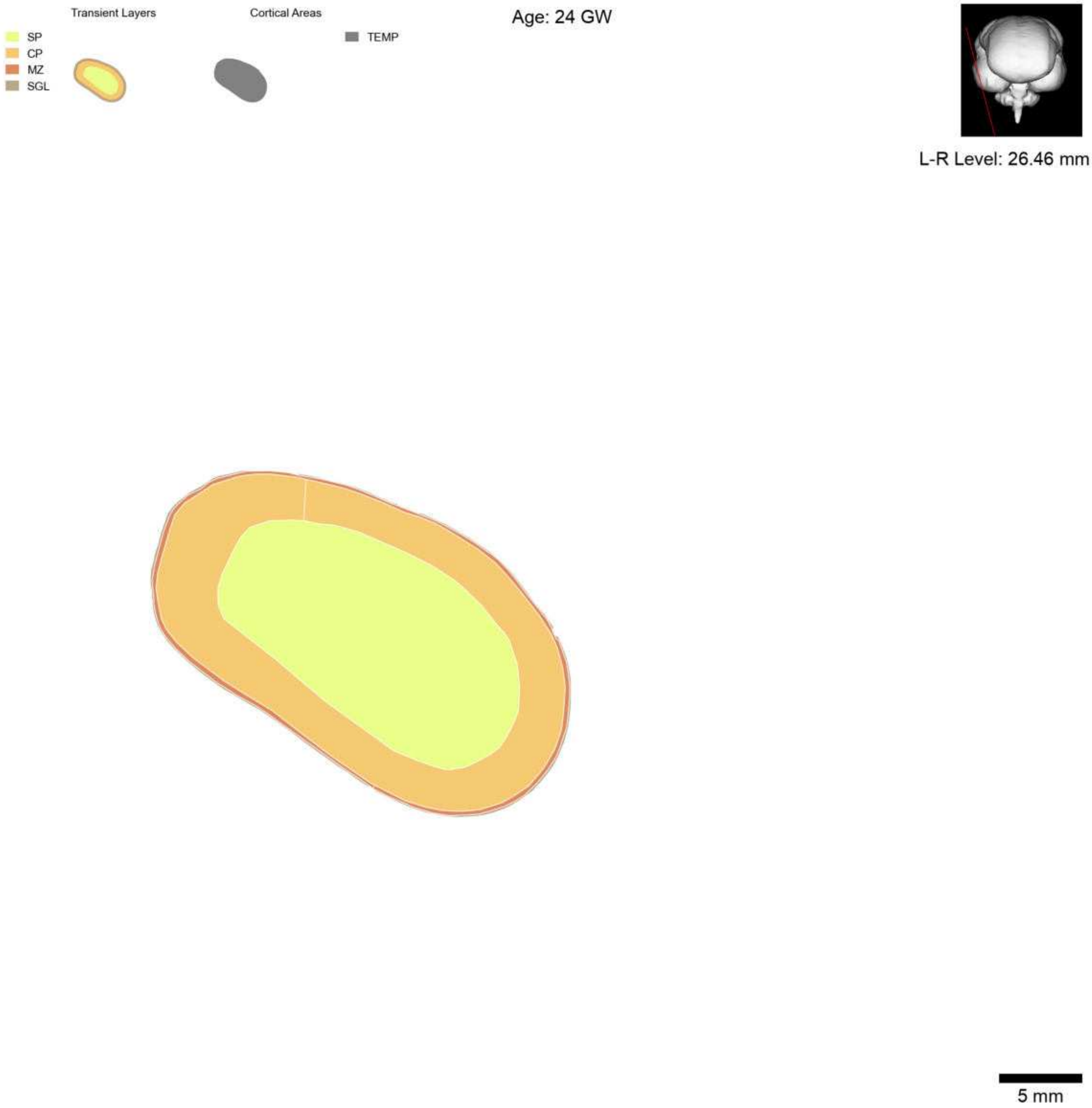
Transient Layers
SP
CP
MZ
SGL
Cortical Areas
TEMP
Age: 24 GW
L-R Level: 26.46 mm
5 mm

Age: 24 GW

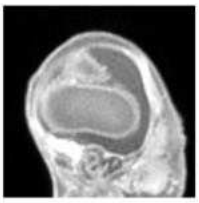

L-R Level: 26.04 mm

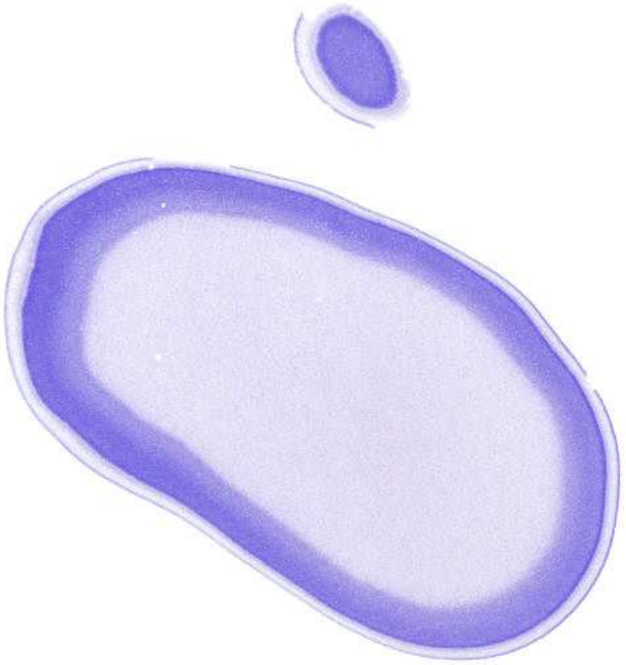

5 mm

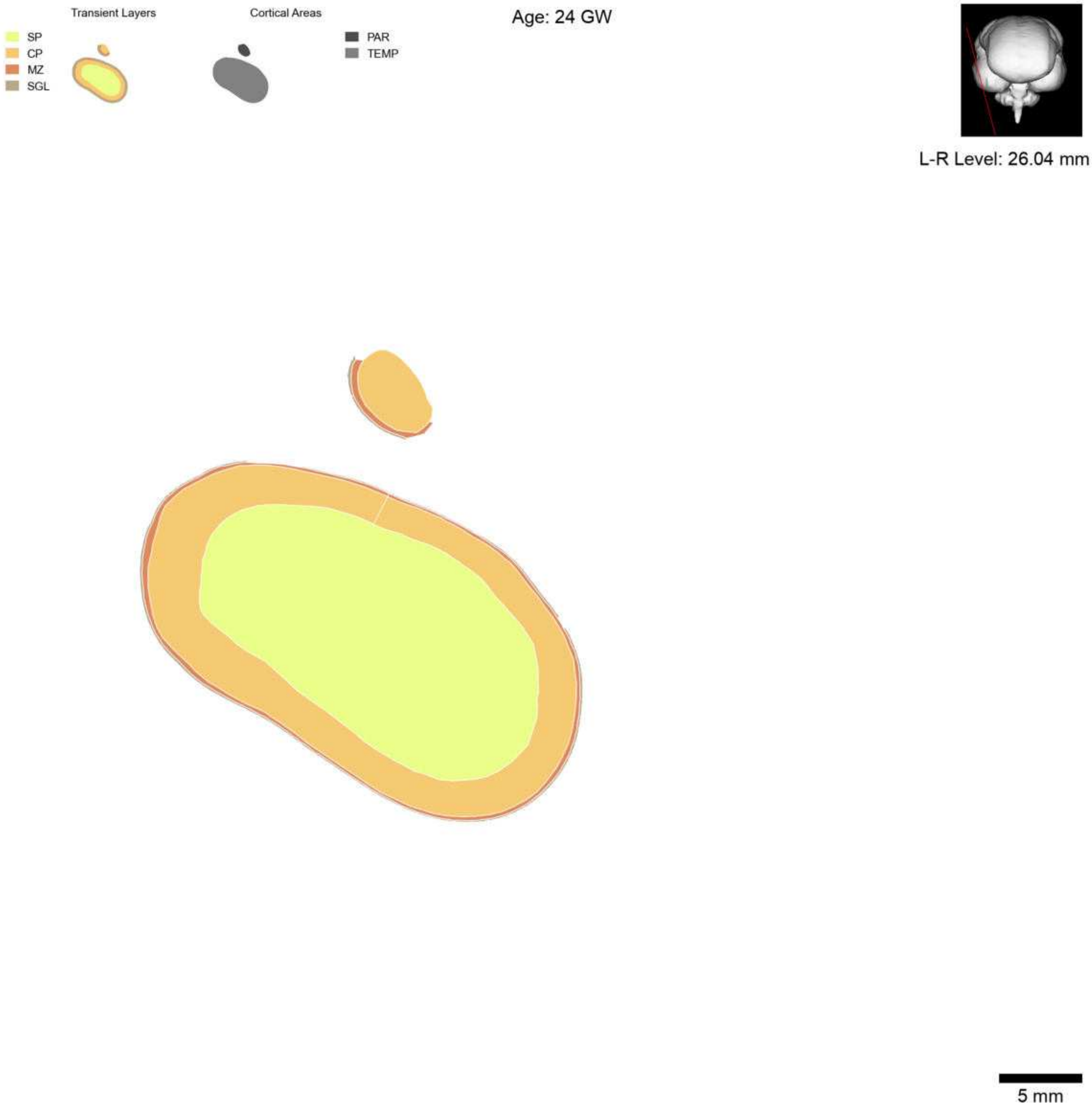
Transient Layers
SP
CP
MZ
SGL
Cortical Areas
PAR
TEMP
Age: 24 GW
L-R Level: 26.04 mm
5 mm

Age: 24 GW

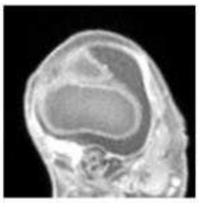

L-R Level: 25.56 mm

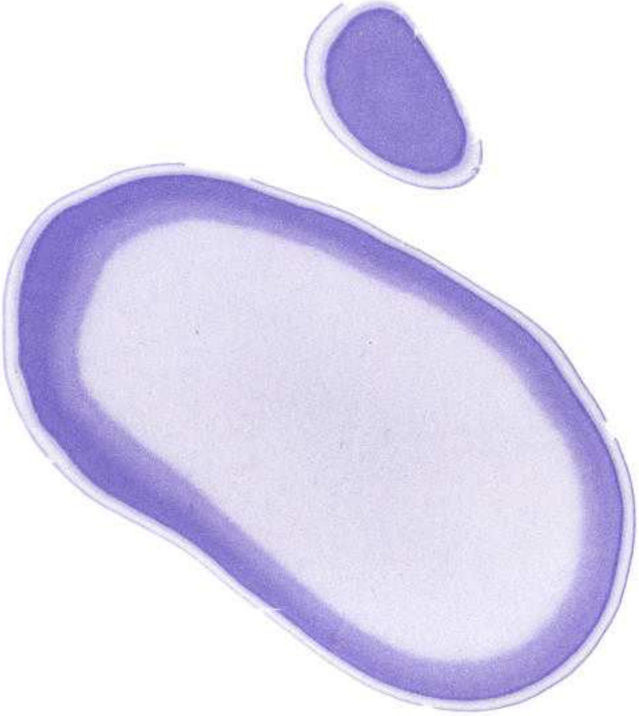

5 mm

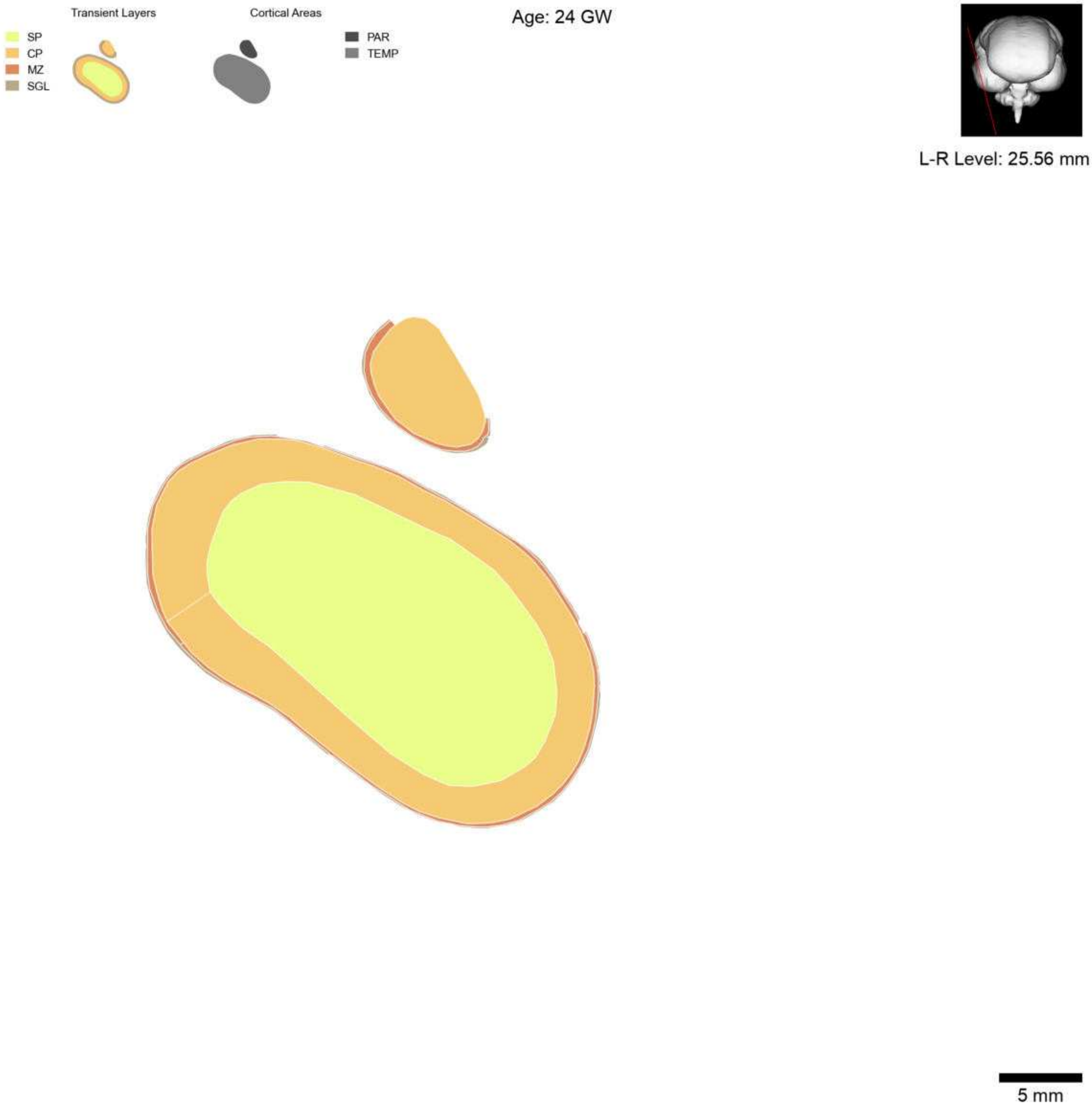
Transient Layers
Cortical Areas
Age: 24 GW
SP
CP
MZ
SGL
PAR
TEMP
L-R Level: 25.56 mm
5 mm

Age: 24 GW

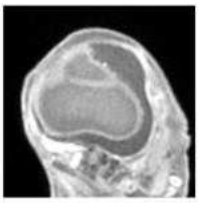

L-R Level: 24.6 mm

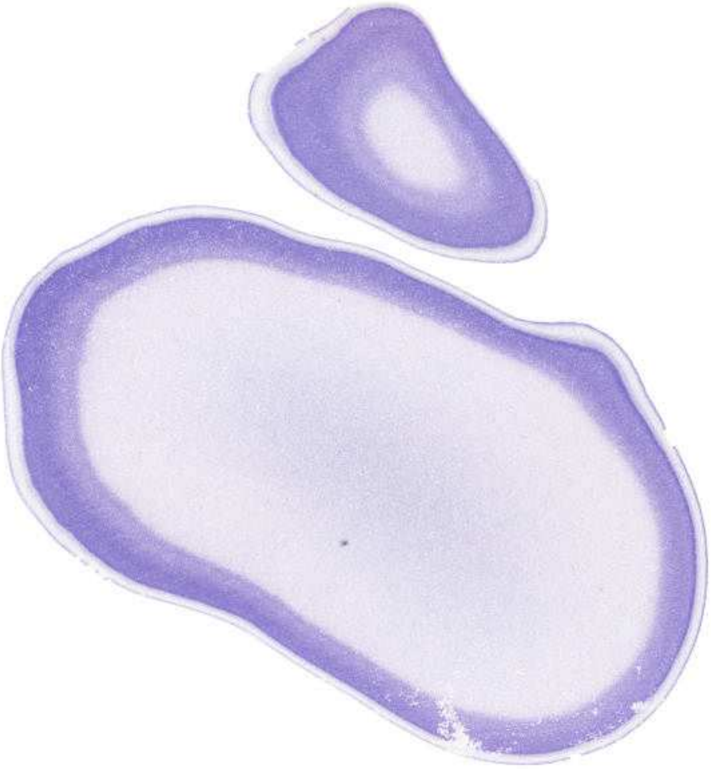

5 mm

Age: 24 GW

Transient Layers

Cortical Areas

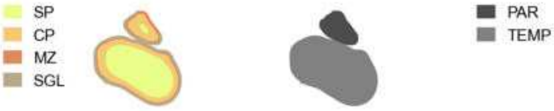

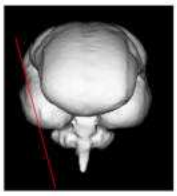

L-R Level: 24.6 mm

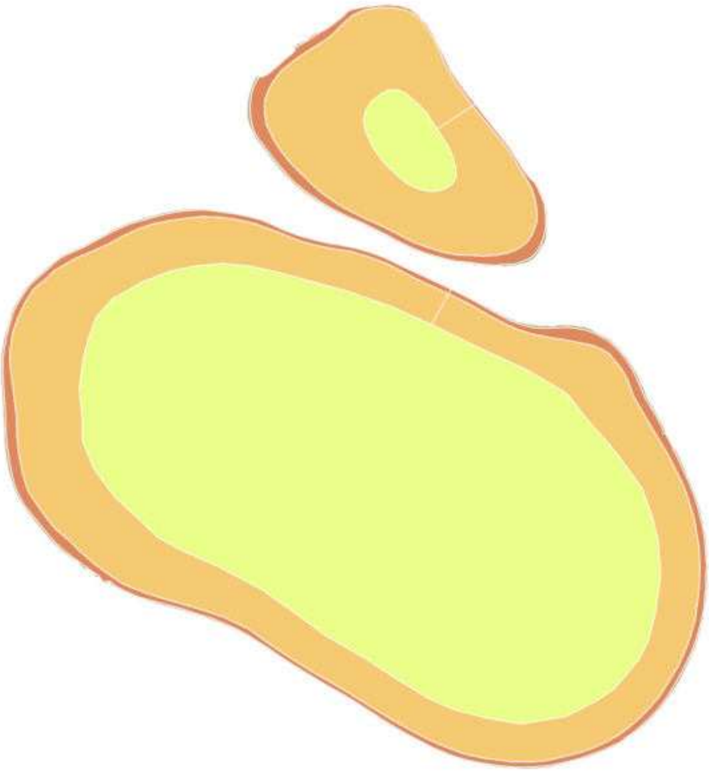

5 mm

Age: 24 GW

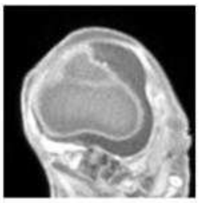

L-R Level: 23.82 mm

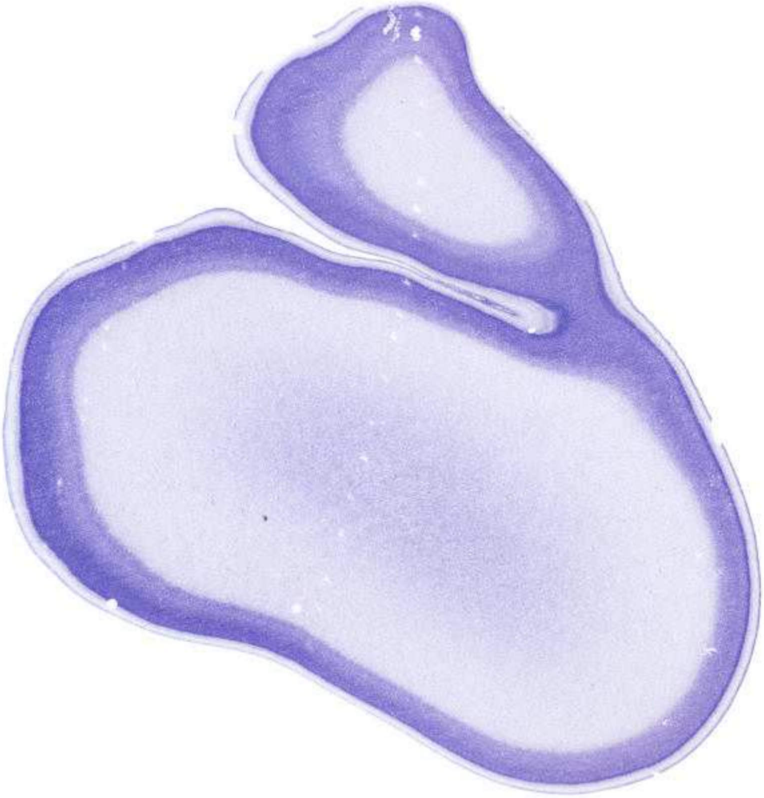

5 mm

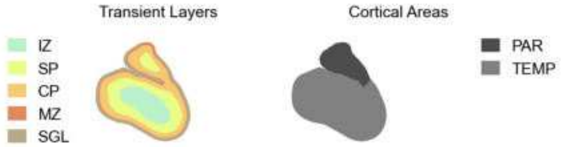

Age: 24 GW

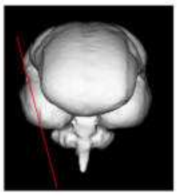

L-R Level: 23.82 mm

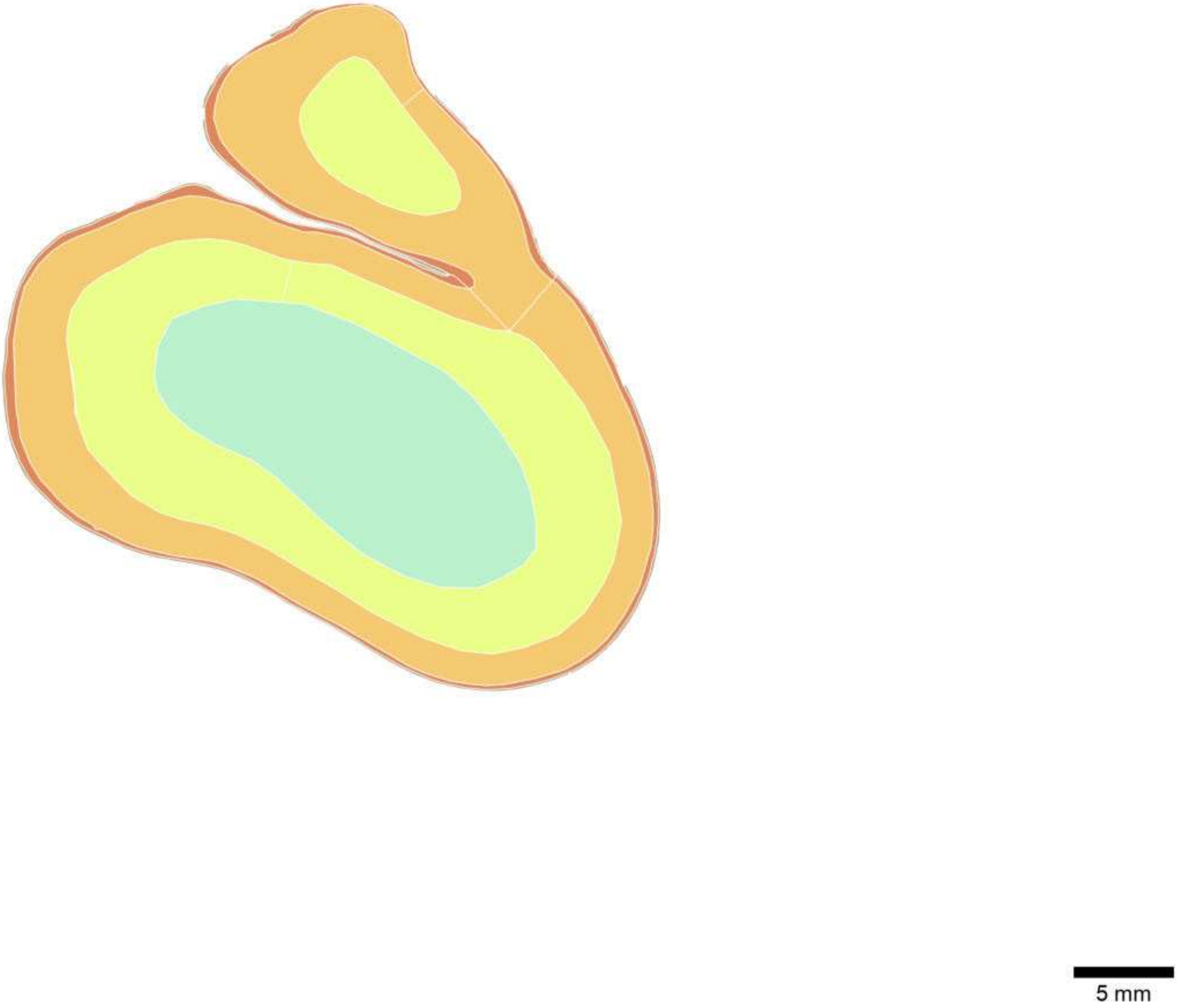

Age: 24 GW

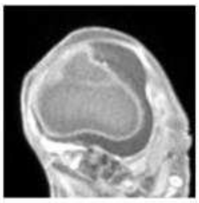

L-R Level: 23.46 mm

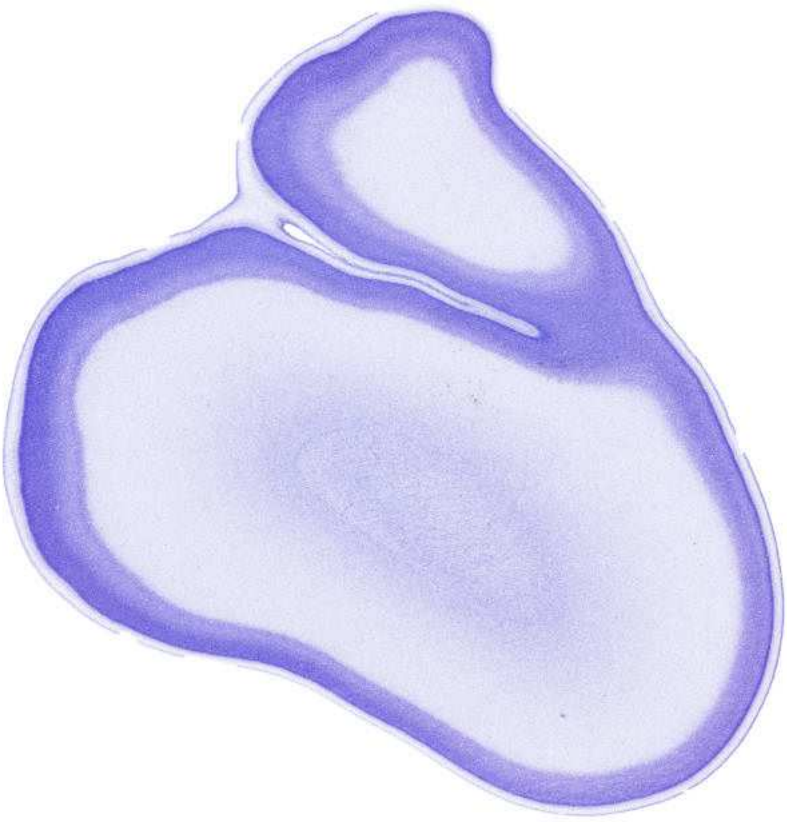

5 mm

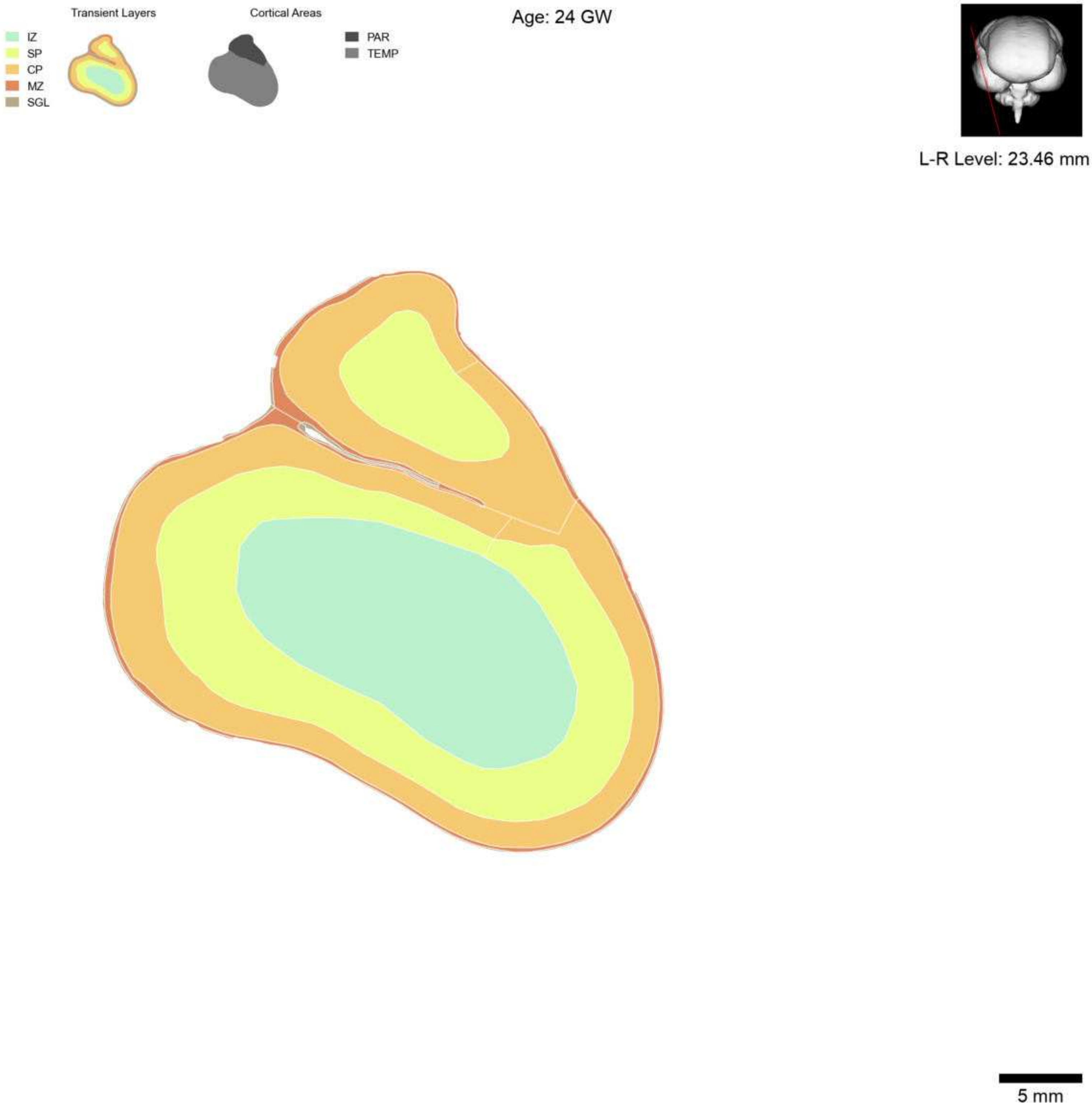
Transient Layers
IZ
SP
CP
MZ
SGL
Cortical Areas
PAR
TEMP
Age: 24 GW
L-R Level: 23.46 mm
5 mm

Age: 24 GW

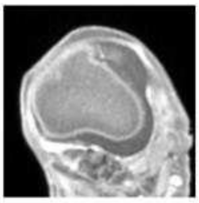

L-R Level: 22.14 mm

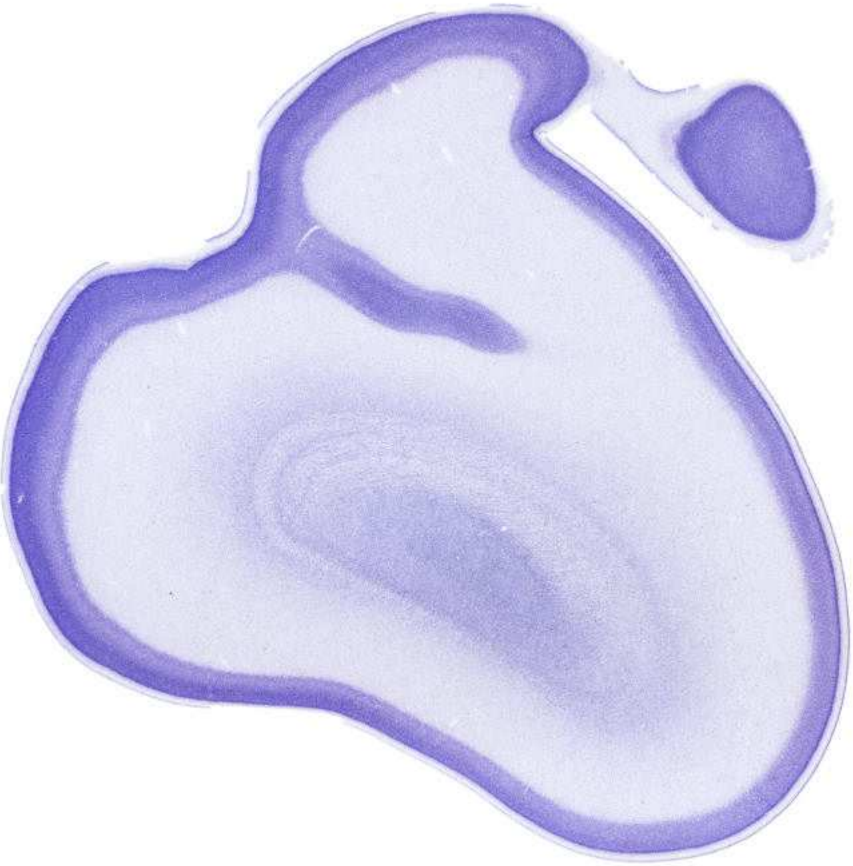

5 mm

Transient Layers

- SVZ
- IZ
- SP
- CP
- MZ
- SGL

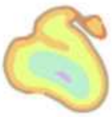

Cortical Areas

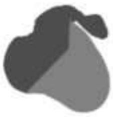

- PAR
- TEMP

Age: 24 GW

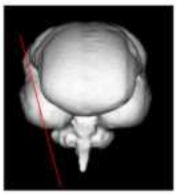

L-R Level: 22.14 mm

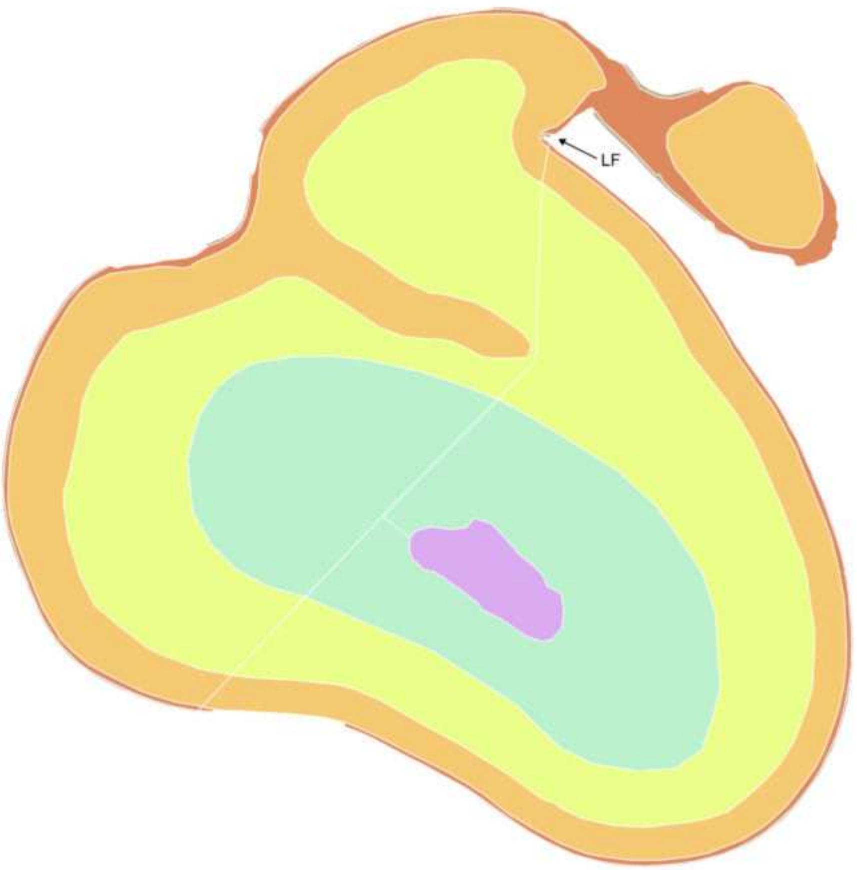

→ LA: Lateral nucleus [amygdala]

5 mm

Age: 24 GW

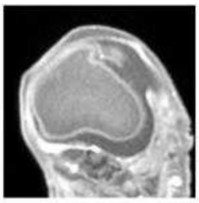

L-R Level: 21.78 mm

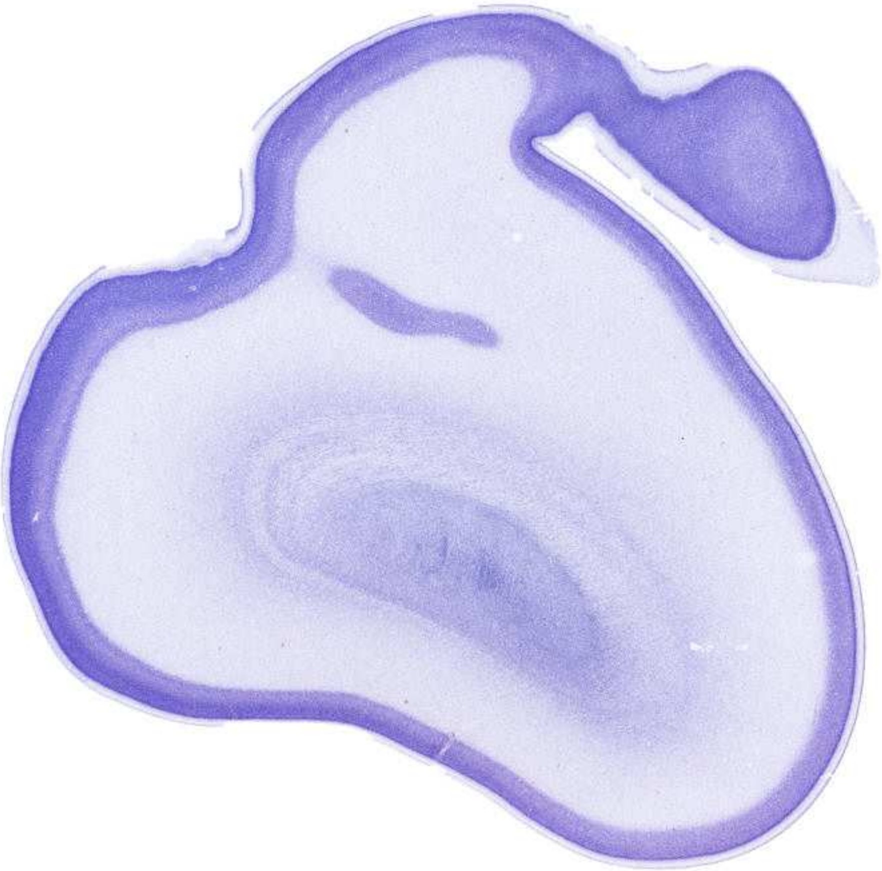

5 mm

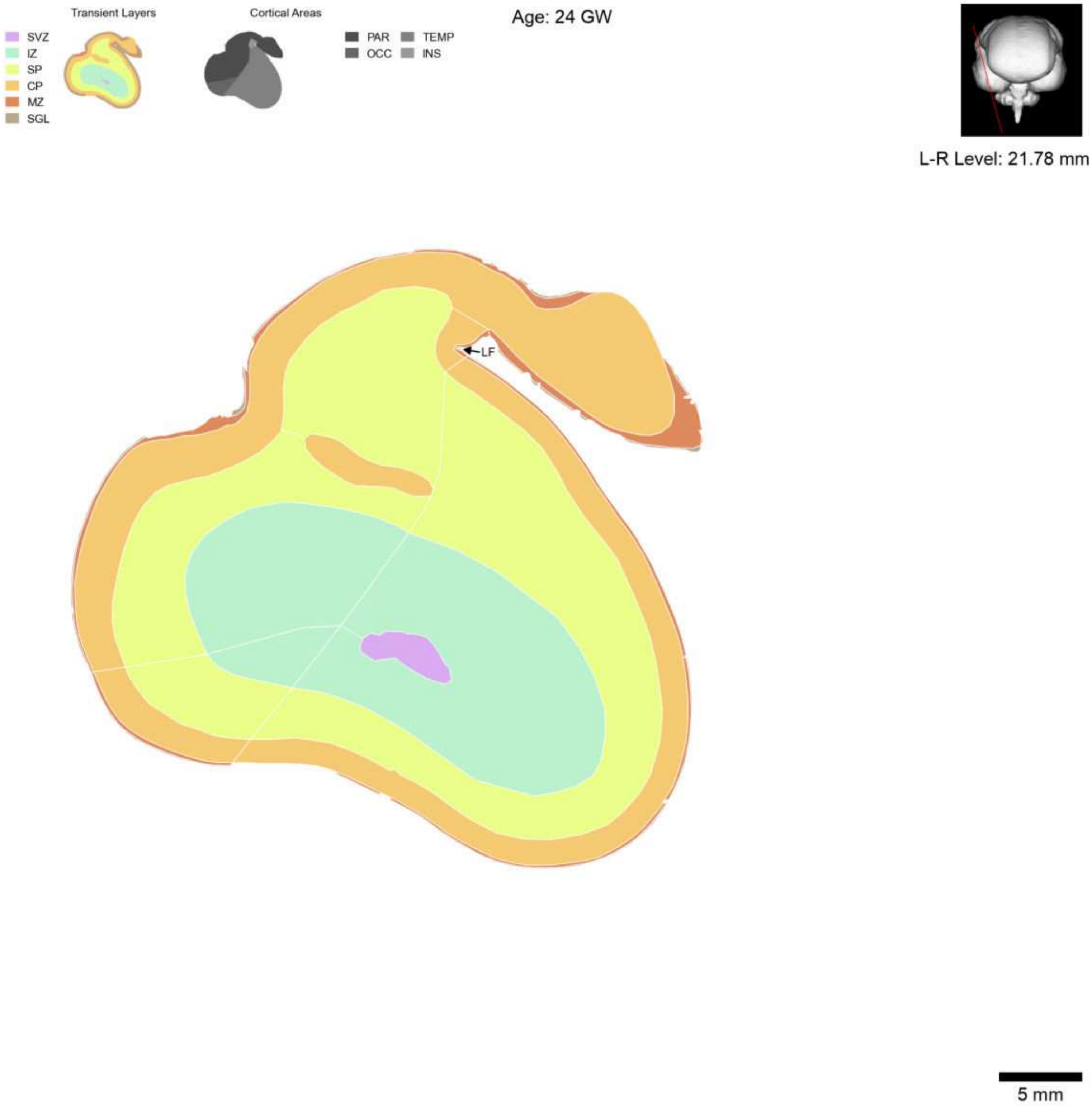

LF: Lateral Fissure

Age: 24 GW

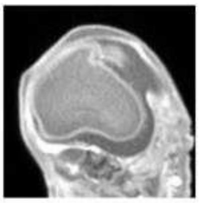

L-R Level: 21.18 mm

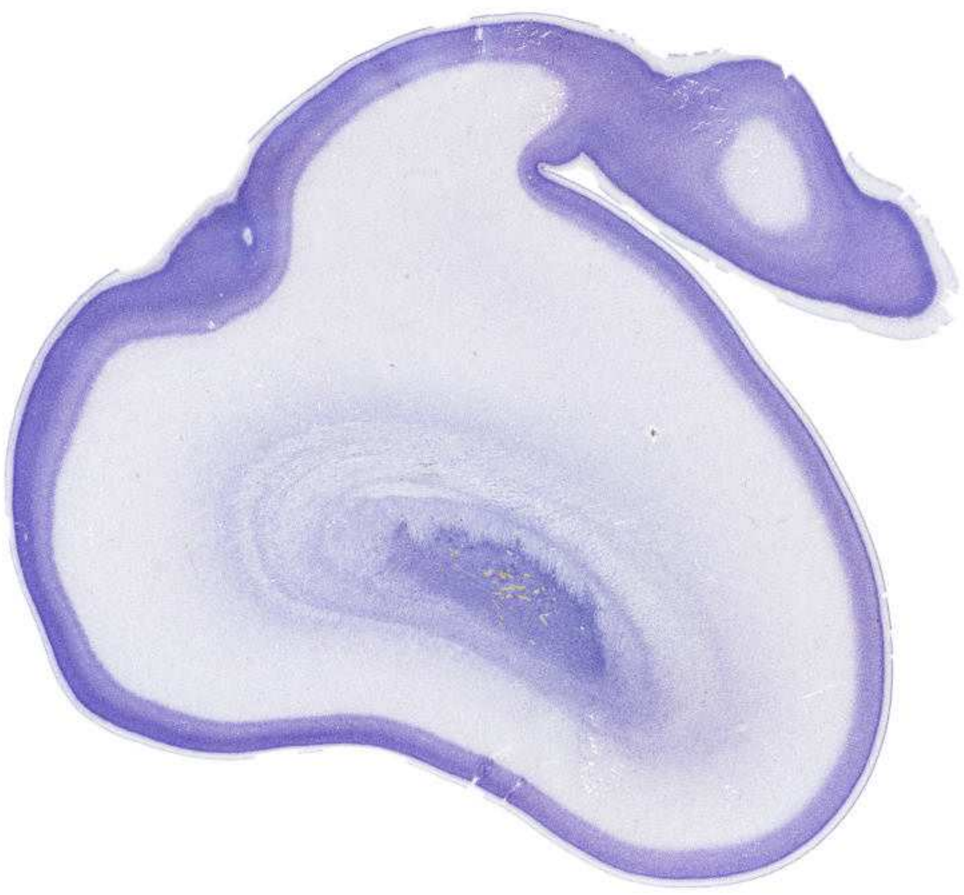

5 mm

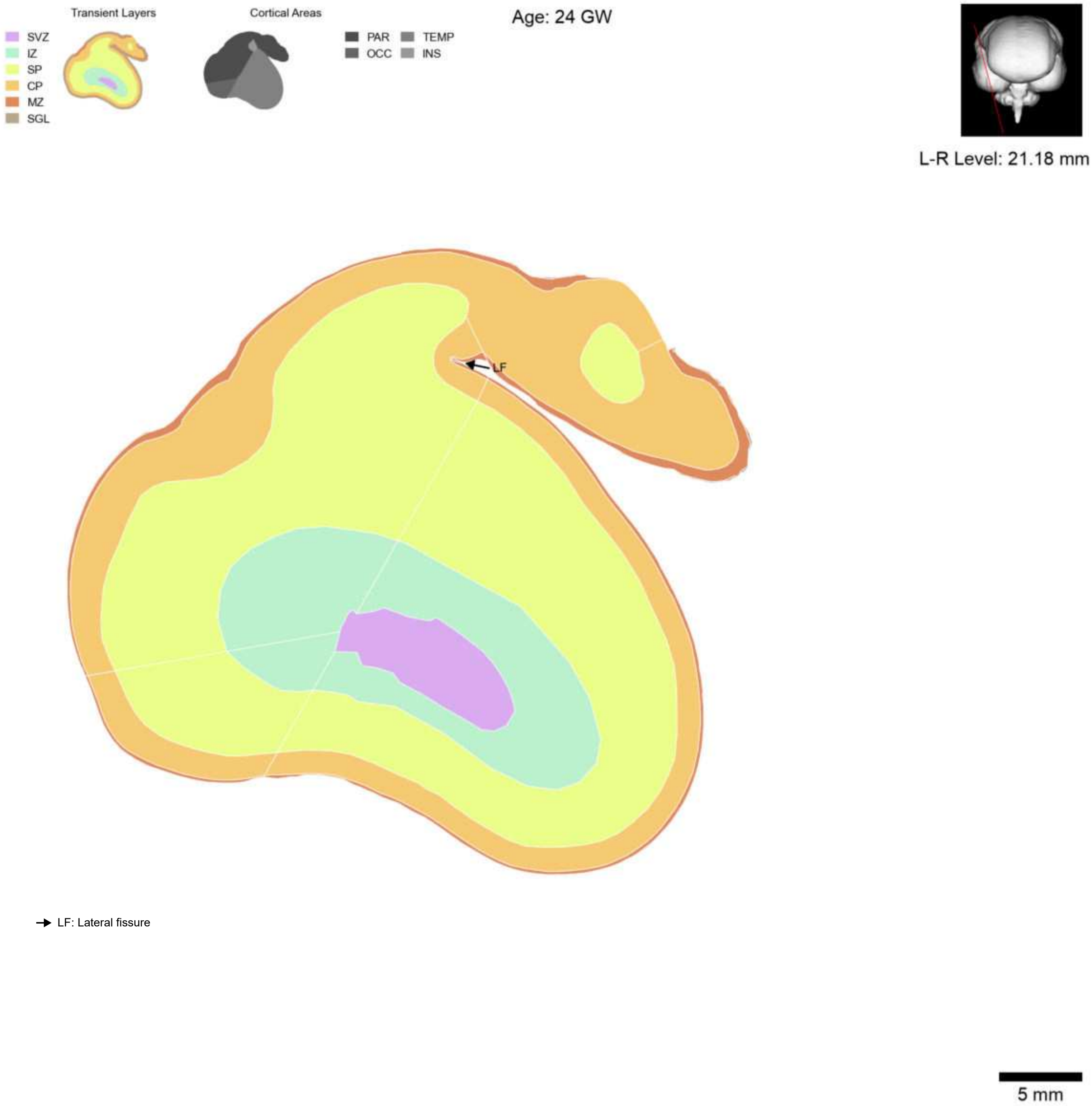

→ LF: Lateral fissure

Age: 24 GW

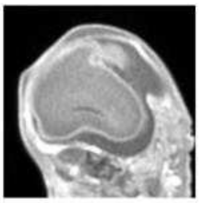

L-R Level: 20.28 mm

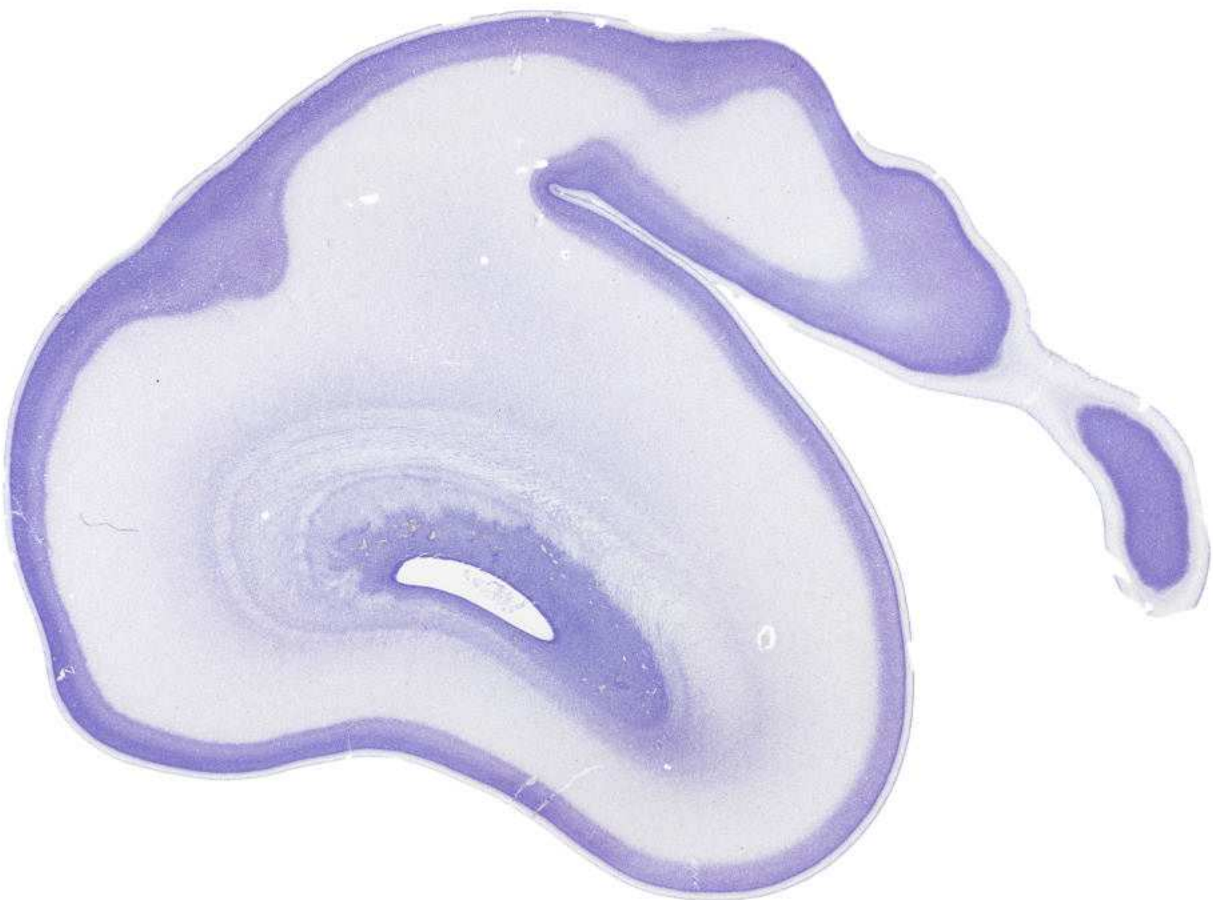

5 mm

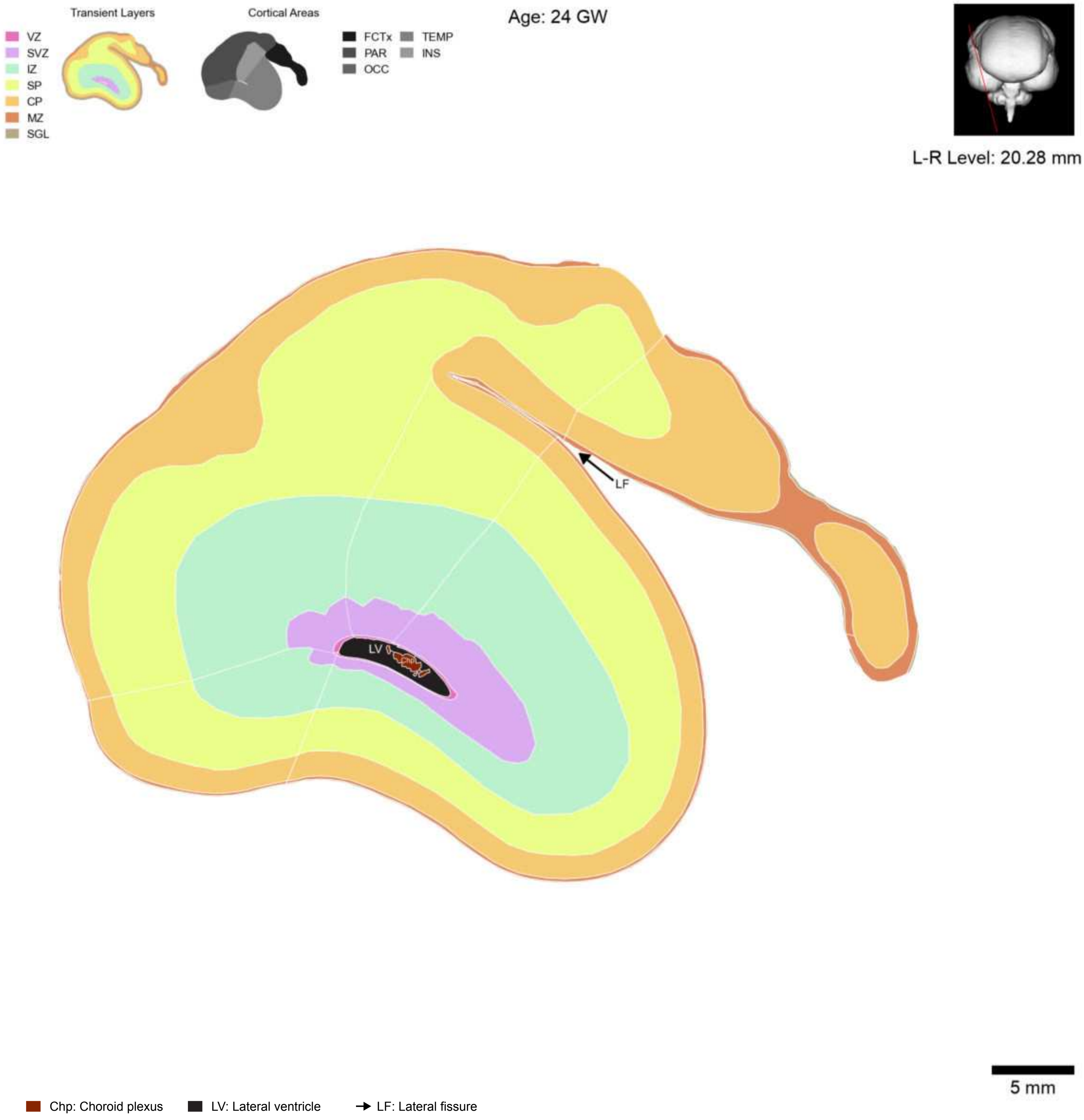
Transient Layers
Cortical Areas
VZ
SVZ
IZ
SP
CP
MZ
SGL
FCTx
PAR
OCC
TEMP
INS
Age: 24 GW
L-R Level: 20.28 mm
LV
Chp
LF
5 mm
Chp: Choroid plexus
LV: Lateral ventricle
LF: Lateral fissure

Age: 24 GW

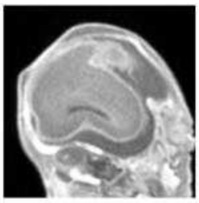

L-R Level: 20.04 mm

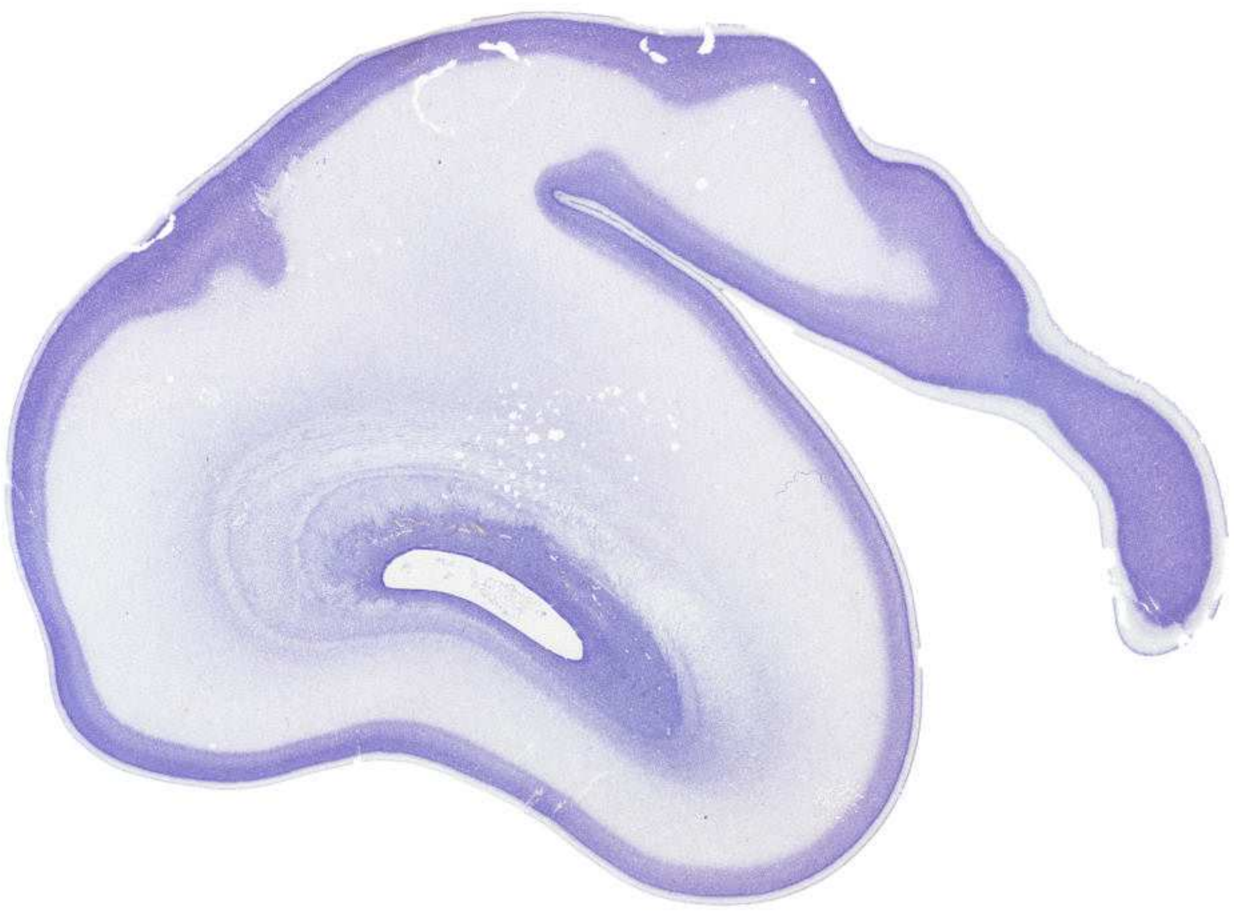

5 mm

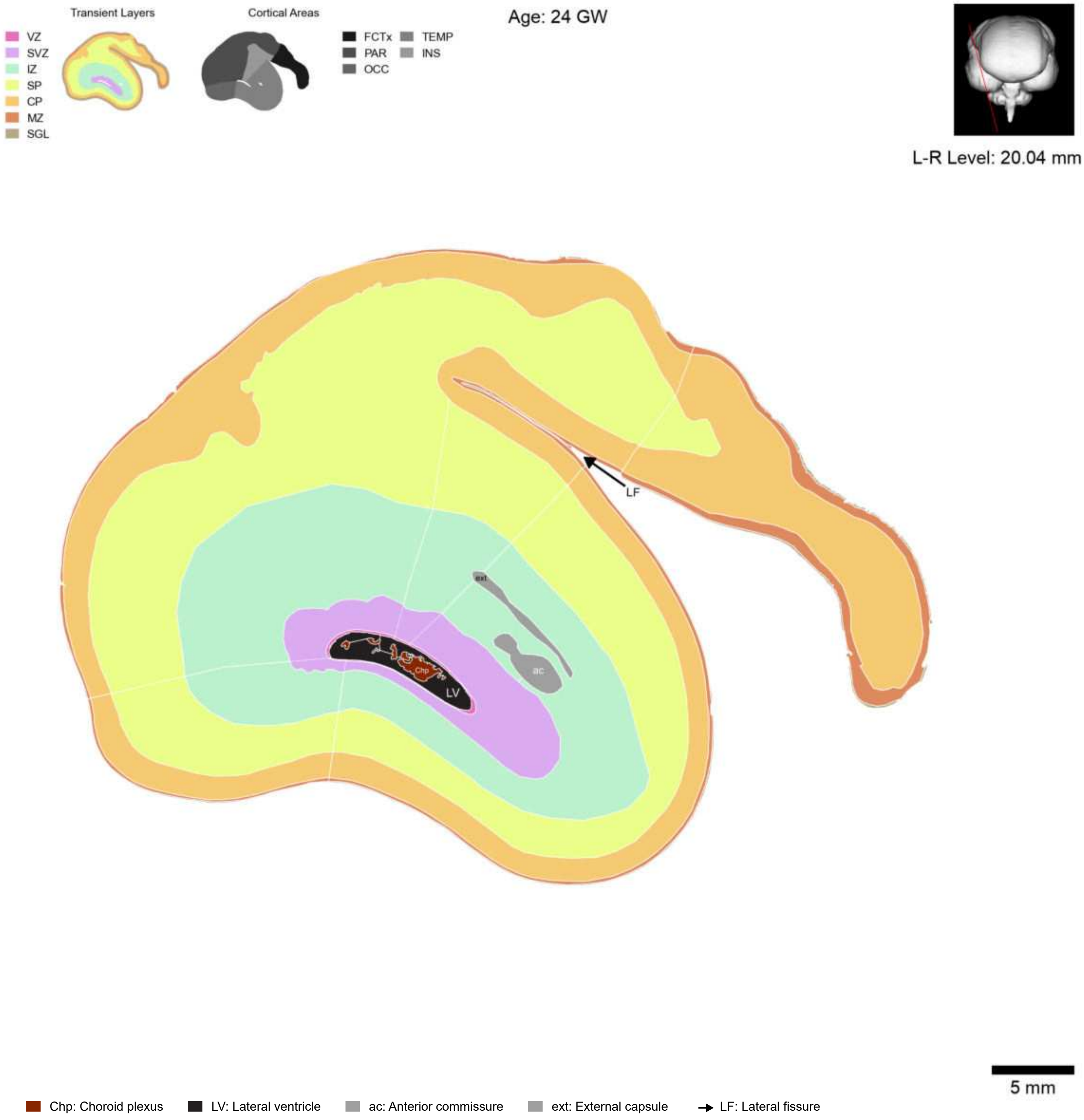

Transient Layers
Cortical Areas
Age: 24 GW
VZ
SVZ
IZ
SP
CP
MZ
SGL
FCTx
PAR
OCC
TEMP
INS
L-R Level: 20.04 mm
LF
ext
ac
Chp
LV
5 mm
Chp: Choroid plexus
LV: Lateral ventricle
ac: Anterior commissure
ext: External capsule
LF: Lateral fissure

Age: 24 GW

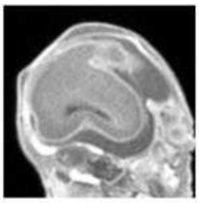

L-R Level: 19.86 mm

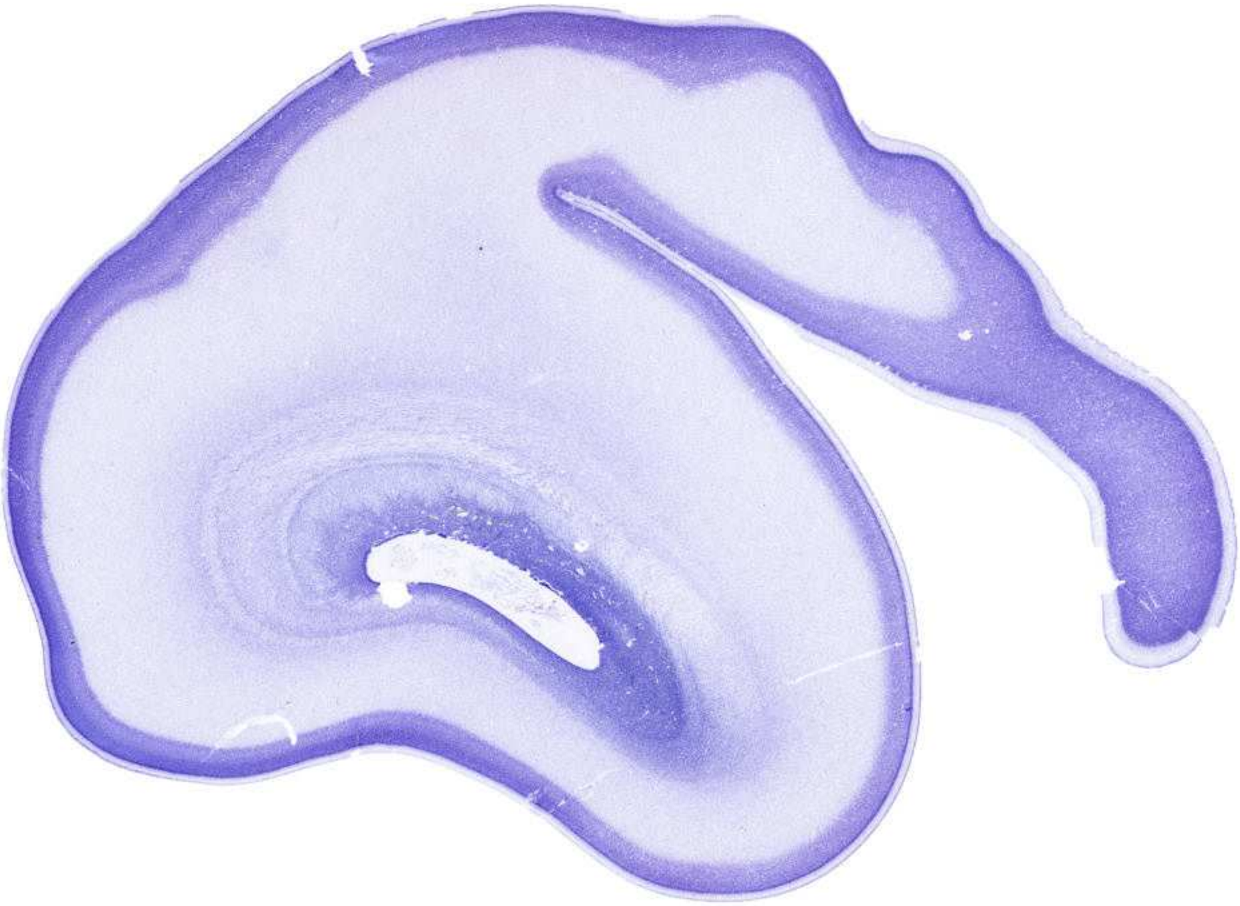

5 mm

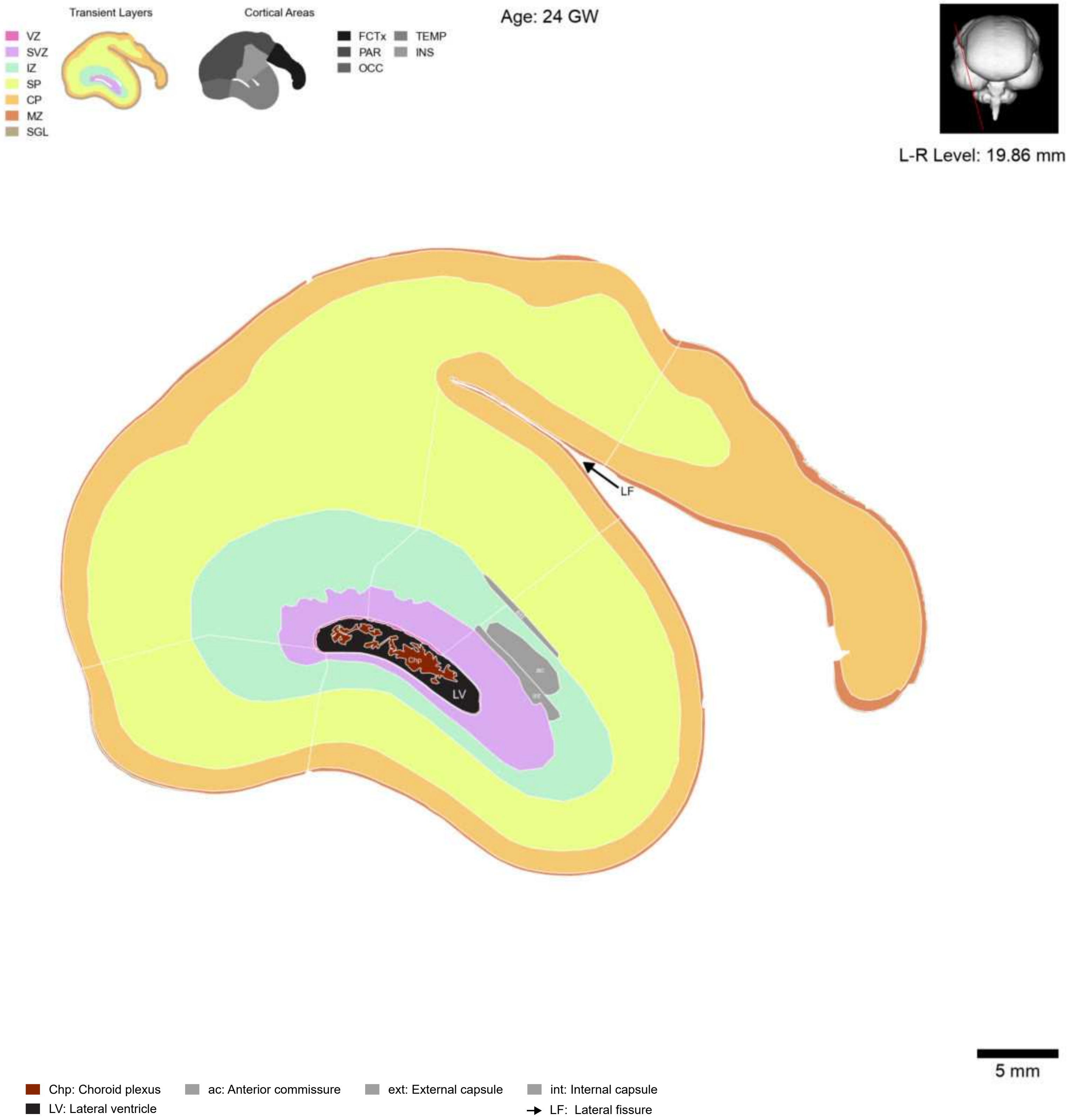
Transient Layers
VZ
SVZ
IZ
SP
CP
MZ
SGL
Cortical Areas
FCTx
PAR
OCC
TEMP
INS
Age: 24 GW
L-R Level: 19.86 mm
LF
Chp
LV
5 mm
Chp: Choroid plexus
LV: Lateral ventricle
ac: Anterior commissure
ext: External capsule
int: Internal capsule
LF: Lateral fissure

Age: 24 GW

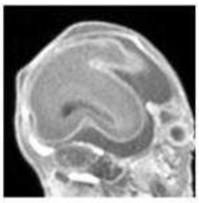

L-R Level: 19.56 mm

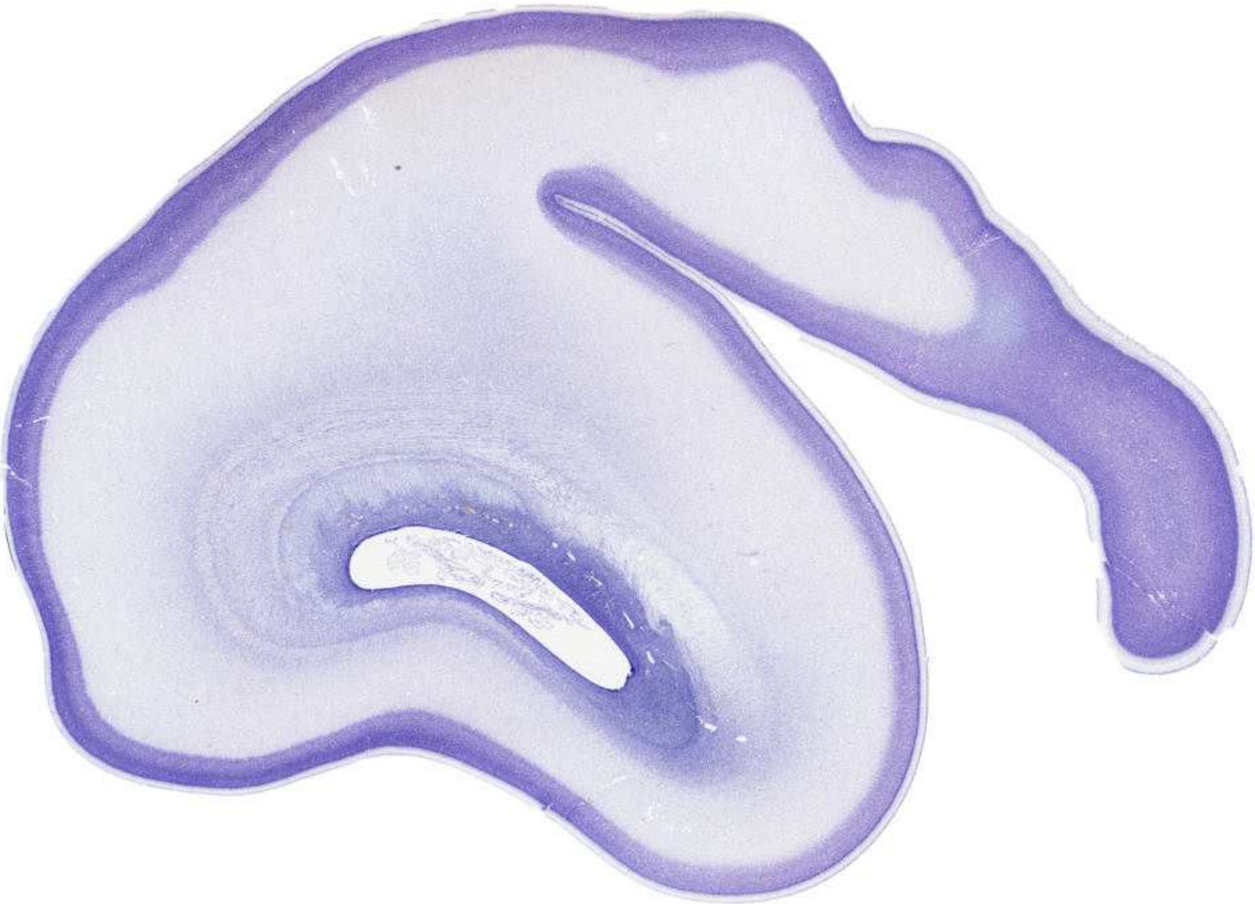

5 mm

Transient Layers

VZ
SVZ
IZ
SP
CP
MZ
SGL

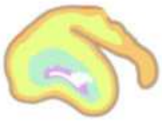

Cortical Areas

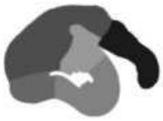

FCTx
PAR
OCC
TEMP
INS

Age: 24 GW

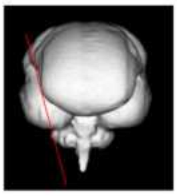

L-R Level: 19.56 mm

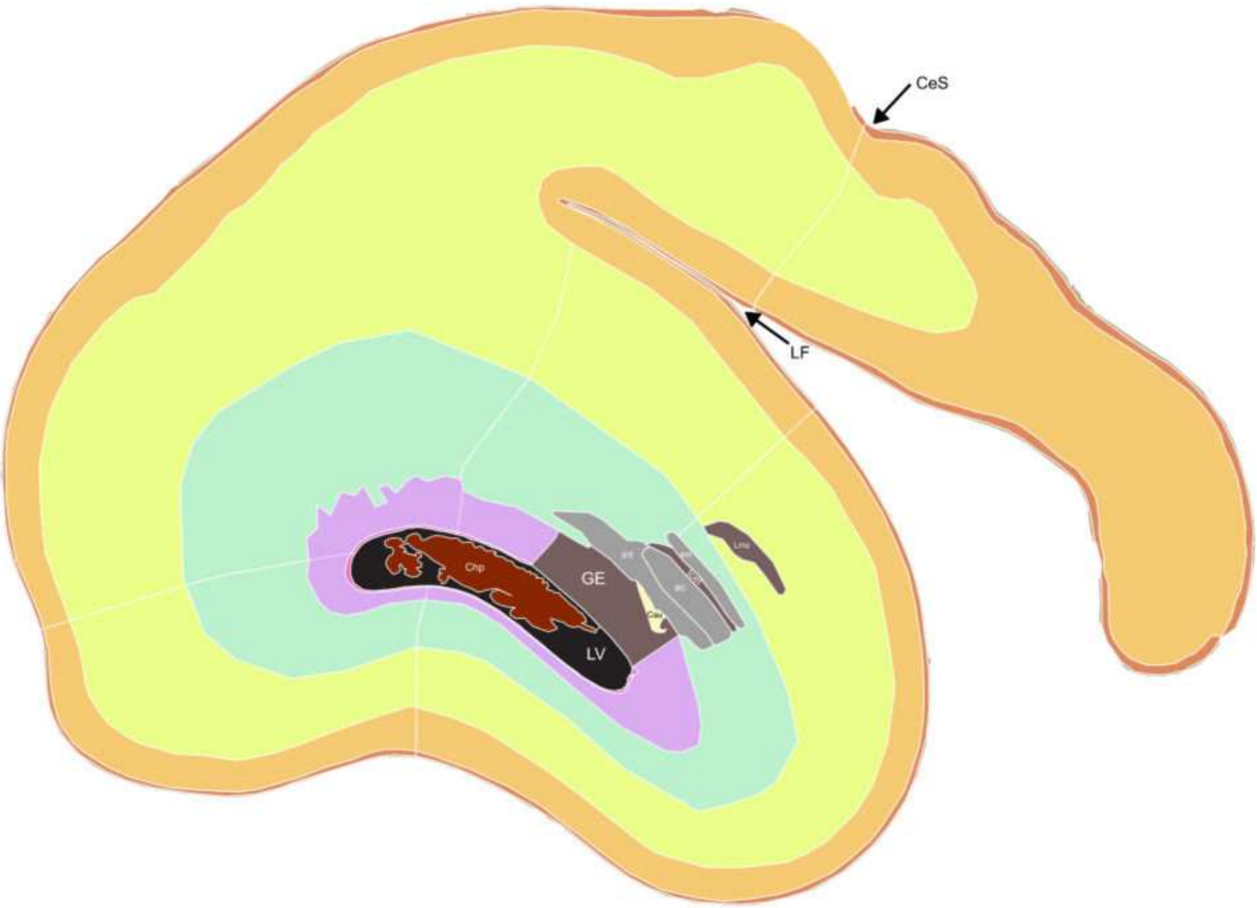

5 mm

Cau: Caudate nucleus
Chp: Choroid plexus
GE: Ganglionic eminence
LV: Lateral ventricle
Lms: Lateral migratory stream
ac: Anterior commissure
ext: External capsule
int: Internal capsule
tcet: Transient cell zone in the external capsule
→ CeS: Central sulcus
→ LF: Lateral fissure

Age: 24 GW

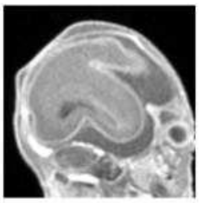

L-R Level: 19.14 mm

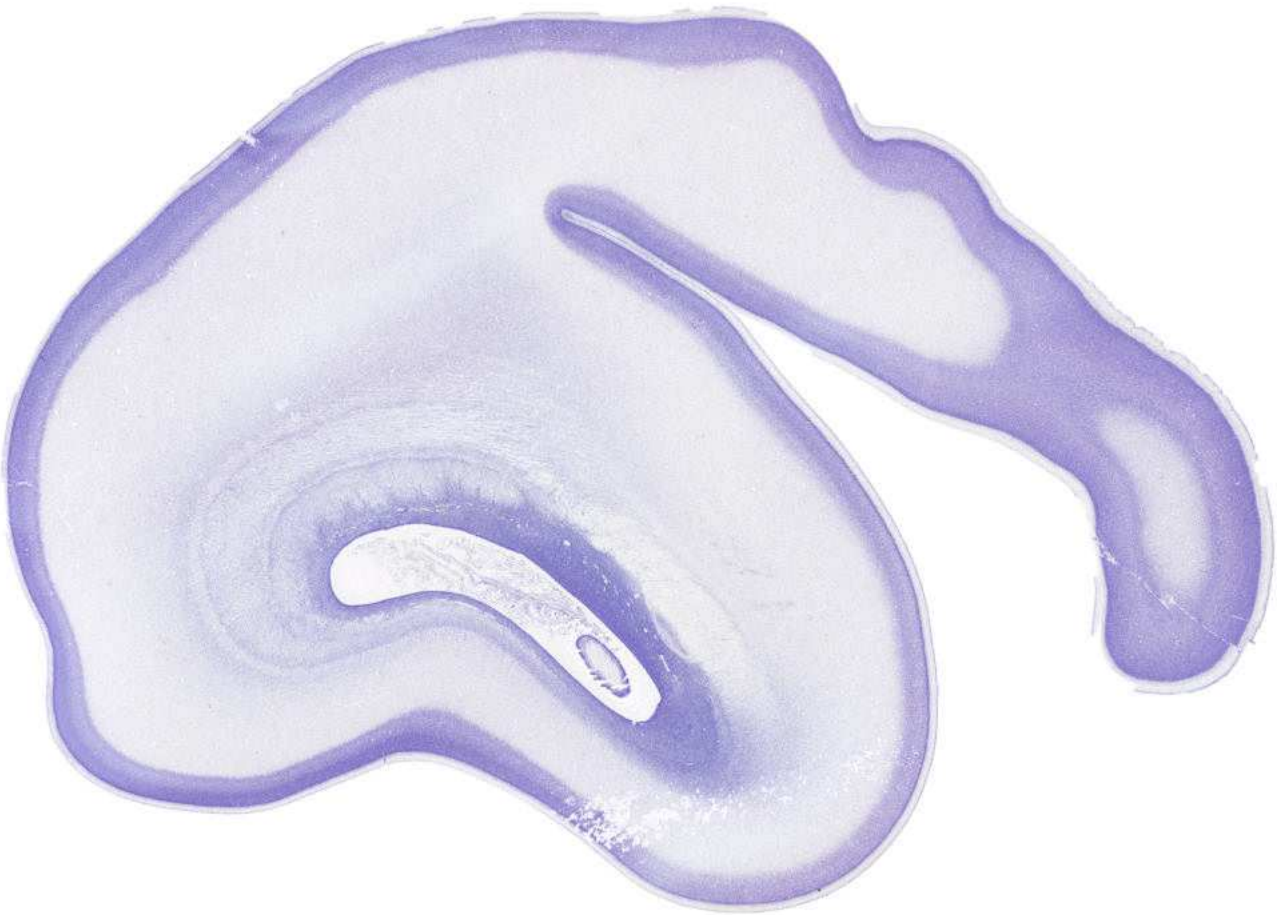

5 mm

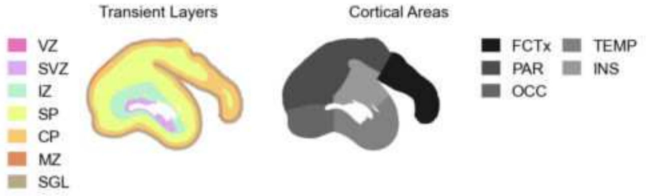

Age: 24 GW

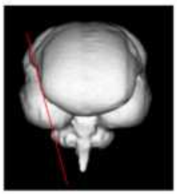

L-R Level: 19.14 mm

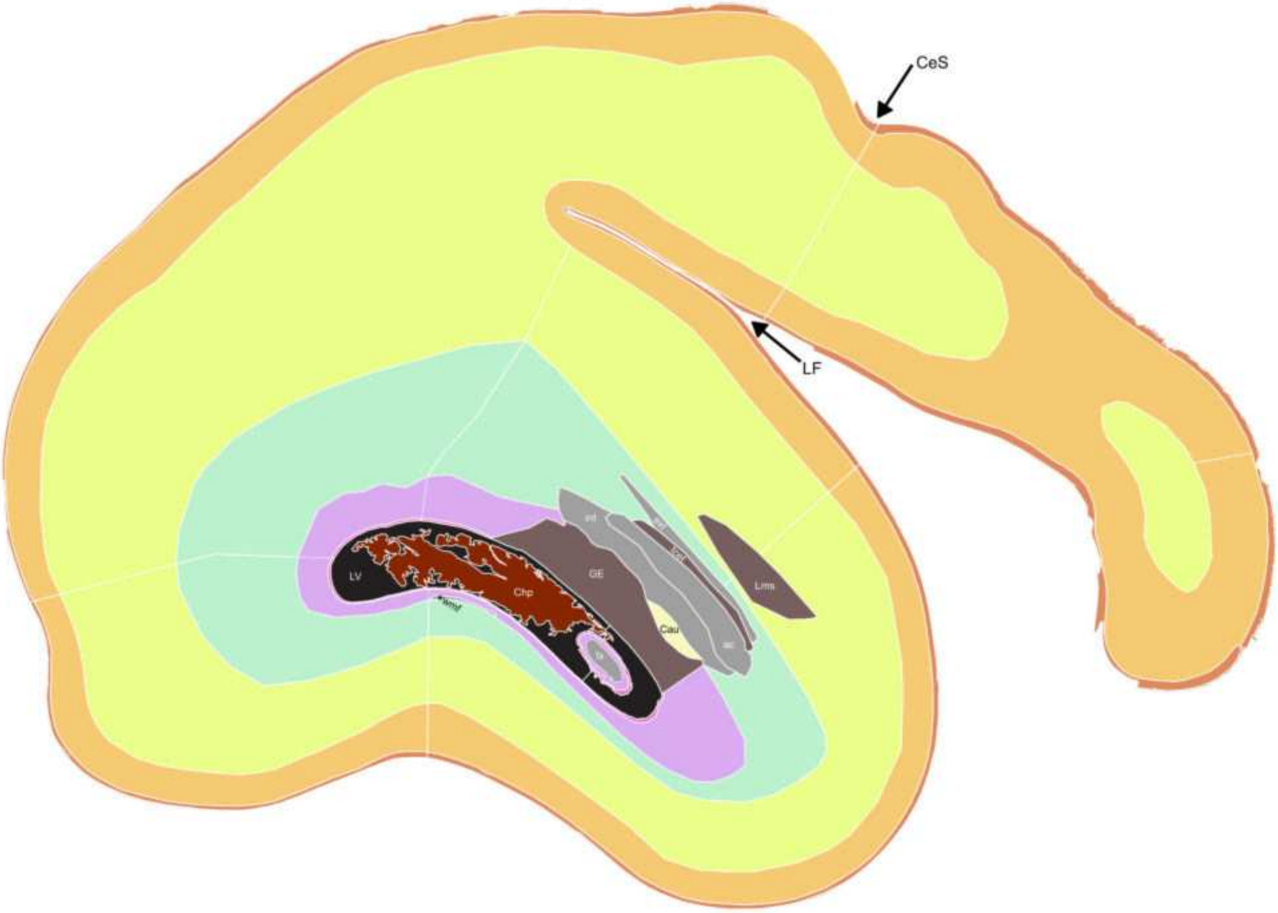

5 mm

Cau: Caudate nucleus	LV: Lateral ventricle	ext: External capsule	tcet: Transient cell zone in the external capsule
Chp: Choroid plexus	Lms: Lateral migratory stream	fx: Fornix	wmf: White matter fibers
GE: Ganglionic eminence	ac: Anterior commissure	int: Internal capsule	CeS: Central sulcus
			LF: Lateral fissure

Age: 24 GW

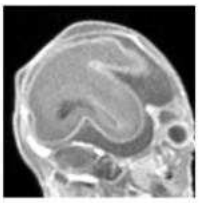

L-R Level: 18.54 mm

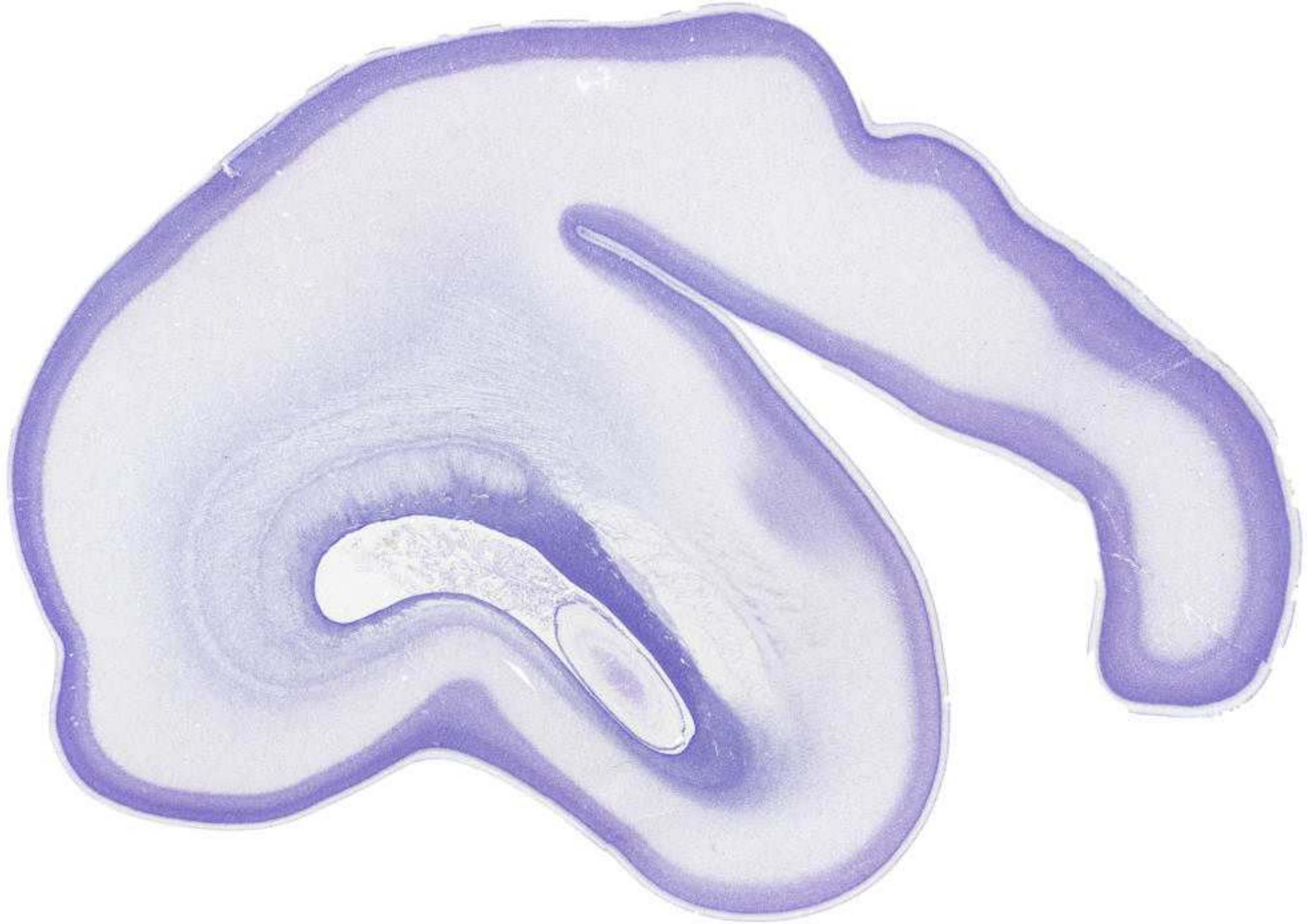

5 mm

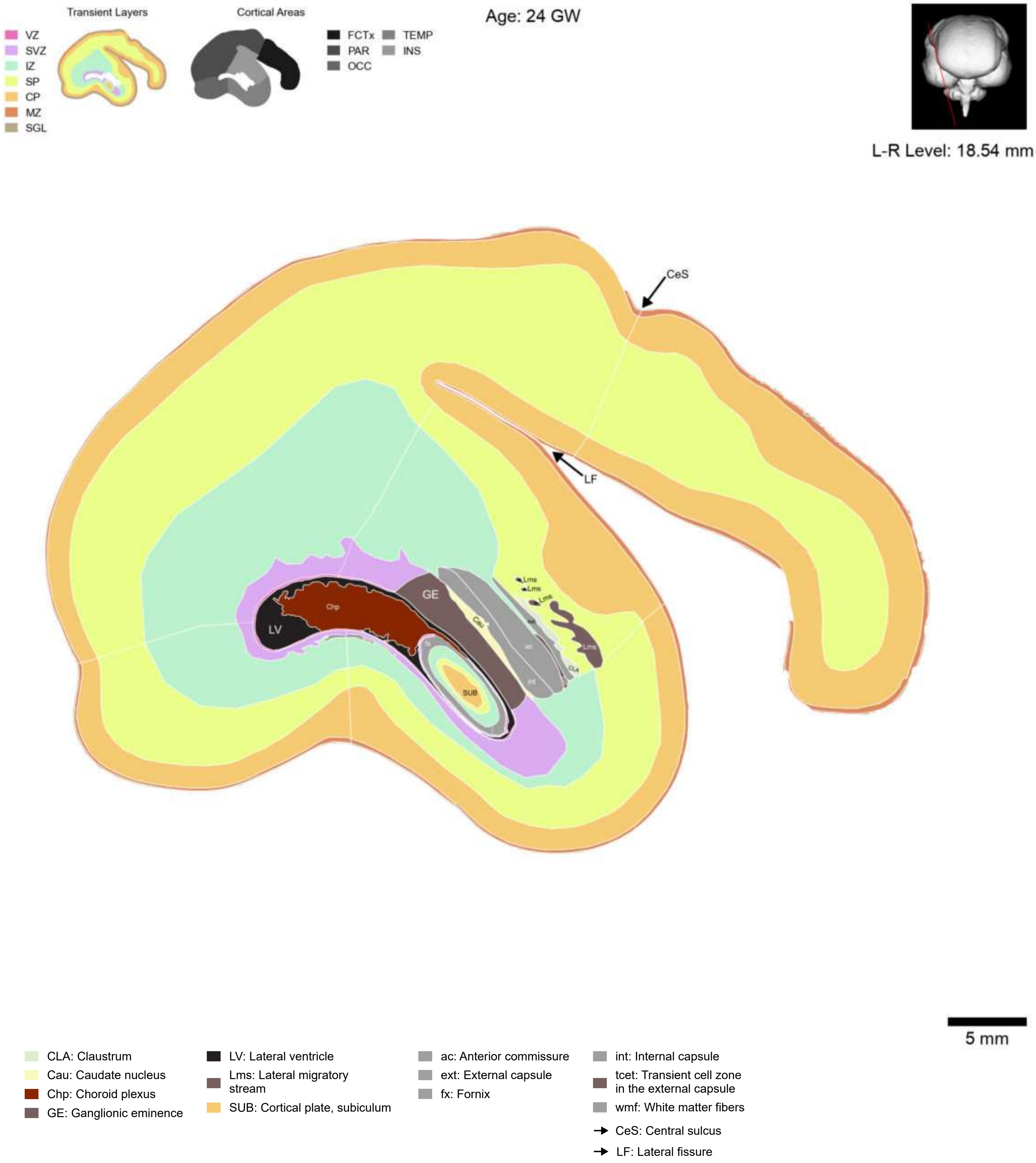

Transient Layers
VZ
SVZ
IZ
SP
CP
MZ
SGL
Cortical Areas
FCTx
PAR
OCC
TEMP
INS
Age: 24 GW
L-R Level: 18.54 mm
CeS
LF
GE
LV
Chp
Cau
SUB
Lms
CLA
5 mm
CLA: Claustrum
Cau: Caudate nucleus
Chp: Choroid plexus
GE: Ganglionic eminence
LV: Lateral ventricle
Lms: Lateral migratory stream
SUB: Cortical plate, subiculum
ac: Anterior commissure
ext: External capsule
fx: Fornix
int: Internal capsule
tcet: Transient cell zone in the external capsule
wmf: White matter fibers
CeS: Central sulcus
LF: Lateral fissure

Age: 24 GW

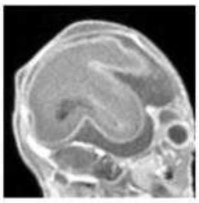

L-R Level: 18.3 mm

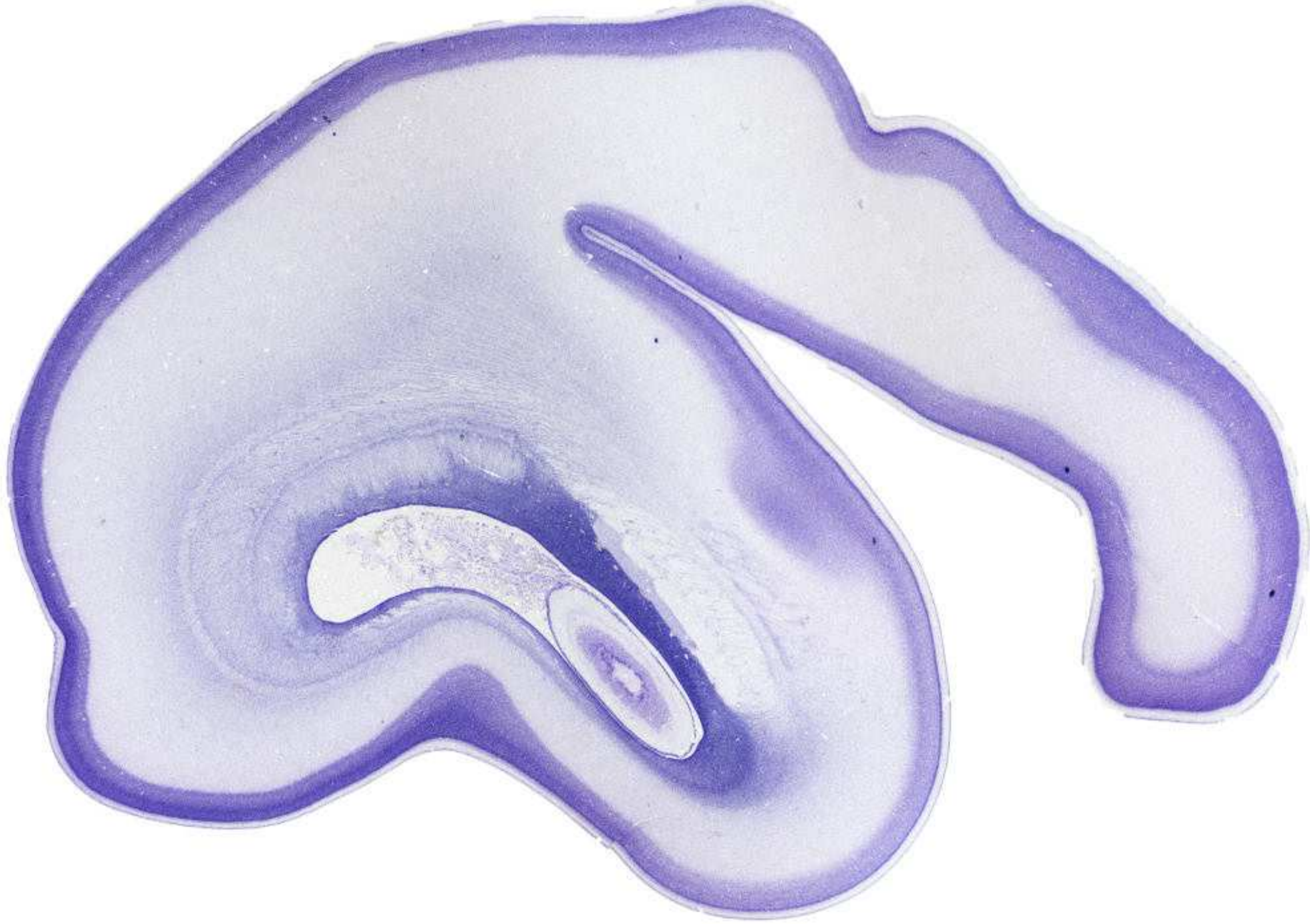

5 mm

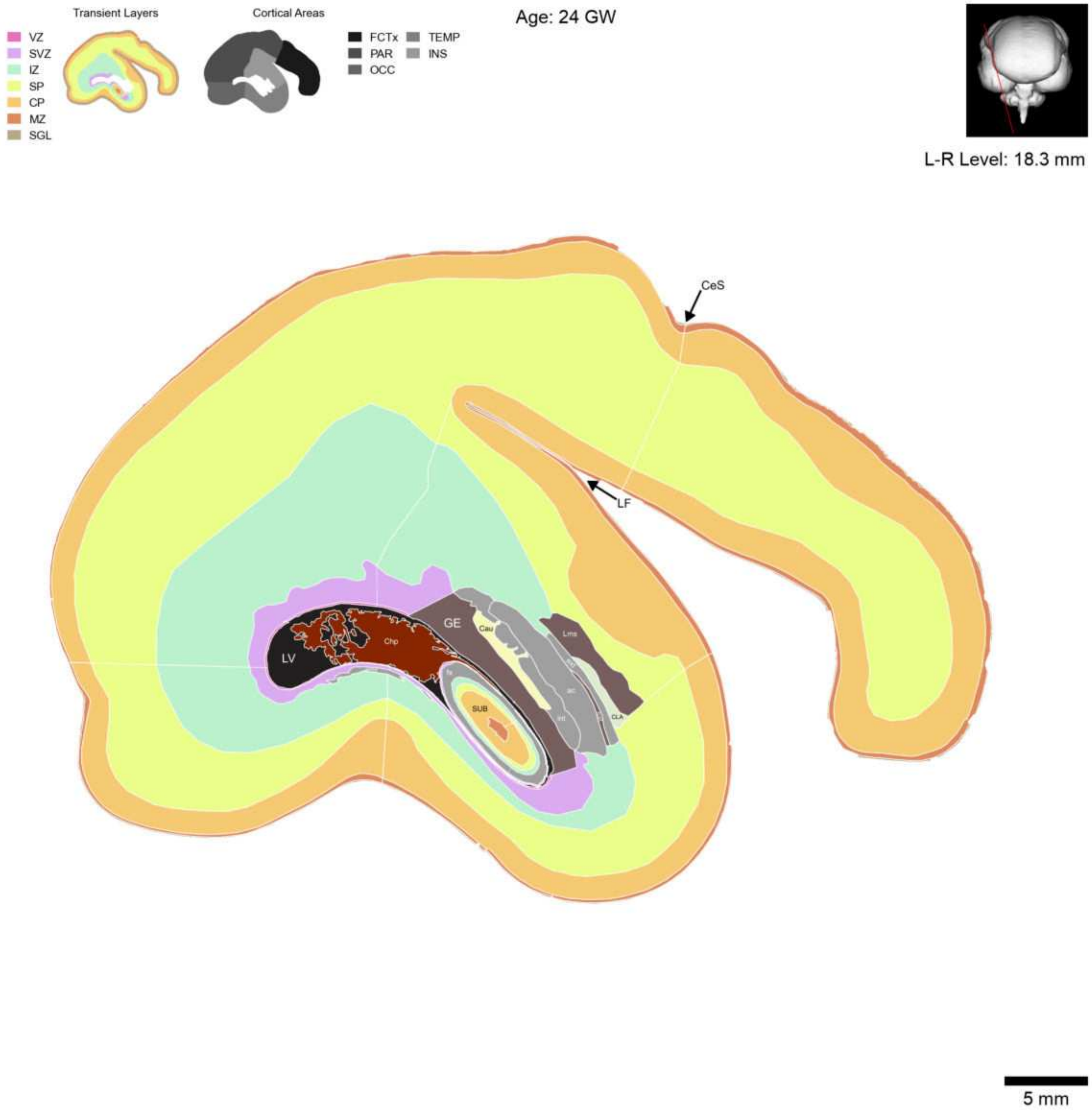

CLA: Claustrum
Cau: Caudate nucleus
Chp: Choroid plexus
GE: Ganglionic eminence

LV: Lateral ventricle
Lms: Lateral migratory stream
SUB: Cortical plate, subiculum

ac: Anterior commissure
ext: External capsule
fx: Fornix
int: Internal capsule

tcet: Transient cell zone in the external capsule
wmf: White matter fibers
CeS: Central sulcus
LF: Lateral fissure

Age: 24 GW

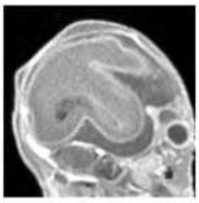

L-R Level: 18.0 mm

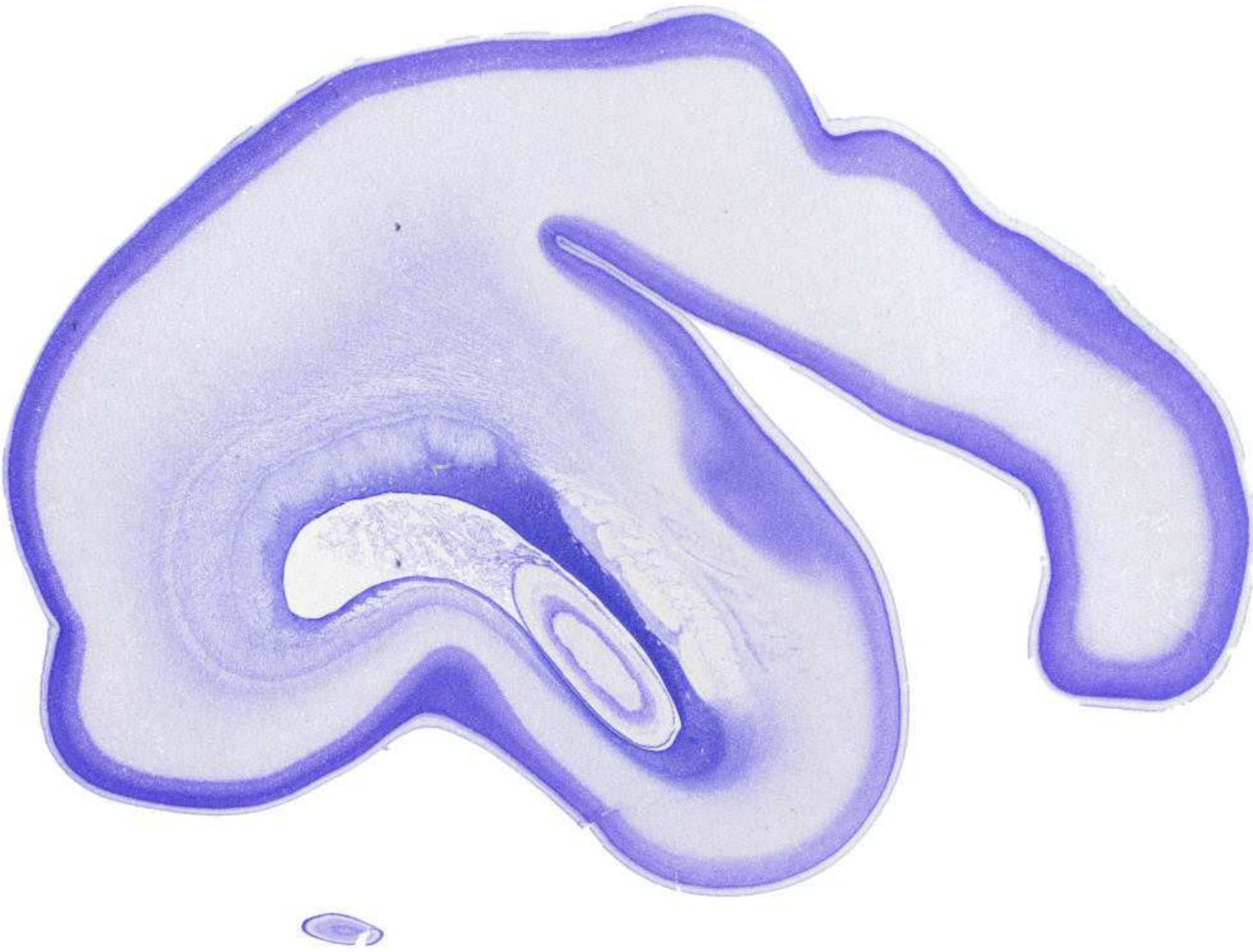

5 mm

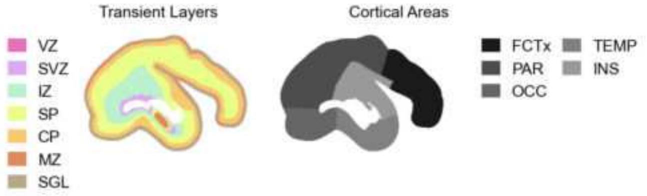

Age: 24 GW

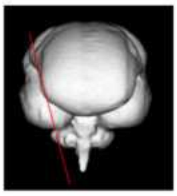

L-R Level: 18.0 mm

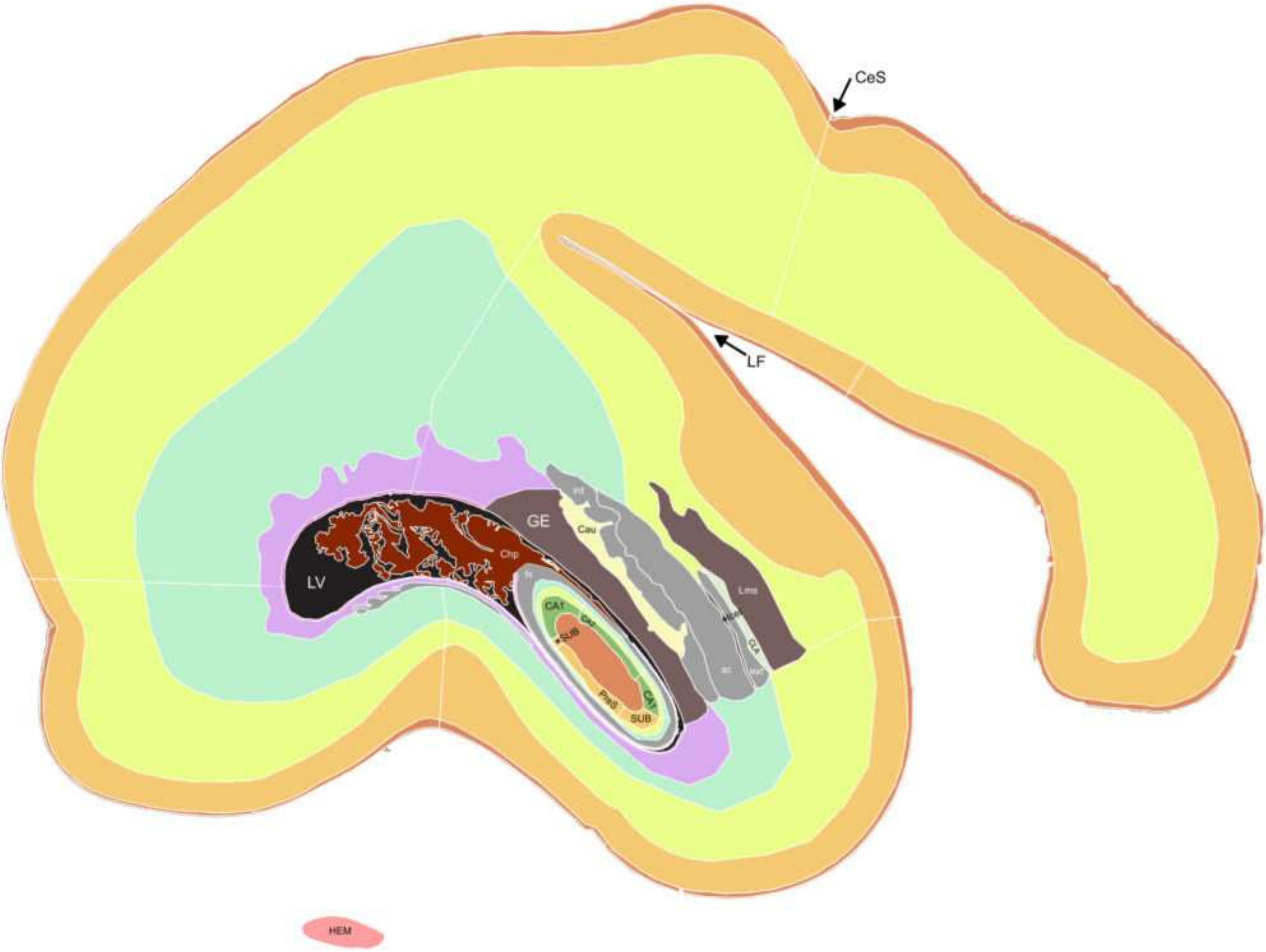

5 mm

- CA1: CA1 field [hippocampus]
- CA2: CA2 field [hippocampus]
- CLA: Claustrum
- Cau: Caudate nucleus
- Chp: Choroid plexus
- GE: Ganglionic eminence
- HEM: Cerebellar hemispheres
- LV: Lateral ventricle
- Lms: Lateral migratory stream
- PreS: Cortical plate, presubiculum
- SUB: Cortical plate, subiculum
- ac: Anterior commissure
- ext: External capsule
- fx: Fornix
- int: Internal capsule
- tcet: Transient cell zone in the external capsule
- wmf: White matter fibers
- LF: Lateral fissure
- CeS: Central sulcus

Age: 24 GW

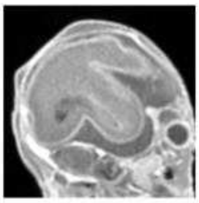

L-R Level: 17.64 mm

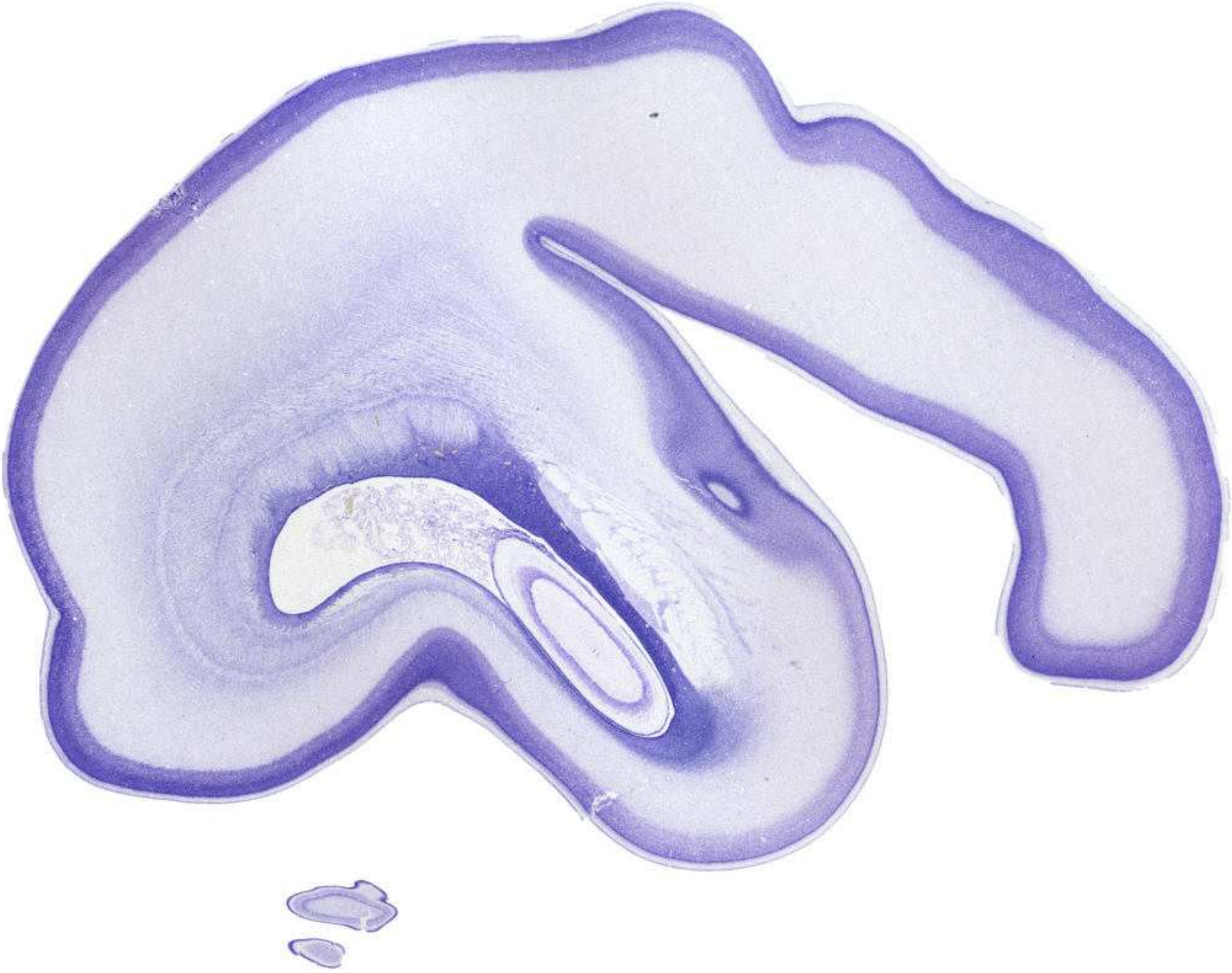

5 mm

Transient Layers

- VZ
- SVZ
- IZ
- SP
- CP
- MZ
- SGL

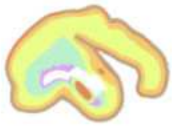

Cortical Areas

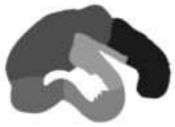

- FCTx
- PAR
- OCC
- TEMP
- INS

Age: 24 GW

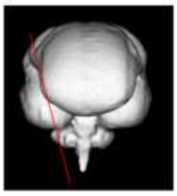

L-R Level: 17.64 mm

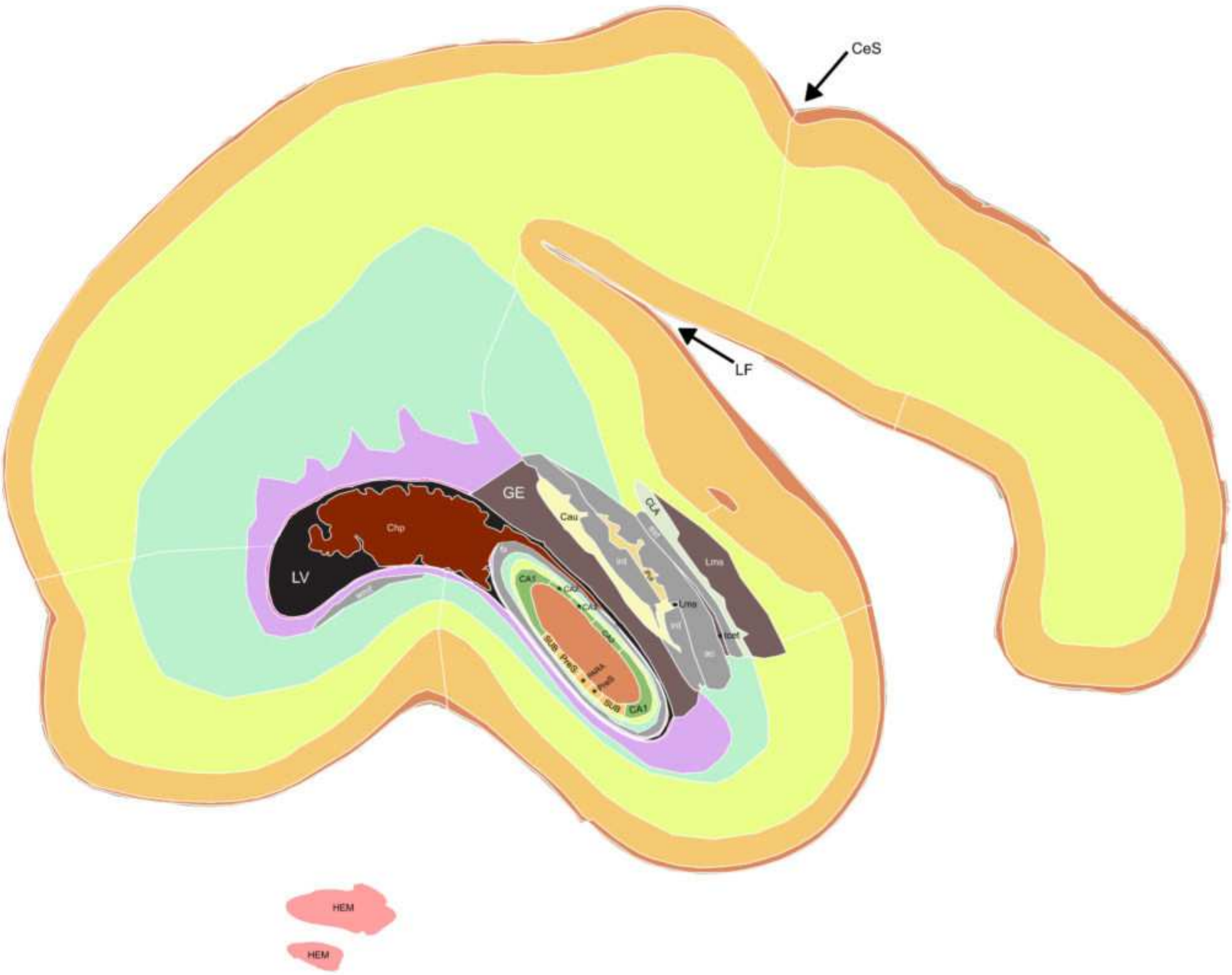

5 mm

- CA1: CA1 field [hippocampus]
- CA2: CA2 field [hippocampus]
- CA3: CA3 field [hippocampus]
- CLA: Claustrum
- Cau: Caudate nucleus
- Chp: Choroid plexus
- GE: Ganglionic eminence
- HEM: Cerebellar hemispheres
- LV: Lateral ventricle
- Lms: Lateral migratory stream
- PARA: Cortical plate, parasubiculum
- PreS: Cortical plate, presubiculum
- Put: Putamen
- SUB: Cortical plate, subiculum
- ac: Anterior commissure
- ext: External capsule
- fx: Fornix
- int: Internal capsule
- tcet: Transient cell zone in the external capsule
- wmf: White matter fibers
- → CeS: Central sulcus
- → LF: Lateral fissure

Age: 24 GW

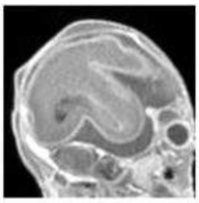

L-R Level: 17.16 mm

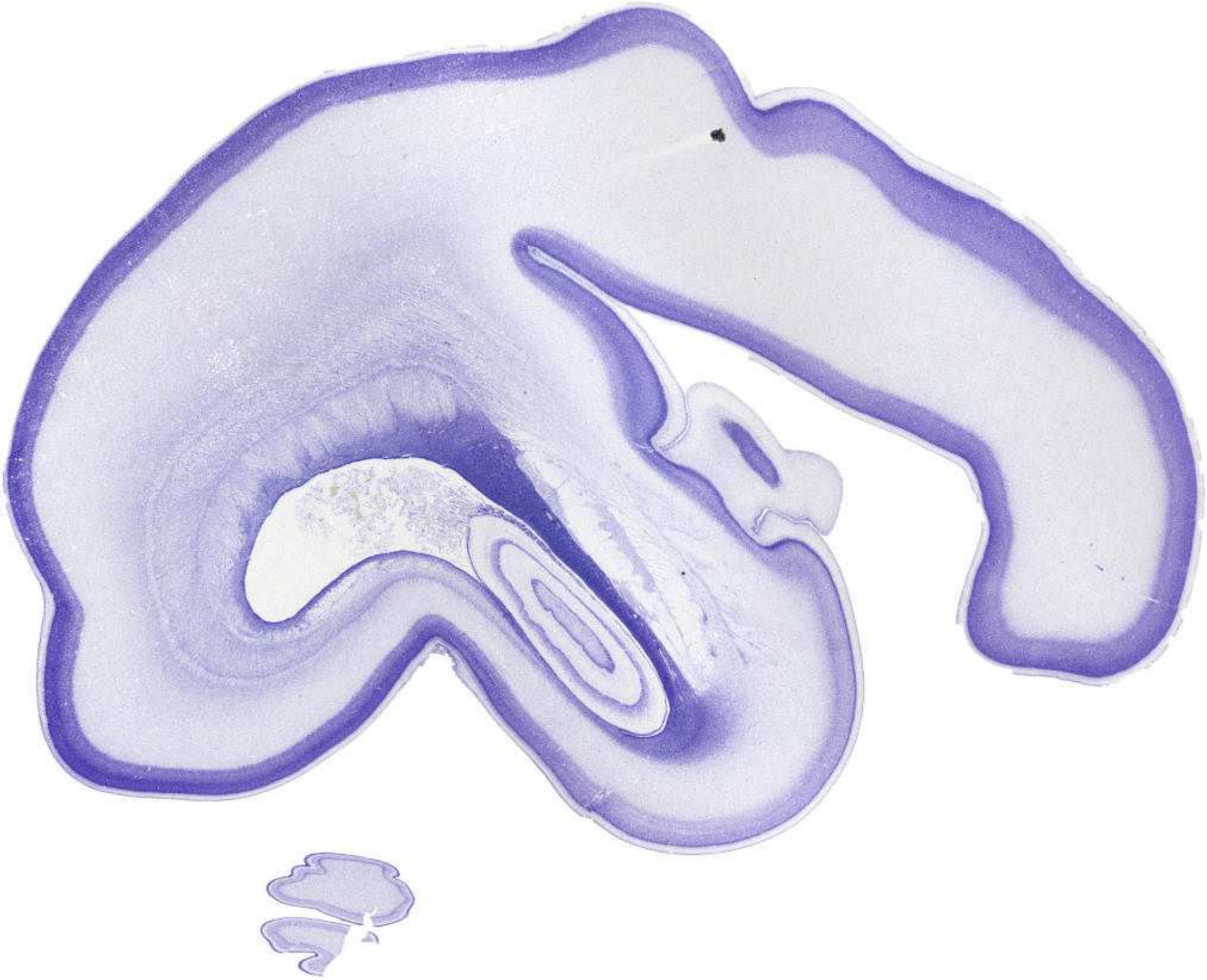

5 mm

Transient Layers

VZ
SVZ
IZ
SP
CP
MZ
SGL

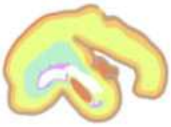

Cortical Areas

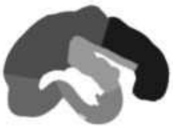

FCTx
PAR
OCC
TEMP
INS
ENT

Age: 24 GW

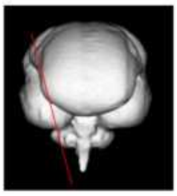

L-R Level: 17.16 mm

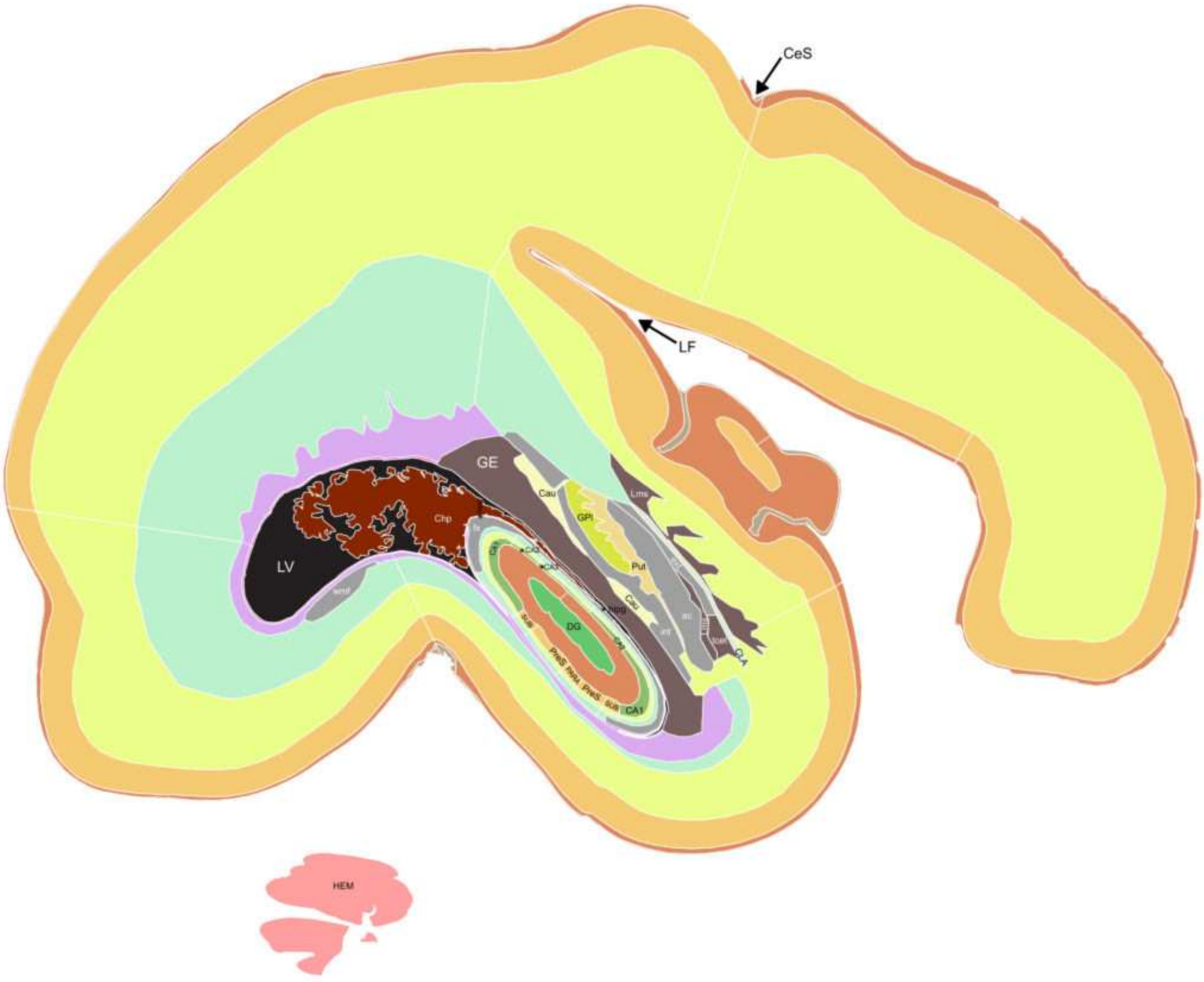

5 mm

- CA1: CA1 field [hippocampus]
- CA2: CA2 field [hippocampus]
- CA3: CA3 field [hippocampus]
- CLA: Claustrum
- Cau: Caudate nucleus
- Chp: Choroid plexus
- DG: Dentate gyrus
- GE: Ganglionic eminence
- GPl: Globus pallidus lateral segment
- HEM: Cerebellar hemispheres
- LV: Lateral ventricle
- Lms: Lateral migratory stream
- PARA: Cortical plate, parasubiculum
- PreS: Cortical plate, presubiculum
- Put: Putamen
- SUB: Cortical plate, subiculum
- ac: Anterior commissure
- ext: External capsule
- fx: Fornix
- hipg: Hippocampal glioepithelium/ependyma
- int: Internal capsule
- tcet: Transient cell zone in the external capsule
- wmf: White matter fibers
- CeS: Central sulcus
- LF: Lateral fissure

Age: 24 GW

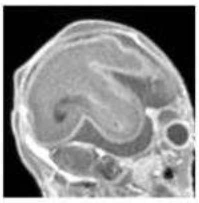

L-R Level: 16.98 mm

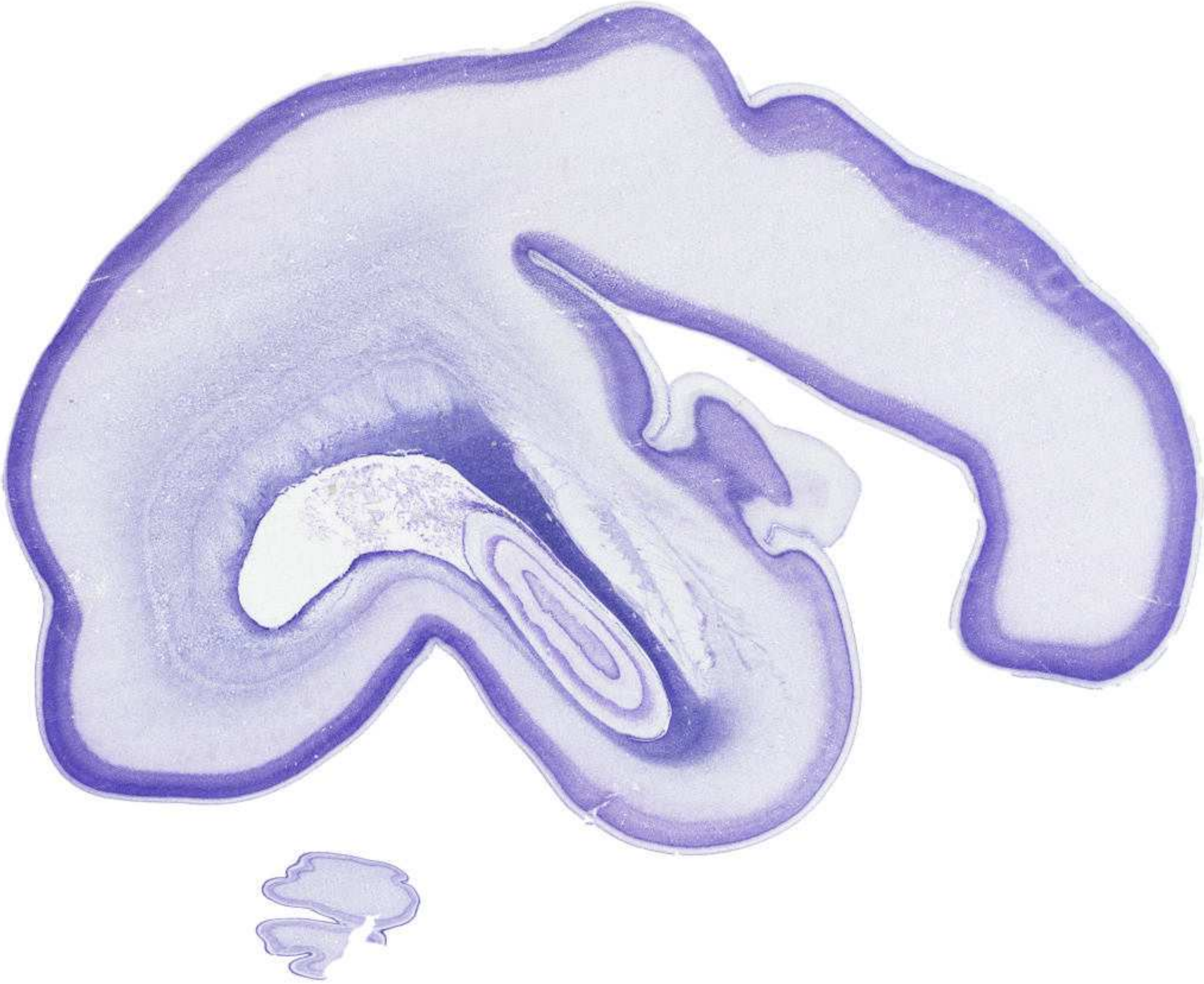

5 mm

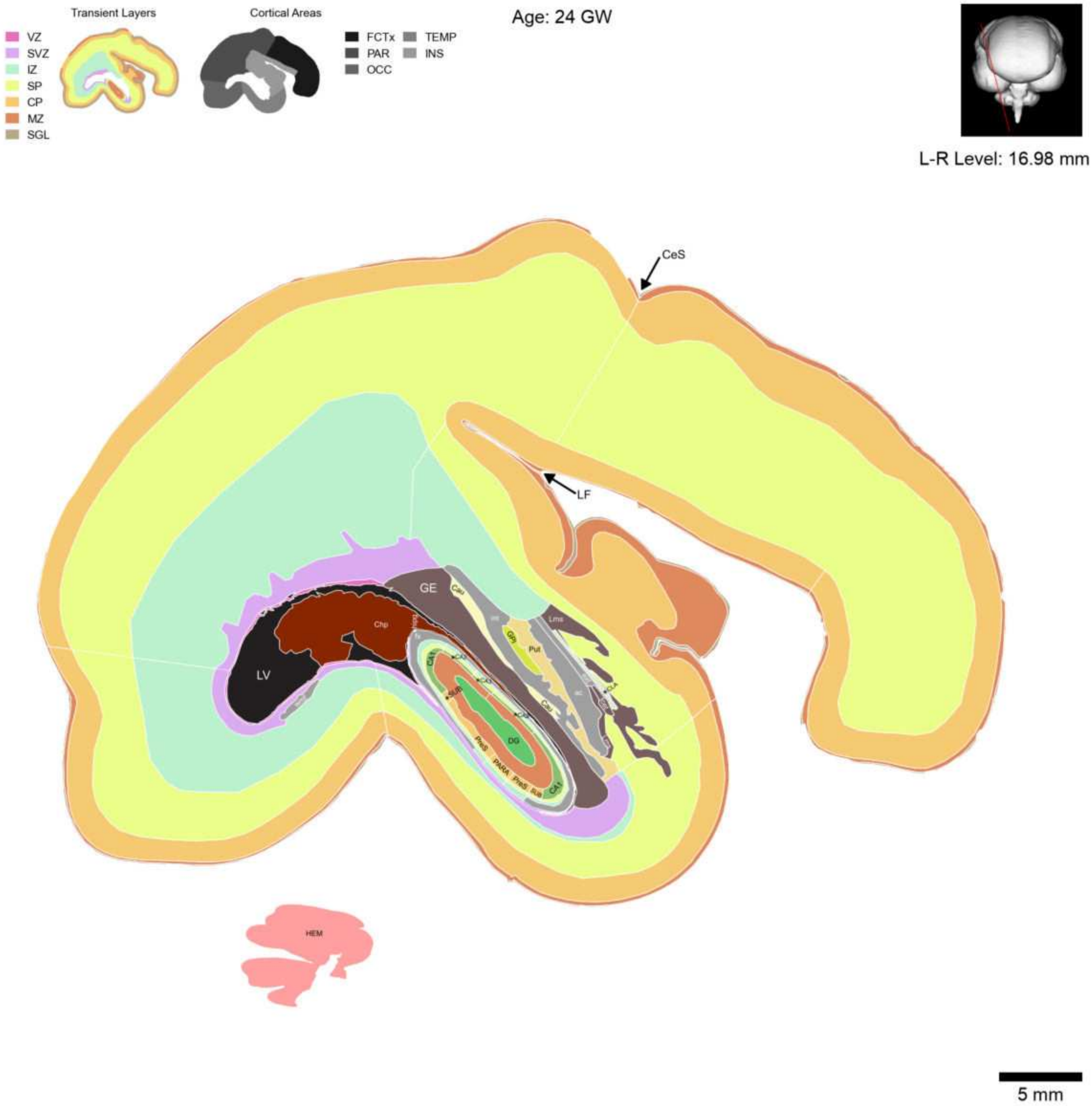

- CA1: CA1 field [hippocampus]
- CA2: CA2 field [hippocampus]
- CA3: CA3 field [hippocampus]
- CLA: Claustrum
- Cau: Caudate nucleus
- Chp: Choroid plexus
- DG: Dentate gyrus
- GE: Ganglionic eminence
- GPl: Globus pallidus lateral segment
- HEM: Cerebellar hemispheres
- LV: Lateral ventricle
- Lms: Lateral migratory stream
- PARA: Cortical plate, parasubiculum
- PreS: Cortical plate, presubiculum
- Put: Putamen
- SUB: Cortical plate, subiculum
- ac: Anterior commissure
- ext: External capsule
- fx: Fornix
- hipg: Hippocampal glioepithelium/ependyma
- int: Internal capsule
- tcet: Transient cell zone in the external capsule
- wmf: White matter fibers
- → LF: Lateral fissure
- → CeS: Central sulcus

Age: 24 GW

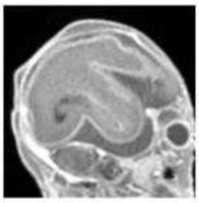

L-R Level: 16.56 mm

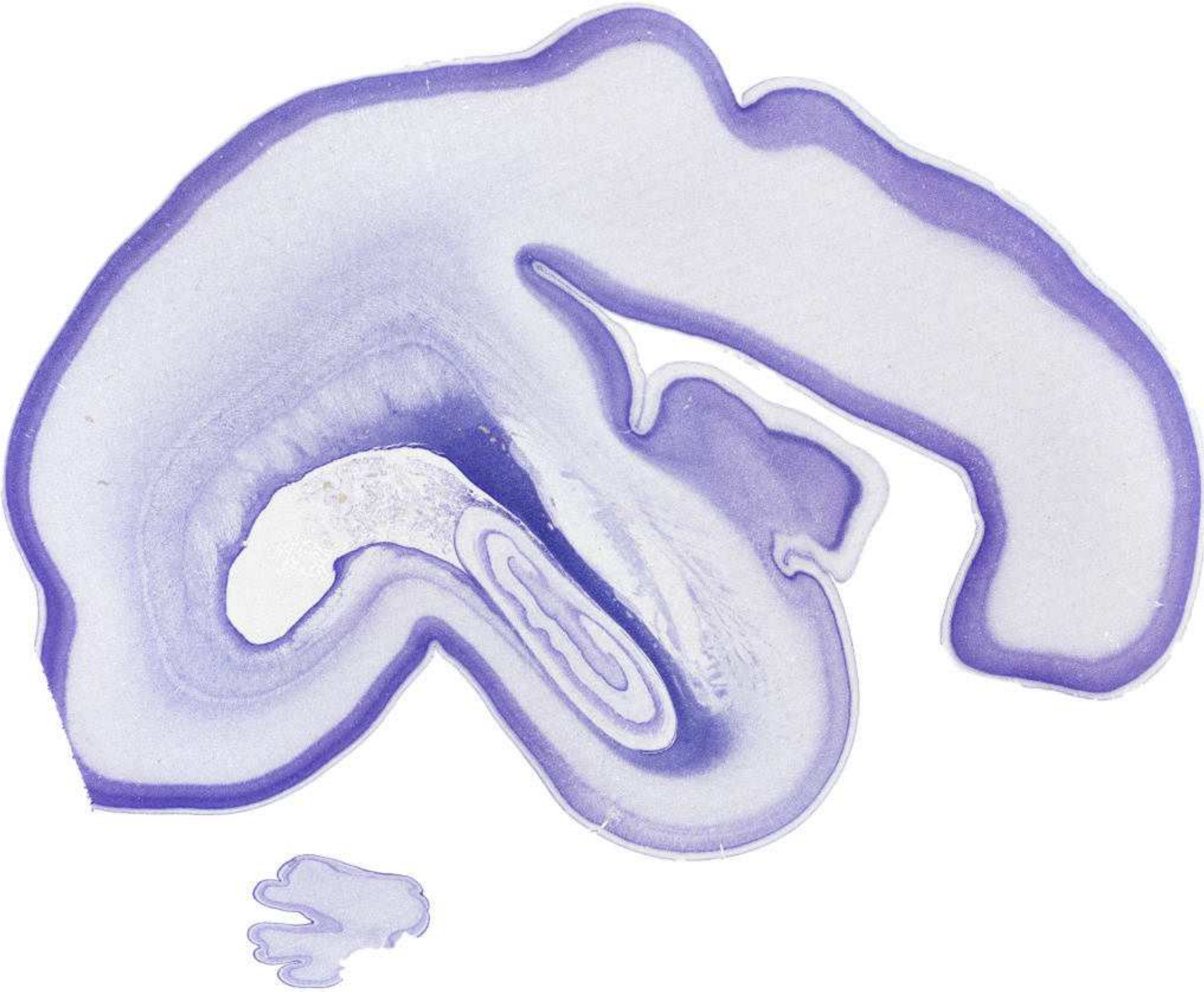

5 mm

Age: 24 GW

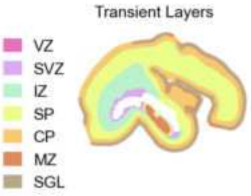

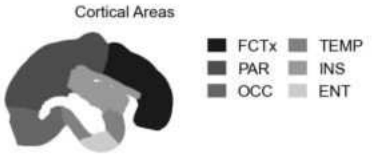

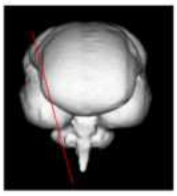

L-R Level: 16.56 mm

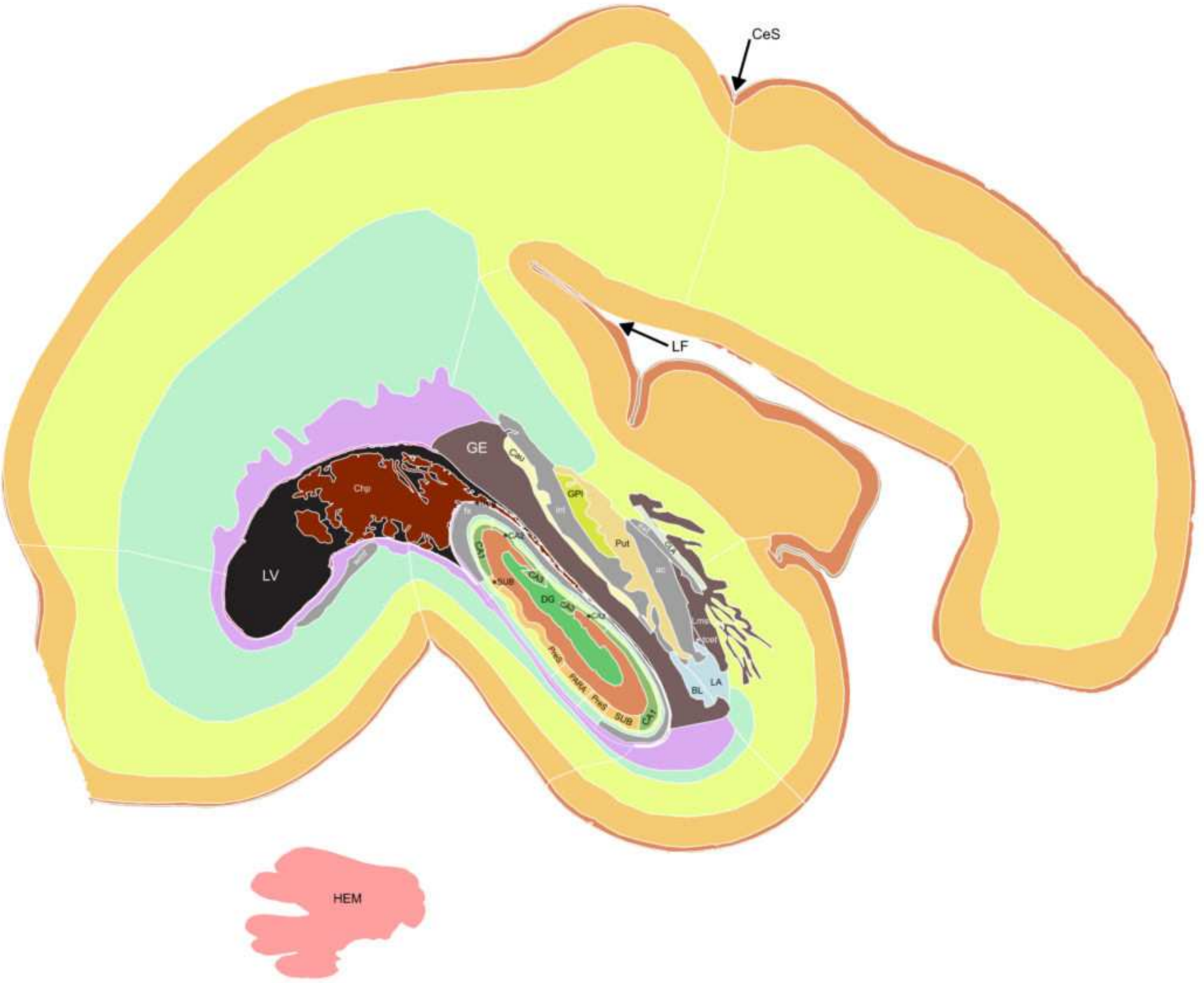

5 mm

- BL: Basal nucleus [amygdala]
- CA1: CA1 field [hippocampus]
- CA2: CA2 field [hippocampus]
- CA3: CA3 field [hippocampus]
- CLA: Claustrum
- Cau: Caudate nucleus
- Chp: Choroid plexus
- DG: Dentate gyrus
- GE: Ganglionic eminence
- GPl: Globus pallidus lateral segment
- HEM: Cerebellar hemispheres
- LA: Lateral nucleus [amygdala]
- LV: Lateral ventricle
- Lms: Lateral migratory stream
- PARA: Cortical plate, parasubiculum
- PreS: Cortical plate, presubiculum
- Put: Putamen
- SUB: Cortical plate, subiculum
- ac: Anterior commissure
- ext: External capsule
- fx: Fornix
- hipg: Hippocampal glioepithelium/ependyma
- int: Internal capsule
- tcet: Transient cell zone in the external capsule
- wmf: White matter fibers
- → CeS: Central sulcus
- → LF: Lateral fissure

Age: 24 GW

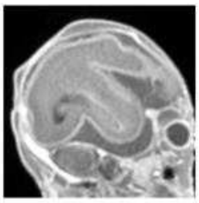

L-R Level: 16.32 mm

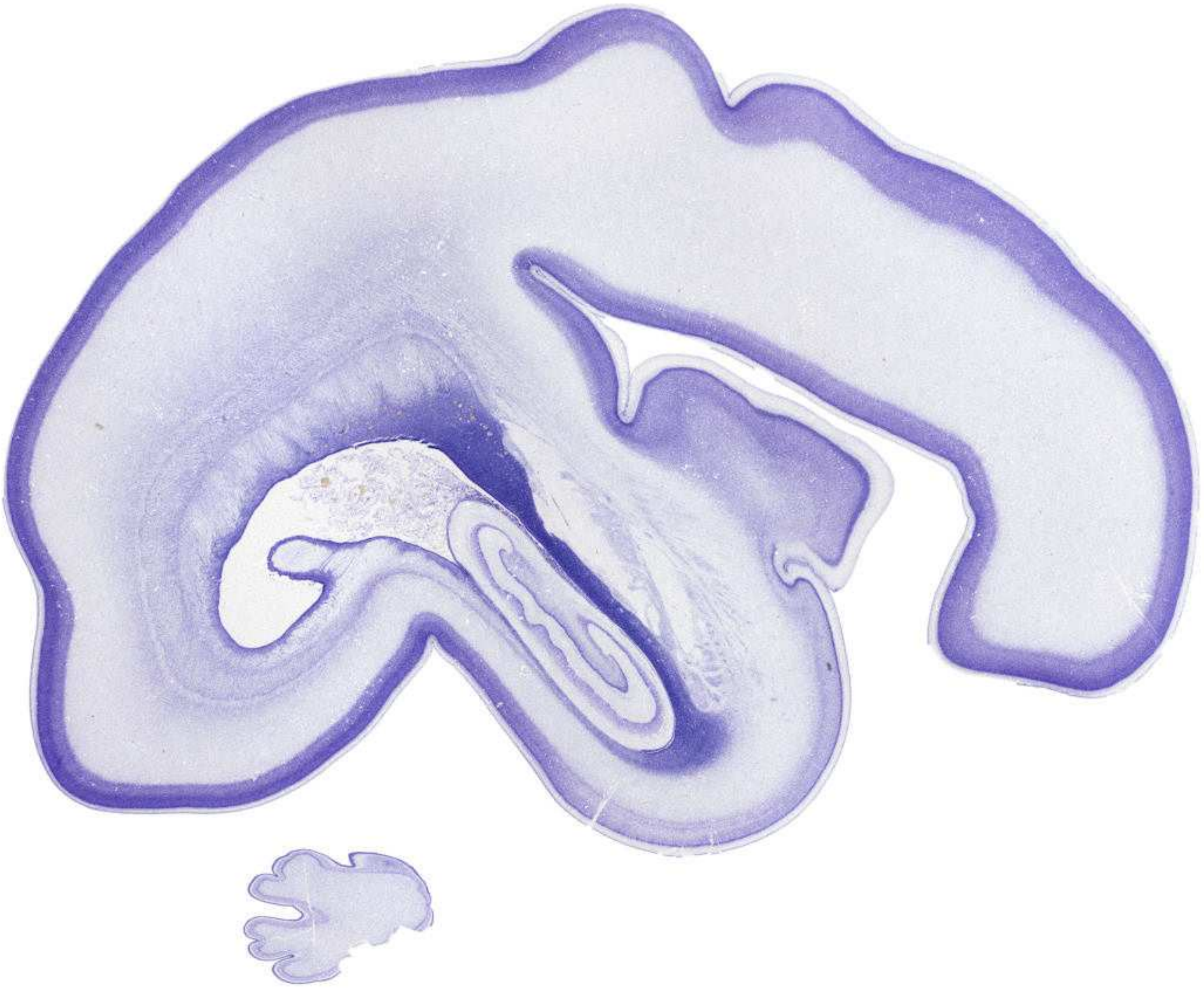

5 mm

Transient Layers

VZ
SVZ
IZ
SP
CP
MZ
SGL

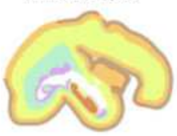

Cortical Areas

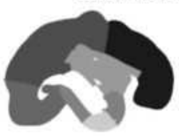

FCTx
PAR
OCC
TEMP
INS
ENT

Age: 24 GW

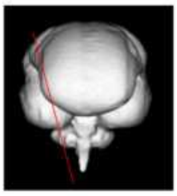

L-R Level: 16.32 mm

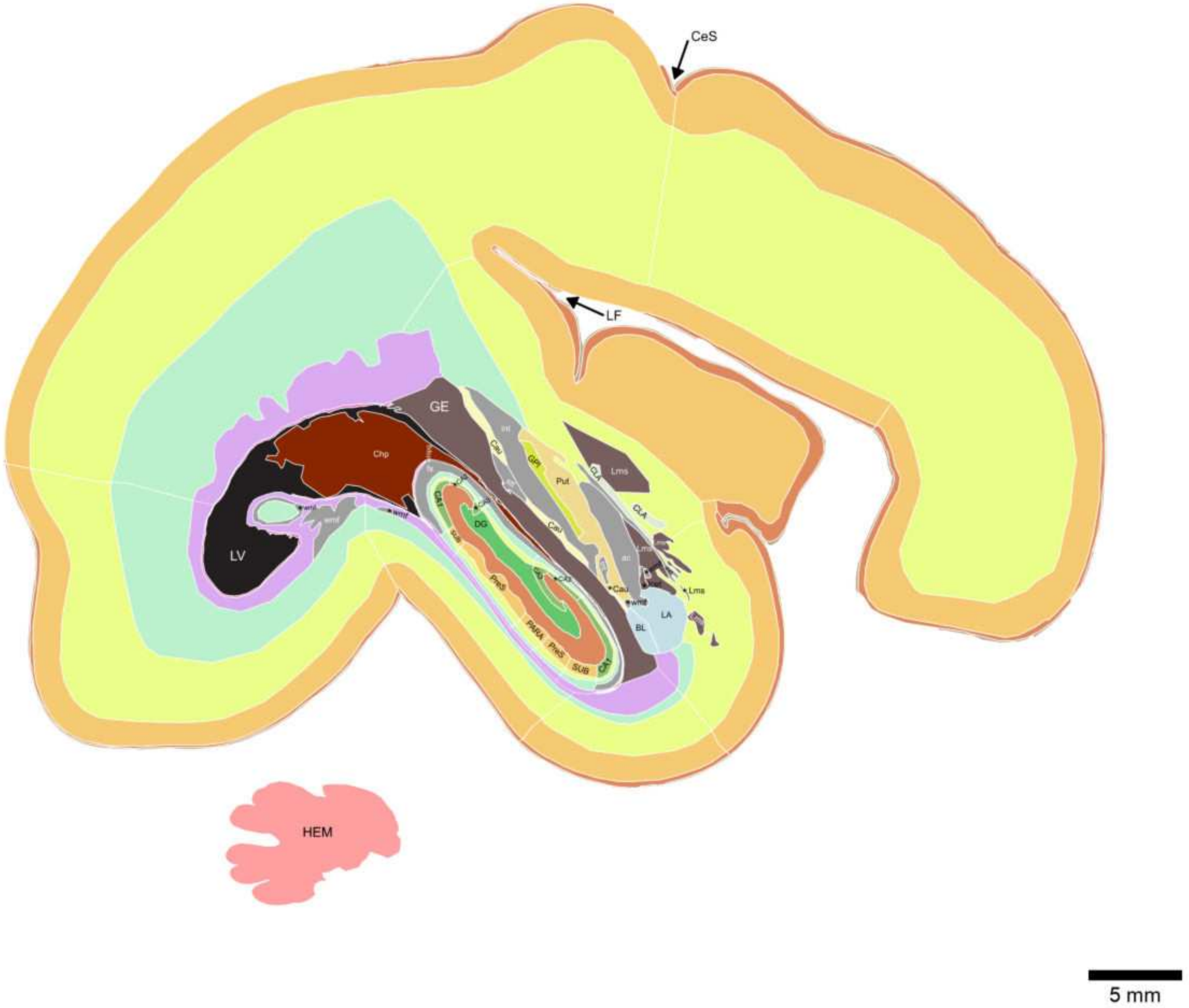

BL: Basal nucleus [amygdala]
CA1: CA1 field [hippocampus]
CA2: CA2 field [hippocampus]
CA3: CA3 field [hippocampus]
CLA: Claustrum
Cau: Caudate nucleus
Chp: Choroid plexus
DG: Dentate gyrus
GE: Ganglionic eminence
GPl: Globus pallidus lateral segment
HEM: Cerebellar hemispheres
LA: Lateral nucleus [amygdala]
LV: Lateral ventricle
Lms: Lateral migratory stream
PARA: Cortical plate, parasubiculum
PreS: Cortical plate, presubiculum
Put: Putamen
SUB: Cortical plate, subiculum
ac: Anterior commissure
ext: External capsule
fx: Fornix
hipg: Hippocampal glioepithelium/ependyma
int: Internal capsule
stt: Stria terminalis
tcet: Transient cell zone in the external capsule
wmf: White matter fibers
→ CeS: Central sulcus
→ LF: Lateral fissure

Age: 24 GW

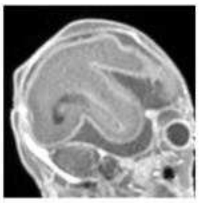

L-R Level: 15.9 mm

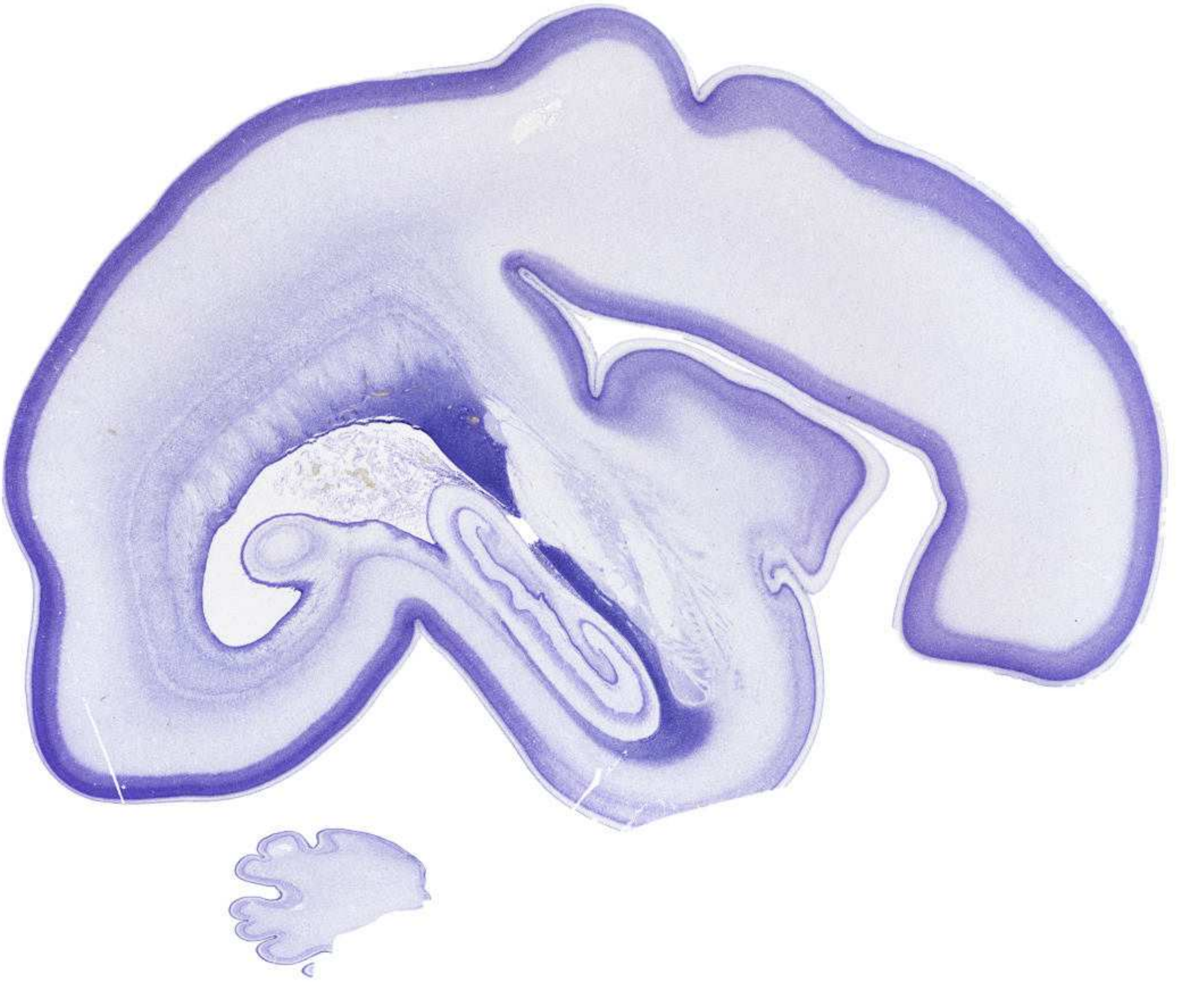

5 mm

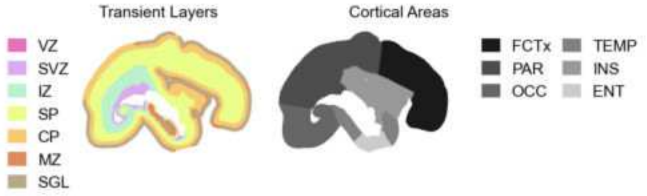

Age: 24 GW

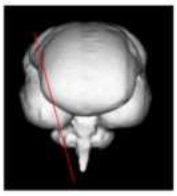

L-R Level: 15.9 mm

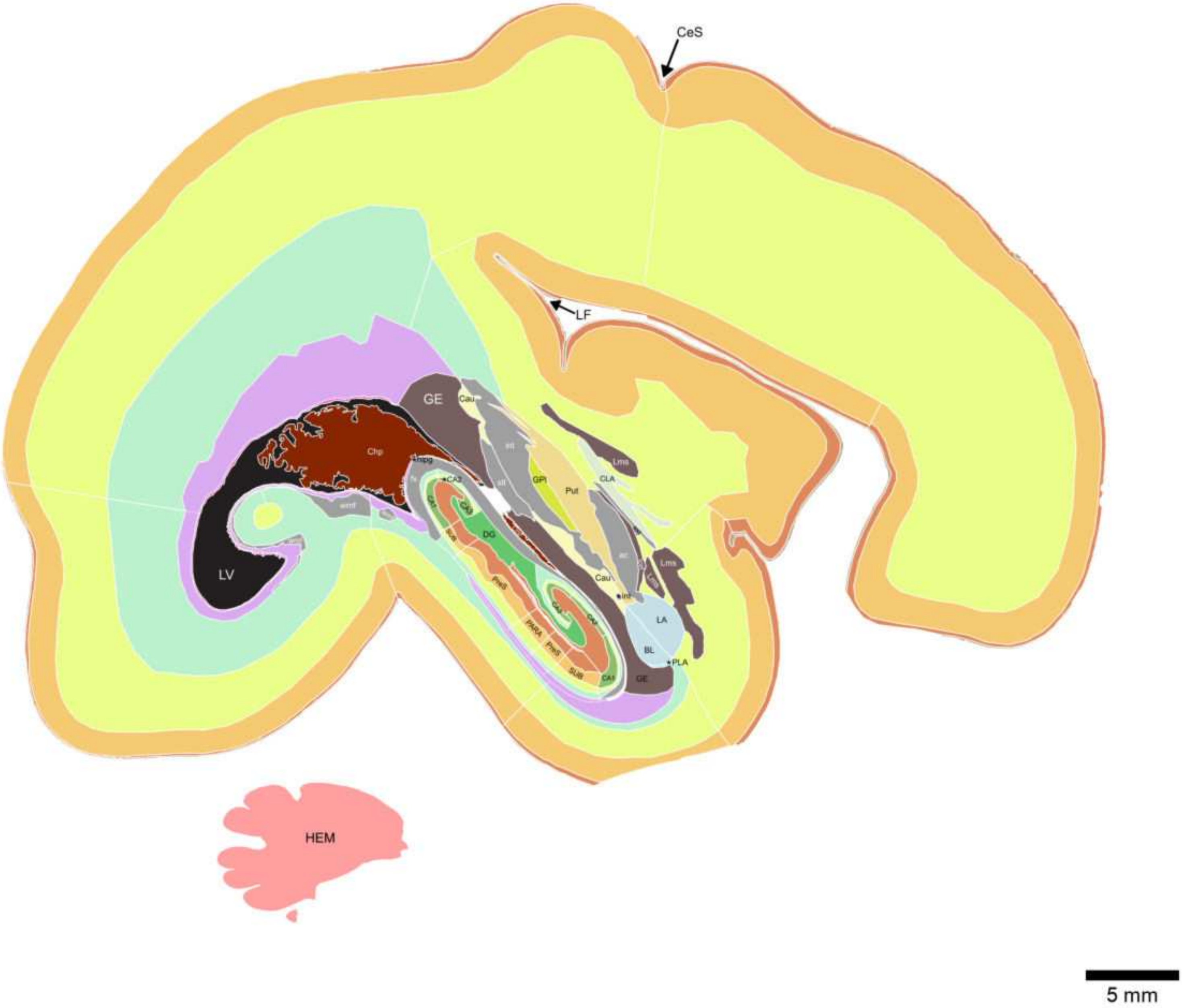

- BL: Basal nucleus [amygdala]
- CA1: CA1 field [hippocampus]
- CA2: CA2 field [hippocampus]
- CA3: CA3 field [hippocampus]
- CLA: Claustrum
- Cau: Caudate nucleus
- Chp: Choroid plexus
- DG: Dentate gyrus
- GE: Ganglionic eminence
- GPl: Globus pallidus lateral segment
- HEM: Cerebellar hemispheres
- LA: Lateral nucleus [amygdala]
- LV: Lateral ventricle
- Lms: Lateral migratory stream
- PARA: Cortical plate, parasubiculum
- PLA: Paralaminar nucleus [amygdala]
- PreS: Cortical plate, presubiculum
- Put: Putamen
- SUB: Cortical plate, subiculum
- ac: Anterior commissure
- ext: External capsule
- fx: Fornix
- hipg: Hippocampal glioepithelium/ependyma
- int: Internal capsule
- stt: Stria terminalis
- tcet: Transient cell zone in the external capsule
- wmf: White matter fibers
- → CeS: Central sulcus
- → LF: Lateral fissure

Age: 24 GW

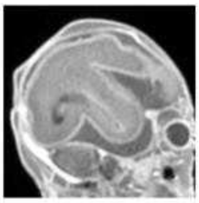

L-R Level: 15.66 mm

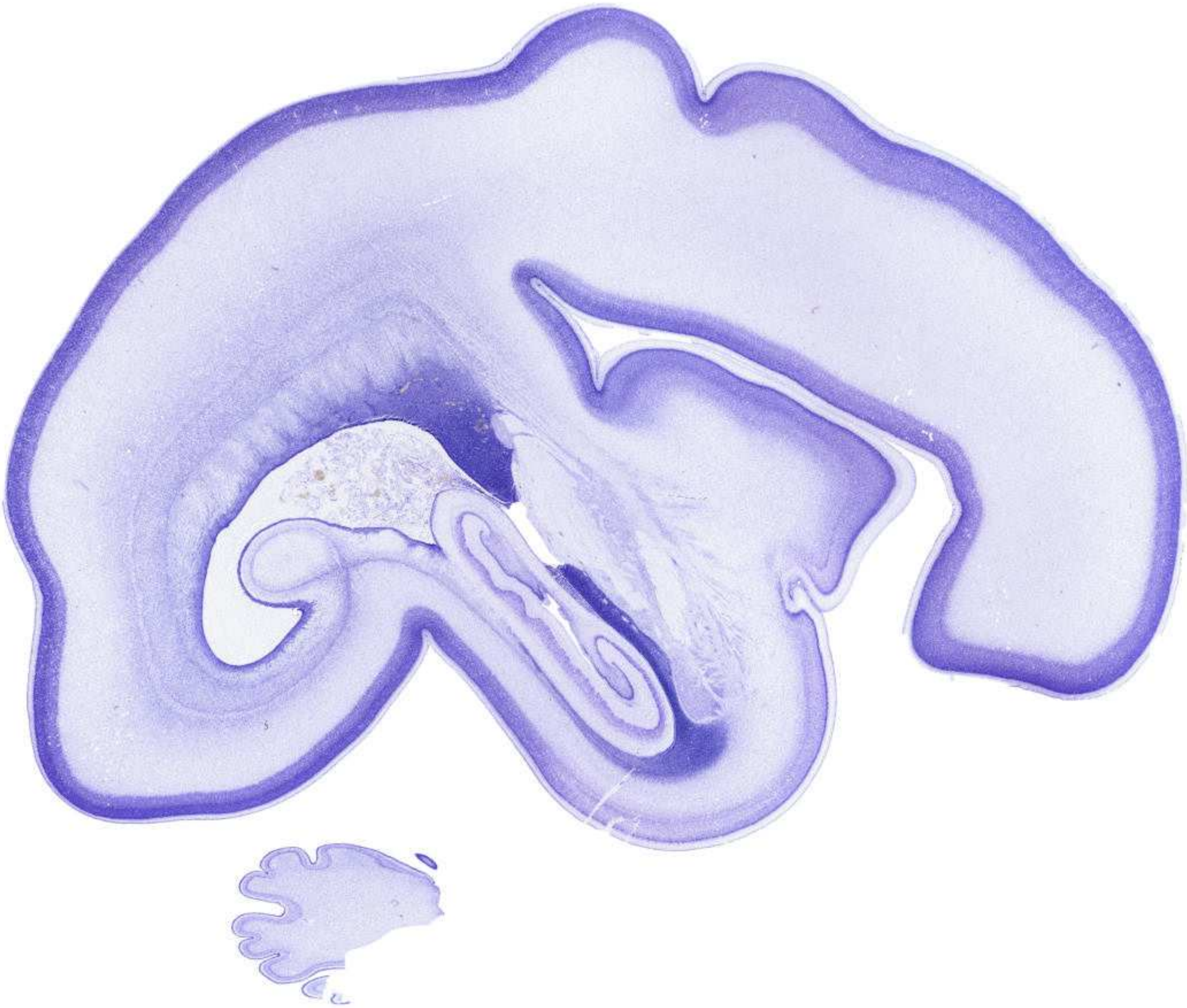

5 mm

Transient Layers

VZ
SVZ
IZ
SP
CP
MZ
SGL

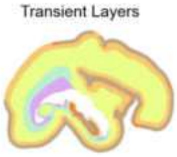

Cortical Areas

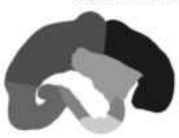

FCTx
PAR
OCC
TEMP
INS
ENT

Age: 24 GW

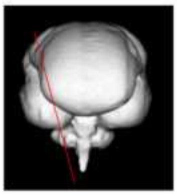

L-R Level: 15.66 mm

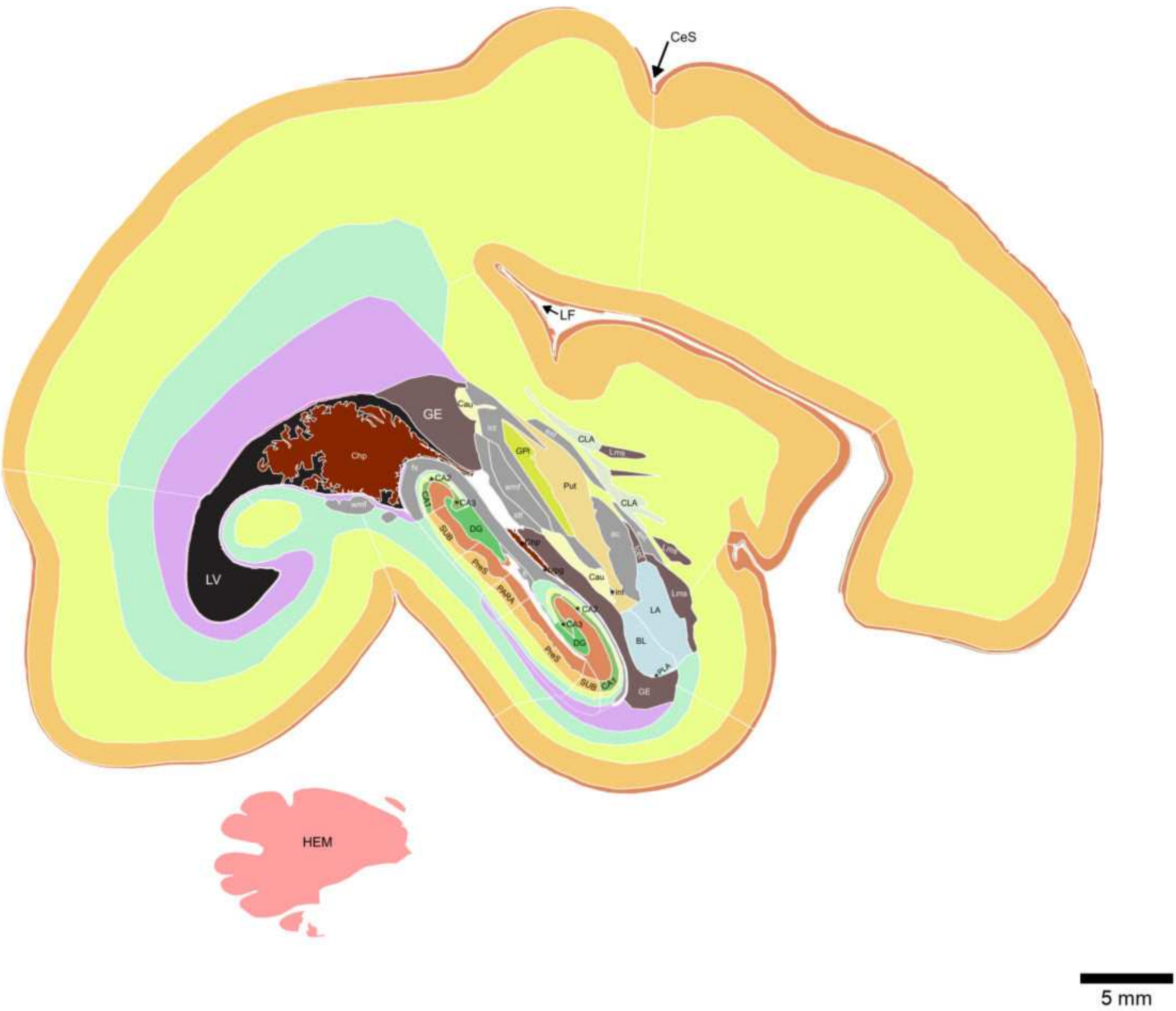

- BL: Basal nucleus [amygdala]
- CA1: CA1 field [hippocampus]
- CA2: CA2 field [hippocampus]
- CA3: CA3 field [hippocampus]
- CLA: Claustrum
- Cau: Caudate nucleus
- Chp: Choroid plexus
- DG: Dentate gyrus
- GE: Ganglionic eminence
- GPl: Globus pallidus lateral segment
- HEM: Cerebellar hemispheres
- LA: Lateral nucleus [amygdala]
- LV: Lateral ventricle
- Lms: Lateral migratory stream
- PARA: Cortical plate, parasubiculum
- PLA: Paralaminar nucleus [amygdala]
- PreS: Cortical plate, presubiculum
- Put: Putamen
- SUB: Cortical plate, subiculum
- ac: Anterior commissure
- ext: External capsule
- fi: Fimbria
- fx: Fornix
- hipg: Hippocampal glioepithelium/ependyma
- int: Internal capsule
- stt: Stria terminalis
- tcet: Transient cell zone in the external capsule
- wmf: White matter fibers
- → LF: Lateral fissure

Age: 24 GW

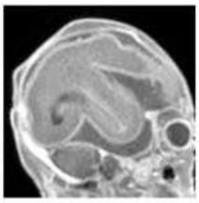

L-R Level: 15.36 mm

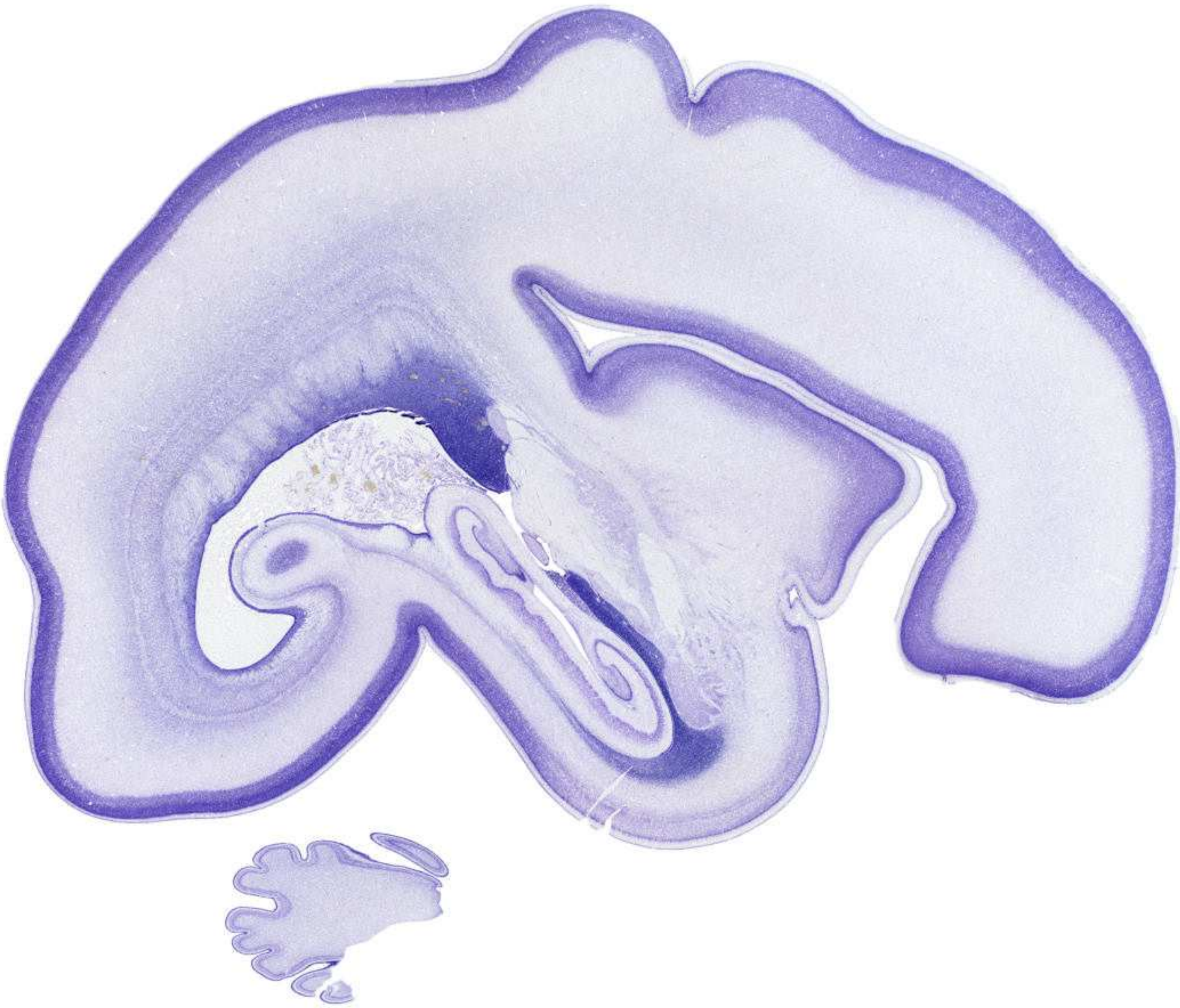

5 mm

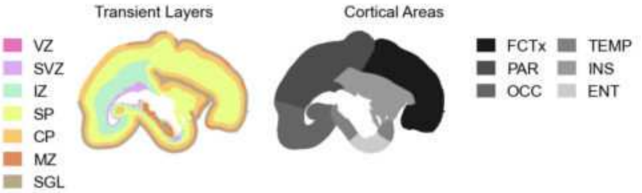

Age: 24 GW

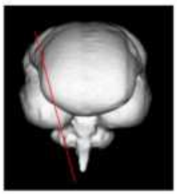

L-R Level: 15.36 mm

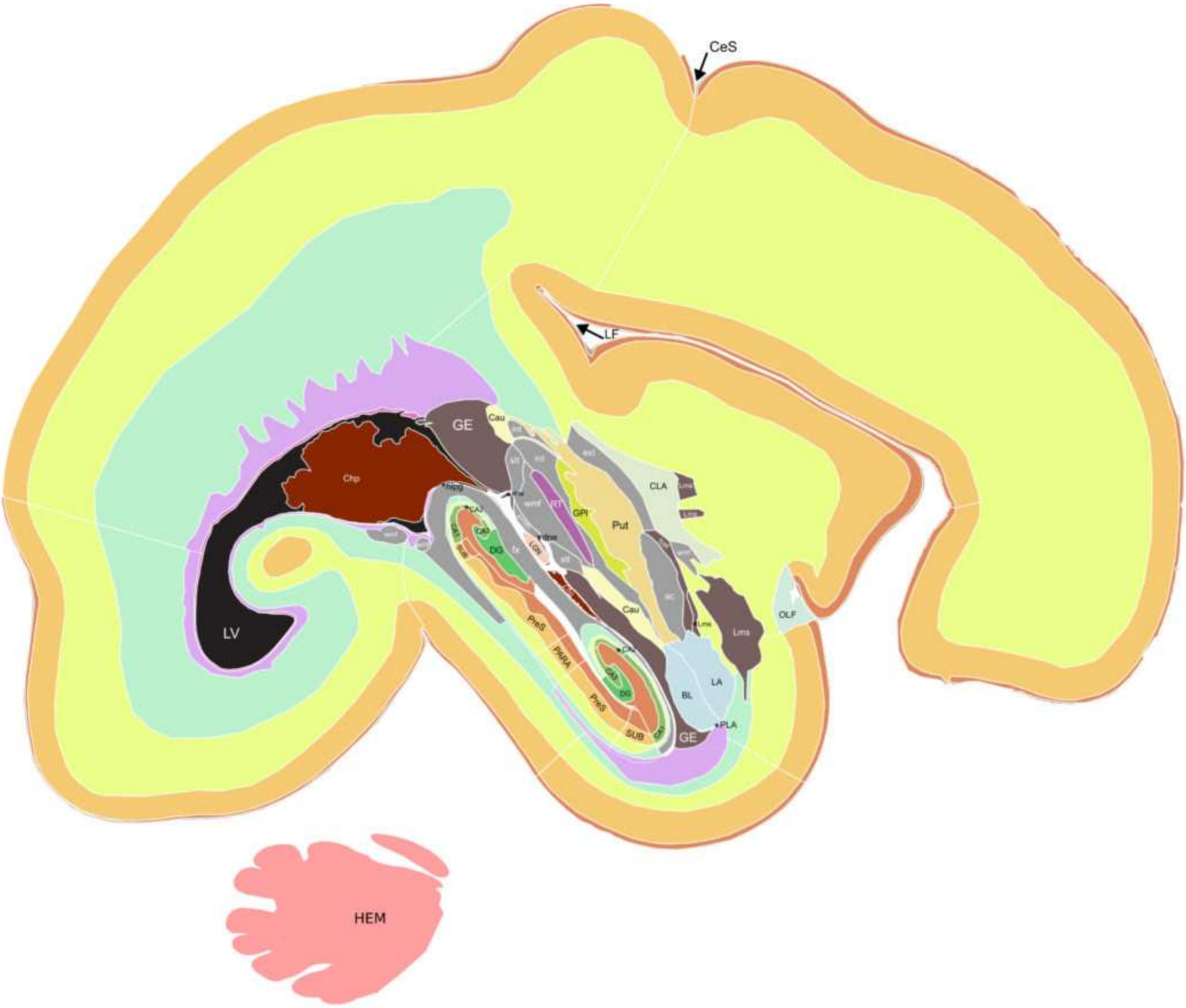

5 mm

BL: Basal nucleus [amygdala]
CA1: CA1 field [hippocampus]
CA2: CA2 field [hippocampus]
CA3: CA3 field [hippocampus]
CLA: Claustrum
Cau: Caudate nucleus
Chp: Choroid plexus
DG: Dentate gyrus

GE: Ganglionic eminence
GPl: Globus pallidus lateral segment
HEM: Cerebellar hemispheres
LA: Lateral nucleus [amygdala]
LGN: Lateral geniculate nucleus
LV: Lateral ventricle
Lms: Lateral migratory stream
PARA: Cortical plate, parasubiculum

PLA: Paralaminar nucleus [amygdala]
PreS: Cortical plate, presubiculum
Put: Putamen
RT: Reticular nucleus [thalamus]
SUB: Cortical plate, subiculum
ac: Anterior commissure
dne: Diencephalic neuroepithelium
emlth: External medullary lamina [thalamus]

ext: External capsule
fx: Fornix
hipg: Hippocampal glioepithelium/ependyma
int: Internal capsule
stt: Stria terminalis
tcet: Transient cell zone in the external capsule
wmf: White matter fibers
CeS: Central sulcus
LF: Lateral fissure

Age: 24 GW

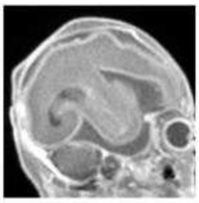

L-R Level: 14.82 mm

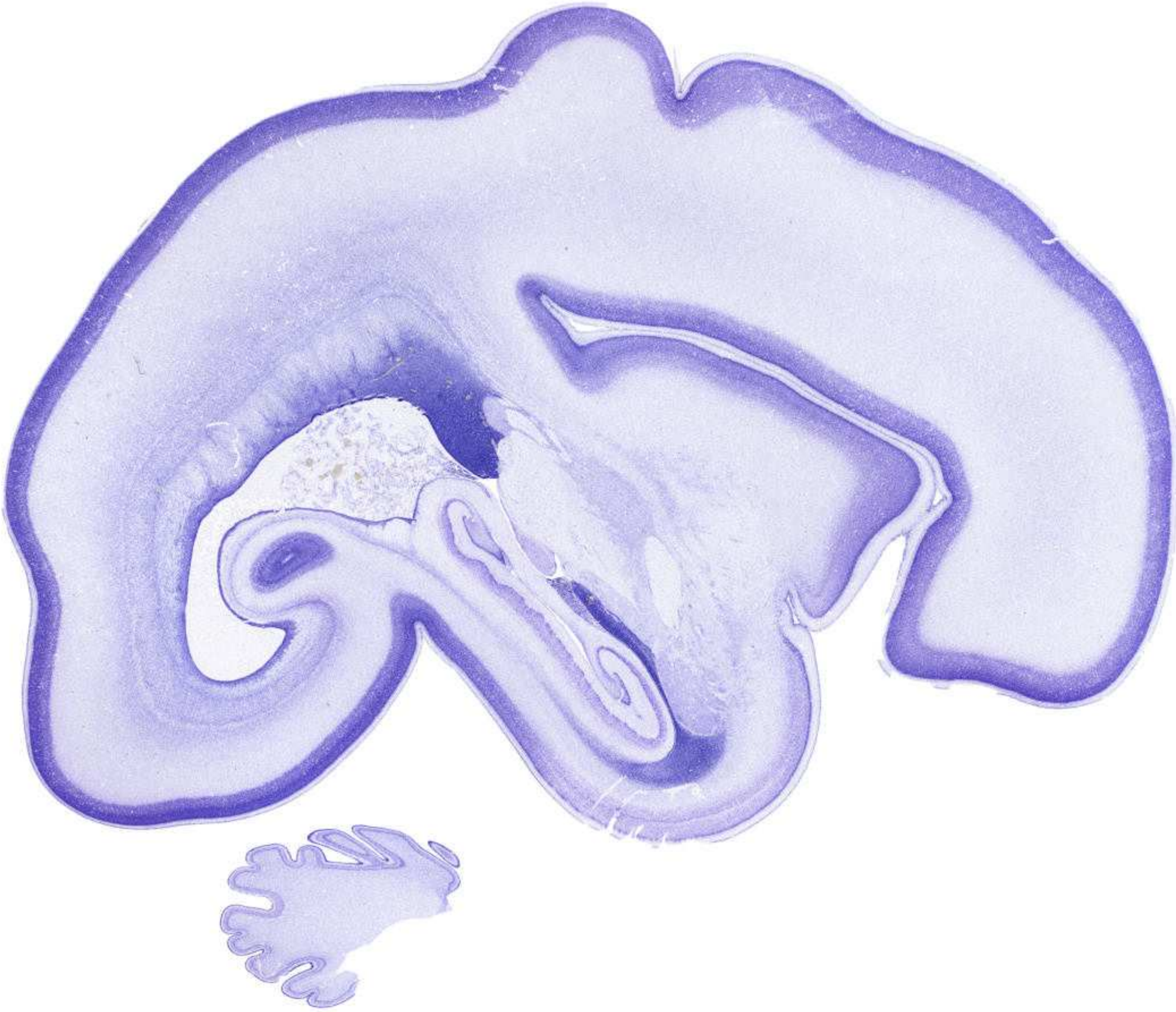

5 mm

Transient Layers

VZ
SVZ
IZ
SP
CP
MZ
SGL

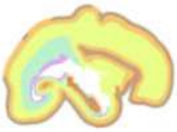

Cortical Areas

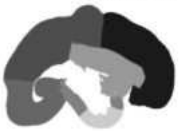

FCTx
PAR
OCC
TEMP
INS
ENT

Age: 24 GW

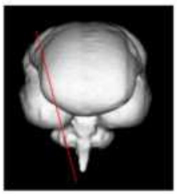

L-R Level: 14.82 mm

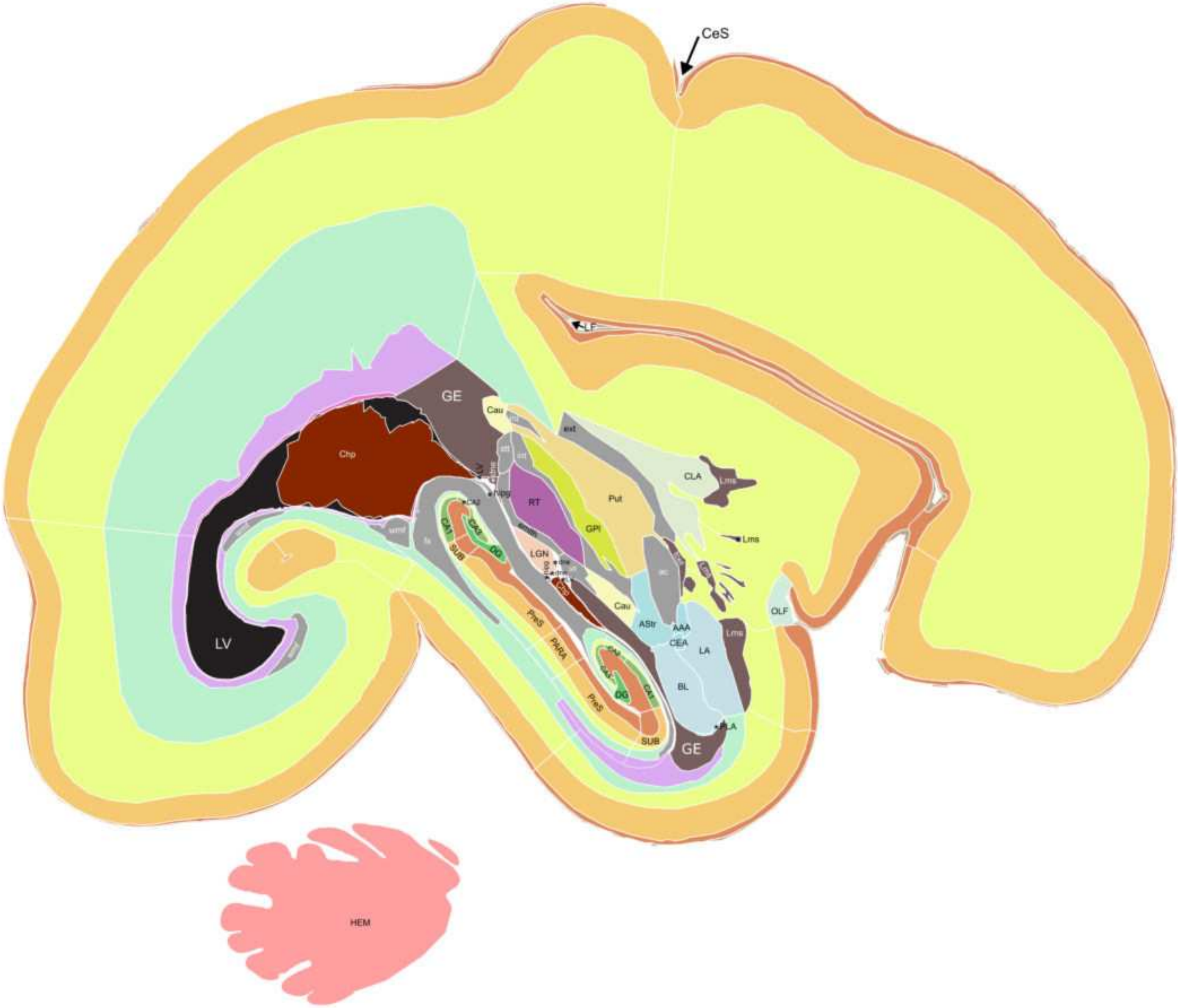

5 mm

- AAA: Anterior amygdaloid area
- AStr: Amygdalo-striatal area
- BL: Basal nucleus [amygdala]
- CA1: CA1 field [hippocampus]
- CA2: CA2 field [hippocampus]
- CA3: CA3 field [hippocampus]
- CEA: Central nucleus [amygdala]
- CLA: Claustrum
- Cau: Caudate nucleus
- Chp: Choroid plexus
- DG: Dentate gyrus
- GE: Ganglionic eminence
- GPl: Globus pallidus lateral segment
- HEM: Cerebellar hemispheres
- LA: Lateral nucleus [amygdala]
- LGN: Lateral geniculate nucleus
- LV: Lateral ventricle
- Lms: Lateral migratory stream
- PARA: Cortical plate, parasubiculum
- PLA: Paralaminar nucleus [amygdala]
- PreS: Cortical plate, presubiculum
- Put: Putamen
- RT: Reticular nucleus [thalamus]
- SUB: Cortical plate, subiculum
- ac: Anterior commissure
- dne: Diencephalic neuroepithelium
- emlth: External medullary lamina [thalamus]
- ext: External capsule
- fx: Fornix
- hipg: Hippocampal glioepithelium/ependyma
- int: Internal capsule
- stt: Stria terminalis
- tcet: Transient cell zone in the external capsule
- wmf: White matter fibers
- → CeS: Central sulcus
- → LF: Lateral fissure

Age: 24 GW

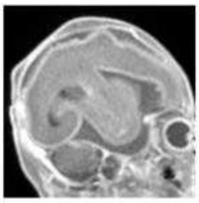

L-R Level: 14.58 mm

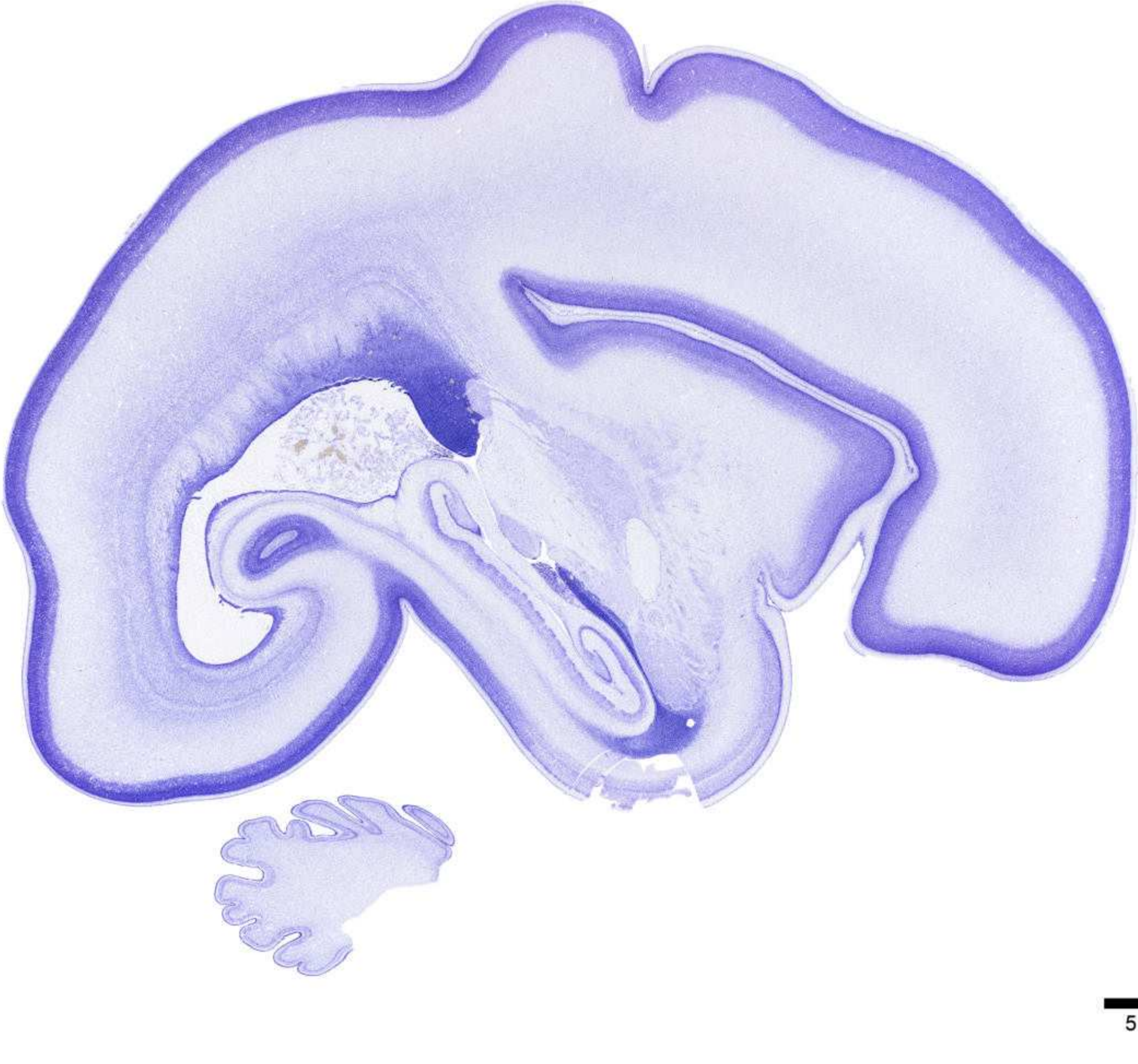

5 mm

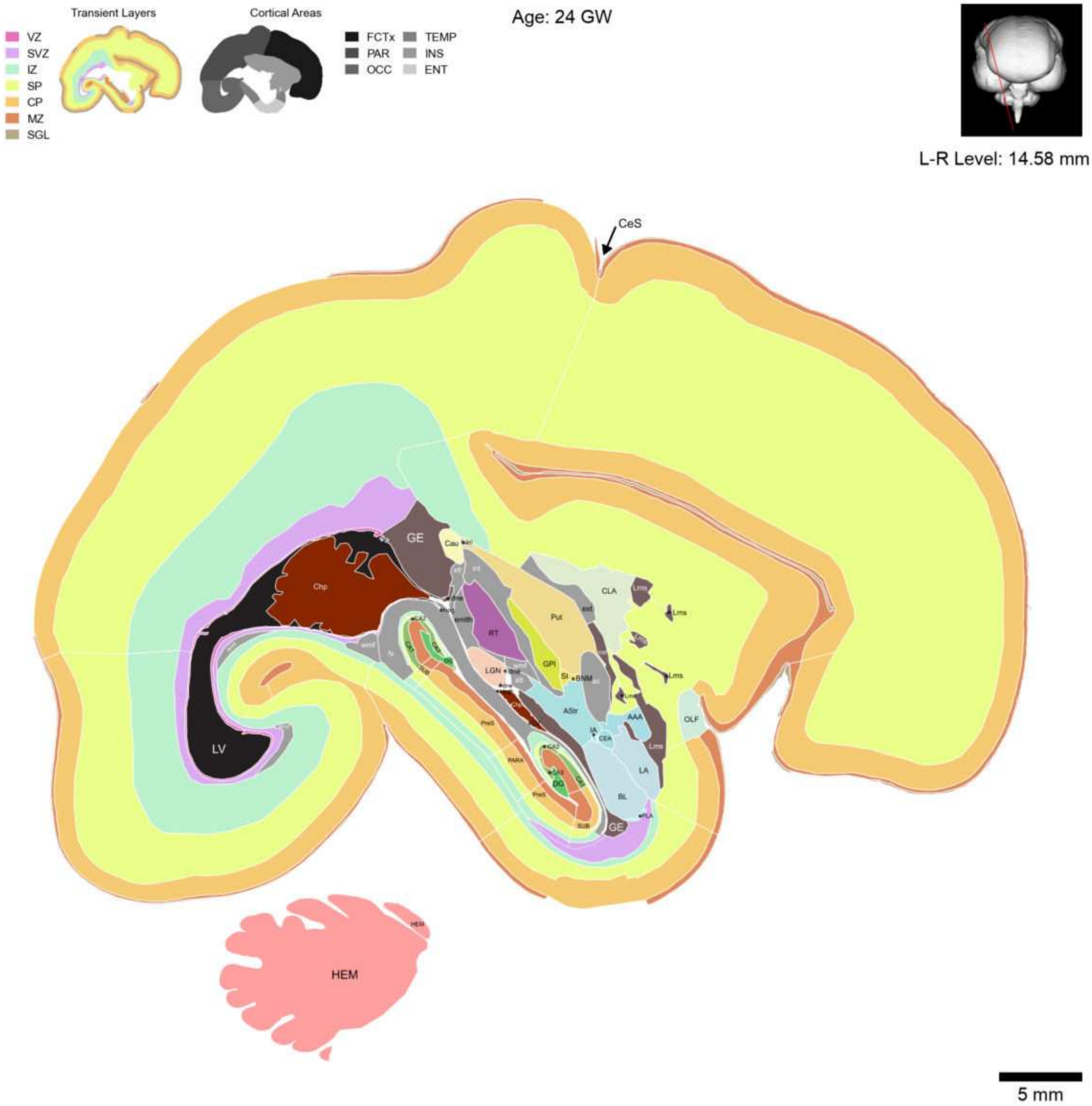

- AAA: Anterior amygdaloid area
- AStr: Amygdalo-striatal area
- BL: Basal nucleus [amygdala]
- BNM: Basal nucleus of Meynert
- CA1: CA1 field [hippocampus]
- CA2: CA2 field [hippocampus]
- CA3: CA3 field [hippocampus]
- CEA: Central nucleus [amygdala]
- CLA: Claustrum
- Cau: Caudate nucleus
- Chp: Choroid plexus
- DG: Dentate gyrus
- GE: Ganglionic eminence
- GPl: Globus pallidus lateral segment
- HEM: Cerebellar hemispheres
- IA: Intercalated cell groups [amygdala]
- LA: Lateral nucleus [amygdala]
- LGN: Lateral geniculate nucleus
- LV: Lateral ventricle
- Lms: Lateral migratory stream
- PARA: Cortical plate, parasubiculum
- PLA: Paralaminar nucleus [amygdala]
- PreS: Cortical plate, presubiculum
- Put: Putamen
- RT: Reticular nucleus [thalamus]
- SI: Substantia innominata
- SUB: Cortical plate, subiculum
- ac: Anterior commissure
- dne: Diencephalic neuroepithelium
- emlth: External medullary lamina [thalamus]
- ext: External capsule
- fx: Fornix
- hipg: Hippocampal glioepithelium/ependyma
- int: Internal capsule
- stt: Stria terminalis
- tcet: Transient cell zone in the external capsule
- wmf: White matter fibers
- → CeS: Central sulcus

Age: 24 GW

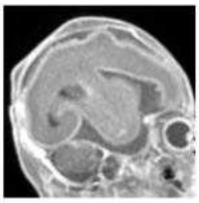

L-R Level: 14.28 mm

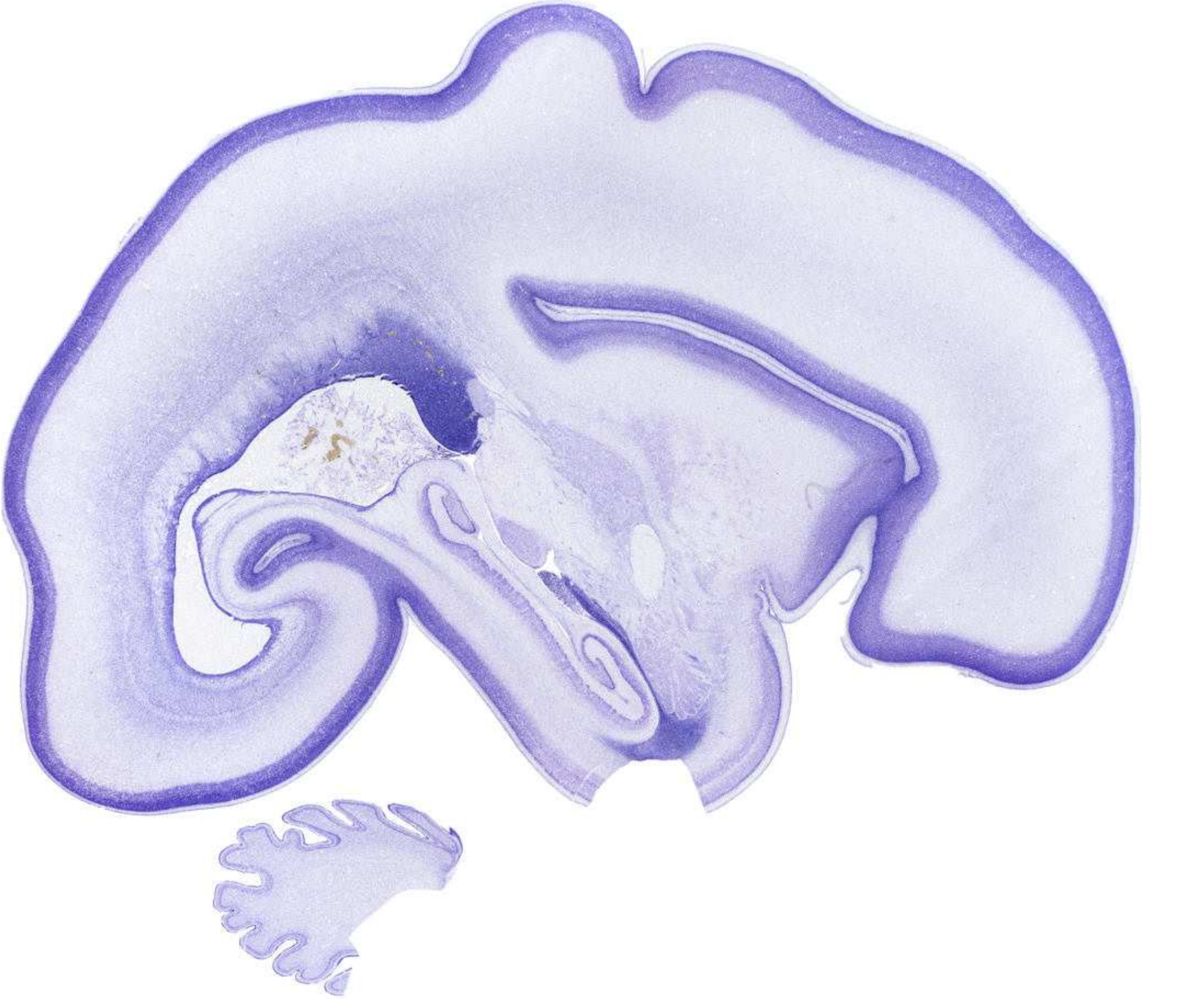

5 mm

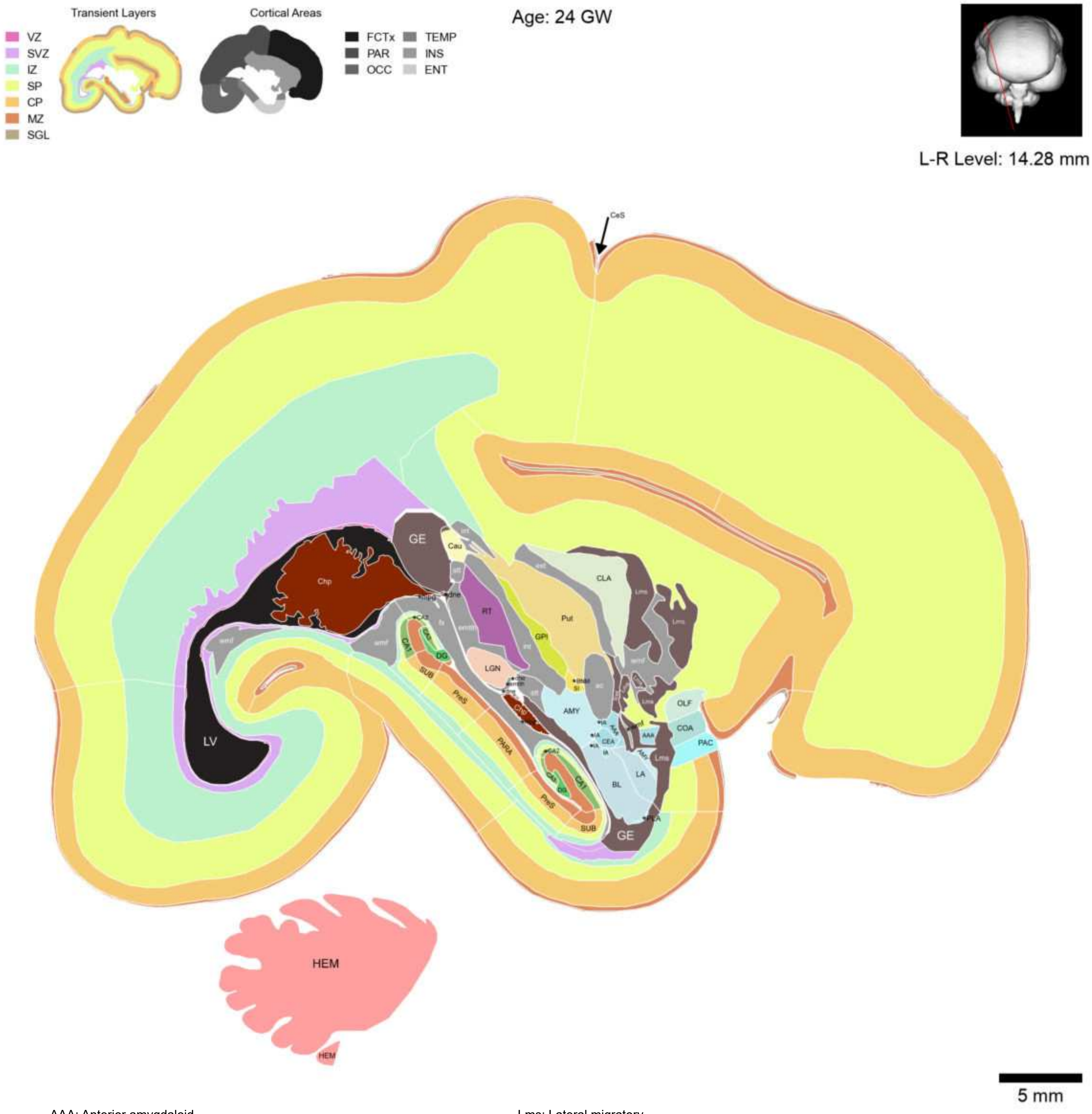

AAA: Anterior amygdaloid area
AMY: Amygdala
BL: Basal nucleus [amygdala]
BNM: Basal nucleus of Meynert
CA1: CA1 field [hippocampus]
CA2: CA2 field [hippocampus]
CA3: CA3 field [hippocampus]
CEA: Central nucleus [amygdala]
CLA: Claustrum
COA: Cortical nucleus [amygdala]
Cau: Caudate nucleus
Chp: Choroid plexus
DG: Dentate gyrus
GE: Ganglionic eminence
GPl: Globus pallidus lateral segment
HEM: Cerebellar hemispheres
IA: Intercalated cell groups [amygdala]
LA: Lateral nucleus [amygdala]
LGN: Lateral geniculate nucleus
LV: Lateral ventricle
Lms: Lateral migratory stream
PARA: Cortical plate, parasubiculum
PLA: Paralaminar nucleus [amygdala]
PreS: Cortical plate, presubiculum
Put: Putamen
RT: Reticular nucleus [thalamus]
SI: Substantia innominata
SUB: Cortical plate, subiculum
ac: Anterior commissure
dne: Diencephalic neuroepithelium
emlth: External medullary lamina [thalamus]
ext: External capsule
fx: Fornix
hipg: Hippocampal glioepithelium/ependyma
int: Internal capsule
stt: Stria terminalis
tcet: Transient cell zone in the external capsule
wmf: White matter fibers
→ CeS: Central sulcus

Age: 24 GW

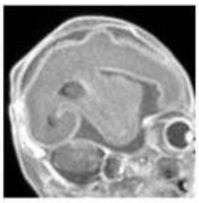

L-R Level: 13.98 mm

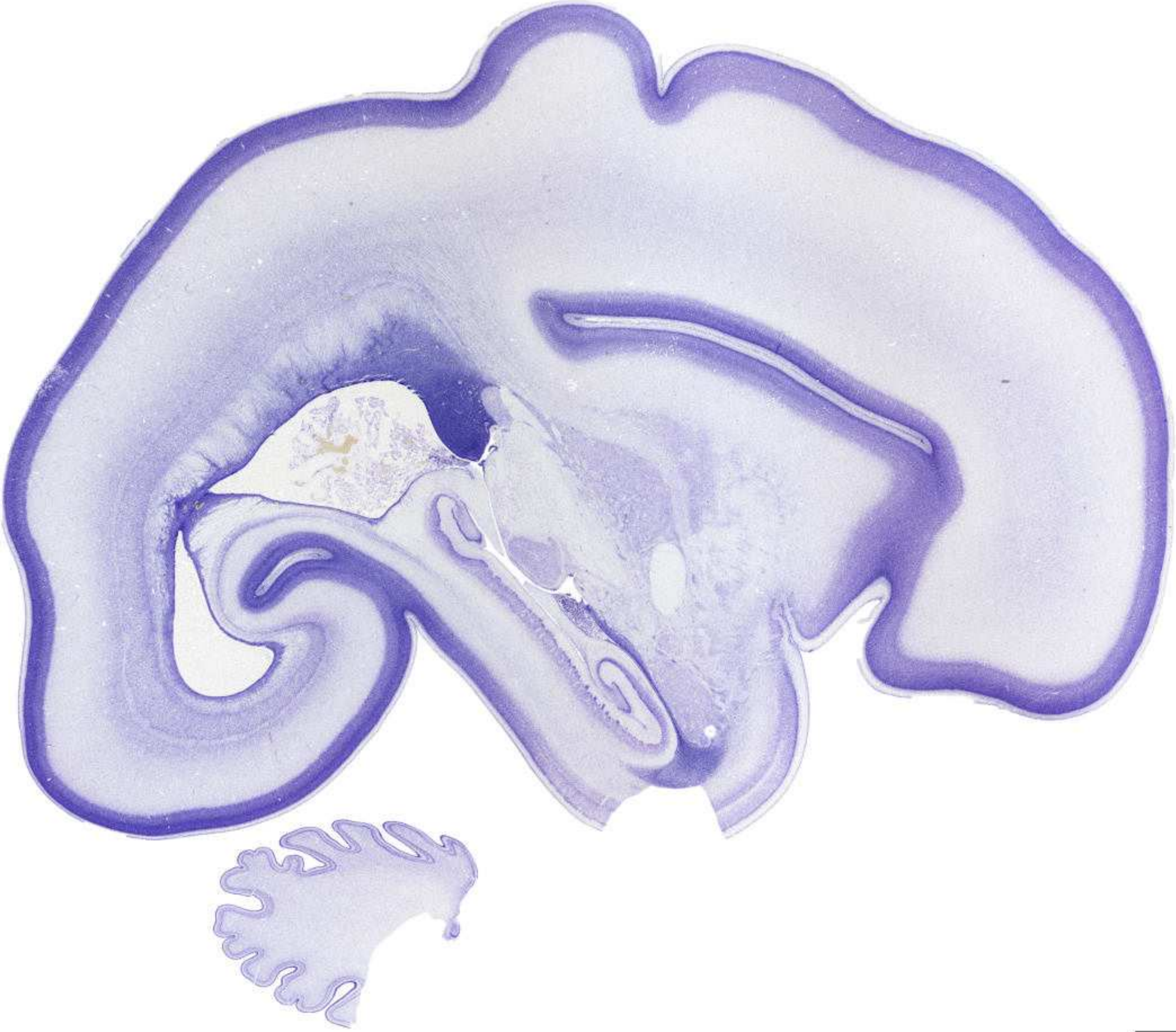

5 mm

Transient Layers

VZ
SVZ
IZ
SP
CP
MZ
SGL

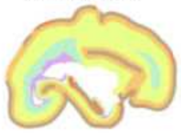

Cortical Areas

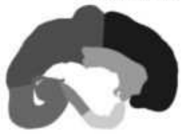

FCTx
PAR
OCC
TEMP
INS
ENT

Age: 24 GW

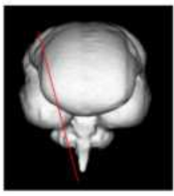

L-R Level: 13.98 mm

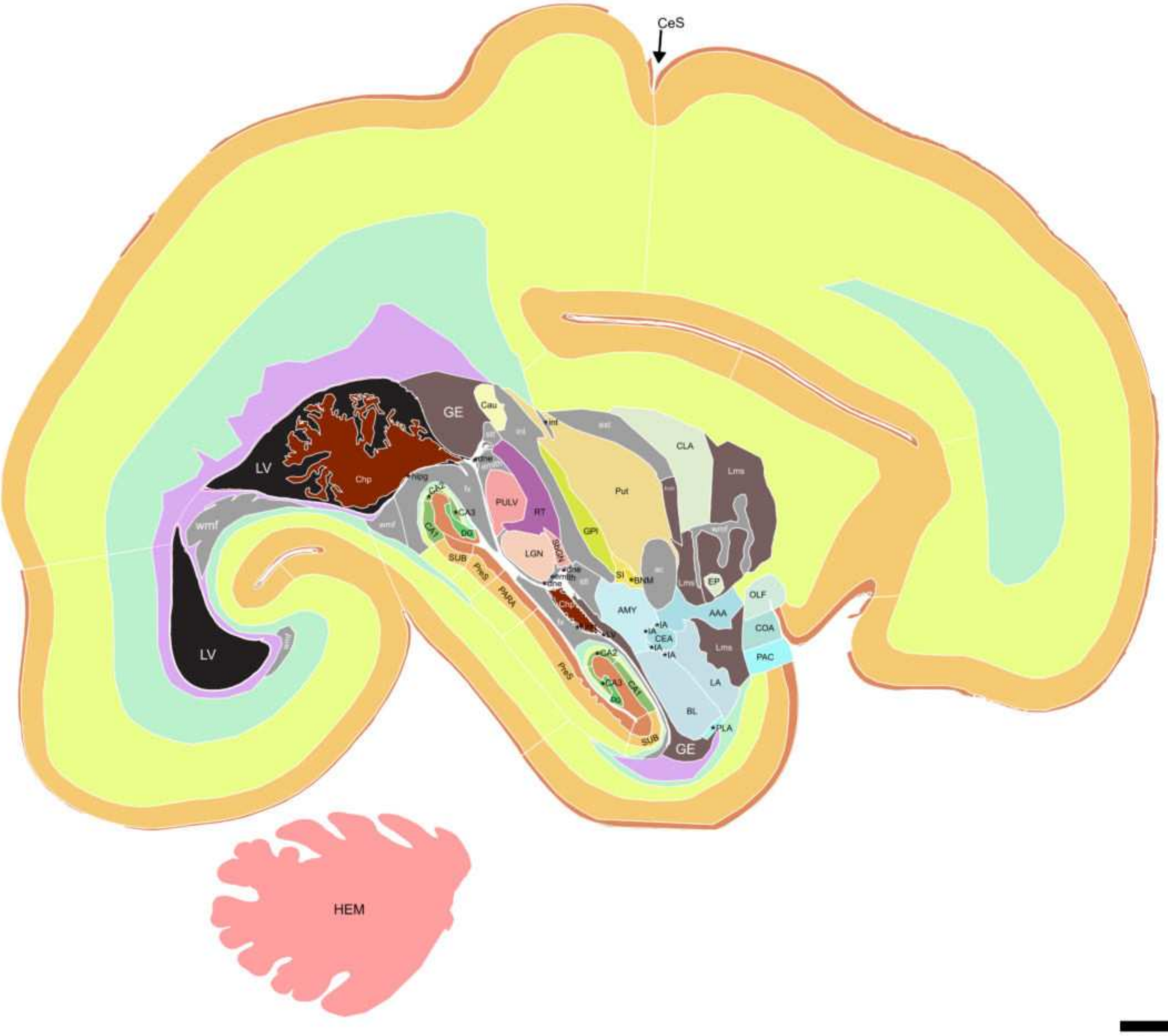

5 mm

AAA: Anterior amygdaloid area
AMY: Amygdala
BL: Basal nucleus [amygdala]
BNM: Basal nucleus of Meynert
CA1: CA1 field [hippocampus]
CA2: CA2 field [hippocampus]
CA3: CA3 field [hippocampus]
CEA: Central nucleus [amygdala]
CLA: Claustrum
COA: Cortical nucleus [amygdala]
Cau: Caudate nucleus

Chp: Choroid plexus
DG: Dentate gyrus
EP: Endopiriform nucleus
GE: Ganglionic eminence
GPl: Globus pallidus lateral segment
HEM: Cerebellar hemispheres
IA: Intercalated cell groups [amygdala]
LA: Lateral nucleus [amygdala]
LGN: Lateral geniculate nucleus
LV: Lateral ventricle

Lms: Lateral migratory stream
PARA: Cortical plate, parasubiculum
PLA: Paralaminar nucleus [amygdala]
PULV: Pulvinar nucleus [thalamus]
PreS: Cortical plate, presubiculum
Put: Putamen
RT: Reticular nucleus [thalamus]
SI: Substantia innominata
SUB: Cortical plate, subiculum
SbGN: Subgeniculate nucleus

ac: Anterior commissure
dne: Diencephalic neuroepithelium
emlth: External medullary lamina [thalamus]
ext: External capsule
fx: Fornix
hipg: Hippocampal glioepithelium/ependyma
int: Internal capsule
stt: Stria terminalis
tcet: Transient cell zone in the external capsule
wmf: White matter fibers
CeS: Central sulcus

Age: 24 GW

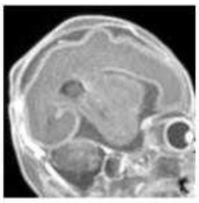

L-R Level: 13.5 mm

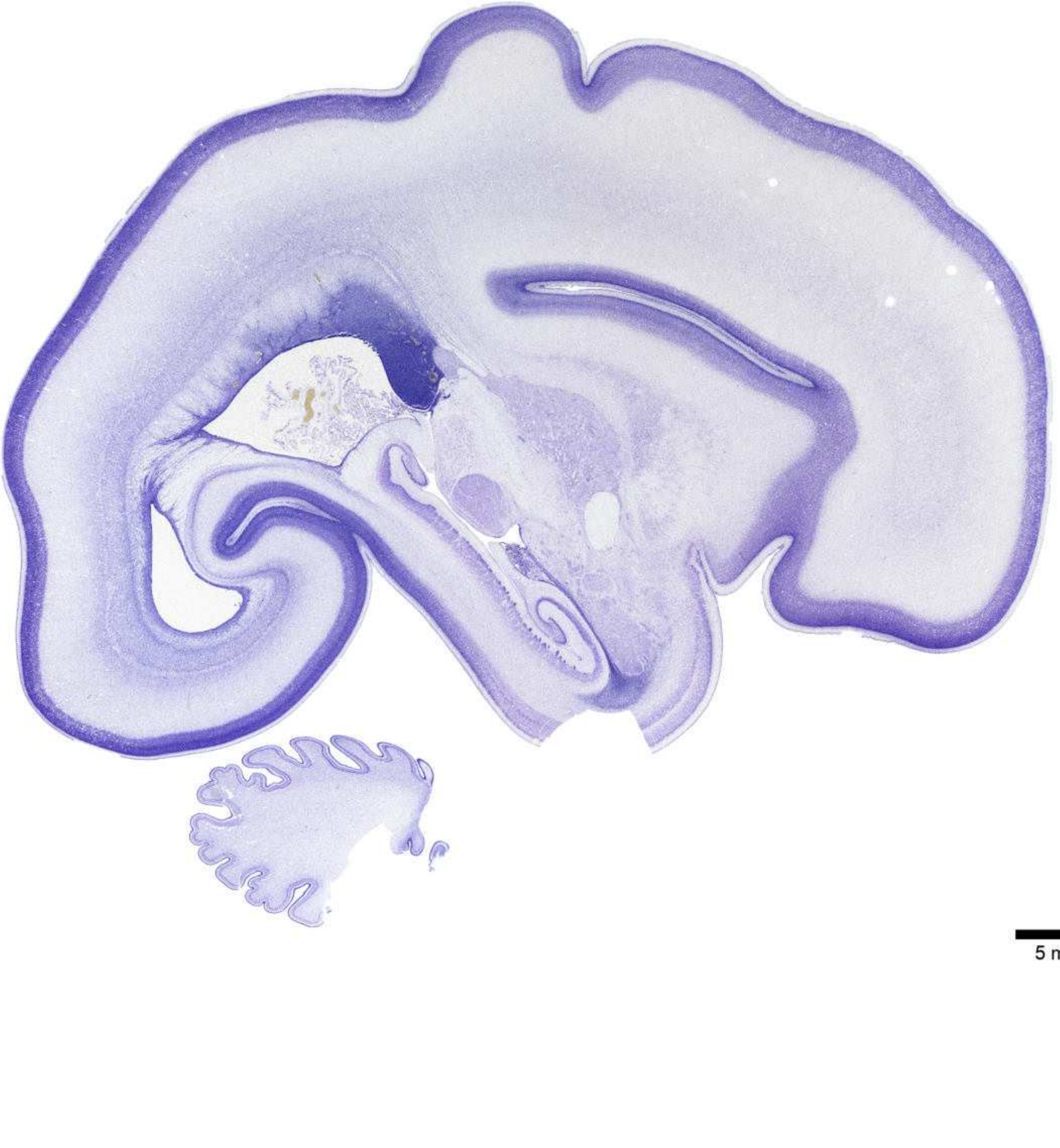

5 mm

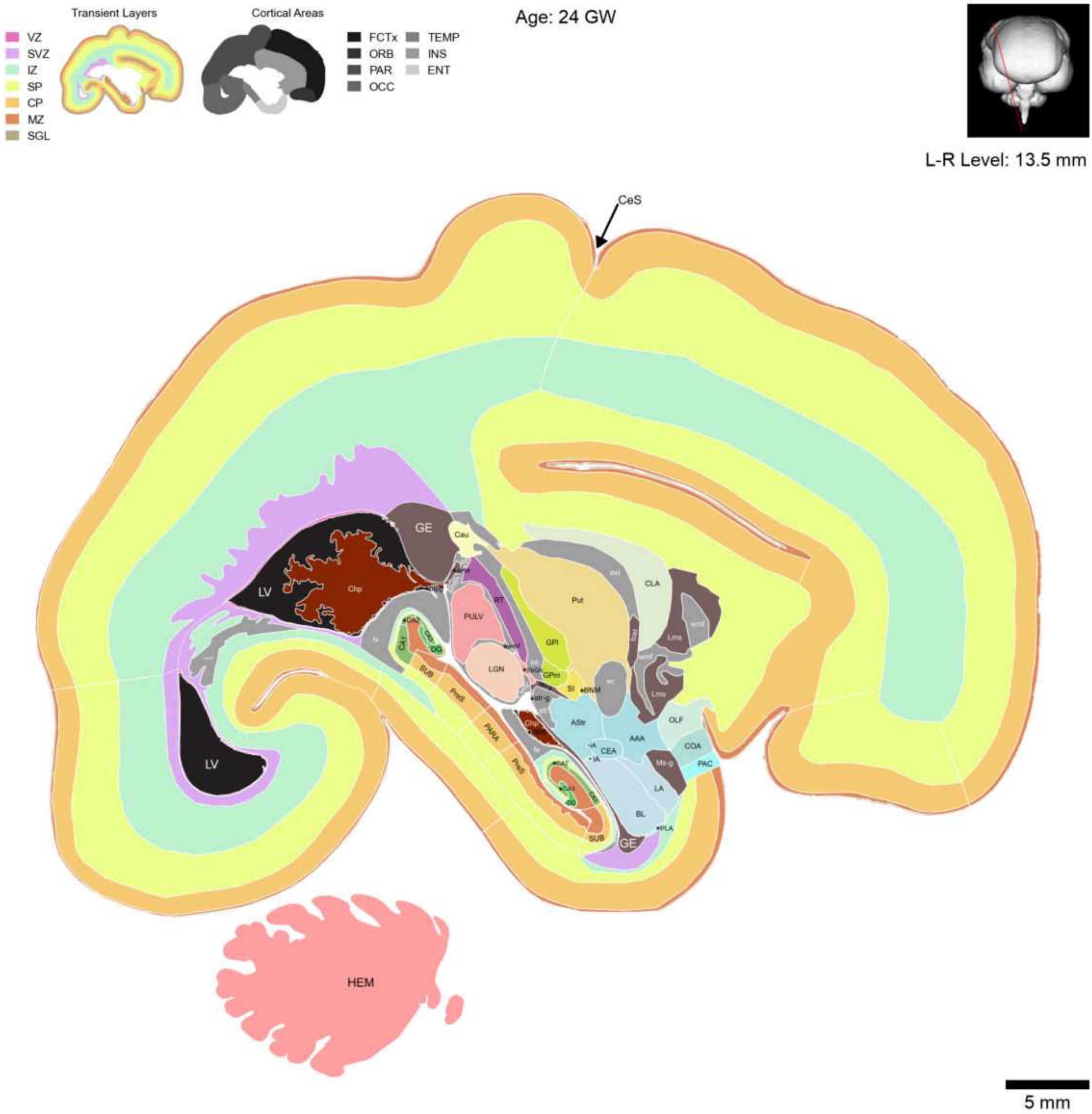

- AAA: Anterior amygdaloid area
- AStr: Amygdalo-striatal area
- BL: Basal nucleus [amygdala]
- BNM: Basal nucleus of Meynert
- CA1: CA1 field [hippocampus]
- CA2: CA2 field [hippocampus]
- CA3: CA3 field [hippocampus]
- CEA: Central nucleus [amygdala]
- CLA: Claustrum
- COA: Cortical nucleus [amygdala]
- Cau: Caudate nucleus
- Chp: Choroid plexus
- DG: Dentate gyrus
- GE: Ganglionic eminence
- GPl: Globus pallidus lateral segment
- GPm: Globus pallidus medial segment
- HEM: Cerebellar hemispheres
- IA: Intercalated cell groups [amygdala]
- LA: Lateral nucleus [amygdala]
- LGN: Lateral geniculate nucleus
- LV: Lateral ventricle
- Lms: Lateral migratory stream
- Ms-g: Migratory stream, general
- PARA: Cortical plate, parasubiculum
- PLA: Paralaminar nucleus [amygdala]
- PULV: Pulvinar nucleus [thalamus]
- PreS: Cortical plate, presubiculum
- Put: Putamen
- RT: Reticular nucleus [thalamus]
- SI: Substantia innominata
- SUB: Cortical plate, subiculum
- SbGN: Subgeniculate nucleus
- ac: Anterior commissure
- dne: Diencephalic neuroepithelium
- emlth: External medullary lamina [thalamus]
- ext: External capsule
- fx: Fornix
- hipg: Hippocampal glioepithelium/ependyma
- int: Internal capsule
- str-g: Strionuclear glioepithelium
- stt: Stria terminalis
- tcet: Transient cell zone in the external capsule
- wmf: White matter fibers
- → CeS: Central sulcus

Age: 24 GW

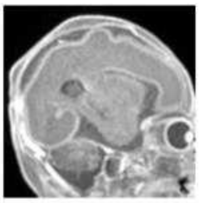

L-R Level: 13.14 mm

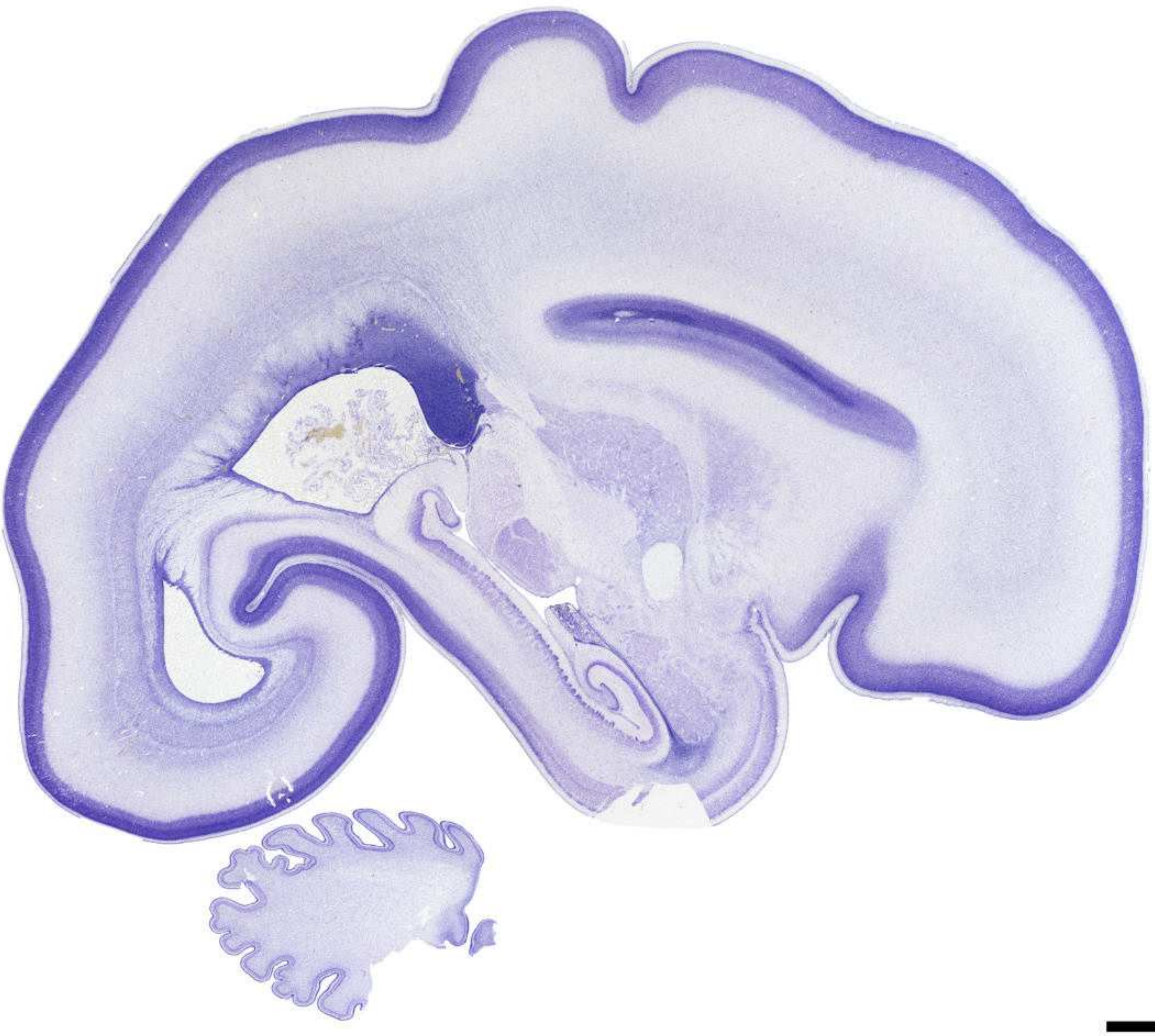

5 mm

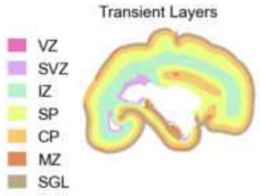

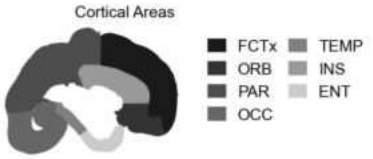

Age: 24 GW

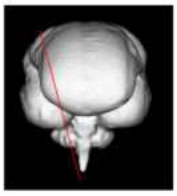

L-R Level: 13.14 mm

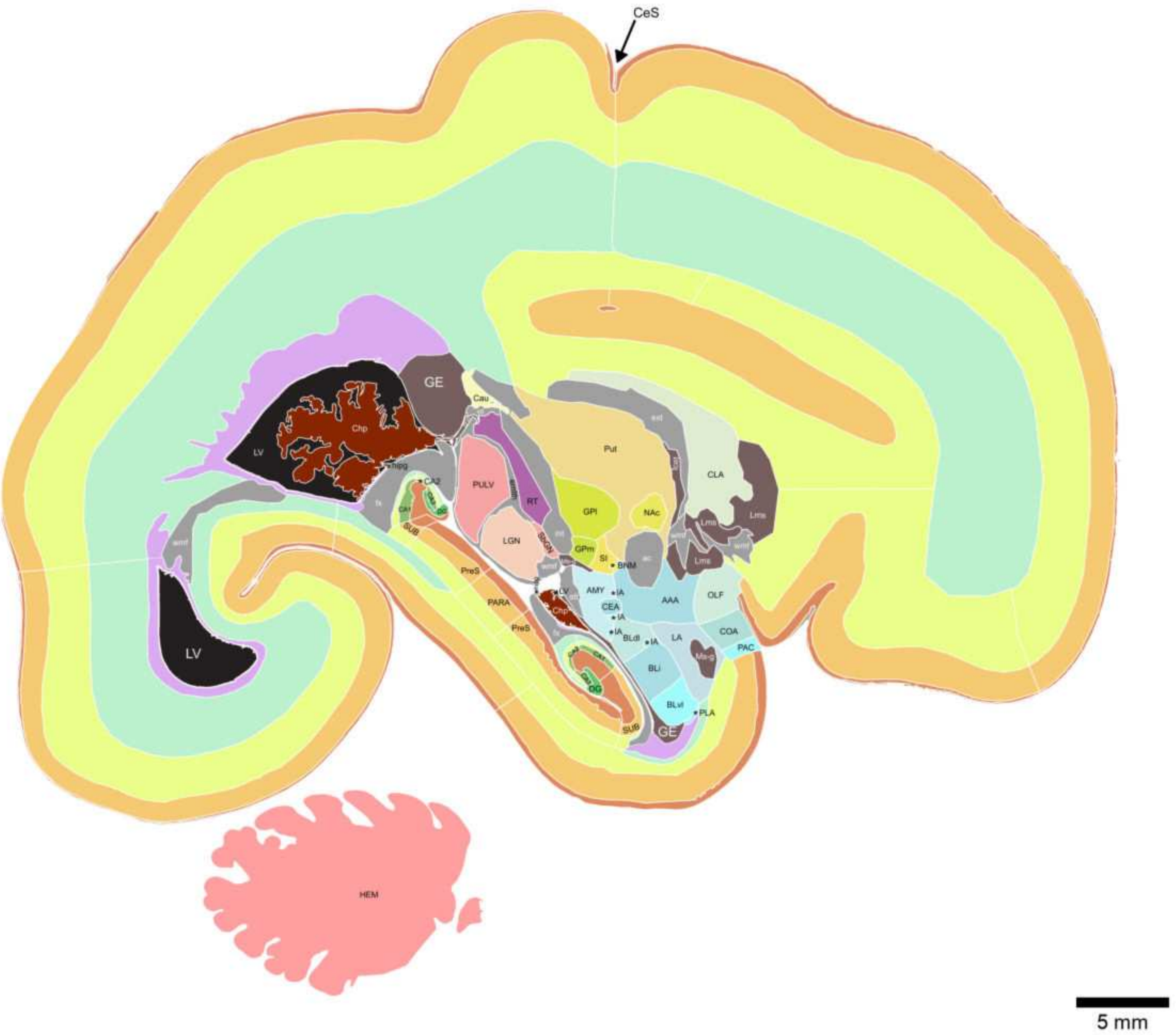

AAA: Anterior amygdaloid area
AMY: Amygdala
BLdl: Basal nucleus [amygdala], dorsolateral part
BLi: Basal nucleus [amygdala], intermediate part
BLvl: Basal nucleus [amygdala], ventrolateral part
BNM: Basal nucleus of Meynert
CA1: CA1 field [hippocampus]
CA2: CA2 field [hippocampus]
CA3: CA3 field [hippocampus]
CEA: Central nucleus [amygdala]
CLA: Claustrum
COA: Cortical nucleus [amygdala]
Cau: Caudate nucleus
Chp: Choroid plexus
DG: Dentate gyrus
GE: Ganglionic eminence
GPl: Globus pallidus lateral segment
GPm: Globus pallidus medial segment
HEM: Cerebellar hemispheres
IA: Intercalated cell groups [amygdala]
LA: Lateral nucleus [amygdala]
LGN: Lateral geniculate nucleus
LV: Lateral ventricle
Lms: Lateral migratory stream
Ms-g: Migratory stream, general
NAc: Nucleus accumbens
PARA: Cortical plate, parasubiculum
PLA: Paralaminar nucleus [amygdala]
PULV: Pulvinar nucleus [thalamus]
PreS: Cortical plate, presubiculum
Put: Putamen
RT: Reticular nucleus [thalamus]
SI: Substantia innominata
SUB: Cortical plate, subiculum
SbGN: Subgeniculate nucleus
ac: Anterior commissure
dne: Diencephalic neuroepithelium
emlth: External medullary lamina [thalamus]
ext: External capsule
fx: Fornix
hipg: Hippocampal glioepithelium/ependyma
int: Internal capsule
stt: Stria terminalis
tcet: Transient cell zone in the external capsule
wmf: White matter fibers
CeS: Central sulcus

Age: 24 GW

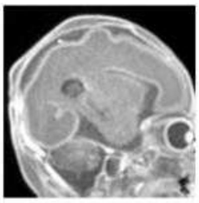

L-R Level: 12.66 mm

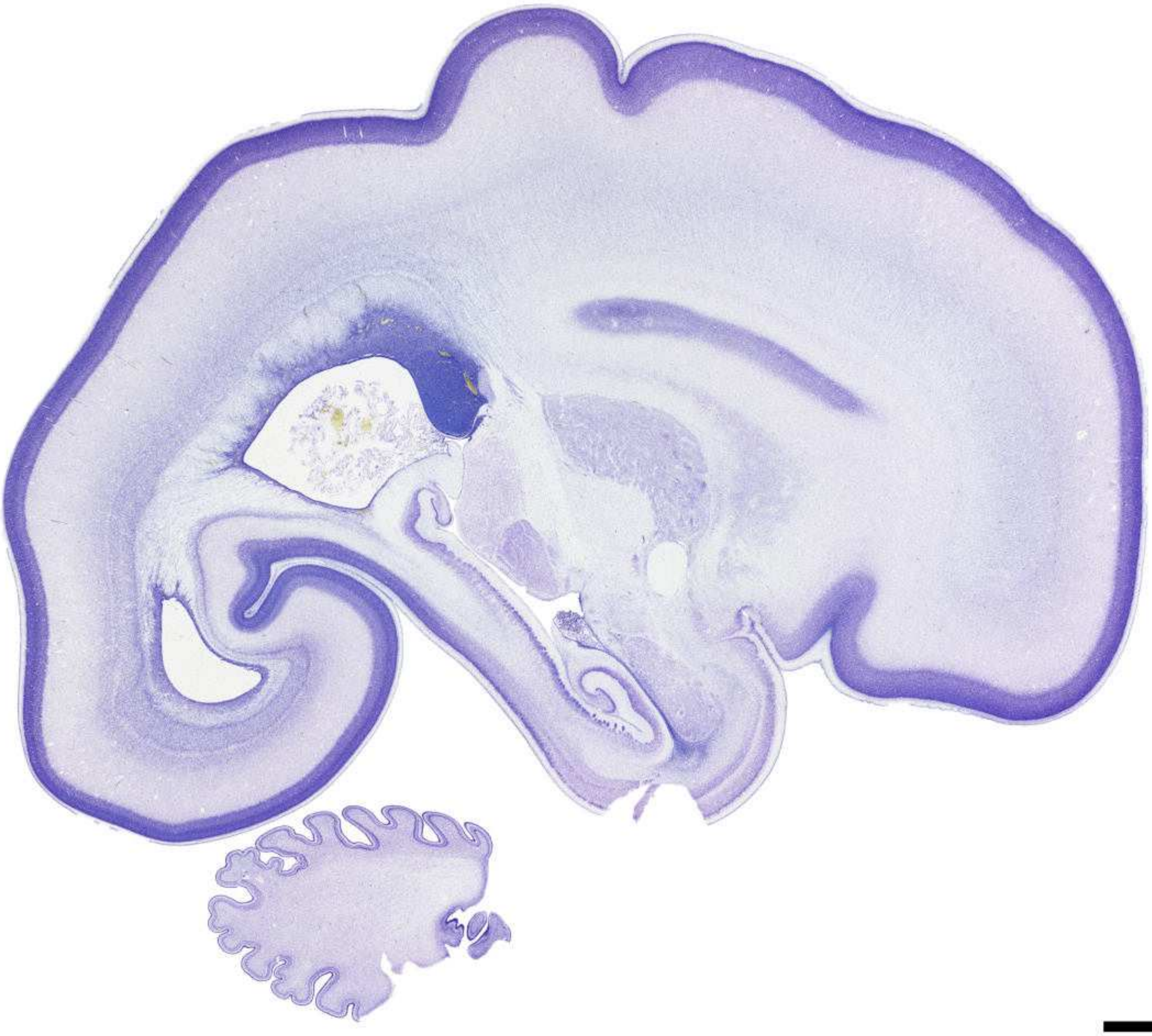

5 mm

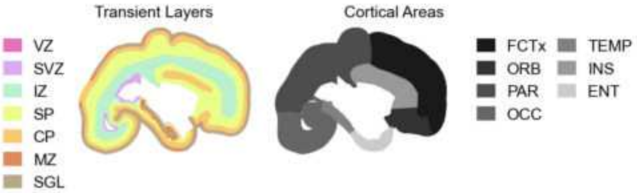

Age: 24 GW

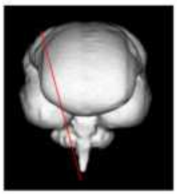

L-R Level: 12.66 mm

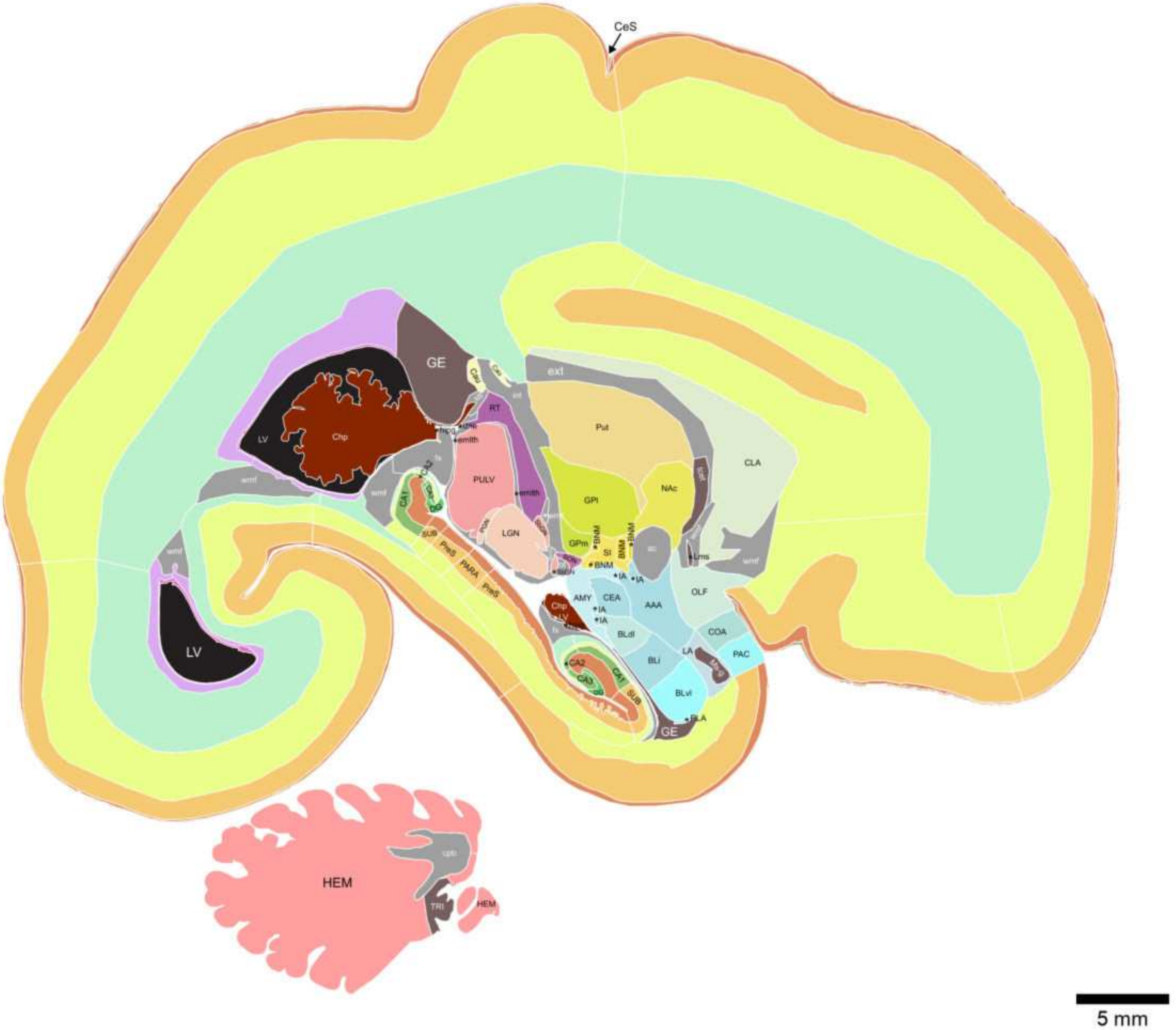

- AAA: Anterior amygdaloid area
- AMY: Amygdala
- BLdl: Basal nucleus [amygdala], dorsolateral part
- BLi: Basal nucleus [amygdala], intermediate part
- BLvl: Basal nucleus [amygdala], ventrolateral part
- BNM: Basal nucleus of Meynert
- CA1: CA1 field [hippocampus]
- CA2: CA2 field [hippocampus]
- CA3: CA3 field [hippocampus]
- CEA: Central nucleus [amygdala]
- CLA: Claustrum
- COA: Cortical nucleus [amygdala]
- Cau: Caudate nucleus
- Chp: Choroid plexus
- DG: Dentate gyrus
- GE: Ganglionic eminence
- GPl: Globus pallidus lateral segment
- GPm: Globus pallidus medial segment
- HEM: Cerebellar hemispheres
- IA: Intercalated cell groups [amygdala]
- LA: Lateral nucleus [amygdala]
- LGN: Lateral geniculate nucleus
- LV: Lateral ventricle
- Lms: Lateral migratory stream
- Ms-g: Migratory stream, general
- NAc: Nucleus accumbens
- PARA: Cortical plate, parasubiculum
- PGN: Pregeniculate nucleus
- PLA: Paralaminar nucleus [amygdala]
- PULV: Pulvinar nucleus [thalamus]
- PreS: Cortical plate, presubiculum
- Put: Putamen
- RT: Reticular nucleus [thalamus]
- SI: Substantia innominata
- SON: Supraoptic nucleus [hypothalamus]
- SUB: Cortical plate, subiculum
- SbGN: Subgeniculate nucleus
- TRI: Germinal trigone
- ac: Anterior commissure
- cpb: Cerebellar peduncles
- dne: Diencephalic neuroepithelium
- emlth: External medullary lamina [thalamus]
- ext: External capsule
- fx: Fornix
- hipg: Hippocampal glioepithelium/ependyma
- int: Internal capsule
- ot: Optic tract
- stt: Stria terminalis
- tcet: Transient cell zone in the external capsule
- wmf: White matter fibers
- → CeS: Central sulcus

Age: 24 GW

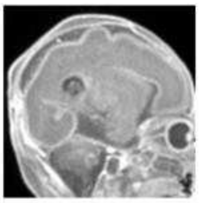

L-R Level: 12.42 mm

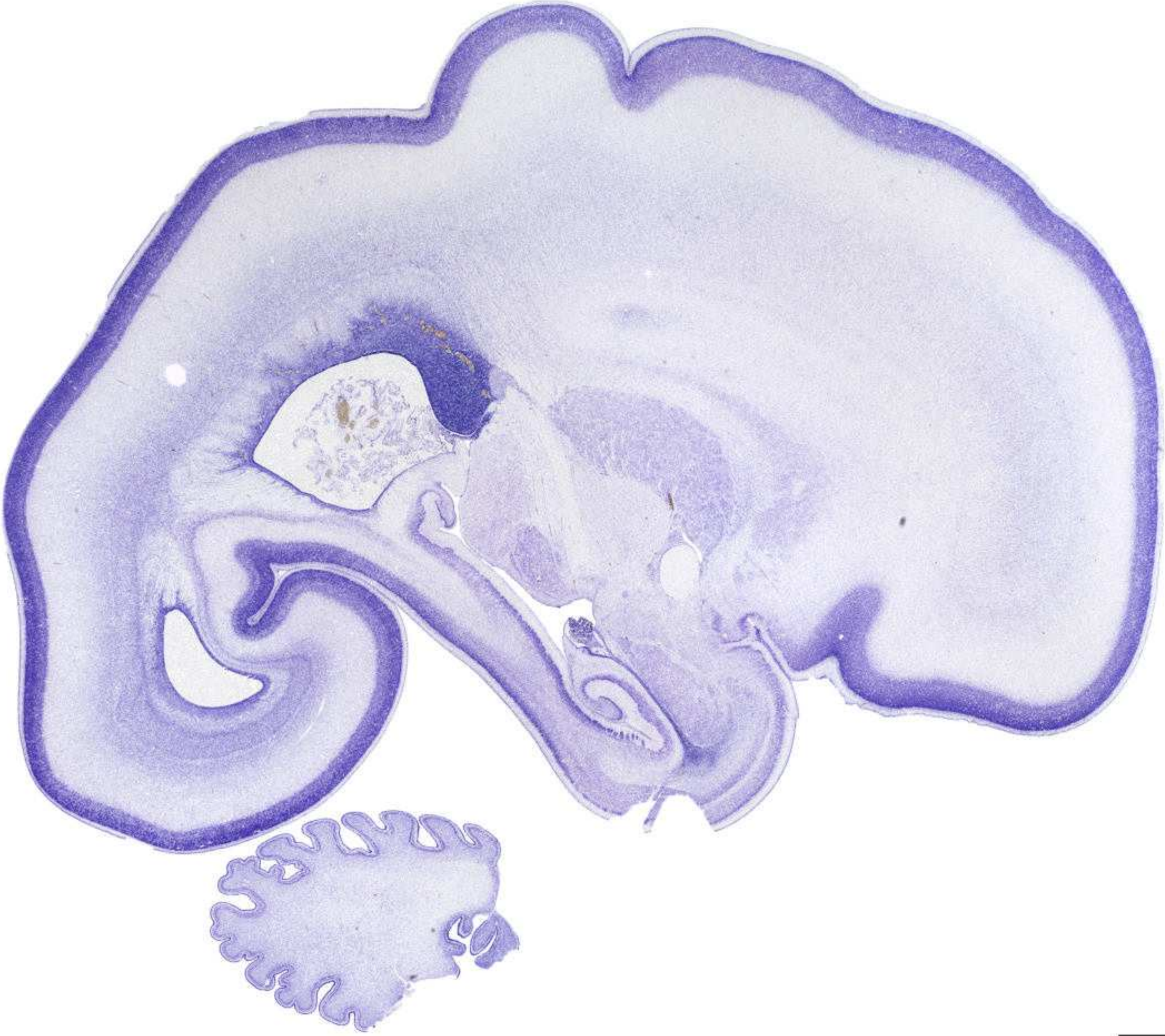

5 mm

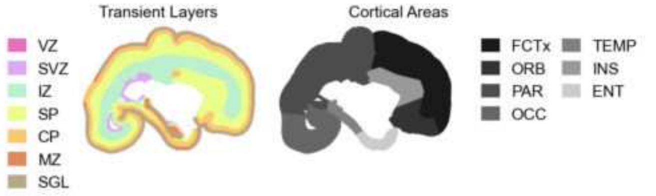

Age: 24 GW

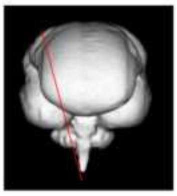

L-R Level: 12.42 mm

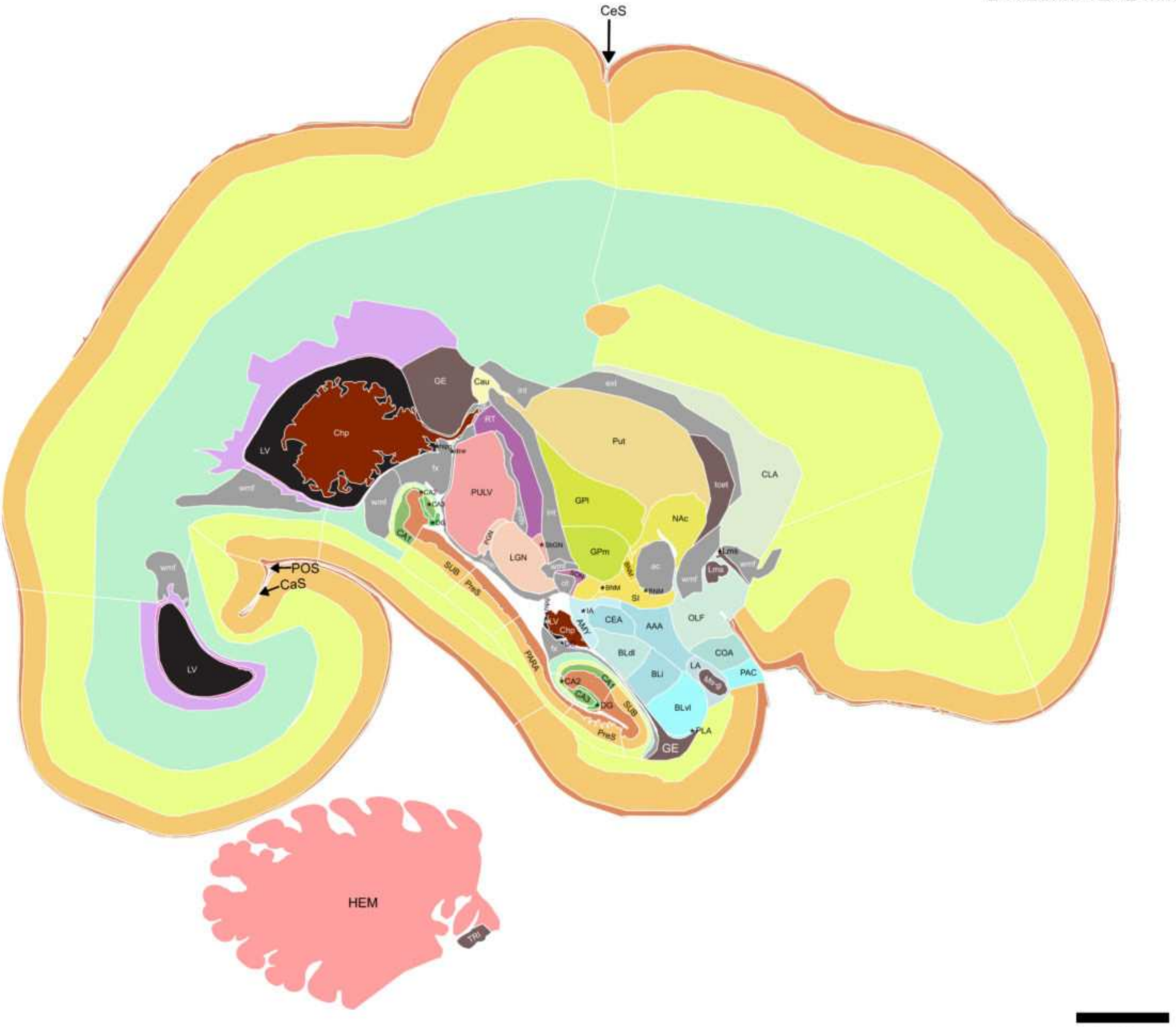

5 mm

- AAA: Anterior amygdaloid area
- AMY: Amygdala
- BLdl: Basal nucleus [amygdala], dorsolateral part
- BLi: Basal nucleus [amygdala], intermediate part
- BLvl: Basal nucleus [amygdala], ventrolateral part
- BNM: Basal nucleus of Meynert
- CA1: CA1 field [hippocampus]
- CA2: CA2 field [hippocampus]
- CA3: CA3 field [hippocampus]
- CEA: Central nucleus [amygdala]
- CLA: Claustrum
- COA: Cortical nucleus [amygdala]
- Cau: Caudate nucleus
- Chp: Choroid plexus
- DG: Dentate gyrus
- GE: Ganglionic eminence
- GPl: Globus pallidus lateral segment
- GPm: Globus pallidus medial segment
- HEM: Cerebellar hemispheres
- IA: Intercalated cell groups [amygdala]
- LA: Lateral nucleus [amygdala]
- LGN: Lateral geniculate nucleus
- LV: Lateral ventricle
- Lms: Lateral migratory stream
- Ms-g: Migratory stream, general
- NAc: Nucleus accumbens
- PARA: Cortical plate, parasubiculum
- PGN: Pregeniculate nucleus
- PLA: Paralaminar nucleus [amygdala]
- PULV: Pulvinar nucleus [thalamus]
- PreS: Cortical plate, presubiculum
- Put: Putamen
- RT: Reticular nucleus [thalamus]
- SI: Substantia innominata
- SON: Supraoptic nucleus [hypothalamus]
- SUB: Cortical plate, subiculum
- SbGN: Subgeniculate nucleus
- TRI: Germinal trigone
- ac: Anterior commissure
- dne: Diencephalic neuroepithelium
- emlth: External medullary lamina [thalamus]
- ext: External capsule
- fx: Fornix
- hipg: Hippocampal glioepithelium/ependyma
- int: Internal capsule
- ot: Optic tract
- stt: Stria terminalis
- tcet: Transient cell zone in the external capsule
- wmf: White matter fibers
- CaS: Calcarine sulcus
- CeS: Central sulcus
- POS: Parieto-occipital sulcus

Age: 24 GW

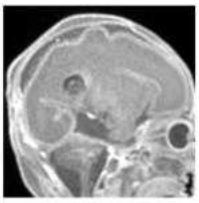

L-R Level: 12.12 mm

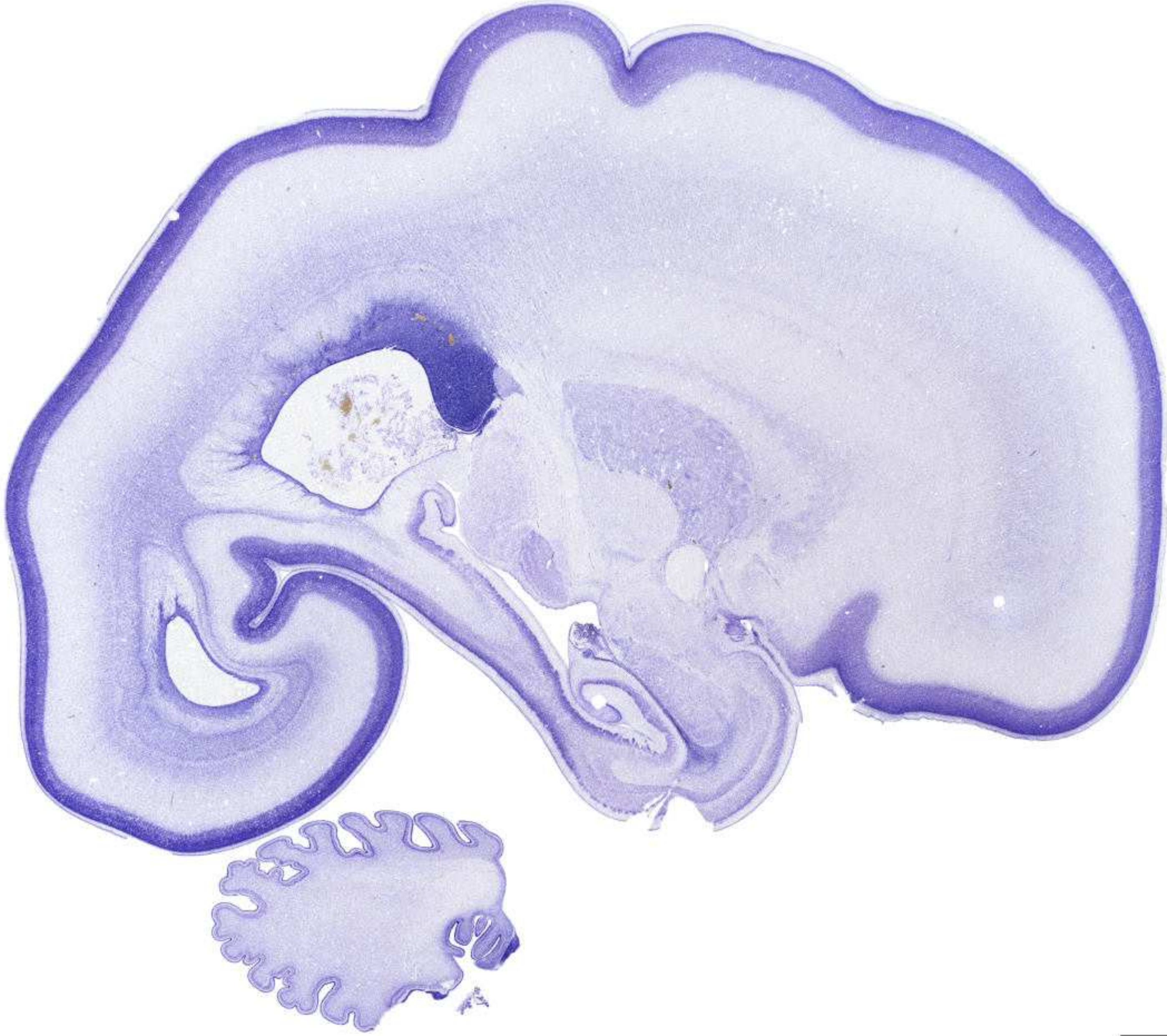

5 mm

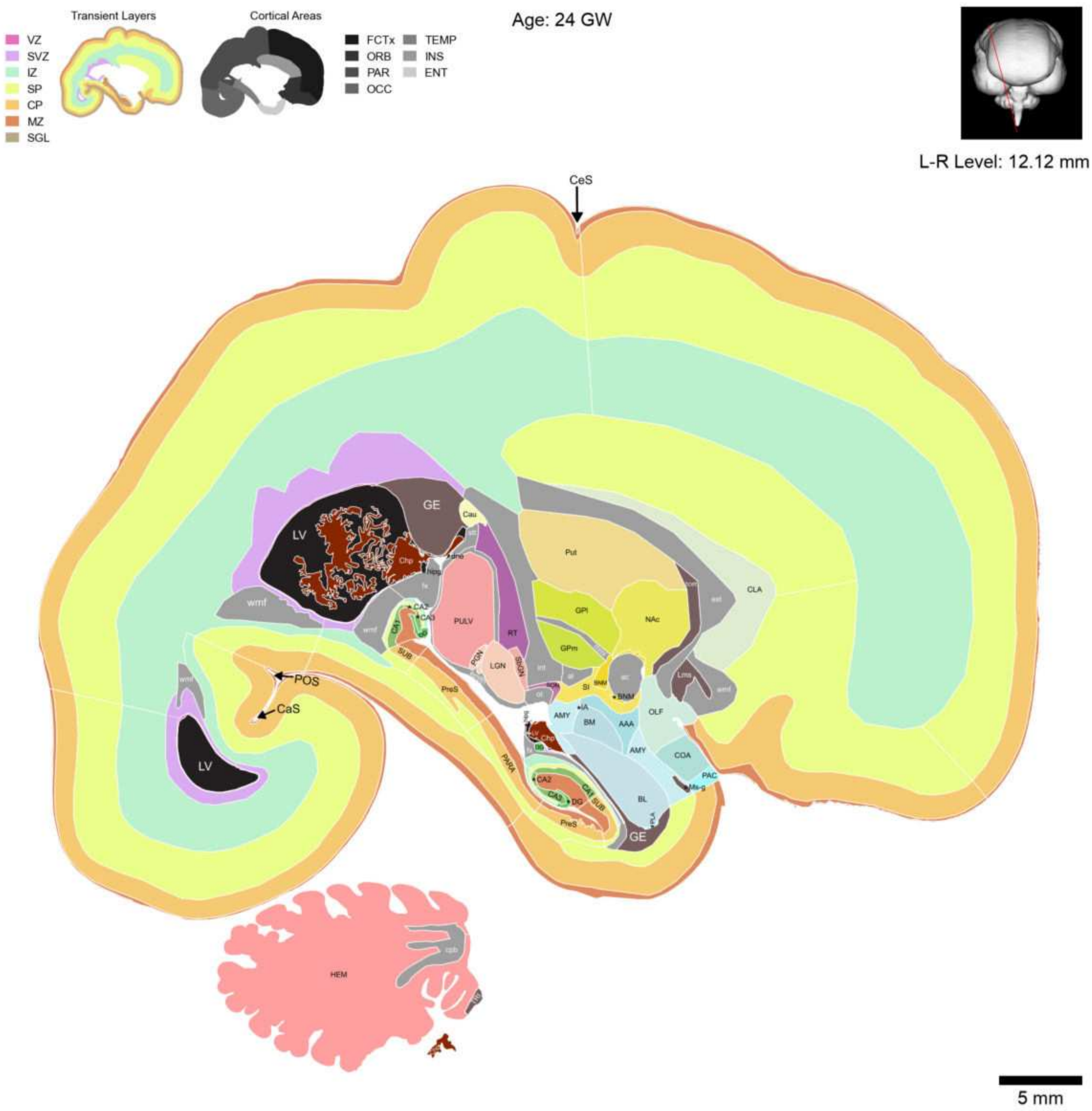

- AAA: Anterior amygdaloid area
- AMY: Amygdala
- BL: Basal nucleus [amygdala]
- BM: Basomedial nucleus [amygdala]
- BNM: Basal nucleus of Meynert
- CA1: CA1 field [hippocampus]
- CA2: CA2 field [hippocampus]
- CA3: CA3 field [hippocampus]
- CLA: Claustrum
- COA: Cortical nucleus [amygdala]
- Cau: Caudate nucleus
- Chp: Choroid plexus
- DG: Dentate gyrus
- GE: Ganglionic eminence
- GPl: Globus pallidus lateral segment
- GPm: Globus pallidus medial segment
- HEM: Cerebellar hemispheres
- IA: Intercalated cell groups [amygdala]
- LGN: Lateral geniculate nucleus
- LV: Lateral ventricle
- Lms: Lateral migratory stream
- Ms-g: Migratory stream, general
- NAc: Nucleus accumbens
- PARA: Cortical plate, parasubiculum
- PGN: Pregeniculate nucleus
- PLA: Paralaminar nucleus [amygdala]
- PULV: Pulvinar nucleus [thalamus]
- PreS: Cortical plate, presubiculum
- Put: Putamen
- RT: Reticular nucleus [thalamus]
- SI: Substantia innominata
- SON: Supraoptic nucleus [hypothalamus]
- SUB: Cortical plate, subiculum
- SbGN: Subgeniculate nucleus
- TRI: Germinal trigone
- ac: Anterior commissure
- al: Ansa lenticularis
- cpb: Cerebellar peduncles
- dne: Diencephalic neuroepithelium
- emlth: External medullary lamina [thalamus]
- ext: External capsule
- fx: Fornix
- hipg: Hippocampal glioepithelium/ependyma
- int: Internal capsule
- mml: Medial medullary lamina
- ot: Optic tract
- stt: Stria terminalis
- tcet: Transient cell zone in the external capsule
- wmf: White matter fibers
- → CaS: Calcarine sulcus
- → CeS: Central sulcus
- → POS: Parieto-occipital sulcus

Age: 24 GW

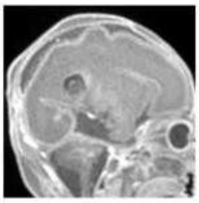

L-R Level: 12.06 mm

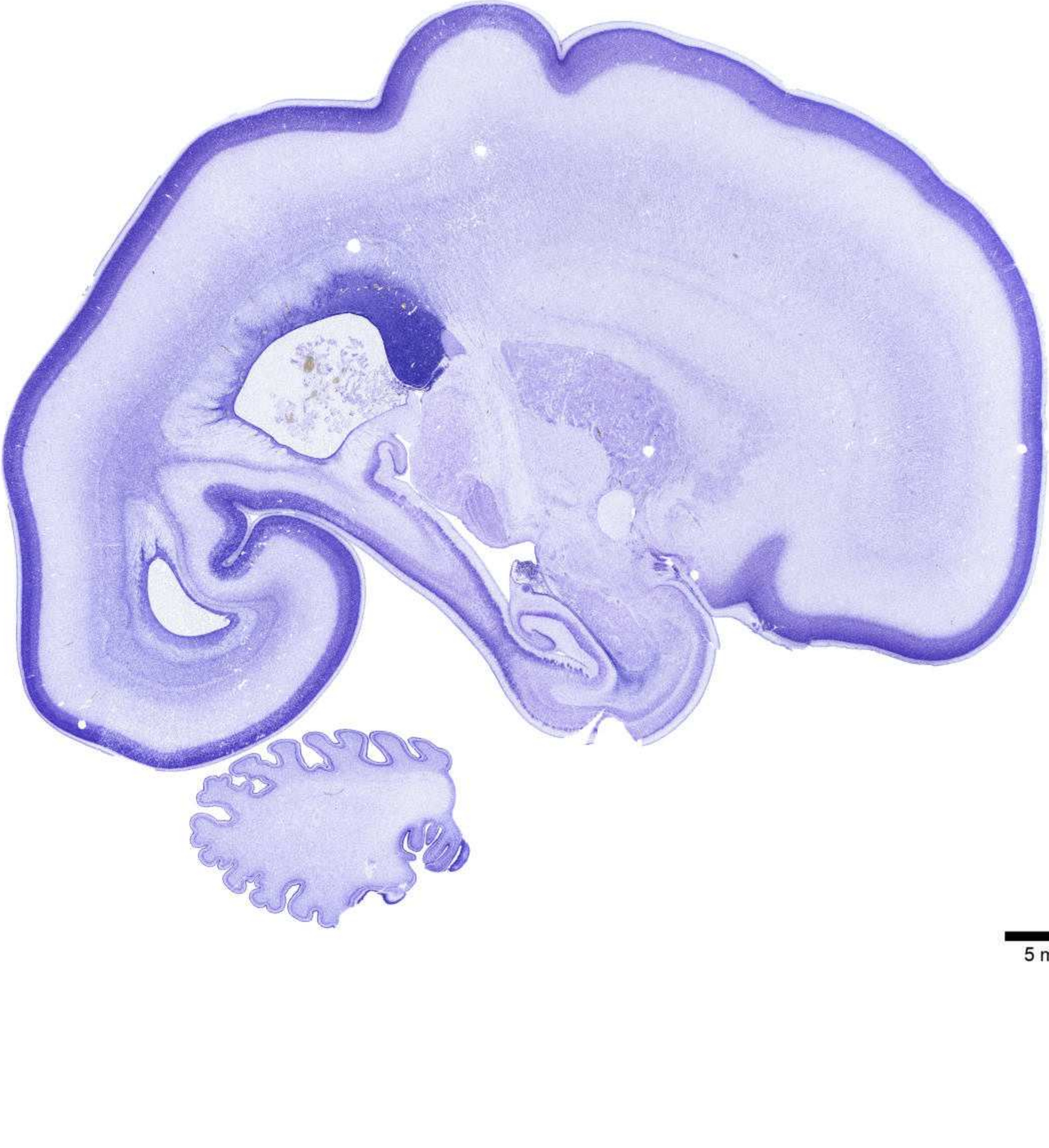

5 mm

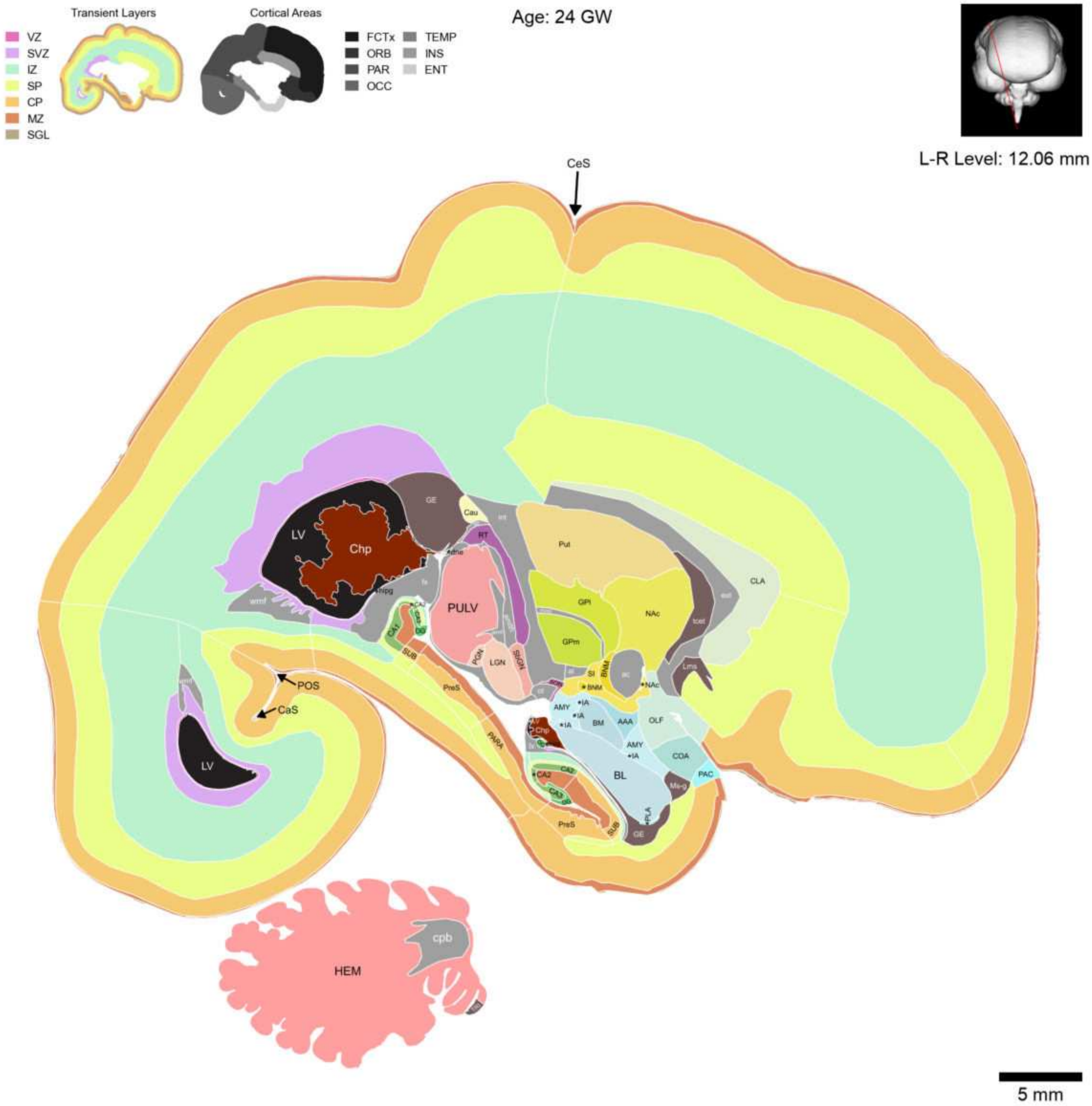

AAA: Anterior amygdaloid area
AMY: Amygdala
BL: Basal nucleus [amygdala]
BM: Basomedial nucleus [amygdala]
BNM: Basal nucleus of Meynert
CA1: CA1 field [hippocampus]
CA2: CA2 field [hippocampus]
CA3: CA3 field [hippocampus]
CLA: Claustrum
COA: Cortical nucleus [amygdala]
Cau: Caudate nucleus
Chp: Choroid plexus
DG: Dentate gyrus
GE: Ganglionic eminence
GPl: Globus pallidus lateral segment
GPm: Globus pallidus medial segment
HEM: Cerebellar hemispheres
IA: Intercalated cell groups [amygdala]
LGN: Lateral geniculate nucleus
LV: Lateral ventricle
Lms: Lateral migratory stream
Ms-g: Migratory stream, general
NAc: Nucleus accumbens
PARA: Cortical plate, parasubiculum
PGN: Pregeniculate nucleus
PLA: Paralaminar nucleus [amygdala]
PULV: Pulvinar nucleus [thalamus]
PreS: Cortical plate, presubiculum
Put: Putamen
RT: Reticular nucleus [thalamus]
SI: Substantia innominata
SON: Supraoptic nucleus [hypothalamus]
SUB: Cortical plate, subiculum
SbGN: Subgeniculate nucleus
TRI: Germinal trigone
ac: Anterior commissure
al: Ansa lenticularis
cpb: Cerebellar peduncles
dne: Diencephalic neuroepithelium
emlth: External medullary lamina [thalamus]
ext: External capsule
fx: Fornix
hipg: Hippocampal glioepithelium/ependyma
int: Internal capsule
mml: Medial medullary lamina
ot: Optic tract
stt: Stria terminalis
tcet: Transient cell zone in the external capsule
wmf: White matter fibers
→ CaS: Calcarine sulcus
→ CeS: Central sulcus
→ POS: Parieto-occipital sulcus

Age: 24 GW

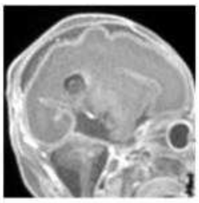

L-R Level: 11.4 mm

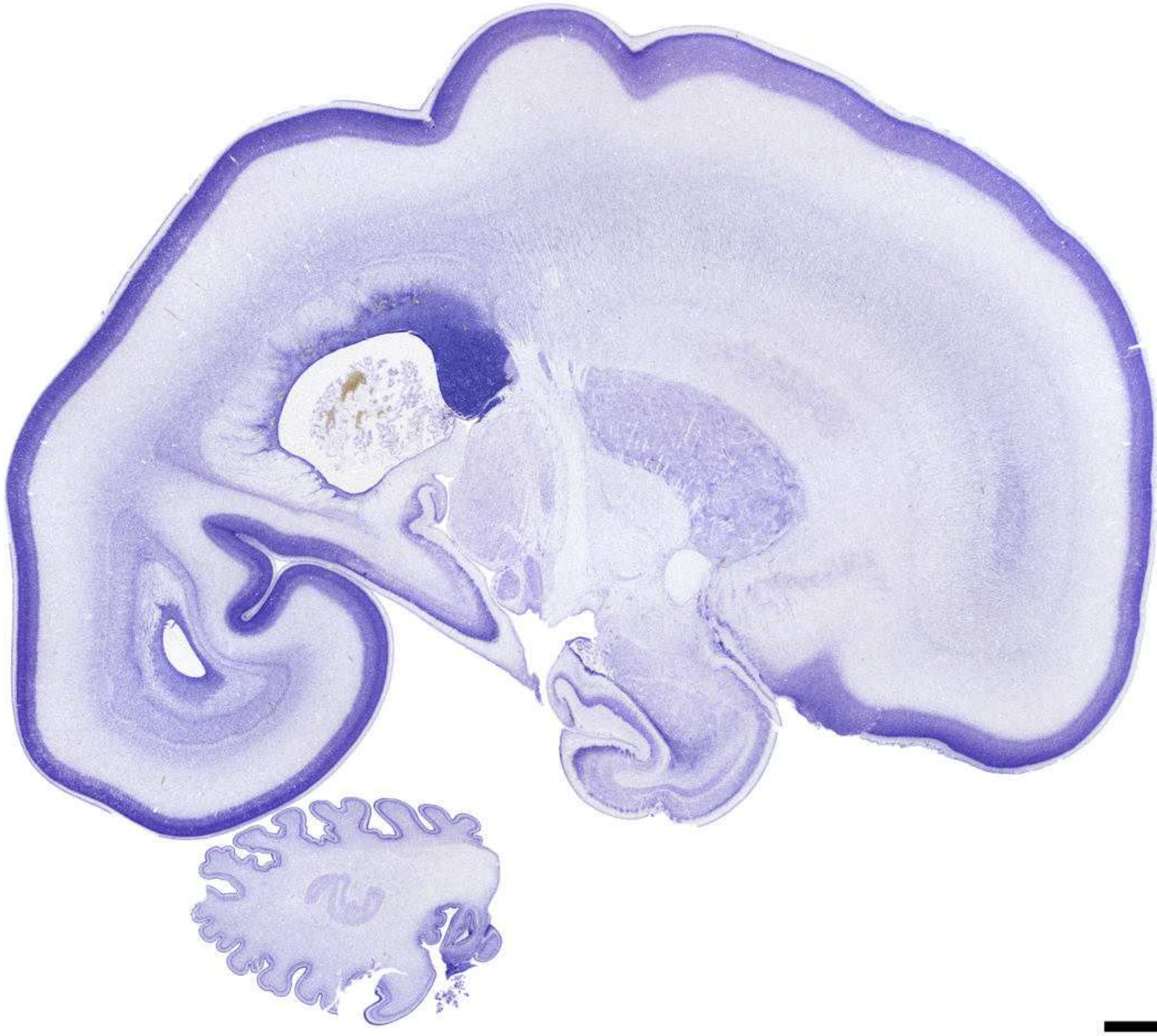

5 mm

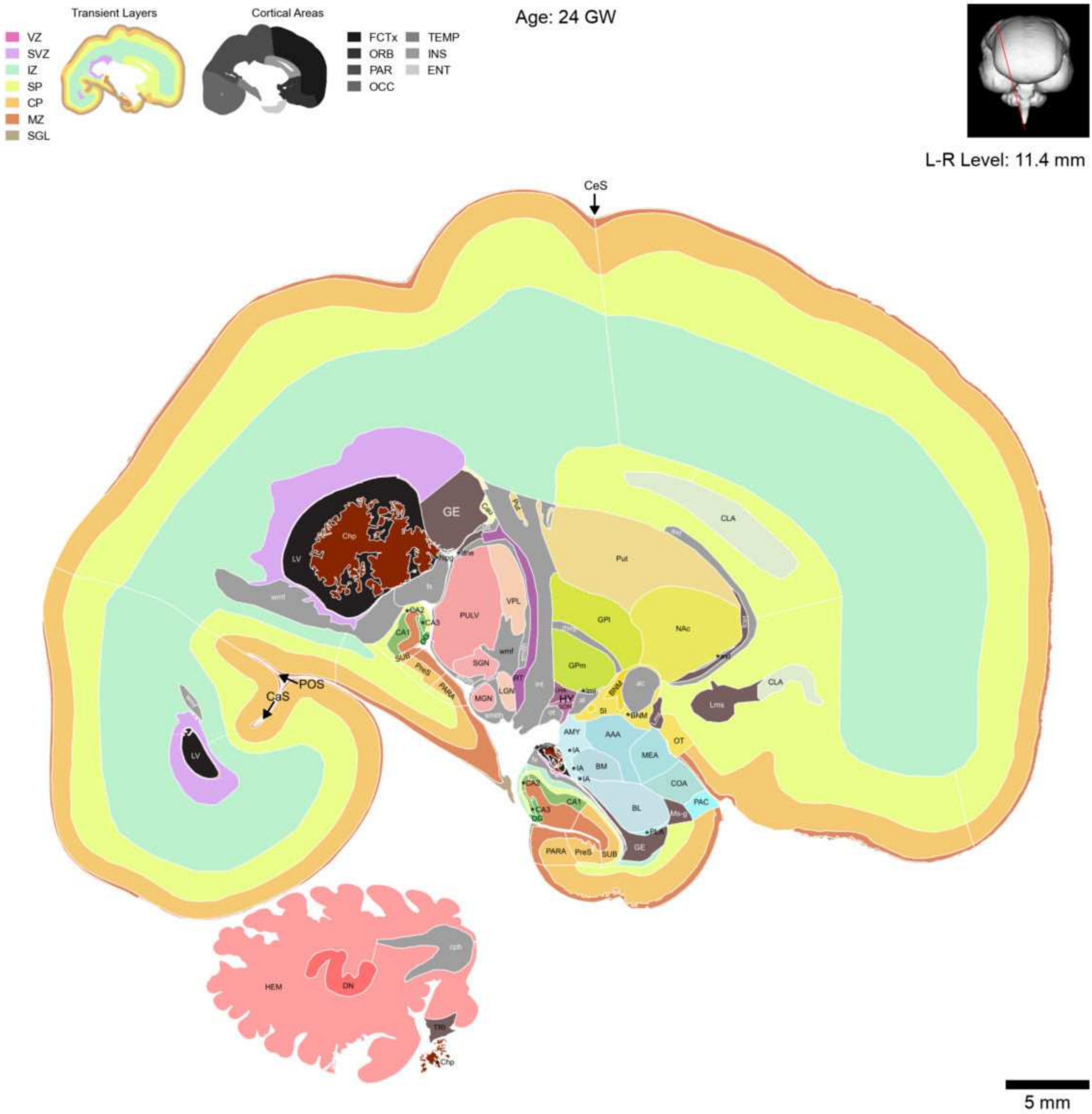

- AAA: Anterior amygdaloid area
- AMY: Amygdala
- BL: Basal nucleus [amygdala]
- BM: Basomedial nucleus [amygdala]
- BNM: Basal nucleus of Meynert
- CA1: CA1 field [hippocampus]
- CA2: CA2 field [hippocampus]
- CA3: CA3 field [hippocampus]
- CLA: Claustrum
- COA: Cortical nucleus [amygdala]
- Cau: Caudate nucleus
- Chp: Choroid plexus
- DG: Dentate gyrus
- DN: Dentate nucleus
- GE: Ganglionic eminence
- GPl: Globus pallidus lateral segment
- GPm: Globus pallidus medial segment
- HEM: Cerebellar hemispheres
- HY: Hypothalamus
- IA: Intercalated cell groups [amygdala]
- LGN: Lateral geniculate nucleus
- LHA: Lateral hypothalamic area
- LV: Lateral ventricle
- Lms: Lateral migratory stream
- MEA: Medial nucleus [amygdala]
- MGN: Medial geniculate nucleus
- Ms-g: Migratory stream, general
- NAc: Nucleus accumbens
- OT: Olfactory tubercle
- PARA: Cortical plate, parasubiculum
- PLA: Paralaminar nucleus [amygdala]
- PULV: Pulvinar nucleus [thalamus]
- PreS: Cortical plate, presubiculum
- Put: Putamen
- RT: Reticular nucleus [thalamus]
- SGN: Suprageniculate nucleus
- SI: Substantia innominata
- SON: Supraoptic nucleus [hypothalamus]
- SUB: Cortical plate, subiculum
- TRI: Germinal trigone
- VPL: Ventral posterolateral nucleus [thalamus]
- ac: Anterior commissure
- al: Ansa lenticularis
- cpb: Cerebellar peduncles
- dne: Diencephalic neuroepithelium
- emlth: External medullary lamina [thalamus]
- ext: External capsule
- fx: Fornix
- hipg: Hippocampal glioepithelium/ependyma
- int: Internal capsule
- lml: Lateral medullary lamina
- mml: Medial medullary lamina
- ot: Optic tract
- stt: Stria terminalis
- tcet: Transient cell zone in the external capsule
- wmf: White matter fibers
- → CaS: Calcarine sulcus
- → CeS: Central sulcus
- → POS: Parieto-occipital sulcus

Age: 24 GW

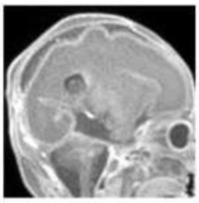

L-R Level: 10.92 mm

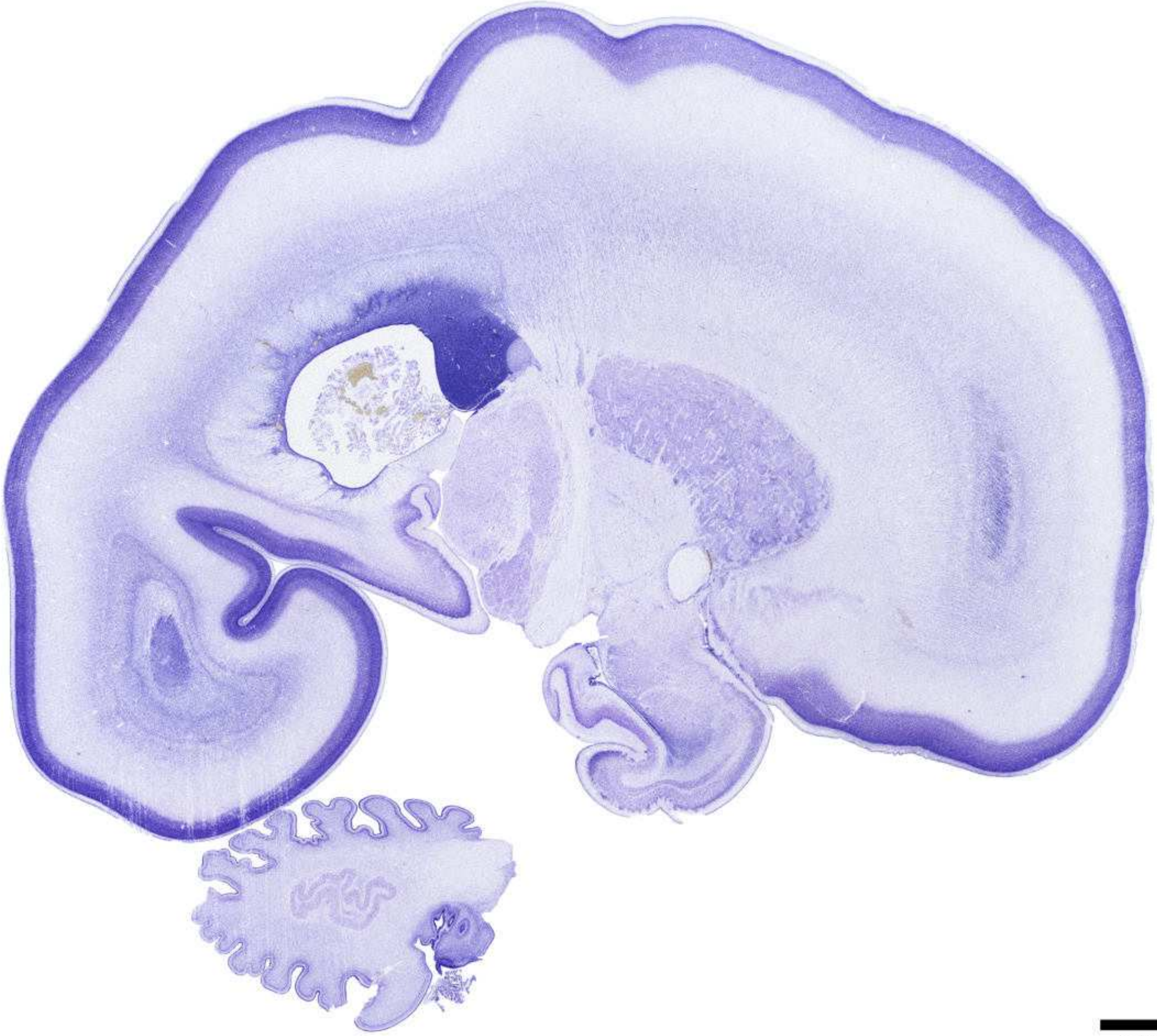

5 mm

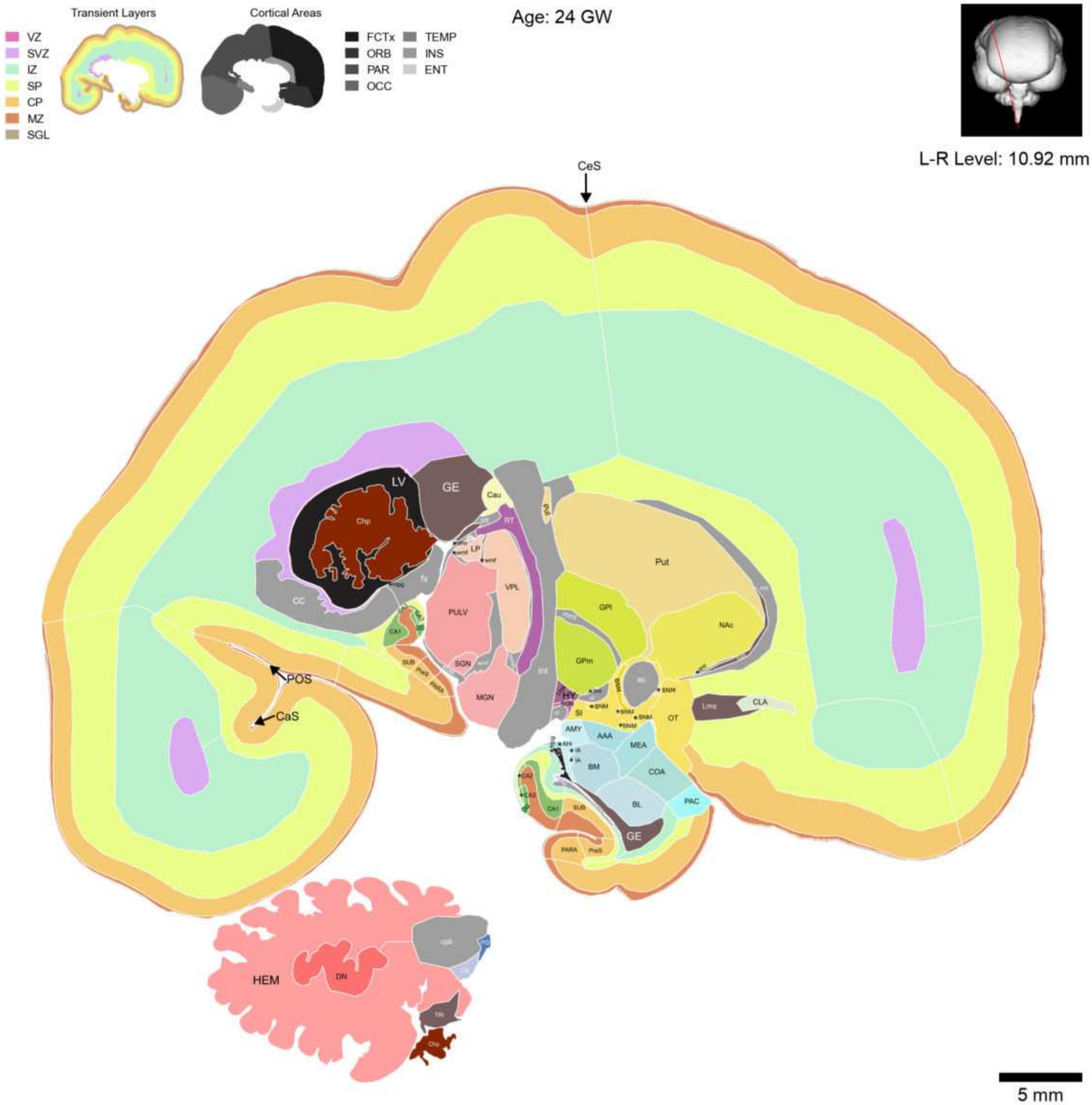

AAA: Anterior amygdaloid area
AHi: Amygdalo-hippocampal area
AMY: Amygdala
BL: Basal nucleus [amygdala]
BM: Basomedial nucleus [amygdala]
BNM: Basal nucleus of Meynert
CA1: CA1 field [hippocampus]
CA2: CA2 field [hippocampus]
CA3: CA3 field [hippocampus]
CLA: Claustrum
CN: Cochlear nuclei
COA: Cortical nucleus [amygdala]
Cau: Caudate nucleus
Chp: Choroid plexus
DG: Dentate gyrus

DN: Dentate nucleus
GE: Ganglionic eminence
GPl: Globus pallidus lateral segment
GPm: Globus pallidus medial segment
HEM: Cerebellar hemispheres
HY: Hypothalamus
IA: Intercalated cell groups [amygdala]
LHA: Lateral hypothalamic area
LP: Lateral posterior nucleus [thalamus]
LV: Lateral ventricle
Lms: Lateral migratory stream
MEA: Medial nucleus [amygdala]
MGN: Medial geniculate nucleus
NAc: Nucleus accumbens
OT: Olfactory tubercle

PARA: Cortical plate, parasubiculum
PG: Pontine gray
PULV: Pulvinar nucleus [thalamus]
PreS: Cortical plate, presubiculum
Put: Putamen
RT: Reticular nucleus [thalamus]
SGN: Suprageniculate nucleus
SI: Substantia innominata
SON: Supraoptic nucleus [hypothalamus]
SUB: Cortical plate, subiculum
TRI: Germinal trigone
VPL: Ventral posterolateral nucleus [thalamus]
ac: Anterior commissure
al: Ansa lenticularis

cc: Corpus callosum
cpb: Cerebellar peduncles
dne: Diencephalic neuroepithelium
emlth: External medullary lamina [thalamus]
ext: External capsule
fx: Fornix
hipg: Hippocampal glioepithelium/ependyma
int: Internal capsule
lml: Lateral medullary lamina
mml: Medial medullary lamina
ot: Optic tract
stt: Stria terminalis
tcet: Transient cell zone in the external capsule
wmf: White matter fibers
→ CaS: Calcarine sulcus
→ CeS: Central sulcus
→ POS: Parieto-occipital sulcus

Age: 24 GW

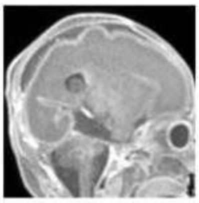

L-R Level: 10.44 mm

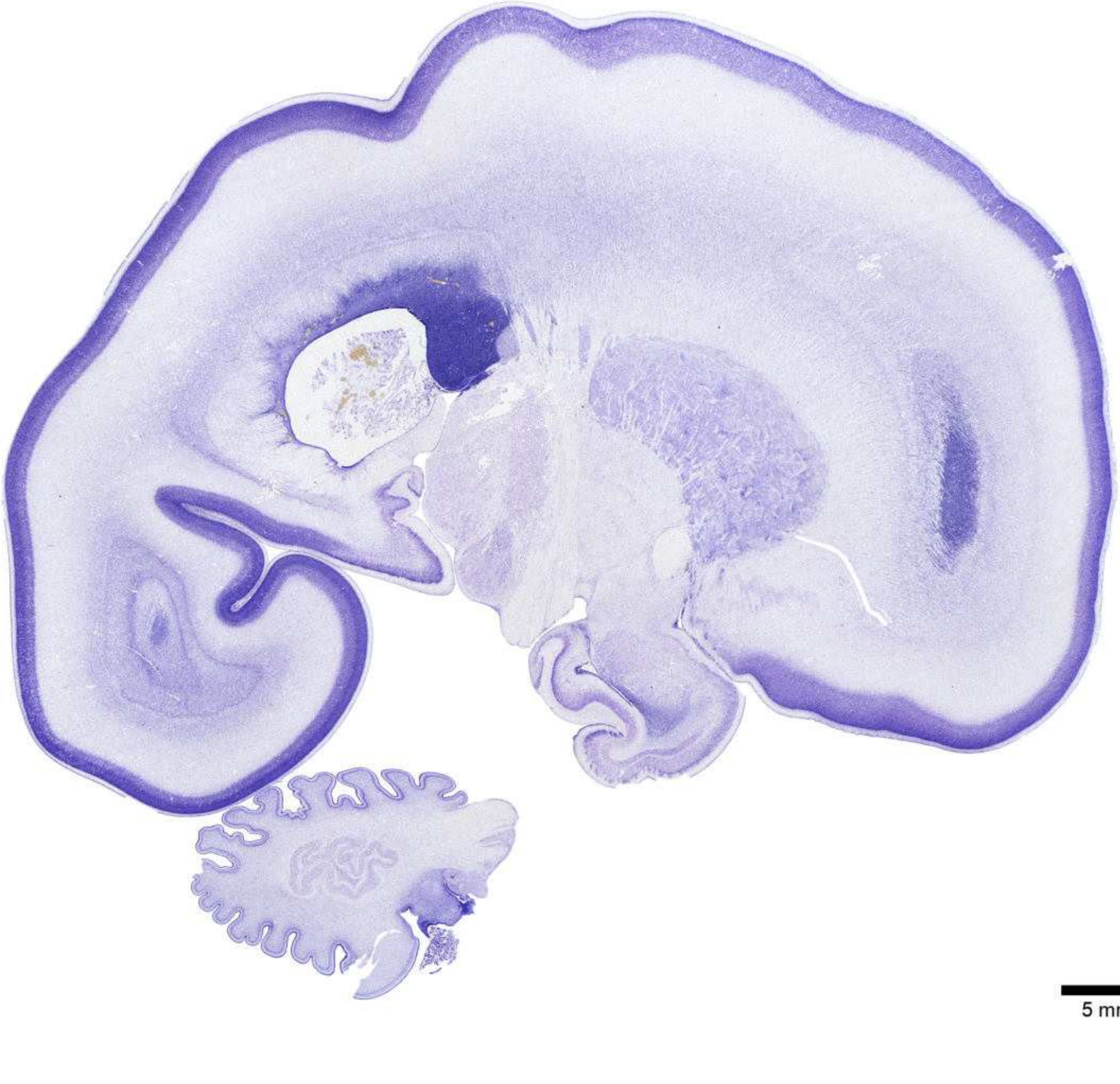

5 mm

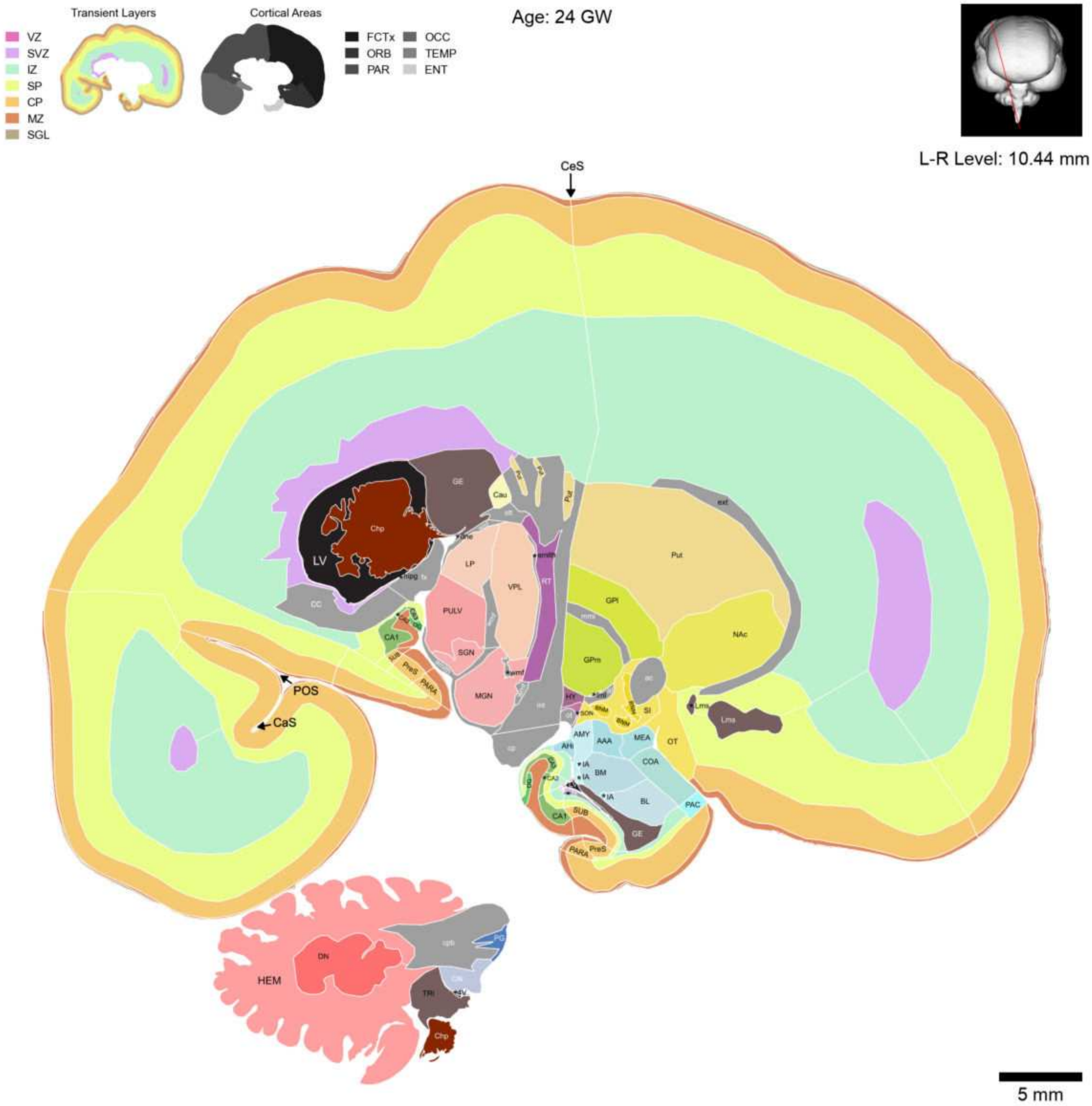

- 4V: Fourth ventricle
- AAA: Anterior amygdaloid area
- AHi: Amygdalo-hippocampal area
- AMY: Amygdala
- BL: Basal nucleus [amygdala]
- BM: Basomedial nucleus [amygdala]
- BNM: Basal nucleus of Meynert
- CA1: CA1 field [hippocampus]
- CA2: CA2 field [hippocampus]
- CA3: CA3 field [hippocampus]
- CN: Cochlear nuclei
- COA: Cortical nucleus [amygdala]
- Cau: Caudate nucleus
- Chp: Choroid plexus
- DG: Dentate gyrus
- DN: Dentate nucleus
- GE: Ganglionic eminence
- GPl: Globus pallidus lateral segment
- GPm: Globus pallidus medial segment
- HEM: Cerebellar hemispheres
- HY: Hypothalamus
- IA: Intercalated cell groups [amygdala]
- LHA: Lateral hypothalamic area
- LP: Lateral posterior nucleus [thalamus]
- LV: Lateral ventricle
- Lms: Lateral migratory stream
- MEA: Medial nucleus [amygdala]
- MGN: Medial geniculate nucleus
- NAc: Nucleus accumbens
- OT: Olfactory tubercle
- PARA: Cortical plate, parasubiculum
- PG: Pontine gray
- PULV: Pulvinar nucleus [thalamus]
- PreS: Cortical plate, presubiculum
- Put: Putamen
- RT: Reticular nucleus [thalamus]
- SGN: Suprageniculate nucleus
- SI: Substantia innominata
- SON: Supraoptic nucleus [hypothalamus]
- SUB: Cortical plate, subiculum
- TRI: Germinal trigone
- VPL: Ventral posterolateral nucleus [thalamus]
- ac: Anterior commissure
- al: Ansa lenticularis
- cc: Corpus callosum
- cp: Cerebral peduncle
- cpb: Cerebellar peduncles
- dne: Diencephalic neuroepithelium
- emlth: External medullary lamina [thalamus]
- ext: External capsule
- fx: Fornix
- hipg: Hippocampal glioepithelium/ependyma
- int: Internal capsule
- lml: Lateral medullary lamina
- mml: Medial medullary lamina
- ot: Optic tract
- stt: Stria terminalis
- wmf: White matter fibers
- → CaS: Calcarine sulcus
- → CeS: Central sulcus
- → POS: Parieto-occipital sulcus

Age: 24 GW

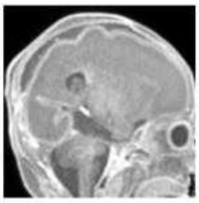

L-R Level: 10.2 mm

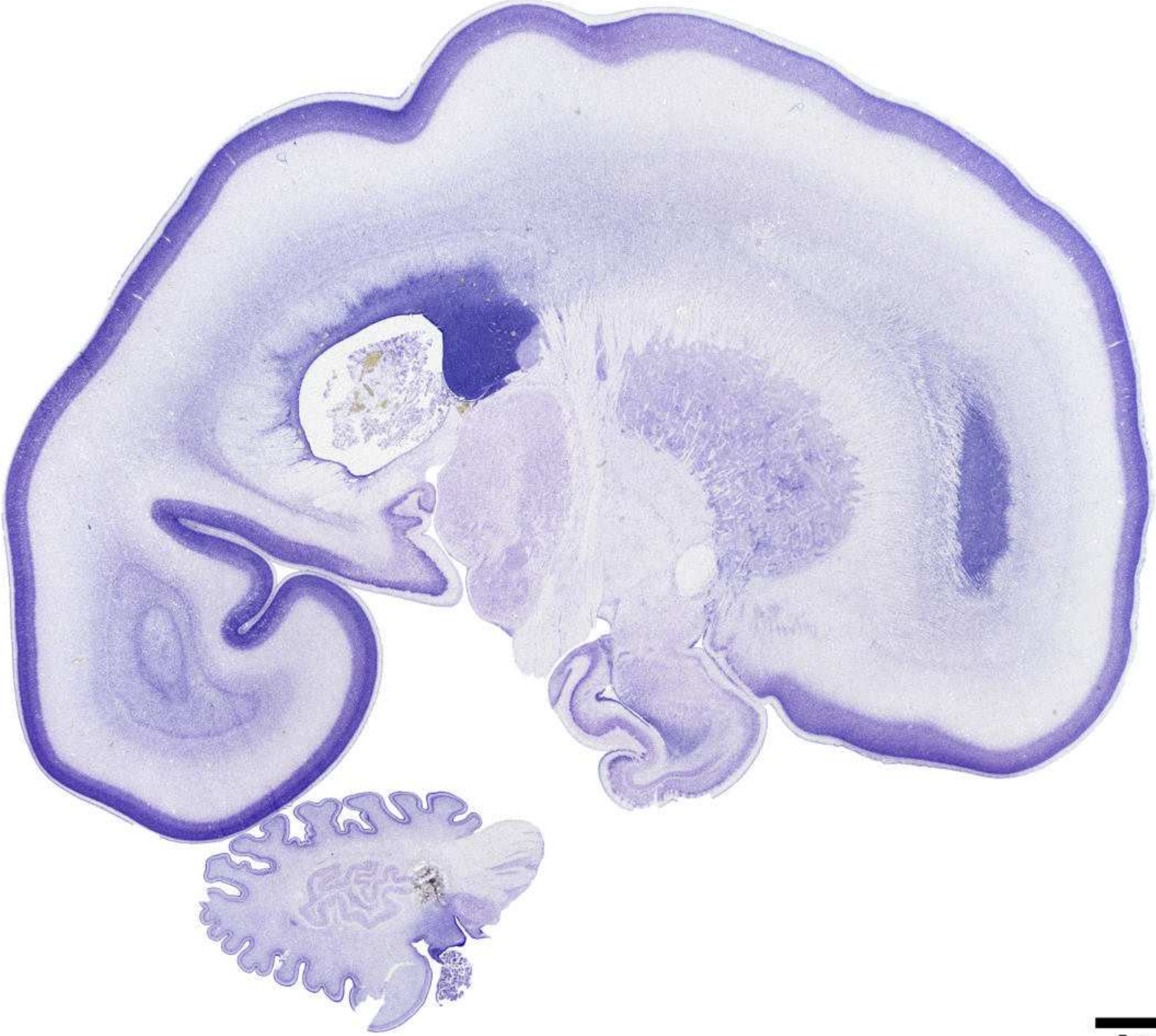

5 mm

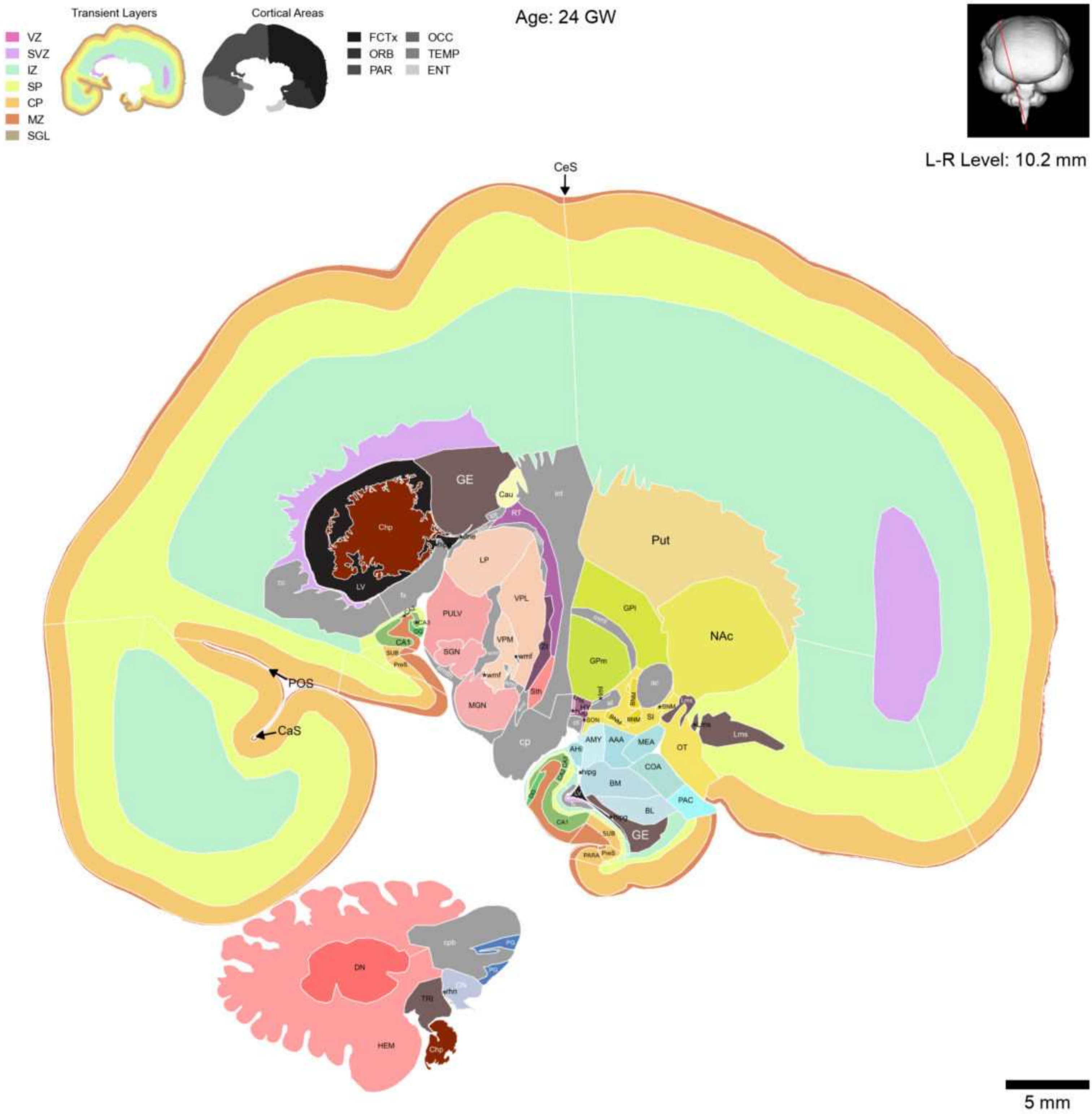

AAA: Anterior amygdaloid area
AHi: Amygdalo-hippocampal area
AMY: Amygdala
BL: Basal nucleus [amygdala]
BM: Basomedial nucleus [amygdala]
BNM: Basal nucleus of Meynert
CA1: CA1 field [hippocampus]
CA2: CA2 field [hippocampus]
CA3: CA3 field [hippocampus]
CN: Cochlear nuclei
COA: Cortical nucleus [amygdala]
Cau: Caudate nucleus
Chp: Choroid plexus
DG: Dentate gyrus
DN: Dentate nucleus
GE: Ganglionic eminence
GPl: Globus pallidus lateral segment
GPm: Globus pallidus medial segment
HEM: Cerebellar hemispheres
HY: Hypothalamus
LHA: Lateral hypothalamic area
LP: Lateral posterior nucleus [thalamus]
LV: Lateral ventricle
Lms: Lateral migratory stream
MEA: Medial nucleus [amygdala]
MGN: Medial geniculate nucleus
NAc: Nucleus accumbens
OT: Olfactory tubercle
PARA: Cortical plate, parasubiculum
PG: Pontine gray
PULV: Pulvinar nucleus [thalamus]
PreS: Cortical plate, presubiculum
Put: Putamen
RT: Reticular nucleus [thalamus]
SGN: Suprageniculate nucleus
SI: Substantia innominata
SON: Supraoptic nucleus [hypothalamus]
SUB: Cortical plate, subiculum
Sth: Subthalamus
TMM: Tuberomammillary nucleus
TRI: Germinal trigone
VPL: Ventral posterolateral nucleus [thalamus]
VPM: Ventral posteromedial nucleus [thalamus]
ZI: Zona incerta
ac: Anterior commissure
al: Ansa lenticularis
cc: Corpus callosum
cp: Cerebral peduncle
cpb: Cerebellar peduncles
dne: Diencephalic neuroepithelium
emlth: External medullary lamina [thalamus]
fx: Fornix
hipg: Hippocampal glioepithelium/ependyma
int: Internal capsule
lml: Lateral medullary lamina
mml: Medial medullary lamina
ot: Optic tract
rhn: Rhombencephalic neuroepithelium
stt: Stria terminalis
wmf: White matter fibers
→ CaS: Calcarine sulcus
→ CeS: Central sulcus
→ POS: Parieto-occipital sulcus

Age: 24 GW

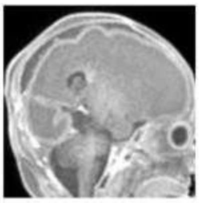

L-R Level: 9.9 mm

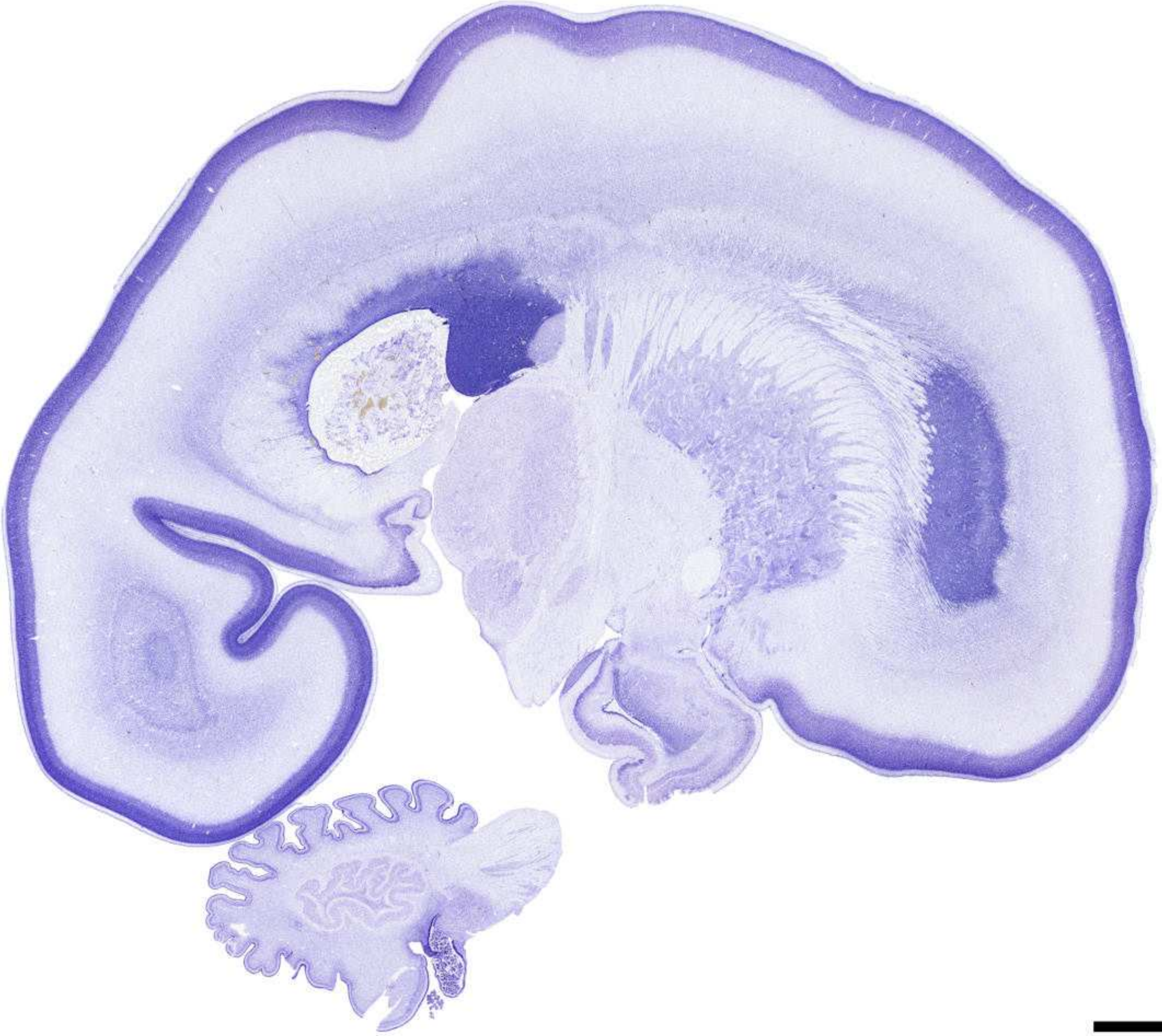

5 mm

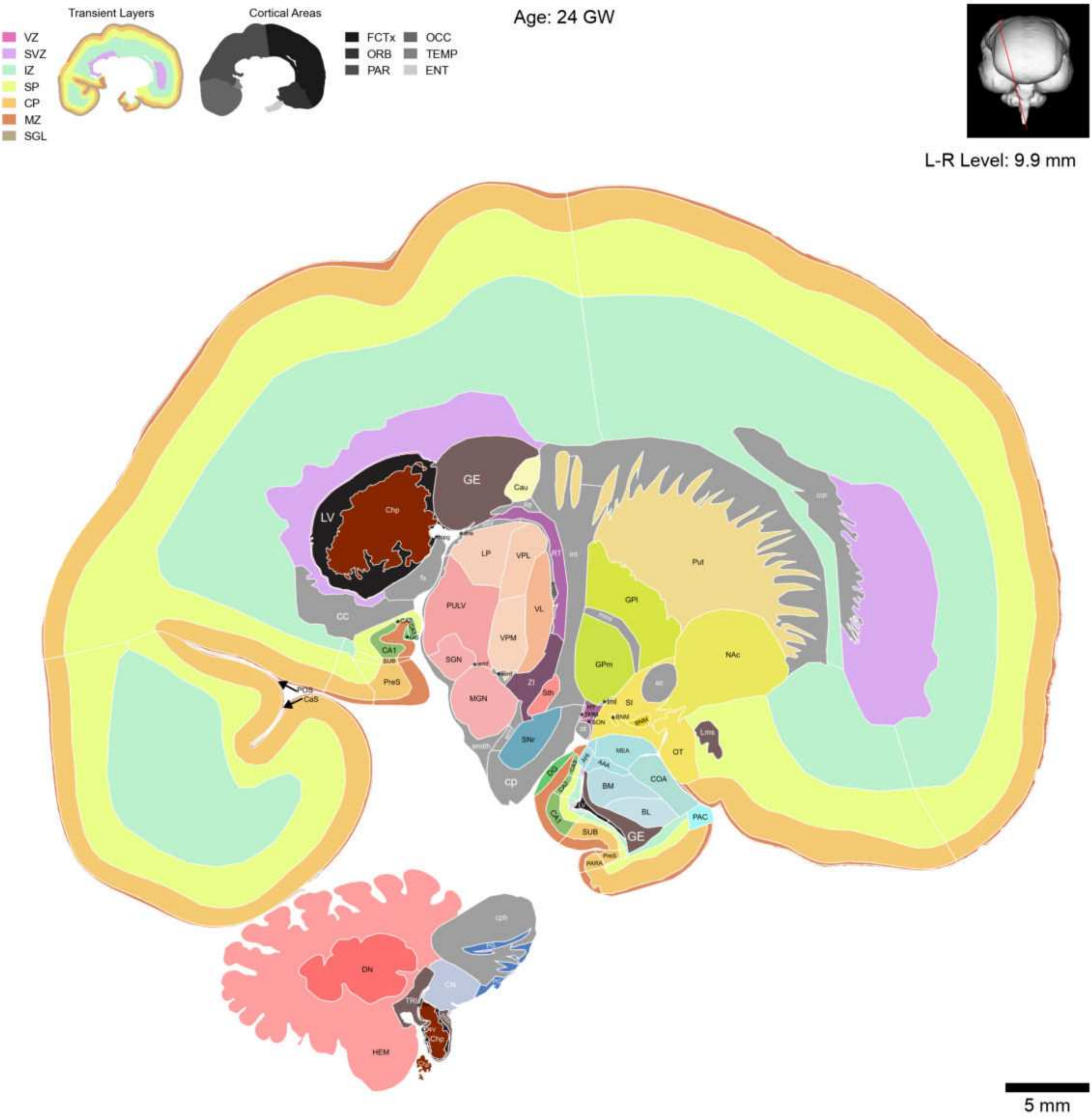

4V: Fourth ventricle
AAA: Anterior amygdaloid area
AHi: Amygdalo-hippocampal area
BL: Basal nucleus [amygdala]
BM: Basomedial nucleus [amygdala]
BNM: Basal nucleus of Meynert
CA1: CA1 field [hippocampus]
CA2: CA2 field [hippocampus]
CA3: CA3 field [hippocampus]
CN: Cochlear nuclei
COA: Cortical nucleus [amygdala]
Cau: Caudate nucleus
Chp: Choroid plexus
DG: Dentate gyrus
DN: Dentate nucleus
GE: Ganglionic eminence
GPl: Globus pallidus lateral segment
GPm: Globus pallidus medial segment
HEM: Cerebellar hemispheres
HY: Hypothalamus
LHA: Lateral hypothalamic area
LP: Lateral posterior nucleus [thalamus]
LV: Lateral ventricle
Lms: Lateral migratory stream
MEA: Medial nucleus [amygdala]
MGN: Medial geniculate nucleus
NAc: Nucleus accumbens
OT: Olfactory tubercle
PARA: Cortical plate, parasubiculum
PG: Pontine gray
PULV: Pulvinar nucleus [thalamus]
PreS: Cortical plate, presubiculum
Put: Putamen
RT: Reticular nucleus [thalamus]
SGN: Suprageniculate nucleus
SI: Substantia innominata
SNr: Substantia nigra pars reticulata
SON: Supraoptic nucleus [hypothalamus]
SUB: Cortical plate, subiculum
Sth: Subthalamus
TMM: Tuberomammillary nucleus
TRI: Germinal trigone
VL: Ventral lateral nucleus [thalamus]
VPL: Ventral posterolateral nucleus [thalamus]
VPM: Ventral posteromedial nucleus [thalamus]
ZI: Zona incerta
ac: Anterior commissure
cc: Corpus callosum
cor: Corona radiata
cp: Cerebral peduncle
cpb: Cerebellar peduncles
dne: Diencephalic neuroepithelium
emlth: External medullary lamina [thalamus]
fx: Fornix
hipg: Hippocampal glioepithelium/ependyma
int: Internal capsule
lml: Lateral medullary lamina
mml: Medial medullary lamina
ot: Optic tract
pcbn: Precerebellar neuroepithelium
rhn: Rhombencephalic neuroepithelium
stt: Stria terminalis
wmf: White matter fibers
→ CaS: Calcarine sulcus
→ POS: Parieto-occipital sulcus

Age: 24 GW

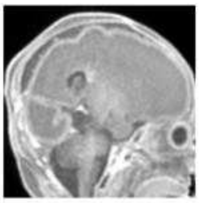

L-R Level: 9.48 mm

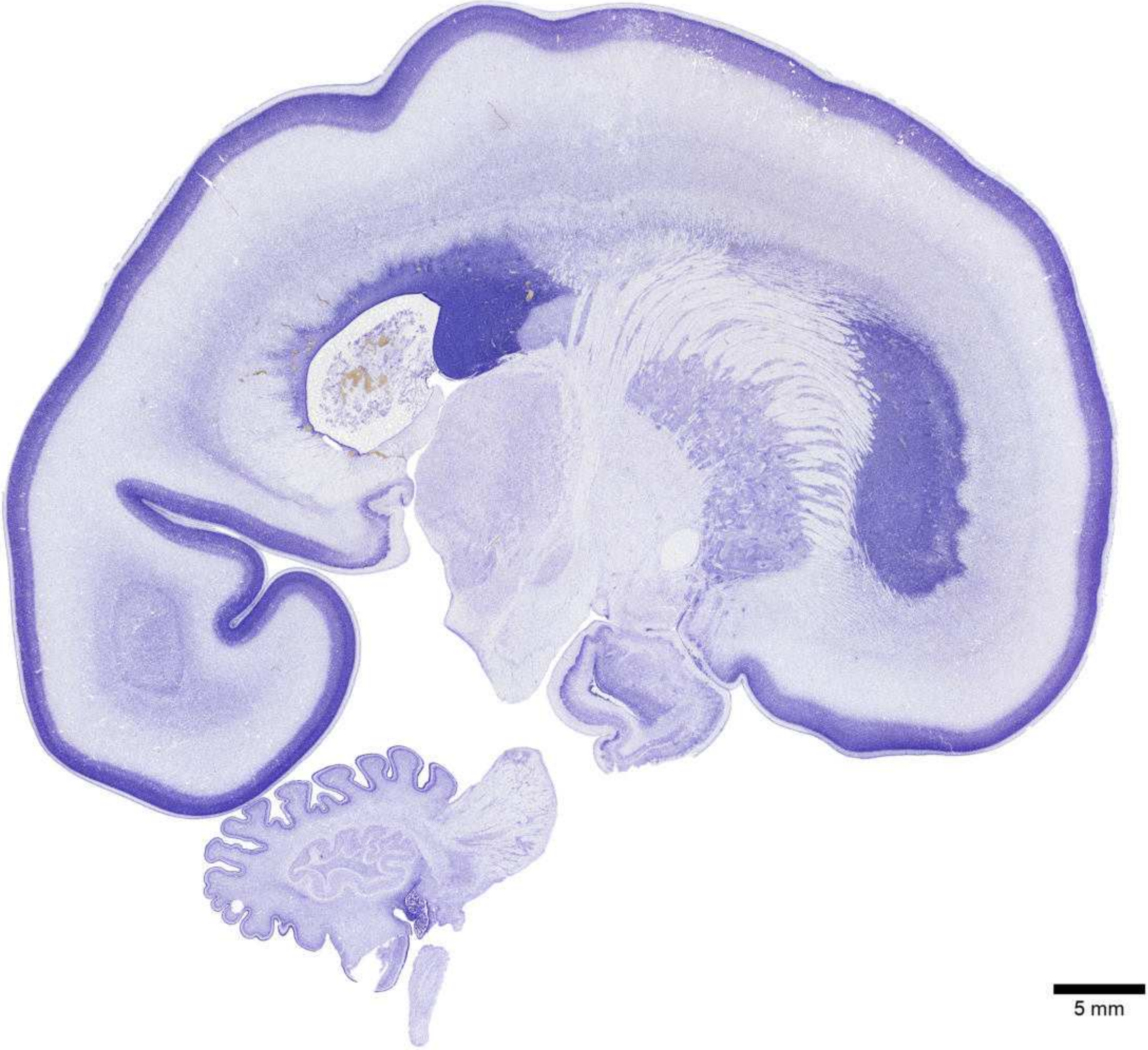

5 mm

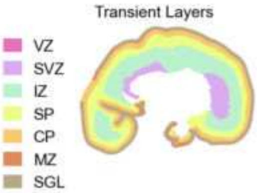

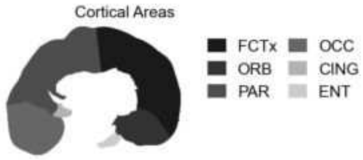

Age: 24 GW

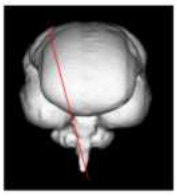

L-R Level: 9.48 mm

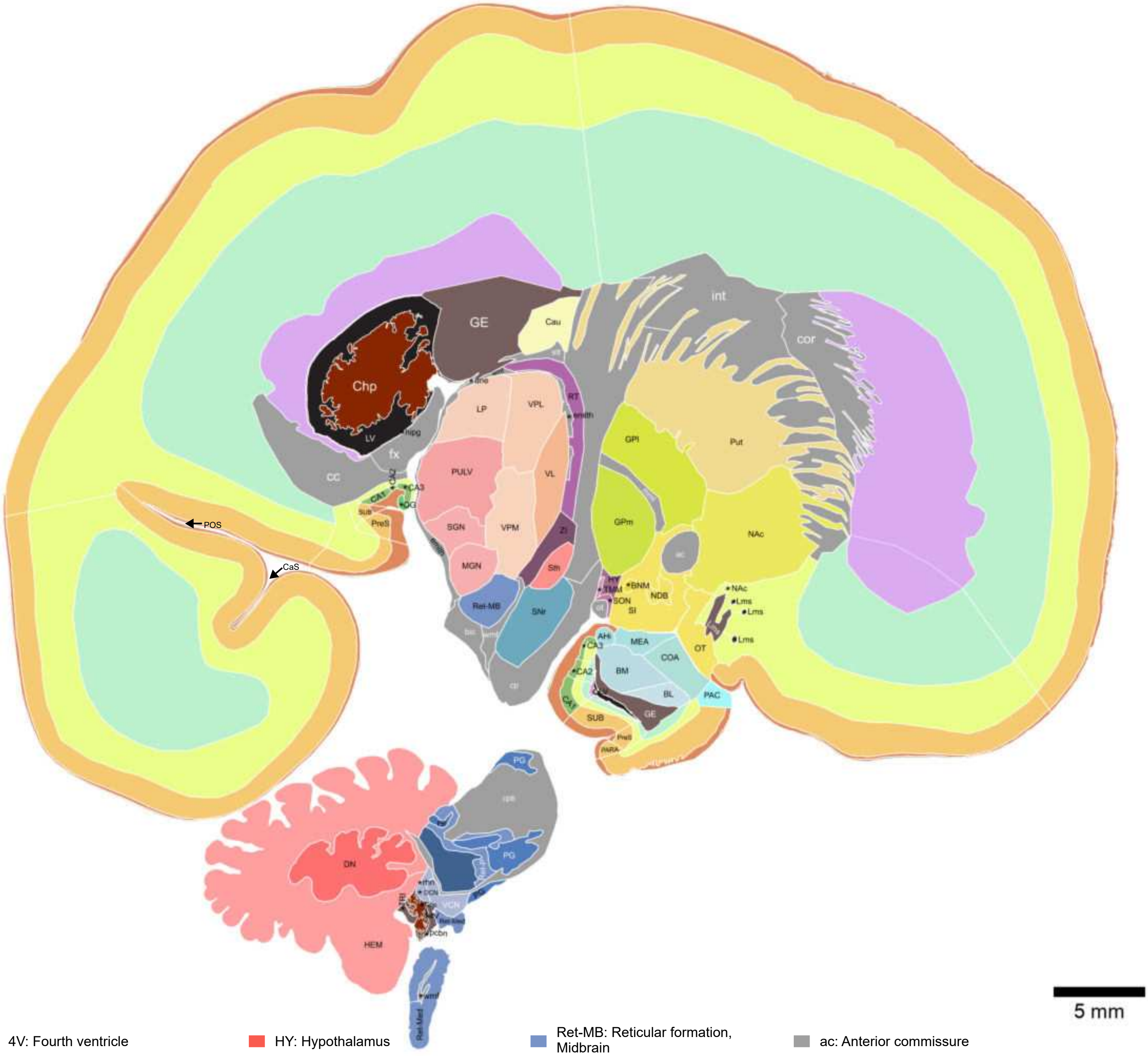

- 4V: Fourth ventricle
- AHi: Amygdalo-hippocampal area
- BL: Basal nucleus [amygdala]
- BM: Basomedial nucleus [amygdala]
- BNM: Basal nucleus of Meynert
- CA1: CA1 field [hippocampus]
- CA2: CA2 field [hippocampus]
- CA3: CA3 field [hippocampus]
- COA: Cortical nucleus [amygdala]
- Cau: Caudate nucleus
- Chp: Choroid plexus
- DCN: Dorsal cochlear nucleus
- DG: Dentate gyrus
- DN: Dentate nucleus
- GE: Ganglionic eminence
- GPl: Globus pallidus lateral segment
- GPm: Globus pallidus medial segment
- HEM: Cerebellar hemispheres
- HY: Hypothalamus
- LHA: Lateral hypothalamic area
- LP: Lateral posterior nucleus [thalamus]
- LV: Lateral ventricle
- Lms: Lateral migratory stream
- MEA: Medial nucleus [amygdala]
- MGN: Medial geniculate nucleus
- NAc: Nucleus accumbens
- NDB: Nucleus of the diagonal band
- OT: Olfactory tubercle
- PARA: Cortical plate, parasubiculum
- PB: Parabrachial nucleus
- PG: Pontine gray
- PULV: Pulvinar nucleus [thalamus]
- PreS: Cortical plate, presubiculum
- Put: Putamen
- RT: Reticular nucleus [thalamus]
- Ret-MB: Reticular formation, Midbrain
- Ret-Med: Reticular formation, Medulla
- Ret-P: Reticular formation, Pons
- SGN: Suprageniculate nucleus
- SI: Substantia innominata
- SNr: Substantia nigra pars reticulata
- SON: Supraoptic nucleus [hypothalamus]
- SPV: Spinal nucleus of the trigeminal
- SUB: Cortical plate, subiculum
- Sth: Subthalamus
- TMM: Tuberomammillary nucleus
- TRI: Germinal trigone
- VCN: Ventral cochlear nucleus
- VL: Ventral lateral nucleus [thalamus]
- VPL: Ventral posterolateral nucleus [thalamus]
- VPM: Ventral posteromedial nucleus [thalamus]
- ZI: Zona incerta
- ac: Anterior commissure
- bic: Brachium of the inferior colliculus
- cc: Corpus callosum
- cor: Corona radiata
- cp: Cerebral peduncle
- cpb: Cerebellar peduncles
- dne: Diencephalic neuroepithelium
- emlth: External medullary lamina [thalamus]
- fx: Fornix
- hipg: Hippocampal glioepithelium/ependyma
- int: Internal capsule
- mml: Medial medullary lamina
- ot: Optic tract
- pcbn: Precerebellar neuroepithelium
- rhn: Rhombencephalic neuroepithelium
- stt: Stria terminalis
- wmf: White matter fibers

→ CaS: Calcarine sulcus

→ POS: Parieto-occipital sulcus

Age: 24 GW

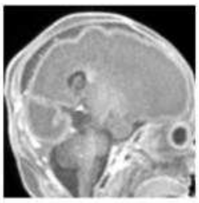

L-R Level: 9.0 mm

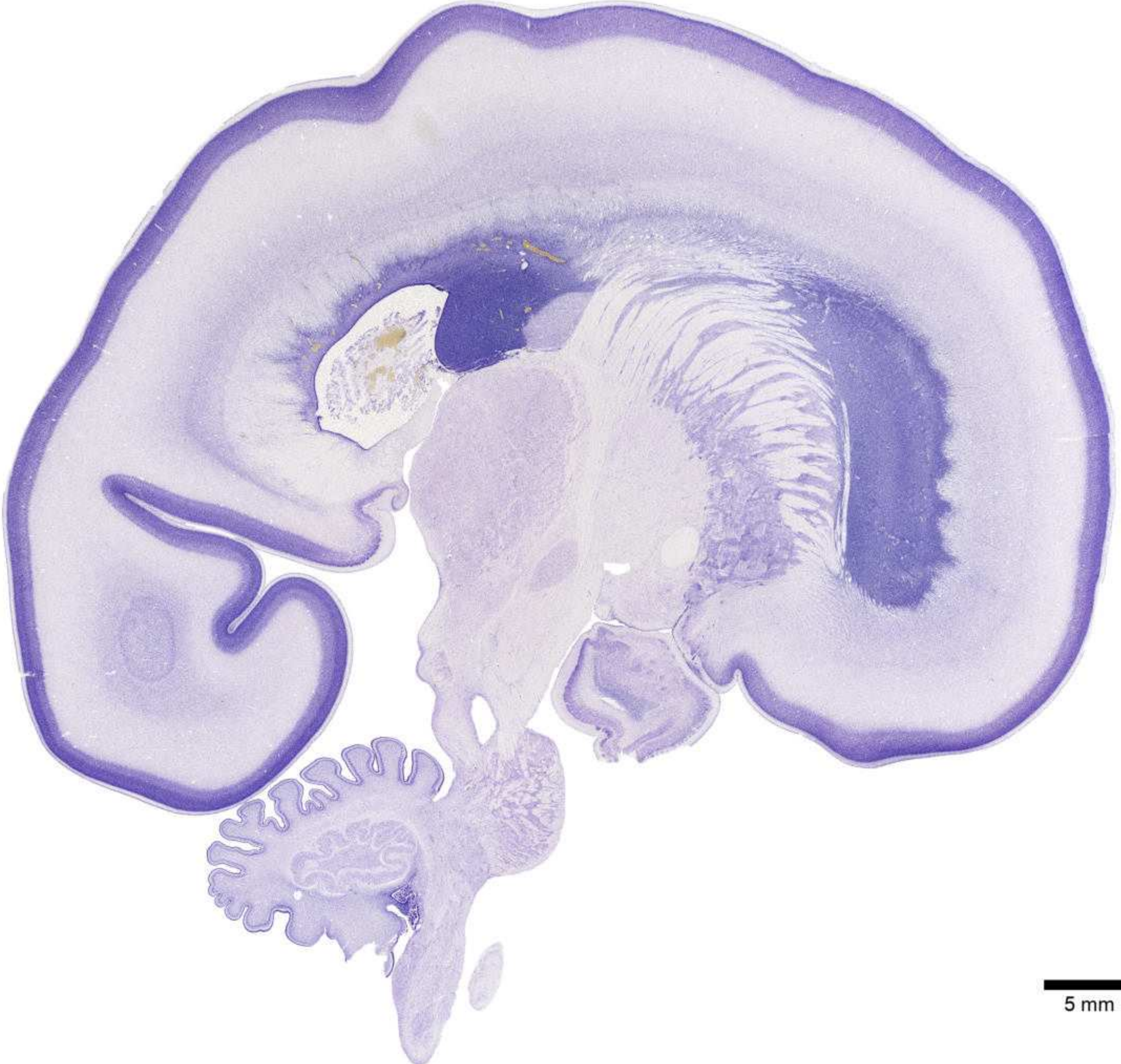

5 mm

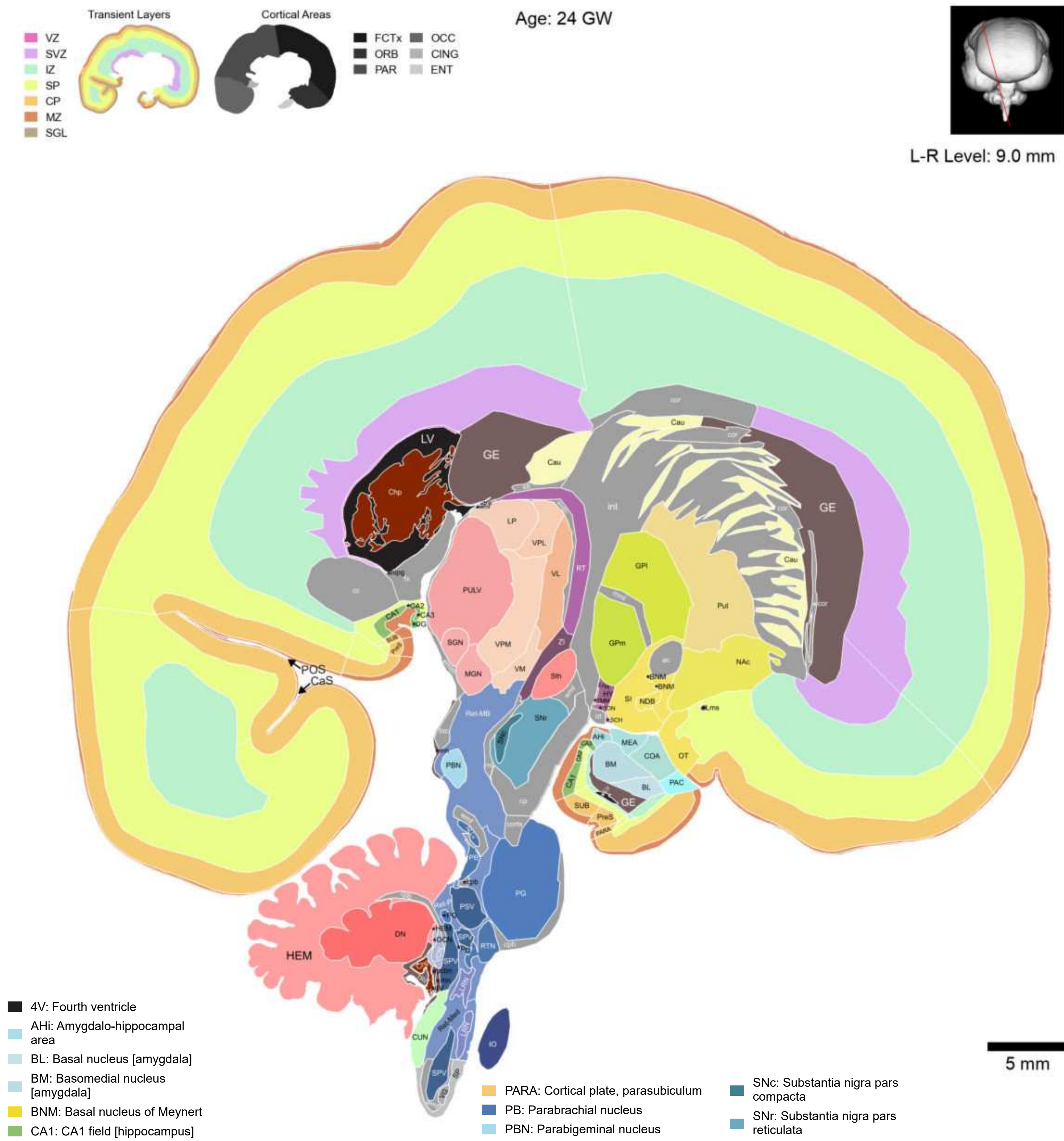

4V: Fourth ventricle
AHi: Amygdalo-hippocampal area
BL: Basal nucleus [amygdala]
BM: Basomedial nucleus [amygdala]
BNM: Basal nucleus of Meynert
CA1: CA1 field [hippocampus]
CA2: CA2 field [hippocampus]
CA3: CA3 field [hippocampus]
COA: Cortical nucleus [amygdala]
CUN: Cuneate nucleus
Cau: Caudate nucleus
Chp: Choroid plexus
DCN: Dorsal cochlear nucleus
DG: Dentate gyrus
DN: Dentate nucleus
GE: Ganglionic eminence
GPl: Globus pallidus lateral segment
GPm: Globus pallidus medial segment
HEM: Cerebellar hemispheres
HY: Hypothalamus
IO: Inferior olive
LHA: Lateral hypothalamic area
LP: Lateral posterior nucleus [thalamus]
LRN: Lateral reticular nucleus
LV: Lateral ventricle
Lms: Lateral migratory stream
MEA: Medial nucleus [amygdala]
MGN: Medial geniculate nucleus
NAc: Nucleus accumbens
NDB: Nucleus of the diagonal band
NLLv: Nucleus of the lateral lemniscus, ventral
OT: Olfactory tubercle
PARA: Cortical plate, parasubiculum
PB: Parabrachial nucleus
PBN: Parabigeminal nucleus
PG: Pontine gray
PSV: Principal sensory nucleus of the trigeminal
PULV: Pulvinar nucleus [thalamus]
PreS: Cortical plate, presubiculum
Put: Putamen
RT: Reticular nucleus [thalamus]
RTN: Reticular tegmental nucleus
Ret-MB: Reticular formation, Midbrain
Ret-Med: Reticular formation, Medulla
Ret-P: Reticular formation, Pons
SCH: Suprachiasmatic nucleus [hypothalamus]
SGN: Suprageniculate nucleus
SI: Substantia innominata
SNc: Substantia nigra pars compacta
SNr: Substantia nigra pars reticulata
SON: Supraoptic nucleus [hypothalamus]
SP: Spinal cord
SPV: Spinal nucleus of the trigeminal
SUB: Cortical plate, subiculum
Sth: Subthalamus
TMM: Tuberomammillary nucleus
TRI: Germinal trigone
VCN: Ventral cochlear nucleus
VG: Ventral gray of the spinal cord
VL: Ventral lateral nucleus [thalamus]
VM: Ventral medial nucleus [thalamus]
VPL: Ventral posterolateral nucleus [thalamus]
VPM: Ventral posteromedial nucleus [thalamus]
ZI: Zona incerta
→ CaS: Calcarine sulcus
→ POS: Parieto-occipital sulcus

Age: 24 GW

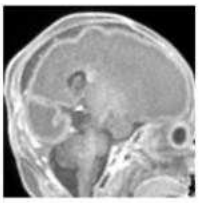

L-R Level: 8.64 mm

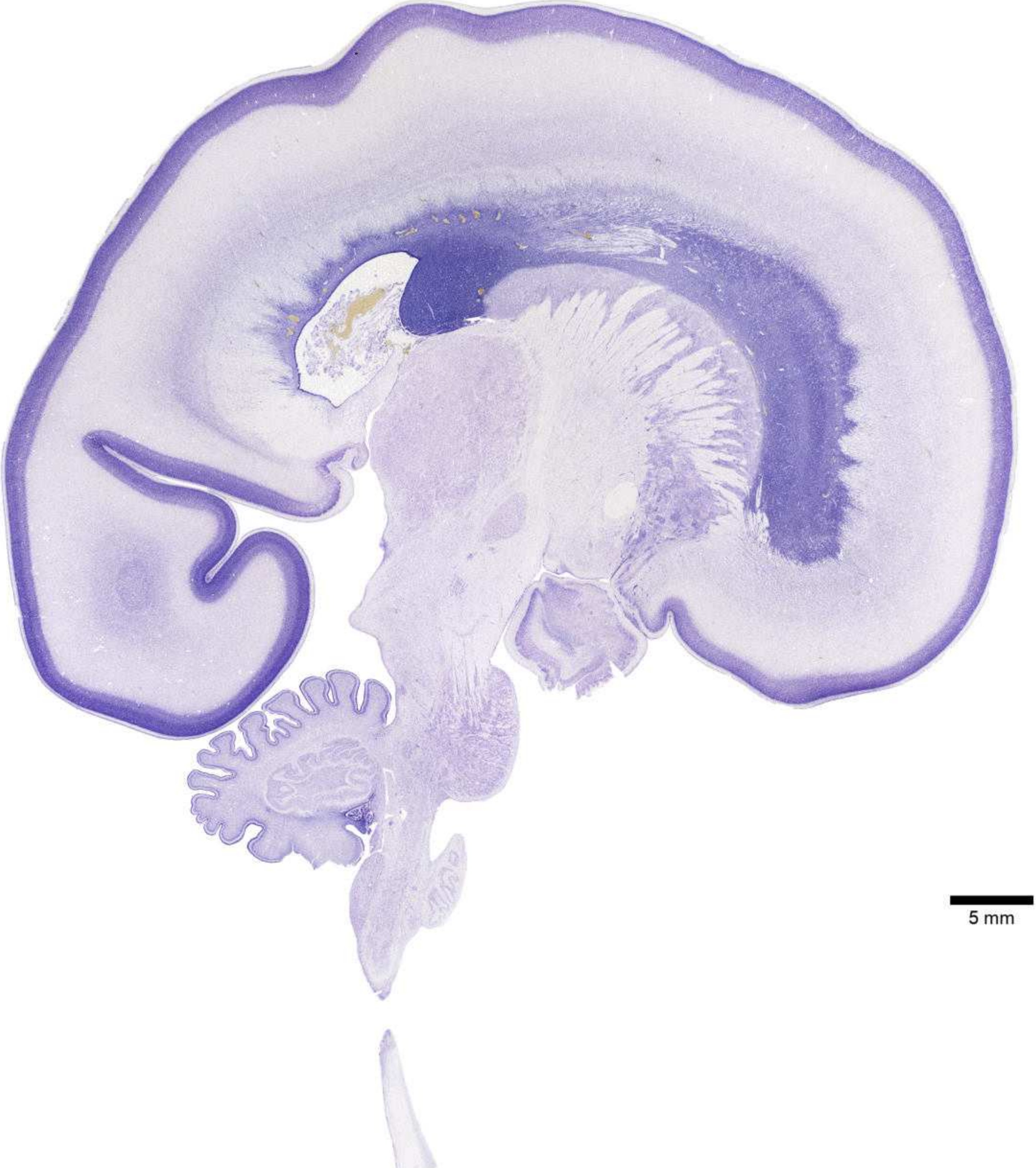

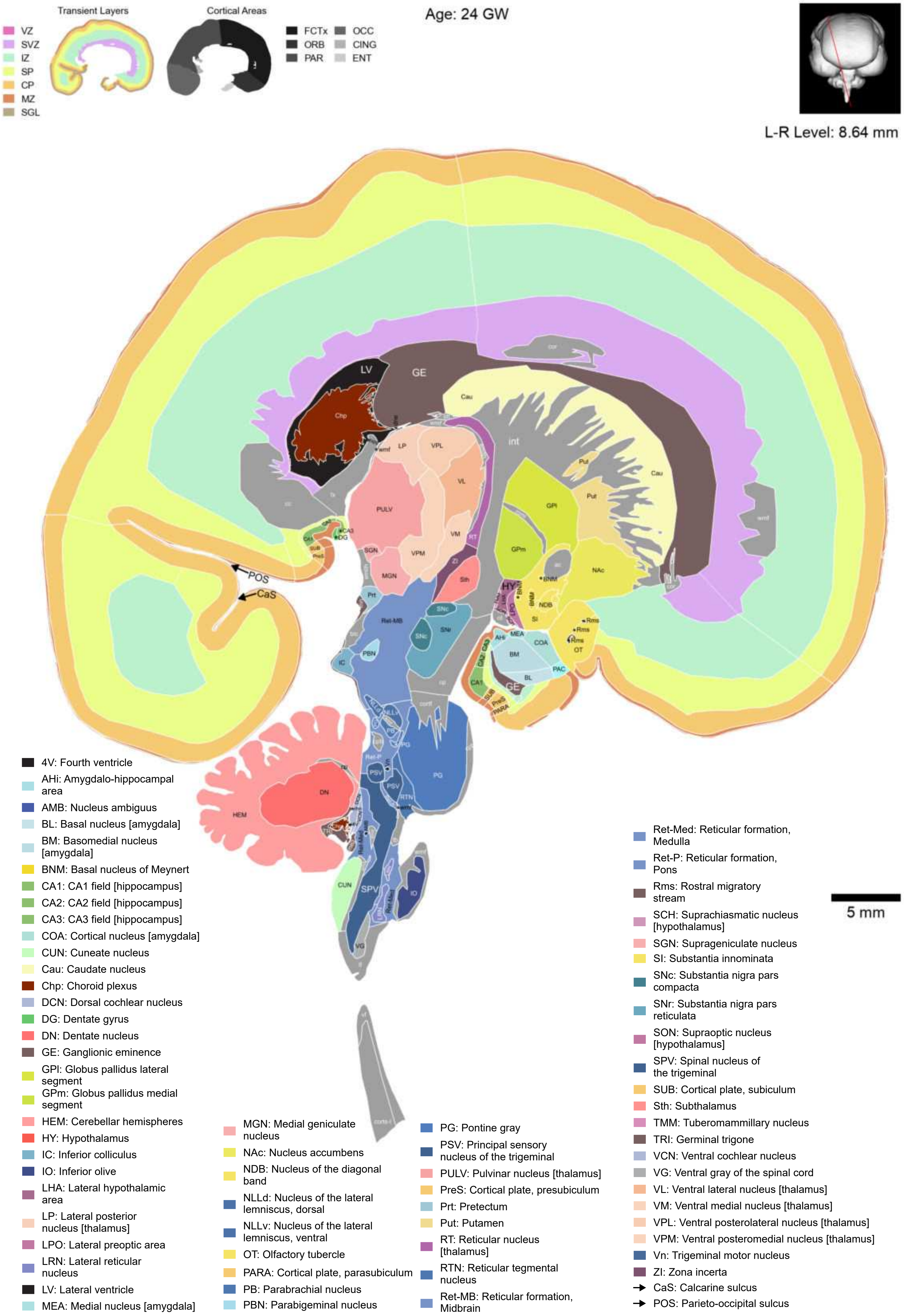

Transient Layers
VZ
SVZ
IZ
SP
CP
MZ
SGL
Cortical Areas
FCTx
ORB
PAR
OCC
CING
ENT
Age: 24 GW
L-R Level: 8.64 mm
5 mm
LV
GE
Chp
Cau
int
LP
VPL
VL
PULV
VM
VPM
SGN
MGN
GPl
GPm
Put
NAc
Sth
ZI
RT
HY
BNM
NDB
SI
Rms
OT
MEA
AHi
COA
BM
BL
PAC
CA1
CA2
CA3
DG
SUB
PreS
PARA
POS
CaS
Prt
Ret-MB
SNc
SNr
PBN
IC
PG
PSV
RTN
DN
HEM
CUN
SPV
IO
VG
4V: Fourth ventricle
AHi: Amygdalo-hippocampal area
AMB: Nucleus ambiguus
BL: Basal nucleus [amygdala]
BM: Basomedial nucleus [amygdala]
BNM: Basal nucleus of Meynert
CA1: CA1 field [hippocampus]
CA2: CA2 field [hippocampus]
CA3: CA3 field [hippocampus]
COA: Cortical nucleus [amygdala]
CUN: Cuneate nucleus
Cau: Caudate nucleus
Chp: Choroid plexus
DCN: Dorsal cochlear nucleus
DG: Dentate gyrus
DN: Dentate nucleus
GE: Ganglionic eminence
GPl: Globus pallidus lateral segment
GPm: Globus pallidus medial segment
HEM: Cerebellar hemispheres
HY: Hypothalamus
IC: Inferior colliculus
IO: Inferior olive
LHA: Lateral hypothalamic area
LP: Lateral posterior nucleus [thalamus]
LPO: Lateral preoptic area
LRN: Lateral reticular nucleus
LV: Lateral ventricle
MEA: Medial nucleus [amygdala]
MGN: Medial geniculate nucleus
NAc: Nucleus accumbens
NDB: Nucleus of the diagonal band
NLLd: Nucleus of the lateral lemniscus, dorsal
NLLv: Nucleus of the lateral lemniscus, ventral
OT: Olfactory tubercle
PARA: Cortical plate, parasubiculum
PB: Parabrachial nucleus
PBN: Parabigeminal nucleus
PG: Pontine gray
PSV: Principal sensory nucleus of the trigeminal
PULV: Pulvinar nucleus [thalamus]
PreS: Cortical plate, presubiculum
Prt: Pretectum
Put: Putamen
RT: Reticular nucleus [thalamus]
RTN: Reticular tegmental nucleus
Ret-MB: Reticular formation, Midbrain
Ret-Med: Reticular formation, Medulla
Ret-P: Reticular formation, Pons
Rms: Rostral migratory stream
SCH: Suprachiasmatic nucleus [hypothalamus]
SGN: Suprageniculate nucleus
SI: Substantia innominata
SNc: Substantia nigra pars compacta
SNr: Substantia nigra pars reticulata
SON: Supraoptic nucleus [hypothalamus]
SPV: Spinal nucleus of the trigeminal
SUB: Cortical plate, subiculum
Sth: Subthalamus
TMM: Tuberomammillary nucleus
TRI: Germinal trigone
VCN: Ventral cochlear nucleus
VG: Ventral gray of the spinal cord
VL: Ventral lateral nucleus [thalamus]
VM: Ventral medial nucleus [thalamus]
VPL: Ventral posterolateral nucleus [thalamus]
VPM: Ventral posteromedial nucleus [thalamus]
Vn: Trigeminal motor nucleus
ZI: Zona incerta
CaS: Calcarine sulcus
POS: Parieto-occipital sulcus

Age: 24 GW

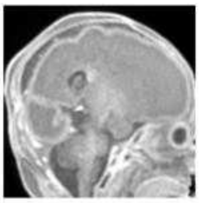

L-R Level: 8.28 mm

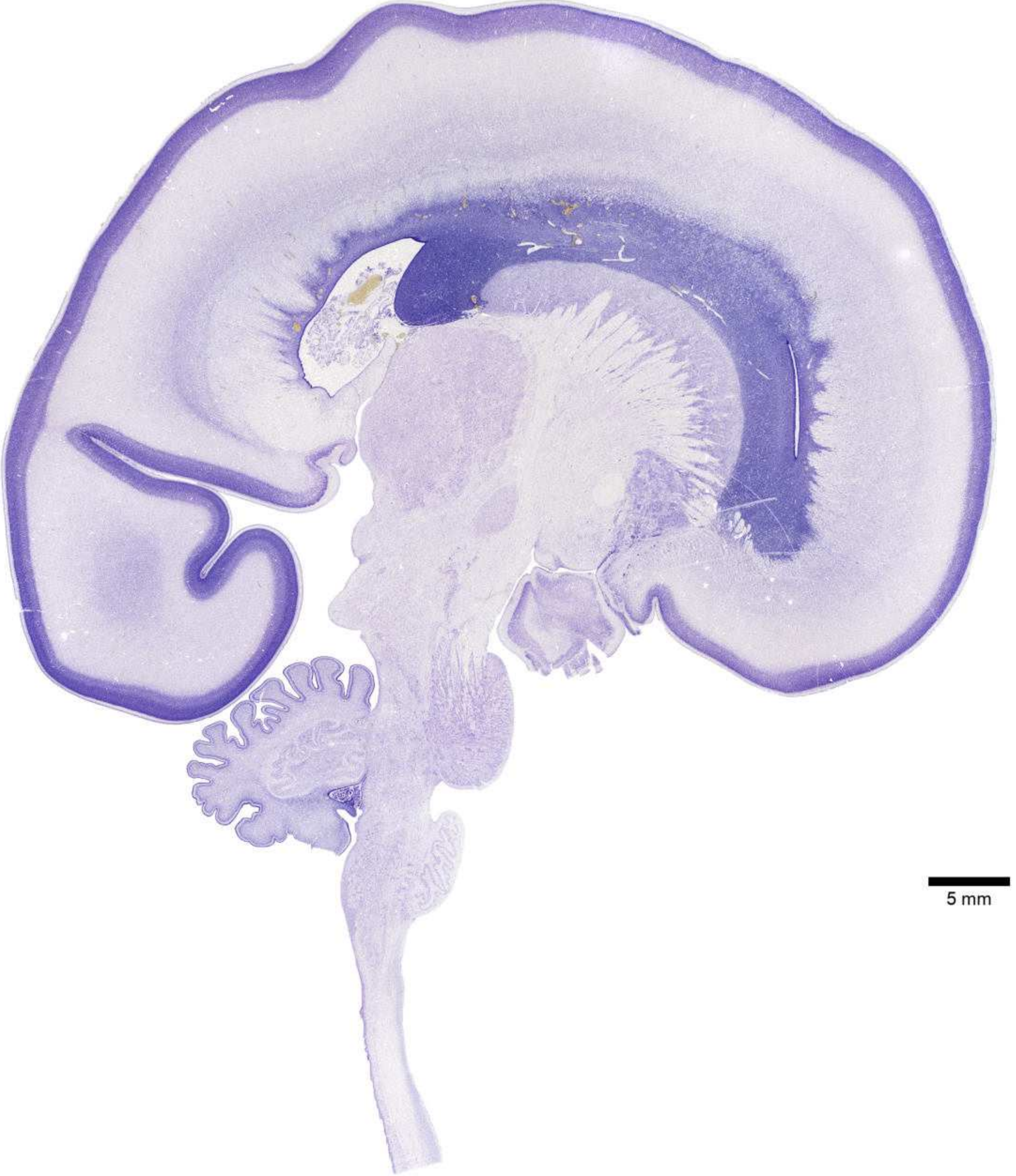

5 mm

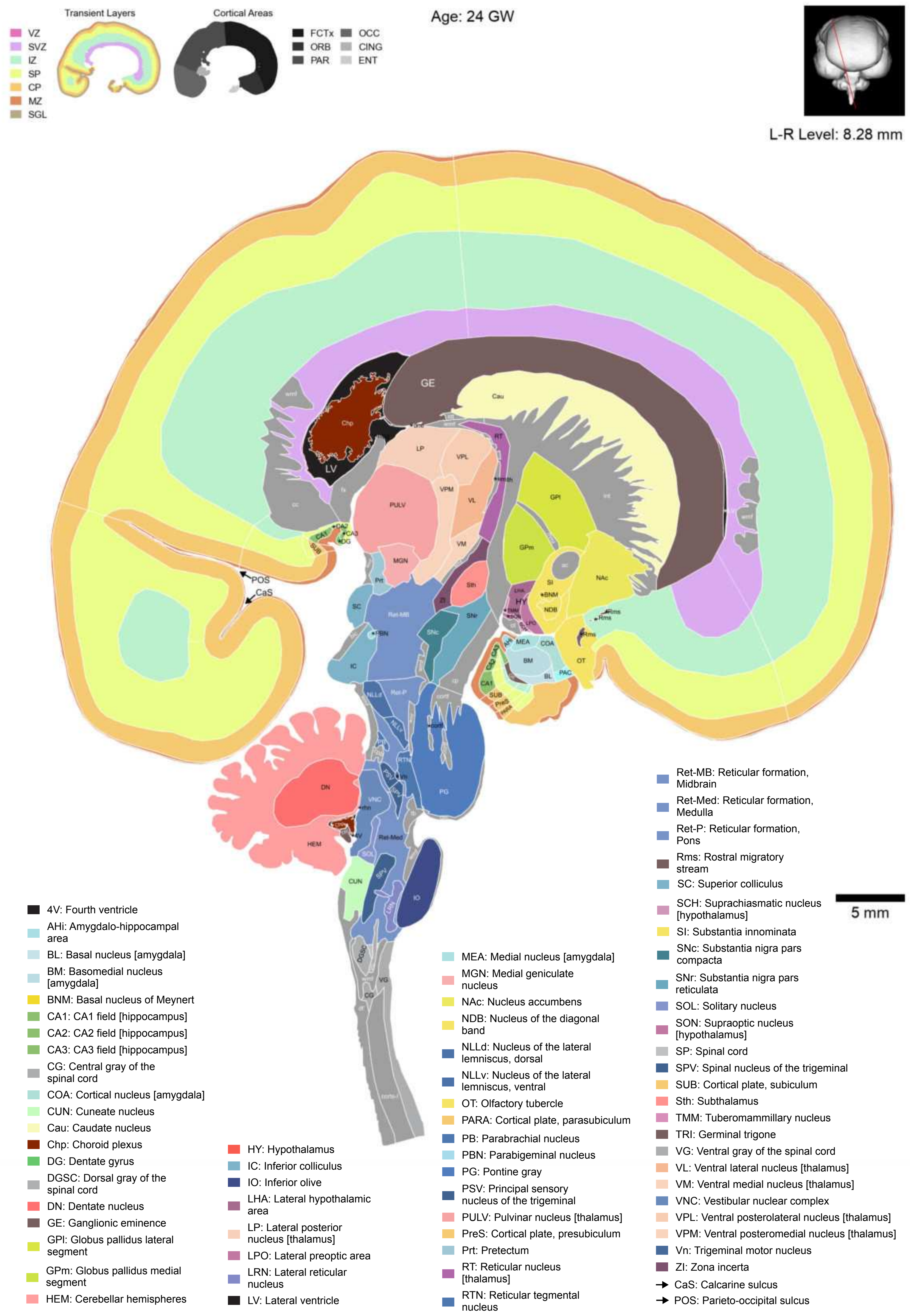

Transient Layers
VZ
SVZ
IZ
SP
CP
MZ
SGL
Cortical Areas
FCTx
ORB
PAR
OCC
CING
ENT
Age: 24 GW
L-R Level: 8.28 mm
5 mm
GE
LV
POS
CaS
4V: Fourth ventricle
AHi: Amygdalo-hippocampal area
BL: Basal nucleus [amygdala]
BM: Basomedial nucleus [amygdala]
BNM: Basal nucleus of Meynert
CA1: CA1 field [hippocampus]
CA2: CA2 field [hippocampus]
CA3: CA3 field [hippocampus]
CG: Central gray of the spinal cord
COA: Cortical nucleus [amygdala]
CUN: Cuneate nucleus
Cau: Caudate nucleus
Chp: Choroid plexus
DG: Dentate gyrus
DGSC: Dorsal gray of the spinal cord
DN: Dentate nucleus
GE: Ganglionic eminence
GPl: Globus pallidus lateral segment
GPm: Globus pallidus medial segment
HEM: Cerebellar hemispheres
HY: Hypothalamus
IC: Inferior colliculus
IO: Inferior olive
LHA: Lateral hypothalamic area
LP: Lateral posterior nucleus [thalamus]
LPO: Lateral preoptic area
LRN: Lateral reticular nucleus
LV: Lateral ventricle
MEA: Medial nucleus [amygdala]
MGN: Medial geniculate nucleus
NAc: Nucleus accumbens
NDB: Nucleus of the diagonal band
NLLd: Nucleus of the lateral lemniscus, dorsal
NLLv: Nucleus of the lateral lemniscus, ventral
OT: Olfactory tubercle
PARA: Cortical plate, parasubiculum
PB: Parabrachial nucleus
PBN: Parabigeminal nucleus
PG: Pontine gray
PSV: Principal sensory nucleus of the trigeminal
PULV: Pulvinar nucleus [thalamus]
PreS: Cortical plate, presubiculum
Prt: Pretectum
RT: Reticular nucleus [thalamus]
RTN: Reticular tegmental nucleus
Ret-MB: Reticular formation, Midbrain
Ret-Med: Reticular formation, Medulla
Ret-P: Reticular formation, Pons
Rms: Rostral migratory stream
SC: Superior colliculus
SCH: Suprachiasmatic nucleus [hypothalamus]
SI: Substantia innominata
SNc: Substantia nigra pars compacta
SNr: Substantia nigra pars reticulata
SOL: Solitary nucleus
SON: Supraoptic nucleus [hypothalamus]
SP: Spinal cord
SPV: Spinal nucleus of the trigeminal
SUB: Cortical plate, subiculum
Sth: Subthalamus
TMM: Tuberomammillary nucleus
TRI: Germinal trigone
VG: Ventral gray of the spinal cord
VL: Ventral lateral nucleus [thalamus]
VM: Ventral medial nucleus [thalamus]
VNC: Vestibular nuclear complex
VPL: Ventral posterolateral nucleus [thalamus]
VPM: Ventral posteromedial nucleus [thalamus]
Vn: Trigeminal motor nucleus
ZI: Zona incerta
CaS: Calcarine sulcus
POS: Parieto-occipital sulcus

Age: 24 GW

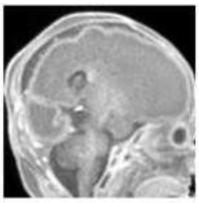

L-R Level: 8.04 mm

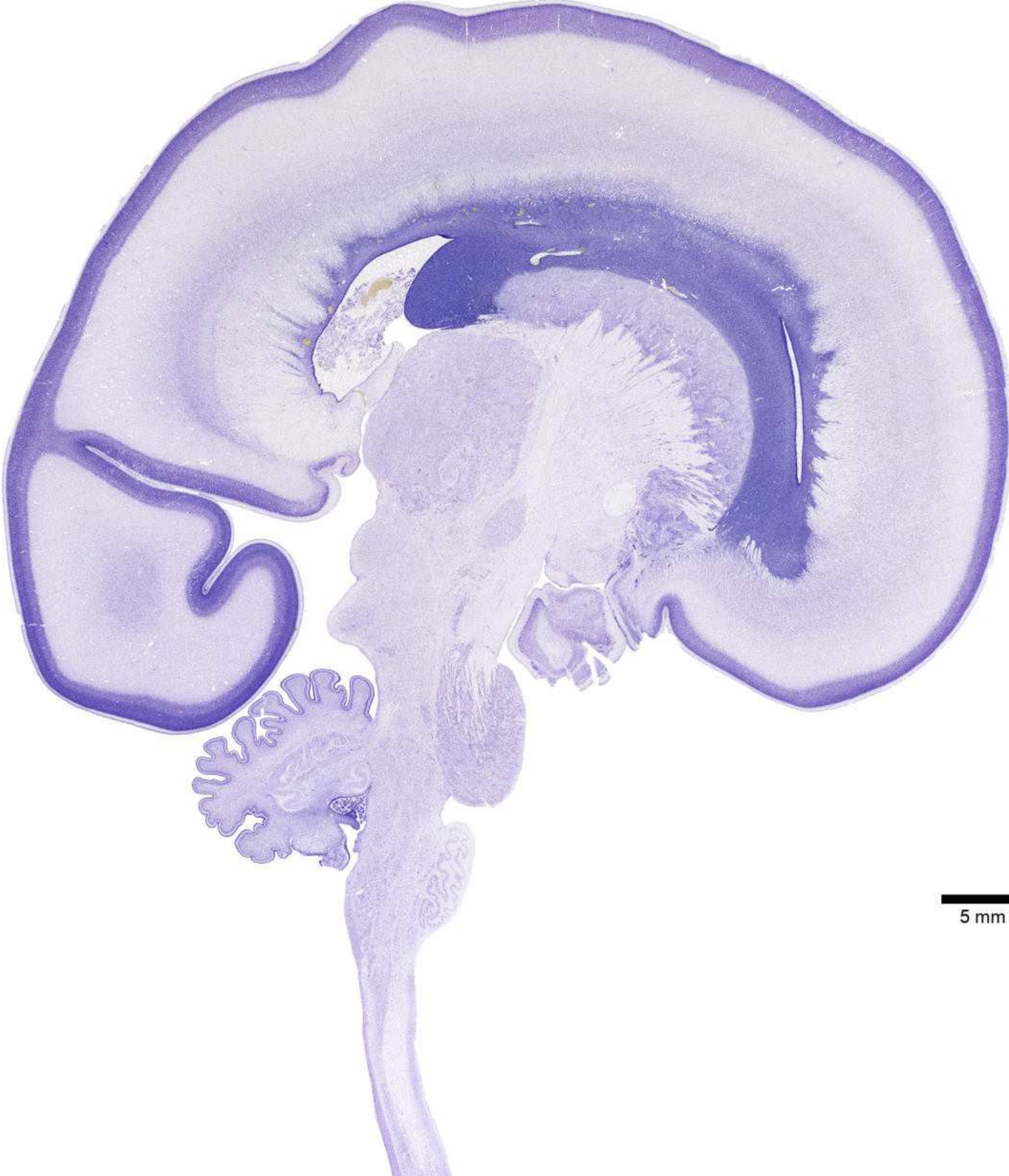

5 mm

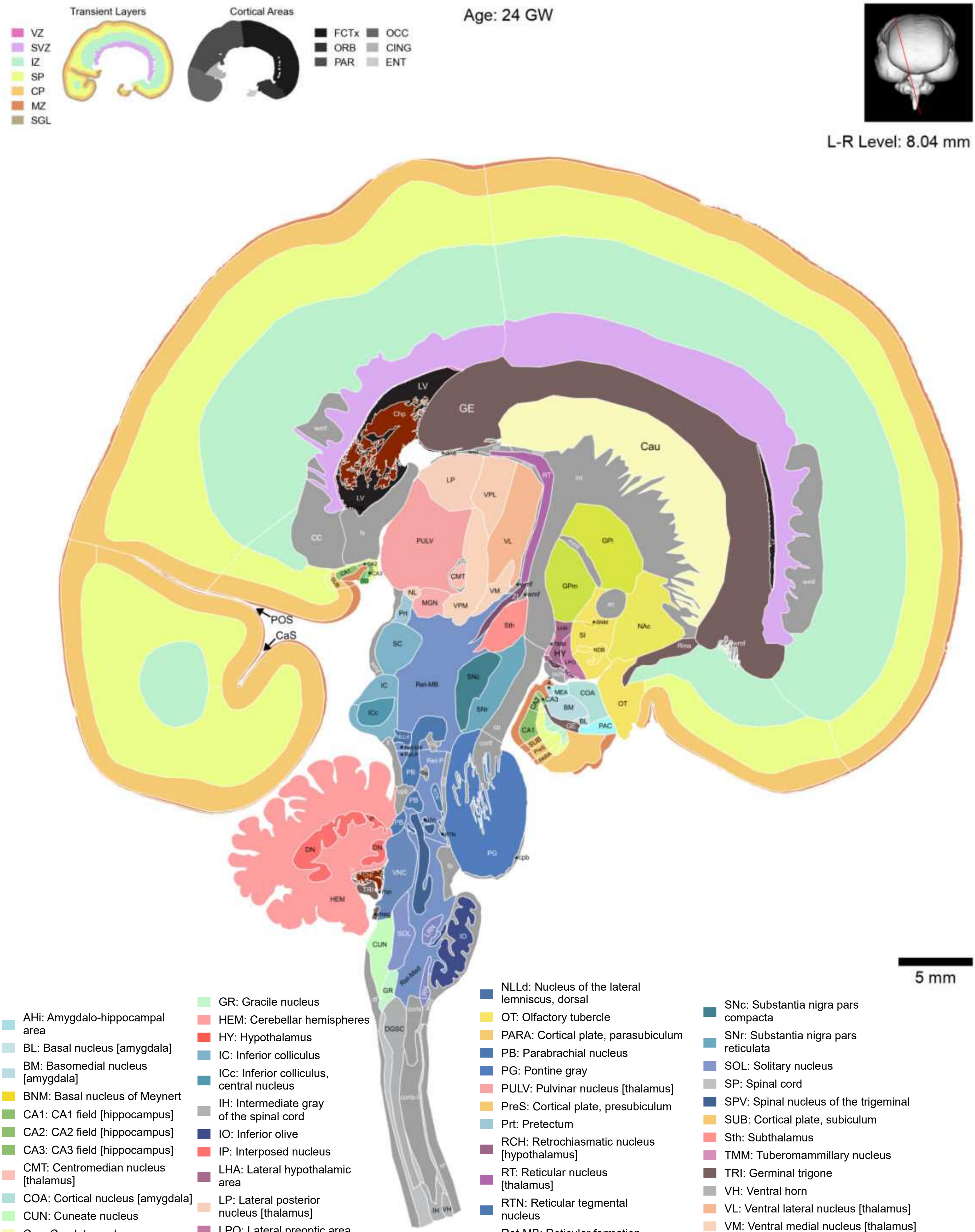

AHi: Amygdalo-hippocampal area
BL: Basal nucleus [amygdala]
BM: Basomedial nucleus [amygdala]
BNM: Basal nucleus of Meynert
CA1: CA1 field [hippocampus]
CA2: CA2 field [hippocampus]
CA3: CA3 field [hippocampus]
CMT: Centromedian nucleus [thalamus]
COA: Cortical nucleus [amygdala]
CUN: Cuneate nucleus
Cau: Caudate nucleus
Chp: Choroid plexus
DG: Dentate gyrus
DGSC: Dorsal gray of the spinal cord
DN: Dentate nucleus
GE: Ganglionic eminence
GPl: Globus pallidus lateral segment
GPm: Globus pallidus medial segment

GR: Gracile nucleus
HEM: Cerebellar hemispheres
HY: Hypothalamus
IC: Inferior colliculus
ICc: Inferior colliculus, central nucleus
IH: Intermediate gray of the spinal cord
IO: Inferior olive
IP: Interposed nucleus
LHA: Lateral hypothalamic area
LP: Lateral posterior nucleus [thalamus]
LPO: Lateral preoptic area
LRN: Lateral reticular nucleus
LV: Lateral ventricle
MEA: Medial nucleus [amygdala]
MGN: Medial geniculate nucleus
NAc: Nucleus accumbens
NDB: Nucleus of the diagonal band
NL: Nucleus limitans [thalamus]

NLLd: Nucleus of the lateral lemniscus, dorsal
OT: Olfactory tubercle
PARA: Cortical plate, parasubiculum
PB: Parabrachial nucleus
PG: Pontine gray
PULV: Pulvinar nucleus [thalamus]
PreS: Cortical plate, presubiculum
Prt: Pretectum
RCH: Retrochiasmatic nucleus [hypothalamus]
RT: Reticular nucleus [thalamus]
RTN: Reticular tegmental nucleus
Ret-MB: Reticular formation, Midbrain
Ret-Med: Reticular formation, Medulla
Ret-P: Reticular formation, Pons
Rms: Rostral migratory stream
SC: Superior colliculus
SCH: Suprachiasmatic nucleus [hypothalamus]
SI: Substantia innominata

SNc: Substantia nigra pars compacta
SNr: Substantia nigra pars reticulata
SOL: Solitary nucleus
SP: Spinal cord
SPV: Spinal nucleus of the trigeminal
SUB: Cortical plate, subiculum
Sth: Subthalamus
TMM: Tuberomammillary nucleus
TRI: Germinal trigone
VH: Ventral horn
VL: Ventral lateral nucleus [thalamus]
VM: Ventral medial nucleus [thalamus]
VNC: Vestibular nuclear complex
VPL: Ventral posterolateral nucleus [thalamus]
VPM: Ventral posteromedial nucleus [thalamus]
Vn: Trigeminal motor nucleus
ZI: Zona incerta
→ CaS: Calcarine sulcus
→ POS: Parieto-occipital sulcus

Age: 24 GW

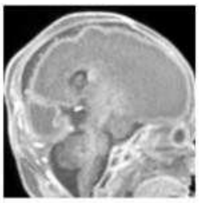

L-R Level: 7.92 mm

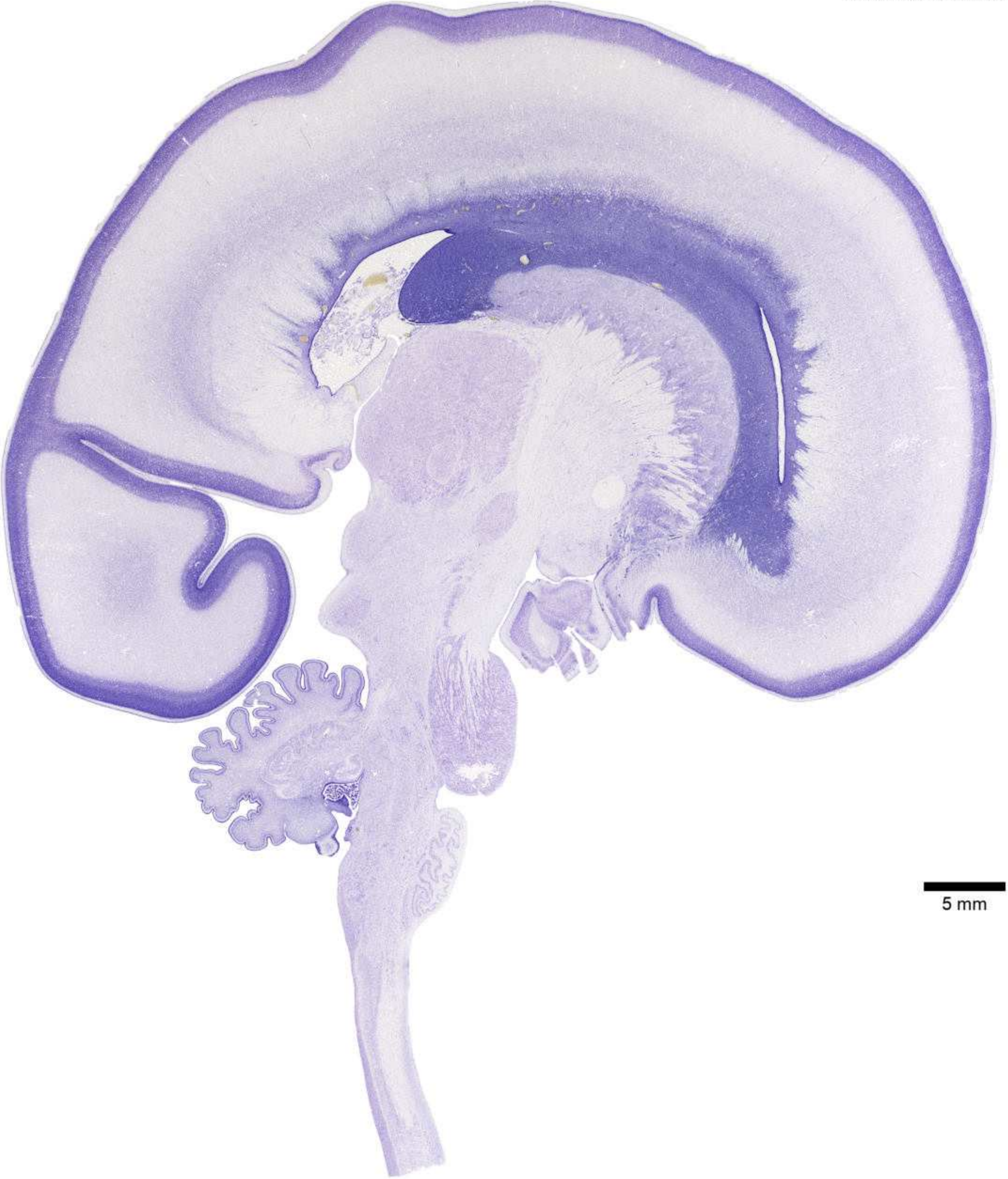

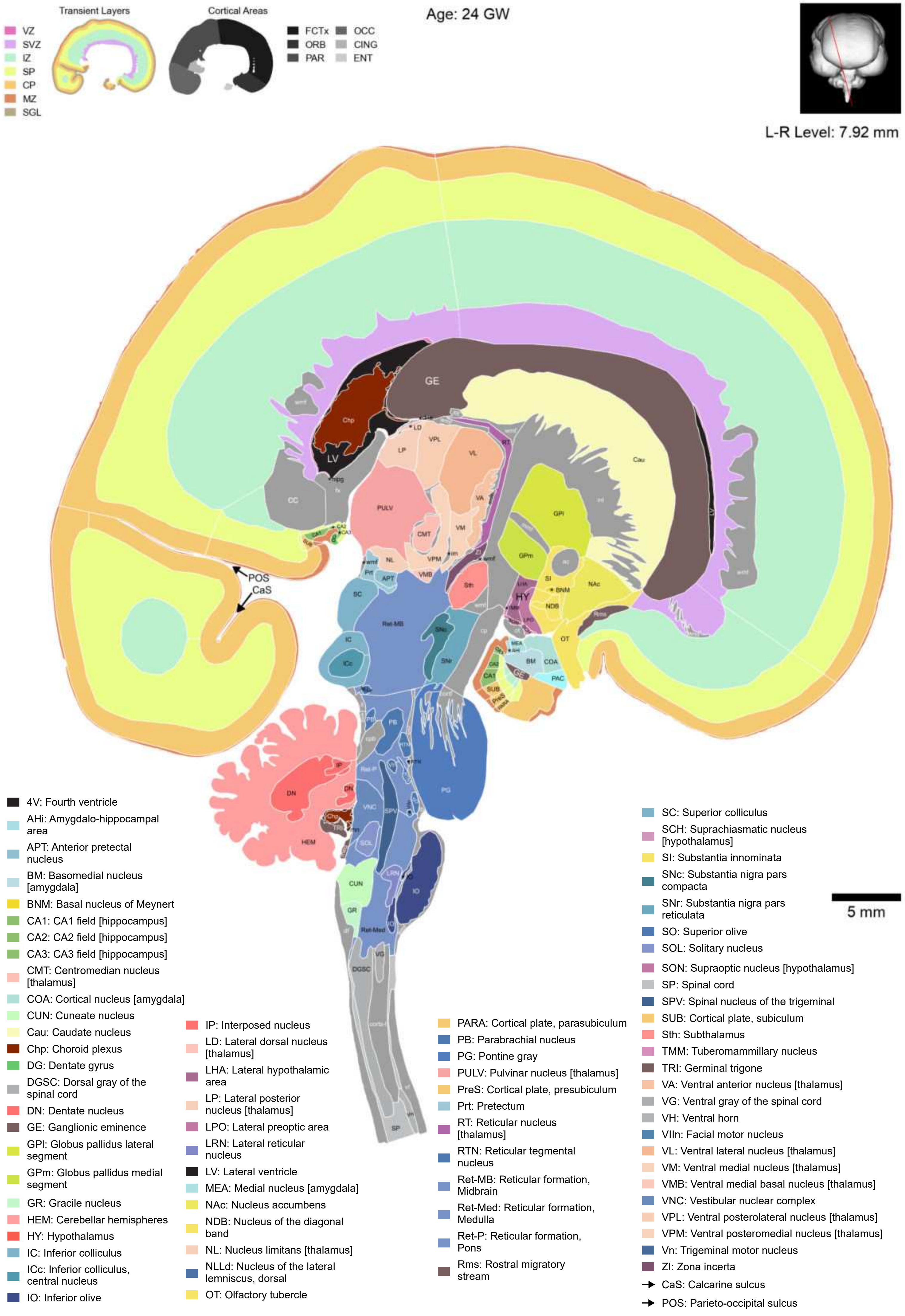
Transient Layers
Cortical Areas
VZ
SVZ
IZ
SP
CP
MZ
SGL
FCTx
ORB
PAR
OCC
CING
ENT
Age: 24 GW
L-R Level: 7.92 mm
GE
Chp
LV
CC
PULV
Cau
GPl
GPm
HY
NAc
OT
POS
CaS
Ret-MB
SC
IC
ICc
PG
DN
HEM
IO
CUN
GR
5 mm
4V: Fourth ventricle
AHi: Amygdalo-hippocampal area
APT: Anterior pretectal nucleus
BM: Basomedial nucleus [amygdala]
BNM: Basal nucleus of Meynert
CA1: CA1 field [hippocampus]
CA2: CA2 field [hippocampus]
CA3: CA3 field [hippocampus]
CMT: Centromedian nucleus [thalamus]
COA: Cortical nucleus [amygdala]
CUN: Cuneate nucleus
Cau: Caudate nucleus
Chp: Choroid plexus
DG: Dentate gyrus
DGSC: Dorsal gray of the spinal cord
DN: Dentate nucleus
GE: Ganglionic eminence
GPl: Globus pallidus lateral segment
GPm: Globus pallidus medial segment
GR: Gracile nucleus
HEM: Cerebellar hemispheres
HY: Hypothalamus
IC: Inferior colliculus
ICc: Inferior colliculus, central nucleus
IO: Inferior olive
IP: Interposed nucleus
LD: Lateral dorsal nucleus [thalamus]
LHA: Lateral hypothalamic area
LP: Lateral posterior nucleus [thalamus]
LPO: Lateral preoptic area
LRN: Lateral reticular nucleus
LV: Lateral ventricle
MEA: Medial nucleus [amygdala]
NAc: Nucleus accumbens
NDB: Nucleus of the diagonal band
NL: Nucleus limitans [thalamus]
NLLd: Nucleus of the lateral lemniscus, dorsal
OT: Olfactory tubercle
PARA: Cortical plate, parasubiculum
PB: Parabrachial nucleus
PG: Pontine gray
PULV: Pulvinar nucleus [thalamus]
PreS: Cortical plate, presubiculum
Prt: Pretectum
RT: Reticular nucleus [thalamus]
RTN: Reticular tegmental nucleus
Ret-MB: Reticular formation, Midbrain
Ret-Med: Reticular formation, Medulla
Ret-P: Reticular formation, Pons
Rms: Rostral migratory stream
SC: Superior colliculus
SCH: Suprachiasmatic nucleus [hypothalamus]
SI: Substantia innominata
SNc: Substantia nigra pars compacta
SNr: Substantia nigra pars reticulata
SO: Superior olive
SOL: Solitary nucleus
SON: Supraoptic nucleus [hypothalamus]
SP: Spinal cord
SPV: Spinal nucleus of the trigeminal
SUB: Cortical plate, subiculum
Sth: Subthalamus
TMM: Tuberomammillary nucleus
TRI: Germinal trigone
VA: Ventral anterior nucleus [thalamus]
VG: Ventral gray of the spinal cord
VH: Ventral horn
VIIn: Facial motor nucleus
VL: Ventral lateral nucleus [thalamus]
VM: Ventral medial nucleus [thalamus]
VMB: Ventral medial basal nucleus [thalamus]
VNC: Vestibular nuclear complex
VPL: Ventral posterolateral nucleus [thalamus]
VPM: Ventral posteromedial nucleus [thalamus]
Vn: Trigeminal motor nucleus
ZI: Zona incerta
CaS: Calcarine sulcus
POS: Parieto-occipital sulcus

Age: 24 GW

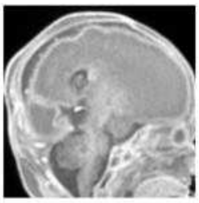

L-R Level: 7.8 mm

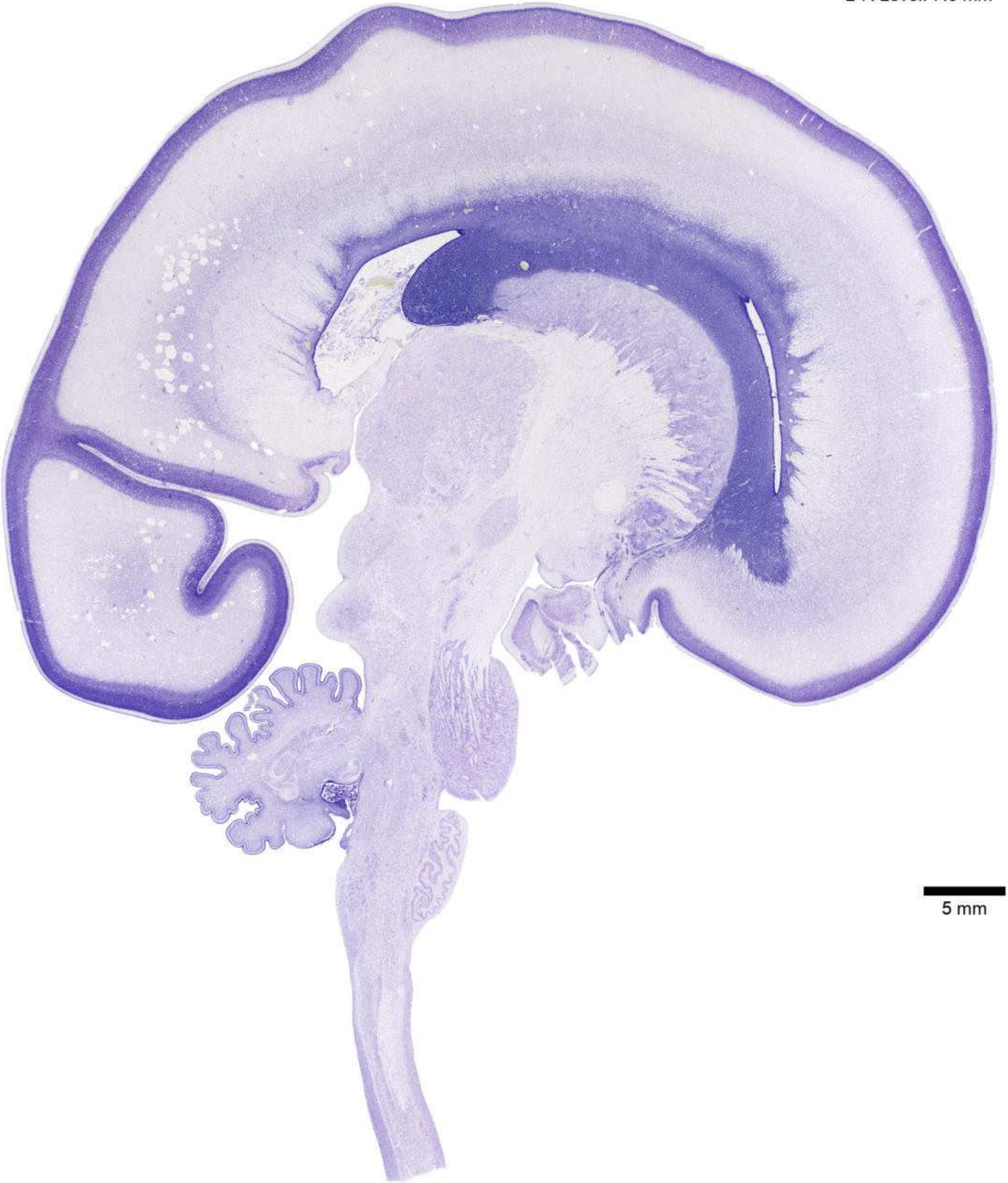

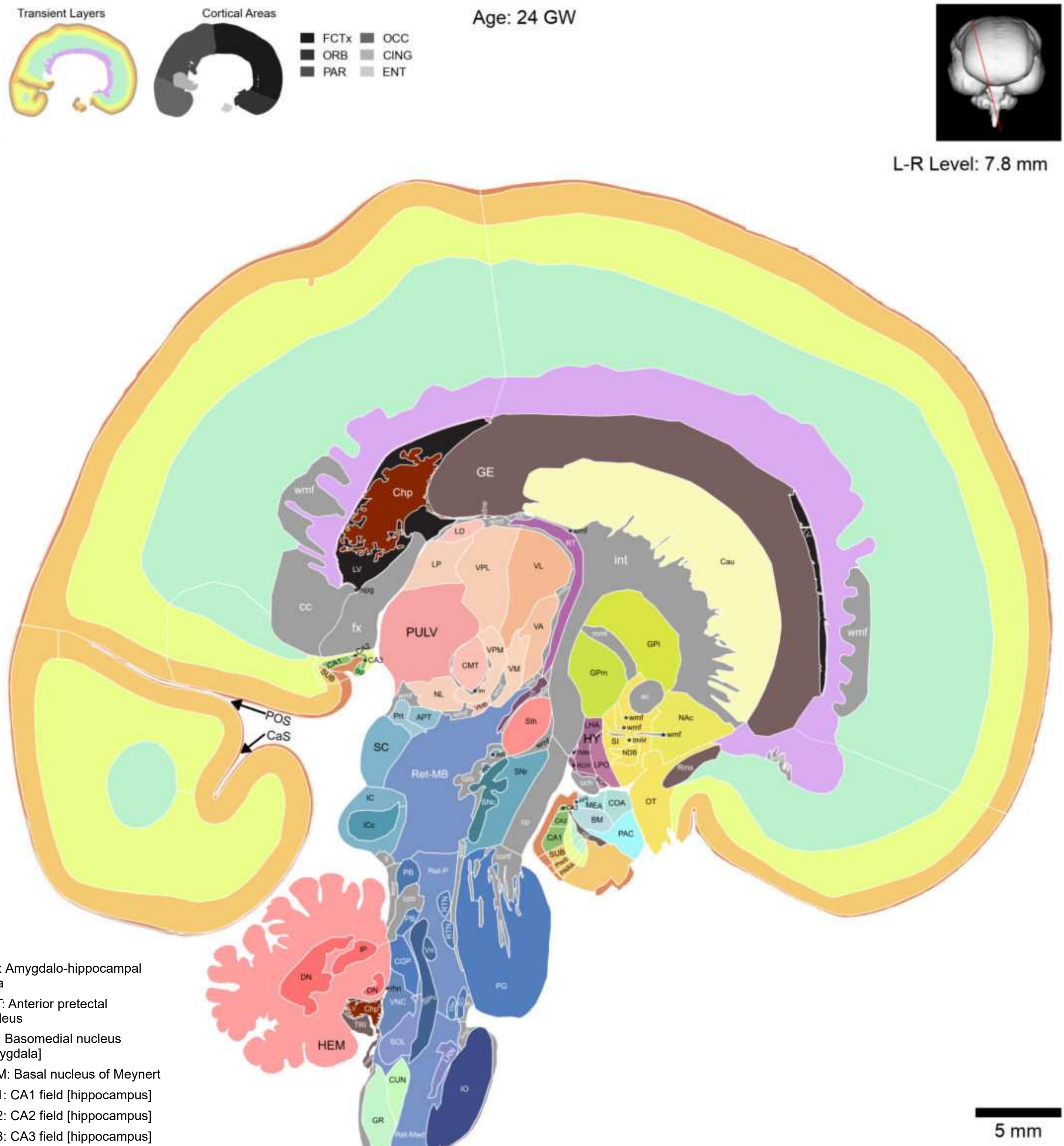

AHi: Amygdalo-hippocampal area
APT: Anterior pretectal nucleus
BM: Basomedial nucleus [amygdala]
BNM: Basal nucleus of Meynert
CA1: CA1 field [hippocampus]
CA2: CA2 field [hippocampus]
CA3: CA3 field [hippocampus]
CGP: Central gray of the pons
CMT: Centromedian nucleus [thalamus]
COA: Cortical nucleus [amygdala]
CUN: Cuneate nucleus
Cau: Caudate nucleus
Chp: Choroid plexus
DG: Dentate gyrus
DGSC: Dorsal gray of the spinal cord
DN: Dentate nucleus
GE: Ganglionic eminence
GPl: Globus pallidus lateral segment
GPm: Globus pallidus medial segment
GR: Gracile nucleus
HEM: Cerebellar hemispheres
HY: Hypothalamus
IC: Inferior colliculus
ICc: Inferior colliculus, central nucleus
IO: Inferior olive
IP: Interposed nucleus
LD: Lateral dorsal nucleus [thalamus]
LHA: Lateral hypothalamic area
LP: Lateral posterior nucleus [thalamus]
LPO: Lateral preoptic area
LRN: Lateral reticular nucleus
LV: Lateral ventricle
MEA: Medial nucleus [amygdala]
NAc: Nucleus accumbens
NDB: Nucleus of the diagonal band
NL: Nucleus limitans [thalamus]
OT: Olfactory tubercle
PARA: Cortical plate, parasubiculum
PB: Parabrachial nucleus
PG: Pontine gray
PULV: Pulvinar nucleus [thalamus]
PreS: Cortical plate, presubiculum
Prt: Pretectum
RCH: Retrochiasmatic nucleus [hypothalamus]
RR: Retrorubral area
RT: Reticular nucleus [thalamus]
RTN: Reticular tegmental nucleus
Ret-MB: Reticular formation, Midbrain
Ret-Med: Reticular formation, Medulla
Ret-P: Reticular formation, Pons
Rms: Rostral migratory stream
SC: Superior colliculus
SCH: Suprachiasmatic nucleus [hypothalamus]
SI: Substantia innominata
SNc: Substantia nigra pars compacta
SNr: Substantia nigra pars reticulata
SO: Superior olive
SOL: Solitary nucleus
SP: Spinal cord
SPV: Spinal nucleus of the trigeminal
SUB: Cortical plate, subiculum
Sth: Subthalamus
TMM: Tuberomammillary nucleus
TRI: Germinal trigone
VA: Ventral anterior nucleus [thalamus]
VG: Ventral gray of the spinal cord
VIIn: Facial motor nucleus
VL: Ventral lateral nucleus [thalamus]
VM: Ventral medial nucleus [thalamus]
VMB: Ventral medial basal nucleus [thalamus]
VNC: Vestibular nuclear complex
VPL: Ventral posterolateral nucleus [thalamus]
VPM: Ventral posteromedial nucleus [thalamus]
Vn: Trigeminal motor nucleus
XIn: Accessory nucleus
ZI: Zona incerta
→ CaS: Calcarine sulcus
→ POS: Parieto-occipital sulcus

Age: 24 GW

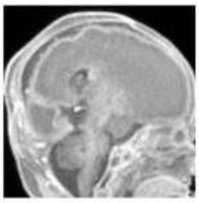

L-R Level: 7.56 mm

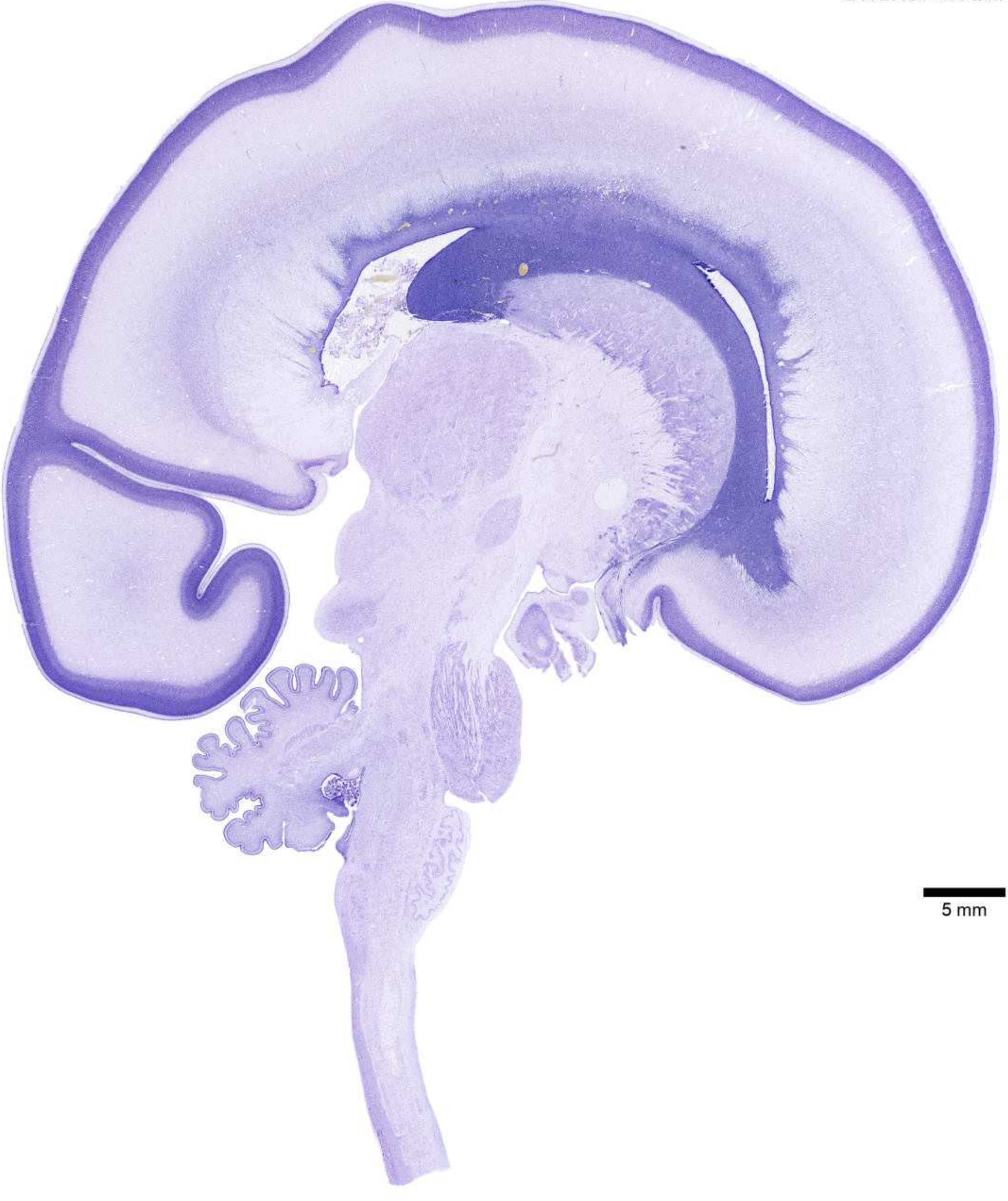

5 mm

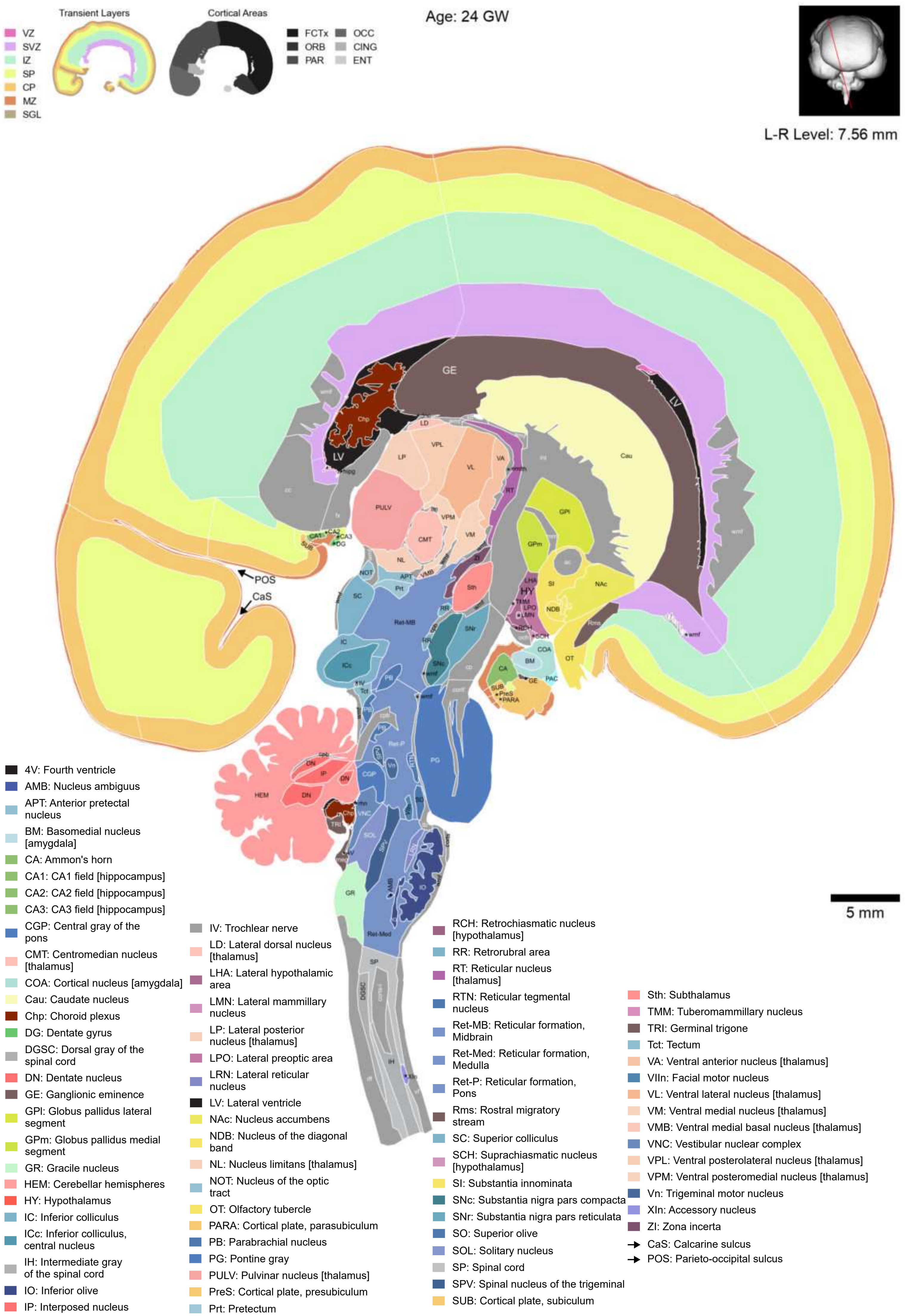

Transient Layers
VZ
SVZ
IZ
SP
CP
MZ
SGL
Cortical Areas
FCTx
ORB
PAR
OCC
CING
ENT
Age: 24 GW
L-R Level: 7.56 mm
5 mm
GE
LV
Chp
Cau
LP
VPL
VL
VA
RT
PULV
VPM
CMT
VM
NL
GPl
GPm
NAc
SI
HY
NDB
NOT
APT
Prt
SC
Ret-MB
Sth
SNr
SNc
RR
IC
ICc
CA
BM
COA
OT
PAC
SUB
PreS
PARA
PB
Tct
PG
HEM
DN
IP
CGP
TRI
VNC
SOL
IO
AMB
GR
Ret-Med
SP
DGSC
IH
POS
CaS
CA1
CA2
CA3
DG
4V: Fourth ventricle
AMB: Nucleus ambiguus
APT: Anterior pretectal nucleus
BM: Basomedial nucleus [amygdala]
CA: Ammon's horn
CA1: CA1 field [hippocampus]
CA2: CA2 field [hippocampus]
CA3: CA3 field [hippocampus]
CGP: Central gray of the pons
CMT: Centromedian nucleus [thalamus]
COA: Cortical nucleus [amygdala]
Cau: Caudate nucleus
Chp: Choroid plexus
DG: Dentate gyrus
DGSC: Dorsal gray of the spinal cord
DN: Dentate nucleus
GE: Ganglionic eminence
GPl: Globus pallidus lateral segment
GPm: Globus pallidus medial segment
GR: Gracile nucleus
HEM: Cerebellar hemispheres
HY: Hypothalamus
IC: Inferior colliculus
ICc: Inferior colliculus, central nucleus
IH: Intermediate gray of the spinal cord
IO: Inferior olive
IP: Interposed nucleus
IV: Trochlear nerve
LD: Lateral dorsal nucleus [thalamus]
LHA: Lateral hypothalamic area
LMN: Lateral mammillary nucleus
LP: Lateral posterior nucleus [thalamus]
LPO: Lateral preoptic area
LRN: Lateral reticular nucleus
LV: Lateral ventricle
NAc: Nucleus accumbens
NDB: Nucleus of the diagonal band
NL: Nucleus limitans [thalamus]
NOT: Nucleus of the optic tract
OT: Olfactory tubercle
PARA: Cortical plate, parasubiculum
PB: Parabrachial nucleus
PG: Pontine gray
PULV: Pulvinar nucleus [thalamus]
PreS: Cortical plate, presubiculum
Prt: Pretectum
RCH: Retrochiasmatic nucleus [hypothalamus]
RR: Retrorubral area
RT: Reticular nucleus [thalamus]
RTN: Reticular tegmental nucleus
Ret-MB: Reticular formation, Midbrain
Ret-Med: Reticular formation, Medulla
Ret-P: Reticular formation, Pons
Rms: Rostral migratory stream
SC: Superior colliculus
SCH: Suprachiasmatic nucleus [hypothalamus]
SI: Substantia innominata
SNc: Substantia nigra pars compacta
SNr: Substantia nigra pars reticulata
SO: Superior olive
SOL: Solitary nucleus
SP: Spinal cord
SPV: Spinal nucleus of the trigeminal
SUB: Cortical plate, subiculum
Sth: Subthalamus
TMM: Tuberomammillary nucleus
TRI: Germinal trigone
Tct: Tectum
VA: Ventral anterior nucleus [thalamus]
VIIn: Facial motor nucleus
VL: Ventral lateral nucleus [thalamus]
VM: Ventral medial nucleus [thalamus]
VMB: Ventral medial basal nucleus [thalamus]
VNC: Vestibular nuclear complex
VPL: Ventral posterolateral nucleus [thalamus]
VPM: Ventral posteromedial nucleus [thalamus]
Vn: Trigeminal motor nucleus
XIn: Accessory nucleus
ZI: Zona incerta
CaS: Calcarine sulcus
POS: Parieto-occipital sulcus

Age: 24 GW

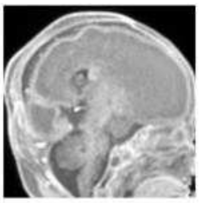

L-R Level: 7.26 mm

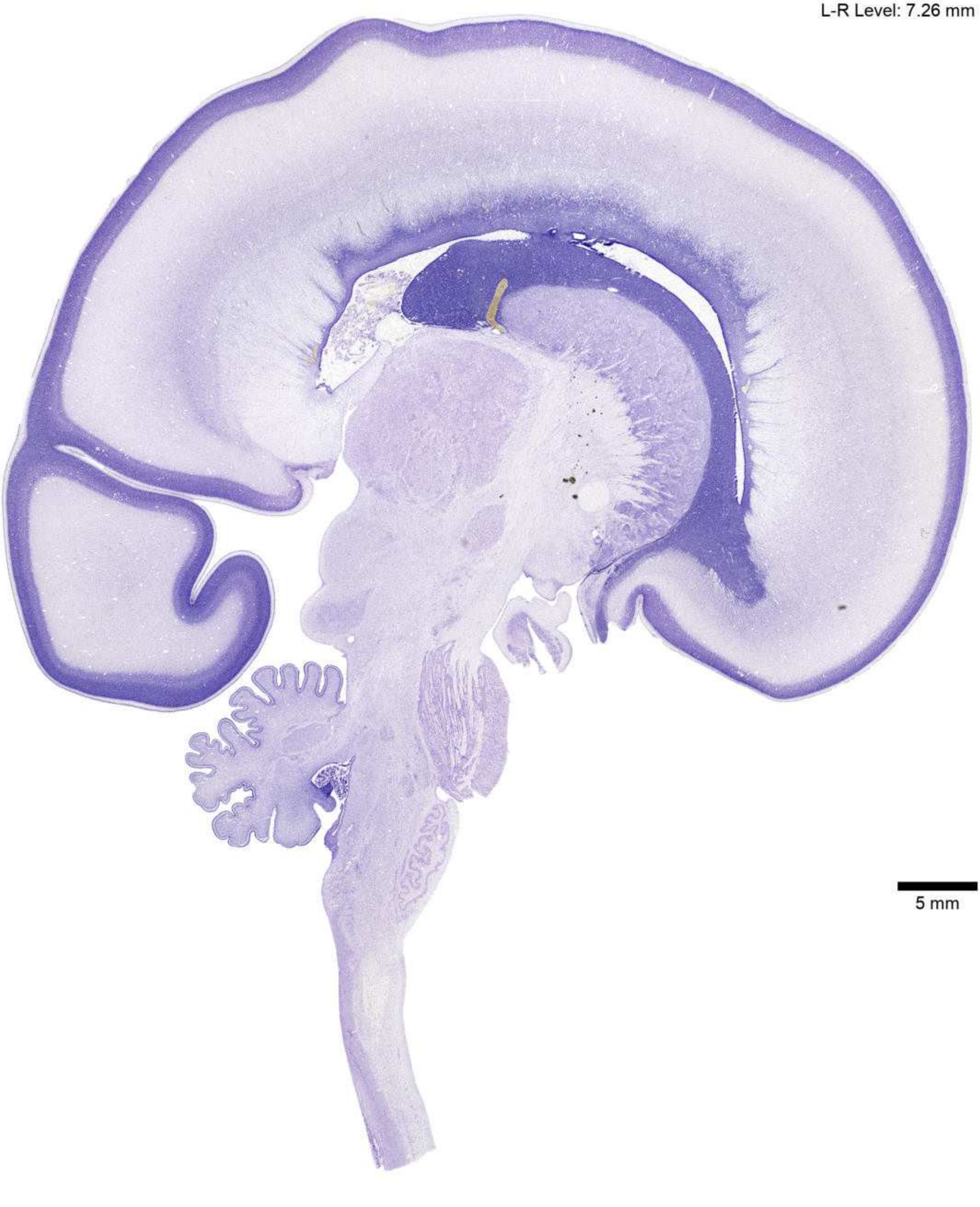

5 mm

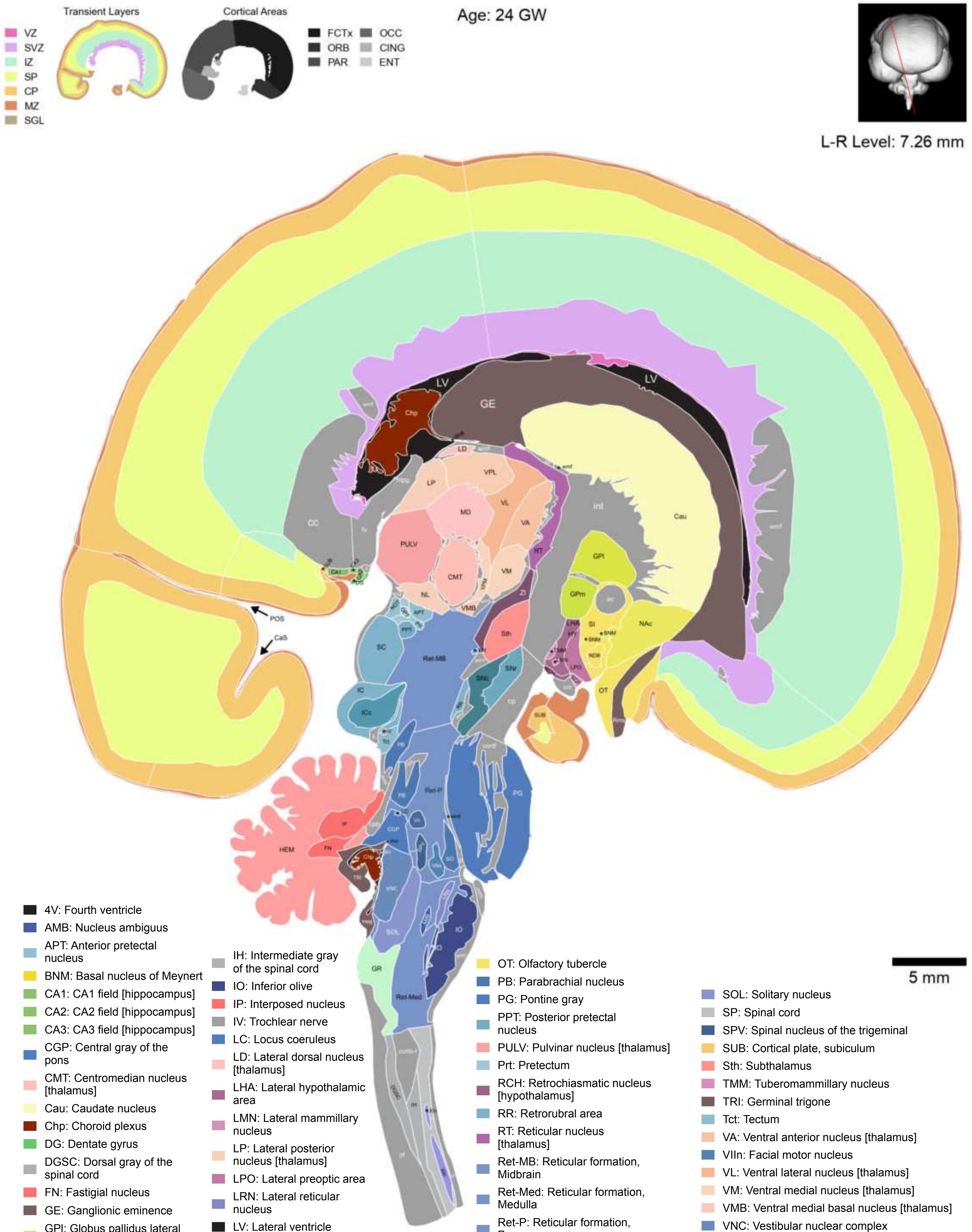

- 4V: Fourth ventricle
- AMB: Nucleus ambiguus
- APT: Anterior pretectal nucleus
- BNM: Basal nucleus of Meynert
- CA1: CA1 field [hippocampus]
- CA2: CA2 field [hippocampus]
- CA3: CA3 field [hippocampus]
- CGP: Central gray of the pons
- CMT: Centromedian nucleus [thalamus]
- Cau: Caudate nucleus
- Chp: Choroid plexus
- DG: Dentate gyrus
- DGSC: Dorsal gray of the spinal cord
- FN: Fastigial nucleus
- GE: Ganglionic eminence
- GPl: Globus pallidus lateral segment
- GPm: Globus pallidus medial segment
- GR: Gracile nucleus
- HEM: Cerebellar hemispheres
- HY: Hypothalamus
- IC: Inferior colliculus
- ICc: Inferior colliculus, central nucleus
- IH: Intermediate gray of the spinal cord
- IO: Inferior olive
- IP: Interposed nucleus
- IV: Trochlear nerve
- LC: Locus coeruleus
- LD: Lateral dorsal nucleus [thalamus]
- LHA: Lateral hypothalamic area
- LMN: Lateral mammillary nucleus
- LP: Lateral posterior nucleus [thalamus]
- LPO: Lateral preoptic area
- LRN: Lateral reticular nucleus
- LV: Lateral ventricle
- MD: Medial dorsal nucleus [thalamus]
- NAc: Nucleus accumbens
- NDB: Nucleus of the diagonal band
- NL: Nucleus limitans [thalamus]
- NOT: Nucleus of the optic tract
- OPT: Olivary pretectal nucleus
- OT: Olfactory tubercle
- PB: Parabrachial nucleus
- PG: Pontine gray
- PPT: Posterior pretectal nucleus
- PULV: Pulvinar nucleus [thalamus]
- Prt: Pretectum
- RCH: Retrochiasmatic nucleus [hypothalamus]
- RR: Retrorubral area
- RT: Reticular nucleus [thalamus]
- Ret-MB: Reticular formation, Midbrain
- Ret-Med: Reticular formation, Medulla
- Ret-P: Reticular formation, Pons
- Rms: Rostral migratory stream
- SC: Superior colliculus
- SCH: Suprachiasmatic nucleus [hypothalamus]
- SI: Substantia innominata
- SNc: Substantia nigra pars compacta
- SNr: Substantia nigra pars reticulata
- SO: Superior olive
- SOL: Solitary nucleus
- SP: Spinal cord
- SPV: Spinal nucleus of the trigeminal
- SUB: Cortical plate, subiculum
- Sth: Subthalamus
- TMM: Tuberomammillary nucleus
- TRI: Germinal trigone
- Tct: Tectum
- VA: Ventral anterior nucleus [thalamus]
- VIIn: Facial motor nucleus
- VL: Ventral lateral nucleus [thalamus]
- VM: Ventral medial nucleus [thalamus]
- VMB: Ventral medial basal nucleus [thalamus]
- VNC: Vestibular nuclear complex
- VPL: Ventral posterolateral nucleus [thalamus]
- VPM: Ventral posteromedial nucleus [thalamus]
- Vn: Trigeminal motor nucleus
- XIn: Accessory nucleus
- ZI: Zona incerta
- → CaS: Calcarine sulcus
- → POS: Parieto-occipital sulcus

Age: 24 GW

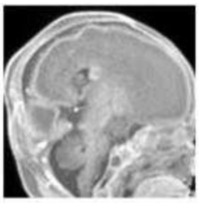

L-R Level: 7.02 mm

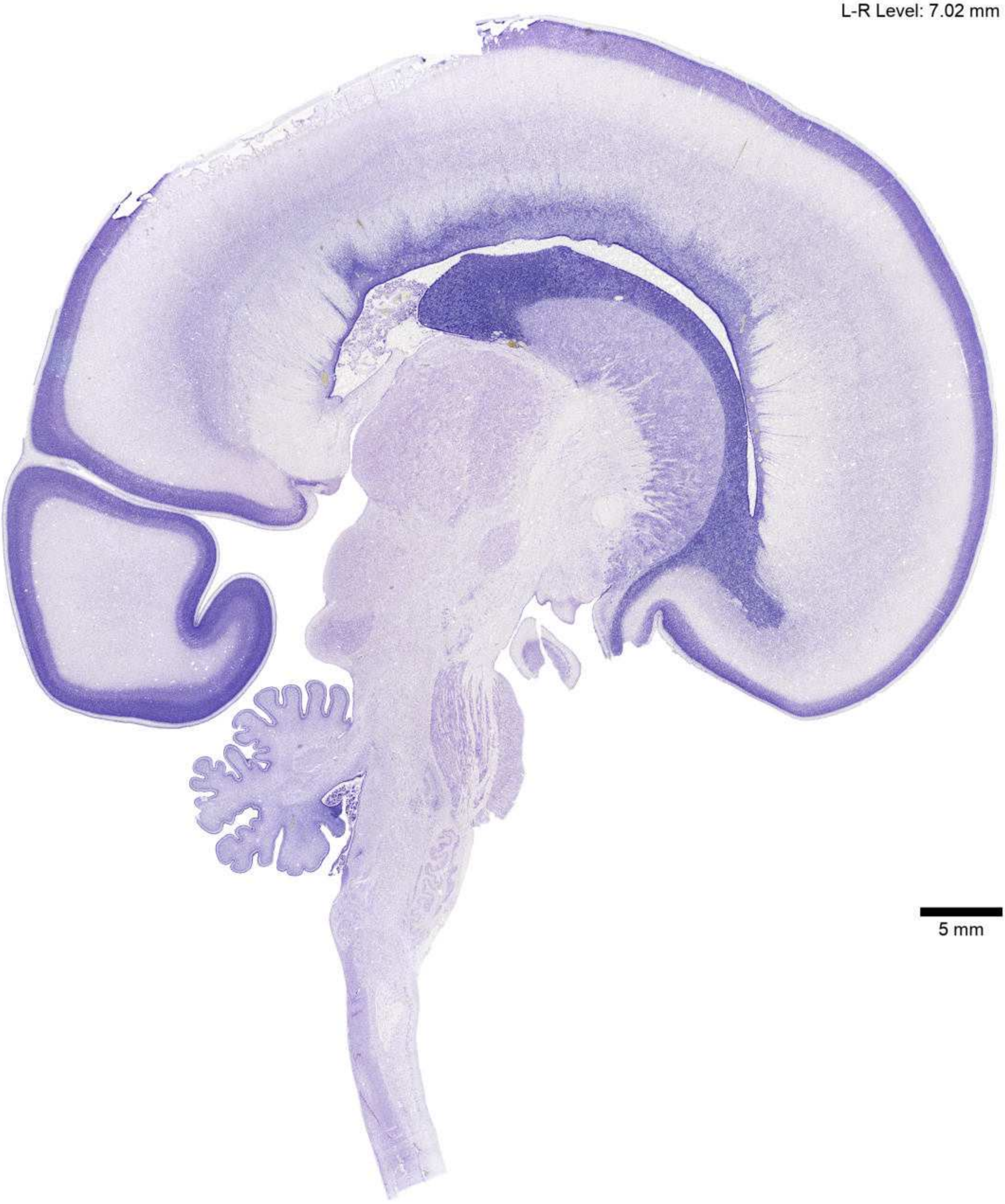

5 mm

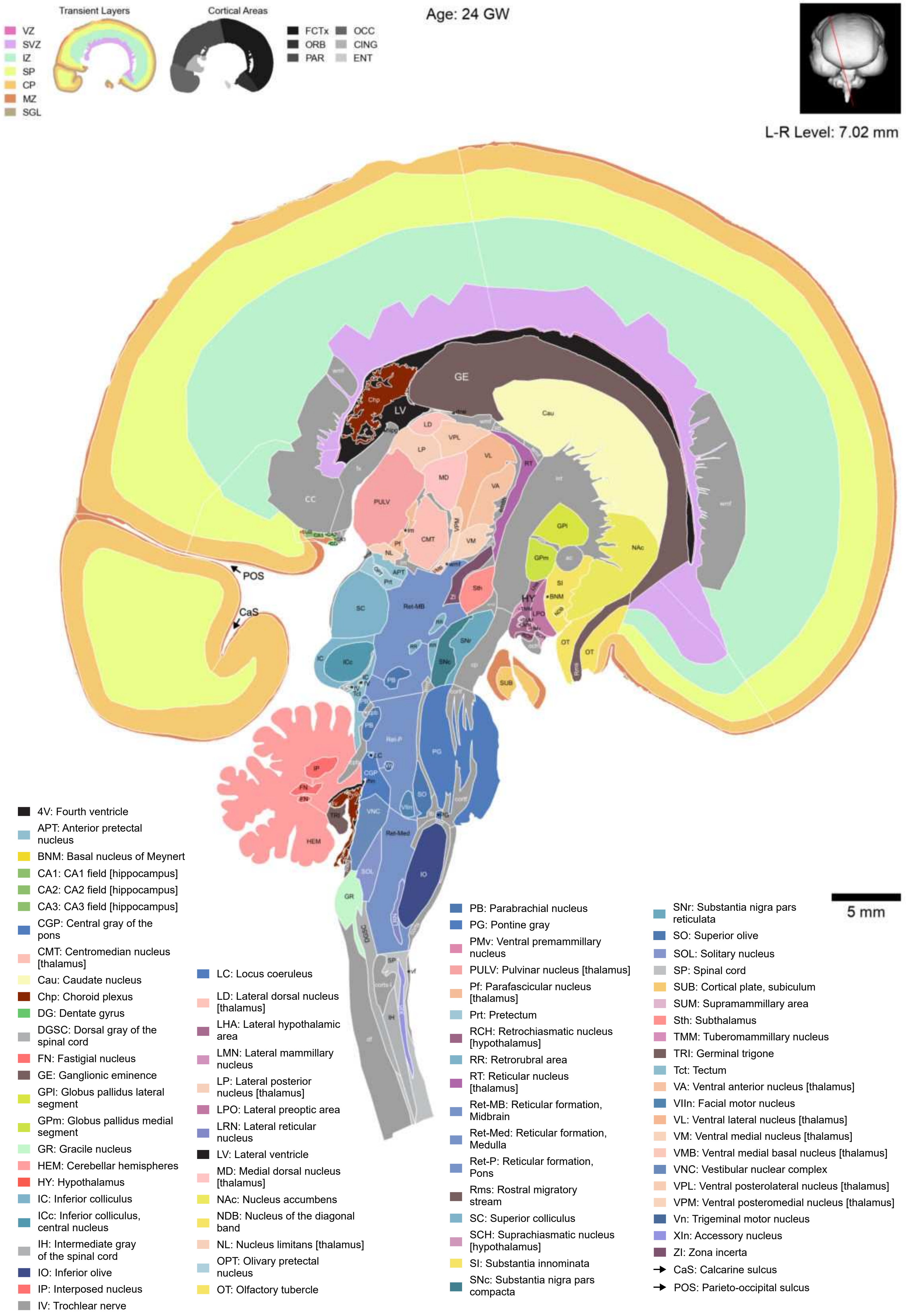
Transient Layers
VZ
SVZ
IZ
SP
CP
MZ
SGL
Cortical Areas
FCTx
ORB
PAR
OCC
CING
ENT
Age: 24 GW
L-R Level: 7.02 mm
GE
LV
Chp
Cau
LD
VPL
LP
VL
MD
VA
RT
PULV
CC
CMT
VM
VPM
GPl
GPm
NAc
POS
CaS
SC
Ret-MB
Sth
HY
SI
BNM
LPO
OT
SNr
SNc
IC
ICc
PB
SUB
Ret-P
PG
IP
FN
SO
HEM
VNC
Ret-Med
IO
SOL
GR
5 mm
4V: Fourth ventricle
APT: Anterior pretectal nucleus
BNM: Basal nucleus of Meynert
CA1: CA1 field [hippocampus]
CA2: CA2 field [hippocampus]
CA3: CA3 field [hippocampus]
CGP: Central gray of the pons
CMT: Centromedian nucleus [thalamus]
Cau: Caudate nucleus
Chp: Choroid plexus
DG: Dentate gyrus
DGSC: Dorsal gray of the spinal cord
FN: Fastigial nucleus
GE: Ganglionic eminence
GPl: Globus pallidus lateral segment
GPm: Globus pallidus medial segment
GR: Gracile nucleus
HEM: Cerebellar hemispheres
HY: Hypothalamus
IC: Inferior colliculus
ICc: Inferior colliculus, central nucleus
IH: Intermediate gray of the spinal cord
IO: Inferior olive
IP: Interposed nucleus
IV: Trochlear nerve
LC: Locus coeruleus
LD: Lateral dorsal nucleus [thalamus]
LHA: Lateral hypothalamic area
LMN: Lateral mammillary nucleus
LP: Lateral posterior nucleus [thalamus]
LPO: Lateral preoptic area
LRN: Lateral reticular nucleus
LV: Lateral ventricle
MD: Medial dorsal nucleus [thalamus]
NAc: Nucleus accumbens
NDB: Nucleus of the diagonal band
NL: Nucleus limitans [thalamus]
OPT: Olivary pretectal nucleus
OT: Olfactory tubercle
PB: Parabrachial nucleus
PG: Pontine gray
PMv: Ventral premammillary nucleus
PULV: Pulvinar nucleus [thalamus]
Pf: Parafascicular nucleus [thalamus]
Prt: Pretectum
RCH: Retrochiasmatic nucleus [hypothalamus]
RR: Retrorubral area
RT: Reticular nucleus [thalamus]
Ret-MB: Reticular formation, Midbrain
Ret-Med: Reticular formation, Medulla
Ret-P: Reticular formation, Pons
Rms: Rostral migratory stream
SC: Superior colliculus
SCH: Suprachiasmatic nucleus [hypothalamus]
SI: Substantia innominata
SNc: Substantia nigra pars compacta
SNr: Substantia nigra pars reticulata
SO: Superior olive
SOL: Solitary nucleus
SP: Spinal cord
SUB: Cortical plate, subiculum
SUM: Supramammillary area
Sth: Subthalamus
TMM: Tuberomammillary nucleus
TRI: Germinal trigone
Tct: Tectum
VA: Ventral anterior nucleus [thalamus]
VIIn: Facial motor nucleus
VL: Ventral lateral nucleus [thalamus]
VM: Ventral medial nucleus [thalamus]
VMB: Ventral medial basal nucleus [thalamus]
VNC: Vestibular nuclear complex
VPL: Ventral posterolateral nucleus [thalamus]
VPM: Ventral posteromedial nucleus [thalamus]
Vn: Trigeminal motor nucleus
XIn: Accessory nucleus
ZI: Zona incerta
CaS: Calcarine sulcus
POS: Parieto-occipital sulcus

Age: 24 GW

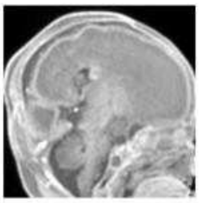

L-R Level: 6.84 mm

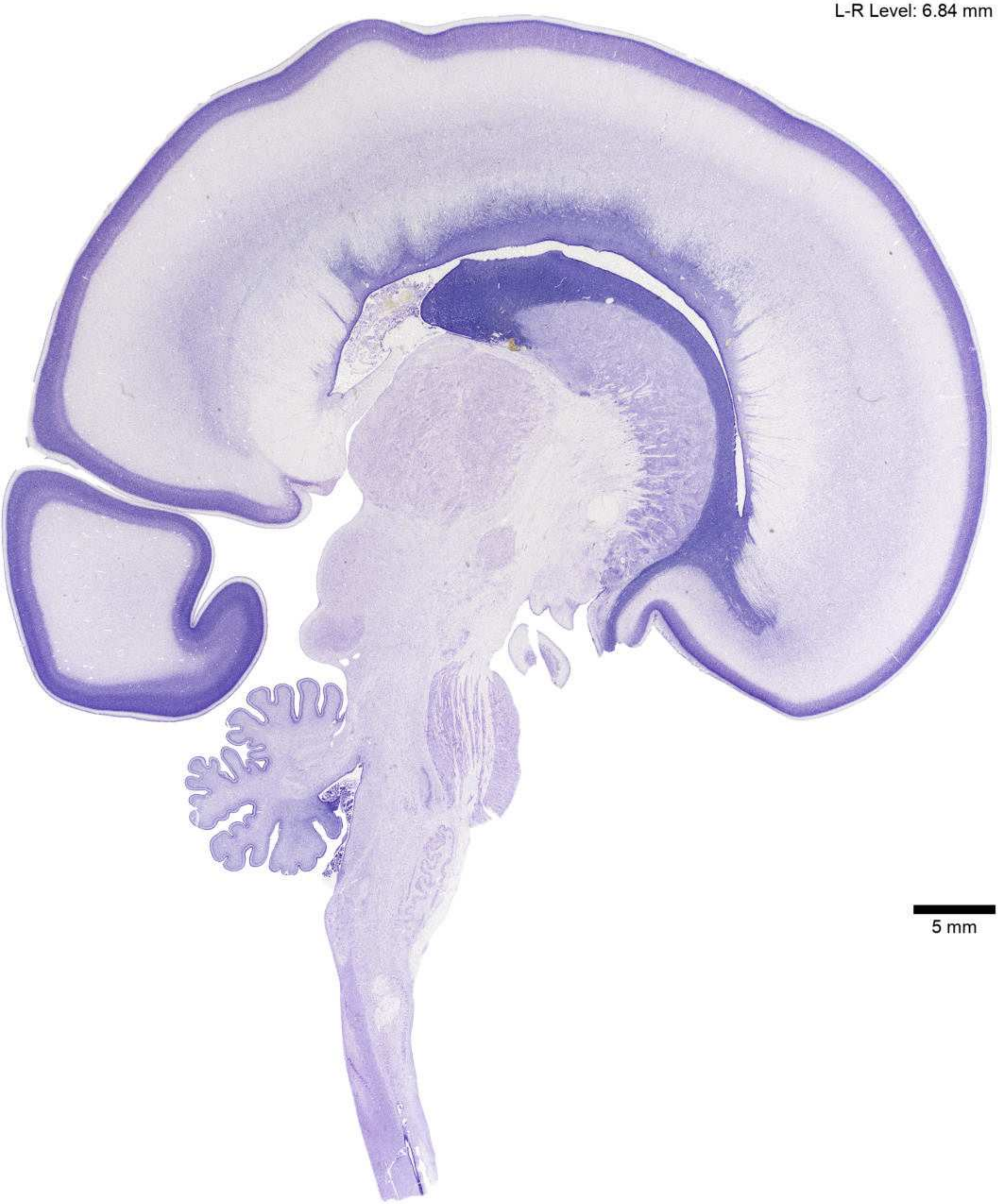

5 mm

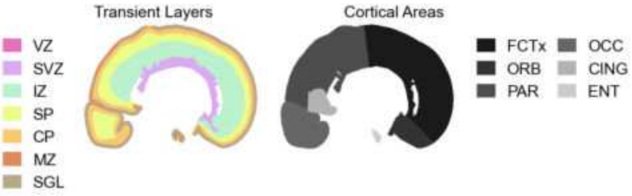

Age: 24 GW

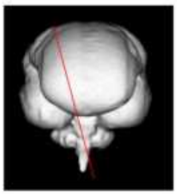

L-R Level: 6.84 mm

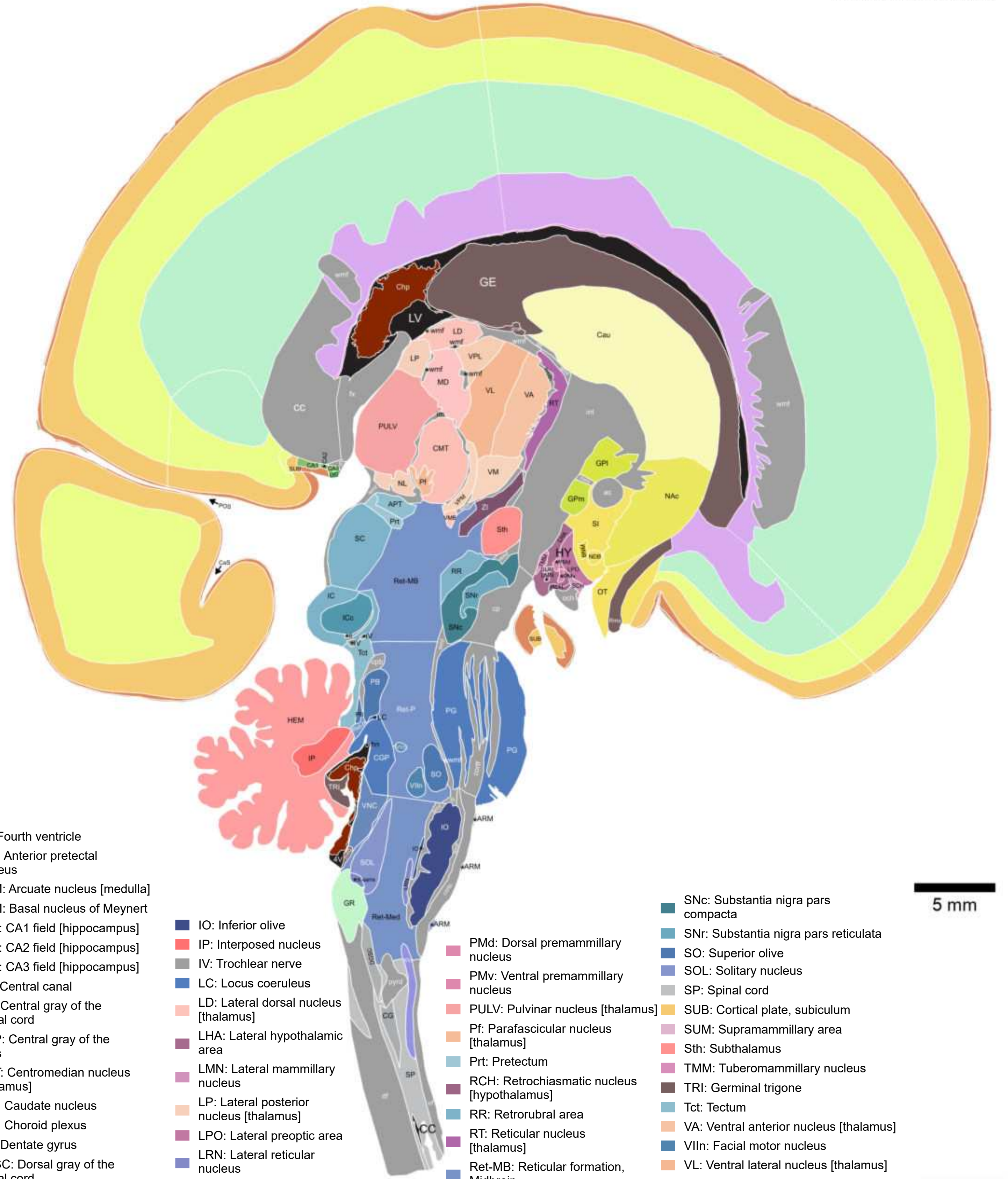

- 4V: Fourth ventricle
- APT: Anterior pretectal nucleus
- ARM: Arcuate nucleus [medulla]
- BNM: Basal nucleus of Meynert
- CA1: CA1 field [hippocampus]
- CA2: CA2 field [hippocampus]
- CA3: CA3 field [hippocampus]
- CC: Central canal
- CG: Central gray of the spinal cord
- CGP: Central gray of the pons
- CMT: Centromedian nucleus [thalamus]
- Cau: Caudate nucleus
- Chp: Choroid plexus
- DG: Dentate gyrus
- DGSC: Dorsal gray of the spinal cord
- GE: Ganglionic eminence
- GPl: Globus pallidus lateral segment
- GPm: Globus pallidus medial segment
- GR: Gracile nucleus
- HEM: Cerebellar hemispheres
- HY: Hypothalamus
- IC: Inferior colliculus
- ICc: Inferior colliculus, central nucleus
- IO: Inferior olive
- IP: Interposed nucleus
- IV: Trochlear nerve
- LC: Locus coeruleus
- LD: Lateral dorsal nucleus [thalamus]
- LHA: Lateral hypothalamic area
- LMN: Lateral mammillary nucleus
- LP: Lateral posterior nucleus [thalamus]
- LPO: Lateral preoptic area
- LRN: Lateral reticular nucleus
- LV: Lateral ventricle
- MD: Medial dorsal nucleus [thalamus]
- NAc: Nucleus accumbens
- NDB: Nucleus of the diagonal band
- NL: Nucleus limitans [thalamus]
- OT: Olfactory tubercle
- PB: Parabrachial nucleus
- PG: Pontine gray
- PMd: Dorsal premammillary nucleus
- PMv: Ventral premammillary nucleus
- PULV: Pulvinar nucleus [thalamus]
- Pf: Parafascicular nucleus [thalamus]
- Prt: Pretectum
- RCH: Retrochiasmatic nucleus [hypothalamus]
- RR: Retrorubral area
- RT: Reticular nucleus [thalamus]
- Ret-MB: Reticular formation, Midbrain
- Ret-Med: Reticular formation, Medulla
- Ret-P: Reticular formation, Pons
- Rms: Rostral migratory stream
- SC: Superior colliculus
- SCH: Suprachiasmatic nucleus [hypothalamus]
- SI: Substantia innominata
- SNc: Substantia nigra pars compacta
- SNr: Substantia nigra pars reticulata
- SO: Superior olive
- SOL: Solitary nucleus
- SP: Spinal cord
- SUB: Cortical plate, subiculum
- SUM: Supramammillary area
- Sth: Subthalamus
- TMM: Tuberomammillary nucleus
- TRI: Germinal trigone
- Tct: Tectum
- VA: Ventral anterior nucleus [thalamus]
- VIIn: Facial motor nucleus
- VL: Ventral lateral nucleus [thalamus]
- VM: Ventral medial nucleus [thalamus]
- VMB: Ventral medial basal nucleus [thalamus]
- VNC: Vestibular nuclear complex
- VPL: Ventral posterolateral nucleus [thalamus]
- VPM: Ventral posteromedial nucleus [thalamus]
- X-sens: Dorsal sensory nucleus X
- XIn: Accessory nucleus
- ZI: Zona incerta
- → CaS: Calcarine sulcus
- → POS: Parieto-occipital sulcus

Age: 24 GW

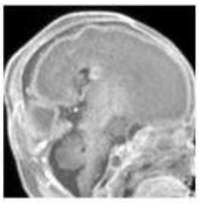

L-R Level: 6.66 mm

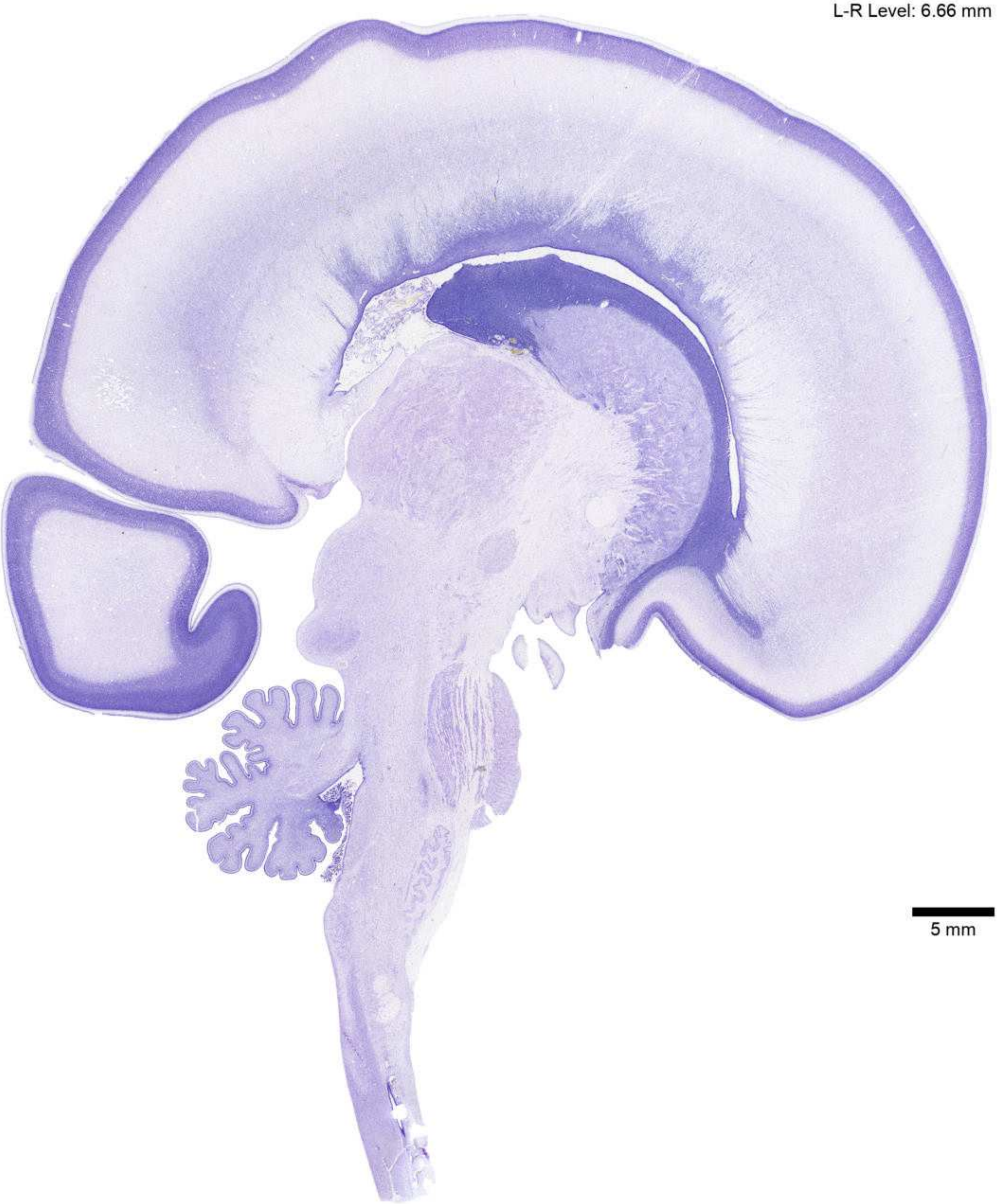

5 mm

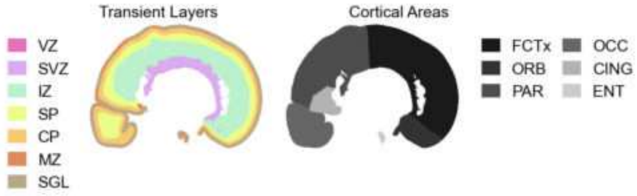

Age: 24 GW

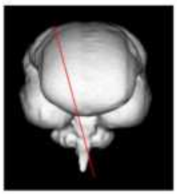

L-R Level: 6.66 mm

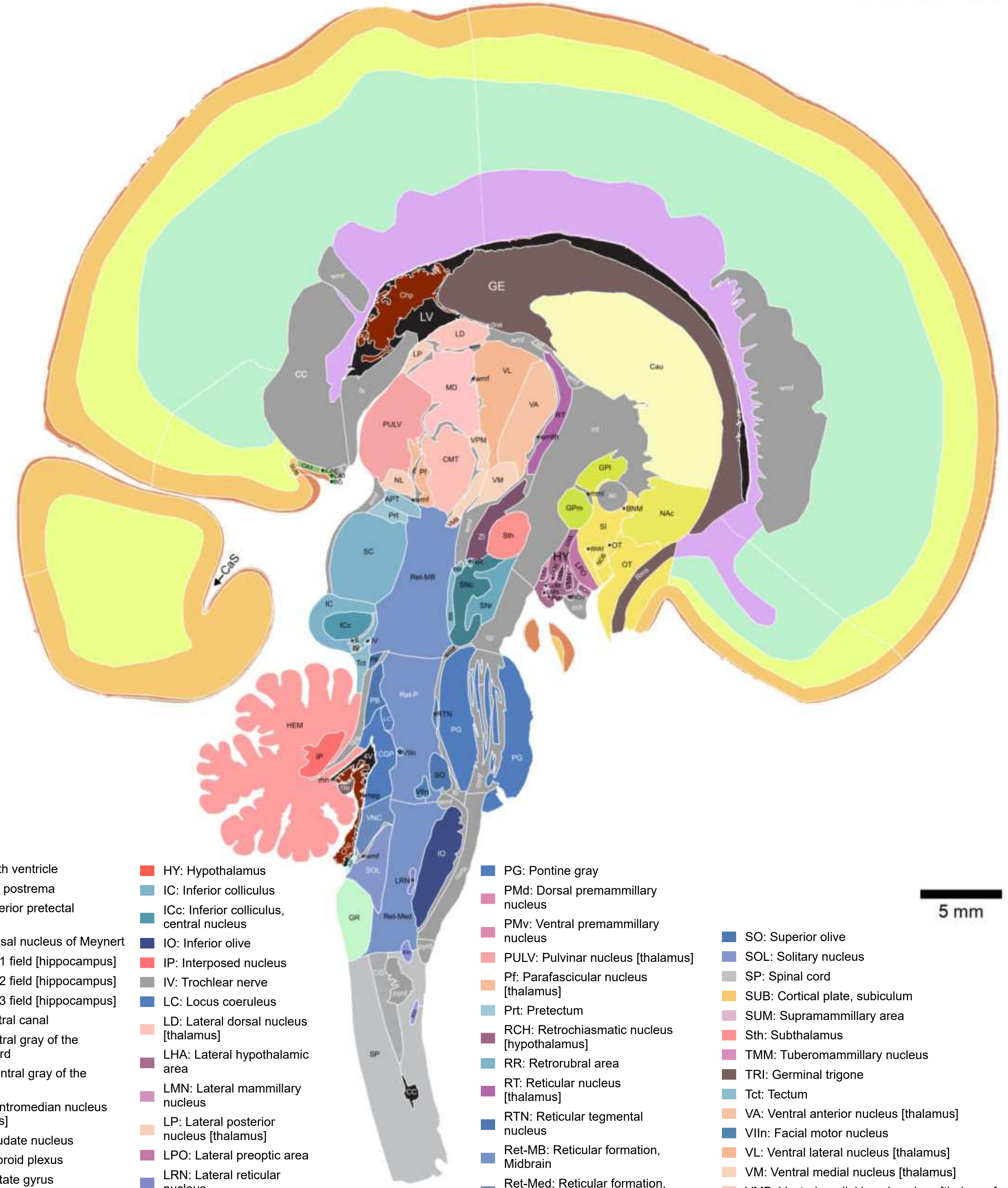

5 mm

- 4V: Fourth ventricle
- AP: Area postrema
- APT: Anterior pretectal nucleus
- BNM: Basal nucleus of Meynert
- CA1: CA1 field [hippocampus]
- CA2: CA2 field [hippocampus]
- CA3: CA3 field [hippocampus]
- CC: Central canal
- CG: Central gray of the spinal cord
- CGP: Central gray of the pons
- CMT: Centromedian nucleus [thalamus]
- Cau: Caudate nucleus
- Chp: Choroid plexus
- DG: Dentate gyrus
- DMH: Dorsomedial nucleus [hypothalamus]
- GE: Ganglionic eminence
- GPl: Globus pallidus lateral segment
- GPm: Globus pallidus medial segment
- GR: Gracile nucleus
- HEM: Cerebellar hemispheres
- HY: Hypothalamus
- IC: Inferior colliculus
- ICc: Inferior colliculus, central nucleus
- IO: Inferior olive
- IP: Interposed nucleus
- IV: Trochlear nerve
- LC: Locus coeruleus
- LD: Lateral dorsal nucleus [thalamus]
- LHA: Lateral hypothalamic area
- LMN: Lateral mammillary nucleus
- LP: Lateral posterior nucleus [thalamus]
- LPO: Lateral preoptic area
- LRN: Lateral reticular nucleus
- LV: Lateral ventricle
- MD: Medial dorsal nucleus [thalamus]
- NAc: Nucleus accumbens
- NDB: Nucleus of the diagonal band
- NL: Nucleus limitans [thalamus]
- OT: Olfactory tubercle
- PB: Parabrachial nucleus
- PG: Pontine gray
- PMd: Dorsal premammillary nucleus
- PMv: Ventral premammillary nucleus
- PULV: Pulvinar nucleus [thalamus]
- Pf: Parafascicular nucleus [thalamus]
- Prt: Pretectum
- RCH: Retrochiasmatic nucleus [hypothalamus]
- RR: Retrorubral area
- RT: Reticular nucleus [thalamus]
- RTN: Reticular tegmental nucleus
- Ret-MB: Reticular formation, Midbrain
- Ret-Med: Reticular formation, Medulla
- Ret-P: Reticular formation, Pons
- Rms: Rostral migratory stream
- SC: Superior colliculus
- SCH: Suprachiasmatic nucleus [hypothalamus]
- SI: Substantia innominata
- SNc: Substantia nigra pars compacta
- SNr: Substantia nigra pars reticulata
- SO: Superior olive
- SOL: Solitary nucleus
- SP: Spinal cord
- SUB: Cortical plate, subiculum
- SUM: Supramammillary area
- Sth: Subthalamus
- TMM: Tuberomammillary nucleus
- TRI: Germinal trigone
- Tct: Tectum
- VA: Ventral anterior nucleus [thalamus]
- VIIn: Facial motor nucleus
- VL: Ventral lateral nucleus [thalamus]
- VM: Ventral medial nucleus [thalamus]
- VMB: Ventral medial basal nucleus [thalamus]
- VMH: Ventromedial nucleus [hypothalamus]
- VNC: Vestibular nuclear complex
- VPM: Ventral posteromedial nucleus [thalamus]
- XIn: Accessory nucleus
- ZI: Zona incerta
- → CaS: Calcarine sulcus

Age: 24 GW

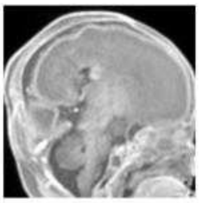

L-R Level: 6.24 mm

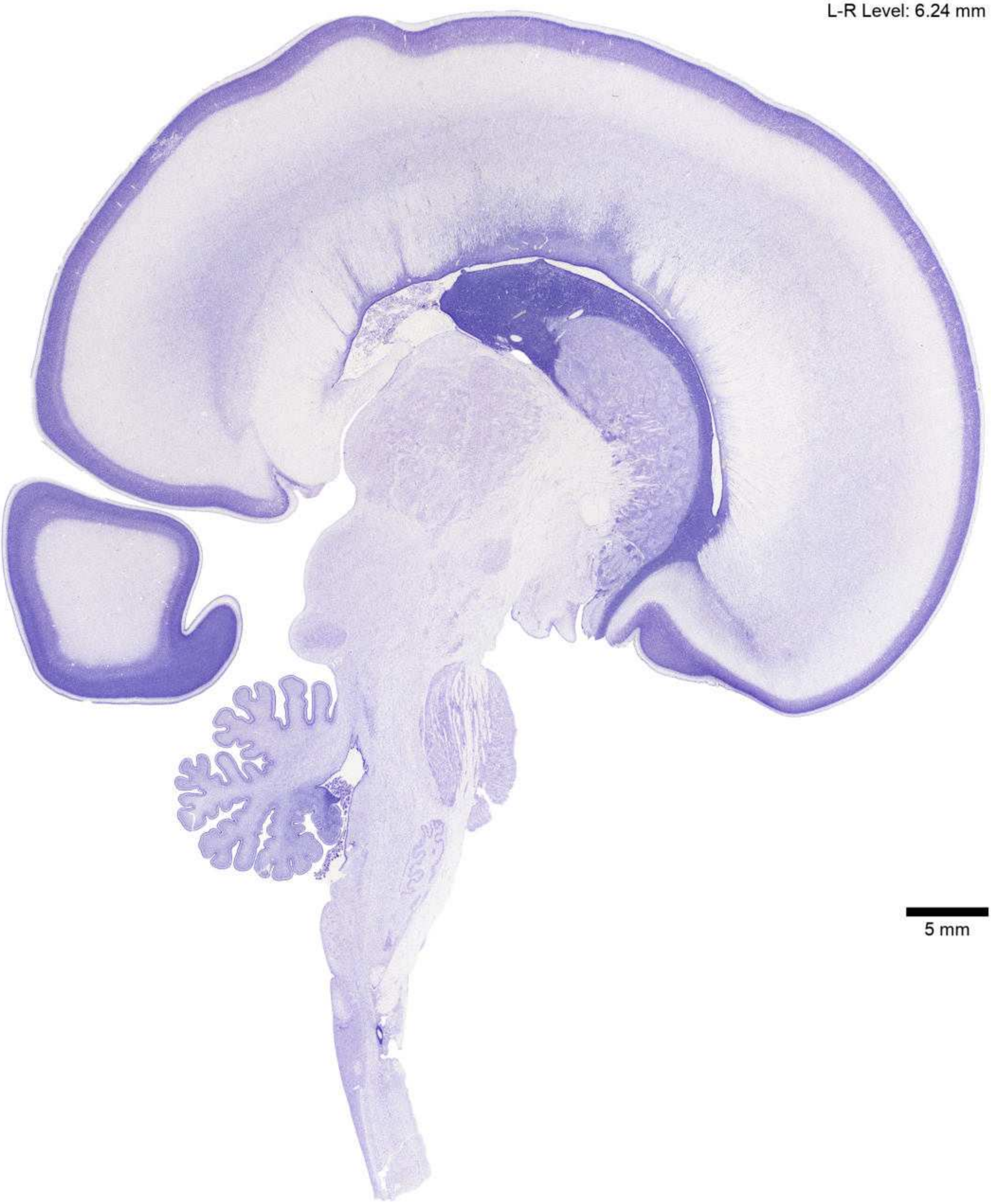

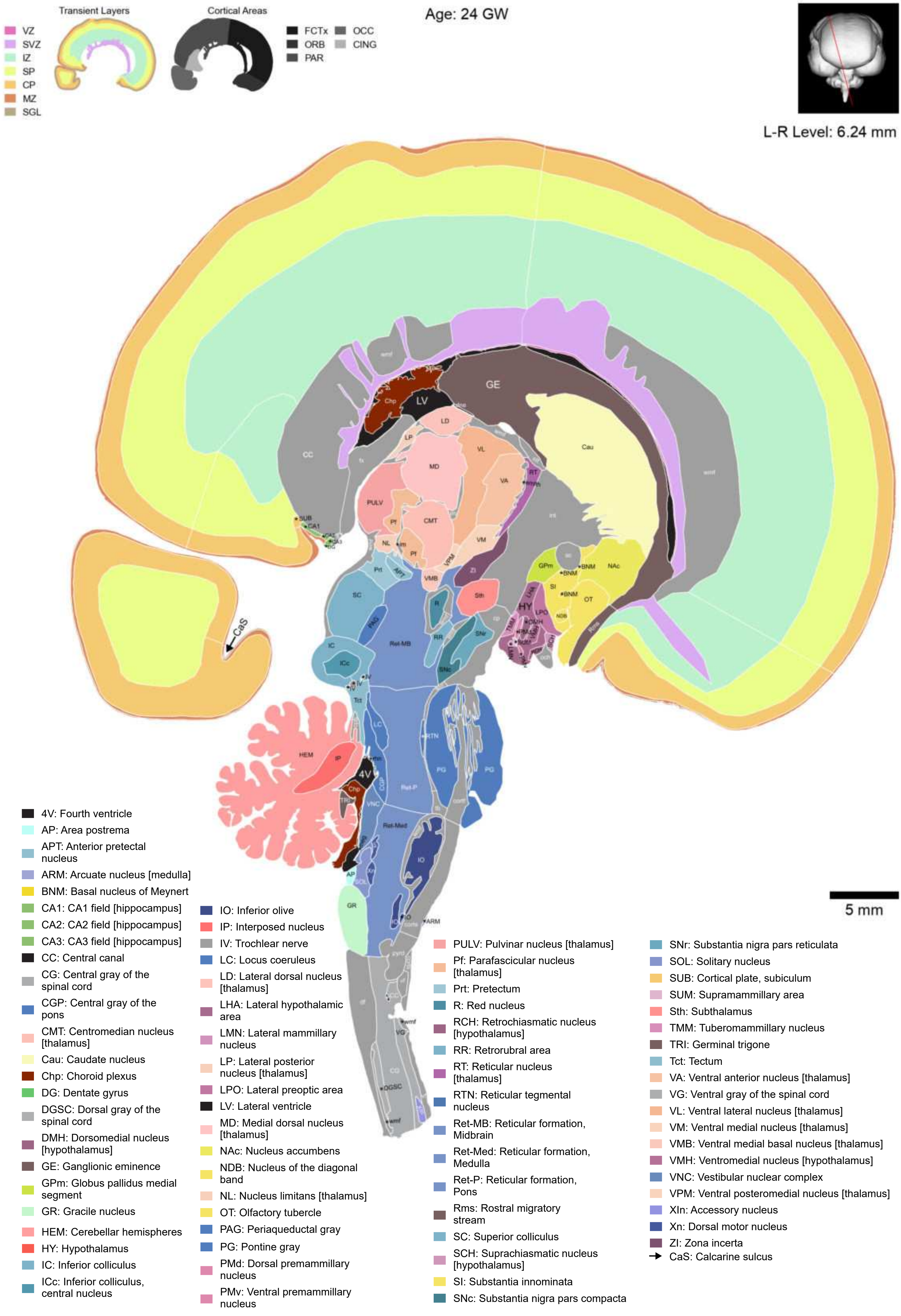
Transient Layers
VZ
SVZ
IZ
SP
CP
MZ
SGL
Cortical Areas
FCTx
ORB
PAR
OCC
CING
Age: 24 GW
L-R Level: 6.24 mm
5 mm
LV
GE
Cau
HY
4V
CaS
4V: Fourth ventricle
AP: Area postrema
APT: Anterior pretectal nucleus
ARM: Arcuate nucleus [medulla]
BNM: Basal nucleus of Meynert
CA1: CA1 field [hippocampus]
CA2: CA2 field [hippocampus]
CA3: CA3 field [hippocampus]
CC: Central canal
CG: Central gray of the spinal cord
CGP: Central gray of the pons
CMT: Centromedian nucleus [thalamus]
Cau: Caudate nucleus
Chp: Choroid plexus
DG: Dentate gyrus
DGSC: Dorsal gray of the spinal cord
DMH: Dorsomedial nucleus [hypothalamus]
GE: Ganglionic eminence
GPm: Globus pallidus medial segment
GR: Gracile nucleus
HEM: Cerebellar hemispheres
HY: Hypothalamus
IC: Inferior colliculus
ICc: Inferior colliculus, central nucleus
IO: Inferior olive
IP: Interposed nucleus
IV: Trochlear nerve
LC: Locus coeruleus
LD: Lateral dorsal nucleus [thalamus]
LHA: Lateral hypothalamic area
LMN: Lateral mammillary nucleus
LP: Lateral posterior nucleus [thalamus]
LPO: Lateral preoptic area
LV: Lateral ventricle
MD: Medial dorsal nucleus [thalamus]
NAc: Nucleus accumbens
NDB: Nucleus of the diagonal band
NL: Nucleus limitans [thalamus]
OT: Olfactory tubercle
PAG: Periaqueductal gray
PG: Pontine gray
PMd: Dorsal premammillary nucleus
PMv: Ventral premammillary nucleus
PULV: Pulvinar nucleus [thalamus]
Pf: Parafascicular nucleus [thalamus]
Prt: Pretectum
R: Red nucleus
RCH: Retrochiasmatic nucleus [hypothalamus]
RR: Retrorubral area
RT: Reticular nucleus [thalamus]
RTN: Reticular tegmental nucleus
Ret-MB: Reticular formation, Midbrain
Ret-Med: Reticular formation, Medulla
Ret-P: Reticular formation, Pons
Rms: Rostral migratory stream
SC: Superior colliculus
SCH: Suprachiasmatic nucleus [hypothalamus]
SI: Substantia innominata
SNc: Substantia nigra pars compacta
SNr: Substantia nigra pars reticulata
SOL: Solitary nucleus
SUB: Cortical plate, subiculum
SUM: Supramammillary area
Sth: Subthalamus
TMM: Tuberomammillary nucleus
TRI: Germinal trigone
Tct: Tectum
VA: Ventral anterior nucleus [thalamus]
VG: Ventral gray of the spinal cord
VL: Ventral lateral nucleus [thalamus]
VM: Ventral medial nucleus [thalamus]
VMB: Ventral medial basal nucleus [thalamus]
VMH: Ventromedial nucleus [hypothalamus]
VNC: Vestibular nuclear complex
VPM: Ventral posteromedial nucleus [thalamus]
XIn: Accessory nucleus
Xn: Dorsal motor nucleus
ZI: Zona incerta
CaS: Calcarine sulcus

Age: 24 GW

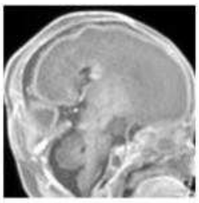

L-R Level: 6.0 mm

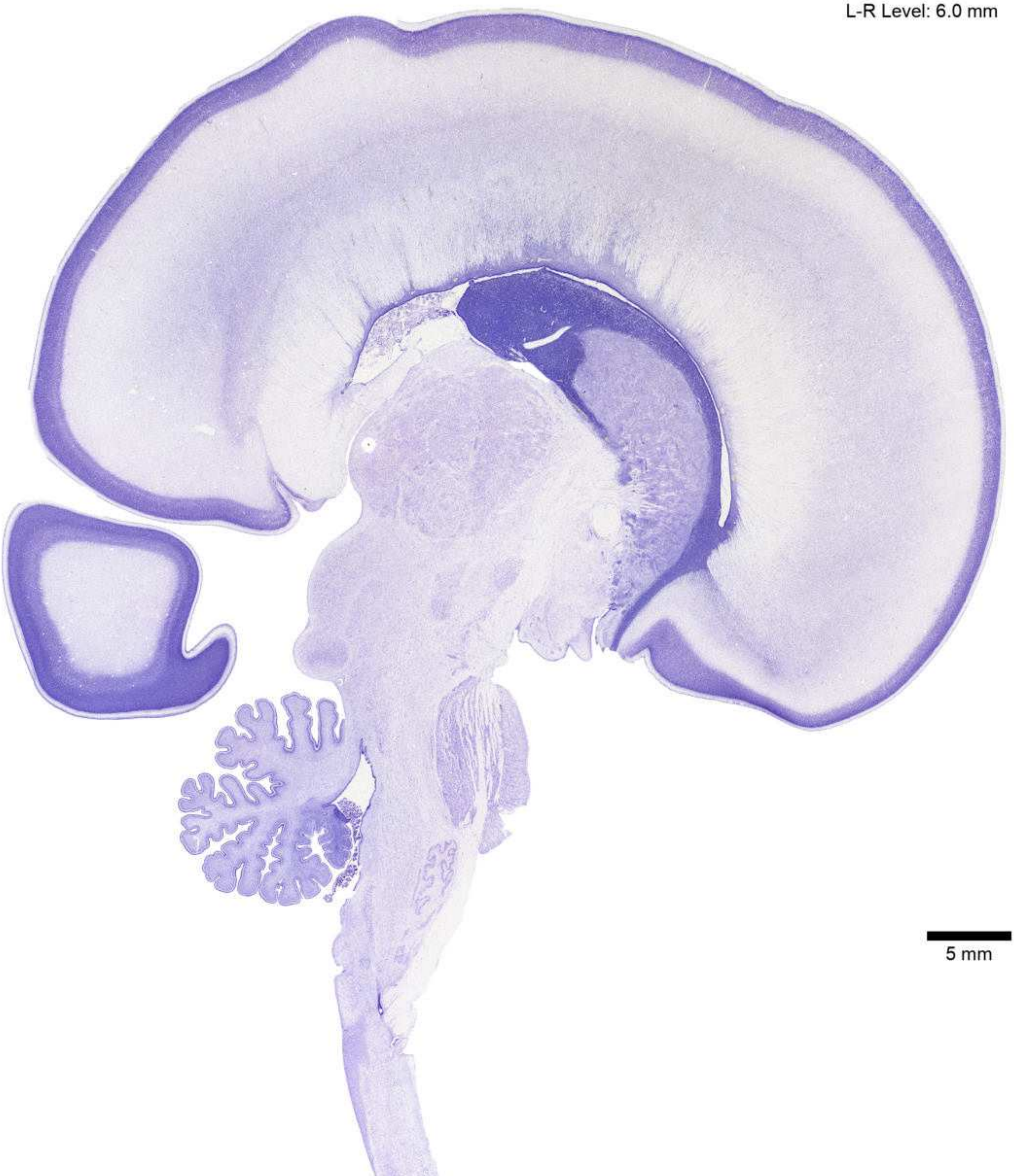

5 mm

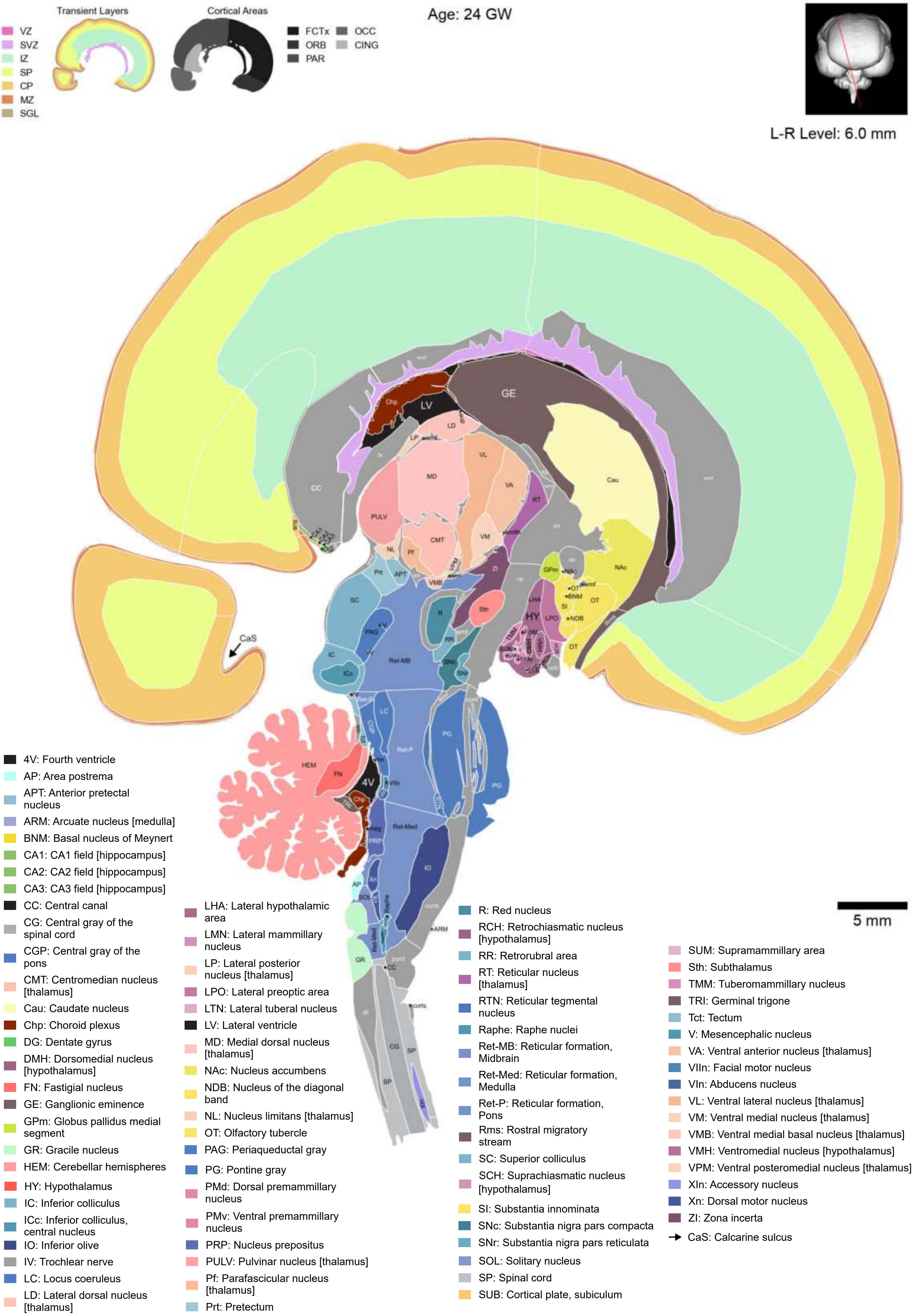
Transient Layers
VZ
SVZ
IZ
SP
CP
MZ
SGL
Cortical Areas
FCTx
ORB
PAR
OCC
CING
Age: 24 GW
L-R Level: 6.0 mm
LV
GE
Chp
CC
Cau
NAc
HY
CaS
HEM
FN
4V
5 mm
4V: Fourth ventricle
AP: Area postrema
APT: Anterior pretectal nucleus
ARM: Arcuate nucleus [medulla]
BNM: Basal nucleus of Meynert
CA1: CA1 field [hippocampus]
CA2: CA2 field [hippocampus]
CA3: CA3 field [hippocampus]
CC: Central canal
CG: Central gray of the spinal cord
CGP: Central gray of the pons
CMT: Centromedian nucleus [thalamus]
Cau: Caudate nucleus
Chp: Choroid plexus
DG: Dentate gyrus
DMH: Dorsomedial nucleus [hypothalamus]
FN: Fastigial nucleus
GE: Ganglionic eminence
GPm: Globus pallidus medial segment
GR: Gracile nucleus
HEM: Cerebellar hemispheres
HY: Hypothalamus
IC: Inferior colliculus
ICc: Inferior colliculus, central nucleus
IO: Inferior olive
IV: Trochlear nerve
LC: Locus coeruleus
LD: Lateral dorsal nucleus [thalamus]
LHA: Lateral hypothalamic area
LMN: Lateral mammillary nucleus
LP: Lateral posterior nucleus [thalamus]
LPO: Lateral preoptic area
LTN: Lateral tuberal nucleus
LV: Lateral ventricle
MD: Medial dorsal nucleus [thalamus]
NAc: Nucleus accumbens
NDB: Nucleus of the diagonal band
NL: Nucleus limitans [thalamus]
OT: Olfactory tubercle
PAG: Periaqueductal gray
PG: Pontine gray
PMd: Dorsal premammillary nucleus
PMv: Ventral premammillary nucleus
PRP: Nucleus prepositus
PULV: Pulvinar nucleus [thalamus]
Pf: Parafascicular nucleus [thalamus]
Prt: Pretectum
R: Red nucleus
RCH: Retrochiasmatic nucleus [hypothalamus]
RR: Retrorubral area
RT: Reticular nucleus [thalamus]
RTN: Reticular tegmental nucleus
Raphe: Raphe nuclei
Ret-MB: Reticular formation, Midbrain
Ret-Med: Reticular formation, Medulla
Ret-P: Reticular formation, Pons
Rms: Rostral migratory stream
SC: Superior colliculus
SCH: Suprachiasmatic nucleus [hypothalamus]
SI: Substantia innominata
SNc: Substantia nigra pars compacta
SNr: Substantia nigra pars reticulata
SOL: Solitary nucleus
SP: Spinal cord
SUB: Cortical plate, subiculum
SUM: Supramammillary area
Sth: Subthalamus
TMM: Tuberomammillary nucleus
TRI: Germinal trigone
Tct: Tectum
V: Mesencephalic nucleus
VA: Ventral anterior nucleus [thalamus]
VIIn: Facial motor nucleus
VIn: Abducens nucleus
VL: Ventral lateral nucleus [thalamus]
VM: Ventral medial nucleus [thalamus]
VMB: Ventral medial basal nucleus [thalamus]
VMH: Ventromedial nucleus [hypothalamus]
VPM: Ventral posteromedial nucleus [thalamus]
XIn: Accessory nucleus
Xn: Dorsal motor nucleus
ZI: Zona incerta
CaS: Calcarine sulcus

Age: 24 GW

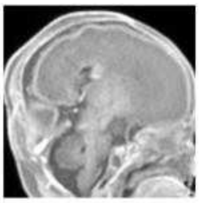

L-R Level: 5.52 mm

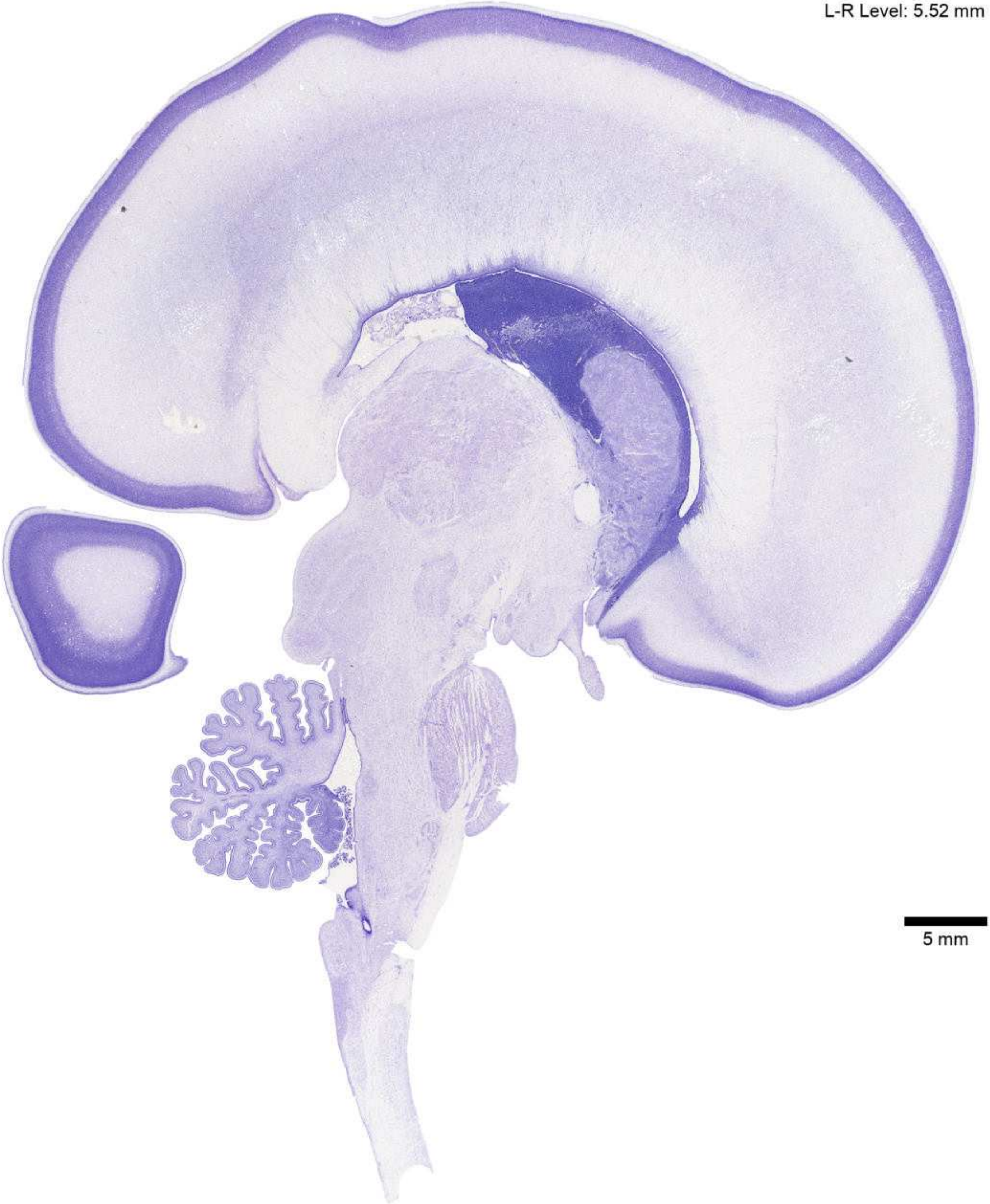

5 mm

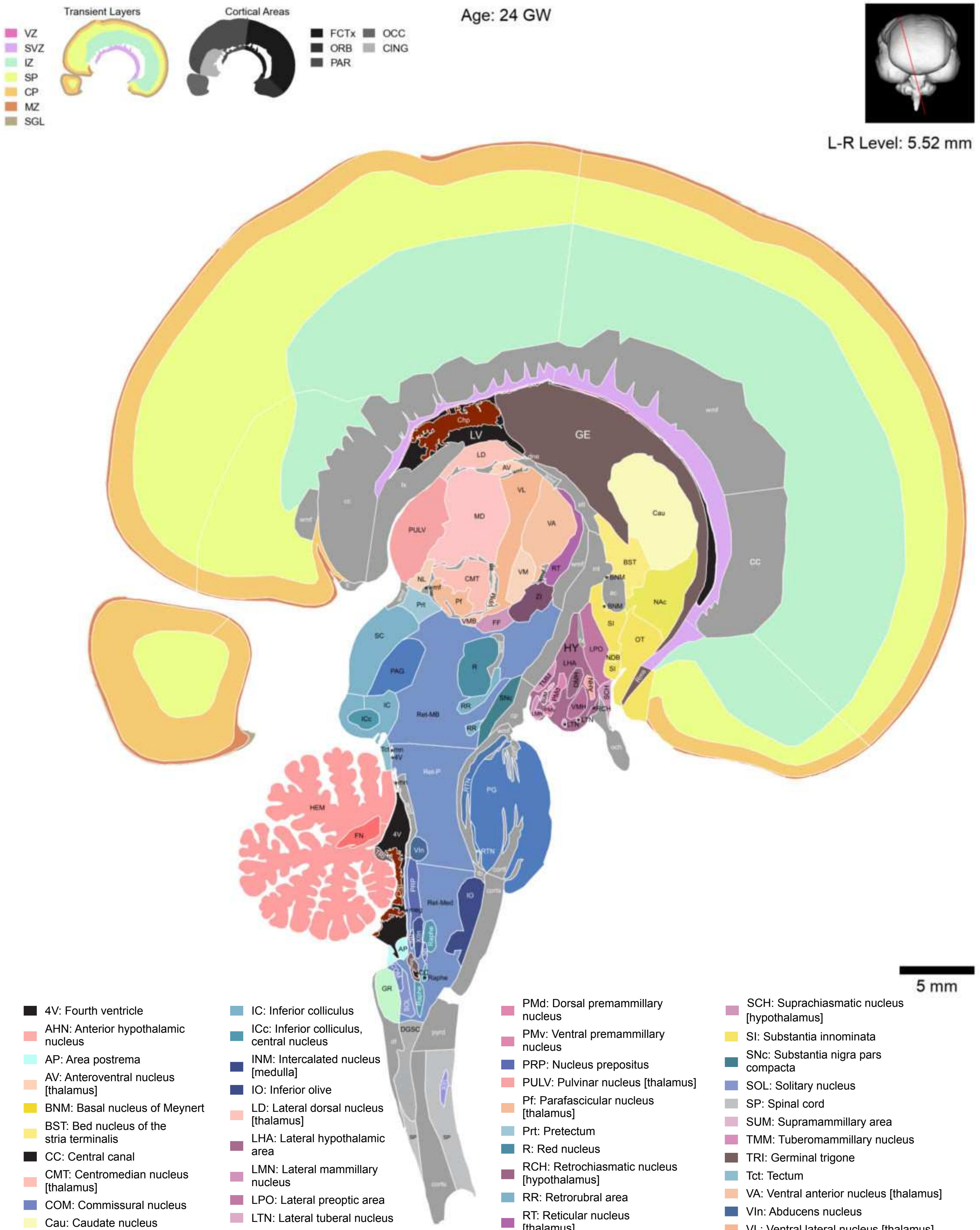

- 4V: Fourth ventricle
- AHN: Anterior hypothalamic nucleus
- AP: Area postrema
- AV: Anteroventral nucleus [thalamus]
- BNM: Basal nucleus of Meynert
- BST: Bed nucleus of the stria terminalis
- CC: Central canal
- CMT: Centromedian nucleus [thalamus]
- COM: Commissural nucleus
- Cau: Caudate nucleus
- Chp: Choroid plexus
- DGSC: Dorsal gray of the spinal cord
- DMH: Dorsomedial nucleus [hypothalamus]
- FF: Field of Forel
- FN: Fastigial nucleus
- GE: Ganglionic eminence
- GR: Gracile nucleus
- HEM: Cerebellar hemispheres
- HY: Hypothalamus
- IC: Inferior colliculus
- ICc: Inferior colliculus, central nucleus
- INM: Intercalated nucleus [medulla]
- IO: Inferior olive
- LD: Lateral dorsal nucleus [thalamus]
- LHA: Lateral hypothalamic area
- LMN: Lateral mammillary nucleus
- LPO: Lateral preoptic area
- LTN: Lateral tuberal nucleus
- LV: Lateral ventricle
- MD: Medial dorsal nucleus [thalamus]
- NAc: Nucleus accumbens
- NDB: Nucleus of the diagonal band
- NL: Nucleus limitans [thalamus]
- NR: Nucleus of Roller
- OT: Olfactory tubercle
- PAG: Periaqueductal gray
- PG: Pontine gray
- PMd: Dorsal premammillary nucleus
- PMv: Ventral premammillary nucleus
- PRP: Nucleus prepositus
- PULV: Pulvinar nucleus [thalamus]
- Pf: Parafascicular nucleus [thalamus]
- Prt: Pretectum
- R: Red nucleus
- RCH: Retrochiasmatic nucleus [hypothalamus]
- RR: Retrorubral area
- RT: Reticular nucleus [thalamus]
- RTN: Reticular tegmental nucleus
- Raphe: Raphe nuclei
- Ret-MB: Reticular formation, Midbrain
- Ret-Med: Reticular formation, Medulla
- Ret-P: Reticular formation, Pons
- Rms: Rostral migratory stream
- SC: Superior colliculus
- SCH: Suprachiasmatic nucleus [hypothalamus]
- SI: Substantia innominata
- SNc: Substantia nigra pars compacta
- SOL: Solitary nucleus
- SP: Spinal cord
- SUM: Supramammillary area
- TMM: Tuberomammillary nucleus
- TRI: Germinal trigone
- Tct: Tectum
- VA: Ventral anterior nucleus [thalamus]
- VIn: Abducens nucleus
- VL: Ventral lateral nucleus [thalamus]
- VM: Ventral medial nucleus [thalamus]
- VMB: Ventral medial basal nucleus [thalamus]
- VMH: Ventromedial nucleus [hypothalamus]
- VPM: Ventral posteromedial nucleus [thalamus]
- XIIn: Hypoglossal nucleus
- XIn: Accessory nucleus
- ZI: Zona incerta

Age: 24 GW

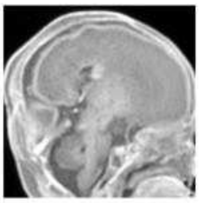

L-R Level: 5.28 mm

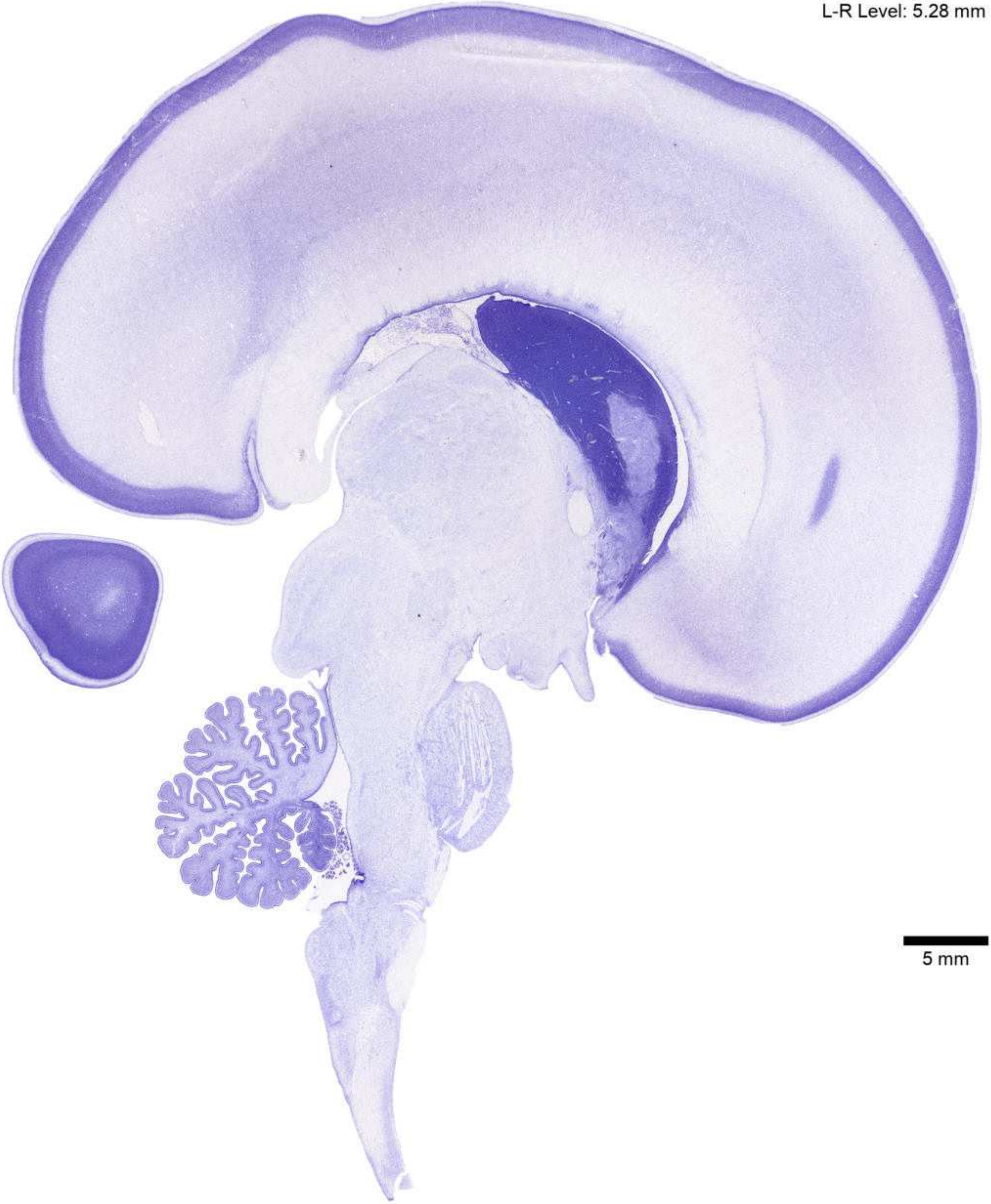

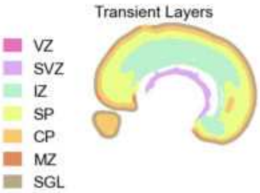

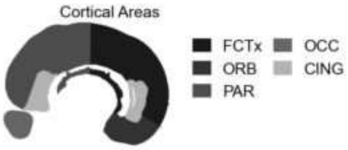

Age: 24 GW

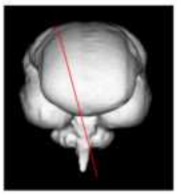

L-R Level: 5.28 mm

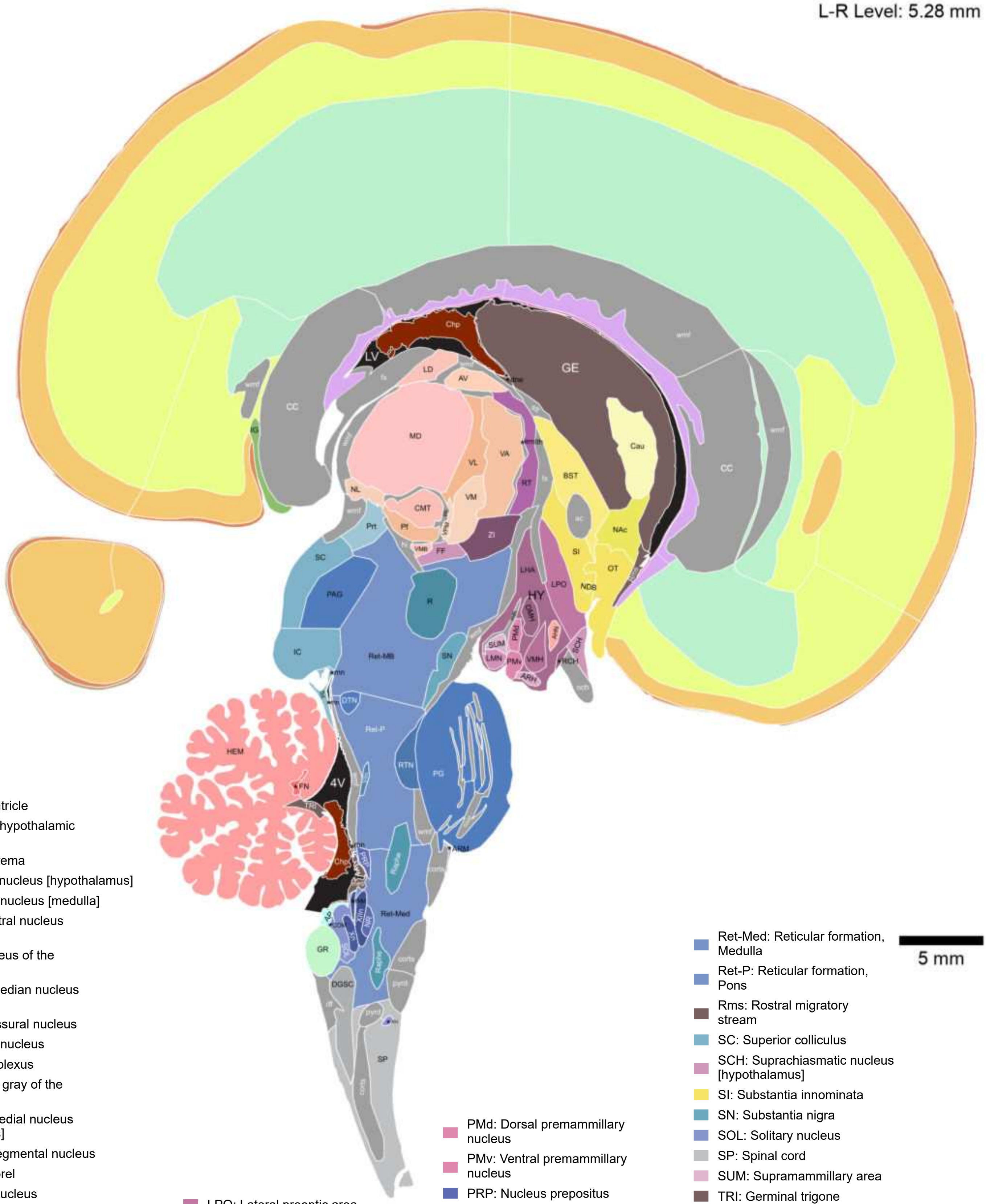

- 4V: Fourth ventricle
- AHN: Anterior hypothalamic nucleus
- AP: Area postrema
- ARH: Arcuate nucleus [hypothalamus]
- ARM: Arcuate nucleus [medulla]
- AV: Anteroventral nucleus [thalamus]
- BST: Bed nucleus of the stria terminalis
- CMT: Centromedian nucleus [thalamus]
- COM: Commissural nucleus
- Cau: Caudate nucleus
- Chp: Choroid plexus
- DGSC: Dorsal gray of the spinal cord
- DMH: Dorsomedial nucleus [hypothalamus]
- DTN: Dorsal tegmental nucleus
- FF: Field of Forel
- FN: Fastigial nucleus
- GE: Ganglionic eminence
- GR: Gracile nucleus
- HEM: Cerebellar hemispheres
- HY: Hypothalamus
- IC: Inferior colliculus
- IG: Induseum griseum
- INM: Intercalated nucleus [medulla]
- LD: Lateral dorsal nucleus [thalamus]
- LHA: Lateral hypothalamic area
- LMN: Lateral mammillary nucleus
- LPO: Lateral preoptic area
- LV: Lateral ventricle
- MD: Medial dorsal nucleus [thalamus]
- NAc: Nucleus accumbens
- NDB: Nucleus of the diagonal band
- NL: Nucleus limitans [thalamus]
- NR: Nucleus of Roller
- OT: Olfactory tubercle
- PAG: Periaqueductal gray
- PG: Pontine gray
- PMd: Dorsal premammillary nucleus
- PMv: Ventral premammillary nucleus
- PRP: Nucleus prepositus
- Pf: Parafascicular nucleus [thalamus]
- Prt: Pretectum
- R: Red nucleus
- RCH: Retrochiasmatic nucleus [hypothalamus]
- RT: Reticular nucleus [thalamus]
- RTN: Reticular tegmental nucleus
- Raphe: Raphe nuclei
- Ret-MB: Reticular formation, Midbrain
- Ret-Med: Reticular formation, Medulla
- Ret-P: Reticular formation, Pons
- Rms: Rostral migratory stream
- SC: Superior colliculus
- SCH: Suprachiasmatic nucleus [hypothalamus]
- SI: Substantia innominata
- SN: Substantia nigra
- SOL: Solitary nucleus
- SP: Spinal cord
- SUM: Supramammillary area
- TRI: Germinal trigone
- Tct: Tectum
- VA: Ventral anterior nucleus [thalamus]
- VIIn: Facial motor nucleus
- VL: Ventral lateral nucleus [thalamus]
- VM: Ventral medial nucleus [thalamus]
- VMB: Ventral medial basal nucleus [thalamus]
- VMH: Ventromedial nucleus [hypothalamus]
- VPM: Ventral posteromedial nucleus [thalamus]
- XIIn: Hypoglossal nucleus
- XIn: Accessory nucleus
- Xn: Dorsal motor nucleus
- ZI: Zona incerta

Age: 24 GW

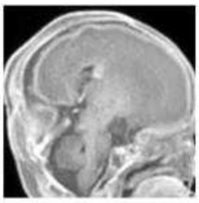

L-R Level: 5.04 mm

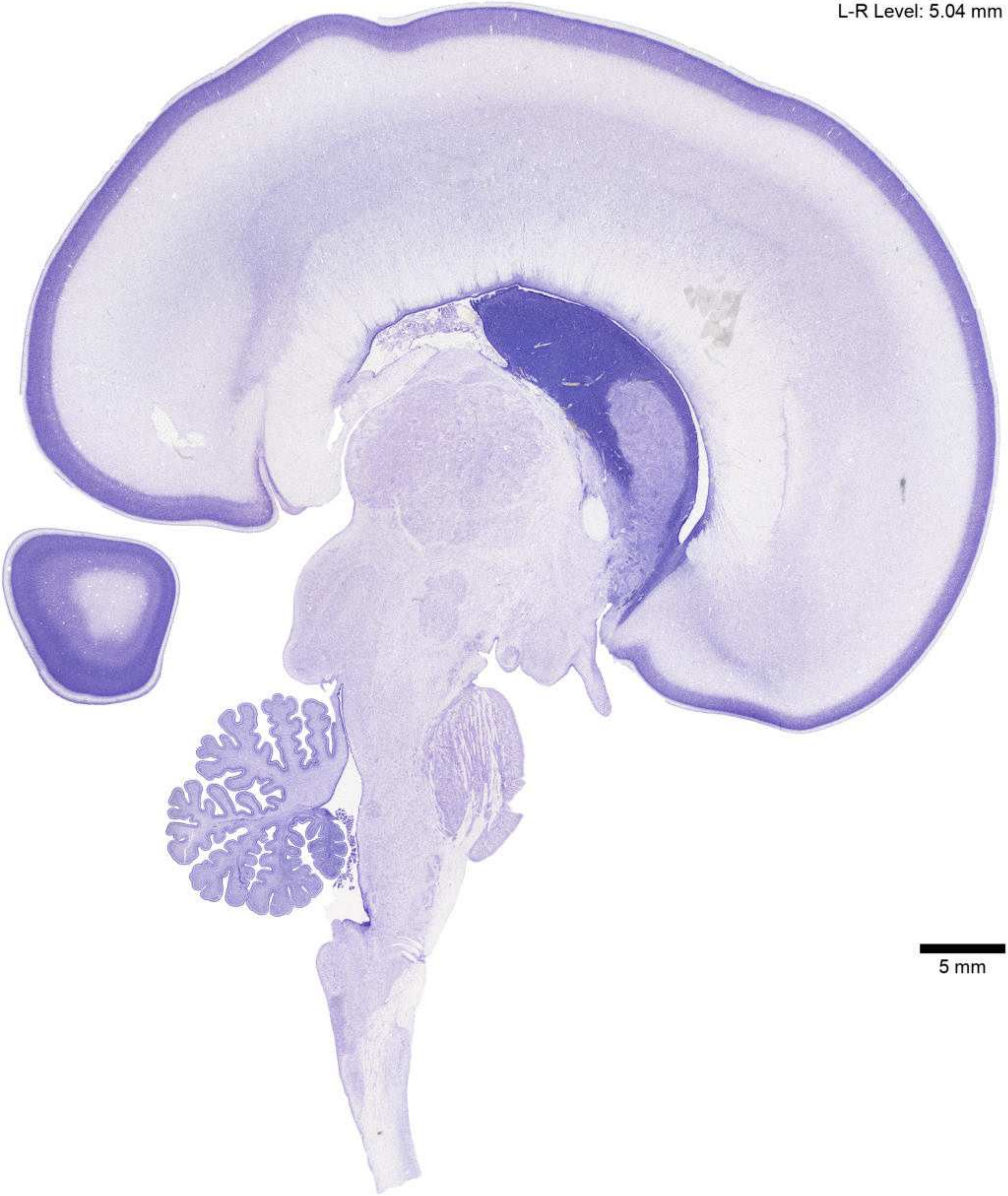

5 mm

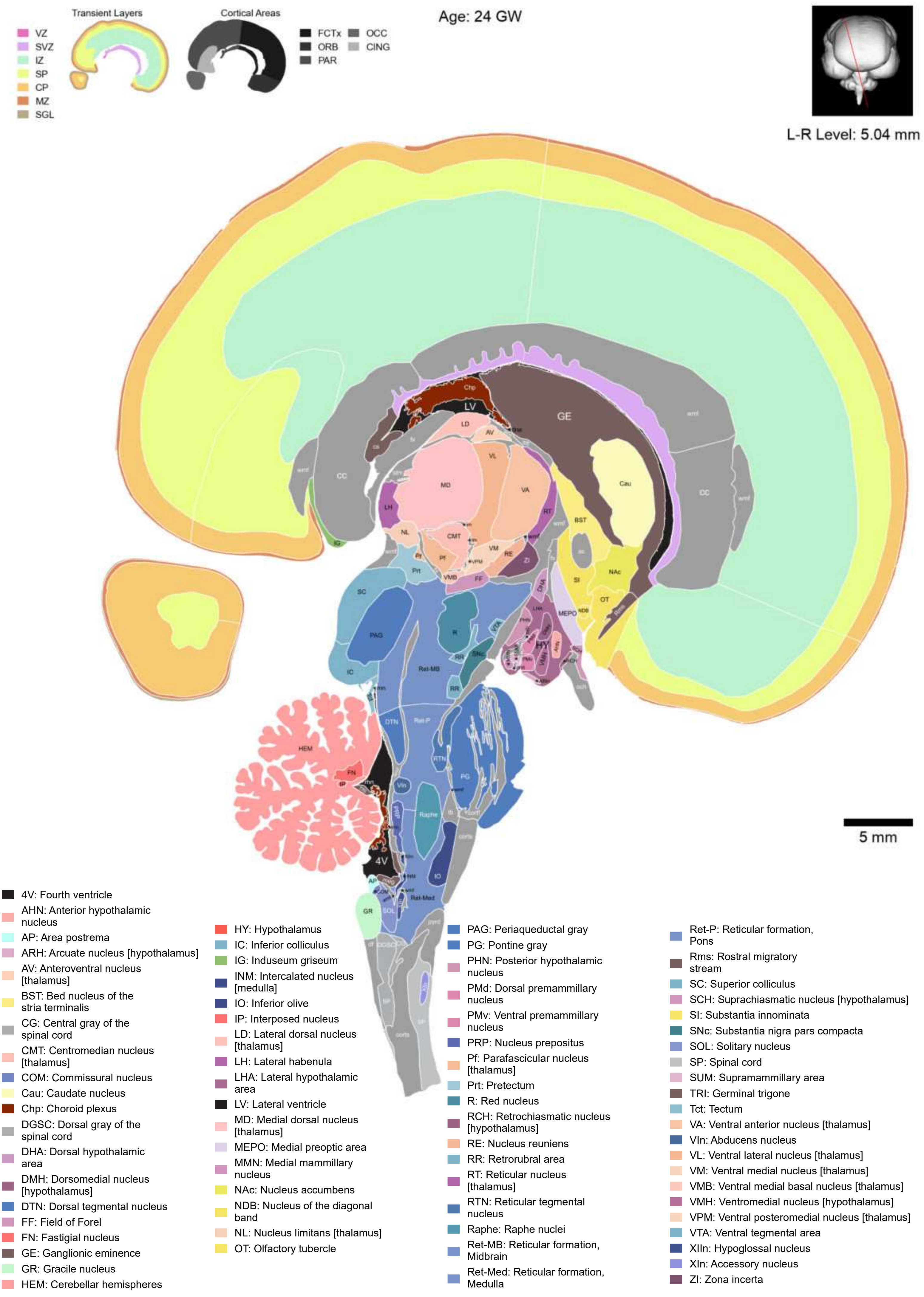
Transient Layers
VZ
SVZ
IZ
SP
CP
MZ
SGL
Cortical Areas
FCTx
ORB
PAR
OCC
CING
Age: 24 GW
L-R Level: 5.04 mm
5 mm
LV
GE
Chp
LD
AV
VL
VA
MD
LH
RT
BST
Cau
CC
NL
CMT
VM
RE
ZI
Pf
Prt
VMB
FF
SC
PAG
R
SNc
RR
Ret-MB
IC
DHA
MEPO
SI
NAc
OT
NDB
Rms
HY
IG
HEM
FN
IP
DTN
Ret-P
RTN
PG
VIn
Raphe
IO
4V
AP
GR
SOL
Ret-Med
4V: Fourth ventricle
AHN: Anterior hypothalamic nucleus
AP: Area postrema
ARH: Arcuate nucleus [hypothalamus]
AV: Anteroventral nucleus [thalamus]
BST: Bed nucleus of the stria terminalis
CG: Central gray of the spinal cord
CMT: Centromedian nucleus [thalamus]
COM: Commissural nucleus
Cau: Caudate nucleus
Chp: Choroid plexus
DGSC: Dorsal gray of the spinal cord
DHA: Dorsal hypothalamic area
DMH: Dorsomedial nucleus [hypothalamus]
DTN: Dorsal tegmental nucleus
FF: Field of Forel
FN: Fastigial nucleus
GE: Ganglionic eminence
GR: Gracile nucleus
HEM: Cerebellar hemispheres
HY: Hypothalamus
IC: Inferior colliculus
IG: Induseum griseum
INM: Intercalated nucleus [medulla]
IO: Inferior olive
IP: Interposed nucleus
LD: Lateral dorsal nucleus [thalamus]
LH: Lateral habenula
LHA: Lateral hypothalamic area
LV: Lateral ventricle
MD: Medial dorsal nucleus [thalamus]
MEPO: Medial preoptic area
MMN: Medial mammillary nucleus
NAc: Nucleus accumbens
NDB: Nucleus of the diagonal band
NL: Nucleus limitans [thalamus]
OT: Olfactory tubercle
PAG: Periaqueductal gray
PG: Pontine gray
PHN: Posterior hypothalamic nucleus
PMd: Dorsal premammillary nucleus
PMv: Ventral premammillary nucleus
PRP: Nucleus prepositus
Pf: Parafascicular nucleus [thalamus]
Prt: Pretectum
R: Red nucleus
RCH: Retrochiasmatic nucleus [hypothalamus]
RE: Nucleus reuniens
RR: Retrorubral area
RT: Reticular nucleus [thalamus]
RTN: Reticular tegmental nucleus
Raphe: Raphe nuclei
Ret-MB: Reticular formation, Midbrain
Ret-Med: Reticular formation, Medulla
Ret-P: Reticular formation, Pons
Rms: Rostral migratory stream
SC: Superior colliculus
SCH: Suprachiasmatic nucleus [hypothalamus]
SI: Substantia innominata
SNc: Substantia nigra pars compacta
SOL: Solitary nucleus
SP: Spinal cord
SUM: Supramammillary area
TRI: Germinal trigone
Tct: Tectum
VA: Ventral anterior nucleus [thalamus]
VIn: Abducens nucleus
VL: Ventral lateral nucleus [thalamus]
VM: Ventral medial nucleus [thalamus]
VMB: Ventral medial basal nucleus [thalamus]
VMH: Ventromedial nucleus [hypothalamus]
VPM: Ventral posteromedial nucleus [thalamus]
VTA: Ventral tegmental area
XIIn: Hypoglossal nucleus
XIn: Accessory nucleus
ZI: Zona incerta

Age: 24 GW

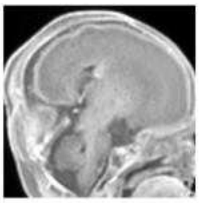

L-R Level: 4.8 mm

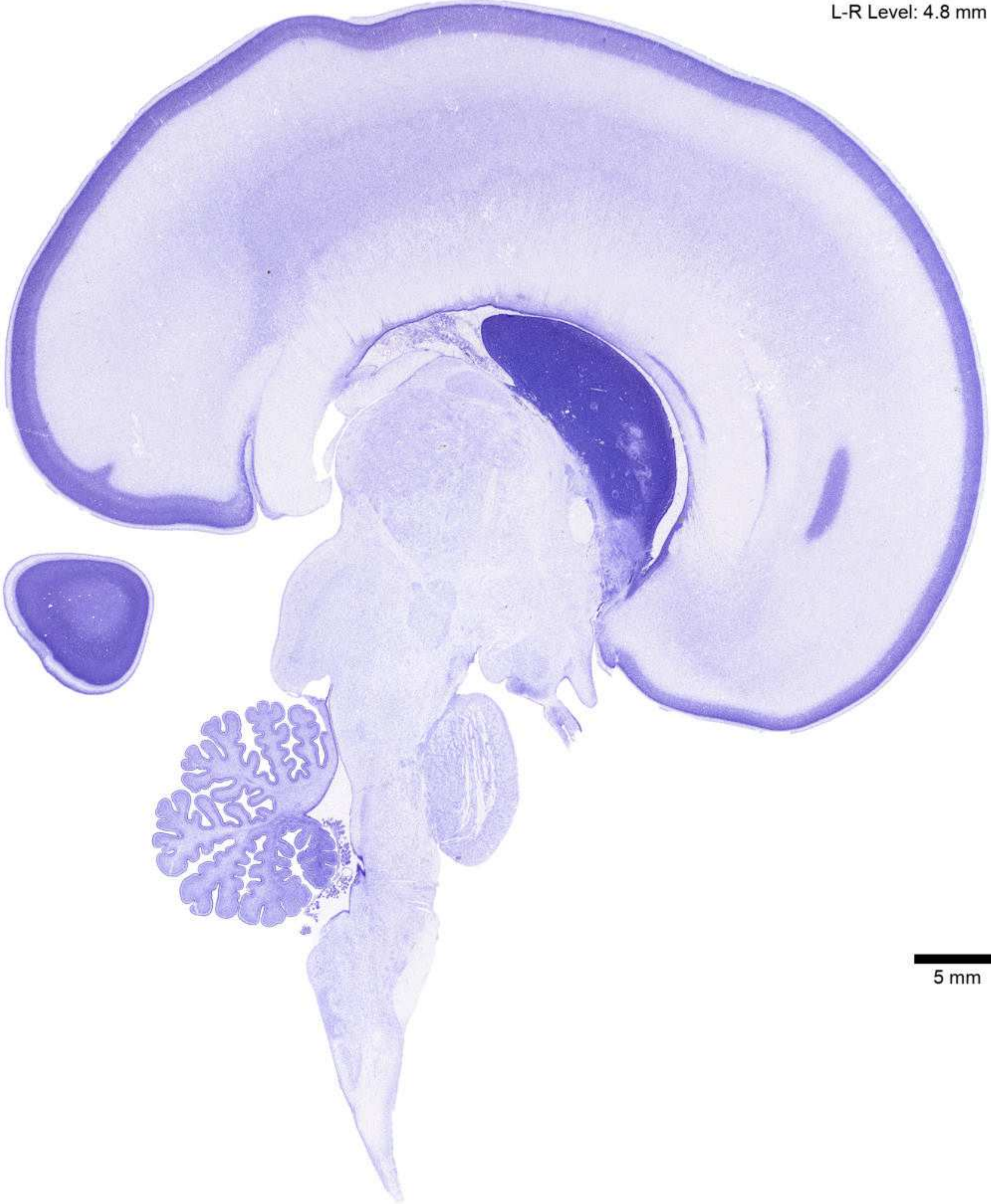

5 mm

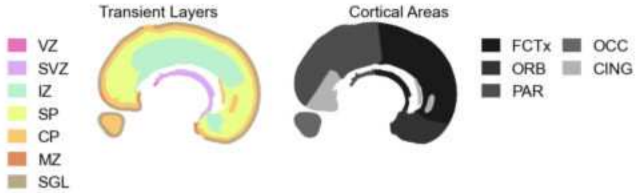

Age: 24 GW

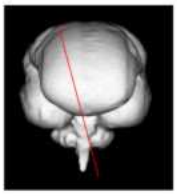

L-R Level: 4.8 mm

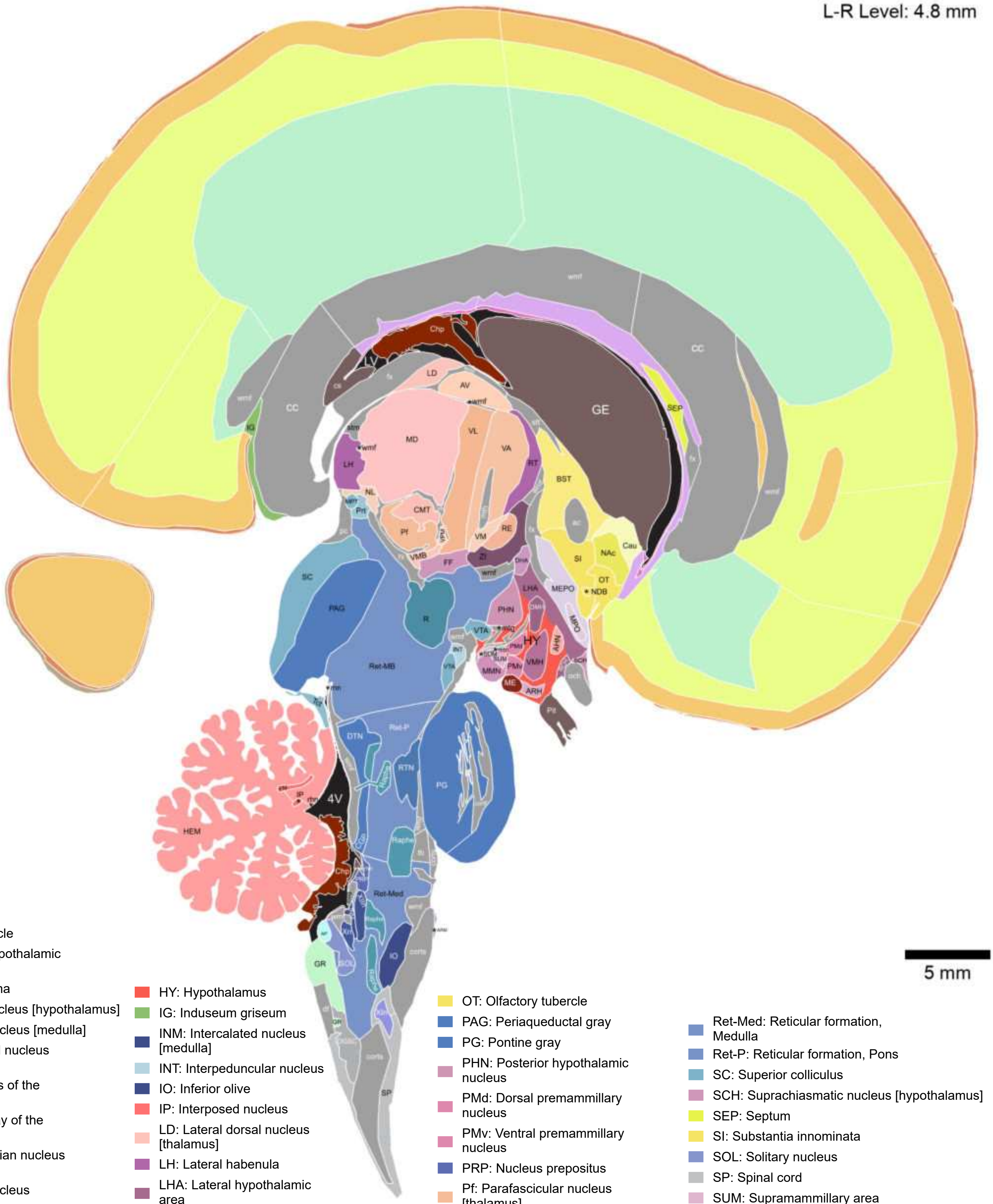

- 4V: Fourth ventricle
- AHN: Anterior hypothalamic nucleus
- AP: Area postrema
- ARH: Arcuate nucleus [hypothalamus]
- ARM: Arcuate nucleus [medulla]
- AV: Anteroventral nucleus [thalamus]
- BST: Bed nucleus of the stria terminalis
- CGP: Central gray of the pons
- CMT: Centromedian nucleus [thalamus]
- Cau: Caudate nucleus
- Chp: Choroid plexus
- DGSC: Dorsal gray of the spinal cord
- DHA: Dorsal hypothalamic area
- DMH: Dorsomedial nucleus [hypothalamus]
- DTN: Dorsal tegmental nucleus
- FF: Field of Forel
- FN: Fastigial nucleus
- GE: Ganglionic eminence
- GR: Gracile nucleus
- HEM: Cerebellar hemispheres
- HY: Hypothalamus
- IG: Induseum griseum
- INM: Intercalated nucleus [medulla]
- INT: Interpeduncular nucleus
- IO: Inferior olive
- IP: Interposed nucleus
- LD: Lateral dorsal nucleus [thalamus]
- LH: Lateral habenula
- LHA: Lateral hypothalamic area
- LV: Lateral ventricle
- MD: Medial dorsal nucleus [thalamus]
- ME: Median eminence
- MEPO: Medial preoptic area
- MMN: Medial mammillary nucleus
- MPO: Medial preoptic nucleus
- MPT: Medial pretectal nucleus
- NAc: Nucleus accumbens
- NDB: Nucleus of the diagonal band
- NL: Nucleus limitans [thalamus]
- OT: Olfactory tubercle
- PAG: Periaqueductal gray
- PG: Pontine gray
- PHN: Posterior hypothalamic nucleus
- PMd: Dorsal premammillary nucleus
- PMv: Ventral premammillary nucleus
- PRP: Nucleus prepositus
- Pf: Parafascicular nucleus [thalamus]
- Pit: Pituitary gland
- Prt: Pretectum
- R: Red nucleus
- RCH: Retrochiasmatic nucleus [hypothalamus]
- RE: Nucleus reuniens
- RT: Reticular nucleus [thalamus]
- RTN: Reticular tegmental nucleus
- Raphe: Raphe nuclei
- Ret-MB: Reticular formation, Midbrain
- Ret-Med: Reticular formation, Medulla
- Ret-P: Reticular formation, Pons
- SC: Superior colliculus
- SCH: Suprachiasmatic nucleus [hypothalamus]
- SEP: Septum
- SI: Substantia innominata
- SOL: Solitary nucleus
- SP: Spinal cord
- SUM: Supramammillary area
- Tct: Tectum
- VA: Ventral anterior nucleus [thalamus]
- VL: Ventral lateral nucleus [thalamus]
- VM: Ventral medial nucleus [thalamus]
- VMB: Ventral medial basal nucleus [thalamus]
- VMH: Ventromedial nucleus [hypothalamus]
- VPM: Ventral posteromedial nucleus [thalamus]
- VTA: Ventral tegmental area
- XIIn: Hypoglossal nucleus
- XIn: Accessory nucleus
- Xn: Dorsal motor nucleus
- ZI: Zona incerta

Age: 24 GW

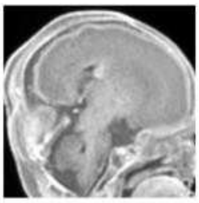

L-R Level: 4.44 mm

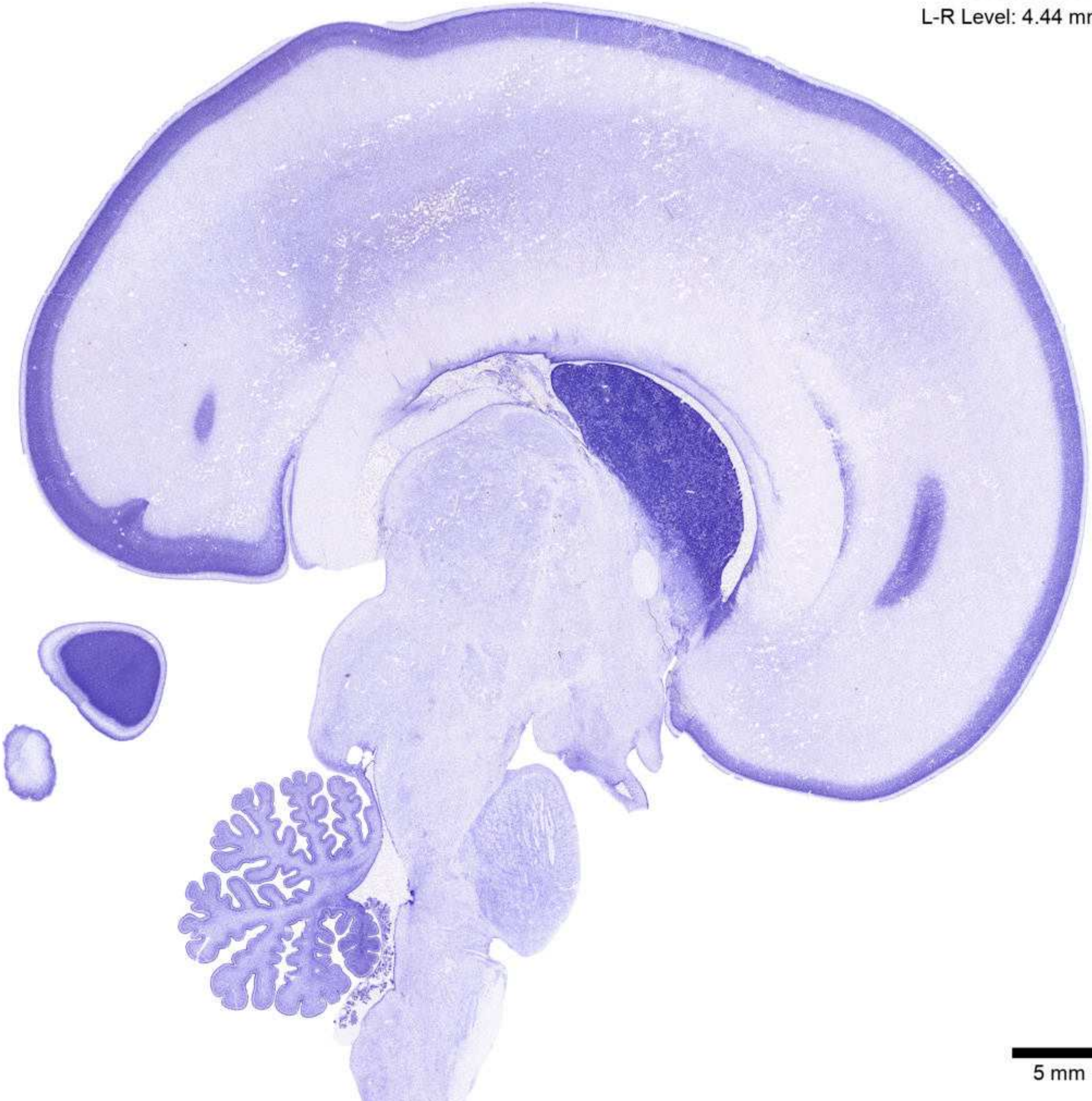

5 mm

Transient Layers

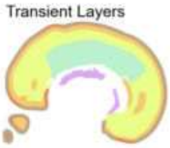

- VZ
- SVZ
- IZ
- SP
- CP
- MZ
- SGL

Cortical Areas

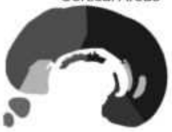

- FCTx
- ORB
- PAR
- OCC
- CING

Age: 24 GW

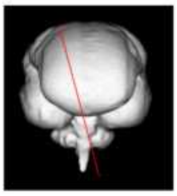

L-R Level: 4.44 mm

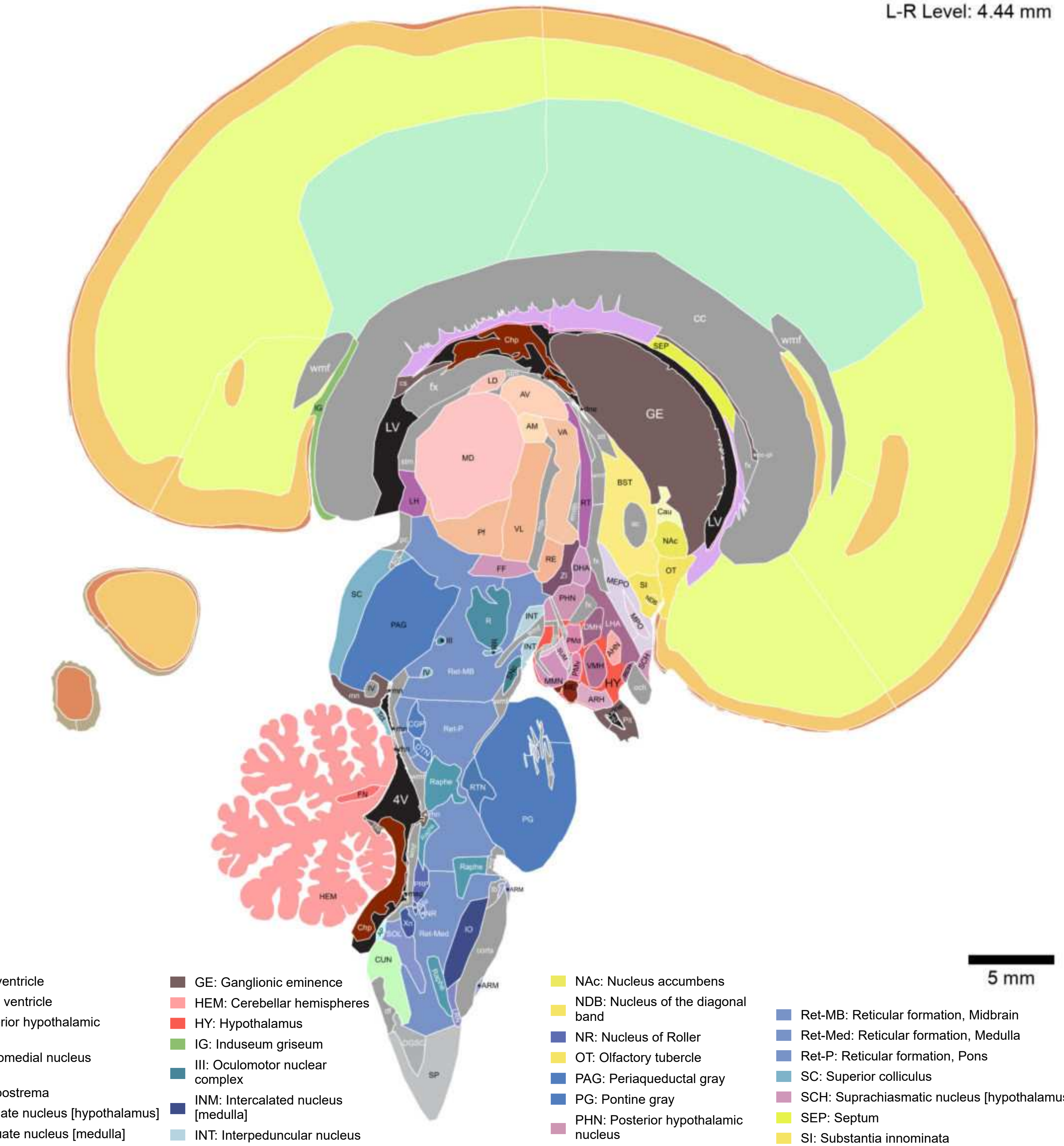

5 mm

- 3V: Third ventricle
- 4V: Fourth ventricle
- AHN: Anterior hypothalamic nucleus
- AM: Anteromedial nucleus [thalamus]
- AP: Area postrema
- ARH: Arcuate nucleus [hypothalamus]
- ARM: Arcuate nucleus [medulla]
- AV: Anteroventral nucleus [thalamus]
- BST: Bed nucleus of the stria terminalis
- CGP: Central gray of the pons
- CUN: Cuneate nucleus
- Cau: Caudate nucleus
- Chp: Choroid plexus
- DGSC: Dorsal gray of the spinal cord
- DHA: Dorsal hypothalamic area
- DMH: Dorsomedial nucleus [hypothalamus]
- DTN: Dorsal tegmental nucleus
- FF: Field of Forel
- FN: Fastigial nucleus
- GE: Ganglionic eminence
- HEM: Cerebellar hemispheres
- HY: Hypothalamus
- IG: Induseum griseum
- III: Oculomotor nuclear complex
- INM: Intercalated nucleus [medulla]
- INT: Interpeduncular nucleus
- IO: Inferior olive
- IV: Trochlear nucleus
- LD: Lateral dorsal nucleus [thalamus]
- LH: Lateral habenula
- LHA: Lateral hypothalamic area
- LRN: Lateral reticular nucleus
- LV: Lateral ventricle
- MD: Medial dorsal nucleus [thalamus]
- ME: Median eminence
- MEPO: Medial preoptic area
- MMN: Medial mammillary nucleus
- MPO: Medial preoptic nucleus
- NAc: Nucleus accumbens
- NDB: Nucleus of the diagonal band
- NR: Nucleus of Roller
- OT: Olfactory tubercle
- PAG: Periaqueductal gray
- PG: Pontine gray
- PHN: Posterior hypothalamic nucleus
- PMd: Dorsal premammillary nucleus
- PMv: Ventral premammillary nucleus
- PRP: Nucleus prepositus
- Pf: Parafascicular nucleus [thalamus]
- Pit: Pituitary gland
- R: Red nucleus
- RCH: Retrochiasmatic nucleus [hypothalamus]
- RE: Nucleus reuniens
- RR: Retrorubral area
- RT: Reticular nucleus [thalamus]
- RTN: Reticular tegmental nucleus
- Raphe: Raphe nuclei
- Ret-MB: Reticular formation, Midbrain
- Ret-Med: Reticular formation, Medulla
- Ret-P: Reticular formation, Pons
- SC: Superior colliculus
- SCH: Suprachiasmatic nucleus [hypothalamus]
- SEP: Septum
- SI: Substantia innominata
- SNc: Substantia nigra pars compacta
- SOL: Solitary nucleus
- SP: Spinal cord
- SUM: Supramammillary area
- TRI: Germinal trigone
- Tct: Tectum
- VA: Ventral anterior nucleus [thalamus]
- VL: Ventral lateral nucleus [thalamus]
- VMH: Ventromedial nucleus [hypothalamus]
- Xn: Dorsal motor nucleus
- ZI: Zona incerta

Age: 24 GW

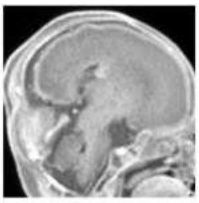

L-R Level: 4.14 mm

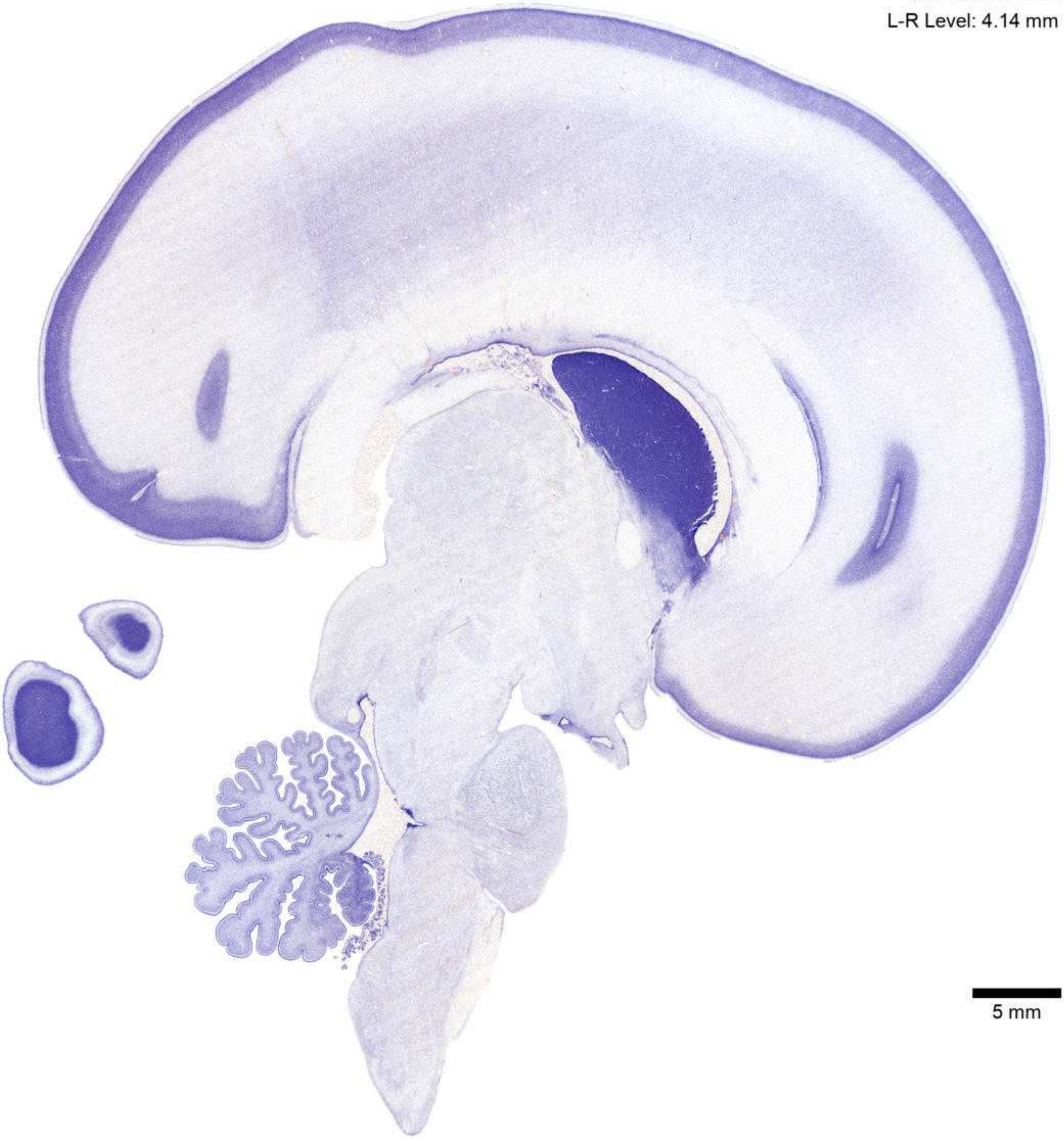

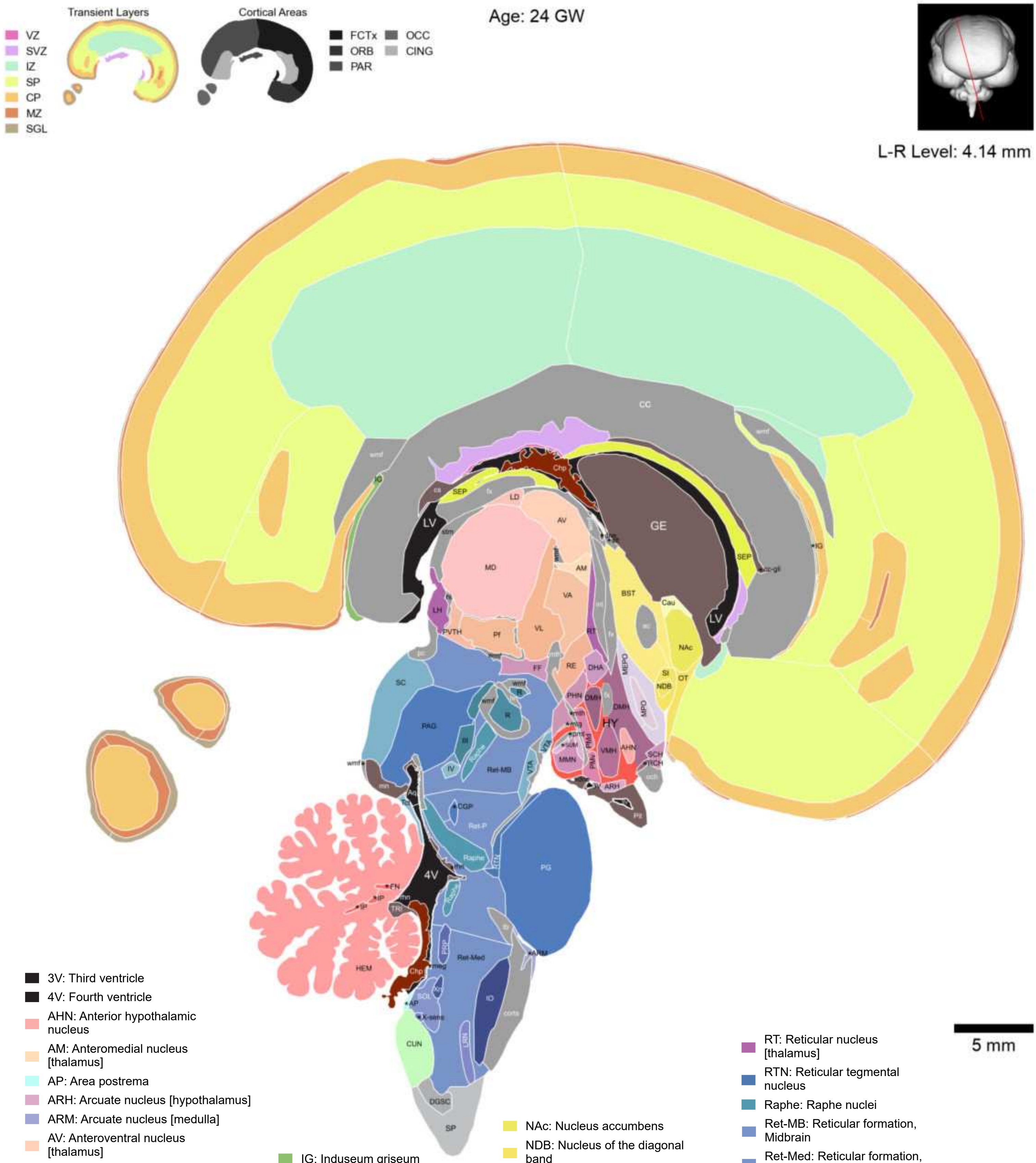

3V: Third ventricle
4V: Fourth ventricle
AHN: Anterior hypothalamic nucleus
AM: Anteromedial nucleus [thalamus]
AP: Area postrema
ARH: Arcuate nucleus [hypothalamus]
ARM: Arcuate nucleus [medulla]
AV: Anteroventral nucleus [thalamus]
Aq: Aqueduct
BST: Bed nucleus of the stria terminalis
CGP: Central gray of the pons
CUN: Cuneate nucleus
Cau: Caudate nucleus
Chp: Choroid plexus
DGSC: Dorsal gray of the spinal cord
DHA: Dorsal hypothalamic area
DMH: Dorsomedial nucleus [hypothalamus]
FF: Field of Forel
FN: Fastigial nucleus
GE: Ganglionic eminence
HEM: Cerebellar hemispheres
HY: Hypothalamus
IG: Induseum griseum
III: Oculomotor nuclear complex
IO: Inferior olive
IP: Interposed nucleus
IV: Trochlear nucleus
LD: Lateral dorsal nucleus [thalamus]
LH: Lateral habenula
LHA: Lateral hypothalamic area
LRN: Lateral reticular nucleus
LV: Lateral ventricle
MD: Medial dorsal nucleus [thalamus]
MEPO: Medial preoptic area
MMN: Medial mammillary nucleus
MPO: Medial preoptic nucleus
NAc: Nucleus accumbens
NDB: Nucleus of the diagonal band
OT: Olfactory tubercle
PAG: Periaqueductal gray
PG: Pontine gray
PHN: Posterior hypothalamic nucleus
PMd: Dorsal premammillary nucleus
PMv: Ventral premammillary nucleus
PRP: Nucleus prepositus
PVTH: Periventricular complex [thalamus]
Pf: Parafascicular nucleus [thalamus]
Pit: Pituitary gland
R: Red nucleus
RCH: Retrochiasmatic nucleus [hypothalamus]
RE: Nucleus reuniens
RT: Reticular nucleus [thalamus]
RTN: Reticular tegmental nucleus
Raphe: Raphe nuclei
Ret-MB: Reticular formation, Midbrain
Ret-Med: Reticular formation, Medulla
Ret-P: Reticular formation, Pons
SC: Superior colliculus
SCH: Suprachiasmatic nucleus [hypothalamus]
SEP: Septum
SI: Substantia innominata
SOL: Solitary nucleus
SP: Spinal cord
SUM: Supramammillary area
TRI: Germinal trigone
Tct: Tectum
VA: Ventral anterior nucleus [thalamus]
VL: Ventral lateral nucleus [thalamus]
VMH: Ventromedial nucleus [hypothalamus]
VTA: Ventral tegmental area
X-sens: Dorsal sensory nucleus X
Xn: Dorsal motor nucleus

Age: 24 GW

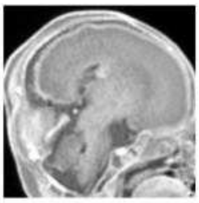

L-R Level: 3.84 mm

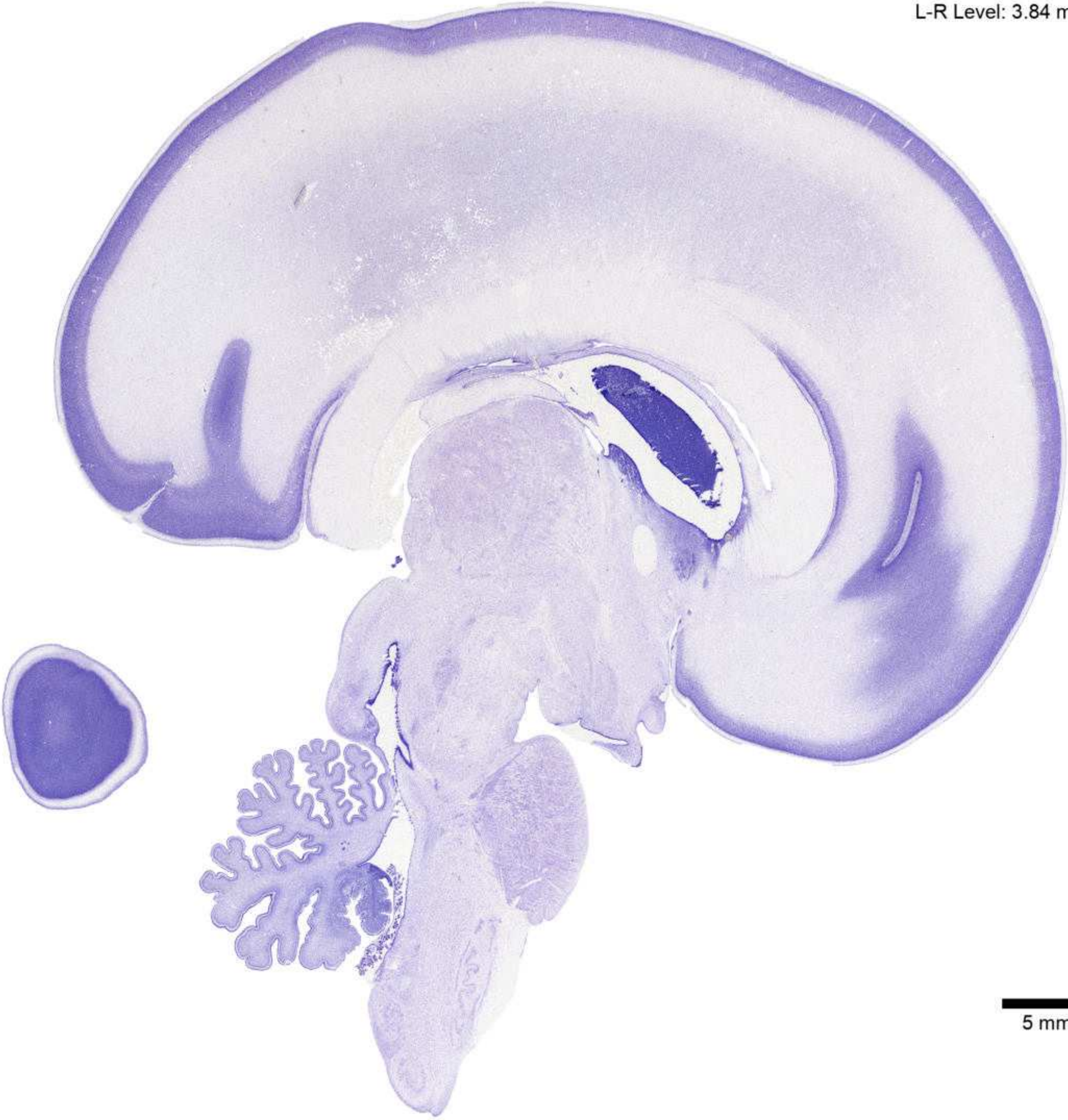

5 mm

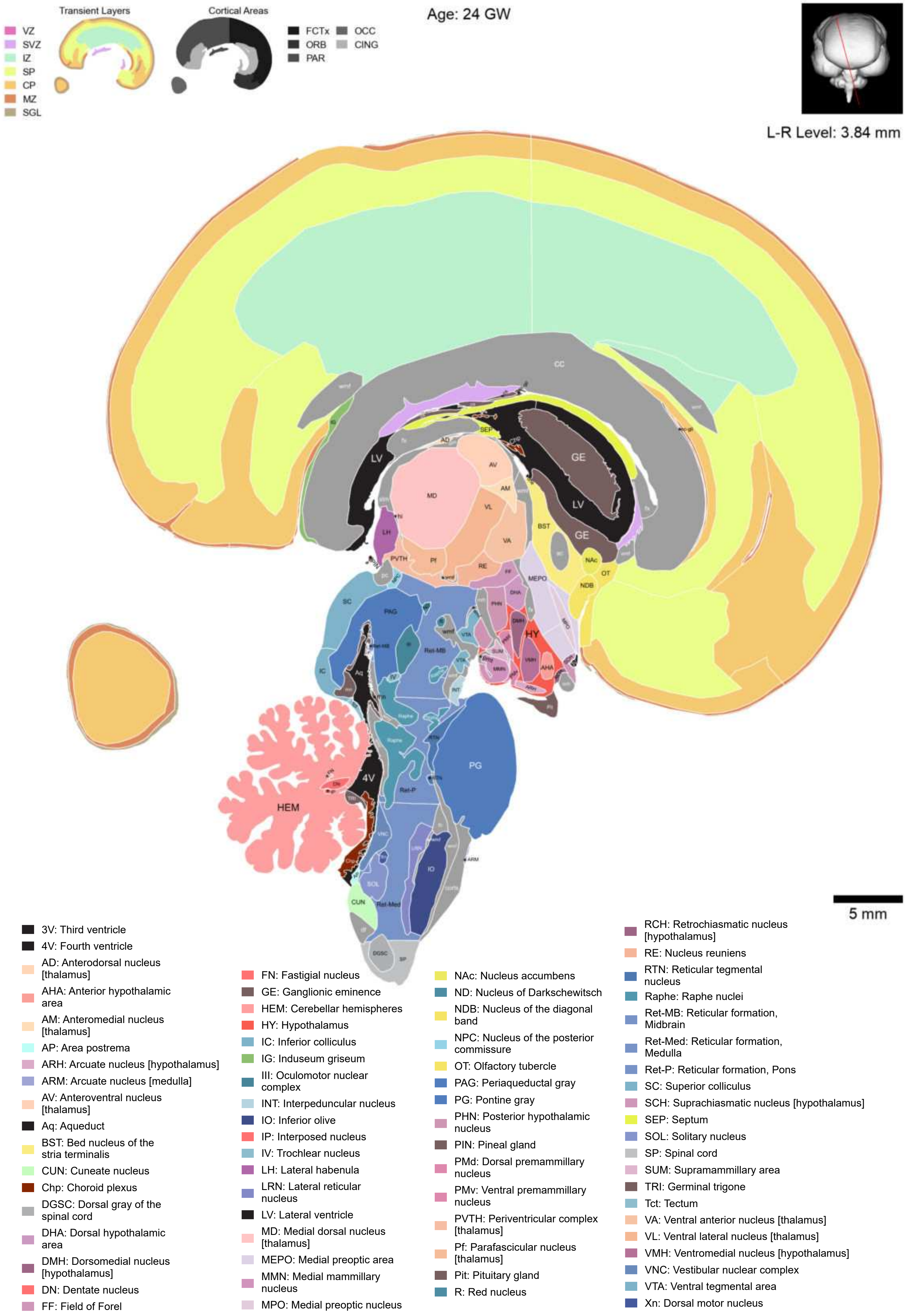

Transient Layers
VZ
SVZ
IZ
SP
CP
MZ
SGL
Cortical Areas
FCTx
ORB
PAR
OCC
CING
Age: 24 GW
L-R Level: 3.84 mm
CC
LV
GE
LV
GE
MD
AV
AM
VL
VA
LH
PVTH
Pf
RE
BST
NAc
NDB
OT
MEPO
SEP
AD
SC
PAG
IC
Aq
HY
AHA
VMH
DMH
Ret-MB
4V
HEM
PG
Ret-P
IO
CUN
SOL
Ret-Med
DGSC
SP
5 mm
3V: Third ventricle
4V: Fourth ventricle
AD: Anterodorsal nucleus [thalamus]
AHA: Anterior hypothalamic area
AM: Anteromedial nucleus [thalamus]
AP: Area postrema
ARH: Arcuate nucleus [hypothalamus]
ARM: Arcuate nucleus [medulla]
AV: Anteroventral nucleus [thalamus]
Aq: Aqueduct
BST: Bed nucleus of the stria terminalis
CUN: Cuneate nucleus
Chp: Choroid plexus
DGSC: Dorsal gray of the spinal cord
DHA: Dorsal hypothalamic area
DMH: Dorsomedial nucleus [hypothalamus]
DN: Dentate nucleus
FF: Field of Forel
FN: Fastigial nucleus
GE: Ganglionic eminence
HEM: Cerebellar hemispheres
HY: Hypothalamus
IC: Inferior colliculus
IG: Induseum griseum
III: Oculomotor nuclear complex
INT: Interpeduncular nucleus
IO: Inferior olive
IP: Interposed nucleus
IV: Trochlear nucleus
LH: Lateral habenula
LRN: Lateral reticular nucleus
LV: Lateral ventricle
MD: Medial dorsal nucleus [thalamus]
MEPO: Medial preoptic area
MMN: Medial mammillary nucleus
MPO: Medial preoptic nucleus
NAc: Nucleus accumbens
ND: Nucleus of Darkschewitsch
NDB: Nucleus of the diagonal band
NPC: Nucleus of the posterior commissure
OT: Olfactory tubercle
PAG: Periaqueductal gray
PG: Pontine gray
PHN: Posterior hypothalamic nucleus
PIN: Pineal gland
PMd: Dorsal premammillary nucleus
PMv: Ventral premammillary nucleus
PVTH: Periventricular complex [thalamus]
Pf: Parafascicular nucleus [thalamus]
Pit: Pituitary gland
R: Red nucleus
RCH: Retrochiasmatic nucleus [hypothalamus]
RE: Nucleus reuniens
RTN: Reticular tegmental nucleus
Raphe: Raphe nuclei
Ret-MB: Reticular formation, Midbrain
Ret-Med: Reticular formation, Medulla
Ret-P: Reticular formation, Pons
SC: Superior colliculus
SCH: Suprachiasmatic nucleus [hypothalamus]
SEP: Septum
SOL: Solitary nucleus
SP: Spinal cord
SUM: Supramammillary area
TRI: Germinal trigone
Tct: Tectum
VA: Ventral anterior nucleus [thalamus]
VL: Ventral lateral nucleus [thalamus]
VMH: Ventromedial nucleus [hypothalamus]
VNC: Vestibular nuclear complex
VTA: Ventral tegmental area
Xn: Dorsal motor nucleus

Age: 24 GW

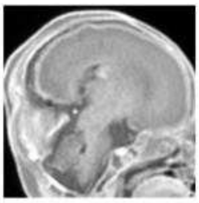

L-R Level: 3.6 mm

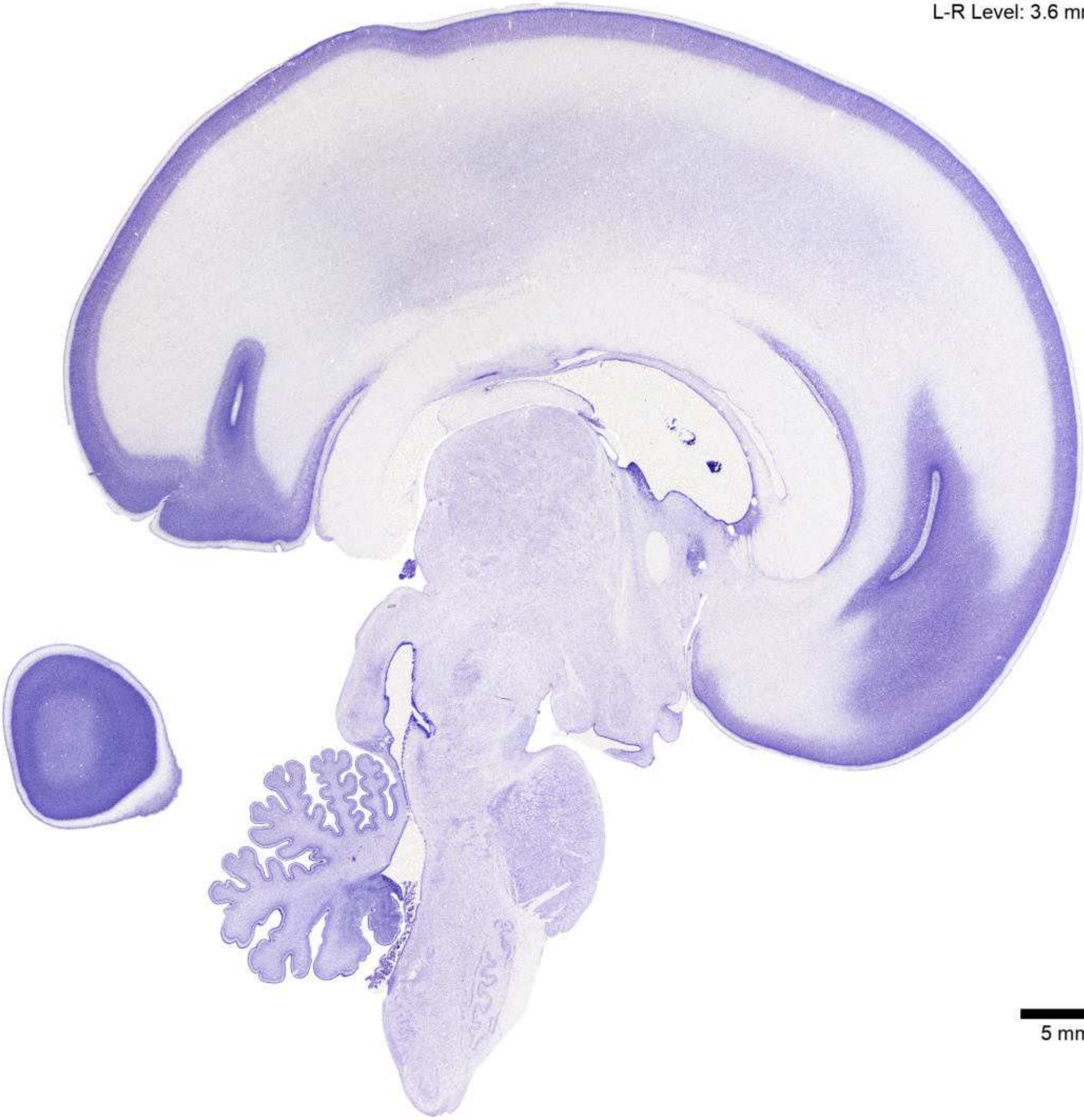

5 mm

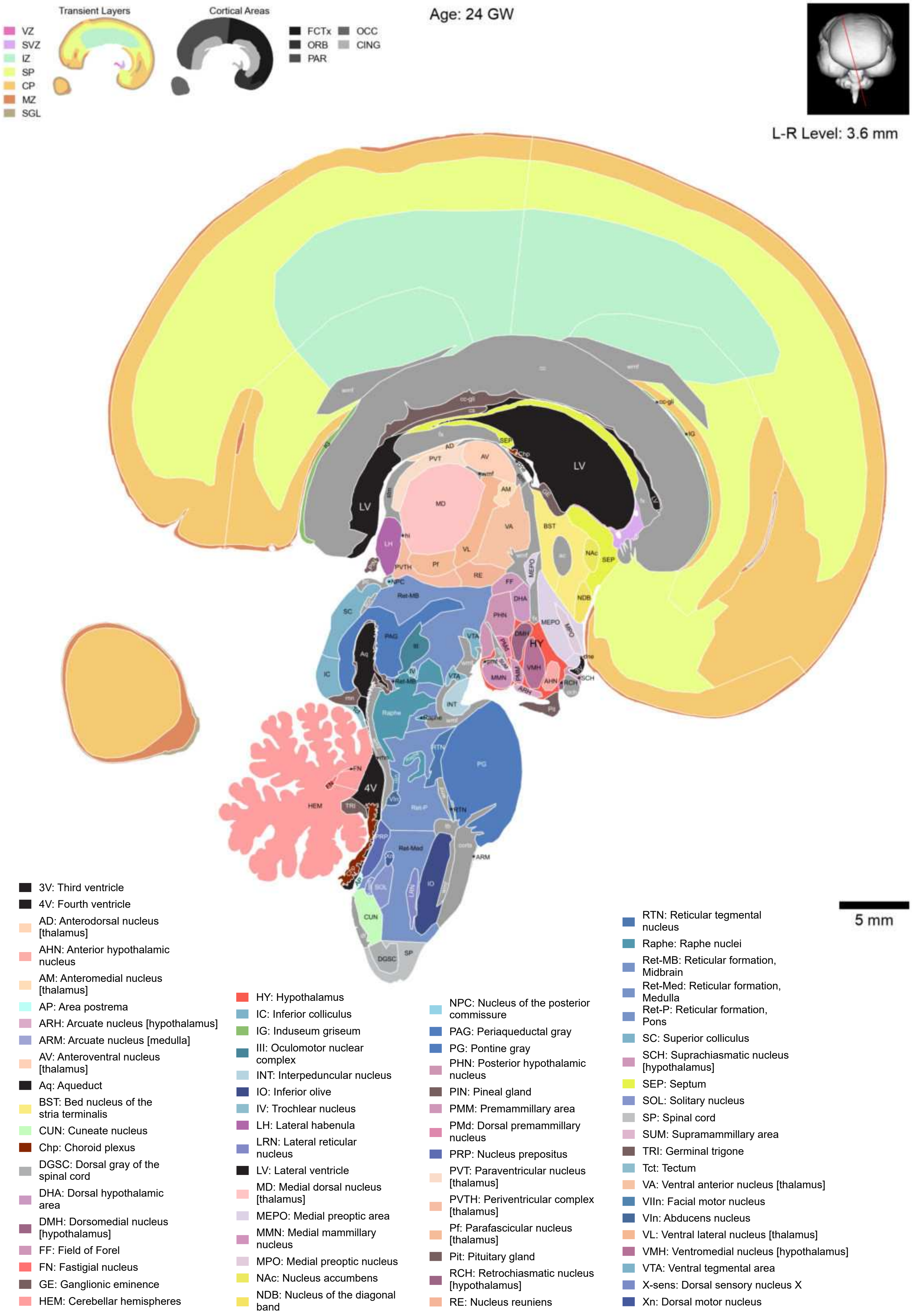

Transient Layers
VZ
SVZ
IZ
SP
CP
MZ
SGL
Cortical Areas
FCTx
ORB
PAR
OCC
CING
Age: 24 GW
L-R Level: 3.6 mm
5 mm
LV
HY
4V
3V: Third ventricle
4V: Fourth ventricle
AD: Anterodorsal nucleus [thalamus]
AHN: Anterior hypothalamic nucleus
AM: Anteromedial nucleus [thalamus]
AP: Area postrema
ARH: Arcuate nucleus [hypothalamus]
ARM: Arcuate nucleus [medulla]
AV: Anteroventral nucleus [thalamus]
Aq: Aqueduct
BST: Bed nucleus of the stria terminalis
CUN: Cuneate nucleus
Chp: Choroid plexus
DGSC: Dorsal gray of the spinal cord
DHA: Dorsal hypothalamic area
DMH: Dorsomedial nucleus [hypothalamus]
FF: Field of Forel
FN: Fastigial nucleus
GE: Ganglionic eminence
HEM: Cerebellar hemispheres
HY: Hypothalamus
IC: Inferior colliculus
IG: Induseum griseum
III: Oculomotor nuclear complex
INT: Interpeduncular nucleus
IO: Inferior olive
IV: Trochlear nucleus
LH: Lateral habenula
LRN: Lateral reticular nucleus
LV: Lateral ventricle
MD: Medial dorsal nucleus [thalamus]
MEPO: Medial preoptic area
MMN: Medial mammillary nucleus
MPO: Medial preoptic nucleus
NAc: Nucleus accumbens
NDB: Nucleus of the diagonal band
NPC: Nucleus of the posterior commissure
PAG: Periaqueductal gray
PG: Pontine gray
PHN: Posterior hypothalamic nucleus
PIN: Pineal gland
PMM: Premammillary area
PMd: Dorsal premammillary nucleus
PRP: Nucleus prepositus
PVT: Paraventricular nucleus [thalamus]
PVTH: Periventricular complex [thalamus]
Pf: Parafascicular nucleus [thalamus]
Pit: Pituitary gland
RCH: Retrochiasmatic nucleus [hypothalamus]
RE: Nucleus reuniens
RTN: Reticular tegmental nucleus
Raphe: Raphe nuclei
Ret-MB: Reticular formation, Midbrain
Ret-Med: Reticular formation, Medulla
Ret-P: Reticular formation, Pons
SC: Superior colliculus
SCH: Suprachiasmatic nucleus [hypothalamus]
SEP: Septum
SOL: Solitary nucleus
SP: Spinal cord
SUM: Supramammillary area
TRI: Germinal trigone
Tct: Tectum
VA: Ventral anterior nucleus [thalamus]
VIIn: Facial motor nucleus
VIn: Abducens nucleus
VL: Ventral lateral nucleus [thalamus]
VMH: Ventromedial nucleus [hypothalamus]
VTA: Ventral tegmental area
X-sens: Dorsal sensory nucleus X
Xn: Dorsal motor nucleus

Age: 24 GW

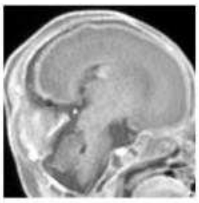

L-R Level: 3.24 mm

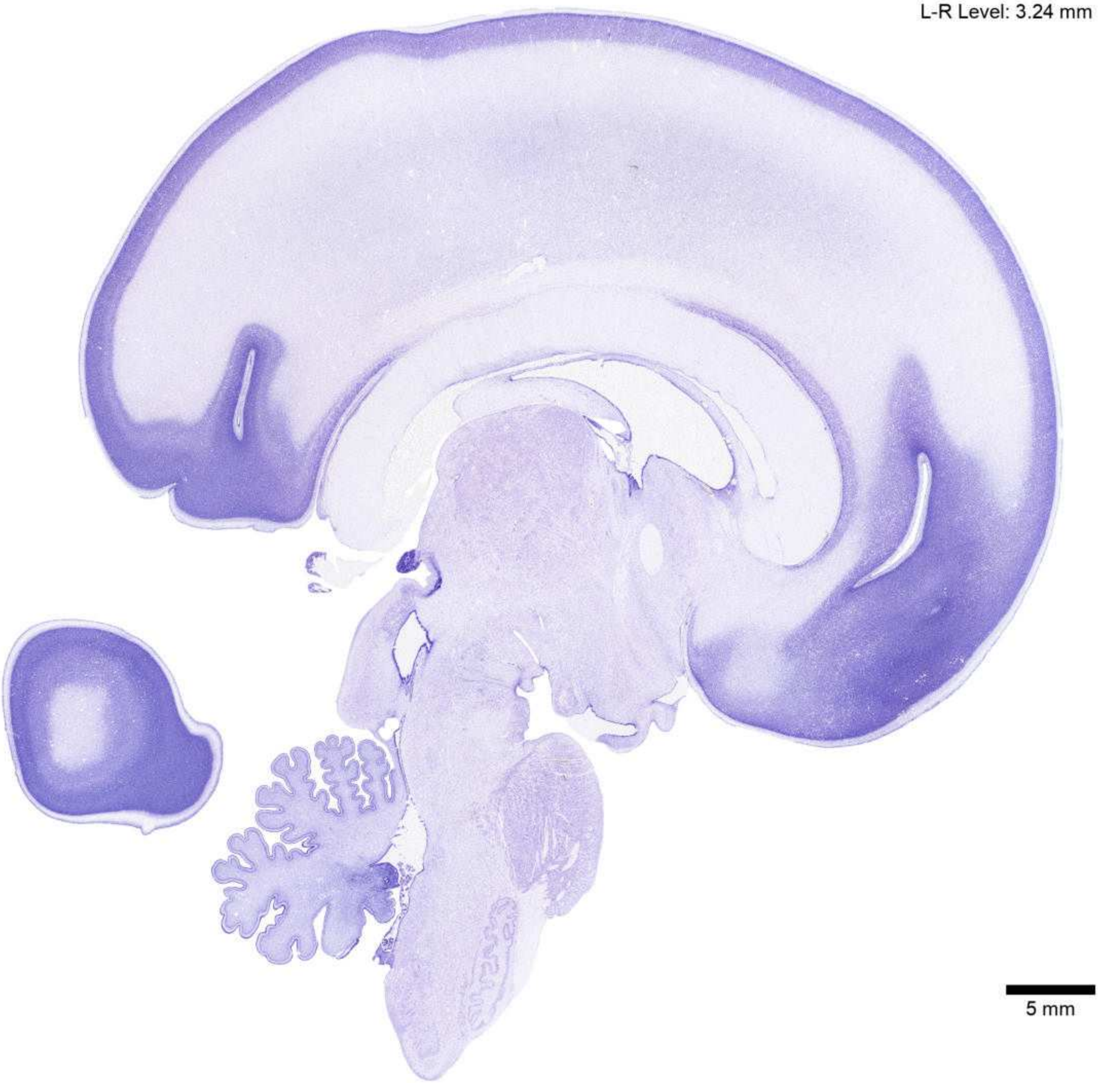

5 mm

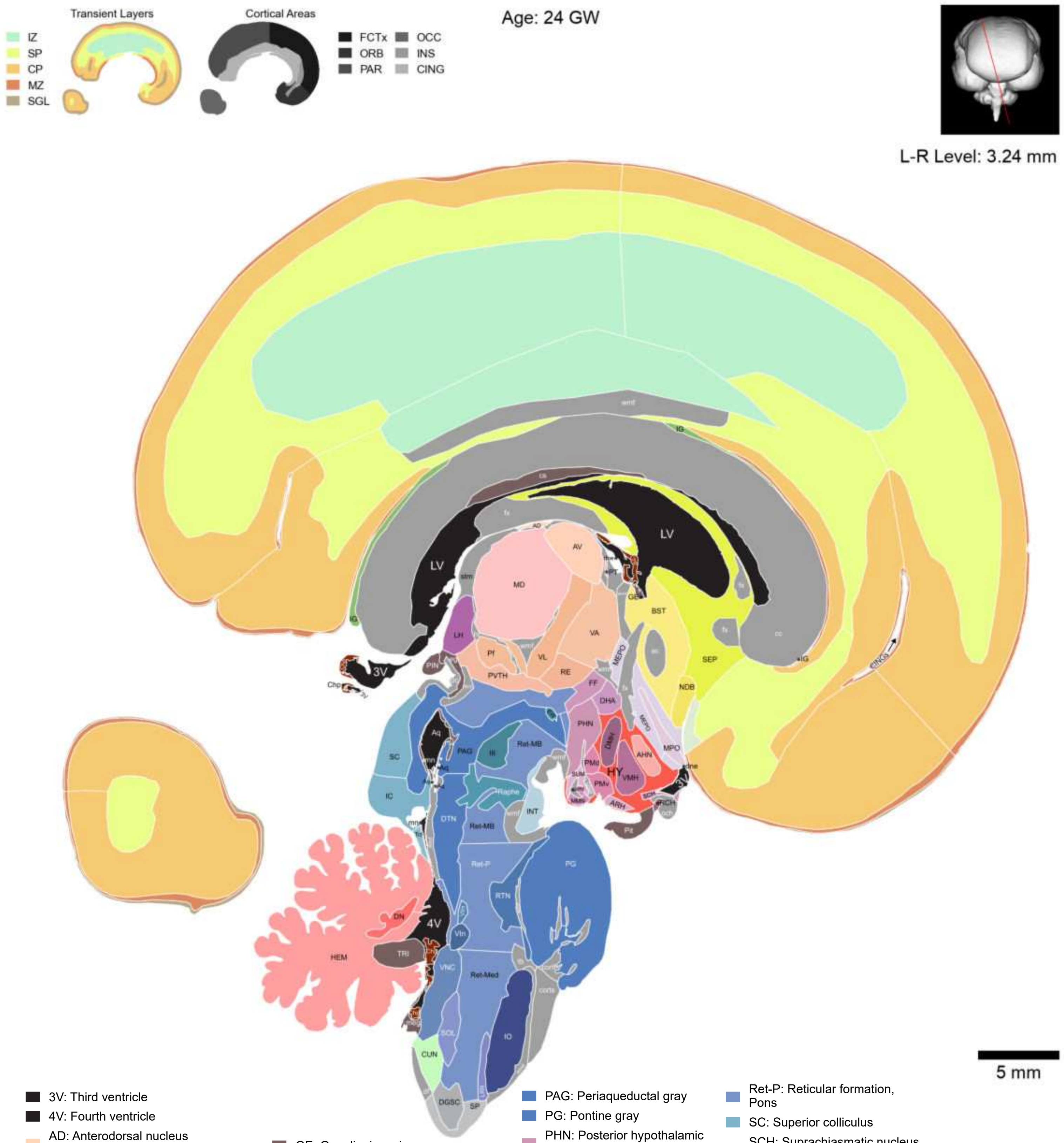

- 3V: Third ventricle
- 4V: Fourth ventricle
- AD: Anterodorsal nucleus [thalamus]
- AHN: Anterior hypothalamic nucleus
- ARH: Arcuate nucleus [hypothalamus]
- AV: Anteroventral nucleus [thalamus]
- Aq: Aqueduct
- BST: Bed nucleus of the stria terminalis
- CUN: Cuneate nucleus
- Chp: Choroid plexus
- DGSC: Dorsal gray of the spinal cord
- DHA: Dorsal hypothalamic area
- DMH: Dorsomedial nucleus [hypothalamus]
- DN: Dentate nucleus
- DTN: Dorsal tegmental nucleus
- EW: Edinger-Westphal nucleus
- FF: Field of Forel
- GE: Ganglionic eminence
- HEM: Cerebellar hemispheres
- HY: Hypothalamus
- IC: Inferior colliculus
- IG: Induseum griseum
- III: Oculomotor nuclear complex
- INT: Interpeduncular nucleus
- IO: Inferior olive
- LH: Lateral habenula
- LRN: Lateral reticular nucleus
- LV: Lateral ventricle
- MD: Medial dorsal nucleus [thalamus]
- MEPO: Medial preoptic area
- MMN: Medial mammillary nucleus
- MPO: Medial preoptic nucleus
- NDB: Nucleus of the diagonal band
- PAG: Periaqueductal gray
- PG: Pontine gray
- PHN: Posterior hypothalamic nucleus
- PIN: Pineal gland
- PMd: Dorsal premammillary nucleus
- PMv: Ventral premammillary nucleus
- PT: Paratenial nucleus [thalamus]
- PVTH: Periventricular complex [thalamus]
- Pf: Parafascicular nucleus [thalamus]
- Pit: Pituitary gland
- RCH: Retrochiasmatic nucleus [hypothalamus]
- RE: Nucleus reuniens
- RTN: Reticular tegmental nucleus
- Raphe: Raphe nuclei
- Ret-MB: Reticular formation, Midbrain
- Ret-Med: Reticular formation, Medulla
- Ret-P: Reticular formation, Pons
- SC: Superior colliculus
- SCH: Suprachiasmatic nucleus [hypothalamus]
- SEP: Septum
- SOL: Solitary nucleus
- SP: Spinal cord
- SUM: Supramammillary area
- TRI: Germinal trigone
- TT: Tenia tecta
- Tct: Tectum
- VA: Ventral anterior nucleus [thalamus]
- VIIn: Facial motor nucleus
- VIn: Abducens nucleus
- VL: Ventral lateral nucleus [thalamus]
- VMH: Ventromedial nucleus [hypothalamus]
- VNC: Vestibular nuclear complex
- → CINGs: Cingulate sulcus

Age: 24 GW

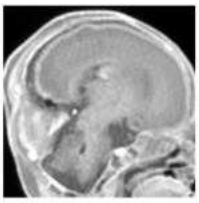

L-R Level: 2.94 mm

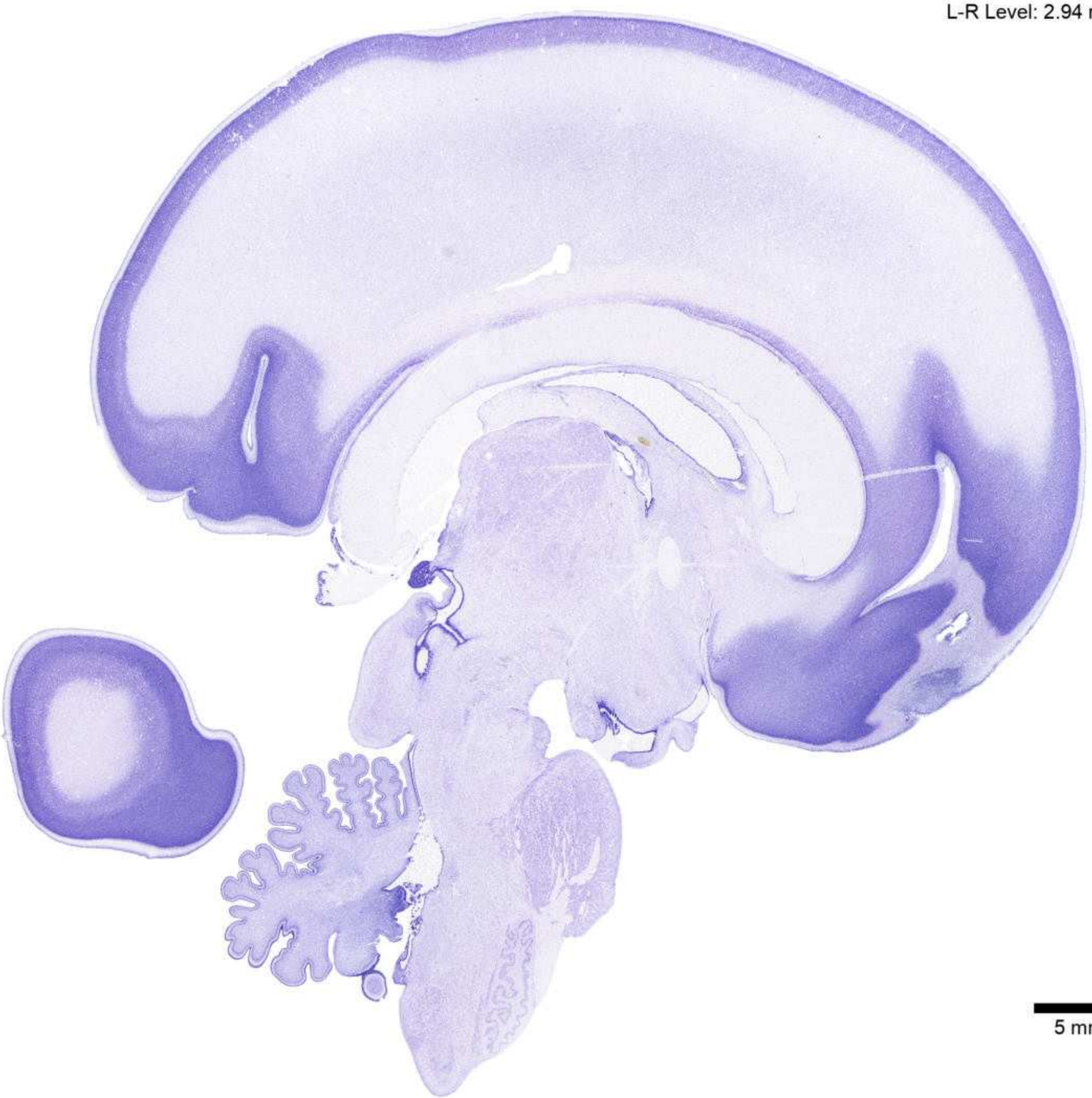

5 mm

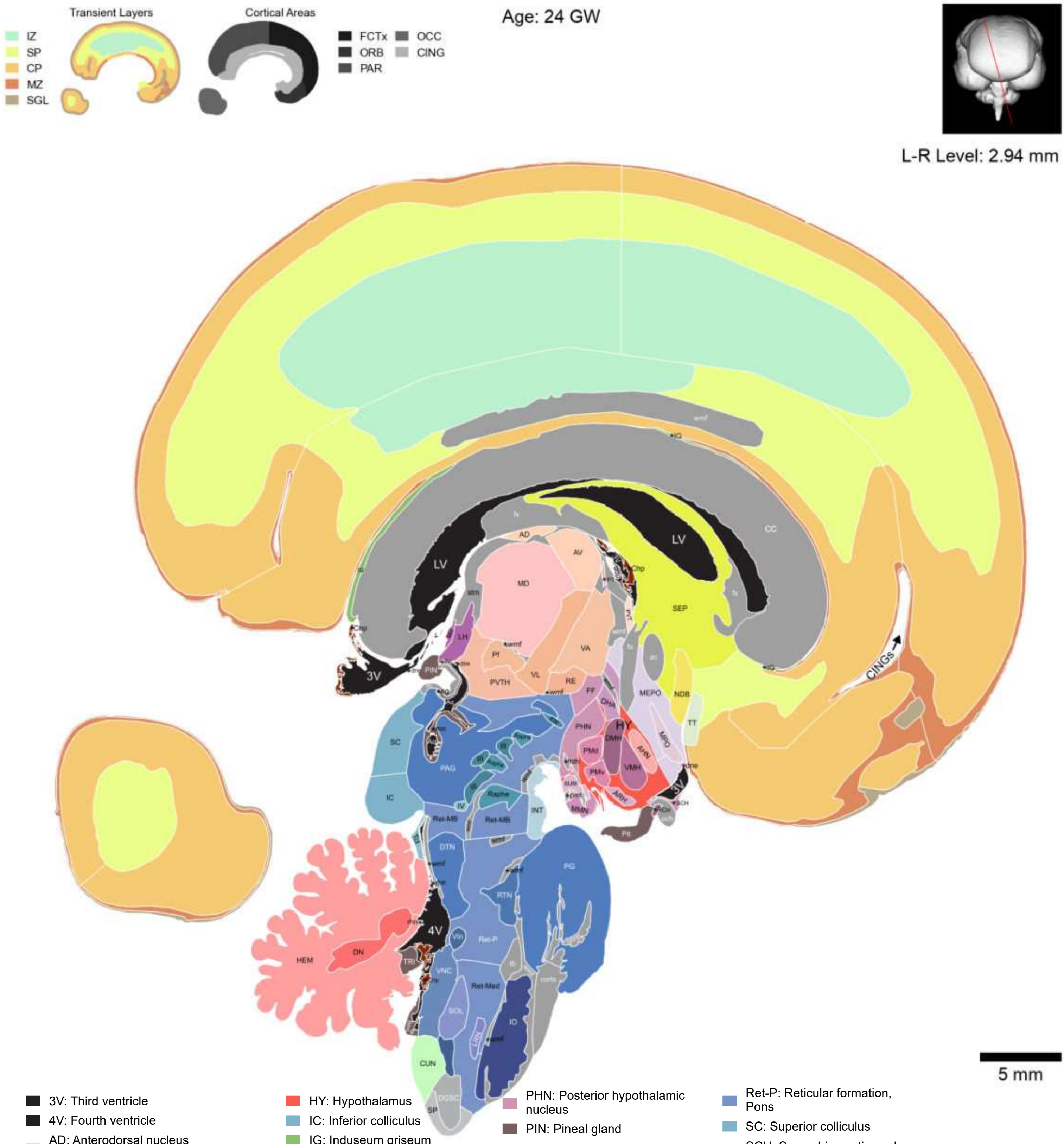

- 3V: Third ventricle
- 4V: Fourth ventricle
- AD: Anterodorsal nucleus [thalamus]
- AHN: Anterior hypothalamic nucleus
- ARH: Arcuate nucleus [hypothalamus]
- AV: Anteroventral nucleus [thalamus]
- Aq: Aqueduct
- CUN: Cuneate nucleus
- Chp: Choroid plexus
- DGSC: Dorsal gray of the spinal cord
- DHA: Dorsal hypothalamic area
- DMH: Dorsomedial nucleus [hypothalamus]
- DN: Dentate nucleus
- DTN: Dorsal tegmental nucleus
- EW: Edinger-Westphal nucleus
- FF: Field of Forel
- HEM: Cerebellar hemispheres
- HY: Hypothalamus
- IC: Inferior colliculus
- IG: Induseum griseum
- III: Oculomotor nuclear complex
- INT: Interpeduncular nucleus
- IO: Inferior olive
- IV: Trochlear nucleus
- LH: Lateral habenula
- LRN: Lateral reticular nucleus
- LV: Lateral ventricle
- MD: Medial dorsal nucleus [thalamus]
- MEPO: Medial preoptic area
- MH: Medial habenula
- MMN: Medial mammillary nucleus
- MPO: Medial preoptic nucleus
- NDB: Nucleus of the diagonal band
- PAG: Periaqueductal gray
- PG: Pontine gray
- PHN: Posterior hypothalamic nucleus
- PIN: Pineal gland
- PMd: Dorsal premammillary nucleus
- PMv: Ventral premammillary nucleus
- PT: Paratenial nucleus [thalamus]
- PVT: Paraventricular nucleus [thalamus]
- PVTH: Periventricular complex [thalamus]
- Pf: Parafascicular nucleus [thalamus]
- Pit: Pituitary gland
- RCH: Retrochiasmatic nucleus [hypothalamus]
- RE: Nucleus reuniens
- RTN: Reticular tegmental nucleus
- Raphe: Raphe nuclei
- Ret-MB: Reticular formation, Midbrain
- Ret-Med: Reticular formation, Medulla
- Ret-P: Reticular formation, Pons
- SC: Superior colliculus
- SCH: Suprachiasmatic nucleus [hypothalamus]
- SEP: Septum
- SOL: Solitary nucleus
- SP: Spinal cord
- SPV: Spinal nucleus of the trigeminal
- SUM: Supramammillary area
- TRI: Germinal trigone
- TT: Tenia tecta
- Tct: Tectum
- VA: Ventral anterior nucleus [thalamus]
- VIn: Abducens nucleus
- VL: Ventral lateral nucleus [thalamus]
- VMH: Ventromedial nucleus [hypothalamus]
- VNC: Vestibular nuclear complex
- → CINGs: Cingulate sulcus

Age: 24 GW

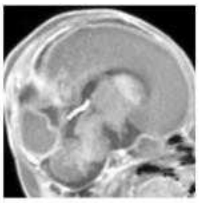

L-R Level: 2.76 mm

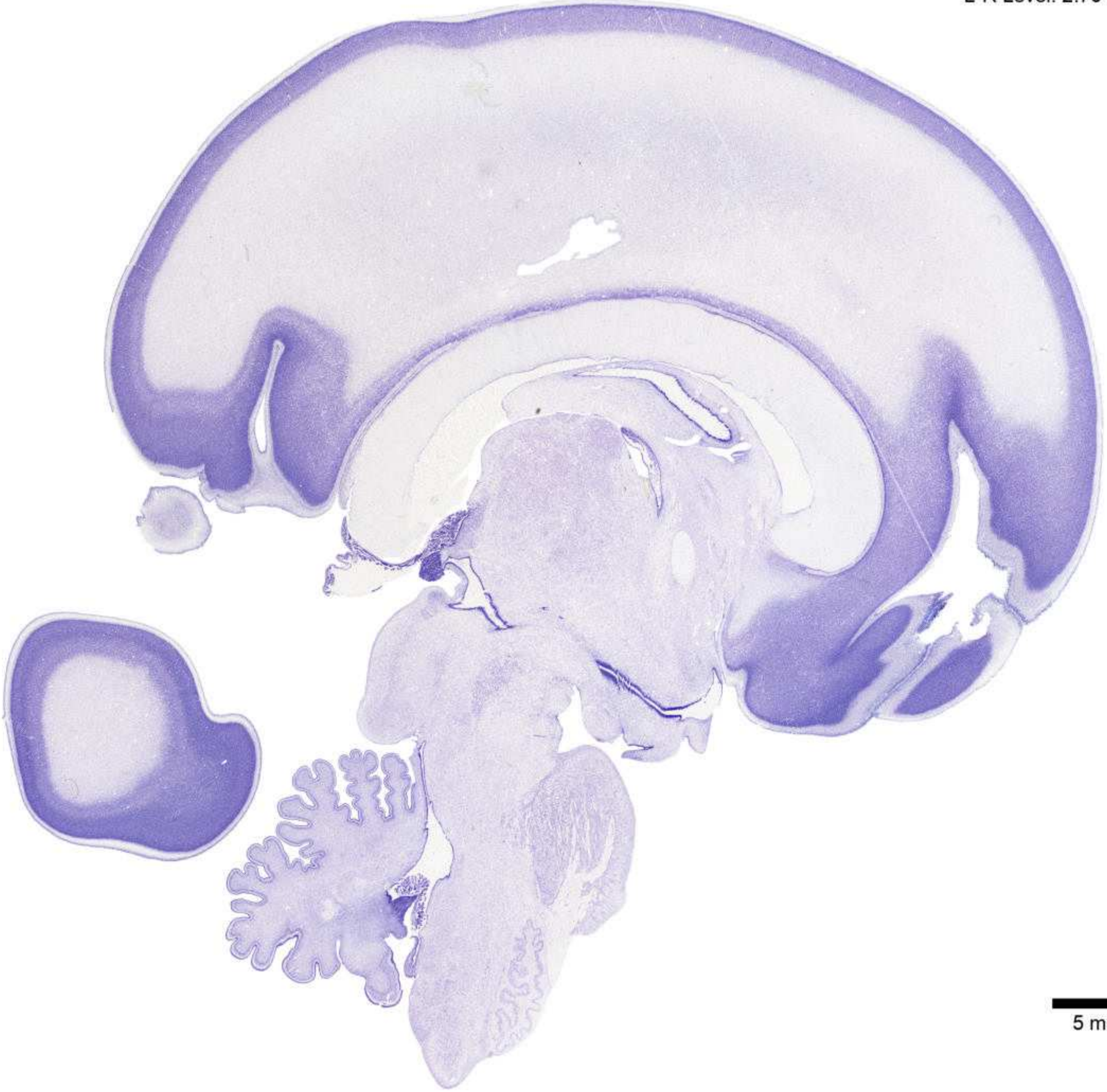

5 mm

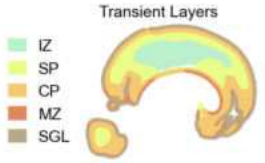

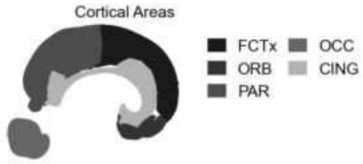

Age: 24 GW

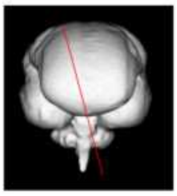

L-R Level: 2.76 mm

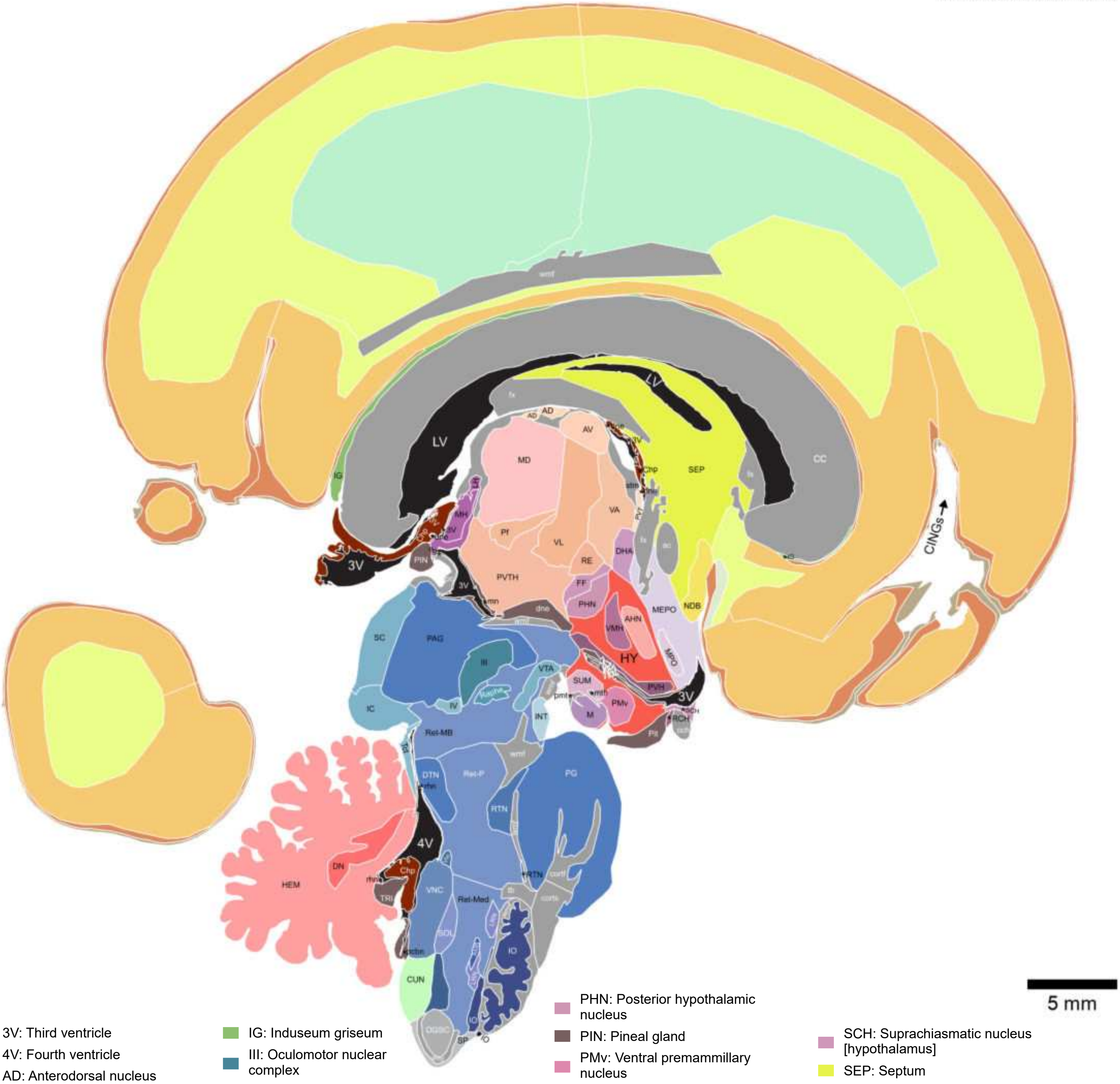

- 3V: Third ventricle
- 4V: Fourth ventricle
- AD: Anterodorsal nucleus [thalamus]
- AHN: Anterior hypothalamic nucleus
- AMB: Nucleus ambiguus
- AV: Anteroventral nucleus [thalamus]
- CUN: Cuneate nucleus
- Chp: Choroid plexus
- DGSC: Dorsal gray of the spinal cord
- DHA: Dorsal hypothalamic area
- DN: Dentate nucleus
- DTN: Dorsal tegmental nucleus
- FF: Field of Forel
- HEM: Cerebellar hemispheres
- HY: Hypothalamus
- IC: Inferior colliculus
- IG: Induseum griseum
- III: Oculomotor nuclear complex
- INT: Interpeduncular nucleus
- IO: Inferior olive
- IV: Trochlear nucleus
- LH: Lateral habenula
- LRN: Lateral reticular nucleus
- LV: Lateral ventricle
- M: Mammillary body
- MD: Medial dorsal nucleus [thalamus]
- MEPO: Medial preoptic area
- MH: Medial habenula
- MPO: Medial preoptic nucleus
- NDB: Nucleus of the diagonal band
- PAG: Periaqueductal gray
- PG: Pontine gray
- PHN: Posterior hypothalamic nucleus
- PIN: Pineal gland
- PMv: Ventral premammillary nucleus
- PVH: Paraventricular nucleus [hypothalamus]
- PVT: Paraventricular nucleus [thalamus]
- PVTH: Periventricular complex [thalamus]
- Pf: Parafascicular nucleus [thalamus]
- Pit: Pituitary gland
- RCH: Retrochiasmatic nucleus [hypothalamus]
- RE: Nucleus reuniens
- RTN: Reticular tegmental nucleus
- Raphe: Raphe nuclei
- Ret-MB: Reticular formation, Midbrain
- Ret-Med: Reticular formation, Medulla
- Ret-P: Reticular formation, Pons
- SC: Superior colliculus
- SCH: Suprachiasmatic nucleus [hypothalamus]
- SEP: Septum
- SOL: Solitary nucleus
- SP: Spinal cord
- SPV: Spinal nucleus of the trigeminal
- SUM: Supramammillary area
- TRI: Germinal trigone
- TT: Tenia tecta
- Tct: Tectum
- VA: Ventral anterior nucleus [thalamus]
- VIn: Abducens nucleus
- VL: Ventral lateral nucleus [thalamus]
- VMH: Ventromedial nucleus [hypothalamus]
- VNC: Vestibular nuclear complex
- VTA: Ventral tegmental area
- → CINGs: Cingulate sulcus

Age: 24 GW

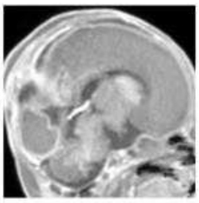

L-R Level: 1.68 mm

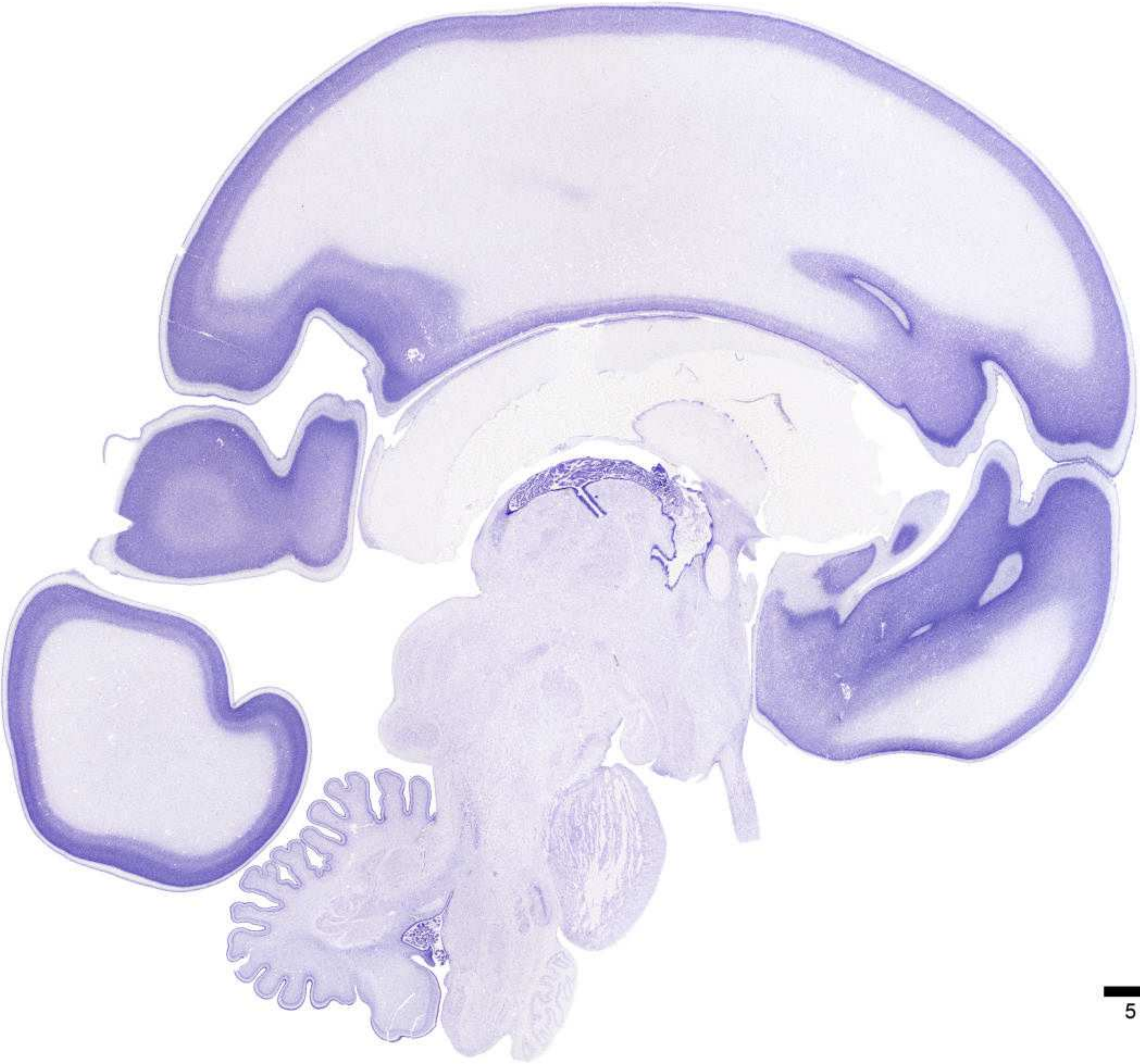

5 mm

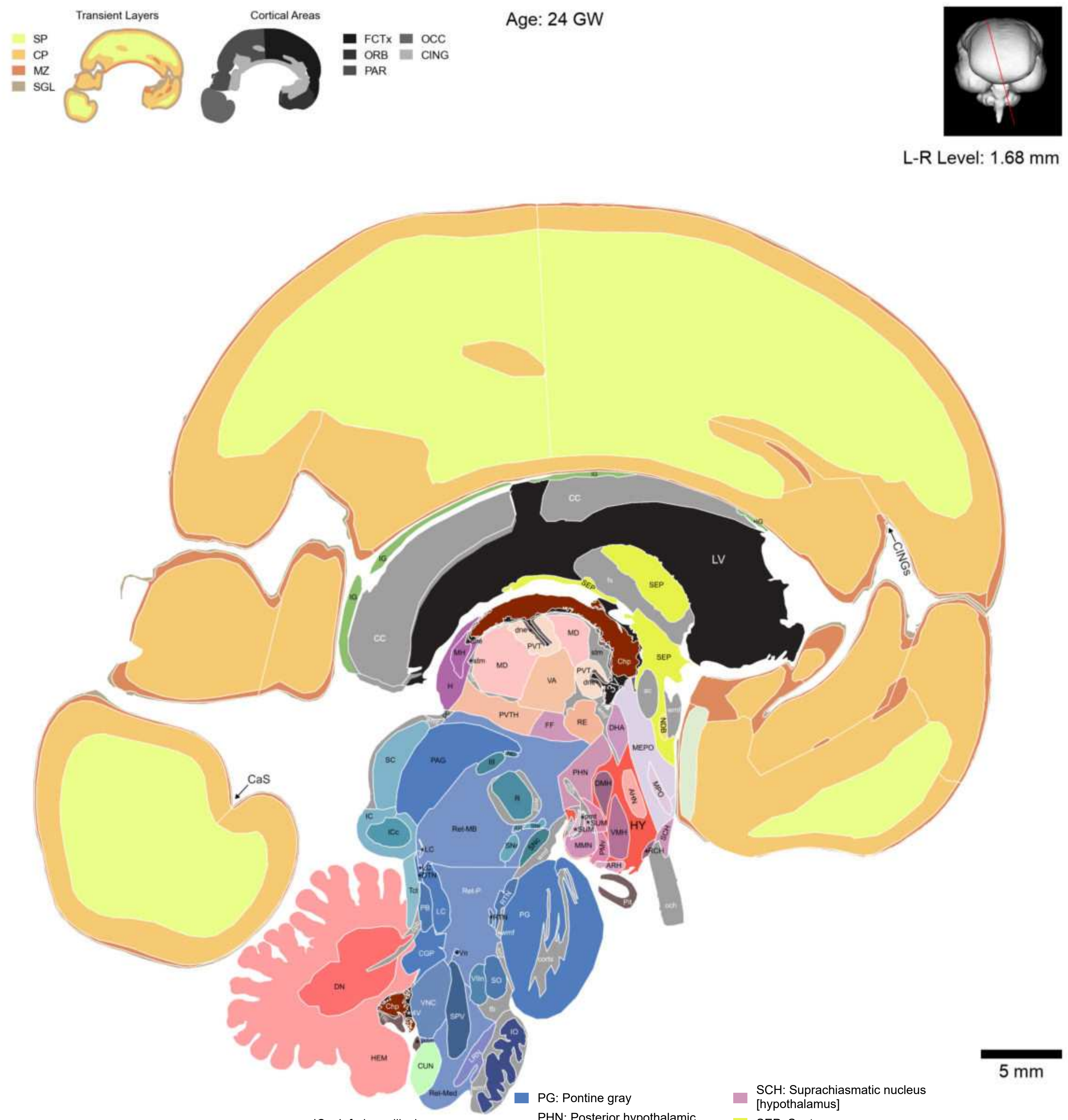

- 3V: Third ventricle
- 4V: Fourth ventricle
- AHN: Anterior hypothalamic nucleus
- ARH: Arcuate nucleus [hypothalamus]
- CGP: Central gray of the pons
- CUN: Cuneate nucleus
- Chp: Choroid plexus
- DHA: Dorsal hypothalamic area
- DMH: Dorsomedial nucleus [hypothalamus]
- DN: Dentate nucleus
- DTN: Dorsal tegmental nucleus
- FF: Field of Forel
- H: Habenula
- HEM: Cerebellar hemispheres
- HY: Hypothalamus
- IC: Inferior colliculus
- ICc: Inferior colliculus, central nucleus
- IG: Induseum griseum
- III: Oculomotor nuclear complex
- IO: Inferior olive
- LC: Locus coeruleus
- LRN: Lateral reticular nucleus
- LV: Lateral ventricle
- MD: Medial dorsal nucleus [thalamus]
- MEPO: Medial preoptic area
- MH: Medial habenula
- MMN: Medial mammillary nucleus
- MPO: Medial preoptic nucleus
- ND: Nucleus of Darkschewitsch
- NDB: Nucleus of the diagonal band
- PAG: Periaqueductal gray
- PB: Parabrachial nucleus
- PG: Pontine gray
- PHN: Posterior hypothalamic nucleus
- PMd: Dorsal premammillary nucleus
- PMv: Ventral premammillary nucleus
- PVT: Paraventricular nucleus [thalamus]
- PVTH: Periventricular complex [thalamus]
- Pit: Pituitary gland
- R: Red nucleus
- RCH: Retrochiasmatic nucleus [hypothalamus]
- RE: Nucleus reuniens
- RR: Retrorubral area
- RTN: Reticular tegmental nucleus
- Ret-MB: Reticular formation, Midbrain
- Ret-Med: Reticular formation, Medulla
- Ret-P: Reticular formation, Pons
- SC: Superior colliculus
- SCH: Suprachiasmatic nucleus [hypothalamus]
- SEP: Septum
- SNc: Substantia nigra pars compacta
- SNr: Substantia nigra pars reticulata
- SO: Superior olive
- SPV: Spinal nucleus of the trigeminal
- SUM: Supramammillary area
- TRI: Germinal trigone
- TT: Tenia tecta
- Tct: Tectum
- VA: Ventral anterior nucleus [thalamus]
- VIIn: Facial motor nucleus
- VMH: Ventromedial nucleus [hypothalamus]
- VNC: Vestibular nuclear complex
- Vn: Trigeminal motor nucleus
- → CaS: Calcarine sulcus
- → CINGs: Cingulate sulcus

Age: 24 GW

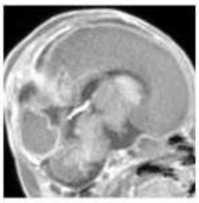

L-R Level: 1.32 mm

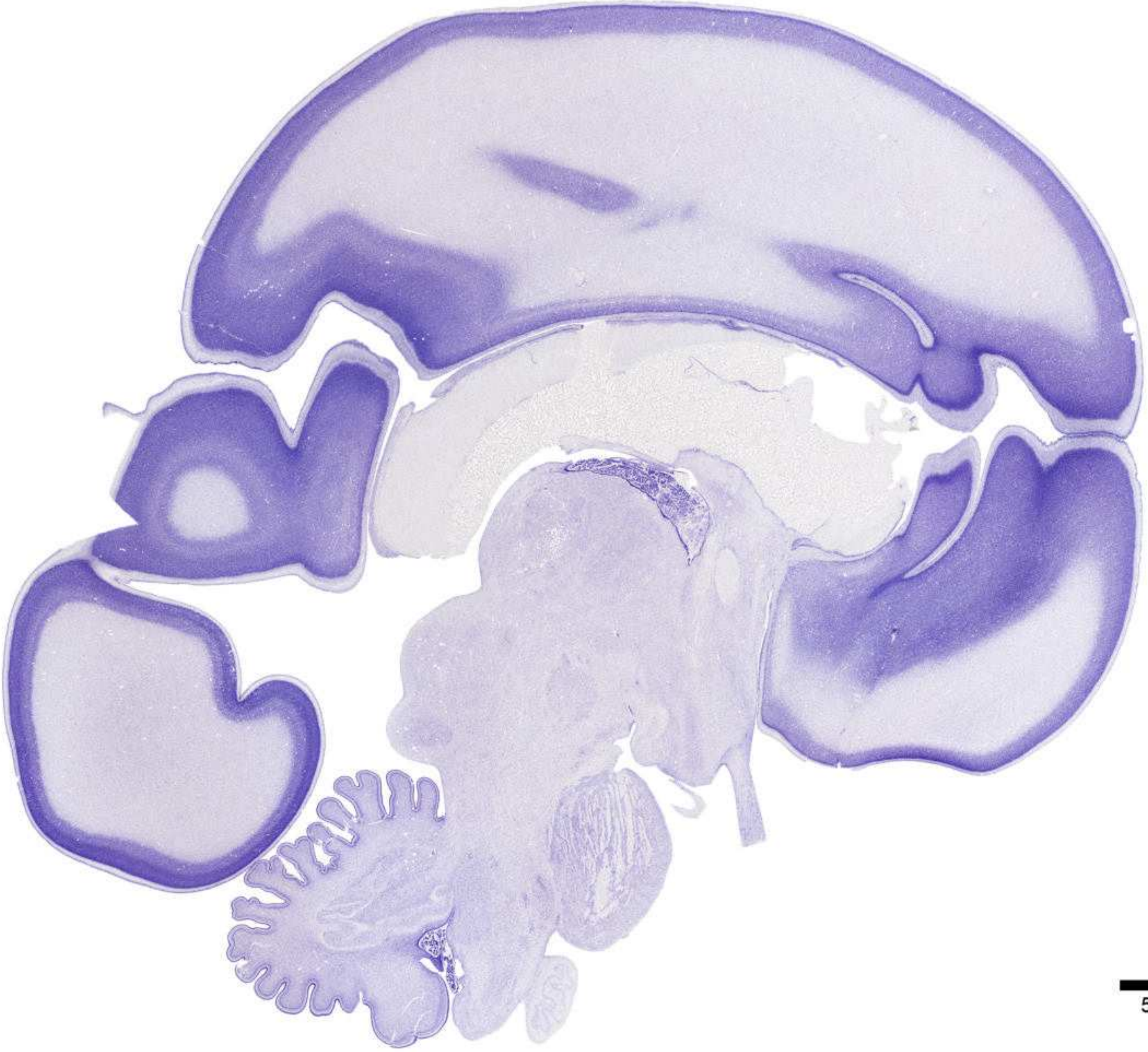

5 mm

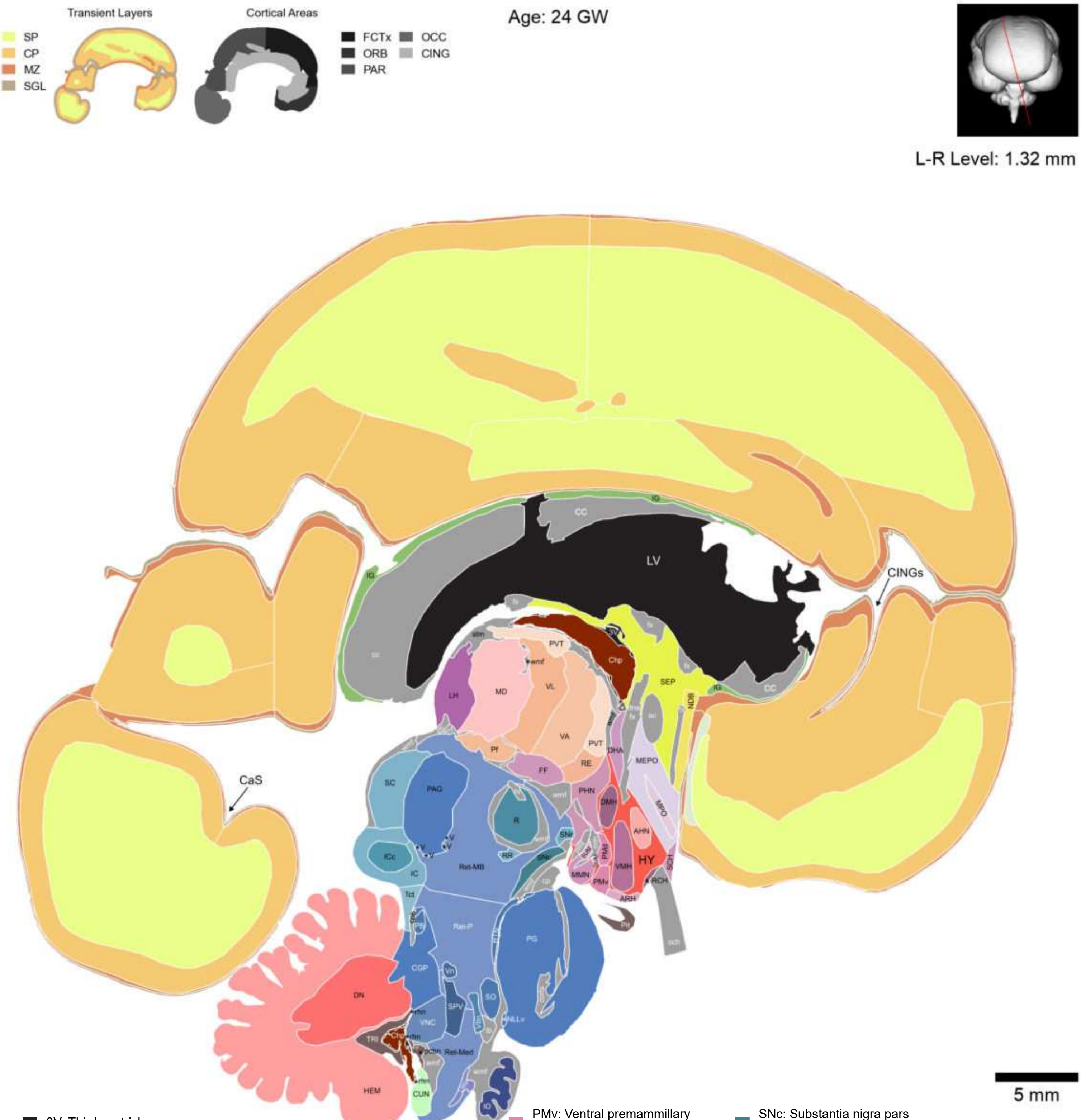

■ 3V: Third ventricle
■ 4V: Fourth ventricle
■ AHN: Anterior hypothalamic nucleus
■ ARH: Arcuate nucleus [hypothalamus]
■ CGP: Central gray of the pons
■ CUN: Cuneate nucleus
■ Chp: Choroid plexus
■ DHA: Dorsal hypothalamic area
■ DMH: Dorsomedial nucleus [hypothalamus]
■ DN: Dentate nucleus
■ FF: Field of Forel
■ HEM: Cerebellar hemispheres
■ HY: Hypothalamus
■ IC: Inferior colliculus
■ ICc: Inferior colliculus, central nucleus
■ IG: Induseum griseum
■ IO: Inferior olive
■ LH: Lateral habenula
■ LRN: Lateral reticular nucleus
■ LV: Lateral ventricle
■ MD: Medial dorsal nucleus [thalamus]
■ MEPO: Medial preoptic area
■ MMN: Medial mammillary nucleus
■ MPO: Medial preoptic nucleus
■ NDB: Nucleus of the diagonal band
■ NLLv: Nucleus of the lateral lemniscus, ventral
■ PAG: Periaqueductal gray
■ PB: Parabrachial nucleus
■ PG: Pontine gray
■ PHN: Posterior hypothalamic nucleus
■ PMd: Dorsal premammillary nucleus
■ PMv: Ventral premammillary nucleus
■ PVT: Paraventricular nucleus [thalamus]
■ Pf: Parafascicular nucleus [thalamus]
■ Pit: Pituitary gland
■ R: Red nucleus
■ RCH: Retrochiasmatic nucleus [hypothalamus]
■ RE: Nucleus reuniens
■ RR: Retrorubral area
■ RTN: Reticular tegmental nucleus
■ Ret-MB: Reticular formation, Midbrain
■ Ret-Med: Reticular formation, Medulla
■ Ret-P: Reticular formation, Pons
■ SC: Superior colliculus
■ SCH: Suprachiasmatic nucleus [hypothalamus]
■ SEP: Septum
■ SNc: Substantia nigra pars compacta
■ SNr: Substantia nigra pars reticulata
■ SO: Superior olive
■ SPV: Spinal nucleus of the trigeminal
■ SUM: Supramammillary area
■ TRI: Germinal trigone
■ TT: Tenia tecta
■ Tct: Tectum
■ V: Mesencephalic nucleus
■ VA: Ventral anterior nucleus [thalamus]
■ VIIn: Facial motor nucleus
■ VL: Ventral lateral nucleus [thalamus]
■ VMH: Ventromedial nucleus [hypothalamus]
■ VNC: Vestibular nuclear complex
■ Vn: Trigeminal motor nucleus
→ CaS: Calcarine sulcus
→ CINGs: Cingulate sulcus

Age: 24 GW

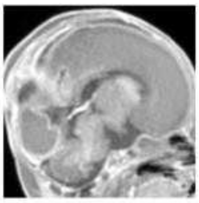

L-R Level: 0.96 mm

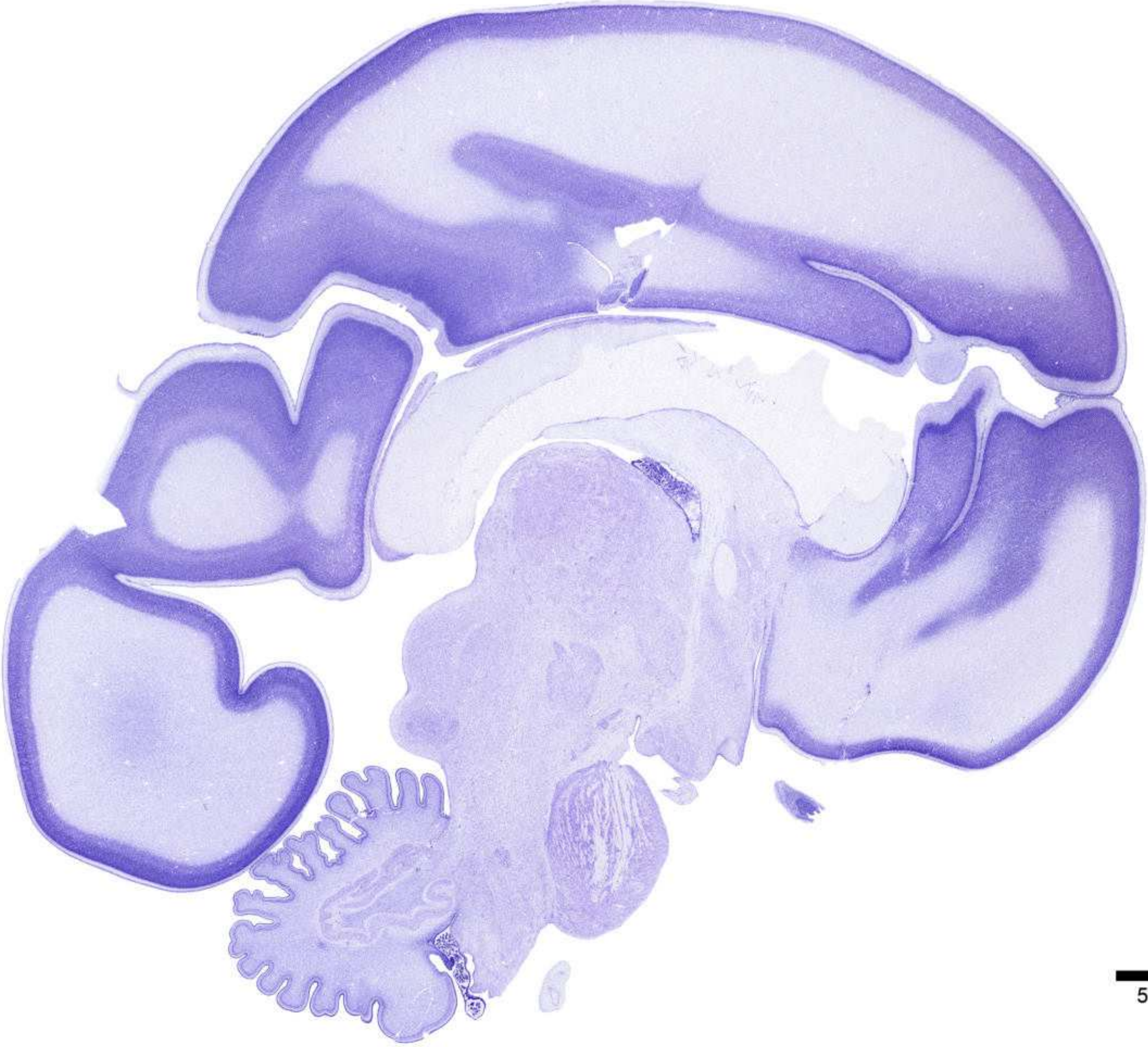

5 mm

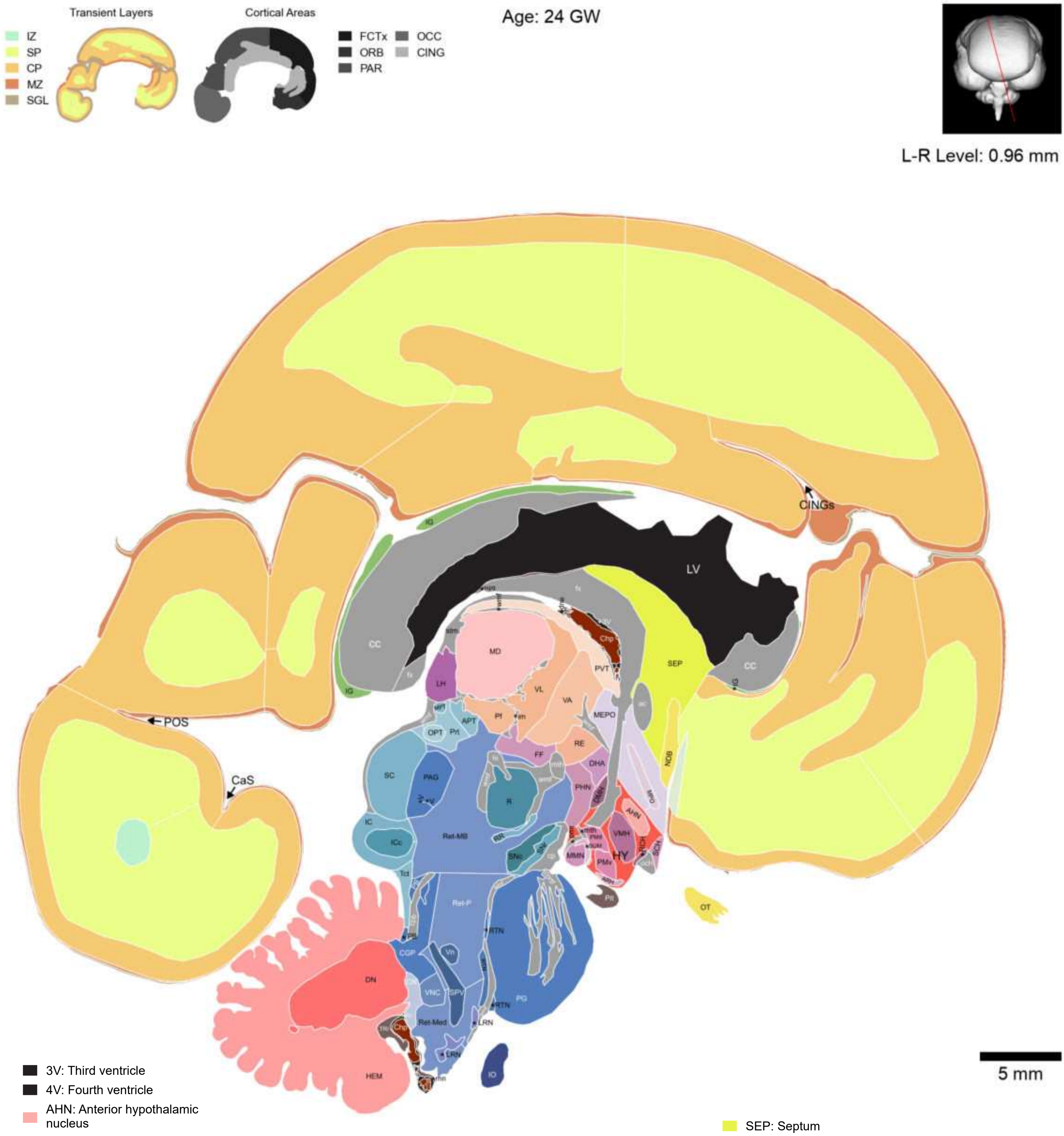

- 3V: Third ventricle
- 4V: Fourth ventricle
- AHN: Anterior hypothalamic nucleus
- APT: Anterior pretectal nucleus
- ARH: Arcuate nucleus [hypothalamus]
- CGP: Central gray of the pons
- CN: Cochlear nuclei
- Chp: Choroid plexus
- DHA: Dorsal hypothalamic area
- DMH: Dorsomedial nucleus [hypothalamus]
- DN: Dentate nucleus
- FF: Field of Forel
- HEM: Cerebellar hemispheres
- HY: Hypothalamus
- IC: Inferior colliculus
- ICc: Inferior colliculus, central nucleus
- IG: Induseum griseum
- IO: Inferior olive
- LH: Lateral habenula
- LRN: Lateral reticular nucleus
- LV: Lateral ventricle
- MD: Medial dorsal nucleus [thalamus]
- MEPO: Medial preoptic area
- MMN: Medial mammillary nucleus
- MPO: Medial preoptic nucleus
- MPT: Medial pretectal nucleus
- NDB: Nucleus of the diagonal band
- OPT: Olivary pretectal nucleus
- OT: Olfactory tubercle
- PAG: Periaqueductal gray
- PB: Parabrachial nucleus
- PG: Pontine gray
- PHN: Posterior hypothalamic nucleus
- PMd: Dorsal premammillary nucleus
- PMv: Ventral premammillary nucleus
- PVT: Paraventricular nucleus [thalamus]
- Pf: Parafascicular nucleus [thalamus]
- Pit: Pituitary gland
- Prt: Pretectum
- R: Red nucleus
- RCH: Retrochiasmatic nucleus [hypothalamus]
- RE: Nucleus reuniens
- RR: Retrorubral area
- RTN: Reticular tegmental nucleus
- Ret-MB: Reticular formation, Midbrain
- Ret-Med: Reticular formation, Medulla
- Ret-P: Reticular formation, Pons
- SC: Superior colliculus
- SCH: Suprachiasmatic nucleus [hypothalamus]
- SEP: Septum
- SNc: Substantia nigra pars compacta
- SNr: Substantia nigra pars reticulata
- SPV: Spinal nucleus of the trigeminal
- SUM: Supramammillary area
- TRI: Germinal trigone
- TT: Tenia tecta
- Tct: Tectum
- V: Mesencephalic nucleus
- VA: Ventral anterior nucleus [thalamus]
- VL: Ventral lateral nucleus [thalamus]
- VMH: Ventromedial nucleus [hypothalamus]
- VNC: Vestibular nuclear complex
- Vn: Trigeminal motor nucleus
- → CaS: Calcarine sulcus
- → CINGs: Cingulate sulcus
- → POS: Parieto-occipital sulcus

Age: 24 GW

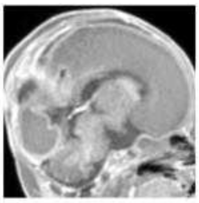

L-R Level: 0.72 mm

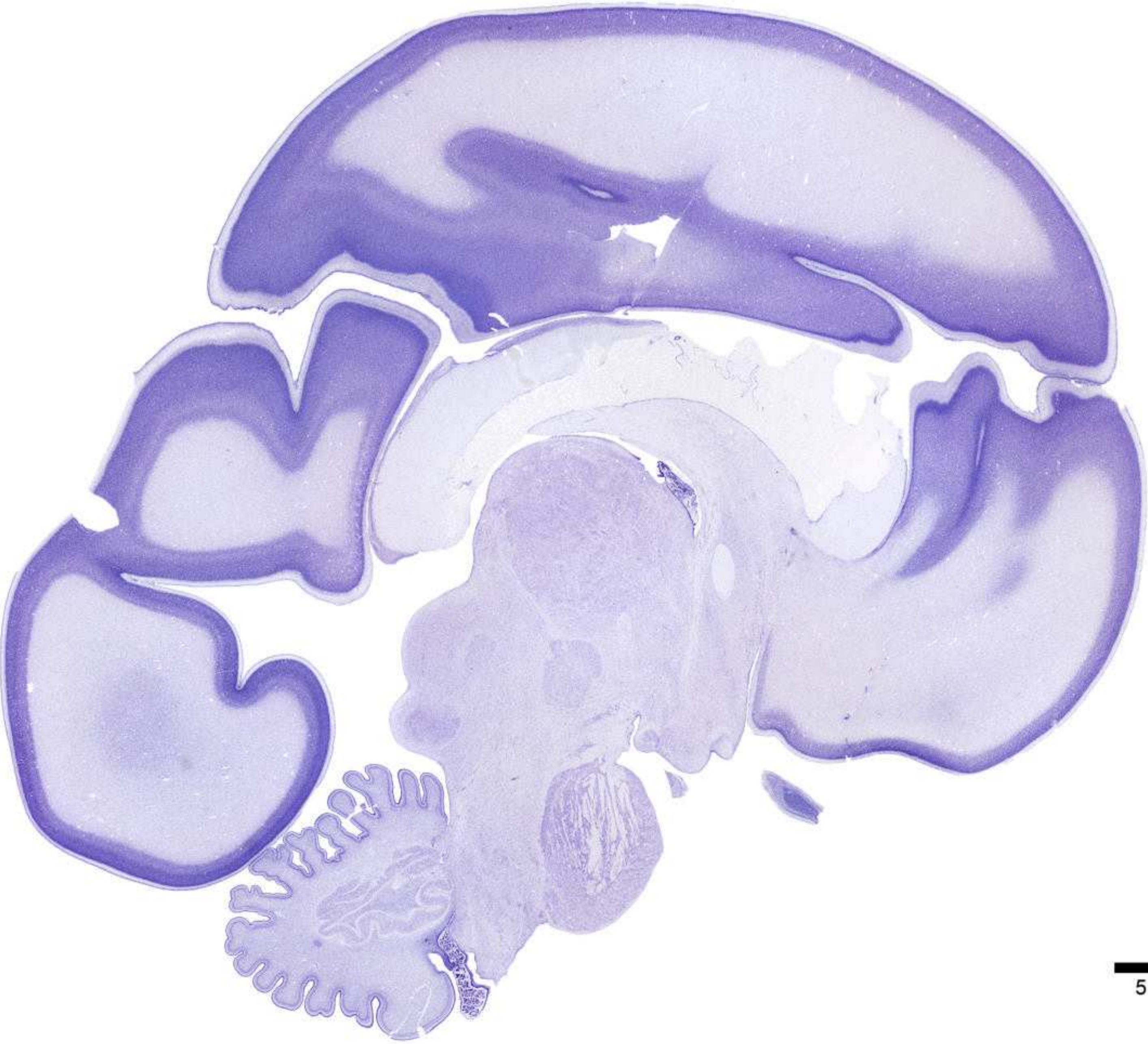

5 mm

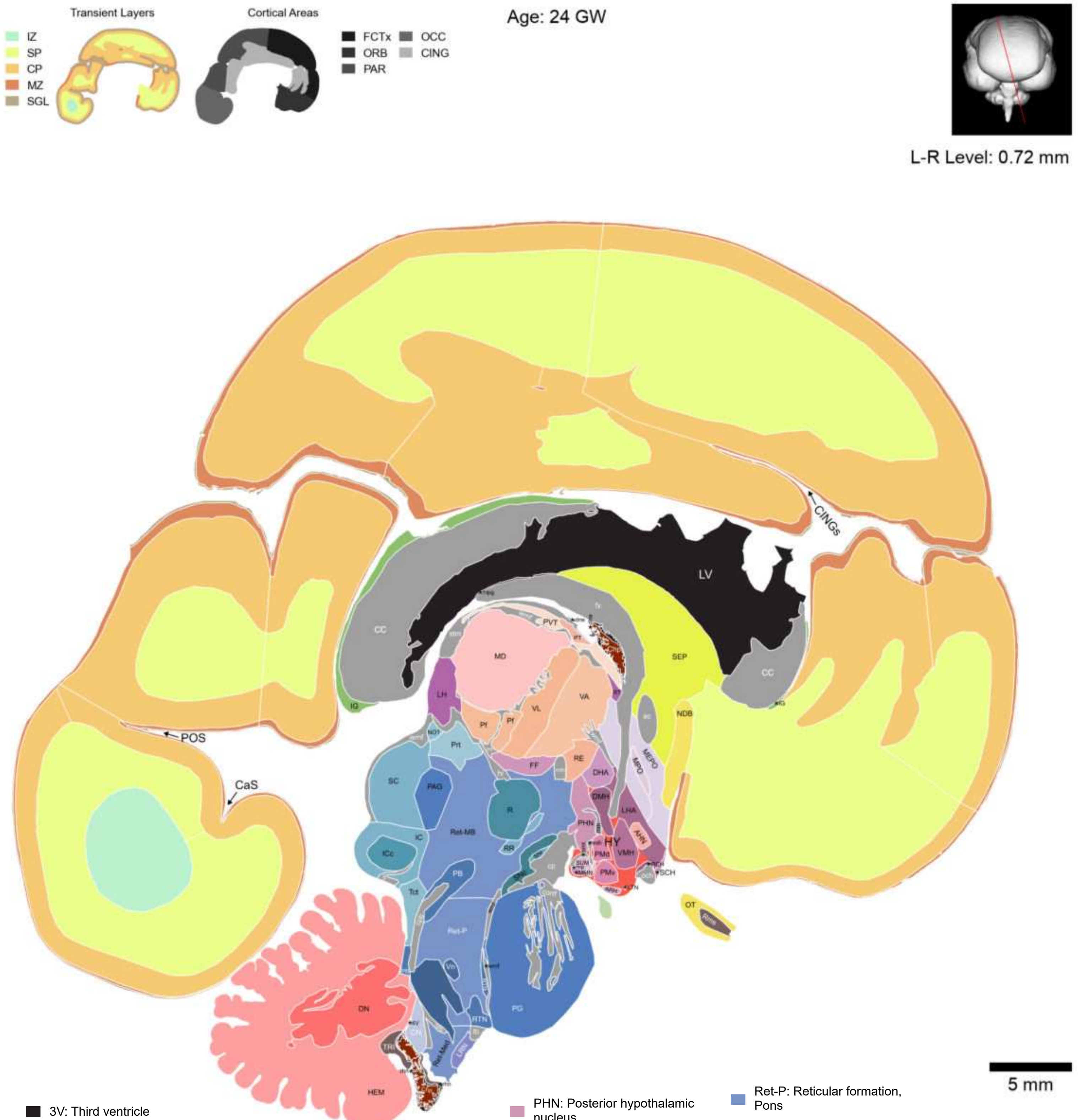

- 3V: Third ventricle
- 4V: Fourth ventricle
- AHN: Anterior hypothalamic nucleus
- ARH: Arcuate nucleus [hypothalamus]
- CN: Cochlear nuclei
- Chp: Choroid plexus
- DHA: Dorsal hypothalamic area
- DMH: Dorsomedial nucleus [hypothalamus]
- DN: Dentate nucleus
- FF: Field of Forel
- HEM: Cerebellar hemispheres
- HY: Hypothalamus
- IC: Inferior colliculus
- ICc: Inferior colliculus, central nucleus
- IG: Induseum griseum
- LH: Lateral habenula
- LHA: Lateral hypothalamic area
- LRN: Lateral reticular nucleus
- LTN: Lateral tuberal nucleus
- LV: Lateral ventricle
- MD: Medial dorsal nucleus [thalamus]
- MEPO: Medial preoptic area
- MMN: Medial mammillary nucleus
- MPO: Medial preoptic nucleus
- NDB: Nucleus of the diagonal band
- NOT: Nucleus of the optic tract
- OT: Olfactory tubercle
- PAG: Periaqueductal gray
- PARG: Parahippocampal gyrus
- PB: Parabrachial nucleus
- PG: Pontine gray
- PHN: Posterior hypothalamic nucleus
- PMd: Dorsal premammillary nucleus
- PMv: Ventral premammillary nucleus
- PT: Paratenial nucleus [thalamus]
- PVT: Paraventricular nucleus [thalamus]
- Pf: Parafascicular nucleus [thalamus]
- Prt: Pretectum
- R: Red nucleus
- RCH: Retrochiasmatic nucleus [hypothalamus]
- RE: Nucleus reuniens
- RR: Retrorubral area
- RT: Reticular nucleus [thalamus]
- RTN: Reticular tegmental nucleus
- Ret-MB: Reticular formation, Midbrain
- Ret-Med: Reticular formation, Medulla
- Ret-P: Reticular formation, Pons
- Rms: Rostral migratory stream
- SC: Superior colliculus
- SCH: Suprachiasmatic nucleus [hypothalamus]
- SEP: Septum
- SNc: Substantia nigra pars compacta
- SNr: Substantia nigra pars reticulata
- SPV: Spinal nucleus of the trigeminal
- SUM: Supramammillary area
- TRI: Germinal trigone
- Tct: Tectum
- VA: Ventral anterior nucleus [thalamus]
- VL: Ventral lateral nucleus [thalamus]
- VMH: Ventromedial nucleus [hypothalamus]
- Vn: Trigeminal motor nucleus
- → CaS: Calcarine sulcus
- → CINGs: Cingulate sulcus
- → POS: Parieto-occipital sulcus

Age: 24 GW

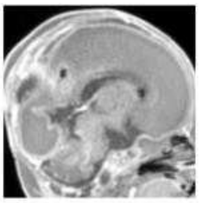

L-R Level: 0.48 mm

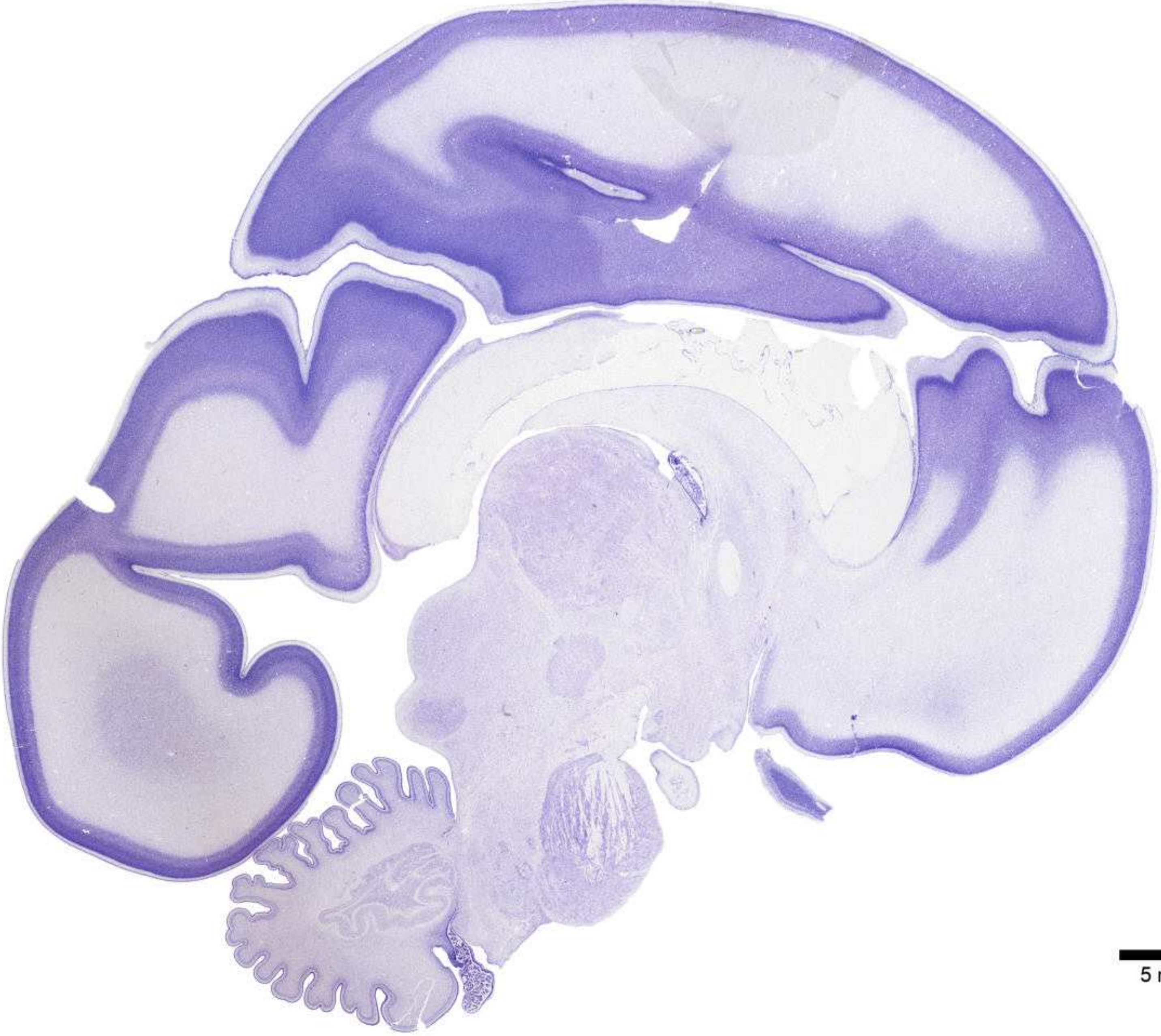

5 mm

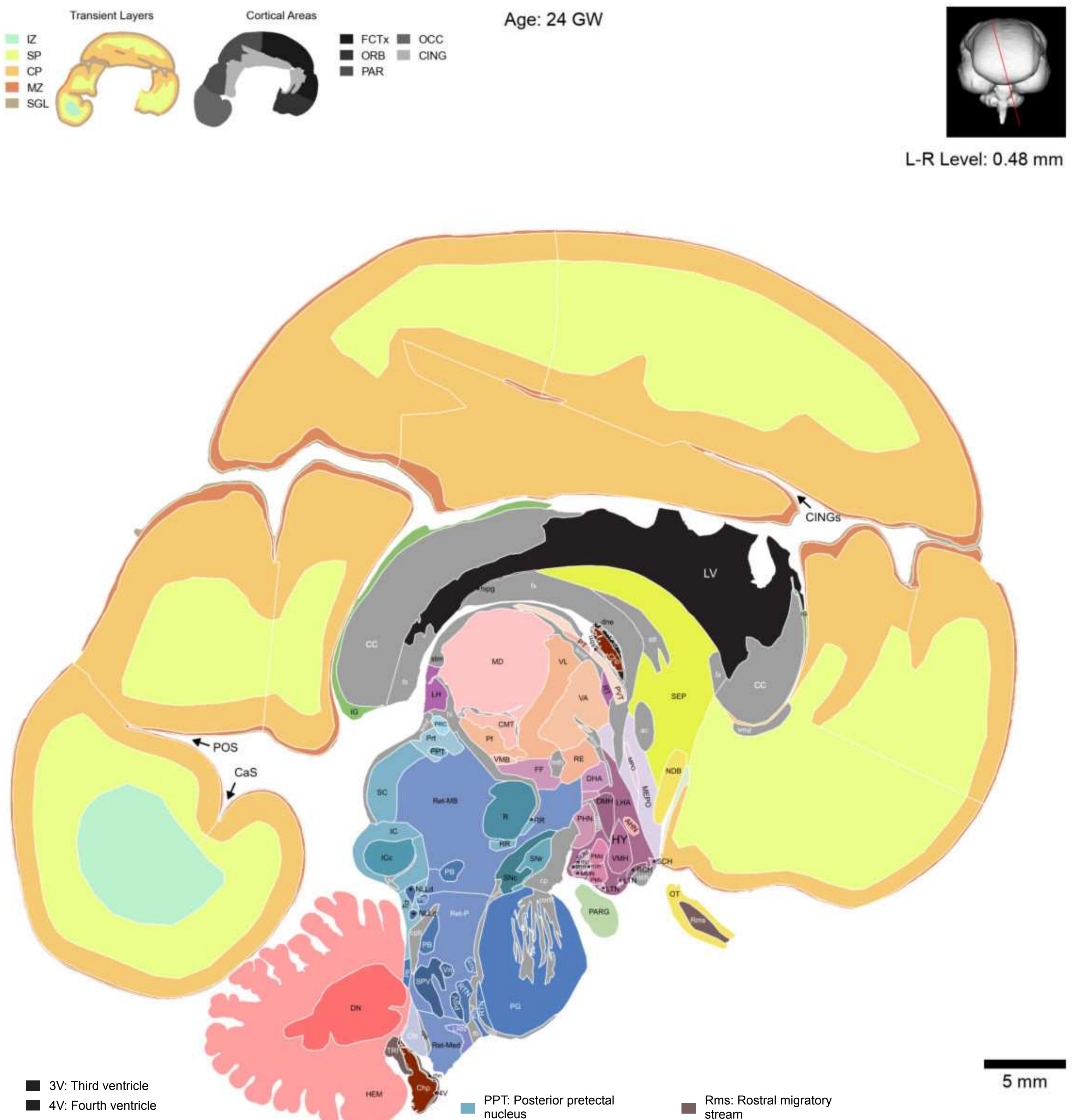

- 3V: Third ventricle
- 4V: Fourth ventricle
- AHN: Anterior hypothalamic nucleus
- CMT: Centromedian nucleus [thalamus]
- CN: Cochlear nuclei
- Chp: Choroid plexus
- DHA: Dorsal hypothalamic area
- DMH: Dorsomedial nucleus [hypothalamus]
- DN: Dentate nucleus
- FF: Field of Forel
- HEM: Cerebellar hemispheres
- HY: Hypothalamus
- IC: Inferior colliculus
- ICc: Inferior colliculus, central nucleus
- IG: Induseum griseum
- LH: Lateral habenula
- LHA: Lateral hypothalamic area
- LRN: Lateral reticular nucleus
- LTN: Lateral tuberal nucleus
- LV: Lateral ventricle
- MD: Medial dorsal nucleus [thalamus]
- MEPO: Medial preoptic area
- MMN: Medial mammillary nucleus
- MPO: Medial preoptic nucleus
- NDB: Nucleus of the diagonal band
- NLLd: Nucleus of the lateral lemniscus, dorsal
- OT: Olfactory tubercle
- PARG: Parahippocampal gyrus
- PB: Parabrachial nucleus
- PG: Pontine gray
- PHN: Posterior hypothalamic nucleus
- PMd: Dorsal premammillary nucleus
- PMv: Ventral premammillary nucleus
- PPT: Posterior pretectal nucleus
- PRC: Precommissural nucleus
- PSV: Principal sensory nucleus of the trigeminal
- PT: Paratenial nucleus [thalamus]
- PVT: Paraventricular nucleus [thalamus]
- Pf: Parafascicular nucleus [thalamus]
- Prt: Pretectum
- R: Red nucleus
- RCH: Retrochiasmatic nucleus [hypothalamus]
- RE: Nucleus reuniens
- RR: Retrorubral area
- RT: Reticular nucleus [thalamus]
- RTN: Reticular tegmental nucleus
- Ret-MB: Reticular formation, Midbrain
- Ret-Med: Reticular formation, Medulla
- Ret-P: Reticular formation, Pons
- Rms: Rostral migratory stream
- SC: Superior colliculus
- SCH: Suprachiasmatic nucleus [hypothalamus]
- SEP: Septum
- SNc: Substantia nigra pars compacta
- SNr: Substantia nigra pars reticulata
- SPV: Spinal nucleus of the trigeminal
- SUM: Supramammillary area
- TRI: Germinal trigone
- TT: Tenia tecta
- Tct: Tectum
- VA: Ventral anterior nucleus [thalamus]
- VL: Ventral lateral nucleus [thalamus]
- VMB: Ventral medial basal nucleus [thalamus]
- VMH: Ventromedial nucleus [hypothalamus]
- Vn: Trigeminal motor nucleus
- → CaS: Calcarine sulcus
- → CINGs: Cingulate sulcus
- → POS: Parieto-occipital sulcus

Age: 24 GW

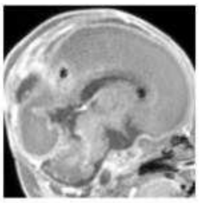

L-R Level: 0.24 mm

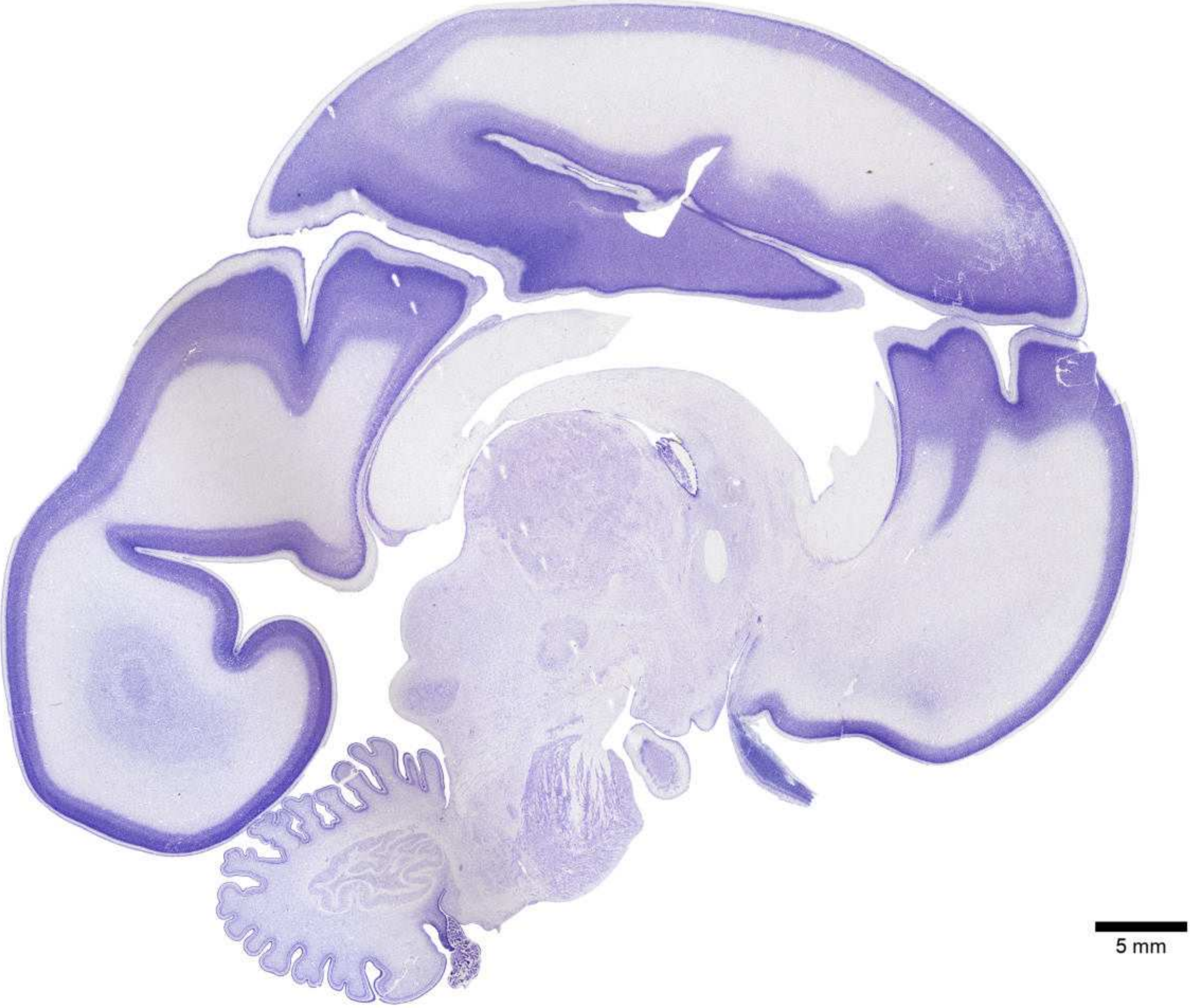

5 mm

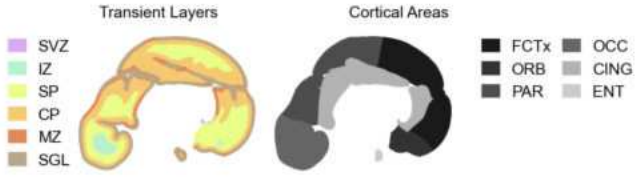

Age: 24 GW

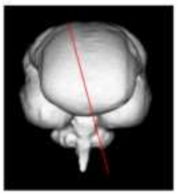

L-R Level: 0.24 mm

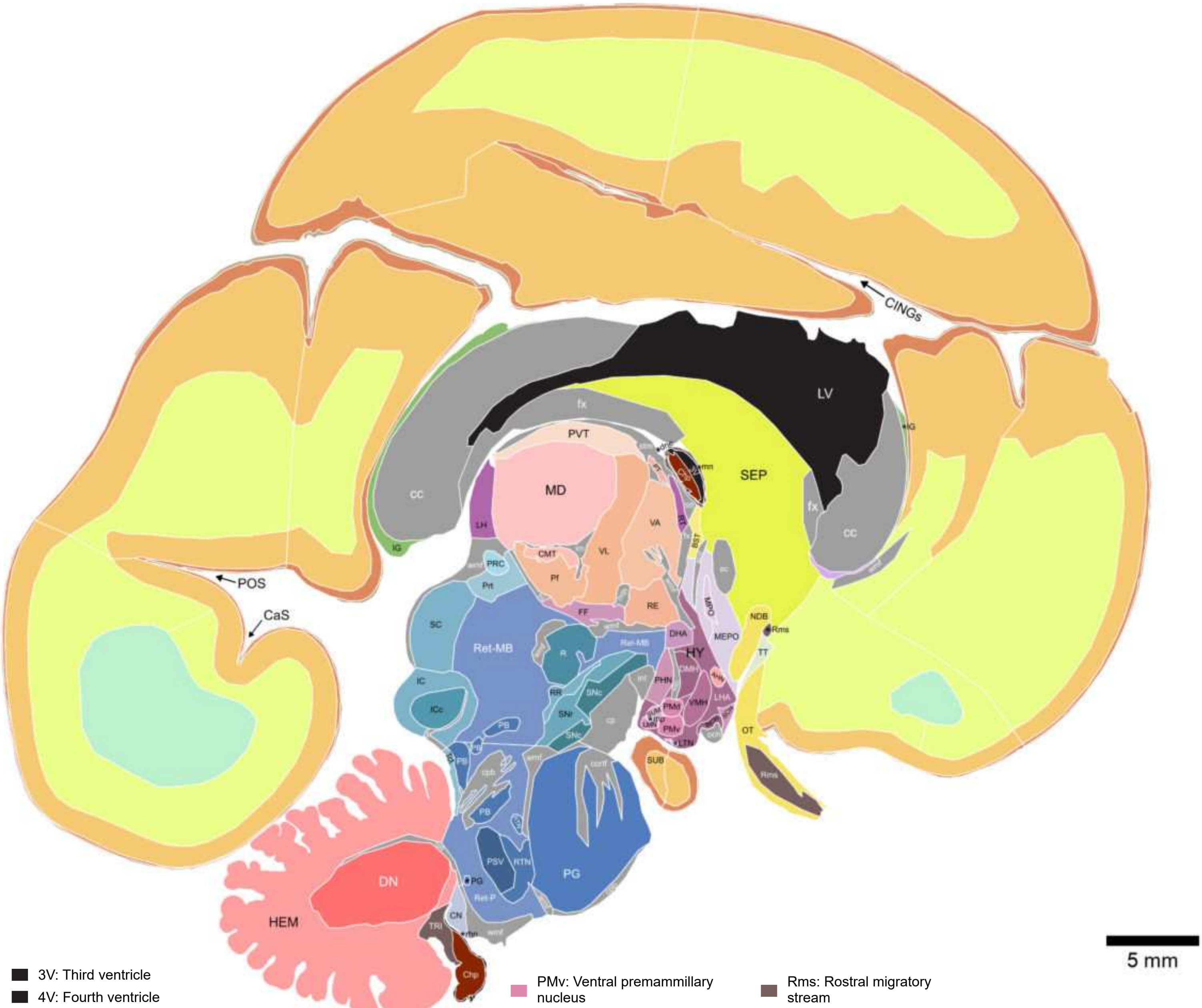

- 3V: Third ventricle
- 4V: Fourth ventricle
- AHN: Anterior hypothalamic nucleus
- BST: Bed nucleus of the stria terminalis
- CMT: Centromedian nucleus [thalamus]
- CN: Cochlear nuclei
- Chp: Choroid plexus
- DHA: Dorsal hypothalamic area
- DMH: Dorsomedial nucleus [hypothalamus]
- DN: Dentate nucleus
- FF: Field of Forel
- HEM: Cerebellar hemispheres
- HY: Hypothalamus
- IC: Inferior colliculus
- ICc: Inferior colliculus, central nucleus
- IG: Induseum griseum
- LH: Lateral habenula
- LHA: Lateral hypothalamic area
- LMN: Lateral mammillary nucleus
- LTN: Lateral tuberal nucleus
- LV: Lateral ventricle
- MD: Medial dorsal nucleus [thalamus]
- MEPO: Medial preoptic area
- MPO: Medial preoptic nucleus
- NDB: Nucleus of the diagonal band
- OT: Olfactory tubercle
- PB: Parabrachial nucleus
- PG: Pontine gray
- PHN: Posterior hypothalamic nucleus
- PMd: Dorsal premammillary nucleus
- PMv: Ventral premammillary nucleus
- PRC: Precommissural nucleus
- PSV: Principal sensory nucleus of the trigeminal
- PT: Paratenial nucleus [thalamus]
- PVT: Paraventricular nucleus [thalamus]
- Pf: Parafascicular nucleus [thalamus]
- Prt: Pretectum
- R: Red nucleus
- RCH: Retrochiasmatic nucleus [hypothalamus]
- RE: Nucleus reuniens
- RR: Retrorubral area
- RT: Reticular nucleus [thalamus]
- RTN: Reticular tegmental nucleus
- Ret-MB: Reticular formation, Midbrain
- Ret-P: Reticular formation, Pons
- Rms: Rostral migratory stream
- SC: Superior colliculus
- SCH: Suprachiasmatic nucleus [hypothalamus]
- SEP: Septum
- SNc: Substantia nigra pars compacta
- SNr: Substantia nigra pars reticulata
- SUB: Cortical plate, subiculum
- SUM: Supramammillary area
- TRI: Germinal trigone
- TT: Tenia tecta
- Tct: Tectum
- VA: Ventral anterior nucleus [thalamus]
- VL: Ventral lateral nucleus [thalamus]
- VMH: Ventromedial nucleus [hypothalamus]

→ CaS: Calcarine sulcus
→ CINGs: Cingulate sulcus
→ POS: Parieto-occipital sulcus

Age: 24 GW

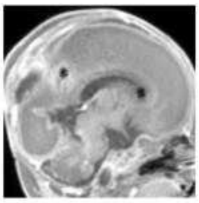

L-R Level: -0.12 mm

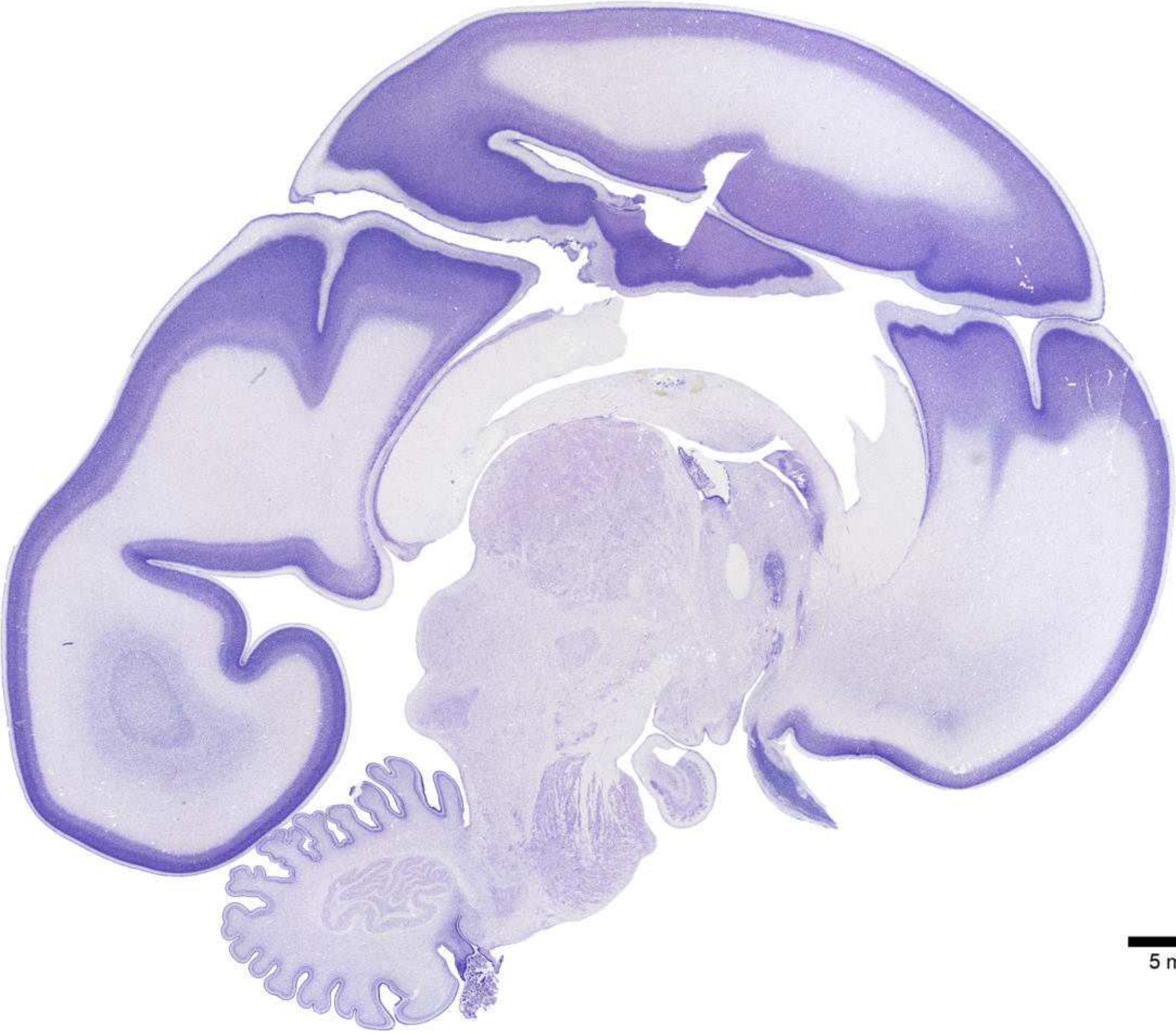

5 mm

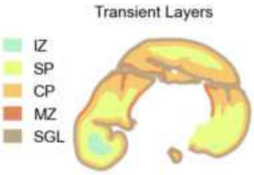

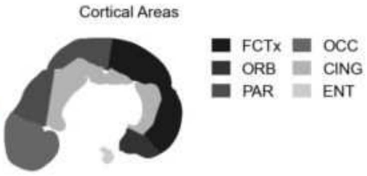

Age: 24 GW

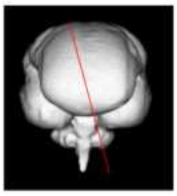

L-R Level: -0.12 mm

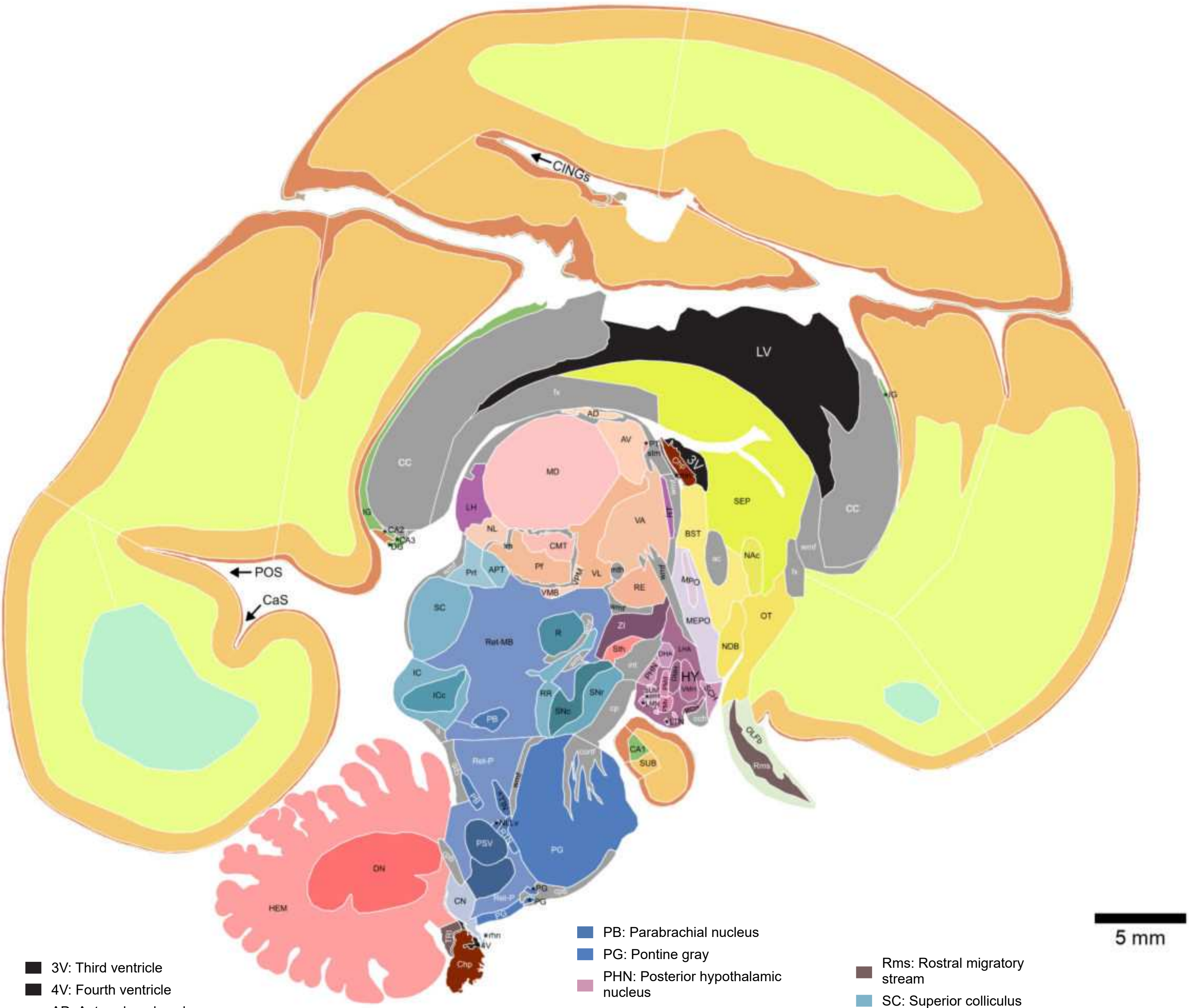

3V: Third ventricle
4V: Fourth ventricle
AD: Anterodorsal nucleus [thalamus]
APT: Anterior pretectal nucleus
AV: Anteroventral nucleus [thalamus]
BST: Bed nucleus of the stria terminalis
CA1: CA1 field [hippocampus]
CA2: CA2 field [hippocampus]
CA3: CA3 field [hippocampus]
CMT: Centromedian nucleus [thalamus]
CN: Cochlear nuclei
Chp: Choroid plexus
DG: Dentate gyrus
DHA: Dorsal hypothalamic area
DMH: Dorsomedial nucleus [hypothalamus]
DN: Dentate nucleus
HEM: Cerebellar hemispheres
HY: Hypothalamus
IC: Inferior colliculus
ICc: Inferior colliculus, central nucleus
IG: Induseum griseum
LH: Lateral habenula
LHA: Lateral hypothalamic area
LMN: Lateral mammillary nucleus
LTN: Lateral tuberal nucleus
LV: Lateral ventricle
MD: Medial dorsal nucleus [thalamus]
MEPO: Medial preoptic area
MPO: Medial preoptic nucleus
NAc: Nucleus accumbens
NDB: Nucleus of the diagonal band
NL: Nucleus limitans [thalamus]
NLLv: Nucleus of the lateral lemniscus, ventral
OLFb: Olfactory bulb
OT: Olfactory tubercle
PB: Parabrachial nucleus
PG: Pontine gray
PHN: Posterior hypothalamic nucleus
PMd: Dorsal premammillary nucleus
PMv: Ventral premammillary nucleus
PSV: Principal sensory nucleus of the trigeminal
PT: Paratenial nucleus [thalamus]
Pf: Parafascicular nucleus [thalamus]
Prt: Pretectum
R: Red nucleus
RCH: Retrochiasmatic nucleus [hypothalamus]
RE: Nucleus reuniens
RR: Retrorubral area
RT: Reticular nucleus [thalamus]
RTN: Reticular tegmental nucleus
Ret-MB: Reticular formation, Midbrain
Ret-P: Reticular formation, Pons
Rms: Rostral migratory stream
SC: Superior colliculus
SCH: Suprachiasmatic nucleus [hypothalamus]
SEP: Septum
SNc: Substantia nigra pars compacta
SNr: Substantia nigra pars reticulata
SPV: Spinal nucleus of the trigeminal
SUB: Cortical plate, subiculum
SUM: Supramammillary area
Sth: Subthalamus
TRI: Germinal trigone
VA: Ventral anterior nucleus [thalamus]
VL: Ventral lateral nucleus [thalamus]
VMB: Ventral medial basal nucleus [thalamus]
VMH: Ventromedial nucleus [hypothalamus]
VPM: Ventral posteromedial nucleus [thalamus]
ZI: Zona incerta
→ CaS: Calcarine sulcus
→ CINGs: Cingulate sulcus
→ POS: Parieto-occipital sulcus

Age: 24 GW

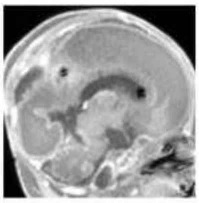

L-R Level: -0.36 mm

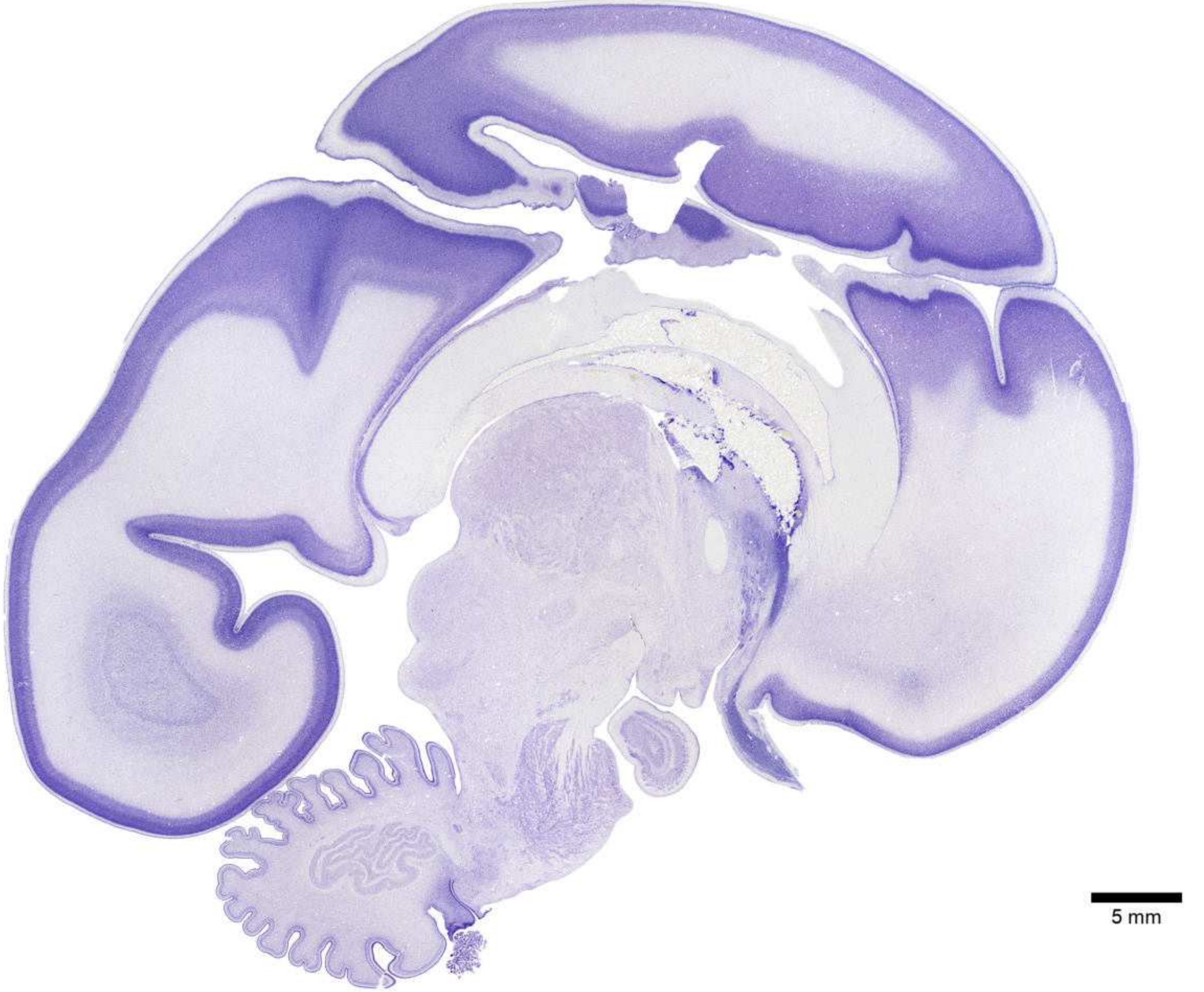

Transient Layers

- SVZ
- IZ
- SP
- CP
- MZ
- SGL

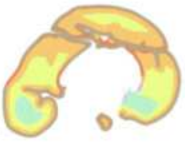

Cortical Areas

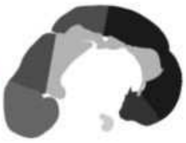

- FCTx
- ORB
- PAR
- OCC
- CING
- ENT

Age: 24 GW

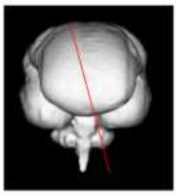

L-R Level: -0.36 mm

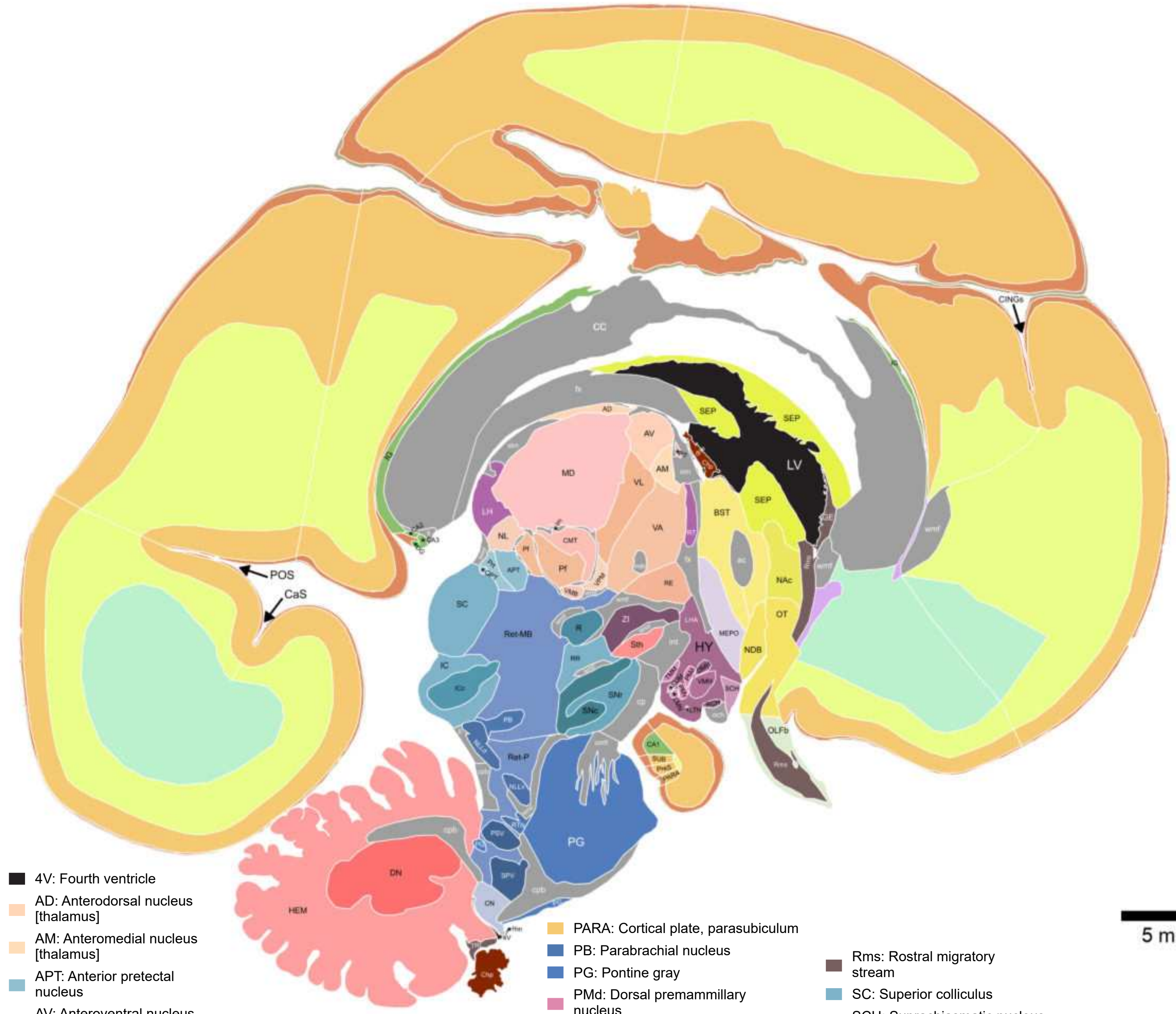

5 mm

- 4V: Fourth ventricle
- AD: Anterodorsal nucleus [thalamus]
- AM: Anteromedial nucleus [thalamus]
- APT: Anterior pretectal nucleus
- AV: Anteroventral nucleus [thalamus]
- BST: Bed nucleus of the stria terminalis
- CA1: CA1 field [hippocampus]
- CA2: CA2 field [hippocampus]
- CA3: CA3 field [hippocampus]
- CMT: Centromedian nucleus [thalamus]
- CN: Cochlear nuclei
- Chp: Choroid plexus
- DG: Dentate gyrus
- DMH: Dorsomedial nucleus [hypothalamus]
- DN: Dentate nucleus
- GE: Ganglionic eminence
- HEM: Cerebellar hemispheres
- HY: Hypothalamus
- IC: Inferior colliculus
- ICc: Inferior colliculus, central nucleus
- IG: Induseum griseum
- LH: Lateral habenula
- LHA: Lateral hypothalamic area
- LMN: Lateral mammillary nucleus
- LTN: Lateral tuberal nucleus
- LV: Lateral ventricle
- MD: Medial dorsal nucleus [thalamus]
- MEPO: Medial preoptic area
- NAc: Nucleus accumbens
- NDB: Nucleus of the diagonal band
- NL: Nucleus limitans [thalamus]
- NLLd: Nucleus of the lateral lemniscus, dorsal
- NLLv: Nucleus of the lateral lemniscus, ventral
- OLFb: Olfactory bulb
- OPT: Olivary pretectal nucleus
- OT: Olfactory tubercle
- PARA: Cortical plate, parasubiculum
- PB: Parabrachial nucleus
- PG: Pontine gray
- PMd: Dorsal premammillary nucleus
- PMv: Ventral premammillary nucleus
- PSV: Principal sensory nucleus of the trigeminal
- PT: Paratenial nucleus [thalamus]
- Pf: Parafascicular nucleus [thalamus]
- PreS: Cortical plate, presubiculum
- Prt: Pretectum
- R: Red nucleus
- RCH: Retrochiasmatic nucleus [hypothalamus]
- RE: Nucleus reuniens
- RR: Retrorubral area
- RT: Reticular nucleus [thalamus]
- RTN: Reticular tegmental nucleus
- Ret-MB: Reticular formation, Midbrain
- Ret-P: Reticular formation, Pons
- Rms: Rostral migratory stream
- SC: Superior colliculus
- SCH: Suprachiasmatic nucleus [hypothalamus]
- SEP: Septum
- SNc: Substantia nigra pars compacta
- SNr: Substantia nigra pars reticulata
- SPV: Spinal nucleus of the trigeminal
- SUB: Cortical plate, subiculum
- SUM: Supramammillary area
- Sth: Subthalamus
- TMM: Tuberomammillary nucleus
- TRI: Germinal trigone
- VA: Ventral anterior nucleus [thalamus]
- VL: Ventral lateral nucleus [thalamus]
- VMB: Ventral medial basal nucleus [thalamus]
- VMH: Ventromedial nucleus [hypothalamus]
- VPM: Ventral posteromedial nucleus [thalamus]
- ZI: Zona incerta
- → CaS: Calcarine sulcus
- → CINGs: Cingulate sulcus
- → POS: Parieto-occipital sulcus

Age: 24 GW

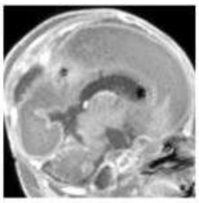

L-R Level: -0.72 mm

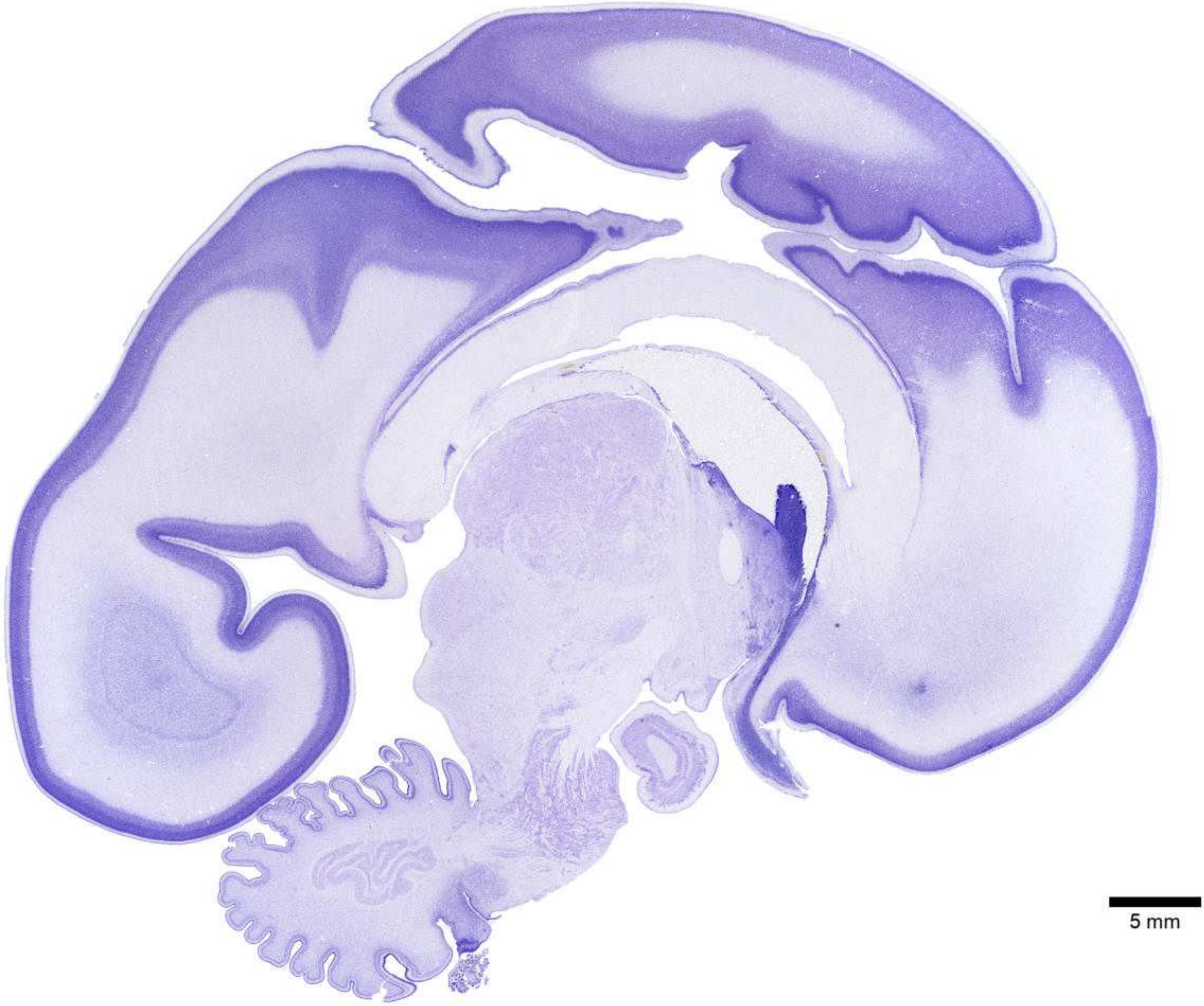

5 mm

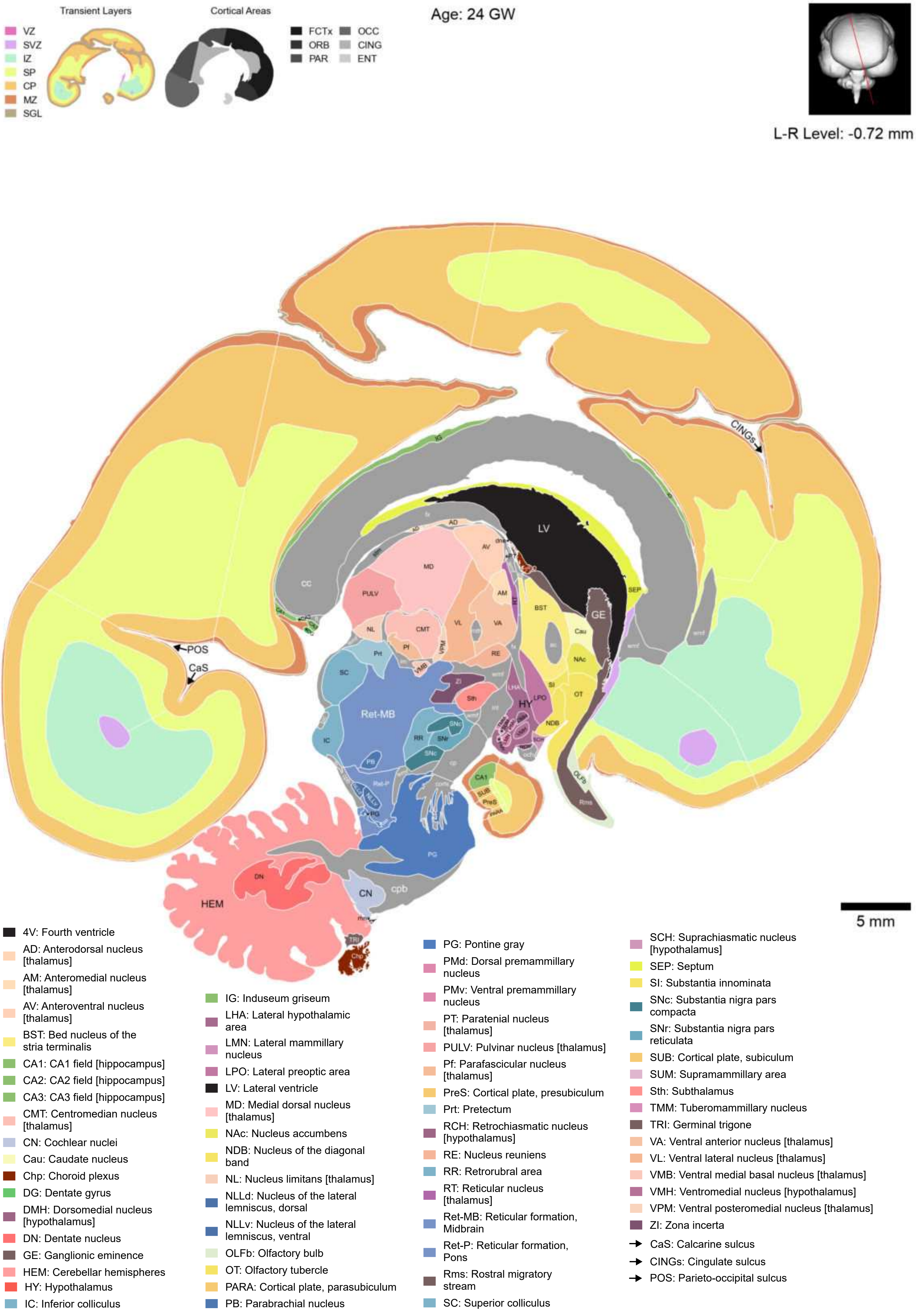

Transient Layers
VZ
SVZ
IZ
SP
CP
MZ
SGL
Cortical Areas
FCTx
ORB
PAR
OCC
CING
ENT
Age: 24 GW
L-R Level: -0.72 mm
CINGs
POS
CaS
LV
CC
GE
SEP
BST
Cau
NAc
OT
NDB
HY
LPO
LHA
MD
PULV
AD
AV
AM
NL
CMT
Pf
VL
VA
RE
VPM
Prt
SC
IC
Ret-MB
ZI
Sth
SNc
SNr
RR
PB
CA1
SUB
PreS
PARA
OLFb
Rms
PG
HEM
DN
CN
cpb
TRI
Chp
5 mm
4V: Fourth ventricle
AD: Anterodorsal nucleus [thalamus]
AM: Anteromedial nucleus [thalamus]
AV: Anteroventral nucleus [thalamus]
BST: Bed nucleus of the stria terminalis
CA1: CA1 field [hippocampus]
CA2: CA2 field [hippocampus]
CA3: CA3 field [hippocampus]
CMT: Centromedian nucleus [thalamus]
CN: Cochlear nuclei
Cau: Caudate nucleus
Chp: Choroid plexus
DG: Dentate gyrus
DMH: Dorsomedial nucleus [hypothalamus]
DN: Dentate nucleus
GE: Ganglionic eminence
HEM: Cerebellar hemispheres
HY: Hypothalamus
IC: Inferior colliculus
IG: Induseum griseum
LHA: Lateral hypothalamic area
LMN: Lateral mammillary nucleus
LPO: Lateral preoptic area
LV: Lateral ventricle
MD: Medial dorsal nucleus [thalamus]
NAc: Nucleus accumbens
NDB: Nucleus of the diagonal band
NL: Nucleus limitans [thalamus]
NLLd: Nucleus of the lateral lemniscus, dorsal
NLLv: Nucleus of the lateral lemniscus, ventral
OLFb: Olfactory bulb
OT: Olfactory tubercle
PARA: Cortical plate, parasubiculum
PB: Parabrachial nucleus
PG: Pontine gray
PMd: Dorsal premammillary nucleus
PMv: Ventral premammillary nucleus
PT: Paratenial nucleus [thalamus]
PULV: Pulvinar nucleus [thalamus]
Pf: Parafascicular nucleus [thalamus]
PreS: Cortical plate, presubiculum
Prt: Pretectum
RCH: Retrochiasmatic nucleus [hypothalamus]
RE: Nucleus reuniens
RR: Retrorubral area
RT: Reticular nucleus [thalamus]
Ret-MB: Reticular formation, Midbrain
Ret-P: Reticular formation, Pons
Rms: Rostral migratory stream
SC: Superior colliculus
SCH: Suprachiasmatic nucleus [hypothalamus]
SEP: Septum
SI: Substantia innominata
SNc: Substantia nigra pars compacta
SNr: Substantia nigra pars reticulata
SUB: Cortical plate, subiculum
SUM: Supramammillary area
Sth: Subthalamus
TMM: Tuberomammillary nucleus
TRI: Germinal trigone
VA: Ventral anterior nucleus [thalamus]
VL: Ventral lateral nucleus [thalamus]
VMB: Ventral medial basal nucleus [thalamus]
VMH: Ventromedial nucleus [hypothalamus]
VPM: Ventral posteromedial nucleus [thalamus]
ZI: Zona incerta
CaS: Calcarine sulcus
CINGs: Cingulate sulcus
POS: Parieto-occipital sulcus

Age: 24 GW

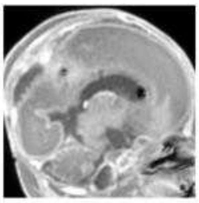

L-R Level: -1.08 mm

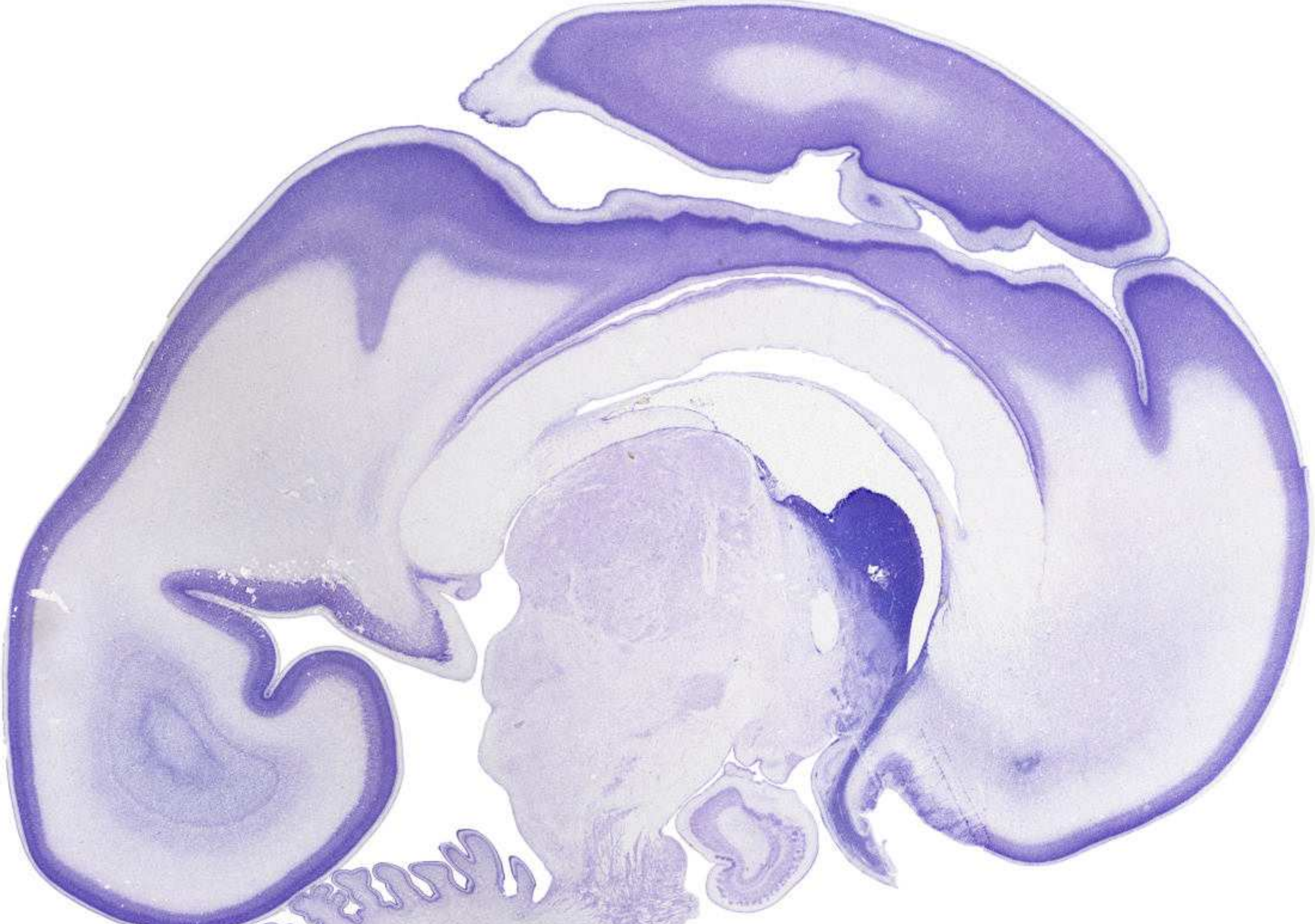

5 mm

Transient Layers

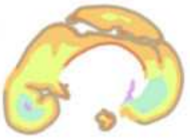

- VZ
- SVZ
- IZ
- SP
- CP
- MZ
- SGL

Cortical Areas

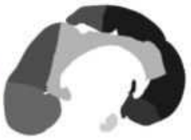

- FCTx
- ORB
- PAR
- OCC
- CING
- ENT

Age: 24 GW

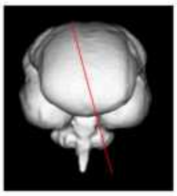

L-R Level: -1.08 mm

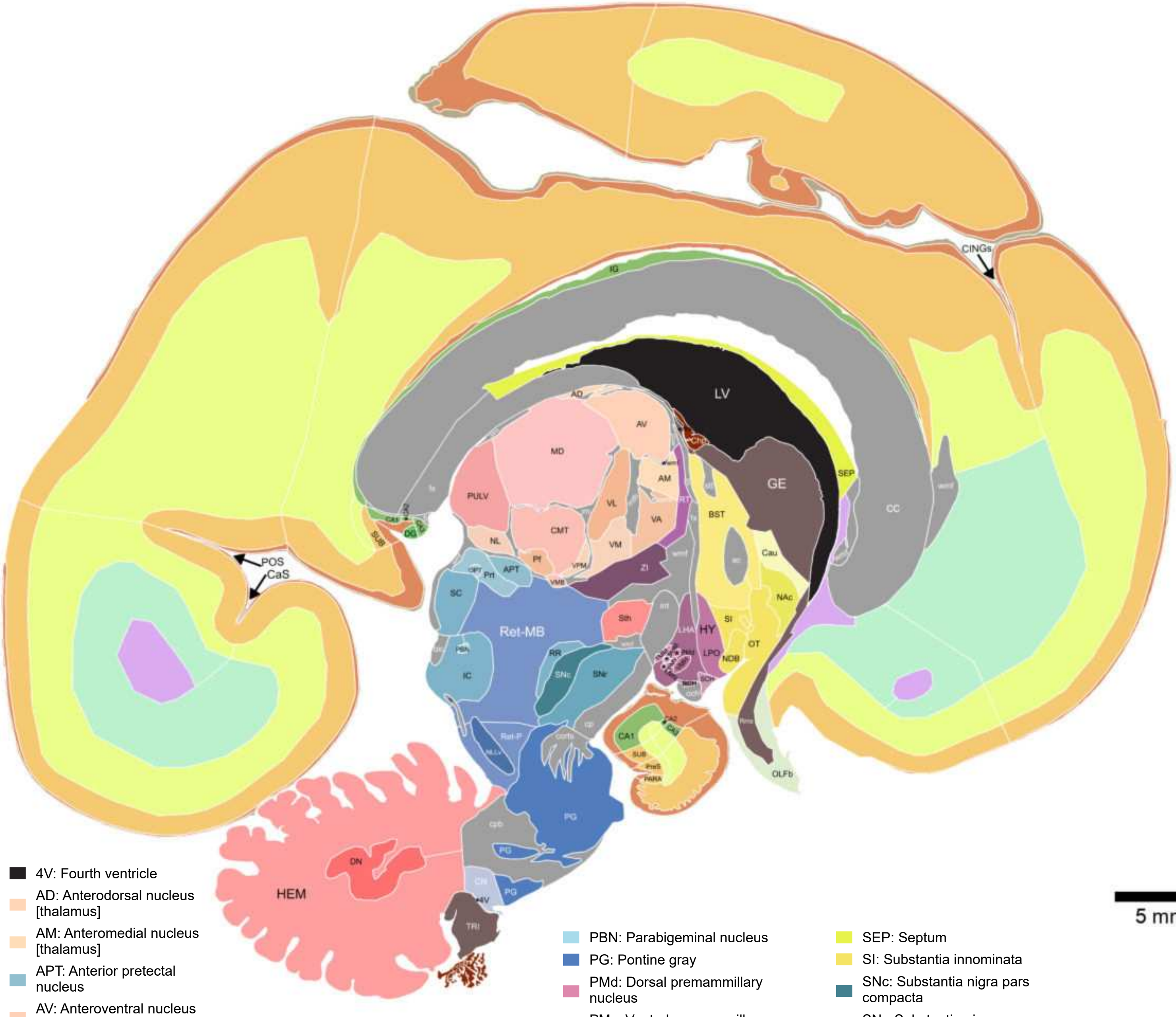

5 mm

- 4V: Fourth ventricle
- AD: Anterodorsal nucleus [thalamus]
- AM: Anteromedial nucleus [thalamus]
- APT: Anterior pretectal nucleus
- AV: Anteroventral nucleus [thalamus]
- BST: Bed nucleus of the stria terminalis
- CA1: CA1 field [hippocampus]
- CA2: CA2 field [hippocampus]
- CA3: CA3 field [hippocampus]
- CMT: Centromedian nucleus [thalamus]
- CN: Cochlear nuclei
- Cau: Caudate nucleus
- Chp: Choroid plexus
- DG: Dentate gyrus
- DN: Dentate nucleus
- GE: Ganglionic eminence
- HEM: Cerebellar hemispheres
- HY: Hypothalamus
- IC: Inferior colliculus
- IG: Induseum griseum
- LHA: Lateral hypothalamic area
- LMN: Lateral mammillary nucleus
- LPO: Lateral preoptic area
- LV: Lateral ventricle
- MD: Medial dorsal nucleus [thalamus]
- NAc: Nucleus accumbens
- NDB: Nucleus of the diagonal band
- NL: Nucleus limitans [thalamus]
- NLLv: Nucleus of the lateral lemniscus, ventral
- OLFb: Olfactory bulb
- OPT: Olivary pretectal nucleus
- OT: Olfactory tubercle
- PARA: Cortical plate, parasubiculum
- PBN: Parabigeminal nucleus
- PG: Pontine gray
- PMd: Dorsal premammillary nucleus
- PMv: Ventral premammillary nucleus
- PULV: Pulvinar nucleus [thalamus]
- Pf: Parafascicular nucleus [thalamus]
- PreS: Cortical plate, presubiculum
- Prt: Pretectum
- RCH: Retrochiasmatic nucleus [hypothalamus]
- RR: Retrorubral area
- RT: Reticular nucleus [thalamus]
- Ret-MB: Reticular formation, Midbrain
- Ret-P: Reticular formation, Pons
- Rms: Rostral migratory stream
- SC: Superior colliculus
- SCH: Suprachiasmatic nucleus [hypothalamus]
- SEP: Septum
- SI: Substantia innominata
- SNc: Substantia nigra pars compacta
- SNr: Substantia nigra pars reticulata
- SUB: Cortical plate, subiculum
- SUM: Supramammillary area
- Sth: Subthalamus
- TMM: Tuberomammillary nucleus
- TRI: Germinal trigone
- VA: Ventral anterior nucleus [thalamus]
- VL: Ventral lateral nucleus [thalamus]
- VM: Ventral medial nucleus [thalamus]
- VMB: Ventral medial basal nucleus [thalamus]
- VMH: Ventromedial nucleus [hypothalamus]
- VPM: Ventral posteromedial nucleus [thalamus]
- ZI: Zona incerta
- → CaS: Calcarine sulcus
- → POS: Parieto-occipital sulcus

Age: 24 GW

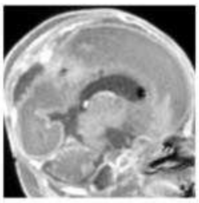

L-R Level: -1.32 mm

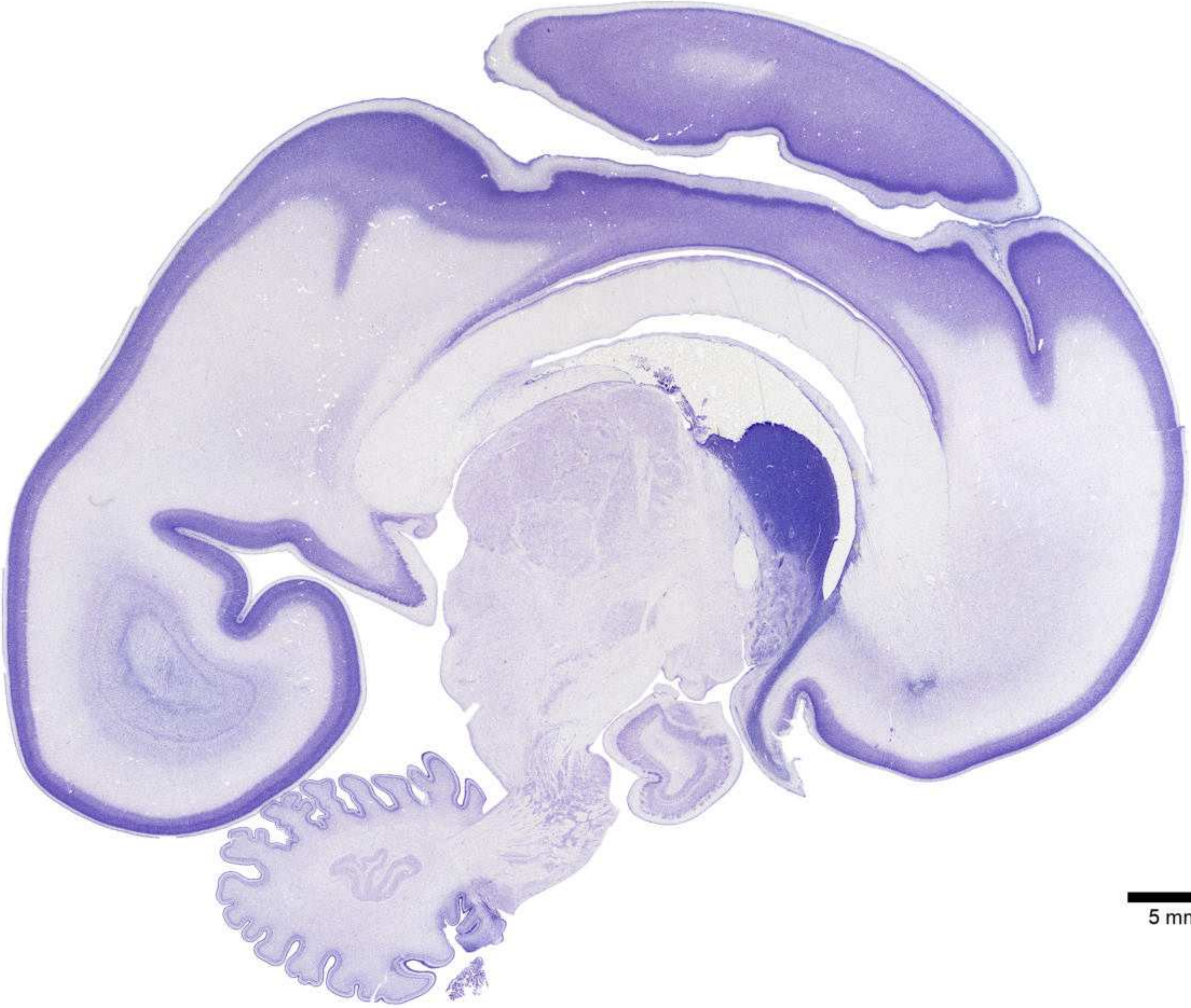

5 mm

Transient Layers

- VZ
- SVZ
- IZ
- SP
- CP
- MZ
- SGL

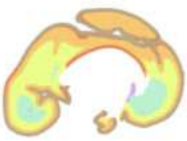

Cortical Areas

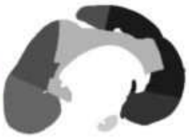

- FCTx
- ORB
- PAR
- OCC
- CING
- ENT

Age: 24 GW

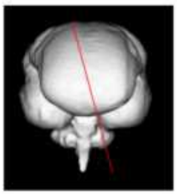

L-R Level: -1.32 mm

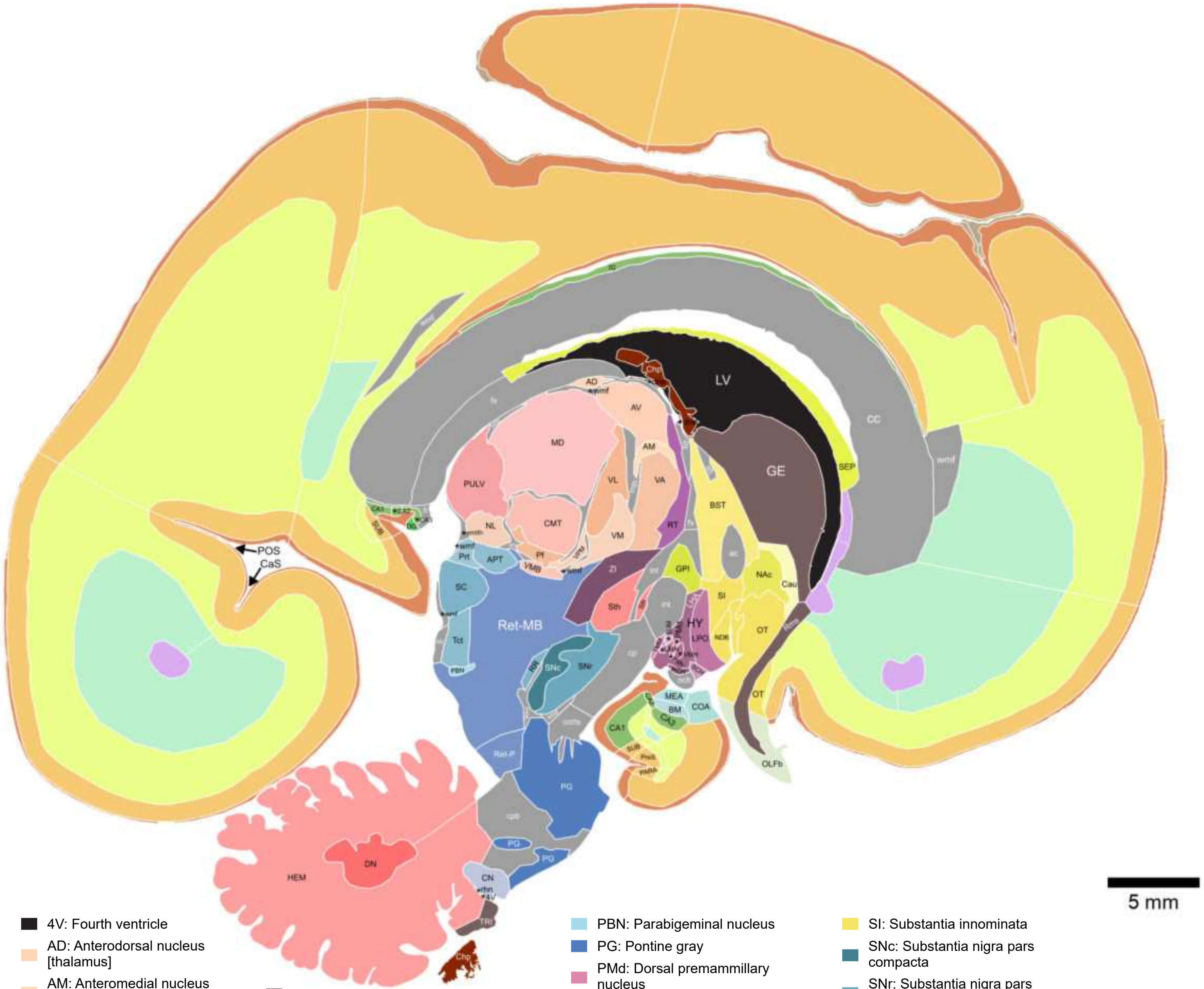

- 4V: Fourth ventricle
- AD: Anterodorsal nucleus [thalamus]
- AM: Anteromedial nucleus [thalamus]
- APT: Anterior pretectal nucleus
- AV: Anteroventral nucleus [thalamus]
- BM: Basomedial nucleus [amygdala]
- BST: Bed nucleus of the stria terminalis
- CA1: CA1 field [hippocampus]
- CA2: CA2 field [hippocampus]
- CA3: CA3 field [hippocampus]
- CMT: Centromedian nucleus [thalamus]
- CN: Cochlear nuclei
- COA: Cortical nucleus [amygdala]
- Cau: Caudate nucleus
- Chp: Choroid plexus
- DG: Dentate gyrus
- DN: Dentate nucleus
- GE: Ganglionic eminence
- GPl: Globus pallidus lateral segment
- HEM: Cerebellar hemispheres
- HY: Hypothalamus
- IG: Induseum griseum
- LHA: Lateral hypothalamic area
- LMN: Lateral mammillary nucleus
- LPO: Lateral preoptic area
- LV: Lateral ventricle
- MD: Medial dorsal nucleus [thalamus]
- MEA: Medial nucleus [amygdala]
- NAc: Nucleus accumbens
- NDB: Nucleus of the diagonal band
- NL: Nucleus limitans [thalamus]
- OLFb: Olfactory bulb
- OT: Olfactory tubercle
- PARA: Cortical plate, parasubiculum
- PBN: Parabigeminal nucleus
- PG: Pontine gray
- PMd: Dorsal premammillary nucleus
- PMv: Ventral premammillary nucleus
- PULV: Pulvinar nucleus [thalamus]
- Pf: Parafascicular nucleus [thalamus]
- PreS: Cortical plate, presubiculum
- Prt: Pretectum
- RCH: Retrochiasmatic nucleus [hypothalamus]
- RR: Retrorubral area
- RT: Reticular nucleus [thalamus]
- Ret-MB: Reticular formation, Midbrain
- Ret-P: Reticular formation, Pons
- Rms: Rostral migratory stream
- SC: Superior colliculus
- SCH: Suprachiasmatic nucleus [hypothalamus]
- SEP: Septum
- SI: Substantia innominata
- SNc: Substantia nigra pars compacta
- SNr: Substantia nigra pars reticulata
- SUB: Cortical plate, subiculum
- SUM: Supramammillary area
- Sth: Subthalamus
- TMM: Tuberomammillary nucleus
- TRI: Germinal trigone
- Tct: Tectum
- VA: Ventral anterior nucleus [thalamus]
- VL: Ventral lateral nucleus [thalamus]
- VM: Ventral medial nucleus [thalamus]
- VMB: Ventral medial basal nucleus [thalamus]
- VMH: Ventromedial nucleus [hypothalamus]
- VPM: Ventral posteromedial nucleus [thalamus]
- ZI: Zona incerta
- → CaS: Calcarine sulcus
- → POS: Parieto-occipital sulcus

Age: 24 GW

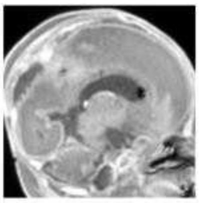

L-R Level: -1.5 mm

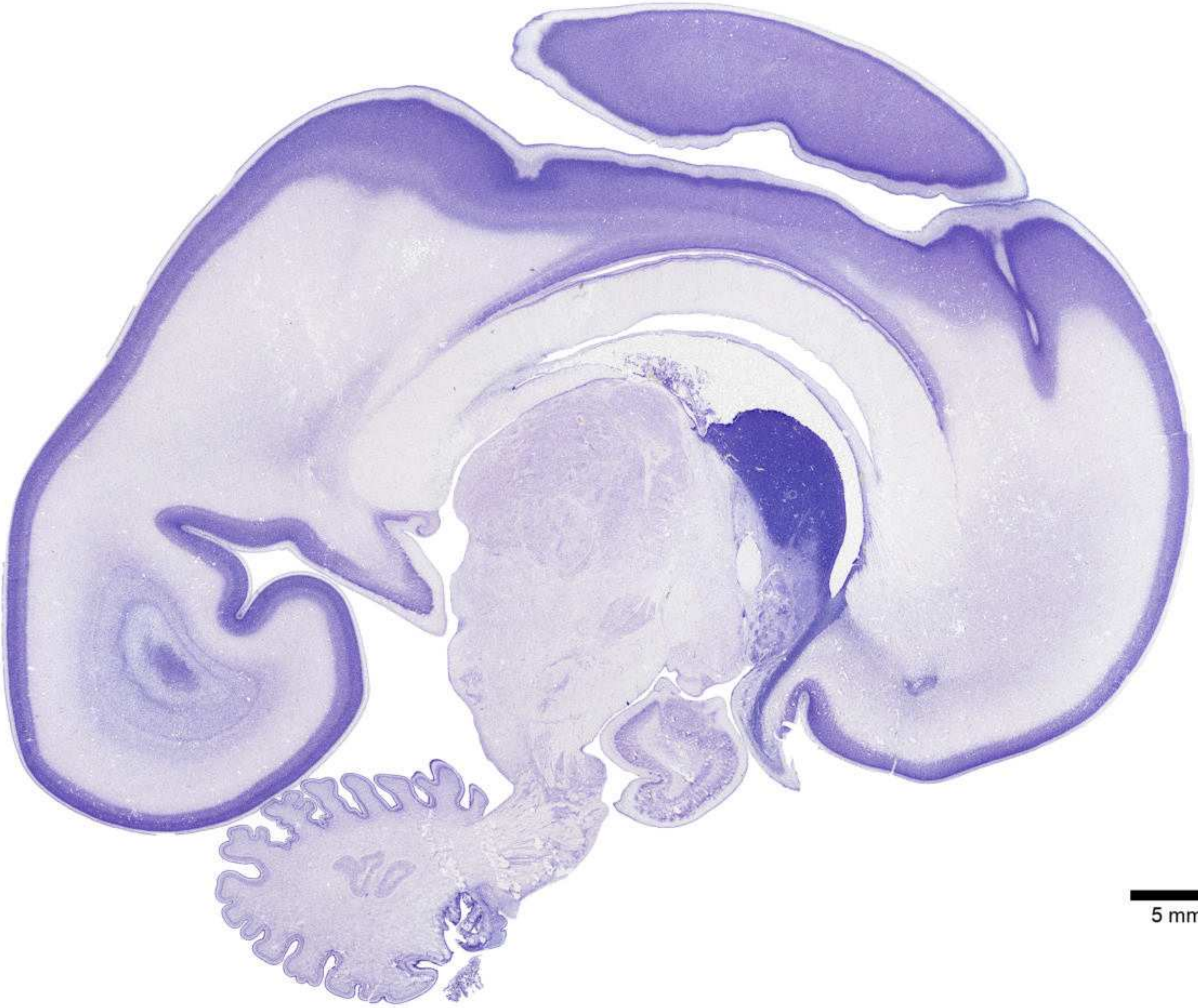

5 mm

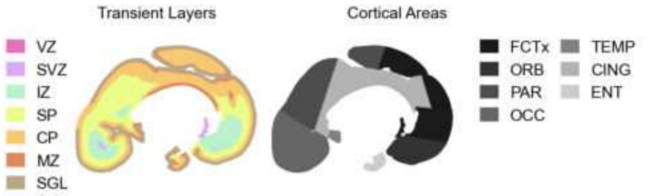

Age: 24 GW

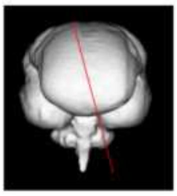

L-R Level: -1.5 mm

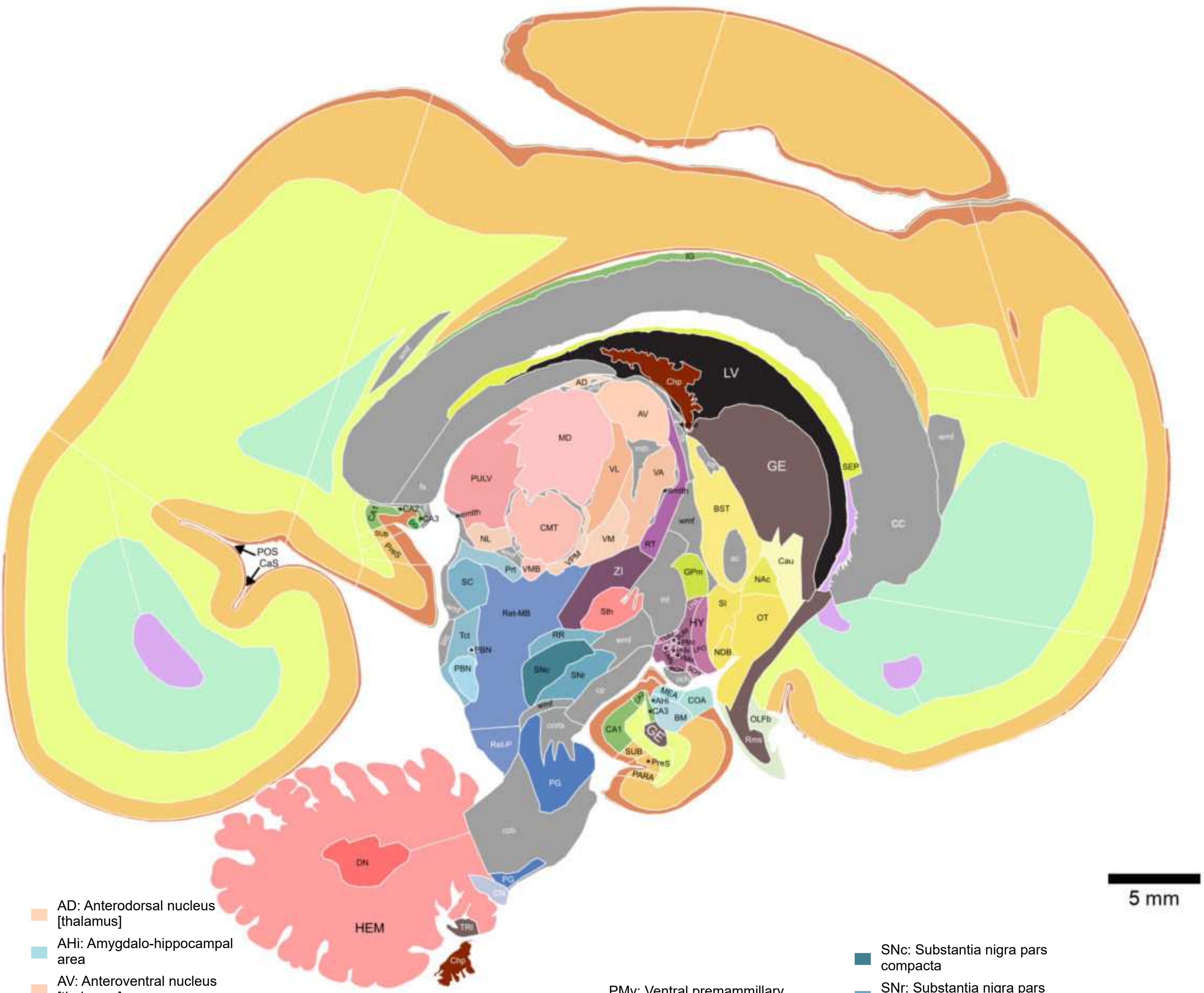

AD: Anterodorsal nucleus [thalamus]
AHi: Amygdalo-hippocampal area
AV: Anteroventral nucleus [thalamus]
BM: Basomedial nucleus [amygdala]
BST: Bed nucleus of the stria terminalis
CA1: CA1 field [hippocampus]
CA2: CA2 field [hippocampus]
CA3: CA3 field [hippocampus]
CMT: Centromedian nucleus [thalamus]
CN: Cochlear nuclei
COA: Cortical nucleus [amygdala]
Cau: Caudate nucleus
Chp: Choroid plexus
DG: Dentate gyrus
DN: Dentate nucleus
GE: Ganglionic eminence
GPm: Globus pallidus medial segment
HEM: Cerebellar hemispheres
HY: Hypothalamus

IG: Induseum griseum
LHA: Lateral hypothalamic area
LMN: Lateral mammillary nucleus
LPO: Lateral preoptic area
LV: Lateral ventricle
MD: Medial dorsal nucleus [thalamus]
MEA: Medial nucleus [amygdala]
NAc: Nucleus accumbens
NDB: Nucleus of the diagonal band
NL: Nucleus limitans [thalamus]
OLFb: Olfactory bulb
OT: Olfactory tubercle
PARA: Cortical plate, parasubiculum
PBN: Parabigeminal nucleus
PG: Pontine gray
PMd: Dorsal premammillary nucleus

PMv: Ventral premammillary nucleus
PULV: Pulvinar nucleus [thalamus]
PreS: Cortical plate, presubiculum
Prt: Pretectum
RCH: Retrochiasmatic nucleus [hypothalamus]
RR: Retrorubral area
RT: Reticular nucleus [thalamus]
Ret-MB: Reticular formation, Midbrain
Ret-P: Reticular formation, Pons
Rms: Rostral migratory stream
SC: Superior colliculus
SCH: Suprachiasmatic nucleus [hypothalamus]
SEP: Septum
SI: Substantia innominata

SNc: Substantia nigra pars compacta
SNr: Substantia nigra pars reticulata
SUB: Cortical plate, subiculum
SUM: Supramammillary area
Sth: Subthalamus
TMM: Tuberomammillary nucleus
TRI: Germinal trigone
Tct: Tectum
VA: Ventral anterior nucleus [thalamus]
VL: Ventral lateral nucleus [thalamus]
VM: Ventral medial nucleus [thalamus]
VMB: Ventral medial basal nucleus [thalamus]
VMH: Ventromedial nucleus [hypothalamus]
VPM: Ventral posteromedial nucleus [thalamus]
ZI: Zona incerta
CaS: Calcarine sulcus
POS: Parieto-occipital sulcus

Age: 24 GW

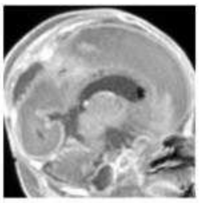

L-R Level: -1.98 mm

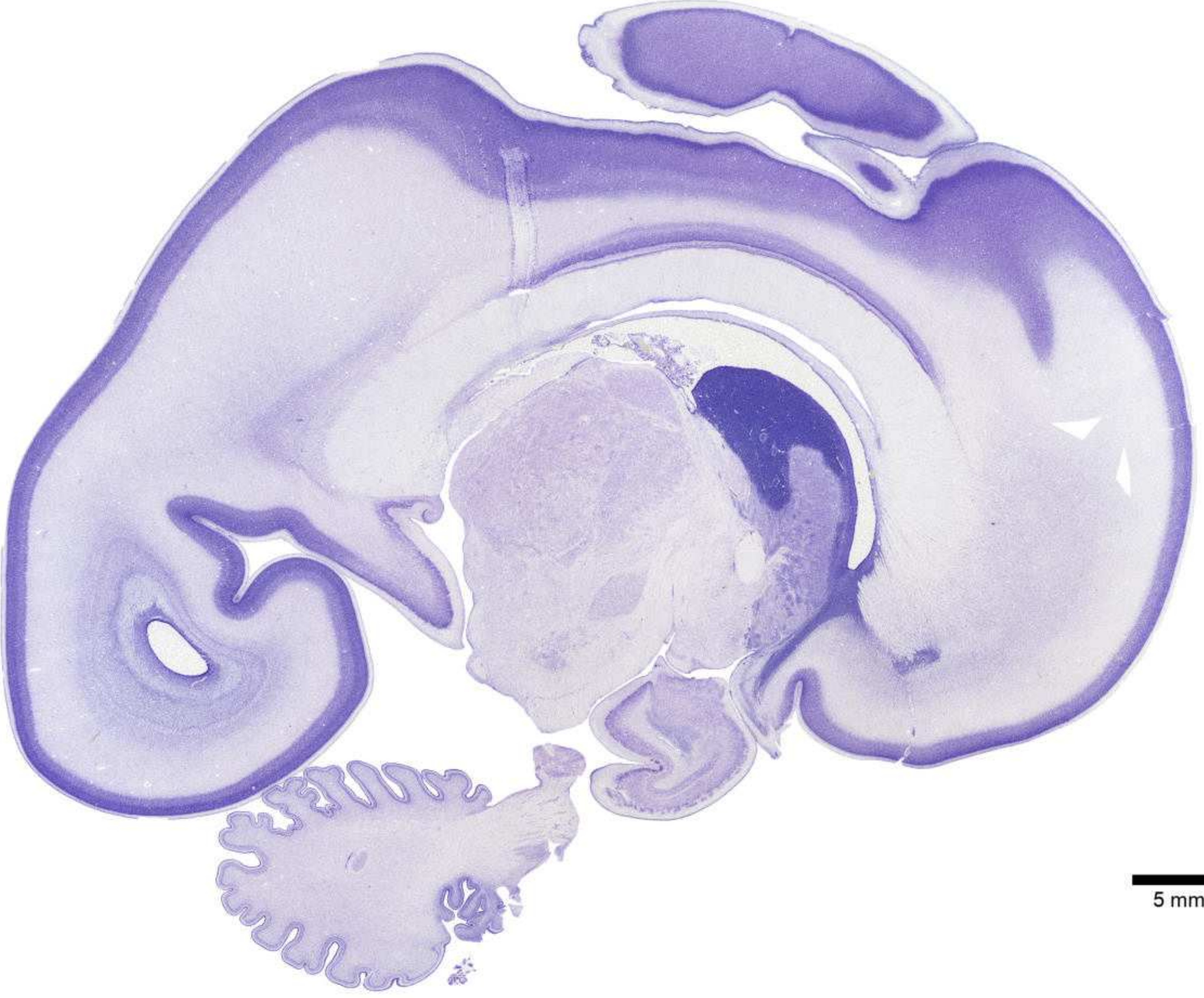

5 mm

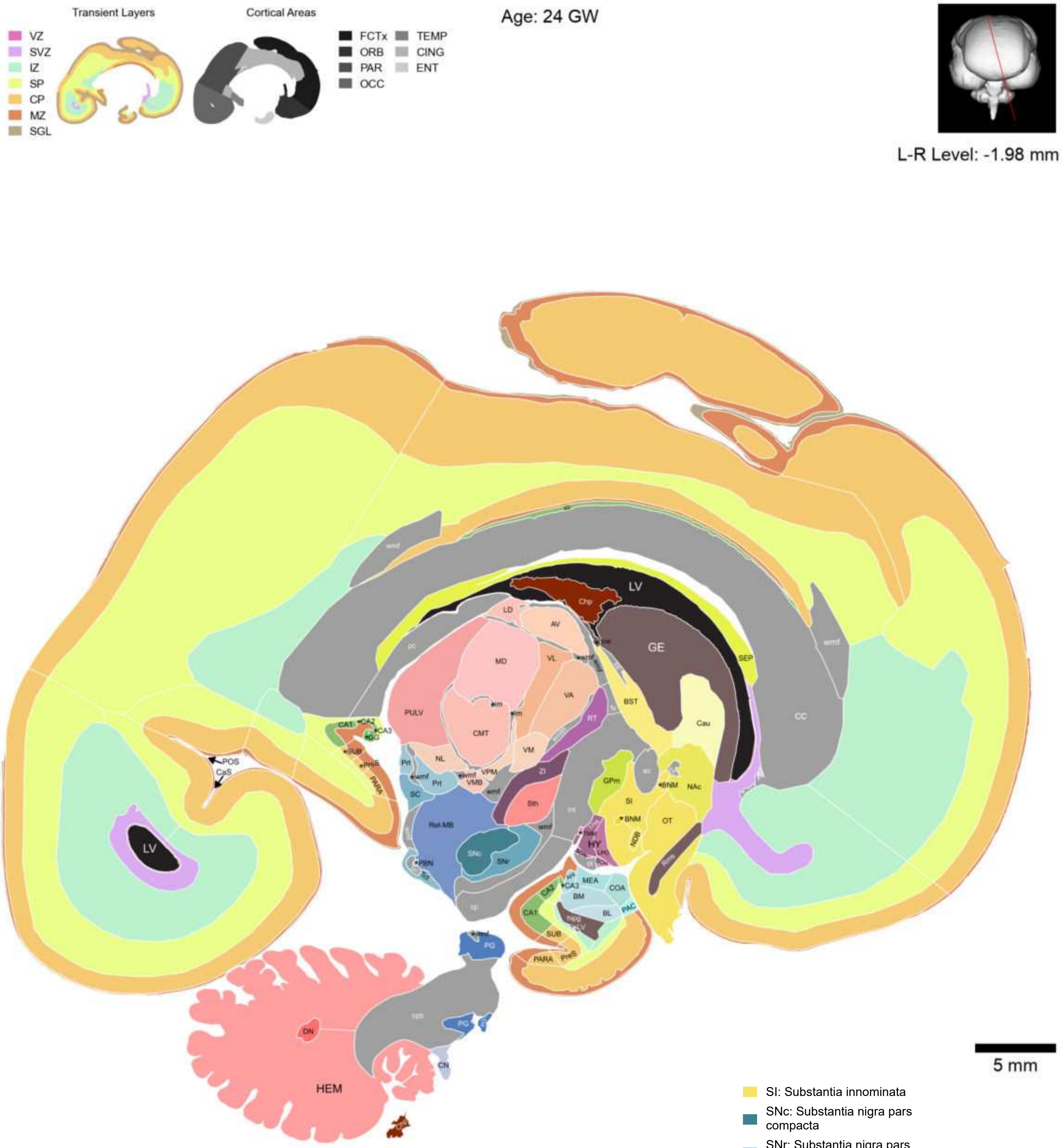

AHi: Amygdalo-hippocampal area
AV: Anteroventral nucleus [thalamus]
BL: Basal nucleus [amygdala]
BM: Basomedial nucleus [amygdala]
BNM: Basal nucleus of Meynert
BST: Bed nucleus of the stria terminalis
CA1: CA1 field [hippocampus]
CA2: CA2 field [hippocampus]
CA3: CA3 field [hippocampus]
CMT: Centromedian nucleus [thalamus]
CN: Cochlear nuclei
COA: Cortical nucleus [amygdala]
Cau: Caudate nucleus
Chp: Choroid plexus
DG: Dentate gyrus
DN: Dentate nucleus
GE: Ganglionic eminence
GPm: Globus pallidus medial segment
HEM: Cerebellar hemispheres
HY: Hypothalamus
IG: Induseum griseum
LD: Lateral dorsal nucleus [thalamus]
LHA: Lateral hypothalamic area
LPO: Lateral preoptic area
LV: Lateral ventricle
MD: Medial dorsal nucleus [thalamus]
MEA: Medial nucleus [amygdala]
NAc: Nucleus accumbens
NDB: Nucleus of the diagonal band
NL: Nucleus limitans [thalamus]
OT: Olfactory tubercle
PARA: Cortical plate, parasubiculum
PBN: Parabigeminal nucleus
PG: Pontine gray
PULV: Pulvinar nucleus [thalamus]
PreS: Cortical plate, presubiculum
Prt: Pretectum
RT: Reticular nucleus [thalamus]
Ret-MB: Reticular formation, Midbrain
Rms: Rostral migratory stream
SC: Superior colliculus
SCH: Suprachiasmatic nucleus [hypothalamus]
SEP: Septum
SI: Substantia innominata
SNc: Substantia nigra pars compacta
SNr: Substantia nigra pars reticulata
SON: Supraoptic nucleus [hypothalamus]
SUB: Cortical plate, subiculum
Sth: Subthalamus
TMM: Tuberomammillary nucleus
Tct: Tectum
VA: Ventral anterior nucleus [thalamus]
VL: Ventral lateral nucleus [thalamus]
VM: Ventral medial nucleus [thalamus]
VMB: Ventral medial basal nucleus [thalamus]
VPM: Ventral posteromedial nucleus [thalamus]
ZI: Zona incerta
→ CaS: Calcarine sulcus
→ POS: Parieto-occipital sulcus

Age: 24 GW

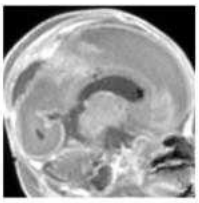

L-R Level: -2.52 mm

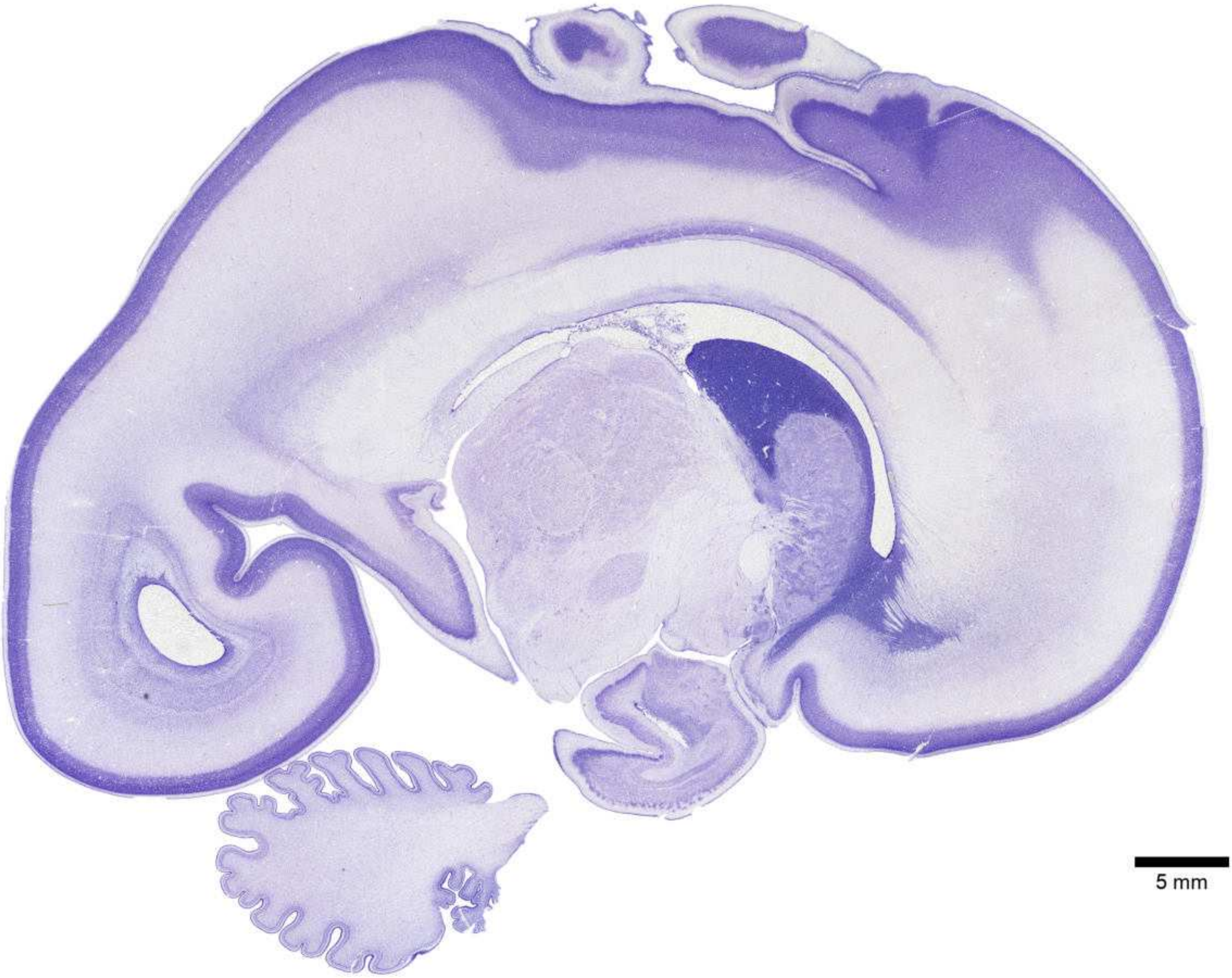

5 mm

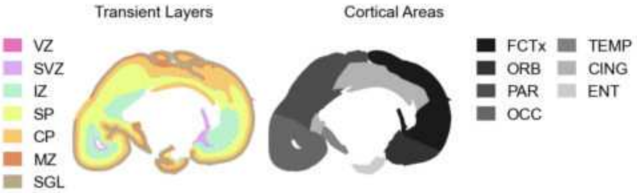

Age: 24 GW

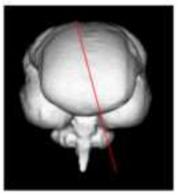

L-R Level: -2.52 mm

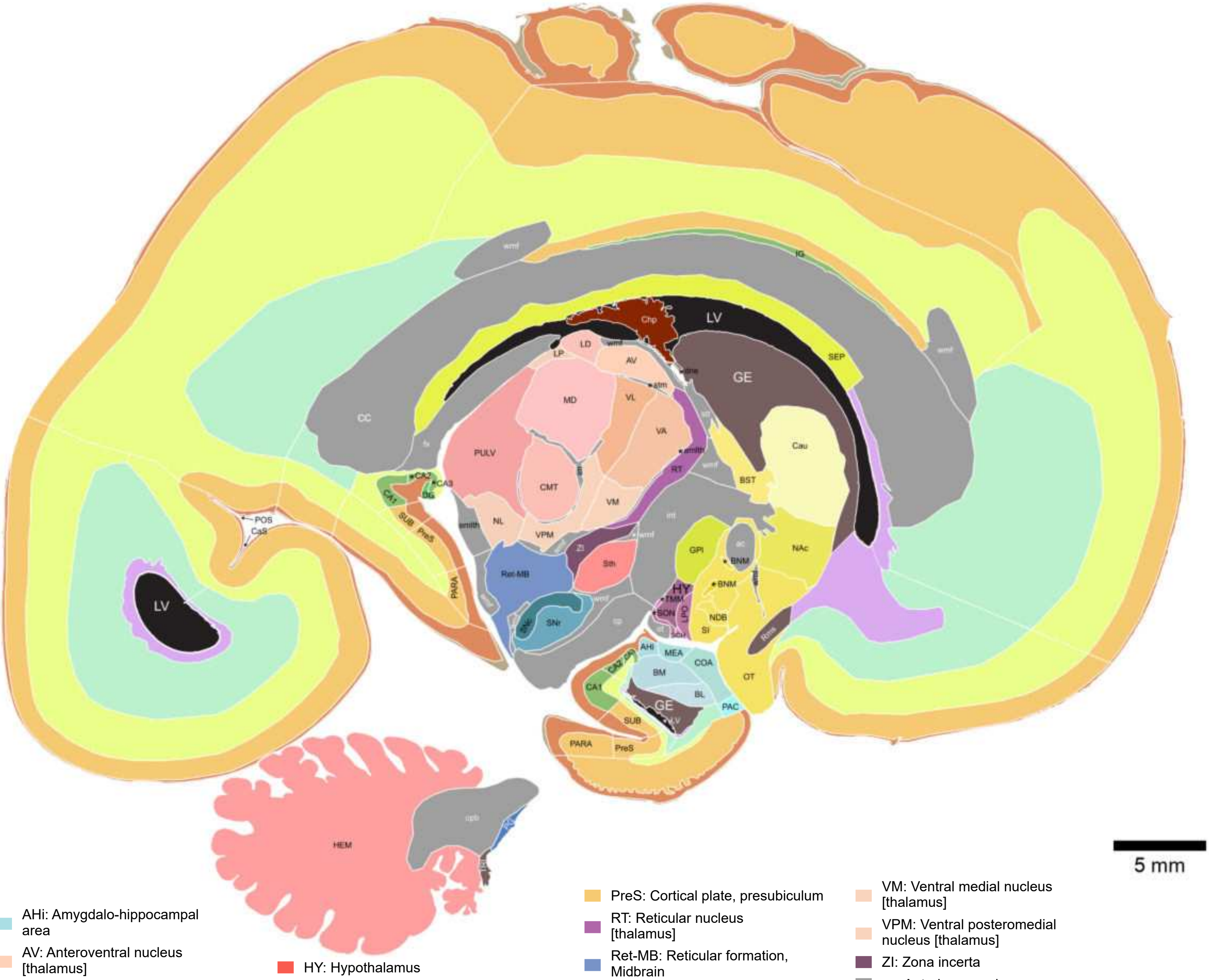

- AHi: Amygdalo-hippocampal area
- AV: Anteroventral nucleus [thalamus]
- BL: Basal nucleus [amygdala]
- BM: Basomedial nucleus [amygdala]
- BNM: Basal nucleus of Meynert
- BST: Bed nucleus of the stria terminalis
- CA1: CA1 field [hippocampus]
- CA2: CA2 field [hippocampus]
- CA3: CA3 field [hippocampus]
- CMT: Centromedian nucleus [thalamus]
- COA: Cortical nucleus [amygdala]
- Cau: Caudate nucleus
- Chp: Choroid plexus
- DG: Dentate gyrus
- GE: Ganglionic eminence
- GPI: Globus pallidus lateral segment
- HEM: Cerebellar hemispheres
- HY: Hypothalamus
- IG: Induseum griseum
- LD: Lateral dorsal nucleus [thalamus]
- LHA: Lateral hypothalamic area
- LP: Lateral posterior nucleus [thalamus]
- LPO: Lateral preoptic area
- LV: Lateral ventricle
- MD: Medial dorsal nucleus [thalamus]
- MEA: Medial nucleus [amygdala]
- NAc: Nucleus accumbens
- NDB: Nucleus of the diagonal band
- NL: Nucleus limitans [thalamus]
- OT: Olfactory tubercle
- PARA: Cortical plate, parasubiculum
- PG: Pontine gray
- PULV: Pulvinar nucleus [thalamus]
- PreS: Cortical plate, presubiculum
- RT: Reticular nucleus [thalamus]
- Ret-MB: Reticular formation, Midbrain
- Rms: Rostral migratory stream
- SCH: Suprachiasmatic nucleus [hypothalamus]
- SEP: Septum
- SI: Substantia innominata
- SNc: Substantia nigra pars compacta
- SNr: Substantia nigra pars reticulata
- SON: Supraoptic nucleus [hypothalamus]
- SUB: Cortical plate, subiculum
- Sth: Subthalamus
- TMM: Tuberomammillary nucleus
- TRI: Germinal trigone
- VA: Ventral anterior nucleus [thalamus]
- VL: Ventral lateral nucleus [thalamus]
- VM: Ventral medial nucleus [thalamus]
- VPM: Ventral posteromedial nucleus [thalamus]
- ZI: Zona incerta
- ac: Anterior commissure
- cc: Corpus callosum
- cp: Cerebral peduncle
- cpb: Cerebellar peduncles
- dne: Diencephalic neuroepithelium
- emlth: External medullary lamina [thalamus]
- fx: Fornix
- im: Internal medullary lamina [thalamus]
- int: Internal capsule
- ot: Optic tract
- stm: Stria medullaris
- stt: Stria terminalis
- wmf: White matter fibers
- → CaS: Calcarine sulcus
- → POS: Parieto-occipital sulcus

Age: 24 GW

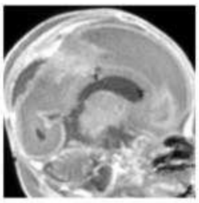

L-R Level: -3.06 mm

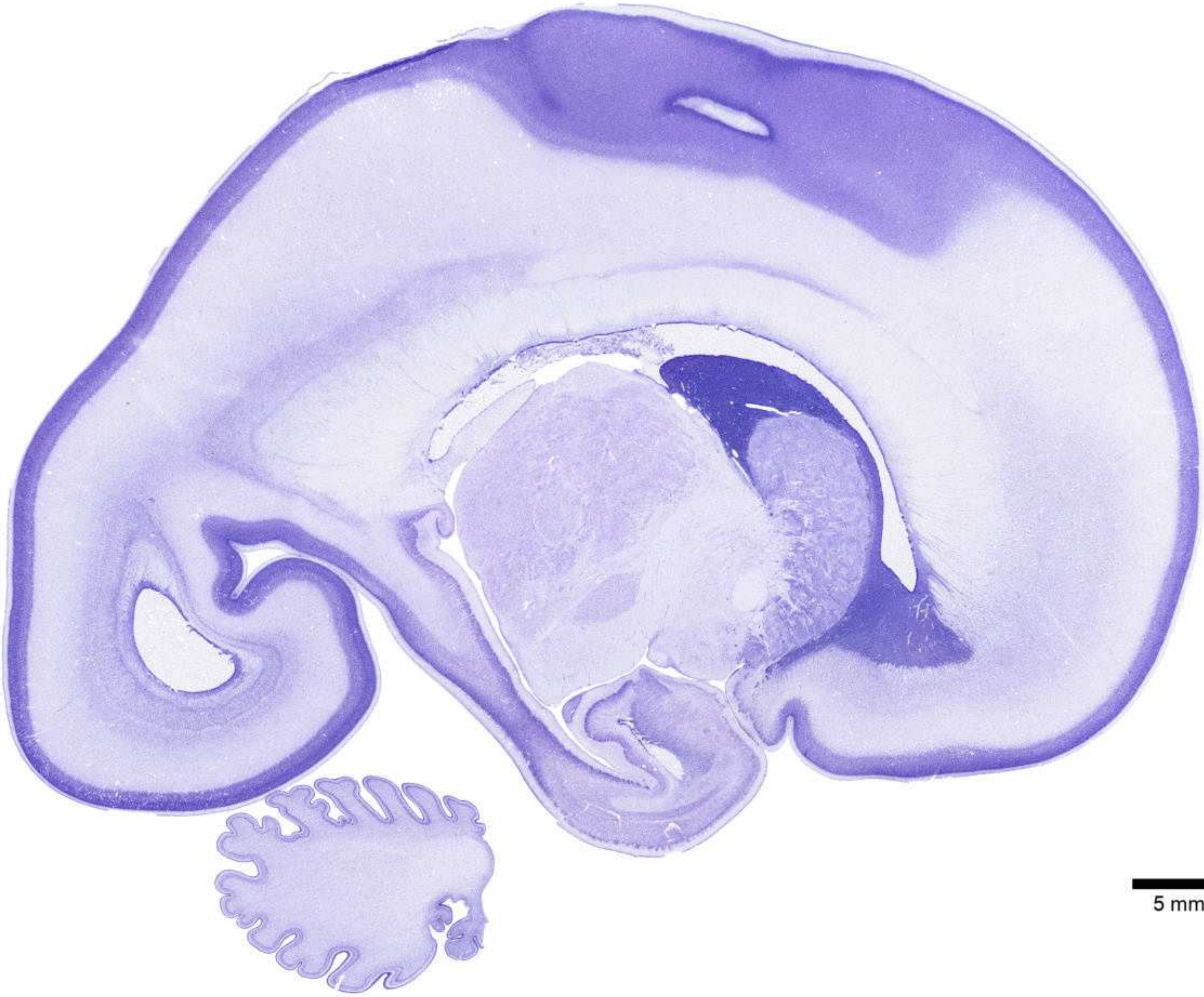

5 mm

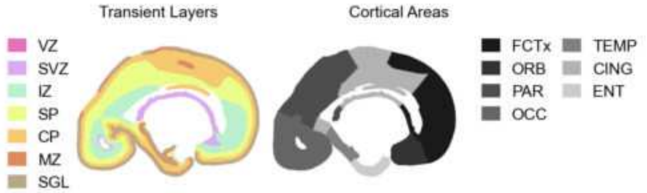

Age: 24 GW

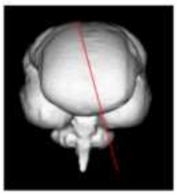

L-R Level: -3.06 mm

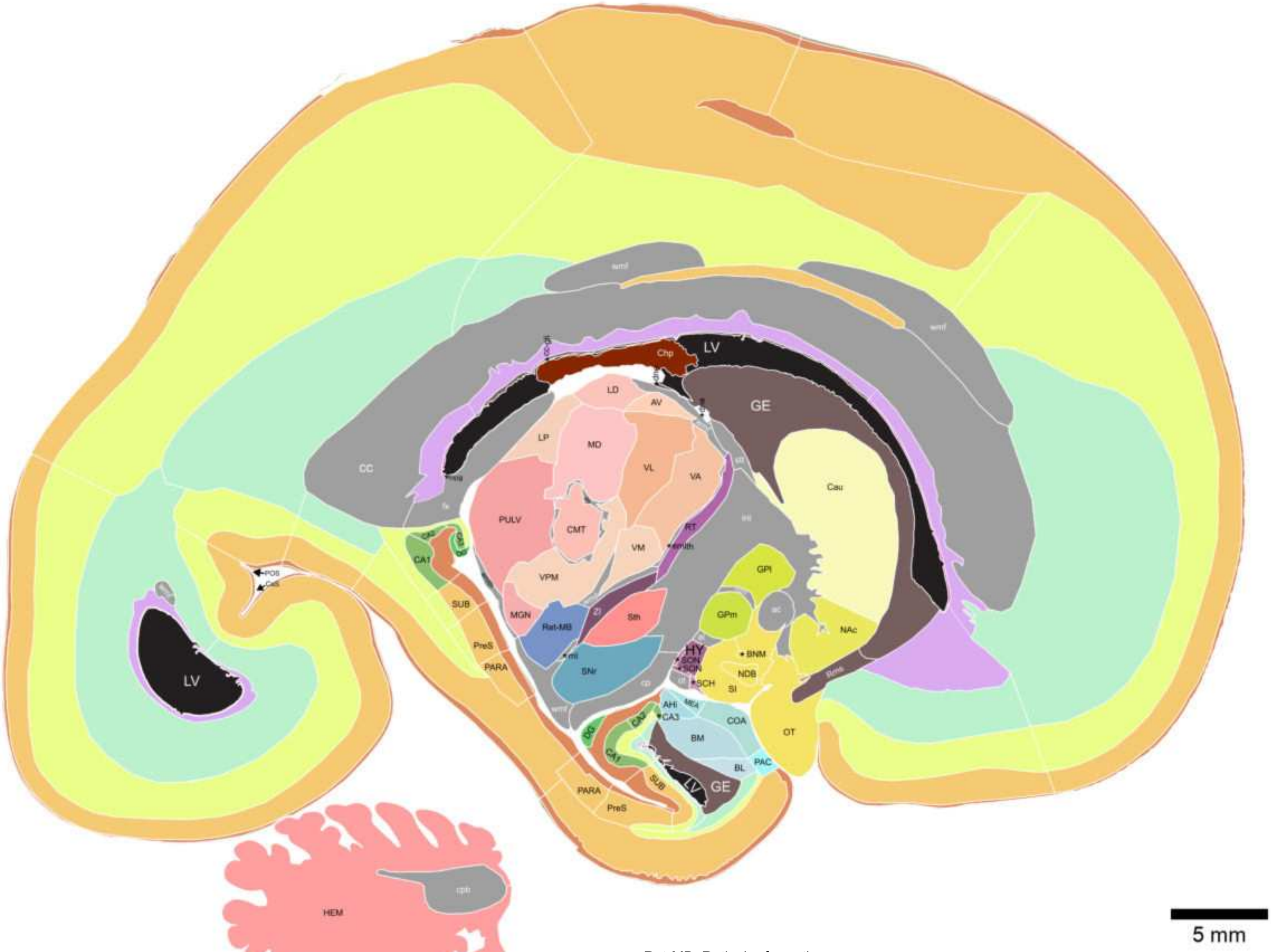

AHi: Amygdalo-hippocampal area
AV: Anteroventral nucleus [thalamus]
BL: Basal nucleus [amygdala]
BM: Basomedial nucleus [amygdala]
BNM: Basal nucleus of Meynert
CA1: CA1 field [hippocampus]
CA2: CA2 field [hippocampus]
CA3: CA3 field [hippocampus]
CMT: Centromedian nucleus [thalamus]
COA: Cortical nucleus [amygdala]
Cau: Caudate nucleus
Chp: Choroid plexus
DG: Dentate gyrus
GE: Ganglionic eminence
GPl: Globus pallidus lateral segment
GPm: Globus pallidus medial segment

HEM: Cerebellar hemispheres
HY: Hypothalamus
LD: Lateral dorsal nucleus [thalamus]
LHA: Lateral hypothalamic area
LP: Lateral posterior nucleus [thalamus]
LV: Lateral ventricle
MD: Medial dorsal nucleus [thalamus]
MEA: Medial nucleus [amygdala]
MGN: Medial geniculate nucleus
NAc: Nucleus accumbens
NDB: Nucleus of the diagonal band
OT: Olfactory tubercle
PARA: Cortical plate, parasubiculum
PULV: Pulvinar nucleus [thalamus]
PreS: Cortical plate, presubiculum
RT: Reticular nucleus [thalamus]

Ret-MB: Reticular formation, Midbrain
Rms: Rostral migratory stream
SCH: Suprachiasmatic nucleus [hypothalamus]
SI: Substantia innominata
SNr: Substantia nigra pars reticulata
SON: Supraoptic nucleus [hypothalamus]
SUB: Cortical plate, subiculum
Sth: Subthalamus
VA: Ventral anterior nucleus [thalamus]
VL: Ventral lateral nucleus [thalamus]
VM: Ventral medial nucleus [thalamus]
VPM: Ventral posteromedial nucleus [thalamus]
ZI: Zona incerta
ac: Anterior commissure
al: Ansa lenticularis

cc: Corpus callosum
cc-gli: Callosal glioepithelium
cp: Cerebral peduncle
cpb: Cerebellar peduncles
dne: Diencephalic neuroepithelium
emlth: External medullary lamina [thalamus]
fx: Fornix
hipg: Hippocampal glioepithelium/ependyma
im: Internal medullary lamina [thalamus]
int: Internal capsule
ml: Medial lemniscus
mml: Medial medullary lamina
ot: Optic tract
stt: Stria terminalis
wmf: White matter fibers
CaS: Calcarine sulcus
POS: Parieto-occipital sulcus

Age: 24 GW

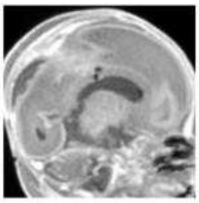

L-R Level: -3.3 mm

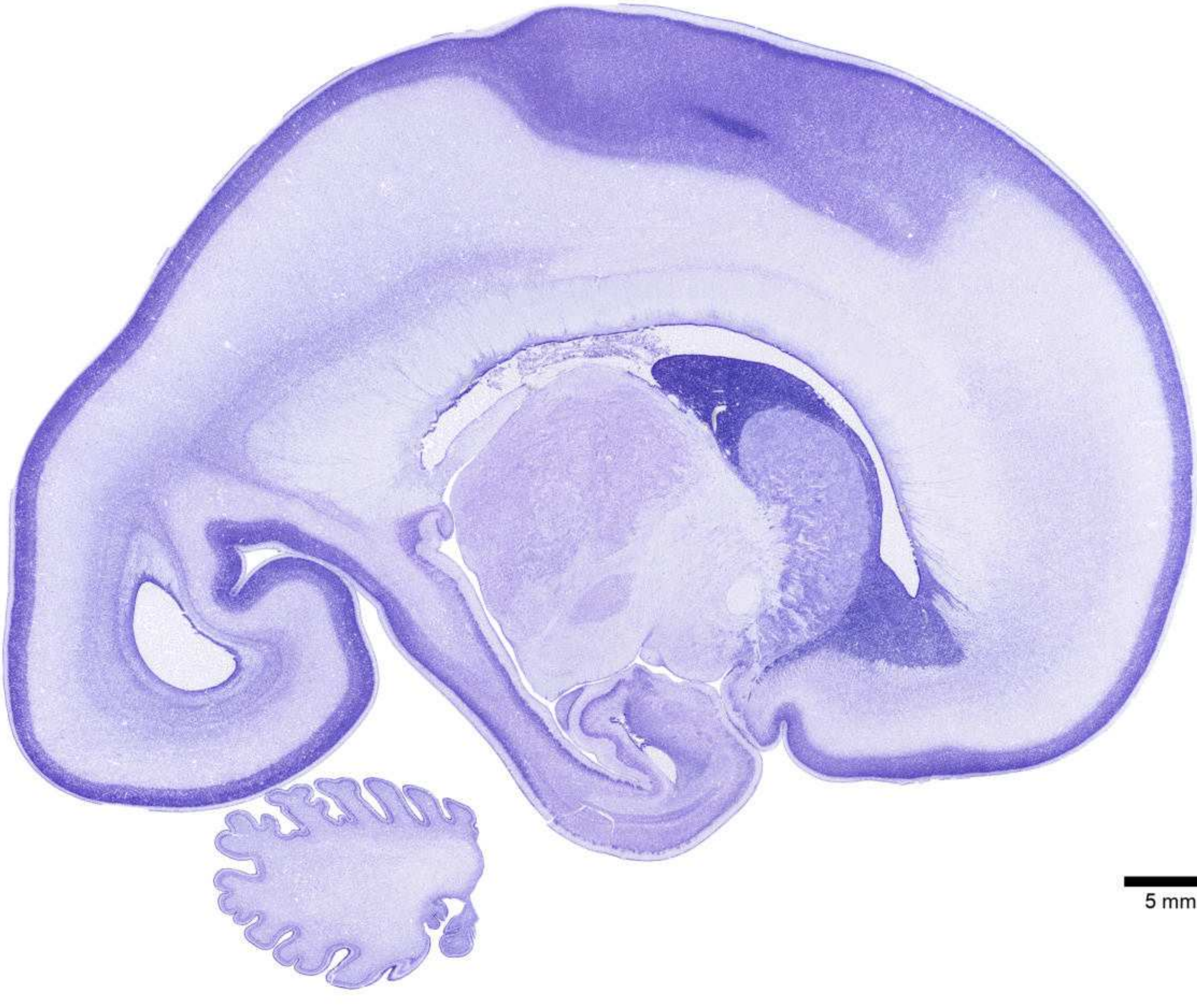

5 mm

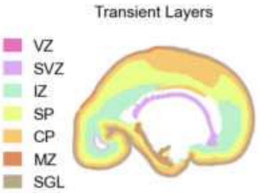

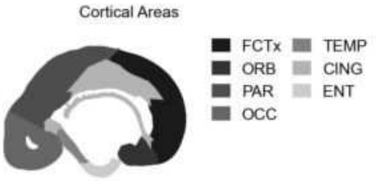

Age: 24 GW

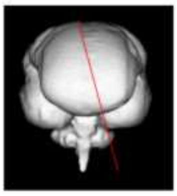

L-R Level: -3.3 mm

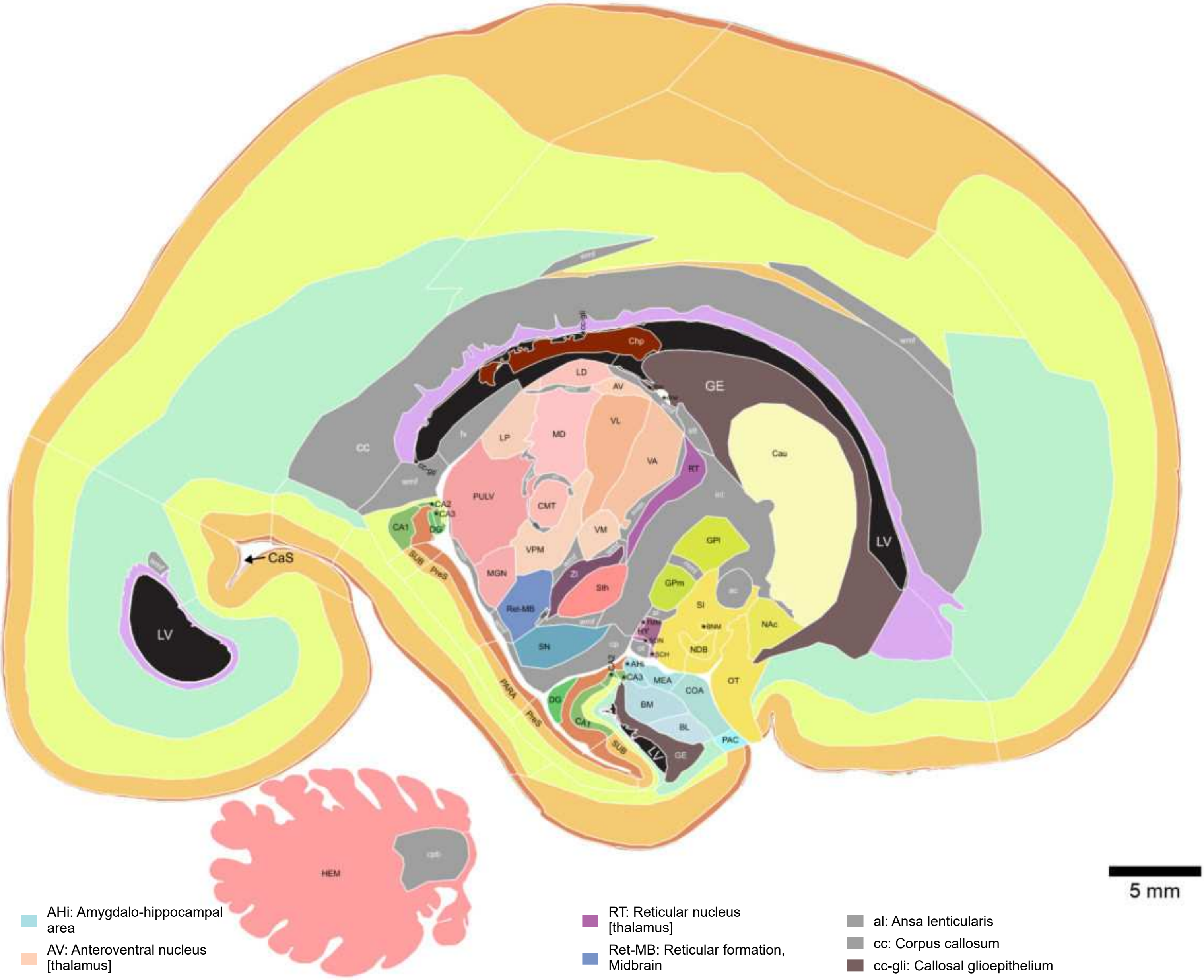

- AHi: Amygdalo-hippocampal area
- AV: Anteroventral nucleus [thalamus]
- BL: Basal nucleus [amygdala]
- BM: Basomedial nucleus [amygdala]
- BNM: Basal nucleus of Meynert
- CA1: CA1 field [hippocampus]
- CA2: CA2 field [hippocampus]
- CA3: CA3 field [hippocampus]
- CMT: Centromedian nucleus [thalamus]
- COA: Cortical nucleus [amygdala]
- Cau: Caudate nucleus
- Chp: Choroid plexus
- DG: Dentate gyrus
- GE: Ganglionic eminence
- GPl: Globus pallidus lateral segment
- GPm: Globus pallidus medial segment
- HEM: Cerebellar hemispheres
- HY: Hypothalamus
- LD: Lateral dorsal nucleus [thalamus]
- LHA: Lateral hypothalamic area
- LP: Lateral posterior nucleus [thalamus]
- LV: Lateral ventricle
- MD: Medial dorsal nucleus [thalamus]
- MEA: Medial nucleus [amygdala]
- MGN: Medial geniculate nucleus
- NAc: Nucleus accumbens
- NDB: Nucleus of the diagonal band
- OT: Olfactory tubercle
- PARA: Cortical plate, parasubiculum
- PULV: Pulvinar nucleus [thalamus]
- PreS: Cortical plate, presubiculum
- RT: Reticular nucleus [thalamus]
- Ret-MB: Reticular formation, Midbrain
- SCH: Suprachiasmatic nucleus [hypothalamus]
- SI: Substantia innominata
- SN: Substantia nigra
- SON: Supraoptic nucleus [hypothalamus]
- SUB: Cortical plate, subiculum
- Sth: Subthalamus
- TMM: Tuberomammillary nucleus
- VA: Ventral anterior nucleus [thalamus]
- VL: Ventral lateral nucleus [thalamus]
- VM: Ventral medial nucleus [thalamus]
- VPM: Ventral posteromedial nucleus [thalamus]
- ZI: Zona incerta
- ac: Anterior commissure
- al: Ansa lenticularis
- cc: Corpus callosum
- cc-gli: Callosal glioepithelium
- cp: Cerebral peduncle
- cpb: Cerebellar peduncles
- dne: Diencephalic neuroepithelium
- emlth: External medullary lamina [thalamus]
- fx: Fornix
- im: Internal medullary lamina [thalamus]
- int: Internal capsule
- ml: Medial lemniscus
- mml: Medial medullary lamina
- ot: Optic tract
- stt: Stria terminalis
- wmf: White matter fibers
- → CaS: Calcarine sulcus

Age: 24 GW

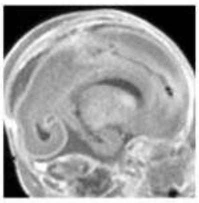

L-R Level: -3.78 mm

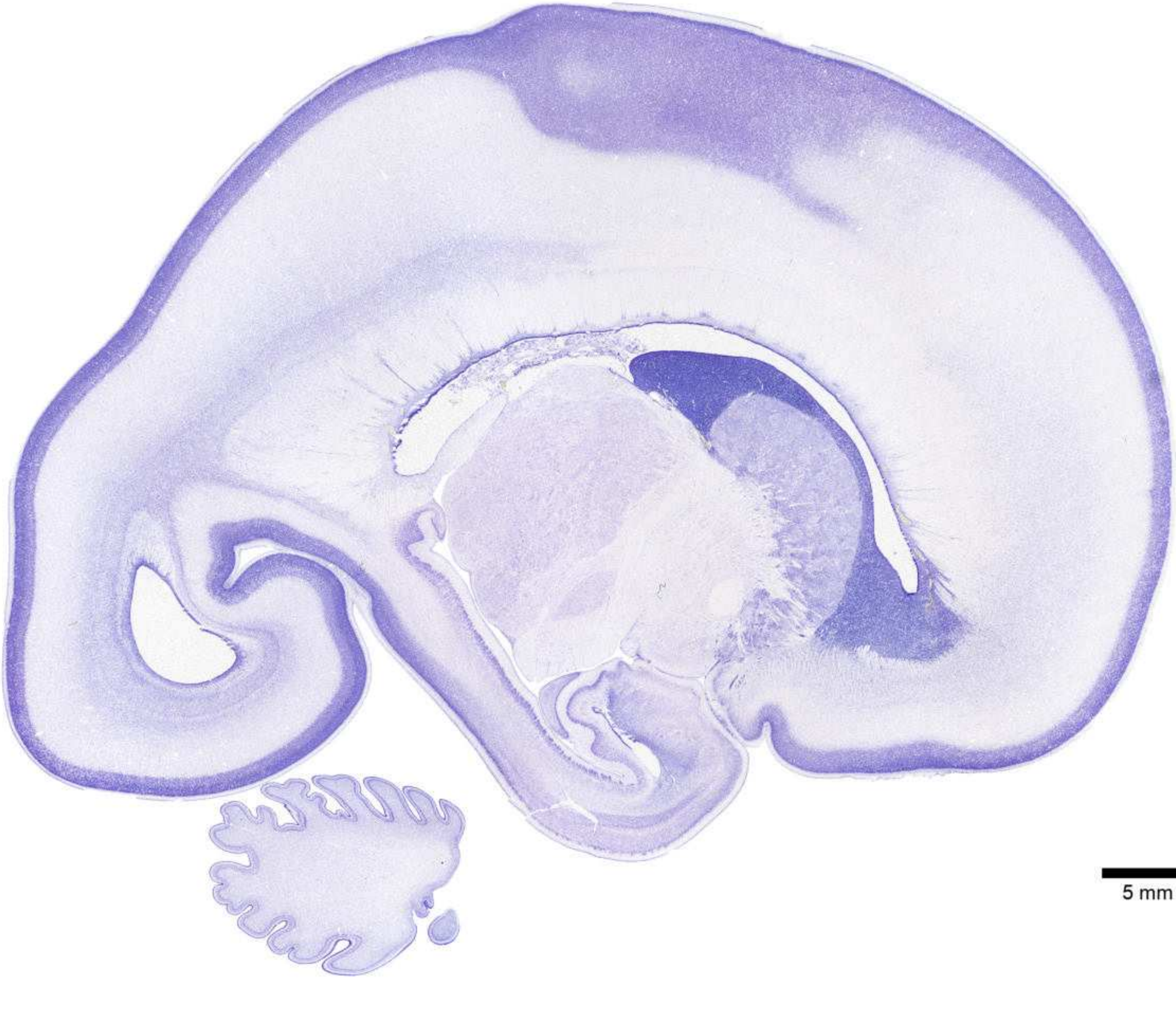

5 mm

Transient Layers

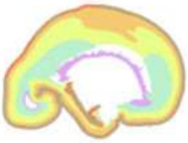

- VZ
- SVZ
- IZ
- SP
- CP
- MZ
- SGL

Cortical Areas

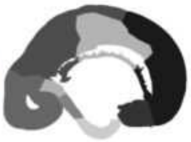

- FCTx
- ORB
- PAR
- OCC
- TEMP
- CING
- ENT

Age: 24 GW

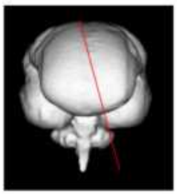

L-R Level: -3.78 mm

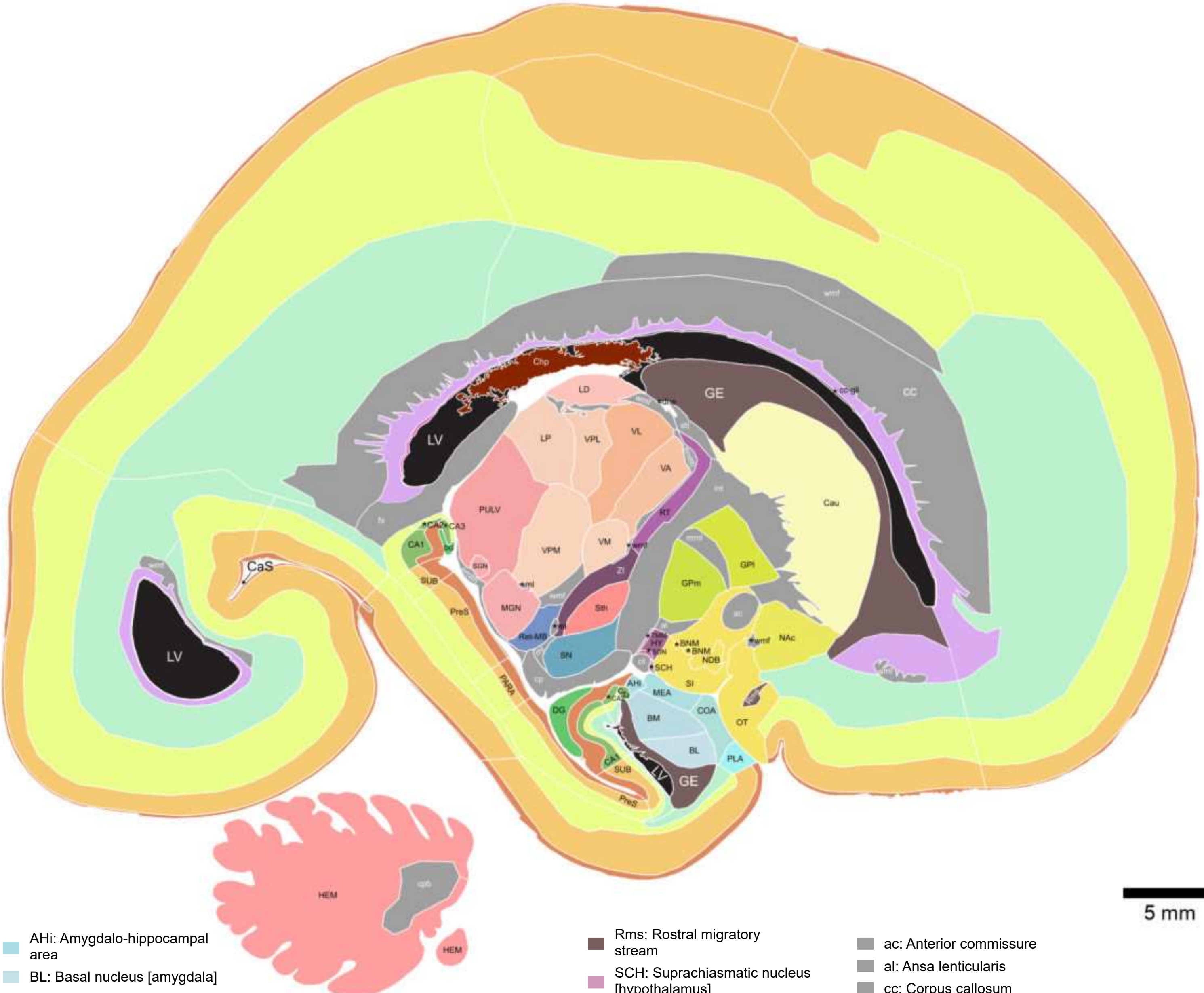

5 mm

- AHi: Amygdalo-hippocampal area
- BL: Basal nucleus [amygdala]
- BM: Basomedial nucleus [amygdala]
- BNM: Basal nucleus of Meynert
- CA1: CA1 field [hippocampus]
- CA2: CA2 field [hippocampus]
- CA3: CA3 field [hippocampus]
- COA: Cortical nucleus [amygdala]
- Cau: Caudate nucleus
- Chp: Choroid plexus
- DG: Dentate gyrus
- GE: Ganglionic eminence
- GPl: Globus pallidus lateral segment
- GPm: Globus pallidus medial segment
- HEM: Cerebellar hemispheres
- HY: Hypothalamus
- LD: Lateral dorsal nucleus [thalamus]
- LHA: Lateral hypothalamic area
- LP: Lateral posterior nucleus [thalamus]
- LV: Lateral ventricle
- MEA: Medial nucleus [amygdala]
- MGN: Medial geniculate nucleus
- NAc: Nucleus accumbens
- NDB: Nucleus of the diagonal band
- OT: Olfactory tubercle
- PARA: Cortical plate, parasubiculum
- PLA: Paralaminar nucleus [amygdala]
- PULV: Pulvinar nucleus [thalamus]
- PreS: Cortical plate, presubiculum
- RT: Reticular nucleus [thalamus]
- Ret-MB: Reticular formation, Midbrain
- Rms: Rostral migratory stream
- SCH: Suprachiasmatic nucleus [hypothalamus]
- SGN: Suprageniculate nucleus
- SI: Substantia innominata
- SN: Substantia nigra
- SON: Supraoptic nucleus [hypothalamus]
- SUB: Cortical plate, subiculum
- Sth: Subthalamus
- TMM: Tuberomammillary nucleus
- VA: Ventral anterior nucleus [thalamus]
- VL: Ventral lateral nucleus [thalamus]
- VM: Ventral medial nucleus [thalamus]
- VPL: Ventral posterolateral nucleus [thalamus]
- VPM: Ventral posteromedial nucleus [thalamus]
- ZI: Zona incerta
- ac: Anterior commissure
- al: Ansa lenticularis
- cc: Corpus callosum
- cc-gli: Callosal glioepithelium
- cp: Cerebral peduncle
- cpb: Cerebellar peduncles
- dne: Diencephalic neuroepithelium
- emlth: External medullary lamina [thalamus]
- fx: Fornix
- int: Internal capsule
- ml: Medial lemniscus
- mml: Medial medullary lamina
- ot: Optic tract
- stt: Stria terminalis
- wmf: White matter fibers
- → CaS: Calcarine sulcus

Age: 24 GW

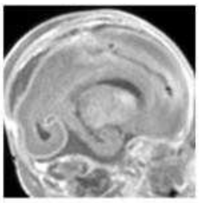

L-R Level: -4.02 mm

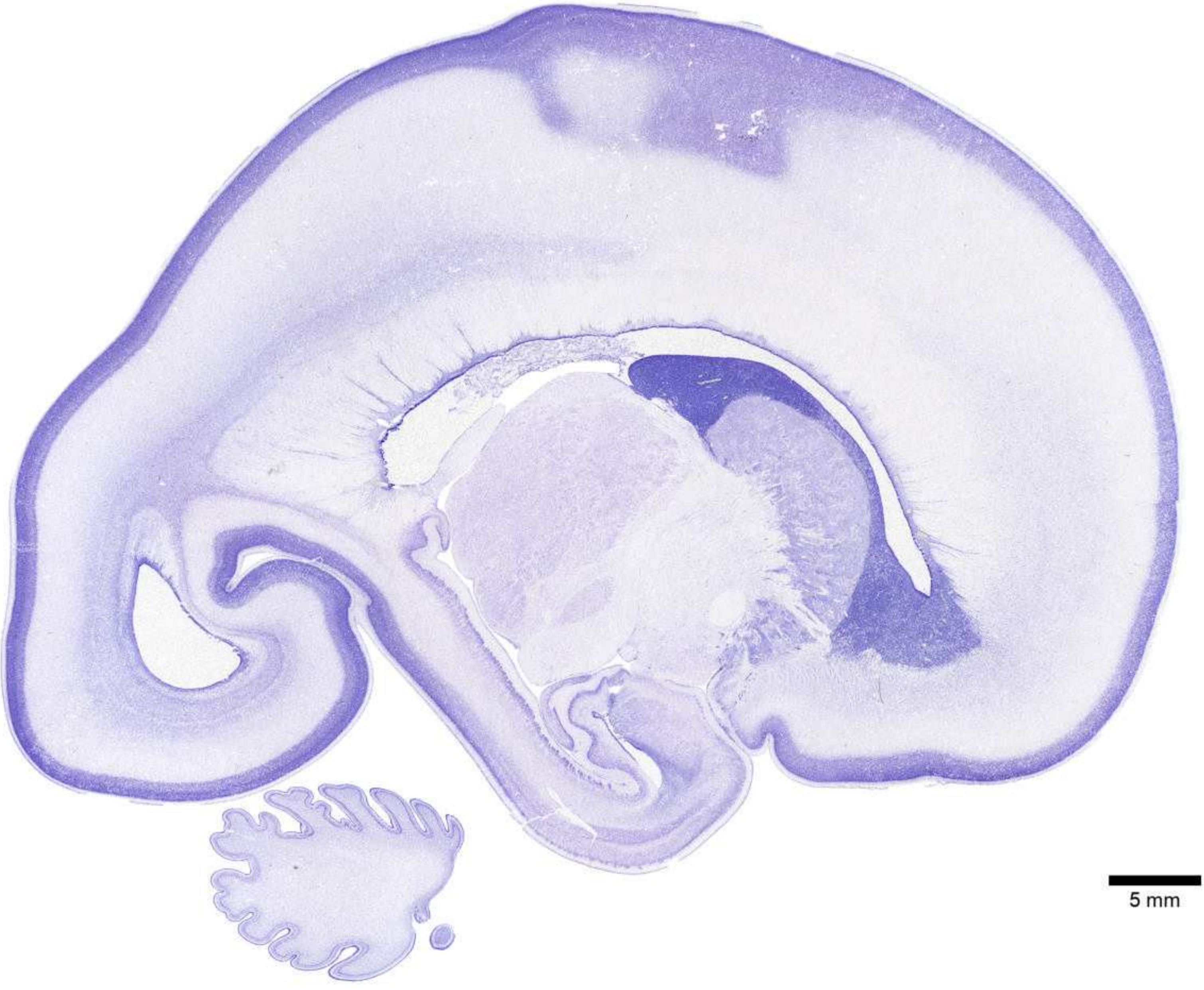

5 mm

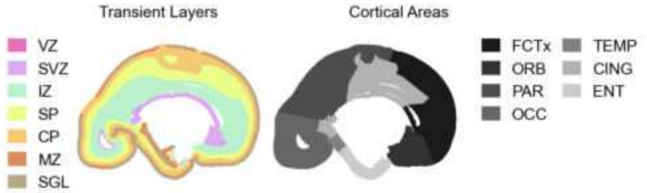

Age: 24 GW

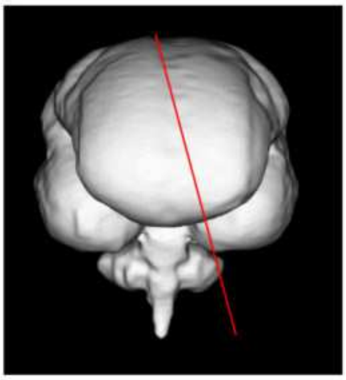

L-R Level: -4.02 mm

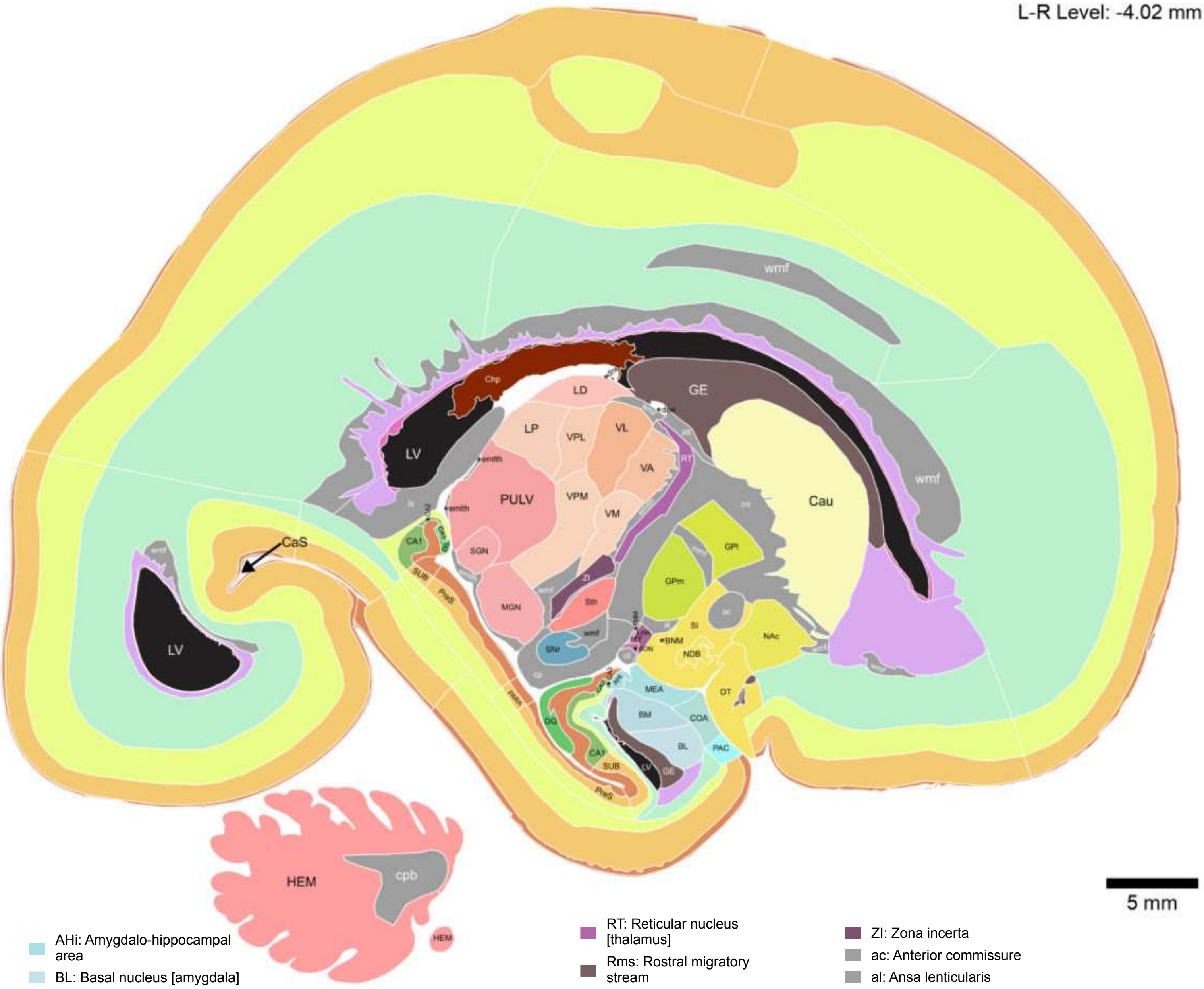

- AHi: Amygdalo-hippocampal area
- BL: Basal nucleus [amygdala]
- BM: Basomedial nucleus [amygdala]
- BNM: Basal nucleus of Meynert
- CA1: CA1 field [hippocampus]
- CA2: CA2 field [hippocampus]
- CA3: CA3 field [hippocampus]
- COA: Cortical nucleus [amygdala]
- Cau: Caudate nucleus
- Chp: Choroid plexus
- DG: Dentate gyrus
- GE: Ganglionic eminence
- GPl: Globus pallidus lateral segment
- GPm: Globus pallidus medial segment
- HEM: Cerebellar hemispheres
- HY: Hypothalamus
- LD: Lateral dorsal nucleus [thalamus]
- LHA: Lateral hypothalamic area
- LP: Lateral posterior nucleus [thalamus]
- LV: Lateral ventricle
- MEA: Medial nucleus [amygdala]
- MGN: Medial geniculate nucleus
- NAc: Nucleus accumbens
- NDB: Nucleus of the diagonal band
- OT: Olfactory tubercle
- PARA: Cortical plate, parasubiculum
- PULV: Pulvinar nucleus [thalamus]
- PreS: Cortical plate, presubiculum
- RT: Reticular nucleus [thalamus]
- Rms: Rostral migratory stream
- SGN: Suprageniculate nucleus
- SI: Substantia innominata
- SNr: Substantia nigra pars reticulata
- SON: Supraoptic nucleus [hypothalamus]
- SUB: Cortical plate, subiculum
- Sth: Subthalamus
- TMM: Tuberomammillary nucleus
- VA: Ventral anterior nucleus [thalamus]
- VL: Ventral lateral nucleus [thalamus]
- VM: Ventral medial nucleus [thalamus]
- VPL: Ventral posterolateral nucleus [thalamus]
- VPM: Ventral posteromedial nucleus [thalamus]
- ZI: Zona incerta
- ac: Anterior commissure
- al: Ansa lenticularis
- cp: Cerebral peduncle
- cpb: Cerebellar peduncles
- dne: Diencephalic neuroepithelium
- emlth: External medullary lamina [thalamus]
- fx: Fornix
- int: Internal capsule
- mml: Medial medullary lamina
- ot: Optic tract
- stt: Stria terminalis
- wmf: White matter fibers
- → CaS: Calcarine sulcus

Age: 24 GW

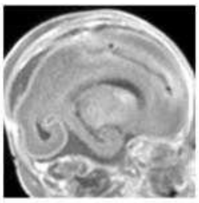

L-R Level: -4.2 mm

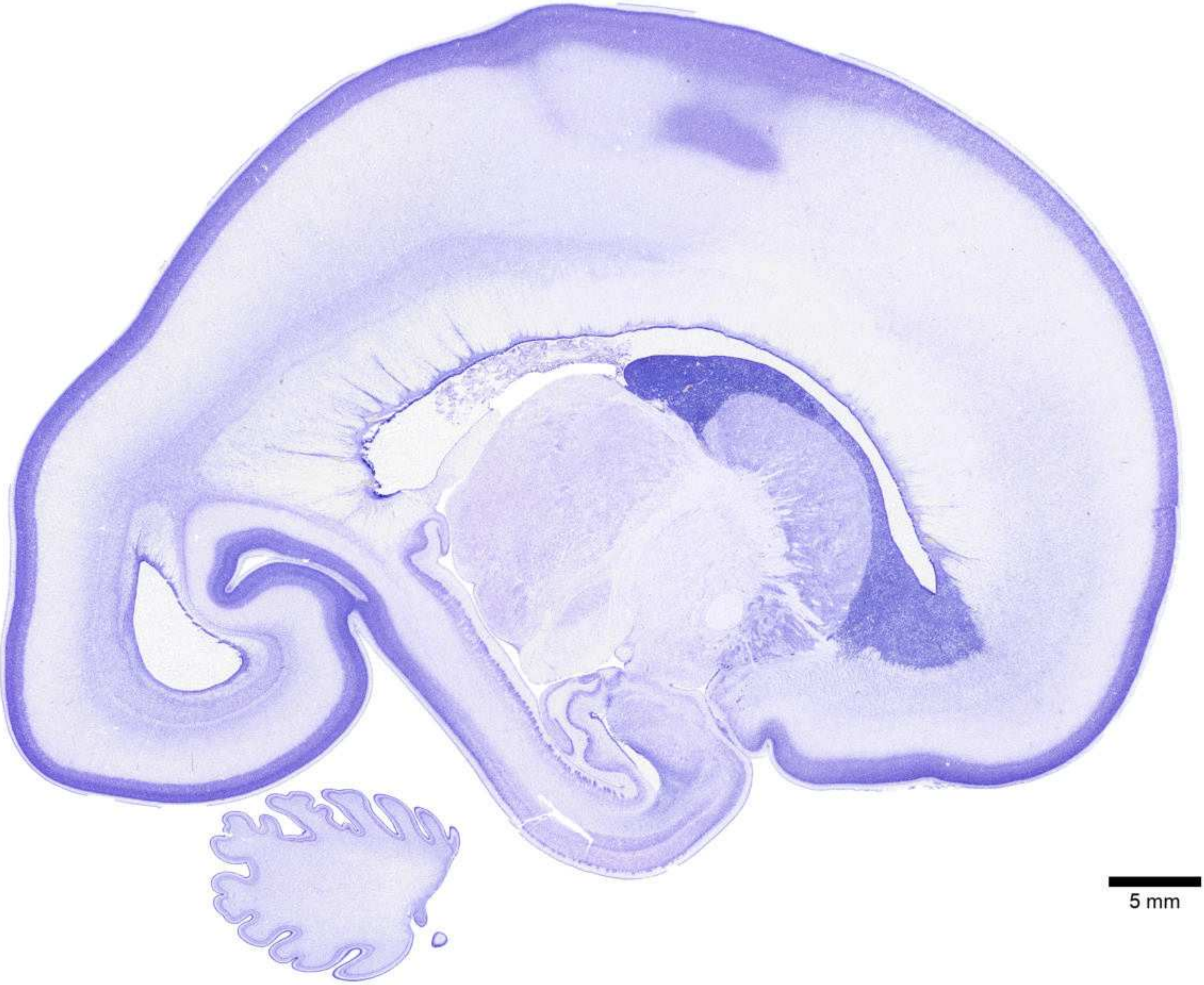

5 mm

Age: 24 GW

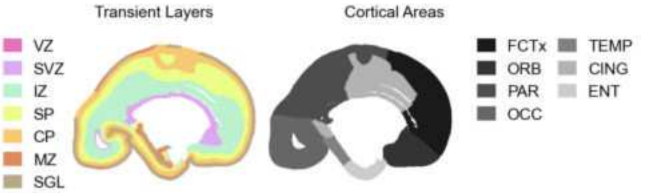

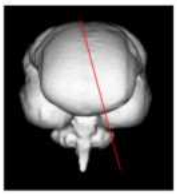

L-R Level: -4.2 mm

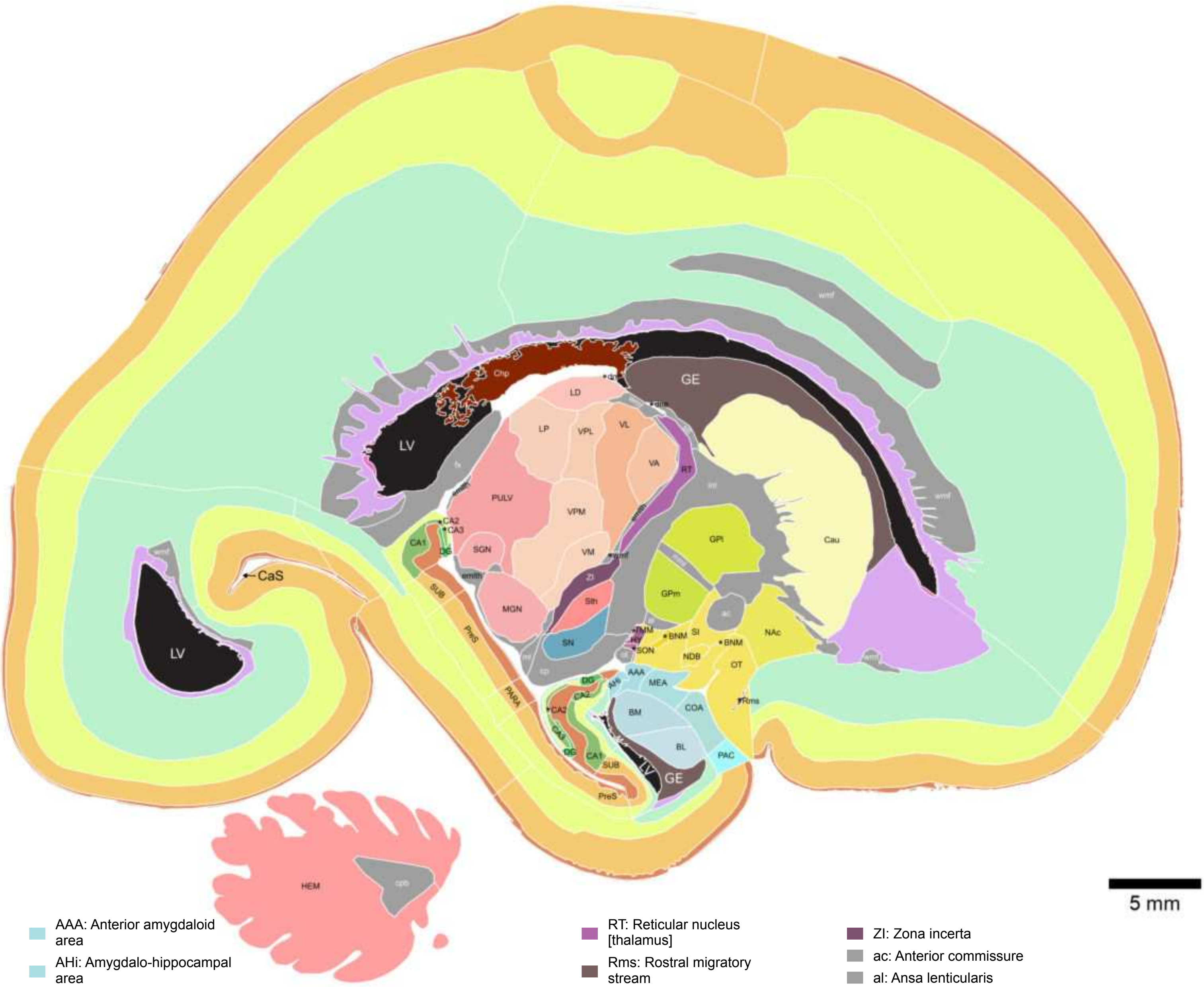

- AAA: Anterior amygdaloid area
- AHi: Amygdalo-hippocampal area
- BL: Basal nucleus [amygdala]
- BM: Basomedial nucleus [amygdala]
- BNM: Basal nucleus of Meynert
- CA1: CA1 field [hippocampus]
- CA2: CA2 field [hippocampus]
- CA3: CA3 field [hippocampus]
- COA: Cortical nucleus [amygdala]
- Cau: Caudate nucleus
- Chp: Choroid plexus
- DG: Dentate gyrus
- GE: Ganglionic eminence
- GPl: Globus pallidus lateral segment
- GPm: Globus pallidus medial segment
- HEM: Cerebellar hemispheres
- HY: Hypothalamus
- LD: Lateral dorsal nucleus [thalamus]
- LHA: Lateral hypothalamic area
- LP: Lateral posterior nucleus [thalamus]
- LV: Lateral ventricle
- MEA: Medial nucleus [amygdala]
- MGN: Medial geniculate nucleus
- NAc: Nucleus accumbens
- NDB: Nucleus of the diagonal band
- OT: Olfactory tubercle
- PARA: Cortical plate, parasubiculum
- PULV: Pulvinar nucleus [thalamus]
- PreS: Cortical plate, presubiculum
- RT: Reticular nucleus [thalamus]
- Rms: Rostral migratory stream
- SGN: Suprageniculate nucleus
- SI: Substantia innominata
- SN: Substantia nigra
- SON: Supraoptic nucleus [hypothalamus]
- SUB: Cortical plate, subiculum
- Sth: Subthalamus
- TMM: Tuberomammillary nucleus
- VA: Ventral anterior nucleus [thalamus]
- VL: Ventral lateral nucleus [thalamus]
- VM: Ventral medial nucleus [thalamus]
- VPL: Ventral posterolateral nucleus [thalamus]
- VPM: Ventral posteromedial nucleus [thalamus]
- ZI: Zona incerta
- ac: Anterior commissure
- al: Ansa lenticularis
- cp: Cerebral peduncle
- cpb: Cerebellar peduncles
- dne: Diencephalic neuroepithelium
- emlth: External medullary lamina [thalamus]
- fx: Fornix
- int: Internal capsule
- ml: Medial lemniscus
- mml: Medial medullary lamina
- ot: Optic tract
- stt: Stria terminalis
- wmf: White matter fibers
- → CaS: Calcarine sulcus

Age: 24 GW

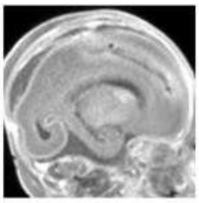

L-R Level: -4.44 mm

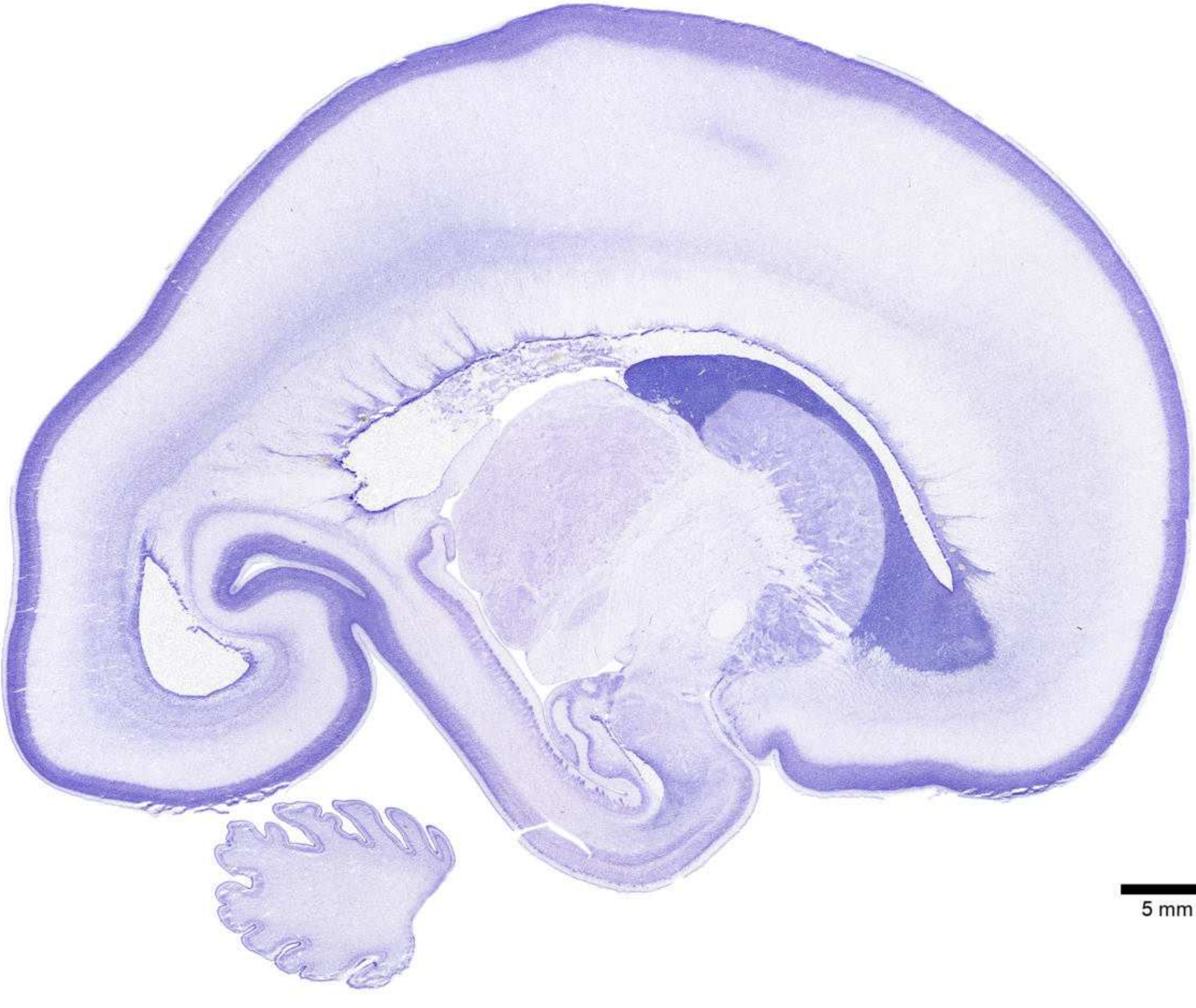

5 mm

Age: 24 GW

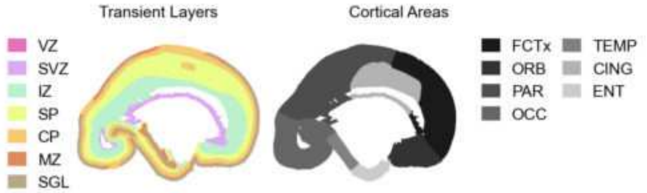

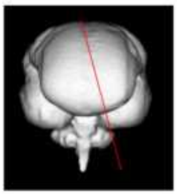

L-R Level: -4.44 mm

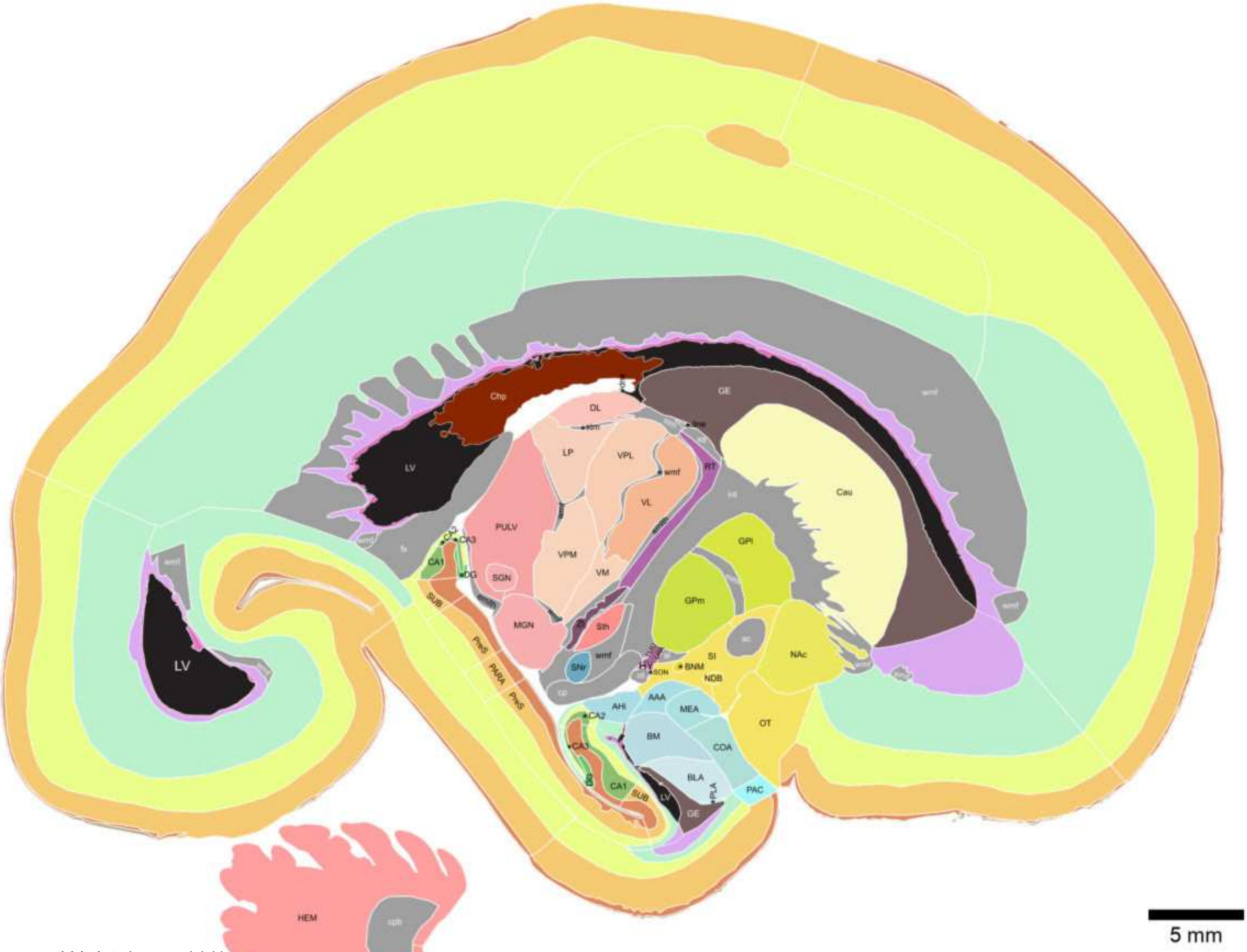

AAA: Anterior amygdaloid area
AHi: Amygdalo-hippocampal area
BL: Basal nucleus [amygdala]
BM: Basomedial nucleus [amygdala]
BNM: Basal nucleus of Meynert
CA1: CA1 field [hippocampus]
CA2: CA2 field [hippocampus]
CA3: CA3 field [hippocampus]
COA: Cortical nucleus [amygdala]
Cau: Caudate nucleus
Chp: Choroid plexus
DG: Dentate gyrus
GE: Ganglionic eminence
GPl: Globus pallidus lateral segment
GPm: Globus pallidus medial segment
HEM: Cerebellar hemispheres
HY: Hypothalamus
LD: Lateral dorsal nucleus [thalamus]
LHA: Lateral hypothalamic area

LP: Lateral posterior nucleus [thalamus]
LV: Lateral ventricle
MEA: Medial nucleus [amygdala]
MGN: Medial geniculate nucleus
NAc: Nucleus accumbens
NDB: Nucleus of the diagonal band
OT: Olfactory tubercle
PARA: Cortical plate, parasubiculum
PLA: Paralaminar nucleus [amygdala]

PULV: Pulvinar nucleus [thalamus]
PreS: Cortical plate, presubiculum
RT: Reticular nucleus [thalamus]
SGN: Suprageniculate nucleus
SI: Substantia innominata
SNr: Substantia nigra pars reticulata
SON: Supraoptic nucleus [hypothalamus]
SUB: Cortical plate, subiculum
Sth: Subthalamus
TMM: Tuberomammillary nucleus
VL: Ventral lateral nucleus [thalamus]
VM: Ventral medial nucleus [thalamus]
VPL: Ventral posterolateral nucleus [thalamus]
VPM: Ventral posteromedial nucleus [thalamus]

ZI: Zona incerta
ac: Anterior commissure
al: Ansa lenticularis
cp: Cerebral peduncle
cpb: Cerebellar peduncles
dne: Diencephalic neuroepithelium
emlth: External medullary lamina [thalamus]
fx: Fornix
int: Internal capsule
mml: Medial medullary lamina
ot: Optic tract
stt: Stria terminalis
wmf: White matter fibers

Age: 24 GW

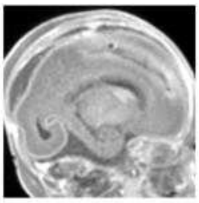

L-R Level: -4.8 mm

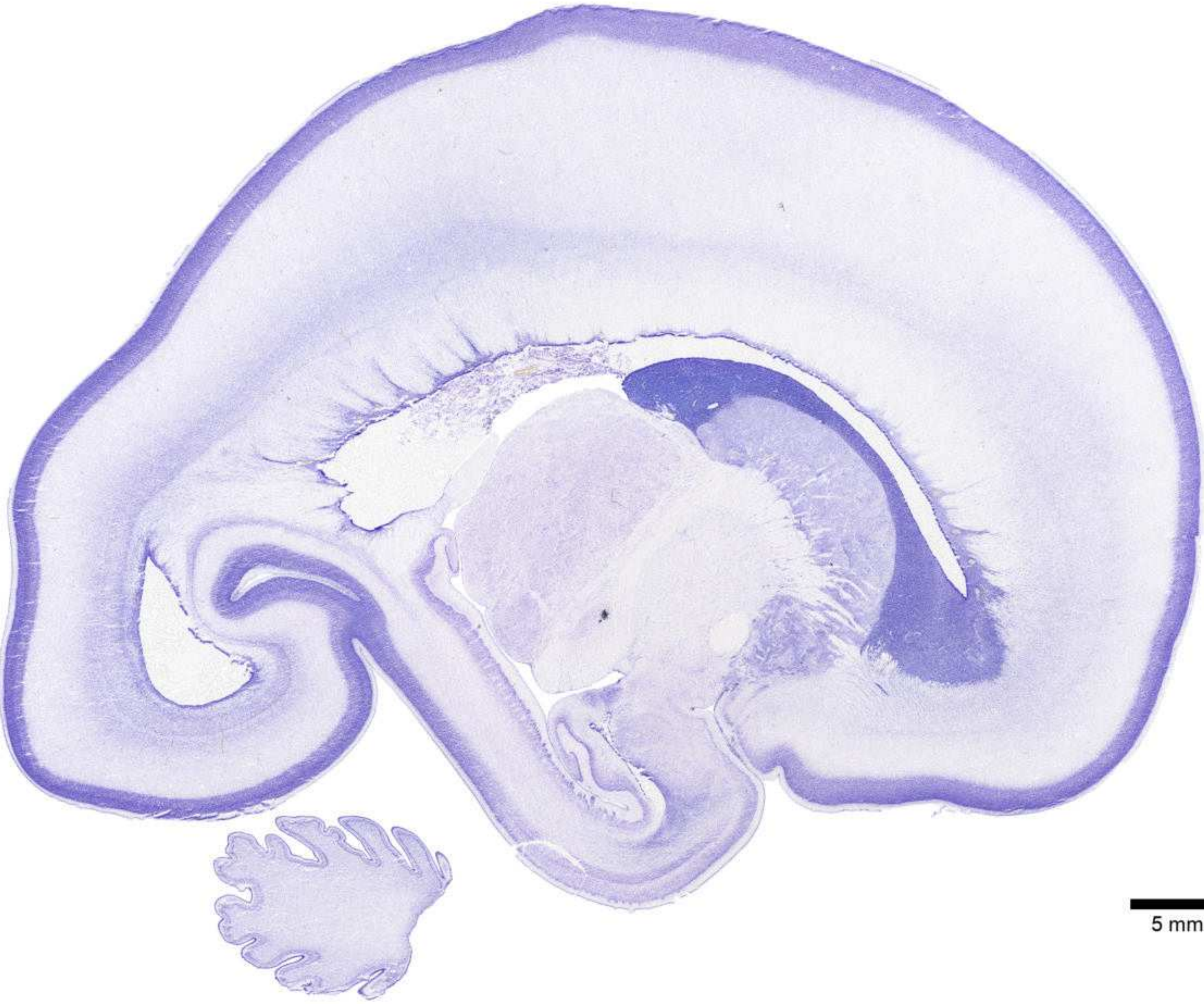

5 mm

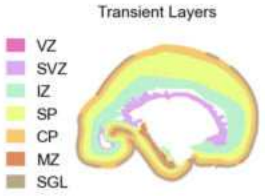

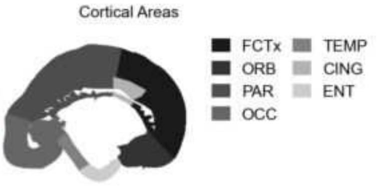

Age: 24 GW

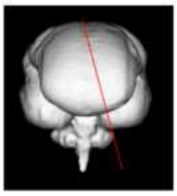

L-R Level: -4.8 mm

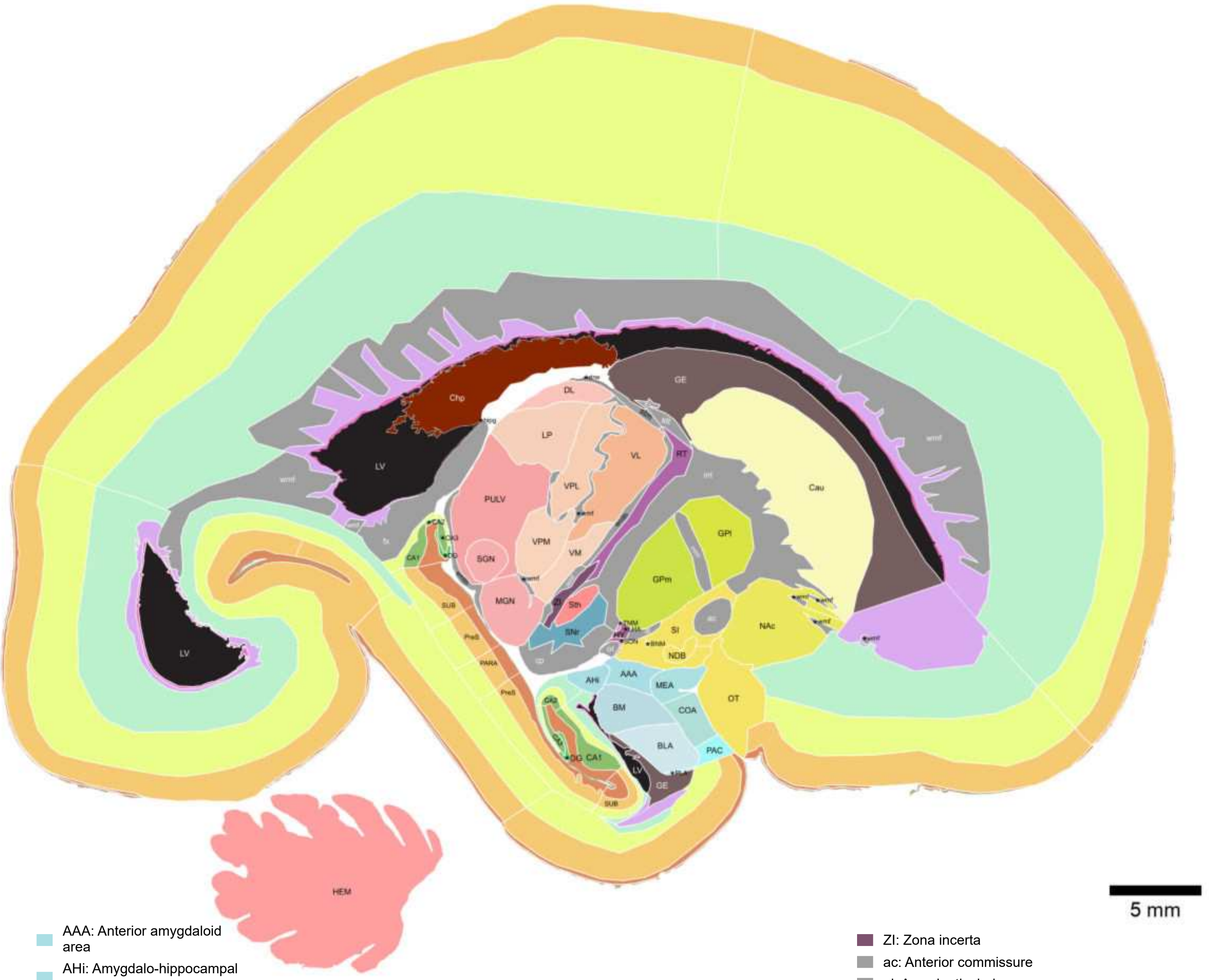

- AAA: Anterior amygdaloid area
- AHi: Amygdalo-hippocampal area
- BL: Basal nucleus [amygdala]
- BM: Basomedial nucleus [amygdala]
- BNM: Basal nucleus of Meynert
- CA1: CA1 field [hippocampus]
- CA2: CA2 field [hippocampus]
- CA3: CA3 field [hippocampus]
- COA: Cortical nucleus [amygdala]
- Cau: Caudate nucleus
- Chp: Choroid plexus
- DG: Dentate gyrus
- GE: Ganglionic eminence
- GPl: Globus pallidus lateral segment
- GPm: Globus pallidus medial segment
- HEM: Cerebellar hemispheres
- HY: Hypothalamus
- LD: Lateral dorsal nucleus [thalamus]
- LHA: Lateral hypothalamic area
- LP: Lateral posterior nucleus [thalamus]
- LV: Lateral ventricle
- MEA: Medial nucleus [amygdala]
- MGN: Medial geniculate nucleus
- NAc: Nucleus accumbens
- NDB: Nucleus of the diagonal band
- OT: Olfactory tubercle
- PARA: Cortical plate, parasubiculum
- PLA: Paralaminar nucleus [amygdala]
- PULV: Pulvinar nucleus [thalamus]
- PreS: Cortical plate, presubiculum
- RT: Reticular nucleus [thalamus]
- SGN: Suprageniculate nucleus
- SI: Substantia innominata
- SNr: Substantia nigra pars reticulata
- SON: Supraoptic nucleus [hypothalamus]
- SUB: Cortical plate, subiculum
- Sth: Subthalamus
- TMM: Tuberomammillary nucleus
- VL: Ventral lateral nucleus [thalamus]
- VM: Ventral medial nucleus [thalamus]
- VPL: Ventral posterolateral nucleus [thalamus]
- VPM: Ventral posteromedial nucleus [thalamus]
- ZI: Zona incerta
- ac: Anterior commissure
- al: Ansa lenticularis
- cp: Cerebral peduncle
- dne: Diencephalic neuroepithelium
- emlth: External medullary lamina [thalamus]
- fx: Fornix
- hipg: Hippocampal glioepithelium/ependyma
- int: Internal capsule
- mml: Medial medullary lamina
- ot: Optic tract
- stt: Stria terminalis
- wmf: White matter fibers

Age: 24 GW

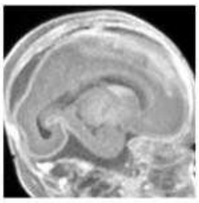

L-R Level: -6.0 mm

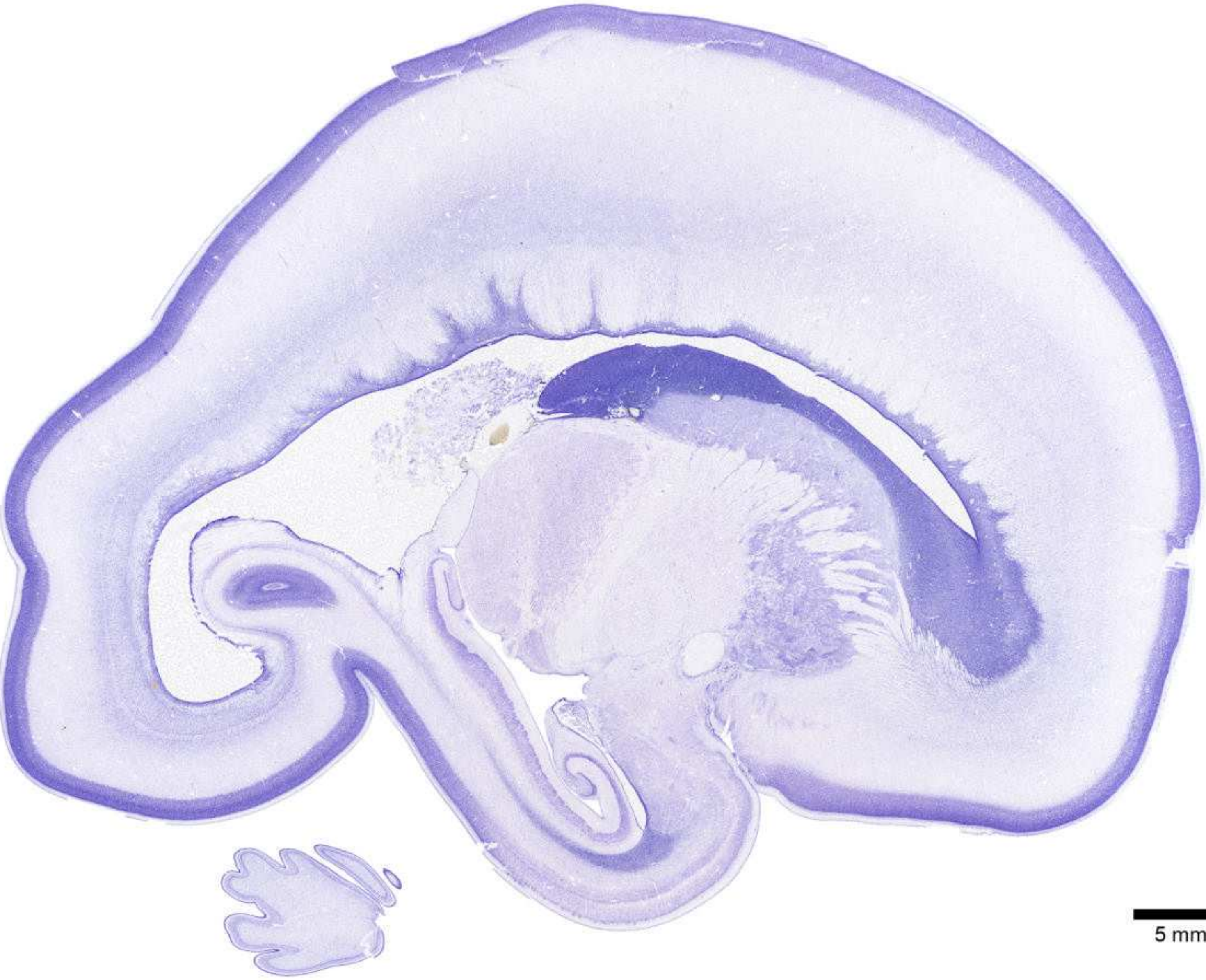

5 mm

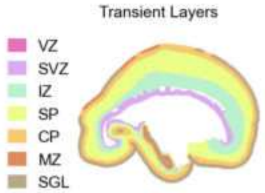

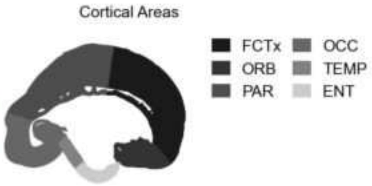

Age: 24 GW

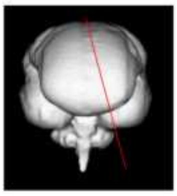

L-R Level: -6.0 mm

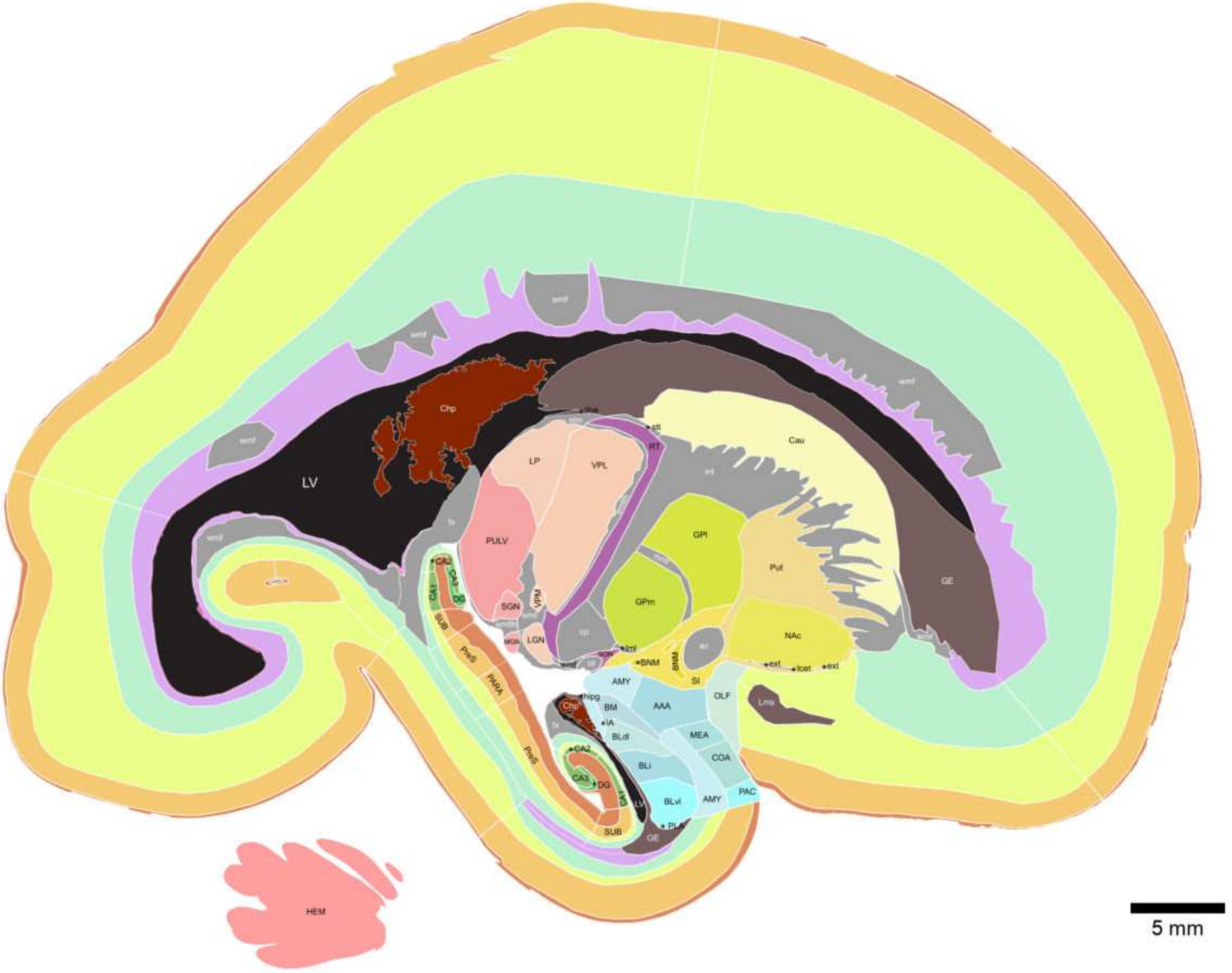

- AAA: Anterior amygdaloid area
- AMY: Amygdala
- BLdl: Basal nucleus [amygdala], dorsolateral part
- BLi: Basal nucleus [amygdala], intermediate part
- BLvl: Basal nucleus [amygdala], ventrolateral part
- BM: Basomedial nucleus [amygdala]
- BNM: Basal nucleus of Meynert
- CA1: CA1 field [hippocampus]
- CA2: CA2 field [hippocampus]
- CA3: CA3 field [hippocampus]
- COA: Cortical nucleus [amygdala]
- Cau: Caudate nucleus
- Chp: Choroid plexus
- DG: Dentate gyrus
- GE: Ganglionic eminence
- GPl: Globus pallidus lateral segment
- GPm: Globus pallidus medial segment
- HEM: Cerebellar hemispheres
- IA: Intercalated cell groups [amygdala]
- LGN: Lateral geniculate nucleus
- LP: Lateral posterior nucleus [thalamus]
- LV: Lateral ventricle
- Lms: Lateral migratory stream
- MEA: Medial nucleus [amygdala]
- MGN: Medial geniculate nucleus
- NAc: Nucleus accumbens
- PARA: Cortical plate, parasubiculum
- PLA: Paralaminar nucleus [amygdala]
- PULV: Pulvinar nucleus [thalamus]
- PreS: Cortical plate, presubiculum
- Put: Putamen
- RT: Reticular nucleus [thalamus]
- SGN: Suprageniculate nucleus
- SI: Substantia innominata
- SON: Supraoptic nucleus [hypothalamus]
- SUB: Cortical plate, subiculum
- VPL: Ventral posterolateral nucleus [thalamus]
- VPM: Ventral posteromedial nucleus [thalamus]
- ac: Anterior commissure
- cp: Cerebral peduncle
- dne: Diencephalic neuroepithelium
- emlth: External medullary lamina [thalamus]
- ext: External capsule
- fx: Fornix
- hipg: Hippocampal glioepithelium/ependyma
- int: Internal capsule
- lml: Lateral medullary lamina
- mml: Medial medullary lamina
- ot: Optic tract
- stt: Stria terminalis
- tcet: Transient cell zone in the external capsule
- wmf: White matter fibers

Age: 24 GW

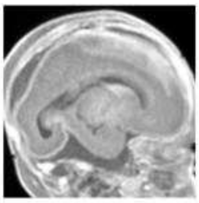

L-R Level: -6.48 mm

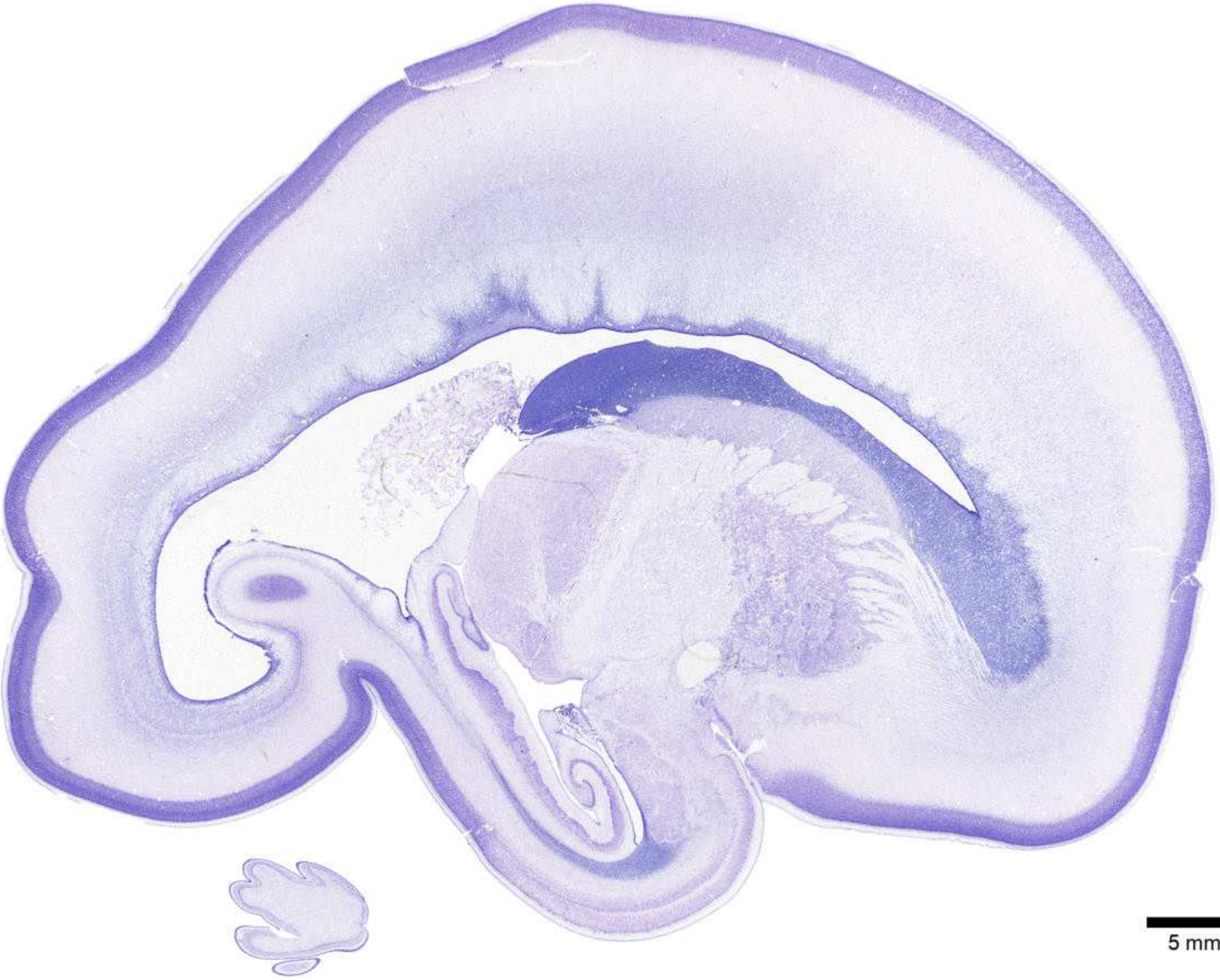

5 mm

Transient Layers

- VZ
- SVZ
- IZ
- SP
- CP
- MZ
- SGL

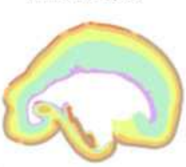

Cortical Areas

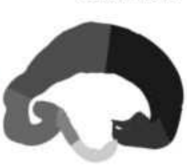

- FCTx
- ORB
- PAR
- OCC
- TEMP
- ENT

Age: 24 GW

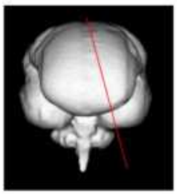

L-R Level: -6.48 mm

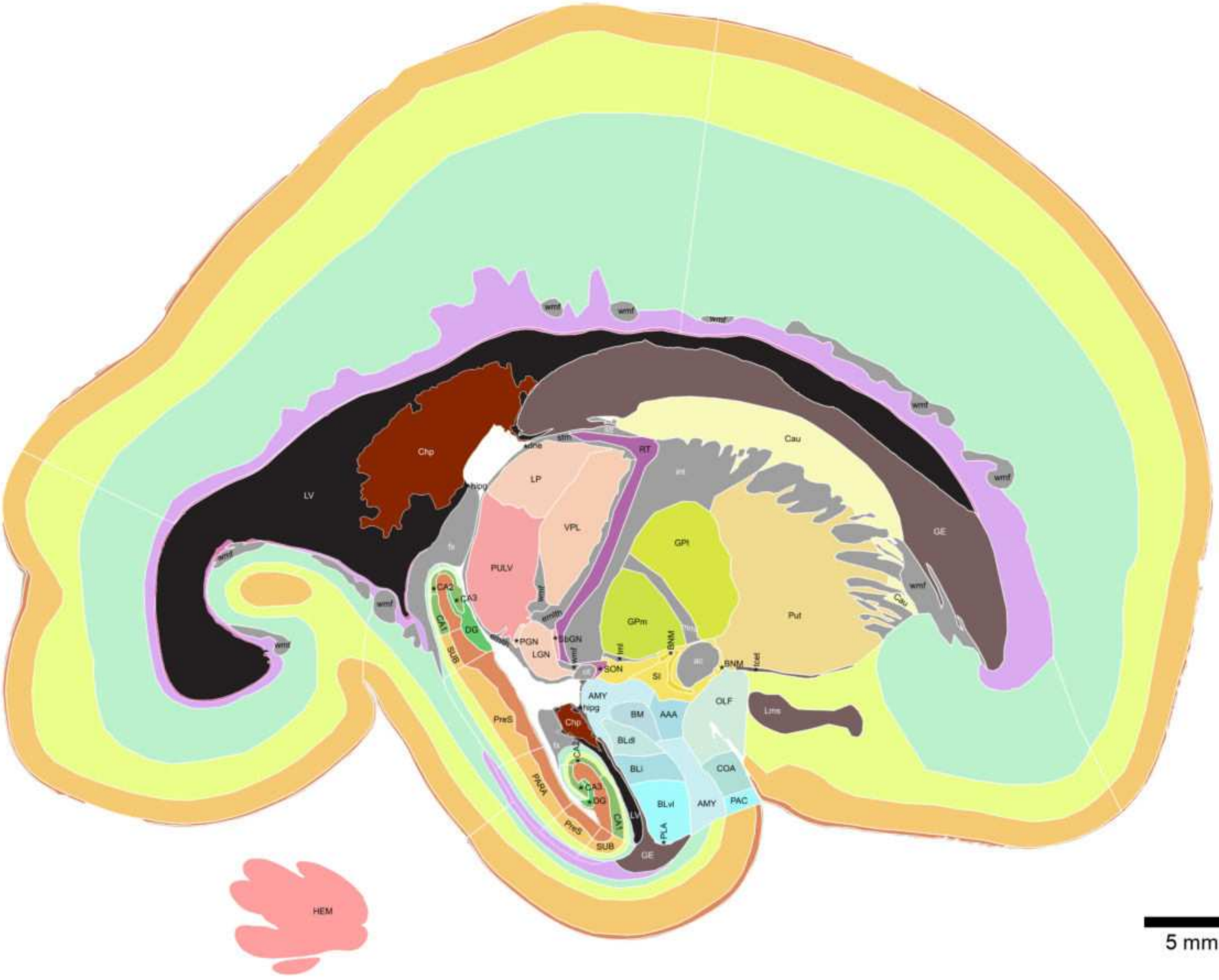

5 mm

- AAA: Anterior amygdaloid area
- AMY: Amygdala
- BLdl: Basal nucleus [amygdala], dorsolateral part
- BLi: Basal nucleus [amygdala], intermediate part
- BLvl: Basal nucleus [amygdala], ventrolateral part
- BM: Basomedial nucleus [amygdala]
- BNM: Basal nucleus of Meynert
- CA1: CA1 field [hippocampus]
- CA2: CA2 field [hippocampus]
- CA3: CA3 field [hippocampus]
- COA: Cortical nucleus [amygdala]
- Cau: Caudate nucleus
- Chp: Choroid plexus
- DG: Dentate gyrus
- GE: Ganglionic eminence
- GPl: Globus pallidus lateral segment
- GPm: Globus pallidus medial segment
- HEM: Cerebellar hemispheres
- LGN: Lateral geniculate nucleus
- LP: Lateral posterior nucleus [thalamus]
- LV: Lateral ventricle
- Lms: Lateral migratory stream
- PARA: Cortical plate, parasubiculum
- PGN: Pregeniculate nucleus
- PLA: Paralaminar nucleus [amygdala]
- PULV: Pulvinar nucleus [thalamus]
- PreS: Cortical plate, presubiculum
- Put: Putamen
- RT: Reticular nucleus [thalamus]
- SI: Substantia innominata
- SON: Supraoptic nucleus [hypothalamus]
- SUB: Cortical plate, subiculum
- SbGN: Subgeniculate nucleus
- VPL: Ventral posterolateral nucleus [thalamus]
- ac: Anterior commissure
- dne: Diencephalic neuroepithelium
- emlth: External medullary lamina [thalamus]
- fx: Fornix
- hipg: Hippocampal glioepithelium/ependyma
- int: Internal capsule
- lml: Lateral medullary lamina
- mml: Medial medullary lamina
- ot: Optic tract
- stt: Stria terminalis
- tcet: Transient cell zone in the external capsule
- wmf: White matter fibers

Age: 24 GW

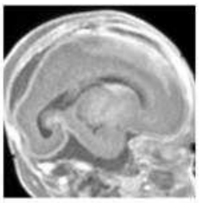

L-R Level: -6.72 mm

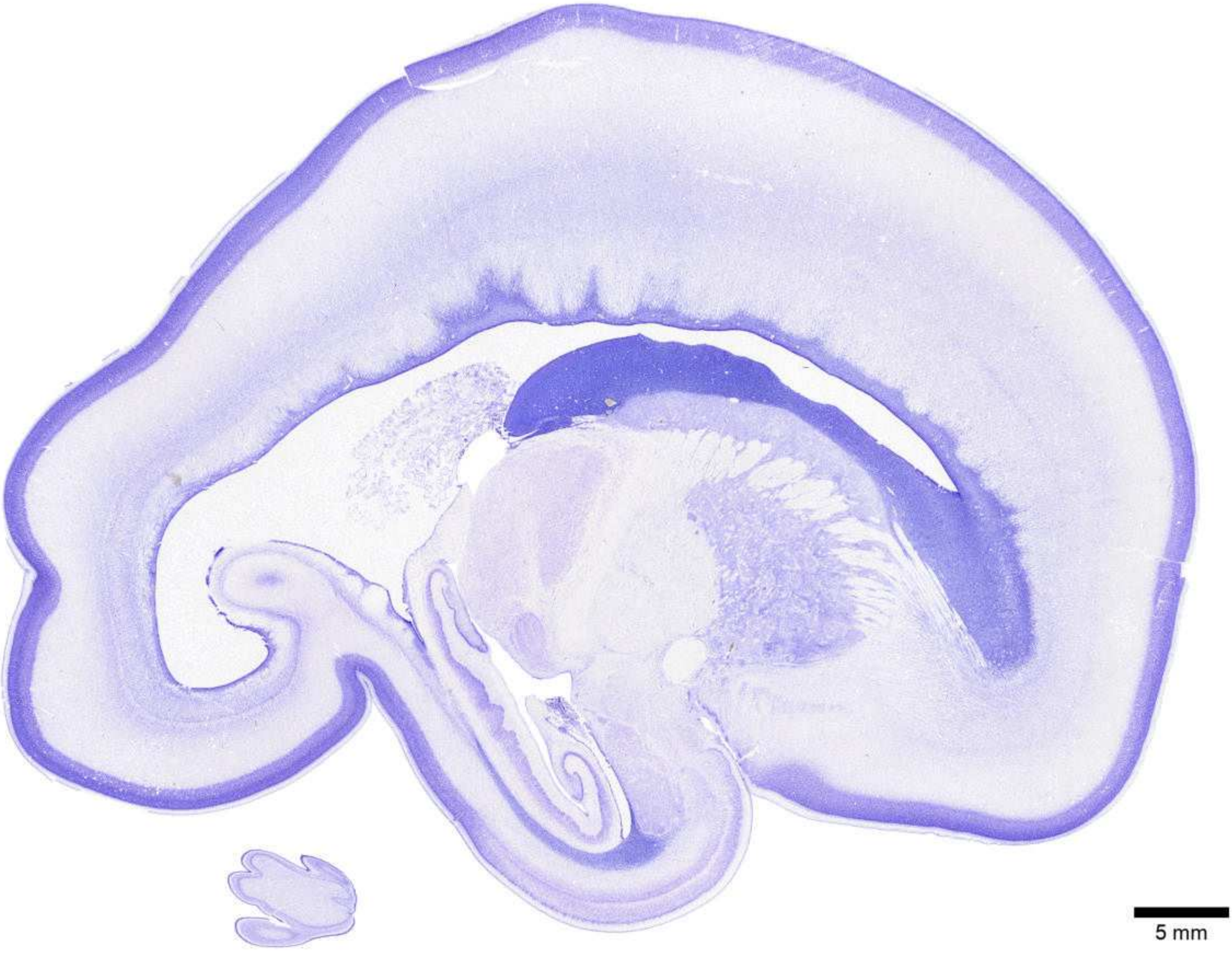

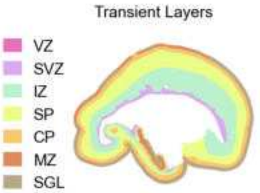

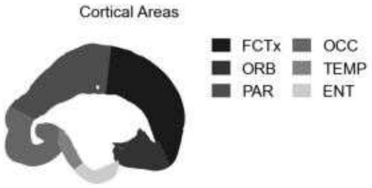

Age: 24 GW

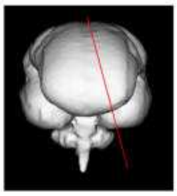

L-R Level: -6.72 mm

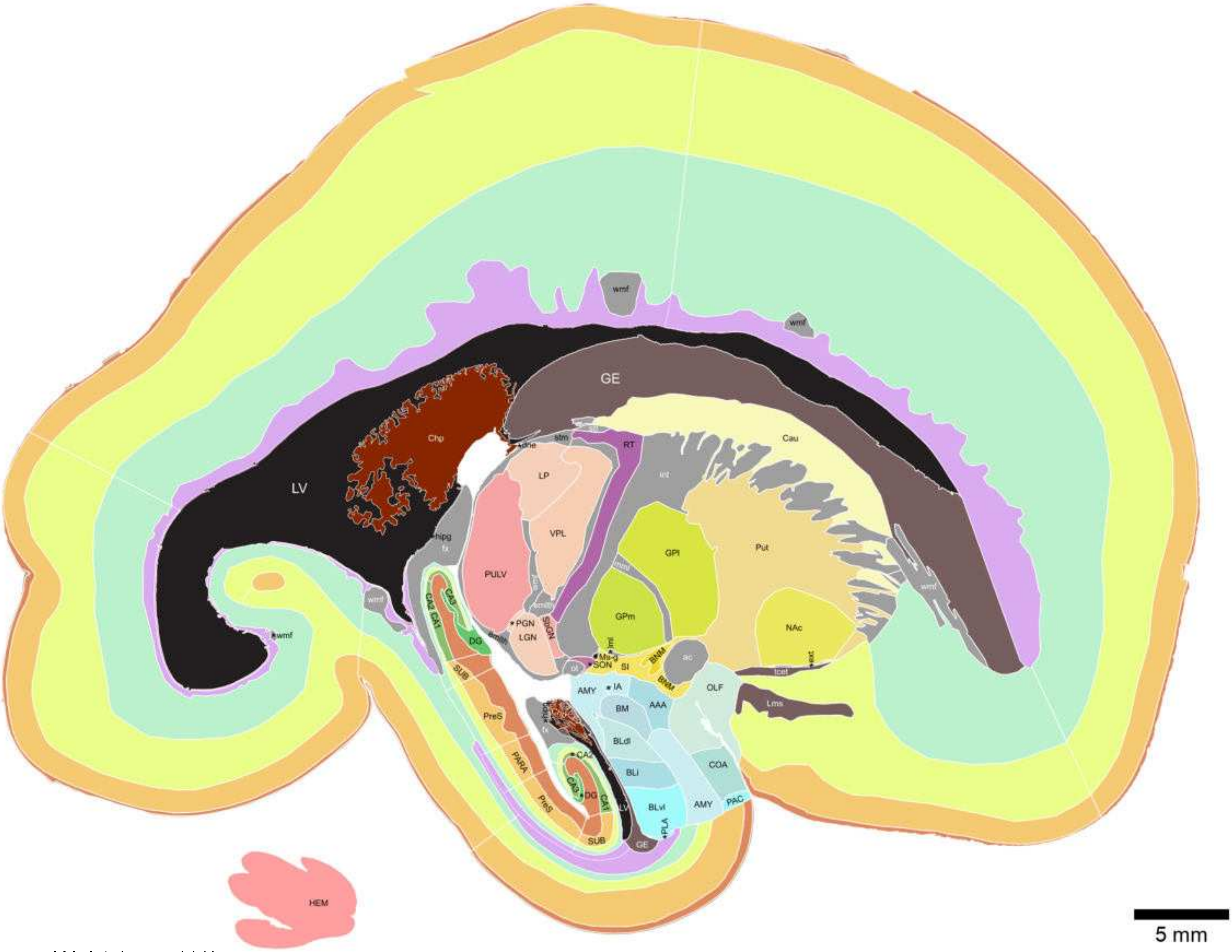

- AAA: Anterior amygdaloid area
- AMY: Amygdala
- BLdl: Basal nucleus [amygdala], dorsolateral part
- BLi: Basal nucleus [amygdala], intermediate part
- BLvl: Basal nucleus [amygdala], ventrolateral part
- BM: Basomedial nucleus [amygdala]
- BNM: Basal nucleus of Meynert
- CA1: CA1 field [hippocampus]
- CA2: CA2 field [hippocampus]
- CA3: CA3 field [hippocampus]
- COA: Cortical nucleus [amygdala]
- Cau: Caudate nucleus
- Chp: Choroid plexus
- DG: Dentate gyrus
- GE: Ganglionic eminence
- GPl: Globus pallidus lateral segment
- GPm: Globus pallidus medial segment
- HEM: Cerebellar hemispheres
- IA: Intercalated cell groups [amygdala]
- LGN: Lateral geniculate nucleus
- LP: Lateral posterior nucleus [thalamus]
- LV: Lateral ventricle
- Lms: Lateral migratory stream
- Ms-g: Migratory stream, general
- NAc: Nucleus accumbens
- PARA: Cortical plate, parasubiculum
- PGN: Pregeniculate nucleus
- PLA: Paralaminar nucleus [amygdala]
- PULV: Pulvinar nucleus [thalamus]
- PreS: Cortical plate, presubiculum
- Put: Putamen
- RT: Reticular nucleus [thalamus]
- SI: Substantia innominata
- SON: Supraoptic nucleus [hypothalamus]
- SUB: Cortical plate, subiculum
- SbGN: Subgeniculate nucleus
- VPL: Ventral posterolateral nucleus [thalamus]
- ac: Anterior commissure
- dne: Diencephalic neuroepithelium
- emlth: External medullary lamina [thalamus]
- ext: External capsule
- fx: Fornix
- hipg: Hippocampal glioepithelium/ependyma
- int: Internal capsule
- lml: Lateral medullary lamina
- mml: Medial medullary lamina
- ot: Optic tract
- stt: Stria terminalis
- tcet: Transient cell zone in the external capsule
- wmf: White matter fibers

Age: 24 GW

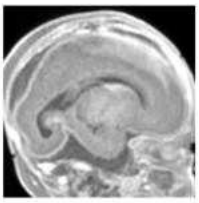

L-R Level: -7.2 mm

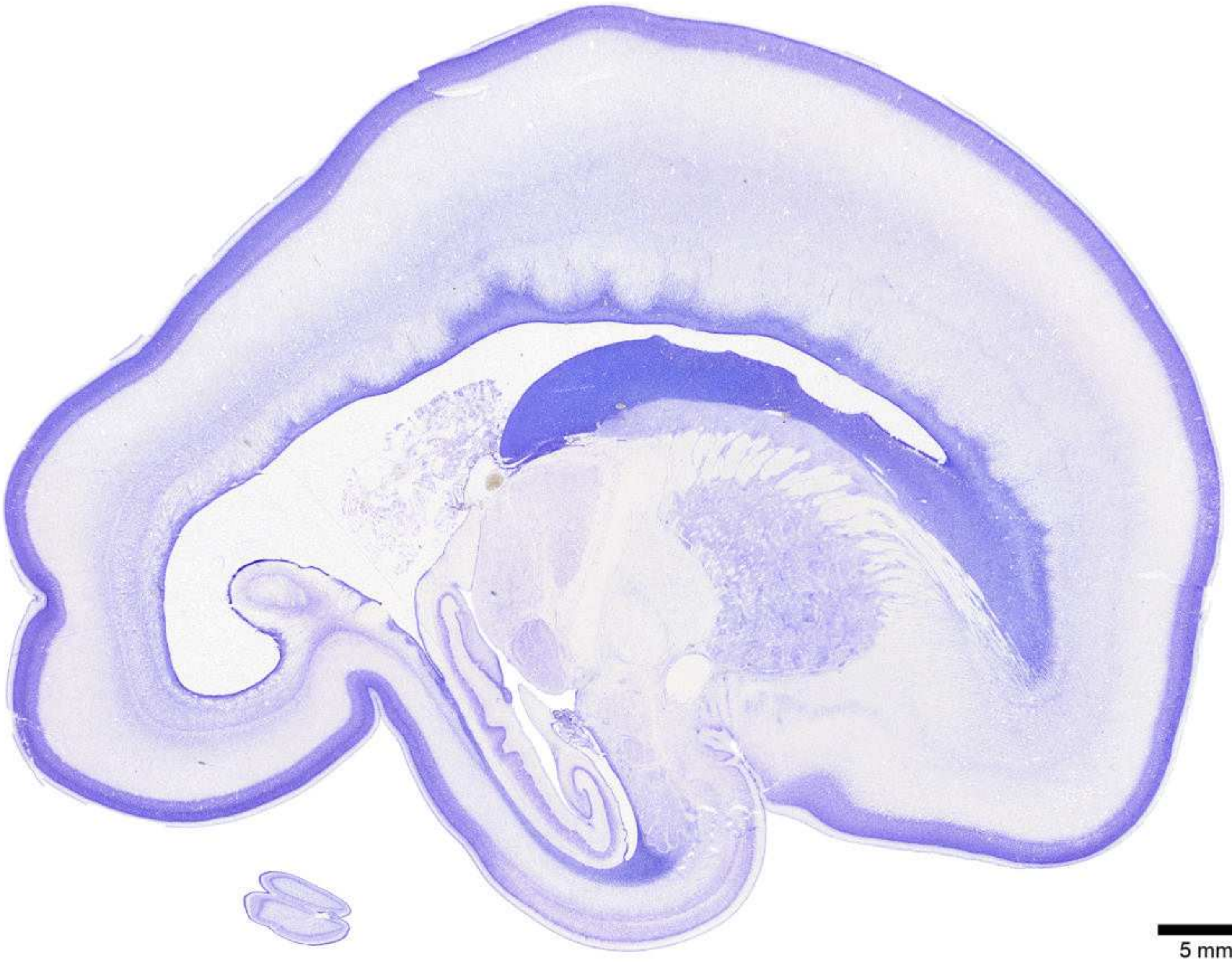

5 mm

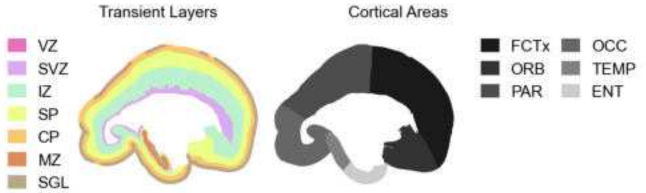

Age: 24 GW

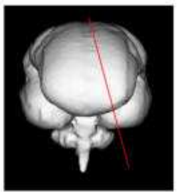

L-R Level: -7.2 mm

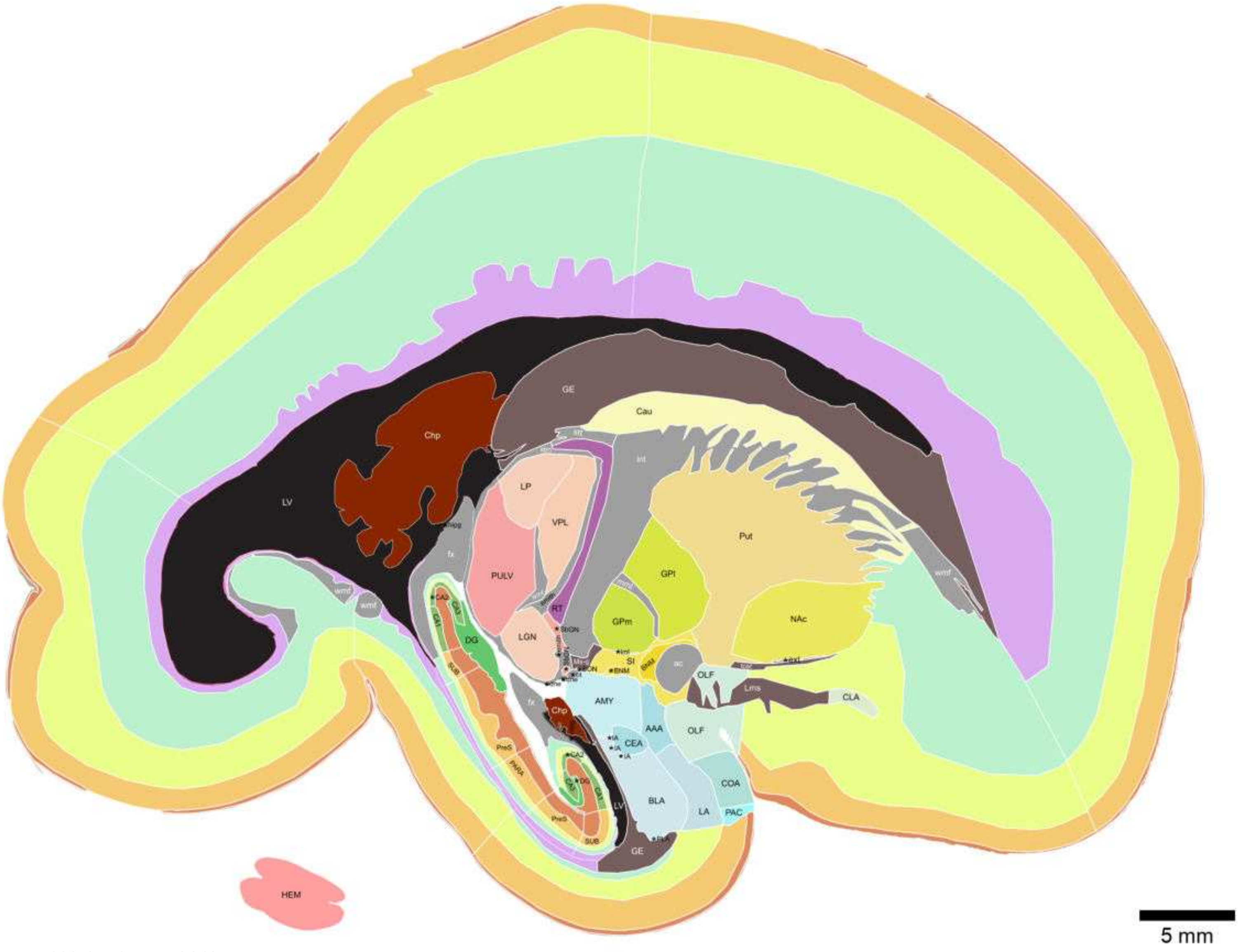

- AAA: Anterior amygdaloid area
- AMY: Amygdala
- BL: Basal nucleus [amygdala]
- BNM: Basal nucleus of Meynert
- CA1: CA1 field [hippocampus]
- CA2: CA2 field [hippocampus]
- CA3: CA3 field [hippocampus]
- CEA: Central nucleus [amygdala]
- CLA: Claustrum
- COA: Cortical nucleus [amygdala]
- Cau: Caudate nucleus
- Chp: Choroid plexus
- DG: Dentate gyrus
- GE: Ganglionic eminence
- GPl: Globus pallidus lateral segment
- GPm: Globus pallidus medial segment
- HEM: Cerebellar hemispheres
- IA: Intercalated cell groups [amygdala]
- LA: Lateral nucleus [amygdala]
- LGN: Lateral geniculate nucleus
- LP: Lateral posterior nucleus [thalamus]
- LV: Lateral ventricle
- Lms: Lateral migratory stream
- Ms-g: Migratory stream, general
- NAc: Nucleus accumbens
- PARA: Cortical plate, parasubiculum
- PLA: Paralaminar nucleus [amygdala]
- PULV: Pulvinar nucleus [thalamus]
- PreS: Cortical plate, presubiculum
- Put: Putamen
- RT: Reticular nucleus [thalamus]
- SI: Substantia innominata
- SON: Supraoptic nucleus [hypothalamus]
- SUB: Cortical plate, subiculum
- SbGN: Subgeniculate nucleus
- VPL: Ventral posterolateral nucleus [thalamus]
- ac: Anterior commissure
- dne: Diencephalic neuroepithelium
- emlth: External medullary lamina [thalamus]
- ext: External capsule
- fx: Fornix
- hipg: Hippocampal glioepithelium/ependyma
- int: Internal capsule
- lml: Lateral medullary lamina
- mml: Medial medullary lamina
- ot: Optic tract
- stt: Stria terminalis
- tcet: Transient cell zone in the external capsule
- wmf: White matter fibers

Age: 24 GW

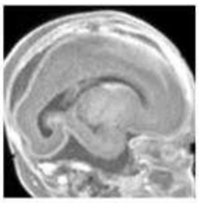

L-R Level: -7.62 mm

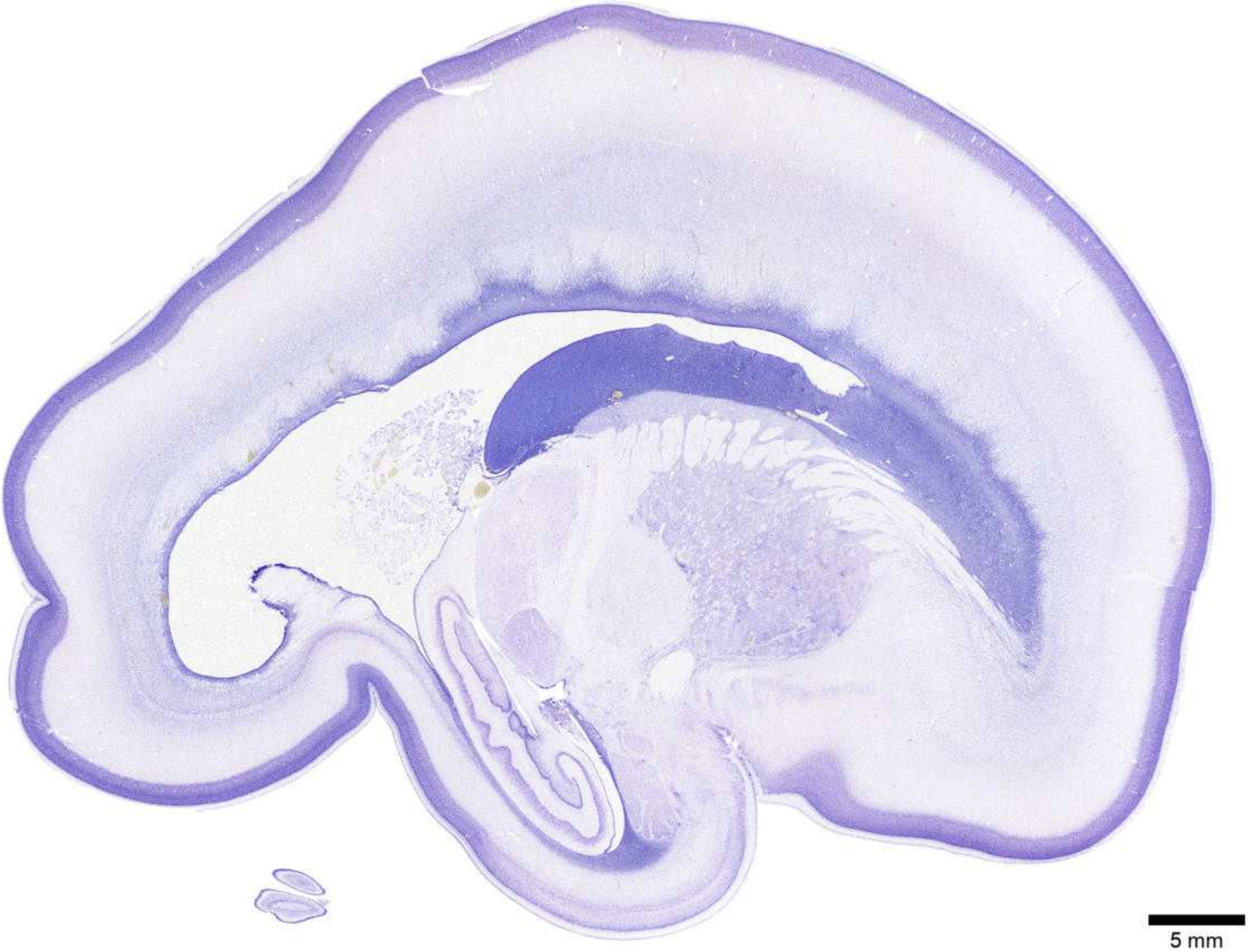

5 mm

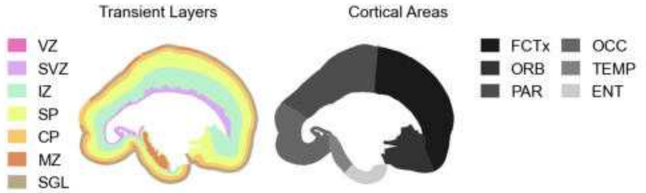

Age: 24 GW

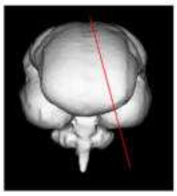

L-R Level: -7.62 mm

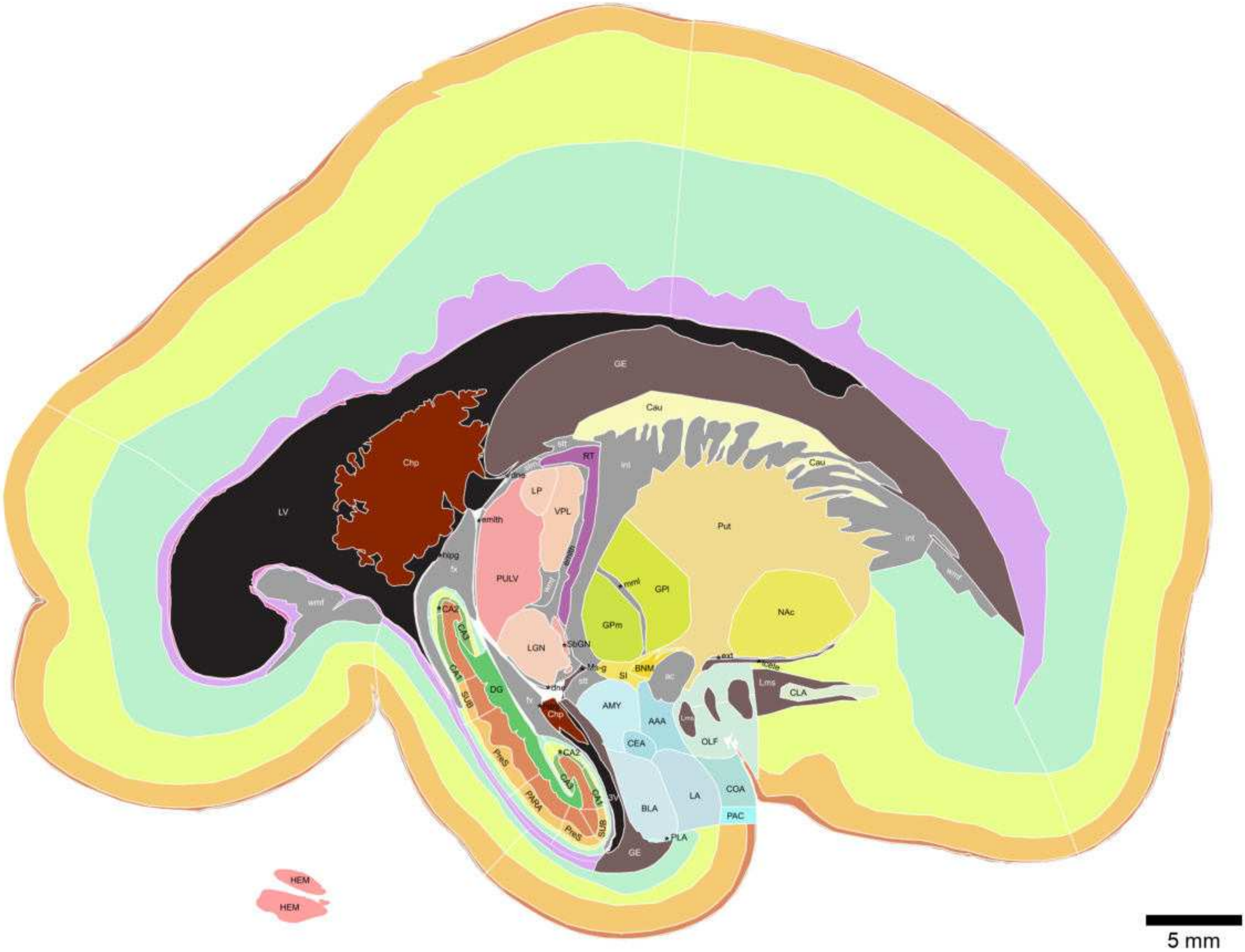

- 3V: Third ventricle
- AAA: Anterior amygdaloid area
- AMY: Amygdala
- BLA: Basolateral complex [amygdala]
- BNM: Basal nucleus of Meynert
- CA1: CA1 field [hippocampus]
- CA2: CA2 field [hippocampus]
- CA3: CA3 field [hippocampus]
- CEA: Central nucleus [amygdala]
- CLA: Claustrum
- COA: Cortical nucleus [amygdala]
- Cau: Caudate nucleus
- Chp: Choroid plexus
- DG: Dentate gyrus
- GE: Ganglionic eminence
- GPl: Globus pallidus lateral segment
- GPm: Globus pallidus medial segment
- HEM: Cerebellar hemispheres
- LA: Lateral nucleus [amygdala]
- LGN: Lateral geniculate nucleus
- LP: Lateral posterior nucleus [thalamus]
- LV: Lateral ventricle
- Lms: Lateral migratory stream
- Ms-g: Migratory stream, general
- NAc: Nucleus accumbens
- PARA: Cortical plate, parasubiculum
- PLA: Paralaminar nucleus [amygdala]
- PULV: Pulvinar nucleus [thalamus]
- PreS: Cortical plate, presubiculum
- Put: Putamen
- RT: Reticular nucleus [thalamus]
- SI: Substantia innominata
- SUB: Cortical plate, subiculum
- SbGN: Subgeniculate nucleus
- VPL: Ventral posterolateral nucleus [thalamus]
- ac: Anterior commissure
- dne: Diencephalic neuroepithelium
- emlth: External medullary lamina [thalamus]
- ext: External capsule
- fx: Fornix
- hipg: Hippocampal glioepithelium/ependyma
- int: Internal capsule
- mml: Medial medullary lamina
- stt: Stria terminalis
- tcete: Transient cell zone in the extreme capsule
- wmf: White matter fibers

Age: 24 GW

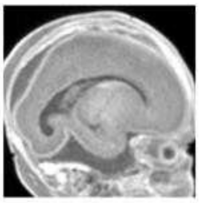

L-R Level: -8.16 mm

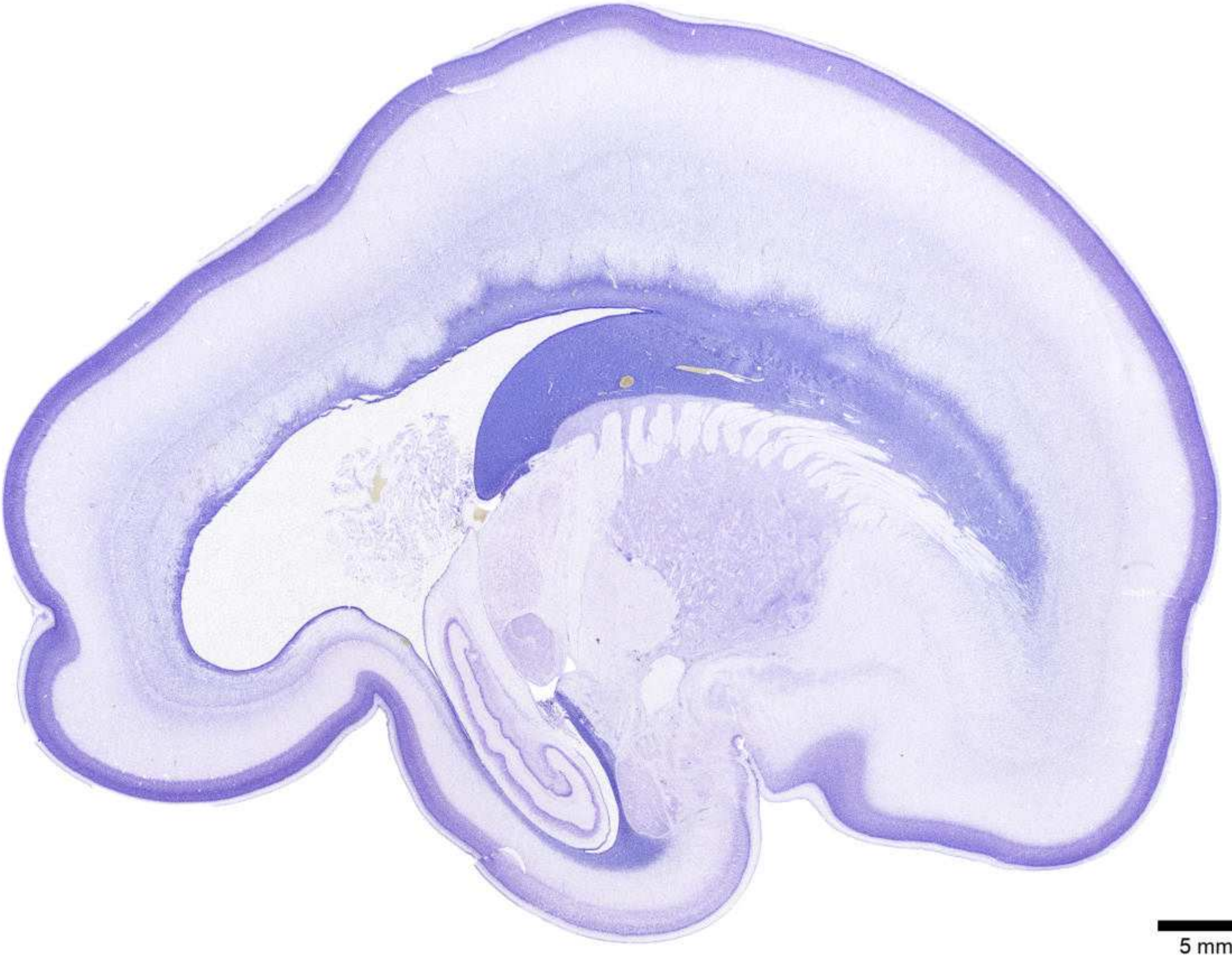

5 mm

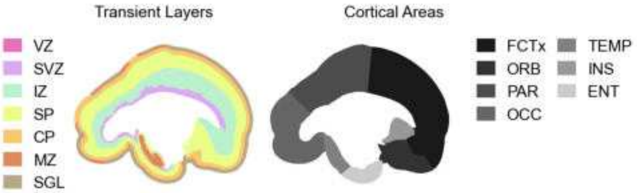

Age: 24 GW

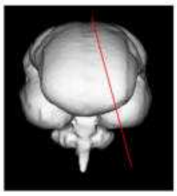

L-R Level: -8.16 mm

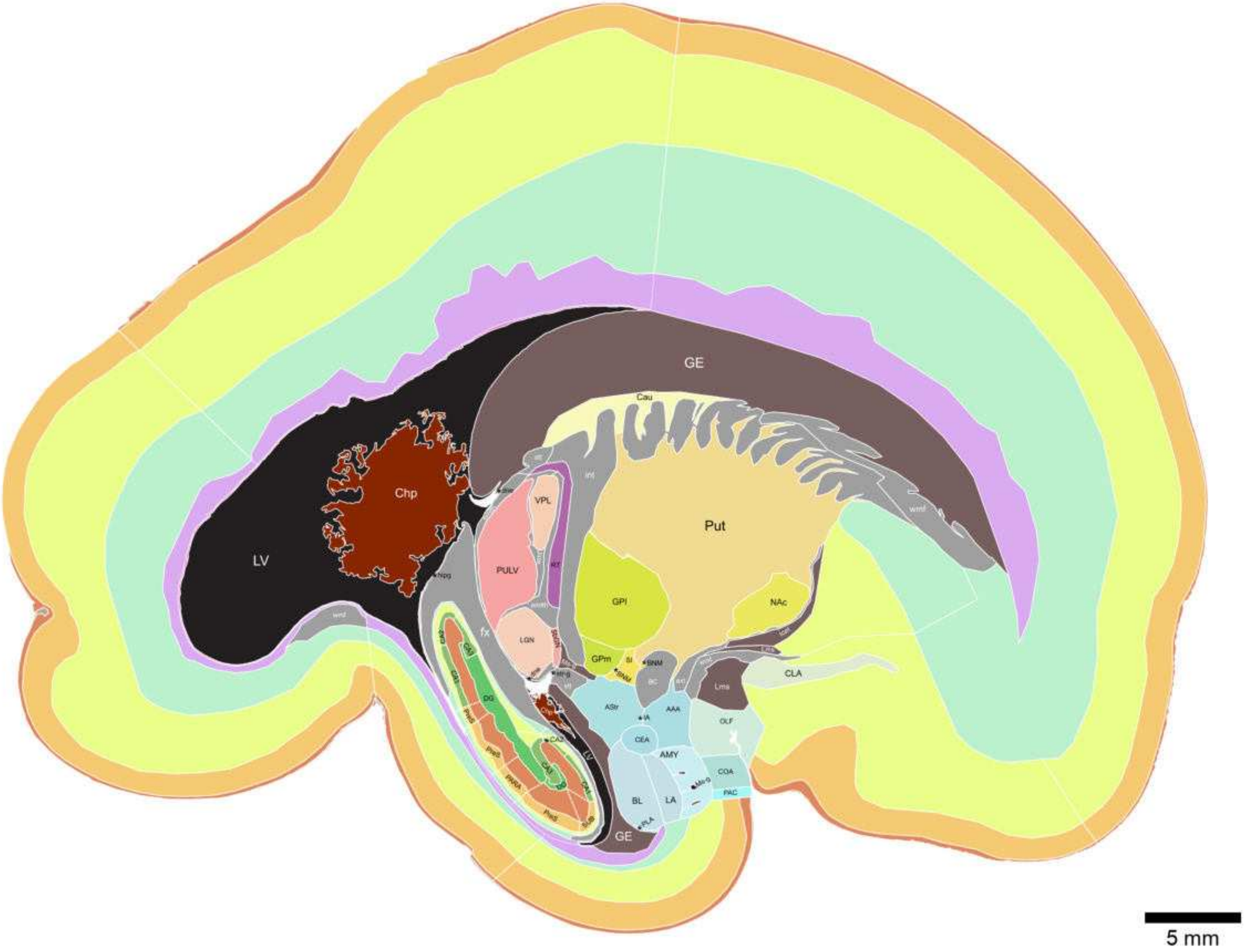

- AAA: Anterior amygdaloid area
- AMY: Amygdala
- AStr: Amygdalo-striatal area
- BL: Basal nucleus [amygdala]
- BNM: Basal nucleus of Meynert
- CA1: CA1 field [hippocampus]
- CA2: CA2 field [hippocampus]
- CA3: CA3 field [hippocampus]
- CEA: Central nucleus [amygdala]
- CLA: Claustrum
- COA: Cortical nucleus [amygdala]
- Cau: Caudate nucleus
- Chp: Choroid plexus
- DG: Dentate gyrus
- GE: Ganglionic eminence
- GPl: Globus pallidus lateral segment
- GPm: Globus pallidus medial segment
- IA: Intercalated cell groups [amygdala]
- LA: Lateral nucleus [amygdala]
- LGN: Lateral geniculate nucleus
- LV: Lateral ventricle
- Lms: Lateral migratory stream
- Ms-g: Migratory stream, general
- NAc: Nucleus accumbens
- PARA: Cortical plate, parasubiculum
- PLA: Paralaminar nucleus [amygdala]
- PULV: Pulvinar nucleus [thalamus]
- PreS: Cortical plate, presubiculum
- Put: Putamen
- RT: Reticular nucleus [thalamus]
- SI: Substantia innominata
- SUB: Cortical plate, subiculum
- SbGN: Subgeniculate nucleus
- VPL: Ventral posterolateral nucleus [thalamus]
- ac: Anterior commissure
- dne: Diencephalic neuroepithelium
- emlth: External medullary lamina [thalamus]
- ext: External capsule
- fx: Fornix
- hipg: Hippocampal glioepithelium/ependyma
- int: Internal capsule
- str-g: Strionuclear glioepithelium
- stt: Stria terminalis
- tcet: Transient cell zone in the external capsule
- wmf: White matter fibers

Age: 24 GW

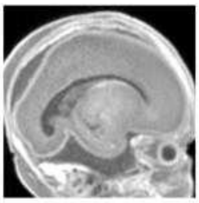

L-R Level: -8.52 mm

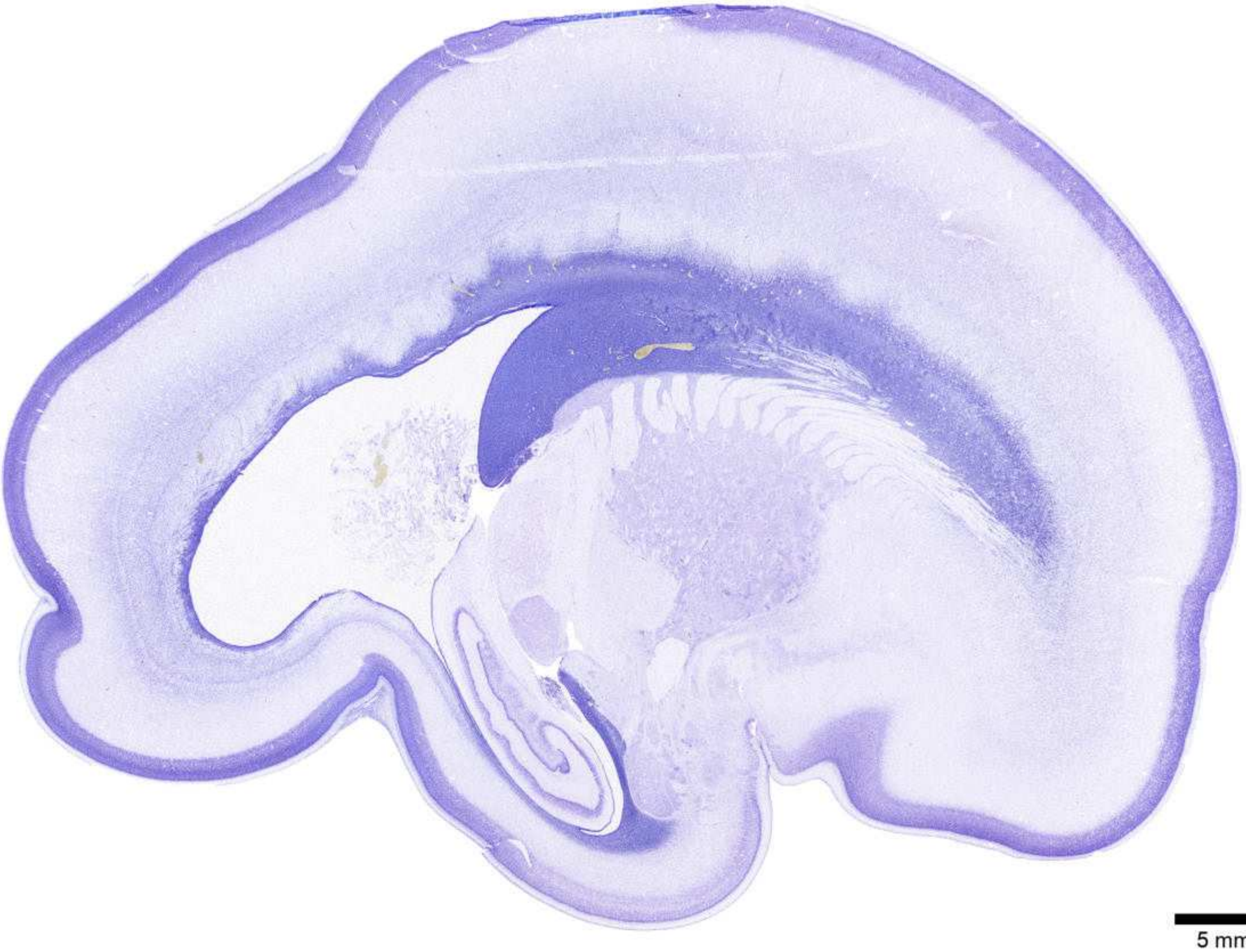

5 mm

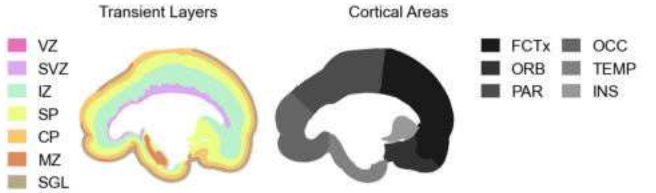

Age: 24 GW

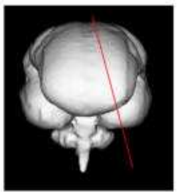

L-R Level: -8.52 mm

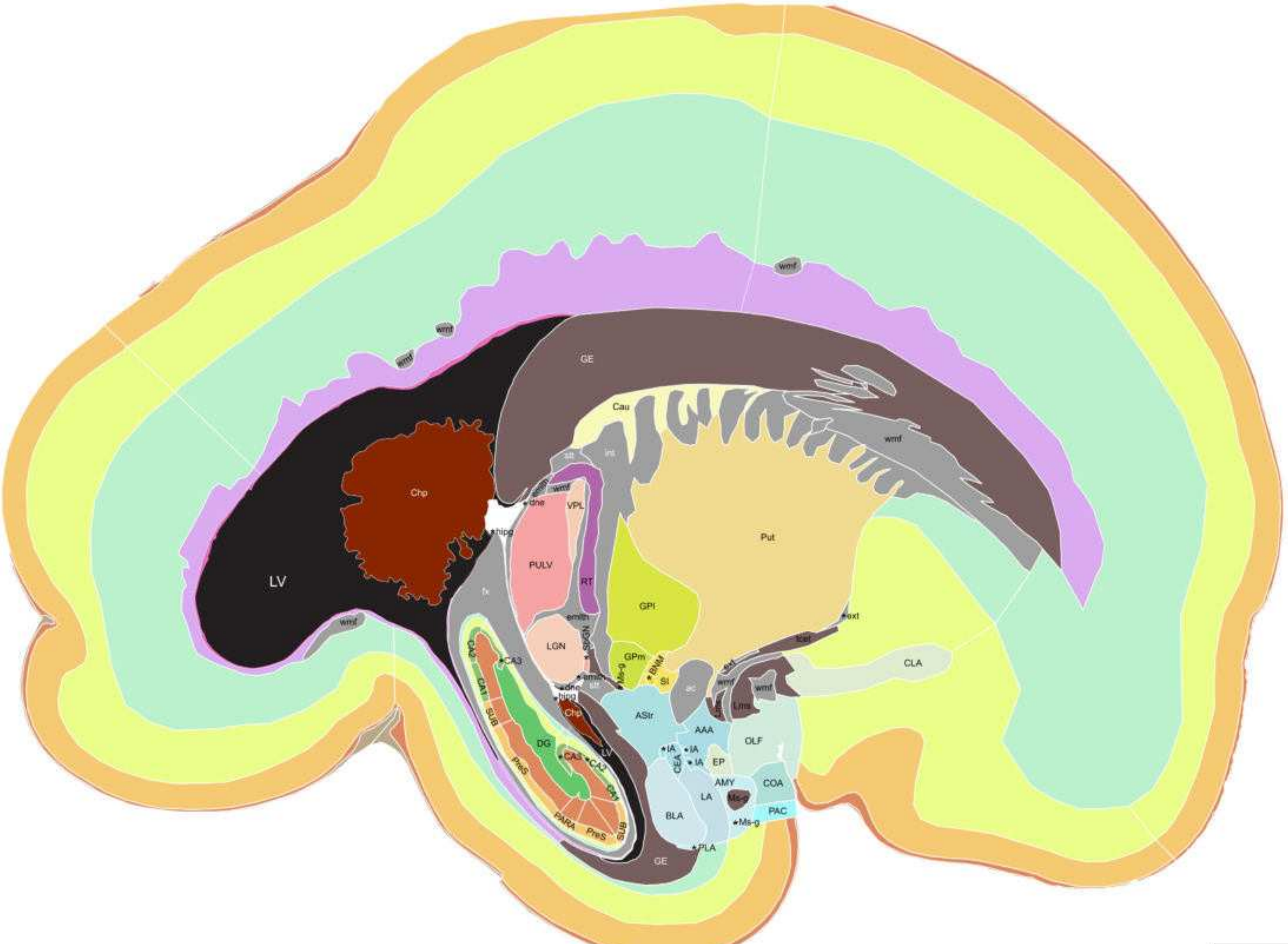

5 mm

- AAA: Anterior amygdaloid area
- AMY: Amygdala
- AStr: Amygdalo-striatal area
- BL: Basal nucleus [amygdala]
- BNM: Basal nucleus of Meynert
- CA1: CA1 field [hippocampus]
- CA2: CA2 field [hippocampus]
- CA3: CA3 field [hippocampus]
- CEA: Central nucleus [amygdala]
- CLA: Claustrum
- COA: Cortical nucleus [amygdala]
- Cau: Caudate nucleus
- Chp: Choroid plexus
- DG: Dentate gyrus
- EP: Endopiriform nucleus
- GE: Ganglionic eminence
- GPl: Globus pallidus lateral segment
- GPm: Globus pallidus medial segment
- IA: Intercalated cell groups [amygdala]
- LA: Lateral nucleus [amygdala]
- LGN: Lateral geniculate nucleus
- LV: Lateral ventricle
- Lms: Lateral migratory stream
- Ms-g: Migratory stream, general
- PARA: Cortical plate, parasubiculum
- PLA: Paralaminar nucleus [amygdala]
- PULV: Pulvinar nucleus [thalamus]
- PreS: Cortical plate, presubiculum
- Put: Putamen
- RT: Reticular nucleus [thalamus]
- SI: Substantia innominata
- SUB: Cortical plate, subiculum
- SbGN: Subgeniculate nucleus
- VPL: Ventral posterolateral nucleus [thalamus]
- ac: Anterior commissure
- dne: Diencephalic neuroepithelium
- emlth: External medullary lamina [thalamus]
- ext: External capsule
- fx: Fornix
- hipg: Hippocampal glioepithelium/ependyma
- int: Internal capsule
- stt: Stria terminalis
- tcet: Transient cell zone in the external capsule
- wmf: White matter fibers

Age: 24 GW

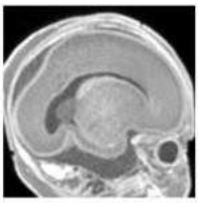

L-R Level: -8.82 mm

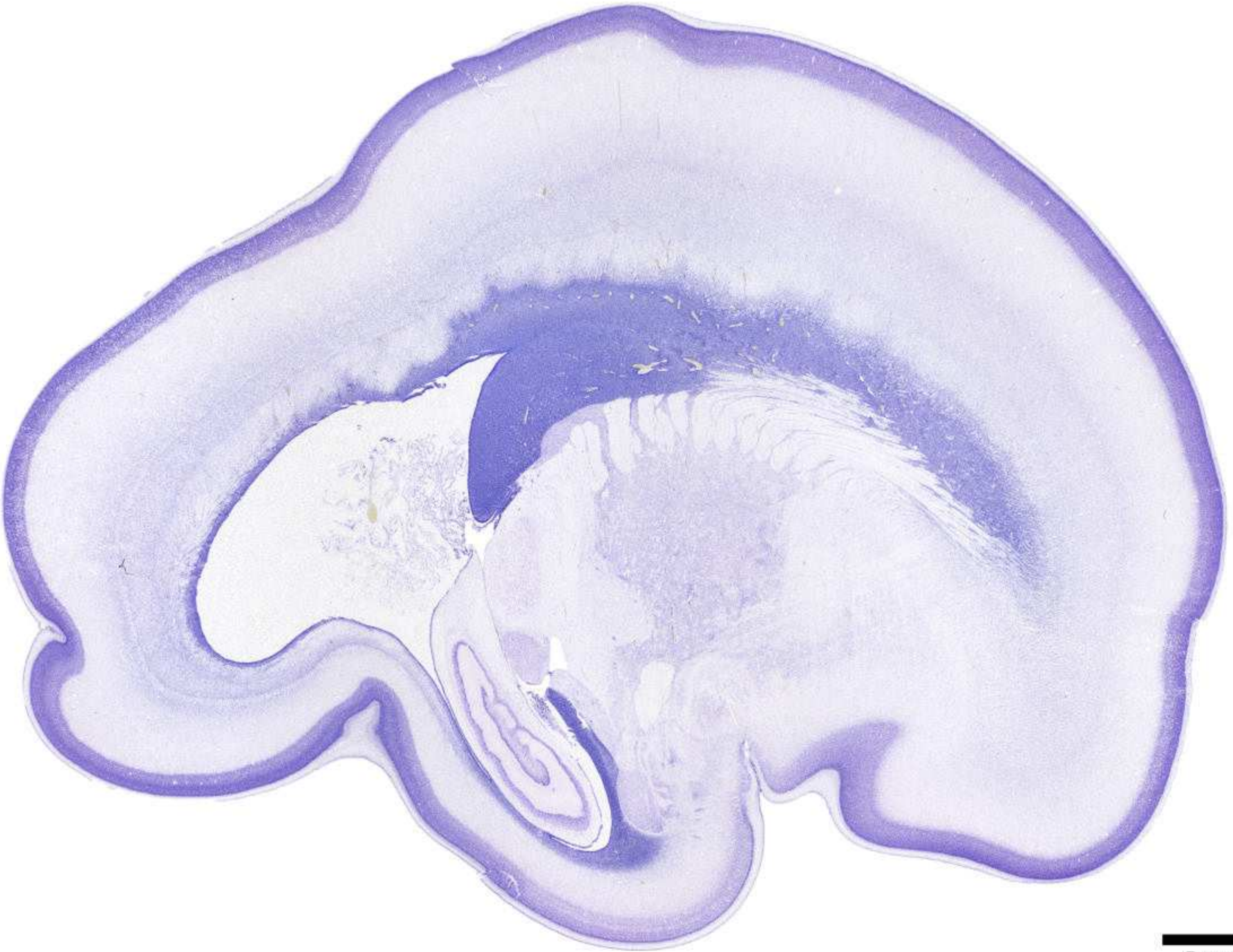

5 mm

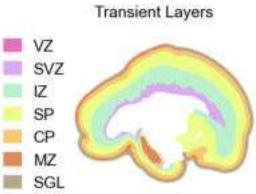

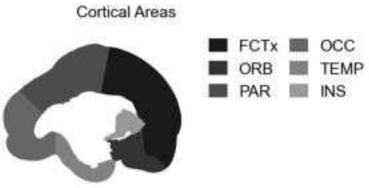

Age: 24 GW

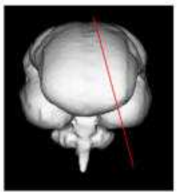

L-R Level: -8.82 mm

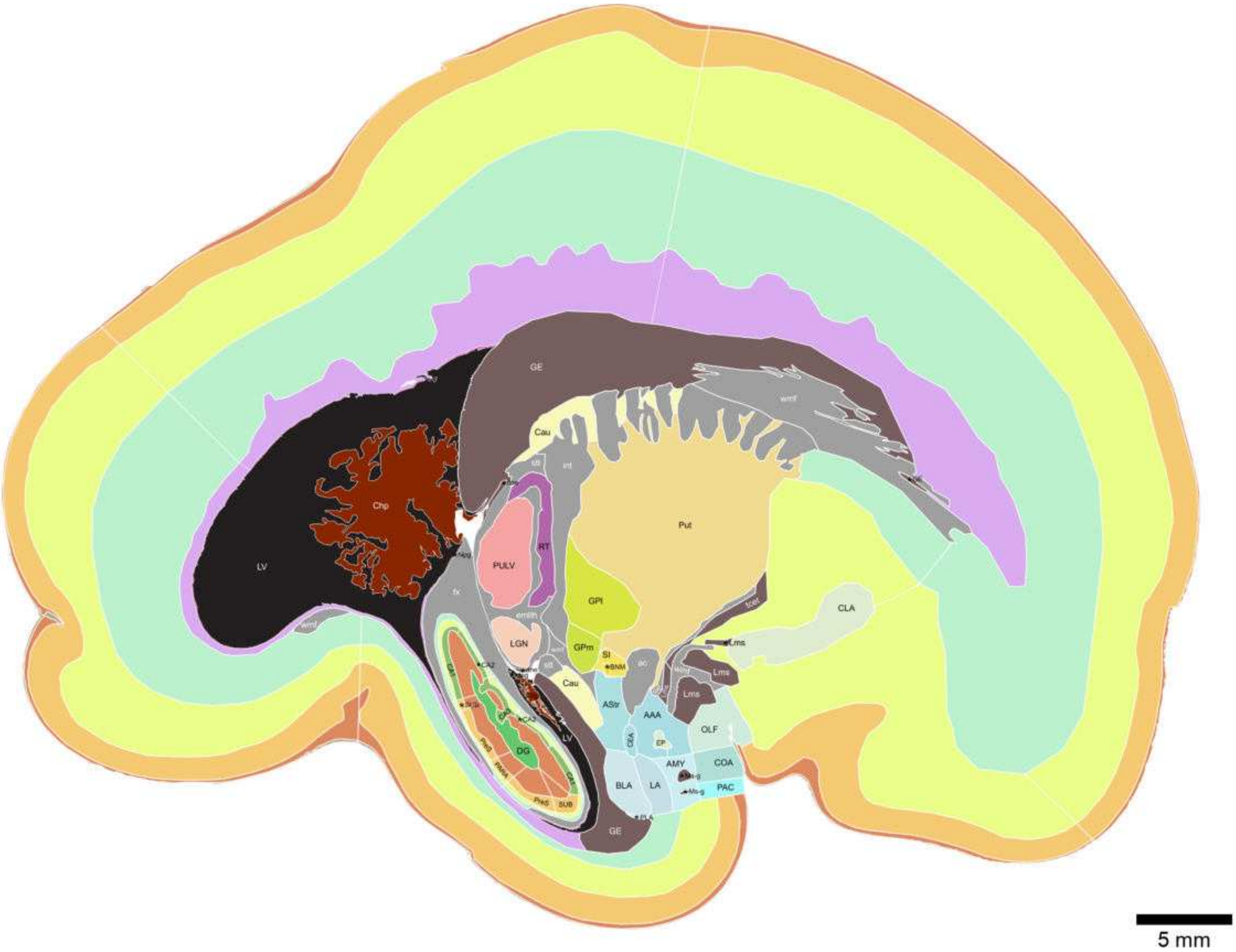

AAA: Anterior amygdaloid area
AMY: Amygdala
AStr: Amygdalo-striatal area
BL: Basal nucleus [amygdala]
BNM: Basal nucleus of Meynert
CA1: CA1 field [hippocampus]
CA2: CA2 field [hippocampus]
CA3: CA3 field [hippocampus]
CEA: Central nucleus [amygdala]
CLA: Claustrum
COA: Cortical nucleus [amygdala]
Cau: Caudate nucleus
Chp: Choroid plexus
DG: Dentate gyrus
EP: Endopiriform nucleus
GE: Ganglionic eminence
GPl: Globus pallidus lateral segment
GPm: Globus pallidus medial segment
LA: Lateral nucleus [amygdala]
LGN: Lateral geniculate nucleus
LV: Lateral ventricle
Lms: Lateral migratory stream
Ms-g: Migratory stream, general
PARA: Cortical plate, parasubiculum
PLA: Paralaminar nucleus [amygdala]
PULV: Pulvinar nucleus [thalamus]
PreS: Cortical plate, presubiculum
Put: Putamen
RT: Reticular nucleus [thalamus]
SI: Substantia innominata
SUB: Cortical plate, subiculum
ac: Anterior commissure
dne: Diencephalic neuroepithelium
emlth: External medullary lamina [thalamus]
ext: External capsule
fx: Fornix
hipg: Hippocampal glioepithelium/ependyma
int: Internal capsule
stt: Stria terminalis
tcet: Transient cell zone in the external capsule
wmf: White matter fibers

Age: 24 GW

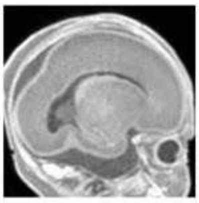

L-R Level: -9.0 mm

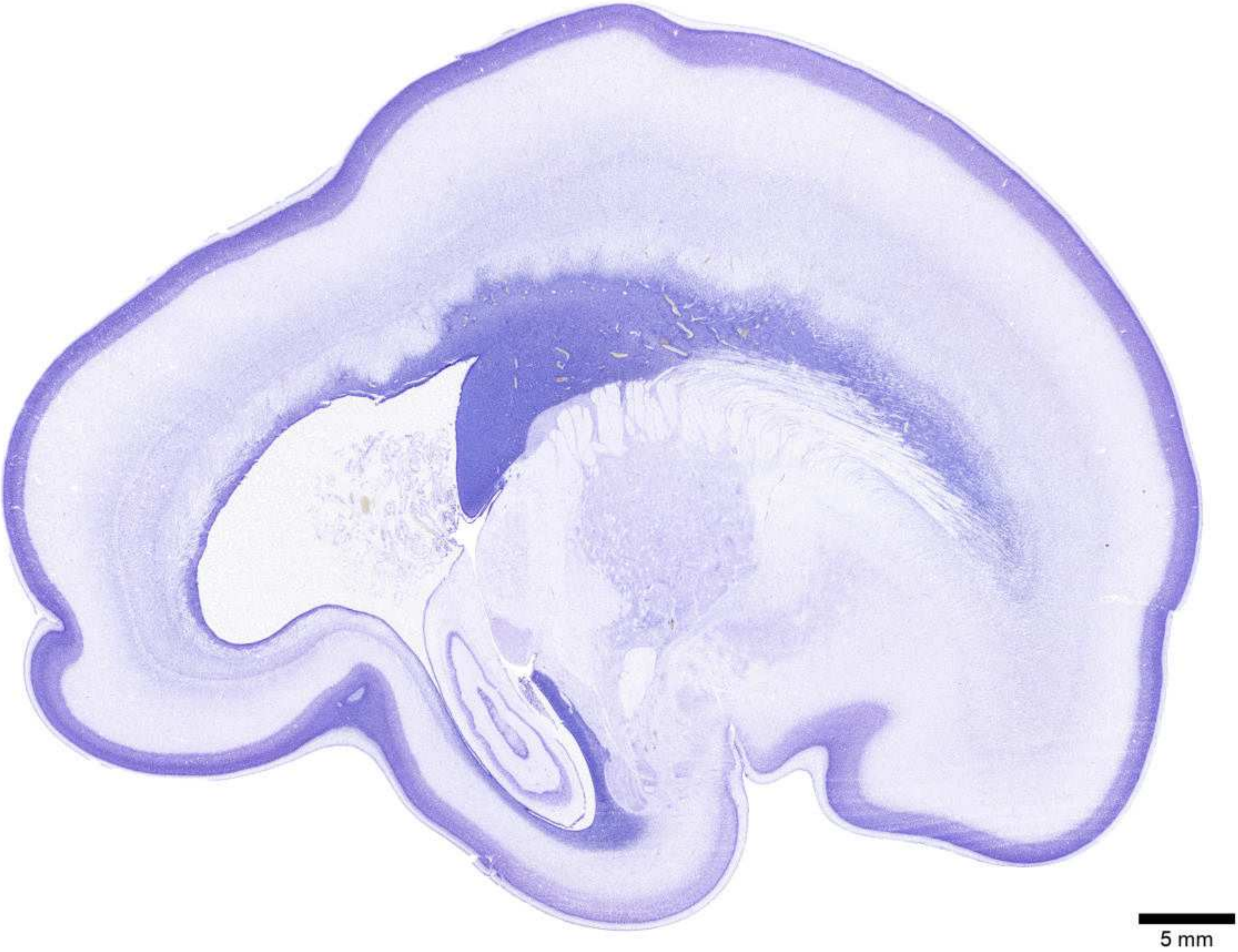

5 mm

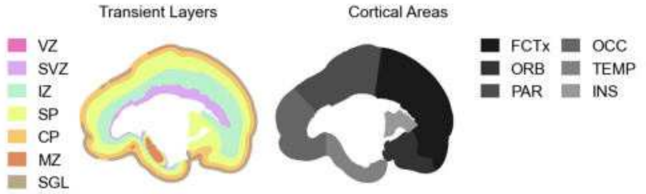

Age: 24 GW

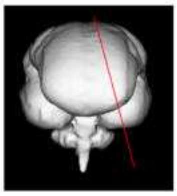

L-R Level: -9.0 mm

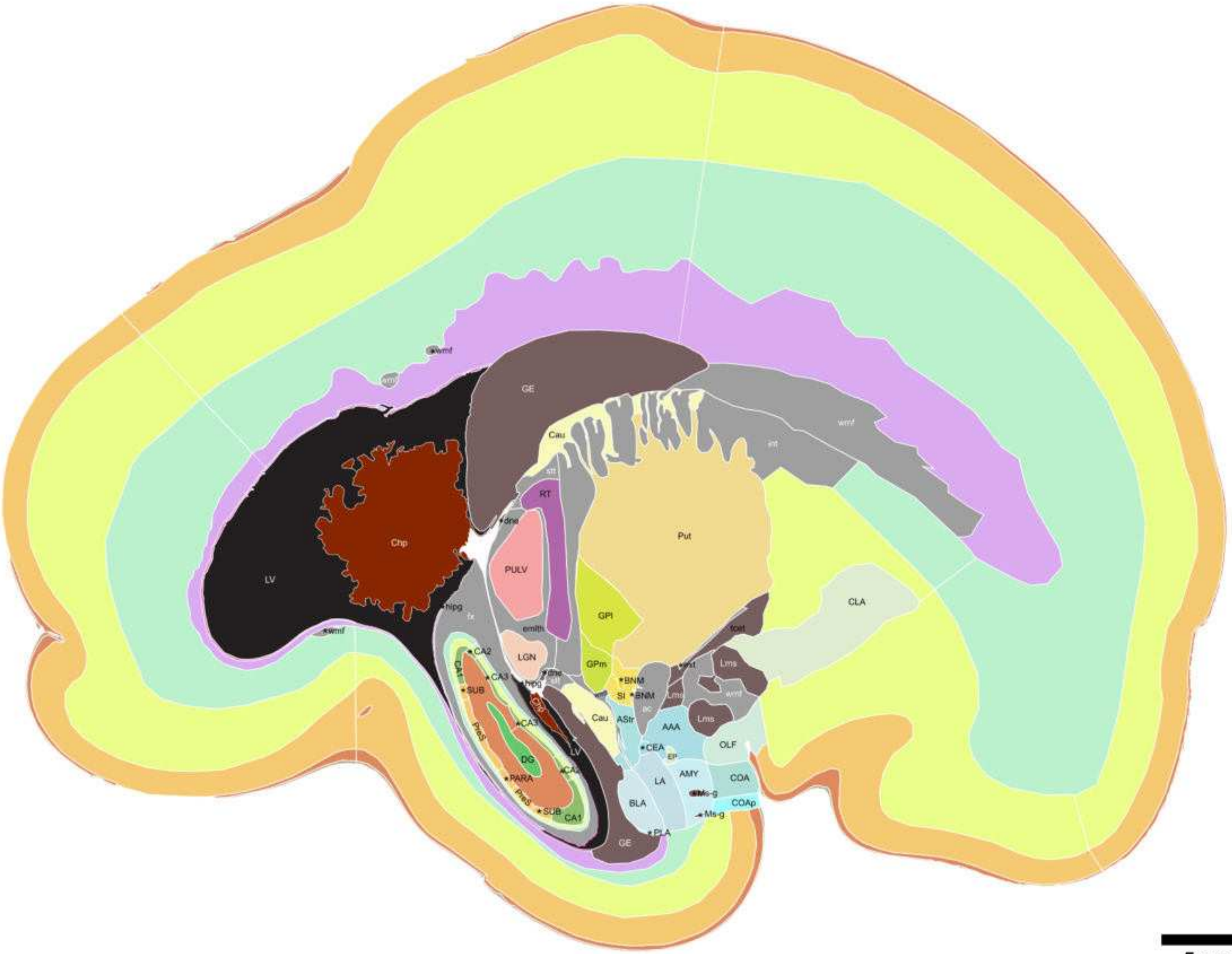

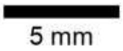

- AAA: Anterior amygdaloid area
- AMY: Amygdala
- AStr: Amygdalo-striatal area
- BL: Basal nucleus [amygdala]
- BNM: Basal nucleus of Meynert
- CA1: CA1 field [hippocampus]
- CA2: CA2 field [hippocampus]
- CA3: CA3 field [hippocampus]
- CEA: Central nucleus [amygdala]
- CLA: Claustrum
- COA: Cortical nucleus [amygdala]
- COAp: Cortical nucleus [amygdala], posterior part
- Cau: Caudate nucleus
- Chp: Choroid plexus
- DG: Dentate gyrus
- EP: Endopiriform nucleus
- GE: Ganglionic eminence
- GPl: Globus pallidus lateral segment
- GPm: Globus pallidus medial segment
- LA: Lateral nucleus [amygdala]
- LGN: Lateral geniculate nucleus
- LV: Lateral ventricle
- Lms: Lateral migratory stream
- Ms-g: Migratory stream, general
- PARA: Cortical plate, parasubiculum
- PLA: Paralaminar nucleus [amygdala]
- PULV: Pulvinar nucleus [thalamus]
- PreS: Cortical plate, presubiculum
- Put: Putamen
- RT: Reticular nucleus [thalamus]
- SI: Substantia innominata
- SUB: Cortical plate, subiculum
- ac: Anterior commissure
- dne: Diencephalic neuroepithelium
- emlth: External medullary lamina [thalamus]
- ext: External capsule
- fx: Fornix
- hipg: Hippocampal glioepithelium/ependyma
- int: Internal capsule
- stt: Stria terminalis
- tcet: Transient cell zone in the external capsule
- wmf: White matter fibers

Age: 24 GW

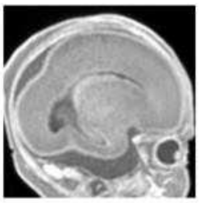

L-R Level: -9.24 mm

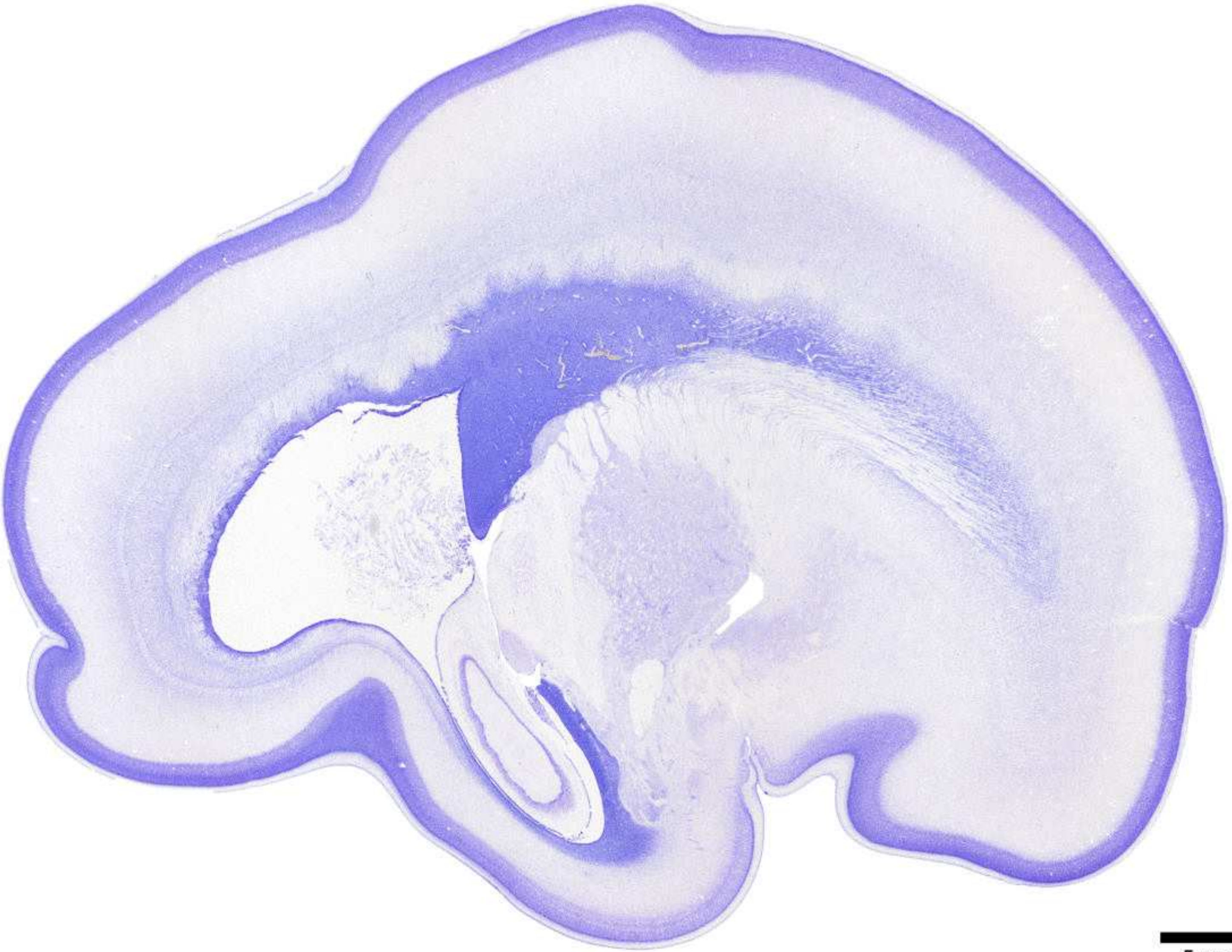

5 mm

Transient Layers

- VZ
- SVZ
- IZ
- SP
- CP
- MZ
- SGL

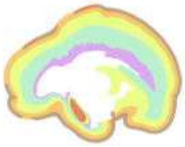

Cortical Areas

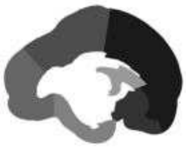

- FCTx
- ORB
- PAR
- OCC
- TEMP
- INS

Age: 24 GW

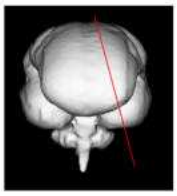

L-R Level: -9.24 mm

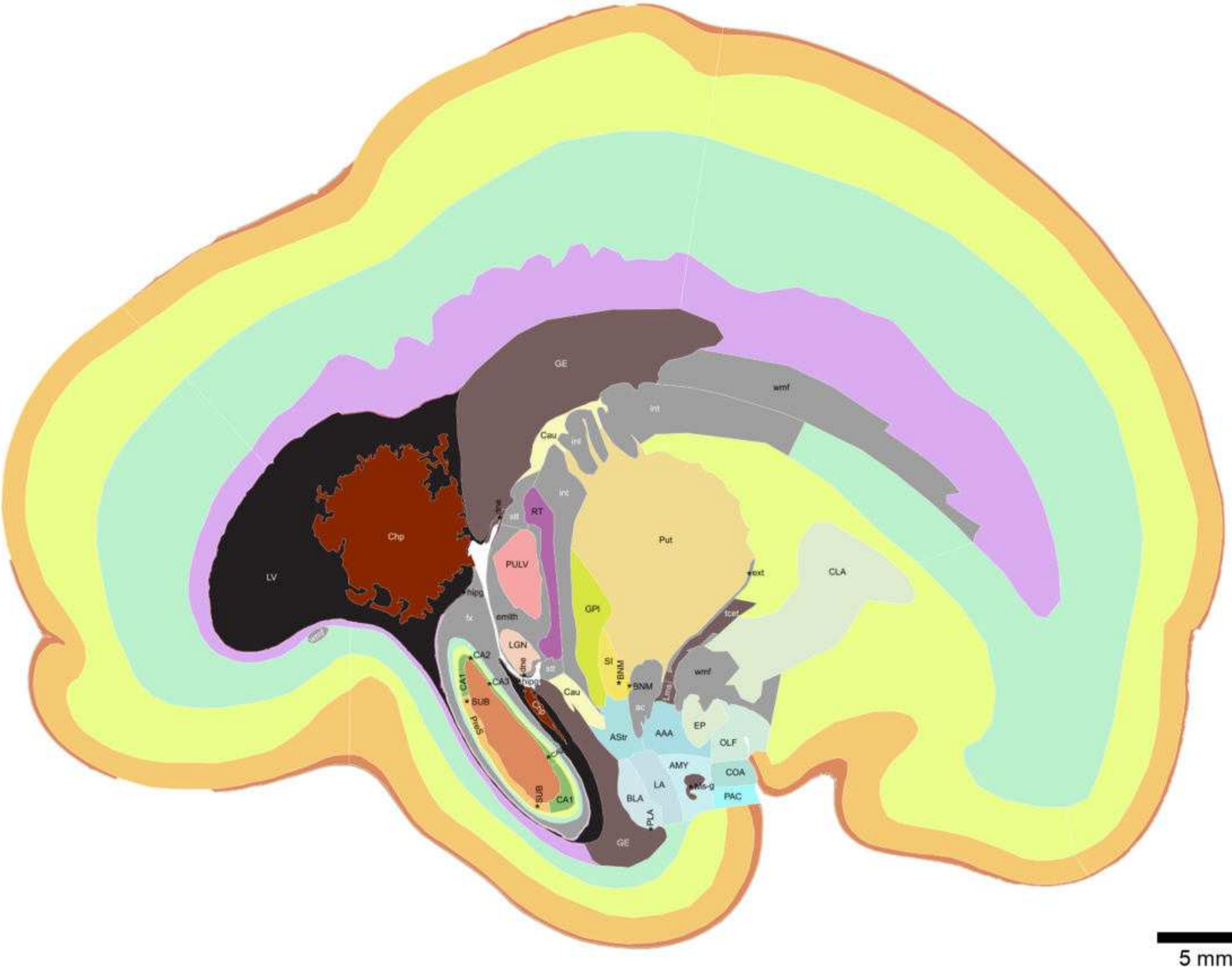

5 mm

- AAA: Anterior amygdaloid area
- AMY: Amygdala
- AStr: Amygdalo-striatal area
- BL: Basal nucleus [amygdala]
- BNM: Basal nucleus of Meynert
- CA1: CA1 field [hippocampus]
- CA2: CA2 field [hippocampus]
- CA3: CA3 field [hippocampus]
- CLA: Claustrum
- COA: Cortical nucleus [amygdala]
- Cau: Caudate nucleus
- Chp: Choroid plexus
- EP: Endopiriform nucleus
- GE: Ganglionic eminence
- GPl: Globus pallidus lateral segment
- LA: Lateral nucleus [amygdala]
- LGN: Lateral geniculate nucleus
- LV: Lateral ventricle
- Lms: Lateral migratory stream
- Ms-g: Migratory stream, general
- PLA: Paralaminar nucleus [amygdala]
- PULV: Pulvinar nucleus [thalamus]
- PreS: Cortical plate, presubiculum
- Put: Putamen
- RT: Reticular nucleus [thalamus]
- SI: Substantia innominata
- SUB: Cortical plate, subiculum
- ac: Anterior commissure
- dne: Diencephalic neuroepithelium
- emlth: External medullary lamina [thalamus]
- ext: External capsule
- fx: Fornix
- hipg: Hippocampal glioepithelium/ependyma
- int: Internal capsule
- stt: Stria terminalis
- tcet: Transient cell zone in the external capsule
- wmf: White matter fibers

Age: 24 GW

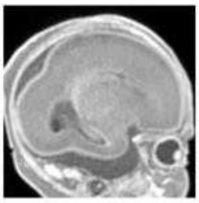

L-R Level: -9.66 mm

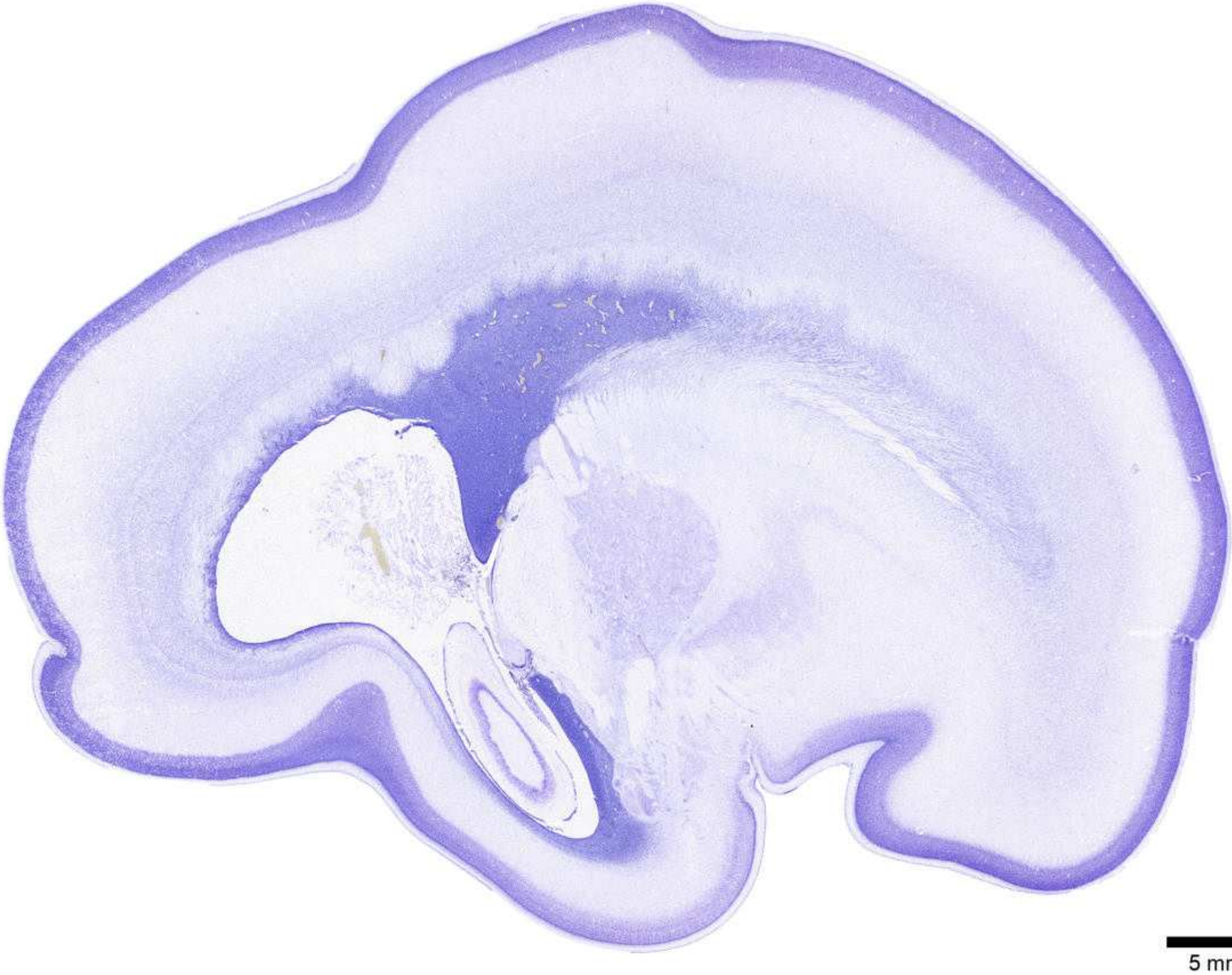

5 mm

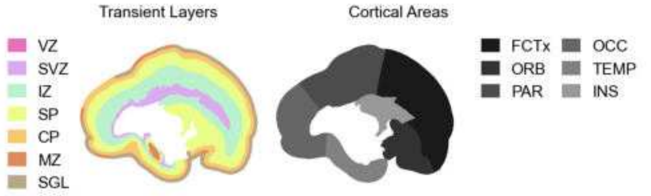

Age: 24 GW

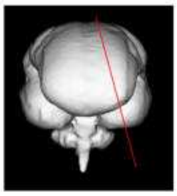

L-R Level: -9.66 mm

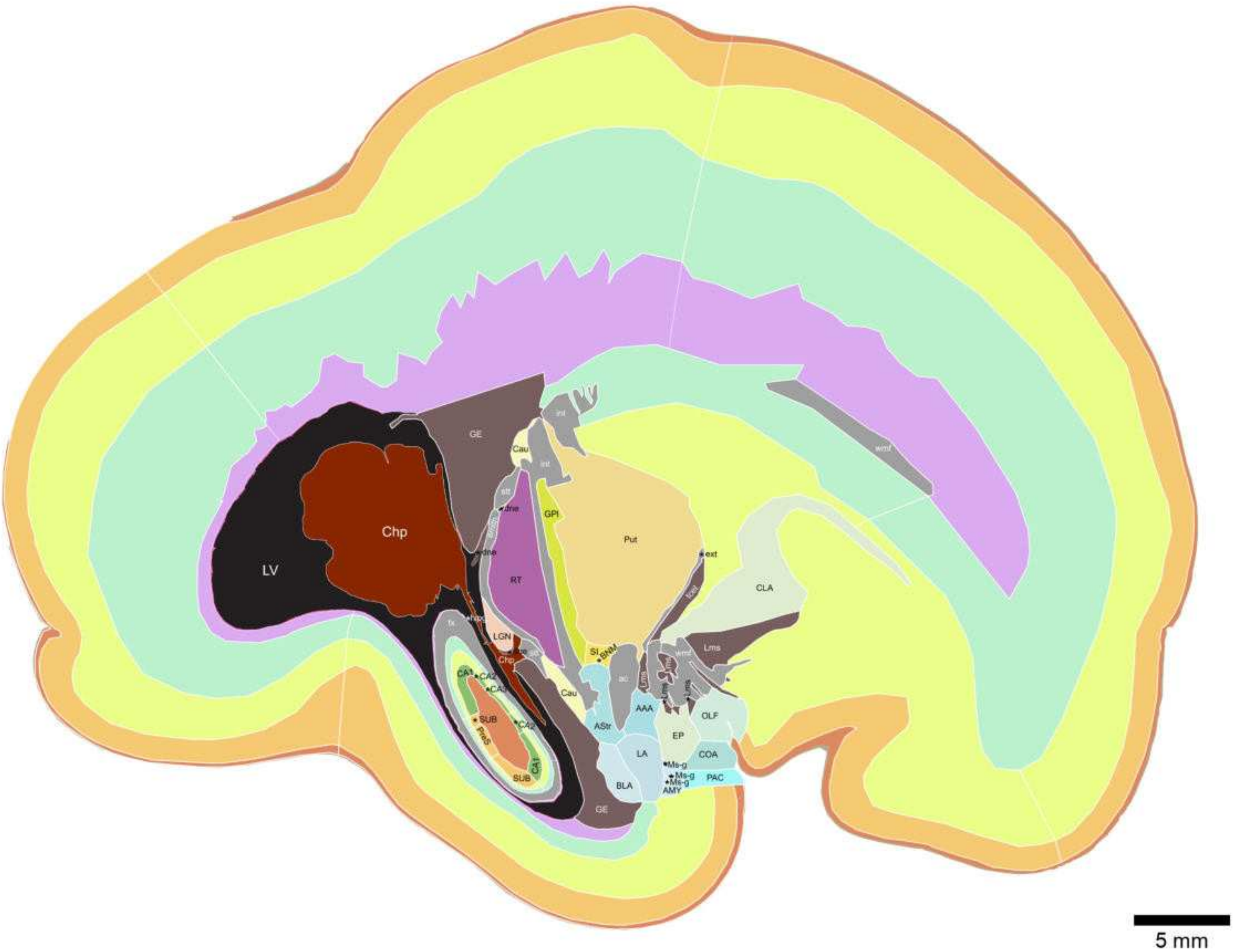

- AAA: Anterior amygdaloid area
- AMY: Amygdala
- AStr: Amygdalo-striatal area
- BL: Basal nucleus [amygdala]
- BNM: Basal nucleus of Meynert
- CA1: CA1 field [hippocampus]
- CA2: CA2 field [hippocampus]
- CA3: CA3 field [hippocampus]
- CLA: Claustrum
- COA: Cortical nucleus [amygdala]
- Cau: Caudate nucleus
- Chp: Choroid plexus
- EP: Endopiriform nucleus
- GE: Ganglionic eminence
- GPl: Globus pallidus lateral segment
- LA: Lateral nucleus [amygdala]
- LGN: Lateral geniculate nucleus
- LV: Lateral ventricle
- Lms: Lateral migratory stream
- Ms-g: Migratory stream, general
- PreS: Cortical plate, presubiculum
- Put: Putamen
- RT: Reticular nucleus [thalamus]
- SI: Substantia innominata
- SUB: Cortical plate, subiculum
- ac: Anterior commissure
- dne: Diencephalic neuroepithelium
- emlth: External medullary lamina [thalamus]
- ext: External capsule
- fx: Fornix
- hipg: Hippocampal glioepithelium/ependyma
- int: Internal capsule
- stt: Stria terminalis
- tcet: Transient cell zone in the external capsule
- wmf: White matter fibers

Age: 24 GW

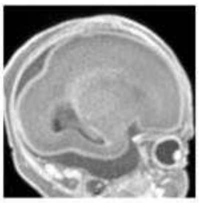

L-R Level: -9.96 mm

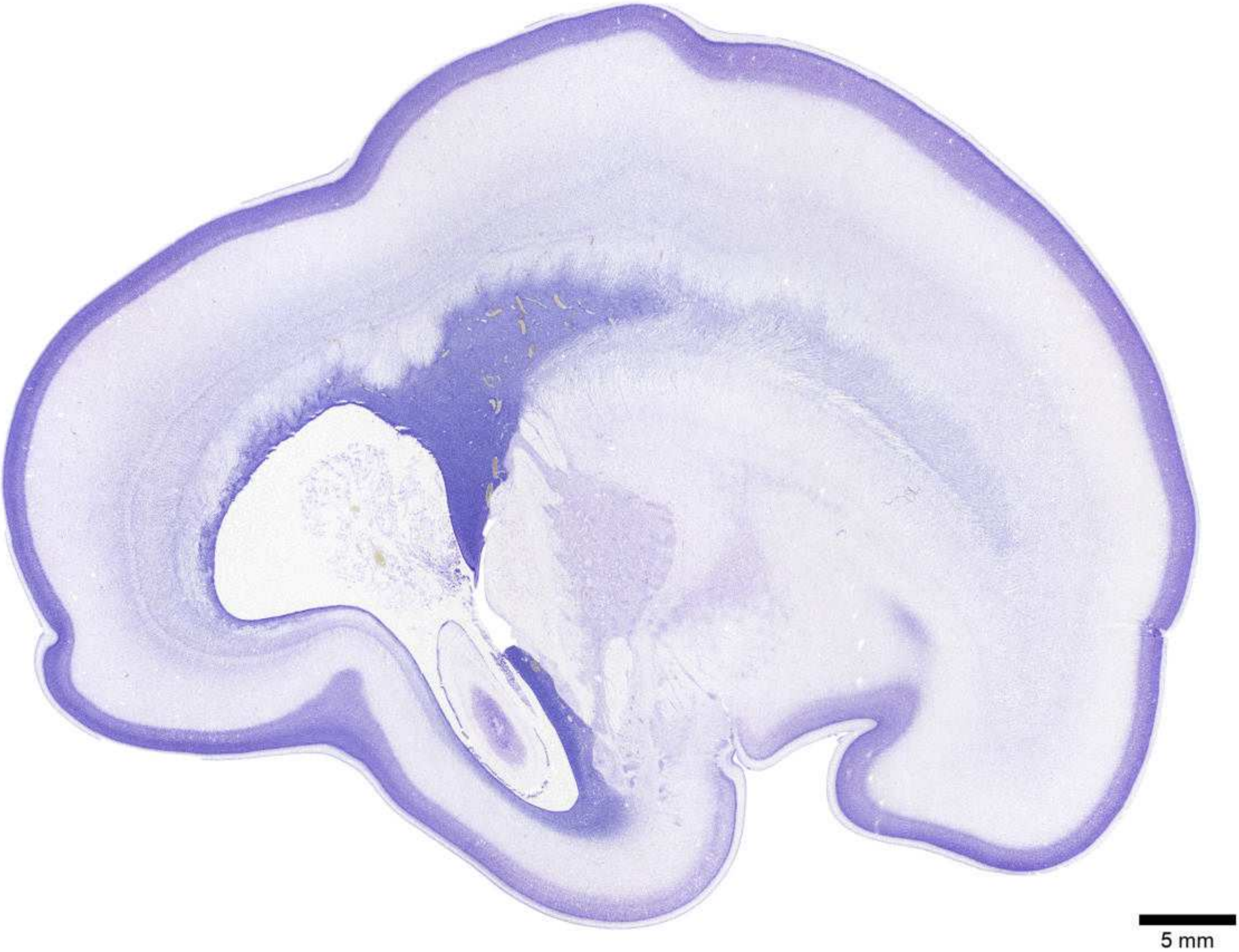

5 mm

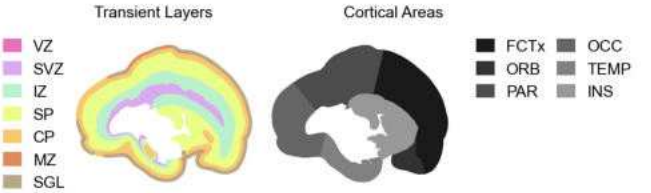

Age: 24 GW

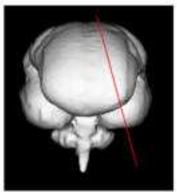

L-R Level: -9.96 mm

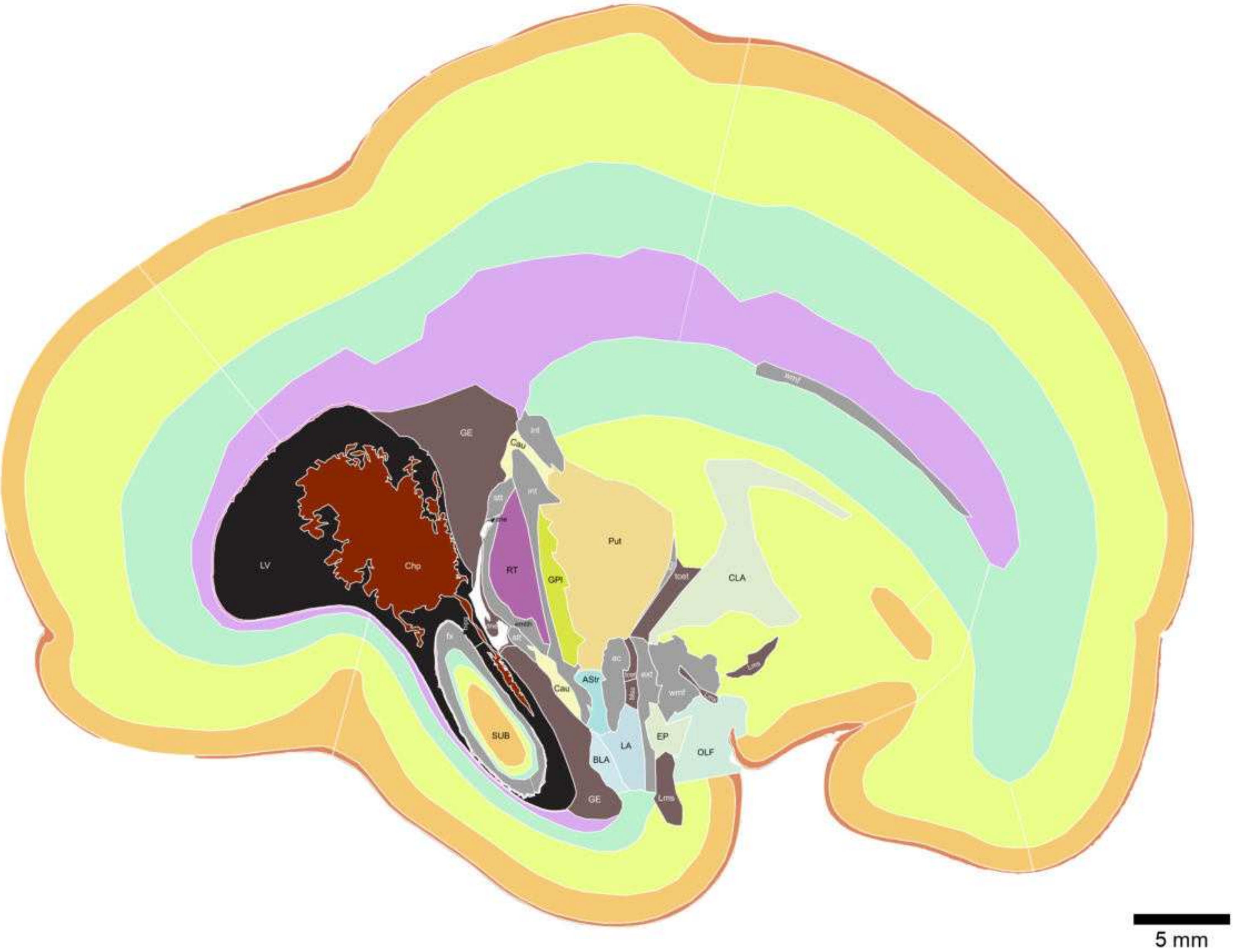

- AStr: Amygdalo-striatal area
- BL: Basal nucleus [amygdala]
- CLA: Claustrum
- Cau: Caudate nucleus
- Chp: Choroid plexus
- EP: Endopiriform nucleus
- GE: Ganglionic eminence
- GPl: Globus pallidus lateral segment
- LA: Lateral nucleus [amygdala]
- LV: Lateral ventricle
- Lms: Lateral migratory stream
- Mss: Migratory streams
- Put: Putamen
- RT: Reticular nucleus [thalamus]
- SUB: Cortical plate, subiculum
- ac: Anterior commissure
- dne: Diencephalic neuroepithelium
- emlth: External medullary lamina [thalamus]
- ext: External capsule
- fx: Fornix
- hipg: Hippocampal glioepithelium/ependyma
- int: Internal capsule
- stt: Stria terminalis
- tcet: Transient cell zone in the external capsule
- wmf: White matter fibers

Age: 24 GW

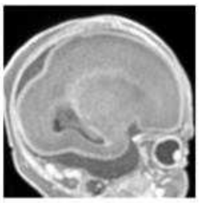

L-R Level: -10.08 mm

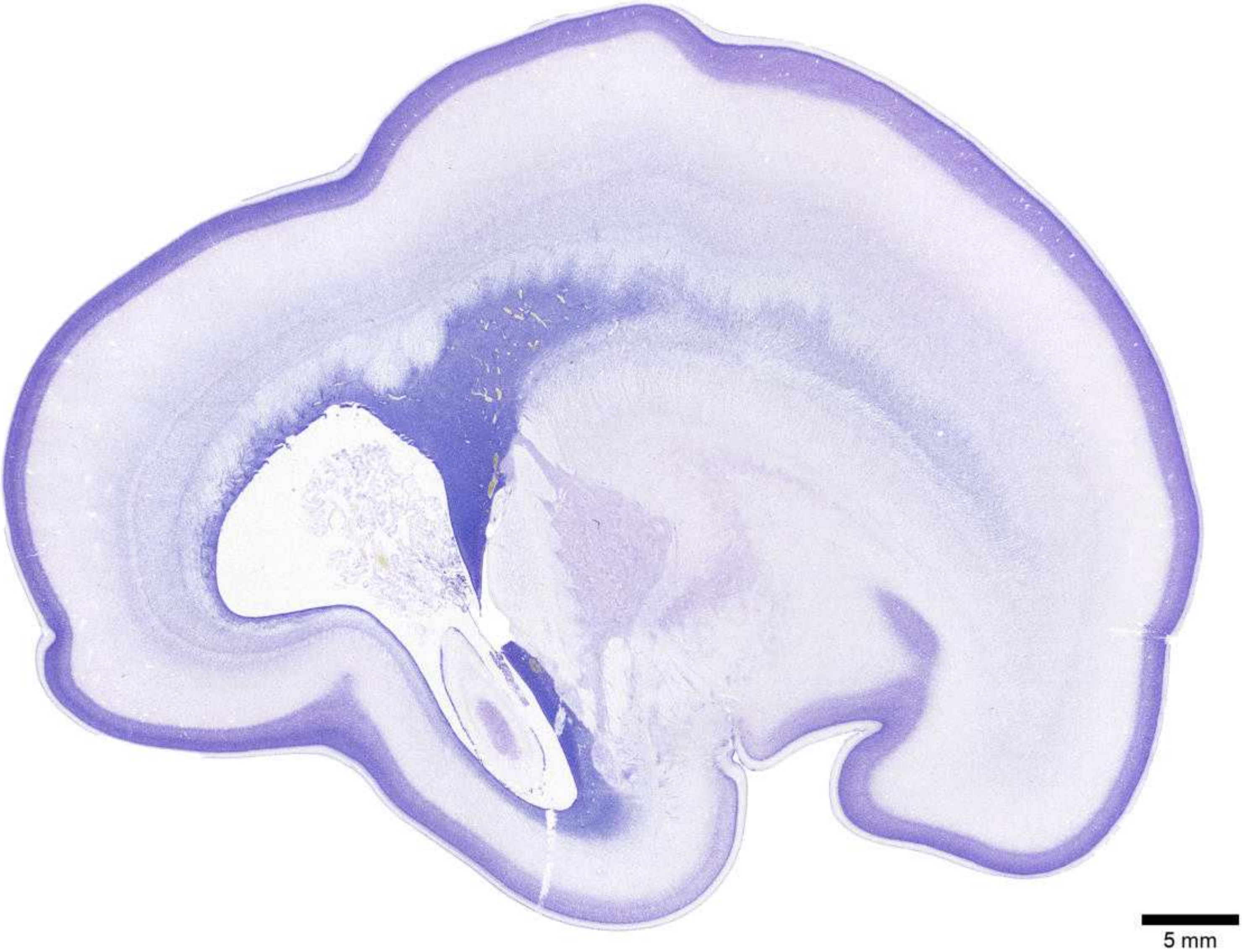

5 mm

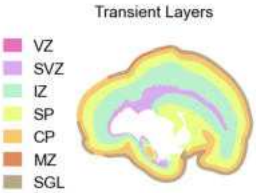

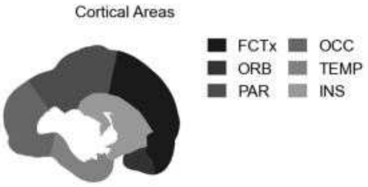

Age: 24 GW

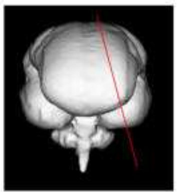

L-R Level: -10.08 mm

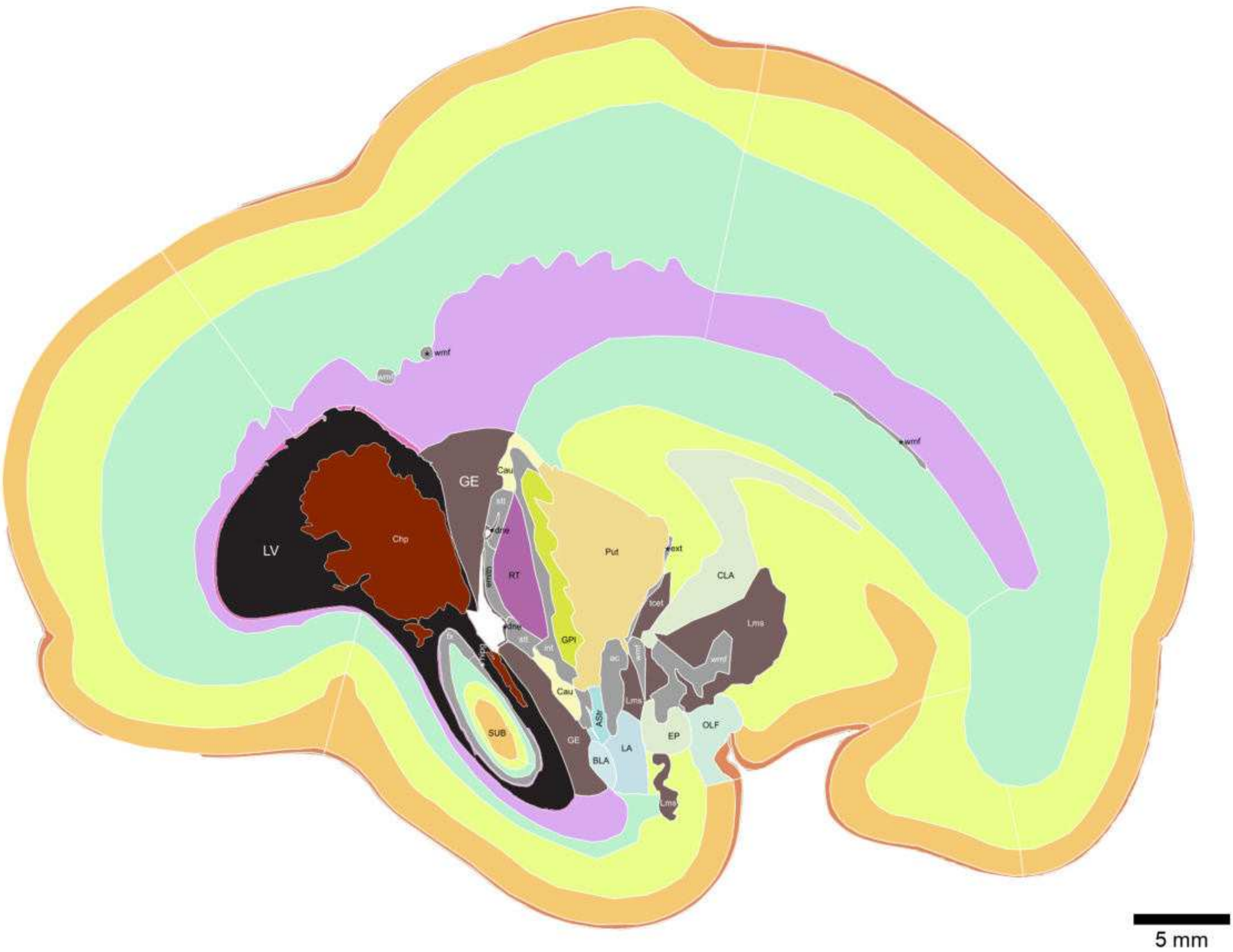

- AStr: Amygdalo-striatal area
- BL: Basal nucleus [amygdala]
- CLA: Claustrum
- Cau: Caudate nucleus
- Chp: Choroid plexus
- EP: Endopiriform nucleus
- GE: Ganglionic eminence
- GPl: Globus pallidus lateral segment
- LA: Lateral nucleus [amygdala]
- LV: Lateral ventricle
- Lms: Lateral migratory stream
- Put: Putamen
- RT: Reticular nucleus [thalamus]
- SUB: Cortical plate, subiculum
- ac: Anterior commissure
- dne: Diencephalic neuroepithelium
- emlth: External medullary lamina [thalamus]
- ext: External capsule
- fx: Fornix
- hipg: Hippocampal glioepithelium/ependyma
- int: Internal capsule
- stt: Stria terminalis
- tcet: Transient cell zone in the external capsule
- wmf: White matter fibers

Age: 24 GW

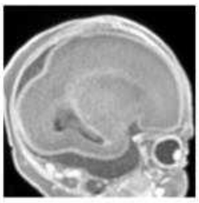

L-R Level: -10.5 mm

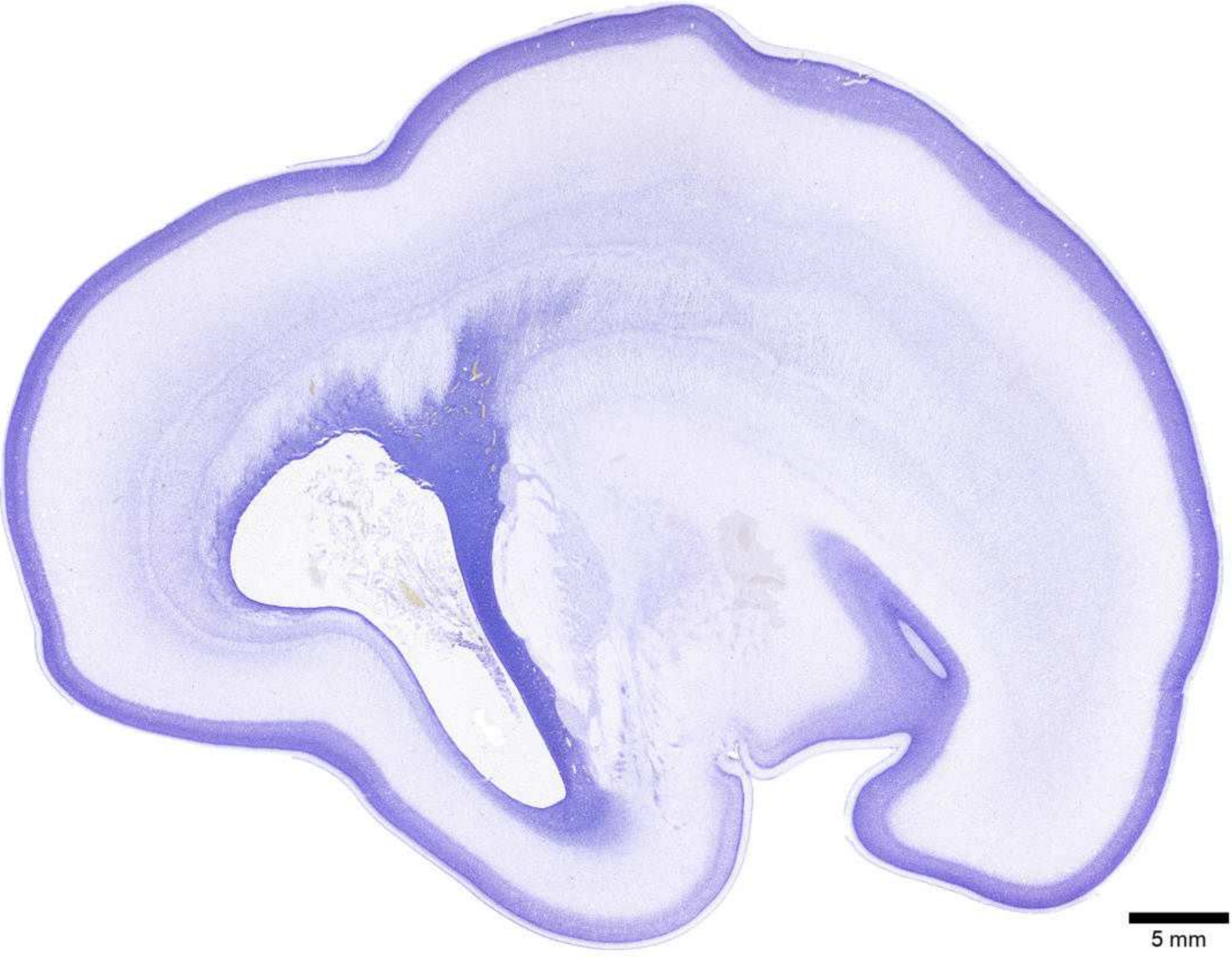

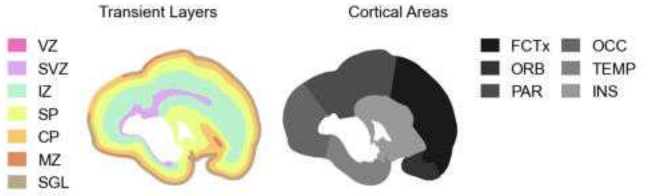

Age: 24 GW

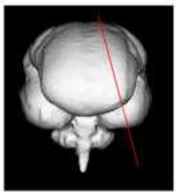

L-R Level: -10.5 mm

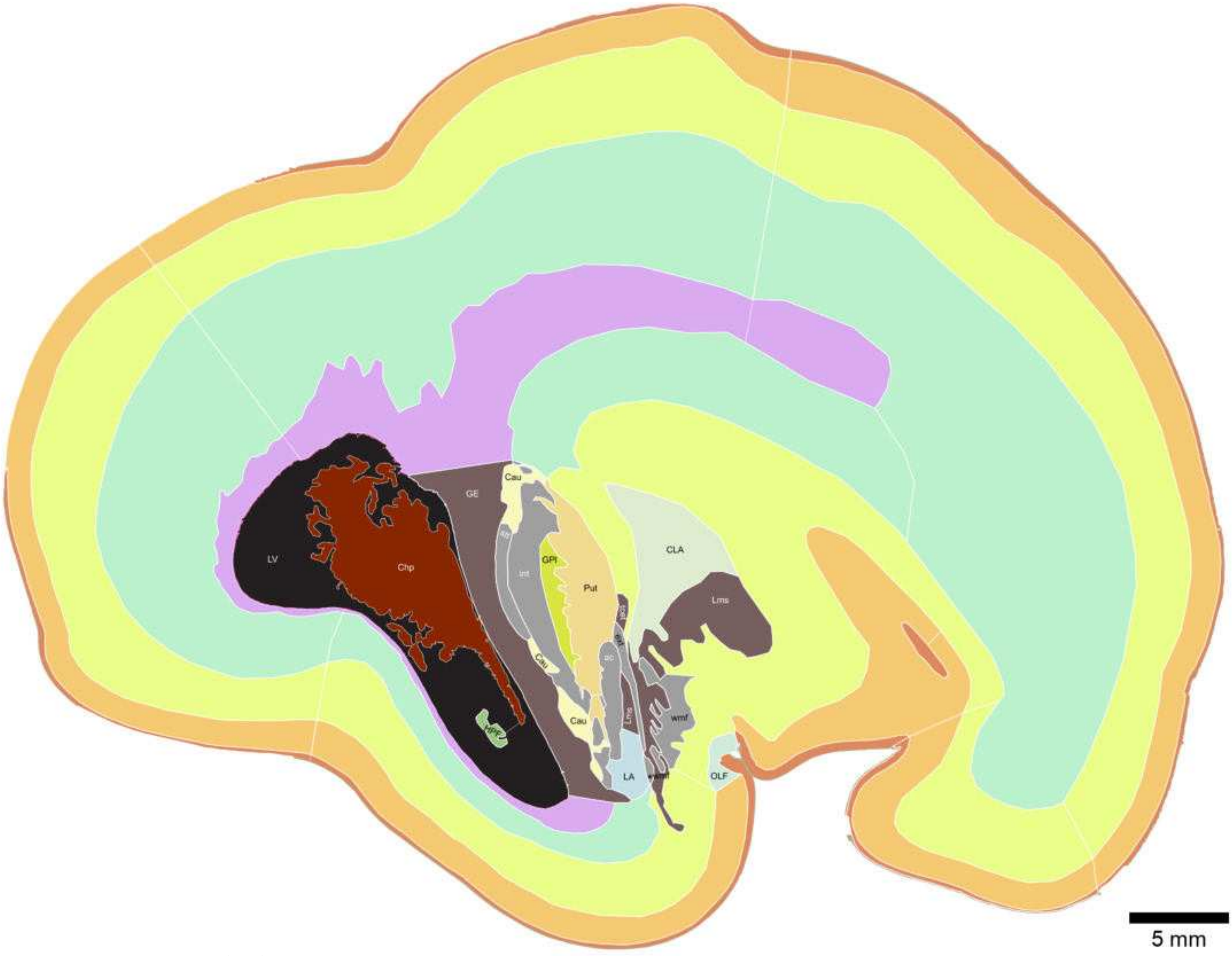

- CLA: Claustrum
- Cau: Caudate nucleus
- Chp: Choroid plexus
- GE: Ganglionic eminence
- GPl: Globus pallidus lateral segment
- HPF: Hippocampal formation
- LA: Lateral nucleus [amygdala]
- LV: Lateral ventricle
- Lms: Lateral migratory stream
- Put: Putamen
- ac: Anterior commissure
- ext: External capsule
- int: Internal capsule
- stt: Stria terminalis
- tcet: Transient cell zone in the external capsule
- wmf: White matter fibers

Age: 24 GW

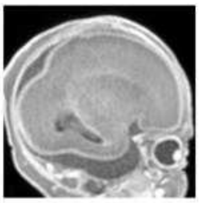

L-R Level: -11.34 mm

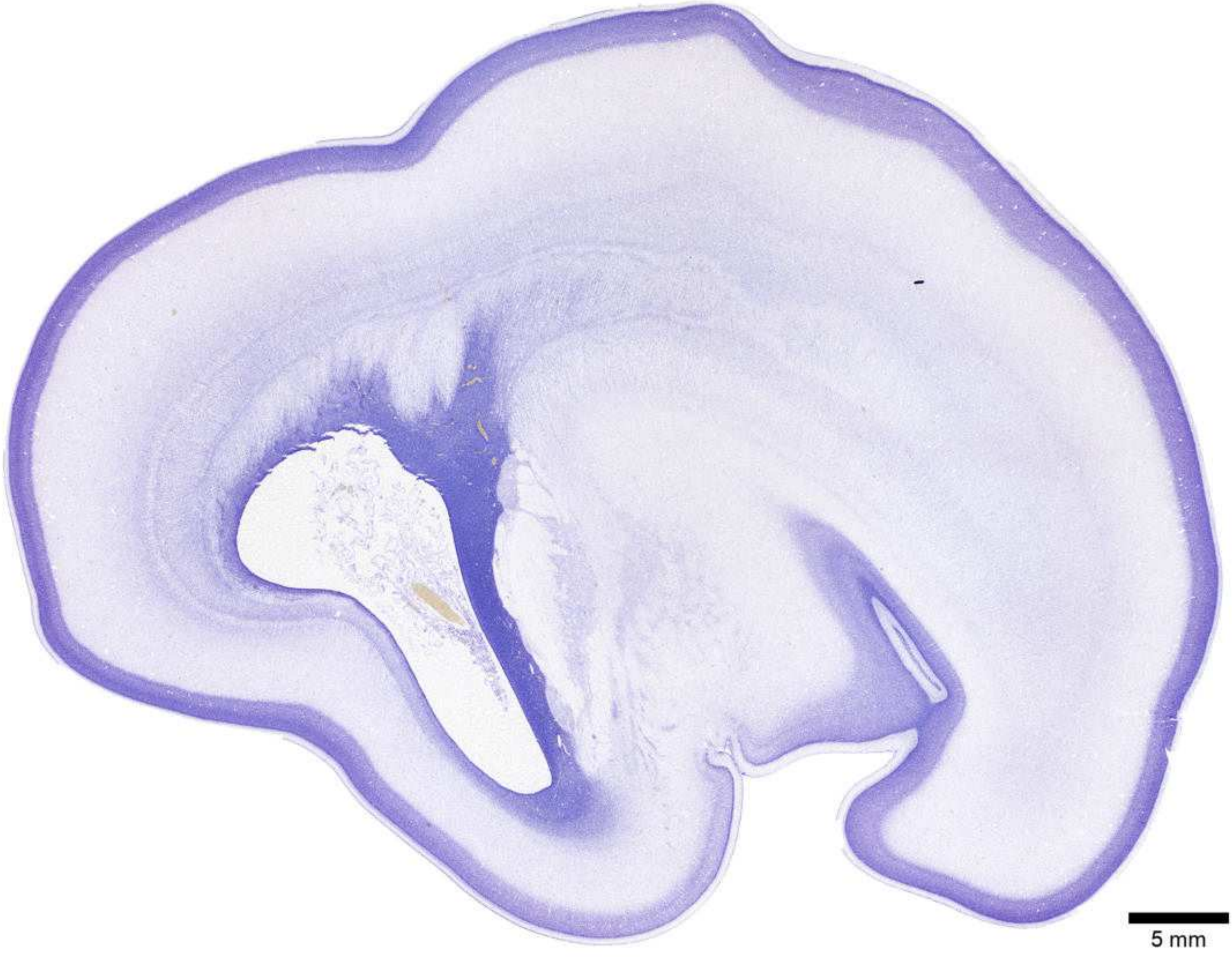

Transient Layers

- VZ
- SVZ
- IZ
- SP
- CP
- MZ
- SGL

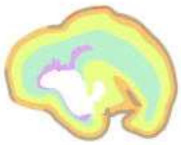

Cortical Areas

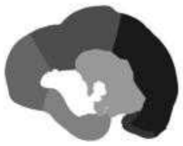

- FCTx
- ORB
- PAR
- OCC
- TEMP
- INS

Age: 24 GW

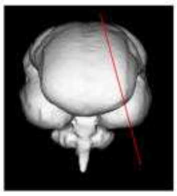

L-R Level: -11.34 mm

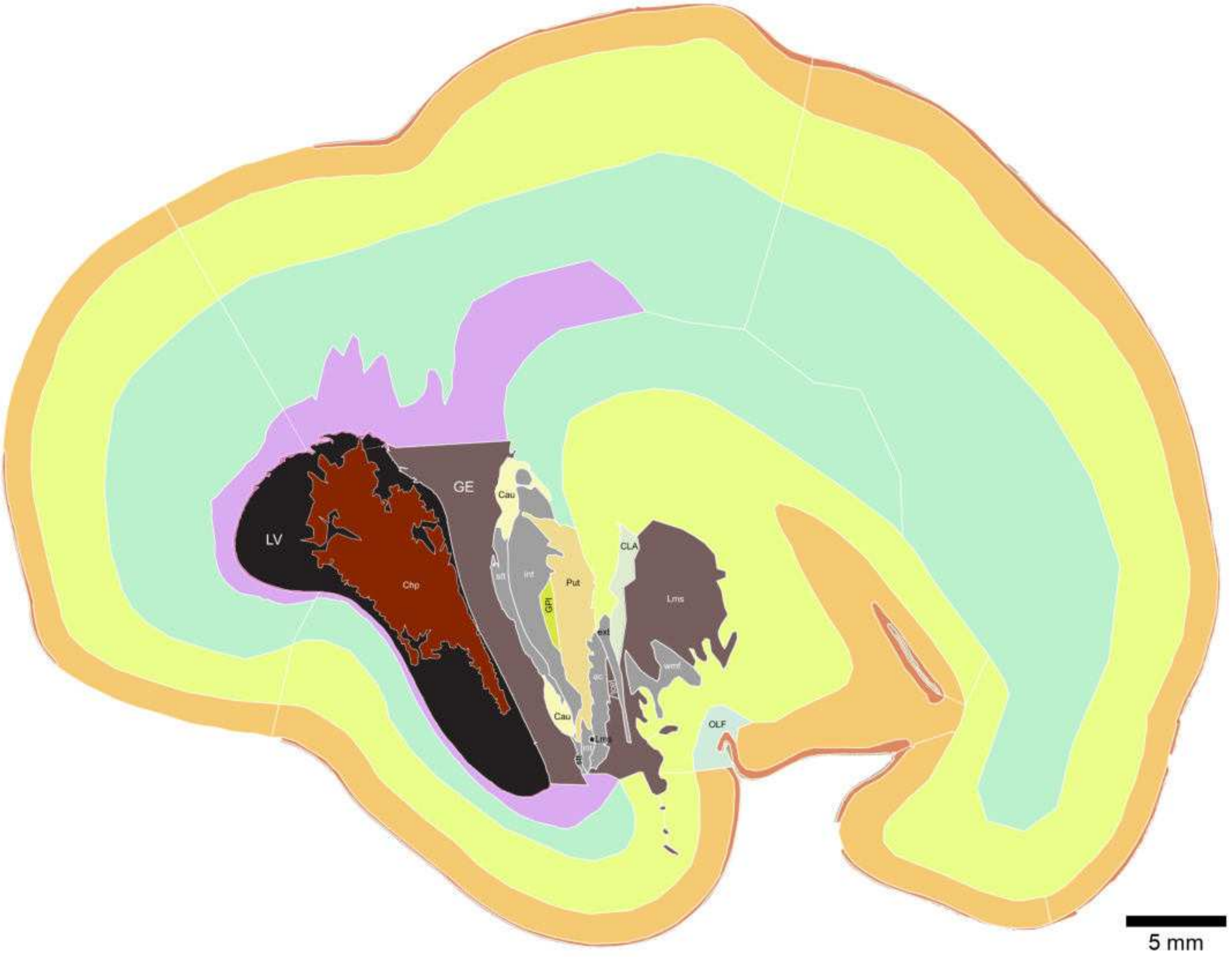

- CLA: Claustrum
- Cau: Caudate nucleus
- Chp: Choroid plexus
- GE: Ganglionic eminence
- GPl: Globus pallidus lateral segment
- LV: Lateral ventricle
- Lms: Lateral migratory stream
- Put: Putamen
- ac: Anterior commissure
- ext: External capsule
- int: Internal capsule
- stt: Stria terminalis
- tcet: Transient cell zone in the external capsule
- wmf: White matter fibers

Age: 24 GW

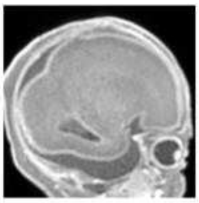

L-R Level: -11.7 mm

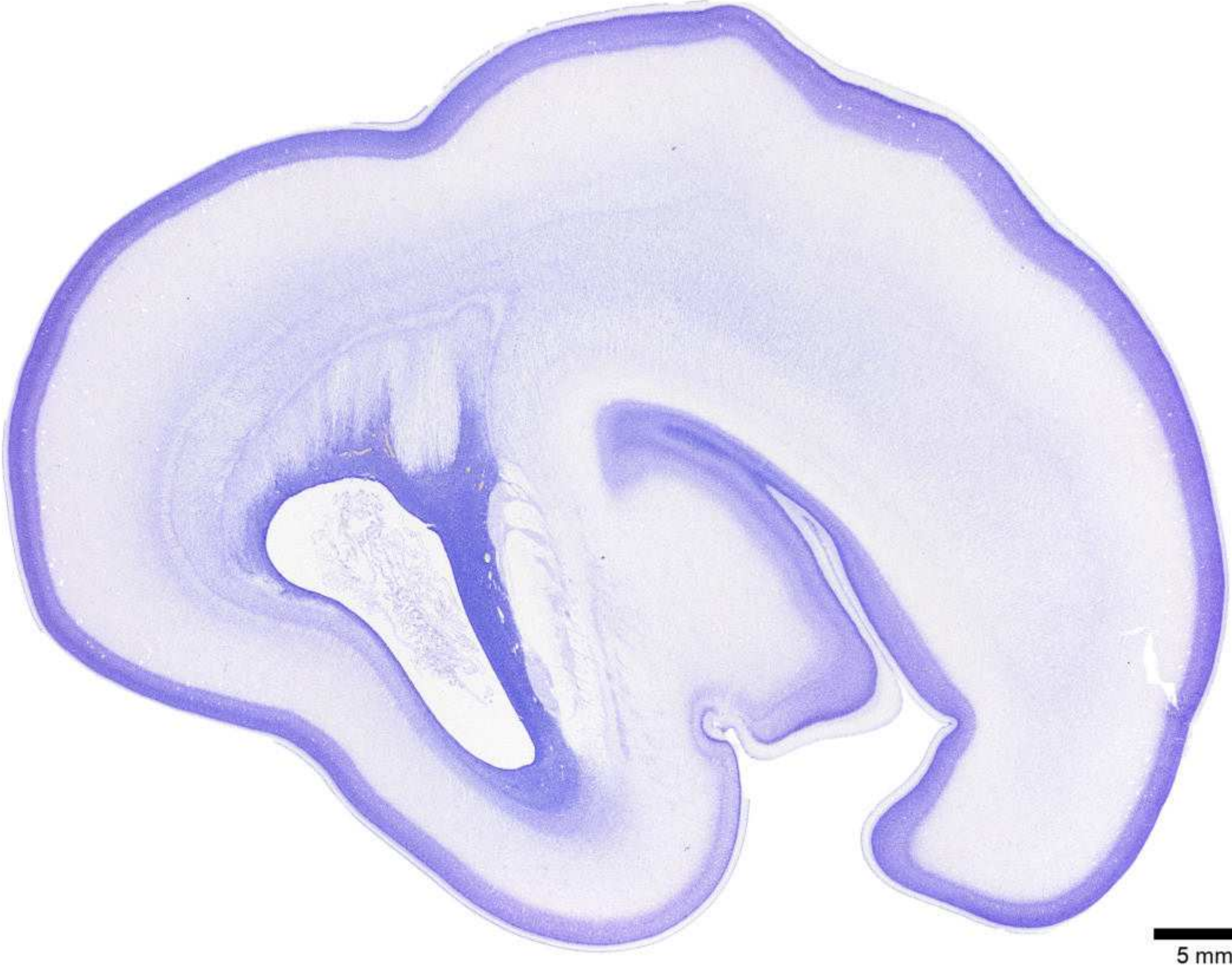

5 mm

Transient Layers

- VZ
- SVZ
- IZ
- SP
- CP
- MZ
- SGL

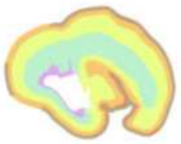

Cortical Areas

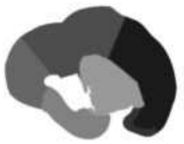

- FCTx
- ORB
- PAR
- OCC
- TEMP
- INS

Age: 24 GW

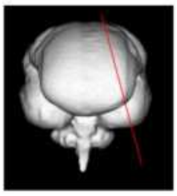

L-R Level: -11.7 mm

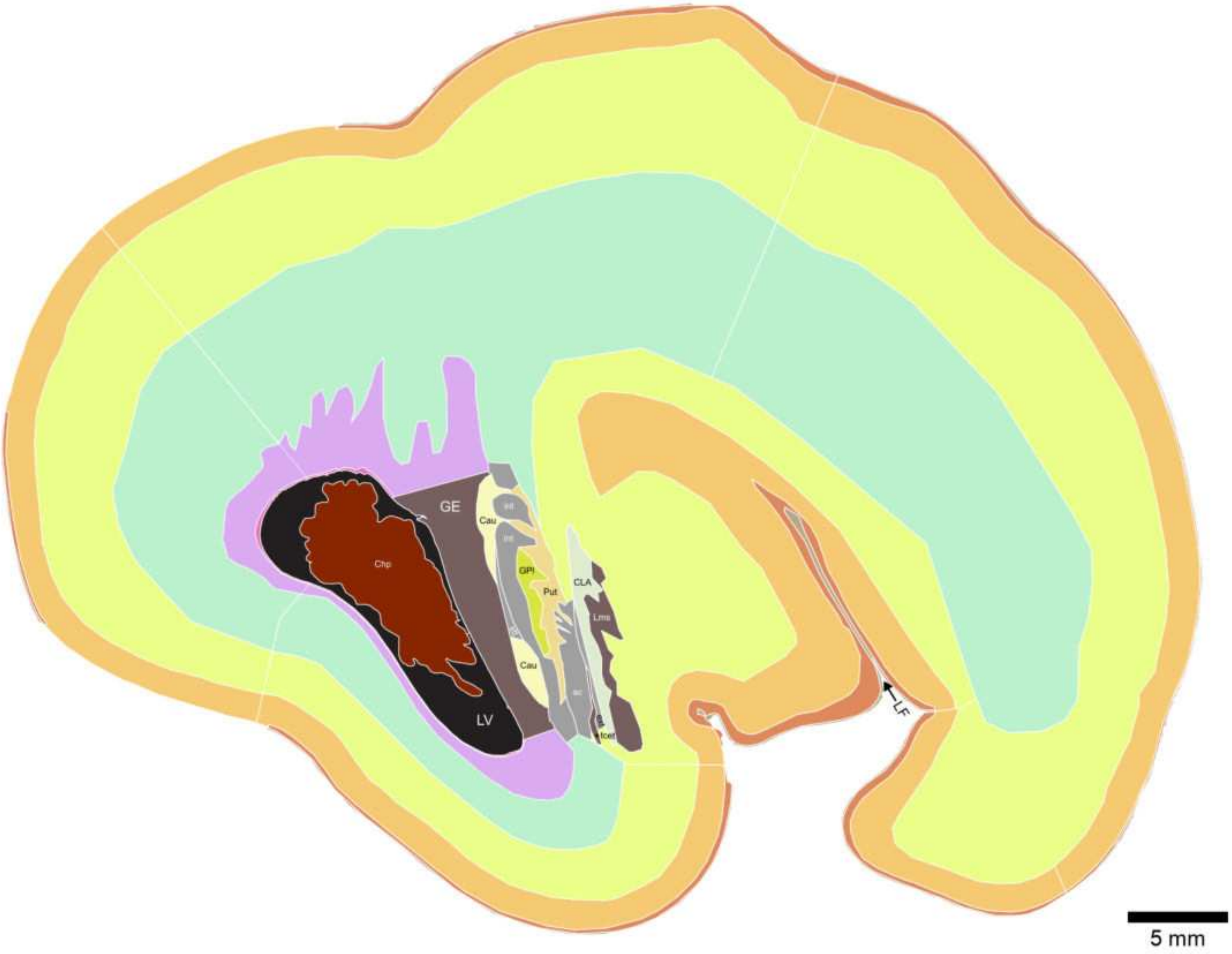

- CLA: Claustrum
- Cau: Caudate nucleus
- Chp: Choroid plexus
- GE: Ganglionic eminence
- GPl: Globus pallidus lateral segment
- LV: Lateral ventricle
- Lms: Lateral migratory stream
- Put: Putamen
- ac: Anterior commissure
- ext: External capsule
- int: Internal capsule
- stt: Stria terminalis
- tcet: Transient cell zone in the external capsule
- → LF: Lateral fissure

Age: 24 GW

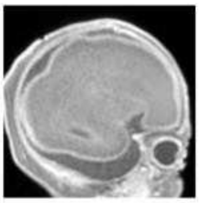

L-R Level: -12.3 mm

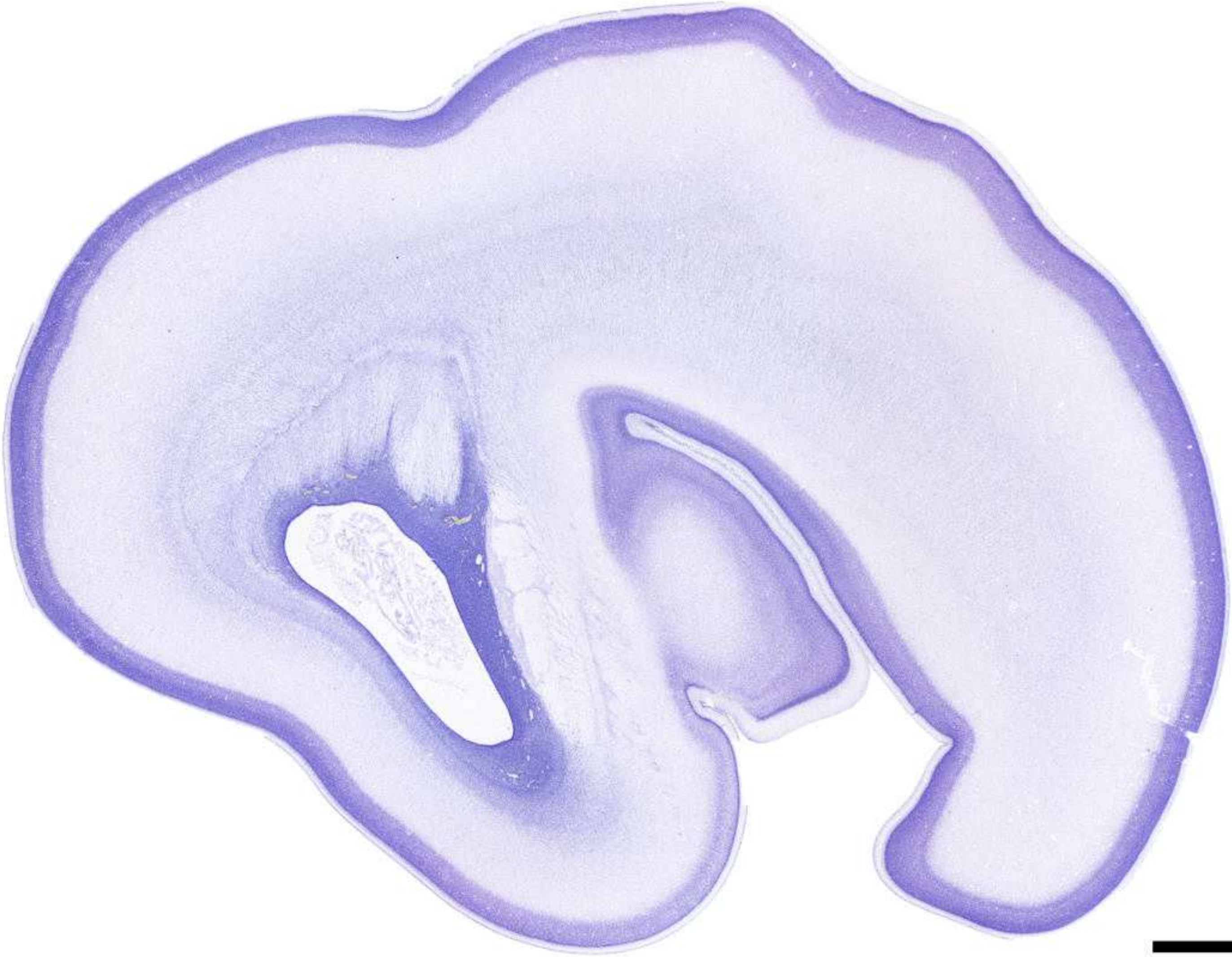

5 mm

Age: 24 GW

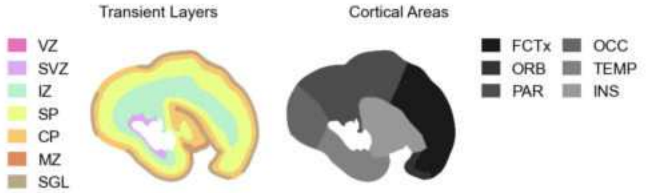

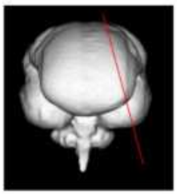

L-R Level: -12.3 mm

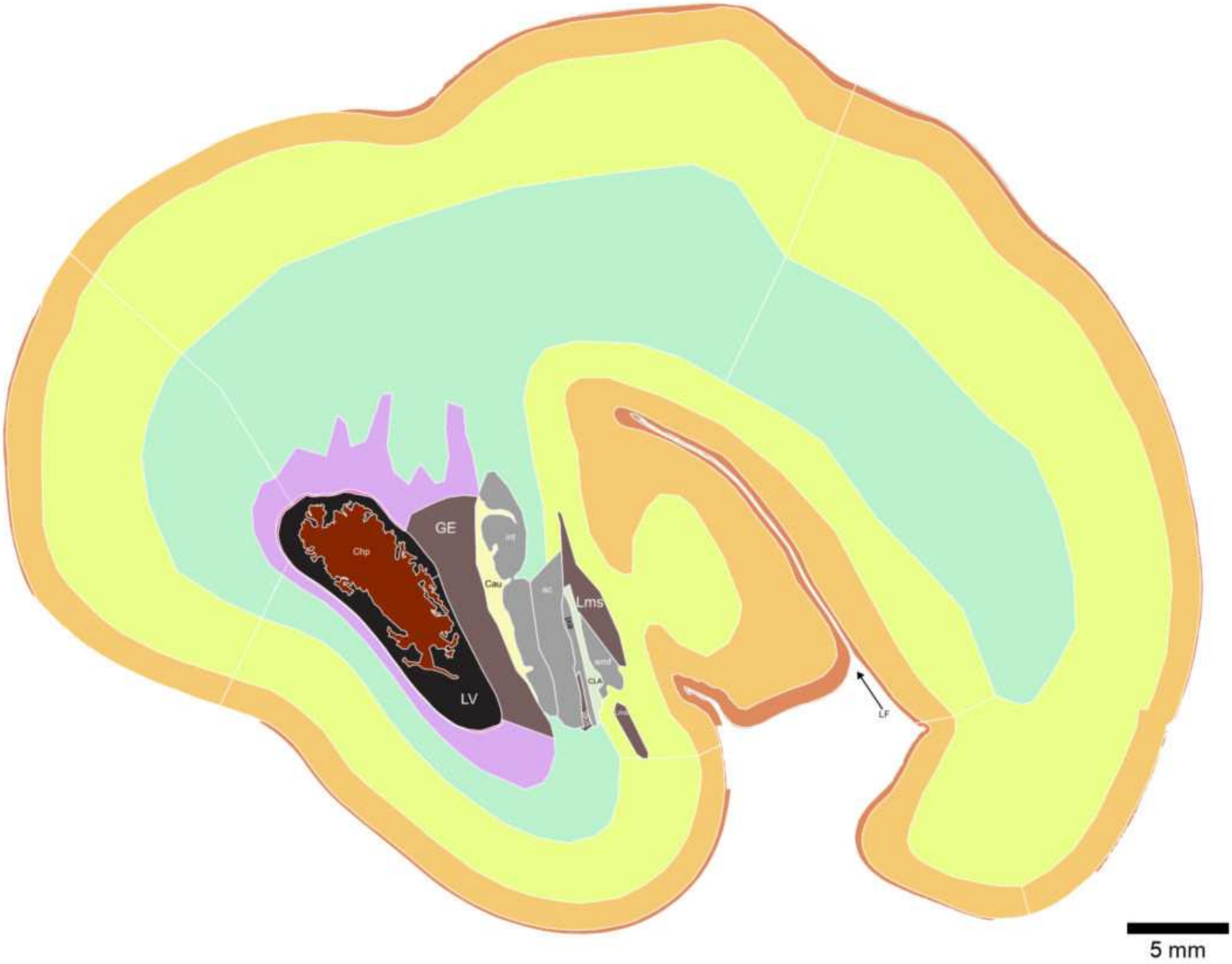

CLA: Claustrum
Cau: Caudate nucleus
Chp: Choroid plexus
GE: Ganglionic eminence
LV: Lateral ventricle
Lms: Lateral migratory stream
ac: Anterior commissure
ext: External capsule
int: Internal capsule
tcet: Transient cell zone in the external capsule
wmf: White matter fibers
LF: Lateral fissure

Age: 24 GW

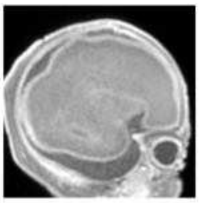

L-R Level: -13.02 mm

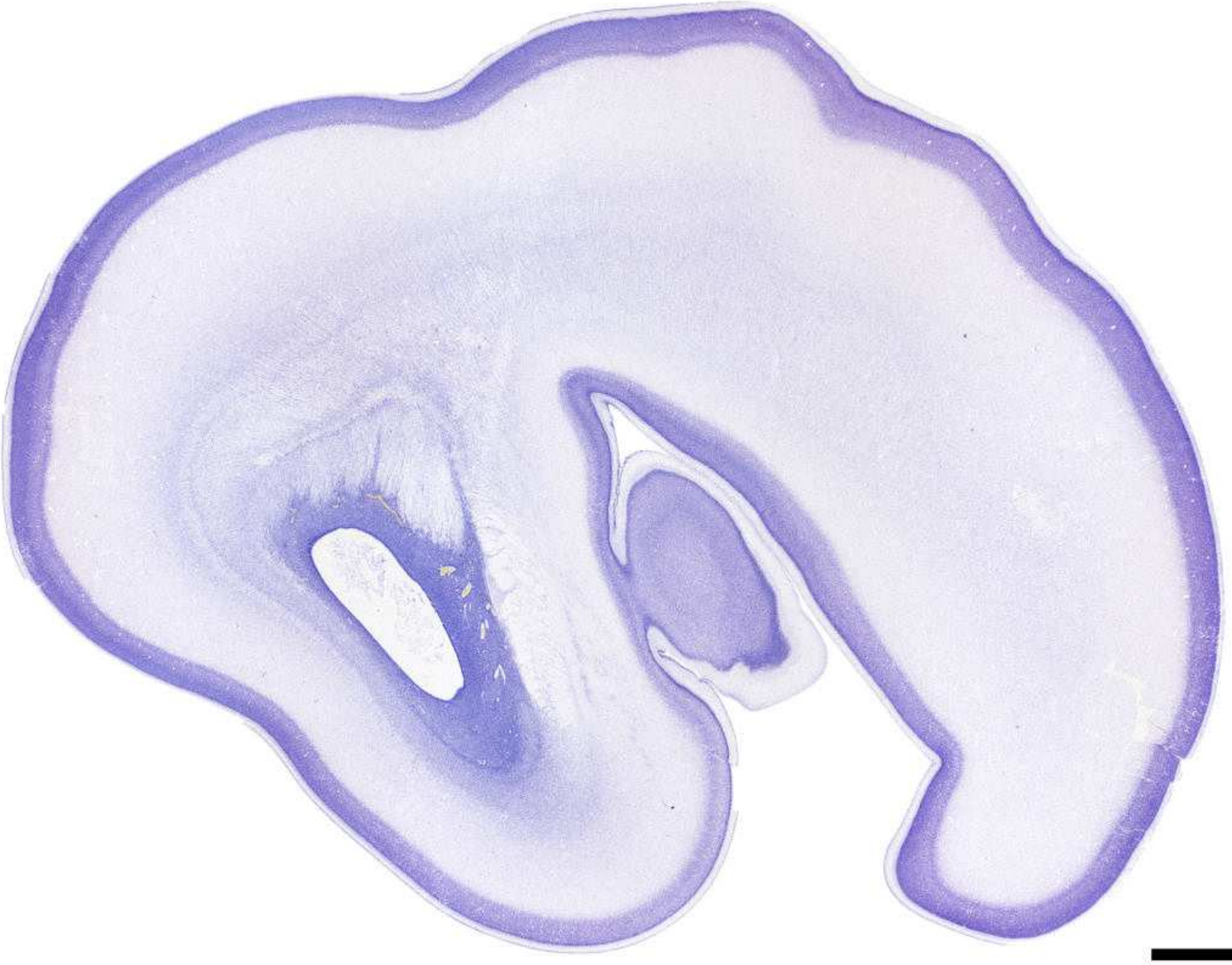

5 mm

Transient Layers

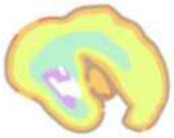

- VZ
- SVZ
- IZ
- SP
- CP
- MZ
- SGL

Cortical Areas

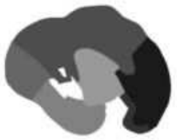

- FCTx
- ORB
- PAR
- OCC
- TEMP
- INS

Age: 24 GW

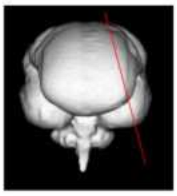

L-R Level: -13.02 mm

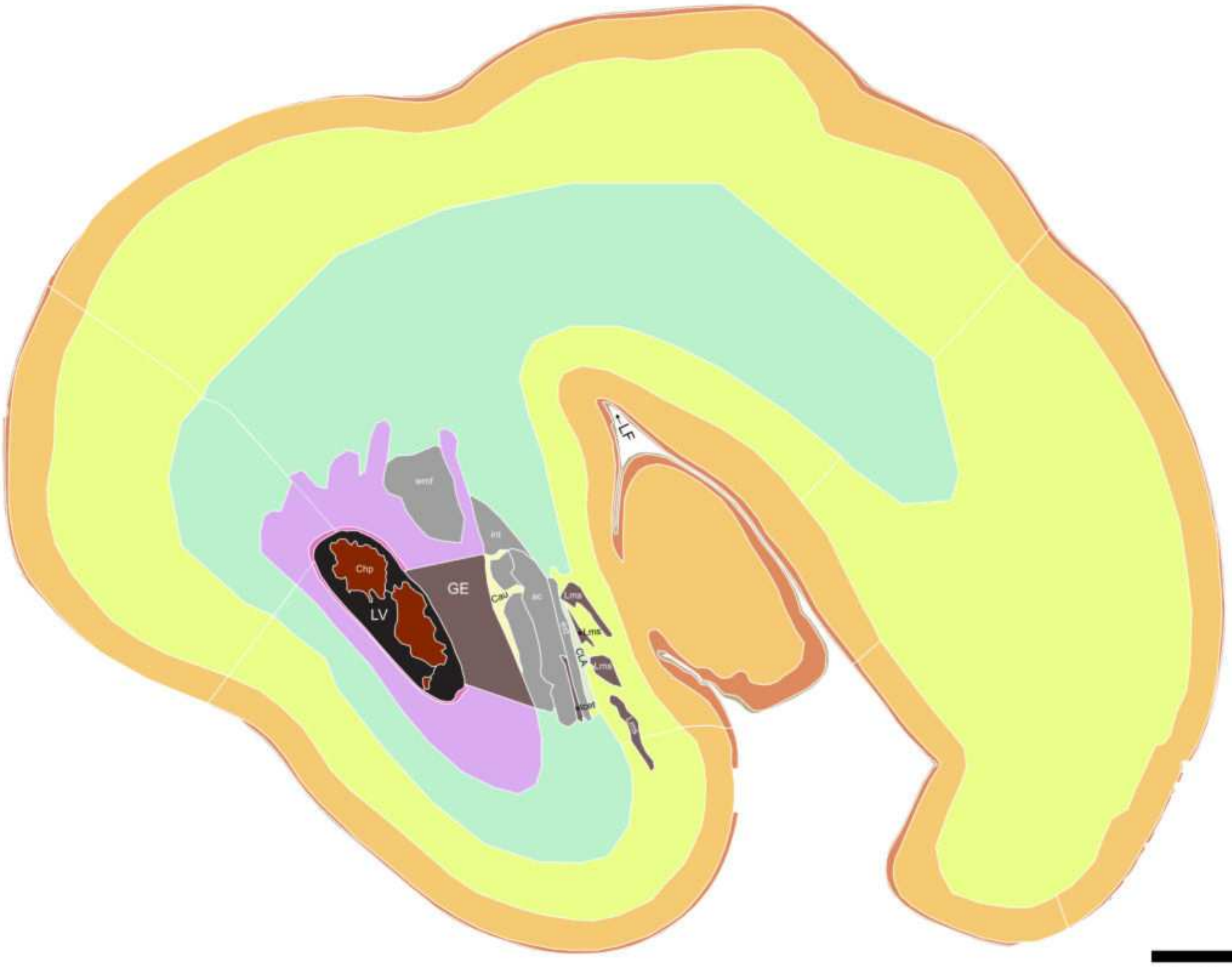

5 mm

- CLA: Claustrum
- Cau: Caudate nucleus
- Chp: Choroid plexus
- GE: Ganglionic eminence
- LV: Lateral ventricle
- Lms: Lateral migratory stream
- ac: Anterior commissure
- ext: External capsule
- int: Internal capsule
- tcet: Transient cell zone in the external capsule
- wmf: White matter fibers
- → LF: Lateral fissure

Age: 24 GW

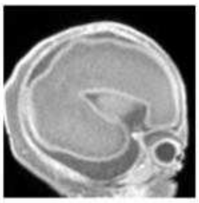

L-R Level: -13.8 mm

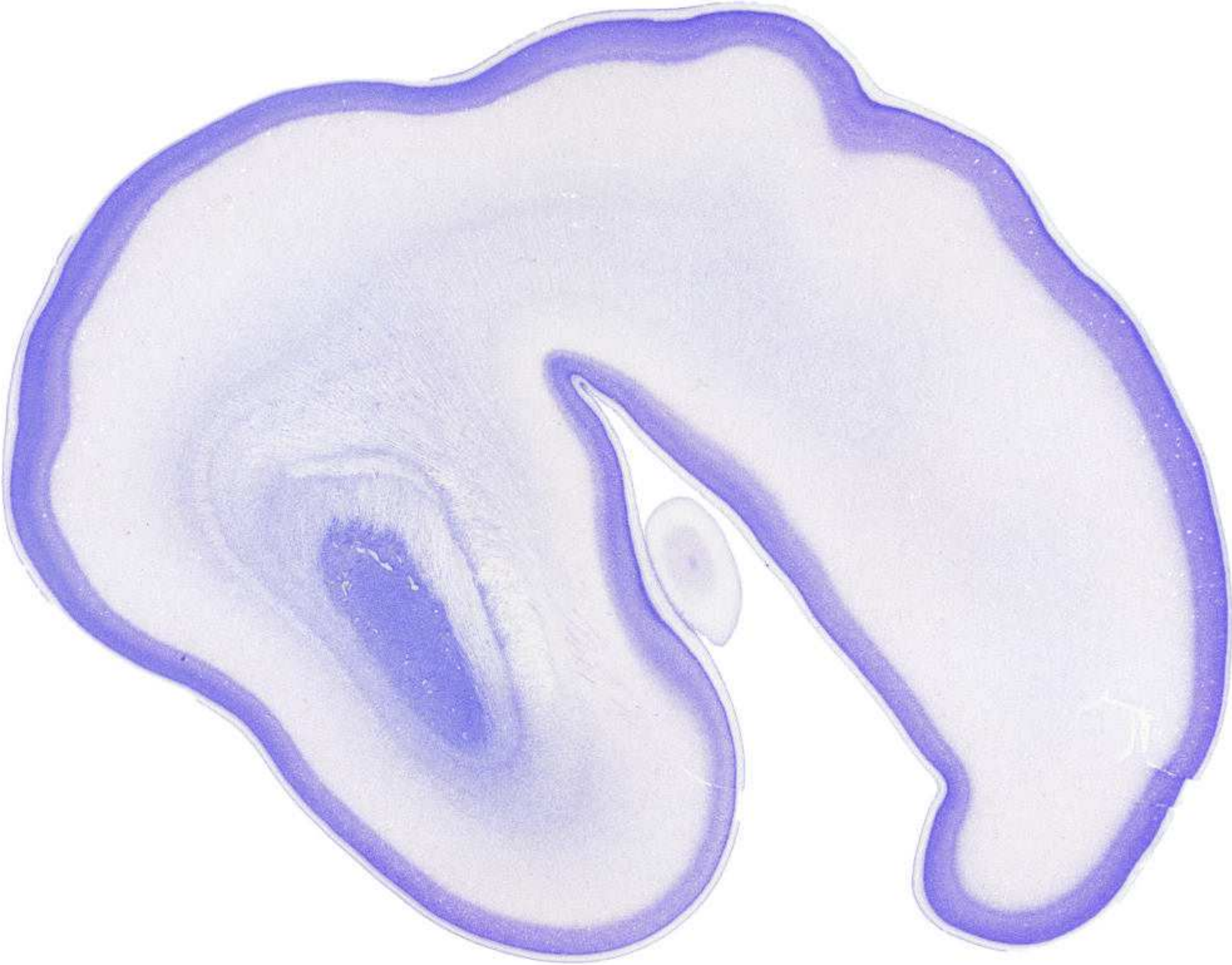

5 mm

Transient Layers

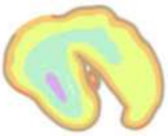

- SVZ
- IZ
- SP
- CP
- MZ
- SGL

Cortical Areas

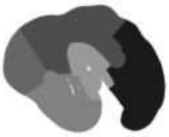

- FCTx
- PAR
- OCC
- TEMP
- INS

Age: 24 GW

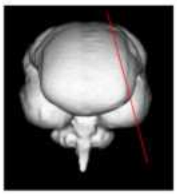

L-R Level: -13.8 mm

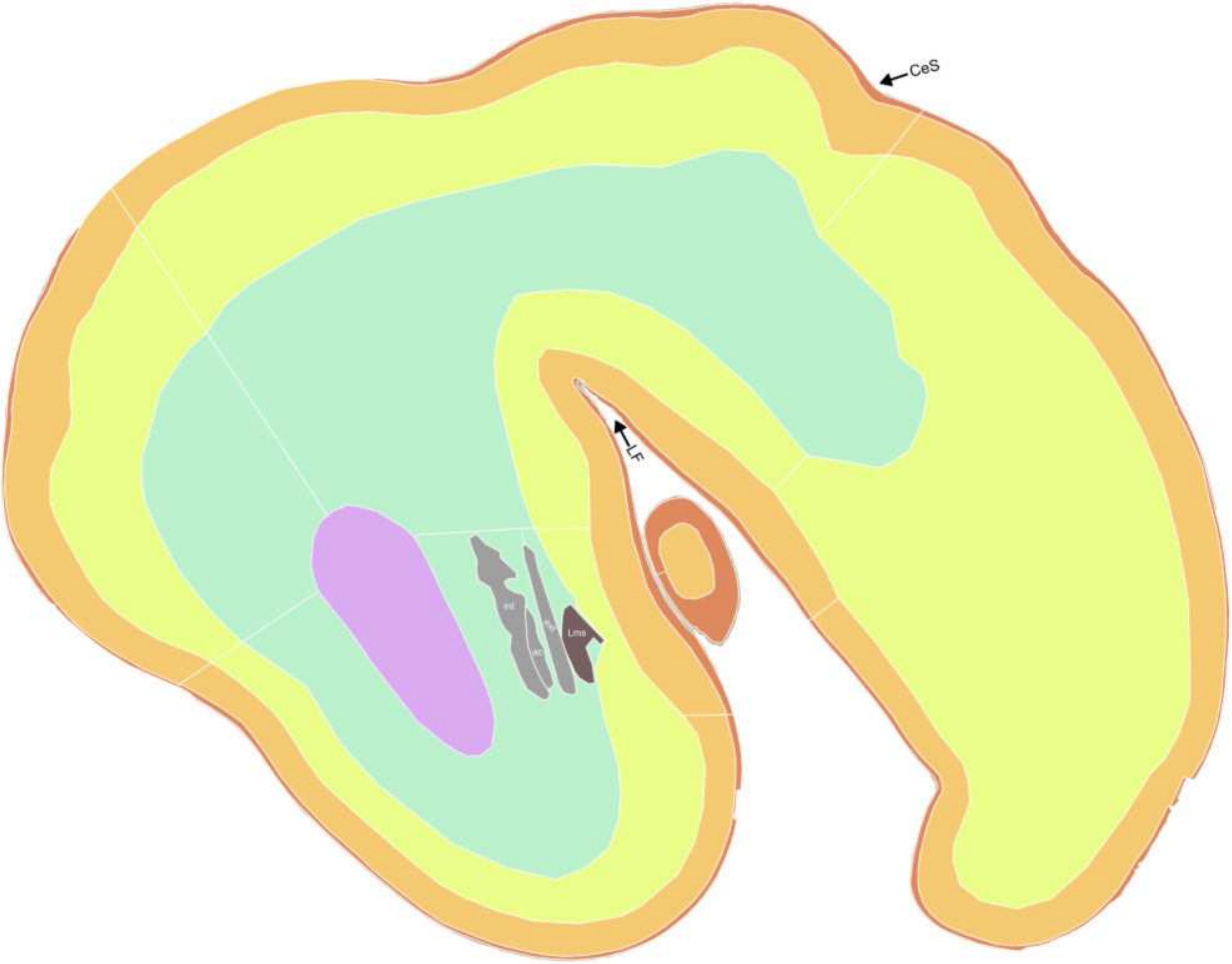

5 mm

- Lms: Lateral migratory stream
- ac: Anterior commissure
- ext: External capsule
- int: Internal capsule
- → LF: Lateral fissure
- → CeS: Central sulcus

Age: 24 GW

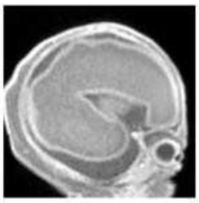

L-R Level: -14.28 mm

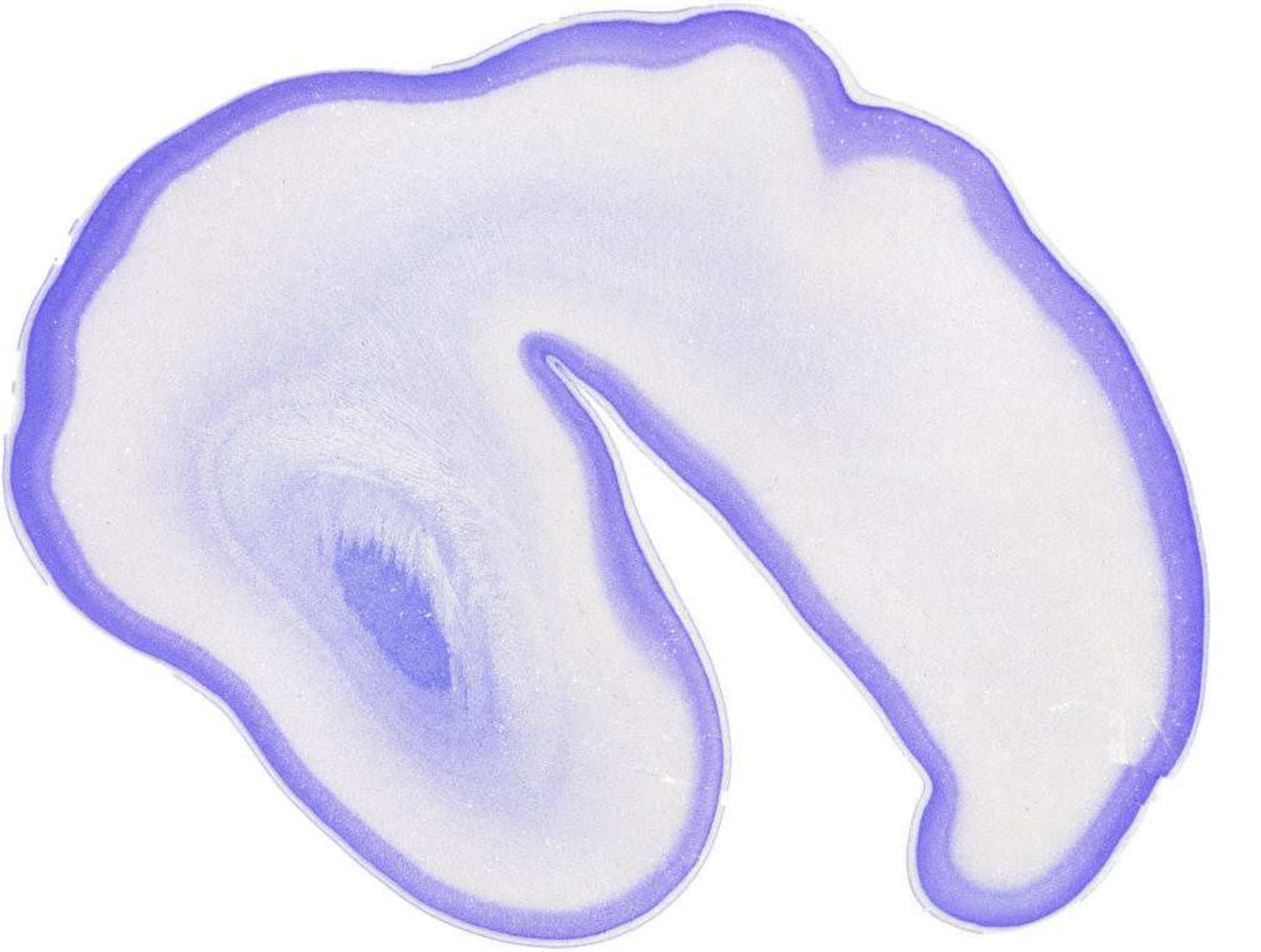

5 mm

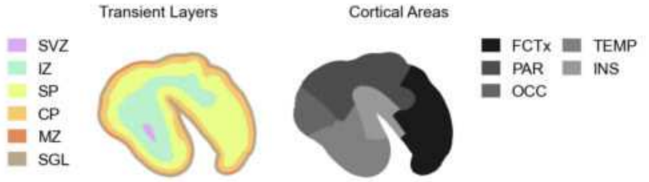

Age: 24 GW

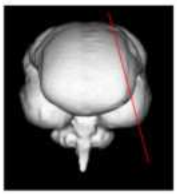

L-R Level: -14.28 mm

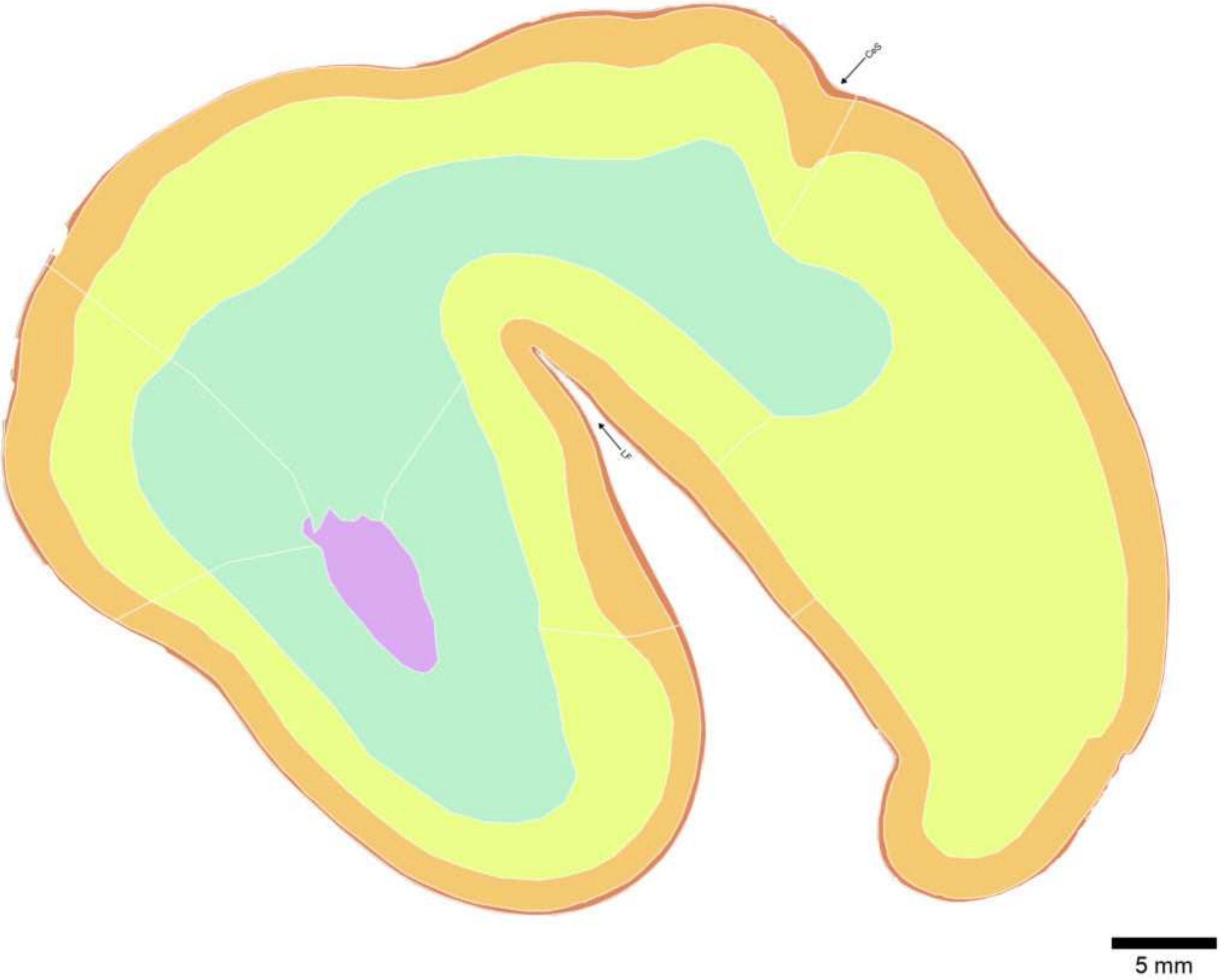

→ CeS: Central sulcus

→ LF: Lateral fissure

Age: 24 GW

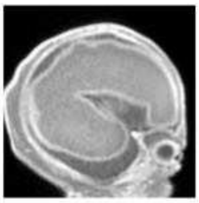

L-R Level: -14.46 mm

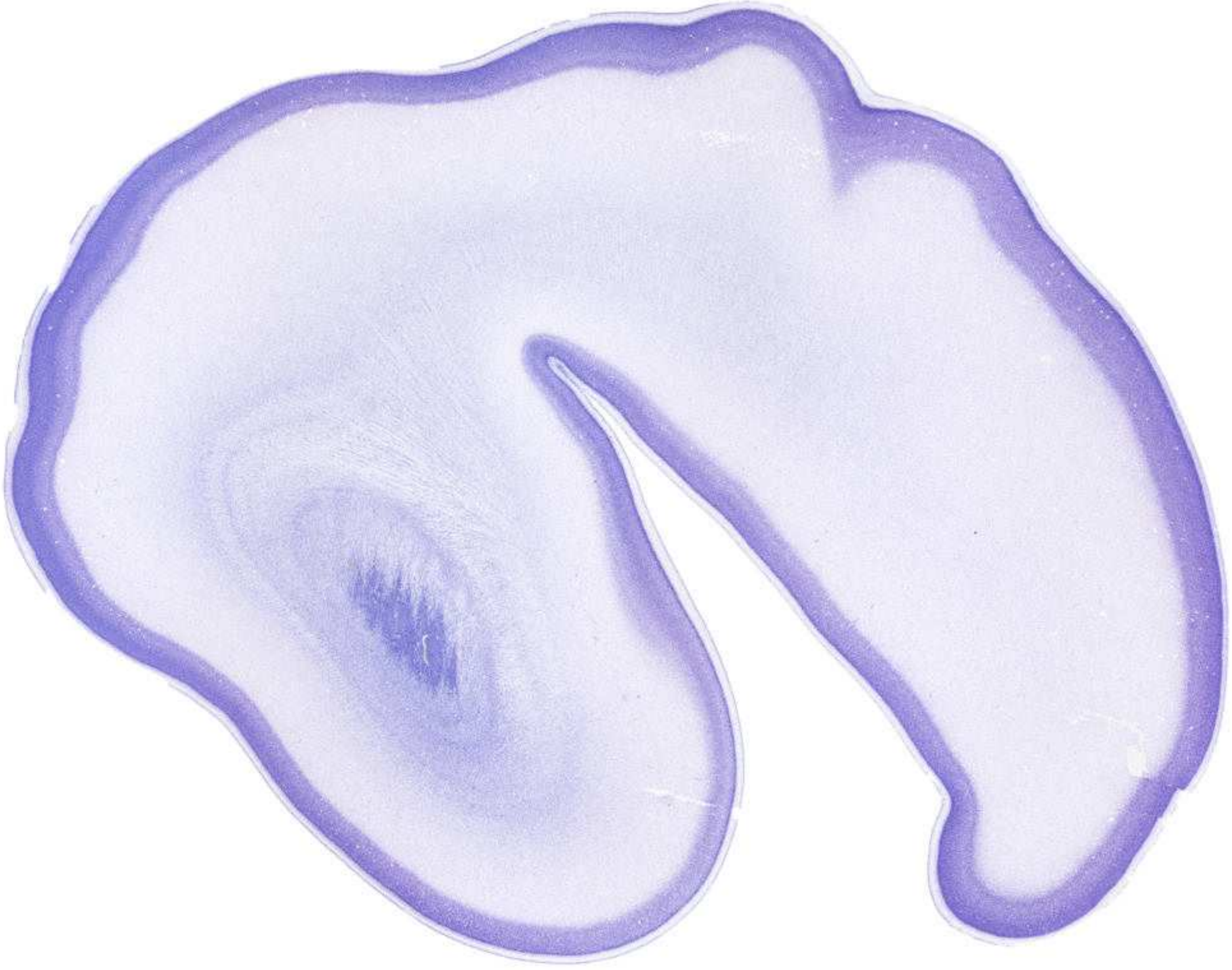

5 mm

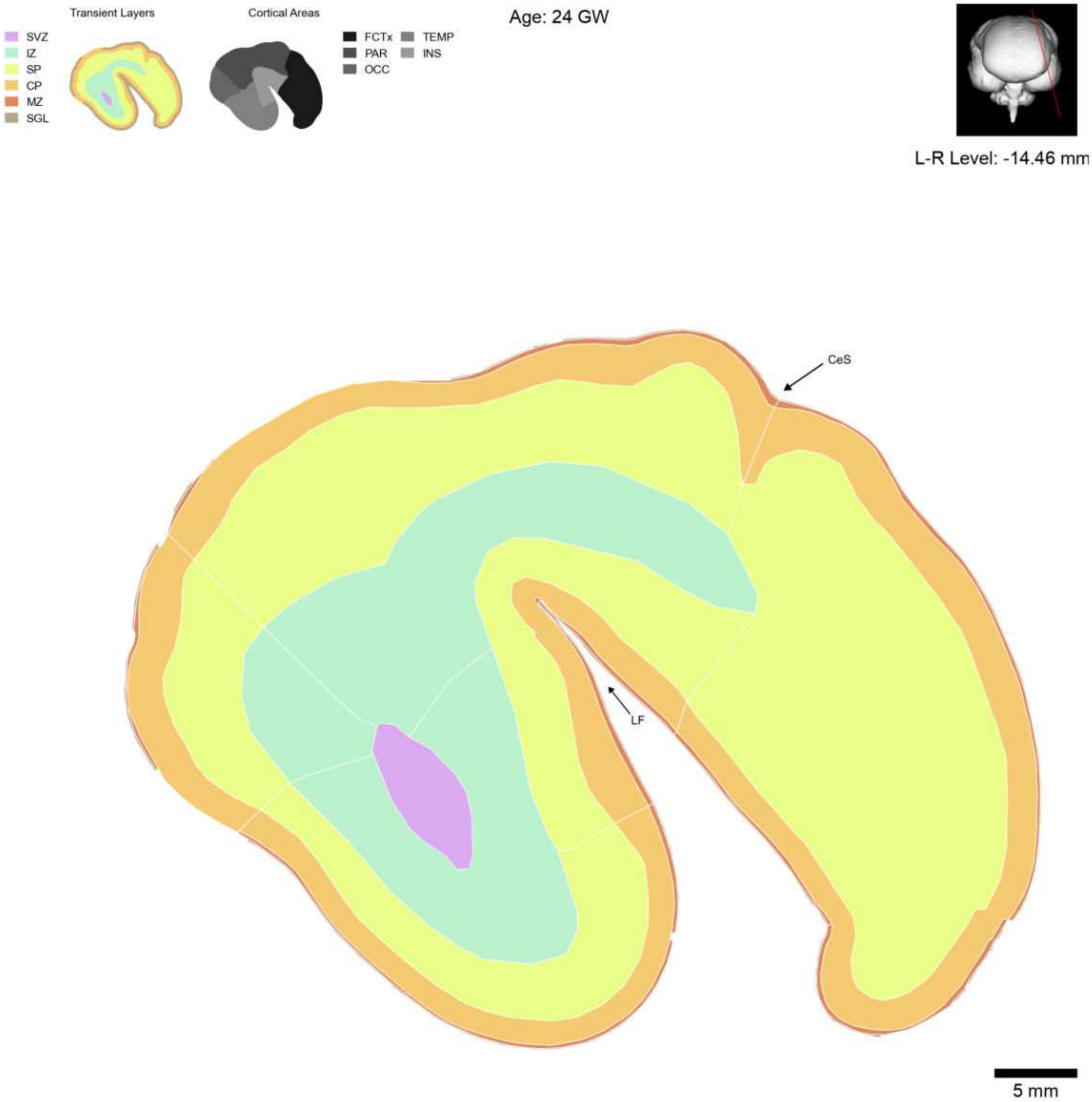

→ LF: Lateral fissure → CeS: Central sulcus

Age: 24 GW

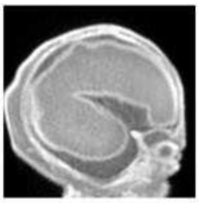

L-R Level: -15.36 mm

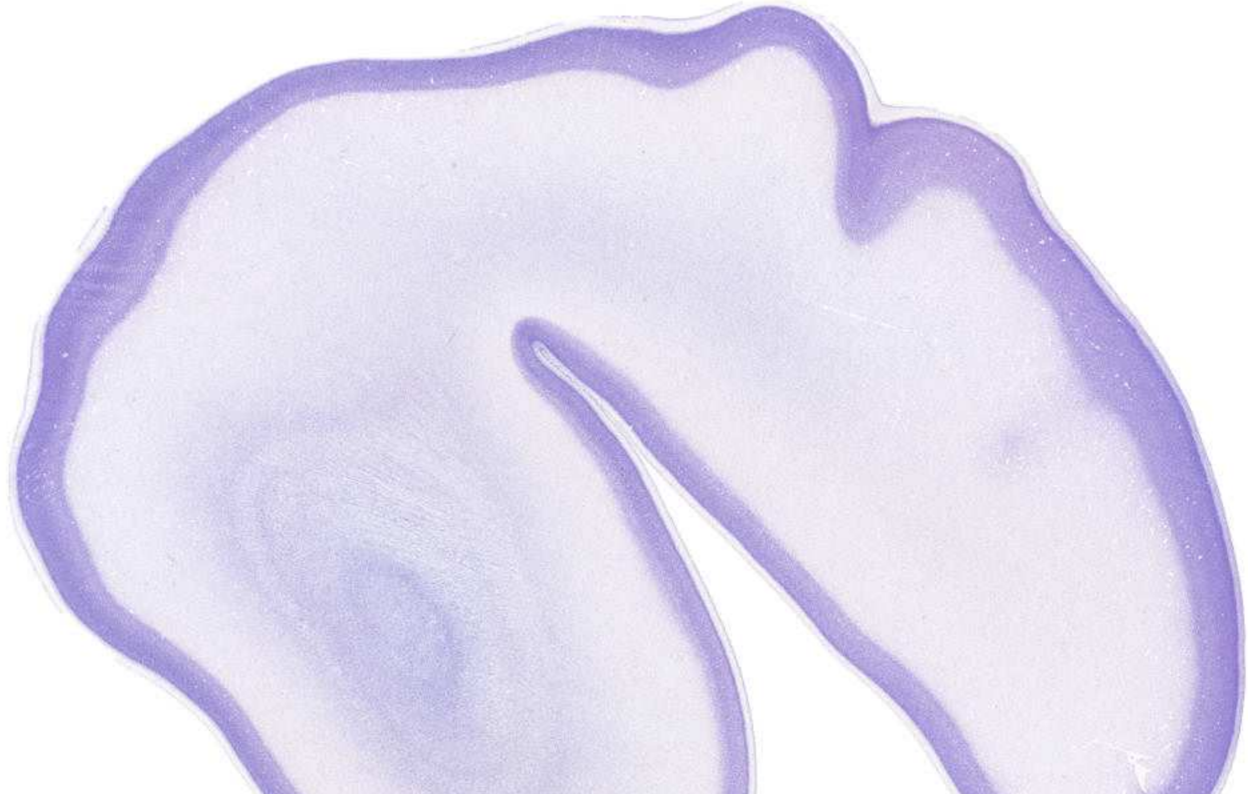

5 mm

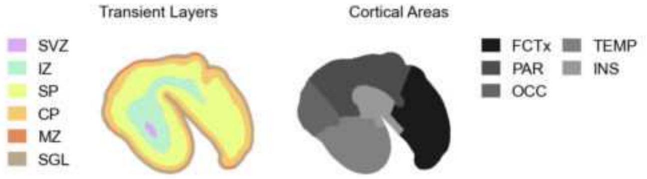

Age: 24 GW

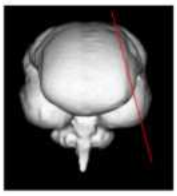

L-R Level: -15.36 mm

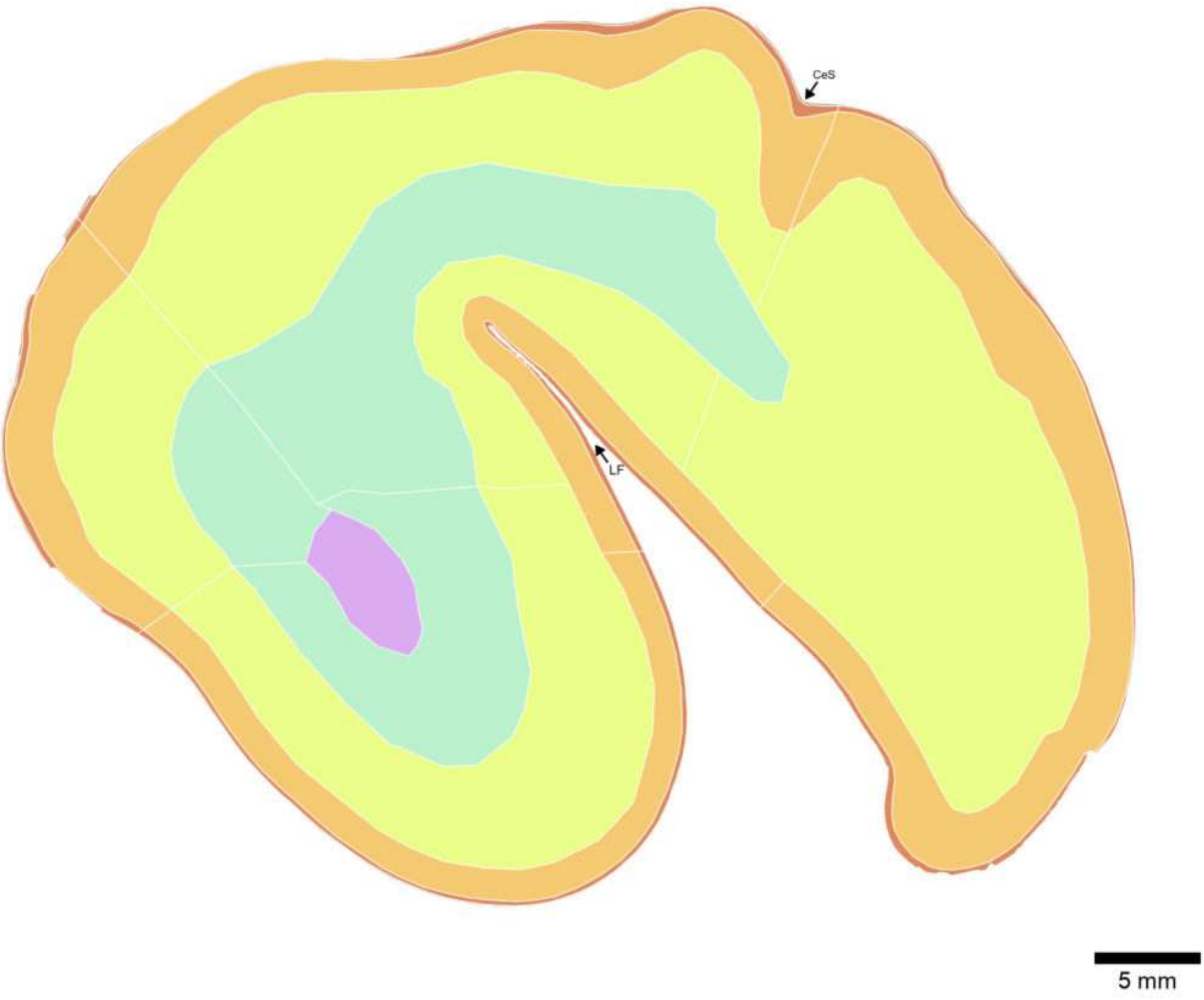

→ CeS: Central sulcus

→ LF: Lateral fissure

Age: 24 GW

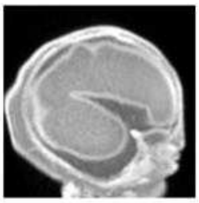

L-R Level: -16.14 mm

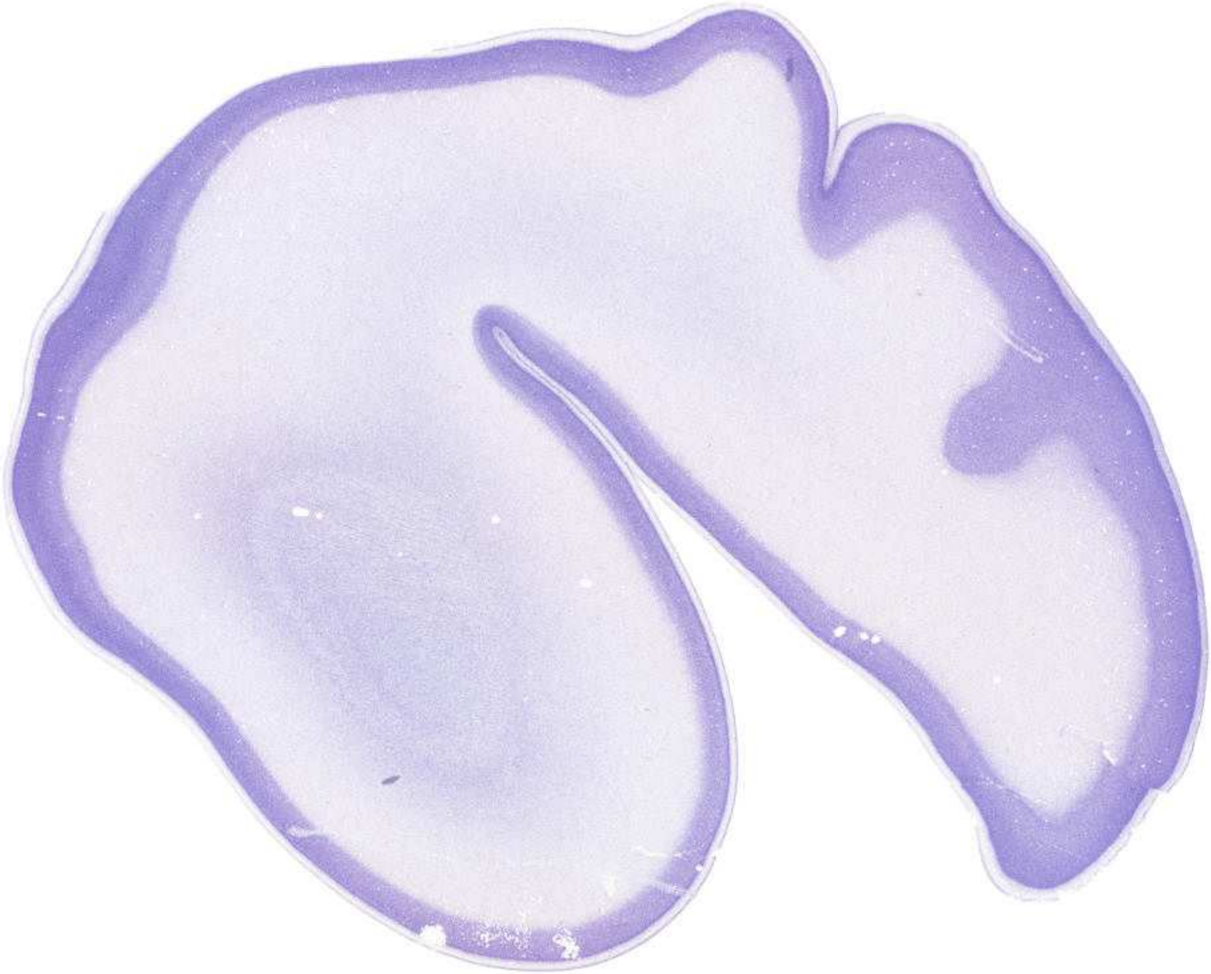

5 mm

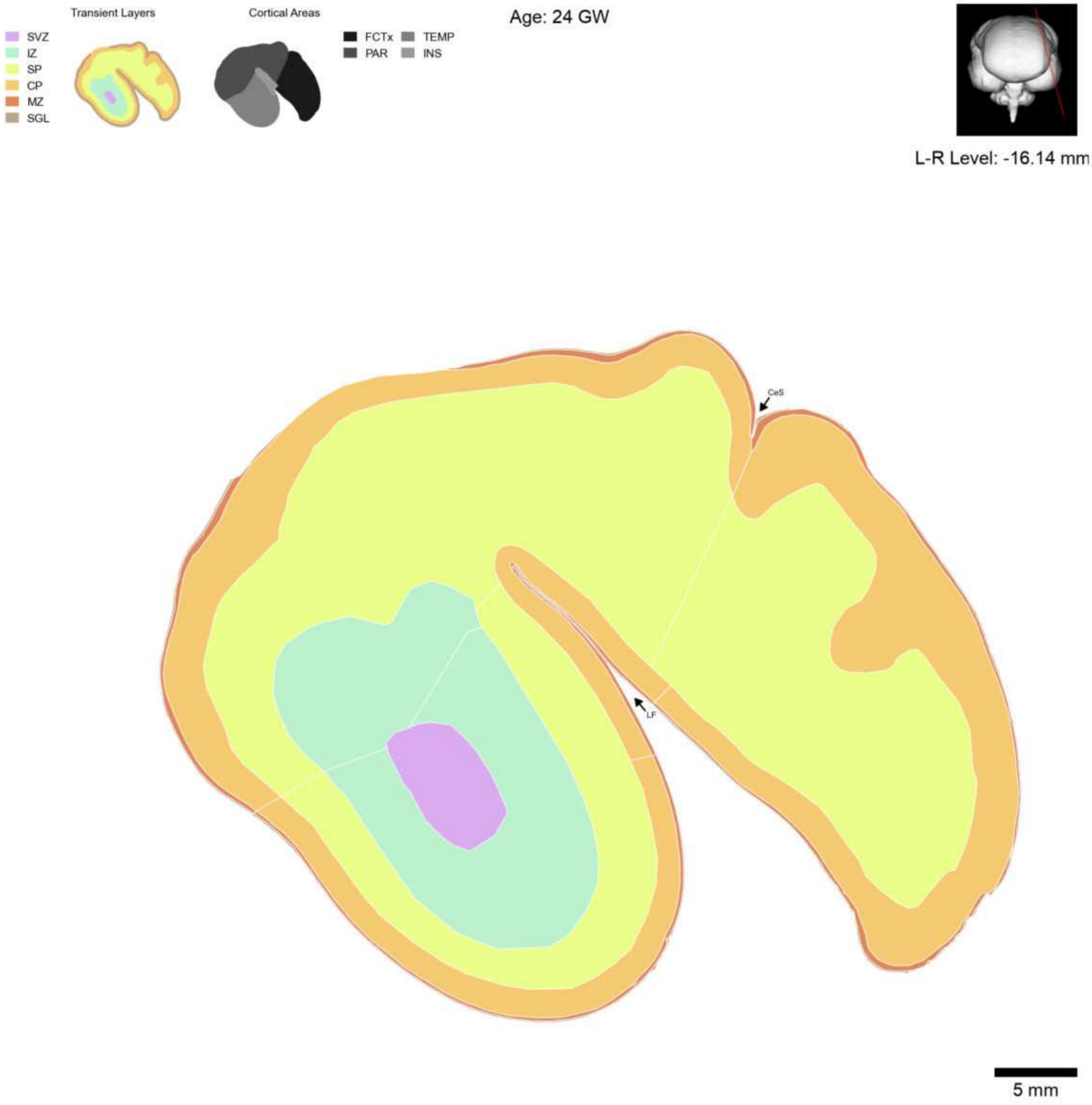

→ CeS: Central sulcus

→ LF: Lateral fissure

Age: 24 GW

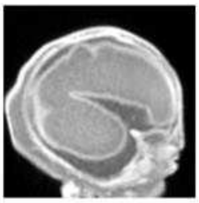

L-R Level: -16.62 mm

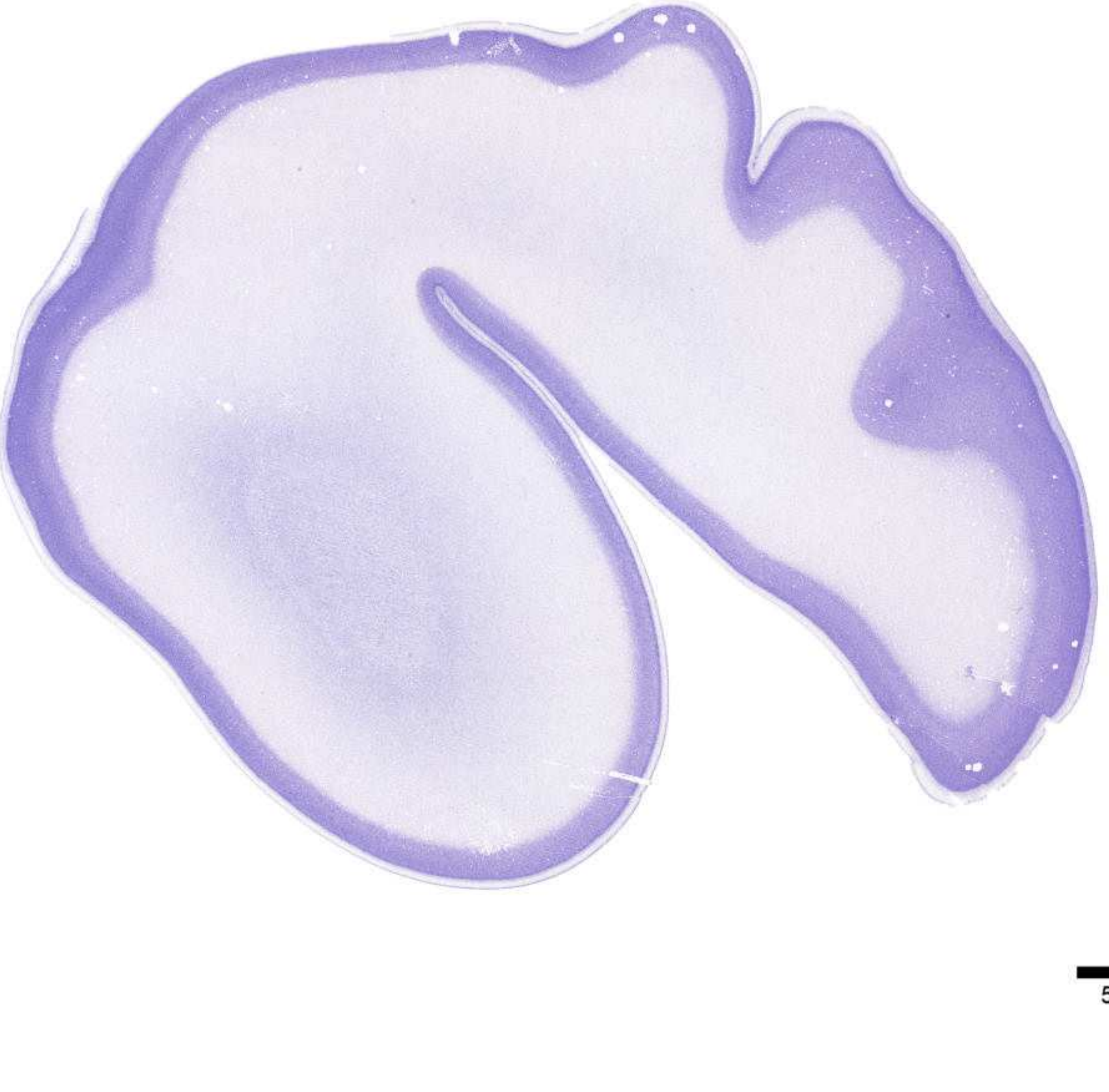

5 mm

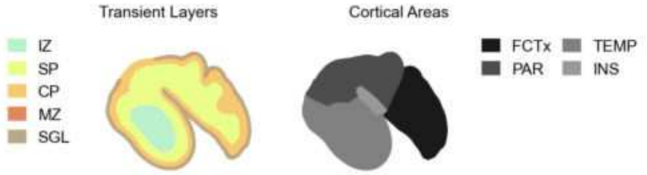

Age: 24 GW

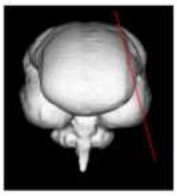

L-R Level: -16.62 mm

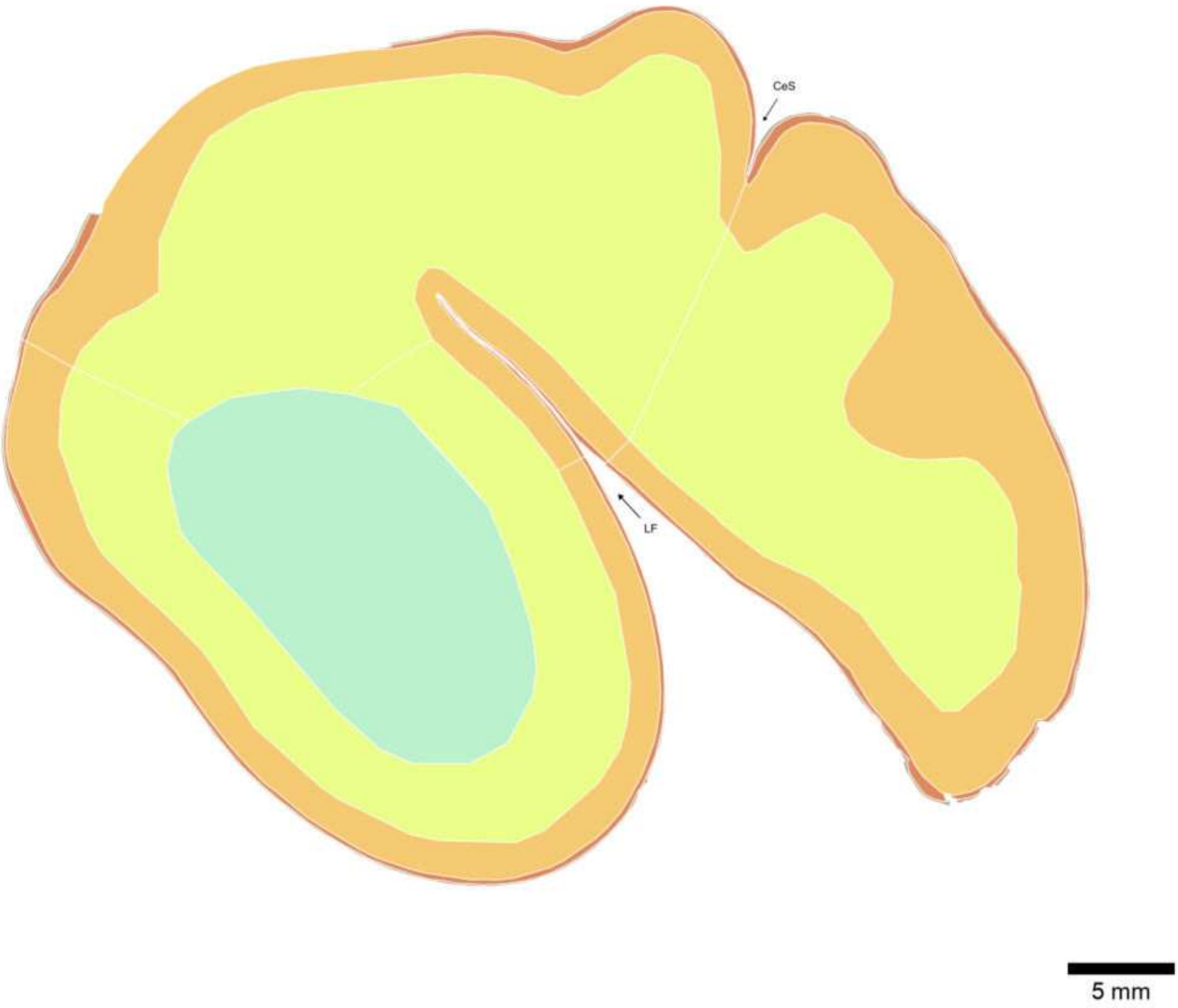

→ CeS: Central sulcus

→ LF: Lateral fissure

Age: 24 GW

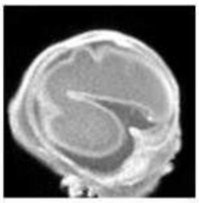

L-R Level: -18.3 mm

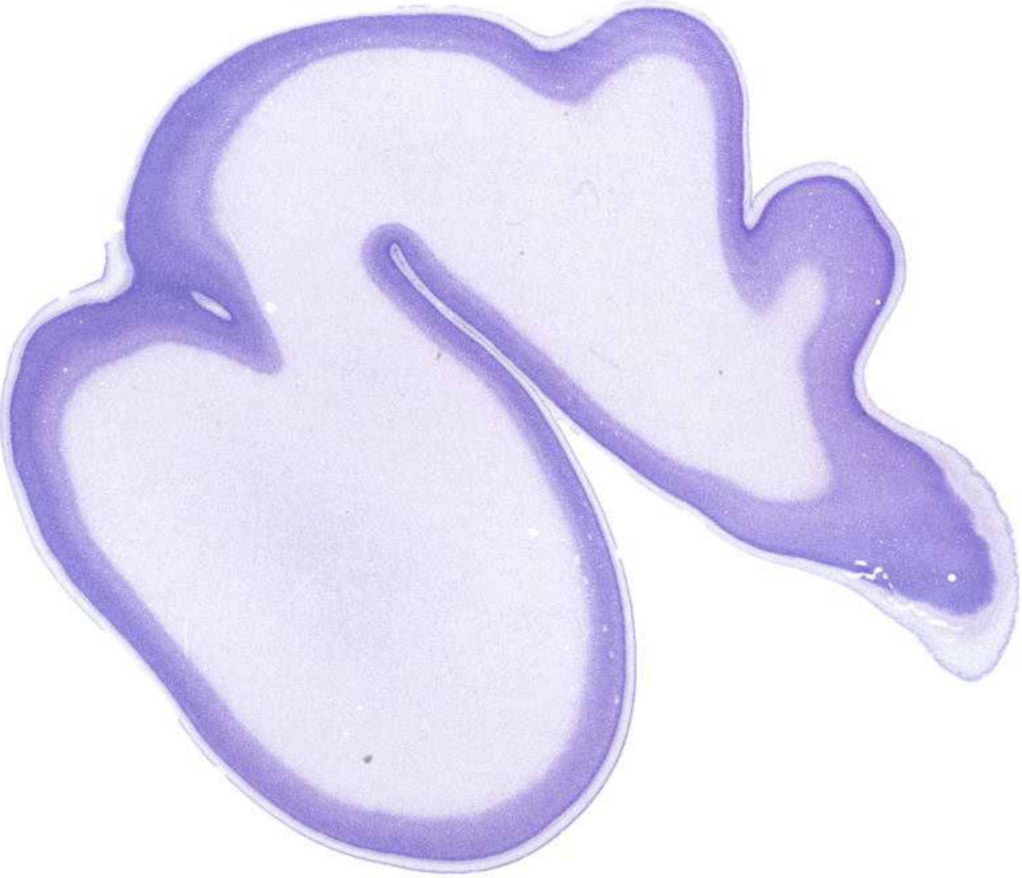

5 mm

Transient Layers

- SP
- CP
- MZ
- SGL

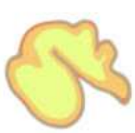

Cortical Areas

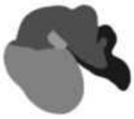

- FCTx
- PAR
- TEMP
- INS

Age: 24 GW

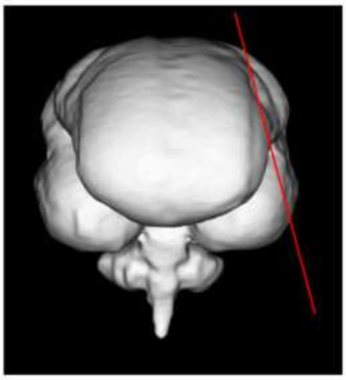

L-R Level: -18.3 mm

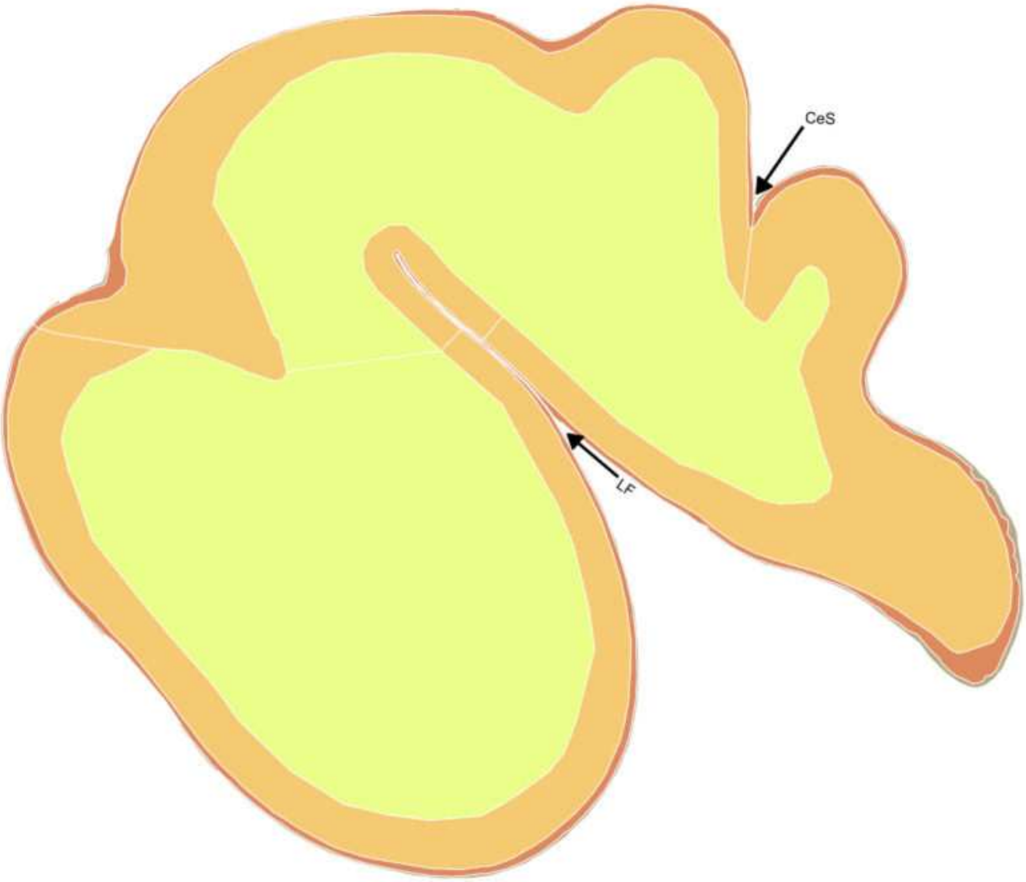

5 mm

→ LF: Lateral fissure → CeS: Central sulcus

Age: 24 GW

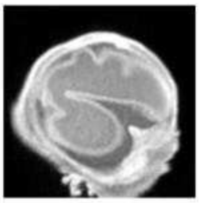

L-R Level: -19.8 mm

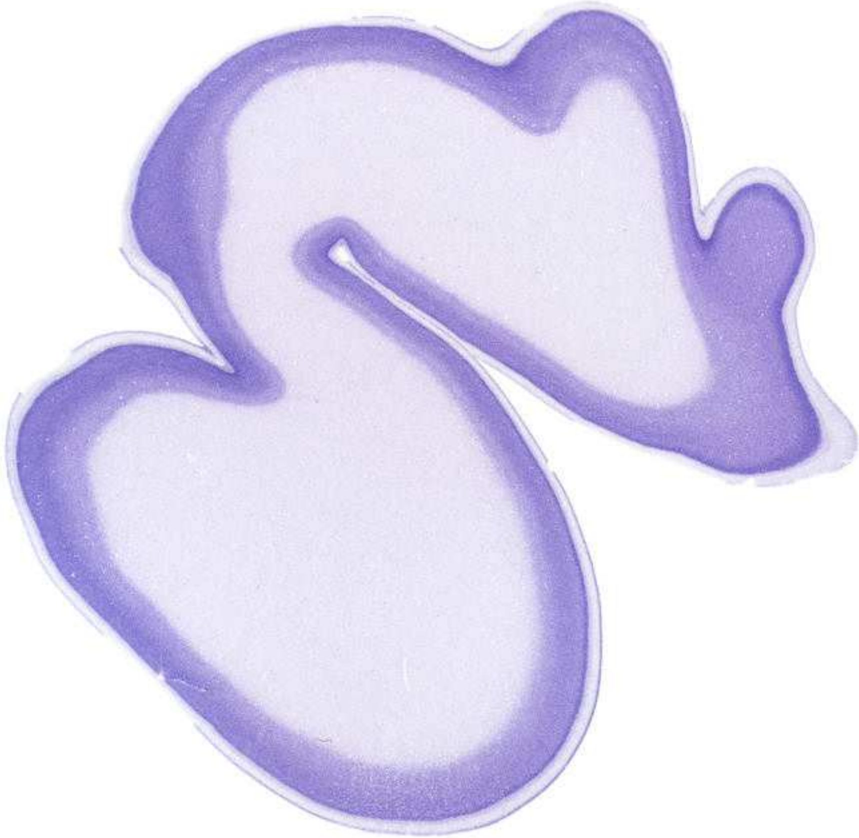

5 mm

Transient Layers

SP
CP
MZ
SGL

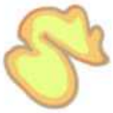

Cortical Areas

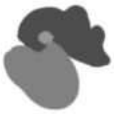

PAR
TEMP
INS

Age: 24 GW

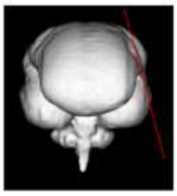

L-R Level: -19.8 mm

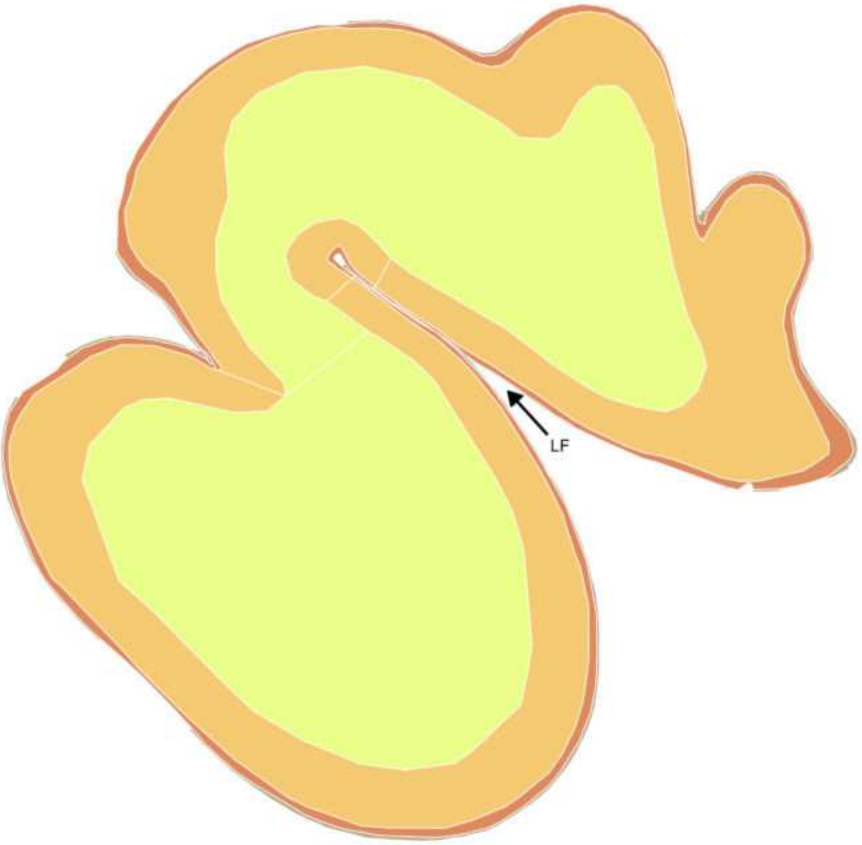

→ Lateral Fissure

5 mm

Age: 24 GW

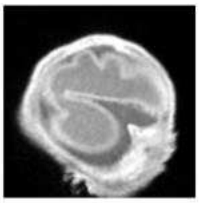

L-R Level: -21.24 mm

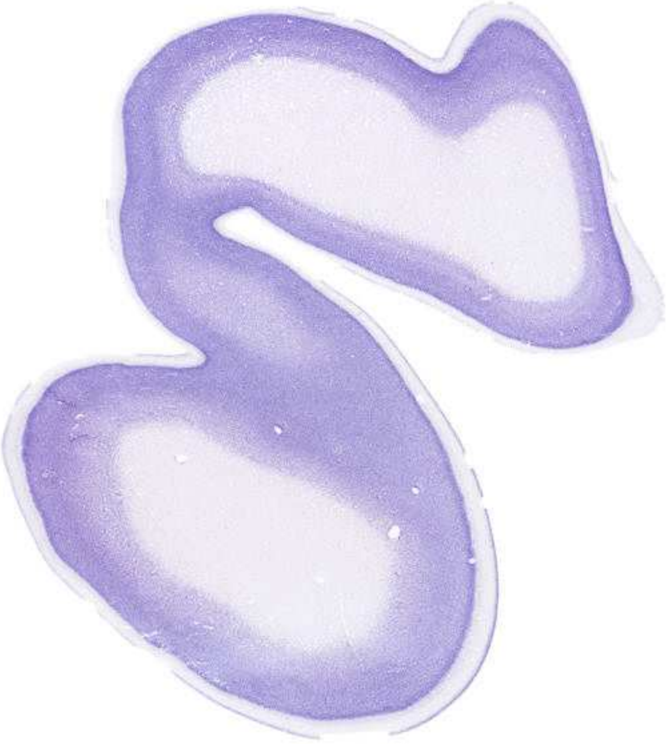

5 mm

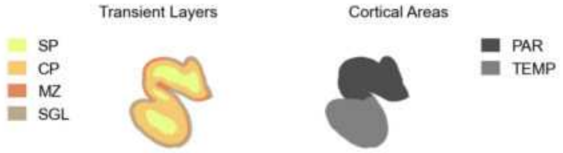

Age: 24 GW

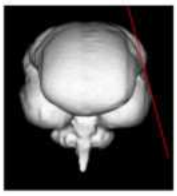

L-R Level: -21.24 mm

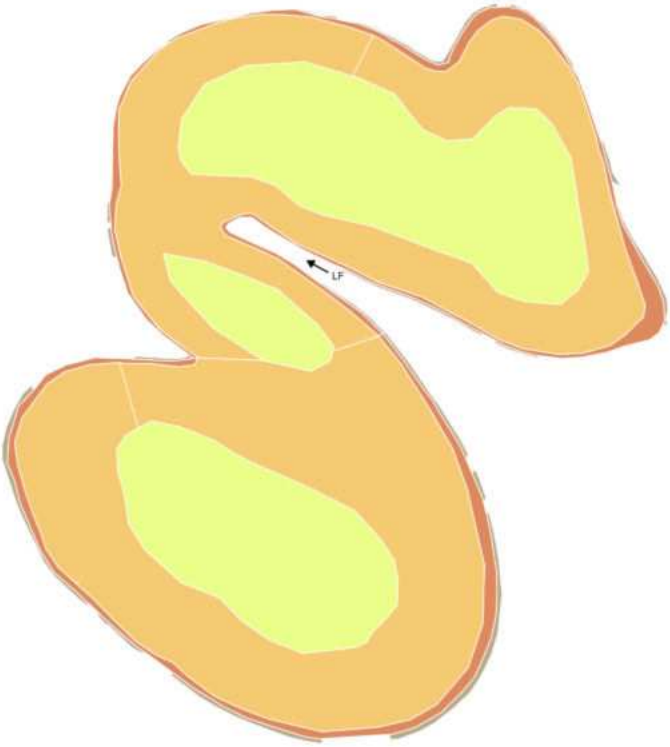

5 mm

➔ LF: Lateral fissure

Age: 24 GW

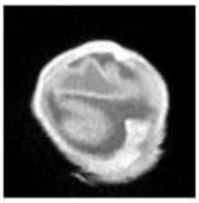

L-R Level: -22.38 mm

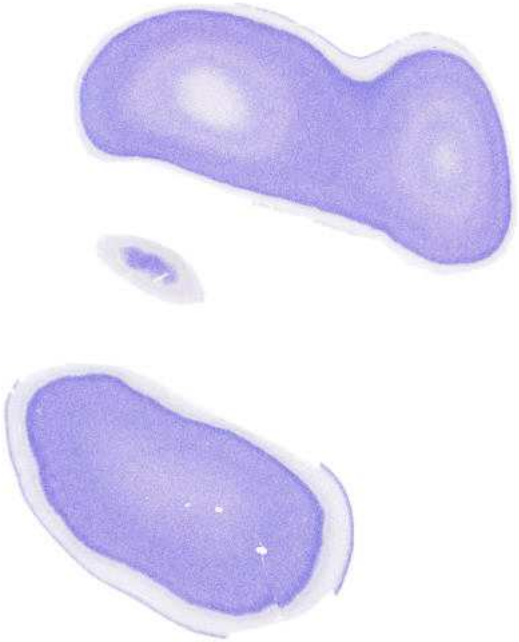

5 mm

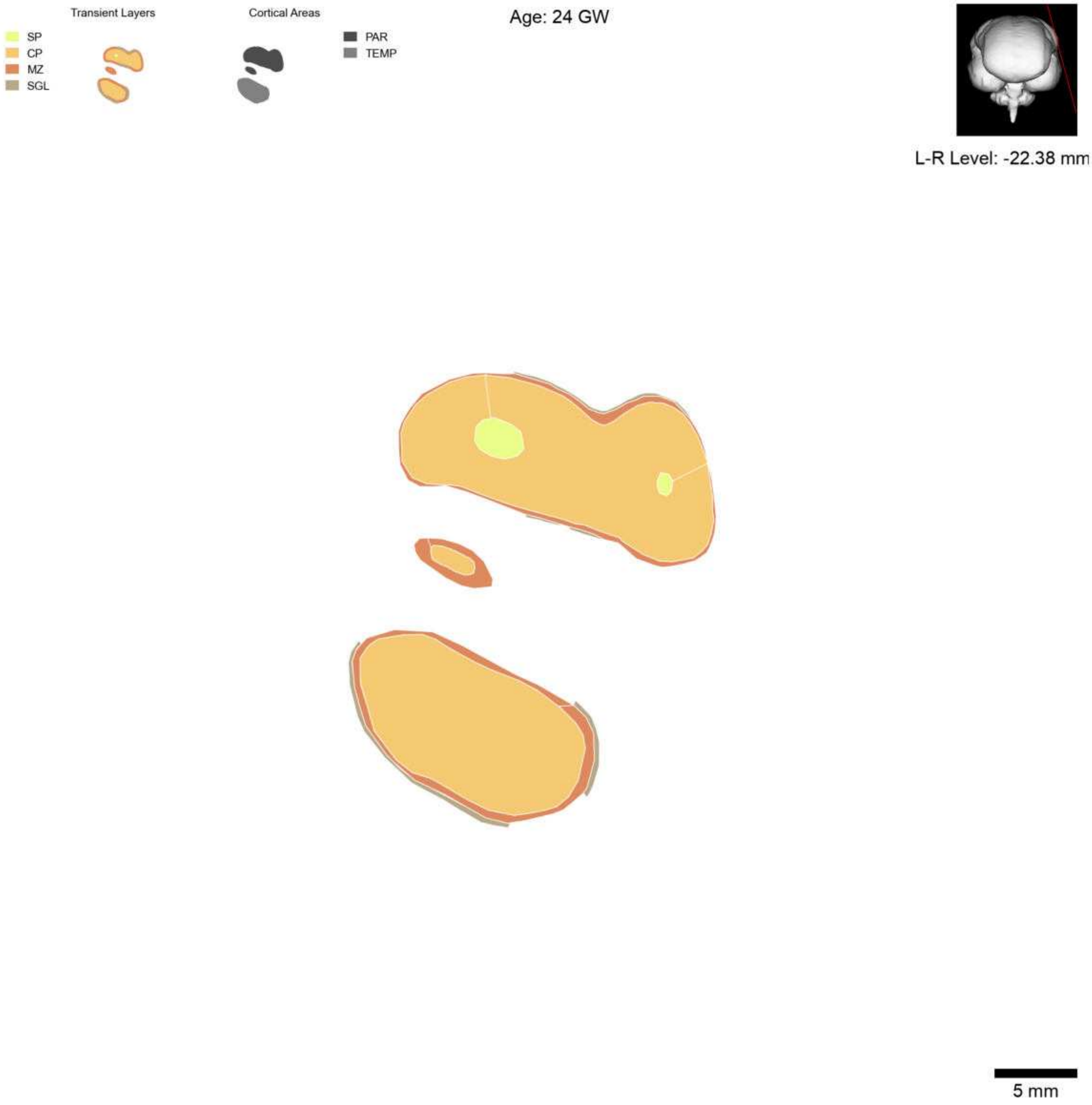
Transient Layers
SP
CP
MZ
SGL
Cortical Areas
PAR
TEMP
Age: 24 GW
L-R Level: -22.38 mm
5 mm

Age: 24 GW

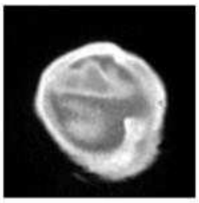

L-R Level: -22.98 mm

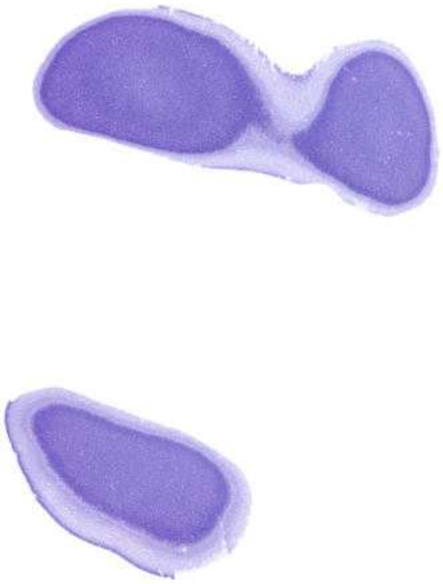

5 mm

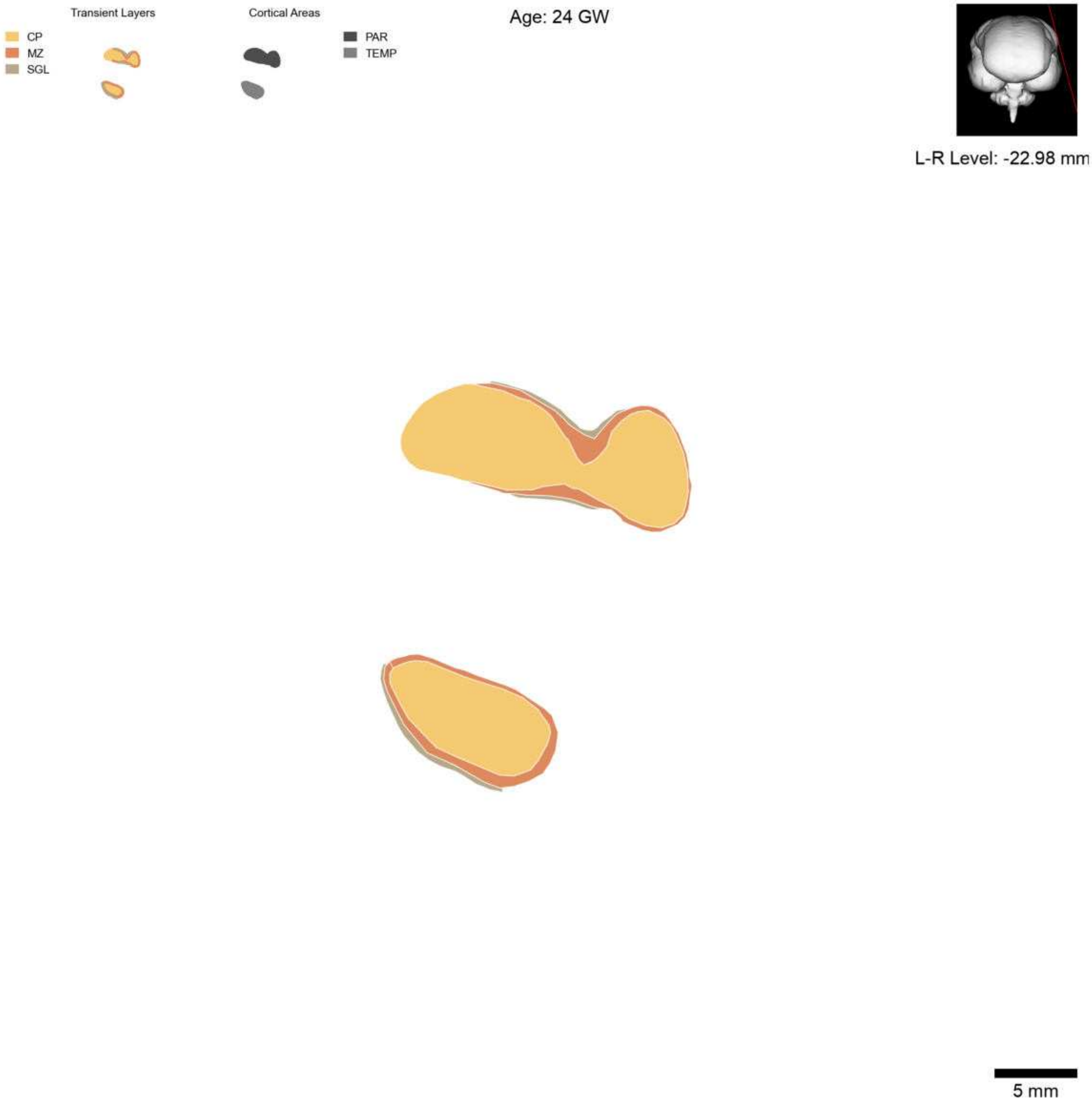
Transient Layers
CP
MZ
SGL
Cortical Areas
PAR
TEMP
Age: 24 GW
L-R Level: -22.98 mm
5 mm

Age: 24 GW

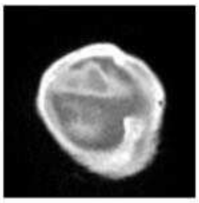

L-R Level: -23.22 mm

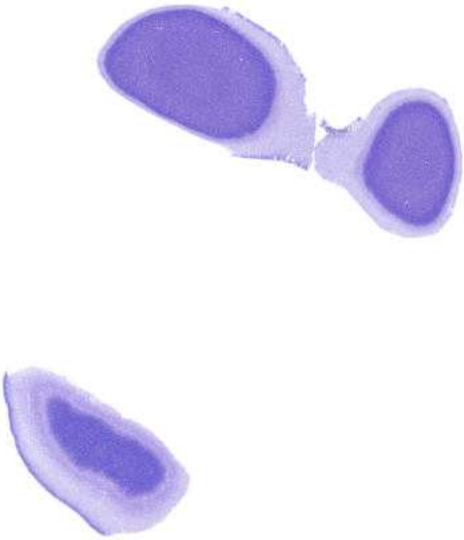

5 mm

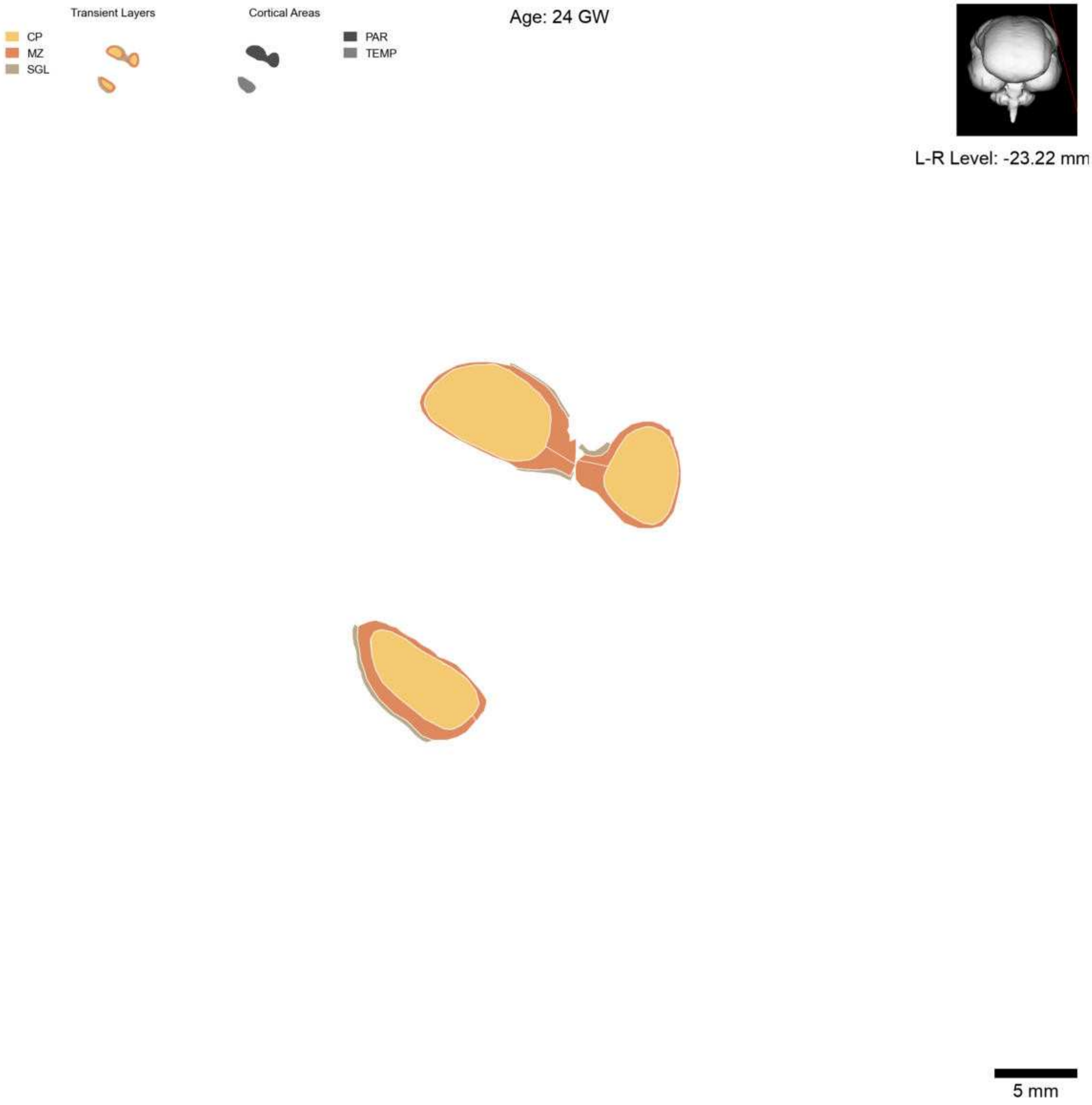

Transient Layers
CP
MZ
SGL
Cortical Areas
PAR
TEMP
Age: 24 GW
L-R Level: -23.22 mm
5 mm

NAME	ACRONYM	COLOR	ID
Abducens nerve	VI		466
Abducens nucleus	VIn		274
Accessory nucleus	XIn		373
Accumbent neuroepithelium	acbn		1024
Allocortex	AlCTx		64
Ammon's horn	CA		83
Amygdala	AMY		135
Amygdalo-hippocampal area	AHi		138
Amygdalo-striatal area	AStr		393
Anisform lobule, crus II	CRUSII		362
Ansa lenticularis	al		337
Ansiform lobule (HVIIA)	AN		360
Ansiform lobule, crus I	CRUSI		361
Anterior amygdaloid area	AAA		289
Anterior commissure	ac		243
Anterior complex of the thalamus	ANT		256
Anterior hypothalamic area	AHA		413
Anterior hypothalamic nucleus	AHN		384
Anterior lobe of cerebellum (H_V)	CBX-Ant		348
Anterior pretectal nucleus	APT		322
Anterodorsal nucleus of the thalamus	AD		163
Anteromedial nucleus of the thalamus	AM		164
Anteroventral nucleus of the thalamus	AV		165
Aqueduct	Aq		227
Archicortex	ArCTx		65
Arcuate nucleus of the hypothalamus	ARH		181
Arcuate nucleus of the medulla	ARM		336
Area postrema	AP		316
Auditory radiation	audr		345
Basal forebrain	BFB		148
Basal nuclei	BN		140
Basal nucleus of Meynert	BNM		313
Basal nucleus of the amygdala	BL		286
Basal nucleus of the amygdala, dorsal part	BLd		433
Basal nucleus of the amygdala, dorsolateral part	BLdl		434
Basal nucleus of the amygdala, intermediate part	BLi		435
Basal nucleus of the amygdala, ventrolateral part	BLvl		436
Basolateral complex of the amygdala	BLA		136
Basomedial nucleus of the amygdala	BM		287

NAME	ACRONYM	COLOR	ID
Basomedial nucleus of the amygdala, magnocellular	BMmg		437
Basomedial nucleus of the amygdala, parvocellular	BMpv		438
Bed nucleus of the stria terminalis	BST		152
Biventral lobule (HVIII)	BVL		364
Brachium of the inferior colliculus	bic		343
Brainff	Br		1
CA1 field of hippocampus	CA1		386
CA2 field of hippocampus	CA2		387
CA3 field of hippocampus	CA3		388
CA4 field of hippocampus	CA4		389
Calcarine sulcus	CaS		46
Callosal glioepithelium	cc-gli		1014
Calossal sling	cs		1028
Caudal linear raphe nucleus	CLI		446
Caudate nucleus	Cau		142
Central canal	CC		230
Central complex of the thalamus	CTH		257
Central gray of the pons	CGP		429
Central gray of the spinal cord	CG		369
Central lateral nucleus of the thalamus	CL		166
Central lobe of cerebellum	CBX-Cent		352
Central medial nucleus of the thalamus	CM		395
Central nucleus of the amygdala	CEA		295
Central sulcus	CeS		20
Centralis (III)	CENT		350
Centromedian nucleus of the thalamus	CMT		258
Cerebellar cortex	CBX		216
Cerebellar glioepithelium/ependym	CB-g		1010
Cerebellar hemispheres	HEM		219
Cerebellar nuclei	CBN		217
Cerebellar peduncles	cpb		250
Cerebellum	CB		215
Cerebral cortex	Ctx		4
Cerebral peduncle	cp		242
Choroid plexus	Chp		229
Cingulate cortex	CING		120
Cingulate gyrus	CINGg		129
Cingulate neuroepithelium	cingn		1015
Cingulate sulcus	CINGs		128

NAME	ACRONYM	COLOR	ID
Claustrum	CLA		131
Cochlear nuclei	CN		374
Commissural nucleus	COM		272
Corona radiata	cor		237
Corpus callosum	cc		238
Corpus callosum, body	cc-b		240
Corpus callosum, genu	cc-g		239
Corpus callosum, splenium	cc-s		241
Cortical nucleus of the amygdala	COA		290
Cortical nucleus of the amygdala, anterior part	COAa		291
Cortical nucleus of the amygdala, posterior part	COAp		292
Cortical plate, cerebral cortex	CP-CTX		7
Cortical plate, cingulate cortex	CP-CING		123
Cortical plate, entorhinal cortex	CP-ENT		59
Cortical plate, frontal cortex	CP-FCTx		15
Cortical plate, hippocampal formation	CP-HPF		69
Cortical plate, hippocampus	CP-HIP		77
Cortical plate, insula	CP-INS		502
Cortical plate, occipital cortex	CP-OCC		41
Cortical plate, orbital cortex	CP-ORB		24
Cortical plate, parasubiculum	CP-PARA		104
Cortical plate, parietal cortex	CP-PAR		32
Cortical plate, postsubiculum	CP-POST		112
Cortical plate, presubiculum	CP-PRESUB		96
Cortical plate, subiculum	CP-SUB		88
Cortical plate, temporal cortex	CP-TEMP		50
Corticofugal tract	cortf		232
Corticomedial complex of the amygdala	CMX		137
Corticospinal tract	corts		233
Culmen (IV-V)	CUL		351
Cuneate nucleus	CUN		314
Declive (VI)	DEC		353
Dentate gyrus	DG		82
Dentate nucleus	DN		222
Developmental (transient) structures	dev		5000
Diencephalic neuroepithelium	dne		1000
Diencephalon	Di		159
Dorsal cochlear nucleus	DCN		375
Dorsal complex of the thalamus	DOR		254

NAME	ACRONYM	COLOR	ID
Dorsal funiculus	df		311
Dorsal gray of the spinal cord	DGSC		317
Dorsal horn	DH		367
Dorsal hypothalamic area	DHA		308
Dorsal motor nucleus	Xn		278
Dorsal premammillary nucleus	PMd		409
Dorsal raphe nucleus	DR		448
Dorsal sensory nucleus X	X-sens		271
Dorsal tegmental decussation	dtgx		457
Dorsal tegmental nucleus	DTN		267
Dorsomedial nucleus of the hypothalamus	DMH		183
Edinger-Westphal nucleus	EW		403
Endopiriform nucleus	EP		132
Entorhinal cortex	ENT		56
Epithalamic glioepithelium/ependyma	eg		1002
Epithalamus	EPI		173
External capsule	ext		244
External germinal layer, cerebellum	CBXext		1009
External medullary lamina of the thalamus	emlth		417
Facial motor nucleus	VIIn		275
Facial nerve	VII		467
Fasciola cinereum	fc		344
Fastigial nucleus	FN		220
Fiber tracts	ft		3000
Field of Forel	FF		175
Fimbria	fi		339
Flocculus (HX)	FLO		365
Folium (VIIa)	FOL		354
Forebrain	FB		2
Fornix	fx		247
Fourth ventricle	4V		228
Frontal cortex	FCtx		12
Frontal neuroepithelium	ftctxn		1023
Ganglionic eminence	GE		158
Germinal trigone	TRI		223
Globus pallidus	GP		145
Globus pallidus lateral segment	GPl		146
Globus pallidus medial segment	GPm		147
Gracile nucleus	GR		315

NAME	ACRONYM	COLOR	ID
Habenula	H		172
Habenulo-interpenduncular tract	hi		310
Hindbrain	HB		204
Hippocampal formation	HPF		66
Hippocampal glioepithelium/ependyma	hipg		1016
Hippocampus	HIP		74
Hypoglossal nucleus	XIIn		279
Hypothalamic glioepithelium/ependyma	hg		1003
Hypothalamus	HY		177
Induseum griseum	IG		84
Inferior colliculus	IC		195
Inferior colliculus, central nucleus	ICc		318
Inferior colliculus, dorsal nucleus	ICd		385
Inferior colliculus, external nucleus	ICe		319
Inferior olive	IO		212
Infundibulum	Inf		190
Insula	INS		118
Intercalated cell groups of the amygdala	IA		296
Intercalated nucleus of the medulla	INM		430
Intermediate gray of the spinal cord	IH		368
Intermediate zone, CA1 field	IZ-CA1		421
Intermediate zone, CA2 field	IZ-CA2		423
Intermediate zone, CA3 field	IZ-CA3		425
Intermediate zone, CA4 field	IZ-CA4		427
Intermediate zone, cerebral cortex	IZ-CTX		9
Intermediate zone, cingulate cortex	IZ-CING		125
Intermediate zone, entorhinal cortex	IZ-ENT		61
Intermediate zone, frontal cortex	IZ-FCTx		17
Intermediate zone, hippocampal formation	IZ-HPF		71
Intermediate zone, hippocampus	IZ-HIP		79
Intermediate zone, insula	IZ-INS		504
Intermediate zone, occipital cortex	IZ-OCC		43
Intermediate zone, orbital cortex	IZ-ORB		26
Intermediate zone, parasubiculum	IZ-PARA		106
Intermediate zone, parietal cortex	IZ-PAR		34
Intermediate zone, postsubiculum	IZ-POST		114
Intermediate zone, presubiculum	IZ-PRESUB		98
Intermediate zone, subiculum	IZ-SUB		90
Intermediate zone, temporal cortex	IZ-TEMP		52

NAME	ACRONYM	COLOR	ID
Internal capsule	int		236
Internal medullary lamina of the thalamus	im		334
Interpeduncular fossa	IpF		443
Interpeduncular nucleu, caudal part	IPNc		452
Interpeduncular nucleu, dorsolateral part	IPNdl		450
Interpeduncular nucleu, dorsomedial part	IPNdm		451
Interpeduncular nucleus	INT		283
Interposed nucleus	IP		221
Interstitial nucleus of Cajal	INC		444
Islands of Calleja	IsCj		439
Lateral corticospinal tract	corts-l		234
Lateral dorsal nucleus of the thalamus	LD		162
Lateral fissure	LF		117
Lateral funiculus	lf		346
Lateral geniculate nucleus	LGN		167
Lateral habenula	LH		394
Lateral hypothalamic area	LHA		184
Lateral lemniscus	ll		327
Lateral mammillary nucleus	LMN		407
Lateral medullary lamina	lml		415
Lateral migratory stream	Lms		156
Lateral nucleus of the amygdala	LA		288
Lateral posterior nucleus of the thalamus	LP		416
Lateral preoptic area	LPO		300
Lateral reticular nucleus	LRN		305
Lateral septal nucleus	LS		150
Lateral tuberal nucleus	LTN		302
Lateral ventricle	LV		226
Lingula (I-II)	LING		349
Locus coeruleus	LC		209
Magnocellular preoptic nucleus	MgPO		411
Mammillary body	M		185
Mammillary glioepithelium/ependyma	mg		1004
Mammillary peduncle	mp		398
Mammillotegmental tract	mtg		312
Mammillothalamic tract	mth		284
Marginal zone, cerebral cortex	MZ-CTX		6
Marginal zone, cingulate cortex	MZ-CING		122
Marginal zone, entorhinal cortex	MZ-ENT		58

NAME	ACRONYM	COLOR	ID
Marginal zone, frontal cortex	MZ-FCTx		14
Marginal zone, hippocampal formation	MZ-HPF		68
Marginal zone, hippocampus	MZ-HIP		76
Marginal zone, insula	MZ-INS		501
Marginal zone, occipital cortex	MZ-OCC		40
Marginal zone, orbital cortex	MZ-ORB		23
Marginal zone, parasubiculum	MZ-PARA		103
Marginal zone, parietal cortex	MZ-PAR		31
Marginal zone, postsubiculum	MZ-POST		111
Marginal zone, presubiculum	MZ-PRESUB		95
Marginal zone, subiculum	MZ-SUB		87
Marginal zone, temporal cortex	MZ-TEMP		49
Medial dorsal nucleus of the thalamus	MD		161
Medial forebrain bundle	mfb		235
Medial geniculate nucleus	MGN		168
Medial habenula	MH		399
Medial lemniscus	ml		341
Medial mammillary nucleus	MMN		408
Medial medullary lamina	mml		414
Medial nucleus of the amygdala	MEA		294
Medial preoptic area	MEPO		301
Medial preoptic nucleus	MPO		187
Medial pretectal nucleus	MPT		323
Medial septal nucleus	MS		151
Median eminence	ME		191
Median raphe nucleus	MrN		447
Medulla	Med		211
Medullary glioepithelium/ependyma	meg		1007
Mesencephalic neuroepithelium	mn		1006
Mesencephalic nucleus	V		261
Midbrain	MB		192
Migratory stream, general	Ms-g		441
Migratory streams	Mss		442
Nodulus (X)	NOD		358
Nucleus accumbens	NAc		144
Nucleus ambiguus	AMB		277
Nucleus fasciculosus of the thalamus	FA		459
Nucleus limitans of the thalamus	NL		405
Nucleus of Darkschewitsch	ND		404

NAME	ACRONYM	COLOR	ID
Nucleus of Roller	NR		213
Nucleus of the diagonal band	NDB		154
Nucleus of the lateral lemniscus, dorsal	NLLd		264
Nucleus of the lateral lemniscus, ventral	NLLv		265
Nucleus of the optic tract	NOT		325
Nucleus of the posterior commissure	NPC		321
Nucleus prepositus	PRP		214
Nucleus reuniens	RE		170
Occipital cortex	OCC		38
Occipital lobe neuroepithelium and subventricular zc	ocn		1020
Oculomotor nerve	III		455
Oculomotor nuclear complex	III		263
Olfactory bulb	OLFb		133
Olfactory cortex	OLF		134
Olfactory cortex	OLF		134
Olfactory penduncle	olfp		252
Olfactory tubercle	OT		155
Olivary pretectal nucleus	OPT		326
Optic chiasm	och		249
Optic tract	ot		251
Orbital cortex	ORB		21
Orbitofrontal neuroepithelium	orbn		1022
Parabigeminal nucleus	PBN		320
Parabrachial nucleus	PB		206
Parabrachial nucleus, lateral part	PBl		464
Parabrachial nucleus, medial part	PBm		465
Parafascicular nucleus of the thalamus	Pf		171
Paraflocculus (HIX)	PFLO		366
Parahippocampal gyrus	PARG		55
Parahippocampal neuroepithelium	phipn		1018
Paralaminar nucleus of the amygdala	PLA		297
Paramedian lobules (HVIIB)	PRM		363
Paranigral nucleus of the VTA	PnVTA		449
Parapeduncular nucleus	PaP		453
Parasubiculum	PARA		101
Paratenial nucleus of the thalamus	PT		391
Paraventricular nucleus of the hypothalamus	PVH		180
Paraventricular nucleus of the thalamus	PVT		333
Parietal cortex	PAR		29

NAME	ACRONYM	COLOR	ID
Parietal lobe neuroepithelium and subventricular zo	parn		1021
Parieto-occipital sulcus	POS		37
Periamygdaloid cortex	PAC		293
Periaqueductal gray	PAG		285
Perifornical nucleus	PeF		303
Periventricular complex of the thalamus	PVTH		260
Pineal gland	PIN		280
Pituitary gland	Pit		189
Pons	P		205
Pontine gray	PG		208
Posterior commissure	pc		248
Posterior complex of the thalamus	POT		259
Posterior hypothalamic nucleus	PHN		307
Posterior pretectal nucleus	PPT		324
Posteromedial hypothalamic nucleus	PHM		309
Postsubiculum	POST		109
Precerebellar neuroepithelium	pcbn		1008
Pregeniculate nucleus	PGN		419
Premammillary area	PMM		299
Preoptic area	PO		186
Preoptic glioepithelium/ependyma	prgl		1005
Preoptic periventricular nucleus	PVPO		304
Presubiculum	PRESUB		93
Pretectum	Prt		203
Principal mammillary tract	pmt		396
Principal sensory nucleus of the trigeminal	PSV		266
Pulvinar nucleus of the thalamus	PULV		169
Putamen	Put		143
Pyramidal decussation	pyrd		281
Pyramis (VIII)	PYR		356
Raphe interpositus nucleus	RIP		461
Raphe magnus nucleus	RM		460
Raphe nuclei	Raphe		210
Raphe obscurus nucleus	RO		463
Raphe pallidus nucleus	RPA		462
Red nucleus	R		200
Reticular formation, Medulla	Ret-Med		378
Reticular formation, Midbrain	Ret-MB		377
Reticular formation, Pons	Ret-P		372

NAME	ACRONYM	COLOR	ID
Reticular nucleus of the thalamus	RT		176
Reticular tegmental nucleus	RTN		268
Retrochiasmatic nucleus of the hypothalamus	RCH		418
Retrorubral area	RR		428
Rhabdoid nucleus	RbN		454
Rhombencephalic neuroepithelium	rhn		1025
Rhomboid nucleus of the thalamus	RH		390
Rostral linear raphe nucleus	RLI		445
Rostral migratory stream	Rms		157
Septum	SEP		149
Simplex lobule (HVI)	SIM		359
Solitary nucleus	SOL		270
Spinal cord	SP		231
Spinal nucleus of the trigeminal	SPV		273
Stria medullaris	stm		246
Stria terminalis	stt		245
Striatum	Str		141
Strionuclear glioepithelium	str-g		1013
Subfornical organ	SFO		255
Subgeniculate nucleus	SbGN		432
Subiculum	SUB		85
Subparafascicular nucleus	SPF		380
Subparafascicular nucleus, magnocellular part	SPFm		381
Subparafascicular nucleus, parvocellular part	SPFp		382
Subpial granular layer, cerebral cortex	SGL-CTX		5
Subpial granular layer, cingulate cortex	SGL-CING		121
Subpial granular layer, entorhinal cortex	SGL-ENT		57
Subpial granular layer, frontal cortex	SGL-FCTx		13
Subpial granular layer, hippocampal formation	SGL-HPF		67
Subpial granular layer, hippocampus	SGL-HIP		75
Subpial granular layer, insula	SGL-INS		500
Subpial granular layer, occipital cortex	SGL-OCC		39
Subpial granular layer, orbital cortex	SGL-ORB		22
Subpial granular layer, parasubiculum	SGL-PARA		102
Subpial granular layer, parietal cortex	SGL-PAR		30
Subpial granular layer, postsubiculum	SGL-POST		110
Subpial granular layer, presubiculum	SGL-PRESUB		94
Subpial granular layer, subiculum	SGL-SUB		86
Subpial granular layer, temporal cortex	SGL-TEMP		48

NAME	ACRONYM	COLOR	ID
Subplate zone, CA1 field	SP-CA1		420
Subplate zone, CA2 field	SP-CA2		422
Subplate zone, CA3 field	SP-CA3		424
Subplate zone, CA4 field	SP-CA4		426
Subplate zone, cerebral cortex	SP-CTX		8
Subplate zone, cingulate cortex	SP-CING		124
Subplate zone, entorhinal cortex	SP-ENT		60
Subplate zone, frontal cortex	SP-FCTx		16
Subplate zone, hippocampal formation	SP-HPF		70
Subplate zone, hippocampus	SP-HIP		78
Subplate zone, insula	SP-INS		503
Subplate zone, occipital cortex	SP-OCC		42
Subplate zone, orbital cortex	SP-ORB		25
Subplate zone, parasubiculum	SP-PARA		105
Subplate zone, parietal cortex	SP-PAR		33
Subplate zone, postsubiculum	SP-POST		113
Subplate zone, presubiculum	SP-PRESUB		97
Subplate zone, subiculum	SP-SUB		89
Subplate zone, temporal cortex	SP-TEMP		51
Substantia innominata	SI		153
Substantia nigra	SN		197
Substantia nigra pars compacta	SNc		198
Substantia nigra pars reticulata	SNr		199
Subthalamus	Sth		188
Subventricular zone, cerebral cortex	SVZ-CTX		10
Subventricular zone, cingulate cortex	SVZ-CING		126
Subventricular zone, entorhinal cortex	SVZ-ENT		62
Subventricular zone, frontal cortex	SVZ-FCTx		18
Subventricular zone, hippocampal formation	SVZ-HPF		72
Subventricular zone, hippocampus	SVZ-HIP		80
Subventricular zone, insula	SVZ-INS		505
Subventricular zone, occipital cortex	SVZ-OCC		44
Subventricular zone, orbital cortex	SVZ-ORB		27
Subventricular zone, parasubiculum	SVZ-PARA		107
Subventricular zone, parietal cortex	SVZ-PAR		35
Subventricular zone, postsubiculum	SVZ-POST		115
Subventricular zone, presubiculum	SVZ-PRESUB		99
Subventricular zone, subiculum	SVZ-SUB		91
Subventricular zone, temporal cortex	SVZ-TEMP		53

NAME	ACRONYM	COLOR	ID
Superior cerebellar peduncle decussation	scpx		458
Superior colliculus	SC		194
Superior colliculus commissure	scc		431
Superior olive	SO		207
Suprachiasmatic nucleus of the hypothalamus	SCH		179
Suprageniculate nucleus	SGN		400
Supramammillary area	SUM		298
Supramammillary decussation	smd		397
Supraoptic nucleus of the hypothalamus	SON		178
Tectum	Tct		193
Tegmentum	Tgt		196
Telencephalon	Tel		3
Temporal cortex	TEMP		47
Temporal lobe neuroepithelium	tempn		1019
Tenia tecta	TT		130
Thalamic glioepithelium/ependyma	tg		1001
Thalamus	TH		160
Third ventricle	3V		225
Transient cell zone in the external capsule	tcet		1026
Transient cell zone in the extreme capsule	tcete		1027
Trapezoid body	tb		342
Trigeminal motor nucleus	Vn		269
Trigeminal nerve	V		469
Trochlear nerve	IV		468
Trochlear nucleus	IV		262
Tuber (VIIb)	TUB		355
Tuberal area	TU		412
Tuberomammillary nucleus	TMM		306
Uncinate fasciculus (forebrain)	Unc-f		338
Uvula (IX)	UVU		357
Ventral anterior nucleus of the thalamus	VA		329
Ventral cochlear nucleus	VCN		376
Ventral complex of the thalamus	VEN		282
Ventral funiculus	vf		347
Ventral gray of the spinal cord	VG		370
Ventral horn	VH		371
Ventral lateral nucleus of the thalamus	VL		330
Ventral medial basal nucleus of the thalamus	VMB		402
Ventral medial nucleus of the thalamus	VM		383

NAME	ACRONYM	COLOR	ID
Ventral posteroinferior nucleus of the thalamus	VPI		401
Ventral posterolateral nucleus of the thalamus	VPL		331
Ventral posteromedial nucleus of the thalamus	VPM		332
Ventral premammillary nucleus	PMv		410
Ventral tegmental area	VTA		201
Ventral tegmental decussation	vtgx		456
Ventricles	Vs		6000
Ventricular zone, cerebral cortex	VZ-CTX		11
Ventricular zone, cingulate cortex	VZ-CING		127
Ventricular zone, entorhinal cortex	VZ-ENT		63
Ventricular zone, frontal cortex	VZ-FCTx		19
Ventricular zone, hippocampal formation	VZ-HPF		73
Ventricular zone, hippocampus	VZ-HIP		81
Ventricular zone, insula	VZ-INS		506
Ventricular zone, occipital cortex	VZ-OCC		45
Ventricular zone, orbital cortex	VZ-ORB		28
Ventricular zone, parasubiculum	VZ-PARA		108
Ventricular zone, parietal cortex	VZ-PAR		36
Ventricular zone, postsubiculum	VZ-POST		116
Ventricular zone, presubiculum	VZ-PRESUB		100
Ventricular zone, subiculum	VZ-SUB		92
Ventricular zone, temporal cortex	VZ-TEMP		54
Ventromedial nucleus of the hypothalamus	VMH		182
Vermis	VERM		218
Vestibular nuclear complex	VNC		276
Visual radiation	visr		340
White matter fibers	wmf		253
Zona incerta	ZI		174
